# Tumor progression and chromatin landscape of lung cancer are regulated by the lineage factor GATA6

**DOI:** 10.1038/s41388-020-1246-z

**Published:** 2020-03-10

**Authors:** Anna Arnal-Estapé, Wesley L. Cai, Alexandra E. Albert, Minghui Zhao, Laura E. Stevens, Francesc López-Giráldez, Kiran D. Patel, Siddhartha Tyagi, Earlene M. Schmitt, Thomas F. Westbrook, Don X. Nguyen

**Affiliations:** 1 Department of Pathology, Yale University School of Medicine, New Haven, CT USA; 2 Yale Cancer Center, Yale University School of Medicine, New Haven, CT USA; 3 Department of Cell Biology, Yale University School of Medicine, New Haven, CT USA; 4 Yale Center for Genome Analysis, Yale University School of Medicine, New Haven, CT USA; 5 Department of Biochemistry & Molecular Biology, Baylor College of Medicine, Houston, TX USA; 6 Department of Molecular & Human Genetics, Baylor College of Medicine, Houston, TX USA; 7 Therapeutic Innovation Center (THINC), Baylor College of Medicine, Houston, TX USA; 8 Department of Medicine (Medical Oncology), Yale University School of Medicine, New Haven, CT USA; 9 Present Address: Department of Medical Oncology, Dana-Farber Cancer Institute, Boston, MA USA

**Keywords:** Non-small-cell lung cancer, Epigenetics

## Abstract

Lineage selective transcription factors (TFs) are important regulators of tumorigenesis, but their biological functions are often context dependent with undefined epigenetic mechanisms of action. In this study, we uncover a conditional role for the endodermal and pulmonary specifying TF GATA6 in lung adenocarcinoma (LUAD) progression. Impairing *Gata6* in genetically engineered mouse models reduces the proliferation and increases the differentiation of Kras mutant LUAD tumors. These effects are influenced by the epithelial cell type that is targeted for transformation and genetic context of Kras-mediated tumor initiation. In LUAD cells derived from surfactant protein C expressing progenitors, we identify multiple genomic loci that are bound by GATA6. Moreover, suppression of *Gata6* in these cells significantly alters chromatin accessibility, particularly at distal enhancer elements. Analogous to its paradoxical activity in lung development, GATA6 expression fluctuates during different stages of LUAD progression and can epigenetically control diverse transcriptional programs associated with bone morphogenetic protein signaling, alveolar specification, and tumor suppression. These findings reveal how GATA6 can modulate the chromatin landscape of lung cancer cells to control their proliferation and divergent lineage dependencies during tumor progression.

## Introduction

Lung epithelial differentiation is coordinated by a network of transcription factors (TFs) whose activities are cell lineage specific. The contextual regulation of these networks is required for pulmonary development and homeostasis while their perturbations are linked to chronic diseases of the airways [1] and lung tumorigenesis [2]. The genetic and cellular determinants under which lineage TFs function in lung cancer are of significant interest, as thoracic malignancies account for most cancer-related deaths, have various cell lineage ontogenies, and are histologically diverse [3].

The GATA family of zinc finger DNA binding TFs is conserved mediators of cell fate. During endodermal patterning, GATAs can activate or repress transcription [4]. GATAs may establish transcriptional competence of lineage genes by recruiting protein complexes which remodel heterochromatin [5]. GATA6 in particular is required for lung development and morphogenesis in a dose and temporal dependent manner. In developing murine lungs, loss of *Gata6* blocks terminal differentiation, whereas *Gata6* gain of function impairs alveolarization [6, 7]. In adult lungs, loss of GATA6 causes an imbalance in progenitor lineage expansion and aberrant epithelial differentiation [8]. In human pluripotent stem cells, low levels of *GATA6* favor lung epithelial specification and proliferation, whereas increased *GATA6* levels may activate more mature markers of the distal lung epithelium [9].

In human lung cancers, *GATA6* is rarely mutated, but its expression is increased in early stage non small cell lung cancer (NSCLC) relative to normal tissue and may correlate with tumor promoting genes [10, 11]. However, *GATA6* is decreased in high-grade NSCLC [12, 13], and this reduction can enhance metastatic competence [14]. The mechanisms of GATA6’s paradoxical functions during malignant transformation in the lungs are unknown and may reflect the conditional requirement for lineage TFs during various stages of lung development. In this study, we uncover a previously unrecognized role for GATA6 during the early stages of lung tumorigenesis and reveal broad epigenomic functions of this lineage factor in lung cancer cells.

## Results

### *Gata6* regulates tumor grade and proliferation of NSCLC

**Fig. 1:**
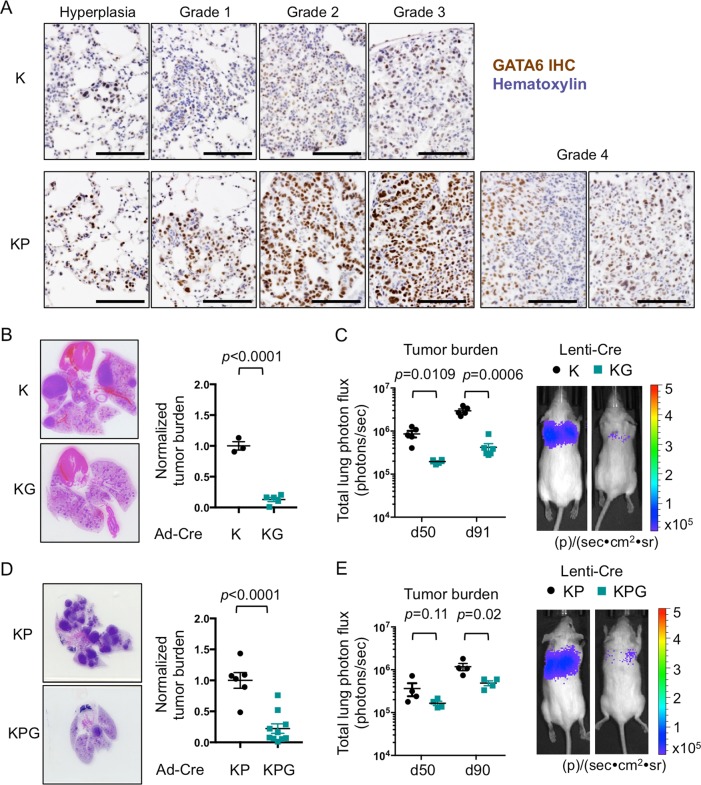
*Gata6* deletion impairs LUAD progression in *Kras*^*LSL-G12D*^ (K) and *Kras*^*LSL-G12D*^*p53* ^*fl/fl*^ (KP) mouse models. **a** Immunohistochemistry of GATA6 in K and KP GEMMs at different stages of LUAD progression. Scale bar = 100 μm. **b** Left, H&E of tumor-bearing lungs from K and KG mice at 50 weeks post infection with Ad-Cre. Right, quantification of tumor burden (total tumor area) per lung (*n* = 3–5 mice). **c** Left, lung tumor burden of K and KG mice measured by bioluminescence at 50 and 91 days post infection with Lenti-Cre (*n* = 5–6 mice). Right, representative bioluminescent pictures from day 91. **d** Left, H&E of tumor-bearing lungs from KP and KPG mice at 12 weeks post infection with Ad-Cre. Right, quantification of tumor burden (total tumor area) per lung (*n* = 6–10 mice). **e** Left, lung tumor burden of KP and KPG mice measured by bioluminescence at 50 and 90 days post infection with Lenti-Cre (*n* = 4 mice). Right, representative bioluminescent pictures from day 90. Unless indicated, data were plotted with standard error of the mean (SEM). *P* value was calculated by unpaired *t*-test, except for day 50 of **e** (Mann–Whitney) and **c** (Welch’s *t*-test). Total tumor area was measured per lung and per mouse, and was normalized to K or KP controls.

**Fig. 2:**
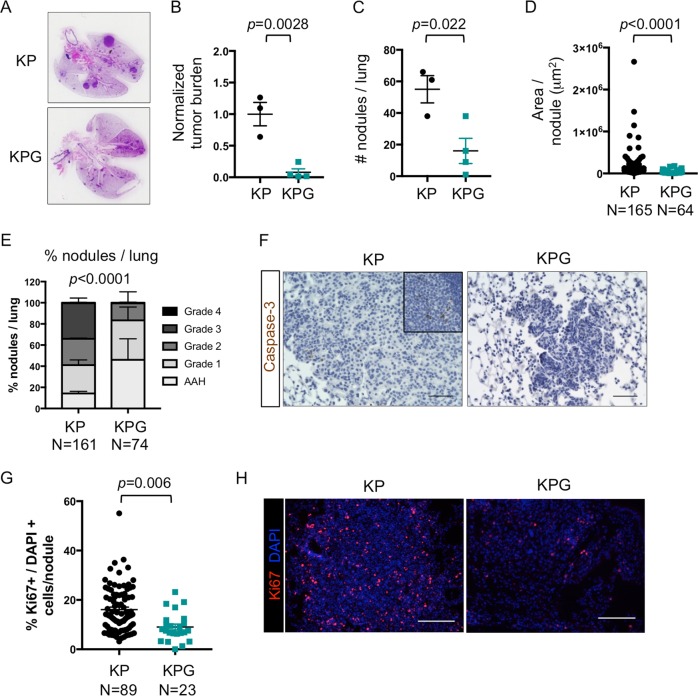
*Gata6* loss impairs cell proliferation and tumor grade of KP tumors. **a** H&E of tumor-bearing lungs from KP and KPG mice from Fig. 1e. **b**–**d** Quantification/measurement of tumor nodules of mice from Fig. 1e (*n* = 3–4 mice). **b** Tumor burden was quantified by measuring the total tumor area per lung and normalized to the KP group. **c** Number of nodules per lung was quantified. **d** Nodule area was measured for each individual nodule (*n* = 64–165 nodules). **e** Tumors from Fig. 1e were graded as previously described [42] (*n* = 74–161 nodules). AAH, atypical adenomatous hyperplasia. *P* value by chi-square. **f** Representative images of cleaved caspase-3 immunohistochemistry in mice from Fig. 1e. Top inset shows staining in the thymus as a positive control for Caspase-3+ apoptotic cells. Scale bar = 50 μm. **g** The percentage of Ki67+ cells relative to all DAPI+ cells was calculated per nodule from animals in Fig. 1e (*n* = 23–89 nodules). **h** Representative immunofluorescence of Ki67 (red) and DAPI (blue) from each group quantified in **g**. Scale bar = 100 μm. Unless indicated, data were plotted with standard error of the mean (SEM). *P* value was calculated by unpaired *t*-test, except for **d**, where *P* value was calculated by Mann–Whitney.

Malignancies from endodermal tissues frequently harbor *KRAS* mutations [15], and GATA6 expression correlates with *KRAS* mutations in human lung cancers [12]. In the lox-stop-lox *Kras*^*LSL-G12D*^ genetically engineered mouse model (GEMM) (referred to herein as K), low-grade adenomas, and lung adenocarcinoma (LUAD) arise by expression of a mutant *Kras* allele (*Kras*^*LSL-G12D*^) in the proximal-distal airways [16]. *Kras*^*LSL-G12D*^ expression in conjunction with loss of *Tp53* using a floxed null allele (exon 10) of *p53 (Kras*^*LSL-G12D*^ mice; *p53*^*fl/fl*^) (referred to herein as KP) generates more heterogeneous disease with higher grade tumors [17]. Across K and KP models, GATA6 was predominantly expressed in epithelial hyperplasia, with its levels increasing as adenomas progress (Grades 1–3) and heterogeneously decreasing in late stage adenocarcinomas (Grade 4) (Fig. 1a). To assess the requirement for GATA6 during LUAD progression, we crossed K and KP mice with mice expressing a floxed null allele (exon 2) of *Gata6* [18] to generate *Kras*^*LSL-G12D*^*Gata6*^*fl/fl*^ (KG) and *Kras*^*LSL-G12D*^*p53*^*fl/fl*^ *Gata6*^*fl/fl*^ (KPG) mice, respectively, with impaired GATA6 expression (Supplementary Fig. 1a, b). Tumors were then initiated via intratracheal delivery of a Cre-expressing adenovirus (Ad-Cre) or lentivirus (Lenti-Cre). Suppression of *Gata6* via Ad-Cre in KG mice significantly reduced lung tumor burden when compared with K mice (Fig. 1b). Similarly, Lenti-Cre infection impaired tumor progression in KG mice over 91 days (Fig. 1c, Supplementary Fig. 1c). Lung tumor burden and LUAD progression were also reduced in Ad-Cre and Lenti-Cre infected KPG mice relative to KP mice (Figs. 1d, e, 2a, b and Supplementary Fig. 1d). Altogether, impairing *Gata6* decreased Kras-mediated tumorigenesis across multiple background strains and animals (Supplementary Table 1).

Epithelial lineage plasticity can dictate the ability of solid tumor cells to modulate their proliferative potential, evade cell death, and/or bypass multiple differentiation cues. Hence, we assessed the biological role(s) of GATA6 in KP mice, which can generate higher grade LUADs. KPG mice harbored reduced tumor burden and number of tumors (Fig. 2a–c). These tumors were of smaller size (Fig. 2d), were well differentiated, and of lower grade when compared with KP mice (Fig. 2e). KPG lesions contained few apoptotic cells, but tumor cell proliferation was significantly decreased relative to KP lesions (Fig. 2f–h). Thus, GATA6 primarily regulates the proliferative capacity and differentiation/grade of KRAS mutant lung tumor cells.

### *Gata6* promotes tumorigenesis in a cell type and genotype dependent manner

**Fig. 3:**
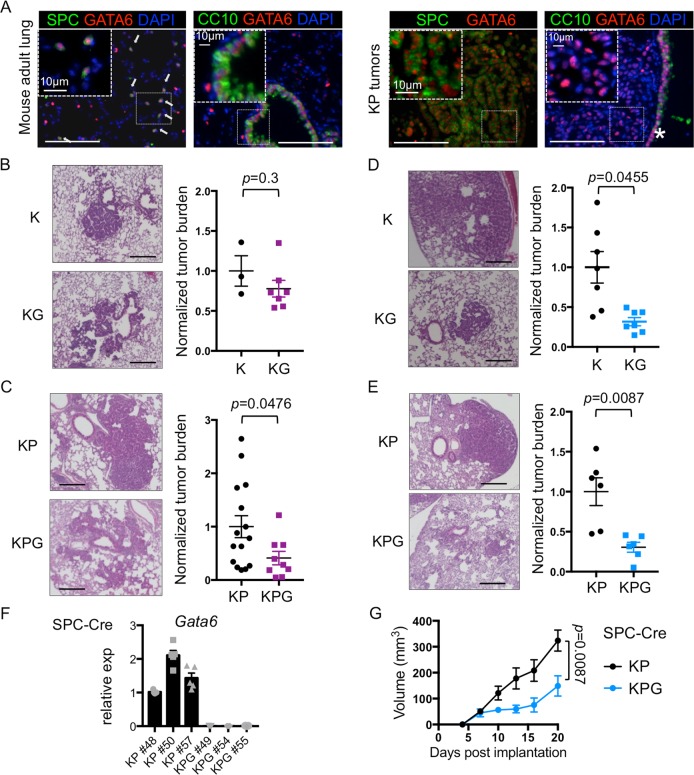
Regulation of lung tumorigenesis by *Gata6* is progenitor cell type dependent. **a** Immunofluorescence of GATA6 (red), SPC or CC10 (green), and DAPI (blue) in mouse adult lung (left) and KP tumors (right). *proximal nonmalignant airway. Scale bar = 100 μm. Arrows indicate SPC+ GATA6+ cells. **b** H&E of tumor-bearing lungs (left) and tumor burden (right) from K or KG mice at 13 weeks post infection with CC10-Cre (*n* = 3–7 mice). **c** KP or KPG mice were infected with CC10-Cre and analyzed as in **b** at 13 weeks post infection (*n* = 9–15 mice). **d** H&E of tumor-bearing lungs (left), and tumor burden (right) from K or KG mice at 22 weeks post infection with SPC-Cre (*n* = 7 mice). **e** KP or KPG mice were infected with SPC-Cre and analyzed as in **b** at 13 weeks (*n* = 6 mice). Scale bar for H&Es = 250 μm. Normalized tumor burden was plotted and analyzed as in Fig. 1b. *P* value was calculated by unpaired *t*-test, except for **c**, where the *P* value was calculated by Mann–Whitney and **d**, **e** with Welch’s *t*-test. **f** Quantitative Real Time PCR (qRT-PCR) for *Gata6* normalized to *b-actin* on tumor cell lines derived from SPC-Cre KP or KPG mice (*n* = 5 biological replicates). **g** Cells in **f** were injected subcutaneously and tumor volumes were measured (*n* = 3 mice per group, 6 tumors total). Data were plotted with SEM. *P* value was calculated by Mann–Whitney test using area under the curve.

NSCLC progression is dependent on a specific mutation(s) and the cell of origin in which it is expressed. Expression of *Kras*^*G12D*^ alone can transform surfactant protein C positive (SPC+) alveolar type II (AT2) cells, which generate LUADs in the distal airways [19]. Alternatively, activation of *Kras*^*G12D*^ in CC10 positive (CC10+) club cells at the proximal duct junctions results in hyperplasia, which can progress to SPC+ tumors at later stages [20]. GATA6 expression was observed in both CC10+ and SPC+ cells in normal lung tissue and was expressed in SPC+ cells within malignant KP tumors (Fig. 3a). As such, we used Ad-Cre under the control of the *CC10* or *SPC* promoters [20] to target *Gata6* specifically in these different progenitor cell populations (Supplementary Fig. 2a–c). GATA6 suppression did not affect CC10+ derived hyperplasia in K mice, while it modestly reduced the burden of CC10-Cre KP lesions (Fig. 3b, c). As previously described [20], SPC-Cre tumors in K mice predominantly remain SPC+ and are located in the distal airways (data not shown). However, we observed a higher proportion of lesions at the proximal duct junctions with a mix of SPC+ and CC10+ cells, preferentially in SPC-Cre infected KG mice (Supplementary Fig. 2d–f), suggesting that suppression of GATA6 differentially perturbs progenitors in this region. Nevertheless, GATA6 suppression in SPC+ cells restrained overall LUAD progression, particularly in the distal airways of both K and KP mice (Fig. 3d, e). Finally, we isolated cell lines from SPC-derived lung tumors of KP and KPG mice (Fig. 3f), which had the strongest phenotypic differences. SPC-derived malignant cells transplanted into syngeneic mice recapitulated the deficiency in tumor initiation and progression of KPG tumors relative to KP tumors arising in the GEMMs (Fig. 3g). Knockdown of GATA6 after transplanted KP tumors were fully established (150 mm^3^) did not cause tumor regression (Supplementary Fig. 2g). In sum, GATA6 is expressed in multiple epithelial cell types but preferentially enhances the early stages of malignant progression from SPC+ cells and/or tumors harboring both Kras and p53 mutations.

### Differential regulation of promoter and enhancer regions by *Gata6* in lung cancer cells

**Fig. 4:**
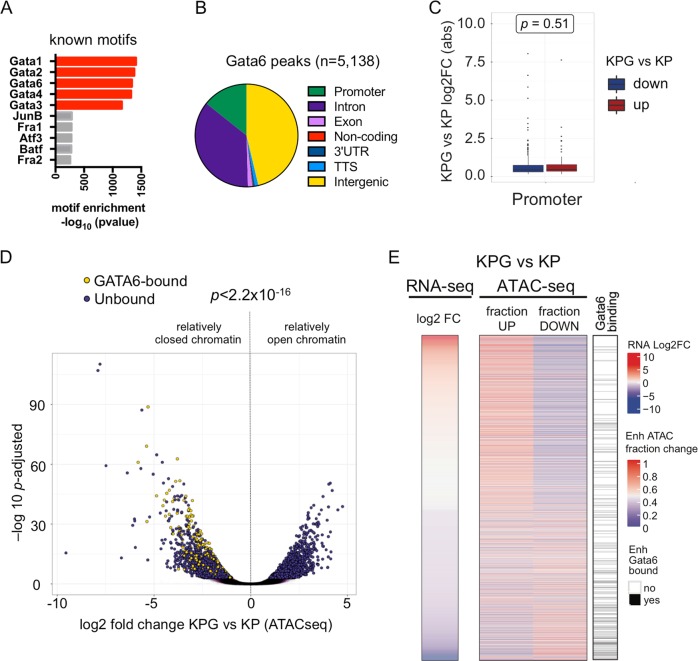
The GATA6 dependent chromatin and epigenomic landscape of LUAD cells. **a** Transcription factor (TF) binding motif analysis for GATA6 ChIP-seq peaks in KP tumor. **b** Annotation of the genomic loci overlapping with GATA6 peaks. Promoter = 0–2 Kb upstream and downstream of transcription start site (TSS). **c** Box plot of absolute log2 fold change of genes differentially expressed in KPG versus KP cells with GATA6 bound at their promoters. No bias was detected between activated (red) or repressed (blue) genes. *P* value was calculated by Mann–Whitney test. **d** Volcano plot of significant ATAC peaks with annotation of GATA6 binding (bound, yellow; unbound, purple). *P* value was calculated by chi-square. **e** Heatmap of differential gene expression, chromatin accessibility, and GATA6 binding in putative enhancers (defined as H3K27ac peaks within 100Kb of TSS). Left: each row is a gene that is differentially expressed in KPG versus KP cells. Middle: for each gene, ATAC peaks within 100 Kb of their TSS were determined, and the fraction of newly open (up) or closed (down) regions in KPG cells is plotted. Right: GATA6 binding (black) is based on whether one or more GATA6 ChIP-seq peaks overlap with significantly changed ATAC peaks within the same region in KP cells.

Cell lineage specification is ultimately controlled by epigenetic mechanisms and chromatin dynamics [21, 22]. Hence, we integrated RNA sequencing, chromatin immunoprecipitation sequencing (ChIP-seq), and assay for transposase-accessible chromatin using sequencing (ATAC-seq) to assess the genomic targets of GATA6 in relation to chromatin accessibility and the distribution of acetylated histone H3 lysine 27 (H3K27ac) or histone H3 lysine 4 trimethylation (H3K4me3) residues, which mark active enhancers or promoter elements, respectively [23] (Supplementary Tables 2–4). In SPC-Cre KP cells, genomic regions directly bound by GATA6 were enriched for canonical GATA motifs and were located across various genetic elements (Fig. 4a, b). Fifty-four percent of GATA6 bound regions had relatively “open” chromatin. Of these, 27.7% corresponded to promoters, whereas a greater proportion, 51.2%, were likely enhancer elements (Fig. 4b and Supplementary Fig. 3e).

Next, we compared the epigenome of KPG cells to KP cells and observed significant alterations in gene expression, histone modifications, and chromatin accessibility (Supplementary Fig. 3a–d). At promoters, chromatin accessibility correlated with differential gene expression when *Gata6* was impaired (Supplementary Fig. 3f). For 5.14% of the differentially expressed genes, GATA6 directly bound to their promoters in KP cells. This correlated with differential chromatin accessibility and both transcriptional activation or repression (Fig. 4c). Strikingly, however, the remaining majority of GATA6 bound regions in KP cells were primarily located near genes with relatively closed chromatin in KPG cells and their expression was decreased when *Gata6* is inhibited (Fig. 4d). A more specific analysis of putative enhancer regions indicated that GATA6 binding correlated most positively with chromatin accessibility and transcriptional activation at these distal loci (Fig. 4e). Thus, in SPC-derived KP cells, GATA6 binding may activate or repress promoters, but predominantly correlates with enhancer activation.

### Suppressing *Gata6* reprograms chromatin at distal enhancers

**Fig. 5:**
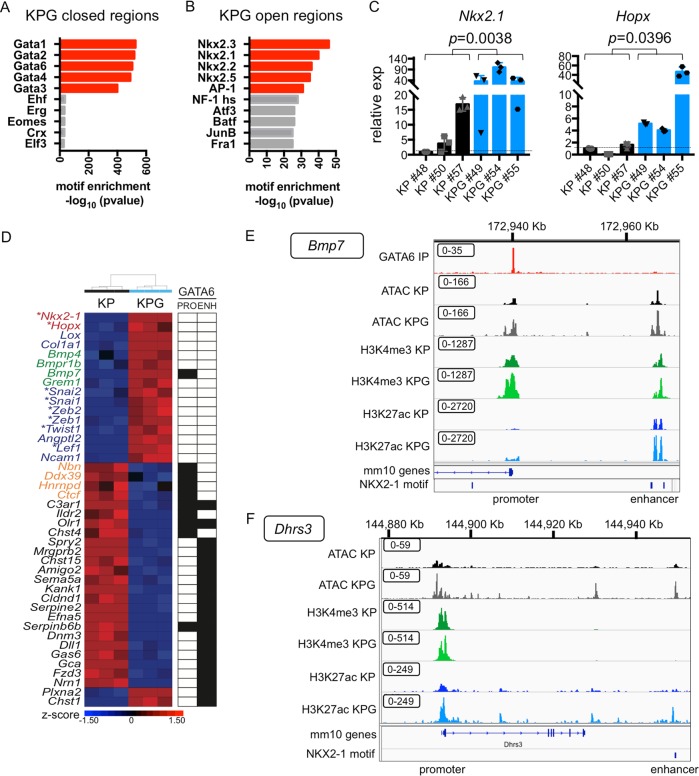
GATA6 deficiency reprograms distal enhancers that are enriched for NKX2-1 motifs. **a** TF motif analysis was performed for regions with newly closed chromatin in KPG cells. Log2 fold change < −1. *P* adjusted < 0.05. **b** TF motif analysis was performed for regions with newly open chromatin in KPG cells. Log2 fold change > 1. *P* adjusted < 0.05. **c** qRT-PCR of the indicated genes in cell lines from Fig. 3f (*n* = 3 biological replicates). Gene expression was normalized to *b-actin*. SEM is plotted and *P* value was calculated by Welch’s *t*-test. **d** Heatmap of representative genes differentially expressed in KPG versus KP cells (*P* adjusted < 0.05) clustered by Pearson average (*z*-score depicted). Genes involved in alveolar differentiation (red), BMP signaling (green), EMT (blue), and E2F targets (orange) are annotated. *TFs. For each gene, GATA6 binding (black box) in KP cells was indicated for their promoter (PRO) or enhancer (ENH). **e** IGV track for the upstream TSS of *Bmp7* (Chr 2 172,918,000–172,970,000 of the mm10 genome) with overlapping GATA6 binding peaks at the promoter and an NKX2-1 motif overlapping a distal enhancer. **f** IGV track at TSS of *Dhrs3* (Chr 4 144,878,000–144,950,000 of the mm10 genome) with an NKX2-1 motif overlapping a distal enhancer. Annotation of the tracks: KP GATA6 IP peaks (red), KP ATAC peaks (black), KPG ATAC peaks (gray), KP H3K4me3 peaks (dark green), KPG H3K4me3 peaks (light green), KP H3K27ac peaks (dark blue), KPG H3K27ac (light blue). Boxed values = data range for each track.

Consistently, closed chromatin regions in KPG cells were enriched for GATA6 motifs (Fig. 5a). These regions encompass many enhancers linked to genes such as *Delta-like 1* (Fig. 5d and Supplementary Fig. 4a) and promoters including *oxidized LDL receptor 1* (Fig. 5d and Supplementary Fig. 4b), which mediate cell proliferation and transformation [24]. A significant number of enhancers that were linked to upregulated genes in KPG cells were less likely to be bound by GATA6 in KP cells (*P* = 6.75 × 10^−10^ by chi-square), suggesting that loss of GATA6 can also indirectly induce activation of chromatin at other distal loci (Fig. 4e). Significantly, the newly open chromatin regions in KPG cells were enriched for NKX2-1 binding motifs (Fig. 5b). Furthermore, when GATA6 was reduced, the expression of the alveolar lineage TFs *Nkx2-1* and *Hopx* [25, 26] were themselves activated (Fig. 5c), and we identified NKX2-1 motifs in putative enhancers and promoters, respectively, for these genes (Supplementary Fig. 4c, d). We also observed NKX2-1 motifs in putative enhancers of other genes upregulated in KPG, such as *Bmp7* and *Dhrs3* (Fig. 5e, f).

We and others have previously identified different molecular subtypes of LUAD, which have distinct developmental gene expression profiles [14, 27, 28]. High *GATA6* expression in particular may be a context-dependent marker of KRAS mutant tumors that express markers of the distal airways [12] and committed alveolar cells (alveolar-like or alveolar-high tumors) [14, 29]. *NKX2-1* and *HOPX* can inhibit LUAD progression [14, 30], and their expression was partially inversely correlated with *GATA6* in “alveolar-high” tumors but less so in other LUAD subtypes (Supplementary Fig. 5).

We conclude that inhibition of GATA6 causes compensatory reprogramming and activation of chromatin at distal enhancers linked to the expression of known lineage-specific tumor suppressive genes.

### GATA6 constrains bone morphogenetic protein (BMP) signaling

**Fig. 6:**
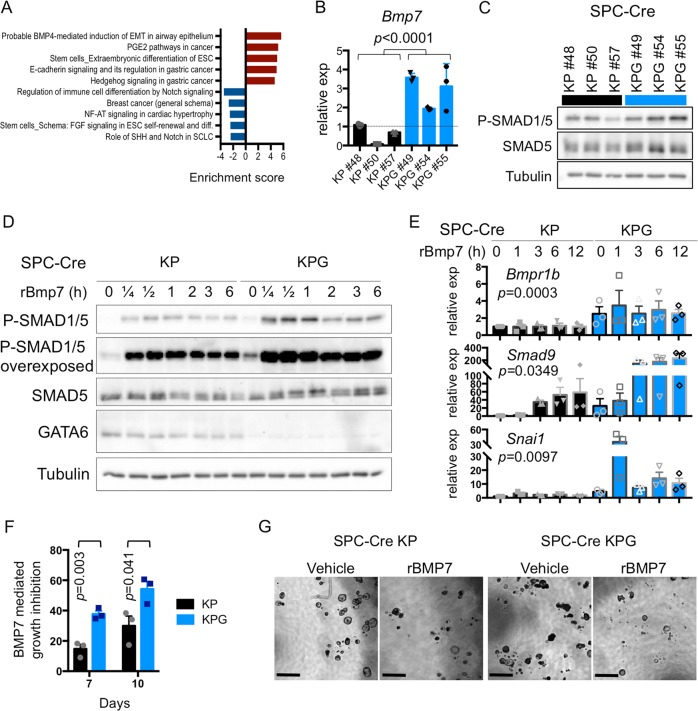
GATA6 inhibits BMP signaling. **a** Top 5 upregulated and downregulated pathways in SPC KPG cells relative to KP cells as defined by Metacore analysis using genes from Supplementary Fig. 3a. Enrichment score = –log10 *P* value (corrected for directionality). **b** qRT-PCR of the *Bmp7* in the indicated cell lines (*n* = 3 biological replicates). Gene expression was normalized to *b-actin*. SEM is plotted and *P* value was calculated by Welch’s *t*-test. **c** Western blot of phospho-SMAD1/5 Ser463/465, SMAD5, and Tubulin in the indicated samples. **d** KP and KPG cells were stimulated with recombinant murine BMP7 (100 ng/mL) and harvested at the indicated time points (h = hours). Western blots for the indicated proteins were performed on cell lysates. Depicted is a representative of three independent experiments. **e** *Bmpr1b, Smad9*, and *Snai1* mRNA were measured by qRT-PCR in KP and KPG cells that were stimulated with BMP7 for the indicated time points (h = hours) as in **d** (*n* = 3 biological replicates). Expression was normalized to *b-actin* and SEM was plotted. For *Bmpr1b*, *P* value was calculated comparing all values for each cell line using unpaired *t*-test. For other genes, *P* value was calculated by two-way ANOVA (cell line and time point). **f** Quantification of KP and KPG cells grown as organoids in the presence of BMP7 or vehicle. BMP7 growth inhibition was calculated by normalizing BMP7 treated cell growth relative to vehicle control and plotted as an absolute value. Mean and SEM plotted from three independent experiments and *P* value was calculated by unpaired *t*-test. **g** Representative bright field images of the organoids are depicted. Scale bar = 1000 μm.

Next, we examined how the epigenomic activity of GATA6 correlates with its biological functions. When compared with KP cells, the most significantly activated gene expression pathway(s) in KPG cells included stem cell differentiation and BMP signaling (Fig. 6a and Supplementary Fig. 6a). In the lungs, stromal BMP inhibits AT2 cell self-renewal [31] and it can induce partial epithelial to mesenchymal transition (EMT) of nonmalignant epithelial airway cells [32].

Across independently generated SPC+ derived LUAD cells, we found that reducing GATA6 by Cre recombination or short hairpin RNAs (shRNAs) activated multiple mediators of BMP signaling, such as *Bmp7* (Fig. 6b and Supplementary Fig. 6e, f). Also, the EMT pathway was upregulated in KPG cells (Supplementary Fig. 6b) and some EMT TFs (e.g., *Snai1*) were coactivated (Fig. 5d and Supplementary Fig. 6c, f). In KPG cells, steady-state BMP signaling, as assessed by phospho-SMAD5, was increased (Fig. 6c). Moreover, suppression of GATA6 induced expression of the Bmp receptor (*Bmpr1b*) (Fig. 6e) and rendered these tumor cells more sensitive to exogenous Bmp7 stimulation and downstream *Smad9* expression (Fig. 6d, e). Analogously, *Bmpr1b* and *Bmp7* levels were generally higher in KG tumor nodules when compared with K tumor nodules in vivo, with increases in *Bmpr1b* expression being most significant (Supplementary Fig. 6d). Exogenous stimulation of cell lines with BMP7 also caused downstream activation of *Snai1* which was further potentiated in KPG cells relative to KP cells (Fig. 6e). Despite activation of EMT markers such as Vimentin, KPG cells retained E-cadherin expression (Fig. 5d and Supplementary Fig. 6g, h). Moreover, KPG cells did not undergo significant morphological changes (Supplementary Fig. 6i). The outgrowth of KPG cells was not significantly different to KP cells when cultured as tumor organoids under standard cell culture conditions (Supplementary Fig. 6j). However, KPG organoids were more responsive to BMP7-mediated growth inhibition (Fig. 6f, g), consistent with the hyperactivation of this pathway when GATA6 is reduced. As such, in KP tumor cells grown as organoids, BMP activation functions as a cytostatic signal, and this pathway can be constrained by GATA6.

In summary, our study identifies novel epigenomic mechanisms by which GATA6 modulates LUAD proliferation, differentiation, and the activation of lineage-specific tumor suppressive pathways.

## Discussion

By combining GEMMs with progenitor-specific gene targeting and integrated epigenomic analysis, we uncovered a novel requirement for GATA6-mediated transcriptional programming in lung cancer. GATA6 can also regulate tumorigenesis in other endodermal tissues and has seemingly paradoxical functions in the same cancer type, which parallels the dose and temporal requirement of this TF during development. In the *Apc*-null mouse model of colorectal cancer, GATA6 is required for tumor initiation [33]. In human esophageal and pancreatic adenocarcinomas, *GATA6* is amplified [34, 35] and may function as a prosurvival oncogene. Conversely, in pancreatic cancer models driven by *Kras* mutations, GATA6 inhibits tumor progression by regulating epithelial differentiation [36]. We demonstrate that in lung cancer, GATA6 deficiency initially impairs cancer cell proliferation and prevents tumor progression to high-grade lesions.

Although GATA6 may be expressed in several epithelial progenitor cell types in the lungs, GATA6 deficiency significantly reduced *Kras*^*G12D*^-mediated transformation of SPC+ progenitors in the presence or absence of p53. It has been suggested that Kras-mediated transformation of CC10+ cells involves the differentiation of CC10+ progenitors into SPC+ tumor cells, and that loss of p53 accelerates tumor progression in this context [20]. While GATA6 suppression did not affect CC10+ derived hyperplasia in K mice, it reduced the burden of CC10-Cre KP lesions, which may be due to the requirement for SPC+ cells at later stages of CC10-Cre tumor progression, and/or a requirement for *Gata6* in CC10-derived tumors that also have lost p53. A prior study concluded that GATA6 inhibits the expansion of double SPC+/ CC10+ cells in the proximal airway following loco-regional injury [8]. Interestingly, we noted that a proportion of lesions at the proximal duct junctions consisted of a mix of SPC+ and CC10+ cells when KG mice were infected with SPC-Cre. These data suggest that *Gata6* loss of function may also perturb the differentiation of progenitors within this region when mutant Kras is activated, but this effect was not sufficient to alter the reduced tumor burden when compared with control mice.

The mechanisms by which GATA factors regulate transcription during mammalian development are contextual as these TFs have nonredundant genomic targets. The molecular activities of GATA6 during pulmonary specification and lung cancer in particular are not fully understood. We demonstrate that GATA6 binding predominantly correlates with enhancer activation in SPC+ progenitor derived cancer cells, consistent with it being a key regulator of epithelial lineage identity. Our epigenomic analysis also revealed that the loss of GATA6 indirectly influences chromatin accessibility at a broad range of aberrantly activated enhancers associated with novel gene networks. Although several of these genes are likely to contribute to intrinsic cell proliferation, responses to stromal- and/or autocrine-derived signals may also account for the ability of GATA6 to enhance Kras-mediated tumorigenesis in vivo. For instance, suppression of *Gata6* sensitized tumor cell organoids to BMP-mediated growth inhibition. Mesenchymal BMP signaling is required for AT2 differentiation [31], and the ability of GATA6 to constrain this pathway via multiple gene targets in alveolar-derived lung cancer cells is reminiscent of its role in the colon stem cell niche [33].

Inhibiting GATA6 in KP cells also induced the expression of several EMT associated TFs, but these cells did not display overt mesenchymal phenotypes despite GATA6 directly targeting E-cadherin in other cell types [37]. In LUAD cells, loss of GATA6 also caused activation of *Hopx* and *Nkx2-1*, which encode for TFs that are essential for alveolar cell identity but can constrain LUAD progression [14, 30]. Moreover, in a distinct molecular subset of human LUADs, the expression of *GATA6* partially inversely correlated with *NKX2-1* and *HOPX*. The compensatory activation of *HOPX* and *NKX2-1* may also explain the increased latency and differentiation of tumors initiated by Kras when GATA6 is inhibited. It is also possible that precocious activation of EMT TFs in this context supports a tumor suppressive state as reported in pancreatic cancer [38]. Aberrant levels of NKX2-1 in particular may target newly open chromatin regions in KPG tumor cells to activate *de-novo* tumor suppressive gene networks. Paradoxically, in late stage human LUAD, *GATA6* is repressed along with other lineage TFs such as NKX2-1 and HOPX [12, 13], and the cooperative reduction of these factors promotes metastasis in experimental models [14, 39]. *GATA6* is also rarely mutated in human lung cancers. Consequently, although GATA6 expression is initially required for the proliferation of LUAD cells, reducing GATA6 at later stages of tumor progression in conjunction with other epigenetic alterations may activate invasive programs and metastatic dissemination. Altogether, our findings demonstrate how a lineage TF can reprogram the chromatin landscape of lung tumor cells in a manner that potentiates seemingly divergent functions during malignant progression.

## Materials and methods

Additional experimental procedures are provided in Supplementary Information.

### Animal studies

*Kras*^*LSL-G12D*^ (JAX #008179 (ref. [16])) (Jackson Laboratory, Bar Harbor, ME, USA), *p53*^*flox*^ (JAX #008462 (ref. [40])), FVB *Kras*^*LSL-G12D*^*p53*^*flox*^ (gift from F. Slack), and *Gata6*^*flox*^ (JAX #008196 (ref. [18])) mice were backcrossed to FVB or C57Bl/6 background for ten generations to obtain FVB and C57Bl/6 KP, KPG, K, and KG lines. Tumors were initiated by intratracheal infection of mice with viral vectors expressing Cre. Additional details are included in Supplementary Table 5. For syngeneic grafts, 6-week-old C57Bl/6 (JAX #00064) mice were injected subcutaneously with SPC-Cre tumor cells. For delayed expression of shRNAs, 7-week-old Athymic NCr-nu/nu (Charles River NCI #553) mice were injected subcutaneously with SPC-Cre cells containing doxycycline inducible shRNAs. Tumor growth was measured by bioluminescent imaging using an IVIS Spectrum or calipers. All animal studies were approved by the Yale University Institutional Animal Care and Use Committee.

### Derivation of cell lines and cell culture

Cell lines from SPC-Cre KP and KPG and Lenti-Cre KP were isolated from bulk lung of tumor-bearing mice 13–16 weeks after tumor initiation, sorted by EpCAM positive expression, and grown in RPMI media. All cell lines were tested for *Mycoplasma* every 6 months using the ATCC Universal *Mycoplasma* Detection Kit (Manassas, VA, USA). Prior to injection into mice, cells were tested for *Mycoplasma* and murine viral contamination by the Yale University Molecular Diagnostics Laboratory.

#### RNA sequencing

SPC-Cre KP and KPG cells lines were grown in plates for 3 days. Three biological replicates were collected. Total RNA was extracted using the RNeasy kit (QIAGEN, Hilden, Germany) with an on-column DNase treatment. Samples were sequenced on a HiSeq 2500 (Illumina, San Diego, CA, USA) with single-end 76 base reads. Data were analyzed using HISAT2 and Deseq2.

#### ChIP and ATAC sequencing

SPC-Cre KP and KPG cells lines were grown for 3 days. Two biological replicates were collected. For ChIP-seq, 20 μg of chromatin was subjected to immunoprecipitation overnight with corresponding antibodies listed in Supplementary Data. The ATAC-seq protocol was performed as previously described [41]. Samples were pooled and sequenced on a HiSeq 4000 (Illumina) using 2 × 101 reads.

All sequencing data are deposited in NCBI’s Gene Expression Omnibus under accession number GSE124604.

#### Western blot and immunostaining

For Western blotting, total protein lysate was harvested from cells using RIPA lysis buffer. For immunostaining, tissue was fixed overnight in 4% PFA at 4 °C and embedded into OCT or paraffin. Images were acquired using a Keyence (BZ-X710, Osaka, Japan) or Olympus microscope (IX-71, Tokyo, Japan) and analyzed using ImageJ (NIH). All antibodies and concentrations are listed in Supplementary Materials and methods.

#### Quantitative real time PCR

SPC-Cre K and KG tumor nodules were macrodissected and immediately flash frozen in liquid nitrogen. Tissue was grinded by mechanical dissociation using a motor/pestel instrument. Total RNA was extracted using RNeasy Mini Kit for mRNA (QIAGEN) with an on-column DNase treatment. 1 μg of mRNA was reverse transcribed into cDNA using the iScript cDNA Synthesis Kit (Bio-Rad, Hercules, CA, USA). cDNA qRT-PCR was performed using SYBR Green Master Mix (Applied Biosystems, Foster City, CA, USA). Reactions were run in quadruplicate. Data were normalized to housekeeping genes *b-actin* and represented as mean ± SEM. Relevant primers are listed in Supplementary Materials and methods.

### Statistical analysis

In vivo and in vitro experimental data were presented as mean ± SEM. *P* values were calculated by two-tailed Student’s *t* test, or by Mann–Whitney if samples did not follow Gaussian distribution. Variation was estimated using *F* test, and if variances were significantly different, *P* value was calculated using Welch’s *t*-test. Tumor burden was analyzed by Mann–Whitney test using specific time points or area under the curve. Grading of KP and KPG tumors was analyzed using chi-square. Quantification of SPC and CC10+ tumors was analyzed using Fisher’s exact test. All statistics were performed using Prism software (GraphPad, San Diego, CA, USA).

## Supplementary information

Supplementary Material

Supplementary Table 1

Supplementary Table 2

Supplementary Table 3

Supplementary Table 4

Supplementary Table 5
