# Supplementary material for: Tumor progression and chromatin landscape of lung cancer are regulated by the lineage factor GATA6
Source: Oncogene. 2020 Mar 10;39(18):3726–37. doi: 10.1038/s41388-020-1246-z (PMC7190573; doi:10.1038/s41388-020-1246-z)

Supplementary Materials and methods

Animal studies. Tumors were induced by viral delivery of Cre using lentivirus or adenovirus (full detailed list in Supplementary Table 5): Lentiviral pHAGE Cre-Luc vector (Lenti-Cre), Adenovirus Cre-eGFP (Iowa University), or Adenovirus CC10-Cre and SPC-Cre (Iowa University). To assess Cre recombination, ROSA26 LSL Tomato mice (gift from V. Greco.) mice were used. Viral inhalation was performed as previously described [1]. Briefly, mice were anesthetized by intraperitoneal injection of a ketamine/xylazine solution at 100 and 10mg/kg, respectively. One at a time, mice were placed on an intubation platform by hanging by their front teeth. A 24G catheter without the needle was inserted into the trachea, and 50µl of virus were pipetted into the catheter. Once the entire volume traveled down the catheter, the catheter was removed, and the mouse was placed on a heating pad until it recovered from anesthesia. For syngeneic grafts, 6 weeks old C57Bl/6 (JAX #00064) mice were anesthetized as described above and injected subcutaneously with $1x10^6$ cells of 3 different cells lines from SPC-Cre KP and from SPC-Cre KPG (1 line per mouse). Cells were resuspended in 100µl of 1:1 PBS:complete Matrigel (Corning, New York, NY, USA) and injected in both flanks of each animal. For delayed expression of shGata6, 7 week-old Athymic NCr-nu/nu (Charles River NCI #553) mice were injected as described above. When tumors reached $150mm^3$, mice were allocated to a mock group (regular mouse chow) or doxycycline group (mouse chow containing 625 ppm doxycycline; Envigo #TD00426). Tumor dimensions were measured with calipers after tumors were palpable, every 3 days, and tumor volume calculated using the formula ($vol=\pi*length*width^2/6$). Mice that did not show any luminescence after LentiCRE-Luciferase infection were not included in the analysis (misinjections). Sample size for mouse experiments was estimated based on power calculation (Lamorte's power calculation) using the results of an initial pilot experiment comparing K and KG phenotypes (Fig. 1b).

Derivation of cell lines and cell culture. To derive cell lines from SPC-Cre KP, SPC-Cre KPG (13 weeks post-induction), and Lenti-Cre KP mice (14 weeks post-induction), lungs were resected, rinsed with PBS, and kept on ice in RPMI and antibiotics (penicillin-streptomycin). The lungs were dissected and the tissues were minced using scissors. Enzymatic dissociation was performed at 37ºC for 30-40 min using a solution of hyaluronidase (0.5mg/mL, Worthington #LS002592), collagenase IV (2mg/mL, Sigma #C5138), and DNase I (1µg/mL, Worthington #LS002006, Lakewood, NJ, USA) in Hank's balanced salt solution (HBSS) buffer. The cell suspension was filtered through a cell strainer (100µm) and lysed with ACK solution to remove red blood cells. Cells were pelleted and washed several times with HBSS. Finally, cells were plated in RPMI media supplemented with 10% fetal bovine serum (FBS) in flasks for 3 days before media was changed. After 3 to 5 passages, cells were sorted on a BD FACS ARIA II for EpCAM expression using EpCAM-APC-eFluor780 (Ebioscience #47-5791-80, LOT 4295256, Santa Clara, CA, USA) excluding DAPI positive cells. Murine cells were cultured using RPMI supplemented with 10% FBS, penicillin-streptomycin and amphotericin. Doxycycline treatment for shRNA expression was performed for 5 days at 1 µg/mL in complete media.

For mouse recombinant Bmp7 experiments, cells were serum starved for 16 hours prior to stimulation with rBmp7 (100ng/mL R&D, Minneapolis, MN, USA) in RPMI with 0.2% FBS. For organoid experiments, 5000 cells were cultured in 24 well ultra-low attachment plates (Corning, #3473) in RPMI with 2% FBS, penicillin-streptomycin and amphotericin and 5% growth factor reduced Matrigel (Corning, #356231). Cells were treated with 100ng/ml rBmp7 one day after seeding and then daily. Growth was measured by fluorescence using an IVIS Spectrum.

RNA sequencing analysis. For purified total RNA samples, the six strand-specific sequencing libraries were produced following the Illumina TruSeq Stranded protocol. The libraries underwent 76-bp single-end sequencing using an Illumina HiSeq 2500 generating an average of 30 million reads per library. For each read, the first 6 and the last nucleotides were trimmed to the point where the Phred score of an examined base fell below 20 using in-house scripts. If, after trimming, the read was shorter than 45bp, the whole read was discarded. Trimmed reads were mapped to the mouse reference genome (mm10) with HISAT2 v2.1. [2] indicating that reads correspond to the reverse complement of the transcripts and reporting alignments tailored for transcript assemblers. Alignments with quality score below 20 were excluded from further analysis. Gene counts were produced with StringTie v1.3.3b [3] and the Python script "prepDE.py" provided in the package. StringTie was limited to reads matching the reference annotation GENCODE v15 [4]. After obtaining the matrix of read counts, differential expression analysis was conducted and normalized counts were produced using DESeq2 [5]. *P*-values were adjusted for multiple testing using the Benjamini-Hochberg procedure. Genes considered to be differentially expressed were those that had an adjusted *p*-value < 0.05. Hierarchical clustering (regularized log counts) and heatmaps were generated in R and Partek® software, Copyright ©; 2018, Partek Inc. (St. Louis, MO, USA).

For determining whether a gene was expressed in any sample, raw counts were first converted to transcript per million (TPM) to normalize for sequencing depth and gene length. Any gene that had a TPM < 6e-7 (which corresponds to a read count of 10 in WT replicate 1 sample) was considered not expressed.

A complete list of genes differentially expressed in KPG compared to KP is available in Supplementary Table 2.

Pathway analysis. Pathway analysis was performed using MetaCore (Clarivate, Philadelphia, PA, USA) with genes significantly differentially expressed (Log2FC 1<x<-1 and *p*-adjusted < 0.05 *n* = 1254 genes). For Gene Set Enrichment Analysis (GSEA)[6] significant genes (*p*-adjusted <0.05. *n* = 4,304 genes) were pre-ranked and Hallmarks was run on GSEA using 1000 permutations and Classic (statistics) parameters. Complete results of the analysis are available in Supplementary Table 2. GREAT [7] analysis on newly open ATAC regions (*p*-adjusted < 0.05) was performed using default parameters and all ATAC peaks as background.

ChIP sequencing protocol. Cells growing in a monolayer were washed with ice-cold PBS and fixed in 1% formaldehyde in DMEM for 10min at room temperature while swirling. The reaction was quenched with glycine to a final concentration of 0.125M for 5

min. Cells were scraped and transferred immediately to ice. The pellet was washed 3 times with ice-cold PBS and resuspended in complete sonication buffer (20mM Tris pH 8.0, 2mM EDTA, 0.5mM EGTA, 1x protease inhibitors (Roche), 0.5% SDS, and 0.5mM PMSF) in 1mL for $1x10^8$ cells. Samples were diluted in sonication buffer and sonicated using a Qsonica Q800 (Newtown, CT, USA) (70% amplitude, 15sec ON, 45sec OFF for 45 min). DNA was extracted and run through agarose gels for fragment size confirmation (200-500bp). Chromatin was measured with Qubit (ThermoFisher Scientific, Waltham, MA, USA). For immunoprecipitation (IP) Protein A/G beads were used. 20% of input material was aliquoted. ChIP material was pre-cleared in batch for 1 hour at 4ºC with IP buffer (20mM Tris pH 8.0, 2mM EDTA, 0.5% Triton X-100, 150mM NaCl, and 10% Glycerol) and Protein A/G slurry. Samples were split into 3 IPs, anti-GATA6 (20µg), anti-H3K4me3 (10µg), and anti-H3K27ac (5µg). Drosophila spike-in chromatin (40ng) and Drosophila spike-in antibody (4µg) were added to normalize across samples. IPs were performed overnight rotating at 4ºC. The next day, Protein A/G beads were added and incubated for 2 more hours. IPs were washed and eluted in a 100mM $NaHC0_3$, 1% SDS solution. IP samples and input were reverse crosslinked, and DNA was purified. 1-25ng of DNA were used for library preparation using ThruPLEX® kit (Takara) and quantified using NEBNext® Library Quant Kit (NEB, Ipswich, MA, USA). Samples were mixed into 2 separate groups (Gata6+Input and H3K4me3+H3K27ac). Prior to submission fragment size selection was performed using AmPURE XP beads (Beckman Coulter, Brea, CA, USA).

Antibody target	Catalog number	Company	Lot
Gata6	AF1700	R&D Systems	KWT031608
Histone H3 (tri methyl K4)	ab8580	Abcam	GR288375-1
Histone H3 (acetyl K27)	ab4729	Abcam	GR276934-1
Drosophila Spike-in Chromatin	53083	Active motif	34217007
Drosophila Spike-in Antibody	61686	Active motif	06418006

ChIP sequencing analysis. For ChIP-seq, two biological replicates and input controls were used. Sequencing was carried out on an Illumina HiSeq 4000 generating on average 44.2M pair of reads of 101 bp long. For each read, we trimmed the first six nucleotides and the last nucleotides at the point where the Phred score of an examined base fell below 20 using in-house scripts. If, after trimming, the read was shorter than 45 bp, the entire pair was discarded. Trimmed reads were mapped to the mouse reference genome (mm10) using BWA-MEM v0.7.17 [8]. Only reads with mapping quality scores equal or higher than 20 were kept.

Duplicated read pairs were removed to allow only unique fragments using Picard MarkDuplicates v2.8.2 (unpublished, http://broadinstitute.github.io/picard). Peak and motif finding was performed using HOMER v4.10 [9]. Before using any peak file for downstream analysis, blacklisted regions were removed using the mm10 blacklist track [10]. For GATA6 peak finding, transcription factor mode was used with default parameters. In addition, only peaks that were significant in the merged replicates compared with merged input were reported. Motifs for GATA6 were found using the option size -given.

To annotate GATA6 binding in the genome, HOMER annotatePeaks.pl was first used with the output of GATA6 peak calls. The resulting file was further modified whereby GATA6 peaks falling within 2Kb upstream or downstream of a gene were deemed "promoter." To capture all potential enhancers, all replicates of H3K27ac were merged across KP and KPG samples. This merged file and the merged input from all replicates and conditions were used with HOMER findPeaks and the parameters -region -size 200 -minDist 1000. Peaks were then filtered for regions that fall greater than 2Kb upstream or downstream of the TSS of an expressed gene (see RNA sequencing analysis methods section) and for blacklisted regions. This gave the consensus enhancer H3K27ac bed file.

To generate bigwig files and visualize genome-wide binding with heatmaps, deepTools2 v3.1.1 [11] was used. For heatmaps of differential histone signal, reads falling within each peak across conditions and replicates were determined using featureCounts and used for input with DESeq2. Significantly changed peaks were called using a *p*-adjusted cutoff of < 0.05. To generate a heatmap, variance-stabilized transformed reads of significant peaks were plotted.

Complete list of ChIP-seq peaks is available in Supplementary Table 3.

ATAC sequencing protocol. The Omni-ATAC-seq method was followed as described in Corces et al [12]. Briefly, SPC-Cre KP and KPG cell lines were checked for viability above 95% prior to experiment. $5x10^4$. viable cells were pelleted and resuspended in 50µl of cold resuspension buffer (10mM Tris-HCl pH 7.4, 10mM NaCl, 3mM $MgCl_2$ 0.1% Tween-20, 0.1% NP40, and 0.01% digitonin). Pellet was washed in resuspension buffer without NP40 or digitonin, samples were treated with transposition mixture (100nM transposase in 20mM Tris-HCl pH 7.6, 10mM $MgCl_2$ 20% dimethyl formamide, 0.01%, and 0.1% Tween-20). DNA of transposed fragments was extracted (Zymo DNA clean and Concentrator 5- kit D4014) and pre-amplified using NEBNext® 2x MasterMix (NEB). Final quantification of the library prep was performed using NEBNext® Library Quant Kit (NEB). Samples were pooled and sequenced on a HiSeq 4000 (Illumina) using pair-end 101 reads.

ATAC sequencing analysis. For ATAC-seq, reads were trimmed of adapters using Trimmomatic v0.32 and aligned using Bowtie2 v2.3.4.3 to mm10 using standard parameters and a maximum fragment length of 2000 [13, 14]. Duplicate reads were determined using Picard tools v2.18.21 and subsequent bam files were filtered using samtools -F 1804 [8]. Reads were then processed as described in Ref [15], where only the most 5' base pair for each read was kept, and forward strand reads were shifted +4bp, and reverse strand reads were shifted -5bp. To determine a consensus ATAC-seq peak set, all replicates across all conditions were first merged into one bam file. Peak calling was performed using macs2 with parameters --shift -75 --extsize 150 --call-summits. Peaks were then split evenly using summits. Read counts that fell within consensus peaks were obtained using featureCounts across replicates, and differential accessibility was determined using DESeq2 [5, 16]. For motif analysis, significant (*p*-adjusted < 0.05) differentially accessible peaks were used as target regions with the entire peak set as background. These peaks were then used in Homer to find enrichment of *de novo* and known motifs [9].

To find the fraction of enhancer accessibility that significantly changed around each gene, we first determined all enhancer peaks (using the consensus enhancer H3K27ac bed file described above) within 100kb window of the putative TSS and only selected consensus ATAC-seq peaks that fell within these enhancers. Of these ATAC-seq peaks the number that increased or decreased significantly (*p*-adjusted < 0.05) was then determined to calculate the fraction. GATA6 peaks were then used to determine whether at least one GATA6 peak fell within the significantly differentially accessible enhancers around each gene. Chi-square test was used to determine whether GATA6 is more likely to bind around down-regulated compared to up-regulated genes.

Complete list of ATAC-seq peaks is available in Supplementary Table 4.

ATAC sequencing library read counts

Sample	Total Reads	Aligning to any chr (%)	Aligning to chrM (%)	Within peaks (%)
KO_ATAC_R1	69531144	98.51	1.49	40.69
KO_ATAC_R2	96899077	98.36	1.64	51.66
WT_ATAC_R1	81471016	99.20	0.80	32.93
WT_ATAC_R2	92198180	99.48	0.52	39.19

Sequencing was performed at the Yale Center for Genome Analysis (YCGA) and analyses were performed using the research computing infrastructure of the Yale Center for Research Computing.

Clustering analysis of TCGA cohorts. For the alveolar-like classification, TCGA cohort were clustered based on a Alveolar lineage signature as previously described[17]. This classification segregated the cohort into 3 clusters (alveolar-high, alveolar-medium, and alveolar-low). Pearson correlation was calculated using Partek® software.

Western Blot (WB). Gels were run using the Mini-PROTEAN electrophoresis system (Bio-Rad). Gels were transferred to 0.2µm nitrocellulose membranes and blocked with 5% milk-TBS-Tween (0.1%) for 1h at room temperature. Antibodies were incubated overnight in 1% BSA TBS-T (0.1%). Antibodies and concentrations are listed below. Membranes were developed using SuperSignal West Pico (Thermo Scientific) and imaged using a BioRad ChemiDoc MP Imaging System. Uncropped gels can be found in *Western blot data*.

Immunohistochemistry (IHC) and immunofluorescence (IF). Paraffin and frozen blocks were sectioned to 5µm and 20µm sections, respectively. For paraffin slides, antigen retrieval was performed using citrate buffer pH=6.0. For immunostaining, slides were blocked in 3% BSA/TBS 0.1% Tween and 0.5% Triton-X100 for 30 minutes at room temperature. Primary antibodies were incubated overnight at 4°C in 0.3% BSA/TBST with DAPI (0.5µg/mL). For immunohistochemistry, peroxidase activity was blocked using 3% H_2O_2 in PBS after antigen retrieval. For Caspase-3 staining, slides

were blocked using 10% goat serum, and primary antibody was incubated in SignalStain® Antibody diluent (#8112 Cell Signaling, Danvers, MA, USA). Slides were developed using DAB (DAKO, Carpinteria, CA, USA), and counterstained with Hematoxylin, and mounted in Permount mounting media (#17986-01 Electronic Microscopy Science, Hatfield, PA, USA). For immunofluorescence, secondary antibodies were incubated at room temperature for 1 hour in 0.3% BSA/TBST. For E-Cadherin staining, cells were grown in monolayer on a coverslip. Cells were fixed in 4% PFA for 15 min, block with 3% BSA and TBS-Tween (0.1%) -Triton X-100 (0.5%) for 1h and then stained with isotype control or antibody for 1h at room temperature in 0.3% BSA-TBS-Tween buffer with DAPI. Rhodamine anti-rabbit was used as a secondary. Slides for immunofluorescence were mounted in ProLong® Gold Antifade Reagent (#9071 Cell Signaling).

Antibodies list. List of antibodies used for Western Blot, immunohistochemistry and immunofluorescence.

Antibody name	Catalog number	Company	Lot	Dilution	Description	Application
Gata6 (D61E4)	5851	Cell Signaling	4	1:500	Primary	WB
Tubulin	T5168	Sigma	086M4773 V	1:5000	Primary	WB
TTF1 (Nkx2.1) [EP1584Y]	ab76013	Abcam	GR76790-24	1:2000	Primary	WB
Phospho Smad1/5 (Ser463/465) (41D10)	9516	Cell Signaling	9	1:1000	Primary	WB
Smad5 (D4G2)	12534	Cell Signaling	2	1:1000	Primary	WB
Vimentin (D21H3) XP	5741	Cell Signaling	1	1:1000	Primary	WB
Ecadherin (24E10)	3195	Cell Signaling	10	1:1000	Primary	WB
Ecadherin (24E10)	3195	Cell Signaling	10	1:200	Primary	IF
Anti-rabbit HRP	31458	Thermo Scientific	-	1:5000	Secondary	WB
Anti-mouse HRP	31437	Thermo Scientific	ML1490539	1:5000	Secondary	WB
Cleaved Caspase-3 Asp175	9661	Cell Signaling	45	1:100	Primary	IHC
Gata6 (D61E4)	5851	Cell Signaling	4	1:50	Primary	IHC
SignalStain® Boost IHC Detection Reagent	8114	Cell Signaling	-	undiluted	Secondary	IHC
Gata6 (D61E4)	5851	Cell Signaling	4	1:500	Primary	IF
SP-C (proSP-C)	AB3786	Milipore	2710270	1:1000	Primary	IF
CC10 (T-18)	sc-9772	Santa Cruz	B0108	1:1000	Primary	IF
TTF1 (Nkx2.1) [EP1584Y]	ab76013	Abcam	GR76790-24	1:200	Primary	IF
Ki67 Alexa 660	50-5698-80	Ebioscience	4283678	1:100	Primary conjugated	IF
EpCAM-PE	12-5791-80	Ebioscience	E01777-1632	1:200	Primary conjugated	IF
EpCAM-FITC	11-5791-80	Ebioscience	4317683	1:200	Primary conjugated	IF
Alexa Fluor® 488-	705-545-	Jackson	126935	1:200	Secondary	IF

AffiniPure Donkey Anti-Goat IgG (H+L)	003	ImmunoResearch				
Alexa Fluor® 647 AffiniPure Donkey Anti-Rabbit IgG (H+L)	711-605-152	Jackson ImmunoResearch	125197	1:200	Secondary	IF
Rhodamine Red-X-AffiniPure Donkey Anti-Rabbit IgG (H+L)	711-295-152	Jackson ImmunoResearch	130068	1:200	Secondary	IF
Rhodamine Red™-X (RRX) AffiniPure Donkey Anti-Goat IgG (H+L)	705-295-147	Jackson ImmunoResearch	138674	1:200	Secondary	IF
Alexa Fluor® 488 AffiniPure Donkey Anti-Rabbit IgG (H+L)	711-545-152	Jackson ImmunoResearch	136422	1:200	Secondary	IF

Processing and analysis of immunofluorescent and bright-field images. Ki67 images were acquired using a Keyence microscope and quantified using ImageJ software (NIH). Nodules were identified using TTF1 or SPC as a marker and ROIs were drawn. Separate masks for DAPI and Ki67 channels were generated, and cells were counted using particle analyzer. Only images with nodules of more than 50 DAPI positive cells were analyzed. For SPC and CC10 staining, a composite of SPC, CC10, and DAPI was created. Separate thresholds for SPC and CC10 were determined based on isotype controls. Tumor nodules were analyzed manually and were annotated "SPC only" if only SPC+ cells were present or "SPC+ CC10+" if both SPC+ and CC10+ cells were mixed in the tumor or if some SPC+ and CC10+ cells were co-detected within the nodule.

For tumor burden, images from Hematoxylin & eosin were acquired and tumor area was measured for each nodule using ImageJ. Data was normalized to K or KP group. For quantifications of tumor burden, tissue blocks and slides were de-identified for the phenotype and an arbitrary number was assigned to each mouse tissue block. No information of the phenotype was present in the slides when outcome was assessed.

Quantitative Real Time-PCR. Total RNA was extracted using RNeasy Mini Kit for mRNA (QIAGEN) with an on-column DNase treatment. 1 µg of mRNA was reverse transcribed into cDNA using the iScript cDNA Synthesis Kit (Bio-Rad). cDNA qRT-PCR was performed using SYBR Green Master Mix (Applied Biosystems). Reactions were run in quadruplicate. Data were normalized to housekeeping genes *b-actin* and represented as mean ± SEM. Relevant primers are listed below.

Primers

**Primer name**	**Sequence**	**Application**
Gata6 ex2 F	AGTCAAGGCCATCCACTGTC	Sybergreen
Gata6 ex2 R	ACTAGCAGACCCCGGACTC	Sybergreen

Snai1 F	CACACGCTGCCTTGTGTCT	Sybergreen
Snai1 R	GGTCAGCAAAAGCACGGTT	Sybergreen
Smad9 F	CTTCCGAGTCAGACAGTCCCT	Sybergreen
Smad9 R	GGTTGTTTAGTTCGTAGTAGGCA	Sybergreen
Bmpr1b F	CCCTCGGCCCAAGATCCTA	Sybergreen
Bmpr1b R	CAACAGGCATTCCAGAGTCATC	Sybergreen
Hopx F	ACCAGGTGGAGATCCTGGAG	Sybergreen
Hopx R	TCCGTAACAGATCTGCATTCC	Sybergreen
Bmp7 F	ACGGACAGGGCTTCTCCTAC	Sybergreen
Bmp7 R	ATGGTGGTATCGAGGGTGGAA	Sybergreen
Gata6 F	TTGCTCCGGTAACAGCAGTG	Sybergreen
Gata6 R	GTGGTCGCTTGTGTAGAAGGA	Sybergreen
b-actin F	CCTCCCTGGAGAAGAGCTATG	Sybergreen
b-actin R	TTACGGATGTCAACGTCACAC	Sybergreen
Gata6 k/o2 F	AGTCTCCCTGTCATTCTTCCTGCTC	PCR
Gata6 k/o2 R	TGATCAAACCTGGGTCTACACTCCTA	PCR
Gata6 gt4 F	GTGGTTGTAAGGCGGTTTGT	PCR
Gata6 gt4 R	ACGCGAGCTCCAGAAAAAGT	PCR

Uncropped Western Blot Data (separate file)

Supplementary Table 1 (separate file)

Summary of the numbers and phenotype of all GEMM experiments**.**

Supplementary Table 2 (separate file)

Significant differentially expressed genes and pathways in SPC-Cre KPG and KP cell lines**.** SPC-Cre KPG-KP: List of genes differentially expressed in SPC-KPG cells compared to SPC-KP cells (*p*-adjusted < 0.05 and |log2 fold change| > 1; *n* = 1254 genes). MetaCore: Top 10 upregulated and downregulated canonical pathways in KPG cells using all significant genes listed in SPC-Cre KPG-KP tab. GSEA: Top 25 Hallmarks Pathways upregulated and downregulated of a pre-ranked list of significantly expressed genes (*p*-adjusted < 0.05; *n* = 4,304) in KPG compared to KP cells.

Supplementary Table 3 (separate file)

ChIP-seq peaks and Motif analysis**.** GATA6 peaks: List of peaks in SPC-Cre KP cells from GATA6 immunoprecipitation (IP) compared to input. Merged H3K4me3 Peaks: List of merged peaks in SPC-Cre KP and KPG cells from H3K4me3 IP compared to input. Merged H3K27ac Peaks: List of merged peaks in SPC-Cre KP and KPG cells from H3K27ac IP compared to input. Motif analysis Gata6 peaks: List of known motifs from Homer (*P*-value < 0.05) using list in GATA6 peaks.

Supplementary Table 4 (separate file)

ATAC-seq peaks and Motif analysis**.** ATAC-seq Peaks: List of all ATAC peaks in SPC-Cre KP and KPG cells. Motif analysis "down" regions: List of known motifs from Homer (*P*-value < 0.05) using peaks downregulated in KPG compared to KP. Motif analysis "up" regions: List of known motifs from Homer (*P*-value < 0.05) using peaks upregulated in KPG compared to KP.

Supplementary Table 5 (separate file)

Summary of GEMM experiments**.** Summary of mouse experiments including strain, background, age at infection, type of viral delivery and titer, sex of the mice, and end time point for each experiment.

Supplementary References

1 DuPage M, Dooley AL, Jacks T. Conditional mouse lung cancer models using adenoviral or lentiviral delivery of Cre recombinase. Nat Protoc 2009; 4: 1064-1072.

2 Kim D, Langmead B, Salzberg SL. HISAT: a fast spliced aligner with low memory requirements. Nat Methods 2015; 12: 357-360.

3 Pertea M, Pertea GM, Antonescu CM, Chang TC, Mendell JT, Salzberg SL. StringTie enables improved reconstruction of a transcriptome from RNA-seq reads. Nat Biotechnol 2015; 33: 290-295.

4 Harrow J, Frankish A, Gonzalez JM, Tapanari E, Diekhans M, Kokocinski F *et al*. GENCODE: the reference human genome annotation for The ENCODE Project. Genome Res 2012; 22: 1760-1774.

5 Love MI, Huber W, Anders S. Moderated estimation of fold change and dispersion for RNA-seq data with DESeq2. Genome Biol 2014; 15: 550.

6 Subramanian A, Tamayo P, Mootha VK, Mukherjee S, Ebert BL, Gillette MA *et al*. Gene set enrichment analysis: a knowledge-based approach for interpreting genome-wide expression profiles. Proc Natl Acad Sci U S A 2005; 102: 15545-15550.

7 McLean CY, Bristor D, Hiller M, Clarke SL, Schaar BT, Lowe CB *et al*. GREAT improves functional interpretation of cis-regulatory regions. Nat Biotechnol 2010; 28: 495-501.

8 Li H, Handsaker B, Wysoker A, Fennell T, Ruan J, Homer N *et al*. The Sequence Alignment/Map format and SAMtools. Bioinformatics 2009; 25: 2078-2079.

9 Heinz S, Benner C, Spann N, Bertolino E, Lin YC, Laslo P *et al*. Simple combinations of lineage-determining transcription factors prime cis-regulatory elements required for macrophage and B cell identities. Mol Cell 2010; 38: 576-589.

10 Consortium EP. An integrated encyclopedia of DNA elements in the human genome. Nature 2012; 489: 57-74.

11 Ramirez F, Ryan DP, Gruning B, Bhardwaj V, Kilpert F, Richter AS *et al*. deepTools2: a next generation web server for deep-sequencing data analysis. Nucleic Acids Res 2016; 44: W160-165.

12 Corces MR, Trevino AE, Hamilton EG, Greenside PG, Sinnott-Armstrong NA, Vesuna S *et al*. An improved ATAC-seq protocol reduces background and enables interrogation of frozen tissues. Nat Methods 2017; 14: 959-962.

13 Langmead B, Salzberg SL. Fast gapped-read alignment with Bowtie 2. Nat Methods 2012; 9: 357-359.

14 Bolger AM, Lohse M, Usadel B. Trimmomatic: a flexible trimmer for Illumina sequence data. Bioinformatics 2014; 30: 2114-2120.

15 Buenrostro JD, Giresi PG, Zaba LC, Chang HY, Greenleaf WJ. Transposition of native chromatin for fast and sensitive epigenomic profiling of open chromatin, DNA-binding proteins and nucleosome position. Nat Methods 2013; 10: 1213-1218.

16 Liao Y, Smyth GK, Shi W. featureCounts: an efficient general purpose program for assigning sequence reads to genomic features. Bioinformatics 2014; 30: 923-930.

17 Cheung WK, Zhao M, Liu Z, Stevens LE, Cao PD, Fang JE *et al*. Control of alveolar differentiation by the lineage transcription factors GATA6 and HOPX inhibits lung adenocarcinoma metastasis. Cancer Cell 2013; 23: 725-738.

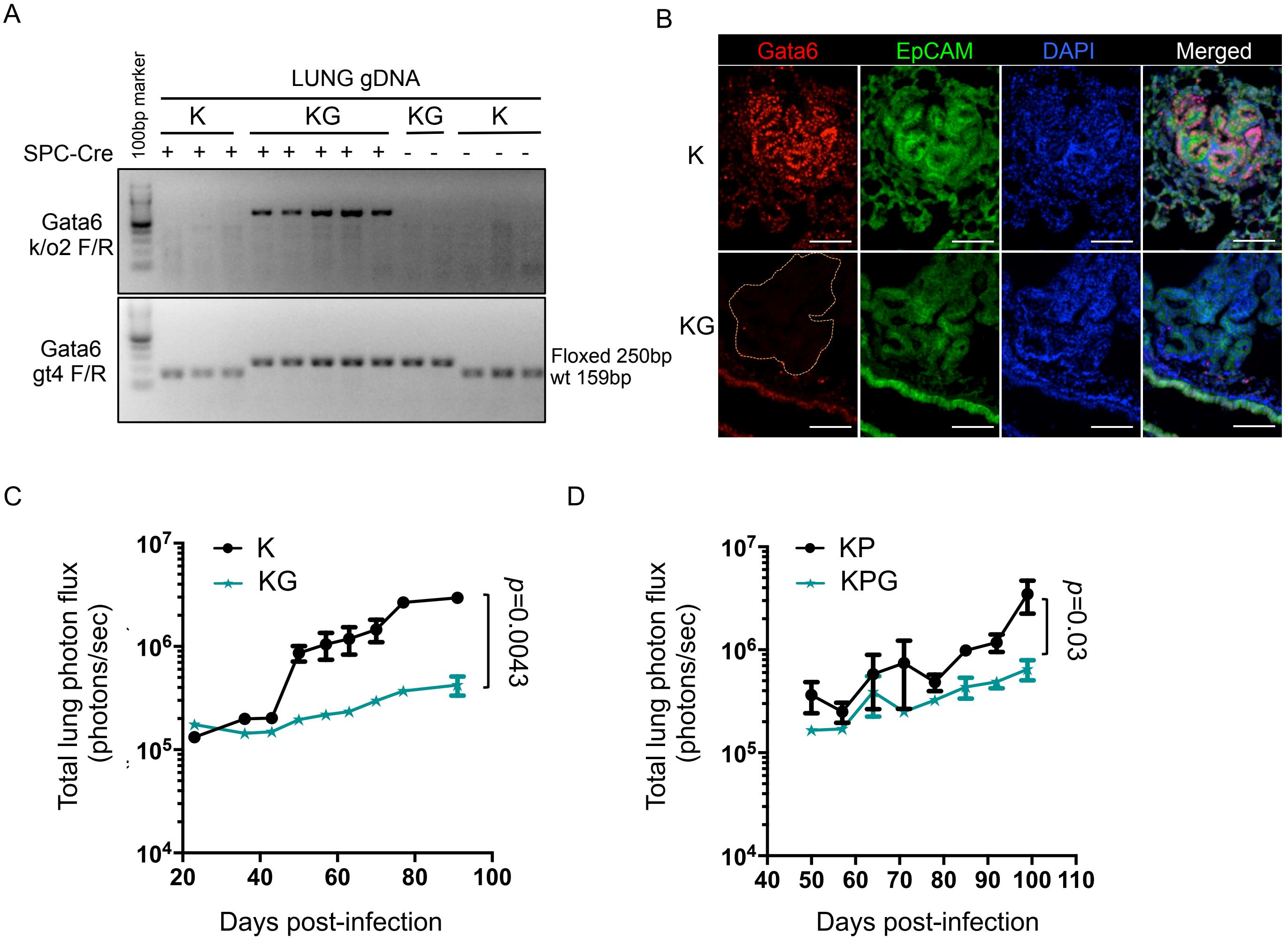

Supplementary Fig. 1 Effects of suppressing *Gata6* in *Kras*^{G12D} (K) and *Kras*^{G12D} *p53*$^{-/-}$ (KP) GEMMs. **a** Cre mediated recombination at exon 2 of *Gata6* was validated by PCR using genomic DNA extracted from lung tumor bearing mice and control uninfected mice as described in Sodhi et al. Ladder is 100bp. **b** Immunofluorescence of GATA6 (red), the epithelial marker EpCAM (green), and DAPI (blue) of representative tumor nodules from K and KG mice 90 days post-infection with Ad-Cre. Scale bar=100µm. **c** Lung tumor burden measured by luminescence (photons/sec) in K and KG mice from Fig. 1 c. **d** Lung tumor burden measured by luminescence (photons/sec) in KP and KPG mice from Fig. 1 e. Data was plotted as SEM and *p*-value calculated by Mann Whitney test using area under the curve across all time points.

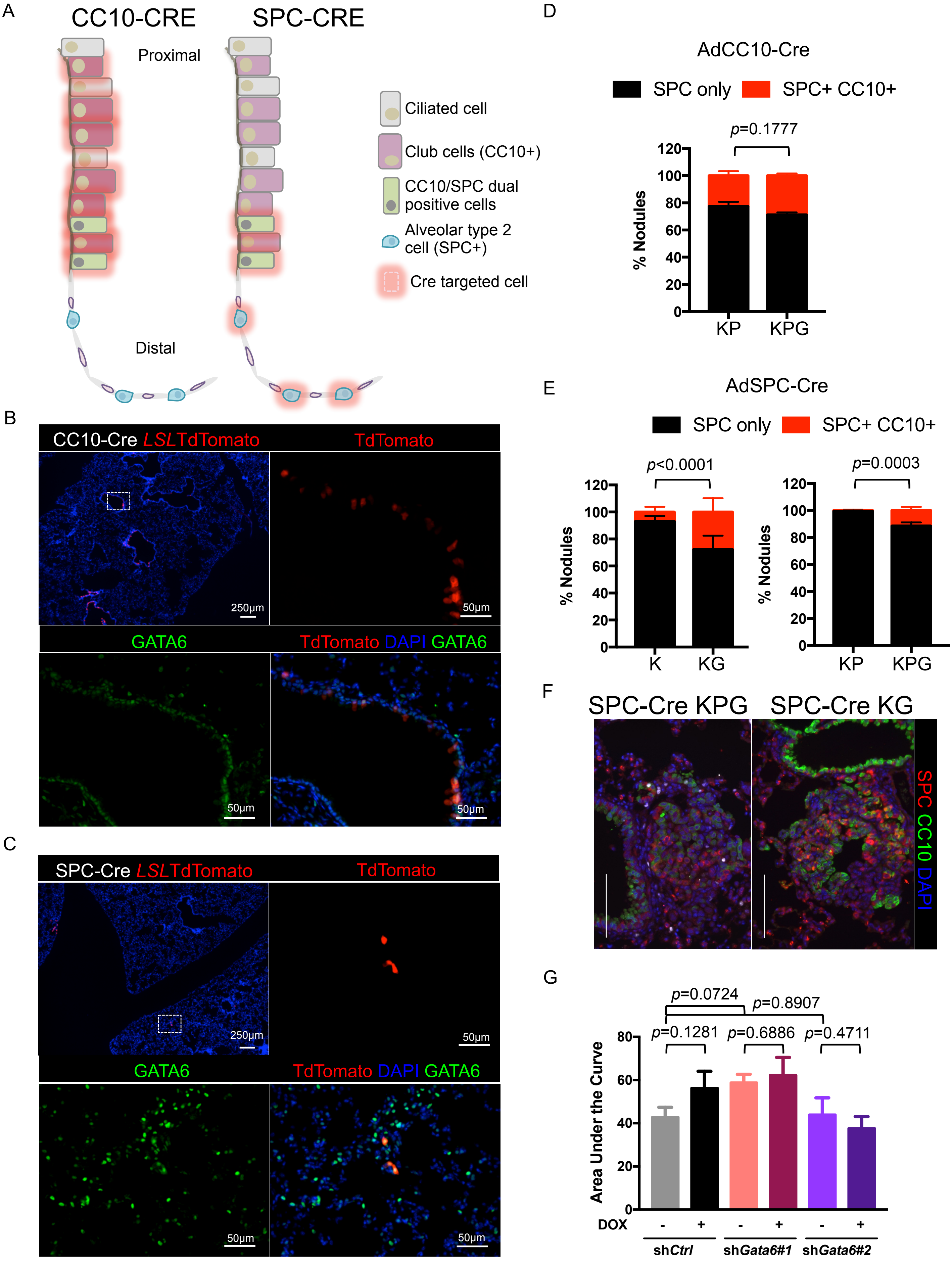
A
CC10-CRE
SPC-CRE
Proximal
Distal
Ciliated cell
Club cells (CC10+)
CC10/SPC dual positive cells
Alveolar type 2 cell (SPC+)
Cre targeted cell
B
CC10-Cre LSLTdTomato
TdTomato
250µm
50µm
GATA6
TdTomato DAPI GATA6
50µm
50µm
C
SPC-Cre LSLTdTomato
TdTomato
250µm
50µm
GATA6
TdTomato DAPI GATA6
50µm
50µm
D
AdCC10-Cre
SPC only
SPC+ CC10+
p=0.1777
% Nodules
120
100
80
60
40
20
0
KP
KPG
E
AdSPC-Cre
SPC only
SPC+ CC10+
p<0.0001
p=0.0003
% Nodules
K
KG
KP
KPG
F
SPC-Cre KPG
SPC-Cre KG
SPC CC10 DAPI
G
p=0.0724
p=0.8907
p=0.1281
p=0.6886
p=0.4711
Area Under the Curve
80
60
40
20
0
DOX
-
+
-
+
-
+
shCtrl
shGata6#1
shGata6#2

Supplementary Fig. 2 Specific targeting of Cre and GATA6 expression in CC10 or SPC positive cells. **a** Scheme of CC10-Cre and SPC-Cre infection of lungs for Fig. 3 **b-c** and **d-e** respectively. **b** Immunofluorescence staining was performed for GATA6 in the lungs of Tomato reporter mice (Rosa26 ^{LSLTdTomato}) 10 days post infection with CC10-Cre. Cre recombination (red) in GATA6 expressing cells (green) at the proximal airway junctions was confirmed. Scale bar=250μm (left panel) or 50μm (right high magnification panels). **c** Similar control experiments were performed as in **b** but using SPC-Cre. Cre recombination (red) in GATA6 expressing cells (green) at the distal airway was confirmed. **d** Quantification (%) of nodules with CC10+ cells and SPC+ cells, or SPC+ only cells in CC10-Cre infected KP or KPG mice. (n = 157-319 nodules, from 4-5 mice per group). **e** Quantification of nodules was performed as in **c** but in SPC-Cre infected K, KG, KP, and KPG mice. (n = 74-171 nodules, from 3-7 mice per group). *P*-value was calculated using Fisher's exact test. **f** Representative immunofluorescence images of SPC (red), CC10 (green) and DAPI (blue) from mixed SPC+ CC10+ nodules in SPC-Cre KG and KPG mice. Scale bar=100μm. **g** Growth of established subcutaneous tumors derived from SPC-Cre KP cell lines expressing the indicated shRNAs. Once tumors reached 150mm^3, each mouse was allocated to no doxycycline group or switched to doxycycline diet to induce shRNA expression. Tumor growth was then monitored up to 13 days and plotted as area under the curve using all time points. *P*-values were calculated using ANOVA with SEM is plotted (n = 10 tumors, 5 mice per group).

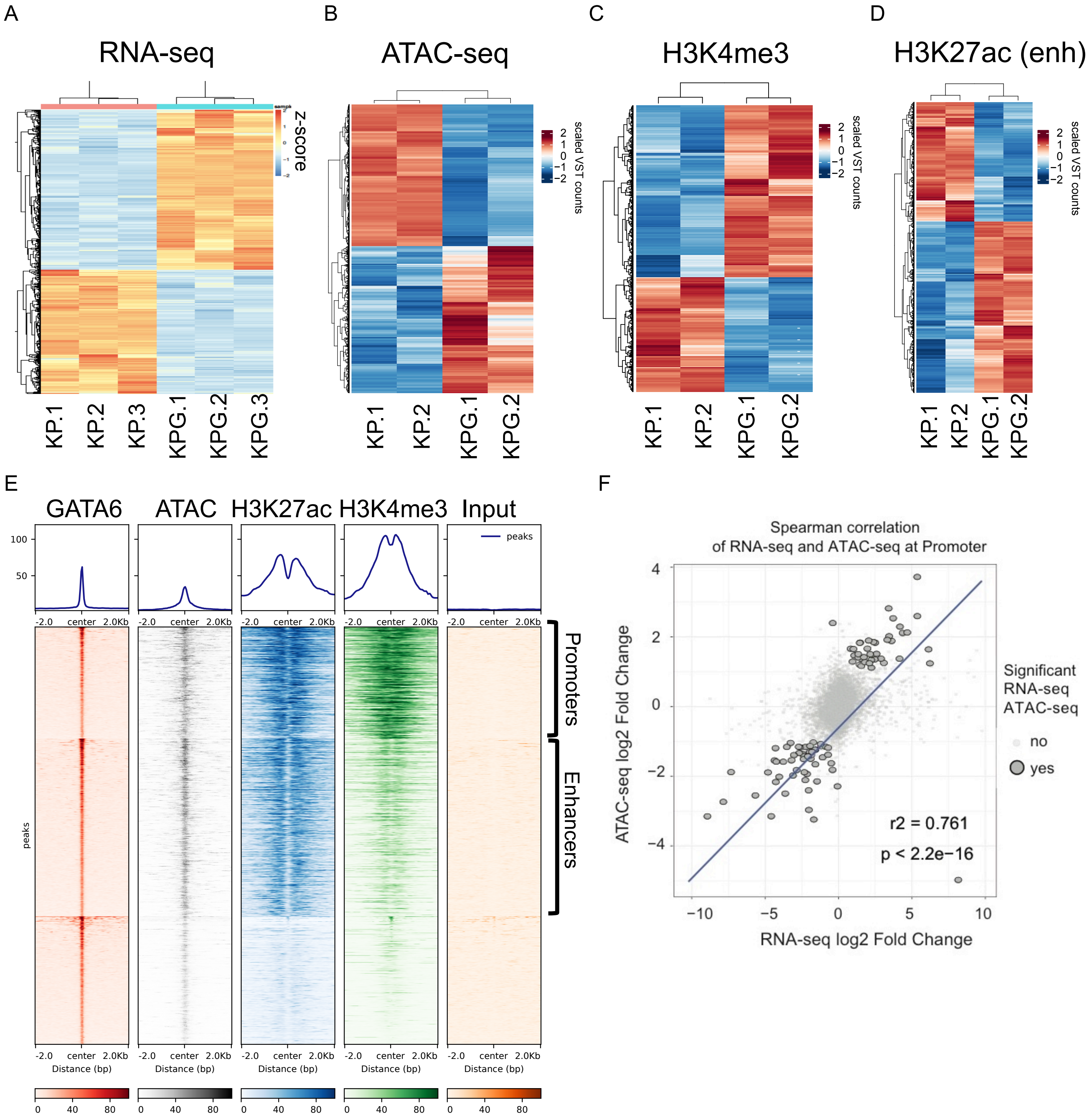
A
RNA-seq
z-score
KP.1
KP.2
KP.3
KPG.1
KPG.2
KPG.3
B
ATAC-seq
scaled VST counts
KP.1
KP.2
KPG.1
KPG.2
C
H3K4me3
scaled VST counts
KP.1
KP.2
KPG.1
KPG.2
D
H3K27ac (enh)
scaled VST counts
KP.1
KP.2
KPG.1
KPG.2
E
GATA6
ATAC
H3K27ac
H3K4me3
Input
peaks
-2.0 center 2.0Kb
Promoters
Enhancers
Distance (bp)
F
Spearman correlation
of RNA-seq and ATAC-seq at Promoter
ATAC-seq log2 Fold Change
RNA-seq log2 Fold Change
Significant
RNA-seq
ATAC-seq
no
yes
r2 = 0.761
p < 2.2e−16

Supplementary Fig. 3 Epigenomic consequences of *Gata6* impairment in SPC derived LUAD cells. **a-d** Hierarchical clustering heatmap of SPC-Cre KPG cells and KP cells, based on RNA-seq, ATAC-seq, and ChIP-seq data. Each column is a biological replicate. **a** RNA-seq heatmap based on all differentially expressed genes (n = 1,254) with |log2 fold change| >1, *P*-adjusted < 0.05. Regularized log counts plotted. **b** ATAC-seq heatmap based on all significant regions differentially open or closed (n = 5,257). **c** ChIP-seq heatmap based on all significant peaks for H3K4me3 (n = 1,713). **d** ChIP-seq heatmap based on H3K27ac peaks defined as enhancers (more than 2Kb upstream and downstream of TSS of all expressed genes) (n = 3,762). **e** Heatmaps of GATA6 binding sites (orange) with significant ATAC peaks (black) and the indicated histone modifications, H3K27ac (blue) and H3K4me3 (green), at each site in the KP genome stratified by promoters, enhancers, and other regions. 2Kb region around the center of each GATA6 binding site are depicted. **f** Correlation of gene expression vs differential accessibility at the cognate promoter for each gene in KPG vs KP cells. Spearman correlation was performed using significant accessible regions and genes. Bordered dot = significant genes by RNA-seq and ATAC-seq.

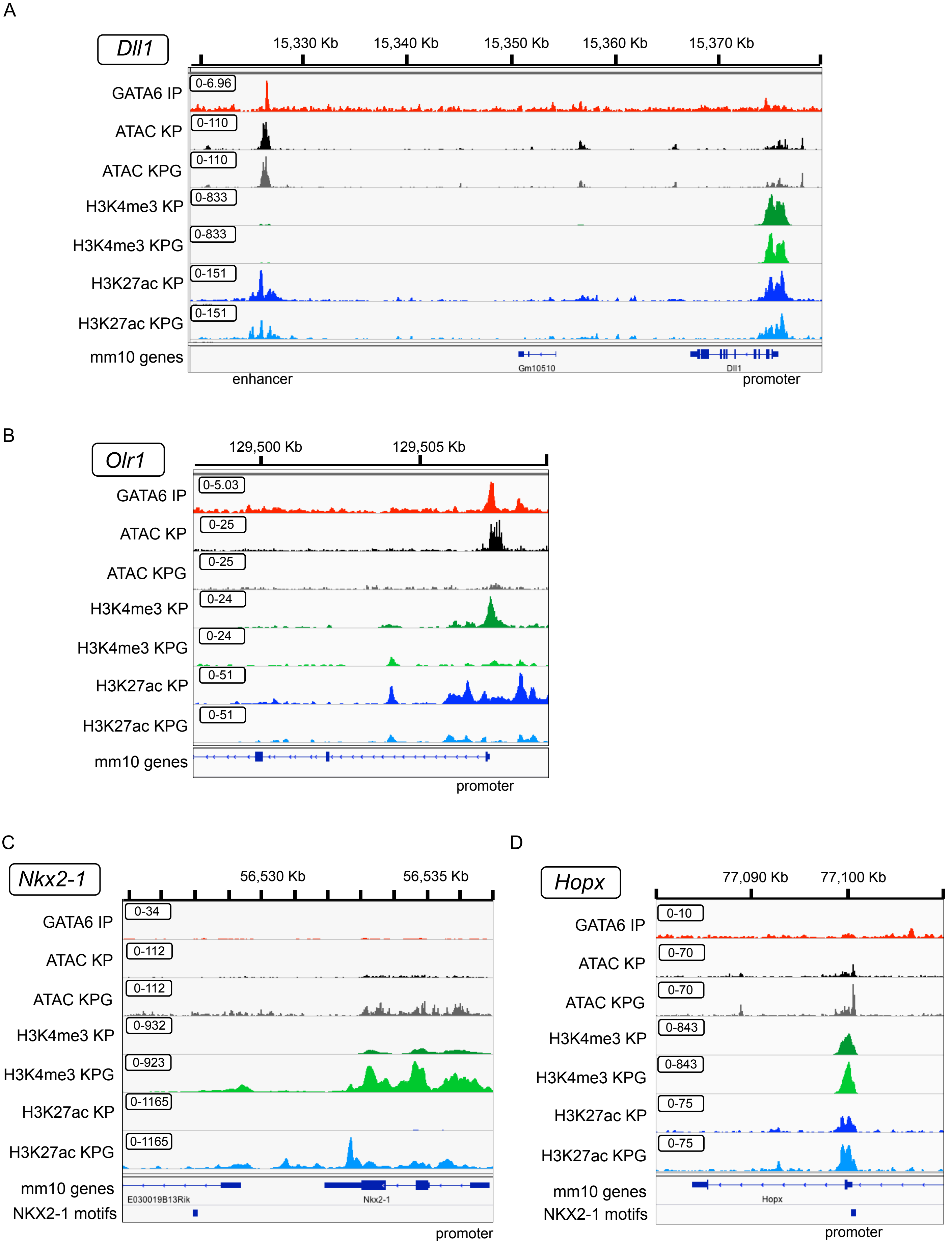
A
Dll1
15,330 Kb
15,340 Kb
15,350 Kb
15,360 Kb
15,370 Kb
GATA6 IP
0-6.96
ATAC KP
0-110
ATAC KPG
0-110
H3K4me3 KP
0-833
H3K4me3 KPG
0-833
H3K27ac KP
0-151
H3K27ac KPG
0-151
mm10 genes
Gm10510
Dll1
enhancer
promoter
B
Olr1
129,500 Kb
129,505 Kb
GATA6 IP
0-5.03
ATAC KP
0-25
ATAC KPG
0-25
H3K4me3 KP
0-24
H3K4me3 KPG
0-24
H3K27ac KP
0-51
H3K27ac KPG
0-51
mm10 genes
promoter
C
Nkx2-1
56,530 Kb
56,535 Kb
GATA6 IP
0-34
ATAC KP
0-112
ATAC KPG
0-112
H3K4me3 KP
0-932
H3K4me3 KPG
0-923
H3K27ac KP
0-1165
H3K27ac KPG
0-1165
mm10 genes
E030019B13Rik
Nkx2-1
NKX2-1 motifs
promoter
D
Hopx
77,090 Kb
77,100 Kb
GATA6 IP
0-10
ATAC KP
0-70
ATAC KPG
0-70
H3K4me3 KP
0-843
H3K4me3 KPG
0-843
H3K27ac KP
0-75
H3K27ac KPG
0-75
mm10 genes
Hopx
NKX2-1 motifs
promoter

Supplementary Fig. 4 Examples of epigenomic effects of GATA6. **a** IGV track at TSS of *Dll1* (Chr 17 15,320,000-15,380,000 of the mm10 genome) with GATA6 binding at an enhancer region. **b** IGV track at TSS of *Olr1* (Chr 6 129,498,000-129,508,000 of mm10 genome) with GATA6 binding at the promoter. **c** IGV track at TSS of *Nkx2-1* (Chr 12 56,526,000-56,537,000 of the mm10 genome) with NKX2-1 motif in the promoter. **d** IGV track at TSS of *Hopx* (Chr 5 77,080,000-77,110,000 of the mm10 genome) with NKX2-1 motif in the promoter. Annotation of the tracks: KP GATA6 IP peaks (red), KP ATAC peaks (black), KPG ATAC peaks (grey), KP H3K4me3 peaks (dark green), KPG H3K4me3 peaks (light green), KP H3K27ac peaks (dark blue), KPG H3K27ac (light blue). Boxed values: data range for each track.

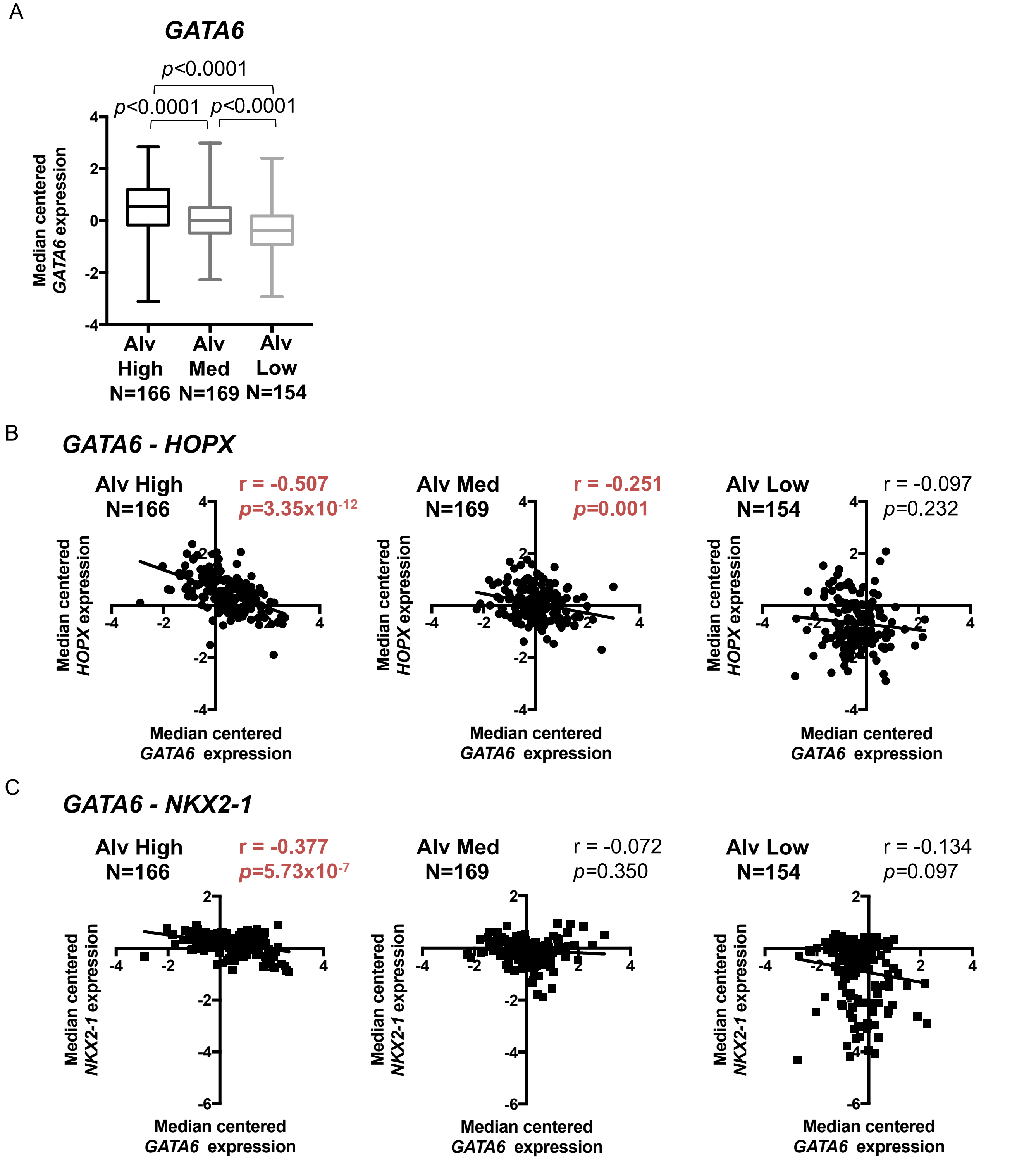

Supplementary Fig. 5 *GATA6* expression anti-correlates with *HOPX* and *NKX2-1* in Alveolar-high tumors from TCGA. **a** Median centered expression of *GATA6* in TCGA tumors (N=489) clustered into 3 groups (Alveolar-high, alveolar-medium or alveolar-low). **b** Pearson correlation of *GATA6* with *HOPX* in tumors from TCGA classified as **a**. **c** Pearson correlation of *GATA6* with *NKX2-1* in tumors from TCGA classified as **a**.

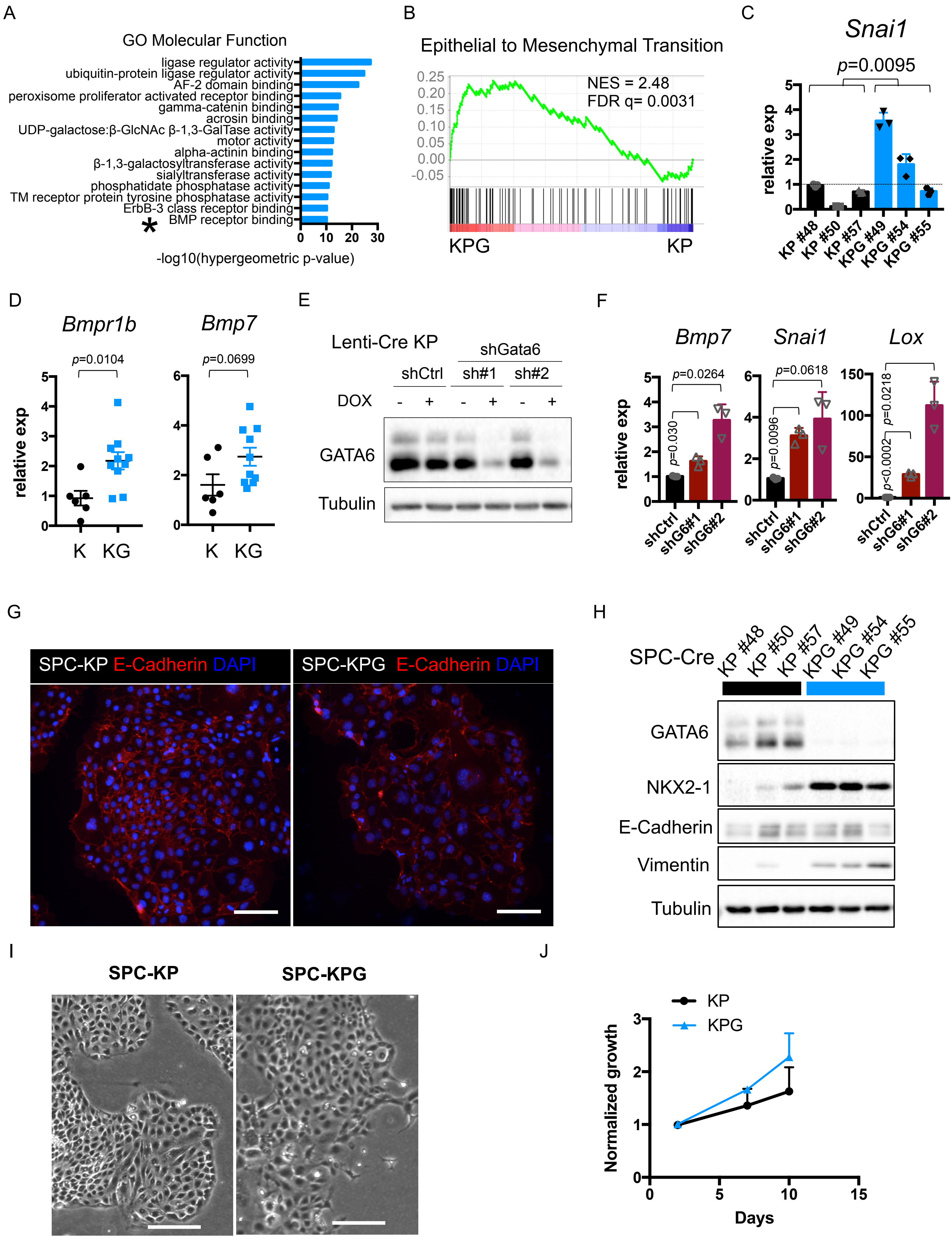
A
GO Molecular Function
ligase regulator activity
ubiquitin-protein ligase regulator activity
AF-2 domain binding
peroxisome proliferator activated receptor binding
gamma-catenin binding
acrosin binding
UDP-galactose:β-GlcNAc β-1,3-GalTase activity
motor activity
alpha-actinin binding
β-1,3-galactosyltransferase activity
sialyltransferase activity
phosphatidate phosphatase activity
TM receptor protein tyrosine phosphatase activity
ErbB-3 class receptor binding
* BMP receptor binding
0 10 20 30
-log10(hypergeometric p-value)
B
Epithelial to Mesenchymal Transition
NES = 2.48
FDR q= 0.0031
0.25 0.20 0.15 0.10 0.05 0.00 -0.05
KPG
KP
C
Snai1
p=0.0095
relative exp
KP #48
KP #50
KP #57
KPG #49
KPG #54
KPG #55
D
Bmpr1b
Bmp7
p=0.0104
p=0.0699
relative exp
K KG
K KG
E
Lenti-Cre KP
shGata6
shCtrl sh#1 sh#2
DOX - + - + - +
GATA6
Tubulin
F
Bmp7
Snai1
Lox
p=0.0264
p=0.030
p=0.0618
p=0.0096
p=0.0218
p<0.0002
relative exp
shCtrl shG6#1 shG6#2
G
SPC-KP E-Cadherin DAPI
SPC-KPG E-Cadherin DAPI
H
SPC-Cre
KP #48 KP #50 KP #57 KPG #49 KPG #54 KPG #55
GATA6
NKX2-1
E-Cadherin
Vimentin
Tubulin
I
SPC-KP
SPC-KPG
J
KP
KPG
Normalized growth
Days
0 5 10 15

Supplementary Fig. 6 *Gata6* restricts BMP signaling in SPC derived lung cancer cells. **a** Top 15 significant GO Molecular function pathways from GREAT analysis using newly open/closed chromatin regions in KPG cells relative to KP cells based on ATAC-seq peaks from Supplementary Fig. 3 **b**. Hypergeometric *p*-value (-log10) is plotted. **b** Gene Set Enrichment Analysis (GSEA) plot of Epithelial to Mesenchymal Transition (EMT) pathway in KPG versus KP cells. NES = normalized enrichment score. **c** qRT-PCR of *Snai1* in cell lines from Fig. 3**f**. (*n* = 3 biological replicates). **d** qRT-PCR of *Bmpr1b* and *Bmp7* in macro-dissected nodules from K and KG animals at 22 weeks post-infection with SPC-Cre (*n* = 3-5 mice, 2 nodules/mouse). SEM is plotted and *p*-value calculated by unpaired t-test. **e-f** Doxycycline-inducible knockdown of GATA6 in Lenti-Cre KP cells using 2 independent hairpins (sh#1, sh#2) and a non-targeting control shRNA (shCtrl). **e** Representative Western-blot of GATA6 and Tubulin in the indicated samples. **f** qRT-PCR of *Bmp7, Snai1* and *Lox* in doxycycline-treated cells. (*n* = 3-5 biological replicates). **g** Immunofluorescence of E-Cadherin (red) and DAPI (blue/nuclei) in SPC-KP or KPG cells grown in a monolayer. Scale bar=100μm. **h** Western blot of GATA6, NKX2-1, E-Cadherin, Vimentin, and Tubulin (loading control) in the SPC-Cre KP and KPG cells. **i** Phase contrast picture of SPC-KP and KPG cells grown in a monolayer. Scale bar=200μm. **j** Growth of KP and KPG organoids was measured by fluorescence over the indicated days. SEM is plotted (*n* = 3 biological replicates). All gene expression data was normalized to *b-actin*. SEM is plotted and *p*-value was calculated by Welch's t-test, except if specified.

Full unedited gels for **Fig. 6c**

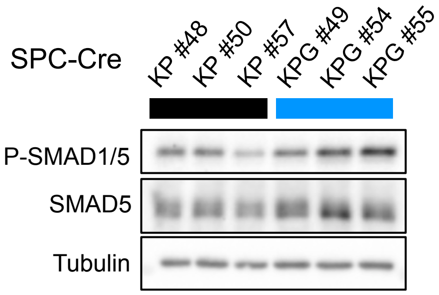

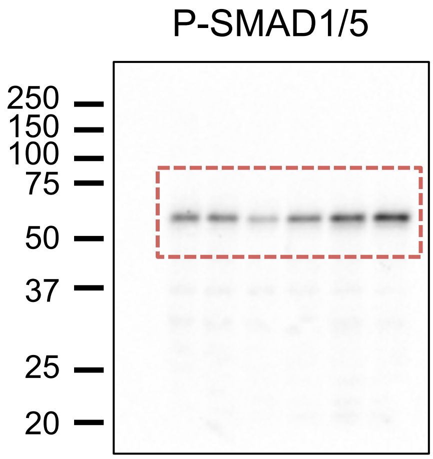

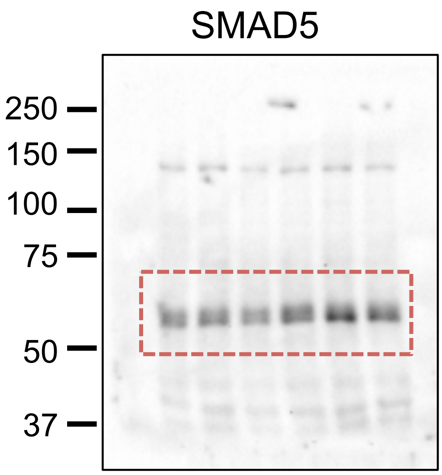

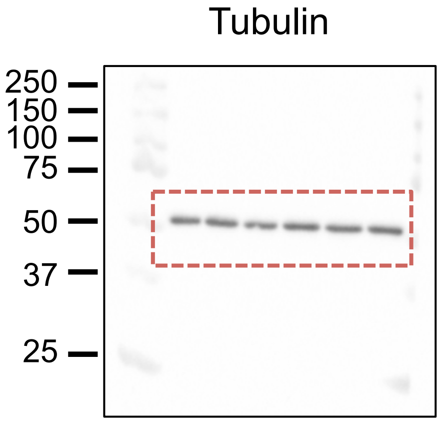

Full unedited gels for **Fig. 6d**

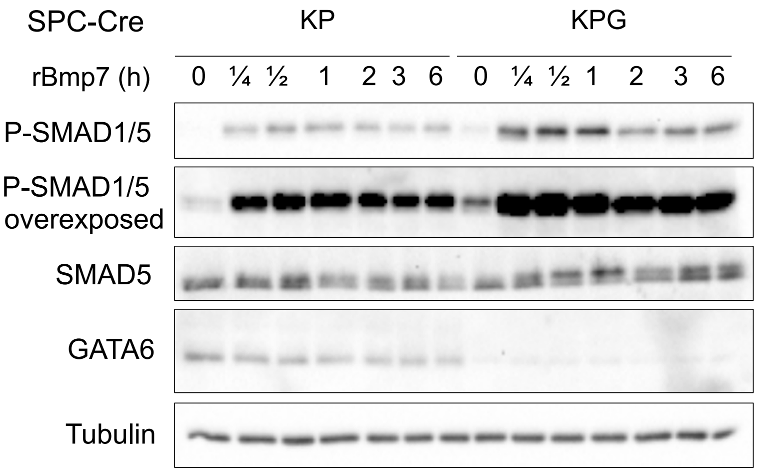

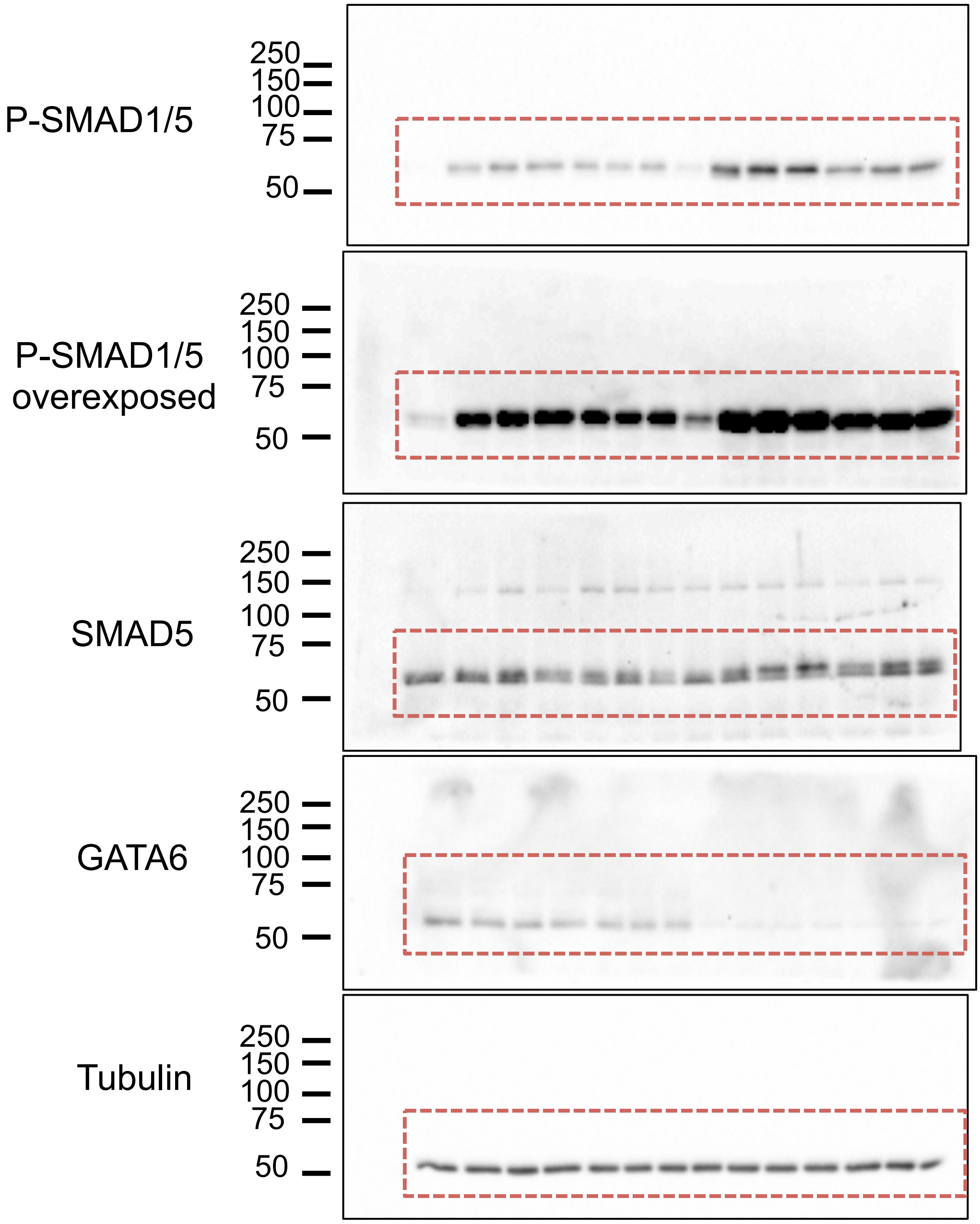

Full unedited gels for **Supplementary Fig. 6e**

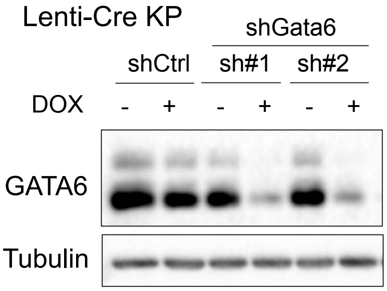

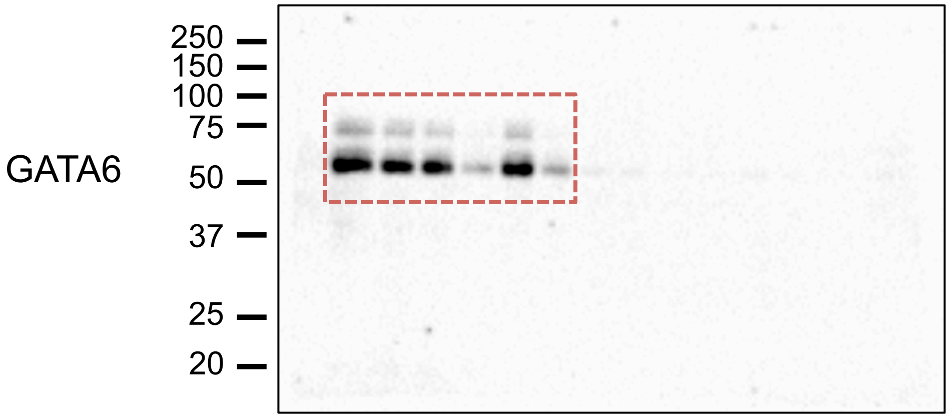

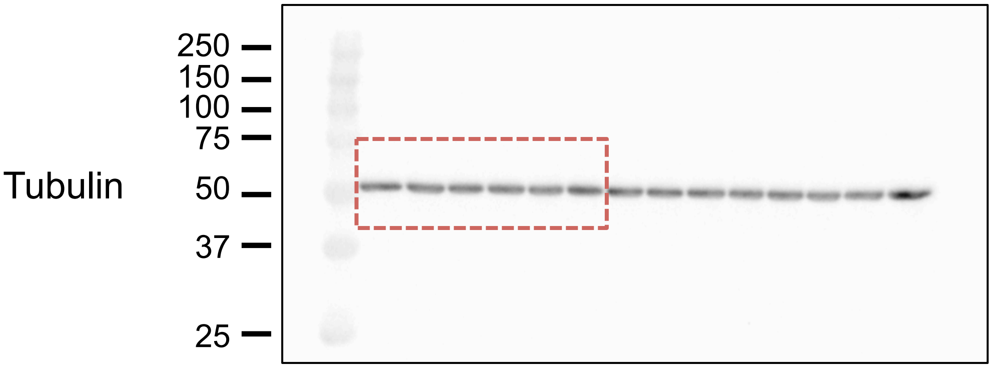

Full unedited gels for **Supplementary Fig. 6h**

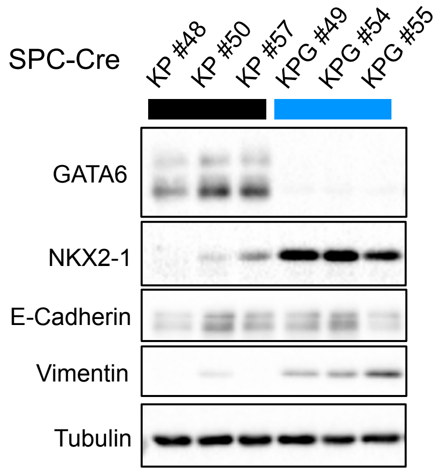

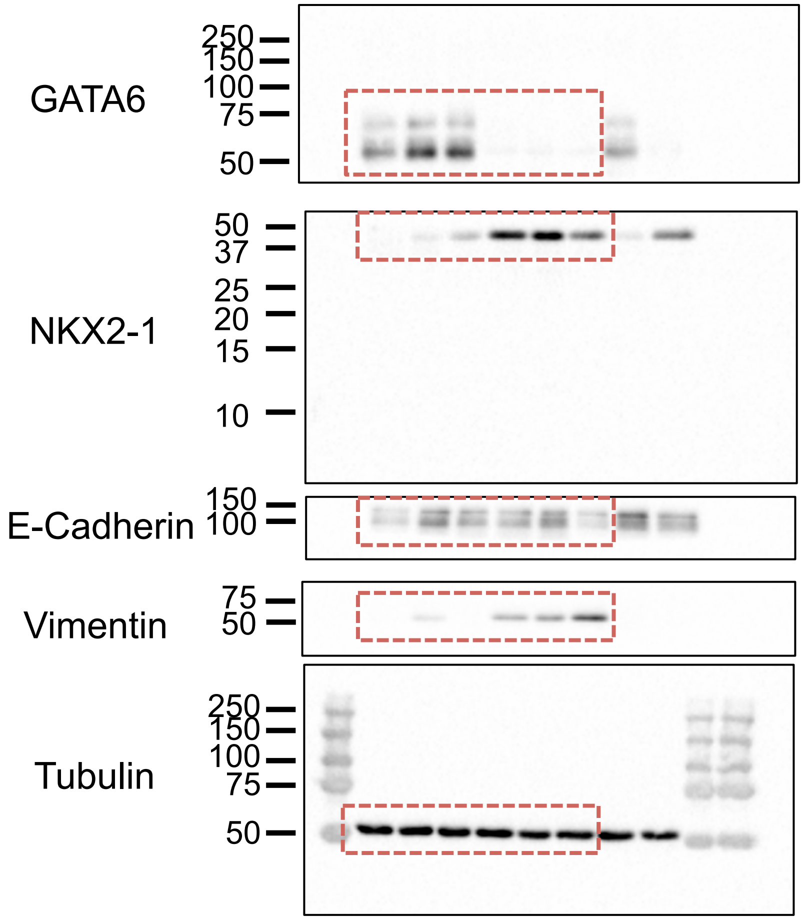

Supplement: Supplementary file 1 — Supplementary Material [file 41388_2020_1246_MOESM1_ESM.pdf]
