# Supplementary material for: Tumor progression and chromatin landscape of lung cancer are regulated by the lineage factor GATA6
Source: Oncogene. 2020 Mar 10;39(18):3726–37. doi: 10.1038/s41388-020-1246-z (PMC7190573; doi:10.1038/s41388-020-1246-z)
Supplement: Supplementary file 2 — Supplementary Table 1 [file 41388_2020_1246_MOESM2_ESM.pdf]

Supplementary Table 1. Summary of the numbers and phenotype of GEMM experiments

Experiment	Sample size		Summary Phenotype (Gata6$^{-/-}$)
	WT	Gata6 -/-	
AdCRE K/KG	3	5	Decrease tumor burden
AdCRE KP/KPG	6	10	Decrease tumor burden
Lenti-CRE K/KG	5	6	Decrease tumor burden and progression
Lenti-CRE KP/KPG	3	4	Decrease tumor burden, progression and proliferation
CC10-CRE K/KG	3	7	Decrease tumor burden
CC10-CRE KP/KPG	15	9	Decrease tumor burden
SPC-CRE K/KG	7	7	Decrease tumor burden
SPC-CRE KP/KPG	6	6	Decrease tumor burden
**TOTAL MICE**	**48**	**54**	
