# Supplementary material for: Tumor progression and chromatin landscape of lung cancer are regulated by the lineage factor GATA6
Source: Oncogene. 2020 Mar 10;39(18):3726–37. doi: 10.1038/s41388-020-1246-z (PMC7190573; doi:10.1038/s41388-020-1246-z)
Supplement: Supplementary file 3 — Supplementary Table 2 [file 41388_2020_1246_MOESM3_ESM.pdf]

Supplementary Table 2. Significant differentially expressed genes and pathways in SPC-Cre KPG and KP cell lines. <u>SPC-Cre KPG-KP</u>: List of genes differentially expressed in SPC-KPG cells compared to SPC-KP cells (*P*-adjusted < 0.05 and |log2 fold change| > 1; n = 1254 genes).

genes	GeneVer	GeneID	GeneType	GeneName	SPC-KPG_mean	SPC-KP_mean	log2FoldChange	lfcSE	stat	pvalue	padj
ENSMUSG00000001555.10	ENSMUSG00000001555.10	ENSMUSG00000001555	protein_coding	Fkbp10	40.96588091	9.773265436	2.08515945	0.4969764	4.1956909	2.72E-05	0.0002606
ENSMUSG00000030411.16	ENSMUSG00000030411.16	ENSMUSG00000030411	protein_coding	Nova2	14.57232272	40.06567917	-1.462806538	0.4016254	-3.642216	0.0002703	0.0020059
ENSMUSG00000027985.14	ENSMUSG00000027985.14	ENSMUSG00000027985	protein_coding	Lef1	174.661979	27.00933123	2.696379311	0.2624569	10.273606	9.267E-25	6.25E-23
ENSMUSG00000043110.2	ENSMUSG00000043110.2	ENSMUSG00000043110	protein_coding	Lrrn4	10.04219936	30.04458855	-1.589030173	0.5546902	-2.864716	0.0041738	0.0213877
ENSMUSG00000094635.2	ENSMUSG00000094635.2	ENSMUSG00000094635	processed_pseudogene	Gm7104	4.915419514	0	4.65379365	1.7555932	2.6508383	0.0080292	0.0368332
ENSMUSG00000020940.13	ENSMUSG00000020940.13	ENSMUSG00000020940	protein_coding	1700023F06Rik	9.61011276	0	5.619058727	1.4910554	3.7685111	0.0001642	0.0012902
ENSMUSG00000087201.1	ENSMUSG00000087201.1	ENSMUSG00000087201	antisense_RNA	Gm15261	5.486215212	0	4.813712691	1.7363305	2.7723481	0.0055653	0.0273501
ENSMUSG00000018479.12	ENSMUSG00000018479.12	ENSMUSG00000018479	protein_coding	1700125H20Rik	21.22438347	42.82420637	-1.017043844	0.3893913	-2.611881	0.0090046	0.0404608
ENSMUSG00000059336.14	ENSMUSG00000059336.14	ENSMUSG00000059336	protein_coding	Slc14a1	6.675188198	501.1138929	-6.261417746	0.3785676	-16.53976	1.898E-61	5.395E-59
ENSMUSG00000039347.7	ENSMUSG00000039347.7	ENSMUSG00000039347	protein_coding	Atp6v0e2	48.0269008	97.68737058	-1.024777019	0.2393411	-4.28166	1.855E-05	0.0001849
ENSMUSG00000029727.7	ENSMUSG00000029727.7	ENSMUSG00000029727	protein_coding	Cyp3a13	458.4556718	145.6274498	1.654622968	0.1356079	12.201523	3.051E-34	3.132E-32
ENSMUSG00000112716.1	ENSMUSG00000112716.1	ENSMUSG00000112716	lincRNA	AC134528.2	33.63900654	10.76240278	1.653724637	0.5230868	3.1614726	0.0015697	0.0092996
ENSMUSG00000079484.12	ENSMUSG00000079484.12	ENSMUSG00000079484	protein_coding	Phyhd1	150.1553741	61.12797013	1.298935402	0.3039976	4.2728471	1.93E-05	0.0001914
ENSMUSG00000092981.1	ENSMUSG00000092981.1	ENSMUSG00000092981	miRNA	Mir5125	4.577449357	49.83226357	-3.430203033	0.5169813	-6.635062	3.244E-11	7.866E-10
ENSMUSG00000046352.7	ENSMUSG00000046352.7	ENSMUSG00000046352	protein_coding	Gjb2	52.93022105	18.74958963	1.497610934	0.345292	4.3372298	1.443E-05	0.0001467
ENSMUSG00000031596.15	ENSMUSG00000031596.15	ENSMUSG00000031596	protein_coding	Slc7a2	36.30914772	1.49239033	4.664677777	0.9002105	5.1817634	2.198E-07	3.139E-06
ENSMUSG00000042312.9	ENSMUSG00000042312.9	ENSMUSG00000042312	protein_coding	S100a13	357.8503372	101.1053227	1.825832809	0.1534974	11.894876	1.258E-32	1.217E-30
ENSMUSG00000004267.16	ENSMUSG00000004267.16	ENSMUSG00000004267	protein_coding	Eno2	16.72728904	34.03519528	-1.024333999	0.391023	-2.619626	0.0088026	0.0397204
ENSMUSG00000030693.10	ENSMUSG00000030693.10	ENSMUSG00000030693	protein_coding	Klk10	263.0487939	632.5140748	-1.26729094	0.1220346	-10.38468	2.911E-25	2.022E-23
ENSMUSG00000031303.8	ENSMUSG00000031303.8	ENSMUSG00000031303	protein_coding	Map3k15	28.66689509	79.00830072	-1.467414306	0.3132769	-4.684081	2.812E-06	3.308E-05
ENSMUSG00000044349.15	ENSMUSG00000044349.15	ENSMUSG00000044349	protein_coding	Snhg11	33.10617089	14.99927834	1.145228682	0.4382547	2.6131577	0.008971	0.0403422
ENSMUSG00000013584.5	ENSMUSG00000013584.5	ENSMUSG00000013584	protein_coding	Aldh1a2	352.0910274	9.798163813	5.155248965	0.3186683	16.177475	7.272E-59	1.892E-56
ENSMUSG00000085183.1	ENSMUSG00000085183.1	ENSMUSG00000085183	lincRNA	Gm12603	405.1782258	869.3325249	-1.101992852	0.1041375	-10.58209	3.608E-26	2.615E-24
ENSMUSG00000106933.1	ENSMUSG00000106933.1	ENSMUSG00000106933	TEC	Gm43621	20.025509	4.950811403	2.012772684	0.6533465	3.0807123	0.0020651	0.0117795
ENSMUSG00000091055.2	ENSMUSG00000091055.2	ENSMUSG00000091055	protein_coding	Siglec15	6.639905879	68.50176397	-3.357006997	0.7733161	-4.341054	1.418E-05	0.0001444
ENSMUSG00000061397.7	ENSMUSG00000061397.7	ENSMUSG00000061397	protein_coding	Krt79	38.9171303	0	7.637678548	1.2587068	6.0678773	1.296E-09	2.572E-08
ENSMUSG00000041658.12	ENSMUSG00000041658.12	ENSMUSG00000041658	protein_coding	Rragb	28.51436888	0	7.190440417	1.2992859	5.5341478	3.127E-08	5.163E-07
ENSMUSG00000087660.1	ENSMUSG00000087660.1	ENSMUSG00000087660	antisense_RNA	B230398E01Rik	5.009000407	0	4.677075676	1.7620222	2.6543796	0.0079454	0.0365395
ENSMUSG00000017446.14	ENSMUSG00000017446.14	ENSMUSG00000017446	protein_coding	C1qtnf1	17.09882368	1.29659816	3.644087376	1.0967267	3.3226942	0.0008915	0.0056688
ENSMUSG00000042797.9	ENSMUSG00000042797.9	ENSMUSG00000042797	protein_coding	Aqp11	44.0422479	20.92630323	1.067060139	0.3816926	2.7956007	0.0051803	0.025829
ENSMUSG00000041390.18	ENSMUSG00000041390.18	ENSMUSG00000041390	protein_coding	Mdfic	619.1043254	159.1904051	1.959157774	0.1692623	11.574686	5.537E-31	5.016E-29
ENSMUSG00000002076.11	ENSMUSG00000002076.11	ENSMUSG00000002076	protein_coding	Hsf2bp	26.61755334	10.87963433	1.285591895	0.4877793	2.6356014	0.0083988	0.0382156
ENSMUSG00000059742.10	ENSMUSG00000059742.10	ENSMUSG00000059742	protein_coding	Kcnh7	84.11918228	195.7493951	-1.223103581	0.2244384	-5.449618	5.048E-08	7.993E-07
ENSMUSG00000030268.17	ENSMUSG00000030268.17	ENSMUSG00000030268	protein_coding	Bcat1	121.9579901	378.3618871	-1.631783064	0.1488187	-10.96491	5.636E-28	4.394E-26
ENSMUSG00000103292.1	ENSMUSG00000103292.1	ENSMUSG00000103292	TEC	Gm35048	5.229726675	43.79621677	-3.058897419	0.541447	-5.649486	1.609E-08	2.764E-07
ENSMUSG00000020566.19	ENSMUSG00000020566.19	ENSMUSG00000020566	protein_coding	Atp6v1c2	25.04387534	1.6207477	3.868517542	0.958912	4.0342778	5.477E-05	0.0004854
ENSMUSG00000035356.16	ENSMUSG00000035356.16	ENSMUSG00000035356	protein_coding	Nfkbiz	965.0742692	2321.302572	-1.267262735	0.09227	-13.7343	6.327E-43	9.505E-41
ENSMUSG00000079173.11	ENSMUSG00000079173.11	ENSMUSG00000079173	protein_coding	Zan	54.47910935	156.5026394	-1.519268724	0.213684	-7.109885	1.161E-12	3.317E-11
ENSMUSG00000036181.2	ENSMUSG00000036181.2	ENSMUSG00000036181	protein_coding	Hist1h1c	639.3563961	298.1781812	1.099810977	0.1245786	8.8282514	1.063E-18	5.038E-17
ENSMUSG00000025810.9	ENSMUSG00000025810.9	ENSMUSG00000025810	protein_coding	Nrp1	203.1340478	705.247252	-1.794641222	0.1240773	-14.4639	2.049E-47	3.863E-45
ENSMUSG00000049511.5	ENSMUSG00000049511.5	ENSMUSG00000049511	protein_coding	Htr1b	48.29441752	7.297300671	2.747750351	0.5763097	4.7678361	1.862E-06	2.267E-05
ENSMUSG00000112468.1	ENSMUSG00000112468.1	ENSMUSG00000112468	TEC	AC154734.2	12.4173564	0	5.988504216	1.4246148	4.2035955	2.627E-05	0.0002523
ENSMUSG00000059246.4	ENSMUSG00000059246.4	ENSMUSG00000059246	protein_coding	Foxb1	14.38682317	0	6.199354604	1.4080375	4.4028334	1.068E-05	0.000112
ENSMUSG00000110631.1	ENSMUSG00000110631.1	ENSMUSG00000110631	lincRNA	Gm42047	770.0843117	1637.354511	-1.089062622	0.1182212	-9.212079	3.199E-20	1.642E-18
ENSMUSG00000037108.13	ENSMUSG00000037108.13	ENSMUSG00000037108	protein_coding	Zcwpw1	65.07176589	21.57460231	1.58201564	0.3782072	4.1829333	2.878E-05	0.0002734
ENSMUSG00000038146.7	ENSMUSG00000038146.7	ENSMUSG00000038146	protein_coding	Notch3	14.17653394	54.32449406	-1.940259324	0.4866189	-3.987225	6.685E-05	0.0005799
ENSMUSG00000041642.18	ENSMUSG00000041642.18	ENSMUSG00000041642	protein_coding	Kif21b	102.4564308	36.78276548	1.471832737	0.3538631	4.1593285	3.192E-05	0.0002989
ENSMUSG00000087263.1	ENSMUSG00000087263.1	ENSMUSG00000087263	antisense_RNA	Gm15726	5.102581299	21.21442858	-2.076438393	0.6698004	-3.100085	0.0019346	0.0111124
ENSMUSG00000036528.15	ENSMUSG00000036528.15	ENSMUSG00000036528	protein_coding	Ppfibp2	66.0564911	24.64059033	1.42215455	0.3281059	4.3344377	1.461E-05	0.0001484
ENSMUSG00000045083.14	ENSMUSG00000045083.14	ENSMUSG00000045083	protein_coding	Lingo2	23.73932071	3.793696429	2.623967161	0.6575263	3.990665	6.589E-05	0.0005721
ENSMUSG00000018865.8	ENSMUSG00000018865.8	ENSMUSG00000018865	protein_coding	Sult4a1	36.66804754	342.4072318	-3.218440614	0.2071183	-15.53914	1.885E-54	4.409E-52
ENSMUSG00000021281.15	ENSMUSG00000021281.15	ENSMUSG00000021281	protein_coding	Tnfaip2	940.8550298	1883.848705	-1.001361862	0.2557172	-3.915896	9.007E-05	0.0007596
ENSMUSG00000001804.9	ENSMUSG00000001804.9	ENSMUSG00000001804	protein_coding	Dsg4	7.234420171	21.29007303	-1.567614032	0.5620447	-2.789127	0.005285	0.0262378
ENSMUSG00000022123.8	ENSMUSG00000022123.8	ENSMUSG00000022123	protein_coding	Scel	523.8917093	1745.569547	-1.73750642	0.1077018	-16.13256	1.506E-58	3.866E-56
ENSMUSG00000032285.15	ENSMUSG00000032285.15	ENSMUSG00000032285	protein_coding	Dnaja4	316.6256588	129.2545691	1.289674049	0.1667214	7.7355056	1.03E-14	3.524E-13
ENSMUSG00000071793.12	ENSMUSG00000071793.12	ENSMUSG00000071793	transcribed_unprocessed_pseudogene	2610005L07Rik	4.996309991	46.93804579	-3.218495771	1.1531276	-2.791101	0.0052529	0.0261203
ENSMUSG00000001496.15	ENSMUSG00000001496.15	ENSMUSG00000001496	protein_coding	Nkx2-1	535.4774088	8.10177166	6.04246478	0.3354542	18.012787	1.546E-72	5.292E-70
ENSMUSG00000015243.4	ENSMUSG00000015243.4	ENSMUSG00000015243	protein_coding	Abca1	22.07313291	1.49239033	3.938444092	1.0322865	3.8152627	0.000136	0.0010924
ENSMUSG00000026787.3	ENSMUSG00000026787.3	ENSMUSG00000026787	protein_coding	Gad2	89.91964905	20.64177395	2.126188517	0.3503295	6.0691099	1.286E-09	2.556E-08
ENSMUSG00000002617.14	ENSMUSG00000002617.14	ENSMUSG00000002617	protein_coding	Zfp40	77.09113572	259.8509923	-1.753136639	0.1800837	-9.735123	2.136E-22	1.241E-20
ENSMUSG00000082051.1	ENSMUSG00000082051.1	ENSMUSG00000082051	processed_pseudogene	Gm16072	10.61245951	1.394494245	2.912473696	1.0578892	2.753099	0.0059034	0.0287136
ENSMUSG00000026834.13	ENSMUSG00000026834.13	ENSMUSG00000026834	protein_coding	Acvr1c	0.908230308	34.99539996	-5.241365179	1.0063035	-5.208533	1.903E-07	2.735E-06
ENSMUSG00000024235.10	ENSMUSG00000024235.10	ENSMUSG00000024235	protein_coding	Map3k8	73.28213648	28.67902698	1.353246626	0.3021252	4.4790916	7.496E-06	8.119E-05
ENSMUSG00000026832.12	ENSMUSG00000026832.12	ENSMUSG00000026832	protein_coding	Cytip	4.682593974	928.3414178	-7.643865934	0.3938445	-19.40833	6.562E-84	3.031E-81
ENSMUSG00000027297.14	ENSMUSG00000027297.14	ENSMUSG00000027297	protein_coding	Ltk	27.42010348	82.9185488	-1.596841497	0.3297388	-4.842746	1.281E-06	1.6E-05
ENSMUSG00000013367.5	ENSMUSG00000013367.5	ENSMUSG00000013367	protein_coding	Iglon5	2.760453193	11.93329032	-2.107278272	0.8045297	-2.619267	0.0088119	0.0397525
ENSMUSG00000047496.6	ENSMUSG00000047496.6	ENSMUSG00000047496	protein_coding	Rnf152	71.50914183	34.34642097	1.054043249	0.2955806	3.5660093	0.0003625	0.0025879
ENSMUSG00000035142.18	ENSMUSG00000035142.18	ENSMUSG00000035142	protein_coding	Nubpl	230.2464768	113.3151014	1.020630457	0.2041752	4.9987974	5.769E-07	7.736E-06
ENSMUSG00000112949.1	ENSMUSG00000112949.1	ENSMUSG00000112949	processed_pseudogene	AC211878.1	0	51.52421094	-8.234938425	1.2410327	-6.635553	3.233E-11	7.85E-10
ENSMUSG00000037902.18	ENSMUSG00000037902.18	ENSMUSG00000037902	protein_coding	Sirpa	28.44611322	106.5766551	-1.906944296	0.3439418	-5.544381	2.95E-08	4.893E-07
ENSMUSG00000018593.13	ENSMUSG00000018593.13	ENSMUSG00000018593	protein_coding	Sparc	6845.534773	1998.641169	1.776087664	0.1046673	16.968894	1.395E-64	4.227E-62
ENSMUSG00000074300.4	ENSMUSG00000074300.4	ENSMUSG00000074300	protein_coding	BC030870	11.39134666	0	5.865886206	1.4729708	3.9823506	6.824E-05	0.0005897
ENSMUSG00000052760.15	ENSMUSG00000052760.15	ENSMUSG00000052760	protein_coding	A630001G21Rik	30.30829319	9.177679272	1.722670206	0.4998474	3.4463921	0.0005681	0.0038132
ENSMUSG00000029298.15	ENSMUSG00000029298.15	ENSMUSG00000029298	protein_coding	Gbp9	210.2236063	72.30770434	1.538290586	0.234913	6.5483405	5.818E-11	1.361E-09
ENSMUSG00000036103.8	ENSMUSG00000036103.8	ENSMUSG00000036103	protein_coding	Colec12	18.660458	5.103218438	1.888767277	0.7327082	2.5777892	0.0099435	0.0439033
ENSMUSG00000056004.16	ENSMUSG00000056004.16	ENSMUSG00000056004	protein_coding	9330182L06Rik	8.584638563	30.69956867	-1.831988747	0.478726	-3.8268	0.0001298	0.0010494
ENSMUSG00000005397.8	ENSMUSG00000005397.8	ENSMUSG00000005397	protein_coding	Nid1	1312.468278	6281.137754	-2.258768763	0.0639342	-35.32956	2.07E-273	9.54E-270
ENSMUSG00000087187.1	ENSMUSG00000087187.1	ENSMUSG00000087187	lincRNA	Gm13431	27.95787024	11.70703686	1.256610226	0.487726	2.5764677	0.0099816	0.0440084
ENSMUSG00000044921.5	ENSMUSG00000044921.5	ENSMUSG00000044921	protein_coding	Rassf9	74.01364203	29.68861792	1.314084403	0.418837	3.1374604	0.0017042	0.0099936
ENSMUSG00000102440.1	ENSMUSG00000102440.1	ENSMUSG00000102440	protein_coding	Pcdhga9	39.53530759	14.86356002	1.409930971	0.4075211	3.4597744	0.0005406	0.0036619
ENSMUSG00000034127.15	ENSMUSG00000034127.15	ENSMUSG00000034127	protein_coding	Tspan8	1636.88464	3415.345414	-1.060556367	0.0869011	-12.20418	2.953E-34	3.048E-32
ENSMUSG00000028631.7	ENSMUSG00000028631.7	ENSMUSG00000028631	protein_coding	Kcnq4	17.64761862	59.34162213	-1.753835836	0.3750867	-4.675815	2.928E-06	3.433E-05
ENSMUSG00000004552.16	ENSMUSG00000004552.16	ENSMUSG00000004552	protein_coding	Ctse	44.10167317	493.5871485	-3.482211316	0.1903733	-18.29149	9.673E-75	3.437E-72
ENSMUSG00000006567.11	ENSMUSG00000006567.11	ENSMUSG00000006567	protein_coding	Atp7b	18.19255354	51.32037793	-1.491746396	0.5067661	-2.943658	0.0032436	0.0172872
ENSMUSG00000043157.8	ENSMUSG00000043157.8	ENSMUSG00000043157	protein_coding	Arl11	18.64996537	1.718643785	3.410169597	0.8925177	3.8208426	0.000133	0.0010717
ENSMUSG00000023443.13	ENSMUSG00000023443.13	ENSMUSG00000023443	protein_coding	Esx1	2.224884217	14.77122685	-2.753523427	0.9047178	-3.043516	0.0023383	0.0131413
ENSMUSG00000005883.15	ENSMUSG00000005883.15	ENSMUSG00000005883	protein_coding	Spo11	75.80922858	31.21659422	1.274107995	0.3125156	4.0769417	4.563E-05	0.0004139
ENSMUSG00000000386.14	ENSMUSG00000000386.14	ENSMUSG00000000386	protein_coding	Mx1	41.72490949	2.415890907	4.083753256	0.6954886	5.871776	4.312E-09	7.999E-08
ENSMUSG00000027500.10	ENSMUSG00000027500.10	ENSMUSG00000027500	protein_coding	Stmn2	52.49914995	6.592692606	2.983794072	0.6943208	4.2974284	1.728E-05	0.0001731
ENSMUSG00000032135.15	ENSMUSG00000032135.15	ENSMUSG00000032135	protein_coding	Mcam	179.8768731	1008.692326	-2.488924567	0.1123874	-22.14595	1.14E-108	9.59E-106
ENSMUSG00000008658.15	ENSMUSG00000008658.15	ENSMUSG00000008658	protein_coding	Rbfox1	5.21816295	0	4.740206298	1.7347931	2.7324332	0.0062868	0.0302207
ENSMUSG00000046688.14	ENSMUSG00000046688.14	ENSMUSG00000046688	protein_coding	Tifa	144.8617873	71.46514171	1.013702912	0.2201589	4.6044155	4.136E-06	4.706E-05
ENSMUSG00000059325.14	ENSMUSG00000059325.14	ENSMUSG00000059325	protein_coding	Hopx	35.91009006	1.837673373	4.325879933	0.9141286	4.7322444	2.221E-06	2.659E-05
ENSMUSG00000050936.5	ENSMUSG00000050936.5	ENSMUSG00000050936	lincRNA	Gm42743	4.927518786	0	4.656591402	1.8073034	2.5765411	0.0099794	0.0440084
ENSMUSG00000046794.9	ENSMUSG00000046794.9	ENSMUSG00000046794	protein_coding	Ppp1r3b	91.62490724	15.94879542	2.511039912	0.4074573	6.1627068	7.151E-10	1.465E-08
ENSMUSG00000107369.1	ENSMUSG00000107369.1	ENSMUSG00000107369	processed_pseudogene	Gstm2-ps1	85.06969627	21.54414103	1.981619265	0.3447612	5.747802	9.041E-09	1.606E-07
ENSMUSG00000047250.13	ENSMUSG00000047250.13	ENSMUSG00000047250	protein_coding	Ptgs1	1879.639654	290.3524597	2.692497635	0.1231009	21.872284	4.77E-106	3.83E-103
ENSMUSG00000064373.11	ENSMUSG00000064373.11	ENSMUSG00000064373	protein_coding	Selenop	38.3000797	5.286086759	2.85458501	0.5368542	5.3172443	1.054E-07	1.58E-06
ENSMUSG00000028558.14	ENSMUSG00000028558.14	ENSMUSG00000028558	protein_coding	Calr4	187.8841389	89.82674307	1.06094279	0.2052234	5.1696967	2.345E-07	3.317E-06
ENSMUSG00000024743.15	ENSMUSG00000024743.15	ENSMUSG00000024743	protein_coding	Syt7	152.7127782	32.66851557	2.226803456	0.271963	8.1878915	2.658E-16	1.063E-14
ENSMUSG00000063730.13	ENSMUSG00000063730.13	ENSMUSG00000063730	protein_coding	Hsd3b2	9.574886039	0	5.614592108	1.491926	3.7633181	0.0001677	0.0013139
ENSMUSG00000059974.10	ENSMUSG00000059974.10	ENSMUSG00000059974	protein_coding	Ntm	131.044728	21.05910538	2.630486102	0.5250537	5.0099377	5.445E-07	7.387E-06
ENSMUSG00000040265.16	ENSMUSG00000040265.16	ENSMUSG00000040265	protein_coding	Dnm3	3.04006918	115.8963232	-5.231583444	0.5352861	-9.773434	1.464E-22	8.615E-21
ENSMUSG00000039814.15	ENSMUSG00000039814.15	ENSMUSG00000039814	protein_coding	Xkr5	6.103265809	0	4.967372721	1.6804462	2.9559845	0.0031167	0.0166979
ENSMUSG00000017639.13	ENSMUSG00000017639.13	ENSMUSG00000017639	protein_coding	Rab11fip4	31.56712848	85.33247286	-1.435685956	0.2865084	-5.010974	5.416E-07	7.353E-06
ENSMUSG00000026676.7	ENSMUSG00000026676.7	ENSMUSG00000026676	protein_coding	Ccdc3	30.85644139	6.07010415	2.355863037	0.610779	3.8571449	0.0001147	0.0009396
ENSMUSG00000092130.2	ENSMUSG00000092130.2	ENSMUSG00000092130	antisense_RNA	D030025P21Rik	70.24029386	28.26895588	1.306117316	0.4581446	2.8508843	0.0043598	0.0222235
ENSMUSG00000090793.2	ENSMUSG00000090793.2	ENSMUSG00000090793	unprocessed_pseudogene	Gm6650	170.255141	35.68752239	2.247538237	0.2726545	8.2431732	1.677E-16	6.826E-15

ENSMUSG00000078949.2	ENSMUSG00000078949.2	ENSMUSG00000078949	protein_coding	R3hdml	16.63370815	0	6.411964832	1.3656267	4.6952545	2.663E-06	3.142E-05
ENSMUSG00000032470.16	ENSMUSG00000032470.16	ENSMUSG00000032470	protein_coding	Mras	301.9817723	608.8289298	-1.013518019	0.1264554	-8.014827	1.103E-15	4.151E-14
ENSMUSG00000071256.3	ENSMUSG00000071256.3	ENSMUSG00000071256	protein_coding	Zfp213	68.05963355	191.628633	-1.493744365	0.2122512	-7.037626	1.955E-12	5.466E-11
ENSMUSG00000034796.14	ENSMUSG00000034796.14	ENSMUSG00000034796	protein_coding	Cpne7	84.55516815	22.25139584	1.924954754	0.3099221	6.2110915	5.262E-10	1.096E-08
ENSMUSG00000002688.8	ENSMUSG00000002688.8	ENSMUSG00000002688	protein_coding	Prkd1	125.1477966	321.3429011	-1.358242455	0.1504448	-9.028177	1.746E-19	8.67E-18
ENSMUSG00000014158.12	ENSMUSG00000014158.12	ENSMUSG00000014158	protein_coding	Trpv4	174.0295602	374.713675	-1.107999107	0.1306233	-8.482399	2.206E-17	9.546E-16
ENSMUSG00000097681.1	ENSMUSG00000097681.1	ENSMUSG00000097681	lincRNA	Gm26643	0	6.383127875	-5.220940486	1.6518393	-3.160683	0.001574	0.0093219
ENSMUSG00000066258.13	ENSMUSG00000066258.13	ENSMUSG00000066258	protein_coding	Trim12a	70.5117818	32.17903515	1.123558008	0.3261743	3.444655	0.0005718	0.0038364
ENSMUSG00000032596.14	ENSMUSG00000032596.14	ENSMUSG00000032596	protein_coding	Uba7	58.2023426	13.38785842	2.120251843	0.4309825	4.9195781	8.673E-07	1.121E-05
ENSMUSG00000047143.3	ENSMUSG00000047143.3	ENSMUSG00000047143	protein_coding	Dmrta2	43.00687222	17.57313919	1.299252905	0.4291917	3.0272088	0.0024682	0.013783
ENSMUSG00000029121.15	ENSMUSG00000029121.15	ENSMUSG00000029121	protein_coding	Crmp1	31.21759459	4.735683764	2.745674521	0.6374284	4.3074242	1.652E-05	0.0001661
ENSMUSG00000040875.12	ENSMUSG00000040875.12	ENSMUSG00000040875	protein_coding	Osbpl10	116.0148986	35.42677336	1.707925944	0.2835551	6.0232595	1.709E-09	3.328E-08
ENSMUSG00000032291.8	ENSMUSG00000032291.8	ENSMUSG00000032291	protein_coding	Crabp1	13.66409241	0	6.126422803	1.4066285	4.355395	1.328E-05	0.0001357
ENSMUSG00000087088.2	ENSMUSG00000087088.2	ENSMUSG00000087088	antisense_RNA	Gm16638	36.98466368	6.971353097	2.399815507	0.8104012	2.9612686	0.0030637	0.0164522
ENSMUSG00000006611.15	ENSMUSG00000006611.15	ENSMUSG00000006611	protein_coding	Hfe	16.7030905	3.561880066	2.235920581	0.7257624	3.0807887	0.0020645	0.0117795
ENSMUSG00000044393.15	ENSMUSG00000044393.15	ENSMUSG00000044393	protein_coding	Dsg2	1879.378106	4689.041707	-1.319263743	0.077574	-17.00651	7.348E-65	2.263E-62
ENSMUSG00000020836.15	ENSMUSG00000020836.15	ENSMUSG00000020836	protein_coding	Coro6	795.2208473	160.1025834	2.313546545	0.1755087	13.181945	1.115E-39	1.493E-37
ENSMUSG00000020577.17	ENSMUSG00000020577.17	ENSMUSG00000020577	protein_coding	Tspan13	86.721587	254.573284	-1.551584188	0.1988981	-7.800901	6.147E-15	2.163E-13
ENSMUSG00000027692.16	ENSMUSG00000027692.16	ENSMUSG00000027692	protein_coding	Tnik	1.910577056	11.95554195	-2.673119328	0.9093662	-2.939541	0.003287	0.0174732
ENSMUSG00000045441.5	ENSMUSG00000045441.5	ENSMUSG00000045441	protein_coding	Gprin3	123.5531889	6.383127875	4.278800275	0.412933	10.361973	3.693E-25	2.537E-23
ENSMUSG00000110935.1	ENSMUSG00000110935.1	ENSMUSG00000110935	processed_pseudogene	AC138292.1	39.46479855	7.219858183	2.429203311	0.5255898	4.6218612	3.803E-06	4.359E-05
ENSMUSG00000024274.9	ENSMUSG00000024274.9	ENSMUSG00000024274	transcribed_processed_pseudogene	Zscan30	7.431483442	0	5.248486531	1.586983	3.3072103	0.0009423	0.0059507
ENSMUSG00000028565.18	ENSMUSG00000028565.18	ENSMUSG00000028565	protein_coding	Nfia	365.6295974	153.6622249	1.247184254	0.1558923	8.0002957	1.241E-15	4.643E-14
ENSMUSG00000034780.6	ENSMUSG00000034780.6	ENSMUSG00000034780	protein_coding	B3galt1	193.683071	38.97043609	2.312401072	0.3186234	7.2574734	3.944E-13	1.168E-11
ENSMUSG00000044317.7	ENSMUSG00000044317.7	ENSMUSG00000044317	protein_coding	Gpr4	25.13965402	82.84982706	-1.730819441	0.3276372	-5.282731	1.273E-07	1.881E-06
ENSMUSG00000030930.14	ENSMUSG00000030930.14	ENSMUSG00000030930	protein_coding	Chst15	28.12136903	113.8548897	-2.023381672	0.2866	-7.05995	1.666E-12	4.699E-11
ENSMUSG00000104861.1	ENSMUSG00000104861.1	ENSMUSG00000104861	lincRNA	3110039M20Rik	48.56963793	283.1867749	-2.535770929	0.2390062	-10.60965	2.688E-26	1.971E-24
ENSMUSG00000027669.14	ENSMUSG00000027669.14	ENSMUSG00000027669	protein_coding	Gnb4	155.0553862	36.45108619	2.08832223	0.2401505	8.6958883	3.441E-18	1.555E-16
ENSMUSG00000031189.12	ENSMUSG00000031189.12	ENSMUSG00000031189	protein_coding	Aff2	154.6936975	387.3199939	-1.324819726	0.1362899	-9.720601	2.463E-22	1.427E-20
ENSMUSG00000043542.12	ENSMUSG00000043542.12	ENSMUSG00000043542	protein_coding	Zc2hc1a	961.7593869	396.5812579	1.278502283	0.2551092	5.0115892	5.398E-07	7.334E-06
ENSMUSG00000006221.7	ENSMUSG00000006221.7	ENSMUSG00000006221	protein_coding	Hspb7	0	5.139242631	-4.914877024	1.8315507	-2.683451	0.0072867	0.0341387
ENSMUSG00000110423.1	ENSMUSG00000110423.1	ENSMUSG00000110423	protein_coding	Gm45736	318.3560312	1.064781797	8.227772619	1.0413067	7.9013919	2.758E-15	1.003E-13
ENSMUSG00000028184.12	ENSMUSG00000028184.12	ENSMUSG00000028184	protein_coding	Adgrl2	82.94787007	458.0644783	-2.464388429	0.1770911	-13.91593	5.069E-44	7.938E-42
ENSMUSG00000110411.1	ENSMUSG00000110411.1	ENSMUSG00000110411	TEC	Gm45457	42.62323831	118.1105214	-1.470304219	0.2979291	-4.935082	8.012E-07	1.043E-05
ENSMUSG00000071847.12	ENSMUSG00000071847.12	ENSMUSG00000071847	protein_coding	Apcdd1	74.42485456	22.01957948	1.758237225	0.3302873	5.3233577	1.019E-07	1.533E-06
ENSMUSG00000113909.1	ENSMUSG00000113909.1	ENSMUSG00000113909	lincRNA	AC113508.4	131.007705	38.3948653	1.767349136	0.3089314	5.7208463	1.06E-08	1.86E-07
ENSMUSG00000008999.7	ENSMUSG00000008999.7	ENSMUSG00000008999	protein_coding	Bmp7	2575.103553	500.3186444	2.361861288	0.0932522	25.327663	1.58E-141	2.25E-138
ENSMUSG00000111511.1	ENSMUSG00000111511.1	ENSMUSG00000111511	processed_transcript	AC160966.2	3.809590387	22.59959504	-2.585627253	0.6907531	-3.7432	0.0001817	0.0014142
ENSMUSG00000050558.13	ENSMUSG00000050558.13	ENSMUSG00000050558	protein_coding	Prokr2	18.71875657	47.98722926	-1.356375328	0.3888049	-3.488576	0.0004856	0.0033344
ENSMUSG00000031881.6	ENSMUSG00000031881.6	ENSMUSG00000031881	protein_coding	Cdh16	54.8496285	8.708483639	2.675207263	0.5010221	5.3394992	9.32E-08	1.417E-06
ENSMUSG00000035493.10	ENSMUSG00000035493.10	ENSMUSG00000035493	protein_coding	Tgfbi	42.65406946	2.956966121	3.89494262	0.7128348	5.4640183	4.655E-08	7.447E-07
ENSMUSG00000020589.17	ENSMUSG00000020589.17	ENSMUSG00000020589	protein_coding	Fam49a	71.06715375	4.588839636	3.951641737	0.5000245	7.902896	2.725E-15	9.931E-14
ENSMUSG00000034786.17	ENSMUSG00000034786.17	ENSMUSG00000034786	protein_coding	Gpsm3	30.09030026	6.10236347	2.31017833	0.5877242	3.9307186	8.469E-05	0.0007195
ENSMUSG00000058056.16	ENSMUSG00000058056.16	ENSMUSG00000058056	protein_coding	Palld	2330.086319	7925.441907	-1.765792608	0.1819099	-9.706961	2.816E-22	1.616E-20
ENSMUSG00000082352.1	ENSMUSG00000082352.1	ENSMUSG00000082352	processed_pseudogene	Gm13228	8.166313476	0	5.381564406	1.5706986	3.4262234	0.000612	0.0040725
ENSMUSG00000029082.17	ENSMUSG00000029082.17	ENSMUSG00000029082	protein_coding	Bst1	2118.539417	307.8977844	2.781329603	0.1354247	20.537837	9.887E-94	6.207E-91
ENSMUSG00000073987.4	ENSMUSG00000073987.4	ENSMUSG00000073987	protein_coding	Ggh	104.5701289	349.2682031	-1.73938603	0.1649563	-10.54453	5.384E-26	3.871E-24
ENSMUSG00000072721.8	ENSMUSG00000072721.8	ENSMUSG00000072721	transcribed_unprocessed_pseudogene	Klra14-ps	28.08507121	59.57035353	-1.09015111	0.3456823	-3.153621	0.0016126	0.009523
ENSMUSG00000042810.14	ENSMUSG00000042810.14	ENSMUSG00000042810	protein_coding	Krba1	127.4327528	376.2158313	-1.563299836	0.1584489	-9.866271	5.829E-23	3.555E-21
ENSMUSG00000051669.5	ENSMUSG00000051669.5	ENSMUSG00000051669	protein_coding	AU021092	85.38298793	11.47898537	2.879794476	0.3701809	7.7794251	7.285E-15	2.53E-13
ENSMUSG00000023036.14	ENSMUSG00000023036.14	ENSMUSG00000023036	protein_coding	Pcdhgc4	304.6566868	8.052823617	5.240335785	1.3498085	3.882281	0.0001035	0.0008575
ENSMUSG00000068154.5	ENSMUSG00000068154.5	ENSMUSG00000068154	protein_coding	Insm1	420.2733159	102.3549396	2.03442401	0.1502499	13.540271	9.045E-42	1.306E-39
ENSMUSG00000026399.12	ENSMUSG00000026399.12	ENSMUSG00000026399	protein_coding	Cd55	66.5496652	197.5994535	-1.571480557	0.2396543	-6.557282	5.48E-11	1.285E-09
ENSMUSG00000036533.8	ENSMUSG00000036533.8	ENSMUSG00000036533	protein_coding	Cdc42ep3	487.4047102	1348.842485	-1.468873638	0.107908	-13.61228	3.385E-42	5.004E-40
ENSMUSG00000102543.5	ENSMUSG00000102543.5	ENSMUSG00000102543	protein_coding	Pcdhgc5	48.3674931	8.994810952	2.436022267	0.6313485	3.8584433	0.0001141	0.0009363
ENSMUSG00000046959.16	ENSMUSG00000046959.16	ENSMUSG00000046959	protein_coding	Slc26a1	15.92420335	51.80565526	-1.706946547	0.3859789	-4.422383	9.762E-06	0.0001031
ENSMUSG00000036111.8	ENSMUSG00000036111.8	ENSMUSG00000036111	protein_coding	Lmo1	76.47905549	5.800465561	3.745775827	0.5276746	7.0986476	1.26E-12	3.592E-11
ENSMUSG00000027376.15	ENSMUSG00000027376.15	ENSMUSG00000027376	protein_coding	Prom2	162.0761927	55.54331212	1.549671229	0.2216622	6.9911385	2.727E-12	7.52E-11
ENSMUSG00000059668.5	ENSMUSG00000059668.5	ENSMUSG00000059668	protein_coding	Krt4	11.27517386	0	5.852942796	1.4850796	3.9411644	8.109E-05	0.0006917
ENSMUSG00000026890.19	ENSMUSG00000026890.19	ENSMUSG00000026890	protein_coding	Lhx6	70.2409406	32.04219871	1.125409272	0.3568599	3.1536444	0.0016125	0.009523
ENSMUSG00000102692.1	ENSMUSG00000102692.1	ENSMUSG00000102692	protein_coding	Dchs2	0	6.939093778	-5.345943573	1.6534989	-3.23311	0.0012245	0.0074789
ENSMUSG00000004151.17	ENSMUSG00000004151.17	ENSMUSG00000004151	protein_coding	Etv1	34.353978	8.360284434	2.02753558	0.5747978	3.5273893	0.0004197	0.0029275
ENSMUSG00000027377.2	ENSMUSG00000027377.2	ENSMUSG00000027377	protein_coding	Mall	210.7130315	46.35202909	2.180050751	0.2569051	8.4858202	2.142E-17	9.313E-16
ENSMUSG00000029108.14	ENSMUSG00000029108.14	ENSMUSG00000029108	protein_coding	Pcdh7	1233.3271	419.2512316	1.556341002	0.3494597	4.4535638	8.446E-06	9.031E-05
ENSMUSG00000042817.15	ENSMUSG00000042817.15	ENSMUSG00000042817	protein_coding	Flt3	1.164718845	16.07630408	-3.742217777	1.3065867	-2.864117	0.0041817	0.0214163
ENSMUSG00000042793.13	ENSMUSG00000042793.13	ENSMUSG00000042793	protein_coding	Lgr6	1573.767485	604.1190937	1.380821084	0.091966	15.014475	5.903E-51	1.239E-48
ENSMUSG00000043587.15	ENSMUSG00000043587.15	ENSMUSG00000043587	protein_coding	Pxylp1	66.95481992	27.71658604	1.27689019	0.3067153	4.1631118	3.139E-05	0.0002946
ENSMUSG00000097745.1	ENSMUSG00000097745.1	ENSMUSG00000097745	antisense_RNA	AI115009	4.938546964	0	4.659618434	1.7911397	2.6014824	0.0092822	0.0414791
ENSMUSG00000027913.5	ENSMUSG00000027913.5	ENSMUSG00000027913	protein_coding	Crct1	62.3642002	30.35052075	1.045353888	0.3289931	3.1774342	0.0014858	0.0088537
ENSMUSG00000022144.3	ENSMUSG00000022144.3	ENSMUSG00000022144	protein_coding	Gdnf	198.6089112	38.13635253	2.378554023	0.2282577	10.420477	1.999E-25	1.405E-23
ENSMUSG00000054146.8	ENSMUSG00000054146.8	ENSMUSG00000054146	protein_coding	Krt15	0	5.604673391	-5.035965899	1.7126947	-2.940376	0.0032781	0.0174363
ENSMUSG00000022490.6	ENSMUSG00000022490.6	ENSMUSG00000022490	protein_coding	Ppp1r1a	12.35900223	37.58038662	-1.602277222	0.4119767	-3.889243	0.0001006	0.0008355
ENSMUSG00000003418.11	ENSMUSG00000003418.11	ENSMUSG00000003418	protein_coding	St8sia6	1332.505383	21.48226914	5.931429261	0.2252952	26.327372	9.32E-153	1.57E-149
ENSMUSG00000051431.7	ENSMUSG00000051431.7	ENSMUSG00000051431	protein_coding	Gpr87	961.2944225	343.3261869	1.48445403	0.0978589	15.169334	5.645E-52	1.227E-49
ENSMUSG00000026817.14	ENSMUSG00000026817.14	ENSMUSG00000026817	protein_coding	Ak1	38.50983339	12.78294447	1.592998316	0.4214185	3.7800859	0.0001568	0.0012406
ENSMUSG00000109244.1	ENSMUSG00000109244.1	ENSMUSG00000109244	lincRNA	Gm44751	43.34377128	15.65314032	1.47384522	0.3830691	3.8474656	0.0001193	0.0009741
ENSMUSG00000031239.5	ENSMUSG00000031239.5	ENSMUSG00000031239	protein_coding	Itm2a	155.0640891	44.4827763	1.802979271	0.3132052	5.7565434	8.585E-09	1.533E-07
ENSMUSG00000019987.9	ENSMUSG00000019987.9	ENSMUSG00000019987	protein_coding	Arg1	91.91340922	8.216356468	3.494429965	0.4477551	7.8043339	5.982E-15	2.109E-13
ENSMUSG00000051439.6	ENSMUSG00000051439.6	ENSMUSG00000051439	protein_coding	Cd14	640.6961218	195.2263684	1.716398736	0.1283698	13.370738	8.965E-41	1.253E-38
ENSMUSG00000026430.16	ENSMUSG00000026430.16	ENSMUSG00000026430	protein_coding	Rassf5	142.8653331	364.926637	-1.351167507	0.1374282	-9.831804	8.213E-23	4.928E-21
ENSMUSG00000105703.2	ENSMUSG00000105703.2	ENSMUSG00000105703	processed_transcript	Gm43305	3.553637397	16.54882636	-2.249446552	0.8135152	-2.765095	0.0056906	0.0278473
ENSMUSG00000025608.9	ENSMUSG00000025608.9	ENSMUSG00000025608	protein_coding	Podxl	1377.585133	3659.422302	-1.409912763	0.06763	-20.84744	1.608E-96	1.1E-93
ENSMUSG00000050534.8	ENSMUSG00000050534.8	ENSMUSG00000050534	protein_coding	Htr5b	48.90097549	4.07069596	3.626906803	0.6473767	5.6024676	2.113E-08	3.569E-07
ENSMUSG00000040987.14	ENSMUSG00000040987.14	ENSMUSG00000040987	protein_coding	Mill2	11.4618001	0	5.8734973	1.4526237	4.0433716	5.269E-05	0.0004685
ENSMUSG00000024256.6	ENSMUSG00000024256.6	ENSMUSG00000024256	protein_coding	Adcyap1	18.07916967	3.567442974	2.347012658	0.6964255	3.3700842	0.0007515	0.0048823
ENSMUSG00000104252.5	ENSMUSG00000104252.5	ENSMUSG00000104252	protein_coding	Pcdha4	15.55154202	0	6.312785424	1.3917023	4.5360169	5.733E-06	6.37E-05
ENSMUSG00000040537.17	ENSMUSG00000040537.17	ENSMUSG00000040537	protein_coding	Adam22	27.9799266	62.05411745	-1.152487434	0.3402023	-3.387653	0.0007049	0.0046093
ENSMUSG00000040713.12	ENSMUSG00000040713.12	ENSMUSG00000040713	protein_coding	Creg1	569.1112782	206.8357057	1.459834678	0.1954753	7.4681282	8.134E-14	2.574E-12
ENSMUSG00000028943.18	ENSMUSG00000028943.18	ENSMUSG00000028943	protein_coding	Espn	5.812086098	38.7635181	-2.728018534	0.5094232	-5.355112	8.55E-08	1.305E-06
ENSMUSG00000094786.1	ENSMUSG00000094786.1	ENSMUSG00000094786	protein_coding	Gm14403	44.26190308	7.404524537	2.579222537	0.478465	5.390619	7.022E-08	1.083E-06
ENSMUSG00000001120.15	ENSMUSG00000001120.15	ENSMUSG00000001120	protein_coding	Pcbp3	58.92775109	135.5894288	-1.199070095	0.2316809	-5.175525	2.273E-07	3.23E-06
ENSMUSG00000051043.16	ENSMUSG00000051043.16	ENSMUSG00000051043	protein_coding	Gprc5c	179.8939427	36.28755334	2.307595593	0.2332841	9.8917814	4.519E-23	2.783E-21
ENSMUSG00000108912.1	ENSMUSG00000108912.1	ENSMUSG00000108912	TEC	E230020D15Rik	26.31540104	75.58641494	-1.525274297	0.3659512	-4.167972	3.073E-05	0.0002887
ENSMUSG00000046093.9	ENSMUSG00000046093.9	ENSMUSG00000046093	protein_coding	Hpcal4	67.84934432	220.9608514	-1.702256323	0.6366758	-2.673663	0.0075028	0.0349563
ENSMUSG00000029153.11	ENSMUSG00000029153.11	ENSMUSG00000029153	protein_coding	Ociad2	301.4766493	100.7481333	1.576553841	0.2600214	6.0631702	1.335E-09	2.638E-08
ENSMUSG00000029185.14	ENSMUSG00000029185.14	ENSMUSG00000029185	protein_coding	Fam114a1	134.2961183	289.781092	-1.112393828	0.1870072	-5.9484	2.708E-09	5.158E-08
ENSMUSG00000070546.2	ENSMUSG00000070546.2	ENSMUSG00000070546	protein_coding	Mrgprb3	16.12013993	53.71761327	-1.727900187	0.4055063	-4.261093	2.034E-05	0.0002007
ENSMUSG00000067158.9	ENSMUSG00000067158.9	ENSMUSG00000067158	protein_coding	Col4a4	358.6489325	877.3958673	-1.291395272	0.0992141	-13.01624	9.892E-39	1.261E-36
ENSMUSG00000017002.14	ENSMUSG00000017002.14	ENSMUSG00000017002	protein_coding	Slpi	39.30135536	7.783185028	2.33680272	0.4724545	4.9460901	7.572E-07	9.93E-06
ENSMUSG00000110218.1	ENSMUSG00000110218.1	ENSMUSG00000110218	protein_coding	Gm20219	107.8024022	342.6748029	-1.673045414	0.2087191	-8.015777	1.094E-15	4.127E-14
ENSMUSG00000071037.5	ENSMUSG00000071037.5	ENSMUSG00000071037	protein_coding	Camkmt	20.85065104	44.7791113	-1.107127544	0.3692519	-2.998299	0.0027149	0.0149127
ENSMUSG00000112206.1	ENSMUSG00000112206.1	ENSMUSG00000112206	lincRNA	AC153544.1	0	5.048707488	-4.879178642	1.7948975	-2.718361	0.0065606	0.0312603
ENSMUSG00000026065.8	ENSMUSG00000026065.8	ENSMUSG00000026065	protein_coding	Slc9a4	2.981179462	136.0609336	-5.465749879	0.5457239	-10.0156	1.302E-23	8.266E-22
ENSMUSG00000040690.15	ENSMUSG00000040690.15	ENSMUSG00000040690	protein_coding	Col16a1	69.39486889	220.7690841	-1.671867502	0.2841042	-5.884698	3.988E-09	7.443E-08
ENSMUSG00000055435.6	ENSMUSG00000055435.6	ENSMUSG00000055435	protein_coding	Maf	37.96323624	5.928822931	2.66453197	0.5228996	5.0956859	3.475E-07	4.839E-06
ENSMUSG00000110276.1	ENSMUSG00000110276.1	ENSMUSG00000110276	lincRNA	Gm45330	14.15287094	0.97244862	3.775609924	1.1585228	3.2589863	0.0011181	0.0069237
ENSMUSG00000021747.12	ENSMUSG00000021747.12	ENSMUSG00000021747	protein_coding	4930452B06Rik	46.57100225	256.2577017	-2.463379721	0.1963469	-12.54606	4.178E-36	4.736E-34
ENSMUSG00000018217.12	ENSMUSG00000018217.12	ENSMUSG00000018217	protein_coding	Pmp22	210.0684023	518.1814375	-1.303585648	0.1253805	-10.39703	2.558E-25	1.79E-23
ENSMUSG00000030162.14	ENSMUSG00000030162.14	ENSMUSG00000030162	protein_coding	Olr1	0	45.36177362	-8.051975083	1.2525675	-6.428376	1.29E-10	2.892E-09
ENSMUSG00000024909.14	ENSMUSG00000024909.14	ENSMUSG00000024909	protein_coding	Efemp2	331.9432093	128.8405643	1.363833174	0.1623744	8.3993093	4.491E-17	1.917E-15
ENSMUSG00000053626.5	ENSMUSG00000053626.5	ENSMUSG00000053626	protein_coding	Tll1	6.977396087	109.4871788	-3.986008425	0.6173758	-6.456373	1.072E-10	2.428E-09
ENSMUSG00000067860.11	ENSMUSG00000067860.11	ENSMUSG00000067860	protein_coding	Zic3	11.97370608	128.9048135	-3.422836306	0.3687824	-9.281451	1.672E-20	8.727E-19

ENSMUSG00000031927.9	ENSMUSG00000031927.9	ENSMUSG00000031927	protein_coding	1700012B09Rik	16.27427278	0	6.377168474	1.3949494	4.5716129	4.84E-06	5.433E-05
ENSMUSG00000058571.10	ENSMUSG00000058571.10	ENSMUSG00000058571	protein_coding	Gpc6	67.92253109	11.19265806	2.59102286	0.3893013	6.6555723	2.822E-11	6.907E-10
ENSMUSG00000005267.13	ENSMUSG00000005267.13	ENSMUSG00000005267	protein_coding	Zfp287	28.50280516	62.77205986	-1.132892999	0.3338323	-3.393599	0.0006898	0.0045179
ENSMUSG00000061411.12	ENSMUSG00000061411.12	ENSMUSG00000061411	protein_coding	Nol4l	1433.930419	428.6541144	1.741010995	0.1256367	13.857509	1.146E-43	1.764E-41
ENSMUSG00000029673.17	ENSMUSG00000029673.17	ENSMUSG00000029673	protein_coding	Auts2	12.87037266	109.362828	-3.077800859	0.3603942	-8.540096	1.341E-17	5.914E-16
ENSMUSG00000041849.7	ENSMUSG00000041849.7	ENSMUSG00000041849	protein_coding	Card6	451.6935124	192.8275767	1.22745178	0.1606462	7.640714	2.16E-14	7.271E-13
ENSMUSG00000087813.1	ENSMUSG00000087813.1	ENSMUSG00000087813	snoRNA	Gm22490	5.894103265	0	4.913673787	1.6682993	2.9453191	0.0032262	0.0172096
ENSMUSG00000026389.16	ENSMUSG00000026389.16	ENSMUSG00000026389	protein_coding	Steap3	27.19996836	10.9608417	1.307797576	0.4995166	2.6181263	0.0088414	0.0398572
ENSMUSG00000027962.14	ENSMUSG00000027962.14	ENSMUSG00000027962	protein_coding	Vcam1	1083.951059	390.2239048	1.473122342	0.1153947	12.765942	2.541E-37	3.068E-35
ENSMUSG00000048218.5	ENSMUSG00000048218.5	ENSMUSG00000048218	protein_coding	Amigo2	11.19428338	36.78636154	-1.719829365	0.4382588	-3.924233	8.701E-05	0.0007371
ENSMUSG00000027068.6	ENSMUSG00000027068.6	ENSMUSG00000027068	protein_coding	Dhrs9	392.3890568	156.7162384	1.325061312	0.1472138	9.0009336	2.238E-19	1.103E-17
ENSMUSG00000084989.3	ENSMUSG00000084989.3	ENSMUSG00000084989	protein_coding	Crocc2	46.99863768	11.75710303	2.004121534	0.6152816	3.257243	0.001125	0.0069601
ENSMUSG00000027221.5	ENSMUSG00000027221.5	ENSMUSG00000027221	protein_coding	Chst1	127.5250958	20.14408388	2.663322187	0.2901526	9.1790382	4.35E-20	2.214E-18
ENSMUSG00000001119.7	ENSMUSG00000001119.7	ENSMUSG00000001119	protein_coding	Col6a1	167.4209655	37.92498976	2.137447766	0.2318257	9.2200625	2.969E-20	1.537E-18
ENSMUSG00000097216.3	ENSMUSG00000097216.3	ENSMUSG00000097216	processed_transcript	4932441J04Rik	25.7676772	6.388690783	2.013951883	0.5654798	3.561492	0.0003688	0.0026237
ENSMUSG00000098172.7	ENSMUSG00000098172.7	ENSMUSG00000098172	antisense_RNA	Gm26973	6.999396845	0	5.164938034	1.7421751	2.9646492	0.0030303	0.0162959
ENSMUSG00000053930.13	ENSMUSG00000053930.13	ENSMUSG00000053930	protein_coding	Shisa6	133.7720018	0	9.4188393	1.2068498	7.8044837	5.975E-15	2.109E-13
ENSMUSG00000061897.5	ENSMUSG00000061897.5	ENSMUSG00000061897	processed_pseudogene	Gm14292	11.46233565	0	5.87346609	1.4464795	4.060525	4.896E-05	0.0004403
ENSMUSG00000033213.16	ENSMUSG00000033213.16	ENSMUSG00000033213	protein_coding	AA467197	359.826381	124.500668	1.529328458	0.1890173	8.0909457	5.92E-16	2.284E-14
ENSMUSG00000027919.5	ENSMUSG00000027919.5	ENSMUSG00000027919	protein_coding	Lce1g	69.83696817	17.09658261	2.03242051	0.3846551	5.2837474	1.266E-07	1.874E-06
ENSMUSG00000053898.12	ENSMUSG00000053898.12	ENSMUSG00000053898	protein_coding	Ech1	321.6561637	147.1173622	1.128253948	0.1872134	6.0265665	1.675E-09	3.271E-08
ENSMUSG00000110205.1	ENSMUSG00000110205.1	ENSMUSG00000110205	lincRNA	Gm33594	0	4.621098955	-4.754462747	1.8278335	-2.601147	0.0092913	0.0415052
ENSMUSG00000020886.16	ENSMUSG00000020886.16	ENSMUSG00000020886	protein_coding	Dlg4	58.29202422	28.35932222	1.038477421	0.3371623	3.0800517	0.0020696	0.0117961
ENSMUSG00000019982.15	ENSMUSG00000019982.15	ENSMUSG00000019982	protein_coding	Myb	37.01918806	139.9967599	-1.91664181	0.2620385	-7.314352	2.586E-13	7.821E-12
ENSMUSG00000022099.16	ENSMUSG00000022099.16	ENSMUSG00000022099	protein_coding	Dmtn	8.586300801	25.56409091	-1.592459233	0.5828442	-2.732221	0.0062909	0.0302323
ENSMUSG00000027238.17	ENSMUSG00000027238.17	ENSMUSG00000027238	protein_coding	Frmd5	77.31576126	165.3382212	-1.092275022	0.2947028	-3.706361	0.0002103	0.0016068
ENSMUSG00000087179.8	ENSMUSG00000087179.8	ENSMUSG00000087179	lincRNA	Gm14230	123.915302	361.330289	-1.541996903	0.1606849	-9.596404	8.278E-22	4.635E-20
ENSMUSG00000026207.16	ENSMUSG00000026207.16	ENSMUSG00000026207	protein_coding	Speg	51.39498306	19.79391783	1.38476494	0.442694	3.128041	0.0017598	0.0102528
ENSMUSG00000024030.6	ENSMUSG00000024030.6	ENSMUSG00000024030	protein_coding	Abcg1	62.03940041	21.57083744	1.525198516	0.3240495	4.706684	2.518E-06	2.982E-05
ENSMUSG00000022456.15	ENSMUSG00000022456.15	ENSMUSG00000022456	protein_coding	Sept3	7.292774342	70.66846988	-3.298084975	0.4821499	-6.840372	7.899E-12	2.059E-10
ENSMUSG00000037709.13	ENSMUSG00000037709.13	ENSMUSG00000037709	protein_coding	Fam13a	6.82599657	0	5.125396246	1.6083614	3.1867193	0.001439	0.0086272
ENSMUSG00000030123.15	ENSMUSG00000030123.15	ENSMUSG00000030123	protein_coding	Plxnd1	231.7061797	80.25038793	1.527783301	0.1849599	8.2600768	1.456E-16	5.938E-15
ENSMUSG00000021214.14	ENSMUSG00000021214.14	ENSMUSG00000021214	protein_coding	Akr1c18	0	5.604673391	-5.035965899	1.7126947	-2.940376	0.0032781	0.0174363
ENSMUSG00000056671.6	ENSMUSG00000056671.6	ENSMUSG00000056671	protein_coding	Prelid2	152.0675578	58.28599931	1.384073695	0.2077553	6.6620386	2.701E-11	6.636E-10
ENSMUSG00000044522.8	ENSMUSG00000044522.8	ENSMUSG00000044522	lincRNA	A730020M07Rik	40.90913338	19.49022189	1.073297152	0.3841939	2.7936341	0.0052119	0.0259537
ENSMUSG00000039577.17	ENSMUSG00000039577.17	ENSMUSG00000039577	protein_coding	Nphp4	6.371318071	37.79816101	-2.557949004	0.6127966	-4.174222	2.99E-05	0.0002816
ENSMUSG00000041372.10	ENSMUSG00000041372.10	ENSMUSG00000041372	protein_coding	B4galnt3	1206.652118	567.4345944	1.089711103	0.1209491	9.009665	2.067E-19	1.021E-17
ENSMUSG00000040033.16	ENSMUSG00000040033.16	ENSMUSG00000040033	protein_coding	Stat2	141.1468489	348.6088107	-1.303531075	0.2584951	-5.042769	4.588E-07	6.271E-06
ENSMUSG00000069255.13	ENSMUSG00000069255.13	ENSMUSG00000069255	protein_coding	Dusp22	82.89219364	192.8234418	-1.218994417	0.3309484	-3.683337	0.0002302	0.0017433
ENSMUSG00000099759.1	ENSMUSG00000099759.1	ENSMUSG00000099759	processed_transcript	1700030C10Rik	78.20023373	9.170318331	3.067268853	0.5451783	5.6261759	1.842E-08	3.141E-07
ENSMUSG00000001383.8	ENSMUSG00000001383.8	ENSMUSG00000001383	protein_coding	Zmat2	2769.380671	1099.056817	1.333104525	0.0960441	13.880124	8.36E-44	1.298E-41
ENSMUSG00000019122.8	ENSMUSG00000019122.8	ENSMUSG00000019122	protein_coding	Ccl9	255.6585786	119.9280788	1.090593316	0.1582351	6.8922329	5.492E-12	1.46E-10
ENSMUSG00000074682.4	ENSMUSG00000074682.4	ENSMUSG00000074682	protein_coding	Zcchc3	61.91166389	189.9707429	-1.614696097	0.2105868	-7.667604	1.752E-14	5.931E-13
ENSMUSG00000024331.11	ENSMUSG00000024331.11	ENSMUSG00000024331	protein_coding	Dsc2	99.92998542	379.5344998	-1.924012278	0.1538283	-12.50753	6.79E-36	7.604E-34
ENSMUSG00000034981.9	ENSMUSG00000034981.9	ENSMUSG00000034981	protein_coding	Parm1	54.39928997	109.5561423	-1.009651729	0.2382716	-4.237399	2.261E-05	0.0002212
ENSMUSG00000022602.14	ENSMUSG00000022602.14	ENSMUSG00000022602	protein_coding	Arc	50.61395372	138.5488729	-1.454352351	0.2268015	-6.412445	1.432E-10	3.18E-09
ENSMUSG00000034336.3	ENSMUSG00000034336.3	ENSMUSG00000034336	protein_coding	Ina	32.62949163	87.12452485	-1.425096976	0.3103232	-4.592299	4.384E-06	4.961E-05
ENSMUSG00000020871.8	ENSMUSG00000020871.8	ENSMUSG00000020871	protein_coding	Dlx4	13.77913852	2.442587319	2.488161662	0.9185746	2.7087203	0.0067543	0.0319894
ENSMUSG00000016024.9	ENSMUSG00000016024.9	ENSMUSG00000016024	protein_coding	Lbp	4.903855789	0	4.65082653	1.7631373	2.637813	0.0083443	0.0380047
ENSMUSG00000030207.15	ENSMUSG00000030207.15	ENSMUSG00000030207	protein_coding	Fam234b	66.67579508	145.9952539	-1.130302787	0.2109711	-5.35762	8.433E-08	1.289E-06
ENSMUSG00000030351.5	ENSMUSG00000030351.5	ENSMUSG00000030351	protein_coding	Tspan11	17.75163655	0	6.507008846	1.3716163	4.7440446	2.095E-06	2.527E-05
ENSMUSG00000040649.15	ENSMUSG00000040649.15	ENSMUSG00000040649	protein_coding	Rimklb	18.09126894	67.8801613	-1.913766405	0.3420871	-5.594383	2.214E-08	3.716E-07
ENSMUSG00000026012.2	ENSMUSG00000026012.2	ENSMUSG00000026012	protein_coding	Cd28	22.15627677	7.161582359	1.635388207	0.5920485	2.7622536	0.0057404	0.0280388
ENSMUSG00000031618.13	ENSMUSG00000031618.13	ENSMUSG00000031618	protein_coding	Nr3c2	421.0698639	143.7261065	1.548448827	0.1623755	9.5362225	1.481E-21	8.122E-20
ENSMUSG00000024357.9	ENSMUSG00000024357.9	ENSMUSG00000024357	protein_coding	Sil1	570.9047945	282.7388815	1.013391594	0.1066579	9.5013256	2.072E-21	1.12E-19
ENSMUSG00000025089.15	ENSMUSG00000025089.15	ENSMUSG00000025089	protein_coding	Gfra1	51.62127084	7.491294807	2.794506387	0.5156124	5.4197815	5.967E-08	9.368E-07
ENSMUSG00000072621.13	ENSMUSG00000072621.13	ENSMUSG00000072621	transcribed_unprocessed_pseudogene	Slfn10-ps	16.8192077	3.047501264	2.436542706	0.8774751	2.7767657	0.0054903	0.0270604
ENSMUSG00000021314.12	ENSMUSG00000021314.12	ENSMUSG00000021314	protein_coding	Amph	15.24826304	0.735069349	4.418467594	1.2385114	3.5675632	0.0003603	0.0025754
ENSMUSG00000038305.14	ENSMUSG00000038305.14	ENSMUSG00000038305	protein_coding	Spats2l	4.741483692	0	4.596023006	1.8122803	2.5360442	0.0112113	0.048527
ENSMUSG00000024014.7	ENSMUSG00000024014.7	ENSMUSG00000024014	protein_coding	Pim1	1292.360239	2997.294802	-1.21356973	0.0935075	-12.97832	1.624E-38	2.014E-36
ENSMUSG00000027583.13	ENSMUSG00000027583.13	ENSMUSG00000027583	protein_coding	Zbtb46	34.75030233	81.01798601	-1.224934857	0.3543693	-3.456662	0.0005469	0.003695
ENSMUSG00000021057.15	ENSMUSG00000021057.15	ENSMUSG00000021057	protein_coding	Akap5	28.87879096	636.8690584	-4.478312572	0.2019861	-22.17139	6.49E-109	5.71E-106
ENSMUSG00000020598.16	ENSMUSG00000020598.16	ENSMUSG00000020598	protein_coding	Nrcam	134.972483	343.0427758	-1.344863801	0.1628125	-8.260199	1.454E-16	5.938E-15
ENSMUSG00000036333.11	ENSMUSG00000036333.11	ENSMUSG00000036333	protein_coding	Kidins220	2638.981391	1153.153551	1.19447256	0.2419728	4.9363924	7.958E-07	1.038E-05
ENSMUSG00000039747.11	ENSMUSG00000039747.11	ENSMUSG00000039747	protein_coding	Orai2	191.9170614	92.25667595	1.058046896	0.1911892	5.534031	3.13E-08	5.163E-07
ENSMUSG00000106290.1	ENSMUSG00000106290.1	ENSMUSG00000106290	TEC	Gm43429	6.476462688	21.96062375	-1.758384805	0.5564037	-3.160268	0.0015762	0.0093322
ENSMUSG00000051065.8	ENSMUSG00000051065.8	ENSMUSG00000051065	protein_coding	Mb21d2	69.02669798	22.48877511	1.614934953	0.3226175	5.0057259	5.565E-07	7.517E-06
ENSMUSG00000114511.1	ENSMUSG00000114511.1	ENSMUSG00000114511	antisense_RNA	AC121094.1	16.53022577	4.643350587	1.83685487	0.7217293	2.5450744	0.0109254	0.047546
ENSMUSG00000029819.6	ENSMUSG00000029819.6	ENSMUSG00000029819	protein_coding	Npy	13.01240624	1.102604024	3.588947262	1.3095065	2.7406868	0.0061311	0.0296416
ENSMUSG00000034686.8	ENSMUSG00000034686.8	ENSMUSG00000034686	protein_coding	Prr7	22.21516649	49.19132543	-1.150380368	0.376572	-3.054875	0.0022515	0.0127035
ENSMUSG00000102976.5	ENSMUSG00000102976.5	ENSMUSG00000102976	protein_coding	Zc3h11a	1045.852801	2713.737136	-1.375645307	0.2364762	-5.817269	5.982E-09	1.094E-07
ENSMUSG00000040616.3	ENSMUSG00000040616.3	ENSMUSG00000040616	protein_coding	Tmem51	116.950652	250.3337617	-1.098964556	0.1560381	-7.042922	1.882E-12	5.278E-11
ENSMUSG00000097789.2	ENSMUSG00000097789.2	ENSMUSG00000097789	protein_coding	Gm2115	110.6018702	24.86128088	2.146176558	0.2842657	7.5498963	4.356E-14	1.44E-12
ENSMUSG00000025815.13	ENSMUSG00000025815.13	ENSMUSG00000025815	protein_coding	Dhtkd1	25.4517634	62.0723348	-1.290955585	0.3273215	-3.943999	8.013E-05	0.0006845
ENSMUSG00000039952.15	ENSMUSG00000039952.15	ENSMUSG00000039952	protein_coding	Dag1	1800.928718	6309.880668	-1.808843488	0.435166	-4.156675	3.229E-05	0.000302
ENSMUSG00000031555.15	ENSMUSG00000031555.15	ENSMUSG00000031555	protein_coding	Adam9	6462.000296	15780.47264	-1.28807335	0.084564	-15.23193	2.171E-52	4.833E-50
ENSMUSG00000086755.1	ENSMUSG00000086755.1	ENSMUSG00000086755	lincRNA	Gm11216	162.863871	12.53820426	3.6835309	0.341948	10.772195	4.658E-27	3.542E-25
ENSMUSG00000032841.15	ENSMUSG00000032841.15	ENSMUSG00000032841	protein_coding	Prr5l	320.0197686	157.7700632	1.016979062	0.1606726	6.3295116	2.459E-10	5.328E-09
ENSMUSG00000032769.4	ENSMUSG00000032769.4	ENSMUSG00000032769	protein_coding	Trpa1	108.9191876	6.658329372	4.024313076	0.4382573	9.1825343	4.211E-20	2.155E-18
ENSMUSG00000039109.16	ENSMUSG00000039109.16	ENSMUSG00000039109	protein_coding	F13a1	6.371318071	0	5.030237102	1.6923157	2.9723987	0.0029548	0.0159881
ENSMUSG00000071648.3	ENSMUSG00000071648.3	ENSMUSG00000071648	protein_coding	Rom1	288.2555213	141.9447421	1.019348467	0.1521018	6.7017532	2.059E-11	5.155E-10
ENSMUSG00000087143.9	ENSMUSG00000087143.9	ENSMUSG00000087143	antisense_RNA	A830082K12Rik	68.99479574	12.95468698	2.403389633	0.3744798	6.4179428	1.381E-10	3.075E-09
ENSMUSG00000088378.1	ENSMUSG00000088378.1	ENSMUSG00000088378	snRNA	Gm25184	11.30986503	2.129563594	2.416688243	0.8892155	2.7177758	0.0065722	0.0312994
ENSMUSG00000036908.16	ENSMUSG00000036908.16	ENSMUSG00000036908	protein_coding	Unc93b1	26.37316407	9.490702997	1.46926186	0.5010544	2.93234	0.0033642	0.0177966
ENSMUSG00000113472.1	ENSMUSG00000113472.1	ENSMUSG00000113472	TEC	CT009757.4	8.036970315	22.10746787	-1.456109946	0.5684628	-2.561487	0.0104225	0.0456585
ENSMUSG00000025892.16	ENSMUSG00000025892.16	ENSMUSG00000025892	protein_coding	Gria4	0.582359423	8.853529732	-3.861573571	1.39156	-2.774996	0.0055202	0.0271935
ENSMUSG00000075028.11	ENSMUSG00000075028.11	ENSMUSG00000075028	protein_coding	Prdm11	36.37740337	11.29055414	1.687724347	0.432357	3.9035438	9.479E-05	0.0007937
ENSMUSG00000031891.6	ENSMUSG00000031891.6	ENSMUSG00000031891	protein_coding	Hsd11b2	13.47859287	51.61098121	-1.94651615	0.4030495	-4.829472	1.369E-06	1.707E-05
ENSMUSG00000037469.9	ENSMUSG00000037469.9	ENSMUSG00000037469	protein_coding	Acp7	20.10532838	96.32428694	-2.257643263	0.3192881	-7.070867	1.54E-12	4.357E-11
ENSMUSG00000007021.4	ENSMUSG00000007021.4	ENSMUSG00000007021	protein_coding	Syngr3	10.52990679	90.10818738	-3.098099767	0.3774301	-8.208407	2.241E-16	9.043E-15
ENSMUSG00000037254.18	ENSMUSG00000037254.18	ENSMUSG00000037254	protein_coding	Itih2	118.5055817	0	9.245329807	1.2201656	7.577111	3.533E-14	1.174E-12
ENSMUSG00000032224.15	ENSMUSG00000032224.15	ENSMUSG00000032224	protein_coding	Fam81a	109.6193428	325.5423926	-1.574016819	0.2094774	-7.514016	5.734E-14	1.856E-12
ENSMUSG00000053024.14	ENSMUSG00000053024.14	ENSMUSG00000053024	protein_coding	Cntn2	950.6640864	102.1332998	3.212422576	0.1964876	16.349234	4.405E-60	1.197E-57
ENSMUSG00000002057.4	ENSMUSG00000002057.4	ENSMUSG00000002057	protein_coding	Foxn1	75.59010895	284.0795447	-1.913278864	0.1852861	-10.32608	5.371E-25	3.662E-23
ENSMUSG00000007035.15	ENSMUSG00000007035.15	ENSMUSG00000007035	protein_coding	Msh5	85.08678225	37.78011247	1.163254246	0.4568775	2.5460965	0.0108935	0.047442
ENSMUSG00000035277.15	ENSMUSG00000035277.15	ENSMUSG00000035277	protein_coding	Arx	0	5.680317845	-5.052534294	1.7595569	-2.871481	0.0040855	0.0210227
ENSMUSG00000031137.17	ENSMUSG00000031137.17	ENSMUSG00000031137	protein_coding	Fgf13	793.9545822	363.1441544	1.128197145	0.1406106	8.0235556	1.027E-15	3.89E-14
ENSMUSG00000031635.14	ENSMUSG00000031635.14	ENSMUSG00000031635	protein_coding	Anxa10	2.365791103	61.30483732	-4.766907773	0.8605587	-5.539317	3.037E-08	5.023E-07
ENSMUSG00000028977.16	ENSMUSG00000028977.16	ENSMUSG00000028977	protein_coding	Casz1	33.197554	139.4452388	-2.07132401	0.2884984	-7.179672	6.988E-13	2.024E-11
ENSMUSG00000113642.1	ENSMUSG00000113642.1	ENSMUSG00000113642	transcribed_unprocessed_pseudogene	CT030170.6	43.15607395	17.05319748	1.344256991	0.3908827	3.4390295	0.0005838	0.0039085
ENSMUSG00000026728.9	ENSMUSG00000026728.9	ENSMUSG00000026728	protein_coding	Vim	4362.845636	1469.453258	1.570003252	0.1084745	14.473481	1.782E-47	3.395E-45
ENSMUSG00000035891.16	ENSMUSG00000035891.16	ENSMUSG00000035891	protein_coding	Cerk	130.9968272	479.4793808	-1.872945695	0.1429082	-13.10593	3.045E-39	4.019E-37
ENSMUSG00000021390.6	ENSMUSG00000021390.6	ENSMUSG00000021390	protein_coding	Ogn	6.628397752	19.16607235	-1.543718663	0.6069544	-2.543385	0.0109784	0.0477279
ENSMUSG00000031373.4	ENSMUSG00000031373.4	ENSMUSG00000031373	protein_coding	Car5b	674.5524353	223.905733	1.588350714	0.1404433	11.30955	1.177E-29	9.976E-28
ENSMUSG00000085705.1	ENSMUSG00000085705.1	ENSMUSG00000085705	processed_transcript	Gm16046	26.65278006	295.8564215	-3.474552267	0.2285828	-15.20041	3.514E-52	7.73E-50
ENSMUSG00000022744.12	ENSMUSG00000022744.12	ENSMUSG00000022744	protein_coding	Cldnd1	440.7540782	1582.436588	-1.843746982	0.1462535	-12.60652	1.944E-36	2.231E-34
ENSMUSG00000090272.8	ENSMUSG00000090272.8	ENSMUSG00000090272	protein_coding	Mndal	15.67868739	1.102604024	3.869705005	1.1369376	3.4036214	0.000665	0.0043759
ENSMUSG00000029092.9	ENSMUSG00000029092.9	ENSMUSG00000029092	lincRNA	D5Ertd615e	2.76098874	45.94999884	-4.050255931	0.6849122	-5.91354	3.348E-09	6.32E-08
ENSMUSG00000109291.1	ENSMUSG00000109291.1	ENSMUSG00000109291	lincRNA	Gm2814	38.45308586	3.150960257	3.598329294	0.6403765	5.6190842	1.92E-08	3.267E-07
ENSMUSG00000053279.7	ENSMUSG00000053279.7	ENSMUSG00000053279	protein_coding	Aldh1a1	131.3171366	643.2182977	-2.296366283	0.1413202	-16.24938	2.256E-59	6.042E-57
ENSMUSG00000026601.14	ENSMUSG00000026601.14	ENSMUSG00000026601	protein_coding	Axdnd1	16.22641124	47.33251856	-1.547633049	0.4004746	-3.864498	0.0001113	0.0009154
ENSMUSG00000031375.17	ENSMUSG00000031375.17	ENSMUSG00000031375	protein_coding	Bgn	438.0238977	121.8765721	1.846342802	0.1457098	12.671369	8.521E-37	9.966E-35

ENSMUSG00000025347.4	ENSMUSG00000025347.4	ENSMUSG00000025347	protein_coding	Mettl7b	141.7434891	16.77799598	3.088281663	0.3350741	9.2167134	3.063E-20	1.581E-18
ENSMUSG00000000693.10	ENSMUSG00000000693.10	ENSMUSG00000000693	protein_coding	Loxl3	54.38499291	15.46291106	1.812185184	0.3771801	4.8045624	1.551E-06	1.914E-05
ENSMUSG00000050052.12	ENSMUSG00000050052.12	ENSMUSG00000050052	protein_coding	Tdrp	89.43750313	362.8497863	-2.020825386	0.1505743	-13.42079	4.568E-41	6.494E-39
ENSMUSG00000059901.12	ENSMUSG00000059901.12	ENSMUSG00000059901	protein_coding	Adamts14	40.29385621	4.681172813	3.117369549	0.6518489	4.7823501	1.733E-06	2.126E-05
ENSMUSG00000074604.9	ENSMUSG00000074604.9	ENSMUSG00000074604	protein_coding	Mgst2	136.176439	7.6363409	4.137545837	0.4024504	10.280884	8.594E-25	5.817E-23
ENSMUSG00000027070.14	ENSMUSG00000027070.14	ENSMUSG00000027070	protein_coding	Lrp2	16.34199289	3017.521397	-7.524571161	0.2541152	-29.61087	1.08E-192	2.22E-189
ENSMUSG00000038253.6	ENSMUSG00000038253.6	ENSMUSG00000038253	protein_coding	Hoxa5	6.779206124	0	5.116956495	1.6112426	3.1757827	0.0014943	0.0088957
ENSMUSG00000005580.11	ENSMUSG00000005580.11	ENSMUSG00000005580	protein_coding	Adcy9	12.03313134	30.75407962	-1.352053723	0.4532215	-2.983207	0.0028524	0.0155159
ENSMUSG00000060738.4	ENSMUSG00000060738.4	ENSMUSG00000060738	protein_coding	Prl7c1	0	9.765904494	-5.838010198	1.5136331	-3.856952	0.0001148	0.0009399
ENSMUSG00000006386.15	ENSMUSG00000006386.15	ENSMUSG00000006386	protein_coding	Tek	0	49.68653757	-8.182865409	1.2453658	-6.570652	5.01E-11	1.182E-09
ENSMUSG00000027230.9	ENSMUSG00000027230.9	ENSMUSG00000027230	protein_coding	Creb3l1	14.36316018	0.97244862	3.793111909	1.1497596	3.2990479	0.0009701	0.0061035
ENSMUSG00000039529.8	ENSMUSG00000039529.8	ENSMUSG00000039529	protein_coding	Atp8b1	1428.09097	468.0200685	1.60760544	0.1213184	13.251128	4.445E-40	6.039E-38
ENSMUSG00000048482.14	ENSMUSG00000048482.14	ENSMUSG00000048482	protein_coding	Bdnf	615.447401	1274.68578	-1.051388402	0.1123702	-9.356469	8.245E-21	4.378E-19
ENSMUSG00000031758.10	ENSMUSG00000031758.10	ENSMUSG00000031758	protein_coding	Cdyl2	112.8003025	33.52749751	1.750823443	0.3245256	5.395024	6.851E-08	1.058E-06
ENSMUSG00000034353.14	ENSMUSG00000034353.14	ENSMUSG00000034353	protein_coding	Ramp1	136.675119	38.86894393	1.807588488	0.3412303	5.2972677	1.175E-07	1.749E-06
ENSMUSG00000044927.6	ENSMUSG00000044927.6	ENSMUSG00000044927	protein_coding	H1fx	171.501397	0	9.776525226	1.1983472	8.1583409	3.397E-16	1.347E-14
ENSMUSG00000020160.18	ENSMUSG00000020160.18	ENSMUSG00000020160	protein_coding	Meis1	8.060097764	72.21981594	-3.165534892	0.4603194	-6.876823	6.12E-12	1.618E-10
ENSMUSG00000097754.1	ENSMUSG00000097754.1	ENSMUSG00000097754	lincRNA	Ptgs2os2	786.7917487	216.4051833	1.863478499	0.1259012	14.801122	1.441E-49	2.893E-47
ENSMUSG00000027797.15	ENSMUSG00000027797.15	ENSMUSG00000027797	protein_coding	Dclk1	262.5097892	102.1316023	1.361937204	0.1679278	8.1102562	5.051E-16	1.961E-14
ENSMUSG00000026249.10	ENSMUSG00000026249.10	ENSMUSG00000026249	protein_coding	Serpine2	497.1669477	1704.017283	-1.776081	0.0944131	-18.8118	6.045E-79	2.376E-76
ENSMUSG00000070280.13	ENSMUSG00000070280.13	ENSMUSG00000070280	protein_coding	Slc22a14	5.998121192	0	4.944517021	1.8055041	2.7385797	0.0061705	0.0298011
ENSMUSG00000031750.15	ENSMUSG00000031750.15	ENSMUSG00000031750	protein_coding	Il34	431.0388764	105.2647558	2.034096122	0.156631	12.986553	1.458E-38	1.821E-36
ENSMUSG00000097165.1	ENSMUSG00000097165.1	ENSMUSG00000097165	lincRNA	2210008F06Rik	84.04219107	37.4391054	1.165333801	0.2872995	4.056164	4.989E-05	0.0004468
ENSMUSG00000047253.4	ENSMUSG00000047253.4	ENSMUSG00000047253	protein_coding	Krtap1-5	21.24638423	6.388690783	1.738634506	0.5666857	3.0680754	0.0021544	0.012219
ENSMUSG00000093908.2	ENSMUSG00000093908.2	ENSMUSG00000093908	lincRNA	Gm5784	21.15280333	0.735069349	4.896021656	1.1927117	4.1049498	4.044E-05	0.0003708
ENSMUSG00000024593.15	ENSMUSG00000024593.15	ENSMUSG00000024593	protein_coding	Megf10	236.4365797	20.99750291	3.484610441	0.2761376	12.61911	1.657E-36	1.913E-34
ENSMUSG00000113348.1	ENSMUSG00000113348.1	ENSMUSG00000113348	unprocessed_pseudogene	AC079644.5	0	15.05378929	-6.456535066	1.4125656	-4.570786	4.859E-06	5.451E-05
ENSMUSG00000057143.15	ENSMUSG00000057143.15	ENSMUSG00000057143	protein_coding	Trim12c	78.07597213	26.09960322	1.580163296	0.3243222	4.8722018	1.104E-06	1.406E-05
ENSMUSG00000030000.10	ENSMUSG00000030000.10	ENSMUSG00000030000	protein_coding	Add2	41.06989883	126.0248794	-1.612004838	0.2520468	-6.395658	1.599E-10	3.521E-09
ENSMUSG00000090125.3	ENSMUSG00000090125.3	ENSMUSG00000090125	protein_coding	Pou3f1	2.795679915	43.91208852	-3.980725098	0.6512783	-6.112173	9.828E-10	1.978E-08
ENSMUSG00000025790.14	ENSMUSG00000025790.14	ENSMUSG00000025790	protein_coding	Slco3a1	658.7923938	204.9368173	1.687098541	0.2657499	6.348444	2.175E-10	4.734E-09
ENSMUSG00000024600.8	ENSMUSG00000024600.8	ENSMUSG00000024600	protein_coding	Slc27a6	32.264534	13.21611591	1.2960638	0.4770101	2.7170572	0.0065865	0.0313271
ENSMUSG00000105366.1	ENSMUSG00000105366.1	ENSMUSG00000105366	TEC	Gm43719	47.63330981	22.55997478	1.074502685	0.377802	2.8440893	0.0044539	0.0226219
ENSMUSG00000012017.3	ENSMUSG00000012017.3	ENSMUSG00000012017	protein_coding	Scarf2	267.2947945	540.9026684	-1.015806043	0.1213867	-8.368347	5.843E-17	2.454E-15
ENSMUSG00000055415.7	ENSMUSG00000055415.7	ENSMUSG00000055415	protein_coding	Atp10b	10.79742351	317.010945	-4.873167675	0.3042813	-16.01534	9.987E-58	2.494E-55
ENSMUSG00000104781.1	ENSMUSG00000104781.1	ENSMUSG00000104781	lincRNA	Gm43303	0	26.65292237	-7.282482651	1.3197232	-5.518189	3.425E-08	5.616E-07
ENSMUSG00000106662.1	ENSMUSG00000106662.1	ENSMUSG00000106662	TEC	Gm43034	31.67227309	9.863800579	1.678960864	0.4648072	3.6121661	0.0003036	0.0022206
ENSMUSG00000050357.9	ENSMUSG00000050357.9	ENSMUSG00000050357	protein_coding	Carmil2	6.82599657	18.84192281	-1.464549992	0.5642633	-2.595508	0.0094451	0.0420385
ENSMUSG00000019102.10	ENSMUSG00000019102.10	ENSMUSG00000019102	protein_coding	Aldh3a1	23.77288519	54.19596788	-1.180250393	0.3696023	-3.193299	0.0014066	0.008455
ENSMUSG00000030559.8	ENSMUSG00000030559.8	ENSMUSG00000030559	protein_coding	Rab38	338.6141524	76.67249905	2.142835664	0.1730322	12.384027	3.189E-35	3.446E-33
ENSMUSG00000043924.16	ENSMUSG00000043924.16	ENSMUSG00000043924	protein_coding	Ncmap	210.3656791	96.45281312	1.123572488	0.1833633	6.127577	8.923E-10	1.81E-08
ENSMUSG00000000409.14	ENSMUSG00000000409.14	ENSMUSG00000000409	protein_coding	Lck	23.7167844	5.195551616	2.21191157	0.8647609	2.5578302	0.0105328	0.0460976
ENSMUSG00000016494.9	ENSMUSG00000016494.9	ENSMUSG00000016494	protein_coding	Cd34	191.6904048	74.24771858	1.367185908	0.1976535	6.9170851	4.61E-12	1.24E-10
ENSMUSG00000042256.4	ENSMUSG00000042256.4	ENSMUSG00000042256	protein_coding	Ptchd4	8.445929462	148.3668104	-4.147829959	0.3595441	-11.53636	8.651E-31	7.76E-29
ENSMUSG00000040612.13	ENSMUSG00000040612.13	ENSMUSG00000040612	protein_coding	Ildr2	0	17.00869421	-6.635113824	1.3960884	-4.752646	2.008E-06	2.431E-05
ENSMUSG00000035799.6	ENSMUSG00000035799.6	ENSMUSG00000035799	protein_coding	Twist1	176.5825131	68.14603501	1.374623679	0.2041746	6.7325906	1.667E-11	4.225E-10
ENSMUSG00000108905.1	ENSMUSG00000108905.1	ENSMUSG00000108905	lincRNA	Gm44618	0	6.068306116	-5.155759311	1.8840586	-2.736517	0.0062093	0.0299606
ENSMUSG00000024529.13	ENSMUSG00000024529.13	ENSMUSG00000024529	protein_coding	Lox	552.168472	47.88933317	3.5173253	0.1866078	18.848755	3.008E-79	1.208E-76
ENSMUSG00000024766.14	ENSMUSG00000024766.14	ENSMUSG00000024766	protein_coding	Lipo3	87.5384898	42.7325531	1.039209278	0.2607572	3.9853518	6.738E-05	0.0005835
ENSMUSG00000053414.7	ENSMUSG00000053414.7	ENSMUSG00000053414	protein_coding	Hunk	189.8552516	37.08449458	2.363008541	0.2343384	10.083744	6.519E-24	4.287E-22
ENSMUSG00000048572.4	ENSMUSG00000048572.4	ENSMUSG00000048572	protein_coding	Tmem252	97.30000207	0	8.958970535	1.2114088	7.3954975	1.409E-13	4.331E-12
ENSMUSG00000024885.8	ENSMUSG00000024885.8	ENSMUSG00000024885	protein_coding	Aldh3b1	92.19350515	42.92474921	1.103249494	0.2903939	3.7991487	0.0001452	0.0011589
ENSMUSG00000041515.9	ENSMUSG00000041515.9	ENSMUSG00000041515	protein_coding	Irf8	14.0824175	3.507369116	2.002434407	0.7916906	2.5293143	0.0114286	0.0492965
ENSMUSG00000022759.13	ENSMUSG00000022759.13	ENSMUSG00000022759	protein_coding	Lrrc74b	5.288616393	0	4.756638392	1.7195353	2.7662348	0.0056708	0.0277869
ENSMUSG00000036585.15	ENSMUSG00000036585.15	ENSMUSG00000036585	protein_coding	Fgf1	255.8472914	57.57206346	2.151116195	0.1850313	11.62569	3.051E-31	2.805E-29
ENSMUSG00000047735.14	ENSMUSG00000047735.14	ENSMUSG00000047735	protein_coding	Samd9l	734.1935839	349.0316726	1.072231548	0.110834	9.6742092	3.881E-22	2.213E-20
ENSMUSG00000022332.7	ENSMUSG00000022332.7	ENSMUSG00000022332	protein_coding	Khdrbs3	8.211441684	129.9117577	-3.967378642	0.3825305	-10.37141	3.346E-25	2.307E-23
ENSMUSG00000097159.1	ENSMUSG00000097159.1	ENSMUSG00000097159	lincRNA	Gm26517	48.4363955	12.82453157	1.903142536	0.4471692	4.2559789	2.081E-05	0.0002049
ENSMUSG00000020689.4	ENSMUSG00000020689.4	ENSMUSG00000020689	protein_coding	Itgb3	531.0375582	118.6239509	2.166089597	0.1405096	15.415959	1.279E-53	2.917E-51
ENSMUSG00000033705.16	ENSMUSG00000033705.16	ENSMUSG00000033705	protein_coding	Stard9	96.11156464	249.1605698	-1.371879396	0.2105504	-6.515682	7.236E-11	1.673E-09
ENSMUSG00000107134.1	ENSMUSG00000107134.1	ENSMUSG00000107134	TEC	Gm42528	13.18521537	29.29951152	-1.146573454	0.4401371	-2.605037	0.0091864	0.0411408
ENSMUSG00000026535.9	ENSMUSG00000026535.9	ENSMUSG00000026535	protein_coding	Ifi202b	4.413950568	147.4172933	-5.00782552	0.49674	-10.08138	6.678E-24	4.356E-22
ENSMUSG00000055421.8	ENSMUSG00000055421.8	ENSMUSG00000055421	protein_coding	Pcdh9	114.0041081	508.5646308	-2.156862133	0.2106057	-10.24123	1.296E-24	8.707E-23
ENSMUSG00000029659.16	ENSMUSG00000029659.16	ENSMUSG00000029659	protein_coding	Medag	125.1681351	48.83311854	1.36085387	0.2548681	5.3394438	9.323E-08	1.417E-06
ENSMUSG00000024810.16	ENSMUSG00000024810.16	ENSMUSG00000024810	protein_coding	Il33	160.4677123	80.05775361	1.00665564	0.2447432	4.1131102	3.904E-05	0.0003587
ENSMUSG00000092060.4	ENSMUSG00000092060.4	ENSMUSG00000092060	protein_coding	Bend4	153.1463995	918.4954565	-2.580388984	0.1516842	-17.01158	6.739E-65	2.111E-62
ENSMUSG00000051279.2	ENSMUSG00000051279.2	ENSMUSG00000051279	protein_coding	Gdf6	14.05929005	0	6.170757281	1.4267842	4.3249409	1.526E-05	0.0001544
ENSMUSG00000033590.8	ENSMUSG00000033590.8	ENSMUSG00000033590	protein_coding	Myo5c	31.43612308	877.5150657	-4.788783251	0.194652	-24.60176	1.21E-133	1.6E-130
ENSMUSG00000114442.1	ENSMUSG00000114442.1	ENSMUSG00000114442	lincRNA	AC159264.1	5.765295651	22.9764575	-1.979858988	0.5925972	-3.340986	0.0008348	0.005358
ENSMUSG00000037664.13	ENSMUSG00000037664.13	ENSMUSG00000037664	protein_coding	Cdkn1c	211.3690413	69.29287289	1.607086365	0.2132581	7.5358766	4.851E-14	1.584E-12
ENSMUSG00000090362.1	ENSMUSG00000090362.1	ENSMUSG00000090362	protein_coding	Vmn2r79	0	6.420950102	-5.228394164	1.6985755	-3.078105	0.0020832	0.0118588
ENSMUSG00000105776.1	ENSMUSG00000105776.1	ENSMUSG00000105776	TEC	Gm43292	8.562637804	31.87072562	-1.906365849	0.6112986	-3.118551	0.0018174	0.0105142
ENSMUSG00000086807.8	ENSMUSG00000086807.8	ENSMUSG00000086807	processed_transcript	Platr21	9.994873364	26.39705635	-1.409529672	0.4832166	-2.916973	0.0035345	0.0185276
ENSMUSG00000092232.1	ENSMUSG00000092232.1	ENSMUSG00000092232	protein_coding	Gm20521	107.5641492	17.65254851	2.607548081	0.3919739	6.6523511	2.884E-11	7.041E-10
ENSMUSG00000057098.14	ENSMUSG00000057098.14	ENSMUSG00000057098	protein_coding	Ebf1	58.28598275	21.85536672	1.405480156	0.50765	2.7686008	0.0056298	0.0276152
ENSMUSG00000090451.1	ENSMUSG00000090451.1	ENSMUSG00000090451	protein_coding	Gm6133	8.795463344	24.11688375	-1.464465295	0.5228788	-2.800774	0.005098	0.0254667
ENSMUSG00000040856.17	ENSMUSG00000040856.17	ENSMUSG00000040856	protein_coding	Dlk1	20.40807182	2.594994354	3.01709695	0.8237676	3.6625582	0.0002497	0.0018703
ENSMUSG00000029759.9	ENSMUSG00000029759.9	ENSMUSG00000029759	protein_coding	Pon3	785.8907422	337.1248093	1.220806199	0.1049153	11.636116	2.7E-31	2.495E-29
ENSMUSG00000026580.16	ENSMUSG00000026580.16	ENSMUSG00000026580	protein_coding	Selp	2.178629318	22.9920281	-3.405577667	0.8072504	-4.218738	2.457E-05	0.0002382
ENSMUSG00000100075.1	ENSMUSG00000100075.1	ENSMUSG00000100075	antisense_RNA	1700018L02Rik	9.318397502	25.38302063	-1.444298442	0.5412677	-2.668362	0.0076222	0.035361
ENSMUSG00000050397.4	ENSMUSG00000050397.4	ENSMUSG00000050397	protein_coding	Foxl2	47.29373301	16.58776672	1.508432709	0.3823583	3.9450761	7.977E-05	0.0006818
ENSMUSG00000020105.9	ENSMUSG00000020105.9	ENSMUSG00000020105	protein_coding	Lrig3	132.4092042	8.525615319	3.982729621	0.6050008	6.5830158	4.61E-11	1.092E-09
ENSMUSG00000056888.11	ENSMUSG00000056888.11	ENSMUSG00000056888	protein_coding	Glipr1	125.4411741	613.411403	-2.288815084	0.1394766	-16.41002	1.621E-60	4.539E-58
ENSMUSG00000090264.1	ENSMUSG00000090264.1	ENSMUSG00000090264	protein_coding	Eif4ebp3	52.06769375	4.166794011	3.636462621	0.9238727	3.936108	8.281E-05	0.0007052
ENSMUSG00000046668.8	ENSMUSG00000046668.8	ENSMUSG00000046668	protein_coding	Cxxc5	35.35519806	121.2587343	-1.779535652	0.2960613	-6.010699	1.847E-09	3.578E-08
ENSMUSG00000037681.13	ENSMUSG00000037681.13	ENSMUSG00000037681	protein_coding	Esyt3	8.152551966	28.28924067	-1.779627485	0.5683351	-3.1313	0.0017403	0.0101477
ENSMUSG00000053897.15	ENSMUSG00000053897.15	ENSMUSG00000053897	protein_coding	Slc39a8	105.2422648	382.2870536	-1.859741941	0.1543435	-12.04937	1.954E-33	1.931E-31
ENSMUSG00000046546.3	ENSMUSG00000046546.3	ENSMUSG00000046546	protein_coding	Fam43a	11.4381371	51.14999523	-2.160720583	0.4363666	-4.951618	7.36E-07	9.666E-06
ENSMUSG00000036264.9	ENSMUSG00000036264.9	ENSMUSG00000036264	protein_coding	Fstl4	27.02377915	7.12932304	1.931542984	0.5362223	3.602131	0.0003156	0.0022961
ENSMUSG00000027834.15	ENSMUSG00000027834.15	ENSMUSG00000027834	protein_coding	Serpini1	25.01025526	81.53277531	-1.703212944	0.3428641	-4.967604	6.779E-07	8.979E-06
ENSMUSG00000032908.9	ENSMUSG00000032908.9	ENSMUSG00000032908	protein_coding	Sgpp2	11.72985236	38.73193869	-1.719660076	0.4204847	-4.089709	4.319E-05	0.0003931
ENSMUSG00000072844.6	ENSMUSG00000072844.6	ENSMUSG00000072844	processed_transcript	G530011O06Rik	4.030316656	17.3662212	-2.108007519	0.6549962	-3.218351	0.0012893	0.0078342
ENSMUSG00000032625.14	ENSMUSG00000032625.14	ENSMUSG00000032625	protein_coding	Thsd7a	40.91962601	926.7153916	-4.497203427	0.2225144	-20.21085	7.859E-91	4.271E-88
ENSMUSG00000037335.13	ENSMUSG00000037335.13	ENSMUSG00000037335	protein_coding	Hand1	7.244321657	0	5.216101634	1.6848354	3.0959117	0.0019621	0.011256
ENSMUSG00000030107.10	ENSMUSG00000030107.10	ENSMUSG00000030107	protein_coding	Usp18	83.44601454	32.5087476	1.354943902	0.3101827	4.3682119	1.253E-05	0.000129
ENSMUSG00000020334.6	ENSMUSG00000020334.6	ENSMUSG00000020334	protein_coding	Slc22a4	118.0141255	58.86130068	1.002083412	0.2874096	3.4866038	0.0004892	0.0033579
ENSMUSG00000054136.5	ENSMUSG00000054136.5	ENSMUSG00000054136	protein_coding	Adm2	116.8543377	56.44720781	1.049729043	0.2315132	4.5342088	5.782E-06	6.417E-05
ENSMUSG00000024008.15	ENSMUSG00000024008.15	ENSMUSG00000024008	protein_coding	Cpne5	9.10757272	25.78657949	-1.490743688	0.5715858	-2.608084	0.0091051	0.0408259
ENSMUSG00000029864.7	ENSMUSG00000029864.7	ENSMUSG00000029864	protein_coding	Gstk1	0	14.14962418	-6.369927681	1.4159877	-4.498576	6.841E-06	7.458E-05
ENSMUSG00000077349.1	ENSMUSG00000077349.1	ENSMUSG00000077349	snRNA	Gm24867	2.166530046	10.97196751	-2.340105153	0.8532877	-2.742457	0.0060981	0.0295055
ENSMUSG00000024827.9	ENSMUSG00000024827.9	ENSMUSG00000024827	protein_coding	Gldc	7.244857204	24.01342476	-1.713964833	0.6009358	-2.85216	0.0043423	0.0221651
ENSMUSG00000034463.4	ENSMUSG00000034463.4	ENSMUSG00000034463	protein_coding	Scara3	182.9752964	862.1858737	-2.238427629	0.1278205	-17.51227	1.155E-68	3.744E-66
ENSMUSG00000024910.4	ENSMUSG00000024910.4	ENSMUSG00000024910	protein_coding	Ctsw	341.5175991	85.37918465	1.99905823	0.1623383	12.314149	7.601E-35	8.072E-33
ENSMUSG00000061186.15	ENSMUSG00000061186.15	ENSMUSG00000061186	protein_coding	Sfmbt2	176.7888311	45.26944044	1.960243175	0.2223347	8.8166318	1.18E-18	5.546E-17
ENSMUSG00000050520.4	ENSMUSG00000050520.4	ENSMUSG00000050520	protein_coding	Cldn8	22.10782409	8.474869242	1.385465902	0.5279893	2.6240415	0.0086893	0.0393051
ENSMUSG00000058248.12	ENSMUSG00000058248.12	ENSMUSG00000058248	protein_coding	Kcnh1	12.35846668	1.102604024	3.522634284	1.2040266	2.9257113	0.0034367	0.0180767
ENSMUSG00000096403.2	ENSMUSG00000096403.2	ENSMUSG00000096403	processed_pseudogene	Gm9825	531.5491903	21.61422257	4.622944112	0.2414645	19.145437	1.056E-81	4.54E-79
ENSMUSG00000085595.1	ENSMUSG00000085595.1	ENSMUSG00000085595	antisense_RNA	Gm16090	2.154966321	11.46785956	-2.41323438	0.8928601	-2.702814	0.0068755	0.0324428
ENSMUSG00000022906.15	ENSMUSG00000022906.15	ENSMUSG00000022906	protein_coding	Parp9	296.5566838	103.7011657	1.514523204	0.1585896	9.5499542	1.298E-21	7.136E-20
ENSMUSG00000005124.9	ENSMUSG00000005124.9	ENSMUSG00000005124	protein_coding	Wisp1	186.678008	68.58297133	1.446696528	0.2500986	5.784504	7.273E-09	1.309E-07
ENSMUSG00000032198.9	ENSMUSG00000032198.9	ENSMUSG00000032198	protein_coding	Dock6	52.80034234	138.9973732	-1.395819373	0.2428156	-5.748474	9.005E-09	1.602E-07
ENSMUSG00000029070.9	ENSMUSG00000029070.9	ENSMUSG00000029070	protein_coding	Mxra8	169.2500609	55.08582159	1.615263571	0.3347366	4.8254764	1.397E-06	1.737E-05

ENSMUSG00000027820.12	ENSMUSG00000027820.12	ENSMUSG00000027820	protein_coding	Mme	17805.92186	274.0145174	6.02443731	0.1326758	45.407222	0	0
ENSMUSG00000035638.14	ENSMUSG00000035638.14	ENSMUSG00000035638	protein_coding	Muc20	1075.263682	47.97722157	4.47895229	0.2126814	21.059443	1.874E-98	1.443E-95
ENSMUSG00000052914.4	ENSMUSG00000052914.4	ENSMUSG00000052914	protein_coding	Cyp2j6	29.91137772	3.415035938	3.10546187	0.7568666	4.1030506	4.077E-05	0.0003735
ENSMUSG00000028176.11	ENSMUSG00000028176.11	ENSMUSG00000028176	protein_coding	Lrrc7	68.11022842	2.962529029	4.575215248	0.6260091	7.3085439	2.701E-13	8.154E-12
ENSMUSG00000100798.1	ENSMUSG00000100798.1	ENSMUSG00000100798	lincRNA	Gm19589	59.17219596	131.6871487	-1.154044749	0.247367	-4.665315	3.081E-06	3.601E-05
ENSMUSG00000039410.16	ENSMUSG00000039410.16	ENSMUSG00000039410	protein_coding	Prdm16	48.33065974	2.837936532	4.09413117	0.7313525	5.5980275	2.168E-08	3.649E-07
ENSMUSG00000028654.13	ENSMUSG00000028654.13	ENSMUSG00000028654	protein_coding	Mycl	84.99715623	191.60723	-1.173808923	0.1956461	-5.999656	1.977E-09	3.817E-08
ENSMUSG00000044080.9	ENSMUSG00000044080.9	ENSMUSG00000044080	protein_coding	S100a1	414.6650696	120.3863175	1.77944036	0.2244125	7.9293292	2.203E-15	8.11E-14
ENSMUSG00000021508.11	ENSMUSG00000021508.11	ENSMUSG00000021508	protein_coding	Cxcl14	3.04006918	28.06118918	-3.191311857	0.6581393	-4.848991	1.241E-06	1.558E-05
ENSMUSG00000042985.7	ENSMUSG00000042985.7	ENSMUSG00000042985	protein_coding	Upk3b	14.98074632	38.6544962	-1.375716433	0.4053084	-3.394246	0.0006882	0.0045093
ENSMUSG00000026640.12	ENSMUSG00000026640.12	ENSMUSG00000026640	protein_coding	Plxna2	656.9081019	320.365159	1.036753475	0.120478	8.6053327	7.61E-18	3.38E-16
ENSMUSG00000113726.1	ENSMUSG00000113726.1	ENSMUSG00000113726	antisense_RNA	AL589871.1	4.403513535	32.80688063	-2.91372171	0.570875	-5.103957	3.326E-07	4.639E-06
ENSMUSG00000028539.14	ENSMUSG00000028539.14	ENSMUSG00000028539	protein_coding	Artn	39.40864216	19.04147985	1.046892147	0.4110194	2.5470626	0.0108634	0.0473736
ENSMUSG00000015962.5	ENSMUSG00000015962.5	ENSMUSG00000015962	protein_coding	1700016C15Rik	189.9697621	32.1040706	2.563669292	0.2466115	10.39558	2.597E-25	1.811E-23
ENSMUSG00000020154.10	ENSMUSG00000020154.10	ENSMUSG00000020154	protein_coding	Ptprb	39.67508778	193.9660639	-2.289541616	0.2280846	-10.03813	1.036E-23	6.625E-22
ENSMUSG00000026204.15	ENSMUSG00000026204.15	ENSMUSG00000026204	protein_coding	Ptprn	429.9399702	133.1172289	1.688176613	0.1674406	10.082241	6.62E-24	4.338E-22
ENSMUSG00000026062.12	ENSMUSG00000026062.12	ENSMUSG00000026062	protein_coding	Slc9a2	320.8898721	764.1500669	-1.251042396	0.1004985	-12.44837	1.427E-35	1.579E-33
ENSMUSG00000022464.13	ENSMUSG00000022464.13	ENSMUSG00000022464	protein_coding	Slc38a4	14.00206257	30.66062831	-1.135484406	0.4475954	-2.536855	0.0111853	0.0484447
ENSMUSG00000041552.7	ENSMUSG00000041552.7	ENSMUSG00000041552	protein_coding	Ptchd1	73.7796898	23.22496259	1.666432726	0.3515253	4.7405768	2.131E-06	2.567E-05
ENSMUSG00000028488.15	ENSMUSG00000028488.15	ENSMUSG00000028488	protein_coding	Sh3gl2	91.89408619	192.2270116	-1.062737742	0.3437711	-3.091411	0.0019921	0.0114138
ENSMUSG00000103979.1	ENSMUSG00000103979.1	ENSMUSG00000103979	processed_pseudogene	Gm20305	7.279012832	0	5.222098232	1.7023714	3.0675435	0.0021583	0.012237
ENSMUSG00000022321.15	ENSMUSG00000022321.15	ENSMUSG00000022321	protein_coding	Cdh10	43.87987581	5.770852988	2.901986761	0.6903377	4.2037207	2.626E-05	0.0002523
ENSMUSG00000053216.15	ENSMUSG00000053216.15	ENSMUSG00000053216	protein_coding	Btn2a2	31.24345538	8.205230652	1.932699559	0.5336892	3.6213953	0.000293	0.0021537
ENSMUSG00000020646.17	ENSMUSG00000020646.17	ENSMUSG00000020646	protein_coding	Mboat2	207.788746	554.7927704	-1.416639115	0.2409298	-5.879884	4.106E-09	7.647E-08
ENSMUSG00000025348.9	ENSMUSG00000025348.9	ENSMUSG00000025348	protein_coding	Itga7	59.67527154	20.81351646	1.518250861	0.3381996	4.489215	7.149E-06	7.761E-05
ENSMUSG00000099512.1	ENSMUSG00000099512.1	ENSMUSG00000099512	lincRNA	Gm28703	6.697188956	27.9428395	-2.048008828	0.5403237	-3.790337	0.0001504	0.0011967
ENSMUSG00000034275.18	ENSMUSG00000034275.18	ENSMUSG00000034275	protein_coding	Igsf9b	193.5288662	446.7163282	-1.206244361	0.1646821	-7.324683	2.395E-13	7.254E-12
ENSMUSG00000022941.8	ENSMUSG00000022941.8	ENSMUSG00000022941	protein_coding	Ripply3	8.68978318	22.07409043	-1.34419932	0.5184863	-2.592545	0.0095269	0.0423472
ENSMUSG00000056124.5	ENSMUSG00000056124.5	ENSMUSG00000056124	protein_coding	B4galt6	55.28112395	549.304681	-3.306258768	0.1661819	-19.89541	4.459E-88	2.289E-85
ENSMUSG00000030562.17	ENSMUSG00000030562.17	ENSMUSG00000030562	protein_coding	Nox4	299.0643973	770.6221736	-1.365535045	0.1073011	-12.72619	4.23E-37	5.042E-35
ENSMUSG00000022265.6	ENSMUSG00000022265.6	ENSMUSG00000022265	protein_coding	Ank	724.3526995	169.5080724	2.096559788	0.1229489	17.052279	3.362E-65	1.071E-62
ENSMUSG00000032473.13	ENSMUSG00000032473.13	ENSMUSG00000032473	protein_coding	Cldn18	6.535352406	382.6561169	-5.879751901	0.3523554	-16.68699	1.63E-62	4.78E-60
ENSMUSG00000060600.15	ENSMUSG00000060600.15	ENSMUSG00000060600	protein_coding	Eno3	2319.641514	941.8879261	1.300148774	0.1425368	9.1214951	7.409E-20	3.72E-18
ENSMUSG00000045725.5	ENSMUSG00000045725.5	ENSMUSG00000045725	protein_coding	Prr15	7.25754762	25.24173941	-1.81029085	0.5559157	-3.256412	0.0011283	0.0069681
ENSMUSG00000036334.12	ENSMUSG00000036334.12	ENSMUSG00000036334	protein_coding	Igsf10	16.19225561	0	6.370786709	1.3818312	4.6103943	4.019E-06	4.584E-05
ENSMUSG00000069874.7	ENSMUSG00000069874.7	ENSMUSG00000069874	protein_coding	Irgm2	50.35301402	18.47815301	1.442207595	0.4274886	3.3736751	0.0007417	0.0048225
ENSMUSG00000062168.12	ENSMUSG00000062168.12	ENSMUSG00000062168	protein_coding	Ppef1	44.18481703	201.4145528	-2.193180614	0.2246707	-9.761758	1.643E-22	9.637E-21
ENSMUSG00000042821.7	ENSMUSG00000042821.7	ENSMUSG00000042821	protein_coding	Snai1	76.10201493	19.14938362	1.991722507	0.3390616	5.8742206	4.248E-09	7.89E-08
ENSMUSG00000048960.13	ENSMUSG00000048960.13	ENSMUSG00000048960	protein_coding	Prex2	201.7722101	690.2083083	-1.773673407	0.1269692	-13.96932	2.399E-44	3.855E-42
ENSMUSG00000038630.8	ENSMUSG00000038630.8	ENSMUSG00000038630	protein_coding	Zkscan16	0	7.491294807	-5.450657955	1.6038866	-3.398406	0.0006778	0.0044491
ENSMUSG00000042439.12	ENSMUSG00000042439.12	ENSMUSG00000042439	protein_coding	Zfp532	131.9688064	43.76952036	1.597537233	0.2897569	5.5133713	3.52E-08	5.761E-07
ENSMUSG00000042474.6	ENSMUSG00000042474.6	ENSMUSG00000042474	protein_coding	Fcmr	12.06835806	2.031667509	2.535966626	0.9862967	2.5712007	0.0101347	0.0445558
ENSMUSG00000025017.9	ENSMUSG00000025017.9	ENSMUSG00000025017	protein_coding	Pik3ap1	2.68999975	72.73795962	-4.71977733	0.631236	-7.477041	7.601E-14	2.426E-12
ENSMUSG00000027784.10	ENSMUSG00000027784.10	ENSMUSG00000027784	protein_coding	Ppm1l	847.0108841	364.6432258	1.217103855	0.1005442	12.105164	9.917E-34	9.905E-32
ENSMUSG00000027227.7	ENSMUSG00000027227.7	ENSMUSG00000027227	protein_coding	Sord	112.4618524	41.19609773	1.447742475	0.2756317	5.2524521	1.501E-07	2.192E-06
ENSMUSG00000026463.17	ENSMUSG00000026463.17	ENSMUSG00000026463	protein_coding	Atp2b4	52.03296332	21.34458398	1.291551948	0.3499517	3.6906577	0.0002237	0.0016967
ENSMUSG00000100980.1	ENSMUSG00000100980.1	ENSMUSG00000100980	antisense_RNA	Gm29100	8.737109173	1.015833755	3.060246442	1.205422	2.5387345	0.0111254	0.0482345
ENSMUSG00000090211.2	ENSMUSG00000090211.2	ENSMUSG00000090211	lincRNA	Gm16050	0	5.544599533	-5.022155838	1.8071993	-2.778972	0.0054531	0.0269204
ENSMUSG00000041577.5	ENSMUSG00000041577.5	ENSMUSG00000041577	protein_coding	Prelp	5.218698497	0	4.739987797	1.7955644	2.6398317	0.0082947	0.037871
ENSMUSG00000063873.10	ENSMUSG00000063873.10	ENSMUSG00000063873	protein_coding	Slc24a3	56.47283027	19.51247352	1.534688641	0.3565801	4.3039104	1.678E-05	0.0001686
ENSMUSG00000063632.6	ENSMUSG00000063632.6	ENSMUSG00000063632	protein_coding	Sox11	1058.271287	211.1298119	2.327453334	0.1133303	20.536911	1.008E-93	6.207E-91
ENSMUSG00000021846.8	ENSMUSG00000021846.8	ENSMUSG00000021846	protein_coding	Peli2	71.54329746	7.491294807	3.265608169	0.4322119	7.5555716	4.17E-14	1.381E-12
ENSMUSG00000000142.15	ENSMUSG00000000142.15	ENSMUSG00000000142	protein_coding	Axin2	14.88716543	2.766736859	2.410052943	0.8663295	2.7819126	0.005404	0.0267085
ENSMUSG00000019772.5	ENSMUSG00000019772.5	ENSMUSG00000019772	protein_coding	Vip	40.44074897	0.97244862	5.272406648	0.9751665	5.4066732	6.421E-08	9.978E-07
ENSMUSG00000041323.6	ENSMUSG00000041323.6	ENSMUSG00000041323	protein_coding	Ak7	0	6.761788365	-5.303253948	1.6257244	-3.262087	0.001106	0.0068531
ENSMUSG00000028583.14	ENSMUSG00000028583.14	ENSMUSG00000028583	protein_coding	Pdpn	4.950646236	38.2286857	-2.948645951	0.5217818	-5.651109	1.594E-08	2.743E-07
ENSMUSG00000030465.19	ENSMUSG00000030465.19	ENSMUSG00000030465	protein_coding	Psd3	537.0175436	1323.334281	-1.300839188	0.4894121	-2.657963	0.0078614	0.0362344
ENSMUSG00000110103.1	ENSMUSG00000110103.1	ENSMUSG00000110103	protein_coding	Zscan4-ps1	35.04865019	15.94503055	1.140025736	0.4257161	2.6779012	0.0074085	0.0346042
ENSMUSG00000047230.6	ENSMUSG00000047230.6	ENSMUSG00000047230	protein_coding	Cldn2	14.19966139	36.9035931	-1.378678977	0.4132161	-3.33646	0.0008485	0.0054385
ENSMUSG00000037375.16	ENSMUSG00000037375.16	ENSMUSG00000037375	protein_coding	Hhat	38.08764828	3.047501264	3.605842464	0.7268337	4.9610283	7.012E-07	9.262E-06
ENSMUSG00000046442.4	ENSMUSG00000046442.4	ENSMUSG00000046442	protein_coding	Ppm1e	15.22513559	32.76529353	-1.105534953	0.4223822	-2.617381	0.0088607	0.0399241
ENSMUSG00000034648.9	ENSMUSG00000034648.9	ENSMUSG00000034648	protein_coding	Lrrn1	24.59026794	57.04655885	-1.212038408	0.37438	-3.237455	0.001206	0.0073881
ENSMUSG00000098030.1	ENSMUSG00000098030.1	ENSMUSG00000098030	processed_pseudogene	Gm6771	2.78411619	11.93885322	-2.102929181	0.7975945	-2.636589	0.0083744	0.0381233
ENSMUSG00000044807.13	ENSMUSG00000044807.13	ENSMUSG00000044807	protein_coding	Zfp354c	24.0326982	58.85309103	-1.298123372	0.3559756	-3.646664	0.0002657	0.0019778
ENSMUSG00000062077.14	ENSMUSG00000062077.14	ENSMUSG00000062077	protein_coding	Trim54	155.4296542	76.88121507	1.017661054	0.2150969	4.7311759	2.232E-06	2.671E-05
ENSMUSG00000049502.16	ENSMUSG00000049502.16	ENSMUSG00000049502	protein_coding	Dtx3l	379.8501722	89.31707846	2.091079293	0.1579473	13.239095	5.217E-40	7.037E-38
ENSMUSG00000071551.13	ENSMUSG00000071551.13	ENSMUSG00000071551	protein_coding	Akr1c19	21.88656227	97.32651694	-2.149345674	0.2904494	-7.400069	1.361E-13	4.199E-12
ENSMUSG00000030607.7	ENSMUSG00000030607.7	ENSMUSG00000030607	protein_coding	Acan	655.5832643	2739.043344	-2.063126152	0.1075188	-19.18851	4.617E-82	2.031E-79
ENSMUSG00000042842.11	ENSMUSG00000042842.11	ENSMUSG00000042842	protein_coding	Serpinb6b	397.8358721	1758.362243	-2.144062092	0.1080014	-19.85217	1.055E-87	5.271E-85
ENSMUSG00000040488.17	ENSMUSG00000040488.17	ENSMUSG00000040488	protein_coding	Ltbp4	170.8562879	505.5990271	-1.564920529	0.1434757	-10.90722	1.065E-27	8.231E-26
ENSMUSG00000005973.6	ENSMUSG00000005973.6	ENSMUSG00000005973	protein_coding	Rcn1	62.47202255	8.491557966	2.879966278	0.4633462	6.2155819	5.113E-10	1.07E-08
ENSMUSG00000071553.10	ENSMUSG00000071553.10	ENSMUSG00000071553	protein_coding	Cpa2	3.249231723	16.92192395	-2.352394169	0.8815541	-2.668463	0.0076199	0.0353593
ENSMUSG00000031906.9	ENSMUSG00000031906.9	ENSMUSG00000031906	protein_coding	Smpd3	38.82295826	122.8647602	-1.657628409	0.2935609	-5.646626	1.636E-08	2.802E-07
ENSMUSG00000041012.4	ENSMUSG00000041012.4	ENSMUSG00000041012	protein_coding	Cmtm8	191.2181048	20.23641705	3.24493371	0.2643306	12.276042	1.218E-34	1.286E-32
ENSMUSG00000008734.9	ENSMUSG00000008734.9	ENSMUSG00000008734	protein_coding	Gprc5b	535.1978811	1529.244229	-1.516237045	0.0933304	-16.24591	2.388E-59	6.303E-57
ENSMUSG00000022415.12	ENSMUSG00000022415.12	ENSMUSG00000022415	protein_coding	Syngr1	109.6767207	301.7570194	-1.45958619	0.2135878	-6.833659	8.278E-12	2.151E-10
ENSMUSG00000025104.13	ENSMUSG00000025104.13	ENSMUSG00000025104	protein_coding	Hdgfl3	19.37269613	1.6207477	3.506821301	1.0608408	3.3057	0.0009474	0.0059788
ENSMUSG00000026904.17	ENSMUSG00000026904.17	ENSMUSG00000026904	protein_coding	Slc4a10	27.25773138	61.6098202	-1.178759324	0.3176982	-3.710311	0.000207	0.0015852
ENSMUSG00000062737.13	ENSMUSG00000062737.13	ENSMUSG00000062737	protein_coding	Prl3d2	6.709288228	45.49928996	-2.747382536	0.4857371	-5.656111	1.548E-08	2.669E-07
ENSMUSG00000062480.11	ENSMUSG00000062480.11	ENSMUSG00000062480	protein_coding	Acat3	11.92964896	40.15604551	-1.755614868	0.5354689	-3.278649	0.0010431	0.0065145
ENSMUSG00000113481.1	ENSMUSG00000113481.1	ENSMUSG00000113481	unprocessed_pseudogene	CT954323.3	7.793116597	0	5.315530427	1.5637942	3.3991239	0.000676	0.0044402
ENSMUSG00000107881.1	ENSMUSG00000107881.1	ENSMUSG00000107881	TEC	Gm44250	1075.931334	371.9402848	1.532089561	0.1171956	13.07293	4.702E-39	6.076E-37
ENSMUSG00000028545.13	ENSMUSG00000028545.13	ENSMUSG00000028545	protein_coding	Bend5	0.291179711	10.22577235	-4.940384198	1.4401956	-3.430356	0.0006028	0.0040153
ENSMUSG00000109446.1	ENSMUSG00000109446.1	ENSMUSG00000109446	protein_coding	Gm9195	72.78890678	332.4558804	-2.186755255	0.1765931	-12.38301	3.23E-35	3.47E-33
ENSMUSG00000021506.7	ENSMUSG00000021506.7	ENSMUSG00000021506	protein_coding	Pitx1	13.82592896	0	6.145783122	1.4110156	4.3555741	1.327E-05	0.0001356
ENSMUSG00000110598.1	ENSMUSG00000110598.1	ENSMUSG00000110598	TEC	Gm45854	40.69066049	84.11108094	-1.052111952	0.2988371	-3.520687	0.0004304	0.0029934
ENSMUSG00000034362.7	ENSMUSG00000034362.7	ENSMUSG00000034362	protein_coding	Csta1	33.68686808	3.134271533	3.411685251	0.6930457	4.922742	8.534E-07	1.106E-05
ENSMUSG00000021464.14	ENSMUSG00000021464.14	ENSMUSG00000021464	protein_coding	Ror2	38.0226615	11.85388099	1.690023509	0.4660312	3.6264171	0.0002874	0.002119
ENSMUSG00000028108.15	ENSMUSG00000028108.15	ENSMUSG00000028108	protein_coding	Ecm1	1199.741214	534.8239164	1.166321298	0.1187647	9.8204397	9.194E-23	5.48E-21
ENSMUSG00000031451.6	ENSMUSG00000031451.6	ENSMUSG00000031451	protein_coding	Gas6	39.29743974	204.0019446	-2.368581267	0.356786	-6.638661	3.165E-11	7.696E-10
ENSMUSG00000028370.7	ENSMUSG00000028370.7	ENSMUSG00000028370	protein_coding	Pappa	9.283706327	44.73820411	-2.265439376	0.4674101	-4.846791	1.255E-06	1.572E-05
ENSMUSG00000082082.1	ENSMUSG00000082082.1	ENSMUSG00000082082	transcribed_unprocessed_pseudogene	Gm13230	131.6341215	317.0914724	-1.271433685	0.1975611	-6.435648	1.229E-10	2.767E-09
ENSMUSG00000034917.8	ENSMUSG00000034917.8	ENSMUSG00000034917	protein_coding	Tjp3	70.78480443	266.0051471	-1.907658675	0.2039847	-9.351967	8.603E-21	4.555E-19
ENSMUSG00000020656.16	ENSMUSG00000020656.16	ENSMUSG00000020656	protein_coding	Grhl1	240.2881013	115.4887301	1.053624856	0.1676056	6.2863327	3.251E-10	6.928E-09
ENSMUSG00000029372.7	ENSMUSG00000029372.7	ENSMUSG00000029372	protein_coding	Ppbp	2019.566735	711.5901526	1.505339395	0.1268344	11.868545	1.724E-32	1.651E-30
ENSMUSG00000047810.8	ENSMUSG00000047810.8	ENSMUSG00000047810	protein_coding	Ccdc88b	28.40981541	4.815093091	2.537457612	0.6056775	4.1894536	2.796E-05	0.0002667
ENSMUSG00000031119.4	ENSMUSG00000031119.4	ENSMUSG00000031119	protein_coding	Gpc4	573.8596725	215.6673943	1.410329931	0.1440019	9.7938268	1.197E-22	7.111E-21
ENSMUSG00000062713.7	ENSMUSG00000062713.7	ENSMUSG00000062713	protein_coding	Sim2	87.09591057	14.33985344	2.614557095	0.3634247	7.194219	6.282E-13	1.825E-11
ENSMUSG00000068748.7	ENSMUSG00000068748.7	ENSMUSG00000068748	protein_coding	Ptprz1	55.35490187	8.908040683	2.640981109	0.4561963	5.7891329	7.075E-09	1.274E-07
ENSMUSG00000064125.14	ENSMUSG00000064125.14	ENSMUSG00000064125	protein_coding	Prr36	47.51386813	11.47898537	2.037559648	0.4264798	4.7776227	1.774E-06	2.169E-05
ENSMUSG00000105954.1	ENSMUSG00000105954.1	ENSMUSG00000105954	TEC	Gm42793	73.76042238	31.79481175	1.207155616	0.325668	3.7067063	0.00021	0.0016052
ENSMUSG00000021211.4	ENSMUSG00000021211.4	ENSMUSG00000021211	protein_coding	Akr1c12	48.64341585	105.1486423	-1.112327529	0.2480986	-4.483409	7.346E-06	7.966E-05
ENSMUSG00000055980.2	ENSMUSG00000055980.2	ENSMUSG00000055980	protein_coding	Irs1	1392.713625	466.0157091	1.579327891	0.0850371	18.572224	5.392E-77	2.033E-74
ENSMUSG00000047759.6	ENSMUSG00000047759.6	ENSMUSG00000047759	protein_coding	Hs3st3a1	10.68071517	1.718643785	2.615613314	1.0298326	2.5398432	0.0110902	0.0481158
ENSMUSG00000026828.11	ENSMUSG00000026828.11	ENSMUSG00000026828	protein_coding	Galnt5	0	10.61735669	-5.951011029	1.6586602	-3.587842	0.0003334	0.0024057
ENSMUSG00000030220.13	ENSMUSG00000030220.13	ENSMUSG00000030220	protein_coding	Arhgdib	8.351277476	35.60879297	-2.089678887	0.5150227	-4.05745	4.961E-05	0.0004448
ENSMUSG00000108700.1	ENSMUSG00000108700.1	ENSMUSG00000108700	processed_pseudogene	Gm7482	10.00643709	0.746195165	3.799834793	1.3427683	2.8298515	0.004657	0.0235306
ENSMUSG00000086494.1	ENSMUSG00000086494.1	ENSMUSG00000086494	lincRNA	2210417A02Rik	24.75323118	10.59330702	1.223544164	0.4821274	2.5378028	0.0111551	0.0483458
ENSMUSG00000089922.2	ENSMUSG00000089922.2	ENSMUSG00000089922	protein_coding	Gm43517	17.64649193	94.39248236	-2.406681998	0.3505718	-6.86502	6.648E-12	1.75E-10
ENSMUSG00000035239.6	ENSMUSG00000035239.6	ENSMUSG00000035239	protein_coding	Neu3	34.45638928	82.3578687	-1.262501242	0.3440249	-3.669796	0.0002427	0.0018256
ENSMUSG00000064339.1	ENSMUSG00000064339.1	ENSMUSG00000064339	Mt_rRNA	mt-Rnr2	90798.54808	44344.65903	1.033908847	0.1594164	6.4855859	8.839E-11	2.021E-09
ENSMUSG00000039943.16	ENSMUSG00000039943.16	ENSMUSG00000039943	protein_coding	Plcb4	353.9844792	55.55640477	2.667819924	0.1965412	13.573846	5.724E-42	8.394E-40

ENSMUSG00000027792.11	ENSMUSG00000027792.11	ENSMUSG00000027792	protein_coding	Bche	0	7.56249448	-5.462713274	1.6553785	-3.299978	0.0009669	0.0060875
ENSMUSG00000060445.11	ENSMUSG00000060445.11	ENSMUSG00000060445	protein_coding	Sycp2	0	4.907426268	-4.844187437	1.7690703	-2.738267	0.0061764	0.0298216
ENSMUSG00000024039.14	ENSMUSG00000024039.14	ENSMUSG00000024039	protein_coding	Cbs	30.38971922	144.6906149	-2.245153967	0.2570577	-8.734048	2.457E-18	1.127E-16
ENSMUSG00000026872.17	ENSMUSG00000026872.17	ENSMUSG00000026872	protein_coding	Zeb2	282.7731268	68.81366956	2.036606459	0.2365431	8.609875	7.314E-18	3.257E-16
ENSMUSG00000026664.7	ENSMUSG00000026664.7	ENSMUSG00000026664	protein_coding	Phyh	152.4556429	62.28926047	1.287791716	0.2076212	6.2026009	5.554E-10	1.153E-08
ENSMUSG00000031621.9	ENSMUSG00000031621.9	ENSMUSG00000031621	protein_coding	Isx	7.21016603	0	5.20889513	1.8029616	2.8890772	0.0038637	0.0200264
ENSMUSG00000029561.17	ENSMUSG00000029561.17	ENSMUSG00000029561	protein_coding	Oasl2	34.39690842	8.755633647	1.969034587	0.4873454	4.0403267	5.338E-05	0.0004737
ENSMUSG00000054752.16	ENSMUSG00000054752.16	ENSMUSG00000054752	protein_coding	Fsd1l	98.33982893	382.3943503	-1.963539143	0.176691	-11.11284	1.086E-28	8.729E-27
ENSMUSG00000034422.14	ENSMUSG00000034422.14	ENSMUSG00000034422	protein_coding	Parp14	157.310566	32.0673665	2.291901842	0.2609418	8.7831931	1.589E-18	7.396E-17
ENSMUSG00000045106.12	ENSMUSG00000045106.12	ENSMUSG00000045106	protein_coding	Ccdc73	42.91168469	91.75633913	-1.094622414	0.286409	-3.821886	0.0001324	0.0010682
ENSMUSG00000086813.1	ENSMUSG00000086813.1	ENSMUSG00000086813	processed_transcript	Gm13657	8.013307318	27.43958651	-1.774434571	0.555401	-3.194871	0.0013989	0.0084173
ENSMUSG00000026442.14	ENSMUSG00000026442.14	ENSMUSG00000026442	protein_coding	Nfasc	196.5203715	66.16443368	1.574610648	0.2232041	7.0545776	1.731E-12	4.877E-11
ENSMUSG00000007989.7	ENSMUSG00000007989.7	ENSMUSG00000007989	protein_coding	Fzd3	100.5293752	287.8269676	-1.519383215	0.1793691	-8.470705	2.439E-17	1.053E-15
ENSMUSG00000071324.11	ENSMUSG00000071324.11	ENSMUSG00000071324	protein_coding	Armc2	9.738920374	29.0547713	-1.590221716	0.5072532	-3.134966	0.0017187	0.0100598
ENSMUSG00000097861.1	ENSMUSG00000097861.1	ENSMUSG00000097861	lincRNA	Gm26858	48.49094524	0	7.955000604	1.2493537	6.3672925	1.924E-10	4.192E-09
ENSMUSG00000027339.15	ENSMUSG00000027339.15	ENSMUSG00000027339	protein_coding	Rassf2	204.1451138	39.28166178	2.37305777	0.2407489	9.8569836	6.394E-23	3.887E-21
ENSMUSG00000033579.16	ENSMUSG00000033579.16	ENSMUSG00000033579	protein_coding	Fa2h	8.305022576	31.18168815	-1.903210634	0.4884576	-3.896368	9.765E-05	0.0008142
ENSMUSG00000028949.13	ENSMUSG00000028949.13	ENSMUSG00000028949	protein_coding	Smarcd3	122.2358883	270.4375732	-1.144446537	0.2409133	-4.75045	2.03E-06	2.454E-05
ENSMUSG00000045648.15	ENSMUSG00000045648.15	ENSMUSG00000045648	protein_coding	Vwc2l	0	4.950811403	-4.854948582	1.7601131	-2.758316	0.00581	0.0283189
ENSMUSG00000005364.11	ENSMUSG00000005364.11	ENSMUSG00000005364	protein_coding	Il5ra	229.6250077	106.0879552	1.114840656	0.2455286	4.5405726	5.61E-06	6.241E-05
ENSMUSG00000021403.3	ENSMUSG00000021403.3	ENSMUSG00000021403	protein_coding	Serpinb9b	636.699386	3598.558859	-2.49854696	0.0772694	-32.33552	2.22E-229	6.83E-226
ENSMUSG00000038286.11	ENSMUSG00000038286.11	ENSMUSG00000038286	protein_coding	Bphl	170.5623748	78.66369758	1.11882558	0.204996	5.4577917	4.821E-08	7.666E-07
ENSMUSG00000111343.1	ENSMUSG00000111343.1	ENSMUSG00000111343	TEC	AC159819.3	14.51396855	4.215742054	1.779177026	0.6878873	2.586437	0.0096974	0.0429738
ENSMUSG00000045518.8	ENSMUSG00000045518.8	ENSMUSG00000045518	protein_coding	Onecut3	3.72757322	162.7807796	-5.453418436	0.4710405	-11.57739	5.366E-31	4.884E-29
ENSMUSG00000058427.10	ENSMUSG00000058427.10	ENSMUSG00000058427	protein_coding	Cxcl2	46.69921872	12.62052975	1.882628571	0.4713804	3.9938627	6.501E-05	0.0005648
ENSMUSG00000102997.1	ENSMUSG00000102997.1	ENSMUSG00000102997	TEC	Gm17244	4.100770099	27.82397872	-2.779745612	0.8093815	-3.434407	0.0005939	0.0039643
ENSMUSG00000032281.11	ENSMUSG00000032281.11	ENSMUSG00000032281	protein_coding	Acsbg1	2171.71999	1047.3538	1.052647834	0.1008844	10.434195	1.731E-25	1.225E-23
ENSMUSG00000033066.15	ENSMUSG00000033066.15	ENSMUSG00000033066	protein_coding	Gas7	259.5831595	75.50161151	1.784017341	0.1722856	10.354997	3.972E-25	2.718E-23
ENSMUSG00000001506.10	ENSMUSG00000001506.10	ENSMUSG00000001506	protein_coding	Col1a1	431.8896732	35.39939705	3.59632379	0.2566949	14.010112	1.352E-44	2.25E-42
ENSMUSG00000032854.12	ENSMUSG00000032854.12	ENSMUSG00000032854	protein_coding	Ugt8a	3.716009495	18.79921758	-2.334779491	0.7982876	-2.924735	0.0034475	0.0181283
ENSMUSG00000034793.15	ENSMUSG00000034793.15	ENSMUSG00000034793	protein_coding	G6pc3	290.363106	104.3256846	1.47683223	0.1571254	9.3990667	5.505E-21	2.948E-19
ENSMUSG00000043811.5	ENSMUSG00000043811.5	ENSMUSG00000043811	protein_coding	Rtn4r	83.94079529	40.81811715	1.042085369	0.27428	3.7993487	0.0001451	0.0011585
ENSMUSG00000043782.8	ENSMUSG00000043782.8	ENSMUSG00000043782	protein_coding	Bicdl2	37.97147548	80.49401001	-1.078663728	0.2870034	-3.758366	0.000171	0.0013385
ENSMUSG00000037892.13	ENSMUSG00000037892.13	ENSMUSG00000037892	protein_coding	Pcdh18	284.1953538	51.28974784	2.471918041	0.3151265	7.8442087	4.357E-15	1.563E-13
ENSMUSG00000044005.13	ENSMUSG00000044005.13	ENSMUSG00000044005	protein_coding	Gls2	86.52243714	207.3304519	-1.261328928	0.1948182	-6.474389	9.52E-11	2.169E-09
ENSMUSG00000044162.12	ENSMUSG00000044162.12	ENSMUSG00000044162	protein_coding	Tnip3	110.7664957	32.2130925	1.781838331	0.2572833	6.9255879	4.342E-12	1.173E-10
ENSMUSG00000039934.12	ENSMUSG00000039934.12	ENSMUSG00000039934	protein_coding	Gsap	62.70436814	12.57714462	2.309977973	0.467611	4.9399569	7.814E-07	1.022E-05
ENSMUSG00000110710.1	ENSMUSG00000110710.1	ENSMUSG00000110710	lincRNA	C78859	13.5116218	32.92146544	-1.279021413	0.419612	-3.048105	0.0023029	0.0129616
ENSMUSG00000039114.16	ENSMUSG00000039114.16	ENSMUSG00000039114	protein_coding	Nrn1	417.0098591	1333.162351	-1.677823155	0.1130753	-14.8381	8.307E-50	1.687E-47
ENSMUSG00000026620.11	ENSMUSG00000026620.11	ENSMUSG00000026620	protein_coding	Mark1	0	5.182627766	-4.92570947	1.788932	-2.753436	0.0058973	0.0286917
ENSMUSG00000106086.1	ENSMUSG00000106086.1	ENSMUSG00000106086	antisense_RNA	Gm43352	1.86378661	10.93414528	-2.557060148	0.8945852	-2.858375	0.0042582	0.0217595
ENSMUSG00000002997.15	ENSMUSG00000002997.15	ENSMUSG00000002997	protein_coding	Prkar2b	596.9887052	264.5131673	1.174271282	0.1869173	6.2823053	3.336E-10	7.093E-09
ENSMUSG00000020159.8	ENSMUSG00000020159.8	ENSMUSG00000020159	protein_coding	Gabrp	96.95479182	12.98874433	2.91413797	0.4622256	6.3045789	2.89E-10	6.202E-09
ENSMUSG00000041482.16	ENSMUSG00000041482.16	ENSMUSG00000041482	protein_coding	Piezo2	282.5803708	118.169646	1.260386262	0.1673134	7.5330853	4.956E-14	1.612E-12
ENSMUSG00000022157.8	ENSMUSG00000022157.8	ENSMUSG00000022157	protein_coding	Mcpt8	428.7826255	80.78769827	2.410410531	0.1597752	15.086266	1.994E-51	4.285E-49
ENSMUSG00000011589.8	ENSMUSG00000011589.8	ENSMUSG00000011589	protein_coding	Fsd1	1.816460617	34.49950791	-4.227665529	0.9513705	-4.443763	8.84E-06	9.42E-05
ENSMUSG00000025232.8	ENSMUSG00000025232.8	ENSMUSG00000025232	protein_coding	Hexa	340.3716121	79.67328852	2.093941808	0.1797185	11.651233	2.262E-31	2.1E-29
ENSMUSG00000022425.15	ENSMUSG00000022425.15	ENSMUSG00000022425	protein_coding	Enpp2	72.08817678	5.814238122	3.610354498	0.6325248	5.7078462	1.144E-08	2.006E-07
ENSMUSG00000030748.9	ENSMUSG00000030748.9	ENSMUSG00000030748	protein_coding	Il4ra	1863.750488	892.1363843	1.062354146	0.2146991	4.9481061	7.494E-07	9.835E-06
ENSMUSG00000057170.6	ENSMUSG00000057170.6	ENSMUSG00000057170	protein_coding	Prl3d1	1.747078268	19.40901453	-3.431630683	1.0907932	-3.145996	0.0016552	0.0097467
ENSMUSG00000044367.7	ENSMUSG00000044367.7	ENSMUSG00000044367	protein_coding	Slc16a13	151.1209038	41.91627639	1.850976445	0.2658788	6.9617313	3.361E-12	9.188E-11
ENSMUSG00000051022.7	ENSMUSG00000051022.7	ENSMUSG00000051022	protein_coding	Hs3st1	89.06543294	278.2697514	-1.645058314	0.1688293	-9.743917	1.959E-22	1.142E-20
ENSMUSG00000005045.16	ENSMUSG00000005045.16	ENSMUSG00000005045	protein_coding	Chd5	21.45667346	52.77186106	-1.303588114	0.3403906	-3.829683	0.0001283	0.001038
ENSMUSG00000057967.12	ENSMUSG00000057967.12	ENSMUSG00000057967	protein_coding	Fgf18	7.3147751	25.37009678	-1.784121607	0.5312311	-3.358466	0.0007838	0.0050655
ENSMUSG00000028451.12	ENSMUSG00000028451.12	ENSMUSG00000028451	protein_coding	1700022I11Rik	3.016406183	13.85885209	-2.178496541	0.8046783	-2.707289	0.0067835	0.032078
ENSMUSG00000104718.1	ENSMUSG00000104718.1	ENSMUSG00000104718	lincRNA	Gm31752	17.31077516	63.46862708	-1.886386162	0.3744294	-5.038029	4.704E-07	6.424E-06
ENSMUSG00000105053.1	ENSMUSG00000105053.1	ENSMUSG00000105053	protein_coding	Gm43064	171.5778919	346.8292848	-1.012947165	0.1884182	-5.376057	7.613E-08	1.169E-06
ENSMUSG00000049422.7	ENSMUSG00000049422.7	ENSMUSG00000049422	protein_coding	Chchd10	439.5275466	209.6320274	1.066984795	0.1382937	7.7153523	1.206E-14	4.113E-13
ENSMUSG00000091007.1	ENSMUSG00000091007.1	ENSMUSG00000091007	protein_coding	D630036H23Rik	0	4.259127188	-4.63700759	1.8356218	-2.526124	0.0115329	0.0496748
ENSMUSG00000032702.16	ENSMUSG00000032702.16	ENSMUSG00000032702	protein_coding	Kank1	349.8209201	766.3893323	-1.132156279	0.107094	-10.57162	4.035E-26	2.912E-24
ENSMUSG00000063767.4	ENSMUSG00000063767.4	ENSMUSG00000063767	protein_coding	S100a7a	1506.333044	261.0833413	2.530123115	0.1074157	23.554497	1.13E-122	1.3E-119
ENSMUSG00000034855.13	ENSMUSG00000034855.13	ENSMUSG00000034855	protein_coding	Cxcl10	47.86340202	110.5128515	-1.203504193	0.2553386	-4.713366	2.437E-06	2.892E-05
ENSMUSG00000062345.10	ENSMUSG00000062345.10	ENSMUSG00000062345	protein_coding	Serpinb2	593.0598702	1526.833327	-1.365305597	0.143359	-9.52368	1.672E-21	9.138E-20
ENSMUSG00000039601.15	ENSMUSG00000039601.15	ENSMUSG00000039601	protein_coding	Rcan2	3.750700669	50.94154863	-3.77408726	0.5584281	-6.758412	1.395E-11	3.556E-10
ENSMUSG00000051596.13	ENSMUSG00000051596.13	ENSMUSG00000051596	protein_coding	Otop1	14.40828839	0	6.205750322	1.4042606	4.4192297	9.905E-06	0.0001042
ENSMUSG00000025921.7	ENSMUSG00000025921.7	ENSMUSG00000025921	protein_coding	Rdh10	1914.760142	5779.959738	-1.59332635	0.0817448	-19.49148	1.297E-84	6.144E-82
ENSMUSG00000034159.12	ENSMUSG00000034159.12	ENSMUSG00000034159	protein_coding	2310007B03Rik	3669.46757	1427.101697	1.362183372	0.0860747	15.825602	2.072E-56	4.909E-54
ENSMUSG00000029168.14	ENSMUSG00000029168.14	ENSMUSG00000029168	protein_coding	Dpysl5	8.106297066	102.3419753	-3.642494572	0.6406983	-5.685194	1.307E-08	2.273E-07
ENSMUSG00000048915.12	ENSMUSG00000048915.12	ENSMUSG00000048915	protein_coding	Efna5	1078.644116	2302.044025	-1.093768799	0.0655262	-16.6921	1.496E-62	4.459E-60
ENSMUSG00000079092.4	ENSMUSG00000079092.4	ENSMUSG00000079092	protein_coding	Prl2c2	2.421947489	32.337685	-3.724811892	1.0399915	-3.581579	0.0003415	0.0024536
ENSMUSG00000062380.4	ENSMUSG00000062380.4	ENSMUSG00000062380	protein_coding	Tubb3	47.710987	135.5073727	-1.506433996	0.2399453	-6.278239	3.424E-10	7.265E-09
ENSMUSG00000035775.2	ENSMUSG00000035775.2	ENSMUSG00000035775	protein_coding	Krt20	331.0202969	135.079933	1.289900501	0.1730366	7.4544942	9.021E-14	2.845E-12
ENSMUSG00000026888.14	ENSMUSG00000026888.14	ENSMUSG00000026888	protein_coding	Grb14	522.1756126	235.0575845	1.152945521	0.1211773	9.5145361	1.825E-21	9.949E-20
ENSMUSG00000069793.12	ENSMUSG00000069793.12	ENSMUSG00000069793	protein_coding	Slfn9	69.76485249	12.37758757	2.493341388	0.3896393	6.3991017	1.563E-10	3.459E-09
ENSMUSG00000028532.14	ENSMUSG00000028532.14	ENSMUSG00000028532	protein_coding	Cachd1	166.3464082	80.2697234	1.047018041	0.238929	4.3821299	1.175E-05	0.0001217
ENSMUSG00000075316.11	ENSMUSG00000075316.11	ENSMUSG00000075316	protein_coding	Scn9a	36.99498952	13.90223722	1.419134984	0.4232256	3.3531409	0.000799	0.005155
ENSMUSG00000029001.15	ENSMUSG00000029001.15	ENSMUSG00000029001	protein_coding	Fbxo44	29.83048724	97.43347139	-1.706335757	0.3060257	-5.575793	2.464E-08	4.117E-07
ENSMUSG00000073408.2	ENSMUSG00000073408.2	ENSMUSG00000073408	protein_coding	Dpcr1	119.0433648	283.5544506	-1.254318538	0.2377746	-5.275242	1.326E-07	1.949E-06
ENSMUSG00000021342.14	ENSMUSG00000021342.14	ENSMUSG00000021342	protein_coding	Prl	441.9674329	2782.935574	-2.656037307	0.0978626	-27.14047	3.28E-162	6.06E-159
ENSMUSG00000074637.7	ENSMUSG00000074637.7	ENSMUSG00000074637	protein_coding	Sox2	27.03866735	1.750903104	3.935691216	0.8703704	4.521858	6.13E-06	6.759E-05
ENSMUSG00000064165.8	ENSMUSG00000064165.8	ENSMUSG00000064165	protein_coding	Krt39	5.870975816	0	4.908759676	1.6813898	2.9194656	0.0035063	0.0184114
ENSMUSG00000020473.13	ENSMUSG00000020473.13	ENSMUSG00000020473	protein_coding	Aebp1	74.28228544	13.81546695	2.428440889	0.3632467	6.6853771	2.303E-11	5.728E-10
ENSMUSG00000042500.11	ENSMUSG00000042500.11	ENSMUSG00000042500	protein_coding	Ago4	41.03413657	11.54350401	1.826143319	0.5477198	3.3340829	0.0008558	0.0054757
ENSMUSG00000053219.15	ENSMUSG00000053219.15	ENSMUSG00000053219	protein_coding	Raet1e	157.1569131	8.277279038	4.236555228	1.2322965	3.4379349	0.0005862	0.0039187
ENSMUSG00000051124.6	ENSMUSG00000051124.6	ENSMUSG00000051124	protein_coding	Gimap9	81.10009836	37.09938527	1.124976304	0.3201622	3.5137696	0.0004418	0.0030655
ENSMUSG00000029379.10	ENSMUSG00000029379.10	ENSMUSG00000029379	protein_coding	Cxcl3	702.7008081	198.018953	1.82991281	0.1294764	14.133176	2.372E-45	3.985E-43
ENSMUSG00000070867.4	ENSMUSG00000070867.4	ENSMUSG00000070867	protein_coding	Trabd2b	13.15052419	0.64829908	4.245484131	1.2880761	3.2959885	0.0009808	0.0061578
ENSMUSG00000041245.13	ENSMUSG00000041245.13	ENSMUSG00000041245	protein_coding	Wnk3	18.23827289	48.85828633	-1.414189054	0.5440446	-2.599399	0.0093387	0.0416782
ENSMUSG00000085101.7	ENSMUSG00000085101.7	ENSMUSG00000085101	processed_transcript	Platr16	17.42635681	46.95206003	-1.434524067	0.3629487	-3.952416	7.737E-05	0.0006628
ENSMUSG00000067855.7	ENSMUSG00000067855.7	ENSMUSG00000067855	protein_coding	Speer3	209.2377936	39.38580068	2.413263552	0.2168189	11.130319	8.932E-29	7.238E-27
ENSMUSG00000044017.16	ENSMUSG00000044017.16	ENSMUSG00000044017	protein_coding	Adgrd1	20.44270739	0	6.710726791	1.384721	4.8462663	1.258E-06	1.575E-05
ENSMUSG00000086854.1	ENSMUSG00000086854.1	ENSMUSG00000086854	antisense_RNA	Gm13781	0	8.231927064	-5.58602144	1.5778128	-3.540357	0.0003996	0.0028097
ENSMUSG00000058806.14	ENSMUSG00000058806.14	ENSMUSG00000058806	protein_coding	Col13a1	35.48287898	1.119292747	5.046997359	1.0409847	4.8482916	1.245E-06	1.562E-05
ENSMUSG00000093800.2	ENSMUSG00000093800.2	ENSMUSG00000093800	processed_pseudogene	Gm20796	0	16.32813582	-6.575749339	1.38132	-4.760482	1.931E-06	2.342E-05
ENSMUSG00000021728.8	ENSMUSG00000021728.8	ENSMUSG00000021728	protein_coding	Emb	160.8079359	387.8550957	-1.269075069	0.1398535	-9.07432	1.144E-19	5.728E-18
ENSMUSG00000045326.13	ENSMUSG00000045326.13	ENSMUSG00000045326	protein_coding	Fndc7	5.859412091	0	4.906253105	1.7112286	2.8670939	0.0041426	0.021269
ENSMUSG00000064032.2	ENSMUSG00000064032.2	ENSMUSG00000064032	processed_transcript	Gm10143	21.5133098	147.1466269	-2.770606014	0.796041	-3.480481	0.0005005	0.0034317
ENSMUSG00000032184.5	ENSMUSG00000032184.5	ENSMUSG00000032184	protein_coding	Lysmd2	46.74708026	19.31111844	1.271155823	0.3973909	3.1987543	0.0013802	0.0083183
ENSMUSG00000045362.8	ENSMUSG00000045362.8	ENSMUSG00000045362	protein_coding	Tnfrsf26	243.0925626	1125.605034	-2.209570282	0.3218182	-6.865895	6.608E-12	1.742E-10
ENSMUSG00000079559.4	ENSMUSG00000079559.4	ENSMUSG00000079559	protein_coding	Colca2	10.53044234	29.30507442	-1.47794392	0.4545086	-3.251741	0.001147	0.0070695
ENSMUSG00000074934.3	ENSMUSG00000074934.3	ENSMUSG00000074934	protein_coding	Grem1	70.74353624	19.58811797	1.855846128	0.341523	5.4340303	5.51E-08	8.686E-07
ENSMUSG00000034295.9	ENSMUSG00000034295.9	ENSMUSG00000034295	protein_coding	Fhod3	142.0584037	63.15380532	1.163797131	0.2384976	4.8797017	1.062E-06	1.359E-05
ENSMUSG00000015090.13	ENSMUSG00000015090.13	ENSMUSG00000015090	protein_coding	Ptgds	17.2039683	37.03195047	-1.106156864	0.4139552	-2.672165	0.0075363	0.0350816
ENSMUSG00000058665.8	ENSMUSG00000058665.8	ENSMUSG00000058665	protein_coding	En1	0	8.912485463	-5.701677756	1.5977852	-3.568488	0.000359	0.0025675
ENSMUSG00000016194.14	ENSMUSG00000016194.14	ENSMUSG00000016194	protein_coding	Hsd11b1	33.30210747	12.26856567	1.435724696	0.4364171	3.2897994	0.0010026	0.0062821
ENSMUSG00000032313.11	ENSMUSG00000032313.11	ENSMUSG00000032313	protein_coding	Tmem266	16.40088261	0.97244862	3.98200251	1.104759	3.6044083	0.0003129	0.002281
ENSMUSG00000040552.8	ENSMUSG00000040552.8	ENSMUSG00000040552	protein_coding	C3ar1	17.59039114	91.52048847	-2.391384604	0.3281801	-7.286805	3.174E-13	9.475E-12
ENSMUSG00000027007.16	ENSMUSG00000027007.16	ENSMUSG00000027007	protein_coding	Ssfa2	1924.724615	904.228085	1.089690792	0.1518183	7.1776003	7.095E-13	2.052E-11
ENSMUSG00000027204.13	ENSMUSG00000027204.13	ENSMUSG00000027204	protein_coding	Fbn1	5.555597561	0	4.829461244	1.8226764	2.6496537	0.0080574	0.0369351
ENSMUSG00000023966.8	ENSMUSG00000023966.8	ENSMUSG00000023966	protein_coding	Rsph9	3.609793783	25.97501072	-2.832122329	0.9080633	-3.11886	0.0018155	0.0105065
ENSMUSG00000041301.15	ENSMUSG00000041301.15	ENSMUSG00000041301	protein_coding	Cftr	46.89847977	191.1340279	-2.030727145	0.2399547	-8.46296	2.607E-17	1.12E-15
ENSMUSG00000063623.3	ENSMUSG00000063623.3	ENSMUSG00000063623	processed_transcript	C230062I16Rik	38.40468877	2.485972453	3.953791133	0.7273532	5.4358612	5.453E-08	8.612E-07

ENSMUSG00000110633.1	ENSMUSG00000110633.1	ENSMUSG00000110633	lincRNA	Gm32122	40.94495124	19.22502808	1.093749449	0.4123016	2.6527899	0.007983	0.0366664
ENSMUSG00000052396.7	ENSMUSG00000052396.7	ENSMUSG00000052396	protein_coding	Mogat2	16.95957903	46.68618632	-1.461123427	0.3808746	-3.836232	0.0001249	0.0010143
ENSMUSG00000108801.1	ENSMUSG00000108801.1	ENSMUSG00000108801	lincRNA	Gm39090	0	7.036989863	-5.363365139	1.6064708	-3.338601	0.000842	0.0054005
ENSMUSG00000030484.5	ENSMUSG00000030484.5	ENSMUSG00000030484	protein_coding	Lypd5	60.8526808	26.33878052	1.211376359	0.3162088	3.8309386	0.0001277	0.0010337
ENSMUSG00000035283.4	ENSMUSG00000035283.4	ENSMUSG00000035283	protein_coding	Adrb1	1.886914059	23.8231955	-3.675486573	0.8512327	-4.31784	1.576E-05	0.000159
ENSMUSG00000096764.1	ENSMUSG00000096764.1	ENSMUSG00000096764	protein_coding	Gm21985	0	73.85640365	-8.753313282	1.250654	-6.998989	2.578E-12	7.142E-11
ENSMUSG00000086806.1	ENSMUSG00000086806.1	ENSMUSG00000086806	antisense_RNA	Gm13054	0	6.010030292	-5.134738532	1.6893669	-3.039445	0.0023701	0.0133024
ENSMUSG00000097373.1	ENSMUSG00000097373.1	ENSMUSG00000097373	antisense_RNA	Gm26877	32.64875906	7.394516849	2.141657007	0.8133039	2.63328	0.0084565	0.0384305
ENSMUSG00000024164.15	ENSMUSG00000024164.15	ENSMUSG00000024164	protein_coding	C3	511.969101	212.561179	1.268334203	0.1324052	9.5791854	9.781E-22	5.432E-20
ENSMUSG00000113959.1	ENSMUSG00000113959.1	ENSMUSG00000113959	TEC	AC124739.5	16.63370815	0.64829908	4.577335461	1.2249945	3.7366172	0.0001865	0.001445
ENSMUSG00000001510.8	ENSMUSG00000001510.8	ENSMUSG00000001510	protein_coding	Dlx3	43.22706294	21.55526684	1.004423998	0.3758898	2.6721233	0.0075373	0.0350816
ENSMUSG00000039384.8	ENSMUSG00000039384.8	ENSMUSG00000039384	protein_coding	Dusp10	369.6770001	773.7514338	-1.067051954	0.157702	-6.766256	1.322E-11	3.382E-10
ENSMUSG00000050737.13	ENSMUSG00000050737.13	ENSMUSG00000050737	protein_coding	Ptges	847.9070936	345.8457334	1.29349233	0.1597717	8.0958788	5.685E-16	2.198E-14
ENSMUSG00000068196.4	ENSMUSG00000068196.4	ENSMUSG00000068196	protein_coding	Col8a1	12315.49213	5195.207192	1.245091463	0.0678316	18.355639	2.976E-75	1.1E-72
ENSMUSG00000067786.16	ENSMUSG00000067786.16	ENSMUSG00000067786	protein_coding	Nnat	55.24000621	215.6115687	-1.963155332	0.4493908	-4.368482	1.251E-05	0.0001289
ENSMUSG00000078503.9	ENSMUSG00000078503.9	ENSMUSG00000078503	protein_coding	Zfp990	50.92938757	0	8.023464793	1.2520534	6.4082448	1.472E-10	3.265E-09
ENSMUSG00000044216.6	ENSMUSG00000044216.6	ENSMUSG00000044216	protein_coding	Kcnj4	9.016189613	26.93745165	-1.584973762	0.5541798	-2.860035	0.0042359	0.0216699
ENSMUSG00000035395.11	ENSMUSG00000035395.11	ENSMUSG00000035395	protein_coding	Pet2	0	5.767088114	-5.072007684	1.8369848	-2.76105	0.0057616	0.0281349
ENSMUSG00000022514.14	ENSMUSG00000022514.14	ENSMUSG00000022514	protein_coding	Il1rap	349.6397998	117.1749457	1.580313801	0.1573804	10.041361	1.003E-23	6.434E-22
ENSMUSG00000079051.5	ENSMUSG00000079051.5	ENSMUSG00000079051	protein_coding	Gm14025	35.09651173	4.637787679	2.929911479	0.5795324	5.0556478	4.289E-07	5.884E-06
ENSMUSG00000085289.1	ENSMUSG00000085289.1	ENSMUSG00000085289	antisense_RNA	Gm15337	32.40656758	3.518494931	3.196420168	0.6280204	5.0896758	3.587E-07	4.976E-06
ENSMUSG00000073413.9	ENSMUSG00000073413.9	ENSMUSG00000073413	protein_coding	Ly6g6d	7.699535704	0	5.300693546	1.5658947	3.3850895	0.0007116	0.004641
ENSMUSG00000100782.1	ENSMUSG00000100782.1	ENSMUSG00000100782	lincRNA	Gm28231	9.680566203	0	5.628078351	1.5090198	3.7296251	0.0001918	0.0014814
ENSMUSG00000049929.7	ENSMUSG00000049929.7	ENSMUSG00000049929	protein_coding	Lpar4	10.07522829	0	5.690415164	1.5018356	3.7889734	0.0001513	0.0012027
ENSMUSG00000043430.5	ENSMUSG00000043430.5	ENSMUSG00000043430	protein_coding	Psapl1	402.1629137	69.49534609	2.532577363	0.2141468	11.826359	2.852E-32	2.703E-30
ENSMUSG00000024087.3	ENSMUSG00000024087.3	ENSMUSG00000024087	protein_coding	Cyp1b1	796.4437045	143.0144798	2.480315609	0.1778113	13.949148	3.184E-44	5.072E-42
ENSMUSG00000047414.6	ENSMUSG00000047414.6	ENSMUSG00000047414	protein_coding	Flrt2	582.897306	189.2116794	1.62284861	0.4114807	3.9439241	8.016E-05	0.0006845
ENSMUSG00000031734.13	ENSMUSG00000031734.13	ENSMUSG00000031734	protein_coding	Irx3	560.6003243	261.0110235	1.102519344	0.2507104	4.3975817	1.095E-05	0.0001145
ENSMUSG00000048764.16	ENSMUSG00000048764.16	ENSMUSG00000048764	protein_coding	Tmprss11f	54.08444726	6.939093778	2.945136	0.4775974	6.1665659	6.979E-10	1.434E-08
ENSMUSG00000029007.8	ENSMUSG00000029007.8	ENSMUSG00000029007	protein_coding	Agtrap	177.1323628	364.7681559	-1.041292947	0.1517694	-6.861019	6.837E-12	1.795E-10
ENSMUSG00000073591.4	ENSMUSG00000073591.4	ENSMUSG00000073591	protein_coding	Pcdhb22	22.22506798	5.648058526	1.975964667	0.5755595	3.4331194	0.0005967	0.0039789
ENSMUSG00000028031.6	ENSMUSG00000028031.6	ENSMUSG00000028031	protein_coding	Dkk2	10.21559963	38.93705864	-1.930275247	0.4258627	-4.532624	5.826E-06	6.457E-05
ENSMUSG00000002006.12	ENSMUSG00000002006.12	ENSMUSG00000002006	protein_coding	Pdzd4	82.35448247	180.5910287	-1.134133688	0.1797666	-6.308922	2.81E-10	6.051E-09
ENSMUSG00000060988.12	ENSMUSG00000060988.12	ENSMUSG00000060988	protein_coding	Galnt13	8.736573626	0.746195165	3.602848511	1.3747054	2.620815	0.008772	0.0396014
ENSMUSG00000019817.18	ENSMUSG00000019817.18	ENSMUSG00000019817	protein_coding	Plagl1	86.32093904	173.4704264	-1.010141092	0.2396899	-4.214367	2.505E-05	0.0002426
ENSMUSG00000017688.14	ENSMUSG00000017688.14	ENSMUSG00000017688	protein_coding	Hnf4g	1.619397345	13.41455483	-3.092067217	0.9750722	-3.171116	0.0015185	0.0090282
ENSMUSG00000029086.15	ENSMUSG00000029086.15	ENSMUSG00000029086	protein_coding	Prom1	101.4768427	456.9576665	-2.171076601	0.2070257	-10.48699	9.913E-26	7.073E-24
ENSMUSG00000048078.16	ENSMUSG00000048078.16	ENSMUSG00000048078	protein_coding	Tenm4	193.0829625	53.24088789	1.865937037	0.223077	8.3645407	6.035E-17	2.523E-15
ENSMUSG00000020607.7	ENSMUSG00000020607.7	ENSMUSG00000020607	protein_coding	Fam84a	536.2150834	248.1484003	1.112846752	0.1240385	8.9717884	2.917E-19	1.419E-17
ENSMUSG00000019990.15	ENSMUSG00000019990.15	ENSMUSG00000019990	protein_coding	Pde7b	9.622212032	0.746195165	3.743480461	1.3692357	2.7339928	0.0062571	0.0300936
ENSMUSG00000041801.5	ENSMUSG00000041801.5	ENSMUSG00000041801	protein_coding	Phlda3	53.95237077	110.380898	-1.026797883	0.254856	-4.028933	5.603E-05	0.0004951
ENSMUSG00000032584.12	ENSMUSG00000032584.12	ENSMUSG00000032584	protein_coding	Mst1r	1.164718845	34.13865427	-4.803386412	0.9188128	-5.227819	1.715E-07	2.484E-06
ENSMUSG00000024388.10	ENSMUSG00000024388.10	ENSMUSG00000024388	protein_coding	Myo7b	5.368435776	78.97868815	-3.851368463	0.5908496	-6.518356	7.108E-11	1.646E-09
ENSMUSG00000062309.8	ENSMUSG00000062309.8	ENSMUSG00000062309	protein_coding	Rpp25	34.62203026	104.9016659	-1.603767459	0.3097997	-5.176788	2.257E-07	3.211E-06
ENSMUSG00000085022.2	ENSMUSG00000085022.2	ENSMUSG00000085022	lincRNA	Gm5860	8.072732583	76.01419228	-3.238792477	0.3975626	-8.146623	3.742E-16	1.471E-14
ENSMUSG00000063851.12	ENSMUSG00000063851.12	ENSMUSG00000063851	protein_coding	Rnf183	102.1350667	38.13258765	1.425526286	0.2659582	5.3599638	8.324E-08	1.274E-06
ENSMUSG00000014773.13	ENSMUSG00000014773.13	ENSMUSG00000014773	protein_coding	Dll1	102.9854227	245.9685288	-1.256425193	0.1699867	-7.391317	1.454E-13	4.462E-12
ENSMUSG00000002578.17	ENSMUSG00000002578.17	ENSMUSG00000002578	protein_coding	Ikzf4	24.29855268	116.0253605	-2.251677475	0.2802065	-8.035778	9.299E-16	3.535E-14
ENSMUSG00000035868.8	ENSMUSG00000035868.8	ENSMUSG00000035868	protein_coding	Zfp983	67.37373615	15.14988734	2.150444519	0.3533815	6.0853349	1.162E-09	2.32E-08
ENSMUSG00000022340.14	ENSMUSG00000022340.14	ENSMUSG00000022340	protein_coding	Sybu	19.9870134	45.60471579	-1.189038116	0.4083525	-2.911794	0.0035936	0.0188109
ENSMUSG00000041180.13	ENSMUSG00000041180.13	ENSMUSG00000041180	protein_coding	Hectd2	25.20571189	64.78798797	-1.355640887	0.414003	-3.274471	0.0010586	0.0066062
ENSMUSG00000021216.7	ENSMUSG00000021216.7	ENSMUSG00000021216	protein_coding	Tubal3	0	16.32257291	-6.575372365	1.3894001	-4.732526	2.217E-06	2.657E-05
ENSMUSG00000043969.4	ENSMUSG00000043969.4	ENSMUSG00000043969	protein_coding	Emx2	50.76642433	24.81233284	1.024103906	0.3624372	2.8256039	0.0047192	0.0237993
ENSMUSG00000045991.18	ENSMUSG00000045991.18	ENSMUSG00000045991	protein_coding	Onecut2	270.3254977	122.8443066	1.135348263	0.1563103	7.263424	3.774E-13	1.119E-11
ENSMUSG00000038508.7	ENSMUSG00000038508.7	ENSMUSG00000038508	protein_coding	Gdf15	45.71839278	5.653621433	3.01560393	0.5200918	5.7982151	6.702E-09	1.215E-07
ENSMUSG00000025889.13	ENSMUSG00000025889.13	ENSMUSG00000025889	protein_coding	Snca	19.5934224	53.81995414	-1.463250581	0.3491387	-4.191029	2.777E-05	0.0002652
ENSMUSG00000051457.7	ENSMUSG00000051457.7	ENSMUSG00000051457	protein_coding	Spn	78.53338396	29.23499288	1.422435668	0.312157	4.5567956	5.194E-06	5.806E-05
ENSMUSG00000027955.16	ENSMUSG00000027955.16	ENSMUSG00000027955	protein_coding	Fam198b	2027.253014	677.0200751	1.581132609	0.1093726	14.456389	2.285E-47	4.264E-45
ENSMUSG00000067377.12	ENSMUSG00000067377.12	ENSMUSG00000067377	protein_coding	Tspan6	76.44720884	11.79937004	2.705660961	0.3852561	7.0230193	2.171E-12	6.051E-11
ENSMUSG00000047907.11	ENSMUSG00000047907.11	ENSMUSG00000047907	protein_coding	Tshz2	97.00904067	13.06567572	2.882077148	0.8901432	3.2377679	0.0012047	0.0073866
ENSMUSG00000081375.1	ENSMUSG00000081375.1	ENSMUSG00000081375	transcribed_processed_pseudogene	Gm14686	12.35733999	2.453713134	2.332518866	0.9243785	2.5233374	0.0116247	0.0499769
ENSMUSG00000027296.7	ENSMUSG00000027296.7	ENSMUSG00000027296	protein_coding	Itpka	5.194499954	0	4.73441036	1.7943202	2.6385537	0.0083261	0.0379499
ENSMUSG00000032086.12	ENSMUSG00000032086.12	ENSMUSG00000032086	protein_coding	Bace1	2672.102151	1248.053596	1.098632297	0.0760067	14.454411	2.351E-47	4.345E-45
ENSMUSG00000039145.16	ENSMUSG00000039145.16	ENSMUSG00000039145	protein_coding	Camk1d	120.2719994	905.2864321	-2.913429094	0.1239996	-23.49547	4.54E-122	4.93E-119
ENSMUSG00000000296.8	ENSMUSG00000000296.8	ENSMUSG00000000296	protein_coding	Tpd52l1	1575.527309	726.4978689	1.115760115	0.1053004	10.595972	3.111E-26	2.263E-24
ENSMUSG00000067199.4	ENSMUSG00000067199.4	ENSMUSG00000067199	protein_coding	Frat1	33.8833958	12.83745542	1.407238904	0.4662165	3.0184233	0.0025409	0.014125
ENSMUSG00000032968.4	ENSMUSG00000032968.4	ENSMUSG00000032968	protein_coding	Inha	27.75973588	55.92282132	-1.017216421	0.325922	-3.121043	0.0018021	0.010442
ENSMUSG00000036218.15	ENSMUSG00000036218.15	ENSMUSG00000036218	protein_coding	Pdzrn4	105.3171138	9.637547124	3.45868907	0.3821719	9.0500876	1.429E-19	7.115E-18
ENSMUSG00000034324.16	ENSMUSG00000034324.16	ENSMUSG00000034324	protein_coding	Tmem132c	26.69796386	78.61474954	-1.559693323	0.2966789	-5.257176	1.463E-07	2.142E-06
ENSMUSG00000018334.17	ENSMUSG00000018334.17	ENSMUSG00000018334	protein_coding	Ksr1	104.0070925	260.0151322	-1.321526861	0.1698599	-7.780099	7.247E-15	2.522E-13
ENSMUSG00000021069.17	ENSMUSG00000021069.17	ENSMUSG00000021069	protein_coding	Pygl	18.46285918	0.64829908	4.728666295	1.2636107	3.742186	0.0001824	0.0014181
ENSMUSG00000039632.13	ENSMUSG00000039632.13	ENSMUSG00000039632	protein_coding	Ccdc151	0	6.767351273	-5.304296922	1.6406471	-3.233052	0.0012248	0.0074789
ENSMUSG00000103897.1	ENSMUSG00000103897.1	ENSMUSG00000103897	protein_coding	Pcdhga8	26.61975112	4.632224771	2.525959853	0.675783	3.7378267	0.0001856	0.001439
ENSMUSG00000096803.1	ENSMUSG00000096803.1	ENSMUSG00000096803	protein_coding	Gm6803	0	7.323317176	-5.422582071	1.6346988	-3.317175	0.0009093	0.0057661
ENSMUSG00000052373.14	ENSMUSG00000052373.14	ENSMUSG00000052373	protein_coding	Mpp3	3.809590387	49.74729134	-3.732705547	0.5646581	-6.610559	3.829E-11	9.152E-10
ENSMUSG00000054203.7	ENSMUSG00000054203.7	ENSMUSG00000054203	protein_coding	Ifi205	33.97965443	68.1764963	-1.004180066	0.3166386	-3.171376	0.0015172	0.009023
ENSMUSG00000040147.14	ENSMUSG00000040147.14	ENSMUSG00000040147	protein_coding	Maob	0	5.274960943	-4.94768307	1.7322874	-2.856156	0.004288	0.021894
ENSMUSG00000029380.11	ENSMUSG00000029380.11	ENSMUSG00000029380	protein_coding	Cxcl1	69.61345297	297.8356177	-2.099655365	0.1842079	-11.39829	4.264E-30	3.699E-28
ENSMUSG00000039850.16	ENSMUSG00000039850.16	ENSMUSG00000039850	protein_coding	Endov	352.2170625	136.393731	1.369652656	0.1409634	9.7163699	2.568E-22	1.478E-20
ENSMUSG00000039542.16	ENSMUSG00000039542.16	ENSMUSG00000039542	protein_coding	Ncam1	363.3363464	146.7340881	1.308844341	0.1629113	8.0340915	9.427E-16	3.577E-14
ENSMUSG00000032548.14	ENSMUSG00000032548.14	ENSMUSG00000032548	protein_coding	Slco2a1	1574.624778	421.2826297	1.901538144	0.1013944	18.753882	1.8E-78	6.928E-76
ENSMUSG00000026475.7	ENSMUSG00000026475.7	ENSMUSG00000026475	protein_coding	Rgs16	714.2353478	210.6672695	1.762002856	0.1888661	9.3293743	1.065E-20	5.622E-19
ENSMUSG00000075284.10	ENSMUSG00000075284.10	ENSMUSG00000075284	protein_coding	Wipf1	393.0056275	125.984141	1.638791296	0.1480378	11.070085	1.752E-28	1.378E-26
ENSMUSG00000000290.13	ENSMUSG00000000290.13	ENSMUSG00000000290	protein_coding	Itgb2	426.7430904	197.9225126	1.106146546	0.1486781	7.4398772	1.008E-13	3.162E-12
ENSMUSG00000097823.2	ENSMUSG00000097823.2	ENSMUSG00000097823	lincRNA	Gm16701	1.013910473	11.39957604	-3.568745136	1.2460043	-2.864152	0.0041813	0.0214163
ENSMUSG00000103309.1	ENSMUSG00000103309.1	ENSMUSG00000103309	antisense_RNA	BC037039	34.43599517	5.410679255	2.683211873	0.612329	4.3819773	1.176E-05	0.0001217
ENSMUSG00000091709.1	ENSMUSG00000091709.1	ENSMUSG00000091709	antisense_RNA	Gm17189	5.346970564	0	4.770388056	1.743584	2.7359668	0.0062197	0.0299995
ENSMUSG00000043613.8	ENSMUSG00000043613.8	ENSMUSG00000043613	protein_coding	Mmp3	150.2676313	23.47679432	2.674075804	0.3474858	7.6954962	1.409E-14	4.796E-13
ENSMUSG00000098050.1	ENSMUSG00000098050.1	ENSMUSG00000098050	processed_pseudogene	Gm5345	4.635803528	0	4.568084367	1.8058203	2.529645	0.0114178	0.0492747
ENSMUSG00000035984.14	ENSMUSG00000035984.14	ENSMUSG00000035984	protein_coding	Nme5	20.33981616	3.047501264	2.704439711	0.841788	3.2127324	0.0013148	0.0079707
ENSMUSG00000089900.1	ENSMUSG00000089900.1	ENSMUSG00000089900	antisense_RNA	Tbc1d22bos	0	5.459627297	-4.991012589	1.8340751	-2.721269	0.0065032	0.0310265
ENSMUSG00000063060.6	ENSMUSG00000063060.6	ENSMUSG00000063060	protein_coding	Sox7	194.5696529	95.61984769	1.0212512	0.1984273	5.1467264	2.651E-07	3.736E-06
ENSMUSG00000097336.8	ENSMUSG00000097336.8	ENSMUSG00000097336	processed_transcript	Fendrr	33.81787348	0	7.432353487	1.2901546	5.760824	8.37E-09	1.496E-07
ENSMUSG00000028399.18	ENSMUSG00000028399.18	ENSMUSG00000028399	protein_coding	Ptprd	24.47474188	130.5932654	-2.425944867	0.2871736	-8.44766	2.972E-17	1.271E-15
ENSMUSG00000044854.7	ENSMUSG00000044854.7	ENSMUSG00000044854	protein_coding	1700056E22Rik	12.18453086	31.74389687	-1.384418989	0.4664831	-2.967779	0.0029996	0.0161695
ENSMUSG00000028927.6	ENSMUSG00000028927.6	ENSMUSG00000028927	protein_coding	Padi2	7219.825457	3097.242484	1.221142006	0.06021	20.281368	1.878E-91	1.052E-88
ENSMUSG00000056271.13	ENSMUSG00000056271.13	ENSMUSG00000056271	protein_coding	Lman1l	246.3754471	11.3922151	4.412283411	0.3539513	12.465792	1.147E-35	1.277E-33
ENSMUSG00000029260.15	ENSMUSG00000029260.15	ENSMUSG00000029260	protein_coding	Ugt2b34	67.98142081	165.7319134	-1.288652043	0.1981729	-6.502667	7.891E-11	1.816E-09
ENSMUSG00000059256.6	ENSMUSG00000059256.6	ENSMUSG00000059256	protein_coding	Gzmd	7.74632615	0	5.30812458	1.5602766	3.4020408	0.0006688	0.0043966
ENSMUSG00000104784.1	ENSMUSG00000104784.1	ENSMUSG00000104784	antisense_RNA	Gm42984	5.323843115	0	4.764896008	1.7419856	2.7353246	0.0062319	0.0300346
ENSMUSG00000113796.1	ENSMUSG00000113796.1	ENSMUSG00000113796	lincRNA	AC113508.3	27.61716675	10.9608417	1.336304786	0.4824436	2.7698676	0.0056079	0.0275227
ENSMUSG00000020601.8	ENSMUSG00000020601.8	ENSMUSG00000020601	protein_coding	Trib2	95.08223041	5.274960943	4.167955673	0.4756779	8.7621388	1.916E-18	8.828E-17
ENSMUSG00000024501.19	ENSMUSG00000024501.19	ENSMUSG00000024501	protein_coding	Dpysl3	446.0305724	193.7234593	1.200314333	0.1594446	7.5280974	5.148E-14	1.672E-12
ENSMUSG00000057315.14	ENSMUSG00000057315.14	ENSMUSG00000057315	protein_coding	Arhgap24	98.01283135	196.018326	-1.003212142	0.2032053	-4.936939	7.936E-07	1.036E-05
ENSMUSG00000031712.10	ENSMUSG00000031712.10	ENSMUSG00000031712	protein_coding	Il15	26.88292786	10.09561694	1.404394598	0.5473449	2.5658311	0.0102929	0.0451334
ENSMUSG00000041556.8	ENSMUSG00000041556.8	ENSMUSG00000041556	protein_coding	Fbxo2	22.3302126	104.9521702	-2.235249176	0.3158366	-7.077233	1.471E-12	4.168E-11
ENSMUSG00000001281.9	ENSMUSG00000001281.9	ENSMUSG00000001281	protein_coding	Itgb7	34.90934995	5.182627766	2.734687283	0.587115	4.6578395	3.195E-06	3.714E-05
ENSMUSG00000068699.12	ENSMUSG00000068699.12	ENSMUSG00000068699	protein_coding	Flnc	3089.693002	7012.962277	-1.18246638	0.0975603	-12.12036	8.239E-34	8.274E-32
ENSMUSG00000097222.1	ENSMUSG00000097222.1	ENSMUSG00000097222	lincRNA	1010001N08Rik	100.3406068	47.77474836	1.064348462	0.3004871	3.5420774	0.000397	0.0027966
ENSMUSG00000071856.10	ENSMUSG00000071856.10	ENSMUSG00000071856	protein_coding	Mcc	247.5445615	566.8536018	-1.193487204	0.1295387	-9.213366	3.161E-20	1.627E-18
ENSMUSG00000066026.14	ENSMUSG00000066026.14	ENSMUSG00000066026	protein_coding	Dhrs3	343.3212417	51.59361258	2.731524508	0.2463141	11.089599	1.409E-28	1.122E-26
ENSMUSG00000032262.13	ENSMUSG00000032262.13	ENSMUSG00000032262	protein_coding	Elovl4	27.87311974	115.2090838	-2.041068179	0.279719	-7.296853	2.946E-13	8.851E-12

ENSMUSG00000033965.10	ENSMUSG00000033965.10	ENSMUSG00000033965	protein_coding	Slc16a2	37.46342953	93.55771889	-1.326497809	0.2682113	-4.945719	7.586E-07	9.942E-06
ENSMUSG00000023982.7	ENSMUSG00000023982.7	ENSMUSG00000023982	protein_coding	Guca1a	0	11.33770415	-6.055402565	1.5382522	-3.936547	8.266E-05	0.0007042
ENSMUSG00000079442.12	ENSMUSG00000079442.12	ENSMUSG00000079442	protein_coding	St6galnac4	296.7250974	85.08107935	1.801632227	0.3980186	4.526503	5.997E-06	6.631E-05
ENSMUSG00000032118.16	ENSMUSG00000032118.16	ENSMUSG00000032118	protein_coding	Fez1	61.80330599	205.6939648	-1.730514489	0.2095003	-8.2602	1.454E-16	5.938E-15
ENSMUSG00000024339.12	ENSMUSG00000024339.12	ENSMUSG00000024339	protein_coding	Tap2	17.04213175	2.589431446	2.754757264	0.8696459	3.1676771	0.0015366	0.0091239
ENSMUSG00000046982.10	ENSMUSG00000046982.10	ENSMUSG00000046982	protein_coding	Tshz1	1119.48418	529.9343375	1.078353825	0.0943989	11.423373	3.196E-30	2.799E-28
ENSMUSG00000078864.10	ENSMUSG00000078864.10	ENSMUSG00000078864	protein_coding	Gm14322	29.37854208	6.345305648	2.208793146	0.5627915	3.9247094	8.683E-05	0.000736
ENSMUSG00000033752.7	ENSMUSG00000033752.7	ENSMUSG00000033752	protein_coding	Mnd1	287.4501266	119.4504041	1.265276366	0.1622654	7.7975714	6.311E-15	2.213E-13
ENSMUSG00000002384.2	ENSMUSG00000002384.2	ENSMUSG00000002384	protein_coding	Bmp8b	27.11789559	642.3783136	-4.561719419	0.1986877	-22.95925	1.19E-116	1.22E-113
ENSMUSG00000076435.3	ENSMUSG00000076435.3	ENSMUSG00000076435	protein_coding	Acsf2	295.433785	102.5618576	1.523555464	0.1652205	9.2213477	2.934E-20	1.523E-18
ENSMUSG00000022040.8	ENSMUSG00000022040.8	ENSMUSG00000022040	protein_coding	Ephx2	54.49950347	7.895971802	2.775603472	0.7346068	3.7783527	0.0001579	0.0012466
ENSMUSG00000031860.17	ENSMUSG00000031860.17	ENSMUSG00000031860	protein_coding	Pbx4	77.46605043	156.0808632	-1.013954005	0.2277176	-4.452683	8.48E-06	9.063E-05
ENSMUSG00000036904.5	ENSMUSG00000036904.5	ENSMUSG00000036904	protein_coding	Fzd8	37.99739186	9.209938591	2.044221665	0.4765192	4.2899045	1.787E-05	0.0001787
ENSMUSG00000020431.5	ENSMUSG00000020431.5	ENSMUSG00000020431	protein_coding	Adcy1	451.2835607	1015.144594	-1.169463612	0.2708477	-4.31779	1.576E-05	0.000159
ENSMUSG00000039058.11	ENSMUSG00000039058.11	ENSMUSG00000039058	protein_coding	Ak5	292.573582	103.0070036	1.502180872	0.1902082	7.8975604	2.844E-15	1.032E-13
ENSMUSG00000096956.2	ENSMUSG00000096956.2	ENSMUSG00000096956	lincRNA	Snhg18	31.26438504	152.9111467	-2.291572299	0.2412145	-9.500142	2.096E-21	1.129E-19
ENSMUSG00000050240.14	ENSMUSG00000050240.14	ENSMUSG00000050240	protein_coding	Hic2	110.8506387	54.22531104	1.032760169	0.3011031	3.429922	0.0006038	0.0040202
ENSMUSG00000024066.8	ENSMUSG00000024066.8	ENSMUSG00000024066	protein_coding	Xdh	190.8239782	7.868157263	4.56276833	0.3773197	12.092578	1.156E-33	1.148E-31
ENSMUSG00000030584.14	ENSMUSG00000030584.14	ENSMUSG00000030584	protein_coding	Dpf1	25.66146149	69.50158891	-1.4357937	0.302997	-4.738639	2.152E-06	2.587E-05
ENSMUSG00000112343.1	ENSMUSG00000112343.1	ENSMUSG00000112343	processed_transcript	AC154734.1	115.4868273	3.335626611	5.160770377	0.6551902	7.8767517	3.36E-15	1.215E-13
ENSMUSG00000026347.13	ENSMUSG00000026347.13	ENSMUSG00000026347	protein_coding	Tmem163	25.12642806	58.00703294	-1.206207143	0.3265644	-3.693627	0.0002211	0.0016811
ENSMUSG00000020300.14	ENSMUSG00000020300.14	ENSMUSG00000020300	protein_coding	Cpeb4	3040.403578	834.5606429	1.865494234	0.2137813	8.7261797	2.634E-18	1.205E-16
ENSMUSG00000042678.16	ENSMUSG00000042678.16	ENSMUSG00000042678	protein_coding	Myo15	11.97536832	1.29659816	3.13711707	1.236543	2.537006	0.0111805	0.0484392
ENSMUSG00000022324.14	ENSMUSG00000022324.14	ENSMUSG00000022324	protein_coding	Matn2	239.0335341	75.36965807	1.663962479	0.188642	8.8207443	1.137E-18	5.36E-17
ENSMUSG00000114138.1	ENSMUSG00000114138.1	ENSMUSG00000114138	antisense_RNA	AC153144.3	0	7.208732367	-5.401820562	1.7555399	-3.077014	0.0020909	0.0118913
ENSMUSG00000048330.14	ENSMUSG00000048330.14	ENSMUSG00000048330	protein_coding	Ric3	21.20072047	7.112634316	1.577191934	0.6005604	2.6262004	0.0086344	0.0391045
ENSMUSG00000031169.13	ENSMUSG00000031169.13	ENSMUSG00000031169	protein_coding	Porcn	577.0977419	228.2396559	1.336049055	0.1315586	10.155542	3.131E-24	2.088E-22
ENSMUSG00000009633.3	ENSMUSG00000009633.3	ENSMUSG00000009633	protein_coding	G0s2	16.00402273	0	6.356471887	1.3855893	4.5875586	4.485E-06	5.068E-05
ENSMUSG00000044254.6	ENSMUSG00000044254.6	ENSMUSG00000044254	protein_coding	Pcsk9	650.19241	1434.410056	-1.14065306	0.1145053	-9.961578	2.245E-23	1.416E-21
ENSMUSG00000021294.7	ENSMUSG00000021294.7	ENSMUSG00000021294	protein_coding	Kif26a	1.35188063	20.40259665	-3.997355894	1.0586486	-3.775904	0.0001594	0.0012573
ENSMUSG00000078490.9	ENSMUSG00000078490.9	ENSMUSG00000078490	protein_coding	Cfap74	12.37276374	36.57944356	-1.578040295	0.518494	-3.043507	0.0023384	0.0131413
ENSMUSG00000045868.12	ENSMUSG00000045868.12	ENSMUSG00000045868	protein_coding	Gvin1	47.85130274	20.54387787	1.222069565	0.3487466	3.5041767	0.000458	0.0031639
ENSMUSG00000019230.14	ENSMUSG00000019230.14	ENSMUSG00000019230	protein_coding	Lhx9	66.59983572	754.7851474	-3.511327618	0.1993601	-17.61299	1.958E-69	6.461E-67
ENSMUSG00000049288.4	ENSMUSG00000049288.4	ENSMUSG00000049288	protein_coding	Lix1l	176.8538179	74.65127745	1.243320798	0.2023311	6.1449804	7.997E-10	1.629E-08
ENSMUSG00000050212.4	ENSMUSG00000050212.4	ENSMUSG00000050212	protein_coding	Eva1b	62.52710784	16.64672245	1.912037118	0.362379	5.276347	1.318E-07	1.94E-06
ENSMUSG00000073008.11	ENSMUSG00000073008.11	ENSMUSG00000073008	protein_coding	Gpr174	5.521441933	0	4.82160619	1.7386651	2.7731655	0.0055514	0.0273106
ENSMUSG00000072620.3	ENSMUSG00000072620.3	ENSMUSG00000072620	protein_coding	Slfn2	36.73742989	9.123168322	2.004689015	0.4845646	4.1370937	3.517E-05	0.0003264
ENSMUSG00000076928.5	ENSMUSG00000076928.5	ENSMUSG00000076928	TR_C_gene	Trac	54.64427038	186.557505	-1.772897361	0.2036925	-8.703791	3.21E-18	1.454E-16
ENSMUSG00000022091.5	ENSMUSG00000022091.5	ENSMUSG00000022091	protein_coding	Sorbs3	1112.0961	335.1173602	1.729054577	0.1266328	13.654078	1.909E-42	2.845E-40
ENSMUSG00000090919.6	ENSMUSG00000090919.6	ENSMUSG00000090919	protein_coding	Pabpc4l	0	9.063094464	-5.730230497	1.5575365	-3.679035	0.0002341	0.0017686
ENSMUSG00000041351.16	ENSMUSG00000041351.16	ENSMUSG00000041351	protein_coding	Rap1gap	490.7602361	240.3446888	1.029180055	0.145181	7.0889464	1.351E-12	3.848E-11
ENSMUSG00000023367.14	ENSMUSG00000023367.14	ENSMUSG00000023367	protein_coding	Tmem176a	1902.129277	812.9299615	1.225881074	0.0773543	15.847609	1.46E-56	3.551E-54
ENSMUSG00000039683.16	ENSMUSG00000039683.16	ENSMUSG00000039683	protein_coding	Sdk1	7.25754762	0	5.210689686	1.634114	3.1886941	0.0014292	0.0085797
ENSMUSG00000024121.13	ENSMUSG00000024121.13	ENSMUSG00000024121	protein_coding	Atp6v0c	166.8914935	827.1046852	-2.306562878	0.1456534	-15.83597	1.757E-56	4.217E-54
ENSMUSG00000010461.15	ENSMUSG00000010461.15	ENSMUSG00000010461	protein_coding	Eya4	548.57034	2021.74616	-1.882917229	0.2606701	-7.223372	5.071E-13	1.487E-11
ENSMUSG00000041731.13	ENSMUSG00000041731.13	ENSMUSG00000041731	protein_coding	Pgm5	19.42938806	0.746195165	4.769037383	1.1935426	3.9956992	6.45E-05	0.000562
ENSMUSG00000027356.8	ENSMUSG00000027356.8	ENSMUSG00000027356	protein_coding	Fermt1	2502.47775	5082.877655	-1.02188512	0.0698505	-14.62961	1.818E-48	3.574E-46
ENSMUSG00000044345.9	ENSMUSG00000044345.9	ENSMUSG00000044345	protein_coding	Marveld1	325.3061153	157.524716	1.048042544	0.1664071	6.2980629	3.014E-10	6.461E-09
ENSMUSG00000025813.14	ENSMUSG00000025813.14	ENSMUSG00000025813	protein_coding	Homer2	165.3517324	35.23946026	2.231021099	0.4238741	5.2634051	1.414E-07	2.074E-06
ENSMUSG00000004043.14	ENSMUSG00000004043.14	ENSMUSG00000004043	protein_coding	Stat5a	99.42033282	47.61592971	1.059237698	0.2510373	4.219444	2.449E-05	0.0002377
ENSMUSG00000013921.15	ENSMUSG00000013921.15	ENSMUSG00000013921	protein_coding	Clip3	19.82737463	64.38836278	-1.710008069	0.3588562	-4.765162	1.887E-06	2.294E-05
ENSMUSG00000039976.4	ENSMUSG00000039976.4	ENSMUSG00000039976	protein_coding	Tbc1d16	232.8985287	520.8162209	-1.161057485	0.2835617	-4.094549	4.23E-05	0.000386
ENSMUSG00000037031.10	ENSMUSG00000037031.10	ENSMUSG00000037031	protein_coding	Tspan15	83.61224667	634.2317693	-2.924145467	0.1483653	-19.70909	1.802E-86	8.762E-84
ENSMUSG00000025867.8	ENSMUSG00000025867.8	ENSMUSG00000025867	protein_coding	Cplx2	72.67884739	1.102604024	6.09173766	0.9155826	6.6534008	2.864E-11	7E-10
ENSMUSG00000013523.13	ENSMUSG00000013523.13	ENSMUSG00000013523	protein_coding	Bcas1	27.4316672	9.64867294	1.515945764	0.520454	2.9127375	0.0035828	0.0187648
ENSMUSG00000046523.4	ENSMUSG00000046523.4	ENSMUSG00000046523	protein_coding	Kctd4	17.61405414	2.956966121	2.602017189	0.9044121	2.8770261	0.0040144	0.0207145
ENSMUSG00000035818.14	ENSMUSG00000035818.14	ENSMUSG00000035818	protein_coding	Plekhs1	566.9136038	231.2941077	1.293186574	0.1541496	8.3891638	4.896E-17	2.08E-15
ENSMUSG00000089727.2	ENSMUSG00000089727.2	ENSMUSG00000089727	protein_coding	Klra8	18.46173249	44.60068776	-1.268473072	0.3787101	-3.349456	0.0008097	0.0052168
ENSMUSG00000039476.13	ENSMUSG00000039476.13	ENSMUSG00000039476	protein_coding	Prrx2	188.2908839	36.21190888	2.375198041	0.2404241	9.8792011	5.124E-23	3.135E-21
ENSMUSG00000027368.6	ENSMUSG00000027368.6	ENSMUSG00000027368	protein_coding	Dusp2	30.61323441	5.925058057	2.389719532	0.8036244	2.9736771	0.0029425	0.015931
ENSMUSG00000025161.16	ENSMUSG00000025161.16	ENSMUSG00000025161	protein_coding	Slc16a3	1257.203606	497.861267	1.337022585	0.143323	9.3287383	1.071E-20	5.64E-19
ENSMUSG00000030796.17	ENSMUSG00000030796.17	ENSMUSG00000030796	protein_coding	Tead2	550.2373463	1377.210136	-1.323789709	0.2036183	-6.501331	7.961E-11	1.83E-09
ENSMUSG00000038517.15	ENSMUSG00000038517.15	ENSMUSG00000038517	protein_coding	Tbkbp1	67.29498786	20.53831496	1.712554715	0.3376096	5.0725893	3.924E-07	5.408E-06
ENSMUSG00000079550.9	ENSMUSG00000079550.9	ENSMUSG00000079550	protein_coding	Mpp4	3.098423351	27.29223128	-3.144513489	1.049719	-2.995576	0.0027393	0.0150107
ENSMUSG00000028270.12	ENSMUSG00000028270.12	ENSMUSG00000028270	protein_coding	Gbp2	62.64322504	10.45758871	2.572674043	0.4187141	6.1442257	8.035E-10	1.635E-08
ENSMUSG00000091697.1	ENSMUSG00000091697.1	ENSMUSG00000091697	processed_pseudogene	Eif3s6-ps2	164.644553	1.345546202	6.868112643	0.7634467	8.9961917	2.337E-19	1.148E-17
ENSMUSG00000064225.6	ENSMUSG00000064225.6	ENSMUSG00000064225	protein_coding	Paqr9	10.92676667	52.85863133	-2.283022005	0.3987676	-5.725194	1.033E-08	1.818E-07
ENSMUSG00000020435.17	ENSMUSG00000020435.17	ENSMUSG00000020435	protein_coding	Osbp2	5.998712336	29.59140174	-2.310572734	0.841134	-2.746973	0.0060148	0.0291404
ENSMUSG00000022156.8	ENSMUSG00000022156.8	ENSMUSG00000022156	protein_coding	Gzme	165.2659991	18.47995104	3.166484486	0.2789335	11.352114	7.239E-30	6.222E-28
ENSMUSG00000113009.1	ENSMUSG00000113009.1	ENSMUSG00000113009	TEC	AC159807.1	17.92723461	0	6.519211371	1.3555958	4.8091116	1.516E-06	1.876E-05
ENSMUSG00000032322.14	ENSMUSG00000032322.14	ENSMUSG00000032322	protein_coding	Pstpip1	155.2711258	68.76163655	1.178975933	0.2495795	4.723849	2.314E-06	2.761E-05
ENSMUSG00000048387.8	ENSMUSG00000048387.8	ENSMUSG00000048387	protein_coding	Osr1	49.01988161	16.84627949	1.533401441	0.3990439	3.842689	0.0001217	0.0009915
ENSMUSG00000005836.8	ENSMUSG00000005836.8	ENSMUSG00000005836	protein_coding	Gata6	3056.076939	1303.69598	1.228913018	0.1078768	11.391817	4.593E-30	3.966E-28
ENSMUSG00000044217.16	ENSMUSG00000044217.16	ENSMUSG00000044217	protein_coding	Aqp5	1490.778609	618.6723497	1.269233642	0.0882346	14.384753	6.451E-47	1.157E-44
ENSMUSG00000025915.14	ENSMUSG00000025915.14	ENSMUSG00000025915	protein_coding	Sgk3	209.3054418	422.5207832	-1.010746645	0.2286779	-4.419959	9.872E-06	0.000104
ENSMUSG00000040253.15	ENSMUSG00000040253.15	ENSMUSG00000040253	protein_coding	Gbp7	11.67316043	0	5.896430155	1.4890205	3.9599388	7.497E-05	0.0006428
ENSMUSG00000026042.16	ENSMUSG00000026042.16	ENSMUSG00000026042	protein_coding	Col5a2	20.02497345	1.394494245	3.826195482	0.9401207	4.069898	4.703E-05	0.0004244
ENSMUSG00000039070.5	ENSMUSG00000039070.5	ENSMUSG00000039070	protein_coding	Cpa4	380.7645878	1611.430893	-2.082332695	0.1542419	-13.50043	1.555E-41	2.227E-39
ENSMUSG00000079465.8	ENSMUSG00000079465.8	ENSMUSG00000079465	protein_coding	Col4a3	388.3610655	1113.669523	-1.52130645	0.118657	-12.82105	1.25E-37	1.53E-35
ENSMUSG00000027855.13	ENSMUSG00000027855.13	ENSMUSG00000027855	protein_coding	Sycp1	38.93421629	0	7.634860201	1.3188056	5.7892233	7.071E-09	1.274E-07
ENSMUSG00000030762.11	ENSMUSG00000030762.11	ENSMUSG00000030762	protein_coding	Aqp8	387.9130981	81.98151734	2.245335708	0.1946601	11.534651	8.824E-31	7.877E-29
ENSMUSG00000027796.2	ENSMUSG00000027796.2	ENSMUSG00000027796	protein_coding	Smad9	5.300180118	0	4.759376395	1.7204071	2.7664245	0.0056675	0.0277781
ENSMUSG00000050050.17	ENSMUSG00000050050.17	ENSMUSG00000050050	protein_coding	Ccdc158	16.60014367	40.39274487	-1.288259839	0.4627487	-2.783929	0.0053705	0.0265904
ENSMUSG00000034205.16	ENSMUSG00000034205.16	ENSMUSG00000034205	protein_coding	Loxl2	96.40333549	246.1789423	-1.3551891	0.177168	-7.649175	2.023E-14	6.82E-13
ENSMUSG00000024304.14	ENSMUSG00000024304.14	ENSMUSG00000024304	protein_coding	Cdh2	4774.447289	969.5416227	2.29948283	0.1005838	22.861368	1.13E-115	1.1E-112
ENSMUSG00000023206.16	ENSMUSG00000023206.16	ENSMUSG00000023206	protein_coding	Il15ra	40.72256274	20.20415773	1.014034221	0.3747717	2.7057385	0.0068153	0.0322078
ENSMUSG00000064080.12	ENSMUSG00000064080.12	ENSMUSG00000064080	protein_coding	Fbln2	12099.24617	25073.35089	-1.051255764	0.1643609	-6.396022	1.595E-10	3.516E-09
ENSMUSG00000097072.7	ENSMUSG00000097072.7	ENSMUSG00000097072	processed_transcript	Foxl2os	55.77869362	18.57228422	1.588958099	0.4491219	3.5379216	0.0004033	0.0028302
ENSMUSG00000103170.1	ENSMUSG00000103170.1	ENSMUSG00000103170	lincRNA	Gm37170	6.208410426	0	4.988581875	1.6729404	2.9819244	0.0028644	0.0155719
ENSMUSG00000051951.5	ENSMUSG00000051951.5	ENSMUSG00000051951	protein_coding	Xkr4	0.873539134	25.68603666	-4.807863488	1.0661916	-4.50938	6.502E-06	7.121E-05
ENSMUSG00000049907.8	ENSMUSG00000049907.8	ENSMUSG00000049907	protein_coding	Rasl11b	38.03315413	91.75701903	-1.272846625	0.2846034	-4.472353	7.736E-06	8.35E-05
ENSMUSG00000055994.15	ENSMUSG00000055994.15	ENSMUSG00000055994	protein_coding	Nod2	24.76645714	57.2572417	-1.218598251	0.3514163	-3.467677	0.000525	0.0035664
ENSMUSG00000020122.16	ENSMUSG00000020122.16	ENSMUSG00000020122	protein_coding	Egfr	3813.906015	1889.299721	1.01327277	0.0874128	11.591817	4.534E-31	4.148E-29
ENSMUSG00000074203.4	ENSMUSG00000074203.4	ENSMUSG00000074203	protein_coding	G430095P16Rik	9.086643056	28.79249366	-1.671017909	0.4994476	-3.345732	0.0008207	0.0052763
ENSMUSG00000004633.17	ENSMUSG00000004633.17	ENSMUSG00000004633	protein_coding	Chn2	103.2771936	1952.313489	-4.242907916	0.1238673	-34.25365	3.85E-257	1.42E-253
ENSMUSG00000026811.18	ENSMUSG00000026811.18	ENSMUSG00000026811	protein_coding	St6galnac6	100.1606688	44.75394351	1.158643891	0.3275267	3.5375556	0.0004038	0.002832
ENSMUSG00000062044.15	ENSMUSG00000062044.15	ENSMUSG00000062044	protein_coding	Lmtk3	81.27231634	183.7964999	-1.179739157	0.1812834	-6.507707	7.631E-11	1.758E-09
ENSMUSG00000030167.15	ENSMUSG00000030167.15	ENSMUSG00000030167	protein_coding	Klrc1	125.5799388	302.0791015	-1.267295141	0.163129	-7.768669	7.932E-15	2.739E-13
ENSMUSG00000015709.9	ENSMUSG00000015709.9	ENSMUSG00000015709	protein_coding	Arnt2	17.60029263	53.53474495	-1.604744972	0.3551058	-4.519061	6.211E-06	6.832E-05
ENSMUSG00000071669.14	ENSMUSG00000071669.14	ENSMUSG00000071669	protein_coding	Snx29	80.43950985	38.29876725	1.074364738	0.3089894	3.4770275	0.000507	0.0034634
ENSMUSG00000025475.17	ENSMUSG00000025475.17	ENSMUSG00000025475	protein_coding	Adgra1	48.44742368	15.47583491	1.656724345	0.4217068	3.9286168	8.544E-05	0.0007255
ENSMUSG00000032420.8	ENSMUSG00000032420.8	ENSMUSG00000032420	protein_coding	Nt5e	1814.379528	319.0397969	2.507843098	0.1258683	19.924343	2.503E-88	1.321E-85
ENSMUSG00000102428.1	ENSMUSG00000102428.1	ENSMUSG00000102428	protein_coding	Pcdhga12	9.247944059	40.77385557	-2.137690418	0.8301605	-2.575033	0.0100231	0.0441524
ENSMUSG00000017652.16	ENSMUSG00000017652.16	ENSMUSG00000017652	protein_coding	Cd40	9.131235717	0	5.548847394	1.5683832	3.5379411	0.0004033	0.0028302
ENSMUSG00000053835.17	ENSMUSG00000053835.17	ENSMUSG00000053835	protein_coding	H2-T24	3.471084683	199.4370587	-5.86659202	0.5219275	-11.24024	2.587E-29	2.134E-27
ENSMUSG00000042428.6	ENSMUSG00000042428.6	ENSMUSG00000042428	protein_coding	Mgat3	582.8405078	280.4066031	1.055526659	0.1550319	6.80845	9.866E-12	2.546E-10
ENSMUSG00000096953.2	ENSMUSG00000096953.2	ENSMUSG00000096953	antisense_RNA	Gm26571	0.582359423	18.43459907	-4.925118748	1.5357915	-3.206893	0.0013418	0.008113
ENSMUSG00000020811.16	ENSMUSG00000020811.16	ENSMUSG00000020811	protein_coding	Wscd1	205.6709858	9.72251936	4.383315907	0.343784	12.750204	3.109E-37	3.731E-35
ENSMUSG00000039007.9	ENSMUSG00000039007.9	ENSMUSG00000039007	protein_coding	Cpq	24.2726919	3.162086072	2.939235704	0.8527208	3.4468909	0.0005671	0.0038076
ENSMUSG00000020865.16	ENSMUSG00000020865.16	ENSMUSG00000020865	protein_coding	Abcc3	1756.160663	267.8334927	2.712547583	0.120348	22.539204	1.71E-112	1.58E-109
ENSMUSG00000039716.12	ENSMUSG00000039716.12	ENSMUSG00000039716	protein_coding	Dock3	28.66630395	159.8421315	-2.474655957	0.2789699	-8.87069	7.269E-19	3.489E-17
ENSMUSG00000097429.1	ENSMUSG00000097429.1	ENSMUSG00000097429	lincRNA	Gm26520	1.816460617	24.06349093	-3.711300369	1.0756803	-3.450189	0.0005602	0.0037723

ENSMUSG00000057933.10	ENSMUSG00000057933.10	ENSMUSG00000057933	protein_coding	Gsta2	0	6.296357605	-5.204186073	1.6604919	-3.134123	0.0017237	0.0100824
ENSMUSG00000035615.12	ENSMUSG00000035615.12	ENSMUSG00000035615	protein_coding	Frmpd1	7.419919718	19.72203825	-1.411338042	0.5441038	-2.593876	0.0094901	0.0422039
ENSMUSG00000022816.11	ENSMUSG00000022816.11	ENSMUSG00000022816	protein_coding	Fstl1	23.68155768	3.908281237	2.601529176	0.8098189	3.2124827	0.0013159	0.0079724
ENSMUSG00000032766.9	ENSMUSG00000032766.9	ENSMUSG00000032766	protein_coding	Gng11	6.150591802	0	4.976736634	1.7027901	2.9226953	0.0034702	0.0182319
ENSMUSG00000097479.1	ENSMUSG00000097479.1	ENSMUSG00000097479	lincRNA	Gm26582	22.4600913	45.49484518	-1.020801234	0.4035016	-2.529856	0.0114109	0.0492747
ENSMUSG00000070704.10	ENSMUSG00000070704.10	ENSMUSG00000070704	protein_coding	Ugt2b36	1.013910473	99.64672029	-6.735895769	0.9051582	-7.441678	9.941E-14	3.124E-12
ENSMUSG00000025912.16	ENSMUSG00000025912.16	ENSMUSG00000025912	protein_coding	Mybl1	242.3093289	502.9024123	-1.054824726	0.1533676	-6.877753	6.08E-12	1.61E-10
ENSMUSG00000040212.12	ENSMUSG00000040212.12	ENSMUSG00000040212	protein_coding	Emp3	153.9417814	58.83528418	1.386990455	0.2336225	5.9368883	2.905E-09	5.522E-08
ENSMUSG00000020053.18	ENSMUSG00000020053.18	ENSMUSG00000020053	protein_coding	Igf1	21.504535	4.210179146	2.343901351	0.6609898	3.5460477	0.0003911	0.0027643
ENSMUSG00000086531.2	ENSMUSG00000086531.2	ENSMUSG00000086531	lincRNA	Gm11351	0	205.2556687	-10.2287664	1.1954376	-8.556504	1.163E-17	5.155E-16
ENSMUSG00000096856.7	ENSMUSG00000096856.7	ENSMUSG00000096856	protein_coding	Gm10778	0	6.767351273	-5.304296922	1.6406471	-3.233052	0.0012248	0.0074789
ENSMUSG00000026932.14	ENSMUSG00000026932.14	ENSMUSG00000026932	protein_coding	Nacc2	1101.140129	534.1575411	1.044500254	0.1796154	5.8152031	6.056E-09	1.107E-07
ENSMUSG00000113011.1	ENSMUSG00000113011.1	ENSMUSG00000113011	processed_pseudogene	AC211878.2	34.92150482	0.64829908	5.637141479	1.1240742	5.0149195	5.306E-07	7.214E-06
ENSMUSG00000001763.14	ENSMUSG00000001763.14	ENSMUSG00000001763	protein_coding	Tspan33	6.429672242	39.7451257	-2.615943098	0.5236856	-4.995255	5.876E-07	7.862E-06
ENSMUSG00000022899.8	ENSMUSG00000022899.8	ENSMUSG00000022899	protein_coding	Slc15a2	30.41558	0	7.281017409	1.2983522	5.607891	2.048E-08	3.469E-07
ENSMUSG00000020427.11	ENSMUSG00000020427.11	ENSMUSG00000020427	protein_coding	Igfbp3	3620.152987	11134.47349	-1.6211174	0.0794295	-20.40951	1.377E-92	7.949E-90
ENSMUSG00000042190.12	ENSMUSG00000042190.12	ENSMUSG00000042190	protein_coding	Cmklr1	4.962209961	0	4.665444187	1.7470104	2.6705303	0.0075732	0.0351954
ENSMUSG00000055538.7	ENSMUSG00000055538.7	ENSMUSG00000055538	protein_coding	Zcchc24	54.46701008	5.263835127	3.365083509	0.523196	6.4317832	1.261E-10	2.835E-09
ENSMUSG00000050241.8	ENSMUSG00000050241.8	ENSMUSG00000050241	protein_coding	Klre1	3.751236216	14.94853226	-2.007548671	0.7543256	-2.661382	0.0077821	0.0359223
ENSMUSG00000064043.13	ENSMUSG00000064043.13	ENSMUSG00000064043	processed_transcript	Trerf1	28.00192735	156.0505707	-2.474409549	0.2583133	-9.579102	9.789E-22	5.432E-20
ENSMUSG00000022103.9	ENSMUSG00000022103.9	ENSMUSG00000022103	protein_coding	Gfra2	8.283557365	0	5.400306655	1.6434495	3.2859583	0.0010164	0.0063619
ENSMUSG00000112758.1	ENSMUSG00000112758.1	ENSMUSG00000112758	bidirectional_promoter_lncRNA	AC159502.1	4.926983239	0	4.656709478	1.7647579	2.6387242	0.0083219	0.0379401
ENSMUSG00000050822.11	ENSMUSG00000050822.11	ENSMUSG00000050822	protein_coding	Slc29a4	66.98287849	160.8745533	-1.267880095	0.2376315	-5.335488	9.529E-08	1.446E-06
ENSMUSG00000049939.6	ENSMUSG00000049939.6	ENSMUSG00000049939	protein_coding	Lrrc4	16.70362605	3.880466698	2.104082157	0.6983319	3.0130115	0.0025867	0.0143405
ENSMUSG00000047045.17	ENSMUSG00000047045.17	ENSMUSG00000047045	protein_coding	Tmem164	269.0542296	861.2247074	-1.678156027	0.598137	-2.805638	0.0050217	0.0251603
ENSMUSG00000024238.14	ENSMUSG00000024238.14	ENSMUSG00000024238	protein_coding	Zeb1	227.9284524	60.05094439	1.927338977	0.213672	9.0200835	1.879E-19	9.311E-18
ENSMUSG00000090958.2	ENSMUSG00000090958.2	ENSMUSG00000090958	protein_coding	Lrrc32	43.03005527	8.619915336	2.300650721	0.5255642	4.3774877	1.201E-05	0.000124
ENSMUSG00000103749.1	ENSMUSG00000103749.1	ENSMUSG00000103749	protein_coding	Pcdhgb5	94.06835263	11.68590336	3.007632218	0.5911331	5.0879107	3.62E-07	5.015E-06
ENSMUSG00000026764.15	ENSMUSG00000026764.15	ENSMUSG00000026764	protein_coding	Kif5c	83.07881988	184.7253946	-1.152979136	0.1905069	-6.052166	1.429E-09	2.815E-08
ENSMUSG00000023913.17	ENSMUSG00000023913.17	ENSMUSG00000023913	protein_coding	Pla2g7	26.5845244	153.1487954	-2.532903758	0.3141382	-8.063023	7.443E-16	2.847E-14
ENSMUSG00000033788.15	ENSMUSG00000033788.15	ENSMUSG00000033788	protein_coding	Dysf	242.8872928	25.53183159	3.241217257	0.2622656	12.358529	4.381E-35	4.679E-33
ENSMUSG00000054545.17	ENSMUSG00000054545.17	ENSMUSG00000054545	protein_coding	Ugt1a6a	25.54035757	3.946103464	2.706701693	0.865497	3.1273379	0.001764	0.010262
ENSMUSG00000033032.15	ENSMUSG00000033032.15	ENSMUSG00000033032	protein_coding	Afap1l1	70.03953736	9.446199734	2.886520584	0.722679	3.9941949	6.491E-05	0.0005645
ENSMUSG00000027843.13	ENSMUSG00000027843.13	ENSMUSG00000027843	protein_coding	Ptpn22	1907.574775	828.2842888	1.202606976	0.0810437	14.838993	8.198E-50	1.683E-47
ENSMUSG00000028015.3	ENSMUSG00000028015.3	ENSMUSG00000028015	protein_coding	Ctso	84.38005004	23.55732178	1.829887048	0.3564884	5.1330906	2.85E-07	4.014E-06
ENSMUSG00000043639.14	ENSMUSG00000043639.14	ENSMUSG00000043639	protein_coding	Rbm20	2.807779186	22.71126369	-3.022318337	0.8005369	-3.775364	0.0001598	0.0012595
ENSMUSG00000043773.5	ENSMUSG00000043773.5	ENSMUSG00000043773	lincRNA	1700048O20Rik	13.3965757	1.29659816	3.294133159	1.1590136	2.8421867	0.0044805	0.0227448
ENSMUSG00000085992.1	ENSMUSG00000085992.1	ENSMUSG00000085992	lincRNA	Gm11515	5.742168202	0	4.880885782	1.7536191	2.7833216	0.0053805	0.0266175
ENSMUSG00000078713.8	ENSMUSG00000078713.8	ENSMUSG00000078713	protein_coding	Tomm5	154.9406533	349.3885925	-1.172098347	0.2245836	-5.218984	1.799E-07	2.589E-06
ENSMUSG00000020681.14	ENSMUSG00000020681.14	ENSMUSG00000020681	protein_coding	Ace	39.88478587	14.00834296	1.503382132	0.4309195	3.4887776	0.0004852	0.0033331
ENSMUSG00000006777.7	ENSMUSG00000006777.7	ENSMUSG00000006777	protein_coding	Krt23	0	96.12798367	-9.135716997	1.2203184	-7.486339	7.082E-14	2.268E-12
ENSMUSG00000027849.18	ENSMUSG00000027849.18	ENSMUSG00000027849	protein_coding	Syt6	11.40344593	1.481264514	2.989788695	1.0330316	2.8941891	0.0038014	0.0197365
ENSMUSG00000033510.14	ENSMUSG00000033510.14	ENSMUSG00000033510	protein_coding	Otud7a	5.894103265	0	4.913673787	1.6682993	2.9453191	0.0032262	0.0172096
ENSMUSG00000022641.15	ENSMUSG00000022641.15	ENSMUSG00000022641	protein_coding	Bbx	1542.68509	576.9393394	1.420003973	0.1123223	12.642223	1.235E-36	1.435E-34
ENSMUSG00000025584.17	ENSMUSG00000025584.17	ENSMUSG00000025584	protein_coding	Pde8a	46.88412712	21.71211866	1.117267771	0.3907115	2.8595723	0.0042421	0.021692
ENSMUSG00000037025.11	ENSMUSG00000037025.11	ENSMUSG00000037025	protein_coding	Foxa2	217.7282765	78.74310691	1.465493696	0.1944946	7.5348825	4.888E-14	1.593E-12
ENSMUSG00000044322.7	ENSMUSG00000044322.7	ENSMUSG00000044322	protein_coding	Dsc1	0	8.474869242	-5.63078074	1.5393418	-3.657915	0.0002543	0.0018999
ENSMUSG00000034612.7	ENSMUSG00000034612.7	ENSMUSG00000034612	protein_coding	Chst11	99.80723561	19.15050175	2.383022459	0.3382273	7.0456244	1.846E-12	5.185E-11
ENSMUSG00000047712.8	ENSMUSG00000047712.8	ENSMUSG00000047712	protein_coding	Ust	56.48718292	11.72928849	2.268485508	0.4518603	5.0203251	5.158E-07	7.024E-06
ENSMUSG00000050578.10	ENSMUSG00000050578.10	ENSMUSG00000050578	protein_coding	Mmp13	315.9249124	116.6046962	1.43801394	0.2172212	6.6200438	3.591E-11	8.651E-10
ENSMUSG00000108926.1	ENSMUSG00000108926.1	ENSMUSG00000108926	processed_pseudogene	Gm10988	0	5.059833304	-4.882314394	1.8538514	-2.633606	0.0084483	0.0384031
ENSMUSG00000033377.14	ENSMUSG00000033377.14	ENSMUSG00000033377	protein_coding	Palmd	81.06090042	34.55710383	1.224330213	0.3154926	3.8806935	0.0001042	0.0008617
ENSMUSG00000075391.6	ENSMUSG00000075391.6	ENSMUSG00000075391	processed_pseudogene	Gm13443	107.3641466	0.735069349	7.244780335	1.0605749	6.8309933	8.433E-12	2.185E-10
ENSMUSG00000044258.10	ENSMUSG00000044258.10	ENSMUSG00000044258	protein_coding	Ctla2a	116.7234435	1204.657461	-3.366977719	0.2101668	-16.0205	9.191E-58	2.326E-55
ENSMUSG00000026470.14	ENSMUSG00000026470.14	ENSMUSG00000026470	protein_coding	Stx6	226.2563179	501.9896134	-1.148756423	0.3184673	-3.607141	0.0003096	0.0022593
ENSMUSG00000096269.3	ENSMUSG00000096269.3	ENSMUSG00000096269	processed_pseudogene	Rpl31-ps21	24.25342447	1.481264514	4.083313814	0.9160179	4.457679	8.285E-06	8.885E-05
ENSMUSG00000014846.12	ENSMUSG00000014846.12	ENSMUSG00000014846	protein_coding	Tppp3	31.99862393	242.4427458	-2.917173028	0.2611456	-11.17067	5.675E-29	4.64E-27
ENSMUSG00000049946.7	ENSMUSG00000049946.7	ENSMUSG00000049946	protein_coding	BC030500	12.66121012	1.49239033	3.12552665	1.2039143	2.5961372	0.0094278	0.041998
ENSMUSG00000036192.15	ENSMUSG00000036192.15	ENSMUSG00000036192	protein_coding	Rorb	9.342596046	23.46302176	-1.330842267	0.5084521	-2.617439	0.0088592	0.0399241
ENSMUSG00000058656.12	ENSMUSG00000058656.12	ENSMUSG00000058656	protein_coding	Samd12	71.15693021	190.0432296	-1.416089691	0.2194039	-6.454258	1.088E-10	2.457E-09
ENSMUSG00000021998.16	ENSMUSG00000021998.16	ENSMUSG00000021998	protein_coding	Lcp1	699.8522596	10.64781797	6.043444096	0.3158958	19.131132	1.39E-81	5.838E-79
ENSMUSG00000058260.2	ENSMUSG00000058260.2	ENSMUSG00000058260	protein_coding	Serpina9	11.93847936	1.29659816	3.135837255	1.2192627	2.5719127	0.0101138	0.0444855
ENSMUSG00000033871.14	ENSMUSG00000033871.14	ENSMUSG00000033871	protein_coding	Ppargc1b	219.1831039	99.69834281	1.134091744	0.2022674	5.6068935	2.06E-08	3.486E-07
ENSMUSG00000027199.14	ENSMUSG00000027199.14	ENSMUSG00000027199	protein_coding	Gatm	353.4077369	3924.652631	-3.472192599	0.0824265	-42.12469	0	0
ENSMUSG00000037010.7	ENSMUSG00000037010.7	ENSMUSG00000037010	protein_coding	Apln	845.835209	218.1453988	1.954977436	0.1090278	17.930994	6.756E-72	2.27E-69
ENSMUSG00000029816.10	ENSMUSG00000029816.10	ENSMUSG00000029816	protein_coding	Gpnmb	5.21816295	30.73294612	-2.551603875	0.5753143	-4.435148	9.201E-06	9.76E-05
ENSMUSG00000022114.4	ENSMUSG00000022114.4	ENSMUSG00000022114	protein_coding	Spry2	409.3142783	1612.231235	-1.978757321	0.0964439	-20.51718	1.512E-93	9.014E-91
ENSMUSG00000032549.7	ENSMUSG00000032549.7	ENSMUSG00000032549	protein_coding	Rab6b	22.23609616	144.3061221	-2.6977372	0.2755617	-9.789958	1.243E-22	7.341E-21
ENSMUSG00000029576.17	ENSMUSG00000029576.17	ENSMUSG00000029576	protein_coding	Radil	54.86392555	15.19147444	1.837556041	0.4285925	4.2874203	1.808E-05	0.0001806
ENSMUSG00000022900.14	ENSMUSG00000022900.14	ENSMUSG00000022900	protein_coding	Ildr1	138.5609227	57.84573634	1.261176528	0.2542701	4.9599877	7.05E-07	9.305E-06
ENSMUSG00000060568.14	ENSMUSG00000060568.14	ENSMUSG00000060568	protein_coding	Fam78b	17.41532863	55.02910212	-1.666416617	0.418742	-3.979578	6.904E-05	0.0005961
ENSMUSG00000103088.1	ENSMUSG00000103088.1	ENSMUSG00000103088	protein_coding	Pcdhgb6	57.32536779	268.6460046	-2.223342117	0.4378447	-5.077924	3.816E-07	5.266E-06
ENSMUSG00000086537.7	ENSMUSG00000086537.7	ENSMUSG00000086537	antisense_RNA	Nespas	19.60445058	43.61087051	-1.159103137	0.3697116	-3.135155	0.0017176	0.0100565
ENSMUSG00000082361.6	ENSMUSG00000082361.6	ENSMUSG00000082361	protein_coding	Btc	193.4061164	402.2341368	-1.05732069	0.3595685	-2.940527	0.0032765	0.0174363
ENSMUSG00000047238.4	ENSMUSG00000047238.4	ENSMUSG00000047238	protein_coding	Mageh1	39.4406	7.18383399	2.471933967	0.5009211	4.9347773	8.024E-07	1.044E-05
ENSMUSG00000079620.13	ENSMUSG00000079620.13	ENSMUSG00000079620	protein_coding	Muc4	854.5866325	340.2903503	1.329170804	0.2701759	4.9196493	8.67E-07	1.121E-05
ENSMUSG00000042195.9	ENSMUSG00000042195.9	ENSMUSG00000042195	protein_coding	Slc35f2	359.5576983	160.7212976	1.16188153	0.1394247	8.3333969	7.856E-17	3.262E-15
ENSMUSG00000065954.11	ENSMUSG00000065954.11	ENSMUSG00000065954	protein_coding	Tacc1	117.6953835	41.15715738	1.512818825	0.2403072	6.2953532	3.067E-10	6.567E-09
ENSMUSG00000091387.2	ENSMUSG00000091387.2	ENSMUSG00000091387	protein_coding	Gcnt4	778.6436316	259.4131344	1.585443014	0.1238111	12.80534	1.531E-37	1.861E-35
ENSMUSG00000076438.5	ENSMUSG00000076438.5	ENSMUSG00000076438	protein_coding	Oxct2b	3.995089934	26.46337302	-2.71834787	0.6359791	-4.274272	1.918E-05	0.0001907
ENSMUSG00000029661.16	ENSMUSG00000029661.16	ENSMUSG00000029661	protein_coding	Col1a2	107.6399582	2.589431446	5.435462773	0.620183	8.7642882	1.88E-18	8.683E-17
ENSMUSG00000026885.13	ENSMUSG00000026885.13	ENSMUSG00000026885	protein_coding	Ttll11	85.82390492	23.9377803	1.83956272	0.2968276	6.1974108	5.74E-10	1.186E-08
ENSMUSG00000037129.7	ENSMUSG00000037129.7	ENSMUSG00000037129	protein_coding	Tmprss13	16.94855086	3.897155422	2.123801544	0.7063707	3.0066387	0.0026415	0.0145877
ENSMUSG00000032023.7	ENSMUSG00000032023.7	ENSMUSG00000032023	protein_coding	Jhy	0	10.94971588	-6.000374766	1.5117458	-3.969169	7.212E-05	0.0006199
ENSMUSG00000030313.15	ENSMUSG00000030313.15	ENSMUSG00000030313	protein_coding	Dennd5b	87.53575647	189.8168073	-1.113824078	0.1848527	-6.025468	1.686E-09	3.287E-08
ENSMUSG00000112148.1	ENSMUSG00000112148.1	ENSMUSG00000112148	protein_coding	Lilrb4a	10.07742608	0.373097582	4.722684971	1.5175855	3.1119729	0.0018584	0.0107278
ENSMUSG00000032419.8	ENSMUSG00000032419.8	ENSMUSG00000032419	protein_coding	Tbx18	9.644803935	0.97244862	3.230138562	1.2760292	2.5313986	0.0113609	0.0491055
ENSMUSG00000047604.3	ENSMUSG00000047604.3	ENSMUSG00000047604	protein_coding	Frat2	125.3156909	59.18056722	1.080008782	0.2599712	4.1543398	3.262E-05	0.0003048
ENSMUSG00000021567.15	ENSMUSG00000021567.15	ENSMUSG00000021567	protein_coding	Nkd2	102.0426125	23.2679095	2.137441161	0.4212939	5.0735155	3.905E-07	5.385E-06
ENSMUSG00000006360.11	ENSMUSG00000006360.11	ENSMUSG00000006360	protein_coding	Crip1	521.7287164	124.5594549	2.068655043	0.1994329	10.372687	3.301E-25	2.285E-23
ENSMUSG00000044165.12	ENSMUSG00000044165.12	ENSMUSG00000044165	protein_coding	Bcl2l15	1128.572133	3038.628631	-1.42920996	0.0741946	-19.26298	1.099E-82	4.951E-80
ENSMUSG00000029646.3	ENSMUSG00000029646.3	ENSMUSG00000029646	protein_coding	Cdx2	38.39419614	14.76010103	1.374684892	0.410528	3.3485779	0.0008123	0.0052315
ENSMUSG00000024530.7	ENSMUSG00000024530.7	ENSMUSG00000024530	protein_coding	Prelid3a	20.11801879	47.0599638	-1.234365031	0.3953171	-3.122468	0.0017934	0.0104079
ENSMUSG00000037188.7	ENSMUSG00000037188.7	ENSMUSG00000037188	protein_coding	Grhl3	1.781769442	19.66196439	-3.425074032	0.8545984	-4.007817	6.128E-05	0.0005374
ENSMUSG00000036782.13	ENSMUSG00000036782.13	ENSMUSG00000036782	protein_coding	Klhl13	55.43365016	4.183482735	3.715282993	0.6171168	6.020389	1.74E-09	3.384E-08
ENSMUSG00000027399.1	ENSMUSG00000027399.1	ENSMUSG00000027399	protein_coding	Il1a	29.24925451	181.02488	-2.634311881	0.244823	-10.76007	5.313E-27	4.024E-25
ENSMUSG00000006488.17	ENSMUSG00000006488.17	ENSMUSG00000006488	protein_coding	Prl7a1	0	9.620858401	-5.811810062	1.5137928	-3.839238	0.0001234	0.0010042
ENSMUSG00000063558.4	ENSMUSG00000063558.4	ENSMUSG00000063558	protein_coding	Aox1	315.9745638	103.5836648	1.60618162	0.2098873	7.6525903	1.97E-14	6.654E-13
ENSMUSG00000066705.7	ENSMUSG00000066705.7	ENSMUSG00000066705	protein_coding	Fxyd6	5.47411594	22.67344146	-2.03599694	0.5949893	-3.421905	0.0006218	0.0041258
ENSMUSG00000067017.5	ENSMUSG00000067017.5	ENSMUSG00000067017	pseudogene	Gm3608	658.0772556	11.56575564	5.819148106	0.2778832	20.940986	2.267E-97	1.676E-94
ENSMUSG00000061353.11	ENSMUSG00000061353.11	ENSMUSG00000061353	protein_coding	Cxcl12	233.3133666	20.37281527	3.530807684	0.4344783	8.1265457	4.417E-16	1.722E-14
ENSMUSG00000015093.15	ENSMUSG00000015093.15	ENSMUSG00000015093	protein_coding	Clic3	68.77235163	261.4332379	-1.924492903	0.1792774	-10.73472	6.993E-27	5.274E-25
ENSMUSG00000086784.2	ENSMUSG00000086784.2	ENSMUSG00000086784	protein_coding	Isoc2a	13.69819244	0	6.133648185	1.4627963	4.1930981	2.752E-05	0.0002633
ENSMUSG00000047996.16	ENSMUSG00000047996.16	ENSMUSG00000047996	protein_coding	Prrg1	88.55293582	22.13972719	2.007262494	0.3147624	6.3770726	1.805E-10	3.938E-09
ENSMUSG00000033278.10	ENSMUSG00000033278.10	ENSMUSG00000033278	protein_coding	Ptprm	992.9865894	104.3201217	3.251190887	0.1337853	24.301547	1.89E-130	2.33E-127
ENSMUSG00000074272.10	ENSMUSG00000074272.10	ENSMUSG00000074272	protein_coding	Ceacam1	431.0459105	91.90779684	2.225104426	0.1860464	11.95994	5.76E-33	5.602E-31
ENSMUSG00000023092.16	ENSMUSG00000023092.16	ENSMUSG00000023092	protein_coding	Fhl1	223.2655502	453.1041332	-1.02140655	0.2098914	-4.866358	1.137E-06	1.444E-05
ENSMUSG00000029223.12	ENSMUSG00000029223.12	ENSMUSG00000029223	protein_coding	Uchl1	262.3448898	702.8801958	-1.420595317	0.1846401	-7.693862	1.428E-14	4.849E-13
ENSMUSG00000016552.13	ENSMUSG00000016552.13	ENSMUSG00000016552	protein_coding	Foxred2	47.0487526	96.21098907	-1.033616259	0.2429557	-4.25434	2.097E-05	0.0002063
ENSMUSG00000078937.8	ENSMUSG00000078937.8	ENSMUSG00000078937	protein_coding	Cpt1b	13.9663003	67.70929524	-2.278877689	0.6414315	-3.5528	0.0003812	0.0026995
ENSMUSG00000042042.14	ENSMUSG00000042042.14	ENSMUSG00000042042	protein_coding	Csgalnact2	1139.430595	2428.977802	-1.091838835	0.0962377	-11.34523	7.832E-30	6.7E-28
ENSMUSG00000079451.9	ENSMUSG00000079451.9	ENSMUSG00000079451	protein_coding	Tmprss11g	5.252854125	0	4.748433337	1.8288468	2.5964084	0.0094204	0.0419807

ENSMUSG00000049593.8	ENSMUSG00000049593.8	ENSMUSG00000049593	protein_coding	Lce1h	68.73001235	32.26204055	1.089579239	0.3171528	3.4355028	0.0005915	0.0039497
ENSMUSG00000102748.1	ENSMUSG00000102748.1	ENSMUSG00000102748	protein_coding	Pcdhgb2	36.76092609	116.7707474	-1.665086033	0.6355862	-2.619764	0.0087991	0.0397139
ENSMUSG00000034121.12	ENSMUSG00000034121.12	ENSMUSG00000034121	protein_coding	Mks1	97.63850778	45.24830694	1.104256625	0.2581083	4.2782686	1.884E-05	0.0001876
ENSMUSG00000060487.7	ENSMUSG00000060487.7	ENSMUSG00000060487	protein_coding	Samd5	611.357274	4511.471977	-2.884275202	0.072203	-39.94676	0	0
ENSMUSG00000037636.6	ENSMUSG00000037636.6	ENSMUSG00000037636	protein_coding	Slc25a43	52.21851846	25.26843582	1.051920585	0.3398651	3.0951119	0.0019674	0.0112794
ENSMUSG00000042812.5	ENSMUSG00000042812.5	ENSMUSG00000042812	protein_coding	Foxf1	233.3509023	7.556931573	4.970660939	0.3664718	13.563557	6.586E-42	9.583E-40
ENSMUSG00000023827.8	ENSMUSG00000023827.8	ENSMUSG00000023827	protein_coding	Agpat4	81.74460003	397.7186269	-2.277861897	0.1713668	-13.29232	2.565E-40	3.537E-38
ENSMUSG00000073158.4	ENSMUSG00000073158.4	ENSMUSG00000073158	protein_coding	9030624G23Rik	12.73718582	0	6.023049539	2.3356856	2.5787073	0.0099171	0.0437973
ENSMUSG00000048231.14	ENSMUSG00000048231.14	ENSMUSG00000048231	protein_coding	H2-M10.4	0	11.25273192	-6.04074507	1.4583338	-4.142224	3.44E-05	0.0003197
ENSMUSG00000073821.11	ENSMUSG00000073821.11	ENSMUSG00000073821	processed_transcript	8030451A03Rik	153.9066659	30.1658544	2.354250185	0.2722873	8.6461983	5.324E-18	2.394E-16
ENSMUSG00000039936.18	ENSMUSG00000039936.18	ENSMUSG00000039936	protein_coding	Pik3cd	16.79827803	77.2618424	-2.206222616	0.3505315	-6.293936	3.095E-10	6.619E-09
ENSMUSG00000021676.9	ENSMUSG00000021676.9	ENSMUSG00000021676	protein_coding	Iqgap2	417.8694699	52.6025965	2.997998991	0.2140983	14.002911	1.496E-44	2.468E-42
ENSMUSG00000021751.13	ENSMUSG00000021751.13	ENSMUSG00000021751	protein_coding	Acox2	198.2637009	93.21063781	1.087138878	0.1879608	5.7838608	7.301E-09	1.312E-07
ENSMUSG00000032890.17	ENSMUSG00000032890.17	ENSMUSG00000032890	protein_coding	Rims3	8.095860033	45.65742871	-2.504412545	0.4821713	-5.194031	2.058E-07	2.943E-06
ENSMUSG00000039395.8	ENSMUSG00000039395.8	ENSMUSG00000039395	protein_coding	Mreg	23.78557561	87.26136129	-1.874195963	0.286741	-6.536197	6.31E-11	1.467E-09
ENSMUSG00000035390.16	ENSMUSG00000035390.16	ENSMUSG00000035390	protein_coding	Brsk1	108.5267232	270.7975504	-1.317928792	0.1605562	-8.208519	2.239E-16	9.043E-15
ENSMUSG00000075302.10	ENSMUSG00000075302.10	ENSMUSG00000075302	protein_coding	Erich2	88.20511977	32.05512256	1.455876272	0.2982098	4.8820543	1.05E-06	1.345E-05
ENSMUSG00000004105.8	ENSMUSG00000004105.8	ENSMUSG00000004105	protein_coding	Angptl2	384.3862421	66.81358147	2.520738576	0.1766994	14.265686	3.581E-46	6.242E-44
ENSMUSG00000091962.2	ENSMUSG00000091962.2	ENSMUSG00000091962	protein_coding	Vmn2r78	0	4.864041134	-4.833133746	1.8035293	-2.67982	0.0073662	0.0344666
ENSMUSG00000070509.15	ENSMUSG00000070509.15	ENSMUSG00000070509	protein_coding	Rgma	34.77509202	95.76180881	-1.469708575	0.3657994	-4.017799	5.874E-05	0.0005171
ENSMUSG00000031327.10	ENSMUSG00000031327.10	ENSMUSG00000031327	protein_coding	Chic1	61.11746419	14.14962418	2.1120041	0.3821501	5.5266347	3.264E-08	5.371E-07
ENSMUSG00000026483.13	ENSMUSG00000026483.13	ENSMUSG00000026483	protein_coding	Fam129a	2697.171113	364.4683255	2.887216439	0.0931107	31.008427	4.15E-211	1.1E-207
ENSMUSG00000029019.8	ENSMUSG00000029019.8	ENSMUSG00000029019	protein_coding	Nppb	27.59516599	118.2786679	-2.099805878	0.2695583	-7.789804	6.711E-15	2.344E-13
ENSMUSG00000101362.1	ENSMUSG00000101362.1	ENSMUSG00000101362	unprocessed_pseudogene	Mrgprb9-ps	30.74145088	196.6275437	-2.679724103	0.2373292	-11.29117	1.451E-29	1.224E-27
ENSMUSG00000009545.14	ENSMUSG00000009545.14	ENSMUSG00000009545	protein_coding	Kcnq1	193.197473	44.04404195	2.131235479	0.2169448	9.8238589	8.887E-23	5.315E-21
ENSMUSG00000043165.7	ENSMUSG00000043165.7	ENSMUSG00000043165	protein_coding	Lor	23.66886726	49.1895274	-1.050817572	0.337505	-3.113488	0.0018489	0.0106763
ENSMUSG00000037169.15	ENSMUSG00000037169.15	ENSMUSG00000037169	protein_coding	Mycn	34.5851413	81.81662468	-1.243330299	0.3073891	-4.044809	5.237E-05	0.0004661
ENSMUSG00000030074.9	ENSMUSG00000030074.9	ENSMUSG00000030074	protein_coding	Gxylt2	62.7577356	25.80883112	1.284992895	0.3574754	3.5946331	0.0003248	0.0023549
ENSMUSG00000054453.11	ENSMUSG00000054453.11	ENSMUSG00000054453	protein_coding	Sytl5	5.393225464	98.16324725	-4.206351165	0.5601717	-7.509039	5.956E-14	1.921E-12
ENSMUSG00000061601.15	ENSMUSG00000061601.15	ENSMUSG00000061601	protein_coding	Pclo	8.655092006	111.3128777	-3.682772915	0.3872984	-9.508878	1.927E-21	1.047E-19
ENSMUSG00000050425.2	ENSMUSG00000050425.2	ENSMUSG00000050425	protein_coding	Mrgprb2	20.16148476	276.7920101	-3.767351615	0.2612868	-14.41846	3.961E-47	7.176E-45
ENSMUSG00000039376.13	ENSMUSG00000039376.13	ENSMUSG00000039376	protein_coding	Synpo2l	60.78222736	28.76288108	1.074098365	0.3311105	3.2439269	0.0011789	0.0072446
ENSMUSG00000057899.6	ENSMUSG00000057899.6	ENSMUSG00000057899	protein_coding	Adgrf2	2.527627653	14.17187581	-2.501290203	0.9157131	-2.731522	0.0063043	0.0302808
ENSMUSG00000022575.4	ENSMUSG00000022575.4	ENSMUSG00000022575	protein_coding	Gsdmd	137.5578736	15.14612246	3.199225079	0.362154	8.833881	1.011E-18	4.803E-17
ENSMUSG00000001348.15	ENSMUSG00000001348.15	ENSMUSG00000001348	protein_coding	Acp5	26.94122644	0	7.108838031	1.310254	5.4255419	5.778E-08	9.079E-07
ENSMUSG00000020467.15	ENSMUSG00000020467.15	ENSMUSG00000020467	protein_coding	Efemp1	774.110066	2652.186174	-1.7770752	0.1110656	-16.00022	1.273E-57	3.137E-55
ENSMUSG00000066720.6	ENSMUSG00000066720.6	ENSMUSG00000066720	protein_coding	Cldn9	427.4907057	134.3399807	1.667487517	0.1480634	11.26198	2.022E-29	1.683E-27
ENSMUSG00000002233.13	ENSMUSG00000002233.13	ENSMUSG00000002233	protein_coding	Rhoc	681.2585658	1493.502264	-1.132396439	0.102225	-11.07749	1.613E-28	1.279E-26
ENSMUSG00000021364.16	ENSMUSG00000021364.16	ENSMUSG00000021364	protein_coding	Elovl2	0	4.259127188	-4.63700759	1.8356218	-2.526124	0.0115329	0.0496748
ENSMUSG00000074457.10	ENSMUSG00000074457.10	ENSMUSG00000074457	protein_coding	S100a16	975.4825881	458.3463561	1.088710404	0.1574565	6.9143558	4.7E-12	1.26E-10
ENSMUSG00000024059.9	ENSMUSG00000024059.9	ENSMUSG00000024059	protein_coding	Clip4	293.0200212	861.9183073	-1.556160704	0.1100531	-14.14009	2.15E-45	3.645E-43
ENSMUSG00000072964.14	ENSMUSG00000072964.14	ENSMUSG00000072964	protein_coding	Bhlhb9	30.33136504	11.30167996	1.427085767	0.4704561	3.0334092	0.0024181	0.0135439
ENSMUSG00000028527.18	ENSMUSG00000028527.18	ENSMUSG00000028527	protein_coding	Ak4	162.9275087	754.1000725	-2.210512917	0.6632326	-3.332938	0.0008593	0.0054925
ENSMUSG00000028525.16	ENSMUSG00000028525.16	ENSMUSG00000028525	protein_coding	Pde4b	13.10373375	67.43209917	-2.350552272	0.3817473	-6.157351	7.397E-10	1.512E-08
ENSMUSG00000097867.1	ENSMUSG00000097867.1	ENSMUSG00000097867	lincRNA	Lppos	111.9059449	51.82790689	1.112332816	0.2415492	4.6049952	4.125E-06	4.696E-05
ENSMUSG00000085068.1	ENSMUSG00000085068.1	ENSMUSG00000085068	antisense_RNA	Gm15895	24.4262892	5.966645158	2.027618197	0.56525	3.5871176	0.0003344	0.0024105
ENSMUSG00000050730.17	ENSMUSG00000050730.17	ENSMUSG00000050730	protein_coding	Arhgap42	1089.853362	377.8072358	1.528406053	0.1143135	13.370307	9.017E-41	1.253E-38
ENSMUSG00000036019.8	ENSMUSG00000036019.8	ENSMUSG00000036019	protein_coding	Tmtc2	47.30138111	134.1838087	-1.498410839	0.2719737	-5.509395	3.601E-08	5.883E-07
ENSMUSG00000109508.1	ENSMUSG00000109508.1	ENSMUSG00000109508	lincRNA	Gm44956	55.37631148	25.07640852	1.13668417	0.3754218	3.027752	0.0024638	0.0137666
ENSMUSG00000007888.15	ENSMUSG00000007888.15	ENSMUSG00000007888	protein_coding	Crlf1	179.9390153	84.42830776	1.090891159	0.2107123	5.1771597	2.253E-07	3.207E-06
ENSMUSG00000024987.5	ENSMUSG00000024987.5	ENSMUSG00000024987	protein_coding	Cyp26a1	9.527560045	0	5.608447707	1.534317	3.6553383	0.0002568	0.0019173
ENSMUSG00000067212.8	ENSMUSG00000067212.8	ENSMUSG00000067212	protein_coding	H2-T23	80.61612341	34.03339725	1.242606035	0.3129482	3.9706448	7.168E-05	0.0006166
ENSMUSG00000028402.18	ENSMUSG00000028402.18	ENSMUSG00000028402	protein_coding	Mpdz	732.1849584	1641.331824	-1.164369053	0.077492	-15.02566	4.986E-51	1.059E-48
ENSMUSG00000064443.1	ENSMUSG00000064443.1	ENSMUSG00000064443	snRNA	Gm26226	10.75116861	2.08617846	2.353041948	0.9015486	2.6100001	0.0090542	0.0406373
ENSMUSG00000038910.6	ENSMUSG00000038910.6	ENSMUSG00000038910	protein_coding	Plcl2	10.16827364	65.12275222	-2.676607467	0.4188362	-6.390584	1.653E-10	3.635E-09
ENSMUSG00000060594.6	ENSMUSG00000060594.6	ENSMUSG00000060594	protein_coding	Layn	38.94132884	157.0611792	-2.013795994	0.313761	-6.418247	1.379E-10	3.073E-09
ENSMUSG00000051341.5	ENSMUSG00000051341.5	ENSMUSG00000051341	protein_coding	Zfp52	332.6202044	1236.114373	-1.892789623	0.1046115	-18.09352	3.585E-73	1.25E-70
ENSMUSG00000009628.14	ENSMUSG00000009628.14	ENSMUSG00000009628	protein_coding	Tex15	31.78181328	104.3446096	-1.723196245	0.429179	-4.015099	5.942E-05	0.0005228
ENSMUSG00000061527.6	ENSMUSG00000061527.6	ENSMUSG00000061527	protein_coding	Krt5	10.67905293	0	5.774002267	2.1497753	2.6858632	0.0072343	0.0339362
ENSMUSG00000009097.9	ENSMUSG00000009097.9	ENSMUSG00000009097	protein_coding	Tbx1	50.09925881	0	8.002966341	1.2510745	6.3968741	1.586E-10	3.505E-09
ENSMUSG00000035273.14	ENSMUSG00000035273.14	ENSMUSG00000035273	protein_coding	Hpse	59.91692748	272.5829213	-2.181819724	0.2046513	-10.66116	1.547E-26	1.143E-24
ENSMUSG00000024678.6	ENSMUSG00000024678.6	ENSMUSG00000024678	protein_coding	Ms4a4d	2.072949153	30.13247695	-3.814361722	0.7445417	-5.1231	3.006E-07	4.214E-06
ENSMUSG00000085956.1	ENSMUSG00000085956.1	ENSMUSG00000085956	processed_transcript	4930481B07Rik	55.23546019	21.9272463	1.334130643	0.3402519	3.9210081	8.818E-05	0.000745
ENSMUSG00000074280.6	ENSMUSG00000074280.6	ENSMUSG00000074280	processed_pseudogene	Gm6166	1024.105297	2.404765092	8.693895379	0.5639514	15.416038	1.277E-53	2.917E-51
ENSMUSG00000073197.9	ENSMUSG00000073197.9	ENSMUSG00000073197	protein_coding	5730507C01Rik	43.55020049	10.59886993	2.040817524	0.4605456	4.4313043	9.366E-06	9.924E-05
ENSMUSG00000114291.1	ENSMUSG00000114291.1	ENSMUSG00000114291	antisense_RNA	AC139750.1	7.74632615	0	5.30812458	1.5602766	3.4020408	0.0006688	0.0043966
ENSMUSG00000029371.7	ENSMUSG00000029371.7	ENSMUSG00000029371	protein_coding	Cxcl5	1572.409713	578.4518908	1.443026234	0.3108658	4.6419588	3.451E-06	3.983E-05
ENSMUSG00000022817.14	ENSMUSG00000022817.14	ENSMUSG00000022817	protein_coding	Itgb5	1817.373639	770.0630776	1.238980206	0.1324044	9.3575473	8.161E-21	4.346E-19
ENSMUSG00000036353.13	ENSMUSG00000036353.13	ENSMUSG00000036353	protein_coding	P2ry12	1283.018103	347.8018016	1.882855048	0.1775191	10.606491	2.78E-26	2.03E-24
ENSMUSG00000025494.8	ENSMUSG00000025494.8	ENSMUSG00000025494	protein_coding	Sigirr	328.0637795	151.3713647	1.114998566	0.2041303	5.4621913	4.703E-08	7.504E-07
ENSMUSG00000028591.11	ENSMUSG00000028591.11	ENSMUSG00000028591	protein_coding	Pramef12	5.195035501	0	4.734590658	1.7476257	2.709156	0.0067455	0.0319597
ENSMUSG00000044976.17	ENSMUSG00000044976.17	ENSMUSG00000044976	protein_coding	Wdr72	38.12174831	9.692058075	1.988239139	0.5423095	3.6662443	0.0002461	0.0018487
ENSMUSG00000063193.8	ENSMUSG00000063193.8	ENSMUSG00000063193	protein_coding	Cd300lb	89.51518032	3.501806208	4.665541908	0.573231	8.1390256	3.985E-16	1.563E-14
ENSMUSG00000049411.14	ENSMUSG00000049411.14	ENSMUSG00000049411	protein_coding	Tmem241	73.81556326	31.20838456	1.2432451	0.29639	4.1946263	2.733E-05	0.0002617
ENSMUSG00000042097.17	ENSMUSG00000042097.17	ENSMUSG00000042097	protein_coding	Zfp239	54.94309819	116.9276998	-1.086761947	0.3638173	-2.987109	0.0028163	0.0153466
ENSMUSG00000027456.8	ENSMUSG00000027456.8	ENSMUSG00000027456	protein_coding	Sdcbp2	1489.493159	3019.172019	-1.019034386	0.0940688	-10.83286	2.405E-27	1.852E-25
ENSMUSG00000059751.7	ENSMUSG00000059751.7	ENSMUSG00000059751	processed_pseudogene	Rps3a3	1662.515095	14.91250807	6.806022465	0.2676917	25.424857	1.34E-142	2.06E-139
ENSMUSG00000071042.11	ENSMUSG00000071042.11	ENSMUSG00000071042	protein_coding	Rasgrp3	188.4130033	54.81088951	1.782135474	0.1998793	8.9160588	4.832E-19	2.337E-17
ENSMUSG00000038587.9	ENSMUSG00000038587.9	ENSMUSG00000038587	protein_coding	Akap12	567.112842	2375.251792	-2.067744496	0.1085352	-19.05137	6.401E-81	2.628E-78
ENSMUSG00000019796.12	ENSMUSG00000019796.12	ENSMUSG00000019796	protein_coding	Lrp11	465.5509095	218.8290422	1.089504337	0.2232521	4.8801521	1.06E-06	1.357E-05
ENSMUSG00000067916.9	ENSMUSG00000067916.9	ENSMUSG00000067916	protein_coding	Zfp991	13.26723254	46.85860873	-1.819559048	0.4000593	-4.548224	5.41E-06	6.033E-05
ENSMUSG00000039057.11	ENSMUSG00000039057.11	ENSMUSG00000039057	protein_coding	Myo16	4.065543377	384.1362632	-6.577021502	0.4283849	-15.35307	3.378E-53	7.613E-51
ENSMUSG00000022580.12	ENSMUSG00000022580.12	ENSMUSG00000022580	protein_coding	Rhpn1	3.831591146	18.80230255	-2.271805972	0.9002502	-2.523527	0.0116184	0.0499616
ENSMUSG00000029375.6	ENSMUSG00000029375.6	ENSMUSG00000029375	protein_coding	Cxcl15	40.36205627	3.415035938	3.529246428	0.6617175	5.3334637	9.636E-08	1.458E-06
ENSMUSG00000023930.13	ENSMUSG00000023930.13	ENSMUSG00000023930	protein_coding	Crisp2	0	4.858478226	-4.831758246	1.8032326	-2.679498	0.0073733	0.0344832
ENSMUSG00000034245.10	ENSMUSG00000034245.10	ENSMUSG00000034245	protein_coding	Hdac11	123.6279823	304.703092	-1.303073858	0.3587234	-3.632531	0.0002807	0.002076
ENSMUSG00000026893.4	ENSMUSG00000026893.4	ENSMUSG00000026893	protein_coding	Gca	693.8816818	1491.581038	-1.104715233	0.0777352	-14.21127	7.8E-46	1.334E-43
ENSMUSG00000046169.10	ENSMUSG00000046169.10	ENSMUSG00000046169	protein_coding	Adamts6	242.8096156	581.1080504	-1.259300225	0.2412102	-5.220758	1.782E-07	2.566E-06
ENSMUSG00000035930.9	ENSMUSG00000035930.9	ENSMUSG00000035930	protein_coding	Chst4	2.783580643	39.6313896	-3.832341397	0.70115	-5.465794	4.608E-08	7.379E-07
ENSMUSG00000079481.11	ENSMUSG00000079481.11	ENSMUSG00000079481	protein_coding	Nhsl2	5.579796105	66.99336482	-3.587096314	0.4645095	-7.722331	1.142E-14	3.901E-13
ENSMUSG00000011256.16	ENSMUSG00000011256.16	ENSMUSG00000011256	protein_coding	Adam19	395.9977959	187.6965715	1.076447821	0.1459566	7.3751248	1.642E-13	5.015E-12
ENSMUSG00000072774.10	ENSMUSG00000072774.10	ENSMUSG00000072774	protein_coding	Zfp951	263.3800595	95.65237642	1.463210711	0.1727549	8.4698628	2.457E-17	1.058E-15
ENSMUSG00000018008.7	ENSMUSG00000018008.7	ENSMUSG00000018008	protein_coding	Cyth4	317.9022105	35.07592741	3.173508607	0.2177354	14.57507	4.047E-48	7.872E-46
ENSMUSG00000040562.12	ENSMUSG00000040562.12	ENSMUSG00000040562	protein_coding	Gstm2	65.13675266	21.31232467	1.616083033	0.3311741	4.8798587	1.062E-06	1.358E-05
ENSMUSG00000032013.6	ENSMUSG00000032013.6	ENSMUSG00000032013	protein_coding	Trim29	22.07425961	51.50932025	-1.223744097	0.3400407	-3.598817	0.0003197	0.002321
ENSMUSG00000071656.6	ENSMUSG00000071656.6	ENSMUSG00000071656	protein_coding	Lrrn4cl	28.78354783	107.6577151	-1.90502476	0.2691702	-7.0774	1.469E-12	4.168E-11
ENSMUSG00000006575.14	ENSMUSG00000006575.14	ENSMUSG00000006575	protein_coding	Rundc3a	80.10155604	32.5094275	1.297571199	0.360036	3.6040038	0.0003134	0.0022832
ENSMUSG00000035129.6	ENSMUSG00000035129.6	ENSMUSG00000035129	processed_pseudogene	Gm6781	17.37849527	50.91485222	-1.552051797	0.4351248	-3.566912	0.0003612	0.00258
ENSMUSG00000053702.16	ENSMUSG00000053702.16	ENSMUSG00000053702	protein_coding	Nebl	58.49845343	14.52828467	2.011342362	0.3631944	5.5379224	3.061E-08	5.059E-07
ENSMUSG00000021062.15	ENSMUSG00000021062.15	ENSMUSG00000021062	protein_coding	Rab15	55.4892154	13.32222165	2.054890952	0.4240697	4.8456441	1.262E-06	1.579E-05
ENSMUSG00000060985.15	ENSMUSG00000060985.15	ENSMUSG00000060985	protein_coding	Tdrd5	10.76493012	100.5466094	-3.241899608	0.4340695	-7.468619	8.104E-14	2.569E-12
ENSMUSG00000035104.14	ENSMUSG00000035104.14	ENSMUSG00000035104	protein_coding	Eva1a	19.88299547	0.64829908	4.835809109	1.2508571	3.8659965	0.0001106	0.0009102
ENSMUSG00000058135.12	ENSMUSG00000058135.12	ENSMUSG00000058135	protein_coding	Gstm1	522.1181464	118.0725986	2.143833942	0.1560308	13.739811	5.863E-43	8.88E-41
ENSMUSG00000078249.5	ENSMUSG00000078249.5	ENSMUSG00000078249	protein_coding	Hmga1-rs1	1022.192578	21.34458398	5.587854322	0.2675674	20.88391	7.5E-97	5.33E-94
ENSMUSG00000002020.15	ENSMUSG00000002020.15	ENSMUSG00000002020	protein_coding	Ltbp2	937.8408312	359.6267776	1.382724251	0.1059747	13.047681	6.551E-39	8.406E-37
ENSMUSG00000026748.13	ENSMUSG00000026748.13	ENSMUSG00000026748	protein_coding	Plxdc2	29.64338106	2.399202184	3.598159356	0.7953198	4.5241667	6.063E-06	6.697E-05
ENSMUSG00000032014.6	ENSMUSG00000032014.6	ENSMUSG00000032014	protein_coding	Oaf	292.3707945	91.77832134	1.668696141	0.1959933	8.5140489	1.68E-17	7.372E-16
ENSMUSG00000112478.1	ENSMUSG00000112478.1	ENSMUSG00000112478	lincRNA	AC153370.2	3.563538884	25.32182864	-2.801233979	0.8221978	-3.407007	0.0006568	0.0043266
ENSMUSG00000022766.12	ENSMUSG00000022766.12	ENSMUSG00000022766	protein_coding	Serpind1	19.3237079	0	6.629109923	1.3500805	4.9101591	9.1E-07	1.173E-05
ENSMUSG00000054893.15	ENSMUSG00000054893.15	ENSMUSG00000054893	protein_coding	Zfp667	16.37721961	42.63465702	-1.377422072	0.3814941	-3.610599	0.0003055	0.0022329
ENSMUSG00000037440.8	ENSMUSG00000037440.8	ENSMUSG00000037440	protein_coding	Vnn1	70.08959669	239.034318	-1.772416319	0.1831361	-9.67814	3.734E-22	2.136E-20
ENSMUSG00000033880.11	ENSMUSG00000033880.11	ENSMUSG00000033880	protein_coding	Lgals3bp	56.81240706	21.64760002	1.393192652	0.3922247	3.5520272	0.0003823	0.0027064

ENSMUSG00000007805.4	ENSMUSG00000007805.4	ENSMUSG00000007805	protein_coding	Twist2	12.49883802	1.383368429	3.152043387	1.0466883	3.0114443	0.0026001	0.0144061
ENSMUSG00000019890.4	ENSMUSG00000019890.4	ENSMUSG00000019890	protein_coding	Nts	0	62.30218432	-8.508242337	1.2306213	-6.913778	4.719E-12	1.264E-10
ENSMUSG00000023094.14	ENSMUSG00000023094.14	ENSMUSG00000023094	protein_coding	Msrb2	71.75578448	12.1817954	2.544010653	0.3861214	6.5886293	4.439E-11	1.053E-09
ENSMUSG00000003153.10	ENSMUSG00000003153.10	ENSMUSG00000003153	protein_coding	Slc2a3	5.684940722	28.66874987	-2.351604825	0.6721624	-3.498567	0.0004678	0.0032263
ENSMUSG00000039062.15	ENSMUSG00000039062.15	ENSMUSG00000039062	protein_coding	Anpep	12.87090821	2.453713134	2.386599998	0.82605	2.8891713	0.0038626	0.0200261
ENSMUSG00000083282.2	ENSMUSG00000083282.2	ENSMUSG00000083282	protein_coding	Ctsf	6.371318071	0	5.030237102	1.6923157	2.9723987	0.0029548	0.0159881
ENSMUSG00000099568.1	ENSMUSG00000099568.1	ENSMUSG00000099568	antisense_RNA	Gm28513	8.317121848	0.735069349	3.539833351	1.3969974	2.5338869	0.0112805	0.0487924
ENSMUSG00000104231.1	ENSMUSG00000104231.1	ENSMUSG00000104231	antisense_RNA	Gm37592	8.351813023	0	5.417110725	1.5357055	3.5274412	0.0004196	0.0029275
ENSMUSG00000104350.1	ENSMUSG00000104350.1	ENSMUSG00000104350	TEC	Gm38244	39.54628017	17.85655034	1.141252081	0.3900095	2.9262161	0.0034311	0.0180576
ENSMUSG00000027314.6	ENSMUSG00000027314.6	ENSMUSG00000027314	protein_coding	Dll4	18.86849385	65.06071152	-1.77442823	0.3818907	-4.64643	3.377E-06	3.917E-05
ENSMUSG00000097451.10	ENSMUSG00000097451.10	ENSMUSG00000097451	processed_transcript	Rian	29.82005021	5.615799206	2.402012495	0.5919833	4.0575677	4.959E-05	0.0004448
ENSMUSG00000020955.9	ENSMUSG00000020955.9	ENSMUSG00000020955	protein_coding	Ap4s1	198.7547884	81.36676451	1.284167193	0.2067389	6.211541	5.247E-10	1.095E-08
ENSMUSG00000020732.13	ENSMUSG00000020732.13	ENSMUSG00000020732	protein_coding	Rab37	14.00259812	2.46483895	2.502183835	0.9553831	2.6190371	0.0088178	0.0397696
ENSMUSG00000052430.15	ENSMUSG00000052430.15	ENSMUSG00000052430	protein_coding	Bmpr1b	22.03903288	4.432667727	2.337124593	0.8497412	2.7503958	0.0059523	0.0288756
ENSMUSG00000022636.13	ENSMUSG00000022636.13	ENSMUSG00000022636	protein_coding	Alcam	2172.487303	872.7868111	1.315054379	0.0919447	14.302673	2.106E-46	3.705E-44
ENSMUSG00000030041.9	ENSMUSG00000030041.9	ENSMUSG00000030041	protein_coding	M1ap	23.12007232	6.794047684	1.778599125	0.627765	2.8332243	0.0046081	0.0233156
ENSMUSG00000083856.1	ENSMUSG00000083856.1	ENSMUSG00000083856	processed_pseudogene	Gm13886	19.44095179	6.712840323	1.534697052	0.5656144	2.7133273	0.0066611	0.0316087
ENSMUSG00000021301.9	ENSMUSG00000021301.9	ENSMUSG00000021301	protein_coding	Hecw1	52.99237964	16.64227767	1.670024631	0.4197831	3.9783035	6.941E-05	0.000599
ENSMUSG00000041548.4	ENSMUSG00000041548.4	ENSMUSG00000041548	protein_coding	Hspb8	206.794972	95.93466944	1.106389936	0.172338	6.4198837	1.364E-10	3.047E-09
ENSMUSG00000054640.14	ENSMUSG00000054640.14	ENSMUSG00000054640	protein_coding	Slc8a1	12.3474385	34.53844827	-1.479670281	0.4314919	-3.429196	0.0006054	0.0040296
ENSMUSG00000040584.8	ENSMUSG00000040584.8	ENSMUSG00000040584	protein_coding	Abcb1a	748.1426543	285.503921	1.392223841	0.1303372	10.681705	1.24E-26	9.237E-25
ENSMUSG00000020396.8	ENSMUSG00000020396.8	ENSMUSG00000020396	protein_coding	Nefh	8.363912294	28.94378256	-1.788785325	0.5391393	-3.317854	0.0009071	0.0057541
ENSMUSG00000020183.11	ENSMUSG00000020183.11	ENSMUSG00000020183	protein_coding	Cpm	171.7171922	72.7887057	1.238534003	0.2189728	5.6561078	1.548E-08	2.669E-07
ENSMUSG00000035473.10	ENSMUSG00000035473.10	ENSMUSG00000035473	protein_coding	Galm	17.05149769	49.54037335	-1.529368948	0.4180919	-3.657973	0.0002542	0.0018999
ENSMUSG00000031342.17	ENSMUSG00000031342.17	ENSMUSG00000031342	protein_coding	Gpm6b	647.4342102	272.0644273	1.252479372	0.2789041	4.4907164	7.098E-06	7.72E-05
ENSMUSG00000041020.14	ENSMUSG00000041020.14	ENSMUSG00000041020	protein_coding	Map7d2	11.31152727	65.34300455	-2.542187627	0.4189935	-6.067368	1.3E-09	2.578E-08
ENSMUSG00000055675.6	ENSMUSG00000055675.6	ENSMUSG00000055675	protein_coding	Kbtbd11	64.86923595	25.14384332	1.36926211	0.3072339	4.4567412	8.321E-06	8.919E-05
ENSMUSG00000038347.14	ENSMUSG00000038347.14	ENSMUSG00000038347	protein_coding	Tcte2	144.5215638	387.8929908	-1.422213205	0.1748267	-8.134989	4.12E-16	1.612E-14
ENSMUSG00000042429.8	ENSMUSG00000042429.8	ENSMUSG00000042429	protein_coding	Adora1	0	6.241846655	-5.193153502	1.7152696	-3.027602	0.002465	0.0137693
ENSMUSG00000027996.13	ENSMUSG00000027996.13	ENSMUSG00000027996	protein_coding	Sfrp2	0.908230308	142.6468723	-7.266161356	0.8746402	-8.3076	9.766E-17	4.046E-15
ENSMUSG00000002565.16	ENSMUSG00000002565.16	ENSMUSG00000002565	protein_coding	Scin	495.8932405	145.0534354	1.770985907	0.1348862	13.12948	2.232E-39	2.967E-37
ENSMUSG00000032735.14	ENSMUSG00000032735.14	ENSMUSG00000032735	protein_coding	Ablim3	40.55906395	4.215742054	3.26168235	0.5728717	5.6935655	1.244E-08	2.176E-07
ENSMUSG00000110635.1	ENSMUSG00000110635.1	ENSMUSG00000110635	TEC	Gm45853	11.31152727	33.13394633	-1.557709168	0.4838908	-3.219134	0.0012858	0.0078179
ENSMUSG00000029167.13	ENSMUSG00000029167.13	ENSMUSG00000029167	protein_coding	Ppargc1a	42.95954623	13.47839356	1.658250177	0.4505515	3.6804894	0.0002328	0.0017607
ENSMUSG00000038070.15	ENSMUSG00000038070.15	ENSMUSG00000038070	protein_coding	Cntln	118.7957067	14.24195736	3.065371422	0.3464342	8.8483503	8.882E-19	4.23E-17
ENSMUSG00000024846.5	ENSMUSG00000024846.5	ENSMUSG00000024846	protein_coding	Cst6	44.27459349	10.97753042	2.013122393	0.4341573	4.6368501	3.538E-06	4.077E-05
ENSMUSG00000032186.15	ENSMUSG00000032186.15	ENSMUSG00000032186	protein_coding	Tmod2	28.79623825	59.19837407	-1.047265339	0.3291491	-3.181735	0.001464	0.00876
ENSMUSG00000062393.13	ENSMUSG00000062393.13	ENSMUSG00000062393	protein_coding	Dgkk	2115.473555	4772.412812	-1.174162107	0.0849678	-13.8189	1.96E-43	2.994E-41
ENSMUSG00000097791.1	ENSMUSG00000097791.1	ENSMUSG00000097791	lincRNA	Gm17315	8.584638563	29.62990387	-1.778628812	0.539554	-3.296479	0.000979	0.0061533
ENSMUSG00000056457.6	ENSMUSG00000056457.6	ENSMUSG00000056457	protein_coding	Prl2c3	8.885719761	52.61304241	-2.55075891	0.765692	-3.331312	0.0008644	0.0055209
ENSMUSG00000046186.8	ENSMUSG00000046186.8	ENSMUSG00000046186	protein_coding	Cd109	53.97716046	6.854121542	2.997054758	0.48327	6.2016155	5.589E-10	1.159E-08
ENSMUSG00000061013.5	ENSMUSG00000061013.5	ENSMUSG00000061013	protein_coding	Mkx	2.760453193	40.41635632	-3.859714512	0.7006477	-5.508781	3.613E-08	5.898E-07
ENSMUSG00000022323.11	ENSMUSG00000022323.11	ENSMUSG00000022323	protein_coding	Rida	881.2944642	380.7946355	1.210086402	0.1018368	11.8826	1.458E-32	1.403E-30
ENSMUSG00000014813.9	ENSMUSG00000014813.9	ENSMUSG00000014813	protein_coding	Stc1	161.5796549	57.46039482	1.492995864	0.2164111	6.8988861	5.241E-12	1.402E-10
ENSMUSG00000040219.4	ENSMUSG00000040219.4	ENSMUSG00000040219	protein_coding	Ttc12	28.58868235	7.848821793	1.866906772	0.6653494	2.8059044	0.0050176	0.0251523
ENSMUSG00000015222.17	ENSMUSG00000015222.17	ENSMUSG00000015222	protein_coding	Map2	203.5509494	3175.70954	-3.965880091	0.2167117	-18.30026	8.236E-75	2.984E-72
ENSMUSG00000038304.14	ENSMUSG00000038304.14	ENSMUSG00000038304	protein_coding	Cd160	5.602923554	0	4.839892171	1.6992971	2.848173	0.0043971	0.0223828
ENSMUSG00000004558.14	ENSMUSG00000004558.14	ENSMUSG00000004558	protein_coding	Ndrg2	343.2432121	102.5947969	1.743185294	0.1545041	11.282456	1.602E-29	1.346E-27
ENSMUSG00000025496.5	ENSMUSG00000025496.5	ENSMUSG00000025496	protein_coding	Drd4	5.241825947	0	4.745707547	1.7246025	2.7517689	0.0059274	0.0288002
ENSMUSG00000040111.16	ENSMUSG00000040111.16	ENSMUSG00000040111	protein_coding	Gramd1b	62.45059659	17.2571993	1.856899915	0.490975	3.7820664	0.0001555	0.0012333
ENSMUSG00000039997.16	ENSMUSG00000039997.16	ENSMUSG00000039997	protein_coding	Ifi203	34.66549623	7.81920922	2.127706318	0.6272532	3.3921009	0.0006936	0.0045383
ENSMUSG00000039375.16	ENSMUSG00000039375.16	ENSMUSG00000039375	protein_coding	Wdr17	1.816460617	15.26891693	-3.056623805	1.1828407	-2.584138	0.0097623	0.0432065
ENSMUSG00000028076.12	ENSMUSG00000028076.12	ENSMUSG00000028076	protein_coding	Cd1d1	11.99903131	0.97244862	3.540365188	1.2443653	2.8451173	0.0044395	0.0225734
ENSMUSG00000029304.14	ENSMUSG00000029304.14	ENSMUSG00000029304	protein_coding	Spp1	25371.75567	9326.680836	1.443766689	0.1003741	14.383861	6.534E-47	1.161E-44
ENSMUSG00000028217.11	ENSMUSG00000028217.11	ENSMUSG00000028217	protein_coding	Cdh17	112.0044405	23.45009791	2.251129151	0.3373001	6.6739651	2.49E-11	6.159E-10
ENSMUSG00000039217.13	ENSMUSG00000039217.13	ENSMUSG00000039217	protein_coding	Il18	0	4.626661863	-4.755846994	1.7992582	-2.643227	0.008212	0.0375598
ENSMUSG00000097287.2	ENSMUSG00000097287.2	ENSMUSG00000097287	lincRNA	D130017N08Rik	14.10554495	62.0730147	-2.1317142	0.380889	-5.596681	2.185E-08	3.67E-07
ENSMUSG00000039616.9	ENSMUSG00000039616.9	ENSMUSG00000039616	protein_coding	Mocos	174.3950371	85.26880294	1.028746084	0.2419495	4.2519047	2.12E-05	0.0002083
ENSMUSG00000048899.8	ENSMUSG00000048899.8	ENSMUSG00000048899	protein_coding	Rimkla	2.364128865	15.47403688	-2.67565009	0.8856045	-3.02127	0.0025172	0.0140182
ENSMUSG00000002668.7	ENSMUSG00000002668.7	ENSMUSG00000002668	protein_coding	Dennd1c	24.96132263	71.3975381	-1.513548764	0.3179018	-4.761057	1.926E-06	2.338E-05
ENSMUSG00000103313.1	ENSMUSG00000103313.1	ENSMUSG00000103313	TEC	Gm38357	40.87230002	139.6179584	-1.76723069	0.3000146	-5.890482	3.851E-09	7.202E-08
ENSMUSG00000048485.12	ENSMUSG00000048485.12	ENSMUSG00000048485	protein_coding	Zbtb8b	15.88897663	60.37217777	-1.931846459	0.3483054	-5.546415	2.916E-08	4.845E-07
ENSMUSG00000039742.15	ENSMUSG00000039742.15	ENSMUSG00000039742	protein_coding	Fam71f1	117.6739739	238.8343228	-1.023445102	0.1604468	-6.378719	1.786E-10	3.9E-09
ENSMUSG00000020774.9	ENSMUSG00000020774.9	ENSMUSG00000020774	protein_coding	Aspa	14.53656045	65.69985163	-2.177576486	0.3756539	-5.796763	6.761E-09	1.222E-07
ENSMUSG00000051177.16	ENSMUSG00000051177.16	ENSMUSG00000051177	protein_coding	Plcb1	23.00615291	2.723351724	3.043550715	0.7800196	3.90189	9.544E-05	0.0007977
ENSMUSG00000003354.5	ENSMUSG00000003354.5	ENSMUSG00000003354	protein_coding	Ccdc65	65.52537329	28.72617698	1.18237611	0.342948	3.4476836	0.0005654	0.0037988
ENSMUSG00000079363.7	ENSMUSG00000079363.7	ENSMUSG00000079363	protein_coding	Gbp4	46.02215171	12.17066959	1.909805989	0.4512315	4.2324308	2.312E-05	0.0002255
ENSMUSG00000025902.13	ENSMUSG00000025902.13	ENSMUSG00000025902	protein_coding	Sox17	2.446146032	14.4626479	-2.554608136	0.8336148	-3.064495	0.0021804	0.0123472
ENSMUSG00000114554.1	ENSMUSG00000114554.1	ENSMUSG00000114554	lincRNA	CT009711.2	83.04579094	36.73073247	1.174734931	0.2995556	3.9215927	8.797E-05	0.0007436
ENSMUSG00000041324.14	ENSMUSG00000041324.14	ENSMUSG00000041324	protein_coding	Inhba	3138.862527	7348.033589	-1.227196909	0.1040639	-11.79272	4.256E-32	4.012E-30
ENSMUSG00000084350.1	ENSMUSG00000084350.1	ENSMUSG00000084350	unprocessed_pseudogene	Znf41-ps	8.76077217	27.99358558	-1.678644314	0.607598	-2.762755	0.0057316	0.0280106
ENSMUSG00000003355.6	ENSMUSG00000003355.6	ENSMUSG00000003355	protein_coding	Fkbp11	609.7120715	207.197481	1.557859589	0.1242384	12.539278	4.551E-36	5.128E-34
ENSMUSG00000047767.17	ENSMUSG00000047767.17	ENSMUSG00000047767	protein_coding	Atg16l2	49.36609102	105.0792407	-1.086767311	0.244345	-4.447676	8.68E-06	9.266E-05
ENSMUSG00000106104.1	ENSMUSG00000106104.1	ENSMUSG00000106104	TEC	Gm42660	154.8115325	0	9.62837589	1.2023958	8.0076593	1.169E-15	4.391E-14
ENSMUSG00000028033.16	ENSMUSG00000028033.16	ENSMUSG00000028033	protein_coding	Kcnq5	18.2294425	5.323908986	1.779534782	0.6129986	2.9029997	0.0036961	0.01926
ENSMUSG00000000732.9	ENSMUSG00000000732.9	ENSMUSG00000000732	protein_coding	Icosl	4.577449357	23.69483813	-2.3596744	0.5980969	-3.945304	7.97E-05	0.0006815
ENSMUSG00000037621.8	ENSMUSG00000037621.8	ENSMUSG00000037621	protein_coding	Atoh8	26.57135403	5.237138716	2.329562571	0.5982806	3.8937626	9.87E-05	0.0008219
ENSMUSG00000087549.1	ENSMUSG00000087549.1	ENSMUSG00000087549	antisense_RNA	Gm13427	5.253389672	0	4.748465841	1.7169638	2.765618	0.0056815	0.0278174
ENSMUSG00000002885.14	ENSMUSG00000002885.14	ENSMUSG00000002885	protein_coding	Adgre5	403.4054724	901.6352992	-1.15935077	0.1675729	-6.918485	4.565E-12	1.231E-10
ENSMUSG00000079502.8	ENSMUSG00000079502.8	ENSMUSG00000079502	protein_coding	Cfap77	11.37978293	2.502661177	2.196842313	0.8553849	2.5682501	0.0102213	0.0448942
ENSMUSG00000098503.1	ENSMUSG00000098503.1	ENSMUSG00000098503	misc_RNA	Gm27937	26.9914918	2.129563594	3.666467912	1.4180744	2.5855257	0.0097231	0.0430639
ENSMUSG00000102418.1	ENSMUSG00000102418.1	ENSMUSG00000102418	protein_coding	Sh2d1b1	414.0284572	201.894295	1.037355653	0.1564409	6.6309734	3.335E-11	8.066E-10
ENSMUSG00000022747.16	ENSMUSG00000022747.16	ENSMUSG00000022747	protein_coding	St3gal6	474.7870216	2158.239684	-2.184466459	0.1563482	-13.97181	2.317E-44	3.755E-42
ENSMUSG00000073555.1	ENSMUSG00000073555.1	ENSMUSG00000073555	protein_coding	Gm4951	4.6479028	0	4.571155542	1.7867686	2.5583367	0.0105174	0.0460414
ENSMUSG00000080242.5	ENSMUSG00000080242.5	ENSMUSG00000080242	processed_pseudogene	Gm15487	320.7299987	0.697247122	8.826557099	1.0532556	8.3802614	5.281E-17	2.233E-15
ENSMUSG00000056121.15	ENSMUSG00000056121.15	ENSMUSG00000056121	protein_coding	Fez2	1749.898521	4378.004831	-1.322746739	0.0807146	-16.38795	2.332E-60	6.431E-58
ENSMUSG00000027858.13	ENSMUSG00000027858.13	ENSMUSG00000027858	protein_coding	Tspan2	57.42345545	164.061369	-1.50947152	0.2255855	-6.691351	2.211E-11	5.514E-10
ENSMUSG00000035864.14	ENSMUSG00000035864.14	ENSMUSG00000035864	protein_coding	Syt1	2.375692589	14.3169219	-2.563206945	0.9137837	-2.805048	0.0050309	0.0251859
ENSMUSG00000006362.16	ENSMUSG00000006362.16	ENSMUSG00000006362	protein_coding	Cbfa2t3	16.98270648	82.73259551	-2.282573132	0.3238127	-7.049055	1.801E-12	5.066E-11
ENSMUSG00000074817.2	ENSMUSG00000074817.2	ENSMUSG00000074817	protein_coding	Papolb	34.23732525	13.84216336	1.304164134	0.459222	2.8399424	0.0045122	0.0228929
ENSMUSG00000033685.13	ENSMUSG00000033685.13	ENSMUSG00000033685	protein_coding	Ucp2	300.3315994	111.2692231	1.430664308	0.1771911	8.0741337	6.796E-16	2.605E-14
ENSMUSG00000102550.1	ENSMUSG00000102550.1	ENSMUSG00000102550	processed_pseudogene	Gm8276	957.3507991	7.161582359	7.080289164	0.3432727	20.625841	1.609E-94	1.062E-91
ENSMUSG00000068874.13	ENSMUSG00000068874.13	ENSMUSG00000068874	protein_coding	Selenbp1	8.724474355	0	5.478764035	1.5699859	3.48969	0.0004836	0.003323
ENSMUSG00000106116.1	ENSMUSG00000106116.1	ENSMUSG00000106116	processed_pseudogene	Gm18830	54.10596807	0	8.112850849	1.2392692	6.5464801	5.891E-11	1.376E-09
ENSMUSG00000056632.7	ENSMUSG00000056632.7	ENSMUSG00000056632	protein_coding	Dsg3	6.104928047	129.4001263	-4.445562777	0.4379831	-10.15008	3.311E-24	2.201E-22
ENSMUSG00000085683.2	ENSMUSG00000085683.2	ENSMUSG00000085683	protein_coding	9130409J20Rik	11.92911342	53.95499254	-2.190108315	0.4274687	-5.123435	3E-07	4.209E-06
ENSMUSG00000037370.13	ENSMUSG00000037370.13	ENSMUSG00000037370	protein_coding	Enpp1	234.5360709	588.0399417	-1.326805068	0.1666834	-7.960032	1.72E-15	6.408E-14
ENSMUSG00000073590.3	ENSMUSG00000073590.3	ENSMUSG00000073590	lincRNA	3222401L13Rik	119.888421	37.56926081	1.673913313	0.2662136	6.2878578	3.219E-10	6.868E-09
ENSMUSG00000003378.9	ENSMUSG00000003378.9	ENSMUSG00000003378	protein_coding	Grik5	44.41544478	99.0277921	-1.156102587	0.2579274	-4.482278	7.385E-06	8.004E-05
ENSMUSG00000023979.14	ENSMUSG00000023979.14	ENSMUSG00000023979	protein_coding	Guca1b	0	11.11324873	-6.018603289	1.4815464	-4.062379	4.858E-05	0.0004374
ENSMUSG00000022231.9	ENSMUSG00000022231.9	ENSMUSG00000022231	protein_coding	Sema5a	47.02176513	240.7787818	-2.348774673	0.2454694	-9.568504	1.085E-21	5.983E-20
ENSMUSG00000036381.13	ENSMUSG00000036381.13	ENSMUSG00000036381	protein_coding	P2ry14	27.19996836	6.064541243	2.169330327	0.5733586	3.783549	0.0001546	0.0012277
ENSMUSG00000049109.15	ENSMUSG00000049109.15	ENSMUSG00000049109	protein_coding	Themis	5.882539541	24.06417083	-2.028019079	0.595465	-3.405773	0.0006598	0.0043447
ENSMUSG00000112164.1	ENSMUSG00000112164.1	ENSMUSG00000112164	lincRNA	AC114575.1	7.211292721	0	5.202696884	1.6600873	3.13399	0.0017245	0.0100838
ENSMUSG00000030413.7	ENSMUSG00000030413.7	ENSMUSG00000030413	protein_coding	Pglyrp1	45.18335935	15.7454735	1.52913387	0.4023441	3.8005622	0.0001444	0.0011533
ENSMUSG00000020057.2	ENSMUSG00000020057.2	ENSMUSG00000020057	protein_coding	Dram1	2102.005791	242.3450463	3.115980402	0.1005061	31.002892	4.93E-211	1.14E-207
ENSMUSG00000109167.1	ENSMUSG00000109167.1	ENSMUSG00000109167	TEC	Gm44652	15.31871648	32.96485058	-1.111238777	0.4175255	-2.661487	0.0077796	0.03592
ENSMUSG00000030257.16	ENSMUSG00000030257.16	ENSMUSG00000030257	protein_coding	Srgap3	1091.068744	419.3728351	1.379613634	0.2044405	6.7482403	1.496E-11	3.809E-10
ENSMUSG00000069171.14	ENSMUSG00000069171.14	ENSMUSG00000069171	protein_coding	Nr2f1	145.796414	12.54000229	3.551624003	0.3379139	10.510442	7.733E-26	5.538E-24
ENSMUSG00000046259.2	ENSMUSG00000046259.2	ENSMUSG00000046259	protein_coding	Sprr2h	26.03299613	4.215742054	2.623852419	0.6266969	4.1867962	2.829E-05	0.0002696
ENSMUSG00000087042.1	ENSMUSG00000087042.1	ENSMUSG00000087042	sense_intronic	Gm11611	31.4173912	77.47808816	-1.306853575	0.2979538	-4.386094	1.154E-05	0.0001199
ENSMUSG00000020654.15	ENSMUSG00000020654.15	ENSMUSG00000020654	protein_coding	Adcy3	291.146356	131.8028516	1.143213104	0.2640945	4.3288028	1.499E-05	0.000152

ENSMUSG00000079293.11	ENSMUSG00000079293.11	ENSMUSG00000079293	polymorphic_pseudogene	Clec7a	0	5.789339745	-5.076206512	1.7522724	-2.896928	0.0037684	0.019587
ENSMUSG00000029409.7	ENSMUSG00000029409.7	ENSMUSG00000029409	protein_coding	U90926	148.7957898	5.431132852	4.733193185	0.4803759	9.8531036	6.646E-23	4.026E-21
ENSMUSG00000102676.1	ENSMUSG00000102676.1	ENSMUSG00000102676	TEC	Gm37435	0	10.78173825	-5.981259725	1.496826	-3.995962	6.443E-05	0.0005616
ENSMUSG00000022836.10	ENSMUSG00000022836.10	ENSMUSG00000022836	protein_coding	Mylk	217.2863832	46.58452536	2.220635052	0.2081594	10.667956	1.438E-26	1.067E-24
ENSMUSG00000038965.15	ENSMUSG00000038965.15	ENSMUSG00000038965	protein_coding	Ube2l3	1316.622733	487.3787521	1.434227121	0.0979029	14.649479	1.358E-48	2.697E-46
ENSMUSG00000029778.12	ENSMUSG00000029778.12	ENSMUSG00000029778	protein_coding	Adcyap1r1	0	8.237489972	-5.586780134	1.5695518	-3.559475	0.0003716	0.0026409
ENSMUSG00000001995.9	ENSMUSG00000001995.9	ENSMUSG00000001995	protein_coding	Sipa1l2	138.2868126	540.1631543	-1.964048812	0.1499069	-13.10179	3.216E-39	4.214E-37
ENSMUSG00000022037.15	ENSMUSG00000022037.15	ENSMUSG00000022037	protein_coding	Clu	12613.7444	31204.76763	-1.306709261	0.0877525	-14.89085	3.779E-50	7.846E-48
ENSMUSG00000031131.11	ENSMUSG00000031131.11	ENSMUSG00000031131	protein_coding	Vgll1	6.243637148	25.92049977	-2.06369992	0.5571663	-3.703921	0.0002123	0.0016216
ENSMUSG00000039717.16	ENSMUSG00000039717.16	ENSMUSG00000039717	protein_coding	Ralyl	20.8500599	57.62409647	-1.465064322	0.3876176	-3.779664	0.000157	0.0012417
ENSMUSG00000032202.11	ENSMUSG00000032202.11	ENSMUSG00000032202	protein_coding	Rab27a	24.55450567	8.85909264	1.474870911	0.5511816	2.6758346	0.0074543	0.0348007
ENSMUSG00000050071.8	ENSMUSG00000050071.8	ENSMUSG00000050071	protein_coding	Bex1	324.248164	778.7702188	-1.264537178	0.1123321	-11.25713	2.136E-29	1.77E-27
ENSMUSG00000059498.13	ENSMUSG00000059498.13	ENSMUSG00000059498	protein_coding	Fcgr3	0	6.004467385	-5.133603872	1.7311346	-2.965456	0.0030223	0.0162724
ENSMUSG00000043822.18	ENSMUSG00000043822.18	ENSMUSG00000043822	protein_coding	Adamtsl5	154.8143214	492.5691062	-1.673837988	0.1651452	-10.13555	3.842E-24	2.545E-22
ENSMUSG00000063296.4	ENSMUSG00000063296.4	ENSMUSG00000063296	protein_coding	Tmem117	17.58926445	3.064189987	2.494161657	0.8478846	2.9416288	0.0032649	0.0173859
ENSMUSG00000022076.9	ENSMUSG00000022076.9	ENSMUSG00000022076	protein_coding	Klhl1	282.5552124	615.7732489	-1.124027529	0.1052046	-10.68421	1.207E-26	9.065E-25
ENSMUSG00000035557.9	ENSMUSG00000035557.9	ENSMUSG00000035557	protein_coding	Krt17	0	5.879874888	-5.107603534	1.7254069	-2.960231	0.0030741	0.0164933
ENSMUSG00000000794.9	ENSMUSG00000000794.9	ENSMUSG00000000794	protein_coding	Kcnn3	377.4313987	987.8261794	-1.388367228	0.1403503	-9.892158	4.502E-23	2.782E-21
ENSMUSG00000028111.4	ENSMUSG00000028111.4	ENSMUSG00000028111	protein_coding	Ctsk	5.92879444	0	4.921314723	1.7645208	2.7890375	0.0052865	0.0262379
ENSMUSG00000003282.9	ENSMUSG00000003282.9	ENSMUSG00000003282	protein_coding	Plag1	88.83025917	272.4180287	-1.618837451	0.364259	-4.444194	8.822E-06	9.407E-05
ENSMUSG00000000031.15	ENSMUSG00000000031.15	ENSMUSG00000000031	lincRNA	H19	30.38094442	2.908018078	3.40873199	0.7312407	4.6615733	3.138E-06	3.658E-05
ENSMUSG00000025969.15	ENSMUSG00000025969.15	ENSMUSG00000025969	protein_coding	Nrp2	3440.623413	1542.547289	1.157384296	0.086457	13.386825	7.22E-41	1.018E-38
ENSMUSG00000073000.3	ENSMUSG00000073000.3	ENSMUSG00000073000	lincRNA	Gm10451	12.5098106	0	6.002688763	1.4510985	4.1366514	3.524E-05	0.0003267
ENSMUSG00000034057.8	ENSMUSG00000034057.8	ENSMUSG00000034057	protein_coding	Myrfl	0	7.151574671	-5.383570302	1.7158288	-3.137592	0.0017034	0.0099936
ENSMUSG00000026360.9	ENSMUSG00000026360.9	ENSMUSG00000026360	protein_coding	Rgs2	15.16571032	80.103375	-2.399232016	0.3548604	-6.761058	1.37E-11	3.501E-10
ENSMUSG00000029287.14	ENSMUSG00000029287.14	ENSMUSG00000029287	protein_coding	Tgfbr3	74.65269339	26.52344688	1.502798529	0.3400847	4.4188949	9.921E-06	0.0001043

Supplementary Table 2. Significant differentially expressed genes and pathways in SPC-Cre KPG and KP cell lines. MetaCore: Top 10 upregulated and downregulated canonical pathways in KPG cells using all significant genes listed in SPC-Cre KPG-KP tab.

PATHWAY	DIRECTION	TOTAL	pValue	MIN FDR	p-value	FDR	-LOG10	IN DATA	NETWORK OBJECTS FROM ACTIVE DATA
Probable BMP4-mediated induction of EMT in airway epithelium	Upregulated in KPG	21	2.173E-06	2.520E-03	2.173E-06	2.520E-03	2.60	7	COL1A1, SMAD9 (SMAD8), N-cadherin, TCF8, SNAIL1, TWIST1, SIP1 (ZFHX1B)
PGE2 pathways in cancer	Upregulated in KPG	55	6.487E-06	3.406E-03	6.487E-06	3.406E-03	2.47	10	G-protein beta/gamma, COX-1 (PTGS1), Tcf(Lef), PKA-reg (cAMP-dependent), EGFR, Axin, PGES, Lef-1, Adenylate cyclase, SLC21A2
Stem cells_Extraembryonic differentiation of embryonic stem cells	Upregulated in KPG	35	9.388E-06	3.406E-03	9.388E-06	3.406E-03	2.47	8	SMAD9 (SMAD8), SOX2, Gremlin, GATA-6, BMP7, BMP2, NCAM1, Transferrin
E-cadherin signaling and its regulation in gastric cancer	Upregulated in KPG	36	1.175E-05	3.406E-03	1.175E-05	3.406E-03	2.47	8	TCF8, EGFR, SNAIL1, BMP2, Frizzled, TWIST1, SIP1 (ZFHX1B), FGF1
Hedgehog signaling in gastric cancer	Upregulated in KPG	29	2.350E-05	4.378E-03	2.350E-05	4.378E-03	2.36	7	N-cadherin, ROBO1, CDX2, ROR2, HNF3-beta, TWIST2, SIP1 (ZFHX1B)
Stem cells_Hedgehog, BMP and Parathyroid hormone in osteogenesis	Upregulated in KPG	51	2.432E-05	4.378E-03	2.432E-05	4.378E-03	2.36	9	COL1A1, SMAD9 (SMAD8), Osteopontin, PKA-reg (cAMP-dependent), BMP7, Adenylate cyclase, BMPR1B, BMP2, PLC-beta
Stem cells_Role of BMP signaling in embryonic stem cell neural differentiation	Upregulated in KPG	30	2.984E-05	4.378E-03	2.984E-05	4.378E-03	2.36	7	SMAD9 (SMAD8), SOX2, Chordin, BMP7, BMPR1B, BMP2, NCAM1
Hypoxia-induced EMT in cancer and fibrosis	Upregulated in KPG	13	3.019E-05	4.378E-03	3.019E-05	4.378E-03	2.36	5	TCF8, SNAIL1, Lysyl oxidase, TWIST1, SIP1 (ZFHX1B)
Development_TGF-beta-dependent induction of EMT via SMADs	Upregulated in KPG	35	8.616E-05	9.190E-03	8.616E-05	9.190E-03	2.04	7	N-cadherin, Vimentin, TCF8, SNAIL1, Lef-1, TWIST1, SIP1 (ZFHX1B)
Stem cells_Schema: FGF signaling in embryonic stem cell self-renewal and differentiation	Upregulated in KPG	47	9.044E-05	9.190E-03	9.044E-05	9.190E-03	2.04	8	TITF1, SOX2, Gremlin, CD34, HNF3-beta, BMP2, NCAM1, FGF1
Regulation of immune cell differentiation by Notch signaling	Downregulated in KPG	43	0.0003363	0.3204651	0.0003363	0.3204651	0.49	6	Ifi202a, NOTCH3 (3ICD), DLL1, DLL4, PI3K cat class IA, NOTCH3
Breast cancer (general schema)	Downregulated in KPG	41	0.0019784	0.4839065	0.0019784	0.4839065	0.32	5	ESR2, DLL1, DLL4, Prolactin, NOTCH3
NF-AT signaling in cardiac hypertrophy	Downregulated in KPG	65	0.0030426	0.4839065	0.0030426	0.4839065	0.32	6	Cardiotrophin-1, NF-AT3(NFATC4), PKC-mu, PI3K cat class IA, NCX1, Beta-1 adrenergic receptor
Stem cells_Schema: FGF signaling in embryonic stem cell self-renewal and differentiation	Downregulated in KPG	47	0.0036379	0.4839065	0.0036379	0.4839065	0.32	5	SPRY2, Activin A, GATA-2, SOX17, NEFH
Role of SHH and Notch in SCLC	Downregulated in KPG	30	0.0040549	0.4839065	0.0040549	0.4839065	0.32	4	DLL1, CD133, AL1A1, ENO2
Protein folding and maturation_Posttranslational processing of neuroendocrine peptides	Downregulated in KPG	50	0.0047633	0.4839065	0.0047633	0.4839065	0.32	5	NN, NT/NN, NT, LargeNT, LargeNN
wtCFTR and deltaF508 traffic / Late endosome and lysosome (normal and CF)	Downregulated in KPG	16	0.0048201	0.4839065	0.0048201	0.4839065	0.32	3	Syntaxin 6, CFTR, Slp5
Stem cells_Endothelial differentiation during embryonic development	Downregulated in KPG	51	0.0051878	0.4839065	0.0051878	0.4839065	0.32	5	GATA-2, DLL4, TIE2, PI3K cat class IA, Neuropilin-1
Notch signaling in breast cancer	Downregulated in KPG	53	0.0061161	0.4839065	0.0061161	0.4839065	0.32	5	TFF1, NOTCH3 (3ICD), DLL1, DLL4, NOTCH3
Development_Epigenetic and transcriptional regulation of oligodendrocyte precursor cell differentiation and myelination	Downregulated in KPG	34	0.0064054	0.4839065	0.0064054	0.4839065	0.32	4	UGT8, SMRT, SOX17, Frizzled

Supplementary Table 2. Significant differentially expressed genes and pathways in SPC-Cre KPG and KP cell lines. GSEA: Top 25 Hallmarks Pathways upregulated and downregulated of a pre-ranked list of significantly expressed genes (P-adjusted < 0.05; n = 4,304) in KPG compared to KP cells.

NAME	DIRECTION	SIZE	ES	NES	NOM p-val	FDR q-val	FWER p-val	RANK AT MAX	LEADING EDGE
HALLMARK_ADIPOGENESIS	Upregulated in KPG	64	0.3087746	2.8973925	0	0	0	1944	tags=81%, list=51%, signal=163%
HALLMARK_EPITHELIAL_MESENCHYMAL_TRANSITION	Upregulated in KPG	80	0.2393306	2.4799433	0	0.0030972	0.006	680	tags=41%, list=18%, signal=49%
HALLMARK_XENOBIOTIC_METABOLISM	Upregulated in KPG	68	0.246631	2.3704953	0	0.0051904	0.015	1490	tags=63%, list=39%, signal=102%
HALLMARK_HEME_METABOLISM	Upregulated in KPG	55	0.2720178	2.368931	0.0021097	0.0038928	0.015	1337	tags=62%, list=35%, signal=94%
HALLMARK_INTERFERON_ALPHA_RESPONSE	Upregulated in KPG	30	0.3143988	2.0540638	0	0.0176706	0.077	1737	tags=77%, list=45%, signal=139%
HALLMARK_HYPOXIA	Upregulated in KPG	69	0.209919	2.0342999	0.0059172	0.0162403	0.084	1593	tags=62%, list=42%, signal=105%
HALLMARK_BILE_ACID_METABOLISM	Upregulated in KPG	29	0.2875987	1.8590988	0.016129	0.0405127	0.223	1544	tags=69%, list=40%, signal=115%
HALLMARK_OXIDATIVE_PHOSPHORYLATION	Upregulated in KPG	56	0.2105683	1.8454502	0.0061475	0.0384866	0.238	2209	tags=79%, list=58%, signal=183%
HALLMARK_MYOGENESIS	Upregulated in KPG	54	0.2072423	1.7691535	0.012	0.0515419	0.335	846	tags=43%, list=22%, signal=54%
HALLMARK_GLYCOLYSIS	Upregulated in KPG	80	0.1373864	1.4076837	0.1072165	0.2562518	0.895	1587	tags=55%, list=42%, signal=92%
HALLMARK_PROTEIN_SECRETION	Upregulated in KPG	31	0.2156891	1.388687	0.1212766	0.2523617	0.912	2017	tags=74%, list=53%, signal=156%
HALLMARK_ALLOGRAFT_REJECTION	Upregulated in KPG	40	0.1893046	1.387612	0.1133201	0.2325755	0.913	2054	tags=73%, list=54%, signal=155%
HALLMARK_IL2_STAT5_SIGNALING	Upregulated in KPG	69	0.1388686	1.3419819	0.1288981	0.2639448	0.956	918	tags=38%, list=24%, signal=49%
HALLMARK_IL6_JAK_STAT3_SIGNALING	Upregulated in KPG	22	0.2351675	1.3211472	0.1425662	0.2674708	0.967	1885	tags=73%, list=49%, signal=143%
HALLMARK_INTERFERON_GAMMA_RESPONSE	Upregulated in KPG	53	0.1435594	1.2341661	0.203125	0.3435778	0.991	2054	tags=68%, list=54%, signal=145%
HALLMARK_FATTY_ACID_METABOLISM	Upregulated in KPG	52	0.1366048	1.140919	0.2900609	0.4467276	0.997	1248	tags=46%, list=33%, signal=68%
HALLMARK_ANGIOGENESIS	Upregulated in KPG	16	0.2369285	1.104808	0.3380567	0.4706182	0.998	1486	tags=63%, list=39%, signal=102%
HALLMARK_ANDROGEN_RESPONSE	Upregulated in KPG	32	0.152688	1.0580735	0.3723609	0.514609	1	973	tags=41%, list=25%, signal=54%
HALLMARK_COAGULATION	Upregulated in KPG	35	0.1463428	1.0255752	0.4047619	0.5340839	1	646	tags=31%, list=17%, signal=37%
HALLMARK_PEROXISOME	Upregulated in KPG	31	0.1551637	1.0100205	0.4388778	0.531258	1	1630	tags=58%, list=43%, signal=100%
HALLMARK_MTORC1_SIGNALING	Upregulated in KPG	71	0.0970258	0.9532559	0.5129311	0.5937614	1	927	tags=34%, list=24%, signal=44%
HALLMARK_APOPTOSIS	Upregulated in KPG	49	0.1168831	0.9423836	0.5297619	0.5837251	1	572	tags=27%, list=15%, signal=31%
HALLMARK_INFLAMMATORY_RESPONSE	Upregulated in KPG	61	0.0984827	0.9180151	0.5671937	0.5935605	1	1195	tags=41%, list=31%, signal=59%
HALLMARK_SPERMATOGENESIS	Upregulated in KPG	24	0.139613	0.8377472	0.6715177	0.6831927	1	2813	tags=88%, list=74%, signal=329%
HALLMARK_TGF_BETA_SIGNALING	Upregulated in KPG	21	0.1344383	0.7427978	0.7992203	0.789022	1	1854	tags=62%, list=49%, signal=120%
HALLMARK_E2F_TARGETS	Downregulated in KPG	58	-0.289842	-2.590151	0	0	0	1480	tags=67%, list=39%, signal=108%
HALLMARK_G2M_CHECKPOINT	Downregulated in KPG	57	-0.27518	-2.451213	0	0.000864	0.002	1580	tags=68%, list=41%, signal=115%
HALLMARK_MYC_TARGETS_V1	Downregulated in KPG	55	-0.195159	-1.689979	0.028169	0.1725581	0.453	1837	tags=67%, list=48%, signal=128%
HALLMARK_CHOLESTEROL_HOMEOSTASIS	Downregulated in KPG	35	-0.236969	-1.674563	0.0226415	0.1399269	0.477	1506	tags=63%, list=39%, signal=103%
HALLMARK_TNFA_SIGNALING_VIA_NFKB	Downregulated in KPG	65	-0.148523	-1.407578	0.1078067	0.3877018	0.899	913	tags=38%, list=24%, signal=50%
HALLMARK_ESTROGEN_RESPONSE_EARLY	Downregulated in KPG	78	-0.136752	-1.396809	0.107943	0.3389201	0.907	518	tags=27%, list=14%, signal=31%
HALLMARK_KRAS_SIGNALING_DN	Downregulated in KPG	35	-0.196462	-1.376531	0.1284585	0.3163018	0.923	1987	tags=71%, list=52%, signal=147%
HALLMARK_KRAS_SIGNALING_UP	Downregulated in KPG	79	-0.130563	-1.313629	0.1729622	0.3606851	0.966	238	tags=19%, list=6%, signal=20%
HALLMARK_COMPLEMENT	Downregulated in KPG	55	-0.133765	-1.159425	0.2620545	0.5715651	0.998	331	tags=22%, list=9%, signal=24%
HALLMARK_UNFOLDED_PROTEIN_RESPONSE	Downregulated in KPG	43	-0.141787	-1.088666	0.3514563	0.6399371	1	2665	tags=84%, list=70%, signal=273%
HALLMARK_UV_RESPONSE_UP	Downregulated in KPG	48	-0.134638	-1.086468	0.3527132	0.5862522	1	2439	tags=77%, list=64%, signal=210%
HALLMARK_ESTROGEN_RESPONSE_LATE	Downregulated in KPG	71	-0.109334	-1.072391	0.3466136	0.5611009	1	829	tags=32%, list=22%, signal=41%
HALLMARK_P53_PATHWAY	Downregulated in KPG	63	-0.09781	-0.884625	0.5783898	0.8654372	1	1514	tags=49%, list=40%, signal=80%
HALLMARK_UV_RESPONSE_DN	Downregulated in KPG	54	-0.09678	-0.834176	0.65286	0.9006521	1	698	tags=28%, list=18%, signal=34%
HALLMARK_DNA_REPAIR	Downregulated in KPG	31	-0.119681	-0.789318	0.7534791	0.9234579	1	3246	tags=97%, list=85%, signal=637%
HALLMARK_WNT_BETA_CATENIN_SIGNALING	Downregulated in KPG	17	-0.155693	-0.782687	0.7391304	0.8767106	1	1432	tags=53%, list=37%, signal=84%
HALLMARK_APICAL_JUNCTION	Downregulated in KPG	65	-0.080543	-0.760303	0.7930368	0.8606477	1	404	tags=18%, list=11%, signal=20%
HALLMARK_PI3K_AKT_MTOR_SIGNALING	Downregulated in KPG	32	-0.104106	-0.691927	0.8742268	0.9010095	1	3309	tags=97%, list=87%, signal=716%
HALLMARK_MITOTIC_SPINDLE	Downregulated in KPG	59	-0.053825	-0.476869	0.9959596	0.9939423	1	3167	tags=88%, list=83%, signal=506%
