# Supplementary material for: Tumor progression and chromatin landscape of lung cancer are regulated by the lineage factor GATA6
Source: Oncogene. 2020 Mar 10;39(18):3726–37. doi: 10.1038/s41388-020-1246-z (PMC7190573; doi:10.1038/s41388-020-1246-z)
Supplement: Supplementary file 6 — Supplementary Table 5 [file 41388_2020_1246_MOESM6_ESM.pdf]

Supplementary Table 5. Summary of GEMM experiments**.** Summary of mouse experiments including strain, background, age at infection, type of viral delivery and titer, sex of the mice, and end time point for each experiment.

Mouse strain	Genotypes	Source	Stock	Reference
LSL K-Ras G12D	B6.129S4-*Kras*^{tm4Tyj}/J	The Jackson laboratory	8179	Jackson et al, 2001. PMID 11751630
p53 floxed	B6.129P2-*Trp53*^{tm1Brn}/J	The Jackson laboratory	8462	Marion et al. PMID 10783170
Gata6 floxed	*Gata6*$^{tm2.1}$Sad/J	The Jackson laboratory	8196	Sodhi et al, 2006. PMID 16611361
R26-LSL Tomato	B6.129-*Gt(ROSA)26Sor*$^{tm65.1(CAG-tdTomato)}$*Hze*/J	Valentina Greco lab		

Background	Genotypes	Viral delivery/injection (Virus type / cells)	Age at inj	Males/females	Titer (mouse)	End timepoint	Figures
C57Bl/6	K / KP	AdCRE	20 weeks	M / F	$2.5x10^7$ PFU	60, 90, 111 days	Fig. 1a
C57Bl/6	K / KG	AdCRE	10 weeks	M & F	$5x10^6$ PFU	365 days	Fig. 1b
FVB	KP / KPG	AdCRE	12 weeks	M & F	$2x10^7$ PFU	90 days	Fig. 1d
FVB	K / KG	Lenti-CRE-Luc	10 weeks	M & F	$3x10^5$ VP	91 days	Fig. 1c, Supplementary Fig. 1c
FVB	KP / KPG	Lenti-CRE-Luc	9-11 weeks	M & F	$5x10^5$ VP	102 days	Fig. 1e, Fig. 2, Supplementary Fig. 1d
FVB	K / KG	AdCRE	11 weeks	M & F	$5x10^6$ PFU	82 days	Supplementary Fig. 1b
C57Bl/6	K / KG	AdSPC-CRE	12-15 weeks	M & F	$2.5x10^8$ PFU	154 days	Fig. 3d, Supplementary Fig. 1a, 2e,f
C57Bl/6	KP / KPG	AdSPC-CRE	14-16 weeks	M & F	$2x10^8$ PFU	90 days	Fig. 3e, Supplementary Fig. 2e, f
C57Bl/6	K / KG	AdCC10-CRE	9-11 weeks	M & F	$2.5x10^8$ PFU	93 days	Fig. 3b
C57Bl/6	KP / KPG	AdCC10-CRE	20 weeks	M & F	$2.5x10^8$ PFU	90 days	Fig. 3c, Supplementary Fig. 2d
B6/129S	R26-LSL Tomato	AdSPC-CRE, AdCC10-CRE	16 weeks	F, M, M	$2.5x10^8$ PFU	11 days	Supplementary Fig. 2b, c
C57BL/6	WT	SPC KP and KPG cells	6 weeks	Matched to sex origin of cell lines	-	20 days	Fig. 3g
Athymic	WT	SPC KPG cells	7 weeks	M	-	13 days post-doxycycline	Supplementary Fig. 2g

Abbreviation	Genotype
K	KrasG12D
KG	KrasG12D Gata6 floxed
KP	Kras G12D p53 floxed
KPG	Kras G12D p53 floxed Gata6 floxed
