# Supplementary material for: Tumor progression and chromatin landscape of lung cancer are regulated by the lineage factor GATA6
Source: Oncogene. 2020 Mar 10;39(18):3726–37. doi: 10.1038/s41388-020-1246-z (PMC7190573; doi:10.1038/s41388-020-1246-z)
Supplement: Supplementary file 4 — Supplementary Table 3 [file 41388_2020_1246_MOESM4_ESM.pdf]

Supplementary Table 3. ChIP-seq peaks and Motif analysis. GATA6 peaks: List of peaks in SPC-Cre KP cells from GATA6 immunoprecipitation (IP) compared to input.

Chr	Start	End	Id
chr1	5000171	5000263	chr1-5000171-5000263
chr1	5170522	5170614	chr1-5170522-5170614
chr1	5586851	5586943	chr1-5586851-5586943
chr1	6008817	6008909	chr1-6008817-6008909
chr1	9228102	9228194	chr1-9228102-9228194
chr1	9755684	9755776	chr1-9755684-9755776
chr1	10285635	10285727	chr1-10285635-10285727
chr1	10493664	10493756	chr1-10493664-10493756
chr1	10898949	10899041	chr1-10898949-10899041
chr1	12175108	12175200	chr1-12175108-12175200
chr1	12644746	12644838	chr1-12644746-12644838
chr1	13421814	13421906	chr1-13421814-13421906
chr1	15936847	15936939	chr1-15936847-15936939
chr1	16235021	16235113	chr1-16235021-16235113
chr1	17007917	17008009	chr1-17007917-17008009
chr1	17014026	17014118	chr1-17014026-17014118
chr1	18067060	18067152	chr1-18067060-18067152
chr1	18958053	18958145	chr1-18958053-18958145
chr1	20863921	20864013	chr1-20863921-20864013
chr1	23271072	23271164	chr1-23271072-23271164
chr1	23802502	23802594	chr1-23802502-23802594
chr1	23840972	23841064	chr1-23840972-23841064
chr1	25551876	25551968	chr1-25551876-25551968
chr1	27403019	27403111	chr1-27403019-27403111
chr1	29751662	29751754	chr1-29751662-29751754
chr1	31899282	31899374	chr1-31899282-31899374
chr1	33039455	33039547	chr1-33039455-33039547
chr1	33990954	33991046	chr1-33990954-33991046
chr1	34041188	34041280	chr1-34041188-34041280
chr1	35858024	35858116	chr1-35858024-35858116
chr1	36274428	36274520	chr1-36274428-36274520
chr1	36721875	36721967	chr1-36721875-36721967
chr1	36920443	36920535	chr1-36920443-36920535
chr1	37063182	37063274	chr1-37063182-37063274
chr1	37712503	37712595	chr1-37712503-37712595
chr1	38030413	38030505	chr1-38030413-38030505
chr1	39018404	39018496	chr1-39018404-39018496
chr1	39759780	39759872	chr1-39759780-39759872

chr1	40234078	40234170	chr1-40234078-40234170
chr1	40565487	40565579	chr1-40565487-40565579
chr1	40614638	40614730	chr1-40614638-40614730
chr1	43152753	43152845	chr1-43152753-43152845
chr1	43415144	43415236	chr1-43415144-43415236
chr1	43715235	43715327	chr1-43715235-43715327
chr1	44022528	44022620	chr1-44022528-44022620
chr1	44101538	44101630	chr1-44101538-44101630
chr1	44951185	44951277	chr1-44951185-44951277
chr1	46016388	46016480	chr1-46016388-46016480
chr1	46880118	46880210	chr1-46880118-46880210
chr1	46970640	46970732	chr1-46970640-46970732
chr1	47014797	47014889	chr1-47014797-47014889
chr1	47039683	47039775	chr1-47039683-47039775
chr1	48630482	48630574	chr1-48630482-48630574
chr1	49752470	49752562	chr1-49752470-49752562
chr1	51448753	51448845	chr1-51448753-51448845
chr1	51849407	51849499	chr1-51849407-51849499
chr1	52775222	52775314	chr1-52775222-52775314
chr1	54604029	54604121	chr1-54604029-54604121
chr1	55827371	55827463	chr1-55827371-55827463
chr1	58753792	58753884	chr1-58753792-58753884
chr1	58803871	58803963	chr1-58803871-58803963
chr1	58859359	58859451	chr1-58859359-58859451
chr1	59616557	59616649	chr1-59616557-59616649
chr1	60450322	60450414	chr1-60450322-60450414
chr1	60513637	60513729	chr1-60513637-60513729
chr1	61809202	61809294	chr1-61809202-61809294
chr1	62333571	62333663	chr1-62333571-62333663
chr1	63550421	63550513	chr1-63550421-63550513
chr1	63630466	63630558	chr1-63630466-63630558
chr1	64111942	64112034	chr1-64111942-64112034
chr1	64188177	64188269	chr1-64188177-64188269
chr1	64941889	64941981	chr1-64941889-64941981
chr1	66133141	66133233	chr1-66133141-66133233
chr1	67450906	67450998	chr1-67450906-67450998
chr1	69401538	69401630	chr1-69401538-69401630
chr1	69461283	69461375	chr1-69461283-69461375
chr1	69567737	69567829	chr1-69567737-69567829
chr1	71242039	71242131	chr1-71242039-71242131
chr1	72075648	72075740	chr1-72075648-72075740
chr1	72244153	72244245	chr1-72244153-72244245

chr1	72255044	72255136	chr1-72255044-72255136
chr1	72360836	72360928	chr1-72360836-72360928
chr1	73061156	73061248	chr1-73061156-73061248
chr1	73716086	73716178	chr1-73716086-73716178
chr1	74061739	74061831	chr1-74061739-74061831
chr1	74240309	74240401	chr1-74240309-74240401
chr1	74295550	74295642	chr1-74295550-74295642
chr1	74719924	74720016	chr1-74719924-74720016
chr1	74854726	74854818	chr1-74854726-74854818
chr1	74949805	74949897	chr1-74949805-74949897
chr1	75026144	75026236	chr1-75026144-75026236
chr1	75618349	75618441	chr1-75618349-75618441
chr1	75883607	75883699	chr1-75883607-75883699
chr1	76737136	76737228	chr1-76737136-76737228
chr1	77008465	77008557	chr1-77008465-77008557
chr1	77402125	77402217	chr1-77402125-77402217
chr1	77460915	77461007	chr1-77460915-77461007
chr1	77462363	77462455	chr1-77462363-77462455
chr1	78322553	78322645	chr1-78322553-78322645
chr1	79641627	79641719	chr1-79641627-79641719
chr1	79740061	79740153	chr1-79740061-79740153
chr1	79776041	79776133	chr1-79776041-79776133
chr1	80171935	80172027	chr1-80171935-80172027
chr1	80447709	80447801	chr1-80447709-80447801
chr1	82780945	82781037	chr1-82780945-82781037
chr1	82787555	82787647	chr1-82787555-82787647
chr1	82834752	82834844	chr1-82834752-82834844
chr1	82972199	82972291	chr1-82972199-82972291
chr1	83142156	83142248	chr1-83142156-83142248
chr1	84579456	84579548	chr1-84579456-84579548
chr1	84695143	84695235	chr1-84695143-84695235
chr1	84696543	84696635	chr1-84696543-84696635
chr1	84839321	84839413	chr1-84839321-84839413
chr1	84962848	84962940	chr1-84962848-84962940
chr1	84963624	84963716	chr1-84963624-84963716
chr1	84993918	84994010	chr1-84993918-84994010
chr1	85090997	85091089	chr1-85090997-85091089
chr1	85099192	85099284	chr1-85099192-85099284
chr1	85147617	85147709	chr1-85147617-85147709
chr1	85164464	85164556	chr1-85164464-85164556
chr1	85164687	85164779	chr1-85164687-85164779
chr1	85165405	85165497	chr1-85165405-85165497

chr1	85167026	85167118	chr1-85167026-85167118
chr1	85170076	85170168	chr1-85170076-85170168
chr1	85179618	85179710	chr1-85179618-85179710
chr1	85179886	85179978	chr1-85179886-85179978
chr1	85180549	85180641	chr1-85180549-85180641
chr1	85184832	85184924	chr1-85184832-85184924
chr1	85198425	85198517	chr1-85198425-85198517
chr1	85231111	85231203	chr1-85231111-85231203
chr1	85231344	85231436	chr1-85231344-85231436
chr1	85270027	85270119	chr1-85270027-85270119
chr1	85304626	85304718	chr1-85304626-85304718
chr1	85305436	85305528	chr1-85305436-85305528
chr1	85312705	85312797	chr1-85312705-85312797
chr1	85340182	85340274	chr1-85340182-85340274
chr1	85481614	85481706	chr1-85481614-85481706
chr1	85485162	85485254	chr1-85485162-85485254
chr1	85494507	85494599	chr1-85494507-85494599
chr1	85495030	85495122	chr1-85495030-85495122
chr1	85499041	85499133	chr1-85499041-85499133
chr1	85506626	85506718	chr1-85506626-85506718
chr1	85536641	85536733	chr1-85536641-85536733
chr1	85551723	85551815	chr1-85551723-85551815
chr1	85553353	85553445	chr1-85553353-85553445
chr1	85555398	85555490	chr1-85555398-85555490
chr1	85607930	85608022	chr1-85607930-85608022
chr1	85608956	85609048	chr1-85608956-85609048
chr1	85617770	85617862	chr1-85617770-85617862
chr1	85627184	85627276	chr1-85627184-85627276
chr1	85634395	85634487	chr1-85634395-85634487
chr1	85864526	85864618	chr1-85864526-85864618
chr1	85943498	85943590	chr1-85943498-85943590
chr1	86278413	86278505	chr1-86278413-86278505
chr1	86313233	86313325	chr1-86313233-86313325
chr1	87180924	87181016	chr1-87180924-87181016
chr1	87492561	87492653	chr1-87492561-87492653
chr1	87873660	87873752	chr1-87873660-87873752
chr1	87961655	87961747	chr1-87961655-87961747
chr1	88455430	88455522	chr1-88455430-88455522
chr1	88475595	88475687	chr1-88475595-88475687
chr1	88560755	88560847	chr1-88560755-88560847
chr1	88645790	88645882	chr1-88645790-88645882
chr1	88646702	88646794	chr1-88646702-88646794

chr1	89122539	89122631	chr1-89122539-89122631
chr1	89191255	89191347	chr1-89191255-89191347
chr1	89271742	89271834	chr1-89271742-89271834
chr1	89566744	89566836	chr1-89566744-89566836
chr1	89600583	89600675	chr1-89600583-89600675
chr1	90289571	90289663	chr1-90289571-90289663
chr1	90519193	90519285	chr1-90519193-90519285
chr1	90624053	90624145	chr1-90624053-90624145
chr1	90923185	90923277	chr1-90923185-90923277
chr1	91041721	91041813	chr1-91041721-91041813
chr1	91085182	91085274	chr1-91085182-91085274
chr1	91109467	91109559	chr1-91109467-91109559
chr1	91250167	91250259	chr1-91250167-91250259
chr1	91763532	91763624	chr1-91763532-91763624
chr1	91962761	91962853	chr1-91962761-91962853
chr1	92052706	92052798	chr1-92052706-92052798
chr1	97071565	97071657	chr1-97071565-97071657
chr1	97661018	97661110	chr1-97661018-97661110
chr1	99642536	99642628	chr1-99642536-99642628
chr1	101126399	101126491	chr1-101126399-101126491
chr1	102097333	102097425	chr1-102097333-102097425
chr1	102628203	102628295	chr1-102628203-102628295
chr1	104908391	104908483	chr1-104908391-104908483
chr1	106187590	106187682	chr1-106187590-106187682
chr1	106400983	106401075	chr1-106400983-106401075
chr1	107114171	107114263	chr1-107114171-107114263
chr1	107511404	107511496	chr1-107511404-107511496
chr1	110038921	110039013	chr1-110038921-110039013
chr1	110483207	110483299	chr1-110483207-110483299
chr1	110954056	110954148	chr1-110954056-110954148
chr1	111191117	111191209	chr1-111191117-111191209
chr1	112457524	112457616	chr1-112457524-112457616
chr1	113466685	113466777	chr1-113466685-113466777
chr1	113904217	113904309	chr1-113904217-113904309
chr1	114041168	114041260	chr1-114041168-114041260
chr1	118311385	118311477	chr1-118311385-118311477
chr1	118479351	118479443	chr1-118479351-118479443
chr1	118707342	118707434	chr1-118707342-118707434
chr1	119064084	119064176	chr1-119064084-119064176
chr1	119344196	119344288	chr1-119344196-119344288
chr1	119649836	119649928	chr1-119649836-119649928
chr1	119920774	119920866	chr1-119920774-119920866

chr1	119990569	119990661	chr1-119990569-119990661
chr1	120825694	120825786	chr1-120825694-120825786
chr1	121767687	121767779	chr1-121767687-121767779
chr1	122357135	122357227	chr1-122357135-122357227
chr1	123222452	123222544	chr1-123222452-123222544
chr1	123673227	123673319	chr1-123673227-123673319
chr1	123823721	123823813	chr1-123823721-123823813
chr1	124530547	124530639	chr1-124530547-124530639
chr1	124813025	124813117	chr1-124813025-124813117
chr1	125519489	125519581	chr1-125519489-125519581
chr1	125700930	125701022	chr1-125700930-125701022
chr1	126030979	126031071	chr1-126030979-126031071
chr1	126404129	126404221	chr1-126404129-126404221
chr1	126649881	126649973	chr1-126649881-126649973
chr1	126984351	126984443	chr1-126984351-126984443
chr1	127622577	127622669	chr1-127622577-127622669
chr1	128399249	128399341	chr1-128399249-128399341
chr1	129451069	129451161	chr1-129451069-129451161
chr1	130741445	130741537	chr1-130741445-130741537
chr1	130761629	130761721	chr1-130761629-130761721
chr1	131126345	131126437	chr1-131126345-131126437
chr1	131281272	131281364	chr1-131281272-131281364
chr1	131511969	131512061	chr1-131511969-131512061
chr1	132095719	132095811	chr1-132095719-132095811
chr1	132150280	132150372	chr1-132150280-132150372
chr1	132151953	132152045	chr1-132151953-132152045
chr1	133118996	133119088	chr1-133118996-133119088
chr1	133161995	133162087	chr1-133161995-133162087
chr1	133211949	133212041	chr1-133211949-133212041
chr1	135181071	135181163	chr1-135181071-135181163
chr1	135540484	135540576	chr1-135540484-135540576
chr1	135818836	135818928	chr1-135818836-135818928
chr1	135844625	135844717	chr1-135844625-135844717
chr1	136110640	136110732	chr1-136110640-136110732
chr1	136685353	136685445	chr1-136685353-136685445
chr1	138304037	138304129	chr1-138304037-138304129
chr1	138537058	138537150	chr1-138537058-138537150
chr1	138617845	138617937	chr1-138617845-138617937
chr1	138735184	138735276	chr1-138735184-138735276
chr1	139107979	139108071	chr1-139107979-139108071
chr1	140672719	140672811	chr1-140672719-140672811
chr1	142740400	142740492	chr1-142740400-142740492

chr1	143355745	143355837	chr1-143355745-143355837
chr1	143871712	143871804	chr1-143871712-143871804
chr1	144020147	144020239	chr1-144020147-144020239
chr1	148474348	148474440	chr1-148474348-148474440
chr1	150710618	150710710	chr1-150710618-150710710
chr1	151647702	151647794	chr1-151647702-151647794
chr1	153202213	153202305	chr1-153202213-153202305
chr1	153304628	153304720	chr1-153304628-153304720
chr1	153683476	153683568	chr1-153683476-153683568
chr1	154309695	154309787	chr1-154309695-154309787
chr1	155167457	155167549	chr1-155167457-155167549
chr1	155523533	155523625	chr1-155523533-155523625
chr1	156474236	156474328	chr1-156474236-156474328
chr1	156919988	156920080	chr1-156919988-156920080
chr1	157061797	157061889	chr1-157061797-157061889
chr1	157851016	157851108	chr1-157851016-157851108
chr1	158064712	158064804	chr1-158064712-158064804
chr1	158523323	158523415	chr1-158523323-158523415
chr1	158804839	158804931	chr1-158804839-158804931
chr1	159544153	159544245	chr1-159544153-159544245
chr1	160868053	160868145	chr1-160868053-160868145
chr1	160878751	160878843	chr1-160878751-160878843
chr1	161250126	161250218	chr1-161250126-161250218
chr1	162225892	162225984	chr1-162225892-162225984
chr1	162449326	162449418	chr1-162449326-162449418
chr1	162509821	162509913	chr1-162509821-162509913
chr1	163441285	163441377	chr1-163441285-163441377
chr1	164279134	164279226	chr1-164279134-164279226
chr1	164566266	164566358	chr1-164566266-164566358
chr1	166009102	166009194	chr1-166009102-166009194
chr1	166255653	166255745	chr1-166255653-166255745
chr1	167350115	167350207	chr1-167350115-167350207
chr1	168502450	168502542	chr1-168502450-168502542
chr1	168675114	168675206	chr1-168675114-168675206
chr1	168784039	168784131	chr1-168784039-168784131
chr1	169253257	169253349	chr1-169253257-169253349
chr1	169853247	169853339	chr1-169853247-169853339
chr1	170482135	170482227	chr1-170482135-170482227
chr1	170757039	170757131	chr1-170757039-170757131
chr1	170962451	170962543	chr1-170962451-170962543
chr1	171064577	171064669	chr1-171064577-171064669
chr1	171065921	171066013	chr1-171065921-171066013

chr1	171077885	171077977	chr1-171077885-171077977
chr1	171078136	171078228	chr1-171078136-171078228
chr1	171094522	171094614	chr1-171094522-171094614
chr1	171247122	171247214	chr1-171247122-171247214
chr1	171441530	171441622	chr1-171441530-171441622
chr1	171470491	171470583	chr1-171470491-171470583
chr1	171493371	171493463	chr1-171493371-171493463
chr1	172514065	172514157	chr1-172514065-172514157
chr1	173152939	173153031	chr1-173152939-173153031
chr1	173973171	173973263	chr1-173973171-173973263
chr1	175092218	175092310	chr1-175092218-175092310
chr1	176059604	176059696	chr1-176059604-176059696
chr1	176818341	176818433	chr1-176818341-176818433
chr1	176834859	176834951	chr1-176834859-176834951
chr1	177960511	177960603	chr1-177960511-177960603
chr1	178348587	178348679	chr1-178348587-178348679
chr1	178943802	178943894	chr1-178943802-178943894
chr1	179042034	179042126	chr1-179042034-179042126
chr1	180015110	180015202	chr1-180015110-180015202
chr1	180015545	180015637	chr1-180015545-180015637
chr1	180850939	180851031	chr1-180850939-180851031
chr1	180917227	180917319	chr1-180917227-180917319
chr1	180942978	180943070	chr1-180942978-180943070
chr1	181401338	181401430	chr1-181401338-181401430
chr1	181415407	181415499	chr1-181415407-181415499
chr1	181444899	181444991	chr1-181444899-181444991
chr1	181842506	181842598	chr1-181842506-181842598
chr1	182025721	182025813	chr1-182025721-182025813
chr1	182372072	182372164	chr1-182372072-182372164
chr1	182517585	182517677	chr1-182517585-182517677
chr1	182866042	182866134	chr1-182866042-182866134
chr1	183144728	183144820	chr1-183144728-183144820
chr1	183413060	183413152	chr1-183413060-183413152
chr1	183506705	183506797	chr1-183506705-183506797
chr1	183680091	183680183	chr1-183680091-183680183
chr1	183803579	183803671	chr1-183803579-183803671
chr1	183865724	183865816	chr1-183865724-183865816
chr1	184016475	184016567	chr1-184016475-184016567
chr1	184051805	184051897	chr1-184051805-184051897
chr1	184057402	184057494	chr1-184057402-184057494
chr1	184261267	184261359	chr1-184261267-184261359
chr1	184275992	184276084	chr1-184275992-184276084

chr1	185209151	185209243	chr1-185209151-185209243
chr1	186568313	186568405	chr1-186568313-186568405
chr1	189623871	189623963	chr1-189623871-189623963
chr1	189661846	189661938	chr1-189661846-189661938
chr1	190483066	190483158	chr1-190483066-190483158
chr1	190761861	190761953	chr1-190761861-190761953
chr1	190790385	190790477	chr1-190790385-190790477
chr1	190870869	190870961	chr1-190870869-190870961
chr1	191133141	191133233	chr1-191133141-191133233
chr1	191183483	191183575	chr1-191183483-191183575
chr1	191193967	191194059	chr1-191193967-191194059
chr1	191446707	191446799	chr1-191446707-191446799
chr1	191454771	191454863	chr1-191454771-191454863
chr1	191534626	191534718	chr1-191534626-191534718
chr1	192710353	192710445	chr1-192710353-192710445
chr1	193151374	193151466	chr1-193151374-193151466
chr1	194090024	194090116	chr1-194090024-194090116
chr1	194544371	194544463	chr1-194544371-194544463
chr1	194660512	194660604	chr1-194660512-194660604
chr1	194675959	194676051	chr1-194675959-194676051
chr1	195100845	195100937	chr1-195100845-195100937
chr10	3112840	3112932	chr10-3112840-3112932
chr10	3114574	3114666	chr10-3114574-3114666
chr10	3236770	3236862	chr10-3236770-3236862
chr10	4108139	4108231	chr10-4108139-4108231
chr10	4167828	4167920	chr10-4167828-4167920
chr10	4583853	4583945	chr10-4583853-4583945
chr10	4616470	4616562	chr10-4616470-4616562
chr10	5646646	5646738	chr10-5646646-5646738
chr10	5689317	5689409	chr10-5689317-5689409
chr10	5707389	5707481	chr10-5707389-5707481
chr10	5804710	5804802	chr10-5804710-5804802
chr10	8959773	8959865	chr10-8959773-8959865
chr10	9258809	9258901	chr10-9258809-9258901
chr10	9529430	9529522	chr10-9529430-9529522
chr10	11492093	11492185	chr10-11492093-11492185
chr10	12181069	12181161	chr10-12181069-12181161
chr10	12867512	12867604	chr10-12867512-12867604
chr10	13324234	13324326	chr10-13324234-13324326
chr10	13369864	13369956	chr10-13369864-13369956
chr10	14686166	14686258	chr10-14686166-14686258
chr10	16159656	16159748	chr10-16159656-16159748

chr10	16507774	16507866	chr10-16507774-16507866
chr10	18615599	18615691	chr10-18615599-18615691
chr10	18988006	18988098	chr10-18988006-18988098
chr10	19167804	19167896	chr10-19167804-19167896
chr10	21532311	21532403	chr10-21532311-21532403
chr10	21588637	21588729	chr10-21588637-21588729
chr10	21844523	21844615	chr10-21844523-21844615
chr10	22223581	22223673	chr10-22223581-22223673
chr10	22698610	22698702	chr10-22698610-22698702
chr10	23457480	23457572	chr10-23457480-23457572
chr10	23609924	23610016	chr10-23609924-23610016
chr10	23988624	23988716	chr10-23988624-23988716
chr10	24446305	24446397	chr10-24446305-24446397
chr10	24540497	24540589	chr10-24540497-24540589
chr10	24581133	24581225	chr10-24581133-24581225
chr10	24629197	24629289	chr10-24629197-24629289
chr10	24636209	24636301	chr10-24636209-24636301
chr10	26079000	26079092	chr10-26079000-26079092
chr10	26652032	26652124	chr10-26652032-26652124
chr10	27597364	27597456	chr10-27597364-27597456
chr10	27632968	27633060	chr10-27632968-27633060
chr10	28216183	28216275	chr10-28216183-28216275
chr10	28498750	28498842	chr10-28498750-28498842
chr10	28618948	28619040	chr10-28618948-28619040
chr10	29381669	29381761	chr10-29381669-29381761
chr10	32237313	32237405	chr10-32237313-32237405
chr10	32984984	32985076	chr10-32984984-32985076
chr10	34096496	34096588	chr10-34096496-34096588
chr10	34508744	34508836	chr10-34508744-34508836
chr10	35039483	35039575	chr10-35039483-35039575
chr10	35885291	35885383	chr10-35885291-35885383
chr10	37263698	37263790	chr10-37263698-37263790
chr10	38444994	38445086	chr10-38444994-38445086
chr10	39342194	39342286	chr10-39342194-39342286
chr10	39614866	39614958	chr10-39614866-39614958
chr10	40868045	40868137	chr10-40868045-40868137
chr10	41749039	41749131	chr10-41749039-41749131
chr10	44006206	44006298	chr10-44006206-44006298
chr10	44079316	44079408	chr10-44079316-44079408
chr10	44520129	44520221	chr10-44520129-44520221
chr10	44607586	44607678	chr10-44607586-44607678
chr10	46516592	46516684	chr10-46516592-46516684

chr10	46647182	46647274	chr10-46647182-46647274
chr10	46785966	46786058	chr10-46785966-46786058
chr10	48018369	48018461	chr10-48018369-48018461
chr10	48067085	48067177	chr10-48067085-48067177
chr10	49640318	49640410	chr10-49640318-49640410
chr10	50584931	50585023	chr10-50584931-50585023
chr10	51949282	51949374	chr10-51949282-51949374
chr10	52219681	52219773	chr10-52219681-52219773
chr10	53410807	53410899	chr10-53410807-53410899
chr10	56563702	56563794	chr10-56563702-56563794
chr10	57644537	57644629	chr10-57644537-57644629
chr10	58224359	58224451	chr10-58224359-58224451
chr10	58224631	58224723	chr10-58224631-58224723
chr10	58224968	58225060	chr10-58224968-58225060
chr10	58231255	58231347	chr10-58231255-58231347
chr10	58231861	58231953	chr10-58231861-58231953
chr10	58232129	58232221	chr10-58232129-58232221
chr10	58232474	58232566	chr10-58232474-58232566
chr10	58239476	58239568	chr10-58239476-58239568
chr10	58371313	58371405	chr10-58371313-58371405
chr10	58386508	58386600	chr10-58386508-58386600
chr10	59152767	59152859	chr10-59152767-59152859
chr10	59430733	59430825	chr10-59430733-59430825
chr10	59430965	59431057	chr10-59430965-59431057
chr10	59557396	59557488	chr10-59557396-59557488
chr10	59943849	59943941	chr10-59943849-59943941
chr10	60081776	60081868	chr10-60081776-60081868
chr10	60332820	60332912	chr10-60332820-60332912
chr10	60831670	60831762	chr10-60831670-60831762
chr10	60877522	60877614	chr10-60877522-60877614
chr10	61419117	61419209	chr10-61419117-61419209
chr10	62221775	62221867	chr10-62221775-62221867
chr10	62413025	62413117	chr10-62413025-62413117
chr10	62413745	62413837	chr10-62413745-62413837
chr10	62855034	62855126	chr10-62855034-62855126
chr10	63158281	63158373	chr10-63158281-63158373
chr10	64396784	64396876	chr10-64396784-64396876
chr10	65970233	65970325	chr10-65970233-65970325
chr10	66946369	66946461	chr10-66946369-66946461
chr10	67300267	67300359	chr10-67300267-67300359
chr10	67934665	67934757	chr10-67934665-67934757
chr10	68008434	68008526	chr10-68008434-68008526

chr10	69533664	69533756	chr10-69533664-69533756
chr10	69895370	69895462	chr10-69895370-69895462
chr10	70120959	70121051	chr10-70120959-70121051
chr10	70195176	70195268	chr10-70195176-70195268
chr10	70222358	70222450	chr10-70222358-70222450
chr10	70330103	70330195	chr10-70330103-70330195
chr10	71238000	71238092	chr10-71238000-71238092
chr10	72015491	72015583	chr10-72015491-72015583
chr10	73060847	73060939	chr10-73060847-73060939
chr10	75119548	75119640	chr10-75119548-75119640
chr10	75300889	75300981	chr10-75300889-75300981
chr10	75434697	75434789	chr10-75434697-75434789
chr10	75684147	75684239	chr10-75684147-75684239
chr10	75827864	75827956	chr10-75827864-75827956
chr10	76032208	76032300	chr10-76032208-76032300
chr10	76332801	76332893	chr10-76332801-76332893
chr10	77487046	77487138	chr10-77487046-77487138
chr10	77614978	77615070	chr10-77614978-77615070
chr10	78006980	78007072	chr10-78006980-78007072
chr10	78018939	78019031	chr10-78018939-78019031
chr10	78590875	78590967	chr10-78590875-78590967
chr10	79937276	79937368	chr10-79937276-79937368
chr10	80033200	80033292	chr10-80033200-80033292
chr10	80053380	80053472	chr10-80053380-80053472
chr10	80104338	80104430	chr10-80104338-80104430
chr10	80137616	80137708	chr10-80137616-80137708
chr10	80180569	80180661	chr10-80180569-80180661
chr10	80260933	80261025	chr10-80260933-80261025
chr10	80261778	80261870	chr10-80261778-80261870
chr10	80282635	80282727	chr10-80282635-80282727
chr10	80433949	80434041	chr10-80433949-80434041
chr10	80552043	80552135	chr10-80552043-80552135
chr10	80717654	80717746	chr10-80717654-80717746
chr10	80827018	80827110	chr10-80827018-80827110
chr10	81106567	81106659	chr10-81106567-81106659
chr10	81136177	81136269	chr10-81136177-81136269
chr10	81137326	81137418	chr10-81137326-81137418
chr10	81144251	81144343	chr10-81144251-81144343
chr10	81178098	81178190	chr10-81178098-81178190
chr10	81178689	81178781	chr10-81178689-81178781
chr10	81182364	81182456	chr10-81182364-81182456
chr10	81407633	81407725	chr10-81407633-81407725

chr10	81627521	81627613	chr10-81627521-81627613
chr10	82409734	82409826	chr10-82409734-82409826
chr10	82811925	82812017	chr10-82811925-82812017
chr10	82858850	82858942	chr10-82858850-82858942
chr10	82907018	82907110	chr10-82907018-82907110
chr10	82932679	82932771	chr10-82932679-82932771
chr10	83360154	83360246	chr10-83360154-83360246
chr10	83875802	83875894	chr10-83875802-83875894
chr10	84376576	84376668	chr10-84376576-84376668
chr10	84389792	84389884	chr10-84389792-84389884
chr10	84409407	84409499	chr10-84409407-84409499
chr10	84494525	84494617	chr10-84494525-84494617
chr10	84503553	84503645	chr10-84503553-84503645
chr10	85830078	85830170	chr10-85830078-85830170
chr10	86525084	86525176	chr10-86525084-86525176
chr10	87511783	87511875	chr10-87511783-87511875
chr10	87880145	87880237	chr10-87880145-87880237
chr10	89527247	89527339	chr10-89527247-89527339
chr10	89594465	89594557	chr10-89594465-89594557
chr10	91156585	91156677	chr10-91156585-91156677
chr10	91171755	91171847	chr10-91171755-91171847
chr10	91361921	91362013	chr10-91361921-91362013
chr10	91559347	91559439	chr10-91559347-91559439
chr10	91695592	91695684	chr10-91695592-91695684
chr10	91891132	91891224	chr10-91891132-91891224
chr10	92388236	92388328	chr10-92388236-92388328
chr10	92581180	92581272	chr10-92581180-92581272
chr10	93287579	93287671	chr10-93287579-93287671
chr10	94072548	94072640	chr10-94072548-94072640
chr10	94116352	94116444	chr10-94116352-94116444
chr10	95084416	95084508	chr10-95084416-95084508
chr10	95631703	95631795	chr10-95631703-95631795
chr10	95644191	95644283	chr10-95644191-95644283
chr10	96177613	96177705	chr10-96177613-96177705
chr10	96871739	96871831	chr10-96871739-96871831
chr10	98292327	98292419	chr10-98292327-98292419
chr10	98629332	98629424	chr10-98629332-98629424
chr10	98951521	98951613	chr10-98951521-98951613
chr10	99198148	99198240	chr10-99198148-99198240
chr10	99858531	99858623	chr10-99858531-99858623
chr10	100152297	100152389	chr10-100152297-100152389
chr10	102135545	102135637	chr10-102135545-102135637

chr10	102629713	102629805	chr10-102629713-102629805
chr10	103071891	103071983	chr10-103071891-103071983
chr10	103602348	103602440	chr10-103602348-103602440
chr10	104339316	104339408	chr10-104339316-104339408
chr10	104630069	104630161	chr10-104630069-104630161
chr10	105270877	105270969	chr10-105270877-105270969
chr10	105425443	105425535	chr10-105425443-105425535
chr10	105574624	105574716	chr10-105574624-105574716
chr10	106557084	106557176	chr10-106557084-106557176
chr10	107112815	107112907	chr10-107112815-107112907
chr10	108113471	108113563	chr10-108113471-108113563
chr10	108162323	108162415	chr10-108162323-108162415
chr10	108445284	108445376	chr10-108445284-108445376
chr10	108531223	108531315	chr10-108531223-108531315
chr10	110115236	110115328	chr10-110115236-110115328
chr10	110674192	110674284	chr10-110674192-110674284
chr10	111009724	111009816	chr10-111009724-111009816
chr10	111423975	111424067	chr10-111423975-111424067
chr10	111576051	111576143	chr10-111576051-111576143
chr10	111583528	111583620	chr10-111583528-111583620
chr10	111619108	111619200	chr10-111619108-111619200
chr10	111621035	111621127	chr10-111621035-111621127
chr10	111672872	111672964	chr10-111672872-111672964
chr10	111997780	111997872	chr10-111997780-111997872
chr10	112883196	112883288	chr10-112883196-112883288
chr10	113102008	113102100	chr10-113102008-113102100
chr10	113826628	113826720	chr10-113826628-113826720
chr10	115146943	115147035	chr10-115146943-115147035
chr10	115170318	115170410	chr10-115170318-115170410
chr10	115262390	115262482	chr10-115262390-115262482
chr10	115764210	115764302	chr10-115764210-115764302
chr10	115780401	115780493	chr10-115780401-115780493
chr10	115817303	115817395	chr10-115817303-115817395
chr10	116261087	116261179	chr10-116261087-116261179
chr10	116266978	116267070	chr10-116266978-116267070
chr10	116460839	116460931	chr10-116460839-116460931
chr10	116733826	116733918	chr10-116733826-116733918
chr10	117023632	117023724	chr10-117023632-117023724
chr10	117177855	117177947	chr10-117177855-117177947
chr10	118509874	118509966	chr10-118509874-118509966
chr10	119221416	119221508	chr10-119221416-119221508
chr10	120012507	120012599	chr10-120012507-120012599

chr10	120433222	120433314	chr10-120433222-120433314
chr10	120481427	120481519	chr10-120481427-120481519
chr10	121455214	121455306	chr10-121455214-121455306
chr10	121455676	121455768	chr10-121455676-121455768
chr10	121739582	121739674	chr10-121739582-121739674
chr10	122787987	122788079	chr10-122787987-122788079
chr10	122808646	122808738	chr10-122808646-122808738
chr10	123092493	123092585	chr10-123092493-123092585
chr10	123674337	123674429	chr10-123674337-123674429
chr10	124705129	124705221	chr10-124705129-124705221
chr10	127266351	127266443	chr10-127266351-127266443
chr10	127290754	127290846	chr10-127290754-127290846
chr10	127738764	127738856	chr10-127738764-127738856
chr10	128507553	128507645	chr10-128507553-128507645
chr10	128771810	128771902	chr10-128771810-128771902
chr10	130333242	130333334	chr10-130333242-130333334
chr11	3112918	3113010	chr11-3112918-3113010
chr11	3124028	3124120	chr11-3124028-3124120
chr11	3151429	3151521	chr11-3151429-3151521
chr11	3332331	3332423	chr11-3332331-3332423
chr11	3514534	3514626	chr11-3514534-3514626
chr11	4102018	4102110	chr11-4102018-4102110
chr11	4121854	4121946	chr11-4121854-4121946
chr11	4297991	4298083	chr11-4297991-4298083
chr11	4746412	4746504	chr11-4746412-4746504
chr11	4951846	4951938	chr11-4951846-4951938
chr11	5230757	5230849	chr11-5230757-5230849
chr11	5490653	5490745	chr11-5490653-5490745
chr11	5522177	5522269	chr11-5522177-5522269
chr11	5542369	5542461	chr11-5542369-5542461
chr11	6618453	6618545	chr11-6618453-6618545
chr11	6937505	6937597	chr11-6937505-6937597
chr11	7019293	7019385	chr11-7019293-7019385
chr11	7239267	7239359	chr11-7239267-7239359
chr11	7794089	7794181	chr11-7794089-7794181
chr11	7794443	7794535	chr11-7794443-7794535
chr11	7795833	7795925	chr11-7795833-7795925
chr11	8635978	8636070	chr11-8635978-8636070
chr11	8675357	8675449	chr11-8675357-8675449
chr11	8710963	8711055	chr11-8710963-8711055
chr11	8821661	8821753	chr11-8821661-8821753
chr11	9143804	9143896	chr11-9143804-9143896

chr11	9651763	9651855	chr11-9651763-9651855
chr11	9788303	9788395	chr11-9788303-9788395
chr11	9989399	9989491	chr11-9989399-9989491
chr11	11681917	11682009	chr11-11681917-11682009
chr11	11811202	11811294	chr11-11811202-11811294
chr11	12409615	12409707	chr11-12409615-12409707
chr11	12677590	12677682	chr11-12677590-12677682
chr11	12823087	12823179	chr11-12823087-12823179
chr11	13391018	13391110	chr11-13391018-13391110
chr11	14749185	14749277	chr11-14749185-14749277
chr11	16584112	16584204	chr11-16584112-16584204
chr11	16739295	16739387	chr11-16739295-16739387
chr11	16770203	16770295	chr11-16770203-16770295
chr11	16795999	16796091	chr11-16795999-16796091
chr11	18024619	18024711	chr11-18024619-18024711
chr11	19257884	19257976	chr11-19257884-19257976
chr11	19848504	19848596	chr11-19848504-19848596
chr11	20306346	20306438	chr11-20306346-20306438
chr11	20452207	20452299	chr11-20452207-20452299
chr11	21105518	21105610	chr11-21105518-21105610
chr11	22728285	22728377	chr11-22728285-22728377
chr11	22992732	22992824	chr11-22992732-22992824
chr11	23391773	23391865	chr11-23391773-23391865
chr11	24211999	24212091	chr11-24211999-24212091
chr11	24520833	24520925	chr11-24520833-24520925
chr11	25100065	25100157	chr11-25100065-25100157
chr11	26588523	26588615	chr11-26588523-26588615
chr11	31057568	31057660	chr11-31057568-31057660
chr11	32146719	32146811	chr11-32146719-32146811
chr11	32284996	32285088	chr11-32284996-32285088
chr11	32489233	32489325	chr11-32489233-32489325
chr11	32762878	32762970	chr11-32762878-32762970
chr11	32963341	32963433	chr11-32963341-32963433
chr11	33143008	33143100	chr11-33143008-33143100
chr11	33738034	33738126	chr11-33738034-33738126
chr11	34809832	34809924	chr11-34809832-34809924
chr11	34987920	34988012	chr11-34987920-34988012
chr11	35452923	35453015	chr11-35452923-35453015
chr11	35945306	35945398	chr11-35945306-35945398
chr11	35970755	35970847	chr11-35970755-35970847
chr11	35971514	35971606	chr11-35971514-35971606
chr11	35982871	35982963	chr11-35982871-35982963

chr11	39143988	39144080	chr11-39143988-39144080
chr11	40197817	40197909	chr11-40197817-40197909
chr11	42418582	42418674	chr11-42418582-42418674
chr11	42906203	42906295	chr11-42906203-42906295
chr11	42944686	42944778	chr11-42944686-42944778
chr11	44469719	44469811	chr11-44469719-44469811
chr11	46675869	46675961	chr11-46675869-46675961
chr11	47118434	47118526	chr11-47118434-47118526
chr11	49779537	49779629	chr11-49779537-49779629
chr11	50252207	50252299	chr11-50252207-50252299
chr11	50377742	50377834	chr11-50377742-50377834
chr11	50500769	50500861	chr11-50500769-50500861
chr11	50669722	50669814	chr11-50669722-50669814
chr11	51208018	51208110	chr11-51208018-51208110
chr11	51584436	51584528	chr11-51584436-51584528
chr11	51651326	51651418	chr11-51651326-51651418
chr11	51808790	51808882	chr11-51808790-51808882
chr11	51916444	51916536	chr11-51916444-51916536
chr11	51949580	51949672	chr11-51949580-51949672
chr11	52525938	52526030	chr11-52525938-52526030
chr11	52546892	52546984	chr11-52546892-52546984
chr11	53483328	53483420	chr11-53483328-53483420
chr11	53483762	53483854	chr11-53483762-53483854
chr11	53721866	53721958	chr11-53721866-53721958
chr11	53795872	53795964	chr11-53795872-53795964
chr11	54061175	54061267	chr11-54061175-54061267
chr11	54249729	54249821	chr11-54249729-54249821
chr11	54257110	54257202	chr11-54257110-54257202
chr11	54269648	54269740	chr11-54269648-54269740
chr11	54769085	54769177	chr11-54769085-54769177
chr11	54807015	54807107	chr11-54807015-54807107
chr11	54866848	54866940	chr11-54866848-54866940
chr11	54934242	54934334	chr11-54934242-54934334
chr11	55924124	55924216	chr11-55924124-55924216
chr11	55947430	55947522	chr11-55947430-55947522
chr11	56163325	56163417	chr11-56163325-56163417
chr11	57323891	57323983	chr11-57323891-57323983
chr11	57913207	57913299	chr11-57913207-57913299
chr11	58931275	58931367	chr11-58931275-58931367
chr11	58962075	58962167	chr11-58962075-58962167
chr11	59283659	59283751	chr11-59283659-59283751
chr11	59384712	59384804	chr11-59384712-59384804

chr11	59632138	59632230	chr11-59632138-59632230
chr11	59636150	59636242	chr11-59636150-59636242
chr11	59637090	59637182	chr11-59637090-59637182
chr11	59655408	59655500	chr11-59655408-59655500
chr11	59750955	59751047	chr11-59750955-59751047
chr11	59826274	59826366	chr11-59826274-59826366
chr11	60046479	60046571	chr11-60046479-60046571
chr11	60125378	60125470	chr11-60125378-60125470
chr11	60308042	60308134	chr11-60308042-60308134
chr11	60341556	60341648	chr11-60341556-60341648
chr11	60454476	60454568	chr11-60454476-60454568
chr11	60935381	60935473	chr11-60935381-60935473
chr11	61267189	61267281	chr11-61267189-61267281
chr11	61310178	61310270	chr11-61310178-61310270
chr11	61494513	61494605	chr11-61494513-61494605
chr11	61930150	61930242	chr11-61930150-61930242
chr11	62552820	62552912	chr11-62552820-62552912
chr11	62814856	62814948	chr11-62814856-62814948
chr11	63634407	63634499	chr11-63634407-63634499
chr11	65056381	65056473	chr11-65056381-65056473
chr11	65560988	65561080	chr11-65560988-65561080
chr11	65598715	65598807	chr11-65598715-65598807
chr11	67468145	67468237	chr11-67468145-67468237
chr11	67608179	67608271	chr11-67608179-67608271
chr11	68103420	68103512	chr11-68103420-68103512
chr11	68652867	68652959	chr11-68652867-68652959
chr11	68838764	68838856	chr11-68838764-68838856
chr11	69067755	69067847	chr11-69067755-69067847
chr11	69073497	69073589	chr11-69073497-69073589
chr11	69366610	69366702	chr11-69366610-69366702
chr11	69917933	69918025	chr11-69917933-69918025
chr11	70082816	70082908	chr11-70082816-70082908
chr11	70171361	70171453	chr11-70171361-70171453
chr11	70239913	70240005	chr11-70239913-70240005
chr11	70259210	70259302	chr11-70259210-70259302
chr11	70654663	70654755	chr11-70654663-70654755
chr11	70655991	70656083	chr11-70655991-70656083
chr11	71165977	71166069	chr11-71165977-71166069
chr11	72094358	72094450	chr11-72094358-72094450
chr11	72472537	72472629	chr11-72472537-72472629
chr11	72490051	72490143	chr11-72490051-72490143
chr11	72568641	72568733	chr11-72568641-72568733

chr11	73047772	73047864	chr11-73047772-73047864
chr11	74450037	74450129	chr11-74450037-74450129
chr11	74724277	74724369	chr11-74724277-74724369
chr11	75169144	75169236	chr11-75169144-75169236
chr11	75564459	75564551	chr11-75564459-75564551
chr11	75678814	75678906	chr11-75678814-75678906
chr11	75687663	75687755	chr11-75687663-75687755
chr11	75844044	75844136	chr11-75844044-75844136
chr11	76017659	76017751	chr11-76017659-76017751
chr11	76477458	76477550	chr11-76477458-76477550
chr11	77003321	77003413	chr11-77003321-77003413
chr11	77053541	77053633	chr11-77053541-77053633
chr11	77145588	77145680	chr11-77145588-77145680
chr11	77766669	77766761	chr11-77766669-77766761
chr11	77783557	77783649	chr11-77783557-77783649
chr11	77784154	77784246	chr11-77784154-77784246
chr11	78198022	78198114	chr11-78198022-78198114
chr11	78254498	78254590	chr11-78254498-78254590
chr11	78260641	78260733	chr11-78260641-78260733
chr11	78536374	78536466	chr11-78536374-78536466
chr11	79085222	79085314	chr11-79085222-79085314
chr11	79112580	79112672	chr11-79112580-79112672
chr11	79214240	79214332	chr11-79214240-79214332
chr11	79254675	79254767	chr11-79254675-79254767
chr11	79256231	79256323	chr11-79256231-79256323
chr11	79368576	79368668	chr11-79368576-79368668
chr11	79629735	79629827	chr11-79629735-79629827
chr11	80818250	80818342	chr11-80818250-80818342
chr11	82930736	82930828	chr11-82930736-82930828
chr11	83079587	83079679	chr11-83079587-83079679
chr11	83511103	83511195	chr11-83511103-83511195
chr11	83550182	83550274	chr11-83550182-83550274
chr11	83882801	83882893	chr11-83882801-83882893
chr11	83946528	83946620	chr11-83946528-83946620
chr11	83993284	83993376	chr11-83993284-83993376
chr11	84520474	84520566	chr11-84520474-84520566
chr11	85266435	85266527	chr11-85266435-85266527
chr11	85321115	85321207	chr11-85321115-85321207
chr11	85857748	85857840	chr11-85857748-85857840
chr11	86257521	86257613	chr11-86257521-86257613
chr11	86581401	86581493	chr11-86581401-86581493
chr11	86587259	86587351	chr11-86587259-86587351

chr11	86962157	86962249	chr11-86962157-86962249
chr11	87043405	87043497	chr11-87043405-87043497
chr11	87074090	87074182	chr11-87074090-87074182
chr11	87309779	87309871	chr11-87309779-87309871
chr11	87459516	87459608	chr11-87459516-87459608
chr11	87519388	87519480	chr11-87519388-87519480
chr11	87610281	87610373	chr11-87610281-87610373
chr11	87689491	87689583	chr11-87689491-87689583
chr11	87926142	87926234	chr11-87926142-87926234
chr11	88047635	88047727	chr11-88047635-88047727
chr11	88173735	88173827	chr11-88173735-88173827
chr11	88352861	88352953	chr11-88352861-88352953
chr11	88496183	88496275	chr11-88496183-88496275
chr11	88553698	88553790	chr11-88553698-88553790
chr11	88614911	88615003	chr11-88614911-88615003
chr11	88638468	88638560	chr11-88638468-88638560
chr11	88791291	88791383	chr11-88791291-88791383
chr11	89206535	89206627	chr11-89206535-89206627
chr11	89303580	89303672	chr11-89303580-89303672
chr11	90225017	90225109	chr11-90225017-90225109
chr11	91977878	91977970	chr11-91977878-91977970
chr11	94327879	94327971	chr11-94327879-94327971
chr11	94365532	94365624	chr11-94365532-94365624
chr11	94370927	94371019	chr11-94370927-94371019
chr11	94509106	94509198	chr11-94509106-94509198
chr11	95079660	95079752	chr11-95079660-95079752
chr11	95105597	95105689	chr11-95105597-95105689
chr11	95135199	95135291	chr11-95135199-95135291
chr11	95318947	95319039	chr11-95318947-95319039
chr11	95358393	95358485	chr11-95358393-95358485
chr11	95712645	95712737	chr11-95712645-95712737
chr11	95778115	95778207	chr11-95778115-95778207
chr11	95792814	95792906	chr11-95792814-95792906
chr11	96403597	96403689	chr11-96403597-96403689
chr11	96840801	96840893	chr11-96840801-96840893
chr11	97065709	97065801	chr11-97065709-97065801
chr11	97192403	97192495	chr11-97192403-97192495
chr11	97812157	97812249	chr11-97812157-97812249
chr11	97821008	97821100	chr11-97821008-97821100
chr11	97932401	97932493	chr11-97932401-97932493
chr11	97994682	97994774	chr11-97994682-97994774
chr11	98355928	98356020	chr11-98355928-98356020

chr11	98419084	98419176	chr11-98419084-98419176
chr11	98905195	98905287	chr11-98905195-98905287
chr11	98963372	98963464	chr11-98963372-98963464
chr11	99024158	99024250	chr11-99024158-99024250
chr11	99047079	99047171	chr11-99047079-99047171
chr11	99101802	99101894	chr11-99101802-99101894
chr11	99657540	99657632	chr11-99657540-99657632
chr11	100147723	100147815	chr11-100147723-100147815
chr11	100395579	100395671	chr11-100395579-100395671
chr11	100442357	100442449	chr11-100442357-100442449
chr11	101442226	101442318	chr11-101442226-101442318
chr11	101604779	101604871	chr11-101604779-101604871
chr11	101733086	101733178	chr11-101733086-101733178
chr11	102643999	102644091	chr11-102643999-102644091
chr11	102691959	102692051	chr11-102691959-102692051
chr11	102881188	102881280	chr11-102881188-102881280
chr11	103093499	103093591	chr11-103093499-103093591
chr11	103387408	103387500	chr11-103387408-103387500
chr11	104289358	104289450	chr11-104289358-104289450
chr11	104517392	104517484	chr11-104517392-104517484
chr11	104520745	104520837	chr11-104520745-104520837
chr11	104521493	104521585	chr11-104521493-104521585
chr11	104522716	104522808	chr11-104522716-104522808
chr11	104528221	104528313	chr11-104528221-104528313
chr11	104534762	104534854	chr11-104534762-104534854
chr11	104536612	104536704	chr11-104536612-104536704
chr11	104550386	104550478	chr11-104550386-104550478
chr11	105029156	105029248	chr11-105029156-105029248
chr11	105412600	105412692	chr11-105412600-105412692
chr11	105794748	105794840	chr11-105794748-105794840
chr11	105948294	105948386	chr11-105948294-105948386
chr11	105955902	105955994	chr11-105955902-105955994
chr11	106066733	106066825	chr11-106066733-106066825
chr11	106135358	106135450	chr11-106135358-106135450
chr11	106500863	106500955	chr11-106500863-106500955
chr11	107044428	107044520	chr11-107044428-107044520
chr11	107088817	107088909	chr11-107088817-107088909
chr11	107247490	107247582	chr11-107247490-107247582
chr11	108101622	108101714	chr11-108101622-108101714
chr11	108307265	108307357	chr11-108307265-108307357
chr11	108365302	108365394	chr11-108365302-108365394
chr11	109312687	109312779	chr11-109312687-109312779

chr11	109321900	109321992	chr11-109321900-109321992
chr11	109473192	109473284	chr11-109473192-109473284
chr11	110528927	110529019	chr11-110528927-110529019
chr11	111313585	111313677	chr11-111313585-111313677
chr11	112653505	112653597	chr11-112653505-112653597
chr11	113180192	113180284	chr11-113180192-113180284
chr11	113458435	113458527	chr11-113458435-113458527
chr11	113636765	113636857	chr11-113636765-113636857
chr11	113960196	113960288	chr11-113960196-113960288
chr11	114273039	114273131	chr11-114273039-114273131
chr11	115564171	115564263	chr11-115564171-115564263
chr11	115656439	115656531	chr11-115656439-115656531
chr11	115709245	115709337	chr11-115709245-115709337
chr11	115842087	115842179	chr11-115842087-115842179
chr11	115884964	115885056	chr11-115884964-115885056
chr11	115965201	115965293	chr11-115965201-115965293
chr11	115971025	115971117	chr11-115971025-115971117
chr11	116023389	116023481	chr11-116023389-116023481
chr11	116077459	116077551	chr11-116077459-116077551
chr11	116595879	116595971	chr11-116595879-116595971
chr11	116825271	116825363	chr11-116825271-116825363
chr11	116842431	116842523	chr11-116842431-116842523
chr11	117580920	117581012	chr11-117580920-117581012
chr11	117843118	117843210	chr11-117843118-117843210
chr11	117986796	117986888	chr11-117986796-117986888
chr11	118133786	118133878	chr11-118133786-118133878
chr11	118216571	118216663	chr11-118216571-118216663
chr11	119023184	119023276	chr11-119023184-119023276
chr11	119215431	119215523	chr11-119215431-119215523
chr11	119428969	119429061	chr11-119428969-119429061
chr11	119817711	119817803	chr11-119817711-119817803
chr11	120231492	120231584	chr11-120231492-120231584
chr11	120274542	120274634	chr11-120274542-120274634
chr11	120319831	120319923	chr11-120319831-120319923
chr11	120353783	120353875	chr11-120353783-120353875
chr11	120672974	120673066	chr11-120672974-120673066
chr11	121085363	121085455	chr11-121085363-121085455
chr11	121244864	121244956	chr11-121244864-121244956
chr11	121373571	121373663	chr11-121373571-121373663
chr11	121670644	121670736	chr11-121670644-121670736
chr11	121672432	121672524	chr11-121672432-121672524
chr12	3151588	3151680	chr12-3151588-3151680

chr12	3427612	3427704	chr12-3427612-3427704
chr12	3734016	3734108	chr12-3734016-3734108
chr12	3806370	3806462	chr12-3806370-3806462
chr12	3884696	3884788	chr12-3884696-3884788
chr12	4234183	4234275	chr12-4234183-4234275
chr12	4417681	4417773	chr12-4417681-4417773
chr12	5055055	5055147	chr12-5055055-5055147
chr12	6353295	6353387	chr12-6353295-6353387
chr12	7897425	7897517	chr12-7897425-7897517
chr12	8643751	8643843	chr12-8643751-8643843
chr12	8784344	8784436	chr12-8784344-8784436
chr12	8809176	8809268	chr12-8809176-8809268
chr12	9029715	9029807	chr12-9029715-9029807
chr12	9856367	9856459	chr12-9856367-9856459
chr12	9914860	9914952	chr12-9914860-9914952
chr12	12549611	12549703	chr12-12549611-12549703
chr12	14302581	14302673	chr12-14302581-14302673
chr12	16015586	16015678	chr12-16015586-16015678
chr12	16841159	16841251	chr12-16841159-16841251
chr12	17239413	17239505	chr12-17239413-17239505
chr12	17313814	17313906	chr12-17313814-17313906
chr12	17420475	17420567	chr12-17420475-17420567
chr12	17569204	17569296	chr12-17569204-17569296
chr12	17727773	17727865	chr12-17727773-17727865
chr12	17731191	17731283	chr12-17731191-17731283
chr12	20534232	20534324	chr12-20534232-20534324
chr12	21144775	21144867	chr12-21144775-21144867
chr12	21478353	21478445	chr12-21478353-21478445
chr12	21607078	21607170	chr12-21607078-21607170
chr12	24514431	24514523	chr12-24514431-24514523
chr12	24530236	24530328	chr12-24530236-24530328
chr12	25098443	25098535	chr12-25098443-25098535
chr12	25105043	25105135	chr12-25105043-25105135
chr12	25266014	25266106	chr12-25266014-25266106
chr12	26782766	26782858	chr12-26782766-26782858
chr12	27342699	27342791	chr12-27342699-27342791
chr12	28805277	28805369	chr12-28805277-28805369
chr12	31167939	31168031	chr12-31167939-31168031
chr12	31271178	31271270	chr12-31271178-31271270
chr12	31271384	31271476	chr12-31271384-31271476
chr12	31352578	31352670	chr12-31352578-31352670
chr12	31567180	31567272	chr12-31567180-31567272

chr12	31997059	31997151	chr12-31997059-31997151
chr12	32351942	32352034	chr12-32351942-32352034
chr12	32451519	32451611	chr12-32451519-32451611
chr12	32952274	32952366	chr12-32952274-32952366
chr12	33164951	33165043	chr12-33164951-33165043
chr12	33174881	33174973	chr12-33174881-33174973
chr12	33182014	33182106	chr12-33182014-33182106
chr12	33218144	33218236	chr12-33218144-33218236
chr12	35562943	35563035	chr12-35562943-35563035
chr12	36136078	36136170	chr12-36136078-36136170
chr12	36780943	36781035	chr12-36780943-36781035
chr12	40559378	40559470	chr12-40559378-40559470
chr12	42942195	42942287	chr12-42942195-42942287
chr12	43924550	43924642	chr12-43924550-43924642
chr12	44248814	44248906	chr12-44248814-44248906
chr12	44930981	44931073	chr12-44930981-44931073
chr12	46402608	46402700	chr12-46402608-46402700
chr12	46449238	46449330	chr12-46449238-46449330
chr12	46939776	46939868	chr12-46939776-46939868
chr12	47121473	47121565	chr12-47121473-47121565
chr12	49393224	49393316	chr12-49393224-49393316
chr12	49503280	49503372	chr12-49503280-49503372
chr12	49564950	49565042	chr12-49564950-49565042
chr12	51522765	51522857	chr12-51522765-51522857
chr12	52493282	52493374	chr12-52493282-52493374
chr12	52500826	52500918	chr12-52500826-52500918
chr12	52504816	52504908	chr12-52504816-52504908
chr12	55595504	55595596	chr12-55595504-55595596
chr12	56119065	56119157	chr12-56119065-56119157
chr12	56288647	56288739	chr12-56288647-56288739
chr12	56299055	56299147	chr12-56299055-56299147
chr12	58409621	58409713	chr12-58409621-58409713
chr12	59040327	59040419	chr12-59040327-59040419
chr12	59040627	59040719	chr12-59040627-59040719
chr12	60086059	60086151	chr12-60086059-60086151
chr12	65838215	65838307	chr12-65838215-65838307
chr12	66780761	66780853	chr12-66780761-66780853
chr12	67404634	67404726	chr12-67404634-67404726
chr12	69304225	69304317	chr12-69304225-69304317
chr12	69879502	69879594	chr12-69879502-69879594
chr12	69919379	69919471	chr12-69919379-69919471
chr12	70557078	70557170	chr12-70557078-70557170

chr12	70633678	70633770	chr12-70633678-70633770
chr12	70649806	70649898	chr12-70649806-70649898
chr12	70707707	70707799	chr12-70707707-70707799
chr12	70913626	70913718	chr12-70913626-70913718
chr12	71309804	71309896	chr12-71309804-71309896
chr12	71747458	71747550	chr12-71747458-71747550
chr12	71899696	71899788	chr12-71899696-71899788
chr12	72696956	72697048	chr12-72696956-72697048
chr12	73118739	73118831	chr12-73118739-73118831
chr12	73842551	73842643	chr12-73842551-73842643
chr12	75686214	75686306	chr12-75686214-75686306
chr12	76318800	76318892	chr12-76318800-76318892
chr12	76404563	76404655	chr12-76404563-76404655
chr12	76404936	76405028	chr12-76404936-76405028
chr12	76459302	76459394	chr12-76459302-76459394
chr12	76493746	76493838	chr12-76493746-76493838
chr12	76532955	76533047	chr12-76532955-76533047
chr12	76552361	76552453	chr12-76552361-76552453
chr12	77261524	77261616	chr12-77261524-77261616
chr12	78350906	78350998	chr12-78350906-78350998
chr12	78910858	78910950	chr12-78910858-78910950
chr12	78914549	78914641	chr12-78914549-78914641
chr12	79294183	79294275	chr12-79294183-79294275
chr12	79499131	79499223	chr12-79499131-79499223
chr12	79553204	79553296	chr12-79553204-79553296
chr12	79677057	79677149	chr12-79677057-79677149
chr12	79764074	79764166	chr12-79764074-79764166
chr12	80084020	80084112	chr12-80084020-80084112
chr12	80220538	80220630	chr12-80220538-80220630
chr12	80271122	80271214	chr12-80271122-80271214
chr12	80587907	80587999	chr12-80587907-80587999
chr12	80660152	80660244	chr12-80660152-80660244
chr12	80689362	80689454	chr12-80689362-80689454
chr12	81485008	81485100	chr12-81485008-81485100
chr12	81749225	81749317	chr12-81749225-81749317
chr12	82189924	82190016	chr12-82189924-82190016
chr12	82286258	82286350	chr12-82286258-82286350
chr12	83874796	83874888	chr12-83874796-83874888
chr12	84416120	84416212	chr12-84416120-84416212
chr12	84773129	84773221	chr12-84773129-84773221
chr12	85733236	85733328	chr12-85733236-85733328
chr12	86726513	86726605	chr12-86726513-86726605

chr12	86744925	86745017	chr12-86744925-86745017
chr12	86795915	86796007	chr12-86795915-86796007
chr12	86826876	86826968	chr12-86826876-86826968
chr12	86894675	86894767	chr12-86894675-86894767
chr12	86945249	86945341	chr12-86945249-86945341
chr12	87114582	87114674	chr12-87114582-87114674
chr12	87186822	87186914	chr12-87186822-87186914
chr12	88633092	88633184	chr12-88633092-88633184
chr12	89204312	89204404	chr12-89204312-89204404
chr12	90206953	90207045	chr12-90206953-90207045
chr12	90781536	90781628	chr12-90781536-90781628
chr12	91668576	91668668	chr12-91668576-91668668
chr12	92936665	92936757	chr12-92936665-92936757
chr12	95407178	95407270	chr12-95407178-95407270
chr12	95692250	95692342	chr12-95692250-95692342
chr12	98714880	98714972	chr12-98714880-98714972
chr12	99644979	99645071	chr12-99644979-99645071
chr12	100982298	100982390	chr12-100982298-100982390
chr12	102143454	102143546	chr12-102143454-102143546
chr12	102426489	102426581	chr12-102426489-102426581
chr12	102652619	102652711	chr12-102652619-102652711
chr12	103457332	103457424	chr12-103457332-103457424
chr12	104644872	104644964	chr12-104644872-104644964
chr12	104791481	104791573	chr12-104791481-104791573
chr12	104898735	104898827	chr12-104898735-104898827
chr12	105554530	105554622	chr12-105554530-105554622
chr12	105885633	105885725	chr12-105885633-105885725
chr12	106681947	106682039	chr12-106681947-106682039
chr12	108276099	108276191	chr12-108276099-108276191
chr12	108392823	108392915	chr12-108392823-108392915
chr12	108393792	108393884	chr12-108393792-108393884
chr12	108793157	108793249	chr12-108793157-108793249
chr12	110599892	110599984	chr12-110599892-110599984
chr12	111139882	111139974	chr12-111139882-111139974
chr12	111327677	111327769	chr12-111327677-111327769
chr12	111377774	111377866	chr12-111377774-111377866
chr12	111429089	111429181	chr12-111429089-111429181
chr12	111461544	111461636	chr12-111461544-111461636
chr12	111531704	111531796	chr12-111531704-111531796
chr12	112588466	112588558	chr12-112588466-112588558
chr12	112599265	112599357	chr12-112599265-112599357
chr12	112628934	112629026	chr12-112628934-112629026

chr12	112674605	112674697	chr12-112674605-112674697
chr12	112794258	112794350	chr12-112794258-112794350
chr12	112806207	112806299	chr12-112806207-112806299
chr12	112829680	112829772	chr12-112829680-112829772
chr12	112875707	112875799	chr12-112875707-112875799
chr12	112950317	112950409	chr12-112950317-112950409
chr12	113121611	113121703	chr12-113121611-113121703
chr12	113128668	113128760	chr12-113128668-113128760
chr12	113224994	113225086	chr12-113224994-113225086
chr12	113424368	113424460	chr12-113424368-113424460
chr12	117390001	117390093	chr12-117390001-117390093
chr12	117432685	117432777	chr12-117432685-117432777
chr12	117450275	117450367	chr12-117450275-117450367
chr12	117481294	117481386	chr12-117481294-117481386
chr13	3865557	3865649	chr13-3865557-3865649
chr13	4160524	4160616	chr13-4160524-4160616
chr13	4588273	4588365	chr13-4588273-4588365
chr13	5769715	5769807	chr13-5769715-5769807
chr13	5826323	5826415	chr13-5826323-5826415
chr13	5837342	5837434	chr13-5837342-5837434
chr13	5845175	5845267	chr13-5845175-5845267
chr13	5863737	5863829	chr13-5863737-5863829
chr13	5870349	5870441	chr13-5870349-5870441
chr13	6009639	6009731	chr13-6009639-6009731
chr13	6077510	6077602	chr13-6077510-6077602
chr13	6255942	6256034	chr13-6255942-6256034
chr13	9011721	9011813	chr13-9011721-9011813
chr13	9012080	9012172	chr13-9012080-9012172
chr13	9834393	9834485	chr13-9834393-9834485
chr13	10636077	10636169	chr13-10636077-10636169
chr13	11549334	11549426	chr13-11549334-11549426
chr13	11716876	11716968	chr13-11716876-11716968
chr13	12813954	12814046	chr13-12813954-12814046
chr13	12864436	12864528	chr13-12864436-12864528
chr13	13464127	13464219	chr13-13464127-13464219
chr13	16456397	16456489	chr13-16456397-16456489
chr13	16847323	16847415	chr13-16847323-16847415
chr13	17163627	17163719	chr13-17163627-17163719
chr13	18195974	18196066	chr13-18195974-18196066
chr13	18218219	18218311	chr13-18218219-18218311
chr13	20163528	20163620	chr13-20163528-20163620
chr13	20382296	20382388	chr13-20382296-20382388

chr13	21419047	21419139	chr13-21419047-21419139
chr13	21456431	21456523	chr13-21456431-21456523
chr13	21716340	21716432	chr13-21716340-21716432
chr13	21735344	21735436	chr13-21735344-21735436
chr13	23180421	23180513	chr13-23180421-23180513
chr13	23463468	23463560	chr13-23463468-23463560
chr13	23682875	23682967	chr13-23682875-23682967
chr13	23761041	23761133	chr13-23761041-23761133
chr13	24104105	24104197	chr13-24104105-24104197
chr13	24272900	24272992	chr13-24272900-24272992
chr13	24565915	24566007	chr13-24565915-24566007
chr13	24611968	24612060	chr13-24611968-24612060
chr13	25165565	25165657	chr13-25165565-25165657
chr13	26940555	26940647	chr13-26940555-26940647
chr13	26976207	26976299	chr13-26976207-26976299
chr13	27081096	27081188	chr13-27081096-27081188
chr13	27113917	27114009	chr13-27113917-27114009
chr13	27149021	27149113	chr13-27149021-27149113
chr13	27206038	27206130	chr13-27206038-27206130
chr13	27730369	27730461	chr13-27730369-27730461
chr13	27734697	27734789	chr13-27734697-27734789
chr13	28313627	28313719	chr13-28313627-28313719
chr13	28545668	28545760	chr13-28545668-28545760
chr13	28633279	28633371	chr13-28633279-28633371
chr13	28680384	28680476	chr13-28680384-28680476
chr13	28751042	28751134	chr13-28751042-28751134
chr13	28830042	28830134	chr13-28830042-28830134
chr13	28845913	28846005	chr13-28845913-28846005
chr13	29218776	29218868	chr13-29218776-29218868
chr13	29230574	29230666	chr13-29230574-29230666
chr13	31567793	31567885	chr13-31567793-31567885
chr13	32337837	32337929	chr13-32337837-32337929
chr13	32925056	32925148	chr13-32925056-32925148
chr13	32926787	32926879	chr13-32926787-32926879
chr13	32964243	32964335	chr13-32964243-32964335
chr13	33051843	33051935	chr13-33051843-33051935
chr13	34192583	34192675	chr13-34192583-34192675
chr13	34192971	34193063	chr13-34192971-34193063
chr13	34332939	34333031	chr13-34332939-34333031
chr13	34461046	34461138	chr13-34461046-34461138
chr13	34771519	34771611	chr13-34771519-34771611
chr13	34784982	34785074	chr13-34784982-34785074

chr13	35711222	35711314	chr13-35711222-35711314
chr13	35765990	35766082	chr13-35765990-35766082
chr13	35874719	35874811	chr13-35874719-35874811
chr13	36696403	36696495	chr13-36696403-36696495
chr13	37432472	37432564	chr13-37432472-37432564
chr13	37506592	37506684	chr13-37506592-37506684
chr13	37716485	37716577	chr13-37716485-37716577
chr13	37825998	37826090	chr13-37825998-37826090
chr13	37826636	37826728	chr13-37826636-37826728
chr13	37910742	37910834	chr13-37910742-37910834
chr13	37994315	37994407	chr13-37994315-37994407
chr13	39185386	39185478	chr13-39185386-39185478
chr13	39423839	39423931	chr13-39423839-39423931
chr13	40663647	40663739	chr13-40663647-40663739
chr13	40921664	40921756	chr13-40921664-40921756
chr13	40989738	40989830	chr13-40989738-40989830
chr13	42042348	42042440	chr13-42042348-42042440
chr13	42301446	42301538	chr13-42301446-42301538
chr13	42810432	42810524	chr13-42810432-42810524
chr13	43193309	43193401	chr13-43193309-43193401
chr13	44739445	44739537	chr13-44739445-44739537
chr13	44869559	44869651	chr13-44869559-44869651
chr13	44869886	44869978	chr13-44869886-44869978
chr13	46016586	46016678	chr13-46016586-46016678
chr13	46745163	46745255	chr13-46745163-46745255
chr13	48502532	48502624	chr13-48502532-48502624
chr13	49188053	49188145	chr13-49188053-49188145
chr13	49307652	49307744	chr13-49307652-49307744
chr13	49326976	49327068	chr13-49326976-49327068
chr13	49593259	49593351	chr13-49593259-49593351
chr13	49666447	49666539	chr13-49666447-49666539
chr13	49768226	49768318	chr13-49768226-49768318
chr13	51970321	51970413	chr13-51970321-51970413
chr13	51976279	51976371	chr13-51976279-51976371
chr13	52233920	52234012	chr13-52233920-52234012
chr13	52282766	52282858	chr13-52282766-52282858
chr13	52623810	52623902	chr13-52623810-52623902
chr13	53110403	53110495	chr13-53110403-53110495
chr13	53721465	53721557	chr13-53721465-53721557
chr13	54101209	54101301	chr13-54101209-54101301
chr13	54190036	54190128	chr13-54190036-54190128
chr13	54483940	54484032	chr13-54483940-54484032

chr13	55189917	55190009	chr13-55189917-55190009
chr13	56404686	56404778	chr13-56404686-56404778
chr13	56521081	56521173	chr13-56521081-56521173
chr13	56703013	56703105	chr13-56703013-56703105
chr13	56703372	56703464	chr13-56703372-56703464
chr13	57695767	57695859	chr13-57695767-57695859
chr13	58768391	58768483	chr13-58768391-58768483
chr13	59770310	59770402	chr13-59770310-59770402
chr13	60549099	60549191	chr13-60549099-60549191
chr13	60877785	60877877	chr13-60877785-60877877
chr13	60953981	60954073	chr13-60953981-60954073
chr13	60957210	60957302	chr13-60957210-60957302
chr13	61476502	61476594	chr13-61476502-61476594
chr13	63272728	63272820	chr13-63272728-63272820
chr13	63682444	63682536	chr13-63682444-63682536
chr13	64740774	64740866	chr13-64740774-64740866
chr13	67113437	67113529	chr13-67113437-67113529
chr13	67512960	67513052	chr13-67512960-67513052
chr13	69488854	69488946	chr13-69488854-69488946
chr13	69709950	69710042	chr13-69709950-69710042
chr13	73178489	73178581	chr13-73178489-73178581
chr13	73299218	73299310	chr13-73299218-73299310
chr13	74468072	74468164	chr13-74468072-74468164
chr13	74640386	74640478	chr13-74640386-74640478
chr13	76127612	76127704	chr13-76127612-76127704
chr13	79989241	79989333	chr13-79989241-79989333
chr13	80820361	80820453	chr13-80820361-80820453
chr13	81096560	81096652	chr13-81096560-81096652
chr13	81184033	81184125	chr13-81184033-81184125
chr13	81332057	81332149	chr13-81332057-81332149
chr13	83285979	83286071	chr13-83285979-83286071
chr13	83710368	83710460	chr13-83710368-83710460
chr13	83865495	83865587	chr13-83865495-83865587
chr13	84628903	84628995	chr13-84628903-84628995
chr13	84824172	84824264	chr13-84824172-84824264
chr13	85296205	85296297	chr13-85296205-85296297
chr13	85578951	85579043	chr13-85578951-85579043
chr13	88226941	88227033	chr13-88226941-88227033
chr13	89570575	89570667	chr13-89570575-89570667
chr13	89710034	89710126	chr13-89710034-89710126
chr13	89828459	89828551	chr13-89828459-89828551
chr13	90401255	90401347	chr13-90401255-90401347

chr13	90489424	90489516	chr13-90489424-90489516
chr13	92068038	92068130	chr13-92068038-92068130
chr13	94250811	94250903	chr13-94250811-94250903
chr13	95003846	95003938	chr13-95003846-95003938
chr13	97021964	97022056	chr13-97021964-97022056
chr13	97071044	97071136	chr13-97071044-97071136
chr13	97419220	97419312	chr13-97419220-97419312
chr13	97492714	97492806	chr13-97492714-97492806
chr13	98693032	98693124	chr13-98693032-98693124
chr13	99085460	99085552	chr13-99085460-99085552
chr13	99802890	99802982	chr13-99802890-99802982
chr13	99948313	99948405	chr13-99948313-99948405
chr13	100138924	100139016	chr13-100138924-100139016
chr13	100516773	100516865	chr13-100516773-100516865
chr13	102894352	102894444	chr13-102894352-102894444
chr13	102906139	102906231	chr13-102906139-102906231
chr13	103260566	103260658	chr13-103260566-103260658
chr13	103557445	103557537	chr13-103557445-103557537
chr13	104037773	104037865	chr13-104037773-104037865
chr13	104565555	104565647	chr13-104565555-104565647
chr13	107523441	107523533	chr13-107523441-107523533
chr13	108139009	108139101	chr13-108139009-108139101
chr13	108714128	108714220	chr13-108714128-108714220
chr13	108964355	108964447	chr13-108964355-108964447
chr13	109651956	109652048	chr13-109651956-109652048
chr13	109659231	109659323	chr13-109659231-109659323
chr13	110724097	110724189	chr13-110724097-110724189
chr13	110924105	110924197	chr13-110924105-110924197
chr13	110967188	110967280	chr13-110967188-110967280
chr13	111295139	111295231	chr13-111295139-111295231
chr13	111346176	111346268	chr13-111346176-111346268
chr13	111967709	111967801	chr13-111967709-111967801
chr13	112030764	112030856	chr13-112030764-112030856
chr13	112578188	112578280	chr13-112578188-112578280
chr13	113130463	113130555	chr13-113130463-113130555
chr13	113495978	113496070	chr13-113495978-113496070
chr13	113580089	113580181	chr13-113580089-113580181
chr13	113917874	113917966	chr13-113917874-113917966
chr13	114228267	114228359	chr13-114228267-114228359
chr13	114610603	114610695	chr13-114610603-114610695
chr13	114619263	114619355	chr13-114619263-114619355
chr13	114699170	114699262	chr13-114699170-114699262

chr13	115387361	115387453	chr13-115387361-115387453
chr13	116136064	116136156	chr13-116136064-116136156
chr13	116564724	116564816	chr13-116564724-116564816
chr13	117053506	117053598	chr13-117053506-117053598
chr13	117143948	117144040	chr13-117143948-117144040
chr13	118253522	118253614	chr13-118253522-118253614
chr13	118586542	118586634	chr13-118586542-118586634
chr14	6429993	6430085	chr14-6429993-6430085
chr14	7808658	7808750	chr14-7808658-7808750
chr14	7953008	7953100	chr14-7953008-7953100
chr14	8463967	8464059	chr14-8463967-8464059
chr14	8733211	8733303	chr14-8733211-8733303
chr14	10764106	10764198	chr14-10764106-10764198
chr14	11238956	11239048	chr14-11238956-11239048
chr14	12380203	12380295	chr14-12380203-12380295
chr14	13876123	13876215	chr14-13876123-13876215
chr14	16034753	16034845	chr14-16034753-16034845
chr14	17589699	17589791	chr14-17589699-17589791
chr14	18026682	18026774	chr14-18026682-18026774
chr14	18455631	18455723	chr14-18455631-18455723
chr14	19302652	19302744	chr14-19302652-19302744
chr14	20351523	20351615	chr14-20351523-20351615
chr14	20767052	20767144	chr14-20767052-20767144
chr14	21065978	21066070	chr14-21065978-21066070
chr14	21083081	21083173	chr14-21083081-21083173
chr14	21510675	21510767	chr14-21510675-21510767
chr14	21824092	21824184	chr14-21824092-21824184
chr14	22356198	22356290	chr14-22356198-22356290
chr14	24206720	24206812	chr14-24206720-24206812
chr14	24601255	24601347	chr14-24601255-24601347
chr14	24902064	24902156	chr14-24902064-24902156
chr14	25533218	25533310	chr14-25533218-25533310
chr14	25797789	25797881	chr14-25797789-25797881
chr14	25857518	25857610	chr14-25857518-25857610
chr14	26534897	26534989	chr14-26534897-26534989
chr14	28359607	28359699	chr14-28359607-28359699
chr14	28985711	28985803	chr14-28985711-28985803
chr14	30498453	30498545	chr14-30498453-30498545
chr14	31404119	31404211	chr14-31404119-31404211
chr14	31926919	31927011	chr14-31926919-31927011
chr14	32854168	32854260	chr14-32854168-32854260
chr14	33214524	33214616	chr14-33214524-33214616

chr14	34549157	34549249	chr14-34549157-34549249
chr14	34764352	34764444	chr14-34764352-34764444
chr14	36347070	36347162	chr14-36347070-36347162
chr14	36442708	36442800	chr14-36442708-36442800
chr14	40294305	40294397	chr14-40294305-40294397
chr14	40684792	40684884	chr14-40684792-40684884
chr14	41620779	41620871	chr14-41620779-41620871
chr14	45329854	45329946	chr14-45329854-45329946
chr14	45849941	45850033	chr14-45849941-45850033
chr14	47021090	47021182	chr14-47021090-47021182
chr14	47356903	47356995	chr14-47356903-47356995
chr14	47367045	47367137	chr14-47367045-47367137
chr14	47634554	47634646	chr14-47634554-47634646
chr14	48224517	48224609	chr14-48224517-48224609
chr14	48836011	48836103	chr14-48836011-48836103
chr14	49036673	49036765	chr14-49036673-49036765
chr14	51653695	51653787	chr14-51653695-51653787
chr14	51957846	51957938	chr14-51957846-51957938
chr14	54107371	54107463	chr14-54107371-54107463
chr14	54185645	54185737	chr14-54185645-54185737
chr14	54227550	54227642	chr14-54227550-54227642
chr14	55502536	55502628	chr14-55502536-55502628
chr14	55721927	55722019	chr14-55721927-55722019
chr14	55724720	55724812	chr14-55724720-55724812
chr14	56319642	56319734	chr14-56319642-56319734
chr14	57760634	57760726	chr14-57760634-57760726
chr14	59614286	59614378	chr14-59614286-59614378
chr14	60302505	60302597	chr14-60302505-60302597
chr14	61172943	61173035	chr14-61172943-61173035
chr14	62383400	62383492	chr14-62383400-62383492
chr14	63983133	63983225	chr14-63983133-63983225
chr14	64250168	64250260	chr14-64250168-64250260
chr14	65343867	65343959	chr14-65343867-65343959
chr14	65878942	65879034	chr14-65878942-65879034
chr14	65907467	65907559	chr14-65907467-65907559
chr14	65958703	65958795	chr14-65958703-65958795
chr14	66106176	66106268	chr14-66106176-66106268
chr14	66277057	66277149	chr14-66277057-66277149
chr14	67008996	67009088	chr14-67008996-67009088
chr14	67158876	67158968	chr14-67158876-67158968
chr14	67234532	67234624	chr14-67234532-67234624
chr14	67635507	67635599	chr14-67635507-67635599

chr14	67716460	67716552	chr14-67716460-67716552
chr14	68744773	68744865	chr14-68744773-68744865
chr14	69778891	69778983	chr14-69778891-69778983
chr14	70096129	70096221	chr14-70096129-70096221
chr14	70516687	70516779	chr14-70516687-70516779
chr14	70653683	70653775	chr14-70653683-70653775
chr14	70689355	70689447	chr14-70689355-70689447
chr14	72446134	72446226	chr14-72446134-72446226
chr14	72463463	72463555	chr14-72463463-72463555
chr14	73708717	73708809	chr14-73708717-73708809
chr14	74482972	74483064	chr14-74482972-74483064
chr14	74910510	74910602	chr14-74910510-74910602
chr14	76171530	76171622	chr14-76171530-76171622
chr14	76520725	76520817	chr14-76520725-76520817
chr14	76594664	76594756	chr14-76594664-76594756
chr14	76883484	76883576	chr14-76883484-76883576
chr14	77392296	77392388	chr14-77392296-77392388
chr14	77690792	77690884	chr14-77690792-77690884
chr14	77768572	77768664	chr14-77768572-77768664
chr14	78355375	78355467	chr14-78355375-78355467
chr14	79426508	79426600	chr14-79426508-79426600
chr14	79481961	79482053	chr14-79481961-79482053
chr14	82710451	82710543	chr14-82710451-82710543
chr14	84912147	84912239	chr14-84912147-84912239
chr14	86699427	86699519	chr14-86699427-86699519
chr14	87597133	87597225	chr14-87597133-87597225
chr14	88901415	88901507	chr14-88901415-88901507
chr14	91461391	91461483	chr14-91461391-91461483
chr14	93062584	93062676	chr14-93062584-93062676
chr14	96170936	96171028	chr14-96170936-96171028
chr14	99624786	99624878	chr14-99624786-99624878
chr14	99647438	99647530	chr14-99647438-99647530
chr14	100346932	100347024	chr14-100346932-100347024
chr14	101798027	101798119	chr14-101798027-101798119
chr14	101832318	101832410	chr14-101832318-101832410
chr14	101870289	101870381	chr14-101870289-101870381
chr14	102329645	102329737	chr14-102329645-102329737
chr14	103422052	103422144	chr14-103422052-103422144
chr14	104336938	104337030	chr14-104336938-104337030
chr14	104715814	104715906	chr14-104715814-104715906
chr14	105802682	105802774	chr14-105802682-105802774
chr14	105803523	105803615	chr14-105803523-105803615

chr14	105923188	105923280	chr14-105923188-105923280
chr14	106230927	106231019	chr14-106230927-106231019
chr14	107858537	107858629	chr14-107858537-107858629
chr14	108763034	108763126	chr14-108763034-108763126
chr14	108992896	108992988	chr14-108992896-108992988
chr14	110888736	110888828	chr14-110888736-110888828
chr14	113489864	113489956	chr14-113489864-113489956
chr14	114228401	114228493	chr14-114228401-114228493
chr14	114865872	114865964	chr14-114865872-114865964
chr14	115633708	115633800	chr14-115633708-115633800
chr14	116191841	116191933	chr14-116191841-116191933
chr14	118189220	118189312	chr14-118189220-118189312
chr14	118643008	118643100	chr14-118643008-118643100
chr14	119453980	119454072	chr14-119453980-119454072
chr14	120478789	120478881	chr14-120478789-120478881
chr14	121037431	121037523	chr14-121037431-121037523
chr14	121104132	121104224	chr14-121104132-121104224
chr14	121399801	121399893	chr14-121399801-121399893
chr14	121435877	121435969	chr14-121435877-121435969
chr14	121617637	121617729	chr14-121617637-121617729
chr14	122471964	122472056	chr14-122471964-122472056
chr14	122909879	122909971	chr14-122909879-122909971
chr14	123242964	123243056	chr14-123242964-123243056
chr15	3464541	3464633	chr15-3464541-3464633
chr15	3905798	3905890	chr15-3905798-3905890
chr15	3995995	3996087	chr15-3995995-3996087
chr15	5195977	5196069	chr15-5195977-5196069
chr15	6386753	6386845	chr15-6386753-6386845
chr15	6646125	6646217	chr15-6646125-6646217
chr15	7350746	7350838	chr15-7350746-7350838
chr15	7931774	7931866	chr15-7931774-7931866
chr15	8532033	8532125	chr15-8532033-8532125
chr15	9421351	9421443	chr15-9421351-9421443
chr15	9522966	9523058	chr15-9522966-9523058
chr15	10690223	10690315	chr15-10690223-10690315
chr15	10763897	10763989	chr15-10763897-10763989
chr15	10828888	10828980	chr15-10828888-10828980
chr15	11082577	11082669	chr15-11082577-11082669
chr15	11155893	11155985	chr15-11155893-11155985
chr15	11552287	11552379	chr15-11552287-11552379
chr15	12118684	12118776	chr15-12118684-12118776
chr15	12134656	12134748	chr15-12134656-12134748

chr15	12233535	12233627	chr15-12233535-12233627
chr15	12435519	12435611	chr15-12435519-12435611
chr15	14519427	14519519	chr15-14519427-14519519
chr15	15245776	15245868	chr15-15245776-15245868
chr15	18681219	18681311	chr15-18681219-18681311
chr15	20101192	20101284	chr15-20101192-20101284
chr15	22733156	22733248	chr15-22733156-22733248
chr15	24677726	24677818	chr15-24677726-24677818
chr15	25602309	25602401	chr15-25602309-25602401
chr15	25915301	25915393	chr15-25915301-25915393
chr15	25932580	25932672	chr15-25932580-25932672
chr15	26395979	26396071	chr15-26395979-26396071
chr15	26487363	26487455	chr15-26487363-26487455
chr15	26877770	26877862	chr15-26877770-26877862
chr15	27369746	27369838	chr15-27369746-27369838
chr15	29030790	29030882	chr15-29030790-29030882
chr15	31101980	31102072	chr15-31101980-31102072
chr15	31286658	31286750	chr15-31286658-31286750
chr15	31396307	31396399	chr15-31396307-31396399
chr15	32322869	32322961	chr15-32322869-32322961
chr15	32333511	32333603	chr15-32333511-32333603
chr15	32580858	32580950	chr15-32580858-32580950
chr15	33021171	33021263	chr15-33021171-33021263
chr15	33913154	33913246	chr15-33913154-33913246
chr15	33916201	33916293	chr15-33916201-33916293
chr15	34064855	34064947	chr15-34064855-34064947
chr15	34098210	34098302	chr15-34098210-34098302
chr15	35071547	35071639	chr15-35071547-35071639
chr15	36283215	36283307	chr15-36283215-36283307
chr15	36318311	36318403	chr15-36318311-36318403
chr15	36375030	36375122	chr15-36375030-36375122
chr15	36419992	36420084	chr15-36419992-36420084
chr15	36762771	36762863	chr15-36762771-36762863
chr15	36793569	36793661	chr15-36793569-36793661
chr15	36826283	36826375	chr15-36826283-36826375
chr15	36876192	36876284	chr15-36876192-36876284
chr15	37243110	37243202	chr15-37243110-37243202
chr15	37566556	37566648	chr15-37566556-37566648
chr15	38570201	38570293	chr15-38570201-38570293
chr15	39731718	39731810	chr15-39731718-39731810
chr15	39801929	39802021	chr15-39801929-39802021
chr15	40355193	40355285	chr15-40355193-40355285

chr15	41276905	41276997	chr15-41276905-41276997
chr15	41483292	41483384	chr15-41483292-41483384
chr15	41615602	41615694	chr15-41615602-41615694
chr15	41651492	41651584	chr15-41651492-41651584
chr15	41693244	41693336	chr15-41693244-41693336
chr15	41693832	41693924	chr15-41693832-41693924
chr15	43282693	43282785	chr15-43282693-43282785
chr15	43383550	43383642	chr15-43383550-43383642
chr15	46025363	46025455	chr15-46025363-46025455
chr15	47924764	47924856	chr15-47924764-47924856
chr15	48430702	48430794	chr15-48430702-48430794
chr15	49224083	49224175	chr15-49224083-49224175
chr15	51308118	51308210	chr15-51308118-51308210
chr15	51854237	51854329	chr15-51854237-51854329
chr15	52055981	52056073	chr15-52055981-52056073
chr15	53676468	53676560	chr15-53676468-53676560
chr15	54372958	54373050	chr15-54372958-54373050
chr15	54535268	54535360	chr15-54535268-54535360
chr15	54574937	54575029	chr15-54574937-54575029
chr15	55141500	55141592	chr15-55141500-55141592
chr15	55209084	55209176	chr15-55209084-55209176
chr15	57605667	57605759	chr15-57605667-57605759
chr15	57730582	57730674	chr15-57730582-57730674
chr15	57864650	57864742	chr15-57864650-57864742
chr15	58254258	58254350	chr15-58254258-58254350
chr15	58517638	58517730	chr15-58517638-58517730
chr15	58547346	58547438	chr15-58547346-58547438
chr15	58567196	58567288	chr15-58567196-58567288
chr15	58594987	58595079	chr15-58594987-58595079
chr15	58874285	58874377	chr15-58874285-58874377
chr15	59727815	59727907	chr15-59727815-59727907
chr15	59792124	59792216	chr15-59792124-59792216
chr15	59956023	59956115	chr15-59956023-59956115
chr15	61609655	61609747	chr15-61609655-61609747
chr15	61864794	61864886	chr15-61864794-61864886
chr15	61987262	61987354	chr15-61987262-61987354
chr15	62119064	62119156	chr15-62119064-62119156
chr15	62328693	62328785	chr15-62328693-62328785
chr15	62953114	62953206	chr15-62953114-62953206
chr15	63067142	63067234	chr15-63067142-63067234
chr15	63325075	63325167	chr15-63325075-63325167
chr15	63340767	63340859	chr15-63340767-63340859

chr15	63545919	63546011	chr15-63545919-63546011
chr15	64383008	64383100	chr15-64383008-64383100
chr15	65039360	65039452	chr15-65039360-65039452
chr15	66357419	66357511	chr15-66357419-66357511
chr15	66443969	66444061	chr15-66443969-66444061
chr15	66508064	66508156	chr15-66508064-66508156
chr15	66555323	66555415	chr15-66555323-66555415
chr15	66679954	66680046	chr15-66679954-66680046
chr15	66808121	66808213	chr15-66808121-66808213
chr15	67129013	67129105	chr15-67129013-67129105
chr15	67130017	67130109	chr15-67130017-67130109
chr15	67145531	67145623	chr15-67145531-67145623
chr15	67923990	67924082	chr15-67923990-67924082
chr15	70880329	70880421	chr15-70880329-70880421
chr15	71452542	71452634	chr15-71452542-71452634
chr15	72801412	72801504	chr15-72801412-72801504
chr15	73243283	73243375	chr15-73243283-73243375
chr15	73318801	73318893	chr15-73318801-73318893
chr15	73434927	73435019	chr15-73434927-73435019
chr15	73478092	73478184	chr15-73478092-73478184
chr15	73515265	73515357	chr15-73515265-73515357
chr15	73645663	73645755	chr15-73645663-73645755
chr15	74613212	74613304	chr15-74613212-74613304
chr15	74879669	74879761	chr15-74879669-74879761
chr15	75603829	75603921	chr15-75603829-75603921
chr15	76035531	76035623	chr15-76035531-76035623
chr15	76082551	76082643	chr15-76082551-76082643
chr15	76103646	76103738	chr15-76103646-76103738
chr15	76294703	76294795	chr15-76294703-76294795
chr15	76607522	76607614	chr15-76607522-76607614
chr15	77799638	77799730	chr15-77799638-77799730
chr15	77820592	77820684	chr15-77820592-77820684
chr15	77820883	77820975	chr15-77820883-77820975
chr15	77971128	77971220	chr15-77971128-77971220
chr15	78088755	78088847	chr15-78088755-78088847
chr15	78091719	78091811	chr15-78091719-78091811
chr15	78136789	78136881	chr15-78136789-78136881
chr15	78210736	78210828	chr15-78210736-78210828
chr15	78450149	78450241	chr15-78450149-78450241
chr15	78845412	78845504	chr15-78845412-78845504
chr15	78877616	78877708	chr15-78877616-78877708
chr15	79424889	79424981	chr15-79424889-79424981

chr15	79927753	79927845	chr15-79927753-79927845
chr15	80054119	80054211	chr15-80054119-80054211
chr15	80083293	80083385	chr15-80083293-80083385
chr15	80720434	80720526	chr15-80720434-80720526
chr15	81193035	81193127	chr15-81193035-81193127
chr15	81522905	81522997	chr15-81522905-81522997
chr15	81730164	81730256	chr15-81730164-81730256
chr15	81732725	81732817	chr15-81732725-81732817
chr15	81802231	81802323	chr15-81802231-81802323
chr15	81872322	81872414	chr15-81872322-81872414
chr15	81991097	81991189	chr15-81991097-81991189
chr15	82016303	82016395	chr15-82016303-82016395
chr15	82218620	82218712	chr15-82218620-82218712
chr15	82351654	82351746	chr15-82351654-82351746
chr15	82898798	82898890	chr15-82898798-82898890
chr15	82928974	82929066	chr15-82928974-82929066
chr15	82963186	82963278	chr15-82963186-82963278
chr15	83411810	83411902	chr15-83411810-83411902
chr15	83432843	83432935	chr15-83432843-83432935
chr15	83512372	83512464	chr15-83512372-83512464
chr15	83698690	83698782	chr15-83698690-83698782
chr15	84213485	84213577	chr15-84213485-84213577
chr15	84239235	84239327	chr15-84239235-84239327
chr15	84610333	84610425	chr15-84610333-84610425
chr15	84697174	84697266	chr15-84697174-84697266
chr15	84713131	84713223	chr15-84713131-84713223
chr15	84754159	84754251	chr15-84754159-84754251
chr15	84829562	84829654	chr15-84829562-84829654
chr15	85177773	85177865	chr15-85177773-85177865
chr15	85347400	85347492	chr15-85347400-85347492
chr15	85390107	85390199	chr15-85390107-85390199
chr15	85542623	85542715	chr15-85542623-85542715
chr15	85544718	85544810	chr15-85544718-85544810
chr15	85553839	85553931	chr15-85553839-85553931
chr15	85568918	85569010	chr15-85568918-85569010
chr15	85970714	85970806	chr15-85970714-85970806
chr15	86025957	86026049	chr15-86025957-86026049
chr15	86182045	86182137	chr15-86182045-86182137
chr15	86554273	86554365	chr15-86554273-86554365
chr15	86762286	86762378	chr15-86762286-86762378
chr15	88163015	88163107	chr15-88163015-88163107
chr15	88313090	88313182	chr15-88313090-88313182

chr15	88430965	88431057	chr15-88430965-88431057
chr15	88865117	88865209	chr15-88865117-88865209
chr15	88893284	88893376	chr15-88893284-88893376
chr15	89476359	89476451	chr15-89476359-89476451
chr15	89488275	89488367	chr15-89488275-89488367
chr15	90184006	90184098	chr15-90184006-90184098
chr15	90402646	90402738	chr15-90402646-90402738
chr15	90607653	90607745	chr15-90607653-90607745
chr15	90609983	90610075	chr15-90609983-90610075
chr15	92102123	92102215	chr15-92102123-92102215
chr15	92311927	92312019	chr15-92311927-92312019
chr15	93744317	93744409	chr15-93744317-93744409
chr15	93827056	93827148	chr15-93827056-93827148
chr15	94891130	94891222	chr15-94891130-94891222
chr15	95824005	95824097	chr15-95824005-95824097
chr15	96003318	96003410	chr15-96003318-96003410
chr15	96078548	96078640	chr15-96078548-96078640
chr15	96524382	96524474	chr15-96524382-96524474
chr15	96660966	96661058	chr15-96660966-96661058
chr15	96744280	96744372	chr15-96744280-96744372
chr15	96839099	96839191	chr15-96839099-96839191
chr15	97036701	97036793	chr15-97036701-97036793
chr15	97088727	97088819	chr15-97088727-97088819
chr15	97315579	97315671	chr15-97315579-97315671
chr15	97587379	97587471	chr15-97587379-97587471
chr15	97786131	97786223	chr15-97786131-97786223
chr15	97790224	97790316	chr15-97790224-97790316
chr15	97831514	97831606	chr15-97831514-97831606
chr15	97837732	97837824	chr15-97837732-97837824
chr15	98156186	98156278	chr15-98156186-98156278
chr15	98181445	98181537	chr15-98181445-98181537
chr15	98494047	98494139	chr15-98494047-98494139
chr15	98664797	98664889	chr15-98664797-98664889
chr15	98951613	98951705	chr15-98951613-98951705
chr15	99033562	99033654	chr15-99033562-99033654
chr15	99438077	99438169	chr15-99438077-99438169
chr15	99474051	99474143	chr15-99474051-99474143
chr15	99499035	99499127	chr15-99499035-99499127
chr15	99664878	99664970	chr15-99664878-99664970
chr15	99866625	99866717	chr15-99866625-99866717
chr15	100556544	100556636	chr15-100556544-100556636
chr15	100950541	100950633	chr15-100950541-100950633

chr15	101237024	101237116	chr15-101237024-101237116
chr15	101320894	101320986	chr15-101320894-101320986
chr15	101372359	101372451	chr15-101372359-101372451
chr15	101379974	101380066	chr15-101379974-101380066
chr15	101387268	101387360	chr15-101387268-101387360
chr15	101941935	101942027	chr15-101941935-101942027
chr15	102004532	102004624	chr15-102004532-102004624
chr15	102014821	102014913	chr15-102014821-102014913
chr15	102016239	102016331	chr15-102016239-102016331
chr15	102025763	102025855	chr15-102025763-102025855
chr15	102065706	102065798	chr15-102065706-102065798
chr15	102376903	102376995	chr15-102376903-102376995
chr15	102406184	102406276	chr15-102406184-102406276
chr15	102510744	102510836	chr15-102510744-102510836
chr15	102942729	102942821	chr15-102942729-102942821
chr15	102966690	102966782	chr15-102966690-102966782
chr15	103220959	103221051	chr15-103220959-103221051
chr16	3236154	3236246	chr16-3236154-3236246
chr16	3254771	3254863	chr16-3254771-3254863
chr16	3354647	3354739	chr16-3354647-3354739
chr16	3420108	3420200	chr16-3420108-3420200
chr16	3490581	3490673	chr16-3490581-3490673
chr16	3700287	3700379	chr16-3700287-3700379
chr16	3820371	3820463	chr16-3820371-3820463
chr16	4559783	4559875	chr16-4559783-4559875
chr16	5515355	5515447	chr16-5515355-5515447
chr16	5996484	5996576	chr16-5996484-5996576
chr16	7383053	7383145	chr16-7383053-7383145
chr16	8174920	8175012	chr16-8174920-8175012
chr16	8423050	8423142	chr16-8423050-8423142
chr16	8525901	8525993	chr16-8525901-8525993
chr16	9751787	9751879	chr16-9751787-9751879
chr16	10728018	10728110	chr16-10728018-10728110
chr16	10734599	10734691	chr16-10734599-10734691
chr16	11193327	11193419	chr16-11193327-11193419
chr16	11199548	11199640	chr16-11199548-11199640
chr16	11235512	11235604	chr16-11235512-11235604
chr16	11511908	11512000	chr16-11511908-11512000
chr16	11813904	11813996	chr16-11813904-11813996
chr16	12964197	12964289	chr16-12964197-12964289
chr16	13312535	13312627	chr16-13312535-13312627
chr16	13419951	13420043	chr16-13419951-13420043

chr16	15294490	15294582	chr16-15294490-15294582
chr16	15498215	15498307	chr16-15498215-15498307
chr16	15642762	15642854	chr16-15642762-15642854
chr16	16224742	16224834	chr16-16224742-16224834
chr16	16552342	16552434	chr16-16552342-16552434
chr16	16787289	16787381	chr16-16787289-16787381
chr16	17222305	17222397	chr16-17222305-17222397
chr16	17260170	17260262	chr16-17260170-17260262
chr16	18248716	18248808	chr16-18248716-18248808
chr16	18471497	18471589	chr16-18471497-18471589
chr16	18617568	18617660	chr16-18617568-18617660
chr16	18958432	18958524	chr16-18958432-18958524
chr16	20476611	20476703	chr16-20476611-20476703
chr16	20477029	20477121	chr16-20477029-20477121
chr16	20789663	20789755	chr16-20789663-20789755
chr16	21239441	21239533	chr16-21239441-21239533
chr16	22081967	22082059	chr16-22081967-22082059
chr16	22578704	22578796	chr16-22578704-22578796
chr16	22847889	22847981	chr16-22847889-22847981
chr16	23231244	23231336	chr16-23231244-23231336
chr16	23403569	23403661	chr16-23403569-23403661
chr16	24362086	24362178	chr16-24362086-24362178
chr16	24364194	24364286	chr16-24364194-24364286
chr16	24364434	24364526	chr16-24364434-24364526
chr16	24365032	24365124	chr16-24365032-24365124
chr16	24431846	24431938	chr16-24431846-24431938
chr16	24434131	24434223	chr16-24434131-24434223
chr16	24733372	24733464	chr16-24733372-24733464
chr16	24780150	24780242	chr16-24780150-24780242
chr16	24801473	24801565	chr16-24801473-24801565
chr16	25483956	25484048	chr16-25483956-25484048
chr16	26163810	26163902	chr16-26163810-26163902
chr16	26591050	26591142	chr16-26591050-26591142
chr16	27053806	27053898	chr16-27053806-27053898
chr16	29816159	29816251	chr16-29816159-29816251
chr16	29851655	29851747	chr16-29851655-29851747
chr16	29886907	29886999	chr16-29886907-29886999
chr16	29946016	29946108	chr16-29946016-29946108
chr16	30065267	30065359	chr16-30065267-30065359
chr16	30069732	30069824	chr16-30069732-30069824
chr16	30502691	30502783	chr16-30502691-30502783
chr16	30835865	30835957	chr16-30835865-30835957

chr16	31162837	31162929	chr16-31162837-31162929
chr16	31275488	31275580	chr16-31275488-31275580
chr16	31353398	31353490	chr16-31353398-31353490
chr16	31460247	31460339	chr16-31460247-31460339
chr16	31597444	31597536	chr16-31597444-31597536
chr16	32062303	32062395	chr16-32062303-32062395
chr16	32088168	32088260	chr16-32088168-32088260
chr16	32645472	32645564	chr16-32645472-32645564
chr16	32670549	32670641	chr16-32670549-32670641
chr16	32677180	32677272	chr16-32677180-32677272
chr16	33115793	33115885	chr16-33115793-33115885
chr16	33550783	33550875	chr16-33550783-33550875
chr16	33580830	33580922	chr16-33580830-33580922
chr16	34391837	34391929	chr16-34391837-34391929
chr16	34494488	34494580	chr16-34494488-34494580
chr16	35456803	35456895	chr16-35456803-35456895
chr16	35588244	35588336	chr16-35588244-35588336
chr16	35981682	35981774	chr16-35981682-35981774
chr16	37046131	37046223	chr16-37046131-37046223
chr16	37620673	37620765	chr16-37620673-37620765
chr16	38408505	38408597	chr16-38408505-38408597
chr16	38414581	38414673	chr16-38414581-38414673
chr16	39253455	39253547	chr16-39253455-39253547
chr16	39315916	39316008	chr16-39315916-39316008
chr16	41497537	41497629	chr16-41497537-41497629
chr16	42724542	42724634	chr16-42724542-42724634
chr16	43392519	43392611	chr16-43392519-43392611
chr16	44166988	44167080	chr16-44166988-44167080
chr16	45103669	45103761	chr16-45103669-45103761
chr16	45110628	45110720	chr16-45110628-45110720
chr16	45837938	45838030	chr16-45837938-45838030
chr16	45844460	45844552	chr16-45844460-45844552
chr16	45849211	45849303	chr16-45849211-45849303
chr16	46232247	46232339	chr16-46232247-46232339
chr16	46807695	46807787	chr16-46807695-46807787
chr16	46858847	46858939	chr16-46858847-46858939
chr16	47474918	47475010	chr16-47474918-47475010
chr16	47850877	47850969	chr16-47850877-47850969
chr16	50414551	50414643	chr16-50414551-50414643
chr16	50418690	50418782	chr16-50418690-50418782
chr16	50689538	50689630	chr16-50689538-50689630
chr16	50803227	50803319	chr16-50803227-50803319

chr16	50841676	50841768	chr16-50841676-50841768
chr16	52482562	52482654	chr16-52482562-52482654
chr16	54153527	54153619	chr16-54153527-54153619
chr16	55298570	55298662	chr16-55298570-55298662
chr16	55532872	55532964	chr16-55532872-55532964
chr16	55555657	55555749	chr16-55555657-55555749
chr16	57241899	57241991	chr16-57241899-57241991
chr16	57257636	57257728	chr16-57257636-57257728
chr16	57307633	57307725	chr16-57307633-57307725
chr16	57398234	57398326	chr16-57398234-57398326
chr16	57419312	57419404	chr16-57419312-57419404
chr16	57453100	57453192	chr16-57453100-57453192
chr16	58352193	58352285	chr16-58352193-58352285
chr16	58457584	58457676	chr16-58457584-58457676
chr16	58494811	58494903	chr16-58494811-58494903
chr16	58548126	58548218	chr16-58548126-58548218
chr16	58584974	58585066	chr16-58584974-58585066
chr16	58670165	58670257	chr16-58670165-58670257
chr16	58684069	58684161	chr16-58684069-58684161
chr16	59394351	59394443	chr16-59394351-59394443
chr16	60696433	60696525	chr16-60696433-60696525
chr16	61056345	61056437	chr16-61056345-61056437
chr16	63461973	63462065	chr16-63461973-63462065
chr16	63952582	63952674	chr16-63952582-63952674
chr16	64830512	64830604	chr16-64830512-64830604
chr16	64942809	64942901	chr16-64942809-64942901
chr16	65689171	65689263	chr16-65689171-65689263
chr16	66481644	66481736	chr16-66481644-66481736
chr16	66883434	66883526	chr16-66883434-66883526
chr16	66905789	66905881	chr16-66905789-66905881
chr16	70178085	70178177	chr16-70178085-70178177
chr16	70992273	70992365	chr16-70992273-70992365
chr16	72804352	72804444	chr16-72804352-72804444
chr16	76444773	76444865	chr16-76444773-76444865
chr16	76509274	76509366	chr16-76509274-76509366
chr16	76814910	76815002	chr16-76814910-76815002
chr16	78318144	78318236	chr16-78318144-78318236
chr16	81514502	81514594	chr16-81514502-81514594
chr16	81541370	81541462	chr16-81541370-81541462
chr16	85054160	85054252	chr16-85054160-85054252
chr16	85099824	85099916	chr16-85099824-85099916
chr16	85128314	85128406	chr16-85128314-85128406

chr16	85473020	85473112	chr16-85473020-85473112
chr16	85704796	85704888	chr16-85704796-85704888
chr16	86252717	86252809	chr16-86252717-86252809
chr16	86704388	86704480	chr16-86704388-86704480
chr16	87348297	87348389	chr16-87348297-87348389
chr16	87443451	87443543	chr16-87443451-87443543
chr16	87842249	87842341	chr16-87842249-87842341
chr16	89461548	89461640	chr16-89461548-89461640
chr16	90065613	90065705	chr16-90065613-90065705
chr16	90110945	90111037	chr16-90110945-90111037
chr16	90197450	90197542	chr16-90197450-90197542
chr16	90541001	90541093	chr16-90541001-90541093
chr16	90830036	90830128	chr16-90830036-90830128
chr16	90856901	90856993	chr16-90856901-90856993
chr16	91399919	91400011	chr16-91399919-91400011
chr16	91534622	91534714	chr16-91534622-91534714
chr16	91551379	91551471	chr16-91551379-91551471
chr16	91729400	91729492	chr16-91729400-91729492
chr16	92301194	92301286	chr16-92301194-92301286
chr16	92333647	92333739	chr16-92333647-92333739
chr16	92341346	92341438	chr16-92341346-92341438
chr16	92654026	92654118	chr16-92654026-92654118
chr16	92661970	92662062	chr16-92661970-92662062
chr16	92662700	92662792	chr16-92662700-92662792
chr16	92697402	92697494	chr16-92697402-92697494
chr16	92786862	92786954	chr16-92786862-92786954
chr16	92879262	92879354	chr16-92879262-92879354
chr16	93123908	93124000	chr16-93123908-93124000
chr16	93297971	93298063	chr16-93297971-93298063
chr16	93381117	93381209	chr16-93381117-93381209
chr16	94317920	94318012	chr16-94317920-94318012
chr16	94725021	94725113	chr16-94725021-94725113
chr16	94854971	94855063	chr16-94854971-94855063
chr16	95682578	95682670	chr16-95682578-95682670
chr16	95958193	95958285	chr16-95958193-95958285
chr16	96938146	96938238	chr16-96938146-96938238
chr16	97373363	97373455	chr16-97373363-97373455
chr16	97594093	97594185	chr16-97594093-97594185
chr16	97697964	97698056	chr16-97697964-97698056
chr16	97717730	97717822	chr16-97717730-97717822
chr16	98076852	98076944	chr16-98076852-98076944
chr17	3005198	3005290	chr17-3005198-3005290

chr17	3006802	3006894	chr17-3006802-3006894
chr17	3007358	3007450	chr17-3007358-3007450
chr17	3012913	3013005	chr17-3012913-3013005
chr17	3018406	3018498	chr17-3018406-3018498
chr17	3020124	3020216	chr17-3020124-3020216
chr17	3020511	3020603	chr17-3020511-3020603
chr17	3022457	3022549	chr17-3022457-3022549
chr17	3022973	3023065	chr17-3022973-3023065
chr17	3026995	3027087	chr17-3026995-3027087
chr17	3029906	3029998	chr17-3029906-3029998
chr17	3033771	3033863	chr17-3033771-3033863
chr17	3038038	3038130	chr17-3038038-3038130
chr17	3077879	3077971	chr17-3077879-3077971
chr17	3081610	3081702	chr17-3081610-3081702
chr17	3082742	3082834	chr17-3082742-3082834
chr17	3762398	3762490	chr17-3762398-3762490
chr17	4626310	4626402	chr17-4626310-4626402
chr17	5883275	5883367	chr17-5883275-5883367
chr17	5977031	5977123	chr17-5977031-5977123
chr17	6079854	6079946	chr17-6079854-6079946
chr17	6128113	6128205	chr17-6128113-6128205
chr17	6154308	6154400	chr17-6154308-6154400
chr17	6173076	6173168	chr17-6173076-6173168
chr17	6710933	6711025	chr17-6710933-6711025
chr17	6808286	6808378	chr17-6808286-6808378
chr17	6814724	6814816	chr17-6814724-6814816
chr17	6829066	6829158	chr17-6829066-6829158
chr17	7055998	7056090	chr17-7055998-7056090
chr17	7173837	7173929	chr17-7173837-7173929
chr17	7598201	7598293	chr17-7598201-7598293
chr17	7755865	7755957	chr17-7755865-7755957
chr17	8311036	8311128	chr17-8311036-8311128
chr17	9538494	9538586	chr17-9538494-9538586
chr17	10040652	10040744	chr17-10040652-10040744
chr17	10378988	10379080	chr17-10378988-10379080
chr17	10413937	10414029	chr17-10413937-10414029
chr17	12123499	12123591	chr17-12123499-12123591
chr17	12195752	12195844	chr17-12195752-12195844
chr17	12740433	12740525	chr17-12740433-12740525
chr17	12745799	12745891	chr17-12745799-12745891
chr17	12750125	12750217	chr17-12750125-12750217
chr17	13043893	13043985	chr17-13043893-13043985

chr17	13062629	13062721	chr17-13062629-13062721
chr17	13177899	13177991	chr17-13177899-13177991
chr17	13421091	13421183	chr17-13421091-13421183
chr17	13669417	13669509	chr17-13669417-13669509
chr17	13683446	13683538	chr17-13683446-13683538
chr17	13699527	13699619	chr17-13699527-13699619
chr17	13709390	13709482	chr17-13709390-13709482
chr17	13709795	13709887	chr17-13709795-13709887
chr17	13732875	13732967	chr17-13732875-13732967
chr17	13743842	13743934	chr17-13743842-13743934
chr17	13761322	13761414	chr17-13761322-13761414
chr17	14375213	14375305	chr17-14375213-14375305
chr17	15299932	15300024	chr17-15299932-15300024
chr17	15326501	15326593	chr17-15326501-15326593
chr17	15374585	15374677	chr17-15374585-15374677
chr17	15545163	15545255	chr17-15545163-15545255
chr17	15572767	15572859	chr17-15572767-15572859
chr17	15705020	15705112	chr17-15705020-15705112
chr17	17849170	17849262	chr17-17849170-17849262
chr17	19938898	19938990	chr17-19938898-19938990
chr17	20945393	20945485	chr17-20945393-20945485
chr17	21107865	21107957	chr17-21107865-21107957
chr17	22876434	22876526	chr17-22876434-22876526
chr17	23993670	23993762	chr17-23993670-23993762
chr17	24000528	24000620	chr17-24000528-24000620
chr17	24035191	24035283	chr17-24035191-24035283
chr17	24163669	24163761	chr17-24163669-24163761
chr17	24206501	24206593	chr17-24206501-24206593
chr17	24318798	24318890	chr17-24318798-24318890
chr17	24479001	24479093	chr17-24479001-24479093
chr17	24628806	24628898	chr17-24628806-24628898
chr17	25184377	25184469	chr17-25184377-25184469
chr17	25269547	25269639	chr17-25269547-25269639
chr17	25451799	25451891	chr17-25451799-25451891
chr17	25755900	25755992	chr17-25755900-25755992
chr17	25756853	25756945	chr17-25756853-25756945
chr17	26258609	26258701	chr17-26258609-26258701
chr17	26368470	26368562	chr17-26368470-26368562
chr17	26535957	26536049	chr17-26535957-26536049
chr17	26584749	26584841	chr17-26584749-26584841
chr17	26596818	26596910	chr17-26596818-26596910
chr17	27038781	27038873	chr17-27038781-27038873

chr17	27056399	27056491	chr17-27056399-27056491
chr17	27592485	27592577	chr17-27592485-27592577
chr17	27922858	27922950	chr17-27922858-27922950
chr17	28076159	28076251	chr17-28076159-28076251
chr17	28269758	28269850	chr17-28269758-28269850
chr17	28270240	28270332	chr17-28270240-28270332
chr17	28278317	28278409	chr17-28278317-28278409
chr17	28328631	28328723	chr17-28328631-28328723
chr17	28345489	28345581	chr17-28345489-28345581
chr17	28375861	28375953	chr17-28375861-28375953
chr17	28391890	28391982	chr17-28391890-28391982
chr17	28633880	28633972	chr17-28633880-28633972
chr17	28691232	28691324	chr17-28691232-28691324
chr17	29103955	29104047	chr17-29103955-29104047
chr17	29165617	29165709	chr17-29165617-29165709
chr17	29437448	29437540	chr17-29437448-29437540
chr17	29438552	29438644	chr17-29438552-29438644
chr17	29440055	29440147	chr17-29440055-29440147
chr17	29451368	29451460	chr17-29451368-29451460
chr17	29582589	29582681	chr17-29582589-29582681
chr17	29994623	29994715	chr17-29994623-29994715
chr17	30027706	30027798	chr17-30027706-30027798
chr17	30068650	30068742	chr17-30068650-30068742
chr17	30072843	30072935	chr17-30072843-30072935
chr17	31305321	31305413	chr17-31305321-31305413
chr17	31332111	31332203	chr17-31332111-31332203
chr17	31537796	31537888	chr17-31537796-31537888
chr17	31587996	31588088	chr17-31587996-31588088
chr17	31659009	31659101	chr17-31659009-31659101
chr17	31693444	31693536	chr17-31693444-31693536
chr17	31693676	31693768	chr17-31693676-31693768
chr17	32354697	32354789	chr17-32354697-32354789
chr17	32815956	32816048	chr17-32815956-32816048
chr17	33342535	33342627	chr17-33342535-33342627
chr17	33543837	33543929	chr17-33543837-33543929
chr17	33629605	33629697	chr17-33629605-33629697
chr17	33704854	33704946	chr17-33704854-33704946
chr17	33712243	33712335	chr17-33712243-33712335
chr17	33746153	33746245	chr17-33746153-33746245
chr17	33998887	33998979	chr17-33998887-33998979
chr17	34023274	34023366	chr17-34023274-34023366
chr17	34430716	34430808	chr17-34430716-34430808

chr17	34605845	34605937	chr17-34605845-34605937
chr17	34894162	34894254	chr17-34894162-34894254
chr17	34977047	34977139	chr17-34977047-34977139
chr17	35048446	35048538	chr17-35048446-35048538
chr17	35068346	35068438	chr17-35068346-35068438
chr17	35149988	35150080	chr17-35149988-35150080
chr17	35156284	35156376	chr17-35156284-35156376
chr17	35184971	35185063	chr17-35184971-35185063
chr17	35242692	35242784	chr17-35242692-35242784
chr17	35638625	35638717	chr17-35638625-35638717
chr17	35819357	35819449	chr17-35819357-35819449
chr17	35827045	35827137	chr17-35827045-35827137
chr17	35910062	35910154	chr17-35910062-35910154
chr17	35922962	35923054	chr17-35922962-35923054
chr17	36002001	36002093	chr17-36002001-36002093
chr17	36020422	36020514	chr17-36020422-36020514
chr17	36927648	36927740	chr17-36927648-36927740
chr17	38414112	38414204	chr17-38414112-38414204
chr17	39438566	39438658	chr17-39438566-39438658
chr17	40885205	40885297	chr17-40885205-40885297
chr17	40963229	40963321	chr17-40963229-40963321
chr17	41067052	41067144	chr17-41067052-41067144
chr17	42905111	42905203	chr17-42905111-42905203
chr17	42939718	42939810	chr17-42939718-42939810
chr17	42975470	42975562	chr17-42975470-42975562
chr17	42981952	42982044	chr17-42981952-42982044
chr17	43219853	43219945	chr17-43219853-43219945
chr17	43252877	43252969	chr17-43252877-43252969
chr17	43329930	43330022	chr17-43329930-43330022
chr17	43396043	43396135	chr17-43396043-43396135
chr17	43811622	43811714	chr17-43811622-43811714
chr17	44022984	44023076	chr17-44022984-44023076
chr17	44203161	44203253	chr17-44203161-44203253
chr17	44767633	44767725	chr17-44767633-44767725
chr17	45693969	45694061	chr17-45693969-45694061
chr17	45873666	45873758	chr17-45873666-45873758
chr17	46162950	46163042	chr17-46162950-46163042
chr17	46553316	46553408	chr17-46553316-46553408
chr17	46857577	46857669	chr17-46857577-46857669
chr17	47039562	47039654	chr17-47039562-47039654
chr17	47195844	47195936	chr17-47195844-47195936
chr17	47653673	47653765	chr17-47653673-47653765

chr17	47980701	47980793	chr17-47980701-47980793
chr17	48932093	48932185	chr17-48932093-48932185
chr17	48972568	48972660	chr17-48972568-48972660
chr17	49013153	49013245	chr17-49013153-49013245
chr17	49851329	49851421	chr17-49851329-49851421
chr17	50349270	50349362	chr17-50349270-50349362
chr17	51414467	51414559	chr17-51414467-51414559
chr17	51882571	51882663	chr17-51882571-51882663
chr17	52857095	52857187	chr17-52857095-52857187
chr17	53046388	53046480	chr17-53046388-53046480
chr17	53478315	53478407	chr17-53478315-53478407
chr17	53478849	53478941	chr17-53478849-53478941
chr17	53583996	53584088	chr17-53583996-53584088
chr17	53637671	53637763	chr17-53637671-53637763
chr17	53688323	53688415	chr17-53688323-53688415
chr17	56200007	56200099	chr17-56200007-56200099
chr17	56276803	56276895	chr17-56276803-56276895
chr17	56291272	56291364	chr17-56291272-56291364
chr17	56511294	56511386	chr17-56511294-56511386
chr17	56625666	56625758	chr17-56625666-56625758
chr17	56717686	56717778	chr17-56717686-56717778
chr17	56822500	56822592	chr17-56822500-56822592
chr17	56935302	56935394	chr17-56935302-56935394
chr17	57001467	57001559	chr17-57001467-57001559
chr17	57060452	57060544	chr17-57060452-57060544
chr17	57192665	57192757	chr17-57192665-57192757
chr17	57219245	57219337	chr17-57219245-57219337
chr17	57249395	57249487	chr17-57249395-57249487
chr17	60099890	60099982	chr17-60099890-60099982
chr17	60250031	60250123	chr17-60250031-60250123
chr17	61589193	61589285	chr17-61589193-61589285
chr17	62460050	62460142	chr17-62460050-62460142
chr17	62461644	62461736	chr17-62461644-62461736
chr17	62690348	62690440	chr17-62690348-62690440
chr17	62691610	62691702	chr17-62691610-62691702
chr17	62784057	62784149	chr17-62784057-62784149
chr17	63755174	63755266	chr17-63755174-63755266
chr17	63799195	63799287	chr17-63799195-63799287
chr17	63993568	63993660	chr17-63993568-63993660
chr17	64407257	64407349	chr17-64407257-64407349
chr17	64550233	64550325	chr17-64550233-64550325
chr17	64568281	64568373	chr17-64568281-64568373

chr17	65773628	65773720	chr17-65773628-65773720
chr17	66323135	66323227	chr17-66323135-66323227
chr17	67264614	67264706	chr17-67264614-67264706
chr17	68804340	68804432	chr17-68804340-68804432
chr17	70709792	70709884	chr17-70709792-70709884
chr17	70739532	70739624	chr17-70739532-70739624
chr17	70755739	70755831	chr17-70755739-70755831
chr17	70963702	70963794	chr17-70963702-70963794
chr17	71458979	71459071	chr17-71458979-71459071
chr17	72064184	72064276	chr17-72064184-72064276
chr17	72147534	72147626	chr17-72147534-72147626
chr17	72795686	72795778	chr17-72795686-72795778
chr17	72828404	72828496	chr17-72828404-72828496
chr17	72836606	72836698	chr17-72836606-72836698
chr17	73142559	73142651	chr17-73142559-73142651
chr17	73335767	73335859	chr17-73335767-73335859
chr17	74068368	74068460	chr17-74068368-74068460
chr17	75105624	75105716	chr17-75105624-75105716
chr17	75199802	75199894	chr17-75199802-75199894
chr17	75277902	75277994	chr17-75277902-75277994
chr17	75905663	75905755	chr17-75905663-75905755
chr17	77856100	77856192	chr17-77856100-77856192
chr17	78392443	78392535	chr17-78392443-78392535
chr17	78401735	78401827	chr17-78401735-78401827
chr17	78404084	78404176	chr17-78404084-78404176
chr17	78684804	78684896	chr17-78684804-78684896
chr17	78961798	78961890	chr17-78961798-78961890
chr17	79542244	79542336	chr17-79542244-79542336
chr17	79615140	79615232	chr17-79615140-79615232
chr17	79896010	79896102	chr17-79896010-79896102
chr17	79950103	79950195	chr17-79950103-79950195
chr17	80062083	80062175	chr17-80062083-80062175
chr17	80480703	80480795	chr17-80480703-80480795
chr17	80489120	80489212	chr17-80489120-80489212
chr17	80727859	80727951	chr17-80727859-80727951
chr17	82056014	82056106	chr17-82056014-82056106
chr17	83312634	83312726	chr17-83312634-83312726
chr17	83983736	83983828	chr17-83983736-83983828
chr17	83998014	83998106	chr17-83998014-83998106
chr17	84026276	84026368	chr17-84026276-84026368
chr17	84116342	84116434	chr17-84116342-84116434
chr17	84153039	84153131	chr17-84153039-84153131

chr17	84304935	84305027	chr17-84304935-84305027
chr17	84333040	84333132	chr17-84333040-84333132
chr17	84855921	84856013	chr17-84855921-84856013
chr17	86330570	86330662	chr17-86330570-86330662
chr17	86720335	86720427	chr17-86720335-86720427
chr17	87143248	87143340	chr17-87143248-87143340
chr17	87446871	87446963	chr17-87446871-87446963
chr17	87476847	87476939	chr17-87476847-87476939
chr17	87598555	87598647	chr17-87598555-87598647
chr17	87882719	87882811	chr17-87882719-87882811
chr17	88066176	88066268	chr17-88066176-88066268
chr17	88460401	88460493	chr17-88460401-88460493
chr17	88629054	88629146	chr17-88629054-88629146
chr17	89627222	89627314	chr17-89627222-89627314
chr17	89868132	89868224	chr17-89868132-89868224
chr17	90941339	90941431	chr17-90941339-90941431
chr17	91872794	91872886	chr17-91872794-91872886
chr17	92012334	92012426	chr17-92012334-92012426
chr17	94397335	94397427	chr17-94397335-94397427
chr18	3115667	3115759	chr18-3115667-3115759
chr18	4099484	4099576	chr18-4099484-4099576
chr18	4913114	4913206	chr18-4913114-4913206
chr18	6237975	6238067	chr18-6237975-6238067
chr18	7045860	7045952	chr18-7045860-7045952
chr18	8022979	8023071	chr18-8022979-8023071
chr18	9533188	9533280	chr18-9533188-9533280
chr18	9562855	9562947	chr18-9562855-9562947
chr18	10052084	10052176	chr18-10052084-10052176
chr18	10680834	10680926	chr18-10680834-10680926
chr18	11051405	11051497	chr18-11051405-11051497
chr18	11052908	11053000	chr18-11052908-11053000
chr18	11974993	11975085	chr18-11974993-11975085
chr18	12042985	12043077	chr18-12042985-12043077
chr18	12636071	12636163	chr18-12636071-12636163
chr18	12735595	12735687	chr18-12735595-12735687
chr18	15391151	15391243	chr18-15391151-15391243
chr18	19900614	19900706	chr18-19900614-19900706
chr18	23039316	23039408	chr18-23039316-23039408
chr18	23547078	23547170	chr18-23547078-23547170
chr18	24095390	24095482	chr18-24095390-24095482
chr18	25414646	25414738	chr18-25414646-25414738
chr18	27948478	27948570	chr18-27948478-27948570

chr18	28195382	28195474	chr18-28195382-28195474
chr18	30543836	30543928	chr18-30543836-30543928
chr18	31883995	31884087	chr18-31883995-31884087
chr18	31950889	31950981	chr18-31950889-31950981
chr18	31998102	31998194	chr18-31998102-31998194
chr18	32162459	32162551	chr18-32162459-32162551
chr18	32335772	32335864	chr18-32335772-32335864
chr18	32343146	32343238	chr18-32343146-32343238
chr18	33849316	33849408	chr18-33849316-33849408
chr18	34137335	34137427	chr18-34137335-34137427
chr18	34768965	34769057	chr18-34768965-34769057
chr18	34989547	34989639	chr18-34989547-34989639
chr18	35065887	35065979	chr18-35065887-35065979
chr18	36130683	36130775	chr18-36130683-36130775
chr18	36384210	36384302	chr18-36384210-36384302
chr18	36404837	36404929	chr18-36404837-36404929
chr18	36965807	36965899	chr18-36965807-36965899
chr18	37935551	37935643	chr18-37935551-37935643
chr18	38151145	38151237	chr18-38151145-38151237
chr18	39488924	39489016	chr18-39488924-39489016
chr18	41816919	41817011	chr18-41816919-41817011
chr18	42766947	42767039	chr18-42766947-42767039
chr18	44472780	44472872	chr18-44472780-44472872
chr18	44668581	44668673	chr18-44668581-44668673
chr18	44946896	44946988	chr18-44946896-44946988
chr18	45056026	45056118	chr18-45056026-45056118
chr18	46327571	46327663	chr18-46327571-46327663
chr18	46642170	46642262	chr18-46642170-46642262
chr18	46778944	46779036	chr18-46778944-46779036
chr18	48126076	48126168	chr18-48126076-48126168
chr18	49653608	49653700	chr18-49653608-49653700
chr18	50063707	50063799	chr18-50063707-50063799
chr18	50922034	50922126	chr18-50922034-50922126
chr18	51106538	51106630	chr18-51106538-51106630
chr18	53254493	53254585	chr18-53254493-53254585
chr18	54906765	54906857	chr18-54906765-54906857
chr18	54911325	54911417	chr18-54911325-54911417
chr18	55200296	55200388	chr18-55200296-55200388
chr18	55622821	55622913	chr18-55622821-55622913
chr18	56303412	56303504	chr18-56303412-56303504
chr18	56390610	56390702	chr18-56390610-56390702
chr18	56549051	56549143	chr18-56549051-56549143

chr18	56621255	56621347	chr18-56621255-56621347
chr18	56704420	56704512	chr18-56704420-56704512
chr18	56723969	56724061	chr18-56723969-56724061
chr18	57837076	57837168	chr18-57837076-57837168
chr18	58502063	58502155	chr18-58502063-58502155
chr18	58825362	58825454	chr18-58825362-58825454
chr18	59529629	59529721	chr18-59529629-59529721
chr18	60249372	60249464	chr18-60249372-60249464
chr18	60502465	60502557	chr18-60502465-60502557
chr18	60675773	60675865	chr18-60675773-60675865
chr18	60937230	60937322	chr18-60937230-60937322
chr18	61010383	61010475	chr18-61010383-61010475
chr18	61089840	61089932	chr18-61089840-61089932
chr18	61262433	61262525	chr18-61262433-61262525
chr18	61362670	61362762	chr18-61362670-61362762
chr18	61637381	61637473	chr18-61637381-61637473
chr18	61674420	61674512	chr18-61674420-61674512
chr18	61934297	61934389	chr18-61934297-61934389
chr18	62017419	62017511	chr18-62017419-62017511
chr18	62166255	62166347	chr18-62166255-62166347
chr18	62653632	62653724	chr18-62653632-62653724
chr18	63200006	63200098	chr18-63200006-63200098
chr18	63229783	63229875	chr18-63229783-63229875
chr18	64185503	64185595	chr18-64185503-64185595
chr18	64474163	64474255	chr18-64474163-64474255
chr18	64489127	64489219	chr18-64489127-64489219
chr18	65000458	65000550	chr18-65000458-65000550
chr18	65071284	65071376	chr18-65071284-65071376
chr18	65427444	65427536	chr18-65427444-65427536
chr18	65427668	65427760	chr18-65427668-65427760
chr18	66150781	66150873	chr18-66150781-66150873
chr18	66635904	66635996	chr18-66635904-66635996
chr18	68979294	68979386	chr18-68979294-68979386
chr18	69925868	69925960	chr18-69925868-69925960
chr18	70586366	70586458	chr18-70586366-70586458
chr18	70769188	70769280	chr18-70769188-70769280
chr18	72574171	72574263	chr18-72574171-72574263
chr18	73533411	73533503	chr18-73533411-73533503
chr18	73706332	73706424	chr18-73706332-73706424
chr18	73811385	73811477	chr18-73811385-73811477
chr18	73846746	73846838	chr18-73846746-73846838
chr18	73898609	73898701	chr18-73898609-73898701

chr18	74216663	74216755	chr18-74216663-74216755
chr18	74551640	74551732	chr18-74551640-74551732
chr18	74778302	74778394	chr18-74778302-74778394
chr18	74778775	74778867	chr18-74778775-74778867
chr18	75363306	75363398	chr18-75363306-75363398
chr18	75386208	75386300	chr18-75386208-75386300
chr18	75493536	75493628	chr18-75493536-75493628
chr18	75513251	75513343	chr18-75513251-75513343
chr18	75565854	75565946	chr18-75565854-75565946
chr18	76307135	76307227	chr18-76307135-76307227
chr18	76699289	76699381	chr18-76699289-76699381
chr18	77601081	77601173	chr18-77601081-77601173
chr18	77845165	77845257	chr18-77845165-77845257
chr18	79022684	79022776	chr18-79022684-79022776
chr18	79286547	79286639	chr18-79286547-79286639
chr18	79350074	79350166	chr18-79350074-79350166
chr18	79769620	79769712	chr18-79769620-79769712
chr18	82959884	82959976	chr18-82959884-82959976
chr18	83374724	83374816	chr18-83374724-83374816
chr18	84641205	84641297	chr18-84641205-84641297
chr18	85416975	85417067	chr18-85416975-85417067
chr18	85698019	85698111	chr18-85698019-85698111
chr19	3237685	3237777	chr19-3237685-3237777
chr19	3820214	3820306	chr19-3820214-3820306
chr19	3832968	3833060	chr19-3832968-3833060
chr19	4208739	4208831	chr19-4208739-4208831
chr19	4213057	4213149	chr19-4213057-4213149
chr19	4244891	4244983	chr19-4244891-4244983
chr19	4477586	4477678	chr19-4477586-4477678
chr19	4478044	4478136	chr19-4478044-4478136
chr19	4686755	4686847	chr19-4686755-4686847
chr19	4878897	4878989	chr19-4878897-4878989
chr19	5013198	5013290	chr19-5013198-5013290
chr19	5349203	5349295	chr19-5349203-5349295
chr19	5425064	5425156	chr19-5425064-5425156
chr19	5430537	5430629	chr19-5430537-5430629
chr19	5547247	5547339	chr19-5547247-5547339
chr19	5635454	5635546	chr19-5635454-5635546
chr19	5724507	5724599	chr19-5724507-5724599
chr19	5743406	5743498	chr19-5743406-5743498
chr19	6060851	6060943	chr19-6060851-6060943
chr19	6061168	6061260	chr19-6061168-6061260

chr19	6137541	6137633	chr19-6137541-6137633
chr19	6554118	6554210	chr19-6554118-6554210
chr19	6952070	6952162	chr19-6952070-6952162
chr19	7120651	7120743	chr19-7120651-7120743
chr19	7348667	7348759	chr19-7348667-7348759
chr19	7597886	7597978	chr19-7597886-7597978
chr19	8252194	8252286	chr19-8252194-8252286
chr19	8884397	8884489	chr19-8884397-8884489
chr19	8983085	8983177	chr19-8983085-8983177
chr19	8994295	8994387	chr19-8994295-8994387
chr19	9080067	9080159	chr19-9080067-9080159
chr19	9106958	9107050	chr19-9106958-9107050
chr19	9132202	9132294	chr19-9132202-9132294
chr19	9870308	9870400	chr19-9870308-9870400
chr19	10506814	10506906	chr19-10506814-10506906
chr19	10561743	10561835	chr19-10561743-10561835
chr19	11274537	11274629	chr19-11274537-11274629
chr19	11400284	11400376	chr19-11400284-11400376
chr19	11403262	11403354	chr19-11403262-11403354
chr19	12472456	12472548	chr19-12472456-12472548
chr19	12562117	12562209	chr19-12562117-12562209
chr19	12805799	12805891	chr19-12805799-12805891
chr19	14085886	14085978	chr19-14085886-14085978
chr19	14186453	14186545	chr19-14186453-14186545
chr19	14272562	14272654	chr19-14272562-14272654
chr19	14486410	14486502	chr19-14486410-14486502
chr19	14655571	14655663	chr19-14655571-14655663
chr19	15576982	15577074	chr19-15576982-15577074
chr19	15633482	15633574	chr19-15633482-15633574
chr19	15925075	15925167	chr19-15925075-15925167
chr19	16493280	16493372	chr19-16493280-16493372
chr19	17289608	17289700	chr19-17289608-17289700
chr19	17335032	17335124	chr19-17335032-17335124
chr19	19427434	19427526	chr19-19427434-19427526
chr19	19696685	19696777	chr19-19696685-19696777
chr19	20924107	20924199	chr19-20924107-20924199
chr19	23397175	23397267	chr19-23397175-23397267
chr19	23910439	23910531	chr19-23910439-23910531
chr19	23956460	23956552	chr19-23956460-23956552
chr19	23968237	23968329	chr19-23968237-23968329
chr19	23969021	23969113	chr19-23969021-23969113
chr19	24225033	24225125	chr19-24225033-24225125

chr19	24280547	24280639	chr19-24280547-24280639
chr19	24476986	24477078	chr19-24476986-24477078
chr19	25267451	25267543	chr19-25267451-25267543
chr19	25636653	25636745	chr19-25636653-25636745
chr19	26021281	26021373	chr19-26021281-26021373
chr19	26022719	26022811	chr19-26022719-26022811
chr19	26466005	26466097	chr19-26466005-26466097
chr19	28878247	28878339	chr19-28878247-28878339
chr19	28887579	28887671	chr19-28887579-28887671
chr19	29241778	29241870	chr19-29241778-29241870
chr19	29667167	29667259	chr19-29667167-29667259
chr19	30125382	30125474	chr19-30125382-30125474
chr19	30222728	30222820	chr19-30222728-30222820
chr19	30377683	30377775	chr19-30377683-30377775
chr19	31539006	31539098	chr19-31539006-31539098
chr19	32242247	32242339	chr19-32242247-32242339
chr19	32292080	32292172	chr19-32292080-32292172
chr19	32319838	32319930	chr19-32319838-32319930
chr19	32396431	32396523	chr19-32396431-32396523
chr19	32602536	32602628	chr19-32602536-32602628
chr19	33027163	33027255	chr19-33027163-33027255
chr19	34241712	34241804	chr19-34241712-34241804
chr19	35368629	35368721	chr19-35368629-35368721
chr19	36129272	36129364	chr19-36129272-36129364
chr19	36129774	36129866	chr19-36129774-36129866
chr19	36291032	36291124	chr19-36291032-36291124
chr19	36863428	36863520	chr19-36863428-36863520
chr19	38382798	38382890	chr19-38382798-38382890
chr19	38813165	38813257	chr19-38813165-38813257
chr19	39264043	39264135	chr19-39264043-39264135
chr19	40226913	40227005	chr19-40226913-40227005
chr19	40247475	40247567	chr19-40247475-40247567
chr19	40614401	40614493	chr19-40614401-40614493
chr19	40675480	40675572	chr19-40675480-40675572
chr19	40676063	40676155	chr19-40676063-40676155
chr19	40723664	40723756	chr19-40723664-40723756
chr19	40761051	40761143	chr19-40761051-40761143
chr19	40996252	40996344	chr19-40996252-40996344
chr19	41102179	41102271	chr19-41102179-41102271
chr19	41389308	41389400	chr19-41389308-41389400
chr19	41552863	41552955	chr19-41552863-41552955
chr19	41790526	41790618	chr19-41790526-41790618

chr19	41857612	41857704	chr19-41857612-41857704
chr19	41984483	41984575	chr19-41984483-41984575
chr19	43232621	43232713	chr19-43232621-43232713
chr19	43524636	43524728	chr19-43524636-43524728
chr19	43614691	43614783	chr19-43614691-43614783
chr19	43947926	43948018	chr19-43947926-43948018
chr19	44246748	44246840	chr19-44246748-44246840
chr19	44284848	44284940	chr19-44284848-44284940
chr19	44294121	44294213	chr19-44294121-44294213
chr19	44912192	44912284	chr19-44912192-44912284
chr19	45001521	45001613	chr19-45001521-45001613
chr19	45064072	45064164	chr19-45064072-45064164
chr19	45163943	45164035	chr19-45163943-45164035
chr19	45661648	45661740	chr19-45661648-45661740
chr19	45737036	45737128	chr19-45737036-45737128
chr19	45783246	45783338	chr19-45783246-45783338
chr19	46005121	46005213	chr19-46005121-46005213
chr19	46338591	46338683	chr19-46338591-46338683
chr19	46350410	46350502	chr19-46350410-46350502
chr19	46416141	46416233	chr19-46416141-46416233
chr19	46490930	46491022	chr19-46490930-46491022
chr19	46502978	46503070	chr19-46502978-46503070
chr19	46509351	46509443	chr19-46509351-46509443
chr19	46523269	46523361	chr19-46523269-46523361
chr19	46558177	46558269	chr19-46558177-46558269
chr19	46610007	46610099	chr19-46610007-46610099
chr19	46900018	46900110	chr19-46900018-46900110
chr19	46988616	46988708	chr19-46988616-46988708
chr19	47219872	47219964	chr19-47219872-47219964
chr19	47537135	47537227	chr19-47537135-47537227
chr19	47562657	47562749	chr19-47562657-47562749
chr19	47938102	47938194	chr19-47938102-47938194
chr19	48650529	48650621	chr19-48650529-48650621
chr19	48947050	48947142	chr19-48947050-48947142
chr19	50230377	50230469	chr19-50230377-50230469
chr19	50436175	50436267	chr19-50436175-50436267
chr19	51299120	51299212	chr19-51299120-51299212
chr19	52663544	52663636	chr19-52663544-52663636
chr19	53032196	53032288	chr19-53032196-53032288
chr19	53043478	53043570	chr19-53043478-53043570
chr19	53080621	53080713	chr19-53080621-53080713
chr19	53185633	53185725	chr19-53185633-53185725

chr19	53404420	53404512	chr19-53404420-53404512
chr19	53435048	53435140	chr19-53435048-53435140
chr19	53449614	53449706	chr19-53449614-53449706
chr19	53451650	53451742	chr19-53451650-53451742
chr19	53464802	53464894	chr19-53464802-53464894
chr19	53502418	53502510	chr19-53502418-53502510
chr19	55111836	55111928	chr19-55111836-55111928
chr19	55232252	55232344	chr19-55232252-55232344
chr19	55258141	55258233	chr19-55258141-55258233
chr19	55262093	55262185	chr19-55262093-55262185
chr19	55769011	55769103	chr19-55769011-55769103
chr19	55865935	55866027	chr19-55865935-55866027
chr19	55962088	55962180	chr19-55962088-55962180
chr19	56120868	56120960	chr19-56120868-56120960
chr19	56750421	56750513	chr19-56750421-56750513
chr19	57098334	57098426	chr19-57098334-57098426
chr19	57196705	57196797	chr19-57196705-57196797
chr19	57197550	57197642	chr19-57197550-57197642
chr19	58113476	58113568	chr19-58113476-58113568
chr19	58828212	58828304	chr19-58828212-58828304
chr19	59121960	59122052	chr19-59121960-59122052
chr19	59201483	59201575	chr19-59201483-59201575
chr19	59345742	59345834	chr19-59345742-59345834
chr19	59634629	59634721	chr19-59634629-59634721
chr19	59963791	59963883	chr19-59963791-59963883
chr19	60556882	60556974	chr19-60556882-60556974
chr2	3239453	3239545	chr2-3239453-3239545
chr2	3659533	3659625	chr2-3659533-3659625
chr2	3682779	3682871	chr2-3682779-3682871
chr2	3964510	3964602	chr2-3964510-3964602
chr2	4260747	4260839	chr2-4260747-4260839
chr2	4497875	4497967	chr2-4497875-4497967
chr2	4596367	4596459	chr2-4596367-4596459
chr2	4830732	4830824	chr2-4830732-4830824
chr2	5081876	5081968	chr2-5081876-5081968
chr2	5769001	5769093	chr2-5769001-5769093
chr2	5769763	5769855	chr2-5769763-5769855
chr2	6072155	6072247	chr2-6072155-6072247
chr2	6915067	6915159	chr2-6915067-6915159
chr2	7081237	7081329	chr2-7081237-7081329
chr2	7533256	7533348	chr2-7533256-7533348
chr2	8305972	8306064	chr2-8305972-8306064

chr2	8781299	8781391	chr2-8781299-8781391
chr2	9149930	9150022	chr2-9149930-9150022
chr2	9409641	9409733	chr2-9409641-9409733
chr2	9635261	9635353	chr2-9635261-9635353
chr2	9671610	9671702	chr2-9671610-9671702
chr2	9818401	9818493	chr2-9818401-9818493
chr2	12053905	12053997	chr2-12053905-12053997
chr2	12699476	12699568	chr2-12699476-12699568
chr2	13294566	13294658	chr2-13294566-13294658
chr2	13372300	13372392	chr2-13372300-13372392
chr2	14073443	14073535	chr2-14073443-14073535
chr2	16683048	16683140	chr2-16683048-16683140
chr2	18264345	18264437	chr2-18264345-18264437
chr2	18943522	18943614	chr2-18943522-18943614
chr2	18992665	18992757	chr2-18992665-18992757
chr2	19349096	19349188	chr2-19349096-19349188
chr2	19442353	19442445	chr2-19442353-19442445
chr2	19814628	19814720	chr2-19814628-19814720
chr2	20446290	20446382	chr2-20446290-20446382
chr2	20529616	20529708	chr2-20529616-20529708
chr2	21043409	21043501	chr2-21043409-21043501
chr2	21203937	21204029	chr2-21203937-21204029
chr2	21259235	21259327	chr2-21259235-21259327
chr2	22353006	22353098	chr2-22353006-22353098
chr2	22744287	22744379	chr2-22744287-22744379
chr2	22745034	22745126	chr2-22745034-22745126
chr2	24439299	24439391	chr2-24439299-24439391
chr2	24774478	24774570	chr2-24774478-24774570
chr2	24923826	24923918	chr2-24923826-24923918
chr2	25111280	25111372	chr2-25111280-25111372
chr2	25236788	25236880	chr2-25236788-25236880
chr2	25296853	25296945	chr2-25296853-25296945
chr2	25332676	25332768	chr2-25332676-25332768
chr2	25456312	25456404	chr2-25456312-25456404
chr2	25552023	25552115	chr2-25552023-25552115
chr2	25599002	25599094	chr2-25599002-25599094
chr2	25655220	25655312	chr2-25655220-25655312
chr2	26206585	26206677	chr2-26206585-26206677
chr2	26409215	26409307	chr2-26409215-26409307
chr2	26619227	26619319	chr2-26619227-26619319
chr2	27327281	27327373	chr2-27327281-27327373
chr2	27388829	27388921	chr2-27388829-27388921

chr2	27699650	27699742	chr2-27699650-27699742
chr2	27778416	27778508	chr2-27778416-27778508
chr2	28365052	28365144	chr2-28365052-28365144
chr2	29253102	29253194	chr2-29253102-29253194
chr2	29417982	29418074	chr2-29417982-29418074
chr2	29619960	29620052	chr2-29619960-29620052
chr2	29729107	29729199	chr2-29729107-29729199
chr2	30439376	30439468	chr2-30439376-30439468
chr2	30587829	30587921	chr2-30587829-30587921
chr2	30754660	30754752	chr2-30754660-30754752
chr2	30758422	30758514	chr2-30758422-30758514
chr2	30827487	30827579	chr2-30827487-30827579
chr2	30918104	30918196	chr2-30918104-30918196
chr2	30920356	30920448	chr2-30920356-30920448
chr2	31061737	31061829	chr2-31061737-31061829
chr2	31455473	31455565	chr2-31455473-31455565
chr2	32587082	32587174	chr2-32587082-32587174
chr2	32694227	32694319	chr2-32694227-32694319
chr2	32870471	32870563	chr2-32870471-32870563
chr2	33506168	33506260	chr2-33506168-33506260
chr2	33632984	33633076	chr2-33632984-33633076
chr2	33684253	33684345	chr2-33684253-33684345
chr2	34081073	34081165	chr2-34081073-34081165
chr2	34610482	34610574	chr2-34610482-34610574
chr2	34708838	34708930	chr2-34708838-34708930
chr2	35003472	35003564	chr2-35003472-35003564
chr2	35018658	35018750	chr2-35018658-35018750
chr2	35509392	35509484	chr2-35509392-35509484
chr2	35691696	35691788	chr2-35691696-35691788
chr2	35720578	35720670	chr2-35720578-35720670
chr2	36278886	36278978	chr2-36278886-36278978
chr2	36473812	36473904	chr2-36473812-36473904
chr2	36814669	36814761	chr2-36814669-36814761
chr2	38412610	38412702	chr2-38412610-38412702
chr2	38533374	38533466	chr2-38533374-38533466
chr2	38904450	38904542	chr2-38904450-38904542
chr2	38950579	38950671	chr2-38950579-38950671
chr2	39931774	39931866	chr2-39931774-39931866
chr2	43075160	43075252	chr2-43075160-43075252
chr2	43228693	43228785	chr2-43228693-43228785
chr2	43540151	43540243	chr2-43540151-43540243
chr2	43654068	43654160	chr2-43654068-43654160

chr2	44920466	44920558	chr2-44920466-44920558
chr2	45095536	45095628	chr2-45095536-45095628
chr2	47435565	47435657	chr2-47435565-47435657
chr2	48624576	48624668	chr2-48624576-48624668
chr2	48814746	48814838	chr2-48814746-48814838
chr2	49742551	49742643	chr2-49742551-49742643
chr2	50687409	50687501	chr2-50687409-50687501
chr2	51736226	51736318	chr2-51736226-51736318
chr2	52336038	52336130	chr2-52336038-52336130
chr2	52406094	52406186	chr2-52406094-52406186
chr2	52952377	52952469	chr2-52952377-52952469
chr2	58181847	58181939	chr2-58181847-58181939
chr2	59161800	59161892	chr2-59161800-59161892
chr2	59670486	59670578	chr2-59670486-59670578
chr2	59917764	59917856	chr2-59917764-59917856
chr2	60003285	60003377	chr2-60003285-60003377
chr2	60086407	60086499	chr2-60086407-60086499
chr2	60402749	60402841	chr2-60402749-60402841
chr2	60689103	60689195	chr2-60689103-60689195
chr2	60806527	60806619	chr2-60806527-60806619
chr2	60872353	60872445	chr2-60872353-60872445
chr2	62651985	62652077	chr2-62651985-62652077
chr2	63245054	63245146	chr2-63245054-63245146
chr2	65191539	65191631	chr2-65191539-65191631
chr2	66011998	66012090	chr2-66011998-66012090
chr2	66096221	66096313	chr2-66096221-66096313
chr2	66436759	66436851	chr2-66436759-66436851
chr2	67967616	67967708	chr2-67967616-67967708
chr2	68471282	68471374	chr2-68471282-68471374
chr2	69440442	69440534	chr2-69440442-69440534
chr2	70291211	70291303	chr2-70291211-70291303
chr2	70661342	70661434	chr2-70661342-70661434
chr2	71542868	71542960	chr2-71542868-71542960
chr2	72037878	72037970	chr2-72037878-72037970
chr2	72838180	72838272	chr2-72838180-72838272
chr2	72979302	72979394	chr2-72979302-72979394
chr2	72997148	72997240	chr2-72997148-72997240
chr2	73012815	73012907	chr2-73012815-73012907
chr2	73867929	73868021	chr2-73867929-73868021
chr2	73949045	73949137	chr2-73949045-73949137
chr2	74704869	74704961	chr2-74704869-74704961
chr2	74836001	74836093	chr2-74836001-74836093

chr2	75611129	75611221	chr2-75611129-75611221
chr2	75642442	75642534	chr2-75642442-75642534
chr2	75771920	75772012	chr2-75771920-75772012
chr2	75777397	75777489	chr2-75777397-75777489
chr2	77777345	77777437	chr2-77777345-77777437
chr2	78059950	78060042	chr2-78059950-78060042
chr2	78234709	78234801	chr2-78234709-78234801
chr2	78580254	78580346	chr2-78580254-78580346
chr2	78788354	78788446	chr2-78788354-78788446
chr2	79908463	79908555	chr2-79908463-79908555
chr2	80638808	80638900	chr2-80638808-80638900
chr2	81892661	81892753	chr2-81892661-81892753
chr2	83056362	83056454	chr2-83056362-83056454
chr2	84476121	84476213	chr2-84476121-84476213
chr2	85248767	85248859	chr2-85248767-85248859
chr2	85561367	85561459	chr2-85561367-85561459
chr2	85654705	85654797	chr2-85654705-85654797
chr2	90560173	90560265	chr2-90560173-90560265
chr2	91584652	91584744	chr2-91584652-91584744
chr2	92027963	92028055	chr2-92027963-92028055
chr2	92678387	92678479	chr2-92678387-92678479
chr2	92921893	92921985	chr2-92921893-92921985
chr2	93195124	93195216	chr2-93195124-93195216
chr2	93212019	93212111	chr2-93212019-93212111
chr2	94010660	94010752	chr2-94010660-94010752
chr2	97527853	97527945	chr2-97527853-97527945
chr2	101665065	101665157	chr2-101665065-101665157
chr2	101722517	101722609	chr2-101722517-101722609
chr2	103073689	103073781	chr2-103073689-103073781
chr2	103325501	103325593	chr2-103325501-103325593
chr2	103656212	103656304	chr2-103656212-103656304
chr2	104525684	104525776	chr2-104525684-104525776
chr2	104705703	104705795	chr2-104705703-104705795
chr2	104717991	104718083	chr2-104717991-104718083
chr2	104746295	104746387	chr2-104746295-104746387
chr2	104864603	104864695	chr2-104864603-104864695
chr2	108622821	108622913	chr2-108622821-108622913
chr2	110186552	110186644	chr2-110186552-110186644
chr2	110994247	110994339	chr2-110994247-110994339
chr2	111098298	111098390	chr2-111098298-111098390
chr2	111497548	111497640	chr2-111497548-111497640
chr2	111961904	111961996	chr2-111961904-111961996

chr2	112245477	112245569	chr2-112245477-112245569
chr2	112451383	112451475	chr2-112451383-112451475
chr2	112489312	112489404	chr2-112489312-112489404
chr2	113217238	113217330	chr2-113217238-113217330
chr2	113736788	113736880	chr2-113736788-113736880
chr2	115474273	115474365	chr2-115474273-115474365
chr2	115586684	115586776	chr2-115586684-115586776
chr2	115881828	115881920	chr2-115881828-115881920
chr2	116065800	116065892	chr2-116065800-116065892
chr2	116628226	116628318	chr2-116628226-116628318
chr2	117139472	117139564	chr2-117139472-117139564
chr2	117637804	117637896	chr2-117637804-117637896
chr2	117745887	117745979	chr2-117745887-117745979
chr2	117999834	117999926	chr2-117999834-117999926
chr2	118110562	118110654	chr2-118110562-118110654
chr2	118179623	118179715	chr2-118179623-118179715
chr2	118426835	118426927	chr2-118426835-118426927
chr2	118677096	118677188	chr2-118677096-118677188
chr2	118870702	118870794	chr2-118870702-118870794
chr2	119251006	119251098	chr2-119251006-119251098
chr2	119263996	119264088	chr2-119263996-119264088
chr2	119288733	119288825	chr2-119288733-119288825
chr2	119350952	119351044	chr2-119350952-119351044
chr2	119477805	119477897	chr2-119477805-119477897
chr2	119809625	119809717	chr2-119809625-119809717
chr2	120094180	120094272	chr2-120094180-120094272
chr2	120134395	120134487	chr2-120134395-120134487
chr2	120546558	120546650	chr2-120546558-120546650
chr2	121448924	121449016	chr2-121448924-121449016
chr2	121457008	121457100	chr2-121457008-121457100
chr2	121628399	121628491	chr2-121628399-121628491
chr2	121629294	121629386	chr2-121629294-121629386
chr2	122682191	122682283	chr2-122682191-122682283
chr2	123187786	123187878	chr2-123187786-123187878
chr2	123327750	123327842	chr2-123327750-123327842
chr2	125788832	125788924	chr2-125788832-125788924
chr2	125859050	125859142	chr2-125859050-125859142
chr2	125941125	125941217	chr2-125941125-125941217
chr2	126147836	126147928	chr2-126147836-126147928
chr2	126696639	126696731	chr2-126696639-126696731
chr2	126759792	126759884	chr2-126759792-126759884
chr2	127008619	127008711	chr2-127008619-127008711

chr2	127644463	127644555	chr2-127644463-127644555
chr2	127652899	127652991	chr2-127652899-127652991
chr2	127714493	127714585	chr2-127714493-127714585
chr2	127917247	127917339	chr2-127917247-127917339
chr2	128239304	128239396	chr2-128239304-128239396
chr2	128379712	128379804	chr2-128379712-128379804
chr2	128436209	128436301	chr2-128436209-128436301
chr2	128458006	128458098	chr2-128458006-128458098
chr2	128544942	128545034	chr2-128544942-128545034
chr2	128567886	128567978	chr2-128567886-128567978
chr2	128967225	128967317	chr2-128967225-128967317
chr2	128993013	128993105	chr2-128993013-128993105
chr2	129408582	129408674	chr2-129408582-129408674
chr2	129834332	129834424	chr2-129834332-129834424
chr2	130038401	130038493	chr2-130038401-130038493
chr2	130526974	130527066	chr2-130526974-130527066
chr2	130702176	130702268	chr2-130702176-130702268
chr2	131037705	131037797	chr2-131037705-131037797
chr2	131210312	131210404	chr2-131210312-131210404
chr2	131404075	131404167	chr2-131404075-131404167
chr2	131491719	131491811	chr2-131491719-131491811
chr2	131562439	131562531	chr2-131562439-131562531
chr2	131691763	131691855	chr2-131691763-131691855
chr2	131909950	131910042	chr2-131909950-131910042
chr2	132340569	132340661	chr2-132340569-132340661
chr2	132923969	132924061	chr2-132923969-132924061
chr2	134644107	134644199	chr2-134644107-134644199
chr2	136163048	136163140	chr2-136163048-136163140
chr2	137160359	137160451	chr2-137160359-137160451
chr2	137566882	137566974	chr2-137566882-137566974
chr2	137601821	137601913	chr2-137601821-137601913
chr2	139817946	139818038	chr2-139817946-139818038
chr2	140104283	140104375	chr2-140104283-140104375
chr2	140249775	140249867	chr2-140249775-140249867
chr2	140759850	140759942	chr2-140759850-140759942
chr2	143197540	143197632	chr2-143197540-143197632
chr2	143768370	143768462	chr2-143768370-143768462
chr2	144300419	144300511	chr2-144300419-144300511
chr2	145396135	145396227	chr2-145396135-145396227
chr2	145675618	145675710	chr2-145675618-145675710
chr2	145866387	145866479	chr2-145866387-145866479
chr2	146022948	146023040	chr2-146022948-146023040

chr2	146533283	146533375	chr2-146533283-146533375
chr2	147379099	147379191	chr2-147379099-147379191
chr2	147794713	147794805	chr2-147794713-147794805
chr2	148041043	148041135	chr2-148041043-148041135
chr2	148185055	148185147	chr2-148185055-148185147
chr2	148504228	148504320	chr2-148504228-148504320
chr2	148505816	148505908	chr2-148505816-148505908
chr2	150913819	150913911	chr2-150913819-150913911
chr2	151039460	151039552	chr2-151039460-151039552
chr2	151530425	151530517	chr2-151530425-151530517
chr2	151530940	151531032	chr2-151530940-151531032
chr2	151619095	151619187	chr2-151619095-151619187
chr2	151976607	151976699	chr2-151976607-151976699
chr2	152741681	152741773	chr2-152741681-152741773
chr2	152789219	152789311	chr2-152789219-152789311
chr2	152792639	152792731	chr2-152792639-152792731
chr2	152817900	152817992	chr2-152817900-152817992
chr2	153291303	153291395	chr2-153291303-153291395
chr2	153450670	153450762	chr2-153450670-153450762
chr2	153597912	153598004	chr2-153597912-153598004
chr2	154679169	154679261	chr2-154679169-154679261
chr2	155591045	155591137	chr2-155591045-155591137
chr2	155749894	155749986	chr2-155749894-155749986
chr2	155809811	155809903	chr2-155809811-155809903
chr2	155864656	155864748	chr2-155864656-155864748
chr2	155923829	155923921	chr2-155923829-155923921
chr2	156474507	156474599	chr2-156474507-156474599
chr2	156474969	156475061	chr2-156474969-156475061
chr2	157078734	157078826	chr2-157078734-157078826
chr2	157377898	157377990	chr2-157377898-157377990
chr2	157913505	157913597	chr2-157913505-157913597
chr2	157935971	157936063	chr2-157935971-157936063
chr2	157952204	157952296	chr2-157952204-157952296
chr2	158258112	158258204	chr2-158258112-158258204
chr2	158332367	158332459	chr2-158332367-158332459
chr2	158409321	158409413	chr2-158409321-158409413
chr2	158839252	158839344	chr2-158839252-158839344
chr2	159044563	159044655	chr2-159044563-159044655
chr2	160114276	160114368	chr2-160114276-160114368
chr2	160454891	160454983	chr2-160454891-160454983
chr2	160630980	160631072	chr2-160630980-160631072
chr2	160928204	160928296	chr2-160928204-160928296

chr2	161594019	161594111	chr2-161594019-161594111
chr2	162287369	162287461	chr2-162287369-162287461
chr2	162660687	162660779	chr2-162660687-162660779
chr2	162821429	162821521	chr2-162821429-162821521
chr2	163010730	163010822	chr2-163010730-163010822
chr2	164403103	164403195	chr2-164403103-164403195
chr2	164433543	164433635	chr2-164433543-164433635
chr2	164519100	164519192	chr2-164519100-164519192
chr2	164598784	164598876	chr2-164598784-164598876
chr2	164884917	164885009	chr2-164884917-164885009
chr2	164971079	164971171	chr2-164971079-164971171
chr2	165234321	165234413	chr2-165234321-165234413
chr2	165464468	165464560	chr2-165464468-165464560
chr2	165547352	165547444	chr2-165547352-165547444
chr2	165701747	165701839	chr2-165701747-165701839
chr2	166128312	166128404	chr2-166128312-166128404
chr2	166793708	166793800	chr2-166793708-166793800
chr2	166845760	166845852	chr2-166845760-166845852
chr2	166894840	166894932	chr2-166894840-166894932
chr2	166908648	166908740	chr2-166908648-166908740
chr2	167089425	167089517	chr2-167089425-167089517
chr2	167263488	167263580	chr2-167263488-167263580
chr2	167383803	167383895	chr2-167383803-167383895
chr2	167828461	167828553	chr2-167828461-167828553
chr2	167866957	167867049	chr2-167866957-167867049
chr2	167887987	167888079	chr2-167887987-167888079
chr2	168011056	168011148	chr2-168011056-168011148
chr2	168073304	168073396	chr2-168073304-168073396
chr2	168078671	168078763	chr2-168078671-168078763
chr2	168080461	168080553	chr2-168080461-168080553
chr2	168085674	168085766	chr2-168085674-168085766
chr2	168131229	168131321	chr2-168131229-168131321
chr2	168200462	168200554	chr2-168200462-168200554
chr2	168458840	168458932	chr2-168458840-168458932
chr2	169526656	169526748	chr2-169526656-169526748
chr2	169586818	169586910	chr2-169586818-169586910
chr2	170287712	170287804	chr2-170287712-170287804
chr2	170373844	170373936	chr2-170373844-170373936
chr2	170385042	170385134	chr2-170385042-170385134
chr2	171299510	171299602	chr2-171299510-171299602
chr2	171395724	171395816	chr2-171395724-171395816
chr2	172627755	172627847	chr2-172627755-172627847

chr2	172940270	172940362	chr2-172940270-172940362
chr2	173266459	173266551	chr2-173266459-173266551
chr2	173586753	173586845	chr2-173586753-173586845
chr2	173673618	173673710	chr2-173673618-173673710
chr2	174076200	174076292	chr2-174076200-174076292
chr2	174747348	174747440	chr2-174747348-174747440
chr2	178434067	178434159	chr2-178434067-178434159
chr2	178883545	178883637	chr2-178883545-178883637
chr2	179783317	179783409	chr2-179783317-179783409
chr2	180162532	180162624	chr2-180162532-180162624
chr2	180233129	180233221	chr2-180233129-180233221
chr2	180277126	180277218	chr2-180277126-180277218
chr2	180842699	180842791	chr2-180842699-180842791
chr2	180963114	180963206	chr2-180963114-180963206
chr2	181520902	181520994	chr2-181520902-181520994
chr3	3001353	3001445	chr3-3001353-3001445
chr3	3001790	3001882	chr3-3001790-3001882
chr3	3003288	3003380	chr3-3003288-3003380
chr3	3011124	3011216	chr3-3011124-3011216
chr3	3013305	3013397	chr3-3013305-3013397
chr3	3015132	3015224	chr3-3015132-3015224
chr3	3015947	3016039	chr3-3015947-3016039
chr3	3024770	3024862	chr3-3024770-3024862
chr3	3124093	3124185	chr3-3124093-3124185
chr3	3147364	3147456	chr3-3147364-3147456
chr3	3170790	3170882	chr3-3170790-3170882
chr3	4689645	4689737	chr3-4689645-4689737
chr3	8857241	8857333	chr3-8857241-8857333
chr3	9251045	9251137	chr3-9251045-9251137
chr3	9634886	9634978	chr3-9634886-9634978
chr3	10081818	10081910	chr3-10081818-10081910
chr3	10741369	10741461	chr3-10741369-10741461
chr3	12217980	12218072	chr3-12217980-12218072
chr3	12802811	12802903	chr3-12802811-12802903
chr3	13703158	13703250	chr3-13703158-13703250
chr3	14284459	14284551	chr3-14284459-14284551
chr3	15160105	15160197	chr3-15160105-15160197
chr3	21268501	21268593	chr3-21268501-21268593
chr3	21962486	21962578	chr3-21962486-21962578
chr3	24786997	24787089	chr3-24786997-24787089
chr3	26600234	26600326	chr3-26600234-26600326
chr3	27607327	27607419	chr3-27607327-27607419

chr3	27616686	27616778	chr3-27616686-27616778
chr3	27718953	27719045	chr3-27718953-27719045
chr3	27995153	27995245	chr3-27995153-27995245
chr3	28374730	28374822	chr3-28374730-28374822
chr3	30222012	30222104	chr3-30222012-30222104
chr3	30509636	30509728	chr3-30509636-30509728
chr3	31070639	31070731	chr3-31070639-31070731
chr3	31134424	31134516	chr3-31134424-31134516
chr3	32909787	32909879	chr3-32909787-32909879
chr3	33401742	33401834	chr3-33401742-33401834
chr3	35249211	35249303	chr3-35249211-35249303
chr3	35409038	35409130	chr3-35409038-35409130
chr3	36391279	36391371	chr3-36391279-36391371
chr3	39930373	39930465	chr3-39930373-39930465
chr3	40002941	40003033	chr3-40002941-40003033
chr3	41375587	41375679	chr3-41375587-41375679
chr3	41377002	41377094	chr3-41377002-41377094
chr3	41522847	41522939	chr3-41522847-41522939
chr3	41527583	41527675	chr3-41527583-41527675
chr3	46180734	46180826	chr3-46180734-46180826
chr3	46253380	46253472	chr3-46253380-46253472
chr3	48997247	48997339	chr3-48997247-48997339
chr3	49067016	49067108	chr3-49067016-49067108
chr3	49915179	49915271	chr3-49915179-49915271
chr3	51223182	51223274	chr3-51223182-51223274
chr3	51761983	51762075	chr3-51761983-51762075
chr3	51766268	51766360	chr3-51766268-51766360
chr3	51766679	51766771	chr3-51766679-51766771
chr3	51908581	51908673	chr3-51908581-51908673
chr3	52102668	52102760	chr3-52102668-52102760
chr3	52103758	52103850	chr3-52103758-52103850
chr3	52124742	52124834	chr3-52124742-52124834
chr3	52653141	52653233	chr3-52653141-52653233
chr3	52944587	52944679	chr3-52944587-52944679
chr3	53304244	53304336	chr3-53304244-53304336
chr3	53375887	53375979	chr3-53375887-53375979
chr3	54322662	54322754	chr3-54322662-54322754
chr3	54585856	54585948	chr3-54585856-54585948
chr3	54633197	54633289	chr3-54633197-54633289
chr3	54895145	54895237	chr3-54895145-54895237
chr3	55834371	55834463	chr3-55834371-55834463
chr3	56186619	56186711	chr3-56186619-56186711

chr3	56187565	56187657	chr3-56187565-56187657
chr3	56216127	56216219	chr3-56216127-56216219
chr3	56369129	56369221	chr3-56369129-56369221
chr3	57019705	57019797	chr3-57019705-57019797
chr3	57344398	57344490	chr3-57344398-57344490
chr3	57554759	57554851	chr3-57554759-57554851
chr3	58172240	58172332	chr3-58172240-58172332
chr3	59050648	59050740	chr3-59050648-59050740
chr3	60501196	60501288	chr3-60501196-60501288
chr3	60601784	60601876	chr3-60601784-60601876
chr3	60750556	60750648	chr3-60750556-60750648
chr3	61050049	61050141	chr3-61050049-61050141
chr3	62159073	62159165	chr3-62159073-62159165
chr3	62159352	62159444	chr3-62159352-62159444
chr3	62339006	62339098	chr3-62339006-62339098
chr3	63976014	63976106	chr3-63976014-63976106
chr3	65111353	65111445	chr3-65111353-65111445
chr3	65487378	65487470	chr3-65487378-65487470
chr3	65633728	65633820	chr3-65633728-65633820
chr3	65691307	65691399	chr3-65691307-65691399
chr3	65904849	65904941	chr3-65904849-65904941
chr3	68505037	68505129	chr3-68505037-68505129
chr3	68566330	68566422	chr3-68566330-68566422
chr3	68668992	68669084	chr3-68668992-68669084
chr3	69046571	69046663	chr3-69046571-69046663
chr3	69324127	69324219	chr3-69324127-69324219
chr3	69759643	69759735	chr3-69759643-69759735
chr3	71355145	71355237	chr3-71355145-71355237
chr3	71801296	71801388	chr3-71801296-71801388
chr3	74664692	74664784	chr3-74664692-74664784
chr3	74666627	74666719	chr3-74666627-74666719
chr3	76624402	76624494	chr3-76624402-76624494
chr3	77752490	77752582	chr3-77752490-77752582
chr3	79851967	79852059	chr3-79851967-79852059
chr3	80318726	80318818	chr3-80318726-80318818
chr3	80422189	80422281	chr3-80422189-80422281
chr3	80883064	80883156	chr3-80883064-80883156
chr3	81200794	81200886	chr3-81200794-81200886
chr3	81482600	81482692	chr3-81482600-81482692
chr3	82057341	82057433	chr3-82057341-82057433
chr3	82474889	82474981	chr3-82474889-82474981
chr3	83446287	83446379	chr3-83446287-83446379

chr3	83450819	83450911	chr3-83450819-83450911
chr3	84319572	84319664	chr3-84319572-84319664
chr3	84426597	84426689	chr3-84426597-84426689
chr3	84479446	84479538	chr3-84479446-84479538
chr3	85176953	85177045	chr3-85176953-85177045
chr3	85245069	85245161	chr3-85245069-85245161
chr3	85693736	85693828	chr3-85693736-85693828
chr3	85813254	85813346	chr3-85813254-85813346
chr3	85899777	85899869	chr3-85899777-85899869
chr3	85971691	85971783	chr3-85971691-85971783
chr3	86253246	86253338	chr3-86253246-86253338
chr3	86659148	86659240	chr3-86659148-86659240
chr3	88141974	88142066	chr3-88141974-88142066
chr3	88491334	88491426	chr3-88491334-88491426
chr3	88519445	88519537	chr3-88519445-88519537
chr3	88591220	88591312	chr3-88591220-88591312
chr3	89233761	89233853	chr3-89233761-89233853
chr3	89459700	89459792	chr3-89459700-89459792
chr3	89526884	89526976	chr3-89526884-89526976
chr3	89565175	89565267	chr3-89565175-89565267
chr3	89683165	89683257	chr3-89683165-89683257
chr3	89767228	89767320	chr3-89767228-89767320
chr3	89901852	89901944	chr3-89901852-89901944
chr3	90004194	90004286	chr3-90004194-90004286
chr3	92034012	92034104	chr3-92034012-92034104
chr3	92281316	92281408	chr3-92281316-92281408
chr3	92609943	92610035	chr3-92609943-92610035
chr3	93011795	93011887	chr3-93011795-93011887
chr3	93537217	93537309	chr3-93537217-93537309
chr3	94649453	94649545	chr3-94649453-94649545
chr3	94710728	94710820	chr3-94710728-94710820
chr3	94718402	94718494	chr3-94718402-94718494
chr3	94868085	94868177	chr3-94868085-94868177
chr3	94957319	94957411	chr3-94957319-94957411
chr3	94987710	94987802	chr3-94987710-94987802
chr3	94990949	94991041	chr3-94990949-94991041
chr3	95028738	95028830	chr3-95028738-95028830
chr3	95312791	95312883	chr3-95312791-95312883
chr3	95666234	95666326	chr3-95666234-95666326
chr3	95696407	95696499	chr3-95696407-95696499
chr3	95710417	95710509	chr3-95710417-95710509
chr3	95930018	95930110	chr3-95930018-95930110

chr3	96580241	96580333	chr3-96580241-96580333
chr3	97658285	97658377	chr3-97658285-97658377
chr3	97659858	97659950	chr3-97659858-97659950
chr3	98027669	98027761	chr3-98027669-98027761
chr3	98112770	98112862	chr3-98112770-98112862
chr3	98772613	98772705	chr3-98772613-98772705
chr3	98908010	98908102	chr3-98908010-98908102
chr3	99188325	99188417	chr3-99188325-99188417
chr3	99278150	99278242	chr3-99278150-99278242
chr3	100027278	100027370	chr3-100027278-100027370
chr3	100166180	100166272	chr3-100166180-100166272
chr3	101188556	101188648	chr3-101188556-101188648
chr3	101346153	101346245	chr3-101346153-101346245
chr3	102372358	102372450	chr3-102372358-102372450
chr3	102659461	102659553	chr3-102659461-102659553
chr3	102756070	102756162	chr3-102756070-102756162
chr3	103256261	103256353	chr3-103256261-103256353
chr3	103593701	103593793	chr3-103593701-103593793
chr3	103949989	103950081	chr3-103949989-103950081
chr3	104577161	104577253	chr3-104577161-104577253
chr3	104638395	104638487	chr3-104638395-104638487
chr3	104706629	104706721	chr3-104706629-104706721
chr3	104796678	104796770	chr3-104796678-104796770
chr3	104857179	104857271	chr3-104857179-104857271
chr3	105215072	105215164	chr3-105215072-105215164
chr3	105687461	105687553	chr3-105687461-105687553
chr3	106788641	106788733	chr3-106788641-106788733
chr3	107336104	107336196	chr3-107336104-107336196
chr3	107412662	107412754	chr3-107412662-107412754
chr3	107733452	107733544	chr3-107733452-107733544
chr3	108807388	108807480	chr3-108807388-108807480
chr3	109011152	109011244	chr3-109011152-109011244
chr3	109011396	109011488	chr3-109011396-109011488
chr3	109117284	109117376	chr3-109117284-109117376
chr3	109168686	109168778	chr3-109168686-109168778
chr3	109286729	109286821	chr3-109286729-109286821
chr3	109363701	109363793	chr3-109363701-109363793
chr3	110002666	110002758	chr3-110002666-110002758
chr3	110076886	110076978	chr3-110076886-110076978
chr3	113629812	113629904	chr3-113629812-113629904
chr3	115641589	115641681	chr3-115641589-115641681
chr3	115932614	115932706	chr3-115932614-115932706

chr3	116251855	116251947	chr3-116251855-116251947
chr3	116508029	116508121	chr3-116508029-116508121
chr3	116950163	116950255	chr3-116950163-116950255
chr3	117070611	117070703	chr3-117070611-117070703
chr3	117169920	117170012	chr3-117169920-117170012
chr3	118111415	118111507	chr3-118111415-118111507
chr3	120367330	120367422	chr3-120367330-120367422
chr3	121713490	121713582	chr3-121713490-121713582
chr3	121736292	121736384	chr3-121736292-121736384
chr3	122391824	122391916	chr3-122391824-122391916
chr3	122463293	122463385	chr3-122463293-122463385
chr3	122471061	122471153	chr3-122471061-122471153
chr3	122620305	122620397	chr3-122620305-122620397
chr3	122620750	122620842	chr3-122620750-122620842
chr3	123470588	123470680	chr3-123470588-123470680
chr3	124462581	124462673	chr3-124462581-124462673
chr3	125228360	125228452	chr3-125228360-125228452
chr3	126540968	126541060	chr3-126540968-126541060
chr3	126611899	126611991	chr3-126611899-126611991
chr3	130106534	130106626	chr3-130106534-130106626
chr3	130123253	130123345	chr3-130123253-130123345
chr3	130172247	130172339	chr3-130172247-130172339
chr3	130709634	130709726	chr3-130709634-130709726
chr3	131803044	131803136	chr3-131803044-131803136
chr3	132791442	132791534	chr3-132791442-132791534
chr3	132964908	132965000	chr3-132964908-132965000
chr3	135498992	135499084	chr3-135498992-135499084
chr3	135522260	135522352	chr3-135522260-135522352
chr3	135767333	135767425	chr3-135767333-135767425
chr3	135825584	135825676	chr3-135825584-135825676
chr3	136626743	136626835	chr3-136626743-136626835
chr3	136830633	136830725	chr3-136830633-136830725
chr3	136903769	136903861	chr3-136903769-136903861
chr3	137150524	137150616	chr3-137150524-137150616
chr3	137838298	137838390	chr3-137838298-137838390
chr3	138452380	138452472	chr3-138452380-138452472
chr3	138745616	138745708	chr3-138745616-138745708
chr3	139030955	139031047	chr3-139030955-139031047
chr3	139276482	139276574	chr3-139276482-139276574
chr3	140602298	140602390	chr3-140602298-140602390
chr3	142191774	142191866	chr3-142191774-142191866
chr3	143157338	143157430	chr3-143157338-143157430

chr3	143690395	143690487	chr3-143690395-143690487
chr3	143964214	143964306	chr3-143964214-143964306
chr3	144058245	144058337	chr3-144058245-144058337
chr3	144091422	144091514	chr3-144091422-144091514
chr3	144099589	144099681	chr3-144099589-144099681
chr3	144272227	144272319	chr3-144272227-144272319
chr3	144310486	144310578	chr3-144310486-144310578
chr3	144411037	144411129	chr3-144411037-144411129
chr3	144416025	144416117	chr3-144416025-144416117
chr3	144663445	144663537	chr3-144663445-144663537
chr3	144669517	144669609	chr3-144669517-144669609
chr3	144887107	144887199	chr3-144887107-144887199
chr3	144971705	144971797	chr3-144971705-144971797
chr3	145195649	145195741	chr3-145195649-145195741
chr3	145684947	145685039	chr3-145684947-145685039
chr3	145685233	145685325	chr3-145685233-145685325
chr3	145690989	145691081	chr3-145690989-145691081
chr3	145876277	145876369	chr3-145876277-145876369
chr3	145964539	145964631	chr3-145964539-145964631
chr3	146268429	146268521	chr3-146268429-146268521
chr3	146396664	146396756	chr3-146396664-146396756
chr3	147079947	147080039	chr3-147079947-147080039
chr3	147863460	147863552	chr3-147863460-147863552
chr3	148483528	148483620	chr3-148483528-148483620
chr3	149112189	149112281	chr3-149112189-149112281
chr3	149116839	149116931	chr3-149116839-149116931
chr3	149317162	149317254	chr3-149317162-149317254
chr3	149487950	149488042	chr3-149487950-149488042
chr3	149696831	149696923	chr3-149696831-149696923
chr3	149768033	149768125	chr3-149768033-149768125
chr3	150807963	150808055	chr3-150807963-150808055
chr3	152171377	152171469	chr3-152171377-152171469
chr3	152308544	152308636	chr3-152308544-152308636
chr3	153123726	153123818	chr3-153123726-153123818
chr3	153835821	153835913	chr3-153835821-153835913
chr3	156584510	156584602	chr3-156584510-156584602
chr3	157053506	157053598	chr3-157053506-157053598
chr3	157799388	157799480	chr3-157799388-157799480
chr3	159080266	159080358	chr3-159080266-159080358
chr3	159883534	159883626	chr3-159883534-159883626
chr3	159885270	159885362	chr3-159885270-159885362
chr4	3050125	3050217	chr4-3050125-3050217

chr4	3050362	3050454	chr4-3050362-3050454
chr4	3052978	3053070	chr4-3052978-3053070
chr4	3062160	3062252	chr4-3062160-3062252
chr4	3064397	3064489	chr4-3064397-3064489
chr4	3067969	3068061	chr4-3067969-3068061
chr4	3164542	3164634	chr4-3164542-3164634
chr4	3165206	3165298	chr4-3165206-3165298
chr4	3176876	3176968	chr4-3176876-3176968
chr4	4493743	4493835	chr4-4493743-4493835
chr4	4614684	4614776	chr4-4614684-4614776
chr4	5619672	5619764	chr4-5619672-5619764
chr4	5725443	5725535	chr4-5725443-5725535
chr4	6452710	6452802	chr4-6452710-6452802
chr4	7024264	7024356	chr4-7024264-7024356
chr4	7199287	7199379	chr4-7199287-7199379
chr4	7391735	7391827	chr4-7391735-7391827
chr4	8519226	8519318	chr4-8519226-8519318
chr4	8761804	8761896	chr4-8761804-8761896
chr4	8982894	8982986	chr4-8982894-8982986
chr4	10137179	10137271	chr4-10137179-10137271
chr4	10340556	10340648	chr4-10340556-10340648
chr4	11147735	11147827	chr4-11147735-11147827
chr4	11161112	11161204	chr4-11161112-11161204
chr4	13635731	13635823	chr4-13635731-13635823
chr4	14777530	14777622	chr4-14777530-14777622
chr4	15758797	15758889	chr4-15758797-15758889
chr4	15958161	15958253	chr4-15958161-15958253
chr4	16739983	16740075	chr4-16739983-16740075
chr4	20727831	20727923	chr4-20727831-20727923
chr4	21949966	21950058	chr4-21949966-21950058
chr4	22491458	22491550	chr4-22491458-22491550
chr4	23085803	23085895	chr4-23085803-23085895
chr4	23587501	23587593	chr4-23587501-23587593
chr4	23954358	23954450	chr4-23954358-23954450
chr4	24723848	24723940	chr4-24723848-24723940
chr4	24842673	24842765	chr4-24842673-24842765
chr4	29310382	29310474	chr4-29310382-29310474
chr4	30829322	30829414	chr4-30829322-30829414
chr4	31088582	31088674	chr4-31088582-31088674
chr4	32288072	32288164	chr4-32288072-32288164
chr4	32337770	32337862	chr4-32337770-32337862
chr4	32388060	32388152	chr4-32388060-32388152

chr4	32441883	32441975	chr4-32441883-32441975
chr4	32482380	32482472	chr4-32482380-32482472
chr4	33551945	33552037	chr4-33551945-33552037
chr4	33994178	33994270	chr4-33994178-33994270
chr4	34854753	34854845	chr4-34854753-34854845
chr4	35756150	35756242	chr4-35756150-35756242
chr4	37084999	37085091	chr4-37084999-37085091
chr4	37261391	37261483	chr4-37261391-37261483
chr4	40850290	40850382	chr4-40850290-40850382
chr4	40908873	40908965	chr4-40908873-40908965
chr4	41026599	41026691	chr4-41026599-41026691
chr4	41108921	41109013	chr4-41108921-41109013
chr4	41569569	41569661	chr4-41569569-41569661
chr4	41619354	41619446	chr4-41619354-41619446
chr4	41628220	41628312	chr4-41628220-41628312
chr4	43398784	43398876	chr4-43398784-43398876
chr4	43477020	43477112	chr4-43477020-43477112
chr4	43558182	43558274	chr4-43558182-43558274
chr4	43605176	43605268	chr4-43605176-43605268
chr4	43728403	43728495	chr4-43728403-43728495
chr4	43765579	43765671	chr4-43765579-43765671
chr4	43971635	43971727	chr4-43971635-43971727
chr4	44098711	44098803	chr4-44098711-44098803
chr4	44205196	44205288	chr4-44205196-44205288
chr4	45342010	45342102	chr4-45342010-45342102
chr4	45475066	45475158	chr4-45475066-45475158
chr4	45492842	45492934	chr4-45492842-45492934
chr4	45586411	45586503	chr4-45586411-45586503
chr4	45812520	45812612	chr4-45812520-45812612
chr4	46033307	46033399	chr4-46033307-46033399
chr4	46498442	46498534	chr4-46498442-46498534
chr4	47788342	47788434	chr4-47788342-47788434
chr4	47932399	47932491	chr4-47932399-47932491
chr4	48043368	48043460	chr4-48043368-48043460
chr4	48592634	48592726	chr4-48592634-48592726
chr4	53165720	53165812	chr4-53165720-53165812
chr4	53453483	53453575	chr4-53453483-53453575
chr4	53517803	53517895	chr4-53517803-53517895
chr4	54356216	54356308	chr4-54356216-54356308
chr4	55141288	55141380	chr4-55141288-55141380
chr4	55172501	55172593	chr4-55172501-55172593
chr4	55174810	55174902	chr4-55174810-55174902

chr4	55399277	55399369	chr4-55399277-55399369
chr4	55579040	55579132	chr4-55579040-55579132
chr4	55927599	55927691	chr4-55927599-55927691
chr4	57154129	57154221	chr4-57154129-57154221
chr4	57312931	57313023	chr4-57312931-57313023
chr4	57606344	57606436	chr4-57606344-57606436
chr4	57843881	57843973	chr4-57843881-57843973
chr4	58479755	58479847	chr4-58479755-58479847
chr4	58555002	58555094	chr4-58555002-58555094
chr4	59315210	59315302	chr4-59315210-59315302
chr4	59438312	59438404	chr4-59438312-59438404
chr4	59717403	59717495	chr4-59717403-59717495
chr4	59814193	59814285	chr4-59814193-59814285
chr4	59919988	59920080	chr4-59919988-59920080
chr4	61242566	61242658	chr4-61242566-61242658
chr4	61447981	61448073	chr4-61447981-61448073
chr4	61855998	61856090	chr4-61855998-61856090
chr4	62200341	62200433	chr4-62200341-62200433
chr4	62220270	62220362	chr4-62220270-62220362
chr4	62560078	62560170	chr4-62560078-62560170
chr4	62695913	62696005	chr4-62695913-62696005
chr4	63068474	63068566	chr4-63068474-63068566
chr4	63594904	63594996	chr4-63594904-63594996
chr4	63667555	63667647	chr4-63667555-63667647
chr4	63892040	63892132	chr4-63892040-63892132
chr4	64097596	64097688	chr4-64097596-64097688
chr4	64438367	64438459	chr4-64438367-64438459
chr4	65242822	65242914	chr4-65242822-65242914
chr4	66881727	66881819	chr4-66881727-66881819
chr4	69613021	69613113	chr4-69613021-69613113
chr4	69980045	69980137	chr4-69980045-69980137
chr4	80832000	80832092	chr4-80832000-80832092
chr4	80917926	80918018	chr4-80917926-80918018
chr4	81830094	81830186	chr4-81830094-81830186
chr4	81926344	81926436	chr4-81926344-81926436
chr4	82436594	82436686	chr4-82436594-82436686
chr4	82447152	82447244	chr4-82447152-82447244
chr4	82447502	82447594	chr4-82447502-82447594
chr4	83362606	83362698	chr4-83362606-83362698
chr4	83567373	83567465	chr4-83567373-83567465
chr4	83647202	83647294	chr4-83647202-83647294
chr4	85560032	85560124	chr4-85560032-85560124

chr4	86575332	86575424	chr4-86575332-86575424
chr4	86686670	86686762	chr4-86686670-86686762
chr4	88192472	88192564	chr4-88192472-88192564
chr4	88641125	88641217	chr4-88641125-88641217
chr4	89041101	89041193	chr4-89041101-89041193
chr4	89044141	89044233	chr4-89044141-89044233
chr4	89124031	89124123	chr4-89124031-89124123
chr4	89149441	89149533	chr4-89149441-89149533
chr4	89446006	89446098	chr4-89446006-89446098
chr4	89486021	89486113	chr4-89486021-89486113
chr4	89523752	89523844	chr4-89523752-89523844
chr4	91095026	91095118	chr4-91095026-91095118
chr4	94094598	94094690	chr4-94094598-94094690
chr4	95052162	95052254	chr4-95052162-95052254
chr4	95052694	95052786	chr4-95052694-95052786
chr4	95143823	95143915	chr4-95143823-95143915
chr4	96491441	96491533	chr4-96491441-96491533
chr4	97499822	97499914	chr4-97499822-97499914
chr4	98474721	98474813	chr4-98474721-98474813
chr4	98825402	98825494	chr4-98825402-98825494
chr4	101894095	101894187	chr4-101894095-101894187
chr4	102087129	102087221	chr4-102087129-102087221
chr4	103099667	103099759	chr4-103099667-103099759
chr4	103249589	103249681	chr4-103249589-103249681
chr4	103927352	103927444	chr4-103927352-103927444
chr4	105257869	105257961	chr4-105257869-105257961
chr4	106464744	106464836	chr4-106464744-106464836
chr4	106529869	106529961	chr4-106529869-106529961
chr4	106550547	106550639	chr4-106550547-106550639
chr4	106859653	106859745	chr4-106859653-106859745
chr4	106875436	106875528	chr4-106875436-106875528
chr4	106959824	106959916	chr4-106959824-106959916
chr4	107374758	107374850	chr4-107374758-107374850
chr4	107756348	107756440	chr4-107756348-107756440
chr4	109090179	109090271	chr4-109090179-109090271
chr4	109127019	109127111	chr4-109127019-109127111
chr4	109136682	109136774	chr4-109136682-109136774
chr4	109280162	109280254	chr4-109280162-109280254
chr4	109406253	109406345	chr4-109406253-109406345
chr4	109445552	109445644	chr4-109445552-109445644
chr4	109673480	109673572	chr4-109673480-109673572
chr4	110460826	110460918	chr4-110460826-110460918

chr4	110465682	110465774	chr4-110465682-110465774
chr4	111425448	111425540	chr4-111425448-111425540
chr4	114908764	114908856	chr4-114908764-114908856
chr4	115056640	115056732	chr4-115056640-115056732
chr4	115331277	115331369	chr4-115331277-115331369
chr4	115545972	115546064	chr4-115545972-115546064
chr4	115969354	115969446	chr4-115969354-115969446
chr4	116097371	116097463	chr4-116097371-116097463
chr4	116167612	116167704	chr4-116167612-116167704
chr4	116243650	116243742	chr4-116243650-116243742
chr4	116484353	116484445	chr4-116484353-116484445
chr4	117501464	117501556	chr4-117501464-117501556
chr4	117823441	117823533	chr4-117823441-117823533
chr4	117887303	117887395	chr4-117887303-117887395
chr4	117997725	117997817	chr4-117997725-117997817
chr4	117998472	117998564	chr4-117998472-117998564
chr4	118085268	118085360	chr4-118085268-118085360
chr4	118260179	118260271	chr4-118260179-118260271
chr4	118272994	118273086	chr4-118272994-118273086
chr4	118687146	118687238	chr4-118687146-118687238
chr4	119082938	119083030	chr4-119082938-119083030
chr4	119338295	119338387	chr4-119338295-119338387
chr4	119637575	119637667	chr4-119637575-119637667
chr4	119785136	119785228	chr4-119785136-119785228
chr4	119813730	119813822	chr4-119813730-119813822
chr4	120204965	120205057	chr4-120204965-120205057
chr4	120225063	120225155	chr4-120225063-120225155
chr4	120516516	120516608	chr4-120516516-120516608
chr4	120652024	120652116	chr4-120652024-120652116
chr4	121024825	121024917	chr4-121024825-121024917
chr4	121649356	121649448	chr4-121649356-121649448
chr4	122023136	122023228	chr4-122023136-122023228
chr4	123093725	123093817	chr4-123093725-123093817
chr4	123422967	123423059	chr4-123422967-123423059
chr4	123455304	123455396	chr4-123455304-123455396
chr4	123572498	123572590	chr4-123572498-123572590
chr4	123664297	123664389	chr4-123664297-123664389
chr4	124108636	124108728	chr4-124108636-124108728
chr4	124584298	124584390	chr4-124584298-124584390
chr4	124646769	124646861	chr4-124646769-124646861
chr4	124705284	124705376	chr4-124705284-124705376
chr4	125106274	125106366	chr4-125106274-125106366

chr4	125266575	125266667	chr4-125266575-125266667
chr4	126007269	126007361	chr4-126007269-126007361
chr4	126103768	126103860	chr4-126103768-126103860
chr4	126117534	126117626	chr4-126117534-126117626
chr4	126163821	126163913	chr4-126163821-126163913
chr4	126256269	126256361	chr4-126256269-126256361
chr4	126494466	126494558	chr4-126494466-126494558
chr4	126677420	126677512	chr4-126677420-126677512
chr4	126788555	126788647	chr4-126788555-126788647
chr4	127896186	127896278	chr4-127896186-127896278
chr4	129038657	129038749	chr4-129038657-129038749
chr4	129234655	129234747	chr4-129234655-129234747
chr4	129263571	129263663	chr4-129263571-129263663
chr4	129416672	129416764	chr4-129416672-129416764
chr4	129794103	129794195	chr4-129794103-129794195
chr4	129811077	129811169	chr4-129811077-129811169
chr4	129960241	129960333	chr4-129960241-129960333
chr4	130133819	130133911	chr4-130133819-130133911
chr4	130163720	130163812	chr4-130163720-130163812
chr4	130576419	130576511	chr4-130576419-130576511
chr4	131429739	131429831	chr4-131429739-131429831
chr4	131431341	131431433	chr4-131431341-131431433
chr4	131703869	131703961	chr4-131703869-131703961
chr4	131828081	131828173	chr4-131828081-131828173
chr4	132076875	132076967	chr4-132076875-132076967
chr4	132342070	132342162	chr4-132342070-132342162
chr4	132757672	132757764	chr4-132757672-132757764
chr4	132776971	132777063	chr4-132776971-132777063
chr4	132941637	132941729	chr4-132941637-132941729
chr4	133122204	133122296	chr4-133122204-133122296
chr4	133144961	133145053	chr4-133144961-133145053
chr4	133595582	133595674	chr4-133595582-133595674
chr4	133756822	133756914	chr4-133756822-133756914
chr4	134494796	134494888	chr4-134494796-134494888
chr4	134924518	134924610	chr4-134924518-134924610
chr4	135599097	135599189	chr4-135599097-135599189
chr4	135851023	135851115	chr4-135851023-135851115
chr4	136178078	136178170	chr4-136178078-136178170
chr4	136187162	136187254	chr4-136187162-136187254
chr4	136310512	136310604	chr4-136310512-136310604
chr4	136311415	136311507	chr4-136311415-136311507
chr4	136451667	136451759	chr4-136451667-136451759

chr4	137048669	137048761	chr4-137048669-137048761
chr4	137506782	137506874	chr4-137506782-137506874
chr4	137688085	137688177	chr4-137688085-137688177
chr4	137735871	137735963	chr4-137735871-137735963
chr4	137758162	137758254	chr4-137758162-137758254
chr4	137954544	137954636	chr4-137954544-137954636
chr4	137955191	137955283	chr4-137955191-137955283
chr4	138496534	138496626	chr4-138496534-138496626
chr4	138508778	138508870	chr4-138508778-138508870
chr4	138911924	138912016	chr4-138911924-138912016
chr4	139192847	139192939	chr4-139192847-139192939
chr4	139422543	139422635	chr4-139422543-139422635
chr4	139549632	139549724	chr4-139549632-139549724
chr4	139723730	139723822	chr4-139723730-139723822
chr4	140125616	140125708	chr4-140125616-140125708
chr4	140455166	140455258	chr4-140455166-140455258
chr4	140702636	140702728	chr4-140702636-140702728
chr4	140773944	140774036	chr4-140773944-140774036
chr4	140803920	140804012	chr4-140803920-140804012
chr4	140816175	140816267	chr4-140816175-140816267
chr4	140843176	140843268	chr4-140843176-140843268
chr4	140947799	140947891	chr4-140947799-140947891
chr4	141147737	141147829	chr4-141147737-141147829
chr4	141287678	141287770	chr4-141287678-141287770
chr4	141287908	141288000	chr4-141287908-141288000
chr4	141309959	141310051	chr4-141309959-141310051
chr4	141310500	141310592	chr4-141310500-141310592
chr4	141453572	141453664	chr4-141453572-141453664
chr4	141773250	141773342	chr4-141773250-141773342
chr4	141875266	141875358	chr4-141875266-141875358
chr4	142048551	142048643	chr4-142048551-142048643
chr4	142098478	142098570	chr4-142098478-142098570
chr4	142167109	142167201	chr4-142167109-142167201
chr4	142189853	142189945	chr4-142189853-142189945
chr4	142264679	142264771	chr4-142264679-142264771
chr4	142291308	142291400	chr4-142291308-142291400
chr4	142409084	142409176	chr4-142409084-142409176
chr4	144528893	144528985	chr4-144528893-144528985
chr4	144921848	144921940	chr4-144921848-144921940
chr4	145250194	145250286	chr4-145250194-145250286
chr4	145585295	145585387	chr4-145585295-145585387
chr4	145679580	145679672	chr4-145679580-145679672

chr4	145825872	145825964	chr4-145825872-145825964
chr4	145837088	145837180	chr4-145837088-145837180
chr4	146067622	146067714	chr4-146067622-146067714
chr4	146158889	146158981	chr4-146158889-146158981
chr4	146167958	146168050	chr4-146167958-146168050
chr4	146168299	146168391	chr4-146168299-146168391
chr4	146242332	146242424	chr4-146242332-146242424
chr4	146437516	146437608	chr4-146437516-146437608
chr4	146474814	146474906	chr4-146474814-146474906
chr4	146475266	146475358	chr4-146475266-146475358
chr4	146514865	146514957	chr4-146514865-146514957
chr4	146546092	146546184	chr4-146546092-146546184
chr4	146710300	146710392	chr4-146710300-146710392
chr4	147026514	147026606	chr4-147026514-147026606
chr4	147095831	147095923	chr4-147095831-147095923
chr4	147102074	147102166	chr4-147102074-147102166
chr4	147116723	147116815	chr4-147116723-147116815
chr4	147117975	147118067	chr4-147117975-147118067
chr4	147124970	147125062	chr4-147124970-147125062
chr4	147176446	147176538	chr4-147176446-147176538
chr4	147195338	147195430	chr4-147195338-147195430
chr4	147257783	147257875	chr4-147257783-147257875
chr4	147275270	147275362	chr4-147275270-147275362
chr4	147305737	147305829	chr4-147305737-147305829
chr4	147382797	147382889	chr4-147382797-147382889
chr4	147738596	147738688	chr4-147738596-147738688
chr4	147985758	147985850	chr4-147985758-147985850
chr4	148163721	148163813	chr4-148163721-148163813
chr4	148166812	148166904	chr4-148166812-148166904
chr4	148444682	148444774	chr4-148444682-148444774
chr4	149016051	149016143	chr4-149016051-149016143
chr4	149132047	149132139	chr4-149132047-149132139
chr4	149678421	149678513	chr4-149678421-149678513
chr4	149953413	149953505	chr4-149953413-149953505
chr4	149956353	149956445	chr4-149956353-149956445
chr4	149989360	149989452	chr4-149989360-149989452
chr4	150258088	150258180	chr4-150258088-150258180
chr4	150708332	150708424	chr4-150708332-150708424
chr4	150748252	150748344	chr4-150748252-150748344
chr4	150748706	150748798	chr4-150748706-150748798
chr4	150782724	150782816	chr4-150782724-150782816
chr4	150807808	150807900	chr4-150807808-150807900

chr4	150808422	150808514	chr4-150808422-150808514
chr4	151101835	151101927	chr4-151101835-151101927
chr4	151141354	151141446	chr4-151141354-151141446
chr4	152530945	152531037	chr4-152530945-152531037
chr4	152555914	152556006	chr4-152555914-152556006
chr4	153113691	153113783	chr4-153113691-153113783
chr4	153975038	153975130	chr4-153975038-153975130
chr4	154510826	154510918	chr4-154510826-154510918
chr4	154764460	154764552	chr4-154764460-154764552
chr4	154908620	154908712	chr4-154908620-154908712
chr4	154946859	154946951	chr4-154946859-154946951
chr4	155173658	155173750	chr4-155173658-155173750
chr4	155623873	155623965	chr4-155623873-155623965
chr4	155763893	155763985	chr4-155763893-155763985
chr4	156186213	156186305	chr4-156186213-156186305
chr5	3152299	3152391	chr5-3152299-3152391
chr5	3363056	3363148	chr5-3363056-3363148
chr5	3365579	3365671	chr5-3365579-3365671
chr5	4273519	4273611	chr5-4273519-4273611
chr5	4311283	4311375	chr5-4311283-4311375
chr5	4325914	4326006	chr5-4325914-4326006
chr5	5211606	5211698	chr5-5211606-5211698
chr5	5342441	5342533	chr5-5342441-5342533
chr5	5344822	5344914	chr5-5344822-5344914
chr5	5691726	5691818	chr5-5691726-5691818
chr5	6530886	6530978	chr5-6530886-6530978
chr5	7060167	7060259	chr5-7060167-7060259
chr5	7482914	7483006	chr5-7482914-7483006
chr5	8413999	8414091	chr5-8413999-8414091
chr5	8737136	8737228	chr5-8737136-8737228
chr5	12398597	12398689	chr5-12398597-12398689
chr5	12937214	12937306	chr5-12937214-12937306
chr5	13153075	13153167	chr5-13153075-13153167
chr5	13959796	13959888	chr5-13959796-13959888
chr5	13987272	13987364	chr5-13987272-13987364
chr5	14728879	14728971	chr5-14728879-14728971
chr5	14961850	14961942	chr5-14961850-14961942
chr5	15004792	15004884	chr5-15004792-15004884
chr5	15011364	15011456	chr5-15011364-15011456
chr5	15458158	15458250	chr5-15458158-15458250
chr5	15512464	15512556	chr5-15512464-15512556
chr5	15552549	15552641	chr5-15552549-15552641

chr5	15600473	15600565	chr5-15600473-15600565
chr5	15691997	15692089	chr5-15691997-15692089
chr5	16399822	16399914	chr5-16399822-16399914
chr5	18307450	18307542	chr5-18307450-18307542
chr5	21145367	21145459	chr5-21145367-21145459
chr5	22855850	22855942	chr5-22855850-22855942
chr5	23518823	23518915	chr5-23518823-23518915
chr5	23543748	23543840	chr5-23543748-23543840
chr5	23560276	23560368	chr5-23560276-23560368
chr5	23590458	23590550	chr5-23590458-23590550
chr5	23761927	23762019	chr5-23761927-23762019
chr5	23863619	23863711	chr5-23863619-23863711
chr5	23942698	23942790	chr5-23942698-23942790
chr5	24602022	24602114	chr5-24602022-24602114
chr5	24907750	24907842	chr5-24907750-24907842
chr5	26006919	26007011	chr5-26006919-26007011
chr5	27478889	27478981	chr5-27478889-27478981
chr5	27708363	27708455	chr5-27708363-27708455
chr5	28071880	28071972	chr5-28071880-28071972
chr5	28835102	28835194	chr5-28835102-28835194
chr5	29083302	29083394	chr5-29083302-29083394
chr5	29467848	29467940	chr5-29467848-29467940
chr5	29735602	29735694	chr5-29735602-29735694
chr5	29816532	29816624	chr5-29816532-29816624
chr5	30238687	30238779	chr5-30238687-30238779
chr5	30343416	30343508	chr5-30343416-30343508
chr5	30461938	30462030	chr5-30461938-30462030
chr5	30582618	30582710	chr5-30582618-30582710
chr5	30921453	30921545	chr5-30921453-30921545
chr5	31050351	31050443	chr5-31050351-31050443
chr5	31077452	31077544	chr5-31077452-31077544
chr5	31568360	31568452	chr5-31568360-31568452
chr5	31596863	31596955	chr5-31596863-31596955
chr5	32209464	32209556	chr5-32209464-32209556
chr5	32706409	32706501	chr5-32706409-32706501
chr5	32727021	32727113	chr5-32727021-32727113
chr5	32730243	32730335	chr5-32730243-32730335
chr5	32737673	32737765	chr5-32737673-32737765
chr5	32738031	32738123	chr5-32738031-32738123
chr5	34582157	34582249	chr5-34582157-34582249
chr5	35740132	35740224	chr5-35740132-35740224
chr5	36133983	36134075	chr5-36133983-36134075

chr5	36160802	36160894	chr5-36160802-36160894
chr5	36433945	36434037	chr5-36433945-36434037
chr5	36701075	36701167	chr5-36701075-36701167
chr5	36840756	36840848	chr5-36840756-36840848
chr5	37000398	37000490	chr5-37000398-37000490
chr5	37522177	37522269	chr5-37522177-37522269
chr5	38733856	38733948	chr5-38733856-38733948
chr5	39573619	39573711	chr5-39573619-39573711
chr5	39716241	39716333	chr5-39716241-39716333
chr5	39762156	39762248	chr5-39762156-39762248
chr5	39833068	39833160	chr5-39833068-39833160
chr5	41085998	41086090	chr5-41085998-41086090
chr5	41645424	41645516	chr5-41645424-41645516
chr5	41855928	41856020	chr5-41855928-41856020
chr5	42463625	42463717	chr5-42463625-42463717
chr5	43007851	43007943	chr5-43007851-43007943
chr5	43537995	43538087	chr5-43537995-43538087
chr5	43820937	43821029	chr5-43820937-43821029
chr5	43915439	43915531	chr5-43915439-43915531
chr5	45292490	45292582	chr5-45292490-45292582
chr5	46009665	46009757	chr5-46009665-46009757
chr5	46361759	46361851	chr5-46361759-46361851
chr5	46363846	46363938	chr5-46363846-46363938
chr5	47480092	47480184	chr5-47480092-47480184
chr5	47780619	47780711	chr5-47780619-47780711
chr5	48004582	48004674	chr5-48004582-48004674
chr5	48091740	48091832	chr5-48091740-48091832
chr5	49987662	49987754	chr5-49987662-49987754
chr5	50049029	50049121	chr5-50049029-50049121
chr5	51511970	51512062	chr5-51511970-51512062
chr5	52655073	52655165	chr5-52655073-52655165
chr5	52680824	52680916	chr5-52680824-52680916
chr5	52908496	52908588	chr5-52908496-52908588
chr5	53199809	53199901	chr5-53199809-53199901
chr5	54581661	54581753	chr5-54581661-54581753
chr5	55089019	55089111	chr5-55089019-55089111
chr5	58908445	58908537	chr5-58908445-58908537
chr5	59174825	59174917	chr5-59174825-59174917
chr5	60800367	60800459	chr5-60800367-60800459
chr5	62702417	62702509	chr5-62702417-62702509
chr5	64227036	64227128	chr5-64227036-64227128
chr5	64243223	64243315	chr5-64243223-64243315

chr5	64371332	64371424	chr5-64371332-64371424
chr5	64588077	64588169	chr5-64588077-64588169
chr5	64610336	64610428	chr5-64610336-64610428
chr5	64667151	64667243	chr5-64667151-64667243
chr5	64750578	64750670	chr5-64750578-64750670
chr5	64812188	64812280	chr5-64812188-64812280
chr5	64851128	64851220	chr5-64851128-64851220
chr5	64867306	64867398	chr5-64867306-64867398
chr5	64992636	64992728	chr5-64992636-64992728
chr5	65128999	65129091	chr5-65128999-65129091
chr5	65335536	65335628	chr5-65335536-65335628
chr5	65454918	65455010	chr5-65454918-65455010
chr5	65484053	65484145	chr5-65484053-65484145
chr5	65492839	65492931	chr5-65492839-65492931
chr5	65834798	65834890	chr5-65834798-65834890
chr5	66166299	66166391	chr5-66166299-66166391
chr5	66466395	66466487	chr5-66466395-66466487
chr5	66524317	66524409	chr5-66524317-66524409
chr5	66550701	66550793	chr5-66550701-66550793
chr5	66814143	66814235	chr5-66814143-66814235
chr5	66896451	66896543	chr5-66896451-66896543
chr5	67131530	67131622	chr5-67131530-67131622
chr5	67298546	67298638	chr5-67298546-67298638
chr5	67410525	67410617	chr5-67410525-67410617
chr5	68107403	68107495	chr5-68107403-68107495
chr5	69098210	69098302	chr5-69098210-69098302
chr5	69361903	69361995	chr5-69361903-69361995
chr5	70000685	70000777	chr5-70000685-70000777
chr5	70997467	70997559	chr5-70997467-70997559
chr5	71065685	71065777	chr5-71065685-71065777
chr5	71234083	71234175	chr5-71234083-71234175
chr5	72740659	72740751	chr5-72740659-72740751
chr5	73127169	73127261	chr5-73127169-73127261
chr5	73288061	73288153	chr5-73288061-73288153
chr5	73400271	73400363	chr5-73400271-73400363
chr5	74041004	74041096	chr5-74041004-74041096
chr5	74137028	74137120	chr5-74137028-74137120
chr5	74160027	74160119	chr5-74160027-74160119
chr5	74208563	74208655	chr5-74208563-74208655
chr5	74626432	74626524	chr5-74626432-74626524
chr5	74944274	74944366	chr5-74944274-74944366
chr5	74959301	74959393	chr5-74959301-74959393

chr5	75081000	75081092	chr5-75081000-75081092
chr5	75826439	75826531	chr5-75826439-75826531
chr5	76375265	76375357	chr5-76375265-76375357
chr5	76382069	76382161	chr5-76382069-76382161
chr5	77265829	77265921	chr5-77265829-77265921
chr5	77407423	77407515	chr5-77407423-77407515
chr5	77662911	77663003	chr5-77662911-77663003
chr5	78631338	78631430	chr5-78631338-78631430
chr5	79093218	79093310	chr5-79093218-79093310
chr5	79511018	79511110	chr5-79511018-79511110
chr5	79928366	79928458	chr5-79928366-79928458
chr5	81536324	81536416	chr5-81536324-81536416
chr5	81894685	81894777	chr5-81894685-81894777
chr5	81946629	81946721	chr5-81946629-81946721
chr5	84251961	84252053	chr5-84251961-84252053
chr5	85031541	85031633	chr5-85031541-85031633
chr5	85146055	85146147	chr5-85146055-85146147
chr5	86172390	86172482	chr5-86172390-86172482
chr5	86638299	86638391	chr5-86638299-86638391
chr5	86741277	86741369	chr5-86741277-86741369
chr5	88302325	88302417	chr5-88302325-88302417
chr5	88634733	88634825	chr5-88634733-88634825
chr5	89265497	89265589	chr5-89265497-89265589
chr5	89277178	89277270	chr5-89277178-89277270
chr5	89390525	89390617	chr5-89390525-89390617
chr5	89536156	89536248	chr5-89536156-89536248
chr5	89747287	89747379	chr5-89747287-89747379
chr5	89748324	89748416	chr5-89748324-89748416
chr5	89880357	89880449	chr5-89880357-89880449
chr5	90612215	90612307	chr5-90612215-90612307
chr5	90691626	90691718	chr5-90691626-90691718
chr5	90754130	90754222	chr5-90754130-90754222
chr5	91037166	91037258	chr5-91037166-91037258
chr5	91071655	91071747	chr5-91071655-91071747
chr5	91098394	91098486	chr5-91098394-91098486
chr5	91110201	91110293	chr5-91110201-91110293
chr5	91200576	91200668	chr5-91200576-91200668
chr5	91302133	91302225	chr5-91302133-91302225
chr5	91641199	91641291	chr5-91641199-91641291
chr5	92220004	92220096	chr5-92220004-92220096
chr5	92267185	92267277	chr5-92267185-92267277
chr5	92660734	92660826	chr5-92660734-92660826

chr5	92745916	92746008	chr5-92745916-92746008
chr5	92763810	92763902	chr5-92763810-92763902
chr5	92824780	92824872	chr5-92824780-92824872
chr5	92888770	92888862	chr5-92888770-92888862
chr5	92962066	92962158	chr5-92962066-92962158
chr5	93038501	93038593	chr5-93038501-93038593
chr5	93127561	93127653	chr5-93127561-93127653
chr5	93267037	93267129	chr5-93267037-93267129
chr5	93595974	93596066	chr5-93595974-93596066
chr5	96072929	96073021	chr5-96072929-96073021
chr5	97867976	97868068	chr5-97867976-97868068
chr5	98076804	98076896	chr5-98076804-98076896
chr5	98805531	98805623	chr5-98805531-98805623
chr5	98946624	98946716	chr5-98946624-98946716
chr5	99242234	99242326	chr5-99242234-99242326
chr5	99294639	99294731	chr5-99294639-99294731
chr5	99420004	99420096	chr5-99420004-99420096
chr5	99978563	99978655	chr5-99978563-99978655
chr5	100207050	100207142	chr5-100207050-100207142
chr5	100331588	100331680	chr5-100331588-100331680
chr5	100798586	100798678	chr5-100798586-100798678
chr5	100861310	100861402	chr5-100861310-100861402
chr5	101217035	101217127	chr5-101217035-101217127
chr5	101426133	101426225	chr5-101426133-101426225
chr5	101857503	101857595	chr5-101857503-101857595
chr5	103132951	103133043	chr5-103132951-103133043
chr5	103365181	103365273	chr5-103365181-103365273
chr5	103519594	103519686	chr5-103519594-103519686
chr5	105523601	105523693	chr5-105523601-105523693
chr5	105685044	105685136	chr5-105685044-105685136
chr5	105699666	105699758	chr5-105699666-105699758
chr5	105700473	105700565	chr5-105700473-105700565
chr5	105779593	105779685	chr5-105779593-105779685
chr5	105831770	105831862	chr5-105831770-105831862
chr5	105916125	105916217	chr5-105916125-105916217
chr5	106821070	106821162	chr5-106821070-106821162
chr5	107121198	107121290	chr5-107121198-107121290
chr5	107212630	107212722	chr5-107212630-107212722
chr5	107295363	107295455	chr5-107295363-107295455
chr5	107311862	107311954	chr5-107311862-107311954
chr5	107691552	107691644	chr5-107691552-107691644
chr5	108367753	108367845	chr5-108367753-108367845

chr5	108599426	108599518	chr5-108599426-108599518
chr5	109343075	109343167	chr5-109343075-109343167
chr5	110284706	110284798	chr5-110284706-110284798
chr5	111096662	111096754	chr5-111096662-111096754
chr5	111122385	111122477	chr5-111122385-111122477
chr5	111501242	111501334	chr5-111501242-111501334
chr5	111545652	111545744	chr5-111545652-111545744
chr5	111790683	111790775	chr5-111790683-111790775
chr5	111911235	111911327	chr5-111911235-111911327
chr5	111944770	111944862	chr5-111944770-111944862
chr5	112060763	112060855	chr5-112060763-112060855
chr5	112065936	112066028	chr5-112065936-112066028
chr5	112295207	112295299	chr5-112295207-112295299
chr5	112324647	112324739	chr5-112324647-112324739
chr5	112497164	112497256	chr5-112497164-112497256
chr5	113139187	113139279	chr5-113139187-113139279
chr5	113278421	113278513	chr5-113278421-113278513
chr5	113989719	113989811	chr5-113989719-113989811
chr5	114173065	114173157	chr5-114173065-114173157
chr5	114187664	114187756	chr5-114187664-114187756
chr5	114378187	114378279	chr5-114378187-114378279
chr5	114636661	114636753	chr5-114636661-114636753
chr5	114646979	114647071	chr5-114646979-114647071
chr5	114691326	114691418	chr5-114691326-114691418
chr5	115008377	115008469	chr5-115008377-115008469
chr5	115211092	115211184	chr5-115211092-115211184
chr5	115532072	115532164	chr5-115532072-115532164
chr5	115872273	115872365	chr5-115872273-115872365
chr5	116009376	116009468	chr5-116009376-116009468
chr5	116040632	116040724	chr5-116040632-116040724
chr5	116925054	116925146	chr5-116925054-116925146
chr5	116934152	116934244	chr5-116934152-116934244
chr5	117115631	117115723	chr5-117115631-117115723
chr5	117361305	117361397	chr5-117361305-117361397
chr5	117441062	117441154	chr5-117441062-117441154
chr5	117976758	117976850	chr5-117976758-117976850
chr5	118435972	118436064	chr5-118435972-118436064
chr5	118634959	118635051	chr5-118634959-118635051
chr5	119679082	119679174	chr5-119679082-119679174
chr5	120171535	120171627	chr5-120171535-120171627
chr5	120195715	120195807	chr5-120195715-120195807
chr5	120329444	120329536	chr5-120329444-120329536

chr5	120466079	120466171	chr5-120466079-120466171
chr5	120644372	120644464	chr5-120644372-120644464
chr5	120648704	120648796	chr5-120648704-120648796
chr5	121102031	121102123	chr5-121102031-121102123
chr5	121102953	121103045	chr5-121102953-121103045
chr5	121191419	121191511	chr5-121191419-121191511
chr5	121269158	121269250	chr5-121269158-121269250
chr5	121823511	121823603	chr5-121823511-121823603
chr5	121837216	121837308	chr5-121837216-121837308
chr5	122054800	122054892	chr5-122054800-122054892
chr5	122136910	122137002	chr5-122136910-122137002
chr5	122372284	122372376	chr5-122372284-122372376
chr5	122431305	122431397	chr5-122431305-122431397
chr5	123006225	123006317	chr5-123006225-123006317
chr5	123136615	123136707	chr5-123136615-123136707
chr5	123138356	123138448	chr5-123138356-123138448
chr5	123684332	123684424	chr5-123684332-123684424
chr5	123749434	123749526	chr5-123749434-123749526
chr5	123880872	123880964	chr5-123880872-123880964
chr5	124007280	124007372	chr5-124007280-124007372
chr5	124025746	124025838	chr5-124025746-124025838
chr5	124052374	124052466	chr5-124052374-124052466
chr5	124145595	124145687	chr5-124145595-124145687
chr5	124225215	124225307	chr5-124225215-124225307
chr5	124328214	124328306	chr5-124328214-124328306
chr5	124389299	124389391	chr5-124389299-124389391
chr5	124446778	124446870	chr5-124446778-124446870
chr5	124451077	124451169	chr5-124451077-124451169
chr5	125206449	125206541	chr5-125206449-125206541
chr5	125272479	125272571	chr5-125272479-125272571
chr5	125372299	125372391	chr5-125372299-125372391
chr5	125671559	125671651	chr5-125671559-125671651
chr5	125931069	125931161	chr5-125931069-125931161
chr5	125991388	125991480	chr5-125991388-125991480
chr5	126002880	126002972	chr5-126002880-126002972
chr5	126555214	126555306	chr5-126555214-126555306
chr5	128107987	128108079	chr5-128107987-128108079
chr5	128643281	128643373	chr5-128643281-128643373
chr5	128661305	128661397	chr5-128661305-128661397
chr5	129657445	129657537	chr5-129657445-129657537
chr5	129715394	129715486	chr5-129715394-129715486
chr5	129724925	129725017	chr5-129724925-129725017

chr5	129978778	129978870	chr5-129978778-129978870
chr5	130023819	130023911	chr5-130023819-130023911
chr5	130255463	130255555	chr5-130255463-130255555
chr5	130553889	130553981	chr5-130553889-130553981
chr5	130858090	130858182	chr5-130858090-130858182
chr5	131519684	131519776	chr5-131519684-131519776
chr5	131613383	131613475	chr5-131613383-131613475
chr5	131665829	131665921	chr5-131665829-131665921
chr5	131732358	131732450	chr5-131732358-131732450
chr5	133127906	133127998	chr5-133127906-133127998
chr5	133620148	133620240	chr5-133620148-133620240
chr5	134064507	134064599	chr5-134064507-134064599
chr5	134450624	134450716	chr5-134450624-134450716
chr5	134594056	134594148	chr5-134594056-134594148
chr5	134933423	134933515	chr5-134933423-134933515
chr5	134947036	134947128	chr5-134947036-134947128
chr5	134951268	134951360	chr5-134951268-134951360
chr5	134960497	134960589	chr5-134960497-134960589
chr5	135538185	135538277	chr5-135538185-135538277
chr5	135688744	135688836	chr5-135688744-135688836
chr5	135962470	135962562	chr5-135962470-135962562
chr5	136307846	136307938	chr5-136307846-136307938
chr5	136394874	136394966	chr5-136394874-136394966
chr5	136801874	136801966	chr5-136801874-136801966
chr5	136830718	136830810	chr5-136830718-136830810
chr5	136854455	136854547	chr5-136854455-136854547
chr5	137078643	137078735	chr5-137078643-137078735
chr5	137152783	137152875	chr5-137152783-137152875
chr5	137288111	137288203	chr5-137288111-137288203
chr5	137417099	137417191	chr5-137417099-137417191
chr5	137502415	137502507	chr5-137502415-137502507
chr5	137613776	137613868	chr5-137613776-137613868
chr5	138171161	138171253	chr5-138171161-138171253
chr5	138344320	138344412	chr5-138344320-138344412
chr5	138575408	138575500	chr5-138575408-138575500
chr5	138707517	138707609	chr5-138707517-138707609
chr5	138846587	138846679	chr5-138846587-138846679
chr5	139073778	139073870	chr5-139073778-139073870
chr5	139379024	139379116	chr5-139379024-139379116
chr5	139480718	139480810	chr5-139480718-139480810
chr5	139505222	139505314	chr5-139505222-139505314
chr5	139591739	139591831	chr5-139591739-139591831

chr5	139730997	139731089	chr5-139730997-139731089
chr5	139797937	139798029	chr5-139797937-139798029
chr5	140444188	140444280	chr5-140444188-140444280
chr5	140562671	140562763	chr5-140562671-140562763
chr5	142426668	142426760	chr5-142426668-142426760
chr5	142439931	142440023	chr5-142439931-142440023
chr5	142460784	142460876	chr5-142460784-142460876
chr5	142569217	142569309	chr5-142569217-142569309
chr5	142912941	142913033	chr5-142912941-142913033
chr5	143036127	143036219	chr5-143036127-143036219
chr5	143036434	143036526	chr5-143036434-143036526
chr5	143203741	143203833	chr5-143203741-143203833
chr5	143218394	143218486	chr5-143218394-143218486
chr5	143389425	143389517	chr5-143389425-143389517
chr5	143434433	143434525	chr5-143434433-143434525
chr5	143467427	143467519	chr5-143467427-143467519
chr5	143658200	143658292	chr5-143658200-143658292
chr5	143696532	143696624	chr5-143696532-143696624
chr5	144008123	144008215	chr5-144008123-144008215
chr5	144100619	144100711	chr5-144100619-144100711
chr5	144289899	144289991	chr5-144289899-144289991
chr5	144305776	144305868	chr5-144305776-144305868
chr5	144317783	144317875	chr5-144317783-144317875
chr5	144546158	144546250	chr5-144546158-144546250
chr5	144633461	144633553	chr5-144633461-144633553
chr5	144806589	144806681	chr5-144806589-144806681
chr5	144898824	144898916	chr5-144898824-144898916
chr5	144968266	144968358	chr5-144968266-144968358
chr5	144978680	144978772	chr5-144978680-144978772
chr5	145096892	145096984	chr5-145096892-145096984
chr5	145173280	145173372	chr5-145173280-145173372
chr5	145231517	145231609	chr5-145231517-145231609
chr5	146703757	146703849	chr5-146703757-146703849
chr5	146716183	146716275	chr5-146716183-146716275
chr5	146799335	146799427	chr5-146799335-146799427
chr5	147292213	147292305	chr5-147292213-147292305
chr5	147405396	147405488	chr5-147405396-147405488
chr5	147652609	147652701	chr5-147652609-147652701
chr5	147694517	147694609	chr5-147694517-147694609
chr5	147793181	147793273	chr5-147793181-147793273
chr5	148307675	148307767	chr5-148307675-148307767
chr5	148361391	148361483	chr5-148361391-148361483

chr5	148563000	148563092	chr5-148563000-148563092
chr5	148626132	148626224	chr5-148626132-148626224
chr5	148634066	148634158	chr5-148634066-148634158
chr5	148706366	148706458	chr5-148706366-148706458
chr5	148783573	148783665	chr5-148783573-148783665
chr5	149184545	149184637	chr5-149184545-149184637
chr5	149247927	149248019	chr5-149247927-149248019
chr5	149282095	149282187	chr5-149282095-149282187
chr5	149392220	149392312	chr5-149392220-149392312
chr5	149441251	149441343	chr5-149441251-149441343
chr5	149525621	149525713	chr5-149525621-149525713
chr5	151054134	151054226	chr5-151054134-151054226
chr5	151112691	151112783	chr5-151112691-151112783
chr5	151154822	151154914	chr5-151154822-151154914
chr5	151196260	151196352	chr5-151196260-151196352
chr5	151251688	151251780	chr5-151251688-151251780
chr5	151346906	151346998	chr5-151346906-151346998
chr6	3050455	3050547	chr6-3050455-3050547
chr6	3050669	3050761	chr6-3050669-3050761
chr6	3051751	3051843	chr6-3051751-3051843
chr6	3122364	3122456	chr6-3122364-3122456
chr6	3133895	3133987	chr6-3133895-3133987
chr6	3137932	3138024	chr6-3137932-3138024
chr6	3141710	3141802	chr6-3141710-3141802
chr6	3182746	3182838	chr6-3182746-3182838
chr6	3183338	3183430	chr6-3183338-3183430
chr6	3191809	3191901	chr6-3191809-3191901
chr6	5164045	5164137	chr6-5164045-5164137
chr6	5442797	5442889	chr6-5442797-5442889
chr6	5574273	5574365	chr6-5574273-5574365
chr6	6149999	6150091	chr6-6149999-6150091
chr6	6398671	6398763	chr6-6398671-6398763
chr6	7035194	7035286	chr6-7035194-7035286
chr6	9349859	9349951	chr6-9349859-9349951
chr6	10186356	10186448	chr6-10186356-10186448
chr6	12812200	12812292	chr6-12812200-12812292
chr6	12813982	12814074	chr6-12813982-12814074
chr6	13383336	13383428	chr6-13383336-13383428
chr6	13860355	13860447	chr6-13860355-13860447
chr6	14526468	14526560	chr6-14526468-14526560
chr6	17171062	17171154	chr6-17171062-17171154
chr6	17240112	17240204	chr6-17240112-17240204

chr6	17403252	17403344	chr6-17403252-17403344
chr6	17464447	17464539	chr6-17464447-17464539
chr6	17601727	17601819	chr6-17601727-17601819
chr6	17764160	17764252	chr6-17764160-17764252
chr6	20564846	20564938	chr6-20564846-20564938
chr6	22447786	22447878	chr6-22447786-22447878
chr6	22875447	22875539	chr6-22875447-22875539
chr6	23234832	23234924	chr6-23234832-23234924
chr6	24313570	24313662	chr6-24313570-24313662
chr6	25684476	25684568	chr6-25684476-25684568
chr6	26469189	26469281	chr6-26469189-26469281
chr6	28202687	28202779	chr6-28202687-28202779
chr6	28814564	28814656	chr6-28814564-28814656
chr6	28895659	28895751	chr6-28895659-28895751
chr6	29043319	29043411	chr6-29043319-29043411
chr6	29064540	29064632	chr6-29064540-29064632
chr6	29089423	29089515	chr6-29089423-29089515
chr6	29192873	29192965	chr6-29192873-29192965
chr6	30174073	30174165	chr6-30174073-30174165
chr6	30307803	30307895	chr6-30307803-30307895
chr6	30370088	30370180	chr6-30370088-30370180
chr6	30537981	30538073	chr6-30537981-30538073
chr6	30563761	30563853	chr6-30563761-30563853
chr6	30697872	30697964	chr6-30697872-30697964
chr6	30815097	30815189	chr6-30815097-30815189
chr6	31081043	31081135	chr6-31081043-31081135
chr6	31108124	31108216	chr6-31108124-31108216
chr6	31568205	31568297	chr6-31568205-31568297
chr6	33853952	33854044	chr6-33853952-33854044
chr6	34302451	34302543	chr6-34302451-34302543
chr6	34317650	34317742	chr6-34317650-34317742
chr6	34865791	34865883	chr6-34865791-34865883
chr6	35256813	35256905	chr6-35256813-35256905
chr6	36776342	36776434	chr6-36776342-36776434
chr6	36848046	36848138	chr6-36848046-36848138
chr6	36899371	36899463	chr6-36899371-36899463
chr6	37144289	37144381	chr6-37144289-37144381
chr6	37389094	37389186	chr6-37389094-37389186
chr6	37496086	37496178	chr6-37496086-37496178
chr6	37723445	37723537	chr6-37723445-37723537
chr6	37856415	37856507	chr6-37856415-37856507
chr6	38126296	38126388	chr6-38126296-38126388

chr6	38883178	38883270	chr6-38883178-38883270
chr6	39243308	39243400	chr6-39243308-39243400
chr6	39515805	39515897	chr6-39515805-39515897
chr6	39546401	39546493	chr6-39546401-39546493
chr6	39696384	39696476	chr6-39696384-39696476
chr6	39996301	39996393	chr6-39996301-39996393
chr6	42964898	42964990	chr6-42964898-42964990
chr6	43337614	43337706	chr6-43337614-43337706
chr6	43525182	43525274	chr6-43525182-43525274
chr6	43649474	43649566	chr6-43649474-43649566
chr6	44680278	44680370	chr6-44680278-44680370
chr6	45475601	45475693	chr6-45475601-45475693
chr6	46776830	46776922	chr6-46776830-46776922
chr6	47328483	47328575	chr6-47328483-47328575
chr6	47682267	47682359	chr6-47682267-47682359
chr6	48291812	48291904	chr6-48291812-48291904
chr6	49236477	49236569	chr6-49236477-49236569
chr6	49314906	49314998	chr6-49314906-49314998
chr6	50368617	50368709	chr6-50368617-50368709
chr6	50826448	50826540	chr6-50826448-50826540
chr6	50895375	50895467	chr6-50895375-50895467
chr6	51760493	51760585	chr6-51760493-51760585
chr6	52821275	52821367	chr6-52821275-52821367
chr6	53207465	53207557	chr6-53207465-53207557
chr6	53497980	53498072	chr6-53497980-53498072
chr6	54028852	54028944	chr6-54028852-54028944
chr6	54203505	54203597	chr6-54203505-54203597
chr6	54443570	54443662	chr6-54443570-54443662
chr6	54719603	54719695	chr6-54719603-54719695
chr6	56235539	56235631	chr6-56235539-56235631
chr6	56579779	56579871	chr6-56579779-56579871
chr6	56611171	56611263	chr6-56611171-56611263
chr6	56797576	56797668	chr6-56797576-56797668
chr6	56825998	56826090	chr6-56825998-56826090
chr6	56919444	56919536	chr6-56919444-56919536
chr6	57255808	57255900	chr6-57255808-57255900
chr6	58540478	58540570	chr6-58540478-58540570
chr6	58578248	58578340	chr6-58578248-58578340
chr6	58594159	58594251	chr6-58594159-58594251
chr6	59269340	59269432	chr6-59269340-59269432
chr6	60674443	60674535	chr6-60674443-60674535
chr6	61246679	61246771	chr6-61246679-61246771

chr6	62567210	62567302	chr6-62567210-62567302
chr6	63499651	63499743	chr6-63499651-63499743
chr6	63764555	63764647	chr6-63764555-63764647
chr6	63843605	63843697	chr6-63843605-63843697
chr6	64116607	64116699	chr6-64116607-64116699
chr6	64263695	64263787	chr6-64263695-64263787
chr6	64275722	64275814	chr6-64275722-64275814
chr6	64762287	64762379	chr6-64762287-64762379
chr6	65996454	65996546	chr6-65996454-65996546
chr6	66347516	66347608	chr6-66347516-66347608
chr6	66706750	66706842	chr6-66706750-66706842
chr6	67037369	67037461	chr6-67037369-67037461
chr6	67139115	67139207	chr6-67139115-67139207
chr6	67199622	67199714	chr6-67199622-67199714
chr6	67266848	67266940	chr6-67266848-67266940
chr6	67533238	67533330	chr6-67533238-67533330
chr6	67691206	67691298	chr6-67691206-67691298
chr6	67903358	67903450	chr6-67903358-67903450
chr6	69516311	69516403	chr6-69516311-69516403
chr6	70104454	70104546	chr6-70104454-70104546
chr6	70784999	70785091	chr6-70784999-70785091
chr6	70982330	70982422	chr6-70982330-70982422
chr6	71270852	71270944	chr6-71270852-71270944
chr6	72004168	72004260	chr6-72004168-72004260
chr6	72293448	72293540	chr6-72293448-72293540
chr6	72329153	72329245	chr6-72329153-72329245
chr6	72493053	72493145	chr6-72493053-72493145
chr6	72927009	72927101	chr6-72927009-72927101
chr6	72956600	72956692	chr6-72956600-72956692
chr6	78820374	78820466	chr6-78820374-78820466
chr6	80769560	80769652	chr6-80769560-80769652
chr6	81658985	81659077	chr6-81658985-81659077
chr6	82380133	82380225	chr6-82380133-82380225
chr6	82725185	82725277	chr6-82725185-82725277
chr6	82730521	82730613	chr6-82730521-82730613
chr6	82756129	82756221	chr6-82756129-82756221
chr6	82762912	82763004	chr6-82762912-82763004
chr6	83078555	83078647	chr6-83078555-83078647
chr6	83248906	83248998	chr6-83248906-83248998
chr6	83309626	83309718	chr6-83309626-83309718
chr6	84008112	84008204	chr6-84008112-84008204
chr6	84508237	84508329	chr6-84508237-84508329

chr6	84697742	84697834	chr6-84697742-84697834
chr6	85331511	85331603	chr6-85331511-85331603
chr6	85366444	85366536	chr6-85366444-85366536
chr6	85382939	85383031	chr6-85382939-85383031
chr6	86148650	86148742	chr6-86148650-86148742
chr6	86182689	86182781	chr6-86182689-86182781
chr6	86219605	86219697	chr6-86219605-86219697
chr6	86300339	86300431	chr6-86300339-86300431
chr6	86319077	86319169	chr6-86319077-86319169
chr6	87229086	87229178	chr6-87229086-87229178
chr6	87883038	87883130	chr6-87883038-87883130
chr6	88012783	88012875	chr6-88012783-88012875
chr6	88054755	88054847	chr6-88054755-88054847
chr6	88189786	88189878	chr6-88189786-88189878
chr6	88228065	88228157	chr6-88228065-88228157
chr6	88935900	88935992	chr6-88935900-88935992
chr6	88966658	88966750	chr6-88966658-88966750
chr6	89087748	89087840	chr6-89087748-89087840
chr6	89412670	89412762	chr6-89412670-89412762
chr6	89457856	89457948	chr6-89457856-89457948
chr6	89651825	89651917	chr6-89651825-89651917
chr6	89782094	89782186	chr6-89782094-89782186
chr6	90359962	90360054	chr6-90359962-90360054
chr6	90518748	90518840	chr6-90518748-90518840
chr6	90909547	90909639	chr6-90909547-90909639
chr6	91210940	91211032	chr6-91210940-91211032
chr6	91389230	91389322	chr6-91389230-91389322
chr6	91686722	91686814	chr6-91686722-91686814
chr6	92596228	92596320	chr6-92596228-92596320
chr6	93131074	93131166	chr6-93131074-93131166
chr6	93938906	93938998	chr6-93938906-93938998
chr6	94117205	94117297	chr6-94117205-94117297
chr6	94180175	94180267	chr6-94180175-94180267
chr6	94203151	94203243	chr6-94203151-94203243
chr6	94494786	94494878	chr6-94494786-94494878
chr6	94514912	94515004	chr6-94514912-94515004
chr6	94743036	94743128	chr6-94743036-94743128
chr6	95069742	95069834	chr6-95069742-95069834
chr6	95364864	95364956	chr6-95364864-95364956
chr6	97207243	97207335	chr6-97207243-97207335
chr6	97258215	97258307	chr6-97258215-97258307
chr6	97523190	97523282	chr6-97523190-97523282

chr6	97585893	97585985	chr6-97585893-97585985
chr6	98383770	98383862	chr6-98383770-98383862
chr6	98746955	98747047	chr6-98746955-98747047
chr6	98843140	98843232	chr6-98843140-98843232
chr6	99481235	99481327	chr6-99481235-99481327
chr6	99934539	99934631	chr6-99934539-99934631
chr6	100571079	100571171	chr6-100571079-100571171
chr6	100610358	100610450	chr6-100610358-100610450
chr6	104628739	104628831	chr6-104628739-104628831
chr6	106876865	106876957	chr6-106876865-106876957
chr6	106877367	106877459	chr6-106877367-106877459
chr6	106962016	106962108	chr6-106962016-106962108
chr6	108447511	108447603	chr6-108447511-108447603
chr6	108510314	108510406	chr6-108510314-108510406
chr6	108627391	108627483	chr6-108627391-108627483
chr6	108704874	108704966	chr6-108704874-108704966
chr6	108705808	108705900	chr6-108705808-108705900
chr6	108835105	108835197	chr6-108835105-108835197
chr6	111564308	111564400	chr6-111564308-111564400
chr6	112278276	112278368	chr6-112278276-112278368
chr6	113076154	113076246	chr6-113076154-113076246
chr6	113191472	113191564	chr6-113191472-113191564
chr6	113385478	113385570	chr6-113385478-113385570
chr6	113690681	113690773	chr6-113690681-113690773
chr6	113809775	113809867	chr6-113809775-113809867
chr6	115313257	115313349	chr6-115313257-115313349
chr6	115313914	115314006	chr6-115313914-115314006
chr6	115315863	115315955	chr6-115315863-115315955
chr6	115480074	115480166	chr6-115480074-115480166
chr6	115480844	115480936	chr6-115480844-115480936
chr6	115501175	115501267	chr6-115501175-115501267
chr6	115539229	115539321	chr6-115539229-115539321
chr6	115592539	115592631	chr6-115592539-115592631
chr6	115722359	115722451	chr6-115722359-115722451
chr6	115776465	115776557	chr6-115776465-115776557
chr6	116022272	116022364	chr6-116022272-116022364
chr6	116024820	116024912	chr6-116024820-116024912
chr6	116337956	116338048	chr6-116337956-116338048
chr6	116791905	116791997	chr6-116791905-116791997
chr6	117972391	117972483	chr6-117972391-117972483
chr6	118031678	118031770	chr6-118031678-118031770
chr6	118128292	118128384	chr6-118128292-118128384

chr6	118215597	118215689	chr6-118215597-118215689
chr6	118626963	118627055	chr6-118626963-118627055
chr6	119273294	119273386	chr6-119273294-119273386
chr6	119329392	119329484	chr6-119329392-119329484
chr6	119332263	119332355	chr6-119332263-119332355
chr6	119449038	119449130	chr6-119449038-119449130
chr6	119456649	119456741	chr6-119456649-119456741
chr6	119642862	119642954	chr6-119642862-119642954
chr6	119663209	119663301	chr6-119663209-119663301
chr6	119902541	119902633	chr6-119902541-119902633
chr6	120073866	120073958	chr6-120073866-120073958
chr6	120482763	120482855	chr6-120482763-120482855
chr6	120594577	120594669	chr6-120594577-120594669
chr6	120932724	120932816	chr6-120932724-120932816
chr6	120987346	120987438	chr6-120987346-120987438
chr6	121159713	121159805	chr6-121159713-121159805
chr6	121160387	121160479	chr6-121160387-121160479
chr6	122825082	122825174	chr6-122825082-122825174
chr6	122826516	122826608	chr6-122826516-122826608
chr6	122856145	122856237	chr6-122856145-122856237
chr6	124128726	124128818	chr6-124128726-124128818
chr6	124130302	124130394	chr6-124130302-124130394
chr6	124683891	124683983	chr6-124683891-124683983
chr6	124740744	124740836	chr6-124740744-124740836
chr6	124805321	124805413	chr6-124805321-124805413
chr6	124903800	124903892	chr6-124903800-124903892
chr6	125084807	125084899	chr6-125084807-125084899
chr6	125109470	125109562	chr6-125109470-125109562
chr6	125318391	125318483	chr6-125318391-125318483
chr6	125323709	125323801	chr6-125323709-125323801
chr6	125473488	125473580	chr6-125473488-125473580
chr6	125617986	125618078	chr6-125617986-125618078
chr6	125876900	125876992	chr6-125876900-125876992
chr6	126351033	126351125	chr6-126351033-126351125
chr6	126483561	126483653	chr6-126483561-126483653
chr6	126493751	126493843	chr6-126493751-126493843
chr6	126792874	126792966	chr6-126792874-126792966
chr6	127109732	127109824	chr6-127109732-127109824
chr6	127330677	127330769	chr6-127330677-127330769
chr6	127404244	127404336	chr6-127404244-127404336
chr6	127719105	127719197	chr6-127719105-127719197
chr6	127935295	127935387	chr6-127935295-127935387

chr6	127944780	127944872	chr6-127944780-127944872
chr6	127991541	127991633	chr6-127991541-127991633
chr6	128330397	128330489	chr6-128330397-128330489
chr6	128349851	128349943	chr6-128349851-128349943
chr6	128435623	128435715	chr6-128435623-128435715
chr6	128835774	128835866	chr6-128835774-128835866
chr6	129231971	129232063	chr6-129231971-129232063
chr6	129284551	129284643	chr6-129284551-129284643
chr6	129507157	129507249	chr6-129507157-129507249
chr6	129514545	129514637	chr6-129514545-129514637
chr6	129701833	129701925	chr6-129701833-129701925
chr6	130135628	130135720	chr6-130135628-130135720
chr6	130475350	130475442	chr6-130475350-130475442
chr6	131197769	131197861	chr6-131197769-131197861
chr6	131377505	131377597	chr6-131377505-131377597
chr6	131951560	131951652	chr6-131951560-131951652
chr6	132463712	132463804	chr6-132463712-132463804
chr6	133341846	133341938	chr6-133341846-133341938
chr6	133643123	133643215	chr6-133643123-133643215
chr6	133986416	133986508	chr6-133986416-133986508
chr6	134614772	134614864	chr6-134614772-134614864
chr6	134808254	134808346	chr6-134808254-134808346
chr6	135025008	135025100	chr6-135025008-135025100
chr6	135026930	135027022	chr6-135026930-135027022
chr6	135040374	135040466	chr6-135040374-135040466
chr6	135290960	135291052	chr6-135290960-135291052
chr6	135322060	135322152	chr6-135322060-135322152
chr6	135471940	135472032	chr6-135471940-135472032
chr6	135920605	135920697	chr6-135920605-135920697
chr6	136465198	136465290	chr6-136465198-136465290
chr6	136492397	136492489	chr6-136492397-136492489
chr6	136645861	136645953	chr6-136645861-136645953
chr6	136672578	136672670	chr6-136672578-136672670
chr6	137165213	137165305	chr6-137165213-137165305
chr6	137506682	137506774	chr6-137506682-137506774
chr6	137784048	137784140	chr6-137784048-137784140
chr6	139537036	139537128	chr6-139537036-139537128
chr6	139854358	139854450	chr6-139854358-139854450
chr6	140200070	140200162	chr6-140200070-140200162
chr6	140448288	140448380	chr6-140448288-140448380
chr6	140707272	140707364	chr6-140707272-140707364
chr6	141540991	141541083	chr6-141540991-141541083

chr6	142647897	142647989	chr6-142647897-142647989
chr6	142684107	142684199	chr6-142684107-142684199
chr6	144444920	144445012	chr6-144444920-144445012
chr6	144716540	144716632	chr6-144716540-144716632
chr6	144780373	144780465	chr6-144780373-144780465
chr6	145742597	145742689	chr6-145742597-145742689
chr6	145977412	145977504	chr6-145977412-145977504
chr6	147290434	147290526	chr6-147290434-147290526
chr6	147346693	147346785	chr6-147346693-147346785
chr6	147738770	147738862	chr6-147738770-147738862
chr6	147760225	147760317	chr6-147760225-147760317
chr6	148281997	148282089	chr6-148281997-148282089
chr6	148769609	148769701	chr6-148769609-148769701
chr6	149238513	149238605	chr6-149238513-149238605
chr7	3629867	3629959	chr7-3629867-3629959
chr7	4170324	4170416	chr7-4170324-4170416
chr7	4507977	4508069	chr7-4507977-4508069
chr7	5015240	5015332	chr7-5015240-5015332
chr7	5260822	5260914	chr7-5260822-5260914
chr7	6155603	6155695	chr7-6155603-6155695
chr7	6924408	6924500	chr7-6924408-6924500
chr7	13408054	13408146	chr7-13408054-13408146
chr7	14190078	14190170	chr7-14190078-14190170
chr7	15895420	15895512	chr7-15895420-15895512
chr7	16295027	16295119	chr7-16295027-16295119
chr7	16582368	16582460	chr7-16582368-16582460
chr7	17352717	17352809	chr7-17352717-17352809
chr7	18183592	18183684	chr7-18183592-18183684
chr7	18482970	18483062	chr7-18482970-18483062
chr7	18500274	18500366	chr7-18500274-18500366
chr7	19327428	19327520	chr7-19327428-19327520
chr7	19332905	19332997	chr7-19332905-19332997
chr7	19516446	19516538	chr7-19516446-19516538
chr7	19748032	19748124	chr7-19748032-19748124
chr7	19825804	19825896	chr7-19825804-19825896
chr7	19921024	19921116	chr7-19921024-19921116
chr7	19932924	19933016	chr7-19932924-19933016
chr7	23485531	23485623	chr7-23485531-23485623
chr7	24112160	24112252	chr7-24112160-24112252
chr7	24449314	24449406	chr7-24449314-24449406
chr7	24546975	24547067	chr7-24546975-24547067
chr7	24940863	24940955	chr7-24940863-24940955

chr7	25816829	25816921	chr7-25816829-25816921
chr7	26196671	26196763	chr7-26196671-26196763
chr7	28577888	28577980	chr7-28577888-28577980
chr7	28595868	28595960	chr7-28595868-28595960
chr7	28810790	28810882	chr7-28810790-28810882
chr7	28970159	28970251	chr7-28970159-28970251
chr7	29134741	29134833	chr7-29134741-29134833
chr7	29420702	29420794	chr7-29420702-29420794
chr7	29512175	29512267	chr7-29512175-29512267
chr7	29980869	29980961	chr7-29980869-29980961
chr7	30183944	30184036	chr7-30183944-30184036
chr7	30524961	30525053	chr7-30524961-30525053
chr7	30602654	30602746	chr7-30602654-30602746
chr7	30990135	30990227	chr7-30990135-30990227
chr7	34759125	34759217	chr7-34759125-34759217
chr7	34840001	34840093	chr7-34840001-34840093
chr7	35319451	35319543	chr7-35319451-35319543
chr7	35344947	35345039	chr7-35344947-35345039
chr7	35348101	35348193	chr7-35348101-35348193
chr7	35425863	35425955	chr7-35425863-35425955
chr7	35432237	35432329	chr7-35432237-35432329
chr7	37007285	37007377	chr7-37007285-37007377
chr7	37208256	37208348	chr7-37208256-37208348
chr7	37705942	37706034	chr7-37705942-37706034
chr7	37869617	37869709	chr7-37869617-37869709
chr7	38030792	38030884	chr7-38030792-38030884
chr7	38206714	38206806	chr7-38206714-38206806
chr7	39805611	39805703	chr7-39805611-39805703
chr7	44443315	44443407	chr7-44443315-44443407
chr7	45018067	45018159	chr7-45018067-45018159
chr7	45623661	45623753	chr7-45623661-45623753
chr7	45957395	45957487	chr7-45957395-45957487
chr7	46036221	46036313	chr7-46036221-46036313
chr7	46236212	46236304	chr7-46236212-46236304
chr7	46798666	46798758	chr7-46798666-46798758
chr7	47008384	47008476	chr7-47008384-47008476
chr7	47018860	47018952	chr7-47018860-47018952
chr7	47113439	47113531	chr7-47113439-47113531
chr7	48658130	48658222	chr7-48658130-48658222
chr7	48718974	48719066	chr7-48718974-48719066
chr7	49919971	49920063	chr7-49919971-49920063
chr7	50757274	50757366	chr7-50757274-50757366

chr7	51568927	51569019	chr7-51568927-51569019
chr7	51724020	51724112	chr7-51724020-51724112
chr7	51750340	51750432	chr7-51750340-51750432
chr7	52017995	52018087	chr7-52017995-52018087
chr7	55412194	55412286	chr7-55412194-55412286
chr7	56019314	56019406	chr7-56019314-56019406
chr7	56303182	56303274	chr7-56303182-56303274
chr7	57728116	57728208	chr7-57728116-57728208
chr7	57800261	57800353	chr7-57800261-57800353
chr7	58623093	58623185	chr7-58623093-58623185
chr7	59767477	59767569	chr7-59767477-59767569
chr7	59768785	59768877	chr7-59768785-59768877
chr7	61151243	61151335	chr7-61151243-61151335
chr7	61597272	61597364	chr7-61597272-61597364
chr7	62397216	62397308	chr7-62397216-62397308
chr7	64015466	64015558	chr7-64015466-64015558
chr7	64073462	64073554	chr7-64073462-64073554
chr7	64073678	64073770	chr7-64073678-64073770
chr7	64073925	64074017	chr7-64073925-64074017
chr7	64078967	64079059	chr7-64078967-64079059
chr7	64080408	64080500	chr7-64080408-64080500
chr7	64082747	64082839	chr7-64082747-64082839
chr7	64086049	64086141	chr7-64086049-64086141
chr7	64086507	64086599	chr7-64086507-64086599
chr7	64089757	64089849	chr7-64089757-64089849
chr7	64155819	64155911	chr7-64155819-64155911
chr7	64249798	64249890	chr7-64249798-64249890
chr7	64345382	64345474	chr7-64345382-64345474
chr7	64468953	64469045	chr7-64468953-64469045
chr7	64494055	64494147	chr7-64494055-64494147
chr7	65115165	65115257	chr7-65115165-65115257
chr7	65467470	65467562	chr7-65467470-65467562
chr7	65817689	65817781	chr7-65817689-65817781
chr7	65831590	65831682	chr7-65831590-65831682
chr7	65873627	65873719	chr7-65873627-65873719
chr7	66163344	66163436	chr7-66163344-66163436
chr7	66238313	66238405	chr7-66238313-66238405
chr7	66309401	66309493	chr7-66309401-66309493
chr7	68545716	68545808	chr7-68545716-68545808
chr7	68684936	68685028	chr7-68684936-68685028
chr7	69226763	69226855	chr7-69226763-69226855
chr7	70429209	70429301	chr7-70429209-70429301

chr7	71084139	71084231	chr7-71084139-71084231
chr7	72324251	72324343	chr7-72324251-72324343
chr7	73069526	73069618	chr7-73069526-73069618
chr7	73972375	73972467	chr7-73972375-73972467
chr7	77516292	77516384	chr7-77516292-77516384
chr7	78995973	78996065	chr7-78995973-78996065
chr7	79710265	79710357	chr7-79710265-79710357
chr7	80166854	80166946	chr7-80166854-80166946
chr7	80209110	80209202	chr7-80209110-80209202
chr7	80375531	80375623	chr7-80375531-80375623
chr7	80435716	80435808	chr7-80435716-80435808
chr7	80552972	80553064	chr7-80552972-80553064
chr7	80789158	80789250	chr7-80789158-80789250
chr7	82011443	82011535	chr7-82011443-82011535
chr7	82894568	82894660	chr7-82894568-82894660
chr7	83627018	83627110	chr7-83627018-83627110
chr7	84564602	84564694	chr7-84564602-84564694
chr7	87217981	87218073	chr7-87217981-87218073
chr7	87281957	87282049	chr7-87281957-87282049
chr7	88056056	88056148	chr7-88056056-88056148
chr7	89782863	89782955	chr7-89782863-89782955
chr7	89787673	89787765	chr7-89787673-89787765
chr7	92538956	92539048	chr7-92538956-92539048
chr7	92811820	92811912	chr7-92811820-92811912
chr7	92874965	92875057	chr7-92874965-92875057
chr7	95602993	95603085	chr7-95602993-95603085
chr7	96386675	96386767	chr7-96386675-96386767
chr7	98059976	98060068	chr7-98059976-98060068
chr7	98163849	98163941	chr7-98163849-98163941
chr7	98775211	98775303	chr7-98775211-98775303
chr7	99094193	99094285	chr7-99094193-99094285
chr7	99316912	99317004	chr7-99316912-99317004
chr7	100227440	100227532	chr7-100227440-100227532
chr7	101058992	101059084	chr7-101058992-101059084
chr7	102259622	102259714	chr7-102259622-102259714
chr7	103883918	103884010	chr7-103883918-103884010
chr7	109782187	109782279	chr7-109782187-109782279
chr7	110432370	110432462	chr7-110432370-110432462
chr7	110625184	110625276	chr7-110625184-110625276
chr7	110773604	110773696	chr7-110773604-110773696
chr7	111792823	111792915	chr7-111792823-111792915
chr7	111874204	111874296	chr7-111874204-111874296

chr7	111880751	111880843	chr7-111880751-111880843
chr7	112040452	112040544	chr7-112040452-112040544
chr7	112202318	112202410	chr7-112202318-112202410
chr7	112202922	112203014	chr7-112202922-112203014
chr7	112316262	112316354	chr7-112316262-112316354
chr7	112408357	112408449	chr7-112408357-112408449
chr7	112455694	112455786	chr7-112455694-112455786
chr7	112660425	112660517	chr7-112660425-112660517
chr7	113140168	113140260	chr7-113140168-113140260
chr7	113419536	113419628	chr7-113419536-113419628
chr7	114117024	114117116	chr7-114117024-114117116
chr7	114203087	114203179	chr7-114203087-114203179
chr7	114879345	114879437	chr7-114879345-114879437
chr7	115036092	115036184	chr7-115036092-115036184
chr7	115355443	115355535	chr7-115355443-115355535
chr7	116444682	116444774	chr7-116444682-116444774
chr7	116462240	116462332	chr7-116462240-116462332
chr7	116716640	116716732	chr7-116716640-116716732
chr7	118096283	118096375	chr7-118096283-118096375
chr7	118481448	118481540	chr7-118481448-118481540
chr7	118940939	118941031	chr7-118940939-118941031
chr7	119962982	119963074	chr7-119962982-119963074
chr7	120875333	120875425	chr7-120875333-120875425
chr7	121192981	121193073	chr7-121192981-121193073
chr7	121245972	121246064	chr7-121245972-121246064
chr7	121308257	121308349	chr7-121308257-121308349
chr7	121355329	121355421	chr7-121355329-121355421
chr7	122772374	122772466	chr7-122772374-122772466
chr7	123108563	123108655	chr7-123108563-123108655
chr7	123377997	123378089	chr7-123377997-123378089
chr7	123781286	123781378	chr7-123781286-123781378
chr7	124048475	124048567	chr7-124048475-124048567
chr7	125125227	125125319	chr7-125125227-125125319
chr7	125472333	125472425	chr7-125472333-125472425
chr7	125535032	125535124	chr7-125535032-125535124
chr7	125801361	125801453	chr7-125801361-125801453
chr7	126281454	126281546	chr7-126281454-126281546
chr7	126465955	126466047	chr7-126465955-126466047
chr7	126780335	126780427	chr7-126780335-126780427
chr7	126977208	126977300	chr7-126977208-126977300
chr7	127511990	127512082	chr7-127511990-127512082
chr7	127513102	127513194	chr7-127513102-127513194

chr7	127683287	127683379	chr7-127683287-127683379
chr7	128172780	128172872	chr7-128172780-128172872
chr7	128364579	128364671	chr7-128364579-128364671
chr7	130362831	130362923	chr7-130362831-130362923
chr7	130692599	130692691	chr7-130692599-130692691
chr7	130834463	130834555	chr7-130834463-130834555
chr7	130965516	130965608	chr7-130965516-130965608
chr7	131717146	131717238	chr7-131717146-131717238
chr7	132344808	132344900	chr7-132344808-132344900
chr7	132931010	132931102	chr7-132931010-132931102
chr7	133007463	133007555	chr7-133007463-133007555
chr7	133041175	133041267	chr7-133041175-133041267
chr7	133041936	133042028	chr7-133041936-133042028
chr7	133382145	133382237	chr7-133382145-133382237
chr7	134287997	134288089	chr7-134287997-134288089
chr7	134647002	134647094	chr7-134647002-134647094
chr7	134775905	134775997	chr7-134775905-134775997
chr7	135716629	135716721	chr7-135716629-135716721
chr7	137698613	137698705	chr7-137698613-137698705
chr7	138171682	138171774	chr7-138171682-138171774
chr7	138210825	138210917	chr7-138210825-138210917
chr7	138950562	138950654	chr7-138950562-138950654
chr7	139258196	139258288	chr7-139258196-139258288
chr7	139353821	139353913	chr7-139353821-139353913
chr7	139371567	139371659	chr7-139371567-139371659
chr7	139498060	139498152	chr7-139498060-139498152
chr7	139928839	139928931	chr7-139928839-139928931
chr7	140121638	140121730	chr7-140121638-140121730
chr7	140221292	140221384	chr7-140221292-140221384
chr7	140274368	140274460	chr7-140274368-140274460
chr7	140750475	140750567	chr7-140750475-140750567
chr7	141297912	141298004	chr7-141297912-141298004
chr7	141398723	141398815	chr7-141398723-141398815
chr7	142434284	142434376	chr7-142434284-142434376
chr7	143008970	143009062	chr7-143008970-143009062
chr7	143016167	143016259	chr7-143016167-143016259
chr7	143368341	143368433	chr7-143368341-143368433
chr7	143625417	143625509	chr7-143625417-143625509
chr7	143649682	143649774	chr7-143649682-143649774
chr7	143785178	143785270	chr7-143785178-143785270
chr7	143798361	143798453	chr7-143798361-143798453
chr7	144233415	144233507	chr7-144233415-144233507

chr7	144233695	144233787	chr7-144233695-144233787
chr7	144456253	144456345	chr7-144456253-144456345
chr7	144747914	144748006	chr7-144747914-144748006
chr7	144780406	144780498	chr7-144780406-144780498
chr7	145025602	145025694	chr7-145025602-145025694
chr7	145103455	145103547	chr7-145103455-145103547
chr8	3412030	3412122	chr8-3412030-3412122
chr8	3473140	3473232	chr8-3473140-3473232
chr8	3515391	3515483	chr8-3515391-3515483
chr8	3606669	3606761	chr8-3606669-3606761
chr8	3754406	3754498	chr8-3754406-3754498
chr8	4325480	4325572	chr8-4325480-4325572
chr8	4582829	4582921	chr8-4582829-4582921
chr8	5283473	5283565	chr8-5283473-5283565
chr8	5495632	5495724	chr8-5495632-5495724
chr8	6053443	6053535	chr8-6053443-6053535
chr8	6739608	6739700	chr8-6739608-6739700
chr8	7650588	7650680	chr8-7650588-7650680
chr8	7709804	7709896	chr8-7709804-7709896
chr8	8061689	8061781	chr8-8061689-8061781
chr8	8275180	8275272	chr8-8275180-8275272
chr8	8602846	8602938	chr8-8602846-8602938
chr8	8603226	8603318	chr8-8603226-8603318
chr8	9631419	9631511	chr8-9631419-9631511
chr8	9809780	9809872	chr8-9809780-9809872
chr8	10396364	10396456	chr8-10396364-10396456
chr8	10646728	10646820	chr8-10646728-10646820
chr8	10683178	10683270	chr8-10683178-10683270
chr8	10946118	10946210	chr8-10946118-10946210
chr8	11258728	11258820	chr8-11258728-11258820
chr8	11259986	11260078	chr8-11259986-11260078
chr8	11323306	11323398	chr8-11323306-11323398
chr8	11371551	11371643	chr8-11371551-11371643
chr8	11502270	11502362	chr8-11502270-11502362
chr8	11550466	11550558	chr8-11550466-11550558
chr8	11558071	11558163	chr8-11558071-11558163
chr8	11601320	11601412	chr8-11601320-11601412
chr8	11776888	11776980	chr8-11776888-11776980
chr8	12058321	12058413	chr8-12058321-12058413
chr8	12087066	12087158	chr8-12087066-12087158
chr8	12768934	12769026	chr8-12768934-12769026
chr8	12779694	12779786	chr8-12779694-12779786

chr8	12782543	12782635	chr8-12782543-12782635
chr8	13380186	13380278	chr8-13380186-13380278
chr8	13531024	13531116	chr8-13531024-13531116
chr8	14055126	14055218	chr8-14055126-14055218
chr8	14440085	14440177	chr8-14440085-14440177
chr8	14818256	14818348	chr8-14818256-14818348
chr8	14890458	14890550	chr8-14890458-14890550
chr8	14994068	14994160	chr8-14994068-14994160
chr8	15905600	15905692	chr8-15905600-15905692
chr8	16541550	16541642	chr8-16541550-16541642
chr8	16759393	16759485	chr8-16759393-16759485
chr8	17408881	17408973	chr8-17408881-17408973
chr8	18594980	18595072	chr8-18594980-18595072
chr8	19317697	19317789	chr8-19317697-19317789
chr8	19697655	19697747	chr8-19697655-19697747
chr8	19698844	19698936	chr8-19698844-19698936
chr8	19703473	19703565	chr8-19703473-19703565
chr8	19709666	19709758	chr8-19709666-19709758
chr8	19781481	19781573	chr8-19781481-19781573
chr8	19784868	19784960	chr8-19784868-19784960
chr8	19909094	19909186	chr8-19909094-19909186
chr8	19912608	19912700	chr8-19912608-19912700
chr8	19919242	19919334	chr8-19919242-19919334
chr8	19934404	19934496	chr8-19934404-19934496
chr8	20122267	20122359	chr8-20122267-20122359
chr8	20122699	20122791	chr8-20122699-20122791
chr8	20259818	20259910	chr8-20259818-20259910
chr8	20266962	20267054	chr8-20266962-20267054
chr8	20267639	20267731	chr8-20267639-20267731
chr8	20300573	20300665	chr8-20300573-20300665
chr8	20331929	20332021	chr8-20331929-20332021
chr8	20339259	20339351	chr8-20339259-20339351
chr8	20353742	20353834	chr8-20353742-20353834
chr8	20380737	20380829	chr8-20380737-20380829
chr8	20382299	20382391	chr8-20382299-20382391
chr8	20389444	20389536	chr8-20389444-20389536
chr8	20395214	20395306	chr8-20395214-20395306
chr8	20408804	20408896	chr8-20408804-20408896
chr8	20424806	20424898	chr8-20424806-20424898
chr8	20436893	20436985	chr8-20436893-20436985
chr8	20437132	20437224	chr8-20437132-20437224
chr8	20495690	20495782	chr8-20495690-20495782

chr8	20514632	20514724	chr8-20514632-20514724
chr8	20516621	20516713	chr8-20516621-20516713
chr8	20524586	20524678	chr8-20524586-20524678
chr8	20529541	20529633	chr8-20529541-20529633
chr8	20544765	20544857	chr8-20544765-20544857
chr8	20545752	20545844	chr8-20545752-20545844
chr8	20550433	20550525	chr8-20550433-20550525
chr8	20588368	20588460	chr8-20588368-20588460
chr8	20590593	20590685	chr8-20590593-20590685
chr8	20593978	20594070	chr8-20593978-20594070
chr8	20597500	20597592	chr8-20597500-20597592
chr8	20630926	20631018	chr8-20630926-20631018
chr8	20638069	20638161	chr8-20638069-20638161
chr8	20678497	20678589	chr8-20678497-20678589
chr8	20812331	20812423	chr8-20812331-20812423
chr8	20813650	20813742	chr8-20813650-20813742
chr8	20822588	20822680	chr8-20822588-20822680
chr8	20946197	20946289	chr8-20946197-20946289
chr8	22185474	22185566	chr8-22185474-22185566
chr8	22209189	22209281	chr8-22209189-22209281
chr8	22858988	22859080	chr8-22858988-22859080
chr8	23051894	23051986	chr8-23051894-23051986
chr8	23058484	23058576	chr8-23058484-23058576
chr8	23167544	23167636	chr8-23167544-23167636
chr8	23348906	23348998	chr8-23348906-23348998
chr8	23541627	23541719	chr8-23541627-23541719
chr8	24403314	24403406	chr8-24403314-24403406
chr8	24431545	24431637	chr8-24431545-24431637
chr8	25010620	25010712	chr8-25010620-25010712
chr8	25592772	25592864	chr8-25592772-25592864
chr8	25654099	25654191	chr8-25654099-25654191
chr8	26144448	26144540	chr8-26144448-26144540
chr8	26485630	26485722	chr8-26485630-26485722
chr8	26500167	26500259	chr8-26500167-26500259
chr8	26781093	26781185	chr8-26781093-26781185
chr8	26816902	26816994	chr8-26816902-26816994
chr8	26850072	26850164	chr8-26850072-26850164
chr8	26862263	26862355	chr8-26862263-26862355
chr8	27413076	27413168	chr8-27413076-27413168
chr8	27474119	27474211	chr8-27474119-27474211
chr8	27648544	27648636	chr8-27648544-27648636
chr8	31040445	31040537	chr8-31040445-31040537

chr8	31226395	31226487	chr8-31226395-31226487
chr8	31373446	31373538	chr8-31373446-31373538
chr8	32556245	32556337	chr8-32556245-32556337
chr8	32885921	32886013	chr8-32885921-32886013
chr8	33978698	33978790	chr8-33978698-33978790
chr8	33993150	33993242	chr8-33993150-33993242
chr8	34137133	34137225	chr8-34137133-34137225
chr8	34763954	34764046	chr8-34763954-34764046
chr8	34809045	34809137	chr8-34809045-34809137
chr8	34954244	34954336	chr8-34954244-34954336
chr8	35589242	35589334	chr8-35589242-35589334
chr8	35591799	35591891	chr8-35591799-35591891
chr8	35630432	35630524	chr8-35630432-35630524
chr8	35645303	35645395	chr8-35645303-35645395
chr8	36155625	36155717	chr8-36155625-36155717
chr8	36340455	36340547	chr8-36340455-36340547
chr8	36569323	36569415	chr8-36569323-36569415
chr8	36676084	36676176	chr8-36676084-36676176
chr8	36727579	36727671	chr8-36727579-36727671
chr8	36770902	36770994	chr8-36770902-36770994
chr8	37717566	37717658	chr8-37717566-37717658
chr8	38478650	38478742	chr8-38478650-38478742
chr8	38691109	38691201	chr8-38691109-38691201
chr8	38870665	38870757	chr8-38870665-38870757
chr8	39056138	39056230	chr8-39056138-39056230
chr8	39155374	39155466	chr8-39155374-39155466
chr8	39405369	39405461	chr8-39405369-39405461
chr8	40334431	40334523	chr8-40334431-40334523
chr8	40486952	40487044	chr8-40486952-40487044
chr8	40511911	40512003	chr8-40511911-40512003
chr8	40634988	40635080	chr8-40634988-40635080
chr8	41374675	41374767	chr8-41374675-41374767
chr8	42542399	42542491	chr8-42542399-42542491
chr8	44377849	44377941	chr8-44377849-44377941
chr8	44776746	44776838	chr8-44776746-44776838
chr8	45505868	45505960	chr8-45505868-45505960
chr8	45607152	45607244	chr8-45607152-45607244
chr8	45610971	45611063	chr8-45610971-45611063
chr8	45611500	45611592	chr8-45611500-45611592
chr8	45637901	45637993	chr8-45637901-45637993
chr8	45830603	45830695	chr8-45830603-45830695
chr8	46162071	46162163	chr8-46162071-46162163

chr8	46210388	46210480	chr8-46210388-46210480
chr8	47927021	47927113	chr8-47927021-47927113
chr8	47932562	47932654	chr8-47932562-47932654
chr8	49717415	49717507	chr8-49717415-49717507
chr8	54548438	54548530	chr8-54548438-54548530
chr8	54600713	54600805	chr8-54600713-54600805
chr8	55346686	55346778	chr8-55346686-55346778
chr8	55347273	55347365	chr8-55347273-55347365
chr8	55347587	55347679	chr8-55347587-55347679
chr8	55360405	55360497	chr8-55360405-55360497
chr8	55363445	55363537	chr8-55363445-55363537
chr8	57576382	57576474	chr8-57576382-57576474
chr8	57652763	57652855	chr8-57652763-57652855
chr8	57706842	57706934	chr8-57706842-57706934
chr8	59944735	59944827	chr8-59944735-59944827
chr8	60859655	60859747	chr8-60859655-60859747
chr8	60954807	60954899	chr8-60954807-60954899
chr8	61247770	61247862	chr8-61247770-61247862
chr8	61248568	61248660	chr8-61248568-61248660
chr8	61276034	61276126	chr8-61276034-61276126
chr8	61341767	61341859	chr8-61341767-61341859
chr8	61451406	61451498	chr8-61451406-61451498
chr8	61475375	61475467	chr8-61475375-61475467
chr8	61574660	61574752	chr8-61574660-61574752
chr8	61629134	61629226	chr8-61629134-61629226
chr8	61773759	61773851	chr8-61773759-61773851
chr8	61832208	61832300	chr8-61832208-61832300
chr8	63550910	63551002	chr8-63550910-63551002
chr8	64586547	64586639	chr8-64586547-64586639
chr8	64867509	64867601	chr8-64867509-64867601
chr8	65062007	65062099	chr8-65062007-65062099
chr8	65369985	65370077	chr8-65369985-65370077
chr8	65587057	65587149	chr8-65587057-65587149
chr8	66312595	66312687	chr8-66312595-66312687
chr8	67649555	67649647	chr8-67649555-67649647
chr8	67802867	67802959	chr8-67802867-67802959
chr8	68105604	68105696	chr8-68105604-68105696
chr8	68260536	68260628	chr8-68260536-68260628
chr8	68478326	68478418	chr8-68478326-68478418
chr8	69544629	69544721	chr8-69544629-69544721
chr8	69547649	69547741	chr8-69547649-69547741
chr8	69849130	69849222	chr8-69849130-69849222

chr8	69894008	69894100	chr8-69894008-69894100
chr8	70405718	70405810	chr8-70405718-70405810
chr8	70422086	70422178	chr8-70422086-70422178
chr8	70502255	70502347	chr8-70502255-70502347
chr8	70594073	70594165	chr8-70594073-70594165
chr8	70673694	70673786	chr8-70673694-70673786
chr8	70701587	70701679	chr8-70701587-70701679
chr8	70777260	70777352	chr8-70777260-70777352
chr8	70802225	70802317	chr8-70802225-70802317
chr8	71142514	71142606	chr8-71142514-71142606
chr8	71148721	71148813	chr8-71148721-71148813
chr8	71367225	71367317	chr8-71367225-71367317
chr8	71591763	71591855	chr8-71591763-71591855
chr8	71703114	71703206	chr8-71703114-71703206
chr8	71814851	71814943	chr8-71814851-71814943
chr8	71857583	71857675	chr8-71857583-71857675
chr8	72239597	72239689	chr8-72239597-72239689
chr8	72548399	72548491	chr8-72548399-72548491
chr8	73618957	73619049	chr8-73618957-73619049
chr8	73619159	73619251	chr8-73619159-73619251
chr8	75087692	75087784	chr8-75087692-75087784
chr8	76264879	76264971	chr8-76264879-76264971
chr8	76448147	76448239	chr8-76448147-76448239
chr8	77093341	77093433	chr8-77093341-77093433
chr8	77231322	77231414	chr8-77231322-77231414
chr8	77237015	77237107	chr8-77237015-77237107
chr8	77470251	77470343	chr8-77470251-77470343
chr8	77556669	77556761	chr8-77556669-77556761
chr8	78796954	78797046	chr8-78796954-78797046
chr8	79067900	79067992	chr8-79067900-79067992
chr8	79356164	79356256	chr8-79356164-79356256
chr8	79417234	79417326	chr8-79417234-79417326
chr8	79550286	79550378	chr8-79550286-79550378
chr8	80870685	80870777	chr8-80870685-80870777
chr8	82417166	82417258	chr8-82417166-82417258
chr8	83594562	83594654	chr8-83594562-83594654
chr8	83716737	83716829	chr8-83716737-83716829
chr8	83757202	83757294	chr8-83757202-83757294
chr8	83803419	83803511	chr8-83803419-83803511
chr8	83899934	83900026	chr8-83899934-83900026
chr8	83966856	83966948	chr8-83966856-83966948
chr8	84157661	84157753	chr8-84157661-84157753

chr8	84210175	84210267	chr8-84210175-84210267
chr8	84931256	84931348	chr8-84931256-84931348
chr8	84985747	84985839	chr8-84985747-84985839
chr8	85033113	85033205	chr8-85033113-85033205
chr8	85037102	85037194	chr8-85037102-85037194
chr8	87730140	87730232	chr8-87730140-87730232
chr8	87837535	87837627	chr8-87837535-87837627
chr8	89782049	89782141	chr8-89782049-89782141
chr8	90310514	90310606	chr8-90310514-90310606
chr8	90445820	90445912	chr8-90445820-90445912
chr8	90647276	90647368	chr8-90647276-90647368
chr8	91263208	91263300	chr8-91263208-91263300
chr8	91312929	91313021	chr8-91312929-91313021
chr8	91529856	91529948	chr8-91529856-91529948
chr8	92299906	92299998	chr8-92299906-92299998
chr8	92349388	92349480	chr8-92349388-92349480
chr8	92763579	92763671	chr8-92763579-92763671
chr8	93868279	93868371	chr8-93868279-93868371
chr8	94180734	94180826	chr8-94180734-94180826
chr8	94214442	94214534	chr8-94214442-94214534
chr8	94375560	94375652	chr8-94375560-94375652
chr8	94386397	94386489	chr8-94386397-94386489
chr8	94528533	94528625	chr8-94528533-94528625
chr8	94568877	94568969	chr8-94568877-94568969
chr8	94857495	94857587	chr8-94857495-94857587
chr8	95146178	95146270	chr8-95146178-95146270
chr8	95358919	95359011	chr8-95358919-95359011
chr8	96005990	96006082	chr8-96005990-96006082
chr8	96550779	96550871	chr8-96550779-96550871
chr8	96589162	96589254	chr8-96589162-96589254
chr8	96836487	96836579	chr8-96836487-96836579
chr8	98115965	98116057	chr8-98115965-98116057
chr8	101753243	101753335	chr8-101753243-101753335
chr8	104591184	104591276	chr8-104591184-104591276
chr8	104933203	104933295	chr8-104933203-104933295
chr8	105171178	105171270	chr8-105171178-105171270
chr8	105289102	105289194	chr8-105289102-105289194
chr8	105636454	105636546	chr8-105636454-105636546
chr8	105993719	105993811	chr8-105993719-105993811
chr8	106007237	106007329	chr8-106007237-106007329
chr8	106138167	106138259	chr8-106138167-106138259
chr8	106150162	106150254	chr8-106150162-106150254

chr8	106198742	106198834	chr8-106198742-106198834
chr8	106378529	106378621	chr8-106378529-106378621
chr8	106577176	106577268	chr8-106577176-106577268
chr8	106588853	106588945	chr8-106588853-106588945
chr8	106608671	106608763	chr8-106608671-106608763
chr8	106616053	106616145	chr8-106616053-106616145
chr8	106641042	106641134	chr8-106641042-106641134
chr8	107114952	107115044	chr8-107114952-107115044
chr8	107255081	107255173	chr8-107255081-107255173
chr8	107437262	107437354	chr8-107437262-107437354
chr8	107643467	107643559	chr8-107643467-107643559
chr8	107798065	107798157	chr8-107798065-107798157
chr8	108059279	108059371	chr8-108059279-108059371
chr8	109608874	109608966	chr8-109608874-109608966
chr8	109707362	109707454	chr8-109707362-109707454
chr8	110039772	110039864	chr8-110039772-110039864
chr8	110040664	110040756	chr8-110040664-110040756
chr8	110498784	110498876	chr8-110498784-110498876
chr8	110658797	110658889	chr8-110658797-110658889
chr8	110965725	110965817	chr8-110965725-110965817
chr8	110997838	110997930	chr8-110997838-110997930
chr8	111031584	111031676	chr8-111031584-111031676
chr8	111059225	111059317	chr8-111059225-111059317
chr8	111231866	111231958	chr8-111231866-111231958
chr8	111258893	111258985	chr8-111258893-111258985
chr8	111520757	111520849	chr8-111520757-111520849
chr8	111637832	111637924	chr8-111637832-111637924
chr8	111746922	111747014	chr8-111746922-111747014
chr8	111865595	111865687	chr8-111865595-111865687
chr8	112850537	112850629	chr8-112850537-112850629
chr8	112854370	112854462	chr8-112854370-112854462
chr8	113384132	113384224	chr8-113384132-113384224
chr8	113635833	113635925	chr8-113635833-113635925
chr8	114417436	114417528	chr8-114417436-114417528
chr8	115217319	115217411	chr8-115217319-115217411
chr8	115705600	115705692	chr8-115705600-115705692
chr8	116004559	116004651	chr8-116004559-116004651
chr8	116426513	116426605	chr8-116426513-116426605
chr8	116827320	116827412	chr8-116827320-116827412
chr8	117085662	117085754	chr8-117085662-117085754
chr8	117256642	117256734	chr8-117256642-117256734
chr8	117470469	117470561	chr8-117470469-117470561

chr8	117801926	117802018	chr8-117801926-117802018
chr8	118833836	118833928	chr8-118833836-118833928
chr8	118903620	118903712	chr8-118903620-118903712
chr8	119206301	119206393	chr8-119206301-119206393
chr8	119432491	119432583	chr8-119432491-119432583
chr8	119500940	119501032	chr8-119500940-119501032
chr8	119582444	119582536	chr8-119582444-119582536
chr8	119670580	119670672	chr8-119670580-119670672
chr8	119709297	119709389	chr8-119709297-119709389
chr8	120109618	120109710	chr8-120109618-120109710
chr8	120267459	120267551	chr8-120267459-120267551
chr8	120370131	120370223	chr8-120370131-120370223
chr8	120488074	120488166	chr8-120488074-120488166
chr8	120537425	120537517	chr8-120537425-120537517
chr8	120563887	120563979	chr8-120563887-120563979
chr8	120686342	120686434	chr8-120686342-120686434
chr8	120846490	120846582	chr8-120846490-120846582
chr8	121036561	121036653	chr8-121036561-121036653
chr8	121051521	121051613	chr8-121051521-121051613
chr8	121164241	121164333	chr8-121164241-121164333
chr8	121225951	121226043	chr8-121225951-121226043
chr8	121316194	121316286	chr8-121316194-121316286
chr8	121603009	121603101	chr8-121603009-121603101
chr8	121688187	121688279	chr8-121688187-121688279
chr8	121696050	121696142	chr8-121696050-121696142
chr8	121753003	121753095	chr8-121753003-121753095
chr8	122114584	122114676	chr8-122114584-122114676
chr8	122236580	122236672	chr8-122236580-122236672
chr8	122284503	122284595	chr8-122284503-122284595
chr8	122284762	122284854	chr8-122284762-122284854
chr8	122444546	122444638	chr8-122444546-122444638
chr8	122824385	122824477	chr8-122824385-122824477
chr8	122989174	122989266	chr8-122989174-122989266
chr8	123278609	123278701	chr8-123278609-123278701
chr8	123617477	123617569	chr8-123617477-123617569
chr8	123717504	123717596	chr8-123717504-123717596
chr8	124078789	124078881	chr8-124078789-124078881
chr8	124231805	124231897	chr8-124231805-124231897
chr8	124722287	124722379	chr8-124722287-124722379
chr8	124832032	124832124	chr8-124832032-124832124
chr8	124922047	124922139	chr8-124922047-124922139
chr8	124954920	124955012	chr8-124954920-124955012

chr8	125253823	125253915	chr8-125253823-125253915
chr8	125395399	125395491	chr8-125395399-125395491
chr8	125637285	125637377	chr8-125637285-125637377
chr8	125746260	125746352	chr8-125746260-125746352
chr8	125779065	125779157	chr8-125779065-125779157
chr8	125964801	125964893	chr8-125964801-125964893
chr8	126024042	126024134	chr8-126024042-126024134
chr8	126478837	126478929	chr8-126478837-126478929
chr8	126725995	126726087	chr8-126725995-126726087
chr8	126742391	126742483	chr8-126742391-126742483
chr8	126847879	126847971	chr8-126847879-126847971
chr8	126867953	126868045	chr8-126867953-126868045
chr8	127144691	127144783	chr8-127144691-127144783
chr8	127962984	127963076	chr8-127962984-127963076
chr8	128218867	128218959	chr8-128218867-128218959
chr8	128219642	128219734	chr8-128219642-128219734
chr8	128399400	128399492	chr8-128399400-128399492
chr9	3258813	3258905	chr9-3258813-3258905
chr9	4260343	4260435	chr9-4260343-4260435
chr9	8104418	8104510	chr9-8104418-8104510
chr9	13430472	13430564	chr9-13430472-13430564
chr9	13442482	13442574	chr9-13442482-13442574
chr9	13461117	13461209	chr9-13461117-13461209
chr9	13559418	13559510	chr9-13559418-13559510
chr9	14452869	14452961	chr9-14452869-14452961
chr9	14530266	14530358	chr9-14530266-14530358
chr9	14676484	14676576	chr9-14676484-14676576
chr9	14891961	14892053	chr9-14891961-14892053
chr9	16996965	16997057	chr9-16996965-16997057
chr9	17987767	17987859	chr9-17987767-17987859
chr9	19575434	19575526	chr9-19575434-19575526
chr9	20492678	20492770	chr9-20492678-20492770
chr9	20975516	20975608	chr9-20975516-20975608
chr9	21029370	21029462	chr9-21029370-21029462
chr9	21199555	21199647	chr9-21199555-21199647
chr9	21265142	21265234	chr9-21265142-21265234
chr9	21329230	21329322	chr9-21329230-21329322
chr9	21340564	21340656	chr9-21340564-21340656
chr9	21761225	21761317	chr9-21761225-21761317
chr9	21924208	21924300	chr9-21924208-21924300
chr9	22047043	22047135	chr9-22047043-22047135
chr9	22097262	22097354	chr9-22097262-22097354

chr9	22209391	22209483	chr9-22209391-22209483
chr9	24418938	24419030	chr9-24418938-24419030
chr9	25173265	25173357	chr9-25173265-25173357
chr9	27163085	27163177	chr9-27163085-27163177
chr9	29423105	29423197	chr9-29423105-29423197
chr9	30073815	30073907	chr9-30073815-30073907
chr9	30272439	30272531	chr9-30272439-30272531
chr9	30794868	30794960	chr9-30794868-30794960
chr9	31176451	31176543	chr9-31176451-31176543
chr9	31254761	31254853	chr9-31254761-31254853
chr9	32562395	32562487	chr9-32562395-32562487
chr9	32629562	32629654	chr9-32629562-32629654
chr9	32706552	32706644	chr9-32706552-32706644
chr9	33858797	33858889	chr9-33858797-33858889
chr9	34478631	34478723	chr9-34478631-34478723
chr9	35249632	35249724	chr9-35249632-35249724
chr9	37479546	37479638	chr9-37479546-37479638
chr9	37806799	37806891	chr9-37806799-37806891
chr9	38001678	38001770	chr9-38001678-38001770
chr9	40681027	40681119	chr9-40681027-40681119
chr9	40776591	40776683	chr9-40776591-40776683
chr9	40795774	40795866	chr9-40795774-40795866
chr9	41035819	41035911	chr9-41035819-41035911
chr9	41170120	41170212	chr9-41170120-41170212
chr9	41390639	41390731	chr9-41390639-41390731
chr9	41481677	41481769	chr9-41481677-41481769
chr9	41488331	41488423	chr9-41488331-41488423
chr9	43725633	43725725	chr9-43725633-43725725
chr9	43750187	43750279	chr9-43750187-43750279
chr9	44881379	44881471	chr9-44881379-44881471
chr9	45125539	45125631	chr9-45125539-45125631
chr9	45240686	45240778	chr9-45240686-45240778
chr9	45300951	45301043	chr9-45300951-45301043
chr9	45384204	45384296	chr9-45384204-45384296
chr9	45580218	45580310	chr9-45580218-45580310
chr9	46018052	46018144	chr9-46018052-46018144
chr9	46081362	46081454	chr9-46081362-46081454
chr9	46694442	46694534	chr9-46694442-46694534
chr9	47545348	47545440	chr9-47545348-47545440
chr9	48028019	48028111	chr9-48028019-48028111
chr9	50080417	50080509	chr9-50080417-50080509
chr9	50444352	50444444	chr9-50444352-50444444

chr9	50515653	50515745	chr9-50515653-50515745
chr9	51175039	51175131	chr9-51175039-51175131
chr9	51255712	51255804	chr9-51255712-51255804
chr9	51911323	51911415	chr9-51911323-51911415
chr9	51964298	51964390	chr9-51964298-51964390
chr9	53279143	53279235	chr9-53279143-53279235
chr9	55921841	55921933	chr9-55921841-55921933
chr9	56318697	56318789	chr9-56318697-56318789
chr9	56566423	56566515	chr9-56566423-56566515
chr9	57071287	57071379	chr9-57071287-57071379
chr9	57299960	57300052	chr9-57299960-57300052
chr9	57328152	57328244	chr9-57328152-57328244
chr9	57354595	57354687	chr9-57354595-57354687
chr9	57402346	57402438	chr9-57402346-57402438
chr9	57402829	57402921	chr9-57402829-57402921
chr9	57420848	57420940	chr9-57420848-57420940
chr9	57505933	57506025	chr9-57505933-57506025
chr9	57600754	57600846	chr9-57600754-57600846
chr9	58213966	58214058	chr9-58213966-58214058
chr9	58214712	58214804	chr9-58214712-58214804
chr9	58247498	58247590	chr9-58247498-58247590
chr9	58453881	58453973	chr9-58453881-58453973
chr9	58554096	58554188	chr9-58554096-58554188
chr9	59486567	59486659	chr9-59486567-59486659
chr9	59578024	59578116	chr9-59578024-59578116
chr9	60024649	60024741	chr9-60024649-60024741
chr9	60545507	60545599	chr9-60545507-60545599
chr9	60794128	60794220	chr9-60794128-60794220
chr9	61010584	61010676	chr9-61010584-61010676
chr9	61224744	61224836	chr9-61224744-61224836
chr9	61946104	61946196	chr9-61946104-61946196
chr9	61947583	61947675	chr9-61947583-61947675
chr9	62016840	62016932	chr9-62016840-62016932
chr9	62608556	62608648	chr9-62608556-62608648
chr9	62810416	62810508	chr9-62810416-62810508
chr9	62818390	62818482	chr9-62818390-62818482
chr9	62827722	62827814	chr9-62827722-62827814
chr9	62990300	62990392	chr9-62990300-62990392
chr9	63657316	63657408	chr9-63657316-63657408
chr9	63674823	63674915	chr9-63674823-63674915
chr9	63678204	63678296	chr9-63678204-63678296
chr9	63745937	63746029	chr9-63745937-63746029

chr9	63793875	63793967	chr9-63793875-63793967
chr9	63941740	63941832	chr9-63941740-63941832
chr9	63942128	63942220	chr9-63942128-63942220
chr9	64050724	64050816	chr9-64050724-64050816
chr9	64062666	64062758	chr9-64062666-64062758
chr9	64271709	64271801	chr9-64271709-64271801
chr9	65021645	65021737	chr9-65021645-65021737
chr9	65199206	65199298	chr9-65199206-65199298
chr9	65215913	65216005	chr9-65215913-65216005
chr9	65550590	65550682	chr9-65550590-65550682
chr9	65827617	65827709	chr9-65827617-65827709
chr9	65827826	65827918	chr9-65827826-65827918
chr9	66124748	66124840	chr9-66124748-66124840
chr9	66125778	66125870	chr9-66125778-66125870
chr9	66213232	66213324	chr9-66213232-66213324
chr9	66673886	66673978	chr9-66673886-66673978
chr9	67025055	67025147	chr9-67025055-67025147
chr9	68270324	68270416	chr9-68270324-68270416
chr9	68589007	68589099	chr9-68589007-68589099
chr9	68820725	68820817	chr9-68820725-68820817
chr9	68913883	68913975	chr9-68913883-68913975
chr9	69180861	69180953	chr9-69180861-69180953
chr9	69397873	69397965	chr9-69397873-69397965
chr9	70046396	70046488	chr9-70046396-70046488
chr9	70625797	70625889	chr9-70625797-70625889
chr9	70688461	70688553	chr9-70688461-70688553
chr9	70806137	70806229	chr9-70806137-70806229
chr9	70826377	70826469	chr9-70826377-70826469
chr9	70833619	70833711	chr9-70833619-70833711
chr9	70926954	70927046	chr9-70926954-70927046
chr9	71693874	71693966	chr9-71693874-71693966
chr9	72060216	72060308	chr9-72060216-72060308
chr9	73208861	73208953	chr9-73208861-73208953
chr9	73965783	73965875	chr9-73965783-73965875
chr9	74874609	74874701	chr9-74874609-74874701
chr9	75347614	75347706	chr9-75347614-75347706
chr9	75410221	75410313	chr9-75410221-75410313
chr9	75437765	75437857	chr9-75437765-75437857
chr9	75547276	75547368	chr9-75547276-75547368
chr9	75827313	75827405	chr9-75827313-75827405
chr9	77530155	77530247	chr9-77530155-77530247
chr9	77543925	77544017	chr9-77543925-77544017

chr9	77937038	77937130	chr9-77937038-77937130
chr9	78480940	78481032	chr9-78480940-78481032
chr9	78520485	78520577	chr9-78520485-78520577
chr9	80019347	80019439	chr9-80019347-80019439
chr9	80697561	80697653	chr9-80697561-80697653
chr9	80780003	80780095	chr9-80780003-80780095
chr9	83576495	83576587	chr9-83576495-83576587
chr9	83806081	83806173	chr9-83806081-83806173
chr9	83866480	83866572	chr9-83866480-83866572
chr9	88553189	88553281	chr9-88553189-88553281
chr9	89902963	89903055	chr9-89902963-89903055
chr9	89915955	89916047	chr9-89915955-89916047
chr9	90035882	90035974	chr9-90035882-90035974
chr9	94136522	94136614	chr9-94136522-94136614
chr9	94287763	94287855	chr9-94287763-94287855
chr9	95511877	95511969	chr9-95511877-95511969
chr9	95671347	95671439	chr9-95671347-95671439
chr9	96381495	96381587	chr9-96381495-96381587
chr9	97324513	97324605	chr9-97324513-97324605
chr9	99702380	99702472	chr9-99702380-99702472
chr9	100458548	100458640	chr9-100458548-100458640
chr9	100643075	100643167	chr9-100643075-100643167
chr9	102563131	102563223	chr9-102563131-102563223
chr9	102726433	102726525	chr9-102726433-102726525
chr9	102761317	102761409	chr9-102761317-102761409
chr9	102772202	102772294	chr9-102772202-102772294
chr9	103029196	103029288	chr9-103029196-103029288
chr9	103078266	103078358	chr9-103078266-103078358
chr9	103779794	103779886	chr9-103779794-103779886
chr9	104579768	104579860	chr9-104579768-104579860
chr9	105116056	105116148	chr9-105116056-105116148
chr9	105190771	105190863	chr9-105190771-105190863
chr9	105494880	105494972	chr9-105494880-105494972
chr9	105512923	105513015	chr9-105512923-105513015
chr9	105764290	105764382	chr9-105764290-105764382
chr9	106887098	106887190	chr9-106887098-106887190
chr9	107278713	107278805	chr9-107278713-107278805
chr9	107307544	107307636	chr9-107307544-107307636
chr9	107704808	107704900	chr9-107704808-107704900
chr9	108252877	108252969	chr9-108252877-108252969
chr9	108264708	108264800	chr9-108264708-108264800
chr9	108377145	108377237	chr9-108377145-108377237

chr9	108783541	108783633	chr9-108783541-108783633
chr9	108832492	108832584	chr9-108832492-108832584
chr9	109010570	109010662	chr9-109010570-109010662
chr9	110264275	110264367	chr9-110264275-110264367
chr9	110567022	110567114	chr9-110567022-110567114
chr9	110661331	110661423	chr9-110661331-110661423
chr9	110685656	110685748	chr9-110685656-110685748
chr9	111859852	111859944	chr9-111859852-111859944
chr9	112836228	112836320	chr9-112836228-112836320
chr9	114579887	114579979	chr9-114579887-114579979
chr9	114808927	114809019	chr9-114808927-114809019
chr9	114830081	114830173	chr9-114830081-114830173
chr9	116254889	116254981	chr9-116254889-116254981
chr9	116383493	116383585	chr9-116383493-116383585
chr9	117134147	117134239	chr9-117134147-117134239
chr9	117750326	117750418	chr9-117750326-117750418
chr9	117882585	117882677	chr9-117882585-117882677
chr9	119002456	119002548	chr9-119002456-119002548
chr9	120521346	120521438	chr9-120521346-120521438
chr9	120596968	120597060	chr9-120596968-120597060
chr9	120826538	120826630	chr9-120826538-120826630
chr9	120906676	120906768	chr9-120906676-120906768
chr9	122648051	122648143	chr9-122648051-122648143
chr9	122950753	122950845	chr9-122950753-122950845
chr9	123366886	123366978	chr9-123366886-123366978
chr9	123461977	123462069	chr9-123461977-123462069
chr9	123928864	123928956	chr9-123928864-123928956
chr9	124138233	124138325	chr9-124138233-124138325
chrX	4899263	4899355	chrX-4899263-4899355
chrX	5459630	5459722	chrX-5459630-5459722
chrX	5495469	5495561	chrX-5495469-5495561
chrX	5794070	5794162	chrX-5794070-5794162
chrX	7279070	7279162	chrX-7279070-7279162
chrX	7318851	7318943	chrX-7318851-7318943
chrX	7421198	7421290	chrX-7421198-7421290
chrX	8069300	8069392	chrX-8069300-8069392
chrX	9753334	9753426	chrX-9753334-9753426
chrX	9849217	9849309	chrX-9849217-9849309
chrX	10630846	10630938	chrX-10630846-10630938
chrX	10694593	10694685	chrX-10694593-10694685
chrX	10717409	10717501	chrX-10717409-10717501
chrX	11086804	11086896	chrX-11086804-11086896

chrX	11664633	11664725	chrX-11664633-11664725
chrX	12007300	12007392	chrX-12007300-12007392
chrX	13395876	13395968	chrX-13395876-13395968
chrX	13462606	13462698	chrX-13462606-13462698
chrX	13977988	13978080	chrX-13977988-13978080
chrX	19067975	19068067	chrX-19067975-19068067
chrX	19168617	19168709	chrX-19168617-19168709
chrX	19236954	19237046	chrX-19236954-19237046
chrX	23513112	23513204	chrX-23513112-23513204
chrX	36598188	36598280	chrX-36598188-36598280
chrX	36930186	36930278	chrX-36930186-36930278
chrX	37110286	37110378	chrX-37110286-37110378
chrX	38727260	38727352	chrX-38727260-38727352
chrX	39146620	39146712	chrX-39146620-39146712
chrX	40414877	40414969	chrX-40414877-40414969
chrX	42932506	42932598	chrX-42932506-42932598
chrX	48285496	48285588	chrX-48285496-48285588
chrX	48439254	48439346	chrX-48439254-48439346
chrX	48466324	48466416	chrX-48466324-48466416
chrX	51807802	51807894	chrX-51807802-51807894
chrX	52771056	52771148	chrX-52771056-52771148
chrX	53162825	53162917	chrX-53162825-53162917
chrX	53955844	53955936	chrX-53955844-53955936
chrX	58004930	58005022	chrX-58004930-58005022
chrX	60404110	60404202	chrX-60404110-60404202
chrX	60929628	60929720	chrX-60929628-60929720
chrX	61659790	61659882	chrX-61659790-61659882
chrX	68495094	68495186	chrX-68495094-68495186
chrX	69269360	69269452	chrX-69269360-69269452
chrX	69331492	69331584	chrX-69331492-69331584
chrX	69619641	69619733	chrX-69619641-69619733
chrX	73476743	73476835	chrX-73476743-73476835
chrX	73659207	73659299	chrX-73659207-73659299
chrX	75863529	75863621	chrX-75863529-75863621
chrX	83111263	83111355	chrX-83111263-83111355
chrX	84018921	84019013	chrX-84018921-84019013
chrX	84088081	84088173	chrX-84088081-84088173
chrX	86053501	86053593	chrX-86053501-86053593
chrX	87238322	87238414	chrX-87238322-87238414
chrX	87899378	87899470	chrX-87899378-87899470
chrX	90200300	90200392	chrX-90200300-90200392
chrX	90215608	90215700	chrX-90215608-90215700

chrX	91698924	91699016	chrX-91698924-91699016
chrX	92350290	92350382	chrX-92350290-92350382
chrX	94118485	94118577	chrX-94118485-94118577
chrX	98149296	98149388	chrX-98149296-98149388
chrX	99205531	99205623	chrX-99205531-99205623
chrX	99378035	99378127	chrX-99378035-99378127
chrX	101739875	101739967	chrX-101739875-101739967
chrX	102402334	102402426	chrX-102402334-102402426
chrX	103215954	103216046	chrX-103215954-103216046
chrX	103254999	103255091	chrX-103254999-103255091
chrX	103264949	103265041	chrX-103264949-103265041
chrX	104014892	104014984	chrX-104014892-104014984
chrX	104015267	104015359	chrX-104015267-104015359
chrX	104737148	104737240	chrX-104737148-104737240
chrX	105684513	105684605	chrX-105684513-105684605
chrX	105800303	105800395	chrX-105800303-105800395
chrX	106006538	106006630	chrX-106006538-106006630
chrX	106040876	106040968	chrX-106040876-106040968
chrX	106044121	106044213	chrX-106044121-106044213
chrX	108732008	108732100	chrX-108732008-108732100
chrX	117576813	117576905	chrX-117576813-117576905
chrX	122831361	122831453	chrX-122831361-122831453
chrX	128240969	128241061	chrX-128240969-128241061
chrX	128636586	128636678	chrX-128636586-128636678
chrX	129109420	129109512	chrX-129109420-129109512
chrX	129756994	129757086	chrX-129756994-129757086
chrX	131019306	131019398	chrX-131019306-131019398
chrX	131381593	131381685	chrX-131381593-131381685
chrX	136705829	136705921	chrX-136705829-136705921
chrX	138723893	138723985	chrX-138723893-138723985
chrX	139862899	139862991	chrX-139862899-139862991
chrX	142555817	142555909	chrX-142555817-142555909
chrX	142567997	142568089	chrX-142567997-142568089
chrX	147295417	147295509	chrX-147295417-147295509
chrX	154407291	154407383	chrX-154407291-154407383
chrX	155352372	155352464	chrX-155352372-155352464
chrX	160745557	160745649	chrX-160745557-160745649
chrX	160991267	160991359	chrX-160991267-160991359
chrX	161241455	161241547	chrX-161241455-161241547
chrX	163535956	163536048	chrX-163535956-163536048
chrX	163591027	163591119	chrX-163591027-163591119
chrX	164124713	164124805	chrX-164124713-164124805

chrX	165654931	165655023	chrX-165654931-165655023
chrX	166114879	166114971	chrX-166114879-166114971
chrX	166563988	166564080	chrX-166563988-166564080
chrX	167329716	167329808	chrX-167329716-167329808
chrX	167332705	167332797	chrX-167332705-167332797
chrX	167921024	167921116	chrX-167921024-167921116
chrX	167951366	167951458	chrX-167951366-167951458
chrX	168505433	168505525	chrX-168505433-168505525
chrX	169911314	169911406	chrX-169911314-169911406
chrX	169986027	169986119	chrX-169986027-169986119
chrX	169987438	169987530	chrX-169987438-169987530
chrX	169993999	169994091	chrX-169993999-169994091
chrX	170005323	170005415	chrX-170005323-170005415
chrX	170673740	170673832	chrX-170673740-170673832
chrX	170735869	170735961	chrX-170735869-170735961
chrX	170736595	170736687	chrX-170736595-170736687
chrX	170736856	170736948	chrX-170736856-170736948
chrX	170810028	170810120	chrX-170810028-170810120
chrX	170810721	170810813	chrX-170810721-170810813
chrY	4245342	4245434	chrY-4245342-4245434
chrY	6737715	6737807	chrY-6737715-6737807
chrY	90731035	90731127	chrY-90731035-90731127
chrY	90761384	90761476	chrY-90761384-90761476
chrY	90788011	90788103	chrY-90788011-90788103
chrY	90788763	90788855	chrY-90788763-90788855

Supplementary Table 3. ChIP-seq peaks and Motif analysis**.**
Merged H3K4me3 Peaks: List of merged peaks in SPC-Cre KP and KPG cells from H3K4me3 IP compared to input.

Chr	Start	End	Id
chr1	3670497	3672686	chr1-3670497-3672686
chr1	4491577	4493871	chr1-4491577-4493871
chr1	4497097	4497619	chr1-4497097-4497619
chr1	4571221	4572328	chr1-4571221-4572328
chr1	4670805	4671111	chr1-4670805-4671111
chr1	4705690	4705891	chr1-4705690-4705891
chr1	4784262	4786674	chr1-4784262-4786674
chr1	4807268	4809147	chr1-4807268-4809147
chr1	4856730	4859460	chr1-4856730-4859460
chr1	5018018	5021055	chr1-5018018-5021055
chr1	5082700	5084108	chr1-5082700-5084108
chr1	5170379	5170789	chr1-5170379-5170789
chr1	5588301	5588741	chr1-5588301-5588741
chr1	5916443	5917529	chr1-5916443-5917529
chr1	6213070	6216278	chr1-6213070-6216278
chr1	6358818	6359678	chr1-6358818-6359678
chr1	6382547	6383508	chr1-6382547-6383508
chr1	6401435	6401636	chr1-6401435-6401636
chr1	6441064	6441673	chr1-6441064-6441673
chr1	7087897	7090839	chr1-7087897-7090839
chr1	7206008	7206989	chr1-7206008-7206989
chr1	7396948	7399030	chr1-7396948-7399030
chr1	7709257	7709458	chr1-7709257-7709458
chr1	8410883	8411084	chr1-8410883-8411084
chr1	9237195	9237448	chr1-9237195-9237448
chr1	9299273	9299474	chr1-9299273-9299474
chr1	9544642	9548963	chr1-9544642-9548963
chr1	9629948	9630760	chr1-9629948-9630760
chr1	9631844	9632045	chr1-9631844-9632045
chr1	9699348	9701445	chr1-9699348-9701445
chr1	9743718	9749059	chr1-9743718-9749059
chr1	9755031	9756475	chr1-9755031-9756475
chr1	9797435	9799043	chr1-9797435-9799043
chr1	9847808	9849216	chr1-9847808-9849216
chr1	9908569	9908770	chr1-9908569-9908770
chr1	9941511	9944889	chr1-9941511-9944889
chr1	9966903	9968649	chr1-9966903-9968649
chr1	10008857	10009338	chr1-10008857-10009338

chr1	10036376	10040881	chr1-10036376-10040881
chr1	10231061	10234446	chr1-10231061-10234446
chr1	10256591	10257807	chr1-10256591-10257807
chr1	10284963	10286491	chr1-10284963-10286491
chr1	10592576	10593360	chr1-10592576-10593360
chr1	10932736	10933345	chr1-10932736-10933345
chr1	10993299	10994694	chr1-10993299-10994694
chr1	11413599	11414774	chr1-11413599-11414774
chr1	12620094	12623370	chr1-12620094-12623370
chr1	12657488	12658025	chr1-12657488-12658025
chr1	12692731	12693478	chr1-12692731-12693478
chr1	12715625	12715826	chr1-12715625-12715826
chr1	12718012	12720047	chr1-12718012-12720047
chr1	12744431	12744917	chr1-12744431-12744917
chr1	12875401	12876007	chr1-12875401-12876007
chr1	12990409	12993083	chr1-12990409-12993083
chr1	13052151	13052654	chr1-13052151-13052654
chr1	13126294	13126716	chr1-13126294-13126716
chr1	13144001	13144307	chr1-13144001-13144307
chr1	13293222	13293899	chr1-13293222-13293899
chr1	13304929	13305930	chr1-13304929-13305930
chr1	13371303	13375045	chr1-13371303-13375045
chr1	13426014	13427113	chr1-13426014-13427113
chr1	13587455	13590479	chr1-13587455-13590479
chr1	13643506	13643707	chr1-13643506-13643707
chr1	13658486	13661632	chr1-13658486-13661632
chr1	14303474	14310721	chr1-14303474-14310721
chr1	14464900	14465101	chr1-14464900-14465101
chr1	14484342	14484543	chr1-14484342-14484543
chr1	14506429	14507298	chr1-14506429-14507298
chr1	14754968	14756288	chr1-14754968-14756288
chr1	14918461	14919130	chr1-14918461-14919130
chr1	15286285	15287523	chr1-15286285-15287523
chr1	15791096	15792146	chr1-15791096-15792146
chr1	15805012	15807464	chr1-15805012-15807464
chr1	15892231	15893192	chr1-15892231-15893192
chr1	16092808	16093564	chr1-16092808-16093564
chr1	16101237	16112339	chr1-16101237-16112339
chr1	16113437	16113742	chr1-16113437-16113742
chr1	16159173	16159445	chr1-16159173-16159445
chr1	16171631	16173020	chr1-16171631-16173020
chr1	16205830	16207114	chr1-16205830-16207114

chr1	16228045	16230016	chr1-16228045-16230016
chr1	16518264	16520340	chr1-16518264-16520340
chr1	16616513	16619911	chr1-16616513-16619911
chr1	16655291	16657906	chr1-16655291-16657906
chr1	16661039	16661658	chr1-16661039-16661658
chr1	16664893	16666954	chr1-16664893-16666954
chr1	16687708	16689725	chr1-16687708-16689725
chr1	17007961	17008162	chr1-17007961-17008162
chr1	17096312	17098353	chr1-17096312-17098353
chr1	17145335	17145847	chr1-17145335-17145847
chr1	17506652	17506853	chr1-17506652-17506853
chr1	17727271	17727572	chr1-17727271-17727572
chr1	18057971	18058896	chr1-18057971-18058896
chr1	19205760	19206319	chr1-19205760-19206319
chr1	19209686	19210904	chr1-19209686-19210904
chr1	19214093	19214461	chr1-19214093-19214461
chr1	19241172	19241785	chr1-19241172-19241785
chr1	20615316	20619379	chr1-20615316-20619379
chr1	20678175	20679455	chr1-20678175-20679455
chr1	20818633	20820780	chr1-20818633-20820780
chr1	20872447	20872698	chr1-20872447-20872698
chr1	20889909	20894101	chr1-20889909-20894101
chr1	20896343	20896781	chr1-20896343-20896781
chr1	20951120	20952092	chr1-20951120-20952092
chr1	21002461	21002928	chr1-21002461-21002928
chr1	21014712	21014913	chr1-21014712-21014913
chr1	21075981	21076401	chr1-21075981-21076401
chr1	21078039	21080042	chr1-21078039-21080042
chr1	21166041	21166917	chr1-21166041-21166917
chr1	21218380	21219593	chr1-21218380-21219593
chr1	21271349	21271962	chr1-21271349-21271962
chr1	21960410	21962492	chr1-21960410-21962492
chr1	22533091	22534203	chr1-22533091-22534203
chr1	22805332	22806605	chr1-22805332-22806605
chr1	23188232	23188737	chr1-23188232-23188737
chr1	23202345	23202994	chr1-23202345-23202994
chr1	23214601	23215020	chr1-23214601-23215020
chr1	23255663	23258229	chr1-23255663-23258229
chr1	23263885	23264086	chr1-23263885-23264086
chr1	23269596	23269797	chr1-23269596-23269797
chr1	23271080	23272567	chr1-23271080-23272567
chr1	23382225	23383804	chr1-23382225-23383804

chr1	23761656	23763612	chr1-23761656-23763612
chr1	23921118	23922816	chr1-23921118-23922816
chr1	23959264	23959567	chr1-23959264-23959567
chr1	24004603	24006022	chr1-24004603-24006022
chr1	24099005	24101284	chr1-24099005-24101284
chr1	24107250	24108585	chr1-24107250-24108585
chr1	24586418	24587567	chr1-24586418-24587567
chr1	24677730	24679844	chr1-24677730-24679844
chr1	25829790	25830675	chr1-25829790-25830675
chr1	30789419	30789872	chr1-30789419-30789872
chr1	30871844	30874942	chr1-30871844-30874942
chr1	30937847	30938317	chr1-30937847-30938317
chr1	30945090	30951548	chr1-30945090-30951548
chr1	31019255	31019906	chr1-31019255-31019906
chr1	31143581	31144084	chr1-31143581-31144084
chr1	31164645	31164896	chr1-31164645-31164896
chr1	32172284	32173291	chr1-32172284-32173291
chr1	33657592	33658308	chr1-33657592-33658308
chr1	33669154	33670934	chr1-33669154-33670934
chr1	33719580	33721357	chr1-33719580-33721357
chr1	33753899	33758322	chr1-33753899-33758322
chr1	33813027	33815112	chr1-33813027-33815112
chr1	33883453	33883911	chr1-33883453-33883911
chr1	33906892	33909155	chr1-33906892-33909155
chr1	33926631	33927706	chr1-33926631-33927706
chr1	34005415	34007872	chr1-34005415-34007872
chr1	34010821	34014269	chr1-34010821-34014269
chr1	34043501	34044935	chr1-34043501-34044935
chr1	34070339	34070689	chr1-34070339-34070689
chr1	34073628	34075064	chr1-34073628-34075064
chr1	34077269	34077470	chr1-34077269-34077470
chr1	34103311	34105675	chr1-34103311-34105675
chr1	34112521	34112722	chr1-34112521-34112722
chr1	34120099	34124764	chr1-34120099-34124764
chr1	34139389	34140673	chr1-34139389-34140673
chr1	34236034	34237499	chr1-34236034-34237499
chr1	34432380	34435733	chr1-34432380-34435733
chr1	34438418	34441281	chr1-34438418-34441281
chr1	34458999	34460706	chr1-34458999-34460706
chr1	34472430	34473390	chr1-34472430-34473390
chr1	34485509	34486094	chr1-34485509-34486094
chr1	34505225	34505639	chr1-34505225-34505639

chr1	34536387	34537805	chr1-34536387-34537805
chr1	34579247	34580061	chr1-34579247-34580061
chr1	34677681	34679373	chr1-34677681-34679373
chr1	34750245	34751196	chr1-34750245-34751196
chr1	34801353	34802625	chr1-34801353-34802625
chr1	34824358	34824614	chr1-34824358-34824614
chr1	34840600	34843877	chr1-34840600-34843877
chr1	34849665	34852960	chr1-34849665-34852960
chr1	35875154	35875355	chr1-35875154-35875355
chr1	35931231	35931543	chr1-35931231-35931543
chr1	35961809	35962655	chr1-35961809-35962655
chr1	36013535	36014366	chr1-36013535-36014366
chr1	36016703	36017578	chr1-36016703-36017578
chr1	36030625	36030929	chr1-36030625-36030929
chr1	36064642	36065697	chr1-36064642-36065697
chr1	36067731	36073634	chr1-36067731-36073634
chr1	36079641	36079897	chr1-36079641-36079897
chr1	36116326	36116683	chr1-36116326-36116683
chr1	36235818	36236069	chr1-36235818-36236069
chr1	36242978	36244914	chr1-36242978-36244914
chr1	36254527	36255550	chr1-36254527-36255550
chr1	36264008	36265812	chr1-36264008-36265812
chr1	36272000	36275188	chr1-36272000-36275188
chr1	36302920	36303676	chr1-36302920-36303676
chr1	36307157	36311544	chr1-36307157-36311544
chr1	36324499	36326652	chr1-36324499-36326652
chr1	36366855	36369684	chr1-36366855-36369684
chr1	36432719	36433125	chr1-36432719-36433125
chr1	36444276	36445938	chr1-36444276-36445938
chr1	36456989	36458503	chr1-36456989-36458503
chr1	36470033	36477047	chr1-36470033-36477047
chr1	36477973	36478334	chr1-36477973-36478334
chr1	36485483	36485848	chr1-36485483-36485848
chr1	36492298	36492933	chr1-36492298-36492933
chr1	36497119	36498968	chr1-36497119-36498968
chr1	36511326	36514951	chr1-36511326-36514951
chr1	36534245	36535311	chr1-36534245-36535311
chr1	36545893	36547854	chr1-36545893-36547854
chr1	36555441	36559108	chr1-36555441-36559108
chr1	36580048	36581053	chr1-36580048-36581053
chr1	36691001	36692767	chr1-36691001-36692767
chr1	36708211	36710506	chr1-36708211-36710506

chr1	36744312	36744927	chr1-36744312-36744927
chr1	36770977	36771178	chr1-36770977-36771178
chr1	36784776	36785816	chr1-36784776-36785816
chr1	36864758	36864959	chr1-36864758-36864959
chr1	36937135	36940738	chr1-36937135-36940738
chr1	37028758	37029811	chr1-37028758-37029811
chr1	37299421	37301154	chr1-37299421-37301154
chr1	37428847	37431941	chr1-37428847-37431941
chr1	37539812	37541678	chr1-37539812-37541678
chr1	37583731	37584368	chr1-37583731-37584368
chr1	37585600	37586612	chr1-37585600-37586612
chr1	37630666	37632206	chr1-37630666-37632206
chr1	37665362	37666945	chr1-37665362-37666945
chr1	37686983	37687664	chr1-37686983-37687664
chr1	37696494	37697310	chr1-37696494-37697310
chr1	37719087	37720711	chr1-37719087-37720711
chr1	37864196	37866598	chr1-37864196-37866598
chr1	37871545	37873712	chr1-37871545-37873712
chr1	37889157	37891879	chr1-37889157-37891879
chr1	37971710	37971911	chr1-37971710-37971911
chr1	37995936	37999956	chr1-37995936-37999956
chr1	38128316	38130532	chr1-38128316-38130532
chr1	38218131	38218599	chr1-38218131-38218599
chr1	38626367	38628190	chr1-38626367-38628190
chr1	38835653	38836701	chr1-38835653-38836701
chr1	38897589	38898392	chr1-38897589-38898392
chr1	38972863	38973810	chr1-38972863-38973810
chr1	38986903	38989645	chr1-38986903-38989645
chr1	39025144	39025674	chr1-39025144-39025674
chr1	39029957	39030447	chr1-39029957-39030447
chr1	39192299	39194604	chr1-39192299-39194604
chr1	39281683	39281935	chr1-39281683-39281935
chr1	39366995	39370241	chr1-39366995-39370241
chr1	39398790	39399667	chr1-39398790-39399667
chr1	39456218	39456865	chr1-39456218-39456865
chr1	39477413	39479332	chr1-39477413-39479332
chr1	39534900	39537083	chr1-39534900-39537083
chr1	39575435	39577952	chr1-39575435-39577952
chr1	39636336	39636919	chr1-39636336-39636919
chr1	39650238	39651433	chr1-39650238-39651433
chr1	39656876	39657077	chr1-39656876-39657077
chr1	39720776	39721284	chr1-39720776-39721284

chr1	39758843	39762540	chr1-39758843-39762540
chr1	39894915	39895687	chr1-39894915-39895687
chr1	39899920	39903522	chr1-39899920-39903522
chr1	39931710	39932123	chr1-39931710-39932123
chr1	39935497	39936865	chr1-39935497-39936865
chr1	39956414	39956827	chr1-39956414-39956827
chr1	40085448	40086205	chr1-40085448-40086205
chr1	40224874	40225541	chr1-40224874-40225541
chr1	40266142	40267678	chr1-40266142-40267678
chr1	40275553	40276185	chr1-40275553-40276185
chr1	40278622	40280354	chr1-40278622-40280354
chr1	40444575	40445540	chr1-40444575-40445540
chr1	40465436	40468379	chr1-40465436-40468379
chr1	40514918	40516786	chr1-40514918-40516786
chr1	40542352	40542553	chr1-40542352-40542553
chr1	40579939	40580703	chr1-40579939-40580703
chr1	40581551	40581752	chr1-40581551-40581752
chr1	40680365	40683755	chr1-40680365-40683755
chr1	40773470	40774070	chr1-40773470-40774070
chr1	40789770	40791296	chr1-40789770-40791296
chr1	40840595	40840915	chr1-40840595-40840915
chr1	42686726	42686989	chr1-42686726-42686989
chr1	42693881	42698992	chr1-42693881-42698992
chr1	42713039	42714803	chr1-42713039-42714803
chr1	42850755	42852493	chr1-42850755-42852493
chr1	42952653	42953809	chr1-42952653-42953809
chr1	43091308	43093463	chr1-43091308-43093463
chr1	43097702	43099898	chr1-43097702-43099898
chr1	43128025	43128828	chr1-43128025-43128828
chr1	43140775	43142351	chr1-43140775-43142351
chr1	43152946	43153266	chr1-43152946-43153266
chr1	43160377	43160649	chr1-43160377-43160649
chr1	43161993	43164469	chr1-43161993-43164469
chr1	43166670	43169416	chr1-43166670-43169416
chr1	43224619	43224924	chr1-43224619-43224924
chr1	43361498	43361917	chr1-43361498-43361917
chr1	43413156	43413940	chr1-43413156-43413940
chr1	43444608	43448236	chr1-43444608-43448236
chr1	43451048	43451305	chr1-43451048-43451305
chr1	43471367	43472762	chr1-43471367-43472762
chr1	43474271	43475071	chr1-43474271-43475071
chr1	43715158	43715857	chr1-43715158-43715857

chr1	43818797	43819730	chr1-43818797-43819730
chr1	43826317	43828632	chr1-43826317-43828632
chr1	43932885	43935836	chr1-43932885-43935836
chr1	44042180	44042657	chr1-44042180-44042657
chr1	44100503	44103240	chr1-44100503-44103240
chr1	44117586	44120740	chr1-44117586-44120740
chr1	44147178	44149013	chr1-44147178-44149013
chr1	44198574	44199334	chr1-44198574-44199334
chr1	44397822	44398023	chr1-44397822-44398023
chr1	44551252	44552770	chr1-44551252-44552770
chr1	45794565	45797430	chr1-45794565-45797430
chr1	45924711	45926677	chr1-45924711-45926677
chr1	46066546	46067412	chr1-46066546-46067412
chr1	46851609	46855074	chr1-46851609-46855074
chr1	46864060	46864261	chr1-46864060-46864261
chr1	47853826	47854280	chr1-47853826-47854280
chr1	50926305	50928382	chr1-50926305-50928382
chr1	51265469	51265722	chr1-51265469-51265722
chr1	51288144	51293447	chr1-51288144-51293447
chr1	51333698	51334159	chr1-51333698-51334159
chr1	51473468	51479394	chr1-51473468-51479394
chr1	51509108	51509444	chr1-51509108-51509444
chr1	51530063	51530264	chr1-51530063-51530264
chr1	51747587	51747883	chr1-51747587-51747883
chr1	51848669	51850253	chr1-51848669-51850253
chr1	51851997	51853967	chr1-51851997-51853967
chr1	51855938	51857248	chr1-51855938-51857248
chr1	51913693	51916338	chr1-51913693-51916338
chr1	52113208	52114171	chr1-52113208-52114171
chr1	52118838	52120631	chr1-52118838-52120631
chr1	52230071	52234411	chr1-52230071-52234411
chr1	52251309	52251620	chr1-52251309-52251620
chr1	52270237	52270602	chr1-52270237-52270602
chr1	52498955	52502283	chr1-52498955-52502283
chr1	52597324	52598239	chr1-52597324-52598239
chr1	52630327	52631723	chr1-52630327-52631723
chr1	52703051	52703252	chr1-52703051-52703252
chr1	52724343	52724750	chr1-52724343-52724750
chr1	52725745	52727968	chr1-52725745-52727968
chr1	52764053	52764312	chr1-52764053-52764312
chr1	52799116	52800070	chr1-52799116-52800070
chr1	52816845	52818058	chr1-52816845-52818058

chr1	52844161	52846152	chr1-52844161-52846152
chr1	52952410	52953550	chr1-52952410-52953550
chr1	53296076	53298324	chr1-53296076-53298324
chr1	53313062	53314551	chr1-53313062-53314551
chr1	53351731	53353303	chr1-53351731-53353303
chr1	53706489	53706899	chr1-53706489-53706899
chr1	53757604	53757805	chr1-53757604-53757805
chr1	53784001	53785755	chr1-53784001-53785755
chr1	53823644	53824102	chr1-53823644-53824102
chr1	53838096	53839194	chr1-53838096-53839194
chr1	53844890	53846316	chr1-53844890-53846316
chr1	54193991	54195338	chr1-54193991-54195338
chr1	54250128	54251548	chr1-54250128-54251548
chr1	54438109	54439322	chr1-54438109-54439322
chr1	54555710	54558473	chr1-54555710-54558473
chr1	54603722	54603975	chr1-54603722-54603975
chr1	54612066	54612482	chr1-54612066-54612482
chr1	54924945	54926996	chr1-54924945-54926996
chr1	55022891	55023142	chr1-55022891-55023142
chr1	55025158	55028099	chr1-55025158-55028099
chr1	55051741	55054608	chr1-55051741-55054608
chr1	55084003	55090286	chr1-55084003-55090286
chr1	55130547	55133602	chr1-55130547-55133602
chr1	55201760	55202613	chr1-55201760-55202613
chr1	55236478	55239441	chr1-55236478-55239441
chr1	55362648	55364129	chr1-55362648-55364129
chr1	55405205	55407246	chr1-55405205-55407246
chr1	55575829	55576481	chr1-55575829-55576481
chr1	55826959	55827214	chr1-55826959-55827214
chr1	56331717	56331918	chr1-56331717-56331918
chr1	56681099	56681934	chr1-56681099-56681934
chr1	56970695	56974166	chr1-56970695-56974166
chr1	56976894	56978551	chr1-56976894-56978551
chr1	56980600	56981312	chr1-56980600-56981312
chr1	57376878	57378667	chr1-57376878-57378667
chr1	57405523	57407869	chr1-57405523-57407869
chr1	57774022	57775874	chr1-57774022-57775874
chr1	57849888	57850189	chr1-57849888-57850189
chr1	57968918	57970357	chr1-57968918-57970357
chr1	57989364	57989668	chr1-57989364-57989668
chr1	57995458	57998178	chr1-57995458-57998178
chr1	58029249	58031450	chr1-58029249-58031450

chr1	58073004	58073546	chr1-58073004-58073546
chr1	58289918	58290326	chr1-58289918-58290326
chr1	58353718	58353973	chr1-58353718-58353973
chr1	58359200	58359725	chr1-58359200-58359725
chr1	58366774	58367098	chr1-58366774-58367098
chr1	58391301	58395212	chr1-58391301-58395212
chr1	58421729	58424559	chr1-58421729-58424559
chr1	58444178	58447332	chr1-58444178-58447332
chr1	58503300	58506051	chr1-58503300-58506051
chr1	58584985	58587562	chr1-58584985-58587562
chr1	58651112	58654079	chr1-58651112-58654079
chr1	58662694	58663451	chr1-58662694-58663451
chr1	58664522	58664778	chr1-58664522-58664778
chr1	58692257	58692509	chr1-58692257-58692509
chr1	58710225	58717299	chr1-58710225-58717299
chr1	58719048	58720399	chr1-58719048-58720399
chr1	58722003	58722262	chr1-58722003-58722262
chr1	58725550	58731090	chr1-58725550-58731090
chr1	58742174	58743356	chr1-58742174-58743356
chr1	58761664	58762121	chr1-58761664-58762121
chr1	58794912	58797883	chr1-58794912-58797883
chr1	58856199	58860294	chr1-58856199-58860294
chr1	58909863	58910064	chr1-58909863-58910064
chr1	58972227	58974625	chr1-58972227-58974625
chr1	59119006	59120844	chr1-59119006-59120844
chr1	59162582	59163573	chr1-59162582-59163573
chr1	59235982	59238236	chr1-59235982-59238236
chr1	59252681	59253812	chr1-59252681-59253812
chr1	59481138	59484685	chr1-59481138-59484685
chr1	59487551	59488116	chr1-59487551-59488116
chr1	59580123	59580860	chr1-59580123-59580860
chr1	59668597	59671994	chr1-59668597-59671994
chr1	59684235	59688442	chr1-59684235-59688442
chr1	59739816	59740200	chr1-59739816-59740200
chr1	59762145	59767110	chr1-59762145-59767110
chr1	59895389	59895699	chr1-59895389-59895699
chr1	59912203	59915788	chr1-59912203-59915788
chr1	59999788	60000405	chr1-59999788-60000405
chr1	60042388	60043729	chr1-60042388-60043729
chr1	60096362	60099391	chr1-60096362-60099391
chr1	60179452	60182420	chr1-60179452-60182420
chr1	60342514	60344803	chr1-60342514-60344803

chr1	60409045	60410903	chr1-60409045-60410903
chr1	60564807	60567670	chr1-60564807-60567670
chr1	60609009	60610059	chr1-60609009-60610059
chr1	60616868	60617120	chr1-60616868-60617120
chr1	60636588	60636789	chr1-60636588-60636789
chr1	61203363	61203726	chr1-61203363-61203726
chr1	61638320	61639840	chr1-61638320-61639840
chr1	62702506	62708408	chr1-62702506-62708408
chr1	62721793	62721994	chr1-62721793-62721994
chr1	62765491	62765814	chr1-62765491-62765814
chr1	62766830	62768970	chr1-62766830-62768970
chr1	62777461	62778190	chr1-62777461-62778190
chr1	62984656	62984907	chr1-62984656-62984907
chr1	62986444	62986695	chr1-62986444-62986695
chr1	63112103	63116134	chr1-63112103-63116134
chr1	63129743	63130432	chr1-63129743-63130432
chr1	63175041	63178926	chr1-63175041-63178926
chr1	63248225	63248648	chr1-63248225-63248648
chr1	63272949	63273977	chr1-63272949-63273977
chr1	63445590	63446714	chr1-63445590-63446714
chr1	63729949	63732383	chr1-63729949-63732383
chr1	63776692	63777602	chr1-63776692-63777602
chr1	63953691	63953946	chr1-63953691-63953946
chr1	64087779	64089159	chr1-64087779-64089159
chr1	64118195	64123102	chr1-64118195-64123102
chr1	64226306	64226976	chr1-64226306-64226976
chr1	64305258	64306108	chr1-64305258-64306108
chr1	64316993	64317244	chr1-64316993-64317244
chr1	64337733	64338829	chr1-64337733-64338829
chr1	64531527	64534879	chr1-64531527-64534879
chr1	64600333	64602117	chr1-64600333-64602117
chr1	64616117	64618516	chr1-64616117-64618516
chr1	64689595	64692565	chr1-64689595-64692565
chr1	64734029	64739497	chr1-64734029-64739497
chr1	64795385	64795691	chr1-64795385-64795691
chr1	64796940	64797846	chr1-64796940-64797846
chr1	64808886	64809138	chr1-64808886-64809138
chr1	64942258	64942734	chr1-64942258-64942734
chr1	64948954	64950342	chr1-64948954-64950342
chr1	64955930	64957355	chr1-64955930-64957355
chr1	65122782	65123248	chr1-65122782-65123248
chr1	65177374	65179998	chr1-65177374-65179998

chr1	65185729	65188055	chr1-65185729-65188055
chr1	65311177	65311919	chr1-65311177-65311919
chr1	65389911	65390112	chr1-65389911-65390112
chr1	65440204	65440716	chr1-65440204-65440716
chr1	65932990	65933242	chr1-65932990-65933242
chr1	66174795	66176630	chr1-66174795-66176630
chr1	66467738	66468713	chr1-66467738-66468713
chr1	66700251	66702467	chr1-66700251-66702467
chr1	66814768	66818393	chr1-66814768-66818393
chr1	66838243	66838559	chr1-66838243-66838559
chr1	66845784	66846632	chr1-66845784-66846632
chr1	66861627	66864245	chr1-66861627-66864245
chr1	67037779	67039528	chr1-67037779-67039528
chr1	68780522	68780824	chr1-68780522-68780824
chr1	69105590	69108720	chr1-69105590-69108720
chr1	69241846	69242410	chr1-69241846-69242410
chr1	69277146	69277972	chr1-69277146-69277972
chr1	69286407	69286784	chr1-69286407-69286784
chr1	69331646	69331900	chr1-69331646-69331900
chr1	69369269	69369639	chr1-69369269-69369639
chr1	69433718	69434506	chr1-69433718-69434506
chr1	69591559	69591760	chr1-69591559-69591760
chr1	69610368	69611550	chr1-69610368-69611550
chr1	69656906	69657778	chr1-69656906-69657778
chr1	69684344	69688942	chr1-69684344-69688942
chr1	70918040	70918301	chr1-70918040-70918301
chr1	71003382	71003635	chr1-71003382-71003635
chr1	71099778	71103927	chr1-71099778-71103927
chr1	71556353	71558788	chr1-71556353-71558788
chr1	71649062	71654622	chr1-71649062-71654622
chr1	71691726	71692135	chr1-71691726-71692135
chr1	71723335	71724300	chr1-71723335-71724300
chr1	71755599	71755857	chr1-71755599-71755857
chr1	71837127	71837551	chr1-71837127-71837551
chr1	71893317	71894343	chr1-71893317-71894343
chr1	72076026	72076227	chr1-72076026-72076227
chr1	72078666	72078867	chr1-72078666-72078867
chr1	72142113	72142314	chr1-72142113-72142314
chr1	72182848	72183421	chr1-72182848-72183421
chr1	72211391	72212701	chr1-72211391-72212701
chr1	72225068	72226303	chr1-72225068-72226303
chr1	72236071	72237139	chr1-72236071-72237139

chr1	72238060	72238441	chr1-72238060-72238441
chr1	72243548	72244322	chr1-72243548-72244322
chr1	72254180	72255108	chr1-72254180-72255108
chr1	72283392	72284847	chr1-72283392-72284847
chr1	72306416	72308533	chr1-72306416-72308533
chr1	72428527	72429621	chr1-72428527-72429621
chr1	72450049	72450577	chr1-72450049-72450577
chr1	72465176	72466368	chr1-72465176-72466368
chr1	72536044	72537565	chr1-72536044-72537565
chr1	72582682	72584263	chr1-72582682-72584263
chr1	72599368	72600138	chr1-72599368-72600138
chr1	72710698	72713610	chr1-72710698-72713610
chr1	72824172	72825204	chr1-72824172-72825204
chr1	72870112	72875108	chr1-72870112-72875108
chr1	72963239	72963725	chr1-72963239-72963725
chr1	73027152	73027801	chr1-73027152-73027801
chr1	73936464	73936994	chr1-73936464-73936994
chr1	73954560	73956040	chr1-73954560-73956040
chr1	73962162	73963409	chr1-73962162-73963409
chr1	73998261	73998522	chr1-73998261-73998522
chr1	74007772	74009231	chr1-74007772-74009231
chr1	74014798	74014999	chr1-74014798-74014999
chr1	74031023	74031274	chr1-74031023-74031274
chr1	74036259	74037541	chr1-74036259-74037541
chr1	74042776	74043540	chr1-74042776-74043540
chr1	74098069	74099527	chr1-74098069-74099527
chr1	74121854	74122376	chr1-74121854-74122376
chr1	74124058	74125816	chr1-74124058-74125816
chr1	74149473	74149828	chr1-74149473-74149828
chr1	74162454	74162716	chr1-74162454-74162716
chr1	74235755	74241327	chr1-74235755-74241327
chr1	74248196	74249945	chr1-74248196-74249945
chr1	74283009	74286147	chr1-74283009-74286147
chr1	74294891	74304907	chr1-74294891-74304907
chr1	74332009	74333305	chr1-74332009-74333305
chr1	74386095	74387956	chr1-74386095-74387956
chr1	74389816	74393957	chr1-74389816-74393957
chr1	74396572	74398413	chr1-74396572-74398413
chr1	74504528	74508208	chr1-74504528-74508208
chr1	74586707	74589184	chr1-74586707-74589184
chr1	74600349	74602170	chr1-74600349-74602170
chr1	74661280	74663401	chr1-74661280-74663401

chr1	74713015	74714077	chr1-74713015-74714077
chr1	74748685	74749906	chr1-74748685-74749906
chr1	74771093	74773621	chr1-74771093-74773621
chr1	74783656	74784847	chr1-74783656-74784847
chr1	74791095	74795635	chr1-74791095-74795635
chr1	74802614	74808828	chr1-74802614-74808828
chr1	74816577	74817956	chr1-74816577-74817956
chr1	74854372	74858801	chr1-74854372-74858801
chr1	74881854	74883101	chr1-74881854-74883101
chr1	74884445	74885727	chr1-74884445-74885727
chr1	74892589	74893684	chr1-74892589-74893684
chr1	74900120	74901517	chr1-74900120-74901517
chr1	74947981	74952168	chr1-74947981-74952168
chr1	75025059	75025311	chr1-75025059-75025311
chr1	75124279	75125846	chr1-75124279-75125846
chr1	75133061	75133923	chr1-75133061-75133923
chr1	75140572	75144090	chr1-75140572-75144090
chr1	75167953	75169949	chr1-75167953-75169949
chr1	75179289	75181012	chr1-75179289-75181012
chr1	75190786	75193666	chr1-75190786-75193666
chr1	75208980	75220370	chr1-75208980-75220370
chr1	75221474	75221729	chr1-75221474-75221729
chr1	75230623	75232349	chr1-75230623-75232349
chr1	75235880	75237769	chr1-75235880-75237769
chr1	75247411	75248888	chr1-75247411-75248888
chr1	75251422	75252078	chr1-75251422-75252078
chr1	75262876	75264552	chr1-75262876-75264552
chr1	75315641	75318603	chr1-75315641-75318603
chr1	75338338	75338589	chr1-75338338-75338589
chr1	75360153	75360887	chr1-75360153-75360887
chr1	75381600	75382871	chr1-75381600-75382871
chr1	75387766	75388858	chr1-75387766-75388858
chr1	75392941	75393414	chr1-75392941-75393414
chr1	75398949	75400676	chr1-75398949-75400676
chr1	75422647	75423536	chr1-75422647-75423536
chr1	75435488	75437085	chr1-75435488-75437085
chr1	75450176	75450863	chr1-75450176-75450863
chr1	75457633	75458891	chr1-75457633-75458891
chr1	75467851	75468052	chr1-75467851-75468052
chr1	75477221	75480372	chr1-75477221-75480372
chr1	75486413	75488346	chr1-75486413-75488346
chr1	75504546	75507402	chr1-75504546-75507402

chr1	75518201	75518616	chr1-75518201-75518616
chr1	75520848	75522613	chr1-75520848-75522613
chr1	75545766	75547463	chr1-75545766-75547463
chr1	75559385	75559636	chr1-75559385-75559636
chr1	75618532	75618839	chr1-75618532-75618839
chr1	75653672	75654063	chr1-75653672-75654063
chr1	77213402	77213709	chr1-77213402-77213709
chr1	77444513	77445500	chr1-77444513-77445500
chr1	77459059	77463669	chr1-77459059-77463669
chr1	77465399	77467014	chr1-77465399-77467014
chr1	77471275	77471476	chr1-77471275-77471476
chr1	77503960	77506994	chr1-77503960-77506994
chr1	77510866	77517469	chr1-77510866-77517469
chr1	77535245	77536695	chr1-77535245-77536695
chr1	78181872	78182337	chr1-78181872-78182337
chr1	78189004	78189994	chr1-78189004-78189994
chr1	78193634	78194146	chr1-78193634-78194146
chr1	78195215	78197486	chr1-78195215-78197486
chr1	78201620	78202034	chr1-78201620-78202034
chr1	78203196	78204429	chr1-78203196-78204429
chr1	78215750	78217929	chr1-78215750-78217929
chr1	78309886	78311334	chr1-78309886-78311334
chr1	78435869	78436277	chr1-78435869-78436277
chr1	78437146	78437473	chr1-78437146-78437473
chr1	78448122	78448414	chr1-78448122-78448414
chr1	78487506	78490146	chr1-78487506-78490146
chr1	78511703	78512197	chr1-78511703-78512197
chr1	78537156	78538178	chr1-78537156-78538178
chr1	78657014	78659717	chr1-78657014-78659717
chr1	78745399	78745600	chr1-78745399-78745600
chr1	78811849	78812459	chr1-78811849-78812459
chr1	78896016	78896217	chr1-78896016-78896217
chr1	79445502	79445754	chr1-79445502-79445754
chr1	79580898	79581728	chr1-79580898-79581728
chr1	79637522	79637723	chr1-79637522-79637723
chr1	79641520	79641721	chr1-79641520-79641721
chr1	79670330	79672744	chr1-79670330-79672744
chr1	79678606	79678919	chr1-79678606-79678919
chr1	79696921	79697740	chr1-79696921-79697740
chr1	79759602	79762254	chr1-79759602-79762254
chr1	79775419	79777126	chr1-79775419-79777126
chr1	79822596	79824747	chr1-79822596-79824747

chr1	79857704	79859491	chr1-79857704-79859491
chr1	80338495	80341992	chr1-80338495-80341992
chr1	80757842	80758866	chr1-80757842-80758866
chr1	82189499	82189750	chr1-82189499-82189750
chr1	82282369	82292370	chr1-82282369-82292370
chr1	82315968	82318023	chr1-82315968-82318023
chr1	82358975	82359230	chr1-82358975-82359230
chr1	82428882	82429136	chr1-82428882-82429136
chr1	82585765	82588925	chr1-82585765-82588925
chr1	82641489	82641852	chr1-82641489-82641852
chr1	82724247	82726056	chr1-82724247-82726056
chr1	82785681	82786010	chr1-82785681-82786010
chr1	82795494	82795925	chr1-82795494-82795925
chr1	82831634	82832564	chr1-82831634-82832564
chr1	82834484	82835025	chr1-82834484-82835025
chr1	82838835	82841089	chr1-82838835-82841089
chr1	82972298	82972499	chr1-82972298-82972499
chr1	83038019	83038636	chr1-83038019-83038636
chr1	83116403	83116984	chr1-83116403-83116984
chr1	83135674	83136046	chr1-83135674-83136046
chr1	83137659	83138078	chr1-83137659-83138078
chr1	83159574	83160085	chr1-83159574-83160085
chr1	84284156	84285019	chr1-84284156-84285019
chr1	84695261	84696379	chr1-84695261-84696379
chr1	84709947	84710310	chr1-84709947-84710310
chr1	84837513	84841056	chr1-84837513-84841056
chr1	84881390	84882340	chr1-84881390-84882340
chr1	84933903	84935892	chr1-84933903-84935892
chr1	85147598	85147986	chr1-85147598-85147986
chr1	85339771	85340214	chr1-85339771-85340214
chr1	85598228	85598818	chr1-85598228-85598818
chr1	85600735	85600936	chr1-85600735-85600936
chr1	85649986	85650187	chr1-85649986-85650187
chr1	85702831	85704182	chr1-85702831-85704182
chr1	85735693	85736689	chr1-85735693-85736689
chr1	85740912	85741532	chr1-85740912-85741532
chr1	85793046	85795685	chr1-85793046-85795685
chr1	85837670	85838085	chr1-85837670-85838085
chr1	85864478	85864679	chr1-85864478-85864679
chr1	85893939	85896207	chr1-85893939-85896207
chr1	86018029	86019053	chr1-86018029-86019053
chr1	86045716	86046864	chr1-86045716-86046864

chr1	86059003	86059638	chr1-86059003-86059638
chr1	86063928	86066186	chr1-86063928-86066186
chr1	86153781	86155611	chr1-86153781-86155611
chr1	86277254	86277563	chr1-86277254-86277563
chr1	86300773	86304542	chr1-86300773-86304542
chr1	86357014	86361280	chr1-86357014-86361280
chr1	86433788	86435332	chr1-86433788-86435332
chr1	86437560	86438434	chr1-86437560-86438434
chr1	86439830	86444030	chr1-86439830-86444030
chr1	86454083	86454284	chr1-86454083-86454284
chr1	86470012	86473305	chr1-86470012-86473305
chr1	86486619	86489258	chr1-86486619-86489258
chr1	86501338	86505964	chr1-86501338-86505964
chr1	86524663	86532348	chr1-86524663-86532348
chr1	86581481	86583491	chr1-86581481-86583491
chr1	86586637	86588028	chr1-86586637-86588028
chr1	86623280	86623745	chr1-86623280-86623745
chr1	86669432	86671024	chr1-86669432-86671024
chr1	86703136	86704739	chr1-86703136-86704739
chr1	87029250	87029451	chr1-87029250-87029451
chr1	87098550	87098907	chr1-87098550-87098907
chr1	87102341	87102542	chr1-87102341-87102542
chr1	87153769	87156472	chr1-87153769-87156472
chr1	87175944	87176198	chr1-87175944-87176198
chr1	87180531	87181000	chr1-87180531-87181000
chr1	87184773	87184974	chr1-87184773-87184974
chr1	87186261	87187335	chr1-87186261-87187335
chr1	87212866	87216171	chr1-87212866-87216171
chr1	87263867	87266031	chr1-87263867-87266031
chr1	87326429	87328508	chr1-87326429-87328508
chr1	87509108	87510833	chr1-87509108-87510833
chr1	87549205	87552082	chr1-87549205-87552082
chr1	87559754	87560075	chr1-87559754-87560075
chr1	87572215	87575622	chr1-87572215-87575622
chr1	87755258	87757558	chr1-87755258-87757558
chr1	87840067	87841100	chr1-87840067-87841100
chr1	87848537	87848888	chr1-87848537-87848888
chr1	87852780	87855359	chr1-87852780-87855359
chr1	87900159	87902517	chr1-87900159-87902517
chr1	87919753	87919954	chr1-87919753-87919954
chr1	88007565	88009059	chr1-88007565-88009059
chr1	88205243	88205903	chr1-88205243-88205903

chr1	88275746	88277975	chr1-88275746-88277975
chr1	88428051	88429157	chr1-88428051-88429157
chr1	88436941	88437726	chr1-88436941-88437726
chr1	88452379	88452784	chr1-88452379-88452784
chr1	88454932	88455288	chr1-88454932-88455288
chr1	88474134	88476376	chr1-88474134-88476376
chr1	88507258	88510428	chr1-88507258-88510428
chr1	88537070	88537556	chr1-88537070-88537556
chr1	88556467	88556981	chr1-88556467-88556981
chr1	88575733	88576305	chr1-88575733-88576305
chr1	88638884	88640756	chr1-88638884-88640756
chr1	88663059	88663315	chr1-88663059-88663315
chr1	88697278	88703060	chr1-88697278-88703060
chr1	88763475	88764132	chr1-88763475-88764132
chr1	88869782	88870180	chr1-88869782-88870180
chr1	89015379	89016304	chr1-89015379-89016304
chr1	89019390	89020632	chr1-89019390-89020632
chr1	89041282	89041483	chr1-89041282-89041483
chr1	89068989	89073721	chr1-89068989-89073721
chr1	89089221	89093454	chr1-89089221-89093454
chr1	89096547	89097006	chr1-89096547-89097006
chr1	89100024	89100439	chr1-89100024-89100439
chr1	89101399	89105519	chr1-89101399-89105519
chr1	89106942	89111612	chr1-89106942-89111612
chr1	89113567	89113838	chr1-89113567-89113838
chr1	89120080	89126367	chr1-89120080-89126367
chr1	89131062	89134305	chr1-89131062-89134305
chr1	89152491	89153544	chr1-89152491-89153544
chr1	89183348	89183716	chr1-89183348-89183716
chr1	89191057	89191695	chr1-89191057-89191695
chr1	89453864	89457796	chr1-89453864-89457796
chr1	89462940	89463401	chr1-89462940-89463401
chr1	89523621	89524174	chr1-89523621-89524174
chr1	89525813	89526014	chr1-89525813-89526014
chr1	89566504	89566771	chr1-89566504-89566771
chr1	89568736	89568937	chr1-89568736-89568937
chr1	89570199	89571688	chr1-89570199-89571688
chr1	89576984	89577242	chr1-89576984-89577242
chr1	89579818	89582256	chr1-89579818-89582256
chr1	89656420	89657321	chr1-89656420-89657321
chr1	89781014	89781215	chr1-89781014-89781215
chr1	89926825	89927808	chr1-89926825-89927808

chr1	89929836	89931889	chr1-89929836-89931889
chr1	89933695	89934101	chr1-89933695-89934101
chr1	89935411	89935719	chr1-89935411-89935719
chr1	89939981	89940694	chr1-89939981-89940694
chr1	89941723	89943409	chr1-89941723-89943409
chr1	89967977	89969000	chr1-89967977-89969000
chr1	90050957	90051158	chr1-90050957-90051158
chr1	90153036	90153812	chr1-90153036-90153812
chr1	90164342	90164810	chr1-90164342-90164810
chr1	90185586	90186825	chr1-90185586-90186825
chr1	90203717	90207129	chr1-90203717-90207129
chr1	90225731	90226532	chr1-90225731-90226532
chr1	90230743	90231120	chr1-90230743-90231120
chr1	90287459	90287711	chr1-90287459-90287711
chr1	90288804	90289977	chr1-90288804-90289977
chr1	90518697	90519937	chr1-90518697-90519937
chr1	90601403	90601604	chr1-90601403-90601604
chr1	90602650	90604559	chr1-90602650-90604559
chr1	90754322	90754523	chr1-90754322-90754523
chr1	90834590	90834791	chr1-90834590-90834791
chr1	90896768	90897207	chr1-90896768-90897207
chr1	90909524	90910456	chr1-90909524-90910456
chr1	90914782	90915602	chr1-90914782-90915602
chr1	90923347	90923548	chr1-90923347-90923548
chr1	90927298	90928630	chr1-90927298-90928630
chr1	90969174	90970220	chr1-90969174-90970220
chr1	90974941	90975659	chr1-90974941-90975659
chr1	90997915	91000707	chr1-90997915-91000707
chr1	91022217	91025287	chr1-91022217-91025287
chr1	91041193	91046053	chr1-91041193-91046053
chr1	91050279	91059086	chr1-91050279-91059086
chr1	91062051	91062862	chr1-91062051-91062862
chr1	91064145	91065270	chr1-91064145-91065270
chr1	91083641	91084670	chr1-91083641-91084670
chr1	91089148	91089349	chr1-91089148-91089349
chr1	91094697	91097430	chr1-91094697-91097430
chr1	91179311	91182078	chr1-91179311-91182078
chr1	91219636	91219837	chr1-91219636-91219837
chr1	91239980	91241385	chr1-91239980-91241385
chr1	91248348	91252475	chr1-91248348-91252475
chr1	91257560	91257892	chr1-91257560-91257892
chr1	91297551	91299710	chr1-91297551-91299710

chr1	91344662	91345827	chr1-91344662-91345827
chr1	91351231	91352929	chr1-91351231-91352929
chr1	91362404	91362659	chr1-91362404-91362659
chr1	91365751	91368149	chr1-91365751-91368149
chr1	91397327	91399322	chr1-91397327-91399322
chr1	91405854	91406172	chr1-91405854-91406172
chr1	91412004	91414690	chr1-91412004-91414690
chr1	91455411	91460416	chr1-91455411-91460416
chr1	91494133	91495874	chr1-91494133-91495874
chr1	91539583	91541675	chr1-91539583-91541675
chr1	91755036	91755237	chr1-91755036-91755237
chr1	91757339	91757713	chr1-91757339-91757713
chr1	91800885	91802148	chr1-91800885-91802148
chr1	91816557	91817351	chr1-91816557-91817351
chr1	91872569	91872770	chr1-91872569-91872770
chr1	92064581	92064835	chr1-92064581-92064835
chr1	92178492	92181579	chr1-92178492-92181579
chr1	92270593	92270848	chr1-92270593-92270848
chr1	92379026	92379693	chr1-92379026-92379693
chr1	92472753	92474707	chr1-92472753-92474707
chr1	92640594	92642539	chr1-92640594-92642539
chr1	92648081	92648388	chr1-92648081-92648388
chr1	92699213	92699468	chr1-92699213-92699468
chr1	92745130	92745594	chr1-92745130-92745594
chr1	92751692	92752394	chr1-92751692-92752394
chr1	92786848	92787852	chr1-92786848-92787852
chr1	92790543	92791540	chr1-92790543-92791540
chr1	92799915	92800218	chr1-92799915-92800218
chr1	92830679	92834095	chr1-92830679-92834095
chr1	92902636	92904588	chr1-92902636-92904588
chr1	92906234	92908326	chr1-92906234-92908326
chr1	92909611	92912923	chr1-92909611-92912923
chr1	92933391	92936013	chr1-92933391-92936013
chr1	92956721	92957233	chr1-92956721-92957233
chr1	93099777	93102317	chr1-93099777-93102317
chr1	93115109	93115864	chr1-93115109-93115864
chr1	93130157	93131272	chr1-93130157-93131272
chr1	93155772	93162972	chr1-93155772-93162972
chr1	93166019	93169009	chr1-93166019-93169009
chr1	93173247	93174559	chr1-93173247-93174559
chr1	93176377	93176922	chr1-93176377-93176922
chr1	93184049	93184302	chr1-93184049-93184302

chr1	93188189	93188780	chr1-93188189-93188780
chr1	93190513	93191215	chr1-93190513-93191215
chr1	93235305	93237100	chr1-93235305-93237100
chr1	93271071	93271272	chr1-93271071-93271272
chr1	93289798	93290049	chr1-93289798-93290049
chr1	93304802	93306455	chr1-93304802-93306455
chr1	93341752	93344757	chr1-93341752-93344757
chr1	93387933	93388134	chr1-93387933-93388134
chr1	93392106	93392416	chr1-93392106-93392416
chr1	93401157	93402047	chr1-93401157-93402047
chr1	93443726	93446304	chr1-93443726-93446304
chr1	93477000	93480825	chr1-93477000-93480825
chr1	93510863	93513517	chr1-93510863-93513517
chr1	93530060	93530261	chr1-93530060-93530261
chr1	93633982	93636765	chr1-93633982-93636765
chr1	93668581	93669671	chr1-93668581-93669671
chr1	93680246	93680880	chr1-93680246-93680880
chr1	93684991	93688294	chr1-93684991-93688294
chr1	93705538	93706135	chr1-93705538-93706135
chr1	93753224	93756095	chr1-93753224-93756095
chr1	93800549	93805516	chr1-93800549-93805516
chr1	93824467	93826680	chr1-93824467-93826680
chr1	94035040	94035707	chr1-94035040-94035707
chr1	95313180	95314851	chr1-95313180-95314851
chr1	95665162	95668467	chr1-95665162-95668467
chr1	96799382	96799687	chr1-96799382-96799687
chr1	96805267	96805518	chr1-96805267-96805518
chr1	96871460	96872716	chr1-96871460-96872716
chr1	97657290	97662730	chr1-97657290-97662730
chr1	97767547	97771544	chr1-97767547-97771544
chr1	97910608	97911466	chr1-97910608-97911466
chr1	97976399	97977645	chr1-97976399-97977645
chr1	97997433	97997687	chr1-97997433-97997687
chr1	98094288	98096435	chr1-98094288-98096435
chr1	99917594	99917960	chr1-99917594-99917960
chr1	100231629	100231933	chr1-100231629-100231933
chr1	104768778	104769112	chr1-104768778-104769112
chr1	104993868	104994545	chr1-104993868-104994545
chr1	105355528	105357209	chr1-105355528-105357209
chr1	105434557	105434858	chr1-105434557-105434858
chr1	105661254	105667056	chr1-105661254-105667056
chr1	105780085	105782222	chr1-105780085-105782222

chr1	105865940	105867400	chr1-105865940-105867400
chr1	105906221	105906574	chr1-105906221-105906574
chr1	105925048	105926706	chr1-105925048-105926706
chr1	105971576	105972032	chr1-105971576-105972032
chr1	105983886	105984187	chr1-105983886-105984187
chr1	105988001	105994756	chr1-105988001-105994756
chr1	106168938	106169200	chr1-106168938-106169200
chr1	106170797	106176186	chr1-106170797-106176186
chr1	106210784	106211094	chr1-106210784-106211094
chr1	106397153	106405214	chr1-106397153-106405214
chr1	106406692	106408561	chr1-106406692-106408561
chr1	106409608	106409927	chr1-106409608-106409927
chr1	106629150	106630691	chr1-106629150-106630691
chr1	106711234	106715462	chr1-106711234-106715462
chr1	106757990	106760535	chr1-106757990-106760535
chr1	106795334	106797427	chr1-106795334-106797427
chr1	106857296	106857497	chr1-106857296-106857497
chr1	106860760	106862725	chr1-106860760-106862725
chr1	107511403	107513146	chr1-107511403-107513146
chr1	107589654	107593022	chr1-107589654-107593022
chr1	111864012	111865364	chr1-111864012-111865364
chr1	118309620	118312250	chr1-118309620-118312250
chr1	118321267	118323380	chr1-118321267-118323380
chr1	118387550	118391145	chr1-118387550-118391145
chr1	118398345	118400140	chr1-118398345-118400140
chr1	118457724	118460319	chr1-118457724-118460319
chr1	118627529	118629375	chr1-118627529-118629375
chr1	118649836	118650836	chr1-118649836-118650836
chr1	118660650	118661585	chr1-118660650-118661585
chr1	118689892	118690260	chr1-118689892-118690260
chr1	119047762	119048481	chr1-119047762-119048481
chr1	119052769	119054430	chr1-119052769-119054430
chr1	119083584	119083785	chr1-119083584-119083785
chr1	119289835	119290996	chr1-119289835-119290996
chr1	119292077	119292278	chr1-119292077-119292278
chr1	119308823	119309024	chr1-119308823-119309024
chr1	119404328	119404785	chr1-119404328-119404785
chr1	119419752	119423080	chr1-119419752-119423080
chr1	119492010	119492777	chr1-119492010-119492777
chr1	119502554	119505458	chr1-119502554-119505458
chr1	119524957	119527240	chr1-119524957-119527240
chr1	119537999	119538200	chr1-119537999-119538200

chr1	119646400	119650108	chr1-119646400-119650108
chr1	119835697	119838122	chr1-119835697-119838122
chr1	119889637	119889838	chr1-119889637-119889838
chr1	119912044	119913735	chr1-119912044-119913735
chr1	119996772	119997692	chr1-119996772-119997692
chr1	120007017	120007729	chr1-120007017-120007729
chr1	120033246	120033447	chr1-120033246-120033447
chr1	120072975	120074352	chr1-120072975-120074352
chr1	120082798	120084427	chr1-120082798-120084427
chr1	120119305	120122355	chr1-120119305-120122355
chr1	120136166	120136367	chr1-120136166-120136367
chr1	120236183	120236890	chr1-120236183-120236890
chr1	120264546	120265834	chr1-120264546-120265834
chr1	120340634	120340835	chr1-120340634-120340835
chr1	120342350	120342551	chr1-120342350-120342551
chr1	120596420	120597136	chr1-120596420-120597136
chr1	120601965	120605053	chr1-120601965-120605053
chr1	120606635	120607469	chr1-120606635-120607469
chr1	120757329	120757530	chr1-120757329-120757530
chr1	120901096	120901347	chr1-120901096-120901347
chr1	120906008	120906209	chr1-120906008-120906209
chr1	121177846	121178431	chr1-121177846-121178431
chr1	121326478	121328923	chr1-121326478-121328923
chr1	121350686	121351091	chr1-121350686-121351091
chr1	121360558	121360759	chr1-121360558-121360759
chr1	121377807	121378496	chr1-121377807-121378496
chr1	121430910	121432360	chr1-121430910-121432360
chr1	121482948	121483203	chr1-121482948-121483203
chr1	121527291	121529008	chr1-121527291-121529008
chr1	121566451	121568357	chr1-121566451-121568357
chr1	122357123	122357324	chr1-122357123-122357324
chr1	124045496	124046162	chr1-124045496-124046162
chr1	124487794	124487995	chr1-124487794-124487995
chr1	125213749	125215524	chr1-125213749-125215524
chr1	125236888	125237558	chr1-125236888-125237558
chr1	125430663	125436686	chr1-125430663-125436686
chr1	125489203	125489404	chr1-125489203-125489404
chr1	125525942	125526491	chr1-125525942-125526491
chr1	125549519	125550647	chr1-125549519-125550647
chr1	125559148	125562387	chr1-125559148-125562387
chr1	125676170	125681151	chr1-125676170-125681151
chr1	125687018	125688762	chr1-125687018-125688762

chr1	125694111	125694668	chr1-125694111-125694668
chr1	125702364	125702682	chr1-125702364-125702682
chr1	125704927	125705228	chr1-125704927-125705228
chr1	125755243	125755763	chr1-125755243-125755763
chr1	125800145	125800930	chr1-125800145-125800930
chr1	125910977	125912939	chr1-125910977-125912939
chr1	126492457	126493007	chr1-126492457-126493007
chr1	126737963	126738269	chr1-126737963-126738269
chr1	126817488	126818058	chr1-126817488-126818058
chr1	126829882	126830874	chr1-126829882-126830874
chr1	127204035	127207186	chr1-127204035-127207186
chr1	127239713	127240553	chr1-127239713-127240553
chr1	127257216	127257820	chr1-127257216-127257820
chr1	127290988	127291600	chr1-127290988-127291600
chr1	127425081	127425599	chr1-127425081-127425599
chr1	127676530	127678456	chr1-127676530-127678456
chr1	127756055	127756256	chr1-127756055-127756256
chr1	127772759	127776862	chr1-127772759-127776862
chr1	127868411	127869801	chr1-127868411-127869801
chr1	127975994	127976401	chr1-127975994-127976401
chr1	128101834	128104696	chr1-128101834-128104696
chr1	128243627	128245584	chr1-128243627-128245584
chr1	128357228	128360187	chr1-128357228-128360187
chr1	128416165	128417889	chr1-128416165-128417889
chr1	128472257	128472985	chr1-128472257-128472985
chr1	128590709	128593473	chr1-128590709-128593473
chr1	128832273	128832752	chr1-128832273-128832752
chr1	129273186	129273868	chr1-129273186-129273868
chr1	130422776	130422977	chr1-130422776-130422977
chr1	130461975	130463293	chr1-130461975-130463293
chr1	130548022	130548486	chr1-130548022-130548486
chr1	130714736	130718962	chr1-130714736-130718962
chr1	130731419	130736212	chr1-130731419-130736212
chr1	130737202	130737822	chr1-130737202-130737822
chr1	130740679	130741425	chr1-130740679-130741425
chr1	130761414	130761615	chr1-130761414-130761615
chr1	130763893	130764164	chr1-130763893-130764164
chr1	130766840	130767438	chr1-130766840-130767438
chr1	130770028	130771452	chr1-130770028-130771452
chr1	130878112	130878684	chr1-130878112-130878684
chr1	130879824	130880767	chr1-130879824-130880767
chr1	130884604	130887802	chr1-130884604-130887802

chr1	130918397	130918759	chr1-130918397-130918759
chr1	131072622	131072873	chr1-131072622-131072873
chr1	131083409	131084582	chr1-131083409-131084582
chr1	131085393	131085594	chr1-131085393-131085594
chr1	131088565	131091492	chr1-131088565-131091492
chr1	131095057	131098371	chr1-131095057-131098371
chr1	131107571	131109124	chr1-131107571-131109124
chr1	131112160	131113496	chr1-131112160-131113496
chr1	131118230	131119250	chr1-131118230-131119250
chr1	131123610	131124385	chr1-131123610-131124385
chr1	131125565	131127256	chr1-131125565-131127256
chr1	131134380	131139469	chr1-131134380-131139469
chr1	131152712	131155044	chr1-131152712-131155044
chr1	131184100	131184940	chr1-131184100-131184940
chr1	131238340	131240401	chr1-131238340-131240401
chr1	131242782	131247050	chr1-131242782-131247050
chr1	131276657	131280678	chr1-131276657-131280678
chr1	131286675	131287759	chr1-131286675-131287759
chr1	131506101	131506749	chr1-131506101-131506749
chr1	131523298	131529860	chr1-131523298-131529860
chr1	131586706	131587009	chr1-131586706-131587009
chr1	131612719	131613030	chr1-131612719-131613030
chr1	131657862	131658063	chr1-131657862-131658063
chr1	131661826	131662233	chr1-131661826-131662233
chr1	131688600	131691567	chr1-131688600-131691567
chr1	131733009	131733671	chr1-131733009-131733671
chr1	131743911	131754145	chr1-131743911-131754145
chr1	131827143	131829573	chr1-131827143-131829573
chr1	131866863	131868375	chr1-131866863-131868375
chr1	131909962	131912729	chr1-131909962-131912729
chr1	131962238	131964566	chr1-131962238-131964566
chr1	131967644	131967897	chr1-131967644-131967897
chr1	131969054	131970109	chr1-131969054-131970109
chr1	131973733	131974923	chr1-131973733-131974923
chr1	131976684	131977780	chr1-131976684-131977780
chr1	132006579	132010827	chr1-132006579-132010827
chr1	132067196	132068459	chr1-132067196-132068459
chr1	132095324	132095835	chr1-132095324-132095835
chr1	132099197	132099587	chr1-132099197-132099587
chr1	132133245	132134200	chr1-132133245-132134200
chr1	132136981	132140249	chr1-132136981-132140249
chr1	132149977	132152709	chr1-132149977-132152709

chr1	132168321	132168572	chr1-132168321-132168572
chr1	132189863	132192679	chr1-132189863-132192679
chr1	132195262	132201866	chr1-132195262-132201866
chr1	132205720	132207712	chr1-132205720-132207712
chr1	132256053	132256254	chr1-132256053-132256254
chr1	132267039	132267240	chr1-132267039-132267240
chr1	132287407	132296518	chr1-132287407-132296518
chr1	132297996	132300817	chr1-132297996-132300817
chr1	132315690	132320083	chr1-132315690-132320083
chr1	132333676	132336063	chr1-132333676-132336063
chr1	132338271	132338579	chr1-132338271-132338579
chr1	132346071	132346754	chr1-132346071-132346754
chr1	132353208	132355652	chr1-132353208-132355652
chr1	132390608	132392211	chr1-132390608-132392211
chr1	132416650	132418759	chr1-132416650-132418759
chr1	132476359	132478857	chr1-132476359-132478857
chr1	132538804	132543213	chr1-132538804-132543213
chr1	132557867	132558654	chr1-132557867-132558654
chr1	132586366	132588065	chr1-132586366-132588065
chr1	132591597	132592686	chr1-132591597-132592686
chr1	132653553	132653977	chr1-132653553-132653977
chr1	132655367	132656660	chr1-132655367-132656660
chr1	132739311	132739684	chr1-132739311-132739684
chr1	132740877	132742156	chr1-132740877-132742156
chr1	132758672	132759178	chr1-132758672-132759178
chr1	132765997	132766590	chr1-132765997-132766590
chr1	132880093	132881569	chr1-132880093-132881569
chr1	133023715	133026521	chr1-133023715-133026521
chr1	133032675	133034593	chr1-133032675-133034593
chr1	133044953	133047922	chr1-133044953-133047922
chr1	133057063	133057860	chr1-133057063-133057860
chr1	133084178	133084488	chr1-133084178-133084488
chr1	133090552	133091123	chr1-133090552-133091123
chr1	133100934	133101670	chr1-133100934-133101670
chr1	133130411	133136284	chr1-133130411-133136284
chr1	133161556	133165752	chr1-133161556-133165752
chr1	133181105	133181745	chr1-133181105-133181745
chr1	133187930	133189583	chr1-133187930-133189583
chr1	133204339	133205691	chr1-133204339-133205691
chr1	133209342	133211094	chr1-133209342-133211094
chr1	133213751	133214068	chr1-133213751-133214068
chr1	133226534	133228410	chr1-133226534-133228410

chr1	133236690	133238154	chr1-133236690-133238154
chr1	133245561	133246627	chr1-133245561-133246627
chr1	133269352	133270547	chr1-133269352-133270547
chr1	133292229	133292691	chr1-133292229-133292691
chr1	133293944	133296668	chr1-133293944-133296668
chr1	133309504	133310388	chr1-133309504-133310388
chr1	133329315	133329671	chr1-133329315-133329671
chr1	133353210	133353679	chr1-133353210-133353679
chr1	133362969	133364575	chr1-133362969-133364575
chr1	133406448	133407321	chr1-133406448-133407321
chr1	133417183	133419099	chr1-133417183-133419099
chr1	133422100	133424824	chr1-133422100-133424824
chr1	133434181	133434499	chr1-133434181-133434499
chr1	133466064	133466320	chr1-133466064-133466320
chr1	133477416	133477785	chr1-133477416-133477785
chr1	133608493	133610745	chr1-133608493-133610745
chr1	133659427	133661859	chr1-133659427-133661859
chr1	133685743	133686166	chr1-133685743-133686166
chr1	133800489	133801619	chr1-133800489-133801619
chr1	133855755	133856542	chr1-133855755-133856542
chr1	133908134	133909482	chr1-133908134-133909482
chr1	133950523	133950923	chr1-133950523-133950923
chr1	133978587	133979127	chr1-133978587-133979127
chr1	134060347	134061728	chr1-134060347-134061728
chr1	134067194	134067545	chr1-134067194-134067545
chr1	134069980	134070960	chr1-134069980-134070960
chr1	134077302	134079724	chr1-134077302-134079724
chr1	134092441	134094010	chr1-134092441-134094010
chr1	134096100	134100744	chr1-134096100-134100744
chr1	134217066	134217317	chr1-134217066-134217317
chr1	134234668	134235942	chr1-134234668-134235942
chr1	134298765	134299472	chr1-134298765-134299472
chr1	134332751	134333303	chr1-134332751-134333303
chr1	134344424	134344919	chr1-134344424-134344919
chr1	134360478	134362765	chr1-134360478-134362765
chr1	134381741	134382502	chr1-134381741-134382502
chr1	134405322	134407369	chr1-134405322-134407369
chr1	134414389	134417891	chr1-134414389-134417891
chr1	134418708	134419512	chr1-134418708-134419512
chr1	134454727	134456600	chr1-134454727-134456600
chr1	134494011	134496580	chr1-134494011-134496580
chr1	134513279	134513743	chr1-134513279-134513743

chr1	134555353	134557505	chr1-134555353-134557505
chr1	134559885	134562342	chr1-134559885-134562342
chr1	134583449	134583650	chr1-134583449-134583650
chr1	134646133	134647456	chr1-134646133-134647456
chr1	134708422	134709834	chr1-134708422-134709834
chr1	134745893	134747732	chr1-134745893-134747732
chr1	134749904	134750844	chr1-134749904-134750844
chr1	134752495	134752849	chr1-134752495-134752849
chr1	134753657	134755188	chr1-134753657-134755188
chr1	134770889	134772239	chr1-134770889-134772239
chr1	134784604	134784805	chr1-134784604-134784805
chr1	134799066	134805007	chr1-134799066-134805007
chr1	134819077	134819757	chr1-134819077-134819757
chr1	134954903	134956292	chr1-134954903-134956292
chr1	134961513	134964089	chr1-134961513-134964089
chr1	134985083	134985685	chr1-134985083-134985685
chr1	135016723	135022811	chr1-135016723-135022811
chr1	135085387	135085588	chr1-135085387-135085588
chr1	135103840	135106215	chr1-135103840-135106215
chr1	135112877	135113078	chr1-135112877-135113078
chr1	135146005	135148727	chr1-135146005-135148727
chr1	135154067	135155056	chr1-135154067-135155056
chr1	135169498	135171214	chr1-135169498-135171214
chr1	135177701	135178005	chr1-135177701-135178005
chr1	135181113	135181314	chr1-135181113-135181314
chr1	135182587	135184632	chr1-135182587-135184632
chr1	135199065	135199680	chr1-135199065-135199680
chr1	135226824	135227977	chr1-135226824-135227977
chr1	135248435	135249228	chr1-135248435-135249228
chr1	135253847	135261794	chr1-135253847-135261794
chr1	135267834	135268628	chr1-135267834-135268628
chr1	135281845	135284447	chr1-135281845-135284447
chr1	135312333	135314646	chr1-135312333-135314646
chr1	135368285	135369144	chr1-135368285-135369144
chr1	135373874	135375681	chr1-135373874-135375681
chr1	135410840	135411910	chr1-135410840-135411910
chr1	135428613	135431165	chr1-135428613-135431165
chr1	135509078	135511267	chr1-135509078-135511267
chr1	135554679	135554880	chr1-135554679-135554880
chr1	135584902	135585911	chr1-135584902-135585911
chr1	135668281	135668533	chr1-135668281-135668533
chr1	135670361	135670562	chr1-135670361-135670562

chr1	135686191	135691902	chr1-135686191-135691902
chr1	135699225	135699871	chr1-135699225-135699871
chr1	135708123	135708625	chr1-135708123-135708625
chr1	135719669	135720375	chr1-135719669-135720375
chr1	135728366	135732974	chr1-135728366-135732974
chr1	135765616	135768063	chr1-135765616-135768063
chr1	135770729	135771613	chr1-135770729-135771613
chr1	135779181	135779484	chr1-135779181-135779484
chr1	135793461	135793874	chr1-135793461-135793874
chr1	135812617	135813327	chr1-135812617-135813327
chr1	135817380	135823088	chr1-135817380-135823088
chr1	135842377	135853002	chr1-135842377-135853002
chr1	135856147	135857751	chr1-135856147-135857751
chr1	135861699	135864297	chr1-135861699-135864297
chr1	135882993	135884232	chr1-135882993-135884232
chr1	135891457	135896025	chr1-135891457-135896025
chr1	135899938	135900682	chr1-135899938-135900682
chr1	135916329	135919779	chr1-135916329-135919779
chr1	135925802	135926456	chr1-135925802-135926456
chr1	135938209	135938786	chr1-135938209-135938786
chr1	135945025	135945226	chr1-135945025-135945226
chr1	135946182	135946433	chr1-135946182-135946433
chr1	136007255	136008449	chr1-136007255-136008449
chr1	136017408	136019167	chr1-136017408-136019167
chr1	136040697	136041118	chr1-136040697-136041118
chr1	136050628	136051661	chr1-136050628-136051661
chr1	136127157	136127408	chr1-136127157-136127408
chr1	136130847	136132384	chr1-136130847-136132384
chr1	136136805	136137523	chr1-136136805-136137523
chr1	136139813	136140899	chr1-136139813-136140899
chr1	136169984	136170185	chr1-136169984-136170185
chr1	136227307	136234935	chr1-136227307-136234935
chr1	136259591	136261126	chr1-136259591-136261126
chr1	136272979	136273282	chr1-136272979-136273282
chr1	136343606	136346924	chr1-136343606-136346924
chr1	136414651	136416124	chr1-136414651-136416124
chr1	136465692	136467448	chr1-136465692-136467448
chr1	136624097	136628025	chr1-136624097-136628025
chr1	136629101	136629302	chr1-136629101-136629302
chr1	136676158	136676880	chr1-136676158-136676880
chr1	136683268	136686062	chr1-136683268-136686062
chr1	136694774	136696533	chr1-136694774-136696533

chr1	136768972	136769223	chr1-136768972-136769223
chr1	136781550	136782239	chr1-136781550-136782239
chr1	136948395	136949574	chr1-136948395-136949574
chr1	136952767	136953240	chr1-136952767-136953240
chr1	137078825	137080006	chr1-137078825-137080006
chr1	137221695	137221998	chr1-137221695-137221998
chr1	137324136	137324438	chr1-137324136-137324438
chr1	137898098	137901311	chr1-137898098-137901311
chr1	138328554	138328969	chr1-138328554-138328969
chr1	138343761	138343962	chr1-138343761-138343962
chr1	138348126	138348553	chr1-138348126-138348553
chr1	138506905	138508085	chr1-138506905-138508085
chr1	138515356	138516395	chr1-138515356-138516395
chr1	138517360	138517694	chr1-138517360-138517694
chr1	138535888	138537960	chr1-138535888-138537960
chr1	138571479	138572237	chr1-138571479-138572237
chr1	138604057	138604258	chr1-138604057-138604258
chr1	138609061	138609546	chr1-138609061-138609546
chr1	138617123	138620562	chr1-138617123-138620562
chr1	138831253	138831673	chr1-138831253-138831673
chr1	138841267	138844422	chr1-138841267-138844422
chr1	138846111	138849250	chr1-138846111-138849250
chr1	138855271	138857082	chr1-138855271-138857082
chr1	138912344	138912696	chr1-138912344-138912696
chr1	138928440	138930039	chr1-138928440-138930039
chr1	138962380	138965524	chr1-138962380-138965524
chr1	138983277	138983478	chr1-138983277-138983478
chr1	139421477	139425330	chr1-139421477-139425330
chr1	139453898	139456468	chr1-139453898-139456468
chr1	140245601	140245802	chr1-140245601-140245802
chr1	143701080	143703699	chr1-143701080-143703699
chr1	143738699	143740798	chr1-143738699-143740798
chr1	143776007	143778868	chr1-143776007-143778868
chr1	144003464	144005098	chr1-144003464-144005098
chr1	144006520	144006848	chr1-144006520-144006848
chr1	144018816	144019017	chr1-144018816-144019017
chr1	146497356	146498847	chr1-146497356-146498847
chr1	146724786	146725190	chr1-146724786-146725190
chr1	149846889	149847353	chr1-149846889-149847353
chr1	149862708	149863231	chr1-149862708-149863231
chr1	149897346	149897599	chr1-149897346-149897599
chr1	149959198	149961791	chr1-149959198-149961791

chr1	150097511	150102880	chr1-150097511-150102880
chr1	150103916	150104117	chr1-150103916-150104117
chr1	150115729	150115981	chr1-150115729-150115981
chr1	150163020	150166025	chr1-150163020-150166025
chr1	150251693	150251949	chr1-150251693-150251949
chr1	150391879	150394789	chr1-150391879-150394789
chr1	150460511	150460712	chr1-150460511-150460712
chr1	150993077	150993681	chr1-150993077-150993681
chr1	151289670	151290092	chr1-151289670-151290092
chr1	151343560	151347273	chr1-151343560-151347273
chr1	151427106	151430472	chr1-151427106-151430472
chr1	151499538	151501641	chr1-151499538-151501641
chr1	151570955	151573310	chr1-151570955-151573310
chr1	151676855	151678216	chr1-151676855-151678216
chr1	151685808	151686059	chr1-151685808-151686059
chr1	151753903	151756761	chr1-151753903-151756761
chr1	152063875	152064383	chr1-152063875-152064383
chr1	152084466	152085458	chr1-152084466-152085458
chr1	152088680	152091398	chr1-152088680-152091398
chr1	152385393	152387079	chr1-152385393-152387079
chr1	152399697	152400782	chr1-152399697-152400782
chr1	152436931	152438150	chr1-152436931-152438150
chr1	152536657	152536858	chr1-152536657-152536858
chr1	152556071	152556954	chr1-152556071-152556954
chr1	152624497	152625648	chr1-152624497-152625648
chr1	152669657	152669908	chr1-152669657-152669908
chr1	152765626	152768019	chr1-152765626-152768019
chr1	152779172	152779587	chr1-152779172-152779587
chr1	152815585	152815786	chr1-152815585-152815786
chr1	152900996	152904869	chr1-152900996-152904869
chr1	152917034	152917675	chr1-152917034-152917675
chr1	152954910	152956407	chr1-152954910-152956407
chr1	153084179	153086524	chr1-153084179-153086524
chr1	153090721	153092955	chr1-153090721-153092955
chr1	153126216	153127631	chr1-153126216-153127631
chr1	153128745	153130999	chr1-153128745-153130999
chr1	153140331	153141893	chr1-153140331-153141893
chr1	153172291	153173075	chr1-153172291-153173075
chr1	153174788	153175665	chr1-153174788-153175665
chr1	153183281	153186945	chr1-153183281-153186945
chr1	153201992	153202603	chr1-153201992-153202603
chr1	153206786	153207758	chr1-153206786-153207758

chr1	153224588	153224839	chr1-153224588-153224839
chr1	153268315	153269893	chr1-153268315-153269893
chr1	153299835	153301276	chr1-153299835-153301276
chr1	153305302	153305553	chr1-153305302-153305553
chr1	153321261	153322096	chr1-153321261-153322096
chr1	153329919	153334260	chr1-153329919-153334260
chr1	153386595	153386796	chr1-153386595-153386796
chr1	153424207	153425711	chr1-153424207-153425711
chr1	153484853	153488254	chr1-153484853-153488254
chr1	153532309	153532719	chr1-153532309-153532719
chr1	153549401	153550015	chr1-153549401-153550015
chr1	153572234	153573513	chr1-153572234-153573513
chr1	153585456	153585964	chr1-153585456-153585964
chr1	153652911	153653390	chr1-153652911-153653390
chr1	153661417	153662153	chr1-153661417-153662153
chr1	153683434	153683635	chr1-153683434-153683635
chr1	153690858	153691179	chr1-153690858-153691179
chr1	153739811	153744714	chr1-153739811-153744714
chr1	153749024	153749965	chr1-153749024-153749965
chr1	153751708	153752887	chr1-153751708-153752887
chr1	153769924	153770125	chr1-153769924-153770125
chr1	153835902	153836153	chr1-153835902-153836153
chr1	153847153	153847406	chr1-153847153-153847406
chr1	153899045	153901852	chr1-153899045-153901852
chr1	153986476	153986827	chr1-153986476-153986827
chr1	154032798	154032999	chr1-154032798-154032999
chr1	154034193	154034541	chr1-154034193-154034541
chr1	154150095	154150682	chr1-154150095-154150682
chr1	154205027	154205228	chr1-154205027-154205228
chr1	154726516	154727023	chr1-154726516-154727023
chr1	154884262	154884773	chr1-154884262-154884773
chr1	155013859	155014450	chr1-155013859-155014450
chr1	155050098	155050776	chr1-155050098-155050776
chr1	155074278	155076030	chr1-155074278-155076030
chr1	155082856	155084413	chr1-155082856-155084413
chr1	155093058	155093959	chr1-155093058-155093959
chr1	155096722	155100106	chr1-155096722-155100106
chr1	155138539	155139372	chr1-155138539-155139372
chr1	155146397	155146769	chr1-155146397-155146769
chr1	155158395	155159820	chr1-155158395-155159820
chr1	155214415	155216119	chr1-155214415-155216119
chr1	155224037	155224388	chr1-155224037-155224388

chr1	155243926	155245106	chr1-155243926-155245106
chr1	155416275	155417731	chr1-155416275-155417731
chr1	155557569	155559500	chr1-155557569-155559500
chr1	155722362	155722678	chr1-155722362-155722678
chr1	155736910	155737967	chr1-155736910-155737967
chr1	155740458	155743403	chr1-155740458-155743403
chr1	155809479	155813565	chr1-155809479-155813565
chr1	155971772	155974496	chr1-155971772-155974496
chr1	156034526	156037978	chr1-156034526-156037978
chr1	156040503	156042707	chr1-156040503-156042707
chr1	156204313	156205231	chr1-156204313-156205231
chr1	156222221	156223078	chr1-156222221-156223078
chr1	156302693	156304101	chr1-156302693-156304101
chr1	156310690	156311150	chr1-156310690-156311150
chr1	156344540	156344741	chr1-156344540-156344741
chr1	156420304	156421629	chr1-156420304-156421629
chr1	156464395	156464714	chr1-156464395-156464714
chr1	156470738	156474679	chr1-156470738-156474679
chr1	156507370	156507696	chr1-156507370-156507696
chr1	156558172	156560252	chr1-156558172-156560252
chr1	156615508	156620810	chr1-156615508-156620810
chr1	156666844	156668227	chr1-156666844-156668227
chr1	156673210	156675168	chr1-156673210-156675168
chr1	156677435	156677787	chr1-156677435-156677787
chr1	156717619	156719918	chr1-156717619-156719918
chr1	156938288	156940906	chr1-156938288-156940906
chr1	156987807	156988108	chr1-156987807-156988108
chr1	157001252	157001554	chr1-157001252-157001554
chr1	157020003	157020259	chr1-157020003-157020259
chr1	157061230	157061431	chr1-157061230-157061431
chr1	157076959	157077318	chr1-157076959-157077318
chr1	157079159	157082037	chr1-157079159-157082037
chr1	157086162	157086930	chr1-157086162-157086930
chr1	157232629	157233130	chr1-157232629-157233130
chr1	157410853	157413371	chr1-157410853-157413371
chr1	157440295	157440660	chr1-157440295-157440660
chr1	157458471	157458886	chr1-157458471-157458886
chr1	157717420	157717621	chr1-157717420-157717621
chr1	158355530	158357039	chr1-158355530-158357039
chr1	158362359	158362781	chr1-158362359-158362781
chr1	159231227	159233920	chr1-159231227-159233920
chr1	159392679	159393553	chr1-159392679-159393553

chr1	159396634	159396886	chr1-159396634-159396886
chr1	159686325	159687167	chr1-159686325-159687167
chr1	159889116	159889632	chr1-159889116-159889632
chr1	160043848	160047149	chr1-160043848-160047149
chr1	160052836	160053373	chr1-160052836-160053373
chr1	160057865	160058232	chr1-160057865-160058232
chr1	160060166	160061099	chr1-160060166-160061099
chr1	160194473	160196509	chr1-160194473-160196509
chr1	160210458	160214250	chr1-160210458-160214250
chr1	160307043	160307545	chr1-160307043-160307545
chr1	160308462	160309477	chr1-160308462-160309477
chr1	160520239	160521510	chr1-160520239-160521510
chr1	160596859	160597225	chr1-160596859-160597225
chr1	160791452	160793530	chr1-160791452-160793530
chr1	160905706	160908056	chr1-160905706-160908056
chr1	161033384	161039528	chr1-161033384-161039528
chr1	161069812	161073526	chr1-161069812-161073526
chr1	161129987	161131930	chr1-161129987-161131930
chr1	161140634	161140835	chr1-161140634-161140835
chr1	161142676	161142877	chr1-161142676-161142877
chr1	161170500	161171179	chr1-161170500-161171179
chr1	161195345	161196576	chr1-161195345-161196576
chr1	161247263	161252024	chr1-161247263-161252024
chr1	161734615	161735632	chr1-161734615-161735632
chr1	161875194	161878045	chr1-161875194-161878045
chr1	161968642	161970204	chr1-161968642-161970204
chr1	162132997	162133198	chr1-162132997-162133198
chr1	162453732	162453933	chr1-162453732-162453933
chr1	162476676	162478277	chr1-162476676-162478277
chr1	162509277	162510071	chr1-162509277-162510071
chr1	162547180	162549211	chr1-162547180-162549211
chr1	162569640	162571930	chr1-162569640-162571930
chr1	162737744	162741301	chr1-162737744-162741301
chr1	162859860	162860136	chr1-162859860-162860136
chr1	163311793	163315228	chr1-163311793-163315228
chr1	163402604	163404265	chr1-163402604-163404265
chr1	163779143	163780492	chr1-163779143-163780492
chr1	163929052	163931413	chr1-163929052-163931413
chr1	163993693	163995797	chr1-163993693-163995797
chr1	164135318	164136174	chr1-164135318-164136174
chr1	164224451	164224652	chr1-164224451-164224652
chr1	164247844	164251272	chr1-164247844-164251272

chr1	164275264	164276244	chr1-164275264-164276244
chr1	164306155	164308584	chr1-164306155-164308584
chr1	164454439	164459539	chr1-164454439-164459539
chr1	164503541	164503742	chr1-164503541-164503742
chr1	164628827	164629028	chr1-164628827-164629028
chr1	164898801	164899458	chr1-164898801-164899458
chr1	164975684	164975935	chr1-164975684-164975935
chr1	164993417	164994523	chr1-164993417-164994523
chr1	165046022	165047054	chr1-165046022-165047054
chr1	165191512	165195284	chr1-165191512-165195284
chr1	165235580	165237760	chr1-165235580-165237760
chr1	165281198	165282965	chr1-165281198-165282965
chr1	165302683	165303336	chr1-165302683-165303336
chr1	165432533	165433001	chr1-165432533-165433001
chr1	165458940	165462303	chr1-165458940-165462303
chr1	165617414	165617717	chr1-165617414-165617717
chr1	165629557	165635679	chr1-165629557-165635679
chr1	165640983	165643824	chr1-165640983-165643824
chr1	165702695	165703067	chr1-165702695-165703067
chr1	165707608	165708427	chr1-165707608-165708427
chr1	165762983	165764983	chr1-165762983-165764983
chr1	165769009	165769413	chr1-165769009-165769413
chr1	165780097	165780909	chr1-165780097-165780909
chr1	165840775	165841394	chr1-165840775-165841394
chr1	165844321	165845837	chr1-165844321-165845837
chr1	165880052	165880364	chr1-165880052-165880364
chr1	166000524	166004046	chr1-166000524-166004046
chr1	166043384	166045960	chr1-166043384-166045960
chr1	166047419	166049376	chr1-166047419-166049376
chr1	166130173	166131891	chr1-166130173-166131891
chr1	166137807	166138458	chr1-166137807-166138458
chr1	166154108	166154831	chr1-166154108-166154831
chr1	166253813	166254510	chr1-166253813-166254510
chr1	166308700	166309772	chr1-166308700-166309772
chr1	166378622	166380686	chr1-166378622-166380686
chr1	166408903	166410273	chr1-166408903-166410273
chr1	166944763	166945033	chr1-166944763-166945033
chr1	167000350	167002767	chr1-167000350-167002767
chr1	167231428	167232846	chr1-167231428-167232846
chr1	167242349	167243518	chr1-167242349-167243518
chr1	167269096	167272042	chr1-167269096-167272042
chr1	167278858	167279317	chr1-167278858-167279317

chr1	167282060	167285786	chr1-167282060-167285786
chr1	167307134	167309884	chr1-167307134-167309884
chr1	167339978	167340847	chr1-167339978-167340847
chr1	167349389	167351084	chr1-167349389-167351084
chr1	167386853	167388160	chr1-167386853-167388160
chr1	167392025	167394822	chr1-167392025-167394822
chr1	167598268	167598792	chr1-167598268-167598792
chr1	167660919	167661459	chr1-167660919-167661459
chr1	167688371	167689535	chr1-167688371-167689535
chr1	167691941	167692467	chr1-167691941-167692467
chr1	167802607	167803475	chr1-167802607-167803475
chr1	167929720	167930139	chr1-167929720-167930139
chr1	168220880	168221820	chr1-168220880-168221820
chr1	168283197	168284319	chr1-168283197-168284319
chr1	168335123	168335715	chr1-168335123-168335715
chr1	168391018	168393199	chr1-168391018-168393199
chr1	168426415	168433122	chr1-168426415-168433122
chr1	168501413	168502440	chr1-168501413-168502440
chr1	168518504	168518916	chr1-168518504-168518916
chr1	168708294	168710146	chr1-168708294-168710146
chr1	169530058	169532265	chr1-169530058-169532265
chr1	169747488	169747792	chr1-169747488-169747792
chr1	169934180	169934481	chr1-169934180-169934481
chr1	169968025	169970716	chr1-169968025-169970716
chr1	170006390	170006641	chr1-170006390-170006641
chr1	170008794	170009045	chr1-170008794-170009045
chr1	170109915	170110860	chr1-170109915-170110860
chr1	170147580	170148540	chr1-170147580-170148540
chr1	170173484	170175835	chr1-170173484-170175835
chr1	170188492	170189106	chr1-170188492-170189106
chr1	170214201	170215731	chr1-170214201-170215731
chr1	170254714	170256305	chr1-170254714-170256305
chr1	170276911	170279012	chr1-170276911-170279012
chr1	170392874	170393306	chr1-170392874-170393306
chr1	170588334	170590405	chr1-170588334-170590405
chr1	170643809	170645377	chr1-170643809-170645377
chr1	170766802	170767054	chr1-170766802-170767054
chr1	170770267	170772084	chr1-170770267-170772084
chr1	170829469	170829670	chr1-170829469-170829670
chr1	170865427	170869342	chr1-170865427-170869342
chr1	170883888	170885932	chr1-170883888-170885932
chr1	170901055	170902087	chr1-170901055-170902087

chr1	170907440	170908767	chr1-170907440-170908767
chr1	171019343	171020223	chr1-171019343-171020223
chr1	171032635	171034781	chr1-171032635-171034781
chr1	171035714	171038723	chr1-171035714-171038723
chr1	171043686	171045554	chr1-171043686-171045554
chr1	171063584	171066686	chr1-171063584-171066686
chr1	171069829	171071826	chr1-171069829-171071826
chr1	171073712	171074422	chr1-171073712-171074422
chr1	171077488	171079486	chr1-171077488-171079486
chr1	171081314	171081953	chr1-171081314-171081953
chr1	171083440	171083641	chr1-171083440-171083641
chr1	171085189	171086247	chr1-171085189-171086247
chr1	171088532	171088913	chr1-171088532-171088913
chr1	171096210	171096719	chr1-171096210-171096719
chr1	171101465	171103899	chr1-171101465-171103899
chr1	171110614	171114623	chr1-171110614-171114623
chr1	171118568	171118769	chr1-171118568-171118769
chr1	171122855	171123407	chr1-171122855-171123407
chr1	171149615	171151463	chr1-171149615-171151463
chr1	171159468	171160281	chr1-171159468-171160281
chr1	171184245	171184446	chr1-171184245-171184446
chr1	171195491	171196430	chr1-171195491-171196430
chr1	171220961	171223514	chr1-171220961-171223514
chr1	171245735	171248790	chr1-171245735-171248790
chr1	171262403	171263590	chr1-171262403-171263590
chr1	171269597	171272729	chr1-171269597-171272729
chr1	171279750	171282344	chr1-171279750-171282344
chr1	171286521	171288457	chr1-171286521-171288457
chr1	171293407	171295799	chr1-171293407-171295799
chr1	171319994	171320498	chr1-171319994-171320498
chr1	171327465	171331072	chr1-171327465-171331072
chr1	171342648	171342849	chr1-171342648-171342849
chr1	171343916	171347477	chr1-171343916-171347477
chr1	171348816	171349171	chr1-171348816-171349171
chr1	171369789	171378782	chr1-171369789-171378782
chr1	171410681	171413471	chr1-171410681-171413471
chr1	171424362	171424946	chr1-171424362-171424946
chr1	171436623	171443295	chr1-171436623-171443295
chr1	171469167	171471703	chr1-171469167-171471703
chr1	171477356	171479150	chr1-171477356-171479150
chr1	171501354	171502342	chr1-171501354-171502342
chr1	171503302	171504981	chr1-171503302-171504981

chr1	172026209	172027938	chr1-172026209-172027938
chr1	172053697	172054448	chr1-172053697-172054448
chr1	172081500	172084436	chr1-172081500-172084436
chr1	172126073	172128038	chr1-172126073-172128038
chr1	172147346	172149852	chr1-172147346-172149852
chr1	172204828	172207193	chr1-172204828-172207193
chr1	172217039	172217342	chr1-172217039-172217342
chr1	172307834	172308733	chr1-172307834-172308733
chr1	172309777	172313377	chr1-172309777-172313377
chr1	172325780	172326831	chr1-172325780-172326831
chr1	172340619	172341577	chr1-172340619-172341577
chr1	172375611	172378435	chr1-172375611-172378435
chr1	172390214	172391095	chr1-172390214-172391095
chr1	172424023	172424224	chr1-172424023-172424224
chr1	172457757	172457958	chr1-172457757-172457958
chr1	172458783	172459086	chr1-172458783-172459086
chr1	172462124	172462967	chr1-172462124-172462967
chr1	172481013	172486738	chr1-172481013-172486738
chr1	172490056	172490619	chr1-172490056-172490619
chr1	172499213	172504547	chr1-172499213-172504547
chr1	172513872	172514260	chr1-172513872-172514260
chr1	172519791	172521495	chr1-172519791-172521495
chr1	172548701	172551073	chr1-172548701-172551073
chr1	172561942	172563810	chr1-172561942-172563810
chr1	172632263	172633582	chr1-172632263-172633582
chr1	173367164	173367990	chr1-173367164-173367990
chr1	173631058	173632452	chr1-173631058-173632452
chr1	173766524	173767653	chr1-173766524-173767653
chr1	173910742	173913670	chr1-173910742-173913670
chr1	173981181	173981483	chr1-173981181-173981483
chr1	174031379	174031962	chr1-174031379-174031962
chr1	174501686	174503719	chr1-174501686-174503719
chr1	174921443	174921907	chr1-174921443-174921907
chr1	175491759	175493031	chr1-175491759-175493031
chr1	175624298	175626232	chr1-175624298-175626232
chr1	175691377	175693767	chr1-175691377-175693767
chr1	175725744	175725945	chr1-175725744-175725945
chr1	175880225	175882180	chr1-175880225-175882180
chr1	175883087	175883288	chr1-175883087-175883288
chr1	175936368	175936619	chr1-175936368-175936619
chr1	176274676	176275710	chr1-176274676-176275710
chr1	176811391	176815575	chr1-176811391-176815575

chr1	176817878	176819083	chr1-176817878-176819083
chr1	176827300	176827888	chr1-176827300-176827888
chr1	176831851	176836589	chr1-176831851-176836589
chr1	177014148	177015094	chr1-177014148-177015094
chr1	177071369	177071640	chr1-177071369-177071640
chr1	177256957	177259485	chr1-177256957-177259485
chr1	177322165	177323304	chr1-177322165-177323304
chr1	177338501	177339422	chr1-177338501-177339422
chr1	177440532	177444419	chr1-177440532-177444419
chr1	177453532	177454115	chr1-177453532-177454115
chr1	177470015	177471129	chr1-177470015-177471129
chr1	177479263	177479914	chr1-177479263-177479914
chr1	177555570	177555873	chr1-177555570-177555873
chr1	177623102	177623950	chr1-177623102-177623950
chr1	177649306	177649507	chr1-177649306-177649507
chr1	177702525	177705007	chr1-177702525-177705007
chr1	177711212	177711969	chr1-177711212-177711969
chr1	177718948	177719204	chr1-177718948-177719204
chr1	177740775	177742651	chr1-177740775-177742651
chr1	177744400	177744601	chr1-177744400-177744601
chr1	177792802	177797170	chr1-177792802-177797170
chr1	177982822	177984594	chr1-177982822-177984594
chr1	178186612	178189589	chr1-178186612-178189589
chr1	178275694	178276859	chr1-178275694-178276859
chr1	178317291	178321443	chr1-178317291-178321443
chr1	178333077	178338879	chr1-178333077-178338879
chr1	178368608	178369694	chr1-178368608-178369694
chr1	178405245	178407261	chr1-178405245-178407261
chr1	178492732	178492933	chr1-178492732-178492933
chr1	178527230	178531101	chr1-178527230-178531101
chr1	179041766	179041967	chr1-179041766-179041967
chr1	179136415	179137280	chr1-179136415-179137280
chr1	179516298	179518479	chr1-179516298-179518479
chr1	179544988	179547647	chr1-179544988-179547647
chr1	179570755	179571164	chr1-179570755-179571164
chr1	179573933	179574185	chr1-179573933-179574185
chr1	179645841	179646105	chr1-179645841-179646105
chr1	179667582	179669597	chr1-179667582-179669597
chr1	179801061	179804610	chr1-179801061-179804610
chr1	179828789	179829602	chr1-179828789-179829602
chr1	179847335	179849152	chr1-179847335-179849152
chr1	179944821	179945339	chr1-179944821-179945339

chr1	179946361	179946622	chr1-179946361-179946622
chr1	179958788	179962826	chr1-179958788-179962826
chr1	179969358	179970237	chr1-179969358-179970237
chr1	179984003	179984418	chr1-179984003-179984418
chr1	180014711	180014912	chr1-180014711-180014912
chr1	180015784	180016101	chr1-180015784-180016101
chr1	180195042	180196627	chr1-180195042-180196627
chr1	180225765	180226670	chr1-180225765-180226670
chr1	180250150	180250408	chr1-180250150-180250408
chr1	180254994	180257065	chr1-180254994-180257065
chr1	180279399	180280267	chr1-180279399-180280267
chr1	180288957	180290112	chr1-180288957-180290112
chr1	180300835	180301467	chr1-180300835-180301467
chr1	180313000	180314017	chr1-180313000-180314017
chr1	180329947	180332695	chr1-180329947-180332695
chr1	180363829	180365944	chr1-180363829-180365944
chr1	180386602	180388100	chr1-180386602-180388100
chr1	180403558	180404847	chr1-180403558-180404847
chr1	180407158	180407673	chr1-180407158-180407673
chr1	180462701	180463677	chr1-180462701-180463677
chr1	180483084	180483812	chr1-180483084-180483812
chr1	180568312	180570423	chr1-180568312-180570423
chr1	180627561	180628529	chr1-180627561-180628529
chr1	180639772	180642731	chr1-180639772-180642731
chr1	180696426	180697470	chr1-180696426-180697470
chr1	180725249	180728193	chr1-180725249-180728193
chr1	180763770	180764075	chr1-180763770-180764075
chr1	180767883	180769080	chr1-180767883-180769080
chr1	180776751	180778908	chr1-180776751-180778908
chr1	180783279	180783666	chr1-180783279-180783666
chr1	180794077	180794488	chr1-180794077-180794488
chr1	180811145	180814851	chr1-180811145-180814851
chr1	180847710	180852520	chr1-180847710-180852520
chr1	180903637	180905844	chr1-180903637-180905844
chr1	180936621	180937232	chr1-180936621-180937232
chr1	180941184	180944591	chr1-180941184-180944591
chr1	181010200	181010579	chr1-181010200-181010579
chr1	181015432	181017773	chr1-181015432-181017773
chr1	181142603	181146207	chr1-181142603-181146207
chr1	181150715	181152303	chr1-181150715-181152303
chr1	181209714	181213170	chr1-181209714-181213170
chr1	181230307	181232883	chr1-181230307-181232883

chr1	181246267	181247704	chr1-181246267-181247704
chr1	181255470	181257911	chr1-181255470-181257911
chr1	181352004	181354036	chr1-181352004-181354036
chr1	181425296	181426101	chr1-181425296-181426101
chr1	181521507	181522009	chr1-181521507-181522009
chr1	181552479	181553667	chr1-181552479-181553667
chr1	181616011	181616212	chr1-181616011-181616212
chr1	181839305	181843692	chr1-181839305-181843692
chr1	181848053	181848254	chr1-181848053-181848254
chr1	181852637	181853853	chr1-181852637-181853853
chr1	181860398	181861223	chr1-181860398-181861223
chr1	181881423	181881782	chr1-181881423-181881782
chr1	181912090	181912291	chr1-181912090-181912291
chr1	181921659	181922045	chr1-181921659-181922045
chr1	182018373	182020909	chr1-182018373-182020909
chr1	182057885	182058624	chr1-182057885-182058624
chr1	182091231	182092683	chr1-182091231-182092683
chr1	182094756	182095736	chr1-182094756-182095736
chr1	182115466	182115667	chr1-182115466-182115667
chr1	182124380	182126464	chr1-182124380-182126464
chr1	182255153	182257146	chr1-182255153-182257146
chr1	182281284	182283491	chr1-182281284-182283491
chr1	182340019	182342059	chr1-182340019-182342059
chr1	182368268	182369993	chr1-182368268-182369993
chr1	182407884	182410380	chr1-182407884-182410380
chr1	182489938	182490394	chr1-182489938-182490394
chr1	182503867	182504939	chr1-182503867-182504939
chr1	182506333	182506898	chr1-182506333-182506898
chr1	182509102	182520827	chr1-182509102-182520827
chr1	182527787	182528990	chr1-182527787-182528990
chr1	182539223	182539574	chr1-182539223-182539574
chr1	182560559	182560760	chr1-182560559-182560760
chr1	182574536	182574861	chr1-182574536-182574861
chr1	182585363	182585824	chr1-182585363-182585824
chr1	182592820	182593188	chr1-182592820-182593188
chr1	182670564	182670903	chr1-182670564-182670903
chr1	182679285	182681141	chr1-182679285-182681141
chr1	182686971	182687172	chr1-182686971-182687172
chr1	182728465	182728666	chr1-182728465-182728666
chr1	182763368	182765664	chr1-182763368-182765664
chr1	182907975	182908898	chr1-182907975-182908898
chr1	182954502	182956801	chr1-182954502-182956801

chr1	182959435	182960455	chr1-182959435-182960455
chr1	182982830	182983603	chr1-182982830-182983603
chr1	183113444	183113746	chr1-183113444-183113746
chr1	183144288	183144705	chr1-183144288-183144705
chr1	183220533	183221758	chr1-183220533-183221758
chr1	183295664	183298809	chr1-183295664-183298809
chr1	183344617	183346471	chr1-183344617-183346471
chr1	183368418	183370206	chr1-183368418-183370206
chr1	183388383	183390436	chr1-183388383-183390436
chr1	183441864	183442167	chr1-183441864-183442167
chr1	183600838	183601095	chr1-183600838-183601095
chr1	183679870	183680884	chr1-183679870-183680884
chr1	183778564	183778871	chr1-183778564-183778871
chr1	184032554	184037626	chr1-184032554-184037626
chr1	184048923	184049270	chr1-184048923-184049270
chr1	184050602	184052795	chr1-184050602-184052795
chr1	184057771	184057972	chr1-184057771-184057972
chr1	184260655	184261344	chr1-184260655-184261344
chr1	184275534	184275896	chr1-184275534-184275896
chr1	184276881	184277142	chr1-184276881-184277142
chr1	184455495	184456591	chr1-184455495-184456591
chr1	184675364	184676073	chr1-184675364-184676073
chr1	184695183	184695492	chr1-184695183-184695492
chr1	184704888	184705324	chr1-184704888-184705324
chr1	184707835	184708140	chr1-184707835-184708140
chr1	184730613	184733515	chr1-184730613-184733515
chr1	184782456	184782657	chr1-184782456-184782657
chr1	184824417	184824618	chr1-184824417-184824618
chr1	184844343	184847106	chr1-184844343-184847106
chr1	184858465	184858818	chr1-184858465-184858818
chr1	184861188	184861504	chr1-184861188-184861504
chr1	184862493	184862804	chr1-184862493-184862804
chr1	184881249	184883451	chr1-184881249-184883451
chr1	184908672	184908873	chr1-184908672-184908873
chr1	184911958	184912263	chr1-184911958-184912263
chr1	184998966	185000237	chr1-184998966-185000237
chr1	185203016	185205459	chr1-185203016-185205459
chr1	185327936	185329860	chr1-185327936-185329860
chr1	185331528	185333374	chr1-185331528-185333374
chr1	185362009	185365382	chr1-185362009-185365382
chr1	185454638	185455734	chr1-185454638-185455734
chr1	186116557	186117569	chr1-186116557-186117569

chr1	186182823	186183024	chr1-186182823-186183024
chr1	186448783	186449251	chr1-186448783-186449251
chr1	186478497	186478748	chr1-186478497-186478748
chr1	186567977	186568178	chr1-186567977-186568178
chr1	186581401	186581653	chr1-186581401-186581653
chr1	186585668	186587772	chr1-186585668-186587772
chr1	186609621	186610483	chr1-186609621-186610483
chr1	186675978	186676179	chr1-186675978-186676179
chr1	186703666	186706247	chr1-186703666-186706247
chr1	186747485	186750401	chr1-186747485-186750401
chr1	186833410	186834732	chr1-186833410-186834732
chr1	187120827	187122024	chr1-187120827-187122024
chr1	187134128	187134795	chr1-187134128-187134795
chr1	187214773	187216512	chr1-187214773-187216512
chr1	187608818	187609747	chr1-187608818-187609747
chr1	187651641	187652361	chr1-187651641-187652361
chr1	187997492	187998101	chr1-187997492-187998101
chr1	189005416	189008286	chr1-189005416-189008286
chr1	189012776	189013666	chr1-189012776-189013666
chr1	189213631	189215576	chr1-189213631-189215576
chr1	189343322	189344107	chr1-189343322-189344107
chr1	189685912	189689481	chr1-189685912-189689481
chr1	189716556	189716875	chr1-189716556-189716875
chr1	189726978	189730725	chr1-189726978-189730725
chr1	189761149	189762294	chr1-189761149-189762294
chr1	189773177	189773452	chr1-189773177-189773452
chr1	189784418	189785410	chr1-189784418-189785410
chr1	189792146	189793004	chr1-189792146-189793004
chr1	189860374	189860629	chr1-189860374-189860629
chr1	189866832	189868385	chr1-189866832-189868385
chr1	189875004	189876228	chr1-189875004-189876228
chr1	189920715	189923177	chr1-189920715-189923177
chr1	189972854	189973235	chr1-189972854-189973235
chr1	190168875	190171234	chr1-190168875-190171234
chr1	190174789	190175509	chr1-190174789-190175509
chr1	190910645	190912552	chr1-190910645-190912552
chr1	190927688	190929834	chr1-190927688-190929834
chr1	190977133	190980066	chr1-190977133-190980066
chr1	191024454	191027096	chr1-191024454-191027096
chr1	191062191	191064197	chr1-191062191-191064197
chr1	191097276	191099213	chr1-191097276-191099213
chr1	191125785	191126474	chr1-191125785-191126474

chr1	191132267	191136062	chr1-191132267-191136062
chr1	191179977	191186958	chr1-191179977-191186958
chr1	191193388	191194327	chr1-191193388-191194327
chr1	191204962	191205230	chr1-191204962-191205230
chr1	191215989	191216924	chr1-191215989-191216924
chr1	191222210	191222565	chr1-191222210-191222565
chr1	191223826	191225575	chr1-191223826-191225575
chr1	191277324	191278657	chr1-191277324-191278657
chr1	191280892	191281669	chr1-191280892-191281669
chr1	191292640	191292942	chr1-191292640-191292942
chr1	191312300	191313572	chr1-191312300-191313572
chr1	191317046	191318552	chr1-191317046-191318552
chr1	191325580	191327500	chr1-191325580-191327500
chr1	191394173	191398259	chr1-191394173-191398259
chr1	191432553	191432812	chr1-191432553-191432812
chr1	191498135	191498850	chr1-191498135-191498850
chr1	191573759	191578215	chr1-191573759-191578215
chr1	191661996	191662407	chr1-191661996-191662407
chr1	191717111	191720760	chr1-191717111-191720760
chr1	191784588	191785892	chr1-191784588-191785892
chr1	191801466	191803776	chr1-191801466-191803776
chr1	191817560	191818287	chr1-191817560-191818287
chr1	191820806	191822774	chr1-191820806-191822774
chr1	191905556	191908797	chr1-191905556-191908797
chr1	191989312	191990677	chr1-191989312-191990677
chr1	192061894	192062155	chr1-192061894-192062155
chr1	192091738	192093172	chr1-192091738-192093172
chr1	192135918	192139215	chr1-192135918-192139215
chr1	192190460	192191568	chr1-192190460-192191568
chr1	192770178	192771488	chr1-192770178-192771488
chr1	192834454	192835324	chr1-192834454-192835324
chr1	192854097	192856168	chr1-192854097-192856168
chr1	193035293	193036209	chr1-193035293-193036209
chr1	193128949	193131268	chr1-193128949-193131268
chr1	193149708	193149909	chr1-193149708-193149909
chr1	193150873	193151363	chr1-193150873-193151363
chr1	193152528	193154721	chr1-193152528-193154721
chr1	193172393	193174845	chr1-193172393-193174845
chr1	193272686	193273481	chr1-193272686-193273481
chr1	193298510	193298769	chr1-193298510-193298769
chr1	193300848	193305727	chr1-193300848-193305727
chr1	193319396	193319725	chr1-193319396-193319725

chr1	193382490	193383008	chr1-193382490-193383008
chr1	193413197	193414399	chr1-193413197-193414399
chr1	194618792	194621773	chr1-194618792-194621773
chr1	194660574	194660840	chr1-194660574-194660840
chr1	194675881	194676258	chr1-194675881-194676258
chr1	194878772	194880325	chr1-194878772-194880325
chr1	194938833	194939190	chr1-194938833-194939190
chr1	194975964	194977846	chr1-194975964-194977846
chr1	195016859	195018225	chr1-195016859-195018225
chr1	195091632	195092770	chr1-195091632-195092770
chr1	195130369	195132006	chr1-195130369-195132006
chr1	195176372	195176948	chr1-195176372-195176948
chr10	3236713	3236914	chr10-3236713-3236914
chr10	3365970	3367341	chr10-3365970-3367341
chr10	3740077	3741333	chr10-3740077-3741333
chr10	3972513	3974979	chr10-3972513-3974979
chr10	4105825	4108597	chr10-4105825-4108597
chr10	4120711	4121275	chr10-4120711-4121275
chr10	4132506	4132707	chr10-4132506-4132707
chr10	4265911	4267798	chr10-4265911-4267798
chr10	4333961	4335043	chr10-4333961-4335043
chr10	4346593	4347305	chr10-4346593-4347305
chr10	4385834	4390178	chr10-4385834-4390178
chr10	4395564	4395925	chr10-4395564-4395925
chr10	4430861	4434311	chr10-4430861-4434311
chr10	4610131	4610332	chr10-4610131-4610332
chr10	4611240	4613135	chr10-4611240-4613135
chr10	4620668	4621833	chr10-4620668-4621833
chr10	4709878	4710559	chr10-4709878-4710559
chr10	4712060	4713088	chr10-4712060-4713088
chr10	5181698	5182114	chr10-5181698-5182114
chr10	5192775	5195169	chr10-5192775-5195169
chr10	5207879	5208863	chr10-5207879-5208863
chr10	5212355	5213426	chr10-5212355-5213426
chr10	5287972	5290030	chr10-5287972-5290030
chr10	5367382	5367790	chr10-5367382-5367790
chr10	5550125	5551065	chr10-5550125-5551065
chr10	5801837	5806894	chr10-5801837-5806894
chr10	5822481	5825114	chr10-5822481-5825114
chr10	5920941	5923195	chr10-5920941-5923195
chr10	7117851	7120230	chr10-7117851-7120230
chr10	7145981	7146182	chr10-7145981-7146182

chr10	7158468	7159652	chr10-7158468-7159652
chr10	7188840	7189667	chr10-7188840-7189667
chr10	7210211	7213028	chr10-7210211-7213028
chr10	7390196	7393831	chr10-7390196-7393831
chr10	7470118	7474551	chr10-7470118-7474551
chr10	7491557	7492697	chr10-7491557-7492697
chr10	7589106	7591249	chr10-7589106-7591249
chr10	7661620	7664294	chr10-7661620-7664294
chr10	7667045	7668584	chr10-7667045-7668584
chr10	7679762	7683439	chr10-7679762-7683439
chr10	7725554	7727811	chr10-7725554-7727811
chr10	7779753	7781812	chr10-7779753-7781812
chr10	7791716	7794052	chr10-7791716-7794052
chr10	7832136	7832445	chr10-7832136-7832445
chr10	7841642	7842302	chr10-7841642-7842302
chr10	7954004	7957505	chr10-7954004-7957505
chr10	8287284	8287538	chr10-8287284-8287538
chr10	8499526	8499940	chr10-8499526-8499940
chr10	8505609	8506488	chr10-8505609-8506488
chr10	8517544	8519578	chr10-8517544-8519578
chr10	8768396	8768903	chr10-8768396-8768903
chr10	8883509	8887484	chr10-8883509-8887484
chr10	8898963	8899659	chr10-8898963-8899659
chr10	9504092	9504425	chr10-9504092-9504425
chr10	9672720	9677256	chr10-9672720-9677256
chr10	9899023	9902251	chr10-9899023-9902251
chr10	10556578	10558388	chr10-10556578-10558388
chr10	11148841	11150819	chr10-11148841-11150819
chr10	11279981	11283054	chr10-11279981-11283054
chr10	11342965	11344410	chr10-11342965-11344410
chr10	12298679	12298937	chr10-12298679-12298937
chr10	12694911	12695288	chr10-12694911-12695288
chr10	12787853	12788054	chr10-12787853-12788054
chr10	12826355	12827793	chr10-12826355-12827793
chr10	12852773	12853024	chr10-12852773-12853024
chr10	12858221	12858850	chr10-12858221-12858850
chr10	12866525	12869924	chr10-12866525-12869924
chr10	12874477	12875302	chr10-12874477-12875302
chr10	12878689	12879198	chr10-12878689-12879198
chr10	12884383	12884809	chr10-12884383-12884809
chr10	12888419	12889028	chr10-12888419-12889028
chr10	12893155	12893356	chr10-12893155-12893356

chr10	12919043	12919333	chr10-12919043-12919333
chr10	12920888	12923861	chr10-12920888-12923861
chr10	12949317	12949928	chr10-12949317-12949928
chr10	12963153	12964924	chr10-12963153-12964924
chr10	13007754	13010037	chr10-13007754-13010037
chr10	13060874	13061389	chr10-13060874-13061389
chr10	13090201	13091823	chr10-13090201-13091823
chr10	13191947	13193918	chr10-13191947-13193918
chr10	13323017	13324622	chr10-13323017-13324622
chr10	13328330	13328643	chr10-13328330-13328643
chr10	13369423	13370162	chr10-13369423-13370162
chr10	13473482	13475053	chr10-13473482-13475053
chr10	13489888	13490089	chr10-13489888-13490089
chr10	13500519	13502266	chr10-13500519-13502266
chr10	13552147	13554187	chr10-13552147-13554187
chr10	13775876	13776077	chr10-13775876-13776077
chr10	13805303	13805504	chr10-13805303-13805504
chr10	13867744	13869964	chr10-13867744-13869964
chr10	13964047	13968550	chr10-13964047-13968550
chr10	13982269	13984100	chr10-13982269-13984100
chr10	14017836	14019662	chr10-14017836-14019662
chr10	14085177	14086686	chr10-14085177-14086686
chr10	14313319	14314921	chr10-14313319-14314921
chr10	14533855	14534166	chr10-14533855-14534166
chr10	14543658	14546412	chr10-14543658-14546412
chr10	14686505	14686756	chr10-14686505-14686756
chr10	14691710	14691911	chr10-14691710-14691911
chr10	14704016	14706185	chr10-14704016-14706185
chr10	14759670	14760701	chr10-14759670-14760701
chr10	16508152	16508353	chr10-16508152-16508353
chr10	16589212	16589519	chr10-16589212-16589519
chr10	17531122	17531387	chr10-17531122-17531387
chr10	17548784	17550189	chr10-17548784-17550189
chr10	17721423	17727944	chr10-17721423-17727944
chr10	17946096	17948764	chr10-17946096-17948764
chr10	18005401	18005602	chr10-18005401-18005602
chr10	18021720	18023623	chr10-18021720-18023623
chr10	18055033	18057607	chr10-18055033-18057607
chr10	18139317	18139633	chr10-18139317-18139633
chr10	18197278	18197479	chr10-18197278-18197479
chr10	18210671	18210975	chr10-18210671-18210975
chr10	18234182	18235939	chr10-18234182-18235939

chr10	18318007	18320526	chr10-18318007-18320526
chr10	18323179	18324264	chr10-18323179-18324264
chr10	18328545	18330191	chr10-18328545-18330191
chr10	18370573	18371301	chr10-18370573-18371301
chr10	18406596	18408531	chr10-18406596-18408531
chr10	18544988	18546380	chr10-18544988-18546380
chr10	18549943	18550200	chr10-18549943-18550200
chr10	18613114	18613315	chr10-18613114-18613315
chr10	18742897	18744278	chr10-18742897-18744278
chr10	18844352	18848280	chr10-18844352-18848280
chr10	18876595	18876910	chr10-18876595-18876910
chr10	19008001	19017039	chr10-19008001-19017039
chr10	19042851	19047364	chr10-19042851-19047364
chr10	19068253	19068876	chr10-19068253-19068876
chr10	19119623	19119874	chr10-19119623-19119874
chr10	19140996	19141248	chr10-19140996-19141248
chr10	19148979	19149434	chr10-19148979-19149434
chr10	19150602	19150803	chr10-19150602-19150803
chr10	19167542	19168677	chr10-19167542-19168677
chr10	19185625	19189884	chr10-19185625-19189884
chr10	19192152	19192651	chr10-19192152-19192651
chr10	19352488	19353233	chr10-19352488-19353233
chr10	19356693	19357687	chr10-19356693-19357687
chr10	19361761	19362098	chr10-19361761-19362098
chr10	19464632	19464941	chr10-19464632-19464941
chr10	19591211	19595079	chr10-19591211-19595079
chr10	19712244	19713064	chr10-19712244-19713064
chr10	19849406	19852073	chr10-19849406-19852073
chr10	19907010	19907963	chr10-19907010-19907963
chr10	19933124	19937021	chr10-19933124-19937021
chr10	19956559	19956986	chr10-19956559-19956986
chr10	19991453	19992218	chr10-19991453-19992218
chr10	20076655	20077976	chr10-20076655-20077976
chr10	20091108	20091597	chr10-20091108-20091597
chr10	20123247	20123502	chr10-20123247-20123502
chr10	20148022	20150760	chr10-20148022-20150760
chr10	20220573	20221025	chr10-20220573-20221025
chr10	20290526	20290936	chr10-20290526-20290936
chr10	20310993	20315413	chr10-20310993-20315413
chr10	20347154	20349015	chr10-20347154-20349015
chr10	20724864	20725216	chr10-20724864-20725216
chr10	20951744	20954717	chr10-20951744-20954717

chr10	21158213	21161811	chr10-21158213-21161811
chr10	21188715	21189285	chr10-21188715-21189285
chr10	21295537	21297351	chr10-21295537-21297351
chr10	21335280	21335481	chr10-21335280-21335481
chr10	21686145	21686405	chr10-21686145-21686405
chr10	21776317	21777168	chr10-21776317-21777168
chr10	21789184	21790808	chr10-21789184-21790808
chr10	21853393	21853701	chr10-21853393-21853701
chr10	21882043	21882548	chr10-21882043-21882548
chr10	21991419	21991886	chr10-21991419-21991886
chr10	21992705	21996751	chr10-21992705-21996751
chr10	21998785	21998986	chr10-21998785-21998986
chr10	22058068	22059412	chr10-22058068-22059412
chr10	22140727	22141196	chr10-22140727-22141196
chr10	22147576	22151828	chr10-22147576-22151828
chr10	22154051	22154666	chr10-22154051-22154666
chr10	22158099	22159529	chr10-22158099-22159529
chr10	22163329	22163930	chr10-22163329-22163930
chr10	22223646	22223847	chr10-22223646-22223847
chr10	22273256	22273712	chr10-22273256-22273712
chr10	22361672	22361977	chr10-22361672-22361977
chr10	22644909	22645618	chr10-22644909-22645618
chr10	22701758	22702431	chr10-22701758-22702431
chr10	22729526	22732468	chr10-22729526-22732468
chr10	22813730	22813931	chr10-22813730-22813931
chr10	22815842	22816824	chr10-22815842-22816824
chr10	22819222	22819480	chr10-22819222-22819480
chr10	23077024	23077225	chr10-23077024-23077225
chr10	23347949	23351010	chr10-23347949-23351010
chr10	23567773	23569346	chr10-23567773-23569346
chr10	23726440	23726811	chr10-23726440-23726811
chr10	23784097	23788930	chr10-23784097-23788930
chr10	23796495	23797940	chr10-23796495-23797940
chr10	23894273	23895338	chr10-23894273-23895338
chr10	24131557	24133316	chr10-24131557-24133316
chr10	24148942	24150887	chr10-24148942-24150887
chr10	24164208	24165317	chr10-24164208-24165317
chr10	24173303	24173822	chr10-24173303-24173822
chr10	24210090	24211345	chr10-24210090-24211345
chr10	24222894	24224561	chr10-24222894-24224561
chr10	24312181	24312585	chr10-24312181-24312585
chr10	24366874	24367735	chr10-24366874-24367735

chr10	24396325	24396797	chr10-24396325-24396797
chr10	24446489	24447343	chr10-24446489-24447343
chr10	24478842	24479471	chr10-24478842-24479471
chr10	24493269	24494225	chr10-24493269-24494225
chr10	24591679	24591930	chr10-24591679-24591930
chr10	24594949	24599041	chr10-24594949-24599041
chr10	24628457	24629744	chr10-24628457-24629744
chr10	24635284	24636140	chr10-24635284-24636140
chr10	24710806	24712607	chr10-24710806-24712607
chr10	24753321	24754833	chr10-24753321-24754833
chr10	24869436	24870923	chr10-24869436-24870923
chr10	24972727	24973858	chr10-24972727-24973858
chr10	25200001	25200333	chr10-25200001-25200333
chr10	25272033	25272754	chr10-25272033-25272754
chr10	25296318	25298332	chr10-25296318-25298332
chr10	25314171	25314731	chr10-25314171-25314731
chr10	25359203	25362231	chr10-25359203-25362231
chr10	25431116	25435972	chr10-25431116-25435972
chr10	26077807	26079282	chr10-26077807-26079282
chr10	26372852	26376506	chr10-26372852-26376506
chr10	26695227	26695840	chr10-26695227-26695840
chr10	26753293	26753494	chr10-26753293-26753494
chr10	26771995	26774816	chr10-26771995-26774816
chr10	26776895	26777096	chr10-26776895-26777096
chr10	26828201	26828668	chr10-26828201-26828668
chr10	26995771	26996462	chr10-26995771-26996462
chr10	27966900	27969212	chr10-27966900-27969212
chr10	27984530	27986166	chr10-27984530-27986166
chr10	27988459	27988804	chr10-27988459-27988804
chr10	28073603	28078135	chr10-28073603-28078135
chr10	28113341	28113542	chr10-28113341-28113542
chr10	28193182	28193788	chr10-28193182-28193788
chr10	28954712	28954913	chr10-28954712-28954913
chr10	29143497	29144396	chr10-29143497-29144396
chr10	29146512	29147834	chr10-29146512-29147834
chr10	29312361	29314406	chr10-29312361-29314406
chr10	29360597	29363067	chr10-29360597-29363067
chr10	29532976	29536288	chr10-29532976-29536288
chr10	29708581	29708940	chr10-29708581-29708940
chr10	30199006	30200901	chr10-30199006-30200901
chr10	30599269	30601431	chr10-30599269-30601431
chr10	30617519	30619206	chr10-30617519-30619206

chr10	30728989	30730031	chr10-30728989-30730031
chr10	30778143	30778499	chr10-30778143-30778499
chr10	30801906	30804173	chr10-30801906-30804173
chr10	30810206	30811151	chr10-30810206-30811151
chr10	30841964	30844086	chr10-30841964-30844086
chr10	31034618	31036279	chr10-31034618-31036279
chr10	31312816	31314771	chr10-31312816-31314771
chr10	31444057	31447818	chr10-31444057-31447818
chr10	31467540	31468063	chr10-31467540-31468063
chr10	31606386	31610167	chr10-31606386-31610167
chr10	32888589	32891012	chr10-32888589-32891012
chr10	33624071	33624652	chr10-33624071-33624652
chr10	33904748	33905624	chr10-33904748-33905624
chr10	33950226	33951583	chr10-33950226-33951583
chr10	34018082	34020964	chr10-34018082-34020964
chr10	34033803	34034217	chr10-34033803-34034217
chr10	34070776	34071682	chr10-34070776-34071682
chr10	34096077	34096738	chr10-34096077-34096738
chr10	34127261	34128036	chr10-34127261-34128036
chr10	34206201	34208430	chr10-34206201-34208430
chr10	34280689	34284747	chr10-34280689-34284747
chr10	34296586	34298456	chr10-34296586-34298456
chr10	34328496	34328697	chr10-34328496-34328697
chr10	34417366	34419209	chr10-34417366-34419209
chr10	34483188	34485657	chr10-34483188-34485657
chr10	34512637	34512998	chr10-34512637-34512998
chr10	36506455	36508187	chr10-36506455-36508187
chr10	36973812	36976177	chr10-36973812-36976177
chr10	37135820	37141992	chr10-37135820-37141992
chr10	37337123	37337374	chr10-37337123-37337374
chr10	37357571	37357772	chr10-37357571-37357772
chr10	38965293	38966194	chr10-38965293-38966194
chr10	39133334	39135253	chr10-39133334-39135253
chr10	39169920	39172035	chr10-39169920-39172035
chr10	39239963	39240328	chr10-39239963-39240328
chr10	39266618	39266819	chr10-39266618-39266819
chr10	39369083	39371692	chr10-39369083-39371692
chr10	39385933	39386459	chr10-39385933-39386459
chr10	39412851	39413396	chr10-39412851-39413396
chr10	39449292	39449677	chr10-39449292-39449677
chr10	39612195	39615512	chr10-39612195-39615512
chr10	39658934	39660254	chr10-39658934-39660254

chr10	39730539	39734304	chr10-39730539-39734304
chr10	39898082	39900057	chr10-39898082-39900057
chr10	39926469	39927233	chr10-39926469-39927233
chr10	39959531	39960743	chr10-39959531-39960743
chr10	40024627	40025740	chr10-40024627-40025740
chr10	40141257	40142682	chr10-40141257-40142682
chr10	40245896	40247752	chr10-40245896-40247752
chr10	40256291	40258569	chr10-40256291-40258569
chr10	40259498	40259835	chr10-40259498-40259835
chr10	40297261	40303206	chr10-40297261-40303206
chr10	40328078	40328338	chr10-40328078-40328338
chr10	40338900	40340031	chr10-40338900-40340031
chr10	40347668	40351189	chr10-40347668-40351189
chr10	40354199	40354501	chr10-40354199-40354501
chr10	40371828	40372482	chr10-40371828-40372482
chr10	40379958	40380159	chr10-40379958-40380159
chr10	40417039	40417240	chr10-40417039-40417240
chr10	40510426	40512439	chr10-40510426-40512439
chr10	40521743	40521994	chr10-40521743-40521994
chr10	40527248	40528347	chr10-40527248-40528347
chr10	40682972	40683840	chr10-40682972-40683840
chr10	40713328	40713649	chr10-40713328-40713649
chr10	40881173	40884939	chr10-40881173-40884939
chr10	40969398	40969701	chr10-40969398-40969701
chr10	41151034	41151820	chr10-41151034-41151820
chr10	41209820	41210021	chr10-41209820-41210021
chr10	41301928	41304672	chr10-41301928-41304672
chr10	41449353	41451728	chr10-41449353-41451728
chr10	41476222	41477938	chr10-41476222-41477938
chr10	41488896	41491325	chr10-41488896-41491325
chr10	41518219	41521799	chr10-41518219-41521799
chr10	41564946	41566006	chr10-41564946-41566006
chr10	41678768	41679025	chr10-41678768-41679025
chr10	41684480	41684996	chr10-41684480-41684996
chr10	41690007	41690674	chr10-41690007-41690674
chr10	41808674	41813036	chr10-41808674-41813036
chr10	41886871	41888080	chr10-41886871-41888080
chr10	41934478	41935331	chr10-41934478-41935331
chr10	41997536	41998199	chr10-41997536-41998199
chr10	42017828	42019010	chr10-42017828-42019010
chr10	42089044	42090021	chr10-42089044-42090021
chr10	42098102	42098303	chr10-42098102-42098303

chr10	42107644	42107904	chr10-42107644-42107904
chr10	42130975	42131229	chr10-42130975-42131229
chr10	42134897	42135664	chr10-42134897-42135664
chr10	42177792	42178939	chr10-42177792-42178939
chr10	42231744	42231945	chr10-42231744-42231945
chr10	42269097	42270268	chr10-42269097-42270268
chr10	42273337	42279060	chr10-42273337-42279060
chr10	42308250	42308555	chr10-42308250-42308555
chr10	42477401	42479135	chr10-42477401-42479135
chr10	42501233	42503962	chr10-42501233-42503962
chr10	42572684	42572989	chr10-42572684-42572989
chr10	42579389	42580469	chr10-42579389-42580469
chr10	42581932	42584905	chr10-42581932-42584905
chr10	42591154	42592496	chr10-42591154-42592496
chr10	42613817	42614572	chr10-42613817-42614572
chr10	42631767	42634169	chr10-42631767-42634169
chr10	42635346	42636272	chr10-42635346-42636272
chr10	42637188	42637389	chr10-42637188-42637389
chr10	42678496	42680183	chr10-42678496-42680183
chr10	42760453	42764023	chr10-42760453-42764023
chr10	42860510	42860889	chr10-42860510-42860889
chr10	42862406	42864467	chr10-42862406-42864467
chr10	42950181	42950952	chr10-42950181-42950952
chr10	42957937	42958633	chr10-42957937-42958633
chr10	43002612	43003063	chr10-43002612-43003063
chr10	43020898	43022442	chr10-43020898-43022442
chr10	43172985	43175534	chr10-43172985-43175534
chr10	43220245	43222930	chr10-43220245-43222930
chr10	43452265	43452727	chr10-43452265-43452727
chr10	43477498	43480976	chr10-43477498-43480976
chr10	43489676	43489986	chr10-43489676-43489986
chr10	43539494	43541491	chr10-43539494-43541491
chr10	43577578	43581898	chr10-43577578-43581898
chr10	43689065	43690215	chr10-43689065-43690215
chr10	43694151	43694969	chr10-43694151-43694969
chr10	43723071	43724005	chr10-43723071-43724005
chr10	43900824	43903007	chr10-43900824-43903007
chr10	43943242	43943546	chr10-43943242-43943546
chr10	43977801	43978002	chr10-43977801-43978002
chr10	43998264	43999093	chr10-43998264-43999093
chr10	44000520	44007240	chr10-44000520-44007240
chr10	44015335	44015857	chr10-44015335-44015857

chr10	44037992	44038704	chr10-44037992-44038704
chr10	44043600	44044159	chr10-44043600-44044159
chr10	44078765	44079946	chr10-44078765-44079946
chr10	44112143	44112574	chr10-44112143-44112574
chr10	44148292	44149302	chr10-44148292-44149302
chr10	44266841	44269813	chr10-44266841-44269813
chr10	44424990	44425535	chr10-44424990-44425535
chr10	44457036	44459234	chr10-44457036-44459234
chr10	44526847	44527615	chr10-44526847-44527615
chr10	44528854	44530305	chr10-44528854-44530305
chr10	44877294	44877545	chr10-44877294-44877545
chr10	45000554	45000979	chr10-45000554-45000979
chr10	45048583	45049091	chr10-45048583-45049091
chr10	45051346	45051547	chr10-45051346-45051547
chr10	45066125	45068643	chr10-45066125-45068643
chr10	45289110	45290020	chr10-45289110-45290020
chr10	45335354	45336275	chr10-45335354-45336275
chr10	45468989	45470291	chr10-45468989-45470291
chr10	45473741	45474096	chr10-45473741-45474096
chr10	45485394	45487078	chr10-45485394-45487078
chr10	45577588	45579047	chr10-45577588-45579047
chr10	49788380	49788938	chr10-49788380-49788938
chr10	50591772	50593915	chr10-50591772-50593915
chr10	50894127	50895506	chr10-50894127-50895506
chr10	50898083	50898336	chr10-50898083-50898336
chr10	50900886	50901389	chr10-50900886-50901389
chr10	50910382	50911284	chr10-50910382-50911284
chr10	51417512	51417846	chr10-51417512-51417846
chr10	51553396	51554009	chr10-51553396-51554009
chr10	51571234	51572012	chr10-51571234-51572012
chr10	51590236	51590437	chr10-51590236-51590437
chr10	51677416	51678340	chr10-51677416-51678340
chr10	52021918	52023480	chr10-52021918-52023480
chr10	52027155	52028221	chr10-52027155-52028221
chr10	52029488	52029741	chr10-52029488-52029741
chr10	52219801	52220167	chr10-52219801-52220167
chr10	52232976	52235158	chr10-52232976-52235158
chr10	52277528	52278540	chr10-52277528-52278540
chr10	52380687	52383471	chr10-52380687-52383471
chr10	52416664	52419830	chr10-52416664-52419830
chr10	52690241	52691733	chr10-52690241-52691733
chr10	52704140	52705012	chr10-52704140-52705012

chr10	53378821	53380428	chr10-53378821-53380428
chr10	53394986	53395393	chr10-53394986-53395393
chr10	53595902	53599261	chr10-53595902-53599261
chr10	53628701	53630783	chr10-53628701-53630783
chr10	53749771	53751845	chr10-53749771-53751845
chr10	54072959	54076851	chr10-54072959-54076851
chr10	56227717	56229595	chr10-56227717-56229595
chr10	56377214	56381520	chr10-56377214-56381520
chr10	56486335	56486739	chr10-56486335-56486739
chr10	56781987	56783055	chr10-56781987-56783055
chr10	56944626	56944931	chr10-56944626-56944931
chr10	57448107	57448460	chr10-57448107-57448460
chr10	57485803	57488071	chr10-57485803-57488071
chr10	57530993	57532845	chr10-57530993-57532845
chr10	57631909	57632525	chr10-57631909-57632525
chr10	57743623	57743926	chr10-57743623-57743926
chr10	57794517	57795554	chr10-57794517-57795554
chr10	58224447	58225103	chr10-58224447-58225103
chr10	58227930	58228131	chr10-58227930-58228131
chr10	58254569	58256623	chr10-58254569-58256623
chr10	58310697	58310898	chr10-58310697-58310898
chr10	58321828	58326185	chr10-58321828-58326185
chr10	58333316	58333517	chr10-58333316-58333517
chr10	58364208	58365858	chr10-58364208-58365858
chr10	58371119	58372964	chr10-58371119-58372964
chr10	58445665	58448368	chr10-58445665-58448368
chr10	58496935	58498883	chr10-58496935-58498883
chr10	58675178	58675852	chr10-58675178-58675852
chr10	58812115	58814080	chr10-58812115-58814080
chr10	59220550	59224218	chr10-59220550-59224218
chr10	59240833	59241308	chr10-59240833-59241308
chr10	59322639	59325254	chr10-59322639-59325254
chr10	59382011	59382262	chr10-59382011-59382262
chr10	59383235	59383436	chr10-59383235-59383436
chr10	59425263	59425676	chr10-59425263-59425676
chr10	59429856	59431660	chr10-59429856-59431660
chr10	59503652	59504003	chr10-59503652-59504003
chr10	59508952	59510052	chr10-59508952-59510052
chr10	59537348	59540002	chr10-59537348-59540002
chr10	59577407	59579913	chr10-59577407-59579913
chr10	59589674	59590587	chr10-59589674-59590587
chr10	59593725	59594723	chr10-59593725-59594723

chr10	59614241	59617374	chr10-59614241-59617374
chr10	59621405	59621665	chr10-59621405-59621665
chr10	59636531	59637420	chr10-59636531-59637420
chr10	59642180	59643319	chr10-59642180-59643319
chr10	59667497	59667857	chr10-59667497-59667857
chr10	59701992	59704074	chr10-59701992-59704074
chr10	59724295	59725167	chr10-59724295-59725167
chr10	59727213	59727414	chr10-59727213-59727414
chr10	59821646	59821847	chr10-59821646-59821847
chr10	59878722	59881392	chr10-59878722-59881392
chr10	59896343	59897558	chr10-59896343-59897558
chr10	59898388	59902658	chr10-59898388-59902658
chr10	59906894	59911952	chr10-59906894-59911952
chr10	59916287	59916848	chr10-59916287-59916848
chr10	59920279	59921776	chr10-59920279-59921776
chr10	59938874	59939176	chr10-59938874-59939176
chr10	59942123	59944177	chr10-59942123-59944177
chr10	59948520	59953717	chr10-59948520-59953717
chr10	59969955	59970359	chr10-59969955-59970359
chr10	60001388	60004565	chr10-60001388-60004565
chr10	60106208	60107876	chr10-60106208-60107876
chr10	60125694	60126421	chr10-60125694-60126421
chr10	60185852	60186974	chr10-60185852-60186974
chr10	60191755	60192268	chr10-60191755-60192268
chr10	60193636	60194323	chr10-60193636-60194323
chr10	60207239	60207440	chr10-60207239-60207440
chr10	60208873	60210050	chr10-60208873-60210050
chr10	60218413	60219681	chr10-60218413-60219681
chr10	60277022	60280265	chr10-60277022-60280265
chr10	60290733	60291087	chr10-60290733-60291087
chr10	60346524	60347462	chr10-60346524-60347462
chr10	60350726	60350990	chr10-60350726-60350990
chr10	60571468	60571831	chr10-60571468-60571831
chr10	60685854	60687912	chr10-60685854-60687912
chr10	60751550	60753296	chr10-60751550-60753296
chr10	60762745	60763088	chr10-60762745-60763088
chr10	60807956	60808212	chr10-60807956-60808212
chr10	60813584	60821020	chr10-60813584-60821020
chr10	60822110	60823972	chr10-60822110-60823972
chr10	60825495	60832974	chr10-60825495-60832974
chr10	60876560	60877745	chr10-60876560-60877745
chr10	60885149	60886688	chr10-60885149-60886688

chr10	60932118	60935283	chr10-60932118-60935283
chr10	61069064	61069265	chr10-61069064-61069265
chr10	61088960	61090361	chr10-61088960-61090361
chr10	61145463	61148527	chr10-61145463-61148527
chr10	61175627	61176290	chr10-61175627-61176290
chr10	61272089	61273982	chr10-61272089-61273982
chr10	61286582	61286996	chr10-61286582-61286996
chr10	61381986	61384199	chr10-61381986-61384199
chr10	61394605	61395067	chr10-61394605-61395067
chr10	61418032	61419458	chr10-61418032-61419458
chr10	61450681	61453467	chr10-61450681-61453467
chr10	61461927	61462128	chr10-61461927-61462128
chr10	61464931	61465240	chr10-61464931-61465240
chr10	61475123	61477448	chr10-61475123-61477448
chr10	61535958	61536908	chr10-61535958-61536908
chr10	61569844	61570552	chr10-61569844-61570552
chr10	61594928	61595587	chr10-61594928-61595587
chr10	61602428	61603042	chr10-61602428-61603042
chr10	61625502	61626297	chr10-61625502-61626297
chr10	61647603	61650313	chr10-61647603-61650313
chr10	61679546	61681911	chr10-61679546-61681911
chr10	61694845	61697815	chr10-61694845-61697815
chr10	61714643	61716604	chr10-61714643-61716604
chr10	61756800	61757105	chr10-61756800-61757105
chr10	61759459	61759763	chr10-61759459-61759763
chr10	61782572	61784400	chr10-61782572-61784400
chr10	61943430	61944836	chr10-61943430-61944836
chr10	61948979	61949594	chr10-61948979-61949594
chr10	61965146	61966070	chr10-61965146-61966070
chr10	61977508	61979659	chr10-61977508-61979659
chr10	62110406	62111558	chr10-62110406-62111558
chr10	62221757	62223397	chr10-62221757-62223397
chr10	62230195	62231716	chr10-62230195-62231716
chr10	62247511	62248547	chr10-62247511-62248547
chr10	62299667	62301020	chr10-62299667-62301020
chr10	62320565	62321133	chr10-62320565-62321133
chr10	62329415	62330768	chr10-62329415-62330768
chr10	62331920	62333534	chr10-62331920-62333534
chr10	62336244	62341538	chr10-62336244-62341538
chr10	62412535	62413895	chr10-62412535-62413895
chr10	62448176	62450993	chr10-62448176-62450993
chr10	62484700	62487848	chr10-62484700-62487848

chr10	62577134	62579022	chr10-62577134-62579022
chr10	62599990	62603503	chr10-62599990-62603503
chr10	62649570	62652252	chr10-62649570-62652252
chr10	62725481	62726691	chr10-62725481-62726691
chr10	62790709	62793505	chr10-62790709-62793505
chr10	62865122	62866068	chr10-62865122-62866068
chr10	62885790	62887585	chr10-62885790-62887585
chr10	62919784	62921794	chr10-62919784-62921794
chr10	62946443	62948143	chr10-62946443-62948143
chr10	62979689	62981423	chr10-62979689-62981423
chr10	63021231	63025701	chr10-63021231-63025701
chr10	63099712	63100873	chr10-63099712-63100873
chr10	63128138	63128865	chr10-63128138-63128865
chr10	63177092	63177963	chr10-63177092-63177963
chr10	63242558	63245330	chr10-63242558-63245330
chr10	63336968	63339754	chr10-63336968-63339754
chr10	63381466	63382974	chr10-63381466-63382974
chr10	63386183	63387242	chr10-63386183-63387242
chr10	63428433	63430688	chr10-63428433-63430688
chr10	64075694	64077212	chr10-64075694-64077212
chr10	64087784	64088351	chr10-64087784-64088351
chr10	64090008	64090419	chr10-64090008-64090419
chr10	66354500	66354701	chr10-66354500-66354701
chr10	66813910	66814707	chr10-66813910-66814707
chr10	66849877	66850296	chr10-66849877-66850296
chr10	66864030	66864674	chr10-66864030-66864674
chr10	66891713	66891914	chr10-66891713-66891914
chr10	66911178	66911442	chr10-66911178-66911442
chr10	66919819	66922121	chr10-66919819-66922121
chr10	66928367	66929071	chr10-66928367-66929071
chr10	67002421	67003666	chr10-67002421-67003666
chr10	67048787	67049496	chr10-67048787-67049496
chr10	67095441	67101035	chr10-67095441-67101035
chr10	67105635	67107721	chr10-67105635-67107721
chr10	67126230	67127928	chr10-67126230-67127928
chr10	67156726	67157308	chr10-67156726-67157308
chr10	67185494	67189473	chr10-67185494-67189473
chr10	67219021	67219273	chr10-67219021-67219273
chr10	67281783	67282378	chr10-67281783-67282378
chr10	67283204	67285890	chr10-67283204-67285890
chr10	67318033	67318234	chr10-67318033-67318234
chr10	67458696	67459157	chr10-67458696-67459157

chr10	67535079	67539337	chr10-67535079-67539337
chr10	67546672	67550021	chr10-67546672-67550021
chr10	67846020	67846662	chr10-67846020-67846662
chr10	67865689	67866034	chr10-67865689-67866034
chr10	67911825	67912984	chr10-67911825-67912984
chr10	67978949	67980415	chr10-67978949-67980415
chr10	67984049	67984341	chr10-67984049-67984341
chr10	68008807	68009414	chr10-68008807-68009414
chr10	68039367	68040492	chr10-68039367-68040492
chr10	68133598	68138089	chr10-68133598-68138089
chr10	68238361	68239311	chr10-68238361-68239311
chr10	68263624	68263825	chr10-68263624-68263825
chr10	68274925	68281480	chr10-68274925-68281480
chr10	68282284	68283535	chr10-68282284-68283535
chr10	68354364	68354670	chr10-68354364-68354670
chr10	68355789	68356405	chr10-68355789-68356405
chr10	68541662	68541863	chr10-68541662-68541863
chr10	68723675	68724087	chr10-68723675-68724087
chr10	68969019	68969220	chr10-68969019-68969220
chr10	69185066	69186492	chr10-69185066-69186492
chr10	69211841	69214367	chr10-69211841-69214367
chr10	69321357	69321957	chr10-69321357-69321957
chr10	69348617	69353512	chr10-69348617-69353512
chr10	69398079	69400960	chr10-69398079-69400960
chr10	69507298	69507843	chr10-69507298-69507843
chr10	69533416	69535845	chr10-69533416-69535845
chr10	69570733	69572850	chr10-69570733-69572850
chr10	69577184	69578049	chr10-69577184-69578049
chr10	69579512	69579774	chr10-69579512-69579774
chr10	69611938	69612390	chr10-69611938-69612390
chr10	69652447	69656016	chr10-69652447-69656016
chr10	69670752	69671566	chr10-69670752-69671566
chr10	69716881	69717135	chr10-69716881-69717135
chr10	69872031	69873254	chr10-69872031-69873254
chr10	69916671	69917107	chr10-69916671-69917107
chr10	69926446	69927188	chr10-69926446-69927188
chr10	69928272	69928900	chr10-69928272-69928900
chr10	70094496	70099475	chr10-70094496-70099475
chr10	70120653	70121462	chr10-70120653-70121462
chr10	70244621	70246068	chr10-70244621-70246068
chr10	70287791	70288303	chr10-70287791-70288303
chr10	70296516	70297007	chr10-70296516-70297007

chr10	70440235	70441478	chr10-70440235-70441478
chr10	70519930	70521183	chr10-70519930-70521183
chr10	70598480	70600415	chr10-70598480-70600415
chr10	71058685	71059292	chr10-71058685-71059292
chr10	71158894	71160192	chr10-71158894-71160192
chr10	71236541	71238628	chr10-71236541-71238628
chr10	71283916	71285952	chr10-71283916-71285952
chr10	71292096	71292694	chr10-71292096-71292694
chr10	71306424	71306678	chr10-71306424-71306678
chr10	71343581	71345704	chr10-71343581-71345704
chr10	71346684	71349349	chr10-71346684-71349349
chr10	72634348	72634605	chr10-72634348-72634605
chr10	72654010	72656652	chr10-72654010-72656652
chr10	72778438	72778639	chr10-72778438-72778639
chr10	73096783	73097091	chr10-73096783-73097091
chr10	73098762	73100540	chr10-73098762-73100540
chr10	73821757	73822069	chr10-73821757-73822069
chr10	73826333	73826911	chr10-73826333-73826911
chr10	74644079	74644280	chr10-74644079-74644280
chr10	74679971	74681185	chr10-74679971-74681185
chr10	74921019	74921395	chr10-74921019-74921395
chr10	74925336	74925745	chr10-74925336-74925745
chr10	74966489	74968209	chr10-74966489-74968209
chr10	74987815	74988745	chr10-74987815-74988745
chr10	75036397	75037635	chr10-75036397-75037635
chr10	75059339	75065950	chr10-75059339-75065950
chr10	75103465	75106122	chr10-75103465-75106122
chr10	75107244	75107445	chr10-75107244-75107445
chr10	75111585	75112091	chr10-75111585-75112091
chr10	75114673	75115839	chr10-75114673-75115839
chr10	75116746	75117486	chr10-75116746-75117486
chr10	75118355	75119948	chr10-75118355-75119948
chr10	75120900	75121206	chr10-75120900-75121206
chr10	75150114	75151346	chr10-75150114-75151346
chr10	75169126	75171218	chr10-75169126-75171218
chr10	75173886	75174618	chr10-75173886-75174618
chr10	75211524	75213505	chr10-75211524-75213505
chr10	75316605	75317933	chr10-75316605-75317933
chr10	75428935	75429528	chr10-75428935-75429528
chr10	75505053	75505320	chr10-75505053-75505320
chr10	75515946	75519594	chr10-75515946-75519594
chr10	75559468	75560679	chr10-75559468-75560679

chr10	75585962	75586163	chr10-75585962-75586163
chr10	75667147	75668309	chr10-75667147-75668309
chr10	75684255	75684534	chr10-75684255-75684534
chr10	75762823	75765485	chr10-75762823-75765485
chr10	75772386	75773944	chr10-75772386-75773944
chr10	75780044	75781537	chr10-75780044-75781537
chr10	75797196	75798739	chr10-75797196-75798739
chr10	75834141	75837986	chr10-75834141-75837986
chr10	75857686	75861028	chr10-75857686-75861028
chr10	75872667	75873214	chr10-75872667-75873214
chr10	75883161	75883414	chr10-75883161-75883414
chr10	75892935	75894014	chr10-75892935-75894014
chr10	75899109	75899310	chr10-75899109-75899310
chr10	75920388	75922570	chr10-75920388-75922570
chr10	75929812	75933152	chr10-75929812-75933152
chr10	75935314	75937884	chr10-75935314-75937884
chr10	75950895	75951723	chr10-75950895-75951723
chr10	76031804	76034286	chr10-76031804-76034286
chr10	76122109	76122362	chr10-76122109-76122362
chr10	76203736	76204244	chr10-76203736-76204244
chr10	76236465	76238649	chr10-76236465-76238649
chr10	76257868	76258518	chr10-76257868-76258518
chr10	76261283	76262668	chr10-76261283-76262668
chr10	76306963	76307373	chr10-76306963-76307373
chr10	76336965	76337217	chr10-76336965-76337217
chr10	76343793	76346867	chr10-76343793-76346867
chr10	76441233	76443802	chr10-76441233-76443802
chr10	76467777	76469832	chr10-76467777-76469832
chr10	76504184	76504597	chr10-76504184-76504597
chr10	76530723	76534153	chr10-76530723-76534153
chr10	76537072	76538342	chr10-76537072-76538342
chr10	76584142	76584343	chr10-76584142-76584343
chr10	76623037	76623860	chr10-76623037-76623860
chr10	76634330	76634768	chr10-76634330-76634768
chr10	76650770	76651045	chr10-76650770-76651045
chr10	76725646	76726063	chr10-76725646-76726063
chr10	76730438	76730689	chr10-76730438-76730689
chr10	76902569	76902770	chr10-76902569-76902770
chr10	76930960	76931161	chr10-76930960-76931161
chr10	76961092	76962598	chr10-76961092-76962598
chr10	77016528	77017166	chr10-77016528-77017166
chr10	77032376	77035553	chr10-77032376-77035553

chr10	77082398	77082599	chr10-77082398-77082599
chr10	77089527	77089883	chr10-77089527-77089883
chr10	77129715	77131275	chr10-77129715-77131275
chr10	77141645	77144618	chr10-77141645-77144618
chr10	77145447	77148406	chr10-77145447-77148406
chr10	77149263	77152963	chr10-77149263-77152963
chr10	77164173	77167139	chr10-77164173-77167139
chr10	77206451	77207319	chr10-77206451-77207319
chr10	77211034	77212130	chr10-77211034-77212130
chr10	77237683	77238364	chr10-77237683-77238364
chr10	77250582	77252992	chr10-77250582-77252992
chr10	77258389	77260833	chr10-77258389-77260833
chr10	77402355	77402556	chr10-77402355-77402556
chr10	77416819	77419218	chr10-77416819-77419218
chr10	77514392	77517592	chr10-77514392-77517592
chr10	77530352	77532245	chr10-77530352-77532245
chr10	77541930	77542579	chr10-77541930-77542579
chr10	77568121	77572847	chr10-77568121-77572847
chr10	77581006	77584850	chr10-77581006-77584850
chr10	77605538	77608333	chr10-77605538-77608333
chr10	77621581	77625123	chr10-77621581-77625123
chr10	77666202	77666740	chr10-77666202-77666740
chr10	77677547	77678109	chr10-77677547-77678109
chr10	77685900	77686893	chr10-77685900-77686893
chr10	77864605	77865157	chr10-77864605-77865157
chr10	77901903	77903681	chr10-77901903-77903681
chr10	77969355	77970117	chr10-77969355-77970117
chr10	77977894	77979567	chr10-77977894-77979567
chr10	78007697	78010951	chr10-78007697-78010951
chr10	78018905	78019261	chr10-78018905-78019261
chr10	78043353	78043840	chr10-78043353-78043840
chr10	78068543	78070288	chr10-78068543-78070288
chr10	78135095	78135498	chr10-78135095-78135498
chr10	78159256	78159763	chr10-78159256-78159763
chr10	78168714	78171904	chr10-78168714-78171904
chr10	78183581	78185944	chr10-78183581-78185944
chr10	78243096	78245712	chr10-78243096-78245712
chr10	78297578	78298502	chr10-78297578-78298502
chr10	78338324	78338777	chr10-78338324-78338777
chr10	78350422	78352992	chr10-78350422-78352992
chr10	78392029	78392332	chr10-78392029-78392332
chr10	78411260	78413533	chr10-78411260-78413533

chr10	78425010	78427223	chr10-78425010-78427223
chr10	78457618	78458174	chr10-78457618-78458174
chr10	78463694	78465457	chr10-78463694-78465457
chr10	78470750	78471425	chr10-78470750-78471425
chr10	78481819	78482446	chr10-78481819-78482446
chr10	78485853	78486104	chr10-78485853-78486104
chr10	78487660	78487861	chr10-78487660-78487861
chr10	78525753	78526061	chr10-78525753-78526061
chr10	78573877	78576228	chr10-78573877-78576228
chr10	78589949	78592451	chr10-78589949-78592451
chr10	78752257	78753129	chr10-78752257-78753129
chr10	78779786	78780858	chr10-78779786-78780858
chr10	79096612	79097883	chr10-79096612-79097883
chr10	79322017	79322523	chr10-79322017-79322523
chr10	79416879	79417861	chr10-79416879-79417861
chr10	79531267	79534371	chr10-79531267-79534371
chr10	79553287	79555861	chr10-79553287-79555861
chr10	79612230	79614439	chr10-79612230-79614439
chr10	79637204	79639182	chr10-79637204-79639182
chr10	79668723	79671081	chr10-79668723-79671081
chr10	79680684	79684873	chr10-79680684-79684873
chr10	79695426	79696893	chr10-79695426-79696893
chr10	79700978	79702095	chr10-79700978-79702095
chr10	79703896	79709354	chr10-79703896-79709354
chr10	79716102	79718143	chr10-79716102-79718143
chr10	79735127	79735782	chr10-79735127-79735782
chr10	79739234	79740393	chr10-79739234-79740393
chr10	79745438	79746779	chr10-79745438-79746779
chr10	79754945	79755360	chr10-79754945-79755360
chr10	79764099	79768126	chr10-79764099-79768126
chr10	79776933	79778816	chr10-79776933-79778816
chr10	79779796	79781579	chr10-79779796-79781579
chr10	79793035	79795073	chr10-79793035-79795073
chr10	79822360	79825064	chr10-79822360-79825064
chr10	79840388	79841937	chr10-79840388-79841937
chr10	79850409	79850610	chr10-79850409-79850610
chr10	79851500	79859037	chr10-79851500-79859037
chr10	79873823	79874940	chr10-79873823-79874940
chr10	79881986	79882352	chr10-79881986-79882352
chr10	79883829	79884091	chr10-79883829-79884091
chr10	79907840	79909761	chr10-79907840-79909761
chr10	79913914	79919764	chr10-79913914-79919764

chr10	79925932	79929258	chr10-79925932-79929258
chr10	79936392	79937648	chr10-79936392-79937648
chr10	79945091	79946365	chr10-79945091-79946365
chr10	79958255	79961372	chr10-79958255-79961372
chr10	79970811	79971325	chr10-79970811-79971325
chr10	79982434	79991525	chr10-79982434-79991525
chr10	79996024	79998421	chr10-79996024-79998421
chr10	80014323	80019419	chr10-80014323-80019419
chr10	80023672	80025610	chr10-80023672-80025610
chr10	80031280	80031481	chr10-80031280-80031481
chr10	80032350	80034441	chr10-80032350-80034441
chr10	80037383	80040265	chr10-80037383-80040265
chr10	80051341	80056770	chr10-80051341-80056770
chr10	80070729	80076563	chr10-80070729-80076563
chr10	80096945	80106706	chr10-80096945-80106706
chr10	80107531	80108176	chr10-80107531-80108176
chr10	80114589	80120388	chr10-80114589-80120388
chr10	80136272	80139969	chr10-80136272-80139969
chr10	80140964	80169366	chr10-80140964-80169366
chr10	80171735	80173882	chr10-80171735-80173882
chr10	80177319	80178001	chr10-80177319-80178001
chr10	80179023	80180305	chr10-80179023-80180305
chr10	80189953	80191378	chr10-80189953-80191378
chr10	80225671	80228388	chr10-80225671-80228388
chr10	80248165	80250849	chr10-80248165-80250849
chr10	80253465	80253775	chr10-80253465-80253775
chr10	80259852	80262689	chr10-80259852-80262689
chr10	80264144	80269110	chr10-80264144-80269110
chr10	80270632	80270833	chr10-80270632-80270833
chr10	80280565	80284088	chr10-80280565-80284088
chr10	80291926	80296468	chr10-80291926-80296468
chr10	80304591	80305357	chr10-80304591-80305357
chr10	80318280	80324345	chr10-80318280-80324345
chr10	80329002	80332408	chr10-80329002-80332408
chr10	80341849	80345862	chr10-80341849-80345862
chr10	80347126	80349557	chr10-80347126-80349557
chr10	80350516	80350717	chr10-80350516-80350717
chr10	80356136	80358621	chr10-80356136-80358621
chr10	80371455	80372064	chr10-80371455-80372064
chr10	80382969	80388725	chr10-80382969-80388725
chr10	80396777	80401095	chr10-80396777-80401095
chr10	80404927	80407849	chr10-80404927-80407849

chr10	80431639	80436722	chr10-80431639-80436722
chr10	80446345	80446699	chr10-80446345-80446699
chr10	80471589	80472354	chr10-80471589-80472354
chr10	80474576	80476083	chr10-80474576-80476083
chr10	80489176	80490849	chr10-80489176-80490849
chr10	80494419	80497243	chr10-80494419-80497243
chr10	80509448	80510116	chr10-80509448-80510116
chr10	80535261	80535788	chr10-80535261-80535788
chr10	80538005	80539636	chr10-80538005-80539636
chr10	80559344	80562277	chr10-80559344-80562277
chr10	80569842	80570205	chr10-80569842-80570205
chr10	80573988	80579649	chr10-80573988-80579649
chr10	80586711	80591010	chr10-80586711-80591010
chr10	80602255	80605299	chr10-80602255-80605299
chr10	80606260	80607081	chr10-80606260-80607081
chr10	80621106	80624851	chr10-80621106-80624851
chr10	80655304	80657685	chr10-80655304-80657685
chr10	80659121	80659857	chr10-80659121-80659857
chr10	80663939	80670675	chr10-80663939-80670675
chr10	80673318	80679011	chr10-80673318-80679011
chr10	80700256	80704130	chr10-80700256-80704130
chr10	80740098	80742996	chr10-80740098-80742996
chr10	80743924	80744688	chr10-80743924-80744688
chr10	80750693	80753074	chr10-80750693-80753074
chr10	80754387	80759260	chr10-80754387-80759260
chr10	80763875	80764447	chr10-80763875-80764447
chr10	80766117	80767180	chr10-80766117-80767180
chr10	80796066	80800533	chr10-80796066-80800533
chr10	80807117	80807469	chr10-80807117-80807469
chr10	80825705	80828974	chr10-80825705-80828974
chr10	80834622	80834931	chr10-80834622-80834931
chr10	80843388	80844604	chr10-80843388-80844604
chr10	80848565	80848820	chr10-80848565-80848820
chr10	80853564	80856587	chr10-80853564-80856587
chr10	80898869	80901846	chr10-80898869-80901846
chr10	80917182	80918909	chr10-80917182-80918909
chr10	80928404	80941327	chr10-80928404-80941327
chr10	80946375	80950001	chr10-80946375-80950001
chr10	80954756	80956379	chr10-80954756-80956379
chr10	80960773	80963981	chr10-80960773-80963981
chr10	80985106	80988258	chr10-80985106-80988258
chr10	80995005	80995419	chr10-80995005-80995419

chr10	80999546	81000463	chr10-80999546-81000463
chr10	81014455	81015220	chr10-81014455-81015220
chr10	81025016	81025767	chr10-81025016-81025767
chr10	81027491	81027743	chr10-81027491-81027743
chr10	81036455	81037748	chr10-81036455-81037748
chr10	81058897	81060865	chr10-81058897-81060865
chr10	81069370	81071518	chr10-81069370-81071518
chr10	81095078	81095986	chr10-81095078-81095986
chr10	81101726	81102745	chr10-81101726-81102745
chr10	81104865	81107732	chr10-81104865-81107732
chr10	81128235	81130429	chr10-81128235-81130429
chr10	81134048	81147254	chr10-81134048-81147254
chr10	81166349	81169938	chr10-81166349-81169938
chr10	81173171	81185687	chr10-81173171-81185687
chr10	81191769	81193703	chr10-81191769-81193703
chr10	81232290	81233859	chr10-81232290-81233859
chr10	81244476	81244837	chr10-81244476-81244837
chr10	81253089	81253340	chr10-81253089-81253340
chr10	81257092	81258332	chr10-81257092-81258332
chr10	81265451	81269615	chr10-81265451-81269615
chr10	81284749	81286501	chr10-81284749-81286501
chr10	81289397	81291599	chr10-81289397-81291599
chr10	81292413	81295142	chr10-81292413-81295142
chr10	81297402	81298382	chr10-81297402-81298382
chr10	81318697	81322878	chr10-81318697-81322878
chr10	81332483	81332861	chr10-81332483-81332861
chr10	81342840	81343802	chr10-81342840-81343802
chr10	81346250	81347146	chr10-81346250-81347146
chr10	81348753	81351563	chr10-81348753-81351563
chr10	81356671	81360355	chr10-81356671-81360355
chr10	81362514	81363085	chr10-81362514-81363085
chr10	81376463	81379664	chr10-81376463-81379664
chr10	81383623	81386406	chr10-81383623-81386406
chr10	81407092	81413065	chr10-81407092-81413065
chr10	81416742	81417510	chr10-81416742-81417510
chr10	81423967	81427676	chr10-81423967-81427676
chr10	81429140	81433518	chr10-81429140-81433518
chr10	81452178	81453414	chr10-81452178-81453414
chr10	81461923	81462530	chr10-81461923-81462530
chr10	81463961	81466029	chr10-81463961-81466029
chr10	81481718	81483041	chr10-81481718-81483041
chr10	81494282	81497319	chr10-81494282-81497319

chr10	81502651	81503370	chr10-81502651-81503370
chr10	81523456	81524376	chr10-81523456-81524376
chr10	81543164	81546296	chr10-81543164-81546296
chr10	81551436	81552169	chr10-81551436-81552169
chr10	81558716	81564368	chr10-81558716-81564368
chr10	81570352	81572750	chr10-81570352-81572750
chr10	81574772	81576179	chr10-81574772-81576179
chr10	81600578	81601519	chr10-81600578-81601519
chr10	81604931	81607501	chr10-81604931-81607501
chr10	81626360	81628654	chr10-81626360-81628654
chr10	81633222	81634338	chr10-81633222-81634338
chr10	81642220	81644858	chr10-81642220-81644858
chr10	81646279	81646530	chr10-81646279-81646530
chr10	81648582	81649922	chr10-81648582-81649922
chr10	81705469	81705670	chr10-81705469-81705670
chr10	81812035	81812490	chr10-81812035-81812490
chr10	81863310	81864749	chr10-81863310-81864749
chr10	81978671	81978872	chr10-81978671-81978872
chr10	81997142	81997950	chr10-81997142-81997950
chr10	82047416	82049388	chr10-82047416-82049388
chr10	82082855	82084228	chr10-82082855-82084228
chr10	82127412	82128841	chr10-82127412-82128841
chr10	82177637	82179194	chr10-82177637-82179194
chr10	82240526	82241801	chr10-82240526-82241801
chr10	82353569	82355231	chr10-82353569-82355231
chr10	82514829	82515082	chr10-82514829-82515082
chr10	82621778	82623975	chr10-82621778-82623975
chr10	82628837	82631745	chr10-82628837-82631745
chr10	82666404	82666990	chr10-82666404-82666990
chr10	82690491	82690800	chr10-82690491-82690800
chr10	82698548	82700173	chr10-82698548-82700173
chr10	82761183	82764873	chr10-82761183-82764873
chr10	82780541	82780742	chr10-82780541-82780742
chr10	82794838	82796021	chr10-82794838-82796021
chr10	82798382	82798644	chr10-82798382-82798644
chr10	82811834	82812738	chr10-82811834-82812738
chr10	82814479	82814733	chr10-82814479-82814733
chr10	82857955	82864130	chr10-82857955-82864130
chr10	82899540	82900860	chr10-82899540-82900860
chr10	82954364	82954565	chr10-82954364-82954565
chr10	82984548	82986712	chr10-82984548-82986712
chr10	83336940	83338206	chr10-83336940-83338206

chr10	83359765	83362038	chr10-83359765-83362038
chr10	83533142	83534479	chr10-83533142-83534479
chr10	83543314	83545184	chr10-83543314-83545184
chr10	83644734	83649529	chr10-83644734-83649529
chr10	83722476	83723649	chr10-83722476-83723649
chr10	83788676	83788877	chr10-83788676-83788877
chr10	83984999	83985531	chr10-83984999-83985531
chr10	84060787	84061040	chr10-84060787-84061040
chr10	84391461	84393039	chr10-84391461-84393039
chr10	84409068	84409370	chr10-84409068-84409370
chr10	84437745	84442463	chr10-84437745-84442463
chr10	84516391	84516698	chr10-84516391-84516698
chr10	84530046	84534772	chr10-84530046-84534772
chr10	84571279	84571691	chr10-84571279-84571691
chr10	84575823	84577881	chr10-84575823-84577881
chr10	84584367	84585386	chr10-84584367-84585386
chr10	84621475	84623933	chr10-84621475-84623933
chr10	84754695	84757152	chr10-84754695-84757152
chr10	84760043	84760525	chr10-84760043-84760525
chr10	84886174	84886693	chr10-84886174-84886693
chr10	84916728	84918628	chr10-84916728-84918628
chr10	85101812	85104042	chr10-85101812-85104042
chr10	85127175	85128810	chr10-85127175-85128810
chr10	85183156	85186267	chr10-85183156-85186267
chr10	85367044	85367296	chr10-85367044-85367296
chr10	85371941	85372761	chr10-85371941-85372761
chr10	85379116	85379317	chr10-85379116-85379317
chr10	85386261	85391183	chr10-85386261-85391183
chr10	85399626	85399827	chr10-85399626-85399827
chr10	85420635	85421574	chr10-85420635-85421574
chr10	85458926	85459465	chr10-85458926-85459465
chr10	85481039	85481290	chr10-85481039-85481290
chr10	85526668	85528235	chr10-85526668-85528235
chr10	85598320	85599140	chr10-85598320-85599140
chr10	85624844	85625111	chr10-85624844-85625111
chr10	85639338	85640598	chr10-85639338-85640598
chr10	85648423	85648867	chr10-85648423-85648867
chr10	85651695	85651896	chr10-85651695-85651896
chr10	85738737	85740275	chr10-85738737-85740275
chr10	85829171	85829840	chr10-85829171-85829840
chr10	85871311	85873745	chr10-85871311-85873745
chr10	85915473	85917752	chr10-85915473-85917752

chr10	85927683	85929661	chr10-85927683-85929661
chr10	85956259	85958206	chr10-85956259-85958206
chr10	85973072	85973273	chr10-85973072-85973273
chr10	86021448	86023196	chr10-86021448-86023196
chr10	86173093	86173294	chr10-86173093-86173294
chr10	86300117	86301445	chr10-86300117-86301445
chr10	86339743	86340263	chr10-86339743-86340263
chr10	86497954	86499368	chr10-86497954-86499368
chr10	86612108	86612479	chr10-86612108-86612479
chr10	86636895	86637599	chr10-86636895-86637599
chr10	86684863	86686536	chr10-86684863-86686536
chr10	86703171	86707152	chr10-86703171-86707152
chr10	86730760	86731364	chr10-86730760-86731364
chr10	86777858	86784590	chr10-86777858-86784590
chr10	86826201	86826402	chr10-86826201-86826402
chr10	87058194	87058812	chr10-87058194-87058812
chr10	87486261	87486723	chr10-87486261-87486723
chr10	87488675	87489036	chr10-87488675-87489036
chr10	87492355	87495026	chr10-87492355-87495026
chr10	87859809	87861103	chr10-87859809-87861103
chr10	88145095	88147995	chr10-88145095-88147995
chr10	88200597	88202116	chr10-88200597-88202116
chr10	88345999	88347294	chr10-88345999-88347294
chr10	88350474	88351224	chr10-88350474-88351224
chr10	88352925	88357752	chr10-88352925-88357752
chr10	88378631	88381516	chr10-88378631-88381516
chr10	88503113	88504522	chr10-88503113-88504522
chr10	88622479	88623044	chr10-88622479-88623044
chr10	88728015	88728479	chr10-88728015-88728479
chr10	88730286	88732732	chr10-88730286-88732732
chr10	88825610	88827695	chr10-88825610-88827695
chr10	88885739	88886749	chr10-88885739-88886749
chr10	89344426	89345294	chr10-89344426-89345294
chr10	89442429	89444551	chr10-89442429-89444551
chr10	89462082	89462283	chr10-89462082-89462283
chr10	89526867	89527682	chr10-89526867-89527682
chr10	89531111	89531412	chr10-89531111-89531412
chr10	89533270	89534011	chr10-89533270-89534011
chr10	89684940	89686952	chr10-89684940-89686952
chr10	89731233	89733192	chr10-89731233-89733192
chr10	89744501	89747392	chr10-89744501-89747392
chr10	89764499	89764934	chr10-89764499-89764934

chr10	89765773	89766030	chr10-89765773-89766030
chr10	89768466	89769876	chr10-89768466-89769876
chr10	89872462	89874374	chr10-89872462-89874374
chr10	90069351	90069552	chr10-90069351-90069552
chr10	90728525	90728987	chr10-90728525-90728987
chr10	90828777	90830001	chr10-90828777-90830001
chr10	90969359	90970466	chr10-90969359-90970466
chr10	91081182	91083855	chr10-91081182-91083855
chr10	91093967	91094168	chr10-91093967-91094168
chr10	91121065	91124822	chr10-91121065-91124822
chr10	91138305	91138506	chr10-91138305-91138506
chr10	91158304	91159089	chr10-91158304-91159089
chr10	91166751	91172801	chr10-91166751-91172801
chr10	91177004	91178169	chr10-91177004-91178169
chr10	91179530	91184300	chr10-91179530-91184300
chr10	91187571	91188174	chr10-91187571-91188174
chr10	91194337	91194538	chr10-91194337-91194538
chr10	91240368	91243830	chr10-91240368-91243830
chr10	91271424	91271737	chr10-91271424-91271737
chr10	92161637	92162987	chr10-92161637-92162987
chr10	92721166	92722953	chr10-92721166-92722953
chr10	93080970	93081780	chr10-93080970-93081780
chr10	93083165	93083519	chr10-93083165-93083519
chr10	93095006	93095415	chr10-93095006-93095415
chr10	93138313	93138718	chr10-93138313-93138718
chr10	93144847	93145571	chr10-93144847-93145571
chr10	93156821	93157022	chr10-93156821-93157022
chr10	93159922	93163099	chr10-93159922-93163099
chr10	93165508	93165973	chr10-93165508-93165973
chr10	93188241	93188855	chr10-93188241-93188855
chr10	93211881	93212082	chr10-93211881-93212082
chr10	93213087	93214492	chr10-93213087-93214492
chr10	93276425	93276859	chr10-93276425-93276859
chr10	93287440	93288473	chr10-93287440-93288473
chr10	93305575	93311941	chr10-93305575-93311941
chr10	93326014	93326215	chr10-93326014-93326215
chr10	93332128	93332634	chr10-93332128-93332634
chr10	93343074	93344015	chr10-93343074-93344015
chr10	93357613	93357814	chr10-93357613-93357814
chr10	93360219	93362145	chr10-93360219-93362145
chr10	93370541	93370742	chr10-93370541-93370742
chr10	93421726	93425792	chr10-93421726-93425792

chr10	93432574	93433014	chr10-93432574-93433014
chr10	93452266	93455027	chr10-93452266-93455027
chr10	93534220	93534638	chr10-93534220-93534638
chr10	93539546	93540867	chr10-93539546-93540867
chr10	93587866	93590356	chr10-93587866-93590356
chr10	93594080	93594501	chr10-93594080-93594501
chr10	93596082	93596283	chr10-93596082-93596283
chr10	93597771	93599230	chr10-93597771-93599230
chr10	93620352	93621146	chr10-93620352-93621146
chr10	93640393	93643444	chr10-93640393-93643444
chr10	93749442	93749744	chr10-93749442-93749744
chr10	93757484	93757685	chr10-93757484-93757685
chr10	93783145	93783886	chr10-93783145-93783886
chr10	93813305	93813506	chr10-93813305-93813506
chr10	93833293	93834756	chr10-93833293-93834756
chr10	93887872	93890507	chr10-93887872-93890507
chr10	93932885	93933186	chr10-93932885-93933186
chr10	93944420	93944876	chr10-93944420-93944876
chr10	94034342	94038898	chr10-94034342-94038898
chr10	94039756	94039957	chr10-94039756-94039957
chr10	94046918	94049990	chr10-94046918-94049990
chr10	94056921	94057122	chr10-94056921-94057122
chr10	94059842	94060420	chr10-94059842-94060420
chr10	94067097	94067404	chr10-94067097-94067404
chr10	94072048	94073846	chr10-94072048-94073846
chr10	94081867	94082665	chr10-94081867-94082665
chr10	94087422	94087991	chr10-94087422-94087991
chr10	94116262	94118383	chr10-94116262-94118383
chr10	94147419	94152708	chr10-94147419-94152708
chr10	94198086	94200146	chr10-94198086-94200146
chr10	94311482	94312815	chr10-94311482-94312815
chr10	94378474	94378840	chr10-94378474-94378840
chr10	94380676	94381601	chr10-94380676-94381601
chr10	94408796	94409215	chr10-94408796-94409215
chr10	94512209	94517876	chr10-94512209-94517876
chr10	94523708	94524209	chr10-94523708-94524209
chr10	94544796	94547256	chr10-94544796-94547256
chr10	94550611	94552569	chr10-94550611-94552569
chr10	94561514	94561774	chr10-94561514-94561774
chr10	94567962	94568494	chr10-94567962-94568494
chr10	94687588	94689904	chr10-94687588-94689904
chr10	94829328	94829787	chr10-94829328-94829787

chr10	94942849	94945668	chr10-94942849-94945668
chr10	94991496	94992361	chr10-94991496-94992361
chr10	95048484	95048735	chr10-95048484-95048735
chr10	95264442	95265332	chr10-95264442-95265332
chr10	95266289	95266490	chr10-95266289-95266490
chr10	95322069	95324478	chr10-95322069-95324478
chr10	95371899	95372100	chr10-95371899-95372100
chr10	95388559	95389656	chr10-95388559-95389656
chr10	95394877	95395594	chr10-95394877-95395594
chr10	95398572	95398824	chr10-95398572-95398824
chr10	95413638	95418091	chr10-95413638-95418091
chr10	95439549	95439750	chr10-95439549-95439750
chr10	95457848	95458472	chr10-95457848-95458472
chr10	95465680	95465943	chr10-95465680-95465943
chr10	95500363	95502164	chr10-95500363-95502164
chr10	95513788	95516926	chr10-95513788-95516926
chr10	95525500	95525701	chr10-95525500-95525701
chr10	95561488	95565003	chr10-95561488-95565003
chr10	95630474	95631212	chr10-95630474-95631212
chr10	95762789	95763355	chr10-95762789-95763355
chr10	95863091	95863350	chr10-95863091-95863350
chr10	95864718	95865138	chr10-95864718-95865138
chr10	95894388	95894955	chr10-95894388-95894955
chr10	95918889	95919245	chr10-95918889-95919245
chr10	95939505	95942459	chr10-95939505-95942459
chr10	96016167	96018411	chr10-96016167-96018411
chr10	96309337	96309538	chr10-96309337-96309538
chr10	96616091	96624609	chr10-96616091-96624609
chr10	96626629	96629766	chr10-96626629-96629766
chr10	96632453	96633231	chr10-96632453-96633231
chr10	96686951	96687152	chr10-96686951-96687152
chr10	96690424	96691029	chr10-96690424-96691029
chr10	96697428	96698185	chr10-96697428-96698185
chr10	96751489	96752060	chr10-96751489-96752060
chr10	96806821	96807073	chr10-96806821-96807073
chr10	96871518	96871984	chr10-96871518-96871984
chr10	97312807	97314133	chr10-97312807-97314133
chr10	98333641	98333897	chr10-98333641-98333897
chr10	98684118	98684684	chr10-98684118-98684684
chr10	98744718	98744919	chr10-98744718-98744919
chr10	98913336	98917439	chr10-98913336-98917439
chr10	98950524	98952243	chr10-98950524-98952243

chr10	98954449	98954775	chr10-98954449-98954775
chr10	98973545	98974489	chr10-98973545-98974489
chr10	99062128	99063301	chr10-99062128-99063301
chr10	99106180	99109229	chr10-99106180-99109229
chr10	99171962	99172708	chr10-99171962-99172708
chr10	99202060	99205130	chr10-99202060-99205130
chr10	99211673	99212078	chr10-99211673-99212078
chr10	99237089	99237788	chr10-99237089-99237788
chr10	99240491	99245254	chr10-99240491-99245254
chr10	99260208	99269422	chr10-99260208-99269422
chr10	99297525	99297777	chr10-99297525-99297777
chr10	99396310	99397088	chr10-99396310-99397088
chr10	99555992	99556395	chr10-99555992-99556395
chr10	99599416	99600365	chr10-99599416-99600365
chr10	100015292	100017963	chr10-100015292-100017963
chr10	100486190	100488394	chr10-100486190-100488394
chr10	100588300	100589553	chr10-100588300-100589553
chr10	101583107	101583308	chr10-101583107-101583308
chr10	102512359	102512560	chr10-102512359-102512560
chr10	102622319	102622520	chr10-102622319-102622520
chr10	103028165	103029064	chr10-103028165-103029064
chr10	103235972	103236427	chr10-103235972-103236427
chr10	103367461	103369474	chr10-103367461-103369474
chr10	105522636	105524076	chr10-105522636-105524076
chr10	105572671	105575610	chr10-105572671-105575610
chr10	105840782	105842933	chr10-105840782-105842933
chr10	106469892	106470803	chr10-106469892-106470803
chr10	107123370	107123738	chr10-107123370-107123738
chr10	107271627	107272762	chr10-107271627-107272762
chr10	107493253	107493623	chr10-107493253-107493623
chr10	108113174	108113927	chr10-108113174-108113927
chr10	108160968	108164196	chr10-108160968-108164196
chr10	108173142	108173343	chr10-108173142-108173343
chr10	108307574	108308040	chr10-108307574-108308040
chr10	108330759	108334743	chr10-108330759-108334743
chr10	108335811	108336064	chr10-108335811-108336064
chr10	108337943	108338465	chr10-108337943-108338465
chr10	108372337	108373197	chr10-108372337-108373197
chr10	108377356	108378821	chr10-108377356-108378821
chr10	108434486	108435785	chr10-108434486-108435785
chr10	108444573	108447192	chr10-108444573-108447192
chr10	108531619	108531870	chr10-108531619-108531870

chr10	108538315	108538616	chr10-108538315-108538616
chr10	108581248	108582353	chr10-108581248-108582353
chr10	108697202	108697456	chr10-108697202-108697456
chr10	109010413	109012128	chr10-109010413-109012128
chr10	109137226	109137427	chr10-109137226-109137427
chr10	110455105	110456405	chr10-110455105-110456405
chr10	110566343	110566759	chr10-110566343-110566759
chr10	110743641	110747140	chr10-110743641-110747140
chr10	110749799	110750103	chr10-110749799-110750103
chr10	110919460	110921013	chr10-110919460-110921013
chr10	110927364	110927892	chr10-110927364-110927892
chr10	111008850	111011497	chr10-111008850-111011497
chr10	111031517	111031983	chr10-111031517-111031983
chr10	111108777	111108978	chr10-111108777-111108978
chr10	111164120	111166474	chr10-111164120-111166474
chr10	111297920	111299384	chr10-111297920-111299384
chr10	111336114	111336315	chr10-111336114-111336315
chr10	111389731	111390382	chr10-111389731-111390382
chr10	111472609	111476009	chr10-111472609-111476009
chr10	111504676	111511218	chr10-111504676-111511218
chr10	111514461	111517735	chr10-111514461-111517735
chr10	111519637	111520120	chr10-111519637-111520120
chr10	111528557	111528876	chr10-111528557-111528876
chr10	111542753	111544092	chr10-111542753-111544092
chr10	111556353	111556989	chr10-111556353-111556989
chr10	111557876	111559812	chr10-111557876-111559812
chr10	111571089	111571367	chr10-111571089-111571367
chr10	111575078	111577098	chr10-111575078-111577098
chr10	111583212	111584099	chr10-111583212-111584099
chr10	111600022	111600953	chr10-111600022-111600953
chr10	111636411	111637806	chr10-111636411-111637806
chr10	111640193	111640394	chr10-111640193-111640394
chr10	111641588	111641789	chr10-111641588-111641789
chr10	111672308	111673381	chr10-111672308-111673381
chr10	111713940	111715819	chr10-111713940-111715819
chr10	111769929	111770130	chr10-111769929-111770130
chr10	111936400	111937086	chr10-111936400-111937086
chr10	111972415	111974562	chr10-111972415-111974562
chr10	111995842	111998183	chr10-111995842-111998183
chr10	111999735	111999936	chr10-111999735-111999936
chr10	112001491	112002594	chr10-112001491-112002594
chr10	112269826	112272368	chr10-112269826-112272368

chr10	112810041	112810347	chr10-112810041-112810347
chr10	112882898	112883515	chr10-112882898-112883515
chr10	112926229	112929523	chr10-112926229-112929523
chr10	114801483	114802076	chr10-114801483-114802076
chr10	114987959	114988340	chr10-114987959-114988340
chr10	115175003	115176318	chr10-115175003-115176318
chr10	115241487	115241688	chr10-115241487-115241688
chr10	115247988	115248492	chr10-115247988-115248492
chr10	115249759	115252745	chr10-115249759-115252745
chr10	115263024	115263326	chr10-115263024-115263326
chr10	115281020	115281701	chr10-115281020-115281701
chr10	115312777	115316759	chr10-115312777-115316759
chr10	115361316	115363394	chr10-115361316-115363394
chr10	115382663	115388286	chr10-115382663-115388286
chr10	115586000	115588219	chr10-115586000-115588219
chr10	115618210	115618673	chr10-115618210-115618673
chr10	115780106	115780435	chr10-115780106-115780435
chr10	115814395	115822158	chr10-115814395-115822158
chr10	116017781	116018971	chr10-116017781-116018971
chr10	116177208	116177915	chr10-116177208-116177915
chr10	116192126	116193002	chr10-116192126-116193002
chr10	116196719	116197498	chr10-116196719-116197498
chr10	116266414	116267559	chr10-116266414-116267559
chr10	116297558	116298612	chr10-116297558-116298612
chr10	116301178	116301884	chr10-116301178-116301884
chr10	116472752	116474699	chr10-116472752-116474699
chr10	116529082	116529384	chr10-116529082-116529384
chr10	116536656	116537514	chr10-116536656-116537514
chr10	116579947	116583221	chr10-116579947-116583221
chr10	116733518	116733719	chr10-116733518-116733719
chr10	116751038	116751452	chr10-116751038-116751452
chr10	116929243	116929597	chr10-116929243-116929597
chr10	116930528	116930929	chr10-116930528-116930929
chr10	116948319	116951330	chr10-116948319-116951330
chr10	116995985	116996395	chr10-116995985-116996395
chr10	117021309	117021651	chr10-117021309-117021651
chr10	117023789	117024601	chr10-117023789-117024601
chr10	117033767	117033968	chr10-117033767-117033968
chr10	117043580	117044153	chr10-117043580-117044153
chr10	117059587	117060275	chr10-117059587-117060275
chr10	117062526	117064762	chr10-117062526-117064762
chr10	117146404	117150637	chr10-117146404-117150637

chr10	117209353	117209861	chr10-117209353-117209861
chr10	117222905	117225444	chr10-117222905-117225444
chr10	117274882	117276412	chr10-117274882-117276412
chr10	117373970	117377918	chr10-117373970-117377918
chr10	117392955	117393897	chr10-117392955-117393897
chr10	117432513	117432814	chr10-117432513-117432814
chr10	117435209	117435950	chr10-117435209-117435950
chr10	117493952	117494934	chr10-117493952-117494934
chr10	117516103	117516354	chr10-117516103-117516354
chr10	117521406	117522176	chr10-117521406-117522176
chr10	117556322	117556792	chr10-117556322-117556792
chr10	117571145	117572036	chr10-117571145-117572036
chr10	117628699	117630144	chr10-117628699-117630144
chr10	117662697	117664493	chr10-117662697-117664493
chr10	117708964	117711681	chr10-117708964-117711681
chr10	117717457	117718741	chr10-117717457-117718741
chr10	117721668	117722023	chr10-117721668-117722023
chr10	117744268	117746976	chr10-117744268-117746976
chr10	117791156	117793609	chr10-117791156-117793609
chr10	117842958	117847072	chr10-117842958-117847072
chr10	117860107	117862864	chr10-117860107-117862864
chr10	117899484	117899794	chr10-117899484-117899794
chr10	117905631	117906669	chr10-117905631-117906669
chr10	117940836	117941364	chr10-117940836-117941364
chr10	118102813	118103706	chr10-118102813-118103706
chr10	118141023	118142773	chr10-118141023-118142773
chr10	118866433	118870568	chr10-118866433-118870568
chr10	118987883	118988642	chr10-118987883-118988642
chr10	118991413	118991770	chr10-118991413-118991770
chr10	119111563	119111764	chr10-119111563-119111764
chr10	119238038	119241555	chr10-119238038-119241555
chr10	119453290	119455772	chr10-119453290-119455772
chr10	119610368	119610686	chr10-119610368-119610686
chr10	119614702	119615063	chr10-119614702-119615063
chr10	119692283	119692596	chr10-119692283-119692596
chr10	119819264	119819924	chr10-119819264-119819924
chr10	119877001	119877202	chr10-119877001-119877202
chr10	120060437	120062019	chr10-120060437-120062019
chr10	120112166	120113489	chr10-120112166-120113489
chr10	120201064	120202412	chr10-120201064-120202412
chr10	120208484	120210244	chr10-120208484-120210244
chr10	120226583	120228914	chr10-120226583-120228914

chr10	120422283	120422765	chr10-120422283-120422765
chr10	120468609	120478827	chr10-120468609-120478827
chr10	120551884	120553486	chr10-120551884-120553486
chr10	120557567	120558944	chr10-120557567-120558944
chr10	120582139	120583008	chr10-120582139-120583008
chr10	120606095	120607232	chr10-120606095-120607232
chr10	120609951	120612555	chr10-120609951-120612555
chr10	120616122	120616323	chr10-120616122-120616323
chr10	120623680	120624353	chr10-120623680-120624353
chr10	120632400	120633550	chr10-120632400-120633550
chr10	120635247	120636463	chr10-120635247-120636463
chr10	120637349	120637550	chr10-120637349-120637550
chr10	120716799	120718136	chr10-120716799-120718136
chr10	120873614	120874127	chr10-120873614-120874127
chr10	120875841	120877042	chr10-120875841-120877042
chr10	120898147	120899579	chr10-120898147-120899579
chr10	120976623	120980431	chr10-120976623-120980431
chr10	121033035	121034794	chr10-121033035-121034794
chr10	121294169	121294687	chr10-121294169-121294687
chr10	121309509	121311484	chr10-121309509-121311484
chr10	121336681	121337092	chr10-121336681-121337092
chr10	121350283	121352009	chr10-121350283-121352009
chr10	121364394	121366507	chr10-121364394-121366507
chr10	121377163	121377465	chr10-121377163-121377465
chr10	121426079	121430614	chr10-121426079-121430614
chr10	121432171	121433510	chr10-121432171-121433510
chr10	121435359	121436209	chr10-121435359-121436209
chr10	121443507	121444480	chr10-121443507-121444480
chr10	121454676	121455826	chr10-121454676-121455826
chr10	121461308	121462832	chr10-121461308-121462832
chr10	121465589	121465790	chr10-121465589-121465790
chr10	121473069	121477169	chr10-121473069-121477169
chr10	121504906	121505944	chr10-121504906-121505944
chr10	121509968	121511100	chr10-121509968-121511100
chr10	121520819	121521904	chr10-121520819-121521904
chr10	121533282	121533599	chr10-121533282-121533599
chr10	121585361	121587831	chr10-121585361-121587831
chr10	121624205	121627443	chr10-121624205-121627443
chr10	121640985	121642696	chr10-121640985-121642696
chr10	121693118	121693319	chr10-121693118-121693319
chr10	121739287	121741016	chr10-121739287-121741016
chr10	121763193	121763444	chr10-121763193-121763444

chr10	121771105	121771512	chr10-121771105-121771512
chr10	121795692	121796780	chr10-121795692-121796780
chr10	121822877	121823224	chr10-121822877-121823224
chr10	122045993	122048260	chr10-122045993-122048260
chr10	122095782	122098002	chr10-122095782-122098002
chr10	122105167	122105368	chr10-122105167-122105368
chr10	122448483	122449525	chr10-122448483-122449525
chr10	122544182	122544497	chr10-122544182-122544497
chr10	122678339	122680629	chr10-122678339-122680629
chr10	122777654	122778212	chr10-122777654-122778212
chr10	122794360	122794991	chr10-122794360-122794991
chr10	122807350	122807551	chr10-122807350-122807551
chr10	122886005	122886970	chr10-122886005-122886970
chr10	122912845	122913046	chr10-122912845-122913046
chr10	122982941	122988629	chr10-122982941-122988629
chr10	123074739	123077326	chr10-123074739-123077326
chr10	123195172	123197862	chr10-123195172-123197862
chr10	123263977	123265927	chr10-123263977-123265927
chr10	124288257	124288983	chr10-124288257-124288983
chr10	125388892	125389702	chr10-125388892-125389702
chr10	125917248	125917664	chr10-125917248-125917664
chr10	125965705	125968061	chr10-125965705-125968061
chr10	126900418	126901917	chr10-126900418-126901917
chr10	126905164	126906358	chr10-126905164-126906358
chr10	126927829	126929201	chr10-126927829-126929201
chr10	126935383	126936436	chr10-126935383-126936436
chr10	126962362	126962563	chr10-126962362-126962563
chr10	126977900	126981999	chr10-126977900-126981999
chr10	126984228	126985238	chr10-126984228-126985238
chr10	127029750	127031082	chr10-127029750-127031082
chr10	127040715	127043271	chr10-127040715-127043271
chr10	127049003	127050068	chr10-127049003-127050068
chr10	127058107	127060969	chr10-127058107-127060969
chr10	127062921	127065599	chr10-127062921-127065599
chr10	127067823	127071563	chr10-127067823-127071563
chr10	127078642	127081819	chr10-127078642-127081819
chr10	127090661	127092956	chr10-127090661-127092956
chr10	127093919	127096236	chr10-127093919-127096236
chr10	127103237	127103692	chr10-127103237-127103692
chr10	127120060	127121605	chr10-127120060-127121605
chr10	127122862	127123689	chr10-127122862-127123689
chr10	127144023	127145060	chr10-127144023-127145060

chr10	127150341	127151511	chr10-127150341-127151511
chr10	127162794	127163269	chr10-127162794-127163269
chr10	127164598	127168735	chr10-127164598-127168735
chr10	127170553	127171170	chr10-127170553-127171170
chr10	127176607	127178909	chr10-127176607-127178909
chr10	127180086	127180546	chr10-127180086-127180546
chr10	127183665	127184367	chr10-127183665-127184367
chr10	127187790	127190087	chr10-127187790-127190087
chr10	127194738	127196555	chr10-127194738-127196555
chr10	127210184	127212281	chr10-127210184-127212281
chr10	127262473	127263483	chr10-127262473-127263483
chr10	127265831	127267729	chr10-127265831-127267729
chr10	127286595	127292469	chr10-127286595-127292469
chr10	127309984	127312744	chr10-127309984-127312744
chr10	127325999	127327185	chr10-127325999-127327185
chr10	127332485	127332686	chr10-127332485-127332686
chr10	127336933	127337188	chr10-127336933-127337188
chr10	127339003	127342588	chr10-127339003-127342588
chr10	127350507	127351040	chr10-127350507-127351040
chr10	127379876	127382105	chr10-127379876-127382105
chr10	127481247	127482086	chr10-127481247-127482086
chr10	127500794	127501408	chr10-127500794-127501408
chr10	127508441	127509997	chr10-127508441-127509997
chr10	127511375	127513898	chr10-127511375-127513898
chr10	127520035	127522638	chr10-127520035-127522638
chr10	127526173	127527138	chr10-127526173-127527138
chr10	127530949	127531150	chr10-127530949-127531150
chr10	127533775	127535345	chr10-127533775-127535345
chr10	127562742	127563020	chr10-127562742-127563020
chr10	127583501	127584329	chr10-127583501-127584329
chr10	127619873	127622531	chr10-127619873-127622531
chr10	127641887	127644752	chr10-127641887-127644752
chr10	127661297	127663291	chr10-127661297-127663291
chr10	127664933	127669412	chr10-127664933-127669412
chr10	127674964	127678193	chr10-127674964-127678193
chr10	127738267	127740712	chr10-127738267-127740712
chr10	127922154	127922355	chr10-127922154-127922355
chr10	127924497	127925718	chr10-127924497-127925718
chr10	128014857	128016035	chr10-128014857-128016035
chr10	128034587	128038724	chr10-128034587-128038724
chr10	128057678	128062743	chr10-128057678-128062743
chr10	128082038	128085451	chr10-128082038-128085451

chr10	128091031	128095410	chr10-128091031-128095410
chr10	128096341	128098336	chr10-128096341-128098336
chr10	128143752	128144217	chr10-128143752-128144217
chr10	128149527	128149928	chr10-128149527-128149928
chr10	128151153	128151354	chr10-128151153-128151354
chr10	128166963	128167629	chr10-128166963-128167629
chr10	128175068	128177525	chr10-128175068-128177525
chr10	128179851	128180516	chr10-128179851-128180516
chr10	128193605	128195835	chr10-128193605-128195835
chr10	128210504	128213573	chr10-128210504-128213573
chr10	128231145	128233257	chr10-128231145-128233257
chr10	128253931	128254132	chr10-128253931-128254132
chr10	128255977	128256846	chr10-128255977-128256846
chr10	128269967	128271252	chr10-128269967-128271252
chr10	128296361	128299138	chr10-128296361-128299138
chr10	128302285	128304636	chr10-128302285-128304636
chr10	128321553	128323990	chr10-128321553-128323990
chr10	128337020	128339718	chr10-128337020-128339718
chr10	128344193	128346002	chr10-128344193-128346002
chr10	128367663	128369770	chr10-128367663-128369770
chr10	128376180	128378861	chr10-128376180-128378861
chr10	128408505	128413076	chr10-128408505-128413076
chr10	128458237	128460783	chr10-128458237-128460783
chr10	128490925	128495472	chr10-128490925-128495472
chr10	128498055	128499936	chr10-128498055-128499936
chr10	128504654	128504910	chr10-128504654-128504910
chr10	128523916	128526975	chr10-128523916-128526975
chr10	128536274	128536975	chr10-128536274-128536975
chr10	128545290	128550087	chr10-128545290-128550087
chr10	128563826	128567223	chr10-128563826-128567223
chr10	128583131	128591000	chr10-128583131-128591000
chr10	128612791	128613275	chr10-128612791-128613275
chr10	128624540	128627193	chr10-128624540-128627193
chr10	128641902	128642420	chr10-128641902-128642420
chr10	128645534	128646186	chr10-128645534-128646186
chr10	128655870	128656071	chr10-128655870-128656071
chr10	128668690	128669799	chr10-128668690-128669799
chr10	128672517	128674778	chr10-128672517-128674778
chr10	128694949	128696994	chr10-128694949-128696994
chr10	128703286	128705678	chr10-128703286-128705678
chr10	128737465	128737816	chr10-128737465-128737816
chr10	128742714	128745046	chr10-128742714-128745046

chr10	128747216	128749769	chr10-128747216-128749769
chr10	128771707	128772008	chr10-128771707-128772008
chr10	128779027	128779622	chr10-128779027-128779622
chr10	128802985	128809177	chr10-128802985-128809177
chr10	128820251	128823558	chr10-128820251-128823558
chr10	128890167	128892563	chr10-128890167-128892563
chr10	128909142	128912322	chr10-128909142-128912322
chr10	128920661	128924578	chr10-128920661-128924578
chr10	128933442	128934575	chr10-128933442-128934575
chr10	128980112	128981084	chr10-128980112-128981084
chr10	130470269	130470790	chr10-130470269-130470790
chr10	130541526	130543669	chr10-130541526-130543669
chr11	3122980	3126216	chr11-3122980-3126216
chr11	3151413	3151714	chr11-3151413-3151714
chr11	3201344	3204479	chr11-3201344-3204479
chr11	3264794	3267436	chr11-3264794-3267436
chr11	3288311	3294566	chr11-3288311-3294566
chr11	3300951	3301260	chr11-3300951-3301260
chr11	3302657	3303024	chr11-3302657-3303024
chr11	3330472	3332989	chr11-3330472-3332989
chr11	3347319	3349747	chr11-3347319-3349747
chr11	3369132	3372198	chr11-3369132-3372198
chr11	3373903	3375566	chr11-3373903-3375566
chr11	3390213	3393686	chr11-3390213-3393686
chr11	3406815	3409742	chr11-3406815-3409742
chr11	3450866	3453808	chr11-3450866-3453808
chr11	3481746	3482258	chr11-3481746-3482258
chr11	3504414	3504931	chr11-3504414-3504931
chr11	3514167	3515464	chr11-3514167-3515464
chr11	3516832	3517910	chr11-3516832-3517910
chr11	3535094	3540086	chr11-3535094-3540086
chr11	3647535	3651956	chr11-3647535-3651956
chr11	3761573	3762640	chr11-3761573-3762640
chr11	3774359	3775123	chr11-3774359-3775123
chr11	3860137	3860338	chr11-3860137-3860338
chr11	3863214	3864342	chr11-3863214-3864342
chr11	3887065	3887755	chr11-3887065-3887755
chr11	3893231	3904002	chr11-3893231-3904002
chr11	3904829	3916547	chr11-3904829-3916547
chr11	3931198	3932522	chr11-3931198-3932522
chr11	3963146	3965674	chr11-3963146-3965674
chr11	3983253	3984585	chr11-3983253-3984585

chr11	4094515	4097825	chr11-4094515-4097825
chr11	4117781	4119222	chr11-4117781-4119222
chr11	4134701	4136747	chr11-4134701-4136747
chr11	4159428	4162051	chr11-4159428-4162051
chr11	4186134	4188831	chr11-4186134-4188831
chr11	4190240	4191194	chr11-4190240-4191194
chr11	4217547	4219677	chr11-4217547-4219677
chr11	4256872	4271935	chr11-4256872-4271935
chr11	4296760	4300513	chr11-4296760-4300513
chr11	4302304	4303788	chr11-4302304-4303788
chr11	4308131	4309875	chr11-4308131-4309875
chr11	4317454	4317826	chr11-4317454-4317826
chr11	4335692	4337295	chr11-4335692-4337295
chr11	4401423	4402101	chr11-4401423-4402101
chr11	4593185	4595411	chr11-4593185-4595411
chr11	4637195	4639234	chr11-4637195-4639234
chr11	4702743	4705575	chr11-4702743-4705575
chr11	4745774	4747655	chr11-4745774-4747655
chr11	4847136	4850423	chr11-4847136-4850423
chr11	4873587	4875191	chr11-4873587-4875191
chr11	4894556	4896460	chr11-4894556-4896460
chr11	4946758	4948357	chr11-4946758-4948357
chr11	4984096	4984297	chr11-4984096-4984297
chr11	4986361	4988167	chr11-4986361-4988167
chr11	5057638	5059211	chr11-5057638-5059211
chr11	5060974	5065973	chr11-5060974-5065973
chr11	5096038	5101102	chr11-5096038-5101102
chr11	5151683	5152652	chr11-5151683-5152652
chr11	5227265	5227619	chr11-5227265-5227619
chr11	5239655	5245703	chr11-5239655-5245703
chr11	5260092	5262505	chr11-5260092-5262505
chr11	5296454	5297639	chr11-5296454-5297639
chr11	5442701	5445715	chr11-5442701-5445715
chr11	5485479	5486338	chr11-5485479-5486338
chr11	5520129	5522844	chr11-5520129-5522844
chr11	5540490	5542673	chr11-5540490-5542673
chr11	5705902	5708479	chr11-5705902-5708479
chr11	5734290	5735293	chr11-5734290-5735293
chr11	5736852	5737925	chr11-5736852-5737925
chr11	5738784	5742285	chr11-5738784-5742285
chr11	5761197	5763213	chr11-5761197-5763213
chr11	5766067	5766318	chr11-5766067-5766318

chr11	5787067	5790088	chr11-5787067-5790088
chr11	5834599	5838533	chr11-5834599-5838533
chr11	5861445	5862741	chr11-5861445-5862741
chr11	5876569	5879459	chr11-5876569-5879459
chr11	5892021	5892618	chr11-5892021-5892618
chr11	5895077	5898991	chr11-5895077-5898991
chr11	5901067	5902661	chr11-5901067-5902661
chr11	5954850	5957560	chr11-5954850-5957560
chr11	5999749	6003127	chr11-5999749-6003127
chr11	6004508	6007964	chr11-6004508-6007964
chr11	6010083	6010699	chr11-6010083-6010699
chr11	6064861	6066185	chr11-6064861-6066185
chr11	6068208	6068595	chr11-6068208-6068595
chr11	6198407	6201261	chr11-6198407-6201261
chr11	6266272	6268734	chr11-6266272-6268734
chr11	6273474	6275507	chr11-6273474-6275507
chr11	6290954	6293069	chr11-6290954-6293069
chr11	6388559	6390820	chr11-6388559-6390820
chr11	6405779	6406807	chr11-6405779-6406807
chr11	6413145	6413431	chr11-6413145-6413431
chr11	6414556	6419418	chr11-6414556-6419418
chr11	6443166	6445740	chr11-6443166-6445740
chr11	6470826	6477200	chr11-6470826-6477200
chr11	6525677	6529261	chr11-6525677-6529261
chr11	6545824	6548613	chr11-6545824-6548613
chr11	6604996	6606111	chr11-6604996-6606111
chr11	6623590	6627321	chr11-6623590-6627321
chr11	6658066	6659611	chr11-6658066-6659611
chr11	6697735	6698872	chr11-6697735-6698872
chr11	6894620	6896207	chr11-6894620-6896207
chr11	6994895	6995147	chr11-6994895-6995147
chr11	7018937	7019239	chr11-7018937-7019239
chr11	7062874	7065184	chr11-7062874-7065184
chr11	7105659	7105860	chr11-7105659-7105860
chr11	7210610	7215003	chr11-7210610-7215003
chr11	7237312	7239704	chr11-7237312-7239704
chr11	7423052	7424597	chr11-7423052-7424597
chr11	7793736	7793937	chr11-7793736-7793937
chr11	8424453	8424756	chr11-8424453-8424756
chr11	8443022	8446369	chr11-8443022-8446369
chr11	8450649	8453032	chr11-8450649-8453032
chr11	8462450	8470147	chr11-8462450-8470147

chr11	8503734	8503988	chr11-8503734-8503988
chr11	8579562	8579932	chr11-8579562-8579932
chr11	8582322	8582923	chr11-8582322-8582923
chr11	8589080	8590385	chr11-8589080-8590385
chr11	8623937	8625005	chr11-8623937-8625005
chr11	8628790	8629316	chr11-8628790-8629316
chr11	8634160	8636637	chr11-8634160-8636637
chr11	8662085	8665301	chr11-8662085-8665301
chr11	8674668	8676056	chr11-8674668-8676056
chr11	8692355	8693587	chr11-8692355-8693587
chr11	8830413	8831116	chr11-8830413-8831116
chr11	8886007	8886908	chr11-8886007-8886908
chr11	9009969	9011902	chr11-9009969-9011902
chr11	9048266	9049388	chr11-9048266-9049388
chr11	9116118	9119551	chr11-9116118-9119551
chr11	11115807	11116066	chr11-11115807-11116066
chr11	11461632	11462611	chr11-11461632-11462611
chr11	11685205	11686616	chr11-11685205-11686616
chr11	11807783	11809813	chr11-11807783-11809813
chr11	11812279	11812480	chr11-11812279-11812480
chr11	12025625	12026568	chr11-12025625-12026568
chr11	12036390	12038027	chr11-12036390-12038027
chr11	12231650	12232873	chr11-12231650-12232873
chr11	12409323	12412218	chr11-12409323-12412218
chr11	12424183	12424595	chr11-12424183-12424595
chr11	12434620	12434821	chr11-12434620-12434821
chr11	12442045	12442464	chr11-12442045-12442464
chr11	12463140	12465648	chr11-12463140-12465648
chr11	16257410	16258063	chr11-16257410-16258063
chr11	16260829	16261456	chr11-16260829-16261456
chr11	16506856	16509021	chr11-16506856-16509021
chr11	16525992	16526696	chr11-16525992-16526696
chr11	16559287	16559748	chr11-16559287-16559748
chr11	16590723	16593968	chr11-16590723-16593968
chr11	16751534	16755397	chr11-16751534-16755397
chr11	16769187	16770185	chr11-16769187-16770185
chr11	16788198	16791824	chr11-16788198-16791824
chr11	16793049	16793518	chr11-16793049-16793518
chr11	16796098	16796663	chr11-16796098-16796663
chr11	16800207	16800865	chr11-16800207-16800865
chr11	16830199	16831201	chr11-16830199-16831201
chr11	16843910	16844541	chr11-16843910-16844541

chr11	16949880	16952364	chr11-16949880-16952364
chr11	17051922	17052342	chr11-17051922-17052342
chr11	17158301	17161028	chr11-17158301-17161028
chr11	17210292	17213101	chr11-17210292-17213101
chr11	17256886	17258804	chr11-17256886-17258804
chr11	17952652	17954208	chr11-17952652-17954208
chr11	18871324	18872006	chr11-18871324-18872006
chr11	18876871	18878375	chr11-18876871-18878375
chr11	18879392	18879593	chr11-18879392-18879593
chr11	19014348	19015030	chr11-19014348-19015030
chr11	19016123	19016324	chr11-19016123-19016324
chr11	19018531	19020969	chr11-19018531-19020969
chr11	19026499	19027673	chr11-19026499-19027673
chr11	19835064	19835696	chr11-19835064-19835696
chr11	19849264	19849773	chr11-19849264-19849773
chr11	19919030	19921095	chr11-19919030-19921095
chr11	19923298	19927254	chr11-19923298-19927254
chr11	19929367	19929776	chr11-19929367-19929776
chr11	19961029	19961280	chr11-19961029-19961280
chr11	19978867	19979956	chr11-19978867-19979956
chr11	19992408	19993184	chr11-19992408-19993184
chr11	20110860	20114505	chr11-20110860-20114505
chr11	20116517	20117476	chr11-20116517-20117476
chr11	20126740	20127626	chr11-20126740-20127626
chr11	20132826	20133390	chr11-20132826-20133390
chr11	20200501	20203371	chr11-20200501-20203371
chr11	20242776	20243127	chr11-20242776-20243127
chr11	20248221	20250452	chr11-20248221-20250452
chr11	20329734	20334166	chr11-20329734-20334166
chr11	20347613	20348623	chr11-20347613-20348623
chr11	20358025	20358484	chr11-20358025-20358484
chr11	20400543	20401103	chr11-20400543-20401103
chr11	20445105	20446114	chr11-20445105-20446114
chr11	20455018	20456110	chr11-20455018-20456110
chr11	20476641	20478159	chr11-20476641-20478159
chr11	20531305	20533717	chr11-20531305-20533717
chr11	20541691	20545560	chr11-20541691-20545560
chr11	20561204	20561757	chr11-20561204-20561757
chr11	20594361	20594807	chr11-20594361-20594807
chr11	20630953	20633990	chr11-20630953-20633990
chr11	20635645	20636004	chr11-20635645-20636004
chr11	20712142	20712343	chr11-20712142-20712343

chr11	20740005	20742862	chr11-20740005-20742862
chr11	20829885	20831970	chr11-20829885-20831970
chr11	20873011	20873321	chr11-20873011-20873321
chr11	20886712	20887038	chr11-20886712-20887038
chr11	20896055	20897100	chr11-20896055-20897100
chr11	20937529	20937730	chr11-20937529-20937730
chr11	20954754	20960200	chr11-20954754-20960200
chr11	20982147	20982701	chr11-20982147-20982701
chr11	21090814	21094002	chr11-21090814-21094002
chr11	21238230	21241061	chr11-21238230-21241061
chr11	21369137	21371908	chr11-21369137-21371908
chr11	21570756	21573217	chr11-21570756-21573217
chr11	21992840	21994714	chr11-21992840-21994714
chr11	21996367	21997492	chr11-21996367-21997492
chr11	22000393	22003679	chr11-22000393-22003679
chr11	22247205	22247613	chr11-22247205-22247613
chr11	22286021	22288122	chr11-22286021-22288122
chr11	22376616	22376963	chr11-22376616-22376963
chr11	22511779	22513259	chr11-22511779-22513259
chr11	22610466	22612029	chr11-22610466-22612029
chr11	22617273	22617837	chr11-22617273-22617837
chr11	22621730	22622359	chr11-22621730-22622359
chr11	22629470	22630328	chr11-22629470-22630328
chr11	22644311	22644512	chr11-22644311-22644512
chr11	22669471	22669774	chr11-22669471-22669774
chr11	22719389	22719590	chr11-22719389-22719590
chr11	22733209	22733461	chr11-22733209-22733461
chr11	22799830	22800670	chr11-22799830-22800670
chr11	22842324	22844505	chr11-22842324-22844505
chr11	22847507	22848926	chr11-22847507-22848926
chr11	22851476	22851677	chr11-22851476-22851677
chr11	22854711	22861644	chr11-22854711-22861644
chr11	22971775	22974600	chr11-22971775-22974600
chr11	22975568	22975769	chr11-22975568-22975769
chr11	22980866	22982772	chr11-22980866-22982772
chr11	22989811	22992604	chr11-22989811-22992604
chr11	23007272	23009052	chr11-23007272-23009052
chr11	23067115	23068677	chr11-23067115-23068677
chr11	23124232	23125145	chr11-23124232-23125145
chr11	23211259	23211460	chr11-23211259-23211460
chr11	23254494	23259346	chr11-23254494-23259346
chr11	23305353	23309232	chr11-23305353-23309232

chr11	23496309	23498918	chr11-23496309-23498918
chr11	23632549	23634284	chr11-23632549-23634284
chr11	23664270	23667882	chr11-23664270-23667882
chr11	23730102	23730303	chr11-23730102-23730303
chr11	23769273	23772277	chr11-23769273-23772277
chr11	23894111	23895674	chr11-23894111-23895674
chr11	23928009	23928426	chr11-23928009-23928426
chr11	24049359	24049760	chr11-24049359-24049760
chr11	24075758	24082198	chr11-24075758-24082198
chr11	26209986	26211018	chr11-26209986-26211018
chr11	26386705	26387975	chr11-26386705-26387975
chr11	26592330	26594112	chr11-26592330-26594112
chr11	26640668	26640869	chr11-26640668-26640869
chr11	28339657	28339908	chr11-28339657-28339908
chr11	28583144	28584507	chr11-28583144-28584507
chr11	28812285	28813206	chr11-28812285-28813206
chr11	28851768	28854384	chr11-28851768-28854384
chr11	29130035	29131807	chr11-29130035-29131807
chr11	29171351	29174658	chr11-29171351-29174658
chr11	29246072	29248033	chr11-29246072-29248033
chr11	29373057	29375984	chr11-29373057-29375984
chr11	29475863	29476164	chr11-29475863-29476164
chr11	29513847	29515620	chr11-29513847-29515620
chr11	29525566	29527572	chr11-29525566-29527572
chr11	29546237	29549337	chr11-29546237-29549337
chr11	29692103	29697124	chr11-29692103-29697124
chr11	29698799	29699000	chr11-29698799-29699000
chr11	29824620	29825502	chr11-29824620-29825502
chr11	29840305	29840576	chr11-29840305-29840576
chr11	30024202	30026793	chr11-30024202-30026793
chr11	30182593	30183796	chr11-30182593-30183796
chr11	30195234	30199415	chr11-30195234-30199415
chr11	30211597	30212205	chr11-30211597-30212205
chr11	30266038	30269434	chr11-30266038-30269434
chr11	30471597	30472512	chr11-30471597-30472512
chr11	30648856	30649828	chr11-30648856-30649828
chr11	30754712	30755625	chr11-30754712-30755625
chr11	30771121	30773618	chr11-30771121-30773618
chr11	30885094	30885731	chr11-30885094-30885731
chr11	30952948	30955377	chr11-30952948-30955377
chr11	30985078	30987255	chr11-30985078-30987255
chr11	31169258	31169926	chr11-31169258-31169926

chr11	31368234	31371913	chr11-31368234-31371913
chr11	31670206	31672771	chr11-31670206-31672771
chr11	31726948	31727744	chr11-31726948-31727744
chr11	31807483	31808902	chr11-31807483-31808902
chr11	31822385	31822586	chr11-31822385-31822586
chr11	31824393	31825314	chr11-31824393-31825314
chr11	31869742	31875670	chr11-31869742-31875670
chr11	31948537	31949946	chr11-31948537-31949946
chr11	31965773	31965974	chr11-31965773-31965974
chr11	32000282	32000600	chr11-32000282-32000600
chr11	32103055	32103357	chr11-32103055-32103357
chr11	32137966	32138338	chr11-32137966-32138338
chr11	32161687	32162609	chr11-32161687-32162609
chr11	32204456	32206554	chr11-32204456-32206554
chr11	32218429	32223375	chr11-32218429-32223375
chr11	32224465	32227574	chr11-32224465-32227574
chr11	32251040	32251515	chr11-32251040-32251515
chr11	32262611	32262812	chr11-32262611-32262812
chr11	32265944	32268444	chr11-32265944-32268444
chr11	32286889	32287395	chr11-32286889-32287395
chr11	32300055	32300467	chr11-32300055-32300467
chr11	32345066	32350100	chr11-32345066-32350100
chr11	32355166	32355424	chr11-32355166-32355424
chr11	32357371	32358592	chr11-32357371-32358592
chr11	32359826	32360234	chr11-32359826-32360234
chr11	32362841	32364246	chr11-32362841-32364246
chr11	32435217	32436197	chr11-32435217-32436197
chr11	32453998	32457253	chr11-32453998-32457253
chr11	32532163	32535093	chr11-32532163-32535093
chr11	32542606	32542866	chr11-32542606-32542866
chr11	32552460	32554662	chr11-32552460-32554662
chr11	32558327	32558528	chr11-32558327-32558528
chr11	32604636	32605863	chr11-32604636-32605863
chr11	32641444	32645385	chr11-32641444-32645385
chr11	32646754	32648159	chr11-32646754-32648159
chr11	32662112	32662472	chr11-32662112-32662472
chr11	32763156	32763561	chr11-32763156-32763561
chr11	32925518	32926265	chr11-32925518-32926265
chr11	33100055	33100668	chr11-33100055-33100668
chr11	33122734	33123302	chr11-33122734-33123302
chr11	33142672	33142923	chr11-33142672-33142923
chr11	33145882	33148661	chr11-33145882-33148661

chr11	33158797	33163970	chr11-33158797-33163970
chr11	33200206	33204517	chr11-33200206-33204517
chr11	33512628	33514169	chr11-33512628-33514169
chr11	33601550	33601803	chr11-33601550-33601803
chr11	33659882	33660239	chr11-33659882-33660239
chr11	33843304	33843912	chr11-33843304-33843912
chr11	34832290	34834662	chr11-34832290-34834662
chr11	35120967	35121829	chr11-35120967-35121829
chr11	35768733	35771676	chr11-35768733-35771676
chr11	35797777	35798745	chr11-35797777-35798745
chr11	35831916	35834963	chr11-35831916-35834963
chr11	35837909	35838943	chr11-35837909-35838943
chr11	35877887	35878603	chr11-35877887-35878603
chr11	35915636	35916008	chr11-35915636-35916008
chr11	35920058	35923277	chr11-35920058-35923277
chr11	35924713	35926891	chr11-35924713-35926891
chr11	35932853	35934345	chr11-35932853-35934345
chr11	35941876	35943228	chr11-35941876-35943228
chr11	35947927	35952203	chr11-35947927-35952203
chr11	35963743	35963995	chr11-35963743-35963995
chr11	35969890	35973802	chr11-35969890-35973802
chr11	35974786	35981328	chr11-35974786-35981328
chr11	35982775	35982976	chr11-35982775-35982976
chr11	35985562	35986534	chr11-35985562-35986534
chr11	36000958	36001859	chr11-36000958-36001859
chr11	36045712	36046277	chr11-36045712-36046277
chr11	37235940	37236923	chr11-37235940-37236923
chr11	40402621	40402884	chr11-40402621-40402884
chr11	40690613	40695457	chr11-40690613-40695457
chr11	40731831	40735643	chr11-40731831-40735643
chr11	40741853	40742054	chr11-40741853-40742054
chr11	40754366	40755812	chr11-40754366-40755812
chr11	40790609	40790912	chr11-40790609-40790912
chr11	40927275	40927627	chr11-40927275-40927627
chr11	42181952	42182681	chr11-42181952-42182681
chr11	42420515	42421044	chr11-42420515-42421044
chr11	43155613	43157037	chr11-43155613-43157037
chr11	43384951	43386025	chr11-43384951-43386025
chr11	43425306	43426903	chr11-43425306-43426903
chr11	43433194	43435015	chr11-43433194-43435015
chr11	43474129	43474695	chr11-43474129-43474695
chr11	43528213	43529672	chr11-43528213-43529672

chr11	43642951	43643674	chr11-43642951-43643674
chr11	43681066	43683546	chr11-43681066-43683546
chr11	43746713	43748429	chr11-43746713-43748429
chr11	43766825	43767755	chr11-43766825-43767755
chr11	43775681	43776499	chr11-43775681-43776499
chr11	43835784	43836950	chr11-43835784-43836950
chr11	43895577	43895829	chr11-43895577-43895829
chr11	43919340	43919678	chr11-43919340-43919678
chr11	43929367	43929759	chr11-43929367-43929759
chr11	43933247	43934048	chr11-43933247-43934048
chr11	43998028	43998945	chr11-43998028-43998945
chr11	44049159	44049461	chr11-44049159-44049461
chr11	44398868	44399232	chr11-44398868-44399232
chr11	44469187	44471999	chr11-44469187-44471999
chr11	44517989	44520967	chr11-44517989-44520967
chr11	44610985	44611729	chr11-44610985-44611729
chr11	44612582	44613708	chr11-44612582-44613708
chr11	44616591	44618678	chr11-44616591-44618678
chr11	44619792	44620274	chr11-44619792-44620274
chr11	45805420	45805780	chr11-45805420-45805780
chr11	45851398	45853810	chr11-45851398-45853810
chr11	45883678	45883879	chr11-45883678-45883879
chr11	45926539	45927100	chr11-45926539-45927100
chr11	45943124	45945157	chr11-45943124-45945157
chr11	45954755	45955817	chr11-45954755-45955817
chr11	45979884	45981161	chr11-45979884-45981161
chr11	45991604	45991975	chr11-45991604-45991975
chr11	46055753	46057600	chr11-46055753-46057600
chr11	46165741	46166798	chr11-46165741-46166798
chr11	46246182	46246433	chr11-46246182-46246433
chr11	46311482	46313002	chr11-46311482-46313002
chr11	46436132	46438484	chr11-46436132-46438484
chr11	46461931	46462132	chr11-46461931-46462132
chr11	47379056	47379482	chr11-47379056-47379482
chr11	47988327	47989466	chr11-47988327-47989466
chr11	48774527	48774832	chr11-48774527-48774832
chr11	48788349	48789169	chr11-48788349-48789169
chr11	48798441	48803578	chr11-48798441-48803578
chr11	48815369	48818539	chr11-48815369-48818539
chr11	48822649	48827365	chr11-48822649-48827365
chr11	48833218	48834929	chr11-48833218-48834929
chr11	48836956	48839015	chr11-48836956-48839015

chr11	48852600	48859187	chr11-48852600-48859187
chr11	48869321	48873898	chr11-48869321-48873898
chr11	48973693	48973894	chr11-48973693-48973894
chr11	48975170	48975421	chr11-48975170-48975421
chr11	48976882	48978242	chr11-48976882-48978242
chr11	48979562	48979983	chr11-48979562-48979983
chr11	49045541	49045900	chr11-49045541-49045900
chr11	49047029	49047230	chr11-49047029-49047230
chr11	49048662	49050042	chr11-49048662-49050042
chr11	49051468	49051824	chr11-49051468-49051824
chr11	49084868	49085121	chr11-49084868-49085121
chr11	49086499	49088694	chr11-49086499-49088694
chr11	49169086	49169702	chr11-49169086-49169702
chr11	49201971	49205565	chr11-49201971-49205565
chr11	49243038	49247758	chr11-49243038-49247758
chr11	49248586	49252466	chr11-49248586-49252466
chr11	49260499	49261653	chr11-49260499-49261653
chr11	49267955	49268358	chr11-49267955-49268358
chr11	49608823	49612595	chr11-49608823-49612595
chr11	49613573	49613833	chr11-49613573-49613833
chr11	49616503	49617435	chr11-49616503-49617435
chr11	49652928	49653280	chr11-49652928-49653280
chr11	49710439	49713910	chr11-49710439-49713910
chr11	49793857	49795000	chr11-49793857-49795000
chr11	49845831	49848465	chr11-49845831-49848465
chr11	49901360	49902528	chr11-49901360-49902528
chr11	49980955	49981274	chr11-49980955-49981274
chr11	50024654	50026598	chr11-50024654-50026598
chr11	50113785	50114086	chr11-50113785-50114086
chr11	50130585	50132700	chr11-50130585-50132700
chr11	50174174	50175683	chr11-50174174-50175683
chr11	50207125	50214020	chr11-50207125-50214020
chr11	50222833	50231946	chr11-50222833-50231946
chr11	50234741	50234942	chr11-50234741-50234942
chr11	50236062	50238945	chr11-50236062-50238945
chr11	50251802	50253081	chr11-50251802-50253081
chr11	50275531	50276387	chr11-50275531-50276387
chr11	50289918	50293177	chr11-50289918-50293177
chr11	50322840	50326993	chr11-50322840-50326993
chr11	50358806	50359984	chr11-50358806-50359984
chr11	50371533	50371784	chr11-50371533-50371784
chr11	50376219	50380938	chr11-50376219-50380938

chr11	50429756	50432128	chr11-50429756-50432128
chr11	50433356	50433711	chr11-50433356-50433711
chr11	50439312	50440254	chr11-50439312-50440254
chr11	50565476	50566048	chr11-50565476-50566048
chr11	50601571	50603642	chr11-50601571-50603642
chr11	50674248	50674984	chr11-50674248-50674984
chr11	50769212	50769668	chr11-50769212-50769668
chr11	50826980	50828194	chr11-50826980-50828194
chr11	50841206	50841658	chr11-50841206-50841658
chr11	50850260	50851719	chr11-50850260-50851719
chr11	50887133	50887743	chr11-50887133-50887743
chr11	50915438	50916660	chr11-50915438-50916660
chr11	50930702	50932014	chr11-50930702-50932014
chr11	50952247	50952835	chr11-50952247-50952835
chr11	51058971	51060482	chr11-51058971-51060482
chr11	51226537	51226738	chr11-51226537-51226738
chr11	51262091	51265790	chr11-51262091-51265790
chr11	51289550	51290860	chr11-51289550-51290860
chr11	51583296	51586635	chr11-51583296-51586635
chr11	51603446	51607763	chr11-51603446-51607763
chr11	51614778	51616423	chr11-51614778-51616423
chr11	51618950	51621991	chr11-51618950-51621991
chr11	51634712	51637007	chr11-51634712-51637007
chr11	51648527	51652746	chr11-51648527-51652746
chr11	51657161	51657524	chr11-51657161-51657524
chr11	51659054	51659357	chr11-51659054-51659357
chr11	51668726	51669030	chr11-51668726-51669030
chr11	51673392	51673649	chr11-51673392-51673649
chr11	51681335	51681755	chr11-51681335-51681755
chr11	51687523	51689920	chr11-51687523-51689920
chr11	51755000	51757476	chr11-51755000-51757476
chr11	51762957	51765134	chr11-51762957-51765134
chr11	51800801	51801896	chr11-51800801-51801896
chr11	51805258	51806132	chr11-51805258-51806132
chr11	51807266	51807578	chr11-51807266-51807578
chr11	51832471	51832780	chr11-51832471-51832780
chr11	51854790	51859266	chr11-51854790-51859266
chr11	51866117	51866548	chr11-51866117-51866548
chr11	51873803	51876263	chr11-51873803-51876263
chr11	51878422	51878778	chr11-51878422-51878778
chr11	51880817	51881612	chr11-51880817-51881612
chr11	51892587	51892926	chr11-51892587-51892926

chr11	51914679	51917537	chr11-51914679-51917537
chr11	51922233	51922839	chr11-51922233-51922839
chr11	51940315	51941144	chr11-51940315-51941144
chr11	51944889	51946525	chr11-51944889-51946525
chr11	51949344	51949720	chr11-51949344-51949720
chr11	51967261	51969691	chr11-51967261-51969691
chr11	51998097	52001361	chr11-51998097-52001361
chr11	52002990	52005798	chr11-52002990-52005798
chr11	52035867	52036119	chr11-52035867-52036119
chr11	52080194	52080808	chr11-52080194-52080808
chr11	52097669	52101557	chr11-52097669-52101557
chr11	52104321	52104522	chr11-52104321-52104522
chr11	52166205	52169041	chr11-52166205-52169041
chr11	52220186	52220387	chr11-52220186-52220387
chr11	52223779	52225207	chr11-52223779-52225207
chr11	52230858	52234645	chr11-52230858-52234645
chr11	52281974	52284064	chr11-52281974-52284064
chr11	52332941	52334246	chr11-52332941-52334246
chr11	52337583	52337784	chr11-52337583-52337784
chr11	52341148	52341349	chr11-52341148-52341349
chr11	52346038	52347008	chr11-52346038-52347008
chr11	52359676	52362696	chr11-52359676-52362696
chr11	52395839	52397785	chr11-52395839-52397785
chr11	52448827	52449028	chr11-52448827-52449028
chr11	52655330	52655634	chr11-52655330-52655634
chr11	52656435	52656885	chr11-52656435-52656885
chr11	52671757	52672822	chr11-52671757-52672822
chr11	52764163	52766197	chr11-52764163-52766197
chr11	53031865	53032220	chr11-53031865-53032220
chr11	53297732	53301903	chr11-53297732-53301903
chr11	53324356	53325986	chr11-53324356-53325986
chr11	53341280	53342435	chr11-53341280-53342435
chr11	53350181	53353068	chr11-53350181-53353068
chr11	53429192	53431876	chr11-53429192-53431876
chr11	53456327	53458430	chr11-53456327-53458430
chr11	53469691	53470580	chr11-53469691-53470580
chr11	53478499	53480505	chr11-53478499-53480505
chr11	53483361	53484156	chr11-53483361-53484156
chr11	53486461	53487809	chr11-53486461-53487809
chr11	53500485	53501379	chr11-53500485-53501379
chr11	53503889	53505454	chr11-53503889-53505454
chr11	53519294	53521950	chr11-53519294-53521950

chr11	53566951	53568557	chr11-53566951-53568557
chr11	53636040	53636763	chr11-53636040-53636763
chr11	53706028	53708129	chr11-53706028-53708129
chr11	53735693	53736150	chr11-53735693-53736150
chr11	53755207	53755462	chr11-53755207-53755462
chr11	53761327	53764452	chr11-53761327-53764452
chr11	53769354	53772676	chr11-53769354-53772676
chr11	53794066	53797366	chr11-53794066-53797366
chr11	53803026	53804325	chr11-53803026-53804325
chr11	53840264	53840465	chr11-53840264-53840465
chr11	53851727	53852031	chr11-53851727-53852031
chr11	53858727	53859737	chr11-53858727-53859737
chr11	53890461	53892494	chr11-53890461-53892494
chr11	53904058	53904372	chr11-53904058-53904372
chr11	53979035	53980673	chr11-53979035-53980673
chr11	54003104	54003305	chr11-54003104-54003305
chr11	54019152	54019764	chr11-54019152-54019764
chr11	54026461	54029506	chr11-54026461-54029506
chr11	54060090	54061126	chr11-54060090-54061126
chr11	54061975	54062920	chr11-54061975-54062920
chr11	54068660	54069420	chr11-54068660-54069420
chr11	54100250	54102302	chr11-54100250-54102302
chr11	54106987	54107487	chr11-54106987-54107487
chr11	54108916	54109227	chr11-54108916-54109227
chr11	54112812	54113447	chr11-54112812-54113447
chr11	54141805	54142484	chr11-54141805-54142484
chr11	54155660	54155981	chr11-54155660-54155981
chr11	54214273	54217657	chr11-54214273-54217657
chr11	54221653	54222377	chr11-54221653-54222377
chr11	54223765	54224355	chr11-54223765-54224355
chr11	54246936	54250336	chr11-54246936-54250336
chr11	54303642	54304855	chr11-54303642-54304855
chr11	54306286	54306691	chr11-54306286-54306691
chr11	54437622	54440660	chr11-54437622-54440660
chr11	54521644	54524342	chr11-54521644-54524342
chr11	54786071	54788340	chr11-54786071-54788340
chr11	54859605	54861713	chr11-54859605-54861713
chr11	54865567	54869416	chr11-54865567-54869416
chr11	54933978	54937454	chr11-54933978-54937454
chr11	54952417	54956723	chr11-54952417-54956723
chr11	54958366	54959595	chr11-54958366-54959595
chr11	54961023	54963402	chr11-54961023-54963402

chr11	55021914	55023991	chr11-55021914-55023991
chr11	55027120	55034074	chr11-55027120-55034074
chr11	55039477	55039735	chr11-55039477-55039735
chr11	55077162	55078460	chr11-55077162-55078460
chr11	55096918	55100515	chr11-55096918-55100515
chr11	55101512	55102116	chr11-55101512-55102116
chr11	55204099	55205209	chr11-55204099-55205209
chr11	55274429	55275315	chr11-55274429-55275315
chr11	55290297	55290498	chr11-55290297-55290498
chr11	55291303	55292433	chr11-55291303-55292433
chr11	55313718	55314206	chr11-55313718-55314206
chr11	55400862	55401063	chr11-55400862-55401063
chr11	55416620	55420256	chr11-55416620-55420256
chr11	55453312	55455986	chr11-55453312-55455986
chr11	55459611	55461904	chr11-55459611-55461904
chr11	55467904	55474251	chr11-55467904-55474251
chr11	55505444	55505695	chr11-55505444-55505695
chr11	57091514	57091773	chr11-57091514-57091773
chr11	57501817	57502018	chr11-57501817-57502018
chr11	57517061	57519732	chr11-57517061-57519732
chr11	57644038	57646816	chr11-57644038-57646816
chr11	57688503	57689184	chr11-57688503-57689184
chr11	57719433	57720407	chr11-57719433-57720407
chr11	57791089	57791472	chr11-57791089-57791472
chr11	57800132	57803289	chr11-57800132-57803289
chr11	57827435	57828219	chr11-57827435-57828219
chr11	57857462	57858069	chr11-57857462-57858069
chr11	57899072	57899650	chr11-57899072-57899650
chr11	57911883	57914129	chr11-57911883-57914129
chr11	57915823	57916216	chr11-57915823-57916216
chr11	57934471	57934873	chr11-57934471-57934873
chr11	57962167	57963646	chr11-57962167-57963646
chr11	57970620	57971160	chr11-57970620-57971160
chr11	57972848	57973960	chr11-57972848-57973960
chr11	58007342	58012453	chr11-58007342-58012453
chr11	58016083	58016470	chr11-58016083-58016470
chr11	58024427	58024782	chr11-58024427-58024782
chr11	58092936	58093240	chr11-58092936-58093240
chr11	58097625	58098330	chr11-58097625-58098330
chr11	58103245	58105780	chr11-58103245-58105780
chr11	58167082	58169223	chr11-58167082-58169223
chr11	58171422	58172715	chr11-58171422-58172715

chr11	58198806	58200072	chr11-58198806-58200072
chr11	58214306	58215565	chr11-58214306-58215565
chr11	58239157	58239910	chr11-58239157-58239910
chr11	58300969	58304077	chr11-58300969-58304077
chr11	58306559	58308388	chr11-58306559-58308388
chr11	58322152	58324295	chr11-58322152-58324295
chr11	58328945	58332617	chr11-58328945-58332617
chr11	58334078	58335369	chr11-58334078-58335369
chr11	58380155	58381107	chr11-58380155-58381107
chr11	58640392	58640958	chr11-58640392-58640958
chr11	58785058	58785320	chr11-58785058-58785320
chr11	58801787	58802378	chr11-58801787-58802378
chr11	58846726	58848168	chr11-58846726-58848168
chr11	58866832	58867943	chr11-58866832-58867943
chr11	58903426	58904725	chr11-58903426-58904725
chr11	58914220	58914928	chr11-58914220-58914928
chr11	58927922	58928660	chr11-58927922-58928660
chr11	58937098	58939497	chr11-58937098-58939497
chr11	58948010	58949837	chr11-58948010-58949837
chr11	58953353	58957200	chr11-58953353-58957200
chr11	58959541	58960662	chr11-58959541-58960662
chr11	58976735	58982463	chr11-58976735-58982463
chr11	58994117	58995094	chr11-58994117-58995094
chr11	59134420	59136125	chr11-59134420-59136125
chr11	59141899	59142252	chr11-59141899-59142252
chr11	59162451	59164571	chr11-59162451-59164571
chr11	59176396	59177311	chr11-59176396-59177311
chr11	59190809	59192653	chr11-59190809-59192653
chr11	59201173	59203558	chr11-59201173-59203558
chr11	59207880	59209713	chr11-59207880-59209713
chr11	59217940	59219355	chr11-59217940-59219355
chr11	59225037	59229454	chr11-59225037-59229454
chr11	59263744	59264756	chr11-59263744-59264756
chr11	59282924	59283233	chr11-59282924-59283233
chr11	59290267	59291263	chr11-59290267-59291263
chr11	59305945	59309709	chr11-59305945-59309709
chr11	59312231	59313643	chr11-59312231-59313643
chr11	59323814	59324015	chr11-59323814-59324015
chr11	59350139	59350391	chr11-59350139-59350391
chr11	59383827	59385942	chr11-59383827-59385942
chr11	59412251	59412900	chr11-59412251-59412900
chr11	59421443	59422395	chr11-59421443-59422395

chr11	59448626	59451621	chr11-59448626-59451621
chr11	59458517	59459836	chr11-59458517-59459836
chr11	59471059	59472679	chr11-59471059-59472679
chr11	59504353	59507676	chr11-59504353-59507676
chr11	59600501	59601506	chr11-59600501-59601506
chr11	59633751	59634610	chr11-59633751-59634610
chr11	59635466	59637286	chr11-59635466-59637286
chr11	59642535	59642951	chr11-59642535-59642951
chr11	59654857	59656990	chr11-59654857-59656990
chr11	59660861	59665636	chr11-59660861-59665636
chr11	59787014	59787888	chr11-59787014-59787888
chr11	59808491	59811771	chr11-59808491-59811771
chr11	59838132	59840708	chr11-59838132-59840708
chr11	59847232	59849221	chr11-59847232-59849221
chr11	59947631	59949628	chr11-59947631-59949628
chr11	59953054	59953255	chr11-59953054-59953255
chr11	59963862	59965921	chr11-59963862-59965921
chr11	60045451	60046889	chr11-60045451-60046889
chr11	60104582	60116223	chr11-60104582-60116223
chr11	60125599	60125800	chr11-60125599-60125800
chr11	60139474	60143715	chr11-60139474-60143715
chr11	60175203	60179460	chr11-60175203-60179460
chr11	60208199	60211435	chr11-60208199-60211435
chr11	60218818	60223334	chr11-60218818-60223334
chr11	60229177	60230401	chr11-60229177-60230401
chr11	60301503	60302361	chr11-60301503-60302361
chr11	60307904	60309253	chr11-60307904-60309253
chr11	60311378	60311646	chr11-60311378-60311646
chr11	60351454	60353923	chr11-60351454-60353923
chr11	60415903	60418974	chr11-60415903-60418974
chr11	60454041	60456070	chr11-60454041-60456070
chr11	60492890	60493745	chr11-60492890-60493745
chr11	60508954	60509981	chr11-60508954-60509981
chr11	60514787	60515327	chr11-60514787-60515327
chr11	60536101	60541366	chr11-60536101-60541366
chr11	60698601	60703609	chr11-60698601-60703609
chr11	60706488	60708681	chr11-60706488-60708681
chr11	60725833	60730212	chr11-60725833-60730212
chr11	60769289	60770135	chr11-60769289-60770135
chr11	60774944	60782757	chr11-60774944-60782757
chr11	60808466	60811878	chr11-60808466-60811878
chr11	60830144	60831892	chr11-60830144-60831892

chr11	60876521	60880295	chr11-60876521-60880295
chr11	60912544	60915219	chr11-60912544-60915219
chr11	60930290	60937248	chr11-60930290-60937248
chr11	60938446	60941664	chr11-60938446-60941664
chr11	61021974	61023199	chr11-61021974-61023199
chr11	61104189	61104390	chr11-61104189-61104390
chr11	61147271	61147687	chr11-61147271-61147687
chr11	61173277	61176993	chr11-61173277-61176993
chr11	61203014	61203272	chr11-61203014-61203272
chr11	61207955	61208748	chr11-61207955-61208748
chr11	61220326	61220684	chr11-61220326-61220684
chr11	61266194	61268014	chr11-61266194-61268014
chr11	61342283	61343151	chr11-61342283-61343151
chr11	61352508	61352709	chr11-61352508-61352709
chr11	61377656	61378485	chr11-61377656-61378485
chr11	61407324	61409235	chr11-61407324-61409235
chr11	61477016	61477489	chr11-61477016-61477489
chr11	61492695	61495447	chr11-61492695-61495447
chr11	61504538	61506098	chr11-61504538-61506098
chr11	61562594	61565069	chr11-61562594-61565069
chr11	61573315	61573516	chr11-61573315-61573516
chr11	61576897	61579882	chr11-61576897-61579882
chr11	61597405	61598135	chr11-61597405-61598135
chr11	61603175	61603376	chr11-61603175-61603376
chr11	61615721	61616558	chr11-61615721-61616558
chr11	61619530	61620947	chr11-61619530-61620947
chr11	61644318	61645116	chr11-61644318-61645116
chr11	61683424	61688080	chr11-61683424-61688080
chr11	61760714	61762595	chr11-61760714-61762595
chr11	61853760	61855675	chr11-61853760-61855675
chr11	61928911	61931161	chr11-61928911-61931161
chr11	61954854	61955549	chr11-61954854-61955549
chr11	61956419	61957690	chr11-61956419-61957690
chr11	62005259	62005714	chr11-62005259-62005714
chr11	62075074	62080833	chr11-62075074-62080833
chr11	62120220	62120596	chr11-62120220-62120596
chr11	62148288	62148591	chr11-62148288-62148591
chr11	62217981	62218182	chr11-62217981-62218182
chr11	62247989	62250001	chr11-62247989-62250001
chr11	62280606	62282508	chr11-62280606-62282508
chr11	62455630	62460367	chr11-62455630-62460367
chr11	62537643	62539830	chr11-62537643-62539830

chr11	62549163	62554724	chr11-62549163-62554724
chr11	62558883	62559402	chr11-62558883-62559402
chr11	62575293	62576307	chr11-62575293-62576307
chr11	62601908	62606279	chr11-62601908-62606279
chr11	62613887	62614273	chr11-62613887-62614273
chr11	62623349	62625114	chr11-62623349-62625114
chr11	62642463	62643847	chr11-62642463-62643847
chr11	62645857	62650170	chr11-62645857-62650170
chr11	62675596	62676000	chr11-62675596-62676000
chr11	62731232	62732188	chr11-62731232-62732188
chr11	62745969	62746808	chr11-62745969-62746808
chr11	62756647	62757476	chr11-62756647-62757476
chr11	62788974	62789698	chr11-62788974-62789698
chr11	62814143	62815364	chr11-62814143-62815364
chr11	62819355	62826703	chr11-62819355-62826703
chr11	62878616	62880577	chr11-62878616-62880577
chr11	62967118	62967319	chr11-62967118-62967319
chr11	62996612	62996889	chr11-62996612-62996889
chr11	63026748	63026949	chr11-63026748-63026949
chr11	63129336	63133423	chr11-63129336-63133423
chr11	63264562	63265094	chr11-63264562-63265094
chr11	63914025	63915001	chr11-63914025-63915001
chr11	63920333	63923751	chr11-63920333-63923751
chr11	63925047	63925725	chr11-63925047-63925725
chr11	63952159	63952563	chr11-63952159-63952563
chr11	64078521	64080476	chr11-64078521-64080476
chr11	64434895	64436773	chr11-64434895-64436773
chr11	64977981	64980304	chr11-64977981-64980304
chr11	65160982	65163213	chr11-65160982-65163213
chr11	65209332	65209675	chr11-65209332-65209675
chr11	65214837	65215657	chr11-65214837-65215657
chr11	65270028	65270304	chr11-65270028-65270304
chr11	65590255	65590456	chr11-65590255-65590456
chr11	65784823	65785024	chr11-65784823-65785024
chr11	65786386	65788691	chr11-65786386-65788691
chr11	65802129	65802383	chr11-65802129-65802383
chr11	65806119	65808258	chr11-65806119-65808258
chr11	65818513	65818888	chr11-65818513-65818888
chr11	66167652	66168665	chr11-66167652-66168665
chr11	66525113	66527082	chr11-66525113-66527082
chr11	66956558	66957068	chr11-66956558-66957068
chr11	66966506	66966872	chr11-66966506-66966872

chr11	66971969	66972170	chr11-66971969-66972170
chr11	66984364	66985828	chr11-66984364-66985828
chr11	66998464	66998829	chr11-66998464-66998829
chr11	67024712	67025650	chr11-67024712-67025650
chr11	67051518	67053746	chr11-67051518-67053746
chr11	67081107	67081308	chr11-67081107-67081308
chr11	67323024	67323277	chr11-67323024-67323277
chr11	67454734	67456242	chr11-67454734-67456242
chr11	67515344	67515616	chr11-67515344-67515616
chr11	67526876	67527229	chr11-67526876-67527229
chr11	67532120	67533483	chr11-67532120-67533483
chr11	67545050	67545507	chr11-67545050-67545507
chr11	67549561	67550579	chr11-67549561-67550579
chr11	67563004	67563815	chr11-67563004-67563815
chr11	67819719	67820080	chr11-67819719-67820080
chr11	67828412	67828983	chr11-67828412-67828983
chr11	67861214	67861839	chr11-67861214-67861839
chr11	67896784	67897981	chr11-67896784-67897981
chr11	67911487	67911862	chr11-67911487-67911862
chr11	67919379	67923770	chr11-67919379-67923770
chr11	67948323	67948524	chr11-67948323-67948524
chr11	67965254	67968169	chr11-67965254-67968169
chr11	68030843	68031044	chr11-68030843-68031044
chr11	68036287	68036641	chr11-68036287-68036641
chr11	68089545	68092121	chr11-68089545-68092121
chr11	68092929	68093130	chr11-68092929-68093130
chr11	68313374	68314467	chr11-68313374-68314467
chr11	68384702	68387589	chr11-68384702-68387589
chr11	68399341	68400882	chr11-68399341-68400882
chr11	68431680	68432807	chr11-68431680-68432807
chr11	68458387	68458640	chr11-68458387-68458640
chr11	68468364	68469105	chr11-68468364-68469105
chr11	68532073	68532455	chr11-68532073-68532455
chr11	68555777	68556888	chr11-68555777-68556888
chr11	68566503	68567217	chr11-68566503-68567217
chr11	68623394	68623804	chr11-68623394-68623804
chr11	68690969	68693630	chr11-68690969-68693630
chr11	68850813	68854581	chr11-68850813-68854581
chr11	68859868	68860273	chr11-68859868-68860273
chr11	68871491	68872224	chr11-68871491-68872224
chr11	68873345	68873598	chr11-68873345-68873598
chr11	68892489	68893207	chr11-68892489-68893207

chr11	68900799	68904132	chr11-68900799-68904132
chr11	68939420	68940875	chr11-68939420-68940875
chr11	68946859	68947466	chr11-68946859-68947466
chr11	68954389	68955157	chr11-68954389-68955157
chr11	68966795	68968929	chr11-68966795-68968929
chr11	68972660	68974376	chr11-68972660-68974376
chr11	69007165	69008997	chr11-69007165-69008997
chr11	69011064	69012831	chr11-69011064-69012831
chr11	69015295	69017384	chr11-69015295-69017384
chr11	69035529	69041959	chr11-69035529-69041959
chr11	69045021	69048009	chr11-69045021-69048009
chr11	69058180	69064709	chr11-69058180-69064709
chr11	69065968	69067961	chr11-69065968-69067961
chr11	69069850	69073430	chr11-69069850-69073430
chr11	69074238	69074680	chr11-69074238-69074680
chr11	69087713	69090193	chr11-69087713-69090193
chr11	69094381	69101246	chr11-69094381-69101246
chr11	69109653	69111692	chr11-69109653-69111692
chr11	69117425	69119470	chr11-69117425-69119470
chr11	69120296	69126955	chr11-69120296-69126955
chr11	69133875	69134668	chr11-69133875-69134668
chr11	69150550	69151207	chr11-69150550-69151207
chr11	69196982	69197917	chr11-69196982-69197917
chr11	69235134	69236597	chr11-69235134-69236597
chr11	69322037	69327455	chr11-69322037-69327455
chr11	69331813	69332528	chr11-69331813-69332528
chr11	69334067	69335334	chr11-69334067-69335334
chr11	69340140	69341397	chr11-69340140-69341397
chr11	69364583	69371228	chr11-69364583-69371228
chr11	69382905	69383438	chr11-69382905-69383438
chr11	69394234	69398769	chr11-69394234-69398769
chr11	69401511	69401813	chr11-69401511-69401813
chr11	69407487	69412345	chr11-69407487-69412345
chr11	69414391	69420830	chr11-69414391-69420830
chr11	69472324	69473273	chr11-69472324-69473273
chr11	69546041	69547923	chr11-69546041-69547923
chr11	69577017	69582753	chr11-69577017-69582753
chr11	69621935	69622602	chr11-69621935-69622602
chr11	69631902	69635682	chr11-69631902-69635682
chr11	69661404	69664148	chr11-69661404-69664148
chr11	69665524	69666188	chr11-69665524-69666188
chr11	69668768	69673187	chr11-69668768-69673187

chr11	69679673	69683366	chr11-69679673-69683366
chr11	69695458	69696224	chr11-69695458-69696224
chr11	69706604	69706970	chr11-69706604-69706970
chr11	69755377	69760680	chr11-69755377-69760680
chr11	69765387	69768025	chr11-69765387-69768025
chr11	69794849	69796578	chr11-69794849-69796578
chr11	69797860	69798234	chr11-69797860-69798234
chr11	69800795	69805880	chr11-69800795-69805880
chr11	69833853	69834584	chr11-69833853-69834584
chr11	69835893	69839635	chr11-69835893-69839635
chr11	69845300	69848517	chr11-69845300-69848517
chr11	69856823	69859454	chr11-69856823-69859454
chr11	69876983	69885186	chr11-69876983-69885186
chr11	69895144	69895468	chr11-69895144-69895468
chr11	69899467	69903593	chr11-69899467-69903593
chr11	69912727	69917531	chr11-69912727-69917531
chr11	69918959	69922982	chr11-69918959-69922982
chr11	69934221	69936970	chr11-69934221-69936970
chr11	69947663	69949174	chr11-69947663-69949174
chr11	69960598	69962310	chr11-69960598-69962310
chr11	69964359	69968088	chr11-69964359-69968088
chr11	69978894	69983507	chr11-69978894-69983507
chr11	69990833	69993260	chr11-69990833-69993260
chr11	69995170	69997892	chr11-69995170-69997892
chr11	70000096	70002308	chr11-70000096-70002308
chr11	70014625	70015639	chr11-70014625-70015639
chr11	70018137	70018961	chr11-70018137-70018961
chr11	70020755	70022040	chr11-70020755-70022040
chr11	70023847	70024048	chr11-70023847-70024048
chr11	70028773	70029872	chr11-70028773-70029872
chr11	70189497	70190084	chr11-70189497-70190084
chr11	70210900	70211235	chr11-70210900-70211235
chr11	70213821	70214073	chr11-70213821-70214073
chr11	70219596	70222230	chr11-70219596-70222230
chr11	70225894	70227178	chr11-70225894-70227178
chr11	70228910	70230745	chr11-70228910-70230745
chr11	70232615	70232920	chr11-70232615-70232920
chr11	70236745	70240851	chr11-70236745-70240851
chr11	70254759	70255494	chr11-70254759-70255494
chr11	70351590	70352265	chr11-70351590-70352265
chr11	70408525	70410786	chr11-70408525-70410786
chr11	70431655	70433914	chr11-70431655-70433914

chr11	70451228	70453958	chr11-70451228-70453958
chr11	70455304	70460892	chr11-70455304-70460892
chr11	70465692	70466164	chr11-70465692-70466164
chr11	70468066	70468470	chr11-70468066-70468470
chr11	70510044	70510245	chr11-70510044-70510245
chr11	70520206	70520407	chr11-70520206-70520407
chr11	70524536	70526676	chr11-70524536-70526676
chr11	70539153	70541448	chr11-70539153-70541448
chr11	70561739	70565283	chr11-70561739-70565283
chr11	70572208	70572459	chr11-70572208-70572459
chr11	70573617	70574219	chr11-70573617-70574219
chr11	70576374	70578741	chr11-70576374-70578741
chr11	70579565	70580564	chr11-70579565-70580564
chr11	70585161	70586384	chr11-70585161-70586384
chr11	70645515	70659599	chr11-70645515-70659599
chr11	70667156	70671607	chr11-70667156-70671607
chr11	70686891	70688848	chr11-70686891-70688848
chr11	70699496	70703127	chr11-70699496-70703127
chr11	70716112	70716469	chr11-70716112-70716469
chr11	70747681	70747882	chr11-70747681-70747882
chr11	70763650	70766722	chr11-70763650-70766722
chr11	70843997	70846634	chr11-70843997-70846634
chr11	70968232	70971430	chr11-70968232-70971430
chr11	70980542	70983495	chr11-70980542-70983495
chr11	71002367	71005318	chr11-71002367-71005318
chr11	71018248	71021906	chr11-71018248-71021906
chr11	71727308	71729038	chr11-71727308-71729038
chr11	71750504	71752138	chr11-71750504-71752138
chr11	71871242	71871493	chr11-71871242-71871493
chr11	71930001	71930544	chr11-71930001-71930544
chr11	71995523	71996612	chr11-71995523-71996612
chr11	72029267	72029522	chr11-72029267-72029522
chr11	72041068	72045328	chr11-72041068-72045328
chr11	72080234	72080897	chr11-72080234-72080897
chr11	72093996	72095447	chr11-72093996-72095447
chr11	72129534	72137057	chr11-72129534-72137057
chr11	72140329	72140530	chr11-72140329-72140530
chr11	72142547	72144030	chr11-72142547-72144030
chr11	72153169	72153835	chr11-72153169-72153835
chr11	72186678	72187081	chr11-72186678-72187081
chr11	72196780	72196981	chr11-72196780-72196981
chr11	72205245	72208693	chr11-72205245-72208693

chr11	72214013	72216065	chr11-72214013-72216065
chr11	72266043	72267494	chr11-72266043-72267494
chr11	72301114	72302460	chr11-72301114-72302460
chr11	72314331	72314732	chr11-72314331-72314732
chr11	72355088	72355496	chr11-72355088-72355496
chr11	72361627	72362405	chr11-72361627-72362405
chr11	72406362	72406625	chr11-72406362-72406625
chr11	72409953	72412112	chr11-72409953-72412112
chr11	72423665	72423974	chr11-72423665-72423974
chr11	72428758	72429650	chr11-72428758-72429650
chr11	72435149	72435908	chr11-72435149-72435908
chr11	72440450	72444443	chr11-72440450-72444443
chr11	72466491	72467583	chr11-72466491-72467583
chr11	72468532	72476325	chr11-72468532-72476325
chr11	72477337	72479579	chr11-72477337-72479579
chr11	72482452	72483193	chr11-72482452-72483193
chr11	72484296	72491973	chr11-72484296-72491973
chr11	72500536	72504619	chr11-72500536-72504619
chr11	72506444	72506695	chr11-72506444-72506695
chr11	72518592	72520634	chr11-72518592-72520634
chr11	72536490	72536747	chr11-72536490-72536747
chr11	72570485	72571103	chr11-72570485-72571103
chr11	72575913	72577390	chr11-72575913-72577390
chr11	72601869	72602383	chr11-72601869-72602383
chr11	72606332	72609436	chr11-72606332-72609436
chr11	72689146	72691703	chr11-72689146-72691703
chr11	72748277	72748528	chr11-72748277-72748528
chr11	72792298	72797302	chr11-72792298-72797302
chr11	72846105	72846356	chr11-72846105-72846356
chr11	72847482	72848444	chr11-72847482-72848444
chr11	72946192	72946967	chr11-72946192-72946967
chr11	72951215	72951781	chr11-72951215-72951781
chr11	72960743	72961864	chr11-72960743-72961864
chr11	73018269	73020094	chr11-73018269-73020094
chr11	73023078	73023279	chr11-73023078-73023279
chr11	73027144	73027864	chr11-73027144-73027864
chr11	73030779	73031030	chr11-73030779-73031030
chr11	73037057	73037308	chr11-73037057-73037308
chr11	73047299	73049539	chr11-73047299-73049539
chr11	73134872	73138796	chr11-73134872-73138796
chr11	73160222	73161615	chr11-73160222-73161615
chr11	73174903	73179032	chr11-73174903-73179032

chr11	73198394	73200420	chr11-73198394-73200420
chr11	73267393	73267803	chr11-73267393-73267803
chr11	73281543	73282057	chr11-73281543-73282057
chr11	73283401	73284453	chr11-73283401-73284453
chr11	73326408	73327086	chr11-73326408-73327086
chr11	74371578	74371884	chr11-74371578-74371884
chr11	74438813	74439914	chr11-74438813-74439914
chr11	74442185	74442505	chr11-74442185-74442505
chr11	74483693	74484148	chr11-74483693-74484148
chr11	74521479	74523610	chr11-74521479-74523610
chr11	74526781	74527211	chr11-74526781-74527211
chr11	74536320	74536621	chr11-74536320-74536621
chr11	74547006	74547270	chr11-74547006-74547270
chr11	74572413	74572985	chr11-74572413-74572985
chr11	74589800	74590509	chr11-74589800-74590509
chr11	74610481	74611249	chr11-74610481-74611249
chr11	74619275	74620065	chr11-74619275-74620065
chr11	74637411	74639339	chr11-74637411-74639339
chr11	74648694	74651998	chr11-74648694-74651998
chr11	74721278	74725392	chr11-74721278-74725392
chr11	74770059	74772672	chr11-74770059-74772672
chr11	74826066	74826656	chr11-74826066-74826656
chr11	74829125	74834018	chr11-74829125-74834018
chr11	74836775	74839640	chr11-74836775-74839640
chr11	74847953	74848946	chr11-74847953-74848946
chr11	74859804	74860107	chr11-74859804-74860107
chr11	74862285	74863040	chr11-74862285-74863040
chr11	74863936	74864582	chr11-74863936-74864582
chr11	74870853	74871729	chr11-74870853-74871729
chr11	74876775	74877194	chr11-74876775-74877194
chr11	74895782	74900432	chr11-74895782-74900432
chr11	74924529	74927230	chr11-74924529-74927230
chr11	75053801	75054002	chr11-75053801-75054002
chr11	75165485	75171100	chr11-75165485-75171100
chr11	75172034	75174413	chr11-75172034-75174413
chr11	75177595	75179401	chr11-75177595-75179401
chr11	75189224	75190981	chr11-75189224-75190981
chr11	75192924	75195474	chr11-75192924-75195474
chr11	75213635	75214378	chr11-75213635-75214378
chr11	75220657	75221040	chr11-75220657-75221040
chr11	75254304	75254857	chr11-75254304-75254857
chr11	75279166	75279776	chr11-75279166-75279776

chr11	75346646	75349318	chr11-75346646-75349318
chr11	75403399	75404204	chr11-75403399-75404204
chr11	75453545	75455366	chr11-75453545-75455366
chr11	75458506	75465508	chr11-75458506-75465508
chr11	75486101	75488704	chr11-75486101-75488704
chr11	75508615	75511064	chr11-75508615-75511064
chr11	75530421	75533029	chr11-75530421-75533029
chr11	75541202	75541499	chr11-75541202-75541499
chr11	75552133	75552384	chr11-75552133-75552384
chr11	75562719	75564755	chr11-75562719-75564755
chr11	75570842	75571622	chr11-75570842-75571622
chr11	75587654	75589668	chr11-75587654-75589668
chr11	75592321	75592834	chr11-75592321-75592834
chr11	75605804	75606167	chr11-75605804-75606167
chr11	75619935	75621959	chr11-75619935-75621959
chr11	75629731	75632567	chr11-75629731-75632567
chr11	75650188	75659219	chr11-75650188-75659219
chr11	75677678	75681190	chr11-75677678-75681190
chr11	75687162	75688220	chr11-75687162-75688220
chr11	75732257	75737525	chr11-75732257-75737525
chr11	75769090	75769727	chr11-75769090-75769727
chr11	75794843	75796425	chr11-75794843-75796425
chr11	75857531	75858828	chr11-75857531-75858828
chr11	75860824	75861977	chr11-75860824-75861977
chr11	75935520	75936341	chr11-75935520-75936341
chr11	75937265	75937863	chr11-75937265-75937863
chr11	75965754	75967630	chr11-75965754-75967630
chr11	75999464	76000467	chr11-75999464-76000467
chr11	76026519	76028423	chr11-76026519-76028423
chr11	76135323	76136305	chr11-76135323-76136305
chr11	76158195	76158498	chr11-76158195-76158498
chr11	76159813	76160812	chr11-76159813-76160812
chr11	76178295	76180604	chr11-76178295-76180604
chr11	76201486	76203152	chr11-76201486-76203152
chr11	76216084	76218150	chr11-76216084-76218150
chr11	76242522	76244891	chr11-76242522-76244891
chr11	76260930	76261834	chr11-76260930-76261834
chr11	76285582	76287106	chr11-76285582-76287106
chr11	76337622	76338091	chr11-76337622-76338091
chr11	76397503	76400104	chr11-76397503-76400104
chr11	76406449	76408406	chr11-76406449-76408406
chr11	76469450	76470109	chr11-76469450-76470109

chr11	76476350	76477791	chr11-76476350-76477791
chr11	76479260	76480293	chr11-76479260-76480293
chr11	76492857	76495764	chr11-76492857-76495764
chr11	76497626	76498085	chr11-76497626-76498085
chr11	76570733	76571941	chr11-76570733-76571941
chr11	76612568	76613307	chr11-76612568-76613307
chr11	76620964	76624146	chr11-76620964-76624146
chr11	76645881	76646183	chr11-76645881-76646183
chr11	76647326	76647829	chr11-76647326-76647829
chr11	76666318	76666687	chr11-76666318-76666687
chr11	76672279	76673279	chr11-76672279-76673279
chr11	76699837	76700617	chr11-76699837-76700617
chr11	76709804	76710266	chr11-76709804-76710266
chr11	76762636	76764060	chr11-76762636-76764060
chr11	76845105	76847712	chr11-76845105-76847712
chr11	76916555	76917413	chr11-76916555-76917413
chr11	76924529	76925196	chr11-76924529-76925196
chr11	76945052	76946755	chr11-76945052-76946755
chr11	76997159	76999183	chr11-76997159-76999183
chr11	77077029	77079330	chr11-77077029-77079330
chr11	77145184	77145543	chr11-77145184-77145543
chr11	77159988	77161026	chr11-77159988-77161026
chr11	77201128	77201788	chr11-77201128-77201788
chr11	77214843	77218316	chr11-77214843-77218316
chr11	77279354	77279555	chr11-77279354-77279555
chr11	77347816	77349561	chr11-77347816-77349561
chr11	77383871	77384669	chr11-77383871-77384669
chr11	77461691	77468159	chr11-77461691-77468159
chr11	77486818	77487124	chr11-77486818-77487124
chr11	77488090	77498135	chr11-77488090-77498135
chr11	77500184	77501303	chr11-77500184-77501303
chr11	77508116	77508734	chr11-77508116-77508734
chr11	77512284	77516104	chr11-77512284-77516104
chr11	77605369	77608730	chr11-77605369-77608730
chr11	77624318	77625337	chr11-77624318-77625337
chr11	77630183	77630687	chr11-77630183-77630687
chr11	77685297	77689006	chr11-77685297-77689006
chr11	77693845	77694154	chr11-77693845-77694154
chr11	77762669	77767557	chr11-77762669-77767557
chr11	77771700	77772555	chr11-77771700-77772555
chr11	77782453	77787082	chr11-77782453-77787082
chr11	77790474	77793994	chr11-77790474-77793994

chr11	77894143	77894344	chr11-77894143-77894344
chr11	77896926	77897300	chr11-77896926-77897300
chr11	77930647	77931790	chr11-77930647-77931790
chr11	77981201	77985699	chr11-77981201-77985699
chr11	78031490	78033598	chr11-78031490-78033598
chr11	78037466	78039601	chr11-78037466-78039601
chr11	78079539	78081256	chr11-78079539-78081256
chr11	78094037	78096108	chr11-78094037-78096108
chr11	78114646	78116482	chr11-78114646-78116482
chr11	78146517	78146718	chr11-78146517-78146718
chr11	78162092	78166958	chr11-78162092-78166958
chr11	78169219	78169522	chr11-78169219-78169522
chr11	78174552	78184430	chr11-78174552-78184430
chr11	78187945	78190109	chr11-78187945-78190109
chr11	78194427	78195242	chr11-78194427-78195242
chr11	78244162	78248621	chr11-78244162-78248621
chr11	78260852	78263257	chr11-78260852-78263257
chr11	78300893	78302764	chr11-78300893-78302764
chr11	78322470	78323832	chr11-78322470-78323832
chr11	78327756	78329580	chr11-78327756-78329580
chr11	78343158	78344515	chr11-78343158-78344515
chr11	78360161	78360645	chr11-78360161-78360645
chr11	78363106	78364298	chr11-78363106-78364298
chr11	78369844	78372054	chr11-78369844-78372054
chr11	78377831	78378082	chr11-78377831-78378082
chr11	78382453	78383148	chr11-78382453-78383148
chr11	78384785	78387064	chr11-78384785-78387064
chr11	78396894	78399144	chr11-78396894-78399144
chr11	78411971	78412311	chr11-78411971-78412311
chr11	78462682	78466895	chr11-78462682-78466895
chr11	78487956	78488157	chr11-78487956-78488157
chr11	78490555	78491268	chr11-78490555-78491268
chr11	78496853	78498492	chr11-78496853-78498492
chr11	78511061	78513579	chr11-78511061-78513579
chr11	78531378	78531579	chr11-78531378-78531579
chr11	78534500	78537790	chr11-78534500-78537790
chr11	78548326	78551544	chr11-78548326-78551544
chr11	78695471	78698409	chr11-78695471-78698409
chr11	78750553	78752189	chr11-78750553-78752189
chr11	78826256	78827825	chr11-78826256-78827825
chr11	78886774	78887578	chr11-78886774-78887578
chr11	78924777	78925596	chr11-78924777-78925596

chr11	78952697	78952999	chr11-78952697-78952999
chr11	78983517	78985585	chr11-78983517-78985585
chr11	79002519	79002971	chr11-79002519-79002971
chr11	79030930	79031131	chr11-79030930-79031131
chr11	79059463	79060104	chr11-79059463-79060104
chr11	79071435	79072081	chr11-79071435-79072081
chr11	79083290	79083873	chr11-79083290-79083873
chr11	79084980	79085730	chr11-79084980-79085730
chr11	79094156	79094571	chr11-79094156-79094571
chr11	79103327	79103832	chr11-79103327-79103832
chr11	79111923	79113357	chr11-79111923-79113357
chr11	79131724	79132389	chr11-79131724-79132389
chr11	79141437	79141733	chr11-79141437-79141733
chr11	79145117	79146928	chr11-79145117-79146928
chr11	79196849	79197287	chr11-79196849-79197287
chr11	79251225	79255231	chr11-79251225-79255231
chr11	79264685	79265044	chr11-79264685-79265044
chr11	79338586	79341032	chr11-79338586-79341032
chr11	79590708	79591873	chr11-79590708-79591873
chr11	79660037	79661471	chr11-79660037-79661471
chr11	79702760	79705494	chr11-79702760-79705494
chr11	79710626	79715790	chr11-79710626-79715790
chr11	79729735	79729936	chr11-79729735-79729936
chr11	79737242	79739904	chr11-79737242-79739904
chr11	79746826	79747183	chr11-79746826-79747183
chr11	79749723	79750196	chr11-79749723-79750196
chr11	79752131	79753622	chr11-79752131-79753622
chr11	79790173	79790498	chr11-79790173-79790498
chr11	79961016	79963535	chr11-79961016-79963535
chr11	79973331	79974690	chr11-79973331-79974690
chr11	79991827	79994946	chr11-79991827-79994946
chr11	80079054	80081687	chr11-80079054-80081687
chr11	80085580	80085997	chr11-80085580-80085997
chr11	80088850	80090940	chr11-80088850-80090940
chr11	80140969	80143441	chr11-80140969-80143441
chr11	80145810	80146225	chr11-80145810-80146225
chr11	80152474	80154935	chr11-80152474-80154935
chr11	80183232	80185773	chr11-80183232-80185773
chr11	80208593	80211097	chr11-80208593-80211097
chr11	80300273	80303025	chr11-80300273-80303025
chr11	80376811	80378702	chr11-80376811-80378702
chr11	80382177	80387168	chr11-80382177-80387168

chr11	80428130	80430813	chr11-80428130-80430813
chr11	80476221	80478228	chr11-80476221-80478228
chr11	80538829	80539080	chr11-80538829-80539080
chr11	80629084	80629385	chr11-80629084-80629385
chr11	80711228	80711549	chr11-80711228-80711549
chr11	80713462	80713784	chr11-80713462-80713784
chr11	80719828	80720272	chr11-80719828-80720272
chr11	80758934	80759495	chr11-80758934-80759495
chr11	80772979	80775550	chr11-80772979-80775550
chr11	80777905	80780374	chr11-80777905-80780374
chr11	80784153	80784877	chr11-80784153-80784877
chr11	80809643	80811873	chr11-80809643-80811873
chr11	80817990	80818795	chr11-80817990-80818795
chr11	80819599	80820394	chr11-80819599-80820394
chr11	80949235	80949486	chr11-80949235-80949486
chr11	81151829	81153794	chr11-81151829-81153794
chr11	81212336	81212657	chr11-81212336-81212657
chr11	81612344	81612595	chr11-81612344-81612595
chr11	82388565	82389812	chr11-82388565-82389812
chr11	82434564	82435077	chr11-82434564-82435077
chr11	82618508	82618709	chr11-82618508-82618709
chr11	82763314	82765748	chr11-82763314-82765748
chr11	82779688	82782683	chr11-82779688-82782683
chr11	82845617	82846269	chr11-82845617-82846269
chr11	82869799	82871452	chr11-82869799-82871452
chr11	82888669	82890766	chr11-82888669-82890766
chr11	82907133	82908692	chr11-82907133-82908692
chr11	82914855	82915159	chr11-82914855-82915159
chr11	82930206	82933587	chr11-82930206-82933587
chr11	82970964	82971271	chr11-82970964-82971271
chr11	82991422	82992165	chr11-82991422-82992165
chr11	83019756	83020220	chr11-83019756-83020220
chr11	83270617	83271484	chr11-83270617-83271484
chr11	83290902	83293362	chr11-83290902-83293362
chr11	83297479	83300202	chr11-83297479-83300202
chr11	83301647	83304673	chr11-83301647-83304673
chr11	83409150	83410358	chr11-83409150-83410358
chr11	83418504	83419040	chr11-83418504-83419040
chr11	83427157	83427613	chr11-83427157-83427613
chr11	83439745	83441277	chr11-83439745-83441277
chr11	83442553	83443070	chr11-83442553-83443070
chr11	83444729	83445194	chr11-83444729-83445194

chr11	83454608	83454809	chr11-83454608-83454809
chr11	83455653	83455978	chr11-83455653-83455978
chr11	83458281	83458482	chr11-83458281-83458482
chr11	83462296	83463524	chr11-83462296-83463524
chr11	83472042	83474990	chr11-83472042-83474990
chr11	83534360	83535406	chr11-83534360-83535406
chr11	83581197	83583342	chr11-83581197-83583342
chr11	83597616	83598812	chr11-83597616-83598812
chr11	83624139	83624340	chr11-83624139-83624340
chr11	83728952	83729723	chr11-83728952-83729723
chr11	83730727	83731017	chr11-83730727-83731017
chr11	83753070	83755110	chr11-83753070-83755110
chr11	83788902	83789595	chr11-83788902-83789595
chr11	83819665	83820524	chr11-83819665-83820524
chr11	83843611	83843901	chr11-83843611-83843901
chr11	83848648	83856241	chr11-83848648-83856241
chr11	83882641	83883114	chr11-83882641-83883114
chr11	83891630	83892149	chr11-83891630-83892149
chr11	83893443	83893770	chr11-83893443-83893770
chr11	83896679	83896880	chr11-83896679-83896880
chr11	83902930	83903407	chr11-83902930-83903407
chr11	83935991	83936250	chr11-83935991-83936250
chr11	83941401	83943409	chr11-83941401-83943409
chr11	83963901	83965223	chr11-83963901-83965223
chr11	84042879	84043554	chr11-84042879-84043554
chr11	84066032	84069656	chr11-84066032-84069656
chr11	84079156	84080576	chr11-84079156-84080576
chr11	84088280	84088683	chr11-84088280-84088683
chr11	84128285	84130304	chr11-84128285-84130304
chr11	84178643	84181650	chr11-84178643-84181650
chr11	84442379	84442941	chr11-84442379-84442941
chr11	84511909	84514398	chr11-84511909-84514398
chr11	84520121	84520571	chr11-84520121-84520571
chr11	84522420	84526602	chr11-84522420-84526602
chr11	84529140	84529546	chr11-84529140-84529546
chr11	84560610	84561902	chr11-84560610-84561902
chr11	84642213	84642986	chr11-84642213-84642986
chr11	84794476	84794727	chr11-84794476-84794727
chr11	84818045	84820426	chr11-84818045-84820426
chr11	84827706	84830005	chr11-84827706-84830005
chr11	84868517	84872013	chr11-84868517-84872013
chr11	84878880	84881772	chr11-84878880-84881772

chr11	84914950	84917078	chr11-84914950-84917078
chr11	84918639	84918950	chr11-84918639-84918950
chr11	84957587	84958406	chr11-84957587-84958406
chr11	85138036	85141490	chr11-85138036-85141490
chr11	85157158	85157516	chr11-85157158-85157516
chr11	85170213	85170570	chr11-85170213-85170570
chr11	85177944	85178624	chr11-85177944-85178624
chr11	85182872	85183383	chr11-85182872-85183383
chr11	85233094	85236209	chr11-85233094-85236209
chr11	85266747	85266998	chr11-85266747-85266998
chr11	85310167	85313095	chr11-85310167-85313095
chr11	85352078	85353961	chr11-85352078-85353961
chr11	85367929	85368283	chr11-85367929-85368283
chr11	85792413	85793239	chr11-85792413-85793239
chr11	85830064	85830442	chr11-85830064-85830442
chr11	85831669	85833782	chr11-85831669-85833782
chr11	85836966	85838616	chr11-85836966-85838616
chr11	85841653	85845118	chr11-85841653-85845118
chr11	85848998	85849255	chr11-85848998-85849255
chr11	85890004	85890568	chr11-85890004-85890568
chr11	85894739	85895586	chr11-85894739-85895586
chr11	85925132	85925793	chr11-85925132-85925793
chr11	85986199	85986880	chr11-85986199-85986880
chr11	85989621	85990642	chr11-85989621-85990642
chr11	86199877	86203021	chr11-86199877-86203021
chr11	86256028	86258374	chr11-86256028-86258374
chr11	86347134	86347495	chr11-86347134-86347495
chr11	86351881	86352132	chr11-86351881-86352132
chr11	86355806	86358524	chr11-86355806-86358524
chr11	86380756	86382226	chr11-86380756-86382226
chr11	86483839	86485999	chr11-86483839-86485999
chr11	86543316	86546008	chr11-86543316-86546008
chr11	86576962	86595856	chr11-86576962-86595856
chr11	86645824	86654029	chr11-86645824-86654029
chr11	86682386	86685983	chr11-86682386-86685983
chr11	86755285	86759099	chr11-86755285-86759099
chr11	86806675	86808050	chr11-86806675-86808050
chr11	86813219	86815997	chr11-86813219-86815997
chr11	86828310	86828511	chr11-86828310-86828511
chr11	86852499	86853539	chr11-86852499-86853539
chr11	86894013	86894473	chr11-86894013-86894473
chr11	86908067	86908268	chr11-86908067-86908268

chr11	86953054	86953362	chr11-86953054-86953362
chr11	86961465	86962688	chr11-86961465-86962688
chr11	86992439	86994400	chr11-86992439-86994400
chr11	87072973	87075300	chr11-87072973-87075300
chr11	87084706	87087458	chr11-87084706-87087458
chr11	87107258	87111303	chr11-87107258-87111303
chr11	87126201	87129327	chr11-87126201-87129327
chr11	87309541	87310207	chr11-87309541-87310207
chr11	87357900	87359550	chr11-87357900-87359550
chr11	87403472	87405307	chr11-87403472-87405307
chr11	87421778	87422838	chr11-87421778-87422838
chr11	87425915	87426710	chr11-87425915-87426710
chr11	87442521	87443184	chr11-87442521-87443184
chr11	87444931	87445343	chr11-87444931-87445343
chr11	87447859	87448592	chr11-87447859-87448592
chr11	87453496	87453697	chr11-87453496-87453697
chr11	87461665	87462193	chr11-87461665-87462193
chr11	87467606	87468192	chr11-87467606-87468192
chr11	87470694	87471251	chr11-87470694-87471251
chr11	87574706	87575438	chr11-87574706-87575438
chr11	87577960	87578980	chr11-87577960-87578980
chr11	87589535	87597022	chr11-87589535-87597022
chr11	87598790	87600129	chr11-87598790-87600129
chr11	87638815	87639641	chr11-87638815-87639641
chr11	87662479	87666970	chr11-87662479-87666970
chr11	87678961	87679264	chr11-87678961-87679264
chr11	87689268	87690255	chr11-87689268-87690255
chr11	87697035	87697286	chr11-87697035-87697286
chr11	87709507	87710921	chr11-87709507-87710921
chr11	87736270	87738680	chr11-87736270-87738680
chr11	87763459	87765007	chr11-87763459-87765007
chr11	87777989	87779362	chr11-87777989-87779362
chr11	87816893	87817627	chr11-87816893-87817627
chr11	87852719	87855184	chr11-87852719-87855184
chr11	87967728	87968828	chr11-87967728-87968828
chr11	87985451	87988200	chr11-87985451-87988200
chr11	88046153	88050462	chr11-88046153-88050462
chr11	88067252	88070400	chr11-88067252-88070400
chr11	88086843	88087517	chr11-88086843-88087517
chr11	88098329	88100535	chr11-88098329-88100535
chr11	88107289	88107541	chr11-88107289-88107541
chr11	88151193	88152068	chr11-88151193-88152068

chr11	88160385	88166772	chr11-88160385-88166772
chr11	88169816	88170017	chr11-88169816-88170017
chr11	88171087	88174182	chr11-88171087-88174182
chr11	88179922	88181633	chr11-88179922-88181633
chr11	88187039	88188129	chr11-88187039-88188129
chr11	88203211	88205665	chr11-88203211-88205665
chr11	88213614	88216719	chr11-88213614-88216719
chr11	88235348	88236377	chr11-88235348-88236377
chr11	88262588	88262949	chr11-88262588-88262949
chr11	88268149	88268499	chr11-88268149-88268499
chr11	88286058	88286549	chr11-88286058-88286549
chr11	88461854	88462262	chr11-88461854-88462262
chr11	88467812	88468064	chr11-88467812-88468064
chr11	88494846	88496695	chr11-88494846-88496695
chr11	88497930	88498653	chr11-88497930-88498653
chr11	88524600	88525313	chr11-88524600-88525313
chr11	88550704	88551299	chr11-88550704-88551299
chr11	88557002	88558397	chr11-88557002-88558397
chr11	88560964	88561319	chr11-88560964-88561319
chr11	88612361	88612744	chr11-88612361-88612744
chr11	88613552	88615760	chr11-88613552-88615760
chr11	88618951	88619152	chr11-88618951-88619152
chr11	88629308	88629972	chr11-88629308-88629972
chr11	88634905	88635106	chr11-88634905-88635106
chr11	88690827	88691131	chr11-88690827-88691131
chr11	88715867	88719570	chr11-88715867-88719570
chr11	88817172	88818310	chr11-88817172-88818310
chr11	88822586	88823301	chr11-88822586-88823301
chr11	88862249	88865382	chr11-88862249-88865382
chr11	88873075	88873986	chr11-88873075-88873986
chr11	88952408	88952609	chr11-88952408-88952609
chr11	88953705	88955988	chr11-88953705-88955988
chr11	88973461	88975089	chr11-88973461-88975089
chr11	88996563	88996868	chr11-88996563-88996868
chr11	88998856	89003338	chr11-88998856-89003338
chr11	89023148	89024142	chr11-89023148-89024142
chr11	89027966	89028270	chr11-89027966-89028270
chr11	89058100	89061265	chr11-89058100-89061265
chr11	89100534	89101579	chr11-89100534-89101579
chr11	89175553	89175813	chr11-89175553-89175813
chr11	89185801	89188170	chr11-89185801-89188170
chr11	89206197	89206824	chr11-89206197-89206824

chr11	89239773	89240128	chr11-89239773-89240128
chr11	89297686	89303726	chr11-89297686-89303726
chr11	90001898	90003352	chr11-90001898-90003352
chr11	90029367	90030962	chr11-90029367-90030962
chr11	90248883	90250968	chr11-90248883-90250968
chr11	90352516	90352996	chr11-90352516-90352996
chr11	90355106	90355838	chr11-90355106-90355838
chr11	90389110	90391456	chr11-90389110-90391456
chr11	90417539	90418533	chr11-90417539-90418533
chr11	90558284	90558485	chr11-90558284-90558485
chr11	90637234	90639369	chr11-90637234-90639369
chr11	90679195	90679396	chr11-90679195-90679396
chr11	90686231	90688325	chr11-90686231-90688325
chr11	90697617	90697975	chr11-90697617-90697975
chr11	92592728	92592987	chr11-92592728-92592987
chr11	93098134	93100250	chr11-93098134-93100250
chr11	93884249	93887706	chr11-93884249-93887706
chr11	93941185	93941438	chr11-93941185-93941438
chr11	93952383	93956882	chr11-93952383-93956882
chr11	93963209	93963647	chr11-93963209-93963647
chr11	93966776	93969128	chr11-93966776-93969128
chr11	93987339	93989121	chr11-93987339-93989121
chr11	93994495	93998108	chr11-93994495-93998108
chr11	94133403	94133969	chr11-94133403-94133969
chr11	94141500	94141804	chr11-94141500-94141804
chr11	94145010	94145727	chr11-94145010-94145727
chr11	94148814	94149596	chr11-94148814-94149596
chr11	94160197	94161825	chr11-94160197-94161825
chr11	94209626	94214615	chr11-94209626-94214615
chr11	94226177	94226534	chr11-94226177-94226534
chr11	94231541	94232514	chr11-94231541-94232514
chr11	94265334	94265747	chr11-94265334-94265747
chr11	94276929	94277712	chr11-94276929-94277712
chr11	94318196	94322996	chr11-94318196-94322996
chr11	94326762	94329456	chr11-94326762-94329456
chr11	94334595	94334796	chr11-94334595-94334796
chr11	94340984	94342255	chr11-94340984-94342255
chr11	94364936	94365249	chr11-94364936-94365249
chr11	94386435	94386954	chr11-94386435-94386954
chr11	94387784	94388096	chr11-94387784-94388096
chr11	94391251	94393214	chr11-94391251-94393214
chr11	94403778	94404998	chr11-94403778-94404998

chr11	94472557	94475014	chr11-94472557-94475014
chr11	94485980	94486181	chr11-94485980-94486181
chr11	94491063	94491525	chr11-94491063-94491525
chr11	94493888	94500906	chr11-94493888-94500906
chr11	94520697	94521956	chr11-94520697-94521956
chr11	94531842	94532748	chr11-94531842-94532748
chr11	94538133	94539493	chr11-94538133-94539493
chr11	94542250	94544125	chr11-94542250-94544125
chr11	94547322	94549744	chr11-94547322-94549744
chr11	94564860	94565307	chr11-94564860-94565307
chr11	94600943	94602476	chr11-94600943-94602476
chr11	94629269	94631786	chr11-94629269-94631786
chr11	94652562	94655043	chr11-94652562-94655043
chr11	94676113	94678064	chr11-94676113-94678064
chr11	94870241	94870512	chr11-94870241-94870512
chr11	94882671	94882974	chr11-94882671-94882974
chr11	94913880	94914392	chr11-94913880-94914392
chr11	94915468	94917084	chr11-94915468-94917084
chr11	94927504	94929388	chr11-94927504-94929388
chr11	94936356	94938569	chr11-94936356-94938569
chr11	94957799	94958882	chr11-94957799-94958882
chr11	94979022	94979426	chr11-94979022-94979426
chr11	94988801	94994877	chr11-94988801-94994877
chr11	95006524	95007545	chr11-95006524-95007545
chr11	95008964	95010787	chr11-95008964-95010787
chr11	95040264	95042426	chr11-95040264-95042426
chr11	95069625	95081307	chr11-95069625-95081307
chr11	95083479	95090244	chr11-95083479-95090244
chr11	95092448	95093265	chr11-95092448-95093265
chr11	95103613	95107272	chr11-95103613-95107272
chr11	95119941	95122087	chr11-95119941-95122087
chr11	95126443	95127111	chr11-95126443-95127111
chr11	95142988	95143459	chr11-95142988-95143459
chr11	95144833	95146580	chr11-95144833-95146580
chr11	95153425	95153941	chr11-95153425-95153941
chr11	95157679	95158478	chr11-95157679-95158478
chr11	95202088	95202499	chr11-95202088-95202499
chr11	95210383	95211370	chr11-95210383-95211370
chr11	95223262	95225746	chr11-95223262-95225746
chr11	95231761	95232382	chr11-95231761-95232382
chr11	95258383	95260094	chr11-95258383-95260094
chr11	95308223	95311848	chr11-95308223-95311848

chr11	95336132	95338433	chr11-95336132-95338433
chr11	95384018	95386029	chr11-95384018-95386029
chr11	95403428	95404563	chr11-95403428-95404563
chr11	95413522	95416596	chr11-95413522-95416596
chr11	95513356	95515327	chr11-95513356-95515327
chr11	95520470	95521846	chr11-95520470-95521846
chr11	95533966	95534167	chr11-95533966-95534167
chr11	95575766	95576175	chr11-95575766-95576175
chr11	95585243	95587980	chr11-95585243-95587980
chr11	95645414	95645979	chr11-95645414-95645979
chr11	95650799	95651431	chr11-95650799-95651431
chr11	95665883	95668314	chr11-95665883-95668314
chr11	95711937	95714943	chr11-95711937-95714943
chr11	95777457	95779296	chr11-95777457-95779296
chr11	95796383	95797095	chr11-95796383-95797095
chr11	95823515	95825389	chr11-95823515-95825389
chr11	95830960	95831311	chr11-95830960-95831311
chr11	95837020	95837221	chr11-95837020-95837221
chr11	95857648	95860639	chr11-95857648-95860639
chr11	95914186	95916181	chr11-95914186-95916181
chr11	95980526	95983366	chr11-95980526-95983366
chr11	95988017	95988960	chr11-95988017-95988960
chr11	95991039	95991751	chr11-95991039-95991751
chr11	96003183	96008275	chr11-96003183-96008275
chr11	96017743	96018650	chr11-96017743-96018650
chr11	96033978	96036090	chr11-96033978-96036090
chr11	96062483	96066042	chr11-96062483-96066042
chr11	96073518	96076792	chr11-96073518-96076792
chr11	96124125	96124955	chr11-96124125-96124955
chr11	96140715	96141401	chr11-96140715-96141401
chr11	96145780	96146304	chr11-96145780-96146304
chr11	96173081	96173350	chr11-96173081-96173350
chr11	96198784	96198985	chr11-96198784-96198985
chr11	96200097	96200298	chr11-96200097-96200298
chr11	96203350	96203764	chr11-96203350-96203764
chr11	96260401	96260657	chr11-96260401-96260657
chr11	96270829	96272826	chr11-96270829-96272826
chr11	96279533	96279971	chr11-96279533-96279971
chr11	96282964	96285565	chr11-96282964-96285565
chr11	96286462	96287451	chr11-96286462-96287451
chr11	96295420	96295621	chr11-96295420-96295621
chr11	96299024	96301046	chr11-96299024-96301046

chr11	96303570	96304250	chr11-96303570-96304250
chr11	96311085	96311971	chr11-96311085-96311971
chr11	96314791	96314992	chr11-96314791-96314992
chr11	96318261	96320412	chr11-96318261-96320412
chr11	96332693	96333262	chr11-96332693-96333262
chr11	96340744	96342395	chr11-96340744-96342395
chr11	96345403	96346310	chr11-96345403-96346310
chr11	96352744	96353530	chr11-96352744-96353530
chr11	96366768	96367025	chr11-96366768-96367025
chr11	96407800	96408212	chr11-96407800-96408212
chr11	96413490	96414401	chr11-96413490-96414401
chr11	96464314	96464778	chr11-96464314-96464778
chr11	96579063	96579264	chr11-96579063-96579264
chr11	96603180	96603546	chr11-96603180-96603546
chr11	96735775	96737474	chr11-96735775-96737474
chr11	96740006	96749985	chr11-96740006-96749985
chr11	96776109	96778494	chr11-96776109-96778494
chr11	96788600	96791213	chr11-96788600-96791213
chr11	96821427	96831257	chr11-96821427-96831257
chr11	96849447	96850657	chr11-96849447-96850657
chr11	96859558	96859812	chr11-96859558-96859812
chr11	96860903	96863671	chr11-96860903-96863671
chr11	96875509	96876491	chr11-96875509-96876491
chr11	96895746	96896053	chr11-96895746-96896053
chr11	96915236	96917206	chr11-96915236-96917206
chr11	96919195	96920119	chr11-96919195-96920119
chr11	96928570	96930894	chr11-96928570-96930894
chr11	96934592	96935784	chr11-96934592-96935784
chr11	96937372	96937626	chr11-96937372-96937626
chr11	96942838	96945453	chr11-96942838-96945453
chr11	96966639	96968061	chr11-96966639-96968061
chr11	96976091	96978893	chr11-96976091-96978893
chr11	96982212	96982577	chr11-96982212-96982577
chr11	96987890	96988563	chr11-96987890-96988563
chr11	96997206	96997615	chr11-96997206-96997615
chr11	97017871	97019806	chr11-97017871-97019806
chr11	97021126	97023021	chr11-97021126-97023021
chr11	97025594	97026410	chr11-97025594-97026410
chr11	97029419	97031226	chr11-97029419-97031226
chr11	97040789	97042917	chr11-97040789-97042917
chr11	97050243	97053256	chr11-97050243-97053256
chr11	97058392	97058593	chr11-97058392-97058593

chr11	97065377	97066060	chr11-97065377-97066060
chr11	97083604	97084280	chr11-97083604-97084280
chr11	97114363	97115983	chr11-97114363-97115983
chr11	97126661	97127903	chr11-97126661-97127903
chr11	97138809	97139337	chr11-97138809-97139337
chr11	97149891	97151997	chr11-97149891-97151997
chr11	97184322	97189901	chr11-97184322-97189901
chr11	97207004	97207574	chr11-97207004-97207574
chr11	97277933	97281446	chr11-97277933-97281446
chr11	97302456	97303011	chr11-97302456-97303011
chr11	97308288	97311848	chr11-97308288-97311848
chr11	97314890	97316979	chr11-97314890-97316979
chr11	97322117	97322318	chr11-97322117-97322318
chr11	97338740	97339255	chr11-97338740-97339255
chr11	97343628	97344031	chr11-97343628-97344031
chr11	97361767	97364639	chr11-97361767-97364639
chr11	97400104	97401621	chr11-97400104-97401621
chr11	97404224	97405484	chr11-97404224-97405484
chr11	97415054	97421247	chr11-97415054-97421247
chr11	97427603	97430069	chr11-97427603-97430069
chr11	97434607	97442180	chr11-97434607-97442180
chr11	97449323	97451850	chr11-97449323-97451850
chr11	97500194	97500892	chr11-97500194-97500892
chr11	97501801	97503076	chr11-97501801-97503076
chr11	97523049	97524523	chr11-97523049-97524523
chr11	97531583	97533266	chr11-97531583-97533266
chr11	97536956	97537364	chr11-97536956-97537364
chr11	97546157	97547569	chr11-97546157-97547569
chr11	97562708	97565116	chr11-97562708-97565116
chr11	97573708	97576805	chr11-97573708-97576805
chr11	97603770	97604204	chr11-97603770-97604204
chr11	97611034	97611457	chr11-97611034-97611457
chr11	97623494	97623795	chr11-97623494-97623795
chr11	97626363	97630589	chr11-97626363-97630589
chr11	97657364	97657719	chr11-97657364-97657719
chr11	97658649	97665805	chr11-97658649-97665805
chr11	97685013	97687647	chr11-97685013-97687647
chr11	97697698	97705029	chr11-97697698-97705029
chr11	97728165	97728938	chr11-97728165-97728938
chr11	97735506	97735868	chr11-97735506-97735868
chr11	97736910	97738068	chr11-97736910-97738068
chr11	97742238	97745721	chr11-97742238-97745721

chr11	97748462	97748873	chr11-97748462-97748873
chr11	97765044	97767469	chr11-97765044-97767469
chr11	97779052	97783525	chr11-97779052-97783525
chr11	97798200	97819012	chr11-97798200-97819012
chr11	97820439	97821664	chr11-97820439-97821664
chr11	97883251	97885148	chr11-97883251-97885148
chr11	97935690	97936108	chr11-97935690-97936108
chr11	97985701	97990256	chr11-97985701-97990256
chr11	97994383	97995181	chr11-97994383-97995181
chr11	98002256	98004256	chr11-98002256-98004256
chr11	98021627	98023200	chr11-98021627-98023200
chr11	98025816	98029097	chr11-98025816-98029097
chr11	98035734	98036751	chr11-98035734-98036751
chr11	98043758	98043959	chr11-98043758-98043959
chr11	98051827	98053694	chr11-98051827-98053694
chr11	98057260	98058566	chr11-98057260-98058566
chr11	98061835	98062471	chr11-98061835-98062471
chr11	98148122	98150812	chr11-98148122-98150812
chr11	98191722	98194011	chr11-98191722-98194011
chr11	98201509	98206639	chr11-98201509-98206639
chr11	98294211	98295421	chr11-98294211-98295421
chr11	98313919	98314221	chr11-98313919-98314221
chr11	98326974	98329055	chr11-98326974-98329055
chr11	98334050	98334935	chr11-98334050-98334935
chr11	98338303	98339706	chr11-98338303-98339706
chr11	98342063	98343172	chr11-98342063-98343172
chr11	98345348	98345839	chr11-98345348-98345839
chr11	98348177	98349355	chr11-98348177-98349355
chr11	98356785	98361370	chr11-98356785-98361370
chr11	98399631	98401140	chr11-98399631-98401140
chr11	98411823	98414762	chr11-98411823-98414762
chr11	98418071	98419684	chr11-98418071-98419684
chr11	98437617	98439359	chr11-98437617-98439359
chr11	98445324	98450710	chr11-98445324-98450710
chr11	98457091	98458699	chr11-98457091-98458699
chr11	98461237	98461490	chr11-98461237-98461490
chr11	98545407	98546226	chr11-98545407-98546226
chr11	98550511	98551824	chr11-98550511-98551824
chr11	98585664	98589370	chr11-98585664-98589370
chr11	98598849	98599574	chr11-98598849-98599574
chr11	98602084	98602585	chr11-98602084-98602585
chr11	98673124	98673325	chr11-98673124-98673325

chr11	98681560	98684135	chr11-98681560-98684135
chr11	98699740	98703594	chr11-98699740-98703594
chr11	98704717	98711238	chr11-98704717-98711238
chr11	98728506	98729625	chr11-98728506-98729625
chr11	98740417	98743311	chr11-98740417-98743311
chr11	98747224	98747486	chr11-98747224-98747486
chr11	98750485	98751814	chr11-98750485-98751814
chr11	98754165	98754677	chr11-98754165-98754677
chr11	98762619	98763870	chr11-98762619-98763870
chr11	98771752	98777558	chr11-98771752-98777558
chr11	98782828	98784094	chr11-98782828-98784094
chr11	98785430	98787526	chr11-98785430-98787526
chr11	98788506	98790237	chr11-98788506-98790237
chr11	98793812	98798260	chr11-98793812-98798260
chr11	98808955	98811506	chr11-98808955-98811506
chr11	98836287	98837868	chr11-98836287-98837868
chr11	98839299	98839675	chr11-98839299-98839675
chr11	98858900	98859256	chr11-98858900-98859256
chr11	98862801	98865187	chr11-98862801-98865187
chr11	98873399	98874346	chr11-98873399-98874346
chr11	98902413	98903238	chr11-98902413-98903238
chr11	98904221	98910213	chr11-98904221-98910213
chr11	98930943	98933987	chr11-98930943-98933987
chr11	98935319	98944091	chr11-98935319-98944091
chr11	98947667	98947981	chr11-98947667-98947981
chr11	98958983	98964997	chr11-98958983-98964997
chr11	98981619	98983357	chr11-98981619-98983357
chr11	99022100	99024997	chr11-99022100-99024997
chr11	99039805	99045571	chr11-99039805-99045571
chr11	99076290	99076727	chr11-99076290-99076727
chr11	99080835	99090209	chr11-99080835-99090209
chr11	99101843	99109045	chr11-99101843-99109045
chr11	99109971	99110501	chr11-99109971-99110501
chr11	99128072	99128395	chr11-99128072-99128395
chr11	99199456	99200730	chr11-99199456-99200730
chr11	99228866	99231770	chr11-99228866-99231770
chr11	99268804	99269546	chr11-99268804-99269546
chr11	99341643	99341844	chr11-99341643-99341844
chr11	99346164	99346468	chr11-99346164-99346468
chr11	99385003	99387066	chr11-99385003-99387066
chr11	99437615	99438488	chr11-99437615-99438488
chr11	99471864	99472185	chr11-99471864-99472185

chr11	99473600	99473801	chr11-99473600-99473801
chr11	99491108	99493221	chr11-99491108-99493221
chr11	99508383	99508746	chr11-99508383-99508746
chr11	99580522	99581556	chr11-99580522-99581556
chr11	99583148	99583349	chr11-99583148-99583349
chr11	99634425	99635034	chr11-99634425-99635034
chr11	99658336	99659067	chr11-99658336-99659067
chr11	99850928	99851600	chr11-99850928-99851600
chr11	99858194	99858712	chr11-99858194-99858712
chr11	100055753	100056597	chr11-100055753-100056597
chr11	100067908	100068109	chr11-100067908-100068109
chr11	100113098	100114297	chr11-100113098-100114297
chr11	100138884	100139141	chr11-100138884-100139141
chr11	100140932	100148520	chr11-100140932-100148520
chr11	100157385	100158601	chr11-100157385-100158601
chr11	100167561	100169540	chr11-100167561-100169540
chr11	100208582	100208892	chr11-100208582-100208892
chr11	100248728	100249083	chr11-100248728-100249083
chr11	100277671	100278729	chr11-100277671-100278729
chr11	100286630	100287750	chr11-100286630-100287750
chr11	100288570	100291681	chr11-100288570-100291681
chr11	100296114	100296756	chr11-100296114-100296756
chr11	100297992	100298780	chr11-100297992-100298780
chr11	100300693	100300944	chr11-100300693-100300944
chr11	100318978	100322982	chr11-100318978-100322982
chr11	100354687	100357123	chr11-100354687-100357123
chr11	100386364	100398672	chr11-100386364-100398672
chr11	100402083	100402973	chr11-100402083-100402973
chr11	100411731	100415916	chr11-100411731-100415916
chr11	100440124	100441685	chr11-100440124-100441685
chr11	100470899	100472980	chr11-100470899-100472980
chr11	100524392	100529040	chr11-100524392-100529040
chr11	100536387	100536588	chr11-100536387-100536588
chr11	100544936	100546447	chr11-100544936-100546447
chr11	100555845	100556096	chr11-100555845-100556096
chr11	100569604	100570595	chr11-100569604-100570595
chr11	100574312	100575940	chr11-100574312-100575940
chr11	100617623	100620607	chr11-100617623-100620607
chr11	100622616	100624540	chr11-100622616-100624540
chr11	100639961	100640716	chr11-100639961-100640716
chr11	100692196	100692656	chr11-100692196-100692656
chr11	100702624	100702985	chr11-100702624-100702985

chr11	100711351	100712686	chr11-100711351-100712686
chr11	100735549	100739168	chr11-100735549-100739168
chr11	100749483	100749787	chr11-100749483-100749787
chr11	100758724	100760237	chr11-100758724-100760237
chr11	100761463	100762368	chr11-100761463-100762368
chr11	100769851	100771509	chr11-100769851-100771509
chr11	100849216	100851670	chr11-100849216-100851670
chr11	100859320	100861073	chr11-100859320-100861073
chr11	100893638	100893839	chr11-100893638-100893839
chr11	100931115	100932215	chr11-100931115-100932215
chr11	100935611	100935862	chr11-100935611-100935862
chr11	100938095	100940332	chr11-100938095-100940332
chr11	100960772	100971645	chr11-100960772-100971645
chr11	100974583	100974784	chr11-100974583-100974784
chr11	100983113	100983727	chr11-100983113-100983727
chr11	100993502	100993753	chr11-100993502-100993753
chr11	101008357	101011985	chr11-101008357-101011985
chr11	101052773	101053658	chr11-101052773-101053658
chr11	101055459	101055710	chr11-101055459-101055710
chr11	101056589	101057552	chr11-101056589-101057552
chr11	101058643	101058938	chr11-101058643-101058938
chr11	101059957	101060841	chr11-101059957-101060841
chr11	101062162	101064202	chr11-101062162-101064202
chr11	101066774	101067239	chr11-101066774-101067239
chr11	101069400	101071375	chr11-101069400-101071375
chr11	101081683	101084001	chr11-101081683-101084001
chr11	101086249	101088452	chr11-101086249-101088452
chr11	101094456	101096318	chr11-101094456-101096318
chr11	101118008	101121328	chr11-101118008-101121328
chr11	101130084	101130285	chr11-101130084-101130285
chr11	101138414	101140951	chr11-101138414-101140951
chr11	101155695	101156580	chr11-101155695-101156580
chr11	101168365	101174595	chr11-101168365-101174595
chr11	101175787	101176710	chr11-101175787-101176710
chr11	101225521	101226958	chr11-101225521-101226958
chr11	101245876	101246929	chr11-101245876-101246929
chr11	101252735	101254982	chr11-101252735-101254982
chr11	101259786	101261570	chr11-101259786-101261570
chr11	101277051	101279591	chr11-101277051-101279591
chr11	101300673	101302744	chr11-101300673-101302744
chr11	101315466	101317506	chr11-101315466-101317506
chr11	101338217	101338595	chr11-101338217-101338595

chr11	101362257	101362519	chr11-101362257-101362519
chr11	101416222	101417846	chr11-101416222-101417846
chr11	101423982	101426799	chr11-101423982-101426799
chr11	101441662	101445028	chr11-101441662-101445028
chr11	101448000	101449419	chr11-101448000-101449419
chr11	101463143	101469052	chr11-101463143-101469052
chr11	101494542	101494743	chr11-101494542-101494743
chr11	101550733	101554576	chr11-101550733-101554576
chr11	101581897	101582916	chr11-101581897-101582916
chr11	101604357	101605708	chr11-101604357-101605708
chr11	101617009	101617210	chr11-101617009-101617210
chr11	101626481	101629809	chr11-101626481-101629809
chr11	101640927	101642783	chr11-101640927-101642783
chr11	101645963	101646758	chr11-101645963-101646758
chr11	101657629	101658264	chr11-101657629-101658264
chr11	101664836	101666186	chr11-101664836-101666186
chr11	101700452	101701013	chr11-101700452-101701013
chr11	101732447	101734381	chr11-101732447-101734381
chr11	101781922	101786175	chr11-101781922-101786175
chr11	101806947	101807200	chr11-101806947-101807200
chr11	101830069	101830374	chr11-101830069-101830374
chr11	101849100	101849739	chr11-101849100-101849739
chr11	101881725	101881976	chr11-101881725-101881976
chr11	101937088	101937755	chr11-101937088-101937755
chr11	101963469	101964484	chr11-101963469-101964484
chr11	101982942	101986295	chr11-101982942-101986295
chr11	101998431	101998632	chr11-101998431-101998632
chr11	102026079	102027644	chr11-102026079-102027644
chr11	102061270	102061835	chr11-102061270-102061835
chr11	102082135	102082807	chr11-102082135-102082807
chr11	102086817	102089495	chr11-102086817-102089495
chr11	102096896	102097505	chr11-102096896-102097505
chr11	102106939	102107410	chr11-102106939-102107410
chr11	102138301	102138613	chr11-102138301-102138613
chr11	102145272	102147677	chr11-102145272-102147677
chr11	102155348	102156831	chr11-102155348-102156831
chr11	102183651	102185845	chr11-102183651-102185845
chr11	102189244	102190746	chr11-102189244-102190746
chr11	102215673	102220374	chr11-102215673-102220374
chr11	102224525	102225253	chr11-102224525-102225253
chr11	102229020	102230831	chr11-102229020-102230831
chr11	102244509	102245803	chr11-102244509-102245803

chr11	102248384	102250048	chr11-102248384-102250048
chr11	102276364	102276994	chr11-102276364-102276994
chr11	102284060	102286399	chr11-102284060-102286399
chr11	102294762	102298444	chr11-102294762-102298444
chr11	102308725	102311670	chr11-102308725-102311670
chr11	102315521	102320993	chr11-102315521-102320993
chr11	102375562	102376185	chr11-102375562-102376185
chr11	102393121	102394835	chr11-102393121-102394835
chr11	102399761	102400015	chr11-102399761-102400015
chr11	102405570	102409218	chr11-102405570-102409218
chr11	102429377	102433009	chr11-102429377-102433009
chr11	102437317	102438719	chr11-102437317-102438719
chr11	102445122	102448045	chr11-102445122-102448045
chr11	102455354	102456777	chr11-102455354-102456777
chr11	102465477	102468018	chr11-102465477-102468018
chr11	102554183	102556969	chr11-102554183-102556969
chr11	102572895	102573258	chr11-102572895-102573258
chr11	102578443	102579170	chr11-102578443-102579170
chr11	102582189	102582869	chr11-102582189-102582869
chr11	102602932	102607365	chr11-102602932-102607365
chr11	102643975	102644590	chr11-102643975-102644590
chr11	102656300	102656723	chr11-102656300-102656723
chr11	102665447	102665699	chr11-102665447-102665699
chr11	102696494	102698588	chr11-102696494-102698588
chr11	102739100	102739853	chr11-102739100-102739853
chr11	102749176	102750420	chr11-102749176-102750420
chr11	102761049	102762448	chr11-102761049-102762448
chr11	102773935	102775364	chr11-102773935-102775364
chr11	102777671	102777973	chr11-102777671-102777973
chr11	102783308	102784082	chr11-102783308-102784082
chr11	102817384	102819995	chr11-102817384-102819995
chr11	102879730	102881997	chr11-102879730-102881997
chr11	102893097	102893713	chr11-102893097-102893713
chr11	102900758	102900959	chr11-102900758-102900959
chr11	102923471	102925829	chr11-102923471-102925829
chr11	102945161	102947240	chr11-102945161-102947240
chr11	102948134	102949097	chr11-102948134-102949097
chr11	102989770	102990537	chr11-102989770-102990537
chr11	102996183	102997013	chr11-102996183-102997013
chr11	103003132	103003818	chr11-103003132-103003818
chr11	103015621	103017624	chr11-103015621-103017624
chr11	103027322	103030465	chr11-103027322-103030465

chr11	103050268	103052400	chr11-103050268-103052400
chr11	103056595	103057606	chr11-103056595-103057606
chr11	103093371	103104101	chr11-103093371-103104101
chr11	103114422	103118806	chr11-103114422-103118806
chr11	103131017	103134893	chr11-103131017-103134893
chr11	103140724	103143205	chr11-103140724-103143205
chr11	103158592	103159623	chr11-103158592-103159623
chr11	103170290	103176144	chr11-103170290-103176144
chr11	103177908	103178563	chr11-103177908-103178563
chr11	103193093	103193294	chr11-103193093-103193294
chr11	103198843	103200282	chr11-103198843-103200282
chr11	103217640	103218843	chr11-103217640-103218843
chr11	103245971	103247735	chr11-103245971-103247735
chr11	103261710	103269589	chr11-103261710-103269589
chr11	103311637	103313633	chr11-103311637-103313633
chr11	103346315	103347232	chr11-103346315-103347232
chr11	103350766	103351067	chr11-103350766-103351067
chr11	103359107	103366071	chr11-103359107-103366071
chr11	103377954	103378205	chr11-103377954-103378205
chr11	103381471	103388469	chr11-103381471-103388469
chr11	103396871	103397333	chr11-103396871-103397333
chr11	103406564	103407740	chr11-103406564-103407740
chr11	103410934	103413567	chr11-103410934-103413567
chr11	103649185	103650452	chr11-103649185-103650452
chr11	103696534	103698745	chr11-103696534-103698745
chr11	103721836	103722037	chr11-103721836-103722037
chr11	103749003	103750381	chr11-103749003-103750381
chr11	103773234	103774614	chr11-103773234-103774614
chr11	103809448	103810770	chr11-103809448-103810770
chr11	103952979	103954567	chr11-103952979-103954567
chr11	103961352	103961777	chr11-103961352-103961777
chr11	103965928	103968409	chr11-103965928-103968409
chr11	104132297	104133425	chr11-104132297-104133425
chr11	104157675	104158304	chr11-104157675-104158304
chr11	104230533	104233251	chr11-104230533-104233251
chr11	104278362	104278718	chr11-104278362-104278718
chr11	104288883	104290189	chr11-104288883-104290189
chr11	104310149	104310722	chr11-104310149-104310722
chr11	104318614	104319895	chr11-104318614-104319895
chr11	104440287	104443628	chr11-104440287-104443628
chr11	104467776	104470129	chr11-104467776-104470129
chr11	104549034	104551300	chr11-104549034-104551300

chr11	104571408	104571609	chr11-104571408-104571609
chr11	104607366	104609017	chr11-104607366-104609017
chr11	104624864	104625663	chr11-104624864-104625663
chr11	105028825	105029511	chr11-105028825-105029511
chr11	105125839	105128086	chr11-105125839-105128086
chr11	105179694	105183521	chr11-105179694-105183521
chr11	105292114	105293365	chr11-105292114-105293365
chr11	105347652	105347963	chr11-105347652-105347963
chr11	105362850	105363548	chr11-105362850-105363548
chr11	105402379	105402994	chr11-105402379-105402994
chr11	105456521	105456845	chr11-105456521-105456845
chr11	105588921	105590854	chr11-105588921-105590854
chr11	105901332	105902182	chr11-105901332-105902182
chr11	105942467	105948802	chr11-105942467-105948802
chr11	105950187	105951122	chr11-105950187-105951122
chr11	105952449	105953862	chr11-105952449-105953862
chr11	105967348	105968755	chr11-105967348-105968755
chr11	106007905	106008361	chr11-106007905-106008361
chr11	106019903	106020894	chr11-106019903-106020894
chr11	106036100	106038538	chr11-106036100-106038538
chr11	106065563	106067657	chr11-106065563-106067657
chr11	106083392	106086271	chr11-106083392-106086271
chr11	106158398	106161195	chr11-106158398-106161195
chr11	106192464	106194383	chr11-106192464-106194383
chr11	106214798	106218713	chr11-106214798-106218713
chr11	106254272	106257770	chr11-106254272-106257770
chr11	106268550	106268751	chr11-106268550-106268751
chr11	106271124	106274203	chr11-106271124-106274203
chr11	106276239	106276948	chr11-106276239-106276948
chr11	106485681	106488614	chr11-106485681-106488614
chr11	106500164	106502245	chr11-106500164-106502245
chr11	106572773	106574142	chr11-106572773-106574142
chr11	106588470	106589376	chr11-106588470-106589376
chr11	106611429	106614348	chr11-106611429-106614348
chr11	106648504	106648705	chr11-106648504-106648705
chr11	106665474	106665675	chr11-106665474-106665675
chr11	106674678	106674931	chr11-106674678-106674931
chr11	106722770	106723182	chr11-106722770-106723182
chr11	106777407	106780326	chr11-106777407-106780326
chr11	106785336	106790387	chr11-106785336-106790387
chr11	106918182	106921469	chr11-106918182-106921469
chr11	106954810	106955011	chr11-106954810-106955011

chr11	106957117	106958113	chr11-106957117-106958113
chr11	106995576	106995882	chr11-106995576-106995882
chr11	106996721	107000114	chr11-106996721-107000114
chr11	107011411	107012936	chr11-107011411-107012936
chr11	107014698	107015155	chr11-107014698-107015155
chr11	107025321	107028953	chr11-107025321-107028953
chr11	107102916	107104148	chr11-107102916-107104148
chr11	107124168	107125303	chr11-107124168-107125303
chr11	107129015	107133087	chr11-107129015-107133087
chr11	107157086	107158182	chr11-107157086-107158182
chr11	107188026	107189808	chr11-107188026-107189808
chr11	107297012	107297213	chr11-107297012-107297213
chr11	107341209	107341961	chr11-107341209-107341961
chr11	107347478	107348579	chr11-107347478-107348579
chr11	107413191	107413697	chr11-107413191-107413697
chr11	107416856	107418058	chr11-107416856-107418058
chr11	107427852	107431974	chr11-107427852-107431974
chr11	107449287	107449538	chr11-107449287-107449538
chr11	107451492	107451743	chr11-107451492-107451743
chr11	107457991	107459073	chr11-107457991-107459073
chr11	107467053	107471539	chr11-107467053-107471539
chr11	107478795	107481420	chr11-107478795-107481420
chr11	107545780	107548953	chr11-107545780-107548953
chr11	107793155	107795075	chr11-107793155-107795075
chr11	107914700	107915255	chr11-107914700-107915255
chr11	108101322	108101988	chr11-108101322-108101988
chr11	108214473	108215973	chr11-108214473-108215973
chr11	108216845	108217531	chr11-108216845-108217531
chr11	108267543	108268668	chr11-108267543-108268668
chr11	108282910	108283392	chr11-108282910-108283392
chr11	108306855	108308224	chr11-108306855-108308224
chr11	108341655	108345189	chr11-108341655-108345189
chr11	108364508	108366048	chr11-108364508-108366048
chr11	108424335	108426116	chr11-108424335-108426116
chr11	108919922	108922792	chr11-108919922-108922792
chr11	108980688	108980998	chr11-108980688-108980998
chr11	109297654	109298369	chr11-109297654-109298369
chr11	109312119	109312734	chr11-109312119-109312734
chr11	109331086	109332124	chr11-109331086-109332124
chr11	109344074	109344416	chr11-109344074-109344416
chr11	109361475	109366013	chr11-109361475-109366013
chr11	109368919	109369175	chr11-109368919-109369175

chr11	109382433	109382788	chr11-109382433-109382788
chr11	109425096	109427423	chr11-109425096-109427423
chr11	109439325	109441054	chr11-109439325-109441054
chr11	109471451	109474783	chr11-109471451-109474783
chr11	109608452	109608653	chr11-109608452-109608653
chr11	109609830	109612459	chr11-109609830-109612459
chr11	109649953	109652768	chr11-109649953-109652768
chr11	109721075	109722533	chr11-109721075-109722533
chr11	109860253	109860824	chr11-109860253-109860824
chr11	109995583	109996040	chr11-109995583-109996040
chr11	110167789	110167990	chr11-110167789-110167990
chr11	110306748	110306949	chr11-110306748-110306949
chr11	110336758	110338353	chr11-110336758-110338353
chr11	110357796	110358462	chr11-110357796-110358462
chr11	110379709	110380308	chr11-110379709-110380308
chr11	110398643	110400111	chr11-110398643-110400111
chr11	110405380	110406140	chr11-110405380-110406140
chr11	110414094	110414295	chr11-110414094-110414295
chr11	110439456	110439710	chr11-110439456-110439710
chr11	110448670	110449142	chr11-110448670-110449142
chr11	110450225	110450653	chr11-110450225-110450653
chr11	110478778	110479551	chr11-110478778-110479551
chr11	111065165	111066327	chr11-111065165-111066327
chr11	112155368	112155741	chr11-112155368-112155741
chr11	112780886	112785514	chr11-112780886-112785514
chr11	112996158	112997944	chr11-112996158-112997944
chr11	112999146	112999972	chr11-112999146-112999972
chr11	113000901	113001673	chr11-113000901-113001673
chr11	113008612	113008919	chr11-113008612-113008919
chr11	113015942	113016208	chr11-113015942-113016208
chr11	113032128	113033522	chr11-113032128-113033522
chr11	113059978	113061674	chr11-113059978-113061674
chr11	113062612	113063641	chr11-113062612-113063641
chr11	113074586	113075099	chr11-113074586-113075099
chr11	113087363	113087564	chr11-113087363-113087564
chr11	113100334	113105509	chr11-113100334-113105509
chr11	113113273	113113706	chr11-113113273-113113706
chr11	113132559	113132760	chr11-113132559-113132760
chr11	113146874	113147771	chr11-113146874-113147771
chr11	113172732	113173044	chr11-113172732-113173044
chr11	113179877	113181044	chr11-113179877-113181044
chr11	113192026	113192822	chr11-113192026-113192822

chr11	113197625	113202101	chr11-113197625-113202101
chr11	113207948	113209750	chr11-113207948-113209750
chr11	113265316	113266212	chr11-113265316-113266212
chr11	113292209	113292410	chr11-113292209-113292410
chr11	113470072	113470489	chr11-113470072-113470489
chr11	113487619	113488161	chr11-113487619-113488161
chr11	113536276	113537816	chr11-113536276-113537816
chr11	113564676	113566100	chr11-113564676-113566100
chr11	113618497	113620319	chr11-113618497-113620319
chr11	113648087	113650778	chr11-113648087-113650778
chr11	113682475	113685631	chr11-113682475-113685631
chr11	113714042	113714593	chr11-113714042-113714593
chr11	113729259	113731306	chr11-113729259-113731306
chr11	113732392	113735803	chr11-113732392-113735803
chr11	113739216	113739684	chr11-113739216-113739684
chr11	113748434	113752905	chr11-113748434-113752905
chr11	113766553	113766754	chr11-113766553-113766754
chr11	114066529	114067212	chr11-114066529-114067212
chr11	114446656	114447011	chr11-114446656-114447011
chr11	114668032	114671466	chr11-114668032-114671466
chr11	114675097	114676728	chr11-114675097-114676728
chr11	114687406	114687860	chr11-114687406-114687860
chr11	114726980	114727862	chr11-114726980-114727862
chr11	114745046	114745495	chr11-114745046-114745495
chr11	114764782	114765840	chr11-114764782-114765840
chr11	114786227	114786751	chr11-114786227-114786751
chr11	114789192	114789551	chr11-114789192-114789551
chr11	114791509	114792421	chr11-114791509-114792421
chr11	114795351	114795705	chr11-114795351-114795705
chr11	114802676	114803038	chr11-114802676-114803038
chr11	114807396	114808039	chr11-114807396-114808039
chr11	114829403	114830636	chr11-114829403-114830636
chr11	114850175	114853401	chr11-114850175-114853401
chr11	114933455	114934417	chr11-114933455-114934417
chr11	115071358	115072659	chr11-115071358-115072659
chr11	115151795	115152097	chr11-115151795-115152097
chr11	115153456	115154549	chr11-115153456-115154549
chr11	115159744	115160750	chr11-115159744-115160750
chr11	115162088	115166371	chr11-115162088-115166371
chr11	115172013	115174481	chr11-115172013-115174481
chr11	115184999	115185200	chr11-115184999-115185200
chr11	115186368	115188418	chr11-115186368-115188418

chr11	115218890	115219935	chr11-115218890-115219935
chr11	115249710	115250812	chr11-115249710-115250812
chr11	115257868	115258436	chr11-115257868-115258436
chr11	115265173	115267530	chr11-115265173-115267530
chr11	115275950	115278460	chr11-115275950-115278460
chr11	115279897	115280424	chr11-115279897-115280424
chr11	115296546	115298072	chr11-115296546-115298072
chr11	115318258	115319037	chr11-115318258-115319037
chr11	115321707	115322535	chr11-115321707-115322535
chr11	115326897	115327098	chr11-115326897-115327098
chr11	115334424	115335040	chr11-115334424-115335040
chr11	115358662	115358863	chr11-115358662-115358863
chr11	115364020	115364301	chr11-115364020-115364301
chr11	115366317	115368066	chr11-115366317-115368066
chr11	115373630	115374891	chr11-115373630-115374891
chr11	115376579	115376780	chr11-115376579-115376780
chr11	115381239	115383542	chr11-115381239-115383542
chr11	115403187	115404807	chr11-115403187-115404807
chr11	115411447	115414511	chr11-115411447-115414511
chr11	115418655	115421357	chr11-115418655-115421357
chr11	115461977	115463809	chr11-115461977-115463809
chr11	115474952	115476905	chr11-115474952-115476905
chr11	115490435	115492877	chr11-115490435-115492877
chr11	115509963	115514908	chr11-115509963-115514908
chr11	115534355	115537067	chr11-115534355-115537067
chr11	115563531	115566085	chr11-115563531-115566085
chr11	115602196	115606229	chr11-115602196-115606229
chr11	115611547	115613841	chr11-115611547-115613841
chr11	115626819	115629204	chr11-115626819-115629204
chr11	115631110	115631720	chr11-115631110-115631720
chr11	115640860	115641683	chr11-115640860-115641683
chr11	115699189	115700051	chr11-115699189-115700051
chr11	115707019	115709834	chr11-115707019-115709834
chr11	115722728	115723192	chr11-115722728-115723192
chr11	115747312	115747768	chr11-115747312-115747768
chr11	115764719	115766936	chr11-115764719-115766936
chr11	115778980	115779550	chr11-115778980-115779550
chr11	115812108	115815991	chr11-115812108-115815991
chr11	115823319	115832746	chr11-115823319-115832746
chr11	115838711	115838912	chr11-115838711-115838912
chr11	115859003	115860221	chr11-115859003-115860221
chr11	115868839	115869605	chr11-115868839-115869605

chr11	115872674	115872927	chr11-115872674-115872927
chr11	115904255	115905726	chr11-115904255-115905726
chr11	115911364	115913025	chr11-115911364-115913025
chr11	115932229	115934977	chr11-115932229-115934977
chr11	115954398	115958715	chr11-115954398-115958715
chr11	115964332	115965489	chr11-115964332-115965489
chr11	115969872	115971829	chr11-115969872-115971829
chr11	115973060	115977158	chr11-115973060-115977158
chr11	116011414	116013226	chr11-116011414-116013226
chr11	116021275	116024668	chr11-116021275-116024668
chr11	116029499	116031898	chr11-116029499-116031898
chr11	116071314	116073766	chr11-116071314-116073766
chr11	116075742	116080440	chr11-116075742-116080440
chr11	116085523	116088151	chr11-116085523-116088151
chr11	116103895	116104649	chr11-116103895-116104649
chr11	116107974	116110721	chr11-116107974-116110721
chr11	116129624	116132850	chr11-116129624-116132850
chr11	116137564	116139488	chr11-116137564-116139488
chr11	116142235	116143326	chr11-116142235-116143326
chr11	116145187	116147013	chr11-116145187-116147013
chr11	116166231	116168792	chr11-116166231-116168792
chr11	116195542	116195743	chr11-116195542-116195743
chr11	116197466	116200164	chr11-116197466-116200164
chr11	116215646	116220055	chr11-116215646-116220055
chr11	116236826	116238800	chr11-116236826-116238800
chr11	116254225	116256510	chr11-116254225-116256510
chr11	116272539	116275064	chr11-116272539-116275064
chr11	116281011	116283207	chr11-116281011-116283207
chr11	116305788	116307489	chr11-116305788-116307489
chr11	116318970	116319931	chr11-116318970-116319931
chr11	116332069	116336537	chr11-116332069-116336537
chr11	116387272	116387749	chr11-116387272-116387749
chr11	116388983	116391184	chr11-116388983-116391184
chr11	116411631	116413742	chr11-116411631-116413742
chr11	116431146	116437469	chr11-116431146-116437469
chr11	116445790	116446815	chr11-116445790-116446815
chr11	116488625	116491318	chr11-116488625-116491318
chr11	116501101	116501514	chr11-116501101-116501514
chr11	116518447	116518648	chr11-116518447-116518648
chr11	116521974	116524658	chr11-116521974-116524658
chr11	116530467	116534956	chr11-116530467-116534956
chr11	116579989	116581888	chr11-116579989-116581888

chr11	116586914	116587318	chr11-116586914-116587318
chr11	116614766	116624765	chr11-116614766-116624765
chr11	116643491	116643742	chr11-116643491-116643742
chr11	116645920	116646121	chr11-116645920-116646121
chr11	116653459	116654624	chr11-116653459-116654624
chr11	116657411	116658238	chr11-116657411-116658238
chr11	116670961	116674698	chr11-116670961-116674698
chr11	116682283	116682980	chr11-116682283-116682980
chr11	116693944	116695110	chr11-116693944-116695110
chr11	116782772	116783212	chr11-116782772-116783212
chr11	116811330	116811870	chr11-116811330-116811870
chr11	116817068	116817424	chr11-116817068-116817424
chr11	116827395	116828602	chr11-116827395-116828602
chr11	116840418	116845235	chr11-116840418-116845235
chr11	116849913	116855095	chr11-116849913-116855095
chr11	116869507	116869816	chr11-116869507-116869816
chr11	116885447	116886165	chr11-116885447-116886165
chr11	116905448	116906716	chr11-116905448-116906716
chr11	116918465	116919577	chr11-116918465-116919577
chr11	116921973	116922225	chr11-116921973-116922225
chr11	116940114	116940424	chr11-116940114-116940424
chr11	117038120	117038811	chr11-117038120-117038811
chr11	117072310	117072614	chr11-117072310-117072614
chr11	117076097	117083003	chr11-117076097-117083003
chr11	117091733	117092358	chr11-117091733-117092358
chr11	117113908	117118184	chr11-117113908-117118184
chr11	117176162	117176566	chr11-117176162-117176566
chr11	117194644	117194845	chr11-117194644-117194845
chr11	117198175	117203421	chr11-117198175-117203421
chr11	117204286	117206728	chr11-117204286-117206728
chr11	117207958	117208797	chr11-117207958-117208797
chr11	117216755	117216956	chr11-117216755-117216956
chr11	117231381	117232473	chr11-117231381-117232473
chr11	117235391	117235592	chr11-117235391-117235592
chr11	117254230	117256836	chr11-117254230-117256836
chr11	117259315	117259977	chr11-117259315-117259977
chr11	117263169	117270532	chr11-117263169-117270532
chr11	117275890	117276241	chr11-117275890-117276241
chr11	117305705	117306007	chr11-117305705-117306007
chr11	117307768	117315834	chr11-117307768-117315834
chr11	117384658	117385370	chr11-117384658-117385370
chr11	117390004	117390427	chr11-117390004-117390427

chr11	117394389	117394905	chr11-117394389-117394905
chr11	117407813	117408124	chr11-117407813-117408124
chr11	117414714	117415427	chr11-117414714-117415427
chr11	117475825	117476132	chr11-117475825-117476132
chr11	117482401	117483840	chr11-117482401-117483840
chr11	117535046	117535247	chr11-117535046-117535247
chr11	117536773	117537379	chr11-117536773-117537379
chr11	117568275	117569147	chr11-117568275-117569147
chr11	117598658	117599950	chr11-117598658-117599950
chr11	117605962	117606431	chr11-117605962-117606431
chr11	117611029	117611686	chr11-117611029-117611686
chr11	117653530	117656677	chr11-117653530-117656677
chr11	117685382	117686680	chr11-117685382-117686680
chr11	117778541	117782365	chr11-117778541-117782365
chr11	117788360	117793034	chr11-117788360-117793034
chr11	117809109	117811855	chr11-117809109-117811855
chr11	117817196	117817397	chr11-117817196-117817397
chr11	117820282	117820708	chr11-117820282-117820708
chr11	117821528	117833087	chr11-117821528-117833087
chr11	117838682	117838883	chr11-117838682-117838883
chr11	117843111	117844043	chr11-117843111-117844043
chr11	117847331	117851396	chr11-117847331-117851396
chr11	117870528	117870729	chr11-117870528-117870729
chr11	117872637	117873913	chr11-117872637-117873913
chr11	117912532	117913139	chr11-117912532-117913139
chr11	117930811	117931237	chr11-117930811-117931237
chr11	117935935	117936809	chr11-117935935-117936809
chr11	117940653	117942349	chr11-117940653-117942349
chr11	117949172	117952673	chr11-117949172-117952673
chr11	117958302	117958765	chr11-117958302-117958765
chr11	117962052	117970370	chr11-117962052-117970370
chr11	117986127	117996294	chr11-117986127-117996294
chr11	117998399	117998875	chr11-117998399-117998875
chr11	118003047	118004196	chr11-118003047-118004196
chr11	118007317	118008068	chr11-118007317-118008068
chr11	118009034	118010229	chr11-118009034-118010229
chr11	118012350	118014610	chr11-118012350-118014610
chr11	118135369	118136355	chr11-118135369-118136355
chr11	118145728	118146689	chr11-118145728-118146689
chr11	118161015	118161489	chr11-118161015-118161489
chr11	118193156	118195132	chr11-118193156-118195132
chr11	118201431	118201682	chr11-118201431-118201682

chr11	118211497	118213270	chr11-118211497-118213270
chr11	118215523	118216828	chr11-118215523-118216828
chr11	118246658	118249570	chr11-118246658-118249570
chr11	118287681	118291139	chr11-118287681-118291139
chr11	118327006	118328407	chr11-118327006-118328407
chr11	118347894	118356162	chr11-118347894-118356162
chr11	118359770	118366841	chr11-118359770-118366841
chr11	118379193	118379812	chr11-118379193-118379812
chr11	118401927	118402348	chr11-118401927-118402348
chr11	118409495	118410308	chr11-118409495-118410308
chr11	118415676	118419819	chr11-118415676-118419819
chr11	118423386	118424222	chr11-118423386-118424222
chr11	118430006	118431820	chr11-118430006-118431820
chr11	118476444	118477768	chr11-118476444-118477768
chr11	118539946	118541205	chr11-118539946-118541205
chr11	118609498	118610610	chr11-118609498-118610610
chr11	118744431	118744733	chr11-118744431-118744733
chr11	118908956	118909560	chr11-118908956-118909560
chr11	118975664	118976282	chr11-118975664-118976282
chr11	118998750	118999819	chr11-118998750-118999819
chr11	119021601	119025199	chr11-119021601-119025199
chr11	119034058	119034828	chr11-119034058-119034828
chr11	119037156	119037357	chr11-119037156-119037357
chr11	119039752	119043235	chr11-119039752-119043235
chr11	119046493	119048398	chr11-119046493-119048398
chr11	119052114	119059201	chr11-119052114-119059201
chr11	119082942	119087857	chr11-119082942-119087857
chr11	119089797	119091845	chr11-119089797-119091845
chr11	119125396	119129076	chr11-119125396-119129076
chr11	119152210	119152411	chr11-119152210-119152411
chr11	119165357	119166446	chr11-119165357-119166446
chr11	119186691	119187728	chr11-119186691-119187728
chr11	119190672	119192641	chr11-119190672-119192641
chr11	119213120	119216606	chr11-119213120-119216606
chr11	119227561	119229626	chr11-119227561-119229626
chr11	119237812	119238796	chr11-119237812-119238796
chr11	119248809	119249165	chr11-119248809-119249165
chr11	119258179	119259379	chr11-119258179-119259379
chr11	119267405	119269194	chr11-119267405-119269194
chr11	119298004	119301240	chr11-119298004-119301240
chr11	119304974	119305503	chr11-119304974-119305503
chr11	119316716	119319046	chr11-119316716-119319046

chr11	119330043	119331526	chr11-119330043-119331526
chr11	119338860	119339061	chr11-119338860-119339061
chr11	119350376	119351306	chr11-119350376-119351306
chr11	119354508	119356819	chr11-119354508-119356819
chr11	119389569	119395890	chr11-119389569-119395890
chr11	119401163	119403846	chr11-119401163-119403846
chr11	119490451	119493079	chr11-119490451-119493079
chr11	119517304	119517505	chr11-119517304-119517505
chr11	119524540	119524997	chr11-119524540-119524997
chr11	119545343	119549782	chr11-119545343-119549782
chr11	119550984	119551726	chr11-119550984-119551726
chr11	119552623	119556208	chr11-119552623-119556208
chr11	119602346	119604622	chr11-119602346-119604622
chr11	119653285	119653486	chr11-119653285-119653486
chr11	119657352	119658383	chr11-119657352-119658383
chr11	119676369	119676854	chr11-119676369-119676854
chr11	119695576	119696538	chr11-119695576-119696538
chr11	119777146	119777399	chr11-119777146-119777399
chr11	119788734	119789730	chr11-119788734-119789730
chr11	119796081	119796992	chr11-119796081-119796992
chr11	119816867	119818272	chr11-119816867-119818272
chr11	119856284	119857225	chr11-119856284-119857225
chr11	119863379	119863856	chr11-119863379-119863856
chr11	119894632	119895374	chr11-119894632-119895374
chr11	119912700	119918307	chr11-119912700-119918307
chr11	119928270	119928629	chr11-119928270-119928629
chr11	119932580	119934596	chr11-119932580-119934596
chr11	119941174	119949502	chr11-119941174-119949502
chr11	119951948	119957016	chr11-119951948-119957016
chr11	119967316	119968581	chr11-119967316-119968581
chr11	119969549	119970546	chr11-119969549-119970546
chr11	119973990	119974299	chr11-119973990-119974299
chr11	119975355	119977232	chr11-119975355-119977232
chr11	119979445	119980142	chr11-119979445-119980142
chr11	119986925	119991586	chr11-119986925-119991586
chr11	119998726	119999389	chr11-119998726-119999389
chr11	120011789	120012334	chr11-120011789-120012334
chr11	120013259	120013816	chr11-120013259-120013816
chr11	120046099	120047495	chr11-120046099-120047495
chr11	120085709	120087300	chr11-120085709-120087300
chr11	120097418	120100223	chr11-120097418-120100223
chr11	120122251	120122572	chr11-120122251-120122572

chr11	120149547	120151936	chr11-120149547-120151936
chr11	120176083	120176284	chr11-120176083-120176284
chr11	120185263	120190790	chr11-120185263-120190790
chr11	120229797	120240667	chr11-120229797-120240667
chr11	120250953	120257619	chr11-120250953-120257619
chr11	120261269	120262567	chr11-120261269-120262567
chr11	120304685	120306228	chr11-120304685-120306228
chr11	120308146	120308347	chr11-120308146-120308347
chr11	120310529	120311813	chr11-120310529-120311813
chr11	120315224	120317770	chr11-120315224-120317770
chr11	120345008	120354625	chr11-120345008-120354625
chr11	120377223	120379392	chr11-120377223-120379392
chr11	120435880	120438199	chr11-120435880-120438199
chr11	120456796	120459605	chr11-120456796-120459605
chr11	120466010	120468844	chr11-120466010-120468844
chr11	120483630	120486321	chr11-120483630-120486321
chr11	120489499	120489700	chr11-120489499-120489700
chr11	120491026	120493784	chr11-120491026-120493784
chr11	120500209	120500801	chr11-120500209-120500801
chr11	120507221	120510157	chr11-120507221-120510157
chr11	120519579	120520201	chr11-120519579-120520201
chr11	120530323	120531244	chr11-120530323-120531244
chr11	120539034	120539974	chr11-120539034-120539974
chr11	120548357	120550918	chr11-120548357-120550918
chr11	120554949	120555567	chr11-120554949-120555567
chr11	120570975	120575030	chr11-120570975-120575030
chr11	120579213	120582849	chr11-120579213-120582849
chr11	120596319	120600149	chr11-120596319-120600149
chr11	120615668	120618445	chr11-120615668-120618445
chr11	120623711	120625838	chr11-120623711-120625838
chr11	120629179	120635068	chr11-120629179-120635068
chr11	120641425	120644179	chr11-120641425-120644179
chr11	120647834	120648423	chr11-120647834-120648423
chr11	120658948	120661452	chr11-120658948-120661452
chr11	120672433	120674621	chr11-120672433-120674621
chr11	120692728	120692979	chr11-120692728-120692979
chr11	120697465	120698652	chr11-120697465-120698652
chr11	120704145	120704747	chr11-120704145-120704747
chr11	120709031	120709303	chr11-120709031-120709303
chr11	120712586	120715118	chr11-120712586-120715118
chr11	120720638	120723011	chr11-120720638-120723011
chr11	120726273	120727815	chr11-120726273-120727815

chr11	120729656	120729907	chr11-120729656-120729907
chr11	120753591	120753896	chr11-120753591-120753896
chr11	120763262	120763677	chr11-120763262-120763677
chr11	120783005	120786646	chr11-120783005-120786646
chr11	120794620	120797032	chr11-120794620-120797032
chr11	120800054	120800788	chr11-120800054-120800788
chr11	120820515	120825696	chr11-120820515-120825696
chr11	120850426	120851412	chr11-120850426-120851412
chr11	120931909	120933503	chr11-120931909-120933503
chr11	120938549	120939054	chr11-120938549-120939054
chr11	120948627	120951774	chr11-120948627-120951774
chr11	120954681	120955518	chr11-120954681-120955518
chr11	120957780	120958354	chr11-120957780-120958354
chr11	120983937	120984455	chr11-120983937-120984455
chr11	120988521	120992349	chr11-120988521-120992349
chr11	121014656	121015397	chr11-121014656-121015397
chr11	121063471	121064493	chr11-121063471-121064493
chr11	121091105	121091566	chr11-121091105-121091566
chr11	121156299	121157101	chr11-121156299-121157101
chr11	121203647	121205680	chr11-121203647-121205680
chr11	121227765	121229898	chr11-121227765-121229898
chr11	121236140	121238295	chr11-121236140-121238295
chr11	121259109	121261898	chr11-121259109-121261898
chr11	121352584	121355477	chr11-121352584-121355477
chr11	121387467	121388995	chr11-121387467-121388995
chr11	121420589	121422406	chr11-121420589-121422406
chr11	121434420	121435451	chr11-121434420-121435451
chr11	121451260	121453152	chr11-121451260-121453152
chr11	121543267	121545298	chr11-121543267-121545298
chr11	121647068	121647752	chr11-121647068-121647752
chr11	121654499	121654956	chr11-121654499-121654956
chr11	121672182	121674024	chr11-121672182-121674024
chr11	121697253	121698611	chr11-121697253-121698611
chr11	121701328	121707637	chr11-121701328-121707637
chr11	121712408	121713367	chr11-121712408-121713367
chr11	121717620	121717943	chr11-121717620-121717943
chr11	121740662	121741676	chr11-121740662-121741676
chr11	121847581	121848432	chr11-121847581-121848432
chr11	121951877	121952336	chr11-121951877-121952336
chr11	121956277	121956835	chr11-121956277-121956835
chr12	3110695	3111102	chr12-3110695-3111102
chr12	3234751	3237392	chr12-3234751-3237392

chr12	3306624	3311348	chr12-3306624-3311348
chr12	3323156	3323697	chr12-3323156-3323697
chr12	3349166	3353002	chr12-3349166-3353002
chr12	3356858	3357059	chr12-3356858-3357059
chr12	3359391	3359710	chr12-3359391-3359710
chr12	3364741	3369430	chr12-3364741-3369430
chr12	3370488	3372311	chr12-3370488-3372311
chr12	3389595	3389796	chr12-3389595-3389796
chr12	3424522	3428879	chr12-3424522-3428879
chr12	3436443	3436754	chr12-3436443-3436754
chr12	3571389	3575190	chr12-3571389-3575190
chr12	3582288	3582489	chr12-3582288-3582489
chr12	3791324	3792570	chr12-3791324-3792570
chr12	3805811	3809020	chr12-3805811-3809020
chr12	3854260	3854619	chr12-3854260-3854619
chr12	3891206	3892064	chr12-3891206-3892064
chr12	3959637	3960823	chr12-3959637-3960823
chr12	3981092	3982465	chr12-3981092-3982465
chr12	4037833	4039512	chr12-4037833-4039512
chr12	4081968	4083431	chr12-4081968-4083431
chr12	4132864	4134735	chr12-4132864-4134735
chr12	4170628	4171871	chr12-4170628-4171871
chr12	4172882	4173863	chr12-4172882-4173863
chr12	4188186	4188565	chr12-4188186-4188565
chr12	4232300	4235244	chr12-4232300-4235244
chr12	4430705	4431249	chr12-4430705-4431249
chr12	4475647	4477811	chr12-4475647-4477811
chr12	4587884	4588135	chr12-4587884-4588135
chr12	4590363	4595269	chr12-4590363-4595269
chr12	4600002	4600448	chr12-4600002-4600448
chr12	4768511	4769672	chr12-4768511-4769672
chr12	4816666	4818813	chr12-4816666-4818813
chr12	4840565	4845002	chr12-4840565-4845002
chr12	4863873	4864176	chr12-4863873-4864176
chr12	4873829	4874761	chr12-4873829-4874761
chr12	4905938	4908468	chr12-4905938-4908468
chr12	4916609	4919824	chr12-4916609-4919824
chr12	5119629	5120037	chr12-5119629-5120037
chr12	5213442	5214105	chr12-5213442-5214105
chr12	5276929	5277231	chr12-5276929-5277231
chr12	5373784	5376309	chr12-5373784-5376309
chr12	7919961	7920220	chr12-7919961-7920220

chr12	7967753	7968082	chr12-7967753-7968082
chr12	8207373	8208978	chr12-8207373-8208978
chr12	8297808	8299203	chr12-8297808-8299203
chr12	8301345	8302546	chr12-8301345-8302546
chr12	8313110	8314868	chr12-8313110-8314868
chr12	8338811	8339012	chr12-8338811-8339012
chr12	8357351	8357685	chr12-8357351-8357685
chr12	8379370	8380488	chr12-8379370-8380488
chr12	8386631	8387372	chr12-8386631-8387372
chr12	8418868	8419527	chr12-8418868-8419527
chr12	8450827	8451188	chr12-8450827-8451188
chr12	8457005	8457541	chr12-8457005-8457541
chr12	8487817	8488178	chr12-8487817-8488178
chr12	8496397	8501077	chr12-8496397-8501077
chr12	8521344	8521845	chr12-8521344-8521845
chr12	8571768	8572616	chr12-8571768-8572616
chr12	8638461	8638834	chr12-8638461-8638834
chr12	8639945	8641801	chr12-8639945-8641801
chr12	8661294	8663052	chr12-8661294-8663052
chr12	8673105	8677658	chr12-8673105-8677658
chr12	8760975	8761574	chr12-8760975-8761574
chr12	8770398	8787978	chr12-8770398-8787978
chr12	8804972	8805794	chr12-8804972-8805794
chr12	8806905	8807619	chr12-8806905-8807619
chr12	8808889	8812187	chr12-8808889-8812187
chr12	8826797	8827467	chr12-8826797-8827467
chr12	8854061	8854993	chr12-8854061-8854993
chr12	8875524	8877829	chr12-8875524-8877829
chr12	8920781	8924163	chr12-8920781-8924163
chr12	8935546	8935960	chr12-8935546-8935960
chr12	8947506	8948570	chr12-8947506-8948570
chr12	8969531	8970158	chr12-8969531-8970158
chr12	8973309	8974987	chr12-8973309-8974987
chr12	9021717	9022129	chr12-9021717-9022129
chr12	9028926	9032998	chr12-9028926-9032998
chr12	9066219	9066838	chr12-9066219-9066838
chr12	9569941	9571157	chr12-9569941-9571157
chr12	9574126	9575104	chr12-9574126-9575104
chr12	9580470	9581490	chr12-9580470-9581490
chr12	9584537	9585677	chr12-9584537-9585677
chr12	9839512	9840469	chr12-9839512-9840469
chr12	9855925	9856295	chr12-9855925-9856295

chr12	9874559	9874815	chr12-9874559-9874815
chr12	9978600	9979092	chr12-9978600-9979092
chr12	10390422	10392183	chr12-10390422-10392183
chr12	11149259	11150789	chr12-11149259-11150789
chr12	11196212	11196710	chr12-11196212-11196710
chr12	11264275	11269010	chr12-11264275-11269010
chr12	11391443	11391644	chr12-11391443-11391644
chr12	11435860	11438283	chr12-11435860-11438283
chr12	11455540	11456557	chr12-11455540-11456557
chr12	12261874	12263071	chr12-12261874-12263071
chr12	12323715	12324219	chr12-12323715-12324219
chr12	12390361	12390966	chr12-12390361-12390966
chr12	12401448	12401855	chr12-12401448-12401855
chr12	12420873	12421443	chr12-12420873-12421443
chr12	12509493	12509694	chr12-12509493-12509694
chr12	12527387	12527588	chr12-12527387-12527588
chr12	12530637	12530838	chr12-12530637-12530838
chr12	12810064	12811512	chr12-12810064-12811512
chr12	12902444	12902645	chr12-12902444-12902645
chr12	12910412	12910714	chr12-12910412-12910714
chr12	12938944	12943035	chr12-12938944-12943035
chr12	13202045	13202885	chr12-13202045-13202885
chr12	13217384	13218400	chr12-13217384-13218400
chr12	13247947	13249962	chr12-13247947-13249962
chr12	13268478	13270262	chr12-13268478-13270262
chr12	14148961	14152951	chr12-14148961-14152951
chr12	14179260	14179461	chr12-14179260-14179461
chr12	14285988	14286294	chr12-14285988-14286294
chr12	14415107	14415793	chr12-14415107-14415793
chr12	15801181	15801585	chr12-15801181-15801585
chr12	15814530	15817655	chr12-15814530-15817655
chr12	16532270	16533358	chr12-16532270-16533358
chr12	16588188	16592436	chr12-16588188-16592436
chr12	16618915	16619281	chr12-16618915-16619281
chr12	16653469	16654113	chr12-16653469-16654113
chr12	16800208	16801326	chr12-16800208-16801326
chr12	16810510	16812344	chr12-16810510-16812344
chr12	16840846	16841148	chr12-16840846-16841148
chr12	16849595	16851823	chr12-16849595-16851823
chr12	16853030	16854549	chr12-16853030-16854549
chr12	16893339	16898505	chr12-16893339-16898505
chr12	16899512	16899872	chr12-16899512-16899872

chr12	16999024	17001429	chr12-16999024-17001429
chr12	17011385	17011844	chr12-17011385-17011844
chr12	17134443	17134804	chr12-17134443-17134804
chr12	17136212	17136413	chr12-17136212-17136413
chr12	17175414	17176921	chr12-17175414-17176921
chr12	17183864	17185060	chr12-17183864-17185060
chr12	17220880	17221343	chr12-17220880-17221343
chr12	17265795	17268065	chr12-17265795-17268065
chr12	17288413	17289340	chr12-17288413-17289340
chr12	17324194	17325357	chr12-17324194-17325357
chr12	17347410	17349843	chr12-17347410-17349843
chr12	17373950	17374403	chr12-17373950-17374403
chr12	17419960	17420217	chr12-17419960-17420217
chr12	17509851	17510930	chr12-17509851-17510930
chr12	17542667	17547856	chr12-17542667-17547856
chr12	17689315	17695446	chr12-17689315-17695446
chr12	17709861	17712468	chr12-17709861-17712468
chr12	17722566	17723368	chr12-17722566-17723368
chr12	17726602	17730088	chr12-17726602-17730088
chr12	17731853	17734099	chr12-17731853-17734099
chr12	17737138	17740974	chr12-17737138-17740974
chr12	17749575	17750755	chr12-17749575-17750755
chr12	17754174	17754375	chr12-17754174-17754375
chr12	17756419	17757563	chr12-17756419-17757563
chr12	17772967	17774395	chr12-17772967-17774395
chr12	17873626	17873880	chr12-17873626-17873880
chr12	17881077	17883652	chr12-17881077-17883652
chr12	17887102	17888304	chr12-17887102-17888304
chr12	17992295	17994288	chr12-17992295-17994288
chr12	17997943	17998837	chr12-17997943-17998837
chr12	18135784	18136415	chr12-18135784-18136415
chr12	18387367	18387762	chr12-18387367-18387762
chr12	18514015	18515691	chr12-18514015-18515691
chr12	18609595	18611526	chr12-18609595-18611526
chr12	18853472	18853903	chr12-18853472-18853903
chr12	19175814	19176245	chr12-19175814-19176245
chr12	19288130	19289752	chr12-19288130-19289752
chr12	19387527	19388566	chr12-19387527-19388566
chr12	19606506	19606972	chr12-19606506-19606972
chr12	19704149	19704403	chr12-19704149-19704403
chr12	19882281	19882706	chr12-19882281-19882706
chr12	19954080	19954744	chr12-19954080-19954744

chr12	20071802	20072055	chr12-20071802-20072055
chr12	20196237	20197707	chr12-20196237-20197707
chr12	20312094	20312697	chr12-20312094-20312697
chr12	20415582	20416095	chr12-20415582-20416095
chr12	20614483	20616201	chr12-20614483-20616201
chr12	20814463	20816377	chr12-20814463-20816377
chr12	20845169	20845525	chr12-20845169-20845525
chr12	20918991	20922001	chr12-20918991-20922001
chr12	20989795	20991831	chr12-20989795-20991831
chr12	21111033	21115342	chr12-21111033-21115342
chr12	21116457	21117895	chr12-21116457-21117895
chr12	21131433	21133469	chr12-21131433-21133469
chr12	21135191	21137101	chr12-21135191-21137101
chr12	21144385	21145581	chr12-21144385-21145581
chr12	21146722	21146923	chr12-21146722-21146923
chr12	21160119	21160678	chr12-21160119-21160678
chr12	21166037	21166238	chr12-21166037-21166238
chr12	21167221	21167472	chr12-21167221-21167472
chr12	21226300	21227841	chr12-21226300-21227841
chr12	21285071	21287785	chr12-21285071-21287785
chr12	21315932	21317702	chr12-21315932-21317702
chr12	21371965	21374919	chr12-21371965-21374919
chr12	21414762	21419216	chr12-21414762-21419216
chr12	21424654	21425342	chr12-21424654-21425342
chr12	21426214	21426634	chr12-21426214-21426634
chr12	21542955	21543862	chr12-21542955-21543862
chr12	21777599	21779268	chr12-21777599-21779268
chr12	21783200	21784983	chr12-21783200-21784983
chr12	21898658	21899464	chr12-21898658-21899464
chr12	22030537	22031025	chr12-22030537-22031025
chr12	22156459	22156767	chr12-22156459-22156767
chr12	22622772	22623544	chr12-22622772-22623544
chr12	22745109	22745943	chr12-22745109-22745943
chr12	22905949	22906649	chr12-22905949-22906649
chr12	23009712	23010490	chr12-23009712-23010490
chr12	23189036	23189947	chr12-23189036-23189947
chr12	23315108	23315524	chr12-23315108-23315524
chr12	23634523	23634724	chr12-23634523-23634724
chr12	23800425	23801091	chr12-23800425-23801091
chr12	23968587	23969327	chr12-23968587-23969327
chr12	24096331	24097588	chr12-24096331-24097588
chr12	24251253	24252870	chr12-24251253-24252870

chr12	24463641	24463972	chr12-24463641-24463972
chr12	24498117	24499491	chr12-24498117-24499491
chr12	24571569	24573440	chr12-24571569-24573440
chr12	24592764	24593949	chr12-24592764-24593949
chr12	24650485	24652832	chr12-24650485-24652832
chr12	24680384	24681444	chr12-24680384-24681444
chr12	24706590	24710923	chr12-24706590-24710923
chr12	24724036	24724821	chr12-24724036-24724821
chr12	24734653	24735371	chr12-24734653-24735371
chr12	24830524	24832884	chr12-24830524-24832884
chr12	24844018	24844219	chr12-24844018-24844219
chr12	24894108	24894534	chr12-24894108-24894534
chr12	24895600	24895955	chr12-24895600-24895955
chr12	24974347	24976046	chr12-24974347-24976046
chr12	25011840	25013033	chr12-25011840-25013033
chr12	25092075	25101699	chr12-25092075-25101699
chr12	25132037	25132525	chr12-25132037-25132525
chr12	25154968	25156097	chr12-25154968-25156097
chr12	25181088	25182185	chr12-25181088-25182185
chr12	25208816	25209794	chr12-25208816-25209794
chr12	25216908	25217235	chr12-25216908-25217235
chr12	25245293	25245703	chr12-25245293-25245703
chr12	25311659	25312107	chr12-25311659-25312107
chr12	25438622	25439024	chr12-25438622-25439024
chr12	26414480	26415541	chr12-26414480-26415541
chr12	26456078	26456779	chr12-26456078-26456779
chr12	26468933	26470401	chr12-26468933-26470401
chr12	27338608	27343848	chr12-27338608-27343848
chr12	27357227	27357534	chr12-27357227-27357534
chr12	27390472	27390792	chr12-27390472-27390792
chr12	27491633	27492815	chr12-27491633-27492815
chr12	27825951	27826404	chr12-27825951-27826404
chr12	28432120	28432321	chr12-28432120-28432321
chr12	28533753	28534208	chr12-28533753-28534208
chr12	28574032	28574233	chr12-28574032-28574233
chr12	28589390	28590094	chr12-28589390-28590094
chr12	28632300	28637035	chr12-28632300-28637035
chr12	28649067	28651056	chr12-28649067-28651056
chr12	28674420	28677029	chr12-28674420-28677029
chr12	28698275	28698798	chr12-28698275-28698798
chr12	28749112	28752737	chr12-28749112-28752737
chr12	29015693	29015894	chr12-29015693-29015894

chr12	29022902	29023103	chr12-29022902-29023103
chr12	29290341	29291747	chr12-29290341-29291747
chr12	29527316	29527517	chr12-29527316-29527517
chr12	29749655	29749906	chr12-29749655-29749906
chr12	29907351	29907864	chr12-29907351-29907864
chr12	29933183	29934573	chr12-29933183-29934573
chr12	29937450	29940357	chr12-29937450-29940357
chr12	29961205	29961685	chr12-29961205-29961685
chr12	29999248	29999449	chr12-29999248-29999449
chr12	30003459	30004275	chr12-30003459-30004275
chr12	30006480	30006783	chr12-30006480-30006783
chr12	30372917	30373583	chr12-30372917-30373583
chr12	30583696	30585771	chr12-30583696-30585771
chr12	30883867	30885080	chr12-30883867-30885080
chr12	30909956	30913265	chr12-30909956-30913265
chr12	31057243	31058476	chr12-31057243-31058476
chr12	31059340	31060700	chr12-31059340-31060700
chr12	31062363	31062614	chr12-31062363-31062614
chr12	31073328	31077637	chr12-31073328-31077637
chr12	31264245	31273415	chr12-31264245-31273415
chr12	31350112	31352218	chr12-31350112-31352218
chr12	31444106	31444626	chr12-31444106-31444626
chr12	31497876	31500704	chr12-31497876-31500704
chr12	31558655	31560117	chr12-31558655-31560117
chr12	31567497	31568155	chr12-31567497-31568155
chr12	31633344	31635501	chr12-31633344-31635501
chr12	31653497	31655943	chr12-31653497-31655943
chr12	31848905	31849786	chr12-31848905-31849786
chr12	31948522	31951406	chr12-31948522-31951406
chr12	32017813	32018014	chr12-32017813-32018014
chr12	32059729	32061884	chr12-32059729-32061884
chr12	32208147	32208615	chr12-32208147-32208615
chr12	32291959	32292326	chr12-32291959-32292326
chr12	32378372	32382405	chr12-32378372-32382405
chr12	32819245	32822033	chr12-32819245-32822033
chr12	32953084	32956743	chr12-32953084-32956743
chr12	32989569	32990604	chr12-32989569-32990604
chr12	32994652	32995440	chr12-32994652-32995440
chr12	33008346	33008979	chr12-33008346-33008979
chr12	33019808	33020880	chr12-33019808-33020880
chr12	33057200	33057554	chr12-33057200-33057554
chr12	33100626	33100939	chr12-33100626-33100939

chr12	33102408	33102961	chr12-33102408-33102961
chr12	33144509	33150592	chr12-33144509-33150592
chr12	33157583	33160858	chr12-33157583-33160858
chr12	33169799	33177550	chr12-33169799-33177550
chr12	33180847	33182842	chr12-33180847-33182842
chr12	33193512	33193875	chr12-33193512-33193875
chr12	33220526	33220888	chr12-33220526-33220888
chr12	33285285	33285947	chr12-33285285-33285947
chr12	33301748	33303489	chr12-33301748-33303489
chr12	33379647	33380383	chr12-33379647-33380383
chr12	33429420	33431008	chr12-33429420-33431008
chr12	33928734	33929243	chr12-33928734-33929243
chr12	33955977	33959551	chr12-33955977-33959551
chr12	33966769	33968909	chr12-33966769-33968909
chr12	34040041	34040603	chr12-34040041-34040603
chr12	34075606	34076325	chr12-34075606-34076325
chr12	34087522	34087826	chr12-34087522-34087826
chr12	34225196	34226300	chr12-34225196-34226300
chr12	34714156	34714357	chr12-34714156-34714357
chr12	34906429	34907729	chr12-34906429-34907729
chr12	35045943	35049112	chr12-35045943-35049112
chr12	35246521	35246942	chr12-35246521-35246942
chr12	35292181	35292382	chr12-35292181-35292382
chr12	35532275	35535592	chr12-35532275-35535592
chr12	35672398	35672761	chr12-35672398-35672761
chr12	35712239	35713188	chr12-35712239-35713188
chr12	35751409	35751610	chr12-35751409-35751610
chr12	36041657	36043001	chr12-36041657-36043001
chr12	36154908	36158841	chr12-36154908-36158841
chr12	36316932	36317648	chr12-36316932-36317648
chr12	36381152	36382433	chr12-36381152-36382433
chr12	37108157	37109769	chr12-37108157-37109769
chr12	37880564	37881005	chr12-37880564-37881005
chr12	38778034	38781271	chr12-38778034-38781271
chr12	38788591	38789296	chr12-38788591-38789296
chr12	38928993	38929408	chr12-38928993-38929408
chr12	39761708	39762179	chr12-39761708-39762179
chr12	39912452	39912653	chr12-39912452-39912653
chr12	40035830	40038714	chr12-40035830-40038714
chr12	40132698	40134724	chr12-40132698-40134724
chr12	40220029	40224043	chr12-40220029-40224043
chr12	40239436	40239637	chr12-40239436-40239637

chr12	40243568	40244279	chr12-40243568-40244279
chr12	40247745	40248462	chr12-40247745-40248462
chr12	40253208	40253409	chr12-40253208-40253409
chr12	40444716	40449971	chr12-40444716-40449971
chr12	40459444	40459950	chr12-40459444-40459950
chr12	40471476	40473694	chr12-40471476-40473694
chr12	40490958	40491212	chr12-40490958-40491212
chr12	40499375	40499735	chr12-40499375-40499735
chr12	40558964	40559387	chr12-40558964-40559387
chr12	40569286	40569641	chr12-40569286-40569641
chr12	40888312	40889286	chr12-40888312-40889286
chr12	41023616	41025099	chr12-41023616-41025099
chr12	41485707	41486174	chr12-41485707-41486174
chr12	41949639	41950346	chr12-41949639-41950346
chr12	44208562	44210954	chr12-44208562-44210954
chr12	44221572	44222346	chr12-44221572-44222346
chr12	44268337	44270093	chr12-44268337-44270093
chr12	44328517	44330379	chr12-44328517-44330379
chr12	44640047	44640248	chr12-44640047-44640248
chr12	45059589	45061743	chr12-45059589-45061743
chr12	45072296	45075833	chr12-45072296-45075833
chr12	46399240	46399772	chr12-46399240-46399772
chr12	46818151	46820121	chr12-46818151-46820121
chr12	49374253	49374962	chr12-49374253-49374962
chr12	49381518	49395416	chr12-49381518-49395416
chr12	49399838	49402228	chr12-49399838-49402228
chr12	49512717	49513074	chr12-49512717-49513074
chr12	50545008	50545265	chr12-50545008-50545265
chr12	50647768	50649805	chr12-50647768-50649805
chr12	50943815	50944137	chr12-50943815-50944137
chr12	51346172	51351026	chr12-51346172-51351026
chr12	51376925	51378308	chr12-51376925-51378308
chr12	51592824	51594596	chr12-51592824-51594596
chr12	51688881	51695560	chr12-51688881-51695560
chr12	51699830	51700389	chr12-51699830-51700389
chr12	51722416	51722617	chr12-51722416-51722617
chr12	51733524	51735203	chr12-51733524-51735203
chr12	51812157	51812358	chr12-51812157-51812358
chr12	51827147	51831243	chr12-51827147-51831243
chr12	51843894	51845577	chr12-51843894-51845577
chr12	51847813	51848064	chr12-51847813-51848064
chr12	51864715	51866807	chr12-51864715-51866807

chr12	51887301	51888921	chr12-51887301-51888921
chr12	51947751	51948059	chr12-51947751-51948059
chr12	51969571	51972031	chr12-51969571-51972031
chr12	52005548	52007018	chr12-52005548-52007018
chr12	52097347	52098623	chr12-52097347-52098623
chr12	52337118	52337557	chr12-52337118-52337557
chr12	52376869	52377461	chr12-52376869-52377461
chr12	52475955	52476258	chr12-52475955-52476258
chr12	52478636	52478887	chr12-52478636-52478887
chr12	52502643	52505825	chr12-52502643-52505825
chr12	52602292	52605849	chr12-52602292-52605849
chr12	52636261	52636836	chr12-52636261-52636836
chr12	52643979	52644283	chr12-52643979-52644283
chr12	52702025	52703032	chr12-52702025-52703032
chr12	52750106	52750425	chr12-52750106-52750425
chr12	52782793	52783571	chr12-52782793-52783571
chr12	52818658	52818859	chr12-52818658-52818859
chr12	52835203	52836566	chr12-52835203-52836566
chr12	52951000	52951263	chr12-52951000-52951263
chr12	53027438	53028127	chr12-53027438-53028127
chr12	53246547	53249094	chr12-53246547-53249094
chr12	53251774	53253838	chr12-53251774-53253838
chr12	53888330	53888688	chr12-53888330-53888688
chr12	54067747	54068843	chr12-54067747-54068843
chr12	54162467	54163395	chr12-54162467-54163395
chr12	54191775	54192527	chr12-54191775-54192527
chr12	54197740	54198196	chr12-54197740-54198196
chr12	54202641	54204048	chr12-54202641-54204048
chr12	54312879	54313809	chr12-54312879-54313809
chr12	54334440	54334981	chr12-54334440-54334981
chr12	54620238	54620549	chr12-54620238-54620549
chr12	54654574	54657157	chr12-54654574-54657157
chr12	54694781	54696769	chr12-54694781-54696769
chr12	54697922	54698175	chr12-54697922-54698175
chr12	54709883	54710236	chr12-54709883-54710236
chr12	54714078	54714329	chr12-54714078-54714329
chr12	54733295	54733496	chr12-54733295-54733496
chr12	54738722	54739078	chr12-54738722-54739078
chr12	54793782	54796325	chr12-54793782-54796325
chr12	54834817	54835734	chr12-54834817-54835734
chr12	54861269	54863604	chr12-54861269-54863604
chr12	54875052	54875253	chr12-54875052-54875253

chr12	54983863	54987073	chr12-54983863-54987073
chr12	54998506	55000110	chr12-54998506-55000110
chr12	55016796	55016997	chr12-55016796-55016997
chr12	55026880	55027184	chr12-55026880-55027184
chr12	55079141	55082029	chr12-55079141-55082029
chr12	55155389	55155893	chr12-55155389-55155893
chr12	55230486	55230912	chr12-55230486-55230912
chr12	55300873	55304427	chr12-55300873-55304427
chr12	55397598	55400552	chr12-55397598-55400552
chr12	55433835	55434145	chr12-55433835-55434145
chr12	55464282	55467094	chr12-55464282-55467094
chr12	55490185	55493481	chr12-55490185-55493481
chr12	55495354	55495607	chr12-55495354-55495607
chr12	55499002	55499253	chr12-55499002-55499253
chr12	55560188	55560700	chr12-55560188-55560700
chr12	55598718	55600746	chr12-55598718-55600746
chr12	55819839	55821674	chr12-55819839-55821674
chr12	55835773	55837515	chr12-55835773-55837515
chr12	56256220	56256421	chr12-56256220-56256421
chr12	56275308	56275878	chr12-56275308-56275878
chr12	56288180	56288739	chr12-56288180-56288739
chr12	56296615	56297069	chr12-56296615-56297069
chr12	56298118	56300016	chr12-56298118-56300016
chr12	56301751	56302104	chr12-56301751-56302104
chr12	56344541	56346328	chr12-56344541-56346328
chr12	56508555	56509461	chr12-56508555-56509461
chr12	56511307	56514000	chr12-56511307-56514000
chr12	56517993	56525614	chr12-56517993-56525614
chr12	56526687	56537774	chr12-56526687-56537774
chr12	56611665	56613646	chr12-56611665-56613646
chr12	56636746	56637361	chr12-56636746-56637361
chr12	56680902	56681831	chr12-56680902-56681831
chr12	56691311	56692045	chr12-56691311-56692045
chr12	56694751	56698127	chr12-56694751-56698127
chr12	57159780	57160087	chr12-57159780-57160087
chr12	57211588	57212295	chr12-57211588-57212295
chr12	57229919	57232751	chr12-57229919-57232751
chr12	57534443	57548898	chr12-57534443-57548898
chr12	57571901	57572156	chr12-57571901-57572156
chr12	57575960	57576511	chr12-57575960-57576511
chr12	58214583	58214784	chr12-58214583-58214784
chr12	59010844	59014478	chr12-59010844-59014478

chr12	59039803	59040454	chr12-59039803-59040454
chr12	59060218	59061747	chr12-59060218-59061747
chr12	59066037	59068593	chr12-59066037-59068593
chr12	59077674	59077931	chr12-59077674-59077931
chr12	59095238	59097005	chr12-59095238-59097005
chr12	59098222	59098527	chr12-59098222-59098527
chr12	59128965	59133355	chr12-59128965-59133355
chr12	59218062	59220524	chr12-59218062-59220524
chr12	61522726	61523482	chr12-61522726-61523482
chr12	64917392	64918882	chr12-64917392-64918882
chr12	64964366	64971651	chr12-64964366-64971651
chr12	64975922	64976438	chr12-64975922-64976438
chr12	65035847	65039401	chr12-65035847-65039401
chr12	65072601	65077190	chr12-65072601-65077190
chr12	65170622	65173801	chr12-65170622-65173801
chr12	67220958	67222735	chr12-67220958-67222735
chr12	69129873	69130147	chr12-69129873-69130147
chr12	69157302	69160036	chr12-69157302-69160036
chr12	69167731	69169919	chr12-69167731-69169919
chr12	69182829	69187467	chr12-69182829-69187467
chr12	69195321	69199309	chr12-69195321-69199309
chr12	69227013	69228887	chr12-69227013-69228887
chr12	69241103	69242630	chr12-69241103-69242630
chr12	69295961	69298622	chr12-69295961-69298622
chr12	69356299	69357881	chr12-69356299-69357881
chr12	69360880	69364920	chr12-69360880-69364920
chr12	69370881	69376471	chr12-69370881-69376471
chr12	69388216	69388569	chr12-69388216-69388569
chr12	69390379	69390681	chr12-69390379-69390681
chr12	69416947	69417211	chr12-69416947-69417211
chr12	69418709	69419704	chr12-69418709-69419704
chr12	69422670	69423438	chr12-69422670-69423438
chr12	69437256	69437507	chr12-69437256-69437507
chr12	69442430	69442631	chr12-69442430-69442631
chr12	69446354	69448977	chr12-69446354-69448977
chr12	69450027	69451399	chr12-69450027-69451399
chr12	69456857	69457058	chr12-69456857-69457058
chr12	69457997	69458837	chr12-69457997-69458837
chr12	69461795	69462366	chr12-69461795-69462366
chr12	69474726	69475689	chr12-69474726-69475689
chr12	69501423	69503670	chr12-69501423-69503670
chr12	69510320	69510873	chr12-69510320-69510873

chr12	69513134	69513443	chr12-69513134-69513443
chr12	69515338	69515539	chr12-69515338-69515539
chr12	69516376	69516745	chr12-69516376-69516745
chr12	69536727	69539342	chr12-69536727-69539342
chr12	69574045	69574687	chr12-69574045-69574687
chr12	69581555	69583446	chr12-69581555-69583446
chr12	69680340	69683991	chr12-69680340-69683991
chr12	69723391	69726285	chr12-69723391-69726285
chr12	69742107	69742358	chr12-69742107-69742358
chr12	69753747	69754054	chr12-69753747-69754054
chr12	69757605	69758568	chr12-69757605-69758568
chr12	69790187	69791520	chr12-69790187-69791520
chr12	69879065	69880176	chr12-69879065-69880176
chr12	69884501	69885699	chr12-69884501-69885699
chr12	69886952	69893843	chr12-69886952-69893843
chr12	69985170	69987879	chr12-69985170-69987879
chr12	69998774	69999037	chr12-69998774-69999037
chr12	70110079	70112446	chr12-70110079-70112446
chr12	70148914	70149174	chr12-70148914-70149174
chr12	70227147	70228290	chr12-70227147-70228290
chr12	70234960	70235525	chr12-70234960-70235525
chr12	70346418	70348134	chr12-70346418-70348134
chr12	70452493	70454443	chr12-70452493-70454443
chr12	70553772	70554023	chr12-70553772-70554023
chr12	70554931	70555132	chr12-70554931-70555132
chr12	70616591	70617565	chr12-70616591-70617565
chr12	70628445	70629434	chr12-70628445-70629434
chr12	70633035	70634341	chr12-70633035-70634341
chr12	70695224	70695475	chr12-70695224-70695475
chr12	70704779	70707798	chr12-70704779-70707798
chr12	70712064	70713542	chr12-70712064-70713542
chr12	70719083	70720822	chr12-70719083-70720822
chr12	70794785	70795627	chr12-70794785-70795627
chr12	70799276	70799967	chr12-70799276-70799967
chr12	70812755	70813672	chr12-70812755-70813672
chr12	70824009	70828707	chr12-70824009-70828707
chr12	70831153	70831354	chr12-70831153-70831354
chr12	70835364	70835665	chr12-70835364-70835665
chr12	70848640	70849059	chr12-70848640-70849059
chr12	70909862	70910266	chr12-70909862-70910266
chr12	70937013	70938892	chr12-70937013-70938892
chr12	70967541	70970307	chr12-70967541-70970307

chr12	70973884	70976840	chr12-70973884-70976840
chr12	71013790	71017677	chr12-71013790-71017677
chr12	71047973	71048274	chr12-71047973-71048274
chr12	71113957	71114208	chr12-71113957-71114208
chr12	71135475	71138560	chr12-71135475-71138560
chr12	71309255	71310854	chr12-71309255-71310854
chr12	71333387	71333640	chr12-71333387-71333640
chr12	71577009	71577724	chr12-71577009-71577724
chr12	71725297	71726009	chr12-71725297-71726009
chr12	71747901	71748954	chr12-71747901-71748954
chr12	71768680	71768931	chr12-71768680-71768931
chr12	71830151	71832917	chr12-71830151-71832917
chr12	71882561	71883887	chr12-71882561-71883887
chr12	71884940	71890219	chr12-71884940-71890219
chr12	71897995	71898704	chr12-71897995-71898704
chr12	72014053	72014695	chr12-72014053-72014695
chr12	72069749	72071476	chr12-72069749-72071476
chr12	72084349	72087262	chr12-72084349-72087262
chr12	72236220	72237241	chr12-72236220-72237241
chr12	72363467	72363668	chr12-72363467-72363668
chr12	72407830	72409004	chr12-72407830-72409004
chr12	72441861	72442475	chr12-72441861-72442475
chr12	72535673	72537569	chr12-72535673-72537569
chr12	72663564	72665580	chr12-72663564-72665580
chr12	72759891	72763135	chr12-72759891-72763135
chr12	72820908	72821683	chr12-72820908-72821683
chr12	72831702	72832415	chr12-72831702-72832415
chr12	72917306	72917976	chr12-72917306-72917976
chr12	72939885	72940138	chr12-72939885-72940138
chr12	72944830	72946154	chr12-72944830-72946154
chr12	73039492	73039819	chr12-73039492-73039819
chr12	73041144	73041345	chr12-73041144-73041345
chr12	73044156	73047184	chr12-73044156-73047184
chr12	73053279	73054501	chr12-73053279-73054501
chr12	73110736	73113954	chr12-73110736-73113954
chr12	73123457	73124877	chr12-73123457-73124877
chr12	73285689	73287813	chr12-73285689-73287813
chr12	73295374	73295871	chr12-73295374-73295871
chr12	73382985	73383664	chr12-73382985-73383664
chr12	73416120	73416658	chr12-73416120-73416658
chr12	73448333	73449429	chr12-73448333-73449429
chr12	73537697	73538337	chr12-73537697-73538337

chr12	73545237	73546954	chr12-73545237-73546954
chr12	73565101	73565399	chr12-73565101-73565399
chr12	73584050	73586720	chr12-73584050-73586720
chr12	73610848	73611293	chr12-73610848-73611293
chr12	73663625	73663929	chr12-73663625-73663929
chr12	73687607	73687860	chr12-73687607-73687860
chr12	73755900	73756156	chr12-73755900-73756156
chr12	73779322	73780289	chr12-73779322-73780289
chr12	73790385	73791489	chr12-73790385-73791489
chr12	73794111	73794521	chr12-73794111-73794521
chr12	73846809	73847215	chr12-73846809-73847215
chr12	73888355	73888608	chr12-73888355-73888608
chr12	73907059	73910358	chr12-73907059-73910358
chr12	73949491	73950099	chr12-73949491-73950099
chr12	73963893	73966338	chr12-73963893-73966338
chr12	73997378	73998391	chr12-73997378-73998391
chr12	74075922	74076187	chr12-74075922-74076187
chr12	74093124	74095355	chr12-74093124-74095355
chr12	74283858	74284888	chr12-74283858-74284888
chr12	74316386	74317592	chr12-74316386-74317592
chr12	74535864	74536447	chr12-74535864-74536447
chr12	74824664	74825948	chr12-74824664-74825948
chr12	75176603	75177353	chr12-75176603-75177353
chr12	75307796	75309102	chr12-75307796-75309102
chr12	75528865	75529584	chr12-75528865-75529584
chr12	75531732	75532085	chr12-75531732-75532085
chr12	75545791	75546151	chr12-75545791-75546151
chr12	75557792	75558744	chr12-75557792-75558744
chr12	75593951	75597238	chr12-75593951-75597238
chr12	75668440	75671158	chr12-75668440-75671158
chr12	75701993	75703320	chr12-75701993-75703320
chr12	75733213	75736210	chr12-75733213-75736210
chr12	75817329	75820348	chr12-75817329-75820348
chr12	75826733	75828551	chr12-75826733-75828551
chr12	75853007	75854276	chr12-75853007-75854276
chr12	75866649	75867427	chr12-75866649-75867427
chr12	75992517	75992718	chr12-75992517-75992718
chr12	76004911	76005576	chr12-76004911-76005576
chr12	76013323	76016729	chr12-76013323-76016729
chr12	76085414	76085665	chr12-76085414-76085665
chr12	76176827	76177900	chr12-76176827-76177900
chr12	76195853	76197009	chr12-76195853-76197009

chr12	76254596	76257202	chr12-76254596-76257202
chr12	76318112	76319513	chr12-76318112-76319513
chr12	76324123	76325608	chr12-76324123-76325608
chr12	76367634	76373285	chr12-76367634-76373285
chr12	76402973	76406126	chr12-76402973-76406126
chr12	76417234	76418006	chr12-76417234-76418006
chr12	76435118	76435481	chr12-76435118-76435481
chr12	76458864	76459623	chr12-76458864-76459623
chr12	76479972	76480240	chr12-76479972-76480240
chr12	76530619	76538669	chr12-76530619-76538669
chr12	76540559	76542233	chr12-76540559-76542233
chr12	76545029	76552879	chr12-76545029-76552879
chr12	76553815	76554967	chr12-76553815-76554967
chr12	76556170	76558915	chr12-76556170-76558915
chr12	76560051	76560531	chr12-76560051-76560531
chr12	76593542	76594643	chr12-76593542-76594643
chr12	76640809	76641010	chr12-76640809-76641010
chr12	76694944	76695372	chr12-76694944-76695372
chr12	76709337	76711144	chr12-76709337-76711144
chr12	76765125	76766743	chr12-76765125-76766743
chr12	76793761	76795820	chr12-76793761-76795820
chr12	76805223	76805798	chr12-76805223-76805798
chr12	76818203	76818404	chr12-76818203-76818404
chr12	76821827	76823042	chr12-76821827-76823042
chr12	76837003	76838947	chr12-76837003-76838947
chr12	76926949	76927982	chr12-76926949-76927982
chr12	76957776	76958293	chr12-76957776-76958293
chr12	76960785	76963232	chr12-76960785-76963232
chr12	76981610	76982068	chr12-76981610-76982068
chr12	77019468	77020229	chr12-77019468-77020229
chr12	77060140	77060399	chr12-77060140-77060399
chr12	77131201	77131928	chr12-77131201-77131928
chr12	77216540	77217113	chr12-77216540-77217113
chr12	77237530	77240231	chr12-77237530-77240231
chr12	77781289	77781642	chr12-77781289-77781642
chr12	77789542	77789743	chr12-77789542-77789743
chr12	77949410	77950042	chr12-77949410-77950042
chr12	78089453	78089977	chr12-78089453-78089977
chr12	78225093	78228404	chr12-78225093-78228404
chr12	78350893	78351144	chr12-78350893-78351144
chr12	78747859	78750545	chr12-78747859-78750545
chr12	78860037	78863823	chr12-78860037-78863823

chr12	78905216	78908721	chr12-78905216-78908721
chr12	78910265	78912446	chr12-78910265-78912446
chr12	78918417	78918718	chr12-78918417-78918718
chr12	78982151	78982507	chr12-78982151-78982507
chr12	79006239	79007740	chr12-79006239-79007740
chr12	79028500	79030420	chr12-79028500-79030420
chr12	79046935	79047301	chr12-79046935-79047301
chr12	79088785	79090069	chr12-79088785-79090069
chr12	79130269	79131675	chr12-79130269-79131675
chr12	79171228	79173201	chr12-79171228-79173201
chr12	79190148	79192187	chr12-79190148-79192187
chr12	79208852	79209103	chr12-79208852-79209103
chr12	79294996	79298414	chr12-79294996-79298414
chr12	79383533	79383880	chr12-79383533-79383880
chr12	79472199	79472400	chr12-79472199-79472400
chr12	79507335	79507598	chr12-79507335-79507598
chr12	79547612	79548053	chr12-79547612-79548053
chr12	79558843	79561919	chr12-79558843-79561919
chr12	79864208	79865042	chr12-79864208-79865042
chr12	79907420	79907738	chr12-79907420-79907738
chr12	79965496	79965847	chr12-79965496-79965847
chr12	80026213	80026726	chr12-80026213-80026726
chr12	80032095	80033859	chr12-80032095-80033859
chr12	80048856	80049302	chr12-80048856-80049302
chr12	80089441	80093709	chr12-80089441-80093709
chr12	80099091	80099541	chr12-80099091-80099541
chr12	80102779	80104347	chr12-80102779-80104347
chr12	80108789	80117698	chr12-80108789-80117698
chr12	80130378	80133092	chr12-80130378-80133092
chr12	80141307	80141610	chr12-80141307-80141610
chr12	80209754	80212111	chr12-80209754-80212111
chr12	80216347	80216696	chr12-80216347-80216696
chr12	80218131	80221489	chr12-80218131-80221489
chr12	80222515	80226674	chr12-80222515-80226674
chr12	80227779	80236077	chr12-80227779-80236077
chr12	80239809	80240010	chr12-80239809-80240010
chr12	80244522	80246170	chr12-80244522-80246170
chr12	80253997	80261909	chr12-80253997-80261909
chr12	80291511	80292244	chr12-80291511-80292244
chr12	80318908	80319290	chr12-80318908-80319290
chr12	80329792	80330130	chr12-80329792-80330130
chr12	80344342	80344865	chr12-80344342-80344865

chr12	80373975	80374176	chr12-80373975-80374176
chr12	80374996	80375248	chr12-80374996-80375248
chr12	80380335	80380739	chr12-80380335-80380739
chr12	80434323	80437359	chr12-80434323-80437359
chr12	80462305	80464157	chr12-80462305-80464157
chr12	80518274	80519980	chr12-80518274-80519980
chr12	80603474	80603675	chr12-80603474-80603675
chr12	80642259	80646696	chr12-80642259-80646696
chr12	80691993	80693420	chr12-80691993-80693420
chr12	80757326	80760757	chr12-80757326-80760757
chr12	80789779	80792160	chr12-80789779-80792160
chr12	80895067	80895323	chr12-80895067-80895323
chr12	80944604	80948412	chr12-80944604-80948412
chr12	81026471	81027365	chr12-81026471-81027365
chr12	81331935	81333667	chr12-81331935-81333667
chr12	81483500	81485767	chr12-81483500-81485767
chr12	81530678	81533708	chr12-81530678-81533708
chr12	81593599	81595765	chr12-81593599-81595765
chr12	81616720	81617806	chr12-81616720-81617806
chr12	81619164	81619468	chr12-81619164-81619468
chr12	81630184	81633620	chr12-81630184-81633620
chr12	81668610	81668862	chr12-81668610-81668862
chr12	81690637	81691352	chr12-81690637-81691352
chr12	81695307	81699291	chr12-81695307-81699291
chr12	81748226	81750483	chr12-81748226-81750483
chr12	81778292	81783944	chr12-81778292-81783944
chr12	81788898	81789403	chr12-81788898-81789403
chr12	81807038	81807874	chr12-81807038-81807874
chr12	81859454	81862015	chr12-81859454-81862015
chr12	81971019	81972318	chr12-81971019-81972318
chr12	82085863	82086536	chr12-82085863-82086536
chr12	82122412	82123286	chr12-82122412-82123286
chr12	82154541	82155639	chr12-82154541-82155639
chr12	82168456	82171827	chr12-82168456-82171827
chr12	82187558	82188926	chr12-82187558-82188926
chr12	82469918	82470934	chr12-82469918-82470934
chr12	82616550	82617865	chr12-82616550-82617865
chr12	83485723	83489072	chr12-83485723-83489072
chr12	83519940	83521639	chr12-83519940-83521639
chr12	83595964	83597785	chr12-83595964-83597785
chr12	83631315	83634087	chr12-83631315-83634087
chr12	83687585	83689926	chr12-83687585-83689926

chr12	83705222	83705473	chr12-83705222-83705473
chr12	83763323	83764465	chr12-83763323-83764465
chr12	83874842	83875261	chr12-83874842-83875261
chr12	83920522	83923291	chr12-83920522-83923291
chr12	83949863	83952899	chr12-83949863-83952899
chr12	83987223	83988946	chr12-83987223-83988946
chr12	83998904	83999617	chr12-83998904-83999617
chr12	84009297	84010150	chr12-84009297-84010150
chr12	84020484	84020739	chr12-84020484-84020739
chr12	84038204	84039225	chr12-84038204-84039225
chr12	84053351	84054222	chr12-84053351-84054222
chr12	84069102	84070525	chr12-84069102-84070525
chr12	84100637	84101543	chr12-84100637-84101543
chr12	84113822	84115220	chr12-84113822-84115220
chr12	84173809	84181678	chr12-84173809-84181678
chr12	84188020	84195259	chr12-84188020-84195259
chr12	84201507	84201825	chr12-84201507-84201825
chr12	84203213	84204193	chr12-84203213-84204193
chr12	84216729	84219843	chr12-84216729-84219843
chr12	84222301	84222972	chr12-84222301-84222972
chr12	84284409	84286473	chr12-84284409-84286473
chr12	84316051	84318315	chr12-84316051-84318315
chr12	84361098	84363130	chr12-84361098-84363130
chr12	84407755	84409571	chr12-84407755-84409571
chr12	84417017	84418185	chr12-84417017-84418185
chr12	84450051	84452782	chr12-84450051-84452782
chr12	84569684	84572206	chr12-84569684-84572206
chr12	84589794	84590930	chr12-84589794-84590930
chr12	84614215	84615420	chr12-84614215-84615420
chr12	84616332	84618274	chr12-84616332-84618274
chr12	84698366	84699225	chr12-84698366-84699225
chr12	84762657	84763213	chr12-84762657-84763213
chr12	84766486	84766687	chr12-84766486-84766687
chr12	84767813	84774769	chr12-84767813-84774769
chr12	84784734	84784935	chr12-84784734-84784935
chr12	84801633	84802552	chr12-84801633-84802552
chr12	84873885	84877850	chr12-84873885-84877850
chr12	84878931	84883308	chr12-84878931-84883308
chr12	84884530	84886223	chr12-84884530-84886223
chr12	84969152	84971981	chr12-84969152-84971981
chr12	84995435	84999157	chr12-84995435-84999157
chr12	85051047	85051837	chr12-85051047-85051837

chr12	85093791	85094844	chr12-85093791-85094844
chr12	85109863	85112788	chr12-85109863-85112788
chr12	85136189	85136876	chr12-85136189-85136876
chr12	85150843	85151310	chr12-85150843-85151310
chr12	85169332	85169761	chr12-85169332-85169761
chr12	85175159	85177861	chr12-85175159-85177861
chr12	85185928	85186562	chr12-85185928-85186562
chr12	85191933	85193214	chr12-85191933-85193214
chr12	85200804	85202404	chr12-85200804-85202404
chr12	85218689	85220910	chr12-85218689-85220910
chr12	85269806	85271195	chr12-85269806-85271195
chr12	85279424	85280970	chr12-85279424-85280970
chr12	85287853	85289719	chr12-85287853-85289719
chr12	85305349	85305711	chr12-85305349-85305711
chr12	85337933	85340240	chr12-85337933-85340240
chr12	85348141	85348393	chr12-85348141-85348393
chr12	85372742	85375880	chr12-85372742-85375880
chr12	85429645	85430780	chr12-85429645-85430780
chr12	85449980	85450396	chr12-85449980-85450396
chr12	85451566	85453057	chr12-85451566-85453057
chr12	85454712	85456211	chr12-85454712-85456211
chr12	85471640	85476727	chr12-85471640-85476727
chr12	85485041	85488191	chr12-85485041-85488191
chr12	85492083	85492334	chr12-85492083-85492334
chr12	85541033	85541408	chr12-85541033-85541408
chr12	85569411	85570401	chr12-85569411-85570401
chr12	85598300	85602525	chr12-85598300-85602525
chr12	85623424	85624447	chr12-85623424-85624447
chr12	85705300	85706186	chr12-85705300-85706186
chr12	85745812	85747199	chr12-85745812-85747199
chr12	85792947	85793148	chr12-85792947-85793148
chr12	85823131	85826093	chr12-85823131-85826093
chr12	85868850	85869988	chr12-85868850-85869988
chr12	85904099	85904300	chr12-85904099-85904300
chr12	85958293	85960978	chr12-85958293-85960978
chr12	85969430	85971195	chr12-85969430-85971195
chr12	85973288	85974047	chr12-85973288-85974047
chr12	86000433	86001108	chr12-86000433-86001108
chr12	86011971	86012337	chr12-86011971-86012337
chr12	86076349	86079847	chr12-86076349-86079847
chr12	86082238	86083708	chr12-86082238-86083708
chr12	86220763	86220964	chr12-86220763-86220964

chr12	86241253	86243108	chr12-86241253-86243108
chr12	86363998	86364258	chr12-86363998-86364258
chr12	86402332	86403109	chr12-86402332-86403109
chr12	86421370	86422956	chr12-86421370-86422956
chr12	86433359	86433820	chr12-86433359-86433820
chr12	86652730	86653116	chr12-86652730-86653116
chr12	86678264	86681033	chr12-86678264-86681033
chr12	86689654	86689969	chr12-86689654-86689969
chr12	86694197	86695029	chr12-86694197-86695029
chr12	86698730	86699196	chr12-86698730-86699196
chr12	86725287	86726607	chr12-86725287-86726607
chr12	86772281	86772544	chr12-86772281-86772544
chr12	86790170	86791028	chr12-86790170-86791028
chr12	86793792	86794557	chr12-86793792-86794557
chr12	86798701	86803226	chr12-86798701-86803226
chr12	86806258	86806767	chr12-86806258-86806767
chr12	86823033	86825306	chr12-86823033-86825306
chr12	86826138	86827682	chr12-86826138-86827682
chr12	86831468	86834667	chr12-86831468-86834667
chr12	86836383	86837450	chr12-86836383-86837450
chr12	86867253	86868967	chr12-86867253-86868967
chr12	86871892	86872872	chr12-86871892-86872872
chr12	86878228	86897422	chr12-86878228-86897422
chr12	86899316	86899829	chr12-86899316-86899829
chr12	86912412	86913035	chr12-86912412-86913035
chr12	86940869	86941660	chr12-86940869-86941660
chr12	86946036	86949872	chr12-86946036-86949872
chr12	86977762	86978065	chr12-86977762-86978065
chr12	86989863	86990635	chr12-86989863-86990635
chr12	87026191	87027163	chr12-87026191-87027163
chr12	87102074	87102604	chr12-87102074-87102604
chr12	87146442	87149028	chr12-87146442-87149028
chr12	87199022	87200834	chr12-87199022-87200834
chr12	87265108	87268577	chr12-87265108-87268577
chr12	87299128	87300783	chr12-87299128-87300783
chr12	87384993	87385194	chr12-87384993-87385194
chr12	87386054	87389318	chr12-87386054-87389318
chr12	87405489	87405690	chr12-87405489-87405690
chr12	87407669	87408760	chr12-87407669-87408760
chr12	87419680	87420221	chr12-87419680-87420221
chr12	87441776	87445472	chr12-87441776-87445472
chr12	87470475	87472529	chr12-87470475-87472529

chr12	88354329	88354741	chr12-88354329-88354741
chr12	88360336	88361558	chr12-88360336-88361558
chr12	89811973	89812749	chr12-89811973-89812749
chr12	91029112	91029517	chr12-91029112-91029517
chr12	91383577	91385202	chr12-91383577-91385202
chr12	91400255	91401383	chr12-91400255-91401383
chr12	91587190	91591441	chr12-91587190-91591441
chr12	91667917	91668864	chr12-91667917-91668864
chr12	91684255	91686590	chr12-91684255-91686590
chr12	91778064	91779614	chr12-91778064-91779614
chr12	91845920	91846584	chr12-91845920-91846584
chr12	91847491	91849749	chr12-91847491-91849749
chr12	91879009	91879529	chr12-91879009-91879529
chr12	93685540	93685799	chr12-93685540-93685799
chr12	95691981	95697368	chr12-95691981-95697368
chr12	96511236	96511437	chr12-96511236-96511437
chr12	98255438	98255698	chr12-98255438-98255698
chr12	98257609	98260036	chr12-98257609-98260036
chr12	98282408	98283551	chr12-98282408-98283551
chr12	98573937	98575545	chr12-98573937-98575545
chr12	98577671	98578351	chr12-98577671-98578351
chr12	98627408	98629167	chr12-98627408-98629167
chr12	98674836	98675037	chr12-98674836-98675037
chr12	98712905	98715326	chr12-98712905-98715326
chr12	98722918	98723169	chr12-98722918-98723169
chr12	98733382	98738277	chr12-98733382-98738277
chr12	98746689	98748994	chr12-98746689-98748994
chr12	98900222	98901939	chr12-98900222-98901939
chr12	98919933	98921314	chr12-98919933-98921314
chr12	98996477	98996678	chr12-98996477-98996678
chr12	99335735	99337439	chr12-99335735-99337439
chr12	99338394	99339295	chr12-99338394-99339295
chr12	99340543	99341534	chr12-99340543-99341534
chr12	99351613	99353650	chr12-99351613-99353650
chr12	99355937	99357635	chr12-99355937-99357635
chr12	99362029	99362230	chr12-99362029-99362230
chr12	99391240	99394038	chr12-99391240-99394038
chr12	99406778	99407766	chr12-99406778-99407766
chr12	99562057	99564118	chr12-99562057-99564118
chr12	99627307	99628399	chr12-99627307-99628399
chr12	99882472	99885971	chr12-99882472-99885971
chr12	99906813	99907068	chr12-99906813-99907068

chr12	99964375	99966528	chr12-99964375-99966528
chr12	100109454	100111863	chr12-100109454-100111863
chr12	100158541	100160151	chr12-100158541-100160151
chr12	100168059	100168523	chr12-100168059-100168523
chr12	100186366	100188571	chr12-100186366-100188571
chr12	100198759	100202354	chr12-100198759-100202354
chr12	100210834	100211138	chr12-100210834-100211138
chr12	100275942	100277428	chr12-100275942-100277428
chr12	100279626	100279988	chr12-100279626-100279988
chr12	100281050	100281960	chr12-100281050-100281960
chr12	100326544	100326860	chr12-100326544-100326860
chr12	100353428	100354173	chr12-100353428-100354173
chr12	100519663	100521300	chr12-100519663-100521300
chr12	100723896	100726094	chr12-100723896-100726094
chr12	100778956	100779327	chr12-100778956-100779327
chr12	100898389	100899812	chr12-100898389-100899812
chr12	100907868	100908335	chr12-100907868-100908335
chr12	100952432	100952633	chr12-100952432-100952633
chr12	100982466	100982829	chr12-100982466-100982829
chr12	100997733	100999048	chr12-100997733-100999048
chr12	101016722	101017200	chr12-101016722-101017200
chr12	101018028	101018549	chr12-101018028-101018549
chr12	101025484	101029861	chr12-101025484-101029861
chr12	101080374	101084802	chr12-101080374-101084802
chr12	101717751	101719242	chr12-101717751-101719242
chr12	101818281	101819784	chr12-101818281-101819784
chr12	101911576	101913977	chr12-101911576-101913977
chr12	101956641	101958752	chr12-101956641-101958752
chr12	101974126	101977639	chr12-101974126-101977639
chr12	102128138	102129608	chr12-102128138-102129608
chr12	102280543	102280744	chr12-102280543-102280744
chr12	102282226	102287512	chr12-102282226-102287512
chr12	102299745	102300007	chr12-102299745-102300007
chr12	102421767	102422469	chr12-102421767-102422469
chr12	102426041	102426242	chr12-102426041-102426242
chr12	102431776	102433400	chr12-102431776-102433400
chr12	102437942	102440334	chr12-102437942-102440334
chr12	102468544	102470126	chr12-102468544-102470126
chr12	102554680	102555353	chr12-102554680-102555353
chr12	102702707	102705963	chr12-102702707-102705963
chr12	102730094	102731784	chr12-102730094-102731784
chr12	102742718	102745089	chr12-102742718-102745089

chr12	102756293	102759491	chr12-102756293-102759491
chr12	102876574	102879253	chr12-102876574-102879253
chr12	102948757	102950024	chr12-102948757-102950024
chr12	103059323	103059581	chr12-103059323-103059581
chr12	103142680	103142931	chr12-103142680-103142931
chr12	103183333	103183534	chr12-103183333-103183534
chr12	103241920	103242864	chr12-103241920-103242864
chr12	103325032	103325741	chr12-103325032-103325741
chr12	103340867	103341804	chr12-103340867-103341804
chr12	103388448	103390056	chr12-103388448-103390056
chr12	103423648	103426406	chr12-103423648-103426406
chr12	103434012	103434751	chr12-103434012-103434751
chr12	103531881	103533492	chr12-103531881-103533492
chr12	104002880	104003277	chr12-104002880-104003277
chr12	104064790	104065595	chr12-104064790-104065595
chr12	104418393	104418647	chr12-104418393-104418647
chr12	104471989	104474605	chr12-104471989-104474605
chr12	104750580	104753376	chr12-104750580-104753376
chr12	104844695	104846785	chr12-104844695-104846785
chr12	104863697	104865615	chr12-104863697-104865615
chr12	104983112	104984927	chr12-104983112-104984927
chr12	104999274	105001786	chr12-104999274-105001786
chr12	105002878	105005971	chr12-105002878-105005971
chr12	105008262	105010706	chr12-105008262-105010706
chr12	105012965	105013527	chr12-105012965-105013527
chr12	105030474	105035105	chr12-105030474-105035105
chr12	105336652	105337418	chr12-105336652-105337418
chr12	105453597	105454679	chr12-105453597-105454679
chr12	105525212	105525790	chr12-105525212-105525790
chr12	105545889	105547321	chr12-105545889-105547321
chr12	105548724	105549444	chr12-105548724-105549444
chr12	105551432	105552609	chr12-105551432-105552609
chr12	105553646	105555759	chr12-105553646-105555759
chr12	105556913	105557768	chr12-105556913-105557768
chr12	105561916	105565906	chr12-105561916-105565906
chr12	105683312	105686821	chr12-105683312-105686821
chr12	105718397	105718995	chr12-105718397-105718995
chr12	105761163	105761465	chr12-105761163-105761465
chr12	105783623	105786942	chr12-105783623-105786942
chr12	105935272	105935896	chr12-105935272-105935896
chr12	105962285	105963719	chr12-105962285-105963719
chr12	105981453	105982016	chr12-105981453-105982016

chr12	106009607	106011629	chr12-106009607-106011629
chr12	106046202	106046655	chr12-106046202-106046655
chr12	106173970	106174893	chr12-106173970-106174893
chr12	106196740	106197463	chr12-106196740-106197463
chr12	107645228	107646642	chr12-107645228-107646642
chr12	107669822	107670378	chr12-107669822-107670378
chr12	107807682	107807989	chr12-107807682-107807989
chr12	107980137	107980904	chr12-107980137-107980904
chr12	108002434	108006469	chr12-108002434-108006469
chr12	108177469	108180891	chr12-108177469-108180891
chr12	108234746	108235459	chr12-108234746-108235459
chr12	108236617	108236818	chr12-108236617-108236818
chr12	108239774	108240099	chr12-108239774-108240099
chr12	108244677	108245429	chr12-108244677-108245429
chr12	108249417	108249720	chr12-108249417-108249720
chr12	108273065	108277041	chr12-108273065-108277041
chr12	108305816	108306976	chr12-108305816-108306976
chr12	108309579	108310040	chr12-108309579-108310040
chr12	108318357	108318795	chr12-108318357-108318795
chr12	108327620	108328571	chr12-108327620-108328571
chr12	108333117	108335010	chr12-108333117-108335010
chr12	108352644	108353320	chr12-108352644-108353320
chr12	108371045	108371622	chr12-108371045-108371622
chr12	108392103	108393880	chr12-108392103-108393880
chr12	108395800	108396179	chr12-108395800-108396179
chr12	108422506	108424125	chr12-108422506-108424125
chr12	108453850	108454237	chr12-108453850-108454237
chr12	108547366	108547617	chr12-108547366-108547617
chr12	108554066	108555956	chr12-108554066-108555956
chr12	108701873	108702817	chr12-108701873-108702817
chr12	108757331	108758547	chr12-108757331-108758547
chr12	108765353	108766708	chr12-108765353-108766708
chr12	108791070	108796098	chr12-108791070-108796098
chr12	108834668	108838517	chr12-108834668-108838517
chr12	108891393	108891667	chr12-108891393-108891667
chr12	108892701	108895287	chr12-108892701-108895287
chr12	108965130	108965707	chr12-108965130-108965707
chr12	109033215	109033859	chr12-109033215-109033859
chr12	109052427	109054009	chr12-109052427-109054009
chr12	109067430	109068657	chr12-109067430-109068657
chr12	109417584	109418058	chr12-109417584-109418058
chr12	109424975	109425176	chr12-109424975-109425176

chr12	109437841	109438195	chr12-109437841-109438195
chr12	109441600	109441801	chr12-109441600-109441801
chr12	109449781	109450191	chr12-109449781-109450191
chr12	109453126	109453735	chr12-109453126-109453735
chr12	109749542	109750268	chr12-109749542-109750268
chr12	110086135	110088464	chr12-110086135-110088464
chr12	110189285	110189544	chr12-110189285-110189544
chr12	110206385	110206586	chr12-110206385-110206586
chr12	110277780	110279339	chr12-110277780-110279339
chr12	110373442	110373907	chr12-110373442-110373907
chr12	110381215	110381564	chr12-110381215-110381564
chr12	110399109	110399310	chr12-110399109-110399310
chr12	110445748	110448129	chr12-110445748-110448129
chr12	110484668	110489236	chr12-110484668-110489236
chr12	110496975	110500195	chr12-110496975-110500195
chr12	110590187	110592846	chr12-110590187-110592846
chr12	110599590	110603598	chr12-110599590-110603598
chr12	110608212	110609238	chr12-110608212-110609238
chr12	110687289	110688673	chr12-110687289-110688673
chr12	110693099	110697464	chr12-110693099-110697464
chr12	110706501	110707294	chr12-110706501-110707294
chr12	110711458	110712075	chr12-110711458-110712075
chr12	110736468	110739794	chr12-110736468-110739794
chr12	110779769	110780032	chr12-110779769-110780032
chr12	110796795	110798463	chr12-110796795-110798463
chr12	110840107	110841450	chr12-110840107-110841450
chr12	110843714	110846480	chr12-110843714-110846480
chr12	110849649	110851660	chr12-110849649-110851660
chr12	110887702	110890819	chr12-110887702-110890819
chr12	110953211	110954986	chr12-110953211-110954986
chr12	110976568	110979854	chr12-110976568-110979854
chr12	110990775	110992917	chr12-110990775-110992917
chr12	111037780	111041382	chr12-111037780-111041382
chr12	111150327	111150528	chr12-111150327-111150528
chr12	111164204	111168415	chr12-111164204-111168415
chr12	111186923	111187232	chr12-111186923-111187232
chr12	111274055	111275963	chr12-111274055-111275963
chr12	111303261	111304225	chr12-111303261-111304225
chr12	111352488	111356122	chr12-111352488-111356122
chr12	111374790	111379192	chr12-111374790-111379192
chr12	111385763	111387518	chr12-111385763-111387518
chr12	111399143	111399344	chr12-111399143-111399344

chr12	111411383	111413642	chr12-111411383-111413642
chr12	111425502	111426237	chr12-111425502-111426237
chr12	111427712	111433473	chr12-111427712-111433473
chr12	111442051	111451304	chr12-111442051-111451304
chr12	111454613	111456540	chr12-111454613-111456540
chr12	111461570	111461893	chr12-111461570-111461893
chr12	111485017	111486291	chr12-111485017-111486291
chr12	111531181	111531588	chr12-111531181-111531588
chr12	111534372	111541536	chr12-111534372-111541536
chr12	111553700	111553901	chr12-111553700-111553901
chr12	111559834	111560144	chr12-111559834-111560144
chr12	111568629	111569375	chr12-111568629-111569375
chr12	111573040	111576916	chr12-111573040-111576916
chr12	111668610	111668811	chr12-111668610-111668811
chr12	111670321	111672933	chr12-111670321-111672933
chr12	111677241	111679929	chr12-111677241-111679929
chr12	111697373	111698804	chr12-111697373-111698804
chr12	111704277	111706027	chr12-111704277-111706027
chr12	111711629	111714324	chr12-111711629-111714324
chr12	111732398	111732707	chr12-111732398-111732707
chr12	111752702	111753658	chr12-111752702-111753658
chr12	111756920	111761286	chr12-111756920-111761286
chr12	111762301	111762502	chr12-111762301-111762502
chr12	111812103	111815739	chr12-111812103-111815739
chr12	111816577	111816778	chr12-111816577-111816778
chr12	111906816	111909119	chr12-111906816-111909119
chr12	111936447	111938125	chr12-111936447-111938125
chr12	111942921	111946606	chr12-111942921-111946606
chr12	111965479	111967502	chr12-111965479-111967502
chr12	112106775	112107026	chr12-112106775-112107026
chr12	112112918	112113119	chr12-112112918-112113119
chr12	112125656	112126815	chr12-112125656-112126815
chr12	112129471	112131619	chr12-112129471-112131619
chr12	112143843	112147252	chr12-112143843-112147252
chr12	112490090	112491561	chr12-112490090-112491561
chr12	112507406	112508614	chr12-112507406-112508614
chr12	112510701	112511381	chr12-112510701-112511381
chr12	112515902	112517624	chr12-112515902-112517624
chr12	112576008	112577463	chr12-112576008-112577463
chr12	112578446	112580465	chr12-112578446-112580465
chr12	112587228	112591143	chr12-112587228-112591143
chr12	112592650	112599800	chr12-112592650-112599800

chr12	112606629	112606991	chr12-112606629-112606991
chr12	112616694	112618067	chr12-112616694-112618067
chr12	112619181	112622752	chr12-112619181-112622752
chr12	112627604	112629928	chr12-112627604-112629928
chr12	112644018	112646765	chr12-112644018-112646765
chr12	112671002	112675920	chr12-112671002-112675920
chr12	112678093	112680373	chr12-112678093-112680373
chr12	112692472	112694388	chr12-112692472-112694388
chr12	112702015	112703538	chr12-112702015-112703538
chr12	112706730	112707541	chr12-112706730-112707541
chr12	112719876	112725061	chr12-112719876-112725061
chr12	112727423	112729418	chr12-112727423-112729418
chr12	112779757	112779958	chr12-112779757-112779958
chr12	112793573	112798638	chr12-112793573-112798638
chr12	112800190	112802918	chr12-112800190-112802918
chr12	112805860	112806758	chr12-112805860-112806758
chr12	112808317	112810481	chr12-112808317-112810481
chr12	112826232	112830928	chr12-112826232-112830928
chr12	112850365	112853286	chr12-112850365-112853286
chr12	112865053	112865414	chr12-112865053-112865414
chr12	112866451	112867531	chr12-112866451-112867531
chr12	112868547	112868962	chr12-112868547-112868962
chr12	112875465	112878696	chr12-112875465-112878696
chr12	112897709	112898296	chr12-112897709-112898296
chr12	112927296	112930490	chr12-112927296-112930490
chr12	112937745	112938595	chr12-112937745-112938595
chr12	112940749	112942610	chr12-112940749-112942610
chr12	112949489	112950993	chr12-112949489-112950993
chr12	112951855	112952645	chr12-112951855-112952645
chr12	112975993	112977732	chr12-112975993-112977732
chr12	112998825	113001795	chr12-112998825-113001795
chr12	113011815	113012016	chr12-113011815-113012016
chr12	113013259	113015936	chr12-113013259-113015936
chr12	113073943	113075316	chr12-113073943-113075316
chr12	113097324	113100279	chr12-113097324-113100279
chr12	113106383	113107192	chr12-113106383-113107192
chr12	113139010	113144557	chr12-113139010-113144557
chr12	113146272	113147046	chr12-113146272-113147046
chr12	113148608	113149021	chr12-113148608-113149021
chr12	113151296	113153560	chr12-113151296-113153560
chr12	113154717	113158206	chr12-113154717-113158206
chr12	113185486	113188465	chr12-113185486-113188465

chr12	113222164	113222575	chr12-113222164-113222575
chr12	114378675	114379409	chr12-114378675-114379409
chr12	116047120	116049350	chr12-116047120-116049350
chr12	116077116	116078328	chr12-116077116-116078328
chr12	116181632	116181883	chr12-116181632-116181883
chr12	116231653	116232015	chr12-116231653-116232015
chr12	116258328	116258656	chr12-116258328-116258656
chr12	116262303	116263486	chr12-116262303-116263486
chr12	116268844	116269963	chr12-116268844-116269963
chr12	116279808	116284085	chr12-116279808-116284085
chr12	116292433	116292634	chr12-116292433-116292634
chr12	116293670	116294634	chr12-116293670-116294634
chr12	116404128	116406913	chr12-116404128-116406913
chr12	116485445	116486556	chr12-116485445-116486556
chr12	117153546	117154483	chr12-117153546-117154483
chr12	117431186	117431387	chr12-117431186-117431387
chr12	117432377	117432578	chr12-117432377-117432578
chr12	117448029	117448558	chr12-117448029-117448558
chr12	117450158	117450359	chr12-117450158-117450359
chr12	117468575	117468827	chr12-117468575-117468827
chr12	117471625	117472811	chr12-117471625-117472811
chr12	117533241	117535708	chr12-117533241-117535708
chr12	117545239	117545440	chr12-117545239-117545440
chr12	117728121	117728322	chr12-117728121-117728322
chr12	117843178	117845537	chr12-117843178-117845537
chr12	118198207	118199293	chr12-118198207-118199293
chr12	118300026	118303078	chr12-118300026-118303078
chr12	118457779	118458186	chr12-118457779-118458186
chr12	118529989	118530787	chr12-118529989-118530787
chr12	118657830	118658234	chr12-118657830-118658234
chr12	118806049	118806474	chr12-118806049-118806474
chr12	118815143	118815344	chr12-118815143-118815344
chr12	118836294	118837752	chr12-118836294-118837752
chr12	118842823	118843437	chr12-118842823-118843437
chr12	118846153	118850096	chr12-118846153-118850096
chr12	118854483	118855451	chr12-118854483-118855451
chr12	119237786	119239348	chr12-119237786-119239348
chr12	119387670	119388181	chr12-119387670-119388181
chr12	119390129	119392652	chr12-119390129-119392652
chr12	119425996	119426297	chr12-119425996-119426297
chr12	119946060	119946312	chr12-119946060-119946312
chr13	3358121	3361781	chr13-3358121-3361781

chr13	3477823	3479090	chr13-3477823-3479090
chr13	3532205	3532456	chr13-3532205-3532456
chr13	3536914	3540519	chr13-3536914-3540519
chr13	3609677	3611878	chr13-3609677-3611878
chr13	3633595	3634912	chr13-3633595-3634912
chr13	3636591	3637336	chr13-3636591-3637336
chr13	3665397	3665648	chr13-3665397-3665648
chr13	3694582	3695462	chr13-3694582-3695462
chr13	3700538	3701216	chr13-3700538-3701216
chr13	3742734	3745722	chr13-3742734-3745722
chr13	3746668	3747174	chr13-3746668-3747174
chr13	3782359	3782957	chr13-3782359-3782957
chr13	3865885	3866432	chr13-3865885-3866432
chr13	3890066	3894093	chr13-3890066-3894093
chr13	3916716	3918487	chr13-3916716-3918487
chr13	4183384	4183646	chr13-4183384-4183646
chr13	4191050	4192304	chr13-4191050-4192304
chr13	4233684	4233947	chr13-4233684-4233947
chr13	4245188	4245546	chr13-4245188-4245546
chr13	4608410	4609639	chr13-4608410-4609639
chr13	4662515	4663075	chr13-4662515-4663075
chr13	4771138	4771552	chr13-4771138-4771552
chr13	4942781	4943345	chr13-4942781-4943345
chr13	5549466	5549667	chr13-5549466-5549667
chr13	5621795	5621996	chr13-5621795-5621996
chr13	5714073	5714692	chr13-5714073-5714692
chr13	5769167	5769741	chr13-5769167-5769741
chr13	5792114	5793589	chr13-5792114-5793589
chr13	5802208	5805466	chr13-5802208-5805466
chr13	5821997	5823535	chr13-5821997-5823535
chr13	5825713	5826772	chr13-5825713-5826772
chr13	5841680	5842281	chr13-5841680-5842281
chr13	5843835	5846385	chr13-5843835-5846385
chr13	5858065	5871908	chr13-5858065-5871908
chr13	5886967	5887380	chr13-5886967-5887380
chr13	5892472	5893744	chr13-5892472-5893744
chr13	6009905	6010261	chr13-6009905-6010261
chr13	6547115	6549291	chr13-6547115-6549291
chr13	6623988	6624849	chr13-6623988-6624849
chr13	6626775	6627026	chr13-6626775-6627026
chr13	6646495	6649323	chr13-6646495-6649323
chr13	8805724	8806025	chr13-8805724-8806025

chr13	8829793	8831814	chr13-8829793-8831814
chr13	8869746	8872167	chr13-8869746-8872167
chr13	8884766	8888239	chr13-8884766-8888239
chr13	8994511	8996895	chr13-8994511-8996895
chr13	9011620	9012296	chr13-9011620-9012296
chr13	9014152	9014796	chr13-9014152-9014796
chr13	9026897	9027306	chr13-9026897-9027306
chr13	9093043	9096002	chr13-9093043-9096002
chr13	9139932	9140396	chr13-9139932-9140396
chr13	9190895	9191785	chr13-9190895-9191785
chr13	9199114	9199315	chr13-9199114-9199315
chr13	9276133	9278073	chr13-9276133-9278073
chr13	9494442	9495359	chr13-9494442-9495359
chr13	9695783	9696212	chr13-9695783-9696212
chr13	9762990	9765706	chr13-9762990-9765706
chr13	9834282	9834533	chr13-9834282-9834533
chr13	10360121	10361141	chr13-10360121-10361141
chr13	11549014	11549267	chr13-11549014-11549267
chr13	11566662	11566966	chr13-11566662-11566966
chr13	12105889	12107131	chr13-12105889-12107131
chr13	12256548	12258332	chr13-12256548-12258332
chr13	12340253	12341049	chr13-12340253-12341049
chr13	12394457	12396720	chr13-12394457-12396720
chr13	12459914	12462350	chr13-12459914-12462350
chr13	12519345	12520640	chr13-12519345-12520640
chr13	12541954	12542613	chr13-12541954-12542613
chr13	12565221	12566940	chr13-12565221-12566940
chr13	12645294	12651254	chr13-12645294-12651254
chr13	12702848	12703725	chr13-12702848-12703725
chr13	12808854	12809245	chr13-12808854-12809245
chr13	13388487	13394466	chr13-13388487-13394466
chr13	13437364	13439640	chr13-13437364-13439640
chr13	13472725	13472976	chr13-13472725-13472976
chr13	13474713	13475078	chr13-13474713-13475078
chr13	13529067	13529706	chr13-13529067-13529706
chr13	13538411	13539160	chr13-13538411-13539160
chr13	13573667	13574023	chr13-13573667-13574023
chr13	13589706	13591493	chr13-13589706-13591493
chr13	13783709	13785750	chr13-13783709-13785750
chr13	13889580	13890051	chr13-13889580-13890051
chr13	13953356	13956186	chr13-13953356-13956186
chr13	14038260	14040822	chr13-14038260-14040822

chr13	14061943	14065594	chr13-14061943-14065594
chr13	14521871	14523291	chr13-14521871-14523291
chr13	14611841	14614344	chr13-14611841-14614344
chr13	14629701	14631513	chr13-14629701-14631513
chr13	15462501	15465100	chr13-15462501-15465100
chr13	15537681	15538281	chr13-15537681-15538281
chr13	15660786	15661091	chr13-15660786-15661091
chr13	15741744	15741945	chr13-15741744-15741945
chr13	15758970	15759750	chr13-15758970-15759750
chr13	15846006	15846420	chr13-15846006-15846420
chr13	16010777	16021395	chr13-16010777-16021395
chr13	16216595	16217074	chr13-16216595-16217074
chr13	16490495	16490696	chr13-16490495-16490696
chr13	16659259	16660253	chr13-16659259-16660253
chr13	16738535	16739499	chr13-16738535-16739499
chr13	16847531	16847890	chr13-16847531-16847890
chr13	17212940	17213192	chr13-17212940-17213192
chr13	17604419	17605434	chr13-17604419-17605434
chr13	17690005	17690206	chr13-17690005-17690206
chr13	17693838	17698500	chr13-17693838-17698500
chr13	17801832	17806228	chr13-17801832-17806228
chr13	17837938	17838438	chr13-17837938-17838438
chr13	17900582	17901838	chr13-17900582-17901838
chr13	17942700	17945287	chr13-17942700-17945287
chr13	17975142	17975343	chr13-17975142-17975343
chr13	17991880	17993908	chr13-17991880-17993908
chr13	18717074	18718275	chr13-18717074-18718275
chr13	18948049	18949161	chr13-18948049-18949161
chr13	19393762	19396532	chr13-19393762-19396532
chr13	19518360	19518561	chr13-19518360-19518561
chr13	19618466	19620051	chr13-19618466-19620051
chr13	19622661	19623728	chr13-19622661-19623728
chr13	19703761	19704012	chr13-19703761-19704012
chr13	20090060	20091839	chr13-20090060-20091839
chr13	20131519	20131771	chr13-20131519-20131771
chr13	20433030	20433655	chr13-20433030-20433655
chr13	21158180	21161828	chr13-21158180-21161828
chr13	21166694	21174639	chr13-21166694-21174639
chr13	21177762	21182807	chr13-21177762-21182807
chr13	21198615	21201095	chr13-21198615-21201095
chr13	21215408	21216320	chr13-21215408-21216320
chr13	21221522	21223837	chr13-21221522-21223837

chr13	21233063	21235373	chr13-21233063-21235373
chr13	21241939	21243829	chr13-21241939-21243829
chr13	21249286	21249538	chr13-21249286-21249538
chr13	21253234	21254363	chr13-21253234-21254363
chr13	21258536	21260682	chr13-21258536-21260682
chr13	21263660	21264704	chr13-21263660-21264704
chr13	21267765	21268475	chr13-21267765-21268475
chr13	21274602	21277075	chr13-21274602-21277075
chr13	21278445	21279809	chr13-21278445-21279809
chr13	21321904	21323385	chr13-21321904-21323385
chr13	21326346	21327989	chr13-21326346-21327989
chr13	21362303	21364200	chr13-21362303-21364200
chr13	21401187	21403101	chr13-21401187-21403101
chr13	21407724	21408990	chr13-21407724-21408990
chr13	21417460	21417732	chr13-21417460-21417732
chr13	21439946	21441326	chr13-21439946-21441326
chr13	21452340	21454141	chr13-21452340-21454141
chr13	21456182	21456602	chr13-21456182-21456602
chr13	21467912	21469143	chr13-21467912-21469143
chr13	21478703	21479809	chr13-21478703-21479809
chr13	21505900	21506956	chr13-21505900-21506956
chr13	21529995	21531561	chr13-21529995-21531561
chr13	21709869	21712038	chr13-21709869-21712038
chr13	21714886	21718818	chr13-21714886-21718818
chr13	21720803	21723358	chr13-21720803-21723358
chr13	21734060	21736101	chr13-21734060-21736101
chr13	21749200	21751174	chr13-21749200-21751174
chr13	21753098	21755356	chr13-21753098-21755356
chr13	21778184	21781221	chr13-21778184-21781221
chr13	21782323	21784318	chr13-21782323-21784318
chr13	21786022	21788426	chr13-21786022-21788426
chr13	21811734	21814344	chr13-21811734-21814344
chr13	21830469	21832274	chr13-21830469-21832274
chr13	21848055	21848610	chr13-21848055-21848610
chr13	21852385	21852740	chr13-21852385-21852740
chr13	21855259	21856418	chr13-21855259-21856418
chr13	21881721	21883848	chr13-21881721-21883848
chr13	21896848	21898387	chr13-21896848-21898387
chr13	21907250	21907924	chr13-21907250-21907924
chr13	21910532	21911608	chr13-21910532-21911608
chr13	21913849	21914805	chr13-21913849-21914805
chr13	21917082	21920240	chr13-21917082-21920240

chr13	21925002	21925716	chr13-21925002-21925716
chr13	21927881	21928873	chr13-21927881-21928873
chr13	21931327	21931694	chr13-21931327-21931694
chr13	21934035	21938111	chr13-21934035-21938111
chr13	21940407	21941758	chr13-21940407-21941758
chr13	21944453	21946349	chr13-21944453-21946349
chr13	21973969	21975136	chr13-21973969-21975136
chr13	21981066	21982191	chr13-21981066-21982191
chr13	21994000	21997899	chr13-21994000-21997899
chr13	22005012	22007717	chr13-22005012-22007717
chr13	22019737	22023268	chr13-22019737-22023268
chr13	22025889	22027096	chr13-22025889-22027096
chr13	22028579	22030065	chr13-22028579-22030065
chr13	22033852	22037126	chr13-22033852-22037126
chr13	22039861	22044302	chr13-22039861-22044302
chr13	22049664	22052085	chr13-22049664-22052085
chr13	22831215	22831416	chr13-22831215-22831416
chr13	23270496	23270848	chr13-23270496-23270848
chr13	23284889	23287483	chr13-23284889-23287483
chr13	23297845	23301848	chr13-23297845-23301848
chr13	23306600	23310514	chr13-23306600-23310514
chr13	23312610	23314055	chr13-23312610-23314055
chr13	23317480	23318416	chr13-23317480-23318416
chr13	23328739	23330241	chr13-23328739-23330241
chr13	23367665	23369943	chr13-23367665-23369943
chr13	23395186	23397488	chr13-23395186-23397488
chr13	23401099	23402842	chr13-23401099-23402842
chr13	23411227	23413735	chr13-23411227-23413735
chr13	23422441	23427791	chr13-23422441-23427791
chr13	23429839	23439313	chr13-23429839-23439313
chr13	23444582	23447130	chr13-23444582-23447130
chr13	23448944	23451248	chr13-23448944-23451248
chr13	23453411	23454473	chr13-23453411-23454473
chr13	23463560	23467369	chr13-23463560-23467369
chr13	23488000	23488965	chr13-23488000-23488965
chr13	23497030	23507293	chr13-23497030-23507293
chr13	23516083	23519940	chr13-23516083-23519940
chr13	23525788	23527632	chr13-23525788-23527632
chr13	23529708	23536931	chr13-23529708-23536931
chr13	23542116	23546127	chr13-23542116-23546127
chr13	23550653	23552437	chr13-23550653-23552437
chr13	23554491	23557159	chr13-23554491-23557159

chr13	23560695	23563390	chr13-23560695-23563390
chr13	23570123	23576967	chr13-23570123-23576967
chr13	23581524	23587074	chr13-23581524-23587074
chr13	23620018	23623212	chr13-23620018-23623212
chr13	23682397	23686477	chr13-23682397-23686477
chr13	23697531	23698936	chr13-23697531-23698936
chr13	23709949	23710954	chr13-23709949-23710954
chr13	23730585	23730945	chr13-23730585-23730945
chr13	23738087	23742169	chr13-23738087-23742169
chr13	23743969	23748090	chr13-23743969-23748090
chr13	23749953	23753111	chr13-23749953-23753111
chr13	23756874	23757957	chr13-23756874-23757957
chr13	23760013	23764422	chr13-23760013-23764422
chr13	23930679	23931356	chr13-23930679-23931356
chr13	24105170	24105371	chr13-24105170-24105371
chr13	24279027	24281823	chr13-24279027-24281823
chr13	24374728	24374929	chr13-24374728-24374929
chr13	24401855	24402056	chr13-24401855-24402056
chr13	24538705	24539116	chr13-24538705-24539116
chr13	24564211	24567585	chr13-24564211-24567585
chr13	24637858	24640788	chr13-24637858-24640788
chr13	24646268	24646571	chr13-24646268-24646571
chr13	24713545	24714097	chr13-24713545-24714097
chr13	24760350	24762901	chr13-24760350-24762901
chr13	24785936	24786392	chr13-24785936-24786392
chr13	24792214	24792974	chr13-24792214-24792974
chr13	24799475	24805101	chr13-24799475-24805101
chr13	24825344	24826529	chr13-24825344-24826529
chr13	24829156	24833858	chr13-24829156-24833858
chr13	24844811	24845531	chr13-24844811-24845531
chr13	24936953	24938427	chr13-24936953-24938427
chr13	24965187	24965962	chr13-24965187-24965962
chr13	25019027	25021020	chr13-25019027-25021020
chr13	25054965	25056634	chr13-25054965-25056634
chr13	25105732	25106395	chr13-25105732-25106395
chr13	25115693	25115894	chr13-25115693-25115894
chr13	25269715	25270319	chr13-25269715-25270319
chr13	25639826	25640088	chr13-25639826-25640088
chr13	26940205	26940406	chr13-26940205-26940406
chr13	27059627	27060741	chr13-27059627-27060741
chr13	28312276	28312733	chr13-28312276-28312733
chr13	28418015	28418350	chr13-28418015-28418350

chr13	28532135	28532445	chr13-28532135-28532445
chr13	28543614	28545718	chr13-28543614-28545718
chr13	28629554	28629820	chr13-28629554-28629820
chr13	28633502	28634128	chr13-28633502-28634128
chr13	28719020	28719221	chr13-28719020-28719221
chr13	28737508	28739561	chr13-28737508-28739561
chr13	28751147	28751627	chr13-28751147-28751627
chr13	28756957	28759384	chr13-28756957-28759384
chr13	28832803	28833684	chr13-28832803-28833684
chr13	28842573	28843235	chr13-28842573-28843235
chr13	28853264	28854822	chr13-28853264-28854822
chr13	28874315	28874890	chr13-28874315-28874890
chr13	28881007	28886150	chr13-28881007-28886150
chr13	28917051	28919571	chr13-28917051-28919571
chr13	28947754	28954445	chr13-28947754-28954445
chr13	28957621	28960043	chr13-28957621-28960043
chr13	29210064	29211075	chr13-29210064-29211075
chr13	29505156	29505557	chr13-29505156-29505557
chr13	29780654	29781363	chr13-29780654-29781363
chr13	29854774	29855953	chr13-29854774-29855953
chr13	29982884	29987149	chr13-29982884-29987149
chr13	30085083	30085284	chr13-30085083-30085284
chr13	30108569	30110197	chr13-30108569-30110197
chr13	30136067	30139234	chr13-30136067-30139234
chr13	30140217	30140470	chr13-30140217-30140470
chr13	30146697	30147003	chr13-30146697-30147003
chr13	30191948	30192431	chr13-30191948-30192431
chr13	30194207	30195490	chr13-30194207-30195490
chr13	30197906	30198670	chr13-30197906-30198670
chr13	30336160	30336978	chr13-30336160-30336978
chr13	30368553	30368860	chr13-30368553-30368860
chr13	30543761	30546044	chr13-30543761-30546044
chr13	30659257	30660922	chr13-30659257-30660922
chr13	30748829	30750964	chr13-30748829-30750964
chr13	30781831	30782851	chr13-30781831-30782851
chr13	30932896	30933407	chr13-30932896-30933407
chr13	30972169	30974808	chr13-30972169-30974808
chr13	30985531	30986000	chr13-30985531-30986000
chr13	31225212	31225413	chr13-31225212-31225413
chr13	31275819	31276176	chr13-31275819-31276176
chr13	31327225	31328159	chr13-31327225-31328159
chr13	31468269	31468583	chr13-31468269-31468583

chr13	31506325	31506943	chr13-31506325-31506943
chr13	31518373	31518574	chr13-31518373-31518574
chr13	31557144	31560570	chr13-31557144-31560570
chr13	31567082	31568199	chr13-31567082-31568199
chr13	31578352	31578553	chr13-31578352-31578553
chr13	31579756	31580021	chr13-31579756-31580021
chr13	31615084	31615441	chr13-31615084-31615441
chr13	31617870	31618957	chr13-31617870-31618957
chr13	31620416	31620681	chr13-31620416-31620681
chr13	31624882	31627411	chr13-31624882-31627411
chr13	31628914	31630203	chr13-31628914-31630203
chr13	31632446	31632647	chr13-31632446-31632647
chr13	31779503	31779704	chr13-31779503-31779704
chr13	31792646	31792904	chr13-31792646-31792904
chr13	31800659	31802326	chr13-31800659-31802326
chr13	31805858	31809245	chr13-31805858-31809245
chr13	31810597	31811812	chr13-31810597-31811812
chr13	32337666	32339644	chr13-32337666-32339644
chr13	32371023	32371224	chr13-32371023-32371224
chr13	32545314	32545515	chr13-32545314-32545515
chr13	32610756	32611818	chr13-32610756-32611818
chr13	32801012	32804257	chr13-32801012-32804257
chr13	32924345	32925069	chr13-32924345-32925069
chr13	32927019	32927880	chr13-32927019-32927880
chr13	32939110	32939761	chr13-32939110-32939761
chr13	32964434	32967149	chr13-32964434-32967149
chr13	33003968	33005110	chr13-33003968-33005110
chr13	33027319	33029163	chr13-33027319-33029163
chr13	33051273	33052140	chr13-33051273-33052140
chr13	33931781	33932032	chr13-33931781-33932032
chr13	33933793	33937885	chr13-33933793-33937885
chr13	33951657	33952334	chr13-33951657-33952334
chr13	33963992	33966285	chr13-33963992-33966285
chr13	34001666	34003623	chr13-34001666-34003623
chr13	34032480	34032988	chr13-34032480-34032988
chr13	34037202	34039165	chr13-34037202-34039165
chr13	34076555	34078886	chr13-34076555-34078886
chr13	34079697	34080078	chr13-34079697-34080078
chr13	34083914	34084575	chr13-34083914-34084575
chr13	34095657	34097316	chr13-34095657-34097316
chr13	34112064	34113344	chr13-34112064-34113344
chr13	34127417	34131881	chr13-34127417-34131881

chr13	34149046	34149355	chr13-34149046-34149355
chr13	34154359	34155096	chr13-34154359-34155096
chr13	34162296	34164026	chr13-34162296-34164026
chr13	34190162	34193965	chr13-34190162-34193965
chr13	34208259	34208460	chr13-34208259-34208460
chr13	34210118	34210534	chr13-34210118-34210534
chr13	34211925	34213833	chr13-34211925-34213833
chr13	34332556	34332807	chr13-34332556-34332807
chr13	34341781	34345853	chr13-34341781-34345853
chr13	34369626	34370655	chr13-34369626-34370655
chr13	34394331	34394659	chr13-34394331-34394659
chr13	34398191	34399478	chr13-34398191-34399478
chr13	34408136	34408489	chr13-34408136-34408489
chr13	34647855	34653946	chr13-34647855-34653946
chr13	34659348	34659871	chr13-34659348-34659871
chr13	34683008	34683430	chr13-34683008-34683430
chr13	34709256	34709986	chr13-34709256-34709986
chr13	34715696	34716004	chr13-34715696-34716004
chr13	34725449	34727069	chr13-34725449-34727069
chr13	34733629	34733883	chr13-34733629-34733883
chr13	34784548	34784801	chr13-34784548-34784801
chr13	34873269	34877707	chr13-34873269-34877707
chr13	34916548	34917163	chr13-34916548-34917163
chr13	34963506	34964707	chr13-34963506-34964707
chr13	34993043	34994978	chr13-34993043-34994978
chr13	35026839	35027199	chr13-35026839-35027199
chr13	35131439	35131640	chr13-35131439-35131640
chr13	35395095	35395871	chr13-35395095-35395871
chr13	35469476	35469677	chr13-35469476-35469677
chr13	35501589	35502664	chr13-35501589-35502664
chr13	35740280	35743270	chr13-35740280-35743270
chr13	35905047	35906668	chr13-35905047-35906668
chr13	35967837	35969840	chr13-35967837-35969840
chr13	36116381	36118979	chr13-36116381-36118979
chr13	36152978	36153501	chr13-36152978-36153501
chr13	36163319	36163520	chr13-36163319-36163520
chr13	36203301	36203502	chr13-36203301-36203502
chr13	36530560	36531070	chr13-36530560-36531070
chr13	36578302	36579368	chr13-36578302-36579368
chr13	36695542	36696814	chr13-36695542-36696814
chr13	36724461	36725440	chr13-36724461-36725440
chr13	36726441	36736879	chr13-36726441-36736879

chr13	37312238	37312699	chr13-37312238-37312699
chr13	37434220	37434833	chr13-37434220-37434833
chr13	37452033	37454797	chr13-37452033-37454797
chr13	37456842	37457446	chr13-37456842-37457446
chr13	37477590	37478543	chr13-37477590-37478543
chr13	37507008	37507670	chr13-37507008-37507670
chr13	37556953	37557556	chr13-37556953-37557556
chr13	37561336	37561854	chr13-37561336-37561854
chr13	37592374	37592575	chr13-37592374-37592575
chr13	37619985	37620239	chr13-37619985-37620239
chr13	37622478	37622948	chr13-37622478-37622948
chr13	37651121	37651376	chr13-37651121-37651376
chr13	37666128	37667713	chr13-37666128-37667713
chr13	37715016	37716198	chr13-37715016-37716198
chr13	37777241	37780255	chr13-37777241-37780255
chr13	37783476	37785068	chr13-37783476-37785068
chr13	37824989	37829173	chr13-37824989-37829173
chr13	37830388	37832038	chr13-37830388-37832038
chr13	37846973	37848215	chr13-37846973-37848215
chr13	37853453	37855320	chr13-37853453-37855320
chr13	37856903	37858778	chr13-37856903-37858778
chr13	37866633	37867351	chr13-37866633-37867351
chr13	37895715	37896149	chr13-37895715-37896149
chr13	37963493	37964098	chr13-37963493-37964098
chr13	37992404	37994828	chr13-37992404-37994828
chr13	38035955	38038388	chr13-38035955-38038388
chr13	38123875	38124076	chr13-38123875-38124076
chr13	38144955	38146076	chr13-38144955-38146076
chr13	38150503	38154010	chr13-38150503-38154010
chr13	38204267	38206550	chr13-38204267-38206550
chr13	38311908	38312977	chr13-38311908-38312977
chr13	38344921	38348298	chr13-38344921-38348298
chr13	38390904	38391105	chr13-38390904-38391105
chr13	38410202	38410867	chr13-38410202-38410867
chr13	38450178	38451090	chr13-38450178-38451090
chr13	38526104	38529076	chr13-38526104-38529076
chr13	38580528	38580784	chr13-38580528-38580784
chr13	38633982	38635746	chr13-38633982-38635746
chr13	38657491	38659499	chr13-38657491-38659499
chr13	38957734	38961379	chr13-38957734-38961379
chr13	39824064	39824538	chr13-39824064-39824538
chr13	40656129	40656767	chr13-40656129-40656767

chr13	40683540	40683855	chr13-40683540-40683855
chr13	40703783	40705506	chr13-40703783-40705506
chr13	40708928	40709403	chr13-40708928-40709403
chr13	40718899	40719640	chr13-40718899-40719640
chr13	40722261	40723998	chr13-40722261-40723998
chr13	40727798	40734911	chr13-40727798-40734911
chr13	40737728	40738880	chr13-40737728-40738880
chr13	40744531	40745061	chr13-40744531-40745061
chr13	40749254	40749506	chr13-40749254-40749506
chr13	40854497	40854809	chr13-40854497-40854809
chr13	40859368	40862873	chr13-40859368-40862873
chr13	40868566	40869487	chr13-40868566-40869487
chr13	40915956	40922711	chr13-40915956-40922711
chr13	40999748	41002747	chr13-40999748-41002747
chr13	41015606	41017896	chr13-41015606-41017896
chr13	41078874	41079878	chr13-41078874-41079878
chr13	41113949	41114475	chr13-41113949-41114475
chr13	41219821	41221065	chr13-41219821-41221065
chr13	41248916	41251117	chr13-41248916-41251117
chr13	41331744	41336534	chr13-41331744-41336534
chr13	41338751	41340410	chr13-41338751-41340410
chr13	41354636	41359921	chr13-41354636-41359921
chr13	41366624	41367682	chr13-41366624-41367682
chr13	41401883	41402190	chr13-41401883-41402190
chr13	41403791	41404391	chr13-41403791-41404391
chr13	41476522	41476773	chr13-41476522-41476773
chr13	41583583	41584139	chr13-41583583-41584139
chr13	41604751	41607393	chr13-41604751-41607393
chr13	41622555	41622862	chr13-41622555-41622862
chr13	41643681	41644294	chr13-41643681-41644294
chr13	41662101	41663021	chr13-41662101-41663021
chr13	41705135	41705780	chr13-41705135-41705780
chr13	41801181	41801433	chr13-41801181-41801433
chr13	41803874	41804176	chr13-41803874-41804176
chr13	41846627	41848004	chr13-41846627-41848004
chr13	42048861	42053948	chr13-42048861-42053948
chr13	42088234	42089287	chr13-42088234-42089287
chr13	42090689	42090952	chr13-42090689-42090952
chr13	42103036	42103842	chr13-42103036-42103842
chr13	42234039	42234527	chr13-42234039-42234527
chr13	42238182	42238551	chr13-42238182-42238551
chr13	42260965	42261267	chr13-42260965-42261267

chr13	42277107	42278525	chr13-42277107-42278525
chr13	42298917	42307172	chr13-42298917-42307172
chr13	42310139	42311076	chr13-42310139-42311076
chr13	42322223	42323747	chr13-42322223-42323747
chr13	42328161	42329423	chr13-42328161-42329423
chr13	42351372	42352603	chr13-42351372-42352603
chr13	42394684	42394935	chr13-42394684-42394935
chr13	42548903	42549282	chr13-42548903-42549282
chr13	42709736	42710170	chr13-42709736-42710170
chr13	42866208	42866990	chr13-42866208-42866990
chr13	42916163	42917170	chr13-42916163-42917170
chr13	43149915	43150166	chr13-43149915-43150166
chr13	43170630	43173409	chr13-43170630-43173409
chr13	43193401	43193758	chr13-43193401-43193758
chr13	43210786	43211046	chr13-43210786-43211046
chr13	43212131	43212963	chr13-43212131-43212963
chr13	43232210	43232569	chr13-43232210-43232569
chr13	43261095	43264326	chr13-43261095-43264326
chr13	43292803	43293341	chr13-43292803-43293341
chr13	43300903	43307278	chr13-43300903-43307278
chr13	43319655	43319961	chr13-43319655-43319961
chr13	43349269	43349673	chr13-43349269-43349673
chr13	43364904	43366751	chr13-43364904-43366751
chr13	43397899	43400109	chr13-43397899-43400109
chr13	43478564	43482211	chr13-43478564-43482211
chr13	43493344	43493770	chr13-43493344-43493770
chr13	43524805	43525365	chr13-43524805-43525365
chr13	43558513	43561343	chr13-43558513-43561343
chr13	43592467	43593223	chr13-43592467-43593223
chr13	43615526	43616491	chr13-43615526-43616491
chr13	43784810	43785536	chr13-43784810-43785536
chr13	43864255	43865201	chr13-43864255-43865201
chr13	44403546	44404263	chr13-44403546-44404263
chr13	44673369	44674340	chr13-44673369-44674340
chr13	44729201	44734470	chr13-44729201-44734470
chr13	44839896	44840832	chr13-44839896-44840832
chr13	44869454	44870028	chr13-44869454-44870028
chr13	44939564	44940384	chr13-44939564-44940384
chr13	45000833	45002691	chr13-45000833-45002691
chr13	45389226	45390817	chr13-45389226-45390817
chr13	45398485	45398738	chr13-45398485-45398738
chr13	45506914	45509199	chr13-45506914-45509199

chr13	45512425	45513859	chr13-45512425-45513859
chr13	45516572	45517341	chr13-45516572-45517341
chr13	45530675	45531118	chr13-45530675-45531118
chr13	45539142	45539406	chr13-45539142-45539406
chr13	45627362	45627625	chr13-45627362-45627625
chr13	45684512	45685847	chr13-45684512-45685847
chr13	45692637	45692904	chr13-45692637-45692904
chr13	45694384	45695833	chr13-45694384-45695833
chr13	45706944	45707351	chr13-45706944-45707351
chr13	45718582	45718961	chr13-45718582-45718961
chr13	45803141	45803467	chr13-45803141-45803467
chr13	45820399	45821241	chr13-45820399-45821241
chr13	45837463	45837714	chr13-45837463-45837714
chr13	45840060	45840319	chr13-45840060-45840319
chr13	45906305	45907796	chr13-45906305-45907796
chr13	45924686	45925369	chr13-45924686-45925369
chr13	45961905	45967219	chr13-45961905-45967219
chr13	45970583	45971403	chr13-45970583-45971403
chr13	46161339	46162225	chr13-46161339-46162225
chr13	46418212	46419320	chr13-46418212-46419320
chr13	46501859	46502712	chr13-46501859-46502712
chr13	46668585	46671127	chr13-46668585-46671127
chr13	46696540	46696952	chr13-46696540-46696952
chr13	46724738	46728800	chr13-46724738-46728800
chr13	46735454	46737087	chr13-46735454-46737087
chr13	46841796	46842701	chr13-46841796-46842701
chr13	46888552	46888856	chr13-46888552-46888856
chr13	46909141	46909453	chr13-46909141-46909453
chr13	46928682	46931163	chr13-46928682-46931163
chr13	47014001	47015224	chr13-47014001-47015224
chr13	47042208	47045016	chr13-47042208-47045016
chr13	47104225	47107178	chr13-47104225-47107178
chr13	47122222	47123256	chr13-47122222-47123256
chr13	47170873	47171724	chr13-47170873-47171724
chr13	47193309	47195829	chr13-47193309-47195829
chr13	47272705	47273851	chr13-47272705-47273851
chr13	47336109	47336310	chr13-47336109-47336310
chr13	48105085	48105391	chr13-48105085-48105391
chr13	48110493	48110808	chr13-48110493-48110808
chr13	48130643	48131052	chr13-48130643-48131052
chr13	48227273	48228406	chr13-48227273-48228406
chr13	48260446	48263205	chr13-48260446-48263205

chr13	48273557	48273932	chr13-48273557-48273932
chr13	48491015	48493390	chr13-48491015-48493390
chr13	48512507	48513900	chr13-48512507-48513900
chr13	48543678	48547110	chr13-48543678-48547110
chr13	48552015	48553291	chr13-48552015-48553291
chr13	48565703	48567576	chr13-48565703-48567576
chr13	48571220	48571720	chr13-48571220-48571720
chr13	48624809	48626206	chr13-48624809-48626206
chr13	48653726	48653927	chr13-48653726-48653927
chr13	48655881	48656082	chr13-48655881-48656082
chr13	48658436	48660057	chr13-48658436-48660057
chr13	48662504	48663623	chr13-48662504-48663623
chr13	48664898	48666479	chr13-48664898-48666479
chr13	48668368	48670030	chr13-48668368-48670030
chr13	48672455	48672781	chr13-48672455-48672781
chr13	48706367	48706941	chr13-48706367-48706941
chr13	48731151	48732915	chr13-48731151-48732915
chr13	48869044	48872370	chr13-48869044-48872370
chr13	48877884	48879170	chr13-48877884-48879170
chr13	48964891	48969391	chr13-48964891-48969391
chr13	49013618	49014072	chr13-49013618-49014072
chr13	49024785	49025252	chr13-49024785-49025252
chr13	49146526	49148400	chr13-49146526-49148400
chr13	49186789	49190520	chr13-49186789-49190520
chr13	49205185	49205439	chr13-49205185-49205439
chr13	49212997	49216758	chr13-49212997-49216758
chr13	49247557	49248460	chr13-49247557-49248460
chr13	49293574	49301743	chr13-49293574-49301743
chr13	49307223	49308269	chr13-49307223-49308269
chr13	49311588	49311789	chr13-49311588-49311789
chr13	49316146	49322316	chr13-49316146-49322316
chr13	49340526	49343312	chr13-49340526-49343312
chr13	49344494	49346649	chr13-49344494-49346649
chr13	49362264	49362742	chr13-49362264-49362742
chr13	49396087	49396602	chr13-49396087-49396602
chr13	49400541	49403242	chr13-49400541-49403242
chr13	49419742	49423343	chr13-49419742-49423343
chr13	49549480	49549784	chr13-49549480-49549784
chr13	49581078	49582159	chr13-49581078-49582159
chr13	49651896	49654589	chr13-49651896-49654589
chr13	49681118	49684451	chr13-49681118-49684451
chr13	50417379	50419165	chr13-50417379-50419165

chr13	50957590	50958845	chr13-50957590-50958845
chr13	51098930	51102768	chr13-51098930-51102768
chr13	51128041	51129090	chr13-51128041-51129090
chr13	51170275	51172004	chr13-51170275-51172004
chr13	51202580	51203138	chr13-51202580-51203138
chr13	51207907	51208215	chr13-51207907-51208215
chr13	51400897	51401180	chr13-51400897-51401180
chr13	51408536	51409169	chr13-51408536-51409169
chr13	51418723	51419094	chr13-51418723-51419094
chr13	51566909	51569870	chr13-51566909-51569870
chr13	51594653	51595317	chr13-51594653-51595317
chr13	51603302	51603625	chr13-51603302-51603625
chr13	51644158	51648856	chr13-51644158-51648856
chr13	51650786	51652818	chr13-51650786-51652818
chr13	51739496	51740347	chr13-51739496-51740347
chr13	51748054	51749207	chr13-51748054-51749207
chr13	51753646	51755298	chr13-51753646-51755298
chr13	51756420	51756762	chr13-51756420-51756762
chr13	51762557	51763776	chr13-51762557-51763776
chr13	51790284	51794270	chr13-51790284-51794270
chr13	51845221	51849962	chr13-51845221-51849962
chr13	51852281	51854468	chr13-51852281-51854468
chr13	51866350	51866725	chr13-51866350-51866725
chr13	51962268	51964995	chr13-51962268-51964995
chr13	51968404	51970667	chr13-51968404-51970667
chr13	51972046	51977120	chr13-51972046-51977120
chr13	52002077	52002614	chr13-52002077-52002614
chr13	52017764	52018022	chr13-52017764-52018022
chr13	52061613	52062049	chr13-52061613-52062049
chr13	52065128	52065392	chr13-52065128-52065392
chr13	52079650	52080346	chr13-52079650-52080346
chr13	52235964	52236647	chr13-52235964-52236647
chr13	52249914	52250115	chr13-52249914-52250115
chr13	52344638	52345603	chr13-52344638-52345603
chr13	52455312	52456062	chr13-52455312-52456062
chr13	52530137	52530952	chr13-52530137-52530952
chr13	52582522	52585480	chr13-52582522-52585480
chr13	52587799	52588000	chr13-52587799-52588000
chr13	52658610	52660163	chr13-52658610-52660163
chr13	52674943	52676651	chr13-52674943-52676651
chr13	52818923	52819182	chr13-52818923-52819182
chr13	52824891	52825092	chr13-52824891-52825092

chr13	52828989	52829446	chr13-52828989-52829446
chr13	52892056	52892723	chr13-52892056-52892723
chr13	52928442	52930291	chr13-52928442-52930291
chr13	52973343	52973597	chr13-52973343-52973597
chr13	52976403	52983239	chr13-52976403-52983239
chr13	53008782	53009440	chr13-53008782-53009440
chr13	53019614	53019959	chr13-53019614-53019959
chr13	53093783	53093984	chr13-53093783-53093984
chr13	53099921	53100385	chr13-53099921-53100385
chr13	53211858	53212439	chr13-53211858-53212439
chr13	53284781	53286532	chr13-53284781-53286532
chr13	53309755	53310165	chr13-53309755-53310165
chr13	53339353	53339765	chr13-53339353-53339765
chr13	53376401	53378079	chr13-53376401-53378079
chr13	53442544	53442745	chr13-53442544-53442745
chr13	53461790	53463504	chr13-53461790-53463504
chr13	53465687	53465888	chr13-53465687-53465888
chr13	53469905	53473212	chr13-53469905-53473212
chr13	53475063	53476639	chr13-53475063-53476639
chr13	53502287	53502790	chr13-53502287-53502790
chr13	53653045	53653297	chr13-53653045-53653297
chr13	53654902	53655218	chr13-53654902-53655218
chr13	53658629	53660231	chr13-53658629-53660231
chr13	53662971	53663479	chr13-53662971-53663479
chr13	53720890	53721531	chr13-53720890-53721531
chr13	53730217	53730701	chr13-53730217-53730701
chr13	54055039	54055929	chr13-54055039-54055929
chr13	54071488	54073990	chr13-54071488-54073990
chr13	54100693	54101801	chr13-54100693-54101801
chr13	54231891	54232323	chr13-54231891-54232323
chr13	54302337	54304061	chr13-54302337-54304061
chr13	54371430	54371791	chr13-54371430-54371791
chr13	54467323	54469579	chr13-54467323-54469579
chr13	54503187	54505539	chr13-54503187-54505539
chr13	54563960	54565992	chr13-54563960-54565992
chr13	54574453	54576119	chr13-54574453-54576119
chr13	54588849	54592049	chr13-54588849-54592049
chr13	54604782	54606250	chr13-54604782-54606250
chr13	54607053	54611916	chr13-54607053-54611916
chr13	54621368	54623691	chr13-54621368-54623691
chr13	54646978	54647179	chr13-54646978-54647179
chr13	54660670	54662158	chr13-54660670-54662158

chr13	54671567	54671856	chr13-54671567-54671856
chr13	54675974	54676349	chr13-54675974-54676349
chr13	54684806	54689221	chr13-54684806-54689221
chr13	54691814	54695248	chr13-54691814-54695248
chr13	54737513	54738509	chr13-54737513-54738509
chr13	54749124	54750317	chr13-54749124-54750317
chr13	54757672	54759146	chr13-54757672-54759146
chr13	54765632	54766767	chr13-54765632-54766767
chr13	54788652	54790679	chr13-54788652-54790679
chr13	54948680	54950186	chr13-54948680-54950186
chr13	54955617	54956686	chr13-54955617-54956686
chr13	55004641	55005238	chr13-55004641-55005238
chr13	55006118	55006432	chr13-55006118-55006432
chr13	55098881	55100717	chr13-55098881-55100717
chr13	55104758	55106558	chr13-55104758-55106558
chr13	55152572	55153342	chr13-55152572-55153342
chr13	55171842	55172214	chr13-55171842-55172214
chr13	55183562	55191970	chr13-55183562-55191970
chr13	55210137	55216042	chr13-55210137-55216042
chr13	55320401	55324509	chr13-55320401-55324509
chr13	55328322	55331129	chr13-55328322-55331129
chr13	55360778	55363346	chr13-55360778-55363346
chr13	55369715	55370167	chr13-55369715-55370167
chr13	55379189	55379705	chr13-55379189-55379705
chr13	55417950	55418527	chr13-55417950-55418527
chr13	55420867	55421744	chr13-55420867-55421744
chr13	55429306	55429791	chr13-55429306-55429791
chr13	55443697	55447026	chr13-55443697-55447026
chr13	55450294	55450495	chr13-55450294-55450495
chr13	55463810	55465651	chr13-55463810-55465651
chr13	55470843	55473495	chr13-55470843-55473495
chr13	55485531	55488432	chr13-55485531-55488432
chr13	55509186	55514132	chr13-55509186-55514132
chr13	55535149	55537322	chr13-55535149-55537322
chr13	55569429	55572437	chr13-55569429-55572437
chr13	55592583	55594315	chr13-55592583-55594315
chr13	55599478	55601331	chr13-55599478-55601331
chr13	55604279	55605314	chr13-55604279-55605314
chr13	55621820	55624426	chr13-55621820-55624426
chr13	55634613	55636694	chr13-55634613-55636694
chr13	55692005	55695396	chr13-55692005-55695396
chr13	55714053	55715975	chr13-55714053-55715975

chr13	55726643	55728264	chr13-55726643-55728264
chr13	55826007	55826425	chr13-55826007-55826425
chr13	55827980	55828984	chr13-55827980-55828984
chr13	55829935	55831883	chr13-55829935-55831883
chr13	55835939	55836454	chr13-55835939-55836454
chr13	55847850	55848283	chr13-55847850-55848283
chr13	55963077	55963538	chr13-55963077-55963538
chr13	55992697	55992898	chr13-55992697-55992898
chr13	56021496	56021850	chr13-56021496-56021850
chr13	56027983	56028184	chr13-56027983-56028184
chr13	56113741	56114011	chr13-56113741-56114011
chr13	56133330	56136453	chr13-56133330-56136453
chr13	56197380	56197645	chr13-56197380-56197645
chr13	56221105	56222479	chr13-56221105-56222479
chr13	56251608	56252684	chr13-56251608-56252684
chr13	56258501	56259533	chr13-56258501-56259533
chr13	56295499	56296910	chr13-56295499-56296910
chr13	56404219	56405397	chr13-56404219-56405397
chr13	56425910	56426806	chr13-56425910-56426806
chr13	56437474	56441452	chr13-56437474-56441452
chr13	56442643	56444229	chr13-56442643-56444229
chr13	56448876	56450018	chr13-56448876-56450018
chr13	56521879	56523223	chr13-56521879-56523223
chr13	56582145	56582777	chr13-56582145-56582777
chr13	56609171	56610140	chr13-56609171-56610140
chr13	56626601	56626987	chr13-56626601-56626987
chr13	56679239	56679551	chr13-56679239-56679551
chr13	56681995	56682883	chr13-56681995-56682883
chr13	56702344	56705308	chr13-56702344-56705308
chr13	56806651	56807327	chr13-56806651-56807327
chr13	57907820	57908446	chr13-57907820-57908446
chr13	58112981	58113870	chr13-58112981-58113870
chr13	58125658	58130258	chr13-58125658-58130258
chr13	58148325	58149658	chr13-58148325-58149658
chr13	58157081	58158889	chr13-58157081-58158889
chr13	58213729	58216758	chr13-58213729-58216758
chr13	58273070	58275256	chr13-58273070-58275256
chr13	58358435	58359773	chr13-58358435-58359773
chr13	58384125	58385679	chr13-58384125-58385679
chr13	58399486	58404135	chr13-58399486-58404135
chr13	58452942	58453459	chr13-58452942-58453459
chr13	58533709	58536302	chr13-58533709-58536302

chr13	58589432	58589633	chr13-58589432-58589633
chr13	58622682	58623144	chr13-58622682-58623144
chr13	58630966	58631605	chr13-58630966-58631605
chr13	58806451	58808315	chr13-58806451-58808315
chr13	59452317	59453892	chr13-59452317-59453892
chr13	59509725	59510100	chr13-59509725-59510100
chr13	59555199	59558242	chr13-59555199-59558242
chr13	59584299	59587255	chr13-59584299-59587255
chr13	59663382	59664342	chr13-59663382-59664342
chr13	59668281	59669567	chr13-59668281-59669567
chr13	59673719	59676373	chr13-59673719-59676373
chr13	59768145	59771625	chr13-59768145-59771625
chr13	59820320	59823821	chr13-59820320-59823821
chr13	59842291	59842771	chr13-59842291-59842771
chr13	59853449	59853946	chr13-59853449-59853946
chr13	60019586	60019947	chr13-60019586-60019947
chr13	60175559	60178682	chr13-60175559-60178682
chr13	60426993	60428156	chr13-60426993-60428156
chr13	60455063	60455529	chr13-60455063-60455529
chr13	60479116	60480141	chr13-60479116-60480141
chr13	60601116	60603426	chr13-60601116-60603426
chr13	60662290	60663260	chr13-60662290-60663260
chr13	60684008	60684571	chr13-60684008-60684571
chr13	60737647	60738671	chr13-60737647-60738671
chr13	60895609	60897639	chr13-60895609-60897639
chr13	61665758	61666174	chr13-61665758-61666174
chr13	61803836	61805170	chr13-61803836-61805170
chr13	61998580	61999053	chr13-61998580-61999053
chr13	62015365	62016902	chr13-62015365-62016902
chr13	62129410	62130746	chr13-62129410-62130746
chr13	62382384	62383695	chr13-62382384-62383695
chr13	62465811	62467432	chr13-62465811-62467432
chr13	62557788	62559143	chr13-62557788-62559143
chr13	62623711	62624550	chr13-62623711-62624550
chr13	62652617	62653710	chr13-62652617-62653710
chr13	62683992	62685484	chr13-62683992-62685484
chr13	62760155	62761023	chr13-62760155-62761023
chr13	62785065	62786342	chr13-62785065-62786342
chr13	62812751	62813316	chr13-62812751-62813316
chr13	62855995	62856690	chr13-62855995-62856690
chr13	62858056	62858772	chr13-62858056-62858772
chr13	62887708	62888346	chr13-62887708-62888346

chr13	62964364	62966051	chr13-62964364-62966051
chr13	63046058	63046336	chr13-63046058-63046336
chr13	63186091	63188659	chr13-63186091-63188659
chr13	63239314	63242990	chr13-63239314-63242990
chr13	63243795	63244050	chr13-63243795-63244050
chr13	63246389	63246651	chr13-63246389-63246651
chr13	63252878	63253503	chr13-63252878-63253503
chr13	63258085	63258399	chr13-63258085-63258399
chr13	63270160	63271051	chr13-63270160-63271051
chr13	63271904	63279143	chr13-63271904-63279143
chr13	63304804	63305583	chr13-63304804-63305583
chr13	63430121	63432621	chr13-63430121-63432621
chr13	63562288	63568683	chr13-63562288-63568683
chr13	63572178	63573845	chr13-63572178-63573845
chr13	63642994	63644018	chr13-63642994-63644018
chr13	63748769	63749430	chr13-63748769-63749430
chr13	63814315	63816359	chr13-63814315-63816359
chr13	63917058	63917743	chr13-63917058-63917743
chr13	64128620	64130009	chr13-64128620-64130009
chr13	64150785	64155967	chr13-64150785-64155967
chr13	64161223	64163595	chr13-64161223-64163595
chr13	64248056	64249450	chr13-64248056-64249450
chr13	64273718	64275930	chr13-64273718-64275930
chr13	64310487	64313281	chr13-64310487-64313281
chr13	64366169	64371364	chr13-64366169-64371364
chr13	64377455	64377820	chr13-64377455-64377820
chr13	64420657	64422883	chr13-64420657-64422883
chr13	64431644	64433447	chr13-64431644-64433447
chr13	64439229	64439753	chr13-64439229-64439753
chr13	65111652	65113590	chr13-65111652-65113590
chr13	65175826	65176405	chr13-65175826-65176405
chr13	65239994	65243149	chr13-65239994-65243149
chr13	65277629	65279991	chr13-65277629-65279991
chr13	65311371	65311640	chr13-65311371-65311640
chr13	65351760	65352338	chr13-65351760-65352338
chr13	65420438	65420639	chr13-65420438-65420639
chr13	65726283	65726535	chr13-65726283-65726535
chr13	66625795	66626099	chr13-66625795-66626099
chr13	66807508	66808169	chr13-66807508-66808169
chr13	66904142	66905851	chr13-66904142-66905851
chr13	66931763	66934528	chr13-66931763-66934528
chr13	66984935	66985136	chr13-66984935-66985136

chr13	67060180	67061815	chr13-67060180-67061815
chr13	67080484	67082125	chr13-67080484-67082125
chr13	67107415	67109301	chr13-67107415-67109301
chr13	67127866	67128981	chr13-67127866-67128981
chr13	67172484	67174309	chr13-67172484-67174309
chr13	67194297	67195164	chr13-67194297-67195164
chr13	67268359	67269722	chr13-67268359-67269722
chr13	67278197	67278555	chr13-67278197-67278555
chr13	67305645	67306983	chr13-67305645-67306983
chr13	67331783	67333208	chr13-67331783-67333208
chr13	67359828	67361042	chr13-67359828-67361042
chr13	67375011	67376101	chr13-67375011-67376101
chr13	67398832	67400295	chr13-67398832-67400295
chr13	67420453	67422028	chr13-67420453-67422028
chr13	67450775	67452217	chr13-67450775-67452217
chr13	67483314	67484798	chr13-67483314-67484798
chr13	67499296	67500878	chr13-67499296-67500878
chr13	67524942	67526742	chr13-67524942-67526742
chr13	67551750	67553889	chr13-67551750-67553889
chr13	67608626	67610201	chr13-67608626-67610201
chr13	67636759	67638365	chr13-67636759-67638365
chr13	67682624	67684246	chr13-67682624-67684246
chr13	67723312	67724848	chr13-67723312-67724848
chr13	67728314	67729739	chr13-67728314-67729739
chr13	67754259	67755631	chr13-67754259-67755631
chr13	67779318	67780399	chr13-67779318-67780399
chr13	67813615	67814934	chr13-67813615-67814934
chr13	67830679	67831408	chr13-67830679-67831408
chr13	67844067	67845055	chr13-67844067-67845055
chr13	68043903	68044264	chr13-68043903-68044264
chr13	68580755	68583805	chr13-68580755-68583805
chr13	68597590	68597791	chr13-68597590-68597791
chr13	68605537	68606205	chr13-68605537-68606205
chr13	68998733	68999798	chr13-68998733-68999798
chr13	69487337	69490583	chr13-69487337-69490583
chr13	69516570	69518285	chr13-69516570-69518285
chr13	69524279	69525757	chr13-69524279-69525757
chr13	69530785	69536649	chr13-69530785-69536649
chr13	69541006	69543378	chr13-69541006-69543378
chr13	69585706	69585960	chr13-69585706-69585960
chr13	69593020	69593767	chr13-69593020-69593767
chr13	69598909	69599110	chr13-69598909-69599110

chr13	69610378	69614054	chr13-69610378-69614054
chr13	69738336	69740851	chr13-69738336-69740851
chr13	69768026	69768333	chr13-69768026-69768333
chr13	69809420	69811587	chr13-69809420-69811587
chr13	70635664	70638837	chr13-70635664-70638837
chr13	70653977	70655416	chr13-70653977-70655416
chr13	70657622	70657992	chr13-70657622-70657992
chr13	70681004	70682575	chr13-70681004-70682575
chr13	70689206	70689617	chr13-70689206-70689617
chr13	70731609	70731941	chr13-70731609-70731941
chr13	70733315	70733595	chr13-70733315-70733595
chr13	70841199	70841804	chr13-70841199-70841804
chr13	71104623	71105404	chr13-71104623-71105404
chr13	71458555	71458949	chr13-71458555-71458949
chr13	71956571	71964869	chr13-71956571-71964869
chr13	72120572	72120831	chr13-72120572-72120831
chr13	72198091	72199259	chr13-72198091-72199259
chr13	72262091	72264486	chr13-72262091-72264486
chr13	72280445	72280856	chr13-72280445-72280856
chr13	72282593	72282948	chr13-72282593-72282948
chr13	72301412	72302256	chr13-72301412-72302256
chr13	72352529	72353311	chr13-72352529-72353311
chr13	72623082	72640855	chr13-72623082-72640855
chr13	73033544	73034674	chr13-73033544-73034674
chr13	73077594	73077795	chr13-73077594-73077795
chr13	73160001	73160817	chr13-73160001-73160817
chr13	73260247	73261068	chr13-73260247-73261068
chr13	73263189	73263806	chr13-73263189-73263806
chr13	73265454	73265979	chr13-73265454-73265979
chr13	73268433	73269176	chr13-73268433-73269176
chr13	73270749	73272263	chr13-73270749-73272263
chr13	73290013	73290324	chr13-73290013-73290324
chr13	73327252	73329304	chr13-73327252-73329304
chr13	73330742	73332420	chr13-73330742-73332420
chr13	73345097	73347509	chr13-73345097-73347509
chr13	73355282	73355594	chr13-73355282-73355594
chr13	73374592	73374793	chr13-73374592-73374793
chr13	73429522	73430088	chr13-73429522-73430088
chr13	73430978	73433032	chr13-73430978-73433032
chr13	73441825	73442083	chr13-73441825-73442083
chr13	73454083	73454708	chr13-73454083-73454708
chr13	73466910	73470762	chr13-73466910-73470762

chr13	73479072	73479797	chr13-73479072-73479797
chr13	73535932	73537259	chr13-73535932-73537259
chr13	73575242	73575600	chr13-73575242-73575600
chr13	73602776	73605665	chr13-73602776-73605665
chr13	73626412	73627946	chr13-73626412-73627946
chr13	73680162	73681004	chr13-73680162-73681004
chr13	73732752	73733476	chr13-73732752-73733476
chr13	73763062	73765453	chr13-73763062-73765453
chr13	73803250	73804200	chr13-73803250-73804200
chr13	73846638	73847924	chr13-73846638-73847924
chr13	73936497	73939002	chr13-73936497-73939002
chr13	73970644	73971291	chr13-73970644-73971291
chr13	74008785	74010717	chr13-74008785-74010717
chr13	74061494	74062869	chr13-74061494-74062869
chr13	74120998	74121757	chr13-74120998-74121757
chr13	74166789	74167194	chr13-74166789-74167194
chr13	74207867	74209558	chr13-74207867-74209558
chr13	74259794	74260361	chr13-74259794-74260361
chr13	74292051	74292611	chr13-74292051-74292611
chr13	74301330	74302118	chr13-74301330-74302118
chr13	74315728	74318122	chr13-74315728-74318122
chr13	74348923	74351491	chr13-74348923-74351491
chr13	74363281	74364241	chr13-74363281-74364241
chr13	74389837	74391015	chr13-74389837-74391015
chr13	74399606	74399912	chr13-74399606-74399912
chr13	74406761	74407064	chr13-74406761-74407064
chr13	74461661	74463752	chr13-74461661-74463752
chr13	74493031	74494926	chr13-74493031-74494926
chr13	74522222	74522998	chr13-74522222-74522998
chr13	74562946	74564950	chr13-74562946-74564950
chr13	74616664	74618359	chr13-74616664-74618359
chr13	74639013	74640791	chr13-74639013-74640791
chr13	74766703	74773127	chr13-74766703-74773127
chr13	74806330	74809043	chr13-74806330-74809043
chr13	75089686	75090859	chr13-75089686-75090859
chr13	75706829	75710741	chr13-75706829-75710741
chr13	75789164	75789365	chr13-75789164-75789365
chr13	75797364	75799477	chr13-75797364-75799477
chr13	75839511	75842624	chr13-75839511-75842624
chr13	75843806	75844718	chr13-75843806-75844718
chr13	75942356	75944884	chr13-75942356-75944884
chr13	76016647	76019518	chr13-76016647-76019518

chr13	76055350	76056959	chr13-76055350-76056959
chr13	76097882	76100175	chr13-76097882-76100175
chr13	76383716	76385463	chr13-76383716-76385463
chr13	76579587	76579890	chr13-76579587-76579890
chr13	77134304	77136361	chr13-77134304-77136361
chr13	77708164	77709961	chr13-77708164-77709961
chr13	78162827	78164310	chr13-78162827-78164310
chr13	78179270	78179782	chr13-78179270-78179782
chr13	78180993	78181937	chr13-78180993-78181937
chr13	78194828	78195939	chr13-78194828-78195939
chr13	78197312	78204301	chr13-78197312-78204301
chr13	78209675	78210953	chr13-78209675-78210953
chr13	80882518	80887220	chr13-80882518-80887220
chr13	80940447	80942370	chr13-80940447-80942370
chr13	80951260	80952103	chr13-80951260-80952103
chr13	80962204	80963886	chr13-80962204-80963886
chr13	81050589	81052001	chr13-81050589-81052001
chr13	81632572	81633526	chr13-81632572-81633526
chr13	81640826	81641910	chr13-81640826-81641910
chr13	81656526	81659011	chr13-81656526-81659011
chr13	81684122	81684566	chr13-81684122-81684566
chr13	81709525	81712606	chr13-81709525-81712606
chr13	81782424	81784801	chr13-81782424-81784801
chr13	82780966	82781249	chr13-82780966-82781249
chr13	83518446	83518647	chr13-83518446-83518647
chr13	83523549	83524998	chr13-83523549-83524998
chr13	83715159	83715414	chr13-83715159-83715414
chr13	83716224	83716425	chr13-83716224-83716425
chr13	83720828	83721323	chr13-83720828-83721323
chr13	83727026	83727590	chr13-83727026-83727590
chr13	83729203	83731700	chr13-83729203-83731700
chr13	83732695	83734020	chr13-83732695-83734020
chr13	83737901	83739044	chr13-83737901-83739044
chr13	83744865	83745521	chr13-83744865-83745521
chr13	84221259	84223653	chr13-84221259-84223653
chr13	84347126	84349643	chr13-84347126-84349643
chr13	85188913	85190523	chr13-85188913-85190523
chr13	85286466	85291387	chr13-85286466-85291387
chr13	85419204	85419405	chr13-85419204-85419405
chr13	85481799	85482107	chr13-85481799-85482107
chr13	86045529	86047048	chr13-86045529-86047048
chr13	86357692	86357893	chr13-86357692-86357893

chr13	88821102	88822451	chr13-88821102-88822451
chr13	88823620	88824292	chr13-88823620-88824292
chr13	89444045	89444297	chr13-89444045-89444297
chr13	89539155	89539870	chr13-89539155-89539870
chr13	89738857	89742616	chr13-89738857-89742616
chr13	90088370	90091168	chr13-90088370-90091168
chr13	90886463	90886929	chr13-90886463-90886929
chr13	90922469	90924752	chr13-90922469-90924752
chr13	91206416	91206773	chr13-91206416-91206773
chr13	91223090	91224772	chr13-91223090-91224772
chr13	91370446	91371746	chr13-91370446-91371746
chr13	91430923	91431175	chr13-91430923-91431175
chr13	91455861	91456217	chr13-91455861-91456217
chr13	91459941	91462566	chr13-91459941-91462566
chr13	91741169	91741946	chr13-91741169-91741946
chr13	91806497	91808802	chr13-91806497-91808802
chr13	92130948	92132190	chr13-92130948-92132190
chr13	92353836	92356620	chr13-92353836-92356620
chr13	92425631	92427248	chr13-92425631-92427248
chr13	92483605	92484483	chr13-92483605-92484483
chr13	92529782	92531914	chr13-92529782-92531914
chr13	92609693	92612362	chr13-92609693-92612362
chr13	92679740	92679941	chr13-92679740-92679941
chr13	92722480	92722889	chr13-92722480-92722889
chr13	92794434	92795113	chr13-92794434-92795113
chr13	92844513	92845639	chr13-92844513-92845639
chr13	93143862	93144981	chr13-93143862-93144981
chr13	93190903	93192978	chr13-93190903-93192978
chr13	93302828	93306146	chr13-93302828-93306146
chr13	93493664	93493918	chr13-93493664-93493918
chr13	93496901	93500445	chr13-93496901-93500445
chr13	93771190	93772878	chr13-93771190-93772878
chr13	93985316	93985517	chr13-93985316-93985517
chr13	93997600	93998694	chr13-93997600-93998694
chr13	94024907	94025213	chr13-94024907-94025213
chr13	94057165	94058861	chr13-94057165-94058861
chr13	94062433	94063471	chr13-94062433-94063471
chr13	94152560	94152761	chr13-94152560-94152761
chr13	94154737	94155046	chr13-94154737-94155046
chr13	94173600	94174875	chr13-94173600-94174875
chr13	94193141	94193342	chr13-94193141-94193342
chr13	94284105	94286589	chr13-94284105-94286589

chr13	94328211	94328522	chr13-94328211-94328522
chr13	94357851	94360227	chr13-94357851-94360227
chr13	94382199	94382586	chr13-94382199-94382586
chr13	94595921	94596330	chr13-94595921-94596330
chr13	94702379	94703420	chr13-94702379-94703420
chr13	94709686	94710701	chr13-94709686-94710701
chr13	94788562	94790429	chr13-94788562-94790429
chr13	94829660	94829861	chr13-94829660-94829861
chr13	94868403	94869503	chr13-94868403-94869503
chr13	94875254	94875558	chr13-94875254-94875558
chr13	94882680	94884071	chr13-94882680-94884071
chr13	94885212	94885881	chr13-94885212-94885881
chr13	94975761	94977159	chr13-94975761-94977159
chr13	95222374	95223431	chr13-95222374-95223431
chr13	95250082	95250341	chr13-95250082-95250341
chr13	95324266	95327835	chr13-95324266-95327835
chr13	95373501	95375919	chr13-95373501-95375919
chr13	95443342	95444445	chr13-95443342-95444445
chr13	95484903	95485321	chr13-95484903-95485321
chr13	95522390	95525939	chr13-95522390-95525939
chr13	95535362	95535680	chr13-95535362-95535680
chr13	95545885	95546657	chr13-95545885-95546657
chr13	95548235	95548758	chr13-95548235-95548758
chr13	95554246	95554447	chr13-95554246-95554447
chr13	95607991	95609269	chr13-95607991-95609269
chr13	95611463	95612210	chr13-95611463-95612210
chr13	95614649	95619411	chr13-95614649-95619411
chr13	95622826	95623133	chr13-95622826-95623133
chr13	95639898	95641432	chr13-95639898-95641432
chr13	95705196	95705825	chr13-95705196-95705825
chr13	95719314	95720240	chr13-95719314-95720240
chr13	95889937	95892766	chr13-95889937-95892766
chr13	95960919	95961745	chr13-95960919-95961745
chr13	96010988	96011344	chr13-96010988-96011344
chr13	96131021	96133079	chr13-96131021-96133079
chr13	96387714	96389654	chr13-96387714-96389654
chr13	96487207	96487408	chr13-96487207-96487408
chr13	96510833	96511367	chr13-96510833-96511367
chr13	96533251	96533860	chr13-96533251-96533860
chr13	96537318	96537571	chr13-96537318-96537571
chr13	96540220	96544554	chr13-96540220-96544554
chr13	96665996	96666197	chr13-96665996-96666197

chr13	96667088	96671704	chr13-96667088-96671704
chr13	96680167	96680898	chr13-96680167-96680898
chr13	96792821	96793072	chr13-96792821-96793072
chr13	96923935	96926842	chr13-96923935-96926842
chr13	96963080	96963281	chr13-96963080-96963281
chr13	97007253	97009148	chr13-97007253-97009148
chr13	97070863	97072460	chr13-97070863-97072460
chr13	97136841	97139194	chr13-97136841-97139194
chr13	97197323	97198705	chr13-97197323-97198705
chr13	97204736	97205853	chr13-97204736-97205853
chr13	97234867	97235270	chr13-97234867-97235270
chr13	97238199	97238400	chr13-97238199-97238400
chr13	97239657	97245258	chr13-97239657-97245258
chr13	97247786	97251016	chr13-97247786-97251016
chr13	97282060	97282525	chr13-97282060-97282525
chr13	97299840	97301012	chr13-97299840-97301012
chr13	97310200	97311519	chr13-97310200-97311519
chr13	97364024	97364225	chr13-97364024-97364225
chr13	97386756	97387214	chr13-97386756-97387214
chr13	97916378	97916579	chr13-97916378-97916579
chr13	97928013	97928214	chr13-97928013-97928214
chr13	98013344	98017451	chr13-98013344-98017451
chr13	98027451	98028685	chr13-98027451-98028685
chr13	98031490	98031894	chr13-98031490-98031894
chr13	98120452	98121197	chr13-98120452-98121197
chr13	98122163	98122433	chr13-98122163-98122433
chr13	98132775	98134075	chr13-98132775-98134075
chr13	98140493	98140950	chr13-98140493-98140950
chr13	98145326	98145747	chr13-98145326-98145747
chr13	98192836	98193142	chr13-98192836-98193142
chr13	98200990	98201242	chr13-98200990-98201242
chr13	98203815	98207016	chr13-98203815-98207016
chr13	98261607	98264505	chr13-98261607-98264505
chr13	98296393	98296594	chr13-98296393-98296594
chr13	98314907	98318018	chr13-98314907-98318018
chr13	98353699	98356260	chr13-98353699-98356260
chr13	98365521	98366773	chr13-98365521-98366773
chr13	98390818	98391742	chr13-98390818-98391742
chr13	98427696	98428163	chr13-98427696-98428163
chr13	98497706	98497907	chr13-98497706-98497907
chr13	98502132	98505692	chr13-98502132-98505692
chr13	98534201	98535280	chr13-98534201-98535280

chr13	98572587	98573358	chr13-98572587-98573358
chr13	98588521	98589740	chr13-98588521-98589740
chr13	98622500	98623766	chr13-98622500-98623766
chr13	98632542	98632846	chr13-98632542-98632846
chr13	98679727	98680260	chr13-98679727-98680260
chr13	98689299	98695935	chr13-98689299-98695935
chr13	98813490	98817096	chr13-98813490-98817096
chr13	98818537	98820030	chr13-98818537-98820030
chr13	98889226	98891873	chr13-98889226-98891873
chr13	98894265	98894466	chr13-98894265-98894466
chr13	98923753	98927297	chr13-98923753-98927297
chr13	98942485	98942844	chr13-98942485-98942844
chr13	98944632	98945087	chr13-98944632-98945087
chr13	98955198	98956004	chr13-98955198-98956004
chr13	99058507	99058875	chr13-99058507-99058875
chr13	99125026	99125227	chr13-99125026-99125227
chr13	99150610	99153274	chr13-99150610-99153274
chr13	99163933	99164361	chr13-99163933-99164361
chr13	99173413	99174277	chr13-99173413-99174277
chr13	99194486	99194891	chr13-99194486-99194891
chr13	99238258	99239417	chr13-99238258-99239417
chr13	99308306	99308608	chr13-99308306-99308608
chr13	99343410	99345874	chr13-99343410-99345874
chr13	99353031	99354183	chr13-99353031-99354183
chr13	99384282	99385084	chr13-99384282-99385084
chr13	99391258	99391509	chr13-99391258-99391509
chr13	99514639	99517314	chr13-99514639-99517314
chr13	99528283	99528484	chr13-99528283-99528484
chr13	99592568	99593360	chr13-99592568-99593360
chr13	99647680	99647989	chr13-99647680-99647989
chr13	99900382	99900640	chr13-99900382-99900640
chr13	99921828	99922029	chr13-99921828-99922029
chr13	100014529	100016359	chr13-100014529-100016359
chr13	100101832	100104792	chr13-100101832-100104792
chr13	100107338	100108957	chr13-100107338-100108957
chr13	100123955	100126197	chr13-100123955-100126197
chr13	100491133	100493107	chr13-100491133-100493107
chr13	100511412	100511663	chr13-100511412-100511663
chr13	100535740	100536658	chr13-100535740-100536658
chr13	100543067	100543931	chr13-100543067-100543931
chr13	100550067	100553704	chr13-100550067-100553704
chr13	100581748	100582331	chr13-100581748-100582331

chr13	100588667	100589069	chr13-100588667-100589069
chr13	100615126	100617249	chr13-100615126-100617249
chr13	100649952	100653065	chr13-100649952-100653065
chr13	100668999	100671292	chr13-100668999-100671292
chr13	100728904	100731604	chr13-100728904-100731604
chr13	100743408	100745152	chr13-100743408-100745152
chr13	100774527	100776757	chr13-100774527-100776757
chr13	100784035	100787312	chr13-100784035-100787312
chr13	100831194	100833910	chr13-100831194-100833910
chr13	101540395	101541158	chr13-101540395-101541158
chr13	101693945	101695201	chr13-101693945-101695201
chr13	101717735	101717936	chr13-101717735-101717936
chr13	101736502	101736881	chr13-101736502-101736881
chr13	101760507	101760708	chr13-101760507-101760708
chr13	101763625	101769373	chr13-101763625-101769373
chr13	101801331	101801931	chr13-101801331-101801931
chr13	102698741	102698942	chr13-102698741-102698942
chr13	102754008	102754263	chr13-102754008-102754263
chr13	102880215	102880499	chr13-102880215-102880499
chr13	102893899	102906905	chr13-102893899-102906905
chr13	102957220	102959487	chr13-102957220-102959487
chr13	102963270	102963577	chr13-102963270-102963577
chr13	103096524	103097603	chr13-103096524-103097603
chr13	103102901	103103155	chr13-103102901-103103155
chr13	103109749	103109950	chr13-103109749-103109950
chr13	103120793	103120994	chr13-103120793-103120994
chr13	103238526	103239000	chr13-103238526-103239000
chr13	103239985	103240186	chr13-103239985-103240186
chr13	103332411	103335367	chr13-103332411-103335367
chr13	103772317	103775214	chr13-103772317-103775214
chr13	103918085	103922074	chr13-103918085-103922074
chr13	103977656	103979083	chr13-103977656-103979083
chr13	104015557	104016231	chr13-104015557-104016231
chr13	104023543	104023938	chr13-104023543-104023938
chr13	104036477	104038816	chr13-104036477-104038816
chr13	104074857	104075217	chr13-104074857-104075217
chr13	104082089	104083285	chr13-104082089-104083285
chr13	104108117	104110833	chr13-104108117-104110833
chr13	104176546	104179834	chr13-104176546-104179834
chr13	104227440	104229944	chr13-104227440-104229944
chr13	104285177	104289624	chr13-104285177-104289624
chr13	104506049	104506250	chr13-104506049-104506250

chr13	104662413	104662664	chr13-104662413-104662664
chr13	104815959	104818601	chr13-104815959-104818601
chr13	104863146	104864341	chr13-104863146-104864341
chr13	105053772	105055028	chr13-105053772-105055028
chr13	105293717	105294808	chr13-105293717-105294808
chr13	106691694	106692051	chr13-106691694-106692051
chr13	106934992	106937712	chr13-106934992-106937712
chr13	106941774	106942077	chr13-106941774-106942077
chr13	106945992	106948874	chr13-106945992-106948874
chr13	107003060	107003314	chr13-107003060-107003314
chr13	107019046	107019247	chr13-107019046-107019247
chr13	107020080	107023203	chr13-107020080-107023203
chr13	107094579	107096035	chr13-107094579-107096035
chr13	107114162	107114424	chr13-107114162-107114424
chr13	107168432	107169302	chr13-107168432-107169302
chr13	107207693	107208585	chr13-107207693-107208585
chr13	107371789	107372251	chr13-107371789-107372251
chr13	107461234	107461435	chr13-107461234-107461435
chr13	107469551	107470174	chr13-107469551-107470174
chr13	107675613	107677456	chr13-107675613-107677456
chr13	107813936	107814192	chr13-107813936-107814192
chr13	107830766	107831408	chr13-107830766-107831408
chr13	107837162	107838749	chr13-107837162-107838749
chr13	107845598	107847946	chr13-107845598-107847946
chr13	107866375	107866959	chr13-107866375-107866959
chr13	107873099	107873510	chr13-107873099-107873510
chr13	107887185	107892529	chr13-107887185-107892529
chr13	108042860	108046608	chr13-108042860-108046608
chr13	108138953	108139154	chr13-108138953-108139154
chr13	108157549	108160167	chr13-108157549-108160167
chr13	108213717	108215918	chr13-108213717-108215918
chr13	108229346	108230499	chr13-108229346-108230499
chr13	108293168	108293730	chr13-108293168-108293730
chr13	108296887	108298255	chr13-108296887-108298255
chr13	108315526	108317367	chr13-108315526-108317367
chr13	108338173	108338529	chr13-108338173-108338529
chr13	109116140	109117222	chr13-109116140-109117222
chr13	109211602	109213202	chr13-109211602-109213202
chr13	109229374	109230940	chr13-109229374-109230940
chr13	109242631	109243950	chr13-109242631-109243950
chr13	109259782	109263208	chr13-109259782-109263208
chr13	109315506	109316340	chr13-109315506-109316340

chr13	109318852	109319354	chr13-109318852-109319354
chr13	109440148	109441708	chr13-109440148-109441708
chr13	109481131	109481779	chr13-109481131-109481779
chr13	109555412	109555769	chr13-109555412-109555769
chr13	109632633	109633199	chr13-109632633-109633199
chr13	109648138	109648589	chr13-109648138-109648589
chr13	109685127	109686313	chr13-109685127-109686313
chr13	109775921	109776590	chr13-109775921-109776590
chr13	109822156	109822938	chr13-109822156-109822938
chr13	109881396	109881799	chr13-109881396-109881799
chr13	109899725	109900360	chr13-109899725-109900360
chr13	109901863	109905038	chr13-109901863-109905038
chr13	110018438	110019677	chr13-110018438-110019677
chr13	110280032	110280654	chr13-110280032-110280654
chr13	110393884	110400664	chr13-110393884-110400664
chr13	110436832	110437291	chr13-110436832-110437291
chr13	110560561	110560929	chr13-110560561-110560929
chr13	110643193	110644137	chr13-110643193-110644137
chr13	110724403	110725917	chr13-110724403-110725917
chr13	110882440	110883203	chr13-110882440-110883203
chr13	110907859	110908060	chr13-110907859-110908060
chr13	110925513	110925841	chr13-110925513-110925841
chr13	110966668	110967990	chr13-110966668-110967990
chr13	111485839	111486867	chr13-111485839-111486867
chr13	111487841	111491211	chr13-111487841-111491211
chr13	111685363	111688061	chr13-111685363-111688061
chr13	111724223	111726510	chr13-111724223-111726510
chr13	111788367	111788774	chr13-111788367-111788774
chr13	111796503	111798430	chr13-111796503-111798430
chr13	111803041	111810350	chr13-111803041-111810350
chr13	112002881	112003192	chr13-112002881-112003192
chr13	112030868	112031345	chr13-112030868-112031345
chr13	112054879	112055282	chr13-112054879-112055282
chr13	112063845	112064096	chr13-112063845-112064096
chr13	112071480	112071681	chr13-112071480-112071681
chr13	112100516	112101484	chr13-112100516-112101484
chr13	112152957	112153525	chr13-112152957-112153525
chr13	112254084	112254893	chr13-112254084-112254893
chr13	112365739	112366049	chr13-112365739-112366049
chr13	112390119	112390320	chr13-112390119-112390320
chr13	112463185	112466102	chr13-112463185-112466102
chr13	112652122	112652374	chr13-112652122-112652374

chr13	112659942	112661661	chr13-112659942-112661661
chr13	112770697	112771552	chr13-112770697-112771552
chr13	112800418	112803810	chr13-112800418-112803810
chr13	112810776	112811433	chr13-112810776-112811433
chr13	112862005	112862362	chr13-112862005-112862362
chr13	112925279	112928799	chr13-112925279-112928799
chr13	112986864	112990019	chr13-112986864-112990019
chr13	112993302	112994593	chr13-112993302-112994593
chr13	112998282	112999333	chr13-112998282-112999333
chr13	113013167	113013977	chr13-113013167-113013977
chr13	113031760	113032513	chr13-113031760-113032513
chr13	113034319	113036105	chr13-113034319-113036105
chr13	113044366	113046752	chr13-113044366-113046752
chr13	113155540	113155741	chr13-113155540-113155741
chr13	113183408	113183659	chr13-113183408-113183659
chr13	113201733	113201984	chr13-113201733-113201984
chr13	113223900	113224304	chr13-113223900-113224304
chr13	113266483	113266836	chr13-113266483-113266836
chr13	113292795	113293807	chr13-113292795-113293807
chr13	113350988	113351444	chr13-113350988-113351444
chr13	113454882	113455220	chr13-113454882-113455220
chr13	113490234	113490544	chr13-113490234-113490544
chr13	113495671	113495872	chr13-113495671-113495872
chr13	113509532	113509783	chr13-113509532-113509783
chr13	113601094	113604116	chr13-113601094-113604116
chr13	113605846	113607238	chr13-113605846-113607238
chr13	113612462	113618801	chr13-113612462-113618801
chr13	113793614	113796272	chr13-113793614-113796272
chr13	113949491	113949742	chr13-113949491-113949742
chr13	114386780	114388606	chr13-114386780-114388606
chr13	114420104	114420305	chr13-114420104-114420305
chr13	114456825	114460957	chr13-114456825-114460957
chr13	114584658	114585154	chr13-114584658-114585154
chr13	114618668	114619022	chr13-114618668-114619022
chr13	114638775	114639319	chr13-114638775-114639319
chr13	114654523	114655034	chr13-114654523-114655034
chr13	114679162	114680950	chr13-114679162-114680950
chr13	114727350	114727654	chr13-114727350-114727654
chr13	114735883	114736198	chr13-114735883-114736198
chr13	114817232	114819464	chr13-114817232-114819464
chr13	114852508	114852709	chr13-114852508-114852709
chr13	114914688	114914992	chr13-114914688-114914992

chr13	114915811	114916222	chr13-114915811-114916222
chr13	114918226	114918588	chr13-114918226-114918588
chr13	114923438	114924337	chr13-114923438-114924337
chr13	114928160	114935039	chr13-114928160-114935039
chr13	115088454	115090973	chr13-115088454-115090973
chr13	115101264	115102430	chr13-115101264-115102430
chr13	116297059	116298045	chr13-116297059-116298045
chr13	116309341	116310320	chr13-116309341-116310320
chr13	116443608	116443911	chr13-116443608-116443911
chr13	116527299	116527550	chr13-116527299-116527550
chr13	116705621	116706282	chr13-116705621-116706282
chr13	116707711	116707912	chr13-116707711-116707912
chr13	116711397	116712304	chr13-116711397-116712304
chr13	117023686	117026231	chr13-117023686-117026231
chr13	117220350	117221718	chr13-117220350-117221718
chr13	117602206	117603346	chr13-117602206-117603346
chr13	118385799	118387812	chr13-118385799-118387812
chr13	118714169	118714829	chr13-118714169-118714829
chr13	119407903	119409869	chr13-119407903-119409869
chr13	119428075	119431167	chr13-119428075-119431167
chr13	119461801	119464287	chr13-119461801-119464287
chr13	119596396	119596647	chr13-119596396-119596647
chr13	119599198	119599550	chr13-119599198-119599550
chr13	119631334	119632350	chr13-119631334-119632350
chr13	119661765	119662175	chr13-119661765-119662175
chr13	119687885	119694883	chr13-119687885-119694883
chr13	119755243	119756366	chr13-119755243-119756366
chr13	119789086	119792474	chr13-119789086-119792474
chr13	119835720	119837531	chr13-119835720-119837531
chr13	119961792	119962329	chr13-119961792-119962329
chr13	119965103	119966615	chr13-119965103-119966615
chr13	120095986	120097298	chr13-120095986-120097298
chr13	120100260	120100632	chr13-120100260-120100632
chr14	3048955	3049883	chr14-3048955-3049883
chr14	3208407	3209155	chr14-3208407-3209155
chr14	3332167	3333245	chr14-3332167-3333245
chr14	3948771	3949517	chr14-3948771-3949517
chr14	4018512	4018951	chr14-4018512-4018951
chr14	4334390	4335023	chr14-4334390-4335023
chr14	4649497	4650101	chr14-4649497-4650101
chr14	4855407	4855902	chr14-4855407-4855902
chr14	5001094	5001761	chr14-5001094-5001761

chr14	5070711	5071129	chr14-5070711-5071129
chr14	5879981	5880449	chr14-5879981-5880449
chr14	5961134	5961975	chr14-5961134-5961975
chr14	6107731	6109019	chr14-6107731-6109019
chr14	6286913	6287642	chr14-6286913-6287642
chr14	6426888	6427249	chr14-6426888-6427249
chr14	6604503	6605397	chr14-6604503-6605397
chr14	6755821	6757706	chr14-6755821-6757706
chr14	6889386	6890232	chr14-6889386-6890232
chr14	7027136	7027757	chr14-7027136-7027757
chr14	7105420	7105725	chr14-7105420-7105725
chr14	7174537	7174838	chr14-7174537-7174838
chr14	7314373	7315413	chr14-7314373-7315413
chr14	7488299	7489000	chr14-7488299-7489000
chr14	7567896	7568762	chr14-7567896-7568762
chr14	7657955	7659136	chr14-7657955-7659136
chr14	7773802	7774789	chr14-7773802-7774789
chr14	7791100	7792093	chr14-7791100-7792093
chr14	7807239	7809714	chr14-7807239-7809714
chr14	7817776	7823235	chr14-7817776-7823235
chr14	7824239	7827664	chr14-7824239-7827664
chr14	7847286	7848771	chr14-7847286-7848771
chr14	7849600	7849801	chr14-7849600-7849801
chr14	7850808	7852113	chr14-7850808-7852113
chr14	7853469	7853903	chr14-7853469-7853903
chr14	7952536	7954909	chr14-7952536-7954909
chr14	7961450	7962369	chr14-7961450-7962369
chr14	7998818	7999633	chr14-7998818-7999633
chr14	8002405	8004206	chr14-8002405-8004206
chr14	8080135	8082065	chr14-8080135-8082065
chr14	8097672	8100305	chr14-8097672-8100305
chr14	8111266	8112327	chr14-8111266-8112327
chr14	8125269	8125880	chr14-8125269-8125880
chr14	8171655	8173909	chr14-8171655-8173909
chr14	8213495	8216248	chr14-8213495-8216248
chr14	8257569	8259333	chr14-8257569-8259333
chr14	8308543	8310121	chr14-8308543-8310121
chr14	8332583	8332890	chr14-8332583-8332890
chr14	8356739	8357699	chr14-8356739-8357699
chr14	8367232	8367433	chr14-8367232-8367433
chr14	8665377	8666614	chr14-8665377-8666614
chr14	10453133	10453535	chr14-10453133-10453535

chr14	11160913	11162580	chr14-11160913-11162580
chr14	11552401	11556835	chr14-11552401-11556835
chr14	11612277	11613159	chr14-11612277-11613159
chr14	11641020	11641272	chr14-11641020-11641272
chr14	12283804	12285271	chr14-12283804-12285271
chr14	12341054	12341733	chr14-12341054-12341733
chr14	12344700	12347161	chr14-12344700-12347161
chr14	12821795	12822862	chr14-12821795-12822862
chr14	13901542	13902051	chr14-13901542-13902051
chr14	13933304	13934085	chr14-13933304-13934085
chr14	13959867	13962211	chr14-13959867-13962211
chr14	14011664	14014381	chr14-14011664-14014381
chr14	14119706	14121144	chr14-14119706-14121144
chr14	14197006	14197361	chr14-14197006-14197361
chr14	14294409	14295147	chr14-14294409-14295147
chr14	14345179	14347394	chr14-14345179-14347394
chr14	14632374	14634303	chr14-14632374-14634303
chr14	14702166	14704695	chr14-14702166-14704695
chr14	14904846	14905047	chr14-14904846-14905047
chr14	15438617	15438987	chr14-15438617-15438987
chr14	16242482	16244757	chr14-16242482-16244757
chr14	16248696	16250193	chr14-16248696-16250193
chr14	16363964	16367322	chr14-16363964-16367322
chr14	16448593	16449057	chr14-16448593-16449057
chr14	16483454	16484074	chr14-16483454-16484074
chr14	16574103	16576367	chr14-16574103-16576367
chr14	17172384	17173147	chr14-17172384-17173147
chr14	17632956	17633326	chr14-17632956-17633326
chr14	17659114	17661621	chr14-17659114-17661621
chr14	17917558	17917813	chr14-17917558-17917813
chr14	18233973	18234701	chr14-18233973-18234701
chr14	18236512	18239958	chr14-18236512-18239958
chr14	18268513	18272256	chr14-18268513-18272256
chr14	18326796	18327460	chr14-18326796-18327460
chr14	18330460	18332645	chr14-18330460-18332645
chr14	18460388	18461438	chr14-18460388-18461438
chr14	18462605	18468509	chr14-18462605-18468509
chr14	18774182	18774531	chr14-18774182-18774531
chr14	18892368	18894539	chr14-18892368-18894539
chr14	19041773	19042836	chr14-19041773-19042836
chr14	19583942	19585857	chr14-19583942-19585857
chr14	19750734	19752052	chr14-19750734-19752052

chr14	19822619	19824737	chr14-19822619-19824737
chr14	19840081	19840282	chr14-19840081-19840282
chr14	19910501	19910938	chr14-19910501-19910938
chr14	19976442	19977457	chr14-19976442-19977457
chr14	20032700	20033680	chr14-20032700-20033680
chr14	20081764	20084037	chr14-20081764-20084037
chr14	20135044	20136159	chr14-20135044-20136159
chr14	20139329	20139736	chr14-20139329-20139736
chr14	20154206	20156541	chr14-20154206-20156541
chr14	20178685	20182135	chr14-20178685-20182135
chr14	20294473	20295436	chr14-20294473-20295436
chr14	20298074	20298326	chr14-20298074-20298326
chr14	20345651	20349206	chr14-20345651-20349206
chr14	20387462	20389641	chr14-20387462-20389641
chr14	20392380	20394485	chr14-20392380-20394485
chr14	20477653	20480646	chr14-20477653-20480646
chr14	20544854	20547212	chr14-20544854-20547212
chr14	20610028	20610798	chr14-20610028-20610798
chr14	20639457	20642115	chr14-20639457-20642115
chr14	20660489	20662097	chr14-20660489-20662097
chr14	20673318	20675839	chr14-20673318-20675839
chr14	20694264	20698271	chr14-20694264-20698271
chr14	20701120	20704116	chr14-20701120-20704116
chr14	20706907	20709403	chr14-20706907-20709403
chr14	20733061	20734950	chr14-20733061-20734950
chr14	20765860	20766061	chr14-20765860-20766061
chr14	20792443	20795033	chr14-20792443-20795033
chr14	20803876	20806792	chr14-20803876-20806792
chr14	20829542	20829866	chr14-20829542-20829866
chr14	20831604	20831805	chr14-20831604-20831805
chr14	20833949	20843180	chr14-20833949-20843180
chr14	20846120	20846678	chr14-20846120-20846678
chr14	20863342	20867162	chr14-20863342-20867162
chr14	20885280	20885481	chr14-20885280-20885481
chr14	20891974	20892175	chr14-20891974-20892175
chr14	20928360	20931696	chr14-20928360-20931696
chr14	20974073	20974274	chr14-20974073-20974274
chr14	21051163	21054009	chr14-21051163-21054009
chr14	21065275	21065980	chr14-21065275-21065980
chr14	21074965	21077792	chr14-21074965-21077792
chr14	21082192	21082512	chr14-21082192-21082512
chr14	21087043	21088818	chr14-21087043-21088818

chr14	21350606	21350917	chr14-21350606-21350917
chr14	21498580	21501941	chr14-21498580-21501941
chr14	21547597	21547798	chr14-21547597-21547798
chr14	21552983	21554311	chr14-21552983-21554311
chr14	21682707	21683380	chr14-21682707-21683380
chr14	21686576	21686955	chr14-21686576-21686955
chr14	21747458	21748754	chr14-21747458-21748754
chr14	21750061	21752101	chr14-21750061-21752101
chr14	21822909	21823218	chr14-21822909-21823218
chr14	21830441	21833770	chr14-21830441-21833770
chr14	21847152	21849307	chr14-21847152-21849307
chr14	21981541	21995994	chr14-21981541-21995994
chr14	22019456	22020006	chr14-22019456-22020006
chr14	23885878	23886130	chr14-23885878-23886130
chr14	24003748	24005116	chr14-24003748-24005116
chr14	24206378	24207700	chr14-24206378-24207700
chr14	24222414	24223029	chr14-24222414-24223029
chr14	24244080	24246433	chr14-24244080-24246433
chr14	24485523	24487505	chr14-24485523-24487505
chr14	24490271	24494856	chr14-24490271-24494856
chr14	24778837	24779882	chr14-24778837-24779882
chr14	24794005	24794206	chr14-24794005-24794206
chr14	25155508	25156640	chr14-25155508-25156640
chr14	25186349	25186661	chr14-25186349-25186661
chr14	25226394	25226996	chr14-25226394-25226996
chr14	25304467	25304729	chr14-25304467-25304729
chr14	25377706	25379668	chr14-25377706-25379668
chr14	25456724	25467005	chr14-25456724-25467005
chr14	25472517	25477950	chr14-25472517-25477950
chr14	25480785	25482041	chr14-25480785-25482041
chr14	25483762	25485078	chr14-25483762-25485078
chr14	25486037	25489900	chr14-25486037-25489900
chr14	25495707	25496712	chr14-25495707-25496712
chr14	25497695	25497896	chr14-25497695-25497896
chr14	25505434	25505742	chr14-25505434-25505742
chr14	25516670	25517604	chr14-25516670-25517604
chr14	25519433	25520436	chr14-25519433-25520436
chr14	25526815	25527953	chr14-25526815-25527953
chr14	25532937	25534497	chr14-25532937-25534497
chr14	25606608	25608371	chr14-25606608-25608371
chr14	25676673	25676874	chr14-25676673-25676874
chr14	25681847	25682213	chr14-25681847-25682213

chr14	25693573	25696407	chr14-25693573-25696407
chr14	25767847	25769561	chr14-25767847-25769561
chr14	25832283	25832792	chr14-25832283-25832792
chr14	25839986	25840237	chr14-25839986-25840237
chr14	25841667	25844689	chr14-25841667-25844689
chr14	25854453	25858106	chr14-25854453-25858106
chr14	25862001	25862415	chr14-25862001-25862415
chr14	26530409	26535749	chr14-26530409-26535749
chr14	26540425	26541115	chr14-26540425-26541115
chr14	26578956	26580635	chr14-26578956-26580635
chr14	26637034	26640610	chr14-26637034-26640610
chr14	26666569	26670905	chr14-26666569-26670905
chr14	26969343	26971240	chr14-26969343-26971240
chr14	26981657	26981969	chr14-26981657-26981969
chr14	27038721	27040375	chr14-27038721-27040375
chr14	27058949	27059376	chr14-27058949-27059376
chr14	27069394	27072549	chr14-27069394-27072549
chr14	27114256	27115672	chr14-27114256-27115672
chr14	27131534	27132006	chr14-27131534-27132006
chr14	27335497	27338608	chr14-27335497-27338608
chr14	27341189	27341966	chr14-27341189-27341966
chr14	27428373	27430689	chr14-27428373-27430689
chr14	27507553	27509212	chr14-27507553-27509212
chr14	27562548	27563027	chr14-27562548-27563027
chr14	27622018	27624077	chr14-27622018-27624077
chr14	27823590	27824342	chr14-27823590-27824342
chr14	27860618	27861087	chr14-27860618-27861087
chr14	28504070	28507790	chr14-28504070-28507790
chr14	28509176	28511719	chr14-28509176-28511719
chr14	29721396	29723256	chr14-29721396-29723256
chr14	29967101	29969881	chr14-29967101-29969881
chr14	29977942	29979966	chr14-29977942-29979966
chr14	30008464	30009632	chr14-30008464-30009632
chr14	30352850	30354208	chr14-30352850-30354208
chr14	30473916	30474117	chr14-30473916-30474117
chr14	30478892	30481023	chr14-30478892-30481023
chr14	30530797	30531070	chr14-30530797-30531070
chr14	30548005	30551936	chr14-30548005-30551936
chr14	30561066	30562201	chr14-30561066-30562201
chr14	30592793	30594141	chr14-30592793-30594141
chr14	30622266	30622467	chr14-30622266-30622467
chr14	30623541	30627108	chr14-30623541-30627108

chr14	30630864	30632096	chr14-30630864-30632096
chr14	30635103	30635862	chr14-30635103-30635862
chr14	30653830	30655370	chr14-30653830-30655370
chr14	30714764	30717339	chr14-30714764-30717339
chr14	30825247	30827285	chr14-30825247-30827285
chr14	30888403	30888806	chr14-30888403-30888806
chr14	30950713	30952267	chr14-30950713-30952267
chr14	30991279	30991480	chr14-30991279-30991480
chr14	31000007	31002634	chr14-31000007-31002634
chr14	31017124	31020764	chr14-31017124-31020764
chr14	31127536	31132253	chr14-31127536-31132253
chr14	31160197	31160724	chr14-31160197-31160724
chr14	31197914	31198406	chr14-31197914-31198406
chr14	31204483	31207494	chr14-31204483-31207494
chr14	31250086	31252710	chr14-31250086-31252710
chr14	31269604	31269805	chr14-31269604-31269805
chr14	31323537	31324217	chr14-31323537-31324217
chr14	31335697	31338220	chr14-31335697-31338220
chr14	31401142	31401401	chr14-31401142-31401401
chr14	31403542	31404793	chr14-31403542-31404793
chr14	31407789	31407990	chr14-31407789-31407990
chr14	31417384	31417585	chr14-31417384-31417585
chr14	31431990	31432621	chr14-31431990-31432621
chr14	31433853	31436718	chr14-31433853-31436718
chr14	31493513	31497052	chr14-31493513-31497052
chr14	31640222	31642172	chr14-31640222-31642172
chr14	31657137	31657338	chr14-31657137-31657338
chr14	31660747	31664266	chr14-31660747-31664266
chr14	31669287	31669899	chr14-31669287-31669899
chr14	31780942	31782244	chr14-31780942-31782244
chr14	31828879	31831866	chr14-31828879-31831866
chr14	31929200	31932384	chr14-31929200-31932384
chr14	31934219	31934420	chr14-31934219-31934420
chr14	31942012	31942524	chr14-31942012-31942524
chr14	31944897	31947793	chr14-31944897-31947793
chr14	31962717	31964404	chr14-31962717-31964404
chr14	32014840	32015796	chr14-32014840-32015796
chr14	32050356	32050738	chr14-32050356-32050738
chr14	32084258	32087613	chr14-32084258-32087613
chr14	32158829	32161449	chr14-32158829-32161449
chr14	32164684	32167128	chr14-32164684-32167128
chr14	32199997	32203239	chr14-32199997-32203239

chr14	32321702	32322823	chr14-32321702-32322823
chr14	32464271	32465390	chr14-32464271-32465390
chr14	32492472	32493644	chr14-32492472-32493644
chr14	32512977	32514827	chr14-32512977-32514827
chr14	32597868	32598069	chr14-32597868-32598069
chr14	32599422	32599796	chr14-32599422-32599796
chr14	32856769	32857121	chr14-32856769-32857121
chr14	33213895	33214908	chr14-33213895-33214908
chr14	33246916	33247932	chr14-33246916-33247932
chr14	33250037	33253097	chr14-33250037-33253097
chr14	33257809	33258117	chr14-33257809-33258117
chr14	33313851	33314309	chr14-33313851-33314309
chr14	33319125	33321521	chr14-33319125-33321521
chr14	33322774	33323086	chr14-33322774-33323086
chr14	33335325	33336681	chr14-33335325-33336681
chr14	33340523	33341595	chr14-33340523-33341595
chr14	33347408	33348798	chr14-33347408-33348798
chr14	33349908	33355747	chr14-33349908-33355747
chr14	33357895	33359698	chr14-33357895-33359698
chr14	33443390	33443591	chr14-33443390-33443591
chr14	33445232	33447805	chr14-33445232-33447805
chr14	33824723	33824924	chr14-33824723-33824924
chr14	33923144	33923920	chr14-33923144-33923920
chr14	34085533	34089826	chr14-34085533-34089826
chr14	34152243	34152610	chr14-34152243-34152610
chr14	34200739	34202016	chr14-34200739-34202016
chr14	34219720	34220327	chr14-34219720-34220327
chr14	34309588	34314417	chr14-34309588-34314417
chr14	34317861	34318579	chr14-34317861-34318579
chr14	34362815	34364241	chr14-34362815-34364241
chr14	34500387	34503968	chr14-34500387-34503968
chr14	34548229	34549872	chr14-34548229-34549872
chr14	34614791	34615464	chr14-34614791-34615464
chr14	34661609	34662580	chr14-34661609-34662580
chr14	34672687	34675777	chr14-34672687-34675777
chr14	34763490	34764410	chr14-34763490-34764410
chr14	34768403	34770024	chr14-34768403-34770024
chr14	34819085	34820754	chr14-34819085-34820754
chr14	34822862	34823117	chr14-34822862-34823117
chr14	36801916	36802626	chr14-36801916-36802626
chr14	36863303	36864359	chr14-36863303-36864359
chr14	36918937	36919541	chr14-36918937-36919541

chr14	36967574	36969399	chr14-36967574-36969399
chr14	37097850	37098731	chr14-37097850-37098731
chr14	37133480	37136147	chr14-37133480-37136147
chr14	39472616	39473526	chr14-39472616-39473526
chr14	40894525	40895135	chr14-40894525-40895135
chr14	40902012	40903518	chr14-40902012-40903518
chr14	40914729	40915417	chr14-40914729-40915417
chr14	40935319	40936213	chr14-40935319-40936213
chr14	40947101	40951009	chr14-40947101-40951009
chr14	40955715	40960047	chr14-40955715-40960047
chr14	40962414	40968502	chr14-40962414-40968502
chr14	40995521	40996736	chr14-40995521-40996736
chr14	41012024	41014337	chr14-41012024-41014337
chr14	41017039	41017979	chr14-41017039-41017979
chr14	41053611	41054569	chr14-41053611-41054569
chr14	41072517	41073177	chr14-41072517-41073177
chr14	41104762	41105481	chr14-41104762-41105481
chr14	41167051	41167313	chr14-41167051-41167313
chr14	41264531	41265286	chr14-41264531-41265286
chr14	43250990	43251191	chr14-43250990-43251191
chr14	43274995	43275450	chr14-43274995-43275450
chr14	43528506	43528761	chr14-43528506-43528761
chr14	43532424	43532682	chr14-43532424-43532682
chr14	43925118	43926206	chr14-43925118-43926206
chr14	44989035	44989289	chr14-44989035-44989289
chr14	45218649	45221414	chr14-45218649-45221414
chr14	45316031	45319292	chr14-45316031-45319292
chr14	45325144	45325606	chr14-45325144-45325606
chr14	45328925	45331220	chr14-45328925-45331220
chr14	45350846	45352536	chr14-45350846-45352536
chr14	45387201	45389529	chr14-45387201-45389529
chr14	45477646	45477901	chr14-45477646-45477901
chr14	45528491	45531101	chr14-45528491-45531101
chr14	45566263	45566770	chr14-45566263-45566770
chr14	45641965	45642476	chr14-45641965-45642476
chr14	45655626	45658949	chr14-45655626-45658949
chr14	45690630	45691238	chr14-45690630-45691238
chr14	45769805	45770006	chr14-45769805-45770006
chr14	45772904	45773159	chr14-45772904-45773159
chr14	45850272	45850593	chr14-45850272-45850593
chr14	45859617	45859818	chr14-45859617-45859818
chr14	45878541	45878742	chr14-45878541-45878742

chr14	45962839	45963659	chr14-45962839-45963659
chr14	46060650	46061541	chr14-46060650-46061541
chr14	46385440	46389732	chr14-46385440-46389732
chr14	46633665	46636426	chr14-46633665-46636426
chr14	46704105	46704932	chr14-46704105-46704932
chr14	46760150	46762035	chr14-46760150-46762035
chr14	46786484	46788698	chr14-46786484-46788698
chr14	46790125	46790813	chr14-46790125-46790813
chr14	46800706	46801430	chr14-46800706-46801430
chr14	46820481	46822749	chr14-46820481-46822749
chr14	46831107	46833478	chr14-46831107-46833478
chr14	46881076	46886583	chr14-46881076-46886583
chr14	46964367	46968267	chr14-46964367-46968267
chr14	46989622	46990192	chr14-46989622-46990192
chr14	47021037	47021347	chr14-47021037-47021347
chr14	47034069	47034270	chr14-47034069-47034270
chr14	47086608	47087227	chr14-47086608-47087227
chr14	47177927	47178439	chr14-47177927-47178439
chr14	47188028	47189907	chr14-47188028-47189907
chr14	47275457	47278425	chr14-47275457-47278425
chr14	47297698	47300271	chr14-47297698-47300271
chr14	47302412	47303419	chr14-47302412-47303419
chr14	47351295	47351496	chr14-47351295-47351496
chr14	47353840	47354708	chr14-47353840-47354708
chr14	47366902	47367963	chr14-47366902-47367963
chr14	47371508	47372409	chr14-47371508-47372409
chr14	47373398	47378650	chr14-47373398-47378650
chr14	47416910	47418927	chr14-47416910-47418927
chr14	47472352	47474885	chr14-47472352-47474885
chr14	47514854	47515665	chr14-47514854-47515665
chr14	47516483	47516845	chr14-47516483-47516845
chr14	47533734	47534369	chr14-47533734-47534369
chr14	47567138	47569092	chr14-47567138-47569092
chr14	47634386	47635272	chr14-47634386-47635272
chr14	47648707	47650750	chr14-47648707-47650750
chr14	48120300	48122195	chr14-48120300-48122195
chr14	48289519	48289941	chr14-48289519-48289941
chr14	48295517	48296106	chr14-48295517-48296106
chr14	48421918	48422493	chr14-48421918-48422493
chr14	48445914	48447725	chr14-48445914-48447725
chr14	48664770	48666551	chr14-48664770-48666551
chr14	48673582	48673886	chr14-48673582-48673886

chr14	48709420	48710057	chr14-48709420-48710057
chr14	49065152	49067891	chr14-49065152-49067891
chr14	49171444	49174220	chr14-49171444-49174220
chr14	49783095	49783456	chr14-49783095-49783456
chr14	50061530	50062801	chr14-50061530-50062801
chr14	50784388	50785959	chr14-50784388-50785959
chr14	50806868	50809043	chr14-50806868-50809043
chr14	50869616	50871550	chr14-50869616-50871550
chr14	50896310	50896985	chr14-50896310-50896985
chr14	50904021	50904660	chr14-50904021-50904660
chr14	50923413	50926597	chr14-50923413-50926597
chr14	50929564	50932438	chr14-50929564-50932438
chr14	50943685	50948237	chr14-50943685-50948237
chr14	50955736	50957091	chr14-50955736-50957091
chr14	51062642	51066673	chr14-51062642-51066673
chr14	51082518	51084183	chr14-51082518-51084183
chr14	51087599	51092544	chr14-51087599-51092544
chr14	51358116	51358674	chr14-51358116-51358674
chr14	51883767	51886296	chr14-51883767-51886296
chr14	51912487	51914390	chr14-51912487-51914390
chr14	51932307	51932508	chr14-51932307-51932508
chr14	51960519	51960874	chr14-51960519-51960874
chr14	51983991	51987280	chr14-51983991-51987280
chr14	52013322	52016560	chr14-52013322-52016560
chr14	52018527	52021211	chr14-52018527-52021211
chr14	52022573	52023899	chr14-52022573-52023899
chr14	52100630	52105047	chr14-52100630-52105047
chr14	52106550	52107144	chr14-52106550-52107144
chr14	52118834	52119698	chr14-52118834-52119698
chr14	52195623	52198454	chr14-52195623-52198454
chr14	52243627	52245372	chr14-52243627-52245372
chr14	52256286	52258598	chr14-52256286-52258598
chr14	52278059	52280634	chr14-52278059-52280634
chr14	52303847	52306207	chr14-52303847-52306207
chr14	52314985	52316880	chr14-52314985-52316880
chr14	52327749	52328371	chr14-52327749-52328371
chr14	52338926	52339179	chr14-52338926-52339179
chr14	53634064	53636281	chr14-53634064-53636281
chr14	53804164	53804365	chr14-53804164-53804365
chr14	54064740	54064993	chr14-54064740-54064993
chr14	54074784	54076270	chr14-54074784-54076270
chr14	54097365	54099045	chr14-54097365-54099045

chr14	54106797	54109555	chr14-54106797-54109555
chr14	54185382	54187605	chr14-54185382-54187605
chr14	54221499	54228780	chr14-54221499-54228780
chr14	54252539	54255497	chr14-54252539-54255497
chr14	54258659	54260324	chr14-54258659-54260324
chr14	54297042	54297511	chr14-54297042-54297511
chr14	54360394	54362350	chr14-54360394-54362350
chr14	54383172	54383425	chr14-54383172-54383425
chr14	54410009	54410618	chr14-54410009-54410618
chr14	54413133	54414606	chr14-54413133-54414606
chr14	54425951	54427973	chr14-54425951-54427973
chr14	54429341	54436210	chr14-54429341-54436210
chr14	54449067	54449620	chr14-54449067-54449620
chr14	54463391	54465782	chr14-54463391-54465782
chr14	54475521	54477240	chr14-54475521-54477240
chr14	54481385	54481586	chr14-54481385-54481586
chr14	54492481	54492786	chr14-54492481-54492786
chr14	54515988	54518420	chr14-54515988-54518420
chr14	54527338	54527777	chr14-54527338-54527777
chr14	54552925	54555081	chr14-54552925-54555081
chr14	54569614	54578886	chr14-54569614-54578886
chr14	54602651	54604100	chr14-54602651-54604100
chr14	54605215	54606585	chr14-54605215-54606585
chr14	54616327	54618512	chr14-54616327-54618512
chr14	54640551	54642110	chr14-54640551-54642110
chr14	54644019	54644322	chr14-54644019-54644322
chr14	54651593	54654105	chr14-54651593-54654105
chr14	54684934	54688591	chr14-54684934-54688591
chr14	54714181	54715193	chr14-54714181-54715193
chr14	54780806	54782268	chr14-54780806-54782268
chr14	54863226	54865034	chr14-54863226-54865034
chr14	54875554	54877998	chr14-54875554-54877998
chr14	54882807	54887396	chr14-54882807-54887396
chr14	54892932	54896047	chr14-54892932-54896047
chr14	54912063	54913427	chr14-54912063-54913427
chr14	54925632	54927120	chr14-54925632-54927120
chr14	54930349	54930717	chr14-54930349-54930717
chr14	55014289	55016422	chr14-55014289-55016422
chr14	55090859	55092781	chr14-55090859-55092781
chr14	55094170	55097098	chr14-55094170-55097098
chr14	55105348	55106875	chr14-55105348-55106875
chr14	55113770	55116063	chr14-55113770-55116063

chr14	55161214	55161519	chr14-55161214-55161519
chr14	55478343	55479551	chr14-55478343-55479551
chr14	55490562	55491882	chr14-55490562-55491882
chr14	55520269	55521352	chr14-55520269-55521352
chr14	55539825	55541844	chr14-55539825-55541844
chr14	55557227	55557974	chr14-55557227-55557974
chr14	55559191	55562197	chr14-55559191-55562197
chr14	55577973	55579386	chr14-55577973-55579386
chr14	55584492	55585903	chr14-55584492-55585903
chr14	55589799	55593701	chr14-55589799-55593701
chr14	55603494	55604695	chr14-55603494-55604695
chr14	55634432	55636072	chr14-55634432-55636072
chr14	55642328	55645424	chr14-55642328-55645424
chr14	55659554	55660842	chr14-55659554-55660842
chr14	55670357	55673168	chr14-55670357-55673168
chr14	55680529	55682482	chr14-55680529-55682482
chr14	55702345	55703351	chr14-55702345-55703351
chr14	55706501	55706990	chr14-55706501-55706990
chr14	55708590	55715931	chr14-55708590-55715931
chr14	55720641	55723244	chr14-55720641-55723244
chr14	55724636	55725229	chr14-55724636-55725229
chr14	55743566	55747420	chr14-55743566-55747420
chr14	55759960	55760419	chr14-55759960-55760419
chr14	55761960	55762922	chr14-55761960-55762922
chr14	55767550	55768550	chr14-55767550-55768550
chr14	55788358	55788731	chr14-55788358-55788731
chr14	55795572	55796337	chr14-55795572-55796337
chr14	55814968	55815940	chr14-55814968-55815940
chr14	55822774	55824059	chr14-55822774-55824059
chr14	55825366	55825707	chr14-55825366-55825707
chr14	55853816	55854428	chr14-55853816-55854428
chr14	55873808	55874603	chr14-55873808-55874603
chr14	55882446	55882969	chr14-55882446-55882969
chr14	55883915	55886267	chr14-55883915-55886267
chr14	55899188	55901391	chr14-55899188-55901391
chr14	56120368	56120678	chr14-56120368-56120678
chr14	56352194	56352446	chr14-56352194-56352446
chr14	56364625	56365394	chr14-56364625-56365394
chr14	56570890	56572436	chr14-56570890-56572436
chr14	56574921	56577020	chr14-56574921-56577020
chr14	56667297	56670259	chr14-56667297-56670259
chr14	56775823	56779228	chr14-56775823-56779228

chr14	56810333	56813388	chr14-56810333-56813388
chr14	56865047	56865298	chr14-56865047-56865298
chr14	56885286	56888805	chr14-56885286-56888805
chr14	57057157	57058373	chr14-57057157-57058373
chr14	57103756	57105178	chr14-57103756-57105178
chr14	57132787	57133650	chr14-57132787-57133650
chr14	57268083	57268940	chr14-57268083-57268940
chr14	57316999	57318021	chr14-57316999-57318021
chr14	57338942	57339262	chr14-57338942-57339262
chr14	57397916	57399409	chr14-57397916-57399409
chr14	57407648	57407999	chr14-57407648-57407999
chr14	57423264	57424996	chr14-57423264-57424996
chr14	57524228	57526518	chr14-57524228-57526518
chr14	57570657	57572222	chr14-57570657-57572222
chr14	57663411	57666013	chr14-57663411-57666013
chr14	57704701	57705173	chr14-57704701-57705173
chr14	57730004	57731604	chr14-57730004-57731604
chr14	57742364	57747617	chr14-57742364-57747617
chr14	57760769	57761696	chr14-57760769-57761696
chr14	57777795	57778113	chr14-57777795-57778113
chr14	57797428	57800983	chr14-57797428-57800983
chr14	57824819	57827570	chr14-57824819-57827570
chr14	57882898	57883202	chr14-57882898-57883202
chr14	57888230	57890810	chr14-57888230-57890810
chr14	57910389	57911098	chr14-57910389-57911098
chr14	57998370	57999731	chr14-57998370-57999731
chr14	58070445	58077155	chr14-58070445-58077155
chr14	58253512	58253713	chr14-58253512-58253713
chr14	58308628	58308947	chr14-58308628-58308947
chr14	59200576	59202434	chr14-59200576-59202434
chr14	59439559	59442103	chr14-59439559-59442103
chr14	59551002	59552181	chr14-59551002-59552181
chr14	59556000	59556356	chr14-59556000-59556356
chr14	59596717	59598733	chr14-59596717-59598733
chr14	59625028	59626574	chr14-59625028-59626574
chr14	60086324	60087275	chr14-60086324-60087275
chr14	60176693	60178048	chr14-60176693-60178048
chr14	60249825	60252865	chr14-60249825-60252865
chr14	60265056	60266481	chr14-60265056-60266481
chr14	60377844	60379473	chr14-60377844-60379473
chr14	60409846	60410047	chr14-60409846-60410047
chr14	60428256	60428941	chr14-60428256-60428941

chr14	60432098	60432412	chr14-60432098-60432412
chr14	60485138	60486976	chr14-60485138-60486976
chr14	60593021	60593222	chr14-60593021-60593222
chr14	60621172	60621373	chr14-60621172-60621373
chr14	60633865	60636238	chr14-60633865-60636238
chr14	60732671	60734194	chr14-60732671-60734194
chr14	60736353	60742542	chr14-60736353-60742542
chr14	60771091	60771443	chr14-60771091-60771443
chr14	60784335	60785564	chr14-60784335-60785564
chr14	60993083	60993611	chr14-60993083-60993611
chr14	61037460	61038279	chr14-61037460-61038279
chr14	61045767	61047032	chr14-61045767-61047032
chr14	61138444	61138797	chr14-61138444-61138797
chr14	61172430	61173831	chr14-61172430-61173831
chr14	61274651	61274947	chr14-61274651-61274947
chr14	61309787	61311308	chr14-61309787-61311308
chr14	61331150	61331407	chr14-61331150-61331407
chr14	61359749	61360991	chr14-61359749-61360991
chr14	61438321	61440777	chr14-61438321-61440777
chr14	61447735	61448152	chr14-61447735-61448152
chr14	61555813	61557797	chr14-61555813-61557797
chr14	61599072	61600279	chr14-61599072-61600279
chr14	61646527	61649835	chr14-61646527-61649835
chr14	61680589	61683760	chr14-61680589-61683760
chr14	61686431	61687520	chr14-61686431-61687520
chr14	61737063	61738058	chr14-61737063-61738058
chr14	61757028	61757822	chr14-61757028-61757822
chr14	62170454	62170774	chr14-62170454-62170774
chr14	62292364	62293251	chr14-62292364-62293251
chr14	62331355	62333330	chr14-62331355-62333330
chr14	62457438	62457639	chr14-62457438-62457639
chr14	62555059	62556486	chr14-62555059-62556486
chr14	62759345	62762608	chr14-62759345-62762608
chr14	62774769	62775617	chr14-62774769-62775617
chr14	62806887	62807200	chr14-62806887-62807200
chr14	62825321	62825784	chr14-62825321-62825784
chr14	62836756	62838885	chr14-62836756-62838885
chr14	62879717	62879918	chr14-62879717-62879918
chr14	62991608	62992469	chr14-62991608-62992469
chr14	63103707	63103965	chr14-63103707-63103965
chr14	63121943	63125024	chr14-63121943-63125024
chr14	63130532	63132809	chr14-63130532-63132809

chr14	63164417	63165766	chr14-63164417-63165766
chr14	63174497	63178830	chr14-63174497-63178830
chr14	63193253	63194264	chr14-63193253-63194264
chr14	63240547	63241209	chr14-63240547-63241209
chr14	63244657	63246065	chr14-63244657-63246065
chr14	63255541	63256267	chr14-63255541-63256267
chr14	63269352	63269708	chr14-63269352-63269708
chr14	63435775	63437127	chr14-63435775-63437127
chr14	63448050	63448601	chr14-63448050-63448601
chr14	63451025	63455142	chr14-63451025-63455142
chr14	63508446	63508999	chr14-63508446-63508999
chr14	63542816	63544800	chr14-63542816-63544800
chr14	63605731	63608581	chr14-63605731-63608581
chr14	63637454	63637655	chr14-63637454-63637655
chr14	63795156	63795357	chr14-63795156-63795357
chr14	63859400	63861729	chr14-63859400-63861729
chr14	63899758	63900009	chr14-63899758-63900009
chr14	63940851	63941434	chr14-63940851-63941434
chr14	63942889	63945328	chr14-63942889-63945328
chr14	63982322	63983850	chr14-63982322-63983850
chr14	64011682	64012336	chr14-64011682-64012336
chr14	64097125	64099454	chr14-64097125-64099454
chr14	64104253	64104507	chr14-64104253-64104507
chr14	64439907	64441654	chr14-64439907-64441654
chr14	64586850	64588377	chr14-64586850-64588377
chr14	64589857	64590399	chr14-64589857-64590399
chr14	64632984	64633643	chr14-64632984-64633643
chr14	64639512	64639995	chr14-64639512-64639995
chr14	64652071	64653692	chr14-64652071-64653692
chr14	64657157	64657408	chr14-64657157-64657408
chr14	64665737	64667144	chr14-64665737-64667144
chr14	64948239	64951136	chr14-64948239-64951136
chr14	64963525	64963827	chr14-64963525-64963827
chr14	65096966	65098894	chr14-65096966-65098894
chr14	65149226	65150467	chr14-65149226-65150467
chr14	65261503	65263000	chr14-65261503-65263000
chr14	65266265	65266785	chr14-65266265-65266785
chr14	65331572	65332583	chr14-65331572-65332583
chr14	65342679	65345332	chr14-65342679-65345332
chr14	65357436	65360076	chr14-65357436-65360076
chr14	65374643	65375750	chr14-65374643-65375750
chr14	65381252	65381752	chr14-65381252-65381752

chr14	65540358	65540660	chr14-65540358-65540660
chr14	65591919	65593648	chr14-65591919-65593648
chr14	65742538	65743251	chr14-65742538-65743251
chr14	65805275	65807364	chr14-65805275-65807364
chr14	65832763	65834174	chr14-65832763-65834174
chr14	65836792	65838929	chr14-65836792-65838929
chr14	65876658	65877440	chr14-65876658-65877440
chr14	65881197	65884138	chr14-65881197-65884138
chr14	65890048	65890901	chr14-65890048-65890901
chr14	65900480	65900943	chr14-65900480-65900943
chr14	65952074	65954247	chr14-65952074-65954247
chr14	65968427	65972177	chr14-65968427-65972177
chr14	66008873	66009498	chr14-66008873-66009498
chr14	66028272	66028473	chr14-66028272-66028473
chr14	66124099	66124712	chr14-66124099-66124712
chr14	66223509	66224200	chr14-66223509-66224200
chr14	66244287	66244538	chr14-66244287-66244538
chr14	66257022	66259883	chr14-66257022-66259883
chr14	66264053	66265149	chr14-66264053-66265149
chr14	66266146	66266563	chr14-66266146-66266563
chr14	66273870	66274277	chr14-66273870-66274277
chr14	66278262	66281439	chr14-66278262-66281439
chr14	66296164	66299505	chr14-66296164-66299505
chr14	66324824	66325137	chr14-66324824-66325137
chr14	66635392	66635706	chr14-66635392-66635706
chr14	66866426	66869812	chr14-66866426-66869812
chr14	66910126	66911815	chr14-66910126-66911815
chr14	66973378	66974406	chr14-66973378-66974406
chr14	66976778	66977721	chr14-66976778-66977721
chr14	66999441	66999702	chr14-66999441-66999702
chr14	67007057	67009955	chr14-67007057-67009955
chr14	67047097	67048535	chr14-67047097-67048535
chr14	67069116	67072929	chr14-67069116-67072929
chr14	67158563	67159541	chr14-67158563-67159541
chr14	67176292	67176675	chr14-67176292-67176675
chr14	67182999	67183302	chr14-67182999-67183302
chr14	67188081	67189416	chr14-67188081-67189416
chr14	67232966	67235928	chr14-67232966-67235928
chr14	67613043	67613784	chr14-67613043-67613784
chr14	67642872	67643223	chr14-67642872-67643223
chr14	67663515	67664751	chr14-67663515-67664751
chr14	67712760	67718247	chr14-67712760-67718247

chr14	67877532	67878776	chr14-67877532-67878776
chr14	67887033	67888119	chr14-67887033-67888119
chr14	67889948	67890254	chr14-67889948-67890254
chr14	67923595	67924143	chr14-67923595-67924143
chr14	67931332	67934447	chr14-67931332-67934447
chr14	67935481	67935682	chr14-67935481-67935682
chr14	67941056	67941704	chr14-67941056-67941704
chr14	68050886	68051824	chr14-68050886-68051824
chr14	68054590	68054791	chr14-68054590-68054791
chr14	68083584	68085300	chr14-68083584-68085300
chr14	68123180	68125306	chr14-68123180-68125306
chr14	69028909	69030513	chr14-69028909-69030513
chr14	69089183	69089598	chr14-69089183-69089598
chr14	69174538	69175426	chr14-69174538-69175426
chr14	69190300	69191263	chr14-69190300-69191263
chr14	69391198	69391509	chr14-69391198-69391509
chr14	69692065	69692321	chr14-69692065-69692321
chr14	69706477	69707753	chr14-69706477-69707753
chr14	69731299	69735091	chr14-69731299-69735091
chr14	69740447	69742180	chr14-69740447-69742180
chr14	69763223	69763956	chr14-69763223-69763956
chr14	69766783	69768962	chr14-69766783-69768962
chr14	69803807	69806184	chr14-69803807-69806184
chr14	69812186	69817685	chr14-69812186-69817685
chr14	69823179	69823930	chr14-69823179-69823930
chr14	69861495	69862072	chr14-69861495-69862072
chr14	69911372	69912587	chr14-69911372-69912587
chr14	69966011	69966751	chr14-69966011-69966751
chr14	69995028	69995444	chr14-69995028-69995444
chr14	70056669	70057050	chr14-70056669-70057050
chr14	70074306	70079277	chr14-70074306-70079277
chr14	70096270	70096577	chr14-70096270-70096577
chr14	70099413	70101270	chr14-70099413-70101270
chr14	70106641	70106895	chr14-70106641-70106895
chr14	70135629	70136496	chr14-70135629-70136496
chr14	70152344	70154380	chr14-70152344-70154380
chr14	70157164	70161475	chr14-70157164-70161475
chr14	70165933	70166343	chr14-70165933-70166343
chr14	70167836	70168143	chr14-70167836-70168143
chr14	70170506	70171612	chr14-70170506-70171612
chr14	70172758	70178270	chr14-70172758-70178270
chr14	70190861	70192878	chr14-70190861-70192878

chr14	70195667	70196190	chr14-70195667-70196190
chr14	70205630	70208462	chr14-70205630-70208462
chr14	70288706	70290355	chr14-70288706-70290355
chr14	70324618	70324819	chr14-70324618-70324819
chr14	70336273	70337023	chr14-70336273-70337023
chr14	70340398	70341828	chr14-70340398-70341828
chr14	70349096	70351926	chr14-70349096-70351926
chr14	70442063	70444347	chr14-70442063-70444347
chr14	70457085	70458160	chr14-70457085-70458160
chr14	70470084	70470337	chr14-70470084-70470337
chr14	70515215	70521097	chr14-70515215-70521097
chr14	70530601	70531256	chr14-70530601-70531256
chr14	70544937	70547294	chr14-70544937-70547294
chr14	70553925	70555694	chr14-70553925-70555694
chr14	70576860	70579202	chr14-70576860-70579202
chr14	70598375	70600251	chr14-70598375-70600251
chr14	70617726	70619208	chr14-70617726-70619208
chr14	70635588	70636397	chr14-70635588-70636397
chr14	70658596	70660548	chr14-70658596-70660548
chr14	70765212	70768226	chr14-70765212-70768226
chr14	70773015	70778479	chr14-70773015-70778479
chr14	70780810	70781733	chr14-70780810-70781733
chr14	70889303	70891241	chr14-70889303-70891241
chr14	71199328	71199783	chr14-71199328-71199783
chr14	72401922	72402123	chr14-72401922-72402123
chr14	72445410	72447185	chr14-72445410-72447185
chr14	72450261	72450573	chr14-72450261-72450573
chr14	72479873	72480461	chr14-72479873-72480461
chr14	72531841	72532984	chr14-72531841-72532984
chr14	72601702	72603618	chr14-72601702-72603618
chr14	72708555	72710957	chr14-72708555-72710957
chr14	72807472	72807829	chr14-72807472-72807829
chr14	72869079	72869881	chr14-72869079-72869881
chr14	72887160	72887412	chr14-72887160-72887412
chr14	72983331	72983636	chr14-72983331-72983636
chr14	73142541	73144320	chr14-73142541-73144320
chr14	73234330	73234751	chr14-73234330-73234751
chr14	73237497	73241125	chr14-73237497-73241125
chr14	73253598	73253854	chr14-73253598-73253854
chr14	73324551	73326836	chr14-73324551-73326836
chr14	73382698	73386207	chr14-73382698-73386207
chr14	73402220	73402523	chr14-73402220-73402523

chr14	73442045	73442355	chr14-73442045-73442355
chr14	73460592	73460893	chr14-73460592-73460893
chr14	73509801	73511424	chr14-73509801-73511424
chr14	73524523	73525491	chr14-73524523-73525491
chr14	73552439	73553887	chr14-73552439-73553887
chr14	74640853	74641864	chr14-74640853-74641864
chr14	74643075	74643567	chr14-74643075-74643567
chr14	74646315	74646878	chr14-74646315-74646878
chr14	74731563	74734589	chr14-74731563-74734589
chr14	74736779	74736980	chr14-74736779-74736980
chr14	74823426	74823677	chr14-74823426-74823677
chr14	74845685	74846132	chr14-74845685-74846132
chr14	74852660	74853655	chr14-74852660-74853655
chr14	74871412	74871613	chr14-74871412-74871613
chr14	74910163	74910719	chr14-74910163-74910719
chr14	74945770	74948465	chr14-74945770-74948465
chr14	75015887	75016857	chr14-75015887-75016857
chr14	75045045	75045679	chr14-75045045-75045679
chr14	75130300	75131766	chr14-75130300-75131766
chr14	75136544	75137053	chr14-75136544-75137053
chr14	75176843	75177208	chr14-75176843-75177208
chr14	75283585	75285646	chr14-75283585-75285646
chr14	75445764	75446015	chr14-75445764-75446015
chr14	75456768	75457471	chr14-75456768-75457471
chr14	75752931	75755055	chr14-75752931-75755055
chr14	75785262	75787587	chr14-75785262-75787587
chr14	75794579	75794980	chr14-75794579-75794980
chr14	75819759	75820235	chr14-75819759-75820235
chr14	75827909	75830550	chr14-75827909-75830550
chr14	75832629	75837140	chr14-75832629-75837140
chr14	75844087	75848151	chr14-75844087-75848151
chr14	75863519	75864553	chr14-75863519-75864553
chr14	75870565	75871343	chr14-75870565-75871343
chr14	75939473	75939674	chr14-75939473-75939674
chr14	75955436	75955637	chr14-75955436-75955637
chr14	76009043	76011624	chr14-76009043-76011624
chr14	76109694	76112378	chr14-76109694-76112378
chr14	76149610	76150453	chr14-76149610-76150453
chr14	76151419	76153720	chr14-76151419-76153720
chr14	76165201	76165553	chr14-76165201-76165553
chr14	76413671	76416706	chr14-76413671-76416706
chr14	76476766	76482710	chr14-76476766-76482710

chr14	76503506	76507772	chr14-76503506-76507772
chr14	76520147	76520519	chr14-76520147-76520519
chr14	76555665	76557483	chr14-76555665-76557483
chr14	76570514	76570715	chr14-76570514-76570715
chr14	76586031	76586283	chr14-76586031-76586283
chr14	76610358	76610813	chr14-76610358-76610813
chr14	76630376	76630683	chr14-76630376-76630683
chr14	76662238	76665711	chr14-76662238-76665711
chr14	76780807	76781458	chr14-76780807-76781458
chr14	76783482	76788179	chr14-76783482-76788179
chr14	76875182	76875750	chr14-76875182-76875750
chr14	76882732	76883521	chr14-76882732-76883521
chr14	76947339	76947796	chr14-76947339-76947796
chr14	77008100	77008354	chr14-77008100-77008354
chr14	77035769	77037718	chr14-77035769-77037718
chr14	77156254	77157908	chr14-77156254-77157908
chr14	77874091	77875300	chr14-77874091-77875300
chr14	77904318	77904672	chr14-77904318-77904672
chr14	78307553	78308221	chr14-78307553-78308221
chr14	78330532	78330834	chr14-78330532-78330834
chr14	78535594	78537532	chr14-78535594-78537532
chr14	78724493	78725562	chr14-78724493-78725562
chr14	78731310	78733364	chr14-78731310-78733364
chr14	78848498	78850262	chr14-78848498-78850262
chr14	79115374	79115680	chr14-79115374-79115680
chr14	79248445	79248950	chr14-79248445-79248950
chr14	79300481	79302079	chr14-79300481-79302079
chr14	79331265	79331466	chr14-79331265-79331466
chr14	79388917	79391271	chr14-79388917-79391271
chr14	79397601	79398670	chr14-79397601-79398670
chr14	79426100	79427568	chr14-79426100-79427568
chr14	79451328	79452627	chr14-79451328-79452627
chr14	79479795	79484432	chr14-79479795-79484432
chr14	79486536	79489362	chr14-79486536-79489362
chr14	79503761	79504817	chr14-79503761-79504817
chr14	79514939	79519763	chr14-79514939-79519763
chr14	79527266	79528521	chr14-79527266-79528521
chr14	79534639	79535888	chr14-79534639-79535888
chr14	79586127	79589205	chr14-79586127-79589205
chr14	79660968	79661761	chr14-79660968-79661761
chr14	79769763	79771816	chr14-79769763-79771816
chr14	84442565	84446404	chr14-84442565-84446404

chr14	87139281	87142061	chr14-87139281-87142061
chr14	87415191	87418472	chr14-87415191-87418472
chr14	87567846	87568726	chr14-87567846-87568726
chr14	88469327	88471151	chr14-88469327-88471151
chr14	89568723	89568976	chr14-89568723-89568976
chr14	89610055	89610465	chr14-89610055-89610465
chr14	93653865	93654066	chr14-93653865-93654066
chr14	93885704	93886062	chr14-93885704-93886062
chr14	93887982	93891877	chr14-93887982-93891877
chr14	96516953	96519316	chr14-96516953-96519316
chr14	98167289	98170343	chr14-98167289-98170343
chr14	98626476	98627148	chr14-98626476-98627148
chr14	99043922	99048102	chr14-99043922-99048102
chr14	99098429	99100851	chr14-99098429-99100851
chr14	99295595	99296408	chr14-99295595-99296408
chr14	99297582	99304805	chr14-99297582-99304805
chr14	99307005	99307256	chr14-99307005-99307256
chr14	99324248	99324647	chr14-99324248-99324647
chr14	99422922	99423123	chr14-99422922-99423123
chr14	99475718	99476074	chr14-99475718-99476074
chr14	99548457	99549323	chr14-99548457-99549323
chr14	99578051	99581038	chr14-99578051-99581038
chr14	99630976	99631177	chr14-99630976-99631177
chr14	99943233	99943434	chr14-99943233-99943434
chr14	100283735	100286608	chr14-100283735-100286608
chr14	100865236	100865437	chr14-100865236-100865437
chr14	101607402	101609475	chr14-101607402-101609475
chr14	101639402	101641025	chr14-101639402-101641025
chr14	101653541	101655007	chr14-101653541-101655007
chr14	101728963	101730683	chr14-101728963-101730683
chr14	101787923	101789393	chr14-101787923-101789393
chr14	101797461	101798523	chr14-101797461-101798523
chr14	101804439	101804794	chr14-101804439-101804794
chr14	101805845	101806300	chr14-101805845-101806300
chr14	101830160	101830617	chr14-101830160-101830617
chr14	101837101	101846336	chr14-101837101-101846336
chr14	101852825	101853026	chr14-101852825-101853026
chr14	101869045	101870534	chr14-101869045-101870534
chr14	101878188	101879011	chr14-101878188-101879011
chr14	102981361	102983425	chr14-102981361-102983425
chr14	103065580	103065781	chr14-103065580-103065781
chr14	103069471	103071349	chr14-103069471-103071349

chr14	103097880	103100280	chr14-103097880-103100280
chr14	103344976	103347422	chr14-103344976-103347422
chr14	103349098	103350358	chr14-103349098-103350358
chr14	103354599	103355246	chr14-103354599-103355246
chr14	103469088	103470087	chr14-103469088-103470087
chr14	103500075	103501321	chr14-103500075-103501321
chr14	103504357	103504863	chr14-103504357-103504863
chr14	103511221	103511492	chr14-103511221-103511492
chr14	103512837	103514419	chr14-103512837-103514419
chr14	103649650	103652100	chr14-103649650-103652100
chr14	103653478	103654403	chr14-103653478-103654403
chr14	104460279	104460847	chr14-104460279-104460847
chr14	104465651	104468574	chr14-104465651-104468574
chr14	104472061	104473504	chr14-104472061-104473504
chr14	104521333	104523066	chr14-104521333-104523066
chr14	104715755	104715956	chr14-104715755-104715956
chr14	105175176	105177917	chr14-105175176-105177917
chr14	105258118	105260024	chr14-105258118-105260024
chr14	105328292	105329073	chr14-105328292-105329073
chr14	105588001	105591455	chr14-105588001-105591455
chr14	105662640	105663784	chr14-105662640-105663784
chr14	105668913	105669324	chr14-105668913-105669324
chr14	105731929	105733315	chr14-105731929-105733315
chr14	105802683	105804313	chr14-105802683-105804313
chr14	105815401	105816276	chr14-105815401-105816276
chr14	105890700	105899533	chr14-105890700-105899533
chr14	105922939	105923583	chr14-105922939-105923583
chr14	108911325	108911578	chr14-108911325-108911578
chr14	108912575	108914322	chr14-108912575-108914322
chr14	111674817	111676762	chr14-111674817-111676762
chr14	111681013	111681834	chr14-111681013-111681834
chr14	115040104	115044512	chr14-115040104-115044512
chr14	115091817	115093044	chr14-115091817-115093044
chr14	116925196	116927307	chr14-116925196-116927307
chr14	118001072	118002213	chr14-118001072-118002213
chr14	118050971	118051774	chr14-118050971-118051774
chr14	118088685	118090799	chr14-118088685-118090799
chr14	118131637	118133163	chr14-118131637-118133163
chr14	118136434	118138304	chr14-118136434-118138304
chr14	118179785	118181098	chr14-118179785-118181098
chr14	118225979	118226235	chr14-118225979-118226235
chr14	118230965	118231925	chr14-118230965-118231925

chr14	118234599	118237618	chr14-118234599-118237618
chr14	118436685	118437852	chr14-118436685-118437852
chr14	118473543	118474401	chr14-118473543-118474401
chr14	118565798	118567269	chr14-118565798-118567269
chr14	118635512	118636060	chr14-118635512-118636060
chr14	118643068	118644064	chr14-118643068-118644064
chr14	118656664	118658999	chr14-118656664-118658999
chr14	118662208	118662517	chr14-118662208-118662517
chr14	118674514	118675177	chr14-118674514-118675177
chr14	118703418	118710522	chr14-118703418-118710522
chr14	118713245	118714289	chr14-118713245-118714289
chr14	118750275	118751767	chr14-118750275-118751767
chr14	118764189	118764390	chr14-118764189-118764390
chr14	118766015	118766326	chr14-118766015-118766326
chr14	118854424	118855463	chr14-118854424-118855463
chr14	118910356	118910821	chr14-118910356-118910821
chr14	118922498	118923341	chr14-118922498-118923341
chr14	118924731	118925661	chr14-118924731-118925661
chr14	118937018	118939147	chr14-118937018-118939147
chr14	119005938	119008431	chr14-119005938-119008431
chr14	119012862	119013373	chr14-119012862-119013373
chr14	119098748	119099854	chr14-119098748-119099854
chr14	119137463	119138878	chr14-119137463-119138878
chr14	119816305	119816557	chr14-119816305-119816557
chr14	120137483	120138128	chr14-120137483-120138128
chr14	120172195	120172396	chr14-120172195-120172396
chr14	120274950	120279974	chr14-120274950-120279974
chr14	120287756	120289329	chr14-120287756-120289329
chr14	120296403	120297572	chr14-120296403-120297572
chr14	120299662	120299863	chr14-120299662-120299863
chr14	120307928	120308659	chr14-120307928-120308659
chr14	120309891	120310092	chr14-120309891-120310092
chr14	120312985	120314016	chr14-120312985-120314016
chr14	120318533	120318891	chr14-120318533-120318891
chr14	120322963	120326719	chr14-120322963-120326719
chr14	120337484	120338091	chr14-120337484-120338091
chr14	120342143	120342444	chr14-120342143-120342444
chr14	120355204	120355556	chr14-120355204-120355556
chr14	120372462	120374237	chr14-120372462-120374237
chr14	120405018	120407272	chr14-120405018-120407272
chr14	120476827	120479808	chr14-120476827-120479808
chr14	120626836	120627251	chr14-120626836-120627251

chr14	120830562	120830969	chr14-120830562-120830969
chr14	120831866	120832911	chr14-120831866-120832911
chr14	120841410	120841711	chr14-120841410-120841711
chr14	120867979	120868586	chr14-120867979-120868586
chr14	120880730	120881463	chr14-120880730-120881463
chr14	120910133	120913188	chr14-120910133-120913188
chr14	121034300	121038901	chr14-121034300-121038901
chr14	121050822	121051525	chr14-121050822-121051525
chr14	121137055	121137745	chr14-121137055-121137745
chr14	121182240	121184051	chr14-121182240-121184051
chr14	121190641	121192049	chr14-121190641-121192049
chr14	121239097	121239508	chr14-121239097-121239508
chr14	121245868	121246069	chr14-121245868-121246069
chr14	121346723	121347391	chr14-121346723-121347391
chr14	121371353	121373322	chr14-121371353-121373322
chr14	121376591	121380351	chr14-121376591-121380351
chr14	121450962	121451214	chr14-121450962-121451214
chr14	121504746	121505549	chr14-121504746-121505549
chr14	121693961	121694871	chr14-121693961-121694871
chr14	121696990	121698831	chr14-121696990-121698831
chr14	121709135	121714270	chr14-121709135-121714270
chr14	121716302	121716940	chr14-121716302-121716940
chr14	121764245	121764446	chr14-121764245-121764446
chr14	121795455	121799603	chr14-121795455-121799603
chr14	121824213	121826554	chr14-121824213-121826554
chr14	121877532	121879862	chr14-121877532-121879862
chr14	122104394	122108800	chr14-122104394-122108800
chr14	122180965	122182295	chr14-122180965-122182295
chr14	122406528	122407133	chr14-122406528-122407133
chr14	122450462	122451523	chr14-122450462-122451523
chr14	122453515	122454027	chr14-122453515-122454027
chr14	122463344	122465914	chr14-122463344-122465914
chr14	122472200	122472401	chr14-122472200-122472401
chr14	122473880	122476706	chr14-122473880-122476706
chr14	122478719	122479288	chr14-122478719-122479288
chr14	122490461	122491151	chr14-122490461-122491151
chr14	122533539	122535407	chr14-122533539-122535407
chr14	122902274	122904637	chr14-122902274-122904637
chr14	122911665	122913943	chr14-122911665-122913943
chr14	122981744	122984167	chr14-122981744-122984167
chr14	123626454	123627227	chr14-123626454-123627227
chr14	124192206	124192686	chr14-124192206-124192686

chr15	3356456	3356766	chr15-3356456-3356766
chr15	3444533	3444857	chr15-3444533-3444857
chr15	3578630	3578881	chr15-3578630-3578881
chr15	3580350	3583756	chr15-3580350-3583756
chr15	3588098	3588405	chr15-3588098-3588405
chr15	3978694	3980019	chr15-3978694-3980019
chr15	3994515	3997206	chr15-3994515-3997206
chr15	4025871	4028265	chr15-4025871-4028265
chr15	4032933	4033134	chr15-4032933-4033134
chr15	4375451	4376225	chr15-4375451-4376225
chr15	5106947	5108678	chr15-5106947-5108678
chr15	5114913	5119520	chr15-5114913-5119520
chr15	5143126	5145749	chr15-5143126-5145749
chr15	5184274	5186935	chr15-5184274-5186935
chr15	5194796	5195097	chr15-5194796-5195097
chr15	5196128	5196642	chr15-5196128-5196642
chr15	5235388	5235864	chr15-5235388-5235864
chr15	5241743	5245410	chr15-5241743-5245410
chr15	5495395	5495977	chr15-5495395-5495977
chr15	5539446	5539798	chr15-5539446-5539798
chr15	6386192	6388567	chr15-6386192-6388567
chr15	6390721	6391024	chr15-6390721-6391024
chr15	6565092	6565598	chr15-6565092-6565598
chr15	6574443	6574985	chr15-6574443-6574985
chr15	6625603	6626010	chr15-6625603-6626010
chr15	6707179	6709994	chr15-6707179-6709994
chr15	6726113	6726866	chr15-6726113-6726866
chr15	6872378	6875141	chr15-6872378-6875141
chr15	7128587	7130514	chr15-7128587-7130514
chr15	7136391	7136592	chr15-7136391-7136592
chr15	7137572	7138229	chr15-7137572-7138229
chr15	7216712	7217279	chr15-7216712-7217279
chr15	7397681	7398865	chr15-7397681-7398865
chr15	7596826	7597471	chr15-7596826-7597471
chr15	7750015	7750611	chr15-7750015-7750611
chr15	7809758	7814236	chr15-7809758-7814236
chr15	7815504	7815893	chr15-7815504-7815893
chr15	7861779	7863906	chr15-7861779-7863906
chr15	7877477	7879122	chr15-7877477-7879122
chr15	7889729	7890622	chr15-7889729-7890622
chr15	7931432	7931890	chr15-7931432-7931890
chr15	8097879	8099570	chr15-8097879-8099570

chr15	8108293	8111112	chr15-8108293-8111112
chr15	8168451	8169915	chr15-8168451-8169915
chr15	8190084	8190547	chr15-8190084-8190547
chr15	8271816	8272201	chr15-8271816-8272201
chr15	8279177	8280327	chr15-8279177-8280327
chr15	8441900	8447722	chr15-8441900-8447722
chr15	8568301	8570273	chr15-8568301-8570273
chr15	8589612	8589932	chr15-8589612-8589932
chr15	8625551	8625752	chr15-8625551-8625752
chr15	8627098	8627817	chr15-8627098-8627817
chr15	8629387	8629853	chr15-8629387-8629853
chr15	8709712	8710933	chr15-8709712-8710933
chr15	9070224	9073131	chr15-9070224-9073131
chr15	9074796	9074997	chr15-9074796-9074997
chr15	9138435	9141619	chr15-9138435-9141619
chr15	9435593	9436295	chr15-9435593-9436295
chr15	9748677	9748878	chr15-9748677-9748878
chr15	10177662	10178266	chr15-10177662-10178266
chr15	10468938	10471287	chr15-10468938-10471287
chr15	10484196	10487807	chr15-10484196-10487807
chr15	10685737	10687143	chr15-10685737-10687143
chr15	10688893	10691520	chr15-10688893-10691520
chr15	10709704	10716385	chr15-10709704-10716385
chr15	10747285	10747953	chr15-10747285-10747953
chr15	10758983	10763905	chr15-10758983-10763905
chr15	10770191	10772026	chr15-10770191-10772026
chr15	10814825	10816875	chr15-10814825-10816875
chr15	10817752	10819612	chr15-10817752-10819612
chr15	10829651	10829962	chr15-10829651-10829962
chr15	10864190	10864553	chr15-10864190-10864553
chr15	10981071	10982683	chr15-10981071-10982683
chr15	11036365	11037462	chr15-11036365-11037462
chr15	11064630	11065471	chr15-11064630-11065471
chr15	11397607	11400450	chr15-11397607-11400450
chr15	11903108	11907503	chr15-11903108-11907503
chr15	11993805	11996765	chr15-11993805-11996765
chr15	12010261	12011451	chr15-12010261-12011451
chr15	12116934	12119607	chr15-12116934-12119607
chr15	12203439	12206925	chr15-12203439-12206925
chr15	12230437	12230960	chr15-12230437-12230960
chr15	12231912	12233014	chr15-12231912-12233014
chr15	12320262	12324373	chr15-12320262-12324373

chr15	12375227	12376140	chr15-12375227-12376140
chr15	12548984	12550386	chr15-12548984-12550386
chr15	12739162	12740596	chr15-12739162-12740596
chr15	12823400	12826293	chr15-12823400-12826293
chr15	12964954	12965689	chr15-12964954-12965689
chr15	13067464	13067665	chr15-13067464-13067665
chr15	13171725	13174220	chr15-13171725-13174220
chr15	13237187	13238302	chr15-13237187-13238302
chr15	13367347	13368147	chr15-13367347-13368147
chr15	13383343	13383615	chr15-13383343-13383615
chr15	18818386	18819113	chr15-18818386-18819113
chr15	23035815	23036887	chr15-23035815-23036887
chr15	23452023	23452542	chr15-23452023-23452542
chr15	25269171	25270279	chr15-25269171-25270279
chr15	25271750	25272062	chr15-25271750-25272062
chr15	25302845	25303153	chr15-25302845-25303153
chr15	25322261	25322512	chr15-25322261-25322512
chr15	25348316	25348881	chr15-25348316-25348881
chr15	25364159	25366547	chr15-25364159-25366547
chr15	25369207	25370705	chr15-25369207-25370705
chr15	25377105	25377642	chr15-25377105-25377642
chr15	25407297	25407498	chr15-25407297-25407498
chr15	25408773	25409429	chr15-25408773-25409429
chr15	25410609	25415249	chr15-25410609-25415249
chr15	25441207	25441611	chr15-25441207-25441611
chr15	25505088	25505339	chr15-25505088-25505339
chr15	25546021	25546540	chr15-25546021-25546540
chr15	25556509	25557139	chr15-25556509-25557139
chr15	25563418	25563619	chr15-25563418-25563619
chr15	25573865	25574399	chr15-25573865-25574399
chr15	25577199	25577912	chr15-25577199-25577912
chr15	25620602	25625800	chr15-25620602-25625800
chr15	25641796	25643385	chr15-25641796-25643385
chr15	25647338	25648056	chr15-25647338-25648056
chr15	25652227	25653656	chr15-25652227-25653656
chr15	25757936	25761103	chr15-25757936-25761103
chr15	25767058	25769006	chr15-25767058-25769006
chr15	25773764	25774273	chr15-25773764-25774273
chr15	25778863	25779187	chr15-25778863-25779187
chr15	25841876	25846074	chr15-25841876-25846074
chr15	25858034	25859148	chr15-25858034-25859148
chr15	25862655	25862856	chr15-25862655-25862856

chr15	25876123	25877761	chr15-25876123-25877761
chr15	25914774	25916477	chr15-25914774-25916477
chr15	25932334	25934297	chr15-25932334-25934297
chr15	25940278	25941328	chr15-25940278-25941328
chr15	25948867	25949068	chr15-25948867-25949068
chr15	25950863	25951229	chr15-25950863-25951229
chr15	25983505	25986985	chr15-25983505-25986985
chr15	26308594	26309757	chr15-26308594-26309757
chr15	26894826	26896045	chr15-26894826-26896045
chr15	27465856	27468038	chr15-27465856-27468038
chr15	27629002	27631656	chr15-27629002-27631656
chr15	27680839	27682172	chr15-27680839-27682172
chr15	27779895	27780196	chr15-27779895-27780196
chr15	27781519	27786090	chr15-27781519-27786090
chr15	27802221	27802422	chr15-27802221-27802422
chr15	27805636	27805896	chr15-27805636-27805896
chr15	27815815	27817300	chr15-27815815-27817300
chr15	27849780	27850031	chr15-27849780-27850031
chr15	27923288	27924421	chr15-27923288-27924421
chr15	27936738	27938223	chr15-27936738-27938223
chr15	27941972	27944492	chr15-27941972-27944492
chr15	27954786	27955973	chr15-27954786-27955973
chr15	27992337	27992595	chr15-27992337-27992595
chr15	28004435	28005058	chr15-28004435-28005058
chr15	28022699	28026751	chr15-28022699-28026751
chr15	28080668	28081189	chr15-28080668-28081189
chr15	28132837	28133914	chr15-28132837-28133914
chr15	28141594	28141795	chr15-28141594-28141795
chr15	28158380	28159740	chr15-28158380-28159740
chr15	28181777	28182229	chr15-28181777-28182229
chr15	29882168	29882480	chr15-29882168-29882480
chr15	30172116	30173951	chr15-30172116-30173951
chr15	30259435	30259688	chr15-30259435-30259688
chr15	30646314	30648540	chr15-30646314-30648540
chr15	31167977	31168328	chr15-31167977-31168328
chr15	31184696	31185422	chr15-31184696-31185422
chr15	31186940	31188232	chr15-31186940-31188232
chr15	31223684	31225798	chr15-31223684-31225798
chr15	31246081	31246332	chr15-31246081-31246332
chr15	31297219	31298357	chr15-31297219-31298357
chr15	31336973	31337224	chr15-31336973-31337224
chr15	31342091	31342752	chr15-31342091-31342752

chr15	31365577	31368305	chr15-31365577-31368305
chr15	31452488	31454415	chr15-31452488-31454415
chr15	31528994	31532405	chr15-31528994-31532405
chr15	31542540	31542855	chr15-31542540-31542855
chr15	31568547	31568982	chr15-31568547-31568982
chr15	31599433	31603494	chr15-31599433-31603494
chr15	32242189	32246779	chr15-32242189-32246779
chr15	32323076	32323646	chr15-32323076-32323646
chr15	32778310	32779664	chr15-32778310-32779664
chr15	32920281	32922538	chr15-32920281-32922538
chr15	33082905	33083727	chr15-33082905-33083727
chr15	33686857	33688076	chr15-33686857-33688076
chr15	34081425	34085736	chr15-34081425-34085736
chr15	34237272	34239822	chr15-34237272-34239822
chr15	34254663	34255019	chr15-34254663-34255019
chr15	34306197	34307612	chr15-34306197-34307612
chr15	34350483	34351096	chr15-34350483-34351096
chr15	34440790	34444852	chr15-34440790-34444852
chr15	34453375	34453932	chr15-34453375-34453932
chr15	34493406	34497128	chr15-34493406-34497128
chr15	34571372	34571885	chr15-34571372-34571885
chr15	34677711	34679531	chr15-34677711-34679531
chr15	34837347	34837986	chr15-34837347-34837986
chr15	35021304	35022106	chr15-35021304-35022106
chr15	35154190	35156187	chr15-35154190-35156187
chr15	35183577	35183778	chr15-35183577-35183778
chr15	35292899	35298038	chr15-35292899-35298038
chr15	35302376	35303052	chr15-35302376-35303052
chr15	35326344	35327009	chr15-35326344-35327009
chr15	35370438	35373298	chr15-35370438-35373298
chr15	35936596	35938358	chr15-35936596-35938358
chr15	36140054	36140670	chr15-36140054-36140670
chr15	36148598	36149061	chr15-36148598-36149061
chr15	36164122	36165464	chr15-36164122-36165464
chr15	36173179	36175114	chr15-36173179-36175114
chr15	36179102	36179840	chr15-36179102-36179840
chr15	36210092	36211052	chr15-36210092-36211052
chr15	36280881	36284000	chr15-36280881-36284000
chr15	36304088	36304605	chr15-36304088-36304605
chr15	36373931	36375820	chr15-36373931-36375820
chr15	36419434	36420661	chr15-36419434-36420661
chr15	36495771	36497151	chr15-36495771-36497151

chr15	36555290	36556009	chr15-36555290-36556009
chr15	36578393	36578797	chr15-36578393-36578797
chr15	36605680	36610202	chr15-36605680-36610202
chr15	36640993	36641194	chr15-36640993-36641194
chr15	36643686	36645298	chr15-36643686-36645298
chr15	36656781	36658029	chr15-36656781-36658029
chr15	36669850	36670051	chr15-36669850-36670051
chr15	36687636	36690712	chr15-36687636-36690712
chr15	36705659	36705911	chr15-36705659-36705911
chr15	36715671	36716862	chr15-36715671-36716862
chr15	36727077	36728200	chr15-36727077-36728200
chr15	36760052	36760253	chr15-36760052-36760253
chr15	36761641	36762815	chr15-36761641-36762815
chr15	36779531	36780679	chr15-36779531-36780679
chr15	36782226	36782480	chr15-36782226-36782480
chr15	36785399	36785600	chr15-36785399-36785600
chr15	36789202	36795211	chr15-36789202-36795211
chr15	36842747	36844215	chr15-36842747-36844215
chr15	36851829	36853041	chr15-36851829-36853041
chr15	36854888	36856321	chr15-36854888-36856321
chr15	36865622	36866282	chr15-36865622-36866282
chr15	36884768	36885436	chr15-36884768-36885436
chr15	36924676	36924930	chr15-36924676-36924930
chr15	36937791	36939495	chr15-36937791-36939495
chr15	37004894	37008555	chr15-37004894-37008555
chr15	37030870	37032082	chr15-37030870-37032082
chr15	37100015	37100527	chr15-37100015-37100527
chr15	37120073	37121010	chr15-37120073-37121010
chr15	37134354	37136598	chr15-37134354-37136598
chr15	37159408	37159760	chr15-37159408-37159760
chr15	37184719	37184972	chr15-37184719-37184972
chr15	37192935	37193342	chr15-37192935-37193342
chr15	37197891	37198463	chr15-37197891-37198463
chr15	37231524	37235765	chr15-37231524-37235765
chr15	37241634	37244504	chr15-37241634-37244504
chr15	37312636	37313988	chr15-37312636-37313988
chr15	37317527	37318639	chr15-37317527-37318639
chr15	37459023	37459480	chr15-37459023-37459480
chr15	37606581	37608340	chr15-37606581-37608340
chr15	37755941	37756347	chr15-37755941-37756347
chr15	37769629	37770089	chr15-37769629-37770089
chr15	37791051	37792661	chr15-37791051-37792661

chr15	37905694	37906449	chr15-37905694-37906449
chr15	37959674	37962002	chr15-37959674-37962002
chr15	38067767	38068075	chr15-38067767-38068075
chr15	38076345	38080504	chr15-38076345-38080504
chr15	38199831	38200188	chr15-38199831-38200188
chr15	38244146	38245495	chr15-38244146-38245495
chr15	38253760	38254218	chr15-38253760-38254218
chr15	38296712	38297016	chr15-38296712-38297016
chr15	38298365	38302786	chr15-38298365-38302786
chr15	38323911	38324432	chr15-38323911-38324432
chr15	38373767	38374268	chr15-38373767-38374268
chr15	38383777	38386078	chr15-38383777-38386078
chr15	38391112	38392560	chr15-38391112-38392560
chr15	38405956	38406824	chr15-38405956-38406824
chr15	38408987	38409392	chr15-38408987-38409392
chr15	38419108	38419374	chr15-38419108-38419374
chr15	38420453	38421271	chr15-38420453-38421271
chr15	38451331	38458993	chr15-38451331-38458993
chr15	38468045	38474014	chr15-38468045-38474014
chr15	38517081	38520554	chr15-38517081-38520554
chr15	38535300	38535822	chr15-38535300-38535822
chr15	38567195	38567396	chr15-38567195-38567396
chr15	38572881	38573082	chr15-38572881-38573082
chr15	38594149	38594952	chr15-38594149-38594952
chr15	38601775	38602520	chr15-38601775-38602520
chr15	38609238	38609543	chr15-38609238-38609543
chr15	38640200	38640401	chr15-38640200-38640401
chr15	38661195	38663231	chr15-38661195-38663231
chr15	38691496	38691750	chr15-38691496-38691750
chr15	38709264	38710772	chr15-38709264-38710772
chr15	38932649	38933340	chr15-38932649-38933340
chr15	39005668	39007653	chr15-39005668-39007653
chr15	39076681	39077404	chr15-39076681-39077404
chr15	39111249	39114110	chr15-39111249-39114110
chr15	39173204	39173515	chr15-39173204-39173515
chr15	39174451	39174703	chr15-39174451-39174703
chr15	39196491	39199726	chr15-39196491-39199726
chr15	39217104	39217409	chr15-39217104-39217409
chr15	39232646	39233417	chr15-39232646-39233417
chr15	39248039	39248712	chr15-39248039-39248712
chr15	39553231	39553432	chr15-39553231-39553432
chr15	39659308	39660159	chr15-39659308-39660159

chr15	39669897	39670098	chr15-39669897-39670098
chr15	39721840	39722041	chr15-39721840-39722041
chr15	39731048	39732352	chr15-39731048-39732352
chr15	39821357	39821617	chr15-39821357-39821617
chr15	39856920	39857545	chr15-39856920-39857545
chr15	39901213	39901472	chr15-39901213-39901472
chr15	39942349	39944882	chr15-39942349-39944882
chr15	40363593	40363997	chr15-40363593-40363997
chr15	40653564	40658153	chr15-40653564-40658153
chr15	40954610	40955442	chr15-40954610-40955442
chr15	41028158	41028986	chr15-41028158-41028986
chr15	41163799	41164319	chr15-41163799-41164319
chr15	41276847	41277098	chr15-41276847-41277098
chr15	41446345	41448927	chr15-41446345-41448927
chr15	41603192	41603444	chr15-41603192-41603444
chr15	41611928	41612330	chr15-41611928-41612330
chr15	41651020	41651437	chr15-41651020-41651437
chr15	41693513	41693714	chr15-41693513-41693714
chr15	41787886	41791686	chr15-41787886-41791686
chr15	42675863	42676658	chr15-42675863-42676658
chr15	43169602	43171035	chr15-43169602-43171035
chr15	43280964	43283138	chr15-43280964-43283138
chr15	43476835	43478117	chr15-43476835-43478117
chr15	43869385	43870394	chr15-43869385-43870394
chr15	44412383	44412638	chr15-44412383-44412638
chr15	44425963	44429310	chr15-44425963-44429310
chr15	44457285	44458041	chr15-44457285-44458041
chr15	44618523	44620545	chr15-44618523-44620545
chr15	44747610	44749255	chr15-44747610-44749255
chr15	44787451	44788274	chr15-44787451-44788274
chr15	45114333	45114820	chr15-45114333-45114820
chr15	45663383	45663686	chr15-45663383-45663686
chr15	48789625	48790833	chr15-48789625-48790833
chr15	50362060	50362261	chr15-50362060-50362261
chr15	50869379	50869986	chr15-50869379-50869986
chr15	50888602	50890733	chr15-50888602-50890733
chr15	51592583	51593161	chr15-51592583-51593161
chr15	51696688	51696889	chr15-51696688-51696889
chr15	51713036	51713558	chr15-51713036-51713558
chr15	51730690	51731370	chr15-51730690-51731370
chr15	51853065	51855481	chr15-51853065-51855481
chr15	51863230	51865874	chr15-51863230-51865874

chr15	51872136	51872337	chr15-51872136-51872337
chr15	51876983	51879224	chr15-51876983-51879224
chr15	51989522	51992552	chr15-51989522-51992552
chr15	52040031	52040747	chr15-52040031-52040747
chr15	52669029	52669393	chr15-52669029-52669393
chr15	52711843	52713501	chr15-52711843-52713501
chr15	53160636	53160887	chr15-53160636-53160887
chr15	53171492	53171991	chr15-53171492-53171991
chr15	53175159	53175713	chr15-53175159-53175713
chr15	53207655	53208012	chr15-53207655-53208012
chr15	53341287	53347440	chr15-53341287-53347440
chr15	53676782	53677033	chr15-53676782-53677033
chr15	53897899	53898937	chr15-53897899-53898937
chr15	53900652	53903085	chr15-53900652-53903085
chr15	54176378	54176579	chr15-54176378-54176579
chr15	54278120	54278861	chr15-54278120-54278861
chr15	54533950	54534664	chr15-54533950-54534664
chr15	54570876	54573710	chr15-54570876-54573710
chr15	54745323	54746627	chr15-54745323-54746627
chr15	54847036	54847459	chr15-54847036-54847459
chr15	54919867	54920423	chr15-54919867-54920423
chr15	54952081	54952888	chr15-54952081-54952888
chr15	55071197	55072919	chr15-55071197-55072919
chr15	55089474	55091274	chr15-55089474-55091274
chr15	55112067	55113745	chr15-55112067-55113745
chr15	55120712	55121676	chr15-55120712-55121676
chr15	55134742	55135551	chr15-55134742-55135551
chr15	55139309	55139510	chr15-55139309-55139510
chr15	55142293	55142656	chr15-55142293-55142656
chr15	55208483	55209008	chr15-55208483-55209008
chr15	55265520	55266493	chr15-55265520-55266493
chr15	55280147	55280838	chr15-55280147-55280838
chr15	55306402	55308579	chr15-55306402-55308579
chr15	55333527	55333829	chr15-55333527-55333829
chr15	55379965	55380166	chr15-55379965-55380166
chr15	55556090	55559068	chr15-55556090-55559068
chr15	55905756	55907379	chr15-55905756-55907379
chr15	56046623	56046978	chr15-56046623-56046978
chr15	56485515	56486070	chr15-56485515-56486070
chr15	56685624	56686042	chr15-56685624-56686042
chr15	56691793	56696601	chr15-56691793-56696601
chr15	56764629	56765930	chr15-56764629-56765930

chr15	56832424	56833107	chr15-56832424-56833107
chr15	56844159	56844486	chr15-56844159-56844486
chr15	57140473	57140674	chr15-57140473-57140674
chr15	57692995	57696643	chr15-57692995-57696643
chr15	57760910	57762946	chr15-57760910-57762946
chr15	57765232	57765892	chr15-57765232-57765892
chr15	57889896	57893403	chr15-57889896-57893403
chr15	57906837	57907089	chr15-57906837-57907089
chr15	57911467	57913516	chr15-57911467-57913516
chr15	57974706	57977382	chr15-57974706-57977382
chr15	58018199	58018719	chr15-58018199-58018719
chr15	58033255	58034942	chr15-58033255-58034942
chr15	58075443	58077538	chr15-58075443-58077538
chr15	58133138	58135835	chr15-58133138-58135835
chr15	58140570	58143125	chr15-58140570-58143125
chr15	58211266	58215957	chr15-58211266-58215957
chr15	58259066	58259317	chr15-58259066-58259317
chr15	58324025	58324605	chr15-58324025-58324605
chr15	58361513	58361714	chr15-58361513-58361714
chr15	58414909	58417549	chr15-58414909-58417549
chr15	58480570	58480771	chr15-58480570-58480771
chr15	58517234	58518761	chr15-58517234-58518761
chr15	58594829	58595312	chr15-58594829-58595312
chr15	58747111	58747366	chr15-58747111-58747366
chr15	58761686	58761887	chr15-58761686-58761887
chr15	58821719	58824485	chr15-58821719-58824485
chr15	58869720	58870296	chr15-58869720-58870296
chr15	58871933	58874153	chr15-58871933-58874153
chr15	58875647	58876716	chr15-58875647-58876716
chr15	58887677	58890908	chr15-58887677-58890908
chr15	58931852	58935344	chr15-58931852-58935344
chr15	58998086	58998616	chr15-58998086-58998616
chr15	59003511	59004911	chr15-59003511-59004911
chr15	59065285	59065668	chr15-59065285-59065668
chr15	59069054	59069314	chr15-59069054-59069314
chr15	59074593	59075632	chr15-59074593-59075632
chr15	59077276	59082814	chr15-59077276-59082814
chr15	59105857	59106461	chr15-59105857-59106461
chr15	59145848	59146162	chr15-59145848-59146162
chr15	59151319	59151520	chr15-59151319-59151520
chr15	59313663	59318853	chr15-59313663-59318853
chr15	59360911	59361355	chr15-59360911-59361355

chr15	59372909	59375794	chr15-59372909-59375794
chr15	59386609	59387743	chr15-59386609-59387743
chr15	59409614	59409888	chr15-59409614-59409888
chr15	59412107	59412866	chr15-59412107-59412866
chr15	59447520	59448822	chr15-59447520-59448822
chr15	59480249	59482336	chr15-59480249-59482336
chr15	59511718	59512203	chr15-59511718-59512203
chr15	59520458	59520762	chr15-59520458-59520762
chr15	59525850	59526237	chr15-59525850-59526237
chr15	59540810	59541011	chr15-59540810-59541011
chr15	59543608	59543809	chr15-59543608-59543809
chr15	59555168	59555369	chr15-59555168-59555369
chr15	59596000	59600871	chr15-59596000-59600871
chr15	59617728	59618939	chr15-59617728-59618939
chr15	59646613	59654008	chr15-59646613-59654008
chr15	59680115	59681049	chr15-59680115-59681049
chr15	59728099	59728865	chr15-59728099-59728865
chr15	59759428	59761076	chr15-59759428-59761076
chr15	59784089	59784851	chr15-59784089-59784851
chr15	59794721	59795940	chr15-59794721-59795940
chr15	59828284	59828485	chr15-59828284-59828485
chr15	59836191	59836827	chr15-59836191-59836827
chr15	59950544	59950900	chr15-59950544-59950900
chr15	60111964	60112432	chr15-60111964-60112432
chr15	60166963	60167480	chr15-60166963-60167480
chr15	60185039	60188395	chr15-60185039-60188395
chr15	60779315	60779849	chr15-60779315-60779849
chr15	60820504	60825376	chr15-60820504-60825376
chr15	61157740	61160199	chr15-61157740-61160199
chr15	61207888	61208142	chr15-61207888-61208142
chr15	61240775	61241762	chr15-61240775-61241762
chr15	61395365	61395566	chr15-61395365-61395566
chr15	61454313	61454709	chr15-61454313-61454709
chr15	61455723	61455924	chr15-61455723-61455924
chr15	61538980	61539285	chr15-61538980-61539285
chr15	61541153	61541354	chr15-61541153-61541354
chr15	61983393	61991212	chr15-61983393-61991212
chr15	61992153	61992354	chr15-61992153-61992354
chr15	62037395	62040611	chr15-62037395-62040611
chr15	62043069	62044081	chr15-62043069-62044081
chr15	62078670	62080655	chr15-62078670-62080655
chr15	62106473	62106877	chr15-62106473-62106877

chr15	62117823	62124036	chr15-62117823-62124036
chr15	62127283	62129675	chr15-62127283-62129675
chr15	62138492	62140106	chr15-62138492-62140106
chr15	62202730	62203038	chr15-62202730-62203038
chr15	62204200	62204842	chr15-62204200-62204842
chr15	62206463	62206716	chr15-62206463-62206716
chr15	62261321	62261522	chr15-62261321-62261522
chr15	62285738	62287175	chr15-62285738-62287175
chr15	62295178	62295777	chr15-62295178-62295777
chr15	62327465	62329544	chr15-62327465-62329544
chr15	62454323	62454524	chr15-62454323-62454524
chr15	62501291	62501593	chr15-62501291-62501593
chr15	62569017	62569683	chr15-62569017-62569683
chr15	63066113	63066425	chr15-63066113-63066425
chr15	63340339	63340805	chr15-63340339-63340805
chr15	63484104	63484920	chr15-63484104-63484920
chr15	63722904	63723566	chr15-63722904-63723566
chr15	63726732	63726983	chr15-63726732-63726983
chr15	63895737	63896103	chr15-63895737-63896103
chr15	63996430	63998969	chr15-63996430-63998969
chr15	64005363	64006549	chr15-64005363-64006549
chr15	64013393	64014063	chr15-64013393-64014063
chr15	64021940	64022788	chr15-64021940-64022788
chr15	64023624	64024440	chr15-64023624-64024440
chr15	64031039	64033812	chr15-64031039-64033812
chr15	64041484	64041685	chr15-64041484-64041685
chr15	64053247	64053969	chr15-64053247-64053969
chr15	64058188	64061048	chr15-64058188-64061048
chr15	64074049	64074353	chr15-64074049-64074353
chr15	64191230	64191893	chr15-64191230-64191893
chr15	64220459	64220713	chr15-64220459-64220713
chr15	64271390	64271698	chr15-64271390-64271698
chr15	64311324	64314215	chr15-64311324-64314215
chr15	64377865	64378066	chr15-64377865-64378066
chr15	64380079	64384490	chr15-64380079-64384490
chr15	64477538	64477739	chr15-64477538-64477739
chr15	64501244	64501599	chr15-64501244-64501599
chr15	64573008	64573209	chr15-64573008-64573209
chr15	64621638	64622897	chr15-64621638-64622897
chr15	64920172	64923404	chr15-64920172-64923404
chr15	65745756	65745957	chr15-65745756-65745957
chr15	65786599	65788222	chr15-65786599-65788222

chr15	66285626	66287139	chr15-66285626-66287139
chr15	66500507	66501113	chr15-66500507-66501113
chr15	66522837	66523105	chr15-66522837-66523105
chr15	66535729	66536034	chr15-66535729-66536034
chr15	66558888	66561408	chr15-66558888-66561408
chr15	66577151	66579454	chr15-66577151-66579454
chr15	66795492	66796157	chr15-66795492-66796157
chr15	66877821	66878153	chr15-66877821-66878153
chr15	66890660	66892471	chr15-66890660-66892471
chr15	66930598	66931091	chr15-66930598-66931091
chr15	66953744	66953996	chr15-66953744-66953996
chr15	66966980	66971008	chr15-66966980-66971008
chr15	66980217	66981121	chr15-66980217-66981121
chr15	66984800	66985102	chr15-66984800-66985102
chr15	66998335	66999092	chr15-66998335-66999092
chr15	67039132	67040719	chr15-67039132-67040719
chr15	67116744	67117454	chr15-67116744-67117454
chr15	67133069	67135307	chr15-67133069-67135307
chr15	67143626	67147553	chr15-67143626-67147553
chr15	67161852	67162182	chr15-67161852-67162182
chr15	67164936	67165187	chr15-67164936-67165187
chr15	67174059	67178960	chr15-67174059-67178960
chr15	67217846	67218152	chr15-67217846-67218152
chr15	68257535	68260221	chr15-68257535-68260221
chr15	68361187	68364150	chr15-68361187-68364150
chr15	68927891	68929726	chr15-68927891-68929726
chr15	72546554	72547862	chr15-72546554-72547862
chr15	72549291	72549492	chr15-72549291-72549492
chr15	72660314	72660515	chr15-72660314-72660515
chr15	72808993	72810970	chr15-72808993-72810970
chr15	72837222	72837587	chr15-72837222-72837587
chr15	72892997	72893554	chr15-72892997-72893554
chr15	73059780	73061915	chr15-73059780-73061915
chr15	73066754	73068307	chr15-73066754-73068307
chr15	73089652	73091540	chr15-73089652-73091540
chr15	73102219	73103140	chr15-73102219-73103140
chr15	73148421	73151406	chr15-73148421-73151406
chr15	73153042	73157284	chr15-73153042-73157284
chr15	73182289	73187050	chr15-73182289-73187050
chr15	73241676	73244271	chr15-73241676-73244271
chr15	73246150	73246924	chr15-73246150-73246924
chr15	73267414	73268462	chr15-73267414-73268462

chr15	73421129	73425510	chr15-73421129-73425510
chr15	73430943	73433245	chr15-73430943-73433245
chr15	73435126	73435638	chr15-73435126-73435638
chr15	73442521	73442722	chr15-73442521-73442722
chr15	73511545	73514779	chr15-73511545-73514779
chr15	73526063	73526581	chr15-73526063-73526581
chr15	73595495	73595822	chr15-73595495-73595822
chr15	73604388	73604639	chr15-73604388-73604639
chr15	73643812	73646370	chr15-73643812-73646370
chr15	73661915	73662404	chr15-73661915-73662404
chr15	73703870	73704242	chr15-73703870-73704242
chr15	73707307	73707717	chr15-73707307-73707717
chr15	73716367	73716973	chr15-73716367-73716973
chr15	73724197	73728422	chr15-73724197-73728422
chr15	73730104	73730878	chr15-73730104-73730878
chr15	73747615	73749778	chr15-73747615-73749778
chr15	73839087	73840207	chr15-73839087-73840207
chr15	73849839	73850040	chr15-73849839-73850040
chr15	73870246	73870960	chr15-73870246-73870960
chr15	73913590	73914052	chr15-73913590-73914052
chr15	73916236	73916915	chr15-73916236-73916915
chr15	73919137	73919839	chr15-73919137-73919839
chr15	74211478	74214758	chr15-74211478-74214758
chr15	74268393	74268877	chr15-74268393-74268877
chr15	74280737	74281192	chr15-74280737-74281192
chr15	74301247	74301550	chr15-74301247-74301550
chr15	74487025	74488326	chr15-74487025-74488326
chr15	74515979	74520075	chr15-74515979-74520075
chr15	74529111	74530032	chr15-74529111-74530032
chr15	74563256	74564598	chr15-74563256-74564598
chr15	74566968	74567288	chr15-74566968-74567288
chr15	74608036	74608566	chr15-74608036-74608566
chr15	74622074	74624244	chr15-74622074-74624244
chr15	74670350	74673663	chr15-74670350-74673663
chr15	74678797	74680188	chr15-74678797-74680188
chr15	74701741	74702820	chr15-74701741-74702820
chr15	74707927	74710339	chr15-74707927-74710339
chr15	74712185	74713052	chr15-74712185-74713052
chr15	74714385	74716756	chr15-74714385-74716756
chr15	74717854	74718717	chr15-74717854-74718717
chr15	74720774	74723207	chr15-74720774-74723207
chr15	74724265	74724818	chr15-74724265-74724818

chr15	74729139	74730246	chr15-74729139-74730246
chr15	74749414	74749729	chr15-74749414-74749729
chr15	74752238	74753447	chr15-74752238-74753447
chr15	74764247	74764682	chr15-74764247-74764682
chr15	74783928	74784559	chr15-74783928-74784559
chr15	74788117	74788424	chr15-74788117-74788424
chr15	74891857	74892529	chr15-74891857-74892529
chr15	74919726	74919979	chr15-74919726-74919979
chr15	74921236	74921691	chr15-74921236-74921691
chr15	74923171	74923478	chr15-74923171-74923478
chr15	74926534	74936304	chr15-74926534-74936304
chr15	74953827	74960829	chr15-74953827-74960829
chr15	74967473	74968669	chr15-74967473-74968669
chr15	74988275	74988914	chr15-74988275-74988914
chr15	74992310	74999776	chr15-74992310-74999776
chr15	75021278	75022279	chr15-75021278-75022279
chr15	75026313	75026727	chr15-75026313-75026727
chr15	75045701	75051313	chr15-75045701-75051313
chr15	75141381	75141582	chr15-75141381-75141582
chr15	75153692	75153966	chr15-75153692-75153966
chr15	75216297	75216498	chr15-75216297-75216498
chr15	75423250	75423451	chr15-75423250-75423451
chr15	75565947	75567654	chr15-75565947-75567654
chr15	75615443	75617726	chr15-75615443-75617726
chr15	75641853	75643004	chr15-75641853-75643004
chr15	75677699	75679533	chr15-75677699-75679533
chr15	75703280	75705108	chr15-75703280-75705108
chr15	75727783	75728460	chr15-75727783-75728460
chr15	75731492	75732202	chr15-75731492-75732202
chr15	75746010	75749195	chr15-75746010-75749195
chr15	75840863	75842992	chr15-75840863-75842992
chr15	75844496	75845040	chr15-75844496-75845040
chr15	75850848	75851291	chr15-75850848-75851291
chr15	75856894	75857431	chr15-75856894-75857431
chr15	75861857	75863068	chr15-75861857-75863068
chr15	75883927	75884385	chr15-75883927-75884385
chr15	75886578	75888232	chr15-75886578-75888232
chr15	75894027	75894438	chr15-75894027-75894438
chr15	75907089	75912006	chr15-75907089-75912006
chr15	75920010	75922041	chr15-75920010-75922041
chr15	75923459	75924356	chr15-75923459-75924356
chr15	75928198	75930348	chr15-75928198-75930348

chr15	75940746	75942668	chr15-75940746-75942668
chr15	75943685	75944046	chr15-75943685-75944046
chr15	75968584	75970613	chr15-75968584-75970613
chr15	75980696	75982585	chr15-75980696-75982585
chr15	75993264	75994224	chr15-75993264-75994224
chr15	75997515	76000601	chr15-75997515-76000601
chr15	76006379	76015543	chr15-76006379-76015543
chr15	76034165	76035146	chr15-76034165-76035146
chr15	76067856	76070484	chr15-76067856-76070484
chr15	76078787	76081925	chr15-76078787-76081925
chr15	76090421	76092176	chr15-76090421-76092176
chr15	76103553	76103754	chr15-76103553-76103754
chr15	76114317	76114965	chr15-76114317-76114965
chr15	76117512	76120180	chr15-76117512-76120180
chr15	76156129	76156436	chr15-76156129-76156436
chr15	76179876	76180933	chr15-76179876-76180933
chr15	76188592	76209266	chr15-76188592-76209266
chr15	76210845	76211233	chr15-76210845-76211233
chr15	76227554	76233503	chr15-76227554-76233503
chr15	76242354	76243899	chr15-76242354-76243899
chr15	76246240	76249156	chr15-76246240-76249156
chr15	76293402	76296238	chr15-76293402-76296238
chr15	76306237	76307817	chr15-76306237-76307817
chr15	76326437	76333282	chr15-76326437-76333282
chr15	76342906	76345269	chr15-76342906-76345269
chr15	76349093	76352617	chr15-76349093-76352617
chr15	76368262	76370837	chr15-76368262-76370837
chr15	76379662	76382076	chr15-76379662-76382076
chr15	76455608	76462604	chr15-76455608-76462604
chr15	76476120	76479465	chr15-76476120-76479465
chr15	76480312	76481295	chr15-76480312-76481295
chr15	76510258	76512738	chr15-76510258-76512738
chr15	76517561	76520239	chr15-76517561-76520239
chr15	76521972	76524061	chr15-76521972-76524061
chr15	76534344	76534545	chr15-76534344-76534545
chr15	76536868	76540677	chr15-76536868-76540677
chr15	76576093	76577672	chr15-76576093-76577672
chr15	76606347	76607810	chr15-76606347-76607810
chr15	76613767	76620515	chr15-76613767-76620515
chr15	76624609	76627039	chr15-76624609-76627039
chr15	76638827	76640341	chr15-76638827-76640341
chr15	76654800	76661693	chr15-76654800-76661693

chr15	76670835	76673181	chr15-76670835-76673181
chr15	76697313	76698917	chr15-76697313-76698917
chr15	76700532	76702504	chr15-76700532-76702504
chr15	76709615	76712052	chr15-76709615-76712052
chr15	76715189	76716600	chr15-76715189-76716600
chr15	76721814	76725182	chr15-76721814-76725182
chr15	76816525	76818598	chr15-76816525-76818598
chr15	76869742	76871983	chr15-76869742-76871983
chr15	76878820	76882057	chr15-76878820-76882057
chr15	76883605	76883806	chr15-76883605-76883806
chr15	76898758	76901658	chr15-76898758-76901658
chr15	76903656	76907183	chr15-76903656-76907183
chr15	76924355	76926322	chr15-76924355-76926322
chr15	76947832	76949984	chr15-76947832-76949984
chr15	76973773	76974526	chr15-76973773-76974526
chr15	76987269	76987926	chr15-76987269-76987926
chr15	76998947	76999451	chr15-76998947-76999451
chr15	77013604	77014211	chr15-77013604-77014211
chr15	77036746	77038256	chr15-77036746-77038256
chr15	77056005	77056761	chr15-77056005-77056761
chr15	77068839	77069094	chr15-77068839-77069094
chr15	77076778	77077186	chr15-77076778-77077186
chr15	77149661	77154632	chr15-77149661-77154632
chr15	77192268	77193107	chr15-77192268-77193107
chr15	77304920	77307754	chr15-77304920-77307754
chr15	77336032	77337147	chr15-77336032-77337147
chr15	77343152	77343967	chr15-77343152-77343967
chr15	77358237	77359167	chr15-77358237-77359167
chr15	77366801	77367419	chr15-77366801-77367419
chr15	77397396	77398460	chr15-77397396-77398460
chr15	77728645	77728846	chr15-77728645-77728846
chr15	77797272	77808388	chr15-77797272-77808388
chr15	77815809	77825700	chr15-77815809-77825700
chr15	77826944	77843942	chr15-77826944-77843942
chr15	77856171	77858986	chr15-77856171-77858986
chr15	77863009	77863534	chr15-77863009-77863534
chr15	77881418	77884095	chr15-77881418-77884095
chr15	77891410	77891611	chr15-77891410-77891611
chr15	77905190	77905765	chr15-77905190-77905765
chr15	77907657	77908311	chr15-77907657-77908311
chr15	77910806	77913633	chr15-77910806-77913633
chr15	77927311	77930278	chr15-77927311-77930278

chr15	77933635	77934092	chr15-77933635-77934092
chr15	77955674	77957240	chr15-77955674-77957240
chr15	77968412	77972233	chr15-77968412-77972233
chr15	78078578	78079524	chr15-78078578-78079524
chr15	78119408	78119879	chr15-78119408-78119879
chr15	78172907	78175249	chr15-78172907-78175249
chr15	78204160	78204512	chr15-78204160-78204512
chr15	78404655	78407804	chr15-78404655-78407804
chr15	78428402	78429427	chr15-78428402-78429427
chr15	78436640	78437662	chr15-78436640-78437662
chr15	78447847	78448463	chr15-78447847-78448463
chr15	78450328	78450856	chr15-78450328-78450856
chr15	78529454	78529965	chr15-78529454-78529965
chr15	78531677	78532809	chr15-78531677-78532809
chr15	78554540	78555073	chr15-78554540-78555073
chr15	78581190	78582268	chr15-78581190-78582268
chr15	78591494	78591746	chr15-78591494-78591746
chr15	78597013	78597379	chr15-78597013-78597379
chr15	78607337	78608501	chr15-78607337-78608501
chr15	78610453	78611319	chr15-78610453-78611319
chr15	78624348	78624812	chr15-78624348-78624812
chr15	78712646	78713221	chr15-78712646-78713221
chr15	78718162	78719266	chr15-78718162-78719266
chr15	78773225	78773680	chr15-78773225-78773680
chr15	78800695	78808325	chr15-78800695-78808325
chr15	78809875	78810076	chr15-78809875-78810076
chr15	78815175	78816265	chr15-78815175-78816265
chr15	78828031	78828890	chr15-78828031-78828890
chr15	78832408	78833208	chr15-78832408-78833208
chr15	78834298	78834499	chr15-78834298-78834499
chr15	78842152	78846633	chr15-78842152-78846633
chr15	78851672	78851975	chr15-78851672-78851975
chr15	78868118	78869572	chr15-78868118-78869572
chr15	78875038	78879597	chr15-78875038-78879597
chr15	78883069	78883454	chr15-78883069-78883454
chr15	78899205	78901863	chr15-78899205-78901863
chr15	78903396	78903705	chr15-78903396-78903705
chr15	78913500	78916330	chr15-78913500-78916330
chr15	78925570	78929905	chr15-78925570-78929905
chr15	78934425	78936610	chr15-78934425-78936610
chr15	78942607	78942964	chr15-78942607-78942964
chr15	78947455	78948606	chr15-78947455-78948606

chr15	78959954	78960535	chr15-78959954-78960535
chr15	78982128	78985215	chr15-78982128-78985215
chr15	78988462	78988872	chr15-78988462-78988872
chr15	79027260	79031764	chr15-79027260-79031764
chr15	79037876	79038346	chr15-79037876-79038346
chr15	79042393	79043707	chr15-79042393-79043707
chr15	79051114	79052149	chr15-79051114-79052149
chr15	79061339	79063783	chr15-79061339-79063783
chr15	79074272	79076906	chr15-79074272-79076906
chr15	79107585	79111803	chr15-79107585-79111803
chr15	79114056	79115897	chr15-79114056-79115897
chr15	79130575	79132201	chr15-79130575-79132201
chr15	79140469	79143515	chr15-79140469-79143515
chr15	79145728	79145929	chr15-79145728-79145929
chr15	79163257	79163942	chr15-79163257-79163942
chr15	79176626	79177225	chr15-79176626-79177225
chr15	79228795	79230687	chr15-79228795-79230687
chr15	79252349	79252550	chr15-79252349-79252550
chr15	79260780	79262377	chr15-79260780-79262377
chr15	79270120	79271435	chr15-79270120-79271435
chr15	79298897	79299340	chr15-79298897-79299340
chr15	79321930	79328940	chr15-79321930-79328940
chr15	79342773	79343532	chr15-79342773-79343532
chr15	79346001	79350246	chr15-79346001-79350246
chr15	79351081	79352219	chr15-79351081-79352219
chr15	79354608	79358565	chr15-79354608-79358565
chr15	79359377	79361800	chr15-79359377-79361800
chr15	79367652	79370461	chr15-79367652-79370461
chr15	79399223	79404279	chr15-79399223-79404279
chr15	79409236	79409692	chr15-79409236-79409692
chr15	79413867	79414657	chr15-79413867-79414657
chr15	79424148	79426379	chr15-79424148-79426379
chr15	79435083	79443302	chr15-79435083-79443302
chr15	79453556	79456259	chr15-79453556-79456259
chr15	79464016	79464681	chr15-79464016-79464681
chr15	79466451	79467074	chr15-79466451-79467074
chr15	79470706	79471025	chr15-79470706-79471025
chr15	79504674	79506518	chr15-79504674-79506518
chr15	79511260	79512225	chr15-79511260-79512225
chr15	79515894	79517909	chr15-79515894-79517909
chr15	79519582	79519783	chr15-79519582-79519783
chr15	79543862	79547616	chr15-79543862-79547616

chr15	79604794	79605410	chr15-79604794-79605410
chr15	79657972	79660071	chr15-79657972-79660071
chr15	79669944	79672394	chr15-79669944-79672394
chr15	79682118	79682319	chr15-79682118-79682319
chr15	79683268	79688816	chr15-79683268-79688816
chr15	79689730	79692594	chr15-79689730-79692594
chr15	79734391	79734794	chr15-79734391-79734794
chr15	79740645	79743669	chr15-79740645-79743669
chr15	79764643	79765851	chr15-79764643-79765851
chr15	79773349	79775420	chr15-79773349-79775420
chr15	79803064	79805367	chr15-79803064-79805367
chr15	79813328	79814161	chr15-79813328-79814161
chr15	79832153	79834921	chr15-79832153-79834921
chr15	79878592	79880400	chr15-79878592-79880400
chr15	79882712	79883899	chr15-79882712-79883899
chr15	79890390	79896591	chr15-79890390-79896591
chr15	79917385	79917972	chr15-79917385-79917972
chr15	79927362	79928339	chr15-79927362-79928339
chr15	79931269	79934119	chr15-79931269-79934119
chr15	79949450	79953146	chr15-79949450-79953146
chr15	79983934	79984348	chr15-79983934-79984348
chr15	80009520	80015574	chr15-80009520-80015574
chr15	80023852	80027621	chr15-80023852-80027621
chr15	80050183	80050546	chr15-80050183-80050546
chr15	80053623	80054577	chr15-80053623-80054577
chr15	80060514	80063361	chr15-80060514-80063361
chr15	80065225	80065640	chr15-80065225-80065640
chr15	80075755	80076265	chr15-80075755-80076265
chr15	80077826	80084390	chr15-80077826-80084390
chr15	80090564	80093091	chr15-80090564-80093091
chr15	80096127	80097679	chr15-80096127-80097679
chr15	80098655	80098959	chr15-80098655-80098959
chr15	80114097	80114905	chr15-80114097-80114905
chr15	80132549	80135006	chr15-80132549-80135006
chr15	80159119	80161191	chr15-80159119-80161191
chr15	80169770	80170202	chr15-80169770-80170202
chr15	80172898	80174829	chr15-80172898-80174829
chr15	80218656	80219900	chr15-80218656-80219900
chr15	80233345	80235134	chr15-80233345-80235134
chr15	80253807	80258787	chr15-80253807-80258787
chr15	80262664	80264935	chr15-80262664-80264935
chr15	80286833	80288388	chr15-80286833-80288388

chr15	80303446	80304372	chr15-80303446-80304372
chr15	80327935	80328599	chr15-80327935-80328599
chr15	80372601	80372802	chr15-80372601-80372802
chr15	80413663	80415164	chr15-80413663-80415164
chr15	80462267	80462528	chr15-80462267-80462528
chr15	80485323	80485836	chr15-80485323-80485836
chr15	80537722	80538010	chr15-80537722-80538010
chr15	80558944	80561668	chr15-80558944-80561668
chr15	80639018	80639388	chr15-80639018-80639388
chr15	80653710	80654065	chr15-80653710-80654065
chr15	80671158	80674810	chr15-80671158-80674810
chr15	80679941	80680208	chr15-80679941-80680208
chr15	80701382	80701753	chr15-80701382-80701753
chr15	80706177	80707424	chr15-80706177-80707424
chr15	80710200	80712633	chr15-80710200-80712633
chr15	80720475	80720676	chr15-80720475-80720676
chr15	80784463	80784839	chr15-80784463-80784839
chr15	80797607	80801306	chr15-80797607-80801306
chr15	80947858	80950518	chr15-80947858-80950518
chr15	80977367	80978792	chr15-80977367-80978792
chr15	81033202	81033814	chr15-81033202-81033814
chr15	81069564	81071805	chr15-81069564-81071805
chr15	81079127	81079671	chr15-81079127-81079671
chr15	81086681	81087290	chr15-81086681-81087290
chr15	81097296	81097863	chr15-81097296-81097863
chr15	81137048	81138069	chr15-81137048-81138069
chr15	81167530	81168997	chr15-81167530-81168997
chr15	81175902	81176426	chr15-81175902-81176426
chr15	81188818	81193195	chr15-81188818-81193195
chr15	81235641	81235893	chr15-81235641-81235893
chr15	81247155	81248491	chr15-81247155-81248491
chr15	81263933	81265230	chr15-81263933-81265230
chr15	81272315	81273097	chr15-81272315-81273097
chr15	81301206	81301561	chr15-81301206-81301561
chr15	81359526	81361252	chr15-81359526-81361252
chr15	81397127	81401489	chr15-81397127-81401489
chr15	81416782	81417440	chr15-81416782-81417440
chr15	81465173	81468306	chr15-81465173-81468306
chr15	81481038	81481653	chr15-81481038-81481653
chr15	81511342	81512537	chr15-81511342-81512537
chr15	81513934	81515304	chr15-81513934-81515304
chr15	81521622	81524456	chr15-81521622-81524456

chr15	81584407	81589413	chr15-81584407-81589413
chr15	81663428	81665349	chr15-81663428-81665349
chr15	81692893	81695312	chr15-81692893-81695312
chr15	81727635	81730820	chr15-81727635-81730820
chr15	81739741	81739993	chr15-81739741-81739993
chr15	81744440	81746717	chr15-81744440-81746717
chr15	81801872	81803313	chr15-81801872-81803313
chr15	81809808	81812677	chr15-81809808-81812677
chr15	81831255	81831899	chr15-81831255-81831899
chr15	81838974	81839175	chr15-81838974-81839175
chr15	81855771	81861297	chr15-81855771-81861297
chr15	81870740	81873586	chr15-81870740-81873586
chr15	81924623	81926865	chr15-81924623-81926865
chr15	81936457	81937538	chr15-81936457-81937538
chr15	81953773	81955064	chr15-81953773-81955064
chr15	81959150	81961552	chr15-81959150-81961552
chr15	81965265	81965517	chr15-81965265-81965517
chr15	81991059	81991260	chr15-81991059-81991260
chr15	82011809	82012832	chr15-82011809-82012832
chr15	82013865	82017753	chr15-82013865-82017753
chr15	82045468	82048263	chr15-82045468-82048263
chr15	82126918	82128853	chr15-82126918-82128853
chr15	82145907	82149866	chr15-82145907-82149866
chr15	82153642	82153843	chr15-82153642-82153843
chr15	82158516	82158717	chr15-82158516-82158717
chr15	82159832	82161571	chr15-82159832-82161571
chr15	82163254	82167047	chr15-82163254-82167047
chr15	82211381	82213085	chr15-82211381-82213085
chr15	82223603	82224662	chr15-82223603-82224662
chr15	82243417	82245372	chr15-82243417-82245372
chr15	82249990	82251031	chr15-82249990-82251031
chr15	82274716	82275987	chr15-82274716-82275987
chr15	82337379	82339505	chr15-82337379-82339505
chr15	82340933	82341495	chr15-82340933-82341495
chr15	82345788	82347799	chr15-82345788-82347799
chr15	82351649	82355226	chr15-82351649-82355226
chr15	82364604	82364963	chr15-82364604-82364963
chr15	82379795	82380698	chr15-82379795-82380698
chr15	82417385	82417691	chr15-82417385-82417691
chr15	82880512	82881073	chr15-82880512-82881073
chr15	82897671	82902029	chr15-82897671-82902029
chr15	82911596	82912256	chr15-82911596-82912256

chr15	82920813	82922522	chr15-82920813-82922522
chr15	82928496	82930127	chr15-82928496-82930127
chr15	82939858	82941856	chr15-82939858-82941856
chr15	82958444	82958695	chr15-82958444-82958695
chr15	82963040	82964202	chr15-82963040-82964202
chr15	82986940	82988458	chr15-82986940-82988458
chr15	83056345	83057068	chr15-83056345-83057068
chr15	83076018	83076373	chr15-83076018-83076373
chr15	83099525	83101068	chr15-83099525-83101068
chr15	83121101	83123307	chr15-83121101-83123307
chr15	83140699	83141234	chr15-83140699-83141234
chr15	83142859	83143060	chr15-83142859-83143060
chr15	83147476	83150323	chr15-83147476-83150323
chr15	83156984	83157961	chr15-83156984-83157961
chr15	83164959	83168032	chr15-83164959-83168032
chr15	83169395	83173410	chr15-83169395-83173410
chr15	83176793	83177045	chr15-83176793-83177045
chr15	83204870	83205385	chr15-83204870-83205385
chr15	83250895	83252028	chr15-83250895-83252028
chr15	83268314	83268738	chr15-83268314-83268738
chr15	83349177	83350916	chr15-83349177-83350916
chr15	83366732	83367701	chr15-83366732-83367701
chr15	83387793	83388744	chr15-83387793-83388744
chr15	83403839	83404190	chr15-83403839-83404190
chr15	83407549	83412985	chr15-83407549-83412985
chr15	83417343	83418156	chr15-83417343-83418156
chr15	83430505	83433684	chr15-83430505-83433684
chr15	83440544	83441612	chr15-83440544-83441612
chr15	83451375	83452191	chr15-83451375-83452191
chr15	83462205	83465328	chr15-83462205-83465328
chr15	83467286	83467963	chr15-83467286-83467963
chr15	83469367	83469752	chr15-83469367-83469752
chr15	83473767	83475320	chr15-83473767-83475320
chr15	83481328	83482711	chr15-83481328-83482711
chr15	83494475	83494726	chr15-83494475-83494726
chr15	83499320	83500021	chr15-83499320-83500021
chr15	83509870	83511526	chr15-83509870-83511526
chr15	83518340	83518645	chr15-83518340-83518645
chr15	83526030	83527866	chr15-83526030-83527866
chr15	83535229	83536100	chr15-83535229-83536100
chr15	83554438	83556286	chr15-83554438-83556286
chr15	83562236	83566647	chr15-83562236-83566647

chr15	83574310	83575846	chr15-83574310-83575846
chr15	83592553	83595947	chr15-83592553-83595947
chr15	83724408	83725721	chr15-83724408-83725721
chr15	83779567	83780315	chr15-83779567-83780315
chr15	84065075	84065477	chr15-84065075-84065477
chr15	84069876	84070502	chr15-84069876-84070502
chr15	84073951	84075039	chr15-84073951-84075039
chr15	84104488	84106101	chr15-84104488-84106101
chr15	84167552	84169203	chr15-84167552-84169203
chr15	84191158	84193488	chr15-84191158-84193488
chr15	84213098	84213401	chr15-84213098-84213401
chr15	84223663	84225078	chr15-84223663-84225078
chr15	84231134	84240623	chr15-84231134-84240623
chr15	84279863	84281352	chr15-84279863-84281352
chr15	84379988	84380604	chr15-84379988-84380604
chr15	84401896	84402150	chr15-84401896-84402150
chr15	84445787	84446803	chr15-84445787-84446803
chr15	84450266	84451150	chr15-84450266-84451150
chr15	84499792	84501341	chr15-84499792-84501341
chr15	84512457	84513546	chr15-84512457-84513546
chr15	84556940	84558049	chr15-84556940-84558049
chr15	84653972	84655238	chr15-84653972-84655238
chr15	84662828	84663947	chr15-84662828-84663947
chr15	84680186	84682919	chr15-84680186-84682919
chr15	84711937	84715486	chr15-84711937-84715486
chr15	84719544	84721170	chr15-84719544-84721170
chr15	84724165	84724418	chr15-84724165-84724418
chr15	84853112	84857044	chr15-84853112-84857044
chr15	84922175	84925758	chr15-84922175-84925758
chr15	84973413	84974096	chr15-84973413-84974096
chr15	84983313	84984450	chr15-84983313-84984450
chr15	84986279	84988883	chr15-84986279-84988883
chr15	85001548	85001749	chr15-85001548-85001749
chr15	85017063	85017366	chr15-85017063-85017366
chr15	85036252	85038365	chr15-85036252-85038365
chr15	85131638	85132316	chr15-85131638-85132316
chr15	85177873	85178074	chr15-85177873-85178074
chr15	85205715	85206649	chr15-85205715-85206649
chr15	85281959	85282210	chr15-85281959-85282210
chr15	85335651	85338041	chr15-85335651-85338041
chr15	85420698	85421382	chr15-85420698-85421382
chr15	85424541	85424999	chr15-85424541-85424999

chr15	85478250	85481089	chr15-85478250-85481089
chr15	85506646	85506971	chr15-85506646-85506971
chr15	85523034	85524321	chr15-85523034-85524321
chr15	85529187	85532276	chr15-85529187-85532276
chr15	85533698	85536087	chr15-85533698-85536087
chr15	85539615	85540593	chr15-85539615-85540593
chr15	85542346	85549349	chr15-85542346-85549349
chr15	85551431	85551683	chr15-85551431-85551683
chr15	85552645	85552846	chr15-85552645-85552846
chr15	85558758	85562272	chr15-85558758-85562272
chr15	85564693	85564894	chr15-85564693-85564894
chr15	85566605	85583166	chr15-85566605-85583166
chr15	85598938	85599478	chr15-85598938-85599478
chr15	85625419	85626506	chr15-85625419-85626506
chr15	85634042	85635827	chr15-85634042-85635827
chr15	85644880	85647946	chr15-85644880-85647946
chr15	85652966	85658890	chr15-85652966-85658890
chr15	85663584	85681251	chr15-85663584-85681251
chr15	85682977	85687482	chr15-85682977-85687482
chr15	85701925	85703834	chr15-85701925-85703834
chr15	85734307	85736456	chr15-85734307-85736456
chr15	85810519	85812417	chr15-85810519-85812417
chr15	85821207	85822120	chr15-85821207-85822120
chr15	85831448	85833193	chr15-85831448-85833193
chr15	85858483	85861306	chr15-85858483-85861306
chr15	85878574	85880701	chr15-85878574-85880701
chr15	85935148	85936307	chr15-85935148-85936307
chr15	85965822	85966746	chr15-85965822-85966746
chr15	85969092	85971801	chr15-85969092-85971801
chr15	85981770	85982591	chr15-85981770-85982591
chr15	85995963	85996688	chr15-85995963-85996688
chr15	86007505	86008451	chr15-86007505-86008451
chr15	86015593	86016740	chr15-86015593-86016740
chr15	86025813	86026324	chr15-86025813-86026324
chr15	86029116	86030712	chr15-86029116-86030712
chr15	86032299	86035545	chr15-86032299-86035545
chr15	86057085	86062021	chr15-86057085-86062021
chr15	86066032	86070384	chr15-86066032-86070384
chr15	86071279	86071540	chr15-86071279-86071540
chr15	86073826	86076752	chr15-86073826-86076752
chr15	86077966	86080536	chr15-86077966-86080536
chr15	86083570	86083850	chr15-86083570-86083850

chr15	86087599	86087800	chr15-86087599-86087800
chr15	86089772	86092553	chr15-86089772-86092553
chr15	86112562	86113717	chr15-86112562-86113717
chr15	86115678	86116426	chr15-86115678-86116426
chr15	86181992	86183300	chr15-86181992-86183300
chr15	86184114	86186657	chr15-86184114-86186657
chr15	86188800	86189512	chr15-86188800-86189512
chr15	86201001	86201202	chr15-86201001-86201202
chr15	86212736	86215396	chr15-86212736-86215396
chr15	86268387	86268638	chr15-86268387-86268638
chr15	86342988	86343719	chr15-86342988-86343719
chr15	87543901	87545028	chr15-87543901-87545028
chr15	87623968	87625785	chr15-87623968-87625785
chr15	88662509	88662760	chr15-88662509-88662760
chr15	88731177	88734956	chr15-88731177-88734956
chr15	88750274	88754001	chr15-88750274-88754001
chr15	88818079	88821244	chr15-88818079-88821244
chr15	88828577	88829175	chr15-88828577-88829175
chr15	88836031	88839256	chr15-88836031-88839256
chr15	88850256	88857070	chr15-88850256-88857070
chr15	88858265	88858621	chr15-88858265-88858621
chr15	88861329	88868439	chr15-88861329-88868439
chr15	88876201	88876829	chr15-88876201-88876829
chr15	88890282	88891890	chr15-88890282-88891890
chr15	88954375	88955188	chr15-88954375-88955188
chr15	88957991	88958192	chr15-88957991-88958192
chr15	89059123	89060689	chr15-89059123-89060689
chr15	89067473	89068649	chr15-89067473-89068649
chr15	89074667	89079056	chr15-89074667-89079056
chr15	89088455	89090728	chr15-89088455-89090728
chr15	89120258	89124229	chr15-89120258-89124229
chr15	89127509	89129386	chr15-89127509-89129386
chr15	89139115	89141602	chr15-89139115-89141602
chr15	89146347	89150484	chr15-89146347-89150484
chr15	89160329	89162555	chr15-89160329-89162555
chr15	89164774	89182651	chr15-89164774-89182651
chr15	89189053	89190575	chr15-89189053-89190575
chr15	89195242	89197480	chr15-89195242-89197480
chr15	89209904	89212655	chr15-89209904-89212655
chr15	89309937	89316190	chr15-89309937-89316190
chr15	89322480	89324442	chr15-89322480-89324442
chr15	89353778	89358341	chr15-89353778-89358341

chr15	89372110	89375016	chr15-89372110-89375016
chr15	89378373	89379494	chr15-89378373-89379494
chr15	89428333	89431031	chr15-89428333-89431031
chr15	89453552	89454693	chr15-89453552-89454693
chr15	89475092	89478322	chr15-89475092-89478322
chr15	89480589	89481129	chr15-89480589-89481129
chr15	89498383	89500795	chr15-89498383-89500795
chr15	89523349	89524529	chr15-89523349-89524529
chr15	89532604	89533104	chr15-89532604-89533104
chr15	89547816	89548346	chr15-89547816-89548346
chr15	89556718	89558740	chr15-89556718-89558740
chr15	89591086	89592702	chr15-89591086-89592702
chr15	89841304	89842182	chr15-89841304-89842182
chr15	90223342	90225457	chr15-90223342-90225457
chr15	90402157	90402967	chr15-90402157-90402967
chr15	90512700	90513040	chr15-90512700-90513040
chr15	90607426	90607784	chr15-90607426-90607784
chr15	90609699	90609951	chr15-90609699-90609951
chr15	90615654	90616736	chr15-90615654-90616736
chr15	90677334	90680597	chr15-90677334-90680597
chr15	90717576	90719785	chr15-90717576-90719785
chr15	91048476	91050462	chr15-91048476-91050462
chr15	91091825	91092801	chr15-91091825-91092801
chr15	91191177	91192483	chr15-91191177-91192483
chr15	91572265	91573636	chr15-91572265-91573636
chr15	91672622	91674219	chr15-91672622-91674219
chr15	91703442	91703643	chr15-91703442-91703643
chr15	92051082	92051853	chr15-92051082-92051853
chr15	92396732	92397649	chr15-92396732-92397649
chr15	92597078	92597894	chr15-92597078-92597894
chr15	92598746	92599260	chr15-92598746-92599260
chr15	92656492	92656796	chr15-92656492-92656796
chr15	92857200	92857401	chr15-92857200-92857401
chr15	93115731	93116038	chr15-93115731-93116038
chr15	93161890	93162372	chr15-93161890-93162372
chr15	93272408	93276752	chr15-93272408-93276752
chr15	93335457	93337927	chr15-93335457-93337927
chr15	93396747	93400112	chr15-93396747-93400112
chr15	93513941	93514356	chr15-93513941-93514356
chr15	93518064	93520160	chr15-93518064-93520160
chr15	93594276	93596721	chr15-93594276-93596721
chr15	93628456	93628793	chr15-93628456-93628793

chr15	93649380	93649682	chr15-93649380-93649682
chr15	93656417	93657440	chr15-93656417-93657440
chr15	94045003	94045635	chr15-94045003-94045635
chr15	94403779	94404638	chr15-94403779-94404638
chr15	94542628	94544707	chr15-94542628-94544707
chr15	94587977	94590492	chr15-94587977-94590492
chr15	94628707	94629760	chr15-94628707-94629760
chr15	94655838	94656147	chr15-94655838-94656147
chr15	94948178	94948480	chr15-94948178-94948480
chr15	95016058	95016331	chr15-95016058-95016331
chr15	95370899	95371359	chr15-95370899-95371359
chr15	95527427	95528822	chr15-95527427-95528822
chr15	95654234	95654707	chr15-95654234-95654707
chr15	95763012	95763684	chr15-95763012-95763684
chr15	95790018	95794906	chr15-95790018-95794906
chr15	95802902	95805773	chr15-95802902-95805773
chr15	95828692	95831802	chr15-95828692-95831802
chr15	95837562	95839202	chr15-95837562-95839202
chr15	95871218	95871872	chr15-95871218-95871872
chr15	96002981	96003781	chr15-96002981-96003781
chr15	96035183	96035591	chr15-96035183-96035591
chr15	96077987	96078936	chr15-96077987-96078936
chr15	96170945	96171819	chr15-96170945-96171819
chr15	96270261	96270723	chr15-96270261-96270723
chr15	96282322	96289854	chr15-96282322-96289854
chr15	96376820	96378059	chr15-96376820-96378059
chr15	96458245	96462375	chr15-96458245-96462375
chr15	96543133	96543858	chr15-96543133-96543858
chr15	96566816	96567469	chr15-96566816-96567469
chr15	96639210	96643632	chr15-96639210-96643632
chr15	96660512	96661541	chr15-96660512-96661541
chr15	96666239	96666916	chr15-96666239-96666916
chr15	96695480	96700843	chr15-96695480-96700843
chr15	96708713	96710934	chr15-96708713-96710934
chr15	96743346	96745123	chr15-96743346-96745123
chr15	96770629	96770830	chr15-96770629-96770830
chr15	96772960	96773636	chr15-96772960-96773636
chr15	96822551	96822752	chr15-96822551-96822752
chr15	96829566	96830842	chr15-96829566-96830842
chr15	96936688	96936939	chr15-96936688-96936939
chr15	96953397	96954146	chr15-96953397-96954146
chr15	96956745	96957100	chr15-96956745-96957100

chr15	96960587	96960788	chr15-96960587-96960788
chr15	97004408	97004818	chr15-97004408-97004818
chr15	97054472	97054910	chr15-97054472-97054910
chr15	97110605	97110806	chr15-97110605-97110806
chr15	97246650	97248855	chr15-97246650-97248855
chr15	97380113	97380365	chr15-97380113-97380365
chr15	97499479	97499786	chr15-97499479-97499786
chr15	97532429	97532784	chr15-97532429-97532784
chr15	97581892	97582152	chr15-97581892-97582152
chr15	97606644	97607197	chr15-97606644-97607197
chr15	97663760	97664428	chr15-97663760-97664428
chr15	97667550	97667851	chr15-97667550-97667851
chr15	97704551	97706702	chr15-97704551-97706702
chr15	97750572	97751168	chr15-97750572-97751168
chr15	97764648	97770815	chr15-97764648-97770815
chr15	97772754	97776648	chr15-97772754-97776648
chr15	97778913	97779230	chr15-97778913-97779230
chr15	97782665	97782866	chr15-97782665-97782866
chr15	97784015	97787891	chr15-97784015-97787891
chr15	97789555	97790842	chr15-97789555-97790842
chr15	97813497	97813748	chr15-97813497-97813748
chr15	97814686	97815061	chr15-97814686-97815061
chr15	97821053	97833023	chr15-97821053-97833023
chr15	97836040	97838640	chr15-97836040-97838640
chr15	97839909	97841012	chr15-97839909-97841012
chr15	97849275	97849951	chr15-97849275-97849951
chr15	97906936	97909083	chr15-97906936-97909083
chr15	97963255	97965164	chr15-97963255-97965164
chr15	97981316	97981729	chr15-97981316-97981729
chr15	98001256	98005033	chr15-98001256-98005033
chr15	98048846	98049098	chr15-98048846-98049098
chr15	98091582	98094140	chr15-98091582-98094140
chr15	98107837	98109287	chr15-98107837-98109287
chr15	98144344	98146366	chr15-98144344-98146366
chr15	98167251	98168526	chr15-98167251-98168526
chr15	98176971	98177172	chr15-98176971-98177172
chr15	98180691	98181813	chr15-98180691-98181813
chr15	98295637	98296547	chr15-98295637-98296547
chr15	98512468	98513324	chr15-98512468-98513324
chr15	98531003	98535361	chr15-98531003-98535361
chr15	98565278	98569607	chr15-98565278-98569607
chr15	98602382	98603614	chr15-98602382-98603614

chr15	98607733	98610690	chr15-98607733-98610690
chr15	98617017	98617632	chr15-98617017-98617632
chr15	98626015	98626856	chr15-98626015-98626856
chr15	98631252	98636145	chr15-98631252-98636145
chr15	98661725	98663471	chr15-98661725-98663471
chr15	98664442	98664812	chr15-98664442-98664812
chr15	98672281	98678382	chr15-98672281-98678382
chr15	98699329	98700091	chr15-98699329-98700091
chr15	98708113	98708543	chr15-98708113-98708543
chr15	98713561	98715746	chr15-98713561-98715746
chr15	98726187	98728405	chr15-98726187-98728405
chr15	98761606	98764016	chr15-98761606-98764016
chr15	98768161	98768484	chr15-98768161-98768484
chr15	98772501	98773276	chr15-98772501-98773276
chr15	98776217	98778988	chr15-98776217-98778988
chr15	98780057	98780875	chr15-98780057-98780875
chr15	98789094	98793017	chr15-98789094-98793017
chr15	98804035	98804386	chr15-98804035-98804386
chr15	98805242	98808531	chr15-98805242-98808531
chr15	98829990	98832131	chr15-98829990-98832131
chr15	98867288	98873369	chr15-98867288-98873369
chr15	98879720	98882171	chr15-98879720-98882171
chr15	98891230	98891535	chr15-98891230-98891535
chr15	98893116	98898855	chr15-98893116-98898855
chr15	98916174	98919080	chr15-98916174-98919080
chr15	98928505	98929086	chr15-98928505-98929086
chr15	98931108	98936143	chr15-98931108-98936143
chr15	98951892	98953930	chr15-98951892-98953930
chr15	98984599	98986343	chr15-98984599-98986343
chr15	98992380	98993929	chr15-98992380-98993929
chr15	99001358	99001908	chr15-99001358-99001908
chr15	99028585	99035016	chr15-99028585-99035016
chr15	99055026	99057389	chr15-99055026-99057389
chr15	99073774	99076628	chr15-99073774-99076628
chr15	99087489	99088701	chr15-99087489-99088701
chr15	99093349	99094282	chr15-99093349-99094282
chr15	99096478	99099837	chr15-99096478-99099837
chr15	99113309	99113916	chr15-99113309-99113916
chr15	99125423	99128182	chr15-99125423-99128182
chr15	99223205	99225613	chr15-99223205-99225613
chr15	99250297	99253341	chr15-99250297-99253341
chr15	99259327	99260665	chr15-99259327-99260665

chr15	99284403	99284760	chr15-99284403-99284760
chr15	99293940	99296245	chr15-99293940-99296245
chr15	99332851	99333730	chr15-99332851-99333730
chr15	99368330	99371012	chr15-99368330-99371012
chr15	99392081	99397130	chr15-99392081-99397130
chr15	99398500	99399838	chr15-99398500-99399838
chr15	99420740	99421227	chr15-99420740-99421227
chr15	99456728	99458822	chr15-99456728-99458822
chr15	99463249	99463500	chr15-99463249-99463500
chr15	99473414	99475288	chr15-99473414-99475288
chr15	99505249	99506245	chr15-99505249-99506245
chr15	99510453	99510861	chr15-99510453-99510861
chr15	99512045	99512365	chr15-99512045-99512365
chr15	99531083	99531587	chr15-99531083-99531587
chr15	99539930	99540131	chr15-99539930-99540131
chr15	99583762	99586842	chr15-99583762-99586842
chr15	99588990	99596561	chr15-99588990-99596561
chr15	99649018	99652247	chr15-99649018-99652247
chr15	99663974	99665437	chr15-99663974-99665437
chr15	99670128	99672245	chr15-99670128-99672245
chr15	99701185	99703375	chr15-99701185-99703375
chr15	99723189	99727240	chr15-99723189-99727240
chr15	99771126	99773275	chr15-99771126-99773275
chr15	99835060	99835965	chr15-99835060-99835965
chr15	99846843	99848160	chr15-99846843-99848160
chr15	99865115	99867171	chr15-99865115-99867171
chr15	99874281	99876217	chr15-99874281-99876217
chr15	99889424	99891567	chr15-99889424-99891567
chr15	99971817	99974885	chr15-99971817-99974885
chr15	100038429	100042600	chr15-100038429-100042600
chr15	100056500	100056701	chr15-100056500-100056701
chr15	100087760	100090324	chr15-100087760-100090324
chr15	100107530	100108901	chr15-100107530-100108901
chr15	100220780	100221087	chr15-100220780-100221087
chr15	100226446	100229852	chr15-100226446-100229852
chr15	100299143	100299732	chr15-100299143-100299732
chr15	100303888	100305511	chr15-100303888-100305511
chr15	100307595	100307796	chr15-100307595-100307796
chr15	100324868	100325341	chr15-100324868-100325341
chr15	100414269	100414521	chr15-100414269-100414521
chr15	100420923	100424335	chr15-100420923-100424335
chr15	100442950	100444229	chr15-100442950-100444229

chr15	100457533	100457734	chr15-100457533-100457734
chr15	100460895	100461147	chr15-100460895-100461147
chr15	100467791	100470001	chr15-100467791-100470001
chr15	100494115	100495821	chr15-100494115-100495821
chr15	100550699	100552858	chr15-100550699-100552858
chr15	100598785	100600766	chr15-100598785-100600766
chr15	100604587	100605061	chr15-100604587-100605061
chr15	100613368	100617145	chr15-100613368-100617145
chr15	100627767	100628021	chr15-100627767-100628021
chr15	100632656	100634014	chr15-100632656-100634014
chr15	100635231	100637954	chr15-100635231-100637954
chr15	100687059	100687477	chr15-100687059-100687477
chr15	100728983	100729802	chr15-100728983-100729802
chr15	100747009	100747466	chr15-100747009-100747466
chr15	100761194	100762636	chr15-100761194-100762636
chr15	100777101	100777608	chr15-100777101-100777608
chr15	100870345	100871575	chr15-100870345-100871575
chr15	100914609	100915271	chr15-100914609-100915271
chr15	100983361	100984489	chr15-100983361-100984489
chr15	101006513	101007013	chr15-101006513-101007013
chr15	101046034	101047614	chr15-101046034-101047614
chr15	101052683	101054178	chr15-101052683-101054178
chr15	101069839	101071993	chr15-101069839-101071993
chr15	101073025	101074481	chr15-101073025-101074481
chr15	101075329	101075641	chr15-101075329-101075641
chr15	101076737	101079636	chr15-101076737-101079636
chr15	101105578	101105883	chr15-101105578-101105883
chr15	101113357	101113711	chr15-101113357-101113711
chr15	101128449	101129398	chr15-101128449-101129398
chr15	101140987	101141342	chr15-101140987-101141342
chr15	101145166	101146303	chr15-101145166-101146303
chr15	101164094	101166414	chr15-101164094-101166414
chr15	101173248	101176027	chr15-101173248-101176027
chr15	101182762	101184570	chr15-101182762-101184570
chr15	101191356	101191718	chr15-101191356-101191718
chr15	101221549	101222213	chr15-101221549-101222213
chr15	101223847	101227518	chr15-101223847-101227518
chr15	101231514	101237046	chr15-101231514-101237046
chr15	101238874	101239492	chr15-101238874-101239492
chr15	101240812	101242836	chr15-101240812-101242836
chr15	101245688	101247545	chr15-101245688-101247545
chr15	101253448	101256545	chr15-101253448-101256545

chr15	101265899	101270378	chr15-101265899-101270378
chr15	101283237	101286056	chr15-101283237-101286056
chr15	101311421	101311622	chr15-101311421-101311622
chr15	101315834	101317706	chr15-101315834-101317706
chr15	101319324	101321461	chr15-101319324-101321461
chr15	101325353	101333718	chr15-101325353-101333718
chr15	101335909	101338665	chr15-101335909-101338665
chr15	101341999	101342720	chr15-101341999-101342720
chr15	101366257	101375329	chr15-101366257-101375329
chr15	101377188	101377465	chr15-101377188-101377465
chr15	101378724	101383598	chr15-101378724-101383598
chr15	101385015	101389271	chr15-101385015-101389271
chr15	101390159	101390412	chr15-101390159-101390412
chr15	101409797	101425190	chr15-101409797-101425190
chr15	101453251	101453656	chr15-101453251-101453656
chr15	101454917	101455504	chr15-101454917-101455504
chr15	101478859	101479723	chr15-101478859-101479723
chr15	101553231	101553432	chr15-101553231-101553432
chr15	101845737	101846503	chr15-101845737-101846503
chr15	101859489	101860427	chr15-101859489-101860427
chr15	101929678	101930030	chr15-101929678-101930030
chr15	101939093	101940153	chr15-101939093-101940153
chr15	101952645	101952846	chr15-101952645-101952846
chr15	101976727	101978128	chr15-101976727-101978128
chr15	101981072	101981273	chr15-101981072-101981273
chr15	101983824	101984914	chr15-101983824-101984914
chr15	101988295	101988600	chr15-101988295-101988600
chr15	101997851	101998604	chr15-101997851-101998604
chr15	102000552	102007789	chr15-102000552-102007789
chr15	102012760	102018767	chr15-102012760-102018767
chr15	102022448	102022908	chr15-102022448-102022908
chr15	102023903	102032109	chr15-102023903-102032109
chr15	102044851	102046793	chr15-102044851-102046793
chr15	102051690	102052896	chr15-102051690-102052896
chr15	102073049	102077400	chr15-102073049-102077400
chr15	102099286	102107521	chr15-102099286-102107521
chr15	102135014	102137509	chr15-102135014-102137509
chr15	102145230	102145642	chr15-102145230-102145642
chr15	102150469	102151495	chr15-102150469-102151495
chr15	102167464	102168218	chr15-102167464-102168218
chr15	102169162	102169363	chr15-102169162-102169363
chr15	102202200	102206132	chr15-102202200-102206132

chr15	102224145	102225121	chr15-102224145-102225121
chr15	102242415	102249728	chr15-102242415-102249728
chr15	102254869	102258723	chr15-102254869-102258723
chr15	102278515	102281085	chr15-102278515-102281085
chr15	102295643	102297376	chr15-102295643-102297376
chr15	102325208	102327368	chr15-102325208-102327368
chr15	102331230	102333178	chr15-102331230-102333178
chr15	102349273	102351463	chr15-102349273-102351463
chr15	102354986	102356226	chr15-102354986-102356226
chr15	102358216	102359069	chr15-102358216-102359069
chr15	102362732	102363040	chr15-102362732-102363040
chr15	102366581	102367093	chr15-102366581-102367093
chr15	102376520	102377722	chr15-102376520-102377722
chr15	102399114	102400500	chr15-102399114-102400500
chr15	102404629	102407574	chr15-102404629-102407574
chr15	102409140	102409566	chr15-102409140-102409566
chr15	102410604	102410905	chr15-102410604-102410905
chr15	102450920	102453186	chr15-102450920-102453186
chr15	102458595	102462488	chr15-102458595-102462488
chr15	102470255	102472421	chr15-102470255-102472421
chr15	102473310	102473562	chr15-102473310-102473562
chr15	102510096	102511457	chr15-102510096-102511457
chr15	102515813	102520974	chr15-102515813-102520974
chr15	102571678	102573300	chr15-102571678-102573300
chr15	102623755	102626382	chr15-102623755-102626382
chr15	102669461	102674717	chr15-102669461-102674717
chr15	102692168	102694263	chr15-102692168-102694263
chr15	102720577	102723822	chr15-102720577-102723822
chr15	102736178	102736646	chr15-102736178-102736646
chr15	102739667	102740185	chr15-102739667-102740185
chr15	102744083	102744284	chr15-102744083-102744284
chr15	102746306	102747659	chr15-102746306-102747659
chr15	102818784	102819037	chr15-102818784-102819037
chr15	102910185	102910386	chr15-102910185-102910386
chr15	102920598	102922227	chr15-102920598-102922227
chr15	102934867	102935273	chr15-102934867-102935273
chr15	102936632	102937386	chr15-102936632-102937386
chr15	102942625	102943541	chr15-102942625-102943541
chr15	102954732	102956576	chr15-102954732-102956576
chr15	102963805	102964006	chr15-102963805-102964006
chr15	102966459	102967022	chr15-102966459-102967022
chr15	102975900	102977897	chr15-102975900-102977897

chr15	102980963	102982447	chr15-102980963-102982447
chr15	102990401	102991073	chr15-102990401-102991073
chr15	102998031	103000926	chr15-102998031-103000926
chr15	103009617	103009818	chr15-103009617-103009818
chr15	103010770	103010971	chr15-103010770-103010971
chr15	103011937	103012253	chr15-103011937-103012253
chr15	103013646	103015208	chr15-103013646-103015208
chr15	103034212	103035208	chr15-103034212-103035208
chr15	103060773	103060974	chr15-103060773-103060974
chr15	103086000	103086304	chr15-103086000-103086304
chr15	103101742	103102851	chr15-103101742-103102851
chr15	103146549	103147273	chr15-103146549-103147273
chr15	103162281	103164014	chr15-103162281-103164014
chr15	103165396	103165597	chr15-103165396-103165597
chr15	103168606	103170532	chr15-103168606-103170532
chr15	103176991	103177403	chr15-103176991-103177403
chr15	103181941	103183590	chr15-103181941-103183590
chr15	103213547	103216586	chr15-103213547-103216586
chr15	103220680	103221618	chr15-103220680-103221618
chr15	103237686	103245190	chr15-103237686-103245190
chr15	103255037	103255593	chr15-103255037-103255593
chr15	103271903	103274240	chr15-103271903-103274240
chr15	103304134	103306082	chr15-103304134-103306082
chr15	103307364	103307615	chr15-103307364-103307615
chr15	103317976	103318351	chr15-103317976-103318351
chr15	103322399	103324271	chr15-103322399-103324271
chr15	103339186	103340700	chr15-103339186-103340700
chr15	103358530	103359529	chr15-103358530-103359529
chr15	103364772	103367454	chr15-103364772-103367454
chr15	103430335	103430536	chr15-103430335-103430536
chr15	103502517	103503746	chr15-103502517-103503746
chr15	103514245	103514446	chr15-103514245-103514446
chr15	103536917	103538344	chr15-103536917-103538344
chr16	3632719	3633245	chr16-3632719-3633245
chr16	3723090	3724088	chr16-3723090-3724088
chr16	3728761	3728962	chr16-3728761-3728962
chr16	3742685	3746382	chr16-3742685-3746382
chr16	3846620	3848227	chr16-3846620-3848227
chr16	3871146	3873743	chr16-3871146-3873743
chr16	3883365	3886082	chr16-3883365-3886082
chr16	3908090	3910035	chr16-3908090-3910035
chr16	3958273	3959618	chr16-3958273-3959618

chr16	4002770	4004624	chr16-4002770-4004624
chr16	4076293	4078649	chr16-4076293-4078649
chr16	4211932	4214957	chr16-4211932-4214957
chr16	4267798	4268610	chr16-4267798-4268610
chr16	4419051	4421089	chr16-4419051-4421089
chr16	4469631	4469940	chr16-4469631-4469940
chr16	4472718	4473381	chr16-4472718-4473381
chr16	4474456	4475348	chr16-4474456-4475348
chr16	4478010	4478315	chr16-4478010-4478315
chr16	4541109	4542206	chr16-4541109-4542206
chr16	4557015	4561242	chr16-4557015-4561242
chr16	4585583	4588885	chr16-4585583-4588885
chr16	4591674	4608260	chr16-4591674-4608260
chr16	4623318	4625349	chr16-4623318-4625349
chr16	4639654	4644200	chr16-4639654-4644200
chr16	4675326	4675724	chr16-4675326-4675724
chr16	4678626	4680185	chr16-4678626-4680185
chr16	4682770	4685140	chr16-4682770-4685140
chr16	4718016	4719627	chr16-4718016-4719627
chr16	4725779	4727836	chr16-4725779-4727836
chr16	4756783	4757084	chr16-4756783-4757084
chr16	4788812	4790828	chr16-4788812-4790828
chr16	4877341	4881932	chr16-4877341-4881932
chr16	4885438	4887820	chr16-4885438-4887820
chr16	4912858	4914217	chr16-4912858-4914217
chr16	4938556	4940627	chr16-4938556-4940627
chr16	4962713	4965630	chr16-4962713-4965630
chr16	5006984	5008622	chr16-5006984-5008622
chr16	5012099	5014393	chr16-5012099-5014393
chr16	5048450	5051619	chr16-5048450-5051619
chr16	5119243	5120090	chr16-5119243-5120090
chr16	5130367	5133069	chr16-5130367-5133069
chr16	5134324	5137450	chr16-5134324-5137450
chr16	5139722	5139923	chr16-5139722-5139923
chr16	5145832	5147543	chr16-5145832-5147543
chr16	5202776	5204662	chr16-5202776-5204662
chr16	5221942	5222507	chr16-5221942-5222507
chr16	5233169	5234704	chr16-5233169-5234704
chr16	5254088	5256866	chr16-5254088-5256866
chr16	5306641	5307305	chr16-5306641-5307305
chr16	5352215	5352520	chr16-5352215-5352520
chr16	7039845	7040156	chr16-7039845-7040156

chr16	8424519	8424825	chr16-8424519-8424825
chr16	8470335	8471819	chr16-8470335-8471819
chr16	8512973	8513975	chr16-8512973-8513975
chr16	8635724	8639483	chr16-8635724-8639483
chr16	8666040	8672953	chr16-8666040-8672953
chr16	8733760	8739683	chr16-8733760-8739683
chr16	8810372	8811987	chr16-8810372-8811987
chr16	8828639	8835017	chr16-8828639-8835017
chr16	8846129	8846743	chr16-8846129-8846743
chr16	8851838	8852094	chr16-8851838-8852094
chr16	8861348	8864958	chr16-8861348-8864958
chr16	9015856	9017025	chr16-9015856-9017025
chr16	9176720	9176921	chr16-9176720-9176921
chr16	9211929	9212130	chr16-9211929-9212130
chr16	9993631	9993884	chr16-9993631-9993884
chr16	10192489	10193805	chr16-10192489-10193805
chr16	10285667	10288209	chr16-10285667-10288209
chr16	10298000	10298564	chr16-10298000-10298564
chr16	10299713	10302238	chr16-10299713-10302238
chr16	10310527	10314748	chr16-10310527-10314748
chr16	10320120	10320405	chr16-10320120-10320405
chr16	10330999	10331251	chr16-10330999-10331251
chr16	10336163	10336679	chr16-10336163-10336679
chr16	10366816	10367071	chr16-10366816-10367071
chr16	10374003	10374954	chr16-10374003-10374954
chr16	10394679	10395542	chr16-10394679-10395542
chr16	10411128	10413172	chr16-10411128-10413172
chr16	10467673	10467874	chr16-10467673-10467874
chr16	10489556	10490289	chr16-10489556-10490289
chr16	10510705	10511013	chr16-10510705-10511013
chr16	10540329	10543540	chr16-10540329-10543540
chr16	10544571	10546539	chr16-10544571-10546539
chr16	10557966	10558167	chr16-10557966-10558167
chr16	10559714	10560129	chr16-10559714-10560129
chr16	10754015	10755442	chr16-10754015-10755442
chr16	10781889	10787144	chr16-10781889-10787144
chr16	10834438	10835886	chr16-10834438-10835886
chr16	10837825	10839598	chr16-10837825-10839598
chr16	10849771	10850786	chr16-10849771-10850786
chr16	10975370	10977169	chr16-10975370-10977169
chr16	10979747	10980143	chr16-10979747-10980143
chr16	10981974	10982654	chr16-10981974-10982654

chr16	10986404	10986605	chr16-10986404-10986605
chr16	10987927	10993759	chr16-10987927-10993759
chr16	11007825	11010521	chr16-11007825-11010521
chr16	11012524	11013243	chr16-11012524-11013243
chr16	11016880	11019005	chr16-11016880-11019005
chr16	11024418	11024880	chr16-11024418-11024880
chr16	11065463	11067983	chr16-11065463-11067983
chr16	11132841	11135787	chr16-11132841-11135787
chr16	11173241	11177685	chr16-11173241-11177685
chr16	11193124	11193325	chr16-11193124-11193325
chr16	11200955	11205021	chr16-11200955-11205021
chr16	11251806	11255344	chr16-11251806-11255344
chr16	11263246	11263760	chr16-11263246-11263760
chr16	11321944	11324364	chr16-11321944-11324364
chr16	11408632	11408944	chr16-11408632-11408944
chr16	11442890	11443142	chr16-11442890-11443142
chr16	11456527	11457378	chr16-11456527-11457378
chr16	11474947	11476350	chr16-11474947-11476350
chr16	11501894	11503427	chr16-11501894-11503427
chr16	11505599	11505959	chr16-11505599-11505959
chr16	11589720	11589921	chr16-11589720-11589921
chr16	11907838	11909811	chr16-11907838-11909811
chr16	11983190	11985066	chr16-11983190-11985066
chr16	13061332	13061585	chr16-13061332-13061585
chr16	13109004	13111131	chr16-13109004-13111131
chr16	13159884	13160748	chr16-13159884-13160748
chr16	13255491	13257848	chr16-13255491-13257848
chr16	13323560	13323967	chr16-13323560-13323967
chr16	13367064	13367653	chr16-13367064-13367653
chr16	13371859	13372060	chr16-13371859-13372060
chr16	13436672	13438640	chr16-13436672-13438640
chr16	13446820	13449016	chr16-13446820-13449016
chr16	13521260	13522098	chr16-13521260-13522098
chr16	13588081	13589184	chr16-13588081-13589184
chr16	13611050	13611455	chr16-13611050-13611455
chr16	13666955	13668693	chr16-13666955-13668693
chr16	13671034	13673244	chr16-13671034-13673244
chr16	13754004	13755453	chr16-13754004-13755453
chr16	13779674	13782085	chr16-13779674-13782085
chr16	13818262	13820720	chr16-13818262-13820720
chr16	13875077	13875475	chr16-13875077-13875475
chr16	13901420	13904081	chr16-13901420-13904081

chr16	13940436	13941604	chr16-13940436-13941604
chr16	13986594	13987536	chr16-13986594-13987536
chr16	14157820	14160315	chr16-14157820-14160315
chr16	14162314	14165360	chr16-14162314-14165360
chr16	14316138	14318092	chr16-14316138-14318092
chr16	14352758	14353215	chr16-14352758-14353215
chr16	14360572	14364218	chr16-14360572-14364218
chr16	14371917	14373106	chr16-14371917-14373106
chr16	14450698	14451115	chr16-14450698-14451115
chr16	14482497	14482748	chr16-14482497-14482748
chr16	14705117	14706648	chr16-14705117-14706648
chr16	14800268	14800675	chr16-14800268-14800675
chr16	14906440	14906760	chr16-14906440-14906760
chr16	15084476	15085399	chr16-15084476-15085399
chr16	15493698	15494030	chr16-15493698-15494030
chr16	15593168	15595076	chr16-15593168-15595076
chr16	15635947	15639226	chr16-15635947-15639226
chr16	15861862	15864398	chr16-15861862-15864398
chr16	15886528	15889242	chr16-15886528-15889242
chr16	15984428	15984679	chr16-15984428-15984679
chr16	15991178	15991379	chr16-15991178-15991379
chr16	15992179	15992380	chr16-15992179-15992380
chr16	16145929	16147363	chr16-16145929-16147363
chr16	16212616	16217583	chr16-16212616-16217583
chr16	16223159	16225068	chr16-16223159-16225068
chr16	16302079	16305133	chr16-16302079-16305133
chr16	16357409	16359612	chr16-16357409-16359612
chr16	16560314	16560623	chr16-16560314-16560623
chr16	16599126	16600686	chr16-16599126-16600686
chr16	16828937	16829588	chr16-16828937-16829588
chr16	16870093	16872128	chr16-16870093-16872128
chr16	16896285	16898051	chr16-16896285-16898051
chr16	16982563	16985495	chr16-16982563-16985495
chr16	17012283	17012550	chr16-17012283-17012550
chr16	17069514	17071259	chr16-17069514-17071259
chr16	17110118	17111606	chr16-17110118-17111606
chr16	17131147	17133388	chr16-17131147-17133388
chr16	17146651	17148848	chr16-17146651-17148848
chr16	17167167	17167368	chr16-17167167-17167368
chr16	17168423	17168624	chr16-17168423-17168624
chr16	17199604	17201822	chr16-17199604-17201822
chr16	17204925	17205126	chr16-17204925-17205126

chr16	17208222	17210810	chr16-17208222-17210810
chr16	17222236	17222487	chr16-17222236-17222487
chr16	17232826	17235790	chr16-17232826-17235790
chr16	17251734	17251988	chr16-17251734-17251988
chr16	17276084	17277723	chr16-17276084-17277723
chr16	17404870	17407332	chr16-17404870-17407332
chr16	17409337	17410675	chr16-17409337-17410675
chr16	17451016	17455984	chr16-17451016-17455984
chr16	17489075	17490437	chr16-17489075-17490437
chr16	17508050	17510514	chr16-17508050-17510514
chr16	17529585	17532179	chr16-17529585-17532179
chr16	17553982	17554654	chr16-17553982-17554654
chr16	17558242	17559260	chr16-17558242-17559260
chr16	17560811	17562385	chr16-17560811-17562385
chr16	17575644	17578280	chr16-17575644-17578280
chr16	17618310	17620691	chr16-17618310-17620691
chr16	17645877	17647479	chr16-17645877-17647479
chr16	17720889	17723468	chr16-17720889-17723468
chr16	17727135	17727436	chr16-17727135-17727436
chr16	17732448	17735290	chr16-17732448-17735290
chr16	17736189	17736454	chr16-17736189-17736454
chr16	17743057	17743570	chr16-17743057-17743570
chr16	17759262	17761284	chr16-17759262-17761284
chr16	17764248	17764663	chr16-17764248-17764663
chr16	17778261	17778581	chr16-17778261-17778581
chr16	17796812	17799335	chr16-17796812-17799335
chr16	17802724	17803497	chr16-17802724-17803497
chr16	17806950	17807632	chr16-17806950-17807632
chr16	17815083	17816299	chr16-17815083-17816299
chr16	17833108	17834502	chr16-17833108-17834502
chr16	17890094	17892296	chr16-17890094-17892296
chr16	17909949	17911524	chr16-17909949-17911524
chr16	17914084	17915365	chr16-17914084-17915365
chr16	17920959	17923507	chr16-17920959-17923507
chr16	17926320	17929316	chr16-17926320-17929316
chr16	17938054	17939123	chr16-17938054-17939123
chr16	18052875	18053076	chr16-18052875-18053076
chr16	18065742	18067516	chr16-18065742-18067516
chr16	18087360	18089883	chr16-18087360-18089883
chr16	18096583	18096959	chr16-18096583-18096959
chr16	18126628	18128878	chr16-18126628-18128878
chr16	18194475	18195726	chr16-18194475-18195726

chr16	18233030	18236826	chr16-18233030-18236826
chr16	18246609	18251620	chr16-18246609-18251620
chr16	18287017	18290315	chr16-18287017-18290315
chr16	18341698	18344581	chr16-18341698-18344581
chr16	18347443	18349323	chr16-18347443-18349323
chr16	18382734	18383670	chr16-18382734-18383670
chr16	18392301	18393839	chr16-18392301-18393839
chr16	18395924	18396329	chr16-18395924-18396329
chr16	18416037	18417025	chr16-18416037-18417025
chr16	18424675	18427339	chr16-18424675-18427339
chr16	18470963	18472249	chr16-18470963-18472249
chr16	18497863	18500340	chr16-18497863-18500340
chr16	18581289	18583031	chr16-18581289-18583031
chr16	18585643	18592047	chr16-18585643-18592047
chr16	18617206	18617918	chr16-18617206-18617918
chr16	18620568	18621734	chr16-18620568-18621734
chr16	18625941	18627209	chr16-18625941-18627209
chr16	18628598	18630551	chr16-18628598-18630551
chr16	18645356	18645671	chr16-18645356-18645671
chr16	18686484	18687653	chr16-18686484-18687653
chr16	18732934	18734139	chr16-18732934-18734139
chr16	18738278	18740505	chr16-18738278-18740505
chr16	18777241	18777853	chr16-18777241-18777853
chr16	18810662	18813673	chr16-18810662-18813673
chr16	18836127	18837489	chr16-18836127-18837489
chr16	18875481	18879151	chr16-18875481-18879151
chr16	19759975	19761645	chr16-19759975-19761645
chr16	19946490	19947292	chr16-19946490-19947292
chr16	20097079	20098669	chr16-20097079-20098669
chr16	20140620	20142816	chr16-20140620-20142816
chr16	20240411	20241777	chr16-20240411-20241777
chr16	20300571	20302727	chr16-20300571-20302727
chr16	20353592	20353793	chr16-20353592-20353793
chr16	20357143	20357876	chr16-20357143-20357876
chr16	20371608	20371909	chr16-20371608-20371909
chr16	20411839	20412460	chr16-20411839-20412460
chr16	20413963	20414164	chr16-20413963-20414164
chr16	20424898	20426964	chr16-20424898-20426964
chr16	20497117	20500088	chr16-20497117-20500088
chr16	20516368	20518689	chr16-20516368-20518689
chr16	20534697	20538422	chr16-20534697-20538422
chr16	20547437	20549703	chr16-20547437-20549703

chr16	20589315	20591695	chr16-20589315-20591695
chr16	20609876	20612612	chr16-20609876-20612612
chr16	20619606	20623014	chr16-20619606-20623014
chr16	20629518	20630323	chr16-20629518-20630323
chr16	20650946	20653025	chr16-20650946-20653025
chr16	20671969	20675653	chr16-20671969-20675653
chr16	20677581	20678663	chr16-20677581-20678663
chr16	20693106	20693812	chr16-20693106-20693812
chr16	20695724	20696338	chr16-20695724-20696338
chr16	20714763	20719069	chr16-20714763-20719069
chr16	20725720	20726959	chr16-20725720-20726959
chr16	20732461	20734430	chr16-20732461-20734430
chr16	20746551	20746959	chr16-20746551-20746959
chr16	20804590	20805096	chr16-20804590-20805096
chr16	20829917	20830219	chr16-20829917-20830219
chr16	21204082	21212654	chr16-21204082-21212654
chr16	21224861	21227086	chr16-21224861-21227086
chr16	21235055	21235505	chr16-21235055-21235505
chr16	21237871	21239493	chr16-21237871-21239493
chr16	21245267	21246865	chr16-21245267-21246865
chr16	21255759	21256060	chr16-21255759-21256060
chr16	21282248	21282449	chr16-21282248-21282449
chr16	21286170	21287059	chr16-21286170-21287059
chr16	21295201	21295525	chr16-21295201-21295525
chr16	21310742	21311400	chr16-21310742-21311400
chr16	21331269	21334333	chr16-21331269-21334333
chr16	21339104	21340061	chr16-21339104-21340061
chr16	21353309	21353952	chr16-21353309-21353952
chr16	21358167	21358977	chr16-21358167-21358977
chr16	21381754	21382991	chr16-21381754-21382991
chr16	21400241	21400442	chr16-21400241-21400442
chr16	21422637	21424144	chr16-21422637-21424144
chr16	21693772	21695624	chr16-21693772-21695624
chr16	21727965	21728485	chr16-21727965-21728485
chr16	21787466	21788130	chr16-21787466-21788130
chr16	21793363	21795464	chr16-21793363-21795464
chr16	21818092	21819603	chr16-21818092-21819603
chr16	21845950	21846775	chr16-21845950-21846775
chr16	21946191	21947938	chr16-21946191-21947938
chr16	21996605	21997743	chr16-21996605-21997743
chr16	22008624	22011164	chr16-22008624-22011164
chr16	22081647	22081951	chr16-22081647-22081951

chr16	22097374	22098548	chr16-22097374-22098548
chr16	22150563	22150816	chr16-22150563-22150816
chr16	22151633	22152360	chr16-22151633-22152360
chr16	22160399	22164760	chr16-22160399-22164760
chr16	22261151	22267090	chr16-22261151-22267090
chr16	22428583	22429704	chr16-22428583-22429704
chr16	22434077	22434998	chr16-22434077-22434998
chr16	22437545	22441130	chr16-22437545-22441130
chr16	22514843	22515198	chr16-22514843-22515198
chr16	22548010	22548934	chr16-22548010-22548934
chr16	22572495	22573338	chr16-22572495-22573338
chr16	22656616	22657468	chr16-22656616-22657468
chr16	22807637	22808024	chr16-22807637-22808024
chr16	22844140	22844901	chr16-22844140-22844901
chr16	22855804	22859042	chr16-22855804-22859042
chr16	22919643	22920788	chr16-22919643-22920788
chr16	23102433	23103364	chr16-23102433-23103364
chr16	23106741	23112116	chr16-23106741-23112116
chr16	23126595	23128948	chr16-23126595-23128948
chr16	23167594	23167795	chr16-23167594-23167795
chr16	23224372	23227394	chr16-23224372-23227394
chr16	23266188	23266492	chr16-23266188-23266492
chr16	23271815	23273265	chr16-23271815-23273265
chr16	23317420	23318597	chr16-23317420-23318597
chr16	23403652	23404111	chr16-23403652-23404111
chr16	23519882	23520890	chr16-23519882-23520890
chr16	23609519	23610326	chr16-23609519-23610326
chr16	23897898	23899034	chr16-23897898-23899034
chr16	23980417	23990180	chr16-23980417-23990180
chr16	24136251	24136502	chr16-24136251-24136502
chr16	24205819	24206875	chr16-24205819-24206875
chr16	24313738	24314158	chr16-24313738-24314158
chr16	24391909	24397268	chr16-24391909-24397268
chr16	24402862	24404647	chr16-24402862-24404647
chr16	24418042	24418300	chr16-24418042-24418300
chr16	24427601	24429110	chr16-24427601-24429110
chr16	24433488	24436664	chr16-24433488-24436664
chr16	24443591	24443916	chr16-24443591-24443916
chr16	24447866	24450851	chr16-24447866-24450851
chr16	24491010	24491569	chr16-24491010-24491569
chr16	24495337	24496176	chr16-24495337-24496176
chr16	24507636	24508295	chr16-24507636-24508295

chr16	24734605	24734806	chr16-24734605-24734806
chr16	24737296	24737848	chr16-24737296-24737848
chr16	24766394	24766645	chr16-24766394-24766645
chr16	24913482	24914186	chr16-24913482-24914186
chr16	25011270	25011529	chr16-25011270-25011529
chr16	25026520	25026772	chr16-25026520-25026772
chr16	25058429	25060301	chr16-25058429-25060301
chr16	25061649	25061850	chr16-25061649-25061850
chr16	25407304	25407909	chr16-25407304-25407909
chr16	25417805	25418580	chr16-25417805-25418580
chr16	25426172	25426373	chr16-25426172-25426373
chr16	25483855	25484220	chr16-25483855-25484220
chr16	25531874	25532184	chr16-25531874-25532184
chr16	25584456	25585476	chr16-25584456-25585476
chr16	25953485	25953800	chr16-25953485-25953800
chr16	26065177	26065937	chr16-26065177-26065937
chr16	26104527	26106346	chr16-26104527-26106346
chr16	26370169	26372004	chr16-26370169-26372004
chr16	26401076	26401277	chr16-26401076-26401277
chr16	26581085	26582740	chr16-26581085-26582740
chr16	26967729	26968446	chr16-26967729-26968446
chr16	27387862	27390622	chr16-27387862-27390622
chr16	28377421	28377622	chr16-28377421-28377622
chr16	28445026	28447067	chr16-28445026-28447067
chr16	28564383	28565137	chr16-28564383-28565137
chr16	28753224	28753425	chr16-28753224-28753425
chr16	28870701	28871111	chr16-28870701-28871111
chr16	28928420	28930479	chr16-28928420-28930479
chr16	29560547	29560748	chr16-29560547-29560748
chr16	29578969	29580855	chr16-29578969-29580855
chr16	29662765	29663991	chr16-29662765-29663991
chr16	29728973	29729527	chr16-29728973-29729527
chr16	29812965	29816241	chr16-29812965-29816241
chr16	29839252	29839606	chr16-29839252-29839606
chr16	29881604	29881861	chr16-29881604-29881861
chr16	29915631	29915940	chr16-29915631-29915940
chr16	29944055	29946953	chr16-29944055-29946953
chr16	29972946	29973585	chr16-29972946-29973585
chr16	29977635	29977900	chr16-29977635-29977900
chr16	30008137	30010073	chr16-30008137-30010073
chr16	30024869	30025329	chr16-30024869-30025329
chr16	30033776	30034966	chr16-30033776-30034966

chr16	30038654	30039947	chr16-30038654-30039947
chr16	30045709	30045991	chr16-30045709-30045991
chr16	30047248	30047857	chr16-30047248-30047857
chr16	30052332	30054603	chr16-30052332-30054603
chr16	30055475	30061070	chr16-30055475-30061070
chr16	30062214	30071523	chr16-30062214-30071523
chr16	30090185	30090548	chr16-30090185-30090548
chr16	30184580	30185448	chr16-30184580-30185448
chr16	30186456	30188611	chr16-30186456-30188611
chr16	30236055	30236661	chr16-30236055-30236661
chr16	30251967	30252729	chr16-30251967-30252729
chr16	30282803	30284395	chr16-30282803-30284395
chr16	30308213	30309498	chr16-30308213-30309498
chr16	30385017	30390676	chr16-30385017-30390676
chr16	30465706	30466117	chr16-30465706-30466117
chr16	30501919	30504159	chr16-30501919-30504159
chr16	30510219	30514359	chr16-30510219-30514359
chr16	30520287	30520744	chr16-30520287-30520744
chr16	30549902	30551225	chr16-30549902-30551225
chr16	30586102	30588661	chr16-30586102-30588661
chr16	30596653	30597825	chr16-30596653-30597825
chr16	30598940	30601445	chr16-30598940-30601445
chr16	30790076	30790327	chr16-30790076-30790327
chr16	30821406	30821657	chr16-30821406-30821657
chr16	30835211	30836444	chr16-30835211-30836444
chr16	30971851	30972624	chr16-30971851-30972624
chr16	31079822	31082050	chr16-31079822-31082050
chr16	31199262	31201769	chr16-31199262-31201769
chr16	31233207	31234512	chr16-31233207-31234512
chr16	31235613	31235918	chr16-31235613-31235918
chr16	31272635	31275663	chr16-31272635-31275663
chr16	31278003	31278989	chr16-31278003-31278989
chr16	31290087	31291843	chr16-31290087-31291843
chr16	31339455	31339916	chr16-31339455-31339916
chr16	31428016	31430527	chr16-31428016-31430527
chr16	31454376	31454577	chr16-31454376-31454577
chr16	31458372	31461430	chr16-31458372-31461430
chr16	31467961	31468383	chr16-31467961-31468383
chr16	31489024	31489276	chr16-31489024-31489276
chr16	31492948	31493375	chr16-31492948-31493375
chr16	31502523	31502992	chr16-31502523-31502992
chr16	31506585	31509437	chr16-31506585-31509437

chr16	31591301	31594480	chr16-31591301-31594480
chr16	31597559	31599722	chr16-31597559-31599722
chr16	31602999	31604108	chr16-31602999-31604108
chr16	31617978	31618283	chr16-31617978-31618283
chr16	31637713	31638811	chr16-31637713-31638811
chr16	31661729	31666875	chr16-31661729-31666875
chr16	31667690	31667999	chr16-31667690-31667999
chr16	31884794	31885164	chr16-31884794-31885164
chr16	31897099	31897572	chr16-31897099-31897572
chr16	31906813	31907215	chr16-31906813-31907215
chr16	31917799	31918662	chr16-31917799-31918662
chr16	31932774	31935755	chr16-31932774-31935755
chr16	31946163	31949888	chr16-31946163-31949888
chr16	32001336	32004071	chr16-32001336-32004071
chr16	32007919	32008963	chr16-32007919-32008963
chr16	32062423	32062734	chr16-32062423-32062734
chr16	32066353	32067442	chr16-32066353-32067442
chr16	32076271	32079727	chr16-32076271-32079727
chr16	32087699	32088322	chr16-32087699-32088322
chr16	32098097	32100733	chr16-32098097-32100733
chr16	32147830	32148515	chr16-32147830-32148515
chr16	32149316	32149742	chr16-32149316-32149742
chr16	32165174	32165836	chr16-32165174-32165836
chr16	32245390	32248261	chr16-32245390-32248261
chr16	32276902	32280480	chr16-32276902-32280480
chr16	32331252	32333693	chr16-32331252-32333693
chr16	32419134	32420971	chr16-32419134-32420971
chr16	32429745	32432201	chr16-32429745-32432201
chr16	32499541	32499893	chr16-32499541-32499893
chr16	32524065	32524316	chr16-32524065-32524316
chr16	32608315	32611177	chr16-32608315-32611177
chr16	32640513	32648268	chr16-32640513-32648268
chr16	32650838	32651686	chr16-32650838-32651686
chr16	32656072	32662381	chr16-32656072-32662381
chr16	32663372	32663835	chr16-32663372-32663835
chr16	32665141	32666871	chr16-32665141-32666871
chr16	32670023	32670310	chr16-32670023-32670310
chr16	32671202	32679024	chr16-32671202-32679024
chr16	32742988	32743635	chr16-32742988-32743635
chr16	32746092	32746499	chr16-32746092-32746499
chr16	32796730	32797715	chr16-32796730-32797715
chr16	32798664	32799013	chr16-32798664-32799013

chr16	32803366	32805352	chr16-32803366-32805352
chr16	32813134	32813335	chr16-32813134-32813335
chr16	32814480	32816192	chr16-32814480-32816192
chr16	32821756	32822903	chr16-32821756-32822903
chr16	32857854	32860228	chr16-32857854-32860228
chr16	32863450	32864198	chr16-32863450-32864198
chr16	32866834	32869086	chr16-32866834-32869086
chr16	32876739	32879722	chr16-32876739-32879722
chr16	32913625	32915679	chr16-32913625-32915679
chr16	33055560	33059952	chr16-33055560-33059952
chr16	33061269	33063874	chr16-33061269-33063874
chr16	33184722	33187322	chr16-33184722-33187322
chr16	33250229	33252768	chr16-33250229-33252768
chr16	33326206	33327356	chr16-33326206-33327356
chr16	33337321	33337981	chr16-33337321-33337981
chr16	33379558	33383319	chr16-33379558-33383319
chr16	33399998	33401302	chr16-33399998-33401302
chr16	33403509	33404641	chr16-33403509-33404641
chr16	33446458	33447293	chr16-33446458-33447293
chr16	33517156	33517668	chr16-33517156-33517668
chr16	33580397	33581310	chr16-33580397-33581310
chr16	33594155	33595049	chr16-33594155-33595049
chr16	33676316	33676517	chr16-33676316-33676517
chr16	33683992	33685770	chr16-33683992-33685770
chr16	33751293	33751494	chr16-33751293-33751494
chr16	33828965	33831265	chr16-33828965-33831265
chr16	33832760	33834470	chr16-33832760-33834470
chr16	33836883	33837718	chr16-33836883-33837718
chr16	33859902	33860364	chr16-33859902-33860364
chr16	33873367	33873976	chr16-33873367-33873976
chr16	33878479	33878680	chr16-33878479-33878680
chr16	33891218	33893477	chr16-33891218-33893477
chr16	33965552	33967593	chr16-33965552-33967593
chr16	34095308	34096282	chr16-34095308-34096282
chr16	34572365	34574353	chr16-34572365-34574353
chr16	34657403	34657923	chr16-34657403-34657923
chr16	34667105	34667699	chr16-34667105-34667699
chr16	34690285	34691908	chr16-34690285-34691908
chr16	34744976	34745985	chr16-34744976-34745985
chr16	34784892	34785146	chr16-34784892-34785146
chr16	34811848	34812348	chr16-34811848-34812348
chr16	34860515	34860974	chr16-34860515-34860974

chr16	34958667	34958868	chr16-34958667-34958868
chr16	35021876	35023763	chr16-35021876-35023763
chr16	35116611	35116919	chr16-35116611-35116919
chr16	35154221	35156648	chr16-35154221-35156648
chr16	35362466	35364696	chr16-35362466-35364696
chr16	35489366	35491313	chr16-35489366-35491313
chr16	35540384	35542642	chr16-35540384-35542642
chr16	35659873	35660793	chr16-35659873-35660793
chr16	35768285	35771876	chr16-35768285-35771876
chr16	35813452	35813653	chr16-35813452-35813653
chr16	35869147	35872096	chr16-35869147-35872096
chr16	35938216	35940162	chr16-35938216-35940162
chr16	35982038	35984964	chr16-35982038-35984964
chr16	36040444	36042216	chr16-36040444-36042216
chr16	36069712	36073224	chr16-36069712-36073224
chr16	36130645	36131213	chr16-36130645-36131213
chr16	36469660	36469861	chr16-36469660-36469861
chr16	36561634	36561835	chr16-36561634-36561835
chr16	36693482	36695050	chr16-36693482-36695050
chr16	36821608	36821972	chr16-36821608-36821972
chr16	36827621	36829353	chr16-36827621-36829353
chr16	36873671	36876435	chr16-36873671-36876435
chr16	37011024	37012961	chr16-37011024-37012961
chr16	37384148	37385599	chr16-37384148-37385599
chr16	37538533	37541138	chr16-37538533-37541138
chr16	37652816	37654922	chr16-37652816-37654922
chr16	37670784	37671211	chr16-37670784-37671211
chr16	37684402	37685560	chr16-37684402-37685560
chr16	37776624	37777597	chr16-37776624-37777597
chr16	37788462	37788766	chr16-37788462-37788766
chr16	37867650	37870331	chr16-37867650-37870331
chr16	37916104	37916859	chr16-37916104-37916859
chr16	38086905	38093965	chr16-38086905-38093965
chr16	38119370	38119571	chr16-38119370-38119571
chr16	38248414	38249115	chr16-38248414-38249115
chr16	38341349	38342162	chr16-38341349-38342162
chr16	38346819	38348715	chr16-38346819-38348715
chr16	38376512	38376919	chr16-38376512-38376919
chr16	38396277	38397093	chr16-38396277-38397093
chr16	38406105	38407286	chr16-38406105-38407286
chr16	38451299	38453473	chr16-38451299-38453473
chr16	38476340	38476853	chr16-38476340-38476853

chr16	38483094	38484480	chr16-38483094-38484480
chr16	38521377	38523988	chr16-38521377-38523988
chr16	38549259	38550730	chr16-38549259-38550730
chr16	38562018	38564457	chr16-38562018-38564457
chr16	38712046	38714500	chr16-38712046-38714500
chr16	38741903	38743135	chr16-38741903-38743135
chr16	38902064	38903200	chr16-38902064-38903200
chr16	41533159	41533519	chr16-41533159-41533519
chr16	42152634	42152986	chr16-42152634-42152986
chr16	42189746	42190944	chr16-42189746-42190944
chr16	42339404	42340574	chr16-42339404-42340574
chr16	42592496	42592697	chr16-42592496-42592697
chr16	42782224	42782425	chr16-42782224-42782425
chr16	42874285	42877008	chr16-42874285-42877008
chr16	43362911	43364926	chr16-43362911-43364926
chr16	43458866	43459341	chr16-43458866-43459341
chr16	43582228	43583302	chr16-43582228-43583302
chr16	43655486	43656732	chr16-43655486-43656732
chr16	43659757	43665306	chr16-43659757-43665306
chr16	43698082	43699999	chr16-43698082-43699999
chr16	43720934	43721186	chr16-43720934-43721186
chr16	43801800	43802258	chr16-43801800-43802258
chr16	43807282	43807483	chr16-43807282-43807483
chr16	43882801	43883002	chr16-43882801-43883002
chr16	43888206	43891052	chr16-43888206-43891052
chr16	43978951	43980422	chr16-43978951-43980422
chr16	44015403	44016968	chr16-44015403-44016968
chr16	44062893	44063713	chr16-44062893-44063713
chr16	44132261	44132462	chr16-44132261-44132462
chr16	44136460	44142859	chr16-44136460-44142859
chr16	44171867	44174630	chr16-44171867-44174630
chr16	44331798	44333267	chr16-44331798-44333267
chr16	44346778	44348570	chr16-44346778-44348570
chr16	44394410	44395271	chr16-44394410-44395271
chr16	44557686	44559245	chr16-44557686-44559245
chr16	44672539	44672851	chr16-44672539-44672851
chr16	44723531	44726535	chr16-44723531-44726535
chr16	44744624	44746817	chr16-44744624-44746817
chr16	45070712	45071499	chr16-45070712-45071499
chr16	45124279	45125078	chr16-45124279-45125078
chr16	45157451	45160788	chr16-45157451-45160788
chr16	45408540	45409070	chr16-45408540-45409070

chr16	45653784	45654492	chr16-45653784-45654492
chr16	45741850	45743578	chr16-45741850-45743578
chr16	45830617	45830818	chr16-45830617-45830818
chr16	45836714	45845751	chr16-45836714-45845751
chr16	45848898	45849149	chr16-45848898-45849149
chr16	45871450	45871962	chr16-45871450-45871962
chr16	45873283	45873588	chr16-45873283-45873588
chr16	45892930	45893181	chr16-45892930-45893181
chr16	45903155	45904014	chr16-45903155-45904014
chr16	45944963	45946569	chr16-45944963-45946569
chr16	45952601	45952968	chr16-45952601-45952968
chr16	45974349	45974668	chr16-45974349-45974668
chr16	46008066	46011233	chr16-46008066-46011233
chr16	46495136	46498462	chr16-46495136-46498462
chr16	48992607	48995992	chr16-48992607-48995992
chr16	49698532	49700188	chr16-49698532-49700188
chr16	49854638	49859322	chr16-49854638-49859322
chr16	49937654	49939038	chr16-49937654-49939038
chr16	49952053	49952732	chr16-49952053-49952732
chr16	50009699	50009900	chr16-50009699-50009900
chr16	50045715	50046288	chr16-50045715-50046288
chr16	50363562	50364830	chr16-50363562-50364830
chr16	50429218	50432958	chr16-50429218-50432958
chr16	50528223	50530557	chr16-50528223-50530557
chr16	50601002	50601535	chr16-50601002-50601535
chr16	50731569	50733269	chr16-50731569-50733269
chr16	50753646	50753847	chr16-50753646-50753847
chr16	52030906	52033349	chr16-52030906-52033349
chr16	52060170	52061683	chr16-52060170-52061683
chr16	52418420	52418787	chr16-52418420-52418787
chr16	52433305	52433913	chr16-52433305-52433913
chr16	52438462	52438807	chr16-52438462-52438807
chr16	52449547	52454508	chr16-52449547-52454508
chr16	55504914	55505368	chr16-55504914-55505368
chr16	55550639	55551110	chr16-55550639-55551110
chr16	55555287	55558239	chr16-55555287-55558239
chr16	55631718	55631970	chr16-55631718-55631970
chr16	55754351	55754552	chr16-55754351-55754552
chr16	55777608	55777914	chr16-55777608-55777914
chr16	55813996	55814247	chr16-55813996-55814247
chr16	55815486	55824218	chr16-55815486-55824218
chr16	55837612	55839477	chr16-55837612-55839477

chr16	55893425	55896190	chr16-55893425-55896190
chr16	55898495	55898696	chr16-55898495-55898696
chr16	55933561	55935200	chr16-55933561-55935200
chr16	55948951	55949406	chr16-55948951-55949406
chr16	55965477	55969782	chr16-55965477-55969782
chr16	55970587	55971010	chr16-55970587-55971010
chr16	55972654	55975786	chr16-55972654-55975786
chr16	56027684	56030865	chr16-56027684-56030865
chr16	56035204	56038466	chr16-56035204-56038466
chr16	56064034	56064896	chr16-56064034-56064896
chr16	56074992	56076849	chr16-56074992-56076849
chr16	56715735	56718416	chr16-56715735-56718416
chr16	56886127	56886436	chr16-56886127-56886436
chr16	57120636	57124196	chr16-57120636-57124196
chr16	57166487	57167892	chr16-57166487-57167892
chr16	57230163	57232020	chr16-57230163-57232020
chr16	57419245	57419706	chr16-57419245-57419706
chr16	57434554	57434855	chr16-57434554-57434855
chr16	57524859	57525274	chr16-57524859-57525274
chr16	57526541	57527605	chr16-57526541-57527605
chr16	57536338	57536604	chr16-57536338-57536604
chr16	57548690	57551378	chr16-57548690-57551378
chr16	57605459	57607789	chr16-57605459-57607789
chr16	57671965	57672703	chr16-57671965-57672703
chr16	57691297	57691717	chr16-57691297-57691717
chr16	57750263	57756147	chr16-57750263-57756147
chr16	58349188	58349889	chr16-58349188-58349889
chr16	58389012	58389527	chr16-58389012-58389527
chr16	58407736	58412194	chr16-58407736-58412194
chr16	58519502	58524394	chr16-58519502-58524394
chr16	58669791	58671980	chr16-58669791-58671980
chr16	58703388	58703926	chr16-58703388-58703926
chr16	58712332	58712533	chr16-58712332-58712533
chr16	58727415	58729233	chr16-58727415-58729233
chr16	59471577	59473228	chr16-59471577-59473228
chr16	59583574	59584073	chr16-59583574-59584073
chr16	59586243	59586651	chr16-59586243-59586651
chr16	59598995	59601674	chr16-59598995-59601674
chr16	59638406	59639908	chr16-59638406-59639908
chr16	60605038	60605368	chr16-60605038-60605368
chr16	60606187	60606488	chr16-60606187-60606488
chr16	62785541	62787624	chr16-62785541-62787624

chr16	62846148	62847545	chr16-62846148-62847545
chr16	62853926	62854794	chr16-62853926-62854794
chr16	62940780	62940981	chr16-62940780-62940981
chr16	63856877	63857426	chr16-63856877-63857426
chr16	63863712	63864476	chr16-63863712-63864476
chr16	64769361	64771710	chr16-64769361-64771710
chr16	64836339	64836700	chr16-64836339-64836700
chr16	64850118	64854139	chr16-64850118-64854139
chr16	64872521	64874422	chr16-64872521-64874422
chr16	65105492	65105799	chr16-65105492-65105799
chr16	65559315	65563439	chr16-65559315-65563439
chr16	65629336	65629758	chr16-65629336-65629758
chr16	65814488	65819498	chr16-65814488-65819498
chr16	67617753	67618357	chr16-67617753-67618357
chr16	67619376	67621623	chr16-67619376-67621623
chr16	70313267	70315435	chr16-70313267-70315435
chr16	72028867	72029149	chr16-72028867-72029149
chr16	72662929	72663895	chr16-72662929-72663895
chr16	74411361	74411994	chr16-74411361-74411994
chr16	75592572	75593910	chr16-75592572-75593910
chr16	75765833	75768161	chr16-75765833-75768161
chr16	76225730	76226191	chr16-76225730-76226191
chr16	76370427	76374341	chr16-76370427-76374341
chr16	76454905	76455364	chr16-76454905-76455364
chr16	76499829	76500190	chr16-76499829-76500190
chr16	76539836	76540209	chr16-76539836-76540209
chr16	76958477	76958878	chr16-76958477-76958878
chr16	77013146	77016314	chr16-77013146-77016314
chr16	77236951	77237307	chr16-77236951-77237307
chr16	77329134	77330230	chr16-77329134-77330230
chr16	77643697	77644846	chr16-77643697-77644846
chr16	78198664	78198968	chr16-78198664-78198968
chr16	78236867	78238079	chr16-78236867-78238079
chr16	78264583	78265562	chr16-78264583-78265562
chr16	78300552	78303335	chr16-78300552-78303335
chr16	78317694	78318770	chr16-78317694-78318770
chr16	78374970	78377499	chr16-78374970-78377499
chr16	78427721	78427922	chr16-78427721-78427922
chr16	78451344	78451645	chr16-78451344-78451645
chr16	78456556	78456757	chr16-78456556-78456757
chr16	78532278	78532479	chr16-78532278-78532479
chr16	78540133	78540334	chr16-78540133-78540334

chr16	78563630	78563895	chr16-78563630-78563895
chr16	78575538	78577773	chr16-78575538-78577773
chr16	78618027	78618380	chr16-78618027-78618380
chr16	78619310	78619561	chr16-78619310-78619561
chr16	78930718	78931579	chr16-78930718-78931579
chr16	79591090	79591768	chr16-79591090-79591768
chr16	79879805	79880067	chr16-79879805-79880067
chr16	80654783	80655038	chr16-80654783-80655038
chr16	81200031	81201296	chr16-81200031-81201296
chr16	84620670	84621674	chr16-84620670-84621674
chr16	84702444	84705224	chr16-84702444-84705224
chr16	84734205	84736192	chr16-84734205-84736192
chr16	84771290	84771491	chr16-84771290-84771491
chr16	84773389	84775330	chr16-84773389-84775330
chr16	84833694	84836678	chr16-84833694-84836678
chr16	85132319	85134450	chr16-85132319-85134450
chr16	85137275	85137940	chr16-85137275-85137940
chr16	85141693	85141946	chr16-85141693-85141946
chr16	85148382	85149220	chr16-85148382-85149220
chr16	85151679	85153055	chr16-85151679-85153055
chr16	85158026	85158392	chr16-85158026-85158392
chr16	85168875	85169685	chr16-85168875-85169685
chr16	85170763	85174310	chr16-85170763-85174310
chr16	85176458	85177171	chr16-85176458-85177171
chr16	85549933	85550507	chr16-85549933-85550507
chr16	85799792	85804803	chr16-85799792-85804803
chr16	85897921	85902037	chr16-85897921-85902037
chr16	86343427	86347082	chr16-86343427-86347082
chr16	86347951	86348203	chr16-86347951-86348203
chr16	87353456	87354862	chr16-87353456-87354862
chr16	87431361	87433136	chr16-87431361-87433136
chr16	87439614	87441687	chr16-87439614-87441687
chr16	87454359	87456571	chr16-87454359-87456571
chr16	87494028	87497481	chr16-87494028-87497481
chr16	87507634	87507835	chr16-87507634-87507835
chr16	87599521	87599931	chr16-87599521-87599931
chr16	87602815	87603177	chr16-87602815-87603177
chr16	87611387	87611764	chr16-87611387-87611764
chr16	87662673	87663706	chr16-87662673-87663706
chr16	87665080	87665952	chr16-87665080-87665952
chr16	87688992	87689923	chr16-87688992-87689923
chr16	87698280	87700703	chr16-87698280-87700703

chr16	87712692	87713784	chr16-87712692-87713784
chr16	87716314	87716615	chr16-87716314-87716615
chr16	87725059	87725363	chr16-87725059-87725363
chr16	87783517	87785360	chr16-87783517-87785360
chr16	88562875	88563340	chr16-88562875-88563340
chr16	89773836	89774568	chr16-89773836-89774568
chr16	89817831	89818844	chr16-89817831-89818844
chr16	89954342	89954968	chr16-89954342-89954968
chr16	89959787	89961242	chr16-89959787-89961242
chr16	90142222	90145047	chr16-90142222-90145047
chr16	90197169	90198457	chr16-90197169-90198457
chr16	90210572	90210876	chr16-90210572-90210876
chr16	90219803	90222907	chr16-90219803-90222907
chr16	90281536	90285614	chr16-90281536-90285614
chr16	90290819	90291917	chr16-90290819-90291917
chr16	90348758	90349992	chr16-90348758-90349992
chr16	90385346	90387713	chr16-90385346-90387713
chr16	90390468	90391192	chr16-90390468-90391192
chr16	90709646	90710467	chr16-90709646-90710467
chr16	90711842	90713024	chr16-90711842-90713024
chr16	90726075	90728339	chr16-90726075-90728339
chr16	90808963	90811059	chr16-90808963-90811059
chr16	90820876	90821287	chr16-90820876-90821287
chr16	90829675	90834274	chr16-90829675-90834274
chr16	90844486	90845974	chr16-90844486-90845974
chr16	90856101	90857733	chr16-90856101-90857733
chr16	90882111	90882475	chr16-90882111-90882475
chr16	90884864	90886818	chr16-90884864-90886818
chr16	90933505	90935571	chr16-90933505-90935571
chr16	91009690	91012087	chr16-91009690-91012087
chr16	91042615	91045550	chr16-91042615-91045550
chr16	91056469	91056670	chr16-91056469-91056670
chr16	91073301	91073768	chr16-91073301-91073768
chr16	91169823	91170290	chr16-91169823-91170290
chr16	91220020	91220618	chr16-91220020-91220618
chr16	91224784	91227493	chr16-91224784-91227493
chr16	91255681	91255989	chr16-91255681-91255989
chr16	91269462	91270703	chr16-91269462-91270703
chr16	91362580	91362880	chr16-91362580-91362880
chr16	91372353	91374226	chr16-91372353-91374226
chr16	91405585	91408260	chr16-91405585-91408260
chr16	91410844	91411045	chr16-91410844-91411045

chr16	91452740	91453009	chr16-91452740-91453009
chr16	91464265	91465797	chr16-91464265-91465797
chr16	91475916	91476117	chr16-91475916-91476117
chr16	91479249	91479510	chr16-91479249-91479510
chr16	91484032	91486577	chr16-91484032-91486577
chr16	91534552	91534853	chr16-91534552-91534853
chr16	91546383	91549103	chr16-91546383-91549103
chr16	91551071	91551877	chr16-91551071-91551877
chr16	91596512	91598341	chr16-91596512-91598341
chr16	91618128	91619371	chr16-91618128-91619371
chr16	91644851	91649682	chr16-91644851-91649682
chr16	91687599	91690081	chr16-91687599-91690081
chr16	91726714	91731009	chr16-91726714-91731009
chr16	91885665	91886094	chr16-91885665-91886094
chr16	91917625	91918236	chr16-91917625-91918236
chr16	91929938	91932319	chr16-91929938-91932319
chr16	91962203	91963288	chr16-91962203-91963288
chr16	91969383	91970854	chr16-91969383-91970854
chr16	92039394	92039656	chr16-92039394-92039656
chr16	92057368	92059647	chr16-92057368-92059647
chr16	92158109	92159234	chr16-92158109-92159234
chr16	92215657	92215858	chr16-92215657-92215858
chr16	92245810	92246113	chr16-92245810-92246113
chr16	92300384	92302412	chr16-92300384-92302412
chr16	92341037	92341598	chr16-92341037-92341598
chr16	92398518	92400801	chr16-92398518-92400801
chr16	92423298	92424001	chr16-92423298-92424001
chr16	92425771	92426695	chr16-92425771-92426695
chr16	92464594	92466697	chr16-92464594-92466697
chr16	92475198	92475751	chr16-92475198-92475751
chr16	92498192	92499514	chr16-92498192-92499514
chr16	92565647	92566187	chr16-92565647-92566187
chr16	92582042	92582452	chr16-92582042-92582452
chr16	92619788	92622518	chr16-92619788-92622518
chr16	92644442	92646410	chr16-92644442-92646410
chr16	92653351	92654862	chr16-92653351-92654862
chr16	92662868	92663069	chr16-92662868-92663069
chr16	92674387	92674742	chr16-92674387-92674742
chr16	92679904	92680710	chr16-92679904-92680710
chr16	92683709	92688163	chr16-92683709-92688163
chr16	92690763	92699482	chr16-92690763-92699482
chr16	92823193	92826598	chr16-92823193-92826598

chr16	92827805	92828769	chr16-92827805-92828769
chr16	92897256	92897573	chr16-92897256-92897573
chr16	92903774	92904731	chr16-92903774-92904731
chr16	92907019	92907220	chr16-92907019-92907220
chr16	92930252	92930713	chr16-92930252-92930713
chr16	92979513	92979864	chr16-92979513-92979864
chr16	93006250	93007094	chr16-93006250-93007094
chr16	93013504	93014250	chr16-93013504-93014250
chr16	93109460	93109818	chr16-93109460-93109818
chr16	93129512	93130283	chr16-93129512-93130283
chr16	93150422	93150725	chr16-93150422-93150725
chr16	93292845	93293453	chr16-93292845-93293453
chr16	93520259	93520460	chr16-93520259-93520460
chr16	93533280	93533481	chr16-93533280-93533481
chr16	93534908	93535517	chr16-93534908-93535517
chr16	93602932	93605333	chr16-93602932-93605333
chr16	93607270	93608702	chr16-93607270-93608702
chr16	93682355	93684145	chr16-93682355-93684145
chr16	93686818	93687169	chr16-93686818-93687169
chr16	93710725	93713258	chr16-93710725-93713258
chr16	93732186	93733586	chr16-93732186-93733586
chr16	93831720	93834298	chr16-93831720-93834298
chr16	93883161	93885274	chr16-93883161-93885274
chr16	93912410	93912661	chr16-93912410-93912661
chr16	93913826	93914259	chr16-93913826-93914259
chr16	93938515	93938870	chr16-93938515-93938870
chr16	93973266	93973794	chr16-93973266-93973794
chr16	94023665	94024021	chr16-94023665-94024021
chr16	94080206	94080607	chr16-94080206-94080607
chr16	94083552	94087643	chr16-94083552-94087643
chr16	94094539	94095494	chr16-94094539-94095494
chr16	94114443	94115521	chr16-94114443-94115521
chr16	94125241	94126049	chr16-94125241-94126049
chr16	94150356	94150896	chr16-94150356-94150896
chr16	94229106	94229415	chr16-94229106-94229415
chr16	94233922	94234275	chr16-94233922-94234275
chr16	94289159	94290788	chr16-94289159-94290788
chr16	94311928	94314184	chr16-94311928-94314184
chr16	94327696	94328957	chr16-94327696-94328957
chr16	94369612	94372296	chr16-94369612-94372296
chr16	94524893	94527892	chr16-94524893-94527892
chr16	94544697	94545003	chr16-94544697-94545003

chr16	94568265	94572587	chr16-94568265-94572587
chr16	94713528	94714193	chr16-94713528-94714193
chr16	94723937	94725313	chr16-94723937-94725313
chr16	94997331	94997987	chr16-94997331-94997987
chr16	95050548	95050972	chr16-95050548-95050972
chr16	95585125	95586106	chr16-95585125-95586106
chr16	95618298	95618499	chr16-95618298-95618499
chr16	95673894	95675940	chr16-95673894-95675940
chr16	95682021	95683308	chr16-95682021-95683308
chr16	95686222	95686423	chr16-95686222-95686423
chr16	95700159	95700410	chr16-95700159-95700410
chr16	95701414	95706365	chr16-95701414-95706365
chr16	95715940	95716986	chr16-95715940-95716986
chr16	95729107	95729412	chr16-95729107-95729412
chr16	95766382	95769965	chr16-95766382-95769965
chr16	95778095	95778400	chr16-95778095-95778400
chr16	95814432	95814964	chr16-95814432-95814964
chr16	95967715	95967967	chr16-95967715-95967967
chr16	95989980	95992065	chr16-95989980-95992065
chr16	96080648	96083645	chr16-96080648-96083645
chr16	96090749	96092263	chr16-96090749-96092263
chr16	96126252	96128982	chr16-96126252-96128982
chr16	96144816	96146518	chr16-96144816-96146518
chr16	96151124	96151425	chr16-96151124-96151425
chr16	96159094	96159717	chr16-96159094-96159717
chr16	96191276	96192802	chr16-96191276-96192802
chr16	96200122	96200764	chr16-96200122-96200764
chr16	96327253	96327560	chr16-96327253-96327560
chr16	96357583	96358294	chr16-96357583-96358294
chr16	96361072	96363188	chr16-96361072-96363188
chr16	97165400	97166041	chr16-97165400-97166041
chr16	97170138	97171047	chr16-97170138-97171047
chr16	97272139	97272603	chr16-97272139-97272603
chr16	97356194	97357856	chr16-97356194-97357856
chr16	97362253	97362748	chr16-97362253-97362748
chr16	97495779	97496139	chr16-97495779-97496139
chr16	97534495	97535725	chr16-97534495-97535725
chr16	97593462	97594123	chr16-97593462-97594123
chr16	97609551	97611670	chr16-97609551-97611670
chr16	97632578	97633297	chr16-97632578-97633297
chr16	97659173	97659593	chr16-97659173-97659593
chr16	97665323	97665947	chr16-97665323-97665947

chr16	97685613	97686645	chr16-97685613-97686645
chr16	97716265	97718031	chr16-97716265-97718031
chr16	97757110	97765134	chr16-97757110-97765134
chr16	97778414	97779026	chr16-97778414-97779026
chr16	97849949	97852680	chr16-97849949-97852680
chr16	97872223	97872992	chr16-97872223-97872992
chr16	97921239	97923039	chr16-97921239-97923039
chr16	97960908	97963499	chr16-97960908-97963499
chr16	97996267	97998697	chr16-97996267-97998697
chr16	98079018	98083376	chr16-98079018-98083376
chr17	3082268	3085505	chr17-3082268-3085505
chr17	3102489	3102853	chr17-3102489-3102853
chr17	3113503	3117765	chr17-3113503-3117765
chr17	3313933	3315001	chr17-3313933-3315001
chr17	3325861	3328299	chr17-3325861-3328299
chr17	3350065	3350487	chr17-3350065-3350487
chr17	3394793	3395483	chr17-3394793-3395483
chr17	3418921	3420422	chr17-3418921-3420422
chr17	3442691	3443044	chr17-3442691-3443044
chr17	3556695	3559062	chr17-3556695-3559062
chr17	4785211	4786407	chr17-4785211-4786407
chr17	4868170	4869364	chr17-4868170-4869364
chr17	4907066	4909261	chr17-4907066-4909261
chr17	4946214	4946415	chr17-4946214-4946415
chr17	4974582	4975132	chr17-4974582-4975132
chr17	4993448	4998696	chr17-4993448-4998696
chr17	4999909	5000592	chr17-4999909-5000592
chr17	5007154	5007664	chr17-5007154-5007664
chr17	5082064	5083410	chr17-5082064-5083410
chr17	5125075	5126395	chr17-5125075-5126395
chr17	5137852	5139734	chr17-5137852-5139734
chr17	5189260	5190766	chr17-5189260-5190766
chr17	5206658	5206960	chr17-5206658-5206960
chr17	5235282	5235483	chr17-5235282-5235483
chr17	5237669	5237870	chr17-5237669-5237870
chr17	5397346	5397877	chr17-5397346-5397877
chr17	5402029	5402873	chr17-5402029-5402873
chr17	5439313	5441149	chr17-5439313-5441149
chr17	5491135	5493981	chr17-5491135-5493981
chr17	5741333	5741688	chr17-5741333-5741688
chr17	5748140	5748401	chr17-5748140-5748401
chr17	5752669	5753383	chr17-5752669-5753383

chr17	5770059	5770923	chr17-5770059-5770923
chr17	5798141	5802501	chr17-5798141-5802501
chr17	5803517	5803869	chr17-5803517-5803869
chr17	5821989	5823303	chr17-5821989-5823303
chr17	5839871	5846473	chr17-5839871-5846473
chr17	5848851	5850043	chr17-5848851-5850043
chr17	5934587	5935645	chr17-5934587-5935645
chr17	5940594	5942841	chr17-5940594-5942841
chr17	5957402	5960227	chr17-5957402-5960227
chr17	5968000	5968672	chr17-5968000-5968672
chr17	5974946	5979430	chr17-5974946-5979430
chr17	5994711	5995118	chr17-5994711-5995118
chr17	6000801	6002612	chr17-6000801-6002612
chr17	6005054	6008547	chr17-6005054-6008547
chr17	6016223	6016529	chr17-6016223-6016529
chr17	6078457	6081454	chr17-6078457-6081454
chr17	6105954	6109649	chr17-6105954-6109649
chr17	6117056	6118330	chr17-6117056-6118330
chr17	6140689	6141768	chr17-6140689-6141768
chr17	6172448	6174097	chr17-6172448-6174097
chr17	6202756	6203012	chr17-6202756-6203012
chr17	6256716	6257339	chr17-6256716-6257339
chr17	6270065	6272270	chr17-6270065-6272270
chr17	6279014	6279687	chr17-6279014-6279687
chr17	6316436	6317866	chr17-6316436-6317866
chr17	6321212	6321593	chr17-6321212-6321593
chr17	6429470	6431304	chr17-6429470-6431304
chr17	6467693	6468655	chr17-6467693-6468655
chr17	6475341	6477582	chr17-6475341-6477582
chr17	6490151	6490352	chr17-6490151-6490352
chr17	6492565	6493271	chr17-6492565-6493271
chr17	6601077	6602560	chr17-6601077-6602560
chr17	6641003	6641319	chr17-6641003-6641319
chr17	6654757	6656457	chr17-6654757-6656457
chr17	6748795	6749185	chr17-6748795-6749185
chr17	6756921	6759039	chr17-6756921-6759039
chr17	6771981	6772234	chr17-6771981-6772234
chr17	6773577	6773898	chr17-6773577-6773898
chr17	6776286	6784665	chr17-6776286-6784665
chr17	6807763	6809866	chr17-6807763-6809866
chr17	6812024	6813227	chr17-6812024-6813227
chr17	6814598	6814900	chr17-6814598-6814900

chr17	6826634	6829527	chr17-6826634-6829527
chr17	6835002	6836045	chr17-6835002-6836045
chr17	6897863	6898535	chr17-6897863-6898535
chr17	6947478	6949570	chr17-6947478-6949570
chr17	6958914	6962027	chr17-6958914-6962027
chr17	6978174	6980472	chr17-6978174-6980472
chr17	7164296	7164497	chr17-7164296-7164497
chr17	7168881	7171878	chr17-7168881-7171878
chr17	7533505	7533813	chr17-7533505-7533813
chr17	7826873	7827532	chr17-7826873-7827532
chr17	7944507	7946986	chr17-7944507-7946986
chr17	8024601	8024802	chr17-8024601-8024802
chr17	8146185	8148561	chr17-8146185-8148561
chr17	8164773	8167777	chr17-8164773-8167777
chr17	8282570	8285197	chr17-8282570-8285197
chr17	8310455	8313095	chr17-8310455-8313095
chr17	8340496	8341667	chr17-8340496-8341667
chr17	8372518	8372719	chr17-8372518-8372719
chr17	8505773	8506389	chr17-8505773-8506389
chr17	8524126	8525722	chr17-8524126-8525722
chr17	8552388	8552639	chr17-8552388-8552639
chr17	8800511	8802483	chr17-8800511-8802483
chr17	8856440	8856767	chr17-8856440-8856767
chr17	8988181	8988807	chr17-8988181-8988807
chr17	9167220	9168655	chr17-9167220-9168655
chr17	9764086	9765389	chr17-9764086-9765389
chr17	9864374	9865865	chr17-9864374-9865865
chr17	10098074	10098436	chr17-10098074-10098436
chr17	10309183	10309457	chr17-10309183-10309457
chr17	10312940	10313191	chr17-10312940-10313191
chr17	10314004	10322240	chr17-10314004-10322240
chr17	10378266	10379643	chr17-10378266-10379643
chr17	10463319	10465629	chr17-10463319-10465629
chr17	10466565	10466996	chr17-10466565-10466996
chr17	10497619	10499103	chr17-10497619-10499103
chr17	10552477	10553024	chr17-10552477-10553024
chr17	10702975	10703228	chr17-10702975-10703228
chr17	10839554	10841252	chr17-10839554-10841252
chr17	12031163	12031470	chr17-12031163-12031470
chr17	12094874	12095623	chr17-12094874-12095623
chr17	12118219	12120219	chr17-12118219-12120219
chr17	12195177	12196171	chr17-12195177-12196171

chr17	12231232	12231538	chr17-12231232-12231538
chr17	12266321	12266927	chr17-12266321-12266927
chr17	12296987	12298108	chr17-12296987-12298108
chr17	12317499	12319445	chr17-12317499-12319445
chr17	12327698	12328099	chr17-12327698-12328099
chr17	12363678	12364587	chr17-12363678-12364587
chr17	12371516	12372050	chr17-12371516-12372050
chr17	12507262	12507463	chr17-12507262-12507463
chr17	12724803	12725055	chr17-12724803-12725055
chr17	12739639	12743771	chr17-12739639-12743771
chr17	12749321	12753310	chr17-12749321-12753310
chr17	12754352	12755474	chr17-12754352-12755474
chr17	12767833	12770300	chr17-12767833-12770300
chr17	12782949	12783880	chr17-12782949-12783880
chr17	12832190	12832391	chr17-12832190-12832391
chr17	12880034	12880287	chr17-12880034-12880287
chr17	12914775	12918285	chr17-12914775-12918285
chr17	12939964	12941226	chr17-12939964-12941226
chr17	12959294	12961579	chr17-12959294-12961579
chr17	12989595	12993413	chr17-12989595-12993413
chr17	13007326	13009482	chr17-13007326-13009482
chr17	13479374	13480109	chr17-13479374-13480109
chr17	13492613	13493079	chr17-13492613-13493079
chr17	13555382	13556564	chr17-13555382-13556564
chr17	13584502	13584811	chr17-13584502-13584811
chr17	13656685	13657160	chr17-13656685-13657160
chr17	13667055	13670415	chr17-13667055-13670415
chr17	13679736	13684365	chr17-13679736-13684365
chr17	13697915	13701802	chr17-13697915-13701802
chr17	13709853	13710306	chr17-13709853-13710306
chr17	13714936	13715939	chr17-13714936-13715939
chr17	13717657	13717858	chr17-13717657-13717858
chr17	13731715	13733330	chr17-13731715-13733330
chr17	13737258	13738514	chr17-13737258-13738514
chr17	13740599	13741121	chr17-13740599-13741121
chr17	13742193	13745277	chr17-13742193-13745277
chr17	13758428	13763632	chr17-13758428-13763632
chr17	13764876	13765239	chr17-13764876-13765239
chr17	13867418	13867728	chr17-13867418-13867728
chr17	14075892	14076145	chr17-14075892-14076145
chr17	14202370	14204501	chr17-14202370-14204501
chr17	14279538	14279739	chr17-14279538-14279739

chr17	14942111	14944837	chr17-14942111-14944837
chr17	14959558	14961401	chr17-14959558-14961401
chr17	14977959	14978486	chr17-14977959-14978486
chr17	14990087	14990625	chr17-14990087-14990625
chr17	15021467	15021837	chr17-15021467-15021837
chr17	15041191	15041623	chr17-15041191-15041623
chr17	15065925	15066126	chr17-15065925-15066126
chr17	15068080	15068281	chr17-15068080-15068281
chr17	15137635	15137910	chr17-15137635-15137910
chr17	15285269	15285623	chr17-15285269-15285623
chr17	15300072	15300273	chr17-15300072-15300273
chr17	15314011	15314212	chr17-15314011-15314212
chr17	15325778	15326922	chr17-15325778-15326922
chr17	15356648	15357133	chr17-15356648-15357133
chr17	15373282	15377121	chr17-15373282-15377121
chr17	15380327	15381055	chr17-15380327-15381055
chr17	15395435	15397744	chr17-15395435-15397744
chr17	15496484	15500966	chr17-15496484-15500966
chr17	15510514	15510879	chr17-15510514-15510879
chr17	15525855	15528348	chr17-15525855-15528348
chr17	15536332	15537036	chr17-15536332-15537036
chr17	15563327	15565356	chr17-15563327-15565356
chr17	15577110	15577670	chr17-15577110-15577670
chr17	15703717	15707125	chr17-15703717-15707125
chr17	15823683	15827759	chr17-15823683-15827759
chr17	15829404	15831640	chr17-15829404-15831640
chr17	16051748	16051999	chr17-16051748-16051999
chr17	16950757	16952598	chr17-16950757-16952598
chr17	17004406	17006327	chr17-17004406-17006327
chr17	17063393	17065213	chr17-17063393-17065213
chr17	17121848	17122163	chr17-17121848-17122163
chr17	17179770	17180078	chr17-17179770-17180078
chr17	17234038	17236208	chr17-17234038-17236208
chr17	17293598	17295975	chr17-17293598-17295975
chr17	17345494	17346411	chr17-17345494-17346411
chr17	17373184	17376071	chr17-17373184-17376071
chr17	17600874	17601075	chr17-17600874-17601075
chr17	17622224	17626042	chr17-17622224-17626042
chr17	17799330	17799531	chr17-17799330-17799531
chr17	17825383	17839305	chr17-17825383-17839305
chr17	17909455	17910936	chr17-17909455-17910936
chr17	20139964	20140165	chr17-20139964-20140165

chr17	20944145	20947149	chr17-20944145-20947149
chr17	21008373	21010428	chr17-21008373-21010428
chr17	21370034	21370744	chr17-21370034-21370744
chr17	21383320	21384455	chr17-21383320-21384455
chr17	21422768	21423853	chr17-21422768-21423853
chr17	21449755	21452132	chr17-21449755-21452132
chr17	21472793	21479720	chr17-21472793-21479720
chr17	21488372	21491363	chr17-21488372-21491363
chr17	21516022	21517459	chr17-21516022-21517459
chr17	21531511	21533241	chr17-21531511-21533241
chr17	21534823	21538168	chr17-21534823-21538168
chr17	21566379	21568694	chr17-21566379-21568694
chr17	21650121	21651016	chr17-21650121-21651016
chr17	21679425	21680687	chr17-21679425-21680687
chr17	21707052	21708991	chr17-21707052-21708991
chr17	21733103	21734733	chr17-21733103-21734733
chr17	21753872	21754073	chr17-21753872-21754073
chr17	21844778	21846264	chr17-21844778-21846264
chr17	21908754	21910172	chr17-21908754-21910172
chr17	21961050	21963595	chr17-21961050-21963595
chr17	22080870	22081622	chr17-22080870-22081622
chr17	22165190	22166390	chr17-22165190-22166390
chr17	22224760	22226188	chr17-22224760-22226188
chr17	22313911	22315698	chr17-22313911-22315698
chr17	22359783	22362790	chr17-22359783-22362790
chr17	22423368	22425435	chr17-22423368-22425435
chr17	22674300	22674623	chr17-22674300-22674623
chr17	22738434	22740374	chr17-22738434-22740374
chr17	22817056	22818254	chr17-22817056-22818254
chr17	22866022	22867494	chr17-22866022-22867494
chr17	23192222	23193945	chr17-23192222-23193945
chr17	23534005	23535388	chr17-23534005-23535388
chr17	23537673	23538834	chr17-23537673-23538834
chr17	23540486	23540872	chr17-23540486-23540872
chr17	23541926	23543872	chr17-23541926-23543872
chr17	23545961	23547504	chr17-23545961-23547504
chr17	23548326	23552015	chr17-23548326-23552015
chr17	23563125	23565208	chr17-23563125-23565208
chr17	23585372	23586702	chr17-23585372-23586702
chr17	23592288	23593544	chr17-23592288-23593544
chr17	23598472	23600320	chr17-23598472-23600320
chr17	23630329	23630885	chr17-23630329-23630885

chr17	23644368	23645549	chr17-23644368-23645549
chr17	23660307	23661308	chr17-23660307-23661308
chr17	23672737	23684195	chr17-23672737-23684195
chr17	23693844	23695988	chr17-23693844-23695988
chr17	23697975	23700676	chr17-23697975-23700676
chr17	23725470	23727749	chr17-23725470-23727749
chr17	23739096	23741106	chr17-23739096-23741106
chr17	23742081	23743030	chr17-23742081-23743030
chr17	23745535	23746238	chr17-23745535-23746238
chr17	23748786	23749701	chr17-23748786-23749701
chr17	23770467	23772038	chr17-23770467-23772038
chr17	23785273	23786999	chr17-23785273-23786999
chr17	23790111	23791323	chr17-23790111-23791323
chr17	23801847	23807295	chr17-23801847-23807295
chr17	23827262	23830296	chr17-23827262-23830296
chr17	23843909	23844261	chr17-23843909-23844261
chr17	23854325	23854526	chr17-23854325-23854526
chr17	23857001	23857355	chr17-23857001-23857355
chr17	23924368	23925910	chr17-23924368-23925910
chr17	23973087	23973497	chr17-23973087-23973497
chr17	23993292	24001310	chr17-23993292-24001310
chr17	24007442	24008265	chr17-24007442-24008265
chr17	24012860	24013061	chr17-24012860-24013061
chr17	24021689	24022202	chr17-24021689-24022202
chr17	24034826	24035821	chr17-24034826-24035821
chr17	24036917	24039498	chr17-24036917-24039498
chr17	24068360	24068614	chr17-24068360-24068614
chr17	24069674	24073741	chr17-24069674-24073741
chr17	24140138	24142472	chr17-24140138-24142472
chr17	24151628	24153025	chr17-24151628-24153025
chr17	24155051	24155654	chr17-24155051-24155654
chr17	24161333	24164009	chr17-24161333-24164009
chr17	24164958	24165316	chr17-24164958-24165316
chr17	24167224	24170562	chr17-24167224-24170562
chr17	24200251	24201330	chr17-24200251-24201330
chr17	24204066	24207138	chr17-24204066-24207138
chr17	24208654	24209648	chr17-24208654-24209648
chr17	24211895	24212355	chr17-24211895-24212355
chr17	24219292	24221325	chr17-24219292-24221325
chr17	24243863	24245060	chr17-24243863-24245060
chr17	24249440	24252450	chr17-24249440-24252450
chr17	24350783	24354534	chr17-24350783-24354534

chr17	24413006	24416982	chr17-24413006-24416982
chr17	24425997	24427829	chr17-24425997-24427829
chr17	24453840	24456189	chr17-24453840-24456189
chr17	24469399	24473231	chr17-24469399-24473231
chr17	24478015	24479777	chr17-24478015-24479777
chr17	24488042	24489704	chr17-24488042-24489704
chr17	24524781	24530904	chr17-24524781-24530904
chr17	24533140	24533862	chr17-24533140-24533862
chr17	24537400	24537713	chr17-24537400-24537713
chr17	24544733	24544934	chr17-24544733-24544934
chr17	24548433	24551726	chr17-24548433-24551726
chr17	24567003	24567492	chr17-24567003-24567492
chr17	24631695	24633722	chr17-24631695-24633722
chr17	24643825	24646102	chr17-24643825-24646102
chr17	24648913	24650856	chr17-24648913-24650856
chr17	24653838	24654203	chr17-24653838-24654203
chr17	24657860	24658671	chr17-24657860-24658671
chr17	24667087	24673401	chr17-24667087-24673401
chr17	24687096	24687813	chr17-24687096-24687813
chr17	24688882	24690326	chr17-24688882-24690326
chr17	24694518	24697191	chr17-24694518-24697191
chr17	24706022	24708567	chr17-24706022-24708567
chr17	24718118	24724891	chr17-24718118-24724891
chr17	24735675	24737742	chr17-24735675-24737742
chr17	24752140	24753648	chr17-24752140-24753648
chr17	24803743	24805057	chr17-24803743-24805057
chr17	24848577	24852052	chr17-24848577-24852052
chr17	24855717	24856989	chr17-24855717-24856989
chr17	24859975	24860236	chr17-24859975-24860236
chr17	24874102	24874354	chr17-24874102-24874354
chr17	24884623	24888181	chr17-24884623-24888181
chr17	24893847	24898073	chr17-24893847-24898073
chr17	24929347	24929762	chr17-24929347-24929762
chr17	24934764	24937434	chr17-24934764-24937434
chr17	24958953	24961441	chr17-24958953-24961441
chr17	25013017	25017600	chr17-25013017-25017600
chr17	25114733	25116447	chr17-25114733-25116447
chr17	25132946	25134874	chr17-25132946-25134874
chr17	25165879	25166830	chr17-25165879-25166830
chr17	25183237	25185901	chr17-25183237-25185901
chr17	25187746	25190048	chr17-25187746-25190048
chr17	25221280	25224025	chr17-25221280-25224025

chr17	25239222	25241640	chr17-25239222-25241640
chr17	25256086	25256402	chr17-25256086-25256402
chr17	25269540	25274945	chr17-25269540-25274945
chr17	25432626	25434569	chr17-25432626-25434569
chr17	25471558	25472143	chr17-25471558-25472143
chr17	25474214	25475116	chr17-25474214-25475116
chr17	25496928	25497288	chr17-25496928-25497288
chr17	25539316	25539980	chr17-25539316-25539980
chr17	25556221	25556977	chr17-25556221-25556977
chr17	25560715	25560916	chr17-25560715-25560916
chr17	25569887	25571697	chr17-25569887-25571697
chr17	25578478	25580103	chr17-25578478-25580103
chr17	25716738	25717998	chr17-25716738-25717998
chr17	25726121	25729123	chr17-25726121-25729123
chr17	25735440	25736164	chr17-25735440-25736164
chr17	25744547	25745085	chr17-25744547-25745085
chr17	25746553	25758528	chr17-25746553-25758528
chr17	25773331	25775209	chr17-25773331-25775209
chr17	25783909	25786010	chr17-25783909-25786010
chr17	25790596	25793424	chr17-25790596-25793424
chr17	25795108	25797701	chr17-25795108-25797701
chr17	25808339	25810291	chr17-25808339-25810291
chr17	25819020	25820047	chr17-25819020-25820047
chr17	25822955	25825531	chr17-25822955-25825531
chr17	25828128	25835329	chr17-25828128-25835329
chr17	25836767	25837322	chr17-25836767-25837322
chr17	25843055	25845279	chr17-25843055-25845279
chr17	25860417	25862235	chr17-25860417-25862235
chr17	25866935	25869428	chr17-25866935-25869428
chr17	25874015	25877147	chr17-25874015-25877147
chr17	25917116	25921308	chr17-25917116-25921308
chr17	25940698	25943051	chr17-25940698-25943051
chr17	25951086	25952616	chr17-25951086-25952616
chr17	25979842	25980310	chr17-25979842-25980310
chr17	25984164	25986689	chr17-25984164-25986689
chr17	26011869	26012281	chr17-26011869-26012281
chr17	26066124	26070418	chr17-26066124-26070418
chr17	26089320	26090757	chr17-26089320-26090757
chr17	26094512	26095891	chr17-26094512-26095891
chr17	26112869	26114295	chr17-26112869-26114295
chr17	26120107	26120644	chr17-26120107-26120644
chr17	26122953	26125681	chr17-26122953-26125681

chr17	26137946	26140712	chr17-26137946-26140712
chr17	26142303	26143693	chr17-26142303-26143693
chr17	26200050	26204207	chr17-26200050-26204207
chr17	26225019	26225903	chr17-26225019-26225903
chr17	26242945	26245038	chr17-26242945-26245038
chr17	26252136	26254797	chr17-26252136-26254797
chr17	26413904	26416154	chr17-26413904-26416154
chr17	26451800	26452152	chr17-26451800-26452152
chr17	26502799	26509792	chr17-26502799-26509792
chr17	26514383	26515284	chr17-26514383-26515284
chr17	26529863	26530376	chr17-26529863-26530376
chr17	26535881	26538622	chr17-26535881-26538622
chr17	26548158	26550248	chr17-26548158-26550248
chr17	26560377	26565097	chr17-26560377-26565097
chr17	26571462	26572139	chr17-26571462-26572139
chr17	26578022	26578656	chr17-26578022-26578656
chr17	26583190	26585649	chr17-26583190-26585649
chr17	26592426	26592987	chr17-26592426-26592987
chr17	26595382	26595583	chr17-26595382-26595583
chr17	26597096	26597297	chr17-26597096-26597297
chr17	26601261	26602592	chr17-26601261-26602592
chr17	26604639	26606469	chr17-26604639-26606469
chr17	26608394	26609093	chr17-26608394-26609093
chr17	26609940	26612539	chr17-26609940-26612539
chr17	26613976	26615398	chr17-26613976-26615398
chr17	26616897	26622150	chr17-26616897-26622150
chr17	26658802	26659966	chr17-26658802-26659966
chr17	26662864	26664066	chr17-26662864-26664066
chr17	26675904	26678505	chr17-26675904-26678505
chr17	26715009	26717250	chr17-26715009-26717250
chr17	26780298	26782103	chr17-26780298-26782103
chr17	26836990	26837299	chr17-26836990-26837299
chr17	26838871	26839895	chr17-26838871-26839895
chr17	26841056	26841670	chr17-26841056-26841670
chr17	26844360	26844983	chr17-26844360-26844983
chr17	26849494	26851232	chr17-26849494-26851232
chr17	26884639	26886598	chr17-26884639-26886598
chr17	26916511	26918848	chr17-26916511-26918848
chr17	26932283	26934463	chr17-26932283-26934463
chr17	26937364	26941607	chr17-26937364-26941607
chr17	26946450	26947068	chr17-26946450-26947068
chr17	26948151	26949493	chr17-26948151-26949493

chr17	26951542	26951743	chr17-26951542-26951743
chr17	26953689	26954057	chr17-26953689-26954057
chr17	26971955	26974477	chr17-26971955-26974477
chr17	27017556	27018986	chr17-27017556-27018986
chr17	27027123	27029184	chr17-27027123-27029184
chr17	27032684	27033344	chr17-27032684-27033344
chr17	27037749	27041858	chr17-27037749-27041858
chr17	27042915	27044439	chr17-27042915-27044439
chr17	27046794	27047239	chr17-27046794-27047239
chr17	27048141	27049440	chr17-27048141-27049440
chr17	27050377	27052495	chr17-27050377-27052495
chr17	27055797	27061346	chr17-27055797-27061346
chr17	27062579	27064248	chr17-27062579-27064248
chr17	27069184	27069707	chr17-27069184-27069707
chr17	27075837	27076038	chr17-27075837-27076038
chr17	27081668	27085087	chr17-27081668-27085087
chr17	27131733	27135464	chr17-27131733-27135464
chr17	27141517	27142797	chr17-27141517-27142797
chr17	27146140	27146505	chr17-27146140-27146505
chr17	27160865	27161273	chr17-27160865-27161273
chr17	27202463	27205599	chr17-27202463-27205599
chr17	27212597	27213055	chr17-27212597-27213055
chr17	27511842	27513972	chr17-27511842-27513972
chr17	27520617	27521126	chr17-27520617-27521126
chr17	27534855	27535176	chr17-27534855-27535176
chr17	27543343	27548106	chr17-27543343-27548106
chr17	27554336	27566690	chr17-27554336-27566690
chr17	27621136	27624383	chr17-27621136-27624383
chr17	27631093	27635973	chr17-27631093-27635973
chr17	27655000	27656268	chr17-27655000-27656268
chr17	27684752	27685958	chr17-27684752-27685958
chr17	27702494	27703011	chr17-27702494-27703011
chr17	27707717	27708783	chr17-27707717-27708783
chr17	27716626	27717560	chr17-27716626-27717560
chr17	27795575	27795832	chr17-27795575-27795832
chr17	27796635	27798130	chr17-27796635-27798130
chr17	27799709	27801298	chr17-27799709-27801298
chr17	27818064	27821699	chr17-27818064-27821699
chr17	27839137	27841312	chr17-27839137-27841312
chr17	27856082	27858605	chr17-27856082-27858605
chr17	27906290	27911618	chr17-27906290-27911618
chr17	27964829	27965694	chr17-27964829-27965694

chr17	27976070	27976872	chr17-27976070-27976872
chr17	27993541	27994756	chr17-27993541-27994756
chr17	28013089	28014048	chr17-28013089-28014048
chr17	28025356	28026499	chr17-28025356-28026499
chr17	28079424	28080945	chr17-28079424-28080945
chr17	28085134	28085486	chr17-28085134-28085486
chr17	28124663	28124915	chr17-28124663-28124915
chr17	28141265	28143706	chr17-28141265-28143706
chr17	28176283	28178345	chr17-28176283-28178345
chr17	28193661	28194009	chr17-28193661-28194009
chr17	28207706	28208263	chr17-28207706-28208263
chr17	28222976	28224674	chr17-28222976-28224674
chr17	28225686	28226498	chr17-28225686-28226498
chr17	28232346	28237238	chr17-28232346-28237238
chr17	28251387	28252178	chr17-28251387-28252178
chr17	28255358	28258185	chr17-28255358-28258185
chr17	28259351	28260895	chr17-28259351-28260895
chr17	28264104	28264509	chr17-28264104-28264509
chr17	28268731	28274932	chr17-28268731-28274932
chr17	28279215	28281150	chr17-28279215-28281150
chr17	28308559	28309020	chr17-28308559-28309020
chr17	28312823	28314709	chr17-28312823-28314709
chr17	28327412	28332708	chr17-28327412-28332708
chr17	28339958	28352261	chr17-28339958-28352261
chr17	28368369	28368994	chr17-28368369-28368994
chr17	28375077	28376255	chr17-28375077-28376255
chr17	28463637	28464482	chr17-28463637-28464482
chr17	28484712	28486765	chr17-28484712-28486765
chr17	28511101	28511302	chr17-28511101-28511302
chr17	28512866	28513067	chr17-28512866-28513067
chr17	28515968	28516271	chr17-28515968-28516271
chr17	28517471	28518839	chr17-28517471-28518839
chr17	28522240	28524257	chr17-28522240-28524257
chr17	28527844	28528106	chr17-28527844-28528106
chr17	28575351	28575933	chr17-28575351-28575933
chr17	28619351	28623056	chr17-28619351-28623056
chr17	28689404	28694043	chr17-28689404-28694043
chr17	28697661	28698137	chr17-28697661-28698137
chr17	28766383	28767064	chr17-28766383-28767064
chr17	28768839	28771638	chr17-28768839-28771638
chr17	28793141	28793342	chr17-28793141-28793342
chr17	28799812	28802726	chr17-28799812-28802726

chr17	28882377	28883026	chr17-28882377-28883026
chr17	28904611	28904812	chr17-28904611-28904812
chr17	28930193	28931248	chr17-28930193-28931248
chr17	28951402	28954107	chr17-28951402-28954107
chr17	28983041	28983242	chr17-28983041-28983242
chr17	29006624	29008634	chr17-29006624-29008634
chr17	29031299	29037376	chr17-29031299-29037376
chr17	29038236	29038682	chr17-29038236-29038682
chr17	29078139	29079391	chr17-29078139-29079391
chr17	29093204	29097776	chr17-29093204-29097776
chr17	29155710	29156006	chr17-29155710-29156006
chr17	29164342	29166815	chr17-29164342-29166815
chr17	29169298	29170039	chr17-29169298-29170039
chr17	29185328	29185794	chr17-29185328-29185794
chr17	29237295	29238759	chr17-29237295-29238759
chr17	29259512	29264915	chr17-29259512-29264915
chr17	29267894	29270531	chr17-29267894-29270531
chr17	29322659	29322860	chr17-29322659-29322860
chr17	29341739	29341940	chr17-29341739-29341940
chr17	29345876	29348616	chr17-29345876-29348616
chr17	29356703	29357012	chr17-29356703-29357012
chr17	29368897	29369564	chr17-29368897-29369564
chr17	29397966	29398326	chr17-29397966-29398326
chr17	29401571	29402431	chr17-29401571-29402431
chr17	29428057	29428503	chr17-29428057-29428503
chr17	29436446	29440649	chr17-29436446-29440649
chr17	29450220	29452153	chr17-29450220-29452153
chr17	29457851	29460500	chr17-29457851-29460500
chr17	29488992	29495166	chr17-29488992-29495166
chr17	29549201	29551348	chr17-29549201-29551348
chr17	29599620	29600627	chr17-29599620-29600627
chr17	29614021	29616916	chr17-29614021-29616916
chr17	29659983	29662061	chr17-29659983-29662061
chr17	29715988	29717657	chr17-29715988-29717657
chr17	29813309	29813615	chr17-29813309-29813615
chr17	29841639	29842629	chr17-29841639-29842629
chr17	29886543	29889991	chr17-29886543-29889991
chr17	29928545	29929525	chr17-29928545-29929525
chr17	29934346	29934612	chr17-29934346-29934612
chr17	29950737	29951270	chr17-29950737-29951270
chr17	29963828	29964029	chr17-29963828-29964029
chr17	29993550	29994654	chr17-29993550-29994654

chr17	30003319	30007561	chr17-30003319-30007561
chr17	30068357	30068685	chr17-30068357-30068685
chr17	30073309	30073568	chr17-30073309-30073568
chr17	30550226	30551200	chr17-30550226-30551200
chr17	30570070	30571666	chr17-30570070-30571666
chr17	30574703	30577738	chr17-30574703-30577738
chr17	30581288	30581639	chr17-30581288-30581639
chr17	30611363	30613097	chr17-30611363-30613097
chr17	30635415	30635709	chr17-30635415-30635709
chr17	30657844	30658752	chr17-30657844-30658752
chr17	30901277	30902198	chr17-30901277-30902198
chr17	30990180	30990381	chr17-30990180-30990381
chr17	31057248	31059282	chr17-31057248-31059282
chr17	31073185	31074462	chr17-31073185-31074462
chr17	31276697	31277666	chr17-31276697-31277666
chr17	31295210	31298099	chr17-31295210-31298099
chr17	31304659	31306569	chr17-31304659-31306569
chr17	31312658	31313269	chr17-31312658-31313269
chr17	31319324	31319802	chr17-31319324-31319802
chr17	31326261	31326858	chr17-31326261-31326858
chr17	31385868	31387079	chr17-31385868-31387079
chr17	31408199	31408400	chr17-31408199-31408400
chr17	31481265	31481518	chr17-31481265-31481518
chr17	31510963	31513177	chr17-31510963-31513177
chr17	31519453	31521367	chr17-31519453-31521367
chr17	31529519	31529923	chr17-31529519-31529923
chr17	31563556	31566722	chr17-31563556-31566722
chr17	31632690	31633299	chr17-31632690-31633299
chr17	31636315	31637541	chr17-31636315-31637541
chr17	31656628	31659445	chr17-31656628-31659445
chr17	31687853	31688324	chr17-31687853-31688324
chr17	31693299	31693877	chr17-31693299-31693877
chr17	31728979	31729341	chr17-31728979-31729341
chr17	31746582	31748042	chr17-31746582-31748042
chr17	31788532	31789445	chr17-31788532-31789445
chr17	31808066	31808780	chr17-31808066-31808780
chr17	31823733	31824086	chr17-31823733-31824086
chr17	31834297	31835386	chr17-31834297-31835386
chr17	31842160	31842531	chr17-31842160-31842531
chr17	31852866	31859067	chr17-31852866-31859067
chr17	31892712	31893321	chr17-31892712-31893321
chr17	31896394	31898195	chr17-31896394-31898195

chr17	31914596	31914904	chr17-31914596-31914904
chr17	31920879	31921140	chr17-31920879-31921140
chr17	31938429	31940125	chr17-31938429-31940125
chr17	31954222	31955520	chr17-31954222-31955520
chr17	31982149	31983083	chr17-31982149-31983083
chr17	32035530	32038309	chr17-32035530-32038309
chr17	32090989	32092338	chr17-32090989-32092338
chr17	32099729	32100789	chr17-32099729-32100789
chr17	32113444	32114467	chr17-32113444-32114467
chr17	32117528	32118660	chr17-32117528-32118660
chr17	32121976	32122292	chr17-32121976-32122292
chr17	32143263	32143770	chr17-32143263-32143770
chr17	32161791	32163150	chr17-32161791-32163150
chr17	32165496	32167238	chr17-32165496-32167238
chr17	32281681	32285971	chr17-32281681-32285971
chr17	32319048	32322455	chr17-32319048-32322455
chr17	32349145	32351458	chr17-32349145-32351458
chr17	32362656	32365333	chr17-32362656-32365333
chr17	32368450	32371664	chr17-32368450-32371664
chr17	32388330	32389920	chr17-32388330-32389920
chr17	32396190	32396862	chr17-32396190-32396862
chr17	32452593	32452949	chr17-32452593-32452949
chr17	32474990	32475381	chr17-32474990-32475381
chr17	32482995	32483505	chr17-32482995-32483505
chr17	32492333	32492821	chr17-32492333-32492821
chr17	32505859	32507514	chr17-32505859-32507514
chr17	32527987	32529212	chr17-32527987-32529212
chr17	32536261	32537493	chr17-32536261-32537493
chr17	32634736	32635347	chr17-32634736-32635347
chr17	32676299	32676500	chr17-32676299-32676500
chr17	32702614	32703152	chr17-32702614-32703152
chr17	32786717	32789278	chr17-32786717-32789278
chr17	32829164	32830716	chr17-32829164-32830716
chr17	32890505	32892324	chr17-32890505-32892324
chr17	32905411	32905718	chr17-32905411-32905718
chr17	32924637	32925300	chr17-32924637-32925300
chr17	32946243	32948026	chr17-32946243-32948026
chr17	32964737	32967553	chr17-32964737-32967553
chr17	32992497	32994277	chr17-32992497-32994277
chr17	33031787	33033879	chr17-33031787-33033879
chr17	33088808	33090232	chr17-33088808-33090232
chr17	33092046	33093495	chr17-33092046-33093495

chr17	33253687	33254333	chr17-33253687-33254333
chr17	33290262	33291055	chr17-33290262-33291055
chr17	33357920	33359533	chr17-33357920-33359533
chr17	33393023	33395245	chr17-33393023-33395245
chr17	33523402	33525521	chr17-33523402-33525521
chr17	33628649	33630480	chr17-33628649-33630480
chr17	33683027	33686022	chr17-33683027-33686022
chr17	33717597	33719291	chr17-33717597-33719291
chr17	33755280	33756398	chr17-33755280-33756398
chr17	33758780	33761057	chr17-33758780-33761057
chr17	33779537	33783570	chr17-33779537-33783570
chr17	33810233	33813223	chr17-33810233-33813223
chr17	33816899	33818533	chr17-33816899-33818533
chr17	33821976	33826316	chr17-33821976-33826316
chr17	33842670	33844379	chr17-33842670-33844379
chr17	33888197	33891262	chr17-33888197-33891262
chr17	33907570	33910951	chr17-33907570-33910951
chr17	33915061	33921248	chr17-33915061-33921248
chr17	33928308	33932232	chr17-33928308-33932232
chr17	33938824	33941928	chr17-33938824-33941928
chr17	33949900	33957015	chr17-33949900-33957015
chr17	33968660	33968929	chr17-33968660-33968929
chr17	33977567	33979425	chr17-33977567-33979425
chr17	33980694	33982342	chr17-33980694-33982342
chr17	33998716	34000882	chr17-33998716-34000882
chr17	34023692	34033849	chr17-34023692-34033849
chr17	34075933	34076341	chr17-34075933-34076341
chr17	34101705	34101956	chr17-34101705-34101956
chr17	34114390	34125675	chr17-34114390-34125675
chr17	34135460	34136026	chr17-34135460-34136026
chr17	34139098	34140476	chr17-34139098-34140476
chr17	34153176	34153478	chr17-34153176-34153478
chr17	34186494	34188496	chr17-34186494-34188496
chr17	34197178	34198855	chr17-34197178-34198855
chr17	34204291	34205723	chr17-34204291-34205723
chr17	34263099	34265288	chr17-34263099-34265288
chr17	34296269	34296470	chr17-34296269-34296470
chr17	34309499	34310129	chr17-34309499-34310129
chr17	34392204	34392722	chr17-34392204-34392722
chr17	34398629	34399353	chr17-34398629-34399353
chr17	34543542	34544324	chr17-34543542-34544324
chr17	34564035	34564683	chr17-34564035-34564683

chr17	34586332	34588537	chr17-34586332-34588537
chr17	34589828	34590029	chr17-34589828-34590029
chr17	34591111	34594496	chr17-34591111-34594496
chr17	34601814	34607697	chr17-34601814-34607697
chr17	34626054	34629165	chr17-34626054-34629165
chr17	34630692	34632762	chr17-34630692-34632762
chr17	34644370	34648814	chr17-34644370-34648814
chr17	34655057	34656523	chr17-34655057-34656523
chr17	34660916	34661789	chr17-34660916-34661789
chr17	34671361	34672577	chr17-34671361-34672577
chr17	34683858	34684574	chr17-34683858-34684574
chr17	34685503	34685704	chr17-34685503-34685704
chr17	34835726	34838720	chr17-34835726-34838720
chr17	34849389	34851619	chr17-34849389-34851619
chr17	34893622	34900547	chr17-34893622-34900547
chr17	34914204	34916193	chr17-34914204-34916193
chr17	34930787	34932967	chr17-34930787-34932967
chr17	34941647	34941970	chr17-34941647-34941970
chr17	34944036	34944494	chr17-34944036-34944494
chr17	34949393	34953409	chr17-34949393-34953409
chr17	34957036	34961687	chr17-34957036-34961687
chr17	34965050	34966216	chr17-34965050-34966216
chr17	34970429	34973080	chr17-34970429-34973080
chr17	34980820	34983657	chr17-34980820-34983657
chr17	34995626	35004688	chr17-34995626-35004688
chr17	35016061	35017379	chr17-35016061-35017379
chr17	35043309	35053206	chr17-35043309-35053206
chr17	35058412	35060591	chr17-35058412-35060591
chr17	35064217	35065953	chr17-35064217-35065953
chr17	35067803	35069192	chr17-35067803-35069192
chr17	35077729	35078350	chr17-35077729-35078350
chr17	35088730	35090715	chr17-35088730-35090715
chr17	35119945	35122654	chr17-35119945-35122654
chr17	35125476	35127967	chr17-35125476-35127967
chr17	35134230	35137667	chr17-35134230-35137667
chr17	35161994	35166302	chr17-35161994-35166302
chr17	35185676	35187084	chr17-35185676-35187084
chr17	35195254	35196576	chr17-35195254-35196576
chr17	35198713	35200626	chr17-35198713-35200626
chr17	35225377	35225936	chr17-35225377-35225936
chr17	35233559	35236887	chr17-35233559-35236887
chr17	35240425	35245386	chr17-35240425-35245386

chr17	35257721	35258616	chr17-35257721-35258616
chr17	35262402	35264714	chr17-35262402-35264714
chr17	35278181	35278599	chr17-35278181-35278599
chr17	35301664	35302611	chr17-35301664-35302611
chr17	35320388	35321500	chr17-35320388-35321500
chr17	35342046	35343591	chr17-35342046-35343591
chr17	35359075	35359858	chr17-35359075-35359858
chr17	35379073	35380767	chr17-35379073-35380767
chr17	35393893	35394997	chr17-35393893-35394997
chr17	35424647	35425994	chr17-35424647-35425994
chr17	35438761	35440333	chr17-35438761-35440333
chr17	35469705	35471191	chr17-35469705-35471191
chr17	35514376	35518735	chr17-35514376-35518735
chr17	35532543	35533252	chr17-35532543-35533252
chr17	35549512	35550265	chr17-35549512-35550265
chr17	35551714	35552468	chr17-35551714-35552468
chr17	35555641	35557903	chr17-35555641-35557903
chr17	35584418	35584831	chr17-35584418-35584831
chr17	35589969	35591906	chr17-35589969-35591906
chr17	35595876	35596077	chr17-35595876-35596077
chr17	35601038	35602385	chr17-35601038-35602385
chr17	35633441	35639403	chr17-35633441-35639403
chr17	35648996	35650680	chr17-35648996-35650680
chr17	35664445	35668635	chr17-35664445-35668635
chr17	35672586	35674301	chr17-35672586-35674301
chr17	35696720	35702557	chr17-35696720-35702557
chr17	35704538	35704739	chr17-35704538-35704739
chr17	35725680	35726242	chr17-35725680-35726242
chr17	35737681	35738086	chr17-35737681-35738086
chr17	35739524	35739827	chr17-35739524-35739827
chr17	35743714	35744528	chr17-35743714-35744528
chr17	35763484	35763914	chr17-35763484-35763914
chr17	35769575	35772425	chr17-35769575-35772425
chr17	35775696	35777949	chr17-35775696-35777949
chr17	35796058	35796259	chr17-35796058-35796259
chr17	35808137	35808722	chr17-35808137-35808722
chr17	35810948	35817578	chr17-35810948-35817578
chr17	35819335	35825212	chr17-35819335-35825212
chr17	35829348	35830293	chr17-35829348-35830293
chr17	35833951	35839559	chr17-35833951-35839559
chr17	35840672	35843189	chr17-35840672-35843189
chr17	35860564	35862209	chr17-35860564-35862209

chr17	35864915	35875456	chr17-35864915-35875456
chr17	35879135	35881376	chr17-35879135-35881376
chr17	35895677	35898332	chr17-35895677-35898332
chr17	35908887	35910444	chr17-35908887-35910444
chr17	35914740	35920637	chr17-35914740-35920637
chr17	35968220	35970491	chr17-35968220-35970491
chr17	35978183	35981529	chr17-35978183-35981529
chr17	35993718	35995851	chr17-35993718-35995851
chr17	35997491	35998149	chr17-35997491-35998149
chr17	36000821	36002656	chr17-36000821-36002656
chr17	36023714	36024384	chr17-36023714-36024384
chr17	36031192	36033043	chr17-36031192-36033043
chr17	36041715	36043790	chr17-36041715-36043790
chr17	36082717	36084488	chr17-36082717-36084488
chr17	36110723	36111925	chr17-36110723-36111925
chr17	36120712	36122039	chr17-36120712-36122039
chr17	36144795	36146084	chr17-36144795-36146084
chr17	36157795	36158331	chr17-36157795-36158331
chr17	36161032	36162137	chr17-36161032-36162137
chr17	36167213	36168685	chr17-36167213-36168685
chr17	36178910	36179417	chr17-36178910-36179417
chr17	36205223	36206478	chr17-36205223-36206478
chr17	36235247	36235551	chr17-36235247-36235551
chr17	36237339	36238054	chr17-36237339-36238054
chr17	36241562	36241867	chr17-36241562-36241867
chr17	36252219	36252575	chr17-36252219-36252575
chr17	36256553	36258247	chr17-36256553-36258247
chr17	36270404	36273819	chr17-36270404-36273819
chr17	36314648	36315912	chr17-36314648-36315912
chr17	36826002	36826203	chr17-36826002-36826203
chr17	36836262	36838335	chr17-36836262-36838335
chr17	36866011	36867691	chr17-36866011-36867691
chr17	36874523	36874776	chr17-36874523-36874776
chr17	36915179	36915534	chr17-36915179-36915534
chr17	36919274	36920984	chr17-36919274-36920984
chr17	36927593	36928081	chr17-36927593-36928081
chr17	36931266	36932012	chr17-36931266-36932012
chr17	36937933	36938134	chr17-36937933-36938134
chr17	36942214	36945713	chr17-36942214-36945713
chr17	36947004	36947557	chr17-36947004-36947557
chr17	36949423	36952246	chr17-36949423-36952246
chr17	36957469	36959890	chr17-36957469-36959890

chr17	37002182	37003702	chr17-37002182-37003702
chr17	37028390	37029251	chr17-37028390-37029251
chr17	37044894	37047705	chr17-37044894-37047705
chr17	37050562	37051010	chr17-37050562-37051010
chr17	37196783	37197795	chr17-37196783-37197795
chr17	37211216	37211731	chr17-37211216-37211731
chr17	37246850	37247051	chr17-37246850-37247051
chr17	37269879	37271505	chr17-37269879-37271505
chr17	37995849	37996213	chr17-37995849-37996213
chr17	40875820	40881452	chr17-40875820-40881452
chr17	40883872	40884543	chr17-40883872-40884543
chr17	40885416	40885833	chr17-40885416-40885833
chr17	40933625	40935770	chr17-40933625-40935770
chr17	42315652	42317304	chr17-42315652-42317304
chr17	42576928	42577129	chr17-42576928-42577129
chr17	42580423	42580887	chr17-42580423-42580887
chr17	42873133	42877288	chr17-42873133-42877288
chr17	42883372	42883623	chr17-42883372-42883623
chr17	42891922	42892798	chr17-42891922-42892798
chr17	42894262	42895694	chr17-42894262-42895694
chr17	42905138	42905556	chr17-42905138-42905556
chr17	42908008	42910803	chr17-42908008-42910803
chr17	42933679	42933880	chr17-42933679-42933880
chr17	42938193	42940604	chr17-42938193-42940604
chr17	42982117	42982423	chr17-42982117-42982423
chr17	43016319	43018346	chr17-43016319-43018346
chr17	43023498	43024191	chr17-43023498-43024191
chr17	43077180	43077431	chr17-43077180-43077431
chr17	43130534	43130798	chr17-43130534-43130798
chr17	43138629	43138830	chr17-43138629-43138830
chr17	43225319	43225520	chr17-43225319-43225520
chr17	43226432	43226696	chr17-43226432-43226696
chr17	43265340	43265658	chr17-43265340-43265658
chr17	43326670	43327725	chr17-43326670-43327725
chr17	43329746	43329947	chr17-43329746-43329947
chr17	43360284	43361564	chr17-43360284-43361564
chr17	43388620	43392987	chr17-43388620-43392987
chr17	43406519	43409036	chr17-43406519-43409036
chr17	43518856	43519173	chr17-43518856-43519173
chr17	43567726	43569165	chr17-43567726-43569165
chr17	43629249	43630478	chr17-43629249-43630478
chr17	43666077	43669707	chr17-43666077-43669707

chr17	43801194	43802555	chr17-43801194-43802555
chr17	43916356	43916660	chr17-43916356-43916660
chr17	44013780	44013981	chr17-44013780-44013981
chr17	44075733	44076095	chr17-44075733-44076095
chr17	44078411	44079555	chr17-44078411-44079555
chr17	44100881	44101434	chr17-44100881-44101434
chr17	44104865	44106484	chr17-44104865-44106484
chr17	44136091	44136292	chr17-44136091-44136292
chr17	44162623	44162943	chr17-44162623-44162943
chr17	44165745	44165946	chr17-44165745-44165946
chr17	44188298	44189392	chr17-44188298-44189392
chr17	44238701	44240425	chr17-44238701-44240425
chr17	44300856	44301926	chr17-44300856-44301926
chr17	44350289	44350825	chr17-44350289-44350825
chr17	44458848	44459358	chr17-44458848-44459358
chr17	44500237	44500540	chr17-44500237-44500540
chr17	44502443	44502750	chr17-44502443-44502750
chr17	44623918	44624169	chr17-44623918-44624169
chr17	44709634	44711267	chr17-44709634-44711267
chr17	44733492	44738615	chr17-44733492-44738615
chr17	44767715	44768019	chr17-44767715-44768019
chr17	44776528	44778480	chr17-44776528-44778480
chr17	45251367	45251673	chr17-45251367-45251673
chr17	45334929	45335184	chr17-45334929-45335184
chr17	45359779	45360036	chr17-45359779-45360036
chr17	45431966	45434982	chr17-45431966-45434982
chr17	45474388	45475297	chr17-45474388-45475297
chr17	45506448	45507800	chr17-45506448-45507800
chr17	45543913	45546002	chr17-45543913-45546002
chr17	45548849	45550263	chr17-45548849-45550263
chr17	45555448	45558732	chr17-45555448-45558732
chr17	45563391	45566749	chr17-45563391-45566749
chr17	45568233	45568485	chr17-45568233-45568485
chr17	45569470	45574813	chr17-45569470-45574813
chr17	45593471	45597334	chr17-45593471-45597334
chr17	45599141	45600051	chr17-45599141-45600051
chr17	45626572	45627075	chr17-45626572-45627075
chr17	45630062	45630775	chr17-45630062-45630775
chr17	45684360	45690286	chr17-45684360-45690286
chr17	45691228	45697215	chr17-45691228-45697215
chr17	45723860	45724576	chr17-45723860-45724576
chr17	45726168	45726576	chr17-45726168-45726576

chr17	45733821	45737610	chr17-45733821-45737610
chr17	45766544	45768486	chr17-45766544-45768486
chr17	45776088	45777963	chr17-45776088-45777963
chr17	45784658	45785600	chr17-45784658-45785600
chr17	45795108	45795409	chr17-45795108-45795409
chr17	45798621	45799052	chr17-45798621-45799052
chr17	45850683	45852972	chr17-45850683-45852972
chr17	45867380	45867686	chr17-45867380-45867686
chr17	45872370	45874523	chr17-45872370-45874523
chr17	45889354	45889768	chr17-45889354-45889768
chr17	45943411	45943814	chr17-45943411-45943814
chr17	45944912	45945456	chr17-45944912-45945456
chr17	46003740	46004760	chr17-46003740-46004760
chr17	46026014	46033290	chr17-46026014-46033290
chr17	46036447	46036838	chr17-46036447-46036838
chr17	46073656	46074721	chr17-46073656-46074721
chr17	46110256	46112185	chr17-46110256-46112185
chr17	46132753	46133071	chr17-46132753-46133071
chr17	46143560	46144760	chr17-46143560-46144760
chr17	46151849	46154015	chr17-46151849-46154015
chr17	46159553	46164030	chr17-46159553-46164030
chr17	46201173	46204720	chr17-46201173-46204720
chr17	46246692	46249436	chr17-46246692-46249436
chr17	46252904	46254795	chr17-46252904-46254795
chr17	46265630	46268407	chr17-46265630-46268407
chr17	46269805	46270467	chr17-46269805-46270467
chr17	46273325	46274869	chr17-46273325-46274869
chr17	46277705	46278635	chr17-46277705-46278635
chr17	46280769	46283523	chr17-46280769-46283523
chr17	46297226	46299183	chr17-46297226-46299183
chr17	46327088	46329105	chr17-46327088-46329105
chr17	46383000	46385079	chr17-46383000-46385079
chr17	46428969	46429438	chr17-46428969-46429438
chr17	46433655	46434342	chr17-46433655-46434342
chr17	46437015	46437216	chr17-46437015-46437216
chr17	46445141	46445505	chr17-46445141-46445505
chr17	46454221	46456039	chr17-46454221-46456039
chr17	46460532	46461447	chr17-46460532-46461447
chr17	46483563	46484506	chr17-46483563-46484506
chr17	46487082	46487846	chr17-46487082-46487846
chr17	46496468	46498878	chr17-46496468-46498878
chr17	46507631	46507832	chr17-46507631-46507832

chr17	46545907	46546562	chr17-46545907-46546562
chr17	46552545	46557047	chr17-46552545-46557047
chr17	46592433	46593785	chr17-46592433-46593785
chr17	46609228	46612170	chr17-46609228-46612170
chr17	46618057	46619940	chr17-46618057-46619940
chr17	46623980	46625055	chr17-46623980-46625055
chr17	46626986	46630449	chr17-46626986-46630449
chr17	46636986	46637287	chr17-46636986-46637287
chr17	46644276	46647256	chr17-46644276-46647256
chr17	46649708	46652038	chr17-46649708-46652038
chr17	46672797	46675179	chr17-46672797-46675179
chr17	46677934	46682338	chr17-46677934-46682338
chr17	46698036	46699469	chr17-46698036-46699469
chr17	46702943	46706021	chr17-46702943-46706021
chr17	46710616	46713536	chr17-46710616-46713536
chr17	46728440	46729513	chr17-46728440-46729513
chr17	46750714	46752910	chr17-46750714-46752910
chr17	46771570	46774274	chr17-46771570-46774274
chr17	46780877	46783589	chr17-46780877-46783589
chr17	46854545	46859236	chr17-46854545-46859236
chr17	46866972	46867640	chr17-46866972-46867640
chr17	46873446	46874848	chr17-46873446-46874848
chr17	46875952	46878065	chr17-46875952-46878065
chr17	46879618	46880411	chr17-46879618-46880411
chr17	46888726	46892959	chr17-46888726-46892959
chr17	46933264	46933515	chr17-46933264-46933515
chr17	47008536	47011330	chr17-47008536-47011330
chr17	47038977	47039543	chr17-47038977-47039543
chr17	47059877	47060268	chr17-47059877-47060268
chr17	47064762	47065366	chr17-47064762-47065366
chr17	47139192	47142368	chr17-47139192-47142368
chr17	47145148	47145405	chr17-47145148-47145405
chr17	47293647	47294675	chr17-47293647-47294675
chr17	47296570	47297498	chr17-47296570-47297498
chr17	47368228	47370371	chr17-47368228-47370371
chr17	47394522	47396262	chr17-47394522-47396262
chr17	47409852	47410869	chr17-47409852-47410869
chr17	47436100	47447251	chr17-47436100-47447251
chr17	47448083	47453925	chr17-47448083-47453925
chr17	47460400	47463314	chr17-47460400-47463314
chr17	47480303	47481355	chr17-47480303-47481355
chr17	47484185	47484926	chr17-47484185-47484926

chr17	47500983	47502724	chr17-47500983-47502724
chr17	47504415	47506376	chr17-47504415-47506376
chr17	47507611	47508799	chr17-47507611-47508799
chr17	47510971	47512142	chr17-47510971-47512142
chr17	47517867	47518693	chr17-47517867-47518693
chr17	47592811	47596670	chr17-47592811-47596670
chr17	47609708	47613100	chr17-47609708-47613100
chr17	47628225	47630965	chr17-47628225-47630965
chr17	47652037	47655008	chr17-47652037-47655008
chr17	47661905	47662106	chr17-47661905-47662106
chr17	47664579	47665500	chr17-47664579-47665500
chr17	47671373	47676704	chr17-47671373-47676704
chr17	47686651	47689904	chr17-47686651-47689904
chr17	47694084	47696105	chr17-47694084-47696105
chr17	47736833	47739560	chr17-47736833-47739560
chr17	47748419	47749311	chr17-47748419-47749311
chr17	47754459	47754710	chr17-47754459-47754710
chr17	47759601	47759802	chr17-47759601-47759802
chr17	47832850	47835150	chr17-47832850-47835150
chr17	47841907	47842485	chr17-47841907-47842485
chr17	47851802	47852516	chr17-47851802-47852516
chr17	47864297	47864760	chr17-47864297-47864760
chr17	47887929	47888332	chr17-47887929-47888332
chr17	47909349	47910098	chr17-47909349-47910098
chr17	47921315	47926580	chr17-47921315-47926580
chr17	47957645	47959185	chr17-47957645-47959185
chr17	47973537	47976604	chr17-47973537-47976604
chr17	48001176	48002878	chr17-48001176-48002878
chr17	48039511	48040426	chr17-48039511-48040426
chr17	48041250	48042782	chr17-48041250-48042782
chr17	48096799	48098886	chr17-48096799-48098886
chr17	48101002	48101464	chr17-48101002-48101464
chr17	48407226	48411677	chr17-48407226-48411677
chr17	48454765	48454966	chr17-48454765-48454966
chr17	48466985	48467711	chr17-48466985-48467711
chr17	48738253	48739649	chr17-48738253-48739649
chr17	48931623	48932187	chr17-48931623-48932187
chr17	49427864	49429724	chr17-49427864-49429724
chr17	49449057	49449426	chr17-49449057-49449426
chr17	49563630	49564662	chr17-49563630-49564662
chr17	49614577	49615957	chr17-49614577-49615957
chr17	49690646	49691931	chr17-49690646-49691931

chr17	49750491	49750908	chr17-49750491-49750908
chr17	49951923	49952124	chr17-49951923-49952124
chr17	50189695	50190853	chr17-50189695-50190853
chr17	50292500	50293356	chr17-50292500-50293356
chr17	50470377	50470578	chr17-50470377-50470578
chr17	50508470	50510295	chr17-50508470-50510295
chr17	50634176	50634377	chr17-50634176-50634377
chr17	50679466	50679771	chr17-50679466-50679771
chr17	50698425	50698889	chr17-50698425-50698889
chr17	51146638	51146994	chr17-51146638-51146994
chr17	51177654	51179929	chr17-51177654-51179929
chr17	51583216	51583621	chr17-51583216-51583621
chr17	51811852	51814002	chr17-51811852-51814002
chr17	51831463	51833713	chr17-51831463-51833713
chr17	52600733	52602918	chr17-52600733-52602918
chr17	53462671	53463911	chr17-53462671-53463911
chr17	53477985	53481492	chr17-53477985-53481492
chr17	53566122	53570224	chr17-53566122-53570224
chr17	53586900	53587778	chr17-53586900-53587778
chr17	53688155	53690299	chr17-53688155-53690299
chr17	54039813	54040075	chr17-54039813-54040075
chr17	54298142	54298868	chr17-54298142-54298868
chr17	55445315	55446302	chr17-55445315-55446302
chr17	55711715	55713104	chr17-55711715-55713104
chr17	55877162	55880110	chr17-55877162-55880110
chr17	55891069	55892851	chr17-55891069-55892851
chr17	55931609	55932734	chr17-55931609-55932734
chr17	55944236	55946241	chr17-55944236-55946241
chr17	55955871	55960409	chr17-55955871-55960409
chr17	55970247	55970877	chr17-55970247-55970877
chr17	55976167	55976914	chr17-55976167-55976914
chr17	55986248	55987059	chr17-55986248-55987059
chr17	56004732	56006369	chr17-56004732-56006369
chr17	56007198	56011134	chr17-56007198-56011134
chr17	56026180	56027028	chr17-56026180-56027028
chr17	56030784	56037454	chr17-56030784-56037454
chr17	56039671	56042118	chr17-56039671-56042118
chr17	56074003	56075663	chr17-56074003-56075663
chr17	56078296	56082087	chr17-56078296-56082087
chr17	56117053	56117722	chr17-56117053-56117722
chr17	56119832	56120963	chr17-56119832-56120963
chr17	56123830	56125205	chr17-56123830-56125205

chr17	56132617	56135129	chr17-56132617-56135129
chr17	56137912	56142624	chr17-56137912-56142624
chr17	56148636	56149301	chr17-56148636-56149301
chr17	56161830	56164706	chr17-56161830-56164706
chr17	56182673	56184785	chr17-56182673-56184785
chr17	56202232	56203231	chr17-56202232-56203231
chr17	56206207	56207379	chr17-56206207-56207379
chr17	56217095	56219861	chr17-56217095-56219861
chr17	56251328	56251529	chr17-56251328-56251529
chr17	56255433	56259741	chr17-56255433-56259741
chr17	56261164	56261492	chr17-56261164-56261492
chr17	56268970	56269786	chr17-56268970-56269786
chr17	56272587	56272788	chr17-56272587-56272788
chr17	56275765	56277838	chr17-56275765-56277838
chr17	56287403	56291817	chr17-56287403-56291817
chr17	56302683	56306764	chr17-56302683-56306764
chr17	56308107	56308510	chr17-56308107-56308510
chr17	56325055	56327565	chr17-56325055-56327565
chr17	56425008	56425966	chr17-56425008-56425966
chr17	56461361	56463688	chr17-56461361-56463688
chr17	56471961	56476697	chr17-56471961-56476697
chr17	56477645	56479596	chr17-56477645-56479596
chr17	56487469	56488051	chr17-56487469-56488051
chr17	56506491	56508324	chr17-56506491-56508324
chr17	56511146	56512055	chr17-56511146-56512055
chr17	56549888	56554984	chr17-56549888-56554984
chr17	56582181	56587605	chr17-56582181-56587605
chr17	56608285	56610736	chr17-56608285-56610736
chr17	56611915	56616231	chr17-56611915-56616231
chr17	56624888	56628325	chr17-56624888-56628325
chr17	56647801	56648152	chr17-56647801-56648152
chr17	56664064	56664478	chr17-56664064-56664478
chr17	56667801	56669231	chr17-56667801-56669231
chr17	56672359	56675111	chr17-56672359-56675111
chr17	56715530	56719830	chr17-56715530-56719830
chr17	56751411	56752000	chr17-56751411-56752000
chr17	56753517	56754161	chr17-56753517-56754161
chr17	56756701	56758358	chr17-56756701-56758358
chr17	56761586	56763083	chr17-56761586-56763083
chr17	56763903	56766039	chr17-56763903-56766039
chr17	56810081	56811009	chr17-56810081-56811009
chr17	56814136	56814536	chr17-56814136-56814536

chr17	56819279	56820711	chr17-56819279-56820711
chr17	56822016	56823176	chr17-56822016-56823176
chr17	56829289	56831881	chr17-56829289-56831881
chr17	56877136	56877702	chr17-56877136-56877702
chr17	56883592	56883974	chr17-56883592-56883974
chr17	56905476	56908732	chr17-56905476-56908732
chr17	56915812	56916448	chr17-56915812-56916448
chr17	56933364	56936217	chr17-56933364-56936217
chr17	56989331	56991595	chr17-56989331-56991595
chr17	56996720	56998185	chr17-56996720-56998185
chr17	57001054	57001467	chr17-57001054-57001467
chr17	57010140	57012566	chr17-57010140-57012566
chr17	57029349	57033093	chr17-57029349-57033093
chr17	57038253	57038955	chr17-57038253-57038955
chr17	57058772	57064015	chr17-57058772-57064015
chr17	57070557	57070860	chr17-57070557-57070860
chr17	57087169	57087789	chr17-57087169-57087789
chr17	57104484	57109375	chr17-57104484-57109375
chr17	57149021	57149763	chr17-57149021-57149763
chr17	57166974	57167225	chr17-57166974-57167225
chr17	57227623	57228404	chr17-57227623-57228404
chr17	57246532	57253674	chr17-57246532-57253674
chr17	57254888	57255222	chr17-57254888-57255222
chr17	57264469	57268246	chr17-57264469-57268246
chr17	57271040	57277259	chr17-57271040-57277259
chr17	57311722	57311977	chr17-57311722-57311977
chr17	59012384	59013666	chr17-59012384-59013666
chr17	59076088	59077695	chr17-59076088-59077695
chr17	59270019	59270326	chr17-59270019-59270326
chr17	62415308	62416230	chr17-62415308-62416230
chr17	62510703	62510967	chr17-62510703-62510967
chr17	62687010	62688045	chr17-62687010-62688045
chr17	62782878	62785458	chr17-62782878-62785458
chr17	62878914	62884501	chr17-62878914-62884501
chr17	63483413	63483614	chr17-63483413-63483614
chr17	63497970	63501288	chr17-63497970-63501288
chr17	63801237	63801938	chr17-63801237-63801938
chr17	63849200	63850022	chr17-63849200-63850022
chr17	63862813	63865178	chr17-63862813-63865178
chr17	64003707	64003908	chr17-64003707-64003908
chr17	64330648	64333136	chr17-64330648-64333136
chr17	64559195	64559614	chr17-64559195-64559614

chr17	64600127	64603182	chr17-64600127-64603182
chr17	65540644	65541261	chr17-65540644-65541261
chr17	65558411	65558774	chr17-65558411-65558774
chr17	65563434	65563921	chr17-65563434-65563921
chr17	65611224	65615023	chr17-65611224-65615023
chr17	65658077	65658974	chr17-65658077-65658974
chr17	65663307	65664633	chr17-65663307-65664633
chr17	65666259	65667220	chr17-65666259-65667220
chr17	65701889	65702592	chr17-65701889-65702592
chr17	65703948	65704478	chr17-65703948-65704478
chr17	65736851	65738299	chr17-65736851-65738299
chr17	65770885	65773508	chr17-65770885-65773508
chr17	65781879	65786459	chr17-65781879-65786459
chr17	65883247	65886583	chr17-65883247-65886583
chr17	65939609	65940566	chr17-65939609-65940566
chr17	65949993	65951616	chr17-65949993-65951616
chr17	66075406	66078029	chr17-66075406-66078029
chr17	66100169	66102614	chr17-66100169-66102614
chr17	66110752	66112898	chr17-66110752-66112898
chr17	66120229	66120980	chr17-66120229-66120980
chr17	66123119	66125382	chr17-66123119-66125382
chr17	66224200	66224638	chr17-66224200-66224638
chr17	66242400	66242705	chr17-66242400-66242705
chr17	66322717	66323286	chr17-66322717-66323286
chr17	66351595	66354476	chr17-66351595-66354476
chr17	66356428	66359625	chr17-66356428-66359625
chr17	66365094	66366541	chr17-66365094-66366541
chr17	66371046	66371999	chr17-66371046-66371999
chr17	66404999	66405685	chr17-66404999-66405685
chr17	66446165	66451368	chr17-66446165-66451368
chr17	66485799	66486367	chr17-66485799-66486367
chr17	66507511	66508047	chr17-66507511-66508047
chr17	66517545	66520341	chr17-66517545-66520341
chr17	66692822	66694091	chr17-66692822-66694091
chr17	66738709	66739182	chr17-66738709-66739182
chr17	66749964	66750650	chr17-66749964-66750650
chr17	67123583	67123860	chr17-67123583-67123860
chr17	67125990	67126710	chr17-67125990-67126710
chr17	67290525	67290726	chr17-67290525-67290726
chr17	67351716	67355300	chr17-67351716-67355300
chr17	67696883	67697955	chr17-67696883-67697955
chr17	68003564	68004630	chr17-68003564-68004630

chr17	68273520	68274287	chr17-68273520-68274287
chr17	68836949	68837528	chr17-68836949-68837528
chr17	68841066	68842093	chr17-68841066-68842093
chr17	69075569	69076520	chr17-69075569-69076520
chr17	69156619	69157748	chr17-69156619-69157748
chr17	69382038	69385243	chr17-69382038-69385243
chr17	69396162	69396754	chr17-69396162-69396754
chr17	69416272	69418526	chr17-69416272-69418526
chr17	69438723	69440179	chr17-69438723-69440179
chr17	69475351	69475919	chr17-69475351-69475919
chr17	69495898	69496151	chr17-69495898-69496151
chr17	69968966	69969966	chr17-69968966-69969966
chr17	70706147	70708128	chr17-70706147-70708128
chr17	70709269	70714437	chr17-70709269-70714437
chr17	70720129	70724209	chr17-70720129-70724209
chr17	70726282	70726483	chr17-70726282-70726483
chr17	70736865	70739867	chr17-70736865-70739867
chr17	70740962	70742052	chr17-70740962-70742052
chr17	70745764	70749407	chr17-70745764-70749407
chr17	70750239	70758371	chr17-70750239-70758371
chr17	70778639	70780454	chr17-70778639-70780454
chr17	70834483	70834836	chr17-70834483-70834836
chr17	70843538	70855285	chr17-70843538-70855285
chr17	70888731	70889090	chr17-70888731-70889090
chr17	70957571	70958503	chr17-70957571-70958503
chr17	70986834	70994977	chr17-70986834-70994977
chr17	70996037	71003725	chr17-70996037-71003725
chr17	71088566	71090588	chr17-71088566-71090588
chr17	71102648	71103138	chr17-71102648-71103138
chr17	71182596	71185818	chr17-71182596-71185818
chr17	71193327	71193630	chr17-71193327-71193630
chr17	71195237	71196778	chr17-71195237-71196778
chr17	71256404	71257314	chr17-71256404-71257314
chr17	71285053	71285378	chr17-71285053-71285378
chr17	71310233	71311705	chr17-71310233-71311705
chr17	71355924	71356175	chr17-71355924-71356175
chr17	71473826	71476554	chr17-71473826-71476554
chr17	71485678	71486424	chr17-71485678-71486424
chr17	71525463	71527823	chr17-71525463-71527823
chr17	71551461	71553184	chr17-71551461-71553184
chr17	71595311	71599766	chr17-71595311-71599766
chr17	71615525	71618744	chr17-71615525-71618744

chr17	71662457	71663286	chr17-71662457-71663286
chr17	71691551	71691752	chr17-71691551-71691752
chr17	71780736	71783845	chr17-71780736-71783845
chr17	71826648	71827635	chr17-71826648-71827635
chr17	71869373	71870191	chr17-71869373-71870191
chr17	72604040	72605143	chr17-72604040-72605143
chr17	72795318	72795917	chr17-72795318-72795917
chr17	72828062	72829364	chr17-72828062-72829364
chr17	72835622	72838753	chr17-72835622-72838753
chr17	72917202	72919824	chr17-72917202-72919824
chr17	72959551	72960478	chr17-72959551-72960478
chr17	73032180	73032541	chr17-73032180-73032541
chr17	73107317	73109191	chr17-73107317-73109191
chr17	73263292	73263493	chr17-73263292-73263493
chr17	73709289	73710867	chr17-73709289-73710867
chr17	73804591	73805309	chr17-73804591-73805309
chr17	73949678	73950803	chr17-73949678-73950803
chr17	74047326	74048349	chr17-74047326-74048349
chr17	74089540	74089793	chr17-74089540-74089793
chr17	74159408	74159863	chr17-74159408-74159863
chr17	74279002	74279354	chr17-74279002-74279354
chr17	74288500	74289338	chr17-74288500-74289338
chr17	74292586	74296619	chr17-74292586-74296619
chr17	74315093	74317290	chr17-74315093-74317290
chr17	74337975	74340739	chr17-74337975-74340739
chr17	74394812	74397142	chr17-74394812-74397142
chr17	74488895	74490953	chr17-74488895-74490953
chr17	74526537	74531045	chr17-74526537-74531045
chr17	74630531	74630943	chr17-74630531-74630943
chr17	74716875	74718722	chr17-74716875-74718722
chr17	74869326	74869577	chr17-74869326-74869577
chr17	75004827	75007124	chr17-75004827-75007124
chr17	75110053	75111668	chr17-75110053-75111668
chr17	75177805	75182663	chr17-75177805-75182663
chr17	75258120	75258627	chr17-75258120-75258627
chr17	75273116	75273499	chr17-75273116-75273499
chr17	75344991	75345192	chr17-75344991-75345192
chr17	75465045	75465818	chr17-75465045-75465818
chr17	75550038	75552604	chr17-75550038-75552604
chr17	75936941	75937989	chr17-75936941-75937989
chr17	78199331	78203296	chr17-78199331-78203296
chr17	78206165	78206495	chr17-78206165-78206495

chr17	78218407	78219181	chr17-78218407-78219181
chr17	78320070	78320436	chr17-78320070-78320436
chr17	78321697	78323358	chr17-78321697-78323358
chr17	78350683	78351143	chr17-78350683-78351143
chr17	78384188	78388870	chr17-78384188-78388870
chr17	78392070	78393164	chr17-78392070-78393164
chr17	78414872	78418519	chr17-78414872-78418519
chr17	78562843	78563203	chr17-78562843-78563203
chr17	78724623	78724926	chr17-78724623-78724926
chr17	78726732	78727338	chr17-78726732-78727338
chr17	78734725	78737388	chr17-78734725-78737388
chr17	78834413	78836340	chr17-78834413-78836340
chr17	78881220	78883353	chr17-78881220-78883353
chr17	78895603	78895854	chr17-78895603-78895854
chr17	78915688	78918006	chr17-78915688-78918006
chr17	78936002	78938046	chr17-78936002-78938046
chr17	79019356	79021430	chr17-79019356-79021430
chr17	79051971	79053046	chr17-79051971-79053046
chr17	79285228	79285792	chr17-79285228-79285792
chr17	79298677	79298944	chr17-79298677-79298944
chr17	79300200	79300833	chr17-79300200-79300833
chr17	79350667	79356672	chr17-79350667-79356672
chr17	79372682	79373392	chr17-79372682-79373392
chr17	79578654	79578855	chr17-79578654-79578855
chr17	79598981	79600477	chr17-79598981-79600477
chr17	79614391	79616241	chr17-79614391-79616241
chr17	79713037	79716392	chr17-79713037-79716392
chr17	79811010	79811710	chr17-79811010-79811710
chr17	79893328	79896693	chr17-79893328-79896693
chr17	79898070	79898722	chr17-79898070-79898722
chr17	79949296	79950728	chr17-79949296-79950728
chr17	79964648	79965141	chr17-79964648-79965141
chr17	80019164	80019365	chr17-80019164-80019365
chr17	80059794	80063272	chr17-80059794-80063272
chr17	80095897	80096476	chr17-80095897-80096476
chr17	80127138	80128403	chr17-80127138-80128403
chr17	80148085	80148759	chr17-80148085-80148759
chr17	80167860	80168061	chr17-80167860-80168061
chr17	80205228	80208258	chr17-80205228-80208258
chr17	80223793	80225511	chr17-80223793-80225511
chr17	80255122	80255687	chr17-80255122-80255687
chr17	80288857	80290774	chr17-80288857-80290774

chr17	80372853	80374150	chr17-80372853-80374150
chr17	80391598	80392152	chr17-80391598-80392152
chr17	80468773	80469157	chr17-80468773-80469157
chr17	80478614	80481175	chr17-80478614-80481175
chr17	80482295	80483572	chr17-80482295-80483572
chr17	80553031	80554261	chr17-80553031-80554261
chr17	80560637	80561001	chr17-80560637-80561001
chr17	80708817	80710776	chr17-80708817-80710776
chr17	80722044	80722520	chr17-80722044-80722520
chr17	80726211	80729402	chr17-80726211-80729402
chr17	80753317	80753518	chr17-80753317-80753518
chr17	80756036	80756346	chr17-80756036-80756346
chr17	80765454	80765806	chr17-80765454-80765806
chr17	80772715	80773177	chr17-80772715-80773177
chr17	80801194	80801990	chr17-80801194-80801990
chr17	80833551	80834448	chr17-80833551-80834448
chr17	80944279	80945304	chr17-80944279-80945304
chr17	81064236	81065362	chr17-81064236-81065362
chr17	81669167	81670748	chr17-81669167-81670748
chr17	83122881	83123288	chr17-83122881-83123288
chr17	83214309	83216412	chr17-83214309-83216412
chr17	83267377	83267751	chr17-83267377-83267751
chr17	83275743	83278013	chr17-83275743-83278013
chr17	83312228	83313004	chr17-83312228-83313004
chr17	83322874	83324599	chr17-83322874-83324599
chr17	83350279	83353408	chr17-83350279-83353408
chr17	83512971	83515271	chr17-83512971-83515271
chr17	83545782	83546194	chr17-83545782-83546194
chr17	83630786	83633156	chr17-83630786-83633156
chr17	83705452	83708230	chr17-83705452-83708230
chr17	83731827	83732581	chr17-83731827-83732581
chr17	83854167	83854844	chr17-83854167-83854844
chr17	83861030	83863379	chr17-83861030-83863379
chr17	83979110	83979726	chr17-83979110-83979726
chr17	83989972	83991231	chr17-83989972-83991231
chr17	83992864	83993732	chr17-83992864-83993732
chr17	83999089	83999390	chr17-83999089-83999390
chr17	84025567	84026785	chr17-84025567-84026785
chr17	84028470	84029204	chr17-84028470-84029204
chr17	84043646	84046121	chr17-84043646-84046121
chr17	84071294	84071610	chr17-84071294-84071610
chr17	84081867	84082068	chr17-84081867-84082068

chr17	84083942	84086825	chr17-84083942-84086825
chr17	84096330	84097106	chr17-84096330-84097106
chr17	84109873	84110141	chr17-84109873-84110141
chr17	84115579	84116873	chr17-84115579-84116873
chr17	84117782	84118584	chr17-84117782-84118584
chr17	84119464	84119757	chr17-84119464-84119757
chr17	84148440	84149881	chr17-84148440-84149881
chr17	84155998	84158002	chr17-84155998-84158002
chr17	84180582	84189400	chr17-84180582-84189400
chr17	84196008	84196209	chr17-84196008-84196209
chr17	84215939	84216140	chr17-84215939-84216140
chr17	84464967	84467091	chr17-84464967-84467091
chr17	84510014	84513244	chr17-84510014-84513244
chr17	84529460	84529774	chr17-84529460-84529774
chr17	84626228	84627488	chr17-84626228-84627488
chr17	84675921	84676480	chr17-84675921-84676480
chr17	84788730	84792353	chr17-84788730-84792353
chr17	84878055	84878307	chr17-84878055-84878307
chr17	84955732	84960549	chr17-84955732-84960549
chr17	85089020	85091749	chr17-85089020-85091749
chr17	85189290	85189642	chr17-85189290-85189642
chr17	85464596	85465569	chr17-85464596-85465569
chr17	85485292	85487037	chr17-85485292-85487037
chr17	85596014	85596345	chr17-85596014-85596345
chr17	85608695	85609161	chr17-85608695-85609161
chr17	85615046	85615247	chr17-85615046-85615247
chr17	85617918	85618616	chr17-85617918-85618616
chr17	85620604	85622237	chr17-85620604-85622237
chr17	85623229	85623953	chr17-85623229-85623953
chr17	85645627	85645968	chr17-85645627-85645968
chr17	85664757	85665218	chr17-85664757-85665218
chr17	85687271	85689552	chr17-85687271-85689552
chr17	85799479	85800892	chr17-85799479-85800892
chr17	86143994	86146045	chr17-86143994-86146045
chr17	86165910	86169424	chr17-86165910-86169424
chr17	86187702	86188005	chr17-86187702-86188005
chr17	86286965	86287317	chr17-86286965-86287317
chr17	86293198	86293663	chr17-86293198-86293663
chr17	86329438	86329885	chr17-86329438-86329885
chr17	86436970	86438036	chr17-86436970-86438036
chr17	86444599	86445065	chr17-86444599-86445065
chr17	86535944	86537018	chr17-86535944-86537018

chr17	86732079	86732546	chr17-86732079-86732546
chr17	86752668	86755566	chr17-86752668-86755566
chr17	86769465	86770348	chr17-86769465-86770348
chr17	86877200	86877401	chr17-86877200-86877401
chr17	86903612	86903863	chr17-86903612-86903863
chr17	86937468	86938296	chr17-86937468-86938296
chr17	86961091	86965455	chr17-86961091-86965455
chr17	87024243	87026500	chr17-87024243-87026500
chr17	87102580	87103918	chr17-87102580-87103918
chr17	87105585	87109951	chr17-87105585-87109951
chr17	87142140	87142914	chr17-87142140-87142914
chr17	87169934	87170548	chr17-87169934-87170548
chr17	87174811	87175012	chr17-87174811-87175012
chr17	87203329	87203650	chr17-87203329-87203650
chr17	87206666	87206917	chr17-87206666-87206917
chr17	87236631	87237664	chr17-87236631-87237664
chr17	87264350	87266588	chr17-87264350-87266588
chr17	87275234	87275835	chr17-87275234-87275835
chr17	87281312	87284908	chr17-87281312-87284908
chr17	87302223	87302738	chr17-87302223-87302738
chr17	87306331	87307104	chr17-87306331-87307104
chr17	87314732	87315116	chr17-87314732-87315116
chr17	87350925	87351915	chr17-87350925-87351915
chr17	87443564	87447837	chr17-87443564-87447837
chr17	87457942	87460673	chr17-87457942-87460673
chr17	87472574	87474631	chr17-87472574-87474631
chr17	87523528	87524256	chr17-87523528-87524256
chr17	87530180	87532293	chr17-87530180-87532293
chr17	87570806	87571898	chr17-87570806-87571898
chr17	87608780	87610347	chr17-87608780-87610347
chr17	87612195	87614147	chr17-87612195-87614147
chr17	87619867	87620323	chr17-87619867-87620323
chr17	87635467	87640562	chr17-87635467-87640562
chr17	87671687	87674472	chr17-87671687-87674472
chr17	87699103	87699563	chr17-87699103-87699563
chr17	87745291	87747004	chr17-87745291-87747004
chr17	87796869	87799038	chr17-87796869-87799038
chr17	87851856	87852273	chr17-87851856-87852273
chr17	87971787	87972067	chr17-87971787-87972067
chr17	87974145	87977090	chr17-87974145-87977090
chr17	88062975	88067845	chr17-88062975-88067845
chr17	88111972	88112716	chr17-88111972-88112716

chr17	88255586	88257455	chr17-88255586-88257455
chr17	88258335	88258895	chr17-88258335-88258895
chr17	88439889	88443527	chr17-88439889-88443527
chr17	88460123	88460528	chr17-88460123-88460528
chr17	88508669	88509654	chr17-88508669-88509654
chr17	88510893	88511450	chr17-88510893-88511450
chr17	88529411	88531195	chr17-88529411-88531195
chr17	88596480	88599753	chr17-88596480-88599753
chr17	88625690	88631726	chr17-88625690-88631726
chr17	88668553	88668854	chr17-88668553-88668854
chr17	90088384	90088585	chr17-90088384-90088585
chr17	90454837	90455800	chr17-90454837-90455800
chr17	91092658	91093061	chr17-91092658-91093061
chr17	93068723	93068980	chr17-93068723-93068980
chr17	93200813	93204141	chr17-93200813-93204141
chr17	93321686	93322242	chr17-93321686-93322242
chr17	93526025	93526333	chr17-93526025-93526333
chr17	94748773	94751444	chr17-94748773-94751444
chr17	94833416	94835588	chr17-94833416-94835588
chr17	94864101	94865685	chr17-94864101-94865685
chr18	3115437	3115638	chr18-3115437-3115638
chr18	3280304	3281589	chr18-3280304-3281589
chr18	3335797	3338874	chr18-3335797-3338874
chr18	3382561	3384320	chr18-3382561-3384320
chr18	3506918	3508658	chr18-3506918-3508658
chr18	3767466	3767717	chr18-3767466-3767717
chr18	4351332	4354767	chr18-4351332-4354767
chr18	4375238	4377305	chr18-4375238-4377305
chr18	4522952	4523388	chr18-4522952-4523388
chr18	4634647	4636597	chr18-4634647-4636597
chr18	4656880	4657707	chr18-4656880-4657707
chr18	4737640	4737841	chr18-4737640-4737841
chr18	4919836	4923779	chr18-4919836-4923779
chr18	4931384	4931685	chr18-4931384-4931685
chr18	4958759	4959943	chr18-4958759-4959943
chr18	4970174	4971087	chr18-4970174-4971087
chr18	5162065	5163290	chr18-5162065-5163290
chr18	5333501	5335407	chr18-5333501-5335407
chr18	5414645	5414846	chr18-5414645-5414846
chr18	5427615	5429270	chr18-5427615-5429270
chr18	5590902	5593762	chr18-5590902-5593762
chr18	5603899	5604100	chr18-5603899-5604100

chr18	5817247	5817448	chr18-5817247-5817448
chr18	5819553	5820517	chr18-5819553-5820517
chr18	5989028	5990547	chr18-5989028-5990547
chr18	6134336	6137220	chr18-6134336-6137220
chr18	6238004	6242879	chr18-6238004-6242879
chr18	6281332	6282563	chr18-6281332-6282563
chr18	6419391	6419849	chr18-6419391-6419849
chr18	6488901	6491655	chr18-6488901-6491655
chr18	6514587	6517134	chr18-6514587-6517134
chr18	6666752	6667005	chr18-6666752-6667005
chr18	6760610	6761296	chr18-6760610-6761296
chr18	6764522	6766399	chr18-6764522-6766399
chr18	7001335	7005604	chr18-7001335-7005604
chr18	7297727	7298186	chr18-7297727-7298186
chr18	7608513	7609937	chr18-7608513-7609937
chr18	7614514	7614767	chr18-7614514-7614767
chr18	7625316	7627421	chr18-7625316-7627421
chr18	7692714	7693593	chr18-7692714-7693593
chr18	7867760	7871102	chr18-7867760-7871102
chr18	8693633	8694539	chr18-8693633-8694539
chr18	9211666	9214972	chr18-9211666-9214972
chr18	9448187	9451338	chr18-9448187-9451338
chr18	9552215	9553051	chr18-9552215-9553051
chr18	9554926	9555288	chr18-9554926-9555288
chr18	9707193	9708468	chr18-9707193-9708468
chr18	9957249	9959354	chr18-9957249-9959354
chr18	10027637	10030541	chr18-10027637-10030541
chr18	10051798	10052896	chr18-10051798-10052896
chr18	10179527	10183000	chr18-10179527-10183000
chr18	10289635	10289888	chr18-10289635-10289888
chr18	10300054	10300307	chr18-10300054-10300307
chr18	10324634	10326152	chr18-10324634-10326152
chr18	10532916	10533117	chr18-10532916-10533117
chr18	10608598	10610836	chr18-10608598-10610836
chr18	10617423	10620337	chr18-10617423-10620337
chr18	10625885	10626262	chr18-10625885-10626262
chr18	10705898	10707447	chr18-10705898-10707447
chr18	10725027	10728296	chr18-10725027-10728296
chr18	10915217	10915418	chr18-10915217-10915418
chr18	10935454	10936914	chr18-10935454-10936914
chr18	11046061	11059982	chr18-11046061-11059982
chr18	11239624	11240182	chr18-11239624-11240182

chr18	11351685	11352606	chr18-11351685-11352606
chr18	11656627	11658573	chr18-11656627-11658573
chr18	11838935	11841055	chr18-11838935-11841055
chr18	11856857	11857503	chr18-11856857-11857503
chr18	11910475	11911873	chr18-11910475-11911873
chr18	12120311	12122162	chr18-12120311-12122162
chr18	12127723	12131980	chr18-12127723-12131980
chr18	12167953	12170406	chr18-12167953-12170406
chr18	12224068	12224665	chr18-12224068-12224665
chr18	12234332	12237230	chr18-12234332-12237230
chr18	12255500	12256161	chr18-12255500-12256161
chr18	12273879	12275911	chr18-12273879-12275911
chr18	12305107	12306028	chr18-12305107-12306028
chr18	12333522	12334927	chr18-12333522-12334927
chr18	12462145	12462346	chr18-12462145-12462346
chr18	12480936	12481137	chr18-12480936-12481137
chr18	12483652	12484926	chr18-12483652-12484926
chr18	12498109	12498411	chr18-12498109-12498411
chr18	12502611	12506682	chr18-12502611-12506682
chr18	12611475	12611779	chr18-12611475-12611779
chr18	12643068	12645234	chr18-12643068-12645234
chr18	12726338	12726539	chr18-12726338-12726539
chr18	12740871	12741893	chr18-12740871-12741893
chr18	12861746	12862968	chr18-12861746-12862968
chr18	12940844	12942292	chr18-12940844-12942292
chr18	12946748	12948021	chr18-12946748-12948021
chr18	12955392	12956955	chr18-12955392-12956955
chr18	12972024	12974805	chr18-12972024-12974805
chr18	13786758	13787634	chr18-13786758-13787634
chr18	13969895	13973374	chr18-13969895-13973374
chr18	14680891	14683715	chr18-14680891-14683715
chr18	14781953	14785037	chr18-14781953-14785037
chr18	14794575	14794776	chr18-14794575-14794776
chr18	15062448	15065235	chr18-15062448-15065235
chr18	15149589	15151525	chr18-15149589-15151525
chr18	15313160	15313361	chr18-15313160-15313361
chr18	15759652	15760467	chr18-15759652-15760467
chr18	16745009	16745260	chr18-16745009-16745260
chr18	16806012	16810293	chr18-16806012-16810293
chr18	19960298	19960553	chr18-19960298-19960553
chr18	20002118	20002319	chr18-20002118-20002319
chr18	20058335	20059764	chr18-20058335-20059764

chr18	20188054	20188768	chr18-20188054-20188768
chr18	20552843	20553587	chr18-20552843-20553587
chr18	20554584	20554974	chr18-20554584-20554974
chr18	20557559	20559700	chr18-20557559-20559700
chr18	20713223	20713554	chr18-20713223-20713554
chr18	20744958	20746810	chr18-20744958-20746810
chr18	20782152	20782558	chr18-20782152-20782558
chr18	20895041	20897338	chr18-20895041-20897338
chr18	20944427	20944736	chr18-20944427-20944736
chr18	20961019	20962550	chr18-20961019-20962550
chr18	21000622	21003619	chr18-21000622-21003619
chr18	21195865	21196131	chr18-21195865-21196131
chr18	21215252	21215571	chr18-21215252-21215571
chr18	21298970	21301158	chr18-21298970-21301158
chr18	21651374	21654072	chr18-21651374-21654072
chr18	22343482	22344996	chr18-22343482-22344996
chr18	22984972	22985374	chr18-22984972-22985374
chr18	23038535	23042309	chr18-23038535-23042309
chr18	23309740	23311210	chr18-23309740-23311210
chr18	23414918	23415912	chr18-23414918-23415912
chr18	23497225	23498668	chr18-23497225-23498668
chr18	23751789	23754855	chr18-23751789-23754855
chr18	23803406	23805789	chr18-23803406-23805789
chr18	23953901	23956148	chr18-23953901-23956148
chr18	23986940	23987391	chr18-23986940-23987391
chr18	23988909	23990546	chr18-23988909-23990546
chr18	24018964	24022020	chr18-24018964-24022020
chr18	24050628	24051894	chr18-24050628-24051894
chr18	24094692	24095570	chr18-24094692-24095570
chr18	24101304	24102388	chr18-24101304-24102388
chr18	24110646	24112966	chr18-24110646-24112966
chr18	24116361	24117320	chr18-24116361-24117320
chr18	24120670	24122559	chr18-24120670-24122559
chr18	24152008	24152303	chr18-24152008-24152303
chr18	24204708	24208216	chr18-24204708-24208216
chr18	24419974	24420440	chr18-24419974-24420440
chr18	24466184	24468000	chr18-24466184-24468000
chr18	24470433	24471765	chr18-24470433-24471765
chr18	24528713	24530832	chr18-24528713-24530832
chr18	24602563	24605310	chr18-24602563-24605310
chr18	24652864	24654810	chr18-24652864-24654810
chr18	24708386	24710624	chr18-24708386-24710624

chr18	24714263	24714849	chr18-24714263-24714849
chr18	24855622	24855874	chr18-24855622-24855874
chr18	25167645	25170229	chr18-25167645-25170229
chr18	25486052	25487723	chr18-25486052-25487723
chr18	25504130	25505851	chr18-25504130-25505851
chr18	25509997	25511332	chr18-25509997-25511332
chr18	25537948	25538937	chr18-25537948-25538937
chr18	25621562	25622090	chr18-25621562-25622090
chr18	25630157	25630574	chr18-25630157-25630574
chr18	25678801	25679827	chr18-25678801-25679827
chr18	25713965	25716268	chr18-25713965-25716268
chr18	25753013	25755848	chr18-25753013-25755848
chr18	26015178	26015862	chr18-26015178-26015862
chr18	30272294	30273888	chr18-30272294-30273888
chr18	31441501	31441702	chr18-31441501-31441702
chr18	31608666	31610367	chr18-31608666-31610367
chr18	31633258	31636332	chr18-31633258-31636332
chr18	31704697	31704958	chr18-31704697-31704958
chr18	31758903	31761842	chr18-31758903-31761842
chr18	31788125	31790749	chr18-31788125-31790749
chr18	31803294	31806094	chr18-31803294-31806094
chr18	31909618	31911915	chr18-31909618-31911915
chr18	31930647	31932578	chr18-31930647-31932578
chr18	31939065	31939517	chr18-31939065-31939517
chr18	31998639	31999089	chr18-31998639-31999089
chr18	32019890	32020479	chr18-32019890-32020479
chr18	32066915	32069436	chr18-32066915-32069436
chr18	32138039	32140351	chr18-32138039-32140351
chr18	32146990	32148571	chr18-32146990-32148571
chr18	32151901	32155851	chr18-32151901-32155851
chr18	32162005	32165729	chr18-32162005-32165729
chr18	32172680	32172935	chr18-32172680-32172935
chr18	32239275	32241559	chr18-32239275-32241559
chr18	32315662	32316022	chr18-32315662-32316022
chr18	32376381	32379308	chr18-32376381-32379308
chr18	32445890	32448579	chr18-32445890-32448579
chr18	32559355	32560264	chr18-32559355-32560264
chr18	32647458	32647709	chr18-32647458-32647709
chr18	32816370	32817349	chr18-32816370-32817349
chr18	32836764	32839068	chr18-32836764-32839068
chr18	32938728	32939948	chr18-32938728-32939948
chr18	33211455	33214569	chr18-33211455-33214569

chr18	33463110	33464765	chr18-33463110-33464765
chr18	33794207	33796989	chr18-33794207-33796989
chr18	33848842	33849369	chr18-33848842-33849369
chr18	33850696	33850949	chr18-33850696-33850949
chr18	33888546	33892392	chr18-33888546-33892392
chr18	33908060	33908388	chr18-33908060-33908388
chr18	33947005	33947418	chr18-33947005-33947418
chr18	33998040	34000130	chr18-33998040-34000130
chr18	34004287	34007922	chr18-34004287-34007922
chr18	34102893	34103300	chr18-34102893-34103300
chr18	34152678	34153030	chr18-34152678-34153030
chr18	34220318	34223025	chr18-34220318-34223025
chr18	34246966	34248369	chr18-34246966-34248369
chr18	34329826	34333106	chr18-34329826-34333106
chr18	34371657	34374028	chr18-34371657-34374028
chr18	34408788	34409871	chr18-34408788-34409871
chr18	34500267	34502043	chr18-34500267-34502043
chr18	34503749	34510085	chr18-34503749-34510085
chr18	34510901	34512901	chr18-34510901-34512901
chr18	34519775	34520097	chr18-34519775-34520097
chr18	34538430	34538681	chr18-34538430-34538681
chr18	34547237	34547521	chr18-34547237-34547521
chr18	34578527	34579287	chr18-34578527-34579287
chr18	34623215	34626834	chr18-34623215-34626834
chr18	34650330	34652318	chr18-34650330-34652318
chr18	34663037	34663338	chr18-34663037-34663338
chr18	34719933	34720556	chr18-34719933-34720556
chr18	34750219	34752689	chr18-34750219-34752689
chr18	34758624	34761096	chr18-34758624-34761096
chr18	34776561	34780783	chr18-34776561-34780783
chr18	34782030	34783774	chr18-34782030-34783774
chr18	34858952	34863243	chr18-34858952-34863243
chr18	34886910	34888423	chr18-34886910-34888423
chr18	34897758	34897959	chr18-34897758-34897959
chr18	34929160	34934428	chr18-34929160-34934428
chr18	34951560	34956151	chr18-34951560-34956151
chr18	34958016	34959643	chr18-34958016-34959643
chr18	34989166	34989581	chr18-34989166-34989581
chr18	35021715	35023244	chr18-35021715-35023244
chr18	35065469	35066067	chr18-35065469-35066067
chr18	35087293	35087708	chr18-35087293-35087708
chr18	35093885	35094320	chr18-35093885-35094320

chr18	35103503	35103704	chr18-35103503-35103704
chr18	35116901	35122551	chr18-35116901-35122551
chr18	35189908	35190109	chr18-35189908-35190109
chr18	35192392	35192747	chr18-35192392-35192747
chr18	35296184	35296536	chr18-35296184-35296536
chr18	35299390	35299910	chr18-35299390-35299910
chr18	35383609	35383810	chr18-35383609-35383810
chr18	35497816	35499369	chr18-35497816-35499369
chr18	35552855	35557669	chr18-35552855-35557669
chr18	35561530	35564346	chr18-35561530-35564346
chr18	35598320	35600258	chr18-35598320-35600258
chr18	35652346	35655456	chr18-35652346-35655456
chr18	35661442	35662549	chr18-35661442-35662549
chr18	35702181	35703897	chr18-35702181-35703897
chr18	35729668	35731228	chr18-35729668-35731228
chr18	35740226	35740480	chr18-35740226-35740480
chr18	35751503	35752588	chr18-35751503-35752588
chr18	35770760	35774342	chr18-35770760-35774342
chr18	35822997	35823648	chr18-35822997-35823648
chr18	35829257	35832194	chr18-35829257-35832194
chr18	35841418	35841829	chr18-35841418-35841829
chr18	35847721	35848670	chr18-35847721-35848670
chr18	35873150	35874383	chr18-35873150-35874383
chr18	35887634	35890332	chr18-35887634-35890332
chr18	35918230	35920694	chr18-35918230-35920694
chr18	35930283	35931078	chr18-35930283-35931078
chr18	35932988	35933731	chr18-35932988-35933731
chr18	35938207	35940054	chr18-35938207-35940054
chr18	35958968	35959685	chr18-35958968-35959685
chr18	35964694	35965443	chr18-35964694-35965443
chr18	36017723	36019147	chr18-36017723-36019147
chr18	36067957	36069116	chr18-36067957-36069116
chr18	36195557	36197995	chr18-36195557-36197995
chr18	36224948	36225303	chr18-36224948-36225303
chr18	36280202	36282898	chr18-36280202-36282898
chr18	36285593	36288881	chr18-36285593-36288881
chr18	36314215	36314525	chr18-36314215-36314525
chr18	36319531	36321138	chr18-36319531-36321138
chr18	36328339	36329269	chr18-36328339-36329269
chr18	36348002	36350933	chr18-36348002-36350933
chr18	36374912	36375273	chr18-36374912-36375273
chr18	36383526	36385035	chr18-36383526-36385035

chr18	36385950	36387029	chr18-36385950-36387029
chr18	36391306	36393321	chr18-36391306-36393321
chr18	36399590	36400383	chr18-36399590-36400383
chr18	36402337	36405584	chr18-36402337-36405584
chr18	36408774	36409077	chr18-36408774-36409077
chr18	36414612	36417662	chr18-36414612-36417662
chr18	36449663	36455193	chr18-36449663-36455193
chr18	36467419	36467723	chr18-36467419-36467723
chr18	36472077	36473050	chr18-36472077-36473050
chr18	36492338	36498240	chr18-36492338-36498240
chr18	36508208	36517397	chr18-36508208-36517397
chr18	36518824	36519435	chr18-36518824-36519435
chr18	36521291	36522357	chr18-36521291-36522357
chr18	36524625	36525802	chr18-36524625-36525802
chr18	36534513	36534932	chr18-36534513-36534932
chr18	36541662	36541863	chr18-36541662-36541863
chr18	36558308	36562393	chr18-36558308-36562393
chr18	36668920	36670756	chr18-36668920-36670756
chr18	36677977	36680745	chr18-36677977-36680745
chr18	36687942	36688320	chr18-36687942-36688320
chr18	36699391	36700173	chr18-36699391-36700173
chr18	36707837	36709195	chr18-36707837-36709195
chr18	36724198	36727430	chr18-36724198-36727430
chr18	36733964	36736213	chr18-36733964-36736213
chr18	36743306	36745841	chr18-36743306-36745841
chr18	36759517	36761631	chr18-36759517-36761631
chr18	36781905	36784520	chr18-36781905-36784520
chr18	36793220	36795021	chr18-36793220-36795021
chr18	36801143	36803008	chr18-36801143-36803008
chr18	36930314	36930575	chr18-36930314-36930575
chr18	36946243	36946444	chr18-36946243-36946444
chr18	36952727	36953149	chr18-36952727-36953149
chr18	37005344	37005545	chr18-37005344-37005545
chr18	37020172	37020643	chr18-37020172-37020643
chr18	37143353	37144500	chr18-37143353-37144500
chr18	37264886	37265087	chr18-37264886-37265087
chr18	37643092	37645010	chr18-37643092-37645010
chr18	37655425	37656438	chr18-37655425-37656438
chr18	37668952	37669317	chr18-37668952-37669317
chr18	37674298	37674808	chr18-37674298-37674808
chr18	37680197	37680724	chr18-37680197-37680724
chr18	37685198	37685753	chr18-37685198-37685753

chr18	37689790	37690347	chr18-37689790-37690347
chr18	37694302	37695136	chr18-37694302-37695136
chr18	37707029	37707588	chr18-37707029-37707588
chr18	37714682	37715318	chr18-37714682-37715318
chr18	37720348	37720903	chr18-37720348-37720903
chr18	37725904	37726699	chr18-37725904-37726699
chr18	37730929	37731330	chr18-37730929-37731330
chr18	37736826	37737591	chr18-37736826-37737591
chr18	37742017	37742737	chr18-37742017-37742737
chr18	37743982	37744239	chr18-37743982-37744239
chr18	37746961	37747636	chr18-37746961-37747636
chr18	37751546	37752204	chr18-37751546-37752204
chr18	37755838	37756207	chr18-37755838-37756207
chr18	37766038	37766953	chr18-37766038-37766953
chr18	37805755	37809591	chr18-37805755-37809591
chr18	37814069	37816180	chr18-37814069-37816180
chr18	37821987	37822952	chr18-37821987-37822952
chr18	37824708	37826612	chr18-37824708-37826612
chr18	37879643	37880355	chr18-37879643-37880355
chr18	37883748	37884006	chr18-37883748-37884006
chr18	37932344	37935668	chr18-37932344-37935668
chr18	37953610	37956489	chr18-37953610-37956489
chr18	37968733	37970632	chr18-37968733-37970632
chr18	37998242	37999496	chr18-37998242-37999496
chr18	38013816	38015803	chr18-38013816-38015803
chr18	38024924	38025750	chr18-38024924-38025750
chr18	38061486	38062045	chr18-38061486-38062045
chr18	38067506	38068863	chr18-38067506-38068863
chr18	38070018	38070428	chr18-38070018-38070428
chr18	38080037	38083589	chr18-38080037-38083589
chr18	38143938	38145973	chr18-38143938-38145973
chr18	38170553	38172341	chr18-38170553-38172341
chr18	38183638	38186712	chr18-38183638-38186712
chr18	38188750	38191275	chr18-38188750-38191275
chr18	38198828	38200449	chr18-38198828-38200449
chr18	38204560	38206951	chr18-38204560-38206951
chr18	38207832	38213029	chr18-38207832-38213029
chr18	38216564	38217235	chr18-38216564-38217235
chr18	38221634	38221887	chr18-38221634-38221887
chr18	38228546	38228747	chr18-38228546-38228747
chr18	38239121	38239687	chr18-38239121-38239687
chr18	38248573	38251485	chr18-38248573-38251485

chr18	38276343	38276544	chr18-38276343-38276544
chr18	38296229	38298929	chr18-38296229-38298929
chr18	38337980	38339589	chr18-38337980-38339589
chr18	38348541	38348842	chr18-38348541-38348842
chr18	38349897	38350613	chr18-38349897-38350613
chr18	38366972	38367913	chr18-38366972-38367913
chr18	38383975	38385376	chr18-38383975-38385376
chr18	38418293	38420857	chr18-38418293-38420857
chr18	38483226	38484039	chr18-38483226-38484039
chr18	38495904	38496253	chr18-38495904-38496253
chr18	38518346	38518648	chr18-38518346-38518648
chr18	38599587	38603050	chr18-38599587-38603050
chr18	38604575	38605279	chr18-38604575-38605279
chr18	38631293	38632575	chr18-38631293-38632575
chr18	38754407	38755118	chr18-38754407-38755118
chr18	38792277	38792631	chr18-38792277-38792631
chr18	38798105	38798408	chr18-38798105-38798408
chr18	38866035	38867041	chr18-38866035-38867041
chr18	38928864	38930295	chr18-38928864-38930295
chr18	38940578	38940831	chr18-38940578-38940831
chr18	38992220	38994681	chr18-38992220-38994681
chr18	39017539	39017962	chr18-39017539-39017962
chr18	39035071	39035475	chr18-39035071-39035475
chr18	39050784	39051139	chr18-39050784-39051139
chr18	39052346	39052886	chr18-39052346-39052886
chr18	39319536	39319892	chr18-39319536-39319892
chr18	39389089	39389924	chr18-39389089-39389924
chr18	39390744	39391552	chr18-39390744-39391552
chr18	39396688	39398074	chr18-39396688-39398074
chr18	39482679	39492084	chr18-39482679-39492084
chr18	39732059	39735730	chr18-39732059-39735730
chr18	39738006	39738309	chr18-39738006-39738309
chr18	39797419	39797944	chr18-39797419-39797944
chr18	39801256	39801457	chr18-39801256-39801457
chr18	39873551	39873752	chr18-39873551-39873752
chr18	40218339	40220007	chr18-40218339-40220007
chr18	40256543	40257336	chr18-40256543-40257336
chr18	41950408	41951917	chr18-41950408-41951917
chr18	42043432	42043633	chr18-42043432-42043633
chr18	42052247	42057259	chr18-42052247-42057259
chr18	42061554	42061806	chr18-42061554-42061806
chr18	42073596	42073898	chr18-42073596-42073898

chr18	42260477	42262832	chr18-42260477-42262832
chr18	42274144	42277064	chr18-42274144-42277064
chr18	42385944	42386407	chr18-42385944-42386407
chr18	42394284	42395507	chr18-42394284-42395507
chr18	42401226	42401681	chr18-42401226-42401681
chr18	42416440	42417284	chr18-42416440-42417284
chr18	42467323	42467856	chr18-42467323-42467856
chr18	42510955	42513880	chr18-42510955-42513880
chr18	42589429	42589630	chr18-42589429-42589630
chr18	42898412	42899679	chr18-42898412-42899679
chr18	43060693	43060894	chr18-43060693-43060894
chr18	43207144	43207999	chr18-43207144-43207999
chr18	43392123	43394014	chr18-43392123-43394014
chr18	43437685	43438590	chr18-43437685-43438590
chr18	43477484	43477936	chr18-43477484-43477936
chr18	44379735	44382485	chr18-44379735-44382485
chr18	44472289	44473895	chr18-44472289-44473895
chr18	44542574	44542828	chr18-44542574-44542828
chr18	44660480	44663010	chr18-44660480-44663010
chr18	44811707	44812859	chr18-44811707-44812859
chr18	44827609	44829613	chr18-44827609-44829613
chr18	45268787	45269088	chr18-45268787-45269088
chr18	45558088	45560775	chr18-45558088-45560775
chr18	46197998	46199803	chr18-46197998-46199803
chr18	46211601	46212851	chr18-46211601-46212851
chr18	46279329	46281343	chr18-46279329-46281343
chr18	46310378	46312319	chr18-46310378-46312319
chr18	46325038	46325690	chr18-46325038-46325690
chr18	46334313	46334778	chr18-46334313-46334778
chr18	46378886	46379216	chr18-46378886-46379216
chr18	46380907	46381584	chr18-46380907-46381584
chr18	46389718	46390558	chr18-46389718-46390558
chr18	46484126	46484327	chr18-46484126-46484327
chr18	46521448	46526984	chr18-46521448-46526984
chr18	46573967	46574662	chr18-46573967-46574662
chr18	46595283	46601010	chr18-46595283-46601010
chr18	46601822	46602123	chr18-46601822-46602123
chr18	46636522	46637016	chr18-46636522-46637016
chr18	46740451	46743291	chr18-46740451-46743291
chr18	46849281	46850529	chr18-46849281-46850529
chr18	46957967	46959775	chr18-46957967-46959775
chr18	47030885	47031150	chr18-47030885-47031150

chr18	47330022	47331444	chr18-47330022-47331444
chr18	47358978	47359179	chr18-47358978-47359179
chr18	47365875	47371736	chr18-47365875-47371736
chr18	49500956	49501269	chr18-49500956-49501269
chr18	49551376	49551577	chr18-49551376-49551577
chr18	49754725	49756667	chr18-49754725-49756667
chr18	49771054	49771374	chr18-49771054-49771374
chr18	49831523	49834494	chr18-49831523-49834494
chr18	49978365	49980913	chr18-49978365-49980913
chr18	49986130	49987472	chr18-49986130-49987472
chr18	50010649	50012097	chr18-50010649-50012097
chr18	50023747	50024213	chr18-50023747-50024213
chr18	50030938	50033048	chr18-50030938-50033048
chr18	50052691	50056252	chr18-50052691-50056252
chr18	50061445	50063490	chr18-50061445-50063490
chr18	50096229	50098139	chr18-50096229-50098139
chr18	50127849	50129180	chr18-50127849-50129180
chr18	51117249	51118706	chr18-51117249-51118706
chr18	52465087	52467393	chr18-52465087-52467393
chr18	52528059	52530089	chr18-52528059-52530089
chr18	52767773	52769152	chr18-52767773-52769152
chr18	52909082	52909487	chr18-52909082-52909487
chr18	53160670	53161812	chr18-53160670-53161812
chr18	53175685	53177879	chr18-53175685-53177879
chr18	53198586	53198848	chr18-53198586-53198848
chr18	53244652	53250906	chr18-53244652-53250906
chr18	53256283	53256484	chr18-53256283-53256484
chr18	53263849	53264834	chr18-53263849-53264834
chr18	53267673	53268856	chr18-53267673-53268856
chr18	53415667	53418574	chr18-53415667-53418574
chr18	53462451	53465598	chr18-53462451-53465598
chr18	53473037	53473999	chr18-53473037-53473999
chr18	53552098	53552665	chr18-53552098-53552665
chr18	53627674	53628459	chr18-53627674-53628459
chr18	53743056	53745905	chr18-53743056-53745905
chr18	53861137	53864670	chr18-53861137-53864670
chr18	53903331	53904034	chr18-53903331-53904034
chr18	54860502	54861190	chr18-54860502-54861190
chr18	54899656	54903021	chr18-54899656-54903021
chr18	54905589	54908786	chr18-54905589-54908786
chr18	54910443	54912062	chr18-54910443-54912062
chr18	54946572	54946929	chr18-54946572-54946929

chr18	54952286	54952870	chr18-54952286-54952870
chr18	54957135	54958216	chr18-54957135-54958216
chr18	54984832	54993460	chr18-54984832-54993460
chr18	56359019	56359327	chr18-56359019-56359327
chr18	56399671	56401466	chr18-56399671-56401466
chr18	56431015	56433269	chr18-56431015-56433269
chr18	56561089	56563366	chr18-56561089-56563366
chr18	56572054	56574723	chr18-56572054-56574723
chr18	56608803	56609056	chr18-56608803-56609056
chr18	56614073	56614606	chr18-56614073-56614606
chr18	56620609	56621720	chr18-56620609-56621720
chr18	56622593	56622898	chr18-56622593-56622898
chr18	56649275	56649577	chr18-56649275-56649577
chr18	56665448	56665649	chr18-56665448-56665649
chr18	56687317	56688119	chr18-56687317-56688119
chr18	56706456	56711027	chr18-56706456-56711027
chr18	56722586	56724830	chr18-56722586-56724830
chr18	56900237	56900438	chr18-56900237-56900438
chr18	56924343	56926504	chr18-56924343-56926504
chr18	56974456	56975866	chr18-56974456-56975866
chr18	56987622	56988252	chr18-56987622-56988252
chr18	57059337	57059859	chr18-57059337-57059859
chr18	57084615	57086118	chr18-57084615-57086118
chr18	57132720	57133763	chr18-57132720-57133763
chr18	57353995	57356010	chr18-57353995-57356010
chr18	57434692	57435000	chr18-57434692-57435000
chr18	57746718	57747789	chr18-57746718-57747789
chr18	57786129	57786436	chr18-57786129-57786436
chr18	57805377	57805879	chr18-57805377-57805879
chr18	57836496	57837255	chr18-57836496-57837255
chr18	57845389	57845590	chr18-57845389-57845590
chr18	57856621	57856822	chr18-57856621-57856822
chr18	57877004	57882234	chr18-57877004-57882234
chr18	57976800	57977109	chr18-57976800-57977109
chr18	58057267	58057468	chr18-58057267-58057468
chr18	58209556	58210709	chr18-58209556-58210709
chr18	58555661	58556159	chr18-58555661-58556159
chr18	58658722	58660670	chr18-58658722-58660670
chr18	58836223	58837719	chr18-58836223-58837719
chr18	59174645	59176440	chr18-59174645-59176440
chr18	60238725	60238989	chr18-60238725-60238989
chr18	60480949	60481150	chr18-60480949-60481150

chr18	60499657	60503498	chr18-60499657-60503498
chr18	60525331	60527818	chr18-60525331-60527818
chr18	60559600	60561924	chr18-60559600-60561924
chr18	60595477	60596807	chr18-60595477-60596807
chr18	60605749	60607603	chr18-60605749-60607603
chr18	60609049	60610822	chr18-60609049-60610822
chr18	60612431	60613965	chr18-60612431-60613965
chr18	60622064	60624664	chr18-60622064-60624664
chr18	60714337	60715724	chr18-60714337-60715724
chr18	60718060	60719332	chr18-60718060-60719332
chr18	60722155	60724173	chr18-60722155-60724173
chr18	60727145	60727859	chr18-60727145-60727859
chr18	60729322	60733664	chr18-60729322-60733664
chr18	60746146	60752459	chr18-60746146-60752459
chr18	60756437	60757007	chr18-60756437-60757007
chr18	60773951	60777368	chr18-60773951-60777368
chr18	60847231	60849606	chr18-60847231-60849606
chr18	60889900	60890240	chr18-60889900-60890240
chr18	60891726	60892393	chr18-60891726-60892393
chr18	60896759	60897081	chr18-60896759-60897081
chr18	60911618	60912270	chr18-60911618-60912270
chr18	60951970	60953364	chr18-60951970-60953364
chr18	61013869	61014376	chr18-61013869-61014376
chr18	61035753	61036300	chr18-61035753-61036300
chr18	61045098	61045299	chr18-61045098-61045299
chr18	61065699	61065900	chr18-61065699-61065900
chr18	61089437	61089894	chr18-61089437-61089894
chr18	61096164	61097325	chr18-61096164-61097325
chr18	61175611	61178400	chr18-61175611-61178400
chr18	61210383	61212164	chr18-61210383-61212164
chr18	61224065	61224675	chr18-61224065-61224675
chr18	61278939	61279140	chr18-61278939-61279140
chr18	61360479	61361565	chr18-61360479-61361565
chr18	61362364	61363442	chr18-61362364-61363442
chr18	61364733	61364988	chr18-61364733-61364988
chr18	61395042	61395350	chr18-61395042-61395350
chr18	61397478	61401769	chr18-61397478-61401769
chr18	61489180	61489840	chr18-61489180-61489840
chr18	61535232	61537044	chr18-61535232-61537044
chr18	61554059	61558455	chr18-61554059-61558455
chr18	61626077	61627141	chr18-61626077-61627141
chr18	61643513	61644180	chr18-61643513-61644180

chr18	61647411	61647897	chr18-61647411-61647897
chr18	61675691	61676192	chr18-61675691-61676192
chr18	61706220	61708164	chr18-61706220-61708164
chr18	61719619	61720247	chr18-61719619-61720247
chr18	61724316	61728017	chr18-61724316-61728017
chr18	61786098	61787000	chr18-61786098-61787000
chr18	61838015	61838350	chr18-61838015-61838350
chr18	61910793	61912040	chr18-61910793-61912040
chr18	61921194	61921395	chr18-61921194-61921395
chr18	61933863	61934227	chr18-61933863-61934227
chr18	61946785	61947050	chr18-61946785-61947050
chr18	62001710	62002443	chr18-62001710-62002443
chr18	62016580	62016831	chr18-62016580-62016831
chr18	62101076	62101614	chr18-62101076-62101614
chr18	62168976	62170016	chr18-62168976-62170016
chr18	62177335	62177611	chr18-62177335-62177611
chr18	62178509	62180758	chr18-62178509-62180758
chr18	62203338	62204354	chr18-62203338-62204354
chr18	62323743	62324564	chr18-62323743-62324564
chr18	62547238	62550037	chr18-62547238-62550037
chr18	62691387	62691639	chr18-62691387-62691639
chr18	62921823	62923421	chr18-62921823-62923421
chr18	62977413	62979270	chr18-62977413-62979270
chr18	63150392	63150593	chr18-63150392-63150593
chr18	63208032	63208518	chr18-63208032-63208518
chr18	63385655	63387445	chr18-63385655-63387445
chr18	63487306	63487507	chr18-63487306-63487507
chr18	63689228	63693275	chr18-63689228-63693275
chr18	63707989	63709678	chr18-63707989-63709678
chr18	63901156	63902138	chr18-63901156-63902138
chr18	64265042	64266729	chr18-64265042-64266729
chr18	64332097	64333739	chr18-64332097-64333739
chr18	64338518	64343802	chr18-64338518-64343802
chr18	64488000	64489690	chr18-64488000-64489690
chr18	64513826	64517825	chr18-64513826-64517825
chr18	64618270	64620052	chr18-64618270-64620052
chr18	64659406	64661886	chr18-64659406-64661886
chr18	64699932	64700237	chr18-64699932-64700237
chr18	64878527	64878728	chr18-64878527-64878728
chr18	64886466	64890130	chr18-64886466-64890130
chr18	64920980	64921748	chr18-64920980-64921748
chr18	64944980	64945481	chr18-64944980-64945481

chr18	64971956	64972263	chr18-64971956-64972263
chr18	64986688	64986998	chr18-64986688-64986998
chr18	65023488	65024193	chr18-65023488-65024193
chr18	65025637	65026329	chr18-65025637-65026329
chr18	65036928	65037129	chr18-65036928-65037129
chr18	65050559	65051505	chr18-65050559-65051505
chr18	65053392	65054064	chr18-65053392-65054064
chr18	65069315	65070005	chr18-65069315-65070005
chr18	65070814	65072312	chr18-65070814-65072312
chr18	65381440	65382331	chr18-65381440-65382331
chr18	65429934	65433269	chr18-65429934-65433269
chr18	65445155	65445356	chr18-65445155-65445356
chr18	65489895	65490651	chr18-65489895-65490651
chr18	65520670	65520922	chr18-65520670-65520922
chr18	65580279	65581879	chr18-65580279-65581879
chr18	65737937	65740783	chr18-65737937-65740783
chr18	65744771	65744972	chr18-65744771-65744972
chr18	65794992	65795402	chr18-65794992-65795402
chr18	65799800	65803601	chr18-65799800-65803601
chr18	65805034	65806376	chr18-65805034-65806376
chr18	65873248	65873828	chr18-65873248-65873828
chr18	65924778	65925441	chr18-65924778-65925441
chr18	65934538	65935719	chr18-65934538-65935719
chr18	65937954	65939244	chr18-65937954-65939244
chr18	66001481	66003255	chr18-66001481-66003255
chr18	66222874	66224097	chr18-66222874-66224097
chr18	66251149	66251474	chr18-66251149-66251474
chr18	66286751	66287226	chr18-66286751-66287226
chr18	66288688	66293242	chr18-66288688-66293242
chr18	66366866	66367067	chr18-66366866-66367067
chr18	66457628	66461419	chr18-66457628-66461419
chr18	66470396	66472807	chr18-66470396-66472807
chr18	66503806	66505833	chr18-66503806-66505833
chr18	66860326	66860639	chr18-66860326-66860639
chr18	67088029	67089152	chr18-67088029-67089152
chr18	67133093	67134241	chr18-67133093-67134241
chr18	67204712	67208850	chr18-67204712-67208850
chr18	67244845	67246359	chr18-67244845-67246359
chr18	67278593	67279012	chr18-67278593-67279012
chr18	67287802	67290729	chr18-67287802-67290729
chr18	67343099	67344391	chr18-67343099-67344391
chr18	67379851	67380487	chr18-67379851-67380487

chr18	67390019	67394438	chr18-67390019-67394438
chr18	67447640	67449934	chr18-67447640-67449934
chr18	67460732	67461260	chr18-67460732-67461260
chr18	67464320	67465786	chr18-67464320-67465786
chr18	67500470	67500774	chr18-67500470-67500774
chr18	67590144	67590345	chr18-67590144-67590345
chr18	67609666	67611387	chr18-67609666-67611387
chr18	67640334	67642617	chr18-67640334-67642617
chr18	67722736	67725318	chr18-67722736-67725318
chr18	67773909	67777426	chr18-67773909-67777426
chr18	67799134	67801759	chr18-67799134-67801759
chr18	67931882	67936198	chr18-67931882-67936198
chr18	67945702	67946061	chr18-67945702-67946061
chr18	67984020	67986666	chr18-67984020-67986666
chr18	67988617	67988876	chr18-67988617-67988876
chr18	68015973	68016174	chr18-68015973-68016174
chr18	68052586	68053605	chr18-68052586-68053605
chr18	68057120	68060517	chr18-68057120-68060517
chr18	68156402	68157576	chr18-68156402-68157576
chr18	68159531	68160209	chr18-68159531-68160209
chr18	68189862	68190215	chr18-68189862-68190215
chr18	68192005	68192206	chr18-68192005-68192206
chr18	68194938	68195454	chr18-68194938-68195454
chr18	68298528	68301986	chr18-68298528-68301986
chr18	68337116	68337855	chr18-68337116-68337855
chr18	68474793	68475205	chr18-68474793-68475205
chr18	68794107	68794998	chr18-68794107-68794998
chr18	68976709	68977063	chr18-68976709-68977063
chr18	69152518	69152883	chr18-69152518-69152883
chr18	69192506	69192809	chr18-69192506-69192809
chr18	69197981	69198999	chr18-69197981-69198999
chr18	69343663	69348509	chr18-69343663-69348509
chr18	69415569	69415770	chr18-69415569-69415770
chr18	69547492	69547899	chr18-69547492-69547899
chr18	69570873	69571333	chr18-69570873-69571333
chr18	69592180	69594013	chr18-69592180-69594013
chr18	69918832	69919555	chr18-69918832-69919555
chr18	69923215	69923630	chr18-69923215-69923630
chr18	69924849	69927168	chr18-69924849-69927168
chr18	70051787	70053956	chr18-70051787-70053956
chr18	70062046	70062418	chr18-70062046-70062418
chr18	70140931	70141628	chr18-70140931-70141628

chr18	70241892	70244876	chr18-70241892-70244876
chr18	70471352	70473236	chr18-70471352-70473236
chr18	70529159	70532071	chr18-70529159-70532071
chr18	70567742	70571138	chr18-70567742-70571138
chr18	70643325	70646127	chr18-70643325-70646127
chr18	70652269	70652741	chr18-70652269-70652741
chr18	73533303	73533607	chr18-73533303-73533607
chr18	73541327	73542546	chr18-73541327-73542546
chr18	73570994	73576325	chr18-73570994-73576325
chr18	73622610	73623594	chr18-73622610-73623594
chr18	73626502	73627387	chr18-73626502-73627387
chr18	73701188	73704991	chr18-73701188-73704991
chr18	73753745	73755240	chr18-73753745-73755240
chr18	73762188	73762913	chr18-73762188-73762913
chr18	73814301	73816822	chr18-73814301-73816822
chr18	73863513	73864175	chr18-73863513-73864175
chr18	73947123	73948986	chr18-73947123-73948986
chr18	73962808	73963522	chr18-73962808-73963522
chr18	73996858	73997437	chr18-73996858-73997437
chr18	74002056	74002417	chr18-74002056-74002417
chr18	74063521	74065672	chr18-74063521-74065672
chr18	74206871	74208299	chr18-74206871-74208299
chr18	74215169	74217631	chr18-74215169-74217631
chr18	74267562	74270011	chr18-74267562-74270011
chr18	74347020	74348899	chr18-74347020-74348899
chr18	74430727	74431502	chr18-74430727-74431502
chr18	74441952	74445712	chr18-74441952-74445712
chr18	74551194	74552940	chr18-74551194-74552940
chr18	74777388	74781338	chr18-74777388-74781338
chr18	74783137	74783868	chr18-74783137-74783868
chr18	74810450	74810752	chr18-74810450-74810752
chr18	74824493	74824806	chr18-74824493-74824806
chr18	74829707	74830557	chr18-74829707-74830557
chr18	74850376	74850577	chr18-74850376-74850577
chr18	74890216	74891138	chr18-74890216-74891138
chr18	74921897	74922255	chr18-74921897-74922255
chr18	74958381	74963644	chr18-74958381-74963644
chr18	74990947	74991359	chr18-74990947-74991359
chr18	74999695	75006935	chr18-74999695-75006935
chr18	75018364	75022714	chr18-75018364-75022714
chr18	75295165	75295366	chr18-75295165-75295366
chr18	75319216	75319996	chr18-75319216-75319996

chr18	75325058	75326048	chr18-75325058-75326048
chr18	75341600	75345921	chr18-75341600-75345921
chr18	75362384	75391824	chr18-75362384-75391824
chr18	75404922	75405932	chr18-75404922-75405932
chr18	75410144	75410739	chr18-75410144-75410739
chr18	75446248	75448492	chr18-75446248-75448492
chr18	75455332	75457723	chr18-75455332-75457723
chr18	75464663	75465974	chr18-75464663-75465974
chr18	75492937	75495920	chr18-75492937-75495920
chr18	75498613	75499529	chr18-75498613-75499529
chr18	75562317	75562573	chr18-75562317-75562573
chr18	75584320	75586213	chr18-75584320-75586213
chr18	75597709	75598129	chr18-75597709-75598129
chr18	75607425	75608107	chr18-75607425-75608107
chr18	75648655	75650149	chr18-75648655-75650149
chr18	75658474	75660515	chr18-75658474-75660515
chr18	75695089	75698515	chr18-75695089-75698515
chr18	75716089	75716702	chr18-75716089-75716702
chr18	75818990	75821105	chr18-75818990-75821105
chr18	76034798	76035419	chr18-76034798-76035419
chr18	76059171	76060633	chr18-76059171-76060633
chr18	76150824	76151284	chr18-76150824-76151284
chr18	76160796	76161658	chr18-76160796-76161658
chr18	76222526	76222727	chr18-76222526-76222727
chr18	76240557	76243656	chr18-76240557-76243656
chr18	76378811	76380659	chr18-76378811-76380659
chr18	76543670	76544960	chr18-76543670-76544960
chr18	76844198	76844611	chr18-76844198-76844611
chr18	76859263	76860784	chr18-76859263-76860784
chr18	76929339	76931476	chr18-76929339-76931476
chr18	76943190	76945430	chr18-76943190-76945430
chr18	77046607	77047865	chr18-77046607-77047865
chr18	77054726	77055488	chr18-77054726-77055488
chr18	77064507	77067099	chr18-77064507-77067099
chr18	77185000	77186327	chr18-77185000-77186327
chr18	77484043	77484449	chr18-77484043-77484449
chr18	77564687	77566186	chr18-77564687-77566186
chr18	77600447	77602066	chr18-77600447-77602066
chr18	77663616	77663817	chr18-77663616-77663817
chr18	77711911	77714866	chr18-77711911-77714866
chr18	77742451	77742909	chr18-77742451-77742909
chr18	77766412	77767984	chr18-77766412-77767984

chr18	77772927	77776785	chr18-77772927-77776785
chr18	77793845	77797583	chr18-77793845-77797583
chr18	77799587	77800177	chr18-77799587-77800177
chr18	77870218	77870570	chr18-77870218-77870570
chr18	77937925	77941005	chr18-77937925-77941005
chr18	77944756	77945086	chr18-77944756-77945086
chr18	77948412	77949306	chr18-77948412-77949306
chr18	78047177	78050794	chr18-78047177-78050794
chr18	78053220	78054605	chr18-78053220-78054605
chr18	78056911	78057360	chr18-78056911-78057360
chr18	78072098	78073432	chr18-78072098-78073432
chr18	78097591	78097845	chr18-78097591-78097845
chr18	78122402	78124367	chr18-78122402-78124367
chr18	79108640	79110534	chr18-79108640-79110534
chr18	80046421	80048201	chr18-80046421-80048201
chr18	80147982	80151888	chr18-80147982-80151888
chr18	80169292	80170580	chr18-80169292-80170580
chr18	80199605	80201185	chr18-80199605-80201185
chr18	80206176	80209459	chr18-80206176-80209459
chr18	80254664	80258105	chr18-80254664-80258105
chr18	80295611	80296290	chr18-80295611-80296290
chr18	80322375	80323157	chr18-80322375-80323157
chr18	80362946	80363919	chr18-80362946-80363919
chr18	80467877	80471011	chr18-80467877-80471011
chr18	80535671	80535872	chr18-80535671-80535872
chr18	80550690	80551245	chr18-80550690-80551245
chr18	80554371	80555918	chr18-80554371-80555918
chr18	80588185	80588487	chr18-80588185-80588487
chr18	80628847	80629406	chr18-80628847-80629406
chr18	80654472	80657040	chr18-80654472-80657040
chr18	80706657	80709142	chr18-80706657-80709142
chr18	80712124	80715875	chr18-80712124-80715875
chr18	80932694	80934544	chr18-80932694-80934544
chr18	80937975	80938279	chr18-80937975-80938279
chr18	80974000	80974677	chr18-80974000-80974677
chr18	80985660	80988059	chr18-80985660-80988059
chr18	81005365	81005897	chr18-81005365-81005897
chr18	81033580	81033983	chr18-81033580-81033983
chr18	81916218	81916734	chr18-81916218-81916734
chr18	82390776	82391191	chr18-82390776-82391191
chr18	82406591	82407367	chr18-82406591-82407367
chr18	82470092	82470574	chr18-82470092-82470574

chr18	82474470	82477770	chr18-82474470-82477770
chr18	82479766	82480178	chr18-82479766-82480178
chr18	82492421	82494468	chr18-82492421-82494468
chr18	82512387	82512711	chr18-82512387-82512711
chr18	82514824	82515081	chr18-82514824-82515081
chr18	82517462	82520451	chr18-82517462-82520451
chr18	82522487	82523237	chr18-82522487-82523237
chr18	82524611	82525065	chr18-82524611-82525065
chr18	82534504	82534880	chr18-82534504-82534880
chr18	82691212	82693701	chr18-82691212-82693701
chr18	82716946	82718261	chr18-82716946-82718261
chr18	82737603	82738653	chr18-82737603-82738653
chr18	82750816	82751644	chr18-82750816-82751644
chr18	82763597	82765602	chr18-82763597-82765602
chr18	82812097	82812625	chr18-82812097-82812625
chr18	82821591	82822266	chr18-82821591-82822266
chr18	82825292	82826515	chr18-82825292-82826515
chr18	82854923	82856794	chr18-82854923-82856794
chr18	82867774	82868827	chr18-82867774-82868827
chr18	82909323	82918300	chr18-82909323-82918300
chr18	82953813	82954327	chr18-82953813-82954327
chr18	82971320	82972680	chr18-82971320-82972680
chr18	82974661	82975658	chr18-82974661-82975658
chr18	83004266	83004865	chr18-83004266-83004865
chr18	83059960	83060318	chr18-83059960-83060318
chr18	83827115	83827371	chr18-83827115-83827371
chr18	84049885	84050876	chr18-84049885-84050876
chr18	84084355	84092866	chr18-84084355-84092866
chr18	84103093	84103349	chr18-84103093-84103349
chr18	84413339	84413743	chr18-84413339-84413743
chr18	84588266	84591036	chr18-84588266-84591036
chr18	84680586	84683044	chr18-84680586-84683044
chr18	84683898	84686213	chr18-84683898-84686213
chr18	84719708	84720831	chr18-84719708-84720831
chr18	84799046	84799504	chr18-84799046-84799504
chr18	84850473	84853239	chr18-84850473-84853239
chr18	84857919	84859328	chr18-84857919-84859328
chr18	84934284	84934485	chr18-84934284-84934485
chr18	84950199	84952527	chr18-84950199-84952527
chr18	85697979	85698280	chr18-85697979-85698280
chr18	86394114	86395769	chr18-86394114-86395769
chr18	86710693	86711901	chr18-86710693-86711901

chr18	86712969	86713274	chr18-86712969-86713274
chr18	88835453	88835654	chr18-88835453-88835654
chr18	88892230	88894809	chr18-88892230-88894809
chr18	88899962	88900369	chr18-88899962-88900369
chr18	88971092	88972741	chr18-88971092-88972741
chr18	89768926	89769802	chr18-89768926-89769802
chr18	90508399	90511219	chr18-90508399-90511219
chr18	90578695	90580711	chr18-90578695-90580711
chr19	3083267	3084338	chr19-3083267-3084338
chr19	3223245	3223813	chr19-3223245-3223813
chr19	3225229	3225586	chr19-3225229-3225586
chr19	3281195	3284236	chr19-3281195-3284236
chr19	3321498	3326465	chr19-3321498-3326465
chr19	3334295	3334661	chr19-3334295-3334661
chr19	3388544	3389676	chr19-3388544-3389676
chr19	3405721	3406029	chr19-3405721-3406029
chr19	3414133	3414851	chr19-3414133-3414851
chr19	3419159	3419938	chr19-3419159-3419938
chr19	3519820	3520021	chr19-3519820-3520021
chr19	3574109	3577081	chr19-3574109-3577081
chr19	3625125	3625326	chr19-3625125-3625326
chr19	3634776	3636207	chr19-3634776-3636207
chr19	3638590	3639017	chr19-3638590-3639017
chr19	3667544	3670210	chr19-3667544-3670210
chr19	3681269	3682015	chr19-3681269-3682015
chr19	3684183	3687186	chr19-3684183-3687186
chr19	3692647	3693292	chr19-3692647-3693292
chr19	3707462	3709734	chr19-3707462-3709734
chr19	3719145	3719950	chr19-3719145-3719950
chr19	3743931	3744588	chr19-3743931-3744588
chr19	3752610	3753124	chr19-3752610-3753124
chr19	3765378	3770183	chr19-3765378-3770183
chr19	3810114	3811742	chr19-3810114-3811742
chr19	3829642	3829944	chr19-3829642-3829944
chr19	3832847	3834503	chr19-3832847-3834503
chr19	3837334	3839085	chr19-3837334-3839085
chr19	3839991	3843442	chr19-3839991-3843442
chr19	3850001	3859412	chr19-3850001-3859412
chr19	3903479	3908182	chr19-3903479-3908182
chr19	3911538	3914268	chr19-3911538-3914268
chr19	3922623	3923152	chr19-3922623-3923152
chr19	3928825	3930377	chr19-3928825-3930377

chr19	3935165	3936462	chr19-3935165-3936462
chr19	3943349	3943701	chr19-3943349-3943701
chr19	3986454	3987271	chr19-3986454-3987271
chr19	3999380	4002218	chr19-3999380-4002218
chr19	4011487	4013494	chr19-4011487-4013494
chr19	4036172	4038604	chr19-4036172-4038604
chr19	4041970	4042222	chr19-4041970-4042222
chr19	4045488	4046511	chr19-4045488-4046511
chr19	4064521	4064835	chr19-4064521-4064835
chr19	4093768	4094358	chr19-4093768-4094358
chr19	4096089	4101778	chr19-4096089-4101778
chr19	4113279	4114218	chr19-4113279-4114218
chr19	4120477	4122038	chr19-4120477-4122038
chr19	4125192	4127846	chr19-4125192-4127846
chr19	4135053	4136314	chr19-4135053-4136314
chr19	4148082	4150495	chr19-4148082-4150495
chr19	4162239	4164000	chr19-4162239-4164000
chr19	4168907	4170454	chr19-4168907-4170454
chr19	4171482	4171996	chr19-4171482-4171996
chr19	4191393	4194699	chr19-4191393-4194699
chr19	4196625	4197106	chr19-4196625-4197106
chr19	4199508	4202738	chr19-4199508-4202738
chr19	4208788	4209432	chr19-4208788-4209432
chr19	4211405	4217699	chr19-4211405-4217699
chr19	4229472	4233523	chr19-4229472-4233523
chr19	4244368	4244931	chr19-4244368-4244931
chr19	4256917	4258313	chr19-4256917-4258313
chr19	4263453	4264505	chr19-4263453-4264505
chr19	4267614	4269744	chr19-4267614-4269744
chr19	4282423	4283990	chr19-4282423-4283990
chr19	4300338	4300861	chr19-4300338-4300861
chr19	4304354	4306841	chr19-4304354-4306841
chr19	4331780	4334498	chr19-4331780-4334498
chr19	4393650	4393851	chr19-4393650-4393851
chr19	4394708	4398666	chr19-4394708-4398666
chr19	4438086	4442154	chr19-4438086-4442154
chr19	4475729	4478462	chr19-4475729-4478462
chr19	4509519	4511468	chr19-4509519-4511468
chr19	4604319	4604520	chr19-4604319-4604520
chr19	4612083	4616798	chr19-4612083-4616798
chr19	4624359	4627214	chr19-4624359-4627214
chr19	4686422	4686623	chr19-4686422-4686623

chr19	4697946	4699184	chr19-4697946-4699184
chr19	4700955	4701940	chr19-4700955-4701940
chr19	4711461	4712920	chr19-4711461-4712920
chr19	4718425	4722093	chr19-4718425-4722093
chr19	4755597	4757934	chr19-4755597-4757934
chr19	4791782	4795037	chr19-4791782-4795037
chr19	4808943	4812698	chr19-4808943-4812698
chr19	4838175	4840245	chr19-4838175-4840245
chr19	4854734	4855901	chr19-4854734-4855901
chr19	4865219	4865580	chr19-4865219-4865580
chr19	4877657	4880115	chr19-4877657-4880115
chr19	4906043	4906827	chr19-4906043-4906827
chr19	4926508	4929125	chr19-4926508-4929125
chr19	4942390	4943849	chr19-4942390-4943849
chr19	4961694	4963590	chr19-4961694-4963590
chr19	4989918	4990174	chr19-4989918-4990174
chr19	4992871	4993173	chr19-4992871-4993173
chr19	5001598	5002359	chr19-5001598-5002359
chr19	5012625	5013865	chr19-5012625-5013865
chr19	5015057	5019239	chr19-5015057-5019239
chr19	5022782	5025385	chr19-5022782-5025385
chr19	5037402	5042778	chr19-5037402-5042778
chr19	5049970	5055170	chr19-5049970-5055170
chr19	5057114	5059768	chr19-5057114-5059768
chr19	5060757	5061122	chr19-5060757-5061122
chr19	5067682	5068855	chr19-5067682-5068855
chr19	5071394	5073817	chr19-5071394-5073817
chr19	5084699	5086055	chr19-5084699-5086055
chr19	5087359	5089766	chr19-5087359-5089766
chr19	5097809	5098842	chr19-5097809-5098842
chr19	5105734	5107762	chr19-5105734-5107762
chr19	5117003	5119069	chr19-5117003-5119069
chr19	5149042	5149296	chr19-5149042-5149296
chr19	5229905	5230106	chr19-5229905-5230106
chr19	5256707	5257751	chr19-5256707-5257751
chr19	5272013	5273785	chr19-5272013-5273785
chr19	5293975	5296455	chr19-5293975-5296455
chr19	5307196	5307816	chr19-5307196-5307816
chr19	5327985	5328186	chr19-5327985-5328186
chr19	5345382	5346594	chr19-5345382-5346594
chr19	5347900	5349601	chr19-5347900-5349601
chr19	5364947	5368633	chr19-5364947-5368633

chr19	5386611	5391073	chr19-5386611-5391073
chr19	5392520	5392986	chr19-5392520-5392986
chr19	5405977	5407373	chr19-5405977-5407373
chr19	5423012	5427353	chr19-5423012-5427353
chr19	5429133	5430727	chr19-5429133-5430727
chr19	5431722	5434000	chr19-5431722-5434000
chr19	5444656	5445379	chr19-5444656-5445379
chr19	5447073	5451812	chr19-5447073-5451812
chr19	5454948	5458207	chr19-5454948-5458207
chr19	5459922	5461833	chr19-5459922-5461833
chr19	5466329	5468669	chr19-5466329-5468669
chr19	5473591	5475871	chr19-5473591-5475871
chr19	5477497	5478034	chr19-5477497-5478034
chr19	5485858	5493719	chr19-5485858-5493719
chr19	5509982	5511488	chr19-5509982-5511488
chr19	5529406	5530272	chr19-5529406-5530272
chr19	5543923	5544347	chr19-5543923-5544347
chr19	5558842	5562183	chr19-5558842-5562183
chr19	5566469	5569529	chr19-5566469-5569529
chr19	5572207	5573115	chr19-5572207-5573115
chr19	5580692	5581302	chr19-5580692-5581302
chr19	5585563	5586260	chr19-5585563-5586260
chr19	5601558	5603527	chr19-5601558-5603527
chr19	5608736	5611147	chr19-5608736-5611147
chr19	5636657	5639218	chr19-5636657-5639218
chr19	5653775	5655717	chr19-5653775-5655717
chr19	5662423	5664176	chr19-5662423-5664176
chr19	5687019	5692987	chr19-5687019-5692987
chr19	5704344	5705114	chr19-5704344-5705114
chr19	5713411	5713979	chr19-5713411-5713979
chr19	5723910	5732365	chr19-5723910-5732365
chr19	5740163	5747668	chr19-5740163-5747668
chr19	5769386	5772045	chr19-5769386-5772045
chr19	5794821	5807302	chr19-5794821-5807302
chr19	5808670	5812853	chr19-5808670-5812853
chr19	5817567	5819135	chr19-5817567-5819135
chr19	5834229	5852691	chr19-5834229-5852691
chr19	5870953	5872022	chr19-5870953-5872022
chr19	5872933	5875900	chr19-5872933-5875900
chr19	5877272	5878526	chr19-5877272-5878526
chr19	5893589	5894614	chr19-5893589-5894614
chr19	5910451	5913986	chr19-5910451-5913986

chr19	5921785	5921986	chr19-5921785-5921986
chr19	5923844	5925453	chr19-5923844-5925453
chr19	5928959	5929210	chr19-5928959-5929210
chr19	5940733	5940991	chr19-5940733-5940991
chr19	5955829	5956333	chr19-5955829-5956333
chr19	5962709	5965386	chr19-5962709-5965386
chr19	5988639	5989261	chr19-5988639-5989261
chr19	5991605	5992129	chr19-5991605-5992129
chr19	6000132	6000394	chr19-6000132-6000394
chr19	6013922	6016449	chr19-6013922-6016449
chr19	6018636	6018946	chr19-6018636-6018946
chr19	6045445	6048487	chr19-6045445-6048487
chr19	6056625	6063299	chr19-6056625-6063299
chr19	6066362	6069118	chr19-6066362-6069118
chr19	6075987	6077978	chr19-6075987-6077978
chr19	6083652	6086567	chr19-6083652-6086567
chr19	6116397	6119404	chr19-6116397-6119404
chr19	6127008	6128797	chr19-6127008-6128797
chr19	6130950	6131379	chr19-6130950-6131379
chr19	6133526	6137422	chr19-6133526-6137422
chr19	6139766	6142016	chr19-6139766-6142016
chr19	6234258	6236748	chr19-6234258-6236748
chr19	6241079	6243481	chr19-6241079-6243481
chr19	6254414	6255341	chr19-6254414-6255341
chr19	6274906	6280357	chr19-6274906-6280357
chr19	6295942	6298466	chr19-6295942-6298466
chr19	6299435	6299737	chr19-6299435-6299737
chr19	6305585	6308946	chr19-6305585-6308946
chr19	6331634	6332162	chr19-6331634-6332162
chr19	6334282	6337000	chr19-6334282-6337000
chr19	6340573	6342512	chr19-6340573-6342512
chr19	6362630	6366131	chr19-6362630-6366131
chr19	6398380	6401953	chr19-6398380-6401953
chr19	6418084	6419168	chr19-6418084-6419168
chr19	6427758	6429050	chr19-6427758-6429050
chr19	6477162	6477513	chr19-6477162-6477513
chr19	6497245	6498643	chr19-6497245-6498643
chr19	6502029	6502863	chr19-6502029-6502863
chr19	6530921	6532221	chr19-6530921-6532221
chr19	6631905	6632217	chr19-6631905-6632217
chr19	6838864	6841749	chr19-6838864-6841749
chr19	6847692	6848727	chr19-6847692-6848727

chr19	6869115	6870135	chr19-6869115-6870135
chr19	6877248	6877552	chr19-6877248-6877552
chr19	6892389	6892849	chr19-6892389-6892849
chr19	6907172	6912070	chr19-6907172-6912070
chr19	6919944	6922900	chr19-6919944-6922900
chr19	6924612	6925256	chr19-6924612-6925256
chr19	6927001	6927252	chr19-6927001-6927252
chr19	6933585	6934462	chr19-6933585-6934462
chr19	6937865	6943360	chr19-6937865-6943360
chr19	6950151	6953234	chr19-6950151-6953234
chr19	6967902	6972195	chr19-6967902-6972195
chr19	6973244	6981964	chr19-6973244-6981964
chr19	6986136	6988412	chr19-6986136-6988412
chr19	6991489	6993826	chr19-6991489-6993826
chr19	6994626	6997044	chr19-6994626-6997044
chr19	7038283	7041218	chr19-7038283-7041218
chr19	7044423	7044900	chr19-7044423-7044900
chr19	7054601	7058562	chr19-7054601-7058562
chr19	7108478	7108679	chr19-7108478-7108679
chr19	7143610	7144530	chr19-7143610-7144530
chr19	7164865	7166491	chr19-7164865-7166491
chr19	7176184	7176385	chr19-7176184-7176385
chr19	7182312	7182822	chr19-7182312-7182822
chr19	7204327	7207607	chr19-7204327-7207607
chr19	7214840	7218507	chr19-7214840-7218507
chr19	7239805	7241632	chr19-7239805-7241632
chr19	7264430	7264631	chr19-7264430-7264631
chr19	7266880	7269984	chr19-7266880-7269984
chr19	7293736	7296230	chr19-7293736-7296230
chr19	7301777	7302908	chr19-7301777-7302908
chr19	7308041	7308399	chr19-7308041-7308399
chr19	7310433	7311707	chr19-7310433-7311707
chr19	7335724	7336401	chr19-7335724-7336401
chr19	7338376	7342595	chr19-7338376-7342595
chr19	7381500	7383910	chr19-7381500-7383910
chr19	7416435	7419496	chr19-7416435-7419496
chr19	7422573	7424000	chr19-7422573-7424000
chr19	7425522	7425877	chr19-7425522-7425877
chr19	7481451	7484424	chr19-7481451-7484424
chr19	7493482	7496100	chr19-7493482-7496100
chr19	7557277	7558328	chr19-7557277-7558328
chr19	7606018	7607265	chr19-7606018-7607265

chr19	7611933	7612907	chr19-7611933-7612907
chr19	8251933	8252796	chr19-8251933-8252796
chr19	8517923	8519142	chr19-8517923-8519142
chr19	8661787	8662648	chr19-8661787-8662648
chr19	8679203	8679404	chr19-8679203-8679404
chr19	8710082	8714526	chr19-8710082-8714526
chr19	8721493	8726705	chr19-8721493-8726705
chr19	8735154	8736992	chr19-8735154-8736992
chr19	8740643	8743056	chr19-8740643-8743056
chr19	8755478	8758882	chr19-8755478-8758882
chr19	8770297	8775718	chr19-8770297-8775718
chr19	8785148	8787047	chr19-8785148-8787047
chr19	8797279	8799184	chr19-8797279-8799184
chr19	8801754	8803483	chr19-8801754-8803483
chr19	8816803	8823090	chr19-8816803-8823090
chr19	8836863	8838478	chr19-8836863-8838478
chr19	8840329	8841047	chr19-8840329-8841047
chr19	8870496	8872979	chr19-8870496-8872979
chr19	8879788	8881478	chr19-8879788-8881478
chr19	8887119	8890190	chr19-8887119-8890190
chr19	8892533	8894203	chr19-8892533-8894203
chr19	8896846	8899441	chr19-8896846-8899441
chr19	8919848	8922123	chr19-8919848-8922123
chr19	8926576	8931912	chr19-8926576-8931912
chr19	8940833	8945620	chr19-8940833-8945620
chr19	8952937	8955557	chr19-8952937-8955557
chr19	8965646	8969629	chr19-8965646-8969629
chr19	8980453	8986367	chr19-8980453-8986367
chr19	8987931	8999250	chr19-8987931-8999250
chr19	9065502	9066504	chr19-9065502-9066504
chr19	9071837	9072164	chr19-9071837-9072164
chr19	9079536	9080268	chr19-9079536-9080268
chr19	9081761	9083248	chr19-9081761-9083248
chr19	9106103	9107801	chr19-9106103-9107801
chr19	9134478	9135940	chr19-9134478-9135940
chr19	9836406	9837182	chr19-9836406-9837182
chr19	9897103	9900854	chr19-9897103-9900854
chr19	9931094	9931295	chr19-9931094-9931295
chr19	9938820	9939129	chr19-9938820-9939129
chr19	9974714	9975395	chr19-9974714-9975395
chr19	9978087	9979185	chr19-9978087-9979185
chr19	9981549	9985495	chr19-9981549-9985495

chr19	9992999	9993200	chr19-9992999-9993200
chr19	10017801	10018860	chr19-10017801-10018860
chr19	10038888	10047711	chr19-10038888-10047711
chr19	10056267	10056573	chr19-10056267-10056573
chr19	10099654	10102943	chr19-10099654-10102943
chr19	10182239	10184741	chr19-10182239-10184741
chr19	10202657	10205117	chr19-10202657-10205117
chr19	10240100	10241018	chr19-10240100-10241018
chr19	10266714	10267435	chr19-10266714-10267435
chr19	10275841	10280312	chr19-10275841-10280312
chr19	10286363	10286616	chr19-10286363-10286616
chr19	10289508	10294607	chr19-10289508-10294607
chr19	10296421	10297997	chr19-10296421-10297997
chr19	10302798	10305397	chr19-10302798-10305397
chr19	10388471	10390312	chr19-10388471-10390312
chr19	10417172	10417840	chr19-10417172-10417840
chr19	10435445	10435646	chr19-10435445-10435646
chr19	10456314	10458425	chr19-10456314-10458425
chr19	10495993	10496543	chr19-10495993-10496543
chr19	10497411	10498354	chr19-10497411-10498354
chr19	10523388	10526748	chr19-10523388-10526748
chr19	10554905	10556789	chr19-10554905-10556789
chr19	10561103	10562250	chr19-10561103-10562250
chr19	10576216	10578893	chr19-10576216-10578893
chr19	10602390	10607257	chr19-10602390-10607257
chr19	10687944	10691158	chr19-10687944-10691158
chr19	10740545	10741793	chr19-10740545-10741793
chr19	10781805	10782595	chr19-10781805-10782595
chr19	10818268	10819095	chr19-10818268-10819095
chr19	10867960	10870403	chr19-10867960-10870403
chr19	10879769	10882419	chr19-10879769-10882419
chr19	10894679	10897036	chr19-10894679-10897036
chr19	10946428	10949989	chr19-10946428-10949989
chr19	10979817	10980180	chr19-10979817-10980180
chr19	11044762	11045589	chr19-11044762-11045589
chr19	11092191	11092558	chr19-11092191-11092558
chr19	11120949	11121813	chr19-11120949-11121813
chr19	11274485	11274686	chr19-11274485-11274686
chr19	11769865	11771578	chr19-11769865-11771578
chr19	11816812	11820364	chr19-11816812-11820364
chr19	11868792	11869094	chr19-11868792-11869094
chr19	11911202	11914100	chr19-11911202-11914100

chr19	11964605	11967686	chr19-11964605-11967686
chr19	12002709	12013717	chr19-12002709-12013717
chr19	12336415	12336668	chr19-12336415-12336668
chr19	12463663	12464554	chr19-12463663-12464554
chr19	12475422	12475990	chr19-12475422-12475990
chr19	12488199	12488872	chr19-12488199-12488872
chr19	12491798	12492364	chr19-12491798-12492364
chr19	12499535	12502382	chr19-12499535-12502382
chr19	12517863	12518540	chr19-12517863-12518540
chr19	12572985	12575956	chr19-12572985-12575956
chr19	12595541	12596920	chr19-12595541-12596920
chr19	12791776	12792031	chr19-12791776-12792031
chr19	12793022	12797457	chr19-12793022-12797457
chr19	14596071	14599467	chr19-14596071-14599467
chr19	14655886	14656179	chr19-14655886-14656179
chr19	15456993	15457441	chr19-15456993-15457441
chr19	15801340	15802112	chr19-15801340-15802112
chr19	15922950	15925942	chr19-15922950-15925942
chr19	15941326	15941792	chr19-15941326-15941792
chr19	15946763	15947273	chr19-15946763-15947273
chr19	15983754	15985633	chr19-15983754-15985633
chr19	16028886	16029713	chr19-16028886-16029713
chr19	16049961	16050714	chr19-16049961-16050714
chr19	16064064	16064523	chr19-16064064-16064523
chr19	16131267	16134571	chr19-16131267-16134571
chr19	16204899	16205781	chr19-16204899-16205781
chr19	16250612	16250865	chr19-16250612-16250865
chr19	16253396	16253597	chr19-16253396-16253597
chr19	16254952	16255318	chr19-16254952-16255318
chr19	16272855	16273868	chr19-16272855-16273868
chr19	16325423	16326574	chr19-16325423-16326574
chr19	16327424	16328691	chr19-16327424-16328691
chr19	16372149	16372350	chr19-16372149-16372350
chr19	16386204	16386463	chr19-16386204-16386463
chr19	16435032	16436985	chr19-16435032-16436985
chr19	16778567	16781857	chr19-16778567-16781857
chr19	16871687	16874978	chr19-16871687-16874978
chr19	16876124	16876516	chr19-16876124-16876516
chr19	16955626	16956799	chr19-16955626-16956799
chr19	17011347	17011749	chr19-17011347-17011749
chr19	17111743	17111944	chr19-17111743-17111944
chr19	17136601	17137215	chr19-17136601-17137215

chr19	17163753	17163954	chr19-17163753-17163954
chr19	17256293	17257088	chr19-17256293-17257088
chr19	17334842	17335724	chr19-17334842-17335724
chr19	17339423	17339674	chr19-17339423-17339674
chr19	17355459	17356926	chr19-17355459-17356926
chr19	17393251	17395939	chr19-17393251-17395939
chr19	17836788	17837894	chr19-17836788-17837894
chr19	18629693	18632524	chr19-18629693-18632524
chr19	18658659	18658928	chr19-18658659-18658928
chr19	18670128	18673568	chr19-18670128-18673568
chr19	18712164	18715799	chr19-18712164-18715799
chr19	18758325	18759255	chr19-18758325-18759255
chr19	18766657	18766963	chr19-18766657-18766963
chr19	19040674	19040875	chr19-19040674-19040875
chr19	19109791	19111836	chr19-19109791-19111836
chr19	19246530	19247351	chr19-19246530-19247351
chr19	20387740	20392224	chr19-20387740-20392224
chr19	20393785	20394599	chr19-20393785-20394599
chr19	20402805	20403258	chr19-20402805-20403258
chr19	20404936	20405191	chr19-20404936-20405191
chr19	20406038	20407077	chr19-20406038-20407077
chr19	20529855	20530624	chr19-20529855-20530624
chr19	20600847	20602296	chr19-20600847-20602296
chr19	21077686	21078041	chr19-21077686-21078041
chr19	21103781	21107124	chr19-21103781-21107124
chr19	21144281	21144748	chr19-21144281-21144748
chr19	21271109	21276099	chr19-21271109-21276099
chr19	21471968	21473075	chr19-21471968-21473075
chr19	21637302	21637957	chr19-21637302-21637957
chr19	21646127	21646495	chr19-21646127-21646495
chr19	21651226	21655615	chr19-21651226-21655615
chr19	21774036	21774340	chr19-21774036-21774340
chr19	21777873	21781270	chr19-21777873-21781270
chr19	21783977	21785070	chr19-21783977-21785070
chr19	21862594	21863806	chr19-21862594-21863806
chr19	21881907	21883331	chr19-21881907-21883331
chr19	22138781	22139405	chr19-22138781-22139405
chr19	22179121	22179372	chr19-22179121-22179372
chr19	22304160	22304515	chr19-22304160-22304515
chr19	22915909	22918005	chr19-22915909-22918005
chr19	23091710	23092015	chr19-23091710-23092015
chr19	23133594	23137030	chr19-23133594-23137030

chr19	23139556	23145073	chr19-23139556-23145073
chr19	23149489	23151757	chr19-23149489-23151757
chr19	23272265	23274395	chr19-23272265-23274395
chr19	23447051	23448538	chr19-23447051-23448538
chr19	23686820	23688973	chr19-23686820-23688973
chr19	23757882	23759782	chr19-23757882-23759782
chr19	23892755	23893780	chr19-23892755-23893780
chr19	24016996	24017247	chr19-24016996-24017247
chr19	24029626	24031667	chr19-24029626-24031667
chr19	24163988	24164683	chr19-24163988-24164683
chr19	24166041	24169780	chr19-24166041-24169780
chr19	24170636	24170897	chr19-24170636-24170897
chr19	24171712	24176796	chr19-24171712-24176796
chr19	24210654	24213236	chr19-24210654-24213236
chr19	24215924	24216336	chr19-24215924-24216336
chr19	24220876	24226139	chr19-24220876-24226139
chr19	24278907	24281241	chr19-24278907-24281241
chr19	24293063	24293621	chr19-24293063-24293621
chr19	24301074	24301429	chr19-24301074-24301429
chr19	24426772	24427588	chr19-24426772-24427588
chr19	24450899	24451100	chr19-24450899-24451100
chr19	24476158	24478356	chr19-24476158-24478356
chr19	24514386	24515282	chr19-24514386-24515282
chr19	24553529	24557784	chr19-24553529-24557784
chr19	24571647	24572193	chr19-24571647-24572193
chr19	24615380	24616441	chr19-24615380-24616441
chr19	24642127	24642478	chr19-24642127-24642478
chr19	24861325	24862191	chr19-24861325-24862191
chr19	24871235	24871436	chr19-24871235-24871436
chr19	24897235	24897958	chr19-24897235-24897958
chr19	24899225	24900973	chr19-24899225-24900973
chr19	24960212	24961836	chr19-24960212-24961836
chr19	24998617	25001069	chr19-24998617-25001069
chr19	25096964	25097165	chr19-25096964-25097165
chr19	25236107	25238543	chr19-25236107-25238543
chr19	25267321	25268896	chr19-25267321-25268896
chr19	25343763	25344074	chr19-25343763-25344074
chr19	25399740	25401031	chr19-25399740-25401031
chr19	25406110	25408461	chr19-25406110-25408461
chr19	25505404	25507005	chr19-25505404-25507005
chr19	25605659	25606513	chr19-25605659-25606513
chr19	25609991	25611639	chr19-25609991-25611639

chr19	25622378	25622798	chr19-25622378-25622798
chr19	25635751	25636113	chr19-25635751-25636113
chr19	25671960	25674189	chr19-25671960-25674189
chr19	26604036	26608161	chr19-26604036-26608161
chr19	26746835	26750253	chr19-26746835-26750253
chr19	26752695	26754982	chr19-26752695-26754982
chr19	26845181	26846421	chr19-26845181-26846421
chr19	27031147	27031449	chr19-27031147-27031449
chr19	27134665	27134866	chr19-27134665-27134866
chr19	27216196	27219682	chr19-27216196-27219682
chr19	27351546	27351747	chr19-27351546-27351747
chr19	27364355	27364829	chr19-27364355-27364829
chr19	27369469	27369670	chr19-27369469-27369670
chr19	27428454	27431260	chr19-27428454-27431260
chr19	28009699	28012657	chr19-28009699-28012657
chr19	28389468	28389722	chr19-28389468-28389722
chr19	28558475	28559686	chr19-28558475-28559686
chr19	28677198	28680742	chr19-28677198-28680742
chr19	28834463	28835723	chr19-28834463-28835723
chr19	28963459	28965185	chr19-28963459-28965185
chr19	28974936	28975757	chr19-28974936-28975757
chr19	28989465	28991932	chr19-28989465-28991932
chr19	29046095	29048646	chr19-29046095-29048646
chr19	29063717	29064712	chr19-29063717-29064712
chr19	29067304	29067505	chr19-29067304-29067505
chr19	29100273	29103301	chr19-29100273-29103301
chr19	29215789	29216330	chr19-29215789-29216330
chr19	29238656	29239572	chr19-29238656-29239572
chr19	29241385	29242407	chr19-29241385-29242407
chr19	29249163	29254288	chr19-29249163-29254288
chr19	29271136	29271604	chr19-29271136-29271604
chr19	29324933	29325798	chr19-29324933-29325798
chr19	29334131	29334744	chr19-29334131-29334744
chr19	29354961	29355837	chr19-29354961-29355837
chr19	29361147	29362557	chr19-29361147-29362557
chr19	29366249	29367958	chr19-29366249-29367958
chr19	29371580	29372356	chr19-29371580-29372356
chr19	29470608	29470809	chr19-29470608-29470809
chr19	29501128	29501479	chr19-29501128-29501479
chr19	29521118	29524576	chr19-29521118-29524576
chr19	29640880	29641891	chr19-29640880-29641891
chr19	29647386	29648745	chr19-29647386-29648745

chr19	29707979	29708483	chr19-29707979-29708483
chr19	29803987	29807336	chr19-29803987-29807336
chr19	29811994	29813702	chr19-29811994-29813702
chr19	29945687	29945992	chr19-29945687-29945992
chr19	29948434	29948712	chr19-29948434-29948712
chr19	30029837	30032966	chr19-30029837-30032966
chr19	30113602	30113904	chr19-30113602-30113904
chr19	30115106	30115409	chr19-30115106-30115409
chr19	30119169	30119491	chr19-30119169-30119491
chr19	30125355	30125633	chr19-30125355-30125633
chr19	30174748	30175615	chr19-30174748-30175615
chr19	30539453	30539855	chr19-30539453-30539855
chr19	30554472	30554673	chr19-30554472-30554673
chr19	31082218	31084818	chr19-31082218-31084818
chr19	31663864	31664929	chr19-31663864-31664929
chr19	31762457	31765356	chr19-31762457-31765356
chr19	32106773	32108421	chr19-32106773-32108421
chr19	32179290	32182305	chr19-32179290-32182305
chr19	32188962	32189325	chr19-32188962-32189325
chr19	32196878	32198128	chr19-32196878-32198128
chr19	32202791	32202992	chr19-32202791-32202992
chr19	32209477	32211540	chr19-32209477-32211540
chr19	32241959	32242792	chr19-32241959-32242792
chr19	32291529	32293373	chr19-32291529-32293373
chr19	32303918	32305079	chr19-32303918-32305079
chr19	32319490	32320649	chr19-32319490-32320649
chr19	32321735	32323339	chr19-32321735-32323339
chr19	32343994	32344775	chr19-32343994-32344775
chr19	32345826	32346738	chr19-32345826-32346738
chr19	32386426	32390481	chr19-32386426-32390481
chr19	32484939	32487628	chr19-32484939-32487628
chr19	32595265	32596496	chr19-32595265-32596496
chr19	32710577	32713085	chr19-32710577-32713085
chr19	32755442	32761023	chr19-32755442-32761023
chr19	32956940	32957215	chr19-32956940-32957215
chr19	33391504	33392634	chr19-33391504-33392634
chr19	33589531	33590036	chr19-33589531-33590036
chr19	34165667	34166071	chr19-34165667-34166071
chr19	34171504	34171705	chr19-34171504-34171705
chr19	34191720	34195148	chr19-34191720-34195148
chr19	34197839	34198040	chr19-34197839-34198040
chr19	34290115	34291533	chr19-34290115-34291533

chr19	34445567	34445818	chr19-34445567-34445818
chr19	34474200	34475919	chr19-34474200-34475919
chr19	34526554	34527706	chr19-34526554-34527706
chr19	34550331	34552214	chr19-34550331-34552214
chr19	34553173	34553426	chr19-34553173-34553426
chr19	34607559	34607827	chr19-34607559-34607827
chr19	34640559	34640911	chr19-34640559-34640911
chr19	34662125	34662631	chr19-34662125-34662631
chr19	34746710	34747539	chr19-34746710-34747539
chr19	34846474	34846675	chr19-34846474-34846675
chr19	34877355	34880023	chr19-34877355-34880023
chr19	34921025	34923810	chr19-34921025-34923810
chr19	36056617	36057948	chr19-36056617-36057948
chr19	36083006	36084880	chr19-36083006-36084880
chr19	36112104	36112305	chr19-36112104-36112305
chr19	36113307	36120840	chr19-36113307-36120840
chr19	36128562	36131280	chr19-36128562-36131280
chr19	36207737	36208153	chr19-36207737-36208153
chr19	36347426	36349929	chr19-36347426-36349929
chr19	36408971	36410956	chr19-36408971-36410956
chr19	36477535	36477736	chr19-36477535-36477736
chr19	36496903	36497259	chr19-36496903-36497259
chr19	36534749	36535217	chr19-36534749-36535217
chr19	36553667	36555549	chr19-36553667-36555549
chr19	36736305	36737274	chr19-36736305-36737274
chr19	36815941	36816142	chr19-36815941-36816142
chr19	36832901	36836835	chr19-36832901-36836835
chr19	36904351	36904552	chr19-36904351-36904552
chr19	36918142	36919865	chr19-36918142-36919865
chr19	36925030	36928238	chr19-36925030-36928238
chr19	37018228	37018952	chr19-37018228-37018952
chr19	37173753	37175852	chr19-37173753-37175852
chr19	37206451	37209847	chr19-37206451-37209847
chr19	37233696	37234407	chr19-37233696-37234407
chr19	37263341	37265106	chr19-37263341-37265106
chr19	37347489	37347786	chr19-37347489-37347786
chr19	37375227	37378465	chr19-37375227-37378465
chr19	37433858	37436168	chr19-37433858-37436168
chr19	37514440	37515680	chr19-37514440-37515680
chr19	37520927	37521405	chr19-37520927-37521405
chr19	37549910	37551506	chr19-37549910-37551506
chr19	37673331	37673582	chr19-37673331-37673582

chr19	37676432	37676633	chr19-37676432-37676633
chr19	37692664	37693475	chr19-37692664-37693475
chr19	37696438	37699350	chr19-37696438-37699350
chr19	37814019	37815010	chr19-37814019-37815010
chr19	37820985	37821942	chr19-37820985-37821942
chr19	37886426	37886978	chr19-37886426-37886978
chr19	37956270	37960682	chr19-37956270-37960682
chr19	37970025	37970276	chr19-37970025-37970276
chr19	37985711	37986779	chr19-37985711-37986779
chr19	38004381	38005839	chr19-38004381-38005839
chr19	38015318	38017598	chr19-38015318-38017598
chr19	38021402	38031588	chr19-38021402-38031588
chr19	38032571	38044094	chr19-38032571-38044094
chr19	38053429	38056725	chr19-38053429-38056725
chr19	38083210	38083518	chr19-38083210-38083518
chr19	38096672	38097892	chr19-38096672-38097892
chr19	38124181	38125354	chr19-38124181-38125354
chr19	38134514	38134767	chr19-38134514-38134767
chr19	38223008	38224812	chr19-38223008-38224812
chr19	38395337	38397495	chr19-38395337-38397495
chr19	38480595	38481793	chr19-38480595-38481793
chr19	38645111	38645312	chr19-38645111-38645312
chr19	38817505	38819896	chr19-38817505-38819896
chr19	38835980	38838447	chr19-38835980-38838447
chr19	38930102	38932840	chr19-38930102-38932840
chr19	40267437	40272198	chr19-40267437-40272198
chr19	40307120	40308279	chr19-40307120-40308279
chr19	40366596	40366797	chr19-40366596-40366797
chr19	40400900	40402595	chr19-40400900-40402595
chr19	40487643	40487965	chr19-40487643-40487965
chr19	40512368	40514322	chr19-40512368-40514322
chr19	40586832	40589986	chr19-40586832-40589986
chr19	40611252	40612671	chr19-40611252-40612671
chr19	40626108	40626415	chr19-40626108-40626415
chr19	40658718	40664910	chr19-40658718-40664910
chr19	40666707	40667010	chr19-40666707-40667010
chr19	40675902	40676749	chr19-40675902-40676749
chr19	40719915	40720451	chr19-40719915-40720451
chr19	40723607	40723858	chr19-40723607-40723858
chr19	40748480	40748898	chr19-40748480-40748898
chr19	40762014	40762544	chr19-40762014-40762544
chr19	40830358	40833214	chr19-40830358-40833214

chr19	40834287	40834644	chr19-40834287-40834644
chr19	40872945	40873958	chr19-40872945-40873958
chr19	40894056	40895802	chr19-40894056-40895802
chr19	40993841	40996133	chr19-40993841-40996133
chr19	40998518	40998775	chr19-40998518-40998775
chr19	41016145	41016346	chr19-41016145-41016346
chr19	41088506	41088811	chr19-41088506-41088811
chr19	41205973	41207526	chr19-41205973-41207526
chr19	41261527	41265081	chr19-41261527-41265081
chr19	41270029	41270736	chr19-41270029-41270736
chr19	41310829	41311689	chr19-41310829-41311689
chr19	41346944	41347145	chr19-41346944-41347145
chr19	41384499	41385606	chr19-41384499-41385606
chr19	41454739	41455092	chr19-41454739-41455092
chr19	41477674	41477976	chr19-41477674-41477976
chr19	41481129	41484715	chr19-41481129-41484715
chr19	41485646	41485900	chr19-41485646-41485900
chr19	41495025	41495278	chr19-41495025-41495278
chr19	41596980	41597664	chr19-41596980-41597664
chr19	41620546	41620967	chr19-41620546-41620967
chr19	41743050	41743810	chr19-41743050-41743810
chr19	41793775	41793976	chr19-41793775-41793976
chr19	41800419	41802638	chr19-41800419-41802638
chr19	41829255	41831674	chr19-41829255-41831674
chr19	41837806	41838007	chr19-41837806-41838007
chr19	41846060	41849381	chr19-41846060-41849381
chr19	41856863	41858278	chr19-41856863-41858278
chr19	41894606	41896803	chr19-41894606-41896803
chr19	41911161	41915179	chr19-41911161-41915179
chr19	41931990	41934859	chr19-41931990-41934859
chr19	41979978	41983197	chr19-41979978-41983197
chr19	41993535	41994496	chr19-41993535-41994496
chr19	42016836	42018287	chr19-42016836-42018287
chr19	42040050	42040530	chr19-42040050-42040530
chr19	42045257	42046375	chr19-42045257-42046375
chr19	42058032	42058829	chr19-42058032-42058829
chr19	42079740	42079995	chr19-42079740-42079995
chr19	42085551	42086621	chr19-42085551-42086621
chr19	42088992	42092279	chr19-42088992-42092279
chr19	42125188	42131621	chr19-42125188-42131621
chr19	42146979	42149276	chr19-42146979-42149276
chr19	42165939	42166458	chr19-42165939-42166458

chr19	42169850	42172082	chr19-42169850-42172082
chr19	42201460	42202299	chr19-42201460-42202299
chr19	42255463	42256275	chr19-42255463-42256275
chr19	42257972	42258918	chr19-42257972-42258918
chr19	42268079	42268846	chr19-42268079-42268846
chr19	42377311	42377612	chr19-42377311-42377612
chr19	42431009	42432716	chr19-42431009-42432716
chr19	42518010	42520276	chr19-42518010-42520276
chr19	42611101	42615185	chr19-42611101-42615185
chr19	42631270	42632841	chr19-42631270-42632841
chr19	42659526	42660736	chr19-42659526-42660736
chr19	42661710	42661911	chr19-42661710-42661911
chr19	42689486	42690517	chr19-42689486-42690517
chr19	42741699	42742422	chr19-42741699-42742422
chr19	42752122	42754032	chr19-42752122-42754032
chr19	42769436	42771685	chr19-42769436-42771685
chr19	42778765	42780672	chr19-42778765-42780672
chr19	42795073	42795632	chr19-42795073-42795632
chr19	42855847	42856255	chr19-42855847-42856255
chr19	43386606	43386807	chr19-43386606-43386807
chr19	43440167	43441755	chr19-43440167-43441755
chr19	43498720	43499029	chr19-43498720-43499029
chr19	43522811	43525564	chr19-43522811-43525564
chr19	43530328	43531318	chr19-43530328-43531318
chr19	43550987	43551662	chr19-43550987-43551662
chr19	43564348	43564751	chr19-43564348-43564751
chr19	43571050	43571760	chr19-43571050-43571760
chr19	43584796	43585248	chr19-43584796-43585248
chr19	43599664	43600219	chr19-43599664-43600219
chr19	43606784	43607057	chr19-43606784-43607057
chr19	43607957	43608158	chr19-43607957-43608158
chr19	43610763	43610964	chr19-43610763-43610964
chr19	43611995	43617046	chr19-43611995-43617046
chr19	43620073	43620374	chr19-43620073-43620374
chr19	43655148	43655349	chr19-43655148-43655349
chr19	43672985	43676922	chr19-43672985-43676922
chr19	43689017	43690877	chr19-43689017-43690877
chr19	43751930	43753965	chr19-43751930-43753965
chr19	43875849	43881040	chr19-43875849-43881040
chr19	43884671	43891195	chr19-43884671-43891195
chr19	43894242	43895357	chr19-43894242-43895357
chr19	43896745	43897069	chr19-43896745-43897069

chr19	43916322	43918013	chr19-43916322-43918013
chr19	43938301	43940798	chr19-43938301-43940798
chr19	43959922	43960324	chr19-43959922-43960324
chr19	43962745	43962996	chr19-43962745-43962996
chr19	43985996	43986503	chr19-43985996-43986503
chr19	44014905	44015247	chr19-44014905-44015247
chr19	44068388	44070301	chr19-44068388-44070301
chr19	44106322	44108344	chr19-44106322-44108344
chr19	44134453	44136511	chr19-44134453-44136511
chr19	44145401	44146926	chr19-44145401-44146926
chr19	44237420	44238080	chr19-44237420-44238080
chr19	44246048	44249789	chr19-44246048-44249789
chr19	44266511	44268183	chr19-44266511-44268183
chr19	44279153	44279408	chr19-44279153-44279408
chr19	44280751	44285219	chr19-44280751-44285219
chr19	44292428	44301488	chr19-44292428-44301488
chr19	44314119	44315599	chr19-44314119-44315599
chr19	44378078	44378500	chr19-44378078-44378500
chr19	44387811	44389329	chr19-44387811-44389329
chr19	44405850	44409048	chr19-44405850-44409048
chr19	44511140	44512182	chr19-44511140-44512182
chr19	44545103	44545965	chr19-44545103-44545965
chr19	44554350	44555857	chr19-44554350-44555857
chr19	44562366	44564421	chr19-44562366-44564421
chr19	44640096	44640418	chr19-44640096-44640418
chr19	44670420	44672614	chr19-44670420-44672614
chr19	44683853	44684608	chr19-44683853-44684608
chr19	44692571	44693096	chr19-44692571-44693096
chr19	44719646	44720738	chr19-44719646-44720738
chr19	44723878	44724576	chr19-44723878-44724576
chr19	44725961	44726840	chr19-44725961-44726840
chr19	44734333	44735150	chr19-44734333-44735150
chr19	44741136	44742510	chr19-44741136-44742510
chr19	44746613	44747018	chr19-44746613-44747018
chr19	44750226	44751300	chr19-44750226-44751300
chr19	44755059	44757609	chr19-44755059-44757609
chr19	44758841	44760136	chr19-44758841-44760136
chr19	44835417	44838111	chr19-44835417-44838111
chr19	44882502	44882853	chr19-44882502-44882853
chr19	44898410	44899943	chr19-44898410-44899943
chr19	44927673	44927983	chr19-44927673-44927983
chr19	44929933	44933031	chr19-44929933-44933031

chr19	44988756	44989826	chr19-44988756-44989826
chr19	45005253	45010904	chr19-45005253-45010904
chr19	45013618	45019572	chr19-45013618-45019572
chr19	45020443	45021759	chr19-45020443-45021759
chr19	45030587	45031045	chr19-45030587-45031045
chr19	45045443	45049964	chr19-45045443-45049964
chr19	45055142	45055694	chr19-45055142-45055694
chr19	45057306	45060022	chr19-45057306-45060022
chr19	45073918	45077526	chr19-45073918-45077526
chr19	45079293	45081322	chr19-45079293-45081322
chr19	45143438	45143899	chr19-45143438-45143899
chr19	45150499	45151561	chr19-45150499-45151561
chr19	45154025	45154329	chr19-45154025-45154329
chr19	45160605	45161336	chr19-45160605-45161336
chr19	45163101	45163302	chr19-45163101-45163302
chr19	45220925	45221766	chr19-45220925-45221766
chr19	45223706	45224337	chr19-45223706-45224337
chr19	45229253	45237026	chr19-45229253-45237026
chr19	45301035	45302526	chr19-45301035-45302526
chr19	45310955	45311333	chr19-45310955-45311333
chr19	45362998	45364649	chr19-45362998-45364649
chr19	45541082	45542206	chr19-45541082-45542206
chr19	45544180	45545414	chr19-45544180-45545414
chr19	45559505	45561350	chr19-45559505-45561350
chr19	45573953	45575373	chr19-45573953-45575373
chr19	45658864	45661057	chr19-45658864-45661057
chr19	45675678	45677003	chr19-45675678-45677003
chr19	45736862	45737650	chr19-45736862-45737650
chr19	45742058	45743508	chr19-45742058-45743508
chr19	45747320	45750201	chr19-45747320-45750201
chr19	45780320	45784710	chr19-45780320-45784710
chr19	45790364	45791146	chr19-45790364-45791146
chr19	45805201	45805455	chr19-45805201-45805455
chr19	45811993	45813304	chr19-45811993-45813304
chr19	45997430	45999306	chr19-45997430-45999306
chr19	46002942	46005526	chr19-46002942-46005526
chr19	46024809	46025552	chr19-46024809-46025552
chr19	46037154	46046392	chr19-46037154-46046392
chr19	46055734	46058835	chr19-46055734-46058835
chr19	46075286	46078118	chr19-46075286-46078118
chr19	46131356	46132346	chr19-46131356-46132346
chr19	46135350	46137588	chr19-46135350-46137588

chr19	46147424	46149392	chr19-46147424-46149392
chr19	46151707	46153699	chr19-46151707-46153699
chr19	46173209	46173465	chr19-46173209-46173465
chr19	46300239	46300440	chr19-46300239-46300440
chr19	46303558	46307141	chr19-46303558-46307141
chr19	46308879	46309284	chr19-46308879-46309284
chr19	46314234	46315154	chr19-46314234-46315154
chr19	46317399	46319679	chr19-46317399-46319679
chr19	46326477	46330196	chr19-46326477-46330196
chr19	46337688	46339532	chr19-46337688-46339532
chr19	46347747	46350730	chr19-46347747-46350730
chr19	46356233	46358679	chr19-46356233-46358679
chr19	46394012	46398713	chr19-46394012-46398713
chr19	46454228	46455325	chr19-46454228-46455325
chr19	46458867	46460414	chr19-46458867-46460414
chr19	46470924	46472014	chr19-46470924-46472014
chr19	46477325	46480473	chr19-46477325-46480473
chr19	46481367	46486321	chr19-46481367-46486321
chr19	46499392	46512137	chr19-46499392-46512137
chr19	46518030	46519012	chr19-46518030-46519012
chr19	46520949	46525326	chr19-46520949-46525326
chr19	46528161	46529712	chr19-46528161-46529712
chr19	46532036	46532583	chr19-46532036-46532583
chr19	46571795	46574398	chr19-46571795-46574398
chr19	46598322	46600885	chr19-46598322-46600885
chr19	46641762	46642076	chr19-46641762-46642076
chr19	46689467	46690780	chr19-46689467-46690780
chr19	46707278	46708040	chr19-46707278-46708040
chr19	46760785	46763252	chr19-46760785-46763252
chr19	46959050	46964177	chr19-46959050-46964177
chr19	46968616	46971439	chr19-46968616-46971439
chr19	46979087	46979985	chr19-46979087-46979985
chr19	46981939	46985911	chr19-46981939-46985911
chr19	46986774	46990263	chr19-46986774-46990263
chr19	47014151	47015934	chr19-47014151-47015934
chr19	47049082	47051402	chr19-47049082-47051402
chr19	47066937	47069076	chr19-47066937-47069076
chr19	47088935	47092304	chr19-47088935-47092304
chr19	47151538	47152097	chr19-47151538-47152097
chr19	47168418	47169157	chr19-47168418-47169157
chr19	47177869	47180026	chr19-47177869-47180026
chr19	47219030	47220292	chr19-47219030-47220292

chr19	47232861	47233577	chr19-47232861-47233577
chr19	47252989	47253799	chr19-47252989-47253799
chr19	47312275	47316713	chr19-47312275-47316713
chr19	47330567	47331432	chr19-47330567-47331432
chr19	47343136	47344010	chr19-47343136-47344010
chr19	47346216	47346834	chr19-47346216-47346834
chr19	47356250	47356451	chr19-47356250-47356451
chr19	47359305	47360041	chr19-47359305-47360041
chr19	47443653	47443954	chr19-47443653-47443954
chr19	47463109	47465328	chr19-47463109-47465328
chr19	47483840	47484041	chr19-47483840-47484041
chr19	47491749	47492160	chr19-47491749-47492160
chr19	47506920	47511164	chr19-47506920-47511164
chr19	47512065	47516275	chr19-47512065-47516275
chr19	47535949	47538998	chr19-47535949-47538998
chr19	47578416	47582909	chr19-47578416-47582909
chr19	47730475	47732541	chr19-47730475-47732541
chr19	47836730	47837950	chr19-47836730-47837950
chr19	47851206	47852195	chr19-47851206-47852195
chr19	47853760	47858700	chr19-47853760-47858700
chr19	47864935	47866295	chr19-47864935-47866295
chr19	47867807	47868110	chr19-47867807-47868110
chr19	47869619	47870556	chr19-47869619-47870556
chr19	47886930	47887294	chr19-47886930-47887294
chr19	47901746	47903346	chr19-47901746-47903346
chr19	47909727	47910092	chr19-47909727-47910092
chr19	47915861	47920610	chr19-47915861-47920610
chr19	47928672	47929994	chr19-47928672-47929994
chr19	47934245	47935319	chr19-47934245-47935319
chr19	47937227	47938188	chr19-47937227-47938188
chr19	48001832	48002131	chr19-48001832-48002131
chr19	48205108	48205574	chr19-48205108-48205574
chr19	48946995	48947196	chr19-48946995-48947196
chr19	49274889	49275253	chr19-49274889-49275253
chr19	50677956	50679092	chr19-50677956-50679092
chr19	52923198	52923505	chr19-52923198-52923505
chr19	53007806	53008969	chr19-53007806-53008969
chr19	53031971	53032668	chr19-53031971-53032668
chr19	53036933	53041964	chr19-53036933-53041964
chr19	53140415	53144117	chr19-53140415-53144117
chr19	53181539	53181846	chr19-53181539-53181846
chr19	53276964	53277165	chr19-53276964-53277165

chr19	53310159	53314819	chr19-53310159-53314819
chr19	53325276	53326237	chr19-53325276-53326237
chr19	53329045	53330651	chr19-53329045-53330651
chr19	53365129	53365330	chr19-53365129-53365330
chr19	53388963	53392284	chr19-53388963-53392284
chr19	53404120	53406329	chr19-53404120-53406329
chr19	53411828	53412926	chr19-53411828-53412926
chr19	53414017	53414319	chr19-53414017-53414319
chr19	53420246	53424307	chr19-53420246-53424307
chr19	53433991	53434350	chr19-53433991-53434350
chr19	53447360	53454087	chr19-53447360-53454087
chr19	53458973	53462394	chr19-53458973-53462394
chr19	53464163	53465314	chr19-53464163-53465314
chr19	53484706	53484907	chr19-53484706-53484907
chr19	53490778	53491507	chr19-53490778-53491507
chr19	53500374	53500959	chr19-53500374-53500959
chr19	53517461	53518029	chr19-53517461-53518029
chr19	53527985	53534223	chr19-53527985-53534223
chr19	53535577	53535778	chr19-53535577-53535778
chr19	53536728	53536929	chr19-53536728-53536929
chr19	53552487	53558432	chr19-53552487-53558432
chr19	53564157	53564358	chr19-53564157-53564358
chr19	53565748	53566377	chr19-53565748-53566377
chr19	53572485	53572906	chr19-53572485-53572906
chr19	53599872	53601902	chr19-53599872-53601902
chr19	53676191	53677731	chr19-53676191-53677731
chr19	53704919	53705541	chr19-53704919-53705541
chr19	53727405	53727978	chr19-53727405-53727978
chr19	53792533	53793688	chr19-53792533-53793688
chr19	53800389	53801111	chr19-53800389-53801111
chr19	53824805	53825616	chr19-53824805-53825616
chr19	53859308	53859560	chr19-53859308-53859560
chr19	53902482	53904610	chr19-53902482-53904610
chr19	53942863	53946351	chr19-53942863-53946351
chr19	54044449	54047516	chr19-54044449-54047516
chr19	54114648	54115637	chr19-54114648-54115637
chr19	54982538	54982739	chr19-54982538-54982739
chr19	55061229	55062104	chr19-55061229-55062104
chr19	55098134	55100409	chr19-55098134-55100409
chr19	55218181	55219759	chr19-55218181-55219759
chr19	55244462	55245627	chr19-55244462-55245627
chr19	55252774	55259606	chr19-55252774-55259606

chr19	55314453	55317396	chr19-55314453-55317396
chr19	55423471	55424375	chr19-55423471-55424375
chr19	55440703	55440955	chr19-55440703-55440955
chr19	55542257	55543007	chr19-55542257-55543007
chr19	55620142	55621405	chr19-55620142-55621405
chr19	55643218	55644107	chr19-55643218-55644107
chr19	55712218	55712861	chr19-55712218-55712861
chr19	55732804	55733111	chr19-55732804-55733111
chr19	55741064	55744952	chr19-55741064-55744952
chr19	55754513	55755023	chr19-55754513-55755023
chr19	55795878	55796079	chr19-55795878-55796079
chr19	55895196	55895758	chr19-55895196-55895758
chr19	56037465	56037724	chr19-56037465-56037724
chr19	56122027	56122278	chr19-56122027-56122278
chr19	56320148	56320401	chr19-56320148-56320401
chr19	56356638	56356999	chr19-56356638-56356999
chr19	56396378	56398127	chr19-56396378-56398127
chr19	56400102	56400455	chr19-56400102-56400455
chr19	56452154	56452355	chr19-56452154-56452355
chr19	56455956	56456157	chr19-56455956-56456157
chr19	56461045	56462936	chr19-56461045-56462936
chr19	56510544	56510745	chr19-56510544-56510745
chr19	56546531	56550199	chr19-56546531-56550199
chr19	56588137	56588826	chr19-56588137-56588826
chr19	56703394	56703713	chr19-56703394-56703713
chr19	56705227	56705634	chr19-56705227-56705634
chr19	56708578	56708779	chr19-56708578-56708779
chr19	56721292	56724575	chr19-56721292-56724575
chr19	56820606	56822929	chr19-56820606-56822929
chr19	56826056	56826568	chr19-56826056-56826568
chr19	56873767	56875227	chr19-56873767-56875227
chr19	56905157	56905857	chr19-56905157-56905857
chr19	56914305	56915796	chr19-56914305-56915796
chr19	56934856	56935940	chr19-56934856-56935940
chr19	56936907	56937171	chr19-56936907-56937171
chr19	56939624	56940050	chr19-56939624-56940050
chr19	56990126	56991579	chr19-56990126-56991579
chr19	56994085	56994286	chr19-56994085-56994286
chr19	57007351	57008915	chr19-57007351-57008915
chr19	57014568	57014821	chr19-57014568-57014821
chr19	57055584	57057149	chr19-57055584-57057149
chr19	57098558	57098759	chr19-57098558-57098759

chr19	57118742	57119005	chr19-57118742-57119005
chr19	57180486	57183505	chr19-57180486-57183505
chr19	57194730	57199763	chr19-57194730-57199763
chr19	57203685	57204692	chr19-57203685-57204692
chr19	57237666	57240030	chr19-57237666-57240030
chr19	57240849	57244038	chr19-57240849-57244038
chr19	57272470	57275715	chr19-57272470-57275715
chr19	57313732	57315551	chr19-57313732-57315551
chr19	57328842	57329043	chr19-57328842-57329043
chr19	57358087	57363196	chr19-57358087-57363196
chr19	57404436	57404689	chr19-57404436-57404689
chr19	57418213	57418414	chr19-57418213-57418414
chr19	57452578	57454260	chr19-57452578-57454260
chr19	57600288	57600758	chr19-57600288-57600758
chr19	57609488	57612871	chr19-57609488-57612871
chr19	57627076	57627885	chr19-57627076-57627885
chr19	58104415	58104719	chr19-58104415-58104719
chr19	58113310	58113511	chr19-58113310-58113511
chr19	58165479	58166245	chr19-58165479-58166245
chr19	58453510	58456322	chr19-58453510-58456322
chr19	58860306	58861332	chr19-58860306-58861332
chr19	58903906	58904260	chr19-58903906-58904260
chr19	58911131	58911544	chr19-58911131-58911544
chr19	58918758	58919075	chr19-58918758-58919075
chr19	58942193	58944453	chr19-58942193-58944453
chr19	59071064	59076518	chr19-59071064-59076518
chr19	59114275	59114727	chr19-59114275-59114727
chr19	59165948	59167088	chr19-59165948-59167088
chr19	59169005	59171876	chr19-59169005-59171876
chr19	59193591	59194717	chr19-59193591-59194717
chr19	59260670	59261685	chr19-59260670-59261685
chr19	59290055	59290256	chr19-59290055-59290256
chr19	59341047	59346954	chr19-59341047-59346954
chr19	59388864	59389284	chr19-59388864-59389284
chr19	59400390	59400766	chr19-59400390-59400766
chr19	59451403	59451916	chr19-59451403-59451916
chr19	59457661	59460366	chr19-59457661-59460366
chr19	59467194	59469677	chr19-59467194-59469677
chr19	59471098	59471299	chr19-59471098-59471299
chr19	59877186	59879689	chr19-59877186-59879689
chr19	59941684	59944382	chr19-59941684-59944382
chr19	60144420	60144989	chr19-60144420-60144989

chr19	60225451	60227266	chr19-60225451-60227266
chr19	60467050	60467781	chr19-60467050-60467781
chr19	60578681	60582243	chr19-60578681-60582243
chr19	60616924	60617175	chr19-60616924-60617175
chr19	60755245	60757996	chr19-60755245-60757996
chr19	60785963	60791644	chr19-60785963-60791644
chr19	60810940	60812873	chr19-60810940-60812873
chr19	60860784	60862034	chr19-60860784-60862034
chr19	60873071	60874991	chr19-60873071-60874991
chr19	60889081	60891567	chr19-60889081-60891567
chr19	60950175	60950479	chr19-60950175-60950479
chr19	60956534	60956735	chr19-60956534-60956735
chr19	61139685	61141517	chr19-61139685-61141517
chr19	61151295	61152834	chr19-61151295-61152834
chr19	61224907	61228663	chr19-61224907-61228663
chr2	3117856	3120000	chr2-3117856-3120000
chr2	3140678	3141775	chr2-3140678-3141775
chr2	3178228	3179007	chr2-3178228-3179007
chr2	3239573	3239970	chr2-3239573-3239970
chr2	3277632	3277950	chr2-3277632-3277950
chr2	3282897	3286086	chr2-3282897-3286086
chr2	3330842	3333655	chr2-3330842-3333655
chr2	3335383	3336562	chr2-3335383-3336562
chr2	3418542	3419646	chr2-3418542-3419646
chr2	3423488	3425318	chr2-3423488-3425318
chr2	3473350	3476126	chr2-3473350-3476126
chr2	3511039	3513866	chr2-3511039-3513866
chr2	3542209	3542410	chr2-3542209-3542410
chr2	3657703	3659576	chr2-3657703-3659576
chr2	3682829	3683130	chr2-3682829-3683130
chr2	3711718	3718794	chr2-3711718-3718794
chr2	3720879	3722550	chr2-3720879-3722550
chr2	3723427	3724152	chr2-3723427-3724152
chr2	3748176	3750557	chr2-3748176-3750557
chr2	3752392	3753811	chr2-3752392-3753811
chr2	3799758	3800270	chr2-3799758-3800270
chr2	3958340	3958541	chr2-3958340-3958541
chr2	4152843	4153715	chr2-4152843-4153715
chr2	4300493	4300694	chr2-4300493-4300694
chr2	4400643	4403264	chr2-4400643-4403264
chr2	4421073	4422593	chr2-4421073-4422593
chr2	4445553	4446420	chr2-4445553-4446420

chr2	4476861	4477112	chr2-4476861-4477112
chr2	4480414	4480869	chr2-4480414-4480869
chr2	4558480	4562797	chr2-4558480-4562797
chr2	4650955	4652359	chr2-4650955-4652359
chr2	4717197	4718931	chr2-4717197-4718931
chr2	4824019	4824429	chr2-4824019-4824429
chr2	4829483	4829941	chr2-4829483-4829941
chr2	4880617	4884186	chr2-4880617-4884186
chr2	4917645	4919739	chr2-4917645-4919739
chr2	5011437	5013277	chr2-5011437-5013277
chr2	5062495	5064553	chr2-5062495-5064553
chr2	5068382	5068583	chr2-5068382-5068583
chr2	5137387	5138549	chr2-5137387-5138549
chr2	5286770	5287125	chr2-5286770-5287125
chr2	5613692	5614998	chr2-5613692-5614998
chr2	5618837	5619144	chr2-5618837-5619144
chr2	5712754	5715566	chr2-5712754-5715566
chr2	5730267	5731055	chr2-5730267-5731055
chr2	5769221	5769422	chr2-5769221-5769422
chr2	5843956	5846944	chr2-5843956-5846944
chr2	5894124	5895921	chr2-5894124-5895921
chr2	5942267	5943020	chr2-5942267-5943020
chr2	5949852	5953502	chr2-5949852-5953502
chr2	6071646	6072598	chr2-6071646-6072598
chr2	6074868	6075484	chr2-6074868-6075484
chr2	6077728	6078088	chr2-6077728-6078088
chr2	6079803	6082594	chr2-6079803-6082594
chr2	6096950	6097559	chr2-6096950-6097559
chr2	6098984	6099343	chr2-6098984-6099343
chr2	6100233	6100667	chr2-6100233-6100667
chr2	6117946	6119482	chr2-6117946-6119482
chr2	6125258	6130488	chr2-6125258-6130488
chr2	6131306	6131559	chr2-6131306-6131559
chr2	6157758	6157959	chr2-6157758-6157959
chr2	6160468	6165439	chr2-6160468-6165439
chr2	6211364	6214213	chr2-6211364-6214213
chr2	6256184	6256972	chr2-6256184-6256972
chr2	6264698	6265263	chr2-6264698-6265263
chr2	6267138	6268171	chr2-6267138-6268171
chr2	6271607	6271959	chr2-6271607-6271959
chr2	6293474	6295486	chr2-6293474-6295486
chr2	6320350	6320604	chr2-6320350-6320604

chr2	6321577	6328548	chr2-6321577-6328548
chr2	6341764	6341965	chr2-6341764-6341965
chr2	6348937	6351329	chr2-6348937-6351329
chr2	6352572	6353380	chr2-6352572-6353380
chr2	6358702	6360080	chr2-6358702-6360080
chr2	6366399	6366910	chr2-6366399-6366910
chr2	6377989	6381342	chr2-6377989-6381342
chr2	6463825	6464026	chr2-6463825-6464026
chr2	6593600	6595172	chr2-6593600-6595172
chr2	6721792	6722043	chr2-6721792-6722043
chr2	6871013	6873342	chr2-6871013-6873342
chr2	6903633	6904965	chr2-6903633-6904965
chr2	6906996	6907248	chr2-6906996-6907248
chr2	7079734	7081917	chr2-7079734-7081917
chr2	7089264	7089823	chr2-7089264-7089823
chr2	8729052	8729775	chr2-8729052-8729775
chr2	9482008	9482379	chr2-9482008-9482379
chr2	9574302	9574503	chr2-9574302-9574503
chr2	9620856	9621210	chr2-9620856-9621210
chr2	9792216	9792699	chr2-9792216-9792699
chr2	9876851	9880223	chr2-9876851-9880223
chr2	9881217	9884762	chr2-9881217-9884762
chr2	9888744	9890269	chr2-9888744-9890269
chr2	9895925	9896469	chr2-9895925-9896469
chr2	9955762	9956635	chr2-9955762-9956635
chr2	10047137	10049923	chr2-10047137-10049923
chr2	10078327	10082003	chr2-10078327-10082003
chr2	10094530	10094731	chr2-10094530-10094731
chr2	10153348	10154133	chr2-10153348-10154133
chr2	10199777	10200418	chr2-10199777-10200418
chr2	10300827	10301130	chr2-10300827-10301130
chr2	10370141	10370342	chr2-10370141-10370342
chr2	10371541	10375719	chr2-10371541-10375719
chr2	10674622	10675121	chr2-10674622-10675121
chr2	10825666	10826183	chr2-10825666-10826183
chr2	11171906	11172817	chr2-11171906-11172817
chr2	11192513	11193067	chr2-11192513-11193067
chr2	11375202	11377458	chr2-11375202-11377458
chr2	11397074	11399259	chr2-11397074-11399259
chr2	11425709	11425975	chr2-11425709-11425975
chr2	11457866	11458591	chr2-11457866-11458591
chr2	11461539	11461740	chr2-11461539-11461740

chr2	11499261	11503779	chr2-11499261-11503779
chr2	11530064	11530315	chr2-11530064-11530315
chr2	11534910	11536127	chr2-11534910-11536127
chr2	11552871	11554350	chr2-11552871-11554350
chr2	11601663	11603479	chr2-11601663-11603479
chr2	11651906	11652107	chr2-11651906-11652107
chr2	11705101	11707216	chr2-11705101-11707216
chr2	11767395	11767596	chr2-11767395-11767596
chr2	11776515	11780015	chr2-11776515-11780015
chr2	12301334	12302190	chr2-12301334-12302190
chr2	12411870	12412433	chr2-12411870-12412433
chr2	12417420	12419963	chr2-12417420-12419963
chr2	12923696	12924971	chr2-12923696-12924971
chr2	13010019	13011840	chr2-13010019-13011840
chr2	13270412	13272509	chr2-13270412-13272509
chr2	13371283	13372861	chr2-13371283-13372861
chr2	13385701	13386113	chr2-13385701-13386113
chr2	13409547	13411015	chr2-13409547-13411015
chr2	13414008	13414260	chr2-13414008-13414260
chr2	13451223	13451683	chr2-13451223-13451683
chr2	13456670	13456921	chr2-13456670-13456921
chr2	13526419	13526788	chr2-13526419-13526788
chr2	13543714	13545051	chr2-13543714-13545051
chr2	13554227	13554428	chr2-13554227-13554428
chr2	13557420	13557944	chr2-13557420-13557944
chr2	13573451	13576299	chr2-13573451-13576299
chr2	13792508	13794228	chr2-13792508-13794228
chr2	14054069	14056522	chr2-14054069-14056522
chr2	14072849	14076061	chr2-14072849-14076061
chr2	14279467	14281599	chr2-14279467-14281599
chr2	14603441	14606447	chr2-14603441-14606447
chr2	14744318	14744942	chr2-14744318-14744942
chr2	15048233	15049931	chr2-15048233-15049931
chr2	15054281	15057283	chr2-15054281-15057283
chr2	16355595	16357374	chr2-16355595-16357374
chr2	16406836	16407158	chr2-16406836-16407158
chr2	17729997	17731863	chr2-17729997-17731863
chr2	17741195	17742811	chr2-17741195-17742811
chr2	18005313	18005615	chr2-18005313-18005615
chr2	18025679	18029414	chr2-18025679-18029414
chr2	18031190	18032334	chr2-18031190-18032334
chr2	18045523	18051004	chr2-18045523-18051004

chr2	18055118	18058665	chr2-18055118-18058665
chr2	18063183	18066517	chr2-18063183-18066517
chr2	18391391	18393285	chr2-18391391-18393285
chr2	18586830	18588767	chr2-18586830-18588767
chr2	18607899	18610009	chr2-18607899-18610009
chr2	18671605	18673563	chr2-18671605-18673563
chr2	18675811	18678625	chr2-18675811-18678625
chr2	18680474	18681552	chr2-18680474-18681552
chr2	18688565	18690849	chr2-18688565-18690849
chr2	18693606	18695309	chr2-18693606-18695309
chr2	18698581	18699291	chr2-18698581-18699291
chr2	18765706	18768154	chr2-18765706-18768154
chr2	18799734	18800900	chr2-18799734-18800900
chr2	18821320	18822337	chr2-18821320-18822337
chr2	18996468	18999049	chr2-18996468-18999049
chr2	19199170	19199491	chr2-19199170-19199491
chr2	19276036	19276497	chr2-19276036-19276497
chr2	19327272	19327577	chr2-19327272-19327577
chr2	19371265	19372440	chr2-19371265-19372440
chr2	19414662	19415124	chr2-19414662-19415124
chr2	19430721	19432519	chr2-19430721-19432519
chr2	19445406	19446545	chr2-19445406-19446545
chr2	19448149	19448696	chr2-19448149-19448696
chr2	19553638	19554405	chr2-19553638-19554405
chr2	19566179	19566485	chr2-19566179-19566485
chr2	19647424	19647778	chr2-19647424-19647778
chr2	19656775	19661899	chr2-19656775-19661899
chr2	19664050	19664824	chr2-19664050-19664824
chr2	19665820	19666830	chr2-19665820-19666830
chr2	19685682	19686142	chr2-19685682-19686142
chr2	19909438	19910492	chr2-19909438-19910492
chr2	20339419	20340333	chr2-20339419-20340333
chr2	20445885	20446140	chr2-20445885-20446140
chr2	20518260	20522201	chr2-20518260-20522201
chr2	20526048	20526658	chr2-20526048-20526658
chr2	20546277	20550279	chr2-20546277-20550279
chr2	20555900	20556154	chr2-20555900-20556154
chr2	20559137	20560309	chr2-20559137-20560309
chr2	20583803	20584004	chr2-20583803-20584004
chr2	20653825	20655041	chr2-20653825-20655041
chr2	20735275	20738564	chr2-20735275-20738564
chr2	20752976	20753976	chr2-20752976-20753976

chr2	20925160	20925361	chr2-20925160-20925361
chr2	20926599	20926928	chr2-20926599-20926928
chr2	20937091	20937394	chr2-20937091-20937394
chr2	20943790	20944042	chr2-20943790-20944042
chr2	20961908	20962681	chr2-20961908-20962681
chr2	20964570	20970156	chr2-20964570-20970156
chr2	21029395	21029702	chr2-21029395-21029702
chr2	21141415	21142633	chr2-21141415-21142633
chr2	21204752	21207077	chr2-21204752-21207077
chr2	21234156	21238295	chr2-21234156-21238295
chr2	21258881	21259466	chr2-21258881-21259466
chr2	21367318	21367991	chr2-21367318-21367991
chr2	21909506	21909807	chr2-21909506-21909807
chr2	21918259	21918513	chr2-21918259-21918513
chr2	22227256	22227815	chr2-22227256-22227815
chr2	22338414	22338882	chr2-22338414-22338882
chr2	22621666	22624229	chr2-22621666-22624229
chr2	22652122	22652426	chr2-22652122-22652426
chr2	22654458	22654659	chr2-22654458-22654659
chr2	22723989	22724240	chr2-22723989-22724240
chr2	22774126	22775542	chr2-22774126-22775542
chr2	22894401	22897193	chr2-22894401-22897193
chr2	22997443	22997644	chr2-22997443-22997644
chr2	23006168	23006538	chr2-23006168-23006538
chr2	23011394	23013420	chr2-23011394-23013420
chr2	23014223	23015921	chr2-23014223-23015921
chr2	23034126	23035794	chr2-23034126-23035794
chr2	23036720	23040461	chr2-23036720-23040461
chr2	23067152	23070125	chr2-23067152-23070125
chr2	23154604	23157948	chr2-23154604-23157948
chr2	23234098	23234858	chr2-23234098-23234858
chr2	23321150	23321457	chr2-23321150-23321457
chr2	23570700	23572593	chr2-23570700-23572593
chr2	24294919	24295120	chr2-24294919-24295120
chr2	24336534	24337258	chr2-24336534-24337258
chr2	24366844	24368890	chr2-24366844-24368890
chr2	24385275	24385680	chr2-24385275-24385680
chr2	24422842	24423287	chr2-24422842-24423287
chr2	24438583	24438893	chr2-24438583-24438893
chr2	24440486	24442078	chr2-24440486-24442078
chr2	24453452	24453653	chr2-24453452-24453653
chr2	24474169	24474526	chr2-24474169-24474526

chr2	24479766	24479967	chr2-24479766-24479967
chr2	24521291	24522153	chr2-24521291-24522153
chr2	24562461	24563453	chr2-24562461-24563453
chr2	24608631	24608974	chr2-24608631-24608974
chr2	24678970	24679581	chr2-24678970-24679581
chr2	24719689	24720292	chr2-24719689-24720292
chr2	24761882	24763604	chr2-24761882-24763604
chr2	24917566	24920557	chr2-24917566-24920557
chr2	24926694	24930358	chr2-24926694-24930358
chr2	24933168	24936355	chr2-24933168-24936355
chr2	24941325	24941579	chr2-24941325-24941579
chr2	24948933	24952102	chr2-24948933-24952102
chr2	24962053	24963635	chr2-24962053-24963635
chr2	24973815	24976755	chr2-24973815-24976755
chr2	25029252	25030106	chr2-25029252-25030106
chr2	25054040	25056093	chr2-25054040-25056093
chr2	25110824	25111981	chr2-25110824-25111981
chr2	25123568	25125158	chr2-25123568-25125158
chr2	25126029	25126498	chr2-25126029-25126498
chr2	25140645	25150415	chr2-25140645-25150415
chr2	25167803	25168678	chr2-25167803-25168678
chr2	25170326	25171130	chr2-25170326-25171130
chr2	25172177	25173526	chr2-25172177-25173526
chr2	25175916	25178766	chr2-25175916-25178766
chr2	25179820	25183904	chr2-25179820-25183904
chr2	25185415	25186649	chr2-25185415-25186649
chr2	25193087	25197847	chr2-25193087-25197847
chr2	25198972	25199290	chr2-25198972-25199290
chr2	25209165	25211932	chr2-25209165-25211932
chr2	25221707	25226221	chr2-25221707-25226221
chr2	25228207	25229234	chr2-25228207-25229234
chr2	25236311	25243495	chr2-25236311-25243495
chr2	25254217	25256751	chr2-25254217-25256751
chr2	25261806	25267996	chr2-25261806-25267996
chr2	25270629	25274042	chr2-25270629-25274042
chr2	25285633	25292959	chr2-25285633-25292959
chr2	25304514	25305755	chr2-25304514-25305755
chr2	25310886	25311801	chr2-25310886-25311801
chr2	25318598	25319458	chr2-25318598-25319458
chr2	25331461	25334253	chr2-25331461-25334253
chr2	25355010	25357416	chr2-25355010-25357416
chr2	25362524	25366539	chr2-25362524-25366539

chr2	25371342	25374661	chr2-25371342-25374661
chr2	25394336	25396699	chr2-25394336-25396699
chr2	25400545	25401005	chr2-25400545-25401005
chr2	25402339	25404550	chr2-25402339-25404550
chr2	25428232	25430187	chr2-25428232-25430187
chr2	25455746	25461859	chr2-25455746-25461859
chr2	25465195	25466382	chr2-25465195-25466382
chr2	25468996	25470089	chr2-25468996-25470089
chr2	25497814	25498015	chr2-25497814-25498015
chr2	25500179	25503365	chr2-25500179-25503365
chr2	25540944	25541145	chr2-25540944-25541145
chr2	25545168	25547647	chr2-25545168-25547647
chr2	25548925	25550186	chr2-25548925-25550186
chr2	25556827	25559304	chr2-25556827-25559304
chr2	25573562	25576180	chr2-25573562-25576180
chr2	25577213	25580253	chr2-25577213-25580253
chr2	25581145	25581777	chr2-25581145-25581777
chr2	25606933	25609037	chr2-25606933-25609037
chr2	25616015	25616593	chr2-25616015-25616593
chr2	25621159	25622226	chr2-25621159-25622226
chr2	25661007	25661208	chr2-25661007-25661208
chr2	25706461	25707594	chr2-25706461-25707594
chr2	25863595	25864405	chr2-25863595-25864405
chr2	25877756	25878219	chr2-25877756-25878219
chr2	25981240	25984462	chr2-25981240-25984462
chr2	26020133	26022282	chr2-26020133-26022282
chr2	26066993	26067346	chr2-26066993-26067346
chr2	26090552	26092699	chr2-26090552-26092699
chr2	26118556	26118911	chr2-26118556-26118911
chr2	26121689	26123646	chr2-26121689-26123646
chr2	26132778	26133694	chr2-26132778-26133694
chr2	26138386	26141424	chr2-26138386-26141424
chr2	26198052	26198821	chr2-26198052-26198821
chr2	26202121	26202650	chr2-26202121-26202650
chr2	26206239	26206766	chr2-26206239-26206766
chr2	26236364	26238033	chr2-26236364-26238033
chr2	26298181	26298537	chr2-26298181-26298537
chr2	26305203	26305559	chr2-26305203-26305559
chr2	26312646	26312847	chr2-26312646-26312847
chr2	26315027	26317151	chr2-26315027-26317151
chr2	26350622	26353301	chr2-26350622-26353301
chr2	26379357	26381263	chr2-26379357-26381263

chr2	26387581	26391133	chr2-26387581-26391133
chr2	26405966	26409665	chr2-26405966-26409665
chr2	26417867	26418281	chr2-26417867-26418281
chr2	26443745	26447315	chr2-26443745-26447315
chr2	26458131	26460659	chr2-26458131-26460659
chr2	26481417	26483874	chr2-26481417-26483874
chr2	26492651	26495138	chr2-26492651-26495138
chr2	26500786	26504627	chr2-26500786-26504627
chr2	26507236	26508276	chr2-26507236-26508276
chr2	26525545	26525866	chr2-26525545-26525866
chr2	26531732	26531933	chr2-26531732-26531933
chr2	26534389	26534853	chr2-26534389-26534853
chr2	26585925	26587539	chr2-26585925-26587539
chr2	26600020	26600737	chr2-26600020-26600737
chr2	26602488	26605059	chr2-26602488-26605059
chr2	26607060	26607910	chr2-26607060-26607910
chr2	26610348	26610549	chr2-26610348-26610549
chr2	26611869	26612121	chr2-26611869-26612121
chr2	26627807	26630013	chr2-26627807-26630013
chr2	26637345	26641003	chr2-26637345-26641003
chr2	26642114	26644821	chr2-26642114-26644821
chr2	26649050	26650246	chr2-26649050-26650246
chr2	26661335	26662340	chr2-26661335-26662340
chr2	26901083	26904394	chr2-26901083-26904394
chr2	26908669	26918405	chr2-26908669-26918405
chr2	26930602	26934448	chr2-26930602-26934448
chr2	26962947	26965640	chr2-26962947-26965640
chr2	26977006	26977207	chr2-26977006-26977207
chr2	27009234	27011285	chr2-27009234-27011285
chr2	27027540	27028638	chr2-27027540-27028638
chr2	27142153	27142674	chr2-27142153-27142674
chr2	27246725	27247747	chr2-27246725-27247747
chr2	27304622	27305223	chr2-27304622-27305223
chr2	27326888	27328816	chr2-27326888-27328816
chr2	27331377	27331731	chr2-27331377-27331731
chr2	27413663	27415049	chr2-27413663-27415049
chr2	27416428	27416772	chr2-27416428-27416772
chr2	27418216	27419256	chr2-27418216-27419256
chr2	27424671	27427997	chr2-27424671-27427997
chr2	27438133	27438790	chr2-27438133-27438790
chr2	27441624	27443521	chr2-27441624-27443521
chr2	27473108	27477063	chr2-27473108-27477063

chr2	27504508	27506631	chr2-27504508-27506631
chr2	27513942	27517549	chr2-27513942-27517549
chr2	27539110	27541933	chr2-27539110-27541933
chr2	27572505	27573751	chr2-27572505-27573751
chr2	27671572	27672329	chr2-27671572-27672329
chr2	27674268	27679157	chr2-27674268-27679157
chr2	27718163	27718828	chr2-27718163-27718828
chr2	27740134	27740501	chr2-27740134-27740501
chr2	27785809	27786150	chr2-27785809-27786150
chr2	27802458	27803091	chr2-27802458-27803091
chr2	27843030	27843282	chr2-27843030-27843282
chr2	27844527	27845574	chr2-27844527-27845574
chr2	27858319	27859196	chr2-27858319-27859196
chr2	27885551	27888820	chr2-27885551-27888820
chr2	27915968	27917021	chr2-27915968-27917021
chr2	27936231	27937322	chr2-27936231-27937322
chr2	28192816	28193584	chr2-28192816-28193584
chr2	28205332	28206422	chr2-28205332-28206422
chr2	28446053	28447940	chr2-28446053-28447940
chr2	28465958	28466628	chr2-28465958-28466628
chr2	28467689	28469524	chr2-28467689-28469524
chr2	28512563	28515336	chr2-28512563-28515336
chr2	28517396	28518332	chr2-28517396-28518332
chr2	28523515	28526869	chr2-28523515-28526869
chr2	28532953	28534019	chr2-28532953-28534019
chr2	28581983	28583792	chr2-28581983-28583792
chr2	28640488	28642387	chr2-28640488-28642387
chr2	28699274	28700801	chr2-28699274-28700801
chr2	28838410	28842476	chr2-28838410-28842476
chr2	28935231	28935585	chr2-28935231-28935585
chr2	28939481	28940296	chr2-28939481-28940296
chr2	29053939	29055484	chr2-29053939-29055484
chr2	29059820	29061730	chr2-29059820-29061730
chr2	29089116	29089545	chr2-29089116-29089545
chr2	29100524	29101574	chr2-29100524-29101574
chr2	29124378	29126725	chr2-29124378-29126725
chr2	29194172	29195695	chr2-29194172-29195695
chr2	29250239	29253797	chr2-29250239-29253797
chr2	29346438	29348279	chr2-29346438-29348279
chr2	29530554	29531190	chr2-29530554-29531190
chr2	29619128	29622032	chr2-29619128-29622032
chr2	29623730	29625273	chr2-29623730-29625273

chr2	29627887	29629908	chr2-29627887-29629908
chr2	29632787	29633992	chr2-29632787-29633992
chr2	29670312	29671023	chr2-29670312-29671023
chr2	29711030	29712925	chr2-29711030-29712925
chr2	29715800	29717711	chr2-29715800-29717711
chr2	29750728	29752110	chr2-29750728-29752110
chr2	29767964	29768286	chr2-29767964-29768286
chr2	29771140	29772308	chr2-29771140-29772308
chr2	29786421	29789313	chr2-29786421-29789313
chr2	29802425	29803940	chr2-29802425-29803940
chr2	29816766	29818151	chr2-29816766-29818151
chr2	29820082	29820815	chr2-29820082-29820815
chr2	29826025	29828759	chr2-29826025-29828759
chr2	29857122	29858189	chr2-29857122-29858189
chr2	29868686	29870020	chr2-29868686-29870020
chr2	29888915	29891306	chr2-29888915-29891306
chr2	29934905	29936546	chr2-29934905-29936546
chr2	29964308	29969328	chr2-29964308-29969328
chr2	30048073	30049852	chr2-30048073-30049852
chr2	30061099	30069319	chr2-30061099-30069319
chr2	30077069	30079358	chr2-30077069-30079358
chr2	30091912	30095451	chr2-30091912-30095451
chr2	30123456	30125276	chr2-30123456-30125276
chr2	30132670	30135502	chr2-30132670-30135502
chr2	30171143	30172749	chr2-30171143-30172749
chr2	30177083	30179001	chr2-30177083-30179001
chr2	30204603	30206914	chr2-30204603-30206914
chr2	30236269	30240231	chr2-30236269-30240231
chr2	30250539	30253272	chr2-30250539-30253272
chr2	30257608	30262051	chr2-30257608-30262051
chr2	30284983	30288229	chr2-30284983-30288229
chr2	30323340	30323701	chr2-30323340-30323701
chr2	30355935	30359673	chr2-30355935-30359673
chr2	30363654	30365845	chr2-30363654-30365845
chr2	30391663	30393995	chr2-30391663-30393995
chr2	30414291	30417587	chr2-30414291-30417587
chr2	30437352	30437666	chr2-30437352-30437666
chr2	30439495	30448033	chr2-30439495-30448033
chr2	30453479	30453680	chr2-30453479-30453680
chr2	30462759	30464923	chr2-30462759-30464923
chr2	30467530	30467731	chr2-30467530-30467731
chr2	30469272	30476742	chr2-30469272-30476742

chr2	30495767	30496569	chr2-30495767-30496569
chr2	30527632	30528035	chr2-30527632-30528035
chr2	30544767	30545787	chr2-30544767-30545787
chr2	30547791	30548105	chr2-30547791-30548105
chr2	30554013	30554365	chr2-30554013-30554365
chr2	30594670	30595409	chr2-30594670-30595409
chr2	30600448	30601058	chr2-30600448-30601058
chr2	30618689	30619112	chr2-30618689-30619112
chr2	30640039	30640505	chr2-30640039-30640505
chr2	30641590	30643104	chr2-30641590-30643104
chr2	30657531	30659212	chr2-30657531-30659212
chr2	30676710	30677726	chr2-30676710-30677726
chr2	30690234	30690798	chr2-30690234-30690798
chr2	30692270	30694169	chr2-30692270-30694169
chr2	30699938	30700344	chr2-30699938-30700344
chr2	30706818	30713622	chr2-30706818-30713622
chr2	30716137	30721261	chr2-30716137-30721261
chr2	30743075	30744456	chr2-30743075-30744456
chr2	30753005	30753739	chr2-30753005-30753739
chr2	30756500	30757016	chr2-30756500-30757016
chr2	30758188	30761193	chr2-30758188-30761193
chr2	30771141	30774054	chr2-30771141-30774054
chr2	30783433	30783738	chr2-30783433-30783738
chr2	30794468	30797011	chr2-30794468-30797011
chr2	30800970	30802076	chr2-30800970-30802076
chr2	30806822	30809957	chr2-30806822-30809957
chr2	30826470	30828830	chr2-30826470-30828830
chr2	30844929	30847032	chr2-30844929-30847032
chr2	30848440	30848859	chr2-30848440-30848859
chr2	30850504	30851374	chr2-30850504-30851374
chr2	30864485	30865702	chr2-30864485-30865702
chr2	30896903	30903674	chr2-30896903-30903674
chr2	30918259	30918996	chr2-30918259-30918996
chr2	30925443	30925868	chr2-30925443-30925868
chr2	30928385	30930249	chr2-30928385-30930249
chr2	30933601	30934050	chr2-30933601-30934050
chr2	30942294	30943007	chr2-30942294-30943007
chr2	30952615	30955313	chr2-30952615-30955313
chr2	30966284	30968604	chr2-30966284-30968604
chr2	30980688	30983574	chr2-30980688-30983574
chr2	30984894	30986479	chr2-30984894-30986479
chr2	30995625	30996288	chr2-30995625-30996288

chr2	31026814	31027389	chr2-31026814-31027389
chr2	31036458	31037252	chr2-31036458-31037252
chr2	31042361	31043426	chr2-31042361-31043426
chr2	31140770	31142792	chr2-31140770-31142792
chr2	31151438	31153617	chr2-31151438-31153617
chr2	31239355	31239771	chr2-31239355-31239771
chr2	31244968	31247817	chr2-31244968-31247817
chr2	31256048	31256984	chr2-31256048-31256984
chr2	31258588	31259890	chr2-31258588-31259890
chr2	31297815	31298845	chr2-31297815-31298845
chr2	31313996	31314731	chr2-31313996-31314731
chr2	31442350	31443450	chr2-31442350-31443450
chr2	31453405	31453664	chr2-31453405-31453664
chr2	31459667	31460734	chr2-31459667-31460734
chr2	31469529	31471867	chr2-31469529-31471867
chr2	31541614	31541950	chr2-31541614-31541950
chr2	31571696	31574541	chr2-31571696-31574541
chr2	31635316	31638246	chr2-31635316-31638246
chr2	31640068	31641472	chr2-31640068-31641472
chr2	31653754	31655425	chr2-31653754-31655425
chr2	31670365	31672576	chr2-31670365-31672576
chr2	31687582	31691086	chr2-31687582-31691086
chr2	31759169	31762611	chr2-31759169-31762611
chr2	31768879	31771479	chr2-31768879-31771479
chr2	31809668	31811976	chr2-31809668-31811976
chr2	31822486	31823415	chr2-31822486-31823415
chr2	31845405	31847140	chr2-31845405-31847140
chr2	31887071	31887741	chr2-31887071-31887741
chr2	31925094	31925644	chr2-31925094-31925644
chr2	31934872	31936111	chr2-31934872-31936111
chr2	31948507	31954276	chr2-31948507-31954276
chr2	31973481	31975905	chr2-31973481-31975905
chr2	32083138	32085023	chr2-32083138-32085023
chr2	32088559	32089019	chr2-32088559-32089019
chr2	32137049	32138605	chr2-32137049-32138605
chr2	32150506	32154658	chr2-32150506-32154658
chr2	32164467	32165372	chr2-32164467-32165372
chr2	32166357	32168155	chr2-32166357-32168155
chr2	32173955	32176094	chr2-32173955-32176094
chr2	32218465	32218760	chr2-32218465-32218760
chr2	32235302	32238092	chr2-32235302-32238092
chr2	32258459	32260659	chr2-32258459-32260659

chr2	32286384	32289709	chr2-32286384-32289709
chr2	32295517	32295819	chr2-32295517-32295819
chr2	32311513	32313010	chr2-32311513-32313010
chr2	32325314	32325880	chr2-32325314-32325880
chr2	32342552	32343214	chr2-32342552-32343214
chr2	32352009	32353821	chr2-32352009-32353821
chr2	32360720	32364534	chr2-32360720-32364534
chr2	32379964	32382687	chr2-32379964-32382687
chr2	32394727	32397844	chr2-32394727-32397844
chr2	32421837	32425157	chr2-32421837-32425157
chr2	32427634	32429272	chr2-32427634-32429272
chr2	32449693	32454351	chr2-32449693-32454351
chr2	32458118	32458473	chr2-32458118-32458473
chr2	32470989	32472162	chr2-32470989-32472162
chr2	32502924	32504126	chr2-32502924-32504126
chr2	32524288	32524915	chr2-32524288-32524915
chr2	32533960	32538904	chr2-32533960-32538904
chr2	32546878	32548673	chr2-32546878-32548673
chr2	32570582	32572582	chr2-32570582-32572582
chr2	32575135	32576003	chr2-32575135-32576003
chr2	32583114	32583675	chr2-32583114-32583675
chr2	32586654	32590153	chr2-32586654-32590153
chr2	32602551	32603107	chr2-32602551-32603107
chr2	32606479	32608885	chr2-32606479-32608885
chr2	32610549	32611159	chr2-32610549-32611159
chr2	32628928	32629418	chr2-32628928-32629418
chr2	32673276	32673977	chr2-32673276-32673977
chr2	32686109	32687328	chr2-32686109-32687328
chr2	32691181	32694976	chr2-32691181-32694976
chr2	32709654	32709855	chr2-32709654-32709855
chr2	32710974	32715862	chr2-32710974-32715862
chr2	32720749	32721003	chr2-32720749-32721003
chr2	32727049	32728147	chr2-32727049-32728147
chr2	32740936	32742595	chr2-32740936-32742595
chr2	32750668	32752473	chr2-32750668-32752473
chr2	32756770	32759787	chr2-32756770-32759787
chr2	32768823	32769281	chr2-32768823-32769281
chr2	32770540	32770894	chr2-32770540-32770894
chr2	32771793	32772259	chr2-32771793-32772259
chr2	32774538	32778325	chr2-32774538-32778325
chr2	32833854	32835494	chr2-32833854-32835494
chr2	32845528	32847777	chr2-32845528-32847777

chr2	32852035	32852791	chr2-32852035-32852791
chr2	32854161	32854362	chr2-32854161-32854362
chr2	32857812	32858117	chr2-32857812-32858117
chr2	32866844	32871152	chr2-32866844-32871152
chr2	32873623	32886308	chr2-32873623-32886308
chr2	32887957	32890342	chr2-32887957-32890342
chr2	32891199	32891865	chr2-32891199-32891865
chr2	32893725	32906941	chr2-32893725-32906941
chr2	32913699	32915709	chr2-32913699-32915709
chr2	32917067	32918284	chr2-32917067-32918284
chr2	32926564	32928787	chr2-32926564-32928787
chr2	32960303	32968312	chr2-32960303-32968312
chr2	32980872	32983341	chr2-32980872-32983341
chr2	33129191	33132189	chr2-33129191-33132189
chr2	33194423	33194624	chr2-33194423-33194624
chr2	33203191	33206853	chr2-33203191-33206853
chr2	33215154	33221930	chr2-33215154-33221930
chr2	33225112	33225592	chr2-33225112-33225592
chr2	33319920	33321098	chr2-33319920-33321098
chr2	33359026	33359277	chr2-33359026-33359277
chr2	33369983	33372068	chr2-33369983-33372068
chr2	33429309	33432183	chr2-33429309-33432183
chr2	33465595	33469317	chr2-33465595-33469317
chr2	33543775	33544869	chr2-33543775-33544869
chr2	33547085	33547441	chr2-33547085-33547441
chr2	33569042	33569352	chr2-33569042-33569352
chr2	33580510	33581346	chr2-33580510-33581346
chr2	33594830	33595154	chr2-33594830-33595154
chr2	33616059	33617223	chr2-33616059-33617223
chr2	33636042	33636937	chr2-33636042-33636937
chr2	33639328	33643339	chr2-33639328-33643339
chr2	33717737	33718147	chr2-33717737-33718147
chr2	33852833	33853990	chr2-33852833-33853990
chr2	33886252	33888261	chr2-33886252-33888261
chr2	34028770	34032007	chr2-34028770-34032007
chr2	34249226	34250406	chr2-34249226-34250406
chr2	34370288	34373772	chr2-34370288-34373772
chr2	34406195	34408078	chr2-34406195-34408078
chr2	34546349	34546656	chr2-34546349-34546656
chr2	34638980	34639598	chr2-34638980-34639598
chr2	34645669	34646740	chr2-34645669-34646740
chr2	34668690	34669051	chr2-34668690-34669051

chr2	34671400	34671970	chr2-34671400-34671970
chr2	34752923	34755620	chr2-34752923-34755620
chr2	34769939	34775281	chr2-34769939-34775281
chr2	34776694	34777053	chr2-34776694-34777053
chr2	34798646	34800765	chr2-34798646-34800765
chr2	34801656	34804150	chr2-34801656-34804150
chr2	34824516	34827496	chr2-34824516-34827496
chr2	34832650	34833929	chr2-34832650-34833929
chr2	34868995	34872081	chr2-34868995-34872081
chr2	34874111	34875371	chr2-34874111-34875371
chr2	34902841	34903242	chr2-34902841-34903242
chr2	34904548	34905745	chr2-34904548-34905745
chr2	34912675	34915194	chr2-34912675-34915194
chr2	34956816	34959259	chr2-34956816-34959259
chr2	34960259	34964518	chr2-34960259-34964518
chr2	34965417	34965776	chr2-34965417-34965776
chr2	35015756	35016727	chr2-35015756-35016727
chr2	35069413	35069823	chr2-35069413-35069823
chr2	35108806	35110421	chr2-35108806-35110421
chr2	35131735	35133472	chr2-35131735-35133472
chr2	35198727	35202168	chr2-35198727-35202168
chr2	35214816	35215343	chr2-35214816-35215343
chr2	35229956	35231150	chr2-35229956-35231150
chr2	35255551	35260213	chr2-35255551-35260213
chr2	35263562	35272424	chr2-35263562-35272424
chr2	35333668	35337877	chr2-35333668-35337877
chr2	35342441	35342798	chr2-35342441-35342798
chr2	35445665	35446309	chr2-35445665-35446309
chr2	35447694	35447959	chr2-35447694-35447959
chr2	35460372	35463115	chr2-35460372-35463115
chr2	35478213	35478712	chr2-35478213-35478712
chr2	35490280	35491094	chr2-35490280-35491094
chr2	35501101	35502227	chr2-35501101-35502227
chr2	35549064	35549265	chr2-35549064-35549265
chr2	35550279	35550588	chr2-35550279-35550588
chr2	35581359	35583801	chr2-35581359-35583801
chr2	35589489	35590429	chr2-35589489-35590429
chr2	35621638	35622605	chr2-35621638-35622605
chr2	35646314	35646807	chr2-35646314-35646807
chr2	35660705	35664110	chr2-35660705-35664110
chr2	35675929	35676629	chr2-35675929-35676629
chr2	35680227	35681449	chr2-35680227-35681449

chr2	35689552	35698599	chr2-35689552-35698599
chr2	35701292	35704289	chr2-35701292-35704289
chr2	35841137	35842008	chr2-35841137-35842008
chr2	35874285	35874543	chr2-35874285-35874543
chr2	35880017	35882904	chr2-35880017-35882904
chr2	35884230	35884532	chr2-35884230-35884532
chr2	35978038	35980301	chr2-35978038-35980301
chr2	36011143	36012033	chr2-36011143-36012033
chr2	36021703	36022256	chr2-36021703-36022256
chr2	36048064	36049859	chr2-36048064-36049859
chr2	36090669	36092114	chr2-36090669-36092114
chr2	36099679	36100137	chr2-36099679-36100137
chr2	36102692	36105962	chr2-36102692-36105962
chr2	36135474	36137834	chr2-36135474-36137834
chr2	36184766	36184967	chr2-36184766-36184967
chr2	36190895	36191212	chr2-36190895-36191212
chr2	36200661	36203924	chr2-36200661-36203924
chr2	36213000	36214508	chr2-36213000-36214508
chr2	36216395	36217855	chr2-36216395-36217855
chr2	36226850	36227982	chr2-36226850-36227982
chr2	36229019	36232432	chr2-36229019-36232432
chr2	36234704	36234905	chr2-36234704-36234905
chr2	37357872	37360324	chr2-37357872-37360324
chr2	37421597	37423537	chr2-37421597-37423537
chr2	37429724	37432205	chr2-37429724-37432205
chr2	37441692	37444901	chr2-37441692-37444901
chr2	37451270	37453434	chr2-37451270-37453434
chr2	37701806	37704536	chr2-37701806-37704536
chr2	37748727	37749571	chr2-37748727-37749571
chr2	37771138	37772317	chr2-37771138-37772317
chr2	37780292	37780777	chr2-37780292-37780777
chr2	37786318	37786986	chr2-37786318-37786986
chr2	37793145	37793867	chr2-37793145-37793867
chr2	38164442	38164936	chr2-38164442-38164936
chr2	38286242	38287828	chr2-38286242-38287828
chr2	38338726	38340058	chr2-38338726-38340058
chr2	38347515	38348779	chr2-38347515-38348779
chr2	38350404	38352332	chr2-38350404-38352332
chr2	38354472	38355359	chr2-38354472-38355359
chr2	38356338	38357171	chr2-38356338-38357171
chr2	38368622	38369384	chr2-38368622-38369384
chr2	38379080	38379678	chr2-38379080-38379678

chr2	38485758	38486678	chr2-38485758-38486678
chr2	38511262	38515666	chr2-38511262-38515666
chr2	38522047	38522248	chr2-38522047-38522248
chr2	38523489	38525750	chr2-38523489-38525750
chr2	38529390	38540085	chr2-38529390-38540085
chr2	38545838	38547571	chr2-38545838-38547571
chr2	38574208	38574510	chr2-38574208-38574510
chr2	38621745	38622110	chr2-38621745-38622110
chr2	38642712	38644623	chr2-38642712-38644623
chr2	38658158	38658818	chr2-38658158-38658818
chr2	38669259	38669624	chr2-38669259-38669624
chr2	38688798	38689376	chr2-38688798-38689376
chr2	38710214	38712540	chr2-38710214-38712540
chr2	38780599	38780800	chr2-38780599-38780800
chr2	38782054	38785493	chr2-38782054-38785493
chr2	38822898	38825821	chr2-38822898-38825821
chr2	38924699	38927926	chr2-38924699-38927926
chr2	38931798	38932267	chr2-38931798-38932267
chr2	38961919	38962120	chr2-38961919-38962120
chr2	38987537	38987957	chr2-38987537-38987957
chr2	38997390	38998963	chr2-38997390-38998963
chr2	39001947	39009820	chr2-39001947-39009820
chr2	39064104	39066345	chr2-39064104-39066345
chr2	39189601	39191252	chr2-39189601-39191252
chr2	39224643	39227113	chr2-39224643-39227113
chr2	39325543	39326128	chr2-39325543-39326128
chr2	42652634	42653146	chr2-42652634-42653146
chr2	44409683	44410245	chr2-44409683-44410245
chr2	44556838	44557343	chr2-44556838-44557343
chr2	44925904	44928477	chr2-44925904-44928477
chr2	44988565	44989123	chr2-44988565-44989123
chr2	45019463	45020237	chr2-45019463-45020237
chr2	45108542	45111492	chr2-45108542-45111492
chr2	45112855	45113466	chr2-45112855-45113466
chr2	45115720	45117611	chr2-45115720-45117611
chr2	45184813	45185431	chr2-45184813-45185431
chr2	45514784	45515132	chr2-45514784-45515132
chr2	45581246	45581696	chr2-45581246-45581696
chr2	46055828	46056029	chr2-46055828-46056029
chr2	46443445	46443908	chr2-46443445-46443908
chr2	48332699	48333214	chr2-48332699-48333214
chr2	48441043	48442168	chr2-48441043-48442168

chr2	48456223	48456583	chr2-48456223-48456583
chr2	48624040	48624616	chr2-48624040-48624616
chr2	48813025	48816777	chr2-48813025-48816777
chr2	48947345	48950869	chr2-48947345-48950869
chr2	49014492	49014693	chr2-49014492-49014693
chr2	49312773	49313451	chr2-49312773-49313451
chr2	49349430	49349873	chr2-49349430-49349873
chr2	49450571	49453495	chr2-49450571-49453495
chr2	49570773	49571750	chr2-49570773-49571750
chr2	49618995	49620221	chr2-49618995-49620221
chr2	49741903	49742724	chr2-49741903-49742724
chr2	49744345	49744810	chr2-49744345-49744810
chr2	49787183	49788391	chr2-49787183-49788391
chr2	49811379	49812640	chr2-49811379-49812640
chr2	49813893	49814144	chr2-49813893-49814144
chr2	50065866	50067309	chr2-50065866-50067309
chr2	50249742	50249943	chr2-50249742-50249943
chr2	50265582	50266737	chr2-50265582-50266737
chr2	50295379	50297680	chr2-50295379-50297680
chr2	50376456	50376760	chr2-50376456-50376760
chr2	50877750	50878365	chr2-50877750-50878365
chr2	51139530	51143566	chr2-51139530-51143566
chr2	51144515	51149340	chr2-51144515-51149340
chr2	51179252	51179505	chr2-51179252-51179505
chr2	51304700	51305670	chr2-51304700-51305670
chr2	51758187	51758442	chr2-51758187-51758442
chr2	51934069	51935551	chr2-51934069-51935551
chr2	51972187	51973809	chr2-51972187-51973809
chr2	52037670	52038417	chr2-52037670-52038417
chr2	52072053	52074551	chr2-52072053-52074551
chr2	52318985	52319712	chr2-52318985-52319712
chr2	52422333	52426122	chr2-52422333-52426122
chr2	52442175	52442550	chr2-52442175-52442550
chr2	52448974	52449175	chr2-52448974-52449175
chr2	52455748	52456201	chr2-52455748-52456201
chr2	52619611	52619912	chr2-52619611-52619912
chr2	52674916	52676808	chr2-52674916-52676808
chr2	52741123	52743601	chr2-52741123-52743601
chr2	52857246	52859760	chr2-52857246-52859760
chr2	52861736	52862812	chr2-52861736-52862812
chr2	52867936	52868788	chr2-52867936-52868788
chr2	52888177	52888378	chr2-52888177-52888378

chr2	52938714	52938965	chr2-52938714-52938965
chr2	52952135	52952336	chr2-52952135-52952336
chr2	52954882	52955527	chr2-52954882-52955527
chr2	53000010	53000211	chr2-53000010-53000211
chr2	53189584	53193647	chr2-53189584-53193647
chr2	53686935	53687798	chr2-53686935-53687798
chr2	54084523	54085804	chr2-54084523-54085804
chr2	54435829	54437382	chr2-54435829-54437382
chr2	56999956	57000286	chr2-56999956-57000286
chr2	57102240	57103035	chr2-57102240-57103035
chr2	57113609	57118146	chr2-57113609-57118146
chr2	57122276	57124732	chr2-57122276-57124732
chr2	57180832	57183277	chr2-57180832-57183277
chr2	57236998	57240243	chr2-57236998-57240243
chr2	57251615	57251969	chr2-57251615-57251969
chr2	57301577	57301962	chr2-57301577-57301962
chr2	57353115	57354498	chr2-57353115-57354498
chr2	57453934	57454461	chr2-57453934-57454461
chr2	57616812	57617169	chr2-57616812-57617169
chr2	57698668	57698979	chr2-57698668-57698979
chr2	58175453	58175988	chr2-58175453-58175988
chr2	58324415	58324733	chr2-58324415-58324733
chr2	58356751	58358260	chr2-58356751-58358260
chr2	58480838	58481163	chr2-58480838-58481163
chr2	58483905	58486341	chr2-58483905-58486341
chr2	58546095	58546513	chr2-58546095-58546513
chr2	58561917	58562530	chr2-58561917-58562530
chr2	58564877	58568771	chr2-58564877-58568771
chr2	58682765	58683116	chr2-58682765-58683116
chr2	58830335	58830593	chr2-58830335-58830593
chr2	59042570	59043086	chr2-59042570-59043086
chr2	59159778	59163226	chr2-59159778-59163226
chr2	59170721	59172060	chr2-59170721-59172060
chr2	59175818	59176833	chr2-59175818-59176833
chr2	59220077	59220392	chr2-59220077-59220392
chr2	59286269	59286634	chr2-59286269-59286634
chr2	59416493	59416854	chr2-59416493-59416854
chr2	59431421	59432307	chr2-59431421-59432307
chr2	59438036	59438891	chr2-59438036-59438891
chr2	59522342	59522745	chr2-59522342-59522745
chr2	59587634	59587888	chr2-59587634-59587888
chr2	59611267	59614159	chr2-59611267-59614159

chr2	59628238	59630219	chr2-59628238-59630219
chr2	59633957	59634208	chr2-59633957-59634208
chr2	59664867	59665068	chr2-59664867-59665068
chr2	59669957	59672037	chr2-59669957-59672037
chr2	59678951	59680899	chr2-59678951-59680899
chr2	59878206	59878510	chr2-59878206-59878510
chr2	59881448	59883401	chr2-59881448-59883401
chr2	60036749	60037888	chr2-60036749-60037888
chr2	60123765	60126374	chr2-60123765-60126374
chr2	60208885	60213045	chr2-60208885-60213045
chr2	60239578	60239883	chr2-60239578-60239883
chr2	60283729	60284380	chr2-60283729-60284380
chr2	60369015	60369726	chr2-60369015-60369726
chr2	60382145	60383880	chr2-60382145-60383880
chr2	60504681	60505309	chr2-60504681-60505309
chr2	60601039	60601240	chr2-60601039-60601240
chr2	60627647	60628057	chr2-60627647-60628057
chr2	60632643	60632894	chr2-60632643-60632894
chr2	60639282	60640812	chr2-60639282-60640812
chr2	60671290	60673888	chr2-60671290-60673888
chr2	60688944	60689597	chr2-60688944-60689597
chr2	60692294	60696670	chr2-60692294-60696670
chr2	60697683	60699737	chr2-60697683-60699737
chr2	60702645	60703964	chr2-60702645-60703964
chr2	60720414	60720668	chr2-60720414-60720668
chr2	60807198	60810967	chr2-60807198-60810967
chr2	60867505	60867813	chr2-60867505-60867813
chr2	60870734	60872981	chr2-60870734-60872981
chr2	60875961	60882482	chr2-60875961-60882482
chr2	60901119	60902689	chr2-60901119-60902689
chr2	60910744	60912032	chr2-60910744-60912032
chr2	60939954	60940963	chr2-60939954-60940963
chr2	60961123	60964144	chr2-60961123-60964144
chr2	61095932	61096284	chr2-61095932-61096284
chr2	61138429	61140117	chr2-61138429-61140117
chr2	61246813	61247281	chr2-61246813-61247281
chr2	61285338	61285539	chr2-61285338-61285539
chr2	61401551	61402834	chr2-61401551-61402834
chr2	61454192	61454646	chr2-61454192-61454646
chr2	61579597	61579798	chr2-61579597-61579798
chr2	61592889	61595008	chr2-61592889-61595008
chr2	61655714	61657147	chr2-61655714-61657147

chr2	61710936	61713517	chr2-61710936-61713517
chr2	61804954	61805929	chr2-61804954-61805929
chr2	61811495	61812617	chr2-61811495-61812617
chr2	61814898	61816272	chr2-61814898-61816272
chr2	62046425	62047987	chr2-62046425-62047987
chr2	62411266	62412246	chr2-62411266-62412246
chr2	62645299	62646743	chr2-62645299-62646743
chr2	62663897	62665935	chr2-62663897-62665935
chr2	63183886	63184697	chr2-63183886-63184697
chr2	63702403	63702772	chr2-63702403-63702772
chr2	64097288	64099043	chr2-64097288-64099043
chr2	65021648	65023465	chr2-65021648-65023465
chr2	65170717	65172032	chr2-65170717-65172032
chr2	65182499	65182854	chr2-65182499-65182854
chr2	65191350	65191713	chr2-65191350-65191713
chr2	65222618	65223637	chr2-65222618-65223637
chr2	65227487	65227849	chr2-65227487-65227849
chr2	65233197	65233500	chr2-65233197-65233500
chr2	65236734	65239972	chr2-65236734-65239972
chr2	65363916	65364172	chr2-65363916-65364172
chr2	65413849	65414641	chr2-65413849-65414641
chr2	65668253	65669055	chr2-65668253-65669055
chr2	65931772	65932228	chr2-65931772-65932228
chr2	65940472	65940673	chr2-65940472-65940673
chr2	66122499	66125659	chr2-66122499-66125659
chr2	66145237	66145438	chr2-66145237-66145438
chr2	66180508	66180823	chr2-66180508-66180823
chr2	66255743	66257382	chr2-66255743-66257382
chr2	66409473	66410215	chr2-66409473-66410215
chr2	66597087	66597343	chr2-66597087-66597343
chr2	66634443	66635114	chr2-66634443-66635114
chr2	67565000	67567724	chr2-67565000-67567724
chr2	68085564	68086401	chr2-68085564-68086401
chr2	68222520	68222934	chr2-68222520-68222934
chr2	68274230	68274431	chr2-68274230-68274431
chr2	68434983	68436810	chr2-68434983-68436810
chr2	68465758	68466696	chr2-68465758-68466696
chr2	68470556	68472882	chr2-68470556-68472882
chr2	68845078	68845279	chr2-68845078-68845279
chr2	68860846	68863313	chr2-68860846-68863313
chr2	69204973	69206505	chr2-69204973-69206505
chr2	69376999	69377473	chr2-69376999-69377473

chr2	69379754	69381457	chr2-69379754-69381457
chr2	69397038	69397341	chr2-69397038-69397341
chr2	69401141	69401756	chr2-69401141-69401756
chr2	69584185	69588378	chr2-69584185-69588378
chr2	69635057	69635677	chr2-69635057-69635677
chr2	69646628	69648326	chr2-69646628-69648326
chr2	69712075	69713814	chr2-69712075-69713814
chr2	69722037	69724342	chr2-69722037-69724342
chr2	69788864	69790802	chr2-69788864-69790802
chr2	69802992	69803358	chr2-69802992-69803358
chr2	69822224	69823703	chr2-69822224-69823703
chr2	69860692	69863320	chr2-69860692-69863320
chr2	69884193	69886295	chr2-69884193-69886295
chr2	69896851	69899562	chr2-69896851-69899562
chr2	70037013	70037474	chr2-70037013-70037474
chr2	70069936	70070443	chr2-70069936-70070443
chr2	70471788	70472520	chr2-70471788-70472520
chr2	70473700	70478408	chr2-70473700-70478408
chr2	70508110	70510131	chr2-70508110-70510131
chr2	70558341	70559696	chr2-70558341-70559696
chr2	70561123	70564056	chr2-70561123-70564056
chr2	70568069	70568786	chr2-70568069-70568786
chr2	70660778	70663547	chr2-70660778-70663547
chr2	70704465	70704933	chr2-70704465-70704933
chr2	70823379	70826587	chr2-70823379-70826587
chr2	71039709	71039910	chr2-71039709-71039910
chr2	71054002	71056907	chr2-71054002-71056907
chr2	71117714	71118685	chr2-71117714-71118685
chr2	71211376	71213215	chr2-71211376-71213215
chr2	71366560	71368453	chr2-71366560-71368453
chr2	71383766	71384174	chr2-71383766-71384174
chr2	71388604	71390590	chr2-71388604-71390590
chr2	71452830	71454243	chr2-71452830-71454243
chr2	71528497	71531127	chr2-71528497-71531127
chr2	71532196	71532760	chr2-71532196-71532760
chr2	71542233	71547321	chr2-71542233-71547321
chr2	71551787	71552195	chr2-71551787-71552195
chr2	71580752	71581561	chr2-71580752-71581561
chr2	71594474	71594675	chr2-71594474-71594675
chr2	71626203	71627857	chr2-71626203-71627857
chr2	71629344	71630044	chr2-71629344-71630044
chr2	71640360	71640618	chr2-71640360-71640618

chr2	71651637	71652841	chr2-71651637-71652841
chr2	71665598	71665919	chr2-71665598-71665919
chr2	71739528	71740164	chr2-71739528-71740164
chr2	71757135	71757436	chr2-71757135-71757436
chr2	71758363	71759018	chr2-71758363-71759018
chr2	71786426	71790902	chr2-71786426-71790902
chr2	71800729	71802659	chr2-71800729-71802659
chr2	71805111	71806812	chr2-71805111-71806812
chr2	71811883	71814935	chr2-71811883-71814935
chr2	71872207	71874623	chr2-71872207-71874623
chr2	71980694	71981725	chr2-71980694-71981725
chr2	72025847	72026766	chr2-72025847-72026766
chr2	72203770	72204075	chr2-72203770-72204075
chr2	72242076	72242437	chr2-72242076-72242437
chr2	72284894	72290411	chr2-72284894-72290411
chr2	72297387	72300427	chr2-72297387-72300427
chr2	72308650	72309863	chr2-72308650-72309863
chr2	72318404	72319171	chr2-72318404-72319171
chr2	72475469	72478111	chr2-72475469-72478111
chr2	72837917	72838949	chr2-72837917-72838949
chr2	72976885	72982203	chr2-72976885-72982203
chr2	72996896	72997257	chr2-72996896-72997257
chr2	73012632	73013012	chr2-73012632-73013012
chr2	73059590	73060543	chr2-73059590-73060543
chr2	73213033	73215204	chr2-73213033-73215204
chr2	73263620	73264599	chr2-73263620-73264599
chr2	73270872	73275006	chr2-73270872-73275006
chr2	73277253	73279835	chr2-73277253-73279835
chr2	73311213	73313774	chr2-73311213-73313774
chr2	73385639	73387407	chr2-73385639-73387407
chr2	73485297	73485709	chr2-73485297-73485709
chr2	73526952	73527401	chr2-73526952-73527401
chr2	73528462	73530303	chr2-73528462-73530303
chr2	73538714	73538977	chr2-73538714-73538977
chr2	73584546	73586058	chr2-73584546-73586058
chr2	73592754	73593203	chr2-73592754-73593203
chr2	73607798	73608352	chr2-73607798-73608352
chr2	73609337	73609699	chr2-73609337-73609699
chr2	73774518	73776113	chr2-73774518-73776113
chr2	73867855	73868498	chr2-73867855-73868498
chr2	73890476	73894148	chr2-73890476-73894148
chr2	73909815	73912493	chr2-73909815-73912493

chr2	74577712	74579699	chr2-74577712-74579699
chr2	74655321	74656494	chr2-74655321-74656494
chr2	74657463	74659378	chr2-74657463-74659378
chr2	74667247	74669037	chr2-74667247-74669037
chr2	74672779	74674015	chr2-74672779-74674015
chr2	74679940	74680141	chr2-74679940-74680141
chr2	74681683	74683307	chr2-74681683-74683307
chr2	74691378	74692995	chr2-74691378-74692995
chr2	74694283	74694484	chr2-74694283-74694484
chr2	74697480	74699091	chr2-74697480-74699091
chr2	74704566	74706103	chr2-74704566-74706103
chr2	74711710	74712634	chr2-74711710-74712634
chr2	74715884	74716242	chr2-74715884-74716242
chr2	74727217	74727884	chr2-74727217-74727884
chr2	74732636	74733298	chr2-74732636-74733298
chr2	74735454	74736576	chr2-74735454-74736576
chr2	74740327	74740742	chr2-74740327-74740742
chr2	74746535	74747303	chr2-74746535-74747303
chr2	74749332	74750265	chr2-74749332-74750265
chr2	74752715	74753444	chr2-74752715-74753444
chr2	74762389	74763773	chr2-74762389-74763773
chr2	74825085	74827038	chr2-74825085-74827038
chr2	75054897	75055098	chr2-75054897-75055098
chr2	75137637	75138149	chr2-75137637-75138149
chr2	75584821	75585501	chr2-75584821-75585501
chr2	75608001	75615052	chr2-75608001-75615052
chr2	75625565	75625766	chr2-75625565-75625766
chr2	75628597	75628798	chr2-75628597-75628798
chr2	75630072	75634753	chr2-75630072-75634753
chr2	75637727	75644457	chr2-75637727-75644457
chr2	75657357	75663500	chr2-75657357-75663500
chr2	75683167	75683527	chr2-75683167-75683527
chr2	75685192	75686516	chr2-75685192-75686516
chr2	75695732	75706221	chr2-75695732-75706221
chr2	75771129	75771380	chr2-75771129-75771380
chr2	75772531	75772962	chr2-75772531-75772962
chr2	75776586	75778139	chr2-75776586-75778139
chr2	75780888	75781302	chr2-75780888-75781302
chr2	75831102	75834875	chr2-75831102-75834875
chr2	75869905	75870156	chr2-75869905-75870156
chr2	75937643	75939345	chr2-75937643-75939345
chr2	75964649	75965165	chr2-75964649-75965165

chr2	75981119	75982300	chr2-75981119-75982300
chr2	75991098	75991299	chr2-75991098-75991299
chr2	76065334	76065535	chr2-76065334-76065535
chr2	76070262	76070922	chr2-76070262-76070922
chr2	76097913	76098114	chr2-76097913-76098114
chr2	76338228	76339373	chr2-76338228-76339373
chr2	76369699	76371462	chr2-76369699-76371462
chr2	76405760	76407631	chr2-76405760-76407631
chr2	76646754	76649117	chr2-76646754-76649117
chr2	76671773	76677113	chr2-76671773-76677113
chr2	77169731	77170947	chr2-77169731-77170947
chr2	77176437	77176858	chr2-77176437-77176858
chr2	77201220	77201471	chr2-77201220-77201471
chr2	77267949	77270111	chr2-77267949-77270111
chr2	77279423	77281319	chr2-77279423-77281319
chr2	77303020	77303360	chr2-77303020-77303360
chr2	77341618	77342227	chr2-77341618-77342227
chr2	77519433	77519690	chr2-77519433-77519690
chr2	77703080	77703442	chr2-77703080-77703442
chr2	77816200	77817380	chr2-77816200-77817380
chr2	77944754	77946555	chr2-77944754-77946555
chr2	78048659	78049442	chr2-78048659-78049442
chr2	78059736	78060726	chr2-78059736-78060726
chr2	78326362	78326563	chr2-78326362-78326563
chr2	78410214	78414112	chr2-78410214-78414112
chr2	78572880	78573520	chr2-78572880-78573520
chr2	78867824	78871058	chr2-78867824-78871058
chr2	79255200	79256139	chr2-79255200-79256139
chr2	79428479	79429209	chr2-79428479-79429209
chr2	79453860	79454622	chr2-79453860-79454622
chr2	79456618	79457173	chr2-79456618-79457173
chr2	79511863	79512120	chr2-79511863-79512120
chr2	79618995	79619196	chr2-79618995-79619196
chr2	79634661	79636839	chr2-79634661-79636839
chr2	80314697	80316848	chr2-80314697-80316848
chr2	80446220	80448529	chr2-80446220-80448529
chr2	80578581	80582029	chr2-80578581-80582029
chr2	80616588	80618256	chr2-80616588-80618256
chr2	80638432	80640249	chr2-80638432-80640249
chr2	80688154	80688355	chr2-80688154-80688355
chr2	80850318	80850627	chr2-80850318-80850627
chr2	81025806	81026007	chr2-81025806-81026007

chr2	82052707	82054087	chr2-82052707-82054087
chr2	83622841	83623211	chr2-83622841-83623211
chr2	83643825	83646533	chr2-83643825-83646533
chr2	83700275	83701414	chr2-83700275-83701414
chr2	83709704	83710246	chr2-83709704-83710246
chr2	83714744	83715047	chr2-83714744-83715047
chr2	83723119	83730332	chr2-83723119-83730332
chr2	83732261	83732691	chr2-83732261-83732691
chr2	83734055	83734435	chr2-83734055-83734435
chr2	83743804	83745378	chr2-83743804-83745378
chr2	83758879	83760374	chr2-83758879-83760374
chr2	83812203	83813405	chr2-83812203-83813405
chr2	83940859	83941357	chr2-83940859-83941357
chr2	84424968	84425629	chr2-84424968-84425629
chr2	84504792	84505349	chr2-84504792-84505349
chr2	84518778	84520257	chr2-84518778-84520257
chr2	84539260	84539566	chr2-84539260-84539566
chr2	84549865	84550292	chr2-84549865-84550292
chr2	84632068	84639091	chr2-84632068-84639091
chr2	84646167	84651495	chr2-84646167-84651495
chr2	84669124	84671734	chr2-84669124-84671734
chr2	84676993	84679479	chr2-84676993-84679479
chr2	84703979	84705909	chr2-84703979-84705909
chr2	84712598	84717259	chr2-84712598-84717259
chr2	84725085	84728136	chr2-84725085-84728136
chr2	84733916	84734786	chr2-84733916-84734786
chr2	84738344	84738907	chr2-84738344-84738907
chr2	84740999	84744658	chr2-84740999-84744658
chr2	84798044	84799862	chr2-84798044-84799862
chr2	84811011	84811467	chr2-84811011-84811467
chr2	84826270	84828348	chr2-84826270-84828348
chr2	84839284	84841017	chr2-84839284-84841017
chr2	84851186	84851490	chr2-84851186-84851490
chr2	84865732	84866644	chr2-84865732-84866644
chr2	84871714	84872824	chr2-84871714-84872824
chr2	84882709	84883058	chr2-84882709-84883058
chr2	84886099	84889947	chr2-84886099-84889947
chr2	84936571	84936994	chr2-84936571-84936994
chr2	85019215	85019696	chr2-85019215-85019696
chr2	85036333	85038914	chr2-85036333-85038914
chr2	85046789	85053804	chr2-85046789-85053804
chr2	85059149	85062365	chr2-85059149-85062365

chr2	85063507	85063844	chr2-85063507-85063844
chr2	85065564	85066176	chr2-85065564-85066176
chr2	85067503	85068791	chr2-85067503-85068791
chr2	85098470	85099516	chr2-85098470-85099516
chr2	85105489	85107107	chr2-85105489-85107107
chr2	90474904	90475408	chr2-90474904-90475408
chr2	90490777	90491089	chr2-90490777-90491089
chr2	90496519	90496720	chr2-90496519-90496720
chr2	90497958	90498209	chr2-90497958-90498209
chr2	90526743	90527456	chr2-90526743-90527456
chr2	90548442	90549713	chr2-90548442-90549713
chr2	90553990	90554254	chr2-90553990-90554254
chr2	90560071	90560548	chr2-90560071-90560548
chr2	90562640	90563046	chr2-90562640-90563046
chr2	90565175	90566489	chr2-90565175-90566489
chr2	90574269	90574616	chr2-90574269-90574616
chr2	90578479	90581627	chr2-90578479-90581627
chr2	90582916	90584943	chr2-90582916-90584943
chr2	90611916	90612716	chr2-90611916-90612716
chr2	90624966	90625217	chr2-90624966-90625217
chr2	90641182	90641759	chr2-90641182-90641759
chr2	90678259	90680826	chr2-90678259-90680826
chr2	90744270	90748041	chr2-90744270-90748041
chr2	90749558	90750034	chr2-90749558-90750034
chr2	90846160	90848859	chr2-90846160-90848859
chr2	90885485	90886348	chr2-90885485-90886348
chr2	90889197	90890694	chr2-90889197-90890694
chr2	90903527	90906239	chr2-90903527-90906239
chr2	90915708	90918824	chr2-90915708-90918824
chr2	90939739	90942382	chr2-90939739-90942382
chr2	91052968	91055774	chr2-91052968-91055774
chr2	91062026	91062382	chr2-91062026-91062382
chr2	91068217	91071456	chr2-91068217-91071456
chr2	91078502	91079153	chr2-91078502-91079153
chr2	91112457	91113045	chr2-91112457-91113045
chr2	91115024	91115836	chr2-91115024-91115836
chr2	91157896	91158252	chr2-91157896-91158252
chr2	91181897	91184519	chr2-91181897-91184519
chr2	91194630	91194994	chr2-91194630-91194994
chr2	91201709	91204268	chr2-91201709-91204268
chr2	91236097	91238297	chr2-91236097-91238297
chr2	91241999	91242875	chr2-91241999-91242875

chr2	91255811	91258800	chr2-91255811-91258800
chr2	91264537	91266564	chr2-91264537-91266564
chr2	91298962	91299669	chr2-91298962-91299669
chr2	91444013	91444834	chr2-91444013-91444834
chr2	91457077	91459508	chr2-91457077-91459508
chr2	91463144	91463518	chr2-91463144-91463518
chr2	91473908	91474933	chr2-91473908-91474933
chr2	91476246	91476628	chr2-91476246-91476628
chr2	91526385	91528337	chr2-91526385-91528337
chr2	91537682	91537991	chr2-91537682-91537991
chr2	91647874	91652098	chr2-91647874-91652098
chr2	91656191	91656578	chr2-91656191-91656578
chr2	91709426	91712016	chr2-91709426-91712016
chr2	91729620	91731688	chr2-91729620-91731688
chr2	91921332	91924352	chr2-91921332-91924352
chr2	91961755	91963988	chr2-91961755-91963988
chr2	91975442	91976569	chr2-91975442-91976569
chr2	92007071	92009367	chr2-92007071-92009367
chr2	92023691	92024859	chr2-92023691-92024859
chr2	92047729	92047983	chr2-92047729-92047983
chr2	92055231	92056256	chr2-92055231-92056256
chr2	92058280	92060353	chr2-92058280-92060353
chr2	92095940	92096292	chr2-92095940-92096292
chr2	92182178	92186534	chr2-92182178-92186534
chr2	92369803	92372246	chr2-92369803-92372246
chr2	92373943	92376820	chr2-92373943-92376820
chr2	92388251	92390068	chr2-92388251-92390068
chr2	92400680	92401707	chr2-92400680-92401707
chr2	92427734	92428507	chr2-92427734-92428507
chr2	92429689	92434962	chr2-92429689-92434962
chr2	92437697	92438164	chr2-92437697-92438164
chr2	92456764	92461063	chr2-92456764-92461063
chr2	92484364	92485301	chr2-92484364-92485301
chr2	92529009	92529966	chr2-92529009-92529966
chr2	92599234	92600818	chr2-92599234-92600818
chr2	92678406	92678866	chr2-92678406-92678866
chr2	92738239	92745354	chr2-92738239-92745354
chr2	92747334	92748564	chr2-92747334-92748564
chr2	92749815	92752441	chr2-92749815-92752441
chr2	92769779	92770277	chr2-92769779-92770277
chr2	92776426	92776627	chr2-92776426-92776627
chr2	92914864	92915541	chr2-92914864-92915541

chr2	92975079	92975280	chr2-92975079-92975280
chr2	93014038	93015518	chr2-93014038-93015518
chr2	93029279	93029806	chr2-93029279-93029806
chr2	93045006	93046690	chr2-93045006-93046690
chr2	93124769	93124970	chr2-93124769-93124970
chr2	93176709	93177453	chr2-93176709-93177453
chr2	93180339	93180540	chr2-93180339-93180540
chr2	93187229	93189107	chr2-93187229-93189107
chr2	93214032	93214774	chr2-93214032-93214774
chr2	93333308	93335042	chr2-93333308-93335042
chr2	93404957	93405439	chr2-93404957-93405439
chr2	93458539	93459971	chr2-93458539-93459971
chr2	93461019	93463355	chr2-93461019-93463355
chr2	93470050	93470927	chr2-93470050-93470927
chr2	93642444	93643686	chr2-93642444-93643686
chr2	93645747	93647435	chr2-93645747-93647435
chr2	93821143	93822913	chr2-93821143-93822913
chr2	93848809	93850334	chr2-93848809-93850334
chr2	93881258	93881923	chr2-93881258-93881923
chr2	93886219	93886548	chr2-93886219-93886548
chr2	93893403	93894019	chr2-93893403-93894019
chr2	93902669	93903854	chr2-93902669-93903854
chr2	93927833	93928337	chr2-93927833-93928337
chr2	93954815	93957516	chr2-93954815-93957516
chr2	94009610	94011577	chr2-94009610-94011577
chr2	94052381	94052670	chr2-94052381-94052670
chr2	94153959	94159039	chr2-94153959-94159039
chr2	94177849	94179171	chr2-94177849-94179171
chr2	94240479	94244297	chr2-94240479-94244297
chr2	94246900	94247252	chr2-94246900-94247252
chr2	94263286	94265583	chr2-94263286-94265583
chr2	94272795	94274112	chr2-94272795-94274112
chr2	94287935	94289508	chr2-94287935-94289508
chr2	94405104	94408534	chr2-94405104-94408534
chr2	94435818	94438687	chr2-94435818-94438687
chr2	94449804	94450550	chr2-94449804-94450550
chr2	94478357	94478844	chr2-94478357-94478844
chr2	97466232	97467651	chr2-97466232-97467651
chr2	101442865	101443229	chr2-101442865-101443229
chr2	101628156	101629390	chr2-101628156-101629390
chr2	101678057	101679884	chr2-101678057-101679884
chr2	101739460	101740060	chr2-101739460-101740060

chr2	101750879	101751521	chr2-101750879-101751521
chr2	101795581	101798468	chr2-101795581-101798468
chr2	101862530	101863443	chr2-101862530-101863443
chr2	101871186	101871437	chr2-101871186-101871437
chr2	101885906	101887132	chr2-101885906-101887132
chr2	101905958	101906373	chr2-101905958-101906373
chr2	101986789	101986990	chr2-101986789-101986990
chr2	102146632	102146964	chr2-102146632-102146964
chr2	102185191	102187001	chr2-102185191-102187001
chr2	102259183	102259562	chr2-102259183-102259562
chr2	102398448	102401836	chr2-102398448-102401836
chr2	102449639	102452475	chr2-102449639-102452475
chr2	102544470	102544671	chr2-102544470-102544671
chr2	102549739	102550407	chr2-102549739-102550407
chr2	102658177	102659645	chr2-102658177-102659645
chr2	102758211	102758412	chr2-102758211-102758412
chr2	102764643	102765320	chr2-102764643-102765320
chr2	102805434	102805737	chr2-102805434-102805737
chr2	102873545	102874516	chr2-102873545-102874516
chr2	102893805	102902233	chr2-102893805-102902233
chr2	102943372	102946431	chr2-102943372-102946431
chr2	102947965	102949321	chr2-102947965-102949321
chr2	103061787	103062367	chr2-103061787-103062367
chr2	103072010	103074643	chr2-103072010-103074643
chr2	103117153	103117458	chr2-103117153-103117458
chr2	103283808	103284091	chr2-103283808-103284091
chr2	103284936	103286301	chr2-103284936-103286301
chr2	103293745	103296235	chr2-103293745-103296235
chr2	103300661	103304235	chr2-103300661-103304235
chr2	103347573	103347829	chr2-103347573-103347829
chr2	103372625	103372826	chr2-103372625-103372826
chr2	103464252	103464603	chr2-103464252-103464603
chr2	103483398	103485917	chr2-103483398-103485917
chr2	103564823	103570580	chr2-103564823-103570580
chr2	103599978	103601222	chr2-103599978-103601222
chr2	103632311	103632690	chr2-103632311-103632690
chr2	103654972	103656713	chr2-103654972-103656713
chr2	103657604	103658338	chr2-103657604-103658338
chr2	103661944	103663365	chr2-103661944-103663365
chr2	103669447	103670904	chr2-103669447-103670904
chr2	103705844	103707032	chr2-103705844-103707032
chr2	103759604	103762183	chr2-103759604-103762183

chr2	103794746	103798571	chr2-103794746-103798571
chr2	103817284	103818068	chr2-103817284-103818068
chr2	103837129	103837383	chr2-103837129-103837383
chr2	103969335	103971208	chr2-103969335-103971208
chr2	104027307	104029255	chr2-104027307-104029255
chr2	104069628	104070363	chr2-104069628-104070363
chr2	104095862	104096285	chr2-104095862-104096285
chr2	104122271	104123574	chr2-104122271-104123574
chr2	104207864	104208191	chr2-104207864-104208191
chr2	104409633	104411000	chr2-104409633-104411000
chr2	104479759	104479960	chr2-104479759-104479960
chr2	104492485	104495138	chr2-104492485-104495138
chr2	104589248	104592459	chr2-104589248-104592459
chr2	104705289	104706589	chr2-104705289-104706589
chr2	104711070	104712886	chr2-104711070-104712886
chr2	104741244	104743609	chr2-104741244-104743609
chr2	104763335	104763637	chr2-104763335-104763637
chr2	104814713	104819085	chr2-104814713-104819085
chr2	104847769	104850347	chr2-104847769-104850347
chr2	104851574	104852119	chr2-104851574-104852119
chr2	104855580	104855845	chr2-104855580-104855845
chr2	104864160	104864412	chr2-104864160-104864412
chr2	105015555	105018163	chr2-105015555-105018163
chr2	105122739	105123351	chr2-105122739-105123351
chr2	105126130	105128853	chr2-105126130-105128853
chr2	105224159	105224831	chr2-105224159-105224831
chr2	105398516	105399840	chr2-105398516-105399840
chr2	105612362	105612718	chr2-105612362-105612718
chr2	105616376	105616577	chr2-105616376-105616577
chr2	105666820	105670300	chr2-105666820-105670300
chr2	105672277	105672957	chr2-105672277-105672957
chr2	105674641	105676980	chr2-105674641-105676980
chr2	105681963	105683141	chr2-105681963-105683141
chr2	105687451	105690058	chr2-105687451-105690058
chr2	105903355	105905608	chr2-105903355-105905608
chr2	106002305	106004079	chr2-106002305-106004079
chr2	106194204	106194405	chr2-106194204-106194405
chr2	106650074	106650611	chr2-106650074-106650611
chr2	106692899	106695405	chr2-106692899-106695405
chr2	106972815	106974821	chr2-106972815-106974821
chr2	107041237	107041488	chr2-107041237-107041488
chr2	107290195	107291476	chr2-107290195-107291476

chr2	108764596	108765015	chr2-108764596-108765015
chr2	108827490	108827741	chr2-108827490-108827741
chr2	109277469	109278577	chr2-109277469-109278577
chr2	109280077	109282280	chr2-109280077-109282280
chr2	109406198	109407182	chr2-109406198-109407182
chr2	109419677	109420085	chr2-109419677-109420085
chr2	109650386	109650587	chr2-109650386-109650587
chr2	109673595	109675004	chr2-109673595-109675004
chr2	109675862	109679018	chr2-109675862-109679018
chr2	109680931	109681132	chr2-109680931-109681132
chr2	109692620	109696021	chr2-109692620-109696021
chr2	109839713	109840224	chr2-109839713-109840224
chr2	109842542	109843232	chr2-109842542-109843232
chr2	109889997	109892523	chr2-109889997-109892523
chr2	109916999	109919441	chr2-109916999-109919441
chr2	110016960	110018860	chr2-110016960-110018860
chr2	110157118	110157614	chr2-110157118-110157614
chr2	110176932	110177239	chr2-110176932-110177239
chr2	110186127	110187273	chr2-110186127-110187273
chr2	110192147	110192977	chr2-110192147-110192977
chr2	110362839	110363100	chr2-110362839-110363100
chr2	112238620	112247053	chr2-112238620-112247053
chr2	112254191	112255883	chr2-112254191-112255883
chr2	112261769	112264337	chr2-112261769-112264337
chr2	112265141	112267314	chr2-112265141-112267314
chr2	112284284	112285455	chr2-112284284-112285455
chr2	112309044	112309554	chr2-112309044-112309554
chr2	112366854	112368958	chr2-112366854-112368958
chr2	112378827	112380921	chr2-112378827-112380921
chr2	112443019	112443434	chr2-112443019-112443434
chr2	112454167	112456241	chr2-112454167-112456241
chr2	112492067	112494087	chr2-112492067-112494087
chr2	113216417	113217576	chr2-113216417-113217576
chr2	113233290	113233542	chr2-113233290-113233542
chr2	113322927	113324007	chr2-113322927-113324007
chr2	113326966	113328231	chr2-113326966-113328231
chr2	113397631	113397882	chr2-113397631-113397882
chr2	113404905	113405628	chr2-113404905-113405628
chr2	113439984	113444865	chr2-113439984-113444865
chr2	113757236	113759235	chr2-113757236-113759235
chr2	113845942	113849706	chr2-113845942-113849706
chr2	114013144	114014019	chr2-114013144-114014019

chr2	114174106	114175521	chr2-114174106-114175521
chr2	114199978	114201870	chr2-114199978-114201870
chr2	114260653	114261005	chr2-114260653-114261005
chr2	114653735	114655562	chr2-114653735-114655562
chr2	115093050	115093251	chr2-115093050-115093251
chr2	115580541	115582697	chr2-115580541-115582697
chr2	115608647	115610844	chr2-115608647-115610844
chr2	115780288	115780640	chr2-115780288-115780640
chr2	115852430	115853189	chr2-115852430-115853189
chr2	115854784	115856670	chr2-115854784-115856670
chr2	116009142	116009556	chr2-116009142-116009556
chr2	116061195	116068176	chr2-116061195-116068176
chr2	116071477	116072238	chr2-116071477-116072238
chr2	116075441	116076492	chr2-116075441-116076492
chr2	116893759	116894452	chr2-116893759-116894452
chr2	116899948	116900461	chr2-116899948-116900461
chr2	116942254	116942866	chr2-116942254-116942866
chr2	116972196	116972966	chr2-116972196-116972966
chr2	116974220	116977474	chr2-116974220-116977474
chr2	117119450	117124103	chr2-117119450-117124103
chr2	117249324	117251093	chr2-117249324-117251093
chr2	117342020	117343373	chr2-117342020-117343373
chr2	117540877	117541133	chr2-117540877-117541133
chr2	117749056	117749631	chr2-117749056-117749631
chr2	117780747	117781351	chr2-117780747-117781351
chr2	117785638	117785839	chr2-117785638-117785839
chr2	117838631	117839117	chr2-117838631-117839117
chr2	118094144	118095336	chr2-118094144-118095336
chr2	118101828	118102131	chr2-118101828-118102131
chr2	118109645	118116984	chr2-118109645-118116984
chr2	118118139	118118403	chr2-118118139-118118403
chr2	118179256	118180843	chr2-118179256-118180843
chr2	118256318	118257486	chr2-118256318-118257486
chr2	118287609	118287810	chr2-118287609-118287810
chr2	118372514	118374407	chr2-118372514-118374407
chr2	118388042	118390364	chr2-118388042-118390364
chr2	118408289	118408590	chr2-118408289-118408590
chr2	118478344	118480207	chr2-118478344-118480207
chr2	118490138	118490774	chr2-118490138-118490774
chr2	118510275	118510686	chr2-118510275-118510686
chr2	118540285	118541465	chr2-118540285-118541465
chr2	118543445	118546244	chr2-118543445-118546244

chr2	118547219	118550587	chr2-118547219-118550587
chr2	118554124	118555464	chr2-118554124-118555464
chr2	118585938	118586594	chr2-118585938-118586594
chr2	118597775	118600465	chr2-118597775-118600465
chr2	118658423	118660441	chr2-118658423-118660441
chr2	118662604	118666262	chr2-118662604-118666262
chr2	118669324	118670051	chr2-118669324-118670051
chr2	118675975	118678455	chr2-118675975-118678455
chr2	118700258	118700828	chr2-118700258-118700828
chr2	118702256	118704725	chr2-118702256-118704725
chr2	118710854	118711721	chr2-118710854-118711721
chr2	118744949	118748142	chr2-118744949-118748142
chr2	118758775	118767401	chr2-118758775-118767401
chr2	118779060	118780094	chr2-118779060-118780094
chr2	118790437	118791330	chr2-118790437-118791330
chr2	118813706	118815838	chr2-118813706-118815838
chr2	118822910	118823111	chr2-118822910-118823111
chr2	118828816	118829316	chr2-118828816-118829316
chr2	118842083	118842284	chr2-118842083-118842284
chr2	118861455	118863043	chr2-118861455-118863043
chr2	118870950	118871252	chr2-118870950-118871252
chr2	118876235	118878005	chr2-118876235-118878005
chr2	118883033	118883687	chr2-118883033-118883687
chr2	118896011	118897337	chr2-118896011-118897337
chr2	118899818	118903785	chr2-118899818-118903785
chr2	118912929	118913811	chr2-118912929-118913811
chr2	118925835	118928131	chr2-118925835-118928131
chr2	118972523	118973244	chr2-118972523-118973244
chr2	118975028	118976949	chr2-118975028-118976949
chr2	119028457	119029841	chr2-119028457-119029841
chr2	119034322	119036272	chr2-119034322-119036272
chr2	119045989	119048679	chr2-119045989-119048679
chr2	119111642	119114359	chr2-119111642-119114359
chr2	119155426	119157810	chr2-119155426-119157810
chr2	119166540	119168461	chr2-119166540-119168461
chr2	119172119	119177854	chr2-119172119-119177854
chr2	119188989	119189703	chr2-119188989-119189703
chr2	119207509	119210006	chr2-119207509-119210006
chr2	119235918	119240581	chr2-119235918-119240581
chr2	119242104	119243010	chr2-119242104-119243010
chr2	119249498	119251507	chr2-119249498-119251507
chr2	119262810	119263839	chr2-119262810-119263839

chr2	119270533	119271438	chr2-119270533-119271438
chr2	119288175	119290394	chr2-119288175-119290394
chr2	119299895	119300865	chr2-119299895-119300865
chr2	119322621	119322897	chr2-119322621-119322897
chr2	119324494	119327568	chr2-119324494-119327568
chr2	119337374	119338609	chr2-119337374-119338609
chr2	119344844	119345045	chr2-119344844-119345045
chr2	119350068	119354106	chr2-119350068-119354106
chr2	119475788	119479540	chr2-119475788-119479540
chr2	119491740	119492364	chr2-119491740-119492364
chr2	119546309	119549320	chr2-119546309-119549320
chr2	119558722	119559475	chr2-119558722-119559475
chr2	119593677	119595837	chr2-119593677-119595837
chr2	119617033	119620922	chr2-119617033-119620922
chr2	119661683	119663503	chr2-119661683-119663503
chr2	119674257	119676415	chr2-119674257-119676415
chr2	119741404	119744555	chr2-119741404-119744555
chr2	119757370	119759471	chr2-119757370-119759471
chr2	119786437	119788121	chr2-119786437-119788121
chr2	119798986	119800822	chr2-119798986-119800822
chr2	119872286	119873638	chr2-119872286-119873638
chr2	119896469	119898984	chr2-119896469-119898984
chr2	119971702	119973529	chr2-119971702-119973529
chr2	120026862	120028840	chr2-120026862-120028840
chr2	120032699	120034111	chr2-120032699-120034111
chr2	120044208	120044561	chr2-120044208-120044561
chr2	120049628	120050583	chr2-120049628-120050583
chr2	120075982	120077066	chr2-120075982-120077066
chr2	120093458	120094405	chr2-120093458-120094405
chr2	120097999	120100087	chr2-120097999-120100087
chr2	120104850	120105441	chr2-120104850-120105441
chr2	120126194	120126859	chr2-120126194-120126859
chr2	120128099	120130395	chr2-120128099-120130395
chr2	120146918	120148589	chr2-120146918-120148589
chr2	120151856	120155372	chr2-120151856-120155372
chr2	120244982	120245537	chr2-120244982-120245537
chr2	120268655	120269018	chr2-120268655-120269018
chr2	120351938	120353728	chr2-120351938-120353728
chr2	120402292	120405695	chr2-120402292-120405695
chr2	120562094	120564383	chr2-120562094-120564383
chr2	120566872	120568921	chr2-120566872-120568921
chr2	120608195	120610802	chr2-120608195-120610802

chr2	120628335	120629718	chr2-120628335-120629718
chr2	120729532	120732278	chr2-120729532-120732278
chr2	120849317	120851274	chr2-120849317-120851274
chr2	120969466	120971445	chr2-120969466-120971445
chr2	120976373	120978009	chr2-120976373-120978009
chr2	121007851	121009641	chr2-121007851-121009641
chr2	121090736	121091200	chr2-121090736-121091200
chr2	121092070	121092404	chr2-121092070-121092404
chr2	121138441	121141506	chr2-121138441-121141506
chr2	121169205	121172360	chr2-121169205-121172360
chr2	121270221	121272148	chr2-121270221-121272148
chr2	121288926	121290437	chr2-121288926-121290437
chr2	121296036	121296641	chr2-121296036-121296641
chr2	121354173	121355707	chr2-121354173-121355707
chr2	121358084	121359206	chr2-121358084-121359206
chr2	121412653	121416638	chr2-121412653-121416638
chr2	121441631	121443309	chr2-121441631-121443309
chr2	121447835	121453748	chr2-121447835-121453748
chr2	121456515	121458336	chr2-121456515-121458336
chr2	121472693	121475027	chr2-121472693-121475027
chr2	121505588	121508226	chr2-121505588-121508226
chr2	121509334	121510098	chr2-121509334-121510098
chr2	121594448	121595141	chr2-121594448-121595141
chr2	121600323	121601400	chr2-121600323-121601400
chr2	121787621	121788180	chr2-121787621-121788180
chr2	121805274	121807962	chr2-121805274-121807962
chr2	121865941	121868420	chr2-121865941-121868420
chr2	121955054	121958646	chr2-121955054-121958646
chr2	121965849	121966050	chr2-121965849-121966050
chr2	122027218	122030903	chr2-122027218-122030903
chr2	122117244	122119138	chr2-122117244-122119138
chr2	122147063	122149559	chr2-122147063-122149559
chr2	122156019	122156220	chr2-122156019-122156220
chr2	122161286	122161603	chr2-122161286-122161603
chr2	122164297	122164498	chr2-122164297-122164498
chr2	122233774	122235404	chr2-122233774-122235404
chr2	122296564	122299303	chr2-122296564-122299303
chr2	122301092	122302029	chr2-122301092-122302029
chr2	122305495	122306768	chr2-122305495-122306768
chr2	122312800	122313945	chr2-122312800-122313945
chr2	122327821	122328182	chr2-122327821-122328182
chr2	122345381	122346065	chr2-122345381-122346065

chr2	122347998	122350050	chr2-122347998-122350050
chr2	122367872	122369961	chr2-122367872-122369961
chr2	122375021	122379159	chr2-122375021-122379159
chr2	122380688	122381250	chr2-122380688-122381250
chr2	122467071	122468308	chr2-122467071-122468308
chr2	122609472	122611765	chr2-122609472-122611765
chr2	122630086	122632299	chr2-122630086-122632299
chr2	122637481	122639294	chr2-122637481-122639294
chr2	122651827	122657170	chr2-122651827-122657170
chr2	122665141	122666118	chr2-122665141-122666118
chr2	122697744	122703217	chr2-122697744-122703217
chr2	122737496	122739743	chr2-122737496-122739743
chr2	122764333	122766334	chr2-122764333-122766334
chr2	123754482	123754839	chr2-123754482-123754839
chr2	124089771	124091122	chr2-124089771-124091122
chr2	124609555	124611248	chr2-124609555-124611248
chr2	124684536	124684893	chr2-124684536-124684893
chr2	124719170	124719371	chr2-124719170-124719371
chr2	124858050	124860660	chr2-124858050-124860660
chr2	124971998	124972587	chr2-124971998-124972587
chr2	125033493	125033694	chr2-125033493-125033694
chr2	125057250	125057601	chr2-125057250-125057601
chr2	125118831	125119032	chr2-125118831-125119032
chr2	125121627	125124210	chr2-125121627-125124210
chr2	125135988	125136798	chr2-125135988-125136798
chr2	125246214	125249466	chr2-125246214-125249466
chr2	125505169	125507029	chr2-125505169-125507029
chr2	125623148	125625821	chr2-125623148-125625821
chr2	125667307	125667781	chr2-125667307-125667781
chr2	125672486	125674261	chr2-125672486-125674261
chr2	125722947	125725044	chr2-125722947-125725044
chr2	125731820	125732383	chr2-125731820-125732383
chr2	125781029	125783353	chr2-125781029-125783353
chr2	125789007	125789650	chr2-125789007-125789650
chr2	125791537	125793430	chr2-125791537-125793430
chr2	125857973	125860083	chr2-125857973-125860083
chr2	125865918	125867297	chr2-125865918-125867297
chr2	125934245	125934446	chr2-125934245-125934446
chr2	126031239	126031770	chr2-126031239-126031770
chr2	126151544	126153745	chr2-126151544-126153745
chr2	126351623	126351977	chr2-126351623-126351977
chr2	126552476	126553777	chr2-126552476-126553777

chr2	126597175	126598062	chr2-126597175-126598062
chr2	126673059	126678043	chr2-126673059-126678043
chr2	126706401	126708683	chr2-126706401-126708683
chr2	126874427	126877016	chr2-126874427-126877016
chr2	126931754	126933952	chr2-126931754-126933952
chr2	126945681	126946095	chr2-126945681-126946095
chr2	127006740	127007342	chr2-127006740-127007342
chr2	127008273	127010166	chr2-127008273-127010166
chr2	127069912	127071611	chr2-127069912-127071611
chr2	127132339	127134813	chr2-127132339-127134813
chr2	127142201	127143898	chr2-127142201-127143898
chr2	127182561	127183511	chr2-127182561-127183511
chr2	127207375	127209660	chr2-127207375-127209660
chr2	127246366	127249281	chr2-127246366-127249281
chr2	127251670	127252284	chr2-127251670-127252284
chr2	127269393	127271610	chr2-127269393-127271610
chr2	127332774	127334076	chr2-127332774-127334076
chr2	127335186	127337909	chr2-127335186-127337909
chr2	127362574	127364619	chr2-127362574-127364619
chr2	127443334	127445218	chr2-127443334-127445218
chr2	127481683	127484926	chr2-127481683-127484926
chr2	127508106	127508477	chr2-127508106-127508477
chr2	127521530	127522343	chr2-127521530-127522343
chr2	127537228	127537429	chr2-127537228-127537429
chr2	127540577	127541781	chr2-127540577-127541781
chr2	127583853	127585316	chr2-127583853-127585316
chr2	127586442	127589149	chr2-127586442-127589149
chr2	127625094	127626715	chr2-127625094-127626715
chr2	127646573	127650541	chr2-127646573-127650541
chr2	127651745	127657354	chr2-127651745-127657354
chr2	127662496	127663014	chr2-127662496-127663014
chr2	127683892	127684093	chr2-127683892-127684093
chr2	127718768	127719469	chr2-127718768-127719469
chr2	127721689	127723705	chr2-127721689-127723705
chr2	127728467	127730211	chr2-127728467-127730211
chr2	127788135	127789854	chr2-127788135-127789854
chr2	127790703	127792996	chr2-127790703-127792996
chr2	127830813	127832762	chr2-127830813-127832762
chr2	127890744	127891223	chr2-127890744-127891223
chr2	127922220	127923192	chr2-127922220-127923192
chr2	128105700	128106384	chr2-128105700-128106384
chr2	128122996	128124301	chr2-128122996-128124301

chr2	128125354	128129005	chr2-128125354-128129005
chr2	128166694	128166947	chr2-128166694-128166947
chr2	128172644	128172945	chr2-128172644-128172945
chr2	128179090	128180616	chr2-128179090-128180616
chr2	128210024	128211574	chr2-128210024-128211574
chr2	128238988	128242450	chr2-128238988-128242450
chr2	128264978	128265862	chr2-128264978-128265862
chr2	128284966	128285219	chr2-128284966-128285219
chr2	128287127	128287972	chr2-128287127-128287972
chr2	128388077	128388336	chr2-128388077-128388336
chr2	128391902	128393900	chr2-128391902-128393900
chr2	128395246	128395669	chr2-128395246-128395669
chr2	128400987	128401410	chr2-128400987-128401410
chr2	128403113	128404423	chr2-128403113-128404423
chr2	128408261	128409041	chr2-128408261-128409041
chr2	128412554	128412810	chr2-128412554-128412810
chr2	128415678	128417466	chr2-128415678-128417466
chr2	128418757	128419018	chr2-128418757-128419018
chr2	128419934	128421278	chr2-128419934-128421278
chr2	128424856	128430783	chr2-128424856-128430783
chr2	128457945	128458146	chr2-128457945-128458146
chr2	128461089	128461290	chr2-128461089-128461290
chr2	128465273	128465590	chr2-128465273-128465590
chr2	128470045	128470720	chr2-128470045-128470720
chr2	128544253	128545506	chr2-128544253-128545506
chr2	128548352	128548657	chr2-128548352-128548657
chr2	128552160	128553522	chr2-128552160-128553522
chr2	128565628	128566790	chr2-128565628-128566790
chr2	128568381	128568687	chr2-128568381-128568687
chr2	128575814	128576120	chr2-128575814-128576120
chr2	128683809	128684061	chr2-128683809-128684061
chr2	128685428	128687837	chr2-128685428-128687837
chr2	128698123	128699766	chr2-128698123-128699766
chr2	128737703	128739350	chr2-128737703-128739350
chr2	128817134	128819979	chr2-128817134-128819979
chr2	128863565	128864536	chr2-128863565-128864536
chr2	128892173	128892783	chr2-128892173-128892783
chr2	128942861	128944690	chr2-128942861-128944690
chr2	128966248	128968809	chr2-128966248-128968809
chr2	129037996	129039746	chr2-129037996-129039746
chr2	129065296	129067626	chr2-129065296-129067626
chr2	129100426	129102573	chr2-129100426-129102573

chr2	129109321	129109522	chr2-129109321-129109522
chr2	129128848	129131085	chr2-129128848-129131085
chr2	129178312	129179303	chr2-129178312-129179303
chr2	129197038	129201376	chr2-129197038-129201376
chr2	129245612	129246725	chr2-129245612-129246725
chr2	129262873	129263757	chr2-129262873-129263757
chr2	129295461	129298078	chr2-129295461-129298078
chr2	129309478	129309679	chr2-129309478-129309679
chr2	129330003	129331554	chr2-129330003-129331554
chr2	129334473	129334875	chr2-129334473-129334875
chr2	129592556	129594400	chr2-129592556-129594400
chr2	129632944	129634334	chr2-129632944-129634334
chr2	129799750	129803220	chr2-129799750-129803220
chr2	129947746	129948413	chr2-129947746-129948413
chr2	130176629	130180290	chr2-130176629-130180290
chr2	130272976	130278323	chr2-130272976-130278323
chr2	130283265	130285309	chr2-130283265-130285309
chr2	130294654	130296575	chr2-130294654-130296575
chr2	130365311	130365713	chr2-130365311-130365713
chr2	130397230	130398045	chr2-130397230-130398045
chr2	130423360	130425858	chr2-130423360-130425858
chr2	130449533	130452064	chr2-130449533-130452064
chr2	130496405	130496994	chr2-130496405-130496994
chr2	130562853	130565358	chr2-130562853-130565358
chr2	130579804	130581439	chr2-130579804-130581439
chr2	130628707	130630574	chr2-130628707-130630574
chr2	130641835	130643464	chr2-130641835-130643464
chr2	130663278	130664987	chr2-130663278-130664987
chr2	130667066	130669035	chr2-130667066-130669035
chr2	130689246	130699183	chr2-130689246-130699183
chr2	130837883	130840831	chr2-130837883-130840831
chr2	130873317	130873635	chr2-130873317-130873635
chr2	130905332	130908047	chr2-130905332-130908047
chr2	131028992	131030029	chr2-131028992-131030029
chr2	131037145	131038155	chr2-131037145-131038155
chr2	131039920	131041961	chr2-131039920-131041961
chr2	131053720	131056709	chr2-131053720-131056709
chr2	131063320	131063755	chr2-131063320-131063755
chr2	131095404	131097455	chr2-131095404-131097455
chr2	131127079	131127754	chr2-131127079-131127754
chr2	131158781	131161152	chr2-131158781-131161152
chr2	131174448	131175154	chr2-131174448-131175154

chr2	131176141	131180716	chr2-131176141-131180716
chr2	131186036	131190065	chr2-131186036-131190065
chr2	131203893	131205458	chr2-131203893-131205458
chr2	131209504	131211612	chr2-131209504-131211612
chr2	131233404	131235758	chr2-131233404-131235758
chr2	131261504	131265432	chr2-131261504-131265432
chr2	131321088	131321497	chr2-131321088-131321497
chr2	131351195	131353954	chr2-131351195-131353954
chr2	131386568	131387125	chr2-131386568-131387125
chr2	131392153	131394536	chr2-131392153-131394536
chr2	131395700	131396206	chr2-131395700-131396206
chr2	131401786	131402789	chr2-131401786-131402789
chr2	131403732	131404866	chr2-131403732-131404866
chr2	131422809	131423830	chr2-131422809-131423830
chr2	131427125	131427894	chr2-131427125-131427894
chr2	131436248	131436639	chr2-131436248-131436639
chr2	131447465	131448328	chr2-131447465-131448328
chr2	131490705	131493916	chr2-131490705-131493916
chr2	131495862	131496519	chr2-131495862-131496519
chr2	131504862	131505885	chr2-131504862-131505885
chr2	131508397	131508654	chr2-131508397-131508654
chr2	131509572	131510497	chr2-131509572-131510497
chr2	131545497	131549094	chr2-131545497-131549094
chr2	131561395	131563267	chr2-131561395-131563267
chr2	131820295	131820708	chr2-131820295-131820708
chr2	131902531	131903528	chr2-131902531-131903528
chr2	131909608	131911920	chr2-131909608-131911920
chr2	131944511	131944852	chr2-131944511-131944852
chr2	131950476	131951064	chr2-131950476-131951064
chr2	132006098	132007486	chr2-132006098-132007486
chr2	132028802	132030639	chr2-132028802-132030639
chr2	132093946	132094970	chr2-132093946-132094970
chr2	132099119	132099633	chr2-132099119-132099633
chr2	132102617	132103620	chr2-132102617-132103620
chr2	132130749	132131895	chr2-132130749-132131895
chr2	132143759	132145904	chr2-132143759-132145904
chr2	132152874	132153075	chr2-132152874-132153075
chr2	132206575	132206827	chr2-132206575-132206827
chr2	132211847	132212626	chr2-132211847-132212626
chr2	132246667	132249092	chr2-132246667-132249092
chr2	132250681	132254384	chr2-132250681-132254384
chr2	132262675	132265038	chr2-132262675-132265038

chr2	132282806	132283060	chr2-132282806-132283060
chr2	132286129	132287211	chr2-132286129-132287211
chr2	132323470	132323774	chr2-132323470-132323774
chr2	132339702	132340449	chr2-132339702-132340449
chr2	132384353	132385774	chr2-132384353-132385774
chr2	132507215	132507679	chr2-132507215-132507679
chr2	132576644	132578731	chr2-132576644-132578731
chr2	132686163	132687763	chr2-132686163-132687763
chr2	132781322	132782029	chr2-132781322-132782029
chr2	132814362	132817428	chr2-132814362-132817428
chr2	132846353	132848591	chr2-132846353-132848591
chr2	132879001	132880238	chr2-132879001-132880238
chr2	132941899	132942100	chr2-132941899-132942100
chr2	132943024	132947038	chr2-132943024-132947038
chr2	132948252	132948821	chr2-132948252-132948821
chr2	132951738	132952168	chr2-132951738-132952168
chr2	132954702	132955006	chr2-132954702-132955006
chr2	132992580	132994085	chr2-132992580-132994085
chr2	133498918	133499271	chr2-133498918-133499271
chr2	133551873	133554978	chr2-133551873-133554978
chr2	134527653	134528499	chr2-134527653-134528499
chr2	134643081	134644509	chr2-134643081-134644509
chr2	134785865	134786938	chr2-134785865-134786938
chr2	135439850	135440051	chr2-135439850-135440051
chr2	135658205	135660290	chr2-135658205-135660290
chr2	136387600	136387973	chr2-136387600-136387973
chr2	136531641	136532595	chr2-136531641-136532595
chr2	136648818	136649121	chr2-136648818-136649121
chr2	136678129	136680277	chr2-136678129-136680277
chr2	136712079	136714376	chr2-136712079-136714376
chr2	136890236	136893163	chr2-136890236-136893163
chr2	136919293	136921458	chr2-136919293-136921458
chr2	137049540	137049954	chr2-137049540-137049954
chr2	137062539	137063120	chr2-137062539-137063120
chr2	137113371	137118041	chr2-137113371-137118041
chr2	137225180	137225505	chr2-137225180-137225505
chr2	137413973	137414174	chr2-137413973-137414174
chr2	137419773	137420026	chr2-137419773-137420026
chr2	137715882	137716083	chr2-137715882-137716083
chr2	138040421	138040842	chr2-138040421-138040842
chr2	138256221	138258431	chr2-138256221-138258431
chr2	139676724	139680441	chr2-139676724-139680441

chr2	139723190	139723443	chr2-139723190-139723443
chr2	140065688	140067883	chr2-140065688-140067883
chr2	140103807	140104109	chr2-140103807-140104109
chr2	140168730	140171794	chr2-140168730-140171794
chr2	140394770	140396554	chr2-140394770-140396554
chr2	140670144	140671662	chr2-140670144-140671662
chr2	140743214	140743518	chr2-140743214-140743518
chr2	140759270	140759987	chr2-140759270-140759987
chr2	140763301	140763558	chr2-140763301-140763558
chr2	141288659	141288915	chr2-141288659-141288915
chr2	141337800	141338001	chr2-141337800-141338001
chr2	141629795	141630046	chr2-141629795-141630046
chr2	142757943	142758301	chr2-142757943-142758301
chr2	142900318	142903812	chr2-142900318-142903812
chr2	143062444	143064255	chr2-143062444-143064255
chr2	143545328	143546755	chr2-143545328-143546755
chr2	143846955	143847270	chr2-143846955-143847270
chr2	143852270	143853812	chr2-143852270-143853812
chr2	143862368	143863402	chr2-143862368-143863402
chr2	143864371	143865140	chr2-143864371-143865140
chr2	143867361	143867931	chr2-143867361-143867931
chr2	143899751	143900353	chr2-143899751-143900353
chr2	143903256	143905182	chr2-143903256-143905182
chr2	143913811	143919125	chr2-143913811-143919125
chr2	143924145	143924403	chr2-143924145-143924403
chr2	144002518	144003449	chr2-144002518-144003449
chr2	144005178	144005379	chr2-144005178-144005379
chr2	144009053	144011904	chr2-144009053-144011904
chr2	144164812	144166786	chr2-144164812-144166786
chr2	144172251	144172973	chr2-144172251-144172973
chr2	144239823	144240074	chr2-144239823-144240074
chr2	144265946	144272448	chr2-144265946-144272448
chr2	144329709	144333238	chr2-144329709-144333238
chr2	144368117	144370835	chr2-144368117-144370835
chr2	144441156	144442076	chr2-144441156-144442076
chr2	144458704	144459723	chr2-144458704-144459723
chr2	144526870	144528937	chr2-144526870-144528937
chr2	144549981	144551070	chr2-144549981-144551070
chr2	144555105	144557387	chr2-144555105-144557387
chr2	144593653	144595263	chr2-144593653-144595263
chr2	144599713	144600885	chr2-144599713-144600885
chr2	144738337	144741359	chr2-144738337-144741359

chr2	145167011	145168897	chr2-145167011-145168897
chr2	145674407	145676888	chr2-145674407-145676888
chr2	145680725	145681031	chr2-145680725-145681031
chr2	145681983	145682184	chr2-145681983-145682184
chr2	145785417	145788251	chr2-145785417-145788251
chr2	145842941	145843511	chr2-145842941-145843511
chr2	145859384	145864668	chr2-145859384-145864668
chr2	145871252	145873922	chr2-145871252-145873922
chr2	145902860	145905563	chr2-145902860-145905563
chr2	145933416	145935431	chr2-145933416-145935431
chr2	146218238	146218817	chr2-146218238-146218817
chr2	146220725	146224277	chr2-146220725-146224277
chr2	146295349	146295804	chr2-146295349-146295804
chr2	146299040	146299241	chr2-146299040-146299241
chr2	146307137	146307501	chr2-146307137-146307501
chr2	146510612	146512779	chr2-146510612-146512779
chr2	146612689	146613197	chr2-146612689-146613197
chr2	146810739	146810940	chr2-146810739-146810940
chr2	146834147	146835538	chr2-146834147-146835538
chr2	146839361	146839662	chr2-146839361-146839662
chr2	146855449	146856737	chr2-146855449-146856737
chr2	147011780	147015246	chr2-147011780-147015246
chr2	147083732	147085780	chr2-147083732-147085780
chr2	147177885	147179103	chr2-147177885-147179103
chr2	147183874	147187473	chr2-147183874-147187473
chr2	147193564	147195558	chr2-147193564-147195558
chr2	147361625	147362633	chr2-147361625-147362633
chr2	147364815	147366381	chr2-147364815-147366381
chr2	147373339	147373993	chr2-147373339-147373993
chr2	147708785	147709648	chr2-147708785-147709648
chr2	147744247	147744448	chr2-147744247-147744448
chr2	148030412	148030764	chr2-148030412-148030764
chr2	148039487	148041121	chr2-148039487-148041121
chr2	148043487	148047229	chr2-148043487-148047229
chr2	148048309	148049277	chr2-148048309-148049277
chr2	148196200	148197884	chr2-148196200-148197884
chr2	148395201	148395993	chr2-148395201-148395993
chr2	148402173	148409188	chr2-148402173-148409188
chr2	148446307	148446734	chr2-148446307-148446734
chr2	148478152	148478457	chr2-148478152-148478457
chr2	148519925	148520737	chr2-148519925-148520737
chr2	148537149	148537400	chr2-148537149-148537400

chr2	148571206	148571407	chr2-148571206-148571407
chr2	148671729	148674595	chr2-148671729-148674595
chr2	148677546	148682594	chr2-148677546-148682594
chr2	148731137	148732893	chr2-148731137-148732893
chr2	148742983	148744194	chr2-148742983-148744194
chr2	148752091	148752544	chr2-148752091-148752544
chr2	148851286	148851538	chr2-148851286-148851538
chr2	148856458	148857217	chr2-148856458-148857217
chr2	148873527	148875972	chr2-148873527-148875972
chr2	148902358	148902559	chr2-148902358-148902559
chr2	149830121	149831798	chr2-149830121-149831798
chr2	150135393	150137242	chr2-150135393-150137242
chr2	150217634	150219269	chr2-150217634-150219269
chr2	150310735	150312929	chr2-150310735-150312929
chr2	150361813	150363201	chr2-150361813-150363201
chr2	150439094	150439418	chr2-150439094-150439418
chr2	150450191	150452095	chr2-150450191-150452095
chr2	150500500	150500906	chr2-150500500-150500906
chr2	150525699	150526311	chr2-150525699-150526311
chr2	150595614	150595931	chr2-150595614-150595931
chr2	150607320	150609247	chr2-150607320-150609247
chr2	150667314	150668818	chr2-150667314-150668818
chr2	150688402	150689014	chr2-150688402-150689014
chr2	150748793	150750977	chr2-150748793-150750977
chr2	150766715	150767040	chr2-150766715-150767040
chr2	150770239	150771236	chr2-150770239-150771236
chr2	150786037	150789343	chr2-150786037-150789343
chr2	150839909	150840364	chr2-150839909-150840364
chr2	150903506	150905523	chr2-150903506-150905523
chr2	150909199	150911713	chr2-150909199-150911713
chr2	151008088	151009612	chr2-151008088-151009612
chr2	151037680	151040329	chr2-151037680-151040329
chr2	151493944	151495486	chr2-151493944-151495486
chr2	151529527	151531221	chr2-151529527-151531221
chr2	151535952	151537204	chr2-151535952-151537204
chr2	151541477	151545882	chr2-151541477-151545882
chr2	151562952	151563306	chr2-151562952-151563306
chr2	151571399	151576929	chr2-151571399-151576929
chr2	151605420	151606202	chr2-151605420-151606202
chr2	151631772	151632888	chr2-151631772-151632888
chr2	151670226	151670598	chr2-151670226-151670598
chr2	151701639	151703412	chr2-151701639-151703412

chr2	151739347	151742039	chr2-151739347-151742039
chr2	151776002	151776255	chr2-151776002-151776255
chr2	151842566	151843327	chr2-151842566-151843327
chr2	151876272	151876583	chr2-151876272-151876583
chr2	151959003	151959935	chr2-151959003-151959935
chr2	151961145	151963545	chr2-151961145-151963545
chr2	151970015	151980658	chr2-151970015-151980658
chr2	151994340	151997249	chr2-151994340-151997249
chr2	151999389	152000254	chr2-151999389-152000254
chr2	152029203	152030158	chr2-152029203-152030158
chr2	152048281	152049479	chr2-152048281-152049479
chr2	152080843	152082471	chr2-152080843-152082471
chr2	152092894	152094088	chr2-152092894-152094088
chr2	152095030	152095408	chr2-152095030-152095408
chr2	152104961	152107110	chr2-152104961-152107110
chr2	152132580	152133227	chr2-152132580-152133227
chr2	152141896	152144358	chr2-152141896-152144358
chr2	152177352	152177553	chr2-152177352-152177553
chr2	152225637	152228427	chr2-152225637-152228427
chr2	152293208	152295943	chr2-152293208-152295943
chr2	152323687	152324595	chr2-152323687-152324595
chr2	152326186	152326891	chr2-152326186-152326891
chr2	152330451	152333287	chr2-152330451-152333287
chr2	152335176	152340134	chr2-152335176-152340134
chr2	152341815	152344725	chr2-152341815-152344725
chr2	152349142	152351097	chr2-152349142-152351097
chr2	152363526	152363879	chr2-152363526-152363879
chr2	152395431	152399551	chr2-152395431-152399551
chr2	152405550	152406871	chr2-152405550-152406871
chr2	152413454	152415748	chr2-152413454-152415748
chr2	152437258	152437868	chr2-152437258-152437868
chr2	152480683	152480988	chr2-152480683-152480988
chr2	152626598	152627722	chr2-152626598-152627722
chr2	152633401	152633704	chr2-152633401-152633704
chr2	152669184	152674681	chr2-152669184-152674681
chr2	152686273	152688505	chr2-152686273-152688505
chr2	152693413	152693979	chr2-152693413-152693979
chr2	152699641	152701327	chr2-152699641-152701327
chr2	152707208	152708360	chr2-152707208-152708360
chr2	152709697	152710718	chr2-152709697-152710718
chr2	152727069	152727270	chr2-152727069-152727270
chr2	152734619	152742630	chr2-152734619-152742630

chr2	152787893	152788094	chr2-152787893-152788094
chr2	152788902	152790019	chr2-152788902-152790019
chr2	152792773	152792974	chr2-152792773-152792974
chr2	152803645	152806963	chr2-152803645-152806963
chr2	152809854	152812941	chr2-152809854-152812941
chr2	152814642	152814895	chr2-152814642-152814895
chr2	152815832	152831940	chr2-152815832-152831940
chr2	152847528	152850206	chr2-152847528-152850206
chr2	152889045	152889301	chr2-152889045-152889301
chr2	152903770	152903971	chr2-152903770-152903971
chr2	152914143	152915018	chr2-152914143-152915018
chr2	152942800	152943591	chr2-152942800-152943591
chr2	152951038	152951994	chr2-152951038-152951994
chr2	152962117	152962842	chr2-152962117-152962842
chr2	153013899	153015616	chr2-153013899-153015616
chr2	153031751	153032470	chr2-153031751-153032470
chr2	153071551	153072255	chr2-153071551-153072255
chr2	153108176	153109088	chr2-153108176-153109088
chr2	153121984	153122286	chr2-153121984-153122286
chr2	153160749	153162997	chr2-153160749-153162997
chr2	153175879	153176080	chr2-153175879-153176080
chr2	153178792	153179176	chr2-153178792-153179176
chr2	153239029	153242525	chr2-153239029-153242525
chr2	153290501	153292675	chr2-153290501-153292675
chr2	153344544	153348849	chr2-153344544-153348849
chr2	153350705	153351009	chr2-153350705-153351009
chr2	153428525	153429226	chr2-153428525-153429226
chr2	153434975	153437656	chr2-153434975-153437656
chr2	153441549	153446410	chr2-153441549-153446410
chr2	153483579	153483780	chr2-153483579-153483780
chr2	153491857	153493615	chr2-153491857-153493615
chr2	153527718	153530677	chr2-153527718-153530677
chr2	153560693	153561239	chr2-153560693-153561239
chr2	153586428	153586893	chr2-153586428-153586893
chr2	153592129	153592925	chr2-153592129-153592925
chr2	153598020	153598221	chr2-153598020-153598221
chr2	153631694	153633314	chr2-153631694-153633314
chr2	153648870	153651061	chr2-153648870-153651061
chr2	153740647	153743412	chr2-153740647-153743412
chr2	153935641	153935842	chr2-153935641-153935842
chr2	153954037	153954338	chr2-153954037-153954338
chr2	154218044	154218245	chr2-154218044-154218245

chr2	154223293	154223859	chr2-154223293-154223859
chr2	154294638	154294995	chr2-154294638-154294995
chr2	154353885	154355921	chr2-154353885-154355921
chr2	154371866	154373574	chr2-154371866-154373574
chr2	154389874	154390821	chr2-154389874-154390821
chr2	154402774	154403265	chr2-154402774-154403265
chr2	154407114	154408680	chr2-154407114-154408680
chr2	154427778	154428493	chr2-154427778-154428493
chr2	154435785	154438209	chr2-154435785-154438209
chr2	154459931	154460234	chr2-154459931-154460234
chr2	154549169	154550225	chr2-154549169-154550225
chr2	154552149	154552898	chr2-154552149-154552898
chr2	154558720	154558974	chr2-154558720-154558974
chr2	154567737	154570806	chr2-154567737-154570806
chr2	154603061	154604459	chr2-154603061-154604459
chr2	154612491	154614495	chr2-154612491-154614495
chr2	154656259	154659652	chr2-154656259-154659652
chr2	154677456	154677657	chr2-154677456-154677657
chr2	154712280	154712848	chr2-154712280-154712848
chr2	154789945	154793611	chr2-154789945-154793611
chr2	154807512	154807902	chr2-154807512-154807902
chr2	154890201	154893547	chr2-154890201-154893547
chr2	154901775	154902092	chr2-154901775-154902092
chr2	154942928	154943337	chr2-154942928-154943337
chr2	155018328	155020088	chr2-155018328-155020088
chr2	155027032	155027547	chr2-155027032-155027547
chr2	155050124	155051748	chr2-155050124-155051748
chr2	155072989	155075801	chr2-155072989-155075801
chr2	155132985	155134741	chr2-155132985-155134741
chr2	155235668	155238070	chr2-155235668-155238070
chr2	155275935	155278016	chr2-155275935-155278016
chr2	155293426	155293884	chr2-155293426-155293884
chr2	155295248	155295936	chr2-155295248-155295936
chr2	155356509	155358348	chr2-155356509-155358348
chr2	155381020	155383343	chr2-155381020-155383343
chr2	155472356	155474588	chr2-155472356-155474588
chr2	155514484	155515139	chr2-155514484-155515139
chr2	155517307	155519229	chr2-155517307-155519229
chr2	155538563	155538764	chr2-155538563-155538764
chr2	155591035	155593401	chr2-155591035-155593401
chr2	155613155	155614006	chr2-155613155-155614006
chr2	155629073	155629740	chr2-155629073-155629740

chr2	155690677	155692685	chr2-155690677-155692685
chr2	155727806	155730178	chr2-155727806-155730178
chr2	155742534	155743381	chr2-155742534-155743381
chr2	155747238	155747535	chr2-155747238-155747535
chr2	155750461	155753731	chr2-155750461-155753731
chr2	155774721	155776369	chr2-155774721-155776369
chr2	155803231	155813150	chr2-155803231-155813150
chr2	155816812	155821027	chr2-155816812-155821027
chr2	155823780	155827834	chr2-155823780-155827834
chr2	155877948	155879467	chr2-155877948-155879467
chr2	155882640	155883947	chr2-155882640-155883947
chr2	155886949	155887206	chr2-155886949-155887206
chr2	155928875	155930847	chr2-155928875-155930847
chr2	155955844	155958151	chr2-155955844-155958151
chr2	155979028	155980247	chr2-155979028-155980247
chr2	156007261	156009257	chr2-156007261-156009257
chr2	156047917	156048341	chr2-156047917-156048341
chr2	156052777	156053139	chr2-156052777-156053139
chr2	156064783	156066774	chr2-156064783-156066774
chr2	156109448	156112482	chr2-156109448-156112482
chr2	156119599	156119852	chr2-156119599-156119852
chr2	156143089	156145988	chr2-156143089-156145988
chr2	156177017	156181340	chr2-156177017-156181340
chr2	156195982	156199815	chr2-156195982-156199815
chr2	156202471	156203513	chr2-156202471-156203513
chr2	156223184	156223862	chr2-156223184-156223862
chr2	156311288	156313571	chr2-156311288-156313571
chr2	156362062	156363129	chr2-156362062-156363129
chr2	156391339	156393336	chr2-156391339-156393336
chr2	156415559	156417555	chr2-156415559-156417555
chr2	156420152	156423051	chr2-156420152-156423051
chr2	156443370	156443571	chr2-156443370-156443571
chr2	156446120	156448610	chr2-156446120-156448610
chr2	156451141	156452092	chr2-156451141-156452092
chr2	156455361	156457766	chr2-156455361-156457766
chr2	156474148	156477435	chr2-156474148-156477435
chr2	156487461	156488653	chr2-156487461-156488653
chr2	156546928	156549041	chr2-156546928-156549041
chr2	156612952	156615157	chr2-156612952-156615157
chr2	156683110	156683311	chr2-156683110-156683311
chr2	156716755	156723532	chr2-156716755-156723532
chr2	156726181	156726436	chr2-156726181-156726436

chr2	156730129	156731000	chr2-156730129-156731000
chr2	156741158	156741359	chr2-156741158-156741359
chr2	156775053	156775735	chr2-156775053-156775735
chr2	156838773	156843035	chr2-156838773-156843035
chr2	156861731	156864963	chr2-156861731-156864963
chr2	156882403	156882923	chr2-156882403-156882923
chr2	156990762	156992573	chr2-156990762-156992573
chr2	157005821	157007836	chr2-157005821-157007836
chr2	157065591	157066215	chr2-157065591-157066215
chr2	157069484	157074472	chr2-157069484-157074472
chr2	157076542	157080001	chr2-157076542-157080001
chr2	157099760	157100877	chr2-157099760-157100877
chr2	157130831	157131503	chr2-157130831-157131503
chr2	157133641	157135723	chr2-157133641-157135723
chr2	157169850	157170891	chr2-157169850-157170891
chr2	157203332	157205475	chr2-157203332-157205475
chr2	157278126	157280792	chr2-157278126-157280792
chr2	157366742	157369228	chr2-157366742-157369228
chr2	157373603	157378656	chr2-157373603-157378656
chr2	157410720	157412188	chr2-157410720-157412188
chr2	157418258	157421104	chr2-157418258-157421104
chr2	157421903	157432791	chr2-157421903-157432791
chr2	157433719	157434961	chr2-157433719-157434961
chr2	157436612	157436813	chr2-157436612-157436813
chr2	157438306	157439499	chr2-157438306-157439499
chr2	157450169	157452438	chr2-157450169-157452438
chr2	157456415	157458647	chr2-157456415-157458647
chr2	157500696	157501418	chr2-157500696-157501418
chr2	157551767	157553953	chr2-157551767-157553953
chr2	157559854	157562065	chr2-157559854-157562065
chr2	157563459	157567975	chr2-157563459-157567975
chr2	157596842	157597479	chr2-157596842-157597479
chr2	157696204	157696680	chr2-157696204-157696680
chr2	157715183	157716398	chr2-157715183-157716398
chr2	157736959	157738392	chr2-157736959-157738392
chr2	157914308	157915055	chr2-157914308-157915055
chr2	157935585	157936053	chr2-157935585-157936053
chr2	157940656	157941472	chr2-157940656-157941472
chr2	157943405	157944722	chr2-157943405-157944722
chr2	158027107	158031405	chr2-158027107-158031405
chr2	158098496	158098802	chr2-158098496-158098802
chr2	158113132	158114333	chr2-158113132-158114333

chr2	158121345	158122432	chr2-158121345-158122432
chr2	158137782	158138038	chr2-158137782-158138038
chr2	158139592	158140081	chr2-158139592-158140081
chr2	158144946	158146614	chr2-158144946-158146614
chr2	158153557	158154770	chr2-158153557-158154770
chr2	158159836	158160165	chr2-158159836-158160165
chr2	158167597	158169470	chr2-158167597-158169470
chr2	158228755	158229685	chr2-158228755-158229685
chr2	158358109	158362126	chr2-158358109-158362126
chr2	158375442	158376139	chr2-158375442-158376139
chr2	158409134	158411567	chr2-158409134-158411567
chr2	158417224	158417525	chr2-158417224-158417525
chr2	158501449	158501650	chr2-158501449-158501650
chr2	158509658	158516196	chr2-158509658-158516196
chr2	158518415	158519514	chr2-158518415-158519514
chr2	158522328	158523973	chr2-158522328-158523973
chr2	158555829	158556617	chr2-158555829-158556617
chr2	158609750	158611429	chr2-158609750-158611429
chr2	158613773	158615090	chr2-158613773-158615090
chr2	158616097	158616558	chr2-158616097-158616558
chr2	158624697	158626683	chr2-158624697-158626683
chr2	158666988	158668572	chr2-158666988-158668572
chr2	158720933	158721303	chr2-158720933-158721303
chr2	158767574	158770689	chr2-158767574-158770689
chr2	158793766	158795731	chr2-158793766-158795731
chr2	159116871	159117175	chr2-159116871-159117175
chr2	160264179	160264648	chr2-160264179-160264648
chr2	160364696	160368293	chr2-160364696-160368293
chr2	160451468	160451885	chr2-160451468-160451885
chr2	160603249	160603682	chr2-160603249-160603682
chr2	160619219	160622524	chr2-160619219-160622524
chr2	160645320	160648320	chr2-160645320-160648320
chr2	160730681	160733541	chr2-160730681-160733541
chr2	160871971	160873803	chr2-160871971-160873803
chr2	160887224	160889165	chr2-160887224-160889165
chr2	160911541	160912668	chr2-160911541-160912668
chr2	160921751	160922006	chr2-160921751-160922006
chr2	161107479	161109993	chr2-161107479-161109993
chr2	161177202	161178127	chr2-161177202-161178127
chr2	161511424	161511892	chr2-161511424-161511892
chr2	161593656	161594712	chr2-161593656-161594712
chr2	161704650	161705007	chr2-161704650-161705007

chr2	162660298	162661607	chr2-162660298-162661607
chr2	162867395	162867816	chr2-162867395-162867816
chr2	162930143	162933208	chr2-162930143-162933208
chr2	162942945	162943920	chr2-162942945-162943920
chr2	162960730	162961615	chr2-162960730-162961615
chr2	162986141	162987707	chr2-162986141-162987707
chr2	163016397	163018607	chr2-163016397-163018607
chr2	163054091	163057292	chr2-163054091-163057292
chr2	163202739	163204998	chr2-163202739-163204998
chr2	163339086	163340024	chr2-163339086-163340024
chr2	163345070	163345577	chr2-163345070-163345577
chr2	163346814	163347015	chr2-163346814-163347015
chr2	163386627	163387184	chr2-163386627-163387184
chr2	163417014	163421372	chr2-163417014-163421372
chr2	163438212	163439040	chr2-163438212-163439040
chr2	163466744	163467328	chr2-163466744-163467328
chr2	163471091	163473278	chr2-163471091-163473278
chr2	163481545	163482223	chr2-163481545-163482223
chr2	163493091	163494779	chr2-163493091-163494779
chr2	163535040	163535460	chr2-163535040-163535460
chr2	163601755	163603567	chr2-163601755-163603567
chr2	163610930	163611232	chr2-163610930-163611232
chr2	163620625	163620826	chr2-163620625-163620826
chr2	163642783	163645572	chr2-163642783-163645572
chr2	163657895	163659462	chr2-163657895-163659462
chr2	163735954	163736549	chr2-163735954-163736549
chr2	163749363	163750823	chr2-163749363-163750823
chr2	163853505	163854137	chr2-163853505-163854137
chr2	163918232	163919410	chr2-163918232-163919410
chr2	163994448	163997280	chr2-163994448-163997280
chr2	164006930	164007181	chr2-164006930-164007181
chr2	164047124	164048324	chr2-164047124-164048324
chr2	164068945	164071708	chr2-164068945-164071708
chr2	164073981	164076222	chr2-164073981-164076222
chr2	164140420	164140691	chr2-164140420-164140691
chr2	164167815	164168954	chr2-164167815-164168954
chr2	164170768	164171486	chr2-164170768-164171486
chr2	164355972	164356937	chr2-164355972-164356937
chr2	164385856	164387204	chr2-164385856-164387204
chr2	164389373	164390028	chr2-164389373-164390028
chr2	164404361	164405024	chr2-164404361-164405024
chr2	164407877	164408182	chr2-164407877-164408182

chr2	164430884	164443832	chr2-164430884-164443832
chr2	164452072	164452486	chr2-164452072-164452486
chr2	164454902	164459173	chr2-164454902-164459173
chr2	164460766	164462501	chr2-164460766-164462501
chr2	164469308	164470426	chr2-164469308-164470426
chr2	164485136	164488314	chr2-164485136-164488314
chr2	164496672	164498470	chr2-164496672-164498470
chr2	164512180	164514263	chr2-164512180-164514263
chr2	164517974	164519662	chr2-164517974-164519662
chr2	164520719	164526987	chr2-164520719-164526987
chr2	164547889	164551724	chr2-164547889-164551724
chr2	164557810	164559649	chr2-164557810-164559649
chr2	164561187	164567617	chr2-164561187-164567617
chr2	164678878	164679631	chr2-164678878-164679631
chr2	164701644	164701945	chr2-164701644-164701945
chr2	164745007	164747820	chr2-164745007-164747820
chr2	164749286	164749638	chr2-164749286-164749638
chr2	164768734	164772528	chr2-164768734-164772528
chr2	164784561	164786977	chr2-164784561-164786977
chr2	164803614	164806447	chr2-164803614-164806447
chr2	164822335	164824137	chr2-164822335-164824137
chr2	164832460	164835054	chr2-164832460-164835054
chr2	164856355	164858314	chr2-164856355-164858314
chr2	164878041	164881062	chr2-164878041-164881062
chr2	164909761	164912794	chr2-164909761-164912794
chr2	164938383	164939392	chr2-164938383-164939392
chr2	164949162	164949637	chr2-164949162-164949637
chr2	164953111	164953539	chr2-164953111-164953539
chr2	164960381	164961541	chr2-164960381-164961541
chr2	164967840	164968757	chr2-164967840-164968757
chr2	164997532	164998292	chr2-164997532-164998292
chr2	165033249	165035431	chr2-165033249-165035431
chr2	165055094	165056254	chr2-165055094-165056254
chr2	165112032	165112673	chr2-165112032-165112673
chr2	165180868	165181337	chr2-165180868-165181337
chr2	165201703	165202530	chr2-165201703-165202530
chr2	165233435	165235306	chr2-165233435-165235306
chr2	165286172	165288923	chr2-165286172-165288923
chr2	165321390	165322427	chr2-165321390-165322427
chr2	165324067	165327513	chr2-165324067-165327513
chr2	165364225	165364594	chr2-165364225-165364594
chr2	165367011	165367478	chr2-165367011-165367478

chr2	165386935	165388542	chr2-165386935-165388542
chr2	165402119	165402320	chr2-165402119-165402320
chr2	165473050	165473305	chr2-165473050-165473305
chr2	165491907	165494192	chr2-165491907-165494192
chr2	165503789	165504403	chr2-165503789-165504403
chr2	165559997	165560353	chr2-165559997-165560353
chr2	165563026	165564033	chr2-165563026-165564033
chr2	165594740	165596123	chr2-165594740-165596123
chr2	165600722	165602179	chr2-165600722-165602179
chr2	165627983	165628719	chr2-165627983-165628719
chr2	165630450	165633797	chr2-165630450-165633797
chr2	165649177	165649378	chr2-165649177-165649378
chr2	165653186	165658255	chr2-165653186-165658255
chr2	165666913	165667281	chr2-165666913-165667281
chr2	165693188	165693389	chr2-165693188-165693389
chr2	165855784	165859882	chr2-165855784-165859882
chr2	165880428	165880932	chr2-165880428-165880932
chr2	165881772	165885451	chr2-165881772-165885451
chr2	165914443	165914853	chr2-165914443-165914853
chr2	165919598	165919853	chr2-165919598-165919853
chr2	165924967	165925429	chr2-165924967-165925429
chr2	165969654	165970011	chr2-165969654-165970011
chr2	165986822	165987788	chr2-165986822-165987788
chr2	165991809	165994229	chr2-165991809-165994229
chr2	166029992	166031871	chr2-166029992-166031871
chr2	166153901	166155797	chr2-166153901-166155797
chr2	166170168	166170768	chr2-166170168-166170768
chr2	166235514	166235715	chr2-166235514-166235715
chr2	166237406	166238211	chr2-166237406-166238211
chr2	166426850	166427323	chr2-166426850-166427323
chr2	166433456	166433761	chr2-166433456-166433761
chr2	166564388	166564740	chr2-166564388-166564740
chr2	166712876	166714737	chr2-166712876-166714737
chr2	166758187	166758439	chr2-166758187-166758439
chr2	166785896	166787384	chr2-166785896-166787384
chr2	166792918	166795265	chr2-166792918-166795265
chr2	166804949	166807814	chr2-166804949-166807814
chr2	166822178	166822495	chr2-166822178-166822495
chr2	166905327	166907881	chr2-166905327-166907881
chr2	166994195	166997204	chr2-166994195-166997204
chr2	167014493	167017081	chr2-167014493-167017081
chr2	167061233	167066754	chr2-167061233-167066754

chr2	167071187	167072347	chr2-167071187-167072347
chr2	167077389	167080803	chr2-167077389-167080803
chr2	167081670	167081871	chr2-167081670-167081871
chr2	167118090	167118503	chr2-167118090-167118503
chr2	167128901	167129790	chr2-167128901-167129790
chr2	167167669	167168035	chr2-167167669-167168035
chr2	167188240	167190470	chr2-167188240-167190470
chr2	167230419	167230729	chr2-167230419-167230729
chr2	167240066	167240722	chr2-167240066-167240722
chr2	167280547	167280806	chr2-167280547-167280806
chr2	167291990	167293199	chr2-167291990-167293199
chr2	167313603	167314502	chr2-167313603-167314502
chr2	167319122	167319392	chr2-167319122-167319392
chr2	167347726	167350201	chr2-167347726-167350201
chr2	167380541	167380965	chr2-167380541-167380965
chr2	167420396	167423141	chr2-167420396-167423141
chr2	167485672	167487039	chr2-167485672-167487039
chr2	167490752	167494030	chr2-167490752-167494030
chr2	167502394	167505475	chr2-167502394-167505475
chr2	167537415	167540536	chr2-167537415-167540536
chr2	167542763	167544643	chr2-167542763-167544643
chr2	167552784	167553357	chr2-167552784-167553357
chr2	167562158	167562461	chr2-167562158-167562461
chr2	167570037	167570506	chr2-167570037-167570506
chr2	167578389	167579300	chr2-167578389-167579300
chr2	167585858	167589827	chr2-167585858-167589827
chr2	167617222	167618333	chr2-167617222-167618333
chr2	167628729	167635001	chr2-167628729-167635001
chr2	167659003	167662428	chr2-167659003-167662428
chr2	167683643	167683894	chr2-167683643-167683894
chr2	167685323	167685676	chr2-167685323-167685676
chr2	167687789	167692771	chr2-167687789-167692771
chr2	167697734	167698647	chr2-167697734-167698647
chr2	167727070	167727639	chr2-167727070-167727639
chr2	167730984	167731491	chr2-167730984-167731491
chr2	167764874	167765633	chr2-167764874-167765633
chr2	167774479	167782057	chr2-167774479-167782057
chr2	167788599	167790546	chr2-167788599-167790546
chr2	167800276	167801316	chr2-167800276-167801316
chr2	167805773	167806080	chr2-167805773-167806080
chr2	167823380	167824200	chr2-167823380-167824200
chr2	167828090	167828951	chr2-167828090-167828951

chr2	167833030	167834863	chr2-167833030-167834863
chr2	167837550	167837803	chr2-167837550-167837803
chr2	167839294	167839831	chr2-167839294-167839831
chr2	167856861	167857427	chr2-167856861-167857427
chr2	167887545	167888439	chr2-167887545-167888439
chr2	167894870	167895652	chr2-167894870-167895652
chr2	167920116	167920606	chr2-167920116-167920606
chr2	167924964	167925583	chr2-167924964-167925583
chr2	167931174	167934134	chr2-167931174-167934134
chr2	167939753	167940123	chr2-167939753-167940123
chr2	167941852	167943189	chr2-167941852-167943189
chr2	167948598	167951895	chr2-167948598-167951895
chr2	167958505	167959662	chr2-167958505-167959662
chr2	167960666	167964917	chr2-167960666-167964917
chr2	167982917	167983232	chr2-167982917-167983232
chr2	167984273	167984760	chr2-167984273-167984760
chr2	168001782	168004283	chr2-168001782-168004283
chr2	168005093	168006744	chr2-168005093-168006744
chr2	168050021	168051076	chr2-168050021-168051076
chr2	168063805	168064006	chr2-168063805-168064006
chr2	168065279	168073576	chr2-168065279-168073576
chr2	168076550	168087287	chr2-168076550-168087287
chr2	168095095	168095450	chr2-168095095-168095450
chr2	168103491	168105574	chr2-168103491-168105574
chr2	168114752	168115586	chr2-168114752-168115586
chr2	168117016	168118126	chr2-168117016-168118126
chr2	168149885	168150750	chr2-168149885-168150750
chr2	168154479	168154781	chr2-168154479-168154781
chr2	168158941	168159725	chr2-168158941-168159725
chr2	168161641	168162326	chr2-168161641-168162326
chr2	168191203	168192769	chr2-168191203-168192769
chr2	168204318	168207847	chr2-168204318-168207847
chr2	168228887	168232201	chr2-168228887-168232201
chr2	168252255	168252581	chr2-168252255-168252581
chr2	168268442	168268887	chr2-168268442-168268887
chr2	168280445	168281819	chr2-168280445-168281819
chr2	168458437	168458638	chr2-168458437-168458638
chr2	168472876	168473384	chr2-168472876-168473384
chr2	168517386	168518991	chr2-168517386-168518991
chr2	168524604	168526086	chr2-168524604-168526086
chr2	168531647	168533023	chr2-168531647-168533023
chr2	168547892	168550013	chr2-168547892-168550013

chr2	168579320	168580353	chr2-168579320-168580353
chr2	168589124	168590860	chr2-168589124-168590860
chr2	168601055	168602298	chr2-168601055-168602298
chr2	168627985	168628301	chr2-168627985-168628301
chr2	168633307	168633777	chr2-168633307-168633777
chr2	168721384	168722031	chr2-168721384-168722031
chr2	168728377	168728660	chr2-168728377-168728660
chr2	168732514	168734731	chr2-168732514-168734731
chr2	168737472	168738508	chr2-168737472-168738508
chr2	168740184	168742786	chr2-168740184-168742786
chr2	168765025	168767296	chr2-168765025-168767296
chr2	168827228	168827429	chr2-168827228-168827429
chr2	168839819	168840577	chr2-168839819-168840577
chr2	168842295	168842546	chr2-168842295-168842546
chr2	168872227	168872585	chr2-168872227-168872585
chr2	168911900	168913740	chr2-168911900-168913740
chr2	168953566	168956777	chr2-168953566-168956777
chr2	169492927	169494393	chr2-169492927-169494393
chr2	169588504	169590374	chr2-169588504-169590374
chr2	169608190	169608443	chr2-169608190-169608443
chr2	169611622	169611823	chr2-169611622-169611823
chr2	169632402	169634515	chr2-169632402-169634515
chr2	169790797	169791100	chr2-169790797-169791100
chr2	169929624	169930165	chr2-169929624-169930165
chr2	170002711	170003221	chr2-170002711-170003221
chr2	170127424	170134775	chr2-170127424-170134775
chr2	170138620	170140141	chr2-170138620-170140141
chr2	170152827	170155966	chr2-170152827-170155966
chr2	170157939	170158834	chr2-170157939-170158834
chr2	170184028	170185423	chr2-170184028-170185423
chr2	170205053	170206642	chr2-170205053-170206642
chr2	170208740	170209945	chr2-170208740-170209945
chr2	170266360	170267910	chr2-170266360-170267910
chr2	170287299	170288342	chr2-170287299-170288342
chr2	170318950	170320049	chr2-170318950-170320049
chr2	170324080	170324391	chr2-170324080-170324391
chr2	170364500	170365311	chr2-170364500-170365311
chr2	170401277	170401679	chr2-170401277-170401679
chr2	170424363	170424631	chr2-170424363-170424631
chr2	170445958	170446328	chr2-170445958-170446328
chr2	170469365	170470538	chr2-170469365-170470538
chr2	170496280	170498171	chr2-170496280-170498171

chr2	170508986	170509339	chr2-170508986-170509339
chr2	170510914	170513147	chr2-170510914-170513147
chr2	170525986	170527552	chr2-170525986-170527552
chr2	170731704	170732061	chr2-170731704-170732061
chr2	172132861	172133171	chr2-172132861-172133171
chr2	172344836	172346807	chr2-172344836-172346807
chr2	172367490	172372355	chr2-172367490-172372355
chr2	172393335	172394079	chr2-172393335-172394079
chr2	172439374	172442167	chr2-172439374-172442167
chr2	172547197	172551945	chr2-172547197-172551945
chr2	172613768	172614458	chr2-172613768-172614458
chr2	172643578	172645019	chr2-172643578-172645019
chr2	172661560	172661761	chr2-172661560-172661761
chr2	172728112	172728929	chr2-172728112-172728929
chr2	172731436	172731803	chr2-172731436-172731803
chr2	172787628	172787894	chr2-172787628-172787894
chr2	172824955	172825464	chr2-172824955-172825464
chr2	172841157	172841674	chr2-172841157-172841674
chr2	172861812	172864073	chr2-172861812-172864073
chr2	172872679	172872880	chr2-172872679-172872880
chr2	172935207	172935620	chr2-172935207-172935620
chr2	172937129	172941439	chr2-172937129-172941439
chr2	172964220	172967615	chr2-172964220-172967615
chr2	172976736	172977765	chr2-172976736-172977765
chr2	172999632	173002039	chr2-172999632-173002039
chr2	173019406	173023925	chr2-173019406-173023925
chr2	173044352	173045387	chr2-173044352-173045387
chr2	173047637	173049063	chr2-173047637-173049063
chr2	173057524	173057776	chr2-173057524-173057776
chr2	173083552	173083806	chr2-173083552-173083806
chr2	173171514	173171715	chr2-173171514-173171715
chr2	173173511	173173976	chr2-173173511-173173976
chr2	173186452	173187418	chr2-173186452-173187418
chr2	173229847	173230048	chr2-173229847-173230048
chr2	173235709	173236079	chr2-173235709-173236079
chr2	173237043	173237385	chr2-173237043-173237385
chr2	173238414	173238786	chr2-173238414-173238786
chr2	173243977	173244178	chr2-173243977-173244178
chr2	173249590	173249791	chr2-173249590-173249791
chr2	173250696	173277864	chr2-173250696-173277864
chr2	173281113	173281754	chr2-173281113-173281754
chr2	173305148	173305401	chr2-173305148-173305401

chr2	173307069	173308098	chr2-173307069-173308098
chr2	173321120	173321423	chr2-173321120-173321423
chr2	173381981	173382853	chr2-173381981-173382853
chr2	173384249	173387300	chr2-173384249-173387300
chr2	173451581	173452171	chr2-173451581-173452171
chr2	173546183	173546541	chr2-173546183-173546541
chr2	173577767	173578678	chr2-173577767-173578678
chr2	173585450	173587035	chr2-173585450-173587035
chr2	173646915	173647921	chr2-173646915-173647921
chr2	173658604	173661366	chr2-173658604-173661366
chr2	173662640	173662841	chr2-173662640-173662841
chr2	173679948	173680508	chr2-173679948-173680508
chr2	173730009	173731555	chr2-173730009-173731555
chr2	173737136	173739638	chr2-173737136-173739638
chr2	173743955	173744737	chr2-173743955-173744737
chr2	173770564	173771427	chr2-173770564-173771427
chr2	173790131	173790440	chr2-173790131-173790440
chr2	173949841	173950042	chr2-173949841-173950042
chr2	174047149	174047465	chr2-174047149-174047465
chr2	174074939	174078656	chr2-174074939-174078656
chr2	174109804	174112136	chr2-174109804-174112136
chr2	174158886	174159648	chr2-174158886-174159648
chr2	174284707	174285353	chr2-174284707-174285353
chr2	174292413	174300686	chr2-174292413-174300686
chr2	174326302	174334731	chr2-174326302-174334731
chr2	174344008	174344267	chr2-174344008-174344267
chr2	174415229	174416991	chr2-174415229-174416991
chr2	174437090	174439460	chr2-174437090-174439460
chr2	174441633	174443513	chr2-174441633-174443513
chr2	174445366	174445629	chr2-174445366-174445629
chr2	174462511	174464510	chr2-174462511-174464510
chr2	174471198	174473735	chr2-174471198-174473735
chr2	174673481	174674473	chr2-174673481-174674473
chr2	174760515	174761190	chr2-174760515-174761190
chr2	175141159	175142202	chr2-175141159-175142202
chr2	176708005	176709479	chr2-176708005-176709479
chr2	176798553	176799844	chr2-176798553-176799844
chr2	177205041	177206837	chr2-177205041-177206837
chr2	177397021	177398581	chr2-177397021-177398581
chr2	177464650	177465951	chr2-177464650-177465951
chr2	177498087	177498899	chr2-177498087-177498899
chr2	177759076	177760046	chr2-177759076-177760046

chr2	177838838	177840761	chr2-177838838-177840761
chr2	177897102	177898503	chr2-177897102-177898503
chr2	177925091	177926234	chr2-177925091-177926234
chr2	177956103	177957622	chr2-177956103-177957622
chr2	178022911	178024721	chr2-178022911-178024721
chr2	178077176	178078626	chr2-178077176-178078626
chr2	178118884	178119549	chr2-178118884-178119549
chr2	178142247	178142766	chr2-178142247-178142766
chr2	178413398	178415482	chr2-178413398-178415482
chr2	178423492	178423743	chr2-178423492-178423743
chr2	178433756	178434026	chr2-178433756-178434026
chr2	178728587	178728845	chr2-178728587-178728845
chr2	179442052	179443748	chr2-179442052-179443748
chr2	179648384	179649261	chr2-179648384-179649261
chr2	179662401	179662705	chr2-179662401-179662705
chr2	179939638	179940349	chr2-179939638-179940349
chr2	179973797	179977878	chr2-179973797-179977878
chr2	179979634	179980708	chr2-179979634-179980708
chr2	179997975	179998944	chr2-179997975-179998944
chr2	180006236	180006539	chr2-180006236-180006539
chr2	180024010	180028036	chr2-180024010-180028036
chr2	180032791	180034893	chr2-180032791-180034893
chr2	180040307	180044173	chr2-180040307-180044173
chr2	180069716	180072814	chr2-180069716-180072814
chr2	180099498	180099964	chr2-180099498-180099964
chr2	180103091	180105321	chr2-180103091-180105321
chr2	180113873	180120680	chr2-180113873-180120680
chr2	180162087	180163207	chr2-180162087-180163207
chr2	180169967	180176054	chr2-180169967-180176054
chr2	180211714	180212056	chr2-180211714-180212056
chr2	180213100	180229358	chr2-180213100-180229358
chr2	180232575	180236312	chr2-180232575-180236312
chr2	180249257	180249929	chr2-180249257-180249929
chr2	180255360	180259553	chr2-180255360-180259553
chr2	180271676	180274577	chr2-180271676-180274577
chr2	180275936	180279813	chr2-180275936-180279813
chr2	180288777	180290287	chr2-180288777-180290287
chr2	180314348	180314549	chr2-180314348-180314549
chr2	180333705	180335263	chr2-180333705-180335263
chr2	180385973	180386651	chr2-180385973-180386651
chr2	180455518	180458369	chr2-180455518-180458369
chr2	180581131	180581332	chr2-180581131-180581332

chr2	180582513	180584045	chr2-180582513-180584045
chr2	180588749	180590850	chr2-180588749-180590850
chr2	180598131	180598332	chr2-180598131-180598332
chr2	180641779	180643084	chr2-180641779-180643084
chr2	180700151	180703059	chr2-180700151-180703059
chr2	180708124	180711881	chr2-180708124-180711881
chr2	180731817	180732849	chr2-180731817-180732849
chr2	180776340	180776988	chr2-180776340-180776988
chr2	180892594	180894865	chr2-180892594-180894865
chr2	180919323	180921601	chr2-180919323-180921601
chr2	180954043	180955170	chr2-180954043-180955170
chr2	180966176	180969460	chr2-180966176-180969460
chr2	181038612	181039639	chr2-181038612-181039639
chr2	181134606	181135492	chr2-181134606-181135492
chr2	181147492	181148738	chr2-181147492-181148738
chr2	181156583	181157053	chr2-181156583-181157053
chr2	181186515	181189658	chr2-181186515-181189658
chr2	181212246	181213427	chr2-181212246-181213427
chr2	181219275	181220866	chr2-181219275-181220866
chr2	181240697	181242666	chr2-181240697-181242666
chr2	181286543	181289464	chr2-181286543-181289464
chr2	181313676	181314323	chr2-181313676-181314323
chr2	181319125	181320999	chr2-181319125-181320999
chr2	181363920	181368202	chr2-181363920-181368202
chr2	181458215	181460687	chr2-181458215-181460687
chr2	181483514	181483822	chr2-181483514-181483822
chr2	181496098	181498975	chr2-181496098-181498975
chr2	181519126	181527091	chr2-181519126-181527091
chr2	181580218	181582596	chr2-181580218-181582596
chr2	181591246	181593419	chr2-181591246-181593419
chr2	181597437	181602387	chr2-181597437-181602387
chr2	181670240	181671913	chr2-181670240-181671913
chr2	181673533	181673788	chr2-181673533-181673788
chr2	181675383	181676041	chr2-181675383-181676041
chr2	181679485	181681312	chr2-181679485-181681312
chr2	181692618	181694594	chr2-181692618-181694594
chr2	181837391	181839032	chr2-181837391-181839032
chr2	181863396	181866028	chr2-181863396-181866028
chr2	181986482	181986734	chr2-181986482-181986734
chr3	3308697	3309172	chr3-3308697-3309172
chr3	3507689	3508550	chr3-3507689-3508550
chr3	5211121	5211847	chr3-5211121-5211847

chr3	5215114	5215761	chr3-5215114-5215761
chr3	5218223	5221747	chr3-5218223-5221747
chr3	5228416	5229209	chr3-5228416-5229209
chr3	5271756	5272446	chr3-5271756-5272446
chr3	5574142	5577105	chr3-5574142-5577105
chr3	7365855	7367988	chr3-7365855-7367988
chr3	7501699	7505098	chr3-7501699-7505098
chr3	7513487	7513788	chr3-7513487-7513788
chr3	7612439	7613823	chr3-7612439-7613823
chr3	8510039	8511580	chr3-8510039-8511580
chr3	8664523	8668442	chr3-8664523-8668442
chr3	8685315	8685566	chr3-8685315-8685566
chr3	8717335	8717911	chr3-8717335-8717911
chr3	8766539	8768006	chr3-8766539-8768006
chr3	8866321	8866580	chr3-8866321-8866580
chr3	8922832	8924499	chr3-8922832-8924499
chr3	8959102	8959407	chr3-8959102-8959407
chr3	8963683	8965105	chr3-8963683-8965105
chr3	8969770	8971012	chr3-8969770-8971012
chr3	8992529	8994550	chr3-8992529-8994550
chr3	9001186	9005302	chr3-9001186-9005302
chr3	9006942	9007143	chr3-9006942-9007143
chr3	9033692	9034597	chr3-9033692-9034597
chr3	9038418	9038825	chr3-9038418-9038825
chr3	9053515	9053945	chr3-9053515-9053945
chr3	9154165	9154366	chr3-9154165-9154366
chr3	9173821	9174517	chr3-9173821-9174517
chr3	9249457	9252272	chr3-9249457-9252272
chr3	9334216	9334686	chr3-9334216-9334686
chr3	9340744	9341099	chr3-9340744-9341099
chr3	9346823	9347805	chr3-9346823-9347805
chr3	9398433	9398693	chr3-9398433-9398693
chr3	9608396	9610478	chr3-9608396-9610478
chr3	9629781	9630361	chr3-9629781-9630361
chr3	9666053	9666359	chr3-9666053-9666359
chr3	9750775	9753461	chr3-9750775-9753461
chr3	9832449	9835096	chr3-9832449-9835096
chr3	9974351	9974757	chr3-9974351-9974757
chr3	10011866	10015005	chr3-10011866-10015005
chr3	10022706	10023186	chr3-10022706-10023186
chr3	10211408	10211962	chr3-10211408-10211962
chr3	10329791	10331650	chr3-10329791-10331650

chr3	10342166	10343377	chr3-10342166-10343377
chr3	10350460	10352054	chr3-10350460-10352054
chr3	10366315	10369297	chr3-10366315-10369297
chr3	10376328	10376629	chr3-10376328-10376629
chr3	10439076	10440494	chr3-10439076-10440494
chr3	10442774	10443259	chr3-10442774-10443259
chr3	10446333	10446810	chr3-10446333-10446810
chr3	11213203	11213404	chr3-11213203-11213404
chr3	13471286	13473703	chr3-13471286-13473703
chr3	13474599	13474800	chr3-13474599-13474800
chr3	14381717	14381918	chr3-14381717-14381918
chr3	14533306	14534849	chr3-14533306-14534849
chr3	14578169	14580346	chr3-14578169-14580346
chr3	14609945	14611836	chr3-14609945-14611836
chr3	14641235	14642212	chr3-14641235-14642212
chr3	14863079	14863364	chr3-14863079-14863364
chr3	14869631	14870053	chr3-14869631-14870053
chr3	14885815	14890098	chr3-14885815-14890098
chr3	14948413	14948714	chr3-14948413-14948714
chr3	14959418	14959619	chr3-14959418-14959619
chr3	14978714	14979353	chr3-14978714-14979353
chr3	15159906	15160308	chr3-15159906-15160308
chr3	16182472	16184881	chr3-16182472-16184881
chr3	17786040	17787129	chr3-17786040-17787129
chr3	18051634	18052212	chr3-18051634-18052212
chr3	18054124	18056006	chr3-18054124-18056006
chr3	18242889	18243967	chr3-18242889-18243967
chr3	18624676	18625095	chr3-18624676-18625095
chr3	19161737	19164172	chr3-19161737-19164172
chr3	19187183	19190034	chr3-19187183-19190034
chr3	19203758	19204644	chr3-19203758-19204644
chr3	19308260	19312909	chr3-19308260-19312909
chr3	19425939	19426140	chr3-19425939-19426140
chr3	19429236	19430399	chr3-19429236-19430399
chr3	19627636	19630159	chr3-19627636-19630159
chr3	19693864	19694877	chr3-19693864-19694877
chr3	19706277	19707473	chr3-19706277-19707473
chr3	19894167	19896290	chr3-19894167-19896290
chr3	20034197	20035830	chr3-20034197-20035830
chr3	20056888	20058997	chr3-20056888-20058997
chr3	20154248	20155453	chr3-20154248-20155453
chr3	21935488	21938664	chr3-21935488-21938664

chr3	21952745	21953566	chr3-21952745-21953566
chr3	22075149	22078698	chr3-22075149-22078698
chr3	22090842	22091559	chr3-22090842-22091559
chr3	24782813	24783846	chr3-24782813-24783846
chr3	25143517	25144674	chr3-25143517-25144674
chr3	26152663	26153779	chr3-26152663-26153779
chr3	26331367	26331988	chr3-26331367-26331988
chr3	26333205	26333876	chr3-26333205-26333876
chr3	27151666	27156165	chr3-27151666-27156165
chr3	27182635	27184358	chr3-27182635-27184358
chr3	27209560	27209911	chr3-27209560-27209911
chr3	27283146	27283621	chr3-27283146-27283621
chr3	27316709	27317471	chr3-27316709-27317471
chr3	27371661	27372208	chr3-27371661-27372208
chr3	27584390	27585155	chr3-27584390-27585155
chr3	27590688	27591485	chr3-27590688-27591485
chr3	27606965	27608736	chr3-27606965-27608736
chr3	27616016	27618546	chr3-27616016-27618546
chr3	27651245	27653810	chr3-27651245-27653810
chr3	27689656	27692121	chr3-27689656-27692121
chr3	27694031	27696898	chr3-27694031-27696898
chr3	27707819	27711684	chr3-27707819-27711684
chr3	27766402	27766603	chr3-27766402-27766603
chr3	27778192	27778601	chr3-27778192-27778601
chr3	27858305	27859612	chr3-27858305-27859612
chr3	27861642	27861967	chr3-27861642-27861967
chr3	27862829	27864010	chr3-27862829-27864010
chr3	27882392	27882593	chr3-27882392-27882593
chr3	27886465	27887189	chr3-27886465-27887189
chr3	27899353	27899676	chr3-27899353-27899676
chr3	27904962	27905163	chr3-27904962-27905163
chr3	27938091	27939808	chr3-27938091-27939808
chr3	27982571	27982838	chr3-27982571-27982838
chr3	27983839	27984602	chr3-27983839-27984602
chr3	28262318	28264334	chr3-28262318-28264334
chr3	28721644	28722054	chr3-28721644-28722054
chr3	28735064	28735265	chr3-28735064-28735265
chr3	28780778	28782256	chr3-28780778-28782256
chr3	28804775	28806870	chr3-28804775-28806870
chr3	29082547	29082891	chr3-29082547-29082891
chr3	29903609	29903916	chr3-29903609-29903916
chr3	30005113	30005488	chr3-30005113-30005488

chr3	30009550	30015335	chr3-30009550-30015335
chr3	30018125	30020605	chr3-30018125-30020605
chr3	30048365	30048725	chr3-30048365-30048725
chr3	30050991	30051510	chr3-30050991-30051510
chr3	30097160	30097474	chr3-30097160-30097474
chr3	30103915	30108674	chr3-30103915-30108674
chr3	30120214	30121113	chr3-30120214-30121113
chr3	30213855	30214107	chr3-30213855-30214107
chr3	30217278	30217622	chr3-30217278-30217622
chr3	30219068	30220151	chr3-30219068-30220151
chr3	30226948	30227268	chr3-30226948-30227268
chr3	30244636	30244997	chr3-30244636-30244997
chr3	30245872	30247944	chr3-30245872-30247944
chr3	30260944	30261391	chr3-30260944-30261391
chr3	30324842	30325043	chr3-30324842-30325043
chr3	30421984	30422235	chr3-30421984-30422235
chr3	30450382	30451301	chr3-30450382-30451301
chr3	30503997	30515710	chr3-30503997-30515710
chr3	30594910	30596267	chr3-30594910-30596267
chr3	30599500	30603827	chr3-30599500-30603827
chr3	30647532	30647986	chr3-30647532-30647986
chr3	30649957	30651159	chr3-30649957-30651159
chr3	30766203	30766454	chr3-30766203-30766454
chr3	30792599	30794626	chr3-30792599-30794626
chr3	30855024	30856890	chr3-30855024-30856890
chr3	30967978	30970003	chr3-30967978-30970003
chr3	30981725	30982237	chr3-30981725-30982237
chr3	30994579	31000672	chr3-30994579-31000672
chr3	31018268	31019091	chr3-31018268-31019091
chr3	31023082	31024504	chr3-31023082-31024504
chr3	31056153	31057571	chr3-31056153-31057571
chr3	31060958	31062023	chr3-31060958-31062023
chr3	31093121	31100435	chr3-31093121-31100435
chr3	31134231	31135162	chr3-31134231-31135162
chr3	31149560	31150377	chr3-31149560-31150377
chr3	32364291	32366771	chr3-32364291-32366771
chr3	32395781	32399218	chr3-32395781-32399218
chr3	32400056	32400257	chr3-32400056-32400257
chr3	32405020	32405275	chr3-32405020-32405275
chr3	32408452	32409611	chr3-32408452-32409611
chr3	32495987	32496827	chr3-32495987-32496827
chr3	32508903	32512296	chr3-32508903-32512296

chr3	32528838	32530465	chr3-32528838-32530465
chr3	32615539	32617275	chr3-32615539-32617275
chr3	32658525	32659217	chr3-32658525-32659217
chr3	32708037	32710413	chr3-32708037-32710413
chr3	32735689	32738260	chr3-32735689-32738260
chr3	32817258	32818223	chr3-32817258-32818223
chr3	33142563	33143482	chr3-33142563-33143482
chr3	33799009	33801756	chr3-33799009-33801756
chr3	33838644	33839207	chr3-33838644-33839207
chr3	33843655	33844755	chr3-33843655-33844755
chr3	33955920	33956486	chr3-33955920-33956486
chr3	34019129	34021739	chr3-34019129-34021739
chr3	34079902	34082092	chr3-34079902-34082092
chr3	34333147	34333348	chr3-34333147-34333348
chr3	34334976	34335612	chr3-34334976-34335612
chr3	34442969	34444146	chr3-34442969-34444146
chr3	34642030	34643012	chr3-34642030-34643012
chr3	34648670	34651484	chr3-34648670-34651484
chr3	34663295	34665435	chr3-34663295-34665435
chr3	35149280	35149481	chr3-35149280-35149481
chr3	35246078	35246625	chr3-35246078-35246625
chr3	35340995	35341308	chr3-35340995-35341308
chr3	35720624	35721287	chr3-35720624-35721287
chr3	35753426	35755976	chr3-35753426-35755976
chr3	35931010	35933921	chr3-35931010-35933921
chr3	35999652	36001184	chr3-35999652-36001184
chr3	36065174	36068026	chr3-36065174-36068026
chr3	36150531	36152843	chr3-36150531-36152843
chr3	36221816	36222575	chr3-36221816-36222575
chr3	36340629	36341592	chr3-36340629-36341592
chr3	36342610	36342865	chr3-36342610-36342865
chr3	36449097	36450970	chr3-36449097-36450970
chr3	36466940	36477614	chr3-36466940-36477614
chr3	36481533	36482851	chr3-36481533-36482851
chr3	36488841	36489658	chr3-36488841-36489658
chr3	36495041	36495616	chr3-36495041-36495616
chr3	36521038	36521901	chr3-36521038-36521901
chr3	36551601	36553553	chr3-36551601-36553553
chr3	36569680	36572903	chr3-36569680-36572903
chr3	36612698	36613889	chr3-36612698-36613889
chr3	36688925	36690465	chr3-36688925-36690465
chr3	36862521	36864232	chr3-36862521-36864232

chr3	37204023	37204785	chr3-37204023-37204785
chr3	37311786	37314160	chr3-37311786-37314160
chr3	37348020	37349494	chr3-37348020-37349494
chr3	37360530	37361205	chr3-37360530-37361205
chr3	37418457	37421733	chr3-37418457-37421733
chr3	37639519	37645966	chr3-37639519-37645966
chr3	37647116	37647317	chr3-37647116-37647317
chr3	37654010	37655226	chr3-37654010-37655226
chr3	37686666	37687847	chr3-37686666-37687847
chr3	37722499	37725377	chr3-37722499-37725377
chr3	37753475	37753729	chr3-37753475-37753729
chr3	37845630	37845883	chr3-37845630-37845883
chr3	37875408	37875896	chr3-37875408-37875896
chr3	37899204	37899405	chr3-37899204-37899405
chr3	37906797	37907999	chr3-37906797-37907999
chr3	37938566	37938767	chr3-37938566-37938767
chr3	37962200	37963041	chr3-37962200-37963041
chr3	38255425	38255676	chr3-38255425-38255676
chr3	38277729	38278286	chr3-38277729-38278286
chr3	38282481	38285115	chr3-38282481-38285115
chr3	38383051	38383252	chr3-38383051-38383252
chr3	38460211	38460412	chr3-38460211-38460412
chr3	38479694	38487173	chr3-38479694-38487173
chr3	38790687	38790888	chr3-38790687-38790888
chr3	38820302	38821113	chr3-38820302-38821113
chr3	38884684	38888483	chr3-38884684-38888483
chr3	40522620	40522930	chr3-40522620-40522930
chr3	40531101	40532218	chr3-40531101-40532218
chr3	40540374	40541483	chr3-40540374-40541483
chr3	40744413	40746870	chr3-40744413-40746870
chr3	40799226	40801307	chr3-40799226-40801307
chr3	40845374	40847882	chr3-40845374-40847882
chr3	40892824	40894986	chr3-40892824-40894986
chr3	40948715	40952318	chr3-40948715-40952318
chr3	41081363	41084401	chr3-41081363-41084401
chr3	41195098	41196022	chr3-41195098-41196022
chr3	41228704	41229040	chr3-41228704-41229040
chr3	41285023	41285291	chr3-41285023-41285291
chr3	41290343	41290596	chr3-41290343-41290596
chr3	41374678	41376041	chr3-41374678-41376041
chr3	41408357	41409146	chr3-41408357-41409146
chr3	41521876	41522986	chr3-41521876-41522986

chr3	41531519	41532475	chr3-41531519-41532475
chr3	41562995	41566824	chr3-41562995-41566824
chr3	41605645	41606573	chr3-41605645-41606573
chr3	41741292	41743530	chr3-41741292-41743530
chr3	45375992	45379986	chr3-45375992-45379986
chr3	45380888	45381396	chr3-45380888-45381396
chr3	45681434	45681738	chr3-45681434-45681738
chr3	46447485	46448297	chr3-46447485-46448297
chr3	49066903	49067210	chr3-49066903-49067210
chr3	49756028	49758056	chr3-49756028-49758056
chr3	50025984	50026285	chr3-50025984-50026285
chr3	50033128	50033475	chr3-50033128-50033475
chr3	50441004	50444266	chr3-50441004-50444266
chr3	50906051	50906917	chr3-50906051-50906917
chr3	51073789	51074101	chr3-51073789-51074101
chr3	51200401	51200815	chr3-51200401-51200815
chr3	51201752	51202314	chr3-51201752-51202314
chr3	51209919	51210429	chr3-51209919-51210429
chr3	51221064	51227741	chr3-51221064-51227741
chr3	51231189	51231573	chr3-51231189-51231573
chr3	51275544	51278442	chr3-51275544-51278442
chr3	51327109	51327310	chr3-51327109-51327310
chr3	51332422	51334405	chr3-51332422-51334405
chr3	51338479	51342433	chr3-51338479-51342433
chr3	51353275	51353526	chr3-51353275-51353526
chr3	51357110	51359902	chr3-51357110-51359902
chr3	51364836	51365552	chr3-51364836-51365552
chr3	51377982	51378854	chr3-51377982-51378854
chr3	51396172	51397130	chr3-51396172-51397130
chr3	51407836	51409359	chr3-51407836-51409359
chr3	51414762	51418352	chr3-51414762-51418352
chr3	51482672	51485654	chr3-51482672-51485654
chr3	51511110	51511311	chr3-51511110-51511311
chr3	51552845	51554192	chr3-51552845-51554192
chr3	51559203	51562142	chr3-51559203-51562142
chr3	51564214	51565323	chr3-51564214-51565323
chr3	51578000	51578301	chr3-51578000-51578301
chr3	51612034	51612235	chr3-51612034-51612235
chr3	51652379	51653162	chr3-51652379-51653162
chr3	51661075	51663044	chr3-51661075-51663044
chr3	51701294	51701956	chr3-51701294-51701956
chr3	51788913	51789214	chr3-51788913-51789214

chr3	51825751	51825952	chr3-51825751-51825952
chr3	51828337	51828642	chr3-51828337-51828642
chr3	51907382	51908998	chr3-51907382-51908998
chr3	51931104	51931305	chr3-51931104-51931305
chr3	51952845	51953445	chr3-51952845-51953445
chr3	52007586	52007892	chr3-52007586-52007892
chr3	52017471	52017727	chr3-52017471-52017727
chr3	52044378	52047907	chr3-52044378-52047907
chr3	52068898	52072644	chr3-52068898-52072644
chr3	52088038	52089342	chr3-52088038-52089342
chr3	52101312	52105716	chr3-52101312-52105716
chr3	52140256	52140575	chr3-52140256-52140575
chr3	52151337	52151640	chr3-52151337-52151640
chr3	52180816	52181331	chr3-52180816-52181331
chr3	52195923	52196682	chr3-52195923-52196682
chr3	52197732	52199359	chr3-52197732-52199359
chr3	52257764	52258322	chr3-52257764-52258322
chr3	52266712	52272694	chr3-52266712-52272694
chr3	52296744	52297862	chr3-52296744-52297862
chr3	52340633	52340884	chr3-52340633-52340884
chr3	52400858	52401943	chr3-52400858-52401943
chr3	52486749	52488661	chr3-52486749-52488661
chr3	52555239	52555746	chr3-52555239-52555746
chr3	52576607	52577461	chr3-52576607-52577461
chr3	52600621	52601691	chr3-52600621-52601691
chr3	52641313	52642317	chr3-52641313-52642317
chr3	52677033	52677975	chr3-52677033-52677975
chr3	52940983	52941184	chr3-52940983-52941184
chr3	53016136	53017526	chr3-53016136-53017526
chr3	53040895	53043706	chr3-53040895-53043706
chr3	53060178	53060379	chr3-53060178-53060379
chr3	53062899	53066372	chr3-53062899-53066372
chr3	53151411	53151989	chr3-53151411-53151989
chr3	53201451	53202621	chr3-53201451-53202621
chr3	53204474	53204777	chr3-53204474-53204777
chr3	53452259	53452897	chr3-53452259-53452897
chr3	53462112	53466613	chr3-53462112-53466613
chr3	53525765	53526178	chr3-53525765-53526178
chr3	53631127	53631389	chr3-53631127-53631389
chr3	53656496	53657958	chr3-53656496-53657958
chr3	53862644	53864529	chr3-53862644-53864529
chr3	54156013	54156214	chr3-54156013-54156214

chr3	54480603	54480854	chr3-54480603-54480854
chr3	54691861	54694140	chr3-54691861-54694140
chr3	54733701	54736923	chr3-54733701-54736923
chr3	54755153	54756410	chr3-54755153-54756410
chr3	54805841	54808647	chr3-54805841-54808647
chr3	54915682	54916160	chr3-54915682-54916160
chr3	55054629	55055959	chr3-55054629-55055959
chr3	55111881	55115925	chr3-55111881-55115925
chr3	55242246	55243274	chr3-55242246-55243274
chr3	55373819	55374070	chr3-55373819-55374070
chr3	55461414	55462348	chr3-55461414-55462348
chr3	55517156	55518099	chr3-55517156-55518099
chr3	55590532	55591113	chr3-55590532-55591113
chr3	55599750	55600869	chr3-55599750-55600869
chr3	55652261	55652512	chr3-55652261-55652512
chr3	56046167	56046624	chr3-56046167-56046624
chr3	56068121	56068580	chr3-56068121-56068580
chr3	56181156	56185176	chr3-56181156-56185176
chr3	56962286	56962788	chr3-56962286-56962788
chr3	57054238	57054494	chr3-57054238-57054494
chr3	57073495	57074109	chr3-57073495-57074109
chr3	57289113	57289881	chr3-57289113-57289881
chr3	57292858	57295544	chr3-57292858-57295544
chr3	57343933	57344488	chr3-57343933-57344488
chr3	57410847	57411286	chr3-57410847-57411286
chr3	57470401	57471660	chr3-57470401-57471660
chr3	57511860	57512873	chr3-57511860-57512873
chr3	57551063	57551264	chr3-57551063-57551264
chr3	57554273	57556250	chr3-57554273-57556250
chr3	57570686	57577177	chr3-57570686-57577177
chr3	57625352	57627505	chr3-57625352-57627505
chr3	57637265	57638914	chr3-57637265-57638914
chr3	57650496	57652141	chr3-57650496-57652141
chr3	57735073	57737243	chr3-57735073-57737243
chr3	57846389	57848467	chr3-57846389-57848467
chr3	57941424	57941736	chr3-57941424-57941736
chr3	58309468	58309795	chr3-58309468-58309795
chr3	58333829	58334345	chr3-58333829-58334345
chr3	58371044	58374393	chr3-58371044-58374393
chr3	58413607	58420700	chr3-58413607-58420700
chr3	58523955	58527272	chr3-58523955-58527272
chr3	58576293	58578314	chr3-58576293-58578314

chr3	58636308	58637462	chr3-58636308-58637462
chr3	58646209	58646942	chr3-58646209-58646942
chr3	58689780	58693155	chr3-58689780-58693155
chr3	58882806	58883208	chr3-58882806-58883208
chr3	59005081	59007612	chr3-59005081-59007612
chr3	59121153	59121997	chr3-59121153-59121997
chr3	59192668	59195291	chr3-59192668-59195291
chr3	59218535	59220443	chr3-59218535-59220443
chr3	59236642	59236843	chr3-59236642-59236843
chr3	59295826	59296683	chr3-59295826-59296683
chr3	59344178	59344638	chr3-59344178-59344638
chr3	60408337	60409536	chr3-60408337-60409536
chr3	60498990	60505390	chr3-60498990-60505390
chr3	60516092	60516448	chr3-60516092-60516448
chr3	60527563	60528515	chr3-60527563-60528515
chr3	60589324	60589638	chr3-60589324-60589638
chr3	60615207	60616008	chr3-60615207-60616008
chr3	61002086	61003763	chr3-61002086-61003763
chr3	61340291	61340820	chr3-61340291-61340820
chr3	61362706	61368981	chr3-61362706-61368981
chr3	61395701	61395902	chr3-61395701-61395902
chr3	61442270	61442471	chr3-61442270-61442471
chr3	62159178	62159491	chr3-62159178-62159491
chr3	62338467	62340310	chr3-62338467-62340310
chr3	62349304	62349779	chr3-62349304-62349779
chr3	62505274	62507607	chr3-62505274-62507607
chr3	62603404	62604141	chr3-62603404-62604141
chr3	63240928	63244280	chr3-63240928-63244280
chr3	63295199	63296424	chr3-63295199-63296424
chr3	63609665	63610078	chr3-63609665-63610078
chr3	63892298	63894609	chr3-63892298-63894609
chr3	63897695	63899807	chr3-63897695-63899807
chr3	63905183	63905636	chr3-63905183-63905636
chr3	63928781	63929592	chr3-63928781-63929592
chr3	63961952	63965346	chr3-63961952-63965346
chr3	63975927	63978180	chr3-63975927-63978180
chr3	65108998	65111409	chr3-65108998-65111409
chr3	65135194	65135563	chr3-65135194-65135563
chr3	65139050	65139571	chr3-65139050-65139571
chr3	65352727	65353010	chr3-65352727-65353010
chr3	65391032	65393056	chr3-65391032-65393056
chr3	65441664	65442747	chr3-65441664-65442747

chr3	65527330	65530394	chr3-65527330-65530394
chr3	65533621	65533883	chr3-65533621-65533883
chr3	65586495	65586746	chr3-65586495-65586746
chr3	65633836	65634037	chr3-65633836-65634037
chr3	65658336	65660280	chr3-65658336-65660280
chr3	65665535	65667106	chr3-65665535-65667106
chr3	65762447	65762751	chr3-65762447-65762751
chr3	65830928	65831186	chr3-65830928-65831186
chr3	65844095	65845794	chr3-65844095-65845794
chr3	65857938	65859286	chr3-65857938-65859286
chr3	65863612	65863813	chr3-65863612-65863813
chr3	65869391	65873987	chr3-65869391-65873987
chr3	65882285	65882486	chr3-65882285-65882486
chr3	65883524	65885709	chr3-65883524-65885709
chr3	65904844	65905260	chr3-65904844-65905260
chr3	65924764	65930222	chr3-65924764-65930222
chr3	65953128	65959403	chr3-65953128-65959403
chr3	65973715	65975463	chr3-65973715-65975463
chr3	66220542	66221555	chr3-66220542-66221555
chr3	66292212	66293031	chr3-66292212-66293031
chr3	66973196	66974104	chr3-66973196-66974104
chr3	66978687	66979204	chr3-66978687-66979204
chr3	66980597	66983096	chr3-66980597-66983096
chr3	66985167	66986883	chr3-66985167-66986883
chr3	67373718	67374886	chr3-67373718-67374886
chr3	67429577	67431475	chr3-67429577-67431475
chr3	67462590	67464499	chr3-67462590-67464499
chr3	67514815	67515976	chr3-67514815-67515976
chr3	67582260	67584052	chr3-67582260-67584052
chr3	68056485	68057298	chr3-68056485-68057298
chr3	68467684	68471313	chr3-68467684-68471313
chr3	68491662	68497993	chr3-68491662-68497993
chr3	68542998	68543501	chr3-68542998-68543501
chr3	68608774	68609075	chr3-68608774-68609075
chr3	68676578	68677492	chr3-68676578-68677492
chr3	68709227	68709638	chr3-68709227-68709638
chr3	68713421	68713828	chr3-68713421-68713828
chr3	68765201	68765513	chr3-68765201-68765513
chr3	68869060	68870850	chr3-68869060-68870850
chr3	69003680	69007687	chr3-69003680-69007687
chr3	69039573	69046912	chr3-69039573-69046912
chr3	69125204	69128035	chr3-69125204-69128035

chr3	69221983	69223258	chr3-69221983-69223258
chr3	69252723	69253228	chr3-69252723-69253228
chr3	69272003	69272204	chr3-69272003-69272204
chr3	69292509	69292710	chr3-69292509-69292710
chr3	69315697	69319321	chr3-69315697-69319321
chr3	69598177	69599508	chr3-69598177-69599508
chr3	69712194	69712445	chr3-69712194-69712445
chr3	69721547	69724185	chr3-69721547-69724185
chr3	69859811	69861562	chr3-69859811-69861562
chr3	70104024	70104949	chr3-70104024-70104949
chr3	70803013	70807400	chr3-70803013-70807400
chr3	73053470	73053671	chr3-73053470-73053671
chr3	73057221	73057861	chr3-73057221-73057861
chr3	73708002	73708562	chr3-73708002-73708562
chr3	74666660	74666925	chr3-74666660-74666925
chr3	75164841	75165198	chr3-75164841-75165198
chr3	75499153	75499606	chr3-75499153-75499606
chr3	75555551	75558117	chr3-75555551-75558117
chr3	75942137	75943288	chr3-75942137-75943288
chr3	75952773	75957726	chr3-75952773-75957726
chr3	76074201	76074669	chr3-76074201-76074669
chr3	78966623	78967437	chr3-78966623-78967437
chr3	79128845	79129460	chr3-79128845-79129460
chr3	79144644	79144996	chr3-79144644-79144996
chr3	79177553	79182520	chr3-79177553-79182520
chr3	79193215	79193467	chr3-79193215-79193467
chr3	79197020	79197221	chr3-79197020-79197221
chr3	79221927	79222394	chr3-79221927-79222394
chr3	79284965	79288302	chr3-79284965-79288302
chr3	79324877	79325237	chr3-79324877-79325237
chr3	79531577	79532533	chr3-79531577-79532533
chr3	79542320	79543173	chr3-79542320-79543173
chr3	79551965	79552489	chr3-79551965-79552489
chr3	79558775	79560070	chr3-79558775-79560070
chr3	79566460	79568599	chr3-79566460-79568599
chr3	79575980	79576585	chr3-79575980-79576585
chr3	79586975	79587281	chr3-79586975-79587281
chr3	79590267	79593207	chr3-79590267-79593207
chr3	79627724	79630175	chr3-79627724-79630175
chr3	79784437	79784638	chr3-79784437-79784638
chr3	79808865	79809392	chr3-79808865-79809392
chr3	79841356	79843480	chr3-79841356-79843480

chr3	79884353	79889398	chr3-79884353-79889398
chr3	80056508	80057515	chr3-80056508-80057515
chr3	80802773	80803074	chr3-80802773-80803074
chr3	80912979	80914088	chr3-80912979-80914088
chr3	81017258	81017835	chr3-81017258-81017835
chr3	81035990	81039500	chr3-81035990-81039500
chr3	81052512	81052777	chr3-81052512-81052777
chr3	81058177	81058701	chr3-81058177-81058701
chr3	81061743	81061996	chr3-81061743-81061996
chr3	81163780	81164247	chr3-81163780-81164247
chr3	81482261	81482462	chr3-81482261-81482462
chr3	81835369	81836119	chr3-81835369-81836119
chr3	81932307	81933286	chr3-81932307-81933286
chr3	82073728	82074696	chr3-82073728-82074696
chr3	82333493	82333795	chr3-82333493-82333795
chr3	82357269	82358857	chr3-82357269-82358857
chr3	82369691	82370212	chr3-82369691-82370212
chr3	82547925	82548188	chr3-82547925-82548188
chr3	82875859	82876617	chr3-82875859-82876617
chr3	82903612	82904406	chr3-82903612-82904406
chr3	82995223	82995733	chr3-82995223-82995733
chr3	83054750	83056565	chr3-83054750-83056565
chr3	83080556	83080874	chr3-83080556-83080874
chr3	83127165	83129157	chr3-83127165-83129157
chr3	83287038	83287390	chr3-83287038-83287390
chr3	83446589	83446790	chr3-83446589-83446790
chr3	83763925	83764695	chr3-83763925-83764695
chr3	83765647	83767114	chr3-83765647-83767114
chr3	83789434	83790447	chr3-83789434-83790447
chr3	83840837	83842056	chr3-83840837-83842056
chr3	83992952	83994412	chr3-83992952-83994412
chr3	84021882	84023230	chr3-84021882-84023230
chr3	84038112	84040968	chr3-84038112-84040968
chr3	84154642	84156482	chr3-84154642-84156482
chr3	84189831	84190928	chr3-84189831-84190928
chr3	84227636	84229396	chr3-84227636-84229396
chr3	84258666	84260635	chr3-84258666-84260635
chr3	84262742	84262943	chr3-84262742-84262943
chr3	84283506	84284077	chr3-84283506-84284077
chr3	84293684	84293949	chr3-84293684-84293949
chr3	84304008	84305728	chr3-84304008-84305728
chr3	84312707	84313120	chr3-84312707-84313120

chr3	84314802	84315452	chr3-84314802-84315452
chr3	84323883	84325173	chr3-84323883-84325173
chr3	84344933	84345910	chr3-84344933-84345910
chr3	84428109	84429804	chr3-84428109-84429804
chr3	84451085	84451392	chr3-84451085-84451392
chr3	84478110	84481646	chr3-84478110-84481646
chr3	84580012	84583589	chr3-84580012-84583589
chr3	84594797	84595946	chr3-84594797-84595946
chr3	84653968	84654577	chr3-84653968-84654577
chr3	84665889	84666615	chr3-84665889-84666615
chr3	84812960	84817022	chr3-84812960-84817022
chr3	84839660	84840105	chr3-84839660-84840105
chr3	85573559	85575015	chr3-85573559-85575015
chr3	85629825	85630394	chr3-85629825-85630394
chr3	85879997	85881335	chr3-85879997-85881335
chr3	85886052	85888768	chr3-85886052-85888768
chr3	85923889	85924540	chr3-85923889-85924540
chr3	85961072	85961600	chr3-85961072-85961600
chr3	85970478	85974207	chr3-85970478-85974207
chr3	85977719	85980788	chr3-85977719-85980788
chr3	86010734	86012524	chr3-86010734-86012524
chr3	86139965	86143240	chr3-86139965-86143240
chr3	86223234	86225861	chr3-86223234-86225861
chr3	86850812	86851124	chr3-86850812-86851124
chr3	86919425	86921170	chr3-86919425-86921170
chr3	86998683	86999717	chr3-86998683-86999717
chr3	87115865	87116373	chr3-87115865-87116373
chr3	87144611	87144812	chr3-87144611-87144812
chr3	87149951	87152382	chr3-87149951-87152382
chr3	87157555	87159607	chr3-87157555-87159607
chr3	87171292	87175614	chr3-87171292-87175614
chr3	87508478	87510555	chr3-87508478-87510555
chr3	87511668	87513100	chr3-87511668-87513100
chr3	87515244	87516108	chr3-87515244-87516108
chr3	87524836	87528083	chr3-87524836-87528083
chr3	87616642	87621983	chr3-87616642-87621983
chr3	87756056	87756460	chr3-87756056-87756460
chr3	87763675	87765905	chr3-87763675-87765905
chr3	87767207	87769456	chr3-87767207-87769456
chr3	87778352	87778757	chr3-87778352-87778757
chr3	87794657	87797218	chr3-87794657-87797218
chr3	87803750	87804567	chr3-87803750-87804567

chr3	87809313	87810320	chr3-87809313-87810320
chr3	87816629	87816830	chr3-87816629-87816830
chr3	87883757	87886619	chr3-87883757-87886619
chr3	87905667	87911557	chr3-87905667-87911557
chr3	87918799	87920810	chr3-87918799-87920810
chr3	87928683	87932330	chr3-87928683-87932330
chr3	87933215	87933573	chr3-87933215-87933573
chr3	87934635	87934836	chr3-87934635-87934836
chr3	87948147	87949418	chr3-87948147-87949418
chr3	87960375	87961186	chr3-87960375-87961186
chr3	87970009	87974632	chr3-87970009-87974632
chr3	87983208	87983575	chr3-87983208-87983575
chr3	87985592	87988097	chr3-87985592-87988097
chr3	87996781	87997648	chr3-87996781-87997648
chr3	87999334	88000826	chr3-87999334-88000826
chr3	88022394	88024227	chr3-88022394-88024227
chr3	88035772	88036699	chr3-88035772-88036699
chr3	88042469	88044438	chr3-88042469-88044438
chr3	88056875	88058997	chr3-88056875-88058997
chr3	88081496	88083925	chr3-88081496-88083925
chr3	88137799	88146704	chr3-88137799-88146704
chr3	88148867	88150742	chr3-88148867-88150742
chr3	88176784	88178663	chr3-88176784-88178663
chr3	88200305	88201316	chr3-88200305-88201316
chr3	88214353	88216113	chr3-88214353-88216113
chr3	88237564	88238124	chr3-88237564-88238124
chr3	88254064	88255125	chr3-88254064-88255125
chr3	88295320	88298948	chr3-88295320-88298948
chr3	88324617	88326727	chr3-88324617-88326727
chr3	88332248	88338058	chr3-88332248-88338058
chr3	88376075	88376704	chr3-88376075-88376704
chr3	88381849	88382250	chr3-88381849-88382250
chr3	88407575	88407826	chr3-88407575-88407826
chr3	88408656	88411059	chr3-88408656-88411059
chr3	88421483	88422595	chr3-88421483-88422595
chr3	88423869	88425722	chr3-88423869-88425722
chr3	88440702	88441282	chr3-88440702-88441282
chr3	88455455	88455763	chr3-88455455-88455763
chr3	88488191	88498063	chr3-88488191-88498063
chr3	88498951	88504462	chr3-88498951-88504462
chr3	88508215	88514978	chr3-88508215-88514978
chr3	88522057	88522258	chr3-88522057-88522258

chr3	88526139	88526391	chr3-88526139-88526391
chr3	88529589	88534236	chr3-88529589-88534236
chr3	88536097	88537183	chr3-88536097-88537183
chr3	88544856	88545371	chr3-88544856-88545371
chr3	88546512	88550196	chr3-88546512-88550196
chr3	88551438	88555471	chr3-88551438-88555471
chr3	88578944	88581171	chr3-88578944-88581171
chr3	88592148	88595401	chr3-88592148-88595401
chr3	88607079	88607949	chr3-88607079-88607949
chr3	88615172	88618454	chr3-88615172-88618454
chr3	88619488	88626015	chr3-88619488-88626015
chr3	88677424	88677982	chr3-88677424-88677982
chr3	88684745	88688098	chr3-88684745-88688098
chr3	88716572	88719345	chr3-88716572-88719345
chr3	88736518	88736772	chr3-88736518-88736772
chr3	88771075	88772867	chr3-88771075-88772867
chr3	88774692	88776450	chr3-88774692-88776450
chr3	88787917	88788168	chr3-88787917-88788168
chr3	88831206	88832087	chr3-88831206-88832087
chr3	88834761	88836467	chr3-88834761-88836467
chr3	88866679	88866880	chr3-88866679-88866880
chr3	88912624	88915095	chr3-88912624-88915095
chr3	88949095	88952440	chr3-88949095-88952440
chr3	89088901	89092163	chr3-89088901-89092163
chr3	89098489	89102967	chr3-89098489-89102967
chr3	89132262	89132672	chr3-89132262-89132672
chr3	89147514	89148424	chr3-89147514-89148424
chr3	89159180	89160514	chr3-89159180-89160514
chr3	89163396	89166583	chr3-89163396-89166583
chr3	89177030	89179385	chr3-89177030-89179385
chr3	89182306	89184412	chr3-89182306-89184412
chr3	89186738	89186939	chr3-89186738-89186939
chr3	89195746	89196832	chr3-89195746-89196832
chr3	89202700	89204154	chr3-89202700-89204154
chr3	89212545	89216102	chr3-89212545-89216102
chr3	89226862	89235263	chr3-89226862-89235263
chr3	89243459	89247803	chr3-89243459-89247803
chr3	89251252	89251858	chr3-89251252-89251858
chr3	89266130	89267630	chr3-89266130-89267630
chr3	89268932	89272200	chr3-89268932-89272200
chr3	89276106	89282049	chr3-89276106-89282049
chr3	89299397	89300041	chr3-89299397-89300041

chr3	89314863	89316902	chr3-89314863-89316902
chr3	89318224	89323365	chr3-89318224-89323365
chr3	89336477	89339538	chr3-89336477-89339538
chr3	89340377	89340578	chr3-89340377-89340578
chr3	89348574	89350957	chr3-89348574-89350957
chr3	89378251	89379169	chr3-89378251-89379169
chr3	89387718	89387919	chr3-89387718-89387919
chr3	89389792	89396881	chr3-89389792-89396881
chr3	89410767	89412816	chr3-89410767-89412816
chr3	89416296	89425478	chr3-89416296-89425478
chr3	89429640	89433214	chr3-89429640-89433214
chr3	89436180	89437696	chr3-89436180-89437696
chr3	89458164	89460988	chr3-89458164-89460988
chr3	89484982	89485448	chr3-89484982-89485448
chr3	89519715	89523968	chr3-89519715-89523968
chr3	89526241	89526941	chr3-89526241-89526941
chr3	89578932	89579559	chr3-89578932-89579559
chr3	89625814	89627355	chr3-89625814-89627355
chr3	89640995	89641196	chr3-89640995-89641196
chr3	89642459	89642660	chr3-89642459-89642660
chr3	89646657	89647575	chr3-89646657-89647575
chr3	89678898	89679449	chr3-89678898-89679449
chr3	89680385	89687314	chr3-89680385-89687314
chr3	89688810	89689275	chr3-89688810-89689275
chr3	89714488	89716309	chr3-89714488-89716309
chr3	89730281	89731660	chr3-89730281-89731660
chr3	89764141	89764756	chr3-89764141-89764756
chr3	89772348	89775799	chr3-89772348-89775799
chr3	89785337	89785588	chr3-89785337-89785588
chr3	89830903	89832132	chr3-89830903-89832132
chr3	89860750	89861349	chr3-89860750-89861349
chr3	89896651	89897082	chr3-89896651-89897082
chr3	89901223	89902256	chr3-89901223-89902256
chr3	89912073	89913843	chr3-89912073-89913843
chr3	89960043	89961147	chr3-89960043-89961147
chr3	89962012	89964588	chr3-89962012-89964588
chr3	89997222	89999755	chr3-89997222-89999755
chr3	90004369	90004570	chr3-90004369-90004570
chr3	90050667	90054578	chr3-90050667-90054578
chr3	90078829	90084299	chr3-90078829-90084299
chr3	90211264	90214716	chr3-90211264-90214716
chr3	90219901	90221617	chr3-90219901-90221617

chr3	90231059	90233068	chr3-90231059-90233068
chr3	90242992	90243327	chr3-90242992-90243327
chr3	90247493	90249737	chr3-90247493-90249737
chr3	90250721	90250922	chr3-90250721-90250922
chr3	90253389	90256157	chr3-90253389-90256157
chr3	90264515	90266847	chr3-90264515-90266847
chr3	90292044	90295241	chr3-90292044-90295241
chr3	90374211	90374514	chr3-90374211-90374514
chr3	90389112	90390092	chr3-90389112-90390092
chr3	90431311	90434745	chr3-90431311-90434745
chr3	90464675	90466828	chr3-90464675-90466828
chr3	90475316	90477372	chr3-90475316-90477372
chr3	90486835	90487825	chr3-90486835-90487825
chr3	90489199	90491258	chr3-90489199-90491258
chr3	90507612	90510416	chr3-90507612-90510416
chr3	90513631	90516083	chr3-90513631-90516083
chr3	90517458	90518042	chr3-90517458-90518042
chr3	90525664	90529275	chr3-90525664-90529275
chr3	90536716	90539992	chr3-90536716-90539992
chr3	90574773	90575333	chr3-90574773-90575333
chr3	90584683	90590565	chr3-90584683-90590565
chr3	90601149	90602484	chr3-90601149-90602484
chr3	90604249	90605405	chr3-90604249-90605405
chr3	90611772	90616977	chr3-90611772-90616977
chr3	90620612	90621717	chr3-90620612-90621717
chr3	90653998	90656415	chr3-90653998-90656415
chr3	92033514	92034034	chr3-92033514-92034034
chr3	92079891	92082312	chr3-92079891-92082312
chr3	92247682	92248164	chr3-92247682-92248164
chr3	92282843	92283044	chr3-92282843-92283044
chr3	92484214	92485961	chr3-92484214-92485961
chr3	92573542	92573856	chr3-92573542-92573856
chr3	92652728	92652929	chr3-92652728-92652929
chr3	92689187	92689984	chr3-92689187-92689984
chr3	93014129	93015826	chr3-93014129-93015826
chr3	93023873	93024128	chr3-93023873-93024128
chr3	93026458	93026709	chr3-93026458-93026709
chr3	93443355	93448250	chr3-93443355-93448250
chr3	93501779	93502163	chr3-93501779-93502163
chr3	93505751	93507599	chr3-93505751-93507599
chr3	93516392	93517467	chr3-93516392-93517467
chr3	93519819	93525062	chr3-93519819-93525062

chr3	93537556	93537807	chr3-93537556-93537807
chr3	93551899	93553037	chr3-93551899-93553037
chr3	93553844	93559367	chr3-93553844-93559367
chr3	93579382	93579583	chr3-93579382-93579583
chr3	93596829	93597820	chr3-93596829-93597820
chr3	94309328	94311384	chr3-94309328-94311384
chr3	94363292	94364510	chr3-94363292-94364510
chr3	94371559	94374108	chr3-94371559-94374108
chr3	94399240	94399600	chr3-94399240-94399600
chr3	94412179	94413897	chr3-94412179-94413897
chr3	94442793	94444757	chr3-94442793-94444757
chr3	94456617	94456977	chr3-94456617-94456977
chr3	94564666	94564867	chr3-94564666-94564867
chr3	94580969	94583174	chr3-94580969-94583174
chr3	94588174	94588375	chr3-94588174-94588375
chr3	94622219	94622476	chr3-94622219-94622476
chr3	94624136	94624957	chr3-94624136-94624957
chr3	94648560	94651112	chr3-94648560-94651112
chr3	94656110	94660183	chr3-94656110-94660183
chr3	94680363	94681503	chr3-94680363-94681503
chr3	94720672	94720934	chr3-94720672-94720934
chr3	94756002	94759231	chr3-94756002-94759231
chr3	94779743	94787935	chr3-94779743-94787935
chr3	94804174	94804537	chr3-94804174-94804537
chr3	94820997	94821976	chr3-94820997-94821976
chr3	94836941	94839289	chr3-94836941-94839289
chr3	94885592	94887573	chr3-94885592-94887573
chr3	94913699	94913900	chr3-94913699-94913900
chr3	94932809	94933585	chr3-94932809-94933585
chr3	94952433	94955413	chr3-94952433-94955413
chr3	94973762	94976004	chr3-94973762-94976004
chr3	95013593	95017424	chr3-95013593-95017424
chr3	95029056	95029257	chr3-95029056-95029257
chr3	95041028	95043202	chr3-95041028-95043202
chr3	95054373	95055216	chr3-95054373-95055216
chr3	95063937	95064526	chr3-95063937-95064526
chr3	95066912	95067113	chr3-95066912-95067113
chr3	95103522	95112856	chr3-95103522-95112856
chr3	95132866	95135695	chr3-95132866-95135695
chr3	95159624	95161678	chr3-95159624-95161678
chr3	95172610	95174489	chr3-95172610-95174489
chr3	95216540	95218965	chr3-95216540-95218965

chr3	95224221	95233707	chr3-95224221-95233707
chr3	95280669	95284944	chr3-95280669-95284944
chr3	95293553	95294245	chr3-95293553-95294245
chr3	95312018	95319012	chr3-95312018-95319012
chr3	95355647	95357598	chr3-95355647-95357598
chr3	95427622	95428802	chr3-95427622-95428802
chr3	95433391	95435862	chr3-95433391-95435862
chr3	95588111	95590152	chr3-95588111-95590152
chr3	95624425	95626560	chr3-95624425-95626560
chr3	95652961	95653834	chr3-95652961-95653834
chr3	95657637	95668953	chr3-95657637-95668953
chr3	95672381	95676952	chr3-95672381-95676952
chr3	95686509	95688897	chr3-95686509-95688897
chr3	95692949	95697085	chr3-95692949-95697085
chr3	95701767	95702582	chr3-95701767-95702582
chr3	95732568	95734626	chr3-95732568-95734626
chr3	95738786	95739681	chr3-95738786-95739681
chr3	95753395	95755620	chr3-95753395-95755620
chr3	95816887	95820624	chr3-95816887-95820624
chr3	95854659	95856462	chr3-95854659-95856462
chr3	95870413	95872652	chr3-95870413-95872652
chr3	95881030	95883368	chr3-95881030-95883368
chr3	95893204	95895918	chr3-95893204-95895918
chr3	95904639	95907270	chr3-95904639-95907270
chr3	95928307	95931371	chr3-95928307-95931371
chr3	95950077	95952184	chr3-95950077-95952184
chr3	95995016	95996883	chr3-95995016-95996883
chr3	96017590	96017791	chr3-96017590-96017791
chr3	96056904	96059180	chr3-96056904-96059180
chr3	96062725	96063406	chr3-96062725-96063406
chr3	96070264	96070640	chr3-96070264-96070640
chr3	96078172	96078655	chr3-96078172-96078655
chr3	96086770	96087206	chr3-96086770-96087206
chr3	96093299	96094993	chr3-96093299-96094993
chr3	96098174	96099835	chr3-96098174-96099835
chr3	96102029	96102953	chr3-96102029-96102953
chr3	96103754	96107149	chr3-96103754-96107149
chr3	96108523	96108724	chr3-96108523-96108724
chr3	96133499	96133824	chr3-96133499-96133824
chr3	96135050	96135360	chr3-96135050-96135360
chr3	96159467	96162800	chr3-96159467-96162800
chr3	96171048	96175605	chr3-96171048-96175605

chr3	96180946	96181457	chr3-96180946-96181457
chr3	96196333	96198928	chr3-96196333-96198928
chr3	96218652	96228036	chr3-96218652-96228036
chr3	96228991	96229192	chr3-96228991-96229192
chr3	96236449	96241063	chr3-96236449-96241063
chr3	96243976	96249288	chr3-96243976-96249288
chr3	96261863	96264909	chr3-96261863-96264909
chr3	96267714	96270479	chr3-96267714-96270479
chr3	96273436	96274467	chr3-96273436-96274467
chr3	96277555	96278907	chr3-96277555-96278907
chr3	96300550	96301412	chr3-96300550-96301412
chr3	96327264	96329455	chr3-96327264-96329455
chr3	96332072	96335046	chr3-96332072-96335046
chr3	96338962	96341182	chr3-96338962-96341182
chr3	96342580	96344004	chr3-96342580-96344004
chr3	96390463	96390664	chr3-96390463-96390664
chr3	96398658	96398859	chr3-96398658-96398859
chr3	96401335	96402373	chr3-96401335-96402373
chr3	96404186	96404955	chr3-96404186-96404955
chr3	96409206	96410161	chr3-96409206-96410161
chr3	96413583	96415323	chr3-96413583-96415323
chr3	96426883	96428269	chr3-96426883-96428269
chr3	96435356	96436772	chr3-96435356-96436772
chr3	96440694	96441662	chr3-96440694-96441662
chr3	96474717	96475389	chr3-96474717-96475389
chr3	96476712	96477176	chr3-96476712-96477176
chr3	96485159	96490021	chr3-96485159-96490021
chr3	96496562	96497886	chr3-96496562-96497886
chr3	96498834	96503631	chr3-96498834-96503631
chr3	96526929	96527634	chr3-96526929-96527634
chr3	96556503	96560784	chr3-96556503-96560784
chr3	96564185	96564975	chr3-96564185-96564975
chr3	96575772	96577735	chr3-96575772-96577735
chr3	96580321	96580628	chr3-96580321-96580628
chr3	96593240	96594597	chr3-96593240-96594597
chr3	96596123	96598904	chr3-96596123-96598904
chr3	96600620	96601734	chr3-96600620-96601734
chr3	96628887	96631173	chr3-96628887-96631173
chr3	96634801	96636894	chr3-96634801-96636894
chr3	96662669	96664280	chr3-96662669-96664280
chr3	96669712	96670718	chr3-96669712-96670718
chr3	96679103	96679962	chr3-96679103-96679962

chr3	96684807	96685165	chr3-96684807-96685165
chr3	96695971	96697934	chr3-96695971-96697934
chr3	96707915	96708718	chr3-96707915-96708718
chr3	96714098	96714299	chr3-96714098-96714299
chr3	96726365	96729424	chr3-96726365-96729424
chr3	96764504	96764831	chr3-96764504-96764831
chr3	96812847	96813555	chr3-96812847-96813555
chr3	96904185	96906024	chr3-96904185-96906024
chr3	96953732	96954015	chr3-96953732-96954015
chr3	97052405	97052606	chr3-97052405-97052606
chr3	97075931	97076915	chr3-97075931-97076915
chr3	97157928	97159948	chr3-97157928-97159948
chr3	97189164	97189365	chr3-97189164-97189365
chr3	97226184	97228676	chr3-97226184-97228676
chr3	97296894	97298534	chr3-97296894-97298534
chr3	97311885	97312444	chr3-97311885-97312444
chr3	97337423	97337624	chr3-97337423-97337624
chr3	97609142	97610864	chr3-97609142-97610864
chr3	97628219	97629212	chr3-97628219-97629212
chr3	97657362	97659576	chr3-97657362-97659576
chr3	97680671	97684667	chr3-97680671-97684667
chr3	97763172	97768674	chr3-97763172-97768674
chr3	97823928	97824706	chr3-97823928-97824706
chr3	97867200	97868620	chr3-97867200-97868620
chr3	97888042	97889059	chr3-97888042-97889059
chr3	97898370	97898952	chr3-97898370-97898952
chr3	97900591	97902607	chr3-97900591-97902607
chr3	97942567	97942768	chr3-97942567-97942768
chr3	98012912	98015796	chr3-98012912-98015796
chr3	98020308	98021103	chr3-98020308-98021103
chr3	98024130	98026736	chr3-98024130-98026736
chr3	98042411	98042681	chr3-98042411-98042681
chr3	98055836	98058008	chr3-98055836-98058008
chr3	98080508	98080861	chr3-98080508-98080861
chr3	98099069	98099270	chr3-98099069-98099270
chr3	98226988	98227450	chr3-98226988-98227450
chr3	98336800	98340153	chr3-98336800-98340153
chr3	98355248	98356344	chr3-98355248-98356344
chr3	98374423	98375482	chr3-98374423-98375482
chr3	98381603	98384448	chr3-98381603-98384448
chr3	98410774	98410975	chr3-98410774-98410975
chr3	98417143	98418162	chr3-98417143-98418162

chr3	98427074	98428917	chr3-98427074-98428917
chr3	98941189	98941595	chr3-98941189-98941595
chr3	99140516	99142206	chr3-99140516-99142206
chr3	99187420	99187834	chr3-99187420-99187834
chr3	99233878	99234489	chr3-99233878-99234489
chr3	99240167	99241871	chr3-99240167-99241871
chr3	99248493	99249229	chr3-99248493-99249229
chr3	99253114	99254553	chr3-99253114-99254553
chr3	99885279	99885600	chr3-99885279-99885600
chr3	100160980	100163526	chr3-100160980-100163526
chr3	100333383	100333584	chr3-100333383-100333584
chr3	100359241	100359492	chr3-100359241-100359492
chr3	100488123	100490389	chr3-100488123-100490389
chr3	100678917	100679735	chr3-100678917-100679735
chr3	100681429	100686622	chr3-100681429-100686622
chr3	100692372	100692848	chr3-100692372-100692848
chr3	100702984	100703606	chr3-100702984-100703606
chr3	100716216	100716521	chr3-100716216-100716521
chr3	100824964	100825649	chr3-100824964-100825649
chr3	100921270	100923607	chr3-100921270-100923607
chr3	100968523	100970392	chr3-100968523-100970392
chr3	101044469	101045807	chr3-101044469-101045807
chr3	101047299	101049147	chr3-101047299-101049147
chr3	101051586	101051889	chr3-101051586-101051889
chr3	101070425	101074163	chr3-101070425-101074163
chr3	101075619	101076385	chr3-101075619-101076385
chr3	101095885	101096142	chr3-101095885-101096142
chr3	101099341	101100315	chr3-101099341-101100315
chr3	101107938	101110971	chr3-101107938-101110971
chr3	101111993	101112194	chr3-101111993-101112194
chr3	101115769	101117660	chr3-101115769-101117660
chr3	101212956	101213571	chr3-101212956-101213571
chr3	101376630	101378695	chr3-101376630-101378695
chr3	101379529	101380606	chr3-101379529-101380606
chr3	101406343	101406717	chr3-101406343-101406717
chr3	101409046	101409910	chr3-101409046-101409910
chr3	101494111	101494377	chr3-101494111-101494377
chr3	101516894	101517574	chr3-101516894-101517574
chr3	101599773	101605549	chr3-101599773-101605549
chr3	101649720	101649921	chr3-101649720-101649921
chr3	101650953	101652553	chr3-101650953-101652553
chr3	101666894	101667924	chr3-101666894-101667924

chr3	101699969	101700567	chr3-101699969-101700567
chr3	101805686	101806466	chr3-101805686-101806466
chr3	101831278	101831917	chr3-101831278-101831917
chr3	101835862	101836249	chr3-101835862-101836249
chr3	101923158	101924998	chr3-101923158-101924998
chr3	101939333	101939534	chr3-101939333-101939534
chr3	102011407	102013251	chr3-102011407-102013251
chr3	102023440	102023745	chr3-102023440-102023745
chr3	102103696	102104221	chr3-102103696-102104221
chr3	102135383	102136647	chr3-102135383-102136647
chr3	102152503	102153430	chr3-102152503-102153430
chr3	102159442	102159976	chr3-102159442-102159976
chr3	102169076	102169928	chr3-102169076-102169928
chr3	102171044	102171349	chr3-102171044-102171349
chr3	102203041	102205109	chr3-102203041-102205109
chr3	102268240	102268491	chr3-102268240-102268491
chr3	102310012	102310525	chr3-102310012-102310525
chr3	102337728	102338532	chr3-102337728-102338532
chr3	102364023	102364224	chr3-102364023-102364224
chr3	102366184	102366652	chr3-102366184-102366652
chr3	102372595	102372796	chr3-102372595-102372796
chr3	102469512	102472407	chr3-102469512-102472407
chr3	102474428	102475171	chr3-102474428-102475171
chr3	102477466	102478438	chr3-102477466-102478438
chr3	102490335	102491259	chr3-102490335-102491259
chr3	102529101	102529302	chr3-102529101-102529302
chr3	102659086	102659868	chr3-102659086-102659868
chr3	102715144	102715560	chr3-102715144-102715560
chr3	102723764	102724531	chr3-102723764-102724531
chr3	102734249	102735687	chr3-102734249-102735687
chr3	102969991	102970716	chr3-102969991-102970716
chr3	102993306	102993507	chr3-102993306-102993507
chr3	102995076	102997103	chr3-102995076-102997103
chr3	103019493	103023214	chr3-103019493-103023214
chr3	103057697	103059642	chr3-103057697-103059642
chr3	103101773	103103588	chr3-103101773-103103588
chr3	103171047	103173127	chr3-103171047-103173127
chr3	103255935	103256702	chr3-103255935-103256702
chr3	103277978	103281825	chr3-103277978-103281825
chr3	103406159	103406683	chr3-103406159-103406683
chr3	103575117	103576963	chr3-103575117-103576963
chr3	103593916	103594372	chr3-103593916-103594372

chr3	103601136	103601594	chr3-103601136-103601594
chr3	103656704	103658686	chr3-103656704-103658686
chr3	103682663	103683968	chr3-103682663-103683968
chr3	103738036	103738410	chr3-103738036-103738410
chr3	103788936	103792070	chr3-103788936-103792070
chr3	103801440	103802518	chr3-103801440-103802518
chr3	103807853	103811202	chr3-103807853-103811202
chr3	103831292	103837305	chr3-103831292-103837305
chr3	103859102	103863649	chr3-103859102-103863649
chr3	103907389	103907641	chr3-103907389-103907641
chr3	103913489	103917526	chr3-103913489-103917526
chr3	103967147	103969323	chr3-103967147-103969323
chr3	104139101	104139355	chr3-104139101-104139355
chr3	104142477	104142678	chr3-104142477-104142678
chr3	104213835	104214246	chr3-104213835-104214246
chr3	104217875	104221896	chr3-104217875-104221896
chr3	104233545	104234105	chr3-104233545-104234105
chr3	104288735	104289721	chr3-104288735-104289721
chr3	104358848	104360909	chr3-104358848-104360909
chr3	104403305	104404198	chr3-104403305-104404198
chr3	104441767	104442219	chr3-104441767-104442219
chr3	104501217	104501544	chr3-104501217-104501544
chr3	104510612	104512920	chr3-104510612-104512920
chr3	104636899	104641053	chr3-104636899-104641053
chr3	104670331	104670990	chr3-104670331-104670990
chr3	104674051	104676249	chr3-104674051-104676249
chr3	104706605	104708092	chr3-104706605-104708092
chr3	104714627	104714828	chr3-104714627-104714828
chr3	104742233	104743507	chr3-104742233-104743507
chr3	104758451	104758763	chr3-104758451-104758763
chr3	104762092	104762398	chr3-104762092-104762398
chr3	104777304	104777606	chr3-104777304-104777606
chr3	104780519	104783340	chr3-104780519-104783340
chr3	104788345	104791983	chr3-104788345-104791983
chr3	104793029	104795739	chr3-104793029-104795739
chr3	104796782	104797150	chr3-104796782-104797150
chr3	104817067	104819005	chr3-104817067-104819005
chr3	104861823	104866422	chr3-104861823-104866422
chr3	104961140	104962740	chr3-104961140-104962740
chr3	104967517	104968531	chr3-104967517-104968531
chr3	104993719	104996080	chr3-104993719-104996080
chr3	105000193	105001669	chr3-105000193-105001669

chr3	105047624	105048016	chr3-105047624-105048016
chr3	105049036	105056043	chr3-105049036-105056043
chr3	105058395	105060835	chr3-105058395-105060835
chr3	105088601	105089083	chr3-105088601-105089083
chr3	105218783	105219103	chr3-105218783-105219103
chr3	105451059	105453226	chr3-105451059-105453226
chr3	105686279	105688370	chr3-105686279-105688370
chr3	105704282	105705700	chr3-105704282-105705700
chr3	105799350	105801975	chr3-105799350-105801975
chr3	105825807	105826008	chr3-105825807-105826008
chr3	105894654	105895416	chr3-105894654-105895416
chr3	105957817	105961053	chr3-105957817-105961053
chr3	106001203	106001708	chr3-106001203-106001708
chr3	106003749	106004000	chr3-106003749-106004000
chr3	106481983	106486285	chr3-106481983-106486285
chr3	106488652	106489008	chr3-106488652-106489008
chr3	106546085	106549838	chr3-106546085-106549838
chr3	106684734	106685644	chr3-106684734-106685644
chr3	106721321	106723025	chr3-106721321-106723025
chr3	107035635	107037078	chr3-107035635-107037078
chr3	107101360	107102562	chr3-107101360-107102562
chr3	107278561	107280083	chr3-107278561-107280083
chr3	107323703	107323904	chr3-107323703-107323904
chr3	107327749	107328000	chr3-107327749-107328000
chr3	107329336	107335120	chr3-107329336-107335120
chr3	107356911	107357533	chr3-107356911-107357533
chr3	107458399	107460447	chr3-107458399-107460447
chr3	107516833	107518320	chr3-107516833-107518320
chr3	107582764	107583428	chr3-107582764-107583428
chr3	107594840	107596877	chr3-107594840-107596877
chr3	107630401	107632609	chr3-107630401-107632609
chr3	107647561	107647936	chr3-107647561-107647936
chr3	107694358	107697061	chr3-107694358-107697061
chr3	107737507	107737968	chr3-107737507-107737968
chr3	107740683	107740934	chr3-107740683-107740934
chr3	107742823	107743024	chr3-107742823-107743024
chr3	107756573	107760910	chr3-107756573-107760910
chr3	107772882	107773234	chr3-107772882-107773234
chr3	107775352	107776675	chr3-107775352-107776675
chr3	107792844	107794171	chr3-107792844-107794171
chr3	107796114	107797259	chr3-107796114-107797259
chr3	107828267	107829974	chr3-107828267-107829974

chr3	107844190	107845105	chr3-107844190-107845105
chr3	107875117	107875742	chr3-107875117-107875742
chr3	107895262	107896653	chr3-107895262-107896653
chr3	107931529	107931930	chr3-107931529-107931930
chr3	107968939	107969240	chr3-107968939-107969240
chr3	108013121	108015140	chr3-108013121-108015140
chr3	108017237	108018698	chr3-108017237-108018698
chr3	108044212	108045333	chr3-108044212-108045333
chr3	108084112	108087669	chr3-108084112-108087669
chr3	108126150	108126434	chr3-108126150-108126434
chr3	108144313	108147003	chr3-108144313-108147003
chr3	108164888	108165345	chr3-108164888-108165345
chr3	108185571	108187401	chr3-108185571-108187401
chr3	108199504	108201673	chr3-108199504-108201673
chr3	108208920	108211431	chr3-108208920-108211431
chr3	108225899	108227018	chr3-108225899-108227018
chr3	108256102	108259247	chr3-108256102-108259247
chr3	108283263	108284985	chr3-108283263-108284985
chr3	108361095	108361296	chr3-108361095-108361296
chr3	108372389	108372743	chr3-108372389-108372743
chr3	108383201	108384427	chr3-108383201-108384427
chr3	108389434	108389635	chr3-108389434-108389635
chr3	108401852	108402255	chr3-108401852-108402255
chr3	108414823	108416294	chr3-108414823-108416294
chr3	108424019	108424610	chr3-108424019-108424610
chr3	108442311	108445935	chr3-108442311-108445935
chr3	108458260	108459174	chr3-108458260-108459174
chr3	108492917	108493176	chr3-108492917-108493176
chr3	108536210	108537811	chr3-108536210-108537811
chr3	108553713	108554926	chr3-108553713-108554926
chr3	108561454	108563735	chr3-108561454-108563735
chr3	108571286	108573006	chr3-108571286-108573006
chr3	108590897	108592615	chr3-108590897-108592615
chr3	108653279	108655198	chr3-108653279-108655198
chr3	108720161	108722869	chr3-108720161-108722869
chr3	108765124	108765444	chr3-108765124-108765444
chr3	108770847	108771215	chr3-108770847-108771215
chr3	108781268	108781635	chr3-108781268-108781635
chr3	108839131	108840732	chr3-108839131-108840732
chr3	108855570	108856390	chr3-108855570-108856390
chr3	108858261	108858676	chr3-108858261-108858676
chr3	108880579	108882522	chr3-108880579-108882522

chr3	108908322	108912788	chr3-108908322-108912788
chr3	108939929	108940698	chr3-108939929-108940698
chr3	108958192	108958780	chr3-108958192-108958780
chr3	108959858	108960329	chr3-108959858-108960329
chr3	108975194	108975450	chr3-108975194-108975450
chr3	109010097	109011773	chr3-109010097-109011773
chr3	109025359	109028279	chr3-109025359-109028279
chr3	109030976	109031235	chr3-109030976-109031235
chr3	109122344	109125774	chr3-109122344-109125774
chr3	109155649	109156521	chr3-109155649-109156521
chr3	109164438	109165423	chr3-109164438-109165423
chr3	109253850	109254201	chr3-109253850-109254201
chr3	109309281	109310300	chr3-109309281-109310300
chr3	109339790	109342243	chr3-109339790-109342243
chr3	109368279	109369745	chr3-109368279-109369745
chr3	109469677	109469932	chr3-109469677-109469932
chr3	109640630	109640893	chr3-109640630-109640893
chr3	109782550	109782922	chr3-109782550-109782922
chr3	110142239	110144047	chr3-110142239-110144047
chr3	110248615	110251581	chr3-110248615-110251581
chr3	113628996	113631275	chr3-113628996-113631275
chr3	114030028	114032459	chr3-114030028-114032459
chr3	115642027	115643142	chr3-115642027-115643142
chr3	115646739	115648198	chr3-115646739-115648198
chr3	115673538	115674149	chr3-115673538-115674149
chr3	115714120	115715765	chr3-115714120-115715765
chr3	115758395	115759025	chr3-115758395-115759025
chr3	115838295	115838550	chr3-115838295-115838550
chr3	115887114	115889287	chr3-115887114-115889287
chr3	116005841	116009118	chr3-116005841-116009118
chr3	116113554	116115312	chr3-116113554-116115312
chr3	116120763	116121072	chr3-116120763-116121072
chr3	116128504	116129917	chr3-116128504-116129917
chr3	116189478	116190258	chr3-116189478-116190258
chr3	116200298	116200499	chr3-116200298-116200499
chr3	116251241	116252915	chr3-116251241-116252915
chr3	116380048	116380874	chr3-116380048-116380874
chr3	116422203	116425239	chr3-116422203-116425239
chr3	116506709	116508509	chr3-116506709-116508509
chr3	116512565	116514101	chr3-116512565-116514101
chr3	116593656	116596186	chr3-116593656-116596186
chr3	116638609	116638922	chr3-116638609-116638922

chr3	116652194	116652498	chr3-116652194-116652498
chr3	116660526	116663196	chr3-116660526-116663196
chr3	116710870	116713239	chr3-116710870-116713239
chr3	116807157	116809009	chr3-116807157-116809009
chr3	116835788	116836304	chr3-116835788-116836304
chr3	116860920	116865474	chr3-116860920-116865474
chr3	116950433	116951779	chr3-116950433-116951779
chr3	117038438	117039006	chr3-117038438-117039006
chr3	117070808	117071009	chr3-117070808-117071009
chr3	117076994	117077195	chr3-117076994-117077195
chr3	117359662	117360673	chr3-117359662-117360673
chr3	117574966	117575970	chr3-117574966-117575970
chr3	117866667	117869722	chr3-117866667-117869722
chr3	118433547	118434625	chr3-118433547-118434625
chr3	118561897	118562864	chr3-118561897-118562864
chr3	119780911	119784077	chr3-119780911-119784077
chr3	119890573	119891181	chr3-119890573-119891181
chr3	120015863	120016227	chr3-120015863-120016227
chr3	120175413	120176137	chr3-120175413-120176137
chr3	121104901	121105305	chr3-121104901-121105305
chr3	121170932	121172191	chr3-121170932-121172191
chr3	121258844	121259160	chr3-121258844-121259160
chr3	121262038	121263619	chr3-121262038-121263619
chr3	121290744	121293069	chr3-121290744-121293069
chr3	121375081	121375970	chr3-121375081-121375970
chr3	121425340	121429722	chr3-121425340-121429722
chr3	121446324	121446525	chr3-121446324-121446525
chr3	121469300	121469583	chr3-121469300-121469583
chr3	121530402	121533057	chr3-121530402-121533057
chr3	121559547	121561210	chr3-121559547-121561210
chr3	121585029	121585280	chr3-121585029-121585280
chr3	121601787	121602724	chr3-121601787-121602724
chr3	121604238	121604591	chr3-121604238-121604591
chr3	121617584	121618173	chr3-121617584-121618173
chr3	121625885	121626460	chr3-121625885-121626460
chr3	121635408	121636036	chr3-121635408-121636036
chr3	121644423	121644780	chr3-121644423-121644780
chr3	121645817	121647405	chr3-121645817-121647405
chr3	121649915	121652403	chr3-121649915-121652403
chr3	121661731	121661932	chr3-121661731-121661932
chr3	121663131	121663454	chr3-121663131-121663454
chr3	121671852	121672828	chr3-121671852-121672828

chr3	121675221	121675598	chr3-121675221-121675598
chr3	121697191	121697786	chr3-121697191-121697786
chr3	121720902	121727797	chr3-121720902-121727797
chr3	121735994	121736352	chr3-121735994-121736352
chr3	121813433	121815984	chr3-121813433-121815984
chr3	121952849	121954319	chr3-121952849-121954319
chr3	121967330	121969518	chr3-121967330-121969518
chr3	122047752	122048954	chr3-122047752-122048954
chr3	122088084	122088734	chr3-122088084-122088734
chr3	122104250	122104617	chr3-122104250-122104617
chr3	122121640	122123215	chr3-122121640-122123215
chr3	122131067	122131909	chr3-122131067-122131909
chr3	122208662	122209119	chr3-122208662-122209119
chr3	122244889	122248199	chr3-122244889-122248199
chr3	122273484	122278093	chr3-122273484-122278093
chr3	122292168	122294825	chr3-122292168-122294825
chr3	122316582	122317158	chr3-122316582-122317158
chr3	122321324	122324887	chr3-122321324-122324887
chr3	122343419	122344029	chr3-122343419-122344029
chr3	122362737	122363245	chr3-122362737-122363245
chr3	122390120	122394404	chr3-122390120-122394404
chr3	122399912	122400113	chr3-122399912-122400113
chr3	122400924	122401694	chr3-122400924-122401694
chr3	122408152	122409896	chr3-122408152-122409896
chr3	122413838	122414522	chr3-122413838-122414522
chr3	122418492	122422732	chr3-122418492-122422732
chr3	122458006	122459553	chr3-122458006-122459553
chr3	122462425	122463751	chr3-122462425-122463751
chr3	122471274	122471475	chr3-122471274-122471475
chr3	122480275	122484244	chr3-122480275-122484244
chr3	122498133	122498638	chr3-122498133-122498638
chr3	122509099	122510816	chr3-122509099-122510816
chr3	122515133	122517302	chr3-122515133-122517302
chr3	122520938	122521241	chr3-122520938-122521241
chr3	122616884	122622414	chr3-122616884-122622414
chr3	122638516	122638768	chr3-122638516-122638768
chr3	122728631	122731457	chr3-122728631-122731457
chr3	122874381	122874802	chr3-122874381-122874802
chr3	122898158	122898359	chr3-122898158-122898359
chr3	122922964	122926698	chr3-122922964-122926698
chr3	122982018	122986112	chr3-122982018-122986112
chr3	123019266	123019467	chr3-123019266-123019467

chr3	123052774	123054361	chr3-123052774-123054361
chr3	123093375	123093680	chr3-123093375-123093680
chr3	123122846	123123104	chr3-123122846-123123104
chr3	123204835	123205454	chr3-123204835-123205454
chr3	123235763	123236422	chr3-123235763-123236422
chr3	123266761	123268852	chr3-123266761-123268852
chr3	123334737	123335090	chr3-123334737-123335090
chr3	123384745	123386688	chr3-123384745-123386688
chr3	123446476	123448462	chr3-123446476-123448462
chr3	123506163	123509957	chr3-123506163-123509957
chr3	123689935	123691403	chr3-123689935-123691403
chr3	123983974	123984283	chr3-123983974-123984283
chr3	124320679	124321586	chr3-124320679-124321586
chr3	125937545	125939269	chr3-125937545-125939269
chr3	126385250	126385603	chr3-126385250-126385603
chr3	126595793	126599187	chr3-126595793-126599187
chr3	126610132	126611894	chr3-126610132-126611894
chr3	126658846	126661767	chr3-126658846-126661767
chr3	126692827	126693906	chr3-126692827-126693906
chr3	126746291	126746929	chr3-126746291-126746929
chr3	126827619	126828008	chr3-126827619-126828008
chr3	126831280	126832078	chr3-126831280-126832078
chr3	126869817	126870359	chr3-126869817-126870359
chr3	126921768	126922183	chr3-126921768-126922183
chr3	127161266	127162866	chr3-127161266-127162866
chr3	127225703	127226591	chr3-127225703-127226591
chr3	127257305	127258496	chr3-127257305-127258496
chr3	127408244	127409181	chr3-127408244-127409181
chr3	127458189	127458390	chr3-127458189-127458390
chr3	127498353	127499469	chr3-127498353-127499469
chr3	127551806	127554912	chr3-127551806-127554912
chr3	127625203	127626388	chr3-127625203-127626388
chr3	127627759	127628076	chr3-127627759-127628076
chr3	127631839	127634888	chr3-127631839-127634888
chr3	127779488	127780707	chr3-127779488-127780707
chr3	127789282	127791496	chr3-127789282-127791496
chr3	127835653	127838640	chr3-127835653-127838640
chr3	127894674	127896585	chr3-127894674-127896585
chr3	127923485	127923737	chr3-127923485-127923737
chr3	127929743	127931473	chr3-127929743-127931473
chr3	128565251	128565563	chr3-128565251-128565563
chr3	129199500	129200352	chr3-129199500-129200352

chr3	129204179	129205066	chr3-129204179-129205066
chr3	129213337	129216329	chr3-129213337-129216329
chr3	129218582	129218992	chr3-129218582-129218992
chr3	129224176	129225529	chr3-129224176-129225529
chr3	129393223	129393671	chr3-129393223-129393671
chr3	129445573	129446414	chr3-129445573-129446414
chr3	129509534	129510264	chr3-129509534-129510264
chr3	129531792	129537072	chr3-129531792-129537072
chr3	129724502	129725688	chr3-129724502-129725688
chr3	129754649	129755554	chr3-129754649-129755554
chr3	129829127	129832116	chr3-129829127-129832116
chr3	129835982	129836636	chr3-129835982-129836636
chr3	129877946	129879923	chr3-129877946-129879923
chr3	129900987	129902371	chr3-129900987-129902371
chr3	129969055	129970830	chr3-129969055-129970830
chr3	130057682	130062047	chr3-130057682-130062047
chr3	130180201	130181675	chr3-130180201-130181675
chr3	130617520	130617818	chr3-130617520-130617818
chr3	130690992	130691785	chr3-130690992-130691785
chr3	130707823	130710298	chr3-130707823-130710298
chr3	130714862	130715431	chr3-130714862-130715431
chr3	130727786	130731609	chr3-130727786-130731609
chr3	130818849	130819267	chr3-130818849-130819267
chr3	130885555	130886524	chr3-130885555-130886524
chr3	130905033	130905284	chr3-130905033-130905284
chr3	130951168	130951529	chr3-130951168-130951529
chr3	131084750	131085461	chr3-131084750-131085461
chr3	131105821	131107009	chr3-131105821-131107009
chr3	131109427	131112934	chr3-131109427-131112934
chr3	131271090	131272598	chr3-131271090-131272598
chr3	131301804	131303687	chr3-131301804-131303687
chr3	131363269	131365840	chr3-131363269-131365840
chr3	131484638	131491795	chr3-131484638-131491795
chr3	131498945	131499254	chr3-131498945-131499254
chr3	131560083	131560411	chr3-131560083-131560411
chr3	131564024	131566036	chr3-131564024-131566036
chr3	131691336	131691697	chr3-131691336-131691697
chr3	131967175	131967946	chr3-131967175-131967946
chr3	132017743	132018101	chr3-132017743-132018101
chr3	132084905	132087648	chr3-132084905-132087648
chr3	132682600	132685141	chr3-132682600-132685141
chr3	132788169	132788370	chr3-132788169-132788370

chr3	132870971	132872229	chr3-132870971-132872229
chr3	132947538	132950918	chr3-132947538-132950918
chr3	132965084	132965522	chr3-132965084-132965522
chr3	132983884	132984895	chr3-132983884-132984895
chr3	133090151	133093042	chr3-133090151-133093042
chr3	133234447	133235390	chr3-133234447-133235390
chr3	133309689	133311576	chr3-133309689-133311576
chr3	133542947	133545300	chr3-133542947-133545300
chr3	133600570	133601071	chr3-133600570-133601071
chr3	134190060	134190361	chr3-134190060-134190361
chr3	134235453	134236807	chr3-134235453-134236807
chr3	134238969	134240809	chr3-134238969-134240809
chr3	134828922	134829651	chr3-134828922-134829651
chr3	135211719	135214471	chr3-135211719-135214471
chr3	135307341	135308194	chr3-135307341-135308194
chr3	135422055	135424540	chr3-135422055-135424540
chr3	135437261	135441562	chr3-135437261-135441562
chr3	135484666	135486640	chr3-135484666-135486640
chr3	135518919	135520327	chr3-135518919-135520327
chr3	135521739	135523234	chr3-135521739-135523234
chr3	135533886	135534138	chr3-135533886-135534138
chr3	135670343	135671458	chr3-135670343-135671458
chr3	135674683	135675266	chr3-135674683-135675266
chr3	135684119	135684320	chr3-135684119-135684320
chr3	135688917	135692407	chr3-135688917-135692407
chr3	135712143	135712828	chr3-135712143-135712828
chr3	135731754	135732173	chr3-135731754-135732173
chr3	135825069	135827008	chr3-135825069-135827008
chr3	136640855	136641056	chr3-136640855-136641056
chr3	136656876	136657974	chr3-136656876-136657974
chr3	136668630	136672664	chr3-136668630-136672664
chr3	136697985	136698302	chr3-136697985-136698302
chr3	136704455	136705516	chr3-136704455-136705516
chr3	136728412	136728613	chr3-136728412-136728613
chr3	136735934	136736864	chr3-136735934-136736864
chr3	137150670	137150871	chr3-137150670-137150871
chr3	137293226	137293589	chr3-137293226-137293589
chr3	137332307	137332508	chr3-137332307-137332508
chr3	137610779	137611351	chr3-137610779-137611351
chr3	137623339	137624557	chr3-137623339-137624557
chr3	137752842	137753193	chr3-137752842-137753193
chr3	137863414	137868779	chr3-137863414-137868779

chr3	137917227	137919707	chr3-137917227-137919707
chr3	137961704	137962525	chr3-137961704-137962525
chr3	137979939	137981994	chr3-137979939-137981994
chr3	138054096	138054401	chr3-138054096-138054401
chr3	138060642	138065341	chr3-138060642-138065341
chr3	138095742	138095995	chr3-138095742-138095995
chr3	138104774	138105025	chr3-138104774-138105025
chr3	138142180	138145034	chr3-138142180-138145034
chr3	138442064	138444246	chr3-138442064-138444246
chr3	138487420	138490450	chr3-138487420-138490450
chr3	138525835	138529554	chr3-138525835-138529554
chr3	138741547	138744085	chr3-138741547-138744085
chr3	138745010	138746444	chr3-138745010-138746444
chr3	138760594	138760795	chr3-138760594-138760795
chr3	139037985	139038259	chr3-139037985-139038259
chr3	139073435	139076373	chr3-139073435-139076373
chr3	139205114	139206204	chr3-139205114-139206204
chr3	141464567	141466515	chr3-141464567-141466515
chr3	141981714	141982365	chr3-141981714-141982365
chr3	142168546	142169716	chr3-142168546-142169716
chr3	142282929	142283130	chr3-142282929-142283130
chr3	142345903	142346104	chr3-142345903-142346104
chr3	142368583	142368884	chr3-142368583-142368884
chr3	142393129	142396349	chr3-142393129-142396349
chr3	142439615	142439935	chr3-142439615-142439935
chr3	142443048	142443302	chr3-142443048-142443302
chr3	142496773	142497024	chr3-142496773-142497024
chr3	142529814	142531096	chr3-142529814-142531096
chr3	142559833	142561050	chr3-142559833-142561050
chr3	142620212	142621251	chr3-142620212-142621251
chr3	142700153	142702028	chr3-142700153-142702028
chr3	142764381	142766796	chr3-142764381-142766796
chr3	142826958	142827213	chr3-142826958-142827213
chr3	142852605	142852906	chr3-142852605-142852906
chr3	142880079	142883523	chr3-142880079-142883523
chr3	143100627	143101462	chr3-143100627-143101462
chr3	143152290	143152591	chr3-143152290-143152591
chr3	143158419	143158770	chr3-143158419-143158770
chr3	143742547	143744057	chr3-143742547-143744057
chr3	144091056	144091308	chr3-144091056-144091308
chr3	144198199	144206440	chr3-144198199-144206440
chr3	144380910	144381743	chr3-144380910-144381743

chr3	144497211	144497514	chr3-144497211-144497514
chr3	144506148	144506449	chr3-144506148-144506449
chr3	144551138	144551664	chr3-144551138-144551664
chr3	144568442	144572132	chr3-144568442-144572132
chr3	144574058	144575131	chr3-144574058-144575131
chr3	144578816	144579017	chr3-144578816-144579017
chr3	144581680	144582343	chr3-144581680-144582343
chr3	144663802	144665943	chr3-144663802-144665943
chr3	144668988	144669975	chr3-144668988-144669975
chr3	144715991	144716396	chr3-144715991-144716396
chr3	144717702	144721049	chr3-144717702-144721049
chr3	144759249	144761519	chr3-144759249-144761519
chr3	144849177	144849452	chr3-144849177-144849452
chr3	144971509	144972733	chr3-144971509-144972733
chr3	145072024	145072431	chr3-145072024-145072431
chr3	145101774	145104464	chr3-145101774-145104464
chr3	145105341	145105592	chr3-145105341-145105592
chr3	145114418	145114669	chr3-145114418-145114669
chr3	145117753	145119960	chr3-145117753-145119960
chr3	145256797	145257105	chr3-145256797-145257105
chr3	145292389	145293247	chr3-145292389-145293247
chr3	145420876	145421077	chr3-145420876-145421077
chr3	145575769	145577926	chr3-145575769-145577926
chr3	145632015	145633675	chr3-145632015-145633675
chr3	145646151	145654664	chr3-145646151-145654664
chr3	145684316	145685762	chr3-145684316-145685762
chr3	145703247	145703931	chr3-145703247-145703931
chr3	145704899	145705154	chr3-145704899-145705154
chr3	145728196	145730877	chr3-145728196-145730877
chr3	145757472	145760853	chr3-145757472-145760853
chr3	145891242	145892664	chr3-145891242-145892664
chr3	145899670	145899923	chr3-145899670-145899923
chr3	145905234	145905648	chr3-145905234-145905648
chr3	145923411	145925840	chr3-145923411-145925840
chr3	145937622	145940246	chr3-145937622-145940246
chr3	145964109	145964888	chr3-145964109-145964888
chr3	145987213	145990499	chr3-145987213-145990499
chr3	146047743	146048005	chr3-146047743-146048005
chr3	146107272	146108470	chr3-146107272-146108470
chr3	146117198	146117971	chr3-146117198-146117971
chr3	146149554	146150906	chr3-146149554-146150906
chr3	146177444	146178500	chr3-146177444-146178500

chr3	146179497	146179698	chr3-146179497-146179698
chr3	146220644	146221653	chr3-146220644-146221653
chr3	146327758	146329047	chr3-146327758-146329047
chr3	146403875	146407436	chr3-146403875-146407436
chr3	146411751	146413565	chr3-146411751-146413565
chr3	146421985	146422301	chr3-146421985-146422301
chr3	146449920	146452015	chr3-146449920-146452015
chr3	146498584	146502550	chr3-146498584-146502550
chr3	146520079	146522079	chr3-146520079-146522079
chr3	146567450	146568736	chr3-146567450-146568736
chr3	146684835	146685716	chr3-146684835-146685716
chr3	146781173	146781426	chr3-146781173-146781426
chr3	146811690	146813917	chr3-146811690-146813917
chr3	146851858	146853651	chr3-146851858-146853651
chr3	146945883	146946288	chr3-146945883-146946288
chr3	148922584	148922951	chr3-148922584-148922951
chr3	148959284	148959962	chr3-148959284-148959962
chr3	148986885	148990889	chr3-148986885-148990889
chr3	149076635	149077541	chr3-149076635-149077541
chr3	149111616	149112731	chr3-149111616-149112731
chr3	149116573	149117958	chr3-149116573-149117958
chr3	149239457	149239708	chr3-149239457-149239708
chr3	149445225	149445426	chr3-149445225-149445426
chr3	151298548	151298910	chr3-151298548-151298910
chr3	151323895	151324096	chr3-151323895-151324096
chr3	151505840	151506091	chr3-151505840-151506091
chr3	151749727	151750079	chr3-151749727-151750079
chr3	151762549	151763330	chr3-151762549-151763330
chr3	151836818	151837821	chr3-151836818-151837821
chr3	151912105	151912512	chr3-151912105-151912512
chr3	152114267	152115030	chr3-152114267-152115030
chr3	152119585	152119899	chr3-152119585-152119899
chr3	152121412	152123407	chr3-152121412-152123407
chr3	152143328	152143529	chr3-152143328-152143529
chr3	152145106	152145539	chr3-152145106-152145539
chr3	152151657	152152706	chr3-152151657-152152706
chr3	152164199	152166769	chr3-152164199-152166769
chr3	152171157	152171358	chr3-152171157-152171358
chr3	152191042	152194153	chr3-152191042-152194153
chr3	152208693	152213637	chr3-152208693-152213637
chr3	152264892	152266431	chr3-152264892-152266431
chr3	152308785	152309527	chr3-152308785-152309527

chr3	152339363	152340915	chr3-152339363-152340915
chr3	152346091	152348126	chr3-152346091-152348126
chr3	152394482	152398036	chr3-152394482-152398036
chr3	152402167	152403231	chr3-152402167-152403231
chr3	152469606	152469858	chr3-152469606-152469858
chr3	152477518	152479609	chr3-152477518-152479609
chr3	152555211	152555412	chr3-152555211-152555412
chr3	152556465	152556716	chr3-152556465-152556716
chr3	152568547	152569600	chr3-152568547-152569600
chr3	152666989	152668623	chr3-152666989-152668623
chr3	152713707	152715170	chr3-152713707-152715170
chr3	152981095	152981362	chr3-152981095-152981362
chr3	153281983	153282446	chr3-153281983-153282446
chr3	153724530	153725585	chr3-153724530-153725585
chr3	153908440	153913901	chr3-153908440-153913901
chr3	153943209	153945391	chr3-153943209-153945391
chr3	153973285	153973889	chr3-153973285-153973889
chr3	154269985	154270186	chr3-154269985-154270186
chr3	154328086	154331052	chr3-154328086-154331052
chr3	154332046	154332668	chr3-154332046-154332668
chr3	154596247	154598019	chr3-154596247-154598019
chr3	154663906	154664479	chr3-154663906-154664479
chr3	154885493	154885694	chr3-154885493-154885694
chr3	155092510	155093997	chr3-155092510-155093997
chr3	156560537	156562476	chr3-156560537-156562476
chr3	157533531	157535585	chr3-157533531-157535585
chr3	157566713	157568106	chr3-157566713-157568106
chr3	157884330	157884531	chr3-157884330-157884531
chr3	157922649	157925582	chr3-157922649-157925582
chr3	157946357	157949699	chr3-157946357-157949699
chr3	158029640	158033228	chr3-158029640-158033228
chr3	158034216	158037771	chr3-158034216-158037771
chr3	158559576	158562540	chr3-158559576-158562540
chr3	159494776	159496512	chr3-159494776-159496512
chr3	159839208	159842024	chr3-159839208-159842024
chr3	159885405	159885658	chr3-159885405-159885658
chr4	3064218	3064552	chr4-3064218-3064552
chr4	3082565	3082766	chr4-3082565-3082766
chr4	3230795	3231654	chr4-3230795-3231654
chr4	3573632	3576313	chr4-3573632-3576313
chr4	3677608	3679161	chr4-3677608-3679161
chr4	3833253	3836363	chr4-3833253-3836363

chr4	3874533	3875754	chr4-3874533-3875754
chr4	3929239	3929929	chr4-3929239-3929929
chr4	3936323	3940257	chr4-3936323-3940257
chr4	4138045	4139181	chr4-4138045-4139181
chr4	4634721	4635984	chr4-4634721-4635984
chr4	4791642	4793760	chr4-4791642-4793760
chr4	5643750	5645244	chr4-5643750-5645244
chr4	5768388	5769228	chr4-5768388-5769228
chr4	5793513	5794023	chr4-5793513-5794023
chr4	5797681	5798777	chr4-5797681-5798777
chr4	6159300	6159743	chr4-6159300-6159743
chr4	6190567	6192475	chr4-6190567-6192475
chr4	6360920	6361391	chr4-6360920-6361391
chr4	6365117	6367994	chr4-6365117-6367994
chr4	6452282	6454845	chr4-6452282-6454845
chr4	6512826	6513180	chr4-6512826-6513180
chr4	6989434	6991671	chr4-6989434-6991671
chr4	7391679	7391931	chr4-7391679-7391931
chr4	8218944	8219257	chr4-8218944-8219257
chr4	8238425	8239588	chr4-8238425-8239588
chr4	8394872	8395073	chr4-8394872-8395073
chr4	8507237	8507496	chr4-8507237-8507496
chr4	8535173	8537867	chr4-8535173-8537867
chr4	8646180	8648785	chr4-8646180-8648785
chr4	8689446	8692522	chr4-8689446-8692522
chr4	8911098	8912008	chr4-8911098-8912008
chr4	8954264	8955097	chr4-8954264-8955097
chr4	9015372	9016236	chr4-9015372-9016236
chr4	9027853	9028770	chr4-9027853-9028770
chr4	9133025	9133430	chr4-9133025-9133430
chr4	9135915	9136519	chr4-9135915-9136519
chr4	9667947	9670171	chr4-9667947-9670171
chr4	9770359	9771344	chr4-9770359-9771344
chr4	9825871	9826133	chr4-9825871-9826133
chr4	9843568	9845516	chr4-9843568-9845516
chr4	9846633	9847726	chr4-9846633-9847726
chr4	9859019	9860260	chr4-9859019-9860260
chr4	9874608	9875075	chr4-9874608-9875075
chr4	10693700	10694761	chr4-10693700-10694761
chr4	10873103	10875887	chr4-10873103-10875887
chr4	10972876	10973077	chr4-10972876-10973077
chr4	11004919	11008999	chr4-11004919-11008999

chr4	11026441	11026642	chr4-11026441-11026642
chr4	11075065	11076624	chr4-11075065-11076624
chr4	11127573	11127874	chr4-11127573-11127874
chr4	11146599	11148811	chr4-11146599-11148811
chr4	11155530	11157490	chr4-11155530-11157490
chr4	11160758	11161904	chr4-11160758-11161904
chr4	11189674	11193386	chr4-11189674-11193386
chr4	11252432	11254715	chr4-11252432-11254715
chr4	11320963	11323605	chr4-11320963-11323605
chr4	11383946	11389569	chr4-11383946-11389569
chr4	11454826	11455748	chr4-11454826-11455748
chr4	11485115	11487056	chr4-11485115-11487056
chr4	11558144	11560120	chr4-11558144-11560120
chr4	11653860	11654164	chr4-11653860-11654164
chr4	11703871	11705066	chr4-11703871-11705066
chr4	11964956	11967484	chr4-11964956-11967484
chr4	11989657	11991109	chr4-11989657-11991109
chr4	12007932	12008563	chr4-12007932-12008563
chr4	12087310	12090979	chr4-12087310-12090979
chr4	12139925	12141138	chr4-12139925-12141138
chr4	12170596	12172330	chr4-12170596-12172330
chr4	12241065	12241266	chr4-12241065-12241266
chr4	12655355	12655556	chr4-12655355-12655556
chr4	12906613	12907519	chr4-12906613-12907519
chr4	13742788	13744454	chr4-13742788-13744454
chr4	13751275	13751683	chr4-13751275-13751683
chr4	13784920	13785249	chr4-13784920-13785249
chr4	14824798	14827416	chr4-14824798-14827416
chr4	14843084	14844169	chr4-14843084-14844169
chr4	14856837	14857306	chr4-14856837-14857306
chr4	14862663	14866501	chr4-14862663-14866501
chr4	14930101	14931115	chr4-14930101-14931115
chr4	15265086	15267324	chr4-15265086-15267324
chr4	15944687	15946099	chr4-15944687-15946099
chr4	15957664	15959377	chr4-15957664-15959377
chr4	16013131	16014754	chr4-16013131-16014754
chr4	16161903	16164613	chr4-16161903-16164613
chr4	16168205	16169326	chr4-16168205-16169326
chr4	17853137	17853727	chr4-17853137-17853727
chr4	19564236	19564437	chr4-19564236-19564437
chr4	19568433	19570652	chr4-19568433-19570652
chr4	19574629	19576028	chr4-19574629-19576028

chr4	19707258	19709776	chr4-19707258-19709776
chr4	20007987	20008955	chr4-20007987-20008955
chr4	20041425	20042761	chr4-20041425-20042761
chr4	20777425	20778846	chr4-20777425-20778846
chr4	21678190	21679969	chr4-21678190-21679969
chr4	21685621	21685874	chr4-21685621-21685874
chr4	21688063	21689676	chr4-21688063-21689676
chr4	21726923	21728924	chr4-21726923-21728924
chr4	21766198	21767333	chr4-21766198-21767333
chr4	21775737	21777567	chr4-21775737-21777567
chr4	21847313	21849691	chr4-21847313-21849691
chr4	21879140	21880608	chr4-21879140-21880608
chr4	21930636	21932889	chr4-21930636-21932889
chr4	22357008	22358802	chr4-22357008-22358802
chr4	22477340	22478104	chr4-22477340-22478104
chr4	22479225	22479426	chr4-22479225-22479426
chr4	22486845	22491673	chr4-22486845-22491673
chr4	22494586	22495508	chr4-22494586-22495508
chr4	22497713	22498834	chr4-22497713-22498834
chr4	23983173	23983374	chr4-23983173-23983374
chr4	24495556	24498062	chr4-24495556-24498062
chr4	24850303	24851728	chr4-24850303-24851728
chr4	24897122	24899502	chr4-24897122-24899502
chr4	24965926	24967480	chr4-24965926-24967480
chr4	25280673	25282214	chr4-25280673-25282214
chr4	25799375	25800216	chr4-25799375-25800216
chr4	26345603	26347333	chr4-26345603-26347333
chr4	27834190	27834443	chr4-27834190-27834443
chr4	28812460	28816042	chr4-28812460-28816042
chr4	28989918	28990458	chr4-28989918-28990458
chr4	31454789	31455834	chr4-31454789-31455834
chr4	31642151	31643040	chr4-31642151-31643040
chr4	31823825	31824450	chr4-31823825-31824450
chr4	31937429	31938222	chr4-31937429-31938222
chr4	31960046	31965782	chr4-31960046-31965782
chr4	32047103	32047442	chr4-32047103-32047442
chr4	32055143	32055964	chr4-32055143-32055964
chr4	32174675	32175496	chr4-32174675-32175496
chr4	32179945	32180505	chr4-32179945-32180505
chr4	32238040	32240459	chr4-32238040-32240459
chr4	32249771	32251720	chr4-32249771-32251720
chr4	32288240	32288705	chr4-32288240-32288705

chr4	32302225	32306886	chr4-32302225-32306886
chr4	32330773	32331238	chr4-32330773-32331238
chr4	32337530	32339070	chr4-32337530-32339070
chr4	32375729	32375930	chr4-32375729-32375930
chr4	32387548	32387985	chr4-32387548-32387985
chr4	32405472	32406273	chr4-32405472-32406273
chr4	32465805	32466006	chr4-32465805-32466006
chr4	32519304	32519914	chr4-32519304-32519914
chr4	32523547	32523801	chr4-32523547-32523801
chr4	32614229	32616750	chr4-32614229-32616750
chr4	32656210	32658887	chr4-32656210-32658887
chr4	32799501	32801331	chr4-32799501-32801331
chr4	32916081	32916420	chr4-32916081-32916420
chr4	32950106	32952376	chr4-32950106-32952376
chr4	32982420	32984576	chr4-32982420-32984576
chr4	33030220	33033119	chr4-33030220-33033119
chr4	33146947	33147310	chr4-33146947-33147310
chr4	33188610	33189974	chr4-33188610-33189974
chr4	33209045	33209893	chr4-33209045-33209893
chr4	33245835	33249376	chr4-33245835-33249376
chr4	33260552	33262179	chr4-33260552-33262179
chr4	33289103	33290588	chr4-33289103-33290588
chr4	33309496	33311788	chr4-33309496-33311788
chr4	33446520	33446721	chr4-33446520-33446721
chr4	33547597	33547798	chr4-33547597-33547798
chr4	33923643	33925286	chr4-33923643-33925286
chr4	34049757	34050249	chr4-34049757-34050249
chr4	34549800	34553885	chr4-34549800-34553885
chr4	34613515	34616412	chr4-34613515-34616412
chr4	34686366	34688328	chr4-34686366-34688328
chr4	34729603	34730516	chr4-34729603-34730516
chr4	34777496	34778716	chr4-34777496-34778716
chr4	34881201	34884027	chr4-34881201-34884027
chr4	34886006	34887765	chr4-34886006-34887765
chr4	35096178	35097321	chr4-35096178-35097321
chr4	35098372	35100706	chr4-35098372-35100706
chr4	35112261	35112776	chr4-35112261-35112776
chr4	35155269	35157626	chr4-35155269-35157626
chr4	35165877	35166182	chr4-35165877-35166182
chr4	35223697	35226888	chr4-35223697-35226888
chr4	36949711	36953553	chr4-36949711-36953553
chr4	39381283	39381484	chr4-39381283-39381484

chr4	40142029	40145199	chr4-40142029-40145199
chr4	40156159	40156902	chr4-40156159-40156902
chr4	40238399	40240498	chr4-40238399-40240498
chr4	40267202	40270578	chr4-40267202-40270578
chr4	40277867	40280127	chr4-40277867-40280127
chr4	40301106	40301660	chr4-40301106-40301660
chr4	40473177	40473492	chr4-40473177-40473492
chr4	40701570	40704694	chr4-40701570-40704694
chr4	40721059	40725560	chr4-40721059-40725560
chr4	40756503	40758460	chr4-40756503-40758460
chr4	40772712	40773481	chr4-40772712-40773481
chr4	40774487	40774898	chr4-40774487-40774898
chr4	40777068	40777345	chr4-40777068-40777345
chr4	40802178	40802748	chr4-40802178-40802748
chr4	40818049	40818994	chr4-40818049-40818994
chr4	40820787	40821718	chr4-40820787-40821718
chr4	40837141	40838048	chr4-40837141-40838048
chr4	40839025	40839942	chr4-40839025-40839942
chr4	40844207	40847848	chr4-40844207-40847848
chr4	40850537	40855052	chr4-40850537-40855052
chr4	40877011	40877363	chr4-40877011-40877363
chr4	40908123	40908949	chr4-40908123-40908949
chr4	40946590	40949553	chr4-40946590-40949553
chr4	40969570	40972879	chr4-40969570-40972879
chr4	41050820	41052115	chr4-41050820-41052115
chr4	41054674	41055757	chr4-41054674-41055757
chr4	41056842	41057685	chr4-41056842-41057685
chr4	41093597	41095021	chr4-41093597-41095021
chr4	41097986	41098405	chr4-41097986-41098405
chr4	41101865	41102116	chr4-41101865-41102116
chr4	41123069	41125145	chr4-41123069-41125145
chr4	41130421	41130727	chr4-41130421-41130727
chr4	41134502	41137814	chr4-41134502-41137814
chr4	41267073	41267274	chr4-41267073-41267274
chr4	41272267	41276076	chr4-41272267-41276076
chr4	41312917	41315800	chr4-41312917-41315800
chr4	41347641	41350725	chr4-41347641-41350725
chr4	41462917	41466203	chr4-41462917-41466203
chr4	41502051	41503490	chr4-41502051-41503490
chr4	41506348	41507847	chr4-41506348-41507847
chr4	41522548	41522854	chr4-41522548-41522854
chr4	41567943	41570143	chr4-41567943-41570143

chr4	41639208	41640743	chr4-41639208-41640743
chr4	41658170	41659171	chr4-41658170-41659171
chr4	41664334	41667190	chr4-41664334-41667190
chr4	41683379	41684272	chr4-41683379-41684272
chr4	41691921	41696430	chr4-41691921-41696430
chr4	41697391	41697592	chr4-41697391-41697592
chr4	41707857	41708540	chr4-41707857-41708540
chr4	41711821	41714405	chr4-41711821-41714405
chr4	41721959	41723668	chr4-41721959-41723668
chr4	41725662	41726279	chr4-41725662-41726279
chr4	41730875	41731828	chr4-41730875-41731828
chr4	41738123	41738377	chr4-41738123-41738377
chr4	41739308	41741853	chr4-41739308-41741853
chr4	41754864	41756900	chr4-41754864-41756900
chr4	41760084	41761287	chr4-41760084-41761287
chr4	41772860	41774843	chr4-41772860-41774843
chr4	42734575	42735243	chr4-42734575-42735243
chr4	42766682	42767041	chr4-42766682-42767041
chr4	42916593	42917821	chr4-42916593-42917821
chr4	42933556	42934177	chr4-42933556-42934177
chr4	42949036	42954006	chr4-42949036-42954006
chr4	42997718	43001040	chr4-42997718-43001040
chr4	43009473	43011074	chr4-43009473-43011074
chr4	43024456	43026221	chr4-43024456-43026221
chr4	43029878	43032105	chr4-43029878-43032105
chr4	43038662	43041214	chr4-43038662-43041214
chr4	43043944	43046701	chr4-43043944-43046701
chr4	43058410	43060530	chr4-43058410-43060530
chr4	43266785	43267299	chr4-43266785-43267299
chr4	43380366	43387803	chr4-43380366-43387803
chr4	43393876	43396105	chr4-43393876-43396105
chr4	43397922	43400573	chr4-43397922-43400573
chr4	43402164	43402471	chr4-43402164-43402471
chr4	43417911	43418608	chr4-43417911-43418608
chr4	43419429	43420484	chr4-43419429-43420484
chr4	43441890	43444816	chr4-43441890-43444816
chr4	43453180	43453895	chr4-43453180-43453895
chr4	43478319	43478778	chr4-43478319-43478778
chr4	43492440	43494656	chr4-43492440-43494656
chr4	43498291	43500091	chr4-43498291-43500091
chr4	43507008	43509376	chr4-43507008-43509376
chr4	43519564	43524716	chr4-43519564-43524716

chr4	43557248	43564441	chr4-43557248-43564441
chr4	43576562	43580395	chr4-43576562-43580395
chr4	43604535	43605504	chr4-43604535-43605504
chr4	43630077	43632417	chr4-43630077-43632417
chr4	43655576	43657513	chr4-43655576-43657513
chr4	43668693	43670154	chr4-43668693-43670154
chr4	43727267	43729286	chr4-43727267-43729286
chr4	43874890	43876473	chr4-43874890-43876473
chr4	43957177	43958780	chr4-43957177-43958780
chr4	43965220	43965421	chr4-43965220-43965421
chr4	43972076	43972812	chr4-43972076-43972812
chr4	43982160	43982617	chr4-43982160-43982617
chr4	44011605	44014602	chr4-44011605-44014602
chr4	44062298	44062651	chr4-44062298-44062651
chr4	44065411	44067117	chr4-44065411-44067117
chr4	44068706	44069012	chr4-44068706-44069012
chr4	44070706	44073609	chr4-44070706-44073609
chr4	44082788	44084948	chr4-44082788-44084948
chr4	44165117	44168998	chr4-44165117-44168998
chr4	44204783	44205674	chr4-44204783-44205674
chr4	44232473	44235137	chr4-44232473-44235137
chr4	44249795	44250213	chr4-44249795-44250213
chr4	44300490	44302759	chr4-44300490-44302759
chr4	44424952	44425847	chr4-44424952-44425847
chr4	44663962	44664820	chr4-44663962-44664820
chr4	44703106	44704923	chr4-44703106-44704923
chr4	44755556	44757982	chr4-44755556-44757982
chr4	44973565	44974144	chr4-44973565-44974144
chr4	44980930	44983534	chr4-44980930-44983534
chr4	45010196	45013952	chr4-45010196-45013952
chr4	45018033	45020410	chr4-45018033-45020410
chr4	45083576	45085401	chr4-45083576-45085401
chr4	45106037	45108626	chr4-45106037-45108626
chr4	45138471	45138672	chr4-45138471-45138672
chr4	45184178	45185754	chr4-45184178-45185754
chr4	45195977	45196178	chr4-45195977-45196178
chr4	45296784	45298121	chr4-45296784-45298121
chr4	45319142	45321174	chr4-45319142-45321174
chr4	45341490	45344367	chr4-45341490-45344367
chr4	45406883	45409643	chr4-45406883-45409643
chr4	45420706	45420907	chr4-45420706-45420907
chr4	45452490	45455802	chr4-45452490-45455802

chr4	45457874	45458250	chr4-45457874-45458250
chr4	45459589	45460053	chr4-45459589-45460053
chr4	45472502	45473821	chr4-45472502-45473821
chr4	45474624	45476125	chr4-45474624-45476125
chr4	45481942	45482917	chr4-45481942-45482917
chr4	45484016	45484272	chr4-45484016-45484272
chr4	45485450	45496095	chr4-45485450-45496095
chr4	45499479	45502692	chr4-45499479-45502692
chr4	45506624	45508361	chr4-45506624-45508361
chr4	45509315	45511696	chr4-45509315-45511696
chr4	45512758	45515333	chr4-45512758-45515333
chr4	45517938	45518478	chr4-45517938-45518478
chr4	45520344	45522118	chr4-45520344-45522118
chr4	45523453	45523654	chr4-45523453-45523654
chr4	45526500	45532711	chr4-45526500-45532711
chr4	45585148	45586639	chr4-45585148-45586639
chr4	45683086	45683382	chr4-45683086-45683382
chr4	45763953	45764276	chr4-45763953-45764276
chr4	45798787	45799605	chr4-45798787-45799605
chr4	45818951	45821273	chr4-45818951-45821273
chr4	45826288	45827060	chr4-45826288-45827060
chr4	45851650	45851966	chr4-45851650-45851966
chr4	45941732	45941933	chr4-45941732-45941933
chr4	45947742	45949736	chr4-45947742-45949736
chr4	45964304	45968124	chr4-45964304-45968124
chr4	45971730	45973489	chr4-45971730-45973489
chr4	46038714	46040124	chr4-46038714-46040124
chr4	46087948	46088312	chr4-46087948-46088312
chr4	46137253	46140998	chr4-46137253-46140998
chr4	46195472	46196961	chr4-46195472-46196961
chr4	46202615	46202927	chr4-46202615-46202927
chr4	46291478	46292191	chr4-46291478-46292191
chr4	46303584	46305932	chr4-46303584-46305932
chr4	46342519	46345569	chr4-46342519-46345569
chr4	46370415	46372025	chr4-46370415-46372025
chr4	46374866	46375237	chr4-46374866-46375237
chr4	46388256	46390995	chr4-46388256-46390995
chr4	46426964	46427520	chr4-46426964-46427520
chr4	46450215	46455616	chr4-46450215-46455616
chr4	46485546	46486205	chr4-46485546-46486205
chr4	46488797	46490835	chr4-46488797-46490835
chr4	46506556	46508262	chr4-46506556-46508262

chr4	46527327	46528110	chr4-46527327-46528110
chr4	46534255	46536591	chr4-46534255-46536591
chr4	46553740	46554694	chr4-46553740-46554694
chr4	46563633	46564749	chr4-46563633-46564749
chr4	46600323	46602517	chr4-46600323-46602517
chr4	46622237	46624009	chr4-46622237-46624009
chr4	46627272	46630839	chr4-46627272-46630839
chr4	46633160	46636298	chr4-46633160-46636298
chr4	46639255	46639997	chr4-46639255-46639997
chr4	46641598	46641909	chr4-46641598-46641909
chr4	46642840	46644213	chr4-46642840-46644213
chr4	46645950	46651304	chr4-46645950-46651304
chr4	46664243	46665654	chr4-46664243-46665654
chr4	46990562	46992344	chr4-46990562-46992344
chr4	47015020	47015776	chr4-47015020-47015776
chr4	47056525	47058038	chr4-47056525-47058038
chr4	47091476	47093160	chr4-47091476-47093160
chr4	47151886	47152202	chr4-47151886-47152202
chr4	47207965	47208791	chr4-47207965-47208791
chr4	47293695	47294557	chr4-47293695-47294557
chr4	47309943	47310839	chr4-47309943-47310839
chr4	47328773	47329629	chr4-47328773-47329629
chr4	47351744	47354591	chr4-47351744-47354591
chr4	47437249	47438433	chr4-47437249-47438433
chr4	47473164	47475740	chr4-47473164-47475740
chr4	47604015	47604216	chr4-47604015-47604216
chr4	48042439	48048345	chr4-48042439-48048345
chr4	48051637	48052068	chr4-48051637-48052068
chr4	48124260	48125861	chr4-48124260-48125861
chr4	48278443	48280680	chr4-48278443-48280680
chr4	48471019	48473902	chr4-48471019-48473902
chr4	48488853	48489054	chr4-48488853-48489054
chr4	48491259	48491673	chr4-48491259-48491673
chr4	48539573	48542873	chr4-48539573-48542873
chr4	48584400	48587018	chr4-48584400-48587018
chr4	49059019	49059857	chr4-49059019-49059857
chr4	49478539	49478791	chr4-49478539-49478791
chr4	49480302	49480985	chr4-49480302-49480985
chr4	49518716	49522244	chr4-49518716-49522244
chr4	49596718	49598140	chr4-49596718-49598140
chr4	49631043	49633090	chr4-49631043-49633090
chr4	49845242	49845503	chr4-49845242-49845503

chr4	52438201	52440543	chr4-52438201-52440543
chr4	53011338	53012925	chr4-53011338-53012925
chr4	53158678	53160314	chr4-53158678-53160314
chr4	53195997	53196794	chr4-53195997-53196794
chr4	53217469	53218437	chr4-53217469-53218437
chr4	53384809	53385316	chr4-53384809-53385316
chr4	53439213	53441819	chr4-53439213-53441819
chr4	53446043	53446646	chr4-53446043-53446646
chr4	53451966	53452167	chr4-53451966-53452167
chr4	53630896	53632416	chr4-53630896-53632416
chr4	53713543	53715347	chr4-53713543-53715347
chr4	53779136	53780089	chr4-53779136-53780089
chr4	53825694	53827150	chr4-53825694-53827150
chr4	53931532	53932046	chr4-53931532-53932046
chr4	54944440	54950110	chr4-54944440-54950110
chr4	54980228	54980615	chr4-54980228-54980615
chr4	55254124	55254325	chr4-55254124-55254325
chr4	55294238	55294682	chr4-55294238-55294682
chr4	55325634	55325996	chr4-55325634-55325996
chr4	55348392	55353448	chr4-55348392-55353448
chr4	55480213	55480914	chr4-55480213-55480914
chr4	55489382	55489638	chr4-55489382-55489638
chr4	55514517	55515471	chr4-55514517-55515471
chr4	55529387	55534480	chr4-55529387-55534480
chr4	55650516	55652424	chr4-55650516-55652424
chr4	55699969	55700170	chr4-55699969-55700170
chr4	55731847	55732101	chr4-55731847-55732101
chr4	55733200	55733401	chr4-55733200-55733401
chr4	56113857	56114212	chr4-56113857-56114212
chr4	56305709	56306204	chr4-56305709-56306204
chr4	56374004	56374858	chr4-56374004-56374858
chr4	56421676	56422050	chr4-56421676-56422050
chr4	56483862	56484166	chr4-56483862-56484166
chr4	56532564	56534279	chr4-56532564-56534279
chr4	56638713	56639367	chr4-56638713-56639367
chr4	56668443	56669490	chr4-56668443-56669490
chr4	56685328	56685632	chr4-56685328-56685632
chr4	56767659	56769242	chr4-56767659-56769242
chr4	56800453	56803939	chr4-56800453-56803939
chr4	56845674	56846373	chr4-56845674-56846373
chr4	56863028	56865645	chr4-56863028-56865645
chr4	56936727	56939067	chr4-56936727-56939067

chr4	56940344	56941223	chr4-56940344-56941223
chr4	56943060	56948394	chr4-56943060-56948394
chr4	56989799	56990671	chr4-56989799-56990671
chr4	57138962	57140244	chr4-57138962-57140244
chr4	57141387	57144200	chr4-57141387-57144200
chr4	57153275	57154382	chr4-57153275-57154382
chr4	57156534	57157142	chr4-57156534-57157142
chr4	57201845	57203501	chr4-57201845-57203501
chr4	57278494	57279700	chr4-57278494-57279700
chr4	57281855	57283457	chr4-57281855-57283457
chr4	57285104	57285305	chr4-57285104-57285305
chr4	57294854	57295659	chr4-57294854-57295659
chr4	57298679	57302620	chr4-57298679-57302620
chr4	57308258	57309081	chr4-57308258-57309081
chr4	57353429	57353732	chr4-57353429-57353732
chr4	57433739	57435208	chr4-57433739-57435208
chr4	57543656	57544429	chr4-57543656-57544429
chr4	57567596	57568575	chr4-57567596-57568575
chr4	57637114	57638892	chr4-57637114-57638892
chr4	57639919	57640685	chr4-57639919-57640685
chr4	57652483	57652684	chr4-57652483-57652684
chr4	57655818	57656782	chr4-57655818-57656782
chr4	57673514	57673715	chr4-57673514-57673715
chr4	57675369	57675678	chr4-57675369-57675678
chr4	57735299	57736121	chr4-57735299-57736121
chr4	57742995	57743450	chr4-57742995-57743450
chr4	57757974	57758175	chr4-57757974-57758175
chr4	57781878	57784225	chr4-57781878-57784225
chr4	57797595	57799018	chr4-57797595-57799018
chr4	57804709	57805438	chr4-57804709-57805438
chr4	57844887	57846002	chr4-57844887-57846002
chr4	57904369	57904940	chr4-57904369-57904940
chr4	57915580	57915781	chr4-57915580-57915781
chr4	57952617	57958359	chr4-57952617-57958359
chr4	58034659	58035041	chr4-58034659-58035041
chr4	58205889	58206552	chr4-58205889-58206552
chr4	58552029	58554170	chr4-58552029-58554170
chr4	58714195	58714615	chr4-58714195-58714615
chr4	58745707	58746136	chr4-58745707-58746136
chr4	58910458	58913357	chr4-58910458-58913357
chr4	58943181	58944306	chr4-58943181-58944306
chr4	58986101	58987326	chr4-58986101-58987326

chr4	59002568	59004940	chr4-59002568-59004940
chr4	59034463	59036309	chr4-59034463-59036309
chr4	59188579	59196167	chr4-59188579-59196167
chr4	59199352	59200127	chr4-59199352-59200127
chr4	59204082	59204352	chr4-59204082-59204352
chr4	59223197	59223500	chr4-59223197-59223500
chr4	59238889	59240609	chr4-59238889-59240609
chr4	59315219	59315983	chr4-59315219-59315983
chr4	59318722	59320536	chr4-59318722-59320536
chr4	59330592	59332410	chr4-59330592-59332410
chr4	59363083	59363284	chr4-59363083-59363284
chr4	59366039	59366349	chr4-59366039-59366349
chr4	59387568	59388779	chr4-59387568-59388779
chr4	59399249	59399711	chr4-59399249-59399711
chr4	59422161	59422443	chr4-59422161-59422443
chr4	59433945	59434488	chr4-59433945-59434488
chr4	59437284	59439369	chr4-59437284-59439369
chr4	59449652	59451875	chr4-59449652-59451875
chr4	59457434	59458474	chr4-59457434-59458474
chr4	59546405	59550361	chr4-59546405-59550361
chr4	59580157	59582556	chr4-59580157-59582556
chr4	59625307	59627269	chr4-59625307-59627269
chr4	59781694	59784637	chr4-59781694-59784637
chr4	59805162	59809016	chr4-59805162-59809016
chr4	59814158	59815169	chr4-59814158-59815169
chr4	59818064	59820089	chr4-59818064-59820089
chr4	59830698	59831513	chr4-59830698-59831513
chr4	59895007	59895310	chr4-59895007-59895310
chr4	59914787	59915196	chr4-59914787-59915196
chr4	62207507	62209437	chr4-62207507-62209437
chr4	62286085	62287971	chr4-62286085-62287971
chr4	62359370	62361898	chr4-62359370-62361898
chr4	62406879	62410371	chr4-62406879-62410371
chr4	62433766	62435368	chr4-62433766-62435368
chr4	62470234	62471447	chr4-62470234-62471447
chr4	62479354	62481919	chr4-62479354-62481919
chr4	62484839	62485145	chr4-62484839-62485145
chr4	62501376	62502957	chr4-62501376-62502957
chr4	62516654	62520553	chr4-62516654-62520553
chr4	62524021	62525884	chr4-62524021-62525884
chr4	62582680	62586189	chr4-62582680-62586189
chr4	62596339	62596680	chr4-62596339-62596680

chr4	62637622	62638742	chr4-62637622-62638742
chr4	62646819	62647208	chr4-62646819-62647208
chr4	62653930	62655157	chr4-62653930-62655157
chr4	62673535	62676527	chr4-62673535-62676527
chr4	62684955	62685156	chr4-62684955-62685156
chr4	62686526	62686844	chr4-62686526-62686844
chr4	62699251	62701029	chr4-62699251-62701029
chr4	62705267	62705518	chr4-62705267-62705518
chr4	62732332	62733492	chr4-62732332-62733492
chr4	62738065	62738818	chr4-62738065-62738818
chr4	62752997	62753389	chr4-62752997-62753389
chr4	62757170	62757547	chr4-62757170-62757547
chr4	62780525	62781136	chr4-62780525-62781136
chr4	62964972	62966278	chr4-62964972-62966278
chr4	63052088	63052289	chr4-63052088-63052289
chr4	63213811	63216416	chr4-63213811-63216416
chr4	63253967	63254287	chr4-63253967-63254287
chr4	63316091	63316693	chr4-63316091-63316693
chr4	63402338	63403837	chr4-63402338-63403837
chr4	63408319	63411098	chr4-63408319-63411098
chr4	63493496	63496744	chr4-63493496-63496744
chr4	63544071	63547016	chr4-63544071-63547016
chr4	63556626	63560346	chr4-63556626-63560346
chr4	63744621	63745263	chr4-63744621-63745263
chr4	64044379	64047530	chr4-64044379-64047530
chr4	64076713	64077023	chr4-64076713-64077023
chr4	64078408	64078660	chr4-64078408-64078660
chr4	64081941	64084927	chr4-64081941-64084927
chr4	64098242	64098737	chr4-64098242-64098737
chr4	64635526	64635727	chr4-64635526-64635727
chr4	64803953	64804766	chr4-64803953-64804766
chr4	65123605	65127165	chr4-65123605-65127165
chr4	65604435	65605986	chr4-65604435-65605986
chr4	66402760	66404765	chr4-66402760-66404765
chr4	66827181	66830120	chr4-66827181-66830120
chr4	70409082	70411555	chr4-70409082-70411555
chr4	70533633	70536329	chr4-70533633-70536329
chr4	72197966	72202376	chr4-72197966-72202376
chr4	73789011	73791478	chr4-73789011-73791478
chr4	74013274	74013999	chr4-74013274-74013999
chr4	74251066	74253414	chr4-74251066-74253414
chr4	75277444	75278555	chr4-75277444-75278555

chr4	76449132	76450767	chr4-76449132-76450767
chr4	77310165	77311008	chr4-77310165-77311008
chr4	78211436	78212673	chr4-78211436-78212673
chr4	80864567	80865000	chr4-80864567-80865000
chr4	80910348	80914283	chr4-80910348-80914283
chr4	80949943	80950875	chr4-80949943-80950875
chr4	81441850	81443413	chr4-81441850-81443413
chr4	81617220	81617700	chr4-81617220-81617700
chr4	82065469	82066998	chr4-82065469-82066998
chr4	82163991	82164246	chr4-82163991-82164246
chr4	82166500	82167710	chr4-82166500-82167710
chr4	82376394	82376997	chr4-82376394-82376997
chr4	82386941	82387142	chr4-82386941-82387142
chr4	82399744	82401557	chr4-82399744-82401557
chr4	82427670	82427983	chr4-82427670-82427983
chr4	82435523	82436404	chr4-82435523-82436404
chr4	82447008	82447919	chr4-82447008-82447919
chr4	82466210	82467038	chr4-82466210-82467038
chr4	82471120	82471744	chr4-82471120-82471744
chr4	82499779	82514600	chr4-82499779-82514600
chr4	82848590	82849093	chr4-82848590-82849093
chr4	82850534	82850835	chr4-82850534-82850835
chr4	82858280	82860261	chr4-82858280-82860261
chr4	82933688	82934242	chr4-82933688-82934242
chr4	83301710	83303740	chr4-83301710-83303740
chr4	83307647	83309703	chr4-83307647-83309703
chr4	83314748	83316651	chr4-83314748-83316651
chr4	83322478	83324848	chr4-83322478-83324848
chr4	83379275	83380928	chr4-83379275-83380928
chr4	83416593	83419318	chr4-83416593-83419318
chr4	83439049	83439354	chr4-83439049-83439354
chr4	83485070	83487240	chr4-83485070-83487240
chr4	83524406	83526876	chr4-83524406-83526876
chr4	83567287	83568584	chr4-83567287-83568584
chr4	84523351	84523552	chr4-84523351-84523552
chr4	84545218	84546078	chr4-84545218-84546078
chr4	84674341	84676171	chr4-84674341-84676171
chr4	84883946	84885209	chr4-84883946-84885209
chr4	85204955	85206429	chr4-85204955-85206429
chr4	85226689	85227002	chr4-85226689-85227002
chr4	85513851	85515125	chr4-85513851-85515125
chr4	86053816	86054067	chr4-86053816-86054067

chr4	86574788	86578339	chr4-86574788-86578339
chr4	86610169	86612975	chr4-86610169-86612975
chr4	86665944	86672728	chr4-86665944-86672728
chr4	86674419	86675068	chr4-86674419-86675068
chr4	86689888	86690798	chr4-86689888-86690798
chr4	86712661	86713812	chr4-86712661-86713812
chr4	86725419	86725620	chr4-86725419-86725620
chr4	86747807	86751257	chr4-86747807-86751257
chr4	86855534	86858626	chr4-86855534-86858626
chr4	86873780	86875107	chr4-86873780-86875107
chr4	86930335	86932047	chr4-86930335-86932047
chr4	87229841	87230729	chr4-87229841-87230729
chr4	87368262	87368998	chr4-87368262-87368998
chr4	87850225	87850508	chr4-87850225-87850508
chr4	88030944	88035637	chr4-88030944-88035637
chr4	88079231	88080044	chr4-88079231-88080044
chr4	88081425	88081676	chr4-88081425-88081676
chr4	88083894	88084394	chr4-88083894-88084394
chr4	88093923	88095561	chr4-88093923-88095561
chr4	88412605	88412910	chr4-88412605-88412910
chr4	88438376	88439143	chr4-88438376-88439143
chr4	88477076	88477277	chr4-88477076-88477277
chr4	88515369	88516272	chr4-88515369-88516272
chr4	88529940	88530455	chr4-88529940-88530455
chr4	88537658	88537961	chr4-88537658-88537961
chr4	88550127	88551006	chr4-88550127-88551006
chr4	88640477	88641341	chr4-88640477-88641341
chr4	88717773	88724302	chr4-88717773-88724302
chr4	88728357	88728916	chr4-88728357-88728916
chr4	88756503	88758262	chr4-88756503-88758262
chr4	88759512	88761110	chr4-88759512-88761110
chr4	88762481	88763991	chr4-88762481-88763991
chr4	88772735	88773682	chr4-88772735-88773682
chr4	88781646	88782672	chr4-88781646-88782672
chr4	88785811	88786012	chr4-88785811-88786012
chr4	88794026	88794277	chr4-88794026-88794277
chr4	88799234	88799694	chr4-88799234-88799694
chr4	88802151	88802607	chr4-88802151-88802607
chr4	88805775	88806127	chr4-88805775-88806127
chr4	88867489	88868777	chr4-88867489-88868777
chr4	88926337	88939830	chr4-88926337-88939830
chr4	88948353	88952256	chr4-88948353-88952256

chr4	88954270	88960551	chr4-88954270-88960551
chr4	89038148	89038721	chr4-89038148-89038721
chr4	89040260	89041334	chr4-89040260-89041334
chr4	89048416	89058810	chr4-89048416-89058810
chr4	89059798	89059999	chr4-89059798-89059999
chr4	89061263	89061464	chr4-89061263-89061464
chr4	89062603	89062901	chr4-89062603-89062901
chr4	89070071	89071249	chr4-89070071-89071249
chr4	89123007	89129751	chr4-89123007-89129751
chr4	89136500	89141208	chr4-89136500-89141208
chr4	89145671	89146026	chr4-89145671-89146026
chr4	89162848	89163244	chr4-89162848-89163244
chr4	89281871	89282398	chr4-89281871-89282398
chr4	89289321	89289522	chr4-89289321-89289522
chr4	89290491	89297258	chr4-89290491-89297258
chr4	89306768	89311492	chr4-89306768-89311492
chr4	89348705	89349644	chr4-89348705-89349644
chr4	89359586	89360428	chr4-89359586-89360428
chr4	89402427	89402685	chr4-89402427-89402685
chr4	89427527	89429090	chr4-89427527-89429090
chr4	89443224	89443425	chr4-89443224-89443425
chr4	89474891	89475289	chr4-89474891-89475289
chr4	89477514	89481291	chr4-89477514-89481291
chr4	89482133	89483063	chr4-89482133-89483063
chr4	89486056	89486912	chr4-89486056-89486912
chr4	89508263	89508673	chr4-89508263-89508673
chr4	89523333	89525242	chr4-89523333-89525242
chr4	89687853	89689394	chr4-89687853-89689394
chr4	89875912	89876453	chr4-89875912-89876453
chr4	91371219	91372350	chr4-91371219-91372350
chr4	91374501	91376938	chr4-91374501-91376938
chr4	91400490	91400804	chr4-91400490-91400804
chr4	93334398	93336038	chr4-93334398-93336038
chr4	93879433	93880093	chr4-93879433-93880093
chr4	94555066	94557374	chr4-94555066-94557374
chr4	94601439	94604242	chr4-94601439-94604242
chr4	94613883	94615311	chr4-94613883-94615311
chr4	94752394	94752862	chr4-94752394-94752862
chr4	94977719	94980010	chr4-94977719-94980010
chr4	95025801	95026156	chr4-95025801-95026156
chr4	95049123	95054398	chr4-95049123-95054398
chr4	95075769	95076096	chr4-95075769-95076096

chr4	95082198	95083051	chr4-95082198-95083051
chr4	95094533	95094954	chr4-95094533-95094954
chr4	95105210	95106717	chr4-95105210-95106717
chr4	95160862	95162124	chr4-95160862-95162124
chr4	95556957	95558276	chr4-95556957-95558276
chr4	95966521	95968505	chr4-95966521-95968505
chr4	96553115	96553737	chr4-96553115-96553737
chr4	97745685	97746537	chr4-97745685-97746537
chr4	97751070	97752368	chr4-97751070-97752368
chr4	97757104	97758339	chr4-97757104-97758339
chr4	97771431	97773433	chr4-97771431-97773433
chr4	97775034	97779206	chr4-97775034-97779206
chr4	97868393	97869769	chr4-97868393-97869769
chr4	97920526	97921009	chr4-97920526-97921009
chr4	98003758	98004706	chr4-98003758-98004706
chr4	98321733	98322678	chr4-98321733-98322678
chr4	98382129	98383855	chr4-98382129-98383855
chr4	98395285	98398190	chr4-98395285-98398190
chr4	98400804	98402115	chr4-98400804-98402115
chr4	98726838	98727039	chr4-98726838-98727039
chr4	98789547	98790368	chr4-98789547-98790368
chr4	98816841	98818102	chr4-98816841-98818102
chr4	98922038	98926279	chr4-98922038-98926279
chr4	99091527	99091938	chr4-99091527-99091938
chr4	99115844	99116807	chr4-99115844-99116807
chr4	99119088	99121652	chr4-99119088-99121652
chr4	99160006	99164248	chr4-99160006-99164248
chr4	99193416	99195200	chr4-99193416-99195200
chr4	99199917	99200336	chr4-99199917-99200336
chr4	99626110	99626417	chr4-99626110-99626417
chr4	99650523	99651661	chr4-99650523-99651661
chr4	99653735	99654004	chr4-99653735-99654004
chr4	99655727	99658284	chr4-99655727-99658284
chr4	99660236	99661680	chr4-99660236-99661680
chr4	99715045	99716513	chr4-99715045-99716513
chr4	99828453	99830370	chr4-99828453-99830370
chr4	99928744	99931678	chr4-99928744-99931678
chr4	99942168	99942648	chr4-99942168-99942648
chr4	99953920	99954397	chr4-99953920-99954397
chr4	100044921	100045789	chr4-100044921-100045789
chr4	100095495	100097429	chr4-100095495-100097429
chr4	100216422	100216723	chr4-100216422-100216723

chr4	100386258	100386534	chr4-100386258-100386534
chr4	100390235	100391407	chr4-100390235-100391407
chr4	100401762	100402479	chr4-100401762-100402479
chr4	100478706	100479115	chr4-100478706-100479115
chr4	100527579	100527986	chr4-100527579-100527986
chr4	100776149	100778128	chr4-100776149-100778128
chr4	101068655	101069922	chr4-101068655-101069922
chr4	101202318	101202778	chr4-101202318-101202778
chr4	101263831	101265961	chr4-101263831-101265961
chr4	101291971	101293527	chr4-101291971-101293527
chr4	101354947	101357946	chr4-101354947-101357946
chr4	101418839	101421238	chr4-101418839-101421238
chr4	101496175	101497756	chr4-101496175-101497756
chr4	101507864	101509348	chr4-101507864-101509348
chr4	101550621	101550922	chr4-101550621-101550922
chr4	101646576	101648883	chr4-101646576-101648883
chr4	101717233	101718196	chr4-101717233-101718196
chr4	102087228	102088551	chr4-102087228-102088551
chr4	102511522	102511878	chr4-102511522-102511878
chr4	102569924	102571386	chr4-102569924-102571386
chr4	102759072	102760502	chr4-102759072-102760502
chr4	103112643	103115736	chr4-103112643-103115736
chr4	103118806	103121440	chr4-103118806-103121440
chr4	103122848	103123447	chr4-103122848-103123447
chr4	103213572	103215931	chr4-103213572-103215931
chr4	103249670	103250174	chr4-103249670-103250174
chr4	103263571	103264312	chr4-103263571-103264312
chr4	103265364	103265671	chr4-103265364-103265671
chr4	103313423	103314928	chr4-103313423-103314928
chr4	103347131	103347438	chr4-103347131-103347438
chr4	104016007	104016318	chr4-104016007-104016318
chr4	104367021	104367953	chr4-104367021-104367953
chr4	104913154	104913888	chr4-104913154-104913888
chr4	104988332	104988533	chr4-104988332-104988533
chr4	105108884	105110794	chr4-105108884-105110794
chr4	105156650	105159384	chr4-105156650-105159384
chr4	105403062	105404329	chr4-105403062-105404329
chr4	105937366	105937683	chr4-105937366-105937683
chr4	106281115	106282415	chr4-106281115-106282415
chr4	106315302	106318544	chr4-106315302-106318544
chr4	106461598	106465676	chr4-106461598-106465676
chr4	106529595	106530575	chr4-106529595-106530575

chr4	106537611	106538266	chr4-106537611-106538266
chr4	106550740	106550941	chr4-106550740-106550941
chr4	106560040	106564778	chr4-106560040-106564778
chr4	106613878	106614402	chr4-106613878-106614402
chr4	106616882	106617398	chr4-106616882-106617398
chr4	106621928	106623196	chr4-106621928-106623196
chr4	106628407	106629657	chr4-106628407-106629657
chr4	106650644	106652207	chr4-106650644-106652207
chr4	106677898	106679205	chr4-106677898-106679205
chr4	106708250	106708451	chr4-106708250-106708451
chr4	106799753	106800008	chr4-106799753-106800008
chr4	106804479	106805338	chr4-106804479-106805338
chr4	106827540	106828759	chr4-106827540-106828759
chr4	106837863	106841745	chr4-106837863-106841745
chr4	106847166	106849049	chr4-106847166-106849049
chr4	106858043	106860389	chr4-106858043-106860389
chr4	106868577	106870829	chr4-106868577-106870829
chr4	106908985	106913001	chr4-106908985-106913001
chr4	106980647	106981001	chr4-106980647-106981001
chr4	106987576	106988352	chr4-106987576-106988352
chr4	107012081	107012449	chr4-107012081-107012449
chr4	107065474	107067983	chr4-107065474-107067983
chr4	107108548	107108799	chr4-107108548-107108799
chr4	107118289	107119541	chr4-107118289-107119541
chr4	107177294	107180842	chr4-107177294-107180842
chr4	107251956	107254813	chr4-107251956-107254813
chr4	107313538	107315386	chr4-107313538-107315386
chr4	107367284	107369215	chr4-107367284-107369215
chr4	107433902	107435893	chr4-107433902-107435893
chr4	107452969	107453659	chr4-107452969-107453659
chr4	107458825	107459462	chr4-107458825-107459462
chr4	107467914	107469145	chr4-107467914-107469145
chr4	107488404	107489788	chr4-107488404-107489788
chr4	107553945	107554146	chr4-107553945-107554146
chr4	107565586	107565787	chr4-107565586-107565787
chr4	107602454	107603410	chr4-107602454-107603410
chr4	107652718	107653134	chr4-107652718-107653134
chr4	107719081	107719282	chr4-107719081-107719282
chr4	107801370	107805025	chr4-107801370-107805025
chr4	107878786	107880929	chr4-107878786-107880929
chr4	107888768	107890944	chr4-107888768-107890944
chr4	107921905	107924193	chr4-107921905-107924193

chr4	108025411	108025713	chr4-108025411-108025713
chr4	108031017	108032467	chr4-108031017-108032467
chr4	108070387	108071861	chr4-108070387-108071861
chr4	108117869	108118923	chr4-108117869-108118923
chr4	108164952	108166446	chr4-108164952-108166446
chr4	108217220	108218309	chr4-108217220-108218309
chr4	108299781	108301495	chr4-108299781-108301495
chr4	108327199	108329266	chr4-108327199-108329266
chr4	108346735	108347816	chr4-108346735-108347816
chr4	108350713	108351193	chr4-108350713-108351193
chr4	108383117	108383826	chr4-108383117-108383826
chr4	108387445	108388351	chr4-108387445-108388351
chr4	108406245	108406928	chr4-108406245-108406928
chr4	108458726	108461489	chr4-108458726-108461489
chr4	108577733	108581215	chr4-108577733-108581215
chr4	108618622	108621281	chr4-108618622-108621281
chr4	108679134	108679335	chr4-108679134-108679335
chr4	108779465	108781290	chr4-108779465-108781290
chr4	108830945	108836325	chr4-108830945-108836325
chr4	108847037	108850253	chr4-108847037-108850253
chr4	108878803	108879865	chr4-108878803-108879865
chr4	108887246	108888042	chr4-108887246-108888042
chr4	108959646	108959902	chr4-108959646-108959902
chr4	108999565	109002322	chr4-108999565-109002322
chr4	109116281	109118781	chr4-109116281-109118781
chr4	109136200	109137761	chr4-109136200-109137761
chr4	109147187	109148041	chr4-109147187-109148041
chr4	109200807	109203327	chr4-109200807-109203327
chr4	109204885	109205136	chr4-109204885-109205136
chr4	109211594	109212856	chr4-109211594-109212856
chr4	109214082	109216113	chr4-109214082-109216113
chr4	109279555	109283034	chr4-109279555-109283034
chr4	109394111	109394312	chr4-109394111-109394312
chr4	109405828	109408004	chr4-109405828-109408004
chr4	109420253	109420690	chr4-109420253-109420690
chr4	109430451	109431019	chr4-109430451-109431019
chr4	109432747	109434187	chr4-109432747-109434187
chr4	109460159	109460515	chr4-109460159-109460515
chr4	109474242	109477330	chr4-109474242-109477330
chr4	109636535	109636856	chr4-109636535-109636856
chr4	109656828	109658168	chr4-109656828-109658168
chr4	109663709	109667869	chr4-109663709-109667869

chr4	109675790	109678376	chr4-109675790-109678376
chr4	109976883	109980509	chr4-109976883-109980509
chr4	109981590	109984445	chr4-109981590-109984445
chr4	110050717	110051538	chr4-110050717-110051538
chr4	110350864	110352157	chr4-110350864-110352157
chr4	110397502	110398103	chr4-110397502-110398103
chr4	111414704	111415620	chr4-111414704-111415620
chr4	111597284	111597708	chr4-111597284-111597708
chr4	111605404	111605605	chr4-111605404-111605605
chr4	111719529	111721482	chr4-111719529-111721482
chr4	111886133	111886388	chr4-111886133-111886388
chr4	114405712	114407068	chr4-114405712-114407068
chr4	114665881	114667018	chr4-114665881-114667018
chr4	114821501	114822219	chr4-114821501-114822219
chr4	114843216	114843759	chr4-114843216-114843759
chr4	114901984	114902439	chr4-114901984-114902439
chr4	114907184	114909998	chr4-114907184-114909998
chr4	114924870	114926220	chr4-114924870-114926220
chr4	114958272	114959313	chr4-114958272-114959313
chr4	114985752	114988213	chr4-114985752-114988213
chr4	114999292	115002185	chr4-114999292-115002185
chr4	115052432	115053112	chr4-115052432-115053112
chr4	115056366	115058120	chr4-115056366-115058120
chr4	115062859	115063913	chr4-115062859-115063913
chr4	115088496	115089333	chr4-115088496-115089333
chr4	115095555	115097382	chr4-115095555-115097382
chr4	115280937	115281439	chr4-115280937-115281439
chr4	115657204	115657716	chr4-115657204-115657716
chr4	115710037	115710289	chr4-115710037-115710289
chr4	115737111	115739505	chr4-115737111-115739505
chr4	115784239	115785996	chr4-115784239-115785996
chr4	115827496	115829115	chr4-115827496-115829115
chr4	115831961	115832652	chr4-115831961-115832652
chr4	115838242	115840335	chr4-115838242-115840335
chr4	115890356	115890924	chr4-115890356-115890924
chr4	115897895	115898669	chr4-115897895-115898669
chr4	115936287	115936625	chr4-115936287-115936625
chr4	115938944	115940321	chr4-115938944-115940321
chr4	115943951	115944260	chr4-115943951-115944260
chr4	115962493	115963619	chr4-115962493-115963619
chr4	115979372	115979935	chr4-115979372-115979935
chr4	116016586	116018373	chr4-116016586-116018373

chr4	116052658	116054594	chr4-116052658-116054594
chr4	116073061	116077796	chr4-116073061-116077796
chr4	116122478	116124880	chr4-116122478-116124880
chr4	116143308	116145042	chr4-116143308-116145042
chr4	116149513	116152286	chr4-116149513-116152286
chr4	116164785	116167764	chr4-116164785-116167764
chr4	116175158	116177829	chr4-116175158-116177829
chr4	116220214	116223240	chr4-116220214-116223240
chr4	116462114	116464975	chr4-116462114-116464975
chr4	116470932	116471133	chr4-116470932-116471133
chr4	116506764	116508713	chr4-116506764-116508713
chr4	116554886	116559764	chr4-116554886-116559764
chr4	116596351	116598967	chr4-116596351-116598967
chr4	116625479	116628630	chr4-116625479-116628630
chr4	116647990	116652359	chr4-116647990-116652359
chr4	116660113	116660367	chr4-116660113-116660367
chr4	116668108	116668309	chr4-116668108-116668309
chr4	116685021	116689601	chr4-116685021-116689601
chr4	116707388	116709656	chr4-116707388-116709656
chr4	116719971	116721821	chr4-116719971-116721821
chr4	116764119	116764320	chr4-116764119-116764320
chr4	116806189	116808830	chr4-116806189-116808830
chr4	116819988	116822169	chr4-116819988-116822169
chr4	116824189	116825595	chr4-116824189-116825595
chr4	116840035	116840448	chr4-116840035-116840448
chr4	116876539	116878357	chr4-116876539-116878357
chr4	116993356	116996542	chr4-116993356-116996542
chr4	117018910	117020625	chr4-117018910-117020625
chr4	117095672	117096579	chr4-117095672-117096579
chr4	117119673	117121737	chr4-117119673-117121737
chr4	117123910	117127273	chr4-117123910-117127273
chr4	117130652	117134962	chr4-117130652-117134962
chr4	117136175	117136794	chr4-117136175-117136794
chr4	117151478	117157000	chr4-117151478-117157000
chr4	117169824	117170580	chr4-117169824-117170580
chr4	117172112	117172426	chr4-117172112-117172426
chr4	117180956	117183347	chr4-117180956-117183347
chr4	117189235	117189999	chr4-117189235-117189999
chr4	117201910	117203339	chr4-117201910-117203339
chr4	117210524	117210827	chr4-117210524-117210827
chr4	117229581	117229937	chr4-117229581-117229937
chr4	117251280	117252775	chr4-117251280-117252775

chr4	117287785	117290128	chr4-117287785-117290128
chr4	117303799	117305930	chr4-117303799-117305930
chr4	117356318	117356625	chr4-117356318-117356625
chr4	117483777	117485145	chr4-117483777-117485145
chr4	117493214	117497049	chr4-117493214-117497049
chr4	117549605	117551475	chr4-117549605-117551475
chr4	117680950	117682839	chr4-117680950-117682839
chr4	117700790	117700991	chr4-117700790-117700991
chr4	117786905	117787725	chr4-117786905-117787725
chr4	117829720	117830543	chr4-117829720-117830543
chr4	117834746	117837585	chr4-117834746-117837585
chr4	117840437	117841831	chr4-117840437-117841831
chr4	117872407	117872883	chr4-117872407-117872883
chr4	117882164	117884594	chr4-117882164-117884594
chr4	117885874	117887872	chr4-117885874-117887872
chr4	117890490	117892494	chr4-117890490-117892494
chr4	117913101	117916374	chr4-117913101-117916374
chr4	117928321	117930174	chr4-117928321-117930174
chr4	117946867	117947225	chr4-117946867-117947225
chr4	117974458	117975335	chr4-117974458-117975335
chr4	117978072	117978273	chr4-117978072-117978273
chr4	117981753	117982156	chr4-117981753-117982156
chr4	117982960	117983161	chr4-117982960-117983161
chr4	117997194	118000895	chr4-117997194-118000895
chr4	118015762	118016014	chr4-118015762-118016014
chr4	118062181	118062711	chr4-118062181-118062711
chr4	118133909	118135632	chr4-118133909-118135632
chr4	118178281	118180986	chr4-118178281-118180986
chr4	118229084	118231923	chr4-118229084-118231923
chr4	118234767	118235985	chr4-118234767-118235985
chr4	118259854	118262133	chr4-118259854-118262133
chr4	118265790	118265991	chr4-118265790-118265991
chr4	118271837	118273758	chr4-118271837-118273758
chr4	118286309	118286689	chr4-118286309-118286689
chr4	118288530	118291818	chr4-118288530-118291818
chr4	118295446	118296037	chr4-118295446-118296037
chr4	118359367	118360996	chr4-118359367-118360996
chr4	118408212	118410541	chr4-118408212-118410541
chr4	118427210	118430592	chr4-118427210-118430592
chr4	118433895	118438476	chr4-118433895-118438476
chr4	118445742	118446685	chr4-118445742-118446685
chr4	118517514	118517715	chr4-118517514-118517715

chr4	118526725	118528184	chr4-118526725-118528184
chr4	118540535	118545253	chr4-118540535-118545253
chr4	118620112	118622382	chr4-118620112-118622382
chr4	119094168	119094427	chr4-119094168-119094427
chr4	119098411	119098673	chr4-119098411-119098673
chr4	119104933	119107002	chr4-119104933-119107002
chr4	119107961	119112754	chr4-119107961-119112754
chr4	119120761	119122110	chr4-119120761-119122110
chr4	119123897	119126838	chr4-119123897-119126838
chr4	119138089	119138731	chr4-119138089-119138731
chr4	119146312	119147460	chr4-119146312-119147460
chr4	119157924	119158125	chr4-119157924-119158125
chr4	119173002	119174669	chr4-119173002-119174669
chr4	119194876	119196285	chr4-119194876-119196285
chr4	119231898	119234034	chr4-119231898-119234034
chr4	119239423	119239624	chr4-119239423-119239624
chr4	119253947	119254199	chr4-119253947-119254199
chr4	119275538	119275739	chr4-119275538-119275739
chr4	119284719	119295973	chr4-119284719-119295973
chr4	119319063	119321587	chr4-119319063-119321587
chr4	119359518	119359769	chr4-119359518-119359769
chr4	119414827	119415842	chr4-119414827-119415842
chr4	119421029	119423464	chr4-119421029-119423464
chr4	119491944	119493113	chr4-119491944-119493113
chr4	119538722	119541263	chr4-119538722-119541263
chr4	119662813	119663014	chr4-119662813-119663014
chr4	119733014	119734739	chr4-119733014-119734739
chr4	119774050	119774303	chr4-119774050-119774303
chr4	119785202	119786967	chr4-119785202-119786967
chr4	119813780	119815494	chr4-119813780-119815494
chr4	119932861	119933320	chr4-119932861-119933320
chr4	120032260	120033646	chr4-120032260-120033646
chr4	120072030	120072387	chr4-120072030-120072387
chr4	120131790	120132041	chr4-120131790-120132041
chr4	120144613	120146356	chr4-120144613-120146356
chr4	120148388	120149335	chr4-120148388-120149335
chr4	120160921	120162012	chr4-120160921-120162012
chr4	120188739	120188990	chr4-120188739-120188990
chr4	120229843	120230044	chr4-120229843-120230044
chr4	120255816	120256327	chr4-120255816-120256327
chr4	120267921	120269092	chr4-120267921-120269092
chr4	120282301	120283470	chr4-120282301-120283470

chr4	120286533	120288419	chr4-120286533-120288419
chr4	120369899	120370154	chr4-120369899-120370154
chr4	120380843	120381246	chr4-120380843-120381246
chr4	120404652	120406670	chr4-120404652-120406670
chr4	120516100	120519103	chr4-120516100-120519103
chr4	120566394	120571989	chr4-120566394-120571989
chr4	120588533	120590390	chr4-120588533-120590390
chr4	120635076	120635878	chr4-120635076-120635878
chr4	120649522	120650246	chr4-120649522-120650246
chr4	120666249	120667913	chr4-120666249-120667913
chr4	120689008	120689751	chr4-120689008-120689751
chr4	120716197	120716987	chr4-120716197-120716987
chr4	120730726	120732047	chr4-120730726-120732047
chr4	120745896	120748733	chr4-120745896-120748733
chr4	120814157	120816679	chr4-120814157-120816679
chr4	120824199	120826443	chr4-120824199-120826443
chr4	120854453	120855535	chr4-120854453-120855535
chr4	120924013	120925820	chr4-120924013-120925820
chr4	120950587	120952573	chr4-120950587-120952573
chr4	121015844	121017695	chr4-121015844-121017695
chr4	121023505	121024423	chr4-121023505-121024423
chr4	121039409	121040472	chr4-121039409-121040472
chr4	121052965	121053166	chr4-121052965-121053166
chr4	121096615	121098725	chr4-121096615-121098725
chr4	121211277	121215838	chr4-121211277-121215838
chr4	121231297	121231548	chr4-121231297-121231548
chr4	122835519	122837205	chr4-122835519-122837205
chr4	122838195	122838515	chr4-122838195-122838515
chr4	122883722	122887390	chr4-122883722-122887390
chr4	122954846	122956155	chr4-122954846-122956155
chr4	122958837	122961955	chr4-122958837-122961955
chr4	122994995	122997418	chr4-122994995-122997418
chr4	123016156	123019300	chr4-123016156-123019300
chr4	123050924	123051542	chr4-123050924-123051542
chr4	123071559	123071976	chr4-123071559-123071976
chr4	123092639	123094256	chr4-123092639-123094256
chr4	123104416	123107053	chr4-123104416-123107053
chr4	123116125	123117045	chr4-123116125-123117045
chr4	123138301	123140330	chr4-123138301-123140330
chr4	123161608	123162912	chr4-123161608-123162912
chr4	123182492	123184124	chr4-123182492-123184124
chr4	123201163	123201829	chr4-123201163-123201829

chr4	123213839	123214553	chr4-123213839-123214553
chr4	123233369	123234175	chr4-123233369-123234175
chr4	123239328	123240148	chr4-123239328-123240148
chr4	123282498	123286333	chr4-123282498-123286333
chr4	123322802	123323710	chr4-123322802-123323710
chr4	123342019	123343770	chr4-123342019-123343770
chr4	123405255	123413061	chr4-123405255-123413061
chr4	123422606	123422983	chr4-123422606-123422983
chr4	123426932	123427566	chr4-123426932-123427566
chr4	123565520	123565826	chr4-123565520-123565826
chr4	123571167	123573525	chr4-123571167-123573525
chr4	123588515	123589550	chr4-123588515-123589550
chr4	123611037	123611870	chr4-123611037-123611870
chr4	123622289	123622490	chr4-123622289-123622490
chr4	123623604	123624058	chr4-123623604-123624058
chr4	123633565	123633816	chr4-123633565-123633816
chr4	123662927	123666139	chr4-123662927-123666139
chr4	123686253	123687884	chr4-123686253-123687884
chr4	123716752	123718825	chr4-123716752-123718825
chr4	123733262	123733818	chr4-123733262-123733818
chr4	123748540	123751166	chr4-123748540-123751166
chr4	123787419	123789269	chr4-123787419-123789269
chr4	123802469	123804609	chr4-123802469-123804609
chr4	123810601	123811227	chr4-123810601-123811227
chr4	123814016	123814352	chr4-123814016-123814352
chr4	123904056	123906478	chr4-123904056-123906478
chr4	123915928	123919258	chr4-123915928-123919258
chr4	123945972	123946388	chr4-123945972-123946388
chr4	123949375	123950529	chr4-123949375-123950529
chr4	123957177	123957378	chr4-123957177-123957378
chr4	123990586	123991552	chr4-123990586-123991552
chr4	124178558	124178823	chr4-124178558-124178823
chr4	124277506	124277991	chr4-124277506-124277991
chr4	124358496	124358697	chr4-124358496-124358697
chr4	124380932	124381133	chr4-124380932-124381133
chr4	124477625	124478592	chr4-124477625-124478592
chr4	124570834	124571312	chr4-124570834-124571312
chr4	124575237	124576007	chr4-124575237-124576007
chr4	124583913	124584727	chr4-124583913-124584727
chr4	124594726	124595427	chr4-124594726-124595427
chr4	124646195	124647224	chr4-124646195-124647224
chr4	124653019	124653220	chr4-124653019-124653220

chr4	124656375	124660280	chr4-124656375-124660280
chr4	124692059	124694049	chr4-124692059-124694049
chr4	124695885	124696432	chr4-124695885-124696432
chr4	124699965	124710312	chr4-124699965-124710312
chr4	124714280	124716442	chr4-124714280-124716442
chr4	124741271	124742801	chr4-124741271-124742801
chr4	124750753	124753032	chr4-124750753-124753032
chr4	124801638	124803972	chr4-124801638-124803972
chr4	124817605	124817862	chr4-124817605-124817862
chr4	124829383	124829998	chr4-124829383-124829998
chr4	124849419	124852303	chr4-124849419-124852303
chr4	124861567	124862635	chr4-124861567-124862635
chr4	124881317	124882022	chr4-124881317-124882022
chr4	124894396	124895404	chr4-124894396-124895404
chr4	124935297	124938107	chr4-124935297-124938107
chr4	124986105	124987293	chr4-124986105-124987293
chr4	125029402	125031666	chr4-125029402-125031666
chr4	125065377	125068291	chr4-125065377-125068291
chr4	125083861	125086146	chr4-125083861-125086146
chr4	125121763	125128520	chr4-125121763-125128520
chr4	125131607	125132116	chr4-125131607-125132116
chr4	125160442	125160970	chr4-125160442-125160970
chr4	125490217	125490852	chr4-125490217-125490852
chr4	126006798	126006999	chr4-126006798-126006999
chr4	126046550	126048538	chr4-126046550-126048538
chr4	126051656	126051907	chr4-126051656-126051907
chr4	126057124	126059497	chr4-126057124-126059497
chr4	126096096	126097599	chr4-126096096-126097599
chr4	126102199	126106096	chr4-126102199-126106096
chr4	126109575	126112041	chr4-126109575-126112041
chr4	126113297	126114345	chr4-126113297-126114345
chr4	126131343	126132405	chr4-126131343-126132405
chr4	126147729	126149595	chr4-126147729-126149595
chr4	126162306	126163964	chr4-126162306-126163964
chr4	126169069	126169372	chr4-126169069-126169372
chr4	126197947	126203626	chr4-126197947-126203626
chr4	126217328	126218010	chr4-126217328-126218010
chr4	126227079	126228177	chr4-126227079-126228177
chr4	126236576	126237397	chr4-126236576-126237397
chr4	126239825	126240986	chr4-126239825-126240986
chr4	126241998	126242250	chr4-126241998-126242250
chr4	126249625	126257916	chr4-126249625-126257916

chr4	126259090	126263698	chr4-126259090-126263698
chr4	126272168	126272877	chr4-126272168-126272877
chr4	126286272	126287282	chr4-126286272-126287282
chr4	126296325	126298239	chr4-126296325-126298239
chr4	126300133	126301954	chr4-126300133-126301954
chr4	126309532	126310131	chr4-126309532-126310131
chr4	126320275	126322425	chr4-126320275-126322425
chr4	126371369	126371720	chr4-126371369-126371720
chr4	126428345	126431320	chr4-126428345-126431320
chr4	126465785	126466092	chr4-126465785-126466092
chr4	126467473	126469235	chr4-126467473-126469235
chr4	126532419	126534157	chr4-126532419-126534157
chr4	126556212	126558137	chr4-126556212-126558137
chr4	126609450	126610316	chr4-126609450-126610316
chr4	126619810	126620412	chr4-126619810-126620412
chr4	126676952	126679030	chr4-126676952-126679030
chr4	126731907	126732979	chr4-126731907-126732979
chr4	126734303	126736536	chr4-126734303-126736536
chr4	126751820	126755347	chr4-126751820-126755347
chr4	126840912	126841345	chr4-126840912-126841345
chr4	126966584	126968659	chr4-126966584-126968659
chr4	127005100	127005407	chr4-127005100-127005407
chr4	127015483	127016047	chr4-127015483-127016047
chr4	127020459	127025457	chr4-127020459-127025457
chr4	127060171	127061611	chr4-127060171-127061611
chr4	127076707	127078389	chr4-127076707-127078389
chr4	127125653	127126832	chr4-127125653-127126832
chr4	127168115	127169887	chr4-127168115-127169887
chr4	127235660	127237112	chr4-127235660-127237112
chr4	127243514	127245121	chr4-127243514-127245121
chr4	127313518	127314560	chr4-127313518-127314560
chr4	127324435	127332520	chr4-127324435-127332520
chr4	127335049	127335301	chr4-127335049-127335301
chr4	127348940	127355840	chr4-127348940-127355840
chr4	127357743	127358047	chr4-127357743-127358047
chr4	127371745	127372050	chr4-127371745-127372050
chr4	127435042	127435243	chr4-127435042-127435243
chr4	127987917	127988378	chr4-127987917-127988378
chr4	128609070	128611065	chr4-128609070-128611065
chr4	128654299	128655818	chr4-128654299-128655818
chr4	128711194	128713018	chr4-128711194-128713018
chr4	128726904	128738768	chr4-128726904-128738768

chr4	128776721	128777230	chr4-128776721-128777230
chr4	128789767	128790119	chr4-128789767-128790119
chr4	128793408	128794637	chr4-128793408-128794637
chr4	128804656	128807358	chr4-128804656-128807358
chr4	128811178	128812665	chr4-128811178-128812665
chr4	128855636	128856281	chr4-128855636-128856281
chr4	128883239	128884882	chr4-128883239-128884882
chr4	128924653	128925697	chr4-128924653-128925697
chr4	128949597	128949905	chr4-128949597-128949905
chr4	128957090	128958466	chr4-128957090-128958466
chr4	128960838	128963821	chr4-128960838-128963821
chr4	128969754	128970971	chr4-128969754-128970971
chr4	128992887	128995209	chr4-128992887-128995209
chr4	128996674	128998038	chr4-128996674-128998038
chr4	129011876	129012299	chr4-129011876-129012299
chr4	129016983	129017864	chr4-129016983-129017864
chr4	129032114	129036624	chr4-129032114-129036624
chr4	129048479	129049598	chr4-129048479-129049598
chr4	129054249	129056086	chr4-129054249-129056086
chr4	129057074	129061809	chr4-129057074-129061809
chr4	129067338	129069396	chr4-129067338-129069396
chr4	129104968	129106951	chr4-129104968-129106951
chr4	129120812	129121781	chr4-129120812-129121781
chr4	129130326	129131333	chr4-129130326-129131333
chr4	129136360	129137471	chr4-129136360-129137471
chr4	129188274	129192112	chr4-129188274-129192112
chr4	129224157	129224435	chr4-129224157-129224435
chr4	129226570	129249532	chr4-129226570-129249532
chr4	129250757	129252189	chr4-129250757-129252189
chr4	129256315	129257021	chr4-129256315-129257021
chr4	129258803	129261758	chr4-129258803-129261758
chr4	129263053	129264561	chr4-129263053-129264561
chr4	129269940	129270300	chr4-129269940-129270300
chr4	129271246	129273441	chr4-129271246-129273441
chr4	129286633	129288126	chr4-129286633-129288126
chr4	129332896	129337076	chr4-129332896-129337076
chr4	129376492	129378513	chr4-129376492-129378513
chr4	129406516	129406767	chr4-129406516-129406767
chr4	129417538	129417891	chr4-129417538-129417891
chr4	129426491	129427057	chr4-129426491-129427057
chr4	129440083	129441440	chr4-129440083-129441440
chr4	129445174	129445725	chr4-129445174-129445725

chr4	129460485	129462678	chr4-129460485-129462678
chr4	129491525	129491936	chr4-129491525-129491936
chr4	129500336	129501989	chr4-129500336-129501989
chr4	129513204	129515480	chr4-129513204-129515480
chr4	129516671	129516872	chr4-129516671-129516872
chr4	129535814	129536015	chr4-129535814-129536015
chr4	129541464	129543481	chr4-129541464-129543481
chr4	129555505	129557424	chr4-129555505-129557424
chr4	129577048	129577332	chr4-129577048-129577332
chr4	129584718	129585807	chr4-129584718-129585807
chr4	129599172	129602394	chr4-129599172-129602394
chr4	129617846	129619744	chr4-129617846-129619744
chr4	129623438	129623947	chr4-129623438-129623947
chr4	129638968	129642040	chr4-129638968-129642040
chr4	129671496	129673680	chr4-129671496-129673680
chr4	129695636	129697533	chr4-129695636-129697533
chr4	129739567	129743945	chr4-129739567-129743945
chr4	129771281	129772324	chr4-129771281-129772324
chr4	129793504	129796514	chr4-129793504-129796514
chr4	129797827	129798296	chr4-129797827-129798296
chr4	129808514	129813299	chr4-129808514-129813299
chr4	129818594	129823765	chr4-129818594-129823765
chr4	129828082	129828339	chr4-129828082-129828339
chr4	129877026	129878210	chr4-129877026-129878210
chr4	129882107	129883247	chr4-129882107-129883247
chr4	129928341	129928799	chr4-129928341-129928799
chr4	129959533	129960936	chr4-129959533-129960936
chr4	129983772	129988605	chr4-129983772-129988605
chr4	130006605	130007015	chr4-130006605-130007015
chr4	130047366	130048537	chr4-130047366-130048537
chr4	130074867	130075763	chr4-130074867-130075763
chr4	130101300	130103627	chr4-130101300-130103627
chr4	130104440	130105092	chr4-130104440-130105092
chr4	130106711	130110455	chr4-130106711-130110455
chr4	130138245	130139582	chr4-130138245-130139582
chr4	130161365	130164848	chr4-130161365-130164848
chr4	130165932	130179696	chr4-130165932-130179696
chr4	130188795	130189250	chr4-130188795-130189250
chr4	130198908	130199719	chr4-130198908-130199719
chr4	130272929	130277493	chr4-130272929-130277493
chr4	130302182	130302824	chr4-130302182-130302824
chr4	130307756	130309185	chr4-130307756-130309185

chr4	130316185	130316386	chr4-130316185-130316386
chr4	130358940	130361545	chr4-130358940-130361545
chr4	130519219	130520983	chr4-130519219-130520983
chr4	130572480	130574779	chr4-130572480-130574779
chr4	130629451	130630134	chr4-130629451-130630134
chr4	130637706	130638062	chr4-130637706-130638062
chr4	130662658	130666363	chr4-130662658-130666363
chr4	130745192	130745867	chr4-130745192-130745867
chr4	130791854	130793765	chr4-130791854-130793765
chr4	130795623	130796120	chr4-130795623-130796120
chr4	130797494	130797695	chr4-130797494-130797695
chr4	130838756	130839104	chr4-130838756-130839104
chr4	130861286	130862404	chr4-130861286-130862404
chr4	130874665	130874980	chr4-130874665-130874980
chr4	130878436	130878790	chr4-130878436-130878790
chr4	131836291	131838682	chr4-131836291-131838682
chr4	131842790	131844689	chr4-131842790-131844689
chr4	131872342	131875635	chr4-131872342-131875635
chr4	131916330	131916634	chr4-131916330-131916634
chr4	131920183	131922655	chr4-131920183-131922655
chr4	132030412	132030896	chr4-132030412-132030896
chr4	132034387	132034588	chr4-132034387-132034588
chr4	132047909	132050139	chr4-132047909-132050139
chr4	132073482	132077603	chr4-132073482-132077603
chr4	132113339	132114108	chr4-132113339-132114108
chr4	132144042	132145008	chr4-132144042-132145008
chr4	132157301	132157666	chr4-132157301-132157666
chr4	132209035	132212865	chr4-132209035-132212865
chr4	132259882	132262435	chr4-132259882-132262435
chr4	132270152	132270929	chr4-132270152-132270929
chr4	132273240	132275609	chr4-132273240-132275609
chr4	132302914	132303572	chr4-132302914-132303572
chr4	132308358	132310968	chr4-132308358-132310968
chr4	132328281	132330261	chr4-132328281-132330261
chr4	132343338	132345997	chr4-132343338-132345997
chr4	132351109	132354712	chr4-132351109-132354712
chr4	132389554	132389857	chr4-132389554-132389857
chr4	132395112	132398499	chr4-132395112-132398499
chr4	132402634	132403944	chr4-132402634-132403944
chr4	132420103	132423092	chr4-132420103-132423092
chr4	132428082	132428334	chr4-132428082-132428334
chr4	132432932	132433348	chr4-132432932-132433348

chr4	132446281	132446482	chr4-132446281-132446482
chr4	132461643	132464587	chr4-132461643-132464587
chr4	132474491	132475124	chr4-132474491-132475124
chr4	132508046	132511004	chr4-132508046-132511004
chr4	132513620	132516187	chr4-132513620-132516187
chr4	132532172	132537420	chr4-132532172-132537420
chr4	132554434	132556478	chr4-132554434-132556478
chr4	132638204	132640571	chr4-132638204-132640571
chr4	132731379	132733006	chr4-132731379-132733006
chr4	132747467	132748943	chr4-132747467-132748943
chr4	132750158	132751119	chr4-132750158-132751119
chr4	132755937	132758321	chr4-132755937-132758321
chr4	132767583	132769655	chr4-132767583-132769655
chr4	132795918	132796576	chr4-132795918-132796576
chr4	132841477	132844272	chr4-132841477-132844272
chr4	132882672	132885483	chr4-132882672-132885483
chr4	132921075	132923811	chr4-132921075-132923811
chr4	132944858	132947153	chr4-132944858-132947153
chr4	132974762	132975319	chr4-132974762-132975319
chr4	133010518	133017239	chr4-133010518-133017239
chr4	133024585	133025832	chr4-133024585-133025832
chr4	133037391	133044591	chr4-133037391-133044591
chr4	133045856	133046108	chr4-133045856-133046108
chr4	133054885	133057773	chr4-133054885-133057773
chr4	133070338	133071295	chr4-133070338-133071295
chr4	133084531	133085847	chr4-133084531-133085847
chr4	133094889	133097129	chr4-133094889-133097129
chr4	133101466	133104818	chr4-133101466-133104818
chr4	133107613	133108040	chr4-133107613-133108040
chr4	133116672	133118856	chr4-133116672-133118856
chr4	133120699	133122520	chr4-133120699-133122520
chr4	133129357	133133110	chr4-133129357-133133110
chr4	133158881	133159348	chr4-133158881-133159348
chr4	133169408	133170041	chr4-133169408-133170041
chr4	133184558	133185915	chr4-133184558-133185915
chr4	133211625	133214140	chr4-133211625-133214140
chr4	133220520	133221530	chr4-133220520-133221530
chr4	133240200	133242425	chr4-133240200-133242425
chr4	133250457	133252270	chr4-133250457-133252270
chr4	133269877	133270078	chr4-133269877-133270078
chr4	133275814	133278652	chr4-133275814-133278652
chr4	133337871	133339970	chr4-133337871-133339970

chr4	133352299	133354052	chr4-133352299-133354052
chr4	133369531	133373116	chr4-133369531-133373116
chr4	133377990	133379368	chr4-133377990-133379368
chr4	133384909	133385217	chr4-133384909-133385217
chr4	133399222	133400192	chr4-133399222-133400192
chr4	133406576	133407950	chr4-133406576-133407950
chr4	133447283	133447895	chr4-133447283-133447895
chr4	133460993	133461545	chr4-133460993-133461545
chr4	133467782	133468690	chr4-133467782-133468690
chr4	133479641	133485416	chr4-133479641-133485416
chr4	133486811	133487087	chr4-133486811-133487087
chr4	133489101	133490006	chr4-133489101-133490006
chr4	133491497	133492422	chr4-133491497-133492422
chr4	133493228	133494695	chr4-133493228-133494695
chr4	133497098	133499251	chr4-133497098-133499251
chr4	133518157	133520634	chr4-133518157-133520634
chr4	133543967	133546363	chr4-133543967-133546363
chr4	133557434	133557688	chr4-133557434-133557688
chr4	133569993	133570357	chr4-133569993-133570357
chr4	133573865	133575883	chr4-133573865-133575883
chr4	133582940	133585546	chr4-133582940-133585546
chr4	133594994	133596058	chr4-133594994-133596058
chr4	133598083	133602880	chr4-133598083-133602880
chr4	133604088	133606092	chr4-133604088-133606092
chr4	133613594	133615088	chr4-133613594-133615088
chr4	133619712	133620127	chr4-133619712-133620127
chr4	133625278	133627472	chr4-133625278-133627472
chr4	133630308	133634082	chr4-133630308-133634082
chr4	133649550	133650311	chr4-133649550-133650311
chr4	133658384	133661427	chr4-133658384-133661427
chr4	133671247	133673973	chr4-133671247-133673973
chr4	133706642	133708341	chr4-133706642-133708341
chr4	133729553	133730463	chr4-133729553-133730463
chr4	133750370	133758141	chr4-133750370-133758141
chr4	133812603	133816670	chr4-133812603-133816670
chr4	133833120	133833586	chr4-133833120-133833586
chr4	133840978	133841592	chr4-133840978-133841592
chr4	133885431	133888701	chr4-133885431-133888701
chr4	133897443	133897851	chr4-133897443-133897851
chr4	133909857	133911518	chr4-133909857-133911518
chr4	133966467	133969074	chr4-133966467-133969074
chr4	133999858	134001529	chr4-133999858-134001529

chr4	134012787	134013099	chr4-134012787-134013099
chr4	134017882	134018682	chr4-134017882-134018682
chr4	134062113	134062537	chr4-134062113-134062537
chr4	134065324	134066089	chr4-134065324-134066089
chr4	134067996	134068817	chr4-134067996-134068817
chr4	134081314	134083096	chr4-134081314-134083096
chr4	134102265	134103278	chr4-134102265-134103278
chr4	134126944	134129666	chr4-134126944-134129666
chr4	134159338	134165087	chr4-134159338-134165087
chr4	134185809	134187782	chr4-134185809-134187782
chr4	134222898	134223582	chr4-134222898-134223582
chr4	134236749	134239196	chr4-134236749-134239196
chr4	134244076	134246075	chr4-134244076-134246075
chr4	134249532	134252439	chr4-134249532-134252439
chr4	134272806	134273830	chr4-134272806-134273830
chr4	134285804	134289110	chr4-134285804-134289110
chr4	134300261	134300607	chr4-134300261-134300607
chr4	134307530	134307859	chr4-134307530-134307859
chr4	134342924	134343743	chr4-134342924-134343743
chr4	134356021	134357458	chr4-134356021-134357458
chr4	134370937	134373014	chr4-134370937-134373014
chr4	134395660	134397635	chr4-134395660-134397635
chr4	134419362	134419613	chr4-134419362-134419613
chr4	134448018	134448581	chr4-134448018-134448581
chr4	134467323	134470070	chr4-134467323-134470070
chr4	134476446	134477418	chr4-134476446-134477418
chr4	134481170	134483327	chr4-134481170-134483327
chr4	134494640	134494841	chr4-134494640-134494841
chr4	134496418	134498119	chr4-134496418-134498119
chr4	134510815	134516074	chr4-134510815-134516074
chr4	134517129	134517631	chr4-134517129-134517631
chr4	134533516	134536202	chr4-134533516-134536202
chr4	134547169	134548269	chr4-134547169-134548269
chr4	134550944	134552646	chr4-134550944-134552646
chr4	134559145	134559346	chr4-134559145-134559346
chr4	134569762	134569963	chr4-134569762-134569963
chr4	134659676	134659986	chr4-134659676-134659986
chr4	134702609	134705113	chr4-134702609-134705113
chr4	134721306	134721507	chr4-134721306-134721507
chr4	134725206	134725929	chr4-134725206-134725929
chr4	134730314	134730676	chr4-134730314-134730676
chr4	134733921	134735332	chr4-134733921-134735332

chr4	134743854	134745415	chr4-134743854-134745415
chr4	134749623	134751125	chr4-134749623-134751125
chr4	134757342	134768925	chr4-134757342-134768925
chr4	134851677	134854642	chr4-134851677-134854642
chr4	134879455	134880160	chr4-134879455-134880160
chr4	134913197	134915629	chr4-134913197-134915629
chr4	134922432	134926581	chr4-134922432-134926581
chr4	134930073	134931906	chr4-134930073-134931906
chr4	134946155	134946407	chr4-134946155-134946407
chr4	134950738	134951039	chr4-134950738-134951039
chr4	134975579	134975780	chr4-134975579-134975780
chr4	135044871	135045325	chr4-135044871-135045325
chr4	135066912	135067376	chr4-135066912-135067376
chr4	135073583	135073835	chr4-135073583-135073835
chr4	135140566	135140978	chr4-135140566-135140978
chr4	135151984	135154329	chr4-135151984-135154329
chr4	135175325	135175795	chr4-135175325-135175795
chr4	135203168	135203818	chr4-135203168-135203818
chr4	135205987	135206699	chr4-135205987-135206699
chr4	135269362	135274037	chr4-135269362-135274037
chr4	135276355	135277460	chr4-135276355-135277460
chr4	135292700	135293067	chr4-135292700-135293067
chr4	135297882	135299525	chr4-135297882-135299525
chr4	135300782	135302446	chr4-135300782-135302446
chr4	135306717	135307661	chr4-135306717-135307661
chr4	135312663	135314554	chr4-135312663-135314554
chr4	135351085	135354678	chr4-135351085-135354678
chr4	135397010	135399749	chr4-135397010-135399749
chr4	135405990	135406292	chr4-135405990-135406292
chr4	135418824	135419557	chr4-135418824-135419557
chr4	135432278	135434881	chr4-135432278-135434881
chr4	135439157	135440186	chr4-135439157-135440186
chr4	135493118	135496313	chr4-135493118-135496313
chr4	135507693	135508098	chr4-135507693-135508098
chr4	135566933	135567134	chr4-135566933-135567134
chr4	135568980	135570650	chr4-135568980-135570650
chr4	135572861	135573868	chr4-135572861-135573868
chr4	135599259	135599511	chr4-135599259-135599511
chr4	135644421	135645911	chr4-135644421-135645911
chr4	135652888	135653379	chr4-135652888-135653379
chr4	135656199	135656622	chr4-135656199-135656622
chr4	135661436	135661752	chr4-135661436-135661752

chr4	135667374	135667625	chr4-135667374-135667625
chr4	135676013	135677281	chr4-135676013-135677281
chr4	135685014	135688388	chr4-135685014-135688388
chr4	135689456	135691494	chr4-135689456-135691494
chr4	135709247	135709448	chr4-135709247-135709448
chr4	135727116	135728784	chr4-135727116-135728784
chr4	135762360	135762871	chr4-135762360-135762871
chr4	135796385	135796687	chr4-135796385-135796687
chr4	135820099	135820565	chr4-135820099-135820565
chr4	135828318	135829357	chr4-135828318-135829357
chr4	135844168	135844643	chr4-135844168-135844643
chr4	135854663	135858476	chr4-135854663-135858476
chr4	135859568	135859769	chr4-135859568-135859769
chr4	135871498	135874535	chr4-135871498-135874535
chr4	135875908	135877266	chr4-135875908-135877266
chr4	135920246	135922222	chr4-135920246-135922222
chr4	135945918	135947563	chr4-135945918-135947563
chr4	135962240	135965783	chr4-135962240-135965783
chr4	135970818	135973654	chr4-135970818-135973654
chr4	135985780	135988032	chr4-135985780-135988032
chr4	136020347	136022315	chr4-136020347-136022315
chr4	136031031	136031795	chr4-136031031-136031795
chr4	136050598	136053920	chr4-136050598-136053920
chr4	136071209	136071461	chr4-136071209-136071461
chr4	136077138	136077339	chr4-136077138-136077339
chr4	136081366	136082346	chr4-136081366-136082346
chr4	136090143	136090661	chr4-136090143-136090661
chr4	136110390	136111059	chr4-136110390-136111059
chr4	136116715	136117661	chr4-136116715-136117661
chr4	136122791	136124140	chr4-136122791-136124140
chr4	136134966	136135705	chr4-136134966-136135705
chr4	136140352	136141123	chr4-136140352-136141123
chr4	136143246	136148854	chr4-136143246-136148854
chr4	136171729	136175558	chr4-136171729-136175558
chr4	136177121	136178734	chr4-136177121-136178734
chr4	136185240	136187297	chr4-136185240-136187297
chr4	136205806	136207405	chr4-136205806-136207405
chr4	136228531	136228785	chr4-136228531-136228785
chr4	136247598	136250567	chr4-136247598-136250567
chr4	136284750	136288217	chr4-136284750-136288217
chr4	136309484	136313133	chr4-136309484-136313133
chr4	136423934	136425551	chr4-136423934-136425551

chr4	136430823	136431024	chr4-136430823-136431024
chr4	136433142	136434934	chr4-136433142-136434934
chr4	136438639	136439565	chr4-136438639-136439565
chr4	136441385	136441695	chr4-136441385-136441695
chr4	136454907	136455311	chr4-136454907-136455311
chr4	136461432	136463642	chr4-136461432-136463642
chr4	136468971	136471927	chr4-136468971-136471927
chr4	136513025	136513278	chr4-136513025-136513278
chr4	136523123	136523380	chr4-136523123-136523380
chr4	136599068	136599269	chr4-136599068-136599269
chr4	136600578	136603074	chr4-136600578-136603074
chr4	136637107	136638272	chr4-136637107-136638272
chr4	136732801	136733102	chr4-136732801-136733102
chr4	136800989	136805426	chr4-136800989-136805426
chr4	136807774	136808919	chr4-136807774-136808919
chr4	136829631	136830303	chr4-136829631-136830303
chr4	136834010	136837083	chr4-136834010-136837083
chr4	136956376	136957531	chr4-136956376-136957531
chr4	137047581	137049333	chr4-137047581-137049333
chr4	137117895	137118510	chr4-137117895-137118510
chr4	137164350	137165455	chr4-137164350-137165455
chr4	137211829	137212431	chr4-137211829-137212431
chr4	137231213	137232282	chr4-137231213-137232282
chr4	137255599	137256168	chr4-137255599-137256168
chr4	137276710	137278515	chr4-137276710-137278515
chr4	137287024	137287865	chr4-137287024-137287865
chr4	137355192	137358085	chr4-137355192-137358085
chr4	137367380	137368448	chr4-137367380-137368448
chr4	137370099	137370769	chr4-137370099-137370769
chr4	137380923	137381651	chr4-137380923-137381651
chr4	137386808	137389339	chr4-137386808-137389339
chr4	137396678	137397618	chr4-137396678-137397618
chr4	137448049	137448826	chr4-137448049-137448826
chr4	137467963	137471705	chr4-137467963-137471705
chr4	137472940	137477905	chr4-137472940-137477905
chr4	137479612	137481601	chr4-137479612-137481601
chr4	137494609	137496450	chr4-137494609-137496450
chr4	137497330	137499087	chr4-137497330-137499087
chr4	137578141	137578664	chr4-137578141-137578664
chr4	137593346	137596022	chr4-137593346-137596022
chr4	137597210	137597411	chr4-137597210-137597411
chr4	137664214	137665386	chr4-137664214-137665386

chr4	137681382	137681954	chr4-137681382-137681954
chr4	137698083	137698785	chr4-137698083-137698785
chr4	137702883	137703665	chr4-137702883-137703665
chr4	137705134	137708387	chr4-137705134-137708387
chr4	137714151	137714677	chr4-137714151-137714677
chr4	137795749	137796655	chr4-137795749-137796655
chr4	137837858	137838339	chr4-137837858-137838339
chr4	137853987	137854373	chr4-137853987-137854373
chr4	137860933	137863717	chr4-137860933-137863717
chr4	137875321	137875918	chr4-137875321-137875918
chr4	137913126	137914281	chr4-137913126-137914281
chr4	137920896	137921148	chr4-137920896-137921148
chr4	137930339	137931572	chr4-137930339-137931572
chr4	137955712	137956014	chr4-137955712-137956014
chr4	137992721	137996927	chr4-137992721-137996927
chr4	138075360	138075561	chr4-138075360-138075561
chr4	138215521	138219266	chr4-138215521-138219266
chr4	138246561	138252836	chr4-138246561-138252836
chr4	138261690	138263144	chr4-138261690-138263144
chr4	138303404	138306129	chr4-138303404-138306129
chr4	138319340	138319541	chr4-138319340-138319541
chr4	138325140	138326787	chr4-138325140-138326787
chr4	138327752	138327953	chr4-138327752-138327953
chr4	138343102	138343303	chr4-138343102-138343303
chr4	138344812	138345013	chr4-138344812-138345013
chr4	138356253	138357227	chr4-138356253-138357227
chr4	138361408	138363828	chr4-138361408-138363828
chr4	138366327	138368562	chr4-138366327-138368562
chr4	138394881	138396968	chr4-138394881-138396968
chr4	138426863	138427663	chr4-138426863-138427663
chr4	138434074	138436349	chr4-138434074-138436349
chr4	138453439	138457662	chr4-138453439-138457662
chr4	138463173	138465932	chr4-138463173-138465932
chr4	138467043	138469807	chr4-138467043-138469807
chr4	138471058	138471535	chr4-138471058-138471535
chr4	138493402	138493891	chr4-138493402-138493891
chr4	138516428	138516680	chr4-138516428-138516680
chr4	138542977	138544105	chr4-138542977-138544105
chr4	138546386	138546694	chr4-138546386-138546694
chr4	138555572	138555823	chr4-138555572-138555823
chr4	138725032	138725512	chr4-138725032-138725512
chr4	138792376	138792688	chr4-138792376-138792688

chr4	138796296	138797123	chr4-138796296-138797123
chr4	138912699	138914160	chr4-138912699-138914160
chr4	138921877	138922331	chr4-138921877-138922331
chr4	138943404	138944148	chr4-138943404-138944148
chr4	138966548	138967882	chr4-138966548-138967882
chr4	138971858	138974231	chr4-138971858-138974231
chr4	139007175	139008701	chr4-139007175-139008701
chr4	139074173	139076103	chr4-139074173-139076103
chr4	139078665	139078866	chr4-139078665-139078866
chr4	139086507	139093549	chr4-139086507-139093549
chr4	139129463	139131685	chr4-139129463-139131685
chr4	139146948	139147255	chr4-139146948-139147255
chr4	139186048	139186558	chr4-139186048-139186558
chr4	139191204	139195499	chr4-139191204-139195499
chr4	139196417	139198217	chr4-139196417-139198217
chr4	139202341	139204800	chr4-139202341-139204800
chr4	139211910	139212761	chr4-139211910-139212761
chr4	139213654	139215183	chr4-139213654-139215183
chr4	139230377	139234974	chr4-139230377-139234974
chr4	139247839	139251881	chr4-139247839-139251881
chr4	139255405	139256673	chr4-139255405-139256673
chr4	139261195	139261614	chr4-139261195-139261614
chr4	139287724	139288251	chr4-139287724-139288251
chr4	139309590	139312458	chr4-139309590-139312458
chr4	139330651	139331357	chr4-139330651-139331357
chr4	139336744	139337303	chr4-139336744-139337303
chr4	139350522	139353766	chr4-139350522-139353766
chr4	139379432	139382725	chr4-139379432-139382725
chr4	139385934	139387235	chr4-139385934-139387235
chr4	139508985	139509522	chr4-139508985-139509522
chr4	139521077	139523035	chr4-139521077-139523035
chr4	139524506	139525010	chr4-139524506-139525010
chr4	139534444	139537026	chr4-139534444-139537026
chr4	139573780	139582537	chr4-139573780-139582537
chr4	139596198	139602707	chr4-139596198-139602707
chr4	139621788	139624085	chr4-139621788-139624085
chr4	139638063	139638264	chr4-139638063-139638264
chr4	139688373	139688574	chr4-139688373-139688574
chr4	139702549	139703511	chr4-139702549-139703511
chr4	139705718	139706521	chr4-139705718-139706521
chr4	139799783	139800284	chr4-139799783-139800284
chr4	139821300	139822110	chr4-139821300-139822110

chr4	139823597	139823855	chr4-139823597-139823855
chr4	139827712	139828676	chr4-139827712-139828676
chr4	139832509	139833662	chr4-139832509-139833662
chr4	139852207	139852571	chr4-139852207-139852571
chr4	139950187	139950803	chr4-139950187-139950803
chr4	139964263	139968535	chr4-139964263-139968535
chr4	139977263	139979348	chr4-139977263-139979348
chr4	140004389	140004968	chr4-140004389-140004968
chr4	140103430	140103731	chr4-140103430-140103731
chr4	140109109	140109310	chr4-140109109-140109310
chr4	140138935	140139457	chr4-140138935-140139457
chr4	140173917	140174187	chr4-140173917-140174187
chr4	140246185	140247014	chr4-140246185-140247014
chr4	140500762	140501181	chr4-140500762-140501181
chr4	140537128	140538261	chr4-140537128-140538261
chr4	140615434	140617991	chr4-140615434-140617991
chr4	140630041	140631408	chr4-140630041-140631408
chr4	140634710	140635578	chr4-140634710-140635578
chr4	140636467	140636882	chr4-140636467-140636882
chr4	140647424	140649511	chr4-140647424-140649511
chr4	140657252	140658665	chr4-140657252-140658665
chr4	140661244	140666633	chr4-140661244-140666633
chr4	140667683	140668206	chr4-140667683-140668206
chr4	140679517	140680318	chr4-140679517-140680318
chr4	140688034	140688991	chr4-140688034-140688991
chr4	140699108	140704664	chr4-140699108-140704664
chr4	140747679	140748915	chr4-140747679-140748915
chr4	140770398	140774597	chr4-140770398-140774597
chr4	140779924	140780177	chr4-140779924-140780177
chr4	140808701	140809988	chr4-140808701-140809988
chr4	140813684	140814992	chr4-140813684-140814992
chr4	140836259	140836460	chr4-140836259-140836460
chr4	140837486	140838692	chr4-140837486-140838692
chr4	140841116	140841369	chr4-140841116-140841369
chr4	140842208	140846323	chr4-140842208-140846323
chr4	140862236	140862437	chr4-140862236-140862437
chr4	140905677	140908426	chr4-140905677-140908426
chr4	140944555	140949929	chr4-140944555-140949929
chr4	140957487	140958072	chr4-140957487-140958072
chr4	140960152	140963450	chr4-140960152-140963450
chr4	140983131	140983925	chr4-140983131-140983925
chr4	140986460	140987934	chr4-140986460-140987934

chr4	141010403	141010818	chr4-141010403-141010818
chr4	141029144	141029345	chr4-141029144-141029345
chr4	141032390	141034902	chr4-141032390-141034902
chr4	141059445	141061785	chr4-141059445-141061785
chr4	141076567	141078789	chr4-141076567-141078789
chr4	141130447	141133073	chr4-141130447-141133073
chr4	141136313	141140617	chr4-141136313-141140617
chr4	141142182	141143260	chr4-141142182-141143260
chr4	141146928	141149573	chr4-141146928-141149573
chr4	141209989	141210240	chr4-141209989-141210240
chr4	141213388	141214889	chr4-141213388-141214889
chr4	141238808	141241115	chr4-141238808-141241115
chr4	141242328	141243434	chr4-141242328-141243434
chr4	141244772	141248324	chr4-141244772-141248324
chr4	141259550	141260651	chr4-141259550-141260651
chr4	141271597	141292313	chr4-141271597-141292313
chr4	141300515	141317130	chr4-141300515-141317130
chr4	141345017	141345403	chr4-141345017-141345403
chr4	141363103	141363304	chr4-141363103-141363304
chr4	141367709	141369023	chr4-141367709-141369023
chr4	141385226	141385479	chr4-141385226-141385479
chr4	141394792	141395166	chr4-141394792-141395166
chr4	141404622	141404927	chr4-141404622-141404927
chr4	141406842	141407366	chr4-141406842-141407366
chr4	141423840	141424041	chr4-141423840-141424041
chr4	141424905	141427555	chr4-141424905-141427555
chr4	141443752	141446510	chr4-141443752-141446510
chr4	141454356	141455297	chr4-141454356-141455297
chr4	141506209	141507368	chr4-141506209-141507368
chr4	141514243	141514784	chr4-141514243-141514784
chr4	141518114	141518884	chr4-141518114-141518884
chr4	141535290	141539863	chr4-141535290-141539863
chr4	141540738	141540989	chr4-141540738-141540989
chr4	141544706	141548111	chr4-141544706-141548111
chr4	141558046	141560554	chr4-141558046-141560554
chr4	141562118	141562392	chr4-141562118-141562392
chr4	141565063	141565760	chr4-141565063-141565760
chr4	141568658	141575586	chr4-141568658-141575586
chr4	141597139	141601979	chr4-141597139-141601979
chr4	141603720	141606536	chr4-141603720-141606536
chr4	141618286	141618555	chr4-141618286-141618555
chr4	141662975	141665345	chr4-141662975-141665345

chr4	141670173	141670881	chr4-141670173-141670881
chr4	141719914	141724131	chr4-141719914-141724131
chr4	141734506	141735503	chr4-141734506-141735503
chr4	141789671	141791503	chr4-141789671-141791503
chr4	141792860	141794671	chr4-141792860-141794671
chr4	141866733	141868255	chr4-141866733-141868255
chr4	141869505	141875701	chr4-141869505-141875701
chr4	141878666	141879288	chr4-141878666-141879288
chr4	141881568	141883051	chr4-141881568-141883051
chr4	141925945	141926412	chr4-141925945-141926412
chr4	141928634	141930152	chr4-141928634-141930152
chr4	141939730	141939931	chr4-141939730-141939931
chr4	141958426	141958960	chr4-141958426-141958960
chr4	141999824	142000025	chr4-141999824-142000025
chr4	142014543	142015862	chr4-142014543-142015862
chr4	142017686	142018320	chr4-142017686-142018320
chr4	142047216	142047624	chr4-142047216-142047624
chr4	142065611	142067515	chr4-142065611-142067515
chr4	142070845	142072146	chr4-142070845-142072146
chr4	142082972	142085712	chr4-142082972-142085712
chr4	142095393	142096141	chr4-142095393-142096141
chr4	142097562	142098619	chr4-142097562-142098619
chr4	142180609	142181060	chr4-142180609-142181060
chr4	142228399	142230020	chr4-142228399-142230020
chr4	142237457	142239890	chr4-142237457-142239890
chr4	143074193	143074452	chr4-143074193-143074452
chr4	143118860	143120662	chr4-143118860-143120662
chr4	143160250	143162792	chr4-143160250-143162792
chr4	143211550	143213415	chr4-143211550-143213415
chr4	143238303	143238870	chr4-143238303-143238870
chr4	143278515	143278923	chr4-143278515-143278923
chr4	143288291	143289233	chr4-143288291-143289233
chr4	143325197	143325822	chr4-143325197-143325822
chr4	143349537	143350475	chr4-143349537-143350475
chr4	143389244	143389708	chr4-143389244-143389708
chr4	143411681	143413665	chr4-143411681-143413665
chr4	144404214	144404974	chr4-144404214-144404974
chr4	144890803	144895426	chr4-144890803-144895426
chr4	144929935	144930682	chr4-144929935-144930682
chr4	144998037	145000392	chr4-144998037-145000392
chr4	145193578	145195282	chr4-145193578-145195282
chr4	145204946	145205306	chr4-145204946-145205306

chr4	145229601	145230392	chr4-145229601-145230392
chr4	145244836	145247297	chr4-145244836-145247297
chr4	145254329	145254747	chr4-145254329-145254747
chr4	145272530	145273376	chr4-145272530-145273376
chr4	145300048	145300810	chr4-145300048-145300810
chr4	145315073	145315375	chr4-145315073-145315375
chr4	145410279	145410588	chr4-145410279-145410588
chr4	145411459	145411710	chr4-145411459-145411710
chr4	145463616	145464450	chr4-145463616-145464450
chr4	145585101	145585781	chr4-145585101-145585781
chr4	145640867	145641221	chr4-145640867-145641221
chr4	145745041	145745561	chr4-145745041-145745561
chr4	145774299	145774833	chr4-145774299-145774833
chr4	145836953	145837407	chr4-145836953-145837407
chr4	146067117	146067952	chr4-146067117-146067952
chr4	146239480	146239731	chr4-146239480-146239731
chr4	146263199	146263635	chr4-146263199-146263635
chr4	146474768	146475563	chr4-146474768-146475563
chr4	146603824	146604025	chr4-146603824-146604025
chr4	146932284	146932842	chr4-146932284-146932842
chr4	146942272	146942586	chr4-146942272-146942586
chr4	147101680	147102523	chr4-147101680-147102523
chr4	147124732	147125334	chr4-147124732-147125334
chr4	147132308	147133072	chr4-147132308-147133072
chr4	147176406	147176607	chr4-147176406-147176607
chr4	147187462	147187876	chr4-147187462-147187876
chr4	147219347	147219921	chr4-147219347-147219921
chr4	147275170	147276154	chr4-147275170-147276154
chr4	147406757	147407642	chr4-147406757-147407642
chr4	147443036	147443491	chr4-147443036-147443491
chr4	147738476	147739240	chr4-147738476-147739240
chr4	147808562	147810218	chr4-147808562-147810218
chr4	147847367	147849422	chr4-147847367-147849422
chr4	147867139	147870812	chr4-147867139-147870812
chr4	147879181	147880319	chr4-147879181-147880319
chr4	147903098	147906905	chr4-147903098-147906905
chr4	147935923	147937136	chr4-147935923-147937136
chr4	147940136	147943308	chr4-147940136-147943308
chr4	147960449	147960650	chr4-147960449-147960650
chr4	147984924	147986943	chr4-147984924-147986943
chr4	147993059	147994437	chr4-147993059-147994437
chr4	147999750	148000532	chr4-147999750-148000532

chr4	148037463	148042420	chr4-148037463-148042420
chr4	148074879	148075687	chr4-148074879-148075687
chr4	148086950	148088382	chr4-148086950-148088382
chr4	148102129	148102491	chr4-148102129-148102491
chr4	148115493	148115957	chr4-148115493-148115957
chr4	148130238	148131359	chr4-148130238-148131359
chr4	148139414	148141562	chr4-148139414-148141562
chr4	148150306	148152254	chr4-148150306-148152254
chr4	148158716	148161755	chr4-148158716-148161755
chr4	148163450	148164677	chr4-148163450-148164677
chr4	148166952	148167153	chr4-148166952-148167153
chr4	148177541	148178286	chr4-148177541-148178286
chr4	148185198	148185515	chr4-148185198-148185515
chr4	148287467	148288427	chr4-148287467-148288427
chr4	148443083	148445257	chr4-148443083-148445257
chr4	148447546	148449733	chr4-148447546-148449733
chr4	148487895	148488096	chr4-148487895-148488096
chr4	148557832	148559564	chr4-148557832-148559564
chr4	148589563	148593669	chr4-148589563-148593669
chr4	148619365	148619705	chr4-148619365-148619705
chr4	148621293	148627572	chr4-148621293-148627572
chr4	148644070	148644321	chr4-148644070-148644321
chr4	148646459	148646931	chr4-148646459-148646931
chr4	148662249	148662963	chr4-148662249-148662963
chr4	148714968	148715230	chr4-148714968-148715230
chr4	148742461	148743375	chr4-148742461-148743375
chr4	148797360	148798428	chr4-148797360-148798428
chr4	148803881	148807272	chr4-148803881-148807272
chr4	148820074	148820481	chr4-148820074-148820481
chr4	148833286	148833539	chr4-148833286-148833539
chr4	148868157	148868493	chr4-148868157-148868493
chr4	148872059	148873353	chr4-148872059-148873353
chr4	148899978	148902002	chr4-148899978-148902002
chr4	148902998	148903592	chr4-148902998-148903592
chr4	148940628	148941217	chr4-148940628-148941217
chr4	149047301	149047652	chr4-149047301-149047652
chr4	149048725	149050922	chr4-149048725-149050922
chr4	149097806	149100450	chr4-149097806-149100450
chr4	149101768	149102461	chr4-149101768-149102461
chr4	149103876	149105234	chr4-149103876-149105234
chr4	149123283	149123484	chr4-149123283-149123484
chr4	149132234	149132873	chr4-149132234-149132873

chr4	149136249	149137840	chr4-149136249-149137840
chr4	149163942	149168080	chr4-149163942-149168080
chr4	149170056	149172230	chr4-149170056-149172230
chr4	149306071	149308712	chr4-149306071-149308712
chr4	149401065	149401778	chr4-149401065-149401778
chr4	149403155	149403356	chr4-149403155-149403356
chr4	149406075	149406276	chr4-149406075-149406276
chr4	149424487	149427267	chr4-149424487-149427267
chr4	149435645	149435898	chr4-149435645-149435898
chr4	149444953	149446202	chr4-149444953-149446202
chr4	149484031	149486536	chr4-149484031-149486536
chr4	149517462	149519354	chr4-149517462-149519354
chr4	149557847	149558530	chr4-149557847-149558530
chr4	149569954	149570155	chr4-149569954-149570155
chr4	149570976	149572519	chr4-149570976-149572519
chr4	149585842	149588071	chr4-149585842-149588071
chr4	149607435	149608730	chr4-149607435-149608730
chr4	149675212	149676734	chr4-149675212-149676734
chr4	149697959	149698974	chr4-149697959-149698974
chr4	149731051	149731252	chr4-149731051-149731252
chr4	149736216	149738547	chr4-149736216-149738547
chr4	149755555	149756150	chr4-149755555-149756150
chr4	149772192	149774483	chr4-149772192-149774483
chr4	149775350	149777486	chr4-149775350-149777486
chr4	149778669	149781488	chr4-149778669-149781488
chr4	149789768	149790025	chr4-149789768-149790025
chr4	149809043	149809294	chr4-149809043-149809294
chr4	149820364	149822997	chr4-149820364-149822997
chr4	149854461	149854868	chr4-149854461-149854868
chr4	149874944	149878501	chr4-149874944-149878501
chr4	149889734	149890493	chr4-149889734-149890493
chr4	149952207	149957200	chr4-149952207-149957200
chr4	149978050	149981177	chr4-149978050-149981177
chr4	149988510	149991212	chr4-149988510-149991212
chr4	149995058	150003998	chr4-149995058-150003998
chr4	150006943	150009602	chr4-150006943-150009602
chr4	150011653	150012703	chr4-150011653-150012703
chr4	150029553	150031550	chr4-150029553-150031550
chr4	150033197	150033659	chr4-150033197-150033659
chr4	150047638	150051544	chr4-150047638-150051544
chr4	150054553	150054754	chr4-150054553-150054754
chr4	150059533	150062637	chr4-150059533-150062637

chr4	150086837	150088478	chr4-150086837-150088478
chr4	150112771	150113036	chr4-150112771-150113036
chr4	150132683	150133418	chr4-150132683-150133418
chr4	150138176	150139767	chr4-150138176-150139767
chr4	150164869	150165225	chr4-150164869-150165225
chr4	150176686	150177039	chr4-150176686-150177039
chr4	150220664	150221077	chr4-150220664-150221077
chr4	150236003	150242470	chr4-150236003-150242470
chr4	150257835	150258261	chr4-150257835-150258261
chr4	150266705	150267537	chr4-150266705-150267537
chr4	150269200	150270849	chr4-150269200-150270849
chr4	150271935	150273378	chr4-150271935-150273378
chr4	150280397	150284323	chr4-150280397-150284323
chr4	150306549	150306750	chr4-150306549-150306750
chr4	150371188	150374052	chr4-150371188-150374052
chr4	150544262	150545423	chr4-150544262-150545423
chr4	150568286	150571117	chr4-150568286-150571117
chr4	150581180	150581522	chr4-150581180-150581522
chr4	150587647	150588023	chr4-150587647-150588023
chr4	150594221	150594472	chr4-150594221-150594472
chr4	150599533	150599845	chr4-150599533-150599845
chr4	150651826	150652291	chr4-150651826-150652291
chr4	150655865	150656169	chr4-150655865-150656169
chr4	150685073	150685802	chr4-150685073-150685802
chr4	150691135	150691588	chr4-150691135-150691588
chr4	150694628	150695593	chr4-150694628-150695593
chr4	150703033	150703411	chr4-150703033-150703411
chr4	150704619	150708628	chr4-150704619-150708628
chr4	150717340	150717882	chr4-150717340-150717882
chr4	150724377	150726444	chr4-150724377-150726444
chr4	150743634	150743987	chr4-150743634-150743987
chr4	150745485	150746762	chr4-150745485-150746762
chr4	150748206	150748748	chr4-150748206-150748748
chr4	150782166	150782468	chr4-150782166-150782468
chr4	150788056	150789768	chr4-150788056-150789768
chr4	150798341	150800735	chr4-150798341-150800735
chr4	150804243	150811336	chr4-150804243-150811336
chr4	150824215	150826204	chr4-150824215-150826204
chr4	150836809	150837831	chr4-150836809-150837831
chr4	150853690	150861603	chr4-150853690-150861603
chr4	150870260	150872654	chr4-150870260-150872654
chr4	150903323	150904156	chr4-150903323-150904156

chr4	150907918	150910497	chr4-150907918-150910497
chr4	150913446	150915919	chr4-150913446-150915919
chr4	150921237	150921488	chr4-150921237-150921488
chr4	150927528	150928235	chr4-150927528-150928235
chr4	150962056	150962308	chr4-150962056-150962308
chr4	150964589	150965470	chr4-150964589-150965470
chr4	150969178	150969592	chr4-150969178-150969592
chr4	150972871	150973764	chr4-150972871-150973764
chr4	151043343	151045608	chr4-151043343-151045608
chr4	151056802	151058981	chr4-151056802-151058981
chr4	151107129	151109281	chr4-151107129-151109281
chr4	151128152	151129725	chr4-151128152-151129725
chr4	151137639	151139657	chr4-151137639-151139657
chr4	151141218	151141745	chr4-151141218-151141745
chr4	151275102	151275564	chr4-151275102-151275564
chr4	151339120	151340164	chr4-151339120-151340164
chr4	151346574	151347520	chr4-151346574-151347520
chr4	151386364	151387552	chr4-151386364-151387552
chr4	151412033	151412234	chr4-151412033-151412234
chr4	151618474	151618729	chr4-151618474-151618729
chr4	151634994	151635254	chr4-151634994-151635254
chr4	151638718	151639031	chr4-151638718-151639031
chr4	151860293	151863972	chr4-151860293-151863972
chr4	151902839	151903040	chr4-151902839-151903040
chr4	151928953	151929334	chr4-151928953-151929334
chr4	151933218	151935817	chr4-151933218-151935817
chr4	151937515	151938105	chr4-151937515-151938105
chr4	151985595	151989503	chr4-151985595-151989503
chr4	151993810	151997940	chr4-151993810-151997940
chr4	152007239	152013311	chr4-152007239-152013311
chr4	152025052	152029054	chr4-152025052-152029054
chr4	152038906	152041321	chr4-152038906-152041321
chr4	152086157	152087843	chr4-152086157-152087843
chr4	152107033	152109105	chr4-152107033-152109105
chr4	152119616	152121324	chr4-152119616-152121324
chr4	152130476	152132486	chr4-152130476-152132486
chr4	152138141	152139479	chr4-152138141-152139479
chr4	152151887	152154287	chr4-152151887-152154287
chr4	152158022	152159887	chr4-152158022-152159887
chr4	152177257	152180327	chr4-152177257-152180327
chr4	152184703	152188204	chr4-152184703-152188204
chr4	152194438	152195482	chr4-152194438-152195482

chr4	152211813	152212935	chr4-152211813-152212935
chr4	152273766	152274887	chr4-152273766-152274887
chr4	152282762	152283788	chr4-152282762-152283788
chr4	152286139	152286745	chr4-152286139-152286745
chr4	152290176	152290838	chr4-152290176-152290838
chr4	152292364	152292907	chr4-152292364-152292907
chr4	152296274	152299131	chr4-152296274-152299131
chr4	152312625	152314159	chr4-152312625-152314159
chr4	152315485	152316262	chr4-152315485-152316262
chr4	152318533	152319108	chr4-152318533-152319108
chr4	152324889	152328358	chr4-152324889-152328358
chr4	152338354	152339731	chr4-152338354-152339731
chr4	152345214	152345709	chr4-152345214-152345709
chr4	152349912	152350265	chr4-152349912-152350265
chr4	152353841	152354963	chr4-152353841-152354963
chr4	152360694	152361155	chr4-152360694-152361155
chr4	152395804	152396063	chr4-152395804-152396063
chr4	152434958	152435475	chr4-152434958-152435475
chr4	152476492	152478742	chr4-152476492-152478742
chr4	153806417	153806669	chr4-153806417-153806669
chr4	153862391	153862592	chr4-153862391-153862592
chr4	153956677	153958546	chr4-153956677-153958546
chr4	153973720	153976399	chr4-153973720-153976399
chr4	154010918	154013997	chr4-154010918-154013997
chr4	154025436	154026628	chr4-154025436-154026628
chr4	154081124	154081540	chr4-154081124-154081540
chr4	154105466	154105883	chr4-154105466-154105883
chr4	154139398	154143400	chr4-154139398-154143400
chr4	154159156	154162040	chr4-154159156-154162040
chr4	154169680	154173764	chr4-154169680-154173764
chr4	154183428	154184747	chr4-154183428-154184747
chr4	154204158	154205716	chr4-154204158-154205716
chr4	154246591	154247004	chr4-154246591-154247004
chr4	154248211	154248962	chr4-154248211-154248962
chr4	154293296	154295017	chr4-154293296-154295017
chr4	154297158	154301830	chr4-154297158-154301830
chr4	154328583	154328784	chr4-154328583-154328784
chr4	154634578	154638177	chr4-154634578-154638177
chr4	154855655	154856581	chr4-154855655-154856581
chr4	154864235	154864816	chr4-154864235-154864816
chr4	154877839	154878664	chr4-154877839-154878664
chr4	154898381	154899634	chr4-154898381-154899634

chr4	154904319	154905257	chr4-154904319-154905257
chr4	154907910	154908318	chr4-154907910-154908318
chr4	154946233	154946590	chr4-154946233-154946590
chr4	154960487	154961922	chr4-154960487-154961922
chr4	154963560	154965799	chr4-154963560-154965799
chr4	155019225	155019426	chr4-155019225-155019426
chr4	155043050	155043407	chr4-155043050-155043407
chr4	155063597	155064645	chr4-155063597-155064645
chr4	155066153	155068950	chr4-155066153-155068950
chr4	155084498	155087789	chr4-155084498-155087789
chr4	155126266	155126613	chr4-155126266-155126613
chr4	155129479	155130718	chr4-155129479-155130718
chr4	155132407	155135188	chr4-155132407-155135188
chr4	155136019	155136506	chr4-155136019-155136506
chr4	155148392	155151914	chr4-155148392-155151914
chr4	155154904	155155428	chr4-155154904-155155428
chr4	155162501	155165252	chr4-155162501-155165252
chr4	155172339	155174867	chr4-155172339-155174867
chr4	155178035	155180070	chr4-155178035-155180070
chr4	155198020	155198575	chr4-155198020-155198575
chr4	155205519	155206288	chr4-155205519-155206288
chr4	155207591	155208904	chr4-155207591-155208904
chr4	155214861	155224025	chr4-155214861-155224025
chr4	155233859	155236374	chr4-155233859-155236374
chr4	155238036	155238392	chr4-155238036-155238392
chr4	155249250	155251879	chr4-155249250-155251879
chr4	155307942	155308303	chr4-155307942-155308303
chr4	155318240	155318988	chr4-155318240-155318988
chr4	155359692	155363050	chr4-155359692-155363050
chr4	155397499	155398515	chr4-155397499-155398515
chr4	155408732	155409853	chr4-155408732-155409853
chr4	155468923	155469684	chr4-155468923-155469684
chr4	155472786	155474320	chr4-155472786-155474320
chr4	155481306	155482179	chr4-155481306-155482179
chr4	155488077	155489238	chr4-155488077-155489238
chr4	155490317	155495024	chr4-155490317-155495024
chr4	155562397	155566718	chr4-155562397-155566718
chr4	155568077	155568278	chr4-155568077-155568278
chr4	155571268	155571790	chr4-155571268-155571790
chr4	155582149	155583973	chr4-155582149-155583973
chr4	155600344	155603006	chr4-155600344-155603006
chr4	155623428	155625823	chr4-155623428-155625823

chr4	155651265	155652068	chr4-155651265-155652068
chr4	155653820	155655815	chr4-155653820-155655815
chr4	155667922	155670076	chr4-155667922-155670076
chr4	155693924	155695565	chr4-155693924-155695565
chr4	155703829	155706623	chr4-155703829-155706623
chr4	155733709	155736341	chr4-155733709-155736341
chr4	155758862	155762286	chr4-155758862-155762286
chr4	155769446	155770945	chr4-155769446-155770945
chr4	155773927	155775145	chr4-155773927-155775145
chr4	155783827	155784999	chr4-155783827-155784999
chr4	155802429	155804929	chr4-155802429-155804929
chr4	155811596	155814611	chr4-155811596-155814611
chr4	155830648	155834499	chr4-155830648-155834499
chr4	155839434	155841530	chr4-155839434-155841530
chr4	155846356	155853060	chr4-155846356-155853060
chr4	155868106	155870675	chr4-155868106-155870675
chr4	155890112	155895105	chr4-155890112-155895105
chr4	155942504	155945471	chr4-155942504-155945471
chr4	155962107	155962934	chr4-155962107-155962934
chr4	155991143	155994712	chr4-155991143-155994712
chr4	156026230	156026592	chr4-156026230-156026592
chr4	156030321	156032833	chr4-156030321-156032833
chr4	156053508	156060426	chr4-156053508-156060426
chr4	156064026	156064483	chr4-156064026-156064483
chr4	156067119	156068022	chr4-156067119-156068022
chr4	156077118	156078554	chr4-156077118-156078554
chr4	156108159	156111251	chr4-156108159-156111251
chr4	156189209	156189586	chr4-156189209-156189586
chr4	156191312	156198471	chr4-156191312-156198471
chr4	156200345	156201403	chr4-156200345-156201403
chr4	156202648	156204271	chr4-156202648-156204271
chr4	156227081	156229388	chr4-156227081-156229388
chr4	156233266	156237845	chr4-156233266-156237845
chr5	3099996	3100197	chr5-3099996-3100197
chr5	3151334	3152954	chr5-3151334-3152954
chr5	3289939	3290603	chr5-3289939-3290603
chr5	3299958	3302835	chr5-3299958-3302835
chr5	3306105	3306517	chr5-3306105-3306517
chr5	3321729	3322035	chr5-3321729-3322035
chr5	3340279	3346301	chr5-3340279-3346301
chr5	3493809	3495031	chr5-3493809-3495031
chr5	3500981	3503258	chr5-3500981-3503258

chr5	3538950	3539410	chr5-3538950-3539410
chr5	3543461	3545177	chr5-3543461-3545177
chr5	3594933	3597336	chr5-3594933-3597336
chr5	3646221	3648477	chr5-3646221-3648477
chr5	3787958	3788159	chr5-3787958-3788159
chr5	3801287	3804467	chr5-3801287-3804467
chr5	3892866	3894167	chr5-3892866-3894167
chr5	3927384	3929851	chr5-3927384-3929851
chr5	3936956	3937224	chr5-3936956-3937224
chr5	3938673	3938983	chr5-3938673-3938983
chr5	3954490	3954787	chr5-3954490-3954787
chr5	4023267	4023468	chr5-4023267-4023468
chr5	4102645	4105453	chr5-4102645-4105453
chr5	4192075	4193556	chr5-4192075-4193556
chr5	4245943	4246246	chr5-4245943-4246246
chr5	4495575	4497032	chr5-4495575-4497032
chr5	4617721	4618003	chr5-4617721-4618003
chr5	4754791	4759140	chr5-4754791-4759140
chr5	5207999	5208250	chr5-5207999-5208250
chr5	5320130	5321013	chr5-5320130-5321013
chr5	5342312	5343836	chr5-5342312-5343836
chr5	5378975	5381625	chr5-5378975-5381625
chr5	5418361	5420945	chr5-5418361-5420945
chr5	5510201	5515343	chr5-5510201-5515343
chr5	5557846	5560302	chr5-5557846-5560302
chr5	5573559	5574474	chr5-5573559-5574474
chr5	5648920	5649536	chr5-5648920-5649536
chr5	5663415	5664523	chr5-5663415-5664523
chr5	5684692	5695312	chr5-5684692-5695312
chr5	5707598	5708748	chr5-5707598-5708748
chr5	5747919	5750273	chr5-5747919-5750273
chr5	7343943	7345504	chr5-7343943-7345504
chr5	7628615	7628816	chr5-7628615-7628816
chr5	7960139	7960719	chr5-7960139-7960719
chr5	8055068	8057765	chr5-8055068-8057765
chr5	8367129	8368527	chr5-8367129-8368527
chr5	8417256	8418322	chr5-8417256-8418322
chr5	8420398	8424522	chr5-8420398-8424522
chr5	8621727	8623614	chr5-8621727-8623614
chr5	8659850	8660915	chr5-8659850-8660915
chr5	8719790	8720420	chr5-8719790-8720420
chr5	8722417	8722618	chr5-8722417-8722618

chr5	8797780	8799399	chr5-8797780-8799399
chr5	8893141	8894345	chr5-8893141-8894345
chr5	8995807	8997896	chr5-8995807-8997896
chr5	9068794	9069512	chr5-9068794-9069512
chr5	9099960	9101984	chr5-9099960-9101984
chr5	9160360	9162872	chr5-9160360-9162872
chr5	9265607	9266926	chr5-9265607-9266926
chr5	9724981	9726279	chr5-9724981-9726279
chr5	12243250	12243591	chr5-12243250-12243591
chr5	12293709	12294128	chr5-12293709-12294128
chr5	12382934	12384367	chr5-12382934-12384367
chr5	12911199	12911400	chr5-12911199-12911400
chr5	13398577	13400774	chr5-13398577-13400774
chr5	13526956	13527311	chr5-13526956-13527311
chr5	13628637	13629310	chr5-13628637-13629310
chr5	13811422	13811623	chr5-13811422-13811623
chr5	13959714	13960364	chr5-13959714-13960364
chr5	13992939	13993671	chr5-13992939-13993671
chr5	14024941	14028965	chr5-14024941-14028965
chr5	14030246	14030506	chr5-14030246-14030506
chr5	14514257	14515886	chr5-14514257-14515886
chr5	15933938	15935693	chr5-15933938-15935693
chr5	16553440	16553641	chr5-16553440-16553641
chr5	17271497	17272793	chr5-17271497-17272793
chr5	17296544	17297147	chr5-17296544-17297147
chr5	17505735	17506092	chr5-17505735-17506092
chr5	17570408	17577054	chr5-17570408-17577054
chr5	17830268	17831027	chr5-17830268-17831027
chr5	18358834	18360985	chr5-18358834-18360985
chr5	18478593	18478907	chr5-18478593-18478907
chr5	19226120	19228411	chr5-19226120-19228411
chr5	20512450	20513017	chr5-20512450-20513017
chr5	20701558	20703336	chr5-20701558-20703336
chr5	20880494	20884557	chr5-20880494-20884557
chr5	20925660	20925911	chr5-20925660-20925911
chr5	20942496	20942847	chr5-20942496-20942847
chr5	20944908	20945826	chr5-20944908-20945826
chr5	20949569	20953045	chr5-20949569-20953045
chr5	21053162	21057662	chr5-21053162-21057662
chr5	21125086	21125287	chr5-21125086-21125287
chr5	21139632	21139990	chr5-21139632-21139990
chr5	21175228	21175839	chr5-21175228-21175839

chr5	21185860	21186874	chr5-21185860-21186874
chr5	21372450	21373161	chr5-21372450-21373161
chr5	21424445	21427253	chr5-21424445-21427253
chr5	21644941	21647604	chr5-21644941-21647604
chr5	21673211	21673614	chr5-21673211-21673614
chr5	21700769	21702251	chr5-21700769-21702251
chr5	21735052	21735922	chr5-21735052-21735922
chr5	21736865	21738492	chr5-21736865-21738492
chr5	21783928	21788678	chr5-21783928-21788678
chr5	21865372	21866022	chr5-21865372-21866022
chr5	22009196	22009508	chr5-22009196-22009508
chr5	22120480	22120788	chr5-22120480-22120788
chr5	22343945	22345649	chr5-22343945-22345649
chr5	22548869	22551348	chr5-22548869-22551348
chr5	22745675	22746921	chr5-22745675-22746921
chr5	23385773	23387355	chr5-23385773-23387355
chr5	23415924	23419701	chr5-23415924-23419701
chr5	23430721	23437753	chr5-23430721-23437753
chr5	23560984	23561290	chr5-23560984-23561290
chr5	23581351	23581602	chr5-23581351-23581602
chr5	23604610	23604864	chr5-23604610-23604864
chr5	23614813	23617467	chr5-23614813-23617467
chr5	23674573	23676656	chr5-23674573-23676656
chr5	23711454	23713199	chr5-23711454-23713199
chr5	23782028	23784155	chr5-23782028-23784155
chr5	23787291	23788993	chr5-23787291-23788993
chr5	23831132	23831548	chr5-23831132-23831548
chr5	23842406	23844988	chr5-23842406-23844988
chr5	23850190	23853806	chr5-23850190-23853806
chr5	23858793	23859160	chr5-23858793-23859160
chr5	23874385	23874918	chr5-23874385-23874918
chr5	23876210	23879621	chr5-23876210-23879621
chr5	23894103	23894768	chr5-23894103-23894768
chr5	24028992	24031104	chr5-24028992-24031104
chr5	24039255	24039998	chr5-24039255-24039998
chr5	24077154	24077841	chr5-24077154-24077841
chr5	24099917	24101805	chr5-24099917-24101805
chr5	24164304	24166132	chr5-24164304-24166132
chr5	24261145	24261346	chr5-24261145-24261346
chr5	24265869	24266953	chr5-24265869-24266953
chr5	24319916	24320918	chr5-24319916-24320918
chr5	24347476	24352845	chr5-24347476-24352845

chr5	24359280	24361751	chr5-24359280-24361751
chr5	24382995	24384906	chr5-24382995-24384906
chr5	24386259	24395371	chr5-24386259-24395371
chr5	24404522	24405230	chr5-24404522-24405230
chr5	24422517	24429187	chr5-24422517-24429187
chr5	24443706	24448443	chr5-24443706-24448443
chr5	24451135	24455759	chr5-24451135-24455759
chr5	24465186	24465913	chr5-24465186-24465913
chr5	24472808	24477230	chr5-24472808-24477230
chr5	24525914	24527415	chr5-24525914-24527415
chr5	24540531	24541208	chr5-24540531-24541208
chr5	24576133	24578610	chr5-24576133-24578610
chr5	24586114	24588529	chr5-24586114-24588529
chr5	24598035	24599206	chr5-24598035-24599206
chr5	24601053	24605745	chr5-24601053-24605745
chr5	24611724	24612629	chr5-24611724-24612629
chr5	24624079	24624735	chr5-24624079-24624735
chr5	24684551	24688035	chr5-24684551-24688035
chr5	24689678	24690138	chr5-24689678-24690138
chr5	24729775	24730947	chr5-24729775-24730947
chr5	24749791	24752559	chr5-24749791-24752559
chr5	24780035	24780392	chr5-24780035-24780392
chr5	24820737	24821686	chr5-24820737-24821686
chr5	24839750	24843776	chr5-24839750-24843776
chr5	24906867	24909614	chr5-24906867-24909614
chr5	24963623	24963874	chr5-24963623-24963874
chr5	24995097	24996355	chr5-24995097-24996355
chr5	25007094	25007473	chr5-25007094-25007473
chr5	25010592	25011340	chr5-25010592-25011340
chr5	25025810	25026011	chr5-25025810-25026011
chr5	25062370	25063011	chr5-25062370-25063011
chr5	25099317	25101635	chr5-25099317-25101635
chr5	25222597	25224292	chr5-25222597-25224292
chr5	25496754	25500609	chr5-25496754-25500609
chr5	25528749	25531004	chr5-25528749-25531004
chr5	25537028	25537330	chr5-25537028-25537330
chr5	25704518	25706503	chr5-25704518-25706503
chr5	25759401	25760655	chr5-25759401-25760655
chr5	25768894	25769095	chr5-25768894-25769095
chr5	25956686	25956991	chr5-25956686-25956991
chr5	26006924	26007661	chr5-26006924-26007661
chr5	27049544	27049858	chr5-27049544-27049858

chr5	27708196	27708501	chr5-27708196-27708501
chr5	27711516	27711768	chr5-27711516-27711768
chr5	27789494	27792912	chr5-27789494-27792912
chr5	28036834	28038451	chr5-28036834-28038451
chr5	28069646	28074906	chr5-28069646-28074906
chr5	28076172	28076531	chr5-28076172-28076531
chr5	28110486	28111285	chr5-28110486-28111285
chr5	28117658	28118901	chr5-28117658-28118901
chr5	28157968	28158741	chr5-28157968-28158741
chr5	28161666	28162526	chr5-28161666-28162526
chr5	28165107	28169036	chr5-28165107-28169036
chr5	28175067	28175564	chr5-28175067-28175564
chr5	28209529	28210458	chr5-28209529-28210458
chr5	28315492	28318947	chr5-28315492-28318947
chr5	28457823	28459864	chr5-28457823-28459864
chr5	28463930	28465740	chr5-28463930-28465740
chr5	28466589	28466842	chr5-28466589-28466842
chr5	28530307	28530598	chr5-28530307-28530598
chr5	28756908	28757229	chr5-28756908-28757229
chr5	29032869	29033070	chr5-29032869-29033070
chr5	29033952	29034153	chr5-29033952-29034153
chr5	29194648	29196673	chr5-29194648-29196673
chr5	29376926	29379628	chr5-29376926-29379628
chr5	29387754	29388688	chr5-29387754-29388688
chr5	29433981	29437143	chr5-29433981-29437143
chr5	29467521	29468843	chr5-29467521-29468843
chr5	29473710	29474429	chr5-29473710-29474429
chr5	29477271	29479585	chr5-29477271-29479585
chr5	29485260	29485967	chr5-29485260-29485967
chr5	29568500	29571017	chr5-29568500-29571017
chr5	29698238	29700493	chr5-29698238-29700493
chr5	29706513	29706714	chr5-29706513-29706714
chr5	29708151	29708562	chr5-29708151-29708562
chr5	29734593	29738524	chr5-29734593-29738524
chr5	29860967	29862226	chr5-29860967-29862226
chr5	30012820	30013543	chr5-30012820-30013543
chr5	30071672	30073978	chr5-30071672-30073978
chr5	30104612	30105873	chr5-30104612-30105873
chr5	30111034	30111505	chr5-30111034-30111505
chr5	30113828	30115242	chr5-30113828-30115242
chr5	30126957	30127158	chr5-30126957-30127158
chr5	30153650	30156683	chr5-30153650-30156683

chr5	30231441	30234749	chr5-30231441-30234749
chr5	30240678	30240879	chr5-30240678-30240879
chr5	30242685	30242936	chr5-30242685-30242936
chr5	30246096	30246457	chr5-30246096-30246457
chr5	30278990	30279549	chr5-30278990-30279549
chr5	30281427	30281628	chr5-30281427-30281628
chr5	30343141	30343392	chr5-30343141-30343392
chr5	30465825	30466401	chr5-30465825-30466401
chr5	30587830	30589161	chr5-30587830-30589161
chr5	30591561	30591762	chr5-30591561-30591762
chr5	30617441	30618341	chr5-30617441-30618341
chr5	30622053	30623129	chr5-30622053-30623129
chr5	30643689	30643890	chr5-30643689-30643890
chr5	30647364	30650174	chr5-30647364-30650174
chr5	30651008	30651580	chr5-30651008-30651580
chr5	30666193	30669105	chr5-30666193-30669105
chr5	30709958	30710220	chr5-30709958-30710220
chr5	30711146	30713818	chr5-30711146-30713818
chr5	30774912	30775316	chr5-30774912-30775316
chr5	30814032	30815700	chr5-30814032-30815700
chr5	30848964	30851430	chr5-30848964-30851430
chr5	30868953	30870910	chr5-30868953-30870910
chr5	30886663	30890813	chr5-30886663-30890813
chr5	30901733	30902036	chr5-30901733-30902036
chr5	30902858	30903059	chr5-30902858-30903059
chr5	30905546	30908736	chr5-30905546-30908736
chr5	30916182	30916504	chr5-30916182-30916504
chr5	30920424	30922743	chr5-30920424-30922743
chr5	30944934	30945739	chr5-30944934-30945739
chr5	30949356	30951243	chr5-30949356-30951243
chr5	30957794	30960870	chr5-30957794-30960870
chr5	30999856	31000877	chr5-30999856-31000877
chr5	31047091	31049780	chr5-31047091-31049780
chr5	31054023	31056937	chr5-31054023-31056937
chr5	31092473	31095745	chr5-31092473-31095745
chr5	31116209	31117468	chr5-31116209-31117468
chr5	31136740	31138897	chr5-31136740-31138897
chr5	31149722	31150266	chr5-31149722-31150266
chr5	31151574	31155129	chr5-31151574-31155129
chr5	31178692	31181259	chr5-31178692-31181259
chr5	31191888	31196928	chr5-31191888-31196928
chr5	31200265	31203358	chr5-31200265-31203358

chr5	31218285	31221860	chr5-31218285-31221860
chr5	31240310	31242548	chr5-31240310-31242548
chr5	31251066	31255685	chr5-31251066-31255685
chr5	31290253	31292045	chr5-31290253-31292045
chr5	31294970	31296482	chr5-31294970-31296482
chr5	31451916	31454264	chr5-31451916-31454264
chr5	31494204	31496265	chr5-31494204-31496265
chr5	31525760	31528692	chr5-31525760-31528692
chr5	31565223	31565424	chr5-31565223-31565424
chr5	31567822	31568687	chr5-31567822-31568687
chr5	31570934	31571996	chr5-31570934-31571996
chr5	31576157	31577645	chr5-31576157-31577645
chr5	31595601	31600391	chr5-31595601-31600391
chr5	31601935	31603085	chr5-31601935-31603085
chr5	31605650	31606953	chr5-31605650-31606953
chr5	31610437	31610889	chr5-31610437-31610889
chr5	31613522	31615731	chr5-31613522-31615731
chr5	31635064	31635265	chr5-31635064-31635265
chr5	31636362	31637446	chr5-31636362-31637446
chr5	31647654	31647958	chr5-31647654-31647958
chr5	31696786	31699326	chr5-31696786-31699326
chr5	31750614	31750872	chr5-31750614-31750872
chr5	31984870	31985071	chr5-31984870-31985071
chr5	32052418	32052842	chr5-32052418-32052842
chr5	32055831	32056343	chr5-32055831-32056343
chr5	32077437	32077638	chr5-32077437-32077638
chr5	32129492	32129799	chr5-32129492-32129799
chr5	32132359	32141385	chr5-32132359-32141385
chr5	32182934	32183617	chr5-32182934-32183617
chr5	32190854	32193146	chr5-32190854-32193146
chr5	32194981	32196503	chr5-32194981-32196503
chr5	32314961	32315218	chr5-32314961-32315218
chr5	32337851	32338852	chr5-32337851-32338852
chr5	32345878	32346792	chr5-32345878-32346792
chr5	32416787	32417100	chr5-32416787-32417100
chr5	32425274	32426022	chr5-32425274-32426022
chr5	32437723	32438576	chr5-32437723-32438576
chr5	32458146	32461639	chr5-32458146-32461639
chr5	32610182	32612737	chr5-32610182-32612737
chr5	32712264	32714821	chr5-32712264-32714821
chr5	32745734	32746733	chr5-32745734-32746733
chr5	32784239	32786502	chr5-32784239-32786502

chr5	32851863	32854486	chr5-32851863-32854486
chr5	32857448	32858164	chr5-32857448-32858164
chr5	32862729	32864762	chr5-32862729-32864762
chr5	33002945	33003197	chr5-33002945-33003197
chr5	33017772	33022067	chr5-33017772-33022067
chr5	33039595	33039846	chr5-33039595-33039846
chr5	33104113	33104731	chr5-33104113-33104731
chr5	33121663	33121999	chr5-33121663-33121999
chr5	33144317	33144518	chr5-33144317-33144518
chr5	33209062	33209263	chr5-33209062-33209263
chr5	33273175	33275862	chr5-33273175-33275862
chr5	33334691	33338447	chr5-33334691-33338447
chr5	33370294	33370605	chr5-33370294-33370605
chr5	33377429	33379858	chr5-33377429-33379858
chr5	33427177	33428178	chr5-33427177-33428178
chr5	33430559	33435226	chr5-33430559-33435226
chr5	33442947	33443552	chr5-33442947-33443552
chr5	33540137	33540599	chr5-33540137-33540599
chr5	33627791	33631004	chr5-33627791-33631004
chr5	33650192	33653099	chr5-33650192-33653099
chr5	33656396	33660457	chr5-33656396-33660457
chr5	33694764	33696126	chr5-33694764-33696126
chr5	33721067	33723917	chr5-33721067-33723917
chr5	33781444	33783477	chr5-33781444-33783477
chr5	33819165	33822840	chr5-33819165-33822840
chr5	33934870	33937610	chr5-33934870-33937610
chr5	33982611	33984461	chr5-33982611-33984461
chr5	33995561	33998624	chr5-33995561-33998624
chr5	34032802	34033106	chr5-34032802-34033106
chr5	34168216	34170222	chr5-34168216-34170222
chr5	34186221	34188846	chr5-34186221-34188846
chr5	34218315	34219324	chr5-34218315-34219324
chr5	34242384	34242898	chr5-34242384-34242898
chr5	34286891	34288966	chr5-34286891-34288966
chr5	34335214	34338960	chr5-34335214-34338960
chr5	34368801	34371695	chr5-34368801-34371695
chr5	34512846	34514684	chr5-34512846-34514684
chr5	34525625	34529198	chr5-34525625-34529198
chr5	34530754	34530955	chr5-34530754-34530955
chr5	34533871	34536816	chr5-34533871-34536816
chr5	34549012	34551447	chr5-34549012-34551447
chr5	34572723	34575760	chr5-34572723-34575760

chr5	34581788	34582427	chr5-34581788-34582427
chr5	34633856	34634123	chr5-34633856-34634123
chr5	34635073	34637691	chr5-34635073-34637691
chr5	34658292	34661684	chr5-34658292-34661684
chr5	34663030	34663683	chr5-34663030-34663683
chr5	34760667	34763206	chr5-34760667-34763206
chr5	34865244	34865445	chr5-34865244-34865445
chr5	34948557	34950511	chr5-34948557-34950511
chr5	34998092	35006499	chr5-34998092-35006499
chr5	35019349	35021471	chr5-35019349-35021471
chr5	35032972	35034252	chr5-35032972-35034252
chr5	35036642	35039285	chr5-35036642-35039285
chr5	35040476	35043956	chr5-35040476-35043956
chr5	35054607	35058913	chr5-35054607-35058913
chr5	35073860	35074607	chr5-35073860-35074607
chr5	35104782	35106412	chr5-35104782-35106412
chr5	35278871	35280899	chr5-35278871-35280899
chr5	35313938	35314139	chr5-35313938-35314139
chr5	35388409	35389479	chr5-35388409-35389479
chr5	35391477	35392638	chr5-35391477-35392638
chr5	35395646	35396151	chr5-35395646-35396151
chr5	35396980	35398150	chr5-35396980-35398150
chr5	35525406	35525659	chr5-35525406-35525659
chr5	35573701	35576130	chr5-35573701-35576130
chr5	35582251	35584287	chr5-35582251-35584287
chr5	35672721	35674600	chr5-35672721-35674600
chr5	35678952	35679683	chr5-35678952-35679683
chr5	35717468	35719144	chr5-35717468-35719144
chr5	35722467	35723400	chr5-35722467-35723400
chr5	35727970	35729769	chr5-35727970-35729769
chr5	35733139	35735025	chr5-35733139-35735025
chr5	35736875	35741754	chr5-35736875-35741754
chr5	35743651	35744109	chr5-35743651-35744109
chr5	35755193	35755657	chr5-35755193-35755657
chr5	35756814	35758978	chr5-35756814-35758978
chr5	35770902	35771397	chr5-35770902-35771397
chr5	35811687	35811888	chr5-35811687-35811888
chr5	35892925	35895254	chr5-35892925-35895254
chr5	35907138	35909112	chr5-35907138-35909112
chr5	35973641	35974781	chr5-35973641-35974781
chr5	36041755	36041956	chr5-36041755-36041956
chr5	36085754	36086322	chr5-36085754-36086322

chr5	36184167	36184680	chr5-36184167-36184680
chr5	36202998	36205788	chr5-36202998-36205788
chr5	36208203	36209636	chr5-36208203-36209636
chr5	36279832	36280033	chr5-36279832-36280033
chr5	36293825	36294026	chr5-36293825-36294026
chr5	36353372	36354222	chr5-36353372-36354222
chr5	36356608	36356809	chr5-36356608-36356809
chr5	36358194	36359605	chr5-36358194-36359605
chr5	36373354	36373555	chr5-36373354-36373555
chr5	36377691	36378458	chr5-36377691-36378458
chr5	36379639	36380799	chr5-36379639-36380799
chr5	36395130	36399118	chr5-36395130-36399118
chr5	36414689	36415373	chr5-36414689-36415373
chr5	36460567	36461798	chr5-36460567-36461798
chr5	36462769	36466749	chr5-36462769-36466749
chr5	36482675	36485626	chr5-36482675-36485626
chr5	36528615	36530819	chr5-36528615-36530819
chr5	36574163	36574808	chr5-36574163-36574808
chr5	36581049	36583010	chr5-36581049-36583010
chr5	36585877	36586186	chr5-36585877-36586186
chr5	36675133	36675801	chr5-36675133-36675801
chr5	36693457	36699639	chr5-36693457-36699639
chr5	36700877	36701353	chr5-36700877-36701353
chr5	36711824	36715617	chr5-36711824-36715617
chr5	36717914	36718115	chr5-36717914-36718115
chr5	36725999	36726784	chr5-36725999-36726784
chr5	36746792	36749238	chr5-36746792-36749238
chr5	36794909	36797671	chr5-36794909-36797671
chr5	36829529	36831090	chr5-36829529-36831090
chr5	36838952	36842306	chr5-36838952-36842306
chr5	36850303	36850504	chr5-36850303-36850504
chr5	36867256	36871411	chr5-36867256-36871411
chr5	36894572	36894875	chr5-36894572-36894875
chr5	36978579	36979867	chr5-36978579-36979867
chr5	36985512	36991257	chr5-36985512-36991257
chr5	37000484	37001705	chr5-37000484-37001705
chr5	37027939	37029697	chr5-37027939-37029697
chr5	37091077	37091657	chr5-37091077-37091657
chr5	37162933	37163541	chr5-37162933-37163541
chr5	37241717	37242679	chr5-37241717-37242679
chr5	37245430	37246539	chr5-37245430-37246539
chr5	37336482	37337160	chr5-37336482-37337160

chr5	37338300	37338863	chr5-37338300-37338863
chr5	37717188	37717389	chr5-37717188-37717389
chr5	37816956	37817494	chr5-37816956-37817494
chr5	37823024	37824893	chr5-37823024-37824893
chr5	37826642	37827568	chr5-37826642-37827568
chr5	37893614	37893815	chr5-37893614-37893815
chr5	38038268	38040222	chr5-38038268-38040222
chr5	38158681	38159932	chr5-38158681-38159932
chr5	38219228	38222315	chr5-38219228-38222315
chr5	38259500	38261560	chr5-38259500-38261560
chr5	38276831	38277907	chr5-38276831-38277907
chr5	38307637	38308090	chr5-38307637-38308090
chr5	38319233	38319817	chr5-38319233-38319817
chr5	38446626	38446827	chr5-38446626-38446827
chr5	38479171	38480805	chr5-38479171-38480805
chr5	38553613	38554644	chr5-38553613-38554644
chr5	38559462	38563325	chr5-38559462-38563325
chr5	38598379	38598732	chr5-38598379-38598732
chr5	38683509	38685254	chr5-38683509-38685254
chr5	38730690	38731568	chr5-38730690-38731568
chr5	38734228	38734852	chr5-38734228-38734852
chr5	39612262	39612463	chr5-39612262-39612463
chr5	39642814	39645538	chr5-39642814-39645538
chr5	39761775	39762084	chr5-39761775-39762084
chr5	39831607	39831976	chr5-39831607-39831976
chr5	41706742	41708925	chr5-41706742-41708925
chr5	41761234	41762373	chr5-41761234-41762373
chr5	41763208	41764966	chr5-41763208-41764966
chr5	41767183	41768732	chr5-41767183-41768732
chr5	41842784	41845677	chr5-41842784-41845677
chr5	42155849	42156050	chr5-42155849-42156050
chr5	43072615	43073707	chr5-43072615-43073707
chr5	43110008	43110619	chr5-43110008-43110619
chr5	43232127	43236381	chr5-43232127-43236381
chr5	43354648	43355763	chr5-43354648-43355763
chr5	43662388	43663024	chr5-43662388-43663024
chr5	43672161	43673122	chr5-43672161-43673122
chr5	43777950	43778151	chr5-43777950-43778151
chr5	43779136	43783048	chr5-43779136-43783048
chr5	43799496	43799697	chr5-43799496-43799697
chr5	43807498	43810033	chr5-43807498-43810033
chr5	43818692	43821687	chr5-43818692-43821687

chr5	43841431	43842199	chr5-43841431-43842199
chr5	43868678	43869650	chr5-43868678-43869650
chr5	43947311	43949652	chr5-43947311-43949652
chr5	43979194	43983036	chr5-43979194-43983036
chr5	44100104	44102094	chr5-44100104-44102094
chr5	44224405	44228071	chr5-44224405-44228071
chr5	45127616	45128295	chr5-45127616-45128295
chr5	45136929	45137305	chr5-45136929-45137305
chr5	45277997	45278865	chr5-45277997-45278865
chr5	45448848	45450958	chr5-45448848-45450958
chr5	45492800	45495112	chr5-45492800-45495112
chr5	45519650	45521958	chr5-45519650-45521958
chr5	45638774	45640205	chr5-45638774-45640205
chr5	45668633	45671238	chr5-45668633-45671238
chr5	45855755	45858450	chr5-45855755-45858450
chr5	46032922	46033543	chr5-46032922-46033543
chr5	46072972	46073349	chr5-46072972-46073349
chr5	46445820	46446231	chr5-46445820-46446231
chr5	47109024	47109275	chr5-47109024-47109275
chr5	47502983	47503184	chr5-47502983-47503184
chr5	47982312	47987356	chr5-47982312-47987356
chr5	48371823	48373360	chr5-48371823-48373360
chr5	48889896	48890147	chr5-48889896-48890147
chr5	49285501	49285783	chr5-49285501-49285783
chr5	49524343	49525215	chr5-49524343-49525215
chr5	50057520	50059987	chr5-50057520-50059987
chr5	51045375	51045576	chr5-51045375-51045576
chr5	51246301	51246554	chr5-51246301-51246554
chr5	51551791	51553965	chr5-51551791-51553965
chr5	51591672	51591873	chr5-51591672-51591873
chr5	51868375	51868684	chr5-51868375-51868684
chr5	52112729	52116535	chr5-52112729-52116535
chr5	52138102	52139452	chr5-52138102-52139452
chr5	52187316	52191516	chr5-52187316-52191516
chr5	52216319	52216723	chr5-52216319-52216723
chr5	52470459	52472145	chr5-52470459-52472145
chr5	52476204	52476774	chr5-52476204-52476774
chr5	52495562	52496383	chr5-52495562-52496383
chr5	52515531	52516553	chr5-52515531-52516553
chr5	52565484	52566638	chr5-52565484-52566638
chr5	52618280	52619171	chr5-52618280-52619171
chr5	52626482	52626896	chr5-52626482-52626896

chr5	52668659	52670183	chr5-52668659-52670183
chr5	52720889	52721405	chr5-52720889-52721405
chr5	52741105	52744228	chr5-52741105-52744228
chr5	52752905	52753583	chr5-52752905-52753583
chr5	52782587	52784507	chr5-52782587-52784507
chr5	52786105	52787127	chr5-52786105-52787127
chr5	52833177	52835516	chr5-52833177-52835516
chr5	52865366	52866105	chr5-52865366-52866105
chr5	52878737	52879099	chr5-52878737-52879099
chr5	52909666	52910909	chr5-52909666-52910909
chr5	53042212	53042790	chr5-53042212-53042790
chr5	53048889	53049554	chr5-53048889-53049554
chr5	53171925	53172126	chr5-53171925-53172126
chr5	53212215	53214380	chr5-53212215-53214380
chr5	53258097	53258778	chr5-53258097-53258778
chr5	53266451	53268372	chr5-53266451-53268372
chr5	53355247	53355651	chr5-53355247-53355651
chr5	53444160	53444516	chr5-53444160-53444516
chr5	53460084	53460391	chr5-53460084-53460391
chr5	53463055	53463899	chr5-53463055-53463899
chr5	53479744	53482172	chr5-53479744-53482172
chr5	53490506	53490758	chr5-53490506-53490758
chr5	53497110	53497361	chr5-53497110-53497361
chr5	53509714	53510139	chr5-53509714-53510139
chr5	53543569	53544646	chr5-53543569-53544646
chr5	53564246	53564790	chr5-53564246-53564790
chr5	53568789	53569093	chr5-53568789-53569093
chr5	53588130	53596172	chr5-53588130-53596172
chr5	53808961	53810609	chr5-53808961-53810609
chr5	53948340	53949229	chr5-53948340-53949229
chr5	53952828	53954276	chr5-53952828-53954276
chr5	53955960	53956221	chr5-53955960-53956221
chr5	53961845	53962412	chr5-53961845-53962412
chr5	53994799	53996160	chr5-53994799-53996160
chr5	53997539	54000877	chr5-53997539-54000877
chr5	54206957	54207691	chr5-54206957-54207691
chr5	57714126	57724310	chr5-57714126-57724310
chr5	62764191	62766867	chr5-62764191-62766867
chr5	63649625	63650364	chr5-63649625-63650364
chr5	63811808	63813369	chr5-63811808-63813369
chr5	63826084	63827661	chr5-63826084-63827661
chr5	63936803	63937127	chr5-63936803-63937127

chr5	63964307	63964982	chr5-63964307-63964982
chr5	63967389	63970524	chr5-63967389-63970524
chr5	63982162	63982687	chr5-63982162-63982687
chr5	64038059	64038621	chr5-64038059-64038621
chr5	64045390	64046016	chr5-64045390-64046016
chr5	64047378	64047579	chr5-64047378-64047579
chr5	64092514	64094357	chr5-64092514-64094357
chr5	64112782	64113033	chr5-64112782-64113033
chr5	64146364	64146682	chr5-64146364-64146682
chr5	64158621	64162445	chr5-64158621-64162445
chr5	64196743	64197114	chr5-64196743-64197114
chr5	64224981	64225389	chr5-64224981-64225389
chr5	64229465	64231421	chr5-64229465-64231421
chr5	64242954	64243497	chr5-64242954-64243497
chr5	64360303	64360883	chr5-64360303-64360883
chr5	64379002	64379203	chr5-64379002-64379203
chr5	64382750	64383680	chr5-64382750-64383680
chr5	64391367	64393862	chr5-64391367-64393862
chr5	64437623	64438867	chr5-64437623-64438867
chr5	64488146	64488347	chr5-64488146-64488347
chr5	64500528	64501128	chr5-64500528-64501128
chr5	64505144	64506827	chr5-64505144-64506827
chr5	64508464	64509020	chr5-64508464-64509020
chr5	64579454	64580627	chr5-64579454-64580627
chr5	64587807	64588108	chr5-64587807-64588108
chr5	64609791	64611284	chr5-64609791-64611284
chr5	64626144	64626832	chr5-64626144-64626832
chr5	64665138	64667493	chr5-64665138-64667493
chr5	64699958	64700324	chr5-64699958-64700324
chr5	64701142	64702313	chr5-64701142-64702313
chr5	64751312	64751944	chr5-64751312-64751944
chr5	64802226	64814504	chr5-64802226-64814504
chr5	64860822	64862699	chr5-64860822-64862699
chr5	64864241	64865568	chr5-64864241-64865568
chr5	64969240	64971010	chr5-64969240-64971010
chr5	64992765	64992966	chr5-64992765-64992966
chr5	65086672	65087089	chr5-65086672-65087089
chr5	65106891	65108551	chr5-65106891-65108551
chr5	65194432	65194796	chr5-65194432-65194796
chr5	65198834	65200626	chr5-65198834-65200626
chr5	65326282	65326602	chr5-65326282-65326602
chr5	65334659	65336617	chr5-65334659-65336617

chr5	65388417	65392650	chr5-65388417-65392650
chr5	65434307	65437793	chr5-65434307-65437793
chr5	65454149	65455589	chr5-65454149-65455589
chr5	65457532	65457733	chr5-65457532-65457733
chr5	65478699	65480534	chr5-65478699-65480534
chr5	65490009	65493877	chr5-65490009-65493877
chr5	65524698	65525012	chr5-65524698-65525012
chr5	65536004	65539251	chr5-65536004-65539251
chr5	65695287	65699290	chr5-65695287-65699290
chr5	65705325	65705526	chr5-65705325-65705526
chr5	65724319	65724982	chr5-65724319-65724982
chr5	65762581	65765926	chr5-65762581-65765926
chr5	65955862	65956369	chr5-65955862-65956369
chr5	66040770	66041725	chr5-66040770-66041725
chr5	66052733	66053250	chr5-66052733-66053250
chr5	66054467	66054668	chr5-66054467-66054668
chr5	66059063	66059617	chr5-66059063-66059617
chr5	66069134	66070764	chr5-66069134-66070764
chr5	66075265	66075466	chr5-66075265-66075466
chr5	66076831	66078155	chr5-66076831-66078155
chr5	66079466	66083795	chr5-66079466-66083795
chr5	66088334	66090588	chr5-66088334-66090588
chr5	66112148	66112506	chr5-66112148-66112506
chr5	66114310	66115235	chr5-66114310-66115235
chr5	66142954	66152723	chr5-66142954-66152723
chr5	66165181	66166848	chr5-66165181-66166848
chr5	66199837	66200512	chr5-66199837-66200512
chr5	66214598	66214799	chr5-66214598-66214799
chr5	66259473	66260681	chr5-66259473-66260681
chr5	66374193	66374605	chr5-66374193-66374605
chr5	66497187	66498367	chr5-66497187-66498367
chr5	66503612	66504780	chr5-66503612-66504780
chr5	66518389	66519146	chr5-66518389-66519146
chr5	66523484	66524252	chr5-66523484-66524252
chr5	66532943	66533473	chr5-66532943-66533473
chr5	66542451	66544658	chr5-66542451-66544658
chr5	66547233	66547785	chr5-66547233-66547785
chr5	66554519	66554720	chr5-66554519-66554720
chr5	66558218	66560857	chr5-66558218-66560857
chr5	66597175	66597638	chr5-66597175-66597638
chr5	66599581	66601505	chr5-66599581-66601505
chr5	66602313	66604504	chr5-66602313-66604504

chr5	66614471	66621707	chr5-66614471-66621707
chr5	66649222	66649476	chr5-66649222-66649476
chr5	66675612	66677302	chr5-66675612-66677302
chr5	66745295	66746782	chr5-66745295-66746782
chr5	66997021	66997280	chr5-66997021-66997280
chr5	67095936	67096803	chr5-67095936-67096803
chr5	67216488	67216741	chr5-67216488-67216741
chr5	67222466	67222970	chr5-67222466-67222970
chr5	67230131	67231674	chr5-67230131-67231674
chr5	67259945	67262390	chr5-67259945-67262390
chr5	67306196	67308291	chr5-67306196-67308291
chr5	67410560	67411090	chr5-67410560-67411090
chr5	67426214	67428509	chr5-67426214-67428509
chr5	67532558	67533060	chr5-67532558-67533060
chr5	67607122	67609010	chr5-67607122-67609010
chr5	67628529	67628730	chr5-67628529-67628730
chr5	67797004	67797943	chr5-67797004-67797943
chr5	67815457	67816198	chr5-67815457-67816198
chr5	67842256	67842840	chr5-67842256-67842840
chr5	67843822	67848395	chr5-67843822-67848395
chr5	69340787	69341848	chr5-69340787-69341848
chr5	69556432	69558058	chr5-69556432-69558058
chr5	69591173	69592689	chr5-69591173-69592689
chr5	71095160	71096463	chr5-71095160-71096463
chr5	71657471	71658505	chr5-71657471-71658505
chr5	71700632	71701528	chr5-71700632-71701528
chr5	72043549	72043851	chr5-72043549-72043851
chr5	72167069	72168706	chr5-72167069-72168706
chr5	72201787	72201988	chr5-72201787-72201988
chr5	72202955	72204126	chr5-72202955-72204126
chr5	72503516	72504597	chr5-72503516-72504597
chr5	72558106	72560333	chr5-72558106-72560333
chr5	72580096	72581306	chr5-72580096-72581306
chr5	72586862	72587826	chr5-72586862-72587826
chr5	72646813	72650191	chr5-72646813-72650191
chr5	72802921	72803122	chr5-72802921-72803122
chr5	72844486	72844687	chr5-72844486-72844687
chr5	72854244	72854760	chr5-72854244-72854760
chr5	72855604	72856625	chr5-72855604-72856625
chr5	72863228	72864152	chr5-72863228-72864152
chr5	72866590	72869201	chr5-72866590-72869201
chr5	72905189	72905802	chr5-72905189-72905802

chr5	72913093	72916952	chr5-72913093-72916952
chr5	73006669	73007715	chr5-73006669-73007715
chr5	73013307	73014422	chr5-73013307-73014422
chr5	73156575	73156970	chr5-73156575-73156970
chr5	73168274	73168993	chr5-73168274-73168993
chr5	73183090	73183509	chr5-73183090-73183509
chr5	73190121	73190372	chr5-73190121-73190372
chr5	73232852	73233878	chr5-73232852-73233878
chr5	73252066	73252267	chr5-73252066-73252267
chr5	73254514	73258040	chr5-73254514-73258040
chr5	73292512	73295367	chr5-73292512-73295367
chr5	73337827	73339095	chr5-73337827-73339095
chr5	73342911	73343170	chr5-73342911-73343170
chr5	73344354	73344660	chr5-73344354-73344660
chr5	73367808	73368009	chr5-73367808-73368009
chr5	73372807	73373453	chr5-73372807-73373453
chr5	73400125	73401930	chr5-73400125-73401930
chr5	73405653	73408088	chr5-73405653-73408088
chr5	73409752	73410030	chr5-73409752-73410030
chr5	73489832	73493351	chr5-73489832-73493351
chr5	73544203	73544467	chr5-73544203-73544467
chr5	73559428	73560505	chr5-73559428-73560505
chr5	73565973	73567092	chr5-73565973-73567092
chr5	73611634	73611835	chr5-73611634-73611835
chr5	73646858	73648225	chr5-73646858-73648225
chr5	73650522	73652206	chr5-73650522-73652206
chr5	74030128	74030329	chr5-74030128-74030329
chr5	74051879	74052397	chr5-74051879-74052397
chr5	74060945	74070081	chr5-74060945-74070081
chr5	74092406	74095424	chr5-74092406-74095424
chr5	74126316	74127024	chr5-74126316-74127024
chr5	74136479	74136680	chr5-74136479-74136680
chr5	74142167	74142473	chr5-74142167-74142473
chr5	74161286	74162106	chr5-74161286-74162106
chr5	74171842	74172824	chr5-74171842-74172824
chr5	74194498	74196430	chr5-74194498-74196430
chr5	74208648	74209102	chr5-74208648-74209102
chr5	74285191	74285392	chr5-74285191-74285392
chr5	74530110	74532397	chr5-74530110-74532397
chr5	74534893	74537448	chr5-74534893-74537448
chr5	74702389	74704839	chr5-74702389-74704839
chr5	74738670	74738929	chr5-74738670-74738929

chr5	74792238	74792579	chr5-74792238-74792579
chr5	74897242	74897493	chr5-74897242-74897493
chr5	74935505	74936516	chr5-74935505-74936516
chr5	74937730	74937931	chr5-74937730-74937931
chr5	74943782	74944641	chr5-74943782-74944641
chr5	74953823	74954082	chr5-74953823-74954082
chr5	74960452	74960762	chr5-74960452-74960762
chr5	75042010	75045369	chr5-75042010-75045369
chr5	75046581	75047210	chr5-75046581-75047210
chr5	75066842	75067850	chr5-75066842-75067850
chr5	75071062	75071386	chr5-75071062-75071386
chr5	75075330	75076551	chr5-75075330-75076551
chr5	75083178	75083429	chr5-75083178-75083429
chr5	75084841	75085845	chr5-75084841-75085845
chr5	75142967	75143423	chr5-75142967-75143423
chr5	75149834	75157321	chr5-75149834-75157321
chr5	75574510	75576117	chr5-75574510-75576117
chr5	75831765	75832874	chr5-75831765-75832874
chr5	75921758	75923119	chr5-75921758-75923119
chr5	75977480	75979246	chr5-75977480-75979246
chr5	76139555	76141797	chr5-76139555-76141797
chr5	76176500	76176751	chr5-76176500-76176751
chr5	76183171	76186046	chr5-76183171-76186046
chr5	76194110	76194369	chr5-76194110-76194369
chr5	76303161	76306155	chr5-76303161-76306155
chr5	76346414	76347028	chr5-76346414-76347028
chr5	76369058	76369706	chr5-76369058-76369706
chr5	76436910	76437324	chr5-76436910-76437324
chr5	76524591	76524792	chr5-76524591-76524792
chr5	76528908	76530607	chr5-76528908-76530607
chr5	76588440	76589933	chr5-76588440-76589933
chr5	76657296	76658579	chr5-76657296-76658579
chr5	76708067	76708390	chr5-76708067-76708390
chr5	76769693	76770264	chr5-76769693-76770264
chr5	76856222	76858206	chr5-76856222-76858206
chr5	76904431	76906571	chr5-76904431-76906571
chr5	76929868	76930255	chr5-76929868-76930255
chr5	76947746	76948112	chr5-76947746-76948112
chr5	76949331	76953783	chr5-76949331-76953783
chr5	76973762	76976046	chr5-76973762-76976046
chr5	77015511	77016673	chr5-77015511-77016673
chr5	77094153	77095626	chr5-77094153-77095626

chr5	77140225	77140426	chr5-77140225-77140426
chr5	77154772	77155520	chr5-77154772-77155520
chr5	77211181	77211435	chr5-77211181-77211435
chr5	77217292	77218002	chr5-77217292-77218002
chr5	77245533	77246690	chr5-77245533-77246690
chr5	77248208	77248735	chr5-77248208-77248735
chr5	77264772	77268213	chr5-77264772-77268213
chr5	77307998	77311872	chr5-77307998-77311872
chr5	77354729	77354981	chr5-77354729-77354981
chr5	77357339	77359035	chr5-77357339-77359035
chr5	77364759	77365074	chr5-77364759-77365074
chr5	77373275	77373810	chr5-77373275-77373810
chr5	77383633	77387793	chr5-77383633-77387793
chr5	77398032	77399127	chr5-77398032-77399127
chr5	77404963	77408550	chr5-77404963-77408550
chr5	77459956	77460659	chr5-77459956-77460659
chr5	77515274	77515475	chr5-77515274-77515475
chr5	77580804	77581913	chr5-77580804-77581913
chr5	81019408	81023090	chr5-81019408-81023090
chr5	81275975	81276379	chr5-81275975-81276379
chr5	81365333	81365794	chr5-81365333-81365794
chr5	82362004	82362255	chr5-82362004-82362255
chr5	84416055	84417809	chr5-84416055-84417809
chr5	86063650	86067385	chr5-86063650-86067385
chr5	86170933	86173758	chr5-86170933-86173758
chr5	86318113	86318666	chr5-86318113-86318666
chr5	86728338	86728539	chr5-86728338-86728539
chr5	86735784	86736114	chr5-86735784-86736114
chr5	86740836	86746296	chr5-86740836-86746296
chr5	86803329	86806446	chr5-86803329-86806446
chr5	87925516	87925717	chr5-87925516-87925717
chr5	88553145	88556399	chr5-88553145-88556399
chr5	88563979	88566845	chr5-88563979-88566845
chr5	88674480	88676695	chr5-88674480-88676695
chr5	88678820	88679021	chr5-88678820-88679021
chr5	88688711	88689173	chr5-88688711-88689173
chr5	88719690	88722661	chr5-88719690-88722661
chr5	88764311	88766569	chr5-88764311-88766569
chr5	88886180	88889669	chr5-88886180-88889669
chr5	88912484	88912736	chr5-88912484-88912736
chr5	88929399	88930469	chr5-88929399-88930469
chr5	88982753	88983763	chr5-88982753-88983763

chr5	89277139	89278177	chr5-89277139-89278177
chr5	89346195	89346511	chr5-89346195-89346511
chr5	89376924	89380589	chr5-89376924-89380589
chr5	89389801	89390472	chr5-89389801-89390472
chr5	89407154	89407411	chr5-89407154-89407411
chr5	89526742	89527460	chr5-89526742-89527460
chr5	89748544	89748745	chr5-89748544-89748745
chr5	89881875	89883948	chr5-89881875-89883948
chr5	90167211	90167412	chr5-90167211-90167412
chr5	90200054	90200310	chr5-90200054-90200310
chr5	90222827	90225121	chr5-90222827-90225121
chr5	90339665	90339866	chr5-90339665-90339866
chr5	90363670	90367718	chr5-90363670-90367718
chr5	90504872	90505127	chr5-90504872-90505127
chr5	90588745	90589253	chr5-90588745-90589253
chr5	90603520	90603984	chr5-90603520-90603984
chr5	90605456	90605927	chr5-90605456-90605927
chr5	90611309	90613478	chr5-90611309-90613478
chr5	90639133	90641275	chr5-90639133-90641275
chr5	90682257	90682458	chr5-90682257-90682458
chr5	90689485	90690396	chr5-90689485-90690396
chr5	90706658	90706912	chr5-90706658-90706912
chr5	90757911	90762094	chr5-90757911-90762094
chr5	90764335	90764536	chr5-90764335-90764536
chr5	90765891	90766304	chr5-90765891-90766304
chr5	90768080	90768837	chr5-90768080-90768837
chr5	90785577	90787597	chr5-90785577-90787597
chr5	90832471	90832672	chr5-90832471-90832672
chr5	90875772	90877133	chr5-90875772-90877133
chr5	90881695	90882171	chr5-90881695-90882171
chr5	90890780	90892248	chr5-90890780-90892248
chr5	90898108	90901307	chr5-90898108-90901307
chr5	90903000	90904267	chr5-90903000-90904267
chr5	90930033	90932892	chr5-90930033-90932892
chr5	90987565	90987766	chr5-90987565-90987766
chr5	91036828	91037331	chr5-91036828-91037331
chr5	91072221	91077564	chr5-91072221-91077564
chr5	91098343	91098594	chr5-91098343-91098594
chr5	91110215	91110416	chr5-91110215-91110416
chr5	91138961	91142341	chr5-91138961-91142341
chr5	91156850	91157592	chr5-91156850-91157592
chr5	91194512	91194835	chr5-91194512-91194835

chr5	91207280	91207589	chr5-91207280-91207589
chr5	91242057	91242475	chr5-91242057-91242475
chr5	91401044	91403249	chr5-91401044-91403249
chr5	91517313	91519274	chr5-91517313-91519274
chr5	91960228	91964025	chr5-91960228-91964025
chr5	91986975	91989442	chr5-91986975-91989442
chr5	92041818	92044180	chr5-92041818-92044180
chr5	92081277	92084589	chr5-92081277-92084589
chr5	92093778	92093979	chr5-92093778-92093979
chr5	92136801	92140062	chr5-92136801-92140062
chr5	92141922	92142687	chr5-92141922-92142687
chr5	92205419	92205963	chr5-92205419-92205963
chr5	92219111	92221391	chr5-92219111-92221391
chr5	92230315	92230516	chr5-92230315-92230516
chr5	92239807	92240127	chr5-92239807-92240127
chr5	92276856	92278966	chr5-92276856-92278966
chr5	92282589	92283992	chr5-92282589-92283992
chr5	92308408	92310673	chr5-92308408-92310673
chr5	92348641	92349941	chr5-92348641-92349941
chr5	92352707	92354085	chr5-92352707-92354085
chr5	92363158	92363359	chr5-92363158-92363359
chr5	92392254	92393316	chr5-92392254-92393316
chr5	92433063	92435928	chr5-92433063-92435928
chr5	92503555	92506601	chr5-92503555-92506601
chr5	92598704	92599717	chr5-92598704-92599717
chr5	92602332	92604638	chr5-92602332-92604638
chr5	92674744	92676021	chr5-92674744-92676021
chr5	92682776	92683815	chr5-92682776-92683815
chr5	92745389	92746284	chr5-92745389-92746284
chr5	92808554	92813453	chr5-92808554-92813453
chr5	92824033	92824894	chr5-92824033-92824894
chr5	92854913	92858685	chr5-92854913-92858685
chr5	92888812	92889164	chr5-92888812-92889164
chr5	92897195	92899124	chr5-92897195-92899124
chr5	92924346	92926238	chr5-92924346-92926238
chr5	92927620	92928170	chr5-92927620-92928170
chr5	92933087	92934807	chr5-92933087-92934807
chr5	92941896	92942992	chr5-92941896-92942992
chr5	92960939	92963740	chr5-92960939-92963740
chr5	92966357	92968053	chr5-92966357-92968053
chr5	92969979	92970529	chr5-92969979-92970529
chr5	93005119	93006719	chr5-93005119-93006719

chr5	93040002	93046283	chr5-93040002-93046283
chr5	93091267	93095801	chr5-93091267-93095801
chr5	93130188	93132295	chr5-93130188-93132295
chr5	93135591	93135846	chr5-93135591-93135846
chr5	93204382	93207476	chr5-93204382-93207476
chr5	93265940	93270404	chr5-93265940-93270404
chr5	93296124	93296947	chr5-93296124-93296947
chr5	93298139	93298690	chr5-93298139-93298690
chr5	96159944	96164335	chr5-96159944-96164335
chr5	96209038	96211350	chr5-96209038-96211350
chr5	96221367	96222550	chr5-96221367-96222550
chr5	96373558	96375983	chr5-96373558-96375983
chr5	96617396	96617597	chr5-96617396-96617597
chr5	96783493	96784566	chr5-96783493-96784566
chr5	96792223	96808000	chr5-96792223-96808000
chr5	96898084	96899165	chr5-96898084-96899165
chr5	96905946	96906297	chr5-96905946-96906297
chr5	96996222	97000184	chr5-96996222-97000184
chr5	97110328	97112049	chr5-97110328-97112049
chr5	97305345	97305659	chr5-97305345-97305659
chr5	97382750	97383310	chr5-97382750-97383310
chr5	97986800	97987064	chr5-97986800-97987064
chr5	98028396	98032828	chr5-98028396-98032828
chr5	98034098	98034770	chr5-98034098-98034770
chr5	98076552	98077245	chr5-98076552-98077245
chr5	98079036	98079237	chr5-98079036-98079237
chr5	98166807	98169258	chr5-98166807-98169258
chr5	98180510	98181689	chr5-98180510-98181689
chr5	98184917	98186789	chr5-98184917-98186789
chr5	98253489	98255075	chr5-98253489-98255075
chr5	98283913	98284221	chr5-98283913-98284221
chr5	98292923	98293401	chr5-98292923-98293401
chr5	98328849	98329911	chr5-98328849-98329911
chr5	98429686	98430505	chr5-98429686-98430505
chr5	98844245	98844654	chr5-98844245-98844654
chr5	98854352	98855948	chr5-98854352-98855948
chr5	98891015	98891266	chr5-98891015-98891266
chr5	98939251	98944487	chr5-98939251-98944487
chr5	99036378	99037784	chr5-99036378-99037784
chr5	99202511	99202865	chr5-99202511-99202865
chr5	99224048	99224986	chr5-99224048-99224986
chr5	99236528	99237755	chr5-99236528-99237755

chr5	99239209	99242652	chr5-99239209-99242652
chr5	99249902	99253618	chr5-99249902-99253618
chr5	99380017	99380592	chr5-99380017-99380592
chr5	99420110	99420623	chr5-99420110-99420623
chr5	99479226	99480167	chr5-99479226-99480167
chr5	99499646	99499999	chr5-99499646-99499999
chr5	99727965	99729795	chr5-99727965-99729795
chr5	99906065	99907185	chr5-99906065-99907185
chr5	99973931	99980296	chr5-99973931-99980296
chr5	100035526	100041242	chr5-100035526-100041242
chr5	100048059	100048483	chr5-100048059-100048483
chr5	100079084	100079542	chr5-100079084-100079542
chr5	100085288	100085489	chr5-100085288-100085489
chr5	100158563	100159931	chr5-100158563-100159931
chr5	100185959	100186160	chr5-100185959-100186160
chr5	100413523	100417995	chr5-100413523-100417995
chr5	100423648	100430946	chr5-100423648-100430946
chr5	100497717	100501297	chr5-100497717-100501297
chr5	100517214	100519624	chr5-100517214-100519624
chr5	100606096	100606463	chr5-100606096-100606463
chr5	100620890	100623767	chr5-100620890-100623767
chr5	100672378	100675306	chr5-100672378-100675306
chr5	100719014	100720434	chr5-100719014-100720434
chr5	100797382	100799913	chr5-100797382-100799913
chr5	100819855	100821828	chr5-100819855-100821828
chr5	100826381	100826737	chr5-100826381-100826737
chr5	100844830	100848238	chr5-100844830-100848238
chr5	100861559	100862308	chr5-100861559-100862308
chr5	101112439	101112802	chr5-101112439-101112802
chr5	101163012	101164242	chr5-101163012-101164242
chr5	101216708	101217449	chr5-101216708-101217449
chr5	101658945	101659899	chr5-101658945-101659899
chr5	101663238	101665944	chr5-101663238-101665944
chr5	101703482	101704106	chr5-101703482-101704106
chr5	101764359	101766629	chr5-101764359-101766629
chr5	101784550	101784855	chr5-101784550-101784855
chr5	101826528	101826882	chr5-101826528-101826882
chr5	101968745	101968946	chr5-101968745-101968946
chr5	102068582	102070798	chr5-102068582-102070798
chr5	102113195	102113550	chr5-102113195-102113550
chr5	102206961	102207162	chr5-102206961-102207162
chr5	102424622	102425205	chr5-102424622-102425205

chr5	102481051	102483831	chr5-102481051-102483831
chr5	102768473	102769194	chr5-102768473-102769194
chr5	102844315	102845831	chr5-102844315-102845831
chr5	102957723	102958212	chr5-102957723-102958212
chr5	102994332	102994583	chr5-102994332-102994583
chr5	103211383	103211684	chr5-103211383-103211684
chr5	103355684	103356750	chr5-103355684-103356750
chr5	103358079	103361716	chr5-103358079-103361716
chr5	103363231	103369202	chr5-103363231-103369202
chr5	103375867	103377945	chr5-103375867-103377945
chr5	103420407	103420897	chr5-103420407-103420897
chr5	103423590	103428967	chr5-103423590-103428967
chr5	103435071	103439673	chr5-103435071-103439673
chr5	103518870	103519460	chr5-103518870-103519460
chr5	103620278	103621645	chr5-103620278-103621645
chr5	103643982	103644234	chr5-103643982-103644234
chr5	103655144	103656106	chr5-103655144-103656106
chr5	103691559	103693892	chr5-103691559-103693892
chr5	103721145	103721455	chr5-103721145-103721455
chr5	103737108	103738011	chr5-103737108-103738011
chr5	103744423	103744985	chr5-103744423-103744985
chr5	103752395	103756623	chr5-103752395-103756623
chr5	103758023	103758654	chr5-103758023-103758654
chr5	103760016	103761315	chr5-103760016-103761315
chr5	103772843	103773631	chr5-103772843-103773631
chr5	103774903	103780197	chr5-103774903-103780197
chr5	103910163	103911631	chr5-103910163-103911631
chr5	103914316	103914938	chr5-103914316-103914938
chr5	103927524	103928217	chr5-103927524-103928217
chr5	104002423	104003650	chr5-104002423-104003650
chr5	104015802	104016108	chr5-104015802-104016108
chr5	104020517	104022803	chr5-104020517-104022803
chr5	104023618	104023819	chr5-104023618-104023819
chr5	104045757	104048536	chr5-104045757-104048536
chr5	104072361	104072562	chr5-104072361-104072562
chr5	104074595	104075007	chr5-104074595-104075007
chr5	104108936	104109403	chr5-104108936-104109403
chr5	104333250	104333451	chr5-104333250-104333451
chr5	104374322	104374692	chr5-104374322-104374692
chr5	104380431	104382113	chr5-104380431-104382113
chr5	104399133	104399599	chr5-104399133-104399599
chr5	104406805	104408192	chr5-104406805-104408192

chr5	104432810	104433011	chr5-104432810-104433011
chr5	104434892	104437316	chr5-104434892-104437316
chr5	104458873	104461381	chr5-104458873-104461381
chr5	104507855	104509611	chr5-104507855-104509611
chr5	104570790	104573012	chr5-104570790-104573012
chr5	104584252	104584453	chr5-104584252-104584453
chr5	104716861	104717220	chr5-104716861-104717220
chr5	104858386	104860696	chr5-104858386-104860696
chr5	104982626	104983077	chr5-104982626-104983077
chr5	105109765	105110802	chr5-105109765-105110802
chr5	105293333	105294148	chr5-105293333-105294148
chr5	105305783	105305984	chr5-105305783-105305984
chr5	105400191	105400720	chr5-105400191-105400720
chr5	105414645	105417398	chr5-105414645-105417398
chr5	105433673	105433874	chr5-105433673-105433874
chr5	105495299	105496735	chr5-105495299-105496735
chr5	105499653	105499854	chr5-105499653-105499854
chr5	105518156	105525762	chr5-105518156-105525762
chr5	105528062	105528467	chr5-105528062-105528467
chr5	105529867	105530068	chr5-105529867-105530068
chr5	105531060	105531370	chr5-105531060-105531370
chr5	105609559	105610150	chr5-105609559-105610150
chr5	105636466	105637686	chr5-105636466-105637686
chr5	105651206	105652303	chr5-105651206-105652303
chr5	105654469	105654886	chr5-105654469-105654886
chr5	105680900	105681154	chr5-105680900-105681154
chr5	105685050	105685457	chr5-105685050-105685457
chr5	105698295	105703212	chr5-105698295-105703212
chr5	105730938	105733005	chr5-105730938-105733005
chr5	105779175	105779478	chr5-105779175-105779478
chr5	105791988	105793714	chr5-105791988-105793714
chr5	105835848	105836854	chr5-105835848-105836854
chr5	105875814	105878378	chr5-105875814-105878378
chr5	105975656	105976273	chr5-105975656-105976273
chr5	106451795	106452182	chr5-106451795-106452182
chr5	106453193	106453964	chr5-106453193-106453964
chr5	106457135	106459602	chr5-106457135-106459602
chr5	106463638	106464201	chr5-106463638-106464201
chr5	106469615	106471176	chr5-106469615-106471176
chr5	106561034	106561687	chr5-106561034-106561687
chr5	106573737	106575110	chr5-106573737-106575110
chr5	106628368	106628619	chr5-106628368-106628619

chr5	106695013	106697406	chr5-106695013-106697406
chr5	106802110	106802942	chr5-106802110-106802942
chr5	106825326	106825843	chr5-106825326-106825843
chr5	106925521	106926482	chr5-106925521-106926482
chr5	106963550	106965816	chr5-106963550-106965816
chr5	107051808	107052291	chr5-107051808-107052291
chr5	107167144	107168361	chr5-107167144-107168361
chr5	107288455	107290032	chr5-107288455-107290032
chr5	107303460	107303661	chr5-107303460-107303661
chr5	107359256	107360414	chr5-107359256-107360414
chr5	107403057	107404184	chr5-107403057-107404184
chr5	107437362	107438852	chr5-107437362-107438852
chr5	107596354	107598603	chr5-107596354-107598603
chr5	107687657	107688885	chr5-107687657-107688885
chr5	107720919	107727055	chr5-107720919-107727055
chr5	107838980	107839287	chr5-107838980-107839287
chr5	107867643	107869684	chr5-107867643-107869684
chr5	107900096	107903533	chr5-107900096-107903533
chr5	107960701	107961922	chr5-107960701-107961922
chr5	107985817	107988588	chr5-107985817-107988588
chr5	107996164	107998768	chr5-107996164-107998768
chr5	108001009	108001746	chr5-108001009-108001746
chr5	108065141	108067101	chr5-108065141-108067101
chr5	108130832	108133866	chr5-108130832-108133866
chr5	108268022	108271764	chr5-108268022-108271764
chr5	108311573	108314178	chr5-108311573-108314178
chr5	108366359	108369798	chr5-108366359-108369798
chr5	108432912	108435106	chr5-108432912-108435106
chr5	108448368	108449545	chr5-108448368-108449545
chr5	108460551	108464056	chr5-108460551-108464056
chr5	108549335	108550511	chr5-108549335-108550511
chr5	108627550	108630682	chr5-108627550-108630682
chr5	108659479	108661331	chr5-108659479-108661331
chr5	108668577	108670594	chr5-108668577-108670594
chr5	108673489	108674167	chr5-108673489-108674167
chr5	108691991	108695588	chr5-108691991-108695588
chr5	108903149	108903709	chr5-108903149-108903709
chr5	109466709	109467456	chr5-109466709-109467456
chr5	109552609	109559573	chr5-109552609-109559573
chr5	109689949	109691586	chr5-109689949-109691586
chr5	109750126	109752501	chr5-109750126-109752501
chr5	109795260	109796826	chr5-109795260-109796826

chr5	109844545	109845268	chr5-109844545-109845268
chr5	109866121	109867961	chr5-109866121-109867961
chr5	109922633	109924799	chr5-109922633-109924799
chr5	109995497	109997691	chr5-109995497-109997691
chr5	110023028	110023980	chr5-110023028-110023980
chr5	110057247	110058551	chr5-110057247-110058551
chr5	110093446	110095834	chr5-110093446-110095834
chr5	110100302	110102270	chr5-110100302-110102270
chr5	110105965	110108773	chr5-110105965-110108773
chr5	110109737	110110935	chr5-110109737-110110935
chr5	110135061	110136649	chr5-110135061-110136649
chr5	110176107	110178119	chr5-110176107-110178119
chr5	110229936	110232163	chr5-110229936-110232163
chr5	110243875	110244291	chr5-110243875-110244291
chr5	110268015	110270138	chr5-110268015-110270138
chr5	110284372	110287741	chr5-110284372-110287741
chr5	110326792	110326993	chr5-110326792-110326993
chr5	110342505	110343465	chr5-110342505-110343465
chr5	110362892	110363799	chr5-110362892-110363799
chr5	110386339	110387563	chr5-110386339-110387563
chr5	110446272	110449739	chr5-110446272-110449739
chr5	110543984	110544955	chr5-110543984-110544955
chr5	110565184	110565871	chr5-110565184-110565871
chr5	110639497	110639698	chr5-110639497-110639698
chr5	110652116	110655111	chr5-110652116-110655111
chr5	110768745	110771265	chr5-110768745-110771265
chr5	110778494	110781386	chr5-110778494-110781386
chr5	110807647	110810340	chr5-110807647-110810340
chr5	110838634	110841198	chr5-110838634-110841198
chr5	110869297	110869699	chr5-110869297-110869699
chr5	110879286	110880670	chr5-110879286-110880670
chr5	110978808	110980624	chr5-110978808-110980624
chr5	111122086	111123761	chr5-111122086-111123761
chr5	111205265	111206190	chr5-111205265-111206190
chr5	111228059	111228932	chr5-111228059-111228932
chr5	111247188	111248606	chr5-111247188-111248606
chr5	111251135	111252270	chr5-111251135-111252270
chr5	111329706	111332345	chr5-111329706-111332345
chr5	111408732	111409134	chr5-111408732-111409134
chr5	111416359	111420390	chr5-111416359-111420390
chr5	111508879	111509868	chr5-111508879-111509868
chr5	111675610	111676068	chr5-111675610-111676068

chr5	111701648	111702205	chr5-111701648-111702205
chr5	111761586	111761787	chr5-111761586-111761787
chr5	111790290	111794083	chr5-111790290-111794083
chr5	111796681	111796941	chr5-111796681-111796941
chr5	111799029	111800851	chr5-111799029-111800851
chr5	111805342	111805605	chr5-111805342-111805605
chr5	111877264	111877792	chr5-111877264-111877792
chr5	111879837	111882071	chr5-111879837-111882071
chr5	111890843	111891584	chr5-111890843-111891584
chr5	112045097	112045696	chr5-112045097-112045696
chr5	112153593	112154048	chr5-112153593-112154048
chr5	112179691	112179892	chr5-112179691-112179892
chr5	112200163	112200415	chr5-112200163-112200415
chr5	112214560	112214815	chr5-112214560-112214815
chr5	112227185	112229211	chr5-112227185-112229211
chr5	112237421	112237897	chr5-112237421-112237897
chr5	112240604	112241016	chr5-112240604-112241016
chr5	112260262	112260463	chr5-112260262-112260463
chr5	112264335	112264536	chr5-112264335-112264536
chr5	112274733	112282877	chr5-112274733-112282877
chr5	112294654	112295704	chr5-112294654-112295704
chr5	112323914	112327999	chr5-112323914-112327999
chr5	112341826	112343980	chr5-112341826-112343980
chr5	112394041	112395544	chr5-112394041-112395544
chr5	112403804	112404932	chr5-112403804-112404932
chr5	112411615	112411991	chr5-112411615-112411991
chr5	112431145	112431655	chr5-112431145-112431655
chr5	112576526	112577404	chr5-112576526-112577404
chr5	112640826	112641487	chr5-112640826-112641487
chr5	112663426	112664226	chr5-112663426-112664226
chr5	113014987	113015720	chr5-113014987-113015720
chr5	113024333	113024703	chr5-113024333-113024703
chr5	113036219	113036420	chr5-113036219-113036420
chr5	113106718	113108066	chr5-113106718-113108066
chr5	113121247	113123375	chr5-113121247-113123375
chr5	113134523	113140214	chr5-113134523-113140214
chr5	113150998	113151853	chr5-113150998-113151853
chr5	113153246	113159127	chr5-113153246-113159127
chr5	113172307	113172508	chr5-113172307-113172508
chr5	113178252	113178656	chr5-113178252-113178656
chr5	113179706	113181257	chr5-113179706-113181257
chr5	113204432	113208348	chr5-113204432-113208348

chr5	113209588	113209789	chr5-113209588-113209789
chr5	113214509	113221531	chr5-113214509-113221531
chr5	113223994	113224617	chr5-113223994-113224617
chr5	113226316	113227171	chr5-113226316-113227171
chr5	113249247	113249888	chr5-113249247-113249888
chr5	113279131	113281071	chr5-113279131-113281071
chr5	113286683	113287493	chr5-113286683-113287493
chr5	113297757	113298545	chr5-113297757-113298545
chr5	113306811	113307534	chr5-113306811-113307534
chr5	113310283	113311139	chr5-113310283-113311139
chr5	113323727	113324739	chr5-113323727-113324739
chr5	113335037	113335547	chr5-113335037-113335547
chr5	113354117	113354646	chr5-113354117-113354646
chr5	113490514	113490867	chr5-113490514-113490867
chr5	113685203	113686069	chr5-113685203-113686069
chr5	113734957	113737281	chr5-113734957-113737281
chr5	113769574	113773976	chr5-113769574-113773976
chr5	113796657	113797626	chr5-113796657-113797626
chr5	113800246	113800695	chr5-113800246-113800695
chr5	113873018	113875198	chr5-113873018-113875198
chr5	113899312	113899513	chr5-113899312-113899513
chr5	113901176	113902424	chr5-113901176-113902424
chr5	113905462	113909641	chr5-113905462-113909641
chr5	113954370	113954571	chr5-113954370-113954571
chr5	113974453	113977831	chr5-113974453-113977831
chr5	113989218	113994616	chr5-113989218-113994616
chr5	113996225	113996582	chr5-113996225-113996582
chr5	114025022	114025273	chr5-114025022-114025273
chr5	114041597	114041798	chr5-114041597-114041798
chr5	114051682	114052998	chr5-114051682-114052998
chr5	114091020	114091685	chr5-114091020-114091685
chr5	114099717	114101603	chr5-114099717-114101603
chr5	114126655	114128537	chr5-114126655-114128537
chr5	114129533	114132352	chr5-114129533-114132352
chr5	114153434	114153897	chr5-114153434-114153897
chr5	114154764	114156214	chr5-114154764-114156214
chr5	114174126	114176710	chr5-114174126-114176710
chr5	114248308	114248618	chr5-114248308-114248618
chr5	114269694	114270454	chr5-114269694-114270454
chr5	114273455	114274825	chr5-114273455-114274825
chr5	114297071	114297340	chr5-114297071-114297340
chr5	114309316	114309870	chr5-114309316-114309870

chr5	114314995	114315577	chr5-114314995-114315577
chr5	114323848	114324049	chr5-114323848-114324049
chr5	114356590	114358279	chr5-114356590-114358279
chr5	114365477	114365728	chr5-114365477-114365728
chr5	114369970	114376361	chr5-114369970-114376361
chr5	114377210	114384023	chr5-114377210-114384023
chr5	114407214	114407890	chr5-114407214-114407890
chr5	114411864	114413781	chr5-114411864-114413781
chr5	114442767	114445639	chr5-114442767-114445639
chr5	114467314	114467774	chr5-114467314-114467774
chr5	114512750	114513057	chr5-114512750-114513057
chr5	114560231	114561536	chr5-114560231-114561536
chr5	114564922	114565857	chr5-114564922-114565857
chr5	114566975	114568770	chr5-114566975-114568770
chr5	114570905	114571904	chr5-114570905-114571904
chr5	114620511	114620762	chr5-114620511-114620762
chr5	114639114	114640004	chr5-114639114-114640004
chr5	114647539	114649619	chr5-114647539-114649619
chr5	114650528	114651899	chr5-114650528-114651899
chr5	114657270	114658875	chr5-114657270-114658875
chr5	114664898	114665150	chr5-114664898-114665150
chr5	114666950	114668027	chr5-114666950-114668027
chr5	114678924	114679125	chr5-114678924-114679125
chr5	114689504	114691678	chr5-114689504-114691678
chr5	114707304	114708912	chr5-114707304-114708912
chr5	114718574	114719772	chr5-114718574-114719772
chr5	114743122	114745960	chr5-114743122-114745960
chr5	114771507	114776585	chr5-114771507-114776585
chr5	114812501	114814369	chr5-114812501-114814369
chr5	114822471	114824527	chr5-114822471-114824527
chr5	114825775	114827007	chr5-114825775-114827007
chr5	114859045	114859650	chr5-114859045-114859650
chr5	114897130	114897978	chr5-114897130-114897978
chr5	114922920	114924365	chr5-114922920-114924365
chr5	114926069	114926711	chr5-114926069-114926711
chr5	114941622	114943237	chr5-114941622-114943237
chr5	114978887	114980552	chr5-114978887-114980552
chr5	115010355	115013666	chr5-115010355-115013666
chr5	115118323	115119936	chr5-115118323-115119936
chr5	115125475	115126904	chr5-115125475-115126904
chr5	115133319	115135357	chr5-115133319-115135357
chr5	115155024	115159647	chr5-115155024-115159647

chr5	115181387	115181706	chr5-115181387-115181706
chr5	115187076	115187713	chr5-115187076-115187713
chr5	115193170	115194977	chr5-115193170-115194977
chr5	115203616	115204990	chr5-115203616-115204990
chr5	115214993	115215194	chr5-115214993-115215194
chr5	115235052	115236268	chr5-115235052-115236268
chr5	115237406	115239384	chr5-115237406-115239384
chr5	115269583	115273583	chr5-115269583-115273583
chr5	115279304	115280782	chr5-115279304-115280782
chr5	115298753	115302234	chr5-115298753-115302234
chr5	115313014	115313979	chr5-115313014-115313979
chr5	115319243	115320108	chr5-115319243-115320108
chr5	115326128	115328975	chr5-115326128-115328975
chr5	115340091	115342672	chr5-115340091-115342672
chr5	115347226	115350032	chr5-115347226-115350032
chr5	115359054	115359255	chr5-115359054-115359255
chr5	115374080	115374281	chr5-115374080-115374281
chr5	115398287	115398853	chr5-115398287-115398853
chr5	115404185	115404804	chr5-115404185-115404804
chr5	115429061	115431054	chr5-115429061-115431054
chr5	115436207	115437128	chr5-115436207-115437128
chr5	115441394	115441595	chr5-115441394-115441595
chr5	115447030	115447388	chr5-115447030-115447388
chr5	115482242	115485311	chr5-115482242-115485311
chr5	115488634	115493048	chr5-115488634-115493048
chr5	115494470	115494722	chr5-115494470-115494722
chr5	115503269	115503585	chr5-115503269-115503585
chr5	115506386	115510096	chr5-115506386-115510096
chr5	115514003	115516010	chr5-115514003-115516010
chr5	115519308	115525619	chr5-115519308-115525619
chr5	115527733	115530382	chr5-115527733-115530382
chr5	115531252	115536306	chr5-115531252-115536306
chr5	115537804	115539199	chr5-115537804-115539199
chr5	115542093	115545264	chr5-115542093-115545264
chr5	115559277	115567027	chr5-115559277-115567027
chr5	115572860	115573653	chr5-115572860-115573653
chr5	115630404	115633466	chr5-115630404-115633466
chr5	115651933	115653509	chr5-115651933-115653509
chr5	115710129	115710382	chr5-115710129-115710382
chr5	115730358	115733288	chr5-115730358-115733288
chr5	115844121	115847451	chr5-115844121-115847451
chr5	115871850	115872804	chr5-115871850-115872804

chr5	116008860	116010532	chr5-116008860-116010532
chr5	116022182	116025207	chr5-116022182-116025207
chr5	116420610	116423057	chr5-116420610-116423057
chr5	116446111	116447422	chr5-116446111-116447422
chr5	116453427	116453883	chr5-116453427-116453883
chr5	117087658	117088107	chr5-117087658-117088107
chr5	117089645	117089902	chr5-117089645-117089902
chr5	117114409	117116718	chr5-117114409-117116718
chr5	117118907	117123986	chr5-117118907-117123986
chr5	117133334	117136342	chr5-117133334-117136342
chr5	117157927	117159634	chr5-117157927-117159634
chr5	117278716	117281174	chr5-117278716-117281174
chr5	117285299	117288394	chr5-117285299-117288394
chr5	117307796	117308113	chr5-117307796-117308113
chr5	117313256	117315093	chr5-117313256-117315093
chr5	117317972	117321195	chr5-117317972-117321195
chr5	117324296	117324622	chr5-117324296-117324622
chr5	117330240	117331158	chr5-117330240-117331158
chr5	117356317	117364296	chr5-117356317-117364296
chr5	117387397	117389414	chr5-117387397-117389414
chr5	117412996	117415220	chr5-117412996-117415220
chr5	117744557	117745131	chr5-117744557-117745131
chr5	117938366	117938824	chr5-117938366-117938824
chr5	117976192	117978457	chr5-117976192-117978457
chr5	118027096	118029986	chr5-118027096-118029986
chr5	118036961	118038378	chr5-118036961-118038378
chr5	118154063	118155978	chr5-118154063-118155978
chr5	118160860	118161170	chr5-118160860-118161170
chr5	118169045	118172861	chr5-118169045-118172861
chr5	118174598	118174949	chr5-118174598-118174949
chr5	118213995	118215322	chr5-118213995-118215322
chr5	118243971	118246993	chr5-118243971-118246993
chr5	118268981	118269182	chr5-118268981-118269182
chr5	118293936	118294847	chr5-118293936-118294847
chr5	118326762	118328312	chr5-118326762-118328312
chr5	118331397	118332423	chr5-118331397-118332423
chr5	118340178	118340605	chr5-118340178-118340605
chr5	118375142	118375445	chr5-118375142-118375445
chr5	118380175	118381931	chr5-118380175-118381931
chr5	118409885	118410237	chr5-118409885-118410237
chr5	118413648	118413949	chr5-118413648-118413949
chr5	118425141	118425618	chr5-118425141-118425618

chr5	118436844	118437045	chr5-118436844-118437045
chr5	118458626	118460216	chr5-118458626-118460216
chr5	118464186	118466830	chr5-118464186-118466830
chr5	118513414	118515792	chr5-118513414-118515792
chr5	118559409	118562965	chr5-118559409-118562965
chr5	118574550	118574801	chr5-118574550-118574801
chr5	118613333	118613793	chr5-118613333-118613793
chr5	118633240	118633708	chr5-118633240-118633708
chr5	118637775	118638183	chr5-118637775-118638183
chr5	118647849	118648050	chr5-118647849-118648050
chr5	118649746	118650055	chr5-118649746-118650055
chr5	118757941	118758142	chr5-118757941-118758142
chr5	119669790	119671839	chr5-119669790-119671839
chr5	119680470	119680671	chr5-119680470-119680671
chr5	119830990	119831902	chr5-119830990-119831902
chr5	120116024	120117678	chr5-120116024-120117678
chr5	120210989	120211513	chr5-120210989-120211513
chr5	120302214	120302737	chr5-120302214-120302737
chr5	120431757	120432381	chr5-120431757-120432381
chr5	120435357	120438382	chr5-120435357-120438382
chr5	120439772	120441050	chr5-120439772-120441050
chr5	120466720	120466980	chr5-120466720-120466980
chr5	120501948	120503779	chr5-120501948-120503779
chr5	120505468	120505871	chr5-120505468-120505871
chr5	120510638	120512419	chr5-120510638-120512419
chr5	120515080	120515675	chr5-120515080-120515675
chr5	120561009	120561260	chr5-120561009-120561260
chr5	120566881	120567143	chr5-120566881-120567143
chr5	120582210	120582514	chr5-120582210-120582514
chr5	120587437	120589507	chr5-120587437-120589507
chr5	120605037	120606003	chr5-120605037-120606003
chr5	120610994	120615069	chr5-120610994-120615069
chr5	120622141	120622342	chr5-120622141-120622342
chr5	120643862	120644120	chr5-120643862-120644120
chr5	120648540	120649764	chr5-120648540-120649764
chr5	120666100	120666351	chr5-120666100-120666351
chr5	120680000	120680251	chr5-120680000-120680251
chr5	120694523	120694837	chr5-120694523-120694837
chr5	120710736	120711876	chr5-120710736-120711876
chr5	120777295	120777990	chr5-120777295-120777990
chr5	120811894	120813348	chr5-120811894-120813348
chr5	121189080	121192227	chr5-121189080-121192227

chr5	121204041	121208947	chr5-121204041-121208947
chr5	121219825	121221886	chr5-121219825-121221886
chr5	121340151	121340522	chr5-121340151-121340522
chr5	121384434	121386966	chr5-121384434-121386966
chr5	121397080	121400398	chr5-121397080-121400398
chr5	121450794	121454990	chr5-121450794-121454990
chr5	121543912	121547030	chr5-121543912-121547030
chr5	121564485	121564945	chr5-121564485-121564945
chr5	121583725	121584865	chr5-121583725-121584865
chr5	121592088	121594623	chr5-121592088-121594623
chr5	121618020	121620431	chr5-121618020-121620431
chr5	121659560	121661768	chr5-121659560-121661768
chr5	121685853	121686054	chr5-121685853-121686054
chr5	121700485	121700853	chr5-121700485-121700853
chr5	121704618	121705258	chr5-121704618-121705258
chr5	121710291	121714146	chr5-121710291-121714146
chr5	121819243	121820309	chr5-121819243-121820309
chr5	121833108	121839963	chr5-121833108-121839963
chr5	121842338	121844065	chr5-121842338-121844065
chr5	121847235	121850671	chr5-121847235-121850671
chr5	121864682	121864940	chr5-121864682-121864940
chr5	121948411	121948955	chr5-121948411-121948955
chr5	122049041	122050905	chr5-122049041-122050905
chr5	122054658	122054965	chr5-122054658-122054965
chr5	122059752	122060157	chr5-122059752-122060157
chr5	122131340	122132703	chr5-122131340-122132703
chr5	122136189	122137052	chr5-122136189-122137052
chr5	122157332	122161993	chr5-122157332-122161993
chr5	122162797	122163075	chr5-122162797-122163075
chr5	122166146	122167069	chr5-122166146-122167069
chr5	122209722	122210031	chr5-122209722-122210031
chr5	122263415	122265000	chr5-122263415-122265000
chr5	122268781	122270173	chr5-122268781-122270173
chr5	122280102	122281469	chr5-122280102-122281469
chr5	122284073	122287784	chr5-122284073-122287784
chr5	122299617	122300671	chr5-122299617-122300671
chr5	122302381	122304963	chr5-122302381-122304963
chr5	122324311	122325923	chr5-122324311-122325923
chr5	122353444	122355705	chr5-122353444-122355705
chr5	122370667	122374118	chr5-122370667-122374118
chr5	122391640	122393227	chr5-122391640-122393227
chr5	122419568	122423972	chr5-122419568-122423972

chr5	122430285	122432066	chr5-122430285-122432066
chr5	122498547	122498748	chr5-122498547-122498748
chr5	122499838	122503348	chr5-122499838-122503348
chr5	122511338	122511864	chr5-122511338-122511864
chr5	122518239	122519719	chr5-122518239-122519719
chr5	122524786	122525745	chr5-122524786-122525745
chr5	122526797	122527466	chr5-122526797-122527466
chr5	122547196	122547710	chr5-122547196-122547710
chr5	122613583	122614980	chr5-122613583-122614980
chr5	122636910	122637373	chr5-122636910-122637373
chr5	122644298	122644499	chr5-122644298-122644499
chr5	122693479	122693901	chr5-122693479-122693901
chr5	122706787	122708840	chr5-122706787-122708840
chr5	122770407	122771696	chr5-122770407-122771696
chr5	122778068	122780242	chr5-122778068-122780242
chr5	122820075	122822186	chr5-122820075-122822186
chr5	122849143	122851476	chr5-122849143-122851476
chr5	122898738	122901215	chr5-122898738-122901215
chr5	122925605	122925906	chr5-122925605-122925906
chr5	122951180	122952713	chr5-122951180-122952713
chr5	122978775	122980000	chr5-122978775-122980000
chr5	122987146	122991570	chr5-122987146-122991570
chr5	123014429	123019915	chr5-123014429-123019915
chr5	123035633	123035989	chr5-123035633-123035989
chr5	123038922	123039669	chr5-123038922-123039669
chr5	123049360	123049870	chr5-123049360-123049870
chr5	123057835	123059077	chr5-123057835-123059077
chr5	123075513	123077562	chr5-123075513-123077562
chr5	123115891	123116309	chr5-123115891-123116309
chr5	123129056	123145319	chr5-123129056-123145319
chr5	123149504	123151340	chr5-123149504-123151340
chr5	123171788	123172269	chr5-123171788-123172269
chr5	123227663	123229602	chr5-123227663-123229602
chr5	123251951	123252412	chr5-123251951-123252412
chr5	123317954	123318763	chr5-123317954-123318763
chr5	123326293	123326546	chr5-123326293-123326546
chr5	123343419	123346829	chr5-123343419-123346829
chr5	123355595	123356438	chr5-123355595-123356438
chr5	123393914	123397402	chr5-123393914-123397402
chr5	123489240	123489692	chr5-123489240-123489692
chr5	123498067	123498490	chr5-123498067-123498490
chr5	123507741	123509251	chr5-123507741-123509251

chr5	123522796	123524996	chr5-123522796-123524996
chr5	123572026	123573664	chr5-123572026-123573664
chr5	123670599	123671656	chr5-123670599-123671656
chr5	123681997	123685331	chr5-123681997-123685331
chr5	123700613	123701011	chr5-123700613-123701011
chr5	123719194	123721743	chr5-123719194-123721743
chr5	123746219	123750706	chr5-123746219-123750706
chr5	123854025	123854922	chr5-123854025-123854922
chr5	123862003	123863498	chr5-123862003-123863498
chr5	123906128	123909486	chr5-123906128-123909486
chr5	123929800	123931756	chr5-123929800-123931756
chr5	123973130	123976727	chr5-123973130-123976727
chr5	123977805	123978006	chr5-123977805-123978006
chr5	123980318	123981588	chr5-123980318-123981588
chr5	123991786	123992979	chr5-123991786-123992979
chr5	124006081	124007852	chr5-124006081-124007852
chr5	124016123	124017798	chr5-124016123-124017798
chr5	124025801	124032650	chr5-124025801-124032650
chr5	124046639	124048502	chr5-124046639-124048502
chr5	124084038	124085040	chr5-124084038-124085040
chr5	124094672	124096544	chr5-124094672-124096544
chr5	124110804	124113917	chr5-124110804-124113917
chr5	124115340	124117863	chr5-124115340-124117863
chr5	124248086	124250673	chr5-124248086-124250673
chr5	124262267	124262468	chr5-124262267-124262468
chr5	124326689	124329213	chr5-124326689-124329213
chr5	124342014	124342265	chr5-124342014-124342265
chr5	124350981	124355841	chr5-124350981-124355841
chr5	124376917	124377220	chr5-124376917-124377220
chr5	124424042	124426675	chr5-124424042-124426675
chr5	124438755	124441123	chr5-124438755-124441123
chr5	124442442	124442693	chr5-124442442-124442693
chr5	124443869	124450496	chr5-124443869-124450496
chr5	124474667	124478878	chr5-124474667-124478878
chr5	124482172	124484801	chr5-124482172-124484801
chr5	124488686	124489457	chr5-124488686-124489457
chr5	124496669	124497410	chr5-124496669-124497410
chr5	124506538	124507367	chr5-124506538-124507367
chr5	124509554	124510425	chr5-124509554-124510425
chr5	124528822	124532114	chr5-124528822-124532114
chr5	124536794	124537150	chr5-124536794-124537150
chr5	124540038	124542736	chr5-124540038-124542736

chr5	124551789	124553978	chr5-124551789-124553978
chr5	124577892	124580621	chr5-124577892-124580621
chr5	124598203	124599945	chr5-124598203-124599945
chr5	124628135	124630770	chr5-124628135-124630770
chr5	124729386	124730212	chr5-124729386-124730212
chr5	124861195	124864537	chr5-124861195-124864537
chr5	124899853	124900671	chr5-124899853-124900671
chr5	124919940	124920351	chr5-124919940-124920351
chr5	124922707	124923317	chr5-124922707-124923317
chr5	124963796	124964575	chr5-124963796-124964575
chr5	125003070	125004156	chr5-125003070-125004156
chr5	125056912	125058813	chr5-125056912-125058813
chr5	125069855	125074329	chr5-125069855-125074329
chr5	125079646	125082413	chr5-125079646-125082413
chr5	125099678	125100199	chr5-125099678-125100199
chr5	125105860	125106634	chr5-125105860-125106634
chr5	125134008	125135559	chr5-125134008-125135559
chr5	125139385	125142425	chr5-125139385-125142425
chr5	125158741	125159753	chr5-125158741-125159753
chr5	125175519	125180296	chr5-125175519-125180296
chr5	125188690	125189518	chr5-125188690-125189518
chr5	125203012	125203592	chr5-125203012-125203592
chr5	125215958	125216159	chr5-125215958-125216159
chr5	125245450	125246068	chr5-125245450-125246068
chr5	125252196	125252898	chr5-125252196-125252898
chr5	125268322	125268776	chr5-125268322-125268776
chr5	125271748	125272101	chr5-125271748-125272101
chr5	125339126	125341735	chr5-125339126-125341735
chr5	125357895	125358585	chr5-125357895-125358585
chr5	125384403	125395319	chr5-125384403-125395319
chr5	125400901	125401439	chr5-125400901-125401439
chr5	125403655	125410345	chr5-125403655-125410345
chr5	125432647	125434599	chr5-125432647-125434599
chr5	125440663	125443381	chr5-125440663-125443381
chr5	125449572	125449827	chr5-125449572-125449827
chr5	125464999	125466759	chr5-125464999-125466759
chr5	125475122	125479681	chr5-125475122-125479681
chr5	125531667	125533776	chr5-125531667-125533776
chr5	125588908	125589937	chr5-125588908-125589937
chr5	126733535	126733846	chr5-126733535-126733846
chr5	126787915	126788116	chr5-126787915-126788116
chr5	127241438	127242790	chr5-127241438-127242790

chr5	127537284	127537690	chr5-127537284-127537690
chr5	127610862	127611495	chr5-127610862-127611495
chr5	127614817	127618196	chr5-127614817-127618196
chr5	127631554	127633797	chr5-127631554-127633797
chr5	128432728	128433043	chr5-128432728-128433043
chr5	128444291	128444764	chr5-128444291-128444764
chr5	128579204	128579832	chr5-128579204-128579832
chr5	128599990	128601554	chr5-128599990-128601554
chr5	128952456	128952657	chr5-128952456-128952657
chr5	129007206	129009100	chr5-129007206-129009100
chr5	129018819	129022980	chr5-129018819-129022980
chr5	129063478	129063839	chr5-129063478-129063839
chr5	129066010	129066265	chr5-129066010-129066265
chr5	129163592	129164571	chr5-129163592-129164571
chr5	129178100	129178509	chr5-129178100-129178509
chr5	129217575	129217837	chr5-129217575-129217837
chr5	129244402	129245171	chr5-129244402-129245171
chr5	129415371	129415733	chr5-129415371-129415733
chr5	129454844	129455496	chr5-129454844-129455496
chr5	129500305	129502988	chr5-129500305-129502988
chr5	129516076	129516277	chr5-129516076-129516277
chr5	129518379	129518732	chr5-129518379-129518732
chr5	129583373	129586531	chr5-129583373-129586531
chr5	129594377	129594634	chr5-129594377-129594634
chr5	129602123	129602382	chr5-129602123-129602382
chr5	129669182	129670545	chr5-129669182-129670545
chr5	129715035	129716688	chr5-129715035-129716688
chr5	129724492	129726207	chr5-129724492-129726207
chr5	129729309	129729561	chr5-129729309-129729561
chr5	129777651	129779869	chr5-129777651-129779869
chr5	129785915	129789494	chr5-129785915-129789494
chr5	129846656	129848461	chr5-129846656-129848461
chr5	129885308	129888116	chr5-129885308-129888116
chr5	129895154	129896635	chr5-129895154-129896635
chr5	129907872	129909708	chr5-129907872-129909708
chr5	129940719	129943383	chr5-129940719-129943383
chr5	130001221	130003417	chr5-130001221-130003417
chr5	130019444	130019946	chr5-130019444-130019946
chr5	130022790	130024941	chr5-130022790-130024941
chr5	130028788	130030597	chr5-130028788-130030597
chr5	130063769	130064021	chr5-130063769-130064021
chr5	130078915	130080798	chr5-130078915-130080798

chr5	130089450	130089884	chr5-130089450-130089884
chr5	130143892	130146242	chr5-130143892-130146242
chr5	130168735	130169734	chr5-130168735-130169734
chr5	130170763	130174213	chr5-130170763-130174213
chr5	130182230	130183081	chr5-130182230-130183081
chr5	130184071	130186284	chr5-130184071-130186284
chr5	130221463	130224322	chr5-130221463-130224322
chr5	130253589	130258047	chr5-130253589-130258047
chr5	130264290	130264699	chr5-130264290-130264699
chr5	131307178	131308103	chr5-131307178-131308103
chr5	131536976	131539076	chr5-131536976-131539076
chr5	131600387	131600794	chr5-131600387-131600794
chr5	131612999	131613511	chr5-131612999-131613511
chr5	131814771	131815027	chr5-131814771-131815027
chr5	132541344	132544117	chr5-132541344-132544117
chr5	134098740	134103480	chr5-134098740-134103480
chr5	134131513	134131770	chr5-134131513-134131770
chr5	134169127	134169559	chr5-134169127-134169559
chr5	134175266	134177463	chr5-134175266-134177463
chr5	134182320	134185096	chr5-134182320-134185096
chr5	134221923	134222186	chr5-134221923-134222186
chr5	134246614	134246923	chr5-134246614-134246923
chr5	134249406	134249669	chr5-134249406-134249669
chr5	134312222	134316353	chr5-134312222-134316353
chr5	134350529	134351129	chr5-134350529-134351129
chr5	134436626	134438067	chr5-134436626-134438067
chr5	134439659	134440570	chr5-134439659-134440570
chr5	134444325	134444846	chr5-134444325-134444846
chr5	134445956	134446312	chr5-134445956-134446312
chr5	134454240	134457289	chr5-134454240-134457289
chr5	134539731	134540548	chr5-134539731-134540548
chr5	134549979	134553145	chr5-134549979-134553145
chr5	134555335	134556012	chr5-134555335-134556012
chr5	134571639	134572809	chr5-134571639-134572809
chr5	134582239	134584083	chr5-134582239-134584083
chr5	134611702	134612815	chr5-134611702-134612815
chr5	134620614	134620924	chr5-134620614-134620924
chr5	134637053	134640005	chr5-134637053-134640005
chr5	134644224	134644872	chr5-134644224-134644872
chr5	134675724	134676924	chr5-134675724-134676924
chr5	134680109	134680662	chr5-134680109-134680662
chr5	134687175	134689722	chr5-134687175-134689722

chr5	134742515	134742716	chr5-134742515-134742716
chr5	134746296	134747726	chr5-134746296-134747726
chr5	134769182	134769536	chr5-134769182-134769536
chr5	134836404	134836655	chr5-134836404-134836655
chr5	134907409	134907660	chr5-134907409-134907660
chr5	134931516	134934069	chr5-134931516-134934069
chr5	134935299	134936129	chr5-134935299-134936129
chr5	134942937	134948454	chr5-134942937-134948454
chr5	134950890	134953423	chr5-134950890-134953423
chr5	134955329	134959281	chr5-134955329-134959281
chr5	134965176	134967876	chr5-134965176-134967876
chr5	134980688	134982468	chr5-134980688-134982468
chr5	134984635	134991228	chr5-134984635-134991228
chr5	135000756	135002658	chr5-135000756-135002658
chr5	135007874	135013385	chr5-135007874-135013385
chr5	135022951	135025372	chr5-135022951-135025372
chr5	135028582	135030258	chr5-135028582-135030258
chr5	135063029	135065652	chr5-135063029-135065652
chr5	135077355	135078888	chr5-135077355-135078888
chr5	135089549	135090141	chr5-135089549-135090141
chr5	135106538	135108491	chr5-135106538-135108491
chr5	135133713	135134946	chr5-135133713-135134946
chr5	135148215	135151166	chr5-135148215-135151166
chr5	135167834	135169865	chr5-135167834-135169865
chr5	135186214	135190198	chr5-135186214-135190198
chr5	135250028	135251655	chr5-135250028-135251655
chr5	135264142	135264734	chr5-135264142-135264734
chr5	135274584	135274888	chr5-135274584-135274888
chr5	135276534	135277093	chr5-135276534-135277093
chr5	135368740	135371495	chr5-135368740-135371495
chr5	135392600	135395581	chr5-135392600-135395581
chr5	135488094	135490218	chr5-135488094-135490218
chr5	135531532	135531834	chr5-135531532-135531834
chr5	135537645	135538156	chr5-135537645-135538156
chr5	135543662	135546177	chr5-135543662-135546177
chr5	135623093	135624714	chr5-135623093-135624714
chr5	135632297	135634011	chr5-135632297-135634011
chr5	135643844	135644324	chr5-135643844-135644324
chr5	135651166	135652655	chr5-135651166-135652655
chr5	135669562	135673316	chr5-135669562-135673316
chr5	135682644	135683422	chr5-135682644-135683422
chr5	135688240	135691778	chr5-135688240-135691778

chr5	135693269	135693470	chr5-135693269-135693470
chr5	135695398	135695649	chr5-135695398-135695649
chr5	135710668	135710928	chr5-135710668-135710928
chr5	135712689	135714317	chr5-135712689-135714317
chr5	135724639	135727443	chr5-135724639-135727443
chr5	135742777	135745083	chr5-135742777-135745083
chr5	135777456	135780499	chr5-135777456-135780499
chr5	135794312	135795083	chr5-135794312-135795083
chr5	135806832	135807100	chr5-135806832-135807100
chr5	135834788	135836310	chr5-135834788-135836310
chr5	135852080	135852608	chr5-135852080-135852608
chr5	135856947	135857414	chr5-135856947-135857414
chr5	135867570	135869258	chr5-135867570-135869258
chr5	135877256	135878383	chr5-135877256-135878383
chr5	135879252	135880266	chr5-135879252-135880266
chr5	135884454	135885101	chr5-135884454-135885101
chr5	135887386	135889441	chr5-135887386-135889441
chr5	135903340	135904144	chr5-135903340-135904144
chr5	135909256	135909457	chr5-135909256-135909457
chr5	135911332	135914960	chr5-135911332-135914960
chr5	135918544	135919168	chr5-135918544-135919168
chr5	135930115	135930316	chr5-135930115-135930316
chr5	135931183	135935544	chr5-135931183-135935544
chr5	135945693	135946513	chr5-135945693-135946513
chr5	135956989	135958676	chr5-135956989-135958676
chr5	135960866	135963831	chr5-135960866-135963831
chr5	135965045	135966174	chr5-135965045-135966174
chr5	135994658	135996677	chr5-135994658-135996677
chr5	136016283	136016484	chr5-136016283-136016484
chr5	136038555	136038897	chr5-136038555-136038897
chr5	136048671	136057651	chr5-136048671-136057651
chr5	136083433	136085867	chr5-136083433-136085867
chr5	136094687	136095163	chr5-136094687-136095163
chr5	136102005	136102840	chr5-136102005-136102840
chr5	136108971	136109273	chr5-136108971-136109273
chr5	136115721	136118295	chr5-136115721-136118295
chr5	136134513	136137568	chr5-136134513-136137568
chr5	136143726	136143927	chr5-136143726-136143927
chr5	136169542	136172188	chr5-136169542-136172188
chr5	136197812	136200012	chr5-136197812-136200012
chr5	136237460	136238120	chr5-136237460-136238120
chr5	136243659	136245729	chr5-136243659-136245729

chr5	136286812	136287063	chr5-136286812-136287063
chr5	136288043	136289110	chr5-136288043-136289110
chr5	136458708	136458959	chr5-136458708-136458959
chr5	136465449	136466269	chr5-136465449-136466269
chr5	136500125	136500931	chr5-136500125-136500931
chr5	136531917	136532218	chr5-136531917-136532218
chr5	136533844	136534282	chr5-136533844-136534282
chr5	136551872	136552124	chr5-136551872-136552124
chr5	136555841	136556146	chr5-136555841-136556146
chr5	136565029	136569430	chr5-136565029-136569430
chr5	136584026	136584290	chr5-136584026-136584290
chr5	136608497	136609306	chr5-136608497-136609306
chr5	136622510	136623603	chr5-136622510-136623603
chr5	136632357	136633211	chr5-136632357-136633211
chr5	136714612	136714917	chr5-136714612-136714917
chr5	136802763	136803119	chr5-136802763-136803119
chr5	136881535	136883150	chr5-136881535-136883150
chr5	136907582	136908963	chr5-136907582-136908963
chr5	136918515	136919994	chr5-136918515-136919994
chr5	136952177	136954555	chr5-136952177-136954555
chr5	136961379	136963293	chr5-136961379-136963293
chr5	136966638	136966839	chr5-136966638-136966839
chr5	136985969	136988888	chr5-136985969-136988888
chr5	137012330	137012641	chr5-137012330-137012641
chr5	137024895	137025735	chr5-137024895-137025735
chr5	137028859	137033469	chr5-137028859-137033469
chr5	137044546	137046536	chr5-137044546-137046536
chr5	137068802	137073070	chr5-137068802-137073070
chr5	137074223	137074478	chr5-137074223-137074478
chr5	137082890	137083198	chr5-137082890-137083198
chr5	137085063	137085325	chr5-137085063-137085325
chr5	137091612	137092033	chr5-137091612-137092033
chr5	137102143	137103835	chr5-137102143-137103835
chr5	137114911	137116936	chr5-137114911-137116936
chr5	137189187	137189905	chr5-137189187-137189905
chr5	137286709	137289412	chr5-137286709-137289412
chr5	137292816	137296175	chr5-137292816-137296175
chr5	137304872	137308899	chr5-137304872-137308899
chr5	137312094	137314895	chr5-137312094-137314895
chr5	137332140	137335249	chr5-137332140-137335249
chr5	137337796	137340226	chr5-137337796-137340226
chr5	137348789	137354579	chr5-137348789-137354579

chr5	137416943	137417470	chr5-137416943-137417470
chr5	137425290	137426372	chr5-137425290-137426372
chr5	137454606	137456573	chr5-137454606-137456573
chr5	137485765	137486068	chr5-137485765-137486068
chr5	137500749	137503680	chr5-137500749-137503680
chr5	137509544	137512006	chr5-137509544-137512006
chr5	137529755	137534125	chr5-137529755-137534125
chr5	137553248	137553873	chr5-137553248-137553873
chr5	137574301	137575047	chr5-137574301-137575047
chr5	137583797	137584150	chr5-137583797-137584150
chr5	137597045	137597402	chr5-137597045-137597402
chr5	137599101	137603706	chr5-137599101-137603706
chr5	137608492	137614131	chr5-137608492-137614131
chr5	137615335	137615536	chr5-137615335-137615536
chr5	137627861	137632618	chr5-137627861-137632618
chr5	137645046	137645510	chr5-137645046-137645510
chr5	137650041	137650300	chr5-137650041-137650300
chr5	137682982	137685260	chr5-137682982-137685260
chr5	137706473	137708305	chr5-137706473-137708305
chr5	137720153	137721522	chr5-137720153-137721522
chr5	137739201	137742274	chr5-137739201-137742274
chr5	137745326	137749708	chr5-137745326-137749708
chr5	137759953	137760262	chr5-137759953-137760262
chr5	137776269	137777102	chr5-137776269-137777102
chr5	137778176	137780381	chr5-137778176-137780381
chr5	137783076	137788214	chr5-137783076-137788214
chr5	137918933	137922020	chr5-137918933-137922020
chr5	137981498	137981699	chr5-137981498-137981699
chr5	138084584	138086371	chr5-138084584-138086371
chr5	138116020	138118619	chr5-138116020-138118619
chr5	138154551	138156015	chr5-138154551-138156015
chr5	138160470	138162466	chr5-138160470-138162466
chr5	138169020	138173187	chr5-138169020-138173187
chr5	138186317	138188877	chr5-138186317-138188877
chr5	138193680	138195441	chr5-138193680-138195441
chr5	138254765	138256663	chr5-138254765-138256663
chr5	138262323	138264893	chr5-138262323-138264893
chr5	138279154	138281088	chr5-138279154-138281088
chr5	138441182	138443104	chr5-138441182-138443104
chr5	138616441	138617431	chr5-138616441-138617431
chr5	138618375	138620269	chr5-138618375-138620269
chr5	138622358	138624100	chr5-138622358-138624100

chr5	138724171	138725333	chr5-138724171-138725333
chr5	138753431	138757165	chr5-138753431-138757165
chr5	138820671	138821687	chr5-138820671-138821687
chr5	138846119	138846577	chr5-138846119-138846577
chr5	138849695	138850483	chr5-138849695-138850483
chr5	138898441	138898925	chr5-138898441-138898925
chr5	138947967	138948591	chr5-138947967-138948591
chr5	138970038	138970369	chr5-138970038-138970369
chr5	138980185	138981030	chr5-138980185-138981030
chr5	138991324	138998559	chr5-138991324-138998559
chr5	139014839	139015511	chr5-139014839-139015511
chr5	139017829	139018030	chr5-139017829-139018030
chr5	139033572	139034359	chr5-139033572-139034359
chr5	139129076	139130139	chr5-139129076-139130139
chr5	139149001	139151947	chr5-139149001-139151947
chr5	139196812	139197447	chr5-139196812-139197447
chr5	139199851	139202373	chr5-139199851-139202373
chr5	139214973	139215755	chr5-139214973-139215755
chr5	139251598	139255282	chr5-139251598-139255282
chr5	139257078	139257581	chr5-139257078-139257581
chr5	139300139	139301342	chr5-139300139-139301342
chr5	139314941	139315466	chr5-139314941-139315466
chr5	139316530	139318209	chr5-139316530-139318209
chr5	139323629	139326116	chr5-139323629-139326116
chr5	139343817	139345904	chr5-139343817-139345904
chr5	139371216	139373027	chr5-139371216-139373027
chr5	139377787	139382953	chr5-139377787-139382953
chr5	139389662	139389925	chr5-139389662-139389925
chr5	139458986	139461448	chr5-139458986-139461448
chr5	139476354	139476555	chr5-139476354-139476555
chr5	139477713	139485262	chr5-139477713-139485262
chr5	139506290	139506491	chr5-139506290-139506491
chr5	139510948	139511149	chr5-139510948-139511149
chr5	139535344	139535768	chr5-139535344-139535768
chr5	139540220	139541842	chr5-139540220-139541842
chr5	139543268	139544967	chr5-139543268-139544967
chr5	139546781	139547869	chr5-139546781-139547869
chr5	139553196	139553447	chr5-139553196-139553447
chr5	139558210	139558726	chr5-139558210-139558726
chr5	139580033	139580746	chr5-139580033-139580746
chr5	139585267	139585849	chr5-139585267-139585849
chr5	139659352	139659718	chr5-139659352-139659718

chr5	139695128	139695381	chr5-139695128-139695381
chr5	139730125	139737057	chr5-139730125-139737057
chr5	139773949	139776176	chr5-139773949-139776176
chr5	139790953	139799423	chr5-139790953-139799423
chr5	139811961	139814728	chr5-139811961-139814728
chr5	139825447	139827526	chr5-139825447-139827526
chr5	139862934	139863192	chr5-139862934-139863192
chr5	139892356	139892557	chr5-139892356-139892557
chr5	139904650	139904851	chr5-139904650-139904851
chr5	139907297	139910566	chr5-139907297-139910566
chr5	139911907	139913285	chr5-139911907-139913285
chr5	139921931	139922132	chr5-139921931-139922132
chr5	139932129	139933158	chr5-139932129-139933158
chr5	139936468	139936878	chr5-139936468-139936878
chr5	139957065	139957317	chr5-139957065-139957317
chr5	140016046	140016458	chr5-140016046-140016458
chr5	140115983	140116304	chr5-140115983-140116304
chr5	140123978	140124179	chr5-140123978-140124179
chr5	140158883	140159141	chr5-140158883-140159141
chr5	140174014	140175474	chr5-140174014-140175474
chr5	140177527	140177881	chr5-140177527-140177881
chr5	140319796	140322940	chr5-140319796-140322940
chr5	140330730	140332957	chr5-140330730-140332957
chr5	140383517	140384257	chr5-140383517-140384257
chr5	140387746	140389855	chr5-140387746-140389855
chr5	140417789	140421993	chr5-140417789-140421993
chr5	140443971	140444280	chr5-140443971-140444280
chr5	140502474	140503769	chr5-140502474-140503769
chr5	140504720	140507089	chr5-140504720-140507089
chr5	140514265	140515792	chr5-140514265-140515792
chr5	140549870	140550071	chr5-140549870-140550071
chr5	140567236	140567643	chr5-140567236-140567643
chr5	140603754	140613153	chr5-140603754-140613153
chr5	140616917	140617118	chr5-140616917-140617118
chr5	140647100	140649875	chr5-140647100-140649875
chr5	140701137	140706359	chr5-140701137-140706359
chr5	140735163	140736494	chr5-140735163-140736494
chr5	140748977	140749904	chr5-140748977-140749904
chr5	140767935	140768186	chr5-140767935-140768186
chr5	140790637	140792461	chr5-140790637-140792461
chr5	140811763	140812114	chr5-140811763-140812114
chr5	140827980	140828181	chr5-140827980-140828181

chr5	140829095	140831404	chr5-140829095-140831404
chr5	141240801	141242286	chr5-141240801-141242286
chr5	142366394	142369076	chr5-142366394-142369076
chr5	142400459	142405265	chr5-142400459-142405265
chr5	142424349	142427280	chr5-142424349-142427280
chr5	142438302	142442222	chr5-142438302-142442222
chr5	142463102	142465515	chr5-142463102-142465515
chr5	142477312	142478209	chr5-142477312-142478209
chr5	142479027	142479283	chr5-142479027-142479283
chr5	142521474	142521675	chr5-142521474-142521675
chr5	142544906	142545956	chr5-142544906-142545956
chr5	142549215	142551600	chr5-142549215-142551600
chr5	142557497	142557698	chr5-142557497-142557698
chr5	142608279	142609118	chr5-142608279-142609118
chr5	142628445	142631707	chr5-142628445-142631707
chr5	142701470	142703169	chr5-142701470-142703169
chr5	142723695	142724096	chr5-142723695-142724096
chr5	142771270	142771727	chr5-142771270-142771727
chr5	142791642	142795605	chr5-142791642-142795605
chr5	142797043	142798440	chr5-142797043-142798440
chr5	142807720	142824425	chr5-142807720-142824425
chr5	142842259	142842610	chr5-142842259-142842610
chr5	142893735	142896465	chr5-142893735-142896465
chr5	142901483	142913044	chr5-142901483-142913044
chr5	142919784	142922682	chr5-142919784-142922682
chr5	142923879	142924798	chr5-142923879-142924798
chr5	142926416	142926617	chr5-142926416-142926617
chr5	142928680	142932968	chr5-142928680-142932968
chr5	142935865	142936740	chr5-142935865-142936740
chr5	142958984	142966253	chr5-142958984-142966253
chr5	143034189	143034445	chr5-143034189-143034445
chr5	143036438	143036853	chr5-143036438-143036853
chr5	143056154	143057143	chr5-143056154-143057143
chr5	143111408	143113654	chr5-143111408-143113654
chr5	143157093	143157610	chr5-143157093-143157610
chr5	143165364	143165853	chr5-143165364-143165853
chr5	143179449	143181216	chr5-143179449-143181216
chr5	143234756	143236303	chr5-143234756-143236303
chr5	143252961	143255380	chr5-143252961-143255380
chr5	143264543	143265188	chr5-143264543-143265188
chr5	143268887	143272774	chr5-143268887-143272774
chr5	143287640	143288536	chr5-143287640-143288536

chr5	143293151	143293783	chr5-143293151-143293783
chr5	143313258	143315818	chr5-143313258-143315818
chr5	143327682	143330482	chr5-143327682-143330482
chr5	143357384	143358016	chr5-143357384-143358016
chr5	143363573	143364747	chr5-143363573-143364747
chr5	143369878	143370434	chr5-143369878-143370434
chr5	143372681	143373453	chr5-143372681-143373453
chr5	143375349	143375806	chr5-143375349-143375806
chr5	143386793	143387709	chr5-143386793-143387709
chr5	143402495	143405539	chr5-143402495-143405539
chr5	143434039	143434602	chr5-143434039-143434602
chr5	143463699	143465865	chr5-143463699-143465865
chr5	143517354	143519245	chr5-143517354-143519245
chr5	143525160	143528897	chr5-143525160-143528897
chr5	143547610	143550436	chr5-143547610-143550436
chr5	143561299	143562133	chr5-143561299-143562133
chr5	143609132	143610810	chr5-143609132-143610810
chr5	143613252	143613882	chr5-143613252-143613882
chr5	143621873	143626403	chr5-143621873-143626403
chr5	143636314	143643276	chr5-143636314-143643276
chr5	143669459	143669881	chr5-143669459-143669881
chr5	143678036	143679454	chr5-143678036-143679454
chr5	143730512	143733284	chr5-143730512-143733284
chr5	143757943	143759189	chr5-143757943-143759189
chr5	143817061	143819359	chr5-143817061-143819359
chr5	143908129	143911582	chr5-143908129-143911582
chr5	144013742	144015722	chr5-144013742-144015722
chr5	144065961	144066162	chr5-144065961-144066162
chr5	144086185	144087167	chr5-144086185-144087167
chr5	144099656	144102224	chr5-144099656-144102224
chr5	144125013	144127124	chr5-144125013-144127124
chr5	144146593	144146847	chr5-144146593-144146847
chr5	144150548	144152424	chr5-144150548-144152424
chr5	144189966	144190494	chr5-144189966-144190494
chr5	144220236	144225174	chr5-144220236-144225174
chr5	144254124	144262079	chr5-144254124-144262079
chr5	144267836	144268620	chr5-144267836-144268620
chr5	144305618	144307096	chr5-144305618-144307096
chr5	144309244	144309500	chr5-144309244-144309500
chr5	144313353	144315561	chr5-144313353-144315561
chr5	144316461	144322135	chr5-144316461-144322135
chr5	144354240	144359165	chr5-144354240-144359165

chr5	144457768	144457969	chr5-144457768-144457969
chr5	144545356	144546684	chr5-144545356-144546684
chr5	144752394	144752957	chr5-144752394-144752957
chr5	144755209	144755889	chr5-144755209-144755889
chr5	144761016	144762038	chr5-144761016-144762038
chr5	144767298	144770018	chr5-144767298-144770018
chr5	144875804	144876056	chr5-144875804-144876056
chr5	144897096	144900302	chr5-144897096-144900302
chr5	144901113	144901424	chr5-144901113-144901424
chr5	144903881	144905577	chr5-144903881-144905577
chr5	144907834	144908181	chr5-144907834-144908181
chr5	144910136	144910487	chr5-144910136-144910487
chr5	144913980	144915186	chr5-144913980-144915186
chr5	144934386	144934587	chr5-144934386-144934587
chr5	144935420	144937492	chr5-144935420-144937492
chr5	144948133	144951323	chr5-144948133-144951323
chr5	144952126	144955741	chr5-144952126-144955741
chr5	144958441	144968621	chr5-144958441-144968621
chr5	144977375	144979513	chr5-144977375-144979513
chr5	145059210	145059515	chr5-145059210-145059515
chr5	145083237	145085514	chr5-145083237-145085514
chr5	145103109	145103310	chr5-145103109-145103310
chr5	145113590	145117537	chr5-145113590-145117537
chr5	145138066	145141384	chr5-145138066-145141384
chr5	145165958	145168706	chr5-145165958-145168706
chr5	145189858	145192058	chr5-145189858-145192058
chr5	145200695	145202061	chr5-145200695-145202061
chr5	145203492	145206727	chr5-145203492-145206727
chr5	145230985	145233895	chr5-145230985-145233895
chr5	145280390	145281923	chr5-145280390-145281923
chr5	146219939	146222288	chr5-146219939-146222288
chr5	146229983	146233452	chr5-146229983-146233452
chr5	146248589	146248894	chr5-146248589-146248894
chr5	146314227	146315887	chr5-146314227-146315887
chr5	146330324	146330625	chr5-146330324-146330625
chr5	146384942	146385754	chr5-146384942-146385754
chr5	146584496	146585307	chr5-146584496-146585307
chr5	146660835	146661983	chr5-146660835-146661983
chr5	146675644	146677425	chr5-146675644-146677425
chr5	146687540	146688432	chr5-146687540-146688432
chr5	146692163	146692999	chr5-146692163-146692999
chr5	146699791	146701165	chr5-146699791-146701165

chr5	146758300	146759459	chr5-146758300-146759459
chr5	146779663	146780425	chr5-146779663-146780425
chr5	146792193	146795493	chr5-146792193-146795493
chr5	146824369	146825293	chr5-146824369-146825293
chr5	146832404	146835752	chr5-146832404-146835752
chr5	146844604	146846119	chr5-146844604-146846119
chr5	146867442	146868013	chr5-146867442-146868013
chr5	146912254	146912726	chr5-146912254-146912726
chr5	146920221	146920657	chr5-146920221-146920657
chr5	146948077	146950420	chr5-146948077-146950420
chr5	146962506	146964356	chr5-146962506-146964356
chr5	146992741	146993980	chr5-146992741-146993980
chr5	147074925	147079101	chr5-147074925-147079101
chr5	147159564	147159765	chr5-147159564-147159765
chr5	147185494	147189460	chr5-147185494-147189460
chr5	147227245	147227596	chr5-147227245-147227596
chr5	147269913	147270672	chr5-147269913-147270672
chr5	147274507	147274919	chr5-147274507-147274919
chr5	147277590	147277966	chr5-147277590-147277966
chr5	147291924	147293865	chr5-147291924-147293865
chr5	147299083	147300785	chr5-147299083-147300785
chr5	147304410	147307630	chr5-147304410-147307630
chr5	147314663	147315981	chr5-147314663-147315981
chr5	147388473	147389475	chr5-147388473-147389475
chr5	147399816	147400987	chr5-147399816-147400987
chr5	147408822	147409085	chr5-147408822-147409085
chr5	147429048	147432865	chr5-147429048-147432865
chr5	147556844	147558625	chr5-147556844-147558625
chr5	147601773	147601974	chr5-147601773-147601974
chr5	147619571	147622041	chr5-147619571-147622041
chr5	147724544	147726824	chr5-147724544-147726824
chr5	147757908	147758826	chr5-147757908-147758826
chr5	147778491	147779141	chr5-147778491-147779141
chr5	147786681	147787541	chr5-147786681-147787541
chr5	147793291	147793492	chr5-147793291-147793492
chr5	147859364	147862048	chr5-147859364-147862048
chr5	147893971	147895235	chr5-147893971-147895235
chr5	147913381	147913582	chr5-147913381-147913582
chr5	147956659	147958297	chr5-147956659-147958297
chr5	147963595	147964778	chr5-147963595-147964778
chr5	147992357	147993353	chr5-147992357-147993353
chr5	148264707	148266039	chr5-148264707-148266039

chr5	148310773	148311342	chr5-148310773-148311342
chr5	148341801	148343059	chr5-148341801-148343059
chr5	148360573	148363259	chr5-148360573-148363259
chr5	148367734	148368165	chr5-148367734-148368165
chr5	148371947	148374672	chr5-148371947-148374672
chr5	148397485	148400512	chr5-148397485-148400512
chr5	148419145	148419514	chr5-148419145-148419514
chr5	148443590	148444492	chr5-148443590-148444492
chr5	148466761	148467349	chr5-148466761-148467349
chr5	148516934	148517428	chr5-148516934-148517428
chr5	148518760	148519061	chr5-148518760-148519061
chr5	148523625	148523927	chr5-148523625-148523927
chr5	148534425	148534832	chr5-148534425-148534832
chr5	148536593	148536844	chr5-148536593-148536844
chr5	148547630	148553282	chr5-148547630-148553282
chr5	148564976	148565177	chr5-148564976-148565177
chr5	148608291	148608613	chr5-148608291-148608613
chr5	148625618	148626695	chr5-148625618-148626695
chr5	148632042	148634563	chr5-148632042-148634563
chr5	148648166	148648433	chr5-148648166-148648433
chr5	148707717	148709949	chr5-148707717-148709949
chr5	148738827	148742487	chr5-148738827-148742487
chr5	148789430	148789887	chr5-148789430-148789887
chr5	148831294	148832102	chr5-148831294-148832102
chr5	148928066	148929620	chr5-148928066-148929620
chr5	148950285	148950595	chr5-148950285-148950595
chr5	148959085	148960072	chr5-148959085-148960072
chr5	148965075	148967081	chr5-148965075-148967081
chr5	148993680	148997052	chr5-148993680-148997052
chr5	149006386	149007588	chr5-149006386-149007588
chr5	149015294	149016032	chr5-149015294-149016032
chr5	149031063	149031932	chr5-149031063-149031932
chr5	149050166	149054158	chr5-149050166-149054158
chr5	149078749	149080034	chr5-149078749-149080034
chr5	149183109	149186373	chr5-149183109-149186373
chr5	149228717	149230236	chr5-149228717-149230236
chr5	149246013	149246441	chr5-149246013-149246441
chr5	149279518	149279774	chr5-149279518-149279774
chr5	149287263	149287709	chr5-149287263-149287709
chr5	149312992	149313560	chr5-149312992-149313560
chr5	149320566	149320923	chr5-149320566-149320923
chr5	149352958	149353782	chr5-149352958-149353782

chr5	149388771	149390171	chr5-149388771-149390171
chr5	149391879	149392391	chr5-149391879-149392391
chr5	149411379	149412551	chr5-149411379-149412551
chr5	149433645	149434850	chr5-149433645-149434850
chr5	149439325	149440609	chr5-149439325-149440609
chr5	149508139	149509209	chr5-149508139-149509209
chr5	149513716	149514682	chr5-149513716-149514682
chr5	149524321	149525037	chr5-149524321-149525037
chr5	149526166	149527166	chr5-149526166-149527166
chr5	149537953	149538154	chr5-149537953-149538154
chr5	149606792	149607354	chr5-149606792-149607354
chr5	149615875	149616365	chr5-149615875-149616365
chr5	149634561	149637711	chr5-149634561-149637711
chr5	149677866	149679631	chr5-149677866-149679631
chr5	149831512	149831878	chr5-149831512-149831878
chr5	150118253	150119367	chr5-150118253-150119367
chr5	150195669	150197261	chr5-150195669-150197261
chr5	150232737	150232938	chr5-150232737-150232938
chr5	150258924	150260289	chr5-150258924-150260289
chr5	150333283	150333904	chr5-150333283-150333904
chr5	150436529	150437157	chr5-150436529-150437157
chr5	150451896	150452097	chr5-150451896-150452097
chr5	150457544	150458659	chr5-150457544-150458659
chr5	150466593	150466794	chr5-150466593-150466794
chr5	150521829	150523815	chr5-150521829-150523815
chr5	150578949	150579309	chr5-150578949-150579309
chr5	150592844	150595145	chr5-150592844-150595145
chr5	150611771	150612430	chr5-150611771-150612430
chr5	150663728	150666204	chr5-150663728-150666204
chr5	150673275	150675477	chr5-150673275-150675477
chr5	150706673	150707211	chr5-150706673-150707211
chr5	150952199	150953718	chr5-150952199-150953718
chr5	150996194	150996799	chr5-150996194-150996799
chr5	151069213	151070418	chr5-151069213-151070418
chr5	151111962	151113414	chr5-151111962-151113414
chr5	151142656	151142907	chr5-151142656-151142907
chr5	151154819	151155190	chr5-151154819-151155190
chr5	151185061	151186098	chr5-151185061-151186098
chr5	151187079	151190653	chr5-151187079-151190653
chr5	151196346	151197384	chr5-151196346-151197384
chr5	151232734	151234344	chr5-151232734-151234344
chr5	151251505	151251706	chr5-151251505-151251706

chr5	151314860	151316946	chr5-151314860-151316946
chr5	151346441	151347169	chr5-151346441-151347169
chr5	151367241	151369873	chr5-151367241-151369873
chr5	151650055	151651424	chr5-151650055-151651424
chr5	151686638	151687200	chr5-151686638-151687200
chr6	3330794	3330995	chr6-3330794-3330995
chr6	3398735	3399629	chr6-3398735-3399629
chr6	3497819	3500006	chr6-3497819-3500006
chr6	3620426	3620728	chr6-3620426-3620728
chr6	3763475	3763978	chr6-3763475-3763978
chr6	4003780	4004436	chr6-4003780-4004436
chr6	4086086	4087418	chr6-4086086-4087418
chr6	4419303	4419580	chr6-4419303-4419580
chr6	4505208	4505748	chr6-4505208-4505748
chr6	4600361	4601977	chr6-4600361-4601977
chr6	4746428	4749503	chr6-4746428-4749503
chr6	4902700	4904568	chr6-4902700-4904568
chr6	5110282	5110583	chr6-5110282-5110583
chr6	5255087	5256676	chr6-5255087-5256676
chr6	5258761	5259889	chr6-5258761-5259889
chr6	5296858	5298764	chr6-5296858-5298764
chr6	5300949	5301257	chr6-5300949-5301257
chr6	5382656	5383119	chr6-5382656-5383119
chr6	5383935	5384312	chr6-5383935-5384312
chr6	5442547	5443726	chr6-5442547-5443726
chr6	5495913	5496446	chr6-5495913-5496446
chr6	5509658	5510020	chr6-5509658-5510020
chr6	5641193	5641497	chr6-5641193-5641497
chr6	5725565	5726337	chr6-5725565-5726337
chr6	5859357	5859765	chr6-5859357-5859765
chr6	5992300	5992750	chr6-5992300-5992750
chr6	6005585	6006577	chr6-6005585-6006577
chr6	6215912	6217547	chr6-6215912-6217547
chr6	6576851	6579199	chr6-6576851-6579199
chr6	6859712	6864384	chr6-6859712-6864384
chr6	6878125	6878429	chr6-6878125-6878429
chr6	6879250	6880169	chr6-6879250-6880169
chr6	6881131	6882256	chr6-6881131-6882256
chr6	6955340	6957122	chr6-6955340-6957122
chr6	7554643	7555761	chr6-7554643-7555761
chr6	7691563	7693533	chr6-7691563-7693533
chr6	7844009	7848668	chr6-7844009-7848668

chr6	7882771	7882972	chr6-7882771-7882972
chr6	7982939	7983737	chr6-7982939-7983737
chr6	8208739	8210519	chr6-8208739-8210519
chr6	8257995	8260364	chr6-8257995-8260364
chr6	8508628	8511354	chr6-8508628-8511354
chr6	8776977	8778834	chr6-8776977-8778834
chr6	8948213	8951969	chr6-8948213-8951969
chr6	8955814	8956189	chr6-8955814-8956189
chr6	11905821	11908626	chr6-11905821-11908626
chr6	11925182	11927396	chr6-11925182-11927396
chr6	12747627	12749707	chr6-12747627-12749707
chr6	12813540	12813842	chr6-12813540-12813842
chr6	13069127	13070982	chr6-13069127-13070982
chr6	13074103	13074361	chr6-13074103-13074361
chr6	13606862	13608834	chr6-13606862-13608834
chr6	13676589	13678591	chr6-13676589-13678591
chr6	13837836	13839458	chr6-13837836-13839458
chr6	13870043	13872432	chr6-13870043-13872432
chr6	13909668	13910292	chr6-13909668-13910292
chr6	14899818	14902079	chr6-14899818-14902079
chr6	15720325	15722207	chr6-15720325-15722207
chr6	17064154	17067241	chr6-17064154-17067241
chr6	17072792	17072993	chr6-17072792-17072993
chr6	17108965	17109320	chr6-17108965-17109320
chr6	17119863	17120167	chr6-17119863-17120167
chr6	17143455	17143656	chr6-17143455-17143656
chr6	17169919	17172790	chr6-17169919-17172790
chr6	17211382	17212576	chr6-17211382-17212576
chr6	17218878	17219234	chr6-17218878-17219234
chr6	17265258	17265459	chr6-17265258-17265459
chr6	17280400	17284049	chr6-17280400-17284049
chr6	17305842	17310073	chr6-17305842-17310073
chr6	17315971	17317928	chr6-17315971-17317928
chr6	17353747	17354725	chr6-17353747-17354725
chr6	17408327	17408789	chr6-17408327-17408789
chr6	17410271	17410680	chr6-17410271-17410680
chr6	17463162	17467098	chr6-17463162-17467098
chr6	17478616	17479769	chr6-17478616-17479769
chr6	17485059	17486208	chr6-17485059-17486208
chr6	17496765	17496966	chr6-17496765-17496966
chr6	17498459	17499426	chr6-17498459-17499426
chr6	17555591	17555792	chr6-17555591-17555792

chr6	17583776	17584337	chr6-17583776-17584337
chr6	17636570	17639848	chr6-17636570-17639848
chr6	17667525	17667726	chr6-17667525-17667726
chr6	17692270	17695346	chr6-17692270-17695346
chr6	17749052	17749466	chr6-17749052-17749466
chr6	17876808	17877159	chr6-17876808-17877159
chr6	18029969	18032250	chr6-18029969-18032250
chr6	18170230	18171922	chr6-18170230-18171922
chr6	18271287	18271590	chr6-18271287-18271590
chr6	18302556	18302814	chr6-18302556-18302814
chr6	18514044	18515290	chr6-18514044-18515290
chr6	18845170	18849960	chr6-18845170-18849960
chr6	21214473	21215807	chr6-21214473-21215807
chr6	21851297	21852791	chr6-21851297-21852791
chr6	21948725	21951009	chr6-21948725-21951009
chr6	21985275	21986585	chr6-21985275-21986585
chr6	22280167	22280753	chr6-22280167-22280753
chr6	22288283	22289591	chr6-22288283-22289591
chr6	22354089	22356660	chr6-22354089-22356660
chr6	22360950	22361482	chr6-22360950-22361482
chr6	22443592	22444105	chr6-22443592-22444105
chr6	22493711	22494111	chr6-22493711-22494111
chr6	22875005	22876253	chr6-22875005-22876253
chr6	23132625	23133072	chr6-23132625-23133072
chr6	23247561	23247989	chr6-23247561-23247989
chr6	23249280	23249821	chr6-23249280-23249821
chr6	23253836	23254191	chr6-23253836-23254191
chr6	23838573	23840100	chr6-23838573-23840100
chr6	24526516	24528581	chr6-24526516-24528581
chr6	24554197	24555153	chr6-24554197-24555153
chr6	24585763	24586396	chr6-24585763-24586396
chr6	24661899	24665878	chr6-24661899-24665878
chr6	24894678	24895185	chr6-24894678-24895185
chr6	24955094	24956494	chr6-24955094-24956494
chr6	25688929	25690458	chr6-25688929-25690458
chr6	25808377	25809784	chr6-25808377-25809784
chr6	28133587	28136192	chr6-28133587-28136192
chr6	28215081	28216040	chr6-28215081-28216040
chr6	28229853	28230054	chr6-28229853-28230054
chr6	28260274	28262926	chr6-28260274-28262926
chr6	28397033	28398196	chr6-28397033-28398196
chr6	28419634	28425281	chr6-28419634-28425281

chr6	28479172	28483588	chr6-28479172-28483588
chr6	28831892	28833712	chr6-28831892-28833712
chr6	28845691	28845892	chr6-28845691-28845892
chr6	28866717	28867328	chr6-28866717-28867328
chr6	28932402	28932658	chr6-28932402-28932658
chr6	28980236	28981990	chr6-28980236-28981990
chr6	29015219	29015641	chr6-29015219-29015641
chr6	29023568	29024417	chr6-29023568-29024417
chr6	29031278	29031529	chr6-29031278-29031529
chr6	29073861	29074062	chr6-29073861-29074062
chr6	29083069	29083378	chr6-29083069-29083378
chr6	29163568	29165122	chr6-29163568-29165122
chr6	29169854	29171706	chr6-29169854-29171706
chr6	29178772	29179877	chr6-29178772-29179877
chr6	29195898	29196462	chr6-29195898-29196462
chr6	29198513	29198765	chr6-29198513-29198765
chr6	29210323	29212820	chr6-29210323-29212820
chr6	29214371	29216796	chr6-29214371-29216796
chr6	29244756	29248946	chr6-29244756-29248946
chr6	29257750	29258001	chr6-29257750-29258001
chr6	29259556	29260360	chr6-29259556-29260360
chr6	29270638	29277076	chr6-29270638-29277076
chr6	29278835	29279036	chr6-29278835-29279036
chr6	29316526	29319732	chr6-29316526-29319732
chr6	29347129	29351286	chr6-29347129-29351286
chr6	29387648	29389241	chr6-29387648-29389241
chr6	29395725	29397866	chr6-29395725-29397866
chr6	29402836	29403037	chr6-29402836-29403037
chr6	29430625	29436996	chr6-29430625-29436996
chr6	29467535	29469293	chr6-29467535-29469293
chr6	29473688	29474610	chr6-29473688-29474610
chr6	29484498	29485559	chr6-29484498-29485559
chr6	29496144	29496802	chr6-29496144-29496802
chr6	29507160	29508301	chr6-29507160-29508301
chr6	29513361	29513662	chr6-29513361-29513662
chr6	29526096	29529579	chr6-29526096-29529579
chr6	29607989	29610612	chr6-29607989-29610612
chr6	29693634	29695116	chr6-29693634-29695116
chr6	29718020	29718221	chr6-29718020-29718221
chr6	29734962	29737604	chr6-29734962-29737604
chr6	29767393	29769899	chr6-29767393-29769899
chr6	29849165	29849366	chr6-29849165-29849366

chr6	29853377	29853848	chr6-29853377-29853848
chr6	29916265	29917880	chr6-29916265-29917880
chr6	29984739	29986056	chr6-29984739-29986056
chr6	30046924	30049999	chr6-30046924-30049999
chr6	30171276	30176506	chr6-30171276-30176506
chr6	30205632	30206159	chr6-30205632-30206159
chr6	30288081	30288332	chr6-30288081-30288332
chr6	30300418	30304994	chr6-30300418-30304994
chr6	30307510	30308177	chr6-30307510-30308177
chr6	30336876	30337077	chr6-30336876-30337077
chr6	30389940	30391727	chr6-30389940-30391727
chr6	30400636	30403054	chr6-30400636-30403054
chr6	30459444	30460402	chr6-30459444-30460402
chr6	30508759	30510594	chr6-30508759-30510594
chr6	30561926	30564042	chr6-30561926-30564042
chr6	30568185	30570886	chr6-30568185-30570886
chr6	30617939	30618376	chr6-30617939-30618376
chr6	30624992	30625355	chr6-30624992-30625355
chr6	30692763	30694355	chr6-30692763-30694355
chr6	30737236	30739826	chr6-30737236-30739826
chr6	30895784	30897423	chr6-30895784-30897423
chr6	30921242	30921670	chr6-30921242-30921670
chr6	30957799	30959556	chr6-30957799-30959556
chr6	31032956	31033277	chr6-31032956-31033277
chr6	31061378	31061885	chr6-31061378-31061885
chr6	31062758	31063332	chr6-31062758-31063332
chr6	31070590	31072177	chr6-31070590-31072177
chr6	31073355	31091120	chr6-31073355-31091120
chr6	31092808	31093009	chr6-31092808-31093009
chr6	31098520	31101749	chr6-31098520-31101749
chr6	31106165	31116890	chr6-31106165-31116890
chr6	31123342	31127640	chr6-31123342-31127640
chr6	31159400	31159855	chr6-31159400-31159855
chr6	31205669	31206699	chr6-31205669-31206699
chr6	31209438	31210258	chr6-31209438-31210258
chr6	31215540	31221743	chr6-31215540-31221743
chr6	31284340	31285452	chr6-31284340-31285452
chr6	31317668	31318187	chr6-31317668-31318187
chr6	31332300	31332816	chr6-31332300-31332816
chr6	31339879	31340080	chr6-31339879-31340080
chr6	31398135	31400463	chr6-31398135-31400463
chr6	31517908	31518268	chr6-31517908-31518268

chr6	31544444	31544697	chr6-31544444-31544697
chr6	31548892	31550214	chr6-31548892-31550214
chr6	31556358	31556559	chr6-31556358-31556559
chr6	31561814	31564672	chr6-31561814-31564672
chr6	31938294	31938847	chr6-31938294-31938847
chr6	32397176	32397493	chr6-32397176-32397493
chr6	32527578	32528826	chr6-32527578-32528826
chr6	32586329	32588729	chr6-32586329-32588729
chr6	33058987	33060435	chr6-33058987-33060435
chr6	33235083	33235334	chr6-33235083-33235334
chr6	33248874	33249952	chr6-33248874-33249952
chr6	33890015	33890216	chr6-33890015-33890216
chr6	33892032	33892335	chr6-33892032-33892335
chr6	34028873	34030263	chr6-34028873-34030263
chr6	34175814	34177472	chr6-34175814-34177472
chr6	34315864	34318696	chr6-34315864-34318696
chr6	34353559	34355918	chr6-34353559-34355918
chr6	34371886	34372191	chr6-34371886-34372191
chr6	34383811	34385443	chr6-34383811-34385443
chr6	34475202	34479897	chr6-34475202-34479897
chr6	34481359	34481622	chr6-34481359-34481622
chr6	34542493	34543952	chr6-34542493-34543952
chr6	34597812	34603922	chr6-34597812-34603922
chr6	34608757	34611707	chr6-34608757-34611707
chr6	34708456	34714183	chr6-34708456-34714183
chr6	34734985	34735186	chr6-34734985-34735186
chr6	34743727	34743978	chr6-34743727-34743978
chr6	34780080	34781595	chr6-34780080-34781595
chr6	34796756	34797007	chr6-34796756-34797007
chr6	34862381	34864300	chr6-34862381-34864300
chr6	34876691	34878629	chr6-34876691-34878629
chr6	34909753	34911361	chr6-34909753-34911361
chr6	34966034	34966489	chr6-34966034-34966489
chr6	34994795	34995621	chr6-34994795-34995621
chr6	35132247	35134990	chr6-35132247-35134990
chr6	35176863	35179008	chr6-35176863-35179008
chr6	35252254	35253705	chr6-35252254-35253705
chr6	35388737	35388938	chr6-35388737-35388938
chr6	35537926	35540100	chr6-35537926-35540100
chr6	35542812	35543013	chr6-35542812-35543013
chr6	36387912	36388786	chr6-36387912-36388786
chr6	37299338	37300394	chr6-37299338-37300394

chr6	37388800	37389051	chr6-37388800-37389051
chr6	37398928	37399340	chr6-37398928-37399340
chr6	37427816	37428877	chr6-37427816-37428877
chr6	37431565	37431920	chr6-37431565-37431920
chr6	37440162	37443337	chr6-37440162-37443337
chr6	37455983	37456287	chr6-37455983-37456287
chr6	37459575	37459840	chr6-37459575-37459840
chr6	37520660	37522160	chr6-37520660-37522160
chr6	37617490	37618284	chr6-37617490-37618284
chr6	37805885	37806200	chr6-37805885-37806200
chr6	37870079	37872711	chr6-37870079-37872711
chr6	38072197	38072452	chr6-38072197-38072452
chr6	38135049	38135250	chr6-38135049-38135250
chr6	38252479	38254885	chr6-38252479-38254885
chr6	38297749	38299816	chr6-38297749-38299816
chr6	38352301	38355796	chr6-38352301-38355796
chr6	38380572	38382303	chr6-38380572-38382303
chr6	38432572	38435833	chr6-38432572-38435833
chr6	38523930	38524295	chr6-38523930-38524295
chr6	38532271	38535874	chr6-38532271-38535874
chr6	38549787	38552994	chr6-38549787-38552994
chr6	38635023	38637856	chr6-38635023-38637856
chr6	38642445	38642850	chr6-38642445-38642850
chr6	38662571	38663804	chr6-38662571-38663804
chr6	38753505	38753962	chr6-38753505-38753962
chr6	38787216	38788546	chr6-38787216-38788546
chr6	38809180	38810606	chr6-38809180-38810606
chr6	38835866	38836176	chr6-38835866-38836176
chr6	38873785	38877146	chr6-38873785-38877146
chr6	38882652	38883011	chr6-38882652-38883011
chr6	39013793	39014103	chr6-39013793-39014103
chr6	39117215	39119106	chr6-39117215-39119106
chr6	39204854	39207728	chr6-39204854-39207728
chr6	39242630	39244263	chr6-39242630-39244263
chr6	39256499	39257578	chr6-39256499-39257578
chr6	39268803	39269893	chr6-39268803-39269893
chr6	39318961	39319753	chr6-39318961-39319753
chr6	39334076	39334389	chr6-39334076-39334389
chr6	39375979	39378073	chr6-39375979-39378073
chr6	39380959	39381932	chr6-39380959-39381932
chr6	39418755	39420844	chr6-39418755-39420844
chr6	39463071	39463447	chr6-39463071-39463447

chr6	39515320	39516129	chr6-39515320-39516129
chr6	39557000	39558427	chr6-39557000-39558427
chr6	39562398	39562802	chr6-39562398-39562802
chr6	39573344	39575204	chr6-39573344-39575204
chr6	39591723	39593810	chr6-39591723-39593810
chr6	39723950	39725872	chr6-39723950-39725872
chr6	39809809	39811429	chr6-39809809-39811429
chr6	39870411	39873502	chr6-39870411-39873502
chr6	40325065	40326476	chr6-40325065-40326476
chr6	40429489	40430110	chr6-40429489-40430110
chr6	40431015	40431681	chr6-40431015-40431681
chr6	40434825	40436720	chr6-40434825-40436720
chr6	40470313	40472896	chr6-40470313-40472896
chr6	41576314	41576797	chr6-41576314-41576797
chr6	41604391	41606417	chr6-41604391-41606417
chr6	42227457	42227767	chr6-42227457-42227767
chr6	42245751	42246419	chr6-42245751-42246419
chr6	42257323	42257642	chr6-42257323-42257642
chr6	42261855	42262464	chr6-42261855-42262464
chr6	42264317	42266787	chr6-42264317-42266787
chr6	42295591	42295792	chr6-42295591-42295792
chr6	42299177	42299694	chr6-42299177-42299694
chr6	42323592	42325416	chr6-42323592-42325416
chr6	42348501	42353740	chr6-42348501-42353740
chr6	42371863	42374178	chr6-42371863-42374178
chr6	42489888	42490209	chr6-42489888-42490209
chr6	42583511	42583712	chr6-42583511-42583712
chr6	42644768	42645633	chr6-42644768-42645633
chr6	42691772	42693382	chr6-42691772-42693382
chr6	43020043	43020244	chr6-43020043-43020244
chr6	43256667	43257126	chr6-43256667-43257126
chr6	43264657	43267445	chr6-43264657-43267445
chr6	43292251	43293444	chr6-43292251-43293444
chr6	43382093	43383378	chr6-43382093-43383378
chr6	43665151	43666837	chr6-43665151-43666837
chr6	47044660	47045112	chr6-47044660-47045112
chr6	47191703	47192011	chr6-47191703-47192011
chr6	47314872	47315292	chr6-47314872-47315292
chr6	47319713	47319968	chr6-47319713-47319968
chr6	47328447	47328648	chr6-47328447-47328648
chr6	47333016	47333428	chr6-47333016-47333428
chr6	47452965	47456167	chr6-47452965-47456167

chr6	47494645	47494896	chr6-47494645-47494896
chr6	47561108	47561898	chr6-47561108-47561898
chr6	47593471	47596437	chr6-47593471-47596437
chr6	47650434	47652098	chr6-47650434-47652098
chr6	47658964	47660082	chr6-47658964-47660082
chr6	47672666	47673730	chr6-47672666-47673730
chr6	47681307	47682376	chr6-47681307-47682376
chr6	47739664	47741189	chr6-47739664-47741189
chr6	47743821	47744768	chr6-47743821-47744768
chr6	47748238	47748766	chr6-47748238-47748766
chr6	47749703	47750005	chr6-47749703-47750005
chr6	47753176	47753627	chr6-47753176-47753627
chr6	47757376	47758501	chr6-47757376-47758501
chr6	47767262	47768375	chr6-47767262-47768375
chr6	47771659	47772211	chr6-47771659-47772211
chr6	47777934	47779406	chr6-47777934-47779406
chr6	47780442	47782163	chr6-47780442-47782163
chr6	47788141	47789604	chr6-47788141-47789604
chr6	47811173	47814493	chr6-47811173-47814493
chr6	47829908	47831091	chr6-47829908-47831091
chr6	47835280	47836941	chr6-47835280-47836941
chr6	47877129	47879545	chr6-47877129-47879545
chr6	47904665	47904866	chr6-47904665-47904866
chr6	47919835	47922362	chr6-47919835-47922362
chr6	47942867	47943642	chr6-47942867-47943642
chr6	47952878	47954528	chr6-47952878-47954528
chr6	47979309	47979560	chr6-47979309-47979560
chr6	48015729	48016542	chr6-48015729-48016542
chr6	48024358	48025405	chr6-48024358-48025405
chr6	48046620	48049974	chr6-48046620-48049974
chr6	48085157	48087299	chr6-48085157-48087299
chr6	48227369	48227699	chr6-48227369-48227699
chr6	48394902	48396646	chr6-48394902-48396646
chr6	48443529	48446495	chr6-48443529-48446495
chr6	48487977	48488228	chr6-48487977-48488228
chr6	48504125	48505102	chr6-48504125-48505102
chr6	48537143	48538225	chr6-48537143-48538225
chr6	48554062	48555760	chr6-48554062-48555760
chr6	48566653	48566854	chr6-48566653-48566854
chr6	48568515	48568988	chr6-48568515-48568988
chr6	48579289	48579695	chr6-48579289-48579695
chr6	48593151	48597753	chr6-48593151-48597753

chr6	48601177	48602975	chr6-48601177-48602975
chr6	48626791	48627771	chr6-48626791-48627771
chr6	48628984	48629964	chr6-48628984-48629964
chr6	48645897	48646098	chr6-48645897-48646098
chr6	48675751	48676646	chr6-48675751-48676646
chr6	48725828	48726079	chr6-48725828-48726079
chr6	48742310	48743414	chr6-48742310-48743414
chr6	48838192	48842831	chr6-48838192-48842831
chr6	48860374	48860575	chr6-48860374-48860575
chr6	49008103	49008715	chr6-49008103-49008715
chr6	49036438	49036968	chr6-49036438-49036968
chr6	49039665	49040122	chr6-49039665-49040122
chr6	49073283	49075386	chr6-49073283-49075386
chr6	49210328	49210861	chr6-49210328-49210861
chr6	49213015	49216046	chr6-49213015-49216046
chr6	49217416	49218728	chr6-49217416-49218728
chr6	49261026	49264674	chr6-49261026-49264674
chr6	49319022	49320050	chr6-49319022-49320050
chr6	49367202	49368648	chr6-49367202-49368648
chr6	49578103	49579099	chr6-49578103-49579099
chr6	49656784	49657104	chr6-49656784-49657104
chr6	49792674	49793132	chr6-49792674-49793132
chr6	49797915	49798229	chr6-49797915-49798229
chr6	49821990	49823159	chr6-49821990-49823159
chr6	49987570	49988314	chr6-49987570-49988314
chr6	50037460	50038517	chr6-50037460-50038517
chr6	50109481	50111683	chr6-50109481-50111683
chr6	50112995	50113196	chr6-50112995-50113196
chr6	50225383	50225638	chr6-50225383-50225638
chr6	50254502	50255766	chr6-50254502-50255766
chr6	50261278	50262299	chr6-50261278-50262299
chr6	50355168	50355645	chr6-50355168-50355645
chr6	50378124	50383614	chr6-50378124-50383614
chr6	50387586	50387996	chr6-50387586-50387996
chr6	50392842	50393043	chr6-50392842-50393043
chr6	50395306	50396437	chr6-50395306-50396437
chr6	50408275	50408694	chr6-50408275-50408694
chr6	50451988	50458398	chr6-50451988-50458398
chr6	50459428	50459629	chr6-50459428-50459629
chr6	50460769	50460970	chr6-50460769-50460970
chr6	50536440	50536796	chr6-50536440-50536796
chr6	50565279	50567530	chr6-50565279-50567530

chr6	50595922	50597242	chr6-50595922-50597242
chr6	50685438	50685740	chr6-50685438-50685740
chr6	50776925	50777435	chr6-50776925-50777435
chr6	50807938	50808299	chr6-50807938-50808299
chr6	51168650	51172088	chr6-51168650-51172088
chr6	51173439	51174655	chr6-51173439-51174655
chr6	51177533	51179470	chr6-51177533-51179470
chr6	51193335	51193782	chr6-51193335-51193782
chr6	51207903	51208104	chr6-51207903-51208104
chr6	51210412	51211549	chr6-51210412-51211549
chr6	51269910	51271782	chr6-51269910-51271782
chr6	51362632	51363303	chr6-51362632-51363303
chr6	51378756	51380227	chr6-51378756-51380227
chr6	51384164	51384605	chr6-51384164-51384605
chr6	51432420	51438273	chr6-51432420-51438273
chr6	51465738	51472930	chr6-51465738-51472930
chr6	51523384	51525418	chr6-51523384-51525418
chr6	51544186	51546208	chr6-51544186-51546208
chr6	51567176	51567377	chr6-51567176-51567377
chr6	51612089	51613308	chr6-51612089-51613308
chr6	51720598	51720901	chr6-51720598-51720901
chr6	51760481	51760789	chr6-51760481-51760789
chr6	51844082	51844333	chr6-51844082-51844333
chr6	52011612	52013808	chr6-52011612-52013808
chr6	52076385	52076854	chr6-52076385-52076854
chr6	52157470	52160950	chr6-52157470-52160950
chr6	52164146	52164760	chr6-52164146-52164760
chr6	52165681	52166057	chr6-52165681-52166057
chr6	52168693	52168894	chr6-52168693-52168894
chr6	52172180	52173156	chr6-52172180-52173156
chr6	52175439	52177739	chr6-52175439-52177739
chr6	52179804	52180005	chr6-52179804-52180005
chr6	52190966	52192617	chr6-52190966-52192617
chr6	52203360	52206692	chr6-52203360-52206692
chr6	52208169	52209142	chr6-52208169-52209142
chr6	52211400	52213875	chr6-52211400-52213875
chr6	52215704	52215963	chr6-52215704-52215963
chr6	52216788	52218044	chr6-52216788-52218044
chr6	52221103	52222166	chr6-52221103-52222166
chr6	52232340	52234964	chr6-52232340-52234964
chr6	52240079	52240926	chr6-52240079-52240926
chr6	52244880	52246886	chr6-52244880-52246886

chr6	52249125	52249681	chr6-52249125-52249681
chr6	52252653	52253979	chr6-52252653-52253979
chr6	52259660	52261555	chr6-52259660-52261555
chr6	52274178	52274489	chr6-52274178-52274489
chr6	52290914	52291665	chr6-52290914-52291665
chr6	52305735	52306557	chr6-52305735-52306557
chr6	52310318	52310847	chr6-52310318-52310847
chr6	52313878	52314181	chr6-52313878-52314181
chr6	52316228	52317238	chr6-52316228-52317238
chr6	52459119	52459320	chr6-52459119-52459320
chr6	52580399	52580600	chr6-52580399-52580600
chr6	52609693	52610443	chr6-52609693-52610443
chr6	52638871	52640873	chr6-52638871-52640873
chr6	52713329	52715143	chr6-52713329-52715143
chr6	52825207	52825646	chr6-52825207-52825646
chr6	53067524	53069138	chr6-53067524-53069138
chr6	53164586	53164888	chr6-53164586-53164888
chr6	53286411	53289227	chr6-53286411-53289227
chr6	53306719	53307463	chr6-53306719-53307463
chr6	53556160	53556361	chr6-53556160-53556361
chr6	53572460	53575662	chr6-53572460-53575662
chr6	53706659	53706860	chr6-53706659-53706860
chr6	53794814	53795015	chr6-53794814-53795015
chr6	53817859	53821180	chr6-53817859-53821180
chr6	54019033	54019234	chr6-54019033-54019234
chr6	54021357	54021558	chr6-54021357-54021558
chr6	54038223	54042005	chr6-54038223-54042005
chr6	54088120	54088400	chr6-54088120-54088400
chr6	54162606	54162857	chr6-54162606-54162857
chr6	54326295	54328120	chr6-54326295-54328120
chr6	54428950	54430438	chr6-54428950-54430438
chr6	54485474	54485955	chr6-54485474-54485955
chr6	54565660	54566667	chr6-54565660-54566667
chr6	54592703	54596297	chr6-54592703-54596297
chr6	54680777	54682753	chr6-54680777-54682753
chr6	54698666	54699990	chr6-54698666-54699990
chr6	54732261	54732916	chr6-54732261-54732916
chr6	54816067	54818839	chr6-54816067-54818839
chr6	54831144	54831614	chr6-54831144-54831614
chr6	54867074	54867554	chr6-54867074-54867554
chr6	54954700	54955106	chr6-54954700-54955106
chr6	54967655	54967907	chr6-54967655-54967907

chr6	54971989	54973344	chr6-54971989-54973344
chr6	54991745	54993551	chr6-54991745-54993551
chr6	55016474	55017179	chr6-55016474-55017179
chr6	55037212	55040603	chr6-55037212-55040603
chr6	55117352	55118265	chr6-55117352-55118265
chr6	55202831	55204213	chr6-55202831-55204213
chr6	55338637	55339849	chr6-55338637-55339849
chr6	55342377	55342628	chr6-55342377-55342628
chr6	55437395	55437704	chr6-55437395-55437704
chr6	55451741	55452791	chr6-55451741-55452791
chr6	55617892	55618419	chr6-55617892-55618419
chr6	55619631	55619885	chr6-55619631-55619885
chr6	56568389	56569511	chr6-56568389-56569511
chr6	56652068	56652701	chr6-56652068-56652701
chr6	56703448	56705238	chr6-56703448-56705238
chr6	56714389	56716400	chr6-56714389-56716400
chr6	56794828	56798755	chr6-56794828-56798755
chr6	56831468	56833246	chr6-56831468-56833246
chr6	56901307	56902558	chr6-56901307-56902558
chr6	56922193	56924948	chr6-56922193-56924948
chr6	57534189	57536533	chr6-57534189-57536533
chr6	57580725	57581880	chr6-57580725-57581880
chr6	57690999	57692520	chr6-57690999-57692520
chr6	57702286	57704070	chr6-57702286-57704070
chr6	57706623	57707032	chr6-57706623-57707032
chr6	57822709	57825661	chr6-57822709-57825661
chr6	58596421	58596723	chr6-58596421-58596723
chr6	58640083	58641559	chr6-58640083-58641559
chr6	58831059	58833142	chr6-58831059-58833142
chr6	59024055	59024876	chr6-59024055-59024876
chr6	59208563	59210112	chr6-59208563-59210112
chr6	59425799	59426708	chr6-59425799-59426708
chr6	60674221	60674583	chr6-60674221-60674583
chr6	60828256	60829923	chr6-60828256-60829923
chr6	61179648	61181987	chr6-61179648-61181987
chr6	61188983	61189234	chr6-61188983-61189234
chr6	61233113	61234096	chr6-61233113-61234096
chr6	61242825	61243214	chr6-61242825-61243214
chr6	63255704	63257989	chr6-63255704-63257989
chr6	64649694	64650095	chr6-64649694-64650095
chr6	64727610	64730365	chr6-64727610-64730365
chr6	64734436	64735066	chr6-64734436-64735066

chr6	64799264	64799950	chr6-64799264-64799950
chr6	65042015	65044424	chr6-65042015-65044424
chr6	65380920	65381643	chr6-65380920-65381643
chr6	65590457	65590729	chr6-65590457-65590729
chr6	65671105	65673281	chr6-65671105-65673281
chr6	65778397	65780042	chr6-65778397-65780042
chr6	66120301	66120615	chr6-66120301-66120615
chr6	66534699	66537690	chr6-66534699-66537690
chr6	66895919	66897947	chr6-66895919-66897947
chr6	66910315	66911659	chr6-66910315-66911659
chr6	66943481	66944955	chr6-66943481-66944955
chr6	66964385	66965266	chr6-66964385-66965266
chr6	67034573	67038071	chr6-67034573-67038071
chr6	67041272	67041577	chr6-67041272-67041577
chr6	67101909	67103618	chr6-67101909-67103618
chr6	67168557	67168827	chr6-67168557-67168827
chr6	67170376	67170904	chr6-67170376-67170904
chr6	67265851	67270388	chr6-67265851-67270388
chr6	67375873	67376392	chr6-67375873-67376392
chr6	67481504	67482011	chr6-67481504-67482011
chr6	67531077	67536424	chr6-67531077-67536424
chr6	70773304	70774433	chr6-70773304-70774433
chr6	70784339	70793360	chr6-70784339-70793360
chr6	70804885	70806211	chr6-70804885-70806211
chr6	70813833	70814656	chr6-70813833-70814656
chr6	70823847	70825385	chr6-70823847-70825385
chr6	70843530	70846253	chr6-70843530-70846253
chr6	70871862	70872266	chr6-70871862-70872266
chr6	70911450	70912541	chr6-70911450-70912541
chr6	70921688	70921989	chr6-70921688-70921989
chr6	70955949	70957348	chr6-70955949-70957348
chr6	71012591	71013763	chr6-71012591-71013763
chr6	71048414	71048968	chr6-71048414-71048968
chr6	71143243	71144567	chr6-71143243-71144567
chr6	71270286	71273342	chr6-71270286-71273342
chr6	71439040	71441909	chr6-71439040-71441909
chr6	71493211	71496324	chr6-71493211-71496324
chr6	71543367	71545324	chr6-71543367-71545324
chr6	71630961	71633720	chr6-71630961-71633720
chr6	71707542	71708591	chr6-71707542-71708591
chr6	71782889	71783090	chr6-71782889-71783090
chr6	71822586	71824297	chr6-71822586-71824297

chr6	71830103	71832698	chr6-71830103-71832698
chr6	71907200	71910225	chr6-71907200-71910225
chr6	71992309	71992510	chr6-71992309-71992510
chr6	72096885	72098453	chr6-72096885-72098453
chr6	72189092	72189395	chr6-72189092-72189395
chr6	72216370	72217571	chr6-72216370-72217571
chr6	72233913	72235879	chr6-72233913-72235879
chr6	72244101	72245321	chr6-72244101-72245321
chr6	72292363	72294097	chr6-72292363-72294097
chr6	72315743	72316014	chr6-72315743-72316014
chr6	72331261	72331575	chr6-72331261-72331575
chr6	72343544	72348765	chr6-72343544-72348765
chr6	72354702	72356718	chr6-72354702-72356718
chr6	72360287	72363387	chr6-72360287-72363387
chr6	72369792	72372301	chr6-72369792-72372301
chr6	72379354	72381085	chr6-72379354-72381085
chr6	72388659	72391222	chr6-72388659-72391222
chr6	72413588	72415639	chr6-72413588-72415639
chr6	72436912	72441065	chr6-72436912-72441065
chr6	72466997	72467413	chr6-72466997-72467413
chr6	72503691	72503892	chr6-72503691-72503892
chr6	72512504	72513067	chr6-72512504-72513067
chr6	72538038	72539350	chr6-72538038-72539350
chr6	72543345	72546003	chr6-72543345-72546003
chr6	72547507	72553751	chr6-72547507-72553751
chr6	72560702	72561493	chr6-72560702-72561493
chr6	72597101	72599468	chr6-72597101-72599468
chr6	72613932	72617984	chr6-72613932-72617984
chr6	72636248	72636502	chr6-72636248-72636502
chr6	72641091	72642122	chr6-72641091-72642122
chr6	72676630	72678478	chr6-72676630-72678478
chr6	72787771	72789992	chr6-72787771-72789992
chr6	72824871	72825397	chr6-72824871-72825397
chr6	72881291	72882077	chr6-72881291-72882077
chr6	72897885	72901603	chr6-72897885-72901603
chr6	72926539	72927398	chr6-72926539-72927398
chr6	72956176	72959275	chr6-72956176-72959275
chr6	72961432	72962183	chr6-72961432-72962183
chr6	72981274	72981683	chr6-72981274-72981683
chr6	73221505	73223423	chr6-73221505-73223423
chr6	73247712	73250351	chr6-73247712-73250351
chr6	73314830	73315664	chr6-73314830-73315664

chr6	73362938	73363255	chr6-73362938-73363255
chr6	73367748	73368766	chr6-73367748-73368766
chr6	74609091	74609342	chr6-74609091-74609342
chr6	77979324	77980086	chr6-77979324-77980086
chr6	81749884	81750199	chr6-81749884-81750199
chr6	81923303	81925294	chr6-81923303-81925294
chr6	81964479	81966440	chr6-81964479-81966440
chr6	82041244	82043259	chr6-82041244-82043259
chr6	82402765	82403017	chr6-82402765-82403017
chr6	82651659	82653620	chr6-82651659-82653620
chr6	82740388	82740798	chr6-82740388-82740798
chr6	82751146	82751875	chr6-82751146-82751875
chr6	82752960	82754428	chr6-82752960-82754428
chr6	82756011	82756708	chr6-82756011-82756708
chr6	82760244	82760515	chr6-82760244-82760515
chr6	82761989	82762635	chr6-82761989-82762635
chr6	82763753	82764707	chr6-82763753-82764707
chr6	82766166	82775315	chr6-82766166-82775315
chr6	82863083	82863868	chr6-82863083-82863868
chr6	82879357	82882049	chr6-82879357-82882049
chr6	82891417	82891961	chr6-82891417-82891961
chr6	82908747	82909488	chr6-82908747-82909488
chr6	82934883	82935084	chr6-82934883-82935084
chr6	82939016	82940069	chr6-82939016-82940069
chr6	82996649	82997398	chr6-82996649-82997398
chr6	83014547	83014901	chr6-83014547-83014901
chr6	83017269	83017622	chr6-83017269-83017622
chr6	83024107	83024414	chr6-83024107-83024414
chr6	83027351	83029397	chr6-83027351-83029397
chr6	83030333	83034891	chr6-83030333-83034891
chr6	83038352	83039069	chr6-83038352-83039069
chr6	83052486	83056429	chr6-83052486-83056429
chr6	83057938	83059025	chr6-83057938-83059025
chr6	83068510	83071595	chr6-83068510-83071595
chr6	83077403	83079782	chr6-83077403-83079782
chr6	83086329	83087997	chr6-83086329-83087997
chr6	83100988	83103395	chr6-83100988-83103395
chr6	83107761	83110189	chr6-83107761-83110189
chr6	83114439	83117316	chr6-83114439-83117316
chr6	83119515	83125868	chr6-83119515-83125868
chr6	83135112	83138644	chr6-83135112-83138644
chr6	83155483	83156882	chr6-83155483-83156882

chr6	83161867	83163124	chr6-83161867-83163124
chr6	83165131	83166803	chr6-83165131-83166803
chr6	83234192	83234758	chr6-83234192-83234758
chr6	83237145	83237400	chr6-83237145-83237400
chr6	83267702	83268914	chr6-83267702-83268914
chr6	83315525	83318022	chr6-83315525-83318022
chr6	83325223	83328090	chr6-83325223-83328090
chr6	83348952	83350712	chr6-83348952-83350712
chr6	83439728	83444289	chr6-83439728-83444289
chr6	83453629	83458196	chr6-83453629-83458196
chr6	83460613	83460814	chr6-83460613-83460814
chr6	83478605	83478861	chr6-83478605-83478861
chr6	83502776	83503034	chr6-83502776-83503034
chr6	83505628	83507635	chr6-83505628-83507635
chr6	83571311	83573246	chr6-83571311-83573246
chr6	83599136	83599337	chr6-83599136-83599337
chr6	83702166	83702992	chr6-83702166-83702992
chr6	83710260	83712254	chr6-83710260-83712254
chr6	83714866	83715067	chr6-83714866-83715067
chr6	83757976	83758611	chr6-83757976-83758611
chr6	83773736	83776529	chr6-83773736-83776529
chr6	83794073	83796302	chr6-83794073-83796302
chr6	83830595	83832609	chr6-83830595-83832609
chr6	83866877	83868441	chr6-83866877-83868441
chr6	83913900	83915673	chr6-83913900-83915673
chr6	84008259	84009221	chr6-84008259-84009221
chr6	84018673	84019894	chr6-84018673-84019894
chr6	84105453	84106057	chr6-84105453-84106057
chr6	84148255	84148456	chr6-84148255-84148456
chr6	84179629	84179830	chr6-84179629-84179830
chr6	84181214	84181888	chr6-84181214-84181888
chr6	84233613	84234056	chr6-84233613-84234056
chr6	84514694	84515072	chr6-84514694-84515072
chr6	84565108	84565724	chr6-84565108-84565724
chr6	84579535	84580103	chr6-84579535-84580103
chr6	84583365	84590506	chr6-84583365-84590506
chr6	84697398	84698293	chr6-84697398-84698293
chr6	84893140	84893543	chr6-84893140-84893543
chr6	85068003	85069715	chr6-85068003-85069715
chr6	85117734	85117985	chr6-85117734-85117985
chr6	85136542	85138468	chr6-85136542-85138468
chr6	85141103	85141466	chr6-85141103-85141466

chr6	85154854	85155508	chr6-85154854-85155508
chr6	85185964	85188861	chr6-85185964-85188861
chr6	85189777	85190899	chr6-85189777-85190899
chr6	85233027	85233442	chr6-85233027-85233442
chr6	85248027	85248387	chr6-85248027-85248387
chr6	85295554	85296103	chr6-85295554-85296103
chr6	85331074	85334172	chr6-85331074-85334172
chr6	85337323	85337677	chr6-85337323-85337677
chr6	85345347	85350199	chr6-85345347-85350199
chr6	85356040	85356293	chr6-85356040-85356293
chr6	85366546	85376336	chr6-85366546-85376336
chr6	85380767	85382935	chr6-85380767-85382935
chr6	85386331	85387114	chr6-85386331-85387114
chr6	85431486	85433330	chr6-85431486-85433330
chr6	85450367	85454289	chr6-85450367-85454289
chr6	85483783	85485047	chr6-85483783-85485047
chr6	85502120	85503451	chr6-85502120-85503451
chr6	85511350	85514107	chr6-85511350-85514107
chr6	85587143	85588810	chr6-85587143-85588810
chr6	85869255	85869508	chr6-85869255-85869508
chr6	85904113	85905112	chr6-85904113-85905112
chr6	85914863	85916865	chr6-85914863-85916865
chr6	85936287	85937883	chr6-85936287-85937883
chr6	85959942	85962887	chr6-85959942-85962887
chr6	85972743	85973230	chr6-85972743-85973230
chr6	86004165	86004810	chr6-86004165-86004810
chr6	86028524	86029659	chr6-86028524-86029659
chr6	86148764	86149817	chr6-86148764-86149817
chr6	86156900	86158086	chr6-86156900-86158086
chr6	86180215	86180574	chr6-86180215-86180574
chr6	86182492	86183224	chr6-86182492-86183224
chr6	86194228	86197214	chr6-86194228-86197214
chr6	86219287	86220024	chr6-86219287-86220024
chr6	86230370	86231601	chr6-86230370-86231601
chr6	86258337	86258798	chr6-86258337-86258798
chr6	86318306	86319600	chr6-86318306-86319600
chr6	86364438	86367043	chr6-86364438-86367043
chr6	86370521	86373175	chr6-86370521-86373175
chr6	86395051	86397889	chr6-86395051-86397889
chr6	86400829	86401381	chr6-86400829-86401381
chr6	86403051	86406326	chr6-86403051-86406326
chr6	86437679	86440210	chr6-86437679-86440210

chr6	86471507	86473811	chr6-86471507-86473811
chr6	86480287	86480896	chr6-86480287-86480896
chr6	86486917	86488483	chr6-86486917-86488483
chr6	86520023	86520224	chr6-86520023-86520224
chr6	86522239	86528710	chr6-86522239-86528710
chr6	86569018	86569437	chr6-86569018-86569437
chr6	86570761	86571186	chr6-86570761-86571186
chr6	86578534	86578785	chr6-86578534-86578785
chr6	86584624	86585834	chr6-86584624-86585834
chr6	86595966	86596388	chr6-86595966-86596388
chr6	86604889	86606866	chr6-86604889-86606866
chr6	86610347	86610912	chr6-86610347-86610912
chr6	86667317	86670099	chr6-86667317-86670099
chr6	86683129	86685144	chr6-86683129-86685144
chr6	86717141	86717460	chr6-86717141-86717460
chr6	86731868	86734280	chr6-86731868-86734280
chr6	86765625	86766284	chr6-86765625-86766284
chr6	86769206	86769407	chr6-86769206-86769407
chr6	86770284	86770485	chr6-86770284-86770485
chr6	86771988	86773366	chr6-86771988-86773366
chr6	86775995	86776247	chr6-86775995-86776247
chr6	86790490	86794281	chr6-86790490-86794281
chr6	86806683	86806884	chr6-86806683-86806884
chr6	86822341	86822663	chr6-86822341-86822663
chr6	86848024	86853826	chr6-86848024-86853826
chr6	87002639	87002940	chr6-87002639-87002940
chr6	87006062	87006325	chr6-87006062-87006325
chr6	87009198	87011344	chr6-87009198-87011344
chr6	87041720	87043895	chr6-87041720-87043895
chr6	87101384	87101688	chr6-87101384-87101688
chr6	87335050	87335928	chr6-87335050-87335928
chr6	87384167	87384594	chr6-87384167-87384594
chr6	87386491	87386897	chr6-87386491-87386897
chr6	87582607	87583127	chr6-87582607-87583127
chr6	87590033	87591271	chr6-87590033-87591271
chr6	87670880	87673024	chr6-87670880-87673024
chr6	87730400	87733155	chr6-87730400-87733155
chr6	87810278	87812733	chr6-87810278-87812733
chr6	87837687	87839848	chr6-87837687-87839848
chr6	87846894	87851891	chr6-87846894-87851891
chr6	87857021	87857650	chr6-87857021-87857650
chr6	87868765	87869645	chr6-87868765-87869645

chr6	87887433	87889236	chr6-87887433-87889236
chr6	87913597	87916423	chr6-87913597-87916423
chr6	87980197	87982545	chr6-87980197-87982545
chr6	88012820	88013021	chr6-88012820-88013021
chr6	88042556	88045785	chr6-88042556-88045785
chr6	88053485	88054503	chr6-88053485-88054503
chr6	88074712	88075674	chr6-88074712-88075674
chr6	88083873	88086068	chr6-88083873-88086068
chr6	88136260	88136768	chr6-88136260-88136768
chr6	88189230	88190470	chr6-88189230-88190470
chr6	88193428	88194649	chr6-88193428-88194649
chr6	88196587	88200037	chr6-88196587-88200037
chr6	88202672	88203955	chr6-88202672-88203955
chr6	88242723	88242974	chr6-88242723-88242974
chr6	88445085	88446867	chr6-88445085-88446867
chr6	88464782	88467022	chr6-88464782-88467022
chr6	88499822	88500536	chr6-88499822-88500536
chr6	88516643	88520021	chr6-88516643-88520021
chr6	88627232	88627910	chr6-88627232-88627910
chr6	88724074	88725365	chr6-88724074-88725365
chr6	88762710	88762911	chr6-88762710-88762911
chr6	88773817	88775051	chr6-88773817-88775051
chr6	88840626	88843406	chr6-88840626-88843406
chr6	88869403	88869706	chr6-88869403-88869706
chr6	88874184	88875492	chr6-88874184-88875492
chr6	88896509	88899402	chr6-88896509-88899402
chr6	88900841	88907003	chr6-88900841-88907003
chr6	88908718	88911122	chr6-88908718-88911122
chr6	88924095	88924747	chr6-88924095-88924747
chr6	88925672	88925873	chr6-88925672-88925873
chr6	88926743	88927688	chr6-88926743-88927688
chr6	88934606	88935443	chr6-88934606-88935443
chr6	88939666	88939976	chr6-88939666-88939976
chr6	88958086	88958287	chr6-88958086-88958287
chr6	88960446	88962465	chr6-88960446-88962465
chr6	88971450	88972382	chr6-88971450-88972382
chr6	88985050	88985412	chr6-88985050-88985412
chr6	89008402	89009327	chr6-89008402-89009327
chr6	89033573	89034107	chr6-89033573-89034107
chr6	89068063	89068369	chr6-89068063-89068369
chr6	89087983	89088462	chr6-89087983-89088462
chr6	89200541	89201316	chr6-89200541-89201316

chr6	89235671	89236133	chr6-89235671-89236133
chr6	89344307	89346350	chr6-89344307-89346350
chr6	89349849	89350688	chr6-89349849-89350688
chr6	89351754	89352009	chr6-89351754-89352009
chr6	89359916	89363430	chr6-89359916-89363430
chr6	89413074	89414739	chr6-89413074-89414739
chr6	89447138	89447339	chr6-89447138-89447339
chr6	89457497	89457806	chr6-89457497-89457806
chr6	89594648	89595860	chr6-89594648-89595860
chr6	89631911	89632213	chr6-89631911-89632213
chr6	89643526	89645767	chr6-89643526-89645767
chr6	89835322	89835577	chr6-89835322-89835577
chr6	89867036	89867292	chr6-89867036-89867292
chr6	89876176	89876494	chr6-89876176-89876494
chr6	90232650	90232851	chr6-90232650-90232851
chr6	90324814	90325368	chr6-90324814-90325368
chr6	90360105	90360306	chr6-90360105-90360306
chr6	90368992	90371773	chr6-90368992-90371773
chr6	90376230	90376543	chr6-90376230-90376543
chr6	90428026	90428989	chr6-90428026-90428989
chr6	90462081	90463972	chr6-90462081-90463972
chr6	90506910	90508748	chr6-90506910-90508748
chr6	90550188	90550971	chr6-90550188-90550971
chr6	90604276	90605562	chr6-90604276-90605562
chr6	90715144	90717094	chr6-90715144-90717094
chr6	90731222	90731705	chr6-90731222-90731705
chr6	90735382	90737706	chr6-90735382-90737706
chr6	90764050	90764361	chr6-90764050-90764361
chr6	90796621	90797042	chr6-90796621-90797042
chr6	90984436	90984637	chr6-90984436-90984637
chr6	90986332	90989621	chr6-90986332-90989621
chr6	90996801	90997170	chr6-90996801-90997170
chr6	91001252	91001453	chr6-91001252-91001453
chr6	91012365	91012672	chr6-91012365-91012672
chr6	91017202	91017517	chr6-91017202-91017517
chr6	91076453	91076951	chr6-91076453-91076951
chr6	91115597	91117982	chr6-91115597-91117982
chr6	91156169	91159179	chr6-91156169-91159179
chr6	91173874	91174407	chr6-91173874-91174407
chr6	91190581	91193657	chr6-91190581-91193657
chr6	91201681	91203324	chr6-91201681-91203324
chr6	91205401	91218927	chr6-91205401-91218927

chr6	91221042	91221243	chr6-91221042-91221243
chr6	91284989	91286509	chr6-91284989-91286509
chr6	91379374	91379707	chr6-91379374-91379707
chr6	91381788	91384692	chr6-91381788-91384692
chr6	91388244	91391152	chr6-91388244-91391152
chr6	91392037	91392238	chr6-91392037-91392238
chr6	91405763	91412338	chr6-91405763-91412338
chr6	91440298	91442148	chr6-91440298-91442148
chr6	91471653	91475167	chr6-91471653-91475167
chr6	91483119	91484761	chr6-91483119-91484761
chr6	91488623	91491848	chr6-91488623-91491848
chr6	91514823	91517274	chr6-91514823-91517274
chr6	91617052	91617357	chr6-91617052-91617357
chr6	91683416	91688248	chr6-91683416-91688248
chr6	91689058	91689416	chr6-91689058-91689416
chr6	91712432	91713680	chr6-91712432-91713680
chr6	91727117	91727375	chr6-91727117-91727375
chr6	91807213	91807515	chr6-91807213-91807515
chr6	91843192	91844063	chr6-91843192-91844063
chr6	91877182	91879288	chr6-91877182-91879288
chr6	91978615	91979629	chr6-91978615-91979629
chr6	92090068	92093287	chr6-92090068-92093287
chr6	92182626	92184579	chr6-92182626-92184579
chr6	92199709	92200117	chr6-92199709-92200117
chr6	92213588	92215512	chr6-92213588-92215512
chr6	92244491	92244904	chr6-92244491-92244904
chr6	92480887	92481565	chr6-92480887-92481565
chr6	92705596	92706555	chr6-92705596-92706555
chr6	92940347	92943938	chr6-92940347-92943938
chr6	93256735	93257943	chr6-93256735-93257943
chr6	93657314	93657883	chr6-93657314-93657883
chr6	93802301	93802720	chr6-93802301-93802720
chr6	94097194	94097650	chr6-94097194-94097650
chr6	94100782	94101679	chr6-94100782-94101679
chr6	94140376	94142085	chr6-94140376-94142085
chr6	94155543	94155798	chr6-94155543-94155798
chr6	94164657	94166644	chr6-94164657-94166644
chr6	94183444	94183785	chr6-94183444-94183785
chr6	94186315	94187506	chr6-94186315-94187506
chr6	94195459	94195913	chr6-94195459-94195913
chr6	94202934	94204850	chr6-94202934-94204850
chr6	94224822	94226573	chr6-94224822-94226573

chr6	94239338	94241501	chr6-94239338-94241501
chr6	94263849	94264270	chr6-94263849-94264270
chr6	94281086	94285163	chr6-94281086-94285163
chr6	94391887	94392088	chr6-94391887-94392088
chr6	94405580	94405885	chr6-94405580-94405885
chr6	94499795	94501290	chr6-94499795-94501290
chr6	94645475	94649062	chr6-94645475-94649062
chr6	94657756	94658497	chr6-94657756-94658497
chr6	94696724	94701983	chr6-94696724-94701983
chr6	94729716	94730636	chr6-94729716-94730636
chr6	94748191	94748503	chr6-94748191-94748503
chr6	94789423	94789624	chr6-94789423-94789624
chr6	95116956	95118954	chr6-95116956-95118954
chr6	95634944	95635145	chr6-95634944-95635145
chr6	95717740	95719303	chr6-95717740-95719303
chr6	96116445	96117312	chr6-96116445-96117312
chr6	97059855	97060733	chr6-97059855-97060733
chr6	97147484	97149396	chr6-97147484-97149396
chr6	97177128	97180047	chr6-97177128-97180047
chr6	97203425	97205782	chr6-97203425-97205782
chr6	97208071	97208708	chr6-97208071-97208708
chr6	97209992	97212746	chr6-97209992-97212746
chr6	97278046	97279543	chr6-97278046-97279543
chr6	97292895	97295757	chr6-97292895-97295757
chr6	97304733	97307002	chr6-97304733-97307002
chr6	97308836	97313001	chr6-97308836-97313001
chr6	97352314	97355858	chr6-97352314-97355858
chr6	97395592	97395951	chr6-97395592-97395951
chr6	97447144	97447499	chr6-97447144-97447499
chr6	97487224	97488188	chr6-97487224-97488188
chr6	97616787	97617786	chr6-97616787-97617786
chr6	97806084	97807953	chr6-97806084-97807953
chr6	97962032	97962233	chr6-97962032-97962233
chr6	98105443	98106024	chr6-98105443-98106024
chr6	98797525	98797781	chr6-98797525-98797781
chr6	98846248	98846665	chr6-98846248-98846665
chr6	98900833	98901199	chr6-98900833-98901199
chr6	98949456	98950512	chr6-98949456-98950512
chr6	98999102	98999456	chr6-98999102-98999456
chr6	99025048	99029312	chr6-99025048-99029312
chr6	99094517	99096640	chr6-99094517-99096640
chr6	99130824	99131025	chr6-99130824-99131025

chr6	99172059	99172260	chr6-99172059-99172260
chr6	99275499	99275700	chr6-99275499-99275700
chr6	99485207	99485609	chr6-99485207-99485609
chr6	99519767	99524047	chr6-99519767-99524047
chr6	99665909	99667172	chr6-99665909-99667172
chr6	99691971	99693669	chr6-99691971-99693669
chr6	99725818	99726927	chr6-99725818-99726927
chr6	99804458	99804761	chr6-99804458-99804761
chr6	99933043	99933244	chr6-99933043-99933244
chr6	100163164	100163466	chr6-100163164-100163466
chr6	100170975	100171399	chr6-100170975-100171399
chr6	100218720	100219080	chr6-100218720-100219080
chr6	100285755	100288811	chr6-100285755-100288811
chr6	100323827	100324659	chr6-100323827-100324659
chr6	100332551	100332752	chr6-100332551-100332752
chr6	100336478	100337295	chr6-100336478-100337295
chr6	100438756	100439171	chr6-100438756-100439171
chr6	100483320	100483573	chr6-100483320-100483573
chr6	100494991	100495529	chr6-100494991-100495529
chr6	100503178	100503756	chr6-100503178-100503756
chr6	100562823	100564163	chr6-100562823-100564163
chr6	100601944	100602145	chr6-100601944-100602145
chr6	100670060	100671769	chr6-100670060-100671769
chr6	100704519	100705856	chr6-100704519-100705856
chr6	100800012	100801117	chr6-100800012-100801117
chr6	100825950	100826321	chr6-100825950-100826321
chr6	100833089	100835993	chr6-100833089-100835993
chr6	100879275	100880084	chr6-100879275-100880084
chr6	100882502	100882977	chr6-100882502-100882977
chr6	101198639	101199025	chr6-101198639-101199025
chr6	101377131	101378321	chr6-101377131-101378321
chr6	102564055	102564710	chr6-102564055-102564710
chr6	106768575	106770791	chr6-106768575-106770791
chr6	106798954	106800449	chr6-106798954-106800449
chr6	106933525	106933776	chr6-106933525-106933776
chr6	107529308	107531241	chr6-107529308-107531241
chr6	108064459	108065942	chr6-108064459-108065942
chr6	108184743	108186000	chr6-108184743-108186000
chr6	108211924	108214716	chr6-108211924-108214716
chr6	108306416	108306668	chr6-108306416-108306668
chr6	108444291	108445131	chr6-108444291-108445131
chr6	108446799	108449277	chr6-108446799-108449277

chr6	108451961	108452873	chr6-108451961-108452873
chr6	108455247	108459006	chr6-108455247-108459006
chr6	108487570	108487771	chr6-108487570-108487771
chr6	108510081	108510392	chr6-108510081-108510392
chr6	108530778	108537243	chr6-108530778-108537243
chr6	108570335	108571971	chr6-108570335-108571971
chr6	108656920	108668969	chr6-108656920-108668969
chr6	108688428	108688837	chr6-108688428-108688837
chr6	108704022	108706354	chr6-108704022-108706354
chr6	108708341	108710389	chr6-108708341-108710389
chr6	108759474	108759675	chr6-108759474-108759675
chr6	108782604	108785223	chr6-108782604-108785223
chr6	108827766	108830814	chr6-108827766-108830814
chr6	112208085	112208286	chr6-112208085-112208286
chr6	112210352	112211173	chr6-112210352-112211173
chr6	112245360	112246184	chr6-112245360-112246184
chr6	112273330	112274773	chr6-112273330-112274773
chr6	112379009	112379621	chr6-112379009-112379621
chr6	112479912	112480113	chr6-112479912-112480113
chr6	112489010	112491191	chr6-112489010-112491191
chr6	112695121	112697315	chr6-112695121-112697315
chr6	112888707	112889747	chr6-112888707-112889747
chr6	112944829	112950981	chr6-112944829-112950981
chr6	112973925	112974339	chr6-112973925-112974339
chr6	113037909	113038661	chr6-113037909-113038661
chr6	113044833	113047804	chr6-113044833-113047804
chr6	113073923	113080773	chr6-113073923-113080773
chr6	113099656	113100145	chr6-113099656-113100145
chr6	113166007	113167814	chr6-113166007-113167814
chr6	113193902	113195659	chr6-113193902-113195659
chr6	113237197	113239473	chr6-113237197-113239473
chr6	113282236	113283701	chr6-113282236-113283701
chr6	113293051	113293478	chr6-113293051-113293478
chr6	113305717	113309454	chr6-113305717-113309454
chr6	113325656	113328648	chr6-113325656-113328648
chr6	113330387	113330854	chr6-113330387-113330854
chr6	113342752	113344752	chr6-113342752-113344752
chr6	113376404	113380083	chr6-113376404-113380083
chr6	113417846	113419985	chr6-113417846-113419985
chr6	113442050	113444784	chr6-113442050-113444784
chr6	113458060	113459702	chr6-113458060-113459702
chr6	113469191	113470021	chr6-113469191-113470021

chr6	113470905	113472512	chr6-113470905-113472512
chr6	113479973	113484728	chr6-113479973-113484728
chr6	113487149	113487831	chr6-113487149-113487831
chr6	113494159	113495425	chr6-113494159-113495425
chr6	113501597	113502110	chr6-113501597-113502110
chr6	113530320	113532454	chr6-113530320-113532454
chr6	113604110	113606407	chr6-113604110-113606407
chr6	113623469	113625591	chr6-113623469-113625591
chr6	113638268	113639225	chr6-113638268-113639225
chr6	113642722	113642984	chr6-113642722-113642984
chr6	113668559	113668933	chr6-113668559-113668933
chr6	113675389	113675796	chr6-113675389-113675796
chr6	113686507	113688228	chr6-113686507-113688228
chr6	113689757	113693118	chr6-113689757-113693118
chr6	113695369	113699675	chr6-113695369-113699675
chr6	113739222	113741110	chr6-113739222-113741110
chr6	113748356	113748726	chr6-113748356-113748726
chr6	113891107	113891649	chr6-113891107-113891649
chr6	113956721	113957130	chr6-113956721-113957130
chr6	114041571	114042314	chr6-114041571-114042314
chr6	114131140	114131591	chr6-114131140-114131591
chr6	114282690	114283204	chr6-114282690-114283204
chr6	114348004	114348482	chr6-114348004-114348482
chr6	114397564	114398973	chr6-114397564-114398973
chr6	114403418	114404472	chr6-114403418-114404472
chr6	114406953	114408912	chr6-114406953-114408912
chr6	114419337	114419591	chr6-114419337-114419591
chr6	114420906	114422375	chr6-114420906-114422375
chr6	114449075	114449276	chr6-114449075-114449276
chr6	114452616	114453047	chr6-114452616-114453047
chr6	114457855	114460188	chr6-114457855-114460188
chr6	114495172	114495533	chr6-114495172-114495533
chr6	114497586	114497937	chr6-114497586-114497937
chr6	114507287	114507488	chr6-114507287-114507488
chr6	114511921	114512228	chr6-114511921-114512228
chr6	114536128	114536329	chr6-114536128-114536329
chr6	114538322	114538523	chr6-114538322-114538523
chr6	114550509	114550813	chr6-114550509-114550813
chr6	114567589	114567790	chr6-114567589-114567790
chr6	114577435	114577737	chr6-114577435-114577737
chr6	114584736	114585092	chr6-114584736-114585092
chr6	114594586	114594787	chr6-114594586-114594787

chr6	114614212	114614413	chr6-114614212-114614413
chr6	114619283	114619591	chr6-114619283-114619591
chr6	114642458	114644227	chr6-114642458-114644227
chr6	114648390	114649232	chr6-114648390-114649232
chr6	114839945	114840612	chr6-114839945-114840612
chr6	114873871	114876373	chr6-114873871-114876373
chr6	114888584	114889463	chr6-114888584-114889463
chr6	114893503	114894714	chr6-114893503-114894714
chr6	114915564	114922266	chr6-114915564-114922266
chr6	114929716	114933096	chr6-114929716-114933096
chr6	114961465	114962031	chr6-114961465-114962031
chr6	114963874	114964453	chr6-114963874-114964453
chr6	114966895	114970732	chr6-114966895-114970732
chr6	114982958	114983349	chr6-114982958-114983349
chr6	114987532	114989379	chr6-114987532-114989379
chr6	115036333	115038259	chr6-115036333-115038259
chr6	115123272	115123628	chr6-115123272-115123628
chr6	115134402	115135739	chr6-115134402-115135739
chr6	115271557	115272532	chr6-115271557-115272532
chr6	115313373	115313755	chr6-115313373-115313755
chr6	115360680	115362417	chr6-115360680-115362417
chr6	115462947	115463407	chr6-115462947-115463407
chr6	115480242	115480936	chr6-115480242-115480936
chr6	115501245	115501547	chr6-115501245-115501547
chr6	115503726	115503984	chr6-115503726-115503984
chr6	115538922	115539497	chr6-115538922-115539497
chr6	115543729	115545591	chr6-115543729-115545591
chr6	115591990	115593123	chr6-115591990-115593123
chr6	115601504	115602988	chr6-115601504-115602988
chr6	115654367	115654668	chr6-115654367-115654668
chr6	115674253	115677630	chr6-115674253-115677630
chr6	115717251	115718535	chr6-115717251-115718535
chr6	115722438	115722962	chr6-115722438-115722962
chr6	115757246	115762675	chr6-115757246-115762675
chr6	115768093	115768804	chr6-115768093-115768804
chr6	115773939	115775102	chr6-115773939-115775102
chr6	115775976	115776278	chr6-115775976-115776278
chr6	115801778	115801979	chr6-115801778-115801979
chr6	115805019	115809654	chr6-115805019-115809654
chr6	115823876	115824459	chr6-115823876-115824459
chr6	115838119	115838581	chr6-115838119-115838581
chr6	115852261	115854420	chr6-115852261-115854420

chr6	115975828	115976129	chr6-115975828-115976129
chr6	115993118	115995847	chr6-115993118-115995847
chr6	116006598	116007157	chr6-116006598-116007157
chr6	116009400	116009756	chr6-116009400-116009756
chr6	116106075	116108410	chr6-116106075-116108410
chr6	116192330	116194500	chr6-116192330-116194500
chr6	116207224	116209807	chr6-116207224-116209807
chr6	116240472	116240808	chr6-116240472-116240808
chr6	116263577	116265306	chr6-116263577-116265306
chr6	116337417	116339398	chr6-116337417-116339398
chr6	116386041	116386345	chr6-116386041-116386345
chr6	116460593	116461304	chr6-116460593-116461304
chr6	116627787	116629409	chr6-116627787-116629409
chr6	116650212	116650932	chr6-116650212-116650932
chr6	116673281	116674281	chr6-116673281-116674281
chr6	117168064	117169979	chr6-117168064-117169979
chr6	117840471	117842832	chr6-117840471-117842832
chr6	117862891	117863809	chr6-117862891-117863809
chr6	117878312	117881492	chr6-117878312-117881492
chr6	117905230	117911195	chr6-117905230-117911195
chr6	117912588	117919676	chr6-117912588-117919676
chr6	118011100	118012034	chr6-118011100-118012034
chr6	118048053	118048310	chr6-118048053-118048310
chr6	118065495	118067282	chr6-118065495-118067282
chr6	118128804	118129381	chr6-118128804-118129381
chr6	118130253	118130659	chr6-118130253-118130659
chr6	118137892	118139604	chr6-118137892-118139604
chr6	118192124	118198593	chr6-118192124-118198593
chr6	118367379	118368212	chr6-118367379-118368212
chr6	118418243	118420334	chr6-118418243-118420334
chr6	118454253	118456271	chr6-118454253-118456271
chr6	118478036	118479895	chr6-118478036-118479895
chr6	118560965	118563385	chr6-118560965-118563385
chr6	118574309	118574666	chr6-118574309-118574666
chr6	118593226	118594312	chr6-118593226-118594312
chr6	119107229	119109362	chr6-119107229-119109362
chr6	119174771	119176475	chr6-119174771-119176475
chr6	119326129	119326601	chr6-119326129-119326601
chr6	119328290	119330750	chr6-119328290-119330750
chr6	119332594	119333695	chr6-119332594-119333695
chr6	119386936	119389650	chr6-119386936-119389650
chr6	119395655	119396544	chr6-119395655-119396544

chr6	119443336	119444347	chr6-119443336-119444347
chr6	119448757	119449369	chr6-119448757-119449369
chr6	119451956	119452627	chr6-119451956-119452627
chr6	119478500	119483531	chr6-119478500-119483531
chr6	119535781	119536259	chr6-119535781-119536259
chr6	119543315	119544847	chr6-119543315-119544847
chr6	119674206	119674512	chr6-119674206-119674512
chr6	119846839	119849234	chr6-119846839-119849234
chr6	119902116	119903823	chr6-119902116-119903823
chr6	120033247	120040444	chr6-120033247-120040444
chr6	120114742	120115934	chr6-120114742-120115934
chr6	120134581	120135825	chr6-120134581-120135825
chr6	120145150	120147775	chr6-120145150-120147775
chr6	120153691	120153942	chr6-120153691-120153942
chr6	120159098	120159965	chr6-120159098-120159965
chr6	120171099	120174808	chr6-120171099-120174808
chr6	120179534	120179787	chr6-120179534-120179787
chr6	120190590	120193842	chr6-120190590-120193842
chr6	120225596	120226327	chr6-120225596-120226327
chr6	120274900	120275832	chr6-120274900-120275832
chr6	120279366	120279675	chr6-120279366-120279675
chr6	120292122	120295345	chr6-120292122-120295345
chr6	120356179	120358132	chr6-120356179-120358132
chr6	120362459	120366270	chr6-120362459-120366270
chr6	120462791	120464548	chr6-120462791-120464548
chr6	120482958	120483588	chr6-120482958-120483588
chr6	120486800	120487420	chr6-120486800-120487420
chr6	120492162	120494312	chr6-120492162-120494312
chr6	120530085	120532350	chr6-120530085-120532350
chr6	120539276	120539577	chr6-120539276-120539577
chr6	120579395	120580761	chr6-120579395-120580761
chr6	120642060	120642366	chr6-120642060-120642366
chr6	120665155	120667653	chr6-120665155-120667653
chr6	120797842	120798305	chr6-120797842-120798305
chr6	120821013	120823400	chr6-120821013-120823400
chr6	120835468	120837504	chr6-120835468-120837504
chr6	120865621	120866655	chr6-120865621-120866655
chr6	120914575	120918095	chr6-120914575-120918095
chr6	120987527	120987945	chr6-120987527-120987945
chr6	120995710	120996092	chr6-120995710-120996092
chr6	121017202	121018101	chr6-121017202-121018101
chr6	121063759	121068069	chr6-121063759-121068069

chr6	121108956	121110859	chr6-121108956-121110859
chr6	121122608	121123226	chr6-121122608-121123226
chr6	121129709	121131244	chr6-121129709-121131244
chr6	121146746	121146947	chr6-121146746-121146947
chr6	121159545	121160326	chr6-121159545-121160326
chr6	121176221	121176546	chr6-121176221-121176546
chr6	121183150	121186025	chr6-121183150-121186025
chr6	121193586	121195985	chr6-121193586-121195985
chr6	121210259	121212299	chr6-121210259-121212299
chr6	121245131	121247312	chr6-121245131-121247312
chr6	121272073	121272886	chr6-121272073-121272886
chr6	122296900	122298048	chr6-122296900-122298048
chr6	122308504	122311020	chr6-122308504-122311020
chr6	122338565	122341065	chr6-122338565-122341065
chr6	122391874	122392127	chr6-122391874-122392127
chr6	122485513	122487173	chr6-122485513-122487173
chr6	122491215	122492975	chr6-122491215-122492975
chr6	122495561	122496025	chr6-122495561-122496025
chr6	122532977	122533543	chr6-122532977-122533543
chr6	122545900	122546101	chr6-122545900-122546101
chr6	122565432	122565683	chr6-122565432-122565683
chr6	122600767	122603149	chr6-122600767-122603149
chr6	122627525	122627828	chr6-122627525-122627828
chr6	122669574	122669983	chr6-122669574-122669983
chr6	122702319	122703058	chr6-122702319-122703058
chr6	122764241	122765526	chr6-122764241-122765526
chr6	122801212	122802380	chr6-122801212-122802380
chr6	122809972	122812503	chr6-122809972-122812503
chr6	122819225	122822255	chr6-122819225-122822255
chr6	122823568	122827174	chr6-122823568-122827174
chr6	122855937	122856189	chr6-122855937-122856189
chr6	122869177	122870929	chr6-122869177-122870929
chr6	122874150	122875864	chr6-122874150-122875864
chr6	123004389	123004590	chr6-123004389-123004590
chr6	124413436	124416551	chr6-124413436-124416551
chr6	124464476	124465107	chr6-124464476-124465107
chr6	124484540	124485698	chr6-124484540-124485698
chr6	124512616	124512817	chr6-124512616-124512817
chr6	124662067	124664483	chr6-124662067-124664483
chr6	124683522	124684533	chr6-124683522-124684533
chr6	124693959	124694992	chr6-124693959-124694992
chr6	124700151	124700454	chr6-124700151-124700454

chr6	124710077	124721787	chr6-124710077-124721787
chr6	124732557	124733318	chr6-124732557-124733318
chr6	124737954	124741963	chr6-124737954-124741963
chr6	124744792	124746566	chr6-124744792-124746566
chr6	124754482	124758208	chr6-124754482-124758208
chr6	124759053	124759558	chr6-124759053-124759558
chr6	124768650	124770127	chr6-124768650-124770127
chr6	124779562	124780099	chr6-124779562-124780099
chr6	124782430	124782934	chr6-124782430-124782934
chr6	124800359	124803097	chr6-124800359-124803097
chr6	124804542	124807127	chr6-124804542-124807127
chr6	124808041	124814809	chr6-124808041-124814809
chr6	124827904	124831548	chr6-124827904-124831548
chr6	124856888	124857961	chr6-124856888-124857961
chr6	124863066	124864360	chr6-124863066-124864360
chr6	124903602	124905152	chr6-124903602-124905152
chr6	124910081	124910389	chr6-124910081-124910389
chr6	124915425	124920519	chr6-124915425-124920519
chr6	124930182	124933161	chr6-124930182-124933161
chr6	124937686	124938053	chr6-124937686-124938053
chr6	124964203	124967196	chr6-124964203-124967196
chr6	124996305	124997563	chr6-124996305-124997563
chr6	125007960	125010468	chr6-125007960-125010468
chr6	125039032	125041299	chr6-125039032-125041299
chr6	125081421	125082341	chr6-125081421-125082341
chr6	125095872	125100363	chr6-125095872-125100363
chr6	125131024	125134626	chr6-125131024-125134626
chr6	125145180	125145773	chr6-125145180-125145773
chr6	125161073	125168021	chr6-125161073-125168021
chr6	125190260	125193707	chr6-125190260-125193707
chr6	125214971	125216857	chr6-125214971-125216857
chr6	125230611	125232424	chr6-125230611-125232424
chr6	125234161	125234738	chr6-125234161-125234738
chr6	125255875	125256571	chr6-125255875-125256571
chr6	125271264	125272099	chr6-125271264-125272099
chr6	125311878	125314739	chr6-125311878-125314739
chr6	125319202	125324243	chr6-125319202-125324243
chr6	125330471	125331376	chr6-125330471-125331376
chr6	125348828	125352133	chr6-125348828-125352133
chr6	125354152	125355467	chr6-125354152-125355467
chr6	125377114	125381229	chr6-125377114-125381229
chr6	125407373	125408140	chr6-125407373-125408140

chr6	125410240	125411950	chr6-125410240-125411950
chr6	125412979	125413993	chr6-125412979-125413993
chr6	125422095	125427206	chr6-125422095-125427206
chr6	125430624	125430825	chr6-125430624-125430825
chr6	125433296	125433700	chr6-125433296-125433700
chr6	125473469	125474179	chr6-125473469-125474179
chr6	125476165	125484666	chr6-125476165-125484666
chr6	125485566	125486957	chr6-125485566-125486957
chr6	125489685	125495361	chr6-125489685-125495361
chr6	125540111	125540743	chr6-125540111-125540743
chr6	125572392	125573697	chr6-125572392-125573697
chr6	125591654	125592509	chr6-125591654-125592509
chr6	125603125	125603579	chr6-125603125-125603579
chr6	125690355	125690613	chr6-125690355-125690613
chr6	126166633	126166895	chr6-126166633-126166895
chr6	126483168	126483470	chr6-126483168-126483470
chr6	126484702	126485006	chr6-126484702-126485006
chr6	126487061	126487363	chr6-126487061-126487363
chr6	126642967	126645977	chr6-126642967-126645977
chr6	126702087	126702388	chr6-126702087-126702388
chr6	126739499	126740692	chr6-126739499-126740692
chr6	126792206	126792457	chr6-126792206-126792457
chr6	126847841	126849935	chr6-126847841-126849935
chr6	126938300	126940963	chr6-126938300-126940963
chr6	127034616	127035124	chr6-127034616-127035124
chr6	127057124	127057905	chr6-127057124-127057905
chr6	127079717	127080679	chr6-127079717-127080679
chr6	127107950	127110119	chr6-127107950-127110119
chr6	127142244	127142552	chr6-127142244-127142552
chr6	127148513	127155609	chr6-127148513-127155609
chr6	127162802	127163003	chr6-127162802-127163003
chr6	127245785	127246306	chr6-127245785-127246306
chr6	127266192	127266845	chr6-127266192-127266845
chr6	127329086	127331442	chr6-127329086-127331442
chr6	127453098	127454618	chr6-127453098-127454618
chr6	127560992	127561711	chr6-127560992-127561711
chr6	127768949	127769460	chr6-127768949-127769460
chr6	127887593	127888136	chr6-127887593-127888136
chr6	127971613	127972593	chr6-127971613-127972593
chr6	127973605	127974622	chr6-127973605-127974622
chr6	127990349	127990550	chr6-127990349-127990550
chr6	127991364	127992387	chr6-127991364-127992387

chr6	128021313	128022027	chr6-128021313-128022027
chr6	128032053	128036057	chr6-128032053-128036057
chr6	128043140	128043391	chr6-128043140-128043391
chr6	128046791	128047093	chr6-128046791-128047093
chr6	128091157	128091521	chr6-128091157-128091521
chr6	128102335	128103331	chr6-128102335-128103331
chr6	128104690	128106354	chr6-128104690-128106354
chr6	128141773	128144683	chr6-128141773-128144683
chr6	128199869	128201558	chr6-128199869-128201558
chr6	128275785	128275986	chr6-128275785-128275986
chr6	128282146	128282457	chr6-128282146-128282457
chr6	128285380	128286868	chr6-128285380-128286868
chr6	128294830	128301968	chr6-128294830-128301968
chr6	128354695	128356494	chr6-128354695-128356494
chr6	128361193	128366707	chr6-128361193-128366707
chr6	128423342	128425396	chr6-128423342-128425396
chr6	128429672	128430932	chr6-128429672-128430932
chr6	128435199	128439918	chr6-128435199-128439918
chr6	128469480	128469735	chr6-128469480-128469735
chr6	128479520	128480326	chr6-128479520-128480326
chr6	128549306	128550072	chr6-128549306-128550072
chr6	128653338	128653539	chr6-128653338-128653539
chr6	128754390	128754591	chr6-128754390-128754591
chr6	128760016	128761648	chr6-128760016-128761648
chr6	128763271	128764970	chr6-128763271-128764970
chr6	128798023	128800399	chr6-128798023-128800399
chr6	128802231	128803722	chr6-128802231-128803722
chr6	128837327	128840642	chr6-128837327-128840642
chr6	128841537	128844453	chr6-128841537-128844453
chr6	128969625	128969928	chr6-128969625-128969928
chr6	129009025	129009335	chr6-129009025-129009335
chr6	129049017	129049680	chr6-129049017-129049680
chr6	129180163	129182856	chr6-129180163-129182856
chr6	129230131	129230383	chr6-129230131-129230383
chr6	129232772	129233117	chr6-129232772-129233117
chr6	129235543	129238701	chr6-129235543-129238701
chr6	129513872	129514400	chr6-129513872-129514400
chr6	129532575	129535985	chr6-129532575-129535985
chr6	129594958	129595592	chr6-129594958-129595592
chr6	129678163	129679091	chr6-129678163-129679091
chr6	129680227	129680642	chr6-129680227-129680642
chr6	130062705	130065747	chr6-130062705-130065747

chr6	131291258	131293817	chr6-131291258-131293817
chr6	131316113	131316921	chr6-131316113-131316921
chr6	131321962	131322756	chr6-131321962-131322756
chr6	131339882	131340350	chr6-131339882-131340350
chr6	131344208	131345041	chr6-131344208-131345041
chr6	131346872	131347124	chr6-131346872-131347124
chr6	131352071	131354088	chr6-131352071-131354088
chr6	131382055	131389126	chr6-131382055-131389126
chr6	131433502	131434111	chr6-131433502-131434111
chr6	133104647	133106501	chr6-133104647-133106501
chr6	133706857	133707173	chr6-133706857-133707173
chr6	134034258	134037707	chr6-134034258-134037707
chr6	134038813	134039161	chr6-134038813-134039161
chr6	134089707	134090606	chr6-134089707-134090606
chr6	134147927	134148631	chr6-134147927-134148631
chr6	134186539	134186740	chr6-134186539-134186740
chr6	134263628	134263888	chr6-134263628-134263888
chr6	134403427	134403628	chr6-134403427-134403628
chr6	134414208	134414605	chr6-134414208-134414605
chr6	134565162	134567748	chr6-134565162-134567748
chr6	134614649	134615546	chr6-134614649-134615546
chr6	134631832	134632848	chr6-134631832-134632848
chr6	134640573	134642145	chr6-134640573-134642145
chr6	134668992	134669261	chr6-134668992-134669261
chr6	134671231	134671903	chr6-134671231-134671903
chr6	134700969	134701597	chr6-134700969-134701597
chr6	134790608	134794418	chr6-134790608-134794418
chr6	134802513	134803162	chr6-134802513-134803162
chr6	134828577	134831507	chr6-134828577-134831507
chr6	134863331	134863662	chr6-134863331-134863662
chr6	134865893	134866342	chr6-134865893-134866342
chr6	134896857	134899544	chr6-134896857-134899544
chr6	134916553	134917559	chr6-134916553-134917559
chr6	134919266	134931786	chr6-134919266-134931786
chr6	134932888	134933089	chr6-134932888-134933089
chr6	134983202	134984941	chr6-134983202-134984941
chr6	134990540	134991312	chr6-134990540-134991312
chr6	134993863	134994120	chr6-134993863-134994120
chr6	134999633	135000839	chr6-134999633-135000839
chr6	135001940	135002141	chr6-135001940-135002141
chr6	135011324	135013225	chr6-135011324-135013225
chr6	135015118	135015382	chr6-135015118-135015382

chr6	135023102	135027432	chr6-135023102-135027432
chr6	135029151	135030134	chr6-135029151-135030134
chr6	135039416	135041167	chr6-135039416-135041167
chr6	135048882	135049343	chr6-135048882-135049343
chr6	135061073	135061427	chr6-135061073-135061427
chr6	135064643	135075075	chr6-135064643-135075075
chr6	135167406	135168587	chr6-135167406-135168587
chr6	135197570	135198781	chr6-135197570-135198781
chr6	135249604	135254474	chr6-135249604-135254474
chr6	135263288	135263606	chr6-135263288-135263606
chr6	135291956	135292948	chr6-135291956-135292948
chr6	135295030	135299132	chr6-135295030-135299132
chr6	135315462	135320479	chr6-135315462-135320479
chr6	135321537	135325541	chr6-135321537-135325541
chr6	135362129	135368603	chr6-135362129-135368603
chr6	135369761	135369962	chr6-135369761-135369962
chr6	135386000	135386268	chr6-135386000-135386268
chr6	135444799	135445050	chr6-135444799-135445050
chr6	135482656	135483077	chr6-135482656-135483077
chr6	136172278	136172635	chr6-136172278-136172635
chr6	136407296	136408460	chr6-136407296-136408460
chr6	136468797	136471318	chr6-136468797-136471318
chr6	136499148	136499760	chr6-136499148-136499760
chr6	136518531	136520453	chr6-136518531-136520453
chr6	136531825	136533353	chr6-136531825-136533353
chr6	136540866	136543851	chr6-136540866-136543851
chr6	136555387	136555588	chr6-136555387-136555588
chr6	136611741	136612042	chr6-136611741-136612042
chr6	136620310	136620867	chr6-136620310-136620867
chr6	136645950	136646151	chr6-136645950-136646151
chr6	136661424	136662035	chr6-136661424-136662035
chr6	136793750	136794640	chr6-136793750-136794640
chr6	136803094	136810740	chr6-136803094-136810740
chr6	136826931	136829160	chr6-136826931-136829160
chr6	136900917	136901344	chr6-136900917-136901344
chr6	136913499	136913804	chr6-136913499-136913804
chr6	136959160	136959616	chr6-136959160-136959616
chr6	137142639	137143230	chr6-137142639-137143230
chr6	137169143	137169851	chr6-137169143-137169851
chr6	137252561	137252825	chr6-137252561-137252825
chr6	137519190	137520816	chr6-137519190-137520816
chr6	137541580	137541781	chr6-137541580-137541781

chr6	137558206	137560753	chr6-137558206-137560753
chr6	137569116	137569531	chr6-137569116-137569531
chr6	137582202	137583230	chr6-137582202-137583230
chr6	137647149	137649952	chr6-137647149-137649952
chr6	137734381	137736851	chr6-137734381-137736851
chr6	137754254	137755967	chr6-137754254-137755967
chr6	137784288	137784700	chr6-137784288-137784700
chr6	137785627	137785828	chr6-137785627-137785828
chr6	137795544	137795745	chr6-137795544-137795745
chr6	137874447	137874814	chr6-137874447-137874814
chr6	138139976	138142231	chr6-138139976-138142231
chr6	139587035	139587288	chr6-139587035-139587288
chr6	140368481	140369509	chr6-140368481-140369509
chr6	140423497	140425741	chr6-140423497-140425741
chr6	140466242	140466549	chr6-140466242-140466549
chr6	140478107	140478364	chr6-140478107-140478364
chr6	140514848	140515157	chr6-140514848-140515157
chr6	140546174	140548679	chr6-140546174-140548679
chr6	140622572	140627060	chr6-140622572-140627060
chr6	141249503	141250287	chr6-141249503-141250287
chr6	142339846	142340375	chr6-142339846-142340375
chr6	142345084	142347254	chr6-142345084-142347254
chr6	142386130	142389334	chr6-142386130-142389334
chr6	142459348	142459549	chr6-142459348-142459549
chr6	142507149	142508429	chr6-142507149-142508429
chr6	142511652	142511912	chr6-142511652-142511912
chr6	142570927	142571840	chr6-142570927-142571840
chr6	142702219	142702420	chr6-142702219-142702420
chr6	142756436	142758343	chr6-142756436-142758343
chr6	142814468	142814834	chr6-142814468-142814834
chr6	142867682	142867985	chr6-142867682-142867985
chr6	142879364	142879647	chr6-142879364-142879647
chr6	142913877	142914184	chr6-142913877-142914184
chr6	142963742	142965293	chr6-142963742-142965293
chr6	143099000	143100696	chr6-143099000-143100696
chr6	143152319	143152677	chr6-143152319-143152677
chr6	143166106	143168940	chr6-143166106-143168940
chr6	143307503	143307756	chr6-143307503-143307756
chr6	143425347	143425652	chr6-143425347-143425652
chr6	143751075	143752007	chr6-143751075-143752007
chr6	144620787	144621849	chr6-144620787-144621849
chr6	144781188	144782959	chr6-144781188-144782959

chr6	144822850	144823051	chr6-144822850-144823051
chr6	144902302	144902803	chr6-144902302-144902803
chr6	145048100	145049133	chr6-145048100-145049133
chr6	145075066	145076332	chr6-145075066-145076332
chr6	145111178	145111999	chr6-145111178-145111999
chr6	145175663	145175864	chr6-145175663-145175864
chr6	145187286	145187982	chr6-145187286-145187982
chr6	145210353	145212522	chr6-145210353-145212522
chr6	145247204	145251357	chr6-145247204-145251357
chr6	145275936	145276959	chr6-145275936-145276959
chr6	145339768	145342084	chr6-145339768-145342084
chr6	145359554	145360237	chr6-145359554-145360237
chr6	145616114	145616904	chr6-145616114-145616904
chr6	145713158	145713997	chr6-145713158-145713997
chr6	145745090	145748663	chr6-145745090-145748663
chr6	145768085	145771771	chr6-145768085-145771771
chr6	145792905	145793963	chr6-145792905-145793963
chr6	145855541	145856201	chr6-145855541-145856201
chr6	145862356	145867065	chr6-145862356-145867065
chr6	145905948	145906365	chr6-145905948-145906365
chr6	145908894	145909203	chr6-145908894-145909203
chr6	145914535	145915499	chr6-145914535-145915499
chr6	145933867	145934935	chr6-145933867-145934935
chr6	145973087	145973904	chr6-145973087-145973904
chr6	145977390	145978334	chr6-145977390-145978334
chr6	146003780	146004521	chr6-146003780-146004521
chr6	146403074	146403670	chr6-146403074-146403670
chr6	146501091	146502942	chr6-146501091-146502942
chr6	146576250	146579940	chr6-146576250-146579940
chr6	146633232	146634923	chr6-146633232-146634923
chr6	146641903	146644131	chr6-146641903-146644131
chr6	146685406	146686505	chr6-146685406-146686505
chr6	146702159	146702782	chr6-146702159-146702782
chr6	146724474	146726616	chr6-146724474-146726616
chr6	146742349	146742550	chr6-146742349-146742550
chr6	146751877	146752078	chr6-146751877-146752078
chr6	146776604	146776805	chr6-146776604-146776805
chr6	146795165	146796923	chr6-146795165-146796923
chr6	146887377	146891501	chr6-146887377-146891501
chr6	146909905	146910473	chr6-146909905-146910473
chr6	146959930	146960233	chr6-146959930-146960233
chr6	147041763	147044084	chr6-147041763-147044084

chr6	147090571	147093627	chr6-147090571-147093627
chr6	147196290	147196949	chr6-147196290-147196949
chr6	147199656	147200184	chr6-147199656-147200184
chr6	147262021	147264670	chr6-147262021-147264670
chr6	147397757	147398615	chr6-147397757-147398615
chr6	147475218	147477139	chr6-147475218-147477139
chr6	147741351	147741753	chr6-147741351-147741753
chr6	148047138	148047617	chr6-148047138-148047617
chr6	148210435	148213471	chr6-148210435-148213471
chr6	148354478	148355558	chr6-148354478-148355558
chr6	148436510	148436916	chr6-148436510-148436916
chr6	148442778	148445011	chr6-148442778-148445011
chr6	148830076	148832932	chr6-148830076-148832932
chr6	148895008	148897018	chr6-148895008-148897018
chr6	148943015	148947403	chr6-148943015-148947403
chr6	149100313	149102166	chr6-149100313-149102166
chr6	149141144	149142885	chr6-149141144-149142885
chr6	149186530	149189194	chr6-149186530-149189194
chr6	149193401	149193655	chr6-149193401-149193655
chr6	149308345	149312033	chr6-149308345-149312033
chr6	149312902	149313212	chr6-149312902-149313212
chr6	149408594	149410362	chr6-149408594-149410362
chr6	149565526	149566797	chr6-149565526-149566797
chr7	3274536	3276146	chr7-3274536-3276146
chr7	3288807	3295382	chr7-3288807-3295382
chr7	3301801	3302258	chr7-3301801-3302258
chr7	3303182	3303872	chr7-3303182-3303872
chr7	3331885	3333308	chr7-3331885-3333308
chr7	3366606	3367976	chr7-3366606-3367976
chr7	3390078	3390334	chr7-3390078-3390334
chr7	3410958	3412608	chr7-3410958-3412608
chr7	3414676	3416050	chr7-3414676-3416050
chr7	3422157	3422694	chr7-3422157-3422694
chr7	3444996	3445197	chr7-3444996-3445197
chr7	3446767	3447278	chr7-3446767-3447278
chr7	3594747	3595699	chr7-3594747-3595699
chr7	3616713	3618230	chr7-3616713-3618230
chr7	3628517	3631032	chr7-3628517-3631032
chr7	3643790	3647733	chr7-3643790-3647733
chr7	3663682	3666265	chr7-3663682-3666265
chr7	3675906	3677934	chr7-3675906-3677934
chr7	3691206	3695937	chr7-3691206-3695937

chr7	3703393	3707434	chr7-3703393-3707434
chr7	4098465	4099518	chr7-4098465-4099518
chr7	4119298	4119964	chr7-4119298-4119964
chr7	4130654	4131816	chr7-4130654-4131816
chr7	4135382	4138956	chr7-4135382-4138956
chr7	4148088	4151761	chr7-4148088-4151761
chr7	4163014	4165500	chr7-4163014-4165500
chr7	4169938	4170290	chr7-4169938-4170290
chr7	4444501	4446124	chr7-4444501-4446124
chr7	4460286	4461579	chr7-4460286-4461579
chr7	4464064	4466716	chr7-4464064-4466716
chr7	4499212	4502903	chr7-4499212-4502903
chr7	4509811	4510062	chr7-4509811-4510062
chr7	4520902	4521487	chr7-4520902-4521487
chr7	4526576	4527112	chr7-4526576-4527112
chr7	4531676	4532968	chr7-4531676-4532968
chr7	4546663	4547661	chr7-4546663-4547661
chr7	4656878	4660200	chr7-4656878-4660200
chr7	4683850	4685705	chr7-4683850-4685705
chr7	4690374	4691882	chr7-4690374-4691882
chr7	4723651	4724004	chr7-4723651-4724004
chr7	4738898	4741638	chr7-4738898-4741638
chr7	4752011	4753178	chr7-4752011-4753178
chr7	4776682	4778489	chr7-4776682-4778489
chr7	4787619	4790346	chr7-4787619-4790346
chr7	4791822	4795424	chr7-4791822-4795424
chr7	4809508	4813972	chr7-4809508-4813972
chr7	4835743	4837138	chr7-4835743-4837138
chr7	4842591	4845019	chr7-4842591-4845019
chr7	4865361	4867431	chr7-4865361-4867431
chr7	4876533	4877954	chr7-4876533-4877954
chr7	4914323	4916748	chr7-4914323-4916748
chr7	4922563	4925798	chr7-4922563-4925798
chr7	4940207	4940458	chr7-4940207-4940458
chr7	4943387	4943844	chr7-4943387-4943844
chr7	4993391	4997330	chr7-4993391-4997330
chr7	5013508	5016519	chr7-5013508-5016519
chr7	5019480	5021802	chr7-5019480-5021802
chr7	5030304	5031889	chr7-5030304-5031889
chr7	5037770	5039314	chr7-5037770-5039314
chr7	5051091	5054060	chr7-5051091-5054060
chr7	5056578	5057301	chr7-5056578-5057301

chr7	5060107	5065753	chr7-5060107-5065753
chr7	5079242	5082187	chr7-5079242-5082187
chr7	5125653	5126262	chr7-5125653-5126262
chr7	6132331	6132654	chr7-6132331-6132654
chr7	6146568	6146882	chr7-6146568-6146882
chr7	6153075	6157277	chr7-6153075-6157277
chr7	6170931	6173664	chr7-6170931-6173664
chr7	6282904	6284089	chr7-6282904-6284089
chr7	6285861	6287321	chr7-6285861-6287321
chr7	6330956	6331642	chr7-6330956-6331642
chr7	6343526	6344088	chr7-6343526-6344088
chr7	6361761	6364166	chr7-6361761-6364166
chr7	6382517	6384314	chr7-6382517-6384314
chr7	6398391	6399202	chr7-6398391-6399202
chr7	6728436	6730679	chr7-6728436-6730679
chr7	7120652	7121924	chr7-7120652-7121924
chr7	7130535	7130793	chr7-7130535-7130793
chr7	7136164	7136977	chr7-7136164-7136977
chr7	7171170	7172469	chr7-7171170-7172469
chr7	7188014	7188624	chr7-7188014-7188624
chr7	7208752	7210573	chr7-7208752-7210573
chr7	7277501	7278629	chr7-7277501-7278629
chr7	7297653	7300928	chr7-7297653-7300928
chr7	8092888	8093089	chr7-8092888-8093089
chr7	8862368	8862619	chr7-8862368-8862619
chr7	10494327	10495733	chr7-10494327-10495733
chr7	12421614	12422921	chr7-12421614-12422921
chr7	12477922	12479514	chr7-12477922-12479514
chr7	12511161	12511937	chr7-12511161-12511937
chr7	12779963	12780972	chr7-12779963-12780972
chr7	12803327	12803735	chr7-12803327-12803735
chr7	12817894	12819250	chr7-12817894-12819250
chr7	12834106	12836033	chr7-12834106-12836033
chr7	12880686	12881899	chr7-12880686-12881899
chr7	12897589	12898943	chr7-12897589-12898943
chr7	12903782	12904084	chr7-12903782-12904084
chr7	12920802	12925057	chr7-12920802-12925057
chr7	12927313	12928430	chr7-12927313-12928430
chr7	12948988	12950517	chr7-12948988-12950517
chr7	12961274	12962318	chr7-12961274-12962318
chr7	12965117	12967430	chr7-12965117-12967430
chr7	12976988	12979134	chr7-12976988-12979134

chr7	13008868	13011045	chr7-13008868-13011045
chr7	13022765	13027237	chr7-13022765-13027237
chr7	13033117	13039368	chr7-13033117-13039368
chr7	13043563	13044304	chr7-13043563-13044304
chr7	13053929	13055273	chr7-13053929-13055273
chr7	13123552	13124368	chr7-13123552-13124368
chr7	13277704	13279790	chr7-13277704-13279790
chr7	14622467	14623413	chr7-14622467-14623413
chr7	15920374	15923168	chr7-15920374-15923168
chr7	15944230	15946975	chr7-15944230-15946975
chr7	15947888	15949397	chr7-15947888-15949397
chr7	15954465	15956663	chr7-15954465-15956663
chr7	15962349	15967943	chr7-15962349-15967943
chr7	16011475	16011676	chr7-16011475-16011676
chr7	16019746	16020052	chr7-16019746-16020052
chr7	16029048	16029314	chr7-16029048-16029314
chr7	16045812	16050050	chr7-16045812-16050050
chr7	16051717	16053491	chr7-16051717-16053491
chr7	16055103	16055304	chr7-16055103-16055304
chr7	16056682	16057750	chr7-16056682-16057750
chr7	16097667	16100740	chr7-16097667-16100740
chr7	16119439	16120920	chr7-16119439-16120920
chr7	16129727	16130794	chr7-16129727-16130794
chr7	16158800	16159154	chr7-16158800-16159154
chr7	16174550	16176206	chr7-16174550-16176206
chr7	16177711	16178126	chr7-16177711-16178126
chr7	16220842	16222574	chr7-16220842-16222574
chr7	16272086	16274505	chr7-16272086-16274505
chr7	16284748	16287271	chr7-16284748-16287271
chr7	16294517	16295941	chr7-16294517-16295941
chr7	16296772	16297493	chr7-16296772-16297493
chr7	16299230	16300418	chr7-16299230-16300418
chr7	16308955	16314762	chr7-16308955-16314762
chr7	16325980	16326353	chr7-16325980-16326353
chr7	16384156	16388290	chr7-16384156-16388290
chr7	16399000	16406667	chr7-16399000-16406667
chr7	16415552	16419068	chr7-16415552-16419068
chr7	16451766	16454150	chr7-16451766-16454150
chr7	16474422	16476944	chr7-16474422-16476944
chr7	16581128	16582169	chr7-16581128-16582169
chr7	16601529	16602054	chr7-16601529-16602054
chr7	16609263	16609624	chr7-16609263-16609624

chr7	16613313	16616017	chr7-16613313-16616017
chr7	16618587	16619201	chr7-16618587-16619201
chr7	16706247	16706728	chr7-16706247-16706728
chr7	16729039	16729786	chr7-16729039-16729786
chr7	16737488	16739840	chr7-16737488-16739840
chr7	16780829	16785895	chr7-16780829-16785895
chr7	16815720	16818067	chr7-16815720-16818067
chr7	16842479	16846277	chr7-16842479-16846277
chr7	16874163	16875983	chr7-16874163-16875983
chr7	16885042	16886257	chr7-16885042-16886257
chr7	16891070	16892331	chr7-16891070-16892331
chr7	16894779	16895646	chr7-16894779-16895646
chr7	16921912	16925115	chr7-16921912-16925115
chr7	16944442	16945206	chr7-16944442-16945206
chr7	16946772	16946973	chr7-16946772-16946973
chr7	16949985	16950493	chr7-16949985-16950493
chr7	16980298	16980701	chr7-16980298-16980701
chr7	17016903	17017595	chr7-17016903-17017595
chr7	17026251	17028502	chr7-17026251-17028502
chr7	17056049	17057013	chr7-17056049-17057013
chr7	17060115	17060316	chr7-17060115-17060316
chr7	17062336	17062752	chr7-17062336-17062752
chr7	18746342	18746595	chr7-18746342-18746595
chr7	18840298	18840752	chr7-18840298-18840752
chr7	18857061	18858789	chr7-18857061-18858789
chr7	18873048	18873624	chr7-18873048-18873624
chr7	18884411	18885303	chr7-18884411-18885303
chr7	18892266	18892771	chr7-18892266-18892771
chr7	18908827	18911369	chr7-18908827-18911369
chr7	18921068	18921437	chr7-18921068-18921437
chr7	18925076	18926701	chr7-18925076-18926701
chr7	18947937	18948733	chr7-18947937-18948733
chr7	18957124	18959450	chr7-18957124-18959450
chr7	18962199	18964021	chr7-18962199-18964021
chr7	18990196	18992753	chr7-18990196-18992753
chr7	19002343	19007993	chr7-19002343-19007993
chr7	19010574	19010775	chr7-19010574-19010775
chr7	19011811	19012582	chr7-19011811-19012582
chr7	19023129	19025598	chr7-19023129-19025598
chr7	19075205	19077771	chr7-19075205-19077771
chr7	19083825	19084792	chr7-19083825-19084792
chr7	19092835	19096473	chr7-19092835-19096473

chr7	19118199	19121120	chr7-19118199-19121120
chr7	19130741	19132235	chr7-19130741-19132235
chr7	19148066	19151461	chr7-19148066-19151461
chr7	19157317	19157618	chr7-19157317-19157618
chr7	19159866	19160782	chr7-19159866-19160782
chr7	19164485	19165242	chr7-19164485-19165242
chr7	19172902	19173205	chr7-19172902-19173205
chr7	19178753	19179715	chr7-19178753-19179715
chr7	19180843	19183111	chr7-19180843-19183111
chr7	19184797	19184998	chr7-19184797-19184998
chr7	19212130	19212989	chr7-19212130-19212989
chr7	19226648	19230198	chr7-19226648-19230198
chr7	19265235	19273097	chr7-19265235-19273097
chr7	19279036	19280160	chr7-19279036-19280160
chr7	19282053	19283626	chr7-19282053-19283626
chr7	19295501	19296013	chr7-19295501-19296013
chr7	19300274	19302053	chr7-19300274-19302053
chr7	19307027	19310948	chr7-19307027-19310948
chr7	19313264	19313465	chr7-19313264-19313465
chr7	19316252	19317311	chr7-19316252-19317311
chr7	19319397	19320733	chr7-19319397-19320733
chr7	19326315	19328359	chr7-19326315-19328359
chr7	19331279	19338848	chr7-19331279-19338848
chr7	19340867	19347221	chr7-19340867-19347221
chr7	19357629	19364642	chr7-19357629-19364642
chr7	19378005	19378577	chr7-19378005-19378577
chr7	19381421	19383527	chr7-19381421-19383527
chr7	19398339	19400390	chr7-19398339-19400390
chr7	19403317	19404628	chr7-19403317-19404628
chr7	19405544	19405852	chr7-19405544-19405852
chr7	19420008	19421858	chr7-19420008-19421858
chr7	19457018	19459678	chr7-19457018-19459678
chr7	19473617	19474839	chr7-19473617-19474839
chr7	19489495	19489950	chr7-19489495-19489950
chr7	19491663	19492150	chr7-19491663-19492150
chr7	19503748	19503949	chr7-19503748-19503949
chr7	19506826	19510161	chr7-19506826-19510161
chr7	19517404	19518278	chr7-19517404-19518278
chr7	19522840	19524739	chr7-19522840-19524739
chr7	19540819	19544102	chr7-19540819-19544102
chr7	19545657	19546861	chr7-19545657-19546861
chr7	19559343	19563579	chr7-19559343-19563579

chr7	19571372	19574471	chr7-19571372-19574471
chr7	19576796	19579091	chr7-19576796-19579091
chr7	19595430	19595631	chr7-19595430-19595631
chr7	19602640	19604909	chr7-19602640-19604909
chr7	19609385	19610137	chr7-19609385-19610137
chr7	19627720	19629986	chr7-19627720-19629986
chr7	19663489	19666381	chr7-19663489-19666381
chr7	19669063	19669641	chr7-19669063-19669641
chr7	19713496	19717407	chr7-19713496-19717407
chr7	19746010	19751545	chr7-19746010-19751545
chr7	19753245	19754576	chr7-19753245-19754576
chr7	19768060	19772128	chr7-19768060-19772128
chr7	19778117	19778824	chr7-19778117-19778824
chr7	19789808	19790323	chr7-19789808-19790323
chr7	19793464	19798036	chr7-19793464-19798036
chr7	19801671	19801872	chr7-19801671-19801872
chr7	19815652	19819257	chr7-19815652-19819257
chr7	19820847	19825472	chr7-19820847-19825472
chr7	19826722	19827170	chr7-19826722-19827170
chr7	19830255	19832458	chr7-19830255-19832458
chr7	19851935	19852607	chr7-19851935-19852607
chr7	19870199	19872133	chr7-19870199-19872133
chr7	19876250	19877475	chr7-19876250-19877475
chr7	19910768	19912103	chr7-19910768-19912103
chr7	19913247	19913873	chr7-19913247-19913873
chr7	19918243	19921633	chr7-19918243-19921633
chr7	19928511	19928985	chr7-19928511-19928985
chr7	19930151	19931070	chr7-19930151-19931070
chr7	19932226	19934591	chr7-19932226-19934591
chr7	19935719	19935920	chr7-19935719-19935920
chr7	19939501	19941354	chr7-19939501-19941354
chr7	19953187	19953388	chr7-19953187-19953388
chr7	24081300	24083531	chr7-24081300-24083531
chr7	24111621	24113542	chr7-24111621-24113542
chr7	24133402	24135291	chr7-24133402-24135291
chr7	24174956	24175358	chr7-24174956-24175358
chr7	24206785	24208343	chr7-24206785-24208343
chr7	24236617	24238023	chr7-24236617-24238023
chr7	24254205	24255255	chr7-24254205-24255255
chr7	24269698	24271159	chr7-24269698-24271159
chr7	24298271	24300407	chr7-24298271-24300407
chr7	24315706	24317382	chr7-24315706-24317382

chr7	24326923	24327476	chr7-24326923-24327476
chr7	24355675	24356357	chr7-24355675-24356357
chr7	24364733	24365979	chr7-24364733-24365979
chr7	24369580	24374438	chr7-24369580-24374438
chr7	24386691	24387971	chr7-24386691-24387971
chr7	24398357	24401182	chr7-24398357-24401182
chr7	24439159	24439414	chr7-24439159-24439414
chr7	24448998	24449308	chr7-24448998-24449308
chr7	24450342	24450787	chr7-24450342-24450787
chr7	24457267	24465768	chr7-24457267-24465768
chr7	24469890	24471383	chr7-24469890-24471383
chr7	24480871	24482858	chr7-24480871-24482858
chr7	24505695	24508908	chr7-24505695-24508908
chr7	24529401	24532705	chr7-24529401-24532705
chr7	24539555	24540299	chr7-24539555-24540299
chr7	24546086	24548327	chr7-24546086-24548327
chr7	24593373	24595388	chr7-24593373-24595388
chr7	24599235	24600110	chr7-24599235-24600110
chr7	24610083	24613026	chr7-24610083-24613026
chr7	24636186	24639128	chr7-24636186-24639128
chr7	24883420	24888616	chr7-24883420-24888616
chr7	24902365	24909123	chr7-24902365-24909123
chr7	24933532	24933949	chr7-24933532-24933949
chr7	24942995	24943248	chr7-24942995-24943248
chr7	24944182	24945142	chr7-24944182-24945142
chr7	24948857	24949058	chr7-24948857-24949058
chr7	24954033	24954897	chr7-24954033-24954897
chr7	24971151	24973140	chr7-24971151-24973140
chr7	25005479	25006146	chr7-25005479-25006146
chr7	25009999	25010821	chr7-25009999-25010821
chr7	25071824	25073325	chr7-25071824-25073325
chr7	25076031	25078959	chr7-25076031-25078959
chr7	25120468	25120669	chr7-25120468-25120669
chr7	25132280	25133684	chr7-25132280-25133684
chr7	25155441	25156134	chr7-25155441-25156134
chr7	25178692	25180127	chr7-25178692-25180127
chr7	25181584	25181785	chr7-25181584-25181785
chr7	25197452	25197704	chr7-25197452-25197704
chr7	25205531	25206646	chr7-25205531-25206646
chr7	25208589	25209851	chr7-25208589-25209851
chr7	25217452	25222918	chr7-25217452-25222918
chr7	25236104	25239638	chr7-25236104-25239638

chr7	25246065	25251702	chr7-25246065-25251702
chr7	25266886	25270213	chr7-25266886-25270213
chr7	25275707	25276333	chr7-25275707-25276333
chr7	25277338	25284632	chr7-25277338-25284632
chr7	25296044	25298458	chr7-25296044-25298458
chr7	25305762	25306649	chr7-25305762-25306649
chr7	25314689	25318162	chr7-25314689-25318162
chr7	25326048	25326511	chr7-25326048-25326511
chr7	25367812	25368217	chr7-25367812-25368217
chr7	25376216	25380209	chr7-25376216-25380209
chr7	25385804	25386990	chr7-25385804-25386990
chr7	25393558	25396995	chr7-25393558-25396995
chr7	25410773	25411084	chr7-25410773-25411084
chr7	25430567	25430768	chr7-25430567-25430768
chr7	25436402	25436812	chr7-25436402-25436812
chr7	25476393	25477929	chr7-25476393-25477929
chr7	25618787	25620479	chr7-25618787-25620479
chr7	25627236	25627815	chr7-25627236-25627815
chr7	25657579	25660371	chr7-25657579-25660371
chr7	25674629	25675386	chr7-25674629-25675386
chr7	25680072	25681473	chr7-25680072-25681473
chr7	25686159	25690247	chr7-25686159-25690247
chr7	25712095	25712567	chr7-25712095-25712567
chr7	25717567	25719910	chr7-25717567-25719910
chr7	25752529	25756349	chr7-25752529-25756349
chr7	25780208	25789582	chr7-25780208-25789582
chr7	25814211	25814482	chr7-25814211-25814482
chr7	25815612	25817191	chr7-25815612-25817191
chr7	25831724	25832605	chr7-25831724-25832605
chr7	25848112	25848522	chr7-25848112-25848522
chr7	25878610	25878967	chr7-25878610-25878967
chr7	27153294	27153652	chr7-27153294-27153652
chr7	27163486	27167701	chr7-27163486-27167701
chr7	27177944	27179623	chr7-27177944-27179623
chr7	27193548	27197376	chr7-27193548-27197376
chr7	27221858	27222111	chr7-27221858-27222111
chr7	27224593	27229564	chr7-27224593-27229564
chr7	27231394	27234052	chr7-27231394-27234052
chr7	27252069	27253148	chr7-27252069-27253148
chr7	27256802	27260139	chr7-27256802-27260139
chr7	27291989	27292190	chr7-27291989-27292190
chr7	27308645	27309453	chr7-27308645-27309453

chr7	27310428	27310783	chr7-27310428-27310783
chr7	27325648	27326010	chr7-27325648-27326010
chr7	27329257	27334971	chr7-27329257-27334971
chr7	27336041	27338147	chr7-27336041-27338147
chr7	27354426	27357490	chr7-27354426-27357490
chr7	27360879	27362412	chr7-27360879-27362412
chr7	27366069	27366832	chr7-27366069-27366832
chr7	27371880	27372550	chr7-27371880-27372550
chr7	27375259	27375625	chr7-27375259-27375625
chr7	27395656	27396912	chr7-27395656-27396912
chr7	27404207	27404985	chr7-27404207-27404985
chr7	27408090	27409363	chr7-27408090-27409363
chr7	27445994	27449267	chr7-27445994-27449267
chr7	27472461	27475519	chr7-27472461-27475519
chr7	27480837	27482189	chr7-27480837-27482189
chr7	27485632	27492431	chr7-27485632-27492431
chr7	27506759	27509145	chr7-27506759-27509145
chr7	27520829	27521030	chr7-27520829-27521030
chr7	27542186	27543428	chr7-27542186-27543428
chr7	27552269	27554659	chr7-27552269-27554659
chr7	27590863	27593376	chr7-27590863-27593376
chr7	27606724	27608956	chr7-27606724-27608956
chr7	27650440	27651119	chr7-27650440-27651119
chr7	27653780	27654493	chr7-27653780-27654493
chr7	27672650	27675749	chr7-27672650-27675749
chr7	27688217	27690115	chr7-27688217-27690115
chr7	27731005	27732816	chr7-27731005-27732816
chr7	27765581	27765782	chr7-27765581-27765782
chr7	27804124	27805764	chr7-27804124-27805764
chr7	27837885	27839860	chr7-27837885-27839860
chr7	27859826	27861468	chr7-27859826-27861468
chr7	27928476	27929751	chr7-27928476-27929751
chr7	27933516	27933868	chr7-27933516-27933868
chr7	27977883	27980021	chr7-27977883-27980021
chr7	28012756	28014669	chr7-28012756-28014669
chr7	28048617	28050296	chr7-28048617-28050296
chr7	28104647	28105266	chr7-28104647-28105266
chr7	28132652	28133173	chr7-28132652-28133173
chr7	28158978	28160130	chr7-28158978-28160130
chr7	28168051	28171473	chr7-28168051-28171473
chr7	28179011	28180791	chr7-28179011-28180791
chr7	28208572	28209470	chr7-28208572-28209470

chr7	28267487	28269071	chr7-28267487-28269071
chr7	28277601	28278648	chr7-28277601-28278648
chr7	28290462	28291332	chr7-28290462-28291332
chr7	28294169	28295233	chr7-28294169-28295233
chr7	28298868	28299222	chr7-28298868-28299222
chr7	28301370	28302415	chr7-28301370-28302415
chr7	28310786	28313132	chr7-28310786-28313132
chr7	28335009	28335687	chr7-28335009-28335687
chr7	28337166	28339474	chr7-28337166-28339474
chr7	28349823	28353151	chr7-28349823-28353151
chr7	28356973	28358707	chr7-28356973-28358707
chr7	28369945	28373489	chr7-28369945-28373489
chr7	28374863	28383145	chr7-28374863-28383145
chr7	28391280	28394276	chr7-28391280-28394276
chr7	28418193	28418394	chr7-28418193-28418394
chr7	28435201	28438006	chr7-28435201-28438006
chr7	28451447	28453173	chr7-28451447-28453173
chr7	28466875	28468035	chr7-28466875-28468035
chr7	28487715	28488666	chr7-28487715-28488666
chr7	28490760	28491014	chr7-28490760-28491014
chr7	28528203	28528642	chr7-28528203-28528642
chr7	28545295	28545551	chr7-28545295-28545551
chr7	28546778	28547097	chr7-28546778-28547097
chr7	28569564	28571533	chr7-28569564-28571533
chr7	28576233	28578610	chr7-28576233-28578610
chr7	28587482	28588266	chr7-28587482-28588266
chr7	28595534	28598769	chr7-28595534-28598769
chr7	28605898	28606332	chr7-28605898-28606332
chr7	28619664	28619865	chr7-28619664-28619865
chr7	28629630	28631323	chr7-28629630-28631323
chr7	28692970	28693841	chr7-28692970-28693841
chr7	28716222	28718509	chr7-28716222-28718509
chr7	28732515	28732983	chr7-28732515-28732983
chr7	28740708	28742834	chr7-28740708-28742834
chr7	28764793	28767882	chr7-28764793-28767882
chr7	28796628	28796829	chr7-28796628-28796829
chr7	28801715	28803631	chr7-28801715-28803631
chr7	28804607	28804808	chr7-28804607-28804808
chr7	28807626	28813549	chr7-28807626-28813549
chr7	28824641	28826332	chr7-28824641-28826332
chr7	28865602	28866600	chr7-28865602-28866600
chr7	28867486	28868142	chr7-28867486-28868142

chr7	28923582	28925551	chr7-28923582-28925551
chr7	28929160	28933031	chr7-28929160-28933031
chr7	28944979	28948128	chr7-28944979-28948128
chr7	28951517	28956127	chr7-28951517-28956127
chr7	28957393	28963091	chr7-28957393-28963091
chr7	28969372	28972848	chr7-28969372-28972848
chr7	28980344	28983145	chr7-28980344-28983145
chr7	29000675	29001763	chr7-29000675-29001763
chr7	29007723	29007976	chr7-29007723-29007976
chr7	29019365	29020284	chr7-29019365-29020284
chr7	29039738	29040934	chr7-29039738-29040934
chr7	29043055	29044082	chr7-29043055-29044082
chr7	29052736	29053442	chr7-29052736-29053442
chr7	29061689	29061890	chr7-29061689-29061890
chr7	29068030	29068231	chr7-29068030-29068231
chr7	29071557	29071979	chr7-29071557-29071979
chr7	29124859	29125316	chr7-29124859-29125316
chr7	29134892	29135093	chr7-29134892-29135093
chr7	29155068	29156893	chr7-29155068-29156893
chr7	29165644	29172504	chr7-29165644-29172504
chr7	29177843	29181056	chr7-29177843-29181056
chr7	29210824	29212385	chr7-29210824-29212385
chr7	29231735	29233065	chr7-29231735-29233065
chr7	29237540	29239663	chr7-29237540-29239663
chr7	29245708	29249420	chr7-29245708-29249420
chr7	29266232	29266433	chr7-29266232-29266433
chr7	29272522	29272723	chr7-29272522-29272723
chr7	29276723	29283025	chr7-29276723-29283025
chr7	29287916	29289861	chr7-29287916-29289861
chr7	29297012	29297213	chr7-29297012-29297213
chr7	29303626	29305547	chr7-29303626-29305547
chr7	29307247	29309848	chr7-29307247-29309848
chr7	29350173	29351056	chr7-29350173-29351056
chr7	29351891	29352334	chr7-29351891-29352334
chr7	29391293	29393845	chr7-29391293-29393845
chr7	29424988	29426174	chr7-29424988-29426174
chr7	29443571	29445699	chr7-29443571-29445699
chr7	29449752	29450254	chr7-29449752-29450254
chr7	29452069	29453687	chr7-29452069-29453687
chr7	29458046	29459779	chr7-29458046-29459779
chr7	29461434	29463077	chr7-29461434-29463077
chr7	29468996	29472131	chr7-29468996-29472131

chr7	29475808	29477912	chr7-29475808-29477912
chr7	29488041	29492850	chr7-29488041-29492850
chr7	29495894	29497341	chr7-29495894-29497341
chr7	29502465	29502720	chr7-29502465-29502720
chr7	29503559	29506761	chr7-29503559-29506761
chr7	29511205	29514261	chr7-29511205-29514261
chr7	29516641	29519849	chr7-29516641-29519849
chr7	29766669	29769920	chr7-29766669-29769920
chr7	29783701	29784855	chr7-29783701-29784855
chr7	29814925	29817393	chr7-29814925-29817393
chr7	29818623	29819744	chr7-29818623-29819744
chr7	29823851	29824052	chr7-29823851-29824052
chr7	29852800	29854017	chr7-29852800-29854017
chr7	29859629	29860906	chr7-29859629-29860906
chr7	29905319	29906987	chr7-29905319-29906987
chr7	29908035	29909551	chr7-29908035-29909551
chr7	29921183	29921888	chr7-29921183-29921888
chr7	29953018	29954816	chr7-29953018-29954816
chr7	29956250	29956613	chr7-29956250-29956613
chr7	29980612	29985862	chr7-29980612-29985862
chr7	30050593	30051753	chr7-30050593-30051753
chr7	30071834	30073345	chr7-30071834-30073345
chr7	30088990	30091351	chr7-30088990-30091351
chr7	30094098	30096556	chr7-30094098-30096556
chr7	30121141	30122954	chr7-30121141-30122954
chr7	30168260	30170992	chr7-30168260-30170992
chr7	30183430	30184526	chr7-30183430-30184526
chr7	30192695	30197094	chr7-30192695-30197094
chr7	30198061	30198940	chr7-30198061-30198940
chr7	30216651	30216956	chr7-30216651-30216956
chr7	30230747	30233631	chr7-30230747-30233631
chr7	30279021	30281517	chr7-30279021-30281517
chr7	30291479	30292190	chr7-30291479-30292190
chr7	30307945	30309920	chr7-30307945-30309920
chr7	30314664	30315557	chr7-30314664-30315557
chr7	30321332	30323197	chr7-30321332-30323197
chr7	30359334	30359729	chr7-30359334-30359729
chr7	30362475	30363349	chr7-30362475-30363349
chr7	30373619	30374187	chr7-30373619-30374187
chr7	30421289	30423589	chr7-30421289-30423589
chr7	30444164	30446009	chr7-30444164-30446009
chr7	30453413	30454315	chr7-30453413-30454315

chr7	30477745	30478209	chr7-30477745-30478209
chr7	30534283	30535803	chr7-30534283-30535803
chr7	30551177	30554431	chr7-30551177-30554431
chr7	30557923	30560276	chr7-30557923-30560276
chr7	30561809	30564642	chr7-30561809-30564642
chr7	30586635	30594285	chr7-30586635-30594285
chr7	30599505	30603366	chr7-30599505-30603366
chr7	30624803	30626905	chr7-30624803-30626905
chr7	30633641	30635186	chr7-30633641-30635186
chr7	30649034	30651276	chr7-30649034-30651276
chr7	30664041	30665837	chr7-30664041-30665837
chr7	30688459	30688717	chr7-30688459-30688717
chr7	30716911	30718112	chr7-30716911-30718112
chr7	30727981	30730854	chr7-30727981-30730854
chr7	30859848	30860203	chr7-30859848-30860203
chr7	30873325	30873526	chr7-30873325-30873526
chr7	30892346	30892805	chr7-30892346-30892805
chr7	30908382	30909355	chr7-30908382-30909355
chr7	30955183	30958638	chr7-30955183-30958638
chr7	30971731	30974755	chr7-30971731-30974755
chr7	31000359	31001113	chr7-31000359-31001113
chr7	31016713	31018154	chr7-31016713-31018154
chr7	31040890	31042917	chr7-31040890-31042917
chr7	31049993	31050346	chr7-31049993-31050346
chr7	31051289	31053160	chr7-31051289-31053160
chr7	31076361	31076801	chr7-31076361-31076801
chr7	31097729	31098289	chr7-31097729-31098289
chr7	31114695	31115798	chr7-31114695-31115798
chr7	31126210	31127666	chr7-31126210-31127666
chr7	31148431	31152049	chr7-31148431-31152049
chr7	31207957	31209240	chr7-31207957-31209240
chr7	34130796	34133893	chr7-34130796-34133893
chr7	34166888	34168974	chr7-34166888-34168974
chr7	34195348	34197214	chr7-34195348-34197214
chr7	34213052	34213408	chr7-34213052-34213408
chr7	34227678	34230849	chr7-34227678-34230849
chr7	34233040	34235516	chr7-34233040-34235516
chr7	34267092	34267293	chr7-34267092-34267293
chr7	34268869	34269312	chr7-34268869-34269312
chr7	34308780	34309887	chr7-34308780-34309887
chr7	34312119	34314179	chr7-34312119-34314179
chr7	34319933	34320134	chr7-34319933-34320134

chr7	34386794	34391289	chr7-34386794-34391289
chr7	34570353	34571142	chr7-34570353-34571142
chr7	34630037	34631040	chr7-34630037-34631040
chr7	34653059	34655958	chr7-34653059-34655958
chr7	34811595	34813320	chr7-34811595-34813320
chr7	34912017	34913238	chr7-34912017-34913238
chr7	35054689	35057400	chr7-35054689-35057400
chr7	35080815	35081660	chr7-35080815-35081660
chr7	35084614	35085122	chr7-35084614-35085122
chr7	35086083	35086997	chr7-35086083-35086997
chr7	35107555	35107813	chr7-35107555-35107813
chr7	35117786	35120811	chr7-35117786-35120811
chr7	35126973	35128076	chr7-35126973-35128076
chr7	35128907	35129640	chr7-35128907-35129640
chr7	35172198	35172449	chr7-35172198-35172449
chr7	35185036	35186585	chr7-35185036-35186585
chr7	35214566	35215752	chr7-35214566-35215752
chr7	35233911	35236000	chr7-35233911-35236000
chr7	35251961	35252604	chr7-35251961-35252604
chr7	35269412	35269971	chr7-35269412-35269971
chr7	35303114	35303417	chr7-35303114-35303417
chr7	35317364	35318996	chr7-35317364-35318996
chr7	35332816	35337206	chr7-35332816-35337206
chr7	35341091	35341906	chr7-35341091-35341906
chr7	35345195	35345396	chr7-35345195-35345396
chr7	35346565	35346766	chr7-35346565-35346766
chr7	35347691	35350412	chr7-35347691-35350412
chr7	35354801	35356142	chr7-35354801-35356142
chr7	35361805	35363445	chr7-35361805-35363445
chr7	35395471	35398388	chr7-35395471-35398388
chr7	35554245	35556333	chr7-35554245-35556333
chr7	35584257	35588019	chr7-35584257-35588019
chr7	35646275	35647978	chr7-35646275-35647978
chr7	35753271	35755114	chr7-35753271-35755114
chr7	35801157	35803287	chr7-35801157-35803287
chr7	36695671	36698749	chr7-36695671-36698749
chr7	37357923	37358124	chr7-37357923-37358124
chr7	37563248	37563449	chr7-37563248-37563449
chr7	37624063	37625007	chr7-37624063-37625007
chr7	37769058	37773096	chr7-37769058-37773096
chr7	38017795	38019912	chr7-38017795-38019912
chr7	38030434	38031217	chr7-38030434-38031217

chr7	38060909	38061916	chr7-38060909-38061916
chr7	38077430	38077948	chr7-38077430-38077948
chr7	38084218	38086204	chr7-38084218-38086204
chr7	38106118	38108586	chr7-38106118-38108586
chr7	38167287	38167540	chr7-38167287-38167540
chr7	38174201	38174622	chr7-38174201-38174622
chr7	38182698	38184583	chr7-38182698-38184583
chr7	38200597	38201159	chr7-38200597-38201159
chr7	38207123	38207324	chr7-38207123-38207324
chr7	38224888	38228611	chr7-38224888-38228611
chr7	38229924	38230586	chr7-38229924-38230586
chr7	38269934	38272738	chr7-38269934-38272738
chr7	39448814	39450500	chr7-39448814-39450500
chr7	39487227	39487894	chr7-39487227-39487894
chr7	39517061	39518853	chr7-39517061-39518853
chr7	39543992	39545561	chr7-39543992-39545561
chr7	39551977	39553649	chr7-39551977-39553649
chr7	39588383	39589104	chr7-39588383-39589104
chr7	39655330	39655944	chr7-39655330-39655944
chr7	40899044	40899475	chr7-40899044-40899475
chr7	41391920	41394448	chr7-41391920-41394448
chr7	41498438	41500682	chr7-41498438-41500682
chr7	41598552	41600480	chr7-41598552-41600480
chr7	41632139	41634488	chr7-41632139-41634488
chr7	42504402	42506110	chr7-42504402-42506110
chr7	42641771	42643395	chr7-42641771-42643395
chr7	42691891	42693728	chr7-42691891-42693728
chr7	43282987	43283293	chr7-43282987-43283293
chr7	43311764	43313865	chr7-43311764-43313865
chr7	43409420	43409723	chr7-43409420-43409723
chr7	43442709	43444939	chr7-43442709-43444939
chr7	43464758	43466716	chr7-43464758-43466716
chr7	43475800	43476001	chr7-43475800-43476001
chr7	43489581	43490640	chr7-43489581-43490640
chr7	43561930	43563847	chr7-43561930-43563847
chr7	43579167	43581585	chr7-43579167-43581585
chr7	43606833	43607603	chr7-43606833-43607603
chr7	43671223	43673406	chr7-43671223-43673406
chr7	43779853	43783747	chr7-43779853-43783747
chr7	43795580	43798841	chr7-43795580-43798841
chr7	43883484	43883796	chr7-43883484-43883796
chr7	44245335	44248353	chr7-44245335-44248353

chr7	44263063	44263365	chr7-44263063-44263365
chr7	44303734	44306209	chr7-44303734-44306209
chr7	44310025	44311635	chr7-44310025-44311635
chr7	44332329	44332530	chr7-44332329-44332530
chr7	44335519	44336249	chr7-44335519-44336249
chr7	44351649	44354169	chr7-44351649-44354169
chr7	44356716	44357017	chr7-44356716-44357017
chr7	44359225	44361200	chr7-44359225-44361200
chr7	44373397	44375249	chr7-44373397-44375249
chr7	44383873	44385141	chr7-44383873-44385141
chr7	44405381	44406198	chr7-44405381-44406198
chr7	44428664	44429503	chr7-44428664-44429503
chr7	44462191	44463040	chr7-44462191-44463040
chr7	44467102	44469363	chr7-44467102-44469363
chr7	44495336	44497841	chr7-44495336-44497841
chr7	44521572	44522206	chr7-44521572-44522206
chr7	44524734	44525583	chr7-44524734-44525583
chr7	44527533	44528533	chr7-44527533-44528533
chr7	44547286	44549051	chr7-44547286-44549051
chr7	44552615	44554831	chr7-44552615-44554831
chr7	44586961	44587567	chr7-44586961-44587567
chr7	44588492	44591998	chr7-44588492-44591998
chr7	44593758	44593959	chr7-44593758-44593959
chr7	44604088	44604706	chr7-44604088-44604706
chr7	44661706	44663707	chr7-44661706-44663707
chr7	44665136	44665388	chr7-44665136-44665388
chr7	44667279	44671437	chr7-44667279-44671437
chr7	44683724	44683979	chr7-44683724-44683979
chr7	44685622	44686418	chr7-44685622-44686418
chr7	44694811	44698647	chr7-44694811-44698647
chr7	44744698	44745467	chr7-44744698-44745467
chr7	44747140	44749847	chr7-44747140-44749847
chr7	44776053	44777722	chr7-44776053-44777722
chr7	44814545	44820830	chr7-44814545-44820830
chr7	44833772	44834479	chr7-44833772-44834479
chr7	44847429	44858974	chr7-44847429-44858974
chr7	44865523	44870810	chr7-44865523-44870810
chr7	44888024	44889166	chr7-44888024-44889166
chr7	44890576	44893030	chr7-44890576-44893030
chr7	44895351	44896922	chr7-44895351-44896922
chr7	44901351	44901713	chr7-44901351-44901713
chr7	44927721	44930730	chr7-44927721-44930730

chr7	44951517	44953477	chr7-44951517-44953477
chr7	44969960	44971412	chr7-44969960-44971412
chr7	44983689	44988835	chr7-44983689-44988835
chr7	44996144	45000422	chr7-44996144-45000422
chr7	45014733	45019858	chr7-45014733-45019858
chr7	45051034	45054935	chr7-45051034-45054935
chr7	45060657	45064509	chr7-45060657-45064509
chr7	45075282	45075957	chr7-45075282-45075957
chr7	45092005	45092363	chr7-45092005-45092363
chr7	45121333	45130315	chr7-45121333-45130315
chr7	45135596	45136720	chr7-45135596-45136720
chr7	45153381	45155626	chr7-45153381-45155626
chr7	45161793	45162770	chr7-45161793-45162770
chr7	45163596	45164363	chr7-45163596-45164363
chr7	45168571	45169076	chr7-45168571-45169076
chr7	45174920	45175172	chr7-45174920-45175172
chr7	45176023	45176759	chr7-45176023-45176759
chr7	45181334	45183056	chr7-45181334-45183056
chr7	45204098	45205387	chr7-45204098-45205387
chr7	45211491	45212272	chr7-45211491-45212272
chr7	45215405	45217408	chr7-45215405-45217408
chr7	45235312	45235571	chr7-45235312-45235571
chr7	45240786	45241038	chr7-45240786-45241038
chr7	45277125	45277784	chr7-45277125-45277784
chr7	45305156	45305412	chr7-45305156-45305412
chr7	45332456	45334157	chr7-45332456-45334157
chr7	45337875	45341465	chr7-45337875-45341465
chr7	45356091	45356393	chr7-45356091-45356393
chr7	45361094	45362264	chr7-45361094-45362264
chr7	45365931	45368445	chr7-45365931-45368445
chr7	45369877	45371144	chr7-45369877-45371144
chr7	45393507	45396575	chr7-45393507-45396575
chr7	45405140	45407236	chr7-45405140-45407236
chr7	45413001	45416142	chr7-45413001-45416142
chr7	45432582	45438303	chr7-45432582-45438303
chr7	45456727	45462003	chr7-45456727-45462003
chr7	45465072	45467449	chr7-45465072-45467449
chr7	45474989	45475547	chr7-45474989-45475547
chr7	45488542	45488998	chr7-45488542-45488998
chr7	45507800	45511252	chr7-45507800-45511252
chr7	45521346	45527245	chr7-45521346-45527245
chr7	45537686	45538917	chr7-45537686-45538917

chr7	45558927	45559282	chr7-45558927-45559282
chr7	45567787	45568678	chr7-45567787-45568678
chr7	45569799	45571808	chr7-45569799-45571808
chr7	45588908	45589704	chr7-45588908-45589704
chr7	45605694	45606406	chr7-45605694-45606406
chr7	45615581	45618278	chr7-45615581-45618278
chr7	45628196	45629432	chr7-45628196-45629432
chr7	45638423	45638842	chr7-45638423-45638842
chr7	45642602	45644566	chr7-45642602-45644566
chr7	45654169	45655032	chr7-45654169-45655032
chr7	45664777	45666573	chr7-45664777-45666573
chr7	45679689	45680885	chr7-45679689-45680885
chr7	45682619	45682921	chr7-45682619-45682921
chr7	45699592	45700960	chr7-45699592-45700960
chr7	45703594	45708526	chr7-45703594-45708526
chr7	45714129	45721577	chr7-45714129-45721577
chr7	45741717	45743003	chr7-45741717-45743003
chr7	45749417	45749880	chr7-45749417-45749880
chr7	45758270	45759933	chr7-45758270-45759933
chr7	45766847	45767102	chr7-45766847-45767102
chr7	45770014	45771314	chr7-45770014-45771314
chr7	45779001	45779615	chr7-45779001-45779615
chr7	45782148	45786717	chr7-45782148-45786717
chr7	45797443	45801551	chr7-45797443-45801551
chr7	45802767	45803237	chr7-45802767-45803237
chr7	45812752	45815343	chr7-45812752-45815343
chr7	45829486	45831481	chr7-45829486-45831481
chr7	45832592	45834147	chr7-45832592-45834147
chr7	45857278	45857687	chr7-45857278-45857687
chr7	45865690	45867358	chr7-45865690-45867358
chr7	45870482	45870969	chr7-45870482-45870969
chr7	45872630	45874600	chr7-45872630-45874600
chr7	45896363	45898361	chr7-45896363-45898361
chr7	45919164	45921367	chr7-45919164-45921367
chr7	45922693	45925056	chr7-45922693-45925056
chr7	46033180	46035274	chr7-46033180-46035274
chr7	46100257	46101014	chr7-46100257-46101014
chr7	46130660	46131290	chr7-46130660-46131290
chr7	46179429	46180216	chr7-46179429-46180216
chr7	46396082	46397255	chr7-46396082-46397255
chr7	46611507	46614649	chr7-46611507-46614649
chr7	46628772	46629251	chr7-46628772-46629251

chr7	46638835	46640575	chr7-46638835-46640575
chr7	46667247	46667656	chr7-46667247-46667656
chr7	46709311	46711089	chr7-46709311-46711089
chr7	46761103	46761623	chr7-46761103-46761623
chr7	46794689	46797574	chr7-46794689-46797574
chr7	46838906	46839916	chr7-46838906-46839916
chr7	46844695	46848973	chr7-46844695-46848973
chr7	46916281	46916584	chr7-46916281-46916584
chr7	46918042	46920859	chr7-46918042-46920859
chr7	46957120	46959260	chr7-46957120-46959260
chr7	46962561	46963708	chr7-46962561-46963708
chr7	47006704	47009989	chr7-47006704-47009989
chr7	47013593	47015941	chr7-47013593-47015941
chr7	47022201	47023292	chr7-47022201-47023292
chr7	47030570	47031462	chr7-47030570-47031462
chr7	47050152	47053150	chr7-47050152-47053150
chr7	47110696	47111572	chr7-47110696-47111572
chr7	47113616	47114089	chr7-47113616-47114089
chr7	47131827	47133548	chr7-47131827-47133548
chr7	48131404	48132249	chr7-48131404-48132249
chr7	48137109	48138681	chr7-48137109-48138681
chr7	48749288	48750205	chr7-48749288-48750205
chr7	48762462	48762933	chr7-48762462-48762933
chr7	48788417	48791061	chr7-48788417-48791061
chr7	48879187	48882993	chr7-48879187-48882993
chr7	48900363	48900619	chr7-48900363-48900619
chr7	48916535	48919483	chr7-48916535-48919483
chr7	48958760	48959434	chr7-48958760-48959434
chr7	49091323	49091648	chr7-49091323-49091648
chr7	49094270	49094533	chr7-49094270-49094533
chr7	49195649	49196004	chr7-49195649-49196004
chr7	49212704	49213074	chr7-49212704-49213074
chr7	49245178	49250312	chr7-49245178-49250312
chr7	49257643	49258011	chr7-49257643-49258011
chr7	49332140	49332929	chr7-49332140-49332929
chr7	49334546	49334797	chr7-49334546-49334797
chr7	49461936	49463123	chr7-49461936-49463123
chr7	49521357	49524239	chr7-49521357-49524239
chr7	49527418	49527865	chr7-49527418-49527865
chr7	49553422	49554204	chr7-49553422-49554204
chr7	49562737	49562938	chr7-49562737-49562938
chr7	49600863	49602504	chr7-49600863-49602504

chr7	49633255	49635477	chr7-49633255-49635477
chr7	49638995	49639638	chr7-49638995-49639638
chr7	49758694	49761140	chr7-49758694-49761140
chr7	49762530	49762833	chr7-49762530-49762833
chr7	49778095	49779738	chr7-49778095-49779738
chr7	49882233	49883353	chr7-49882233-49883353
chr7	49887862	49888678	chr7-49887862-49888678
chr7	49911861	49912423	chr7-49911861-49912423
chr7	49974802	49975474	chr7-49974802-49975474
chr7	49984641	49984994	chr7-49984641-49984994
chr7	51861053	51863304	chr7-51861053-51863304
chr7	51878978	51879693	chr7-51878978-51879693
chr7	51887792	51888095	chr7-51887792-51888095
chr7	51913037	51913914	chr7-51913037-51913914
chr7	52005142	52007034	chr7-52005142-52007034
chr7	52017880	52018206	chr7-52017880-52018206
chr7	55614923	55615246	chr7-55614923-55615246
chr7	55793260	55795218	chr7-55793260-55795218
chr7	55841354	55844083	chr7-55841354-55844083
chr7	55961024	55963288	chr7-55961024-55963288
chr7	55968118	55968840	chr7-55968118-55968840
chr7	56018660	56020147	chr7-56018660-56020147
chr7	56049147	56051519	chr7-56049147-56051519
chr7	56421760	56422012	chr7-56421760-56422012
chr7	56555655	56557888	chr7-56555655-56557888
chr7	57386573	57387264	chr7-57386573-57387264
chr7	57509743	57509944	chr7-57509743-57509944
chr7	57590491	57592100	chr7-57590491-57592100
chr7	58379703	58380144	chr7-58379703-58380144
chr7	58397889	58400157	chr7-58397889-58400157
chr7	58657695	58660899	chr7-58657695-58660899
chr7	58676182	58677797	chr7-58676182-58677797
chr7	58750231	58750497	chr7-58750231-58750497
chr7	58800732	58801800	chr7-58800732-58801800
chr7	58843999	58844480	chr7-58843999-58844480
chr7	59227587	59230872	chr7-59227587-59230872
chr7	60003683	60005295	chr7-60003683-60005295
chr7	60243345	60244756	chr7-60243345-60244756
chr7	60390799	60391771	chr7-60390799-60391771
chr7	60491639	60492917	chr7-60491639-60492917
chr7	60622477	60623045	chr7-60622477-60623045
chr7	61535221	61535472	chr7-61535221-61535472

chr7	62203791	62203992	chr7-62203791-62203992
chr7	62209184	62209640	chr7-62209184-62209640
chr7	62348156	62349091	chr7-62348156-62349091
chr7	62376803	62377866	chr7-62376803-62377866
chr7	62418804	62420612	chr7-62418804-62420612
chr7	62462376	62464644	chr7-62462376-62464644
chr7	63211866	63212790	chr7-63211866-63212790
chr7	63444380	63445393	chr7-63444380-63445393
chr7	63753231	63755802	chr7-63753231-63755802
chr7	63757250	63759517	chr7-63757250-63759517
chr7	63815335	63817286	chr7-63815335-63817286
chr7	63820263	63821831	chr7-63820263-63821831
chr7	63850779	63852439	chr7-63850779-63852439
chr7	63858650	63859060	chr7-63858650-63859060
chr7	63887823	63888129	chr7-63887823-63888129
chr7	63889511	63889712	chr7-63889511-63889712
chr7	63896331	63897924	chr7-63896331-63897924
chr7	63908522	63908946	chr7-63908522-63908946
chr7	63924532	63926138	chr7-63924532-63926138
chr7	63934650	63940353	chr7-63934650-63940353
chr7	64021435	64021907	chr7-64021435-64021907
chr7	64155626	64156033	chr7-64155626-64156033
chr7	64192315	64192516	chr7-64192315-64192516
chr7	64238766	64239811	chr7-64238766-64239811
chr7	64249314	64250524	chr7-64249314-64250524
chr7	64274332	64274823	chr7-64274332-64274823
chr7	64276741	64277042	chr7-64276741-64277042
chr7	64278198	64278399	chr7-64278198-64278399
chr7	64285427	64290668	chr7-64285427-64290668
chr7	64292947	64293148	chr7-64292947-64293148
chr7	64372968	64374534	chr7-64372968-64374534
chr7	64391002	64393688	chr7-64391002-64393688
chr7	64501595	64502618	chr7-64501595-64502618
chr7	64839667	64840624	chr7-64839667-64840624
chr7	64855692	64856252	chr7-64855692-64856252
chr7	64870544	64873640	chr7-64870544-64873640
chr7	64959313	64959621	chr7-64959313-64959621
chr7	65155475	65157009	chr7-65155475-65157009
chr7	65213172	65213797	chr7-65213172-65213797
chr7	65366258	65366459	chr7-65366258-65366459
chr7	65367295	65371942	chr7-65367295-65371942
chr7	65379039	65379615	chr7-65379039-65379615

chr7	65430280	65431479	chr7-65430280-65431479
chr7	65468165	65471173	chr7-65468165-65471173
chr7	65481841	65482042	chr7-65481841-65482042
chr7	65486183	65486739	chr7-65486183-65486739
chr7	65527002	65528164	chr7-65527002-65528164
chr7	65644184	65645719	chr7-65644184-65645719
chr7	65692671	65694681	chr7-65692671-65694681
chr7	65801685	65804367	chr7-65801685-65804367
chr7	65822402	65822823	chr7-65822402-65822823
chr7	65860945	65864369	chr7-65860945-65864369
chr7	65873460	65873712	chr7-65873460-65873712
chr7	65906350	65907163	chr7-65906350-65907163
chr7	65979231	65979535	chr7-65979231-65979535
chr7	66059716	66062074	chr7-66059716-66062074
chr7	66078778	66080832	chr7-66078778-66080832
chr7	66108595	66111808	chr7-66108595-66111808
chr7	66117243	66117494	chr7-66117243-66117494
chr7	66118302	66118704	chr7-66118302-66118704
chr7	66179232	66179600	chr7-66179232-66179600
chr7	66180544	66181027	chr7-66180544-66181027
chr7	66228044	66228676	chr7-66228044-66228676
chr7	66308602	66310038	chr7-66308602-66310038
chr7	66343011	66343579	chr7-66343011-66343579
chr7	66358617	66358818	chr7-66358617-66358818
chr7	66365397	66365713	chr7-66365397-66365713
chr7	66367070	66367271	chr7-66367070-66367271
chr7	66368933	66370648	chr7-66368933-66370648
chr7	66373548	66378648	chr7-66373548-66378648
chr7	66383567	66389483	chr7-66383567-66389483
chr7	66426806	66428141	chr7-66426806-66428141
chr7	66449258	66451093	chr7-66449258-66451093
chr7	66580628	66580829	chr7-66580628-66580829
chr7	66591823	66592482	chr7-66591823-66592482
chr7	66661609	66661918	chr7-66661609-66661918
chr7	66688526	66690993	chr7-66688526-66690993
chr7	66743078	66745844	chr7-66743078-66745844
chr7	66746883	66747507	chr7-66746883-66747507
chr7	66756899	66757281	chr7-66756899-66757281
chr7	66779980	66780824	chr7-66779980-66780824
chr7	66789930	66790635	chr7-66789930-66790635
chr7	66827512	66827713	chr7-66827512-66827713
chr7	66839543	66840970	chr7-66839543-66840970

chr7	67221854	67223528	chr7-67221854-67223528
chr7	67287654	67287955	chr7-67287654-67287955
chr7	67371365	67373859	chr7-67371365-67373859
chr7	67548808	67549528	chr7-67548808-67549528
chr7	67639284	67639485	chr7-67639284-67639485
chr7	67644313	67649348	chr7-67644313-67649348
chr7	67741435	67741840	chr7-67741435-67741840
chr7	67758455	67760106	chr7-67758455-67760106
chr7	67803248	67804159	chr7-67803248-67804159
chr7	67849133	67849648	chr7-67849133-67849648
chr7	67854601	67854887	chr7-67854601-67854887
chr7	67950133	67955634	chr7-67950133-67955634
chr7	67957154	67957763	chr7-67957154-67957763
chr7	67967849	67969795	chr7-67967849-67969795
chr7	67972840	67974092	chr7-67972840-67974092
chr7	68019616	68022815	chr7-68019616-68022815
chr7	68128438	68130644	chr7-68128438-68130644
chr7	68180446	68181626	chr7-68180446-68181626
chr7	68275726	68276499	chr7-68275726-68276499
chr7	68300908	68301384	chr7-68300908-68301384
chr7	68302632	68303427	chr7-68302632-68303427
chr7	68369770	68370226	chr7-68369770-68370226
chr7	68571574	68572402	chr7-68571574-68572402
chr7	68579795	68580078	chr7-68579795-68580078
chr7	68717419	68717732	chr7-68717419-68717732
chr7	68748237	68750145	chr7-68748237-68750145
chr7	70280322	70280783	chr7-70280322-70280783
chr7	70331271	70332196	chr7-70331271-70332196
chr7	70334628	70335130	chr7-70334628-70335130
chr7	70346230	70346431	chr7-70346230-70346431
chr7	70350399	70353511	chr7-70350399-70353511
chr7	70354557	70362901	chr7-70354557-70362901
chr7	70364716	70366955	chr7-70364716-70366955
chr7	70429077	70429578	chr7-70429077-70429578
chr7	70606491	70606794	chr7-70606491-70606794
chr7	70624628	70626031	chr7-70624628-70626031
chr7	71023705	71023906	chr7-71023705-71023906
chr7	71038573	71039015	chr7-71038573-71039015
chr7	71411322	71412427	chr7-71411322-71412427
chr7	71456329	71456601	chr7-71456329-71456601
chr7	71705292	71706022	chr7-71705292-71706022
chr7	71960736	71961109	chr7-71960736-71961109

chr7	72098852	72099161	chr7-72098852-72099161
chr7	72262749	72263155	chr7-72262749-72263155
chr7	72305392	72307640	chr7-72305392-72307640
chr7	72509941	72510195	chr7-72509941-72510195
chr7	72519746	72519997	chr7-72519746-72519997
chr7	73372214	73373116	chr7-73372214-73373116
chr7	73374781	73376745	chr7-73374781-73376745
chr7	73389594	73392672	chr7-73389594-73392672
chr7	73398173	73400200	chr7-73398173-73400200
chr7	73532008	73542415	chr7-73532008-73542415
chr7	73552092	73553267	chr7-73552092-73553267
chr7	73555389	73560113	chr7-73555389-73560113
chr7	73586171	73587155	chr7-73586171-73587155
chr7	73588170	73589941	chr7-73588170-73589941
chr7	73594202	73595778	chr7-73594202-73595778
chr7	73606541	73609170	chr7-73606541-73609170
chr7	73615973	73619905	chr7-73615973-73619905
chr7	73622298	73622707	chr7-73622298-73622707
chr7	73624023	73624436	chr7-73624023-73624436
chr7	73740159	73741042	chr7-73740159-73741042
chr7	74012755	74013995	chr7-74012755-74013995
chr7	74376599	74376800	chr7-74376599-74376800
chr7	74459642	74459843	chr7-74459642-74459843
chr7	74464576	74464777	chr7-74464576-74464777
chr7	74553537	74555836	chr7-74553537-74555836
chr7	75308661	75309122	chr7-75308661-75309122
chr7	75397255	75397813	chr7-75397255-75397813
chr7	75411598	75411799	chr7-75411598-75411799
chr7	75454800	75456972	chr7-75454800-75456972
chr7	75585069	75587782	chr7-75585069-75587782
chr7	75609499	75610768	chr7-75609499-75610768
chr7	75642255	75643177	chr7-75642255-75643177
chr7	75655626	75656019	chr7-75655626-75656019
chr7	75780943	75782963	chr7-75780943-75782963
chr7	75796663	75796864	chr7-75796663-75796864
chr7	75847462	75850084	chr7-75847462-75850084
chr7	76393176	76393377	chr7-76393176-76393377
chr7	76410733	76410934	chr7-76410733-76410934
chr7	76805807	76806008	chr7-76805807-76806008
chr7	78577247	78579178	chr7-78577247-78579178
chr7	78781919	78784173	chr7-78781919-78784173
chr7	78845858	78847548	chr7-78845858-78847548

chr7	78882344	78883508	chr7-78882344-78883508
chr7	78895032	78897937	chr7-78895032-78897937
chr7	78913108	78916653	chr7-78913108-78916653
chr7	78924691	78926193	chr7-78924691-78926193
chr7	78967808	78968009	chr7-78967808-78968009
chr7	78971242	78973384	chr7-78971242-78973384
chr7	78990097	78990973	chr7-78990097-78990973
chr7	78995200	78995977	chr7-78995200-78995977
chr7	79052528	79055614	chr7-79052528-79055614
chr7	79147099	79149486	chr7-79147099-79149486
chr7	79246145	79246447	chr7-79246145-79246447
chr7	79259544	79259853	chr7-79259544-79259853
chr7	79269200	79280330	chr7-79269200-79280330
chr7	79284975	79286406	chr7-79284975-79286406
chr7	79287317	79289179	chr7-79287317-79289179
chr7	79299857	79302029	chr7-79299857-79302029
chr7	79303292	79304823	chr7-79303292-79304823
chr7	79316001	79317500	chr7-79316001-79317500
chr7	79323803	79324613	chr7-79323803-79324613
chr7	79336209	79336514	chr7-79336209-79336514
chr7	79349016	79349322	chr7-79349016-79349322
chr7	79391468	79393403	chr7-79391468-79393403
chr7	79464353	79467935	chr7-79464353-79467935
chr7	79496119	79496530	chr7-79496119-79496530
chr7	79499609	79500374	chr7-79499609-79500374
chr7	79504677	79505626	chr7-79504677-79505626
chr7	79508565	79509039	chr7-79508565-79509039
chr7	79515186	79515823	chr7-79515186-79515823
chr7	79535268	79536584	chr7-79535268-79536584
chr7	79543381	79544070	chr7-79543381-79544070
chr7	79578827	79579129	chr7-79578827-79579129
chr7	79591748	79592873	chr7-79591748-79592873
chr7	79607337	79607742	chr7-79607337-79607742
chr7	79617471	79617777	chr7-79617471-79617777
chr7	79659284	79662593	chr7-79659284-79662593
chr7	79710058	79710518	chr7-79710058-79710518
chr7	79714892	79716352	chr7-79714892-79716352
chr7	79721132	79722047	chr7-79721132-79722047
chr7	79742250	79743872	chr7-79742250-79743872
chr7	79780719	79781081	chr7-79780719-79781081
chr7	79792454	79793619	chr7-79792454-79793619
chr7	79810789	79811583	chr7-79810789-79811583

chr7	79847867	79848538	chr7-79847867-79848538
chr7	79881697	79881898	chr7-79881697-79881898
chr7	79919473	79921564	chr7-79919473-79921564
chr7	79934408	79936230	chr7-79934408-79936230
chr7	80023583	80029842	chr7-80023583-80029842
chr7	80056446	80060729	chr7-80056446-80060729
chr7	80092297	80092600	chr7-80092297-80092600
chr7	80113856	80115956	chr7-80113856-80115956
chr7	80130129	80130767	chr7-80130129-80130767
chr7	80134613	80134922	chr7-80134613-80134922
chr7	80156743	80157328	chr7-80156743-80157328
chr7	80160225	80161128	chr7-80160225-80161128
chr7	80166334	80166535	chr7-80166334-80166535
chr7	80185903	80188057	chr7-80185903-80188057
chr7	80191313	80192991	chr7-80191313-80192991
chr7	80197432	80203817	chr7-80197432-80203817
chr7	80206133	80210004	chr7-80206133-80210004
chr7	80231147	80234406	chr7-80231147-80234406
chr7	80245846	80247030	chr7-80245846-80247030
chr7	80260390	80262758	chr7-80260390-80262758
chr7	80268942	80270851	chr7-80268942-80270851
chr7	80293601	80298570	chr7-80293601-80298570
chr7	80299744	80299945	chr7-80299744-80299945
chr7	80322774	80324660	chr7-80322774-80324660
chr7	80338714	80341273	chr7-80338714-80341273
chr7	80342823	80343899	chr7-80342823-80343899
chr7	80369427	80371897	chr7-80369427-80371897
chr7	80374688	80376026	chr7-80374688-80376026
chr7	80386382	80388944	chr7-80386382-80388944
chr7	80399919	80405533	chr7-80399919-80405533
chr7	80410628	80410880	chr7-80410628-80410880
chr7	80419328	80419967	chr7-80419328-80419967
chr7	80439081	80439341	chr7-80439081-80439341
chr7	80533822	80536021	chr7-80533822-80536021
chr7	80628885	80629086	chr7-80628885-80629086
chr7	80666707	80666959	chr7-80666707-80666959
chr7	80687540	80689596	chr7-80687540-80689596
chr7	80753270	80754512	chr7-80753270-80754512
chr7	80761994	80762895	chr7-80761994-80762895
chr7	80763802	80764003	chr7-80763802-80764003
chr7	80773043	80775956	chr7-80773043-80775956
chr7	80778863	80784320	chr7-80778863-80784320

chr7	80785772	80790878	chr7-80785772-80790878
chr7	80796397	80803896	chr7-80796397-80803896
chr7	80827874	80828994	chr7-80827874-80828994
chr7	80832353	80833934	chr7-80832353-80833934
chr7	80837534	80841464	chr7-80837534-80841464
chr7	80860069	80862152	chr7-80860069-80862152
chr7	80865494	80867731	chr7-80865494-80867731
chr7	80888924	80889227	chr7-80888924-80889227
chr7	80900049	80902251	chr7-80900049-80902251
chr7	80904719	80905287	chr7-80904719-80905287
chr7	80945490	80948461	chr7-80945490-80948461
chr7	80991776	80995104	chr7-80991776-80995104
chr7	81057504	81057855	chr7-81057504-81057855
chr7	81134602	81135059	chr7-81134602-81135059
chr7	81182928	81183301	chr7-81182928-81183301
chr7	81212263	81214458	chr7-81212263-81214458
chr7	81342537	81345948	chr7-81342537-81345948
chr7	81452987	81455669	chr7-81452987-81455669
chr7	81480632	81480993	chr7-81480632-81480993
chr7	81493056	81494006	chr7-81493056-81494006
chr7	81522885	81524910	chr7-81522885-81524910
chr7	81570820	81573110	chr7-81570820-81573110
chr7	81705710	81707461	chr7-81705710-81707461
chr7	81761910	81764407	chr7-81761910-81764407
chr7	81788327	81790325	chr7-81788327-81790325
chr7	81827651	81830089	chr7-81827651-81830089
chr7	81858809	81859819	chr7-81858809-81859819
chr7	81933408	81934901	chr7-81933408-81934901
chr7	81991425	81992964	chr7-81991425-81992964
chr7	82016791	82018284	chr7-82016791-82018284
chr7	82070397	82071481	chr7-82070397-82071481
chr7	82174533	82175652	chr7-82174533-82175652
chr7	82264085	82265037	chr7-82264085-82265037
chr7	82335103	82336511	chr7-82335103-82336511
chr7	82464239	82464440	chr7-82464239-82464440
chr7	82647849	82649789	chr7-82647849-82649789
chr7	82786089	82786290	chr7-82786089-82786290
chr7	82865090	82870017	chr7-82865090-82870017
chr7	82894218	82894419	chr7-82894218-82894419
chr7	83597363	83597564	chr7-83597363-83597564
chr7	83631202	83633112	chr7-83631202-83633112
chr7	83881806	83885612	chr7-83881806-83885612

chr7	83891512	83893812	chr7-83891512-83893812
chr7	84086115	84086887	chr7-84086115-84086887
chr7	84117037	84117946	chr7-84117037-84117946
chr7	84120441	84120693	chr7-84120441-84120693
chr7	84136102	84136785	chr7-84136102-84136785
chr7	84149713	84152788	chr7-84149713-84152788
chr7	84329058	84329259	chr7-84329058-84329259
chr7	84409343	84410632	chr7-84409343-84410632
chr7	84521692	84521893	chr7-84521692-84521893
chr7	84528584	84529642	chr7-84528584-84529642
chr7	84546673	84547177	chr7-84546673-84547177
chr7	84605128	84606888	chr7-84605128-84606888
chr7	84678576	84680392	chr7-84678576-84680392
chr7	84687746	84690649	chr7-84687746-84690649
chr7	84749503	84750352	chr7-84749503-84750352
chr7	84779068	84779945	chr7-84779068-84779945
chr7	86775579	86775901	chr7-86775579-86775901
chr7	87217341	87217592	chr7-87217341-87217592
chr7	87246083	87247799	chr7-87246083-87247799
chr7	87584224	87584735	chr7-87584224-87584735
chr7	88130145	88130506	chr7-88130145-88130506
chr7	88199169	88199714	chr7-88199169-88199714
chr7	88277118	88280105	chr7-88277118-88280105
chr7	88429807	88431608	chr7-88429807-88431608
chr7	89290124	89290325	chr7-89290124-89290325
chr7	89337420	89339377	chr7-89337420-89339377
chr7	89403061	89405742	chr7-89403061-89405742
chr7	89441119	89441320	chr7-89441119-89441320
chr7	89500197	89500398	chr7-89500197-89500398
chr7	89515700	89518789	chr7-89515700-89518789
chr7	89522312	89522728	chr7-89522312-89522728
chr7	89559213	89559618	chr7-89559213-89559618
chr7	89563407	89563608	chr7-89563407-89563608
chr7	89632216	89633529	chr7-89632216-89633529
chr7	89640685	89641231	chr7-89640685-89641231
chr7	89664451	89665121	chr7-89664451-89665121
chr7	89835782	89835983	chr7-89835782-89835983
chr7	89837656	89838392	chr7-89837656-89838392
chr7	89939536	89941963	chr7-89939536-89941963
chr7	89978516	89982346	chr7-89978516-89982346
chr7	90096781	90097389	chr7-90096781-90097389
chr7	90128698	90132888	chr7-90128698-90132888

chr7	90145916	90147112	chr7-90145916-90147112
chr7	90231404	90231998	chr7-90231404-90231998
chr7	90265334	90265799	chr7-90265334-90265799
chr7	90284977	90285570	chr7-90284977-90285570
chr7	90301985	90303443	chr7-90301985-90303443
chr7	90370406	90370607	chr7-90370406-90370607
chr7	90426478	90426962	chr7-90426478-90426962
chr7	90442069	90446450	chr7-90442069-90446450
chr7	90456018	90458054	chr7-90456018-90458054
chr7	90475189	90477107	chr7-90475189-90477107
chr7	91262005	91262965	chr7-91262005-91262965
chr7	91542583	91543346	chr7-91542583-91543346
chr7	92180486	92181427	chr7-92180486-92181427
chr7	92560723	92562544	chr7-92560723-92562544
chr7	92635982	92638294	chr7-92635982-92638294
chr7	92666363	92670919	chr7-92666363-92670919
chr7	92698488	92699564	chr7-92698488-92699564
chr7	92739263	92743397	chr7-92739263-92743397
chr7	92873357	92875969	chr7-92873357-92875969
chr7	93079628	93081327	chr7-93079628-93081327
chr7	94136957	94137158	chr7-94136957-94137158
chr7	96152953	96153359	chr7-96152953-96153359
chr7	96170929	96172108	chr7-96170929-96172108
chr7	96209728	96213930	chr7-96209728-96213930
chr7	96522138	96522602	chr7-96522138-96522602
chr7	96575220	96575523	chr7-96575220-96575523
chr7	96707367	96708512	chr7-96707367-96708512
chr7	96950942	96952745	chr7-96950942-96952745
chr7	97080985	97083259	chr7-97080985-97083259
chr7	97129203	97129762	chr7-97129203-97129762
chr7	97330921	97333648	chr7-97330921-97333648
chr7	97370817	97372941	chr7-97370817-97372941
chr7	97399493	97401528	chr7-97399493-97401528
chr7	97438868	97439174	chr7-97438868-97439174
chr7	97453136	97453955	chr7-97453136-97453955
chr7	97480357	97482265	chr7-97480357-97482265
chr7	97578482	97581827	chr7-97578482-97581827
chr7	97695818	97698057	chr7-97695818-97698057
chr7	97737253	97739102	chr7-97737253-97739102
chr7	97748167	97749690	chr7-97748167-97749690
chr7	97766818	97767210	chr7-97766818-97767210
chr7	97787976	97790777	chr7-97787976-97790777

chr7	97842801	97843523	chr7-97842801-97843523
chr7	98102802	98103516	chr7-98102802-98103516
chr7	98116471	98120970	chr7-98116471-98120970
chr7	98156415	98165863	chr7-98156415-98165863
chr7	98170076	98173719	chr7-98170076-98173719
chr7	98174780	98179588	chr7-98174780-98179588
chr7	98193503	98194782	chr7-98193503-98194782
chr7	98307572	98310193	chr7-98307572-98310193
chr7	98330882	98331190	chr7-98330882-98331190
chr7	98341477	98341984	chr7-98341477-98341984
chr7	98358398	98361972	chr7-98358398-98361972
chr7	98383310	98383986	chr7-98383310-98383986
chr7	98385092	98385443	chr7-98385092-98385443
chr7	98489362	98490485	chr7-98489362-98490485
chr7	98514047	98514248	chr7-98514047-98514248
chr7	98523867	98524295	chr7-98523867-98524295
chr7	98558690	98558948	chr7-98558690-98558948
chr7	98655366	98658153	chr7-98655366-98658153
chr7	98692856	98693523	chr7-98692856-98693523
chr7	98701581	98704878	chr7-98701581-98704878
chr7	98774906	98775219	chr7-98774906-98775219
chr7	98788520	98789476	chr7-98788520-98789476
chr7	98814455	98818589	chr7-98814455-98818589
chr7	98820342	98820543	chr7-98820342-98820543
chr7	98834459	98839549	chr7-98834459-98839549
chr7	98876264	98876465	chr7-98876264-98876465
chr7	98962340	98963548	chr7-98962340-98963548
chr7	98967104	98969209	chr7-98967104-98969209
chr7	98972267	98972532	chr7-98972267-98972532
chr7	98976975	98977400	chr7-98976975-98977400
chr7	99127410	99129330	chr7-99127410-99129330
chr7	99132701	99132902	chr7-99132701-99132902
chr7	99139112	99142309	chr7-99139112-99142309
chr7	99181643	99183686	chr7-99181643-99183686
chr7	99200163	99200542	chr7-99200163-99200542
chr7	99215270	99216212	chr7-99215270-99216212
chr7	99237814	99238903	chr7-99237814-99238903
chr7	99248869	99249810	chr7-99248869-99249810
chr7	99267293	99272068	chr7-99267293-99272068
chr7	99304112	99305621	chr7-99304112-99305621
chr7	99315764	99316957	chr7-99315764-99316957
chr7	99322149	99323728	chr7-99322149-99323728

chr7	99350369	99353673	chr7-99350369-99353673
chr7	99380314	99385016	chr7-99380314-99385016
chr7	99403052	99403604	chr7-99403052-99403604
chr7	99428225	99429859	chr7-99428225-99429859
chr7	99432208	99432409	chr7-99432208-99432409
chr7	99434614	99435529	chr7-99434614-99435529
chr7	99440974	99443674	chr7-99440974-99443674
chr7	99468213	99469089	chr7-99468213-99469089
chr7	99473673	99475310	chr7-99473673-99475310
chr7	99479236	99484289	chr7-99479236-99484289
chr7	99519220	99519577	chr7-99519220-99519577
chr7	99534949	99536491	chr7-99534949-99536491
chr7	99625422	99627331	chr7-99625422-99627331
chr7	99795506	99795818	chr7-99795506-99795818
chr7	99827781	99828913	chr7-99827781-99828913
chr7	99857476	99859565	chr7-99857476-99859565
chr7	99917001	99917878	chr7-99917001-99917878
chr7	99978828	99980887	chr7-99978828-99980887
chr7	100005622	100006382	chr7-100005622-100006382
chr7	100042085	100042393	chr7-100042085-100042393
chr7	100119963	100122307	chr7-100119963-100122307
chr7	100158932	100160400	chr7-100158932-100160400
chr7	100178252	100179419	chr7-100178252-100179419
chr7	100226828	100228510	chr7-100226828-100228510
chr7	100285099	100285936	chr7-100285099-100285936
chr7	100288012	100289140	chr7-100288012-100289140
chr7	100370494	100373271	chr7-100370494-100373271
chr7	100465529	100466076	chr7-100465529-100466076
chr7	100474762	100475050	chr7-100474762-100475050
chr7	100479348	100480354	chr7-100479348-100480354
chr7	100492377	100494606	chr7-100492377-100494606
chr7	100520872	100521123	chr7-100520872-100521123
chr7	100536221	100538093	chr7-100536221-100538093
chr7	100582356	100583486	chr7-100582356-100583486
chr7	100590646	100591777	chr7-100590646-100591777
chr7	100606769	100609663	chr7-100606769-100609663
chr7	100658268	100658469	chr7-100658268-100658469
chr7	100679130	100679805	chr7-100679130-100679805
chr7	100705814	100707700	chr7-100705814-100707700
chr7	100733009	100733664	chr7-100733009-100733664
chr7	100862082	100863761	chr7-100862082-100863761
chr7	100911198	100911634	chr7-100911198-100911634

chr7	100918229	100918480	chr7-100918229-100918480
chr7	100919295	100920169	chr7-100919295-100920169
chr7	100925141	100933618	chr7-100925141-100933618
chr7	100974297	100974857	chr7-100974297-100974857
chr7	100990712	100991385	chr7-100990712-100991385
chr7	101003477	101004157	chr7-101003477-101004157
chr7	101010210	101012829	chr7-101010210-101012829
chr7	101107531	101110584	chr7-101107531-101110584
chr7	101300788	101302773	chr7-101300788-101302773
chr7	101310059	101311295	chr7-101310059-101311295
chr7	101314763	101314964	chr7-101314763-101314964
chr7	101317069	101317322	chr7-101317069-101317322
chr7	101320490	101323790	chr7-101320490-101323790
chr7	101343830	101349933	chr7-101343830-101349933
chr7	101360756	101360957	chr7-101360756-101360957
chr7	101450223	101451768	chr7-101450223-101451768
chr7	101472675	101474793	chr7-101472675-101474793
chr7	101504308	101504932	chr7-101504308-101504932
chr7	101537632	101538659	chr7-101537632-101538659
chr7	101547560	101551434	chr7-101547560-101551434
chr7	101653875	101654076	chr7-101653875-101654076
chr7	101663193	101665278	chr7-101663193-101665278
chr7	101817840	101818764	chr7-101817840-101818764
chr7	101821518	101822425	chr7-101821518-101822425
chr7	101835241	101839077	chr7-101835241-101839077
chr7	101895526	101897666	chr7-101895526-101897666
chr7	101905175	101907927	chr7-101905175-101907927
chr7	101932583	101936144	chr7-101932583-101936144
chr7	101969464	101972794	chr7-101969464-101972794
chr7	102063144	102067016	chr7-102063144-102067016
chr7	102106911	102107199	chr7-102106911-102107199
chr7	102208626	102211344	chr7-102208626-102211344
chr7	102222048	102225331	chr7-102222048-102225331
chr7	102248155	102251478	chr7-102248155-102251478
chr7	102266817	102269742	chr7-102266817-102269742
chr7	102441127	102445013	chr7-102441127-102445013
chr7	102512737	102513657	chr7-102512737-102513657
chr7	102516685	102517127	chr7-102516685-102517127
chr7	102564821	102565835	chr7-102564821-102565835
chr7	102686616	102687876	chr7-102686616-102687876
chr7	104218718	104220546	chr7-104218718-104220546
chr7	104244041	104245314	chr7-104244041-104245314

chr7	104287491	104288148	chr7-104287491-104288148
chr7	104314539	104315534	chr7-104314539-104315534
chr7	104352659	104353713	chr7-104352659-104353713
chr7	104501453	104502570	chr7-104501453-104502570
chr7	104646912	104647385	chr7-104646912-104647385
chr7	105398898	105401095	chr7-105398898-105401095
chr7	105425349	105426270	chr7-105425349-105426270
chr7	105480857	105482753	chr7-105480857-105482753
chr7	105499056	105499407	chr7-105499056-105499407
chr7	105501509	105501990	chr7-105501509-105501990
chr7	105553575	105555773	chr7-105553575-105555773
chr7	105580477	105582043	chr7-105580477-105582043
chr7	105619331	105619532	chr7-105619331-105619532
chr7	105621398	105622252	chr7-105621398-105622252
chr7	105632293	105634173	chr7-105632293-105634173
chr7	105638906	105641878	chr7-105638906-105641878
chr7	105650516	105651647	chr7-105650516-105651647
chr7	105734970	105741720	chr7-105734970-105741720
chr7	105742666	105744852	chr7-105742666-105744852
chr7	105751086	105752429	chr7-105751086-105752429
chr7	105762210	105762866	chr7-105762210-105762866
chr7	105786978	105787996	chr7-105786978-105787996
chr7	105809658	105811501	chr7-105809658-105811501
chr7	107209742	107210607	chr7-107209742-107210607
chr7	107370152	107371565	chr7-107370152-107371565
chr7	107594403	107595668	chr7-107594403-107595668
chr7	107664279	107664544	chr7-107664279-107664544
chr7	107665656	107666610	chr7-107665656-107666610
chr7	107685835	107686190	chr7-107685835-107686190
chr7	107708650	107708903	chr7-107708650-107708903
chr7	107757622	107758189	chr7-107757622-107758189
chr7	108934237	108936133	chr7-108934237-108936133
chr7	108950222	108951434	chr7-108950222-108951434
chr7	109010696	109011404	chr7-109010696-109011404
chr7	109083091	109083445	chr7-109083091-109083445
chr7	109169556	109170232	chr7-109169556-109170232
chr7	109174295	109175608	chr7-109174295-109175608
chr7	109438597	109439525	chr7-109438597-109439525
chr7	109493731	109494034	chr7-109493731-109494034
chr7	109518001	109522268	chr7-109518001-109522268
chr7	109546433	109546789	chr7-109546433-109546789
chr7	109559752	109560003	chr7-109559752-109560003

chr7	109568772	109569215	chr7-109568772-109569215
chr7	109576433	109576634	chr7-109576433-109576634
chr7	109608546	109609473	chr7-109608546-109609473
chr7	109613486	109617198	chr7-109613486-109617198
chr7	109630261	109630573	chr7-109630261-109630573
chr7	109671488	109672419	chr7-109671488-109672419
chr7	109703107	109704640	chr7-109703107-109704640
chr7	109708971	109709222	chr7-109708971-109709222
chr7	109751197	109753367	chr7-109751197-109753367
chr7	109780752	109782298	chr7-109780752-109782298
chr7	109865194	109866203	chr7-109865194-109866203
chr7	109958388	109961002	chr7-109958388-109961002
chr7	109984455	109987298	chr7-109984455-109987298
chr7	110003120	110003636	chr7-110003120-110003636
chr7	110017790	110022159	chr7-110017790-110022159
chr7	110059797	110064052	chr7-110059797-110064052
chr7	110108696	110110023	chr7-110108696-110110023
chr7	110120681	110125351	chr7-110120681-110125351
chr7	110156903	110157501	chr7-110156903-110157501
chr7	110164139	110165299	chr7-110164139-110165299
chr7	110221165	110223225	chr7-110221165-110223225
chr7	110278147	110278414	chr7-110278147-110278414
chr7	110288000	110289172	chr7-110288000-110289172
chr7	110613071	110616188	chr7-110613071-110616188
chr7	110625053	110625305	chr7-110625053-110625305
chr7	110627326	110628544	chr7-110627326-110628544
chr7	110630019	110631040	chr7-110630019-110631040
chr7	110689328	110689784	chr7-110689328-110689784
chr7	110715309	110715561	chr7-110715309-110715561
chr7	110767717	110769949	chr7-110767717-110769949
chr7	110772638	110774575	chr7-110772638-110774575
chr7	110781388	110781640	chr7-110781388-110781640
chr7	110784315	110785896	chr7-110784315-110785896
chr7	110787026	110787886	chr7-110787026-110787886
chr7	110798111	110798374	chr7-110798111-110798374
chr7	110843297	110844609	chr7-110843297-110844609
chr7	110914577	110914878	chr7-110914577-110914878
chr7	110941084	110942438	chr7-110941084-110942438
chr7	110948333	110948747	chr7-110948333-110948747
chr7	111028163	111030301	chr7-111028163-111030301
chr7	111080136	111084266	chr7-111080136-111084266
chr7	111121542	111123522	chr7-111121542-111123522

chr7	111156404	111158066	chr7-111156404-111158066
chr7	111779257	111780444	chr7-111779257-111780444
chr7	111977015	111977490	chr7-111977015-111977490
chr7	112010714	112010915	chr7-112010714-112010915
chr7	112022580	112026600	chr7-112022580-112026600
chr7	112158275	112158530	chr7-112158275-112158530
chr7	112180249	112180762	chr7-112180249-112180762
chr7	112187742	112188410	chr7-112187742-112188410
chr7	112202346	112205138	chr7-112202346-112205138
chr7	112213083	112213919	chr7-112213083-112213919
chr7	112224781	112228309	chr7-112224781-112228309
chr7	112276627	112277329	chr7-112276627-112277329
chr7	112278302	112279476	chr7-112278302-112279476
chr7	112289557	112290008	chr7-112289557-112290008
chr7	112298125	112300841	chr7-112298125-112300841
chr7	112316189	112316609	chr7-112316189-112316609
chr7	112332803	112333056	chr7-112332803-112333056
chr7	112375128	112375329	chr7-112375128-112375329
chr7	112376140	112377220	chr7-112376140-112377220
chr7	112407309	112408138	chr7-112407309-112408138
chr7	112426776	112429566	chr7-112426776-112429566
chr7	112444437	112445112	chr7-112444437-112445112
chr7	112453703	112454056	chr7-112453703-112454056
chr7	112456425	112456626	chr7-112456425-112456626
chr7	112481062	112481317	chr7-112481062-112481317
chr7	112498579	112499920	chr7-112498579-112499920
chr7	112615341	112615704	chr7-112615341-112615704
chr7	112620661	112621180	chr7-112620661-112621180
chr7	112645590	112645791	chr7-112645590-112645791
chr7	112656111	112656312	chr7-112656111-112656312
chr7	112662923	112663875	chr7-112662923-112663875
chr7	112678285	112682199	chr7-112678285-112682199
chr7	112688185	112689347	chr7-112688185-112689347
chr7	112690833	112691134	chr7-112690833-112691134
chr7	112695911	112698536	chr7-112695911-112698536
chr7	112699617	112700233	chr7-112699617-112700233
chr7	112719830	112721336	chr7-112719830-112721336
chr7	112729872	112730127	chr7-112729872-112730127
chr7	112772261	112773385	chr7-112772261-112773385
chr7	112822808	112824559	chr7-112822808-112824559
chr7	112953473	112955591	chr7-112953473-112955591
chr7	113146396	113148418	chr7-113146396-113148418

chr7	113150993	113152038	chr7-113150993-113152038
chr7	113172438	113172639	chr7-113172438-113172639
chr7	113185421	113187162	chr7-113185421-113187162
chr7	113206291	113208919	chr7-113206291-113208919
chr7	113210413	113210714	chr7-113210413-113210714
chr7	113241456	113242961	chr7-113241456-113242961
chr7	113259509	113261675	chr7-113259509-113261675
chr7	113262875	113263126	chr7-113262875-113263126
chr7	113368444	113370468	chr7-113368444-113370468
chr7	113460801	113461077	chr7-113460801-113461077
chr7	113512553	113515262	chr7-113512553-113515262
chr7	113590260	113591786	chr7-113590260-113591786
chr7	113737699	113738448	chr7-113737699-113738448
chr7	113765616	113766989	chr7-113765616-113766989
chr7	114011934	114012622	chr7-114011934-114012622
chr7	114020522	114021028	chr7-114020522-114021028
chr7	114063643	114064014	chr7-114063643-114064014
chr7	114066411	114067123	chr7-114066411-114067123
chr7	114070274	114073058	chr7-114070274-114073058
chr7	114079773	114079974	chr7-114079773-114079974
chr7	114103829	114104362	chr7-114103829-114104362
chr7	114114602	114118506	chr7-114114602-114118506
chr7	114132283	114133025	chr7-114132283-114133025
chr7	114171120	114171913	chr7-114171120-114171913
chr7	114203182	114206196	chr7-114203182-114206196
chr7	114253716	114255318	chr7-114253716-114255318
chr7	114274683	114276877	chr7-114274683-114276877
chr7	114414681	114416695	chr7-114414681-114416695
chr7	114562523	114563182	chr7-114562523-114563182
chr7	114635728	114636478	chr7-114635728-114636478
chr7	114745677	114745878	chr7-114745677-114745878
chr7	115216987	115217188	chr7-115216987-115217188
chr7	115615837	115616260	chr7-115615837-115616260
chr7	116031301	116034851	chr7-116031301-116034851
chr7	116039340	116040535	chr7-116039340-116040535
chr7	116092413	116094603	chr7-116092413-116094603
chr7	116099993	116100297	chr7-116099993-116100297
chr7	116196509	116199173	chr7-116196509-116199173
chr7	116200837	116201459	chr7-116200837-116201459
chr7	116202424	116202625	chr7-116202424-116202625
chr7	116230634	116231379	chr7-116230634-116231379
chr7	116236943	116238002	chr7-116236943-116238002

chr7	116246755	116247056	chr7-116246755-116247056
chr7	116286059	116289300	chr7-116286059-116289300
chr7	116306782	116309864	chr7-116306782-116309864
chr7	116331933	116335305	chr7-116331933-116335305
chr7	116409944	116410145	chr7-116409944-116410145
chr7	116441326	116445433	chr7-116441326-116445433
chr7	116457467	116457888	chr7-116457467-116457888
chr7	116462442	116462755	chr7-116462442-116462755
chr7	116503658	116505343	chr7-116503658-116505343
chr7	116603762	116603963	chr7-116603762-116603963
chr7	116608178	116608483	chr7-116608178-116608483
chr7	117380346	117381852	chr7-117380346-117381852
chr7	117505548	117505950	chr7-117505548-117505950
chr7	118104471	118104774	chr7-118104471-118104774
chr7	118110554	118117150	chr7-118110554-118117150
chr7	118126531	118130590	chr7-118126531-118130590
chr7	118241377	118244725	chr7-118241377-118244725
chr7	118251924	118253292	chr7-118251924-118253292
chr7	118276801	118278113	chr7-118276801-118278113
chr7	118446951	118449054	chr7-118446951-118449054
chr7	118478962	118479163	chr7-118478962-118479163
chr7	118480051	118482435	chr7-118480051-118482435
chr7	118484515	118492557	chr7-118484515-118492557
chr7	118494079	118495714	chr7-118494079-118495714
chr7	118498878	118500384	chr7-118498878-118500384
chr7	118506628	118507371	chr7-118506628-118507371
chr7	118508448	118508649	chr7-118508448-118508649
chr7	118532275	118534120	chr7-118532275-118534120
chr7	118584110	118585176	chr7-118584110-118585176
chr7	118594915	118595736	chr7-118594915-118595736
chr7	118703572	118706107	chr7-118703572-118706107
chr7	118712207	118713413	chr7-118712207-118713413
chr7	118739870	118741903	chr7-118739870-118741903
chr7	118848919	118852099	chr7-118848919-118852099
chr7	118853864	118856849	chr7-118853864-118856849
chr7	118922396	118922751	chr7-118922396-118922751
chr7	118928920	118930752	chr7-118928920-118930752
chr7	118993352	118996398	chr7-118993352-118996398
chr7	119184503	119184704	chr7-119184503-119184704
chr7	119719420	119721312	chr7-119719420-119721312
chr7	119739783	119740387	chr7-119739783-119740387
chr7	119744216	119744680	chr7-119744216-119744680

chr7	119793165	119795333	chr7-119793165-119795333
chr7	119861303	119862139	chr7-119861303-119862139
chr7	119894627	119896791	chr7-119894627-119896791
chr7	120098047	120098299	chr7-120098047-120098299
chr7	120102003	120103785	chr7-120102003-120103785
chr7	120120067	120120414	chr7-120120067-120120414
chr7	120201315	120202120	chr7-120201315-120202120
chr7	120206145	120206458	chr7-120206145-120206458
chr7	120327261	120327819	chr7-120327261-120327819
chr7	120504384	120504689	chr7-120504384-120504689
chr7	120597650	120598165	chr7-120597650-120598165
chr7	120634054	120636627	chr7-120634054-120636627
chr7	120670122	120670661	chr7-120670122-120670661
chr7	120676466	120679435	chr7-120676466-120679435
chr7	120809699	120810204	chr7-120809699-120810204
chr7	120830202	120831878	chr7-120830202-120831878
chr7	120834417	120836763	chr7-120834417-120836763
chr7	120842378	120844986	chr7-120842378-120844986
chr7	120864782	120866472	chr7-120864782-120866472
chr7	120874980	120875577	chr7-120874980-120875577
chr7	120916550	120919541	chr7-120916550-120919541
chr7	120980923	120983374	chr7-120980923-120983374
chr7	121033717	121036107	chr7-121033717-121036107
chr7	121120162	121120413	chr7-121120162-121120413
chr7	121705926	121708012	chr7-121705926-121708012
chr7	121734170	121735186	chr7-121734170-121735186
chr7	121864787	121865610	chr7-121864787-121865610
chr7	121980618	121982449	chr7-121980618-121982449
chr7	122019906	122021550	chr7-122019906-122021550
chr7	122065637	122069250	chr7-122065637-122069250
chr7	122099090	122099291	chr7-122099090-122099291
chr7	122100143	122102469	chr7-122100143-122102469
chr7	122131887	122134174	chr7-122131887-122134174
chr7	122157725	122161789	chr7-122157725-122161789
chr7	122288952	122289927	chr7-122288952-122289927
chr7	122498389	122498643	chr7-122498389-122498643
chr7	122563161	122563564	chr7-122563161-122563564
chr7	122940326	122940786	chr7-122940326-122940786
chr7	122969217	122972967	chr7-122969217-122972967
chr7	123025204	123025455	chr7-123025204-123025455
chr7	123030561	123031496	chr7-123030561-123031496
chr7	123046349	123047087	chr7-123046349-123047087

chr7	123097705	123098599	chr7-123097705-123098599
chr7	123107611	123111086	chr7-123107611-123111086
chr7	123120728	123121221	chr7-123120728-123121221
chr7	123123400	123125508	chr7-123123400-123125508
chr7	123293741	123294686	chr7-123293741-123294686
chr7	123322533	123322947	chr7-123322533-123322947
chr7	123346583	123347041	chr7-123346583-123347041
chr7	123366806	123371258	chr7-123366806-123371258
chr7	123377556	123379091	chr7-123377556-123379091
chr7	123385778	123386322	chr7-123385778-123386322
chr7	123461929	123464202	chr7-123461929-123464202
chr7	123499949	123501173	chr7-123499949-123501173
chr7	123780288	123780593	chr7-123780288-123780593
chr7	123982357	123983901	chr7-123982357-123983901
chr7	125393184	125394306	chr7-125393184-125394306
chr7	125443381	125446409	chr7-125443381-125446409
chr7	125472081	125472836	chr7-125472081-125472836
chr7	125490245	125492755	chr7-125490245-125492755
chr7	125499565	125500312	chr7-125499565-125500312
chr7	125534607	125534914	chr7-125534607-125534914
chr7	125536785	125537576	chr7-125536785-125537576
chr7	125538961	125539399	chr7-125538961-125539399
chr7	125551592	125557363	chr7-125551592-125557363
chr7	125559824	125561887	chr7-125559824-125561887
chr7	125576698	125577055	chr7-125576698-125577055
chr7	125580936	125581692	chr7-125580936-125581692
chr7	125705579	125708930	chr7-125705579-125708930
chr7	125716618	125716819	chr7-125716618-125716819
chr7	125898921	125899725	chr7-125898921-125899725
chr7	125946019	125946387	chr7-125946019-125946387
chr7	126081547	126082693	chr7-126081547-126082693
chr7	126198381	126201260	chr7-126198381-126201260
chr7	126271175	126274423	chr7-126271175-126274423
chr7	126350771	126351722	chr7-126350771-126351722
chr7	126376143	126378394	chr7-126376143-126378394
chr7	126395589	126397182	chr7-126395589-126397182
chr7	126427917	126430045	chr7-126427917-126430045
chr7	126450086	126450839	chr7-126450086-126450839
chr7	126473688	126476463	chr7-126473688-126476463
chr7	126482341	126483231	chr7-126482341-126483231
chr7	126486622	126489093	chr7-126486622-126489093
chr7	126500619	126504573	chr7-126500619-126504573

chr7	126535808	126536416	chr7-126535808-126536416
chr7	126564905	126567072	chr7-126564905-126567072
chr7	126581983	126585834	chr7-126581983-126585834
chr7	126622529	126626411	chr7-126622529-126626411
chr7	126627810	126628747	chr7-126627810-126628747
chr7	126634159	126634924	chr7-126634159-126634924
chr7	126648748	126650236	chr7-126648748-126650236
chr7	126663828	126664366	chr7-126663828-126664366
chr7	126694513	126697636	chr7-126694513-126697636
chr7	126704038	126705268	chr7-126704038-126705268
chr7	126759157	126761800	chr7-126759157-126761800
chr7	126765523	126767078	chr7-126765523-126767078
chr7	126774587	126778124	chr7-126774587-126778124
chr7	126780782	126781353	chr7-126780782-126781353
chr7	126790556	126793561	chr7-126790556-126793561
chr7	126795282	126801033	chr7-126795282-126801033
chr7	126807696	126809677	chr7-126807696-126809677
chr7	126822294	126823847	chr7-126822294-126823847
chr7	126832043	126832602	chr7-126832043-126832602
chr7	126847380	126848455	chr7-126847380-126848455
chr7	126860801	126863336	chr7-126860801-126863336
chr7	126882204	126885375	chr7-126882204-126885375
chr7	126896915	126898865	chr7-126896915-126898865
chr7	126928118	126930040	chr7-126928118-126930040
chr7	126940906	126941715	chr7-126940906-126941715
chr7	126949376	126950290	chr7-126949376-126950290
chr7	126975542	126978419	chr7-126975542-126978419
chr7	127008441	127009609	chr7-127008441-127009609
chr7	127012828	127017611	chr7-127012828-127017611
chr7	127020957	127021534	chr7-127020957-127021534
chr7	127023340	127028458	chr7-127023340-127028458
chr7	127041075	127043155	chr7-127041075-127043155
chr7	127088849	127093135	chr7-127088849-127093135
chr7	127104661	127105227	chr7-127104661-127105227
chr7	127153909	127154218	chr7-127153909-127154218
chr7	127161714	127162727	chr7-127161714-127162727
chr7	127177225	127178670	chr7-127177225-127178670
chr7	127193972	127197191	chr7-127193972-127197191
chr7	127205665	127209837	chr7-127205665-127209837
chr7	127215143	127215445	chr7-127215143-127215445
chr7	127230711	127234721	chr7-127230711-127234721
chr7	127243628	127245964	chr7-127243628-127245964

chr7	127253502	127255586	chr7-127253502-127255586
chr7	127258999	127261380	chr7-127258999-127261380
chr7	127272325	127274567	chr7-127272325-127274567
chr7	127344075	127347197	chr7-127344075-127347197
chr7	127374694	127376384	chr7-127374694-127376384
chr7	127385697	127387402	chr7-127385697-127387402
chr7	127392366	127394554	chr7-127392366-127394554
chr7	127405941	127407061	chr7-127405941-127407061
chr7	127421040	127422906	chr7-127421040-127422906
chr7	127447923	127450808	chr7-127447923-127450808
chr7	127469749	127473063	chr7-127469749-127473063
chr7	127476872	127480718	chr7-127476872-127480718
chr7	127511051	127514373	chr7-127511051-127514373
chr7	127561525	127561903	chr7-127561525-127561903
chr7	127564700	127564951	chr7-127564700-127564951
chr7	127572294	127574844	chr7-127572294-127574844
chr7	127587143	127590107	chr7-127587143-127590107
chr7	127613843	127616195	chr7-127613843-127616195
chr7	127636787	127637662	chr7-127636787-127637662
chr7	127691426	127692038	chr7-127691426-127692038
chr7	127707148	127709545	chr7-127707148-127709545
chr7	127712043	127713519	chr7-127712043-127713519
chr7	127716887	127717479	chr7-127716887-127717479
chr7	127744213	127747886	chr7-127744213-127747886
chr7	127769056	127771202	chr7-127769056-127771202
chr7	127775816	127778861	chr7-127775816-127778861
chr7	127800469	127802481	chr7-127800469-127802481
chr7	127824278	127824739	chr7-127824278-127824739
chr7	127840517	127843073	chr7-127840517-127843073
chr7	127874333	127878216	chr7-127874333-127878216
chr7	127894489	127896162	chr7-127894489-127896162
chr7	127903770	127905289	chr7-127903770-127905289
chr7	127911596	127913746	chr7-127911596-127913746
chr7	127924796	127933290	chr7-127924796-127933290
chr7	127934697	127936622	chr7-127934697-127936622
chr7	127946452	127947342	chr7-127946452-127947342
chr7	127966270	127970420	chr7-127966270-127970420
chr7	128236637	128239571	chr7-128236637-128239571
chr7	128296983	128298707	chr7-128296983-128298707
chr7	128310329	128310580	chr7-128310329-128310580
chr7	128319736	128319937	chr7-128319736-128319937
chr7	128320811	128321074	chr7-128320811-128321074

chr7	128364040	128365116	chr7-128364040-128365116
chr7	128417296	128419019	chr7-128417296-128419019
chr7	128459542	128462835	chr7-128459542-128462835
chr7	128523038	128525840	chr7-128523038-128525840
chr7	128529514	128529980	chr7-128529514-128529980
chr7	128531768	128532656	chr7-128531768-128532656
chr7	128553837	128554138	chr7-128553837-128554138
chr7	128573550	128573944	chr7-128573550-128573944
chr7	128610619	128612475	chr7-128610619-128612475
chr7	128622151	128622707	chr7-128622151-128622707
chr7	128687867	128688789	chr7-128687867-128688789
chr7	128738529	128741106	chr7-128738529-128741106
chr7	128744041	128746533	chr7-128744041-128746533
chr7	129591104	129593068	chr7-129591104-129593068
chr7	129763297	129765048	chr7-129763297-129765048
chr7	129846984	129847185	chr7-129846984-129847185
chr7	129876712	129876972	chr7-129876712-129876972
chr7	130143673	130143874	chr7-130143673-130143874
chr7	130174518	130174821	chr7-130174518-130174821
chr7	130175910	130178286	chr7-130175910-130178286
chr7	130264659	130266786	chr7-130264659-130266786
chr7	130518305	130520953	chr7-130518305-130520953
chr7	130545472	130547708	chr7-130545472-130547708
chr7	130574099	130575554	chr7-130574099-130575554
chr7	130576419	130577802	chr7-130576419-130577802
chr7	130640018	130649551	chr7-130640018-130649551
chr7	130660144	130660345	chr7-130660144-130660345
chr7	130670882	130671899	chr7-130670882-130671899
chr7	130673557	130674242	chr7-130673557-130674242
chr7	130807924	130808247	chr7-130807924-130808247
chr7	130834405	130835820	chr7-130834405-130835820
chr7	130865419	130867671	chr7-130865419-130867671
chr7	130935402	130939926	chr7-130935402-130939926
chr7	130940812	130941237	chr7-130940812-130941237
chr7	130964916	130966139	chr7-130964916-130966139
chr7	130967883	130969072	chr7-130967883-130969072
chr7	130977549	130980301	chr7-130977549-130980301
chr7	130981939	130983491	chr7-130981939-130983491
chr7	131235600	131236065	chr7-131235600-131236065
chr7	131361299	131363748	chr7-131361299-131363748
chr7	131370900	131372178	chr7-131370900-131372178
chr7	131409052	131411724	chr7-131409052-131411724

chr7	131541934	131544817	chr7-131541934-131544817
chr7	131552375	131556155	chr7-131552375-131556155
chr7	131560205	131562576	chr7-131560205-131562576
chr7	132154545	132154808	chr7-132154545-132154808
chr7	132315318	132317708	chr7-132315318-132317708
chr7	132345799	132346177	chr7-132345799-132346177
chr7	132551297	132552087	chr7-132551297-132552087
chr7	132575188	132576847	chr7-132575188-132576847
chr7	132597089	132597873	chr7-132597089-132597873
chr7	132599324	132600001	chr7-132599324-132600001
chr7	132610287	132611147	chr7-132610287-132611147
chr7	132656185	132656644	chr7-132656185-132656644
chr7	132735674	132736232	chr7-132735674-132736232
chr7	132811515	132814541	chr7-132811515-132814541
chr7	132851691	132853110	chr7-132851691-132853110
chr7	132858704	132860394	chr7-132858704-132860394
chr7	132901178	132901379	chr7-132901178-132901379
chr7	132930146	132933168	chr7-132930146-132933168
chr7	133007500	133007802	chr7-133007500-133007802
chr7	133040285	133042695	chr7-133040285-133042695
chr7	133085303	133085504	chr7-133085303-133085504
chr7	133088818	133089279	chr7-133088818-133089279
chr7	133111776	133118238	chr7-133111776-133118238
chr7	133119080	133125855	chr7-133119080-133125855
chr7	133161763	133162509	chr7-133161763-133162509
chr7	133186284	133186485	chr7-133186284-133186485
chr7	133244533	133244886	chr7-133244533-133244886
chr7	133382103	133382609	chr7-133382103-133382609
chr7	133636843	133639156	chr7-133636843-133639156
chr7	133708049	133710633	chr7-133708049-133710633
chr7	133762513	133763200	chr7-133762513-133763200
chr7	133775263	133777315	chr7-133775263-133777315
chr7	133780881	133783376	chr7-133780881-133783376
chr7	133788406	133788657	chr7-133788406-133788657
chr7	133792940	133795365	chr7-133792940-133795365
chr7	133846808	133847576	chr7-133846808-133847576
chr7	134032488	134033172	chr7-134032488-134033172
chr7	134080053	134081113	chr7-134080053-134081113
chr7	134114661	134114932	chr7-134114661-134114932
chr7	134186028	134186229	chr7-134186028-134186229
chr7	134222333	134226491	chr7-134222333-134226491
chr7	134262949	134263851	chr7-134262949-134263851

chr7	134287665	134287866	chr7-134287665-134287866
chr7	134619650	134620548	chr7-134619650-134620548
chr7	134670167	134673374	chr7-134670167-134673374
chr7	134773898	134774530	chr7-134773898-134774530
chr7	134937432	134938507	chr7-134937432-134938507
chr7	135465306	135465839	chr7-135465306-135465839
chr7	135509517	135509771	chr7-135509517-135509771
chr7	135537375	135538797	chr7-135537375-135538797
chr7	135604117	135610789	chr7-135604117-135610789
chr7	135651513	135654267	chr7-135651513-135654267
chr7	135712988	135717360	chr7-135712988-135717360
chr7	135735717	135736567	chr7-135735717-135736567
chr7	136894225	136894792	chr7-136894225-136894792
chr7	137309185	137311260	chr7-137309185-137311260
chr7	137312988	137315567	chr7-137312988-137315567
chr7	137321478	137323163	chr7-137321478-137323163
chr7	137409649	137411692	chr7-137409649-137411692
chr7	137437321	137439315	chr7-137437321-137439315
chr7	137478445	137478849	chr7-137478445-137478849
chr7	138397362	138397840	chr7-138397362-138397840
chr7	138844951	138848631	chr7-138844951-138848631
chr7	138906772	138910523	chr7-138906772-138910523
chr7	138919001	138920025	chr7-138919001-138920025
chr7	138940259	138941622	chr7-138940259-138941622
chr7	139020891	139021201	chr7-139020891-139021201
chr7	139085708	139086809	chr7-139085708-139086809
chr7	139156134	139158137	chr7-139156134-139158137
chr7	139168899	139169691	chr7-139168899-139169691
chr7	139187296	139189122	chr7-139187296-139189122
chr7	139208693	139208955	chr7-139208693-139208955
chr7	139212367	139214042	chr7-139212367-139214042
chr7	139246201	139254139	chr7-139246201-139254139
chr7	139255524	139260877	chr7-139255524-139260877
chr7	139264197	139264467	chr7-139264197-139264467
chr7	139266113	139267938	chr7-139266113-139267938
chr7	139272975	139273946	chr7-139272975-139273946
chr7	139279492	139283908	chr7-139279492-139283908
chr7	139285468	139286218	chr7-139285468-139286218
chr7	139363728	139364950	chr7-139363728-139364950
chr7	139383070	139383397	chr7-139383070-139383397
chr7	139387870	139390986	chr7-139387870-139390986
chr7	139467586	139468160	chr7-139467586-139468160

chr7	139516012	139517000	chr7-139516012-139517000
chr7	139581450	139583256	chr7-139581450-139583256
chr7	139584248	139585492	chr7-139584248-139585492
chr7	139683595	139684882	chr7-139683595-139684882
chr7	139833534	139834827	chr7-139833534-139834827
chr7	139843678	139844308	chr7-139843678-139844308
chr7	139894559	139895478	chr7-139894559-139895478
chr7	139914073	139914941	chr7-139914073-139914941
chr7	139943641	139944887	chr7-139943641-139944887
chr7	139949919	139951555	chr7-139949919-139951555
chr7	139970989	139972554	chr7-139970989-139972554
chr7	139975761	139976116	chr7-139975761-139976116
chr7	139977977	139979410	chr7-139977977-139979410
chr7	139990668	139993015	chr7-139990668-139993015
chr7	140034944	140037656	chr7-140034944-140037656
chr7	140047608	140049957	chr7-140047608-140049957
chr7	140057633	140058648	chr7-140057633-140058648
chr7	140061213	140061523	chr7-140061213-140061523
chr7	140077914	140078243	chr7-140077914-140078243
chr7	140080472	140081194	chr7-140080472-140081194
chr7	140101794	140102655	chr7-140101794-140102655
chr7	140115156	140117388	chr7-140115156-140117388
chr7	140121543	140121744	chr7-140121543-140121744
chr7	140122672	140123027	chr7-140122672-140123027
chr7	140125150	140128116	chr7-140125150-140128116
chr7	140137241	140138730	chr7-140137241-140138730
chr7	140821351	140822469	chr7-140821351-140822469
chr7	140828225	140828578	chr7-140828225-140828578
chr7	140834992	140836055	chr7-140834992-140836055
chr7	140854816	140859228	chr7-140854816-140859228
chr7	140881166	140883522	chr7-140881166-140883522
chr7	140908644	140909268	chr7-140908644-140909268
chr7	140922154	140923256	chr7-140922154-140923256
chr7	140941229	140942698	chr7-140941229-140942698
chr7	140954315	140956485	chr7-140954315-140956485
chr7	141010254	141010980	chr7-141010254-141010980
chr7	141047630	141049028	chr7-141047630-141049028
chr7	141060134	141062258	chr7-141060134-141062258
chr7	141070160	141070963	chr7-141070160-141070963
chr7	141078830	141081370	chr7-141078830-141081370
chr7	141092073	141092838	chr7-141092073-141092838
chr7	141094687	141097314	chr7-141094687-141097314

chr7	141099155	141101061	chr7-141099155-141101061
chr7	141116644	141118628	chr7-141116644-141118628
chr7	141122363	141124092	chr7-141122363-141124092
chr7	141130756	141132704	chr7-141130756-141132704
chr7	141164350	141165507	chr7-141164350-141165507
chr7	141166996	141169407	chr7-141166996-141169407
chr7	141171567	141173707	chr7-141171567-141173707
chr7	141191995	141195490	chr7-141191995-141195490
chr7	141214289	141222610	chr7-141214289-141222610
chr7	141228174	141230972	chr7-141228174-141230972
chr7	141265738	141266804	chr7-141265738-141266804
chr7	141269046	141269466	chr7-141269046-141269466
chr7	141278689	141279626	chr7-141278689-141279626
chr7	141291777	141292485	chr7-141291777-141292485
chr7	141293681	141294919	chr7-141293681-141294919
chr7	141325245	141329325	chr7-141325245-141329325
chr7	141337310	141342143	chr7-141337310-141342143
chr7	141372222	141372783	chr7-141372222-141372783
chr7	141391436	141393943	chr7-141391436-141393943
chr7	141412983	141415121	chr7-141412983-141415121
chr7	141416558	141416759	chr7-141416558-141416759
chr7	141434870	141437975	chr7-141434870-141437975
chr7	141442322	141444425	chr7-141442322-141444425
chr7	141446535	141450506	chr7-141446535-141450506
chr7	141454659	141458043	chr7-141454659-141458043
chr7	141460811	141462961	chr7-141460811-141462961
chr7	141464553	141470725	chr7-141464553-141470725
chr7	141472846	141478583	chr7-141472846-141478583
chr7	141481843	141482355	chr7-141481843-141482355
chr7	141538345	141541480	chr7-141538345-141541480
chr7	141561394	141564050	chr7-141561394-141564050
chr7	141569730	141570089	chr7-141569730-141570089
chr7	141584845	141585441	chr7-141584845-141585441
chr7	141693500	141693904	chr7-141693500-141693904
chr7	141900733	141903113	chr7-141900733-141903113
chr7	141911170	141911731	chr7-141911170-141911731
chr7	141949034	141950884	chr7-141949034-141950884
chr7	142059588	142061924	chr7-142059588-142061924
chr7	142070979	142071648	chr7-142070979-142071648
chr7	142081936	142085728	chr7-142081936-142085728
chr7	142088798	142088999	chr7-142088798-142088999
chr7	142092988	142096523	chr7-142092988-142096523

chr7	142283729	142285540	chr7-142283729-142285540
chr7	142303022	142304359	chr7-142303022-142304359
chr7	142362848	142363279	chr7-142362848-142363279
chr7	142369830	142372977	chr7-142369830-142372977
chr7	142374586	142376618	chr7-142374586-142376618
chr7	142385236	142388361	chr7-142385236-142388361
chr7	142392976	142393694	chr7-142392976-142393694
chr7	142409872	142411189	chr7-142409872-142411189
chr7	142430134	142430778	chr7-142430134-142430778
chr7	142433473	142439207	chr7-142433473-142439207
chr7	142470429	142471314	chr7-142470429-142471314
chr7	142481340	142482520	chr7-142481340-142482520
chr7	142532404	142534636	chr7-142532404-142534636
chr7	142578035	142578385	chr7-142578035-142578385
chr7	142579966	142580874	chr7-142579966-142580874
chr7	142581774	142582075	chr7-142581774-142582075
chr7	142658166	142661053	chr7-142658166-142661053
chr7	142663860	142664299	chr7-142663860-142664299
chr7	142967236	142969527	chr7-142967236-142969527
chr7	143008251	143009870	chr7-143008251-143009870
chr7	143013388	143018175	chr7-143013388-143018175
chr7	143052100	143056150	chr7-143052100-143056150
chr7	143062520	143063193	chr7-143062520-143063193
chr7	143068283	143071187	chr7-143068283-143071187
chr7	143106750	143109434	chr7-143106750-143109434
chr7	143119683	143120791	chr7-143119683-143120791
chr7	143122311	143122565	chr7-143122311-143122565
chr7	143123743	143125284	chr7-143123743-143125284
chr7	143153927	143154381	chr7-143153927-143154381
chr7	143181086	143181348	chr7-143181086-143181348
chr7	143201517	143201792	chr7-143201517-143201792
chr7	143293959	143297153	chr7-143293959-143297153
chr7	143313688	143314168	chr7-143313688-143314168
chr7	143367563	143368218	chr7-143367563-143368218
chr7	143437742	143438558	chr7-143437742-143438558
chr7	143445310	143446183	chr7-143445310-143446183
chr7	143458965	143461651	chr7-143458965-143461651
chr7	143463734	143465532	chr7-143463734-143465532
chr7	143473462	143474563	chr7-143473462-143474563
chr7	143500461	143503762	chr7-143500461-143503762
chr7	143517487	143517770	chr7-143517487-143517770
chr7	143547283	143550107	chr7-143547283-143550107

chr7	143563587	143563944	chr7-143563587-143563944
chr7	143598293	143601263	chr7-143598293-143601263
chr7	143614136	143615270	chr7-143614136-143615270
chr7	143624351	143628628	chr7-143624351-143628628
chr7	143648255	143649870	chr7-143648255-143649870
chr7	143684298	143686033	chr7-143684298-143686033
chr7	143690493	143692152	chr7-143690493-143692152
chr7	143723582	143724985	chr7-143723582-143724985
chr7	143737987	143741436	chr7-143737987-143741436
chr7	143748273	143748882	chr7-143748273-143748882
chr7	143756506	143757742	chr7-143756506-143757742
chr7	143783051	143786552	chr7-143783051-143786552
chr7	143797948	143801535	chr7-143797948-143801535
chr7	143821767	143823697	chr7-143821767-143823697
chr7	143829431	143831717	chr7-143829431-143831717
chr7	143977626	143979752	chr7-143977626-143979752
chr7	144038374	144038783	chr7-144038374-144038783
chr7	144054950	144055554	chr7-144054950-144055554
chr7	144120293	144120574	chr7-144120293-144120574
chr7	144233247	144233500	chr7-144233247-144233500
chr7	144417328	144418376	chr7-144417328-144418376
chr7	144446171	144450342	chr7-144446171-144450342
chr7	144467500	144471217	chr7-144467500-144471217
chr7	144551057	144554627	chr7-144551057-144554627
chr7	144563312	144565161	chr7-144563312-144565161
chr7	144566719	144568007	chr7-144566719-144568007
chr7	144578204	144578508	chr7-144578204-144578508
chr7	144580670	144583648	chr7-144580670-144583648
chr7	144675106	144680093	chr7-144675106-144680093
chr7	144684627	144685799	chr7-144684627-144685799
chr7	144746131	144752517	chr7-144746131-144752517
chr7	144778618	144778870	chr7-144778618-144778870
chr7	144779681	144780797	chr7-144779681-144780797
chr7	144838547	144839745	chr7-144838547-144839745
chr7	144861001	144862321	chr7-144861001-144862321
chr7	144883165	144883469	chr7-144883165-144883469
chr7	144895291	144897426	chr7-144895291-144897426
chr7	144914409	144916459	chr7-144914409-144916459
chr7	144930060	144930311	chr7-144930060-144930311
chr7	144932492	144944155	chr7-144932492-144944155
chr7	144945089	144946033	chr7-144945089-144946033
chr7	144964143	144965378	chr7-144964143-144965378

chr7	144970791	144973852	chr7-144970791-144973852
chr7	144981109	144983341	chr7-144981109-144983341
chr7	145024570	145026072	chr7-145024570-145026072
chr7	145030625	145031349	chr7-145030625-145031349
chr7	145043565	145043766	chr7-145043565-145043766
chr7	145045021	145045842	chr7-145045021-145045842
chr7	145048698	145049075	chr7-145048698-145049075
chr7	145105731	145113283	chr7-145105731-145113283
chr7	145131100	145132006	chr7-145131100-145132006
chr7	145194008	145195911	chr7-145194008-145195911
chr7	145198175	145198814	chr7-145198175-145198814
chr7	145205930	145206883	chr7-145205930-145206883
chr7	145282962	145284711	chr7-145282962-145284711
chr7	145329832	145330033	chr7-145329832-145330033
chr8	3220400	3222245	chr8-3220400-3222245
chr8	3247605	3247806	chr8-3247605-3247806
chr8	3252290	3253450	chr8-3252290-3253450
chr8	3277971	3280277	chr8-3277971-3280277
chr8	3392489	3395656	chr8-3392489-3395656
chr8	3411103	3414647	chr8-3411103-3414647
chr8	3417366	3418715	chr8-3417366-3418715
chr8	3425368	3427533	chr8-3425368-3427533
chr8	3430838	3431039	chr8-3430838-3431039
chr8	3466598	3468567	chr8-3466598-3468567
chr8	3492663	3494268	chr8-3492663-3494268
chr8	3499432	3501721	chr8-3499432-3501721
chr8	3514714	3517961	chr8-3514714-3517961
chr8	3587091	3589519	chr8-3587091-3589519
chr8	3606202	3606917	chr8-3606202-3606917
chr8	3608692	3610524	chr8-3608692-3610524
chr8	3620178	3622665	chr8-3620178-3622665
chr8	3630509	3632522	chr8-3630509-3632522
chr8	3671897	3672722	chr8-3671897-3672722
chr8	3675850	3678076	chr8-3675850-3678076
chr8	3754349	3754600	chr8-3754349-3754600
chr8	3994232	3994586	chr8-3994232-3994586
chr8	4165293	4167242	chr8-4165293-4167242
chr8	4193456	4193707	chr8-4193456-4193707
chr8	4212668	4215241	chr8-4212668-4215241
chr8	4216782	4217513	chr8-4216782-4217513
chr8	4226451	4227518	chr8-4226451-4227518
chr8	4230963	4231769	chr8-4230963-4231769

chr8	4237558	4240511	chr8-4237558-4240511
chr8	4252292	4254586	chr8-4252292-4254586
chr8	4257894	4259772	chr8-4257894-4259772
chr8	4274545	4276736	chr8-4274545-4276736
chr8	4322647	4326792	chr8-4322647-4326792
chr8	4404400	4405341	chr8-4404400-4405341
chr8	4492854	4494046	chr8-4492854-4494046
chr8	4612376	4614567	chr8-4612376-4614567
chr8	4656518	4656920	chr8-4656518-4656920
chr8	4664477	4664678	chr8-4664477-4664678
chr8	4676461	4679179	chr8-4676461-4679179
chr8	4777826	4779759	chr8-4777826-4779759
chr8	8236782	8237161	chr8-8236782-8237161
chr8	8256185	8256539	chr8-8256185-8256539
chr8	8274444	8275040	chr8-8274444-8275040
chr8	8453880	8454081	chr8-8453880-8454081
chr8	8487278	8489263	chr8-8487278-8489263
chr8	8576429	8577439	chr8-8576429-8577439
chr8	8601971	8606346	chr8-8601971-8606346
chr8	8643747	8645701	chr8-8643747-8645701
chr8	8647499	8649016	chr8-8647499-8649016
chr8	8649907	8650108	chr8-8649907-8650108
chr8	8655258	8662308	chr8-8655258-8662308
chr8	8688128	8690997	chr8-8688128-8690997
chr8	8797491	8797981	chr8-8797491-8797981
chr8	8969009	8969384	chr8-8969009-8969384
chr8	8983593	8983794	chr8-8983593-8983794
chr8	8992613	8992814	chr8-8992613-8992814
chr8	9770203	9772071	chr8-9770203-9772071
chr8	9975257	9979380	chr8-9975257-9979380
chr8	10007027	10007621	chr8-10007027-10007621
chr8	10131110	10131423	chr8-10131110-10131423
chr8	10153430	10155116	chr8-10153430-10155116
chr8	10415285	10415486	chr8-10415285-10415486
chr8	10534664	10535032	chr8-10534664-10535032
chr8	10587181	10587913	chr8-10587181-10587913
chr8	10589089	10590437	chr8-10589089-10590437
chr8	10629495	10630052	chr8-10629495-10630052
chr8	10646080	10646416	chr8-10646080-10646416
chr8	10665117	10665689	chr8-10665117-10665689
chr8	10909287	10911079	chr8-10909287-10911079
chr8	10921500	10926848	chr8-10921500-10926848

chr8	10928497	10929083	chr8-10928497-10929083
chr8	10945119	10947106	chr8-10945119-10947106
chr8	10953566	10954142	chr8-10953566-10954142
chr8	10961614	10962480	chr8-10961614-10962480
chr8	10964461	10967171	chr8-10964461-10967171
chr8	10968628	10969571	chr8-10968628-10969571
chr8	10977088	10979276	chr8-10977088-10979276
chr8	10990790	10991201	chr8-10990790-10991201
chr8	10993320	10995054	chr8-10993320-10995054
chr8	10997268	10997899	chr8-10997268-10997899
chr8	11000981	11010271	chr8-11000981-11010271
chr8	11079934	11080195	chr8-11079934-11080195
chr8	11156213	11156566	chr8-11156213-11156566
chr8	11173156	11174028	chr8-11173156-11174028
chr8	11187536	11188400	chr8-11187536-11188400
chr8	11258698	11260911	chr8-11258698-11260911
chr8	11309978	11317459	chr8-11309978-11317459
chr8	11322689	11324155	chr8-11322689-11324155
chr8	11331479	11331870	chr8-11331479-11331870
chr8	11340001	11341164	chr8-11340001-11341164
chr8	11346287	11347399	chr8-11346287-11347399
chr8	11371402	11372267	chr8-11371402-11372267
chr8	11381613	11382759	chr8-11381613-11382759
chr8	11387133	11387537	chr8-11387133-11387537
chr8	11393129	11393743	chr8-11393129-11393743
chr8	11475586	11479485	chr8-11475586-11479485
chr8	11496765	11499174	chr8-11496765-11499174
chr8	11549721	11551463	chr8-11549721-11551463
chr8	11554495	11559505	chr8-11554495-11559505
chr8	11632694	11637222	chr8-11632694-11637222
chr8	11700091	11700392	chr8-11700091-11700392
chr8	11727258	11728971	chr8-11727258-11728971
chr8	11757416	11759985	chr8-11757416-11759985
chr8	11766568	11768873	chr8-11766568-11768873
chr8	11770428	11770686	chr8-11770428-11770686
chr8	11774088	11776256	chr8-11774088-11776256
chr8	11777147	11777509	chr8-11777147-11777509
chr8	11778751	11781050	chr8-11778751-11781050
chr8	11801417	11801773	chr8-11801417-11801773
chr8	11924065	11924421	chr8-11924065-11924421
chr8	12073300	12073781	chr8-12073300-12073781
chr8	12099889	12100141	chr8-12099889-12100141

chr8	12149085	12149336	chr8-12149085-12149336
chr8	12163122	12163530	chr8-12163122-12163530
chr8	12384008	12386519	chr8-12384008-12386519
chr8	12394996	12397661	chr8-12394996-12397661
chr8	12430627	12431376	chr8-12430627-12431376
chr8	12477910	12478111	chr8-12477910-12478111
chr8	12502115	12503115	chr8-12502115-12503115
chr8	12572779	12573439	chr8-12572779-12573439
chr8	12662336	12662537	chr8-12662336-12662537
chr8	12671117	12673025	chr8-12671117-12673025
chr8	12756327	12759205	chr8-12756327-12759205
chr8	12765164	12765365	chr8-12765164-12765365
chr8	12768849	12769207	chr8-12768849-12769207
chr8	12778991	12780164	chr8-12778991-12780164
chr8	12786257	12786508	chr8-12786257-12786508
chr8	12873093	12874879	chr8-12873093-12874879
chr8	12915251	12916016	chr8-12915251-12916016
chr8	12992617	12993029	chr8-12992617-12993029
chr8	12996285	12997680	chr8-12996285-12997680
chr8	13103511	13107542	chr8-13103511-13107542
chr8	13144536	13144737	chr8-13144536-13144737
chr8	13158466	13161559	chr8-13158466-13161559
chr8	13199817	13201577	chr8-13199817-13201577
chr8	13204289	13204552	chr8-13204289-13204552
chr8	13273414	13273818	chr8-13273414-13273818
chr8	13286589	13289269	chr8-13286589-13289269
chr8	13338032	13341684	chr8-13338032-13341684
chr8	13373401	13373666	chr8-13373401-13373666
chr8	13435518	13435846	chr8-13435518-13435846
chr8	13492653	13494950	chr8-13492653-13494950
chr8	13530783	13531106	chr8-13530783-13531106
chr8	13594503	13595384	chr8-13594503-13595384
chr8	13623924	13624525	chr8-13623924-13624525
chr8	13643849	13644840	chr8-13643849-13644840
chr8	13648743	13649158	chr8-13648743-13649158
chr8	13650866	13651067	chr8-13650866-13651067
chr8	13668058	13669017	chr8-13668058-13669017
chr8	13675567	13678646	chr8-13675567-13678646
chr8	13694822	13696840	chr8-13694822-13696840
chr8	13757051	13759310	chr8-13757051-13759310
chr8	13784544	13786748	chr8-13784544-13786748
chr8	13868861	13871016	chr8-13868861-13871016

chr8	13889218	13890773	chr8-13889218-13890773
chr8	13907092	13908994	chr8-13907092-13908994
chr8	13973434	13975686	chr8-13973434-13975686
chr8	14089408	14091068	chr8-14089408-14091068
chr8	14095402	14097386	chr8-14095402-14097386
chr8	14683479	14684192	chr8-14683479-14684192
chr8	14887810	14890828	chr8-14887810-14890828
chr8	14910762	14912929	chr8-14910762-14912929
chr8	14913985	14914237	chr8-14913985-14914237
chr8	14969687	14971044	chr8-14969687-14971044
chr8	14991803	14993418	chr8-14991803-14993418
chr8	15010750	15012311	chr8-15010750-15012311
chr8	15026809	15029209	chr8-15026809-15029209
chr8	17535075	17535480	chr8-17535075-17535480
chr8	18564389	18564842	chr8-18564389-18564842
chr8	18594640	18596640	chr8-18594640-18596640
chr8	18699051	18699811	chr8-18699051-18699811
chr8	18767142	18769519	chr8-18767142-18769519
chr8	18845146	18848773	chr8-18845146-18848773
chr8	18898505	18899649	chr8-18898505-18899649
chr8	18918279	18918970	chr8-18918279-18918970
chr8	18950162	18951530	chr8-18950162-18951530
chr8	19161730	19162300	chr8-19161730-19162300
chr8	19535169	19535734	chr8-19535169-19535734
chr8	19784421	19785599	chr8-19784421-19785599
chr8	19979751	19981035	chr8-19979751-19981035
chr8	20121879	20123931	chr8-20121879-20123931
chr8	20296024	20297819	chr8-20296024-20297819
chr8	20423978	20425172	chr8-20423978-20425172
chr8	20549645	20551735	chr8-20549645-20551735
chr8	20557166	20558253	chr8-20557166-20558253
chr8	20590552	20590753	chr8-20590552-20590753
chr8	22039205	22039943	chr8-22039205-22039943
chr8	22059628	22061866	chr8-22059628-22061866
chr8	22124749	22125166	chr8-22124749-22125166
chr8	22165474	22166942	chr8-22165474-22166942
chr8	22184371	22186501	chr8-22184371-22186501
chr8	22192616	22194679	chr8-22192616-22194679
chr8	22226934	22227893	chr8-22226934-22227893
chr8	22303164	22303470	chr8-22303164-22303470
chr8	22397591	22399490	chr8-22397591-22399490
chr8	22410601	22412594	chr8-22410601-22412594

chr8	22433064	22433526	chr8-22433064-22433526
chr8	22475689	22479633	chr8-22475689-22479633
chr8	22491602	22492782	chr8-22491602-22492782
chr8	22506087	22506667	chr8-22506087-22506667
chr8	22591351	22594799	chr8-22591351-22594799
chr8	22652430	22653966	chr8-22652430-22653966
chr8	22693029	22693995	chr8-22693029-22693995
chr8	22705219	22707275	chr8-22705219-22707275
chr8	22708518	22708719	chr8-22708518-22708719
chr8	22744674	22745234	chr8-22744674-22745234
chr8	22754319	22754638	chr8-22754319-22754638
chr8	22756880	22759745	chr8-22756880-22759745
chr8	22805026	22809263	chr8-22805026-22809263
chr8	22847849	22848100	chr8-22847849-22848100
chr8	22858318	22861141	chr8-22858318-22861141
chr8	22872961	22873262	chr8-22872961-22873262
chr8	22882391	22883158	chr8-22882391-22883158
chr8	22973987	22975866	chr8-22973987-22975866
chr8	23027851	23028412	chr8-23027851-23028412
chr8	23034918	23035647	chr8-23034918-23035647
chr8	23037607	23039246	chr8-23037607-23039246
chr8	23057921	23059245	chr8-23057921-23059245
chr8	23131220	23133141	chr8-23131220-23133141
chr8	23144489	23145058	chr8-23144489-23145058
chr8	23148989	23150546	chr8-23148989-23150546
chr8	23157430	23158263	chr8-23157430-23158263
chr8	23165643	23169904	chr8-23165643-23169904
chr8	23189194	23189826	chr8-23189194-23189826
chr8	23198549	23199851	chr8-23198549-23199851
chr8	23206532	23209261	chr8-23206532-23209261
chr8	23236656	23237919	chr8-23236656-23237919
chr8	23255155	23258054	chr8-23255155-23258054
chr8	23410822	23412684	chr8-23410822-23412684
chr8	23435357	23435558	chr8-23435357-23435558
chr8	23516718	23517128	chr8-23516718-23517128
chr8	23563145	23563682	chr8-23563145-23563682
chr8	23669462	23669921	chr8-23669462-23669921
chr8	23682873	23683354	chr8-23682873-23683354
chr8	24204011	24204728	chr8-24204011-24204728
chr8	24359044	24359416	chr8-24359044-24359416
chr8	24403478	24403679	chr8-24403478-24403679
chr8	24405071	24405384	chr8-24405071-24405384

chr8	24419462	24420828	chr8-24419462-24420828
chr8	24431042	24431973	chr8-24431042-24431973
chr8	24433142	24433543	chr8-24433142-24433543
chr8	24437248	24439548	chr8-24437248-24439548
chr8	24442144	24442420	chr8-24442144-24442420
chr8	24479679	24480065	chr8-24479679-24480065
chr8	24488796	24488997	chr8-24488796-24488997
chr8	24517987	24519547	chr8-24517987-24519547
chr8	24521157	24521417	chr8-24521157-24521417
chr8	24554214	24555164	chr8-24554214-24555164
chr8	24568448	24568802	chr8-24568448-24568802
chr8	24823672	24824174	chr8-24823672-24824174
chr8	25009608	25018679	chr8-25009608-25018679
chr8	25027771	25028339	chr8-25027771-25028339
chr8	25037941	25038991	chr8-25037941-25038991
chr8	25066380	25071171	chr8-25066380-25071171
chr8	25097330	25104172	chr8-25097330-25104172
chr8	25108583	25108835	chr8-25108583-25108835
chr8	25139363	25139746	chr8-25139363-25139746
chr8	25200577	25202286	chr8-25200577-25202286
chr8	25240313	25241274	chr8-25240313-25241274
chr8	25255770	25257162	chr8-25255770-25257162
chr8	25263258	25263939	chr8-25263258-25263939
chr8	25274553	25275625	chr8-25274553-25275625
chr8	25304282	25305840	chr8-25304282-25305840
chr8	25428969	25429322	chr8-25428969-25429322
chr8	25463778	25464091	chr8-25463778-25464091
chr8	25465601	25466559	chr8-25465601-25466559
chr8	25518402	25520501	chr8-25518402-25520501
chr8	25521422	25521859	chr8-25521422-25521859
chr8	25537201	25537666	chr8-25537201-25537666
chr8	25543188	25543866	chr8-25543188-25543866
chr8	25596468	25598198	chr8-25596468-25598198
chr8	25600939	25604420	chr8-25600939-25604420
chr8	25624941	25625194	chr8-25624941-25625194
chr8	25636662	25637190	chr8-25636662-25637190
chr8	25719688	25721305	chr8-25719688-25721305
chr8	25753321	25755801	chr8-25753321-25755801
chr8	25784034	25786819	chr8-25784034-25786819
chr8	25804581	25804782	chr8-25804581-25804782
chr8	25805799	25806698	chr8-25805799-25806698
chr8	25839232	25842419	chr8-25839232-25842419

chr8	25975657	25977390	chr8-25975657-25977390
chr8	25990221	25990422	chr8-25990221-25990422
chr8	25992870	25994558	chr8-25992870-25994558
chr8	26014747	26016298	chr8-26014747-26016298
chr8	26084904	26085514	chr8-26084904-26085514
chr8	26117652	26120644	chr8-26117652-26120644
chr8	26156938	26159702	chr8-26156938-26159702
chr8	26168705	26169368	chr8-26168705-26169368
chr8	26273447	26273648	chr8-26273447-26273648
chr8	26352099	26352300	chr8-26352099-26352300
chr8	26362115	26363095	chr8-26362115-26363095
chr8	26438794	26439148	chr8-26438794-26439148
chr8	26454316	26454618	chr8-26454316-26454618
chr8	26502728	26503192	chr8-26502728-26503192
chr8	26747127	26747488	chr8-26747127-26747488
chr8	26831670	26832001	chr8-26831670-26832001
chr8	26854113	26854865	chr8-26854113-26854865
chr8	26887231	26888797	chr8-26887231-26888797
chr8	26963114	26963492	chr8-26963114-26963492
chr8	26972772	26981855	chr8-26972772-26981855
chr8	27022997	27025910	chr8-27022997-27025910
chr8	27041878	27043894	chr8-27041878-27043894
chr8	27057370	27059163	chr8-27057370-27059163
chr8	27084594	27088897	chr8-27084594-27088897
chr8	27105660	27113654	chr8-27105660-27113654
chr8	27114453	27114654	chr8-27114453-27114654
chr8	27115593	27115794	chr8-27115593-27115794
chr8	27127414	27129094	chr8-27127414-27129094
chr8	27134543	27135260	chr8-27134543-27135260
chr8	27137124	27137443	chr8-27137124-27137443
chr8	27140210	27140511	chr8-27140210-27140511
chr8	27168245	27168515	chr8-27168245-27168515
chr8	27171962	27175496	chr8-27171962-27175496
chr8	27176561	27178288	chr8-27176561-27178288
chr8	27206172	27207136	chr8-27206172-27207136
chr8	27226925	27228684	chr8-27226925-27228684
chr8	27234762	27235038	chr8-27234762-27235038
chr8	27244564	27245620	chr8-27244564-27245620
chr8	27259335	27266328	chr8-27259335-27266328
chr8	27361951	27362359	chr8-27361951-27362359
chr8	29218749	29220132	chr8-29218749-29220132
chr8	30958969	30959662	chr8-30958969-30959662

chr8	31002495	31002696	chr8-31002495-31002696
chr8	31031736	31033604	chr8-31031736-31033604
chr8	31089457	31090278	chr8-31089457-31090278
chr8	31105896	31107777	chr8-31105896-31107777
chr8	31111099	31113634	chr8-31111099-31113634
chr8	31122351	31122667	chr8-31122351-31122667
chr8	31148638	31152747	chr8-31148638-31152747
chr8	31167276	31169697	chr8-31167276-31169697
chr8	31176239	31176710	chr8-31176239-31176710
chr8	31186815	31188030	chr8-31186815-31188030
chr8	31372771	31373248	chr8-31372771-31373248
chr8	31520577	31520895	chr8-31520577-31520895
chr8	32004308	32004509	chr8-32004308-32004509
chr8	32006297	32010394	chr8-32006297-32010394
chr8	32200008	32200430	chr8-32200008-32200430
chr8	32210109	32210310	chr8-32210109-32210310
chr8	32213913	32214484	chr8-32213913-32214484
chr8	32220781	32221763	chr8-32220781-32221763
chr8	32284598	32284799	chr8-32284598-32284799
chr8	32285988	32287669	chr8-32285988-32287669
chr8	32292545	32293810	chr8-32292545-32293810
chr8	32883359	32885152	chr8-32883359-32885152
chr8	32989407	32989758	chr8-32989407-32989758
chr8	33384069	33387091	chr8-33384069-33387091
chr8	33516578	33517937	chr8-33516578-33517937
chr8	33599184	33608762	chr8-33599184-33608762
chr8	33641025	33642743	chr8-33641025-33642743
chr8	33646727	33647039	chr8-33646727-33647039
chr8	33652249	33656274	chr8-33652249-33656274
chr8	33658418	33658619	chr8-33658418-33658619
chr8	33659964	33660808	chr8-33659964-33660808
chr8	33663101	33663742	chr8-33663101-33663742
chr8	33672745	33675037	chr8-33672745-33675037
chr8	33731160	33733947	chr8-33731160-33733947
chr8	33805402	33805769	chr8-33805402-33805769
chr8	33871452	33871923	chr8-33871452-33871923
chr8	33879623	33880517	chr8-33879623-33880517
chr8	33884885	33886257	chr8-33884885-33886257
chr8	33902624	33903526	chr8-33902624-33903526
chr8	33913099	33915991	chr8-33913099-33915991
chr8	33916806	33917439	chr8-33916806-33917439
chr8	33927221	33931991	chr8-33927221-33931991

chr8	33978427	33979219	chr8-33978427-33979219
chr8	33985824	33988195	chr8-33985824-33988195
chr8	34055370	34056402	chr8-34055370-34056402
chr8	34066159	34066472	chr8-34066159-34066472
chr8	34107137	34109289	chr8-34107137-34109289
chr8	34123788	34124655	chr8-34123788-34124655
chr8	34137614	34137866	chr8-34137614-34137866
chr8	34144051	34147970	chr8-34144051-34147970
chr8	34154005	34155589	chr8-34154005-34155589
chr8	34173465	34173716	chr8-34173465-34173716
chr8	34225177	34225774	chr8-34225177-34225774
chr8	34276842	34277043	chr8-34276842-34277043
chr8	34288432	34288917	chr8-34288432-34288917
chr8	34472576	34472986	chr8-34472576-34472986
chr8	34529795	34530599	chr8-34529795-34530599
chr8	34591214	34591926	chr8-34591214-34591926
chr8	34614139	34614527	chr8-34614139-34614527
chr8	34622307	34622929	chr8-34622307-34622929
chr8	34688387	34690684	chr8-34688387-34690684
chr8	34760765	34761779	chr8-34760765-34761779
chr8	34763447	34764257	chr8-34763447-34764257
chr8	34804461	34816740	chr8-34804461-34816740
chr8	34952634	34955037	chr8-34952634-34955037
chr8	34963427	34966525	chr8-34963427-34966525
chr8	35177153	35177726	chr8-35177153-35177726
chr8	35205213	35206231	chr8-35205213-35206231
chr8	35321422	35321824	chr8-35321422-35321824
chr8	35375501	35377461	chr8-35375501-35377461
chr8	35434021	35434567	chr8-35434021-35434567
chr8	35440416	35441010	chr8-35440416-35441010
chr8	35494012	35496331	chr8-35494012-35496331
chr8	35587324	35593688	chr8-35587324-35593688
chr8	35599946	35600366	chr8-35599946-35600366
chr8	35707940	35708444	chr8-35707940-35708444
chr8	35725377	35725578	chr8-35725377-35725578
chr8	35765104	35766166	chr8-35765104-35766166
chr8	35825147	35827280	chr8-35825147-35827280
chr8	35837507	35838049	chr8-35837507-35838049
chr8	35850230	35850879	chr8-35850230-35850879
chr8	35865531	35866369	chr8-35865531-35866369
chr8	35944391	35944762	chr8-35944391-35944762
chr8	35946334	35946846	chr8-35946334-35946846

chr8	35970909	35971669	chr8-35970909-35971669
chr8	36027945	36028346	chr8-36027945-36028346
chr8	36093809	36097344	chr8-36093809-36097344
chr8	36137039	36137727	chr8-36137039-36137727
chr8	36155077	36156038	chr8-36155077-36156038
chr8	36163893	36164282	chr8-36163893-36164282
chr8	36183909	36184160	chr8-36183909-36184160
chr8	36186983	36187571	chr8-36186983-36187571
chr8	36247520	36250151	chr8-36247520-36250151
chr8	36457298	36458708	chr8-36457298-36458708
chr8	36549147	36549348	chr8-36549147-36549348
chr8	36559723	36559974	chr8-36559723-36559974
chr8	36610400	36614755	chr8-36610400-36614755
chr8	36663300	36664178	chr8-36663300-36664178
chr8	36677898	36678251	chr8-36677898-36678251
chr8	36690485	36690686	chr8-36690485-36690686
chr8	36727332	36727585	chr8-36727332-36727585
chr8	36730016	36730337	chr8-36730016-36730337
chr8	36731796	36733201	chr8-36731796-36733201
chr8	36993131	36994656	chr8-36993131-36994656
chr8	38659840	38661269	chr8-38659840-38661269
chr8	39005381	39007264	chr8-39005381-39007264
chr8	40286107	40287050	chr8-40286107-40287050
chr8	40307061	40309021	chr8-40307061-40309021
chr8	40331201	40331402	chr8-40331201-40331402
chr8	40423034	40424947	chr8-40423034-40424947
chr8	40510348	40513719	chr8-40510348-40513719
chr8	40594894	40595203	chr8-40594894-40595203
chr8	40610019	40610479	chr8-40610019-40610479
chr8	40633945	40635524	chr8-40633945-40635524
chr8	40861982	40863110	chr8-40861982-40863110
chr8	40926095	40926919	chr8-40926095-40926919
chr8	41016494	41016849	chr8-41016494-41016849
chr8	41114979	41115243	chr8-41114979-41115243
chr8	41132748	41134659	chr8-41132748-41134659
chr8	41239039	41241379	chr8-41239039-41241379
chr8	41348760	41349230	chr8-41348760-41349230
chr8	41367722	41367977	chr8-41367722-41367977
chr8	41371140	41371341	chr8-41371140-41371341
chr8	41372390	41375833	chr8-41372390-41375833
chr8	41389452	41389708	chr8-41389452-41389708
chr8	41396222	41397069	chr8-41396222-41397069

chr8	41415425	41417558	chr8-41415425-41417558
chr8	43791679	43792184	chr8-43791679-43792184
chr8	44261108	44261309	chr8-44261108-44261309
chr8	44687135	44687336	chr8-44687135-44687336
chr8	44877196	44878395	chr8-44877196-44878395
chr8	44879274	44879475	chr8-44879274-44879475
chr8	44882879	44883249	chr8-44882879-44883249
chr8	44920573	44920774	chr8-44920573-44920774
chr8	44925397	44925598	chr8-44925397-44925598
chr8	44933287	44940369	chr8-44933287-44940369
chr8	44974745	44974946	chr8-44974745-44974946
chr8	44977912	44979081	chr8-44977912-44979081
chr8	44984744	44985121	chr8-44984744-44985121
chr8	45001162	45002041	chr8-45001162-45002041
chr8	45062662	45064457	chr8-45062662-45064457
chr8	45068894	45069357	chr8-45068894-45069357
chr8	45109870	45110616	chr8-45109870-45110616
chr8	45332545	45334077	chr8-45332545-45334077
chr8	45357654	45359176	chr8-45357654-45359176
chr8	45381277	45381843	chr8-45381277-45381843
chr8	45409088	45411775	chr8-45409088-45411775
chr8	45579239	45581560	chr8-45579239-45581560
chr8	45598189	45598598	chr8-45598189-45598598
chr8	45602105	45602306	chr8-45602105-45602306
chr8	45610471	45610672	chr8-45610471-45610672
chr8	45614532	45615639	chr8-45614532-45615639
chr8	45626039	45631634	chr8-45626039-45631634
chr8	45637332	45637688	chr8-45637332-45637688
chr8	45719596	45720515	chr8-45719596-45720515
chr8	45884502	45885831	chr8-45884502-45885831
chr8	45934514	45935026	chr8-45934514-45935026
chr8	45974273	45976581	chr8-45974273-45976581
chr8	45999031	46000880	chr8-45999031-46000880
chr8	46005405	46005828	chr8-46005405-46005828
chr8	46150788	46153132	chr8-46150788-46153132
chr8	46162534	46164862	chr8-46162534-46164862
chr8	46208529	46212021	chr8-46208529-46212021
chr8	46216774	46217327	chr8-46216774-46217327
chr8	46293612	46294992	chr8-46293612-46294992
chr8	46299104	46299492	chr8-46299104-46299492
chr8	46429598	46430624	chr8-46429598-46430624
chr8	46446167	46446884	chr8-46446167-46446884

chr8	46470381	46472179	chr8-46470381-46472179
chr8	46502570	46503181	chr8-46502570-46503181
chr8	46551133	46553400	chr8-46551133-46553400
chr8	46615947	46619625	chr8-46615947-46619625
chr8	46639249	46639554	chr8-46639249-46639554
chr8	46648084	46648340	chr8-46648084-46648340
chr8	46651611	46652379	chr8-46651611-46652379
chr8	46686376	46687463	chr8-46686376-46687463
chr8	46738042	46743073	chr8-46738042-46743073
chr8	46780545	46781873	chr8-46780545-46781873
chr8	46811161	46811420	chr8-46811161-46811420
chr8	46829435	46829636	chr8-46829435-46829636
chr8	46868264	46870260	chr8-46868264-46870260
chr8	46940854	46941717	chr8-46940854-46941717
chr8	47288354	47289530	chr8-47288354-47289530
chr8	47412250	47414198	chr8-47412250-47414198
chr8	47532258	47535013	chr8-47532258-47535013
chr8	47650729	47651156	chr8-47650729-47651156
chr8	47673033	47677434	chr8-47673033-47677434
chr8	47712056	47715240	chr8-47712056-47715240
chr8	47749951	47750354	chr8-47749951-47750354
chr8	47807070	47807321	chr8-47807070-47807321
chr8	47917399	47917702	chr8-47917399-47917702
chr8	47926682	47927350	chr8-47926682-47927350
chr8	47931309	47934116	chr8-47931309-47934116
chr8	47935535	47935880	chr8-47935535-47935880
chr8	47961856	47962216	chr8-47961856-47962216
chr8	47974091	47975996	chr8-47974091-47975996
chr8	47982905	47983209	chr8-47982905-47983209
chr8	47986734	47992217	chr8-47986734-47992217
chr8	48044052	48044253	chr8-48044052-48044253
chr8	48097457	48097774	chr8-48097457-48097774
chr8	48109343	48112662	chr8-48109343-48112662
chr8	48222010	48222416	chr8-48222010-48222416
chr8	48554813	48556140	chr8-48554813-48556140
chr8	48842622	48844315	chr8-48842622-48844315
chr8	50915875	50916558	chr8-50915875-50916558
chr8	53511566	53512687	chr8-53511566-53512687
chr8	53637927	53639413	chr8-53637927-53639413
chr8	54076703	54079410	chr8-54076703-54079410
chr8	54441066	54441368	chr8-54441066-54441368
chr8	54508888	54509548	chr8-54508888-54509548

chr8	54528281	54530593	chr8-54528281-54530593
chr8	54548582	54548891	chr8-54548582-54548891
chr8	54600383	54601247	chr8-54600383-54601247
chr8	54610058	54610385	chr8-54610058-54610385
chr8	54723958	54724834	chr8-54723958-54724834
chr8	54779340	54780383	chr8-54779340-54780383
chr8	56294277	56294888	chr8-56294277-56294888
chr8	56549704	56552799	chr8-56549704-56552799
chr8	57312507	57313212	chr8-57312507-57313212
chr8	57320751	57322419	chr8-57320751-57322419
chr8	57323545	57323949	chr8-57323545-57323949
chr8	57328304	57329297	chr8-57328304-57329297
chr8	57333718	57334557	chr8-57333718-57334557
chr8	57340788	57341349	chr8-57340788-57341349
chr8	57353679	57354200	chr8-57353679-57354200
chr8	57410493	57410694	chr8-57410493-57410694
chr8	57485637	57488889	chr8-57485637-57488889
chr8	57498306	57499907	chr8-57498306-57499907
chr8	57505746	57506080	chr8-57505746-57506080
chr8	57507604	57507805	chr8-57507604-57507805
chr8	57510656	57514254	chr8-57510656-57514254
chr8	57576451	57576721	chr8-57576451-57576721
chr8	57651181	57654470	chr8-57651181-57654470
chr8	57732924	57733125	chr8-57732924-57733125
chr8	57741403	57742066	chr8-57741403-57742066
chr8	57799721	57800326	chr8-57799721-57800326
chr8	58912020	58913564	chr8-58912020-58913564
chr8	60491351	60492759	chr8-60491351-60492759
chr8	60494601	60495152	chr8-60494601-60495152
chr8	60505690	60506333	chr8-60505690-60506333
chr8	60631365	60633925	chr8-60631365-60633925
chr8	60859848	60860049	chr8-60859848-60860049
chr8	60889912	60891922	chr8-60889912-60891922
chr8	60953752	60955450	chr8-60953752-60955450
chr8	60981599	60984281	chr8-60981599-60984281
chr8	60992116	60994648	chr8-60992116-60994648
chr8	61194013	61194783	chr8-61194013-61194783
chr8	61223293	61230105	chr8-61223293-61230105
chr8	61230956	61231309	chr8-61230956-61231309
chr8	61246818	61249035	chr8-61246818-61249035
chr8	61254301	61254745	chr8-61254301-61254745
chr8	61264990	61266184	chr8-61264990-61266184

chr8	61269802	61271793	chr8-61269802-61271793
chr8	61272849	61277226	chr8-61272849-61277226
chr8	61283673	61283874	chr8-61283673-61283874
chr8	61288356	61288616	chr8-61288356-61288616
chr8	61309448	61311075	chr8-61309448-61311075
chr8	61340314	61340515	chr8-61340314-61340515
chr8	61341360	61343026	chr8-61341360-61343026
chr8	61380219	61380420	chr8-61380219-61380420
chr8	61450648	61451980	chr8-61450648-61451980
chr8	61475043	61475942	chr8-61475043-61475942
chr8	61478516	61480614	chr8-61478516-61480614
chr8	61487390	61488958	chr8-61487390-61488958
chr8	61494925	61495751	chr8-61494925-61495751
chr8	61549562	61550182	chr8-61549562-61550182
chr8	61578549	61579216	chr8-61578549-61579216
chr8	61589532	61591842	chr8-61589532-61591842
chr8	61621396	61621597	chr8-61621396-61621597
chr8	61623613	61624027	chr8-61623613-61624027
chr8	61712480	61713798	chr8-61712480-61713798
chr8	61745166	61745854	chr8-61745166-61745854
chr8	61755020	61755342	chr8-61755020-61755342
chr8	61756894	61760483	chr8-61756894-61760483
chr8	61803313	61804558	chr8-61803313-61804558
chr8	61927621	61928794	chr8-61927621-61928794
chr8	62951175	62952163	chr8-62951175-62952163
chr8	63951878	63952389	chr8-63951878-63952389
chr8	64204223	64207046	chr8-64204223-64207046
chr8	64522356	64523678	chr8-64522356-64523678
chr8	64691604	64693486	chr8-64691604-64693486
chr8	64725884	64734124	chr8-64725884-64734124
chr8	64806744	64806945	chr8-64806744-64806945
chr8	64826547	64826869	chr8-64826547-64826869
chr8	64842082	64842446	chr8-64842082-64842446
chr8	64848135	64851005	chr8-64848135-64851005
chr8	64946328	64948153	chr8-64946328-64948153
chr8	65035427	65035832	chr8-65035427-65035832
chr8	65037077	65037536	chr8-65037077-65037536
chr8	65617907	65618576	chr8-65617907-65618576
chr8	66484853	66487111	chr8-66484853-66487111
chr8	66603688	66604150	chr8-66603688-66604150
chr8	66687537	66688299	chr8-66687537-66688299
chr8	66696979	66697884	chr8-66696979-66697884

chr8	66752159	66752417	chr8-66752159-66752417
chr8	66859910	66862893	chr8-66859910-66862893
chr8	67431342	67431595	chr8-67431342-67431595
chr8	67494506	67495763	chr8-67494506-67495763
chr8	67930987	67931324	chr8-67930987-67931324
chr8	68010499	68010700	chr8-68010499-68010700
chr8	68016245	68016602	chr8-68016245-68016602
chr8	68060896	68063058	chr8-68060896-68063058
chr8	68083368	68083885	chr8-68083368-68083885
chr8	68119955	68121734	chr8-68119955-68121734
chr8	68157070	68157271	chr8-68157070-68157271
chr8	68253189	68254006	chr8-68253189-68254006
chr8	68275631	68279783	chr8-68275631-68279783
chr8	68734365	68735882	chr8-68734365-68735882
chr8	68740495	68740696	chr8-68740495-68740696
chr8	68793109	68795153	chr8-68793109-68795153
chr8	68880503	68881753	chr8-68880503-68881753
chr8	69088261	69090265	chr8-69088261-69090265
chr8	69119043	69119345	chr8-69119043-69119345
chr8	69183415	69184884	chr8-69183415-69184884
chr8	69193368	69193633	chr8-69193368-69193633
chr8	69208446	69210584	chr8-69208446-69210584
chr8	69270984	69271487	chr8-69270984-69271487
chr8	69328933	69329436	chr8-69328933-69329436
chr8	69394281	69396553	chr8-69394281-69396553
chr8	69399272	69399884	chr8-69399272-69399884
chr8	69583935	69585182	chr8-69583935-69585182
chr8	69598005	69598206	chr8-69598005-69598206
chr8	69623972	69625982	chr8-69623972-69625982
chr8	69647949	69648355	chr8-69647949-69648355
chr8	69653969	69655360	chr8-69653969-69655360
chr8	69715256	69717838	chr8-69715256-69717838
chr8	69749256	69750599	chr8-69749256-69750599
chr8	69774001	69775421	chr8-69774001-69775421
chr8	69789731	69793180	chr8-69789731-69793180
chr8	69808386	69810041	chr8-69808386-69810041
chr8	69821643	69824467	chr8-69821643-69824467
chr8	69832074	69833667	chr8-69832074-69833667
chr8	69842839	69843334	chr8-69842839-69843334
chr8	69881154	69883297	chr8-69881154-69883297
chr8	69885919	69889319	chr8-69885919-69889319
chr8	69893386	69894832	chr8-69893386-69894832

chr8	69899869	69904333	chr8-69899869-69904333
chr8	69935599	69937166	chr8-69935599-69937166
chr8	69939714	69940659	chr8-69939714-69940659
chr8	69967093	69968902	chr8-69967093-69968902
chr8	69971053	69975133	chr8-69971053-69975133
chr8	69994266	69997716	chr8-69994266-69997716
chr8	70000874	70002229	chr8-70000874-70002229
chr8	70041111	70044372	chr8-70041111-70044372
chr8	70083058	70085096	chr8-70083058-70085096
chr8	70087809	70088627	chr8-70087809-70088627
chr8	70111526	70112897	chr8-70111526-70112897
chr8	70120676	70120985	chr8-70120676-70120985
chr8	70130843	70132904	chr8-70130843-70132904
chr8	70138026	70141260	chr8-70138026-70141260
chr8	70152416	70153003	chr8-70152416-70153003
chr8	70158137	70159792	chr8-70158137-70159792
chr8	70166535	70167298	chr8-70166535-70167298
chr8	70171808	70173630	chr8-70171808-70173630
chr8	70211451	70213553	chr8-70211451-70213553
chr8	70222079	70222340	chr8-70222079-70222340
chr8	70233193	70236294	chr8-70233193-70236294
chr8	70271527	70273125	chr8-70271527-70273125
chr8	70282182	70284672	chr8-70282182-70284672
chr8	70300988	70304119	chr8-70300988-70304119
chr8	70315238	70316663	chr8-70315238-70316663
chr8	70349218	70354488	chr8-70349218-70354488
chr8	70373392	70376812	chr8-70373392-70376812
chr8	70394391	70394923	chr8-70394391-70394923
chr8	70438427	70440422	chr8-70438427-70440422
chr8	70460029	70462784	chr8-70460029-70462784
chr8	70466875	70467415	chr8-70466875-70467415
chr8	70474982	70478385	chr8-70474982-70478385
chr8	70488145	70490093	chr8-70488145-70490093
chr8	70491808	70494840	chr8-70491808-70494840
chr8	70500585	70501485	chr8-70500585-70501485
chr8	70503255	70503565	chr8-70503255-70503565
chr8	70505207	70514195	chr8-70505207-70514195
chr8	70522016	70523880	chr8-70522016-70523880
chr8	70526513	70530047	chr8-70526513-70530047
chr8	70537957	70541996	chr8-70537957-70541996
chr8	70543865	70544655	chr8-70543865-70544655
chr8	70546539	70546794	chr8-70546539-70546794

chr8	70565563	70565764	chr8-70565563-70565764
chr8	70593371	70596093	chr8-70593371-70596093
chr8	70597685	70597886	chr8-70597685-70597886
chr8	70606402	70612209	chr8-70606402-70612209
chr8	70618503	70618869	chr8-70618503-70618869
chr8	70629350	70631869	chr8-70629350-70631869
chr8	70633925	70634723	chr8-70633925-70634723
chr8	70657519	70660836	chr8-70657519-70660836
chr8	70666964	70667539	chr8-70666964-70667539
chr8	70672430	70675344	chr8-70672430-70675344
chr8	70677612	70677813	chr8-70677612-70677813
chr8	70678996	70680528	chr8-70678996-70680528
chr8	70681970	70682945	chr8-70681970-70682945
chr8	70694858	70703551	chr8-70694858-70703551
chr8	70718414	70718870	chr8-70718414-70718870
chr8	70729844	70730419	chr8-70729844-70730419
chr8	70743918	70744638	chr8-70743918-70744638
chr8	70752740	70755634	chr8-70752740-70755634
chr8	70758628	70761586	chr8-70758628-70761586
chr8	70764432	70767466	chr8-70764432-70767466
chr8	70771829	70778666	chr8-70771829-70778666
chr8	70790812	70793540	chr8-70790812-70793540
chr8	70796248	70798600	chr8-70796248-70798600
chr8	70803608	70805522	chr8-70803608-70805522
chr8	70812343	70812605	chr8-70812343-70812605
chr8	70816615	70817421	chr8-70816615-70817421
chr8	70819905	70820868	chr8-70819905-70820868
chr8	70821757	70822011	chr8-70821757-70822011
chr8	70838420	70841094	chr8-70838420-70841094
chr8	70856388	70857403	chr8-70856388-70857403
chr8	70862431	70866481	chr8-70862431-70866481
chr8	70868771	70869079	chr8-70868771-70869079
chr8	70871864	70874264	chr8-70871864-70874264
chr8	70878085	70878641	chr8-70878085-70878641
chr8	70879641	70881565	chr8-70879641-70881565
chr8	70887585	70888047	chr8-70887585-70888047
chr8	70891143	70892979	chr8-70891143-70892979
chr8	70894873	70898896	chr8-70894873-70898896
chr8	70905257	70907344	chr8-70905257-70907344
chr8	71026323	71026828	chr8-71026323-71026828
chr8	71205345	71206261	chr8-71205345-71206261
chr8	71270855	71274697	chr8-71270855-71274697

chr8	71275956	71276708	chr8-71275956-71276708
chr8	71282045	71282353	chr8-71282045-71282353
chr8	71310862	71311114	chr8-71310862-71311114
chr8	71312390	71313628	chr8-71312390-71313628
chr8	71366154	71368926	chr8-71366154-71368926
chr8	71370513	71372293	chr8-71370513-71372293
chr8	71375665	71377042	chr8-71375665-71377042
chr8	71378319	71383705	chr8-71378319-71383705
chr8	71396211	71398123	chr8-71396211-71398123
chr8	71404951	71407619	chr8-71404951-71407619
chr8	71411945	71412247	chr8-71411945-71412247
chr8	71456793	71458236	chr8-71456793-71458236
chr8	71461563	71466105	chr8-71461563-71466105
chr8	71468568	71471067	chr8-71468568-71471067
chr8	71475471	71477711	chr8-71475471-71477711
chr8	71484345	71490532	chr8-71484345-71490532
chr8	71495140	71497308	chr8-71495140-71497308
chr8	71505552	71508415	chr8-71505552-71508415
chr8	71521356	71521610	chr8-71521356-71521610
chr8	71535854	71538772	chr8-71535854-71538772
chr8	71542411	71544381	chr8-71542411-71544381
chr8	71558006	71559646	chr8-71558006-71559646
chr8	71562475	71563161	chr8-71562475-71563161
chr8	71568584	71571629	chr8-71568584-71571629
chr8	71572707	71573312	chr8-71572707-71573312
chr8	71574831	71575702	chr8-71574831-71575702
chr8	71578940	71581051	chr8-71578940-71581051
chr8	71590850	71594022	chr8-71590850-71594022
chr8	71595868	71596386	chr8-71595868-71596386
chr8	71602186	71603937	chr8-71602186-71603937
chr8	71610347	71613111	chr8-71610347-71613111
chr8	71627212	71630563	chr8-71627212-71630563
chr8	71633228	71634827	chr8-71633228-71634827
chr8	71637593	71637850	chr8-71637593-71637850
chr8	71654352	71655251	chr8-71654352-71655251
chr8	71665042	71672566	chr8-71665042-71672566
chr8	71675316	71677815	chr8-71675316-71677815
chr8	71679680	71679881	chr8-71679680-71679881
chr8	71681189	71683262	chr8-71681189-71683262
chr8	71698531	71703712	chr8-71698531-71703712
chr8	71709521	71710328	chr8-71709521-71710328
chr8	71723950	71726344	chr8-71723950-71726344

chr8	71728474	71728727	chr8-71728474-71728727
chr8	71779694	71780796	chr8-71779694-71780796
chr8	71788873	71789440	chr8-71788873-71789440
chr8	71881663	71882632	chr8-71881663-71882632
chr8	71908193	71909322	chr8-71908193-71909322
chr8	71922086	71924250	chr8-71922086-71924250
chr8	71950127	71952398	chr8-71950127-71952398
chr8	71988413	71989035	chr8-71988413-71989035
chr8	72067194	72067446	chr8-72067194-72067446
chr8	72134676	72138714	chr8-72134676-72138714
chr8	72159682	72162795	chr8-72159682-72162795
chr8	72189550	72190098	chr8-72189550-72190098
chr8	72219306	72221178	chr8-72219306-72221178
chr8	72239316	72241289	chr8-72239316-72241289
chr8	72311105	72311460	chr8-72311105-72311460
chr8	72318239	72321597	chr8-72318239-72321597
chr8	72322611	72323042	chr8-72322611-72323042
chr8	72419817	72422484	chr8-72419817-72422484
chr8	72442988	72444701	chr8-72442988-72444701
chr8	72473684	72477041	chr8-72473684-72477041
chr8	72491023	72494489	chr8-72491023-72494489
chr8	72546684	72549834	chr8-72546684-72549834
chr8	72569937	72573165	chr8-72569937-72573165
chr8	72722894	72724606	chr8-72722894-72724606
chr8	73123823	73124161	chr8-73123823-73124161
chr8	73162101	73162464	chr8-73162101-73162464
chr8	73342590	73343233	chr8-73342590-73343233
chr8	73351206	73354988	chr8-73351206-73354988
chr8	74990170	74990473	chr8-74990170-74990473
chr8	74992817	74995106	chr8-74992817-74995106
chr8	75017851	75019085	chr8-75017851-75019085
chr8	75033132	75035063	chr8-75033132-75035063
chr8	75083394	75084222	chr8-75083394-75084222
chr8	75088807	75090267	chr8-75088807-75090267
chr8	75092732	75095695	chr8-75092732-75095695
chr8	75108394	75111381	chr8-75108394-75111381
chr8	75212608	75214183	chr8-75212608-75214183
chr8	76898560	76903541	chr8-76898560-76903541
chr8	76912403	76912824	chr8-76912403-76912824
chr8	77132697	77133340	chr8-77132697-77133340
chr8	77288019	77288220	chr8-77288019-77288220
chr8	77432915	77433353	chr8-77432915-77433353

chr8	77478634	77479045	chr8-77478634-77479045
chr8	77510605	77510857	chr8-77510605-77510857
chr8	77512000	77512444	chr8-77512000-77512444
chr8	77515827	77519777	chr8-77515827-77519777
chr8	77548954	77550493	chr8-77548954-77550493
chr8	77609401	77611219	chr8-77609401-77611219
chr8	78063595	78064549	chr8-78063595-78064549
chr8	78419998	78420620	chr8-78419998-78420620
chr8	78434873	78438449	chr8-78434873-78438449
chr8	78463810	78464082	chr8-78463810-78464082
chr8	78507309	78510727	chr8-78507309-78510727
chr8	78584545	78585352	chr8-78584545-78585352
chr8	78598306	78598507	chr8-78598306-78598507
chr8	78656576	78657182	chr8-78656576-78657182
chr8	78741723	78742959	chr8-78741723-78742959
chr8	78770954	78771949	chr8-78770954-78771949
chr8	78819162	78821668	chr8-78819162-78821668
chr8	79027257	79031561	chr8-79027257-79031561
chr8	79059768	79060019	chr8-79059768-79060019
chr8	79067938	79068404	chr8-79067938-79068404
chr8	79083449	79083650	chr8-79083449-79083650
chr8	79094584	79097528	chr8-79094584-79097528
chr8	79150678	79151085	chr8-79150678-79151085
chr8	79162716	79163034	chr8-79162716-79163034
chr8	79207887	79210588	chr8-79207887-79210588
chr8	79293211	79296196	chr8-79293211-79296196
chr8	79349735	79350733	chr8-79349735-79350733
chr8	79375101	79375302	chr8-79375101-79375302
chr8	79397532	79400071	chr8-79397532-79400071
chr8	79447493	79448106	chr8-79447493-79448106
chr8	79549973	79550819	chr8-79549973-79550819
chr8	79559433	79559634	chr8-79559433-79559634
chr8	79562211	79562518	chr8-79562211-79562518
chr8	79597079	79597280	chr8-79597079-79597280
chr8	79638015	79643839	chr8-79638015-79643839
chr8	79680486	79681047	chr8-79680486-79681047
chr8	79709121	79713182	chr8-79709121-79713182
chr8	79945667	79946019	chr8-79945667-79946019
chr8	80057113	80058520	chr8-80057113-80058520
chr8	80314801	80315881	chr8-80314801-80315881
chr8	80492611	80493846	chr8-80492611-80493846
chr8	80669201	80669719	chr8-80669201-80669719

chr8	80737424	80740703	chr8-80737424-80740703
chr8	80847629	80850155	chr8-80847629-80850155
chr8	80852360	80854108	chr8-80852360-80854108
chr8	80856155	80858926	chr8-80856155-80858926
chr8	80863322	80863580	chr8-80863322-80863580
chr8	80870710	80870911	chr8-80870710-80870911
chr8	80878318	80881245	chr8-80878318-80881245
chr8	80962047	80962913	chr8-80962047-80962913
chr8	81011647	81016356	chr8-81011647-81016356
chr8	81341445	81343042	chr8-81341445-81343042
chr8	81756766	81757922	chr8-81756766-81757922
chr8	81836385	81838236	chr8-81836385-81838236
chr8	81853809	81855447	chr8-81853809-81855447
chr8	81861435	81863414	chr8-81861435-81863414
chr8	81865484	81866514	chr8-81865484-81866514
chr8	82202171	82202372	chr8-82202171-82202372
chr8	82304164	82304626	chr8-82304164-82304626
chr8	82372970	82373231	chr8-82372970-82373231
chr8	82401583	82403460	chr8-82401583-82403460
chr8	82416860	82417114	chr8-82416860-82417114
chr8	82772869	82774878	chr8-82772869-82774878
chr8	82863012	82864742	chr8-82863012-82864742
chr8	83164907	83167313	chr8-83164907-83167313
chr8	83177416	83177776	chr8-83177416-83177776
chr8	83274475	83274809	chr8-83274475-83274809
chr8	83289975	83290751	chr8-83289975-83290751
chr8	83331486	83333100	chr8-83331486-83333100
chr8	83389604	83390406	chr8-83389604-83390406
chr8	83441449	83443259	chr8-83441449-83443259
chr8	83566437	83567994	chr8-83566437-83567994
chr8	83591955	83595390	chr8-83591955-83595390
chr8	83601791	83603467	chr8-83601791-83603467
chr8	83606395	83610295	chr8-83606395-83610295
chr8	83619427	83619986	chr8-83619427-83619986
chr8	83621960	83622832	chr8-83621960-83622832
chr8	83651708	83655027	chr8-83651708-83655027
chr8	83662497	83662850	chr8-83662497-83662850
chr8	83666655	83666856	chr8-83666655-83666856
chr8	83697110	83699982	chr8-83697110-83699982
chr8	83714597	83717598	chr8-83714597-83717598
chr8	83737596	83741892	chr8-83737596-83741892
chr8	83783067	83783370	chr8-83783067-83783370

chr8	83874672	83876861	chr8-83874672-83876861
chr8	83897205	83897613	chr8-83897205-83897613
chr8	83898930	83902184	chr8-83898930-83902184
chr8	83954506	83957168	chr8-83954506-83957168
chr8	83971737	83974556	chr8-83971737-83974556
chr8	83996066	84000224	chr8-83996066-84000224
chr8	84001305	84001609	chr8-84001305-84001609
chr8	84003060	84005467	chr8-84003060-84005467
chr8	84009867	84013120	chr8-84009867-84013120
chr8	84019669	84024562	chr8-84019669-84024562
chr8	84026033	84026234	chr8-84026033-84026234
chr8	84042042	84042853	chr8-84042042-84042853
chr8	84065539	84068686	chr8-84065539-84068686
chr8	84103049	84105540	chr8-84103049-84105540
chr8	84146210	84148813	chr8-84146210-84148813
chr8	84182773	84184560	chr8-84182773-84184560
chr8	84186519	84187593	chr8-84186519-84187593
chr8	84191071	84193489	chr8-84191071-84193489
chr8	84196805	84199105	chr8-84196805-84199105
chr8	84199958	84204582	chr8-84199958-84204582
chr8	84207926	84208127	chr8-84207926-84208127
chr8	84233192	84233393	chr8-84233192-84233393
chr8	84235389	84238345	chr8-84235389-84238345
chr8	84240955	84242127	chr8-84240955-84242127
chr8	84248420	84250227	chr8-84248420-84250227
chr8	84255844	84258024	chr8-84255844-84258024
chr8	84268868	84271195	chr8-84268868-84271195
chr8	84414074	84416030	chr8-84414074-84416030
chr8	84459044	84459707	chr8-84459044-84459707
chr8	84460837	84461092	chr8-84460837-84461092
chr8	84501054	84503084	chr8-84501054-84503084
chr8	84554383	84554949	chr8-84554383-84554949
chr8	84558958	84559762	chr8-84558958-84559762
chr8	84592194	84592566	chr8-84592194-84592566
chr8	84615768	84616394	chr8-84615768-84616394
chr8	84633819	84634020	chr8-84633819-84634020
chr8	84635736	84636760	chr8-84635736-84636760
chr8	84638074	84640666	chr8-84638074-84640666
chr8	84655695	84657866	chr8-84655695-84657866
chr8	84659230	84666338	chr8-84659230-84666338
chr8	84685922	84690772	chr8-84685922-84690772
chr8	84699018	84699345	chr8-84699018-84699345

chr8	84706808	84708079	chr8-84706808-84708079
chr8	84722504	84724161	chr8-84722504-84724161
chr8	84737742	84738050	chr8-84737742-84738050
chr8	84742465	84744697	chr8-84742465-84744697
chr8	84749871	84750143	chr8-84749871-84750143
chr8	84773823	84774272	chr8-84773823-84774272
chr8	84798544	84800895	chr8-84798544-84800895
chr8	84810886	84811393	chr8-84810886-84811393
chr8	84823472	84823930	chr8-84823472-84823930
chr8	84831400	84833678	chr8-84831400-84833678
chr8	84839460	84840877	chr8-84839460-84840877
chr8	84844524	84848331	chr8-84844524-84848331
chr8	84856510	84858673	chr8-84856510-84858673
chr8	84871657	84873026	chr8-84871657-84873026
chr8	84892545	84894541	chr8-84892545-84894541
chr8	84901912	84903965	chr8-84901912-84903965
chr8	84907999	84910072	chr8-84907999-84910072
chr8	84912427	84915832	chr8-84912427-84915832
chr8	84921773	84921974	chr8-84921773-84921974
chr8	84928527	84929564	chr8-84928527-84929564
chr8	84936626	84937785	chr8-84936626-84937785
chr8	84944271	84946763	chr8-84944271-84946763
chr8	84952581	84953504	chr8-84952581-84953504
chr8	84961921	84962384	chr8-84961921-84962384
chr8	84964745	84971583	chr8-84964745-84971583
chr8	84974471	84993426	chr8-84974471-84993426
chr8	85013250	85013815	chr8-85013250-85013815
chr8	85023901	85029747	chr8-85023901-85029747
chr8	85034565	85039520	chr8-85034565-85039520
chr8	85059439	85061666	chr8-85059439-85061666
chr8	85070731	85073627	chr8-85070731-85073627
chr8	85079444	85084494	chr8-85079444-85084494
chr8	85118931	85120196	chr8-85118931-85120196
chr8	85297729	85301526	chr8-85297729-85301526
chr8	85408274	85408578	chr8-85408274-85408578
chr8	85429198	85433428	chr8-85429198-85433428
chr8	85491775	85494091	chr8-85491775-85494091
chr8	85553801	85556403	chr8-85553801-85556403
chr8	85597624	85597883	chr8-85597624-85597883
chr8	85699935	85701505	chr8-85699935-85701505
chr8	85839633	85841956	chr8-85839633-85841956
chr8	86445811	86446122	chr8-86445811-86446122

chr8	86478071	86478476	chr8-86478071-86478476
chr8	86536247	86536549	chr8-86536247-86536549
chr8	86585132	86585947	chr8-86585132-86585947
chr8	86623352	86625617	chr8-86623352-86625617
chr8	86744135	86747016	chr8-86744135-86747016
chr8	86768655	86768931	chr8-86768655-86768931
chr8	86856049	86856353	chr8-86856049-86856353
chr8	86882799	86885997	chr8-86882799-86885997
chr8	87037857	87038476	chr8-87037857-87038476
chr8	87131104	87131803	chr8-87131104-87131803
chr8	87468724	87469124	chr8-87468724-87469124
chr8	87469998	87473816	chr8-87469998-87473816
chr8	87656430	87656908	chr8-87656430-87656908
chr8	87662885	87663086	chr8-87662885-87663086
chr8	87943597	87943966	chr8-87943597-87943966
chr8	87957986	87962354	chr8-87957986-87962354
chr8	87974662	87975034	chr8-87974662-87975034
chr8	88118103	88120382	chr8-88118103-88120382
chr8	88137444	88139213	chr8-88137444-88139213
chr8	88198510	88202012	chr8-88198510-88202012
chr8	88221017	88222460	chr8-88221017-88222460
chr8	88272089	88273116	chr8-88272089-88273116
chr8	88284815	88285224	chr8-88284815-88285224
chr8	88289008	88289383	chr8-88289008-88289383
chr8	88359975	88363219	chr8-88359975-88363219
chr8	88453916	88455834	chr8-88453916-88455834
chr8	88520829	88522359	chr8-88520829-88522359
chr8	88626897	88628110	chr8-88626897-88628110
chr8	88646947	88648412	chr8-88646947-88648412
chr8	88649636	88651021	chr8-88649636-88651021
chr8	88696139	88698942	chr8-88696139-88698942
chr8	88755267	88755680	chr8-88755267-88755680
chr8	89006390	89006861	chr8-89006390-89006861
chr8	89041032	89044325	chr8-89041032-89044325
chr8	89046152	89046522	chr8-89046152-89046522
chr8	89272381	89272683	chr8-89272381-89272683
chr8	90347705	90348765	chr8-90347705-90348765
chr8	90828214	90829663	chr8-90828214-90829663
chr8	90840036	90841008	chr8-90840036-90841008
chr8	90870562	90870763	chr8-90870562-90870763
chr8	90876106	90878193	chr8-90876106-90878193
chr8	90886168	90887466	chr8-90886168-90887466

chr8	90907436	90909838	chr8-90907436-90909838
chr8	90954901	90955764	chr8-90954901-90955764
chr8	91069416	91071448	chr8-91069416-91071448
chr8	91132512	91134977	chr8-91132512-91134977
chr8	91147980	91148181	chr8-91147980-91148181
chr8	91312515	91314777	chr8-91312515-91314777
chr8	91455599	91455871	chr8-91455599-91455871
chr8	91712798	91713154	chr8-91712798-91713154
chr8	91797456	91806925	chr8-91797456-91806925
chr8	91822986	91823239	chr8-91822986-91823239
chr8	92024089	92024639	chr8-92024089-92024639
chr8	92349953	92351704	chr8-92349953-92351704
chr8	92352782	92367216	chr8-92352782-92367216
chr8	92460105	92461150	chr8-92460105-92461150
chr8	92673516	92674799	chr8-92673516-92674799
chr8	92827315	92827784	chr8-92827315-92827784
chr8	92845882	92848012	chr8-92845882-92848012
chr8	92854741	92856496	chr8-92854741-92856496
chr8	92960361	92961366	chr8-92960361-92961366
chr8	92972873	92973140	chr8-92972873-92973140
chr8	93809847	93811663	chr8-93809847-93811663
chr8	93813208	93813714	chr8-93813208-93813714
chr8	94009920	94013298	chr8-94009920-94013298
chr8	94035033	94038363	chr8-94035033-94038363
chr8	94097679	94099249	chr8-94097679-94099249
chr8	94155873	94156074	chr8-94155873-94156074
chr8	94167176	94167491	chr8-94167176-94167491
chr8	94171560	94175100	chr8-94171560-94175100
chr8	94178308	94184932	chr8-94178308-94184932
chr8	94186102	94187351	chr8-94186102-94187351
chr8	94199710	94199962	chr8-94199710-94199962
chr8	94213851	94215734	chr8-94213851-94215734
chr8	94323157	94323358	chr8-94323157-94323358
chr8	94341342	94341543	chr8-94341342-94341543
chr8	94358437	94358638	chr8-94358437-94358638
chr8	94365775	94366090	chr8-94365775-94366090
chr8	94377585	94378291	chr8-94377585-94378291
chr8	94385535	94388712	chr8-94385535-94388712
chr8	94433705	94434827	chr8-94433705-94434827
chr8	94456489	94456690	chr8-94456489-94456690
chr8	94492096	94492760	chr8-94492096-94492760
chr8	94532170	94534342	chr8-94532170-94534342

chr8	94551205	94552152	chr8-94551205-94552152
chr8	94553418	94553723	chr8-94553418-94553723
chr8	94556703	94557838	chr8-94556703-94557838
chr8	94572165	94572366	chr8-94572165-94572366
chr8	94600315	94603293	chr8-94600315-94603293
chr8	94620255	94620561	chr8-94620255-94620561
chr8	94666011	94668082	chr8-94666011-94668082
chr8	94678288	94679857	chr8-94678288-94679857
chr8	94682315	94683360	chr8-94682315-94683360
chr8	94695292	94696716	chr8-94695292-94696716
chr8	94702522	94704940	chr8-94702522-94704940
chr8	94706777	94707353	chr8-94706777-94707353
chr8	94766673	94767191	chr8-94766673-94767191
chr8	94770786	94773322	chr8-94770786-94773322
chr8	94778463	94778821	chr8-94778463-94778821
chr8	94781043	94782233	chr8-94781043-94782233
chr8	94784689	94786117	chr8-94784689-94786117
chr8	94787555	94788664	chr8-94787555-94788664
chr8	94835985	94839525	chr8-94835985-94839525
chr8	94857209	94859251	chr8-94857209-94859251
chr8	94874857	94876754	chr8-94874857-94876754
chr8	94888552	94888964	chr8-94888552-94888964
chr8	94899151	94899503	chr8-94899151-94899503
chr8	94905097	94905465	chr8-94905097-94905465
chr8	94913045	94913718	chr8-94913045-94913718
chr8	94914793	94915132	chr8-94914793-94915132
chr8	94916190	94918551	chr8-94916190-94918551
chr8	94953770	94956138	chr8-94953770-94956138
chr8	94967221	94967673	chr8-94967221-94967673
chr8	94974122	94975090	chr8-94974122-94975090
chr8	94976315	94982077	chr8-94976315-94982077
chr8	94983016	94989299	chr8-94983016-94989299
chr8	94990868	94991184	chr8-94990868-94991184
chr8	94992440	94993056	chr8-94992440-94993056
chr8	94996563	95002436	chr8-94996563-95002436
chr8	95011565	95013180	chr8-95011565-95013180
chr8	95079946	95082273	chr8-95079946-95082273
chr8	95091736	95092198	chr8-95091736-95092198
chr8	95136755	95143629	chr8-95136755-95143629
chr8	95145826	95146198	chr8-95145826-95146198
chr8	95149002	95150388	chr8-95149002-95150388
chr8	95154508	95155229	chr8-95154508-95155229

chr8	95162397	95163190	chr8-95162397-95163190
chr8	95209973	95210275	chr8-95209973-95210275
chr8	95254964	95255373	chr8-95254964-95255373
chr8	95319986	95321207	chr8-95319986-95321207
chr8	95330264	95333568	chr8-95330264-95333568
chr8	95348670	95349107	chr8-95348670-95349107
chr8	95351570	95353811	chr8-95351570-95353811
chr8	95358994	95359363	chr8-95358994-95359363
chr8	95377643	95378139	chr8-95377643-95378139
chr8	95433111	95435541	chr8-95433111-95435541
chr8	95487236	95489375	chr8-95487236-95489375
chr8	95533668	95534484	chr8-95533668-95534484
chr8	95612198	95612449	chr8-95612198-95612449
chr8	95633155	95634771	chr8-95633155-95634771
chr8	95677221	95678878	chr8-95677221-95678878
chr8	95697101	95697302	chr8-95697101-95697302
chr8	95702191	95704291	chr8-95702191-95704291
chr8	95715210	95717514	chr8-95715210-95717514
chr8	95805459	95808511	chr8-95805459-95808511
chr8	95851977	95853904	chr8-95851977-95853904
chr8	95878158	95879202	chr8-95878158-95879202
chr8	95886403	95888903	chr8-95886403-95888903
chr8	95915871	95916529	chr8-95915871-95916529
chr8	95951885	95952239	chr8-95951885-95952239
chr8	102784802	102785434	chr8-102784802-102785434
chr8	104061684	104061885	chr8-104061684-104061885
chr8	104091168	104091591	chr8-104091168-104091591
chr8	104170210	104171471	chr8-104170210-104171471
chr8	104191542	104191903	chr8-104191542-104191903
chr8	104246839	104249097	chr8-104246839-104249097
chr8	104250541	104252192	chr8-104250541-104252192
chr8	104339840	104341627	chr8-104339840-104341627
chr8	104375008	104376201	chr8-104375008-104376201
chr8	104383118	104384566	chr8-104383118-104384566
chr8	104388122	104388323	chr8-104388122-104388323
chr8	104393609	104396441	chr8-104393609-104396441
chr8	104404794	104407353	chr8-104404794-104407353
chr8	104408642	104409102	chr8-104408642-104409102
chr8	104434447	104434704	chr8-104434447-104434704
chr8	104435725	104436236	chr8-104435725-104436236
chr8	104440985	104443546	chr8-104440985-104443546
chr8	104508864	104510096	chr8-104508864-104510096

chr8	104533278	104536096	chr8-104533278-104536096
chr8	104540520	104542369	chr8-104540520-104542369
chr8	104564740	104565146	chr8-104564740-104565146
chr8	104570566	104571377	chr8-104570566-104571377
chr8	104591036	104592817	chr8-104591036-104592817
chr8	104601808	104602240	chr8-104601808-104602240
chr8	104630139	104631986	chr8-104630139-104631986
chr8	104640181	104642802	chr8-104640181-104642802
chr8	105142367	105142823	chr8-105142367-105142823
chr8	105152730	105153543	chr8-105152730-105153543
chr8	105169727	105172870	chr8-105169727-105172870
chr8	105224316	105226667	chr8-105224316-105226667
chr8	105253871	105255222	chr8-105253871-105255222
chr8	105263708	105265774	chr8-105263708-105265774
chr8	105268512	105270323	chr8-105268512-105270323
chr8	105276247	105276705	chr8-105276247-105276705
chr8	105278665	105279764	chr8-105278665-105279764
chr8	105288137	105291044	chr8-105288137-105291044
chr8	105292246	105293182	chr8-105292246-105293182
chr8	105297002	105299695	chr8-105297002-105299695
chr8	105303399	105306928	chr8-105303399-105306928
chr8	105325349	105328407	chr8-105325349-105328407
chr8	105339558	105341621	chr8-105339558-105341621
chr8	105343420	105350579	chr8-105343420-105350579
chr8	105355079	105356118	chr8-105355079-105356118
chr8	105378239	105380970	chr8-105378239-105380970
chr8	105387589	105387890	chr8-105387589-105387890
chr8	105413283	105413843	chr8-105413283-105413843
chr8	105470642	105471992	chr8-105470642-105471992
chr8	105473905	105474901	chr8-105473905-105474901
chr8	105495687	105497305	chr8-105495687-105497305
chr8	105515833	105516678	chr8-105515833-105516678
chr8	105518075	105519840	chr8-105518075-105519840
chr8	105564942	105567455	chr8-105564942-105567455
chr8	105593743	105595251	chr8-105593743-105595251
chr8	105596327	105597089	chr8-105596327-105597089
chr8	105604555	105607414	chr8-105604555-105607414
chr8	105612961	105614827	chr8-105612961-105614827
chr8	105635383	105638563	chr8-105635383-105638563
chr8	105639815	105640178	chr8-105639815-105640178
chr8	105693565	105694586	chr8-105693565-105694586
chr8	105699865	105702456	chr8-105699865-105702456

chr8	105706618	105708647	chr8-105706618-105708647
chr8	105750337	105751492	chr8-105750337-105751492
chr8	105752571	105752772	chr8-105752571-105752772
chr8	105757224	105759370	chr8-105757224-105759370
chr8	105768629	105769158	chr8-105768629-105769158
chr8	105789007	105790800	chr8-105789007-105790800
chr8	105793430	105794618	chr8-105793430-105794618
chr8	105795606	105796493	chr8-105795606-105796493
chr8	105799105	105799848	chr8-105799105-105799848
chr8	105808684	105808953	chr8-105808684-105808953
chr8	105825890	105828457	chr8-105825890-105828457
chr8	105835509	105835975	chr8-105835509-105835975
chr8	105850401	105852369	chr8-105850401-105852369
chr8	105854344	105858551	chr8-105854344-105858551
chr8	105859604	105862732	chr8-105859604-105862732
chr8	105880452	105882443	chr8-105880452-105882443
chr8	105899684	105901780	chr8-105899684-105901780
chr8	105919699	105919900	chr8-105919699-105919900
chr8	105935810	105938591	chr8-105935810-105938591
chr8	105951158	105951487	chr8-105951158-105951487
chr8	105960530	105960907	chr8-105960530-105960907
chr8	105963845	105966630	chr8-105963845-105966630
chr8	106009750	106012352	chr8-106009750-106012352
chr8	106058251	106061975	chr8-106058251-106061975
chr8	106133504	106139510	chr8-106133504-106139510
chr8	106149791	106151466	chr8-106149791-106151466
chr8	106168122	106170646	chr8-106168122-106170646
chr8	106208918	106212172	chr8-106208918-106212172
chr8	106280010	106281174	chr8-106280010-106281174
chr8	106287528	106287983	chr8-106287528-106287983
chr8	106307562	106308591	chr8-106307562-106308591
chr8	106336432	106339139	chr8-106336432-106339139
chr8	106377107	106377308	chr8-106377107-106377308
chr8	106378228	106379154	chr8-106378228-106379154
chr8	106399548	106400803	chr8-106399548-106400803
chr8	106414733	106416742	chr8-106414733-106416742
chr8	106430785	106431742	chr8-106430785-106431742
chr8	106433094	106433501	chr8-106433094-106433501
chr8	106434991	106435728	chr8-106434991-106435728
chr8	106451361	106451720	chr8-106451361-106451720
chr8	106510156	106512834	chr8-106510156-106512834
chr8	106518523	106519679	chr8-106518523-106519679

chr8	106529547	106529748	chr8-106529547-106529748
chr8	106560834	106561813	chr8-106560834-106561813
chr8	106563799	106564625	chr8-106563799-106564625
chr8	106571715	106572489	chr8-106571715-106572489
chr8	106577021	106577760	chr8-106577021-106577760
chr8	106602372	106612381	chr8-106602372-106612381
chr8	106614673	106623370	chr8-106614673-106623370
chr8	106628316	106628517	chr8-106628316-106628517
chr8	106631549	106633905	chr8-106631549-106633905
chr8	106640112	106642015	chr8-106640112-106642015
chr8	106645915	106647345	chr8-106645915-106647345
chr8	106682636	106684040	chr8-106682636-106684040
chr8	106717753	106718234	chr8-106717753-106718234
chr8	106769398	106769708	chr8-106769398-106769708
chr8	106818329	106820266	chr8-106818329-106820266
chr8	106870247	106872726	chr8-106870247-106872726
chr8	106888690	106888991	chr8-106888690-106888991
chr8	106891780	106895123	chr8-106891780-106895123
chr8	106935073	106938169	chr8-106935073-106938169
chr8	106953516	106954192	chr8-106953516-106954192
chr8	106957623	106957973	chr8-106957623-106957973
chr8	106969736	106970087	chr8-106969736-106970087
chr8	106973725	106973926	chr8-106973725-106973926
chr8	106976079	106977492	chr8-106976079-106977492
chr8	107030753	107032785	chr8-107030753-107032785
chr8	107047109	107049730	chr8-107047109-107049730
chr8	107055038	107058304	chr8-107055038-107058304
chr8	107094836	107097268	chr8-107094836-107097268
chr8	107114204	107116200	chr8-107114204-107116200
chr8	107132991	107134354	chr8-107132991-107134354
chr8	107149982	107153635	chr8-107149982-107153635
chr8	107254352	107255679	chr8-107254352-107255679
chr8	107292541	107295380	chr8-107292541-107295380
chr8	107389729	107390093	chr8-107389729-107390093
chr8	107403106	107404060	chr8-107403106-107404060
chr8	107423243	107425403	chr8-107423243-107425403
chr8	107435416	107437657	chr8-107435416-107437657
chr8	107457272	107458467	chr8-107457272-107458467
chr8	107488856	107489057	chr8-107488856-107489057
chr8	107503474	107503808	chr8-107503474-107503808
chr8	107544626	107545364	chr8-107544626-107545364
chr8	107576689	107576890	chr8-107576689-107576890

chr8	107586143	107588954	chr8-107586143-107588954
chr8	108387225	108388114	chr8-108387225-108388114
chr8	108691683	108692649	chr8-108691683-108692649
chr8	108695908	108698055	chr8-108695908-108698055
chr8	108702947	108705375	chr8-108702947-108705375
chr8	108713554	108718989	chr8-108713554-108718989
chr8	108722263	108724003	chr8-108722263-108724003
chr8	108725247	108727162	chr8-108725247-108727162
chr8	108754746	108755068	chr8-108754746-108755068
chr8	108758338	108759831	chr8-108758338-108759831
chr8	108764027	108765647	chr8-108764027-108765647
chr8	108797658	108798563	chr8-108797658-108798563
chr8	108802349	108802607	chr8-108802349-108802607
chr8	108886538	108887577	chr8-108886538-108887577
chr8	108896026	108897962	chr8-108896026-108897962
chr8	108898955	108899866	chr8-108898955-108899866
chr8	109075237	109075438	chr8-109075237-109075438
chr8	109352761	109353071	chr8-109352761-109353071
chr8	109564084	109567112	chr8-109564084-109567112
chr8	109575802	109576159	chr8-109575802-109576159
chr8	109586995	109587247	chr8-109586995-109587247
chr8	109607310	109609648	chr8-109607310-109609648
chr8	109691053	109694565	chr8-109691053-109694565
chr8	109704592	109706919	chr8-109704592-109706919
chr8	109736187	109738170	chr8-109736187-109738170
chr8	109749405	109751455	chr8-109749405-109751455
chr8	109766050	109766688	chr8-109766050-109766688
chr8	109773365	109773716	chr8-109773365-109773716
chr8	109778016	109780793	chr8-109778016-109780793
chr8	109810442	109810762	chr8-109810442-109810762
chr8	109867949	109869881	chr8-109867949-109869881
chr8	109921298	109921604	chr8-109921298-109921604
chr8	109956977	109958938	chr8-109956977-109958938
chr8	109960332	109962529	chr8-109960332-109962529
chr8	109969186	109969387	chr8-109969186-109969387
chr8	110039315	110039928	chr8-110039315-110039928
chr8	110040741	110041012	chr8-110040741-110041012
chr8	110079258	110080578	chr8-110079258-110080578
chr8	110217299	110219287	chr8-110217299-110219287
chr8	110222804	110223422	chr8-110222804-110223422
chr8	110266653	110267486	chr8-110266653-110267486
chr8	110617886	110620120	chr8-110617886-110620120

chr8	110630690	110631153	chr8-110630690-110631153
chr8	110720890	110722717	chr8-110720890-110722717
chr8	110754860	110755483	chr8-110754860-110755483
chr8	110780525	110780815	chr8-110780525-110780815
chr8	110786893	110787194	chr8-110786893-110787194
chr8	110788664	110794841	chr8-110788664-110794841
chr8	110802885	110806169	chr8-110802885-110806169
chr8	110844435	110848487	chr8-110844435-110848487
chr8	110901170	110902678	chr8-110901170-110902678
chr8	110919241	110920881	chr8-110919241-110920881
chr8	110940640	110943089	chr8-110940640-110943089
chr8	110965245	110966702	chr8-110965245-110966702
chr8	110969865	110971293	chr8-110969865-110971293
chr8	110995504	110995705	chr8-110995504-110995705
chr8	110996602	110998652	chr8-110996602-110998652
chr8	111026778	111028518	chr8-111026778-111028518
chr8	111029747	111030376	chr8-111029747-111030376
chr8	111033250	111036202	chr8-111033250-111036202
chr8	111055971	111059594	chr8-111055971-111059594
chr8	111060835	111062722	chr8-111060835-111062722
chr8	111093846	111095961	chr8-111093846-111095961
chr8	111144372	111146346	chr8-111144372-111146346
chr8	111237363	111237881	chr8-111237363-111237881
chr8	111257424	111260180	chr8-111257424-111260180
chr8	111298443	111301175	chr8-111298443-111301175
chr8	111336637	111338932	chr8-111336637-111338932
chr8	111360188	111361356	chr8-111360188-111361356
chr8	111370115	111370544	chr8-111370115-111370544
chr8	111393007	111394259	chr8-111393007-111394259
chr8	111435719	111435920	chr8-111435719-111435920
chr8	111439090	111439346	chr8-111439090-111439346
chr8	111449445	111449853	chr8-111449445-111449853
chr8	111520739	111522690	chr8-111520739-111522690
chr8	111535673	111539876	chr8-111535673-111539876
chr8	111540960	111541161	chr8-111540960-111541161
chr8	111555167	111555571	chr8-111555167-111555571
chr8	111596837	111597093	chr8-111596837-111597093
chr8	111615291	111615542	chr8-111615291-111615542
chr8	111623883	111624652	chr8-111623883-111624652
chr8	111629417	111630831	chr8-111629417-111630831
chr8	111642432	111645020	chr8-111642432-111645020
chr8	111724575	111736755	chr8-111724575-111736755

chr8	111739300	111747362	chr8-111739300-111747362
chr8	111748174	111748561	chr8-111748174-111748561
chr8	111754086	111754358	chr8-111754086-111754358
chr8	111781698	111782996	chr8-111781698-111782996
chr8	111852645	111854772	chr8-111852645-111854772
chr8	111875590	111877100	chr8-111875590-111877100
chr8	111890127	111890639	chr8-111890127-111890639
chr8	111909942	111910507	chr8-111909942-111910507
chr8	111923308	111923811	chr8-111923308-111923811
chr8	111932362	111934116	chr8-111932362-111934116
chr8	111939257	111941866	chr8-111939257-111941866
chr8	111991101	111992812	chr8-111991101-111992812
chr8	112009628	112013344	chr8-112009628-112013344
chr8	113523384	113523648	chr8-113523384-113523648
chr8	113634260	113636843	chr8-113634260-113636843
chr8	113848208	113849040	chr8-113848208-113849040
chr8	114133181	114134673	chr8-114133181-114134673
chr8	114205477	114206135	chr8-114205477-114206135
chr8	114438971	114441031	chr8-114438971-114441031
chr8	114634296	114634877	chr8-114634296-114634877
chr8	114777542	114778009	chr8-114777542-114778009
chr8	115529747	115530062	chr8-115529747-115530062
chr8	115705578	115709246	chr8-115705578-115709246
chr8	115748941	115749192	chr8-115748941-115749192
chr8	115764938	115765241	chr8-115764938-115765241
chr8	115766424	115766677	chr8-115766424-115766677
chr8	115770881	115771791	chr8-115770881-115771791
chr8	116004432	116004701	chr8-116004432-116004701
chr8	116116125	116116377	chr8-116116125-116116377
chr8	116504514	116505095	chr8-116504514-116505095
chr8	116587864	116588065	chr8-116587864-116588065
chr8	116731668	116733820	chr8-116731668-116733820
chr8	116863297	116863928	chr8-116863297-116863928
chr8	116920300	116922860	chr8-116920300-116922860
chr8	116943093	116945207	chr8-116943093-116945207
chr8	116978124	116979645	chr8-116978124-116979645
chr8	116991564	116994316	chr8-116991564-116994316
chr8	117074097	117075398	chr8-117074097-117075398
chr8	117085220	117086375	chr8-117085220-117086375
chr8	117094643	117094844	chr8-117094643-117094844
chr8	117111715	117112514	chr8-117111715-117112514
chr8	117115247	117116431	chr8-117115247-117116431

chr8	117121145	117122017	chr8-117121145-117122017
chr8	117123983	117124703	chr8-117123983-117124703
chr8	117136651	117137769	chr8-117136651-117137769
chr8	117155708	117159857	chr8-117155708-117159857
chr8	117224714	117225140	chr8-117224714-117225140
chr8	117256010	117263351	chr8-117256010-117263351
chr8	117267488	117268461	chr8-117267488-117268461
chr8	117279751	117284422	chr8-117279751-117284422
chr8	117334487	117335714	chr8-117334487-117335714
chr8	117357862	117358468	chr8-117357862-117358468
chr8	117361616	117364488	chr8-117361616-117364488
chr8	117371870	117373371	chr8-117371870-117373371
chr8	117474962	117475163	chr8-117474962-117475163
chr8	117497829	117499604	chr8-117497829-117499604
chr8	117561164	117563052	chr8-117561164-117563052
chr8	117673047	117673992	chr8-117673047-117673992
chr8	117742651	117743070	chr8-117742651-117743070
chr8	117800485	117803106	chr8-117800485-117803106
chr8	118282859	118290940	chr8-118282859-118290940
chr8	118311504	118312082	chr8-118311504-118312082
chr8	118313366	118315165	chr8-118313366-118315165
chr8	118502691	118502949	chr8-118502691-118502949
chr8	118566233	118566535	chr8-118566233-118566535
chr8	118679663	118680023	chr8-118679663-118680023
chr8	119182843	119183094	chr8-119182843-119183094
chr8	119343564	119345937	chr8-119343564-119345937
chr8	119394227	119396070	chr8-119394227-119396070
chr8	119412826	119413385	chr8-119412826-119413385
chr8	119418911	119420097	chr8-119418911-119420097
chr8	119420963	119433252	chr8-119420963-119433252
chr8	119436284	119441718	chr8-119436284-119441718
chr8	119446600	119446976	chr8-119446600-119446976
chr8	119490533	119491299	chr8-119490533-119491299
chr8	119496659	119497024	chr8-119496659-119497024
chr8	119557167	119559603	chr8-119557167-119559603
chr8	119573758	119575838	chr8-119573758-119575838
chr8	119585792	119586400	chr8-119585792-119586400
chr8	119603826	119605713	chr8-119603826-119605713
chr8	119670483	119671309	chr8-119670483-119671309
chr8	119678287	119678708	chr8-119678287-119678708
chr8	119685681	119687920	chr8-119685681-119687920
chr8	119688942	119689356	chr8-119688942-119689356

chr8	119696625	119701906	chr8-119696625-119701906
chr8	119708618	119712826	chr8-119708618-119712826
chr8	119714747	119716860	chr8-119714747-119716860
chr8	119755295	119756140	chr8-119755295-119756140
chr8	119777181	119779178	chr8-119777181-119779178
chr8	119798567	119799039	chr8-119798567-119799039
chr8	119817108	119817700	chr8-119817108-119817700
chr8	119839601	119841297	chr8-119839601-119841297
chr8	119844513	119844877	chr8-119844513-119844877
chr8	119861728	119863089	chr8-119861728-119863089
chr8	119885397	119886182	chr8-119885397-119886182
chr8	119895190	119896267	chr8-119895190-119896267
chr8	119910005	119914022	chr8-119910005-119914022
chr8	119992101	119992737	chr8-119992101-119992737
chr8	120075408	120075949	chr8-120075408-120075949
chr8	120096080	120097283	chr8-120096080-120097283
chr8	120100269	120102853	chr8-120100269-120102853
chr8	120112995	120115171	chr8-120112995-120115171
chr8	120177384	120179353	chr8-120177384-120179353
chr8	120190884	120191464	chr8-120190884-120191464
chr8	120201206	120201761	chr8-120201206-120201761
chr8	120224688	120232429	chr8-120224688-120232429
chr8	120264761	120265235	chr8-120264761-120265235
chr8	120275925	120277477	chr8-120275925-120277477
chr8	120308260	120309029	chr8-120308260-120309029
chr8	120317238	120320072	chr8-120317238-120320072
chr8	120368572	120369692	chr8-120368572-120369692
chr8	120378275	120378873	chr8-120378275-120378873
chr8	120382333	120383229	chr8-120382333-120383229
chr8	120407825	120409082	chr8-120407825-120409082
chr8	120411789	120412595	chr8-120411789-120412595
chr8	120416170	120417561	chr8-120416170-120417561
chr8	120427964	120428303	chr8-120427964-120428303
chr8	120486455	120491666	chr8-120486455-120491666
chr8	120492513	120492935	chr8-120492513-120492935
chr8	120494435	120495761	chr8-120494435-120495761
chr8	120498123	120501656	chr8-120498123-120501656
chr8	120535141	120540722	chr8-120535141-120540722
chr8	120542815	120544069	chr8-120542815-120544069
chr8	120561685	120565389	chr8-120561685-120565389
chr8	120587692	120590328	chr8-120587692-120590328
chr8	120608248	120608499	chr8-120608248-120608499

chr8	120632013	120632343	chr8-120632013-120632343
chr8	120633510	120635022	chr8-120633510-120635022
chr8	120665770	120669985	chr8-120665770-120669985
chr8	120702000	120702201	chr8-120702000-120702201
chr8	120725108	120725472	chr8-120725108-120725472
chr8	120735590	120737013	chr8-120735590-120737013
chr8	120743899	120744240	chr8-120743899-120744240
chr8	120831902	120832513	chr8-120831902-120832513
chr8	120883385	120883586	chr8-120883385-120883586
chr8	120908992	120909293	chr8-120908992-120909293
chr8	120971735	120972856	chr8-120971735-120972856
chr8	121073193	121073992	chr8-121073193-121073992
chr8	121076129	121076330	chr8-121076129-121076330
chr8	121080689	121080999	chr8-121080689-121080999
chr8	121082541	121086503	chr8-121082541-121086503
chr8	121089176	121089768	chr8-121089176-121089768
chr8	121099949	121100150	chr8-121099949-121100150
chr8	121106411	121109256	chr8-121106411-121109256
chr8	121113507	121114035	chr8-121113507-121114035
chr8	121115072	121118339	chr8-121115072-121118339
chr8	121124749	121126188	chr8-121124749-121126188
chr8	121127849	121129073	chr8-121127849-121129073
chr8	121148632	121148833	chr8-121148632-121148833
chr8	121187098	121188266	chr8-121187098-121188266
chr8	121241601	121242469	chr8-121241601-121242469
chr8	121262811	121263752	chr8-121262811-121263752
chr8	121283404	121283605	chr8-121283404-121283605
chr8	121286008	121286815	chr8-121286008-121286815
chr8	121316032	121316518	chr8-121316032-121316518
chr8	121318713	121318964	chr8-121318713-121318964
chr8	121382598	121383162	chr8-121382598-121383162
chr8	121451383	121451829	chr8-121451383-121451829
chr8	121474433	121475288	chr8-121474433-121475288
chr8	121539903	121540426	chr8-121539903-121540426
chr8	121569874	121571445	chr8-121569874-121571445
chr8	121573039	121573457	chr8-121573039-121573457
chr8	121576856	121580921	chr8-121576856-121580921
chr8	121584640	121586244	chr8-121584640-121586244
chr8	121589764	121592647	chr8-121589764-121592647
chr8	121631396	121632693	chr8-121631396-121632693
chr8	121648577	121654466	chr8-121648577-121654466
chr8	121663642	121664063	chr8-121663642-121664063

chr8	121665169	121665947	chr8-121665169-121665947
chr8	121668150	121670271	chr8-121668150-121670271
chr8	121695862	121696335	chr8-121695862-121696335
chr8	121729708	121731077	chr8-121729708-121731077
chr8	121827742	121830388	chr8-121827742-121830388
chr8	121832696	121833692	chr8-121832696-121833692
chr8	121835306	121835507	chr8-121835306-121835507
chr8	121850489	121851917	chr8-121850489-121851917
chr8	121872083	121872284	chr8-121872083-121872284
chr8	121889097	121891725	chr8-121889097-121891725
chr8	121896887	121899985	chr8-121896887-121899985
chr8	121901471	121908795	chr8-121901471-121908795
chr8	121947580	121953123	chr8-121947580-121953123
chr8	122060212	122060471	chr8-122060212-122060471
chr8	122139736	122140540	chr8-122139736-122140540
chr8	122144387	122146056	chr8-122144387-122146056
chr8	122148815	122150230	chr8-122148815-122150230
chr8	122173930	122174794	chr8-122173930-122174794
chr8	122186565	122187390	chr8-122186565-122187390
chr8	122224767	122225668	chr8-122224767-122225668
chr8	122229666	122231930	chr8-122229666-122231930
chr8	122234008	122234538	chr8-122234008-122234538
chr8	122269123	122269742	chr8-122269123-122269742
chr8	122280599	122285383	chr8-122280599-122285383
chr8	122293429	122295151	chr8-122293429-122295151
chr8	122335454	122337184	chr8-122335454-122337184
chr8	122342563	122344696	chr8-122342563-122344696
chr8	122347623	122347930	chr8-122347623-122347930
chr8	122355393	122356625	chr8-122355393-122356625
chr8	122368284	122369171	chr8-122368284-122369171
chr8	122375419	122378584	chr8-122375419-122378584
chr8	122420008	122420366	chr8-122420008-122420366
chr8	122431517	122433583	chr8-122431517-122433583
chr8	122441184	122445090	chr8-122441184-122445090
chr8	122459049	122464581	chr8-122459049-122464581
chr8	122474626	122477457	chr8-122474626-122477457
chr8	122526393	122527183	chr8-122526393-122527183
chr8	122536351	122537833	chr8-122536351-122537833
chr8	122546249	122552155	chr8-122546249-122552155
chr8	122556278	122557522	chr8-122556278-122557522
chr8	122558716	122559246	chr8-122558716-122559246
chr8	122566221	122570561	chr8-122566221-122570561

chr8	122574525	122577473	chr8-122574525-122577473
chr8	122584898	122586173	chr8-122584898-122586173
chr8	122610458	122612761	chr8-122610458-122612761
chr8	122635230	122637240	chr8-122635230-122637240
chr8	122665836	122666037	chr8-122665836-122666037
chr8	122677064	122679231	chr8-122677064-122679231
chr8	122774854	122776700	chr8-122774854-122776700
chr8	122848196	122848703	chr8-122848196-122848703
chr8	122864524	122865706	chr8-122864524-122865706
chr8	122870265	122871089	chr8-122870265-122871089
chr8	122905501	122907392	chr8-122905501-122907392
chr8	122989810	122990061	chr8-122989810-122990061
chr8	123021427	123021889	chr8-123021427-123021889
chr8	123037597	123037848	chr8-123037597-123037848
chr8	123040022	123044338	chr8-123040022-123044338
chr8	123064197	123067074	chr8-123064197-123067074
chr8	123101574	123105214	chr8-123101574-123105214
chr8	123116873	123118378	chr8-123116873-123118378
chr8	123126206	123127644	chr8-123126206-123127644
chr8	123135116	123136148	chr8-123135116-123136148
chr8	123190940	123192249	chr8-123190940-123192249
chr8	123211290	123214093	chr8-123211290-123214093
chr8	123223754	123226171	chr8-123223754-123226171
chr8	123235033	123236721	chr8-123235033-123236721
chr8	123253085	123256087	chr8-123253085-123256087
chr8	123278205	123278518	chr8-123278205-123278518
chr8	123283911	123284986	chr8-123283911-123284986
chr8	123317365	123319498	chr8-123317365-123319498
chr8	123330658	123334731	chr8-123330658-123334731
chr8	123340967	123342566	chr8-123340967-123342566
chr8	123345332	123346538	chr8-123345332-123346538
chr8	123372483	123375174	chr8-123372483-123375174
chr8	123406710	123407015	chr8-123406710-123407015
chr8	123410918	123412306	chr8-123410918-123412306
chr8	123422097	123423045	chr8-123422097-123423045
chr8	123442732	123444391	chr8-123442732-123444391
chr8	123446215	123448343	chr8-123446215-123448343
chr8	123476874	123479639	chr8-123476874-123479639
chr8	123514202	123515962	chr8-123514202-123515962
chr8	123518282	123519939	chr8-123518282-123519939
chr8	123540503	123540704	chr8-123540503-123540704
chr8	123542149	123542350	chr8-123542149-123542350

chr8	123548549	123548750	chr8-123548549-123548750
chr8	123582822	123583023	chr8-123582822-123583023
chr8	123598013	123598214	chr8-123598013-123598214
chr8	123599833	123601021	chr8-123599833-123601021
chr8	123607449	123607650	chr8-123607449-123607650
chr8	123610088	123610440	chr8-123610088-123610440
chr8	123616885	123617717	chr8-123616885-123617717
chr8	123618890	123619367	chr8-123618890-123619367
chr8	123621936	123622408	chr8-123621936-123622408
chr8	123624105	123624610	chr8-123624105-123624610
chr8	123627989	123629758	chr8-123627989-123629758
chr8	123630658	123631020	chr8-123630658-123631020
chr8	123648451	123649796	chr8-123648451-123649796
chr8	123653169	123655753	chr8-123653169-123655753
chr8	123717153	123719430	chr8-123717153-123719430
chr8	123736778	123737629	chr8-123736778-123737629
chr8	123752891	123753982	chr8-123752891-123753982
chr8	123768719	123769206	chr8-123768719-123769206
chr8	123788507	123788708	chr8-123788507-123788708
chr8	123805223	123807354	chr8-123805223-123807354
chr8	123849709	123850213	chr8-123849709-123850213
chr8	123858106	123860567	chr8-123858106-123860567
chr8	123884449	123884860	chr8-123884449-123884860
chr8	123891654	123892571	chr8-123891654-123892571
chr8	123948108	123950462	chr8-123948108-123950462
chr8	123981484	123984090	chr8-123981484-123984090
chr8	123993591	123994601	chr8-123993591-123994601
chr8	124019065	124022914	chr8-124019065-124022914
chr8	124112931	124113664	chr8-124112931-124113664
chr8	124149874	124150426	chr8-124149874-124150426
chr8	124230816	124233267	chr8-124230816-124233267
chr8	124240519	124242716	chr8-124240519-124242716
chr8	124253716	124254149	chr8-124253716-124254149
chr8	124264015	124266252	chr8-124264015-124266252
chr8	124278081	124279229	chr8-124278081-124279229
chr8	124290458	124291489	chr8-124290458-124291489
chr8	124412359	124412661	chr8-124412359-124412661
chr8	124433151	124435033	chr8-124433151-124435033
chr8	124520287	124522440	chr8-124520287-124522440
chr8	124617167	124617833	chr8-124617167-124617833
chr8	124643022	124644847	chr8-124643022-124644847
chr8	124658176	124659920	chr8-124658176-124659920

chr8	124661103	124664046	chr8-124661103-124664046
chr8	124680844	124682112	chr8-124680844-124682112
chr8	124720279	124723636	chr8-124720279-124723636
chr8	124750999	124752417	chr8-124750999-124752417
chr8	124775676	124775927	chr8-124775676-124775927
chr8	124792062	124794847	chr8-124792062-124794847
chr8	124826616	124827558	chr8-124826616-124827558
chr8	124831533	124832817	chr8-124831533-124832817
chr8	124861851	124864774	chr8-124861851-124864774
chr8	124894511	124899759	chr8-124894511-124899759
chr8	124921446	124923617	chr8-124921446-124923617
chr8	124926142	124927272	chr8-124926142-124927272
chr8	124946083	124951175	chr8-124946083-124951175
chr8	124969477	124969781	chr8-124969477-124969781
chr8	124991236	124991437	chr8-124991236-124991437
chr8	125012455	125014533	chr8-125012455-125014533
chr8	125053399	125054933	chr8-125053399-125054933
chr8	125253314	125255399	chr8-125253314-125255399
chr8	125292892	125293647	chr8-125292892-125293647
chr8	125394987	125395502	chr8-125394987-125395502
chr8	125415774	125416791	chr8-125415774-125416791
chr8	125556136	125559223	chr8-125556136-125559223
chr8	125560646	125561584	chr8-125560646-125561584
chr8	125562799	125563334	chr8-125562799-125563334
chr8	125568164	125570787	chr8-125568164-125570787
chr8	125618671	125620086	chr8-125618671-125620086
chr8	125624466	125624822	chr8-125624466-125624822
chr8	125636726	125637882	chr8-125636726-125637882
chr8	125640448	125640808	chr8-125640448-125640808
chr8	125669585	125670508	chr8-125669585-125670508
chr8	125733723	125735306	chr8-125733723-125735306
chr8	125897973	125898384	chr8-125897973-125898384
chr8	125909695	125911602	chr8-125909695-125911602
chr8	125955565	125955825	chr8-125955565-125955825
chr8	125964164	125964365	chr8-125964164-125964365
chr8	125994747	125998716	chr8-125994747-125998716
chr8	126011170	126011423	chr8-126011170-126011423
chr8	126083361	126083815	chr8-126083361-126083815
chr8	126138562	126139692	chr8-126138562-126139692
chr8	126188549	126188853	chr8-126188549-126188853
chr8	126258303	126258554	chr8-126258303-126258554
chr8	126291946	126292197	chr8-126291946-126292197

chr8	126298155	126299173	chr8-126298155-126299173
chr8	126421647	126423796	chr8-126421647-126423796
chr8	126473219	126475426	chr8-126473219-126475426
chr8	126497590	126498442	chr8-126497590-126498442
chr8	126516856	126517437	chr8-126516856-126517437
chr8	126522913	126523715	chr8-126522913-126523715
chr8	126531545	126531869	chr8-126531545-126531869
chr8	126538906	126540880	chr8-126538906-126540880
chr8	126548127	126548328	chr8-126548127-126548328
chr8	126550611	126551689	chr8-126550611-126551689
chr8	126583882	126597090	chr8-126583882-126597090
chr8	126612970	126616875	chr8-126612970-126616875
chr8	126665289	126667310	chr8-126665289-126667310
chr8	126684212	126684525	chr8-126684212-126684525
chr8	126685506	126685707	chr8-126685506-126685707
chr8	126710943	126711201	chr8-126710943-126711201
chr8	126734852	126736789	chr8-126734852-126736789
chr8	126757988	126758448	chr8-126757988-126758448
chr8	126833383	126833936	chr8-126833383-126833936
chr8	126838665	126840206	chr8-126838665-126840206
chr8	126844234	126845308	chr8-126844234-126845308
chr8	126847124	126850683	chr8-126847124-126850683
chr8	126851961	126853633	chr8-126851961-126853633
chr8	126867072	126868276	chr8-126867072-126868276
chr8	126907863	126909845	chr8-126907863-126909845
chr8	126919520	126919966	chr8-126919520-126919966
chr8	126941577	126941946	chr8-126941577-126941946
chr8	126942926	126946473	chr8-126942926-126946473
chr8	126969854	126971810	chr8-126969854-126971810
chr8	126978729	126979584	chr8-126978729-126979584
chr8	126986322	126986573	chr8-126986322-126986573
chr8	127062905	127065989	chr8-127062905-127065989
chr8	127073001	127074854	chr8-127073001-127074854
chr8	127076413	127076933	chr8-127076413-127076933
chr8	127143523	127145008	chr8-127143523-127145008
chr8	127181573	127182004	chr8-127181573-127182004
chr8	127183915	127184329	chr8-127183915-127184329
chr8	127440874	127441233	chr8-127440874-127441233
chr8	128025307	128025611	chr8-128025307-128025611
chr8	128112992	128113243	chr8-128112992-128113243
[truncated: 509,712 more chars]
