# Supplementary material for: Tumor progression and chromatin landscape of lung cancer are regulated by the lineage factor GATA6
Source: Oncogene. 2020 Mar 10;39(18):3726–37. doi: 10.1038/s41388-020-1246-z (PMC7190573; doi:10.1038/s41388-020-1246-z)
Supplement: Supplementary file 5 — Supplementary Table 4 [file 41388_2020_1246_MOESM5_ESM.pdf]

Supplementary Table 4. ATAC-seq peaks and Motif analysis**.** ATAC-seq Peaks: List of all ATAC peaks in SPC-Cre KP and KPG cells.

Chr	Start	End
chr1	3045792	3045975
chr1	3046415	3046725
chr1	3164926	3165132
chr1	3249335	3249508
chr1	3472092	3472316
chr1	3493475	3494045
chr1	3514631	3515318
chr1	3517047	3517269
chr1	3534806	3535070
chr1	3611117	3611529
chr1	3670682	3670961
chr1	3670961	3671240
chr1	3671310	3671983
chr1	3671983	3672381
chr1	3672381	3672652
chr1	3858985	3859148
chr1	4256446	4256608
chr1	4256713	4257248
chr1	4257343	4257539
chr1	4257539	4257706
chr1	4258219	4258755
chr1	4434873	4435047
chr1	4473908	4474080
chr1	4491821	4492303
chr1	4492303	4492571
chr1	4492571	4492814
chr1	4492940	4493103
chr1	4493103	4493292
chr1	4493292	4493482
chr1	4493482	4493813
chr1	4571240	4571633
chr1	4571633	4572254
chr1	4581998	4582243
chr1	4586165	4586690
chr1	4602397	4602710
chr1	4622507	4622687
chr1	4628385	4628595

chr1	4666003	4666256
chr1	4670656	4670942
chr1	4671272	4671722
chr1	4671722	4672145
chr1	4672393	4672715
chr1	4698549	4699183
chr1	4706078	4706650
chr1	4722232	4722475
chr1	4722475	4722992
chr1	4751319	4751645
chr1	4762337	4762517
chr1	4771042	4771225
chr1	4780169	4780387
chr1	4785254	4785592
chr1	4785592	4785937
chr1	4785949	4786113
chr1	4786113	4786284
chr1	4798442	4798658
chr1	4807404	4807916
chr1	4807916	4808269
chr1	4857374	4857684
chr1	4857684	4857878
chr1	4857878	4858204
chr1	4858204	4858498
chr1	4858517	4858739
chr1	4880600	4880810
chr1	5008031	5008212
chr1	5015363	5015745
chr1	5018730	5018921
chr1	5019083	5019297
chr1	5019297	5019506
chr1	5019506	5019791
chr1	5019803	5019978
chr1	5019978	5020176
chr1	5020204	5020382
chr1	5022803	5023053
chr1	5070292	5070544
chr1	5072053	5072271
chr1	5072386	5072772
chr1	5072772	5072980
chr1	5082728	5082969
chr1	5082969	5083245

chr1	5083245	5083660
chr1	5129596	5129875
chr1	5206784	5206954
chr1	5588300	5588522
chr1	5910025	5910296
chr1	5917035	5917195
chr1	6135221	6135371
chr1	6193651	6193816
chr1	6214105	6214464
chr1	6214464	6214767
chr1	6214767	6215068
chr1	6215068	6215324
chr1	6215352	6215513
chr1	6272291	6272571
chr1	6279690	6279928
chr1	6279928	6280165
chr1	6280209	6280783
chr1	6288040	6288295
chr1	6289690	6290044
chr1	6304875	6305065
chr1	6306690	6307143
chr1	6311446	6311650
chr1	6311650	6311922
chr1	6311922	6312319
chr1	6330701	6330931
chr1	6359026	6359581
chr1	6382613	6383014
chr1	6383014	6383408
chr1	6388094	6388326
chr1	6406477	6406629
chr1	6410167	6410390
chr1	6454033	6454190
chr1	6454220	6454536
chr1	6457653	6458388
chr1	6459810	6460129
chr1	6460164	6460364
chr1	6509974	6510182
chr1	6623908	6624364
chr1	6624424	6624767
chr1	6755385	6755674
chr1	6756934	6757135
chr1	6818572	6818750

chr1	6869155	6869349
chr1	6875522	6875720
chr1	6907168	6907384
chr1	6966389	6966573
chr1	6966573	6966775
chr1	6969620	6969786
chr1	6969825	6970259
chr1	6973987	6974264
chr1	6980427	6980681
chr1	7053430	7053608
chr1	7088413	7088913
chr1	7088913	7089305
chr1	7177645	7177795
chr1	7199618	7199773
chr1	7206195	7206574
chr1	7206675	7206896
chr1	7375804	7376033
chr1	7397586	7397866
chr1	7397866	7398514
chr1	7476612	7476786
chr1	7499102	7499337
chr1	7707469	7707849
chr1	7709172	7709540
chr1	7750774	7750974
chr1	7898854	7899448
chr1	7966745	7966975
chr1	8190162	8190318
chr1	8196842	8197000
chr1	8686019	8686244
chr1	9133089	9133292
chr1	9153926	9154142
chr1	9235199	9235389
chr1	9237128	9237303
chr1	9238155	9238657
chr1	9239926	9240109
chr1	9530528	9530827
chr1	9541334	9541554
chr1	9545068	9545472
chr1	9545472	9545782
chr1	9545782	9546014
chr1	9546014	9546230
chr1	9547958	9548217

chr1	9548554	9549005
chr1	9561514	9561705
chr1	9573305	9573526
chr1	9593096	9593302
chr1	9621465	9621730
chr1	9627393	9627699
chr1	9630263	9630488
chr1	9630552	9630908
chr1	9630927	9631272
chr1	9631540	9631940
chr1	9636403	9636672
chr1	9636846	9637357
chr1	9638708	9638871
chr1	9640874	9641197
chr1	9642176	9642332
chr1	9699886	9700530
chr1	9700530	9700920
chr1	9747432	9747704
chr1	9747772	9748179
chr1	9748179	9748763
chr1	9754997	9755310
chr1	9755310	9755650
chr1	9755650	9755974
chr1	9756053	9756482
chr1	9764592	9764834
chr1	9772800	9773082
chr1	9773088	9773455
chr1	9773455	9773775
chr1	9797816	9798719
chr1	9799111	9799261
chr1	9805505	9805754
chr1	9847709	9847904
chr1	9847904	9848146
chr1	9848146	9848809
chr1	9937333	9937502
chr1	9938652	9939039
chr1	9943649	9943963
chr1	9943963	9944327
chr1	9947237	9947507
chr1	9947580	9947980
chr1	9967019	9967519
chr1	9967519	9967855

chr1	9967855	9968104
chr1	9968104	9968370
chr1	9983030	9983500
chr1	9985747	9985897
chr1	9986753	9986958
chr1	9989896	9990350
chr1	10007290	10007544
chr1	10009002	10009292
chr1	10037467	10037669
chr1	10037800	10038062
chr1	10038062	10038312
chr1	10039486	10039722
chr1	10039722	10039947
chr1	10039947	10040298
chr1	10049475	10049723
chr1	10089603	10089849
chr1	10099687	10099892
chr1	10100494	10100692
chr1	10100692	10101147
chr1	10101184	10101500
chr1	10101588	10101893
chr1	10113768	10114089
chr1	10114207	10114358
chr1	10114773	10115273
chr1	10131927	10132077
chr1	10132611	10133046
chr1	10139407	10139612
chr1	10165760	10166003
chr1	10170248	10170510
chr1	10170510	10170811
chr1	10177971	10178171
chr1	10232022	10232473
chr1	10232473	10232841
chr1	10232841	10233230
chr1	10233230	10233499
chr1	10233557	10233720
chr1	10247962	10248206
chr1	10253631	10253875
chr1	10253890	10254207
chr1	10254420	10254654
chr1	10255140	10255334
chr1	10255585	10255810

chr1	10256694	10257080
chr1	10257204	10257565
chr1	10257565	10257853
chr1	10263814	10264251
chr1	10265293	10265612
chr1	10271995	10272241
chr1	10278774	10279008
chr1	10283300	10283569
chr1	10284651	10284977
chr1	10285140	10285393
chr1	10285393	10285627
chr1	10285627	10285850
chr1	10285850	10286091
chr1	10293029	10293179
chr1	10298045	10298290
chr1	10300438	10300871
chr1	10300871	10301227
chr1	10307491	10307746
chr1	10311211	10311725
chr1	10311875	10312209
chr1	10327757	10328297
chr1	10328297	10328580
chr1	10380854	10381104
chr1	10397994	10398153
chr1	10459962	10460209
chr1	10478334	10478574
chr1	10478574	10478882
chr1	10486361	10486709
chr1	10544710	10545216
chr1	10571008	10571300
chr1	10592530	10593015
chr1	10593151	10593553
chr1	10598264	10598480
chr1	10603297	10603734
chr1	10708937	10709206
chr1	10710022	10710377
chr1	10719898	10720102
chr1	10759519	10759826
chr1	10797703	10797913
chr1	10853691	10853885
chr1	10872305	10872688
chr1	10872706	10873081

chr1	10878242	10878511
chr1	10878511	10878751
chr1	10880042	10880314
chr1	10880369	10880661
chr1	10898774	10899170
chr1	10899176	10899445
chr1	10902632	10903006
chr1	10932456	10932716
chr1	10932766	10932996
chr1	10932996	10933265
chr1	10933265	10933632
chr1	10940304	10940691
chr1	10955231	10955632
chr1	10993349	10993740
chr1	10993740	10994064
chr1	11065259	11065470
chr1	11079095	11079508
chr1	11097345	11097528
chr1	11110566	11110718
chr1	11145774	11145946
chr1	11202592	11202818
chr1	11214248	11214403
chr1	11253804	11253964
chr1	11330382	11330532
chr1	11340411	11340561
chr1	11348332	11348584
chr1	11401166	11401396
chr1	11413917	11414342
chr1	11414342	11414582
chr1	11624715	11624873
chr1	11895086	11895326
chr1	11895326	11895555
chr1	11896701	11896898
chr1	11938224	11938415
chr1	11953707	11953885
chr1	12117106	12117434
chr1	12117434	12117651
chr1	12198664	12198884
chr1	12227335	12227629
chr1	12230652	12230894
chr1	12231169	12231460
chr1	12232010	12232185

chr1	12233134	12233761
chr1	12238264	12238462
chr1	12238608	12238850
chr1	12238894	12239205
chr1	12273377	12273585
chr1	12287672	12288216
chr1	12291793	12292143
chr1	12295904	12296096
chr1	12433693	12434145
chr1	12466340	12466505
chr1	12564906	12565273
chr1	12572773	12573207
chr1	12573207	12573719
chr1	12578806	12579044
chr1	12579044	12579541
chr1	12612177	12612445
chr1	12620422	12620822
chr1	12620868	12621283
chr1	12623144	12623718
chr1	12633086	12633433
chr1	12641688	12641960
chr1	12655892	12656284
chr1	12657401	12658160
chr1	12658218	12658745
chr1	12685504	12685714
chr1	12692345	12692604
chr1	12706150	12706308
chr1	12714700	12714973
chr1	12715349	12715787
chr1	12718118	12718446
chr1	12718446	12718744
chr1	12719393	12719597
chr1	12729483	12729647
chr1	12733013	12733198
chr1	12734211	12734784
chr1	12744564	12745051
chr1	12745061	12745359
chr1	12759583	12759935
chr1	12760713	12760902
chr1	12776790	12777303
chr1	12777474	12777723
chr1	12810906	12811316

chr1	12836873	12837045
chr1	12838522	12838700
chr1	12864115	12864348
chr1	12875551	12875757
chr1	12877654	12878009
chr1	12942491	12942737
chr1	12987140	12987290
chr1	12990930	12991396
chr1	12991447	12991707
chr1	12991707	12991906
chr1	12991906	12992105
chr1	12992105	12992367
chr1	12992367	12992628
chr1	12992628	12992913
chr1	12997240	12997541
chr1	13019654	13019838
chr1	13052126	13052300
chr1	13056180	13056332
chr1	13117848	13118015
chr1	13119920	13120338
chr1	13123052	13123287
chr1	13134365	13134664
chr1	13143730	13144139
chr1	13151199	13151381
chr1	13161356	13161922
chr1	13162032	13162182
chr1	13188488	13188757
chr1	13191876	13192097
chr1	13231523	13231786
chr1	13258095	13258273
chr1	13259592	13259805
chr1	13301704	13301854
chr1	13303994	13304238
chr1	13304865	13305242
chr1	13305242	13305535
chr1	13305535	13305799
chr1	13308960	13309240
chr1	13312636	13312835
chr1	13372175	13372704
chr1	13372704	13373002
chr1	13373002	13373276
chr1	13373276	13373502

chr1	13373502	13373679
chr1	13373679	13373891
chr1	13373961	13374212
chr1	13374338	13374671
chr1	13386929	13387340
chr1	13426489	13426717
chr1	13426964	13427120
chr1	13454280	13454520
chr1	13454630	13454820
chr1	13531572	13531803
chr1	13546365	13546619
chr1	13589028	13589633
chr1	13589633	13589957
chr1	13589957	13590353
chr1	13599062	13599261
chr1	13600383	13600701
chr1	13608459	13608751
chr1	13614938	13615183
chr1	13615687	13615877
chr1	13615890	13616237
chr1	13616237	13616538
chr1	13625696	13625906
chr1	13639308	13639482
chr1	13639526	13640011
chr1	13643099	13643502
chr1	13659875	13660419
chr1	13660419	13660970
chr1	13661065	13661242
chr1	13677756	13678020
chr1	13706378	13706624
chr1	13752479	13752931
chr1	13789290	13789515
chr1	13789515	13789697
chr1	13873876	13874322
chr1	13879113	13879483
chr1	13930298	13930498
chr1	14001477	14001773
chr1	14002030	14002454
chr1	14002593	14002930
chr1	14010777	14011088
chr1	14011098	14011258
chr1	14011521	14011944

chr1	14012011	14012237
chr1	14012588	14012935
chr1	14013586	14013796
chr1	14036938	14037217
chr1	14037217	14037449
chr1	14037449	14037737
chr1	14105776	14105928
chr1	14124054	14124248
chr1	14124741	14125051
chr1	14131774	14132118
chr1	14148280	14148519
chr1	14167138	14167379
chr1	14190517	14190687
chr1	14200730	14200937
chr1	14225354	14225741
chr1	14273608	14274346
chr1	14274525	14274886
chr1	14304413	14304863
chr1	14304863	14305142
chr1	14305142	14305473
chr1	14309568	14310038
chr1	14310038	14310412
chr1	14355130	14355414
chr1	14359652	14359827
chr1	14359907	14360442
chr1	14362931	14363171
chr1	14364450	14364662
chr1	14364665	14364945
chr1	14460511	14460718
chr1	14462429	14462915
chr1	14463333	14463520
chr1	14467366	14467722
chr1	14484196	14484536
chr1	14484536	14484969
chr1	14484990	14485145
chr1	14499185	14499378
chr1	14506526	14506863
chr1	14513596	14513871
chr1	14513871	14514181
chr1	14566498	14566724
chr1	14596140	14596306
chr1	14710441	14710599

chr1	14740091	14740335
chr1	14746948	14747144
chr1	14755121	14755638
chr1	14755638	14756006
chr1	14772912	14773169
chr1	14774698	14774855
chr1	14775271	14775630
chr1	14781285	14781761
chr1	14858341	14858725
chr1	14858903	14859240
chr1	14918746	14919018
chr1	14922321	14922501
chr1	14923724	14923927
chr1	14924867	14925239
chr1	14925941	14926132
chr1	15070065	15070321
chr1	15070362	15070682
chr1	15118510	15118974
chr1	15172901	15173053
chr1	15179799	15180006
chr1	15286455	15287119
chr1	15312968	15313151
chr1	15691566	15691754
chr1	15719390	15719970
chr1	15721093	15721266
chr1	15725950	15726394
chr1	15784476	15784628
chr1	15784769	15785173
chr1	15785173	15785375
chr1	15791104	15791410
chr1	15791422	15791919
chr1	15791919	15792156
chr1	15805368	15805809
chr1	15805809	15806167
chr1	15806167	15806439
chr1	15892471	15892691
chr1	15902510	15902660
chr1	15936525	15937180
chr1	15976441	15976695
chr1	15977098	15977272
chr1	15993652	15993840
chr1	16071851	16072106

chr1	16072113	16072263
chr1	16072370	16072721
chr1	16073247	16073582
chr1	16073582	16073859
chr1	16073866	16074060
chr1	16074060	16074238
chr1	16074238	16074431
chr1	16074431	16074617
chr1	16087252	16087455
chr1	16089791	16090209
chr1	16093030	16093254
chr1	16093254	16093474
chr1	16096678	16096864
chr1	16104609	16105081
chr1	16105081	16105581
chr1	16105581	16105960
chr1	16109202	16109629
chr1	16110790	16110952
chr1	16111774	16112009
chr1	16113008	16113269
chr1	16113269	16113571
chr1	16113656	16113904
chr1	16121649	16121846
chr1	16122018	16122206
chr1	16123569	16123748
chr1	16130050	16130355
chr1	16132766	16132916
chr1	16135436	16135826
chr1	16135826	16136087
chr1	16158737	16159096
chr1	16159248	16159415
chr1	16159425	16159840
chr1	16161676	16161970
chr1	16161970	16162207
chr1	16171955	16172354
chr1	16172354	16172946
chr1	16177616	16177938
chr1	16179920	16180387
chr1	16182456	16182799
chr1	16182799	16183097
chr1	16199308	16199517
chr1	16205959	16206343

chr1	16206517	16206833
chr1	16206833	16207148
chr1	16207148	16207449
chr1	16207449	16207770
chr1	16207782	16207979
chr1	16227758	16228225
chr1	16228225	16228512
chr1	16228512	16229033
chr1	16234649	16235113
chr1	16235113	16235559
chr1	16240907	16241416
chr1	16241416	16241662
chr1	16258250	16258808
chr1	16268665	16269249
chr1	16288693	16288993
chr1	16297752	16298013
chr1	16304899	16305201
chr1	16342853	16343174
chr1	16393634	16394020
chr1	16433482	16433699
chr1	16440318	16440512
chr1	16443517	16443697
chr1	16518571	16519000
chr1	16519000	16519466
chr1	16519640	16519883
chr1	16519883	16520213
chr1	16578489	16578707
chr1	16593146	16593318
chr1	16618522	16618775
chr1	16618775	16619116
chr1	16619116	16619621
chr1	16629367	16629580
chr1	16631362	16631547
chr1	16656460	16656819
chr1	16656819	16657231
chr1	16657231	16657624
chr1	16661331	16661607
chr1	16662398	16662562
chr1	16665017	16665308
chr1	16665308	16665693
chr1	16683924	16684129
chr1	16687867	16688248

chr1	16688248	16688570
chr1	16688570	16688852
chr1	16688852	16689100
chr1	16696722	16696920
chr1	16750305	16750455
chr1	16790699	16791248
chr1	16796533	16796767
chr1	16798399	16798641
chr1	16888225	16888409
chr1	16889812	16890110
chr1	16904002	16904235
chr1	17007701	17008288
chr1	17008379	17008651
chr1	17008651	17008983
chr1	17010854	17011239
chr1	17016017	17016230
chr1	17016230	17016462
chr1	17096756	17097083
chr1	17097126	17097347
chr1	17097347	17097672
chr1	17097672	17098239
chr1	17122916	17123078
chr1	17123470	17123755
chr1	17124454	17124750
chr1	17145482	17145823
chr1	17163677	17163827
chr1	17273397	17273617
chr1	17362568	17362718
chr1	17375681	17376348
chr1	17403099	17403283
chr1	17449424	17449574
chr1	17488868	17489245
chr1	17489245	17489740
chr1	17489740	17490116
chr1	17490225	17490437
chr1	17490571	17490794
chr1	17506714	17507106
chr1	17523255	17523502
chr1	17596855	17597084
chr1	17601358	17601508
chr1	17605653	17605889
chr1	17639209	17639404

chr1	18498608	18499034
chr1	19176213	19176409
chr1	19179164	19179328
chr1	19205741	19206016
chr1	19206285	19206490
chr1	19210320	19210498
chr1	19590290	19590620
chr1	19660064	19660306
chr1	19716311	19716568
chr1	19716791	19716953
chr1	19744511	19744686
chr1	19746218	19746499
chr1	19746607	19746830
chr1	19749058	19749424
chr1	19752177	19752577
chr1	19774435	19774925
chr1	19794188	19794482
chr1	19794535	19794766
chr1	19849951	19850363
chr1	19850363	19850652
chr1	19857319	19857541
chr1	19895988	19896143
chr1	19906501	19906830
chr1	19951412	19951620
chr1	19973412	19973669
chr1	19974019	19974268
chr1	20042099	20042279
chr1	20067538	20067688
chr1	20069644	20070077
chr1	20148626	20148858
chr1	20310982	20311271
chr1	20311582	20311802
chr1	20356616	20356788
chr1	20364536	20364740
chr1	20414833	20415039
chr1	20464191	20464374
chr1	20471310	20471638
chr1	20544614	20544766
chr1	20554481	20554715
chr1	20570790	20570944
chr1	20571596	20571858
chr1	20612771	20612946

chr1	20617638	20617901
chr1	20617901	20618432
chr1	20619313	20619751
chr1	20624455	20624646
chr1	20634909	20635153
chr1	20677994	20678394
chr1	20678702	20679246
chr1	20736688	20736916
chr1	20740546	20740898
chr1	20743926	20744124
chr1	20744124	20744331
chr1	20748590	20748908
chr1	20748908	20749194
chr1	20752615	20752874
chr1	20787063	20787413
chr1	20791495	20791812
chr1	20819840	20820198
chr1	20820198	20820596
chr1	20832574	20832733
chr1	20846210	20846422
chr1	20847871	20848303
chr1	20848303	20848562
chr1	20848830	20849108
chr1	20857017	20857187
chr1	20862827	20863024
chr1	20863755	20864239
chr1	20870895	20871355
chr1	20872278	20872520
chr1	20872549	20872951
chr1	20877754	20878166
chr1	20878985	20879251
chr1	20879443	20879631
chr1	20880582	20880742
chr1	20890378	20890678
chr1	20890678	20890956
chr1	20890956	20891218
chr1	20891218	20891593
chr1	20893257	20893409
chr1	20893563	20893785
chr1	20896470	20896928
chr1	20923191	20923410
chr1	20932264	20932414

chr1	20933559	20933801
chr1	20939118	20939359
chr1	20951209	20952109
chr1	20989444	20989951
chr1	21002126	21003097
chr1	21008399	21008661
chr1	21008883	21009251
chr1	21010341	21010578
chr1	21014455	21015038
chr1	21023950	21024165
chr1	21040862	21041477
chr1	21052019	21052174
chr1	21052265	21052435
chr1	21052587	21052966
chr1	21068521	21068855
chr1	21076351	21076513
chr1	21078717	21079077
chr1	21079077	21079375
chr1	21079375	21079572
chr1	21079572	21079850
chr1	21086230	21086721
chr1	21089630	21089842
chr1	21090397	21090547
chr1	21094935	21095132
chr1	21101290	21101555
chr1	21114934	21115170
chr1	21121624	21121868
chr1	21153089	21153276
chr1	21161893	21162055
chr1	21166244	21166747
chr1	21166864	21167057
chr1	21210126	21210533
chr1	21210533	21210914
chr1	21218289	21218688
chr1	21218688	21219106
chr1	21221751	21221923
chr1	21244427	21244587
chr1	21271338	21271536
chr1	21271536	21271778
chr1	21279216	21279606
chr1	21344405	21344643
chr1	21344676	21344897

chr1	21344897	21345134
chr1	21458672	21458896
chr1	21499936	21500124
chr1	21530929	21531081
chr1	21552717	21552986
chr1	21554602	21554761
chr1	21613743	21613920
chr1	21613964	21614320
chr1	21616689	21616980
chr1	21618740	21618990
chr1	21622961	21623379
chr1	21623379	21623671
chr1	21632684	21633043
chr1	21634145	21634299
chr1	21672523	21672837
chr1	21676368	21676559
chr1	21676818	21677039
chr1	21712770	21712989
chr1	21729222	21729429
chr1	21817816	21818021
chr1	21836387	21836750
chr1	21838605	21838867
chr1	21960744	21960973
chr1	21960973	21961272
chr1	21961272	21961665
chr1	21961665	21962000
chr1	21962000	21962176
chr1	21962176	21962342
chr1	22268171	22268485
chr1	22269062	22269295
chr1	22307877	22308180
chr1	22327557	22327816
chr1	22422150	22422471
chr1	22533285	22533648
chr1	22805783	22806206
chr1	22806206	22806511
chr1	23056679	23057095
chr1	23061958	23062327
chr1	23189848	23190197
chr1	23190213	23190661
chr1	23190661	23191094
chr1	23191137	23191407

chr1	23202543	23202841
chr1	23202841	23203332
chr1	23211385	23211545
chr1	23214296	23214795
chr1	23214795	23215061
chr1	23219811	23220116
chr1	23225068	23225222
chr1	23228491	23228863
chr1	23240399	23240617
chr1	23255847	23256495
chr1	23256495	23257057
chr1	23263896	23264069
chr1	23268141	23268398
chr1	23268622	23268796
chr1	23269327	23269525
chr1	23270932	23271233
chr1	23271392	23271869
chr1	23273250	23273480
chr1	23279731	23280184
chr1	23286804	23287035
chr1	23301171	23301336
chr1	23302173	23302323
chr1	23306070	23306225
chr1	23382619	23383071
chr1	23383071	23383319
chr1	23383319	23383622
chr1	23396580	23396847
chr1	23645050	23645558
chr1	23761776	23762023
chr1	23762053	23762233
chr1	23762233	23762447
chr1	23762447	23762764
chr1	23762764	23763283
chr1	23792829	23793068
chr1	23827156	23827406
chr1	23899741	23899920
chr1	23920382	23920577
chr1	23921588	23921954
chr1	23921954	23922203
chr1	23922203	23922591
chr1	23944442	23944642
chr1	23949129	23949335

chr1	23952715	23952870
chr1	23958951	23959153
chr1	23961802	23962054
chr1	24005169	24005350
chr1	24005350	24005572
chr1	24005572	24005974
chr1	24006703	24006899
chr1	24047222	24047403
chr1	24099632	24100168
chr1	24100168	24100388
chr1	24100388	24100822
chr1	24102551	24102870
chr1	24107367	24107737
chr1	24107836	24108390
chr1	24108390	24108734
chr1	24118938	24119133
chr1	24134952	24135182
chr1	24137208	24137432
chr1	24171551	24171777
chr1	24249222	24249385
chr1	24302812	24302971
chr1	24326278	24326510
chr1	24331212	24331442
chr1	24331481	24331797
chr1	24491824	24492018
chr1	24553455	24553852
chr1	24611567	24612174
chr1	24612174	24612732
chr1	24612732	24613078
chr1	24613142	24613501
chr1	24613501	24613755
chr1	24613755	24614037
chr1	24614037	24614248
chr1	24614248	24614444
chr1	24614444	24614663
chr1	24614663	24614922
chr1	24614922	24615170
chr1	24615170	24615643
chr1	24615646	24616184
chr1	24659816	24660131
chr1	24677743	24677963
chr1	24678165	24678411

chr1	24678411	24678571
chr1	24678571	24679012
chr1	24679025	24679417
chr1	24683995	24684324
chr1	24686731	24686922
chr1	24798796	24799454
chr1	25015018	25015250
chr1	25017521	25018024
chr1	25019414	25019564
chr1	25199468	25199662
chr1	25228704	25228881
chr1	26353146	26353338
chr1	26686062	26686318
chr1	26686391	26686590
chr1	26686851	26687194
chr1	26687194	26687445
chr1	26687445	26687699
chr1	26687893	26688337
chr1	28054039	28054338
chr1	28328669	28328899
chr1	28611411	28611590
chr1	28819009	28819248
chr1	28831454	28831704
chr1	28841348	28842006
chr1	28843666	28843985
chr1	28893995	28894204
chr1	29035555	29035705
chr1	29159993	29160259
chr1	29285000	29285303
chr1	29493323	29493521
chr1	29794832	29794992
chr1	29818845	29819307
chr1	30143925	30144150
chr1	30176972	30177145
chr1	30184639	30184825
chr1	30184832	30185168
chr1	30214668	30214829
chr1	30590672	30590943
chr1	30595229	30595400
chr1	30700482	30700696
chr1	30718673	30718897
chr1	30740987	30741154

chr1	30771912	30772062
chr1	30789076	30789329
chr1	30789562	30790082
chr1	30790237	30790606
chr1	30792931	30793318
chr1	30838855	30839223
chr1	30872827	30873081
chr1	30873081	30873305
chr1	30873305	30873576
chr1	30873576	30873851
chr1	30873851	30874279
chr1	30877705	30877963
chr1	30889697	30889866
chr1	30891800	30892064
chr1	30892627	30892819
chr1	30895357	30895751
chr1	30898696	30898982
chr1	30903015	30903186
chr1	30903550	30903849
chr1	30903849	30904274
chr1	30908855	30909280
chr1	30930703	30930918
chr1	30930918	30931154
chr1	30937240	30937398
chr1	30937417	30937996
chr1	30938989	30939417
chr1	30940582	30940980
chr1	30948988	30949222
chr1	30949222	30949591
chr1	30949591	30949977
chr1	30949977	30950390
chr1	31019215	31019753
chr1	31023208	31023445
chr1	31045388	31045668
chr1	31045668	31045878
chr1	31050123	31050710
chr1	31059035	31059217
chr1	31094064	31094413
chr1	31094719	31095242
chr1	31095564	31095782
chr1	31095782	31096012
chr1	31096012	31096244

chr1	31096244	31096461
chr1	31101154	31101444
chr1	31103906	31104114
chr1	31104648	31104914
chr1	31105296	31105553
chr1	31110322	31110524
chr1	31133905	31134062
chr1	31143415	31143925
chr1	31143941	31144177
chr1	31149891	31150132
chr1	31153093	31153348
chr1	31153806	31154283
chr1	31156892	31157276
chr1	31164386	31165134
chr1	31210272	31210432
chr1	31227722	31227976
chr1	31227976	31228412
chr1	31232753	31233015
chr1	31346030	31346206
chr1	31615402	31615558
chr1	31749049	31749253
chr1	32101170	32101562
chr1	32104060	32104579
chr1	32105539	32105724
chr1	32172354	32173207
chr1	32412724	32412919
chr1	33207910	33208127
chr1	33214424	33214597
chr1	33244704	33245027
chr1	33265778	33266051
chr1	33266161	33266347
chr1	33463366	33463522
chr1	33466963	33467302
chr1	33467458	33467847
chr1	33467943	33468140
chr1	33471181	33471341
chr1	33501354	33501562
chr1	33522194	33522563
chr1	33529906	33530175
chr1	33581558	33581819
chr1	33583893	33584172
chr1	33584223	33584685

chr1	33600804	33600994
chr1	33601114	33601314
chr1	33613432	33613594
chr1	33616936	33617137
chr1	33631003	33631274
chr1	33649177	33649332
chr1	33657430	33657910
chr1	33657910	33658193
chr1	33669485	33669696
chr1	33669696	33669933
chr1	33669933	33670237
chr1	33670237	33670687
chr1	33672630	33672804
chr1	33684499	33684771
chr1	33689866	33690083
chr1	33710581	33710982
chr1	33717773	33717966
chr1	33719655	33719998
chr1	33719998	33720251
chr1	33720251	33720503
chr1	33724595	33724887
chr1	33732873	33733460
chr1	33739254	33739655
chr1	33742889	33743170
chr1	33754298	33754461
chr1	33756295	33756639
chr1	33757012	33757405
chr1	33757408	33757678
chr1	33757678	33758115
chr1	33792532	33792820
chr1	33798460	33798643
chr1	33799243	33799409
chr1	33813289	33813500
chr1	33813500	33813807
chr1	33813866	33814260
chr1	33814260	33814751
chr1	33819878	33820124
chr1	33870918	33871134
chr1	33883504	33883687
chr1	33900136	33900527
chr1	33906987	33907294
chr1	33907330	33907798

chr1	33907798	33908386
chr1	33908515	33908674
chr1	33926501	33926875
chr1	33926947	33927239
chr1	33927239	33927482
chr1	33927508	33927748
chr1	33944054	33944277
chr1	33945048	33945255
chr1	33947981	33948132
chr1	33964666	33965074
chr1	33966479	33966649
chr1	33989479	33990052
chr1	33990052	33990338
chr1	33996463	33996663
chr1	33996663	33996861
chr1	34005543	34006205
chr1	34006205	34006699
chr1	34010996	34011382
chr1	34011382	34011739
chr1	34011739	34012184
chr1	34013619	34013834
chr1	34021057	34021264
chr1	34021602	34021867
chr1	34027205	34027601
chr1	34029911	34030073
chr1	34030073	34030263
chr1	34030263	34030511
chr1	34033148	34033382
chr1	34034012	34034295
chr1	34035407	34035687
chr1	34038672	34038909
chr1	34041117	34041415
chr1	34041415	34041688
chr1	34042293	34042642
chr1	34043368	34043548
chr1	34044191	34044389
chr1	34044517	34044819
chr1	34044819	34045048
chr1	34049760	34049910
chr1	34060417	34060637
chr1	34060637	34060921
chr1	34061046	34061506

chr1	34070492	34070798
chr1	34071329	34071557
chr1	34073570	34073872
chr1	34073872	34074277
chr1	34074322	34074570
chr1	34088641	34088791
chr1	34094322	34094661
chr1	34094817	34095153
chr1	34095153	34095445
chr1	34102484	34102648
chr1	34102675	34103071
chr1	34103592	34103960
chr1	34104517	34104757
chr1	34104913	34105201
chr1	34109491	34109887
chr1	34109887	34110272
chr1	34110972	34111250
chr1	34112060	34112345
chr1	34112635	34112915
chr1	34120192	34120364
chr1	34120505	34120967
chr1	34121188	34121552
chr1	34122943	34123273
chr1	34123328	34123716
chr1	34127807	34128083
chr1	34132903	34133141
chr1	34139262	34139714
chr1	34140092	34140504
chr1	34144272	34144429
chr1	34148046	34148400
chr1	34150697	34151104
chr1	34158796	34158949
chr1	34159908	34160202
chr1	34164381	34164531
chr1	34164624	34164891
chr1	34175039	34175204
chr1	34184327	34184621
chr1	34185998	34186480
chr1	34190129	34190310
chr1	34200153	34200316
chr1	34202654	34202960
chr1	34218381	34218687

chr1	34223999	34224177
chr1	34230287	34230495
chr1	34230495	34230846
chr1	34236211	34236770
chr1	34237343	34237706
chr1	34250862	34251156
chr1	34251156	34251507
chr1	34251971	34252144
chr1	34254094	34254289
chr1	34259146	34259313
chr1	34259993	34260213
chr1	34261077	34261250
chr1	34300738	34300997
chr1	34310568	34310754
chr1	34314572	34315047
chr1	34370764	34371037
chr1	34399749	34400019
chr1	34403856	34404006
chr1	34410021	34410171
chr1	34411353	34411560
chr1	34429432	34429717
chr1	34433263	34433413
chr1	34434021	34434191
chr1	34434256	34434443
chr1	34434664	34434949
chr1	34434949	34435191
chr1	34439200	34439597
chr1	34439597	34439950
chr1	34439950	34440234
chr1	34459375	34459967
chr1	34459967	34460376
chr1	34462824	34463107
chr1	34471985	34472263
chr1	34472566	34472802
chr1	34472802	34473069
chr1	34473100	34473307
chr1	34485935	34486182
chr1	34503133	34503295
chr1	34504951	34505273
chr1	34505273	34505623
chr1	34511097	34511304
chr1	34536691	34536943

chr1	34536943	34537180
chr1	34537180	34537480
chr1	34579464	34579983
chr1	34601766	34601917
chr1	34602420	34602724
chr1	34642524	34642787
chr1	34656428	34656795
chr1	34677775	34678037
chr1	34678037	34678285
chr1	34678285	34678460
chr1	34678835	34679061
chr1	34712536	34712905
chr1	34738506	34738658
chr1	34738747	34739009
chr1	34739408	34739623
chr1	34801548	34801838
chr1	34801838	34802236
chr1	34802236	34802626
chr1	34815887	34816184
chr1	34819333	34819532
chr1	34823803	34823954
chr1	34824172	34824545
chr1	34824545	34825024
chr1	34829352	34829555
chr1	34831010	34831281
chr1	34840100	34840252
chr1	34842431	34842883
chr1	34842883	34843180
chr1	34843180	34843445
chr1	34843445	34843662
chr1	34849826	34850096
chr1	34850096	34850314
chr1	34850314	34850550
chr1	34850550	34850821
chr1	34851047	34851295
chr1	34851516	34851960
chr1	34852525	34852754
chr1	34858808	34858992
chr1	34867225	34867592
chr1	34891668	34891889
chr1	34896100	34896380
chr1	34896471	34896734

chr1	34896973	34897223
chr1	34979425	34979678
chr1	35017629	35017779
chr1	35106753	35106983
chr1	35149381	35149610
chr1	35274318	35274534
chr1	35385513	35385732
chr1	35509047	35509328
chr1	35724813	35724973
chr1	35726697	35726908
chr1	35759686	35760100
chr1	35762403	35762553
chr1	35765743	35765939
chr1	35780474	35780660
chr1	35780745	35780922
chr1	35783407	35783667
chr1	35813684	35813879
chr1	35839438	35839852
chr1	35843684	35843968
chr1	35843970	35844151
chr1	35858440	35858754
chr1	35874868	35875253
chr1	35875253	35875741
chr1	35878520	35878755
chr1	35879553	35879915
chr1	35879971	35880126
chr1	35889808	35889958
chr1	35903972	35904516
chr1	35913469	35914025
chr1	35914087	35914261
chr1	35914261	35914442
chr1	35930473	35930671
chr1	35930671	35930888
chr1	35931019	35931583
chr1	35940156	35940552
chr1	35945415	35945581
chr1	35951816	35952086
chr1	35952601	35953060
chr1	35953060	35953357
chr1	35954095	35954386
chr1	35961819	35962492
chr1	35963065	35963324

chr1	35975795	35976195
chr1	35976630	35976945
chr1	35977222	35977442
chr1	36001864	36002047
chr1	36012048	36012411
chr1	36013758	36014061
chr1	36016759	36017156
chr1	36017156	36017627
chr1	36030249	36030801
chr1	36047681	36047893
chr1	36059965	36060277
chr1	36060688	36060873
chr1	36064966	36065193
chr1	36068057	36068494
chr1	36068494	36068786
chr1	36068897	36069240
chr1	36069288	36069534
chr1	36069534	36069788
chr1	36071284	36071608
chr1	36073258	36073517
chr1	36079464	36079757
chr1	36083299	36083507
chr1	36083509	36083986
chr1	36087816	36088025
chr1	36088344	36088587
chr1	36089453	36089804
chr1	36090083	36090233
chr1	36095428	36095749
chr1	36095749	36096159
chr1	36106362	36106964
chr1	36116204	36116534
chr1	36116534	36117116
chr1	36127574	36127920
chr1	36128462	36128646
chr1	36129188	36129390
chr1	36129896	36130050
chr1	36130237	36130731
chr1	36134669	36134921
chr1	36135476	36135672
chr1	36145153	36145347
chr1	36149475	36149727
chr1	36160164	36160654

chr1	36162263	36162437
chr1	36162723	36163101
chr1	36198936	36199206
chr1	36200418	36200667
chr1	36235055	36235477
chr1	36235851	36236232
chr1	36243798	36244553
chr1	36252891	36253149
chr1	36254644	36255016
chr1	36255016	36255304
chr1	36255304	36255521
chr1	36263979	36264155
chr1	36264209	36264539
chr1	36264785	36265020
chr1	36265085	36265378
chr1	36265378	36265724
chr1	36266384	36266696
chr1	36272502	36272979
chr1	36273031	36273372
chr1	36273372	36273685
chr1	36274364	36274926
chr1	36275119	36275290
chr1	36277358	36277508
chr1	36279215	36279522
chr1	36285889	36286043
chr1	36287686	36287979
chr1	36288960	36289206
chr1	36295328	36295600
chr1	36296311	36296822
chr1	36296822	36297030
chr1	36303352	36303893
chr1	36307513	36307912
chr1	36307912	36308220
chr1	36308541	36308911
chr1	36309016	36309468
chr1	36309468	36309809
chr1	36315001	36315296
chr1	36324108	36324365
chr1	36325332	36325597
chr1	36325718	36326146
chr1	36326146	36326511
chr1	36368202	36368367

chr1	36368460	36368729
chr1	36368729	36369019
chr1	36369019	36369304
chr1	36369304	36369560
chr1	36372475	36372770
chr1	36387050	36387272
chr1	36396290	36396596
chr1	36432780	36433073
chr1	36433073	36433400
chr1	36433421	36433603
chr1	36436793	36437127
chr1	36444761	36445123
chr1	36445123	36445531
chr1	36445610	36445822
chr1	36457176	36457649
chr1	36457649	36458263
chr1	36470056	36470441
chr1	36471217	36471867
chr1	36471867	36472231
chr1	36477295	36477447
chr1	36480578	36480820
chr1	36484757	36484927
chr1	36485148	36485534
chr1	36490463	36490860
chr1	36492290	36492867
chr1	36497418	36497654
chr1	36497743	36498403
chr1	36498563	36498717
chr1	36501361	36501530
chr1	36511408	36512023
chr1	36512023	36512355
chr1	36512355	36512607
chr1	36512607	36512816
chr1	36512816	36513058
chr1	36517895	36518189
chr1	36518908	36519097
chr1	36534468	36535159
chr1	36535755	36535907
chr1	36536023	36536331
chr1	36546718	36546877
chr1	36546882	36547702
chr1	36550586	36550797

chr1	36556511	36556873
chr1	36556873	36557122
chr1	36557122	36557395
chr1	36557395	36557697
chr1	36557697	36558072
chr1	36558072	36558368
chr1	36558368	36558582
chr1	36569015	36569175
chr1	36570428	36570807
chr1	36571674	36571889
chr1	36574387	36574577
chr1	36580109	36580368
chr1	36580433	36580665
chr1	36583408	36583770
chr1	36583770	36584067
chr1	36596105	36596441
chr1	36603367	36603649
chr1	36608306	36608504
chr1	36610344	36610601
chr1	36610601	36610891
chr1	36613716	36613866
chr1	36625557	36625867
chr1	36634785	36635033
chr1	36635033	36635413
chr1	36659111	36659263
chr1	36683961	36684113
chr1	36684134	36684764
chr1	36691232	36691634
chr1	36691634	36691890
chr1	36708700	36709009
chr1	36709009	36709316
chr1	36709316	36709563
chr1	36709563	36709839
chr1	36709839	36710257
chr1	36715307	36715629
chr1	36722519	36722743
chr1	36737368	36737630
chr1	36744445	36744961
chr1	36746553	36746762
chr1	36766916	36767145
chr1	36772310	36772466
chr1	36772612	36772840

chr1	36784509	36784669
chr1	36784685	36785155
chr1	36785217	36785500
chr1	36798146	36798504
chr1	36802466	36802799
chr1	36822013	36822224
chr1	36861588	36861780
chr1	36864532	36864820
chr1	36864820	36865170
chr1	36865170	36865473
chr1	36871307	36871568
chr1	36874446	36874760
chr1	36874760	36875002
chr1	36875002	36875267
chr1	36875293	36875443
chr1	36879163	36879474
chr1	36879743	36879920
chr1	36889715	36889914
chr1	36904615	36904829
chr1	36920179	36920417
chr1	36920417	36920684
chr1	36920684	36920977
chr1	36923055	36923413
chr1	36923413	36923758
chr1	36930148	36930302
chr1	36937386	36937564
chr1	36937738	36937963
chr1	36939003	36939426
chr1	36939426	36939805
chr1	36939805	36940244
chr1	36942072	36942227
chr1	36944018	36944217
chr1	36949657	36949875
chr1	36965281	36965514
chr1	36965514	36965838
chr1	36965838	36966243
chr1	36971009	36971239
chr1	37029141	37029582
chr1	37041134	37041453
chr1	37044369	37044538
chr1	37077364	37077628
chr1	37216118	37216415

chr1	37216494	37216714
chr1	37218468	37218683
chr1	37231325	37231499
chr1	37232781	37233163
chr1	37237462	37237786
chr1	37248419	37248867
chr1	37249529	37249886
chr1	37250759	37250909
chr1	37253283	37253575
chr1	37272367	37272517
chr1	37272539	37272823
chr1	37288317	37288528
chr1	37288732	37288883
chr1	37288885	37289280
chr1	37299548	37299823
chr1	37299823	37300037
chr1	37300037	37300494
chr1	37329284	37329434
chr1	37351403	37351596
chr1	37401749	37402083
chr1	37404986	37405455
chr1	37419432	37419786
chr1	37429525	37429876
chr1	37429876	37430492
chr1	37430521	37430771
chr1	37466687	37466844
chr1	37475423	37475636
chr1	37519128	37519324
chr1	37524076	37524465
chr1	37525109	37525327
chr1	37528359	37528584
chr1	37540503	37540932
chr1	37540932	37541550
chr1	37559780	37560037
chr1	37560414	37560857
chr1	37566804	37567096
chr1	37567386	37567630
chr1	37583827	37584147
chr1	37585646	37586100
chr1	37586100	37586496
chr1	37595100	37595271
chr1	37595439	37595971

chr1	37603192	37603773
chr1	37609650	37609833
chr1	37616208	37616459
chr1	37616463	37616786
chr1	37628955	37629136
chr1	37630812	37631304
chr1	37631304	37632009
chr1	37632598	37632840
chr1	37639140	37639489
chr1	37646841	37647003
chr1	37665602	37665904
chr1	37666533	37667016
chr1	37678175	37678462
chr1	37678462	37678734
chr1	37684782	37685016
chr1	37687141	37687462
chr1	37690756	37690958
chr1	37691301	37691485
chr1	37694546	37694747
chr1	37696405	37696830
chr1	37696830	37697333
chr1	37706520	37706747
chr1	37709764	37710030
chr1	37711519	37712003
chr1	37712018	37712297
chr1	37712353	37712812
chr1	37719415	37719743
chr1	37719743	37720049
chr1	37720049	37720508
chr1	37723344	37723494
chr1	37793627	37793795
chr1	37864469	37865137
chr1	37865137	37865603
chr1	37871612	37872031
chr1	37872061	37872300
chr1	37872300	37872536
chr1	37872768	37872966
chr1	37873465	37873660
chr1	37884453	37884796
chr1	37889580	37889864
chr1	37890045	37890442
chr1	37890442	37890809

chr1	37891504	37891708
chr1	37903242	37903560
chr1	37913486	37913655
chr1	37931709	37932217
chr1	37954370	37954713
chr1	37954713	37955225
chr1	37968891	37969220
chr1	37971247	37971605
chr1	37971605	37971927
chr1	37985838	37986095
chr1	37996833	37997139
chr1	37997139	37997512
chr1	37997764	37998324
chr1	37998324	37998683
chr1	38012283	38012452
chr1	38012600	38012842
chr1	38012842	38013080
chr1	38025472	38025660
chr1	38038776	38039333
chr1	38049520	38049847
chr1	38052198	38052559
chr1	38081414	38081648
chr1	38108802	38109107
chr1	38109794	38110168
chr1	38110175	38110339
chr1	38114133	38114285
chr1	38129141	38129458
chr1	38129505	38130316
chr1	38141052	38141202
chr1	38165722	38165920
chr1	38185590	38185828
chr1	38194498	38194668
chr1	38212416	38212707
chr1	38286669	38286822
chr1	38292005	38292246
chr1	38292891	38293179
chr1	38424464	38424865
chr1	38430413	38430609
chr1	38437733	38438103
chr1	38470185	38470422
chr1	38547602	38548058
chr1	38548709	38548948

chr1	38548948	38549159
chr1	38626559	38626724
chr1	38626907	38627294
chr1	38627294	38627677
chr1	38647372	38647793
chr1	38653297	38653491
chr1	38705901	38706117
chr1	38787519	38787776
chr1	38835699	38835849
chr1	38897911	38898193
chr1	38951776	38951969
chr1	38956868	38957159
chr1	38971665	38971970
chr1	38972936	38973276
chr1	38973276	38973593
chr1	38973599	38973789
chr1	38979922	38980362
chr1	38980852	38981032
chr1	38987119	38987512
chr1	38987512	38987834
chr1	38987834	38988153
chr1	38988341	38988501
chr1	38991460	38991611
chr1	39001187	39001453
chr1	39010727	39011005
chr1	39056390	39056603
chr1	39081321	39081531
chr1	39105605	39105807
chr1	39189489	39189689
chr1	39196756	39197118
chr1	39207017	39207194
chr1	39253880	39254056
chr1	39256108	39256434
chr1	39256484	39256886
chr1	39256900	39257103
chr1	39261092	39261271
chr1	39281806	39282236
chr1	39340722	39341055
chr1	39367609	39367954
chr1	39367954	39368213
chr1	39368213	39368443
chr1	39373552	39373754

chr1	39374444	39374778
chr1	39376610	39376884
chr1	39376971	39377133
chr1	39392242	39392500
chr1	39394344	39394506
chr1	39398883	39399203
chr1	39399203	39399812
chr1	39399842	39400028
chr1	39456243	39456630
chr1	39456630	39456952
chr1	39457277	39457468
chr1	39478018	39478302
chr1	39478302	39478614
chr1	39478614	39479102
chr1	39480568	39480764
chr1	39499459	39499786
chr1	39499786	39500033
chr1	39511235	39511385
chr1	39522601	39522879
chr1	39525945	39526095
chr1	39526509	39526670
chr1	39527512	39527766
chr1	39530345	39530582
chr1	39535437	39535892
chr1	39535892	39536392
chr1	39545554	39546025
chr1	39548619	39548906
chr1	39548906	39549163
chr1	39556100	39556414
chr1	39563237	39563486
chr1	39568868	39569141
chr1	39576475	39576841
chr1	39576841	39577137
chr1	39577137	39577422
chr1	39577422	39577674
chr1	39602628	39602801
chr1	39636221	39636600
chr1	39636602	39637024
chr1	39650465	39651105
chr1	39656810	39657282
chr1	39657282	39657528
chr1	39667109	39667406

chr1	39667406	39667684
chr1	39670217	39670616
chr1	39670616	39670887
chr1	39678959	39679256
chr1	39680629	39680881
chr1	39685888	39686157
chr1	39695264	39695543
chr1	39695543	39695808
chr1	39752749	39752958
chr1	39755700	39755947
chr1	39755947	39756242
chr1	39756270	39756446
chr1	39759145	39759490
chr1	39759490	39759820
chr1	39759887	39760059
chr1	39761094	39761414
chr1	39761414	39761715
chr1	39761754	39761921
chr1	39762087	39762304
chr1	39796203	39796403
chr1	39810517	39810916
chr1	39845159	39845343
chr1	39867577	39867729
chr1	39889395	39889782
chr1	39895055	39895734
chr1	39900255	39900557
chr1	39900557	39901082
chr1	39901082	39901494
chr1	39901494	39901725
chr1	39901922	39902081
chr1	39902748	39902993
chr1	39910146	39910363
chr1	39911396	39911822
chr1	39913304	39913656
chr1	39915616	39915915
chr1	39923465	39923730
chr1	39923821	39924001
chr1	39925077	39925493
chr1	39926268	39926434
chr1	39931479	39931656
chr1	39931713	39931911
chr1	39931971	39932342

chr1	39932342	39932709
chr1	39935607	39935799
chr1	39935961	39936434
chr1	39936434	39936834
chr1	39940935	39941261
chr1	39941261	39941603
chr1	39941603	39942116
chr1	39949170	39949490
chr1	39949490	39949697
chr1	39950115	39950469
chr1	39956499	39957104
chr1	39977022	39977182
chr1	39986207	39986505
chr1	40000996	40001233
chr1	40002172	40002330
chr1	40006142	40006387
chr1	40006387	40006597
chr1	40015081	40015380
chr1	40028238	40028450
chr1	40028450	40028655
chr1	40031747	40032034
chr1	40033408	40033589
chr1	40033613	40034091
chr1	40051257	40051418
chr1	40054009	40054165
chr1	40060777	40060998
chr1	40063097	40063262
chr1	40085613	40085772
chr1	40085845	40085997
chr1	40088714	40089106
chr1	40092594	40092813
chr1	40107132	40107497
chr1	40115433	40115586
chr1	40116477	40116789
chr1	40139752	40140256
chr1	40140256	40140429
chr1	40155541	40155718
chr1	40167112	40167348
chr1	40167553	40167879
chr1	40171836	40172074
chr1	40172074	40172350
chr1	40174113	40174581

chr1	40183443	40183623
chr1	40183623	40183787
chr1	40187878	40188080
chr1	40188122	40188443
chr1	40190176	40190326
chr1	40194151	40194815
chr1	40217189	40217342
chr1	40219298	40219504
chr1	40224490	40224892
chr1	40224892	40225403
chr1	40233703	40233937
chr1	40235616	40235831
chr1	40251999	40252149
chr1	40253986	40254289
chr1	40266404	40266699
chr1	40266699	40266917
chr1	40266917	40267372
chr1	40272256	40272640
chr1	40275209	40275671
chr1	40279111	40279696
chr1	40284497	40284670
chr1	40284670	40284978
chr1	40294757	40295205
chr1	40295205	40295470
chr1	40318048	40318226
chr1	40318777	40319108
chr1	40339431	40339626
chr1	40358579	40359233
chr1	40378917	40379077
chr1	40380143	40380494
chr1	40385017	40385524
chr1	40392919	40393591
chr1	40393591	40393905
chr1	40400658	40400949
chr1	40400949	40401323
chr1	40412484	40412767
chr1	40413129	40413406
chr1	40417045	40417557
chr1	40420033	40420321
chr1	40435924	40436348
chr1	40436348	40436653
chr1	40437111	40437393

chr1	40444679	40444998
chr1	40444998	40445266
chr1	40465451	40465747
chr1	40465747	40465994
chr1	40466171	40466326
chr1	40467288	40467775
chr1	40468891	40469343
chr1	40501682	40501838
chr1	40512911	40513080
chr1	40515234	40515508
chr1	40516075	40516270
chr1	40516432	40516709
chr1	40529353	40529634
chr1	40529634	40529928
chr1	40530888	40531092
chr1	40534334	40534595
chr1	40542223	40543136
chr1	40551451	40551601
chr1	40579835	40580194
chr1	40580372	40580558
chr1	40586759	40587359
chr1	40592181	40592567
chr1	40592567	40592782
chr1	40594038	40594470
chr1	40614269	40614972
chr1	40616940	40617236
chr1	40617236	40617491
chr1	40617528	40617786
chr1	40619782	40620200
chr1	40631391	40631615
chr1	40632687	40633034
chr1	40633480	40633738
chr1	40658032	40658349
chr1	40681055	40681465
chr1	40681465	40681906
chr1	40681906	40682335
chr1	40682783	40683423
chr1	40714904	40715054
chr1	40724690	40724969
chr1	40768715	40768866
chr1	40773232	40773756
chr1	40775640	40775941

chr1	40779953	40780221
chr1	40789926	40790132
chr1	40790218	40790589
chr1	40790589	40790826
chr1	40790826	40791045
chr1	40797365	40797667
chr1	40802847	40803070
chr1	40808732	40808925
chr1	40832749	40833162
chr1	40835506	40835761
chr1	40840268	40840510
chr1	40840510	40840949
chr1	40841018	40841344
chr1	40842497	40842729
chr1	40844733	40844889
chr1	40847003	40847199
chr1	40847199	40847396
chr1	40847553	40847978
chr1	40848068	40848219
chr1	40851381	40851544
chr1	40857808	40858010
chr1	40865049	40865367
chr1	40866474	40866733
chr1	40874978	40875584
chr1	40878199	40878386
chr1	40887704	40887915
chr1	40907093	40907267
chr1	40945775	40945964
chr1	40968200	40968402
chr1	41390178	41390401
chr1	42021205	42021454
chr1	42169624	42169774
chr1	42200703	42200857
chr1	42591790	42592330
chr1	42668467	42668623
chr1	42695581	42695826
chr1	42695826	42696072
chr1	42696546	42696766
chr1	42713272	42713450
chr1	42714673	42714857
chr1	42715213	42715450
chr1	42769152	42769338

chr1	42787470	42787626
chr1	42850794	42851016
chr1	42851016	42851321
chr1	42851321	42851774
chr1	42882319	42882622
chr1	42915090	42915351
chr1	42917935	42918262
chr1	42952766	42953017
chr1	42953134	42953452
chr1	43051791	43052410
chr1	43065967	43066240
chr1	43070807	43071029
chr1	43091597	43091753
chr1	43091772	43092083
chr1	43092083	43092397
chr1	43092397	43092619
chr1	43092619	43092827
chr1	43092827	43093079
chr1	43098093	43098295
chr1	43098306	43098775
chr1	43098775	43099165
chr1	43110634	43110865
chr1	43112963	43113204
chr1	43119033	43119279
chr1	43127767	43128219
chr1	43128219	43128732
chr1	43139653	43139952
chr1	43141475	43141782
chr1	43141782	43142106
chr1	43144433	43144718
chr1	43147524	43147820
chr1	43152261	43152630
chr1	43152630	43152965
chr1	43152965	43153231
chr1	43163098	43163456
chr1	43163529	43164297
chr1	43168035	43168325
chr1	43168325	43168636
chr1	43168636	43169209
chr1	43169649	43170030
chr1	43174851	43175197
chr1	43207789	43207939

chr1	43218071	43218302
chr1	43218512	43218715
chr1	43223156	43223516
chr1	43224168	43224603
chr1	43225079	43225257
chr1	43225864	43226017
chr1	43237970	43238199
chr1	43253081	43253235
chr1	43256118	43256305
chr1	43260453	43260876
chr1	43264446	43264705
chr1	43264705	43264920
chr1	43265681	43266209
chr1	43269493	43269731
chr1	43269741	43270021
chr1	43281067	43281305
chr1	43284763	43284935
chr1	43299529	43299796
chr1	43302731	43303115
chr1	43305462	43305630
chr1	43306093	43306307
chr1	43338654	43338888
chr1	43361123	43361402
chr1	43361402	43361922
chr1	43367592	43367874
chr1	43385065	43385537
chr1	43394078	43394333
chr1	43395804	43396213
chr1	43396250	43396588
chr1	43396588	43396849
chr1	43411916	43412142
chr1	43412929	43413206
chr1	43413373	43413740
chr1	43413766	43414155
chr1	43414247	43414497
chr1	43423426	43423692
chr1	43444143	43444429
chr1	43444603	43444889
chr1	43445360	43445566
chr1	43445575	43445847
chr1	43445847	43446094
chr1	43446094	43446429

chr1	43450457	43450730
chr1	43450765	43451359
chr1	43462723	43463032
chr1	43465981	43466180
chr1	43471832	43472176
chr1	43472176	43472655
chr1	43474472	43474855
chr1	43500756	43500993
chr1	43522190	43522360
chr1	43530345	43530503
chr1	43544569	43544947
chr1	43549447	43549903
chr1	43593609	43594057
chr1	43596244	43596413
chr1	43635806	43635981
chr1	43698570	43698769
chr1	43701089	43701497
chr1	43714882	43715255
chr1	43715255	43715771
chr1	43719531	43719732
chr1	43738269	43738497
chr1	43742276	43742467
chr1	43760198	43760355
chr1	43766342	43766710
chr1	43768439	43768713
chr1	43768713	43769007
chr1	43777726	43778001
chr1	43778001	43778241
chr1	43778375	43778962
chr1	43779500	43779708
chr1	43785301	43785677
chr1	43818032	43818243
chr1	43819042	43819414
chr1	43819414	43819791
chr1	43826593	43826866
chr1	43826940	43827197
chr1	43827197	43827560
chr1	43827560	43828247
chr1	43895771	43896225
chr1	43896474	43896631
chr1	43899054	43899293
chr1	43899293	43899461

chr1	43902920	43903254
chr1	43918075	43918448
chr1	43933467	43934080
chr1	43934080	43934486
chr1	43964467	43964629
chr1	43969338	43969705
chr1	43971155	43971428
chr1	44041879	44042099
chr1	44064711	44064928
chr1	44094289	44094666
chr1	44101533	44101900
chr1	44101900	44102286
chr1	44102286	44102844
chr1	44118514	44119096
chr1	44119226	44119538
chr1	44119538	44119808
chr1	44119808	44120095
chr1	44120095	44120323
chr1	44147524	44148004
chr1	44148004	44148471
chr1	44198393	44198579
chr1	44198666	44198840
chr1	44198840	44199079
chr1	44201193	44201391
chr1	44242066	44242386
chr1	44242438	44242786
chr1	44367253	44367407
chr1	44392000	44392530
chr1	44398088	44398385
chr1	44424638	44424896
chr1	44551478	44551754
chr1	44551754	44552320
chr1	44552362	44552652
chr1	44584823	44585114
chr1	45207994	45208170
chr1	45314087	45314283
chr1	45355985	45356274
chr1	45430543	45430693
chr1	45431970	45432236
chr1	45495122	45495288
chr1	45527446	45527997
chr1	45539099	45539690

chr1	45539690	45540024
chr1	45541202	45541502
chr1	45580436	45580894
chr1	45580960	45581161
chr1	45626035	45626274
chr1	45626816	45626994
chr1	45627952	45628225
chr1	45672815	45673118
chr1	45675659	45676317
chr1	45700959	45701219
chr1	45709360	45709467
chr1	45709467	45709512
chr1	45734057	45734306
chr1	45735179	45735426
chr1	45739036	45739205
chr1	45756804	45757118
chr1	45776638	45776995
chr1	45777482	45777965
chr1	45780719	45780944
chr1	45780944	45781243
chr1	45794885	45795308
chr1	45795308	45795603
chr1	45795603	45795966
chr1	45816955	45817185
chr1	45825218	45825496
chr1	45830461	45830776
chr1	45832526	45832808
chr1	45837679	45838125
chr1	45838333	45838597
chr1	45842841	45843021
chr1	45864517	45864767
chr1	45864821	45864986
chr1	45866690	45866978
chr1	45917129	45917491
chr1	45917561	45917747
chr1	45921072	45921252
chr1	45925108	45925383
chr1	45925474	45925798
chr1	45925798	45926076
chr1	45926076	45926495
chr1	46018474	46018687
chr1	46055519	46055711

chr1	46066528	46066951
chr1	46066951	46067292
chr1	46075905	46076249
chr1	46128068	46128431
chr1	46325457	46325607
chr1	46360963	46361247
chr1	46377192	46377438
chr1	46378811	46378984
chr1	46408116	46408356
chr1	46408356	46408653
chr1	46408717	46409045
chr1	46409045	46409266
chr1	46413892	46414096
chr1	46415024	46415232
chr1	46416750	46416992
chr1	46732533	46732689
chr1	46782294	46782563
chr1	46808756	46808931
chr1	46849824	46850101
chr1	46852282	46852491
chr1	46852672	46853197
chr1	46853197	46853590
chr1	46853590	46853930
chr1	46853930	46854309
chr1	46863814	46864165
chr1	46864165	46864506
chr1	46864506	46864804
chr1	46871218	46871480
chr1	46873493	46873780
chr1	46873780	46874164
chr1	46879603	46879889
chr1	46880001	46880357
chr1	46884440	46885034
chr1	46885166	46885829
chr1	46894589	46894830
chr1	46912869	46913076
chr1	46963375	46963686
chr1	46964006	46964229
chr1	46970544	46970750
chr1	47004389	47004817
chr1	47014097	47014527
chr1	47014634	47015099

chr1	47021503	47021666
chr1	47034667	47034857
chr1	47036491	47036721
chr1	47036721	47037045
chr1	47039313	47039844
chr1	47051110	47051490
chr1	47190261	47190499
chr1	47277836	47278044
chr1	47300752	47301140
chr1	47306995	47307254
chr1	47398705	47399312
chr1	47399696	47399925
chr1	47545698	47545914
chr1	48090152	48090331
chr1	48462159	48462353
chr1	50459505	50459812
chr1	50480723	50481020
chr1	50481265	50481474
chr1	50507235	50507471
chr1	50507901	50508261
chr1	50855137	50855532
chr1	50890696	50890946
chr1	50900379	50900674
chr1	50926759	50927031
chr1	50927031	50927327
chr1	50927327	50927702
chr1	50927702	50928210
chr1	50962372	50962613
chr1	50967627	50967779
chr1	50980196	50980566
chr1	51003869	51004052
chr1	51058270	51058585
chr1	51064597	51064931
chr1	51064931	51065142
chr1	51065376	51065689
chr1	51065689	51066091
chr1	51111958	51112499
chr1	51113736	51113969
chr1	51122158	51122851
chr1	51136845	51137049
chr1	51143790	51144120
chr1	51165440	51165634

chr1	51198055	51198277
chr1	51217273	51217583
chr1	51243261	51243682
chr1	51250121	51250648
chr1	51250648	51250923
chr1	51260166	51260528
chr1	51265067	51265692
chr1	51265830	51266279
chr1	51268252	51268607
chr1	51282926	51283204
chr1	51286875	51287285
chr1	51288716	51289033
chr1	51289033	51289336
chr1	51289336	51289623
chr1	51289623	51289926
chr1	51297087	51297258
chr1	51299073	51299233
chr1	51301292	51301557
chr1	51302685	51302884
chr1	51303248	51303465
chr1	51303621	51303806
chr1	51303806	51303993
chr1	51304118	51304325
chr1	51304325	51304539
chr1	51333665	51333865
chr1	51333970	51334285
chr1	51334321	51334489
chr1	51370366	51370583
chr1	51382207	51382607
chr1	51386876	51387329
chr1	51401796	51402014
chr1	51402014	51402201
chr1	51404414	51404694
chr1	51404737	51405283
chr1	51448589	51449381
chr1	51459046	51459357
chr1	51466475	51466913
chr1	51466926	51467107
chr1	51467900	51468407
chr1	51468407	51468675
chr1	51476505	51476703
chr1	51477674	51477868

chr1	51477900	51478081
chr1	51478097	51478437
chr1	51478437	51478884
chr1	51499191	51499455
chr1	51505625	51506034
chr1	51507240	51507460
chr1	51508568	51508828
chr1	51509052	51509424
chr1	51525672	51525874
chr1	51525923	51526217
chr1	51530082	51530621
chr1	51531005	51531256
chr1	51552260	51552450
chr1	51562636	51562921
chr1	51584674	51585414
chr1	51597021	51597321
chr1	51597326	51597483
chr1	51600509	51600748
chr1	51615546	51615821
chr1	51643652	51643972
chr1	51644498	51644669
chr1	51688002	51688181
chr1	51720414	51720569
chr1	51747426	51748021
chr1	51748346	51748608
chr1	51789310	51789835
chr1	51808315	51808511
chr1	51815720	51815935
chr1	51829364	51829576
chr1	51830992	51831191
chr1	51837943	51838093
chr1	51848528	51848982
chr1	51849056	51849289
chr1	51849609	51849865
chr1	51849910	51850087
chr1	51852360	51852538
chr1	51853211	51853937
chr1	51854558	51854754
chr1	51856219	51856545
chr1	51856577	51856843
chr1	51913160	51913343
chr1	51914999	51915443

chr1	51915443	51915858
chr1	51915858	51916210
chr1	51944927	51945155
chr1	51963144	51963380
chr1	51996658	51997244
chr1	52004160	52004342
chr1	52035434	52035655
chr1	52039248	52039398
chr1	52049901	52050051
chr1	52113172	52113634
chr1	52113634	52114090
chr1	52118965	52119218
chr1	52119218	52119622
chr1	52119622	52120100
chr1	52146629	52146905
chr1	52169967	52170493
chr1	52170568	52170719
chr1	52171410	52171608
chr1	52190347	52190637
chr1	52232346	52232550
chr1	52232760	52233740
chr1	52234655	52234936
chr1	52250972	52251367
chr1	52252546	52252706
chr1	52254052	52254235
chr1	52254272	52254519
chr1	52254519	52254833
chr1	52267666	52267887
chr1	52270346	52270729
chr1	52282778	52283021
chr1	52302625	52302880
chr1	52331541	52331733
chr1	52354413	52354616
chr1	52388867	52389176
chr1	52389197	52389359
chr1	52419502	52419719
chr1	52427930	52428195
chr1	52429448	52429692
chr1	52430196	52430392
chr1	52454533	52454770
chr1	52454770	52455230
chr1	52456517	52457133

chr1	52499684	52500178
chr1	52500178	52500641
chr1	52500641	52501065
chr1	52501604	52502085
chr1	52514451	52514651
chr1	52530034	52530213
chr1	52578279	52578529
chr1	52578529	52578821
chr1	52580574	52580816
chr1	52597811	52598172
chr1	52598240	52598395
chr1	52600462	52600619
chr1	52630458	52630935
chr1	52630935	52631294
chr1	52631361	52631604
chr1	52631673	52631905
chr1	52647840	52647993
chr1	52648211	52648436
chr1	52652602	52652908
chr1	52675561	52675887
chr1	52676798	52677022
chr1	52687752	52687919
chr1	52702782	52703104
chr1	52703104	52703422
chr1	52703422	52703685
chr1	52712228	52712434
chr1	52718427	52718674
chr1	52724251	52724514
chr1	52726308	52726706
chr1	52726706	52727126
chr1	52727126	52727585
chr1	52748354	52748506
chr1	52765166	52765471
chr1	52793628	52793888
chr1	52799396	52799761
chr1	52799780	52800100
chr1	52802465	52802788
chr1	52803034	52803184
chr1	52816926	52817290
chr1	52817290	52817600
chr1	52817600	52817989
chr1	52844665	52845348

chr1	52854112	52854348
chr1	52952589	52953056
chr1	52953056	52953452
chr1	53296667	53297444
chr1	53298968	53299137
chr1	53312581	53312753
chr1	53313340	53313756
chr1	53313756	53314031
chr1	53352228	53352567
chr1	53352567	53352934
chr1	53692679	53693005
chr1	53694126	53694643
chr1	53696693	53696856
chr1	53715985	53716201
chr1	53720715	53720880
chr1	53720963	53721328
chr1	53721399	53721713
chr1	53721767	53721919
chr1	53728497	53728820
chr1	53728855	53729177
chr1	53757951	53758341
chr1	53761679	53762072
chr1	53762102	53762413
chr1	53763690	53764027
chr1	53784303	53784514
chr1	53784514	53784926
chr1	53784926	53785490
chr1	53797560	53797743
chr1	53797794	53798188
chr1	53808270	53808547
chr1	53808659	53809049
chr1	53814220	53814374
chr1	53822024	53822330
chr1	53823686	53824144
chr1	53824144	53824388
chr1	53838063	53838514
chr1	53838514	53838916
chr1	53845021	53845179
chr1	53845222	53845671
chr1	53845859	53846271
chr1	53846271	53846507
chr1	53855756	53856064

chr1	53856238	53856530
chr1	53856652	53856919
chr1	53866469	53866780
chr1	53881844	53882049
chr1	53890517	53890690
chr1	53909192	53909454
chr1	53931396	53931624
chr1	53931734	53931898
chr1	53939362	53939620
chr1	53940235	53940619
chr1	54122030	54122190
chr1	54187693	54187856
chr1	54194193	54194380
chr1	54194380	54194634
chr1	54194762	54195054
chr1	54250466	54251150
chr1	54253314	54253464
chr1	54323077	54323254
chr1	54380729	54380879
chr1	54381351	54381566
chr1	54430240	54430400
chr1	54438371	54438730
chr1	54438730	54439177
chr1	54448248	54448493
chr1	54467425	54467698
chr1	54492429	54492760
chr1	54544848	54545225
chr1	54556869	54557072
chr1	54557072	54557291
chr1	54557291	54557528
chr1	54557528	54557814
chr1	54557814	54558167
chr1	54584637	54585015
chr1	54585968	54586222
chr1	54600212	54600728
chr1	54600728	54601035
chr1	54601252	54601427
chr1	54603874	54604329
chr1	54606123	54606489
chr1	54609784	54610098
chr1	54611944	54612332
chr1	54612332	54612674

chr1	54612961	54613260
chr1	54639174	54639328
chr1	54647272	54647820
chr1	54653040	54653340
chr1	54658588	54658870
chr1	54660629	54661204
chr1	54661372	54661699
chr1	54666049	54666292
chr1	54713294	54713551
chr1	54713578	54713734
chr1	54713860	54714120
chr1	54768805	54768955
chr1	54785902	54786062
chr1	54823609	54823795
chr1	54826270	54826667
chr1	54826667	54827091
chr1	54830208	54830599
chr1	54832803	54833041
chr1	54882297	54882498
chr1	54882498	54882685
chr1	54882685	54882843
chr1	54882843	54883050
chr1	54902914	54903072
chr1	54909151	54909303
chr1	54909898	54910177
chr1	54920166	54920469
chr1	54922416	54922581
chr1	54925586	54926241
chr1	54926241	54926539
chr1	54926539	54926761
chr1	54930793	54931107
chr1	54934921	54935164
chr1	54954976	54955152
chr1	54966933	54967170
chr1	54983374	54983653
chr1	55012610	55012825
chr1	55026823	55027068
chr1	55027068	55027323
chr1	55027323	55027875
chr1	55033976	55034136
chr1	55043277	55043427
chr1	55052291	55052931

chr1	55052931	55053366
chr1	55073039	55073333
chr1	55073518	55073673
chr1	55074897	55075126
chr1	55075497	55075663
chr1	55075732	55075970
chr1	55076458	55076609
chr1	55076823	55077127
chr1	55082189	55082410
chr1	55087316	55087715
chr1	55087715	55088039
chr1	55088039	55088669
chr1	55097290	55097537
chr1	55097537	55097790
chr1	55108684	55108834
chr1	55130893	55131171
chr1	55131171	55131516
chr1	55131516	55131982
chr1	55133261	55133416
chr1	55133500	55133664
chr1	55162014	55162220
chr1	55163901	55164452
chr1	55165266	55165429
chr1	55172267	55172417
chr1	55185799	55186030
chr1	55186594	55186795
chr1	55192046	55192223
chr1	55193211	55193668
chr1	55196425	55196604
chr1	55201775	55202111
chr1	55202171	55202448
chr1	55202470	55202704
chr1	55209144	55209330
chr1	55228277	55228553
chr1	55228553	55228827
chr1	55236946	55237445
chr1	55237445	55237844
chr1	55237844	55238114
chr1	55238114	55238280
chr1	55239380	55239583
chr1	55363265	55363656
chr1	55363656	55364012

chr1	55377849	55378007
chr1	55383184	55383445
chr1	55405610	55406087
chr1	55406087	55406463
chr1	55410720	55411017
chr1	55454205	55454399
chr1	55488357	55488548
chr1	55489423	55489786
chr1	55528491	55529245
chr1	55531686	55531888
chr1	55533780	55533954
chr1	55533954	55534138
chr1	55569429	55569588
chr1	55574694	55574890
chr1	55575856	55576546
chr1	55582352	55582611
chr1	55610337	55610606
chr1	55620490	55620682
chr1	55624336	55624652
chr1	55627697	55627884
chr1	55736452	55736680
chr1	55812610	55812814
chr1	55826992	55827267
chr1	55827267	55827770
chr1	55828263	55828514
chr1	55833728	55833895
chr1	55838277	55838428
chr1	55865116	55865806
chr1	55874211	55874485
chr1	55874837	55874992
chr1	55886465	55886685
chr1	56206989	56207191
chr1	56546292	56546473
chr1	56630959	56631119
chr1	56668150	56668435
chr1	56681050	56681337
chr1	56681337	56681623
chr1	56681623	56681839
chr1	56685137	56685499
chr1	56695272	56695607
chr1	56759419	56759814
chr1	56782235	56782454

chr1	56888953	56889149
chr1	56896802	56896952
chr1	56953077	56953371
chr1	56971512	56971858
chr1	56972007	56972446
chr1	56972446	56972715
chr1	56972715	56972974
chr1	56972974	56973334
chr1	56973348	56973617
chr1	56973666	56973849
chr1	56977179	56977448
chr1	56977493	56977687
chr1	56980039	56980261
chr1	56991706	56991873
chr1	57204364	57204670
chr1	57205701	57205871
chr1	57223494	57223649
chr1	57269798	57269973
chr1	57332629	57332851
chr1	57377164	57377325
chr1	57377378	57377723
chr1	57377723	57378270
chr1	57378382	57378624
chr1	57406071	57406563
chr1	57406563	57406824
chr1	57406824	57407317
chr1	57428964	57429222
chr1	57430185	57430446
chr1	57450345	57450596
chr1	57773972	57774302
chr1	57774336	57774659
chr1	57774659	57774888
chr1	57774888	57775093
chr1	57775093	57775396
chr1	57845385	57845628
chr1	57849169	57849462
chr1	57850001	57850220
chr1	57852649	57852870
chr1	57874048	57874428
chr1	57884934	57885084
chr1	57892423	57892612
chr1	57898090	57898409

chr1	57898441	57898743
chr1	57898743	57899186
chr1	57969278	57969512
chr1	57969512	57969838
chr1	57969838	57970363
chr1	57989109	57989687
chr1	57995730	57996071
chr1	57996071	57996401
chr1	57996401	57996705
chr1	58029554	58030196
chr1	58030257	58030508
chr1	58030596	58030934
chr1	58031261	58031491
chr1	58072998	58073263
chr1	58073266	58073646
chr1	58079776	58079926
chr1	58081456	58081676
chr1	58092321	58092489
chr1	58111342	58111685
chr1	58112862	58113218
chr1	58144242	58144544
chr1	58144544	58144736
chr1	58144736	58144937
chr1	58257150	58257602
chr1	58257631	58257919
chr1	58271206	58271493
chr1	58288242	58288451
chr1	58289872	58290110
chr1	58296366	58296689
chr1	58300824	58301210
chr1	58304413	58304724
chr1	58308555	58308929
chr1	58309944	58310137
chr1	58335715	58335878
chr1	58341702	58341899
chr1	58343848	58344254
chr1	58346868	58347172
chr1	58347196	58347387
chr1	58351380	58351808
chr1	58352001	58352224
chr1	58353362	58354034
chr1	58359030	58359536

chr1	58359536	58359922
chr1	58366537	58367102
chr1	58367102	58367361
chr1	58374185	58374546
chr1	58392266	58392621
chr1	58392621	58392926
chr1	58392926	58393231
chr1	58393231	58393550
chr1	58393550	58393842
chr1	58393870	58394109
chr1	58407620	58408001
chr1	58408328	58408614
chr1	58409382	58409558
chr1	58409676	58409789
chr1	58409789	58409852
chr1	58409852	58410036
chr1	58410277	58410427
chr1	58410870	58411061
chr1	58415576	58415789
chr1	58415885	58416149
chr1	58416183	58416396
chr1	58422932	58423221
chr1	58423371	58423581
chr1	58423581	58423899
chr1	58423899	58424224
chr1	58424224	58424440
chr1	58425863	58426148
chr1	58431028	58431272
chr1	58433907	58434127
chr1	58434127	58434457
chr1	58435788	58435945
chr1	58445062	58445382
chr1	58445382	58445871
chr1	58460631	58460937
chr1	58504511	58504854
chr1	58504854	58505280
chr1	58540071	58540490
chr1	58540659	58540876
chr1	58541530	58541799
chr1	58549413	58549756
chr1	58577359	58577564
chr1	58585769	58586133

chr1	58586133	58586448
chr1	58586448	58586713
chr1	58586713	58586905
chr1	58606993	58607198
chr1	58613843	58614087
chr1	58614452	58614936
chr1	58636516	58636666
chr1	58651642	58652127
chr1	58652127	58652373
chr1	58652448	58652926
chr1	58652926	58653315
chr1	58662782	58662933
chr1	58663092	58663348
chr1	58663348	58663893
chr1	58664323	58664615
chr1	58677390	58677913
chr1	58678427	58678580
chr1	58690454	58690632
chr1	58692100	58692292
chr1	58692330	58692874
chr1	58697661	58697970
chr1	58703283	58703640
chr1	58711412	58712163
chr1	58712232	58712408
chr1	58712458	58713039
chr1	58713150	58713341
chr1	58720071	58720537
chr1	58722121	58722422
chr1	58725857	58726106
chr1	58727051	58727405
chr1	58729372	58729838
chr1	58742220	58742878
chr1	58742878	58743101
chr1	58761295	58761934
chr1	58762071	58762303
chr1	58770204	58770365
chr1	58773395	58773560
chr1	58777688	58777845
chr1	58794963	58795243
chr1	58795243	58795562
chr1	58795562	58796021
chr1	58796258	58796433

chr1	58797291	58797628
chr1	58830819	58831215
chr1	58831490	58831751
chr1	58849342	58849536
chr1	58856775	58857144
chr1	58857144	58857399
chr1	58857831	58858123
chr1	58859288	58859497
chr1	58859675	58860229
chr1	58890245	58890414
chr1	58897592	58897912
chr1	58909555	58909930
chr1	58909930	58910171
chr1	58946068	58946607
chr1	58950873	58951252
chr1	58951292	58951648
chr1	58972976	58973709
chr1	58973709	58974104
chr1	58974104	58974452
chr1	58993234	58993453
chr1	59068073	59068301
chr1	59070922	59071175
chr1	59071209	59071365
chr1	59119653	59119991
chr1	59119991	59120598
chr1	59141486	59141650
chr1	59142890	59143055
chr1	59144616	59144831
chr1	59147696	59147920
chr1	59162519	59162886
chr1	59162886	59163569
chr1	59176572	59176795
chr1	59177956	59178166
chr1	59178217	59178514
chr1	59212166	59212333
chr1	59236808	59237086
chr1	59237086	59237292
chr1	59237292	59237578
chr1	59237620	59237838
chr1	59237995	59238269
chr1	59242774	59243021
chr1	59247727	59247953

chr1	59252849	59253070
chr1	59256672	59256970
chr1	59286605	59286760
chr1	59292099	59292329
chr1	59340672	59341205
chr1	59400286	59400677
chr1	59400725	59400885
chr1	59400969	59401144
chr1	59401144	59401258
chr1	59401258	59401309
chr1	59428059	59428209
chr1	59428810	59429159
chr1	59444182	59444645
chr1	59464047	59464401
chr1	59464740	59465366
chr1	59475714	59475872
chr1	59481575	59481805
chr1	59481847	59482263
chr1	59482263	59482665
chr1	59482665	59482970
chr1	59483143	59483362
chr1	59487597	59487828
chr1	59487828	59488158
chr1	59516688	59516885
chr1	59531346	59531563
chr1	59531563	59531805
chr1	59566841	59567233
chr1	59580113	59580778
chr1	59584736	59584943
chr1	59593750	59593986
chr1	59604044	59604238
chr1	59613838	59614061
chr1	59617715	59617872
chr1	59623380	59623697
chr1	59669944	59670250
chr1	59670250	59670616
chr1	59670616	59670939
chr1	59670939	59671289
chr1	59684738	59685312
chr1	59685509	59685714
chr1	59685753	59685907
chr1	59687559	59687825

chr1	59690575	59690745
chr1	59719163	59719352
chr1	59719475	59719718
chr1	59719718	59719985
chr1	59719985	59720304
chr1	59739348	59739524
chr1	59739594	59739905
chr1	59739909	59740217
chr1	59744920	59745072
chr1	59745122	59745382
chr1	59762662	59763187
chr1	59763187	59763605
chr1	59763605	59763820
chr1	59763820	59764044
chr1	59764044	59764305
chr1	59764420	59764570
chr1	59764607	59764975
chr1	59882546	59882761
chr1	59883243	59883583
chr1	59883801	59884049
chr1	59895207	59895533
chr1	59895533	59895807
chr1	59896070	59896239
chr1	59897825	59898194
chr1	59912502	59913136
chr1	59913136	59913463
chr1	59913463	59913715
chr1	59913715	59913967
chr1	59915951	59916197
chr1	59926381	59926555
chr1	59930475	59930674
chr1	59948090	59948434
chr1	59963656	59963972
chr1	59966188	59966495
chr1	59966495	59966775
chr1	59995523	59995878
chr1	59999945	60000292
chr1	60000292	60000776
chr1	60000849	60001099
chr1	60042552	60043090
chr1	60043090	60043480
chr1	60063853	60064066

chr1	60097533	60097806
chr1	60097806	60098091
chr1	60098091	60098379
chr1	60098379	60098804
chr1	60137367	60137957
chr1	60138526	60138880
chr1	60152794	60153011
chr1	60166577	60166821
chr1	60166821	60167109
chr1	60169425	60169824
chr1	60169824	60170045
chr1	60180036	60180448
chr1	60180448	60180676
chr1	60180676	60180939
chr1	60180939	60181205
chr1	60231957	60232177
chr1	60235175	60235484
chr1	60240547	60240718
chr1	60342813	60343115
chr1	60343115	60343411
chr1	60343411	60343843
chr1	60353367	60353556
chr1	60353556	60353732
chr1	60398253	60398449
chr1	60408940	60409095
chr1	60409153	60409540
chr1	60409540	60409871
chr1	60409871	60410283
chr1	60428451	60428605
chr1	60429027	60429204
chr1	60464571	60464936
chr1	60565215	60565608
chr1	60565610	60565760
chr1	60566186	60566400
chr1	60566400	60566642
chr1	60566642	60566924
chr1	60566924	60567352
chr1	60572838	60573043
chr1	60573139	60573408
chr1	60587708	60587954
chr1	60603010	60603287
chr1	60604583	60604831

chr1	60609045	60609199
chr1	60609199	60609423
chr1	60609423	60609802
chr1	60611287	60611531
chr1	60613259	60613628
chr1	60616707	60616863
chr1	60616888	60617230
chr1	60621788	60622155
chr1	60622901	60623183
chr1	60624147	60624515
chr1	60636752	60637108
chr1	60637108	60637408
chr1	60658397	60658743
chr1	60705046	60705225
chr1	60744425	60744600
chr1	60762592	60762745
chr1	60815962	60816199
chr1	60822990	60823267
chr1	60824577	60824786
chr1	60825861	60826255
chr1	60839306	60839493
chr1	60859893	60860188
chr1	60860683	60860879
chr1	60866612	60866841
chr1	60875259	60875680
chr1	60898769	60899009
chr1	60916588	60916810
chr1	60916875	60917114
chr1	60919997	60920181
chr1	60920283	60920866
chr1	60961143	60961350
chr1	60967977	60968309
chr1	60983091	60983254
chr1	60994305	60994477
chr1	61050454	61050604
chr1	61092173	61092360
chr1	61095436	61095869
chr1	61095869	61096175
chr1	61097431	61097652
chr1	61131713	61132097
chr1	61136077	61136534
chr1	61181164	61181417

chr1	61203170	61203566
chr1	61470671	61471079
chr1	61480593	61480809
chr1	61485593	61485781
chr1	61638644	61639037
chr1	61639037	61639365
chr1	61639365	61639627
chr1	61639662	61640066
chr1	61641967	61642128
chr1	61780033	61780221
chr1	61903317	61903514
chr1	61991619	61991774
chr1	62085971	62086156
chr1	62086156	62086348
chr1	62175073	62175365
chr1	62444524	62444674
chr1	62466674	62466872
chr1	62539876	62540169
chr1	62551331	62552046
chr1	62591702	62591889
chr1	62591889	62592084
chr1	62697037	62697264
chr1	62701092	62701375
chr1	62703002	62703793
chr1	62706019	62706644
chr1	62706644	62707068
chr1	62720041	62720590
chr1	62721736	62722338
chr1	62724441	62724609
chr1	62727285	62727448
chr1	62734212	62734392
chr1	62734434	62734854
chr1	62736495	62736875
chr1	62738166	62738532
chr1	62740285	62740530
chr1	62747405	62747746
chr1	62747746	62748147
chr1	62748304	62748456
chr1	62750453	62750919
chr1	62751440	62751627
chr1	62763828	62764153
chr1	62765518	62765721

chr1	62767618	62768228
chr1	62772124	62772343
chr1	62777223	62777615
chr1	62777615	62777944
chr1	62777944	62778177
chr1	62782736	62782937
chr1	62783327	62783829
chr1	62796402	62796572
chr1	62835279	62835512
chr1	62843249	62843531
chr1	62904690	62904905
chr1	62929571	62929811
chr1	62933388	62933546
chr1	62964333	62964630
chr1	62982802	62982993
chr1	62984520	62984761
chr1	62986413	62986578
chr1	62987712	62987891
chr1	63051015	63051282
chr1	63088209	63088398
chr1	63088703	63088871
chr1	63098942	63099117
chr1	63112877	63113221
chr1	63113384	63113629
chr1	63113629	63113904
chr1	63113904	63114250
chr1	63114250	63114564
chr1	63114564	63114858
chr1	63129966	63130145
chr1	63134463	63135087
chr1	63135425	63135695
chr1	63176313	63176550
chr1	63176552	63176941
chr1	63176941	63177222
chr1	63177222	63177456
chr1	63180590	63180763
chr1	63180763	63180954
chr1	63181012	63181392
chr1	63181557	63181729
chr1	63186373	63186540
chr1	63203992	63204203
chr1	63204355	63204547

chr1	63273168	63273328
chr1	63273533	63273743
chr1	63323738	63323948
chr1	63446120	63446445
chr1	63491092	63491298
chr1	63529356	63529537
chr1	63567099	63567426
chr1	63629789	63629965
chr1	63629965	63630299
chr1	63630299	63630694
chr1	63630694	63630950
chr1	63658084	63658344
chr1	63659695	63659914
chr1	63668870	63669129
chr1	63696538	63696898
chr1	63729943	63730208
chr1	63730234	63730465
chr1	63730486	63730833
chr1	63732645	63732883
chr1	63756068	63756230
chr1	63763193	63763375
chr1	63763375	63763561
chr1	63766478	63766777
chr1	63776365	63776762
chr1	63776840	63777281
chr1	63777281	63777591
chr1	63849921	63850220
chr1	63850220	63850544
chr1	63916862	63917212
chr1	63917297	63917678
chr1	63919896	63920316
chr1	63928477	63928686
chr1	63953549	63953837
chr1	63956590	63956740
chr1	63967870	63968202
chr1	63976857	63977280
chr1	63992763	63992948
chr1	63995107	63995259
chr1	63995294	63995457
chr1	63995856	63996173
chr1	63996173	63996411
chr1	63998226	63998386

chr1	63998801	63999226
chr1	64005059	64005706
chr1	64005786	64005979
chr1	64005979	64006207
chr1	64023534	64023732
chr1	64036972	64037443
chr1	64053617	64053877
chr1	64077269	64077614
chr1	64077614	64077909
chr1	64078368	64078675
chr1	64081848	64082109
chr1	64087972	64088257
chr1	64088257	64088895
chr1	64089014	64089318
chr1	64102207	64102684
chr1	64105334	64105732
chr1	64118773	64119187
chr1	64121170	64121511
chr1	64121511	64122015
chr1	64122015	64122497
chr1	64128498	64128686
chr1	64187875	64188532
chr1	64191603	64191885
chr1	64197181	64197642
chr1	64197642	64198035
chr1	64198346	64198584
chr1	64208930	64209645
chr1	64211076	64211328
chr1	64211328	64211630
chr1	64225802	64226087
chr1	64226087	64226464
chr1	64226464	64226791
chr1	64252696	64252851
chr1	64287153	64287389
chr1	64298204	64298391
chr1	64301177	64301676
chr1	64305360	64305640
chr1	64305640	64305981
chr1	64316583	64316738
chr1	64316763	64317087
chr1	64317087	64317509
chr1	64319276	64319438

chr1	64319555	64319807
chr1	64324100	64324305
chr1	64337062	64337265
chr1	64337730	64337954
chr1	64337954	64338442
chr1	64338487	64339003
chr1	64347623	64347911
chr1	64353501	64353673
chr1	64355320	64355570
chr1	64373966	64374448
chr1	64374710	64375092
chr1	64435237	64435399
chr1	64446080	64446356
chr1	64448167	64448325
chr1	64451469	64451674
chr1	64484513	64484778
chr1	64494649	64494853
chr1	64532035	64532324
chr1	64532324	64532657
chr1	64532657	64533357
chr1	64539090	64539311
chr1	64546134	64546348
chr1	64551983	64552295
chr1	64600511	64600718
chr1	64600718	64601008
chr1	64601309	64601566
chr1	64601566	64601859
chr1	64601868	64602041
chr1	64608180	64608586
chr1	64611097	64611328
chr1	64616699	64616863
chr1	64616863	64617097
chr1	64617097	64617386
chr1	64617386	64617658
chr1	64617658	64617934
chr1	64623740	64624318
chr1	64640226	64640580
chr1	64649448	64649656
chr1	64656437	64656753
chr1	64669486	64669748
chr1	64673980	64674366
chr1	64690284	64690589

chr1	64690589	64691108
chr1	64691108	64691546
chr1	64705304	64705562
chr1	64709963	64710146
chr1	64715424	64715798
chr1	64717594	64717838
chr1	64718063	64718357
chr1	64734858	64735014
chr1	64736132	64736584
chr1	64736992	64737403
chr1	64737403	64737817
chr1	64737817	64738131
chr1	64738161	64738477
chr1	64775091	64775406
chr1	64782293	64782546
chr1	64787499	64788145
chr1	64790872	64791024
chr1	64795329	64795665
chr1	64795665	64796085
chr1	64796847	64797196
chr1	64797196	64797591
chr1	64797591	64797886
chr1	64807242	64807462
chr1	64808967	64809554
chr1	64826333	64826762
chr1	64842811	64843038
chr1	64852286	64852519
chr1	64853810	64854296
chr1	64855456	64855824
chr1	64859856	64860246
chr1	64860614	64860994
chr1	64868725	64868913
chr1	64875698	64875848
chr1	64876351	64876628
chr1	64889735	64890025
chr1	64907847	64908091
chr1	64908988	64909225
chr1	64919075	64919230
chr1	64929124	64929371
chr1	64929792	64929949
chr1	64932975	64933327
chr1	64941846	64942402

chr1	64942402	64942894
chr1	64949138	64949500
chr1	64949500	64949756
chr1	64950074	64950313
chr1	64950372	64950686
chr1	64956415	64957079
chr1	64957100	64957271
chr1	64970720	64971032
chr1	64986100	64986481
chr1	64988371	64988540
chr1	65001561	65001793
chr1	65010173	65010499
chr1	65112098	65112329
chr1	65117987	65118260
chr1	65118260	65118501
chr1	65118563	65118836
chr1	65129757	65129920
chr1	65130320	65130490
chr1	65154803	65155171
chr1	65178096	65178605
chr1	65178605	65178968
chr1	65178968	65179519
chr1	65186206	65187220
chr1	65245675	65246001
chr1	65246001	65246234
chr1	65282841	65283051
chr1	65311660	65311810
chr1	65312996	65313189
chr1	65337803	65338308
chr1	65342496	65342679
chr1	65353065	65353342
chr1	65354140	65354635
chr1	65354667	65354906
chr1	65357611	65357777
chr1	65362062	65362284
chr1	65388449	65388607
chr1	65389401	65389573
chr1	65389620	65390073
chr1	65390073	65390465
chr1	65407315	65407558
chr1	65407558	65407838
chr1	65422479	65422710

chr1	65422710	65422989
chr1	65423404	65423614
chr1	65440134	65440762
chr1	65470134	65470416
chr1	65574960	65575207
chr1	65619280	65619469
chr1	65625912	65626117
chr1	65667830	65667989
chr1	65808618	65808996
chr1	65829089	65829278
chr1	65847135	65847315
chr1	65932110	65932418
chr1	65932418	65932899
chr1	65933000	65933263
chr1	65933389	65933553
chr1	66098828	66098986
chr1	66108109	66108355
chr1	66128449	66128698
chr1	66133084	66133400
chr1	66145158	66145523
chr1	66147282	66147434
chr1	66168758	66169217
chr1	66174938	66175378
chr1	66175378	66175749
chr1	66244867	66245369
chr1	66257708	66257916
chr1	66280498	66281056
chr1	66302492	66302719
chr1	66311687	66311936
chr1	66467908	66468329
chr1	66585933	66586255
chr1	66617251	66617477
chr1	66649571	66649919
chr1	66650109	66650339
chr1	66655210	66655398
chr1	66700617	66700966
chr1	66700966	66701544
chr1	66815981	66816314
chr1	66816445	66816742
chr1	66816742	66817018
chr1	66817030	66817251
chr1	66817251	66817466

chr1	66817466	66817648
chr1	66817648	66817836
chr1	66817836	66818056
chr1	66828566	66828736
chr1	66838581	66838758
chr1	66845706	66846274
chr1	66846274	66846588
chr1	66861933	66862087
chr1	66862656	66863105
chr1	66863105	66863544
chr1	66881838	66882064
chr1	66884393	66884573
chr1	66895407	66895623
chr1	66895755	66896061
chr1	66896157	66896445
chr1	66901042	66901263
chr1	66996716	66997284
chr1	66997911	66998135
chr1	67003705	67004002
chr1	67038377	67038681
chr1	67038681	67039071
chr1	67061498	67061770
chr1	67246727	67246882
chr1	67288081	67288241
chr1	67450857	67451195
chr1	67496967	67497161
chr1	67692141	67692337
chr1	68501592	68501835
chr1	68720651	68720948
chr1	68720948	68721264
chr1	68743483	68743769
chr1	68756022	68756218
chr1	68780142	68780327
chr1	69106559	69106784
chr1	69106877	69107296
chr1	69165294	69165530
chr1	69210169	69210590
chr1	69241867	69242277
chr1	69275913	69276109
chr1	69276994	69277183
chr1	69277183	69277427
chr1	69277427	69277801

chr1	69277801	69278110
chr1	69285231	69285572
chr1	69286311	69286768
chr1	69331711	69331976
chr1	69367583	69367950
chr1	69368052	69368367
chr1	69368744	69368926
chr1	69369321	69370052
chr1	69370052	69370386
chr1	69379765	69380069
chr1	69397733	69397991
chr1	69398690	69398990
chr1	69401383	69401826
chr1	69427508	69427759
chr1	69427759	69428226
chr1	69433568	69433999
chr1	69433999	69434500
chr1	69440638	69440912
chr1	69440912	69441407
chr1	69444517	69444765
chr1	69450507	69450833
chr1	69452919	69453242
chr1	69453242	69453519
chr1	69456734	69456913
chr1	69460782	69461116
chr1	69461116	69461566
chr1	69467911	69468336
chr1	69469201	69469439
chr1	69470916	69471227
chr1	69504888	69505073
chr1	69507742	69507958
chr1	69508321	69508562
chr1	69585301	69585548
chr1	69589870	69590081
chr1	69609970	69610261
chr1	69610261	69610702
chr1	69610702	69611209
chr1	69611209	69611645
chr1	69655467	69655753
chr1	69655850	69656116
chr1	69657013	69657190
chr1	69657999	69658523

chr1	69658593	69658815
chr1	69686100	69686286
chr1	69686723	69686880
chr1	69686969	69687417
chr1	69687417	69688049
chr1	69688301	69688486
chr1	69689041	69689343
chr1	69728011	69728322
chr1	69752075	69752226
chr1	69775704	69775856
chr1	69833557	69833888
chr1	70077894	70078284
chr1	70078848	70079215
chr1	70082278	70082540
chr1	70082648	70082972
chr1	70115298	70115471
chr1	70135510	70135777
chr1	70164337	70164579
chr1	70339607	70339849
chr1	70504663	70504850
chr1	70523242	70523425
chr1	70621634	70622179
chr1	70622179	70622401
chr1	70665433	70665593
chr1	70698175	70698390
chr1	70725635	70725895
chr1	70776639	70776943
chr1	70776989	70777450
chr1	70777520	70777693
chr1	70795177	70795432
chr1	70821323	70821635
chr1	70821635	70822115
chr1	70846885	70847173
chr1	70884249	70884443
chr1	70885534	70885724
chr1	70916876	70917145
chr1	70918240	70918673
chr1	70918673	70918919
chr1	70926174	70926509
chr1	70936485	70936645
chr1	70937809	70938210
chr1	70959854	70960012

chr1	71000325	71000847
chr1	71003752	71003984
chr1	71004138	71004662
chr1	71004768	71004971
chr1	71014118	71014416
chr1	71014450	71014664
chr1	71018731	71018978
chr1	71019536	71019869
chr1	71024531	71024835
chr1	71030580	71030801
chr1	71037069	71037278
chr1	71056197	71056534
chr1	71079274	71079482
chr1	71095252	71095609
chr1	71102246	71102799
chr1	71102799	71103367
chr1	71219119	71219318
chr1	71289606	71289817
chr1	71310699	71310891
chr1	71323349	71323499
chr1	71339320	71339536
chr1	71366454	71366771
chr1	71395997	71396464
chr1	71396480	71396668
chr1	71402165	71402360
chr1	71413069	71413387
chr1	71413387	71413698
chr1	71414875	71415120
chr1	71432427	71432579
chr1	71458045	71458308
chr1	71460695	71461342
chr1	71478598	71479021
chr1	71479143	71479315
chr1	71531669	71532004
chr1	71546521	71546696
chr1	71556765	71557290
chr1	71557290	71557689
chr1	71567931	71568162
chr1	71575781	71575992
chr1	71576172	71576419
chr1	71580482	71580791
chr1	71582532	71582801

chr1	71582937	71583180
chr1	71583206	71583454
chr1	71583454	71583693
chr1	71586775	71586978
chr1	71589583	71589788
chr1	71590861	71591018
chr1	71593054	71593204
chr1	71597428	71597578
chr1	71601087	71601347
chr1	71610002	71610219
chr1	71625835	71626004
chr1	71626068	71626246
chr1	71629580	71629776
chr1	71638126	71638389
chr1	71638425	71638575
chr1	71639101	71639333
chr1	71641817	71641988
chr1	71642962	71643139
chr1	71643301	71643661
chr1	71652582	71653046
chr1	71653046	71653949
chr1	71667204	71667855
chr1	71691599	71691879
chr1	71691879	71692185
chr1	71723409	71723741
chr1	71723823	71724262
chr1	71752315	71752740
chr1	71755432	71755805
chr1	71756200	71756560
chr1	71756656	71756902
chr1	71763575	71764012
chr1	71814391	71814669
chr1	71830426	71830694
chr1	71837121	71837338
chr1	71837338	71837641
chr1	71851116	71851784
chr1	71865487	71866055
chr1	71880399	71880958
chr1	71886994	71887189
chr1	71887784	71888302
chr1	71893288	71893603
chr1	71893603	71894033

chr1	71894033	71894300
chr1	71895817	71896092
chr1	71912450	71912671
chr1	71912673	71912837
chr1	71919030	71919414
chr1	71919871	71920301
chr1	71921252	71921427
chr1	71923767	71924083
chr1	71936647	71937019
chr1	71945125	71945487
chr1	71948676	71949088
chr1	71952643	71952795
chr1	71963499	71963667
chr1	71970313	71970885
chr1	71977323	71977743
chr1	71983856	71984088
chr1	72002563	72002746
chr1	72013915	72014263
chr1	72022622	72022936
chr1	72069208	72069395
chr1	72070473	72070976
chr1	72075566	72075943
chr1	72076103	72076841
chr1	72079030	72079473
chr1	72079661	72079880
chr1	72083218	72083532
chr1	72084974	72085248
chr1	72099101	72099516
chr1	72099516	72100023
chr1	72105366	72105956
chr1	72106304	72106607
chr1	72107231	72107403
chr1	72114395	72114594
chr1	72123506	72123732
chr1	72123732	72123983
chr1	72141939	72142175
chr1	72144406	72144974
chr1	72172835	72173216
chr1	72173352	72174028
chr1	72182510	72182714
chr1	72182921	72183268
chr1	72183268	72183549

chr1	72186317	72186485
chr1	72197184	72197334
chr1	72198781	72198938
chr1	72202743	72202961
chr1	72211759	72212214
chr1	72212214	72212483
chr1	72220070	72220301
chr1	72225856	72226105
chr1	72226155	72226477
chr1	72226477	72226668
chr1	72226668	72226980
chr1	72226980	72227452
chr1	72227580	72227981
chr1	72236360	72236597
chr1	72236597	72236828
chr1	72236861	72237307
chr1	72237307	72237708
chr1	72237708	72238044
chr1	72238044	72238359
chr1	72242711	72242901
chr1	72243684	72244088
chr1	72244129	72244614
chr1	72244614	72245281
chr1	72254654	72254871
chr1	72254924	72255370
chr1	72255370	72255867
chr1	72255867	72256360
chr1	72256480	72256771
chr1	72283933	72284525
chr1	72306944	72307226
chr1	72307226	72307835
chr1	72309813	72310071
chr1	72352588	72352811
chr1	72352894	72353108
chr1	72371662	72371856
chr1	72371983	72372366
chr1	72372456	72372705
chr1	72372705	72372955
chr1	72379627	72379796
chr1	72379796	72379964
chr1	72399713	72400085
chr1	72400085	72400364

chr1	72400443	72400606
chr1	72405581	72405770
chr1	72420416	72420641
chr1	72428535	72428983
chr1	72428983	72429567
chr1	72449448	72449713
chr1	72449757	72450061
chr1	72450061	72450316
chr1	72450316	72450551
chr1	72450551	72450758
chr1	72450817	72451061
chr1	72456906	72457158
chr1	72457158	72457379
chr1	72457379	72457665
chr1	72464908	72465311
chr1	72465311	72465555
chr1	72466085	72466639
chr1	72476635	72476956
chr1	72509430	72509606
chr1	72509622	72510095
chr1	72511193	72511402
chr1	72513948	72514099
chr1	72536655	72536954
chr1	72536954	72537314
chr1	72538379	72538625
chr1	72550374	72550663
chr1	72551013	72551182
chr1	72556510	72556816
chr1	72564408	72564981
chr1	72582958	72583372
chr1	72583372	72583821
chr1	72593516	72593763
chr1	72599216	72599569
chr1	72599569	72600156
chr1	72601276	72601506
chr1	72602238	72602535
chr1	72609235	72609489
chr1	72676519	72676721
chr1	72710740	72711084
chr1	72711084	72711357
chr1	72711357	72711580
chr1	72711580	72711894

chr1	72716614	72716767
chr1	72717036	72717279
chr1	72718890	72719237
chr1	72798592	72798754
chr1	72824734	72824884
chr1	72854972	72855237
chr1	72865886	72866042
chr1	72870228	72870503
chr1	72871065	72871344
chr1	72871913	72872063
chr1	72872609	72872765
chr1	72873822	72874010
chr1	72877904	72878101
chr1	72963387	72963856
chr1	73007631	73007797
chr1	73026865	73027266
chr1	73027293	73027779
chr1	73027830	73028126
chr1	73032568	73032848
chr1	73043630	73043871
chr1	73046843	73047094
chr1	73051471	73051964
chr1	73123107	73123326
chr1	73213051	73213206
chr1	73235241	73235608
chr1	73257338	73257582
chr1	73270771	73271062
chr1	73826372	73826623
chr1	73853488	73853656
chr1	73862775	73863081
chr1	73883440	73883752
chr1	73901397	73901587
chr1	73901587	73902035
chr1	73902175	73902391
chr1	73902391	73902643
chr1	73907056	73907631
chr1	73926340	73926662
chr1	73928399	73928762
chr1	73935769	73935998
chr1	73936064	73936333
chr1	73936510	73937199
chr1	73947378	73947944

chr1	73954542	73955140
chr1	73955333	73956048
chr1	73958153	73958555
chr1	73962694	73963016
chr1	73963016	73963582
chr1	73968600	73968833
chr1	73986828	73987011
chr1	73987822	73988158
chr1	73991001	73991256
chr1	73996252	73996572
chr1	73998072	73998369
chr1	73998369	73998846
chr1	73998933	73999113
chr1	73999113	73999347
chr1	74007983	74008137
chr1	74008226	74008536
chr1	74008536	74008979
chr1	74008979	74009314
chr1	74015845	74016145
chr1	74027545	74028006
chr1	74028626	74029206
chr1	74036575	74037407
chr1	74042366	74042789
chr1	74042789	74043136
chr1	74043807	74043957
chr1	74054034	74054365
chr1	74058427	74058750
chr1	74063907	74064063
chr1	74066086	74066272
chr1	74069185	74069378
chr1	74069390	74069848
chr1	74070078	74070542
chr1	74076262	74076671
chr1	74077441	74077688
chr1	74081601	74081766
chr1	74082915	74083159
chr1	74084470	74084746
chr1	74084748	74085116
chr1	74092332	74092719
chr1	74095739	74096105
chr1	74098123	74098479
chr1	74098479	74098856

chr1	74098856	74099117
chr1	74102774	74102937
chr1	74121901	74122198
chr1	74122198	74122594
chr1	74124366	74124739
chr1	74124739	74124990
chr1	74125044	74125638
chr1	74126412	74126588
chr1	74132111	74132267
chr1	74147909	74148119
chr1	74149107	74149720
chr1	74155207	74155577
chr1	74155577	74156002
chr1	74162134	74162842
chr1	74182282	74182529
chr1	74196058	74196284
chr1	74227248	74227626
chr1	74236066	74236216
chr1	74236243	74236449
chr1	74236449	74236812
chr1	74236812	74237222
chr1	74239244	74239407
chr1	74239753	74239944
chr1	74240157	74240432
chr1	74248805	74249188
chr1	74249277	74249653
chr1	74249653	74249907
chr1	74270063	74270235
chr1	74271687	74271951
chr1	74275187	74275517
chr1	74276658	74276825
chr1	74276925	74277204
chr1	74277952	74278224
chr1	74279335	74279510
chr1	74284155	74284537
chr1	74284539	74285159
chr1	74287239	74287476
chr1	74295083	74295451
chr1	74295451	74295799
chr1	74295799	74296129
chr1	74296479	74296637
chr1	74297229	74297706

chr1	74300512	74300851
chr1	74303539	74303851
chr1	74303851	74304066
chr1	74304066	74304307
chr1	74304307	74304526
chr1	74313338	74313677
chr1	74332237	74332902
chr1	74340530	74340874
chr1	74340874	74341204
chr1	74351396	74351587
chr1	74352587	74353045
chr1	74361271	74361421
chr1	74361817	74362284
chr1	74367043	74367306
chr1	74381362	74381755
chr1	74386624	74387041
chr1	74387041	74387362
chr1	74391282	74391783
chr1	74391789	74392176
chr1	74392176	74392413
chr1	74397035	74397478
chr1	74397478	74397770
chr1	74397770	74398063
chr1	74411819	74412250
chr1	74415088	74415560
chr1	74431997	74432271
chr1	74432271	74432568
chr1	74433207	74433475
chr1	74434620	74434977
chr1	74438066	74438501
chr1	74452555	74452827
chr1	74452827	74453053
chr1	74460365	74460515
chr1	74492688	74493010
chr1	74496596	74496813
chr1	74505438	74505715
chr1	74505715	74506107
chr1	74506107	74506526
chr1	74506526	74506867
chr1	74506965	74507115
chr1	74537851	74538043
chr1	74551239	74551473

chr1	74570081	74570411
chr1	74578883	74579615
chr1	74579773	74579959
chr1	74587439	74587689
chr1	74587689	74588045
chr1	74588045	74588557
chr1	74600878	74601068
chr1	74601068	74601299
chr1	74601299	74601570
chr1	74601570	74601890
chr1	74610460	74610745
chr1	74610745	74610997
chr1	74643998	74644235
chr1	74661389	74662223
chr1	74712127	74712323
chr1	74713173	74713338
chr1	74713338	74713582
chr1	74713582	74713867
chr1	74714015	74714246
chr1	74723873	74724030
chr1	74726643	74727247
chr1	74748666	74749064
chr1	74749207	74749708
chr1	74754223	74754405
chr1	74759895	74760169
chr1	74761287	74761639
chr1	74762115	74762267
chr1	74763171	74763416
chr1	74771651	74771801
chr1	74771877	74772076
chr1	74772076	74772296
chr1	74773087	74773513
chr1	74784053	74784294
chr1	74784294	74784531
chr1	74784531	74784781
chr1	74787892	74788184
chr1	74791369	74791859
chr1	74791859	74792131
chr1	74792131	74792332
chr1	74793278	74793644
chr1	74803003	74803561
chr1	74804768	74804977

chr1	74805276	74805840
chr1	74806349	74806617
chr1	74806617	74806928
chr1	74807008	74807442
chr1	74808130	74808495
chr1	74816572	74816877
chr1	74816877	74817322
chr1	74851894	74852320
chr1	74854702	74855175
chr1	74855218	74855437
chr1	74857712	74857967
chr1	74858104	74858366
chr1	74858366	74858550
chr1	74858550	74858715
chr1	74863358	74863538
chr1	74880824	74881101
chr1	74882084	74882546
chr1	74891645	74891818
chr1	74892880	74893486
chr1	74893546	74893724
chr1	74895103	74895279
chr1	74900389	74900698
chr1	74900698	74901380
chr1	74926782	74927009
chr1	74948854	74949166
chr1	74949586	74950036
chr1	74950170	74950384
chr1	74962881	74963093
chr1	74964490	74964772
chr1	74968010	74968330
chr1	74971684	74972009
chr1	74998943	74999153
chr1	75016907	75017176
chr1	75017420	75017583
chr1	75024424	75024667
chr1	75024771	75025365
chr1	75036637	75036934
chr1	75040390	75040873
chr1	75051528	75051755
chr1	75051794	75051944
chr1	75124239	75124441
chr1	75124524	75124844

chr1	75124844	75125095
chr1	75125095	75125375
chr1	75133237	75133482
chr1	75133482	75133901
chr1	75140767	75140970
chr1	75141337	75142011
chr1	75142308	75142552
chr1	75142552	75142752
chr1	75142752	75142939
chr1	75142939	75143307
chr1	75168187	75168469
chr1	75168469	75168801
chr1	75168801	75169103
chr1	75169103	75169337
chr1	75179134	75179589
chr1	75179589	75179870
chr1	75179870	75180086
chr1	75180086	75180317
chr1	75180317	75180625
chr1	75191467	75191899
chr1	75191899	75192500
chr1	75192520	75192794
chr1	75210504	75210654
chr1	75210656	75211114
chr1	75214513	75215007
chr1	75218563	75219030
chr1	75219124	75219469
chr1	75219469	75219709
chr1	75219709	75220026
chr1	75221567	75222006
chr1	75230652	75231214
chr1	75231265	75231581
chr1	75234653	75234940
chr1	75236103	75236401
chr1	75236401	75236682
chr1	75236682	75237068
chr1	75237070	75237226
chr1	75244971	75245154
chr1	75248024	75248467
chr1	75248467	75248823
chr1	75251619	75251939
chr1	75254313	75254542

chr1	75263012	75263301
chr1	75263301	75263605
chr1	75263605	75263929
chr1	75264077	75264445
chr1	75277286	75277492
chr1	75283182	75283334
chr1	75289493	75289670
chr1	75292419	75292671
chr1	75292741	75293253
chr1	75316930	75317244
chr1	75317244	75317539
chr1	75317539	75317847
chr1	75317847	75318161
chr1	75337815	75338125
chr1	75338125	75338438
chr1	75341238	75341436
chr1	75342932	75343174
chr1	75347589	75347777
chr1	75360355	75360513
chr1	75369595	75369773
chr1	75371470	75371704
chr1	75380760	75381085
chr1	75381544	75381700
chr1	75381968	75382178
chr1	75382801	75383025
chr1	75388253	75388445
chr1	75392941	75393494
chr1	75398724	75399077
chr1	75399077	75399356
chr1	75399902	75400216
chr1	75408330	75408483
chr1	75413844	75414021
chr1	75422897	75423138
chr1	75423138	75423482
chr1	75425886	75426043
chr1	75427500	75427777
chr1	75435783	75436095
chr1	75436095	75436399
chr1	75445761	75446084
chr1	75450682	75450855
chr1	75450945	75451095
chr1	75455554	75455793

chr1	75457963	75458153
chr1	75458316	75458577
chr1	75458577	75458778
chr1	75460569	75460849
chr1	75460849	75461199
chr1	75461199	75461662
chr1	75461662	75462157
chr1	75462157	75462511
chr1	75462511	75462757
chr1	75467306	75467463
chr1	75467508	75467954
chr1	75467964	75468148
chr1	75477217	75477384
chr1	75477422	75477814
chr1	75478590	75478814
chr1	75478814	75479085
chr1	75479085	75479405
chr1	75479405	75479714
chr1	75486543	75487066
chr1	75487098	75487334
chr1	75487422	75487657
chr1	75487657	75487923
chr1	75487923	75488224
chr1	75495243	75495517
chr1	75499014	75499244
chr1	75499537	75499734
chr1	75505055	75505416
chr1	75505416	75505809
chr1	75505809	75506202
chr1	75506202	75506575
chr1	75506575	75506914
chr1	75513325	75513487
chr1	75518021	75518420
chr1	75521198	75521637
chr1	75521637	75521879
chr1	75521879	75522089
chr1	75530580	75530840
chr1	75546153	75546989
chr1	75559255	75559532
chr1	75559532	75560011
chr1	75575077	75575259
chr1	75588508	75588676

chr1	75615098	75615321
chr1	75618294	75618573
chr1	75618576	75618735
chr1	75635758	75635918
chr1	75652593	75652812
chr1	75653700	75653994
chr1	75896133	75896310
chr1	76010575	76010752
chr1	76018108	76018293
chr1	76019144	76019573
chr1	76019597	76019897
chr1	76296569	76296861
chr1	76570365	76570706
chr1	76616458	76616632
chr1	76644870	76645320
chr1	76646013	76646404
chr1	76776616	76776842
chr1	76927144	76927312
chr1	76928059	76928390
chr1	76958235	76958403
chr1	76970753	76970993
chr1	76993867	76994070
chr1	77008060	77008326
chr1	77008417	77008704
chr1	77051289	77051591
chr1	77143052	77143431
chr1	77163347	77163668
chr1	77206570	77206869
chr1	77213299	77213505
chr1	77216421	77216583
chr1	77219535	77219781
chr1	77219865	77220231
chr1	77258818	77259019
chr1	77363160	77363484
chr1	77363653	77364342
chr1	77364637	77364837
chr1	77365897	77366073
chr1	77374179	77374361
chr1	77396189	77396339
chr1	77399820	77399997
chr1	77420426	77420576
chr1	77421199	77421482

chr1	77433238	77433466
chr1	77439743	77439991
chr1	77444759	77445038
chr1	77445038	77445401
chr1	77454073	77454345
chr1	77459056	77459307
chr1	77460617	77461518
chr1	77462354	77462510
chr1	77463353	77463667
chr1	77465624	77465779
chr1	77467387	77467938
chr1	77471084	77471508
chr1	77473447	77473754
chr1	77489429	77489779
chr1	77490262	77490513
chr1	77490513	77490764
chr1	77503808	77504067
chr1	77504067	77504363
chr1	77504779	77505116
chr1	77506018	77506273
chr1	77506362	77506525
chr1	77506834	77507037
chr1	77513493	77513692
chr1	77513692	77513889
chr1	77514651	77515032
chr1	77515032	77515331
chr1	77515331	77515589
chr1	77515705	77516049
chr1	77516049	77516314
chr1	77516314	77516649
chr1	77516739	77516999
chr1	77523431	77523691
chr1	77535964	77536251
chr1	77536251	77536533
chr1	77538326	77538575
chr1	77538628	77538995
chr1	77616570	77616822
chr1	77631026	77631197
chr1	77631197	77631356
chr1	77674714	77674895
chr1	77695180	77695726
chr1	77749127	77749279

chr1	77809086	77809252
chr1	77820204	77820423
chr1	77832260	77832570
chr1	77839668	77839906
chr1	77845880	77846611
chr1	77861731	77862017
chr1	77869190	77869491
chr1	77870182	77870399
chr1	77876877	77877077
chr1	77879069	77879377
chr1	77959385	77959615
chr1	77978762	77978997
chr1	78033025	78033175
chr1	78057943	78058232
chr1	78060918	78061215
chr1	78088665	78088944
chr1	78196581	78196886
chr1	78196886	78197367
chr1	78203605	78203755
chr1	78203839	78204027
chr1	78204027	78204213
chr1	78215812	78216145
chr1	78216209	78216373
chr1	78216517	78216810
chr1	78216810	78217181
chr1	78217181	78217714
chr1	78269383	78269624
chr1	78269840	78270031
chr1	78272897	78273292
chr1	78310029	78310474
chr1	78310474	78310796
chr1	78310886	78311036
chr1	78320374	78320614
chr1	78322581	78322742
chr1	78347600	78347841
chr1	78366021	78366272
chr1	78387889	78388196
chr1	78388226	78388379
chr1	78388658	78388875
chr1	78395276	78395568
chr1	78396317	78396639
chr1	78398638	78398881

chr1	78419726	78419910
chr1	78435938	78436389
chr1	78436389	78436863
chr1	78437020	78437581
chr1	78447973	78448265
chr1	78448265	78448760
chr1	78488385	78488974
chr1	78489037	78489291
chr1	78489291	78489647
chr1	78511876	78512176
chr1	78521310	78521516
chr1	78525285	78525446
chr1	78528859	78529035
chr1	78529041	78529374
chr1	78529374	78529628
chr1	78530304	78530733
chr1	78536865	78537065
chr1	78537514	78538040
chr1	78538463	78538747
chr1	78554092	78554573
chr1	78559313	78559953
chr1	78568216	78568452
chr1	78573659	78574267
chr1	78574267	78574722
chr1	78575253	78575462
chr1	78585130	78585679
chr1	78586953	78587310
chr1	78602124	78602297
chr1	78603024	78603243
chr1	78609677	78609827
chr1	78622131	78622513
chr1	78629024	78629596
chr1	78642552	78642824
chr1	78644005	78644518
chr1	78644518	78644968
chr1	78646938	78647123
chr1	78647352	78647791
chr1	78649300	78649590
chr1	78656431	78656830
chr1	78657554	78658120
chr1	78658120	78658549
chr1	78684732	78684902

chr1	78686969	78687165
chr1	78687773	78688042
chr1	78701851	78702047
chr1	78704986	78705318
chr1	78724590	78724842
chr1	78724842	78725139
chr1	78725139	78725427
chr1	78725669	78725894
chr1	78725894	78726112
chr1	78744522	78744776
chr1	78745010	78745479
chr1	78745479	78745832
chr1	78770925	78771098
chr1	78775399	78775567
chr1	78781969	78782226
chr1	78807565	78807715
chr1	78810754	78811175
chr1	78811695	78812179
chr1	78812179	78812626
chr1	78814743	78814957
chr1	78846610	78846761
chr1	78866010	78866337
chr1	78866590	78866814
chr1	78867003	78867162
chr1	78876165	78876470
chr1	78895782	78896089
chr1	78897463	78897809
chr1	78908092	78908326
chr1	78924962	78925206
chr1	78999571	78999875
chr1	79074600	79074801
chr1	79075484	79075666
chr1	79090341	79090523
chr1	79105980	79106307
chr1	79144082	79144514
chr1	79144514	79144746
chr1	79176855	79177325
chr1	79188804	79189027
chr1	79189358	79189679
chr1	79190530	79190806
chr1	79190946	79191160
chr1	79191160	79191446

chr1	79197789	79198018
chr1	79315663	79315843
chr1	79362710	79363007
chr1	79363324	79363502
chr1	79400410	79400634
chr1	79424376	79424708
chr1	79424708	79425019
chr1	79431059	79431398
chr1	79432403	79432669
chr1	79439908	79440322
chr1	79457675	79457841
chr1	79470594	79470915
chr1	79470915	79471252
chr1	79506848	79507250
chr1	79507762	79507956
chr1	79520946	79521225
chr1	79521730	79522088
chr1	79559334	79559734
chr1	79561078	79561230
chr1	79561233	79561560
chr1	79570532	79570736
chr1	79581078	79581397
chr1	79581397	79581761
chr1	79637552	79638103
chr1	79638103	79638360
chr1	79638360	79638641
chr1	79639155	79639381
chr1	79640227	79640632
chr1	79641542	79641792
chr1	79671486	79671852
chr1	79671852	79672089
chr1	79672089	79672366
chr1	79678216	79678413
chr1	79678413	79678723
chr1	79678723	79679195
chr1	79679199	79679475
chr1	79691493	79691881
chr1	79697029	79697366
chr1	79697366	79697793
chr1	79698287	79698445
chr1	79736318	79736807
chr1	79741214	79741411

chr1	79746021	79746484
chr1	79749542	79749728
chr1	79759503	79759905
chr1	79760696	79761080
chr1	79761109	79761563
chr1	79761563	79761915
chr1	79775880	79776512
chr1	79782469	79782688
chr1	79786674	79786833
chr1	79819417	79819715
chr1	79822590	79822849
chr1	79822849	79823135
chr1	79823135	79823456
chr1	79858046	79858763
chr1	79858763	79859016
chr1	79859016	79859242
chr1	79859476	79859725
chr1	79928748	79928905
chr1	80025830	80026151
chr1	80171241	80171391
chr1	80171553	80172388
chr1	80172704	80172890
chr1	80177637	80177947
chr1	80180256	80180406
chr1	80278816	80279048
chr1	80308954	80309174
chr1	80339435	80339646
chr1	80340163	80340512
chr1	80340512	80340896
chr1	80340896	80341414
chr1	80362419	80362852
chr1	80395441	80395596
chr1	80405223	80405534
chr1	80418042	80418313
chr1	80418374	80418652
chr1	80418722	80419002
chr1	80447195	80447413
chr1	80447586	80447925
chr1	80447930	80448239
chr1	80496412	80496638
chr1	80506086	80506415
chr1	80512715	80512883

chr1	80603195	80603386
chr1	80606147	80606566
chr1	80607605	80607781
chr1	80609757	80609911
chr1	80639780	80640073
chr1	80641793	80642212
chr1	80642328	80642497
chr1	80714030	80714190
chr1	80717551	80717727
chr1	80855274	80855426
chr1	81310186	81310409
chr1	81316675	81316879
chr1	81369172	81369358
chr1	81516708	81516863
chr1	81525886	81526128
chr1	81591746	81591962
chr1	81623863	81624174
chr1	81624174	81624508
chr1	81625958	81626109
chr1	81630529	81630769
chr1	81636105	81636308
chr1	81636404	81636556
chr1	81671070	81671473
chr1	81671838	81672096
chr1	81677790	81677974
chr1	81726414	81726724
chr1	81726724	81727019
chr1	81733953	81734254
chr1	81735606	81735885
chr1	81740029	81740355
chr1	81743448	81743708
chr1	81744551	81744981
chr1	81745685	81746007
chr1	81746130	81746322
chr1	81754193	81754359
chr1	81755295	81755632
chr1	81755715	81756120
chr1	81756318	81756479
chr1	81759961	81760235
chr1	81859501	81859940
chr1	81860046	81860314
chr1	81869665	81869831

chr1	81912591	81913006
chr1	81913442	81913824
chr1	81913824	81914018
chr1	81915052	81915220
chr1	81933725	81933923
chr1	81940323	81940582
chr1	81950094	81950407
chr1	81951535	81951937
chr1	81954547	81954918
chr1	81987443	81987804
chr1	81991236	81991429
chr1	82031240	82031525
chr1	82031541	82031838
chr1	82032402	82032802
chr1	82110393	82110543
chr1	82150785	82151221
chr1	82155939	82156454
chr1	82157144	82157294
chr1	82157463	82157661
chr1	82158567	82158803
chr1	82162874	82163253
chr1	82189537	82190163
chr1	82283040	82283750
chr1	82283952	82284148
chr1	82290106	82290261
chr1	82290635	82290964
chr1	82290964	82291353
chr1	82291353	82291864
chr1	82316179	82316810
chr1	82316810	82317147
chr1	82347693	82347876
chr1	82358936	82359324
chr1	82362372	82362567
chr1	82363874	82364179
chr1	82371909	82372189
chr1	82373709	82374029
chr1	82414552	82414738
chr1	82428767	82429092
chr1	82429092	82429419
chr1	82429419	82429639
chr1	82433446	82433650
chr1	82477314	82477787

chr1	82477787	82478098
chr1	82526039	82526409
chr1	82536613	82536766
chr1	82536805	82537043
chr1	82537902	82538084
chr1	82554407	82554676
chr1	82574473	82574713
chr1	82577884	82578215
chr1	82586462	82586752
chr1	82586752	82587094
chr1	82587094	82587403
chr1	82587403	82587649
chr1	82597239	82597627
chr1	82598985	82599564
chr1	82603330	82603506
chr1	82603607	82603758
chr1	82605310	82605756
chr1	82610235	82610478
chr1	82613224	82613387
chr1	82616371	82616521
chr1	82634529	82634679
chr1	82648795	82648983
chr1	82701590	82701774
chr1	82724699	82725005
chr1	82725005	82725279
chr1	82725297	82725533
chr1	82785598	82785883
chr1	82787391	82787842
chr1	82793118	82793441
chr1	82795099	82795597
chr1	82795697	82795975
chr1	82796017	82796356
chr1	82801163	82801428
chr1	82801428	82801713
chr1	82801787	82802061
chr1	82803691	82803918
chr1	82811690	82812206
chr1	82826056	82826461
chr1	82831794	82832266
chr1	82834597	82834911
chr1	82839189	82839606
chr1	82839606	82840442

chr1	82879188	82879668
chr1	82902203	82902379
chr1	82902924	82903222
chr1	82903590	82903750
chr1	82905520	82905786
chr1	82913013	82913182
chr1	82913262	82913448
chr1	82913448	82913675
chr1	82916884	82917116
chr1	82938820	82938975
chr1	82946171	82946370
chr1	82946687	82946906
chr1	82947316	82947503
chr1	82948702	82948935
chr1	82949180	82949421
chr1	82954857	82955066
chr1	82962344	82962540
chr1	82971586	82971806
chr1	82972107	82972415
chr1	82975747	82976227
chr1	82976227	82976630
chr1	82977942	82978093
chr1	82987859	82988105
chr1	83010987	83011254
chr1	83031247	83031478
chr1	83036980	83037188
chr1	83045212	83045447
chr1	83063146	83063333
chr1	83076220	83076443
chr1	83096141	83096395
chr1	83096490	83096892
chr1	83099656	83100096
chr1	83111749	83112071
chr1	83116463	83116980
chr1	83119678	83119924
chr1	83119924	83120605
chr1	83135605	83135900
chr1	83135900	83136201
chr1	83137334	83137591
chr1	83137814	83138170
chr1	83138215	83138427
chr1	83142093	83142353

chr1	83154728	83155157
chr1	83156496	83156646
chr1	83156713	83157258
chr1	83187247	83187483
chr1	83850469	83850737
chr1	83850895	83851192
chr1	83851192	83851455
chr1	83900239	83900448
chr1	83906704	83906884
chr1	83907046	83907202
chr1	84093435	84093663
chr1	84284458	84284699
chr1	84286912	84287126
chr1	84360157	84360460
chr1	84363755	84363939
chr1	84387624	84387947
chr1	84388207	84388366
chr1	84390314	84390464
chr1	84405920	84406247
chr1	84406810	84407217
chr1	84407217	84407560
chr1	84444123	84444518
chr1	84561900	84562177
chr1	84581913	84582118
chr1	84596444	84596665
chr1	84695542	84695755
chr1	84695755	84696140
chr1	84696149	84696470
chr1	84838604	84838865
chr1	84838865	84839177
chr1	84839220	84839667
chr1	84839667	84839983
chr1	84839983	84840202
chr1	84840202	84840440
chr1	84862473	84862685
chr1	84869544	84869879
chr1	84881248	84881502
chr1	84881520	84881867
chr1	84881867	84882215
chr1	84882231	84882393
chr1	84919277	84919492
chr1	84933544	84933758

chr1	84934363	84934734
chr1	84934734	84934956
chr1	84934963	84935145
chr1	84935145	84935339
chr1	84935772	84935981
chr1	84939028	84939276
chr1	84955348	84955545
chr1	84955718	84955942
chr1	84956711	84956878
chr1	84960478	84960635
chr1	84962594	84963089
chr1	84963089	84963301
chr1	84963576	84963860
chr1	84963860	84964037
chr1	84964037	84964220
chr1	84964811	84964979
chr1	84965023	84965240
chr1	84965622	84965950
chr1	84965950	84966257
chr1	84975838	84976332
chr1	84976435	84976893
chr1	84979040	84979302
chr1	84979646	84979852
chr1	84982272	84982446
chr1	84982620	84982823
chr1	84982823	84983055
chr1	84984444	84984748
chr1	84986224	84986438
chr1	84987396	84987693
chr1	84987693	84987888
chr1	84991233	84991398
chr1	84993039	84993199
chr1	84993829	84994247
chr1	84994303	84994546
chr1	84994546	84994841
chr1	84994968	84995206
chr1	84995206	84995418
chr1	85000870	85001196
chr1	85001196	85001506
chr1	85001518	85001719
chr1	85001719	85001907
chr1	85003213	85003413

chr1	85007713	85007864
chr1	85008837	85009042
chr1	85009130	85009344
chr1	85009441	85009622
chr1	85009622	85009821
chr1	85009821	85010084
chr1	85010156	85010374
chr1	85011890	85012061
chr1	85025970	85026142
chr1	85026245	85026780
chr1	85027552	85027816
chr1	85028293	85028536
chr1	85028727	85028877
chr1	85029112	85029342
chr1	85029415	85029700
chr1	85029700	85029958
chr1	85031617	85032072
chr1	85034651	85034837
chr1	85037498	85037653
chr1	85038765	85038931
chr1	85039818	85039992
chr1	85043847	85044072
chr1	85045485	85045769
chr1	85046243	85046674
chr1	85046889	85047046
chr1	85047071	85047237
chr1	85061821	85062065
chr1	85062208	85062383
chr1	85063024	85063281
chr1	85063281	85063604
chr1	85064143	85064527
chr1	85068101	85068367
chr1	85068552	85068704
chr1	85069271	85069433
chr1	85073915	85074078
chr1	85075079	85075388
chr1	85080473	85080665
chr1	85080774	85081019
chr1	85081019	85081260
chr1	85082338	85082557
chr1	85084841	85085081
chr1	85086305	85086621

chr1	85086694	85086844
chr1	85087534	85087813
chr1	85087813	85088043
chr1	85089891	85090161
chr1	85090272	85090553
chr1	85090553	85090869
chr1	85090869	85091140
chr1	85092404	85092567
chr1	85097251	85097454
chr1	85097631	85097803
chr1	85098579	85098883
chr1	85099057	85099286
chr1	85100448	85100643
chr1	85101121	85101304
chr1	85102754	85103034
chr1	85105276	85105483
chr1	85105483	85105734
chr1	85105734	85106012
chr1	85106012	85106280
chr1	85109543	85109812
chr1	85109812	85110051
chr1	85110765	85111042
chr1	85111496	85111799
chr1	85112132	85112291
chr1	85113071	85113334
chr1	85113334	85113551
chr1	85114345	85114542
chr1	85114771	85115044
chr1	85120104	85120369
chr1	85120529	85120715
chr1	85122982	85123180
chr1	85123180	85123417
chr1	85123695	85123876
chr1	85124675	85124828
chr1	85125195	85125477
chr1	85128373	85128701
chr1	85131030	85131252
chr1	85147552	85147751
chr1	85147751	85148057
chr1	85153665	85153885
chr1	85153885	85154121
chr1	85154260	85154439

chr1	85155078	85155427
chr1	85155490	85155693
chr1	85156130	85156333
chr1	85156379	85156534
chr1	85158612	85158780
chr1	85160653	85160841
chr1	85161067	85161263
chr1	85161460	85161656
chr1	85161656	85161843
chr1	85162074	85162349
chr1	85162456	85162581
chr1	85162581	85162661
chr1	85164142	85164560
chr1	85165795	85165967
chr1	85166979	85167363
chr1	85168007	85168175
chr1	85169190	85169340
chr1	85169983	85170208
chr1	85171114	85171323
chr1	85179517	85179889
chr1	85179889	85180132
chr1	85182004	85182195
chr1	85182703	85182941
chr1	85183161	85183363
chr1	85185556	85185826
chr1	85186502	85186727
chr1	85195927	85196077
chr1	85196817	85197017
chr1	85197510	85197681
chr1	85198321	85198613
chr1	85198613	85198904
chr1	85198904	85199131
chr1	85199131	85199347
chr1	85199551	85199710
chr1	85199983	85200174
chr1	85202409	85202698
chr1	85206703	85206976
chr1	85209404	85209679
chr1	85212869	85213101
chr1	85214199	85214552
chr1	85214902	85215108
chr1	85215108	85215457

chr1	85216149	85216423
chr1	85230924	85231194
chr1	85233143	85233417
chr1	85233702	85233982
chr1	85234363	85234566
chr1	85234581	85234858
chr1	85235232	85235407
chr1	85235645	85235834
chr1	85236082	85236481
chr1	85241722	85242014
chr1	85243366	85243568
chr1	85243776	85244023
chr1	85244250	85244423
chr1	85245713	85245863
chr1	85246933	85247270
chr1	85247590	85247882
chr1	85248045	85248246
chr1	85250601	85250901
chr1	85254651	85254990
chr1	85255076	85255254
chr1	85256362	85256545
chr1	85257103	85257367
chr1	85260357	85260573
chr1	85260573	85260763
chr1	85261325	85261523
chr1	85261906	85262088
chr1	85265681	85265867
chr1	85269932	85270158
chr1	85270158	85270456
chr1	85270549	85270770
chr1	85272068	85272477
chr1	85273434	85273858
chr1	85275466	85275621
chr1	85276024	85276204
chr1	85276520	85276686
chr1	85276850	85277201
chr1	85277785	85277980
chr1	85278405	85278560
chr1	85278664	85278828
chr1	85278828	85278971
chr1	85280380	85280662
chr1	85281732	85281982

chr1	85284002	85284169
chr1	85284560	85284716
chr1	85285451	85285742
chr1	85286605	85286758
chr1	85287135	85287398
chr1	85287531	85287825
chr1	85294653	85294816
chr1	85294965	85295127
chr1	85295129	85295309
chr1	85296579	85296756
chr1	85297161	85297382
chr1	85297730	85297931
chr1	85304521	85304777
chr1	85304872	85305051
chr1	85305168	85305336
chr1	85305465	85305680
chr1	85306922	85307206
chr1	85309577	85309761
chr1	85309761	85309956
chr1	85311482	85311666
chr1	85314022	85314262
chr1	85319204	85319410
chr1	85323188	85323587
chr1	85323735	85323927
chr1	85327189	85327428
chr1	85330046	85330233
chr1	85330251	85330415
chr1	85330792	85330947
chr1	85332230	85332400
chr1	85332403	85332649
chr1	85332988	85333240
chr1	85333240	85333686
chr1	85339834	85340048
chr1	85340048	85340408
chr1	85340616	85340841
chr1	85341084	85341395
chr1	85341919	85342102
chr1	85342229	85342588
chr1	85344739	85344973
chr1	85344973	85345231
chr1	85345288	85345687
chr1	85345877	85346027

chr1	85346366	85346827
chr1	85447251	85447401
chr1	85448531	85448702
chr1	85448754	85448936
chr1	85449554	85449738
chr1	85451539	85452170
chr1	85454130	85454392
chr1	85454663	85454841
chr1	85456200	85456351
chr1	85458820	85459045
chr1	85460347	85460762
chr1	85462527	85462677
chr1	85462952	85463208
chr1	85478916	85479173
chr1	85479173	85479550
chr1	85481419	85481606
chr1	85481606	85481806
chr1	85482991	85483166
chr1	85483482	85483756
chr1	85485122	85485328
chr1	85485450	85485627
chr1	85485879	85486208
chr1	85488950	85489232
chr1	85491512	85491832
chr1	85492243	85492405
chr1	85494711	85494947
chr1	85494947	85495263
chr1	85495979	85496140
chr1	85497049	85497199
chr1	85499006	85499258
chr1	85500068	85500279
chr1	85500361	85500511
chr1	85502634	85502907
chr1	85502947	85503205
chr1	85503798	85504007
chr1	85504080	85504360
chr1	85506579	85506743
chr1	85508864	85509080
chr1	85511662	85511835
chr1	85522847	85523423
chr1	85524833	85525034
chr1	85525252	85525457

chr1	85525679	85525875
chr1	85525875	85526073
chr1	85526073	85526265
chr1	85526842	85527168
chr1	85528876	85529066
chr1	85529958	85530221
chr1	85534051	85534379
chr1	85535006	85535170
chr1	85536583	85536831
chr1	85539258	85539551
chr1	85543746	85543974
chr1	85545050	85545376
chr1	85546557	85546799
chr1	85549715	85549894
chr1	85550428	85550627
chr1	85550627	85550867
chr1	85551159	85551347
chr1	85551621	85552112
chr1	85553283	85553440
chr1	85555313	85555614
chr1	85555614	85555811
chr1	85562538	85562690
chr1	85562694	85562866
chr1	85563098	85563391
chr1	85565284	85565507
chr1	85565507	85565831
chr1	85566046	85566210
chr1	85570515	85570675
chr1	85570979	85571171
chr1	85572293	85572445
chr1	85572676	85572857
chr1	85573675	85573868
chr1	85574011	85574356
chr1	85575180	85575365
chr1	85578306	85578488
chr1	85581290	85581444
chr1	85581651	85581897
chr1	85582857	85583046
chr1	85583378	85583534
chr1	85584235	85584438
chr1	85584813	85585254
chr1	85585867	85586220

chr1	85588667	85589143
chr1	85591189	85591445
chr1	85591445	85591797
chr1	85591810	85592019
chr1	85592179	85592443
chr1	85592443	85592634
chr1	85592715	85592981
chr1	85592981	85593354
chr1	85595032	85595198
chr1	85597544	85597662
chr1	85597662	85597813
chr1	85598377	85598689
chr1	85598689	85599029
chr1	85599228	85599378
chr1	85599397	85599632
chr1	85600071	85600277
chr1	85600378	85600729
chr1	85600737	85600913
chr1	85600913	85601105
chr1	85601117	85601323
chr1	85604224	85604427
chr1	85604427	85604649
chr1	85604745	85604962
chr1	85604962	85605170
chr1	85605170	85605366
chr1	85608108	85608263
chr1	85608924	85609236
chr1	85609797	85610002
chr1	85610002	85610248
chr1	85610248	85610482
chr1	85614539	85614821
chr1	85617499	85617682
chr1	85617682	85617886
chr1	85618593	85618840
chr1	85621022	85621341
chr1	85621827	85622238
chr1	85624449	85624784
chr1	85624787	85625230
chr1	85625308	85625646
chr1	85625800	85625952
chr1	85627022	85627368
chr1	85629728	85629927

chr1	85632478	85632737
chr1	85636248	85636474
chr1	85643768	85643954
chr1	85648296	85648461
chr1	85650401	85650587
chr1	85650587	85650812
chr1	85653354	85653671
chr1	85653813	85653979
chr1	85657511	85657825
chr1	85657825	85658074
chr1	85658329	85658582
chr1	85662765	85662973
chr1	85668041	85668394
chr1	85697452	85697769
chr1	85697797	85698004
chr1	85703156	85703585
chr1	85703585	85703868
chr1	85703868	85704125
chr1	85729280	85729503
chr1	85736363	85736532
chr1	85736554	85736804
chr1	85740900	85741358
chr1	85743092	85743400
chr1	85744661	85744920
chr1	85747859	85748118
chr1	85760709	85760859
chr1	85771985	85772224
chr1	85775128	85775521
chr1	85778573	85778723
chr1	85778936	85779281
chr1	85779819	85780078
chr1	85793234	85793535
chr1	85793535	85793759
chr1	85793759	85793937
chr1	85793937	85794225
chr1	85794225	85794577
chr1	85794577	85794828
chr1	85799716	85800095
chr1	85800206	85800375
chr1	85806729	85807021
chr1	85808469	85808869
chr1	85810810	85811099

chr1	85811099	85811323
chr1	85820352	85820555
chr1	85828948	85829387
chr1	85837713	85837900
chr1	85837940	85838416
chr1	85850932	85851090
chr1	85853334	85853635
chr1	85861725	85861895
chr1	85866054	85866247
chr1	85868300	85868616
chr1	85884780	85884938
chr1	85892837	85893028
chr1	85894383	85894671
chr1	85894671	85895018
chr1	85895149	85895822
chr1	85924291	85924565
chr1	85935534	85935780
chr1	85966939	85967129
chr1	86007875	86008148
chr1	86015858	86016035
chr1	86018218	86018478
chr1	86018478	86018750
chr1	86021799	86021978
chr1	86032290	86032458
chr1	86037041	86037324
chr1	86038356	86038601
chr1	86038601	86038843
chr1	86038843	86039051
chr1	86045796	86046053
chr1	86046053	86046260
chr1	86046260	86046711
chr1	86048246	86048583
chr1	86059370	86059782
chr1	86061241	86061477
chr1	86064209	86064472
chr1	86064555	86064774
chr1	86064774	86065285
chr1	86094474	86094760
chr1	86110657	86110821
chr1	86110821	86110995
chr1	86124493	86124690
chr1	86126048	86126352

chr1	86126407	86126622
chr1	86129576	86129788
chr1	86136463	86136760
chr1	86141027	86141253
chr1	86147645	86147933
chr1	86148290	86148478
chr1	86148478	86148662
chr1	86150534	86150756
chr1	86151082	86151319
chr1	86151319	86151742
chr1	86154496	86155094
chr1	86167273	86167624
chr1	86178849	86179366
chr1	86186649	86187257
chr1	86189125	86189479
chr1	86210772	86210957
chr1	86214149	86214601
chr1	86225204	86225623
chr1	86226231	86226788
chr1	86229443	86229789
chr1	86245793	86246033
chr1	86257225	86257583
chr1	86262587	86262768
chr1	86263974	86264203
chr1	86273346	86273512
chr1	86277144	86277355
chr1	86277355	86277569
chr1	86278047	86278212
chr1	86278311	86278606
chr1	86279059	86279289
chr1	86279862	86280025
chr1	86282225	86282496
chr1	86284911	86285342
chr1	86293012	86293532
chr1	86294580	86294943
chr1	86301312	86301740
chr1	86301740	86302031
chr1	86303028	86303307
chr1	86303307	86303580
chr1	86303580	86303927
chr1	86313729	86314038
chr1	86336327	86336675

chr1	86338265	86338480
chr1	86339495	86339740
chr1	86341198	86341487
chr1	86341652	86341860
chr1	86342408	86342909
chr1	86342909	86343540
chr1	86343653	86343989
chr1	86344010	86344276
chr1	86346985	86347172
chr1	86347244	86347396
chr1	86348442	86348592
chr1	86348643	86348822
chr1	86353291	86353462
chr1	86358586	86359238
chr1	86359238	86359737
chr1	86359737	86360151
chr1	86372034	86372294
chr1	86376438	86376856
chr1	86380987	86381404
chr1	86389281	86389600
chr1	86403716	86404246
chr1	86406374	86406693
chr1	86406898	86407172
chr1	86415583	86415834
chr1	86415834	86416207
chr1	86416395	86416560
chr1	86419942	86420118
chr1	86421160	86421324
chr1	86430024	86430368
chr1	86434104	86434412
chr1	86434412	86434926
chr1	86435429	86435765
chr1	86437929	86438186
chr1	86439913	86440219
chr1	86440219	86440512
chr1	86440512	86440868
chr1	86441418	86441751
chr1	86442188	86442751
chr1	86442751	86443007
chr1	86448321	86448655
chr1	86449981	86450362
chr1	86463580	86464134

chr1	86470507	86471269
chr1	86472168	86472586
chr1	86472593	86472961
chr1	86487079	86487308
chr1	86487308	86487588
chr1	86487588	86487848
chr1	86487848	86488192
chr1	86501579	86501913
chr1	86503355	86503872
chr1	86503872	86504702
chr1	86509397	86509797
chr1	86513083	86513360
chr1	86516133	86516314
chr1	86520463	86520690
chr1	86525228	86525526
chr1	86525854	86526105
chr1	86526105	86526406
chr1	86526406	86526975
chr1	86527894	86528160
chr1	86528160	86528446
chr1	86528626	86528935
chr1	86530950	86531211
chr1	86531399	86531549
chr1	86531710	86532202
chr1	86532202	86532448
chr1	86532448	86532737
chr1	86533779	86533941
chr1	86538486	86538712
chr1	86542674	86542902
chr1	86557825	86558200
chr1	86582150	86582432
chr1	86582432	86582709
chr1	86582709	86583017
chr1	86586997	86587245
chr1	86587245	86587490
chr1	86598646	86598803
chr1	86614404	86614567
chr1	86618533	86618761
chr1	86622289	86622547
chr1	86623058	86623309
chr1	86623330	86623649
chr1	86623649	86623899

chr1	86628290	86628657
chr1	86650318	86650641
chr1	86657588	86657930
chr1	86669522	86669880
chr1	86669880	86670321
chr1	86670321	86670736
chr1	86679943	86680487
chr1	86697096	86697255
chr1	86698822	86699040
chr1	86703301	86703695
chr1	86703695	86704016
chr1	86704016	86704290
chr1	86728909	86729156
chr1	86740783	86740950
chr1	86822236	86822749
chr1	86995545	86995765
chr1	87006759	87006965
chr1	87007144	87007390
chr1	87008281	87008583
chr1	87008640	87008851
chr1	87011779	87012051
chr1	87012650	87013013
chr1	87014033	87014246
chr1	87021678	87021862
chr1	87028620	87029171
chr1	87029171	87029539
chr1	87030615	87030969
chr1	87040647	87041035
chr1	87041188	87041431
chr1	87042095	87042403
chr1	87051256	87051860
chr1	87057131	87057285
chr1	87059741	87059992
chr1	87070773	87071042
chr1	87075603	87075711
chr1	87075711	87075758
chr1	87077073	87077739
chr1	87087051	87087280
chr1	87098622	87099026
chr1	87102175	87102489
chr1	87102518	87102794
chr1	87108426	87108596

chr1	87108643	87108846
chr1	87114784	87115121
chr1	87131837	87132034
chr1	87132034	87132225
chr1	87149505	87149800
chr1	87152647	87152835
chr1	87154174	87154465
chr1	87154465	87154748
chr1	87155891	87156168
chr1	87159452	87159790
chr1	87174341	87174733
chr1	87175808	87176078
chr1	87176078	87176343
chr1	87180570	87180721
chr1	87180782	87181314
chr1	87181314	87181686
chr1	87184867	87185092
chr1	87186626	87186839
chr1	87186839	87187063
chr1	87187063	87187337
chr1	87198332	87198548
chr1	87205194	87205501
chr1	87206488	87206653
chr1	87213236	87213537
chr1	87213703	87214096
chr1	87214096	87214591
chr1	87214944	87215094
chr1	87215638	87215852
chr1	87238549	87238726
chr1	87239091	87239465
chr1	87246052	87246209
chr1	87246211	87246665
chr1	87252804	87253128
chr1	87254327	87254889
chr1	87255137	87255631
chr1	87255705	87255877
chr1	87259488	87259740
chr1	87264174	87264463
chr1	87264463	87264756
chr1	87264756	87264996
chr1	87265245	87265492
chr1	87265654	87265848

chr1	87266029	87266181
chr1	87272437	87272804
chr1	87280399	87280602
chr1	87287264	87287450
chr1	87299576	87299777
chr1	87315840	87316250
chr1	87316765	87316945
chr1	87326621	87327063
chr1	87327063	87327355
chr1	87327355	87327545
chr1	87327545	87327758
chr1	87381718	87381921
chr1	87381921	87382168
chr1	87398930	87399182
chr1	87418762	87419010
chr1	87447575	87447791
chr1	87456236	87456481
chr1	87460059	87460344
chr1	87466932	87467130
chr1	87468462	87468728
chr1	87469254	87469442
chr1	87470224	87470443
chr1	87472337	87472547
chr1	87484921	87485196
chr1	87486351	87486869
chr1	87486984	87487239
chr1	87491703	87491908
chr1	87492187	87492682
chr1	87492682	87493153
chr1	87509099	87509473
chr1	87509473	87509728
chr1	87509728	87510006
chr1	87510006	87510267
chr1	87510267	87510668
chr1	87521566	87521889
chr1	87541653	87541965
chr1	87548988	87549392
chr1	87550832	87551233
chr1	87551233	87551527
chr1	87553746	87554124
chr1	87559168	87559622
chr1	87560194	87560576

chr1	87565870	87566087
chr1	87566087	87566327
chr1	87573744	87574291
chr1	87574388	87574725
chr1	87574742	87575276
chr1	87589899	87590298
chr1	87594317	87594676
chr1	87607876	87608128
chr1	87608487	87608722
chr1	87608722	87608965
chr1	87609533	87609683
chr1	87621390	87621794
chr1	87637490	87637938
chr1	87643123	87643355
chr1	87648765	87648915
chr1	87667122	87667292
chr1	87681557	87681807
chr1	87689702	87690010
chr1	87690010	87690306
chr1	87718176	87718331
chr1	87721489	87721680
chr1	87722070	87722310
chr1	87723445	87723638
chr1	87730349	87730566
chr1	87755732	87756144
chr1	87756144	87756558
chr1	87766161	87766554
chr1	87772664	87773035
chr1	87791907	87792097
chr1	87792884	87793123
chr1	87793123	87793363
chr1	87821046	87821253
chr1	87840160	87840775
chr1	87840775	87841196
chr1	87848604	87848988
chr1	87849011	87849292
chr1	87850713	87850893
chr1	87853067	87853371
chr1	87853371	87853677
chr1	87853696	87854034
chr1	87854096	87854438
chr1	87862159	87862525

chr1	87885535	87885875
chr1	87891769	87891991
chr1	87899727	87899877
chr1	87900864	87901563
chr1	87906441	87906907
chr1	87910491	87910753
chr1	87911575	87911735
chr1	87911765	87911939
chr1	87913365	87913586
chr1	87917340	87917692
chr1	87920065	87920357
chr1	87920357	87920601
chr1	87922764	87922949
chr1	87935012	87935266
chr1	87935590	87935896
chr1	87936378	87936545
chr1	87936584	87936868
chr1	87961529	87961851
chr1	87986880	87987291
chr1	87988409	87988731
chr1	88007801	88008122
chr1	88008122	88008444
chr1	88008444	88008695
chr1	88055571	88055736
chr1	88094238	88094658
chr1	88103841	88104053
chr1	88109084	88109363
chr1	88120608	88120889
chr1	88121606	88121806
chr1	88125920	88126322
chr1	88135330	88135620
chr1	88140525	88140858
chr1	88144114	88144266
chr1	88144337	88144640
chr1	88145333	88145497
chr1	88150217	88150560
chr1	88151015	88151193
chr1	88205488	88205895
chr1	88211715	88212214
chr1	88212378	88212656
chr1	88213772	88213994
chr1	88213994	88214228

chr1	88214228	88214445
chr1	88214482	88214655
chr1	88214655	88214810
chr1	88218133	88218288
chr1	88219163	88219413
chr1	88220436	88220587
chr1	88223255	88223485
chr1	88223533	88223755
chr1	88223755	88223917
chr1	88224290	88224444
chr1	88224446	88224613
chr1	88228112	88228283
chr1	88228289	88228480
chr1	88229866	88230047
chr1	88230134	88230350
chr1	88230350	88230565
chr1	88233959	88234150
chr1	88235111	88235362
chr1	88236883	88237095
chr1	88237381	88237772
chr1	88237893	88238044
chr1	88238745	88238906
chr1	88242721	88242918
chr1	88243138	88243288
chr1	88244017	88244179
chr1	88249102	88249262
chr1	88249266	88249468
chr1	88249541	88249714
chr1	88250122	88250277
chr1	88255637	88255864
chr1	88256504	88256786
chr1	88257194	88257610
chr1	88262235	88262683
chr1	88268707	88268914
chr1	88270949	88271199
chr1	88276794	88277033
chr1	88277061	88277442
chr1	88277442	88277858
chr1	88284364	88284592
chr1	88284954	88285223
chr1	88287467	88287648
chr1	88287648	88287887

chr1	88287911	88288130
chr1	88289999	88290252
chr1	88296117	88296372
chr1	88296372	88296558
chr1	88300594	88300834
chr1	88301249	88301555
chr1	88301655	88301913
chr1	88302023	88302200
chr1	88302547	88302699
chr1	88306677	88306882
chr1	88310067	88310217
chr1	88310430	88310704
chr1	88317239	88317761
chr1	88318618	88318843
chr1	88331037	88331346
chr1	88331346	88331553
chr1	88331553	88331747
chr1	88345578	88346029
chr1	88357470	88357962
chr1	88361032	88361408
chr1	88361519	88361730
chr1	88393370	88393684
chr1	88424688	88424928
chr1	88428153	88428412
chr1	88428412	88428754
chr1	88428754	88429096
chr1	88436942	88437684
chr1	88437684	88438043
chr1	88438043	88438507
chr1	88445449	88445724
chr1	88452219	88452772
chr1	88453140	88453444
chr1	88454772	88454963
chr1	88456751	88457014
chr1	88462523	88462734
chr1	88463272	88463557
chr1	88464205	88464456
chr1	88464553	88464857
chr1	88465813	88466034
chr1	88466574	88467019
chr1	88468417	88468868
chr1	88474404	88475110

chr1	88475316	88475952
chr1	88497250	88497504
chr1	88507873	88508247
chr1	88508247	88508552
chr1	88509719	88509951
chr1	88509951	88510356
chr1	88525784	88526167
chr1	88526167	88526619
chr1	88532187	88532453
chr1	88533885	88534135
chr1	88534135	88534455
chr1	88536349	88536570
chr1	88536839	88537142
chr1	88537142	88537382
chr1	88543145	88543365
chr1	88543365	88543662
chr1	88543698	88543889
chr1	88556402	88556981
chr1	88559398	88559648
chr1	88560420	88560580
chr1	88560663	88561146
chr1	88562421	88562655
chr1	88575888	88576083
chr1	88583770	88584204
chr1	88584418	88584934
chr1	88600176	88600504
chr1	88605579	88605916
chr1	88620134	88620457
chr1	88627385	88627856
chr1	88631579	88631771
chr1	88631908	88632093
chr1	88633847	88634061
chr1	88638724	88638968
chr1	88639033	88639193
chr1	88639422	88639815
chr1	88639815	88640215
chr1	88645668	88645956
chr1	88646317	88647065
chr1	88654673	88655046
chr1	88662650	88663079
chr1	88663120	88663291
chr1	88663697	88663979

chr1	88668516	88668747
chr1	88669953	88670103
chr1	88680787	88681115
chr1	88681412	88681673
chr1	88687914	88688097
chr1	88692238	88692751
chr1	88694464	88694848
chr1	88699480	88699630
chr1	88700539	88700703
chr1	88701582	88701939
chr1	88701939	88702306
chr1	88702306	88702769
chr1	88702790	88703014
chr1	88705217	88705386
chr1	88705816	88706134
chr1	88729665	88729843
chr1	88735626	88735943
chr1	88742547	88742698
chr1	88745232	88745695
chr1	88746841	88746991
chr1	88747864	88748147
chr1	88750648	88750899
chr1	88754765	88754915
chr1	88756765	88756923
chr1	88763543	88763744
chr1	88763751	88764326
chr1	88764725	88764911
chr1	88774173	88774435
chr1	88774435	88774675
chr1	88779868	88780082
chr1	88783191	88783425
chr1	88794946	88795313
chr1	88829282	88829459
chr1	88836264	88836436
chr1	88868226	88868590
chr1	88869153	88869407
chr1	88869438	88869765
chr1	88869765	88870202
chr1	88878852	88879229
chr1	88911704	88912073
chr1	88912073	88912316
chr1	88929324	88929496

chr1	88945859	88946119
chr1	88949988	88950221
chr1	88959469	88960096
chr1	88988356	88988678
chr1	88991876	88992178
chr1	88994825	88995116
chr1	88995116	88995401
chr1	89015451	89015786
chr1	89019641	89020360
chr1	89030582	89030816
chr1	89041258	89041648
chr1	89042727	89042997
chr1	89068860	89069128
chr1	89069461	89069791
chr1	89069877	89070278
chr1	89070278	89070505
chr1	89070505	89070761
chr1	89070905	89071063
chr1	89074767	89074994
chr1	89080882	89081125
chr1	89088873	89089258
chr1	89089956	89090171
chr1	89090171	89090428
chr1	89090487	89090719
chr1	89090719	89090977
chr1	89092539	89092793
chr1	89092793	89093064
chr1	89096029	89096315
chr1	89102128	89102636
chr1	89103585	89103810
chr1	89103896	89104199
chr1	89104199	89104459
chr1	89104459	89104861
chr1	89108283	89108664
chr1	89108664	89109078
chr1	89113761	89114076
chr1	89116382	89116664
chr1	89118802	89119209
chr1	89120089	89120300
chr1	89121552	89121791
chr1	89121855	89122071
chr1	89122478	89122789

chr1	89123890	89124070
chr1	89124072	89124406
chr1	89124406	89124630
chr1	89125539	89126049
chr1	89130903	89131178
chr1	89131178	89131504
chr1	89131643	89132128
chr1	89133053	89133288
chr1	89133288	89133699
chr1	89141753	89141917
chr1	89147813	89148020
chr1	89151345	89151522
chr1	89151815	89151986
chr1	89152112	89152446
chr1	89152446	89152703
chr1	89152703	89153130
chr1	89153373	89153543
chr1	89175499	89175656
chr1	89175832	89176339
chr1	89183254	89183897
chr1	89188155	89188520
chr1	89191184	89191451
chr1	89191451	89191795
chr1	89192941	89193091
chr1	89196120	89196393
chr1	89196436	89196594
chr1	89205882	89206142
chr1	89206150	89206305
chr1	89206443	89206869
chr1	89206869	89207316
chr1	89207316	89207620
chr1	89226712	89227036
chr1	89234973	89235259
chr1	89235293	89235878
chr1	89250268	89250448
chr1	89252431	89252909
chr1	89265590	89266257
chr1	89266288	89266466
chr1	89271665	89272166
chr1	89272991	89273407
chr1	89320155	89320430
chr1	89320481	89320661

chr1	89323171	89323408
chr1	89340645	89340936
chr1	89344548	89344868
chr1	89360390	89360552
chr1	89373981	89374239
chr1	89374239	89374699
chr1	89378031	89378230
chr1	89390365	89390503
chr1	89390503	89390647
chr1	89392640	89393325
chr1	89424586	89424902
chr1	89426252	89426549
chr1	89454381	89455191
chr1	89455521	89455748
chr1	89455754	89455937
chr1	89456003	89456156
chr1	89456457	89456833
chr1	89457184	89457420
chr1	89462279	89462509
chr1	89462991	89463163
chr1	89463187	89463489
chr1	89470377	89470620
chr1	89472272	89472482
chr1	89474711	89474877
chr1	89474926	89475146
chr1	89477082	89477288
chr1	89482166	89482555
chr1	89489466	89489840
chr1	89491667	89491943
chr1	89493265	89493483
chr1	89494892	89495196
chr1	89495196	89495534
chr1	89496399	89496579
chr1	89501747	89501911
chr1	89509744	89510054
chr1	89510073	89510223
chr1	89513834	89514149
chr1	89518449	89518650
chr1	89523794	89524145
chr1	89524280	89524745
chr1	89525833	89526193
chr1	89553823	89554125

chr1	89554149	89554391
chr1	89554984	89555356
chr1	89555356	89555853
chr1	89556163	89556313
chr1	89563518	89563676
chr1	89566599	89566952
chr1	89568762	89568991
chr1	89570511	89570991
chr1	89570991	89571460
chr1	89571460	89571813
chr1	89574826	89575037
chr1	89575283	89575469
chr1	89575657	89575910
chr1	89577014	89577360
chr1	89579987	89580381
chr1	89580381	89580784
chr1	89580784	89581045
chr1	89581045	89581264
chr1	89581264	89581573
chr1	89593224	89593747
chr1	89594449	89594704
chr1	89597740	89597991
chr1	89600475	89600803
chr1	89601039	89601252
chr1	89601409	89601688
chr1	89602834	89603299
chr1	89606664	89606877
chr1	89620164	89620333
chr1	89625373	89625537
chr1	89626129	89626295
chr1	89628391	89628556
chr1	89630924	89631165
chr1	89636895	89637056
chr1	89641644	89641842
chr1	89643547	89643753
chr1	89643773	89643955
chr1	89644114	89644442
chr1	89646296	89646471
chr1	89646487	89646637
chr1	89646680	89646973
chr1	89648137	89648287
chr1	89656293	89656671

chr1	89656671	89656960
chr1	89656960	89657225
chr1	89659166	89659415
chr1	89663353	89663503
chr1	89676503	89676653
chr1	89688989	89689323
chr1	89696653	89696943
chr1	89697314	89697571
chr1	89701417	89701635
chr1	89730623	89730878
chr1	89730878	89731359
chr1	89735269	89735451
chr1	89742472	89742645
chr1	89757639	89757849
chr1	89769428	89770046
chr1	89771645	89772033
chr1	89779634	89779814
chr1	89780517	89780724
chr1	89780724	89781245
chr1	89804869	89805534
chr1	89822459	89822750
chr1	89831191	89831367
chr1	89843120	89843274
chr1	89844127	89844338
chr1	89927084	89927304
chr1	89930116	89930398
chr1	89930398	89930751
chr1	89930751	89931118
chr1	89931118	89931611
chr1	89940086	89940243
chr1	89940358	89940730
chr1	89942468	89942724
chr1	89943266	89943549
chr1	89943986	89944179
chr1	89967135	89967451
chr1	89968190	89968786
chr1	90010157	90010404
chr1	90018165	90018315
chr1	90020426	90020576
chr1	90027012	90027264
chr1	90028428	90028652
chr1	90029640	90029875

chr1	90038853	90039100
chr1	90051009	90051705
chr1	90059194	90059419
chr1	90060646	90060959
chr1	90069308	90069593
chr1	90080998	90081387
chr1	90094057	90094223
chr1	90097387	90097644
chr1	90099823	90100168
chr1	90100389	90100885
chr1	90100885	90101285
chr1	90112411	90112599
chr1	90112603	90113200
chr1	90124365	90124653
chr1	90136037	90136371
chr1	90141159	90141464
chr1	90152801	90153109
chr1	90153143	90153713
chr1	90164076	90164472
chr1	90164472	90164774
chr1	90175145	90175391
chr1	90177636	90177939
chr1	90177939	90178443
chr1	90185329	90185887
chr1	90186073	90186619
chr1	90186649	90186874
chr1	90203771	90204075
chr1	90204075	90204328
chr1	90204331	90204611
chr1	90205349	90205664
chr1	90205843	90205993
chr1	90224562	90224799
chr1	90225418	90226276
chr1	90230625	90230977
chr1	90234427	90235130
chr1	90236655	90236984
chr1	90244340	90244585
chr1	90244585	90244938
chr1	90245475	90245857
chr1	90250496	90250762
chr1	90258512	90258836
chr1	90258836	90259201

chr1	90273281	90273446
chr1	90273455	90273929
chr1	90275040	90275353
chr1	90275856	90276160
chr1	90287382	90287539
chr1	90288804	90289276
chr1	90289276	90289519
chr1	90289519	90289745
chr1	90289781	90290162
chr1	90377066	90377222
chr1	90380264	90380818
chr1	90415881	90416128
chr1	90416128	90416394
chr1	90439825	90440077
chr1	90440077	90440447
chr1	90441188	90441560
chr1	90445850	90446142
chr1	90446142	90446456
chr1	90446456	90446846
chr1	90446846	90447345
chr1	90447345	90447783
chr1	90447783	90448193
chr1	90448193	90448690
chr1	90461446	90461651
chr1	90461752	90462055
chr1	90466044	90466318
chr1	90494505	90494664
chr1	90503293	90503614
chr1	90505522	90506035
chr1	90506590	90507042
chr1	90517545	90517786
chr1	90518653	90518895
chr1	90518928	90519329
chr1	90519329	90519557
chr1	90519557	90519869
chr1	90536209	90536417
chr1	90587063	90587301
chr1	90588189	90588677
chr1	90590874	90591113
chr1	90592923	90593172
chr1	90593172	90593437
chr1	90599254	90599600

chr1	90600980	90601493
chr1	90603201	90603610
chr1	90603612	90603773
chr1	90633456	90634199
chr1	90634199	90634700
chr1	90650732	90650971
chr1	90650996	90651157
chr1	90668879	90669029
chr1	90676154	90676361
chr1	90677749	90678106
chr1	90718700	90718990
chr1	90718990	90719255
chr1	90719342	90719521
chr1	90727467	90727635
chr1	90729462	90729861
chr1	90738208	90738467
chr1	90754078	90754511
chr1	90771260	90771419
chr1	90803162	90803473
chr1	90834590	90835197
chr1	90838710	90838887
chr1	90840312	90840497
chr1	90848580	90848985
chr1	90849933	90850191
chr1	90857803	90858080
chr1	90866615	90867254
chr1	90896362	90896544
chr1	90896638	90897084
chr1	90897154	90897396
chr1	90900608	90900816
chr1	90906425	90906691
chr1	90908775	90908956
chr1	90908956	90909105
chr1	90909115	90909571
chr1	90909574	90909902
chr1	90909902	90910211
chr1	90914867	90915235
chr1	90917095	90917435
chr1	90923082	90923455
chr1	90923455	90924014
chr1	90927374	90927749
chr1	90928001	90928260

chr1	90928260	90928512
chr1	90928567	90928853
chr1	90928853	90929088
chr1	90937065	90937374
chr1	90949662	90949812
chr1	90956358	90956575
chr1	90956586	90956741
chr1	90956744	90957045
chr1	90963026	90963195
chr1	90963195	90963362
chr1	90969213	90969576
chr1	90969620	90969830
chr1	90973320	90973590
chr1	90974914	90975631
chr1	90986952	90987107
chr1	90988818	90989062
chr1	90998295	90998608
chr1	90998608	90999017
chr1	90999017	90999385
chr1	90999385	90999564
chr1	91022508	91022849
chr1	91022975	91023264
chr1	91023264	91023575
chr1	91023971	91024278
chr1	91024278	91024538
chr1	91024538	91024749
chr1	91032651	91032878
chr1	91035424	91035625
chr1	91035625	91036001
chr1	91038087	91038329
chr1	91041377	91041619
chr1	91041619	91042103
chr1	91043324	91043681
chr1	91043879	91044029
chr1	91044180	91044587
chr1	91049355	91049616
chr1	91052674	91053222
chr1	91053222	91053580
chr1	91053594	91053992
chr1	91056422	91056765
chr1	91062250	91062716
chr1	91063502	91063713

chr1	91064849	91064999
chr1	91065228	91065407
chr1	91065659	91065981
chr1	91069701	91069973
chr1	91082102	91082268
chr1	91082268	91082410
chr1	91084095	91084284
chr1	91088073	91088385
chr1	91088385	91088739
chr1	91089228	91089569
chr1	91094801	91095011
chr1	91095011	91095209
chr1	91095209	91095705
chr1	91095897	91096082
chr1	91097285	91097735
chr1	91098781	91099180
chr1	91104388	91104679
chr1	91108861	91109302
chr1	91109302	91109722
chr1	91125967	91126332
chr1	91126945	91127236
chr1	91135988	91136145
chr1	91136515	91136729
chr1	91136815	91137523
chr1	91137628	91137822
chr1	91138549	91138797
chr1	91160750	91160900
chr1	91166948	91167202
chr1	91175137	91175306
chr1	91179112	91179703
chr1	91179841	91180305
chr1	91180399	91180601
chr1	91180611	91181082
chr1	91181364	91181529
chr1	91193071	91193730
chr1	91205680	91205988
chr1	91210882	91211475
chr1	91216094	91216266
chr1	91216604	91216807
chr1	91219539	91220081
chr1	91220463	91220636
chr1	91230664	91231126

chr1	91231199	91231465
chr1	91233538	91233719
chr1	91239841	91240125
chr1	91240125	91240360
chr1	91240477	91240673
chr1	91240685	91241280
chr1	91248258	91248507
chr1	91248881	91249081
chr1	91249913	91250362
chr1	91250362	91250683
chr1	91252048	91252251
chr1	91257382	91257604
chr1	91257856	91258192
chr1	91258192	91258493
chr1	91259875	91260134
chr1	91262508	91262950
chr1	91263528	91263862
chr1	91272238	91272690
chr1	91278332	91278840
chr1	91278859	91279182
chr1	91279182	91279442
chr1	91279751	91280019
chr1	91297876	91298662
chr1	91304075	91304416
chr1	91304683	91304889
chr1	91320008	91320159
chr1	91322265	91322459
chr1	91324779	91324972
chr1	91339161	91339337
chr1	91344985	91345226
chr1	91345235	91345673
chr1	91346801	91346995
chr1	91346995	91347268
chr1	91352185	91352490
chr1	91352490	91352698
chr1	91352698	91352942
chr1	91356193	91356811
chr1	91362106	91362525
chr1	91366210	91366584
chr1	91366584	91366916
chr1	91366916	91367237
chr1	91367344	91367510

chr1	91377734	91377897
chr1	91380400	91380808
chr1	91380810	91380980
chr1	91398072	91398453
chr1	91398453	91398666
chr1	91398666	91399164
chr1	91405030	91405387
chr1	91405428	91405675
chr1	91407895	91408076
chr1	91412607	91413061
chr1	91413061	91413668
chr1	91414170	91414352
chr1	91422030	91422247
chr1	91422247	91422432
chr1	91422432	91422583
chr1	91422583	91422792
chr1	91428588	91428951
chr1	91433870	91434105
chr1	91437957	91438107
chr1	91442231	91442515
chr1	91442567	91442805
chr1	91444466	91444651
chr1	91456530	91456798
chr1	91457788	91458045
chr1	91458175	91458850
chr1	91459040	91459504
chr1	91459504	91459870
chr1	91483363	91483645
chr1	91483738	91484056
chr1	91485032	91485326
chr1	91490172	91490792
chr1	91492843	91493042
chr1	91494383	91494738
chr1	91494738	91495008
chr1	91495008	91495139
chr1	91523828	91524077
chr1	91539688	91540077
chr1	91540188	91541024
chr1	91563102	91563561
chr1	91564096	91564324
chr1	91574294	91574540
chr1	91577881	91578188

chr1	91578188	91578476
chr1	91590033	91590214
chr1	91600688	91600978
chr1	91612805	91613351
chr1	91636618	91636895
chr1	91640602	91640811
chr1	91663349	91663707
chr1	91663853	91664138
chr1	91664613	91664879
chr1	91674083	91674347
chr1	91674347	91674603
chr1	91674603	91674835
chr1	91682755	91683035
chr1	91683137	91683287
chr1	91691743	91691924
chr1	91692439	91692718
chr1	91692718	91693035
chr1	91693035	91693308
chr1	91713259	91713409
chr1	91732618	91732924
chr1	91743036	91743235
chr1	91755160	91755397
chr1	91768143	91768357
chr1	91815761	91815911
chr1	91816118	91816412
chr1	91816449	91817187
chr1	91822405	91822720
chr1	91823405	91823959
chr1	91828277	91828441
chr1	91828819	91829061
chr1	91872297	91872754
chr1	91874694	91874992
chr1	91875095	91875285
chr1	91883086	91883312
chr1	91883312	91883519
chr1	91897171	91897399
chr1	91905368	91905603
chr1	91905603	91905794
chr1	91912261	91912557
chr1	91913872	91914276
chr1	91941200	91941420
chr1	91941420	91941836

chr1	91944800	91945261
chr1	91945261	91945707
chr1	91947657	91947814
chr1	91952794	91953105
chr1	91956120	91956627
chr1	91962332	91962586
chr1	91969119	91969335
chr1	91978528	91978783
chr1	92025924	92026116
chr1	92042280	92042562
chr1	92048881	92049263
chr1	92050814	92051067
chr1	92051067	92051452
chr1	92052846	92053159
chr1	92064170	92064859
chr1	92073864	92074077
chr1	92075343	92075591
chr1	92076323	92076609
chr1	92086239	92086472
chr1	92086763	92087352
chr1	92087636	92087786
chr1	92087867	92088079
chr1	92089157	92089369
chr1	92119260	92119551
chr1	92179949	92180436
chr1	92180436	92180712
chr1	92180712	92181089
chr1	92195757	92196273
chr1	92215396	92215697
chr1	92232489	92232800
chr1	92252019	92252230
chr1	92263308	92263556
chr1	92270362	92270650
chr1	92270710	92271065
chr1	92271985	92272317
chr1	92272317	92272660
chr1	92281633	92281887
chr1	92283480	92283630
chr1	92316402	92316649
chr1	92348382	92348651
chr1	92360399	92360616
chr1	92371673	92371925

chr1	92378952	92379471
chr1	92379471	92379775
chr1	92405246	92405427
chr1	92405873	92406080
chr1	92440166	92440424
chr1	92453862	92454012
chr1	92473014	92473200
chr1	92473249	92473461
chr1	92473461	92473659
chr1	92473659	92473839
chr1	92473839	92474043
chr1	92474043	92474321
chr1	92474321	92474583
chr1	92480176	92480369
chr1	92481202	92481354
chr1	92484523	92484797
chr1	92537142	92537297
chr1	92545996	92546180
chr1	92611109	92611330
chr1	92615903	92616111
chr1	92616111	92616469
chr1	92616469	92616826
chr1	92641262	92641450
chr1	92641484	92642227
chr1	92647694	92648121
chr1	92648144	92648352
chr1	92648352	92648545
chr1	92648798	92649081
chr1	92652667	92652864
chr1	92653332	92653482
chr1	92654070	92654236
chr1	92654501	92654781
chr1	92654781	92655089
chr1	92663145	92663348
chr1	92669437	92669796
chr1	92670299	92670449
chr1	92677797	92678035
chr1	92696898	92697164
chr1	92699051	92699629
chr1	92708052	92708279
chr1	92712382	92712944
chr1	92718091	92718448

chr1	92725110	92725386
chr1	92742982	92743244
chr1	92744816	92745184
chr1	92745280	92745608
chr1	92745608	92745901
chr1	92751567	92752351
chr1	92769034	92769572
chr1	92769572	92769923
chr1	92775348	92775501
chr1	92779307	92779470
chr1	92785426	92785777
chr1	92786966	92787661
chr1	92790276	92790501
chr1	92791203	92791353
chr1	92799556	92800248
chr1	92800267	92800449
chr1	92805246	92805636
chr1	92806612	92807200
chr1	92809666	92809922
chr1	92811833	92812230
chr1	92815917	92816217
chr1	92831102	92831473
chr1	92831473	92831865
chr1	92831865	92832157
chr1	92832157	92832336
chr1	92832336	92832554
chr1	92832681	92832961
chr1	92834022	92834294
chr1	92839978	92840129
chr1	92840345	92840583
chr1	92849593	92849766
chr1	92861077	92861235
chr1	92863274	92863432
chr1	92875704	92875869
chr1	92883402	92883776
chr1	92883784	92883934
chr1	92889292	92889529
chr1	92895138	92895439
chr1	92902829	92903083
chr1	92903715	92904176
chr1	92906721	92907181
chr1	92907181	92907601

chr1	92910424	92910899
chr1	92910979	92911234
chr1	92911892	92912066
chr1	92912383	92912606
chr1	92919515	92919667
chr1	92924882	92925079
chr1	92934161	92934525
chr1	92934525	92935042
chr1	92944976	92945126
chr1	92949514	92949749
chr1	92956640	92957140
chr1	92957203	92957404
chr1	92971912	92972205
chr1	92972937	92973175
chr1	92978575	92978894
chr1	93006132	93006363
chr1	93008494	93008726
chr1	93008843	93008996
chr1	93009064	93009518
chr1	93014472	93014785
chr1	93015491	93015704
chr1	93017177	93017428
chr1	93023428	93023641
chr1	93056571	93056748
chr1	93062258	93062544
chr1	93062544	93062778
chr1	93063531	93063846
chr1	93063846	93064195
chr1	93081018	93081258
chr1	93100221	93100539
chr1	93100539	93100900
chr1	93100900	93101151
chr1	93101151	93101496
chr1	93101589	93102175
chr1	93107605	93107798
chr1	93115068	93115507
chr1	93128796	93129022
chr1	93130135	93130604
chr1	93130631	93131172
chr1	93139714	93140099
chr1	93142973	93143501
chr1	93146244	93146493

chr1	93153015	93153355
chr1	93159933	93160218
chr1	93160218	93160523
chr1	93160523	93160811
chr1	93160811	93161148
chr1	93161489	93161684
chr1	93161847	93162310
chr1	93166367	93166719
chr1	93166719	93167098
chr1	93168333	93168627
chr1	93173412	93173685
chr1	93173685	93173985
chr1	93173985	93174419
chr1	93174460	93174740
chr1	93181867	93182145
chr1	93183978	93184529
chr1	93187992	93188611
chr1	93191010	93191274
chr1	93193755	93193935
chr1	93197113	93197384
chr1	93197436	93197662
chr1	93200119	93200552
chr1	93206188	93206353
chr1	93206353	93206557
chr1	93207686	93207880
chr1	93210379	93210548
chr1	93223998	93224324
chr1	93229168	93229934
chr1	93235467	93236077
chr1	93236077	93236541
chr1	93236541	93236805
chr1	93245369	93245542
chr1	93246236	93246406
chr1	93258844	93259212
chr1	93261592	93262030
chr1	93268339	93268590
chr1	93270492	93270904
chr1	93270904	93271299
chr1	93272571	93272818
chr1	93284477	93284627
chr1	93286278	93286471
chr1	93289715	93289930

chr1	93289930	93290168
chr1	93299772	93300183
chr1	93305513	93306102
chr1	93322407	93322686
chr1	93326344	93326578
chr1	93326578	93327015
chr1	93334431	93334921
chr1	93334921	93335237
chr1	93342509	93343010
chr1	93343010	93343385
chr1	93343385	93343943
chr1	93372815	93373026
chr1	93373774	93373961
chr1	93375066	93375216
chr1	93377095	93377340
chr1	93379201	93379417
chr1	93387684	93387998
chr1	93389004	93389219
chr1	93392093	93392566
chr1	93392566	93392811
chr1	93400808	93401092
chr1	93401437	93402190
chr1	93444781	93445082
chr1	93445082	93445322
chr1	93445322	93445551
chr1	93445551	93445822
chr1	93445822	93446036
chr1	93446036	93446202
chr1	93477905	93478211
chr1	93478211	93478666
chr1	93478682	93479447
chr1	93506984	93507259
chr1	93511488	93511820
chr1	93511820	93512238
chr1	93512238	93512658
chr1	93529142	93529492
chr1	93529506	93529658
chr1	93529780	93530203
chr1	93530203	93530531
chr1	93532632	93532952
chr1	93563315	93563553
chr1	93578135	93578344

chr1	93607837	93607988
chr1	93611347	93611594
chr1	93618243	93618417
chr1	93618426	93618682
chr1	93634559	93634777
chr1	93634983	93635544
chr1	93635544	93635935
chr1	93635935	93636241
chr1	93636241	93636511
chr1	93654422	93654609
chr1	93654835	93655162
chr1	93655162	93655407
chr1	93656684	93657198
chr1	93667816	93668052
chr1	93668676	93669014
chr1	93669014	93669455
chr1	93674334	93674560
chr1	93678550	93678702
chr1	93680120	93680708
chr1	93685380	93685735
chr1	93685735	93686136
chr1	93686240	93686495
chr1	93686495	93686972
chr1	93692068	93692304
chr1	93698534	93698762
chr1	93705394	93705732
chr1	93705732	93706065
chr1	93706065	93706384
chr1	93715610	93715768
chr1	93717312	93717759
chr1	93725234	93725566
chr1	93725716	93726081
chr1	93726081	93726460
chr1	93726954	93727322
chr1	93740691	93740964
chr1	93743003	93743201
chr1	93748553	93748961
chr1	93753817	93754151
chr1	93754151	93754443
chr1	93754443	93754778
chr1	93754778	93755021
chr1	93755021	93755479

chr1	93763621	93763782
chr1	93771635	93771835
chr1	93773740	93773977
chr1	93801682	93802148
chr1	93803489	93804126
chr1	93804126	93804492
chr1	93824661	93824829
chr1	93824872	93825028
chr1	93825041	93825364
chr1	93825364	93825722
chr1	93825813	93826007
chr1	93832792	93833032
chr1	93835874	93836259
chr1	93836283	93836494
chr1	93846037	93846285
chr1	93853878	93854077
chr1	93854158	93854414
chr1	93857098	93857417
chr1	93857417	93857789
chr1	93897532	93897707
chr1	93909306	93909531
chr1	93911941	93912193
chr1	93919780	93920117
chr1	93920932	93921217
chr1	93966576	93966932
chr1	93978403	93978630
chr1	93980997	93981244
chr1	94034920	94035219
chr1	94035219	94035648
chr1	94056059	94056254
chr1	94079148	94079438
chr1	94149939	94150360
chr1	94366638	94366950
chr1	94469838	94470195
chr1	94499760	94499968
chr1	94537143	94537408
chr1	94938149	94938350
chr1	94949917	94950266
chr1	95074336	95074608
chr1	95308504	95308716
chr1	95313430	95313737
chr1	95313737	95313974

chr1	95313974	95314501
chr1	95367669	95367869
chr1	95367869	95368073
chr1	95422408	95422558
chr1	95433093	95433340
chr1	95451871	95452119
chr1	95467044	95467251
chr1	95467351	95467650
chr1	95468099	95468606
chr1	95474875	95475256
chr1	95636733	95637011
chr1	95665897	95666067
chr1	95666905	95667183
chr1	95667310	95668009
chr1	95668013	95668170
chr1	95707066	95707285
chr1	95707367	95707775
chr1	95720706	95720969
chr1	95830818	95831044
chr1	96000889	96001111
chr1	96129286	96129462
chr1	96799147	96799450
chr1	96799450	96799757
chr1	96805220	96805897
chr1	96813937	96814237
chr1	96814257	96814407
chr1	96871813	96872095
chr1	96872095	96872591
chr1	97260388	97260547
chr1	97263039	97263222
chr1	97577071	97577465
chr1	97587604	97587924
chr1	97604837	97605059
chr1	97613836	97614228
chr1	97647126	97647362
chr1	97650168	97650360
chr1	97657278	97657552
chr1	97660680	97660947
chr1	97660947	97661175
chr1	97661175	97661362
chr1	97661362	97661580
chr1	97661580	97661868

chr1	97661868	97662304
chr1	97688845	97689087
chr1	97726803	97727035
chr1	97727283	97727467
chr1	97759869	97760216
chr1	97769294	97769571
chr1	97769635	97769921
chr1	97769921	97770253
chr1	97770253	97770572
chr1	97770572	97770774
chr1	97771251	97771436
chr1	97799953	97800183
chr1	97872150	97872394
chr1	97873571	97873975
chr1	97873975	97874289
chr1	97910632	97910939
chr1	97910939	97911234
chr1	97911234	97911490
chr1	97918284	97918537
chr1	97918537	97918819
chr1	97919403	97919822
chr1	97963640	97964090
chr1	97964929	97965157
chr1	97976825	97977492
chr1	97983255	97983540
chr1	97992491	97992744
chr1	97993215	97993426
chr1	97995803	97995971
chr1	97996935	97997568
chr1	98002860	98003125
chr1	98028994	98029424
chr1	98035771	98036087
chr1	98036593	98036825
chr1	98038047	98038651
chr1	98047626	98048170
chr1	98072066	98072377
chr1	98079651	98079993
chr1	98083936	98084265
chr1	98084649	98084915
chr1	98089174	98089389
chr1	98089405	98089704
chr1	98089716	98089918

chr1	98094753	98095290
chr1	98095290	98095977
chr1	98096631	98096877
chr1	98147290	98147720
chr1	98178609	98179020
chr1	98179135	98179363
chr1	98275607	98275812
chr1	98304301	98304587
chr1	98304817	98305107
chr1	98727532	98727686
chr1	98727711	98728220
chr1	98730440	98730795
chr1	98732738	98733237
chr1	98953244	98953590
chr1	99249556	99249757
chr1	99772227	99772438
chr1	99825353	99825515
chr1	99843845	99844249
chr1	99877916	99878263
chr1	99881769	99882397
chr1	99917790	99918183
chr1	100082741	100082920
chr1	100100452	100101005
chr1	100101019	100101456
chr1	100122532	100122731
chr1	100171474	100172009
chr1	100173439	100173594
chr1	100173720	100173950
chr1	100180036	100180491
chr1	100185404	100185793
chr1	100223893	100224193
chr1	100231395	100231839
chr1	100231839	100232227
chr1	100738816	100739020
chr1	100784607	100784828
chr1	101133459	101133730
chr1	101133921	101134101
chr1	101134291	101134472
chr1	101135167	101135317
chr1	102628120	102628397
chr1	103589863	103590104
chr1	104224130	104224285

chr1	104293436	104293744
chr1	104333079	104333347
chr1	104508423	104508601
chr1	104986301	104986527
chr1	104994045	104994222
chr1	105179390	105179721
chr1	105179721	105180128
chr1	105191226	105191395
chr1	105199316	105199665
chr1	105291779	105292230
chr1	105302323	105302541
chr1	105342351	105342753
chr1	105355948	105356378
chr1	105356378	105357145
chr1	105362887	105363037
chr1	105374099	105374436
chr1	105384545	105385105
chr1	105418194	105418381
chr1	105420542	105420983
chr1	105431869	105432237
chr1	105434853	105435418
chr1	105442271	105442570
chr1	105442900	105443158
chr1	105453893	105454419
chr1	105493727	105494020
chr1	105555207	105555597
chr1	105563273	105563545
chr1	105564754	105565069
chr1	105565069	105565420
chr1	105663152	105663500
chr1	105663500	105663805
chr1	105663805	105664293
chr1	105664548	105664713
chr1	105669789	105670041
chr1	105680688	105680906
chr1	105685617	105685783
chr1	105735371	105735528
chr1	105742583	105742809
chr1	105770829	105771136
chr1	105780154	105781124
chr1	105781467	105781678
chr1	105787131	105787302

chr1	105790159	105790540
chr1	105790540	105790847
chr1	105801407	105801717
chr1	105802300	105802518
chr1	105805733	105805916
chr1	105813476	105813626
chr1	105825701	105825854
chr1	105866703	105867214
chr1	105867214	105867532
chr1	105881093	105881249
chr1	105886471	105886687
chr1	105891079	105891359
chr1	105906158	105906464
chr1	105906464	105906998
chr1	105907403	105907721
chr1	105922685	105922867
chr1	105923041	105923301
chr1	105924936	105925179
chr1	105925357	105925819
chr1	105925819	105926079
chr1	105926096	105926298
chr1	105926419	105926569
chr1	105930886	105931239
chr1	105947284	105947570
chr1	105971796	105971946
chr1	105977815	105978040
chr1	105981307	105981595
chr1	105982590	105982831
chr1	105983052	105983332
chr1	105984129	105984545
chr1	105990095	105990895
chr1	105991009	105991201
chr1	105991291	105991729
chr1	105993313	105993519
chr1	105993894	105994153
chr1	106000346	106000496
chr1	106000713	106001149
chr1	106007672	106007875
chr1	106037012	106037451
chr1	106043093	106043451
chr1	106043451	106043844
chr1	106071313	106071564

chr1	106109082	106109313
chr1	106144280	106144526
chr1	106144526	106144942
chr1	106152857	106153039
chr1	106160947	106161346
chr1	106162690	106162886
chr1	106163435	106163776
chr1	106168857	106169169
chr1	106171234	106171564
chr1	106171564	106171895
chr1	106171895	106172203
chr1	106172388	106172656
chr1	106172656	106172870
chr1	106178059	106178229
chr1	106187477	106187768
chr1	106187768	106188029
chr1	106190817	106190967
chr1	106210876	106211169
chr1	106211169	106211578
chr1	106213996	106214151
chr1	106220460	106220784
chr1	106220830	106221236
chr1	106221538	106221715
chr1	106230320	106230606
chr1	106240553	106240830
chr1	106241751	106242019
chr1	106242107	106242446
chr1	106278631	106278878
chr1	106307448	106307635
chr1	106308161	106308329
chr1	106314528	106314767
chr1	106316960	106317207
chr1	106320687	106320933
chr1	106324538	106324983
chr1	106324983	106325432
chr1	106326868	106327032
chr1	106339620	106339818
chr1	106346829	106347025
chr1	106352808	106353090
chr1	106353164	106353384
chr1	106367386	106367608
chr1	106378938	106379209

chr1	106389824	106390019
chr1	106394675	106395189
chr1	106395540	106395825
chr1	106400795	106401225
chr1	106401225	106401633
chr1	106403193	106403811
chr1	106407046	106407391
chr1	106407391	106407701
chr1	106407713	106408424
chr1	106408740	106409014
chr1	106409014	106409237
chr1	106409466	106409750
chr1	106423395	106423573
chr1	106425619	106425870
chr1	106426322	106426697
chr1	106430773	106431150
chr1	106431235	106431744
chr1	106449774	106450123
chr1	106460990	106461317
chr1	106477663	106478524
chr1	106479057	106479262
chr1	106500869	106501309
chr1	106501533	106501755
chr1	106501830	106502027
chr1	106503131	106503404
chr1	106511628	106511929
chr1	106513309	106513552
chr1	106513552	106513859
chr1	106515640	106515790
chr1	106525645	106525903
chr1	106525903	106526174
chr1	106526174	106526595
chr1	106544744	106544977
chr1	106547436	106547763
chr1	106591292	106591706
chr1	106628805	106629030
chr1	106629448	106629861
chr1	106629898	106630159
chr1	106630159	106630618
chr1	106700550	106701031
chr1	106712273	106712485
chr1	106712485	106712706

chr1	106713704	106714942
chr1	106735655	106735865
chr1	106738144	106738381
chr1	106758764	106759463
chr1	106759463	106760061
chr1	106765869	106766069
chr1	106796156	106796507
chr1	106796507	106796907
chr1	106797119	106797367
chr1	106797369	106797583
chr1	106799508	106799687
chr1	106814952	106815142
chr1	106818454	106818635
chr1	106839508	106839796
chr1	106839796	106840135
chr1	106857420	106857805
chr1	106860862	106861274
chr1	106861312	106861486
chr1	106861495	106861645
chr1	106865432	106865587
chr1	106876205	106876422
chr1	106883421	106883633
chr1	106883850	106884208
chr1	106901536	106901782
chr1	106942134	106942455
chr1	106944934	106945168
chr1	107000544	107000735
chr1	107130091	107130343
chr1	107214759	107215013
chr1	107250792	107250960
chr1	107362140	107362290
chr1	107365379	107365838
chr1	107366103	107366253
chr1	107384595	107384829
chr1	107414536	107415025
chr1	107471899	107472116
chr1	107481515	107481826
chr1	107488252	107489024
chr1	107495567	107495839
chr1	107496932	107497118
chr1	107499037	107499373
chr1	107500773	107500957

chr1	107501990	107502198
chr1	107502198	107502392
chr1	107505300	107505521
chr1	107505856	107506467
chr1	107511105	107511610
chr1	107589797	107590150
chr1	107590150	107590454
chr1	107590454	107590685
chr1	107591078	107591431
chr1	107608996	107609213
chr1	107609598	107609875
chr1	107810019	107810346
chr1	107822181	107822331
chr1	107836764	107836951
chr1	107967240	107967484
chr1	108046267	108046533
chr1	108117123	108117435
chr1	108131207	108131549
chr1	108131626	108131886
chr1	108132002	108132398
chr1	108409014	108409185
chr1	108415819	108416019
chr1	109132566	109132766
chr1	109512757	109512919
chr1	110330123	110330375
chr1	110483483	110483851
chr1	110745332	110745572
chr1	110796063	110796403
chr1	111373134	111373397
chr1	111864018	111864247
chr1	111864254	111864746
chr1	111864746	111865193
chr1	112245429	112245688
chr1	112486134	112486304
chr1	112748507	112748855
chr1	112750670	112751082
chr1	113161319	113161522
chr1	113258370	113258525
chr1	113259531	113259687
chr1	113259891	113260041
chr1	113270875	113271287
chr1	113287881	113288214

chr1	113288214	113288914
chr1	113296193	113296351
chr1	113297765	113298339
chr1	113302652	113302809
chr1	113373180	113373582
chr1	113482480	113482800
chr1	113530453	113530864
chr1	113628960	113629169
chr1	114039405	114039770
chr1	114186349	114186573
chr1	114644177	114644372
chr1	115058526	115058769
chr1	115174277	115174475
chr1	115488060	115488225
chr1	115511684	115511961
chr1	115511961	115512234
chr1	115512781	115512976
chr1	116415061	116415574
chr1	116667999	116668194
chr1	118012716	118013042
chr1	118275248	118275470
chr1	118288586	118288817
chr1	118294123	118294586
chr1	118310769	118311659
chr1	118320791	118321005
chr1	118321590	118321955
chr1	118321955	118322383
chr1	118325190	118325547
chr1	118334813	118335029
chr1	118335029	118335272
chr1	118335981	118336131
chr1	118343670	118344009
chr1	118366384	118366754
chr1	118368109	118368298
chr1	118368432	118368594
chr1	118368701	118369117
chr1	118369117	118369623
chr1	118388177	118388363
chr1	118388588	118389117
chr1	118389117	118389504
chr1	118389504	118389939
chr1	118389939	118390308

chr1	118395819	118396038
chr1	118398545	118398834
chr1	118399533	118399943
chr1	118399943	118400248
chr1	118424712	118424985
chr1	118424985	118425328
chr1	118425328	118425655
chr1	118428217	118428437
chr1	118435090	118435271
chr1	118440673	118440848
chr1	118441156	118441479
chr1	118443171	118443404
chr1	118444506	118444726
chr1	118444960	118445194
chr1	118447185	118447458
chr1	118447515	118447665
chr1	118449383	118449785
chr1	118449912	118450155
chr1	118450210	118450476
chr1	118453543	118453866
chr1	118458280	118458630
chr1	118458723	118459380
chr1	118459380	118459701
chr1	118459701	118460001
chr1	118466577	118466753
chr1	118476960	118477207
chr1	118479225	118479641
chr1	118492897	118493136
chr1	118494546	118494938
chr1	118497259	118497461
chr1	118498199	118498399
chr1	118498399	118498684
chr1	118529345	118529495
chr1	118558813	118559010
chr1	118559010	118559275
chr1	118627678	118628127
chr1	118628127	118628528
chr1	118630202	118630528
chr1	118647983	118648450
chr1	118649691	118649983
chr1	118649983	118650289
chr1	118650289	118650707

chr1	118657120	118657614
chr1	118660666	118661102
chr1	118661102	118661395
chr1	118661395	118661614
chr1	118676567	118676856
chr1	118689715	118689971
chr1	118689971	118690444
chr1	118690750	118691065
chr1	118697189	118697353
chr1	118697976	118698157
chr1	118700032	118700309
chr1	118711730	118711894
chr1	118752938	118753089
chr1	118758086	118758306
chr1	118759609	118759915
chr1	118767327	118767610
chr1	118768075	118768289
chr1	118772313	118772636
chr1	118776748	118776951
chr1	118807739	118807962
chr1	118825157	118825517
chr1	118852090	118852283
chr1	118976543	118976969
chr1	118993816	118994078
chr1	119048034	119048309
chr1	119053583	119053838
chr1	119076842	119077021
chr1	119082699	119082907
chr1	119083718	119083972
chr1	119085882	119086071
chr1	119087603	119087907
chr1	119093798	119094067
chr1	119123062	119123357
chr1	119160846	119161002
chr1	119217401	119217712
chr1	119234171	119234552
chr1	119269038	119269297
chr1	119289817	119290015
chr1	119290015	119290210
chr1	119290210	119290558
chr1	119292094	119292613
chr1	119307313	119307521

chr1	119308745	119309073
chr1	119309073	119309432
chr1	119344091	119344344
chr1	119375300	119375773
chr1	119390761	119391073
chr1	119404267	119404764
chr1	119404764	119405127
chr1	119420412	119420577
chr1	119420661	119420833
chr1	119420833	119420999
chr1	119421009	119421292
chr1	119421292	119421565
chr1	119421565	119421799
chr1	119421799	119422003
chr1	119422003	119422231
chr1	119422272	119422425
chr1	119458101	119458254
chr1	119458270	119458539
chr1	119463461	119463703
chr1	119484545	119484773
chr1	119492075	119492485
chr1	119492485	119492780
chr1	119494425	119494641
chr1	119494757	119495058
chr1	119495058	119495333
chr1	119504247	119505146
chr1	119509529	119509851
chr1	119509851	119510091
chr1	119510091	119510292
chr1	119513856	119514029
chr1	119525609	119526008
chr1	119526008	119526255
chr1	119526255	119526608
chr1	119534429	119534692
chr1	119536219	119536574
chr1	119538336	119538778
chr1	119548711	119548979
chr1	119548979	119549182
chr1	119549442	119549603
chr1	119639775	119640182
chr1	119640240	119640391
chr1	119642325	119642698

chr1	119648274	119648550
chr1	119648550	119648848
chr1	119648848	119649221
chr1	119649691	119649973
chr1	119660577	119660811
chr1	119674256	119674538
chr1	119675080	119675277
chr1	119794536	119794788
chr1	119794916	119795090
chr1	119799282	119799600
chr1	119806081	119806293
chr1	119836785	119837004
chr1	119837004	119837325
chr1	119837325	119837597
chr1	119837597	119837896
chr1	119891880	119892035
chr1	119895018	119895321
chr1	119912927	119913654
chr1	119920704	119921109
chr1	119921122	119921295
chr1	119921295	119921475
chr1	119929841	119930056
chr1	119931415	119931744
chr1	119965827	119966140
chr1	119986629	119986885
chr1	119991937	119992100
chr1	119997101	119997358
chr1	119997358	119997601
chr1	119997792	119997972
chr1	120012364	120012687
chr1	120013204	120013428
chr1	120032871	120033524
chr1	120034761	120035105
chr1	120047092	120047315
chr1	120066746	120067000
chr1	120073628	120074132
chr1	120074132	120074619
chr1	120080675	120081099
chr1	120082924	120083275
chr1	120083275	120083553
chr1	120083937	120084252
chr1	120093468	120093866

chr1	120099534	120099856
chr1	120105701	120106005
chr1	120120106	120120762
chr1	120120762	120121403
chr1	120121406	120121575
chr1	120124981	120125201
chr1	120136037	120136586
chr1	120136586	120136856
chr1	120154308	120154542
chr1	120156180	120156337
chr1	120171863	120172115
chr1	120197194	120197690
chr1	120202528	120202691
chr1	120231399	120231618
chr1	120235683	120235897
chr1	120236384	120236929
chr1	120264769	120265590
chr1	120276181	120276331
chr1	120305822	120306120
chr1	120331989	120332189
chr1	120363662	120363931
chr1	120430243	120430478
chr1	120549358	120549613
chr1	120549697	120550253
chr1	120550253	120550489
chr1	120574823	120575002
chr1	120586544	120586774
chr1	120596474	120596670
chr1	120602197	120602385
chr1	120602818	120603277
chr1	120603277	120603586
chr1	120603614	120603995
chr1	120607079	120607293
chr1	120654750	120655515
chr1	120667485	120667699
chr1	120668264	120668590
chr1	120685960	120686134
chr1	120757333	120757816
chr1	120757816	120758182
chr1	120758728	120759150
chr1	120783035	120783197
chr1	120812733	120813103

chr1	120814475	120814719
chr1	120816302	120816561
chr1	120828308	120828493
chr1	120828507	120828728
chr1	120887156	120887357
chr1	120900576	120900998
chr1	120901105	120901314
chr1	120901314	120901505
chr1	120901705	120901858
chr1	120905488	120905890
chr1	120906044	120906372
chr1	120906602	120906761
chr1	120910535	120910803
chr1	120910844	120911372
chr1	120974645	120974896
chr1	121012089	121012456
chr1	121014151	121014439
chr1	121014439	121014656
chr1	121016241	121016498
chr1	121034764	121035118
chr1	121174597	121174758
chr1	121180399	121180611
chr1	121260461	121260684
chr1	121280570	121280861
chr1	121295230	121295517
chr1	121313901	121314090
chr1	121327090	121327248
chr1	121327275	121327618
chr1	121327618	121328224
chr1	121328228	121328467
chr1	121344005	121344335
chr1	121346156	121346380
chr1	121350643	121350862
chr1	121351115	121351397
chr1	121360318	121361086
chr1	121366313	121366849
chr1	121369603	121369771
chr1	121377279	121377771
chr1	121377832	121378233
chr1	121380052	121380387
chr1	121398049	121398201
chr1	121398280	121398553

chr1	121400348	121400577
chr1	121419191	121419387
chr1	121430901	121431193
chr1	121431193	121431485
chr1	121431485	121432017
chr1	121441136	121441389
chr1	121460173	121460360
chr1	121482989	121483754
chr1	121490307	121490571
chr1	121497252	121497454
chr1	121527605	121527907
chr1	121527907	121528313
chr1	121528370	121529017
chr1	121549541	121549698
chr1	121567530	121567839
chr1	121567839	121568138
chr1	121568138	121568403
chr1	121653296	121653555
chr1	121653555	121653761
chr1	121653923	121654202
chr1	121654202	121654470
chr1	122021513	122021802
chr1	122050177	122050387
chr1	122099859	122100122
chr1	122101630	122101916
chr1	122101916	122102272
chr1	122102361	122102847
chr1	122193763	122193956
chr1	122357067	122357315
chr1	123134240	123134441
chr1	123303659	123304044
chr1	123580072	123580528
chr1	123820485	123820645
chr1	124453057	124453575
chr1	124458574	124458859
chr1	124487827	124488328
chr1	124488348	124488915
chr1	124496405	124496827
chr1	124497346	124497519
chr1	124504096	124504407
chr1	124584709	124584916
chr1	124602887	124603239

chr1	124674519	124674721
chr1	124805103	124805572
chr1	124829960	124830275
chr1	124885425	124885599
chr1	124910712	124910936
chr1	125146113	125146263
chr1	125147231	125147489
chr1	125176451	125176664
chr1	125213745	125214134
chr1	125214134	125214812
chr1	125214932	125215084
chr1	125219727	125219937
chr1	125236318	125236882
chr1	125236882	125237589
chr1	125237695	125237961
chr1	125286367	125286691
chr1	125325583	125325733
chr1	125370865	125371109
chr1	125389644	125390192
chr1	125390192	125390662
chr1	125428470	125428715
chr1	125430088	125430305
chr1	125434849	125435001
chr1	125435055	125436110
chr1	125450248	125450532
chr1	125450532	125450916
chr1	125454600	125454765
chr1	125476365	125476552
chr1	125478777	125479018
chr1	125489157	125489862
chr1	125496948	125497107
chr1	125497154	125497315
chr1	125508114	125508504
chr1	125508569	125508867
chr1	125519074	125519753
chr1	125520317	125520638
chr1	125525185	125525353
chr1	125525906	125526333
chr1	125526333	125526635
chr1	125526635	125526972
chr1	125533250	125533495
chr1	125540657	125540892

chr1	125549947	125550308
chr1	125550359	125550897
chr1	125551604	125552006
chr1	125559230	125559544
chr1	125560184	125560663
chr1	125560663	125561443
chr1	125607270	125607481
chr1	125607481	125607768
chr1	125610791	125611016
chr1	125612752	125613191
chr1	125615875	125616076
chr1	125640008	125640176
chr1	125640254	125640479
chr1	125647769	125647980
chr1	125647985	125648523
chr1	125648704	125648854
chr1	125649078	125649330
chr1	125654108	125654273
chr1	125661031	125661195
chr1	125665196	125665377
chr1	125665377	125665459
chr1	125665800	125666111
chr1	125669100	125669363
chr1	125670160	125670332
chr1	125676407	125676574
chr1	125676578	125677191
chr1	125677191	125677535
chr1	125677535	125677782
chr1	125679337	125679821
chr1	125687526	125687733
chr1	125687770	125688021
chr1	125688021	125688683
chr1	125693670	125693860
chr1	125694174	125694784
chr1	125696803	125696968
chr1	125700817	125701369
chr1	125701572	125702151
chr1	125704803	125705032
chr1	125705118	125705304
chr1	125706933	125707173
chr1	125709931	125710273
chr1	125721238	125722023

chr1	125730177	125730464
chr1	125731711	125731893
chr1	125731893	125732040
chr1	125740752	125741042
chr1	125755003	125755333
chr1	125755333	125755657
chr1	125755657	125755955
chr1	125789907	125790092
chr1	125800132	125800898
chr1	125804474	125804693
chr1	125825341	125825624
chr1	125850156	125850381
chr1	125910575	125910762
chr1	125911314	125911577
chr1	125911577	125911877
chr1	125911877	125912168
chr1	125912168	125912519
chr1	125950760	125951527
chr1	126032357	126032544
chr1	126065834	126065989
chr1	126153622	126153772
chr1	126269660	126269868
chr1	126271589	126272090
chr1	126296705	126296921
chr1	126408035	126408195
chr1	126431707	126431937
chr1	126443427	126443857
chr1	126592508	126593011
chr1	126593011	126593383
chr1	126604591	126604854
chr1	126608954	126609204
chr1	126639541	126639790
chr1	126649844	126650114
chr1	126650248	126650569
chr1	126650835	126651160
chr1	126658693	126659109
chr1	126670497	126670902
chr1	126673186	126673636
chr1	126679380	126679762
chr1	126679762	126680142
chr1	126680177	126680337
chr1	126736698	126736884

chr1	126755749	126756072
chr1	126811443	126811593
chr1	126817404	126817687
chr1	126817687	126818061
chr1	126818115	126818390
chr1	126827009	126827371
chr1	126844835	126844985
chr1	126931872	126932204
chr1	126939215	126939391
chr1	126940197	126940537
chr1	126940582	126940737
chr1	126944051	126944243
chr1	126993381	126993531
chr1	127053773	127053975
chr1	127204760	127205099
chr1	127205099	127205624
chr1	127205771	127205932
chr1	127205981	127206255
chr1	127206255	127206616
chr1	127212908	127213116
chr1	127219229	127219422
chr1	127219707	127219964
chr1	127233040	127233658
chr1	127233658	127233965
chr1	127239756	127240363
chr1	127240363	127240611
chr1	127246243	127246510
chr1	127248232	127248488
chr1	127248488	127248797
chr1	127251677	127251887
chr1	127251952	127252192
chr1	127256524	127256703
chr1	127256994	127257467
chr1	127290451	127290640
chr1	127290700	127290949
chr1	127290949	127291222
chr1	127291222	127291792
chr1	127301073	127301468
chr1	127330383	127330563
chr1	127344550	127344958
chr1	127344958	127345455
chr1	127408800	127409003

chr1	127409675	127409871
chr1	127424889	127425554
chr1	127458965	127459244
chr1	127461067	127461230
chr1	127484358	127484545
chr1	127484898	127485130
chr1	127487316	127487466
chr1	127493929	127494382
chr1	127494480	127494828
chr1	127496765	127497175
chr1	127500290	127500535
chr1	127569211	127569465
chr1	127583812	127584039
chr1	127608161	127608435
chr1	127608435	127608762
chr1	127609149	127609409
chr1	127609565	127609828
chr1	127609828	127610049
chr1	127622180	127622419
chr1	127622419	127622976
chr1	127626700	127626954
chr1	127627423	127627905
chr1	127628512	127628665
chr1	127646290	127646440
chr1	127660120	127660435
chr1	127660472	127660784
chr1	127677218	127677621
chr1	127677621	127677965
chr1	127677965	127678257
chr1	127679902	127680086
chr1	127755714	127756180
chr1	127773769	127774083
chr1	127774083	127774279
chr1	127774279	127774513
chr1	127774513	127774872
chr1	127781139	127781438
chr1	127806225	127806463
chr1	127806463	127806684
chr1	127818846	127819111
chr1	127819111	127819353
chr1	127819546	127819699
chr1	127826340	127826490

chr1	127827386	127827624
chr1	127833723	127833932
chr1	127838372	127838603
chr1	127838685	127838947
chr1	127860120	127860298
chr1	127860588	127860854
chr1	127868584	127868895
chr1	127868895	127869342
chr1	127895917	127896158
chr1	127958571	127958726
chr1	127961205	127961468
chr1	127975629	127976306
chr1	127982042	127982335
chr1	127982335	127982644
chr1	127982766	127982955
chr1	127983302	127983641
chr1	127983641	127983922
chr1	127988614	127988873
chr1	127991489	127991651
chr1	127991705	127992106
chr1	128016242	128016409
chr1	128032279	128032461
chr1	128058680	128058976
chr1	128065546	128065756
chr1	128102371	128102567
chr1	128102598	128103020
chr1	128103020	128103436
chr1	128103436	128103881
chr1	128115651	128115870
chr1	128124477	128124702
chr1	128221595	128221889
chr1	128243930	128244261
chr1	128244261	128244633
chr1	128244633	128244995
chr1	128245011	128245224
chr1	128270793	128270944
chr1	128357548	128357784
chr1	128358871	128359288
chr1	128359288	128359575
chr1	128359575	128359970
chr1	128368732	128368964
chr1	128391777	128392235

chr1	128398738	128399658
chr1	128416559	128417001
chr1	128417001	128417224
chr1	128417224	128417743
chr1	128435602	128436218
chr1	128471993	128472500
chr1	128472500	128472942
chr1	128591895	128592054
chr1	128592062	128592296
chr1	128592296	128592507
chr1	128592528	128592764
chr1	128593215	128593386
chr1	128701405	128701605
chr1	128824307	128824575
chr1	128824930	128825485
chr1	128832672	128832914
chr1	129179390	129179736
chr1	129184317	129184559
chr1	129228050	129228247
chr1	129244392	129244553
chr1	129251700	129251963
chr1	129253421	129253709
chr1	129254590	129254742
chr1	129363420	129363748
chr1	129363995	129364347
chr1	129386336	129386919
chr1	129463015	129463166
chr1	129497910	129498104
chr1	130214647	130214837
chr1	130224559	130224868
chr1	130225079	130225288
chr1	130272338	130272556
chr1	130350369	130350524
chr1	130372237	130372387
chr1	130438360	130438521
chr1	130457313	130457534
chr1	130462193	130462438
chr1	130462438	130462661
chr1	130462661	130463174
chr1	130474237	130474727
chr1	130485510	130485725
chr1	130485911	130486337

chr1	130486337	130486581
chr1	130548055	130548284
chr1	130634017	130634313
chr1	130648353	130648679
chr1	130692008	130692189
chr1	130692599	130692785
chr1	130715503	130715776
chr1	130715776	130716255
chr1	130716780	130717240
chr1	130717240	130717673
chr1	130717692	130717851
chr1	130731749	130732189
chr1	130733556	130734090
chr1	130734425	130734777
chr1	130737611	130737936
chr1	130740380	130740539
chr1	130740864	130741239
chr1	130741321	130741588
chr1	130745686	130745858
chr1	130747123	130747649
chr1	130761079	130761504
chr1	130761504	130761958
chr1	130764045	130764268
chr1	130764268	130764488
chr1	130766866	130767136
chr1	130770350	130770914
chr1	130770937	130771430
chr1	130776741	130776979
chr1	130784180	130784429
chr1	130786378	130786586
chr1	130787081	130787265
chr1	130789568	130790007
chr1	130790007	130790654
chr1	130797040	130797257
chr1	130804013	130804227
chr1	130838386	130838608
chr1	130853307	130853487
chr1	130860406	130860780
chr1	130875218	130875436
chr1	130879918	130880691
chr1	130886388	130886794
chr1	130886794	130887036

chr1	130887226	130887634
chr1	130892290	130892901
chr1	130902459	130902919
chr1	130918347	130918644
chr1	130918644	130918957
chr1	130944044	130944336
chr1	130979610	130979782
chr1	130984578	130984772
chr1	130987611	130987803
chr1	130996165	130996480
chr1	131000464	131000668
chr1	131010324	131010550
chr1	131010590	131010774
chr1	131021517	131021878
chr1	131025966	131026116
chr1	131038041	131038660
chr1	131039533	131039805
chr1	131042732	131043034
chr1	131046073	131046240
chr1	131050748	131050898
chr1	131051118	131051305
chr1	131053145	131053611
chr1	131054422	131054572
chr1	131055545	131055750
chr1	131060375	131060603
chr1	131060963	131061124
chr1	131061124	131061283
chr1	131063591	131063778
chr1	131064208	131064563
chr1	131072335	131072667
chr1	131072667	131072909
chr1	131080024	131080228
chr1	131082655	131082973
chr1	131083006	131083239
chr1	131083749	131084013
chr1	131085371	131085553
chr1	131088715	131088901
chr1	131089003	131089217
chr1	131089750	131090168
chr1	131090168	131090549
chr1	131096759	131097394
chr1	131097394	131098067

chr1	131107687	131107994
chr1	131107994	131108279
chr1	131108279	131108551
chr1	131108673	131108965
chr1	131112532	131112920
chr1	131115776	131115944
chr1	131116037	131116243
chr1	131118489	131118976
chr1	131118976	131119474
chr1	131123910	131124226
chr1	131125908	131126377
chr1	131126377	131126757
chr1	131135184	131135521
chr1	131137692	131138154
chr1	131138154	131138663
chr1	131138686	131138942
chr1	131152991	131153512
chr1	131153702	131153858
chr1	131155624	131155798
chr1	131159069	131159280
chr1	131159959	131160191
chr1	131181714	131181949
chr1	131184021	131184234
chr1	131184347	131184640
chr1	131184640	131184984
chr1	131193803	131194037
chr1	131200145	131200322
chr1	131207608	131208027
chr1	131221918	131222200
chr1	131222200	131222533
chr1	131228184	131228482
chr1	131238162	131238554
chr1	131238621	131238936
chr1	131238936	131239302
chr1	131239302	131240012
chr1	131244637	131244810
chr1	131244839	131245316
chr1	131245316	131245615
chr1	131245615	131245926
chr1	131245926	131246224
chr1	131246266	131246421
chr1	131246538	131246688

chr1	131252038	131252288
chr1	131252667	131253044
chr1	131260421	131260694
chr1	131270124	131270558
chr1	131270594	131270751
chr1	131270800	131270969
chr1	131276924	131277103
chr1	131278250	131278516
chr1	131279087	131279489
chr1	131279489	131279803
chr1	131279897	131280138
chr1	131280162	131280592
chr1	131286787	131287062
chr1	131287062	131287343
chr1	131287343	131287743
chr1	131290708	131290938
chr1	131293908	131294340
chr1	131300807	131301073
chr1	131333102	131333387
chr1	131335654	131335823
chr1	131378175	131378379
chr1	131400918	131401152
chr1	131427558	131427815
chr1	131444033	131444201
chr1	131447468	131447618
chr1	131460854	131461257
chr1	131461257	131461528
chr1	131462638	131462894
chr1	131504330	131504665
chr1	131504665	131504884
chr1	131506264	131506662
chr1	131506662	131506992
chr1	131509090	131509362
chr1	131511751	131512137
chr1	131513182	131513437
chr1	131521530	131521948
chr1	131526379	131526732
chr1	131526821	131527251
chr1	131527251	131527599
chr1	131527599	131527761
chr1	131527761	131528137
chr1	131528207	131528401

chr1	131533912	131534084
chr1	131541495	131541652
chr1	131551994	131552243
chr1	131555019	131555236
chr1	131555236	131555563
chr1	131573752	131573943
chr1	131586597	131586879
chr1	131586879	131587400
chr1	131600460	131600614
chr1	131613380	131613826
chr1	131625028	131625366
chr1	131627119	131627291
chr1	131657915	131658128
chr1	131658128	131658432
chr1	131661300	131661450
chr1	131661729	131661975
chr1	131663689	131663924
chr1	131663941	131664245
chr1	131664245	131664510
chr1	131672502	131672696
chr1	131679100	131679265
chr1	131680055	131680447
chr1	131690340	131691014
chr1	131691384	131691794
chr1	131703487	131703853
chr1	131703853	131704180
chr1	131705791	131705973
chr1	131708425	131708654
chr1	131712312	131712736
chr1	131714265	131714614
chr1	131717585	131717855
chr1	131719433	131719655
chr1	131725115	131725720
chr1	131725720	131726107
chr1	131730749	131731019
chr1	131733461	131733882
chr1	131733919	131734181
chr1	131737281	131737648
chr1	131740533	131740910
chr1	131742299	131742533
chr1	131747718	131747984
chr1	131747984	131748274

chr1	131748451	131748601
chr1	131748912	131749363
chr1	131749861	131750074
chr1	131750227	131750449
chr1	131751195	131751579
chr1	131751832	131752097
chr1	131752141	131752405
chr1	131754338	131754495
chr1	131759744	131759902
chr1	131760074	131760547
chr1	131761251	131761975
chr1	131765620	131765901
chr1	131797250	131797442
chr1	131799048	131799198
chr1	131827364	131827536
chr1	131827536	131827798
chr1	131827798	131828201
chr1	131828201	131828627
chr1	131828627	131828955
chr1	131841017	131841373
chr1	131846389	131846613
chr1	131867048	131867322
chr1	131867322	131867583
chr1	131867583	131868145
chr1	131880039	131880234
chr1	131893432	131893603
chr1	131910198	131910593
chr1	131910593	131910943
chr1	131910943	131911264
chr1	131911264	131911517
chr1	131913499	131913667
chr1	131917193	131917346
chr1	131937756	131938003
chr1	131945706	131946028
chr1	131946028	131946295
chr1	131948089	131948448
chr1	131956339	131956518
chr1	131962533	131962900
chr1	131962900	131963122
chr1	131963122	131963339
chr1	131963339	131963637
chr1	131963637	131963993

chr1	131967439	131967626
chr1	131968712	131968909
chr1	131969089	131969482
chr1	131969897	131970133
chr1	131974070	131974326
chr1	131976764	131977251
chr1	131977251	131977493
chr1	131981705	131981963
chr1	131982951	131983354
chr1	131995148	131995391
chr1	131995615	131995765
chr1	131999351	131999615
chr1	132006066	132006419
chr1	132007283	132007808
chr1	132007808	132008419
chr1	132010627	132011054
chr1	132014936	132015092
chr1	132033841	132033991
chr1	132046263	132046457
chr1	132046457	132046861
chr1	132062759	132063023
chr1	132063166	132063410
chr1	132063711	132063962
chr1	132067515	132068250
chr1	132075437	132075778
chr1	132095405	132095634
chr1	132095634	132096026
chr1	132099023	132099403
chr1	132104439	132104732
chr1	132105751	132105924
chr1	132106810	132106960
chr1	132113130	132113676
chr1	132121239	132121735
chr1	132133181	132134046
chr1	132138212	132138392
chr1	132138749	132138983
chr1	132139318	132139619
chr1	132139619	132139985
chr1	132140257	132140417
chr1	132143138	132143327
chr1	132144245	132144619
chr1	132144847	132145133

chr1	132145133	132145540
chr1	132146130	132146410
chr1	132150143	132150550
chr1	132150732	132151191
chr1	132151248	132151473
chr1	132151544	132151756
chr1	132151782	132152317
chr1	132155835	132155992
chr1	132157424	132157884
chr1	132157916	132158111
chr1	132158779	132159078
chr1	132160589	132160745
chr1	132162746	132162897
chr1	132168197	132168464
chr1	132190104	132190330
chr1	132190330	132190568
chr1	132190634	132190974
chr1	132190974	132191161
chr1	132192306	132192471
chr1	132195160	132195381
chr1	132195472	132195780
chr1	132195780	132195972
chr1	132196298	132196517
chr1	132196517	132196720
chr1	132199358	132199615
chr1	132200623	132200982
chr1	132200982	132201297
chr1	132205951	132206365
chr1	132206365	132206755
chr1	132206871	132207250
chr1	132210812	132211262
chr1	132211788	132212008
chr1	132233649	132233886
chr1	132237627	132237866
chr1	132241599	132241751
chr1	132254503	132254810
chr1	132255069	132255342
chr1	132255342	132255597
chr1	132255844	132256107
chr1	132260377	132260642
chr1	132264594	132265014
chr1	132265319	132265489

chr1	132266723	132267146
chr1	132271579	132271896
chr1	132271952	132272344
chr1	132280713	132280901
chr1	132286804	132287122
chr1	132291382	132291590
chr1	132291734	132292062
chr1	132292315	132292610
chr1	132292610	132293174
chr1	132294875	132295115
chr1	132295560	132295863
chr1	132295922	132296072
chr1	132296074	132296273
chr1	132298419	132298733
chr1	132298733	132299023
chr1	132299023	132299433
chr1	132299832	132300013
chr1	132306090	132306273
chr1	132306273	132306499
chr1	132306861	132307127
chr1	132312725	132312981
chr1	132315846	132316366
chr1	132316396	132316585
chr1	132318374	132318702
chr1	132318745	132319022
chr1	132319144	132319771
chr1	132326948	132327135
chr1	132333770	132333928
chr1	132334818	132335199
chr1	132335199	132335624
chr1	132335624	132335877
chr1	132338571	132338727
chr1	132338893	132339252
chr1	132346124	132346500
chr1	132346500	132346878
chr1	132353405	132353789
chr1	132353825	132354106
chr1	132354777	132355004
chr1	132355082	132355377
chr1	132355377	132355645
chr1	132355801	132356139
chr1	132364377	132364663

chr1	132364663	132364878
chr1	132365258	132365490
chr1	132367616	132367903
chr1	132371701	132372060
chr1	132384652	132385011
chr1	132390319	132390504
chr1	132390699	132390943
chr1	132390943	132391226
chr1	132391334	132391993
chr1	132410806	132410958
chr1	132417216	132417685
chr1	132417685	132418066
chr1	132418066	132418270
chr1	132431863	132432204
chr1	132432961	132433246
chr1	132443213	132443528
chr1	132476584	132477187
chr1	132477270	132477499
chr1	132477499	132477733
chr1	132513975	132514135
chr1	132520827	132521168
chr1	132524076	132524323
chr1	132525704	132526166
chr1	132526192	132526359
chr1	132526359	132526544
chr1	132527224	132527458
chr1	132538574	132539107
chr1	132540072	132540492
chr1	132541230	132541672
chr1	132542789	132543049
chr1	132557802	132558113
chr1	132558113	132558441
chr1	132558441	132558720
chr1	132565775	132566072
chr1	132568285	132568873
chr1	132580113	132580376
chr1	132586622	132586915
chr1	132586915	132587254
chr1	132591860	132592373
chr1	132594728	132594946
chr1	132602259	132602475
chr1	132606328	132606766

chr1	132609120	132609365
chr1	132609433	132609602
chr1	132636779	132637215
chr1	132641512	132641684
chr1	132653388	132653593
chr1	132655710	132656190
chr1	132656190	132656666
chr1	132678451	132678607
chr1	132681685	132681975
chr1	132705471	132705646
chr1	132710463	132710683
chr1	132727685	132728002
chr1	132733950	132734179
chr1	132741503	132742002
chr1	132745968	132746134
chr1	132758648	132759004
chr1	132759020	132759303
chr1	132759903	132760226
chr1	132765222	132765600
chr1	132766221	132766459
chr1	132771485	132771734
chr1	132791101	132791345
chr1	132840862	132841014
chr1	132841485	132841793
chr1	132841793	132842000
chr1	132851390	132851545
chr1	132864193	132864466
chr1	132865608	132865838
chr1	132865838	132866034
chr1	132880343	132880521
chr1	132880523	132880734
chr1	132912715	132913174
chr1	132913180	132913531
chr1	132921847	132922105
chr1	132934968	132935209
chr1	132937230	132937380
chr1	132944638	132944810
chr1	132955798	132956004
chr1	132956004	132956316
chr1	132956345	132956504
chr1	132982786	132983060
chr1	133007390	133007567

chr1	133024966	133025262
chr1	133025262	133025645
chr1	133025645	133026013
chr1	133026013	133026208
chr1	133028076	133028283
chr1	133033102	133033320
chr1	133033320	133033541
chr1	133033541	133033903
chr1	133033903	133034466
chr1	133039440	133039939
chr1	133045690	133046250
chr1	133046250	133046485
chr1	133046485	133046684
chr1	133046684	133046916
chr1	133050166	133050513
chr1	133057163	133057681
chr1	133057681	133058209
chr1	133068439	133068637
chr1	133083223	133083389
chr1	133083389	133083548
chr1	133084140	133084555
chr1	133087526	133087682
chr1	133087694	133087957
chr1	133090564	133090989
chr1	133091563	133091875
chr1	133100852	133101362
chr1	133101362	133101721
chr1	133102259	133102461
chr1	133118186	133118559
chr1	133118673	133119176
chr1	133130691	133131024
chr1	133131024	133131384
chr1	133131384	133131825
chr1	133133525	133133751
chr1	133141246	133141467
chr1	133146017	133146406
chr1	133146494	133146809
chr1	133147407	133147661
chr1	133155270	133155420
chr1	133161711	133161924
chr1	133162096	133162447
chr1	133162478	133162688

chr1	133162919	133163495
chr1	133163702	133164103
chr1	133164103	133164387
chr1	133164545	133164830
chr1	133177491	133177790
chr1	133177790	133178098
chr1	133181005	133181462
chr1	133187994	133188300
chr1	133188310	133188591
chr1	133188591	133188869
chr1	133188869	133189230
chr1	133201508	133201732
chr1	133204283	133204911
chr1	133205061	133205295
chr1	133209941	133210723
chr1	133211895	133212208
chr1	133214051	133214328
chr1	133217501	133217880
chr1	133217939	133218190
chr1	133225281	133225603
chr1	133226077	133226293
chr1	133226565	133226853
chr1	133226853	133227383
chr1	133235857	133236234
chr1	133236621	133236808
chr1	133236852	133237480
chr1	133245540	133246229
chr1	133254312	133254523
chr1	133265350	133265715
chr1	133269147	133269421
chr1	133269859	133270352
chr1	133270352	133270982
chr1	133276993	133277332
chr1	133277332	133277610
chr1	133291864	133292286
chr1	133295148	133295422
chr1	133295422	133295630
chr1	133296466	133296817
chr1	133302559	133302858
chr1	133305969	133306202
chr1	133306870	133307154
chr1	133307443	133307682

chr1	133309648	133309935
chr1	133309935	133310191
chr1	133311300	133311583
chr1	133314454	133314734
chr1	133315809	133316109
chr1	133316344	133316622
chr1	133316725	133317007
chr1	133317282	133317432
chr1	133327992	133328278
chr1	133329445	133329672
chr1	133334512	133334706
chr1	133342966	133343187
chr1	133347894	133348176
chr1	133353179	133353559
chr1	133353559	133353867
chr1	133363166	133363682
chr1	133363682	133364174
chr1	133364193	133364408
chr1	133372199	133372516
chr1	133380819	133380983
chr1	133382028	133382368
chr1	133385562	133385815
chr1	133392779	133392932
chr1	133400277	133400744
chr1	133404147	133404308
chr1	133406538	133406815
chr1	133406815	133407228
chr1	133413163	133413465
chr1	133418111	133418726
chr1	133422997	133423156
chr1	133423263	133423564
chr1	133423564	133423981
chr1	133423981	133424310
chr1	133424310	133424719
chr1	133460779	133461021
chr1	133465752	133466052
chr1	133466686	133467011
chr1	133470753	133470993
chr1	133471064	133471588
chr1	133471638	133471893
chr1	133476890	133477243
chr1	133477243	133477494

chr1	133477494	133477711
chr1	133487176	133487536
chr1	133487536	133487888
chr1	133488026	133488218
chr1	133499270	133499554
chr1	133499554	133499843
chr1	133507932	133508091
chr1	133510234	133510465
chr1	133536400	133536571
chr1	133560960	133561454
chr1	133577887	133578146
chr1	133594440	133594998
chr1	133594998	133595589
chr1	133595589	133596146
chr1	133604638	133604907
chr1	133609723	133609935
chr1	133609935	133610182
chr1	133610182	133610591
chr1	133641277	133641445
chr1	133660724	133661229
chr1	133661229	133661620
chr1	133685685	133686187
chr1	133694325	133694535
chr1	133694649	133694812
chr1	133695703	133696475
chr1	133701951	133702108
chr1	133712783	133712960
chr1	133716555	133716798
chr1	133736304	133736604
chr1	133740647	133740851
chr1	133745143	133745362
chr1	133750017	133750542
chr1	133751697	133752182
chr1	133765170	133765929
chr1	133771692	133772323
chr1	133777678	133778079
chr1	133778981	133779614
chr1	133781672	133781862
chr1	133784562	133784990
chr1	133790062	133790298
chr1	133793429	133793647
chr1	133800655	133800830

chr1	133800867	133801355
chr1	133801427	133801600
chr1	133815500	133815749
chr1	133815749	133816214
chr1	133816715	133816946
chr1	133843283	133843655
chr1	133853192	133853434
chr1	133855589	133855974
chr1	133856032	133856472
chr1	133885869	133886071
chr1	133894089	133894269
chr1	133896373	133896660
chr1	133899083	133899250
chr1	133905255	133905608
chr1	133908390	133908633
chr1	133909002	133909267
chr1	133909267	133909527
chr1	133911480	133911824
chr1	133930622	133930772
chr1	133941319	133941527
chr1	133950083	133950470
chr1	133950470	133950831
chr1	133953272	133953656
chr1	133959511	133959698
chr1	133961468	133961776
chr1	133961776	133962189
chr1	133976355	133976509
chr1	133978362	133978771
chr1	133978774	133979061
chr1	133979087	133979237
chr1	133981285	133981637
chr1	134022434	134022683
chr1	134045992	134046247
chr1	134048803	134049086
chr1	134053278	134053536
chr1	134059810	134060046
chr1	134060799	134061191
chr1	134061191	134061515
chr1	134065333	134065784
chr1	134067079	134067546
chr1	134070175	134070442
chr1	134070553	134070707

chr1	134078775	134079249
chr1	134079249	134079596
chr1	134091858	134092027
chr1	134092305	134092662
chr1	134092836	134093506
chr1	134094065	134094244
chr1	134098022	134098242
chr1	134099062	134099725
chr1	134099725	134100290
chr1	134101641	134101861
chr1	134106589	134107083
chr1	134110497	134110749
chr1	134111047	134111346
chr1	134113630	134113828
chr1	134119318	134119471
chr1	134119473	134119634
chr1	134120255	134120486
chr1	134122266	134122453
chr1	134151591	134152018
chr1	134161540	134161900
chr1	134162474	134163013
chr1	134191219	134191578
chr1	134196481	134196781
chr1	134202125	134202280
chr1	134206121	134206321
chr1	134217019	134217412
chr1	134221201	134221459
chr1	134222163	134222467
chr1	134222572	134222722
chr1	134234788	134235083
chr1	134235132	134235351
chr1	134244237	134244588
chr1	134288312	134288594
chr1	134299092	134299334
chr1	134322695	134322905
chr1	134331952	134332215
chr1	134332751	134333059
chr1	134333059	134333329
chr1	134336483	134336768
chr1	134344448	134344944
chr1	134346537	134346705
chr1	134346755	134346947

chr1	134348998	134349302
chr1	134361295	134361445
chr1	134361502	134361851
chr1	134361851	134362218
chr1	134368646	134368796
chr1	134368863	134369096
chr1	134391175	134391377
chr1	134393582	134393750
chr1	134393868	134394092
chr1	134399764	134400358
chr1	134405239	134405469
chr1	134405607	134406127
chr1	134406127	134406449
chr1	134406449	134406743
chr1	134414863	134415125
chr1	134415148	134415633
chr1	134415633	134416003
chr1	134420433	134420800
chr1	134433581	134433741
chr1	134435521	134435677
chr1	134442060	134442334
chr1	134447246	134447404
chr1	134451086	134451287
chr1	134454961	134455270
chr1	134455270	134455616
chr1	134455616	134455900
chr1	134493592	134493766
chr1	134494397	134494767
chr1	134494767	134495031
chr1	134495031	134495315
chr1	134513306	134513469
chr1	134518669	134518939
chr1	134555209	134555524
chr1	134556536	134556883
chr1	134556883	134557254
chr1	134560081	134560416
chr1	134560416	134560750
chr1	134560750	134561039
chr1	134561039	134561259
chr1	134561259	134561486
chr1	134580949	134581225
chr1	134583375	134584005

chr1	134636782	134636932
chr1	134638664	134638849
chr1	134646293	134646856
chr1	134646922	134647072
chr1	134650382	134650594
chr1	134651731	134651949
chr1	134664964	134665124
chr1	134674387	134674666
chr1	134676805	134676955
chr1	134679502	134679710
chr1	134686936	134687124
chr1	134699039	134699427
chr1	134701235	134701589
chr1	134701619	134701769
chr1	134704978	134705211
chr1	134708893	134709181
chr1	134709181	134709556
chr1	134725345	134725624
chr1	134729205	134729432
chr1	134737557	134737848
chr1	134741538	134741784
chr1	134742053	134742234
chr1	134745780	134746023
chr1	134746269	134746496
chr1	134749584	134750310
chr1	134752455	134752614
chr1	134753737	134754270
chr1	134754270	134754657
chr1	134771116	134771952
chr1	134772082	134772311
chr1	134783910	134784366
chr1	134784494	134784785
chr1	134799172	134799353
chr1	134799353	134799479
chr1	134803345	134803664
chr1	134803664	134804139
chr1	134804139	134804533
chr1	134804533	134804764
chr1	134809129	134809406
chr1	134809406	134809799
chr1	134810739	134811135
chr1	134816660	134816984

chr1	134818510	134819103
chr1	134819103	134819645
chr1	134819686	134819874
chr1	134829631	134829880
chr1	134831948	134832109
chr1	134837035	134837326
chr1	134854412	134854637
chr1	134880427	134880767
chr1	134896140	134896315
chr1	134902310	134902524
chr1	134909474	134909830
chr1	134929229	134929384
chr1	134939571	134939728
chr1	134942748	134942944
chr1	134947178	134947331
chr1	134949308	134949568
chr1	134950016	134950307
chr1	134955073	134955499
chr1	134955499	134955846
chr1	134955846	134956192
chr1	134961673	134961856
chr1	134962112	134962374
chr1	134962374	134962861
chr1	134962883	134963154
chr1	134968690	134968895
chr1	134971716	134971995
chr1	134985174	134985754
chr1	134986051	134986306
chr1	134990891	134991055
chr1	134994459	134994728
chr1	134995989	134996272
chr1	134996560	134996747
chr1	135001455	135001605
chr1	135004626	135004832
chr1	135016801	135017322
chr1	135019018	135019228
chr1	135020356	135020591
chr1	135020591	135020828
chr1	135020995	135021469
chr1	135021469	135021790
chr1	135021797	135022023
chr1	135022023	135022436

chr1	135030071	135030444
chr1	135054286	135054490
chr1	135063034	135063268
chr1	135063466	135063793
chr1	135083383	135083819
chr1	135084926	135085083
chr1	135090976	135091319
chr1	135094373	135094659
chr1	135095290	135095455
chr1	135096687	135096916
chr1	135104886	135105597
chr1	135105804	135106014
chr1	135106014	135106304
chr1	135108698	135108936
chr1	135110236	135110401
chr1	135112114	135112374
chr1	135112553	135112765
chr1	135112765	135113033
chr1	135132467	135132759
chr1	135136567	135136849
chr1	135146509	135147013
chr1	135147013	135147386
chr1	135147386	135147930
chr1	135153924	135154190
chr1	135154190	135154467
chr1	135154467	135154743
chr1	135154743	135155013
chr1	135169657	135169832
chr1	135169881	135170257
chr1	135170257	135170620
chr1	135170620	135170951
chr1	135171871	135172280
chr1	135177778	135178178
chr1	135180899	135181305
chr1	135182321	135182515
chr1	135183061	135183330
chr1	135183333	135183806
chr1	135184001	135184569
chr1	135188284	135188516
chr1	135198975	135199354
chr1	135199354	135199814
chr1	135199991	135200173

chr1	135207001	135207153
chr1	135208799	135209024
chr1	135226254	135226404
chr1	135226474	135226978
chr1	135226986	135227216
chr1	135227332	135227626
chr1	135227626	135227928
chr1	135231990	135232155
chr1	135248429	135248866
chr1	135250668	135251046
chr1	135251562	135251712
chr1	135256355	135256615
chr1	135258188	135258872
chr1	135258875	135259042
chr1	135259098	135259340
chr1	135260974	135261257
chr1	135265887	135266218
chr1	135268046	135268436
chr1	135268436	135268729
chr1	135273387	135273707
chr1	135281146	135281340
chr1	135283189	135283468
chr1	135283468	135283836
chr1	135283836	135284374
chr1	135313244	135313663
chr1	135313663	135314152
chr1	135324671	135324872
chr1	135346917	135347234
chr1	135347924	135348109
chr1	135366958	135367533
chr1	135368250	135368524
chr1	135369156	135369373
chr1	135374067	135374410
chr1	135374410	135374664
chr1	135374748	135375674
chr1	135381634	135382003
chr1	135401524	135401719
chr1	135401807	135402061
chr1	135410798	135411030
chr1	135411030	135411308
chr1	135411308	135411546
chr1	135411599	135411749

chr1	135413888	135414298
chr1	135428444	135428634
chr1	135429809	135430152
chr1	135430152	135430373
chr1	135430373	135430647
chr1	135430647	135430928
chr1	135448343	135448497
chr1	135452339	135452599
chr1	135461204	135461620
chr1	135466893	135467428
chr1	135471828	135472102
chr1	135492461	135492780
chr1	135509249	135509474
chr1	135509474	135509716
chr1	135509720	135509980
chr1	135510085	135510235
chr1	135510295	135510582
chr1	135510582	135510844
chr1	135515370	135515607
chr1	135515607	135515845
chr1	135523150	135523422
chr1	135535260	135535656
chr1	135535656	135535816
chr1	135536397	135536637
chr1	135540314	135540607
chr1	135540607	135540803
chr1	135541253	135541582
chr1	135544827	135545101
chr1	135554672	135554918
chr1	135554918	135555218
chr1	135555218	135555540
chr1	135560620	135560886
chr1	135562152	135562411
chr1	135585069	135585396
chr1	135586653	135586813
chr1	135590881	135591073
chr1	135594045	135594338
chr1	135609633	135610072
chr1	135615999	135616258
chr1	135626273	135626455
chr1	135634564	135634755
chr1	135634901	135635108

chr1	135637516	135637847
chr1	135659448	135659819
chr1	135659819	135660065
chr1	135668235	135668690
chr1	135670382	135670736
chr1	135675563	135675863
chr1	135675866	135676099
chr1	135687275	135687496
chr1	135687724	135688091
chr1	135688091	135688559
chr1	135691104	135691374
chr1	135691510	135691748
chr1	135697539	135697767
chr1	135699052	135699334
chr1	135699334	135699703
chr1	135700667	135700949
chr1	135708468	135708698
chr1	135711108	135711584
chr1	135715000	135715282
chr1	135719519	135719869
chr1	135720019	135720328
chr1	135722487	135722764
chr1	135723131	135723315
chr1	135724244	135724468
chr1	135728994	135729341
chr1	135729341	135729683
chr1	135729862	135730152
chr1	135730400	135730695
chr1	135731475	135731739
chr1	135731828	135732272
chr1	135741956	135742221
chr1	135742221	135742506
chr1	135752801	135753277
chr1	135755896	135756398
chr1	135760296	135760599
chr1	135764229	135764395
chr1	135765751	135765906
chr1	135765914	135766276
chr1	135766276	135766783
chr1	135770896	135771239
chr1	135771239	135771522
chr1	135779190	135779552

chr1	135781182	135781601
chr1	135782820	135783067
chr1	135787877	135788149
chr1	135788795	135789003
chr1	135789897	135790151
chr1	135792339	135792585
chr1	135792585	135792851
chr1	135793347	135793563
chr1	135793813	135793989
chr1	135794515	135794976
chr1	135811206	135811588
chr1	135812202	135812497
chr1	135812497	135812715
chr1	135812715	135812984
chr1	135813117	135813338
chr1	135818552	135819053
chr1	135819982	135820469
chr1	135820641	135821346
chr1	135821472	135821904
chr1	135828764	135828981
chr1	135828981	135829267
chr1	135834288	135834530
chr1	135841610	135841793
chr1	135841860	135842060
chr1	135843607	135843778
chr1	135844426	135845009
chr1	135845220	135845468
chr1	135846752	135846920
chr1	135847139	135847408
chr1	135847454	135847616
chr1	135850779	135851046
chr1	135852134	135852301
chr1	135852310	135852730
chr1	135856564	135856770
chr1	135857071	135857654
chr1	135861859	135862281
chr1	135862281	135862752
chr1	135863727	135864145
chr1	135865623	135865853
chr1	135882796	135883048
chr1	135883048	135883319
chr1	135883319	135883614

chr1	135883614	135883895
chr1	135883895	135884153
chr1	135891549	135891856
chr1	135891856	135892302
chr1	135893318	135893637
chr1	135893637	135894109
chr1	135895456	135895699
chr1	135895959	135896188
chr1	135899217	135899553
chr1	135900458	135900770
chr1	135900770	135900975
chr1	135906428	135906758
chr1	135906758	135907126
chr1	135907126	135907462
chr1	135918564	135919036
chr1	135919036	135919534
chr1	135925808	135926438
chr1	135926588	135926793
chr1	135928175	135928355
chr1	135929119	135929329
chr1	135929855	135930194
chr1	135931769	135931958
chr1	135933711	135934024
chr1	135938306	135938943
chr1	135944788	135945124
chr1	135945744	135946038
chr1	135946162	135946733
chr1	135948458	135948647
chr1	135958351	135958819
chr1	135958885	135959035
chr1	135982608	135982789
chr1	136007499	136007935
chr1	136007935	136008201
chr1	136008201	136008462
chr1	136010342	136010531
chr1	136017822	136018109
chr1	136018109	136018335
chr1	136018335	136018533
chr1	136018537	136018819
chr1	136040522	136040821
chr1	136040821	136041218
chr1	136045863	136046091

chr1	136047508	136047659
chr1	136050438	136050894
chr1	136050894	136051402
chr1	136051408	136051864
chr1	136064068	136064396
chr1	136069893	136070078
chr1	136076152	136076417
chr1	136116050	136116464
chr1	136119680	136120183
chr1	136120669	136121005
chr1	136121005	136121328
chr1	136126247	136126886
chr1	136126899	136127280
chr1	136131137	136131609
chr1	136135168	136135326
chr1	136137090	136137331
chr1	136139989	136140293
chr1	136140293	136140661
chr1	136156376	136156601
chr1	136167762	136167970
chr1	136169977	136170453
chr1	136175261	136175672
chr1	136177198	136177366
chr1	136194543	136194801
chr1	136212883	136213128
chr1	136220031	136220492
chr1	136228269	136228795
chr1	136228988	136229309
chr1	136230171	136230596
chr1	136231642	136231923
chr1	136233916	136234199
chr1	136234199	136234438
chr1	136234454	136234661
chr1	136255171	136255626
chr1	136259808	136260145
chr1	136260145	136260954
chr1	136262118	136262362
chr1	136268343	136268503
chr1	136273091	136273249
chr1	136273311	136273828
chr1	136291457	136291683
chr1	136306523	136306705

chr1	136325964	136326378
chr1	136345283	136345961
chr1	136345961	136346346
chr1	136346346	136346562
chr1	136346745	136346991
chr1	136361116	136361268
chr1	136361446	136361898
chr1	136364817	136365037
chr1	136370534	136370692
chr1	136399321	136399528
chr1	136399850	136400166
chr1	136401323	136401554
chr1	136401589	136401739
chr1	136405445	136405900
chr1	136414900	136415412
chr1	136415412	136415761
chr1	136458993	136459143
chr1	136466049	136466458
chr1	136466458	136466750
chr1	136521490	136521864
chr1	136525008	136525247
chr1	136550949	136551198
chr1	136556136	136556293
chr1	136565084	136565313
chr1	136624167	136624453
chr1	136624538	136625242
chr1	136625287	136625461
chr1	136625461	136625666
chr1	136625666	136625896
chr1	136652185	136652393
chr1	136652393	136652686
chr1	136656514	136657049
chr1	136665236	136665719
chr1	136672144	136672378
chr1	136673367	136673636
chr1	136676284	136676848
chr1	136683032	136683515
chr1	136683515	136683776
chr1	136683776	136684094
chr1	136685167	136685650
chr1	136685801	136686025
chr1	136695004	136695168

chr1	136695220	136695401
chr1	136695644	136695846
chr1	136695866	136696281
chr1	136696281	136696732
chr1	136698496	136698742
chr1	136707415	136707633
chr1	136718006	136718158
chr1	136739613	136739835
chr1	136749250	136749459
chr1	136751584	136751777
chr1	136767058	136767607
chr1	136831151	136831311
chr1	136839334	136839521
chr1	136859548	136859743
chr1	136899814	136900223
chr1	136915489	136915702
chr1	136948585	136948906
chr1	136948906	136949270
chr1	137020643	137020811
chr1	137061951	137062261
chr1	137079105	137079700
chr1	137079700	137079953
chr1	137083481	137083784
chr1	137091853	137092147
chr1	137127310	137127623
chr1	137127750	137127999
chr1	137148088	137148276
chr1	137197528	137197757
chr1	137209519	137209725
chr1	137216229	137216483
chr1	137225563	137225814
chr1	137323864	137324234
chr1	137329085	137329237
chr1	137467037	137467200
chr1	137561679	137561846
chr1	137561846	137562008
chr1	137588189	137588584
chr1	137588584	137588814
chr1	137591286	137591640
chr1	137745838	137746001
chr1	137751405	137751578
chr1	137863523	137864003

chr1	137898285	137898799
chr1	137898943	137899093
chr1	137900153	137900620
chr1	137900694	137901198
chr1	137929801	137930110
chr1	137935109	137935302
chr1	137935760	137936223
chr1	138009556	138009774
chr1	138039169	138039478
chr1	138044615	138044855
chr1	138085910	138086068
chr1	138179722	138179938
chr1	138179958	138180395
chr1	138180395	138180728
chr1	138190858	138191200
chr1	138207419	138207569
chr1	138248750	138249110
chr1	138258809	138259086
chr1	138259274	138259513
chr1	138265990	138266423
chr1	138275969	138276181
chr1	138288740	138289207
chr1	138290085	138290550
chr1	138304261	138304421
chr1	138328403	138328565
chr1	138328580	138328759
chr1	138328830	138329013
chr1	138329091	138329635
chr1	138329635	138329889
chr1	138331678	138332020
chr1	138334792	138335269
chr1	138343308	138343829
chr1	138348257	138348637
chr1	138348637	138349075
chr1	138355756	138355975
chr1	138356451	138356770
chr1	138357914	138358189
chr1	138370191	138370341
chr1	138423592	138423934
chr1	138446897	138447195
chr1	138487623	138487925
chr1	138488248	138488546

chr1	138494946	138495310
chr1	138495346	138495499
chr1	138506754	138506904
chr1	138506953	138507542
chr1	138507545	138508041
chr1	138508089	138508359
chr1	138515045	138515781
chr1	138516996	138517266
chr1	138535834	138536104
chr1	138536104	138536474
chr1	138537317	138537550
chr1	138537550	138537776
chr1	138537786	138538045
chr1	138538283	138538533
chr1	138558454	138558610
chr1	138571031	138571642
chr1	138571642	138572154
chr1	138596234	138596668
chr1	138603787	138604039
chr1	138604039	138604302
chr1	138604302	138604591
chr1	138604797	138605052
chr1	138606796	138607219
chr1	138617975	138618311
chr1	138618969	138619525
chr1	138619525	138619913
chr1	138620134	138620334
chr1	138641108	138641367
chr1	138643419	138643774
chr1	138668953	138669205
chr1	138669353	138669613
chr1	138676406	138676630
chr1	138692373	138692523
chr1	138708879	138709167
chr1	138750355	138750538
chr1	138752434	138752666
chr1	138831387	138831600
chr1	138833486	138833670
chr1	138835419	138835642
chr1	138841423	138841687
chr1	138841687	138841920
chr1	138841920	138842118

chr1	138842352	138842603
chr1	138848118	138848506
chr1	138848506	138849093
chr1	138856153	138856440
chr1	138856440	138856720
chr1	138856720	138857020
chr1	138911752	138911914
chr1	138912071	138912679
chr1	138928505	138928812
chr1	138928812	138929121
chr1	138929121	138929434
chr1	138929661	138929856
chr1	138929976	138930349
chr1	138947100	138947373
chr1	138953856	138954127
chr1	138959757	138960202
chr1	138962654	138963050
chr1	138963050	138963451
chr1	138963451	138963725
chr1	138963725	138963876
chr1	138963876	138964094
chr1	138964133	138964307
chr1	138974778	138975641
chr1	138983476	138983873
chr1	138992384	138992692
chr1	138994988	138995179
chr1	139018939	139019276
chr1	139028997	139029154
chr1	139041391	139041547
chr1	139088681	139088842
chr1	139095960	139096383
chr1	139104915	139105105
chr1	139107699	139107879
chr1	139107888	139108314
chr1	139108362	139108563
chr1	139116030	139116225
chr1	139145635	139145931
chr1	139146337	139146628
chr1	139160191	139160380
chr1	139161692	139161924
chr1	139162845	139163186
chr1	139176960	139177303

chr1	139177424	139177667
chr1	139179665	139180167
chr1	139209731	139210064
chr1	139210606	139211012
chr1	139267981	139268134
chr1	139276961	139277289
chr1	139324541	139324911
chr1	139325190	139325411
chr1	139340575	139340799
chr1	139345311	139345471
chr1	139345471	139345664
chr1	139369036	139369267
chr1	139379201	139379662
chr1	139379664	139379918
chr1	139421825	139422093
chr1	139422093	139422361
chr1	139422361	139422885
chr1	139422964	139423114
chr1	139454294	139454555
chr1	139454555	139454828
chr1	139454828	139455059
chr1	139455059	139455290
chr1	139455290	139455531
chr1	139473890	139474121
chr1	139481122	139481429
chr1	139481540	139481702
chr1	139725910	139726108
chr1	141066859	141067153
chr1	141464609	141464896
chr1	142218192	142218400
chr1	142585023	142585228
chr1	142588569	142588775
chr1	142588775	142589028
chr1	142589931	142590136
chr1	142740367	142740632
chr1	142740889	142741168
chr1	142742382	142742645
chr1	142828370	142828616
chr1	143391960	143392113
chr1	143475266	143475746
chr1	143536421	143536714
chr1	143536897	143537349

chr1	143548621	143548831
chr1	143593048	143593215
chr1	143702090	143702286
chr1	143702313	143702639
chr1	143702639	143703319
chr1	143705894	143706170
chr1	143725636	143726078
chr1	143728080	143728378
chr1	143730984	143731269
chr1	143739189	143739864
chr1	143739864	143740443
chr1	143776676	143776936
chr1	143776936	143777168
chr1	143777168	143777471
chr1	143777471	143777763
chr1	143777763	143777975
chr1	143803626	143803862
chr1	143813998	143814234
chr1	143818956	143819388
chr1	143819677	143820006
chr1	143871540	143871733
chr1	143871733	143871900
chr1	143874270	143874441
chr1	143878284	143878621
chr1	143878621	143878935
chr1	143941369	143941868
chr1	144003900	144004435
chr1	144004435	144004743
chr1	144004847	144005071
chr1	144006646	144006903
chr1	144018363	144018702
chr1	144018702	144019052
chr1	144019930	144020419
chr1	144075969	144076427
chr1	144076525	144076824
chr1	144085642	144085854
chr1	144085913	144086151
chr1	144086151	144086468
chr1	144202908	144203147
chr1	144239050	144239528
chr1	144296253	144296529
chr1	144306329	144306656

chr1	144308353	144308586
chr1	144316333	144316579
chr1	144316595	144316801
chr1	144343316	144343639
chr1	144355071	144355387
chr1	144356054	144356315
chr1	144406699	144406981
chr1	144493215	144493413
chr1	144502328	144502507
chr1	144600844	144601148
chr1	144604124	144604355
chr1	144783610	144783828
chr1	144786964	144787204
chr1	145344431	145344753
chr1	145781394	145781737
chr1	145930327	145930564
chr1	145940328	145940495
chr1	145957844	145958078
chr1	146320891	146321146
chr1	146353962	146354175
chr1	146357013	146357378
chr1	146498794	146499165
chr1	146724897	146725115
chr1	147802058	147802350
chr1	147802838	147803099
chr1	148723034	148723355
chr1	148723459	148723663
chr1	149617747	149617934
chr1	149705098	149705606
chr1	149743757	149743909
chr1	149813537	149814001
chr1	149814001	149814398
chr1	149833034	149833296
chr1	149833764	149834125
chr1	149846567	149847231
chr1	149850129	149850283
chr1	149852345	149852514
chr1	149855633	149855895
chr1	149856756	149857099
chr1	149862724	149863447
chr1	149876581	149877205
chr1	149877762	149877968

chr1	149878283	149878475
chr1	149889065	149889306
chr1	149897120	149897866
chr1	149906877	149907077
chr1	149929241	149929552
chr1	149940381	149940663
chr1	149940698	149940861
chr1	149946490	149946653
chr1	149946985	149947360
chr1	149949323	149949784
chr1	149950308	149950631
chr1	149960311	149960466
chr1	149960630	149961096
chr1	149961096	149961409
chr1	149961409	149961656
chr1	149979761	149980132
chr1	150026184	150026483
chr1	150026483	150026732
chr1	150026878	150027061
chr1	150027120	150027724
chr1	150042871	150043605
chr1	150047384	150047698
chr1	150049077	150049396
chr1	150049874	150050029
chr1	150050272	150050551
chr1	150050606	150050882
chr1	150053938	150054255
chr1	150054751	150055039
chr1	150058962	150059271
chr1	150061357	150061730
chr1	150062777	150063144
chr1	150063421	150063578
chr1	150064851	150065193
chr1	150065765	150065950
chr1	150096706	150096943
chr1	150097761	150098007
chr1	150098612	150098790
chr1	150099566	150100036
chr1	150100036	150100412
chr1	150106752	150107008
chr1	150108242	150108481
chr1	150108481	150108704

chr1	150108704	150109081
chr1	150109081	150109545
chr1	150109545	150109873
chr1	150114997	150115981
chr1	150130091	150130372
chr1	150130700	150130971
chr1	150130971	150131234
chr1	150131621	150132221
chr1	150132262	150132541
chr1	150135510	150135660
chr1	150144151	150144335
chr1	150144898	150145511
chr1	150151309	150151814
chr1	150152504	150152889
chr1	150156674	150156898
chr1	150164809	150165317
chr1	150165337	150165549
chr1	150170031	150170253
chr1	150177148	150177482
chr1	150177482	150177727
chr1	150197560	150198115
chr1	150198476	150198627
chr1	150206444	150206687
chr1	150208828	150209245
chr1	150218350	150218886
chr1	150218886	150219373
chr1	150247812	150248140
chr1	150251508	150251768
chr1	150251768	150252197
chr1	150256669	150256905
chr1	150257678	150257912
chr1	150260131	150260358
chr1	150260358	150260698
chr1	150260769	150261140
chr1	150266602	150266848
chr1	150267057	150267213
chr1	150307705	150307913
chr1	150323251	150323635
chr1	150360959	150361169
chr1	150392626	150392984
chr1	150392984	150393230
chr1	150393230	150393497

chr1	150393497	150393847
chr1	150425754	150426055
chr1	150426229	150426555
chr1	150460533	150461124
chr1	150472480	150472881
chr1	150486970	150487173
chr1	150497779	150497969
chr1	150540341	150540587
chr1	150565726	150565952
chr1	150566123	150566279
chr1	150567322	150567512
chr1	150577133	150577348
chr1	150761026	150761331
chr1	150826111	150826330
chr1	150827241	150827490
chr1	151009503	151009712
chr1	151011152	151011348
chr1	151078981	151079234
chr1	151084118	151084294
chr1	151164839	151165031
chr1	151168501	151169049
chr1	151172052	151172384
chr1	151177746	151177920
chr1	151177953	151178109
chr1	151181111	151181342
chr1	151188086	151188295
chr1	151192968	151193201
chr1	151204586	151204952
chr1	151210837	151211005
chr1	151223442	151223812
chr1	151226762	151226970
chr1	151226970	151227152
chr1	151227485	151227884
chr1	151228664	151228899
chr1	151236113	151236381
chr1	151236793	151236953
chr1	151239272	151239592
chr1	151264897	151265101
chr1	151289206	151290157
chr1	151303333	151303528
chr1	151305161	151305364
chr1	151343926	151344607

chr1	151344607	151344907
chr1	151344907	151345184
chr1	151345184	151345474
chr1	151350753	151350943
chr1	151352989	151353331
chr1	151362779	151363056
chr1	151371590	151371811
chr1	151390136	151390286
chr1	151428040	151428345
chr1	151428345	151428686
chr1	151428686	151428950
chr1	151428950	151429172
chr1	151462815	151463251
chr1	151466518	151466973
chr1	151500087	151500330
chr1	151500330	151500569
chr1	151500569	151500841
chr1	151500841	151501337
chr1	151515747	151515980
chr1	151550462	151550647
chr1	151550647	151550843
chr1	151564439	151564732
chr1	151568774	151569026
chr1	151571218	151571605
chr1	151571605	151571937
chr1	151571991	151572174
chr1	151572698	151572923
chr1	151582096	151582288
chr1	151602120	151602389
chr1	151610690	151610928
chr1	151616557	151616885
chr1	151616885	151617186
chr1	151618319	151618604
chr1	151618839	151619085
chr1	151630041	151630191
chr1	151646431	151646589
chr1	151651177	151651442
chr1	151651442	151651718
chr1	151672916	151673121
chr1	151675608	151676129
chr1	151676699	151677042
chr1	151677101	151677275

chr1	151678081	151678489
chr1	151679232	151679582
chr1	151679958	151680143
chr1	151685090	151685406
chr1	151686025	151686317
chr1	151686781	151687098
chr1	151689090	151689467
chr1	151689592	151689909
chr1	151690619	151690893
chr1	151692983	151693156
chr1	151698308	151698570
chr1	151706080	151706445
chr1	151716459	151716610
chr1	151719639	151719809
chr1	151719900	151720050
chr1	151732732	151733044
chr1	151735493	151735651
chr1	151754924	151755416
chr1	151755416	151755717
chr1	151755717	151755918
chr1	151755918	151756105
chr1	151766241	151766466
chr1	151766884	151767177
chr1	151800226	151800923
chr1	151800923	151801531
chr1	151801619	151801858
chr1	151801858	151802214
chr1	151802214	151802560
chr1	151802560	151802820
chr1	151872550	151872731
chr1	151872861	151873459
chr1	151875252	151875562
chr1	151875564	151875750
chr1	151907073	151907636
chr1	151943696	151944027
chr1	152063047	152063354
chr1	152063354	152063773
chr1	152063805	152064118
chr1	152084636	152084980
chr1	152085021	152085210
chr1	152089528	152089726
chr1	152089726	152090076

chr1	152090076	152090569
chr1	152090569	152091055
chr1	152105338	152105587
chr1	152106198	152106445
chr1	152141446	152141616
chr1	152201595	152201755
chr1	152218097	152218427
chr1	152223255	152223426
chr1	152225009	152225200
chr1	152226130	152226298
chr1	152230341	152230543
chr1	152260150	152260419
chr1	152271758	152272026
chr1	152273620	152273990
chr1	152274028	152274379
chr1	152278329	152278618
chr1	152386097	152386561
chr1	152386561	152386866
chr1	152399726	152400031
chr1	152400031	152400277
chr1	152400277	152400536
chr1	152400621	152400794
chr1	152437715	152438066
chr1	152439442	152439748
chr1	152439952	152440250
chr1	152497650	152497966
chr1	152499916	152500136
chr1	152511265	152511601
chr1	152520864	152521121
chr1	152524244	152524402
chr1	152524899	152525334
chr1	152536384	152536915
chr1	152538250	152538563
chr1	152551047	152551341
chr1	152555937	152556560
chr1	152556560	152556886
chr1	152562048	152562484
chr1	152581316	152581472
chr1	152585045	152585278
chr1	152585523	152585920
chr1	152611586	152611746
chr1	152624603	152624965

chr1	152624965	152625471
chr1	152632031	152632198
chr1	152651794	152652045
chr1	152660848	152661050
chr1	152663942	152664154
chr1	152669542	152669813
chr1	152669813	152670189
chr1	152678600	152678771
chr1	152746524	152746799
chr1	152766058	152766490
chr1	152766490	152766847
chr1	152766847	152767236
chr1	152768094	152768463
chr1	152776903	152777182
chr1	152778726	152778876
chr1	152779076	152779370
chr1	152779370	152779677
chr1	152790272	152790550
chr1	152794477	152794682
chr1	152815196	152815488
chr1	152817443	152817722
chr1	152820783	152821224
chr1	152831055	152831347
chr1	152831840	152831990
chr1	152832362	152832520
chr1	152833010	152833243
chr1	152844772	152844923
chr1	152902045	152902321
chr1	152902321	152902573
chr1	152902573	152902984
chr1	152902984	152903309
chr1	152903309	152903532
chr1	152903574	152903850
chr1	152904546	152904878
chr1	152915891	152916094
chr1	152916933	152917113
chr1	152917353	152917537
chr1	152917566	152917932
chr1	152921738	152922336
chr1	152923149	152923317
chr1	152956108	152956289
chr1	152956289	152956540

chr1	153000089	153000320
chr1	153000320	153000570
chr1	153000570	153000874
chr1	153009328	153009676
chr1	153010504	153011028
chr1	153011554	153011930
chr1	153011930	153012231
chr1	153022851	153023083
chr1	153035605	153035755
chr1	153040048	153040656
chr1	153041130	153041344
chr1	153048126	153048464
chr1	153049625	153050327
chr1	153059720	153059967
chr1	153066417	153066749
chr1	153068506	153068673
chr1	153069273	153069576
chr1	153076039	153076214
chr1	153084225	153084530
chr1	153085114	153085718
chr1	153091630	153091985
chr1	153091985	153092294
chr1	153092294	153092613
chr1	153092613	153092928
chr1	153101595	153101777
chr1	153102320	153102894
chr1	153102894	153103167
chr1	153115352	153115505
chr1	153120495	153120818
chr1	153121281	153121738
chr1	153126408	153126758
chr1	153126758	153127077
chr1	153129755	153130059
chr1	153130059	153130355
chr1	153130372	153130733
chr1	153130774	153130924
chr1	153132265	153132419
chr1	153136682	153136917
chr1	153140670	153141407
chr1	153141505	153141655
chr1	153141844	153142143
chr1	153153413	153154009

chr1	153163923	153164190
chr1	153172195	153172648
chr1	153173367	153173661
chr1	153174805	153175223
chr1	153180624	153180909
chr1	153185723	153186379
chr1	153186379	153186730
chr1	153186808	153187044
chr1	153187044	153187398
chr1	153188184	153188375
chr1	153200699	153200889
chr1	153202149	153202584
chr1	153204050	153204485
chr1	153206975	153207561
chr1	153215644	153215837
chr1	153215837	153216024
chr1	153224282	153224651
chr1	153238054	153238385
chr1	153241940	153242127
chr1	153266607	153266844
chr1	153266954	153267154
chr1	153268313	153268736
chr1	153268745	153268895
chr1	153269004	153269229
chr1	153269757	153270020
chr1	153270063	153270316
chr1	153274660	153275000
chr1	153277590	153277871
chr1	153278154	153278384
chr1	153283083	153283288
chr1	153292207	153292776
chr1	153299423	153299793
chr1	153300345	153300861
chr1	153301404	153301554
chr1	153304602	153304849
chr1	153304849	153305183
chr1	153321315	153321817
chr1	153321856	153322009
chr1	153332031	153332550
chr1	153332550	153333034
chr1	153333034	153333325
chr1	153333325	153333673

chr1	153333713	153334093
chr1	153351047	153351413
chr1	153386538	153387047
chr1	153401606	153402125
chr1	153407782	153408000
chr1	153414773	153414940
chr1	153424245	153424782
chr1	153424782	153425462
chr1	153446200	153446401
chr1	153451326	153451553
chr1	153452613	153452782
chr1	153453185	153453444
chr1	153453465	153453701
chr1	153460755	153461048
chr1	153480463	153480709
chr1	153480733	153480976
chr1	153481007	153481290
chr1	153486190	153486387
chr1	153486772	153487019
chr1	153487019	153487277
chr1	153487277	153487581
chr1	153487581	153487925
chr1	153493485	153493636
chr1	153500206	153500357
chr1	153506569	153506855
chr1	153506855	153507191
chr1	153540844	153540999
chr1	153549586	153549872
chr1	153550869	153551146
chr1	153556206	153556521
chr1	153556967	153557235
chr1	153559308	153559584
chr1	153559620	153560103
chr1	153560103	153560408
chr1	153564680	153565033
chr1	153565244	153565368
chr1	153565368	153565496
chr1	153565728	153566097
chr1	153566097	153566465
chr1	153572057	153572710
chr1	153572712	153572980
chr1	153572980	153573252

chr1	153573252	153573557
chr1	153583103	153583696
chr1	153585383	153585690
chr1	153585690	153586077
chr1	153588029	153588258
chr1	153598932	153599094
chr1	153599409	153599754
chr1	153604233	153604506
chr1	153631137	153631577
chr1	153635113	153635898
chr1	153648508	153648781
chr1	153648781	153649056
chr1	153652805	153653069
chr1	153653749	153654028
chr1	153661407	153661710
chr1	153661710	153661990
chr1	153661990	153662298
chr1	153662501	153662657
chr1	153669126	153669363
chr1	153679803	153679974
chr1	153689746	153689977
chr1	153690678	153690828
chr1	153691036	153691570
chr1	153717841	153718013
chr1	153736168	153736396
chr1	153740130	153740569
chr1	153742805	153743121
chr1	153746215	153746453
chr1	153746791	153747045
chr1	153749276	153749608
chr1	153749608	153750003
chr1	153751042	153751257
chr1	153751760	153752312
chr1	153752542	153752762
chr1	153769686	153770005
chr1	153770005	153770394
chr1	153770985	153771184
chr1	153771787	153772182
chr1	153775306	153775546
chr1	153788990	153789175
chr1	153789175	153789368
chr1	153807461	153807668

chr1	153835841	153836069
chr1	153836102	153836429
chr1	153837842	153838088
chr1	153842327	153842534
chr1	153846976	153847255
chr1	153855931	153856105
chr1	153857008	153857358
chr1	153874996	153875286
chr1	153892317	153892643
chr1	153894252	153894645
chr1	153899622	153900035
chr1	153900035	153900381
chr1	153900446	153900725
chr1	153922753	153922905
chr1	153924121	153924632
chr1	153937435	153937676
chr1	153940296	153940458
chr1	153986325	153986617
chr1	153986617	153987021
chr1	153987055	153987313
chr1	153999888	154000096
chr1	154004059	154004234
chr1	154021985	154022507
chr1	154032620	154033016
chr1	154033884	154034192
chr1	154034192	154034682
chr1	154061846	154062196
chr1	154064647	154065008
chr1	154065135	154066007
chr1	154069897	154070283
chr1	154086037	154086393
chr1	154086478	154086944
chr1	154091204	154091360
chr1	154094393	154094710
chr1	154100619	154100867
chr1	154112455	154112844
chr1	154118092	154118358
chr1	154118514	154118704
chr1	154118704	154118964
chr1	154125102	154125341
chr1	154126612	154126822
chr1	154149117	154149269

chr1	154150062	154150439
chr1	154150439	154150914
chr1	154178278	154178543
chr1	154178543	154178874
chr1	154181018	154181277
chr1	154187054	154187232
chr1	154206825	154206983
chr1	154207011	154207173
chr1	154207185	154207386
chr1	154207555	154207782
chr1	154207886	154208153
chr1	154461577	154461857
chr1	154500599	154500799
chr1	154528888	154529206
chr1	154729663	154729889
chr1	154786241	154786575
chr1	154788145	154788379
chr1	154875789	154876273
chr1	154936104	154936309
chr1	154970602	154970937
chr1	155006522	155006911
chr1	155007227	155007410
chr1	155011102	155011295
chr1	155013764	155014164
chr1	155014164	155014516
chr1	155025844	155026137
chr1	155027639	155027893
chr1	155035569	155035719
chr1	155035918	155036142
chr1	155040137	155040710
chr1	155040936	155041086
chr1	155041683	155041910
chr1	155049019	155049325
chr1	155049343	155049509
chr1	155050166	155050654
chr1	155051003	155051486
chr1	155056110	155056421
chr1	155057053	155057236
chr1	155057809	155058301
chr1	155071479	155071807
chr1	155073743	155073992
chr1	155073992	155074217

chr1	155074792	155075345
chr1	155075889	155076319
chr1	155082935	155083476
chr1	155083476	155083986
chr1	155084007	155084270
chr1	155091329	155091711
chr1	155093143	155093865
chr1	155098700	155099271
chr1	155099271	155099894
chr1	155107923	155108131
chr1	155108226	155109096
chr1	155115306	155115640
chr1	155115640	155115827
chr1	155121949	155122303
chr1	155138558	155138881
chr1	155138881	155139427
chr1	155140173	155140406
chr1	155146717	155146961
chr1	155152969	155153142
chr1	155158436	155158914
chr1	155158914	155159335
chr1	155163601	155163770
chr1	155171947	155172097
chr1	155172973	155173139
chr1	155173623	155174069
chr1	155184288	155184515
chr1	155201045	155201331
chr1	155201331	155201632
chr1	155201632	155201922
chr1	155201922	155202190
chr1	155214976	155215393
chr1	155215393	155215843
chr1	155216020	155216199
chr1	155216235	155216523
chr1	155224033	155224858
chr1	155244101	155244452
chr1	155244452	155244732
chr1	155273023	155273354
chr1	155281382	155281578
chr1	155281578	155281858
chr1	155293524	155293714
chr1	155416624	155417186

chr1	155417186	155417668
chr1	155472659	155472874
chr1	155493367	155493671
chr1	155510203	155510798
chr1	155516755	155516923
chr1	155527425	155527684
chr1	155529267	155529569
chr1	155533129	155533438
chr1	155537319	155537573
chr1	155557776	155558073
chr1	155558073	155558412
chr1	155558412	155558661
chr1	155558661	155558918
chr1	155564841	155565062
chr1	155576510	155576735
chr1	155670752	155670913
chr1	155722254	155722482
chr1	155740742	155740975
chr1	155741048	155741368
chr1	155741581	155741788
chr1	155741956	155742358
chr1	155742358	155742643
chr1	155742643	155742939
chr1	155742939	155743190
chr1	155784908	155785132
chr1	155785979	155786206
chr1	155792016	155792194
chr1	155792249	155792542
chr1	155801627	155802035
chr1	155809701	155810086
chr1	155810086	155810433
chr1	155810575	155810820
chr1	155810820	155810991
chr1	155812362	155812754
chr1	155812754	155813061
chr1	155815930	155816141
chr1	155816141	155816400
chr1	155832764	155833093
chr1	155837587	155837891
chr1	155875243	155875476
chr1	155972516	155972680
chr1	155972686	155973116

chr1	155973116	155973484
chr1	155973484	155973782
chr1	155990968	155991288
chr1	155994697	155994867
chr1	156010554	156010794
chr1	156010957	156011339
chr1	156035449	156035883
chr1	156035883	156036319
chr1	156036319	156036775
chr1	156040665	156041009
chr1	156041960	156042119
chr1	156043112	156043337
chr1	156045973	156046163
chr1	156066122	156066435
chr1	156070148	156070523
chr1	156085822	156086154
chr1	156096767	156097046
chr1	156204721	156204926
chr1	156204926	156205092
chr1	156217410	156217740
chr1	156218103	156218325
chr1	156222146	156222967
chr1	156224973	156225200
chr1	156229523	156229758
chr1	156230644	156230996
chr1	156241125	156241548
chr1	156241790	156242044
chr1	156242090	156242241
chr1	156261609	156261773
chr1	156263397	156263635
chr1	156264004	156264425
chr1	156275933	156276083
chr1	156302879	156303470
chr1	156303470	156303812
chr1	156340923	156341162
chr1	156344268	156344937
chr1	156349141	156349385
chr1	156393098	156393301
chr1	156420586	156420995
chr1	156420995	156421329
chr1	156437794	156438053
chr1	156450608	156450818

chr1	156454454	156454735
chr1	156455105	156455308
chr1	156464319	156464539
chr1	156464539	156464765
chr1	156467976	156468223
chr1	156468629	156468938
chr1	156473073	156473311
chr1	156473885	156474706
chr1	156477373	156477523
chr1	156479274	156479652
chr1	156507448	156507760
chr1	156507760	156508329
chr1	156511197	156511409
chr1	156515408	156515568
chr1	156520462	156520683
chr1	156521011	156521173
chr1	156523921	156524158
chr1	156538540	156538690
chr1	156540257	156540463
chr1	156558386	156558629
chr1	156558629	156558972
chr1	156558972	156559283
chr1	156559283	156559675
chr1	156566265	156566464
chr1	156584314	156584464
chr1	156590380	156590645
chr1	156593251	156593699
chr1	156598580	156598802
chr1	156598802	156599043
chr1	156605252	156605414
chr1	156605431	156605581
chr1	156605603	156605815
chr1	156605980	156606257
chr1	156614797	156614952
chr1	156615331	156616158
chr1	156616990	156617266
chr1	156620191	156620391
chr1	156620430	156620715
chr1	156622270	156622541
chr1	156622569	156622733
chr1	156626780	156627032
chr1	156631049	156631283

chr1	156633049	156633508
chr1	156639365	156639838
chr1	156639838	156640111
chr1	156640706	156640901
chr1	156642918	156643092
chr1	156649727	156649880
chr1	156650549	156650837
chr1	156654822	156655006
chr1	156657534	156657731
chr1	156658221	156658521
chr1	156658618	156658930
chr1	156659956	156660145
chr1	156660145	156660339
chr1	156660552	156660702
chr1	156665990	156666257
chr1	156667068	156667279
chr1	156667279	156667498
chr1	156667498	156667991
chr1	156673887	156674183
chr1	156674183	156674524
chr1	156674524	156674907
chr1	156677280	156677703
chr1	156678166	156678398
chr1	156679674	156679929
chr1	156680325	156680488
chr1	156718292	156718873
chr1	156718873	156719322
chr1	156741691	156741926
chr1	156741929	156742192
chr1	156742218	156742399
chr1	156743943	156744113
chr1	156752503	156752653
chr1	156790251	156790460
chr1	156791144	156791334
chr1	156861772	156861991
chr1	156862904	156863059
chr1	156912000	156912270
chr1	156917542	156917771
chr1	156938689	156939146
chr1	156939146	156939467
chr1	156939467	156939729
chr1	156939729	156939960

chr1	156939960	156940202
chr1	156940222	156940508
chr1	156971222	156971656
chr1	156987603	156987944
chr1	156991164	156991485
chr1	156993186	156993400
chr1	156995094	156995646
chr1	156997889	156998389
chr1	157000751	157000941
chr1	157000997	157001512
chr1	157011139	157011844
chr1	157019918	157020177
chr1	157035642	157035809
chr1	157046695	157046986
chr1	157061241	157061493
chr1	157061493	157061770
chr1	157061770	157062088
chr1	157062088	157062378
chr1	157062451	157062741
chr1	157073563	157073995
chr1	157074441	157074634
chr1	157075071	157075314
chr1	157076347	157076604
chr1	157076834	157077143
chr1	157077162	157077381
chr1	157079402	157079700
chr1	157079700	157079938
chr1	157079938	157080157
chr1	157081095	157081372
chr1	157081372	157081897
chr1	157084840	157084994
chr1	157086196	157086410
chr1	157090787	157091397
chr1	157091474	157091662
chr1	157126793	157127104
chr1	157127104	157127365
chr1	157127804	157128051
chr1	157134741	157135110
chr1	157219530	157219832
chr1	157232410	157232754
chr1	157232754	157233145
chr1	157248091	157248395

chr1	157256521	157256951
chr1	157286637	157286872
chr1	157301318	157301609
chr1	157319038	157319203
chr1	157320477	157320702
chr1	157343538	157343890
chr1	157347582	157347789
chr1	157353902	157354052
chr1	157368256	157368721
chr1	157380495	157380755
chr1	157380755	157381167
chr1	157393155	157393602
chr1	157411687	157412095
chr1	157412095	157412498
chr1	157412498	157412877
chr1	157412899	157413111
chr1	157437783	157438287
chr1	157438287	157438591
chr1	157440213	157440475
chr1	157446433	157446583
chr1	157458432	157458641
chr1	157480962	157481218
chr1	157481769	157481970
chr1	157499224	157499595
chr1	157512689	157513011
chr1	157518188	157518505
chr1	157536640	157536807
chr1	157553100	157553422
chr1	157578854	157579075
chr1	157594660	157594867
chr1	157599460	157599615
chr1	157604505	157604798
chr1	157631881	157632118
chr1	157632611	157633109
chr1	157633275	157633483
chr1	157633499	157633929
chr1	157634501	157634746
chr1	157634826	157635086
chr1	157645738	157646405
chr1	157648554	157648787
chr1	157663631	157663807
chr1	157672242	157672887

chr1	157680313	157680588
chr1	157680762	157681061
chr1	157707756	157707925
chr1	157717048	157717608
chr1	157717693	157717855
chr1	157745702	157745921
chr1	157825150	157825310
chr1	157858119	157858377
chr1	157874872	157875607
chr1	157877213	157877389
chr1	157905092	157905247
chr1	158102680	158102830
chr1	158198176	158198583
chr1	158201443	158201749
chr1	158212820	158213001
chr1	158213822	158213999
chr1	158241295	158241567
chr1	158247728	158247904
chr1	158248700	158249075
chr1	158249119	158249305
chr1	158255929	158256375
chr1	158256433	158256583
chr1	158310564	158310714
chr1	158355950	158356551
chr1	158597174	158597324
chr1	158605614	158605892
chr1	158606222	158606580
chr1	158654668	158654884
chr1	158670265	158670507
chr1	158699305	158699625
chr1	158701333	158701827
chr1	158765851	158766175
chr1	158830189	158830499
chr1	158853008	158853189
chr1	158853225	158853591
chr1	158864067	158864217
chr1	158897200	158897711
chr1	158917107	158917414
chr1	158935123	158935335
chr1	158935335	158935802
chr1	158935886	158936125
chr1	158936370	158936603

chr1	158947990	158948376
chr1	158948442	158948687
chr1	158948799	158949044
chr1	158953608	158953861
chr1	159073228	159073448
chr1	159082230	159082537
chr1	159135392	159135587
chr1	159231763	159232049
chr1	159232049	159232439
chr1	159232439	159232825
chr1	159232905	159233088
chr1	159234602	159234839
chr1	159253171	159253321
chr1	159390157	159390321
chr1	159392734	159393168
chr1	159393168	159393548
chr1	159402804	159403072
chr1	159404664	159404909
chr1	159460940	159461157
chr1	159471012	159471261
chr1	159523599	159523812
chr1	159524143	159524384
chr1	159595703	159596168
chr1	159596168	159596517
chr1	159596707	159596909
chr1	159658151	159658435
chr1	159660686	159660881
chr1	159660881	159661129
chr1	159686391	159686865
chr1	159686865	159687274
chr1	159693881	159694068
chr1	159694068	159694286
chr1	159702123	159702290
chr1	159765121	159765271
chr1	159812781	159813229
chr1	159817038	159817420
chr1	159827637	159827871
chr1	159828653	159828835
chr1	159832368	159832597
chr1	159833003	159833572
chr1	159843608	159843863
chr1	159843863	159844317

chr1	159852222	159852408
chr1	159884231	159884413
chr1	159888699	159888986
chr1	159889043	159889386
chr1	159889386	159889999
chr1	159890662	159890905
chr1	159911082	159911354
chr1	159917243	159917602
chr1	159932202	159932373
chr1	159982581	159982810
chr1	159986252	159986479
chr1	159992150	159992494
chr1	159997315	159997888
chr1	160006003	160006760
chr1	160025645	160025878
chr1	160039971	160040372
chr1	160044199	160044762
chr1	160044762	160045043
chr1	160052599	160052878
chr1	160052971	160053276
chr1	160053496	160053671
chr1	160053882	160054202
chr1	160054202	160054425
chr1	160057355	160057605
chr1	160057674	160058059
chr1	160060010	160060318
chr1	160060437	160060794
chr1	160060794	160061060
chr1	160073140	160073649
chr1	160079474	160079664
chr1	160080897	160081131
chr1	160081131	160081407
chr1	160082763	160082956
chr1	160082999	160083149
chr1	160084440	160084608
chr1	160086791	160086997
chr1	160088001	160088379
chr1	160091755	160092018
chr1	160101031	160101215
chr1	160150701	160151260
chr1	160175340	160175585
chr1	160184603	160184834

chr1	160194838	160195329
chr1	160195329	160195638
chr1	160212133	160212397
chr1	160212397	160212761
chr1	160212761	160213163
chr1	160213163	160213467
chr1	160227844	160228136
chr1	160233980	160234285
chr1	160261878	160262168
chr1	160263168	160263353
chr1	160263704	160263884
chr1	160264012	160264213
chr1	160267361	160267724
chr1	160271679	160271851
chr1	160280697	160280891
chr1	160297249	160297422
chr1	160297842	160298087
chr1	160308996	160309422
chr1	160311985	160312158
chr1	160327585	160327854
chr1	160370709	160370895
chr1	160372069	160372229
chr1	160373378	160373688
chr1	160378461	160378622
chr1	160408034	160408296
chr1	160509528	160509693
chr1	160509747	160510420
chr1	160520138	160520381
chr1	160520660	160521172
chr1	160521291	160521567
chr1	160531428	160531818
chr1	160542106	160542609
chr1	160548617	160548767
chr1	160550596	160551038
chr1	160561484	160561653
chr1	160595972	160596282
chr1	160596282	160596633
chr1	160596688	160596932
chr1	160596932	160597185
chr1	160597367	160597746
chr1	160692974	160693444
chr1	160704635	160704981

chr1	160717836	160717986
chr1	160734645	160734925
chr1	160734972	160735158
chr1	160735396	160735655
chr1	160750695	160751138
chr1	160758332	160758542
chr1	160791905	160792230
chr1	160792230	160792654
chr1	160792654	160792985
chr1	160792985	160793276
chr1	160819849	160820010
chr1	160834584	160834734
chr1	160863138	160863340
chr1	160867898	160868236
chr1	160874812	160874986
chr1	160877031	160877358
chr1	160878448	160879085
chr1	160881357	160881672
chr1	160891083	160891667
chr1	160905909	160906312
chr1	160906312	160906504
chr1	160906504	160906713
chr1	160906713	160906954
chr1	160906954	160907206
chr1	160934271	160934451
chr1	160980039	160980290
chr1	161034251	161034925
chr1	161034925	161035310
chr1	161035310	161035641
chr1	161039068	161039259
chr1	161039577	161040001
chr1	161040001	161040436
chr1	161040436	161040846
chr1	161041111	161041262
chr1	161041753	161041915
chr1	161070310	161070566
chr1	161070566	161070929
chr1	161070929	161071247
chr1	161071305	161071641
chr1	161071660	161071865
chr1	161130856	161131093
chr1	161131093	161131335

chr1	161131335	161131689
chr1	161140094	161140386
chr1	161140386	161140796
chr1	161143928	161144180
chr1	161148368	161148713
chr1	161158638	161158839
chr1	161159256	161159510
chr1	161170592	161171100
chr1	161180788	161180938
chr1	161195516	161195810
chr1	161195810	161196053
chr1	161196053	161196365
chr1	161214556	161214806
chr1	161229846	161230411
chr1	161239228	161239405
chr1	161248787	161248985
chr1	161250098	161250358
chr1	161250543	161250775
chr1	161250775	161251056
chr1	161251056	161251372
chr1	161251372	161251695
chr1	161281100	161281413
chr1	161286779	161287025
chr1	161301440	161301649
chr1	161327134	161327284
chr1	161327315	161327753
chr1	161328144	161328342
chr1	161351048	161351292
chr1	161364995	161365339
chr1	161394542	161394855
chr1	161400220	161400438
chr1	161406334	161406825
chr1	161409107	161409381
chr1	161424482	161424635
chr1	161436535	161436756
chr1	161436756	161437109
chr1	161444689	161444929
chr1	161444929	161445246
chr1	161451361	161451693
chr1	161451731	161452323
chr1	161479917	161480100
chr1	161482225	161482693

chr1	161487251	161487465
chr1	161494505	161494655
chr1	161497351	161497677
chr1	161506453	161506662
chr1	161507740	161508060
chr1	161576754	161576917
chr1	161598768	161598954
chr1	161601207	161601406
chr1	161607527	161607757
chr1	161610484	161610657
chr1	161614268	161614456
chr1	161621201	161621731
chr1	161621731	161622055
chr1	161628350	161628532
chr1	161688920	161689251
chr1	161704620	161704772
chr1	161727492	161727678
chr1	161734681	161735205
chr1	161735205	161735493
chr1	161735521	161735776
chr1	161744303	161744609
chr1	161744798	161745199
chr1	161748225	161748439
chr1	161748774	161748966
chr1	161749218	161749402
chr1	161750300	161750571
chr1	161750612	161751032
chr1	161766630	161766982
chr1	161767304	161767509
chr1	161799915	161800089
chr1	161875857	161876041
chr1	161876232	161876589
chr1	161876589	161876864
chr1	161876864	161877113
chr1	161877180	161877432
chr1	161886545	161886995
chr1	161896216	161896394
chr1	161912910	161913218
chr1	161913425	161913575
chr1	161913744	161913901
chr1	161963055	161963260
chr1	161968811	161969535

chr1	161976248	161976451
chr1	161976451	161976765
chr1	161999876	162000031
chr1	162006167	162006465
chr1	162016618	162016821
chr1	162039103	162039500
chr1	162076496	162077005
chr1	162125886	162126386
chr1	162132962	162133297
chr1	162133297	162133596
chr1	162133718	162133973
chr1	162207317	162207544
chr1	162215719	162216148
chr1	162224896	162225176
chr1	162225614	162226186
chr1	162243061	162243255
chr1	162243404	162243747
chr1	162313698	162313927
chr1	162358131	162358339
chr1	162359279	162359525
chr1	162388858	162389030
chr1	162428977	162429245
chr1	162449194	162449521
chr1	162449570	162449750
chr1	162453528	162454008
chr1	162462884	162463261
chr1	162463993	162464321
chr1	162464465	162464796
chr1	162476683	162477188
chr1	162477188	162477452
chr1	162477452	162477664
chr1	162477664	162477887
chr1	162477887	162478253
chr1	162508707	162508915
chr1	162509208	162509709
chr1	162509709	162509968
chr1	162529758	162529982
chr1	162531702	162531915
chr1	162547768	162547931
chr1	162548003	162548208
chr1	162548208	162548441
chr1	162548441	162548725

chr1	162548725	162548938
chr1	162554997	162555147
chr1	162561704	162561963
chr1	162565048	162565292
chr1	162566731	162566934
chr1	162568593	162568788
chr1	162570207	162570882
chr1	162570882	162571282
chr1	162571427	162571657
chr1	162582718	162582920
chr1	162620559	162620709
chr1	162650283	162650511
chr1	162665171	162665464
chr1	162665508	162665700
chr1	162665700	162665877
chr1	162665877	162666070
chr1	162693823	162694058
chr1	162739857	162740197
chr1	162740197	162740438
chr1	162740438	162740793
chr1	162745647	162745802
chr1	162746019	162746449
chr1	162754726	162754886
chr1	162763861	162764128
chr1	162773573	162774081
chr1	162792424	162792825
chr1	162792825	162793230
chr1	162830482	162830659
chr1	162832329	162832538
chr1	162859853	162860408
chr1	162892123	162892296
chr1	162892296	162892486
chr1	162952981	162953451
chr1	162953451	162953773
chr1	163070592	163070805
chr1	163089591	163089811
chr1	163091611	163091847
chr1	163091847	163092110
chr1	163112546	163112950
chr1	163112950	163113285
chr1	163113676	163114388
chr1	163114834	163115067

chr1	163126141	163126381
chr1	163130071	163130275
chr1	163142825	163143062
chr1	163143062	163143416
chr1	163143416	163143713
chr1	163150499	163150834
chr1	163181871	163182195
chr1	163209308	163209543
chr1	163273181	163273553
chr1	163274906	163275253
chr1	163285266	163285463
chr1	163291926	163292410
chr1	163293104	163293476
chr1	163311899	163312225
chr1	163312264	163312704
chr1	163345037	163345197
chr1	163380099	163380282
chr1	163382068	163382408
chr1	163382408	163382985
chr1	163399788	163399975
chr1	163403389	163403822
chr1	163403822	163404124
chr1	163426377	163426651
chr1	163453195	163453412
chr1	163466178	163466401
chr1	163466476	163466752
chr1	163496147	163496312
chr1	163511144	163511427
chr1	163512828	163513231
chr1	163561077	163561402
chr1	163562380	163562640
chr1	163563942	163564182
chr1	163574487	163574779
chr1	163575691	163575941
chr1	163576544	163576729
chr1	163654174	163654369
chr1	163654369	163654685
chr1	163655735	163656144
chr1	163656558	163656800
chr1	163656800	163657024
chr1	163779316	163779781
chr1	163779781	163780132

chr1	163894350	163894557
chr1	163907002	163907282
chr1	163907282	163907533
chr1	163907699	163907881
chr1	163907881	163908095
chr1	163910221	163910371
chr1	163910429	163910658
chr1	163928905	163929157
chr1	163929565	163929858
chr1	163929858	163930148
chr1	163930148	163930443
chr1	163930443	163930862
chr1	163974428	163974688
chr1	163994252	163994560
chr1	163994560	163994929
chr1	163994929	163995199
chr1	163995199	163995410
chr1	163995465	163995617
chr1	164009382	164009532
chr1	164014191	164014433
chr1	164030935	164031153
chr1	164061378	164061528
chr1	164070501	164070666
chr1	164113390	164113754
chr1	164114297	164114714
chr1	164115007	164115279
chr1	164123794	164124165
chr1	164126147	164126554
chr1	164135302	164135911
chr1	164135911	164136353
chr1	164138707	164138947
chr1	164149192	164149378
chr1	164151725	164151986
chr1	164174395	164174704
chr1	164184593	164184854
chr1	164208488	164208776
chr1	164209284	164209606
chr1	164223947	164224146
chr1	164229007	164229166
chr1	164238166	164238332
chr1	164242933	164243143
chr1	164248486	164248956

chr1	164248956	164249341
chr1	164249341	164249720
chr1	164250307	164250716
chr1	164250826	164251026
chr1	164260276	164260426
chr1	164261547	164261887
chr1	164266459	164266749
chr1	164271035	164271185
chr1	164271565	164271889
chr1	164275424	164275744
chr1	164275744	164276008
chr1	164276664	164276951
chr1	164306967	164307367
chr1	164307387	164307904
chr1	164308080	164308230
chr1	164315334	164315536
chr1	164316650	164317026
chr1	164330197	164330363
chr1	164347235	164347611
chr1	164366625	164367056
chr1	164389844	164390002
chr1	164418958	164419406
chr1	164419436	164419871
chr1	164421075	164421282
chr1	164425205	164425383
chr1	164450793	164451037
chr1	164451195	164451777
chr1	164457575	164457776
chr1	164457782	164458194
chr1	164458194	164458606
chr1	164458606	164459048
chr1	164461909	164462082
chr1	164467976	164468262
chr1	164481389	164482123
chr1	164493111	164493356
chr1	164494808	164495116
chr1	164495162	164495441
chr1	164495441	164495703
chr1	164503276	164503665
chr1	164539848	164540009
chr1	164540016	164540215
chr1	164621302	164621586

chr1	164622309	164622792
chr1	164623014	164623266
chr1	164628851	164629455
chr1	164631730	164632050
chr1	164644609	164644894
chr1	164666347	164666628
chr1	164669600	164669884
chr1	164708025	164708196
chr1	164714112	164714365
chr1	164714365	164714594
chr1	164745037	164745240
chr1	164762406	164762566
chr1	164812776	164813046
chr1	164831219	164831386
chr1	164855755	164856009
chr1	164861080	164861390
chr1	164861510	164861760
chr1	164863038	164863250
chr1	164864558	164864953
chr1	164868960	164869121
chr1	164874743	164875000
chr1	164880919	164881083
chr1	164887666	164888164
chr1	164888365	164888613
chr1	164888613	164888924
chr1	164898777	164899055
chr1	164899055	164899374
chr1	164905147	164905357
chr1	164969492	164969642
chr1	164975620	164976148
chr1	164977414	164977691
chr1	164986766	164987022
chr1	164987121	164987335
chr1	164987758	164988387
chr1	164993150	164993522
chr1	164993522	164993826
chr1	164993826	164994147
chr1	164994147	164994485
chr1	164994488	164994682
chr1	164999274	164999581
chr1	164999581	164999844
chr1	165018815	165019113

chr1	165032549	165032870
chr1	165032873	165033609
chr1	165035807	165035970
chr1	165043429	165043606
chr1	165046046	165046480
chr1	165046555	165046952
chr1	165075665	165075888
chr1	165075888	165076149
chr1	165084215	165084523
chr1	165089582	165089867
chr1	165091873	165092033
chr1	165104639	165105029
chr1	165120428	165120663
chr1	165120663	165120855
chr1	165132562	165132718
chr1	165185325	165185610
chr1	165190763	165191144
chr1	165192997	165193247
chr1	165193818	165194280
chr1	165194280	165194587
chr1	165194587	165194847
chr1	165203556	165203813
chr1	165203813	165204061
chr1	165236234	165236825
chr1	165236825	165237403
chr1	165247239	165247407
chr1	165252108	165252258
chr1	165280723	165280900
chr1	165281369	165281581
chr1	165281597	165282233
chr1	165282233	165282742
chr1	165282746	165282896
chr1	165289904	165290146
chr1	165291697	165291884
chr1	165295022	165295278
chr1	165299470	165299832
chr1	165302545	165302812
chr1	165302812	165303200
chr1	165303200	165303603
chr1	165316451	165316619
chr1	165320417	165320885
chr1	165432651	165432909

chr1	165432941	165433205
chr1	165433205	165433417
chr1	165438744	165438941
chr1	165459968	165460358
chr1	165460358	165460799
chr1	165460810	165461516
chr1	165492760	165492916
chr1	165543863	165544051
chr1	165576915	165577078
chr1	165583574	165583840
chr1	165583840	165584039
chr1	165586357	165586693
chr1	165603150	165603632
chr1	165608894	165609102
chr1	165617219	165617469
chr1	165630082	165630362
chr1	165633973	165634334
chr1	165634334	165634883
chr1	165641528	165641764
chr1	165641841	165642171
chr1	165642227	165642479
chr1	165642479	165642742
chr1	165642742	165642940
chr1	165642940	165643125
chr1	165643125	165643358
chr1	165643695	165643982
chr1	165655082	165655330
chr1	165658667	165658961
chr1	165660841	165661083
chr1	165670187	165670404
chr1	165672540	165672700
chr1	165691637	165691853
chr1	165707946	165708134
chr1	165730733	165730966
chr1	165736200	165736471
chr1	165751965	165752216
chr1	165758398	165758580
chr1	165758580	165758751
chr1	165763541	165763878
chr1	165763878	165764419
chr1	165769020	165769260
chr1	165769260	165769493

chr1	165771578	165771905
chr1	165775625	165775777
chr1	165780337	165780565
chr1	165780629	165780831
chr1	165789992	165790207
chr1	165802540	165802765
chr1	165817350	165817619
chr1	165840765	165840994
chr1	165840994	165841128
chr1	165844360	165844681
chr1	165844681	165845002
chr1	165845002	165845349
chr1	165845349	165845801
chr1	165853781	165854070
chr1	165858244	165858462
chr1	165858462	165858716
chr1	165863597	165863751
chr1	165864030	165864256
chr1	165879861	165880123
chr1	165880123	165880450
chr1	165891755	165891945
chr1	166001310	166001460
chr1	166001768	166002072
chr1	166002072	166002398
chr1	166002480	166002880
chr1	166002880	166003191
chr1	166003191	166003613
chr1	166008769	166008924
chr1	166008993	166009304
chr1	166009304	166009643
chr1	166038658	166038823
chr1	166043693	166044137
chr1	166044137	166044474
chr1	166045378	166045598
chr1	166079633	166079892
chr1	166086354	166086628
chr1	166089719	166089963
chr1	166094486	166094742
chr1	166130229	166130524
chr1	166130524	166130779
chr1	166137457	166138060
chr1	166138060	166138557

chr1	166149230	166149595
chr1	166153403	166153731
chr1	166154190	166154508
chr1	166160681	166160941
chr1	166160963	166161122
chr1	166174986	166175146
chr1	166180268	166180418
chr1	166253958	166254300
chr1	166255613	166256052
chr1	166267354	166267525
chr1	166308990	166309251
chr1	166309251	166309530
chr1	166313715	166313937
chr1	166319226	166319403
chr1	166360220	166360514
chr1	166374201	166374454
chr1	166378807	166379079
chr1	166379079	166379370
chr1	166379370	166379723
chr1	166379723	166380019
chr1	166380019	166380235
chr1	166409035	166409332
chr1	166409332	166409661
chr1	166409661	166410153
chr1	166429015	166429222
chr1	166448376	166448533
chr1	166639324	166639512
chr1	166639725	166640142
chr1	166651456	166651760
chr1	166651876	166652171
chr1	166652171	166652542
chr1	166652916	166653073
chr1	166737266	166737478
chr1	166782196	166782378
chr1	166784666	166784851
chr1	166787560	166787925
chr1	166787925	166788421
chr1	166793703	166793993
chr1	166836373	166836546
chr1	166839880	166840052
chr1	166895988	166896138
chr1	166899821	166900084

chr1	166900084	166900338
chr1	166924215	166924542
chr1	166942265	166942542
chr1	166944398	166944898
chr1	166944957	166945432
chr1	166964614	166964933
chr1	166968224	166968685
chr1	166970786	166971093
chr1	166973835	166974027
chr1	166996871	166997341
chr1	166997392	166997663
chr1	167000488	167000890
chr1	167000890	167001157
chr1	167001157	167001406
chr1	167001406	167001642
chr1	167002523	167002819
chr1	167101337	167101719
chr1	167212348	167212625
chr1	167220432	167220622
chr1	167220622	167220872
chr1	167220927	167221249
chr1	167230996	167231305
chr1	167231886	167232251
chr1	167232251	167232556
chr1	167233008	167233206
chr1	167234090	167234267
chr1	167234388	167234676
chr1	167242040	167242665
chr1	167242716	167243019
chr1	167243019	167243237
chr1	167243512	167243761
chr1	167249848	167250167
chr1	167254108	167254570
chr1	167254620	167254772
chr1	167255511	167255890
chr1	167255968	167256164
chr1	167261782	167262096
chr1	167262549	167263028
chr1	167265502	167265662
chr1	167269639	167269794
chr1	167269878	167270318
chr1	167270318	167270692

chr1	167270692	167270956
chr1	167270956	167271197
chr1	167278974	167279557
chr1	167283723	167283957
chr1	167284249	167284737
chr1	167284737	167285369
chr1	167285423	167285709
chr1	167300157	167300344
chr1	167306608	167306764
chr1	167308101	167308820
chr1	167308820	167309080
chr1	167337501	167337779
chr1	167340136	167340582
chr1	167349303	167349529
chr1	167349733	167350061
chr1	167350061	167350313
chr1	167350313	167350563
chr1	167350563	167350819
chr1	167369780	167370267
chr1	167374050	167374327
chr1	167374460	167374656
chr1	167374725	167374925
chr1	167381374	167381683
chr1	167386935	167387288
chr1	167387288	167387673
chr1	167387673	167387988
chr1	167392557	167392827
chr1	167392827	167393146
chr1	167393230	167393460
chr1	167393460	167393685
chr1	167393685	167393971
chr1	167393988	167394271
chr1	167394271	167394523
chr1	167414995	167415159
chr1	167425939	167426170
chr1	167426487	167426687
chr1	167426835	167426995
chr1	167446543	167446856
chr1	167486932	167487275
chr1	167490370	167490656
chr1	167551661	167551877
chr1	167598361	167598592

chr1	167637004	167637327
chr1	167638107	167638300
chr1	167644977	167645222
chr1	167660842	167661115
chr1	167661190	167661457
chr1	167669856	167670110
chr1	167672619	167672955
chr1	167674969	167675155
chr1	167803035	167803259
chr1	167806297	167806447
chr1	167903733	167903913
chr1	168057611	168057771
chr1	168109327	168109515
chr1	168109917	168110077
chr1	168133708	168134075
chr1	168138769	168139025
chr1	168139288	168139551
chr1	168139620	168139926
chr1	168139926	168140232
chr1	168214181	168214467
chr1	168217588	168217748
chr1	168220708	168221035
chr1	168221035	168221333
chr1	168221384	168221533
chr1	168221533	168221675
chr1	168228977	168229150
chr1	168230137	168230498
chr1	168237263	168237507
chr1	168238042	168238225
chr1	168239657	168239955
chr1	168271937	168272087
chr1	168272102	168272481
chr1	168278282	168278453
chr1	168278477	168278770
chr1	168278770	168279118
chr1	168279123	168279506
chr1	168279648	168279882
chr1	168283424	168283791
chr1	168283791	168284131
chr1	168312786	168313020
chr1	168313842	168314056
chr1	168319053	168319288

chr1	168322992	168323197
chr1	168323811	168324087
chr1	168334932	168335207
chr1	168335259	168335525
chr1	168355059	168355219
chr1	168362436	168362909
chr1	168364338	168364494
chr1	168374922	168375307
chr1	168382883	168383104
chr1	168390781	168390999
chr1	168390999	168391245
chr1	168391250	168391615
chr1	168391615	168392061
chr1	168392126	168392404
chr1	168402637	168403027
chr1	168411942	168412098
chr1	168415236	168415538
chr1	168417175	168417425
chr1	168426427	168426640
chr1	168431351	168431549
chr1	168431800	168432297
chr1	168432297	168432792
chr1	168473295	168473464
chr1	168494543	168494890
chr1	168501870	168502202
chr1	168502202	168502843
chr1	168507819	168508142
chr1	168518050	168518318
chr1	168518318	168518570
chr1	168518570	168518868
chr1	168545540	168545708
chr1	168560667	168560895
chr1	168561265	168561590
chr1	168583939	168584638
chr1	168598086	168598791
chr1	168600963	168601413
chr1	168631946	168632397
chr1	168637225	168637494
chr1	168694577	168694727
chr1	168708134	168708456
chr1	168708456	168708667
chr1	168708684	168708876

chr1	168709005	168709230
chr1	168709235	168709509
chr1	168709551	168709735
chr1	168709753	168709924
chr1	168709946	168710221
chr1	168710284	168710476
chr1	168728780	168728930
chr1	168772072	168772226
chr1	168783713	168784235
chr1	168798400	168798567
chr1	168832024	168832316
chr1	168838241	168838554
chr1	168950987	168951205
chr1	168967458	168967652
chr1	168975334	168975779
chr1	169047221	169047472
chr1	169368311	169368546
chr1	169368732	169369107
chr1	169438632	169438810
chr1	169445419	169445635
chr1	169459444	169459720
chr1	169485639	169485988
chr1	169517552	169517865
chr1	169517865	169518352
chr1	169520489	169520658
chr1	169526026	169526288
chr1	169526419	169526604
chr1	169530273	169530489
chr1	169530981	169531137
chr1	169531248	169531749
chr1	169531749	169531995
chr1	169532206	169532393
chr1	169555081	169555375
chr1	169555375	169555534
chr1	169555637	169556154
chr1	169558588	169558813
chr1	169562859	169563016
chr1	169579448	169579876
chr1	169591599	169591802
chr1	169600862	169601053
chr1	169601984	169602209
chr1	169625354	169625790

chr1	169625790	169626283
chr1	169638598	169638900
chr1	169638900	169639117
chr1	169642186	169642390
chr1	169642432	169642684
chr1	169643882	169644285
chr1	169670728	169670936
chr1	169674207	169674428
chr1	169675179	169675329
chr1	169693167	169693355
chr1	169693554	169694141
chr1	169695390	169695643
chr1	169699181	169699535
chr1	169703819	169704137
chr1	169704388	169704736
chr1	169704736	169705150
chr1	169720928	169721143
chr1	169728960	169729123
chr1	169730773	169730963
chr1	169732713	169733238
chr1	169774525	169774703
chr1	169775476	169775672
chr1	169776599	169776926
chr1	169804892	169805390
chr1	169840112	169840432
chr1	169840432	169840701
chr1	169894855	169895102
chr1	169918033	169918360
chr1	169919239	169919517
chr1	169921061	169921295
chr1	169921662	169921883
chr1	169934105	169934310
chr1	169936459	169936635
chr1	169947690	169947951
chr1	169968847	169969554
chr1	169969559	169969754
chr1	169977607	169978007
chr1	169998706	169999165
chr1	170003010	170003341
chr1	170004342	170004655
chr1	170006545	170006936
chr1	170008526	170008854

chr1	170008880	170009077
chr1	170009339	170009953
chr1	170015871	170016200
chr1	170023082	170023281
chr1	170037860	170038045
chr1	170042975	170043182
chr1	170043182	170043371
chr1	170044937	170045280
chr1	170054110	170054312
chr1	170057451	170057668
chr1	170058101	170058448
chr1	170059370	170059783
chr1	170062721	170063105
chr1	170069240	170069489
chr1	170079391	170079785
chr1	170080900	170081254
chr1	170081517	170081760
chr1	170094258	170094495
chr1	170097346	170097496
chr1	170111814	170112259
chr1	170124693	170124984
chr1	170136206	170136455
chr1	170136455	170136921
chr1	170145542	170145957
chr1	170145957	170146247
chr1	170147610	170148025
chr1	170148025	170148442
chr1	170164091	170164598
chr1	170174195	170174509
chr1	170174509	170174883
chr1	170174883	170175490
chr1	170188525	170188994
chr1	170200503	170200660
chr1	170214351	170214587
chr1	170214726	170215169
chr1	170215169	170215555
chr1	170218521	170218803
chr1	170225983	170226216
chr1	170232472	170232760
chr1	170232760	170233043
chr1	170255255	170255890
chr1	170276911	170277366

chr1	170277366	170277700
chr1	170292573	170292952
chr1	170318378	170318803
chr1	170361362	170361568
chr1	170381490	170381812
chr1	170390107	170390426
chr1	170393075	170393258
chr1	170394751	170394964
chr1	170417064	170417270
chr1	170438485	170439095
chr1	170445537	170445834
chr1	170457683	170458100
chr1	170460066	170460568
chr1	170469796	170470083
chr1	170470083	170470319
chr1	170471309	170471610
chr1	170473406	170473900
chr1	170479541	170479741
chr1	170483653	170484033
chr1	170496216	170496643
chr1	170497103	170497348
chr1	170530481	170530674
chr1	170530882	170531424
chr1	170531443	170531611
chr1	170539322	170539472
chr1	170539516	170539842
chr1	170554528	170554909
chr1	170554909	170555373
chr1	170558058	170558254
chr1	170563234	170563384
chr1	170588959	170589146
chr1	170589146	170589419
chr1	170589419	170589728
chr1	170589728	170590190
chr1	170612234	170612390
chr1	170613202	170613568
chr1	170630544	170630804
chr1	170632407	170632754
chr1	170639513	170639741
chr1	170644233	170644840
chr1	170649986	170650225
chr1	170664685	170664904

chr1	170691289	170691873
chr1	170694352	170694536
chr1	170699545	170699741
chr1	170699829	170700046
chr1	170702332	170702566
chr1	170702566	170702823
chr1	170705315	170705530
chr1	170729639	170729887
chr1	170731679	170731963
chr1	170731963	170732213
chr1	170732213	170732492
chr1	170753616	170753895
chr1	170754725	170754987
chr1	170756929	170757377
chr1	170766892	170767336
chr1	170768878	170769066
chr1	170770736	170770952
chr1	170770952	170771182
chr1	170771182	170771427
chr1	170771451	170771917
chr1	170781077	170781388
chr1	170790820	170790990
chr1	170791008	170791391
chr1	170795004	170795252
chr1	170796014	170796362
chr1	170804095	170804265
chr1	170807800	170808263
chr1	170817501	170817722
chr1	170821393	170821544
chr1	170822752	170822902
chr1	170829427	170829710
chr1	170829715	170830377
chr1	170834838	170835056
chr1	170839640	170839971
chr1	170844456	170844704
chr1	170853344	170854087
chr1	170854155	170854334
chr1	170854942	170855189
chr1	170858977	170859188
chr1	170863116	170863266
chr1	170864897	170865102
chr1	170867206	170867397

chr1	170867462	170867681
chr1	170867681	170867979
chr1	170868512	170868948
chr1	170884326	170884707
chr1	170884938	170885275
chr1	170885275	170885650
chr1	170895357	170895507
chr1	170901652	170901915
chr1	170907773	170908178
chr1	170912132	170912656
chr1	170912771	170913062
chr1	170919936	170920265
chr1	170962363	170962666
chr1	170978587	170979007
chr1	170981718	170982020
chr1	170984035	170984244
chr1	170988287	170988546
chr1	170992715	170992900
chr1	170996796	170997076
chr1	171019368	171019652
chr1	171019652	171019945
chr1	171019945	171020189
chr1	171027944	171028174
chr1	171033219	171033409
chr1	171033500	171034101
chr1	171036978	171037227
chr1	171037935	171038191
chr1	171038191	171038442
chr1	171038534	171038740
chr1	171044236	171044592
chr1	171044592	171044854
chr1	171044854	171045147
chr1	171050734	171051072
chr1	171063875	171064027
chr1	171064107	171064546
chr1	171064546	171064915
chr1	171064915	171065232
chr1	171065232	171065596
chr1	171065610	171065867
chr1	171065867	171066100
chr1	171066553	171066915
chr1	171070378	171070589

chr1	171071768	171072182
chr1	171072182	171072550
chr1	171072550	171072870
chr1	171072870	171073224
chr1	171073252	171073543
chr1	171074212	171074562
chr1	171077974	171078264
chr1	171079382	171079796
chr1	171079796	171080166
chr1	171080166	171080486
chr1	171080486	171080845
chr1	171080864	171081157
chr1	171081760	171082170
chr1	171094398	171094778
chr1	171095069	171095227
chr1	171095451	171095716
chr1	171096183	171096440
chr1	171096440	171096767
chr1	171101787	171102127
chr1	171102127	171102556
chr1	171102556	171102911
chr1	171102911	171103208
chr1	171111024	171111330
chr1	171111355	171111588
chr1	171111588	171111856
chr1	171111856	171112102
chr1	171113801	171114312
chr1	171118310	171118581
chr1	171118587	171118829
chr1	171122978	171123386
chr1	171150234	171150484
chr1	171150484	171150788
chr1	171156769	171156942
chr1	171159437	171159768
chr1	171159778	171160013
chr1	171184211	171184695
chr1	171189537	171189695
chr1	171193330	171193491
chr1	171195859	171196128
chr1	171196128	171196422
chr1	171220872	171221125
chr1	171221125	171221317

chr1	171222327	171223072
chr1	171238540	171238820
chr1	171246809	171247033
chr1	171247033	171247221
chr1	171247221	171247515
chr1	171247515	171247872
chr1	171248022	171248224
chr1	171248469	171248701
chr1	171248701	171249119
chr1	171256901	171257053
chr1	171257053	171257238
chr1	171257238	171257491
chr1	171260400	171260741
chr1	171262566	171262779
chr1	171262779	171263067
chr1	171263067	171263405
chr1	171263768	171264009
chr1	171269967	171270178
chr1	171270178	171270491
chr1	171270491	171270953
chr1	171271006	171271178
chr1	171277316	171277568
chr1	171280663	171281016
chr1	171281016	171281447
chr1	171281447	171281700
chr1	171281700	171281897
chr1	171287443	171287779
chr1	171287779	171288123
chr1	171288123	171288348
chr1	171292197	171292420
chr1	171294271	171294468
chr1	171294519	171295119
chr1	171306174	171306497
chr1	171311977	171312166
chr1	171315121	171315312
chr1	171319878	171320074
chr1	171321504	171321696
chr1	171321990	171322331
chr1	171328654	171328848
chr1	171328862	171329390
chr1	171329390	171329708
chr1	171329708	171330086

chr1	171330086	171330340
chr1	171342791	171343102
chr1	171344351	171344633
chr1	171345335	171345555
chr1	171345555	171345924
chr1	171345924	171346474
chr1	171349321	171349543
chr1	171353109	171353529
chr1	171356038	171356276
chr1	171358463	171358724
chr1	171360668	171361025
chr1	171361200	171361479
chr1	171361486	171361767
chr1	171370184	171370492
chr1	171370492	171370755
chr1	171374837	171375035
chr1	171375035	171375230
chr1	171375571	171376175
chr1	171376949	171377121
chr1	171377966	171378498
chr1	171384456	171384726
chr1	171386978	171387281
chr1	171387281	171387502
chr1	171390226	171390456
chr1	171410944	171411458
chr1	171411458	171411881
chr1	171412018	171412317
chr1	171424537	171425098
chr1	171425098	171425457
chr1	171437160	171437460
chr1	171437460	171437674
chr1	171437674	171437931
chr1	171440382	171440589
chr1	171441435	171441966
chr1	171445113	171445381
chr1	171453088	171453264
chr1	171455018	171455214
chr1	171457700	171457893
chr1	171460627	171460819
chr1	171461569	171461788
chr1	171469757	171470053
chr1	171470132	171470395

chr1	171470406	171470613
chr1	171470613	171470848
chr1	171470913	171471446
chr1	171477861	171478323
chr1	171478323	171478608
chr1	171478993	171479318
chr1	171490465	171490618
chr1	171490618	171490759
chr1	171501111	171501563
chr1	171501563	171502074
chr1	171502074	171502429
chr1	171502429	171502604
chr1	171502604	171502769
chr1	171502769	171503239
chr1	171503330	171503639
chr1	171503639	171503906
chr1	171504014	171504168
chr1	171515136	171515296
chr1	171529688	171529846
chr1	171532941	171533152
chr1	171564335	171564669
chr1	171566884	171567166
chr1	171590622	171590772
chr1	171610098	171610339
chr1	171610846	171611105
chr1	171815723	171816105
chr1	171827760	171827990
chr1	171903005	171903160
chr1	171979863	171980373
chr1	172000729	172000984
chr1	172012079	172012368
chr1	172018970	172019235
chr1	172019340	172019524
chr1	172026333	172026888
chr1	172026923	172027252
chr1	172027252	172027603
chr1	172030026	172030225
chr1	172046547	172046731
chr1	172046955	172047223
chr1	172047247	172047402
chr1	172047402	172047540
chr1	172064662	172064817

chr1	172064847	172065042
chr1	172082272	172082894
chr1	172082894	172083411
chr1	172099647	172099854
chr1	172119765	172120000
chr1	172126328	172126538
chr1	172126568	172126850
chr1	172126850	172127062
chr1	172127062	172127261
chr1	172143656	172143890
chr1	172147845	172148283
chr1	172148283	172148674
chr1	172148674	172148981
chr1	172154621	172154892
chr1	172162058	172162330
chr1	172169918	172170184
chr1	172183115	172183295
chr1	172186511	172186688
chr1	172186895	172187065
chr1	172188809	172189237
chr1	172200204	172200486
chr1	172205482	172205731
chr1	172205819	172206087
chr1	172206096	172206508
chr1	172206508	172206945
chr1	172216911	172217162
chr1	172217162	172217486
chr1	172220476	172220797
chr1	172242344	172242945
chr1	172247843	172248035
chr1	172267417	172267571
chr1	172280022	172280172
chr1	172280405	172280818
chr1	172288497	172288682
chr1	172288844	172289220
chr1	172289383	172289954
chr1	172292953	172293115
chr1	172302312	172302680
chr1	172307910	172308385
chr1	172309758	172309976
chr1	172310176	172310470
chr1	172311389	172311868

chr1	172311951	172312494
chr1	172324770	172325049
chr1	172326167	172326561
chr1	172340780	172340941
chr1	172340947	172341236
chr1	172341236	172341546
chr1	172350382	172350862
chr1	172357619	172357872
chr1	172364711	172364963
chr1	172368844	172369083
chr1	172376037	172376681
chr1	172376681	172377072
chr1	172377072	172377362
chr1	172390863	172391252
chr1	172391252	172391567
chr1	172392821	172393047
chr1	172406151	172406340
chr1	172409173	172409325
chr1	172409574	172409724
chr1	172410897	172411118
chr1	172411526	172411817
chr1	172411817	172412177
chr1	172412177	172412553
chr1	172413617	172413821
chr1	172423267	172424025
chr1	172428592	172428946
chr1	172428946	172429230
chr1	172431891	172432129
chr1	172432325	172432507
chr1	172436900	172437128
chr1	172458100	172458771
chr1	172462474	172462944
chr1	172464266	172464585
chr1	172475557	172475823
chr1	172475950	172476157
chr1	172481509	172481844
chr1	172481844	172482091
chr1	172482091	172482354
chr1	172483405	172483648
chr1	172489833	172490312
chr1	172490312	172490809
chr1	172499714	172500022

chr1	172500022	172500572
chr1	172500668	172500963
chr1	172500963	172501526
chr1	172501713	172502036
chr1	172502036	172502432
chr1	172503043	172503193
chr1	172503359	172503649
chr1	172503649	172503915
chr1	172508916	172509091
chr1	172509314	172509513
chr1	172513036	172513227
chr1	172513651	172513889
chr1	172513905	172514246
chr1	172514246	172514487
chr1	172519492	172520126
chr1	172520616	172520878
chr1	172520901	172521233
chr1	172528718	172529069
chr1	172537292	172537631
chr1	172546120	172546407
chr1	172548540	172548753
chr1	172549321	172549685
chr1	172549841	172549991
chr1	172550496	172551007
chr1	172562686	172562980
chr1	172562980	172563247
chr1	172563247	172563548
chr1	172563550	172563705
chr1	172565315	172565623
chr1	172580059	172580598
chr1	172582801	172583047
chr1	172585320	172585599
chr1	172592462	172592834
chr1	172594584	172594734
chr1	172612842	172612994
chr1	172619316	172619551
chr1	172632445	172632817
chr1	172632817	172633379
chr1	172636302	172636590
chr1	172664746	172664888
chr1	172664888	172665008
chr1	172679391	172679546

chr1	172711494	172711693
chr1	172960542	172960773
chr1	173339671	173339822
chr1	173377624	173377780
chr1	173391850	173392077
chr1	173398695	173398850
chr1	173409056	173409420
chr1	173418917	173419079
chr1	173437610	173437900
chr1	173448920	173449116
chr1	173456433	173456668
chr1	173538018	173538317
chr1	173582823	173583148
chr1	173591385	173591633
chr1	173628439	173628681
chr1	173630546	173630961
chr1	173723638	173723980
chr1	173733276	173733574
chr1	173737048	173737259
chr1	173766617	173767282
chr1	173767282	173767778
chr1	173776465	173776827
chr1	173802653	173802913
chr1	173802913	173803325
chr1	173846521	173846875
chr1	173880124	173880674
chr1	173912579	173913281
chr1	173913281	173913767
chr1	173922468	173923016
chr1	173924223	173924418
chr1	173942375	173943022
chr1	173955066	173955432
chr1	173961301	173961771
chr1	173972763	173972975
chr1	173973108	173973528
chr1	173982519	173982861
chr1	173982861	173983251
chr1	173983389	173983609
chr1	174031417	174031819
chr1	174031819	174032262
chr1	174032264	174032514
chr1	174036244	174036407

chr1	174037084	174037435
chr1	174040913	174041349
chr1	174043538	174043743
chr1	174043807	174044031
chr1	174048854	174049333
chr1	174052985	174053447
chr1	174376580	174376819
chr1	174383313	174383537
chr1	174408135	174408319
chr1	174456731	174456911
chr1	174463395	174463610
chr1	174469820	174470044
chr1	174501745	174501952
chr1	174503378	174503665
chr1	174595132	174595289
chr1	174623202	174623406
chr1	174640875	174641465
chr1	174654353	174654633
chr1	174654633	174654958
chr1	174659426	174659796
chr1	174659796	174660089
chr1	174677294	174677444
chr1	174754772	174754985
chr1	174826188	174826395
chr1	174826589	174826943
chr1	174869444	174869622
chr1	174875250	174875441
chr1	174918567	174918762
chr1	174950221	174950467
chr1	175041493	175041738
chr1	175239713	175239888
chr1	175240084	175240346
chr1	175290178	175290336
chr1	175427839	175428294
chr1	175428294	175428864
chr1	175453983	175454566
chr1	175459184	175459404
chr1	175462471	175462639
chr1	175471537	175471732
chr1	175491935	175492244
chr1	175492244	175492470
chr1	175492470	175492985

chr1	175532383	175532628
chr1	175586779	175587145
chr1	175625031	175625507
chr1	175625507	175625964
chr1	175626050	175626301
chr1	175640351	175640571
chr1	175652569	175652772
chr1	175669123	175669315
chr1	175670045	175670364
chr1	175692003	175692606
chr1	175692606	175693360
chr1	175699822	175700141
chr1	175700230	175700770
chr1	175725836	175726159
chr1	175851467	175851784
chr1	175880402	175880654
chr1	175880654	175880904
chr1	175880904	175881110
chr1	175881110	175881306
chr1	175881593	175881781
chr1	175883533	175883703
chr1	175923916	175924181
chr1	175936315	175936506
chr1	175936506	175936778
chr1	176075538	176075749
chr1	176076931	176077142
chr1	176077271	176077442
chr1	176077450	176077603
chr1	176080622	176080836
chr1	176082403	176082627
chr1	176274917	176275104
chr1	176275340	176275553
chr1	176660233	176660637
chr1	176712469	176712744
chr1	176764052	176764223
chr1	176789372	176789596
chr1	176797421	176797587
chr1	176801087	176801254
chr1	176801558	176801745
chr1	176804533	176804824
chr1	176806870	176807192
chr1	176808231	176808490

chr1	176813339	176813569
chr1	176813569	176813895
chr1	176813895	176814234
chr1	176814234	176814471
chr1	176814530	176815052
chr1	176818127	176818513
chr1	176818662	176818877
chr1	176818877	176819108
chr1	176824036	176824186
chr1	176827279	176827529
chr1	176827529	176828034
chr1	176832123	176832334
chr1	176832555	176833075
chr1	176834390	176834540
chr1	176834767	176835229
chr1	176835526	176835832
chr1	176836101	176836284
chr1	176847120	176847416
chr1	176847517	176847918
chr1	176851459	176851701
chr1	176862230	176862482
chr1	176919551	176919829
chr1	177008705	177008975
chr1	177008975	177009250
chr1	177014146	177014350
chr1	177014350	177014532
chr1	177014532	177014721
chr1	177014721	177015037
chr1	177015037	177015395
chr1	177029061	177029325
chr1	177040437	177040768
chr1	177041949	177042117
chr1	177061745	177061960
chr1	177070599	177071225
chr1	177126045	177126302
chr1	177132895	177133146
chr1	177135382	177135623
chr1	177147894	177148044
chr1	177158537	177158918
chr1	177188799	177189093
chr1	177248757	177248947
chr1	177257184	177257710

chr1	177257714	177257969
chr1	177258015	177258291
chr1	177258291	177258520
chr1	177258520	177258763
chr1	177258763	177259221
chr1	177292707	177293385
chr1	177293385	177293616
chr1	177321793	177322266
chr1	177322266	177322558
chr1	177322728	177322938
chr1	177322938	177323192
chr1	177336461	177336807
chr1	177337206	177337458
chr1	177337580	177337823
chr1	177338724	177339136
chr1	177352937	177353116
chr1	177382927	177383078
chr1	177386038	177386275
chr1	177406872	177407090
chr1	177441469	177441767
chr1	177441767	177441996
chr1	177441996	177442395
chr1	177442523	177442769
chr1	177443233	177443499
chr1	177453895	177454280
chr1	177470081	177470779
chr1	177478983	177479349
chr1	177479349	177479907
chr1	177483859	177484009
chr1	177504064	177504214
chr1	177504332	177504665
chr1	177555477	177555801
chr1	177571021	177571315
chr1	177623222	177623811
chr1	177623941	177624091
chr1	177634037	177634192
chr1	177634231	177634553
chr1	177634553	177634783
chr1	177642679	177642950
chr1	177644321	177644539
chr1	177648646	177649562
chr1	177653318	177653552

chr1	177659981	177660260
chr1	177676607	177677298
chr1	177679676	177679845
chr1	177686213	177686486
chr1	177697054	177697289
chr1	177702518	177702728
chr1	177702908	177703393
chr1	177703393	177703870
chr1	177704315	177704476
chr1	177708549	177709138
chr1	177709901	177710206
chr1	177711360	177711731
chr1	177714174	177714453
chr1	177719017	177719671
chr1	177729218	177729467
chr1	177730246	177730477
chr1	177737664	177737943
chr1	177742076	177742461
chr1	177742461	177742701
chr1	177744672	177744993
chr1	177748075	177748731
chr1	177768380	177768551
chr1	177772415	177772664
chr1	177795888	177796442
chr1	177796442	177796951
chr1	177808610	177808805
chr1	177982998	177983285
chr1	177983285	177983616
chr1	177983616	177984154
chr1	178064821	178064972
chr1	178072877	178073081
chr1	178146593	178146749
chr1	178187249	178187714
chr1	178187714	178188387
chr1	178217199	178217581
chr1	178218270	178218864
chr1	178224824	178225153
chr1	178242136	178242415
chr1	178276055	178276331
chr1	178276331	178276593
chr1	178276593	178276831
chr1	178281706	178281899

chr1	178291521	178291780
chr1	178291780	178292066
chr1	178298062	178298261
chr1	178301267	178301603
chr1	178301819	178302111
chr1	178302445	178302626
chr1	178303494	178303699
chr1	178318126	178318371
chr1	178318371	178318634
chr1	178318634	178318944
chr1	178318944	178319365
chr1	178319396	178319635
chr1	178321431	178321583
chr1	178321948	178322339
chr1	178330130	178330379
chr1	178336296	178336720
chr1	178336720	178336917
chr1	178336917	178337204
chr1	178337204	178337588
chr1	178337588	178338078
chr1	178344063	178344421
chr1	178344421	178344744
chr1	178368701	178368915
chr1	178368915	178369263
chr1	178369341	178369623
chr1	178375435	178375655
chr1	178376165	178376420
chr1	178395553	178395791
chr1	178396411	178396744
chr1	178405373	178405888
chr1	178405918	178406283
chr1	178406283	178407064
chr1	178413470	178413826
chr1	178415161	178415426
chr1	178426851	178427048
chr1	178449195	178449513
chr1	178463588	178464105
chr1	178492185	178492415
chr1	178492497	178492841
chr1	178492841	178493113
chr1	178495479	178495700
chr1	178515940	178516134

chr1	178527175	178527418
chr1	178527418	178527626
chr1	178527626	178527834
chr1	178527870	178528039
chr1	178528039	178528449
chr1	178528451	178528810
chr1	178528810	178529108
chr1	178529108	178529312
chr1	178530336	178530575
chr1	178530575	178531003
chr1	178538959	178539234
chr1	178543085	178543471
chr1	178544604	178544971
chr1	178590310	178590486
chr1	178600852	178601211
chr1	178601211	178601474
chr1	178641144	178641480
chr1	178642903	178643230
chr1	178655653	178655921
chr1	178908651	178908836
chr1	178911082	178911329
chr1	178942707	178942983
chr1	178944266	178944457
chr1	178990210	178990503
chr1	178999963	179000128
chr1	179041823	179042370
chr1	179042370	179042840
chr1	179045919	179046233
chr1	179047975	179048256
chr1	179048256	179048604
chr1	179053954	179054332
chr1	179054420	179054742
chr1	179072427	179072711
chr1	179091331	179091635
chr1	179091649	179091804
chr1	179131183	179131725
chr1	179136540	179137363
chr1	179142225	179142513
chr1	179180681	179180946
chr1	179181481	179181954
chr1	179183053	179183205
chr1	179232083	179232346

chr1	179236566	179236814
chr1	179250485	179250698
chr1	179256561	179256713
chr1	179324207	179324680
chr1	179333822	179334056
chr1	179360641	179360835
chr1	179454691	179454963
chr1	179476592	179476799
chr1	179477138	179477618
chr1	179477618	179478018
chr1	179506505	179506889
chr1	179511727	179512380
chr1	179516881	179517274
chr1	179517274	179517662
chr1	179517662	179517907
chr1	179517907	179518193
chr1	179545852	179546142
chr1	179546142	179546485
chr1	179546485	179547003
chr1	179547312	179547502
chr1	179552832	179553046
chr1	179553635	179553857
chr1	179566326	179566758
chr1	179566876	179567226
chr1	179568930	179569085
chr1	179570514	179570905
chr1	179573680	179574174
chr1	179623737	179623987
chr1	179624414	179624696
chr1	179633420	179633679
chr1	179645169	179645441
chr1	179645441	179645713
chr1	179645713	179646171
chr1	179654163	179654391
chr1	179660788	179661009
chr1	179667928	179668447
chr1	179668447	179668755
chr1	179669046	179669205
chr1	179707588	179707813
chr1	179715969	179716119
chr1	179734492	179734778
chr1	179734924	179735112

chr1	179735207	179735359
chr1	179741475	179741639
chr1	179743109	179743330
chr1	179743330	179743684
chr1	179743684	179744203
chr1	179744303	179744489
chr1	179750431	179750627
chr1	179802905	179803188
chr1	179803188	179803500
chr1	179803500	179803780
chr1	179803780	179804083
chr1	179819720	179819922
chr1	179820021	179820213
chr1	179828823	179829328
chr1	179835938	179836109
chr1	179837096	179837393
chr1	179839674	179839915
chr1	179844901	179845389
chr1	179847764	179848508
chr1	179853502	179853718
chr1	179859107	179859260
chr1	179859294	179859535
chr1	179862861	179863300
chr1	179863300	179863837
chr1	179879681	179879846
chr1	179913165	179913816
chr1	179913902	179914061
chr1	179915015	179915170
chr1	179928917	179929165
chr1	179935384	179935621
chr1	179944417	179944612
chr1	179944786	179945090
chr1	179945090	179945426
chr1	179946659	179946972
chr1	179958771	179959035
chr1	179959059	179959266
chr1	179959362	179959728
chr1	179959728	179960128
chr1	179960128	179960389
chr1	179960389	179960627
chr1	179961442	179961660
chr1	179961868	179962124

chr1	179969809	179970114
chr1	179970114	179970462
chr1	179976684	179976956
chr1	179976956	179977199
chr1	179984024	179984880
chr1	179986234	179986444
chr1	179991810	179992153
chr1	180013512	180013762
chr1	180014908	180015390
chr1	180015390	180015825
chr1	180015882	180016056
chr1	180021439	180021648
chr1	180021714	180022019
chr1	180022024	180022174
chr1	180023837	180024110
chr1	180029722	180030209
chr1	180043154	180043358
chr1	180070079	180070259
chr1	180095725	180095935
chr1	180104611	180104926
chr1	180116603	180116822
chr1	180131533	180131683
chr1	180139166	180139558
chr1	180167069	180167275
chr1	180167777	180167941
chr1	180169532	180169786
chr1	180171473	180171623
chr1	180175287	180175462
chr1	180178361	180178539
chr1	180182024	180182178
chr1	180195243	180195471
chr1	180195664	180196332
chr1	180200897	180201065
chr1	180214753	180215066
chr1	180215066	180215413
chr1	180216546	180216696
chr1	180225170	180225495
chr1	180225884	180226174
chr1	180226250	180226627
chr1	180242428	180242678
chr1	180242858	180243158
chr1	180243582	180243909

chr1	180244267	180244454
chr1	180244567	180244784
chr1	180250081	180250408
chr1	180255583	180256061
chr1	180256061	180256683
chr1	180263487	180263657
chr1	180265501	180265699
chr1	180268451	180268601
chr1	180268631	180269107
chr1	180274711	180275113
chr1	180279607	180280148
chr1	180288861	180289194
chr1	180289194	180289493
chr1	180289493	180289769
chr1	180301036	180301342
chr1	180301342	180301638
chr1	180303623	180303900
chr1	180312788	180313349
chr1	180313583	180313808
chr1	180313852	180314150
chr1	180315233	180315408
chr1	180320718	180321094
chr1	180321094	180321491
chr1	180323406	180323668
chr1	180323668	180323878
chr1	180328839	180329029
chr1	180329029	180329229
chr1	180330323	180330657
chr1	180330657	180330906
chr1	180330906	180331139
chr1	180331139	180331567
chr1	180331574	180331791
chr1	180363946	180364402
chr1	180365124	180365478
chr1	180365478	180365743
chr1	180372270	180372558
chr1	180387185	180387480
chr1	180387480	180387886
chr1	180389401	180389662
chr1	180401712	180402086
chr1	180403615	180404170
chr1	180404185	180404363

chr1	180407149	180407380
chr1	180407437	180407661
chr1	180410399	180410720
chr1	180410720	180411001
chr1	180411159	180411479
chr1	180411479	180411677
chr1	180411764	180412426
chr1	180419728	180420167
chr1	180420911	180421143
chr1	180422189	180422439
chr1	180431961	180432111
chr1	180434203	180434502
chr1	180434985	180435237
chr1	180439250	180439404
chr1	180442805	180443098
chr1	180462532	180463004
chr1	180463004	180463447
chr1	180467993	180468377
chr1	180483344	180483600
chr1	180483968	180484318
chr1	180507266	180507515
chr1	180507538	180507882
chr1	180546024	180546698
chr1	180550906	180551071
chr1	180557082	180557232
chr1	180568578	180569042
chr1	180569042	180569484
chr1	180577870	180578221
chr1	180578767	180578952
chr1	180622074	180622322
chr1	180622385	180622730
chr1	180626021	180626533
chr1	180627732	180628106
chr1	180628106	180628421
chr1	180633398	180633599
chr1	180635448	180635756
chr1	180640559	180640890
chr1	180640890	180641188
chr1	180641188	180641451
chr1	180641451	180641798
chr1	180641798	180642189
chr1	180657831	180658022

chr1	180669957	180670121
chr1	180678954	180679166
chr1	180696564	180696818
chr1	180696827	180697012
chr1	180707449	180707755
chr1	180707790	180708190
chr1	180715293	180715459
chr1	180719326	180719530
chr1	180720324	180720645
chr1	180725653	180726181
chr1	180726181	180726611
chr1	180757919	180758177
chr1	180763824	180764216
chr1	180768279	180768526
chr1	180768526	180768840
chr1	180777576	180778050
chr1	180778050	180778673
chr1	180783239	180783604
chr1	180783604	180783908
chr1	180790428	180790588
chr1	180790610	180790901
chr1	180790903	180791079
chr1	180792099	180792408
chr1	180792877	180793152
chr1	180793730	180793880
chr1	180811726	180811940
chr1	180812491	180812660
chr1	180812969	180813162
chr1	180813162	180813435
chr1	180813435	180813696
chr1	180813696	180813936
chr1	180813936	180814162
chr1	180814162	180814492
chr1	180829881	180830107
chr1	180838159	180838424
chr1	180839172	180839559
chr1	180839559	180839887
chr1	180841631	180841836
chr1	180848116	180848421
chr1	180848558	180848905
chr1	180849046	180849196
chr1	180850448	180851020

chr1	180851020	180851294
chr1	180858686	180859084
chr1	180863730	180863889
chr1	180872496	180872716
chr1	180873860	180874436
chr1	180888350	180888593
chr1	180888703	180888854
chr1	180889934	180890124
chr1	180903124	180903288
chr1	180903801	180903984
chr1	180904055	180904370
chr1	180904370	180904683
chr1	180904683	180904977
chr1	180913824	180914346
chr1	180915490	180915694
chr1	180917073	180917427
chr1	180930887	180931191
chr1	180931191	180931514
chr1	180936886	180937038
chr1	180940910	180941112
chr1	180942122	180942369
chr1	180942369	180942598
chr1	180942598	180942831
chr1	180942831	180943264
chr1	180943904	180944221
chr1	180944588	180944919
chr1	180952098	180952454
chr1	180952454	180952828
chr1	180952868	180953137
chr1	180953744	180953906
chr1	180958104	180958411
chr1	180966504	180966998
chr1	180968734	180968976
chr1	180969942	180970161
chr1	180972172	180972524
chr1	180974266	180974480
chr1	180974480	180974738
chr1	180976394	180976617
chr1	180980527	180980794
chr1	180981283	180981650
chr1	180981773	180981984
chr1	180987613	180987783

chr1	180987831	180988126
chr1	180991418	180991706
chr1	180991969	180992186
chr1	180992273	180992481
chr1	181009677	181010213
chr1	181010213	181010561
chr1	181016853	181017128
chr1	181017220	181017437
chr1	181017437	181017709
chr1	181020805	181021127
chr1	181022278	181022618
chr1	181035058	181035282
chr1	181035436	181035772
chr1	181039660	181039886
chr1	181045478	181045922
chr1	181051092	181051247
chr1	181125956	181126297
chr1	181126313	181126463
chr1	181126793	181127053
chr1	181127145	181127427
chr1	181131919	181132464
chr1	181143653	181143865
chr1	181143865	181144134
chr1	181144134	181144554
chr1	181144572	181144754
chr1	181150751	181151051
chr1	181151051	181151271
chr1	181151271	181151523
chr1	181172528	181172788
chr1	181203151	181203482
chr1	181203518	181203702
chr1	181210586	181210857
chr1	181210964	181211152
chr1	181211152	181211335
chr1	181211335	181211726
chr1	181211726	181212170
chr1	181212170	181212670
chr1	181213180	181213459
chr1	181230634	181231093
chr1	181231093	181231302
chr1	181231302	181231623
chr1	181232298	181232603

chr1	181232696	181232933
chr1	181244100	181244474
chr1	181246867	181247106
chr1	181247265	181247627
chr1	181255605	181255917
chr1	181255917	181256181
chr1	181256232	181256462
chr1	181256462	181256667
chr1	181256667	181257396
chr1	181272781	181273368
chr1	181284442	181284600
chr1	181310725	181310935
chr1	181331995	181332169
chr1	181332686	181332961
chr1	181345699	181345933
chr1	181352228	181352829
chr1	181352829	181353229
chr1	181353601	181353898
chr1	181355557	181355768
chr1	181355772	181356078
chr1	181371287	181371620
chr1	181376715	181376900
chr1	181379719	181380111
chr1	181393308	181393531
chr1	181393740	181394160
chr1	181394936	181395146
chr1	181395199	181395500
chr1	181395500	181395763
chr1	181401975	181402187
chr1	181415062	181415336
chr1	181415336	181415758
chr1	181418029	181418205
chr1	181425234	181426000
chr1	181441941	181442141
chr1	181446739	181447148
chr1	181465096	181465305
chr1	181472915	181473104
chr1	181511418	181511592
chr1	181521136	181521296
chr1	181521336	181521691
chr1	181521691	181522067
chr1	181529481	181529645

chr1	181535588	181535878
chr1	181536016	181536166
chr1	181540552	181540766
chr1	181543731	181544022
chr1	181545379	181545747
chr1	181552464	181552804
chr1	181552804	181553142
chr1	181553142	181553473
chr1	181564662	181564844
chr1	181611327	181611502
chr1	181615978	181616222
chr1	181616222	181616503
chr1	181617440	181617590
chr1	181623114	181623621
chr1	181623621	181623918
chr1	181627279	181627438
chr1	181638392	181638636
chr1	181660368	181660547
chr1	181689280	181689445
chr1	181691781	181691968
chr1	181693963	181694214
chr1	181694214	181694499
chr1	181718008	181718158
chr1	181733525	181733823
chr1	181742388	181742580
chr1	181742580	181742901
chr1	181742925	181743313
chr1	181743313	181743596
chr1	181743596	181743873
chr1	181753145	181753429
chr1	181758842	181759010
chr1	181834560	181834766
chr1	181841637	181842212
chr1	181842212	181842797
chr1	181842935	181843152
chr1	181847990	181848240
chr1	181848240	181848573
chr1	181853062	181853395
chr1	181853395	181853841
chr1	181860467	181861210
chr1	181866392	181866578
chr1	181866874	181867026

chr1	181868275	181868463
chr1	181877135	181877277
chr1	181877277	181877530
chr1	181879935	181880085
chr1	181880980	181881237
chr1	181881495	181881981
chr1	181888816	181889331
chr1	181911009	181911195
chr1	181911411	181911731
chr1	181911824	181912381
chr1	181914266	181914450
chr1	181921587	181921745
chr1	181921876	181922042
chr1	181984725	181985139
chr1	181985268	181985576
chr1	181985951	181986105
chr1	181997230	181997470
chr1	182015699	182015970
chr1	182018891	182019051
chr1	182019188	182019346
chr1	182019379	182019600
chr1	182019600	182019838
chr1	182019838	182020107
chr1	182020107	182020399
chr1	182020399	182020667
chr1	182021679	182022000
chr1	182026226	182026505
chr1	182028565	182028715
chr1	182057900	182058332
chr1	182058332	182058645
chr1	182059928	182060087
chr1	182071972	182072188
chr1	182072188	182072426
chr1	182088271	182088565
chr1	182091282	182091652
chr1	182091746	182092298
chr1	182095251	182095441
chr1	182095441	182095717
chr1	182110085	182110316
chr1	182115250	182115562
chr1	182115604	182116036
chr1	182124496	182124987

chr1	182124987	182125388
chr1	182140061	182140261
chr1	182140396	182140726
chr1	182143452	182143602
chr1	182154366	182154521
chr1	182238189	182238572
chr1	182254752	182254963
chr1	182255341	182255746
chr1	182255909	182256118
chr1	182256118	182256361
chr1	182256361	182256671
chr1	182256683	182256844
chr1	182265474	182265754
chr1	182282028	182282405
chr1	182282405	182282662
chr1	182282662	182283019
chr1	182302602	182302911
chr1	182320050	182320200
chr1	182340894	182341426
chr1	182341426	182341833
chr1	182358210	182358509
chr1	182361830	182362241
chr1	182368311	182368760
chr1	182368785	182369138
chr1	182369511	182369805
chr1	182369818	182370202
chr1	182371844	182372223
chr1	182376244	182376514
chr1	182383084	182383483
chr1	182408606	182408871
chr1	182408871	182409069
chr1	182409069	182409354
chr1	182409354	182409668
chr1	182432350	182432587
chr1	182439110	182439324
chr1	182460099	182460311
chr1	182461367	182461614
chr1	182465384	182465641
chr1	182465989	182466451
chr1	182478970	182479135
chr1	182479135	182479332
chr1	182481764	182481931

chr1	182489776	182490104
chr1	182490104	182490525
chr1	182490648	182490871
chr1	182496655	182497133
chr1	182499496	182499827
chr1	182503592	182503824
chr1	182504251	182504618
chr1	182505019	182505169
chr1	182506304	182506592
chr1	182506661	182506952
chr1	182509442	182509778
chr1	182511137	182511294
chr1	182511780	182512113
chr1	182514963	182515128
chr1	182515189	182515540
chr1	182515633	182515783
chr1	182516770	182517246
chr1	182517246	182517658
chr1	182517658	182517930
chr1	182520217	182520607
chr1	182522197	182522461
chr1	182524376	182524670
chr1	182527879	182528285
chr1	182528285	182528581
chr1	182528581	182528857
chr1	182534972	182535257
chr1	182535257	182535516
chr1	182539392	182539629
chr1	182540270	182540528
chr1	182543117	182543646
chr1	182554035	182554396
chr1	182559899	182560109
chr1	182560381	182560720
chr1	182560720	182561075
chr1	182561634	182561866
chr1	182564535	182564702
chr1	182564756	182565079
chr1	182571224	182571509
chr1	182574672	182574951
chr1	182582751	182582993
chr1	182592166	182592529
chr1	182592529	182592783

chr1	182592783	182593210
chr1	182629653	182629976
chr1	182639183	182639826
chr1	182645503	182645665
chr1	182657846	182658075
chr1	182669889	182670053
chr1	182670406	182670900
chr1	182670900	182671165
chr1	182671191	182671515
chr1	182678045	182678311
chr1	182678839	182679430
chr1	182679615	182680038
chr1	182680038	182680437
chr1	182686854	182687151
chr1	182687151	182687526
chr1	182704910	182705186
chr1	182705186	182705410
chr1	182705410	182705637
chr1	182725565	182725818
chr1	182726905	182727186
chr1	182728591	182728941
chr1	182729678	182730014
chr1	182735168	182735528
chr1	182763629	182764096
chr1	182764096	182764542
chr1	182764646	182764969
chr1	182767525	182767797
chr1	182792560	182792718
chr1	182794294	182794504
chr1	182830095	182830288
chr1	182839345	182839500
chr1	182839635	182839894
chr1	182878744	182879010
chr1	182882815	182882999
chr1	182908205	182908755
chr1	182931030	182931204
chr1	182944048	182944251
chr1	182944485	182944977
chr1	182944977	182945245
chr1	182951203	182951694
chr1	182956249	182956653
chr1	182956665	182956897

chr1	182957265	182957670
chr1	182957670	182957857
chr1	182959875	182960075
chr1	182960180	182960412
chr1	182982966	182983379
chr1	182983604	182983900
chr1	182989973	182990125
chr1	182990947	182991419
chr1	183052643	183053236
chr1	183058490	183059066
chr1	183082475	183082817
chr1	183103162	183103548
chr1	183103620	183103936
chr1	183113453	183113768
chr1	183113768	183114174
chr1	183115176	183115404
chr1	183123523	183124126
chr1	183129209	183129451
chr1	183132398	183132548
chr1	183133447	183133631
chr1	183138658	183138808
chr1	183144494	183144872
chr1	183144872	183145116
chr1	183147429	183147657
chr1	183152237	183152442
chr1	183215790	183215948
chr1	183219054	183219410
chr1	183220599	183220953
chr1	183221139	183221412
chr1	183221412	183221683
chr1	183227422	183228042
chr1	183273263	183273450
chr1	183296477	183296683
chr1	183296683	183296908
chr1	183296908	183297169
chr1	183297169	183297446
chr1	183297446	183297857
chr1	183298204	183298448
chr1	183299090	183299631
chr1	183315880	183316085
chr1	183320835	183321132
chr1	183344793	183345089

chr1	183345089	183345349
chr1	183345349	183345641
chr1	183345641	183345929
chr1	183349868	183350194
chr1	183351107	183351402
chr1	183368867	183369131
chr1	183369131	183369489
chr1	183369489	183369958
chr1	183381874	183382141
chr1	183383501	183383727
chr1	183388697	183389043
chr1	183389043	183389316
chr1	183408039	183408210
chr1	183414151	183414355
chr1	183419652	183419808
chr1	183434835	183435350
chr1	183441885	183442447
chr1	183442475	183442668
chr1	183516243	183516448
chr1	183548578	183548806
chr1	183573468	183574110
chr1	183574237	183574583
chr1	183584357	183584692
chr1	183584867	183585155
chr1	183585434	183585873
chr1	183600423	183601017
chr1	183607321	183607665
chr1	183619467	183619669
chr1	183634282	183634474
chr1	183634946	183635165
chr1	183658383	183659076
chr1	183668972	183669220
chr1	183669750	183670009
chr1	183671541	183672077
chr1	183674387	183674684
chr1	183675015	183675196
chr1	183677028	183677188
chr1	183679804	183680391
chr1	183680391	183680735
chr1	183685447	183685795
chr1	183702663	183702912
chr1	183702912	183703291

chr1	183744262	183744462
chr1	183761647	183762004
chr1	183764209	183764496
chr1	183764496	183764849
chr1	183766857	183767073
chr1	183778290	183778647
chr1	183778647	183778921
chr1	183778921	183779281
chr1	183788147	183788342
chr1	183788514	183789079
chr1	183789079	183789293
chr1	183790666	183790857
chr1	183803363	183803781
chr1	183803781	183804210
chr1	183804275	183804633
chr1	183841581	183841824
chr1	183864867	183865475
chr1	183865475	183865999
chr1	183869038	183869300
chr1	183880792	183881389
chr1	183905129	183905692
chr1	183905692	183906030
chr1	183913961	183914291
chr1	183921098	183921673
chr1	183921706	183921959
chr1	183930327	183930820
chr1	183931409	183931612
chr1	183933878	183934263
chr1	183942321	183942633
chr1	183944856	183945298
chr1	183947082	183947469
chr1	183949290	183949464
chr1	183963792	183964020
chr1	183964020	183964534
chr1	183993867	183994141
chr1	183999265	183999495
chr1	184014721	184014915
chr1	184016381	184016707
chr1	184018861	184019020
chr1	184033391	184034099
chr1	184034099	184034737
chr1	184041729	184042247

chr1	184042247	184042509
chr1	184042509	184042667
chr1	184042788	184043081
chr1	184051283	184051662
chr1	184051662	184052296
chr1	184052319	184052592
chr1	184057306	184057831
chr1	184060486	184060764
chr1	184060764	184060977
chr1	184060977	184061169
chr1	184061407	184061693
chr1	184061693	184061962
chr1	184065961	184066452
chr1	184067003	184067229
chr1	184068326	184068526
chr1	184070443	184070766
chr1	184076110	184076328
chr1	184076515	184077273
chr1	184081821	184082031
chr1	184083238	184083518
chr1	184084617	184084931
chr1	184084931	184085341
chr1	184106951	184107242
chr1	184127424	184127682
chr1	184128529	184128683
chr1	184132421	184132651
chr1	184138690	184139105
chr1	184140270	184140462
chr1	184140483	184141023
chr1	184166573	184167355
chr1	184171624	184171810
chr1	184235691	184235920
chr1	184260807	184261126
chr1	184261126	184261751
chr1	184261788	184262094
chr1	184271302	184271553
chr1	184275542	184276369
chr1	184276957	184277412
chr1	184289794	184289945
chr1	184291246	184291449
chr1	184297477	184297694
chr1	184322228	184322471

chr1	184353930	184354090
chr1	184358038	184358247
chr1	184363454	184363618
chr1	184389038	184389377
chr1	184395369	184395536
chr1	184414096	184414312
chr1	184416744	184416939
chr1	184431238	184431621
chr1	184435511	184435836
chr1	184440961	184441278
chr1	184441278	184441645
chr1	184451713	184452083
chr1	184455603	184455808
chr1	184455848	184456146
chr1	184456715	184457232
chr1	184471512	184471667
chr1	184478144	184478308
chr1	184482177	184482371
chr1	184530552	184531110
chr1	184534199	184534415
chr1	184534505	184534804
chr1	184534804	184535044
chr1	184546008	184546321
chr1	184546321	184546676
chr1	184576603	184576833
chr1	184612704	184613218
chr1	184628318	184628516
chr1	184628726	184629004
chr1	184629252	184629427
chr1	184650852	184651006
chr1	184657367	184657673
chr1	184663321	184663567
chr1	184674957	184675204
chr1	184675373	184676295
chr1	184687572	184687722
chr1	184692780	184692995
chr1	184695422	184696027
chr1	184704706	184704994
chr1	184704994	184705282
chr1	184707427	184708066
chr1	184713873	184714063
chr1	184731678	184732097

chr1	184732097	184732510
chr1	184732563	184732829
chr1	184732829	184733186
chr1	184737927	184738194
chr1	184741463	184741707
chr1	184745046	184745270
chr1	184762036	184762294
chr1	184771629	184771920
chr1	184775918	184776180
chr1	184777070	184777479
chr1	184778597	184778821
chr1	184782128	184782561
chr1	184787823	184788012
chr1	184791174	184791423
chr1	184792054	184792360
chr1	184793201	184793410
chr1	184814354	184814751
chr1	184814759	184814951
chr1	184814999	184815213
chr1	184815213	184815427
chr1	184815427	184815629
chr1	184815629	184815746
chr1	184824237	184824509
chr1	184824509	184825026
chr1	184825182	184825426
chr1	184829818	184830085
chr1	184844749	184844940
chr1	184845221	184845709
chr1	184845709	184846083
chr1	184846083	184846416
chr1	184852193	184852703
chr1	184853307	184853819
chr1	184858265	184858645
chr1	184861336	184861848
chr1	184861848	184862139
chr1	184877434	184877751
chr1	184882320	184882702
chr1	184882702	184882934
chr1	184882934	184883321
chr1	184889478	184889639
chr1	184889684	184890067
chr1	184908679	184908830

chr1	184911650	184911920
chr1	184911920	184912349
chr1	184912366	184912699
chr1	184937280	184937453
chr1	184963268	184963542
chr1	184963558	184963708
chr1	184966043	184966256
chr1	184999305	184999455
chr1	184999595	184999827
chr1	185038156	185038413
chr1	185039259	185039409
chr1	185052761	185052921
chr1	185096166	185096363
chr1	185121067	185121217
chr1	185121303	185121572
chr1	185121572	185121903
chr1	185145379	185145616
chr1	185193477	185193915
chr1	185196891	185197469
chr1	185203149	185203576
chr1	185203718	185204044
chr1	185204044	185204306
chr1	185204306	185204621
chr1	185205132	185205379
chr1	185209683	185210175
chr1	185214756	185214925
chr1	185222204	185222413
chr1	185224715	185225017
chr1	185229768	185230360
chr1	185236643	185236836
chr1	185262597	185262837
chr1	185263596	185264218
chr1	185274892	185275089
chr1	185279801	185280008
chr1	185282704	185282866
chr1	185282870	185283233
chr1	185289302	185289468
chr1	185297760	185297946
chr1	185318958	185319392
chr1	185319392	185319736
chr1	185319736	185320016
chr1	185328602	185328849

chr1	185328849	185329066
chr1	185329066	185329264
chr1	185329264	185329564
chr1	185331953	185332465
chr1	185343503	185343746
chr1	185346276	185346521
chr1	185362629	185363343
chr1	185363343	185363885
chr1	185376201	185376795
chr1	185377475	185377777
chr1	185455080	185455214
chr1	185455214	185455302
chr1	185596778	185597055
chr1	185628318	185628784
chr1	185681978	185682137
chr1	185887041	185887206
chr1	185989489	185989660
chr1	186008033	186008217
chr1	186013168	186013326
chr1	186015832	186016116
chr1	186016116	186016358
chr1	186043520	186043906
chr1	186084890	186085113
chr1	186116704	186117083
chr1	186117083	186117493
chr1	186119522	186119721
chr1	186182726	186182929
chr1	186254567	186254800
chr1	186289148	186289571
chr1	186294615	186295100
chr1	186307720	186307981
chr1	186393062	186393363
chr1	186444742	186445162
chr1	186447699	186447981
chr1	186448774	186449054
chr1	186449054	186449259
chr1	186449291	186449650
chr1	186454736	186455027
chr1	186460763	186461230
chr1	186461491	186461659
chr1	186461863	186462143
chr1	186478310	186478610

chr1	186478610	186478912
chr1	186493688	186493908
chr1	186494508	186494704
chr1	186515973	186516331
chr1	186516331	186516724
chr1	186530217	186530528
chr1	186567919	186568631
chr1	186568658	186568902
chr1	186580199	186580359
chr1	186581041	186581708
chr1	186581708	186582316
chr1	186584034	186584277
chr1	186586815	186587702
chr1	186604158	186604524
chr1	186609790	186610371
chr1	186610371	186610699
chr1	186656286	186656637
chr1	186671867	186672088
chr1	186674271	186674507
chr1	186674715	186674916
chr1	186675814	186676054
chr1	186676054	186676283
chr1	186676283	186676592
chr1	186704472	186704747
chr1	186705092	186705418
chr1	186705418	186705812
chr1	186706042	186706228
chr1	186706737	186706941
chr1	186718994	186719144
chr1	186721960	186722286
chr1	186722468	186722667
chr1	186729881	186730269
chr1	186730269	186730792
chr1	186730792	186731176
chr1	186731176	186731454
chr1	186731454	186731886
chr1	186731886	186732458
chr1	186732458	186732816
chr1	186732816	186732930
chr1	186732930	186733140
chr1	186748813	186748965
chr1	186748972	186749258

chr1	186749258	186749679
chr1	186749753	186749967
chr1	186834403	186834622
chr1	186926262	186926584
chr1	186927644	186927828
chr1	186930440	186930899
chr1	186934326	186934637
chr1	186943787	186944041
chr1	186952311	186952533
chr1	186959465	186959625
chr1	187012559	187012836
chr1	187016121	187016444
chr1	187020026	187020369
chr1	187031837	187032094
chr1	187053320	187053683
chr1	187076211	187076515
chr1	187076625	187076786
chr1	187076942	187077243
chr1	187078984	187079149
chr1	187098278	187098640
chr1	187100756	187100960
chr1	187114526	187115091
chr1	187121555	187121822
chr1	187121863	187122028
chr1	187133867	187134171
chr1	187134201	187134558
chr1	187134558	187134833
chr1	187134833	187135228
chr1	187137510	187137725
chr1	187155752	187155916
chr1	187171458	187171872
chr1	187171872	187172284
chr1	187187223	187187418
chr1	187198829	187199036
chr1	187199040	187199766
chr1	187215140	187215703
chr1	187215703	187216029
chr1	187226478	187226725
chr1	187226725	187226970
chr1	187228481	187228781
chr1	187228781	187229108
chr1	187229148	187229305

chr1	187235192	187235479
chr1	187236332	187236635
chr1	187236683	187237068
chr1	187270317	187270662
chr1	187276138	187276427
chr1	187293714	187293892
chr1	187294542	187294830
chr1	187295125	187295405
chr1	187303367	187303604
chr1	187316601	187316826
chr1	187353097	187353278
chr1	187353278	187353521
chr1	187353634	187353812
chr1	187354485	187354708
chr1	187354708	187354935
chr1	187355310	187355473
chr1	187355763	187355927
chr1	187365428	187365756
chr1	187374532	187374872
chr1	187740656	187740850
chr1	187799330	187799641
chr1	187800691	187801085
chr1	187910724	187911136
chr1	187997631	187997872
chr1	188871687	188871893
chr1	188896058	188896340
chr1	188980021	188980532
chr1	188987646	188987808
chr1	188988920	188989139
chr1	188989565	188989759
chr1	188993246	188993557
chr1	189006242	189006606
chr1	189006938	189007237
chr1	189007237	189007648
chr1	189007648	189008042
chr1	189013207	189013535
chr1	189027077	189027242
chr1	189028361	189028555
chr1	189037966	189038126
chr1	189047281	189047520
chr1	189054989	189055183
chr1	189068849	189069106

chr1	189080836	189081157
chr1	189106406	189106605
chr1	189119917	189120209
chr1	189159582	189159774
chr1	189194367	189195130
chr1	189201780	189201942
chr1	189214195	189214400
chr1	189214977	189215199
chr1	189215199	189215472
chr1	189215472	189215760
chr1	189216517	189216669
chr1	189227599	189227810
chr1	189243364	189243647
chr1	189257157	189257372
chr1	189353980	189354230
chr1	189566514	189566873
chr1	189579466	189579673
chr1	189615321	189615618
chr1	189628479	189628727
chr1	189662019	189662169
chr1	189667441	189667699
chr1	189675600	189675797
chr1	189687770	189688019
chr1	189688019	189688635
chr1	189698483	189698747
chr1	189701053	189701349
chr1	189703180	189703456
chr1	189705350	189705640
chr1	189716044	189716224
chr1	189716320	189716859
chr1	189719606	189719830
chr1	189727288	189727761
chr1	189727761	189728820
chr1	189736391	189736544
chr1	189742066	189742280
chr1	189749961	189750163
chr1	189761484	189762157
chr1	189767154	189767450
chr1	189773206	189773516
chr1	189773516	189773860
chr1	189777195	189777613
chr1	189778299	189778729

chr1	189784446	189784901
chr1	189784901	189785251
chr1	189785251	189785407
chr1	189792034	189792286
chr1	189792286	189792580
chr1	189792580	189792852
chr1	189792852	189793120
chr1	189800258	189800514
chr1	189811749	189812345
chr1	189812697	189812936
chr1	189813120	189813270
chr1	189838042	189838368
chr1	189860083	189860853
chr1	189862036	189862250
chr1	189867094	189867467
chr1	189867467	189867897
chr1	189868024	189868466
chr1	189874986	189875381
chr1	189875381	189875648
chr1	189875668	189875935
chr1	189883038	189883243
chr1	189883846	189884181
chr1	189887564	189887845
chr1	189888767	189889129
chr1	189895571	189895732
chr1	189898786	189898947
chr1	189905822	189906039
chr1	189906039	189906270
chr1	189908047	189908239
chr1	189908239	189908446
chr1	189911071	189911259
chr1	189921467	189921626
chr1	189921740	189922155
chr1	189922155	189922699
chr1	189927705	189927996
chr1	189936519	189936789
chr1	189936789	189937139
chr1	189959728	189960182
chr1	189968144	189968398
chr1	189972855	189973090
chr1	189973090	189973402
chr1	190008829	190009028

chr1	190039393	190039671
chr1	190125449	190125630
chr1	190170190	190170350
chr1	190170391	190170833
chr1	190300381	190300681
chr1	190301160	190301333
chr1	190301409	190301645
chr1	190301744	190302057
chr1	190302127	190302465
chr1	190302465	190302840
chr1	190303136	190303394
chr1	190471834	190472052
chr1	190542050	190542282
chr1	190595931	190596339
chr1	190596339	190596612
chr1	190596612	190596859
chr1	190695722	190695874
chr1	190710333	190710525
chr1	190745428	190745643
chr1	190756014	190756320
chr1	190756350	190756610
chr1	190756610	190756840
chr1	190770498	190770835
chr1	190776793	190776943
chr1	190780271	190780506
chr1	190780513	190780740
chr1	190780740	190781040
chr1	190781042	190781276
chr1	190781415	190781629
chr1	190790268	190790444
chr1	190797328	190797480
chr1	190806792	190806942
chr1	190830412	190830670
chr1	190830670	190830944
chr1	190831869	190832126
chr1	190834675	190834866
chr1	190871107	190871264
chr1	190880760	190880919
chr1	190881510	190881740
chr1	190882926	190883125
chr1	190899247	190899485
chr1	190899846	190900200

chr1	190901166	190901405
chr1	190902641	190902968
chr1	190902968	190903276
chr1	190911136	190911310
chr1	190911369	190911688
chr1	190911688	190912288
chr1	190927816	190928006
chr1	190928341	190928649
chr1	190928649	190928980
chr1	190928980	190929302
chr1	190934296	190934473
chr1	190934540	190935226
chr1	190944191	190944384
chr1	190944397	190944724
chr1	190978151	190978312
chr1	190978476	190978677
chr1	190978677	190978849
chr1	190978849	190979043
chr1	190979046	190979662
chr1	190989764	190989969
chr1	190998078	190998611
chr1	191025136	191025299
chr1	191025302	191025676
chr1	191025676	191025992
chr1	191025992	191026409
chr1	191026409	191026740
chr1	191031022	191031409
chr1	191035645	191036039
chr1	191050692	191050916
chr1	191050916	191051167
chr1	191051238	191051581
chr1	191051694	191052122
chr1	191052122	191052420
chr1	191054924	191055115
chr1	191055251	191055533
chr1	191055533	191055776
chr1	191057543	191057764
chr1	191062595	191062868
chr1	191062868	191063214
chr1	191063214	191063515
chr1	191065335	191065521
chr1	191068502	191068698

chr1	191072451	191072877
chr1	191075274	191075650
chr1	191075650	191076187
chr1	191085024	191085266
chr1	191085804	191086018
chr1	191093016	191093185
chr1	191093261	191093450
chr1	191095119	191095285
chr1	191097199	191097717
chr1	191097717	191098165
chr1	191098165	191098858
chr1	191107039	191107248
chr1	191120489	191120683
chr1	191120863	191121169
chr1	191123902	191124104
chr1	191124203	191124428
chr1	191125853	191126273
chr1	191126273	191126558
chr1	191131838	191131990
chr1	191132473	191132728
chr1	191132843	191133395
chr1	191134993	191135192
chr1	191135192	191135392
chr1	191135470	191135852
chr1	191141002	191141371
chr1	191152010	191152232
chr1	191152232	191152382
chr1	191152461	191152756
chr1	191152920	191153411
chr1	191166338	191166778
chr1	191166822	191166973
chr1	191167154	191167333
chr1	191167333	191167648
chr1	191167667	191167822
chr1	191167957	191168184
chr1	191168214	191168396
chr1	191168396	191168576
chr1	191168694	191168988
chr1	191169520	191169918
chr1	191170088	191170291
chr1	191172613	191172773
chr1	191173460	191173640

chr1	191173868	191174133
chr1	191174958	191175117
chr1	191175117	191175271
chr1	191175582	191175902
chr1	191182402	191182653
chr1	191182950	191183381
chr1	191183381	191183983
chr1	191184886	191185246
chr1	191186612	191186764
chr1	191189902	191190235
chr1	191193269	191193692
chr1	191193692	191194114
chr1	191194114	191194388
chr1	191200110	191200330
chr1	191202194	191202444
chr1	191204507	191204834
chr1	191204834	191205385
chr1	191213591	191213767
chr1	191215833	191216120
chr1	191216120	191216447
chr1	191216447	191216880
chr1	191221883	191222453
chr1	191224073	191224280
chr1	191224280	191224620
chr1	191224775	191225085
chr1	191232838	191233233
chr1	191233233	191233520
chr1	191254472	191254798
chr1	191265145	191265297
chr1	191265626	191265815
chr1	191267937	191268266
chr1	191275593	191276055
chr1	191277495	191277887
chr1	191277887	191278193
chr1	191278214	191278483
chr1	191281140	191281580
chr1	191288798	191289063
chr1	191292293	191292940
chr1	191292987	191293559
chr1	191299033	191299543
chr1	191299625	191299950
chr1	191305677	191305932

chr1	191305932	191306139
chr1	191306410	191306624
chr1	191312448	191313009
chr1	191313285	191313453
chr1	191317603	191317985
chr1	191317985	191318299
chr1	191325721	191326171
chr1	191326171	191326598
chr1	191326598	191326885
chr1	191346302	191346476
chr1	191350530	191350680
chr1	191352127	191352382
chr1	191352500	191352794
chr1	191352918	191353076
chr1	191353133	191353338
chr1	191364574	191364839
chr1	191368337	191368489
chr1	191391391	191391671
chr1	191391978	191392180
chr1	191396498	191396994
chr1	191396994	191397457
chr1	191397457	191397883
chr1	191404807	191405017
chr1	191414396	191414763
chr1	191416619	191416943
chr1	191417098	191417432
chr1	191417432	191417630
chr1	191419631	191419831
chr1	191425956	191426191
chr1	191432664	191432953
chr1	191435408	191435734
chr1	191438587	191438877
chr1	191442374	191442703
chr1	191444554	191444803
chr1	191445897	191446244
chr1	191452993	191453214
chr1	191454428	191454581
chr1	191454636	191454921
chr1	191468970	191469125
chr1	191470388	191470657
chr1	191470657	191471011
chr1	191472986	191473204

chr1	191479920	191480115
chr1	191484599	191485262
chr1	191497807	191498163
chr1	191498163	191498695
chr1	191498695	191499086
chr1	191513297	191513461
chr1	191526822	191527141
chr1	191527141	191527628
chr1	191528395	191528649
chr1	191529565	191529832
chr1	191556958	191557220
chr1	191557220	191557530
chr1	191559901	191560055
chr1	191562944	191563150
chr1	191567987	191568161
chr1	191574838	191575197
chr1	191575197	191575461
chr1	191575461	191575848
chr1	191575848	191576242
chr1	191584551	191584773
chr1	191592048	191592387
chr1	191599350	191599547
chr1	191609057	191609261
chr1	191621110	191621355
chr1	191622798	191623167
chr1	191624333	191624532
chr1	191628475	191628771
chr1	191632558	191633016
chr1	191640281	191640680
chr1	191641585	191641828
chr1	191645986	191646145
chr1	191662014	191662264
chr1	191662264	191662558
chr1	191662995	191663183
chr1	191668151	191668411
chr1	191717681	191718114
chr1	191718114	191718483
chr1	191718483	191718825
chr1	191718864	191719270
chr1	191732577	191733210
chr1	191756327	191756485
chr1	191768459	191768699

chr1	191773921	191774208
chr1	191774242	191774420
chr1	191784914	191785516
chr1	191793055	191793316
chr1	191793316	191793565
chr1	191793733	191794274
chr1	191801759	191802037
chr1	191802086	191802428
chr1	191802428	191802833
chr1	191802833	191803153
chr1	191803153	191803405
chr1	191803405	191803725
chr1	191817836	191818170
chr1	191818204	191818354
chr1	191821216	191821708
chr1	191821708	191822088
chr1	191829153	191829401
chr1	191834113	191834341
chr1	191834850	191835305
chr1	191837719	191837869
chr1	191841011	191841232
chr1	191841248	191841415
chr1	191854721	191854902
chr1	191855547	191855802
chr1	191859485	191859662
chr1	191868426	191868751
chr1	191875838	191876043
chr1	191876859	191877127
chr1	191881077	191881260
chr1	191894558	191894761
chr1	191906000	191906459
chr1	191906459	191906812
chr1	191906812	191907190
chr1	191907190	191907502
chr1	191907502	191907815
chr1	191913797	191913947
chr1	191914482	191915069
chr1	191915069	191915296
chr1	191917182	191917423
chr1	191918285	191918548
chr1	191924541	191924990
chr1	191931549	191931701

chr1	191933736	191933973
chr1	191942006	191942247
chr1	191942699	191942854
chr1	191950745	191950984
chr1	191963481	191963715
chr1	191964255	191964748
chr1	191964750	191964933
chr1	191964933	191965128
chr1	191965128	191965398
chr1	191977230	191977488
chr1	191989135	191989303
chr1	191989888	191990404
chr1	191995564	191995797
chr1	191999010	191999359
chr1	192061580	192061982
chr1	192061982	192062236
chr1	192067017	192067257
chr1	192068300	192068466
chr1	192076866	192077057
chr1	192077198	192077686
chr1	192092096	192092836
chr1	192092950	192093235
chr1	192094518	192094755
chr1	192107419	192107655
chr1	192116320	192116590
chr1	192136510	192136767
chr1	192136767	192137082
chr1	192137082	192137441
chr1	192137490	192137867
chr1	192137867	192138664
chr1	192138690	192138873
chr1	192154406	192154677
chr1	192188019	192188451
chr1	192189174	192189351
chr1	192190531	192191051
chr1	192266926	192267203
chr1	192307127	192307398
chr1	192315726	192315995
chr1	192316793	192317067
chr1	192366151	192366577
chr1	192406491	192406652
chr1	192418823	192418973

chr1	192419238	192419518
chr1	192450618	192450789
chr1	192453969	192454308
chr1	192454512	192454876
chr1	192498240	192498503
chr1	192525815	192525989
chr1	192540799	192541001
chr1	192596583	192596834
chr1	192598419	192598705
chr1	192660430	192660590
chr1	192670842	192671162
chr1	192722246	192722450
chr1	192727039	192727330
chr1	192728590	192728769
chr1	192745496	192746155
chr1	192746155	192746523
chr1	192746523	192746906
chr1	192752694	192752993
chr1	192754348	192754556
chr1	192754656	192754853
chr1	192765593	192765795
chr1	192770530	192770861
chr1	192770861	192771142
chr1	192771142	192771335
chr1	192774237	192774421
chr1	192779358	192779557
chr1	192781319	192781663
chr1	192784426	192784779
chr1	192786173	192786774
chr1	192790582	192790761
chr1	192794925	192795378
chr1	192811473	192811654
chr1	192834610	192834830
chr1	192834830	192835022
chr1	192854518	192855029
chr1	192855029	192855262
chr1	192855425	192855869
chr1	192857668	192857985
chr1	192858248	192858478
chr1	192860370	192860639
chr1	192882739	192883332
chr1	192918657	192918842

chr1	193024408	193024585
chr1	193035404	193035992
chr1	193035992	193036292
chr1	193102619	193102954
chr1	193102965	193103247
chr1	193109990	193110172
chr1	193120740	193120900
chr1	193129793	193130095
chr1	193130095	193130295
chr1	193130295	193130499
chr1	193130538	193130835
chr1	193130835	193131184
chr1	193149441	193149800
chr1	193151031	193151262
chr1	193151295	193151545
chr1	193152883	193153229
chr1	193153229	193153498
chr1	193153718	193153916
chr1	193153988	193154138
chr1	193163669	193163971
chr1	193164934	193165297
chr1	193165543	193165760
chr1	193166081	193166262
chr1	193169935	193170281
chr1	193172993	193173333
chr1	193173333	193174031
chr1	193198414	193198665
chr1	193208236	193208507
chr1	193213997	193214210
chr1	193216024	193216328
chr1	193226812	193227045
chr1	193227124	193227426
chr1	193227742	193227994
chr1	193255654	193255821
chr1	193255829	193256094
chr1	193256290	193256477
chr1	193269299	193269691
chr1	193273326	193273543
chr1	193279291	193279565
chr1	193279565	193279871
chr1	193279884	193280048
chr1	193280636	193280786

chr1	193288452	193288737
chr1	193288915	193289142
chr1	193298119	193298624
chr1	193301232	193301555
chr1	193301555	193301824
chr1	193301824	193302264
chr1	193311482	193311617
chr1	193311617	193311756
chr1	193312453	193312669
chr1	193319444	193319704
chr1	193319732	193320068
chr1	193324316	193324546
chr1	193324858	193325112
chr1	193330201	193330592
chr1	193332120	193332376
chr1	193333091	193333294
chr1	193334657	193334971
chr1	193337264	193337449
chr1	193342230	193342600
chr1	193342612	193342915
chr1	193350108	193350258
chr1	193351607	193351867
chr1	193362476	193362696
chr1	193362696	193362974
chr1	193363895	193364112
chr1	193364544	193364885
chr1	193376812	193377096
chr1	193382561	193382711
chr1	193382894	193383119
chr1	193383184	193383336
chr1	193385811	193385965
chr1	193386193	193386492
chr1	193387388	193387581
chr1	193398775	193399120
chr1	193399125	193399373
chr1	193400132	193400748
chr1	193412857	193413028
chr1	193413205	193413684
chr1	193413686	193414076
chr1	193414076	193414512
chr1	193420075	193420225
chr1	193422881	193423218

chr1	193440567	193440817
chr1	193444317	193444593
chr1	193449376	193449907
chr1	193449925	193450112
chr1	193451496	193451858
chr1	193477212	193477672
chr1	193478541	193478979
chr1	193501311	193501663
chr1	193503604	193503901
chr1	193503976	193504242
chr1	193510183	193510366
chr1	193513884	193514256
chr1	193543405	193543625
chr1	193576043	193576337
chr1	193589120	193589395
chr1	193599257	193599480
chr1	193632809	193633003
chr1	193655375	193655777
chr1	193673299	193673502
chr1	193698961	193699196
chr1	193703241	193703395
chr1	193705281	193705524
chr1	193868799	193869158
chr1	193870022	193870249
chr1	193870770	193871161
chr1	193875344	193875672
chr1	193892858	193893023
chr1	193893118	193893395
chr1	193905802	193906097
chr1	193966729	193966987
chr1	193966987	193967246
chr1	193967246	193967547
chr1	194026359	194026570
chr1	194089868	194090227
chr1	194090227	194090610
chr1	194091624	194092003
chr1	194095791	194096363
chr1	194102162	194102504
chr1	194106939	194107111
chr1	194107406	194107575
chr1	194107610	194107944
chr1	194223111	194223314

chr1	194238578	194238926
chr1	194330128	194330319
chr1	194372564	194372787
chr1	194569325	194569602
chr1	194589195	194589455
chr1	194602088	194602468
chr1	194608529	194608686
chr1	194610620	194610793
chr1	194618790	194619019
chr1	194619067	194619280
chr1	194619376	194619699
chr1	194619699	194620085
chr1	194620085	194620478
chr1	194620478	194620802
chr1	194626196	194626438
chr1	194626995	194627145
chr1	194639818	194640008
chr1	194641637	194641822
chr1	194651738	194652151
chr1	194652151	194652529
chr1	194658443	194659046
chr1	194659120	194659277
chr1	194659956	194660108
chr1	194660363	194660949
chr1	194671806	194672146
chr1	194672146	194672394
chr1	194672520	194672692
chr1	194675712	194676229
chr1	194682025	194682322
chr1	194683189	194683415
chr1	194687796	194688173
chr1	194688173	194688535
chr1	194722421	194722664
chr1	194723967	194724137
chr1	194724137	194724309
chr1	194734924	194735171
chr1	194749533	194749861
chr1	194749945	194750113
chr1	194750943	194751104
chr1	194753259	194753413
chr1	194761587	194761772
chr1	194781677	194781923

chr1	194805429	194805579
chr1	194816930	194817239
chr1	194821109	194821328
chr1	194836236	194836520
chr1	194837877	194838106
chr1	194839305	194839543
chr1	194849923	194850181
chr1	194852753	194853205
chr1	194878271	194878512
chr1	194878512	194878828
chr1	194878923	194879168
chr1	194879226	194879676
chr1	194879676	194879902
chr1	194879964	194880114
chr1	194885794	194885979
chr1	194908937	194909338
chr1	194922863	194923018
chr1	194925050	194925262
chr1	194928220	194928380
chr1	194938835	194939055
chr1	194941536	194941862
chr1	194945506	194945761
chr1	194957201	194957351
chr1	194962231	194962534
chr1	194962678	194963072
chr1	194976090	194976491
chr1	194976491	194976790
chr1	194976790	194977144
chr1	194978126	194978281
chr1	194995244	194995521
chr1	195012538	195012867
chr1	195016816	195017068
chr1	195017068	195017295
chr1	195017295	195017556
chr1	195017928	195018090
chr1	195018182	195018430
chr1	195033983	195034323
chr1	195040513	195040765
chr1	195080097	195080371
chr1	195081837	195082058
chr1	195084664	195085086
chr1	195091781	195092240

chr1	195092240	195092497
chr1	195092497	195092741
chr1	195100696	195101240
chr1	195130885	195131203
chr1	195131203	195131473
chr1	195131473	195131754
chr1	195165076	195165292
chr1	195169187	195169460
chr1	195176571	195176906
chr1	195232538	195232941
chr1	195240673	195240848
chr1	195241445	195241968
chr1	195279163	195279349
chr1	195371644	195372040
chr10	3109516	3109695
chr10	3109905	3110398
chr10	3111314	3111559
chr10	3111559	3111802
chr10	3111802	3112137
chr10	3112308	3112490
chr10	3112562	3113138
chr10	3113138	3113640
chr10	3113640	3114008
chr10	3114472	3114694
chr10	3114824	3115094
chr10	3115094	3115325
chr10	3116081	3116529
chr10	3116529	3117125
chr10	3236709	3236987
chr10	3236987	3237434
chr10	3365993	3366216
chr10	3366289	3366794
chr10	3446627	3446826
chr10	3447143	3447337
chr10	3466222	3466596
chr10	3466596	3467008
chr10	3519103	3519314
chr10	3531478	3531720
chr10	3573106	3573437
chr10	3740195	3740491
chr10	3740491	3740812
chr10	3740812	3741158

chr10	3741158	3741458
chr10	3778557	3778833
chr10	3941274	3941799
chr10	3942370	3942749
chr10	3943940	3944129
chr10	3967274	3967559
chr10	3972886	3973475
chr10	3973475	3974139
chr10	3978423	3978680
chr10	3991382	3991532
chr10	3992549	3992721
chr10	3995358	3995532
chr10	3996289	3996453
chr10	3996517	3996802
chr10	3997232	3997382
chr10	4000788	4001026
chr10	4002331	4002779
chr10	4002779	4003041
chr10	4003041	4003251
chr10	4004097	4004335
chr10	4026707	4026905
chr10	4027015	4027172
chr10	4027416	4027969
chr10	4028856	4029215
chr10	4037232	4037392
chr10	4041508	4041668
chr10	4041743	4042138
chr10	4055343	4055653
chr10	4063637	4063920
chr10	4076921	4077268
chr10	4079080	4079404
chr10	4105592	4105761
chr10	4105848	4106022
chr10	4106144	4106387
chr10	4106387	4106596
chr10	4107528	4108304
chr10	4111080	4111373
chr10	4120751	4120973
chr10	4120973	4121218
chr10	4121218	4121713
chr10	4123370	4123545
chr10	4132053	4132431

chr10	4132509	4133182
chr10	4144624	4145049
chr10	4200405	4201101
chr10	4212189	4212339
chr10	4227096	4227331
chr10	4230504	4230842
chr10	4233265	4233465
chr10	4241473	4241643
chr10	4241660	4242008
chr10	4245883	4246057
chr10	4246463	4246628
chr10	4247622	4247871
chr10	4250750	4251018
chr10	4257617	4257853
chr10	4266173	4266890
chr10	4266890	4267242
chr10	4272823	4273010
chr10	4273066	4273345
chr10	4273959	4274271
chr10	4278001	4278401
chr10	4282501	4282721
chr10	4313849	4314058
chr10	4316836	4317106
chr10	4333446	4333667
chr10	4334302	4334788
chr10	4341494	4341813
chr10	4346579	4347157
chr10	4372014	4372287
chr10	4386599	4386865
chr10	4386865	4387084
chr10	4387084	4387335
chr10	4387335	4387645
chr10	4388089	4388284
chr10	4388284	4388458
chr10	4389025	4389381
chr10	4395066	4395894
chr10	4402509	4402674
chr10	4404958	4405356
chr10	4407287	4407514
chr10	4413771	4414000
chr10	4414000	4414285
chr10	4432041	4432386

chr10	4432386	4432662
chr10	4432662	4432963
chr10	4433464	4433650
chr10	4455894	4456117
chr10	4460318	4460505
chr10	4476711	4476874
chr10	4480014	4480399
chr10	4593913	4594143
chr10	4595915	4596081
chr10	4596215	4596569
chr10	4598394	4598570
chr10	4598570	4598752
chr10	4599660	4599963
chr10	4610003	4610327
chr10	4610327	4610670
chr10	4611449	4611690
chr10	4611848	4612130
chr10	4612132	4612292
chr10	4612303	4612586
chr10	4614354	4614528
chr10	4620866	4621246
chr10	4621246	4621672
chr10	4635402	4635609
chr10	4635801	4636036
chr10	4675050	4675208
chr10	4710014	4710255
chr10	4712309	4712475
chr10	4712537	4712943
chr10	4730134	4730562
chr10	4909369	4909608
chr10	4983967	4984325
chr10	5031215	5031449
chr10	5070832	5071137
chr10	5071192	5071488
chr10	5083665	5083850
chr10	5085533	5085695
chr10	5085839	5086262
chr10	5086437	5086651
chr10	5089197	5089372
chr10	5110818	5111268
chr10	5116725	5117070
chr10	5161730	5161890

chr10	5166723	5166919
chr10	5177661	5177875
chr10	5181629	5182221
chr10	5182221	5182493
chr10	5188843	5189349
chr10	5194342	5194667
chr10	5194667	5195182
chr10	5199756	5200171
chr10	5200820	5201159
chr10	5208011	5208252
chr10	5208285	5208810
chr10	5209226	5209407
chr10	5212666	5213146
chr10	5256729	5257058
chr10	5264474	5264685
chr10	5266430	5266622
chr10	5287598	5287877
chr10	5288005	5288160
chr10	5288786	5289023
chr10	5289033	5289399
chr10	5289399	5289882
chr10	5317693	5317919
chr10	5326790	5327028
chr10	5340852	5341111
chr10	5369683	5369893
chr10	5455048	5455243
chr10	5457617	5457823
chr10	5470759	5470981
chr10	5480149	5480368
chr10	5481659	5481811
chr10	5481842	5481999
chr10	5483654	5483866
chr10	5483866	5484086
chr10	5497979	5498134
chr10	5511509	5511673
chr10	5515682	5515839
chr10	5548617	5548979
chr10	5549005	5549313
chr10	5550240	5550502
chr10	5550502	5550967
chr10	5555025	5555262
chr10	5571202	5571598

chr10	5576813	5577098
chr10	5592757	5592963
chr10	5600150	5600490
chr10	5633729	5633998
chr10	5634432	5634657
chr10	5639196	5639414
chr10	5646459	5646796
chr10	5673946	5674384
chr10	5674384	5674653
chr10	5688639	5689114
chr10	5689177	5689401
chr10	5766333	5766500
chr10	5787019	5787243
chr10	5790751	5790959
chr10	5804734	5804884
chr10	5804944	5805744
chr10	5805744	5806030
chr10	5806030	5806267
chr10	5823449	5823620
chr10	5823620	5823832
chr10	5823832	5824166
chr10	5824166	5824569
chr10	5824569	5824848
chr10	5824901	5825178
chr10	5829690	5829879
chr10	5841452	5841837
chr10	5849987	5850262
chr10	5853317	5853649
chr10	5868267	5868610
chr10	5901830	5902033
chr10	5921070	5921241
chr10	5921297	5921449
chr10	5922216	5922630
chr10	5922630	5922873
chr10	5967329	5967532
chr10	5967532	5967760
chr10	5967760	5968044
chr10	6032474	6032974
chr10	6035882	6036150
chr10	6366172	6366562
chr10	6630503	6630921
chr10	6788766	6788999

chr10	6814577	6814800
chr10	6820592	6820805
chr10	6824642	6824971
chr10	6825087	6825547
chr10	6842341	6842570
chr10	6844510	6844885
chr10	6849894	6850174
chr10	6916626	6916848
chr10	6934870	6935106
chr10	6953153	6953503
chr10	6978113	6978291
chr10	7015175	7015403
chr10	7020101	7020404
chr10	7025138	7025357
chr10	7075773	7075935
chr10	7076208	7076492
chr10	7093202	7093352
chr10	7100365	7100653
chr10	7102514	7102667
chr10	7113212	7113454
chr10	7113753	7114153
chr10	7118422	7118758
chr10	7118758	7119025
chr10	7119025	7119284
chr10	7119406	7119566
chr10	7120174	7120438
chr10	7124846	7125269
chr10	7142322	7142714
chr10	7145640	7146161
chr10	7152820	7153019
chr10	7155275	7155506
chr10	7158660	7159018
chr10	7159018	7159539
chr10	7170052	7170287
chr10	7183439	7183668
chr10	7189003	7189621
chr10	7189621	7189911
chr10	7189911	7190219
chr10	7211035	7211403
chr10	7211403	7211673
chr10	7211673	7211880
chr10	7211880	7212115

chr10	7212115	7212448
chr10	7212545	7212895
chr10	7289505	7289993
chr10	7323389	7323560
chr10	7333390	7333713
chr10	7386900	7387052
chr10	7388256	7388452
chr10	7388722	7388940
chr10	7390498	7390922
chr10	7390922	7391130
chr10	7391130	7391644
chr10	7391680	7391866
chr10	7391866	7392256
chr10	7394622	7394814
chr10	7462082	7462311
chr10	7470114	7470454
chr10	7473487	7474086
chr10	7491823	7492091
chr10	7492321	7492862
chr10	7578169	7578326
chr10	7581482	7581799
chr10	7589480	7589928
chr10	7589928	7590190
chr10	7590190	7590640
chr10	7600712	7600990
chr10	7619945	7620182
chr10	7662597	7662890
chr10	7662890	7663174
chr10	7663174	7663471
chr10	7663471	7663816
chr10	7667344	7667615
chr10	7667615	7667871
chr10	7673337	7673602
chr10	7673743	7674012
chr10	7680823	7681080
chr10	7681080	7681396
chr10	7681396	7681747
chr10	7681747	7682024
chr10	7695352	7695627
chr10	7725575	7726364
chr10	7726542	7726712
chr10	7756861	7757045

chr10	7774313	7774623
chr10	7780196	7780523
chr10	7780523	7780821
chr10	7780821	7781145
chr10	7787324	7787720
chr10	7792498	7792686
chr10	7792688	7793004
chr10	7793004	7793221
chr10	7793221	7793407
chr10	7809992	7810299
chr10	7832343	7832519
chr10	7832519	7832684
chr10	7841599	7842222
chr10	7861714	7861882
chr10	7883592	7883845
chr10	7890244	7890522
chr10	7896953	7897280
chr10	7902662	7902823
chr10	7912338	7912529
chr10	7923365	7923591
chr10	7923591	7923851
chr10	7943041	7943367
chr10	7955472	7955982
chr10	7955982	7956864
chr10	7966229	7966454
chr10	7968897	7969133
chr10	7970385	7970650
chr10	7976166	7976379
chr10	8116876	8117193
chr10	8152664	8152910
chr10	8156546	8156698
chr10	8174289	8174488
chr10	8196360	8196642
chr10	8213918	8214330
chr10	8225065	8225266
chr10	8240347	8240559
chr10	8242455	8242669
chr10	8254718	8254994
chr10	8287376	8287603
chr10	8292888	8293041
chr10	8293041	8293208
chr10	8293231	8293552

chr10	8323357	8323507
chr10	8330969	8331354
chr10	8362142	8362403
chr10	8416576	8416779
chr10	8431851	8432306
chr10	8492076	8492330
chr10	8498882	8499172
chr10	8499172	8499428
chr10	8499428	8499848
chr10	8505447	8505722
chr10	8505722	8506068
chr10	8506122	8506364
chr10	8506364	8506683
chr10	8517809	8518137
chr10	8518137	8518374
chr10	8518374	8518682
chr10	8518682	8519206
chr10	8544488	8544735
chr10	8550923	8551262
chr10	8677029	8677254
chr10	8679272	8679436
chr10	8707843	8708013
chr10	8715717	8715994
chr10	8742946	8743200
chr10	8746190	8746385
chr10	8768592	8769130
chr10	8772405	8772793
chr10	8792454	8792741
chr10	8795421	8795661
chr10	8797906	8798128
chr10	8799167	8799539
chr10	8804841	8805052
chr10	8805052	8805346
chr10	8805371	8805679
chr10	8811296	8811619
chr10	8812910	8813120
chr10	8829789	8829949
chr10	8833829	8834045
chr10	8841427	8841672
chr10	8844479	8844689
chr10	8866364	8866573
chr10	8868188	8868401

chr10	8870213	8870802
chr10	8874765	8874957
chr10	8875227	8875438
chr10	8885639	8886214
chr10	8886214	8886727
chr10	8890097	8890367
chr10	8890545	8890959
chr10	8895164	8895361
chr10	8898922	8899162
chr10	8899162	8899439
chr10	8899439	8899685
chr10	8969565	8969934
chr10	8973995	8974292
chr10	8990549	8990787
chr10	8995168	8995434
chr10	9012878	9013114
chr10	9014486	9014646
chr10	9027233	9027449
chr10	9027449	9027678
chr10	9175061	9175331
chr10	9226192	9226434
chr10	9239443	9239837
chr10	9239837	9240394
chr10	9249727	9249963
chr10	9258760	9259026
chr10	9300028	9300178
chr10	9312406	9312662
chr10	9404257	9404442
chr10	9496675	9497523
chr10	9503424	9503822
chr10	9503822	9504156
chr10	9504340	9504621
chr10	9506572	9506730
chr10	9523079	9523281
chr10	9528141	9528445
chr10	9529002	9529178
chr10	9529293	9530062
chr10	9531047	9531227
chr10	9544131	9544330
chr10	9574255	9574519
chr10	9575623	9575939
chr10	9581334	9581506

chr10	9594713	9595315
chr10	9596376	9596532
chr10	9597047	9597248
chr10	9597248	9597455
chr10	9603393	9603632
chr10	9617633	9617906
chr10	9637894	9638055
chr10	9638061	9638348
chr10	9640126	9640598
chr10	9640598	9641055
chr10	9646440	9646621
chr10	9674413	9675015
chr10	9675015	9675527
chr10	9675527	9675971
chr10	9676996	9677270
chr10	9791098	9791248
chr10	9801490	9801776
chr10	9803584	9803834
chr10	9808090	9808348
chr10	9854356	9854527
chr10	9880396	9880697
chr10	9881884	9882248
chr10	9883084	9883469
chr10	9900379	9900775
chr10	9900775	9901115
chr10	9901115	9901491
chr10	9901491	9901913
chr10	9901913	9902290
chr10	10059116	10059673
chr10	10093825	10094149
chr10	10224675	10224896
chr10	10252194	10252455
chr10	10253090	10253376
chr10	10307334	10307814
chr10	10321119	10321445
chr10	10321481	10321676
chr10	10325669	10325831
chr10	10335239	10335431
chr10	10335431	10335631
chr10	10343978	10344342
chr10	10355621	10355886
chr10	10355927	10356078

chr10	10382714	10382941
chr10	10408229	10408481
chr10	10477536	10477694
chr10	10544385	10544539
chr10	10549608	10549787
chr10	10550207	10550500
chr10	10555236	10555607
chr10	10557081	10557278
chr10	10557278	10557479
chr10	10557479	10557815
chr10	10557815	10558128
chr10	10558128	10558434
chr10	10601755	10601941
chr10	11023443	11023655
chr10	11024188	11024338
chr10	11024803	11025149
chr10	11149089	11149679
chr10	11149679	11150037
chr10	11197285	11197699
chr10	11197780	11197995
chr10	11198119	11198273
chr10	11198356	11198690
chr10	11205917	11206134
chr10	11260971	11261121
chr10	11261439	11261795
chr10	11280525	11281080
chr10	11281080	11281396
chr10	11281396	11281690
chr10	11281808	11282014
chr10	11300558	11300714
chr10	11343161	11344095
chr10	11350662	11350865
chr10	11352606	11352915
chr10	11352915	11353273
chr10	11362409	11362571
chr10	11449572	11449723
chr10	11449977	11450189
chr10	11491999	11492310
chr10	11609180	11609382
chr10	11826900	11827267
chr10	11841649	11841923
chr10	11842332	11842520

chr10	11880678	11880838
chr10	12013674	12014027
chr10	12028740	12029114
chr10	12035159	12035693
chr10	12035744	12035976
chr10	12091665	12091862
chr10	12092022	12092185
chr10	12101015	12101274
chr10	12106943	12107476
chr10	12144242	12144417
chr10	12144702	12145070
chr10	12147219	12147463
chr10	12147463	12147671
chr10	12180382	12180735
chr10	12180799	12181327
chr10	12193486	12193880
chr10	12196024	12196193
chr10	12207164	12207683
chr10	12207683	12207906
chr10	12246369	12246586
chr10	12248833	12249351
chr10	12298629	12299089
chr10	12299089	12299518
chr10	12310050	12310200
chr10	12315193	12315457
chr10	12315962	12316137
chr10	12336969	12337174
chr10	12365507	12365678
chr10	12376897	12377047
chr10	12411585	12411764
chr10	12412155	12412506
chr10	12507736	12507969
chr10	12535293	12535486
chr10	12556908	12557082
chr10	12566990	12567140
chr10	12601249	12601522
chr10	12608328	12608530
chr10	12612542	12612783
chr10	12658476	12658678
chr10	12690703	12690929
chr10	12694659	12695207
chr10	12696765	12697153

chr10	12698365	12698654
chr10	12704278	12704719
chr10	12714428	12714819
chr10	12719452	12719704
chr10	12726182	12726693
chr10	12743098	12743313
chr10	12755022	12755227
chr10	12756985	12757152
chr10	12787722	12787996
chr10	12787996	12788457
chr10	12799571	12799728
chr10	12803970	12804412
chr10	12804476	12804662
chr10	12806916	12807270
chr10	12809665	12810004
chr10	12810696	12811374
chr10	12817410	12817751
chr10	12821210	12821761
chr10	12823950	12824148
chr10	12826684	12827339
chr10	12827512	12827674
chr10	12827862	12828045
chr10	12837743	12837991
chr10	12842527	12842874
chr10	12850804	12851079
chr10	12851089	12851373
chr10	12852383	12852807
chr10	12852997	12853194
chr10	12854521	12854684
chr10	12858041	12858402
chr10	12858438	12858814
chr10	12858814	12859072
chr10	12861318	12861709
chr10	12862158	12862390
chr10	12868033	12868487
chr10	12868487	12868845
chr10	12868917	12869373
chr10	12869373	12869707
chr10	12874901	12875161
chr10	12876526	12876751
chr10	12878547	12879101
chr10	12884310	12884615

chr10	12884615	12884942
chr10	12888351	12888711
chr10	12890331	12890680
chr10	12893160	12893776
chr10	12903874	12904113
chr10	12904261	12904411
chr10	12909937	12910295
chr10	12912209	12912456
chr10	12912456	12912644
chr10	12915893	12916136
chr10	12916254	12916464
chr10	12916464	12916681
chr10	12919245	12919657
chr10	12922326	12922604
chr10	12922604	12923003
chr10	12923003	12923361
chr10	12929220	12929386
chr10	12931108	12931308
chr10	12931406	12931800
chr10	12931800	12932084
chr10	12932084	12932303
chr10	12932373	12932612
chr10	12936202	12936385
chr10	12949340	12949882
chr10	12955951	12956141
chr10	12963262	12963568
chr10	12963568	12963784
chr10	12963899	12964161
chr10	12964161	12964426
chr10	12964426	12964784
chr10	12965861	12966099
chr10	12976558	12977075
chr10	12977919	12978195
chr10	12981200	12981453
chr10	12992097	12992262
chr10	12996817	12997417
chr10	13008204	13008526
chr10	13008526	13008772
chr10	13008772	13009095
chr10	13009662	13009913
chr10	13016605	13016922
chr10	13018602	13018769

chr10	13028320	13028542
chr10	13035249	13035586
chr10	13080276	13080546
chr10	13087258	13087517
chr10	13090451	13090993
chr10	13090993	13091319
chr10	13192678	13192983
chr10	13192983	13193386
chr10	13256261	13256411
chr10	13270209	13270614
chr10	13287299	13287449
chr10	13288304	13288531
chr10	13288531	13288808
chr10	13303883	13304238
chr10	13304608	13305061
chr10	13323375	13323557
chr10	13323557	13323731
chr10	13323778	13323991
chr10	13323991	13324475
chr10	13328515	13328936
chr10	13369672	13370009
chr10	13370009	13370387
chr10	13370387	13370576
chr10	13370607	13371041
chr10	13382065	13382292
chr10	13402054	13402205
chr10	13424433	13424833
chr10	13446793	13447275
chr10	13467019	13467169
chr10	13473730	13473959
chr10	13473959	13474195
chr10	13474195	13474528
chr10	13474528	13474843
chr10	13489537	13489762
chr10	13489762	13490302
chr10	13500880	13501221
chr10	13501221	13501547
chr10	13501641	13501793
chr10	13543583	13543863
chr10	13552700	13553507
chr10	13553535	13553692
chr10	13564740	13564952

chr10	13576822	13577001
chr10	13577155	13577546
chr10	13584421	13584624
chr10	13595875	13596617
chr10	13601767	13602052
chr10	13622905	13623138
chr10	13641808	13642026
chr10	13659392	13659634
chr10	13663575	13664080
chr10	13679494	13679703
chr10	13683875	13684307
chr10	13684402	13684815
chr10	13739962	13740118
chr10	13767236	13767388
chr10	13775408	13775894
chr10	13775918	13776469
chr10	13783626	13783826
chr10	13787269	13787436
chr10	13788206	13788481
chr10	13789542	13789766
chr10	13789766	13790121
chr10	13792834	13793141
chr10	13805067	13805448
chr10	13822891	13823090
chr10	13826981	13827148
chr10	13832545	13833108
chr10	13833108	13833499
chr10	13842867	13843125
chr10	13861127	13861322
chr10	13861375	13861756
chr10	13861849	13862194
chr10	13868385	13868720
chr10	13868720	13869285
chr10	13869347	13869702
chr10	13869702	13870043
chr10	13878711	13878984
chr10	13928616	13928804
chr10	13964535	13965254
chr10	13965502	13965896
chr10	13965896	13966191
chr10	13966191	13966449
chr10	13966532	13966712

chr10	13966867	13967167
chr10	13967410	13967750
chr10	13969115	13969270
chr10	13972894	13973071
chr10	13973881	13974264
chr10	13976387	13976557
chr10	13982754	13983148
chr10	13983148	13983427
chr10	13983677	13983993
chr10	13983995	13984163
chr10	13984279	13984533
chr10	14017745	14018438
chr10	14018778	14018977
chr10	14027801	14028007
chr10	14030527	14030744
chr10	14030744	14031196
chr10	14041352	14041529
chr10	14047013	14047230
chr10	14048285	14048526
chr10	14048526	14049051
chr10	14070790	14071024
chr10	14085179	14085399
chr10	14085438	14085725
chr10	14085950	14086221
chr10	14086221	14086543
chr10	14192079	14192324
chr10	14197413	14197572
chr10	14200450	14200659
chr10	14213373	14213600
chr10	14215587	14215763
chr10	14233395	14233686
chr10	14239749	14239961
chr10	14240144	14240459
chr10	14303525	14303893
chr10	14304288	14304440
chr10	14307269	14307518
chr10	14307518	14307969
chr10	14312000	14312208
chr10	14313008	14313293
chr10	14313986	14314481
chr10	14314617	14314966
chr10	14321604	14321816

chr10	14332528	14333000
chr10	14341324	14341793
chr10	14385660	14385845
chr10	14391034	14391307
chr10	14391307	14391596
chr10	14404152	14404307
chr10	14451922	14452107
chr10	14463898	14464160
chr10	14464160	14464780
chr10	14476835	14477043
chr10	14491833	14492118
chr10	14507223	14507519
chr10	14509680	14510059
chr10	14527773	14528167
chr10	14530740	14530903
chr10	14531139	14531573
chr10	14534115	14534303
chr10	14544656	14545175
chr10	14545175	14545509
chr10	14545509	14545750
chr10	14546092	14546364
chr10	14573962	14574189
chr10	14574278	14574606
chr10	14582814	14583269
chr10	14583552	14583765
chr10	14601035	14601453
chr10	14602653	14603158
chr10	14618340	14618536
chr10	14630099	14630253
chr10	14639650	14640011
chr10	14659616	14659845
chr10	14660948	14661197
chr10	14685760	14686024
chr10	14686039	14686412
chr10	14686412	14686681
chr10	14691131	14691504
chr10	14692876	14693116
chr10	14693116	14693422
chr10	14705000	14705407
chr10	14705407	14705747
chr10	14705782	14706007
chr10	14902039	14902248

chr10	14941962	14942212
chr10	14959088	14959375
chr10	15037122	15037514
chr10	15037514	15037977
chr10	15080259	15080481
chr10	15085275	15085441
chr10	15137736	15137935
chr10	15244455	15244713
chr10	15300148	15300383
chr10	15303260	15303443
chr10	15706527	15706827
chr10	15757555	15757791
chr10	15844776	15844960
chr10	15898273	15898584
chr10	16014762	16015160
chr10	16088224	16088568
chr10	16119465	16119636
chr10	16159497	16159939
chr10	16160134	16160303
chr10	16171329	16171542
chr10	16301048	16301267
chr10	16313701	16313885
chr10	16429313	16429564
chr10	16498610	16499073
chr10	16507347	16507578
chr10	16507594	16508024
chr10	16508024	16508298
chr10	16508298	16508573
chr10	16524273	16524509
chr10	16537608	16538104
chr10	16544821	16545007
chr10	16545260	16545522
chr10	16545522	16545870
chr10	16552982	16553195
chr10	16553320	16553603
chr10	16553603	16553863
chr10	16578547	16579102
chr10	16589242	16589395
chr10	16589413	16589878
chr10	16593727	16593916
chr10	16600885	16601283
chr10	16621418	16621586

chr10	16622868	16623242
chr10	16678176	16678472
chr10	16714601	16714815
chr10	16804146	16804356
chr10	16806085	16806258
chr10	16863616	16863987
chr10	16874175	16874438
chr10	16878133	16878610
chr10	16922178	16922425
chr10	16990147	16990536
chr10	17010071	17010336
chr10	17011200	17011350
chr10	17011882	17012163
chr10	17012163	17012459
chr10	17012459	17012959
chr10	17013996	17014158
chr10	17034812	17035411
chr10	17037992	17038239
chr10	17059871	17060270
chr10	17065575	17065767
chr10	17082928	17083320
chr10	17099999	17100443
chr10	17103419	17103790
chr10	17129850	17130008
chr10	17173981	17174131
chr10	17175438	17175734
chr10	17253020	17253370
chr10	17298982	17299391
chr10	17331009	17331236
chr10	17347744	17348339
chr10	17364469	17364627
chr10	17368600	17369134
chr10	17382944	17383140
chr10	17394202	17394402
chr10	17495900	17496324
chr10	17506180	17506380
chr10	17528903	17529216
chr10	17531085	17531349
chr10	17531349	17531587
chr10	17548073	17548317
chr10	17548844	17549545
chr10	17549642	17549916

chr10	17549961	17550314
chr10	17567490	17568189
chr10	17574980	17575248
chr10	17575451	17575817
chr10	17575904	17576176
chr10	17579568	17579750
chr10	17584827	17585219
chr10	17602539	17602843
chr10	17605741	17605957
chr10	17654344	17654675
chr10	17654675	17654957
chr10	17656403	17656619
chr10	17656619	17656872
chr10	17666440	17666637
chr10	17668783	17669178
chr10	17670094	17670441
chr10	17670892	17671128
chr10	17697375	17697684
chr10	17697722	17698287
chr10	17722168	17722583
chr10	17722689	17723066
chr10	17723066	17723381
chr10	17723381	17723805
chr10	17723899	17724240
chr10	17724240	17724613
chr10	17733090	17733406
chr10	17734294	17734464
chr10	17750414	17750682
chr10	17760163	17760458
chr10	17760844	17761333
chr10	17765373	17765536
chr10	17772331	17772750
chr10	17774953	17775106
chr10	17775145	17775450
chr10	17775545	17775814
chr10	17776407	17776734
chr10	17776734	17777006
chr10	17792643	17792897
chr10	17792933	17793249
chr10	17793432	17793891
chr10	17793893	17794452
chr10	17795321	17795523

chr10	17804929	17805355
chr10	17842182	17842367
chr10	17852643	17852910
chr10	17857972	17858144
chr10	17860754	17860962
chr10	17861163	17861436
chr10	17870006	17870311
chr10	17918846	17919515
chr10	17924170	17924466
chr10	17946944	17947236
chr10	17947236	17947506
chr10	17947506	17947732
chr10	17947832	17948334
chr10	17948334	17948671
chr10	17971683	17972098
chr10	17997023	17997173
chr10	17997185	17997668
chr10	17997732	17997959
chr10	17997959	17998185
chr10	18005665	18005868
chr10	18008174	18008430
chr10	18022290	18022526
chr10	18022526	18022754
chr10	18022754	18023046
chr10	18023046	18023445
chr10	18055138	18055689
chr10	18055689	18055977
chr10	18055977	18056223
chr10	18056223	18056442
chr10	18076873	18077524
chr10	18081559	18081893
chr10	18082850	18083101
chr10	18139215	18139411
chr10	18197208	18197616
chr10	18204099	18204366
chr10	18205125	18205308
chr10	18210730	18211096
chr10	18234001	18234189
chr10	18234600	18234868
chr10	18234868	18235140
chr10	18235140	18235435
chr10	18235435	18235689

chr10	18243703	18243870
chr10	18256444	18256606
chr10	18298608	18298835
chr10	18304981	18305669
chr10	18311078	18311274
chr10	18318263	18318820
chr10	18318820	18319195
chr10	18319195	18319471
chr10	18320443	18320665
chr10	18323033	18323489
chr10	18323649	18324163
chr10	18328576	18328816
chr10	18329199	18329477
chr10	18329477	18329757
chr10	18329757	18330152
chr10	18337780	18337993
chr10	18350593	18351267
chr10	18365409	18365710
chr10	18370330	18370738
chr10	18370738	18371319
chr10	18388730	18389049
chr10	18393626	18394042
chr10	18406751	18407462
chr10	18407462	18407984
chr10	18420773	18421176
chr10	18421180	18421335
chr10	18430188	18430482
chr10	18433538	18433822
chr10	18453271	18453462
chr10	18453462	18453734
chr10	18455135	18455503
chr10	18455900	18456058
chr10	18469294	18469536
chr10	18471669	18472051
chr10	18492067	18492257
chr10	18506421	18506610
chr10	18536822	18537025
chr10	18545683	18546400
chr10	18563025	18563246
chr10	18572669	18572882
chr10	18602117	18602360
chr10	18612840	18613319

chr10	18644792	18645020
chr10	18705794	18706105
chr10	18743262	18743614
chr10	18743614	18744160
chr10	18760324	18760551
chr10	18784806	18785046
chr10	18826803	18827099
chr10	18836044	18836210
chr10	18836210	18836383
chr10	18844580	18844821
chr10	18844821	18845499
chr10	18850211	18850473
chr10	18859689	18859988
chr10	18860578	18860813
chr10	18860890	18861163
chr10	18863411	18863766
chr10	18871129	18871279
chr10	18876155	18876595
chr10	18876595	18876886
chr10	18886953	18887230
chr10	18892038	18892339
chr10	18892339	18892779
chr10	18894458	18894741
chr10	18910880	18911265
chr10	18917062	18917372
chr10	18929399	18929642
chr10	18935059	18935539
chr10	18936302	18936517
chr10	18942069	18942260
chr10	18946542	18946755
chr10	18946755	18947096
chr10	18948791	18949116
chr10	18949183	18949625
chr10	18953753	18954074
chr10	18973517	18973721
chr10	18987497	18987701
chr10	18987869	18988219
chr10	18995629	18995852
chr10	18996389	18996818
chr10	18999363	18999806
chr10	19000033	19000241
chr10	19002074	19002226

chr10	19002397	19002748
chr10	19008416	19008644
chr10	19008649	19009014
chr10	19009427	19009678
chr10	19011817	19012121
chr10	19014389	19014778
chr10	19014778	19015128
chr10	19015128	19015440
chr10	19015440	19015679
chr10	19026505	19027169
chr10	19028311	19028756
chr10	19028756	19028998
chr10	19043146	19043308
chr10	19043935	19044296
chr10	19044416	19044598
chr10	19045965	19046397
chr10	19046593	19046888
chr10	19047574	19047741
chr10	19061989	19062667
chr10	19065195	19065370
chr10	19065439	19065595
chr10	19066878	19067076
chr10	19068569	19068830
chr10	19081767	19082466
chr10	19082466	19082720
chr10	19086978	19087391
chr10	19091991	19092469
chr10	19094383	19094633
chr10	19099608	19099766
chr10	19117292	19117686
chr10	19118671	19119175
chr10	19119175	19119653
chr10	19126451	19126937
chr10	19126937	19127373
chr10	19127458	19127608
chr10	19131112	19131317
chr10	19139252	19139598
chr10	19140298	19140616
chr10	19140616	19141062
chr10	19141127	19141297
chr10	19149239	19149549
chr10	19149549	19149777

chr10	19150083	19150505
chr10	19150737	19151143
chr10	19154103	19154331
chr10	19162034	19162596
chr10	19162910	19163090
chr10	19167572	19168041
chr10	19168041	19168392
chr10	19168392	19168697
chr10	19177143	19177419
chr10	19177419	19177804
chr10	19181237	19181416
chr10	19185781	19185939
chr10	19186000	19186254
chr10	19186254	19186605
chr10	19187907	19188242
chr10	19188242	19188547
chr10	19191514	19191842
chr10	19192214	19192597
chr10	19192763	19192924
chr10	19192960	19193207
chr10	19193207	19193571
chr10	19220135	19220292
chr10	19240141	19240340
chr10	19268075	19268357
chr10	19283303	19283501
chr10	19312898	19313266
chr10	19352876	19353027
chr10	19353027	19353187
chr10	19357105	19357435
chr10	19384278	19384473
chr10	19463921	19464367
chr10	19464367	19464757
chr10	19464757	19465016
chr10	19491601	19491752
chr10	19494476	19494743
chr10	19514458	19514874
chr10	19526072	19526237
chr10	19526277	19526689
chr10	19529702	19529966
chr10	19531270	19531884
chr10	19532035	19532268
chr10	19533888	19534097

chr10	19555606	19555795
chr10	19563383	19563578
chr10	19569395	19569630
chr10	19569853	19570369
chr10	19570369	19570865
chr10	19586641	19586868
chr10	19591457	19591891
chr10	19591891	19592128
chr10	19592128	19592377
chr10	19594640	19594797
chr10	19598050	19598498
chr10	19601530	19601727
chr10	19639732	19639898
chr10	19687761	19688130
chr10	19693168	19693466
chr10	19693712	19693938
chr10	19705058	19705552
chr10	19705564	19706001
chr10	19708361	19708594
chr10	19711651	19711864
chr10	19712313	19712611
chr10	19712611	19712835
chr10	19721566	19721926
chr10	19761980	19762369
chr10	19811896	19812108
chr10	19849518	19849691
chr10	19850001	19850330
chr10	19850490	19850890
chr10	19850890	19851256
chr10	19851256	19851487
chr10	19851487	19851742
chr10	19868895	19869048
chr10	19875536	19875762
chr10	19877288	19877623
chr10	19893195	19893685
chr10	19894066	19894301
chr10	19907236	19907545
chr10	19907545	19907806
chr10	19915373	19915665
chr10	19918032	19918240
chr10	19933590	19934044
chr10	19934044	19934328

chr10	19934328	19934629
chr10	19934629	19935034
chr10	19941816	19942415
chr10	19945612	19945850
chr10	19945850	19946092
chr10	19946247	19946446
chr10	19956736	19957148
chr10	19964912	19965405
chr10	19966989	19967197
chr10	19990604	19990867
chr10	19991178	19991530
chr10	19991604	19992346
chr10	20046154	20046387
chr10	20073314	20073543
chr10	20073613	20073817
chr10	20076713	20077215
chr10	20077215	20077520
chr10	20077520	20077947
chr10	20090999	20091315
chr10	20091433	20091906
chr10	20104350	20104704
chr10	20104704	20105052
chr10	20105498	20106070
chr10	20106187	20106465
chr10	20119360	20119900
chr10	20123292	20123756
chr10	20125156	20125571
chr10	20148500	20149089
chr10	20149089	20149336
chr10	20149336	20149842
chr10	20152218	20152493
chr10	20159176	20159690
chr10	20169221	20169741
chr10	20170503	20170825
chr10	20178840	20179156
chr10	20179943	20180103
chr10	20186595	20186919
chr10	20186919	20187318
chr10	20220505	20220814
chr10	20220814	20221191
chr10	20233388	20233795
chr10	20235682	20235985

chr10	20239542	20239741
chr10	20254586	20254810
chr10	20269071	20269271
chr10	20272012	20272354
chr10	20272354	20272635
chr10	20284967	20285210
chr10	20285248	20285670
chr10	20285670	20285963
chr10	20290457	20290703
chr10	20290703	20290918
chr10	20294351	20294582
chr10	20296106	20296266
chr10	20311859	20312190
chr10	20312190	20312640
chr10	20312640	20313140
chr10	20347369	20347644
chr10	20347644	20348150
chr10	20348154	20348330
chr10	20361931	20362184
chr10	20382850	20383166
chr10	20383797	20384069
chr10	20404306	20404486
chr10	20405513	20405730
chr10	20409072	20409561
chr10	20459755	20459959
chr10	20518137	20518706
chr10	20527602	20527761
chr10	20527808	20528080
chr10	20547185	20547378
chr10	20657773	20657948
chr10	20658134	20658309
chr10	20724950	20725133
chr10	20768427	20768615
chr10	20770471	20770733
chr10	20773348	20773498
chr10	20813284	20813483
chr10	20817914	20818069
chr10	20818175	20818407
chr10	20818585	20818844
chr10	20824963	20825159
chr10	20825159	20825416
chr10	20828326	20828820

chr10	20944315	20944651
chr10	20944651	20944932
chr10	20948503	20948653
chr10	20952040	20952384
chr10	20952384	20952979
chr10	20966110	20966517
chr10	20992745	20992971
chr10	21051358	21051728
chr10	21115412	21115618
chr10	21128316	21128536
chr10	21158420	21158596
chr10	21158714	21158874
chr10	21158909	21159495
chr10	21159651	21159900
chr10	21159900	21160110
chr10	21160537	21160919
chr10	21160919	21161396
chr10	21189054	21189257
chr10	21252273	21252506
chr10	21258134	21258317
chr10	21287151	21287583
chr10	21287631	21288068
chr10	21294285	21294653
chr10	21295740	21296118
chr10	21296118	21296357
chr10	21296357	21296674
chr10	21334371	21334814
chr10	21335253	21335531
chr10	21335531	21335943
chr10	21337199	21337388
chr10	21369821	21370174
chr10	21378680	21378914
chr10	21382968	21383215
chr10	21385681	21386028
chr10	21399014	21399564
chr10	21411574	21412092
chr10	21414436	21414698
chr10	21414967	21415351
chr10	21421069	21421372
chr10	21427548	21427715
chr10	21458708	21458891
chr10	21465405	21465727

chr10	21465760	21466091
chr10	21476348	21476749
chr10	21477321	21477597
chr10	21490960	21491211
chr10	21496901	21497186
chr10	21497186	21497585
chr10	21504391	21504764
chr10	21504764	21505013
chr10	21518383	21518636
chr10	21527088	21527345
chr10	21529155	21529340
chr10	21529456	21529786
chr10	21532205	21532711
chr10	21533060	21533258
chr10	21551577	21551794
chr10	21577558	21577900
chr10	21594917	21595091
chr10	21601009	21601247
chr10	21606988	21607252
chr10	21611931	21612326
chr10	21618814	21619209
chr10	21619209	21619652
chr10	21619670	21619820
chr10	21639906	21640258
chr10	21675059	21675252
chr10	21685567	21685774
chr10	21686019	21686485
chr10	21687436	21687586
chr10	21727392	21727554
chr10	21727954	21728235
chr10	21735982	21736224
chr10	21773516	21774137
chr10	21776305	21776464
chr10	21776466	21776799
chr10	21776799	21777076
chr10	21782966	21783335
chr10	21783524	21783828
chr10	21783828	21784165
chr10	21785764	21786224
chr10	21789790	21790102
chr10	21790102	21790853
chr10	21790994	21791377

chr10	21796439	21796861
chr10	21797145	21797511
chr10	21798237	21798452
chr10	21801431	21802068
chr10	21807336	21807816
chr10	21807865	21808145
chr10	21808992	21809406
chr10	21809449	21809620
chr10	21809620	21809861
chr10	21827605	21827791
chr10	21831088	21831325
chr10	21833554	21833787
chr10	21833787	21834225
chr10	21839234	21839672
chr10	21844178	21844404
chr10	21844449	21844764
chr10	21845415	21845623
chr10	21846426	21846824
chr10	21847543	21847704
chr10	21849252	21849607
chr10	21853179	21853412
chr10	21853630	21853821
chr10	21854430	21854622
chr10	21857730	21857950
chr10	21877359	21877669
chr10	21879417	21879624
chr10	21882194	21882351
chr10	21886965	21887218
chr10	21905572	21905919
chr10	21934430	21934672
chr10	21946712	21946907
chr10	21974812	21975106
chr10	21975282	21975707
chr10	21979001	21979230
chr10	21986190	21986465
chr10	21991220	21991534
chr10	21993104	21993362
chr10	21993856	21994145
chr10	21994145	21994401
chr10	21994401	21994935
chr10	22000057	22000579
chr10	22000579	22000799

chr10	22000799	22001187
chr10	22001717	22001940
chr10	22005386	22005540
chr10	22007906	22008067
chr10	22010196	22010457
chr10	22014633	22014795
chr10	22036327	22036594
chr10	22050970	22051162
chr10	22058326	22059155
chr10	22064830	22064985
chr10	22103569	22103723
chr10	22104051	22104240
chr10	22114050	22114240
chr10	22134440	22134738
chr10	22134738	22134970
chr10	22141004	22141258
chr10	22142232	22142798
chr10	22142798	22143115
chr10	22148384	22148714
chr10	22148714	22149032
chr10	22149032	22149381
chr10	22158330	22158746
chr10	22163441	22163703
chr10	22163703	22164047
chr10	22165718	22165875
chr10	22169134	22169633
chr10	22169831	22169993
chr10	22192039	22192191
chr10	22194787	22194987
chr10	22202761	22203133
chr10	22203152	22203309
chr10	22212070	22212721
chr10	22223418	22223803
chr10	22223803	22224128
chr10	22277640	22277804
chr10	22295937	22296113
chr10	22312504	22312701
chr10	22357416	22357732
chr10	22393026	22393360
chr10	22402313	22402934
chr10	22413657	22414017
chr10	22414017	22414341

chr10	22448321	22448473
chr10	22470367	22470660
chr10	22470660	22471059
chr10	22471318	22471612
chr10	22479935	22480151
chr10	22579501	22579729
chr10	22618987	22619149
chr10	22645201	22645456
chr10	22696973	22697150
chr10	22698297	22699042
chr10	22699630	22699800
chr10	22701705	22701860
chr10	22701999	22702225
chr10	22713770	22714083
chr10	22714602	22714866
chr10	22730302	22730561
chr10	22730834	22731044
chr10	22731044	22731312
chr10	22731312	22731636
chr10	22731636	22732158
chr10	22744595	22744874
chr10	22744874	22745148
chr10	22745148	22745367
chr10	22745367	22745629
chr10	22746009	22746164
chr10	22774721	22774876
chr10	22781564	22781715
chr10	22815833	22816420
chr10	22816449	22816624
chr10	22853817	22853972
chr10	22957024	22957443
chr10	23080837	23080997
chr10	23081360	23081604
chr10	23086989	23087144
chr10	23091971	23092437
chr10	23116843	23117096
chr10	23157035	23157193
chr10	23161199	23161363
chr10	23176238	23176535
chr10	23177363	23177619
chr10	23177636	23177973
chr10	23177973	23178273

chr10	23184611	23184761
chr10	23185084	23185845
chr10	23198166	23198382
chr10	23203930	23204328
chr10	23228718	23228876
chr10	23233562	23233831
chr10	23348894	23349130
chr10	23349130	23349328
chr10	23349328	23349517
chr10	23349517	23349725
chr10	23349725	23350013
chr10	23350013	23350367
chr10	23350367	23350702
chr10	23350740	23350952
chr10	23365555	23365732
chr10	23385355	23385516
chr10	23421372	23421686
chr10	23452944	23453151
chr10	23457114	23457783
chr10	23458254	23458690
chr10	23511351	23511506
chr10	23530659	23530988
chr10	23533453	23533639
chr10	23549286	23549493
chr10	23549493	23549933
chr10	23557396	23557789
chr10	23557789	23558257
chr10	23559476	23559656
chr10	23569426	23569862
chr10	23571024	23571423
chr10	23613056	23613427
chr10	23613521	23613949
chr10	23621125	23621419
chr10	23622053	23622374
chr10	23626151	23626444
chr10	23699059	23699296
chr10	23703360	23703655
chr10	23726374	23726560
chr10	23749423	23749645
chr10	23777377	23777551
chr10	23780976	23781191
chr10	23782992	23783316

chr10	23783316	23783650
chr10	23784260	23784410
chr10	23786664	23786986
chr10	23786986	23787233
chr10	23787233	23787505
chr10	23787542	23787751
chr10	23796586	23796933
chr10	23796933	23797387
chr10	23798267	23798417
chr10	23809936	23810130
chr10	23811609	23811762
chr10	23812225	23812392
chr10	23864600	23864757
chr10	23886033	23886440
chr10	23886440	23886790
chr10	23894255	23894494
chr10	23894570	23894765
chr10	23894765	23895019
chr10	23895019	23895248
chr10	23897631	23897916
chr10	23928047	23928287
chr10	23928287	23928478
chr10	23931530	23931802
chr10	23935191	23935366
chr10	23964996	23965296
chr10	23974650	23974899
chr10	23978411	23978877
chr10	23979352	23979715
chr10	23988565	23988757
chr10	24006516	24006761
chr10	24029065	24029510
chr10	24051458	24051618
chr10	24052928	24053133
chr10	24053133	24053438
chr10	24053438	24053759
chr10	24055285	24055435
chr10	24080254	24080480
chr10	24111222	24111437
chr10	24115751	24115978
chr10	24125066	24125515
chr10	24131520	24131747
chr10	24131747	24131954

chr10	24132008	24132313
chr10	24132313	24132683
chr10	24133232	24133442
chr10	24149058	24149411
chr10	24149411	24149824
chr10	24162857	24163109
chr10	24164428	24164855
chr10	24164855	24165218
chr10	24173092	24173639
chr10	24173684	24173956
chr10	24190483	24190680
chr10	24209970	24210510
chr10	24210716	24211035
chr10	24211035	24211316
chr10	24212381	24212613
chr10	24216904	24217102
chr10	24223280	24223723
chr10	24223723	24224113
chr10	24226859	24227054
chr10	24249697	24249876
chr10	24298557	24298864
chr10	24310560	24310824
chr10	24312133	24312408
chr10	24315518	24315795
chr10	24365370	24365752
chr10	24365771	24365964
chr10	24366896	24367291
chr10	24367291	24367749
chr10	24372186	24372450
chr10	24373384	24373751
chr10	24373758	24374499
chr10	24379207	24379649
chr10	24382010	24382221
chr10	24388301	24388785
chr10	24392034	24392420
chr10	24396050	24396377
chr10	24396377	24396694
chr10	24397057	24397726
chr10	24404996	24405250
chr10	24410044	24410307
chr10	24438695	24438962
chr10	24444688	24444878

chr10	24445049	24445327
chr10	24446108	24446680
chr10	24447101	24447521
chr10	24451561	24451771
chr10	24454917	24455132
chr10	24458668	24459022
chr10	24469128	24469340
chr10	24469423	24469992
chr10	24470449	24470615
chr10	24470897	24471075
chr10	24476353	24476577
chr10	24478565	24479112
chr10	24479112	24479633
chr10	24482620	24482810
chr10	24491891	24492235
chr10	24493195	24493456
chr10	24493456	24493773
chr10	24493987	24494282
chr10	24552972	24553151
chr10	24554123	24554493
chr10	24572440	24572747
chr10	24580999	24581382
chr10	24581382	24581763
chr10	24588981	24589249
chr10	24589288	24589661
chr10	24589847	24589997
chr10	24591290	24591440
chr10	24591851	24592060
chr10	24592362	24592648
chr10	24595273	24595528
chr10	24595528	24596089
chr10	24596205	24596674
chr10	24596719	24596960
chr10	24597363	24597531
chr10	24599412	24599726
chr10	24621735	24621885
chr10	24622652	24622825
chr10	24625162	24625387
chr10	24628509	24628762
chr10	24628839	24629597
chr10	24635352	24635575
chr10	24635575	24635929

chr10	24635929	24636432
chr10	24637067	24637270
chr10	24638335	24638638
chr10	24711444	24711862
chr10	24711862	24712343
chr10	24728512	24728708
chr10	24732232	24732402
chr10	24739057	24739570
chr10	24739637	24739955
chr10	24741337	24741500
chr10	24741650	24741863
chr10	24741909	24742097
chr10	24753535	24753858
chr10	24754010	24754190
chr10	24754190	24754420
chr10	24754420	24754695
chr10	24755967	24756364
chr10	24756483	24756712
chr10	24765289	24765755
chr10	24766264	24766414
chr10	24780901	24781139
chr10	24783970	24784365
chr10	24789783	24790034
chr10	24797337	24797554
chr10	24822064	24822323
chr10	24822323	24822642
chr10	24830539	24830745
chr10	24869643	24870101
chr10	24870101	24870473
chr10	24879278	24879582
chr10	24884346	24884686
chr10	24886091	24886422
chr10	24907462	24907623
chr10	24917917	24918204
chr10	24924588	24925335
chr10	24927483	24927757
chr10	24931539	24931869
chr10	24961141	24961409
chr10	24972188	24972546
chr10	24972732	24973345
chr10	24973389	24973707
chr10	24984624	24985209

chr10	25039568	25039797
chr10	25040100	25040278
chr10	25061392	25062111
chr10	25072721	25072969
chr10	25084802	25085147
chr10	25119211	25119386
chr10	25128563	25128893
chr10	25155902	25156187
chr10	25156249	25156561
chr10	25167792	25167954
chr10	25188151	25188553
chr10	25213773	25213928
chr10	25219233	25219395
chr10	25224479	25224691
chr10	25239228	25239489
chr10	25246278	25246497
chr10	25248428	25248587
chr10	25252824	25253344
chr10	25253545	25254175
chr10	25268578	25268819
chr10	25271753	25272065
chr10	25272065	25272494
chr10	25275560	25275716
chr10	25278812	25279206
chr10	25291136	25291402
chr10	25296635	25297000
chr10	25297000	25297359
chr10	25303067	25303337
chr10	25308785	25309131
chr10	25314100	25314417
chr10	25314417	25314667
chr10	25317709	25318014
chr10	25319578	25319871
chr10	25331425	25331651
chr10	25331675	25332181
chr10	25340499	25340722
chr10	25340722	25341004
chr10	25345312	25345524
chr10	25355133	25355312
chr10	25359278	25359626
chr10	25359626	25360134
chr10	25360134	25360472

chr10	25360472	25360703
chr10	25362388	25362595
chr10	25366709	25367009
chr10	25367125	25367348
chr10	25377360	25377575
chr10	25383467	25383922
chr10	25383922	25384146
chr10	25392870	25393172
chr10	25395307	25396054
chr10	25418574	25418739
chr10	25426156	25426311
chr10	25426520	25426672
chr10	25426742	25427073
chr10	25427073	25427348
chr10	25432360	25432678
chr10	25432708	25432960
chr10	25433005	25433305
chr10	25433544	25433945
chr10	25434380	25434681
chr10	25434734	25434933
chr10	25436020	25436170
chr10	25437701	25437889
chr10	25439303	25439480
chr10	25453154	25453325
chr10	25457271	25457556
chr10	25458038	25458336
chr10	25458378	25458683
chr10	25458820	25459179
chr10	25488402	25488814
chr10	25584083	25584264
chr10	25594834	25595034
chr10	25619819	25619969
chr10	25621278	25621430
chr10	25621463	25621732
chr10	25640012	25640185
chr10	25681339	25681545
chr10	25690673	25690949
chr10	25690949	25691242
chr10	25691482	25691968
chr10	25695511	25695838
chr10	25695838	25696137
chr10	25713170	25713320

chr10	25768574	25768725
chr10	25787946	25788486
chr10	25788664	25789019
chr10	25824697	25824910
chr10	25829326	25829543
chr10	25918136	25918606
chr10	25928458	25928689
chr10	25947796	25948012
chr10	25960555	25960705
chr10	25960849	25961435
chr10	25961435	25961824
chr10	26078150	26078522
chr10	26078531	26078876
chr10	26078876	26079208
chr10	26079756	26080171
chr10	26082166	26082316
chr10	26114359	26114808
chr10	26139518	26139732
chr10	26166658	26166900
chr10	26176020	26176198
chr10	26220815	26221027
chr10	26232724	26233056
chr10	26233073	26233381
chr10	26244482	26244781
chr10	26249828	26250036
chr10	26250302	26250456
chr10	26263758	26264591
chr10	26273859	26274019
chr10	26275487	26275751
chr10	26280080	26280332
chr10	26358331	26358486
chr10	26358614	26358764
chr10	26358799	26359040
chr10	26359040	26359425
chr10	26359716	26360063
chr10	26372971	26373227
chr10	26373984	26374252
chr10	26374252	26374497
chr10	26374627	26374905
chr10	26374905	26375322
chr10	26375367	26375588
chr10	26375882	26376174

chr10	26496420	26496661
chr10	26544136	26544397
chr10	26544593	26545232
chr10	26559404	26559720
chr10	26564479	26564874
chr10	26577104	26577439
chr10	26607232	26607456
chr10	26613276	26613705
chr10	26639447	26639597
chr10	26719584	26719804
chr10	26752711	26752889
chr10	26753223	26753775
chr10	26761372	26761636
chr10	26762586	26762784
chr10	26772255	26772696
chr10	26772696	26773033
chr10	26773033	26773350
chr10	26776548	26776729
chr10	26780517	26780816
chr10	26798851	26799435
chr10	26799467	26800081
chr10	26803300	26803620
chr10	26807556	26807784
chr10	26828420	26828750
chr10	26828750	26829196
chr10	26854490	26855025
chr10	26867201	26867393
chr10	26877371	26877681
chr10	26890646	26890827
chr10	26893059	26893560
chr10	26895594	26896183
chr10	26919628	26919878
chr10	26939620	26939868
chr10	26958430	26958924
chr10	26962548	26962755
chr10	26995692	26996043
chr10	26996043	26996525
chr10	27010366	27010903
chr10	27012967	27013229
chr10	27013236	27013513
chr10	27022836	27022986
chr10	27063721	27064020

chr10	27074390	27074576
chr10	27116998	27117282
chr10	27117681	27118352
chr10	27133215	27133439
chr10	27174165	27174619
chr10	27174619	27174846
chr10	27178159	27178630
chr10	27245721	27245917
chr10	27249599	27249908
chr10	27249908	27250178
chr10	27250178	27250383
chr10	27250383	27250537
chr10	27265955	27266122
chr10	27375287	27375575
chr10	27378299	27378612
chr10	27459472	27459704
chr10	27533316	27533569
chr10	27538086	27538279
chr10	27558536	27558686
chr10	27595749	27595993
chr10	27632895	27633238
chr10	27633238	27633771
chr10	27643625	27643812
chr10	27643873	27644161
chr10	27645745	27645993
chr10	27654197	27654841
chr10	27659051	27659279
chr10	27663426	27663578
chr10	27674475	27674650
chr10	27729741	27729957
chr10	27732853	27733030
chr10	27809478	27809902
chr10	27820339	27820587
chr10	27822046	27822330
chr10	27822342	27822492
chr10	27822623	27822781
chr10	27822781	27822941
chr10	27823054	27823225
chr10	27823414	27823565
chr10	27823663	27823814
chr10	27828373	27828637
chr10	27828637	27828899

chr10	27829218	27829437
chr10	27829745	27830170
chr10	27863486	27864062
chr10	27900934	27901261
chr10	27912273	27912423
chr10	27942822	27943037
chr10	27968131	27968549
chr10	27968655	27969044
chr10	27969145	27969375
chr10	27979814	27979986
chr10	27983977	27984352
chr10	27985018	27985456
chr10	27985672	27986040
chr10	27987012	27987351
chr10	27988050	27988447
chr10	28001684	28001899
chr10	28001984	28002452
chr10	28010858	28011066
chr10	28014268	28014494
chr10	28029085	28029255
chr10	28061702	28061904
chr10	28061904	28062336
chr10	28066391	28066893
chr10	28074401	28075078
chr10	28075078	28075524
chr10	28075662	28075886
chr10	28075886	28076126
chr10	28076126	28076456
chr10	28083747	28083943
chr10	28085997	28086267
chr10	28087349	28087694
chr10	28087694	28088016
chr10	28094606	28095009
chr10	28102553	28103136
chr10	28105199	28105461
chr10	28113261	28113472
chr10	28113684	28114382
chr10	28127411	28127651
chr10	28128526	28128716
chr10	28133772	28134076
chr10	28157675	28157838
chr10	28157984	28158288

chr10	28158296	28158680
chr10	28160892	28161194
chr10	28162994	28163187
chr10	28163271	28163471
chr10	28171000	28171241
chr10	28171241	28171616
chr10	28176064	28176259
chr10	28178220	28178464
chr10	28185474	28185662
chr10	28191142	28191360
chr10	28193507	28194002
chr10	28194053	28194289
chr10	28227662	28228154
chr10	28228254	28228607
chr10	28262241	28262566
chr10	28299169	28299493
chr10	28301268	28301455
chr10	28303790	28304245
chr10	28304493	28304810
chr10	28380950	28381189
chr10	28426935	28427139
chr10	28492928	28493083
chr10	28494085	28494241
chr10	28662128	28662503
chr10	28694851	28695407
chr10	28695566	28695936
chr10	28696523	28696724
chr10	28730212	28730442
chr10	28759165	28759366
chr10	28798170	28798394
chr10	28814734	28814884
chr10	28825356	28825560
chr10	28844230	28844392
chr10	28860738	28860984
chr10	28922403	28922592
chr10	29030037	29030292
chr10	29096907	29097282
chr10	29146600	29147098
chr10	29147098	29147317
chr10	29147317	29147511
chr10	29178094	29178320
chr10	29206824	29206989

chr10	29285840	29286094
chr10	29288514	29288679
chr10	29313034	29313358
chr10	29313358	29313700
chr10	29333898	29334092
chr10	29361381	29361963
chr10	29361963	29362476
chr10	29381517	29381984
chr10	29394964	29395195
chr10	29426203	29426353
chr10	29433488	29433687
chr10	29450702	29450994
chr10	29532893	29533119
chr10	29533370	29533645
chr10	29534300	29534509
chr10	29534509	29534696
chr10	29534749	29535109
chr10	29535427	29536019
chr10	29554605	29554791
chr10	29601355	29601562
chr10	29698739	29698961
chr10	29708403	29708760
chr10	29708760	29709043
chr10	29741118	29741471
chr10	29788186	29788374
chr10	29804271	29804438
chr10	29815963	29816131
chr10	29829765	29829916
chr10	29875246	29875399
chr10	29894700	29895049
chr10	29897881	29898329
chr10	29970890	29971105
chr10	30069143	30069433
chr10	30089515	30089775
chr10	30102547	30102826
chr10	30195155	30195315
chr10	30200024	30200772
chr10	30259690	30260253
chr10	30314996	30315242
chr10	30380569	30380839
chr10	30411829	30412026
chr10	30529970	30530220

chr10	30548237	30548414
chr10	30552511	30552695
chr10	30600059	30600308
chr10	30600308	30600583
chr10	30600583	30601042
chr10	30608066	30608442
chr10	30618005	30618301
chr10	30618301	30618813
chr10	30623006	30623210
chr10	30628641	30628865
chr10	30629632	30629820
chr10	30636870	30637076
chr10	30638149	30638554
chr10	30639301	30639456
chr10	30678143	30678314
chr10	30686821	30687200
chr10	30709981	30710242
chr10	30712496	30712958
chr10	30712958	30713256
chr10	30718964	30719346
chr10	30729163	30729475
chr10	30729475	30729806
chr10	30729806	30730140
chr10	30737345	30737541
chr10	30745302	30745494
chr10	30745668	30745987
chr10	30758655	30758821
chr10	30778208	30778687
chr10	30787633	30787933
chr10	30802634	30802971
chr10	30802971	30803224
chr10	30803224	30803527
chr10	30810208	30810453
chr10	30810453	30810689
chr10	30810742	30811281
chr10	30825124	30825357
chr10	30842317	30842691
chr10	30842691	30843097
chr10	30843097	30843328
chr10	30843328	30843542
chr10	30858953	30859144
chr10	30866457	30866788

chr10	30898128	30898387
chr10	30918639	30918938
chr10	30919124	30919415
chr10	30922410	30922565
chr10	31034697	31035311
chr10	31035548	31036168
chr10	31079643	31079995
chr10	31079995	31080321
chr10	31089730	31090013
chr10	31104645	31105109
chr10	31112739	31112957
chr10	31151045	31151322
chr10	31161820	31162115
chr10	31200360	31200615
chr10	31201641	31201987
chr10	31212182	31212630
chr10	31231988	31232483
chr10	31248544	31248730
chr10	31257712	31257990
chr10	31288799	31288956
chr10	31299262	31299523
chr10	31299554	31299772
chr10	31312803	31313179
chr10	31313179	31313511
chr10	31313511	31313948
chr10	31331427	31331660
chr10	31336513	31336760
chr10	31345668	31345974
chr10	31346081	31346284
chr10	31346489	31346639
chr10	31346656	31347351
chr10	31350809	31350986
chr10	31354020	31354757
chr10	31370157	31370502
chr10	31402156	31402330
chr10	31429337	31429534
chr10	31434403	31434580
chr10	31445239	31445461
chr10	31445461	31445751
chr10	31445751	31446189
chr10	31446547	31446719
chr10	31446891	31447362

chr10	31462586	31462956
chr10	31462956	31463528
chr10	31466695	31466845
chr10	31467131	31467567
chr10	31467634	31467807
chr10	31479304	31479527
chr10	31501698	31501998
chr10	31503560	31503778
chr10	31515069	31515369
chr10	31519482	31519790
chr10	31528704	31528963
chr10	31532882	31533045
chr10	31533557	31533781
chr10	31536258	31536628
chr10	31538252	31538626
chr10	31538638	31539033
chr10	31552923	31553085
chr10	31553369	31553550
chr10	31557337	31557904
chr10	31557912	31558090
chr10	31558329	31558571
chr10	31592102	31592436
chr10	31592493	31592645
chr10	31592774	31593054
chr10	31594049	31594215
chr10	31594603	31594852
chr10	31607025	31607194
chr10	31608296	31608510
chr10	31608510	31608817
chr10	31608932	31609288
chr10	31609288	31609577
chr10	31609577	31610049
chr10	31742473	31742745
chr10	31874710	31874875
chr10	32013671	32013955
chr10	32019494	32019740
chr10	32019740	32020070
chr10	32037641	32037863
chr10	32063242	32063578
chr10	32063900	32064274
chr10	32064366	32064777
chr10	32073417	32073647

chr10	32077425	32078137
chr10	32126295	32126636
chr10	32149776	32149936
chr10	32223705	32224227
chr10	32350085	32350294
chr10	32386321	32386588
chr10	32775199	32775370
chr10	32776380	32776780
chr10	32781084	32781387
chr10	32827898	32828168
chr10	32828505	32828742
chr10	32849032	32849345
chr10	32857443	32857896
chr10	32858260	32858499
chr10	32889597	32890099
chr10	32890099	32890481
chr10	32890481	32890788
chr10	32890800	32891116
chr10	32965285	32965600
chr10	32984716	32985364
chr10	32986799	32987051
chr10	33012673	33012828
chr10	33073247	33073508
chr10	33253227	33253388
chr10	33333383	33333806
chr10	33375511	33375724
chr10	33557670	33557878
chr10	33557878	33558133
chr10	33558133	33558539
chr10	33679891	33680043
chr10	33716214	33716373
chr10	33784030	33784285
chr10	33888013	33888224
chr10	33904723	33905158
chr10	33905272	33905524
chr10	33950811	33951446
chr10	33986835	33987154
chr10	34013308	34013681
chr10	34019136	34020053
chr10	34033934	34034255
chr10	34035674	34035907
chr10	34036237	34036593

chr10	34036641	34036896
chr10	34060239	34060435
chr10	34070917	34071119
chr10	34071119	34071336
chr10	34071336	34071638
chr10	34071638	34071966
chr10	34092014	34092278
chr10	34096275	34096708
chr10	34096708	34096917
chr10	34127450	34127769
chr10	34183769	34184126
chr10	34206437	34206764
chr10	34206764	34207068
chr10	34207068	34207364
chr10	34207364	34207609
chr10	34208714	34208993
chr10	34218212	34218441
chr10	34231936	34232189
chr10	34233111	34233349
chr10	34234127	34234363
chr10	34239404	34239660
chr10	34280630	34280853
chr10	34281291	34281509
chr10	34281641	34281991
chr10	34281991	34282418
chr10	34282418	34282791
chr10	34282791	34283032
chr10	34297260	34297554
chr10	34297554	34297959
chr10	34307114	34307376
chr10	34328342	34328631
chr10	34342959	34343360
chr10	34356530	34356875
chr10	34382333	34382817
chr10	34385744	34385953
chr10	34418159	34418848
chr10	34418923	34419185
chr10	34444297	34444571
chr10	34444571	34444946
chr10	34464292	34464668
chr10	34482784	34483026
chr10	34483238	34483686

chr10	34483686	34484114
chr10	34484114	34484490
chr10	34485125	34485385
chr10	34507848	34508212
chr10	34508580	34509003
chr10	34509319	34509596
chr10	34513038	34513241
chr10	34532329	34532672
chr10	34532672	34533020
chr10	34551688	34551899
chr10	34552034	34552235
chr10	34596489	34596719
chr10	34618400	34618879
chr10	34654408	34654674
chr10	34719360	34719672
chr10	34731342	34731576
chr10	34731576	34731805
chr10	34793770	34793989
chr10	34794070	34794269
chr10	34811176	34811438
chr10	34904847	34905095
chr10	35075075	35075341
chr10	35441599	35441800
chr10	35444020	35444241
chr10	35552028	35552268
chr10	35586022	35586196
chr10	35586259	35586654
chr10	35586920	35587071
chr10	35714312	35714672
chr10	35726450	35726877
chr10	35727954	35728218
chr10	35737711	35737919
chr10	35814460	35814675
chr10	35838350	35838605
chr10	35884248	35884458
chr10	35949043	35949434
chr10	35972316	35972692
chr10	36045215	36045400
chr10	36125547	36125742
chr10	36431425	36431671
chr10	36483535	36483768
chr10	36491684	36491895

chr10	36491978	36492301
chr10	36492332	36492562
chr10	36493671	36493953
chr10	36506146	36506325
chr10	36506528	36507135
chr10	36507135	36507698
chr10	36509901	36510114
chr10	36513880	36514030
chr10	36692034	36692255
chr10	36848911	36849073
chr10	36852761	36852992
chr10	36910783	36910945
chr10	36917821	36918566
chr10	36964538	36964884
chr10	36965672	36966161
chr10	36973990	36974407
chr10	36974407	36974663
chr10	36974663	36974884
chr10	36974884	36975227
chr10	37014345	37014583
chr10	37072952	37073102
chr10	37136157	37136374
chr10	37136968	37137446
chr10	37138839	37139088
chr10	37139187	37139339
chr10	37140879	37141260
chr10	37141260	37141537
chr10	37141681	37141896
chr10	37145576	37145726
chr10	37170957	37171303
chr10	37171580	37171925
chr10	37171925	37172220
chr10	37183401	37183599
chr10	37185304	37185574
chr10	37206944	37207344
chr10	37207520	37207675
chr10	37220513	37220835
chr10	37227946	37228256
chr10	37235176	37235326
chr10	37240695	37240983
chr10	37247205	37247459
chr10	37278224	37278437

chr10	37278784	37279047
chr10	37279258	37279570
chr10	37279570	37279817
chr10	37283014	37283224
chr10	37305559	37305775
chr10	37315150	37315385
chr10	37320098	37320395
chr10	37335186	37335570
chr10	37335570	37335988
chr10	37337070	37337657
chr10	37348797	37349217
chr10	37349319	37349645
chr10	37357125	37357673
chr10	37357673	37357999
chr10	37360980	37361307
chr10	37361413	37361684
chr10	37367825	37368217
chr10	37400474	37400770
chr10	37450004	37450190
chr10	37457213	37457504
chr10	37457504	37457793
chr10	37479994	37480295
chr10	37480295	37481000
chr10	37483592	37483888
chr10	37484421	37484571
chr10	37494196	37494419
chr10	37502627	37502863
chr10	37506048	37506326
chr10	37506377	37506604
chr10	37591639	37591913
chr10	37616317	37616659
chr10	37630472	37630738
chr10	37803265	37803728
chr10	37806463	37806649
chr10	37820387	37820603
chr10	37823690	37823911
chr10	38059263	38059450
chr10	38112034	38112352
chr10	38123598	38123815
chr10	38143063	38143372
chr10	38147535	38147751
chr10	38155071	38155227

chr10	38174285	38174738
chr10	38180683	38180891
chr10	38185589	38185884
chr10	38246822	38247174
chr10	38309086	38309246
chr10	38521515	38521665
chr10	38645005	38645174
chr10	38647103	38647308
chr10	38649466	38649724
chr10	38965517	38965667
chr10	38966038	38966199
chr10	38977692	38977921
chr10	38979484	38979703
chr10	38994699	38994978
chr10	39019822	39020235
chr10	39064830	39065009
chr10	39067049	39067308
chr10	39107053	39107203
chr10	39133605	39134106
chr10	39134106	39134524
chr10	39134982	39135145
chr10	39157214	39157734
chr10	39157759	39157982
chr10	39161395	39161749
chr10	39170200	39170514
chr10	39170768	39170929
chr10	39170929	39171084
chr10	39171139	39171660
chr10	39171712	39172069
chr10	39176602	39176836
chr10	39197002	39197182
chr10	39239915	39240168
chr10	39245235	39245467
chr10	39248548	39248807
chr10	39250767	39250940
chr10	39251834	39252494
chr10	39261732	39262107
chr10	39272463	39272654
chr10	39274338	39274587
chr10	39274658	39274823
chr10	39335936	39336267
chr10	39359753	39360025

chr10	39361916	39362196
chr10	39369411	39369963
chr10	39369963	39370637
chr10	39385850	39386218
chr10	39386218	39386553
chr10	39388188	39388418
chr10	39403498	39403649
chr10	39406306	39406576
chr10	39412261	39412510
chr10	39412690	39413074
chr10	39413220	39413637
chr10	39415576	39415957
chr10	39430461	39430682
chr10	39430837	39431150
chr10	39431230	39431508
chr10	39435049	39435495
chr10	39436766	39436979
chr10	39442172	39442373
chr10	39442651	39442943
chr10	39449277	39449531
chr10	39449531	39449742
chr10	39449762	39450347
chr10	39477778	39478030
chr10	39490774	39491119
chr10	39503294	39503589
chr10	39505852	39506112
chr10	39518970	39519148
chr10	39519855	39520005
chr10	39520039	39520189
chr10	39520192	39520416
chr10	39520416	39520652
chr10	39520652	39520850
chr10	39521878	39522089
chr10	39555330	39555482
chr10	39566833	39567476
chr10	39571021	39571236
chr10	39572672	39573133
chr10	39582502	39582664
chr10	39600916	39601092
chr10	39612905	39613350
chr10	39613350	39613588
chr10	39613588	39614034

chr10	39614683	39615040
chr10	39644968	39645132
chr10	39658817	39659370
chr10	39659370	39659754
chr10	39659754	39660116
chr10	39660118	39660283
chr10	39716617	39716771
chr10	39731122	39731745
chr10	39731745	39732026
chr10	39732026	39732279
chr10	39732279	39732555
chr10	39732555	39732893
chr10	39732893	39733243
chr10	39742128	39742347
chr10	39785018	39785219
chr10	39811569	39811832
chr10	39880462	39880614
chr10	39881576	39882010
chr10	39898789	39899486
chr10	39919834	39919984
chr10	39926680	39926905
chr10	39926905	39927127
chr10	39936612	39936937
chr10	39939087	39939358
chr10	39957120	39957275
chr10	39959701	39960115
chr10	39960115	39960526
chr10	39960550	39960728
chr10	39967621	39967781
chr10	39984947	39985142
chr10	40024693	40025143
chr10	40025143	40025629
chr10	40041432	40041648
chr10	40042409	40042663
chr10	40053379	40053605
chr10	40063447	40063634
chr10	40067018	40067267
chr10	40067267	40067533
chr10	40091076	40091333
chr10	40094744	40095267
chr10	40128489	40128715
chr10	40141863	40142558

chr10	40152360	40152562
chr10	40152565	40152929
chr10	40162007	40162244
chr10	40177657	40177937
chr10	40212231	40212381
chr10	40212447	40213032
chr10	40246494	40246930
chr10	40246930	40247207
chr10	40247207	40247373
chr10	40256741	40256995
chr10	40257572	40257837
chr10	40257959	40258274
chr10	40258404	40259194
chr10	40259218	40259468
chr10	40259554	40259785
chr10	40262306	40262511
chr10	40263672	40264072
chr10	40274076	40274258
chr10	40281565	40281889
chr10	40281889	40282250
chr10	40282264	40282430
chr10	40284474	40284705
chr10	40284831	40285088
chr10	40285095	40285245
chr10	40292514	40293037
chr10	40300700	40301144
chr10	40301144	40301505
chr10	40301505	40301765
chr10	40302055	40302653
chr10	40302829	40302984
chr10	40307969	40308618
chr10	40309885	40310263
chr10	40310283	40310584
chr10	40317210	40317471
chr10	40328129	40328603
chr10	40329877	40330278
chr10	40330370	40330530
chr10	40338122	40338351
chr10	40338478	40338718
chr10	40339003	40339215
chr10	40339309	40339665
chr10	40339665	40339943

chr10	40348556	40349029
chr10	40349029	40349481
chr10	40349481	40349814
chr10	40349814	40350139
chr10	40354660	40354955
chr10	40355063	40355347
chr10	40371270	40371498
chr10	40372165	40372766
chr10	40379105	40379373
chr10	40379642	40380045
chr10	40385464	40385698
chr10	40416952	40417266
chr10	40417266	40417625
chr10	40428519	40428839
chr10	40437144	40437491
chr10	40457162	40457453
chr10	40457534	40457715
chr10	40465353	40465835
chr10	40476270	40476571
chr10	40479150	40479403
chr10	40479403	40479701
chr10	40487130	40487622
chr10	40500879	40501210
chr10	40510500	40511121
chr10	40511121	40511754
chr10	40511756	40512139
chr10	40521257	40521480
chr10	40521501	40521988
chr10	40527052	40527421
chr10	40527442	40527958
chr10	40528440	40528632
chr10	40529751	40529954
chr10	40551674	40552209
chr10	40584005	40584171
chr10	40597187	40597524
chr10	40629207	40629466
chr10	40629808	40630037
chr10	40645995	40646308
chr10	40646308	40646633
chr10	40646669	40646973
chr10	40646973	40647214
chr10	40648743	40649115

chr10	40683116	40683419
chr10	40683536	40683771
chr10	40712891	40713405
chr10	40713405	40713701
chr10	40808178	40808328
chr10	40813693	40813960
chr10	40830050	40830415
chr10	40830415	40830632
chr10	40832510	40832749
chr10	40851238	40851477
chr10	40865332	40865482
chr10	40872024	40872499
chr10	40880849	40881107
chr10	40882967	40883185
chr10	40883251	40883627
chr10	40883627	40884077
chr10	40884176	40884518
chr10	40884543	40884809
chr10	40908608	40908898
chr10	40913502	40913710
chr10	41003286	41003730
chr10	41004410	41004687
chr10	41015767	41016003
chr10	41022338	41022537
chr10	41028191	41028370
chr10	41040611	41041041
chr10	41163143	41163305
chr10	41193530	41193709
chr10	41200341	41200606
chr10	41209536	41209902
chr10	41209902	41210191
chr10	41221135	41221520
chr10	41224640	41224877
chr10	41224999	41225295
chr10	41225295	41225571
chr10	41225605	41225997
chr10	41226074	41226315
chr10	41226315	41226528
chr10	41244011	41244225
chr10	41253176	41253394
chr10	41282210	41282390
chr10	41288754	41289149

chr10	41293079	41293409
chr10	41302697	41302929
chr10	41302929	41303140
chr10	41303140	41303397
chr10	41303611	41303771
chr10	41303774	41304082
chr10	41304141	41304336
chr10	41375425	41375633
chr10	41377908	41378323
chr10	41417264	41417488
chr10	41426531	41426696
chr10	41432013	41432240
chr10	41434678	41434971
chr10	41436718	41436993
chr10	41445035	41445243
chr10	41449753	41449949
chr10	41449990	41450743
chr10	41460903	41461055
chr10	41472976	41473186
chr10	41477146	41477474
chr10	41477474	41477755
chr10	41477776	41478032
chr10	41489450	41489638
chr10	41489837	41490265
chr10	41490265	41490750
chr10	41505202	41505340
chr10	41505340	41505486
chr10	41505499	41505782
chr10	41505782	41505994
chr10	41509233	41509450
chr10	41518475	41518754
chr10	41518754	41518992
chr10	41518992	41519189
chr10	41519189	41519378
chr10	41519378	41519683
chr10	41519683	41519998
chr10	41519998	41520204
chr10	41553924	41554076
chr10	41561747	41561974
chr10	41564966	41565837
chr10	41568422	41568633
chr10	41571192	41571345

chr10	41575834	41576139
chr10	41576444	41576844
chr10	41579642	41579871
chr10	41582933	41583353
chr10	41587081	41587259
chr10	41605510	41605787
chr10	41611646	41612138
chr10	41613581	41614070
chr10	41652195	41652350
chr10	41655763	41656009
chr10	41659718	41660071
chr10	41678859	41679149
chr10	41680950	41681168
chr10	41681492	41681854
chr10	41682664	41682846
chr10	41682846	41683108
chr10	41684540	41684690
chr10	41684770	41685040
chr10	41689948	41690213
chr10	41690267	41690626
chr10	41690629	41690781
chr10	41716632	41716904
chr10	41731370	41731552
chr10	41757269	41757479
chr10	41796032	41796197
chr10	41809528	41809803
chr10	41809878	41810333
chr10	41810333	41810676
chr10	41810700	41810851
chr10	41821949	41822145
chr10	41822145	41822329
chr10	41822439	41822647
chr10	41822647	41822884
chr10	41826566	41826794
chr10	41830971	41831166
chr10	41866262	41866591
chr10	41887003	41887873
chr10	41894099	41894395
chr10	41930509	41930661
chr10	41934661	41934953
chr10	41934953	41935226
chr10	41980788	41981072

chr10	41982813	41983060
chr10	41986032	41986539
chr10	41996286	41996608
chr10	41997523	41997919
chr10	41997940	41998207
chr10	42017852	42018290
chr10	42018290	42018615
chr10	42028926	42029275
chr10	42031092	42031327
chr10	42032678	42032867
chr10	42033563	42033933
chr10	42033933	42034223
chr10	42055291	42055531
chr10	42070712	42070931
chr10	42072847	42073019
chr10	42079897	42080113
chr10	42086713	42087107
chr10	42088741	42088901
chr10	42089158	42089682
chr10	42089727	42090017
chr10	42096618	42096820
chr10	42098081	42098305
chr10	42100712	42100938
chr10	42101080	42101494
chr10	42101559	42101868
chr10	42107220	42107817
chr10	42107817	42108171
chr10	42114706	42115104
chr10	42115816	42116252
chr10	42121671	42122033
chr10	42122044	42122397
chr10	42127975	42128487
chr10	42130490	42130838
chr10	42134477	42134632
chr10	42134849	42135173
chr10	42135173	42135742
chr10	42140659	42141133
chr10	42148256	42148457
chr10	42148457	42148681
chr10	42167921	42168080
chr10	42177820	42178230
chr10	42178230	42178676

chr10	42207878	42208072
chr10	42210788	42210951
chr10	42216592	42216956
chr10	42216999	42217231
chr10	42219223	42219453
chr10	42220142	42220420
chr10	42220496	42220880
chr10	42220900	42221058
chr10	42222141	42222334
chr10	42231136	42231564
chr10	42244442	42244860
chr10	42244925	42245221
chr10	42269237	42269604
chr10	42269604	42269834
chr10	42269834	42270124
chr10	42275551	42275860
chr10	42276338	42276586
chr10	42276586	42277074
chr10	42277109	42277288
chr10	42277559	42277985
chr10	42277985	42278300
chr10	42278300	42278520
chr10	42287877	42288030
chr10	42290750	42290942
chr10	42291415	42291681
chr10	42293989	42294239
chr10	42295114	42295333
chr10	42298051	42298263
chr10	42299466	42299791
chr10	42299974	42300410
chr10	42303173	42303372
chr10	42308280	42308432
chr10	42310192	42310342
chr10	42477901	42478289
chr10	42478289	42478781
chr10	42491193	42491699
chr10	42492460	42492678
chr10	42501707	42502275
chr10	42502275	42502549
chr10	42502549	42502787
chr10	42521381	42521600
chr10	42562331	42562615

chr10	42579921	42580071
chr10	42611287	42611509
chr10	42614019	42614489
chr10	42633094	42633345
chr10	42633531	42633835
chr10	42633835	42634139
chr10	42634139	42634435
chr10	42637301	42637585
chr10	42639876	42640191
chr10	42669962	42670409
chr10	42678687	42679075
chr10	42679075	42679484
chr10	42683334	42683529
chr10	42684129	42684410
chr10	42696896	42697108
chr10	42697108	42697391
chr10	42734658	42734864
chr10	42754214	42754460
chr10	42754460	42754836
chr10	42759792	42760121
chr10	42761217	42761751
chr10	42761751	42762178
chr10	42762178	42762474
chr10	42781608	42782199
chr10	42782199	42782555
chr10	42830070	42830277
chr10	42832778	42832952
chr10	42833903	42834268
chr10	42834268	42834678
chr10	42835214	42835448
chr10	42841917	42842072
chr10	42842399	42842694
chr10	42856752	42856929
chr10	42857138	42857329
chr10	42857615	42857858
chr10	42858072	42858373
chr10	42859818	42860107
chr10	42860107	42860423
chr10	42860646	42860896
chr10	42863880	42864040
chr10	42864116	42864464
chr10	42864779	42865028

chr10	42888448	42888656
chr10	42902253	42902446
chr10	42907178	42907389
chr10	42950262	42950629
chr10	42950629	42950860
chr10	42951028	42951371
chr10	42957977	42958180
chr10	42958229	42958504
chr10	42958506	42958662
chr10	42972848	42973117
chr10	42987557	42987793
chr10	42987817	42988060
chr10	42989983	42990226
chr10	42998567	42998758
chr10	43021021	43021171
chr10	43021518	43021960
chr10	43034835	43035139
chr10	43035729	43035936
chr10	43068940	43069097
chr10	43130113	43130271
chr10	43143184	43143345
chr10	43155077	43155392
chr10	43173096	43173330
chr10	43173330	43173553
chr10	43173553	43173796
chr10	43173834	43174004
chr10	43174051	43174358
chr10	43174358	43174627
chr10	43174627	43174848
chr10	43174848	43175111
chr10	43203123	43203313
chr10	43217554	43217835
chr10	43220996	43221247
chr10	43221247	43221664
chr10	43221664	43222123
chr10	43226515	43226683
chr10	43226905	43227155
chr10	43227155	43227606
chr10	43228246	43228563
chr10	43292495	43292760
chr10	43310825	43311065
chr10	43311075	43311348

chr10	43326236	43326426
chr10	43330823	43330988
chr10	43337932	43338151
chr10	43375399	43375606
chr10	43378315	43378604
chr10	43382760	43382913
chr10	43393893	43394136
chr10	43397946	43398176
chr10	43411317	43411494
chr10	43423257	43423465
chr10	43438348	43438880
chr10	43439570	43439758
chr10	43439758	43439925
chr10	43441319	43441729
chr10	43448783	43448946
chr10	43452397	43452864
chr10	43462885	43463218
chr10	43463330	43463864
chr10	43478025	43478241
chr10	43478423	43478691
chr10	43478834	43479134
chr10	43479134	43479597
chr10	43479597	43480179
chr10	43489213	43489376
chr10	43489471	43489856
chr10	43490167	43490321
chr10	43517100	43517314
chr10	43517870	43518139
chr10	43524066	43524225
chr10	43540389	43540743
chr10	43540743	43541237
chr10	43549503	43549735
chr10	43562533	43562870
chr10	43562897	43563283
chr10	43577700	43577990
chr10	43578318	43578901
chr10	43578901	43579379
chr10	43579379	43579741
chr10	43579741	43580023
chr10	43580081	43580231
chr10	43580861	43581086
chr10	43591036	43591373

chr10	43593615	43593848
chr10	43604407	43604761
chr10	43604761	43605124
chr10	43605124	43605342
chr10	43610141	43610306
chr10	43622624	43622794
chr10	43634476	43634694
chr10	43641123	43641417
chr10	43651536	43651875
chr10	43657471	43658087
chr10	43679615	43679871
chr10	43684238	43684574
chr10	43688342	43688592
chr10	43688960	43689145
chr10	43689261	43689584
chr10	43689752	43689982
chr10	43689982	43690300
chr10	43691752	43692013
chr10	43694022	43694321
chr10	43694519	43694768
chr10	43701078	43701287
chr10	43718815	43719199
chr10	43723323	43723774
chr10	43790919	43791085
chr10	43856052	43856238
chr10	43856381	43856868
chr10	43870839	43870993
chr10	43871539	43871824
chr10	43901244	43902096
chr10	43919027	43919279
chr10	43943267	43943780
chr10	43943780	43944146
chr10	43944922	43945136
chr10	43953958	43954370
chr10	43954455	43954740
chr10	43957848	43958058
chr10	43960401	43960698
chr10	43973050	43973657
chr10	43977374	43977705
chr10	43980068	43980284
chr10	43980837	43981049
chr10	43982900	43983211

chr10	43990899	43991119
chr10	43991373	43991524
chr10	43998440	43999004
chr10	44002665	44002872
chr10	44003586	44003768
chr10	44003834	44004055
chr10	44004137	44004347
chr10	44004347	44004715
chr10	44005632	44006013
chr10	44006036	44006321
chr10	44006321	44006673
chr10	44015386	44015675
chr10	44015675	44015974
chr10	44016072	44016315
chr10	44016315	44016822
chr10	44024657	44025043
chr10	44026134	44026416
chr10	44030145	44030788
chr10	44037921	44038212
chr10	44038261	44038490
chr10	44039201	44039554
chr10	44043611	44043889
chr10	44043889	44044325
chr10	44050144	44050438
chr10	44054485	44054653
chr10	44066091	44066257
chr10	44071022	44071612
chr10	44078715	44079153
chr10	44079153	44079519
chr10	44079528	44079737
chr10	44084404	44084643
chr10	44094464	44094778
chr10	44094778	44095217
chr10	44106567	44106777
chr10	44111994	44112315
chr10	44112315	44112564
chr10	44112906	44113179
chr10	44120528	44120678
chr10	44129952	44130200
chr10	44136685	44136852
chr10	44142200	44142399
chr10	44148773	44149192

chr10	44150500	44151223
chr10	44160133	44160403
chr10	44171733	44172132
chr10	44176050	44176539
chr10	44176601	44177171
chr10	44177369	44177671
chr10	44192203	44192584
chr10	44209018	44209238
chr10	44219194	44219380
chr10	44233436	44233657
chr10	44258749	44258984
chr10	44266660	44266870
chr10	44267784	44268089
chr10	44268130	44268565
chr10	44268565	44268840
chr10	44284263	44284528
chr10	44316331	44316649
chr10	44323441	44323695
chr10	44390399	44390557
chr10	44400587	44400873
chr10	44409851	44410042
chr10	44420297	44420483
chr10	44421415	44421565
chr10	44424962	44425370
chr10	44425370	44425620
chr10	44425767	44425947
chr10	44427958	44428422
chr10	44431767	44432023
chr10	44440328	44440479
chr10	44458475	44458949
chr10	44462543	44462783
chr10	44518523	44519365
chr10	44520122	44520437
chr10	44529171	44529660
chr10	44529660	44529889
chr10	44529947	44530170
chr10	44541295	44541597
chr10	44556952	44557181
chr10	44564727	44565002
chr10	44565032	44565456
chr10	44566013	44566389
chr10	44574513	44575000

chr10	44575000	44575258
chr10	44607371	44607749
chr10	44615672	44615876
chr10	44657130	44657318
chr10	44676582	44676844
chr10	44685239	44685611
chr10	44691505	44691977
chr10	44698607	44698809
chr10	44700087	44700732
chr10	44715953	44716276
chr10	44716276	44716518
chr10	44716790	44716966
chr10	44716976	44717565
chr10	44723292	44723634
chr10	44730209	44730398
chr10	44730823	44731077
chr10	44731077	44731282
chr10	44733671	44733989
chr10	44744283	44744551
chr10	44745145	44745424
chr10	44745673	44745937
chr10	44747478	44747816
chr10	44749180	44749594
chr10	44754769	44755000
chr10	44755000	44755314
chr10	44755314	44755885
chr10	44818529	44818931
chr10	44875465	44875722
chr10	44877013	44877404
chr10	44877404	44877797
chr10	44883987	44884539
chr10	44884547	44884791
chr10	44887265	44887970
chr10	44914450	44914735
chr10	44916987	44917153
chr10	44932423	44932591
chr10	44986956	44987245
chr10	44987245	44987575
chr10	44988844	44989140
chr10	45000874	45001305
chr10	45010657	45010815
chr10	45019477	45019697

chr10	45045357	45045577
chr10	45048380	45048530
chr10	45048580	45049157
chr10	45051153	45051468
chr10	45066249	45066435
chr10	45066679	45067342
chr10	45067342	45067717
chr10	45067722	45067969
chr10	45082828	45082994
chr10	45086742	45086892
chr10	45115942	45116102
chr10	45132471	45132682
chr10	45156028	45156181
chr10	45161835	45162047
chr10	45180762	45180963
chr10	45286311	45286494
chr10	45289273	45289501
chr10	45292338	45292528
chr10	45295183	45295349
chr10	45303393	45303605
chr10	45373350	45373588
chr10	45395580	45395768
chr10	45398122	45398628
chr10	45485647	45485923
chr10	45485923	45486244
chr10	45486244	45486626
chr10	45486626	45486952
chr10	45546858	45547043
chr10	45577659	45577966
chr10	45577966	45578228
chr10	45578228	45578561
chr10	45578561	45578861
chr10	45640556	45641113
chr10	45734692	45734861
chr10	45734932	45735197
chr10	45761242	45761485
chr10	45766924	45767172
chr10	45874519	45874812
chr10	45875301	45875596
chr10	45883793	45884107
chr10	45888698	45889205
chr10	45889408	45889578

chr10	45933728	45933981
chr10	46011994	46012266
chr10	46228244	46228446
chr10	46228446	46228712
chr10	46228712	46229010
chr10	46229010	46229292
chr10	46298324	46298689
chr10	46320273	46320542
chr10	46323617	46323782
chr10	46539578	46539842
chr10	46665497	46665740
chr10	46689881	46690220
chr10	46708226	46708509
chr10	46723733	46723979
chr10	46785316	46785551
chr10	46785558	46786257
chr10	46791921	46792479
chr10	46939436	46939586
chr10	47402137	47402422
chr10	47452270	47452607
chr10	47455213	47455740
chr10	47456293	47456610
chr10	47590820	47590973
chr10	48067021	48067490
chr10	48178169	48178406
chr10	48433364	48433693
chr10	48549782	48550109
chr10	48556216	48556452
chr10	48627621	48627904
chr10	48770015	48770404
chr10	49050382	49050648
chr10	50419935	50420144
chr10	50427279	50427478
chr10	50584658	50585153
chr10	50586587	50586914
chr10	50592334	50592804
chr10	50592804	50593288
chr10	50624603	50625055
chr10	50657419	50657650
chr10	50704330	50704492
chr10	50800148	50800376
chr10	50892891	50893041

chr10	50894219	50894462
chr10	50894462	50894764
chr10	50894764	50895080
chr10	50895080	50895358
chr10	50898332	50898517
chr10	50910542	50910750
chr10	51405241	51405460
chr10	51414598	51414768
chr10	51416683	51417036
chr10	51417263	51417587
chr10	51418032	51418341
chr10	51421542	51421717
chr10	51451637	51452014
chr10	51454064	51454346
chr10	51454408	51454777
chr10	51454777	51455086
chr10	51521623	51522024
chr10	51529639	51529863
chr10	51553407	51553679
chr10	51553792	51554019
chr10	51571266	51571641
chr10	51571641	51572014
chr10	51595253	51595410
chr10	51650824	51650992
chr10	51677555	51677837
chr10	51677837	51678079
chr10	51789305	51789594
chr10	51940379	51940529
chr10	51944520	51944853
chr10	51946228	51946550
chr10	51946603	51946931
chr10	51946931	51947283
chr10	51950094	51950339
chr10	51961573	51961733
chr10	51975138	51975491
chr10	51975491	51975806
chr10	51975982	51976278
chr10	52022113	52022263
chr10	52022343	52022597
chr10	52022597	52023048
chr10	52027344	52028062
chr10	52029808	52029997

chr10	52031760	52031923
chr10	52072587	52072795
chr10	52106281	52106464
chr10	52142144	52142561
chr10	52144319	52144906
chr10	52145139	52145371
chr10	52170544	52170842
chr10	52174404	52174760
chr10	52205500	52205866
chr10	52216853	52217099
chr10	52219687	52219937
chr10	52219984	52220234
chr10	52233266	52233504
chr10	52233504	52233754
chr10	52233754	52233987
chr10	52233987	52234340
chr10	52273023	52273391
chr10	52277356	52277628
chr10	52277628	52277885
chr10	52277944	52278228
chr10	52278228	52278560
chr10	52342785	52343068
chr10	52343313	52343559
chr10	52381357	52381542
chr10	52381560	52381784
chr10	52381799	52382335
chr10	52382335	52382877
chr10	52392380	52392546
chr10	52399779	52400104
chr10	52417185	52417657
chr10	52417657	52418034
chr10	52418034	52418373
chr10	52419034	52419235
chr10	52528369	52528522
chr10	52528937	52529128
chr10	52529580	52529730
chr10	52690288	52690620
chr10	52690620	52690960
chr10	52690960	52691241
chr10	52691241	52691466
chr10	52896719	52897123
chr10	52934701	52934956

chr10	53129489	53129810
chr10	53271294	53271492
chr10	53348398	53348714
chr10	53355409	53355713
chr10	53379129	53379734
chr10	53379734	53380240
chr10	53395038	53395251
chr10	53395260	53395859
chr10	53413010	53413189
chr10	53419120	53419469
chr10	53428747	53429019
chr10	53466916	53467166
chr10	53509239	53509398
chr10	53528480	53528693
chr10	53540617	53540777
chr10	53596525	53597102
chr10	53597102	53597516
chr10	53597516	53597872
chr10	53598943	53599176
chr10	53629296	53629722
chr10	53629722	53630086
chr10	53630086	53630349
chr10	53641597	53641790
chr10	53651769	53651935
chr10	53653035	53653187
chr10	53711187	53711340
chr10	53718327	53718477
chr10	53718929	53719201
chr10	53749968	53750410
chr10	53750410	53750649
chr10	53750649	53750994
chr10	53750994	53751630
chr10	53753135	53753368
chr10	53755577	53755761
chr10	53765506	53765864
chr10	53806776	53806941
chr10	53863492	53863775
chr10	53959831	53960127
chr10	54019342	54019510
chr10	54026613	54026890
chr10	54026890	54027272
chr10	54032323	54032900

chr10	54040212	54040649
chr10	54040716	54041006
chr10	54049381	54049850
chr10	54058666	54058816
chr10	54058934	54059528
chr10	54066803	54067002
chr10	54073859	54074184
chr10	54074352	54074573
chr10	54074656	54074896
chr10	54074988	54075384
chr10	54075388	54076163
chr10	54076547	54076784
chr10	54085200	54085352
chr10	54087353	54087754
chr10	54099959	54100510
chr10	54101746	54101978
chr10	54117757	54118226
chr10	54118571	54118823
chr10	54197286	54197700
chr10	54197700	54197973
chr10	54202482	54202632
chr10	54341059	54341400
chr10	54350482	54350726
chr10	54351172	54351399
chr10	54369144	54369499
chr10	54369499	54369734
chr10	54371327	54371558
chr10	54371558	54371864
chr10	54478083	54478308
chr10	54482914	54483217
chr10	54606772	54607042
chr10	54629672	54629880
chr10	54659258	54659505
chr10	54814451	54814667
chr10	54814670	54814937
chr10	54839044	54839377
chr10	54839542	54839981
chr10	55033966	55034181
chr10	55051187	55051398
chr10	55198418	55198650
chr10	55198650	55198902
chr10	55257362	55257661

chr10	55264869	55265050
chr10	55265172	55265349
chr10	55284950	55285249
chr10	55290704	55290871
chr10	55333813	55334311
chr10	55335932	55336376
chr10	55345801	55346027
chr10	55348123	55348310
chr10	55461863	55462337
chr10	55471032	55471216
chr10	55522771	55523052
chr10	55564983	55565313
chr10	55572746	55573090
chr10	55779686	55779836
chr10	55905972	55906267
chr10	55926787	55927053
chr10	55927053	55927318
chr10	55927866	55928124
chr10	55928200	55928527
chr10	55980219	55980435
chr10	56158935	56159163
chr10	56163025	56163213
chr10	56227984	56228138
chr10	56228497	56228853
chr10	56228853	56229146
chr10	56229485	56229638
chr10	56314360	56314515
chr10	56339181	56339421
chr10	56365917	56366187
chr10	56369134	56369464
chr10	56377029	56377529
chr10	56377809	56377986
chr10	56377986	56378226
chr10	56378317	56378491
chr10	56378594	56378899
chr10	56378899	56379363
chr10	56426485	56426756
chr10	56440849	56441006
chr10	56453750	56453993
chr10	56468604	56468822
chr10	56469054	56469253
chr10	56486387	56487050

chr10	56499213	56499389
chr10	56535340	56535694
chr10	56560657	56560807
chr10	56582020	56582570
chr10	56598188	56598544
chr10	56611575	56611896
chr10	56611896	56612181
chr10	56685942	56686194
chr10	56694360	56694624
chr10	56766167	56766494
chr10	56769006	56769253
chr10	56781873	56782152
chr10	56782212	56782638
chr10	56782710	56783078
chr10	56783103	56783301
chr10	56783674	56783852
chr10	56783906	56784286
chr10	56910846	56911174
chr10	56912805	56913067
chr10	56914149	56914367
chr10	56935115	56935726
chr10	56944629	56945174
chr10	56945177	56945443
chr10	56949201	56949380
chr10	56949394	56949794
chr10	56956249	56956620
chr10	56956664	56957132
chr10	56963290	56963606
chr10	56965552	56965790
chr10	56980821	56981333
chr10	56981478	56981639
chr10	56985640	56985908
chr10	56988740	56989198
chr10	56994996	56995518
chr10	57001377	57001687
chr10	57160541	57160912
chr10	57411132	57411389
chr10	57437955	57438127
chr10	57438300	57438468
chr10	57441167	57441359
chr10	57445140	57445290
chr10	57448008	57448249

chr10	57448344	57448501
chr10	57448546	57448780
chr10	57463373	57463640
chr10	57465808	57466109
chr10	57485876	57486463
chr10	57486463	57487039
chr10	57491688	57491838
chr10	57499755	57500066
chr10	57500566	57500768
chr10	57501425	57501636
chr10	57522895	57523104
chr10	57530583	57530844
chr10	57532000	57532712
chr10	57571292	57571508
chr10	57615399	57615583
chr10	57631944	57632199
chr10	57644337	57645076
chr10	57670058	57670495
chr10	57671551	57671767
chr10	57743803	57743982
chr10	57763059	57763394
chr10	57794569	57795239
chr10	57799534	57799791
chr10	57822541	57822730
chr10	57841999	57842155
chr10	57846157	57846359
chr10	58221419	58221656
chr10	58221656	58221928
chr10	58221928	58222185
chr10	58223131	58223476
chr10	58223894	58224181
chr10	58224279	58224532
chr10	58224532	58224833
chr10	58224869	58225180
chr10	58225180	58225650
chr10	58227958	58228076
chr10	58228076	58228136
chr10	58231224	58231647
chr10	58231732	58232075
chr10	58232075	58232349
chr10	58232349	58232631
chr10	58232631	58232908

chr10	58233274	58233715
chr10	58233715	58234045
chr10	58238471	58238623
chr10	58239068	58239369
chr10	58239369	58239789
chr10	58255241	58255558
chr10	58255558	58255940
chr10	58262972	58263137
chr10	58281624	58281905
chr10	58310498	58311235
chr10	58320937	58321139
chr10	58322820	58323172
chr10	58323172	58323443
chr10	58323443	58323933
chr10	58330732	58330961
chr10	58331792	58332075
chr10	58333339	58333916
chr10	58346574	58346794
chr10	58347122	58347581
chr10	58351410	58351983
chr10	58354576	58354732
chr10	58354732	58354899
chr10	58355352	58355660
chr10	58359207	58359425
chr10	58360680	58360979
chr10	58362849	58363039
chr10	58364782	58365120
chr10	58365120	58365633
chr10	58370603	58370847
chr10	58371264	58371539
chr10	58371539	58371924
chr10	58377868	58378123
chr10	58378501	58378711
chr10	58400900	58401138
chr10	58401938	58402149
chr10	58405266	58405852
chr10	58405908	58406285
chr10	58408353	58408517
chr10	58441604	58441793
chr10	58446372	58446567
chr10	58446584	58447105
chr10	58447105	58447515

chr10	58452849	58453016
chr10	58460041	58460194
chr10	58497435	58497783
chr10	58497783	58498043
chr10	58498043	58498347
chr10	58559191	58559432
chr10	58559876	58560300
chr10	58604792	58605145
chr10	58608895	58609156
chr10	58609363	58609740
chr10	58627649	58627929
chr10	58651246	58651612
chr10	58673601	58673765
chr10	58675446	58675899
chr10	58812136	58812410
chr10	58812410	58812896
chr10	58812896	58813414
chr10	58813501	58813909
chr10	59078046	59078232
chr10	59090353	59090503
chr10	59109068	59109386
chr10	59114457	59114788
chr10	59115092	59115242
chr10	59149916	59150169
chr10	59150188	59150445
chr10	59152589	59153000
chr10	59166625	59166861
chr10	59175264	59175613
chr10	59195037	59195187
chr10	59221205	59221437
chr10	59221437	59221722
chr10	59221722	59222158
chr10	59222158	59222617
chr10	59222778	59222928
chr10	59225194	59225401
chr10	59241072	59241696
chr10	59250760	59250974
chr10	59250974	59251198
chr10	59261764	59261918
chr10	59265866	59266430
chr10	59266430	59266673
chr10	59283929	59284140

chr10	59285680	59285846
chr10	59289087	59289414
chr10	59292541	59292923
chr10	59295234	59295568
chr10	59295568	59295848
chr10	59296117	59296643
chr10	59309124	59309331
chr10	59309391	59309677
chr10	59311161	59311349
chr10	59322995	59323540
chr10	59323540	59323949
chr10	59323952	59324165
chr10	59380661	59380863
chr10	59381713	59382045
chr10	59382828	59383143
chr10	59383143	59383417
chr10	59386707	59386925
chr10	59395580	59395776
chr10	59425243	59425758
chr10	59430394	59431318
chr10	59431339	59431531
chr10	59449668	59449852
chr10	59451856	59452228
chr10	59453014	59453194
chr10	59453616	59453910
chr10	59453989	59454225
chr10	59464128	59464349
chr10	59464417	59464622
chr10	59476452	59476718
chr10	59508201	59508478
chr10	59509113	59509926
chr10	59514718	59515101
chr10	59527393	59527545
chr10	59536272	59536622
chr10	59537438	59537930
chr10	59537930	59538183
chr10	59539502	59539753
chr10	59556988	59557155
chr10	59557327	59557707
chr10	59559036	59559189
chr10	59564414	59564668
chr10	59564668	59565022

chr10	59577806	59578104
chr10	59578104	59578451
chr10	59578451	59578774
chr10	59578774	59578991
chr10	59579467	59579693
chr10	59579747	59580016
chr10	59580563	59580845
chr10	59586909	59587358
chr10	59587753	59587939
chr10	59589481	59589651
chr10	59589946	59590160
chr10	59593864	59594739
chr10	59594964	59595157
chr10	59603900	59604162
chr10	59609227	59609430
chr10	59615947	59616215
chr10	59616215	59616534
chr10	59616534	59617061
chr10	59621360	59621644
chr10	59623014	59623211
chr10	59624574	59624776
chr10	59636112	59636368
chr10	59636772	59636981
chr10	59638635	59638996
chr10	59641950	59642279
chr10	59642408	59642706
chr10	59642706	59643081
chr10	59643357	59643531
chr10	59651599	59652096
chr10	59667229	59667863
chr10	59682454	59682702
chr10	59685437	59685591
chr10	59685704	59686032
chr10	59686470	59686632
chr10	59702225	59703073
chr10	59710365	59710628
chr10	59715021	59715410
chr10	59724420	59724591
chr10	59724602	59725042
chr10	59733364	59733625
chr10	59741175	59741372
chr10	59741498	59741716

chr10	59777125	59777773
chr10	59781643	59781824
chr10	59792025	59792348
chr10	59799245	59799689
chr10	59800145	59800537
chr10	59810053	59810269
chr10	59811090	59811467
chr10	59814887	59815145
chr10	59817243	59817433
chr10	59817622	59817852
chr10	59818557	59818754
chr10	59820953	59821227
chr10	59821272	59821710
chr10	59821744	59821902
chr10	59825483	59825870
chr10	59836720	59837145
chr10	59837528	59837830
chr10	59857304	59857491
chr10	59860744	59860923
chr10	59861814	59862010
chr10	59865080	59865401
chr10	59870230	59870509
chr10	59873341	59873517
chr10	59873771	59873978
chr10	59878894	59879118
chr10	59879174	59879732
chr10	59879732	59880024
chr10	59880024	59880244
chr10	59884740	59885370
chr10	59893092	59893299
chr10	59894874	59895115
chr10	59899303	59899962
chr10	59899962	59900323
chr10	59901187	59901337
chr10	59907072	59907638
chr10	59907696	59908088
chr10	59908088	59908466
chr10	59908962	59909323
chr10	59909323	59909623
chr10	59909623	59909944
chr10	59914763	59914913
chr10	59915820	59916084

chr10	59916670	59916843
chr10	59918096	59918557
chr10	59919551	59919765
chr10	59920857	59921617
chr10	59923587	59923742
chr10	59932500	59932946
chr10	59934074	59934279
chr10	59938546	59938761
chr10	59938973	59939484
chr10	59942756	59942978
chr10	59943030	59943451
chr10	59943451	59943734
chr10	59943734	59944036
chr10	59948939	59949266
chr10	59949288	59949621
chr10	59950685	59950940
chr10	59950940	59951153
chr10	59951546	59952191
chr10	59952230	59952392
chr10	59964468	59964638
chr10	59969800	59969985
chr10	59977405	59977557
chr10	59977746	59978054
chr10	59978505	59978661
chr10	59978849	59979205
chr10	59979213	59979379
chr10	59980598	59980976
chr10	59997695	59997868
chr10	60002559	60002915
chr10	60002915	60003354
chr10	60003354	60003717
chr10	60021661	60021870
chr10	60023848	60024080
chr10	60026845	60027080
chr10	60027735	60027894
chr10	60028359	60028520
chr10	60034562	60034920
chr10	60046759	60046973
chr10	60057688	60057873
chr10	60064891	60065057
chr10	60065394	60065618
chr10	60065633	60065916

chr10	60066565	60066842
chr10	60077254	60077516
chr10	60078570	60078885
chr10	60078983	60079274
chr10	60086496	60086776
chr10	60095555	60095800
chr10	60106605	60106992
chr10	60106992	60107218
chr10	60107499	60107797
chr10	60112598	60112775
chr10	60124157	60124388
chr10	60125717	60125974
chr10	60125974	60126244
chr10	60141678	60141846
chr10	60147593	60147801
chr10	60147970	60148120
chr10	60148152	60148459
chr10	60150986	60151188
chr10	60158250	60158464
chr10	60185227	60185425
chr10	60186196	60186580
chr10	60186580	60186818
chr10	60186864	60187166
chr10	60191351	60191506
chr10	60191586	60192223
chr10	60193476	60193641
chr10	60193889	60194230
chr10	60207206	60207611
chr10	60209133	60209840
chr10	60210431	60210796
chr10	60218572	60219007
chr10	60219007	60219277
chr10	60237505	60237739
chr10	60237841	60238078
chr10	60243920	60244219
chr10	60245046	60245459
chr10	60246619	60246877
chr10	60252318	60252694
chr10	60262315	60262908
chr10	60263443	60263711
chr10	60269532	60270057
chr10	60271779	60272050

chr10	60272050	60272285
chr10	60272393	60272570
chr10	60277301	60277752
chr10	60277752	60278002
chr10	60278002	60278244
chr10	60278524	60278800
chr10	60285765	60285985
chr10	60287290	60287546
chr10	60287546	60287964
chr10	60290263	60290680
chr10	60290680	60291066
chr10	60304880	60305118
chr10	60305118	60305339
chr10	60305339	60305619
chr10	60346734	60347060
chr10	60347060	60347318
chr10	60350870	60351092
chr10	60351767	60352163
chr10	60355333	60355612
chr10	60379470	60379839
chr10	60380409	60380745
chr10	60395557	60396063
chr10	60407929	60408235
chr10	60408365	60408531
chr10	60416589	60416821
chr10	60416998	60417206
chr10	60418185	60418427
chr10	60420876	60421039
chr10	60421645	60421923
chr10	60445164	60445597
chr10	60445761	60445912
chr10	60531390	60531684
chr10	60531704	60531859
chr10	60532806	60532966
chr10	60534280	60534541
chr10	60569143	60569460
chr10	60571236	60571713
chr10	60573410	60573687
chr10	60574928	60575078
chr10	60578226	60578707
chr10	60578707	60579153
chr10	60584326	60584613

chr10	60616005	60616260
chr10	60621602	60621787
chr10	60635992	60636332
chr10	60659961	60660116
chr10	60668674	60668847
chr10	60686831	60687215
chr10	60687215	60687536
chr10	60687545	60687868
chr10	60696998	60697471
chr10	60712355	60712592
chr10	60723446	60723599
chr10	60742407	60742558
chr10	60744720	60744870
chr10	60752102	60752386
chr10	60752386	60752678
chr10	60752678	60753159
chr10	60760486	60760782
chr10	60762030	60762522
chr10	60762897	60763112
chr10	60775542	60775795
chr10	60778621	60778929
chr10	60779564	60779958
chr10	60779958	60780167
chr10	60781659	60781843
chr10	60785313	60785509
chr10	60787907	60788104
chr10	60790937	60791381
chr10	60792119	60792349
chr10	60796158	60796324
chr10	60796573	60796816
chr10	60807976	60808230
chr10	60808304	60809006
chr10	60814423	60814767
chr10	60816874	60817332
chr10	60817406	60817761
chr10	60819233	60819496
chr10	60820739	60820986
chr10	60822627	60823168
chr10	60825992	60826382
chr10	60830523	60830673
chr10	60830776	60830974
chr10	60830974	60831314

chr10	60831314	60831813
chr10	60832825	60833157
chr10	60835040	60835277
chr10	60845232	60845449
chr10	60851607	60851757
chr10	60876858	60877127
chr10	60877127	60877413
chr10	60877413	60877672
chr10	60884880	60885260
chr10	60885260	60885655
chr10	60885655	60886049
chr10	60886118	60886289
chr10	60897132	60897371
chr10	60900838	60901529
chr10	60911507	60911708
chr10	60914484	60914675
chr10	60917525	60917774
chr10	60925675	60925953
chr10	60925953	60926164
chr10	60926616	60926955
chr10	60932968	60933191
chr10	60933473	60933744
chr10	60933835	60934228
chr10	60934228	60934867
chr10	60943164	60943371
chr10	60949019	60949604
chr10	60951914	60952099
chr10	60955076	60955665
chr10	60956420	60956636
chr10	60967323	60967473
chr10	60979856	60980028
chr10	60985276	60985557
chr10	61015844	61016013
chr10	61016405	61016668
chr10	61018063	61018250
chr10	61023546	61023769
chr10	61027509	61027681
chr10	61027876	61028108
chr10	61028539	61028947
chr10	61045099	61045312
chr10	61052101	61052306
chr10	61052306	61052569

chr10	61063039	61063229
chr10	61064737	61064894
chr10	61068397	61068853
chr10	61068928	61069083
chr10	61069092	61069615
chr10	61072426	61072680
chr10	61079371	61079688
chr10	61080315	61080564
chr10	61089123	61089490
chr10	61089490	61089804
chr10	61089804	61090119
chr10	61114324	61114562
chr10	61114562	61114768
chr10	61114768	61115010
chr10	61130918	61131313
chr10	61131345	61131585
chr10	61131585	61131820
chr10	61134120	61134354
chr10	61146137	61146383
chr10	61146383	61146760
chr10	61146760	61147123
chr10	61147123	61147443
chr10	61147443	61147727
chr10	61147727	61148077
chr10	61155673	61155834
chr10	61157323	61157708
chr10	61166818	61167081
chr10	61169907	61170149
chr10	61175637	61175846
chr10	61175846	61176180
chr10	61199576	61199971
chr10	61201945	61202090
chr10	61202090	61202255
chr10	61211786	61212002
chr10	61212921	61213239
chr10	61213370	61213580
chr10	61214538	61214906
chr10	61258289	61258456
chr10	61271842	61272126
chr10	61272126	61272484
chr10	61273122	61273485
chr10	61275700	61275855

chr10	61275864	61276110
chr10	61277142	61277292
chr10	61277938	61278453
chr10	61281216	61281428
chr10	61285237	61285390
chr10	61286115	61286330
chr10	61286330	61286719
chr10	61294332	61294554
chr10	61317575	61317758
chr10	61332620	61332836
chr10	61336113	61336426
chr10	61336455	61336607
chr10	61339305	61339519
chr10	61341528	61341833
chr10	61344036	61344289
chr10	61349479	61349635
chr10	61360722	61360995
chr10	61373966	61374162
chr10	61375051	61375258
chr10	61382517	61382722
chr10	61382877	61383053
chr10	61383053	61383284
chr10	61383284	61383850
chr10	61392511	61392806
chr10	61394350	61394696
chr10	61394696	61395069
chr10	61399430	61399725
chr10	61399728	61399894
chr10	61413105	61413277
chr10	61417491	61417692
chr10	61418340	61418547
chr10	61418551	61418701
chr10	61418989	61419333
chr10	61423272	61423499
chr10	61423499	61423806
chr10	61424667	61424822
chr10	61445659	61445829
chr10	61451421	61451643
chr10	61451988	61452255
chr10	61452255	61452607
chr10	61452607	61453204
chr10	61461778	61462086

chr10	61465006	61465389
chr10	61467003	61467215
chr10	61468683	61469200
chr10	61475524	61475895
chr10	61475895	61476439
chr10	61479528	61479916
chr10	61481873	61482182
chr10	61486639	61486865
chr10	61486969	61487392
chr10	61488715	61488993
chr10	61492077	61492548
chr10	61502808	61502996
chr10	61506324	61506710
chr10	61509215	61509420
chr10	61517120	61517308
chr10	61535925	61536462
chr10	61536462	61536726
chr10	61536726	61537043
chr10	61537060	61537368
chr10	61544132	61544465
chr10	61544487	61544950
chr10	61569788	61570172
chr10	61570172	61570559
chr10	61583360	61583538
chr10	61587350	61587500
chr10	61595022	61595412
chr10	61602350	61602601
chr10	61602601	61602836
chr10	61602836	61603078
chr10	61605906	61606161
chr10	61610862	61611182
chr10	61617345	61617549
chr10	61625507	61625689
chr10	61625689	61625976
chr10	61627096	61627334
chr10	61627479	61627721
chr10	61636937	61637424
chr10	61637424	61637773
chr10	61648331	61648789
chr10	61648789	61649138
chr10	61649138	61649523
chr10	61654486	61654754

chr10	61654754	61655020
chr10	61657750	61657923
chr10	61658033	61658266
chr10	61669881	61670410
chr10	61670853	61671061
chr10	61679992	61680382
chr10	61680382	61680791
chr10	61694921	61695077
chr10	61695115	61695639
chr10	61695639	61696049
chr10	61696049	61696544
chr10	61714877	61715562
chr10	61716094	61716281
chr10	61716497	61716668
chr10	61734158	61734342
chr10	61734342	61734544
chr10	61734544	61734804
chr10	61741251	61741567
chr10	61741567	61741833
chr10	61745215	61745439
chr10	61756853	61757518
chr10	61759859	61760262
chr10	61782890	61783080
chr10	61783150	61783582
chr10	61783743	61784116
chr10	61820148	61820298
chr10	61838808	61839050
chr10	61844548	61844761
chr10	61845505	61845809
chr10	61854827	61855063
chr10	61855264	61855534
chr10	61874819	61875057
chr10	61877845	61878252
chr10	61906096	61906467
chr10	61943339	61943814
chr10	61943845	61944093
chr10	61944267	61944826
chr10	61949134	61949857
chr10	61950766	61950958
chr10	61955224	61955380
chr10	61965084	61965495
chr10	61965495	61966024

chr10	61972405	61972669
chr10	61977103	61977393
chr10	61978183	61978348
chr10	61978403	61978619
chr10	61979019	61979207
chr10	61980478	61980768
chr10	62019470	62019628
chr10	62033496	62033692
chr10	62044440	62044608
chr10	62050659	62050809
chr10	62055453	62055793
chr10	62059115	62059290
chr10	62066279	62066540
chr10	62069477	62069746
chr10	62102100	62102399
chr10	62104864	62105209
chr10	62105218	62105496
chr10	62110468	62110707
chr10	62110707	62110926
chr10	62110926	62111220
chr10	62111273	62111552
chr10	62113508	62113687
chr10	62116100	62116374
chr10	62124866	62125067
chr10	62148369	62148879
chr10	62181726	62181992
chr10	62183073	62183447
chr10	62183472	62183622
chr10	62186946	62187239
chr10	62189392	62189577
chr10	62190061	62190314
chr10	62192599	62193314
chr10	62195284	62195807
chr10	62201295	62201535
chr10	62202603	62202864
chr10	62212494	62212928
chr10	62221498	62222230
chr10	62222798	62223014
chr10	62223822	62224031
chr10	62230557	62230961
chr10	62230961	62231626
chr10	62237911	62238317

chr10	62238378	62238576
chr10	62242813	62243273
chr10	62244156	62244320
chr10	62247519	62248234
chr10	62261087	62261505
chr10	62261505	62261849
chr10	62266874	62267033
chr10	62275284	62275534
chr10	62282880	62283041
chr10	62299901	62300270
chr10	62300270	62300867
chr10	62313834	62314106
chr10	62320257	62320736
chr10	62320736	62321045
chr10	62323702	62324189
chr10	62329629	62330026
chr10	62330026	62330389
chr10	62332418	62332876
chr10	62336663	62336922
chr10	62337206	62337364
chr10	62337384	62337648
chr10	62338284	62338485
chr10	62339378	62339541
chr10	62339826	62340113
chr10	62340113	62340436
chr10	62340436	62340744
chr10	62358982	62359138
chr10	62376915	62377200
chr10	62381080	62381258
chr10	62383179	62383341
chr10	62411026	62411253
chr10	62412871	62413496
chr10	62413496	62413999
chr10	62415811	62416020
chr10	62419156	62419331
chr10	62420564	62420750
chr10	62424972	62425189
chr10	62425189	62425450
chr10	62425450	62425713
chr10	62425713	62425905
chr10	62426325	62426475
chr10	62449290	62449590

chr10	62449590	62449865
chr10	62449865	62450115
chr10	62450115	62450326
chr10	62450326	62450565
chr10	62469811	62470088
chr10	62471053	62471441
chr10	62485856	62486092
chr10	62486236	62486621
chr10	62486621	62486861
chr10	62486861	62487138
chr10	62487138	62487427
chr10	62488390	62488584
chr10	62491810	62492068
chr10	62500095	62500283
chr10	62500283	62500534
chr10	62517230	62517411
chr10	62521977	62522139
chr10	62522411	62522640
chr10	62522890	62523057
chr10	62535775	62535967
chr10	62549598	62549854
chr10	62549854	62550107
chr10	62553624	62553822
chr10	62557810	62558067
chr10	62577803	62578082
chr10	62578082	62578356
chr10	62578356	62578699
chr10	62578830	62579015
chr10	62588794	62588944
chr10	62590212	62590590
chr10	62590753	62590918
chr10	62601914	62602188
chr10	62602188	62602460
chr10	62602460	62602685
chr10	62602685	62602921
chr10	62627317	62627653
chr10	62650467	62650650
chr10	62650725	62651147
chr10	62651147	62651445
chr10	62651445	62651768
chr10	62662067	62662221
chr10	62683149	62683386

chr10	62703722	62703872
chr10	62725700	62726003
chr10	62726003	62726348
chr10	62730870	62731252
chr10	62736764	62736978
chr10	62737150	62737325
chr10	62740002	62740246
chr10	62745610	62745774
chr10	62747514	62747723
chr10	62791742	62792241
chr10	62792241	62792815
chr10	62804174	62804412
chr10	62808364	62808621
chr10	62854993	62855372
chr10	62857046	62857196
chr10	62862519	62862740
chr10	62865363	62865562
chr10	62865562	62865740
chr10	62885808	62885995
chr10	62885995	62886196
chr10	62886196	62886462
chr10	62886476	62886804
chr10	62886804	62887196
chr10	62887196	62887527
chr10	62902832	62903176
chr10	62920097	62920297
chr10	62920297	62920533
chr10	62920533	62920789
chr10	62920789	62921186
chr10	62946649	62947490
chr10	62979977	62980340
chr10	62980340	62980962
chr10	62987397	62987624
chr10	63008229	63008509
chr10	63023134	63023583
chr10	63023583	63023963
chr10	63023963	63024511
chr10	63024626	63024865
chr10	63031747	63031910
chr10	63066007	63066462
chr10	63100009	63100219
chr10	63100219	63100540

chr10	63122599	63122776
chr10	63128234	63128636
chr10	63128636	63128897
chr10	63133173	63133383
chr10	63153665	63153821
chr10	63158131	63158583
chr10	63172052	63172401
chr10	63172552	63172828
chr10	63177246	63177556
chr10	63177556	63177860
chr10	63182491	63182780
chr10	63186599	63186959
chr10	63189536	63189872
chr10	63189872	63190221
chr10	63201401	63201831
chr10	63221406	63221920
chr10	63224263	63224659
chr10	63236035	63236189
chr10	63236661	63237064
chr10	63237340	63237550
chr10	63243555	63244009
chr10	63244009	63244249
chr10	63244249	63244527
chr10	63244856	63245063
chr10	63308625	63308930
chr10	63309023	63309337
chr10	63310241	63310549
chr10	63327154	63327466
chr10	63338283	63338628
chr10	63338628	63338979
chr10	63338979	63339593
chr10	63377465	63377848
chr10	63382389	63382673
chr10	63382673	63382967
chr10	63386440	63386848
chr10	63400488	63400714
chr10	63421967	63422185
chr10	63422326	63422589
chr10	63424486	63424737
chr10	63428810	63429018
chr10	63429401	63429739
chr10	63429739	63430001

chr10	63430001	63430343
chr10	63536583	63536882
chr10	63541698	63542187
chr10	63556693	63556843
chr10	63571526	63572009
chr10	63572919	63573225
chr10	63582628	63582910
chr10	63692205	63692355
chr10	63738740	63738890
chr10	63784865	63785041
chr10	63797944	63798298
chr10	63818857	63819095
chr10	63844632	63844892
chr10	63897357	63897628
chr10	63898333	63898515
chr10	63908248	63908440
chr10	63908780	63909305
chr10	63912873	63913242
chr10	63915916	63916300
chr10	63917356	63917839
chr10	63929093	63929256
chr10	63978112	63978329
chr10	64023403	64023627
chr10	64076053	64076314
chr10	64076400	64076792
chr10	64076792	64077093
chr10	64077120	64077327
chr10	64084539	64084699
chr10	64090130	64090420
chr10	64230266	64230567
chr10	64230567	64230725
chr10	64343009	64343403
chr10	64344339	64344729
chr10	64361581	64361988
chr10	64362053	64362223
chr10	64362920	64363292
chr10	64376535	64376751
chr10	64446751	64446927
chr10	64470010	64470256
chr10	64483338	64483779
chr10	64522358	64522587
chr10	64744150	64744527

chr10	64744938	64745230
chr10	64750847	64751010
chr10	64835682	64835979
chr10	64836192	64836706
chr10	64836900	64837188
chr10	64837199	64837389
chr10	64944619	64944999
chr10	65033530	65033804
chr10	65110956	65111168
chr10	65174935	65175233
chr10	65517972	65518273
chr10	65681363	65681570
chr10	65697571	65697836
chr10	65697932	65698263
chr10	65722576	65722816
chr10	65725234	65725444
chr10	65798535	65798744
chr10	65839008	65839555
chr10	65844756	65844988
chr10	65845207	65845515
chr10	65846213	65846453
chr10	65954697	65954973
chr10	65975447	65975785
chr10	66036346	66036496
chr10	66131835	66132324
chr10	66213476	66213685
chr10	66281835	66282156
chr10	66323509	66323878
chr10	66336441	66336627
chr10	66337873	66338298
chr10	66338298	66338528
chr10	66345456	66345771
chr10	66351099	66351271
chr10	66353869	66354625
chr10	66354860	66355254
chr10	66355499	66355763
chr10	66369223	66369543
chr10	66392556	66392916
chr10	66470769	66471010
chr10	66492535	66492815
chr10	66503254	66503566
chr10	66504792	66504947

chr10	66511284	66511699
chr10	66511699	66512317
chr10	66534597	66534758
chr10	66548159	66548328
chr10	66552792	66553190
chr10	66553190	66553534
chr10	66571830	66572073
chr10	66574830	66575090
chr10	66712251	66712455
chr10	66733780	66733953
chr10	66807667	66807853
chr10	66812986	66813274
chr10	66813900	66814097
chr10	66814097	66814336
chr10	66814336	66814552
chr10	66814552	66814822
chr10	66844561	66844737
chr10	66849639	66850217
chr10	66850217	66850704
chr10	66864145	66864952
chr10	66884964	66885164
chr10	66885415	66885821
chr10	66885821	66886063
chr10	66891675	66892357
chr10	66899872	66900069
chr10	66904198	66904371
chr10	66904956	66905325
chr10	66909253	66909506
chr10	66911248	66911704
chr10	66911704	66912110
chr10	66919884	66920283
chr10	66920800	66921012
chr10	66921053	66921497
chr10	66921497	66921883
chr10	66928158	66928562
chr10	66928562	66928993
chr10	66935959	66936113
chr10	66942033	66942224
chr10	66945932	66946716
chr10	66947171	66947333
chr10	66947335	66947515
chr10	66968589	66968847

chr10	66974476	66974648
chr10	66975529	66975791
chr10	66976592	66976768
chr10	66984244	66984757
chr10	66987543	66987886
chr10	66990315	66990558
chr10	66990558	66990963
chr10	66991811	66992083
chr10	66998625	66998775
chr10	67001196	67001410
chr10	67002642	67003102
chr10	67003199	67003615
chr10	67014833	67015036
chr10	67022858	67023236
chr10	67023236	67023598
chr10	67025806	67026265
chr10	67040908	67041184
chr10	67042248	67042521
chr10	67048858	67049249
chr10	67049249	67049610
chr10	67069052	67069470
chr10	67071498	67071733
chr10	67083602	67084096
chr10	67086065	67086289
chr10	67095913	67096194
chr10	67096194	67096705
chr10	67096803	67097087
chr10	67097087	67097338
chr10	67098448	67099108
chr10	67105757	67105960
chr10	67106880	67107562
chr10	67111101	67111259
chr10	67115867	67116215
chr10	67119660	67119810
chr10	67126308	67126632
chr10	67126632	67126975
chr10	67126975	67127336
chr10	67127336	67127600
chr10	67155357	67155564
chr10	67156424	67156734
chr10	67156734	67157312
chr10	67157468	67157845

chr10	67174132	67174366
chr10	67176059	67176408
chr10	67185486	67185826
chr10	67185826	67186133
chr10	67186954	67187200
chr10	67187294	67187476
chr10	67192472	67192924
chr10	67195205	67195464
chr10	67196596	67197065
chr10	67207571	67207736
chr10	67218652	67218915
chr10	67218975	67219217
chr10	67219217	67219545
chr10	67223927	67224283
chr10	67238145	67238313
chr10	67247170	67247516
chr10	67256377	67256582
chr10	67259628	67259924
chr10	67259924	67260196
chr10	67264948	67265168
chr10	67282000	67282269
chr10	67282589	67282866
chr10	67284300	67284471
chr10	67284622	67285109
chr10	67285109	67285649
chr10	67311007	67311185
chr10	67311364	67311612
chr10	67311647	67311862
chr10	67315043	67315372
chr10	67316313	67316608
chr10	67360802	67361269
chr10	67362248	67362496
chr10	67377753	67378364
chr10	67393511	67393661
chr10	67439083	67439617
chr10	67458734	67458962
chr10	67458962	67459431
chr10	67461400	67461632
chr10	67483486	67483669
chr10	67487403	67487813
chr10	67488522	67488774
chr10	67489994	67490223

chr10	67490470	67490987
chr10	67493621	67493892
chr10	67504315	67504508
chr10	67535981	67536425
chr10	67536696	67536882
chr10	67536882	67537080
chr10	67537293	67537644
chr10	67537644	67537943
chr10	67547855	67548381
chr10	67548381	67548674
chr10	67548674	67548921
chr10	67548921	67549228
chr10	67549228	67549508
chr10	67549508	67549805
chr10	67607651	67607849
chr10	67610638	67611081
chr10	67666096	67666300
chr10	67671664	67671995
chr10	67712091	67712327
chr10	67717408	67717931
chr10	67717931	67718258
chr10	67726459	67726688
chr10	67732570	67732720
chr10	67756154	67756365
chr10	67785147	67785455
chr10	67785455	67786144
chr10	67845872	67846576
chr10	67851159	67851448
chr10	67858940	67859211
chr10	67865755	67866273
chr10	67868591	67868970
chr10	67871871	67872151
chr10	67890964	67891284
chr10	67891284	67891679
chr10	67899016	67899283
chr10	67912279	67912883
chr10	67917922	67918175
chr10	67933792	67933952
chr10	67934393	67935043
chr10	67936852	67937170
chr10	67938356	67938554
chr10	67938579	67938836

chr10	67943528	67943765
chr10	67979320	67979777
chr10	67979777	67980144
chr10	67983873	67984043
chr10	67984133	67984623
chr10	67984623	67984919
chr10	67996289	67996451
chr10	67998156	67998434
chr10	67998446	67998649
chr10	68002131	68002312
chr10	68007994	68008262
chr10	68008262	68008582
chr10	68008582	68008902
chr10	68038601	68038831
chr10	68039353	68039503
chr10	68039626	68039962
chr10	68039962	68040275
chr10	68087433	68087590
chr10	68087841	68088035
chr10	68089945	68090501
chr10	68091852	68092266
chr10	68102040	68102295
chr10	68103490	68103803
chr10	68104026	68104266
chr10	68104353	68104526
chr10	68104565	68104918
chr10	68104918	68105227
chr10	68105257	68105409
chr10	68117369	68117582
chr10	68127255	68127522
chr10	68134587	68134880
chr10	68136495	68137081
chr10	68137359	68137869
chr10	68142225	68142524
chr10	68144358	68144804
chr10	68146927	68147179
chr10	68148605	68148792
chr10	68156296	68156618
chr10	68156672	68156971
chr10	68156993	68157345
chr10	68203375	68203800
chr10	68225486	68225718

chr10	68238234	68238503
chr10	68238503	68238691
chr10	68238761	68238958
chr10	68246718	68246868
chr10	68247524	68247783
chr10	68248379	68248734
chr10	68248734	68249139
chr10	68249530	68249840
chr10	68263357	68263671
chr10	68263671	68263973
chr10	68265459	68265771
chr10	68268705	68268986
chr10	68275372	68275624
chr10	68276333	68276639
chr10	68276639	68276927
chr10	68276927	68277250
chr10	68277255	68277411
chr10	68281155	68281339
chr10	68282543	68282735
chr10	68282814	68283479
chr10	68354405	68354720
chr10	68354720	68355126
chr10	68356100	68356481
chr10	68361211	68361442
chr10	68375306	68375515
chr10	68375782	68375952
chr10	68377492	68377765
chr10	68417159	68417321
chr10	68512444	68512817
chr10	68587486	68587705
chr10	68625465	68625637
chr10	68662011	68662161
chr10	68666090	68666317
chr10	68695309	68695649
chr10	68695649	68696299
chr10	68701758	68701936
chr10	68705567	68705770
chr10	68711982	68712144
chr10	68897500	68897866
chr10	68907299	68907560
chr10	68908199	68908349
chr10	68916207	68916704

chr10	68968643	68969176
chr10	68969176	68969556
chr10	68970661	68970821
chr10	68976474	68976746
chr10	68976746	68977015
chr10	68990547	68990860
chr10	69005633	69005787
chr10	69093261	69093479
chr10	69102140	69102298
chr10	69104385	69104571
chr10	69112182	69112382
chr10	69131307	69131616
chr10	69133873	69134102
chr10	69134348	69134853
chr10	69149278	69149614
chr10	69157506	69157831
chr10	69165260	69165419
chr10	69165897	69166241
chr10	69185096	69185246
chr10	69185375	69185850
chr10	69185850	69186283
chr10	69186367	69186558
chr10	69204704	69205026
chr10	69205029	69205561
chr10	69212499	69212895
chr10	69212895	69213380
chr10	69213380	69213886
chr10	69218734	69219316
chr10	69271475	69271691
chr10	69276158	69276432
chr10	69287493	69287644
chr10	69296431	69296791
chr10	69304934	69305084
chr10	69306540	69306998
chr10	69307167	69307438
chr10	69321289	69321567
chr10	69321737	69321956
chr10	69328744	69328973
chr10	69329682	69329832
chr10	69333286	69333497
chr10	69340042	69340519
chr10	69341286	69341574

chr10	69352213	69352429
chr10	69352429	69352728
chr10	69352728	69353367
chr10	69367046	69367213
chr10	69371532	69371765
chr10	69398326	69398637
chr10	69398637	69399117
chr10	69399117	69399635
chr10	69399869	69400073
chr10	69452141	69452487
chr10	69454560	69454847
chr10	69461705	69462012
chr10	69462012	69462270
chr10	69465651	69465855
chr10	69475667	69475881
chr10	69487570	69487767
chr10	69497466	69497725
chr10	69503782	69504031
chr10	69507164	69508123
chr10	69509846	69510127
chr10	69516668	69516863
chr10	69517415	69517637
chr10	69533488	69533888
chr10	69533978	69534179
chr10	69536772	69536928
chr10	69556374	69556620
chr10	69570967	69571274
chr10	69571287	69571480
chr10	69571504	69571834
chr10	69572128	69572302
chr10	69572524	69572726
chr10	69577356	69577746
chr10	69577746	69578057
chr10	69578057	69578335
chr10	69579627	69579899
chr10	69611737	69612059
chr10	69612059	69612413
chr10	69621174	69621378
chr10	69628714	69629163
chr10	69629352	69629506
chr10	69629587	69629869
chr10	69629949	69630151

chr10	69632977	69633247
chr10	69640723	69640896
chr10	69648941	69649163
chr10	69652629	69652994
chr10	69652994	69653327
chr10	69653327	69653566
chr10	69654914	69655288
chr10	69655288	69655778
chr10	69662380	69662864
chr10	69670798	69671048
chr10	69671048	69671376
chr10	69671430	69671762
chr10	69709776	69709967
chr10	69710804	69711057
chr10	69711057	69711370
chr10	69711370	69711794
chr10	69716779	69717065
chr10	69717065	69717441
chr10	69721676	69721896
chr10	69736385	69736636
chr10	69739490	69739852
chr10	69746904	69747148
chr10	69756714	69757510
chr10	69777031	69777754
chr10	69784773	69785038
chr10	69793128	69793440
chr10	69829030	69829180
chr10	69829313	69829553
chr10	69834776	69834932
chr10	69845626	69845824
chr10	69845824	69846257
chr10	69849017	69849324
chr10	69852491	69852678
chr10	69870419	69870654
chr10	69872092	69872327
chr10	69872327	69872917
chr10	69872941	69873164
chr10	69888485	69888727
chr10	69895742	69895892
chr10	69908451	69908686
chr10	69910411	69910960
chr10	69916170	69916472

chr10	69916472	69916759
chr10	69916759	69917037
chr10	69917037	69917244
chr10	69928311	69928770
chr10	69930531	69931072
chr10	69936508	69936685
chr10	69937204	69937557
chr10	69937557	69937864
chr10	69964703	69964873
chr10	70030338	70030599
chr10	70030602	70030806
chr10	70031873	70032327
chr10	70039958	70040233
chr10	70056792	70057031
chr10	70057087	70057470
chr10	70060716	70061015
chr10	70067215	70067405
chr10	70094998	70095254
chr10	70095254	70095524
chr10	70096441	70096671
chr10	70096671	70096927
chr10	70096927	70097379
chr10	70097637	70097824
chr10	70097855	70098297
chr10	70110098	70110332
chr10	70120671	70121289
chr10	70121289	70121688
chr10	70123453	70123822
chr10	70133237	70133543
chr10	70138297	70138447
chr10	70156494	70156754
chr10	70160174	70160326
chr10	70165999	70166163
chr10	70173364	70173615
chr10	70186229	70186459
chr10	70186580	70186776
chr10	70222296	70222537
chr10	70244842	70245185
chr10	70245185	70245626
chr10	70245650	70245899
chr10	70256489	70256662
chr10	70257118	70257469

chr10	70266537	70266853
chr10	70278199	70278405
chr10	70287463	70287741
chr10	70287741	70288039
chr10	70288039	70288287
chr10	70288487	70288653
chr10	70296665	70296876
chr10	70296876	70297143
chr10	70318736	70318967
chr10	70328561	70328829
chr10	70470900	70471169
chr10	70519622	70520174
chr10	70520236	70520522
chr10	70520855	70521011
chr10	70595074	70595366
chr10	70598457	70598627
chr10	70598627	70598896
chr10	70598896	70599237
chr10	70599237	70599519
chr10	70599519	70599972
chr10	70600036	70600198
chr10	70703371	70703550
chr10	70847412	70847870
chr10	70848176	70848448
chr10	70852726	70852877
chr10	70856067	70856225
chr10	70864652	70865079
chr10	70901294	70901864
chr10	70902829	70903157
chr10	70903157	70903413
chr10	70919448	70919828
chr10	70923322	70923544
chr10	70932669	70932955
chr10	70932955	70933282
chr10	70960433	70960585
chr10	71058585	71059296
chr10	71119719	71119879
chr10	71130319	71130611
chr10	71158903	71159094
chr10	71159094	71159322
chr10	71159322	71159539
chr10	71159539	71159761

chr10	71159761	71160014
chr10	71217382	71217652
chr10	71218298	71218563
chr10	71225979	71226198
chr10	71237517	71237899
chr10	71237899	71238439
chr10	71261813	71261965
chr10	71266252	71266489
chr10	71284418	71284693
chr10	71284693	71284932
chr10	71284932	71285150
chr10	71285150	71285593
chr10	71292263	71292639
chr10	71293366	71293555
chr10	71306240	71306461
chr10	71324796	71324981
chr10	71326862	71327104
chr10	71334298	71334569
chr10	71338584	71338762
chr10	71344533	71344826
chr10	71344826	71345232
chr10	71347222	71347710
chr10	71347710	71347937
chr10	71347937	71348287
chr10	71348287	71348609
chr10	71363883	71364301
chr10	71376063	71376326
chr10	71398123	71398769
chr10	71399434	71399765
chr10	71401351	71401561
chr10	71480021	71480251
chr10	71830761	71830916
chr10	71857727	71857936
chr10	71867205	71867694
chr10	71867694	71867992
chr10	71885017	71885371
chr10	71885371	71885671
chr10	71895570	71895788
chr10	71901413	71901645
chr10	71901679	71901966
chr10	71905091	71905341
chr10	71953300	71953520

chr10	71959626	71960179
chr10	71960206	71960464
chr10	71960487	71960719
chr10	71960719	71961055
chr10	71971150	71971445
chr10	71980546	71980929
chr10	71988181	71988342
chr10	72015856	72016260
chr10	72016260	72016556
chr10	72127750	72127957
chr10	72293302	72293482
chr10	72297104	72297411
chr10	72352883	72353300
chr10	72484451	72484722
chr10	72551576	72551986
chr10	72588417	72588631
chr10	72588631	72588954
chr10	72598712	72599202
chr10	72599246	72599484
chr10	72604497	72604716
chr10	72605015	72605214
chr10	72613128	72613409
chr10	72620572	72620870
chr10	72622923	72623182
chr10	72623182	72623365
chr10	72634000	72634662
chr10	72645393	72645545
chr10	72654367	72654662
chr10	72654662	72654987
chr10	72654987	72655256
chr10	72655256	72655591
chr10	72660344	72660799
chr10	72664005	72664203
chr10	72674584	72674739
chr10	72676653	72676826
chr10	72680620	72680832
chr10	72681269	72681522
chr10	72702331	72702515
chr10	72709111	72709379
chr10	72713849	72714004
chr10	72775897	72776180
chr10	72778293	72778541

chr10	72778541	72778827
chr10	72783055	72783362
chr10	72783460	72784034
chr10	72797654	72797932
chr10	72834699	72834978
chr10	72947875	72948125
chr10	72955686	72955883
chr10	72974820	72975045
chr10	72981454	72981670
chr10	72988271	72988554
chr10	72988554	72989116
chr10	72989640	72990096
chr10	73001365	73001572
chr10	73011960	73012139
chr10	73020135	73020316
chr10	73060526	73061012
chr10	73061012	73061328
chr10	73077887	73078087
chr10	73098136	73098387
chr10	73098900	73099353
chr10	73099353	73099748
chr10	73099748	73100037
chr10	73100037	73100287
chr10	73106026	73106186
chr10	73116633	73116825
chr10	73216113	73216294
chr10	73242413	73242659
chr10	73422583	73422834
chr10	73733256	73733570
chr10	73742631	73742907
chr10	73743123	73743495
chr10	73746034	73746254
chr10	73747087	73747339
chr10	73812355	73812618
chr10	73826471	73826621
chr10	73995858	73996035
chr10	74051821	74052142
chr10	74062064	74062257
chr10	74079748	74079982
chr10	74098344	74098705
chr10	74115305	74115559
chr10	74123508	74123756

chr10	74146014	74146504
chr10	74148012	74148343
chr10	74148832	74149141
chr10	74150550	74150882
chr10	74283296	74283454
chr10	74353696	74353883
chr10	74362729	74362930
chr10	74368405	74368718
chr10	74444131	74444285
chr10	74447579	74447951
chr10	74527548	74527951
chr10	74538338	74538775
chr10	74538812	74539301
chr10	74601716	74601938
chr10	74643899	74644179
chr10	74644179	74644481
chr10	74662835	74663209
chr10	74663209	74663650
chr10	74676734	74676886
chr10	74679942	74680150
chr10	74680237	74680571
chr10	74680571	74680885
chr10	74680885	74681161
chr10	74681986	74682138
chr10	74722321	74722471
chr10	74724094	74724269
chr10	74789353	74789555
chr10	74823452	74823730
chr10	74834199	74834755
chr10	74835428	74835610
chr10	74848438	74848682
chr10	74892401	74892846
chr10	74920931	74921240
chr10	74922719	74923385
chr10	74924140	74924396
chr10	74928693	74928935
chr10	74929570	74930088
chr10	74934650	74934812
chr10	74935097	74935606
chr10	74937067	74937357
chr10	74938536	74938802
chr10	74939390	74939597

chr10	74939881	74940058
chr10	74948390	74948540
chr10	74966840	74967191
chr10	74967191	74967504
chr10	74973145	74973298
chr10	74978129	74978404
chr10	74988018	74988678
chr10	74995588	74995833
chr10	75005229	75005453
chr10	75021197	75021454
chr10	75022778	75023016
chr10	75031026	75031267
chr10	75031438	75031687
chr10	75031848	75032088
chr10	75032457	75032651
chr10	75036832	75037545
chr10	75042170	75042394
chr10	75044500	75044690
chr10	75053654	75053981
chr10	75059411	75059692
chr10	75060127	75060391
chr10	75060438	75060846
chr10	75062427	75063031
chr10	75063721	75063942
chr10	75068783	75069074
chr10	75069074	75069303
chr10	75070151	75070347
chr10	75070350	75070655
chr10	75070691	75070966
chr10	75071918	75072338
chr10	75085500	75085797
chr10	75091522	75091756
chr10	75091782	75092030
chr10	75092592	75092901
chr10	75093752	75094049
chr10	75098566	75098761
chr10	75104069	75104481
chr10	75104481	75105054
chr10	75105070	75105428
chr10	75105453	75105636
chr10	75111867	75112176
chr10	75112449	75112722

chr10	75114195	75114482
chr10	75115285	75115620
chr10	75116736	75116955
chr10	75117212	75117398
chr10	75118614	75118824
chr10	75119354	75119788
chr10	75120963	75121443
chr10	75129005	75129787
chr10	75130444	75130604
chr10	75133181	75133367
chr10	75137163	75137436
chr10	75144129	75144376
chr10	75150089	75150424
chr10	75150424	75150918
chr10	75150918	75151293
chr10	75151322	75151654
chr10	75160186	75160370
chr10	75160487	75160701
chr10	75160701	75160943
chr10	75163713	75163897
chr10	75169357	75169718
chr10	75170240	75170981
chr10	75173707	75173953
chr10	75180585	75180745
chr10	75182168	75182404
chr10	75182407	75182683
chr10	75184485	75184842
chr10	75185605	75185888
chr10	75191639	75192053
chr10	75211659	75211921
chr10	75211921	75212227
chr10	75212227	75212469
chr10	75212469	75212703
chr10	75221986	75222392
chr10	75222392	75223044
chr10	75230641	75230906
chr10	75231469	75231719
chr10	75232134	75232302
chr10	75232648	75233250
chr10	75233305	75233502
chr10	75233592	75233742
chr10	75240617	75240896

chr10	75250837	75251089
chr10	75251089	75251318
chr10	75252288	75252688
chr10	75299710	75300126
chr10	75300126	75300509
chr10	75300808	75301088
chr10	75316610	75316953
chr10	75316953	75317209
chr10	75317231	75317465
chr10	75317465	75317723
chr10	75320431	75320587
chr10	75322076	75322361
chr10	75326340	75327059
chr10	75343188	75343352
chr10	75378758	75379106
chr10	75394821	75395025
chr10	75406785	75407007
chr10	75428960	75429517
chr10	75434400	75434951
chr10	75436715	75436962
chr10	75439543	75439775
chr10	75441987	75442274
chr10	75449389	75449626
chr10	75466145	75466626
chr10	75486484	75486700
chr10	75486700	75486899
chr10	75487225	75487416
chr10	75497300	75497546
chr10	75504784	75505155
chr10	75505217	75505382
chr10	75516959	75517603
chr10	75517603	75518118
chr10	75525548	75525811
chr10	75534986	75535175
chr10	75535608	75535942
chr10	75548466	75548958
chr10	75554194	75554482
chr10	75559851	75560113
chr10	75560113	75560432
chr10	75566811	75567122
chr10	75568868	75569166
chr10	75571402	75571600

chr10	75586264	75586567
chr10	75589758	75590061
chr10	75614828	75615040
chr10	75618026	75618228
chr10	75621939	75622099
chr10	75627088	75627239
chr10	75635693	75636027
chr10	75638266	75638421
chr10	75643901	75644247
chr10	75649094	75649328
chr10	75663228	75663508
chr10	75667444	75667869
chr10	75667965	75668221
chr10	75674123	75674311
chr10	75675994	75676339
chr10	75683988	75684483
chr10	75684483	75684974
chr10	75685155	75685378
chr10	75685784	75685939
chr10	75702639	75702897
chr10	75704164	75704517
chr10	75708735	75708980
chr10	75739471	75739743
chr10	75744071	75744365
chr10	75751738	75751950
chr10	75763813	75764115
chr10	75764115	75764496
chr10	75764496	75764767
chr10	75764774	75764981
chr10	75765065	75765454
chr10	75767826	75768009
chr10	75772729	75772879
chr10	75772946	75773266
chr10	75773266	75773726
chr10	75780684	75780958
chr10	75780958	75781275
chr10	75781336	75781566
chr10	75789841	75790184
chr10	75790924	75791110
chr10	75797603	75797917
chr10	75797919	75798247
chr10	75798346	75798703

chr10	75819829	75820212
chr10	75820212	75820471
chr10	75827520	75828040
chr10	75828040	75828341
chr10	75828360	75829050
chr10	75835303	75835498
chr10	75835498	75835713
chr10	75835713	75835892
chr10	75836056	75836643
chr10	75837137	75837385
chr10	75837385	75837691
chr10	75853535	75853783
chr10	75853783	75853993
chr10	75854186	75854401
chr10	75858991	75859155
chr10	75859444	75860065
chr10	75860065	75860437
chr10	75872731	75873246
chr10	75876156	75876377
chr10	75878909	75879080
chr10	75881281	75881548
chr10	75883067	75883522
chr10	75892974	75893709
chr10	75898724	75899394
chr10	75905801	75906139
chr10	75920887	75921410
chr10	75921410	75921867
chr10	75921867	75922209
chr10	75923367	75923553
chr10	75930299	75930870
chr10	75931953	75932170
chr10	75932170	75932379
chr10	75932379	75932670
chr10	75935612	75936094
chr10	75936094	75936465
chr10	75951773	75952105
chr10	75954616	75954783
chr10	75968307	75968605
chr10	75975292	75975564
chr10	75983481	75983640
chr10	75993743	75994011
chr10	75997764	75997928

chr10	76032123	76032790
chr10	76032790	76033116
chr10	76046767	76047054
chr10	76047054	76047266
chr10	76047266	76047489
chr10	76047543	76047772
chr10	76072106	76072489
chr10	76076992	76077172
chr10	76118028	76118279
chr10	76118498	76118734
chr10	76118734	76118948
chr10	76120112	76120318
chr10	76120367	76120699
chr10	76121374	76121881
chr10	76140528	76140736
chr10	76144129	76144489
chr10	76146835	76147213
chr10	76152705	76152856
chr10	76157200	76157462
chr10	76169838	76170364
chr10	76190224	76190428
chr10	76198501	76198674
chr10	76202146	76202543
chr10	76204034	76204293
chr10	76214282	76214503
chr10	76227434	76227597
chr10	76230764	76230924
chr10	76236580	76236823
chr10	76237362	76237697
chr10	76237697	76238022
chr10	76238022	76238283
chr10	76258023	76258399
chr10	76261411	76261864
chr10	76262077	76262423
chr10	76262423	76262769
chr10	76265526	76265803
chr10	76271270	76271732
chr10	76287677	76287896
chr10	76288343	76288655
chr10	76288734	76288943
chr10	76306768	76307082
chr10	76307082	76307395

chr10	76326493	76326678
chr10	76326691	76326965
chr10	76328610	76328882
chr10	76336973	76337709
chr10	76338266	76338468
chr10	76344689	76345083
chr10	76345083	76345374
chr10	76345374	76345585
chr10	76345585	76345854
chr10	76346018	76346284
chr10	76346308	76346531
chr10	76346701	76346929
chr10	76357445	76357922
chr10	76358260	76358677
chr10	76358677	76358910
chr10	76382279	76382439
chr10	76388924	76389126
chr10	76391896	76392120
chr10	76411402	76411565
chr10	76411745	76411899
chr10	76415131	76415314
chr10	76418390	76418639
chr10	76428250	76428559
chr10	76442175	76442583
chr10	76442583	76443117
chr10	76443135	76443313
chr10	76448727	76449276
chr10	76455346	76455632
chr10	76461367	76461517
chr10	76468522	76468780
chr10	76468780	76469205
chr10	76503231	76503425
chr10	76504265	76504658
chr10	76504658	76504984
chr10	76513191	76513710
chr10	76531026	76531327
chr10	76531327	76531943
chr10	76537331	76537581
chr10	76537581	76538108
chr10	76542767	76543180
chr10	76545016	76545312
chr10	76550501	76550677

chr10	76551609	76551799
chr10	76577881	76578045
chr10	76584215	76584413
chr10	76607984	76608383
chr10	76623197	76623729
chr10	76633912	76634466
chr10	76634466	76634791
chr10	76637112	76637355
chr10	76642314	76642482
chr10	76642547	76642846
chr10	76643000	76643200
chr10	76645216	76645534
chr10	76648669	76649021
chr10	76649021	76649277
chr10	76650867	76651275
chr10	76651480	76651655
chr10	76653276	76653539
chr10	76668958	76669223
chr10	76693464	76693792
chr10	76725938	76726265
chr10	76730271	76730581
chr10	76730581	76730890
chr10	76735055	76735385
chr10	76736874	76737040
chr10	76739018	76739213
chr10	76762407	76762574
chr10	76763048	76763266
chr10	76783889	76784039
chr10	76791535	76791732
chr10	76795874	76796100
chr10	76798746	76799071
chr10	76816596	76816750
chr10	76835573	76835778
chr10	76835778	76836189
chr10	76858979	76859172
chr10	76866056	76866212
chr10	76874046	76874243
chr10	76874371	76874702
chr10	76902573	76903032
chr10	76903117	76903453
chr10	76903453	76903731
chr10	76930916	76931089

chr10	76931122	76931744
chr10	76935581	76935788
chr10	76961704	76962374
chr10	76963079	76963536
chr10	76972429	76972632
chr10	76986723	76986985
chr10	76991195	76991345
chr10	76993462	76993921
chr10	76997825	76998103
chr10	77005209	77005465
chr10	77006276	77006457
chr10	77016211	77016391
chr10	77016498	77017165
chr10	77017194	77017388
chr10	77018194	77018497
chr10	77021405	77021711
chr10	77022847	77023073
chr10	77030764	77030963
chr10	77032553	77032953
chr10	77032953	77033537
chr10	77033537	77034157
chr10	77034187	77034361
chr10	77040488	77040643
chr10	77040645	77040846
chr10	77042543	77042741
chr10	77049017	77049251
chr10	77050873	77051042
chr10	77051042	77051229
chr10	77051332	77051496
chr10	77052566	77052892
chr10	77058147	77058305
chr10	77061224	77061419
chr10	77061487	77061683
chr10	77064743	77064989
chr10	77070135	77070523
chr10	77071313	77071482
chr10	77082257	77082512
chr10	77082512	77082878
chr10	77084596	77084885
chr10	77089423	77089799
chr10	77097968	77098318
chr10	77098697	77099065

chr10	77110038	77110311
chr10	77111341	77111498
chr10	77111531	77112175
chr10	77121352	77121617
chr10	77125169	77125376
chr10	77129982	77130420
chr10	77130420	77130919
chr10	77130959	77131109
chr10	77135046	77135206
chr10	77135280	77135678
chr10	77136589	77136887
chr10	77142172	77142685
chr10	77146851	77147629
chr10	77151181	77151331
chr10	77151375	77151987
chr10	77152136	77152425
chr10	77152425	77152762
chr10	77165429	77165754
chr10	77165754	77165992
chr10	77165992	77166341
chr10	77166341	77166813
chr10	77169078	77169253
chr10	77184646	77184917
chr10	77193460	77193764
chr10	77199271	77199684
chr10	77201989	77202278
chr10	77206529	77206824
chr10	77206824	77207133
chr10	77207133	77207430
chr10	77211095	77211583
chr10	77211583	77211877
chr10	77212458	77212692
chr10	77218177	77218345
chr10	77218633	77218869
chr10	77225454	77225604
chr10	77235276	77235530
chr10	77236832	77237178
chr10	77237195	77237435
chr10	77237539	77237705
chr10	77237770	77238215
chr10	77238303	77238565
chr10	77246343	77246733

chr10	77251168	77251573
chr10	77251596	77252150
chr10	77252150	77252602
chr10	77252861	77253156
chr10	77258758	77259079
chr10	77259079	77259396
chr10	77259396	77259745
chr10	77260398	77260558
chr10	77270463	77270738
chr10	77277676	77278153
chr10	77282674	77282888
chr10	77282892	77283148
chr10	77288678	77288854
chr10	77300625	77301193
chr10	77303720	77303936
chr10	77313988	77314153
chr10	77314860	77315020
chr10	77315733	77315911
chr10	77316139	77316289
chr10	77321844	77322106
chr10	77327892	77328361
chr10	77353401	77353725
chr10	77360567	77360722
chr10	77367061	77367250
chr10	77379295	77379565
chr10	77381455	77381791
chr10	77387315	77387654
chr10	77387654	77388011
chr10	77388455	77388613
chr10	77396101	77396456
chr10	77398134	77398464
chr10	77402254	77403025
chr10	77406727	77407003
chr10	77417716	77418055
chr10	77418055	77418322
chr10	77418322	77418649
chr10	77419343	77419661
chr10	77420218	77420681
chr10	77427649	77427983
chr10	77430613	77431113
chr10	77434815	77434965
chr10	77445162	77445498

chr10	77447231	77447576
chr10	77447791	77448008
chr10	77449293	77449608
chr10	77454128	77454352
chr10	77456661	77456829
chr10	77457594	77457854
chr10	77457854	77458100
chr10	77464240	77464691
chr10	77466014	77466327
chr10	77470189	77470436
chr10	77484485	77484818
chr10	77485967	77486123
chr10	77486123	77486296
chr10	77486415	77486610
chr10	77492195	77492520
chr10	77509507	77510192
chr10	77515406	77516072
chr10	77516075	77516248
chr10	77516317	77516603
chr10	77517073	77517537
chr10	77520670	77520856
chr10	77524056	77524232
chr10	77526634	77526840
chr10	77530932	77531492
chr10	77531492	77532208
chr10	77536742	77536919
chr10	77541766	77542054
chr10	77542087	77542525
chr10	77544726	77544989
chr10	77544989	77545288
chr10	77556058	77556217
chr10	77556629	77557028
chr10	77557028	77557413
chr10	77557709	77557991
chr10	77558051	77558332
chr10	77568615	77568878
chr10	77568901	77569270
chr10	77569555	77569809
chr10	77570013	77570654
chr10	77570654	77570963
chr10	77581441	77581880
chr10	77581880	77582291

chr10	77582334	77582513
chr10	77583491	77584108
chr10	77590646	77591244
chr10	77605036	77605274
chr10	77605363	77605636
chr10	77605865	77606144
chr10	77606144	77606448
chr10	77606448	77606826
chr10	77606938	77607137
chr10	77607139	77607328
chr10	77614784	77614997
chr10	77616223	77616469
chr10	77621923	77622739
chr10	77623185	77623461
chr10	77623726	77623924
chr10	77626622	77626956
chr10	77627586	77627841
chr10	77630091	77630321
chr10	77634162	77634402
chr10	77634906	77635127
chr10	77643335	77643492
chr10	77646811	77646961
chr10	77647122	77647272
chr10	77656250	77656469
chr10	77658699	77658852
chr10	77665630	77665782
chr10	77665976	77666389
chr10	77666389	77666809
chr10	77685959	77686264
chr10	77686264	77686711
chr10	77690135	77690359
chr10	77690601	77690751
chr10	77693289	77693700
chr10	77696611	77696888
chr10	77696888	77697217
chr10	77700084	77700309
chr10	77715257	77715531
chr10	77715712	77715985
chr10	77715985	77716275
chr10	77716442	77716667
chr10	77726433	77726600
chr10	77726600	77726793

chr10	77729344	77729541
chr10	77741779	77742052
chr10	77742052	77742353
chr10	77742521	77742689
chr10	77749239	77749476
chr10	77750482	77750839
chr10	77750973	77751149
chr10	77751208	77751416
chr10	77752434	77752584
chr10	77758965	77759413
chr10	77759413	77759728
chr10	77769836	77770089
chr10	77770608	77770882
chr10	77770894	77771224
chr10	77776079	77776496
chr10	77776496	77776835
chr10	77781844	77782109
chr10	77782109	77782356
chr10	77787514	77787749
chr10	77787749	77788071
chr10	77794469	77794766
chr10	77796873	77797078
chr10	77798455	77798700
chr10	77798700	77798918
chr10	77811157	77811428
chr10	77811428	77811681
chr10	77814948	77815336
chr10	77818495	77818667
chr10	77818667	77818857
chr10	77826360	77826659
chr10	77826659	77826932
chr10	77826932	77827167
chr10	77830320	77830680
chr10	77836348	77836613
chr10	77849325	77849498
chr10	77852783	77853020
chr10	77853031	77853262
chr10	77853262	77853550
chr10	77862310	77862584
chr10	77864333	77864943
chr10	77876583	77876769
chr10	77886064	77886266

chr10	77893716	77893994
chr10	77901886	77902668
chr10	77902668	77902931
chr10	77902931	77903227
chr10	77916682	77916906
chr10	77925557	77925732
chr10	77926220	77926450
chr10	77939572	77939971
chr10	77964074	77964242
chr10	77966476	77966738
chr10	77969708	77970024
chr10	77975673	77975934
chr10	77978176	77978721
chr10	77978721	77978961
chr10	77978961	77979199
chr10	77984430	77984749
chr10	77984749	77985032
chr10	77991307	77991558
chr10	77993874	77994048
chr10	77995255	77995428
chr10	77998890	77999040
chr10	77999788	77999943
chr10	78009039	78009597
chr10	78009597	78009973
chr10	78009973	78010231
chr10	78018788	78019225
chr10	78021422	78021612
chr10	78025541	78025756
chr10	78028165	78028475
chr10	78043378	78043690
chr10	78069006	78069479
chr10	78069479	78070031
chr10	78088365	78088594
chr10	78091902	78092162
chr10	78132071	78132381
chr10	78135035	78135230
chr10	78135379	78135655
chr10	78136039	78136264
chr10	78138883	78139080
chr10	78154331	78154543
chr10	78159303	78159484
chr10	78159984	78160275

chr10	78160463	78160671
chr10	78162711	78163011
chr10	78163011	78163317
chr10	78163994	78164243
chr10	78169460	78169879
chr10	78169879	78170137
chr10	78171038	78171545
chr10	78184589	78184974
chr10	78184974	78185391
chr10	78192024	78192219
chr10	78199847	78200060
chr10	78200190	78200483
chr10	78211398	78211647
chr10	78222969	78223279
chr10	78226914	78227117
chr10	78227284	78227482
chr10	78243991	78244457
chr10	78244457	78244943
chr10	78244943	78245475
chr10	78255717	78256100
chr10	78267025	78267206
chr10	78279227	78279659
chr10	78282502	78282748
chr10	78297715	78298519
chr10	78303721	78304142
chr10	78309908	78310115
chr10	78311988	78312162
chr10	78315957	78316521
chr10	78317360	78317548
chr10	78322860	78323124
chr10	78329111	78329839
chr10	78338135	78338691
chr10	78342266	78342624
chr10	78342624	78343034
chr10	78348305	78348540
chr10	78351923	78352375
chr10	78352375	78352672
chr10	78353571	78353901
chr10	78360152	78360466
chr10	78360477	78360783
chr10	78365859	78366174
chr10	78371868	78372252

chr10	78372694	78372939
chr10	78392099	78392394
chr10	78392447	78392624
chr10	78396236	78396591
chr10	78402470	78402620
chr10	78403655	78403805
chr10	78409447	78409721
chr10	78412257	78412721
chr10	78412721	78413239
chr10	78413264	78413414
chr10	78417951	78418191
chr10	78418953	78419235
chr10	78421016	78421335
chr10	78425472	78425763
chr10	78425763	78426059
chr10	78426298	78426704
chr10	78428343	78428909
chr10	78429200	78429554
chr10	78431001	78431168
chr10	78438156	78438412
chr10	78438412	78438628
chr10	78439620	78439915
chr10	78440900	78441176
chr10	78442366	78442545
chr10	78446256	78446597
chr10	78447783	78447997
chr10	78457553	78457744
chr10	78457854	78458229
chr10	78458310	78458727
chr10	78464502	78465363
chr10	78470721	78471648
chr10	78484640	78484902
chr10	78484949	78485179
chr10	78485443	78485849
chr10	78485849	78486267
chr10	78487760	78488067
chr10	78520346	78520553
chr10	78520702	78520853
chr10	78525002	78525568
chr10	78525571	78525846
chr10	78525846	78526055
chr10	78565212	78565409

chr10	78565762	78565970
chr10	78574138	78574561
chr10	78574561	78574856
chr10	78574856	78575106
chr10	78575106	78575351
chr10	78575351	78575594
chr10	78575785	78576027
chr10	78583132	78583304
chr10	78583304	78583496
chr10	78590815	78591064
chr10	78591280	78591622
chr10	78591622	78591831
chr10	78591831	78592223
chr10	78594199	78594515
chr10	78598564	78598915
chr10	78605170	78605348
chr10	78607715	78607940
chr10	78632017	78632181
chr10	78632400	78632611
chr10	78657091	78657288
chr10	78666336	78666642
chr10	78666642	78666940
chr10	78700978	78701159
chr10	78709959	78710110
chr10	78722061	78722288
chr10	78745925	78746094
chr10	78747029	78747203
chr10	78752422	78752849
chr10	78780063	78780470
chr10	79097111	79097506
chr10	79097506	79097891
chr10	79106622	79106785
chr10	79116599	79116801
chr10	79295331	79295488
chr10	79309492	79309740
chr10	79314332	79314600
chr10	79321930	79322197
chr10	79322319	79322619
chr10	79417225	79417438
chr10	79493336	79493549
chr10	79503077	79503799
chr10	79525423	79525664

chr10	79526072	79526222
chr10	79532726	79533103
chr10	79533103	79533567
chr10	79533567	79533996
chr10	79538842	79539260
chr10	79547989	79548297
chr10	79548513	79548870
chr10	79553951	79554112
chr10	79554226	79554376
chr10	79554448	79554714
chr10	79554714	79554989
chr10	79554989	79555454
chr10	79574267	79574585
chr10	79574585	79574855
chr10	79580869	79581134
chr10	79585314	79585480
chr10	79595268	79595547
chr10	79595547	79595790
chr10	79607244	79607479
chr10	79607883	79608191
chr10	79612241	79612591
chr10	79613517	79614055
chr10	79614055	79614499
chr10	79620643	79620796
chr10	79624030	79624250
chr10	79624261	79624421
chr10	79625815	79626095
chr10	79626144	79626520
chr10	79632771	79633057
chr10	79633057	79633386
chr10	79633386	79633699
chr10	79638923	79639092
chr10	79649435	79649713
chr10	79652755	79652984
chr10	79654888	79655380
chr10	79658885	79659164
chr10	79660360	79660608
chr10	79664076	79664376
chr10	79668773	79669019
chr10	79669019	79669270
chr10	79669270	79669606
chr10	79669606	79670076

chr10	79670166	79670326
chr10	79672131	79672368
chr10	79675712	79675862
chr10	79681195	79681439
chr10	79681467	79682490
chr10	79682676	79682873
chr10	79682917	79683159
chr10	79683159	79683387
chr10	79683387	79683630
chr10	79684477	79684691
chr10	79688634	79688846
chr10	79689021	79689313
chr10	79695272	79695541
chr10	79696018	79696271
chr10	79696281	79696777
chr10	79697037	79697361
chr10	79698895	79699153
chr10	79701455	79701806
chr10	79704084	79704629
chr10	79704629	79704961
chr10	79707229	79707570
chr10	79707751	79708189
chr10	79712120	79712338
chr10	79712471	79712729
chr10	79716517	79716725
chr10	79716921	79717138
chr10	79717138	79717356
chr10	79717682	79717868
chr10	79717880	79718058
chr10	79718058	79718279
chr10	79723341	79723496
chr10	79726088	79726490
chr10	79732592	79732812
chr10	79734039	79734189
chr10	79737589	79737785
chr10	79738321	79738532
chr10	79746088	79746254
chr10	79746254	79746484
chr10	79746484	79746803
chr10	79748757	79749024
chr10	79754912	79755389
chr10	79766411	79766825

chr10	79766825	79767379
chr10	79767398	79767810
chr10	79768459	79768710
chr10	79777996	79778267
chr10	79779874	79780349
chr10	79780349	79780557
chr10	79780557	79781028
chr10	79787348	79787527
chr10	79788470	79788633
chr10	79793046	79793222
chr10	79793222	79793421
chr10	79793421	79793934
chr10	79798256	79798462
chr10	79803809	79803973
chr10	79807040	79807342
chr10	79811849	79812059
chr10	79812273	79812730
chr10	79818180	79818617
chr10	79823902	79824389
chr10	79824389	79824786
chr10	79830383	79830568
chr10	79834806	79835285
chr10	79840510	79840955
chr10	79840955	79841303
chr10	79841384	79841564
chr10	79842409	79842658
chr10	79853230	79853412
chr10	79853431	79853728
chr10	79853809	79854176
chr10	79854176	79854701
chr10	79854701	79854995
chr10	79854995	79855313
chr10	79864408	79864776
chr10	79864776	79865057
chr10	79865065	79865368
chr10	79865368	79865706
chr10	79865706	79865956
chr10	79865956	79866116
chr10	79866116	79866391
chr10	79866557	79866809
chr10	79866809	79867059
chr10	79874297	79874492

chr10	79874492	79874681
chr10	79880802	79881025
chr10	79882044	79882304
chr10	79882558	79882708
chr10	79883676	79883956
chr10	79883956	79884164
chr10	79891479	79891662
chr10	79892643	79893096
chr10	79893442	79893785
chr10	79896898	79897210
chr10	79897431	79897581
chr10	79900884	79901343
chr10	79908207	79908505
chr10	79908505	79908789
chr10	79908789	79909029
chr10	79909152	79909338
chr10	79909338	79909574
chr10	79914948	79915243
chr10	79915243	79915694
chr10	79916025	79916440
chr10	79916753	79917058
chr10	79917118	79917287
chr10	79919593	79919764
chr10	79925334	79925656
chr10	79926521	79927091
chr10	79927091	79927337
chr10	79927337	79927616
chr10	79927616	79927889
chr10	79930498	79930702
chr10	79934316	79934652
chr10	79936678	79936902
chr10	79936902	79937186
chr10	79937186	79937635
chr10	79945299	79945533
chr10	79945533	79945887
chr10	79945994	79946381
chr10	79950692	79951003
chr10	79954001	79954205
chr10	79958187	79958479
chr10	79959533	79959683
chr10	79959763	79960023
chr10	79960023	79960474

chr10	79966220	79966370
chr10	79966422	79966585
chr10	79966585	79966736
chr10	79969418	79969624
chr10	79970664	79971090
chr10	79973005	79973213
chr10	79973256	79973433
chr10	79979020	79979290
chr10	79984202	79984441
chr10	79984487	79984814
chr10	79984814	79985301
chr10	79985729	79986029
chr10	79986232	79986595
chr10	79986960	79987139
chr10	79987174	79987345
chr10	79988525	79988675
chr10	79989098	79989288
chr10	79996078	79996238
chr10	79996255	79996584
chr10	79996584	79996792
chr10	79996964	79997232
chr10	79997232	79997508
chr10	80003910	80004091
chr10	80014511	80014946
chr10	80014970	80015226
chr10	80015532	80015907
chr10	80015930	80016140
chr10	80016359	80016738
chr10	80018949	80019240
chr10	80023846	80024053
chr10	80024097	80024504
chr10	80024504	80024779
chr10	80024779	80025039
chr10	80025988	80026220
chr10	80028339	80028489
chr10	80031635	80031788
chr10	80032767	80033120
chr10	80033141	80033497
chr10	80033497	80033924
chr10	80033924	80034381
chr10	80039417	80039706
chr10	80039706	80040027

chr10	80044124	80044369
chr10	80052084	80052367
chr10	80053267	80053776
chr10	80053776	80054083
chr10	80054083	80054374
chr10	80054397	80054843
chr10	80063998	80064180
chr10	80070992	80071263
chr10	80071263	80071603
chr10	80072608	80073407
chr10	80075061	80075319
chr10	80075319	80075653
chr10	80076484	80076730
chr10	80089207	80089409
chr10	80091597	80091764
chr10	80091768	80091921
chr10	80097298	80097639
chr10	80097639	80097892
chr10	80098551	80098701
chr10	80099278	80099737
chr10	80100960	80101417
chr10	80102576	80102830
chr10	80104193	80104569
chr10	80105502	80105671
chr10	80105958	80106252
chr10	80107533	80108302
chr10	80114584	80114875
chr10	80115305	80115951
chr10	80123174	80123447
chr10	80129654	80129877
chr10	80130193	80130348
chr10	80130440	80130718
chr10	80130734	80131069
chr10	80136993	80137312
chr10	80137334	80137673
chr10	80137673	80138022
chr10	80138902	80139152
chr10	80139152	80139516
chr10	80141288	80141676
chr10	80142135	80142498
chr10	80142695	80142888
chr10	80146050	80146205

chr10	80146434	80146711
chr10	80147686	80147989
chr10	80147989	80148197
chr10	80148197	80148370
chr10	80148370	80148582
chr10	80148679	80149164
chr10	80149279	80149787
chr10	80152945	80153174
chr10	80156061	80156310
chr10	80158413	80158970
chr10	80159227	80159635
chr10	80159656	80159919
chr10	80159919	80160366
chr10	80160795	80161112
chr10	80161112	80161427
chr10	80161622	80161798
chr10	80163765	80164106
chr10	80164160	80164381
chr10	80164985	80165345
chr10	80167225	80167411
chr10	80167462	80168006
chr10	80168006	80168384
chr10	80172790	80173048
chr10	80173048	80173299
chr10	80174819	80175032
chr10	80175580	80175805
chr10	80176027	80176195
chr10	80177432	80177764
chr10	80177764	80177997
chr10	80179624	80179774
chr10	80185572	80185834
chr10	80190449	80190788
chr10	80190825	80191047
chr10	80191047	80191253
chr10	80193306	80193524
chr10	80193613	80193813
chr10	80199481	80199930
chr10	80205561	80206001
chr10	80215959	80216112
chr10	80221072	80221264
chr10	80226195	80227092
chr10	80244973	80245123

chr10	80245627	80245942
chr10	80247953	80248119
chr10	80248905	80249141
chr10	80249141	80249760
chr10	80253595	80253911
chr10	80260825	80261078
chr10	80261078	80261285
chr10	80261285	80261588
chr10	80261588	80262023
chr10	80264722	80264945
chr10	80264945	80265212
chr10	80270339	80270489
chr10	80281902	80282056
chr10	80282058	80282446
chr10	80282446	80282771
chr10	80282927	80283698
chr10	80292213	80292541
chr10	80292541	80292828
chr10	80293584	80293749
chr10	80295027	80295352
chr10	80295432	80295813
chr10	80295813	80296086
chr10	80302412	80302692
chr10	80303435	80303645
chr10	80304762	80304960
chr10	80304960	80305258
chr10	80310593	80310805
chr10	80310805	80311161
chr10	80314087	80314237
chr10	80318293	80318625
chr10	80320424	80320726
chr10	80322787	80322949
chr10	80322988	80323293
chr10	80323293	80323654
chr10	80323654	80324056
chr10	80324667	80324817
chr10	80329358	80329749
chr10	80329749	80330433
chr10	80331179	80331448
chr10	80331657	80331906
chr10	80340119	80340367
chr10	80340395	80340632

chr10	80342562	80343035
chr10	80343035	80343462
chr10	80348331	80348873
chr10	80349028	80349247
chr10	80350128	80350341
chr10	80350341	80350651
chr10	80356447	80356626
chr10	80356820	80357139
chr10	80357515	80357711
chr10	80357917	80358194
chr10	80358194	80358580
chr10	80362701	80362983
chr10	80363860	80364027
chr10	80367863	80368022
chr10	80368477	80368721
chr10	80370146	80370540
chr10	80371376	80371655
chr10	80371655	80371903
chr10	80374152	80374422
chr10	80376205	80376531
chr10	80376895	80377062
chr10	80382676	80383063
chr10	80385739	80386063
chr10	80386165	80386383
chr10	80386383	80386767
chr10	80386898	80387123
chr10	80387518	80388074
chr10	80398690	80399370
chr10	80399370	80399995
chr10	80405835	80406123
chr10	80406484	80406725
chr10	80406725	80406990
chr10	80406990	80407376
chr10	80412317	80412670
chr10	80418404	80418611
chr10	80420924	80421079
chr10	80423622	80423889
chr10	80433489	80433867
chr10	80433891	80434370
chr10	80435776	80436208
chr10	80440583	80440810
chr10	80440965	80441161

chr10	80445883	80446220
chr10	80446220	80446733
chr10	80450711	80450929
chr10	80460918	80461080
chr10	80461386	80461719
chr10	80464418	80464601
chr10	80471449	80471975
chr10	80475244	80475597
chr10	80475597	80475802
chr10	80481092	80481252
chr10	80481946	80482186
chr10	80483275	80483651
chr10	80489200	80489598
chr10	80489598	80489903
chr10	80490030	80490362
chr10	80494680	80495144
chr10	80495144	80495567
chr10	80495567	80495929
chr10	80495986	80496181
chr10	80504870	80505136
chr10	80506269	80506541
chr10	80509390	80509676
chr10	80509710	80509896
chr10	80509896	80510083
chr10	80516200	80516488
chr10	80516607	80516767
chr10	80516821	80517029
chr10	80522777	80522928
chr10	80523372	80523531
chr10	80526318	80526481
chr10	80533717	80533973
chr10	80538213	80538522
chr10	80538711	80539036
chr10	80539036	80539512
chr10	80539547	80539736
chr10	80539736	80539916
chr10	80548580	80549022
chr10	80553976	80554192
chr10	80556348	80556609
chr10	80557360	80557641
chr10	80558825	80559001
chr10	80560870	80561145

chr10	80561145	80561443
chr10	80561443	80561694
chr10	80561694	80561957
chr10	80561957	80562184
chr10	80566357	80566668
chr10	80569733	80570020
chr10	80576417	80576684
chr10	80576786	80577108
chr10	80577188	80577652
chr10	80577652	80578118
chr10	80578118	80578380
chr10	80578442	80578749
chr10	80578944	80579227
chr10	80583225	80583395
chr10	80584081	80584383
chr10	80589215	80589662
chr10	80589836	80590002
chr10	80590033	80590237
chr10	80590237	80590509
chr10	80590509	80590857
chr10	80602551	80603010
chr10	80603010	80603368
chr10	80604127	80604283
chr10	80605019	80605249
chr10	80606474	80606672
chr10	80611125	80611379
chr10	80621919	80622301
chr10	80622301	80622699
chr10	80622699	80623095
chr10	80623095	80623280
chr10	80623558	80623778
chr10	80627860	80628029
chr10	80637434	80637677
chr10	80639417	80639577
chr10	80642332	80642522
chr10	80642758	80643103
chr10	80643312	80643521
chr10	80643930	80644219
chr10	80647047	80647230
chr10	80653217	80653531
chr10	80656151	80656513
chr10	80656513	80656881

chr10	80656881	80657107
chr10	80657107	80657329
chr10	80658911	80659086
chr10	80659310	80659462
chr10	80664516	80665072
chr10	80665088	80665355
chr10	80665553	80665845
chr10	80668638	80668992
chr10	80669690	80669902
chr10	80670123	80670425
chr10	80676073	80676597
chr10	80676597	80676904
chr10	80677191	80677477
chr10	80677953	80678496
chr10	80679591	80679841
chr10	80684266	80684572
chr10	80687910	80688208
chr10	80695258	80695560
chr10	80701724	80702100
chr10	80702100	80702433
chr10	80702454	80702853
chr10	80716828	80717348
chr10	80717348	80717708
chr10	80726227	80726509
chr10	80727766	80728075
chr10	80741969	80742215
chr10	80742215	80742667
chr10	80744342	80744623
chr10	80747497	80747719
chr10	80751207	80751861
chr10	80751879	80752361
chr10	80752361	80752679
chr10	80754909	80755294
chr10	80757627	80758076
chr10	80758641	80759065
chr10	80763443	80764093
chr10	80764288	80764637
chr10	80766388	80766697
chr10	80774285	80774606
chr10	80774606	80774934
chr10	80774934	80775115
chr10	80776460	80776895

chr10	80779648	80779938
chr10	80786987	80787198
chr10	80791838	80792087
chr10	80792540	80792700
chr10	80797482	80797659
chr10	80797676	80798172
chr10	80798172	80798446
chr10	80798446	80798747
chr10	80798747	80799041
chr10	80805732	80805956
chr10	80805956	80806387
chr10	80812095	80812404
chr10	80813532	80813795
chr10	80814296	80814451
chr10	80814694	80814865
chr10	80817196	80817466
chr10	80818065	80818273
chr10	80826121	80826776
chr10	80826841	80827171
chr10	80838387	80838863
chr10	80843965	80844219
chr10	80844324	80844475
chr10	80848558	80849084
chr10	80853813	80854017
chr10	80854517	80854667
chr10	80854686	80854876
chr10	80854876	80855142
chr10	80855142	80855554
chr10	80855618	80856070
chr10	80856253	80856534
chr10	80865855	80866005
chr10	80871597	80871906
chr10	80876121	80876346
chr10	80878344	80878663
chr10	80880398	80880670
chr10	80880706	80880867
chr10	80881009	80881210
chr10	80881374	80881613
chr10	80887153	80887595
chr10	80888297	80888471
chr10	80895077	80895263
chr10	80900241	80900481

chr10	80900481	80900758
chr10	80900758	80901200
chr10	80906828	80907022
chr10	80907022	80907237
chr10	80914228	80914394
chr10	80917083	80917642
chr10	80917642	80917912
chr10	80917912	80918144
chr10	80918144	80918404
chr10	80923573	80923739
chr10	80928574	80928970
chr10	80929058	80929352
chr10	80929766	80930163
chr10	80930324	80930599
chr10	80931214	80931518
chr10	80933176	80933550
chr10	80933595	80933978
chr10	80936641	80936844
chr10	80937729	80938067
chr10	80939184	80939488
chr10	80939570	80939972
chr10	80940449	80941058
chr10	80946573	80946907
chr10	80947098	80947388
chr10	80947434	80947747
chr10	80947821	80948135
chr10	80948135	80948510
chr10	80948574	80948963
chr10	80949155	80949402
chr10	80955029	80955402
chr10	80955402	80955700
chr10	80955700	80955985
chr10	80960846	80961175
chr10	80961175	80961865
chr10	80966403	80966564
chr10	80967316	80967686
chr10	80985583	80986125
chr10	80987832	80988416
chr10	80992028	80992259
chr10	80993259	80993516
chr10	80995110	80995260
chr10	80995285	80995552

chr10	80998836	80999158
chr10	80999746	81000226
chr10	81000758	81000910
chr10	81001386	81001539
chr10	81007302	81007546
chr10	81014488	81014647
chr10	81014777	81015258
chr10	81016739	81016960
chr10	81018453	81018638
chr10	81020259	81020532
chr10	81024638	81024870
chr10	81025049	81025273
chr10	81025273	81025491
chr10	81027308	81027692
chr10	81036900	81037602
chr10	81041570	81041722
chr10	81043869	81044033
chr10	81044033	81044197
chr10	81044553	81044885
chr10	81050687	81050969
chr10	81050969	81051184
chr10	81052597	81052903
chr10	81059580	81059851
chr10	81060049	81060282
chr10	81060282	81060576
chr10	81064686	81064975
chr10	81065449	81065797
chr10	81065940	81066177
chr10	81069748	81070021
chr10	81070021	81070304
chr10	81076220	81076752
chr10	81082149	81082608
chr10	81082656	81082816
chr10	81087103	81087264
chr10	81088859	81089065
chr10	81095004	81095235
chr10	81095235	81095804
chr10	81101458	81101808
chr10	81102469	81102835
chr10	81105539	81105710
chr10	81105735	81106096
chr10	81106096	81106409

chr10	81106409	81106809
chr10	81112995	81113365
chr10	81118957	81119181
chr10	81128434	81128932
chr10	81128932	81129284
chr10	81129284	81129576
chr10	81129576	81129748
chr10	81135913	81136271
chr10	81136271	81136667
chr10	81136910	81137126
chr10	81137236	81137687
chr10	81137687	81138094
chr10	81138094	81138339
chr10	81138339	81138529
chr10	81143160	81143581
chr10	81145747	81146054
chr10	81146054	81146352
chr10	81146352	81146697
chr10	81152351	81152634
chr10	81152634	81152954
chr10	81154281	81154453
chr10	81162082	81162303
chr10	81162370	81162565
chr10	81167401	81167645
chr10	81167645	81167988
chr10	81167988	81168301
chr10	81168816	81169226
chr10	81169226	81169620
chr10	81173155	81173418
chr10	81174719	81174879
chr10	81175051	81175342
chr10	81176365	81176575
chr10	81176575	81176774
chr10	81176774	81176968
chr10	81176968	81177174
chr10	81182339	81183385
chr10	81184900	81185170
chr10	81190184	81190442
chr10	81190896	81191169
chr10	81192298	81192611
chr10	81192611	81192984
chr10	81193021	81193291

chr10	81197354	81197541
chr10	81198269	81198528
chr10	81202421	81202749
chr10	81203408	81203615
chr10	81208079	81208242
chr10	81216845	81216998
chr10	81217367	81217585
chr10	81217955	81218401
chr10	81221519	81221672
chr10	81232900	81233612
chr10	81234321	81234548
chr10	81240954	81241191
chr10	81244479	81244683
chr10	81244683	81244932
chr10	81252570	81252726
chr10	81253104	81253433
chr10	81256476	81256638
chr10	81256642	81256858
chr10	81257690	81257948
chr10	81257948	81258196
chr10	81259572	81259802
chr10	81266303	81266453
chr10	81266494	81266788
chr10	81266788	81267056
chr10	81268045	81268421
chr10	81274291	81274475
chr10	81277097	81277259
chr10	81279595	81279774
chr10	81284864	81285014
chr10	81285562	81286070
chr10	81286185	81286417
chr10	81289358	81289590
chr10	81292490	81292781
chr10	81292781	81293090
chr10	81293090	81293569
chr10	81294524	81294677
chr10	81297954	81298244
chr10	81298530	81298684
chr10	81298848	81299015
chr10	81303385	81303540
chr10	81304843	81305190
chr10	81314329	81314521

chr10	81319763	81319974
chr10	81320045	81321066
chr10	81321074	81321342
chr10	81321381	81321688
chr10	81328181	81328478
chr10	81330885	81331035
chr10	81335338	81335606
chr10	81336174	81336307
chr10	81336307	81336500
chr10	81341088	81341285
chr10	81341624	81341900
chr10	81343307	81343519
chr10	81344562	81344750
chr10	81346742	81346946
chr10	81349167	81349439
chr10	81350026	81350769
chr10	81350769	81351082
chr10	81357317	81357618
chr10	81357618	81357837
chr10	81357837	81358173
chr10	81358314	81358472
chr10	81358472	81358616
chr10	81360170	81360482
chr10	81362560	81362711
chr10	81362711	81362870
chr10	81365676	81365994
chr10	81365994	81366309
chr10	81366309	81366664
chr10	81367885	81368223
chr10	81377031	81377202
chr10	81377881	81378116
chr10	81378116	81378342
chr10	81378342	81378627
chr10	81378627	81379142
chr10	81384089	81384576
chr10	81384576	81384915
chr10	81384984	81385313
chr10	81389835	81390270
chr10	81395032	81395182
chr10	81395209	81395503
chr10	81398135	81398292
chr10	81401682	81402026

chr10	81405969	81406124
chr10	81407572	81407906
chr10	81408808	81409082
chr10	81409082	81409320
chr10	81409428	81409783
chr10	81410421	81410676
chr10	81411811	81412002
chr10	81416606	81416779
chr10	81416798	81417152
chr10	81424351	81424545
chr10	81424658	81425118
chr10	81425136	81425307
chr10	81425319	81425666
chr10	81426950	81427255
chr10	81429577	81429844
chr10	81429844	81430088
chr10	81430088	81430297
chr10	81430297	81430488
chr10	81430559	81430736
chr10	81431111	81431287
chr10	81431679	81432077
chr10	81432885	81433252
chr10	81434913	81435270
chr10	81441109	81441327
chr10	81443416	81443704
chr10	81443772	81443941
chr10	81450027	81450504
chr10	81452395	81452755
chr10	81452755	81453443
chr10	81457302	81457572
chr10	81464363	81464834
chr10	81464834	81465248
chr10	81465248	81465697
chr10	81474555	81474713
chr10	81477625	81477864
chr10	81482243	81482393
chr10	81482404	81482822
chr10	81482822	81483054
chr10	81484041	81484319
chr10	81485171	81485372
chr10	81495891	81496316
chr10	81496316	81496683

chr10	81496683	81496947
chr10	81502677	81503117
chr10	81503868	81504132
chr10	81506016	81506174
chr10	81508214	81508400
chr10	81508589	81508795
chr10	81518409	81518559
chr10	81518600	81518875
chr10	81523460	81523876
chr10	81523930	81524343
chr10	81526785	81527094
chr10	81534787	81535129
chr10	81536791	81537037
chr10	81537081	81537378
chr10	81544444	81544768
chr10	81544808	81545285
chr10	81545285	81545794
chr10	81550328	81550479
chr10	81551493	81552135
chr10	81559144	81559371
chr10	81559371	81559616
chr10	81559616	81559951
chr10	81559951	81560289
chr10	81560289	81560545
chr10	81560545	81560781
chr10	81560915	81561382
chr10	81563552	81563734
chr10	81564826	81565051
chr10	81570732	81571159
chr10	81571513	81572077
chr10	81572315	81572466
chr10	81575177	81575401
chr10	81580341	81580502
chr10	81580882	81581032
chr10	81591689	81591928
chr10	81591928	81592347
chr10	81600735	81601229
chr10	81605125	81605507
chr10	81605842	81606455
chr10	81606477	81606735
chr10	81612679	81613185
chr10	81623492	81623676

chr10	81626152	81626329
chr10	81626743	81626893
chr10	81627066	81627508
chr10	81627508	81627853
chr10	81627874	81628045
chr10	81633327	81634046
chr10	81643082	81643505
chr10	81643505	81644108
chr10	81646088	81646316
chr10	81648734	81649139
chr10	81649139	81649401
chr10	81649401	81649731
chr10	81687054	81687484
chr10	81687484	81687746
chr10	81687746	81687942
chr10	81704409	81704901
chr10	81704901	81705274
chr10	81770569	81770814
chr10	81770814	81771080
chr10	81771080	81771416
chr10	81811921	81812266
chr10	81812266	81812667
chr10	81846188	81846590
chr10	81846590	81846863
chr10	81846863	81847047
chr10	81863518	81864008
chr10	81864008	81864393
chr10	81929907	81930149
chr10	81930149	81930421
chr10	81930421	81930768
chr10	81978414	81978750
chr10	81978750	81979062
chr10	81996626	81997115
chr10	81997115	81997526
chr10	82004851	82005136
chr10	82047712	82047874
chr10	82047895	82048227
chr10	82048227	82048515
chr10	82048515	82048757
chr10	82083078	82083421
chr10	82083421	82083696
chr10	82083696	82083928

chr10	82127758	82128122
chr10	82128122	82128599
chr10	82161475	82161639
chr10	82177943	82178206
chr10	82178206	82178482
chr10	82178482	82178982
chr10	82240685	82241172
chr10	82241172	82241515
chr10	82354045	82354391
chr10	82354391	82354676
chr10	82354676	82354894
chr10	82359616	82359802
chr10	82359802	82360049
chr10	82360079	82360407
chr10	82409520	82409969
chr10	82428282	82428452
chr10	82440723	82441104
chr10	82497128	82497310
chr10	82505220	82505386
chr10	82514643	82514859
chr10	82520099	82520286
chr10	82567057	82567260
chr10	82622470	82623030
chr10	82623030	82623253
chr10	82623253	82623571
chr10	82629325	82629551
chr10	82629564	82629993
chr10	82629993	82630356
chr10	82631050	82631287
chr10	82646187	82646373
chr10	82666447	82666847
chr10	82666847	82667174
chr10	82689699	82690356
chr10	82695968	82696270
chr10	82696569	82696720
chr10	82697333	82697483
chr10	82698663	82699224
chr10	82699224	82699669
chr10	82699676	82699984
chr10	82747083	82747293
chr10	82747293	82747493
chr10	82747493	82747680

chr10	82761113	82761397
chr10	82761397	82761701
chr10	82763740	82764386
chr10	82771202	82771373
chr10	82771539	82771694
chr10	82777136	82777354
chr10	82777888	82778056
chr10	82778056	82778225
chr10	82780316	82780696
chr10	82782580	82782736
chr10	82789255	82789520
chr10	82790499	82790885
chr10	82795057	82795369
chr10	82795369	82795734
chr10	82798463	82798926
chr10	82804536	82804701
chr10	82807276	82807498
chr10	82811842	82812375
chr10	82813968	82814584
chr10	82814680	82814839
chr10	82824518	82824935
chr10	82826067	82826279
chr10	82838507	82838782
chr10	82839213	82839420
chr10	82844728	82845160
chr10	82850799	82850966
chr10	82851591	82851819
chr10	82851819	82852146
chr10	82858423	82858821
chr10	82858821	82859562
chr10	82859681	82859875
chr10	82860384	82860690
chr10	82863169	82863484
chr10	82863918	82864166
chr10	82866905	82867055
chr10	82868392	82868692
chr10	82871065	82871220
chr10	82876071	82876386
chr10	82877368	82877520
chr10	82880419	82880604
chr10	82882031	82882252
chr10	82888706	82888880

chr10	82890342	82890590
chr10	82890885	82891039
chr10	82891455	82891660
chr10	82898955	82899142
chr10	82899472	82899642
chr10	82899737	82900007
chr10	82900007	82900347
chr10	82900347	82900697
chr10	82900697	82900952
chr10	82901488	82901773
chr10	82909371	82909603
chr10	82909631	82909785
chr10	82910319	82910480
chr10	82913779	82914023
chr10	82914039	82914249
chr10	82953472	82953763
chr10	82955218	82955423
chr10	82958609	82958801
chr10	82961433	82961607
chr10	82962356	82963094
chr10	82975697	82975979
chr10	82975979	82976273
chr10	82984910	82985422
chr10	82985422	82985995
chr10	82990724	82990972
chr10	83036337	83036579
chr10	83038333	83038527
chr10	83073256	83073416
chr10	83095440	83095614
chr10	83104403	83104623
chr10	83131985	83132192
chr10	83144245	83144553
chr10	83152343	83152682
chr10	83178151	83178334
chr10	83196320	83196480
chr10	83197869	83198035
chr10	83199410	83199626
chr10	83215571	83215756
chr10	83219779	83220010
chr10	83249049	83249241
chr10	83320797	83321189
chr10	83337104	83337675

chr10	83337675	83338014
chr10	83343214	83343705
chr10	83343830	83344122
chr10	83354414	83354645
chr10	83359904	83360310
chr10	83360310	83360576
chr10	83360576	83360875
chr10	83374626	83374776
chr10	83388143	83388297
chr10	83389585	83389778
chr10	83509029	83509222
chr10	83533310	83533621
chr10	83533642	83533947
chr10	83533947	83534396
chr10	83543577	83543795
chr10	83543795	83544080
chr10	83544080	83544447
chr10	83617422	83617661
chr10	83618900	83619432
chr10	83624880	83625231
chr10	83626584	83627112
chr10	83629541	83629731
chr10	83634595	83634809
chr10	83636322	83636482
chr10	83637341	83637641
chr10	83644912	83645324
chr10	83645324	83645589
chr10	83648158	83648982
chr10	83658665	83658854
chr10	83659190	83659374
chr10	83666745	83666898
chr10	83688338	83688498
chr10	83699444	83699691
chr10	83722557	83723143
chr10	83723152	83723305
chr10	83729584	83729819
chr10	83733737	83733965
chr10	83757644	83757931
chr10	83776634	83776784
chr10	83788523	83788822
chr10	83819731	83819961
chr10	83820942	83821473

chr10	83854405	83854601
chr10	83861551	83861808
chr10	83898550	83898722
chr10	83906706	83907123
chr10	83909037	83909344
chr10	83934398	83934898
chr10	83958763	83959017
chr10	83970317	83970521
chr10	83984817	83985088
chr10	83985088	83985721
chr10	83996598	83996789
chr10	84021084	84021781
chr10	84044938	84045115
chr10	84056235	84056702
chr10	84060685	84060950
chr10	84065512	84065716
chr10	84075648	84075926
chr10	84088027	84088235
chr10	84090491	84090692
chr10	84097623	84097926
chr10	84101082	84101676
chr10	84109804	84109998
chr10	84146687	84146930
chr10	84166655	84166815
chr10	84194073	84194656
chr10	84212982	84213303
chr10	84217573	84217908
chr10	84230377	84230606
chr10	84234994	84235317
chr10	84235317	84235528
chr10	84240352	84240572
chr10	84255000	84255164
chr10	84276031	84276449
chr10	84286302	84286452
chr10	84291047	84291214
chr10	84291722	84291971
chr10	84295536	84295693
chr10	84302843	84303169
chr10	84303169	84303447
chr10	84304336	84304516
chr10	84305363	84305633
chr10	84310736	84310992

chr10	84311911	84312126
chr10	84312703	84312873
chr10	84313620	84313784
chr10	84334981	84335148
chr10	84339438	84339967
chr10	84353429	84353773
chr10	84376355	84376728
chr10	84376728	84376977
chr10	84386310	84386510
chr10	84392023	84392384
chr10	84392384	84392955
chr10	84395867	84396205
chr10	84396205	84396458
chr10	84400111	84400365
chr10	84401276	84401548
chr10	84401771	84402092
chr10	84409020	84409767
chr10	84412864	84413125
chr10	84415872	84416022
chr10	84416676	84417055
chr10	84418347	84418516
chr10	84428757	84429103
chr10	84429103	84429459
chr10	84436053	84436484
chr10	84438101	84438332
chr10	84440236	84440498
chr10	84440498	84440948
chr10	84440948	84441469
chr10	84441531	84441682
chr10	84444505	84444710
chr10	84448750	84448928
chr10	84450574	84450781
chr10	84450781	84451090
chr10	84460779	84460975
chr10	84467987	84468208
chr10	84483165	84483463
chr10	84483583	84483974
chr10	84485520	84485812
chr10	84487235	84487467
chr10	84488396	84488614
chr10	84493828	84493991
chr10	84494329	84494837

chr10	84496490	84496788
chr10	84500272	84500686
chr10	84501241	84501444
chr10	84505259	84505442
chr10	84514158	84514426
chr10	84515498	84515760
chr10	84515760	84516004
chr10	84516100	84516386
chr10	84516386	84516756
chr10	84523847	84524023
chr10	84530413	84530753
chr10	84530806	84530999
chr10	84532192	84532539
chr10	84532847	84533380
chr10	84533380	84533778
chr10	84533839	84534356
chr10	84539763	84540149
chr10	84542751	84542997
chr10	84545286	84545508
chr10	84548113	84548487
chr10	84551923	84552162
chr10	84560430	84560612
chr10	84562723	84563152
chr10	84570973	84571515
chr10	84574936	84575155
chr10	84576084	84576407
chr10	84576407	84576687
chr10	84576745	84577036
chr10	84577036	84577337
chr10	84577337	84577641
chr10	84584394	84584821
chr10	84584821	84585274
chr10	84586977	84587145
chr10	84598268	84598482
chr10	84612425	84612614
chr10	84621825	84622023
chr10	84622108	84622440
chr10	84622440	84622727
chr10	84622727	84622944
chr10	84629636	84629837
chr10	84631273	84631514
chr10	84645345	84645705

chr10	84646725	84646963
chr10	84646963	84647229
chr10	84647313	84647492
chr10	84655208	84655438
chr10	84655511	84655693
chr10	84687796	84687961
chr10	84701095	84701458
chr10	84701458	84701691
chr10	84704683	84705024
chr10	84712802	84712964
chr10	84713020	84713492
chr10	84714023	84714173
chr10	84726646	84727018
chr10	84727018	84727451
chr10	84732545	84732888
chr10	84745768	84745923
chr10	84754889	84755060
chr10	84756812	84756996
chr10	84756996	84757214
chr10	84760176	84760348
chr10	84768855	84769088
chr10	84780886	84781038
chr10	84816137	84816353
chr10	84836186	84836458
chr10	84838698	84838958
chr10	84846762	84846912
chr10	84869068	84869222
chr10	84878894	84879053
chr10	84879065	84879322
chr10	84886151	84886441
chr10	84886441	84886724
chr10	84886724	84886952
chr10	84886952	84887134
chr10	84895928	84896088
chr10	84902199	84902629
chr10	84917017	84917422
chr10	84917422	84917905
chr10	84986096	84986350
chr10	85010104	85010388
chr10	85011179	85011559
chr10	85013516	85013798
chr10	85023483	85023843

chr10	85025903	85026139
chr10	85029013	85029215
chr10	85045421	85045627
chr10	85050763	85050996
chr10	85057274	85057899
chr10	85102224	85102451
chr10	85102451	85102675
chr10	85102675	85102886
chr10	85102886	85103150
chr10	85103150	85103428
chr10	85103428	85103646
chr10	85103646	85103887
chr10	85117979	85118230
chr10	85127727	85128488
chr10	85128545	85128725
chr10	85134068	85134258
chr10	85136697	85136897
chr10	85154291	85154496
chr10	85155684	85155847
chr10	85161757	85162270
chr10	85162994	85163182
chr10	85167544	85167892
chr10	85171270	85171609
chr10	85174681	85174856
chr10	85174856	85175117
chr10	85183745	85184034
chr10	85184251	85184514
chr10	85184514	85184849
chr10	85184849	85185221
chr10	85185221	85185702
chr10	85211558	85212050
chr10	85220965	85221313
chr10	85228466	85228633
chr10	85231473	85231666
chr10	85261472	85261627
chr10	85295226	85295623
chr10	85311688	85312389
chr10	85318374	85318845
chr10	85350781	85351093
chr10	85351093	85351557
chr10	85352865	85353024
chr10	85355302	85355507

chr10	85366787	85367258
chr10	85371362	85371847
chr10	85371907	85372156
chr10	85372156	85372487
chr10	85372520	85372698
chr10	85372727	85372906
chr10	85377718	85377923
chr10	85378781	85379305
chr10	85386548	85386956
chr10	85386956	85387225
chr10	85387225	85387433
chr10	85387433	85387670
chr10	85387670	85388033
chr10	85388033	85388346
chr10	85388527	85388757
chr10	85388802	85389068
chr10	85390742	85390947
chr10	85399283	85399688
chr10	85399688	85399991
chr10	85412941	85413156
chr10	85415083	85415452
chr10	85420839	85421506
chr10	85422031	85422276
chr10	85437337	85437499
chr10	85445997	85446310
chr10	85447978	85448142
chr10	85456044	85456284
chr10	85458940	85459363
chr10	85471115	85471284
chr10	85473371	85473674
chr10	85473674	85473926
chr10	85480416	85480648
chr10	85480930	85481415
chr10	85481415	85481644
chr10	85486399	85487098
chr10	85495194	85495485
chr10	85502727	85502967
chr10	85512778	85513221
chr10	85519937	85520188
chr10	85522332	85522591
chr10	85526843	85527104
chr10	85527329	85527685

chr10	85527685	85528132
chr10	85534650	85535193
chr10	85535380	85535934
chr10	85545136	85545404
chr10	85592076	85592349
chr10	85597923	85598520
chr10	85598520	85598923
chr10	85601462	85601744
chr10	85625003	85625180
chr10	85627429	85627689
chr10	85636164	85636514
chr10	85639463	85640194
chr10	85643479	85643797
chr10	85648638	85649090
chr10	85651121	85651655
chr10	85651655	85652111
chr10	85656470	85656774
chr10	85660599	85660752
chr10	85662153	85662312
chr10	85676458	85676683
chr10	85678114	85678280
chr10	85678698	85679012
chr10	85699798	85700083
chr10	85725460	85725816
chr10	85731622	85731777
chr10	85731797	85732077
chr10	85732077	85732316
chr10	85739082	85739321
chr10	85739321	85739602
chr10	85739602	85739958
chr10	85745632	85745926
chr10	85745926	85746154
chr10	85747190	85747400
chr10	85748078	85748233
chr10	85780911	85781061
chr10	85786243	85786469
chr10	85823269	85823464
chr10	85829038	85829188
chr10	85829361	85829539
chr10	85829673	85829872
chr10	85871471	85871703
chr10	85871703	85871968

chr10	85871968	85872364
chr10	85872364	85872808
chr10	85897317	85897665
chr10	85905790	85905940
chr10	85916362	85916817
chr10	85916817	85917366
chr10	85927966	85928134
chr10	85928188	85928688
chr10	85928688	85928999
chr10	85953299	85953948
chr10	85957408	85957668
chr10	85957668	85957979
chr10	85967867	85968042
chr10	85968083	85968367
chr10	85972707	85973356
chr10	85989786	85990048
chr10	85990048	85990250
chr10	85998068	85998302
chr10	85999072	85999222
chr10	86006524	86006949
chr10	86021441	86021718
chr10	86021718	86022018
chr10	86022018	86022377
chr10	86022377	86022670
chr10	86029158	86029419
chr10	86032232	86032471
chr10	86064747	86065322
chr10	86065914	86066106
chr10	86074262	86074545
chr10	86080008	86080162
chr10	86092050	86092204
chr10	86094328	86094478
chr10	86096025	86096183
chr10	86100182	86100626
chr10	86100626	86101081
chr10	86116309	86116679
chr10	86139024	86139174
chr10	86161553	86161983
chr10	86161983	86162223
chr10	86169193	86169349
chr10	86171224	86171668
chr10	86173245	86173784

chr10	86180654	86180814
chr10	86182249	86182505
chr10	86183196	86183487
chr10	86192533	86192797
chr10	86196214	86196364
chr10	86196396	86197068
chr10	86198677	86198982
chr10	86233206	86233361
chr10	86233405	86234013
chr10	86242803	86243060
chr10	86246892	86247098
chr10	86247183	86247335
chr10	86250640	86250818
chr10	86264583	86264810
chr10	86264810	86265132
chr10	86265193	86265442
chr10	86270301	86270802
chr10	86286253	86286966
chr10	86290305	86290502
chr10	86296095	86296508
chr10	86300253	86300437
chr10	86300437	86300646
chr10	86300654	86300925
chr10	86302727	86303046
chr10	86307298	86307549
chr10	86310500	86310976
chr10	86328210	86328385
chr10	86330453	86330693
chr10	86339136	86339290
chr10	86339556	86339902
chr10	86339902	86340157
chr10	86340157	86340352
chr10	86346350	86346504
chr10	86351361	86351629
chr10	86387970	86388386
chr10	86406984	86407322
chr10	86413187	86413409
chr10	86452072	86452224
chr10	86452296	86452893
chr10	86459731	86460022
chr10	86463713	86463891
chr10	86463891	86464142

chr10	86473770	86473925
chr10	86475276	86475660
chr10	86483484	86483701
chr10	86498095	86498416
chr10	86498416	86498753
chr10	86498753	86499070
chr10	86499070	86499368
chr10	86513922	86514122
chr10	86524739	86525328
chr10	86525328	86525732
chr10	86528875	86529250
chr10	86555920	86556159
chr10	86591843	86592006
chr10	86600928	86601202
chr10	86612326	86612664
chr10	86612664	86612970
chr10	86616544	86616724
chr10	86635728	86635886
chr10	86636870	86637155
chr10	86637155	86637482
chr10	86668126	86668335
chr10	86671513	86671843
chr10	86672016	86672536
chr10	86685136	86685418
chr10	86685418	86685740
chr10	86685740	86686258
chr10	86693084	86693263
chr10	86693263	86693457
chr10	86705007	86705157
chr10	86705197	86705886
chr10	86705886	86706201
chr10	86706201	86706437
chr10	86711599	86711773
chr10	86730827	86731013
chr10	86731013	86731236
chr10	86731236	86731496
chr10	86772292	86772734
chr10	86778254	86778813
chr10	86778813	86779157
chr10	86779157	86779372
chr10	86779447	86779686
chr10	86783644	86784016

chr10	86784305	86784679
chr10	86789211	86789526
chr10	86796261	86796526
chr10	86825726	86826119
chr10	86826119	86826386
chr10	86839897	86840322
chr10	86840322	86840604
chr10	86844737	86844914
chr10	86845299	86845558
chr10	86845558	86845819
chr10	86849240	86849523
chr10	86849523	86849892
chr10	86863499	86863730
chr10	86880051	86880280
chr10	86967321	86967638
chr10	86972019	86972238
chr10	86976104	86976278
chr10	87024928	87025211
chr10	87058379	87058553
chr10	87370180	87370440
chr10	87370440	87370796
chr10	87395304	87395775
chr10	87398192	87398396
chr10	87398852	87399280
chr10	87399280	87399517
chr10	87486326	87486584
chr10	87488826	87489109
chr10	87492532	87492780
chr10	87492780	87493061
chr10	87493245	87493523
chr10	87494766	87494992
chr10	87503950	87504262
chr10	87552747	87553004
chr10	87590104	87590256
chr10	87602262	87602527
chr10	87639291	87639637
chr10	87654662	87654968
chr10	87678925	87679481
chr10	87679601	87679848
chr10	87679877	87680313
chr10	87687840	87688228
chr10	87711799	87712087

chr10	87712087	87712308
chr10	87750363	87750641
chr10	87792458	87792701
chr10	87805676	87806226
chr10	87816790	87817234
chr10	87854487	87854735
chr10	87856548	87856764
chr10	87860115	87860363
chr10	87860575	87860852
chr10	87860866	87861051
chr10	87865774	87866134
chr10	87879853	87880536
chr10	87885297	87885690
chr10	87885690	87886120
chr10	87889911	87890243
chr10	87916622	87916794
chr10	87927345	87927614
chr10	88000016	88000284
chr10	88008697	88009063
chr10	88146662	88147151
chr10	88147151	88147386
chr10	88150431	88150753
chr10	88178983	88179217
chr10	88200754	88200904
chr10	88200963	88201268
chr10	88201268	88201722
chr10	88264206	88264551
chr10	88294325	88294513
chr10	88304371	88304622
chr10	88306309	88306501
chr10	88327503	88327760
chr10	88329476	88329663
chr10	88345868	88346095
chr10	88346325	88346832
chr10	88346832	88347100
chr10	88351855	88352084
chr10	88353798	88354008
chr10	88356318	88356471
chr10	88356600	88356973
chr10	88356973	88357621
chr10	88376827	88376991
chr10	88378949	88379314

chr10	88379314	88379552
chr10	88379552	88379960
chr10	88380127	88380374
chr10	88380514	88380713
chr10	88380779	88380968
chr10	88389636	88389993
chr10	88390394	88390625
chr10	88403065	88403247
chr10	88405600	88406118
chr10	88408370	88408535
chr10	88426501	88426742
chr10	88428180	88428389
chr10	88451280	88451507
chr10	88452052	88452394
chr10	88491668	88491925
chr10	88503393	88504398
chr10	88514555	88514835
chr10	88642843	88643025
chr10	88647863	88648032
chr10	88649309	88649489
chr10	88684331	88684561
chr10	88708740	88709028
chr10	88717426	88717914
chr10	88719861	88720193
chr10	88725418	88725682
chr10	88728097	88728317
chr10	88728317	88728587
chr10	88730560	88730870
chr10	88730870	88731176
chr10	88731221	88731499
chr10	88731499	88731715
chr10	88731715	88731913
chr10	88731913	88732117
chr10	88732389	88732569
chr10	88779817	88780091
chr10	88780149	88780323
chr10	88780795	88780945
chr10	88783199	88783456
chr10	88784479	88784639
chr10	88826203	88826464
chr10	88826464	88826701
chr10	88826701	88826980

chr10	88850099	88850271
chr10	88874939	88875246
chr10	88875266	88875416
chr10	88881206	88881419
chr10	88885913	88886065
chr10	89307544	89307890
chr10	89308987	89309194
chr10	89326986	89327351
chr10	89344589	89344831
chr10	89383556	89383794
chr10	89398176	89398349
chr10	89439465	89439716
chr10	89442696	89443088
chr10	89443088	89443517
chr10	89443517	89443807
chr10	89443807	89444216
chr10	89445764	89445918
chr10	89458441	89458782
chr10	89458782	89459078
chr10	89459078	89459346
chr10	89461881	89462186
chr10	89465057	89465453
chr10	89486994	89487326
chr10	89526918	89527518
chr10	89531185	89531699
chr10	89533247	89533513
chr10	89533513	89533857
chr10	89548134	89548356
chr10	89589978	89590182
chr10	89594384	89594658
chr10	89599105	89599255
chr10	89599267	89599890
chr10	89619802	89619970
chr10	89630171	89630410
chr10	89635136	89635355
chr10	89670339	89670726
chr10	89677955	89678242
chr10	89678242	89678464
chr10	89679580	89679757
chr10	89680561	89680837
chr10	89685568	89685840
chr10	89685840	89686182

chr10	89686182	89686467
chr10	89686467	89686740
chr10	89696389	89696549
chr10	89702781	89703051
chr10	89703112	89703402
chr10	89707513	89707677
chr10	89707781	89708001
chr10	89708036	89708231
chr10	89708797	89709282
chr10	89731438	89731644
chr10	89731982	89732214
chr10	89732214	89732475
chr10	89732475	89732844
chr10	89743282	89743568
chr10	89743570	89743820
chr10	89744814	89745118
chr10	89745118	89745410
chr10	89745410	89745725
chr10	89745725	89746018
chr10	89746372	89746674
chr10	89756773	89756933
chr10	89757051	89757412
chr10	89758467	89758633
chr10	89758716	89758915
chr10	89760980	89761191
chr10	89764067	89764250
chr10	89764250	89764444
chr10	89764605	89764773
chr10	89764799	89765056
chr10	89768418	89768666
chr10	89768666	89769124
chr10	89769196	89769381
chr10	89776012	89776345
chr10	89819236	89819552
chr10	89824000	89824390
chr10	89824390	89824622
chr10	89824622	89824675
chr10	89824766	89825061
chr10	89825061	89825388
chr10	89825388	89825608
chr10	89845435	89845753
chr10	89845775	89845970

chr10	89872556	89872856
chr10	89872866	89873166
chr10	89873166	89873408
chr10	89873408	89873890
chr10	89942373	89942523
chr10	89947127	89947743
chr10	89975413	89975607
chr10	89997678	89997848
chr10	90024230	90024718
chr10	90024763	90025299
chr10	90025299	90025545
chr10	90068419	90068988
chr10	90068988	90069396
chr10	90074257	90074652
chr10	90074652	90075039
chr10	90075039	90075291
chr10	90081314	90081464
chr10	90081724	90081905
chr10	90112905	90113111
chr10	90116756	90117105
chr10	90130962	90131226
chr10	90140030	90140274
chr10	90140496	90140722
chr10	90140862	90141311
chr10	90144448	90144854
chr10	90144854	90145171
chr10	90148479	90148733
chr10	90169931	90170256
chr10	90193424	90193644
chr10	90239140	90239467
chr10	90266179	90266389
chr10	90303785	90303950
chr10	90304082	90304266
chr10	90304973	90305170
chr10	90364627	90364946
chr10	90368875	90369025
chr10	90369781	90370040
chr10	90370696	90371088
chr10	90373526	90373811
chr10	90380223	90380389
chr10	90380791	90380987
chr10	90385544	90385750

chr10	90407508	90407894
chr10	90507661	90507861
chr10	90569127	90569449
chr10	90595404	90595628
chr10	90600250	90600522
chr10	90606027	90606219
chr10	90609313	90609605
chr10	90690238	90690395
chr10	90694929	90695137
chr10	90728481	90728592
chr10	90728592	90728640
chr10	90728714	90729063
chr10	90731891	90732094
chr10	90732840	90733167
chr10	90733695	90733971
chr10	90753139	90753418
chr10	90754417	90754666
chr10	90757808	90758242
chr10	90759592	90759838
chr10	90771350	90771832
chr10	90772356	90772506
chr10	90809880	90810456
chr10	90829055	90829353
chr10	90829551	90829789
chr10	90863907	90864127
chr10	90864584	90864838
chr10	90936493	90936644
chr10	90964574	90964936
chr10	90969326	90969556
chr10	90969556	90969795
chr10	90969875	90970166
chr10	91029072	91029295
chr10	91032427	91032708
chr10	91033886	91034439
chr10	91040957	91041206
chr10	91049758	91050029
chr10	91060815	91061174
chr10	91071856	91072064
chr10	91076826	91076985
chr10	91082068	91082258
chr10	91082258	91082538
chr10	91082538	91082853

chr10	91082853	91083169
chr10	91083172	91083374
chr10	91083374	91083599
chr10	91092296	91092622
chr10	91092848	91093168
chr10	91094288	91094570
chr10	91110362	91110581
chr10	91111068	91111287
chr10	91112035	91112218
chr10	91114012	91114240
chr10	91116985	91117327
chr10	91123247	91123466
chr10	91123466	91123808
chr10	91123808	91124343
chr10	91131603	91131913
chr10	91134781	91134988
chr10	91138281	91138646
chr10	91141628	91141897
chr10	91142890	91143137
chr10	91143970	91144126
chr10	91156388	91156718
chr10	91158408	91158985
chr10	91166916	91167293
chr10	91167293	91167684
chr10	91170677	91171001
chr10	91171001	91171418
chr10	91171418	91172095
chr10	91177203	91177545
chr10	91180396	91180546
chr10	91181388	91181967
chr10	91181991	91182252
chr10	91183230	91183890
chr10	91187967	91188238
chr10	91193991	91194689
chr10	91199176	91199556
chr10	91199556	91199888
chr10	91200489	91200649
chr10	91207240	91207646
chr10	91207646	91207942
chr10	91220628	91220872
chr10	91230486	91230731
chr10	91234499	91234752

chr10	91236870	91237227
chr10	91240533	91240697
chr10	91241079	91241580
chr10	91241622	91241799
chr10	91241799	91241981
chr10	91242474	91242685
chr10	91270241	91270644
chr10	91271216	91271470
chr10	91271470	91271695
chr10	91297891	91298041
chr10	91440191	91440492
chr10	91704844	91705025
chr10	91820587	91820756
chr10	91825356	91825728
chr10	91825800	91826024
chr10	92068680	92068869
chr10	92074065	92074297
chr10	92094432	92094865
chr10	92097567	92097958
chr10	92098768	92098928
chr10	92157194	92157670
chr10	92158002	92158342
chr10	92162347	92162835
chr10	92335288	92335486
chr10	92387971	92388197
chr10	92388197	92388451
chr10	92721655	92722264
chr10	92722264	92722875
chr10	92868423	92868642
chr10	92928316	92928585
chr10	92932658	92932964
chr10	92933715	92933927
chr10	92942976	92943142
chr10	93049550	93049874
chr10	93050973	93051425
chr10	93052415	93052721
chr10	93063077	93063286
chr10	93069284	93069673
chr10	93070502	93070858
chr10	93081065	93081316
chr10	93081316	93081540
chr10	93081540	93081858

chr10	93083365	93083612
chr10	93090715	93090956
chr10	93094948	93095279
chr10	93095279	93095770
chr10	93098510	93098750
chr10	93102578	93102728
chr10	93114038	93114211
chr10	93114869	93115061
chr10	93126124	93126405
chr10	93138288	93139198
chr10	93144870	93145087
chr10	93145117	93145494
chr10	93156478	93156725
chr10	93160265	93160502
chr10	93160502	93160772
chr10	93160772	93161014
chr10	93161014	93161366
chr10	93161366	93161796
chr10	93165530	93165713
chr10	93166492	93166725
chr10	93168158	93168475
chr10	93171245	93171442
chr10	93175420	93175626
chr10	93184660	93184933
chr10	93188358	93189045
chr10	93194810	93195112
chr10	93195467	93195649
chr10	93195939	93196183
chr10	93207302	93207464
chr10	93208218	93208509
chr10	93212536	93212767
chr10	93212767	93213094
chr10	93213094	93213391
chr10	93213391	93213734
chr10	93214291	93214658
chr10	93227987	93228241
chr10	93233116	93233309
chr10	93233309	93233528
chr10	93234219	93234699
chr10	93234796	93235203
chr10	93242930	93243107
chr10	93244334	93244484

chr10	93246481	93246871
chr10	93246871	93247348
chr10	93250817	93250967
chr10	93259440	93259714
chr10	93262197	93262484
chr10	93267528	93267792
chr10	93268444	93268656
chr10	93271873	93272161
chr10	93272161	93272447
chr10	93272876	93273087
chr10	93273087	93273357
chr10	93273446	93273705
chr10	93273709	93273980
chr10	93275207	93275403
chr10	93280712	93280894
chr10	93287470	93288197
chr10	93288197	93288689
chr10	93291950	93292112
chr10	93298243	93298641
chr10	93298846	93299011
chr10	93299420	93299583
chr10	93305976	93306400
chr10	93309001	93309563
chr10	93309578	93309840
chr10	93310156	93310394
chr10	93310406	93310639
chr10	93310882	93311318
chr10	93316950	93317162
chr10	93325913	93326455
chr10	93326496	93326752
chr10	93327366	93327569
chr10	93331622	93331783
chr10	93332167	93332415
chr10	93332415	93332784
chr10	93342935	93343392
chr10	93343392	93344062
chr10	93353564	93353877
chr10	93354001	93354331
chr10	93357211	93357584
chr10	93357624	93357884
chr10	93360304	93360534
chr10	93360740	93361017

chr10	93361779	93362206
chr10	93370007	93370335
chr10	93370335	93370831
chr10	93374956	93375177
chr10	93394498	93395043
chr10	93402738	93402941
chr10	93402941	93403114
chr10	93403265	93403796
chr10	93407795	93408025
chr10	93408269	93408451
chr10	93409347	93409553
chr10	93421966	93422441
chr10	93422711	93422926
chr10	93422926	93423361
chr10	93423539	93423876
chr10	93425608	93426030
chr10	93429600	93429773
chr10	93432392	93433119
chr10	93433582	93433824
chr10	93444543	93444917
chr10	93452303	93452512
chr10	93452898	93453197
chr10	93453197	93453518
chr10	93453518	93453942
chr10	93471470	93471792
chr10	93481135	93481288
chr10	93485064	93485356
chr10	93485356	93485629
chr10	93486709	93486919
chr10	93488200	93488685
chr10	93488968	93489135
chr10	93534366	93534679
chr10	93539753	93540180
chr10	93540212	93540655
chr10	93542595	93542864
chr10	93549044	93549336
chr10	93549342	93549495
chr10	93559398	93559560
chr10	93559721	93559924
chr10	93559947	93560180
chr10	93577525	93577820
chr10	93588920	93589090

chr10	93589221	93589576
chr10	93589576	93589873
chr10	93592597	93592899
chr10	93594047	93594245
chr10	93595773	93596255
chr10	93597362	93597643
chr10	93598926	93599171
chr10	93599171	93599459
chr10	93601150	93601308
chr10	93610510	93610766
chr10	93611471	93611696
chr10	93611730	93611880
chr10	93614292	93614478
chr10	93617258	93617432
chr10	93620309	93620847
chr10	93620847	93621277
chr10	93625826	93626029
chr10	93638818	93639030
chr10	93640737	93641097
chr10	93641097	93641345
chr10	93641345	93641628
chr10	93641800	93642163
chr10	93642163	93642411
chr10	93642475	93642654
chr10	93642880	93643170
chr10	93647805	93648436
chr10	93648582	93649066
chr10	93653469	93653665
chr10	93661072	93661287
chr10	93661316	93661744
chr10	93662069	93662264
chr10	93666393	93666558
chr10	93667100	93667301
chr10	93677048	93677372
chr10	93696045	93696310
chr10	93696310	93696541
chr10	93708596	93708774
chr10	93726783	93727121
chr10	93727123	93727410
chr10	93727410	93727661
chr10	93738270	93738553
chr10	93741835	93742103

chr10	93742103	93742384
chr10	93742662	93742819
chr10	93749183	93749570
chr10	93749570	93750022
chr10	93757283	93757817
chr10	93757817	93758397
chr10	93759798	93759948
chr10	93763142	93763392
chr10	93777479	93777629
chr10	93783176	93783927
chr10	93803198	93803608
chr10	93812904	93813075
chr10	93813097	93813339
chr10	93813357	93813656
chr10	93818670	93818962
chr10	93818962	93819222
chr10	93833485	93833999
chr10	93833999	93834435
chr10	93852131	93852268
chr10	93852268	93852439
chr10	93882835	93882995
chr10	93888996	93889332
chr10	93889332	93889667
chr10	93889667	93889963
chr10	93889963	93890255
chr10	93899145	93899711
chr10	93899711	93900035
chr10	93918845	93918995
chr10	93932876	93933302
chr10	93934003	93934401
chr10	93938169	93938435
chr10	93944002	93944810
chr10	93945883	93946309
chr10	93995189	93995391
chr10	94010090	94010248
chr10	94013272	94013496
chr10	94035074	94035235
chr10	94035242	94035576
chr10	94035644	94035989
chr10	94035989	94036200
chr10	94036200	94036410
chr10	94036410	94036619

chr10	94039889	94040229
chr10	94047791	94048210
chr10	94048210	94048518
chr10	94048818	94048968
chr10	94049144	94049555
chr10	94056904	94057148
chr10	94059355	94059998
chr10	94059998	94060445
chr10	94064646	94064858
chr10	94065453	94065767
chr10	94065767	94066239
chr10	94066814	94067251
chr10	94072149	94072435
chr10	94072435	94072951
chr10	94072951	94073478
chr10	94081970	94082373
chr10	94082373	94082668
chr10	94087341	94087704
chr10	94087755	94087960
chr10	94089049	94089250
chr10	94110196	94110499
chr10	94116314	94116642
chr10	94116803	94117309
chr10	94117942	94118294
chr10	94125275	94125507
chr10	94128201	94128614
chr10	94129647	94129885
chr10	94129885	94130171
chr10	94147429	94147711
chr10	94147711	94148095
chr10	94148095	94148586
chr10	94148924	94149108
chr10	94151504	94151916
chr10	94160344	94160494
chr10	94174683	94175083
chr10	94187514	94187822
chr10	94198588	94198864
chr10	94198864	94199169
chr10	94199169	94199472
chr10	94209744	94210069
chr10	94225125	94225386
chr10	94225438	94225672

chr10	94242880	94243144
chr10	94279594	94279951
chr10	94280648	94281031
chr10	94283369	94283674
chr10	94283674	94283931
chr10	94311848	94312388
chr10	94333394	94333634
chr10	94334063	94334230
chr10	94337853	94338136
chr10	94344565	94344928
chr10	94352215	94352477
chr10	94378331	94378644
chr10	94380646	94380976
chr10	94381048	94381545
chr10	94381791	94381941
chr10	94391690	94392071
chr10	94398201	94398473
chr10	94408714	94409202
chr10	94431970	94432230
chr10	94443477	94443801
chr10	94456971	94457192
chr10	94462804	94462996
chr10	94466969	94467223
chr10	94473609	94473842
chr10	94473944	94474271
chr10	94480747	94481226
chr10	94489442	94489741
chr10	94512826	94513215
chr10	94514093	94514339
chr10	94514339	94514593
chr10	94514597	94515092
chr10	94515092	94515593
chr10	94519982	94520233
chr10	94522984	94523173
chr10	94523428	94523649
chr10	94523770	94524139
chr10	94524920	94525371
chr10	94533200	94533371
chr10	94533453	94533667
chr10	94545311	94545631
chr10	94545631	94546278
chr10	94550756	94551042

chr10	94551417	94551615
chr10	94554132	94554468
chr10	94555408	94555899
chr10	94556110	94556439
chr10	94558000	94558150
chr10	94561305	94561724
chr10	94561735	94561911
chr10	94562274	94562501
chr10	94567889	94568487
chr10	94570759	94571056
chr10	94572023	94572231
chr10	94574862	94575019
chr10	94575580	94575745
chr10	94575944	94576297
chr10	94585827	94586143
chr10	94592366	94592610
chr10	94597411	94597850
chr10	94598512	94598707
chr10	94602981	94603196
chr10	94609180	94609768
chr10	94623627	94623934
chr10	94674077	94674523
chr10	94674558	94674708
chr10	94687904	94688054
chr10	94688141	94688505
chr10	94688505	94688824
chr10	94688824	94689154
chr10	94689154	94689452
chr10	94695047	94695198
chr10	94706776	94707012
chr10	94716318	94716671
chr10	94732164	94732385
chr10	94732385	94732620
chr10	94771370	94771892
chr10	94789192	94789639
chr10	94818417	94818722
chr10	94824980	94825140
chr10	94839750	94839919
chr10	94872077	94872290
chr10	94939002	94939152
chr10	94943513	94943891
chr10	94943891	94944110

chr10	94944110	94944533
chr10	94944602	94944958
chr10	94948929	94949085
chr10	94958390	94958628
chr10	94990951	94991301
chr10	94991337	94991489
chr10	94991534	94991769
chr10	94991769	94992016
chr10	94992198	94992486
chr10	95008979	95009284
chr10	95048532	95048811
chr10	95048827	95049108
chr10	95049568	95049789
chr10	95086884	95087199
chr10	95088216	95088446
chr10	95119439	95119596
chr10	95122805	95123008
chr10	95124586	95124809
chr10	95133050	95133250
chr10	95147247	95147402
chr10	95191983	95192216
chr10	95213055	95213346
chr10	95213346	95213681
chr10	95232550	95232869
chr10	95248298	95248685
chr10	95264834	95265000
chr10	95278118	95278545
chr10	95281774	95282030
chr10	95300245	95300395
chr10	95300479	95300749
chr10	95323397	95323785
chr10	95323785	95324042
chr10	95324042	95324296
chr10	95347785	95348264
chr10	95351444	95351808
chr10	95369254	95369574
chr10	95371753	95372364
chr10	95372409	95372567
chr10	95378075	95378319
chr10	95385698	95386361
chr10	95388624	95388827
chr10	95388827	95389107

chr10	95389107	95389566
chr10	95414705	95415096
chr10	95415343	95415596
chr10	95415596	95415856
chr10	95415897	95416152
chr10	95416441	95416940
chr10	95416940	95417213
chr10	95417213	95417464
chr10	95417464	95417642
chr10	95417961	95418141
chr10	95422794	95422988
chr10	95433646	95434028
chr10	95438664	95438826
chr10	95439356	95440072
chr10	95440084	95440301
chr10	95440699	95440882
chr10	95457709	95458032
chr10	95458058	95458359
chr10	95458435	95458779
chr10	95458779	95459080
chr10	95465436	95465982
chr10	95466006	95466245
chr10	95471211	95471675
chr10	95476010	95476304
chr10	95489749	95489949
chr10	95501168	95501346
chr10	95501423	95501620
chr10	95501620	95501831
chr10	95501831	95502136
chr10	95508047	95508467
chr10	95508708	95509005
chr10	95514605	95515257
chr10	95515257	95515623
chr10	95515623	95515878
chr10	95524174	95524403
chr10	95524403	95524714
chr10	95524873	95525166
chr10	95525166	95525737
chr10	95532999	95533252
chr10	95538157	95538330
chr10	95539282	95539596
chr10	95544080	95544324

chr10	95545003	95545378
chr10	95551831	95552076
chr10	95556491	95556798
chr10	95557355	95557607
chr10	95557637	95557831
chr10	95562537	95562693
chr10	95563245	95563463
chr10	95563482	95563865
chr10	95563865	95564170
chr10	95564170	95564406
chr10	95564406	95564606
chr10	95589244	95589421
chr10	95613850	95614015
chr10	95627445	95627696
chr10	95630672	95631023
chr10	95631023	95631374
chr10	95631527	95631859
chr10	95631859	95632171
chr10	95639454	95639998
chr10	95643806	95643962
chr10	95644001	95644435
chr10	95656826	95657094
chr10	95661954	95662105
chr10	95682940	95683413
chr10	95683840	95684061
chr10	95685263	95685434
chr10	95686183	95686333
chr10	95687868	95688070
chr10	95689885	95690145
chr10	95700433	95700846
chr10	95702857	95703255
chr10	95703848	95704051
chr10	95704868	95705236
chr10	95708141	95708452
chr10	95709449	95709692
chr10	95712122	95712560
chr10	95720405	95720751
chr10	95747444	95747781
chr10	95762477	95763100
chr10	95763828	95764057
chr10	95767614	95768304
chr10	95768334	95768524

chr10	95768948	95769260
chr10	95772810	95773170
chr10	95779383	95779872
chr10	95779872	95780162
chr10	95782227	95782381
chr10	95846308	95846643
chr10	95846643	95846886
chr10	95862630	95863134
chr10	95864753	95865102
chr10	95883907	95884534
chr10	95885320	95885537
chr10	95886146	95886367
chr10	95893593	95893805
chr10	95894414	95894722
chr10	95894803	95895060
chr10	95898530	95898810
chr10	95899350	95899571
chr10	95904371	95904605
chr10	95904767	95904963
chr10	95911111	95911393
chr10	95918174	95918345
chr10	95918345	95918530
chr10	95918671	95919184
chr10	95923632	95923898
chr10	95923898	95924231
chr10	95925021	95925239
chr10	95940237	95940852
chr10	95940852	95941474
chr10	95941797	95941969
chr10	95945211	95945361
chr10	95953798	95954099
chr10	95954099	95954323
chr10	95969829	95969979
chr10	96015567	96016122
chr10	96016122	96016544
chr10	96033478	96033628
chr10	96033698	96033982
chr10	96048737	96048952
chr10	96077546	96077914
chr10	96080584	96080760
chr10	96083049	96083199
chr10	96096603	96097214

chr10	96105256	96105458
chr10	96120862	96121062
chr10	96121078	96121621
chr10	96121621	96121939
chr10	96125841	96126088
chr10	96126986	96127198
chr10	96132254	96132483
chr10	96160882	96161108
chr10	96161108	96161505
chr10	96181303	96181800
chr10	96181800	96182131
chr10	96188091	96188532
chr10	96211279	96211456
chr10	96213892	96214334
chr10	96214334	96214641
chr10	96220783	96221060
chr10	96221145	96221374
chr10	96227523	96227883
chr10	96231133	96231288
chr10	96231365	96231763
chr10	96236112	96236357
chr10	96237060	96237349
chr10	96237650	96238080
chr10	96238287	96238619
chr10	96238876	96239248
chr10	96259264	96259590
chr10	96293297	96293492
chr10	96294833	96295007
chr10	96309298	96309660
chr10	96309694	96310281
chr10	96325351	96325793
chr10	96328892	96329070
chr10	96337827	96338525
chr10	96338626	96338967
chr10	96404755	96404997
chr10	96458964	96459140
chr10	96459144	96459588
chr10	96460633	96460891
chr10	96463876	96464071
chr10	96550331	96550606
chr10	96557220	96557708
chr10	96557975	96558182

chr10	96589792	96590233
chr10	96605239	96605450
chr10	96616395	96616712
chr10	96616712	96617202
chr10	96617230	96617414
chr10	96617692	96617849
chr10	96618251	96618623
chr10	96621120	96621285
chr10	96621573	96621885
chr10	96623305	96623501
chr10	96628158	96628665
chr10	96628665	96629044
chr10	96629281	96629697
chr10	96632806	96633178
chr10	96635380	96635815
chr10	96651645	96651833
chr10	96651833	96652023
chr10	96660509	96660683
chr10	96686282	96686485
chr10	96686958	96687145
chr10	96690313	96690623
chr10	96690860	96691352
chr10	96691400	96691637
chr10	96697513	96697789
chr10	96697789	96698336
chr10	96703097	96703257
chr10	96708456	96708949
chr10	96709043	96709226
chr10	96716418	96716794
chr10	96716794	96717078
chr10	96722555	96722803
chr10	96726148	96726638
chr10	96727189	96727537
chr10	96727540	96727795
chr10	96750779	96751239
chr10	96751578	96751876
chr10	96756407	96756604
chr10	96756604	96756791
chr10	96756856	96757006
chr10	96757386	96757664
chr10	96792608	96792911
chr10	96793013	96793163

chr10	96794957	96795203
chr10	96806471	96807097
chr10	96826348	96826514
chr10	96828897	96829234
chr10	96862605	96862827
chr10	96871322	96871516
chr10	96871525	96871831
chr10	96871831	96872217
chr10	96874519	96874728
chr10	96875663	96876013
chr10	96919607	96919912
chr10	96934758	96934951
chr10	96947634	96947965
chr10	96947965	96948372
chr10	97093745	97094338
chr10	97160389	97160554
chr10	97241519	97241815
chr10	97247213	97247485
chr10	97308041	97308201
chr10	97308306	97308736
chr10	97312982	97313317
chr10	97313427	97313682
chr10	97313682	97313975
chr10	97337974	97338343
chr10	97338343	97338730
chr10	97345429	97345649
chr10	97363984	97364145
chr10	97436571	97437122
chr10	97442161	97442311
chr10	97442347	97442767
chr10	97446170	97446332
chr10	97446572	97446816
chr10	97446816	97447074
chr10	97516506	97516766
chr10	97525696	97525913
chr10	97533694	97533908
chr10	97625218	97625473
chr10	97650493	97650694
chr10	97697089	97697425
chr10	97704716	97704866
chr10	97712624	97712938
chr10	97793624	97794154

chr10	97794154	97794373
chr10	97794373	97794557
chr10	97796069	97796257
chr10	97866081	97866372
chr10	97866404	97866733
chr10	97866733	97866998
chr10	97964251	97964490
chr10	98008420	98008735
chr10	98075203	98075373
chr10	98075373	98075545
chr10	98235271	98235476
chr10	98235574	98235823
chr10	98248409	98248602
chr10	98292121	98292358
chr10	98292358	98292566
chr10	98318433	98318782
chr10	98324557	98324988
chr10	98332609	98333079
chr10	98333316	98333948
chr10	98346055	98346231
chr10	98370468	98370941
chr10	98371120	98371299
chr10	98373958	98374230
chr10	98374794	98374961
chr10	98374961	98375133
chr10	98494446	98494673
chr10	98495950	98496251
chr10	98496255	98496646
chr10	98497111	98497329
chr10	98508397	98509054
chr10	98536595	98537010
chr10	98537130	98537303
chr10	98554506	98554688
chr10	98561942	98562541
chr10	98564560	98564829
chr10	98581894	98582124
chr10	98629307	98629566
chr10	98667658	98668051
chr10	98668051	98668318
chr10	98674212	98674472
chr10	98684113	98684648
chr10	98694988	98695336

chr10	98700410	98700662
chr10	98700662	98700897
chr10	98702418	98702862
chr10	98704588	98704968
chr10	98739040	98739339
chr10	98743944	98744235
chr10	98744235	98744660
chr10	98744800	98744971
chr10	98745534	98745728
chr10	98745916	98746282
chr10	98785506	98785692
chr10	98828575	98828860
chr10	98829060	98829690
chr10	98848088	98848522
chr10	98861624	98861829
chr10	98864475	98864724
chr10	98864724	98864985
chr10	98864985	98865305
chr10	98877915	98878162
chr10	98880080	98880428
chr10	98884975	98885224
chr10	98913843	98914143
chr10	98914143	98914358
chr10	98914358	98914770
chr10	98914770	98915287
chr10	98915456	98915664
chr10	98919436	98919599
chr10	98923241	98923520
chr10	98924717	98925030
chr10	98943536	98943963
chr10	98943963	98944430
chr10	98950487	98950717
chr10	98950717	98950956
chr10	98951344	98951689
chr10	98951689	98952149
chr10	98954224	98954658
chr10	98963322	98963604
chr10	98966751	98966970
chr10	98973875	98974277
chr10	98974624	98974948
chr10	98974948	98975219
chr10	98983918	98984140

chr10	98992055	98992286
chr10	99004690	99005310
chr10	99008724	99008950
chr10	99011116	99011287
chr10	99013309	99013534
chr10	99047193	99047506
chr10	99047630	99047835
chr10	99050175	99050483
chr10	99062283	99062671
chr10	99062671	99063275
chr10	99078731	99078905
chr10	99081006	99081158
chr10	99081538	99082248
chr10	99084971	99085230
chr10	99097475	99097625
chr10	99106681	99106959
chr10	99106959	99107301
chr10	99107301	99107796
chr10	99107916	99108229
chr10	99108229	99108608
chr10	99123894	99124096
chr10	99124096	99124371
chr10	99124371	99124732
chr10	99137676	99137857
chr10	99152164	99152325
chr10	99156904	99157114
chr10	99162086	99162275
chr10	99164990	99165398
chr10	99166483	99166680
chr10	99168924	99169090
chr10	99172036	99172528
chr10	99173190	99173462
chr10	99176170	99176697
chr10	99190824	99191142
chr10	99193708	99193924
chr10	99194747	99195091
chr10	99201830	99202260
chr10	99202380	99202807
chr10	99204003	99204203
chr10	99204353	99204510
chr10	99204610	99204874
chr10	99211139	99211289

chr10	99211398	99211847
chr10	99211847	99212137
chr10	99212137	99212399
chr10	99219989	99220198
chr10	99226923	99227207
chr10	99237196	99237719
chr10	99241869	99242416
chr10	99242416	99242797
chr10	99243889	99244178
chr10	99244178	99244445
chr10	99250826	99251009
chr10	99255821	99256158
chr10	99261433	99261707
chr10	99261707	99262020
chr10	99262020	99262401
chr10	99262902	99263404
chr10	99263404	99263781
chr10	99268766	99269170
chr10	99269174	99269762
chr10	99270275	99270516
chr10	99270612	99270839
chr10	99287834	99288090
chr10	99293022	99293438
chr10	99297144	99297434
chr10	99297564	99298114
chr10	99308808	99309345
chr10	99376278	99376722
chr10	99379171	99379443
chr10	99396374	99396876
chr10	99396876	99397224
chr10	99405518	99405723
chr10	99423383	99423736
chr10	99460131	99460323
chr10	99511934	99512117
chr10	99555894	99556628
chr10	99576287	99576835
chr10	99577335	99577600
chr10	99592247	99592411
chr10	99599481	99599872
chr10	99610982	99611229
chr10	99615851	99616130
chr10	99617720	99617872

chr10	99619519	99619800
chr10	99624532	99624697
chr10	99632328	99632616
chr10	99641871	99642090
chr10	99656885	99657156
chr10	99701502	99701692
chr10	99899919	99900070
chr10	99907128	99907287
chr10	99908134	99908365
chr10	99909960	99910346
chr10	99910346	99910647
chr10	99929827	99930104
chr10	99933307	99933494
chr10	99933602	99933793
chr10	99945423	99945799
chr10	99945882	99946177
chr10	99948758	99949100
chr10	99949100	99949413
chr10	99951307	99951639
chr10	99951738	99951938
chr10	99997403	99997643
chr10	100002401	100002705
chr10	100015429	100015956
chr10	100015956	100016410
chr10	100016426	100016728
chr10	100020467	100020791
chr10	100024719	100025081
chr10	100027877	100028088
chr10	100030473	100030671
chr10	100032621	100032896
chr10	100032896	100033204
chr10	100055173	100055330
chr10	100055426	100055581
chr10	100057185	100057400
chr10	100059213	100059692
chr10	100101082	100101511
chr10	100102183	100102507
chr10	100119325	100119548
chr10	100137344	100137677
chr10	100151905	100152158
chr10	100152227	100152868
chr10	100152877	100153193

chr10	100153283	100153790
chr10	100160990	100161507
chr10	100161598	100161753
chr10	100170944	100171122
chr10	100180811	100180961
chr10	100190912	100191153
chr10	100232684	100232885
chr10	100402460	100402692
chr10	100431223	100431438
chr10	100446174	100446328
chr10	100460565	100460896
chr10	100486748	100486996
chr10	100487012	100487908
chr10	100519998	100520376
chr10	100567256	100567455
chr10	100588629	100589136
chr10	100589136	100589424
chr10	100598445	100598621
chr10	100627428	100627616
chr10	100958433	100958654
chr10	100965738	100965930
chr10	100981110	100981356
chr10	100981356	100981780
chr10	100987370	100987521
chr10	100987904	100988168
chr10	100997023	100997231
chr10	101097322	101097580
chr10	101395744	101395915
chr10	101582882	101583078
chr10	101583090	101583476
chr10	101825760	101826054
chr10	102125606	102125895
chr10	102153265	102153471
chr10	102174631	102175180
chr10	102175203	102175446
chr10	102183651	102184001
chr10	102304702	102305109
chr10	102341185	102341426
chr10	102435165	102435525
chr10	102451838	102452073
chr10	102498827	102499067
chr10	102521437	102521629

chr10	102581226	102581577
chr10	102622492	102622949
chr10	102622949	102623380
chr10	102629617	102629968
chr10	102639829	102640011
chr10	102640326	102640659
chr10	102644918	102645173
chr10	102645195	102645345
chr10	102659666	102660164
chr10	102660164	102660678
chr10	102661020	102661389
chr10	102667372	102667903
chr10	102668567	102668771
chr10	102683054	102683369
chr10	102689957	102690407
chr10	102690407	102690798
chr10	102705913	102706081
chr10	102735296	102735526
chr10	102885561	102885776
chr10	102885776	102885985
chr10	103028338	103028500
chr10	103236170	103236425
chr10	103367557	103367762
chr10	103367779	103367968
chr10	103368634	103368819
chr10	103368819	103368990
chr10	103369139	103369410
chr10	103443064	103443347
chr10	103504947	103505299
chr10	103505435	103505590
chr10	103507175	103507336
chr10	103783068	103783224
chr10	104357369	104357596
chr10	104388237	104388599
chr10	104448937	104449115
chr10	104536143	104536360
chr10	104540680	104540885
chr10	104629876	104630420
chr10	104644566	104644736
chr10	104823449	104823621
chr10	105123548	105123698
chr10	105238352	105238590

chr10	105279954	105280269
chr10	105281017	105281335
chr10	105432480	105432732
chr10	105522458	105522746
chr10	105522989	105523139
chr10	105523359	105523743
chr10	105523743	105524197
chr10	105535625	105535818
chr10	105535962	105536148
chr10	105544675	105544898
chr10	105573393	105573613
chr10	105573613	105573866
chr10	105573866	105574119
chr10	105574119	105574373
chr10	105574373	105574640
chr10	105574640	105575044
chr10	105575126	105575597
chr10	105579378	105579536
chr10	105580533	105581055
chr10	105632026	105632219
chr10	105676633	105676806
chr10	105774466	105774725
chr10	105834735	105834900
chr10	105840873	105841236
chr10	105841236	105841523
chr10	105841523	105841906
chr10	105880861	105881011
chr10	105901137	105901299
chr10	106006126	106006453
chr10	106163424	106163614
chr10	106429051	106429397
chr10	106770144	106770294
chr10	106775267	106775674
chr10	106775674	106776055
chr10	106776389	106776620
chr10	106776857	106777014
chr10	107272418	107272615
chr10	107342795	107343118
chr10	107389689	107389839
chr10	107467894	107468133
chr10	107493577	107493827
chr10	107502260	107502503

chr10	107534148	107534381
chr10	107601472	107601624
chr10	107601624	107601768
chr10	107624177	107624645
chr10	107632586	107632868
chr10	107658472	107658810
chr10	107658810	107659137
chr10	107659185	107659492
chr10	107662468	107662727
chr10	107662773	107662996
chr10	107696741	107696915
chr10	107765166	107765337
chr10	107800046	107800288
chr10	107858779	107859009
chr10	107912996	107913314
chr10	107921600	107921898
chr10	107922141	107922370
chr10	107930648	107930934
chr10	107932498	107932823
chr10	107933551	107933946
chr10	107934167	107934474
chr10	107950034	107950213
chr10	107951667	107951919
chr10	107966977	107967591
chr10	107989048	107989252
chr10	107994810	107995028
chr10	107995028	107995229
chr10	107995229	107995494
chr10	108008974	108009446
chr10	108015973	108016494
chr10	108020158	108020346
chr10	108030146	108030303
chr10	108030639	108030815
chr10	108033316	108033569
chr10	108033569	108033825
chr10	108064021	108064333
chr10	108064500	108064667
chr10	108066323	108066587
chr10	108072218	108072646
chr10	108083346	108083497
chr10	108108661	108108979
chr10	108108979	108109470

chr10	108113353	108113856
chr10	108120021	108120202
chr10	108161445	108162383
chr10	108162383	108162790
chr10	108162790	108163026
chr10	108170830	108171128
chr10	108172885	108173218
chr10	108173218	108173613
chr10	108185436	108185705
chr10	108190727	108190877
chr10	108201466	108201664
chr10	108201727	108201946
chr10	108237100	108237371
chr10	108291742	108292016
chr10	108294362	108294690
chr10	108299810	108300233
chr10	108300866	108301275
chr10	108307576	108307849
chr10	108307849	108308246
chr10	108331031	108331452
chr10	108331586	108331819
chr10	108331819	108332018
chr10	108332018	108332244
chr10	108332244	108332525
chr10	108332525	108332942
chr10	108333010	108333186
chr10	108333243	108333457
chr10	108338040	108338484
chr10	108356715	108356919
chr10	108372309	108372593
chr10	108372644	108372889
chr10	108372889	108373235
chr10	108377745	108378100
chr10	108378100	108378432
chr10	108378432	108378709
chr10	108400961	108401111
chr10	108403591	108403791
chr10	108407426	108407702
chr10	108408147	108408305
chr10	108432202	108432603
chr10	108434088	108434247
chr10	108434769	108435085

chr10	108435096	108435292
chr10	108435670	108435977
chr10	108444552	108444792
chr10	108445181	108445470
chr10	108445604	108445999
chr10	108446086	108446343
chr10	108446415	108447071
chr10	108448480	108448721
chr10	108452521	108452727
chr10	108463499	108463820
chr10	108505022	108505313
chr10	108508310	108508622
chr10	108510265	108510459
chr10	108531117	108531492
chr10	108531492	108531817
chr10	108531817	108532157
chr10	108532349	108532609
chr10	108537712	108537953
chr10	108538375	108538644
chr10	108538644	108539100
chr10	108541661	108541830
chr10	108545612	108545971
chr10	108564213	108564420
chr10	108564420	108564683
chr10	108581208	108581619
chr10	108581619	108582043
chr10	108582106	108582399
chr10	108947302	108947469
chr10	109000908	109001131
chr10	109010547	109010778
chr10	109010864	109011183
chr10	109011403	109011803
chr10	109480648	109480819
chr10	109487245	109487581
chr10	109515019	109515256
chr10	109515256	109515698
chr10	109536672	109536984
chr10	109584331	109584596
chr10	109665048	109665210
chr10	109693571	109693733
chr10	109852522	109852672
chr10	109869368	109869824

chr10	109903968	109904173
chr10	109920840	109921054
chr10	110141585	110141815
chr10	110188586	110188790
chr10	110191950	110192165
chr10	110248366	110248594
chr10	110323536	110323996
chr10	110331459	110332026
chr10	110445948	110446167
chr10	110455140	110455393
chr10	110455458	110456302
chr10	110484829	110485255
chr10	110526321	110526471
chr10	110526609	110526791
chr10	110546868	110547154
chr10	110554053	110554361
chr10	110565544	110565932
chr10	110565983	110566259
chr10	110566259	110566715
chr10	110573537	110573880
chr10	110727861	110728174
chr10	110728405	110728943
chr10	110733402	110733655
chr10	110744155	110744450
chr10	110744854	110745233
chr10	110745233	110745964
chr10	110749969	110750273
chr10	110760619	110760884
chr10	110777957	110778199
chr10	110782787	110782994
chr10	110794572	110794820
chr10	110804389	110804880
chr10	110805092	110805250
chr10	110903928	110904205
chr10	110919693	110920031
chr10	110920031	110920380
chr10	110920380	110920682
chr10	110926936	110927142
chr10	110927142	110927371
chr10	110927430	110927689
chr10	110927689	110927962
chr10	110974558	110974710

chr10	110976128	110976376
chr10	111009417	111009883
chr10	111009883	111010158
chr10	111010158	111010575
chr10	111048003	111048247
chr10	111055881	111056042
chr10	111079195	111079445
chr10	111100203	111100356
chr10	111103123	111103397
chr10	111107438	111107668
chr10	111108826	111109059
chr10	111127435	111127857
chr10	111137923	111138472
chr10	111153967	111154165
chr10	111164285	111164718
chr10	111164718	111165015
chr10	111165015	111165324
chr10	111165324	111165528
chr10	111165528	111165740
chr10	111180722	111180939
chr10	111189127	111189288
chr10	111190671	111190941
chr10	111190941	111191194
chr10	111210094	111210244
chr10	111213711	111213914
chr10	111213942	111214468
chr10	111226482	111227206
chr10	111234513	111234766
chr10	111256225	111256482
chr10	111257274	111257787
chr10	111280303	111280806
chr10	111289884	111290143
chr10	111298216	111298768
chr10	111298768	111299080
chr10	111313409	111313629
chr10	111363920	111364149
chr10	111364149	111364406
chr10	111364406	111364658
chr10	111364929	111365463
chr10	111369080	111369387
chr10	111373924	111374528
chr10	111388625	111389003

chr10	111389005	111389223
chr10	111389727	111390324
chr10	111404788	111404971
chr10	111472783	111473401
chr10	111473401	111473763
chr10	111473763	111473951
chr10	111474215	111474402
chr10	111478765	111478931
chr10	111478931	111479137
chr10	111479380	111479544
chr10	111488698	111488885
chr10	111488885	111489052
chr10	111496015	111496165
chr10	111506035	111506777
chr10	111506777	111507167
chr10	111507167	111507446
chr10	111507446	111507726
chr10	111515108	111515756
chr10	111515842	111516228
chr10	111516228	111516675
chr10	111516786	111517224
chr10	111522245	111522578
chr10	111527106	111527357
chr10	111527669	111527978
chr10	111528279	111528793
chr10	111529161	111529545
chr10	111543605	111544368
chr10	111544848	111545076
chr10	111550121	111550352
chr10	111550352	111550662
chr10	111556267	111556513
chr10	111558005	111558592
chr10	111558592	111559016
chr10	111559016	111559366
chr10	111559366	111559647
chr10	111569231	111569478
chr10	111570631	111571401
chr10	111575362	111575583
chr10	111575727	111575880
chr10	111575918	111576300
chr10	111576300	111576661
chr10	111576661	111576997

chr10	111577032	111577329
chr10	111583431	111583870
chr10	111583870	111584257
chr10	111584257	111584496
chr10	111591247	111591524
chr10	111599667	111599871
chr10	111599871	111600063
chr10	111600099	111600596
chr10	111600596	111601177
chr10	111618931	111619212
chr10	111619541	111619783
chr10	111619783	111619979
chr10	111619979	111620450
chr10	111621041	111621257
chr10	111622859	111623121
chr10	111635552	111635858
chr10	111636228	111636491
chr10	111636859	111637359
chr10	111637359	111637731
chr10	111639946	111640240
chr10	111640563	111640790
chr10	111641587	111641983
chr10	111654421	111654771
chr10	111654793	111654954
chr10	111666376	111666578
chr10	111666718	111666885
chr10	111668201	111668416
chr10	111672495	111672690
chr10	111672720	111673461
chr10	111684971	111685306
chr10	111685571	111685721
chr10	111694326	111694607
chr10	111694948	111695295
chr10	111696571	111696721
chr10	111713674	111714083
chr10	111714083	111714525
chr10	111715037	111715786
chr10	111719612	111719890
chr10	111723992	111724194
chr10	111741079	111741229
chr10	111741347	111741630
chr10	111741630	111741898

chr10	111746089	111746514
chr10	111746938	111747109
chr10	111762400	111762597
chr10	111769664	111770014
chr10	111770014	111770423
chr10	111776876	111777026
chr10	111777653	111777872
chr10	111780687	111780993
chr10	111794252	111794489
chr10	111795174	111795415
chr10	111795751	111796245
chr10	111806484	111806822
chr10	111813777	111814128
chr10	111824245	111824482
chr10	111826247	111826726
chr10	111826857	111827025
chr10	111827025	111827196
chr10	111827323	111827557
chr10	111827649	111827985
chr10	111828742	111829061
chr10	111836866	111837144
chr10	111842952	111843237
chr10	111858471	111858685
chr10	111874317	111874690
chr10	111877481	111877698
chr10	111878751	111878976
chr10	111880346	111880733
chr10	111896889	111897175
chr10	111925530	111925854
chr10	111935263	111935594
chr10	111936374	111936832
chr10	111936832	111937227
chr10	111947711	111947865
chr10	111957753	111958044
chr10	111959536	111959717
chr10	111959926	111960231
chr10	111960231	111960520
chr10	111960520	111960874
chr10	111962265	111962607
chr10	111966398	111966695
chr10	111972461	111972831
chr10	111972831	111973287

chr10	111977647	111977910
chr10	111978426	111979043
chr10	111982358	111982509
chr10	111990112	111990377
chr10	111995815	111996155
chr10	111996535	111996924
chr10	111997145	111997371
chr10	111997683	111998080
chr10	111999087	111999328
chr10	112000445	112000633
chr10	112001454	112001614
chr10	112001684	112001847
chr10	112002662	112002832
chr10	112051273	112051542
chr10	112051758	112051938
chr10	112052023	112052234
chr10	112060006	112060302
chr10	112094222	112094473
chr10	112105733	112106099
chr10	112160072	112160532
chr10	112203581	112203736
chr10	112204425	112204651
chr10	112267595	112267767
chr10	112269957	112270135
chr10	112270144	112270434
chr10	112270434	112270688
chr10	112270974	112271201
chr10	112271648	112271815
chr10	112271815	112272001
chr10	112403826	112404000
chr10	112481510	112481793
chr10	112481974	112482236
chr10	112564783	112564948
chr10	112569749	112569917
chr10	112611804	112612023
chr10	112613818	112614046
chr10	112614142	112614641
chr10	112614715	112614865
chr10	112629815	112630097
chr10	112630097	112630353
chr10	112650443	112650690
chr10	112653668	112653993

chr10	112660206	112660356
chr10	112675147	112675690
chr10	112728086	112728236
chr10	112743953	112744298
chr10	112788275	112788622
chr10	112801665	112801883
chr10	112802261	112802546
chr10	112804983	112805133
chr10	112806549	112806761
chr10	112810067	112810647
chr10	112882854	112883100
chr10	112883100	112883410
chr10	112883410	112883839
chr10	112928344	112928641
chr10	112928641	112928910
chr10	112928910	112929225
chr10	112930386	112930542
chr10	113520480	113520670
chr10	113540170	113540459
chr10	113561768	113562081
chr10	113562081	113562328
chr10	113562328	113562593
chr10	113736332	113736637
chr10	113889127	113889472
chr10	113920154	113920436
chr10	114256000	114256150
chr10	114737309	114737513
chr10	114874795	114875036
chr10	114987964	114988170
chr10	114988567	114988731
chr10	115097236	115097473
chr10	115100189	115100484
chr10	115175350	115175987
chr10	115184749	115185124
chr10	115193016	115193229
chr10	115225653	115226112
chr10	115229120	115229276
chr10	115236802	115237067
chr10	115241174	115241445
chr10	115241445	115241685
chr10	115241876	115242190
chr10	115242218	115242639

chr10	115250446	115250720
chr10	115250882	115251088
chr10	115251095	115251326
chr10	115251326	115251779
chr10	115252283	115252718
chr10	115256222	115256519
chr10	115261560	115261751
chr10	115262294	115262658
chr10	115262987	115263275
chr10	115263275	115263596
chr10	115280770	115280981
chr10	115280981	115281217
chr10	115315173	115315493
chr10	115315493	115315787
chr10	115315787	115316232
chr10	115323368	115323615
chr10	115332104	115332298
chr10	115332300	115332731
chr10	115350964	115351289
chr10	115354616	115354867
chr10	115362069	115362675
chr10	115362688	115362873
chr10	115384104	115384675
chr10	115384675	115385117
chr10	115385117	115385507
chr10	115385507	115385707
chr10	115385707	115385908
chr10	115393643	115393803
chr10	115395992	115396387
chr10	115406508	115406693
chr10	115435427	115435709
chr10	115435838	115436204
chr10	115436320	115436606
chr10	115438652	115438830
chr10	115438992	115439349
chr10	115451621	115451862
chr10	115452010	115452186
chr10	115492192	115492409
chr10	115493028	115493290
chr10	115557287	115557498
chr10	115557639	115557815
chr10	115572872	115573230

chr10	115573230	115573472
chr10	115573472	115573650
chr10	115576480	115576758
chr10	115586189	115586483
chr10	115586483	115586692
chr10	115586829	115587271
chr10	115587271	115587640
chr10	115587640	115587985
chr10	115617526	115617778
chr10	115618156	115618322
chr10	115620275	115620496
chr10	115623486	115623742
chr10	115665428	115665638
chr10	115687388	115687576
chr10	115742660	115742939
chr10	115758770	115758925
chr10	115780095	115780817
chr10	115787922	115788095
chr10	115809242	115809440
chr10	115814383	115814637
chr10	115814637	115815107
chr10	115815791	115816073
chr10	115816438	115816903
chr10	115817080	115817395
chr10	115821762	115821969
chr10	115828918	115829253
chr10	115931620	115931793
chr10	116017804	116018154
chr10	116018154	116018781
chr10	116019112	116019420
chr10	116098623	116098778
chr10	116147253	116147428
chr10	116155366	116155848
chr10	116175411	116175877
chr10	116180607	116180780
chr10	116180812	116181466
chr10	116183357	116183561
chr10	116186822	116187063
chr10	116187345	116187679
chr10	116187805	116188013
chr10	116192330	116192698
chr10	116192985	116193135

chr10	116196360	116196656
chr10	116196672	116196971
chr10	116196974	116197351
chr10	116197366	116197569
chr10	116208041	116208321
chr10	116208398	116208548
chr10	116220387	116220733
chr10	116224872	116225022
chr10	116243443	116243685
chr10	116254085	116254357
chr10	116260691	116261005
chr10	116261005	116261250
chr10	116261250	116261472
chr10	116266481	116266893
chr10	116266893	116267161
chr10	116267161	116267396
chr10	116267396	116267652
chr10	116267652	116268025
chr10	116268383	116268598
chr10	116280063	116280256
chr10	116296434	116296888
chr10	116297607	116297771
chr10	116297780	116298234
chr10	116298234	116298575
chr10	116298855	116299059
chr10	116304480	116304733
chr10	116304877	116305278
chr10	116316618	116316817
chr10	116319285	116319555
chr10	116330498	116330747
chr10	116335446	116335629
chr10	116341989	116342169
chr10	116376957	116377574
chr10	116384980	116385151
chr10	116393600	116393761
chr10	116410778	116411117
chr10	116422664	116422842
chr10	116430865	116431276
chr10	116457937	116458184
chr10	116462913	116463307
chr10	116473026	116473600
chr10	116473600	116474179

chr10	116474179	116474593
chr10	116501425	116501767
chr10	116506203	116506452
chr10	116525087	116525284
chr10	116528779	116529348
chr10	116530159	116530381
chr10	116531173	116531383
chr10	116536660	116537063
chr10	116537063	116537429
chr10	116537588	116537768
chr10	116548045	116548334
chr10	116580907	116581380
chr10	116581380	116581774
chr10	116581774	116582168
chr10	116582168	116582442
chr10	116596514	116596671
chr10	116615478	116615628
chr10	116616065	116616395
chr10	116626098	116626248
chr10	116666949	116667227
chr10	116669945	116670143
chr10	116693851	116694104
chr10	116725110	116725450
chr10	116733527	116734253
chr10	116748961	116749282
chr10	116749529	116749836
chr10	116750084	116750401
chr10	116750856	116751404
chr10	116754967	116755117
chr10	116763145	116763390
chr10	116769344	116769629
chr10	116769629	116769871
chr10	116770017	116770270
chr10	116787757	116787924
chr10	116788153	116788354
chr10	116789224	116789479
chr10	116796610	116796846
chr10	116827326	116827669
chr10	116848825	116849071
chr10	116849071	116849256
chr10	116849256	116849406
chr10	116874383	116874539

chr10	116876022	116876194
chr10	116878386	116878622
chr10	116879138	116879473
chr10	116889987	116890210
chr10	116893889	116894045
chr10	116900945	116901204
chr10	116928688	116928891
chr10	116928891	116929186
chr10	116929228	116929383
chr10	116930101	116930445
chr10	116930561	116930821
chr10	116944242	116944437
chr10	116947782	116948013
chr10	116949609	116949897
chr10	116949970	116950281
chr10	116950281	116950475
chr10	116950475	116950856
chr10	116980939	116981206
chr10	116982581	116983020
chr10	116994043	116994196
chr10	116995729	116995938
chr10	116995938	116996229
chr10	116996229	116996697
chr10	116998849	116999010
chr10	116999074	116999431
chr10	117010069	117010480
chr10	117014725	117014928
chr10	117017425	117017969
chr10	117021044	117021410
chr10	117023550	117024350
chr10	117033725	117034328
chr10	117040442	117040667
chr10	117040722	117040917
chr10	117040917	117041160
chr10	117043502	117043840
chr10	117043840	117044056
chr10	117048964	117049114
chr10	117049762	117049950
chr10	117050048	117050281
chr10	117050281	117050576
chr10	117052639	117052868
chr10	117055800	117055970

chr10	117063236	117063475
chr10	117063475	117063719
chr10	117063719	117064080
chr10	117069243	117069419
chr10	117073233	117073440
chr10	117078330	117078641
chr10	117100230	117100515
chr10	117106402	117106579
chr10	117111165	117111483
chr10	117111645	117111842
chr10	117112607	117112853
chr10	117113537	117113762
chr10	117118554	117118730
chr10	117124684	117124862
chr10	117130045	117130332
chr10	117131115	117131297
chr10	117131471	117131833
chr10	117142048	117142217
chr10	117147862	117148096
chr10	117148096	117148386
chr10	117148386	117148704
chr10	117148704	117148972
chr10	117150121	117150510
chr10	117151328	117151566
chr10	117167096	117167358
chr10	117171590	117171839
chr10	117172134	117172370
chr10	117173581	117173856
chr10	117173856	117174090
chr10	117174654	117174953
chr10	117208534	117208740
chr10	117209271	117209462
chr10	117209462	117209662
chr10	117209662	117209948
chr10	117216868	117217157
chr10	117217278	117217620
chr10	117223824	117224330
chr10	117224330	117224622
chr10	117224622	117224863
chr10	117229904	117230099
chr10	117250097	117250254
chr10	117259302	117259605

chr10	117275367	117275687
chr10	117275744	117276031
chr10	117276034	117276192
chr10	117277808	117277991
chr10	117289059	117289232
chr10	117330638	117330834
chr10	117363157	117363410
chr10	117364985	117365524
chr10	117376231	117376619
chr10	117376619	117376869
chr10	117376869	117377236
chr10	117387834	117388072
chr10	117393214	117393684
chr10	117402545	117402730
chr10	117417427	117417717
chr10	117418177	117418338
chr10	117432465	117432677
chr10	117435409	117435708
chr10	117435708	117436039
chr10	117436795	117437035
chr10	117453000	117453249
chr10	117455556	117455810
chr10	117459020	117459261
chr10	117480902	117481052
chr10	117483787	117484004
chr10	117490390	117490649
chr10	117494475	117494625
chr10	117511061	117511283
chr10	117515867	117516094
chr10	117516272	117516504
chr10	117518793	117519118
chr10	117521540	117521913
chr10	117521913	117522185
chr10	117522267	117522542
chr10	117522827	117523046
chr10	117529790	117530054
chr10	117530842	117531010
chr10	117570347	117570499
chr10	117571395	117571631
chr10	117587509	117587659
chr10	117599093	117599378
chr10	117624238	117624398

chr10	117625895	117626045
chr10	117628987	117629568
chr10	117629568	117629808
chr10	117629808	117630008
chr10	117637157	117637346
chr10	117649804	117650050
chr10	117650973	117651422
chr10	117662697	117663059
chr10	117663059	117663507
chr10	117663507	117663862
chr10	117663862	117664143
chr10	117664155	117664360
chr10	117677296	117677821
chr10	117682611	117682770
chr10	117687598	117687903
chr10	117688266	117688575
chr10	117688679	117688841
chr10	117689160	117689368
chr10	117697551	117697783
chr10	117709946	117710279
chr10	117710279	117710565
chr10	117710565	117710785
chr10	117710785	117711025
chr10	117716389	117716579
chr10	117717609	117717950
chr10	117718560	117718792
chr10	117721468	117721829
chr10	117729357	117729884
chr10	117730168	117730366
chr10	117731330	117731526
chr10	117734183	117734441
chr10	117745619	117746079
chr10	117746079	117746766
chr10	117757188	117757489
chr10	117757489	117757697
chr10	117768759	117768969
chr10	117768969	117769245
chr10	117769245	117769509
chr10	117769509	117769702
chr10	117784902	117785117
chr10	117786146	117786429
chr10	117792150	117792500

chr10	117792500	117792823
chr10	117792823	117793152
chr10	117801264	117801481
chr10	117801487	117801864
chr10	117808179	117808340
chr10	117808999	117809220
chr10	117809220	117809485
chr10	117813253	117813482
chr10	117816610	117816773
chr10	117818197	117818384
chr10	117836510	117836697
chr10	117838930	117839284
chr10	117845190	117845590
chr10	117845590	117845919
chr10	117845919	117846192
chr10	117846192	117846646
chr10	117860300	117860512
chr10	117860643	117860927
chr10	117860927	117861225
chr10	117861960	117862480
chr10	117867210	117868094
chr10	117873966	117874298
chr10	117891420	117891604
chr10	117899494	117899711
chr10	117905663	117905831
chr10	117906279	117906589
chr10	117915437	117915707
chr10	117916296	117916657
chr10	117936475	117936735
chr10	117940811	117941247
chr10	117941247	117941486
chr10	117976366	117976668
chr10	117979160	117979352
chr10	117984761	117985191
chr10	117986073	117986233
chr10	117987580	117988067
chr10	117988067	117988576
chr10	117990349	117990517
chr10	117993875	117994213
chr10	118012179	118012382
chr10	118012382	118012581
chr10	118034595	118034770

chr10	118038911	118039352
chr10	118050347	118050499
chr10	118058353	118058508
chr10	118058931	118059240
chr10	118103100	118103753
chr10	118122571	118122867
chr10	118122867	118123182
chr10	118130985	118131166
chr10	118141381	118141847
chr10	118141847	118142210
chr10	118178719	118179150
chr10	118180961	118181462
chr10	118195176	118195546
chr10	118217190	118217364
chr10	118302261	118302496
chr10	118370480	118370807
chr10	118470675	118470960
chr10	118479372	118479717
chr10	118518110	118518320
chr10	118573369	118573574
chr10	118628068	118628342
chr10	118795110	118795302
chr10	118804623	118804919
chr10	118867970	118868295
chr10	118868453	118868674
chr10	118868674	118868947
chr10	118868947	118869244
chr10	118869244	118869624
chr10	118869624	118870081
chr10	118870299	118870603
chr10	118981976	118982208
chr10	118987994	118988342
chr10	118988342	118988864
chr10	118991239	118991589
chr10	118993966	118994123
chr10	118996358	118996629
chr10	118999088	118999397
chr10	119003835	119004172
chr10	119010165	119010385
chr10	119030644	119030982
chr10	119050381	119050608
chr10	119058574	119058775

chr10	119079385	119079585
chr10	119079948	119080108
chr10	119080587	119080789
chr10	119081024	119081292
chr10	119088006	119088179
chr10	119091404	119091652
chr10	119109282	119109518
chr10	119111580	119111763
chr10	119111763	119111993
chr10	119111993	119112258
chr10	119138060	119138286
chr10	119144962	119145233
chr10	119154940	119155214
chr10	119229576	119229736
chr10	119230095	119230354
chr10	119239255	119239678
chr10	119239678	119239937
chr10	119239937	119240141
chr10	119240141	119240392
chr10	119240392	119240731
chr10	119367308	119367460
chr10	119367485	119367813
chr10	119368429	119368898
chr10	119408378	119408608
chr10	119421442	119421769
chr10	119453445	119453831
chr10	119453831	119454101
chr10	119454101	119454604
chr10	119454771	119454935
chr10	119454935	119455089
chr10	119550481	119550728
chr10	119572867	119573053
chr10	119573203	119573378
chr10	119591349	119591511
chr10	119610335	119610597
chr10	119610597	119610825
chr10	119615235	119615641
chr10	119667057	119667248
chr10	119670506	119670868
chr10	119670868	119671336
chr10	119674178	119674334
chr10	119697411	119697701

chr10	119702205	119702360
chr10	119706362	119706564
chr10	119713640	119714098
chr10	119727057	119727246
chr10	119727521	119727708
chr10	119752065	119752395
chr10	119752395	119752914
chr10	119757290	119757688
chr10	119757772	119757926
chr10	119785292	119785462
chr10	119803666	119803953
chr10	119803953	119804231
chr10	119805174	119805544
chr10	119819244	119819641
chr10	119850937	119851107
chr10	119869033	119869338
chr10	119875866	119876070
chr10	119876409	119876715
chr10	119876715	119877238
chr10	119886837	119887147
chr10	119974316	119974473
chr10	119992475	119992644
chr10	120020742	120021434
chr10	120023770	120023973
chr10	120050774	120051008
chr10	120060636	120060899
chr10	120060940	120061204
chr10	120061204	120061448
chr10	120061448	120061784
chr10	120061792	120061988
chr10	120063234	120063394
chr10	120077960	120078110
chr10	120095280	120095435
chr10	120100896	120101051
chr10	120112435	120112799
chr10	120112799	120113362
chr10	120120573	120120824
chr10	120123877	120124073
chr10	120125201	120125455
chr10	120127915	120128071
chr10	120130711	120130934
chr10	120133552	120133713

chr10	120151754	120151910
chr10	120168192	120168460
chr10	120182972	120183188
chr10	120188495	120188882
chr10	120201249	120201477
chr10	120201477	120201887
chr10	120202206	120202427
chr10	120204551	120205035
chr10	120208570	120209056
chr10	120209184	120209418
chr10	120209633	120209788
chr10	120216759	120217375
chr10	120218877	120219501
chr10	120226727	120227152
chr10	120227152	120227391
chr10	120227391	120227654
chr10	120227654	120228008
chr10	120231873	120232032
chr10	120234086	120234295
chr10	120243112	120243294
chr10	120247441	120247721
chr10	120255863	120256073
chr10	120257319	120257626
chr10	120260485	120260722
chr10	120270902	120271071
chr10	120298400	120298681
chr10	120299881	120300097
chr10	120300315	120300497
chr10	120308338	120308558
chr10	120308696	120309104
chr10	120309798	120310110
chr10	120314362	120314569
chr10	120349918	120350221
chr10	120354569	120354816
chr10	120354928	120355131
chr10	120355829	120356020
chr10	120357063	120357244
chr10	120357504	120357845
chr10	120358235	120358739
chr10	120359260	120359566
chr10	120365672	120365842
chr10	120366169	120366575

chr10	120371270	120371506
chr10	120375477	120375770
chr10	120377244	120377519
chr10	120377564	120377772
chr10	120378819	120379054
chr10	120383656	120384040
chr10	120384749	120384926
chr10	120385655	120385905
chr10	120385905	120386125
chr10	120389769	120389942
chr10	120390973	120391177
chr10	120392390	120392664
chr10	120393774	120393930
chr10	120394708	120395068
chr10	120399808	120400170
chr10	120401877	120402194
chr10	120403015	120403346
chr10	120409630	120409803
chr10	120410742	120410938
chr10	120413774	120413979
chr10	120413979	120414193
chr10	120421973	120422525
chr10	120422561	120423210
chr10	120424106	120424410
chr10	120428680	120428908
chr10	120428908	120429181
chr10	120429249	120429476
chr10	120431537	120431711
chr10	120432312	120432470
chr10	120432860	120433141
chr10	120433209	120433439
chr10	120433439	120433641
chr10	120436401	120436612
chr10	120442587	120442772
chr10	120443903	120444187
chr10	120444822	120445130
chr10	120451014	120451312
chr10	120451466	120451660
chr10	120452840	120453134
chr10	120457090	120457245
chr10	120459854	120460004
chr10	120460781	120461183

chr10	120461329	120461642
chr10	120461875	120462162
chr10	120463194	120463462
chr10	120464497	120464675
chr10	120465155	120465403
chr10	120469065	120469540
chr10	120469572	120469871
chr10	120475687	120476093
chr10	120476093	120476599
chr10	120476599	120477038
chr10	120477038	120477421
chr10	120477646	120477853
chr10	120478210	120478360
chr10	120495673	120495823
chr10	120496977	120497169
chr10	120525341	120525841
chr10	120525841	120526171
chr10	120527064	120527341
chr10	120552357	120552670
chr10	120552687	120553131
chr10	120553131	120553493
chr10	120557888	120558231
chr10	120558231	120558614
chr10	120558614	120558861
chr10	120558861	120559046
chr10	120559046	120559316
chr10	120559352	120559524
chr10	120576921	120577390
chr10	120582381	120582816
chr10	120582816	120583110
chr10	120584601	120584869
chr10	120584869	120585058
chr10	120586473	120586674
chr10	120596547	120596762
chr10	120602635	120602797
chr10	120602820	120603509
chr10	120606258	120606564
chr10	120609685	120610019
chr10	120610019	120610470
chr10	120616185	120616593
chr10	120616593	120616980
chr10	120616980	120617233

chr10	120623965	120624630
chr10	120627964	120628571
chr10	120632130	120632272
chr10	120632272	120632444
chr10	120635725	120636102
chr10	120636102	120636575
chr10	120646316	120646477
chr10	120650512	120650674
chr10	120653506	120653675
chr10	120665662	120665957
chr10	120666385	120666786
chr10	120666786	120667030
chr10	120667030	120667312
chr10	120691513	120691749
chr10	120691749	120692014
chr10	120692014	120692382
chr10	120696573	120696865
chr10	120701532	120701730
chr10	120701856	120702265
chr10	120712003	120712211
chr10	120716521	120716698
chr10	120717026	120717814
chr10	120720139	120720290
chr10	120722528	120722806
chr10	120765180	120765466
chr10	120765466	120765681
chr10	120777526	120777907
chr10	120799598	120799772
chr10	120807796	120808038
chr10	120809094	120809252
chr10	120810172	120810414
chr10	120810414	120810671
chr10	120840867	120841051
chr10	120841051	120841256
chr10	120847296	120847547
chr10	120857801	120857988
chr10	120861661	120862032
chr10	120864520	120864786
chr10	120872492	120872680
chr10	120873636	120873862
chr10	120873862	120874161
chr10	120875743	120876014

chr10	120876046	120876198
chr10	120876227	120876495
chr10	120876495	120876701
chr10	120876968	120877198
chr10	120895769	120896095
chr10	120896095	120896278
chr10	120898326	120898799
chr10	120898799	120899479
chr10	120903964	120904183
chr10	120912078	120912269
chr10	120921632	120921792
chr10	120961668	120961833
chr10	120976987	120977303
chr10	120978209	120978531
chr10	120978740	120978998
chr10	120978998	120979207
chr10	120979220	120979757
chr10	120990838	120991041
chr10	120996967	120997333
chr10	120997665	120997825
chr10	121016512	121016762
chr10	121033167	121033396
chr10	121033396	121033690
chr10	121033810	121034080
chr10	121034080	121034296
chr10	121034296	121034525
chr10	121055507	121055809
chr10	121057019	121057282
chr10	121059475	121059625
chr10	121122532	121122682
chr10	121136200	121136420
chr10	121142900	121143100
chr10	121173937	121174361
chr10	121252871	121253056
chr10	121256511	121256765
chr10	121259979	121260230
chr10	121293944	121294533
chr10	121309212	121309680
chr10	121309816	121310230
chr10	121310230	121310517
chr10	121310517	121310737
chr10	121310737	121310943

chr10	121310943	121311205
chr10	121321660	121321865
chr10	121331058	121331262
chr10	121334649	121334945
chr10	121350612	121351022
chr10	121351022	121351314
chr10	121351314	121351623
chr10	121364737	121365181
chr10	121365181	121365412
chr10	121365412	121365696
chr10	121365750	121366027
chr10	121376039	121376549
chr10	121376549	121376894
chr10	121376894	121377305
chr10	121389484	121389829
chr10	121390782	121390944
chr10	121395966	121396116
chr10	121403328	121403550
chr10	121409387	121409537
chr10	121409787	121410053
chr10	121419822	121420088
chr10	121420088	121420629
chr10	121421100	121421310
chr10	121422374	121422555
chr10	121425872	121426059
chr10	121426608	121427107
chr10	121427107	121427456
chr10	121427543	121427745
chr10	121428545	121428720
chr10	121429134	121429410
chr10	121433032	121433347
chr10	121435688	121435881
chr10	121436214	121436477
chr10	121436849	121437057
chr10	121439225	121439440
chr10	121443645	121444489
chr10	121454753	121455151
chr10	121455151	121455510
chr10	121455510	121455832
chr10	121461378	121461868
chr10	121461868	121462111
chr10	121462111	121462301

chr10	121465021	121465445
chr10	121474050	121474290
chr10	121475499	121475742
chr10	121475742	121476073
chr10	121476073	121476399
chr10	121476399	121476667
chr10	121483541	121484002
chr10	121493380	121493719
chr10	121496793	121496943
chr10	121498525	121498675
chr10	121499065	121499328
chr10	121500046	121500430
chr10	121505077	121505636
chr10	121505675	121505832
chr10	121509833	121510057
chr10	121510142	121510389
chr10	121510389	121510664
chr10	121515264	121515415
chr10	121516280	121516569
chr10	121520838	121521678
chr10	121533244	121533465
chr10	121536203	121536512
chr10	121542246	121542497
chr10	121556290	121556574
chr10	121576664	121577073
chr10	121577073	121577371
chr10	121577371	121577632
chr10	121578791	121579241
chr10	121579254	121579622
chr10	121579622	121579957
chr10	121579957	121580252
chr10	121586034	121586346
chr10	121586346	121586666
chr10	121586666	121587120
chr10	121587140	121587342
chr10	121604981	121605281
chr10	121615275	121615666
chr10	121620764	121620946
chr10	121620990	121621445
chr10	121625836	121626235
chr10	121626235	121626887
chr10	121640011	121640240

chr10	121640294	121640444
chr10	121640462	121640758
chr10	121641519	121641793
chr10	121641793	121642177
chr10	121642262	121642520
chr10	121689097	121689257
chr10	121689628	121689907
chr10	121689907	121690213
chr10	121701389	121701549
chr10	121705807	121706052
chr10	121725084	121725244
chr10	121727274	121727436
chr10	121730652	121730869
chr10	121739520	121740132
chr10	121740132	121740507
chr10	121763150	121763323
chr10	121770629	121770893
chr10	121770895	121771213
chr10	121771261	121771411
chr10	121787218	121787437
chr10	121788615	121788865
chr10	121788865	121789138
chr10	121796046	121796408
chr10	121796408	121796899
chr10	121802324	121802774
chr10	121821301	121821497
chr10	121822310	121822637
chr10	121822637	121823227
chr10	121856116	121856483
chr10	121890149	121890315
chr10	121902861	121903028
chr10	121903780	121904773
chr10	121918675	121918993
chr10	121961201	121961425
chr10	121961525	121961856
chr10	121962130	121962541
chr10	121968965	121969184
chr10	121972249	121972675
chr10	121979271	121979588
chr10	121979588	121979818
chr10	121994280	121994452
chr10	122038635	122038884

chr10	122043317	122043571
chr10	122045904	122046179
chr10	122046779	122047169
chr10	122047169	122047649
chr10	122047649	122048142
chr10	122051874	122052164
chr10	122054628	122054858
chr10	122096540	122096782
chr10	122096782	122097086
chr10	122097086	122097794
chr10	122138119	122138269
chr10	122145727	122145973
chr10	122167498	122167766
chr10	122167766	122168104
chr10	122172696	122172936
chr10	122273790	122274185
chr10	122274298	122274465
chr10	122274926	122275222
chr10	122275222	122275488
chr10	122275569	122275772
chr10	122276776	122277014
chr10	122289622	122289815
chr10	122308778	122309014
chr10	122340789	122340939
chr10	122408829	122409004
chr10	122409081	122409327
chr10	122448887	122449104
chr10	122489602	122489814
chr10	122522740	122522998
chr10	122529459	122529609
chr10	122544228	122544378
chr10	122551656	122551884
chr10	122553140	122553412
chr10	122553913	122554156
chr10	122678498	122678883
chr10	122678883	122679201
chr10	122679201	122679715
chr10	122762765	122763043
chr10	122777022	122777324
chr10	122777482	122777900
chr10	122777900	122778260
chr10	122786078	122786238

chr10	122792810	122792966
chr10	122794375	122794618
chr10	122794618	122794995
chr10	122798000	122798227
chr10	122803970	122804233
chr10	122804602	122804752
chr10	122807128	122807401
chr10	122807401	122807703
chr10	122815115	122815457
chr10	122828165	122828510
chr10	122854142	122854494
chr10	122860474	122860670
chr10	122866631	122866783
chr10	122870101	122870416
chr10	122870416	122870655
chr10	122886138	122886483
chr10	122886483	122886928
chr10	122891438	122891906
chr10	122893436	122893856
chr10	122894734	122895101
chr10	122895170	122895339
chr10	122895539	122895764
chr10	122912854	122913234
chr10	122928742	122929063
chr10	122966343	122966915
chr10	122969802	122969962
chr10	122970048	122970215
chr10	122982956	122983223
chr10	122985238	122985443
chr10	122985534	122985983
chr10	122985983	122986284
chr10	122986284	122986641
chr10	122987759	122987988
chr10	123004567	123004720
chr10	123004980	123005228
chr10	123005302	123005530
chr10	123005555	123005747
chr10	123011292	123011697
chr10	123011697	123012106
chr10	123045229	123045412
chr10	123075751	123076018
chr10	123076018	123076288

chr10	123076288	123076606
chr10	123076606	123076850
chr10	123091828	123092107
chr10	123110685	123110835
chr10	123121106	123121315
chr10	123175866	123176081
chr10	123183316	123183558
chr10	123186287	123186441
chr10	123196036	123196218
chr10	123196265	123196640
chr10	123196640	123196846
chr10	123196846	123197133
chr10	123197133	123197399
chr10	123197399	123197620
chr10	123215818	123216021
chr10	123232697	123232942
chr10	123256041	123256210
chr10	123263908	123264244
chr10	123264449	123264731
chr10	123264731	123265219
chr10	123265301	123265615
chr10	123268693	123268943
chr10	123341344	123341606
chr10	123348883	123349190
chr10	123420394	123420549
chr10	123516489	123516639
chr10	123536097	123536456
chr10	123566796	123567147
chr10	123716681	123716963
chr10	123818238	123818448
chr10	124087346	124087581
chr10	124138468	124138863
chr10	124143258	124143538
chr10	124168017	124168260
chr10	124214141	124214299
chr10	124288411	124288584
chr10	124370958	124371246
chr10	124372247	124372440
chr10	124606811	124606982
chr10	124845287	124845494
chr10	124853087	124853239
chr10	124853856	124854147

chr10	124913296	124913446
chr10	125109078	125109252
chr10	125171207	125171479
chr10	125507481	125507685
chr10	125663128	125663278
chr10	125680254	125680455
chr10	125692256	125692486
chr10	125725414	125725609
chr10	125854999	125855196
chr10	125855701	125855871
chr10	125903480	125903677
chr10	125904166	125904416
chr10	125917439	125917772
chr10	125938537	125938775
chr10	125938775	125939057
chr10	125961880	125962086
chr10	125965867	125966277
chr10	125966277	125966491
chr10	125966491	125966695
chr10	125966695	125966934
chr10	125994925	125995159
chr10	126262484	126262704
chr10	126266715	126266873
chr10	126298323	126298500
chr10	126330699	126330864
chr10	126681010	126681343
chr10	126746500	126746650
chr10	126821471	126821723
chr10	126833108	126833327
chr10	126840348	126840568
chr10	126849420	126849684
chr10	126863593	126863870
chr10	126869728	126869988
chr10	126900737	126901171
chr10	126901171	126901573
chr10	126901628	126901778
chr10	126905606	126905857
chr10	126905857	126906151
chr10	126918913	126919150
chr10	126919150	126919437
chr10	126924118	126924561
chr10	126928075	126928300

chr10	126928300	126928572
chr10	126928572	126928905
chr10	126935509	126935789
chr10	126935789	126936139
chr10	126936139	126936421
chr10	126937393	126937649
chr10	126947307	126947580
chr10	126947659	126947948
chr10	126948921	126949190
chr10	126950656	126950855
chr10	126953030	126953257
chr10	126957803	126958155
chr10	126962229	126962637
chr10	126972132	126972337
chr10	126978114	126978406
chr10	126978406	126978725
chr10	126978725	126979041
chr10	126979041	126979320
chr10	126979404	126979643
chr10	126979795	126979984
chr10	126980450	126980682
chr10	126984320	126984494
chr10	126984551	126984987
chr10	126990201	126990709
chr10	127002111	127002328
chr10	127002796	127003328
chr10	127003331	127003637
chr10	127015836	127016044
chr10	127016547	127016840
chr10	127030274	127030627
chr10	127030627	127031001
chr10	127033124	127033301
chr10	127040885	127041058
chr10	127041307	127041700
chr10	127041700	127042047
chr10	127042047	127042342
chr10	127042764	127043009
chr10	127049419	127049815
chr10	127049815	127050073
chr10	127050073	127050274
chr10	127058243	127058506
chr10	127060186	127060541

chr10	127062573	127062822
chr10	127063147	127063453
chr10	127063453	127063925
chr10	127068390	127068636
chr10	127069313	127069547
chr10	127069547	127069808
chr10	127069808	127070110
chr10	127070155	127070493
chr10	127070690	127070915
chr10	127078135	127078395
chr10	127079678	127079950
chr10	127082092	127082291
chr10	127083973	127084208
chr10	127088361	127088620
chr10	127090881	127091131
chr10	127091131	127091337
chr10	127091355	127091521
chr10	127091526	127091885
chr10	127094300	127094832
chr10	127094909	127095128
chr10	127103040	127103456
chr10	127114321	127114610
chr10	127115122	127115429
chr10	127120696	127120890
chr10	127120986	127121468
chr10	127122780	127123044
chr10	127123622	127123784
chr10	127125360	127125545
chr10	127126787	127127150
chr10	127129495	127129811
chr10	127133238	127133528
chr10	127134453	127134721
chr10	127134721	127134972
chr10	127144058	127144323
chr10	127144323	127144584
chr10	127144584	127144878
chr10	127144878	127145148
chr10	127145387	127145668
chr10	127149790	127150122
chr10	127150481	127150916
chr10	127150916	127151185
chr10	127151338	127151602

chr10	127162675	127163233
chr10	127164976	127165371
chr10	127165371	127165676
chr10	127165676	127165885
chr10	127165885	127166111
chr10	127166138	127166523
chr10	127167551	127167922
chr10	127167922	127168233
chr10	127170815	127171055
chr10	127171055	127171309
chr10	127177361	127177514
chr10	127177514	127177741
chr10	127177741	127178284
chr10	127178388	127178538
chr10	127180230	127180464
chr10	127183824	127184100
chr10	127188429	127188705
chr10	127188705	127189210
chr10	127189356	127189554
chr10	127195108	127195369
chr10	127195369	127195609
chr10	127195609	127195845
chr10	127195845	127196071
chr10	127196410	127196686
chr10	127200366	127200535
chr10	127201008	127201206
chr10	127210926	127211191
chr10	127211191	127211492
chr10	127211492	127211887
chr10	127217287	127217495
chr10	127218495	127218646
chr10	127226555	127226828
chr10	127228947	127229236
chr10	127233244	127233470
chr10	127242297	127242500
chr10	127242896	127243128
chr10	127244911	127245063
chr10	127255884	127256139
chr10	127256139	127256395
chr10	127263036	127263222
chr10	127265719	127265934
chr10	127266117	127266613

chr10	127266615	127266908
chr10	127266908	127267177
chr10	127276106	127276278
chr10	127277964	127278266
chr10	127279861	127280023
chr10	127288355	127288527
chr10	127288570	127288826
chr10	127288826	127289156
chr10	127289156	127289664
chr10	127289812	127290083
chr10	127290227	127290519
chr10	127290519	127290825
chr10	127290825	127291080
chr10	127291080	127291298
chr10	127296444	127296612
chr10	127297644	127297954
chr10	127298619	127298945
chr10	127300451	127300613
chr10	127302956	127303192
chr10	127305685	127305957
chr10	127311117	127311515
chr10	127311515	127311984
chr10	127312149	127312344
chr10	127312344	127312613
chr10	127318171	127318331
chr10	127318363	127318533
chr10	127322847	127323037
chr10	127324554	127324885
chr10	127326377	127326631
chr10	127327893	127328076
chr10	127332273	127332432
chr10	127333248	127333401
chr10	127336966	127337241
chr10	127338879	127339195
chr10	127339216	127339576
chr10	127339905	127340155
chr10	127341782	127342136
chr10	127342318	127342602
chr10	127350620	127351018
chr10	127353955	127354176
chr10	127380015	127380727
chr10	127380727	127380988

chr10	127380988	127381216
chr10	127381216	127381467
chr10	127391862	127392088
chr10	127413682	127413995
chr10	127414088	127414516
chr10	127420756	127420934
chr10	127425221	127425542
chr10	127425872	127426053
chr10	127430594	127430798
chr10	127453888	127454085
chr10	127479156	127479315
chr10	127481208	127481487
chr10	127481487	127481757
chr10	127481757	127482004
chr10	127485375	127485559
chr10	127495268	127495506
chr10	127496324	127496506
chr10	127499960	127500174
chr10	127500174	127500445
chr10	127500458	127500733
chr10	127500733	127500917
chr10	127500943	127501266
chr10	127501367	127501599
chr10	127501599	127501765
chr10	127508400	127508591
chr10	127508999	127509286
chr10	127509322	127509559
chr10	127511630	127511784
chr10	127511866	127512144
chr10	127512144	127512458
chr10	127512659	127513207
chr10	127513592	127513779
chr10	127515643	127515830
chr10	127521504	127521721
chr10	127521807	127522052
chr10	127522052	127522274
chr10	127522274	127522608
chr10	127526695	127527035
chr10	127527601	127527827
chr10	127528514	127528719
chr10	127534102	127534252
chr10	127534297	127534794

chr10	127534794	127535156
chr10	127535498	127535815
chr10	127536726	127536887
chr10	127537806	127538162
chr10	127543975	127544257
chr10	127546589	127546797
chr10	127555706	127556246
chr10	127557484	127557728
chr10	127557872	127558156
chr10	127558407	127558585
chr10	127562610	127562786
chr10	127562884	127563446
chr10	127564365	127564536
chr10	127569118	127569403
chr10	127569478	127569806
chr10	127570405	127570591
chr10	127576286	127577004
chr10	127583604	127584056
chr10	127584543	127584698
chr10	127586201	127586571
chr10	127591459	127591867
chr10	127592516	127592738
chr10	127592771	127593025
chr10	127596535	127597289
chr10	127597304	127597598
chr10	127604622	127604936
chr10	127605313	127605538
chr10	127607098	127607373
chr10	127608071	127608266
chr10	127608266	127608430
chr10	127611431	127611677
chr10	127616332	127616482
chr10	127621121	127621639
chr10	127621639	127622024
chr10	127622024	127622367
chr10	127631715	127632236
chr10	127636060	127636210
chr10	127636667	127636911
chr10	127641812	127642280
chr10	127642280	127642777
chr10	127642777	127643340
chr10	127643364	127643530

chr10	127643530	127643718
chr10	127643718	127643936
chr10	127643936	127644195
chr10	127657795	127658129
chr10	127658213	127658429
chr10	127661923	127662160
chr10	127662160	127662442
chr10	127662473	127662668
chr10	127666403	127666897
chr10	127666897	127667164
chr10	127667164	127667474
chr10	127667474	127667745
chr10	127667745	127668093
chr10	127668093	127668478
chr10	127674699	127675140
chr10	127675140	127675355
chr10	127676721	127677170
chr10	127677170	127677491
chr10	127678013	127678258
chr10	127694813	127694968
chr10	127703451	127703646
chr10	127711339	127711492
chr10	127713213	127713426
chr10	127729097	127729306
chr10	127736212	127736449
chr10	127738170	127738330
chr10	127738368	127738625
chr10	127738625	127738953
chr10	127739006	127739266
chr10	127739266	127739670
chr10	127739670	127740122
chr10	127744599	127744837
chr10	127744837	127745081
chr10	127759484	127759774
chr10	127763890	127764086
chr10	127803101	127803251
chr10	127825476	127825712
chr10	127827990	127828284
chr10	127872370	127872788
chr10	127877667	127878069
chr10	127878197	127878786
chr10	127879213	127879465

chr10	127893615	127894079
chr10	127899930	127900202
chr10	127904445	127904595
chr10	127904964	127905514
chr10	127914706	127915001
chr10	127922258	127922726
chr10	127924640	127924992
chr10	127924992	127925455
chr10	127939744	127940062
chr10	127945348	127945816
chr10	127949834	127949995
chr10	127955125	127955357
chr10	127956164	127956479
chr10	127956479	127956774
chr10	127960897	127961178
chr10	127968571	127968785
chr10	128008273	128008597
chr10	128008615	128008805
chr10	128015013	128015284
chr10	128015284	128015576
chr10	128015576	128015836
chr10	128035228	128035763
chr10	128035769	128036030
chr10	128036030	128036278
chr10	128036819	128037115
chr10	128039919	128040161
chr10	128041075	128041301
chr10	128046079	128046327
chr10	128049813	128050080
chr10	128050384	128050829
chr10	128050995	128051150
chr10	128058357	128058935
chr10	128058935	128059517
chr10	128059606	128059975
chr10	128067698	128067909
chr10	128070492	128070718
chr10	128077849	128077999
chr10	128082893	128083166
chr10	128083166	128083384
chr10	128083384	128083629
chr10	128090981	128091292
chr10	128091442	128091692

chr10	128091723	128091901
chr10	128092554	128092856
chr10	128092856	128093190
chr10	128093190	128093481
chr10	128093937	128094093
chr10	128094645	128094949
chr10	128096666	128096982
chr10	128097177	128097363
chr10	128114086	128114251
chr10	128123346	128123568
chr10	128124008	128124165
chr10	128125908	128126058
chr10	128127549	128127732
chr10	128143610	128144290
chr10	128149571	128150023
chr10	128150713	128150945
chr10	128150945	128151323
chr10	128154006	128154272
chr10	128166881	128167488
chr10	128175558	128175819
chr10	128175947	128176148
chr10	128176148	128176453
chr10	128176453	128176799
chr10	128177403	128177677
chr10	128184435	128184619
chr10	128185440	128185949
chr10	128185949	128186293
chr10	128188599	128188816
chr10	128193966	128194221
chr10	128194221	128194503
chr10	128194503	128194890
chr10	128194890	128195257
chr10	128195257	128195465
chr10	128203520	128203905
chr10	128205002	128205362
chr10	128210967	128211327
chr10	128211527	128212152
chr10	128212806	128213079
chr10	128213119	128213281
chr10	128220953	128221215
chr10	128221775	128221935
chr10	128231727	128231882

chr10	128231888	128232193
chr10	128232193	128232448
chr10	128232448	128232702
chr10	128232702	128233007
chr10	128253735	128253980
chr10	128253980	128254226
chr10	128256008	128256310
chr10	128256310	128256717
chr10	128270179	128270610
chr10	128270610	128270927
chr10	128281805	128281995
chr10	128283909	128284120
chr10	128284120	128284451
chr10	128297611	128298060
chr10	128298060	128298450
chr10	128303114	128303470
chr10	128303470	128303795
chr10	128303795	128304037
chr10	128304037	128304234
chr10	128309760	128309953
chr10	128322145	128323148
chr10	128327727	128327909
chr10	128337254	128337501
chr10	128337501	128337835
chr10	128337835	128338348
chr10	128338427	128338726
chr10	128344305	128344455
chr10	128344604	128345440
chr10	128358849	128359134
chr10	128362649	128362856
chr10	128368256	128368838
chr10	128368838	128369343
chr10	128376380	128376602
chr10	128376655	128376904
chr10	128376904	128377169
chr10	128377169	128377425
chr10	128377425	128377666
chr10	128382609	128382843
chr10	128385837	128386030
chr10	128387596	128387842
chr10	128388535	128388697
chr10	128394425	128394582

chr10	128395898	128396100
chr10	128396326	128396481
chr10	128396890	128397086
chr10	128397086	128397261
chr10	128397471	128397722
chr10	128409674	128410080
chr10	128411082	128411410
chr10	128411410	128411767
chr10	128411767	128412105
chr10	128417438	128417663
chr10	128418367	128418868
chr10	128422530	128422745
chr10	128434673	128435099
chr10	128440498	128440738
chr10	128445748	128445910
chr10	128449022	128449218
chr10	128451077	128451227
chr10	128455385	128455569
chr10	128458313	128458891
chr10	128458891	128459341
chr10	128459341	128459986
chr10	128491556	128491707
chr10	128492897	128493171
chr10	128493377	128493731
chr10	128493731	128494091
chr10	128494151	128494498
chr10	128498599	128499068
chr10	128499107	128499297
chr10	128499310	128499460
chr10	128504744	128504958
chr10	128504969	128505253
chr10	128509413	128509568
chr10	128515136	128515367
chr10	128516734	128516987
chr10	128518901	128519202
chr10	128524786	128525077
chr10	128525085	128525417
chr10	128525417	128525792
chr10	128525792	128526298
chr10	128536106	128536335
chr10	128536335	128536681
chr10	128536681	128537010

chr10	128537713	128537998
chr10	128545655	128545831
chr10	128546377	128546533
chr10	128547204	128547667
chr10	128547667	128548098
chr10	128548936	128549491
chr10	128555876	128556083
chr10	128556330	128556775
chr10	128557097	128557251
chr10	128557572	128557738
chr10	128565268	128565448
chr10	128565448	128565656
chr10	128565812	128566216
chr10	128566216	128566620
chr10	128568156	128568340
chr10	128575150	128575389
chr10	128583674	128584044
chr10	128584127	128584447
chr10	128584447	128584787
chr10	128588820	128589007
chr10	128589149	128589344
chr10	128589344	128589582
chr10	128589582	128589840
chr10	128589840	128590098
chr10	128590240	128590484
chr10	128590484	128590715
chr10	128599281	128599604
chr10	128606479	128606893
chr10	128612695	128612971
chr10	128612971	128613174
chr10	128613174	128613537
chr10	128622108	128622301
chr10	128625964	128626157
chr10	128626195	128626557
chr10	128626687	128626964
chr10	128626964	128627185
chr10	128631752	128631912
chr10	128636530	128636713
chr10	128641872	128642388
chr10	128645727	128646048
chr10	128646272	128646424
chr10	128655414	128655569

chr10	128659117	128659279
chr10	128665410	128665622
chr10	128665819	128666163
chr10	128668870	128669128
chr10	128672259	128672422
chr10	128673526	128673798
chr10	128673798	128674060
chr10	128674060	128674451
chr10	128695723	128695958
chr10	128695958	128696244
chr10	128696244	128696771
chr10	128704313	128704713
chr10	128704713	128704972
chr10	128704972	128705298
chr10	128716090	128716285
chr10	128716298	128716518
chr10	128722011	128722237
chr10	128722751	128722956
chr10	128730957	128731386
chr10	128731791	128732110
chr10	128732110	128732339
chr10	128737457	128737792
chr10	128742702	128742895
chr10	128743771	128743952
chr10	128743952	128744156
chr10	128747511	128748121
chr10	128748121	128748651
chr10	128757435	128757590
chr10	128763686	128763862
chr10	128773036	128773204
chr10	128773616	128774018
chr10	128779441	128779886
chr10	128780267	128780674
chr10	128786832	128787109
chr10	128787109	128787351
chr10	128787602	128787796
chr10	128792131	128792342
chr10	128795198	128795392
chr10	128803837	128804257
chr10	128804257	128804604
chr10	128804837	128805215
chr10	128805215	128805521

chr10	128805521	128805851
chr10	128821329	128821754
chr10	128821754	128822068
chr10	128822232	128822402
chr10	128878379	128878533
chr10	128890642	128890827
chr10	128890827	128891002
chr10	128891033	128891555
chr10	128891555	128891794
chr10	128891794	128892068
chr10	128895512	128895769
chr10	128900652	128901037
chr10	128901504	128901659
chr10	128903437	128903742
chr10	128909385	128909995
chr10	128909995	128910296
chr10	128910296	128910602
chr10	128910602	128910909
chr10	128911043	128911260
chr10	128913277	128913568
chr10	128921854	128922191
chr10	128922933	128923318
chr10	128923318	128923718
chr10	128928575	128928696
chr10	128928696	128928769
chr10	128931571	128931786
chr10	128933558	128933986
chr10	128933986	128934334
chr10	128939068	128939265
chr10	128945732	128945986
chr10	128948831	128949099
chr10	128950273	128950733
chr10	128956195	128956568
chr10	128967915	128968261
chr10	128968261	128968619
chr10	128968776	128969003
chr10	128969063	128969217
chr10	128972941	128973236
chr10	128977626	128977834
chr10	128980506	128981001
chr10	128981001	128981541
chr10	128989992	128990195

chr10	128990195	128990438
chr10	128991168	128991355
chr10	128992505	128992731
chr10	128996567	128997213
chr10	129000485	129000791
chr10	129004201	129004552
chr10	129004552	129004811
chr10	129005320	129005580
chr10	129008948	129009132
chr10	129011383	129011819
chr10	129023477	129023646
chr10	129023658	129023827
chr10	129026085	129026331
chr10	129026355	129026580
chr10	129101452	129101608
chr10	129107142	129107378
chr10	129510636	129510944
chr10	129735403	129735607
chr10	129973128	129973278
chr10	130092940	130093090
chr10	130228380	130228539
chr10	130280156	130280313
chr10	130313607	130313819
chr10	130412252	130412506
chr10	130434248	130434534
chr10	130473294	130473490
chr10	130541855	130542150
chr10	130542150	130542661
chr10	130566456	130566792
chr10	130594371	130594688
chr10	130594688	130595061
chr11	3100401	3100615
chr11	3101170	3101378
chr11	3101455	3101647
chr11	3101647	3101786
chr11	3101976	3102195
chr11	3103908	3104102
chr11	3112541	3112702
chr11	3112879	3113064
chr11	3114513	3114737
chr11	3123489	3123773
chr11	3123773	3124025

chr11	3124025	3124272
chr11	3125478	3125761
chr11	3127316	3127631
chr11	3132014	3132222
chr11	3132888	3133194
chr11	3133194	3133397
chr11	3133562	3133779
chr11	3133796	3134072
chr11	3134353	3134509
chr11	3134640	3134930
chr11	3134964	3135413
chr11	3135434	3135845
chr11	3136778	3137040
chr11	3137404	3137556
chr11	3140796	3141205
chr11	3142415	3142785
chr11	3143063	3143477
chr11	3143671	3143848
chr11	3144111	3144338
chr11	3144456	3144623
chr11	3144627	3144798
chr11	3144798	3144985
chr11	3146050	3146324
chr11	3146738	3146973
chr11	3146973	3147171
chr11	3151308	3151710
chr11	3151730	3151974
chr11	3151974	3152228
chr11	3152388	3152627
chr11	3153764	3153919
chr11	3157826	3158026
chr11	3158263	3158419
chr11	3159286	3159651
chr11	3159651	3160066
chr11	3161139	3161615
chr11	3162865	3163050
chr11	3163715	3163982
chr11	3164485	3164647
chr11	3165462	3165683
chr11	3167298	3167465
chr11	3167538	3167705
chr11	3168584	3169063

chr11	3170748	3170933
chr11	3171276	3171523
chr11	3174379	3174545
chr11	3177131	3177320
chr11	3177320	3177489
chr11	3177489	3177663
chr11	3177816	3178100
chr11	3179434	3179730
chr11	3179730	3179927
chr11	3180030	3180377
chr11	3181243	3181480
chr11	3184454	3184607
chr11	3184607	3184791
chr11	3184791	3184952
chr11	3184952	3185141
chr11	3185301	3185600
chr11	3185852	3186060
chr11	3187873	3188065
chr11	3188712	3188935
chr11	3192918	3193266
chr11	3193266	3193639
chr11	3195587	3195958
chr11	3195974	3196315
chr11	3196414	3196673
chr11	3196673	3196902
chr11	3199021	3199363
chr11	3202007	3202206
chr11	3202206	3202467
chr11	3202501	3202805
chr11	3202805	3203095
chr11	3203095	3203358
chr11	3208796	3209029
chr11	3212488	3212863
chr11	3237827	3237985
chr11	3243807	3244165
chr11	3247420	3247724
chr11	3265663	3265918
chr11	3265962	3266318
chr11	3266318	3266830
chr11	3288532	3288728
chr11	3289044	3289292
chr11	3289292	3289475

chr11	3289475	3289807
chr11	3289843	3290142
chr11	3290142	3290411
chr11	3290411	3290653
chr11	3290659	3290887
chr11	3292085	3292337
chr11	3292337	3292555
chr11	3292636	3292958
chr11	3298023	3298304
chr11	3300963	3301483
chr11	3302894	3303222
chr11	3311998	3312247
chr11	3313592	3313742
chr11	3314085	3314559
chr11	3331765	3332204
chr11	3332204	3332698
chr11	3337618	3338122
chr11	3347144	3347411
chr11	3348334	3348828
chr11	3348828	3349232
chr11	3349263	3349576
chr11	3353028	3353279
chr11	3368936	3369241
chr11	3369241	3369537
chr11	3371293	3371665
chr11	3372212	3372473
chr11	3374226	3374497
chr11	3374497	3374739
chr11	3374814	3375104
chr11	3377296	3377516
chr11	3386909	3387508
chr11	3390780	3391198
chr11	3392530	3392817
chr11	3392817	3393127
chr11	3393177	3393439
chr11	3393439	3393670
chr11	3393933	3394091
chr11	3396135	3396285
chr11	3403161	3403343
chr11	3407575	3407906
chr11	3408115	3408283
chr11	3408463	3408692

chr11	3408712	3409042
chr11	3409042	3409402
chr11	3409402	3409643
chr11	3427817	3428006
chr11	3442756	3443032
chr11	3443069	3443611
chr11	3451739	3452206
chr11	3452206	3452805
chr11	3452847	3453062
chr11	3453545	3453783
chr11	3472528	3472814
chr11	3474805	3475030
chr11	3477463	3477660
chr11	3481786	3482044
chr11	3482044	3482339
chr11	3482339	3482697
chr11	3487986	3488208
chr11	3488996	3489173
chr11	3490155	3490378
chr11	3494770	3495153
chr11	3504697	3504978
chr11	3505542	3505699
chr11	3508320	3508504
chr11	3514481	3514762
chr11	3514762	3515155
chr11	3517528	3517782
chr11	3519354	3519507
chr11	3526295	3526465
chr11	3535366	3535671
chr11	3535671	3535944
chr11	3538339	3538651
chr11	3538651	3538897
chr11	3538925	3539533
chr11	3539540	3539772
chr11	3558023	3558266
chr11	3560076	3560649
chr11	3565881	3566430
chr11	3567790	3568278
chr11	3573132	3573453
chr11	3576781	3577166
chr11	3577166	3577645
chr11	3580331	3580661

chr11	3585899	3586606
chr11	3587444	3587627
chr11	3592285	3592440
chr11	3602005	3602299
chr11	3602299	3602617
chr11	3602617	3603000
chr11	3609692	3609896
chr11	3622733	3623228
chr11	3648512	3648917
chr11	3648917	3649469
chr11	3649469	3649953
chr11	3676379	3676676
chr11	3683991	3684180
chr11	3684209	3684686
chr11	3684692	3684865
chr11	3692573	3692786
chr11	3692786	3692963
chr11	3700730	3700880
chr11	3702060	3702540
chr11	3703219	3703490
chr11	3703599	3703749
chr11	3705086	3705263
chr11	3714487	3714772
chr11	3727985	3728185
chr11	3729731	3730080
chr11	3730312	3730575
chr11	3733206	3733373
chr11	3738805	3739188
chr11	3747304	3747517
chr11	3749961	3750173
chr11	3753931	3754081
chr11	3754784	3755012
chr11	3755433	3755585
chr11	3761899	3762440
chr11	3764356	3764711
chr11	3767059	3767218
chr11	3771497	3771659
chr11	3773827	3774099
chr11	3774426	3775111
chr11	3779037	3779245
chr11	3786652	3786911
chr11	3787806	3788160

chr11	3790014	3790243
chr11	3790847	3791015
chr11	3802129	3802403
chr11	3805437	3805664
chr11	3807978	3808167
chr11	3808183	3808473
chr11	3824533	3824929
chr11	3832268	3832561
chr11	3832561	3832871
chr11	3832871	3833129
chr11	3834468	3834624
chr11	3844700	3845095
chr11	3847774	3847924
chr11	3848060	3848359
chr11	3853076	3853252
chr11	3853509	3854057
chr11	3857382	3857545
chr11	3859715	3860234
chr11	3860392	3860676
chr11	3863643	3864095
chr11	3870116	3870396
chr11	3871673	3872078
chr11	3873925	3874158
chr11	3878658	3879037
chr11	3879899	3880182
chr11	3887257	3887761
chr11	3887761	3888019
chr11	3893316	3893477
chr11	3894080	3894366
chr11	3894454	3894875
chr11	3894875	3895455
chr11	3895469	3895821
chr11	3899871	3900208
chr11	3900307	3900709
chr11	3902536	3902798
chr11	3903043	3903420
chr11	3905390	3905788
chr11	3905788	3906101
chr11	3908892	3909128
chr11	3909295	3909484
chr11	3911074	3911249
chr11	3911561	3911739

chr11	3913316	3913624
chr11	3913700	3914154
chr11	3914154	3914528
chr11	3914528	3914841
chr11	3916099	3916409
chr11	3921217	3921342
chr11	3921342	3921531
chr11	3921566	3921841
chr11	3929983	3930137
chr11	3930137	3930307
chr11	3931641	3932225
chr11	3963353	3963547
chr11	3963575	3963826
chr11	3963893	3964100
chr11	3964100	3964311
chr11	3964311	3964541
chr11	3964543	3964694
chr11	3969948	3970163
chr11	3973311	3973461
chr11	3982829	3983006
chr11	3983266	3983522
chr11	3983522	3983906
chr11	3983906	3984359
chr11	3984399	3984666
chr11	3989973	3990123
chr11	3993607	3993887
chr11	3995468	3995901
chr11	4001653	4002014
chr11	4004940	4005230
chr11	4007720	4007873
chr11	4012739	4012899
chr11	4033540	4033718
chr11	4035307	4035813
chr11	4035999	4036294
chr11	4043767	4044027
chr11	4045788	4046210
chr11	4053667	4053989
chr11	4061013	4061192
chr11	4063088	4063370
chr11	4069719	4069949
chr11	4080297	4080563
chr11	4083398	4083679

chr11	4095169	4095495
chr11	4096297	4096478
chr11	4096482	4097008
chr11	4101826	4102299
chr11	4111021	4111293
chr11	4117776	4117946
chr11	4118035	4118549
chr11	4118549	4118949
chr11	4118949	4119227
chr11	4124002	4124394
chr11	4134948	4135309
chr11	4135309	4135882
chr11	4142404	4142669
chr11	4148234	4148405
chr11	4159947	4160448
chr11	4160448	4160754
chr11	4161171	4161459
chr11	4164777	4164997
chr11	4176628	4176868
chr11	4182131	4182410
chr11	4186447	4186936
chr11	4186936	4187358
chr11	4190022	4190224
chr11	4190646	4190861
chr11	4195564	4196047
chr11	4196762	4197058
chr11	4198815	4199428
chr11	4205200	4205371
chr11	4206028	4206266
chr11	4209634	4209880
chr11	4210750	4211052
chr11	4217811	4218696
chr11	4219345	4219538
chr11	4219538	4219784
chr11	4220522	4220780
chr11	4229594	4230117
chr11	4230271	4230431
chr11	4235381	4235603
chr11	4237312	4237576
chr11	4240138	4240536
chr11	4249248	4249550
chr11	4251589	4251977

chr11	4257111	4257461
chr11	4258849	4259089
chr11	4260519	4260933
chr11	4261154	4261730
chr11	4261942	4262350
chr11	4262902	4263235
chr11	4263239	4263399
chr11	4264120	4264401
chr11	4264865	4265300
chr11	4271300	4271455
chr11	4271578	4271823
chr11	4272171	4272406
chr11	4283652	4284190
chr11	4291798	4292047
chr11	4296617	4297174
chr11	4297871	4298146
chr11	4298601	4298953
chr11	4299177	4299571
chr11	4299717	4300071
chr11	4302386	4302571
chr11	4303092	4303493
chr11	4308157	4308518
chr11	4308518	4308785
chr11	4308785	4309044
chr11	4309044	4309401
chr11	4309680	4309942
chr11	4316821	4317078
chr11	4317377	4317708
chr11	4318112	4318334
chr11	4322080	4322230
chr11	4335374	4335704
chr11	4335704	4336068
chr11	4336219	4336599
chr11	4336794	4337238
chr11	4344473	4344704
chr11	4345233	4345542
chr11	4398346	4398553
chr11	4398553	4398839
chr11	4400527	4400726
chr11	4401166	4401594
chr11	4401594	4401820
chr11	4403372	4403762

chr11	4404315	4404644
chr11	4428481	4428976
chr11	4438298	4438473
chr11	4441167	4441362
chr11	4445215	4445365
chr11	4451182	4451443
chr11	4451495	4451647
chr11	4461286	4461501
chr11	4466557	4466746
chr11	4469228	4469392
chr11	4485318	4485631
chr11	4487391	4487553
chr11	4507213	4507389
chr11	4507475	4507799
chr11	4578512	4579043
chr11	4579043	4579415
chr11	4594071	4594372
chr11	4594372	4594716
chr11	4594716	4595061
chr11	4602373	4602591
chr11	4605532	4605730
chr11	4606485	4606950
chr11	4621909	4622195
chr11	4622730	4622919
chr11	4628281	4628511
chr11	4633444	4633765
chr11	4637456	4637908
chr11	4637908	4638249
chr11	4638341	4638656
chr11	4659896	4660165
chr11	4661368	4661769
chr11	4672257	4672547
chr11	4674642	4674893
chr11	4679353	4679568
chr11	4685750	4685974
chr11	4697846	4697996
chr11	4700790	4701136
chr11	4703992	4704224
chr11	4704224	4704490
chr11	4704509	4704804
chr11	4712890	4713159
chr11	4718621	4718889

chr11	4720028	4720235
chr11	4720424	4720668
chr11	4726548	4726756
chr11	4728115	4728283
chr11	4729788	4730005
chr11	4730160	4730471
chr11	4730471	4730768
chr11	4736464	4736626
chr11	4740608	4740858
chr11	4744793	4745041
chr11	4747003	4747227
chr11	4750316	4750655
chr11	4753463	4753846
chr11	4759074	4759293
chr11	4771900	4772078
chr11	4772986	4773159
chr11	4815176	4815369
chr11	4822596	4822773
chr11	4826213	4826664
chr11	4836822	4837103
chr11	4843521	4843793
chr11	4848732	4848968
chr11	4848968	4849376
chr11	4849376	4849905
chr11	4858488	4858759
chr11	4860053	4860340
chr11	4860417	4860608
chr11	4861070	4861328
chr11	4865955	4866193
chr11	4867263	4867418
chr11	4867423	4867676
chr11	4873781	4874140
chr11	4874140	4874481
chr11	4874484	4874919
chr11	4883070	4883344
chr11	4883372	4883527
chr11	4883579	4883756
chr11	4883932	4884118
chr11	4885478	4885647
chr11	4888923	4889075
chr11	4894937	4895185
chr11	4895185	4895481

chr11	4895481	4895844
chr11	4915972	4916185
chr11	4916185	4916450
chr11	4946907	4947323
chr11	4947323	4947681
chr11	4947681	4948055
chr11	4949305	4949644
chr11	4954034	4954219
chr11	4954267	4954452
chr11	4973229	4973520
chr11	4973520	4973760
chr11	4975704	4975944
chr11	4977375	4977617
chr11	4979629	4979858
chr11	4979858	4980084
chr11	4985657	4985888
chr11	4986482	4986632
chr11	4986648	4987060
chr11	4987163	4987396
chr11	4987488	4987643
chr11	4998012	4998355
chr11	4998355	4998690
chr11	4998889	4999085
chr11	5009755	5010064
chr11	5015750	5016096
chr11	5016220	5016720
chr11	5022230	5022382
chr11	5042913	5043531
chr11	5046614	5046871
chr11	5048879	5049071
chr11	5054188	5054449
chr11	5058045	5058195
chr11	5061510	5062226
chr11	5064158	5064354
chr11	5064727	5064950
chr11	5065013	5065219
chr11	5065219	5065494
chr11	5065494	5065852
chr11	5070850	5071000
chr11	5075006	5075196
chr11	5075196	5075346
chr11	5075844	5076113

chr11	5076126	5076305
chr11	5076969	5077128
chr11	5087941	5088155
chr11	5091760	5092044
chr11	5098576	5098928
chr11	5098928	5099246
chr11	5099246	5099534
chr11	5099534	5099750
chr11	5099750	5099981
chr11	5099981	5100236
chr11	5100429	5100616
chr11	5114726	5114920
chr11	5114946	5115412
chr11	5125591	5125949
chr11	5131830	5132139
chr11	5140575	5141014
chr11	5141014	5141335
chr11	5141359	5141509
chr11	5146635	5147063
chr11	5151715	5151887
chr11	5152029	5152329
chr11	5152484	5152668
chr11	5159840	5160030
chr11	5160030	5160209
chr11	5160798	5161031
chr11	5168313	5168553
chr11	5168553	5168785
chr11	5168785	5169008
chr11	5169008	5169309
chr11	5169309	5169658
chr11	5169658	5169890
chr11	5169980	5170432
chr11	5181897	5182195
chr11	5182527	5182695
chr11	5182813	5183155
chr11	5186200	5186425
chr11	5186425	5186601
chr11	5193345	5193561
chr11	5197063	5197278
chr11	5197278	5197491
chr11	5198967	5199177
chr11	5219038	5219254

chr11	5219310	5219704
chr11	5219971	5220170
chr11	5227216	5227471
chr11	5227471	5227985
chr11	5228488	5228710
chr11	5237711	5238145
chr11	5240326	5240627
chr11	5242063	5242492
chr11	5243828	5244015
chr11	5244046	5244293
chr11	5244293	5244573
chr11	5244810	5245185
chr11	5245277	5245508
chr11	5245508	5245758
chr11	5245798	5246005
chr11	5251114	5251375
chr11	5252479	5252788
chr11	5253138	5253351
chr11	5261015	5261362
chr11	5261464	5261787
chr11	5261787	5262151
chr11	5281956	5282151
chr11	5284194	5284410
chr11	5288424	5288646
chr11	5290763	5290995
chr11	5294894	5295228
chr11	5296603	5297019
chr11	5297109	5297501
chr11	5304403	5305043
chr11	5305159	5305365
chr11	5330710	5330871
chr11	5331003	5331158
chr11	5333781	5333948
chr11	5343726	5344002
chr11	5352699	5352869
chr11	5353090	5353328
chr11	5353328	5353633
chr11	5354716	5355144
chr11	5367664	5367966
chr11	5398455	5398619
chr11	5432378	5432685
chr11	5434075	5434414

chr11	5434901	5435051
chr11	5437460	5437623
chr11	5437688	5437858
chr11	5443464	5443979
chr11	5444054	5444314
chr11	5444314	5444656
chr11	5444656	5445015
chr11	5445015	5445229
chr11	5445229	5445433
chr11	5470888	5471058
chr11	5471222	5471376
chr11	5474237	5474511
chr11	5484483	5484701
chr11	5485179	5485612
chr11	5485640	5485810
chr11	5485810	5486046
chr11	5496942	5497092
chr11	5509398	5509622
chr11	5520500	5521037
chr11	5521037	5521289
chr11	5521289	5521507
chr11	5521507	5521761
chr11	5521761	5522037
chr11	5522037	5522330
chr11	5527958	5528139
chr11	5528181	5528448
chr11	5533157	5533414
chr11	5541724	5542134
chr11	5542134	5542565
chr11	5551541	5551691
chr11	5552063	5552261
chr11	5554389	5554938
chr11	5565693	5565912
chr11	5629161	5629428
chr11	5650394	5650641
chr11	5691881	5692052
chr11	5700932	5701090
chr11	5701127	5701321
chr11	5701377	5701730
chr11	5701730	5702070
chr11	5706986	5707155
chr11	5707160	5707490

chr11	5707490	5708089
chr11	5720093	5720250
chr11	5720497	5720665
chr11	5734421	5734686
chr11	5734686	5734907
chr11	5737195	5737565
chr11	5740575	5740996
chr11	5740996	5741548
chr11	5761829	5762247
chr11	5762247	5762604
chr11	5766151	5766443
chr11	5777589	5777879
chr11	5778505	5778769
chr11	5782665	5782887
chr11	5788084	5788654
chr11	5788654	5789103
chr11	5795731	5796063
chr11	5820654	5820970
chr11	5822276	5822600
chr11	5824853	5825158
chr11	5825511	5825701
chr11	5826299	5826471
chr11	5828788	5829041
chr11	5835088	5835518
chr11	5836102	5836613
chr11	5837393	5838277
chr11	5849335	5849705
chr11	5849705	5850014
chr11	5851415	5851631
chr11	5854491	5854649
chr11	5855488	5855762
chr11	5855868	5856293
chr11	5860165	5860504
chr11	5860708	5860907
chr11	5861612	5861826
chr11	5861826	5862022
chr11	5862044	5862289
chr11	5862441	5862654
chr11	5862725	5863086
chr11	5877939	5878583
chr11	5880009	5880235
chr11	5886148	5886558

chr11	5888040	5888195
chr11	5892142	5892577
chr11	5894513	5894839
chr11	5895277	5895674
chr11	5895756	5896130
chr11	5896598	5896827
chr11	5901919	5902330
chr11	5902330	5902730
chr11	5903793	5904013
chr11	5908575	5908972
chr11	5909365	5909762
chr11	5912712	5912960
chr11	5912960	5913292
chr11	5915872	5916136
chr11	5920408	5920623
chr11	5930633	5930848
chr11	5944887	5945107
chr11	5945175	5945501
chr11	5949902	5950338
chr11	5955194	5955444
chr11	5955485	5955891
chr11	5955891	5956239
chr11	5956619	5956917
chr11	5976248	5976428
chr11	5981434	5981588
chr11	5989925	5990138
chr11	5990990	5991226
chr11	5996030	5996299
chr11	5998555	5998972
chr11	6000357	6000858
chr11	6001887	6002370
chr11	6002698	6002965
chr11	6005426	6005605
chr11	6005637	6005978
chr11	6010745	6011253
chr11	6016482	6016646
chr11	6017249	6017419
chr11	6017719	6018052
chr11	6018742	6018954
chr11	6023965	6024152
chr11	6026405	6026685
chr11	6026685	6026881

chr11	6026881	6027032
chr11	6033239	6033413
chr11	6035953	6036103
chr11	6037433	6037743
chr11	6037743	6038221
chr11	6047800	6048054
chr11	6049132	6049403
chr11	6065492	6065817
chr11	6065817	6065965
chr11	6065965	6066143
chr11	6068252	6068484
chr11	6068713	6068872
chr11	6070354	6070536
chr11	6070548	6070747
chr11	6103252	6103445
chr11	6103833	6104066
chr11	6114232	6114523
chr11	6128793	6128945
chr11	6130680	6130851
chr11	6169315	6169599
chr11	6174730	6175239
chr11	6175577	6175798
chr11	6187866	6188327
chr11	6199418	6199607
chr11	6199607	6199812
chr11	6199812	6200059
chr11	6200059	6200313
chr11	6200313	6200595
chr11	6201349	6201600
chr11	6206994	6207241
chr11	6216884	6217185
chr11	6217185	6217394
chr11	6223396	6223580
chr11	6254644	6254970
chr11	6256635	6256931
chr11	6261734	6262030
chr11	6267254	6267652
chr11	6267652	6268142
chr11	6273789	6273965
chr11	6274142	6274296
chr11	6274405	6274780
chr11	6274780	6275118

chr11	6275118	6275423
chr11	6286314	6286509
chr11	6291399	6291750
chr11	6291750	6292071
chr11	6292071	6292402
chr11	6292402	6292678
chr11	6305753	6306072
chr11	6341595	6341794
chr11	6365925	6366291
chr11	6367272	6367450
chr11	6369808	6370126
chr11	6371276	6371715
chr11	6371753	6371910
chr11	6372853	6373079
chr11	6373968	6374118
chr11	6380822	6381421
chr11	6388713	6389175
chr11	6389175	6389595
chr11	6389595	6390009
chr11	6396679	6396838
chr11	6397526	6397789
chr11	6402769	6403061
chr11	6406180	6406628
chr11	6406630	6406843
chr11	6414530	6414754
chr11	6414786	6415135
chr11	6415307	6415582
chr11	6415582	6415981
chr11	6415981	6416449
chr11	6416560	6416802
chr11	6420400	6420715
chr11	6420819	6420999
chr11	6436492	6436795
chr11	6443991	6444388
chr11	6444388	6444856
chr11	6451683	6451948
chr11	6465038	6465489
chr11	6472152	6472339
chr11	6474839	6475336
chr11	6475336	6475719
chr11	6475742	6475892
chr11	6475895	6476277

chr11	6476277	6476514
chr11	6485034	6485291
chr11	6490110	6490452
chr11	6494278	6494582
chr11	6497737	6498028
chr11	6500901	6501095
chr11	6506904	6507229
chr11	6507229	6507541
chr11	6510241	6510449
chr11	6523591	6524002
chr11	6526740	6526941
chr11	6528035	6528246
chr11	6528246	6528554
chr11	6528554	6529088
chr11	6539905	6540083
chr11	6540234	6540555
chr11	6546184	6546632
chr11	6546632	6547004
chr11	6547004	6547388
chr11	6559737	6559947
chr11	6572750	6573033
chr11	6586772	6586997
chr11	6587179	6587598
chr11	6598349	6598678
chr11	6599845	6600018
chr11	6603759	6603923
chr11	6604042	6604274
chr11	6604974	6605136
chr11	6605430	6605654
chr11	6605654	6605910
chr11	6605972	6606255
chr11	6611473	6611633
chr11	6615629	6615792
chr11	6617127	6617277
chr11	6618447	6618688
chr11	6625699	6625948
chr11	6625948	6626464
chr11	6626501	6626660
chr11	6628617	6628871
chr11	6636238	6636567
chr11	6637661	6637852
chr11	6639783	6639940

chr11	6644560	6644900
chr11	6654975	6655213
chr11	6655213	6655424
chr11	6658358	6658652
chr11	6660086	6660285
chr11	6664638	6664919
chr11	6666612	6666988
chr11	6670153	6670382
chr11	6674502	6674979
chr11	6677588	6677866
chr11	6689509	6689761
chr11	6689761	6690228
chr11	6695168	6695720
chr11	6697863	6698129
chr11	6698252	6698654
chr11	6710373	6710581
chr11	6710581	6710798
chr11	6710814	6711084
chr11	6714177	6714547
chr11	6716514	6716676
chr11	6716689	6716898
chr11	6735212	6735500
chr11	6735500	6735770
chr11	6736308	6736545
chr11	6749737	6750080
chr11	6758036	6758343
chr11	6768737	6768889
chr11	6768904	6769181
chr11	6769212	6769362
chr11	6785558	6785751
chr11	6790772	6790941
chr11	6828326	6828739
chr11	6837609	6837790
chr11	6855792	6856372
chr11	6866629	6866779
chr11	6873114	6873308
chr11	6881761	6881912
chr11	6884395	6884712
chr11	6894916	6895636
chr11	6895636	6895964
chr11	6895964	6896212
chr11	6902342	6902592

chr11	6902800	6903032
chr11	6925465	6925816
chr11	6930952	6931284
chr11	6937232	6937441
chr11	6937441	6937764
chr11	6937764	6938073
chr11	6961192	6961463
chr11	6963612	6963869
chr11	6963869	6964142
chr11	6966883	6967140
chr11	6980699	6980907
chr11	6981362	6981638
chr11	6990675	6990910
chr11	6993544	6993694
chr11	6993864	6994110
chr11	6994110	6994673
chr11	6995045	6995242
chr11	7015224	7015498
chr11	7018788	7019200
chr11	7019200	7019565
chr11	7037253	7037433
chr11	7041401	7041589
chr11	7052547	7052798
chr11	7053389	7053837
chr11	7053861	7054058
chr11	7063184	7063513
chr11	7063591	7063896
chr11	7063896	7064302
chr11	7064329	7064734
chr11	7076638	7076793
chr11	7095949	7096277
chr11	7096308	7096478
chr11	7105443	7105887
chr11	7105887	7106461
chr11	7124522	7124785
chr11	7129493	7129733
chr11	7138087	7138318
chr11	7140516	7140837
chr11	7142832	7142998
chr11	7145025	7145262
chr11	7145262	7145631
chr11	7153198	7153368

chr11	7153803	7154012
chr11	7156225	7156672
chr11	7161650	7161825
chr11	7175008	7175316
chr11	7184515	7184747
chr11	7194207	7194661
chr11	7194661	7194917
chr11	7200099	7200338
chr11	7205229	7205672
chr11	7205672	7206026
chr11	7210484	7210660
chr11	7213298	7213765
chr11	7213765	7214003
chr11	7214003	7214507
chr11	7216358	7216545
chr11	7221982	7222325
chr11	7226820	7227130
chr11	7234212	7234442
chr11	7237642	7238221
chr11	7239052	7239456
chr11	7239456	7239766
chr11	7242789	7243125
chr11	7295754	7296108
chr11	7299417	7299604
chr11	7299957	7300439
chr11	7301185	7301354
chr11	7317417	7317764
chr11	7370284	7370474
chr11	7404956	7405155
chr11	7417440	7417667
chr11	7423427	7424541
chr11	7441759	7442111
chr11	7457323	7457563
chr11	7467205	7467362
chr11	7502827	7503091
chr11	7503481	7503696
chr11	7503696	7503939
chr11	7504336	7504491
chr11	7504718	7504944
chr11	7517768	7518116
chr11	7561425	7561887
chr11	7561988	7562197

chr11	7562197	7562420
chr11	7615484	7615687
chr11	7630634	7630785
chr11	7664677	7664970
chr11	7676806	7677048
chr11	7683221	7683460
chr11	7684024	7684213
chr11	7705384	7705537
chr11	7731736	7732240
chr11	7737443	7737655
chr11	7758092	7758257
chr11	7777061	7777309
chr11	7777916	7778091
chr11	7780409	7780941
chr11	7780941	7781250
chr11	7793774	7794290
chr11	7794290	7794769
chr11	7795716	7795997
chr11	7796198	7796684
chr11	7798936	7799297
chr11	7808491	7808924
chr11	7814686	7814842
chr11	7820156	7820335
chr11	7820412	7820562
chr11	7822626	7822816
chr11	7862660	7862863
chr11	7869725	7869905
chr11	7900322	7900683
chr11	7917224	7917397
chr11	7933500	7933704
chr11	7934745	7935072
chr11	7995698	7996057
chr11	7996132	7996297
chr11	7998910	7999120
chr11	8088577	8088781
chr11	8105041	8105247
chr11	8137434	8137708
chr11	8140100	8140453
chr11	8157647	8157823
chr11	8210407	8210597
chr11	8227805	8228204
chr11	8247576	8247776

chr11	8251793	8252031
chr11	8269138	8269545
chr11	8281850	8282001
chr11	8302891	8303141
chr11	8365456	8366015
chr11	8366328	8366619
chr11	8379593	8379779
chr11	8391988	8392138
chr11	8405589	8405905
chr11	8421908	8422095
chr11	8424256	8424752
chr11	8424752	8425235
chr11	8425235	8425522
chr11	8425596	8425824
chr11	8433085	8433319
chr11	8434668	8434858
chr11	8438618	8438796
chr11	8443445	8443834
chr11	8443834	8444231
chr11	8444821	8445058
chr11	8445058	8445398
chr11	8445900	8446057
chr11	8450666	8450952
chr11	8451016	8451452
chr11	8451456	8452123
chr11	8463123	8463639
chr11	8463786	8464247
chr11	8466077	8466408
chr11	8466581	8466793
chr11	8468203	8468493
chr11	8468493	8469121
chr11	8476387	8476566
chr11	8484272	8484486
chr11	8484611	8484880
chr11	8486196	8486353
chr11	8488324	8488487
chr11	8492952	8493349
chr11	8498862	8499074
chr11	8503431	8503776
chr11	8503776	8504109
chr11	8509644	8510128
chr11	8513419	8513655

chr11	8515268	8515418
chr11	8521173	8521323
chr11	8521341	8521750
chr11	8522981	8523152
chr11	8523720	8523916
chr11	8523916	8524153
chr11	8524218	8524368
chr11	8524388	8524538
chr11	8530086	8530312
chr11	8532183	8532421
chr11	8532913	8533128
chr11	8534950	8535220
chr11	8535220	8535555
chr11	8540372	8540626
chr11	8544450	8544622
chr11	8545840	8546095
chr11	8547773	8547993
chr11	8548708	8549079
chr11	8549143	8549382
chr11	8554038	8554225
chr11	8554574	8554759
chr11	8555297	8555478
chr11	8557118	8557275
chr11	8558013	8558174
chr11	8563680	8564013
chr11	8564026	8564360
chr11	8564360	8564717
chr11	8573055	8573305
chr11	8575230	8575631
chr11	8579662	8580400
chr11	8582138	8582431
chr11	8582532	8582956
chr11	8582980	8583153
chr11	8588043	8588282
chr11	8588295	8588445
chr11	8589288	8589809
chr11	8589809	8590184
chr11	8591414	8591566
chr11	8593230	8593489
chr11	8593489	8593738
chr11	8603044	8603250
chr11	8615526	8615832

chr11	8616023	8616204
chr11	8616483	8616646
chr11	8624587	8624892
chr11	8626006	8626282
chr11	8626732	8627053
chr11	8627170	8627415
chr11	8628566	8628716
chr11	8628787	8629037
chr11	8629037	8629315
chr11	8634618	8635034
chr11	8635034	8635423
chr11	8635704	8636286
chr11	8649587	8649789
chr11	8654044	8654230
chr11	8654237	8654398
chr11	8655425	8655661
chr11	8656540	8656962
chr11	8663476	8663762
chr11	8663762	8664005
chr11	8664005	8664232
chr11	8664232	8664462
chr11	8664462	8664726
chr11	8664726	8664982
chr11	8668064	8668391
chr11	8669183	8669496
chr11	8673836	8674123
chr11	8674635	8675204
chr11	8675204	8675490
chr11	8678255	8678585
chr11	8684343	8684526
chr11	8684931	8685207
chr11	8685207	8685558
chr11	8686114	8686349
chr11	8692231	8692505
chr11	8692505	8692812
chr11	8692812	8693146
chr11	8693184	8693490
chr11	8693490	8693705
chr11	8701922	8702334
chr11	8705057	8705625
chr11	8709682	8709988
chr11	8718121	8718339

chr11	8719680	8719926
chr11	8759004	8759173
chr11	8759886	8760185
chr11	8764807	8765102
chr11	8777328	8777615
chr11	8787168	8787372
chr11	8787754	8787909
chr11	8788089	8788319
chr11	8788387	8788585
chr11	8795196	8795469
chr11	8795469	8795713
chr11	8797840	8798013
chr11	8819109	8819337
chr11	8821228	8821955
chr11	8826556	8827053
chr11	8827385	8827735
chr11	8830532	8830866
chr11	8830866	8831114
chr11	8831114	8831328
chr11	8834211	8834614
chr11	8847600	8847942
chr11	8859961	8860315
chr11	8860944	8861125
chr11	8879361	8879574
chr11	8886023	8886505
chr11	8886526	8886752
chr11	8886752	8887099
chr11	8910119	8910463
chr11	8910463	8910809
chr11	8910854	8911007
chr11	8916121	8916466
chr11	8920299	8920613
chr11	8928629	8928896
chr11	8938641	8938886
chr11	8938949	8939141
chr11	8942580	8942808
chr11	8945536	8945725
chr11	8945725	8945902
chr11	8948088	8948734
chr11	8955724	8956063
chr11	8957100	8957338
chr11	8966018	8966229

chr11	8983374	8983529
chr11	8984096	8984281
chr11	8991100	8991362
chr11	8991650	8991833
chr11	9010428	9010884
chr11	9010884	9011369
chr11	9011416	9011599
chr11	9018937	9019150
chr11	9041309	9041479
chr11	9048343	9048748
chr11	9048748	9049246
chr11	9117654	9118093
chr11	9118093	9118361
chr11	9118361	9118656
chr11	9118656	9118906
chr11	9125801	9125982
chr11	9126087	9126342
chr11	9126414	9126746
chr11	9126746	9127070
chr11	9135690	9135995
chr11	9136495	9136834
chr11	9139928	9140221
chr11	9189591	9189805
chr11	9194986	9195302
chr11	9248892	9249046
chr11	9287373	9287543
chr11	9316569	9316753
chr11	9317370	9317581
chr11	9382743	9382932
chr11	9497201	9497416
chr11	9497590	9497923
chr11	9499824	9499992
chr11	9519897	9520126
chr11	9556917	9557186
chr11	9731614	9731764
chr11	9966314	9966482
chr11	9989549	9989726
chr11	10046389	10046818
chr11	10050323	10050643
chr11	10102360	10102668
chr11	10102940	10103099
chr11	10103156	10103712

chr11	10193810	10194089
chr11	10194904	10195070
chr11	10328955	10329163
chr11	11112038	11112197
chr11	11113962	11114119
chr11	11114348	11114511
chr11	11239497	11239653
chr11	11248501	11248651
chr11	11461650	11461845
chr11	11461882	11462154
chr11	11462154	11462408
chr11	11492134	11492377
chr11	11684967	11685149
chr11	11685566	11685805
chr11	11685887	11686156
chr11	11686237	11686408
chr11	11788476	11788763
chr11	11788763	11789098
chr11	11808589	11808885
chr11	11808885	11809187
chr11	11809187	11809462
chr11	11812029	11812257
chr11	11820273	11820772
chr11	11820772	11821153
chr11	11825506	11826250
chr11	11829282	11829525
chr11	11840803	11841103
chr11	11939605	11940001
chr11	11973438	11973711
chr11	11974930	11975198
chr11	12025756	12026120
chr11	12026163	12026584
chr11	12037108	12037382
chr11	12037408	12037599
chr11	12072311	12072717
chr11	12195477	12195755
chr11	12197530	12197807
chr11	12217028	12217443
chr11	12225440	12225592
chr11	12231288	12231752
chr11	12231752	12232117
chr11	12232148	12232365

chr11	12232412	12232726
chr11	12234980	12235272
chr11	12263901	12264061
chr11	12285427	12285689
chr11	12285689	12285933
chr11	12291475	12291683
chr11	12314759	12314909
chr11	12316865	12317022
chr11	12319290	12319460
chr11	12321035	12321278
chr11	12359421	12359651
chr11	12373428	12373716
chr11	12373716	12374024
chr11	12375068	12375254
chr11	12382656	12382889
chr11	12382980	12383527
chr11	12384822	12385039
chr11	12390681	12391017
chr11	12394234	12394498
chr11	12396047	12396640
chr11	12402294	12402574
chr11	12402958	12403214
chr11	12403214	12403494
chr11	12403494	12403789
chr11	12405586	12405736
chr11	12409447	12409855
chr11	12411163	12411496
chr11	12412959	12413293
chr11	12416986	12417270
chr11	12417270	12417496
chr11	12421219	12421687
chr11	12424228	12424630
chr11	12434660	12434926
chr11	12435028	12435420
chr11	12438469	12438770
chr11	12441970	12442288
chr11	12442288	12442796
chr11	12443082	12443247
chr11	12444339	12444636
chr11	12448301	12448512
chr11	12448729	12449084
chr11	12450748	12451110

chr11	12458404	12458786
chr11	12463654	12463972
chr11	12464045	12464256
chr11	12464256	12464521
chr11	12464521	12464880
chr11	12464880	12465239
chr11	12470934	12471150
chr11	12480189	12480425
chr11	12487847	12488010
chr11	12495735	12495951
chr11	12511248	12511428
chr11	12516454	12516699
chr11	12532299	12532734
chr11	12540511	12540797
chr11	12543214	12543375
chr11	12545874	12546150
chr11	12559322	12559565
chr11	12957975	12958155
chr11	13666784	13666963
chr11	13970143	13970303
chr11	15267286	15267474
chr11	15376821	15376971
chr11	15407556	15407835
chr11	15607951	15608132
chr11	16257535	16257714
chr11	16257714	16257870
chr11	16261299	16261555
chr11	16412698	16412936
chr11	16507676	16507996
chr11	16507996	16508620
chr11	16525049	16525242
chr11	16525280	16525625
chr11	16525796	16526300
chr11	16526332	16526536
chr11	16526536	16526855
chr11	16526893	16527059
chr11	16532921	16533236
chr11	16537901	16538250
chr11	16538250	16538543
chr11	16546479	16546731
chr11	16549719	16549949
chr11	16558705	16558855

chr11	16558949	16559270
chr11	16559276	16559755
chr11	16560664	16560814
chr11	16587129	16587403
chr11	16590677	16590856
chr11	16591414	16591952
chr11	16591952	16592380
chr11	16592598	16592753
chr11	16592822	16593069
chr11	16672486	16672803
chr11	16683409	16684189
chr11	16695674	16696160
chr11	16716512	16717212
chr11	16718034	16718485
chr11	16720075	16720244
chr11	16734845	16735205
chr11	16735205	16735607
chr11	16735607	16735880
chr11	16737372	16737688
chr11	16737912	16738136
chr11	16751980	16752411
chr11	16752411	16752717
chr11	16752717	16752954
chr11	16752954	16753216
chr11	16753271	16753454
chr11	16759909	16760396
chr11	16769219	16769478
chr11	16769744	16770547
chr11	16781384	16781631
chr11	16781676	16781918
chr11	16783269	16783470
chr11	16784023	16784194
chr11	16790050	16790337
chr11	16790337	16790606
chr11	16790686	16790838
chr11	16791147	16791721
chr11	16792367	16792576
chr11	16795910	16796254
chr11	16796254	16796627
chr11	16800533	16801071
chr11	16803983	16804520
chr11	16810219	16810422

chr11	16817235	16817672
chr11	16830562	16830887
chr11	16830887	16831147
chr11	16833327	16833564
chr11	16843886	16844201
chr11	16844201	16844682
chr11	16850764	16850961
chr11	16850998	16851354
chr11	16865045	16865229
chr11	16866604	16866930
chr11	16869384	16869811
chr11	16871166	16871469
chr11	16871609	16871780
chr11	16950829	16951849
chr11	16967932	16968090
chr11	17158786	17159562
chr11	17159562	17159937
chr11	17160357	17160598
chr11	17169207	17169464
chr11	17172340	17172809
chr11	17176657	17177000
chr11	17187671	17187919
chr11	17192259	17192409
chr11	17203448	17203662
chr11	17204808	17205076
chr11	17211198	17211451
chr11	17211451	17211735
chr11	17211735	17212008
chr11	17212008	17212223
chr11	17212223	17212431
chr11	17216559	17216736
chr11	17221245	17221403
chr11	17234411	17234653
chr11	17257096	17257267
chr11	17257418	17257889
chr11	17257904	17258082
chr11	17258082	17258247
chr11	17267282	17267433
chr11	17279939	17280164
chr11	17311868	17312057
chr11	17374904	17375115
chr11	17386680	17387194

chr11	17459993	17460228
chr11	17468483	17468816
chr11	17468816	17469190
chr11	17469190	17469658
chr11	17486626	17486785
chr11	17620630	17620785
chr11	17620919	17621162
chr11	17694269	17694457
chr11	17953184	17953531
chr11	17953531	17953767
chr11	17953767	17954109
chr11	18011177	18011327
chr11	18070654	18070828
chr11	18070958	18071307
chr11	18075072	18075312
chr11	18331641	18331951
chr11	18331951	18332396
chr11	18528586	18528915
chr11	18547058	18547440
chr11	18575314	18575657
chr11	18575657	18575893
chr11	18585273	18585533
chr11	18590637	18590859
chr11	18591037	18591224
chr11	18632403	18632730
chr11	18741969	18742132
chr11	18834607	18834875
chr11	18864739	18864985
chr11	18877530	18877690
chr11	18877705	18877968
chr11	18879466	18879733
chr11	18920672	18920946
chr11	18927459	18927630
chr11	18937817	18937993
chr11	18953865	18954259
chr11	18975892	18976331
chr11	18989029	18989325
chr11	19018859	19019217
chr11	19019825	19020037
chr11	19020350	19020626
chr11	19024634	19024786
chr11	19027367	19027552

chr11	19221574	19221757
chr11	19302529	19302755
chr11	19333503	19333693
chr11	19666877	19667032
chr11	19708686	19708861
chr11	19728583	19728833
chr11	19737534	19737694
chr11	19822179	19822452
chr11	19823320	19823470
chr11	19823503	19823720
chr11	19823720	19823956
chr11	19829387	19829528
chr11	19829528	19829693
chr11	19829693	19829882
chr11	19830720	19830956
chr11	19834686	19834977
chr11	19835129	19835486
chr11	19835486	19835833
chr11	19839085	19839257
chr11	19842903	19843509
chr11	19848408	19849030
chr11	19849305	19849860
chr11	19849860	19850243
chr11	19866973	19867222
chr11	19868984	19869179
chr11	19911280	19911465
chr11	19914760	19914929
chr11	19915218	19915383
chr11	19919851	19920124
chr11	19920124	19920415
chr11	19920415	19920722
chr11	19923929	19924288
chr11	19924288	19924563
chr11	19925089	19925432
chr11	19925432	19925816
chr11	19925816	19926152
chr11	19926152	19926385
chr11	19929194	19929359
chr11	19932917	19933181
chr11	19933181	19933404
chr11	19939315	19939488
chr11	19939568	19939768

chr11	19941935	19942082
chr11	19942082	19942220
chr11	19947553	19947855
chr11	19947855	19948086
chr11	19948086	19948342
chr11	19948762	19948947
chr11	19960860	19961589
chr11	19972578	19972740
chr11	19978018	19978185
chr11	19978185	19978369
chr11	19979105	19979379
chr11	19979379	19979896
chr11	19990714	19991067
chr11	19992437	19992686
chr11	19992710	19993477
chr11	19999987	20000174
chr11	20001801	20002034
chr11	20002111	20002288
chr11	20009137	20009343
chr11	20025999	20026263
chr11	20031644	20031861
chr11	20032008	20032272
chr11	20032867	20033029
chr11	20035434	20035678
chr11	20037397	20037604
chr11	20037696	20038123
chr11	20043254	20043483
chr11	20045807	20045968
chr11	20056267	20056474
chr11	20057522	20057730
chr11	20066142	20066392
chr11	20067630	20067791
chr11	20072268	20072459
chr11	20107716	20108003
chr11	20112280	20112753
chr11	20112753	20113405
chr11	20116771	20117061
chr11	20117232	20117391
chr11	20117474	20117721
chr11	20122779	20122940
chr11	20125525	20125681
chr11	20126954	20127708

chr11	20132986	20133439
chr11	20143779	20144002
chr11	20190323	20190503
chr11	20200839	20201205
chr11	20201205	20201480
chr11	20201480	20201773
chr11	20201773	20202076
chr11	20204455	20204620
chr11	20214851	20215070
chr11	20231570	20231750
chr11	20236993	20237452
chr11	20237471	20237671
chr11	20242449	20242725
chr11	20242725	20243068
chr11	20243205	20243486
chr11	20246655	20246891
chr11	20248878	20249276
chr11	20249276	20249896
chr11	20249900	20250051
chr11	20253174	20253384
chr11	20263104	20263262
chr11	20266924	20267267
chr11	20276067	20276217
chr11	20282274	20282605
chr11	20282605	20283265
chr11	20285610	20285791
chr11	20299944	20300233
chr11	20301482	20301647
chr11	20305538	20305722
chr11	20305722	20305958
chr11	20310438	20310808
chr11	20310808	20311175
chr11	20311175	20311486
chr11	20311486	20311655
chr11	20325207	20325475
chr11	20326744	20326951
chr11	20332055	20332299
chr11	20332299	20332540
chr11	20332564	20333089
chr11	20333089	20333467
chr11	20340825	20340991
chr11	20341090	20341584

chr11	20346313	20346789
chr11	20347532	20347757
chr11	20347757	20348015
chr11	20348017	20348259
chr11	20348497	20348842
chr11	20357633	20358269
chr11	20364789	20364980
chr11	20365214	20365466
chr11	20369001	20369184
chr11	20371220	20371517
chr11	20378861	20379256
chr11	20379256	20379594
chr11	20400764	20401206
chr11	20432016	20432166
chr11	20445340	20445644
chr11	20445644	20445962
chr11	20445962	20446447
chr11	20455042	20455300
chr11	20455300	20455532
chr11	20455544	20455810
chr11	20456348	20456613
chr11	20463149	20463668
chr11	20469309	20469765
chr11	20469765	20470039
chr11	20470039	20470301
chr11	20475719	20475906
chr11	20477209	20478056
chr11	20490635	20490823
chr11	20492157	20492530
chr11	20500609	20500820
chr11	20521364	20521576
chr11	20531821	20532243
chr11	20532243	20532564
chr11	20532564	20532948
chr11	20542385	20542932
chr11	20542932	20543279
chr11	20543279	20543574
chr11	20543574	20543752
chr11	20543809	20544055
chr11	20556848	20557062
chr11	20558105	20558576
chr11	20559628	20559831

chr11	20561368	20561547
chr11	20564536	20564820
chr11	20567302	20567670
chr11	20575331	20575501
chr11	20584971	20585268
chr11	20585352	20585591
chr11	20585886	20586056
chr11	20586246	20586612
chr11	20592168	20592320
chr11	20594178	20594554
chr11	20594554	20594915
chr11	20594915	20595255
chr11	20595443	20595761
chr11	20599574	20599749
chr11	20607824	20608038
chr11	20608038	20608300
chr11	20608411	20608767
chr11	20609116	20609284
chr11	20611109	20611547
chr11	20616172	20616560
chr11	20631225	20631668
chr11	20631668	20631917
chr11	20631917	20632166
chr11	20632166	20632620
chr11	20632780	20632930
chr11	20638914	20639204
chr11	20639905	20640128
chr11	20645489	20645979
chr11	20656824	20656998
chr11	20661728	20661979
chr11	20662960	20663132
chr11	20668354	20668504
chr11	20671888	20672165
chr11	20674551	20674892
chr11	20691076	20691359
chr11	20729920	20730228
chr11	20740472	20740750
chr11	20740771	20741201
chr11	20741201	20741498
chr11	20741498	20741735
chr11	20741735	20742056
chr11	20742435	20742599

chr11	20783463	20783823
chr11	20783823	20784130
chr11	20794220	20794587
chr11	20794632	20794913
chr11	20794913	20795155
chr11	20830585	20831135
chr11	20831135	20831682
chr11	20831887	20832082
chr11	20847205	20847383
chr11	20847443	20847634
chr11	20856263	20856521
chr11	20857302	20857465
chr11	20859240	20859660
chr11	20860056	20860213
chr11	20869865	20870348
chr11	20870348	20870596
chr11	20873003	20873296
chr11	20873468	20873719
chr11	20877636	20877914
chr11	20878575	20879020
chr11	20886503	20886705
chr11	20886780	20887614
chr11	20896099	20896570
chr11	20896570	20897030
chr11	20908231	20908409
chr11	20908576	20908802
chr11	20909004	20909303
chr11	20909531	20909852
chr11	20914023	20914301
chr11	20916123	20916339
chr11	20916544	20917032
chr11	20929057	20929341
chr11	20929992	20930453
chr11	20936673	20936869
chr11	20936940	20937320
chr11	20937716	20938312
chr11	20939261	20939503
chr11	20954035	20954214
chr11	20955197	20955623
chr11	20955623	20956087
chr11	20956087	20956516
chr11	20956572	20956839

chr11	20958519	20958734
chr11	20958900	20959193
chr11	20967715	20967939
chr11	20968349	20968759
chr11	20982130	20982504
chr11	20982504	20983037
chr11	20983087	20983246
chr11	20987604	20987779
chr11	20987842	20988198
chr11	20996778	20997196
chr11	21034117	21034397
chr11	21036155	21036595
chr11	21036600	21036764
chr11	21041109	21041290
chr11	21082717	21083113
chr11	21083214	21083391
chr11	21089678	21089919
chr11	21090919	21091555
chr11	21091555	21091960
chr11	21105463	21105733
chr11	21131796	21132064
chr11	21139660	21139951
chr11	21149503	21149823
chr11	21150879	21151159
chr11	21157545	21157870
chr11	21172393	21172543
chr11	21176931	21177087
chr11	21191959	21192109
chr11	21226150	21226497
chr11	21238552	21239116
chr11	21239116	21239449
chr11	21239449	21239730
chr11	21241269	21241472
chr11	21282066	21282283
chr11	21308232	21308427
chr11	21326871	21327040
chr11	21370375	21370774
chr11	21370774	21371124
chr11	21371124	21371486
chr11	21398606	21398781
chr11	21399461	21399667
chr11	21428803	21429152

chr11	21453767	21454032
chr11	21456562	21456804
chr11	21500258	21500416
chr11	21549167	21549358
chr11	21549822	21550077
chr11	21571507	21571819
chr11	21571819	21572493
chr11	21581512	21581844
chr11	21869212	21869462
chr11	21942382	21942587
chr11	21948802	21949060
chr11	21951744	21952000
chr11	21992884	21993034
chr11	21993399	21993583
chr11	21993583	21993768
chr11	21996430	21996722
chr11	22001361	22001583
chr11	22001583	22001847
chr11	22001847	22002314
chr11	22005569	22005719
chr11	22008430	22008610
chr11	22140307	22140531
chr11	22140820	22141087
chr11	22154477	22154844
chr11	22154948	22155160
chr11	22156150	22156549
chr11	22171293	22171592
chr11	22234849	22235073
chr11	22247063	22247412
chr11	22247833	22248032
chr11	22255396	22255822
chr11	22262614	22262778
chr11	22270529	22270758
chr11	22273335	22273768
chr11	22274213	22274486
chr11	22286346	22286628
chr11	22286628	22286915
chr11	22286915	22287201
chr11	22287201	22287531
chr11	22305128	22305359
chr11	22313345	22313635
chr11	22314372	22314679

chr11	22317951	22318379
chr11	22334925	22335136
chr11	22339224	22339379
chr11	22372808	22372969
chr11	22383630	22383974
chr11	22383974	22384295
chr11	22437343	22437493
chr11	22437527	22437878
chr11	22438876	22439122
chr11	22440628	22440879
chr11	22446685	22447084
chr11	22451383	22452069
chr11	22454365	22454544
chr11	22462036	22462249
chr11	22478741	22479009
chr11	22494747	22494992
chr11	22497464	22497984
chr11	22506836	22507041
chr11	22507041	22507288
chr11	22507308	22507490
chr11	22512147	22512995
chr11	22517740	22518175
chr11	22597616	22597819
chr11	22610831	22611167
chr11	22611167	22611514
chr11	22611514	22612131
chr11	22617448	22617920
chr11	22621812	22622228
chr11	22623910	22624060
chr11	22629690	22629851
chr11	22631841	22632164
chr11	22632258	22632772
chr11	22633115	22633297
chr11	22638231	22638428
chr11	22643915	22644318
chr11	22644318	22644602
chr11	22661034	22661547
chr11	22662072	22662609
chr11	22668260	22668542
chr11	22669046	22669302
chr11	22669302	22669787
chr11	22672209	22672392

chr11	22677410	22677674
chr11	22692780	22693061
chr11	22693061	22693381
chr11	22693381	22693701
chr11	22709776	22709929
chr11	22710763	22711007
chr11	22711397	22711662
chr11	22716112	22716366
chr11	22719300	22719998
chr11	22732741	22732970
chr11	22732970	22733271
chr11	22733305	22733655
chr11	22734594	22734802
chr11	22735170	22735433
chr11	22777654	22777804
chr11	22780775	22781115
chr11	22781259	22781547
chr11	22791694	22792159
chr11	22792159	22792470
chr11	22794790	22795078
chr11	22795311	22795612
chr11	22795953	22796160
chr11	22799957	22800633
chr11	22805227	22805498
chr11	22811405	22811787
chr11	22821137	22821616
chr11	22830371	22830612
chr11	22830612	22830837
chr11	22831121	22831317
chr11	22842517	22842781
chr11	22843807	22844424
chr11	22847733	22848049
chr11	22848049	22848336
chr11	22851475	22851660
chr11	22854450	22854903
chr11	22856642	22856875
chr11	22858948	22859280
chr11	22859280	22859618
chr11	22859618	22859920
chr11	22859920	22860312
chr11	22860312	22860742
chr11	22897772	22897983

chr11	22921255	22921460
chr11	22948963	22949606
chr11	22956808	22957254
chr11	22965076	22965242
chr11	22965438	22965932
chr11	22967745	22968034
chr11	22971822	22972130
chr11	22972130	22972412
chr11	22972412	22972721
chr11	22972721	22973039
chr11	22973039	22973261
chr11	22973714	22974088
chr11	22981672	22981937
chr11	22981937	22982497
chr11	22982510	22982660
chr11	22990302	22990686
chr11	22990686	22990936
chr11	22990936	22991152
chr11	23007327	23007606
chr11	23007606	23007969
chr11	23007979	23008235
chr11	23008235	23008746
chr11	23013655	23013895
chr11	23052434	23052700
chr11	23064686	23064850
chr11	23067485	23068204
chr11	23069219	23069443
chr11	23074733	23075235
chr11	23098138	23098280
chr11	23098280	23098439
chr11	23124271	23124508
chr11	23124508	23124938
chr11	23149865	23150080
chr11	23176770	23176985
chr11	23255130	23255760
chr11	23255760	23256185
chr11	23256197	23256451
chr11	23257326	23257684
chr11	23261508	23261808
chr11	23272117	23272388
chr11	23305729	23306257
chr11	23306257	23306683

chr11	23306683	23307033
chr11	23307033	23307269
chr11	23430087	23430310
chr11	23451282	23451612
chr11	23451612	23451982
chr11	23497424	23497781
chr11	23497781	23498098
chr11	23498098	23498470
chr11	23504908	23505181
chr11	23509555	23509815
chr11	23510210	23510583
chr11	23558255	23558405
chr11	23565764	23566123
chr11	23570176	23570432
chr11	23575306	23575476
chr11	23632922	23633390
chr11	23633390	23633985
chr11	23661051	23661201
chr11	23665356	23665798
chr11	23665798	23666186
chr11	23666186	23666701
chr11	23679110	23679322
chr11	23679322	23679541
chr11	23730054	23730727
chr11	23730829	23731042
chr11	23742779	23742947
chr11	23746234	23746430
chr11	23753979	23754282
chr11	23754307	23754676
chr11	23758600	23758861
chr11	23770442	23770982
chr11	23770982	23771264
chr11	23771264	23771569
chr11	23771589	23771757
chr11	23786311	23786669
chr11	23855420	23855727
chr11	23894525	23894846
chr11	23894846	23895139
chr11	23895139	23895472
chr11	23899324	23899693
chr11	23913802	23914135
chr11	23979403	23979636

chr11	24049259	24049558
chr11	24076062	24076283
chr11	24076283	24076615
chr11	24076891	24077131
chr11	24077164	24077387
chr11	24077389	24077646
chr11	24077895	24078079
chr11	24079141	24079291
chr11	24079744	24079942
chr11	24080620	24080812
chr11	24080812	24080974
chr11	24401839	24402050
chr11	24423393	24423637
chr11	25321446	25321814
chr11	25321883	25322106
chr11	25322561	25322725
chr11	25378736	25378952
chr11	25792658	25792928
chr11	25862858	25863079
chr11	25914165	25914336
chr11	26183586	26183839
chr11	26183839	26184118
chr11	26201069	26201314
chr11	26210184	26210612
chr11	26215922	26216118
chr11	26386835	26387320
chr11	26387320	26387773
chr11	26519048	26519252
chr11	26593102	26593476
chr11	26593476	26593852
chr11	26593852	26594122
chr11	26640398	26640831
chr11	26961676	26961917
chr11	27126015	27126242
chr11	27331115	27331404
chr11	27753740	27753895
chr11	28079800	28079952
chr11	28339634	28339935
chr11	28583703	28584179
chr11	28584179	28584484
chr11	28810892	28811126
chr11	28811899	28812213

chr11	28812372	28812557
chr11	28812582	28812984
chr11	28812984	28813353
chr11	28833777	28834356
chr11	28848873	28849116
chr11	28849772	28849958
chr11	28849958	28850177
chr11	28851773	28851950
chr11	28852085	28852571
chr11	28852662	28852819
chr11	28853024	28853846
chr11	28906098	28906810
chr11	28911506	28911872
chr11	28912518	28912809
chr11	28925658	28925825
chr11	29125623	29125791
chr11	29130173	29130353
chr11	29130358	29130605
chr11	29130605	29130971
chr11	29130971	29131342
chr11	29171945	29172380
chr11	29172433	29173112
chr11	29173237	29173691
chr11	29242067	29242254
chr11	29246095	29246374
chr11	29246795	29247240
chr11	29247240	29247640
chr11	29267941	29268164
chr11	29344939	29345288
chr11	29345659	29346178
chr11	29346473	29346658
chr11	29349349	29349500
chr11	29363202	29363404
chr11	29373265	29373900
chr11	29373900	29374216
chr11	29374216	29374404
chr11	29428862	29429064
chr11	29474717	29474991
chr11	29475707	29475946
chr11	29476054	29476328
chr11	29514621	29514910
chr11	29514910	29515195

chr11	29515195	29515451
chr11	29526034	29526524
chr11	29526524	29526784
chr11	29547565	29548046
chr11	29548046	29548564
chr11	29604130	29604567
chr11	29605117	29605416
chr11	29607518	29607737
chr11	29628660	29628938
chr11	29638789	29639168
chr11	29645429	29645957
chr11	29653536	29653739
chr11	29656723	29657046
chr11	29676853	29677008
chr11	29679755	29680258
chr11	29684926	29685128
chr11	29685820	29685970
chr11	29691983	29692411
chr11	29692553	29693314
chr11	29693484	29693882
chr11	29693882	29694121
chr11	29694158	29694353
chr11	29704600	29704847
chr11	29705011	29705197
chr11	29705210	29705618
chr11	29705618	29705955
chr11	29709649	29709799
chr11	29724260	29724505
chr11	29725693	29725895
chr11	29727967	29728250
chr11	29735166	29735364
chr11	29737235	29737437
chr11	29737869	29738104
chr11	29739695	29740048
chr11	29752326	29752488
chr11	29755177	29755402
chr11	29758365	29758536
chr11	29758579	29758933
chr11	29759943	29760181
chr11	29761632	29761830
chr11	29761935	29762103
chr11	29770650	29771241

chr11	29772419	29772650
chr11	29777983	29778356
chr11	29782779	29782929
chr11	29783235	29783389
chr11	29801718	29802037
chr11	29802157	29802325
chr11	29807430	29807626
chr11	29810404	29810604
chr11	29824645	29825479
chr11	29837123	29837350
chr11	29838902	29839127
chr11	29839781	29840253
chr11	29862085	29862318
chr11	29879679	29880055
chr11	29884086	29884392
chr11	29884392	29884759
chr11	29885195	29885505
chr11	29903278	29903458
chr11	29904105	29904271
chr11	30025644	30026487
chr11	30057392	30057604
chr11	30092035	30092213
chr11	30095331	30095486
chr11	30095486	30095660
chr11	30096038	30096275
chr11	30102714	30102947
chr11	30118030	30118507
chr11	30130644	30130802
chr11	30133451	30133650
chr11	30161027	30161365
chr11	30161365	30161651
chr11	30181257	30181523
chr11	30182722	30183115
chr11	30183115	30183366
chr11	30183366	30183646
chr11	30184600	30184909
chr11	30189026	30189176
chr11	30197502	30197784
chr11	30197784	30198132
chr11	30198132	30198439
chr11	30198439	30198769
chr11	30209525	30209988

chr11	30210284	30210665
chr11	30211618	30212042
chr11	30212057	30212246
chr11	30222273	30222655
chr11	30222683	30222959
chr11	30238025	30238250
chr11	30243331	30243625
chr11	30243625	30244042
chr11	30247139	30247557
chr11	30265592	30266052
chr11	30266846	30267398
chr11	30267398	30267760
chr11	30267760	30268009
chr11	30268009	30268269
chr11	30268269	30268581
chr11	30268650	30268876
chr11	30269655	30269855
chr11	30394442	30394674
chr11	30471681	30472255
chr11	30485732	30485887
chr11	30648970	30649296
chr11	30649296	30649630
chr11	30657164	30657409
chr11	30676160	30676474
chr11	30744014	30744203
chr11	30750097	30750486
chr11	30750486	30750786
chr11	30754781	30755542
chr11	30771375	30772083
chr11	30772083	30772593
chr11	30773146	30773322
chr11	30799592	30799817
chr11	30800147	30800468
chr11	30885195	30885805
chr11	30933362	30933552
chr11	30933800	30934041
chr11	30953553	30953808
chr11	30953826	30954234
chr11	30954234	30954622
chr11	30954622	30954930
chr11	30985840	30986173
chr11	30986173	30986619

chr11	30986619	30986981
chr11	30996720	30997216
chr11	31138056	31138236
chr11	31157048	31157341
chr11	31169563	31169740
chr11	31170084	31170312
chr11	31225302	31225774
chr11	31230566	31230873
chr11	31237463	31237665
chr11	31248552	31248782
chr11	31369277	31369870
chr11	31369974	31370491
chr11	31371525	31371656
chr11	31371656	31371733
chr11	31373523	31373799
chr11	31382288	31382480
chr11	31564191	31564349
chr11	31617324	31617537
chr11	31638052	31638339
chr11	31649640	31649845
chr11	31656502	31656703
chr11	31656703	31656922
chr11	31657089	31657702
chr11	31657702	31658117
chr11	31658126	31658370
chr11	31663200	31663500
chr11	31664205	31664629
chr11	31664894	31665106
chr11	31665192	31665371
chr11	31670373	31670667
chr11	31671302	31671711
chr11	31671711	31672162
chr11	31681757	31681937
chr11	31683747	31683972
chr11	31690783	31690935
chr11	31713620	31714012
chr11	31718255	31718632
chr11	31719816	31719966
chr11	31727078	31727737
chr11	31728823	31729057
chr11	31733118	31733319
chr11	31736200	31736484

chr11	31736639	31737069
chr11	31738110	31738306
chr11	31739873	31740055
chr11	31740166	31740795
chr11	31745906	31746152
chr11	31748005	31748165
chr11	31748358	31748812
chr11	31750746	31750984
chr11	31751327	31751542
chr11	31753745	31753938
chr11	31753938	31754134
chr11	31772054	31772370
chr11	31782560	31782734
chr11	31786594	31786826
chr11	31796218	31796637
chr11	31798135	31798496
chr11	31807418	31807753
chr11	31807753	31808136
chr11	31808136	31808504
chr11	31808504	31808967
chr11	31810080	31810267
chr11	31821975	31822486
chr11	31822545	31822835
chr11	31822910	31823139
chr11	31823139	31823412
chr11	31824497	31825259
chr11	31828746	31829030
chr11	31832530	31832735
chr11	31833179	31833537
chr11	31839516	31839683
chr11	31845203	31845746
chr11	31851047	31851438
chr11	31868090	31868341
chr11	31868587	31868799
chr11	31870291	31870561
chr11	31870600	31871034
chr11	31871355	31871543
chr11	31900530	31900774
chr11	31906186	31906364
chr11	31914311	31914477
chr11	31914477	31914633
chr11	31917672	31917877

chr11	31922522	31922931
chr11	31945603	31945794
chr11	31948627	31949145
chr11	31949145	31949479
chr11	31949479	31949732
chr11	31989857	31990213
chr11	32000257	32000633
chr11	32020004	32020210
chr11	32039186	32039544
chr11	32039713	32039914
chr11	32103054	32103261
chr11	32104826	32105035
chr11	32138372	32138544
chr11	32141087	32141344
chr11	32150077	32150262
chr11	32161534	32161869
chr11	32162063	32162517
chr11	32165185	32165461
chr11	32170475	32170625
chr11	32182542	32182740
chr11	32194164	32194332
chr11	32195215	32195441
chr11	32196837	32196987
chr11	32199286	32199466
chr11	32204990	32205750
chr11	32210682	32210905
chr11	32210905	32211136
chr11	32216988	32217182
chr11	32220753	32220920
chr11	32220933	32221362
chr11	32221362	32221743
chr11	32221761	32222085
chr11	32222164	32222458
chr11	32224893	32225056
chr11	32226325	32226624
chr11	32226624	32226857
chr11	32237073	32237366
chr11	32238343	32238832
chr11	32250844	32251306
chr11	32251306	32251820
chr11	32261589	32261928
chr11	32261945	32262156

chr11	32262156	32262445
chr11	32262659	32263150
chr11	32263241	32263493
chr11	32265169	32265374
chr11	32265480	32265666
chr11	32267259	32267532
chr11	32267532	32267834
chr11	32267950	32268219
chr11	32281592	32281826
chr11	32283483	32283721
chr11	32286797	32287153
chr11	32287153	32287454
chr11	32292890	32293186
chr11	32295024	32295261
chr11	32296310	32296540
chr11	32299924	32300205
chr11	32320558	32320794
chr11	32320871	32321171
chr11	32321171	32321520
chr11	32324590	32324970
chr11	32335969	32336125
chr11	32341194	32341344
chr11	32343464	32343645
chr11	32345160	32345515
chr11	32345515	32345894
chr11	32345894	32346242
chr11	32346242	32346567
chr11	32347630	32347951
chr11	32352840	32353016
chr11	32355359	32355701
chr11	32357604	32358144
chr11	32358405	32358742
chr11	32359603	32359934
chr11	32363247	32363558
chr11	32363558	32363858
chr11	32363858	32364204
chr11	32375058	32375286
chr11	32375286	32375577
chr11	32387320	32387470
chr11	32398430	32398683
chr11	32404500	32405260
chr11	32429273	32429491

chr11	32429491	32429735
chr11	32435435	32435758
chr11	32435758	32436089
chr11	32437440	32437755
chr11	32442183	32442333
chr11	32450279	32450431
chr11	32454554	32454721
chr11	32454721	32454929
chr11	32454929	32455223
chr11	32455223	32455680
chr11	32455680	32456099
chr11	32461266	32461416
chr11	32461956	32462125
chr11	32464381	32464763
chr11	32485385	32485590
chr11	32489101	32489415
chr11	32489415	32489659
chr11	32502700	32503000
chr11	32532738	32533041
chr11	32533041	32533408
chr11	32533408	32533811
chr11	32533811	32534305
chr11	32542005	32542223
chr11	32542223	32542456
chr11	32542704	32543013
chr11	32552509	32552969
chr11	32552969	32553254
chr11	32553767	32554114
chr11	32554114	32554390
chr11	32558015	32558503
chr11	32561544	32561741
chr11	32563626	32564145
chr11	32564848	32565004
chr11	32565060	32565322
chr11	32566950	32567131
chr11	32567395	32567786
chr11	32571995	32572220
chr11	32579624	32579835
chr11	32579835	32580039
chr11	32585148	32585488
chr11	32587307	32587575
chr11	32587911	32588318

chr11	32588449	32588688
chr11	32592485	32592867
chr11	32601917	32602156
chr11	32604842	32605021
chr11	32605061	32605387
chr11	32605387	32605892
chr11	32605927	32606159
chr11	32612406	32612649
chr11	32614676	32614875
chr11	32618753	32618912
chr11	32619078	32619250
chr11	32619250	32619406
chr11	32619418	32619613
chr11	32625751	32626021
chr11	32634288	32634450
chr11	32635104	32635277
chr11	32642101	32642601
chr11	32642601	32642908
chr11	32642908	32643273
chr11	32643279	32643454
chr11	32644119	32644290
chr11	32647312	32647549
chr11	32647549	32647775
chr11	32650423	32650714
chr11	32661690	32662047
chr11	32662154	32662359
chr11	32662372	32662628
chr11	32665782	32665943
chr11	32667110	32667295
chr11	32672271	32672435
chr11	32676241	32676520
chr11	32676783	32677086
chr11	32677839	32678124
chr11	32678214	32678364
chr11	32680044	32680327
chr11	32689581	32689990
chr11	32691971	32692199
chr11	32693576	32694130
chr11	32696542	32696741
chr11	32699806	32700013
chr11	32700013	32700226
chr11	32705093	32705571

chr11	32706157	32706380
chr11	32706609	32706970
chr11	32714297	32714536
chr11	32725551	32725701
chr11	32725824	32726058
chr11	32726071	32726393
chr11	32726393	32726673
chr11	32726673	32726977
chr11	32751716	32751892
chr11	32752324	32752544
chr11	32752544	32752818
chr11	32762281	32762723
chr11	32762723	32762976
chr11	32762976	32763313
chr11	32766167	32766539
chr11	32771107	32771376
chr11	32781185	32781676
chr11	32781680	32781882
chr11	32782764	32782971
chr11	32787617	32787778
chr11	32799811	32800053
chr11	32801138	32801288
chr11	32802936	32803161
chr11	32805964	32806284
chr11	32809169	32809473
chr11	32842657	32843146
chr11	32843832	32844026
chr11	32889425	32889596
chr11	32905477	32905679
chr11	32925636	32926079
chr11	32957049	32957308
chr11	32965442	32965671
chr11	32999330	32999507
chr11	33001390	33001540
chr11	33029007	33029225
chr11	33070947	33071291
chr11	33087988	33088363
chr11	33088363	33088638
chr11	33097920	33098140
chr11	33100075	33100331
chr11	33100331	33100601
chr11	33122624	33123104

chr11	33129266	33129693
chr11	33134654	33134819
chr11	33142706	33143265
chr11	33146759	33146955
chr11	33147147	33147580
chr11	33147580	33147900
chr11	33147900	33148158
chr11	33148158	33148432
chr11	33148432	33148721
chr11	33150517	33150761
chr11	33162019	33162265
chr11	33162265	33162555
chr11	33162555	33162915
chr11	33162915	33163608
chr11	33170589	33170830
chr11	33176644	33176877
chr11	33177333	33177517
chr11	33186070	33186290
chr11	33201104	33201282
chr11	33201289	33201657
chr11	33202547	33202724
chr11	33202947	33203103
chr11	33203103	33203388
chr11	33203388	33203812
chr11	33205074	33205326
chr11	33217790	33217965
chr11	33265826	33266000
chr11	33278696	33278846
chr11	33296448	33296772
chr11	33427830	33428146
chr11	33512795	33513151
chr11	33513151	33513396
chr11	33513396	33513628
chr11	33513628	33513898
chr11	33541385	33541787
chr11	33570506	33570730
chr11	33579546	33579847
chr11	33579847	33580037
chr11	33581857	33582203
chr11	33586960	33587463
chr11	33587937	33588141
chr11	33589823	33590071

chr11	33590933	33591248
chr11	33594529	33594763
chr11	33599659	33600104
chr11	33601280	33601826
chr11	33602156	33602567
chr11	33602774	33603013
chr11	33614234	33614585
chr11	33623578	33623762
chr11	33649469	33649681
chr11	33660006	33660550
chr11	33660550	33660878
chr11	33664910	33665233
chr11	33680236	33680407
chr11	33700760	33700896
chr11	33700896	33701035
chr11	33707304	33707454
chr11	33721382	33721630
chr11	33725682	33725935
chr11	33744349	33744960
chr11	33754789	33755114
chr11	33767573	33767847
chr11	33843513	33843709
chr11	33860761	33861213
chr11	33861213	33861509
chr11	33882766	33883036
chr11	33917662	33917897
chr11	33928775	33928953
chr11	33931915	33932079
chr11	33932079	33932221
chr11	34009915	34010224
chr11	34030104	34030367
chr11	34036386	34036658
chr11	34098558	34098710
chr11	34223185	34223480
chr11	34229059	34229216
chr11	34314612	34314819
chr11	34525534	34525810
chr11	34791959	34792126
chr11	34812949	34813125
chr11	34832792	34833039
chr11	34833268	34833800
chr11	34833800	34834085

chr11	34836371	34836583
chr11	34899291	34899802
chr11	35065840	35066403
chr11	35082418	35082687
chr11	35083094	35083341
chr11	35086051	35086254
chr11	35094014	35094218
chr11	35094639	35094791
chr11	35119471	35119703
chr11	35121065	35121231
chr11	35296257	35296407
chr11	35407738	35407893
chr11	35451647	35451936
chr11	35517860	35518078
chr11	35537521	35537851
chr11	35571960	35572153
chr11	35587118	35587492
chr11	35589918	35590273
chr11	35665061	35665224
chr11	35667483	35667728
chr11	35668025	35668436
chr11	35690428	35690580
chr11	35693168	35693484
chr11	35695177	35695343
chr11	35731916	35732414
chr11	35746075	35746383
chr11	35755367	35755574
chr11	35755700	35755907
chr11	35769182	35769644
chr11	35769644	35769938
chr11	35769938	35770184
chr11	35775678	35775941
chr11	35776444	35776846
chr11	35797977	35798197
chr11	35798961	35799212
chr11	35803113	35803316
chr11	35804125	35804350
chr11	35806245	35806445
chr11	35806957	35807143
chr11	35811556	35811713
chr11	35813466	35813659
chr11	35816396	35816649

chr11	35818320	35818471
chr11	35832257	35832511
chr11	35834135	35834355
chr11	35834355	35834737
chr11	35837713	35838231
chr11	35838384	35838772
chr11	35839000	35839285
chr11	35844306	35844906
chr11	35852815	35853077
chr11	35853715	35853952
chr11	35857969	35858164
chr11	35860072	35860264
chr11	35868780	35869201
chr11	35869328	35869483
chr11	35877590	35877975
chr11	35877975	35878253
chr11	35878253	35878540
chr11	35878540	35878836
chr11	35890343	35890544
chr11	35891914	35892128
chr11	35894483	35894682
chr11	35908820	35909044
chr11	35915122	35915425
chr11	35915425	35915791
chr11	35915791	35916092
chr11	35919979	35920189
chr11	35921316	35921593
chr11	35921766	35922102
chr11	35922102	35922458
chr11	35925438	35925965
chr11	35926246	35926485
chr11	35926485	35926687
chr11	35932588	35933166
chr11	35933443	35933697
chr11	35933697	35933981
chr11	35933981	35934199
chr11	35940579	35940947
chr11	35941785	35941999
chr11	35942564	35942802
chr11	35942915	35943163
chr11	35945186	35945615
chr11	35948317	35948979

chr11	35950544	35950998
chr11	35950998	35951291
chr11	35955591	35956053
chr11	35961957	35962508
chr11	35963600	35963961
chr11	35970416	35970661
chr11	35970706	35971242
chr11	35971242	35971594
chr11	35971594	35971882
chr11	35977458	35977691
chr11	35979093	35979279
chr11	35979843	35980148
chr11	35980257	35980813
chr11	35982640	35983241
chr11	35985817	35986126
chr11	35986126	35986421
chr11	35995389	35995653
chr11	35997954	35998401
chr11	36000998	36001285
chr11	36001285	36001695
chr11	36002062	36002232
chr11	36013219	36013538
chr11	36015054	36015206
chr11	36019998	36020293
chr11	36031074	36031230
chr11	36034419	36034632
chr11	36036168	36036873
chr11	36045687	36045960
chr11	36045960	36046197
chr11	36046613	36046958
chr11	36106550	36106702
chr11	36140520	36140687
chr11	36173376	36173535
chr11	36221304	36221565
chr11	36390358	36390674
chr11	36526487	36526639
chr11	37674001	37674160
chr11	38010705	38010875
chr11	38117743	38118121
chr11	38118121	38118428
chr11	38122580	38122730
chr11	38140812	38141030

chr11	38147005	38147155
chr11	38297737	38297979
chr11	38509751	38509995
chr11	38781130	38781436
chr11	40023687	40023898
chr11	40300047	40300198
chr11	40366630	40366899
chr11	40401989	40402269
chr11	40402269	40402577
chr11	40402577	40402924
chr11	40407439	40407665
chr11	40407665	40407976
chr11	40426369	40426598
chr11	40428843	40429365
chr11	40429439	40429728
chr11	40446883	40447281
chr11	40455063	40455392
chr11	40469388	40469659
chr11	40487541	40487812
chr11	40505575	40506039
chr11	40552143	40552423
chr11	40552555	40553134
chr11	40556671	40557042
chr11	40560100	40560550
chr11	40564392	40564708
chr11	40566278	40566448
chr11	40566678	40567065
chr11	40568636	40568895
chr11	40592008	40592463
chr11	40600239	40600406
chr11	40600499	40600680
chr11	40677301	40677451
chr11	40692114	40692305
chr11	40692305	40692551
chr11	40692551	40692881
chr11	40692963	40693244
chr11	40693417	40693845
chr11	40694835	40695000
chr11	40718322	40718472
chr11	40733057	40733369
chr11	40733369	40733741
chr11	40733741	40734005

chr11	40734005	40734303
chr11	40741521	40741853
chr11	40741853	40742321
chr11	40747591	40747960
chr11	40754524	40754820
chr11	40754820	40755160
chr11	40755160	40755566
chr11	40763456	40763738
chr11	40789079	40789263
chr11	40789844	40790094
chr11	40790168	40790615
chr11	40790615	40790897
chr11	40803107	40803423
chr11	40900872	40901129
chr11	40901186	40901393
chr11	40901481	40901631
chr11	40927248	40927424
chr11	41144625	41144828
chr11	41256093	41256292
chr11	41340142	41340403
chr11	41340403	41340824
chr11	41341079	41341388
chr11	41341772	41342016
chr11	41354641	41354854
chr11	41359312	41359505
chr11	41632554	41632812
chr11	42793312	42793659
chr11	42905626	42905938
chr11	42906012	42906373
chr11	42939308	42939714
chr11	42944440	42945002
chr11	42999261	42999420
chr11	43023126	43023515
chr11	43023515	43023923
chr11	43024855	43025060
chr11	43050473	43050730
chr11	43075558	43075720
chr11	43125591	43126064
chr11	43126064	43126510
chr11	43126647	43126909
chr11	43141323	43141520
chr11	43144087	43144624

chr11	43155498	43155771
chr11	43174502	43174740
chr11	43174816	43174972
chr11	43182866	43183198
chr11	43183579	43183881
chr11	43186385	43186750
chr11	43187372	43187598
chr11	43263926	43264159
chr11	43344767	43345140
chr11	43373421	43373859
chr11	43385064	43385396
chr11	43385396	43385670
chr11	43398310	43398464
chr11	43411515	43411767
chr11	43412835	43413085
chr11	43425923	43426421
chr11	43426505	43426666
chr11	43433645	43433864
chr11	43433864	43434083
chr11	43434083	43434334
chr11	43447358	43447508
chr11	43455053	43455392
chr11	43461927	43462180
chr11	43474187	43474491
chr11	43474491	43474737
chr11	43492671	43493000
chr11	43493965	43494312
chr11	43495649	43495943
chr11	43499586	43499880
chr11	43500378	43500597
chr11	43503904	43504066
chr11	43504204	43504366
chr11	43512836	43513071
chr11	43528672	43528930
chr11	43528930	43529130
chr11	43529130	43529320
chr11	43529320	43529515
chr11	43531756	43531911
chr11	43551818	43552116
chr11	43586430	43586650
chr11	43602460	43602615
chr11	43613170	43613418

chr11	43624383	43624610
chr11	43642874	43643141
chr11	43643141	43643627
chr11	43644633	43644785
chr11	43653264	43653490
chr11	43658115	43658295
chr11	43681534	43682048
chr11	43682048	43682368
chr11	43682368	43682581
chr11	43682581	43682813
chr11	43703634	43703936
chr11	43729121	43729370
chr11	43747437	43747865
chr11	43747865	43748228
chr11	43766858	43767146
chr11	43767146	43767499
chr11	43776066	43776340
chr11	43783801	43784046
chr11	43820625	43820812
chr11	43835909	43836138
chr11	43836153	43836440
chr11	43836440	43836717
chr11	43836717	43836992
chr11	43844930	43845084
chr11	43853120	43853285
chr11	43889215	43889840
chr11	43892700	43892987
chr11	43894741	43895203
chr11	43895382	43895729
chr11	43895878	43896145
chr11	43901318	43901645
chr11	43910131	43910520
chr11	43910520	43910984
chr11	43910984	43911299
chr11	43919472	43919862
chr11	43919862	43920338
chr11	43926253	43926504
chr11	43926506	43926680
chr11	43927152	43927404
chr11	43929195	43929553
chr11	43932737	43932941
chr11	43933061	43933442

chr11	43933445	43933962
chr11	43940423	43940645
chr11	43940645	43940875
chr11	43958299	43958556
chr11	43958573	43958883
chr11	43959001	43959203
chr11	43960805	43960991
chr11	43987244	43987503
chr11	43993394	43993598
chr11	43997663	43998065
chr11	43998065	43998380
chr11	43998380	43998694
chr11	43999299	43999493
chr11	44001866	44002181
chr11	44002240	44002521
chr11	44049214	44049613
chr11	44049613	44049950
chr11	44065823	44066026
chr11	44066166	44066492
chr11	44066690	44066957
chr11	44076947	44077097
chr11	44085019	44085245
chr11	44156096	44156424
chr11	44164006	44164586
chr11	44179770	44180141
chr11	44180989	44181184
chr11	44211838	44212072
chr11	44216344	44216810
chr11	44216943	44217189
chr11	44303890	44304158
chr11	44323035	44323326
chr11	44323326	44323738
chr11	44325613	44325883
chr11	44325883	44326077
chr11	44331529	44331762
chr11	44367197	44367428
chr11	44390162	44390541
chr11	44398844	44399036
chr11	44399036	44399278
chr11	44417391	44417710
chr11	44417728	44417882
chr11	44427643	44427899

chr11	44428049	44428287
chr11	44428724	44429068
chr11	44453481	44453888
chr11	44462704	44462870
chr11	44469557	44469881
chr11	44469881	44470142
chr11	44470142	44470395
chr11	44470395	44470744
chr11	44470744	44471043
chr11	44471137	44471318
chr11	44495623	44496000
chr11	44504309	44504584
chr11	44518496	44518836
chr11	44518836	44519140
chr11	44519140	44519429
chr11	44519429	44519689
chr11	44519689	44519987
chr11	44519987	44520363
chr11	44520365	44520745
chr11	44525804	44526012
chr11	44541917	44542072
chr11	44543639	44543898
chr11	44551090	44551293
chr11	44581454	44581708
chr11	44586175	44586326
chr11	44601169	44601327
chr11	44606916	44607217
chr11	44610930	44611185
chr11	44611203	44611567
chr11	44616866	44617410
chr11	44618197	44618558
chr11	44624666	44624866
chr11	44636711	44636873
chr11	44637144	44637429
chr11	44717749	44717940
chr11	44724102	44724377
chr11	44725838	44726137
chr11	44729910	44730302
chr11	44730321	44730508
chr11	44767961	44768247
chr11	44772362	44772547
chr11	44773094	44773259

chr11	44779042	44779232
chr11	44780637	44781024
chr11	44787223	44787397
chr11	44811888	44812077
chr11	44812384	44812779
chr11	44902230	44902529
chr11	44908394	44908719
chr11	44916514	44916664
chr11	44943798	44944115
chr11	44944393	44944715
chr11	44944766	44944953
chr11	44945577	44945798
chr11	44948466	44948955
chr11	44949174	44949474
chr11	44951253	44951446
chr11	44951476	44951981
chr11	45008181	45008436
chr11	45012122	45012456
chr11	45017205	45017441
chr11	45045284	45045532
chr11	45087222	45087439
chr11	45271377	45271571
chr11	45678775	45678935
chr11	45752283	45752537
chr11	45789153	45789479
chr11	45795745	45795986
chr11	45805191	45805540
chr11	45805540	45805868
chr11	45829087	45829288
chr11	45831674	45832092
chr11	45849341	45849515
chr11	45851645	45852122
chr11	45852122	45852481
chr11	45852481	45852773
chr11	45881156	45881336
chr11	45883338	45883628
chr11	45883628	45883898
chr11	45884169	45884415
chr11	45916997	45917152
chr11	45926513	45926864
chr11	45926864	45927149
chr11	45927393	45927710

chr11	45943855	45944073
chr11	45944147	45944575
chr11	45944575	45944922
chr11	45944922	45945224
chr11	45955071	45955373
chr11	45955373	45955683
chr11	45960143	45960300
chr11	45980062	45980235
chr11	45980320	45980596
chr11	45980596	45980869
chr11	46052178	46052384
chr11	46052384	46052564
chr11	46055868	46056462
chr11	46056573	46056723
chr11	46058009	46058335
chr11	46063340	46063625
chr11	46065399	46065656
chr11	46065656	46066091
chr11	46072567	46072736
chr11	46072914	46073139
chr11	46073452	46073643
chr11	46080068	46080310
chr11	46080333	46080751
chr11	46101889	46102476
chr11	46115732	46116046
chr11	46116635	46116791
chr11	46119829	46120020
chr11	46120793	46121017
chr11	46122479	46123043
chr11	46128634	46128867
chr11	46160635	46161088
chr11	46161237	46161408
chr11	46164353	46164715
chr11	46165921	46166234
chr11	46166234	46166708
chr11	46177687	46178070
chr11	46215417	46215691
chr11	46215691	46215837
chr11	46245958	46246325
chr11	46252067	46252303
chr11	46269040	46269265
chr11	46275394	46275672

chr11	46276889	46277061
chr11	46311682	46311882
chr11	46311882	46312172
chr11	46312172	46312565
chr11	46312565	46312899
chr11	46326551	46326842
chr11	46424763	46424981
chr11	46431224	46431514
chr11	46436720	46437471
chr11	46437477	46437656
chr11	46454474	46455037
chr11	46461419	46461629
chr11	46461955	46462340
chr11	46466802	46467331
chr11	46488363	46488515
chr11	46504290	46504494
chr11	46573393	46573563
chr11	46631771	46631942
chr11	46666225	46666432
chr11	46693344	46693689
chr11	46700358	46700508
chr11	46746002	46746415
chr11	47149726	47149876
chr11	47190852	47191074
chr11	47805052	47805418
chr11	47810438	47810588
chr11	47823552	47823711
chr11	47823727	47824262
chr11	47833340	47833636
chr11	47900591	47900808
chr11	47900808	47901029
chr11	47902612	47902867
chr11	47905599	47906039
chr11	47916615	47916874
chr11	47966579	47966742
chr11	47988372	47988524
chr11	47988711	47989008
chr11	48097557	48097787
chr11	48097822	48098128
chr11	48098128	48098415
chr11	48133184	48133422
chr11	48133422	48133784

chr11	48588914	48589121
chr11	48698975	48699127
chr11	48699131	48699564
chr11	48699613	48699844
chr11	48699976	48700136
chr11	48748322	48748482
chr11	48774574	48774750
chr11	48774789	48775198
chr11	48775553	48775816
chr11	48775957	48776258
chr11	48776722	48776965
chr11	48779563	48779717
chr11	48783050	48783257
chr11	48785932	48786146
chr11	48787898	48788080
chr11	48788326	48788476
chr11	48788545	48788704
chr11	48788707	48789051
chr11	48790284	48790543
chr11	48799627	48799918
chr11	48799918	48800183
chr11	48800183	48800450
chr11	48800450	48800849
chr11	48801184	48801391
chr11	48806888	48807234
chr11	48807234	48807577
chr11	48807577	48808156
chr11	48808289	48808448
chr11	48808471	48808690
chr11	48808690	48808825
chr11	48808999	48809237
chr11	48809951	48810101
chr11	48816551	48816741
chr11	48816823	48817222
chr11	48817222	48817685
chr11	48818338	48818656
chr11	48819726	48820154
chr11	48820443	48820623
chr11	48822804	48823135
chr11	48823695	48823971
chr11	48825932	48826188
chr11	48826188	48826465

chr11	48826465	48826763
chr11	48826871	48827042
chr11	48833742	48834028
chr11	48834150	48834639
chr11	48837112	48837330
chr11	48837330	48837594
chr11	48837594	48837872
chr11	48837872	48838147
chr11	48838188	48838615
chr11	48844296	48844520
chr11	48845487	48845709
chr11	48846424	48846632
chr11	48846702	48846852
chr11	48852372	48852557
chr11	48853563	48853806
chr11	48853850	48854139
chr11	48855528	48855778
chr11	48855888	48856103
chr11	48856217	48856493
chr11	48856493	48856773
chr11	48856773	48857180
chr11	48858122	48858302
chr11	48869863	48870018
chr11	48871301	48871619
chr11	48871619	48871991
chr11	48872015	48872526
chr11	48873433	48873590
chr11	48873617	48873817
chr11	48873873	48874039
chr11	48874589	48874765
chr11	48898886	48899550
chr11	48914426	48914741
chr11	48933977	48934275
chr11	48934992	48935199
chr11	48935233	48935649
chr11	48935649	48935969
chr11	48974351	48974725
chr11	48974725	48975057
chr11	48975110	48975280
chr11	48979343	48979706
chr11	48979706	48980047
chr11	48980099	48980350

chr11	48984466	48984686
chr11	49006778	49007180
chr11	49008111	49008527
chr11	49008650	49008840
chr11	49046198	49046569
chr11	49046569	49046904
chr11	49046928	49047109
chr11	49050837	49050992
chr11	49051187	49051543
chr11	49051543	49051893
chr11	49051958	49052196
chr11	49056329	49056565
chr11	49060077	49060348
chr11	49083278	49083460
chr11	49084370	49084594
chr11	49084738	49085233
chr11	49087053	49087432
chr11	49087900	49088127
chr11	49088293	49088627
chr11	49090235	49090460
chr11	49092151	49092375
chr11	49098914	49099068
chr11	49103141	49103379
chr11	49103379	49103648
chr11	49104381	49104538
chr11	49108171	49108448
chr11	49108882	49109061
chr11	49109061	49109240
chr11	49124252	49124513
chr11	49189767	49190055
chr11	49191088	49191556
chr11	49192976	49193149
chr11	49198007	49198243
chr11	49203108	49203847
chr11	49203862	49204350
chr11	49215010	49215207
chr11	49235586	49235792
chr11	49243686	49243936
chr11	49243936	49244174
chr11	49244174	49244632
chr11	49245312	49245523
chr11	49248754	49248943

chr11	49249189	49249415
chr11	49250360	49250685
chr11	49250685	49251094
chr11	49260396	49261007
chr11	49261007	49261432
chr11	49264757	49265119
chr11	49268000	49268295
chr11	49268295	49268527
chr11	49271369	49271624
chr11	49273584	49273921
chr11	49295905	49296202
chr11	49308564	49308774
chr11	49337088	49337456
chr11	49381835	49382213
chr11	49406035	49406316
chr11	49410623	49410855
chr11	49428845	49429112
chr11	49439263	49439660
chr11	49493634	49493789
chr11	49497750	49498016
chr11	49513961	49514159
chr11	49523987	49524305
chr11	49531377	49531918
chr11	49532792	49533119
chr11	49536700	49536959
chr11	49554082	49554335
chr11	49577857	49578226
chr11	49578340	49578816
chr11	49590029	49590298
chr11	49595749	49596035
chr11	49597237	49597479
chr11	49603147	49603297
chr11	49609176	49609352
chr11	49609391	49609964
chr11	49609964	49610453
chr11	49611395	49611568
chr11	49612223	49612450
chr11	49616233	49617138
chr11	49624212	49624446
chr11	49626021	49626127
chr11	49626127	49626249
chr11	49628638	49628865

chr11	49631227	49631477
chr11	49632689	49632887
chr11	49633114	49633301
chr11	49635543	49636035
chr11	49650526	49650704
chr11	49651532	49651780
chr11	49652802	49653066
chr11	49653066	49653315
chr11	49658178	49658397
chr11	49663052	49663316
chr11	49664463	49664633
chr11	49695049	49695317
chr11	49703023	49703291
chr11	49703426	49703768
chr11	49711941	49712288
chr11	49712288	49712627
chr11	49712627	49713053
chr11	49713239	49713544
chr11	49727939	49728177
chr11	49739089	49739300
chr11	49739865	49740076
chr11	49746691	49747109
chr11	49755297	49755535
chr11	49766665	49767156
chr11	49792792	49792942
chr11	49793174	49793402
chr11	49793965	49794378
chr11	49811060	49811573
chr11	49824971	49825225
chr11	49829267	49829661
chr11	49829663	49829972
chr11	49833963	49834153
chr11	49846312	49846927
chr11	49846927	49847305
chr11	49869806	49870098
chr11	49871941	49872195
chr11	49874732	49875062
chr11	49877628	49877810
chr11	49879989	49880229
chr11	49901637	49902308
chr11	49903421	49903661
chr11	49913242	49913392

chr11	49945217	49945578
chr11	49955680	49955854
chr11	49955907	49956355
chr11	49961371	49961719
chr11	49979731	49979899
chr11	49980596	49981143
chr11	49981207	49981693
chr11	49986266	49986520
chr11	49986713	49986974
chr11	49997280	49997737
chr11	50017720	50017905
chr11	50024802	50025094
chr11	50025094	50025343
chr11	50025343	50025840
chr11	50076869	50077020
chr11	50099829	50099997
chr11	50113469	50113919
chr11	50113919	50114292
chr11	50114409	50114786
chr11	50130449	50130795
chr11	50130795	50131110
chr11	50131110	50131802
chr11	50138448	50138839
chr11	50145610	50145965
chr11	50155837	50156254
chr11	50161443	50161602
chr11	50161619	50161867
chr11	50161867	50162044
chr11	50167090	50167335
chr11	50174387	50174561
chr11	50174576	50174949
chr11	50174949	50175170
chr11	50175170	50175365
chr11	50188842	50189097
chr11	50193612	50193809
chr11	50195297	50195476
chr11	50196909	50197355
chr11	50197355	50197599
chr11	50197599	50197912
chr11	50198088	50198662
chr11	50198814	50199093
chr11	50199093	50199366

chr11	50199499	50199650
chr11	50200718	50200877
chr11	50203530	50203783
chr11	50204771	50205133
chr11	50210046	50210385
chr11	50210385	50210658
chr11	50210658	50210864
chr11	50210981	50211180
chr11	50211959	50212305
chr11	50212811	50213072
chr11	50213072	50213374
chr11	50213374	50213769
chr11	50219115	50219314
chr11	50222642	50222803
chr11	50224592	50224918
chr11	50224918	50225166
chr11	50225166	50225578
chr11	50225608	50225998
chr11	50225998	50226193
chr11	50229277	50229485
chr11	50230999	50231411
chr11	50231457	50231710
chr11	50235171	50235710
chr11	50236543	50236806
chr11	50236806	50237113
chr11	50237113	50237445
chr11	50237445	50237671
chr11	50237942	50238173
chr11	50238494	50238644
chr11	50244548	50244783
chr11	50251589	50251825
chr11	50251825	50252024
chr11	50252093	50252447
chr11	50252447	50252729
chr11	50252729	50253060
chr11	50262091	50262344
chr11	50267028	50267368
chr11	50267439	50267814
chr11	50269244	50269453
chr11	50274814	50274999
chr11	50275378	50275893
chr11	50275893	50276230

chr11	50291536	50291889
chr11	50291889	50292262
chr11	50292262	50292623
chr11	50292623	50292887
chr11	50300944	50301124
chr11	50319408	50319628
chr11	50325106	50325572
chr11	50325572	50326227
chr11	50357626	50357811
chr11	50358192	50358416
chr11	50359007	50359276
chr11	50359276	50359493
chr11	50359548	50359828
chr11	50363934	50364226
chr11	50364468	50364642
chr11	50364642	50364889
chr11	50366919	50367157
chr11	50369335	50369686
chr11	50371439	50371629
chr11	50371658	50371820
chr11	50376836	50377095
chr11	50377095	50377412
chr11	50377412	50377705
chr11	50377705	50377894
chr11	50377894	50378104
chr11	50378104	50378355
chr11	50378355	50378653
chr11	50386691	50387048
chr11	50387063	50387284
chr11	50387284	50387580
chr11	50387588	50387753
chr11	50387882	50388321
chr11	50388321	50388628
chr11	50389749	50389929
chr11	50391527	50391835
chr11	50391835	50392010
chr11	50407058	50407215
chr11	50416672	50416822
chr11	50421904	50422270
chr11	50430318	50430557
chr11	50430557	50430778
chr11	50430778	50430984

chr11	50430984	50431250
chr11	50431469	50431684
chr11	50431806	50431967
chr11	50432002	50432436
chr11	50432854	50433326
chr11	50433341	50433553
chr11	50439535	50439792
chr11	50439792	50440324
chr11	50441694	50442019
chr11	50448276	50448427
chr11	50459300	50459508
chr11	50465083	50465248
chr11	50483473	50483671
chr11	50559110	50559379
chr11	50559414	50559656
chr11	50565516	50565769
chr11	50565769	50566187
chr11	50587122	50587344
chr11	50587790	50588106
chr11	50589148	50589298
chr11	50601618	50601792
chr11	50601798	50602291
chr11	50603195	50603439
chr11	50622296	50622531
chr11	50632431	50632647
chr11	50664934	50665185
chr11	50672907	50673303
chr11	50674359	50675028
chr11	50683868	50684069
chr11	50712291	50712608
chr11	50713258	50713426
chr11	50713682	50713928
chr11	50720482	50720670
chr11	50737199	50737364
chr11	50766090	50766311
chr11	50767100	50767319
chr11	50769192	50769484
chr11	50769484	50769809
chr11	50827040	50827220
chr11	50827339	50827515
chr11	50827649	50828049
chr11	50841466	50841712

chr11	50851158	50851467
chr11	50915638	50915845
chr11	50915845	50916060
chr11	50916060	50916396
chr11	50929462	50929782
chr11	50931143	50931342
chr11	50931391	50931931
chr11	50932217	50932417
chr11	50932556	50932791
chr11	50939970	50940244
chr11	50951262	50951412
chr11	50952353	50952655
chr11	50963175	50963356
chr11	51012844	51013063
chr11	51013063	51013305
chr11	51031136	51031287
chr11	51054583	51054791
chr11	51059093	51059365
chr11	51059365	51059743
chr11	51059749	51059939
chr11	51063364	51063536
chr11	51064132	51064531
chr11	51164922	51165126
chr11	51165504	51165774
chr11	51182728	51182955
chr11	51184278	51184456
chr11	51198458	51198652
chr11	51206298	51206586
chr11	51207784	51208336
chr11	51208398	51208572
chr11	51262660	51262936
chr11	51262936	51263284
chr11	51263287	51263528
chr11	51263528	51263813
chr11	51264301	51264793
chr11	51265487	51265649
chr11	51287447	51287602
chr11	51289515	51289756
chr11	51289756	51290090
chr11	51290090	51290465
chr11	51353873	51354111
chr11	51401826	51402082

chr11	51404891	51405257
chr11	51422105	51422280
chr11	51431371	51431661
chr11	51447109	51447307
chr11	51455576	51455883
chr11	51485002	51485211
chr11	51513057	51513331
chr11	51527499	51527675
chr11	51534393	51534543
chr11	51539231	51539601
chr11	51539606	51540024
chr11	51540051	51540205
chr11	51546864	51547019
chr11	51551273	51551534
chr11	51551624	51551774
chr11	51555717	51555867
chr11	51565124	51565274
chr11	51571988	51572140
chr11	51575820	51575970
chr11	51581439	51581631
chr11	51582887	51583053
chr11	51584060	51584306
chr11	51584326	51584831
chr11	51584831	51585260
chr11	51585503	51585750
chr11	51585750	51585941
chr11	51591383	51591542
chr11	51592124	51592291
chr11	51593522	51593734
chr11	51593821	51594013
chr11	51594013	51594186
chr11	51594186	51594358
chr11	51594926	51595312
chr11	51595312	51595598
chr11	51598277	51598432
chr11	51605839	51606215
chr11	51606215	51606664
chr11	51606664	51607088
chr11	51607088	51607357
chr11	51614948	51615125
chr11	51615233	51615517
chr11	51615651	51615955

chr11	51615955	51616149
chr11	51617770	51617994
chr11	51619170	51619360
chr11	51619377	51619854
chr11	51619854	51620089
chr11	51620733	51620945
chr11	51625097	51625304
chr11	51625304	51625647
chr11	51625647	51626004
chr11	51627767	51627929
chr11	51629432	51629658
chr11	51631734	51631998
chr11	51635473	51635829
chr11	51635829	51636151
chr11	51636151	51636401
chr11	51636409	51636646
chr11	51648919	51649127
chr11	51650195	51650645
chr11	51650645	51651116
chr11	51651116	51651507
chr11	51651507	51651744
chr11	51657177	51657482
chr11	51658458	51658774
chr11	51658774	51659438
chr11	51661283	51661453
chr11	51662948	51663297
chr11	51668665	51668872
chr11	51668885	51669189
chr11	51671475	51671736
chr11	51672565	51672715
chr11	51673387	51673648
chr11	51673648	51673861
chr11	51681455	51681659
chr11	51681659	51681945
chr11	51681949	51682320
chr11	51688306	51688573
chr11	51688573	51688953
chr11	51688953	51689325
chr11	51702897	51703178
chr11	51703404	51703664
chr11	51703693	51703944
chr11	51735913	51736173

chr11	51747100	51747317
chr11	51755688	51755879
chr11	51755904	51756271
chr11	51756271	51756879
chr11	51760403	51760568
chr11	51763500	51763960
chr11	51764923	51765128
chr11	51768304	51768630
chr11	51771037	51771438
chr11	51777544	51777849
chr11	51779680	51780005
chr11	51780912	51781212
chr11	51786016	51786280
chr11	51789824	51790027
chr11	51796280	51796656
chr11	51801077	51801563
chr11	51801563	51801910
chr11	51805278	51805691
chr11	51807204	51807466
chr11	51809267	51809603
chr11	51809603	51809903
chr11	51812057	51812345
chr11	51817743	51818111
chr11	51818127	51818308
chr11	51820029	51820267
chr11	51820719	51820893
chr11	51820911	51821168
chr11	51824441	51824647
chr11	51832270	51832510
chr11	51832510	51832880
chr11	51837809	51838140
chr11	51843231	51843477
chr11	51855458	51855733
chr11	51855733	51855971
chr11	51855971	51856291
chr11	51856291	51856871
chr11	51857006	51857408
chr11	51857784	51858115
chr11	51858888	51859038
chr11	51865633	51865934
chr11	51865988	51866511
chr11	51866583	51866733

chr11	51874304	51874871
chr11	51874871	51875372
chr11	51875823	51876113
chr11	51878268	51878493
chr11	51880991	51881386
chr11	51881386	51881676
chr11	51881896	51882089
chr11	51886844	51887038
chr11	51891979	51892282
chr11	51892527	51893180
chr11	51895925	51896197
chr11	51904151	51904718
chr11	51906681	51906891
chr11	51914326	51914522
chr11	51915455	51915993
chr11	51915993	51916451
chr11	51916451	51916938
chr11	51922396	51922949
chr11	51922951	51923134
chr11	51931008	51931441
chr11	51931781	51932083
chr11	51935320	51935625
chr11	51940446	51941151
chr11	51945481	51945992
chr11	51946159	51946558
chr11	51949540	51949809
chr11	51950927	51951152
chr11	51951223	51951475
chr11	51951964	51952190
chr11	51952190	51952399
chr11	51957865	51958221
chr11	51961204	51961458
chr11	51967274	51967761
chr11	51967761	51968047
chr11	51968047	51968387
chr11	51972832	51973084
chr11	51983553	51983734
chr11	51983734	51983939
chr11	51983939	51984136
chr11	51984471	51984669
chr11	51999657	51999881
chr11	51999881	52000085

chr11	52000085	52000326
chr11	52000326	52000642
chr11	52000642	52000997
chr11	52003862	52004582
chr11	52015838	52015989
chr11	52035157	52035431
chr11	52035431	52035688
chr11	52035904	52036279
chr11	52038374	52038602
chr11	52046527	52046771
chr11	52055727	52055923
chr11	52056054	52056287
chr11	52064021	52064243
chr11	52064457	52064731
chr11	52064881	52065043
chr11	52069116	52069308
chr11	52070865	52071137
chr11	52071823	52072055
chr11	52076854	52077316
chr11	52077793	52078315
chr11	52079947	52080532
chr11	52081409	52081610
chr11	52087586	52087907
chr11	52088541	52088836
chr11	52089278	52089586
chr11	52091343	52091587
chr11	52098145	52098596
chr11	52098596	52098992
chr11	52098992	52099444
chr11	52099486	52099636
chr11	52099726	52099935
chr11	52104773	52104923
chr11	52104976	52105332
chr11	52112321	52112586
chr11	52117246	52117470
chr11	52118416	52118573
chr11	52124473	52124821
chr11	52125405	52125686
chr11	52129029	52129223
chr11	52129226	52129518
chr11	52129773	52130123
chr11	52130352	52130515

chr11	52130532	52130796
chr11	52135727	52136001
chr11	52138926	52139253
chr11	52139340	52139646
chr11	52143752	52144018
chr11	52144041	52144193
chr11	52147649	52147887
chr11	52148827	52149374
chr11	52155703	52156034
chr11	52157296	52157520
chr11	52162596	52162746
chr11	52165242	52165410
chr11	52167216	52167659
chr11	52167794	52168474
chr11	52168490	52168734
chr11	52179973	52180226
chr11	52181616	52182002
chr11	52187256	52187471
chr11	52198600	52199042
chr11	52199161	52199507
chr11	52212007	52212280
chr11	52220335	52220622
chr11	52223019	52223232
chr11	52223986	52224349
chr11	52224349	52224756
chr11	52231532	52231780
chr11	52231780	52231987
chr11	52231987	52232248
chr11	52232248	52232526
chr11	52243086	52243243
chr11	52253250	52253414
chr11	52253810	52254023
chr11	52254023	52254268
chr11	52260824	52261268
chr11	52262015	52262271
chr11	52268166	52268424
chr11	52275611	52275767
chr11	52282448	52282773
chr11	52282773	52283136
chr11	52283136	52283366
chr11	52283366	52283593
chr11	52297417	52297717

chr11	52299766	52299946
chr11	52301486	52301644
chr11	52314500	52314744
chr11	52318449	52318645
chr11	52320830	52321038
chr11	52332880	52333154
chr11	52333533	52334103
chr11	52335978	52336207
chr11	52337953	52338212
chr11	52341022	52341257
chr11	52343208	52343484
chr11	52346331	52346624
chr11	52346667	52346903
chr11	52350385	52350537
chr11	52350903	52351216
chr11	52359748	52359951
chr11	52360215	52360490
chr11	52360490	52360850
chr11	52360850	52361279
chr11	52361279	52361591
chr11	52361591	52361821
chr11	52363114	52363329
chr11	52368603	52368773
chr11	52373897	52374224
chr11	52374334	52374788
chr11	52379036	52379430
chr11	52396249	52396561
chr11	52396561	52396762
chr11	52396762	52396962
chr11	52396962	52397276
chr11	52418472	52418765
chr11	52419375	52419525
chr11	52421459	52421610
chr11	52425087	52425355
chr11	52429758	52429978
chr11	52433459	52433728
chr11	52437101	52437417
chr11	52447308	52447536
chr11	52448846	52449426
chr11	52464147	52464359
chr11	52469275	52469528
chr11	52480729	52480958

chr11	52506860	52507114
chr11	52517414	52517637
chr11	52534542	52534882
chr11	52535438	52535712
chr11	52537678	52538173
chr11	52551149	52551440
chr11	52551440	52551852
chr11	52570302	52570460
chr11	52574271	52574669
chr11	52574811	52575039
chr11	52610730	52611151
chr11	52634298	52634488
chr11	52648879	52649274
chr11	52655751	52656073
chr11	52656119	52656459
chr11	52656459	52656699
chr11	52671836	52672367
chr11	52672367	52672617
chr11	52718609	52718864
chr11	52744001	52744300
chr11	52744712	52744872
chr11	52753327	52753789
chr11	52764405	52764805
chr11	52764805	52765128
chr11	52765128	52765365
chr11	52765365	52765561
chr11	52765752	52765985
chr11	52765985	52766245
chr11	52874851	52875056
chr11	52933049	52933396
chr11	53016801	53017038
chr11	53028517	53028677
chr11	53030909	53031304
chr11	53031728	53032185
chr11	53032409	53032674
chr11	53047915	53048215
chr11	53096916	53097213
chr11	53132798	53132948
chr11	53139507	53139705
chr11	53140243	53140495
chr11	53143668	53143845
chr11	53144577	53144861

chr11	53146376	53146620
chr11	53175598	53175832
chr11	53230850	53231000
chr11	53231017	53231409
chr11	53245556	53246050
chr11	53246104	53246368
chr11	53254520	53254739
chr11	53254992	53255168
chr11	53256420	53256633
chr11	53256665	53256815
chr11	53259046	53259276
chr11	53259276	53259521
chr11	53260188	53260382
chr11	53260490	53260640
chr11	53267634	53267935
chr11	53288256	53288426
chr11	53299529	53299740
chr11	53299751	53299979
chr11	53300074	53300407
chr11	53300407	53300815
chr11	53300815	53301118
chr11	53308131	53308398
chr11	53308398	53308636
chr11	53314678	53315233
chr11	53319936	53320290
chr11	53324494	53324995
chr11	53330862	53331082
chr11	53331135	53331597
chr11	53341076	53341405
chr11	53341435	53341887
chr11	53342455	53342838
chr11	53350254	53350613
chr11	53350613	53351273
chr11	53351481	53351642
chr11	53365720	53365880
chr11	53374998	53375255
chr11	53392214	53392448
chr11	53411837	53412070
chr11	53412471	53412664
chr11	53414020	53414173
chr11	53430270	53430617
chr11	53430617	53430936

chr11	53430936	53431142
chr11	53431142	53431390
chr11	53431428	53431598
chr11	53443894	53444230
chr11	53450086	53450321
chr11	53456540	53456788
chr11	53457040	53457370
chr11	53457370	53457642
chr11	53457661	53457923
chr11	53460214	53460687
chr11	53461789	53461975
chr11	53469964	53470261
chr11	53470303	53470611
chr11	53472834	53473021
chr11	53479720	53479941
chr11	53479959	53480287
chr11	53483082	53483520
chr11	53483520	53484148
chr11	53487215	53487668
chr11	53494483	53494719
chr11	53500557	53501001
chr11	53503787	53504073
chr11	53508157	53508431
chr11	53508431	53508664
chr11	53508718	53508994
chr11	53519327	53519527
chr11	53519527	53519738
chr11	53519738	53519979
chr11	53519979	53520182
chr11	53520182	53520384
chr11	53520384	53520661
chr11	53520661	53520951
chr11	53536721	53536943
chr11	53539146	53539525
chr11	53541465	53541690
chr11	53543738	53543924
chr11	53545400	53545560
chr11	53558082	53558352
chr11	53567062	53567493
chr11	53567493	53567707
chr11	53567707	53567997
chr11	53572992	53573282

chr11	53577709	53577876
chr11	53583989	53584240
chr11	53584913	53585130
chr11	53590344	53590496
chr11	53627924	53628228
chr11	53632387	53632762
chr11	53636140	53636489
chr11	53655127	53655387
chr11	53706633	53706950
chr11	53706950	53707228
chr11	53707228	53707667
chr11	53728708	53728914
chr11	53735606	53735902
chr11	53735902	53736072
chr11	53737054	53737396
chr11	53737396	53737662
chr11	53737662	53737933
chr11	53749753	53750062
chr11	53750960	53751176
chr11	53751176	53751383
chr11	53754914	53755507
chr11	53759579	53759734
chr11	53761358	53761566
chr11	53761787	53762287
chr11	53762400	53762867
chr11	53762867	53763219
chr11	53763269	53763539
chr11	53763539	53763859
chr11	53763859	53764195
chr11	53769696	53770180
chr11	53770180	53770559
chr11	53770559	53770836
chr11	53774846	53774996
chr11	53779705	53780187
chr11	53780334	53780523
chr11	53786329	53786690
chr11	53794631	53795203
chr11	53795203	53795595
chr11	53795858	53796250
chr11	53796570	53797166
chr11	53802902	53803136
chr11	53803136	53803483

chr11	53803483	53803824
chr11	53803824	53804167
chr11	53804719	53804940
chr11	53810178	53810461
chr11	53815018	53815181
chr11	53820404	53820861
chr11	53821070	53821226
chr11	53821389	53821907
chr11	53837740	53838045
chr11	53839721	53840099
chr11	53840099	53840592
chr11	53849947	53850236
chr11	53851792	53852117
chr11	53852117	53852495
chr11	53853731	53853961
chr11	53858813	53859287
chr11	53859287	53859804
chr11	53861138	53861288
chr11	53868208	53868622
chr11	53873963	53874234
chr11	53874814	53875047
chr11	53883674	53884006
chr11	53891020	53891522
chr11	53891522	53892169
chr11	53903658	53904138
chr11	53904138	53904501
chr11	53914750	53914939
chr11	53915700	53916001
chr11	53929718	53930053
chr11	53931437	53931690
chr11	53931690	53931860
chr11	53931935	53932181
chr11	53948382	53948540
chr11	53953723	53953889
chr11	53953905	53954077
chr11	53956668	53956952
chr11	53958330	53958495
chr11	53959093	53959292
chr11	53960945	53961107
chr11	53969075	53969445
chr11	53969445	53969839
chr11	53979439	53979779

chr11	53979779	53980099
chr11	53980099	53980612
chr11	53989321	53989480
chr11	54002207	54002442
chr11	54002836	54003050
chr11	54003058	54003253
chr11	54003253	54003450
chr11	54003692	54003848
chr11	54003848	54003995
chr11	54004154	54004631
chr11	54004699	54004977
chr11	54011107	54011313
chr11	54011313	54011862
chr11	54017655	54017973
chr11	54018259	54018585
chr11	54018891	54019253
chr11	54019253	54019755
chr11	54024615	54024799
chr11	54027879	54028431
chr11	54028431	54028929
chr11	54051183	54051343
chr11	54054305	54054592
chr11	54060277	54060774
chr11	54061092	54061446
chr11	54061950	54062583
chr11	54065850	54066049
chr11	54066521	54066738
chr11	54066804	54066977
chr11	54069938	54070154
chr11	54072759	54072954
chr11	54073384	54073594
chr11	54080390	54080778
chr11	54085975	54086128
chr11	54088797	54089194
chr11	54089194	54089411
chr11	54093072	54093381
chr11	54100422	54100572
chr11	54100643	54101081
chr11	54101081	54101478
chr11	54101608	54101891
chr11	54106599	54107164
chr11	54108970	54109383

chr11	54112867	54113307
chr11	54119870	54120023
chr11	54122610	54122797
chr11	54122886	54123251
chr11	54132387	54132609
chr11	54140019	54140388
chr11	54140388	54140702
chr11	54141342	54141530
chr11	54141628	54142007
chr11	54144513	54144890
chr11	54144946	54145201
chr11	54147380	54147640
chr11	54149498	54149806
chr11	54155613	54156444
chr11	54178970	54179216
chr11	54181594	54181998
chr11	54181998	54182306
chr11	54185673	54185823
chr11	54185908	54186394
chr11	54201205	54201567
chr11	54201567	54201854
chr11	54201854	54202272
chr11	54202809	54203028
chr11	54203789	54204036
chr11	54204114	54204323
chr11	54205453	54205684
chr11	54206849	54207410
chr11	54214508	54215059
chr11	54216092	54216691
chr11	54217350	54218022
chr11	54221936	54222304
chr11	54224011	54224437
chr11	54224437	54224656
chr11	54224656	54224902
chr11	54224902	54225133
chr11	54231384	54231627
chr11	54232087	54232292
chr11	54232310	54232525
chr11	54236748	54236905
chr11	54236931	54237504
chr11	54242588	54242740
chr11	54244358	54244614

chr11	54245395	54245593
chr11	54245851	54246042
chr11	54246242	54246517
chr11	54248997	54249413
chr11	54249413	54249867
chr11	54249891	54250068
chr11	54254180	54254428
chr11	54257047	54257335
chr11	54257997	54258225
chr11	54258234	54258441
chr11	54258617	54258804
chr11	54263569	54263724
chr11	54264634	54264872
chr11	54267505	54267774
chr11	54268649	54268843
chr11	54269387	54269854
chr11	54270364	54270527
chr11	54286571	54286740
chr11	54303147	54303349
chr11	54304030	54304298
chr11	54306498	54306960
chr11	54307505	54307719
chr11	54317360	54317606
chr11	54326130	54326520
chr11	54326520	54326797
chr11	54326797	54327155
chr11	54354666	54354944
chr11	54400896	54401086
chr11	54432675	54432909
chr11	54437763	54438083
chr11	54438083	54438318
chr11	54438318	54438834
chr11	54444060	54444285
chr11	54491997	54492267
chr11	54492267	54492532
chr11	54492604	54492856
chr11	54522414	54522937
chr11	54522937	54523257
chr11	54523257	54523499
chr11	54528983	54529250
chr11	54534994	54535235
chr11	54620367	54620602

chr11	54659596	54659774
chr11	54693747	54694087
chr11	54696100	54696290
chr11	54702770	54703006
chr11	54703186	54703336
chr11	54703351	54703631
chr11	54703686	54703873
chr11	54703873	54704105
chr11	54724434	54724676
chr11	54724745	54724935
chr11	54726541	54726735
chr11	54727178	54727410
chr11	54727410	54727638
chr11	54729358	54729576
chr11	54733255	54733492
chr11	54748601	54748848
chr11	54748891	54749456
chr11	54755203	54755439
chr11	54786806	54787021
chr11	54787021	54787260
chr11	54787260	54787540
chr11	54787540	54788106
chr11	54801756	54802127
chr11	54802191	54802599
chr11	54803948	54804363
chr11	54809703	54810327
chr11	54812832	54812985
chr11	54813136	54813466
chr11	54830315	54830595
chr11	54830655	54830921
chr11	54859408	54859603
chr11	54860285	54861165
chr11	54866043	54866504
chr11	54866504	54867034
chr11	54867044	54867281
chr11	54869408	54869641
chr11	54874990	54875306
chr11	54879453	54879738
chr11	54880844	54881273
chr11	54881273	54881632
chr11	54899430	54899604
chr11	54899635	54900078

chr11	54901623	54901843
chr11	54906124	54906342
chr11	54906438	54906931
chr11	54916821	54916971
chr11	54917602	54917752
chr11	54918356	54918616
chr11	54927804	54928081
chr11	54928097	54928247
chr11	54930401	54930616
chr11	54934215	54934509
chr11	54935983	54936405
chr11	54936405	54936842
chr11	54936842	54937213
chr11	54946889	54947263
chr11	54948370	54948676
chr11	54952716	54953146
chr11	54953994	54954412
chr11	54955631	54955998
chr11	54955998	54956333
chr11	54958440	54958737
chr11	54958737	54959006
chr11	54961403	54961949
chr11	54962714	54963160
chr11	54965307	54965571
chr11	54970555	54971112
chr11	54971431	54971591
chr11	54971595	54971919
chr11	54972085	54972378
chr11	54972378	54972728
chr11	54984181	54984427
chr11	54984462	54984616
chr11	54984635	54984934
chr11	54985175	54985383
chr11	54985441	54985631
chr11	54985631	54985861
chr11	54988049	54988277
chr11	54989769	54990019
chr11	54992903	54993073
chr11	54998281	54998672
chr11	54998672	54998962
chr11	54998962	54999144
chr11	55006620	55006977

chr11	55012917	55013139
chr11	55013139	55013360
chr11	55021932	55022226
chr11	55022983	55023473
chr11	55024486	55024706
chr11	55027467	55027724
chr11	55027724	55027964
chr11	55027964	55028254
chr11	55031724	55031934
chr11	55032942	55033120
chr11	55033174	55033690
chr11	55033758	55033924
chr11	55036671	55036920
chr11	55039557	55039985
chr11	55045300	55045741
chr11	55046693	55046909
chr11	55050845	55051208
chr11	55060843	55061207
chr11	55062943	55063208
chr11	55063208	55063426
chr11	55066976	55067497
chr11	55069105	55069265
chr11	55076320	55076486
chr11	55077143	55077565
chr11	55077565	55077883
chr11	55077883	55078250
chr11	55087057	55087345
chr11	55097554	55097919
chr11	55097919	55098432
chr11	55100993	55101470
chr11	55102331	55102639
chr11	55111899	55112049
chr11	55113712	55113888
chr11	55116138	55116409
chr11	55125634	55125803
chr11	55127964	55128275
chr11	55131011	55131352
chr11	55134512	55134734
chr11	55134957	55135158
chr11	55158847	55159287
chr11	55162696	55163062
chr11	55164681	55165053

chr11	55170662	55170841
chr11	55178904	55179060
chr11	55180174	55180581
chr11	55185654	55186075
chr11	55187364	55187531
chr11	55194614	55195145
chr11	55195246	55195485
chr11	55197133	55197396
chr11	55198286	55198504
chr11	55204131	55204408
chr11	55204408	55204713
chr11	55204713	55205003
chr11	55217757	55218510
chr11	55219063	55219355
chr11	55236594	55236869
chr11	55236869	55237190
chr11	55237190	55237448
chr11	55240644	55240952
chr11	55250797	55251045
chr11	55251297	55251541
chr11	55264325	55264670
chr11	55264670	55265203
chr11	55273079	55273258
chr11	55274522	55275241
chr11	55280109	55280499
chr11	55289813	55290392
chr11	55291843	55292315
chr11	55302254	55302952
chr11	55313748	55314165
chr11	55328215	55328370
chr11	55328372	55328643
chr11	55329424	55329733
chr11	55335740	55335985
chr11	55342102	55342317
chr11	55342852	55343057
chr11	55343057	55343489
chr11	55347311	55347595
chr11	55358520	55358718
chr11	55358816	55359182
chr11	55361238	55361734
chr11	55377236	55377432
chr11	55378409	55378729

chr11	55386276	55386523
chr11	55386523	55386865
chr11	55389831	55390115
chr11	55400881	55401255
chr11	55404584	55404807
chr11	55404807	55405024
chr11	55405732	55406543
chr11	55415828	55416057
chr11	55417437	55417638
chr11	55417834	55418197
chr11	55418197	55418470
chr11	55418723	55419034
chr11	55419552	55420120
chr11	55423815	55424030
chr11	55428048	55428865
chr11	55439727	55439956
chr11	55441173	55441341
chr11	55444852	55445141
chr11	55446492	55446963
chr11	55453097	55453377
chr11	55453441	55453672
chr11	55453672	55453983
chr11	55455295	55455582
chr11	55455582	55455858
chr11	55455962	55456122
chr11	55460617	55461024
chr11	55461024	55461633
chr11	55468849	55469059
chr11	55469074	55469430
chr11	55469430	55470140
chr11	55477237	55477575
chr11	55481194	55481433
chr11	55483824	55484092
chr11	55487250	55487450
chr11	55497760	55498252
chr11	55503066	55503244
chr11	55503499	55503833
chr11	55503833	55504118
chr11	55504118	55504315
chr11	55505399	55505824
chr11	55505824	55506061
chr11	55511364	55511562

chr11	55512474	55512723
chr11	55518768	55519117
chr11	55519122	55519272
chr11	55519364	55519514
chr11	55522632	55522833
chr11	55574493	55574806
chr11	55589871	55590047
chr11	55607571	55607739
chr11	55607779	55608080
chr11	55682485	55682661
chr11	55695442	55695610
chr11	55819697	55819939
chr11	55882572	55882819
chr11	56001382	56001696
chr11	56107960	56108218
chr11	56131330	56131556
chr11	56862636	56862995
chr11	56897177	56897333
chr11	56901052	56901279
chr11	56901279	56901510
chr11	56999846	57000001
chr11	57026364	57026605
chr11	57026690	57026965
chr11	57051964	57052359
chr11	57052359	57052810
chr11	57079514	57079814
chr11	57089959	57090272
chr11	57090853	57091036
chr11	57091224	57091614
chr11	57091614	57092009
chr11	57096800	57096997
chr11	57121862	57122071
chr11	57130179	57130589
chr11	57131676	57131872
chr11	57140353	57140554
chr11	57140554	57140858
chr11	57169962	57170212
chr11	57170212	57170432
chr11	57176044	57176262
chr11	57176612	57176797
chr11	57249723	57249994
chr11	57250281	57250492

chr11	57258678	57258930
chr11	57297543	57297693
chr11	57302577	57302757
chr11	57310860	57311072
chr11	57367404	57367617
chr11	57414770	57414920
chr11	57465097	57465361
chr11	57467817	57468082
chr11	57470177	57470407
chr11	57475208	57475447
chr11	57479923	57480281
chr11	57496535	57496730
chr11	57496765	57497028
chr11	57501836	57502116
chr11	57517071	57517430
chr11	57517993	57518294
chr11	57518294	57518522
chr11	57518522	57519003
chr11	57528224	57528421
chr11	57536581	57536745
chr11	57539849	57540267
chr11	57546153	57546350
chr11	57547263	57547561
chr11	57547815	57548106
chr11	57548963	57549240
chr11	57550312	57550539
chr11	57551055	57551292
chr11	57563082	57563346
chr11	57568976	57569459
chr11	57569459	57569785
chr11	57585766	57585945
chr11	57589567	57589838
chr11	57601207	57601546
chr11	57610999	57611268
chr11	57617560	57617887
chr11	57619022	57619232
chr11	57627172	57627555
chr11	57627833	57628050
chr11	57630953	57631125
chr11	57642702	57643006
chr11	57644241	57644445
chr11	57644445	57644639

chr11	57644639	57644828
chr11	57644828	57645030
chr11	57645030	57645294
chr11	57645294	57645801
chr11	57657778	57658087
chr11	57660648	57660953
chr11	57666170	57666417
chr11	57672995	57673390
chr11	57687951	57688232
chr11	57688232	57688602
chr11	57688602	57688993
chr11	57689942	57690162
chr11	57694421	57694942
chr11	57695028	57695178
chr11	57706452	57706816
chr11	57707276	57707721
chr11	57708342	57708509
chr11	57708904	57709064
chr11	57719428	57720044
chr11	57720044	57720401
chr11	57724938	57725094
chr11	57747331	57747701
chr11	57750448	57750674
chr11	57758774	57758955
chr11	57767731	57768013
chr11	57773180	57773380
chr11	57773459	57773609
chr11	57774450	57774680
chr11	57778420	57778675
chr11	57778715	57778886
chr11	57787988	57788385
chr11	57789942	57790151
chr11	57791105	57791524
chr11	57793729	57793879
chr11	57800706	57800856
chr11	57800967	57801153
chr11	57801153	57801397
chr11	57801397	57801852
chr11	57801852	57802386
chr11	57805140	57805315
chr11	57818789	57818954
chr11	57820063	57820289

chr11	57820289	57820514
chr11	57823295	57823519
chr11	57825331	57825578
chr11	57845791	57846180
chr11	57846691	57846990
chr11	57847089	57847332
chr11	57850383	57850701
chr11	57850869	57851130
chr11	57853462	57853690
chr11	57855563	57855992
chr11	57857357	57857556
chr11	57857674	57858038
chr11	57862105	57862454
chr11	57864400	57864623
chr11	57868816	57869078
chr11	57876269	57876419
chr11	57883368	57883670
chr11	57883670	57884208
chr11	57899283	57899949
chr11	57900737	57901081
chr11	57911650	57912275
chr11	57913008	57913399
chr11	57913895	57914110
chr11	57914240	57914561
chr11	57916125	57916287
chr11	57917978	57918236
chr11	57923281	57923499
chr11	57933085	57933303
chr11	57934176	57934616
chr11	57934616	57934933
chr11	57935203	57935358
chr11	57947354	57947789
chr11	57949128	57949293
chr11	57952511	57952725
chr11	57952725	57953088
chr11	57962317	57962659
chr11	57962659	57963104
chr11	57964197	57964368
chr11	57969899	57970076
chr11	57970481	57970954
chr11	57972915	57973645
chr11	57984072	57984264

chr11	57993111	57993598
chr11	57998899	57999222
chr11	58008400	58008607
chr11	58008607	58008924
chr11	58008924	58009240
chr11	58009240	58009449
chr11	58009470	58009800
chr11	58009800	58010104
chr11	58010613	58010763
chr11	58016334	58016911
chr11	58017617	58017786
chr11	58018376	58018593
chr11	58018828	58019007
chr11	58024192	58024575
chr11	58024575	58025046
chr11	58030920	58031077
chr11	58039099	58039331
chr11	58042494	58042698
chr11	58043018	58043398
chr11	58043645	58043879
chr11	58046052	58046332
chr11	58046523	58046736
chr11	58057377	58057548
chr11	58057567	58057803
chr11	58059821	58060018
chr11	58066758	58066925
chr11	58067584	58067734
chr11	58068009	58068232
chr11	58068747	58069312
chr11	58069357	58069517
chr11	58089876	58090106
chr11	58090743	58091139
chr11	58091560	58091710
chr11	58093009	58093240
chr11	58095985	58096141
chr11	58096186	58096390
chr11	58097221	58097502
chr11	58097649	58097874
chr11	58097874	58098187
chr11	58098401	58098649
chr11	58103464	58103820
chr11	58103820	58104373

chr11	58104373	58104852
chr11	58116876	58117443
chr11	58126484	58126649
chr11	58168045	58168360
chr11	58168360	58168689
chr11	58168689	58168974
chr11	58171399	58171740
chr11	58171740	58172028
chr11	58178147	58178360
chr11	58179606	58179964
chr11	58180085	58180239
chr11	58199160	58199428
chr11	58199428	58199677
chr11	58199677	58199959
chr11	58210880	58211091
chr11	58214631	58215061
chr11	58215061	58215345
chr11	58223762	58224014
chr11	58224428	58224618
chr11	58225490	58225705
chr11	58225705	58226096
chr11	58235251	58235527
chr11	58239129	58239541
chr11	58239541	58239851
chr11	58244071	58244261
chr11	58249258	58249514
chr11	58249717	58249982
chr11	58286238	58286468
chr11	58288746	58289183
chr11	58289400	58289733
chr11	58292858	58293118
chr11	58301317	58301478
chr11	58301750	58302087
chr11	58302087	58302394
chr11	58302394	58302735
chr11	58302735	58303055
chr11	58306920	58307285
chr11	58307440	58308141
chr11	58322754	58323202
chr11	58323202	58323687
chr11	58330011	58330196
chr11	58330249	58330486

chr11	58330524	58330847
chr11	58330876	58331135
chr11	58334596	58334917
chr11	58334917	58335174
chr11	58352787	58352965
chr11	58364803	58365005
chr11	58380218	58381008
chr11	58401983	58402183
chr11	58402183	58402424
chr11	58405571	58405771
chr11	58406114	58406266
chr11	58466624	58466774
chr11	58466801	58466955
chr11	58502330	58502646
chr11	58567194	58567465
chr11	58572542	58572771
chr11	58580787	58580953
chr11	58591288	58591446
chr11	58596514	58596733
chr11	58636642	58636910
chr11	58638230	58638626
chr11	58640511	58640802
chr11	58642633	58642866
chr11	58643393	58643752
chr11	58766626	58766969
chr11	58773040	58773287
chr11	58782532	58782718
chr11	58782829	58783011
chr11	58789377	58789873
chr11	58790504	58791018
chr11	58801684	58801844
chr11	58801909	58802196
chr11	58846925	58847650
chr11	58867099	58867425
chr11	58867425	58867645
chr11	58893919	58894101
chr11	58903576	58904159
chr11	58904159	58904393
chr11	58904393	58904594
chr11	58914439	58914756
chr11	58916773	58917270
chr11	58919338	58919571

chr11	58927995	58928344
chr11	58928344	58928706
chr11	58931655	58932048
chr11	58937918	58938092
chr11	58938176	58938763
chr11	58938763	58939236
chr11	58948168	58948453
chr11	58948483	58948972
chr11	58948972	58949355
chr11	58954005	58954203
chr11	58954203	58954431
chr11	58954431	58954733
chr11	58954733	58954961
chr11	58954961	58955370
chr11	58959903	58960237
chr11	58960237	58960521
chr11	58965623	58965834
chr11	58967426	58967658
chr11	58977222	58977513
chr11	58977534	58977727
chr11	58977727	58977969
chr11	58977969	58978217
chr11	58978217	58978490
chr11	58978490	58978900
chr11	58979740	58979932
chr11	58981997	58982248
chr11	58982248	58982503
chr11	58985638	58985850
chr11	58990233	58990392
chr11	58994289	58994819
chr11	59004940	59005215
chr11	59011330	59011507
chr11	59015005	59015447
chr11	59034647	59034842
chr11	59044859	59045130
chr11	59082577	59082747
chr11	59085146	59085350
chr11	59097565	59097741
chr11	59098497	59098648
chr11	59116379	59116695
chr11	59134344	59134687
chr11	59134687	59134934

chr11	59137424	59137579
chr11	59141834	59142161
chr11	59151830	59151989
chr11	59152517	59152727
chr11	59154242	59154577
chr11	59163207	59163634
chr11	59163634	59164025
chr11	59169695	59169977
chr11	59169977	59170265
chr11	59170709	59170907
chr11	59174996	59175197
chr11	59176561	59176721
chr11	59176721	59176884
chr11	59176944	59177157
chr11	59183098	59183400
chr11	59183881	59184138
chr11	59191171	59191429
chr11	59191431	59191732
chr11	59191732	59192210
chr11	59201815	59202020
chr11	59202033	59202351
chr11	59202351	59202595
chr11	59202595	59202814
chr11	59202814	59203012
chr11	59208140	59208534
chr11	59208534	59209000
chr11	59209000	59209642
chr11	59218022	59218188
chr11	59218334	59218678
chr11	59218678	59219046
chr11	59219123	59219385
chr11	59226283	59226579
chr11	59226579	59226817
chr11	59226817	59227023
chr11	59227811	59228178
chr11	59228178	59228597
chr11	59228694	59228853
chr11	59230492	59230705
chr11	59238951	59239245
chr11	59239262	59239459
chr11	59239484	59239728
chr11	59255201	59255381

chr11	59263864	59264089
chr11	59264089	59264437
chr11	59266810	59267064
chr11	59276746	59276897
chr11	59276936	59277170
chr11	59279203	59279446
chr11	59282161	59282476
chr11	59282995	59283282
chr11	59291012	59291172
chr11	59293650	59294281
chr11	59298037	59298276
chr11	59298276	59298552
chr11	59300332	59300484
chr11	59302759	59303035
chr11	59306071	59306237
chr11	59306531	59307076
chr11	59307076	59307358
chr11	59307660	59307811
chr11	59307822	59308150
chr11	59308171	59308515
chr11	59313111	59313558
chr11	59323104	59323312
chr11	59323579	59323746
chr11	59323902	59324252
chr11	59330485	59331022
chr11	59334902	59335161
chr11	59335165	59335552
chr11	59336010	59336239
chr11	59336437	59336750
chr11	59337998	59338186
chr11	59346164	59346576
chr11	59346606	59346802
chr11	59350205	59350447
chr11	59350447	59350742
chr11	59356479	59356755
chr11	59357840	59358053
chr11	59364914	59365093
chr11	59366720	59366881
chr11	59368072	59368292
chr11	59373694	59373884
chr11	59377726	59377899
chr11	59377901	59378344

chr11	59384256	59385031
chr11	59385141	59385728
chr11	59393603	59393852
chr11	59396590	59396793
chr11	59398591	59399055
chr11	59401709	59401864
chr11	59402664	59403163
chr11	59410902	59411165
chr11	59412094	59412448
chr11	59412448	59412734
chr11	59412765	59412869
chr11	59412869	59413032
chr11	59413032	59413308
chr11	59413559	59413792
chr11	59421945	59422305
chr11	59424846	59425287
chr11	59425688	59425919
chr11	59448638	59449039
chr11	59449081	59449549
chr11	59449713	59450158
chr11	59450158	59450599
chr11	59450599	59450978
chr11	59458916	59459566
chr11	59471461	59471630
chr11	59471630	59471832
chr11	59471832	59472090
chr11	59472090	59472372
chr11	59472372	59472739
chr11	59483129	59483302
chr11	59483320	59483568
chr11	59484998	59485148
chr11	59485714	59485909
chr11	59486264	59486584
chr11	59491951	59492383
chr11	59505286	59505538
chr11	59505538	59505924
chr11	59505924	59506433
chr11	59506433	59507007
chr11	59513636	59513895
chr11	59520643	59521033
chr11	59521033	59521285
chr11	59521306	59521456

chr11	59521794	59522232
chr11	59525267	59525614
chr11	59526615	59526814
chr11	59531173	59531552
chr11	59531552	59531893
chr11	59544137	59544379
chr11	59554261	59554527
chr11	59569378	59569608
chr11	59571293	59571526
chr11	59571675	59572087
chr11	59572196	59572435
chr11	59600865	59601435
chr11	59602355	59602605
chr11	59612212	59612595
chr11	59614139	59614452
chr11	59622245	59622662
chr11	59624103	59624574
chr11	59634048	59634401
chr11	59635880	59636139
chr11	59636139	59636535
chr11	59636535	59636987
chr11	59636987	59637357
chr11	59641351	59641503
chr11	59642426	59642823
chr11	59655242	59655705
chr11	59655705	59655957
chr11	59656437	59656686
chr11	59662132	59662796
chr11	59662899	59663389
chr11	59671048	59671217
chr11	59671258	59671427
chr11	59671427	59671627
chr11	59693221	59693476
chr11	59714818	59715010
chr11	59717690	59718061
chr11	59718061	59718364
chr11	59721421	59721700
chr11	59731636	59731796
chr11	59731796	59731992
chr11	59732007	59732206
chr11	59732717	59732939
chr11	59732939	59733126

chr11	59737236	59737454
chr11	59744324	59744680
chr11	59746697	59746931
chr11	59759203	59759414
chr11	59759414	59759693
chr11	59759693	59760087
chr11	59766600	59766781
chr11	59769845	59770028
chr11	59778228	59778592
chr11	59778592	59778919
chr11	59778919	59779200
chr11	59784617	59784864
chr11	59785577	59785809
chr11	59786248	59786449
chr11	59786449	59786630
chr11	59786630	59786880
chr11	59786965	59787487
chr11	59787487	59787835
chr11	59788952	59789102
chr11	59791573	59791743
chr11	59793573	59793726
chr11	59794889	59795088
chr11	59795090	59795313
chr11	59795313	59795514
chr11	59800488	59800713
chr11	59801057	59801209
chr11	59809072	59809315
chr11	59809315	59809619
chr11	59809619	59810056
chr11	59810056	59810417
chr11	59837454	59837690
chr11	59838955	59839213
chr11	59839213	59839589
chr11	59839589	59840022
chr11	59847188	59847459
chr11	59847738	59848614
chr11	59879982	59880453
chr11	59892292	59892452
chr11	59892478	59892692
chr11	59894722	59894997
chr11	59913671	59914178
chr11	59921301	59921570

chr11	59932249	59932453
chr11	59937928	59938153
chr11	59941624	59941860
chr11	59941906	59942188
chr11	59942194	59942356
chr11	59947797	59948391
chr11	59948471	59948714
chr11	59964448	59964598
chr11	59964632	59965192
chr11	59965258	59965566
chr11	59965628	59965804
chr11	59971006	59971194
chr11	59971762	59971948
chr11	59978871	59979324
chr11	59990282	59990460
chr11	60011129	60011369
chr11	60021234	60021384
chr11	60027178	60027637
chr11	60045820	60046000
chr11	60046032	60046396
chr11	60046396	60046773
chr11	60058002	60058277
chr11	60065513	60065748
chr11	60068755	60068969
chr11	60070654	60070899
chr11	60076842	60077151
chr11	60084665	60084902
chr11	60089011	60089494
chr11	60091152	60091330
chr11	60104763	60105024
chr11	60105024	60105442
chr11	60105510	60105940
chr11	60106062	60106263
chr11	60106271	60106485
chr11	60108624	60109009
chr11	60109009	60109379
chr11	60112292	60112461
chr11	60114289	60114718
chr11	60114718	60115115
chr11	60115115	60115666
chr11	60120658	60120948
chr11	60121039	60121199

chr11	60125249	60125543
chr11	60125623	60125917
chr11	60127086	60127473
chr11	60127526	60127697
chr11	60127887	60128225
chr11	60134816	60135028
chr11	60137238	60137478
chr11	60139736	60139952
chr11	60139952	60140187
chr11	60141033	60141316
chr11	60141316	60141599
chr11	60142103	60142266
chr11	60142266	60142474
chr11	60142474	60142754
chr11	60143131	60143374
chr11	60154064	60154351
chr11	60160481	60160725
chr11	60165115	60165411
chr11	60170544	60170918
chr11	60175683	60176040
chr11	60176040	60176339
chr11	60176339	60176668
chr11	60176668	60176976
chr11	60178496	60178872
chr11	60179349	60179622
chr11	60181158	60181319
chr11	60185126	60185335
chr11	60193653	60193888
chr11	60194513	60194689
chr11	60195264	60195414
chr11	60199658	60199952
chr11	60199952	60200298
chr11	60200768	60201025
chr11	60209277	60209697
chr11	60210242	60210829
chr11	60210837	60210987
chr11	60219674	60219982
chr11	60220039	60220242
chr11	60220242	60220511
chr11	60220511	60220819
chr11	60221658	60221850
chr11	60222695	60223001

chr11	60223001	60223254
chr11	60228857	60229229
chr11	60229247	60229508
chr11	60229752	60230211
chr11	60244586	60244883
chr11	60244883	60245192
chr11	60245192	60245464
chr11	60247263	60247413
chr11	60251298	60251589
chr11	60253575	60253872
chr11	60253916	60254103
chr11	60258972	60259243
chr11	60260761	60260993
chr11	60273307	60273538
chr11	60273538	60273804
chr11	60292819	60293074
chr11	60296080	60296613
chr11	60301532	60302347
chr11	60307912	60308445
chr11	60308583	60309089
chr11	60309354	60309574
chr11	60309985	60310166
chr11	60311591	60311944
chr11	60318801	60318981
chr11	60319567	60319809
chr11	60319809	60320027
chr11	60321067	60321261
chr11	60323766	60324062
chr11	60339302	60339804
chr11	60346539	60346936
chr11	60352429	60352794
chr11	60352794	60353487
chr11	60357226	60357384
chr11	60360087	60360239
chr11	60367355	60367727
chr11	60373716	60374246
chr11	60381828	60382055
chr11	60382219	60382474
chr11	60383181	60383352
chr11	60416622	60416990
chr11	60416990	60417352
chr11	60417352	60417718

chr11	60417718	60418023
chr11	60418023	60418239
chr11	60424927	60425247
chr11	60436469	60437243
chr11	60454209	60454447
chr11	60454447	60454734
chr11	60454765	60455124
chr11	60458114	60458352
chr11	60460755	60461054
chr11	60465847	60466189
chr11	60469982	60470132
chr11	60478255	60478496
chr11	60480348	60480555
chr11	60483411	60483612
chr11	60492971	60493412
chr11	60493412	60493718
chr11	60495723	60495886
chr11	60505107	60505450
chr11	60505532	60505721
chr11	60505743	60505945
chr11	60509550	60509818
chr11	60514754	60515311
chr11	60515311	60515646
chr11	60518221	60518667
chr11	60520182	60520378
chr11	60522133	60522376
chr11	60527286	60527570
chr11	60527949	60528210
chr11	60536514	60536790
chr11	60537108	60537395
chr11	60537444	60537836
chr11	60537836	60538357
chr11	60538366	60538581
chr11	60538680	60539079
chr11	60539079	60539295
chr11	60559365	60559739
chr11	60563365	60563592
chr11	60588652	60588809
chr11	60601746	60601900
chr11	60630137	60630354
chr11	60668224	60668437
chr11	60681276	60681477

chr11	60698953	60699105
chr11	60699515	60699863
chr11	60699863	60700190
chr11	60701427	60701877
chr11	60707040	60707603
chr11	60707603	60708155
chr11	60714882	60715498
chr11	60717165	60717372
chr11	60726574	60726792
chr11	60726792	60727104
chr11	60727104	60727417
chr11	60727417	60727639
chr11	60728199	60728737
chr11	60729229	60729485
chr11	60729533	60729704
chr11	60734226	60734570
chr11	60735330	60735480
chr11	60739715	60740023
chr11	60742820	60743008
chr11	60758128	60758310
chr11	60758972	60759125
chr11	60761797	60762029
chr11	60764803	60765121
chr11	60769273	60770178
chr11	60776704	60777100
chr11	60777100	60777540
chr11	60777540	60777855
chr11	60784153	60784443
chr11	60787678	60787910
chr11	60794864	60795171
chr11	60795847	60795997
chr11	60800061	60800249
chr11	60810237	60810811
chr11	60810927	60811608
chr11	60825260	60825555
chr11	60830472	60830782
chr11	60830782	60831188
chr11	60859777	60859962
chr11	60861314	60861476
chr11	60862063	60862316
chr11	60864187	60864381
chr11	60878143	60878687

chr11	60878687	60879385
chr11	60879548	60879764
chr11	60908846	60909211
chr11	60913337	60913959
chr11	60914672	60914952
chr11	60915150	60915690
chr11	60927395	60927581
chr11	60930619	60931266
chr11	60931376	60931544
chr11	60931748	60932187
chr11	60933630	60933823
chr11	60935285	60935651
chr11	60935651	60935895
chr11	60939438	60939863
chr11	60941129	60941398
chr11	60941398	60941759
chr11	60951190	60951354
chr11	60952919	60953076
chr11	60953076	60953245
chr11	60953283	60953664
chr11	60953876	60954228
chr11	60954520	60954712
chr11	60959635	60959816
chr11	60959829	60960047
chr11	60964373	60964576
chr11	60964576	60964795
chr11	60968052	60968271
chr11	60976781	60977012
chr11	60977179	60977516
chr11	60980272	60980563
chr11	60986036	60986564
chr11	61011985	61012251
chr11	61018591	61018743
chr11	61021835	61022064
chr11	61041415	61041577
chr11	61043509	61043994
chr11	61045116	61045330
chr11	61046160	61046352
chr11	61062337	61062566
chr11	61062616	61062861
chr11	61068237	61068551
chr11	61091222	61091503

chr11	61091721	61091949
chr11	61091979	61092134
chr11	61099498	61100025
chr11	61100025	61100391
chr11	61102483	61102845
chr11	61104352	61104619
chr11	61119728	61120393
chr11	61138228	61138411
chr11	61138905	61139154
chr11	61140183	61140366
chr11	61146670	61146920
chr11	61147292	61147641
chr11	61150325	61150602
chr11	61151041	61151280
chr11	61152177	61152355
chr11	61155875	61156054
chr11	61160419	61160687
chr11	61168516	61168671
chr11	61169213	61169364
chr11	61174289	61174487
chr11	61174487	61174831
chr11	61174831	61175167
chr11	61175167	61175653
chr11	61176269	61176759
chr11	61192486	61192777
chr11	61193427	61193698
chr11	61193698	61193891
chr11	61199667	61200025
chr11	61200025	61200317
chr11	61201096	61201532
chr11	61202851	61203168
chr11	61207899	61208247
chr11	61208247	61208904
chr11	61215904	61216121
chr11	61220193	61220494
chr11	61220494	61220888
chr11	61228570	61228798
chr11	61231343	61231730
chr11	61237493	61237645
chr11	61260764	61261189
chr11	61261189	61261465
chr11	61266564	61267010

chr11	61267010	61267296
chr11	61267296	61267654
chr11	61273534	61273746
chr11	61273952	61274339
chr11	61278511	61278805
chr11	61278830	61278985
chr11	61283704	61284126
chr11	61294523	61294732
chr11	61297383	61297641
chr11	61297716	61298402
chr11	61307862	61308158
chr11	61320868	61321109
chr11	61321109	61321404
chr11	61337736	61338088
chr11	61342563	61342763
chr11	61342763	61343064
chr11	61346215	61346368
chr11	61349373	61349697
chr11	61352137	61352614
chr11	61377905	61378191
chr11	61378764	61378914
chr11	61379719	61379961
chr11	61381339	61381593
chr11	61407587	61408190
chr11	61408190	61408470
chr11	61408470	61408741
chr11	61408789	61408949
chr11	61410216	61410470
chr11	61416603	61416759
chr11	61417924	61418342
chr11	61421502	61421713
chr11	61441743	61441995
chr11	61443917	61444162
chr11	61453973	61454163
chr11	61457526	61457759
chr11	61473717	61473909
chr11	61477132	61477351
chr11	61478288	61478524
chr11	61487423	61487884
chr11	61488031	61488249
chr11	61493528	61493956
chr11	61493956	61494408

chr11	61494408	61494677
chr11	61494677	61494917
chr11	61504679	61504906
chr11	61504906	61505090
chr11	61505090	61505541
chr11	61511825	61512020
chr11	61515032	61515360
chr11	61518015	61518327
chr11	61519996	61520227
chr11	61521293	61521576
chr11	61521576	61521912
chr11	61526941	61527362
chr11	61532785	61533343
chr11	61539201	61539470
chr11	61552965	61553197
chr11	61562416	61562729
chr11	61562729	61562988
chr11	61563813	61564052
chr11	61564052	61564388
chr11	61564469	61565003
chr11	61569900	61570072
chr11	61573062	61573273
chr11	61578761	61579033
chr11	61579040	61579518
chr11	61579518	61579916
chr11	61597366	61597724
chr11	61597724	61598202
chr11	61600297	61600551
chr11	61602751	61603141
chr11	61603472	61603749
chr11	61607844	61608475
chr11	61608500	61608655
chr11	61615794	61616023
chr11	61616023	61616179
chr11	61616179	61616537
chr11	61619717	61620286
chr11	61620286	61620684
chr11	61628745	61628971
chr11	61629672	61630026
chr11	61633861	61634189
chr11	61636785	61636966
chr11	61637990	61638465

chr11	61642051	61642541
chr11	61643646	61643879
chr11	61644308	61644479
chr11	61644533	61644930
chr11	61648384	61648637
chr11	61649682	61649912
chr11	61658632	61658794
chr11	61664461	61664834
chr11	61665043	61665195
chr11	61666034	61666341
chr11	61666358	61666745
chr11	61667320	61667530
chr11	61673804	61673989
chr11	61674859	61675088
chr11	61683885	61684227
chr11	61684227	61684720
chr11	61686981	61687225
chr11	61687225	61687512
chr11	61687687	61687921
chr11	61698783	61699331
chr11	61699331	61699761
chr11	61699902	61700275
chr11	61705986	61706262
chr11	61706262	61706565
chr11	61709065	61709230
chr11	61714385	61714681
chr11	61714681	61714977
chr11	61732671	61732906
chr11	61744545	61744780
chr11	61744957	61745215
chr11	61745813	61746130
chr11	61746130	61746416
chr11	61751691	61751922
chr11	61760572	61760984
chr11	61761708	61761976
chr11	61761976	61762492
chr11	61778673	61778912
chr11	61840649	61840850
chr11	61854030	61854461
chr11	61854461	61854808
chr11	61854808	61855180
chr11	61855180	61855460

chr11	61929749	61930080
chr11	61930080	61930652
chr11	61930687	61930850
chr11	61949387	61949613
chr11	61950691	61950999
chr11	61953879	61954069
chr11	61955107	61955602
chr11	61956625	61956912
chr11	61956912	61957179
chr11	61957179	61957403
chr11	61957501	61957702
chr11	61970767	61970963
chr11	61970963	61971281
chr11	61975186	61975353
chr11	61975842	61976193
chr11	61981379	61981605
chr11	61987011	61987271
chr11	61998791	61999021
chr11	61999021	61999339
chr11	62005245	62005573
chr11	62005573	62006030
chr11	62020444	62020630
chr11	62020704	62021037
chr11	62023098	62023443
chr11	62027315	62027470
chr11	62030979	62031182
chr11	62037863	62038100
chr11	62038583	62038979
chr11	62044714	62044996
chr11	62047878	62048123
chr11	62050681	62050901
chr11	62051080	62051240
chr11	62051548	62051857
chr11	62070311	62070515
chr11	62074639	62074907
chr11	62075156	62075425
chr11	62076588	62076945
chr11	62076945	62077212
chr11	62077212	62077481
chr11	62077481	62077696
chr11	62088714	62088957
chr11	62090356	62090618

chr11	62103625	62103805
chr11	62106669	62106819
chr11	62119952	62120136
chr11	62120136	62120326
chr11	62120456	62120606
chr11	62127770	62128015
chr11	62130286	62130449
chr11	62130802	62131017
chr11	62145361	62145598
chr11	62145716	62146094
chr11	62146733	62146986
chr11	62147647	62147848
chr11	62148118	62148392
chr11	62150319	62150489
chr11	62150657	62151016
chr11	62184627	62184909
chr11	62184909	62185422
chr11	62187144	62187360
chr11	62213496	62213656
chr11	62217612	62218163
chr11	62220768	62221094
chr11	62223999	62224309
chr11	62232697	62232858
chr11	62237839	62238521
chr11	62243860	62244146
chr11	62248356	62248629
chr11	62248629	62248864
chr11	62248864	62249236
chr11	62249236	62249636
chr11	62268242	62268827
chr11	62278128	62278405
chr11	62280696	62280905
chr11	62281011	62281278
chr11	62281278	62281595
chr11	62281595	62281988
chr11	62294278	62294451
chr11	62309142	62309709
chr11	62332099	62332555
chr11	62410362	62410522
chr11	62456548	62456874
chr11	62456874	62457210
chr11	62457210	62457560

chr11	62457560	62457874
chr11	62458278	62458880
chr11	62466522	62467028
chr11	62471000	62471246
chr11	62495089	62495464
chr11	62495546	62495841
chr11	62496426	62496651
chr11	62515054	62515230
chr11	62515230	62515455
chr11	62515485	62515695
chr11	62538305	62538664
chr11	62538664	62538915
chr11	62538915	62539123
chr11	62539123	62539656
chr11	62549751	62549968
chr11	62549968	62550211
chr11	62550312	62550808
chr11	62550808	62551288
chr11	62551422	62551619
chr11	62551619	62551840
chr11	62555367	62555530
chr11	62556191	62556341
chr11	62557933	62558153
chr11	62558613	62559036
chr11	62559036	62559284
chr11	62568299	62568512
chr11	62575319	62575617
chr11	62575619	62576128
chr11	62579276	62579714
chr11	62580537	62580861
chr11	62581041	62581322
chr11	62592697	62593089
chr11	62593205	62593434
chr11	62602531	62603195
chr11	62603195	62603603
chr11	62605572	62605839
chr11	62606042	62606244
chr11	62606244	62606463
chr11	62606551	62606701
chr11	62609100	62609338
chr11	62610439	62610739
chr11	62613688	62614082

chr11	62623368	62623518
chr11	62624284	62624506
chr11	62624506	62624727
chr11	62624727	62624988
chr11	62624988	62625272
chr11	62637377	62637764
chr11	62637905	62638059
chr11	62642451	62642670
chr11	62643114	62643694
chr11	62647862	62648134
chr11	62648134	62648491
chr11	62648491	62648897
chr11	62648897	62649129
chr11	62649129	62649317
chr11	62654773	62655025
chr11	62670052	62670282
chr11	62675580	62675816
chr11	62675816	62676057
chr11	62676057	62676277
chr11	62681980	62682234
chr11	62693367	62693632
chr11	62693658	62693914
chr11	62701241	62701572
chr11	62731321	62731789
chr11	62731789	62732149
chr11	62732711	62732987
chr11	62733643	62733887
chr11	62744148	62744413
chr11	62744457	62744642
chr11	62745895	62746297
chr11	62746297	62746570
chr11	62754532	62754754
chr11	62756587	62757237
chr11	62757247	62757397
chr11	62813963	62814478
chr11	62814571	62815017
chr11	62815562	62815748
chr11	62819966	62820577
chr11	62820664	62821127
chr11	62821163	62821414
chr11	62825235	62825553
chr11	62825553	62825883

chr11	62825883	62826226
chr11	62831813	62832360
chr11	62838453	62838603
chr11	62841562	62841712
chr11	62846279	62846470
chr11	62849248	62849406
chr11	62851285	62851517
chr11	62867531	62867774
chr11	62879161	62879598
chr11	62879598	62880049
chr11	62901764	62901997
chr11	62905054	62905255
chr11	62921077	62921369
chr11	62921555	62921769
chr11	62929719	62930206
chr11	62954610	62954764
chr11	62996287	62996849
chr11	63026606	63027062
chr11	63038339	63038589
chr11	63052056	63052359
chr11	63052853	63053412
chr11	63054773	63055009
chr11	63099415	63099683
chr11	63099683	63099911
chr11	63109466	63109797
chr11	63114898	63115313
chr11	63115313	63115694
chr11	63119805	63120057
chr11	63122862	63123296
chr11	63129388	63129686
chr11	63129986	63130282
chr11	63130391	63130916
chr11	63131017	63131216
chr11	63131335	63131568
chr11	63135667	63136008
chr11	63136271	63136600
chr11	63165178	63165405
chr11	63170099	63170278
chr11	63173862	63174012
chr11	63175374	63175546
chr11	63213121	63213357
chr11	63233648	63233803

chr11	63253257	63253573
chr11	63264460	63264668
chr11	63264705	63264966
chr11	63404754	63404904
chr11	63419834	63420039
chr11	63565548	63565774
chr11	63565998	63566201
chr11	63745538	63745836
chr11	63745932	63746140
chr11	63782371	63782664
chr11	63782664	63782863
chr11	63862135	63862318
chr11	63862781	63863110
chr11	63863320	63863455
chr11	63863455	63863591
chr11	63914359	63914736
chr11	63920546	63920793
chr11	63921804	63922026
chr11	63922043	63922642
chr11	63925320	63925582
chr11	63952153	63952367
chr11	63952369	63952560
chr11	63957346	63957630
chr11	63977776	63978289
chr11	64027421	64027826
chr11	64065692	64066506
chr11	64066847	64067006
chr11	64067006	64067189
chr11	64078980	64079280
chr11	64079280	64079712
chr11	64079712	64080152
chr11	64248130	64248320
chr11	64434968	64435386
chr11	64435386	64435619
chr11	64435619	64435856
chr11	64435860	64436170
chr11	64436253	64436501
chr11	64468774	64469132
chr11	64491070	64491296
chr11	64496783	64497068
chr11	64502692	64502919
chr11	64502945	64503419

chr11	64510955	64511105
chr11	64511321	64511528
chr11	64599247	64599505
chr11	64618055	64618205
chr11	64619978	64620363
chr11	64703929	64704089
chr11	64718821	64718981
chr11	64793210	64793409
chr11	64890878	64891097
chr11	64898840	64899070
chr11	64968531	64968882
chr11	64971536	64971772
chr11	64978725	64979186
chr11	64979186	64979556
chr11	64987029	64987244
chr11	65003178	65003328
chr11	65009258	65009526
chr11	65016828	65017086
chr11	65030286	65030626
chr11	65088990	65089235
chr11	65089901	65090199
chr11	65128512	65128687
chr11	65128694	65129075
chr11	65129075	65129364
chr11	65145878	65146054
chr11	65148613	65148822
chr11	65153027	65153337
chr11	65161778	65162049
chr11	65162049	65162354
chr11	65162354	65162631
chr11	65162631	65162843
chr11	65162843	65163105
chr11	65195922	65196136
chr11	65197336	65197637
chr11	65197676	65197991
chr11	65209349	65209862
chr11	65209862	65210184
chr11	65214975	65215346
chr11	65215346	65215893
chr11	65215948	65216119
chr11	65217484	65217701
chr11	65236700	65236930

chr11	65243639	65243811
chr11	65245695	65246036
chr11	65246433	65246746
chr11	65268367	65268683
chr11	65271099	65271303
chr11	65275520	65275883
chr11	65292677	65293013
chr11	65293013	65293336
chr11	65293336	65293565
chr11	65300698	65301091
chr11	65338675	65339024
chr11	65339024	65339357
chr11	65347026	65347221
chr11	65365700	65366047
chr11	65366047	65366477
chr11	65366678	65367184
chr11	65375798	65376008
chr11	65386843	65387187
chr11	65389289	65389500
chr11	65447605	65447755
chr11	65448387	65449025
chr11	65460343	65460597
chr11	65476313	65476479
chr11	65482527	65482758
chr11	65490613	65490992
chr11	65492512	65492662
chr11	65560872	65561228
chr11	65594649	65594854
chr11	65598316	65598503
chr11	65598579	65598960
chr11	65598960	65599217
chr11	65631256	65631457
chr11	65643733	65643999
chr11	65667380	65667677
chr11	65667677	65667874
chr11	65678909	65679164
chr11	65680419	65680760
chr11	65725058	65725209
chr11	65744056	65744245
chr11	65746742	65746924
chr11	65784378	65784786
chr11	65787663	65787942

chr11	65787942	65788214
chr11	65788214	65788561
chr11	65794334	65794655
chr11	65794876	65795144
chr11	65795193	65795398
chr11	65802015	65802236
chr11	65806486	65806739
chr11	65806739	65806992
chr11	65806992	65807268
chr11	65807268	65807601
chr11	65818147	65818626
chr11	65818637	65818788
chr11	65818788	65818934
chr11	65819622	65820179
chr11	65849920	65850156
chr11	65852125	65852399
chr11	65866070	65866482
chr11	65930855	65931085
chr11	65972289	65972439
chr11	66088867	66089130
chr11	66122169	66122324
chr11	66167666	66168065
chr11	66168065	66168372
chr11	66168372	66168746
chr11	66188757	66188952
chr11	66188952	66189130
chr11	66232910	66233158
chr11	66233994	66234198
chr11	66238155	66238468
chr11	66238750	66238942
chr11	66258941	66259099
chr11	66341657	66341886
chr11	66344888	66345205
chr11	66359684	66360247
chr11	66404622	66404893
chr11	66420927	66421311
chr11	66433179	66433455
chr11	66433455	66433759
chr11	66485225	66485530
chr11	66485573	66485956
chr11	66486012	66486264
chr11	66488938	66489327

chr11	66499138	66499484
chr11	66499851	66500059
chr11	66525588	66525843
chr11	66525843	66526064
chr11	66526064	66526277
chr11	66526277	66526511
chr11	66526511	66526808
chr11	66526808	66527222
chr11	66551139	66551339
chr11	66551916	66552223
chr11	66564989	66565219
chr11	66790108	66790367
chr11	66899461	66899724
chr11	66900675	66900854
chr11	66900930	66901326
chr11	66905864	66906176
chr11	66912542	66912827
chr11	66914292	66914577
chr11	66945290	66945642
chr11	66956189	66956798
chr11	66966548	66967195
chr11	66968600	66968860
chr11	66971994	66972377
chr11	66980590	66981049
chr11	66983894	66984131
chr11	66984142	66984488
chr11	66984918	66985158
chr11	66996640	66996866
chr11	66998279	66998835
chr11	66998847	66999066
chr11	67006809	67007039
chr11	67017739	67018207
chr11	67018292	67018466
chr11	67021048	67021209
chr11	67024811	67025461
chr11	67031026	67031374
chr11	67031374	67031628
chr11	67031628	67031884
chr11	67034852	67035022
chr11	67052039	67052217
chr11	67052269	67053115
chr11	67080300	67080542

chr11	67081056	67081206
chr11	67081244	67081666
chr11	67085497	67085754
chr11	67101828	67102029
chr11	67110221	67110569
chr11	67112066	67112363
chr11	67112783	67113079
chr11	67165118	67165299
chr11	67323973	67324123
chr11	67350471	67350707
chr11	67353095	67353301
chr11	67373340	67373648
chr11	67379961	67380190
chr11	67380190	67380390
chr11	67403972	67404504
chr11	67414050	67414202
chr11	67425767	67425936
chr11	67428334	67428489
chr11	67454734	67455175
chr11	67455175	67455455
chr11	67455455	67455763
chr11	67455799	67455949
chr11	67498072	67498223
chr11	67514571	67514762
chr11	67515203	67515652
chr11	67515652	67515984
chr11	67521636	67521872
chr11	67522014	67522406
chr11	67522689	67523009
chr11	67526603	67526922
chr11	67527010	67527326
chr11	67527326	67527579
chr11	67529354	67529586
chr11	67532244	67532645
chr11	67532645	67533253
chr11	67534978	67535229
chr11	67538628	67539063
chr11	67544695	67544890
chr11	67544897	67545428
chr11	67545802	67545952
chr11	67549306	67549826
chr11	67549826	67550447

chr11	67558163	67558344
chr11	67563019	67563728
chr11	67570984	67571166
chr11	67588112	67588307
chr11	67588894	67589215
chr11	67600770	67600999
chr11	67601091	67601448
chr11	67622373	67622647
chr11	67647794	67648112
chr11	67650398	67650573
chr11	67652352	67652600
chr11	67656180	67656340
chr11	67657602	67658091
chr11	67658195	67658394
chr11	67676201	67676489
chr11	67679979	67680352
chr11	67701427	67701670
chr11	67738784	67738935
chr11	67772630	67772864
chr11	67819559	67819826
chr11	67819826	67820047
chr11	67820199	67820514
chr11	67828040	67828277
chr11	67828284	67828655
chr11	67828655	67829045
chr11	67838156	67838416
chr11	67839863	67840175
chr11	67855112	67855370
chr11	67860748	67861091
chr11	67861091	67861414
chr11	67861414	67861892
chr11	67862242	67862441
chr11	67865633	67865833
chr11	67879908	67880392
chr11	67896809	67897317
chr11	67897317	67897616
chr11	67911033	67911557
chr11	67911571	67911736
chr11	67919679	67919988
chr11	67920225	67920537
chr11	67921438	67921801
chr11	67921801	67922127

chr11	67922127	67922434
chr11	67922434	67922696
chr11	67922791	67923099
chr11	67939123	67939313
chr11	67945537	67945831
chr11	67946123	67946273
chr11	67946686	67946867
chr11	67947105	67947476
chr11	67948371	67948660
chr11	67964185	67964423
chr11	67965331	67965550
chr11	67965550	67965787
chr11	67966249	67966577
chr11	67966577	67966797
chr11	67975017	67975282
chr11	67982662	67982836
chr11	68004244	68004494
chr11	68008012	68008183
chr11	68008690	68009083
chr11	68030689	68031080
chr11	68032566	68032869
chr11	68035376	68035913
chr11	68036505	68036867
chr11	68045677	68046038
chr11	68071875	68072208
chr11	68072208	68072487
chr11	68072487	68072784
chr11	68081571	68081893
chr11	68083302	68083653
chr11	68083653	68083919
chr11	68089853	68090448
chr11	68092028	68092384
chr11	68092384	68092699
chr11	68095132	68095288
chr11	68099533	68099763
chr11	68114319	68114541
chr11	68124963	68125118
chr11	68155815	68156187
chr11	68176614	68176837
chr11	68183234	68183487
chr11	68195223	68195440
chr11	68199207	68199428

chr11	68210103	68210258
chr11	68211908	68212207
chr11	68212255	68212553
chr11	68214014	68214234
chr11	68215256	68215478
chr11	68216288	68216608
chr11	68220786	68221153
chr11	68223284	68223453
chr11	68231630	68231860
chr11	68244610	68244881
chr11	68244881	68245359
chr11	68258826	68259164
chr11	68259167	68259367
chr11	68272565	68272720
chr11	68300436	68300657
chr11	68313409	68313804
chr11	68313804	68314188
chr11	68314188	68314457
chr11	68319362	68319635
chr11	68324934	68325161
chr11	68333483	68333904
chr11	68339919	68340091
chr11	68385202	68385777
chr11	68385777	68386319
chr11	68386349	68386550
chr11	68386602	68387419
chr11	68394902	68395056
chr11	68395244	68395501
chr11	68399687	68400056
chr11	68400056	68400345
chr11	68400345	68400666
chr11	68404295	68404466
chr11	68408201	68408484
chr11	68417930	68418114
chr11	68427131	68427424
chr11	68431880	68432180
chr11	68432180	68432403
chr11	68432403	68432626
chr11	68432626	68432845
chr11	68438614	68438848
chr11	68445706	68446180
chr11	68446197	68446399

chr11	68446511	68446906
chr11	68451811	68451974
chr11	68458090	68458550
chr11	68467523	68467883
chr11	68468204	68468550
chr11	68468741	68469337
chr11	68485121	68485272
chr11	68485302	68485553
chr11	68488051	68488446
chr11	68488446	68488744
chr11	68500898	68501137
chr11	68502205	68502373
chr11	68531282	68531486
chr11	68531486	68531762
chr11	68532005	68532436
chr11	68536505	68536765
chr11	68538917	68539560
chr11	68544638	68544863
chr11	68554199	68554349
chr11	68555864	68556574
chr11	68566279	68566429
chr11	68566484	68566736
chr11	68566736	68566965
chr11	68573239	68573396
chr11	68586042	68586255
chr11	68589489	68589748
chr11	68623525	68623863
chr11	68638018	68638216
chr11	68681153	68681347
chr11	68681351	68681676
chr11	68682209	68682431
chr11	68691285	68691660
chr11	68691660	68692390
chr11	68716059	68716366
chr11	68750362	68750512
chr11	68750550	68750708
chr11	68769957	68770591
chr11	68782489	68782724
chr11	68786881	68787358
chr11	68796609	68796847
chr11	68796847	68797141
chr11	68799418	68799528

chr11	68799528	68799572
chr11	68805251	68805420
chr11	68807780	68808181
chr11	68811960	68812207
chr11	68817862	68818189
chr11	68818202	68818428
chr11	68818428	68818619
chr11	68822416	68822602
chr11	68825475	68825625
chr11	68825765	68826149
chr11	68826501	68826765
chr11	68839657	68840014
chr11	68847018	68847210
chr11	68852432	68852849
chr11	68852849	68853414
chr11	68853726	68853905
chr11	68854093	68854496
chr11	68859897	68860499
chr11	68863739	68864071
chr11	68864727	68865262
chr11	68871791	68872164
chr11	68872991	68873506
chr11	68874946	68875173
chr11	68889828	68890073
chr11	68890646	68891001
chr11	68892065	68892418
chr11	68894049	68894412
chr11	68896810	68897081
chr11	68897227	68897377
chr11	68901128	68901487
chr11	68901487	68901681
chr11	68901681	68901911
chr11	68904805	68905039
chr11	68905843	68906183
chr11	68906400	68906963
chr11	68918955	68919125
chr11	68933861	68934005
chr11	68934005	68934158
chr11	68939480	68939708
chr11	68939708	68940163
chr11	68946804	68947340
chr11	68947492	68947642

chr11	68951379	68951569
chr11	68954416	68954789
chr11	68955482	68955703
chr11	68955703	68955973
chr11	68957034	68957184
chr11	68961521	68961919
chr11	68967550	68967918
chr11	68967918	68968253
chr11	68973205	68973522
chr11	68973522	68973760
chr11	68973760	68974033
chr11	68981005	68981257
chr11	68982550	68982949
chr11	68982949	68983208
chr11	68994492	68994696
chr11	69008146	69008925
chr11	69012362	69012665
chr11	69015727	69016194
chr11	69016194	69016482
chr11	69028778	69029179
chr11	69035951	69036210
chr11	69036941	69037141
chr11	69037307	69037618
chr11	69037768	69037992
chr11	69040008	69040513
chr11	69040513	69040797
chr11	69040906	69041172
chr11	69041172	69041399
chr11	69041639	69041922
chr11	69045437	69045784
chr11	69045784	69046148
chr11	69046452	69046634
chr11	69053935	69054127
chr11	69059151	69059359
chr11	69059382	69060002
chr11	69060032	69060388
chr11	69060456	69060865
chr11	69062280	69062446
chr11	69062563	69062757
chr11	69062757	69062942
chr11	69062942	69063263
chr11	69063500	69063702

chr11	69063917	69064098
chr11	69067031	69067195
chr11	69070620	69070907
chr11	69070907	69071178
chr11	69073111	69073283
chr11	69073365	69074117
chr11	69078916	69079205
chr11	69088187	69088654
chr11	69088654	69088924
chr11	69088929	69089178
chr11	69089298	69089577
chr11	69094679	69095033
chr11	69095033	69095373
chr11	69096967	69097338
chr11	69097611	69097801
chr11	69098580	69099034
chr11	69099892	69100235
chr11	69100304	69100509
chr11	69102392	69102699
chr11	69108880	69109174
chr11	69110335	69110730
chr11	69110730	69111086
chr11	69117872	69118255
chr11	69118470	69118620
chr11	69118750	69119041
chr11	69122273	69122684
chr11	69122684	69123023
chr11	69123709	69124003
chr11	69124134	69124459
chr11	69124532	69124726
chr11	69125119	69125368
chr11	69125368	69125680
chr11	69125680	69125970
chr11	69125970	69126273
chr11	69133540	69133906
chr11	69133906	69134222
chr11	69134222	69134455
chr11	69134455	69134710
chr11	69142567	69142743
chr11	69143641	69144103
chr11	69150577	69150783
chr11	69150783	69150997

chr11	69150997	69151364
chr11	69153970	69154566
chr11	69160457	69160954
chr11	69171690	69171894
chr11	69172657	69172951
chr11	69174717	69174962
chr11	69174962	69175288
chr11	69175288	69175618
chr11	69180185	69180404
chr11	69180688	69180861
chr11	69192973	69193179
chr11	69196873	69197159
chr11	69197287	69197664
chr11	69213351	69213610
chr11	69235202	69235495
chr11	69235495	69235719
chr11	69235719	69235936
chr11	69235936	69236188
chr11	69238567	69238752
chr11	69245019	69245252
chr11	69253112	69253469
chr11	69257861	69258014
chr11	69258378	69258660
chr11	69259466	69259805
chr11	69260110	69260478
chr11	69264407	69264581
chr11	69267431	69267644
chr11	69269641	69270019
chr11	69272378	69272734
chr11	69276122	69276339
chr11	69301077	69301331
chr11	69316251	69316580
chr11	69323651	69324274
chr11	69325722	69325948
chr11	69326091	69326403
chr11	69326473	69326716
chr11	69327727	69327904
chr11	69334005	69334248
chr11	69334391	69334785
chr11	69334785	69335226
chr11	69339621	69339867
chr11	69340079	69340549

chr11	69340549	69341168
chr11	69342037	69342251
chr11	69342718	69343006
chr11	69362580	69362847
chr11	69364961	69365305
chr11	69365401	69365789
chr11	69365789	69366057
chr11	69366226	69366456
chr11	69366456	69366938
chr11	69367993	69368278
chr11	69368354	69368619
chr11	69368810	69369092
chr11	69377576	69377835
chr11	69377835	69378248
chr11	69382614	69383228
chr11	69390298	69390620
chr11	69395305	69395563
chr11	69395627	69395928
chr11	69395928	69396224
chr11	69396224	69396454
chr11	69397625	69397869
chr11	69397869	69398152
chr11	69398172	69398341
chr11	69401213	69401821
chr11	69401859	69402107
chr11	69408547	69409116
chr11	69409133	69409445
chr11	69409445	69409770
chr11	69409770	69410018
chr11	69410363	69410669
chr11	69410669	69411095
chr11	69411095	69411464
chr11	69411631	69412010
chr11	69417537	69417855
chr11	69417858	69418309
chr11	69418503	69418811
chr11	69418811	69419178
chr11	69419178	69419481
chr11	69419485	69419645
chr11	69428814	69429085
chr11	69432085	69432401
chr11	69432414	69432609

chr11	69442799	69443072
chr11	69443239	69444002
chr11	69452553	69452713
chr11	69459395	69459707
chr11	69471532	69471795
chr11	69472424	69473210
chr11	69479289	69479502
chr11	69482640	69482879
chr11	69483063	69483221
chr11	69484209	69484542
chr11	69490938	69491154
chr11	69492887	69493152
chr11	69502729	69502912
chr11	69503841	69504146
chr11	69513908	69514171
chr11	69530880	69531091
chr11	69535124	69535294
chr11	69541571	69541778
chr11	69546037	69546348
chr11	69546348	69546737
chr11	69546737	69547101
chr11	69547101	69547410
chr11	69554751	69554952
chr11	69554952	69555146
chr11	69561282	69561695
chr11	69575322	69575490
chr11	69578578	69578777
chr11	69578897	69579619
chr11	69580150	69580490
chr11	69580490	69580776
chr11	69581107	69581367
chr11	69581367	69581688
chr11	69592399	69592718
chr11	69598601	69598919
chr11	69604432	69604657
chr11	69605186	69605339
chr11	69605808	69606140
chr11	69613626	69613849
chr11	69613991	69614262
chr11	69616884	69617056
chr11	69628370	69628596
chr11	69632372	69632959

chr11	69632959	69633470
chr11	69646958	69647126
chr11	69654892	69655064
chr11	69655428	69655680
chr11	69656236	69656419
chr11	69656780	69657192
chr11	69657287	69657682
chr11	69662238	69662452
chr11	69662490	69662936
chr11	69663865	69664156
chr11	69665876	69666641
chr11	69671172	69671683
chr11	69671842	69672028
chr11	69672028	69672354
chr11	69672354	69672778
chr11	69681543	69682306
chr11	69682836	69683069
chr11	69685500	69685910
chr11	69685990	69686181
chr11	69687159	69687341
chr11	69691017	69691294
chr11	69695511	69695725
chr11	69695863	69696166
chr11	69700755	69700975
chr11	69706735	69707239
chr11	69715584	69715815
chr11	69715896	69716279
chr11	69717056	69717258
chr11	69723205	69723367
chr11	69726024	69726320
chr11	69729145	69729383
chr11	69731487	69731708
chr11	69734342	69734614
chr11	69736560	69736761
chr11	69751206	69751374
chr11	69752746	69753007
chr11	69758079	69758518
chr11	69758518	69758926
chr11	69758926	69759290
chr11	69759321	69759668
chr11	69760182	69760401
chr11	69765534	69765858

chr11	69765858	69766141
chr11	69766141	69766378
chr11	69766378	69766812
chr11	69776660	69776948
chr11	69777261	69777485
chr11	69778602	69778848
chr11	69778866	69779057
chr11	69784904	69785097
chr11	69785097	69785299
chr11	69795207	69795724
chr11	69795797	69796099
chr11	69797982	69798300
chr11	69802830	69803107
chr11	69803526	69803809
chr11	69803809	69804142
chr11	69804142	69804450
chr11	69804531	69804853
chr11	69805152	69805320
chr11	69805320	69805440
chr11	69805440	69805528
chr11	69805530	69805873
chr11	69815353	69815539
chr11	69818981	69819165
chr11	69825685	69825854
chr11	69828798	69828973
chr11	69837372	69837944
chr11	69837944	69838265
chr11	69838265	69838729
chr11	69838841	69839016
chr11	69845475	69845686
chr11	69846011	69846418
chr11	69846418	69846736
chr11	69846755	69847043
chr11	69847043	69847299
chr11	69849149	69849374
chr11	69857941	69858115
chr11	69858124	69858391
chr11	69858391	69858656
chr11	69858656	69859068
chr11	69872225	69872456
chr11	69872479	69872847
chr11	69876933	69877202

chr11	69880545	69880896
chr11	69880896	69881295
chr11	69881295	69881684
chr11	69883753	69884087
chr11	69884087	69884337
chr11	69887338	69887544
chr11	69894705	69894925
chr11	69895470	69895683
chr11	69900385	69900837
chr11	69900837	69901205
chr11	69901205	69901541
chr11	69901541	69901988
chr11	69902031	69902202
chr11	69902202	69902302
chr11	69913325	69913573
chr11	69913645	69914140
chr11	69914140	69914446
chr11	69914446	69914735
chr11	69915078	69915296
chr11	69915802	69916019
chr11	69919603	69920110
chr11	69920559	69921056
chr11	69921056	69921511
chr11	69921511	69922071
chr11	69927613	69927849
chr11	69933697	69933979
chr11	69934177	69934625
chr11	69935189	69935516
chr11	69935516	69936127
chr11	69936346	69936505
chr11	69948084	69948410
chr11	69948410	69948696
chr11	69948696	69948945
chr11	69948945	69949232
chr11	69951212	69951362
chr11	69952360	69952584
chr11	69953457	69953626
chr11	69953775	69953933
chr11	69955680	69956000
chr11	69961476	69961719
chr11	69961904	69962203
chr11	69964676	69964973

chr11	69965157	69965729
chr11	69965991	69966365
chr11	69966535	69966969
chr11	69979971	69980414
chr11	69980414	69980653
chr11	69980653	69980894
chr11	69980894	69981172
chr11	69990806	69991114
chr11	69991364	69991713
chr11	69991731	69991924
chr11	69995363	69995635
chr11	69995635	69995922
chr11	69996844	69997123
chr11	69997123	69997587
chr11	70000363	70000888
chr11	70001912	70002192
chr11	70005578	70005849
chr11	70014520	70014682
chr11	70014851	70015063
chr11	70015063	70015281
chr11	70015281	70015590
chr11	70020750	70021049
chr11	70021105	70021462
chr11	70021462	70021981
chr11	70028697	70029148
chr11	70029148	70029491
chr11	70029491	70029874
chr11	70052435	70052784
chr11	70055641	70055890
chr11	70055890	70056081
chr11	70074729	70075128
chr11	70075553	70075890
chr11	70075927	70076092
chr11	70079261	70079460
chr11	70083191	70083406
chr11	70087795	70088024
chr11	70106976	70107150
chr11	70110227	70110460
chr11	70114458	70114638
chr11	70114638	70114839
chr11	70141959	70142141
chr11	70151161	70151422

chr11	70171910	70172178
chr11	70173690	70173898
chr11	70179563	70179713
chr11	70183776	70183995
chr11	70189039	70189772
chr11	70193961	70194279
chr11	70198268	70198846
chr11	70201687	70201900
chr11	70208968	70209410
chr11	70209410	70209698
chr11	70210683	70211142
chr11	70213012	70213221
chr11	70220415	70220663
chr11	70220663	70220969
chr11	70220969	70221498
chr11	70226187	70226342
chr11	70226408	70226780
chr11	70228938	70229193
chr11	70230216	70230783
chr11	70237333	70238084
chr11	70238084	70238337
chr11	70238374	70238524
chr11	70239105	70239299
chr11	70239582	70239912
chr11	70240056	70240212
chr11	70242562	70242795
chr11	70247503	70247790
chr11	70250933	70251157
chr11	70259007	70259424
chr11	70259424	70259682
chr11	70275909	70276189
chr11	70283700	70283888
chr11	70284704	70285147
chr11	70291399	70291730
chr11	70293337	70293487
chr11	70307684	70307866
chr11	70313930	70314156
chr11	70321699	70321958
chr11	70321990	70322140
chr11	70324751	70325060
chr11	70329549	70329713
chr11	70334562	70334820

chr11	70345580	70345867
chr11	70353185	70353345
chr11	70393906	70394086
chr11	70409543	70409994
chr11	70409994	70410574
chr11	70414047	70414277
chr11	70431648	70431891
chr11	70432066	70432414
chr11	70432414	70432946
chr11	70432955	70433184
chr11	70440074	70440353
chr11	70440677	70441069
chr11	70441195	70441362
chr11	70451715	70452041
chr11	70452041	70452645
chr11	70453223	70453387
chr11	70458713	70459072
chr11	70459183	70459515
chr11	70459515	70459869
chr11	70459869	70460321
chr11	70467630	70468364
chr11	70470164	70470391
chr11	70499636	70499921
chr11	70499957	70500222
chr11	70500222	70500453
chr11	70500453	70500745
chr11	70506426	70506710
chr11	70506710	70506922
chr11	70510078	70510383
chr11	70510439	70510593
chr11	70511332	70511533
chr11	70513652	70513907
chr11	70516418	70516706
chr11	70523386	70523592
chr11	70525070	70525457
chr11	70525457	70525682
chr11	70525819	70526057
chr11	70531265	70531520
chr11	70539860	70540318
chr11	70540318	70540682
chr11	70540761	70540912
chr11	70561988	70562274

chr11	70562274	70562654
chr11	70562660	70563104
chr11	70563104	70563410
chr11	70563452	70563673
chr11	70563673	70563909
chr11	70564073	70564275
chr11	70571133	70571396
chr11	70572121	70572283
chr11	70572435	70572930
chr11	70574042	70574335
chr11	70577239	70577709
chr11	70577793	70578058
chr11	70579722	70580042
chr11	70580045	70580246
chr11	70585312	70585801
chr11	70586259	70586530
chr11	70586530	70586771
chr11	70586963	70587113
chr11	70595793	70595943
chr11	70601349	70601592
chr11	70605104	70605510
chr11	70608664	70608928
chr11	70610843	70610994
chr11	70612594	70612799
chr11	70615078	70615309
chr11	70615606	70615906
chr11	70616403	70616614
chr11	70617236	70617417
chr11	70622070	70622216
chr11	70622216	70622374
chr11	70622440	70622839
chr11	70623110	70623401
chr11	70625537	70625769
chr11	70629645	70629869
chr11	70635727	70635892
chr11	70640152	70640312
chr11	70640597	70641000
chr11	70641434	70641608
chr11	70646684	70647106
chr11	70647106	70647382
chr11	70647382	70647646
chr11	70647762	70648048

chr11	70651423	70651691
chr11	70653563	70653958
chr11	70653958	70654200
chr11	70654440	70654711
chr11	70654955	70655247
chr11	70655279	70655548
chr11	70655622	70655830
chr11	70655830	70656010
chr11	70656010	70656253
chr11	70658700	70658863
chr11	70668328	70668812
chr11	70668812	70669133
chr11	70669133	70669522
chr11	70669978	70670159
chr11	70681918	70682249
chr11	70687165	70687631
chr11	70687631	70688030
chr11	70688030	70688291
chr11	70699848	70700051
chr11	70700051	70700319
chr11	70700319	70700632
chr11	70700632	70700936
chr11	70700936	70701172
chr11	70701172	70701370
chr11	70701370	70701603
chr11	70715847	70716150
chr11	70716150	70716351
chr11	70720119	70720292
chr11	70720638	70721016
chr11	70731072	70731309
chr11	70732434	70732607
chr11	70747382	70747878
chr11	70747880	70748314
chr11	70753377	70753537
chr11	70755082	70755241
chr11	70755686	70755961
chr11	70755987	70756146
chr11	70756479	70756726
chr11	70764095	70764804
chr11	70765969	70766192
chr11	70766192	70766501
chr11	70777217	70777541

chr11	70777726	70777920
chr11	70777920	70778149
chr11	70778149	70778363
chr11	70778363	70778610
chr11	70785232	70785458
chr11	70790607	70790787
chr11	70792501	70792744
chr11	70796920	70797076
chr11	70803491	70803765
chr11	70808217	70808417
chr11	70815033	70815219
chr11	70815568	70815812
chr11	70815812	70816060
chr11	70816060	70816267
chr11	70828943	70829263
chr11	70829263	70829604
chr11	70844512	70844853
chr11	70844853	70845075
chr11	70845075	70845261
chr11	70845261	70845498
chr11	70850233	70850401
chr11	70857122	70857505
chr11	70866545	70866919
chr11	70867060	70867336
chr11	70867895	70868185
chr11	70870470	70870760
chr11	70873986	70874348
chr11	70874348	70874646
chr11	70877746	70877985
chr11	70878428	70878732
chr11	70906737	70906968
chr11	70917887	70918263
chr11	70924296	70924552
chr11	70935304	70935549
chr11	70964313	70964577
chr11	70969459	70969786
chr11	70969844	70970318
chr11	70970334	70970594
chr11	70970597	70970956
chr11	70974086	70974280
chr11	70982602	70982885
chr11	70982885	70983241

chr11	71003765	71004051
chr11	71004051	71004334
chr11	71004334	71004755
chr11	71012820	71013000
chr11	71018846	71019124
chr11	71019127	71019611
chr11	71019611	71020011
chr11	71039996	71040259
chr11	71052673	71052824
chr11	71371965	71372118
chr11	71434996	71435212
chr11	71499459	71500003
chr11	71666914	71667101
chr11	71672719	71673017
chr11	71674325	71674662
chr11	71728029	71728450
chr11	71728450	71728867
chr11	71730703	71730860
chr11	71733869	71734122
chr11	71734480	71734751
chr11	71737143	71737448
chr11	71749754	71750035
chr11	71750437	71750882
chr11	71750882	71751225
chr11	71751298	71751554
chr11	71831292	71831620
chr11	71848041	71848279
chr11	71855661	71855883
chr11	71871103	71871280
chr11	71871398	71871578
chr11	71873299	71873486
chr11	71930151	71930961
chr11	71931772	71931975
chr11	71954628	71954804
chr11	71962332	71962536
chr11	71970577	71970867
chr11	71976758	71977150
chr11	71977150	71977555
chr11	71986093	71986260
chr11	71991567	71991822
chr11	71995721	71995968
chr11	71995968	71996252

chr11	71996252	71996542
chr11	72007875	72008053
chr11	72017758	72018186
chr11	72018443	72018612
chr11	72024460	72024786
chr11	72025923	72026086
chr11	72029156	72029466
chr11	72038159	72038321
chr11	72041798	72042165
chr11	72042165	72042623
chr11	72042623	72042991
chr11	72051616	72051829
chr11	72052682	72052962
chr11	72052962	72053310
chr11	72060898	72061048
chr11	72066356	72066816
chr11	72067047	72067217
chr11	72078040	72078276
chr11	72078335	72078664
chr11	72083642	72084340
chr11	72085448	72085716
chr11	72094036	72094475
chr11	72094475	72094873
chr11	72094873	72095356
chr11	72110502	72110817
chr11	72110817	72111143
chr11	72112484	72112764
chr11	72120325	72120604
chr11	72120604	72120817
chr11	72123592	72123897
chr11	72130022	72130440
chr11	72130440	72130854
chr11	72132170	72132421
chr11	72132421	72132678
chr11	72134345	72134898
chr11	72134975	72135162
chr11	72135162	72135361
chr11	72135629	72135975
chr11	72140280	72140531
chr11	72142788	72143333
chr11	72145272	72145501
chr11	72145669	72145838

chr11	72152785	72153446
chr11	72153446	72153880
chr11	72170381	72170696
chr11	72175348	72175498
chr11	72186070	72186291
chr11	72186403	72187043
chr11	72194323	72194621
chr11	72194646	72195020
chr11	72196513	72196838
chr11	72196838	72197163
chr11	72206041	72206369
chr11	72206369	72206907
chr11	72206907	72207408
chr11	72207408	72207881
chr11	72214654	72214906
chr11	72214906	72215139
chr11	72215139	72215432
chr11	72215432	72215755
chr11	72221326	72221753
chr11	72224921	72225219
chr11	72263942	72264118
chr11	72266107	72266557
chr11	72266557	72266942
chr11	72266942	72267302
chr11	72273509	72273661
chr11	72275867	72276053
chr11	72276212	72276372
chr11	72281363	72282158
chr11	72299343	72299574
chr11	72300096	72300287
chr11	72301317	72301689
chr11	72301689	72301947
chr11	72314368	72314639
chr11	72325781	72325952
chr11	72342271	72342584
chr11	72342584	72343034
chr11	72354924	72355329
chr11	72355329	72355632
chr11	72361833	72361999
chr11	72362048	72362198
chr11	72396505	72396707
chr11	72397858	72398054

chr11	72410686	72410845
chr11	72410985	72411472
chr11	72411472	72411968
chr11	72412038	72412261
chr11	72423604	72424260
chr11	72428686	72428959
chr11	72429277	72429704
chr11	72429704	72429913
chr11	72434741	72434916
chr11	72434929	72435339
chr11	72435339	72435716
chr11	72440691	72440935
chr11	72440966	72441450
chr11	72441450	72441908
chr11	72446351	72446701
chr11	72447542	72447795
chr11	72451346	72451662
chr11	72451970	72452274
chr11	72454071	72454240
chr11	72455036	72455190
chr11	72455920	72456090
chr11	72459500	72459666
chr11	72466354	72466632
chr11	72467116	72467358
chr11	72467358	72467653
chr11	72468928	72469171
chr11	72469320	72469600
chr11	72472517	72472911
chr11	72472914	72473269
chr11	72473269	72473477
chr11	72473524	72473882
chr11	72474206	72474416
chr11	72477018	72477184
chr11	72478246	72478822
chr11	72479604	72479800
chr11	72484663	72485133
chr11	72485187	72485717
chr11	72488676	72489098
chr11	72489841	72490258
chr11	72490341	72490501
chr11	72491062	72491531
chr11	72494279	72494603

chr11	72494603	72494822
chr11	72496046	72496267
chr11	72499171	72499321
chr11	72500253	72500502
chr11	72501153	72501441
chr11	72501441	72501868
chr11	72501868	72502281
chr11	72502424	72502604
chr11	72506492	72506822
chr11	72508732	72508950
chr11	72510329	72510530
chr11	72510717	72511075
chr11	72511075	72511351
chr11	72519250	72519593
chr11	72519616	72519974
chr11	72519974	72520569
chr11	72526210	72526459
chr11	72526506	72526992
chr11	72529895	72530103
chr11	72531073	72531429
chr11	72531429	72531693
chr11	72531737	72531961
chr11	72536500	72537020
chr11	72541814	72542095
chr11	72542095	72542328
chr11	72558232	72558398
chr11	72558467	72558736
chr11	72564297	72564646
chr11	72569178	72569343
chr11	72570538	72571088
chr11	72573884	72574143
chr11	72576148	72576419
chr11	72576419	72576690
chr11	72578206	72578370
chr11	72578570	72578832
chr11	72580597	72580821
chr11	72581391	72581561
chr11	72581633	72582004
chr11	72594926	72595281
chr11	72595319	72595623
chr11	72596046	72596291
chr11	72597173	72597533

chr11	72597896	72598118
chr11	72602037	72602211
chr11	72606872	72607521
chr11	72607521	72608142
chr11	72608142	72608681
chr11	72689725	72690108
chr11	72690108	72690712
chr11	72696642	72697009
chr11	72705137	72705706
chr11	72726137	72726330
chr11	72737572	72737863
chr11	72752000	72752338
chr11	72759681	72759885
chr11	72764860	72765071
chr11	72765317	72765583
chr11	72765627	72766118
chr11	72766205	72766384
chr11	72766384	72766568
chr11	72766766	72767161
chr11	72767656	72768073
chr11	72772520	72772737
chr11	72772737	72772846
chr11	72782186	72782399
chr11	72792098	72792480
chr11	72792734	72792951
chr11	72792951	72793236
chr11	72795260	72795500
chr11	72795500	72795693
chr11	72795693	72795904
chr11	72795982	72796366
chr11	72796366	72796673
chr11	72799366	72799536
chr11	72803554	72803722
chr11	72803927	72804155
chr11	72818876	72819040
chr11	72821095	72821372
chr11	72842811	72843068
chr11	72843129	72843404
chr11	72843657	72843896
chr11	72846730	72847012
chr11	72847898	72848221
chr11	72848221	72848566

chr11	72848566	72848860
chr11	72854132	72854337
chr11	72897722	72897898
chr11	72900934	72901333
chr11	72917762	72917912
chr11	72926558	72926842
chr11	72928557	72928855
chr11	72934333	72934533
chr11	72940911	72941157
chr11	72946224	72946694
chr11	72948309	72948534
chr11	72951201	72951426
chr11	72951623	72952070
chr11	72952166	72952345
chr11	72952687	72952933
chr11	72953462	72953713
chr11	72953721	72954078
chr11	72960422	72960580
chr11	72960973	72961567
chr11	72980175	72980387
chr11	72983249	72983624
chr11	72988605	72988756
chr11	72989271	72989457
chr11	73001099	73001370
chr11	73006015	73006165
chr11	73006415	73006688
chr11	73006688	73006899
chr11	73007770	73008013
chr11	73012591	73012749
chr11	73018430	73018750
chr11	73018750	73019187
chr11	73019314	73019467
chr11	73019517	73019781
chr11	73021550	73021803
chr11	73022154	73022460
chr11	73023107	73023451
chr11	73030783	73031180
chr11	73036855	73037384
chr11	73039481	73039708
chr11	73047559	73047922
chr11	73047922	73048297
chr11	73048297	73048571

chr11	73048636	73048786
chr11	73049397	73049646
chr11	73065768	73066005
chr11	73066066	73066514
chr11	73071913	73072113
chr11	73083931	73084284
chr11	73084284	73084769
chr11	73092105	73092399
chr11	73092603	73092860
chr11	73092865	73093329
chr11	73095478	73095911
chr11	73099001	73099221
chr11	73107435	73107694
chr11	73108698	73108851
chr11	73111398	73111809
chr11	73117153	73117313
chr11	73131027	73131428
chr11	73137557	73137873
chr11	73137873	73138190
chr11	73138190	73138590
chr11	73160281	73160693
chr11	73160822	73161295
chr11	73161379	73161664
chr11	73161664	73161944
chr11	73167127	73167345
chr11	73169137	73169682
chr11	73175721	73175966
chr11	73176534	73176702
chr11	73176718	73177301
chr11	73177301	73177564
chr11	73177564	73177890
chr11	73183799	73183976
chr11	73192958	73193353
chr11	73198664	73198925
chr11	73198925	73199261
chr11	73199261	73199795
chr11	73199956	73200151
chr11	73205227	73205523
chr11	73205791	73206056
chr11	73207734	73207959
chr11	73220640	73220999
chr11	73222893	73223142

chr11	73224020	73224347
chr11	73228726	73229061
chr11	73245854	73246017
chr11	73266282	73266436
chr11	73266517	73266755
chr11	73266942	73267162
chr11	73267284	73267726
chr11	73274981	73275164
chr11	73281518	73281753
chr11	73281765	73282094
chr11	73283606	73283972
chr11	73283972	73284670
chr11	73318461	73318923
chr11	73319676	73319857
chr11	73326390	73326932
chr11	73354105	73354302
chr11	73388529	73388679
chr11	73498606	73498791
chr11	74371525	74371735
chr11	74371735	74372055
chr11	74374893	74375043
chr11	74376876	74377263
chr11	74379168	74379391
chr11	74382396	74382620
chr11	74385446	74385666
chr11	74387299	74387449
chr11	74402204	74402485
chr11	74408760	74409232
chr11	74409737	74410124
chr11	74419673	74420041
chr11	74438444	74438611
chr11	74438807	74438976
chr11	74439147	74439674
chr11	74442397	74442771
chr11	74443923	74444102
chr11	74447980	74448538
chr11	74452219	74452602
chr11	74464033	74464233
chr11	74465700	74466137
chr11	74475783	74476062
chr11	74483037	74483437
chr11	74483437	74483830

chr11	74483830	74484237
chr11	74502157	74502428
chr11	74502474	74503064
chr11	74522043	74522249
chr11	74522415	74522881
chr11	74522881	74523286
chr11	74526275	74526504
chr11	74526504	74526787
chr11	74529895	74530122
chr11	74536294	74536449
chr11	74541234	74541405
chr11	74546242	74546480
chr11	74547233	74547451
chr11	74547451	74547725
chr11	74548279	74548449
chr11	74548449	74548540
chr11	74548738	74549141
chr11	74550786	74550995
chr11	74554624	74554987
chr11	74555691	74556371
chr11	74557512	74557763
chr11	74566340	74566745
chr11	74572120	74572384
chr11	74572659	74573054
chr11	74583879	74584082
chr11	74584139	74584342
chr11	74584980	74585244
chr11	74586013	74586163
chr11	74590014	74590199
chr11	74595663	74595912
chr11	74597410	74597560
chr11	74610831	74611144
chr11	74619158	74619536
chr11	74634564	74634725
chr11	74637910	74638369
chr11	74638369	74638923
chr11	74649097	74649622
chr11	74649622	74649925
chr11	74649925	74650227
chr11	74650379	74650617
chr11	74656021	74656256
chr11	74660413	74660623

chr11	74670336	74670580
chr11	74671135	74671306
chr11	74673277	74673427
chr11	74673643	74673872
chr11	74678724	74678920
chr11	74710774	74710963
chr11	74713259	74713437
chr11	74723395	74723750
chr11	74723750	74723987
chr11	74723987	74724240
chr11	74724240	74724551
chr11	74724551	74724819
chr11	74727495	74727812
chr11	74747999	74748363
chr11	74764175	74764706
chr11	74770453	74771002
chr11	74771002	74771278
chr11	74771342	74771537
chr11	74821587	74821737
chr11	74826210	74826456
chr11	74829855	74830144
chr11	74830144	74830548
chr11	74830548	74830850
chr11	74830850	74831027
chr11	74831262	74831412
chr11	74831675	74832287
chr11	74832287	74832577
chr11	74832830	74833093
chr11	74837494	74837680
chr11	74837680	74838056
chr11	74838056	74838443
chr11	74838443	74838821
chr11	74839024	74839252
chr11	74840051	74840238
chr11	74847905	74848216
chr11	74848216	74848668
chr11	74848747	74848979
chr11	74859637	74859911
chr11	74860270	74860558
chr11	74862053	74862574
chr11	74863654	74863825
chr11	74864050	74864292

chr11	74870807	74871343
chr11	74871343	74871683
chr11	74876821	74877302
chr11	74881029	74881295
chr11	74896019	74896192
chr11	74896263	74896514
chr11	74896514	74896856
chr11	74896856	74897163
chr11	74897163	74897643
chr11	74897688	74897909
chr11	74897909	74898206
chr11	74910784	74911099
chr11	74911099	74911318
chr11	74911947	74912099
chr11	74912226	74912381
chr11	74912458	74912858
chr11	74914760	74914910
chr11	74925416	74925866
chr11	74925866	74926289
chr11	74926289	74926596
chr11	74926706	74926877
chr11	74946405	74946641
chr11	74953297	74953452
chr11	74954833	74955303
chr11	74955359	74955509
chr11	74965590	74965825
chr11	74973290	74973701
chr11	74981471	74981822
chr11	74990741	74991201
chr11	75008453	75008645
chr11	75027094	75027301
chr11	75027667	75027904
chr11	75045503	75045772
chr11	75045856	75046188
chr11	75052970	75053242
chr11	75053242	75053516
chr11	75053744	75053947
chr11	75054170	75054533
chr11	75056784	75057078
chr11	75059645	75059988
chr11	75072344	75072720
chr11	75097819	75098064

chr11	75102812	75102962
chr11	75128825	75129026
chr11	75137514	75137887
chr11	75140488	75140705
chr11	75142831	75143093
chr11	75147913	75148077
chr11	75153236	75153549
chr11	75155993	75156187
chr11	75159674	75159935
chr11	75165397	75165647
chr11	75166951	75167221
chr11	75168598	75168837
chr11	75169096	75169362
chr11	75170154	75170443
chr11	75170443	75170778
chr11	75172899	75173451
chr11	75173451	75173935
chr11	75178525	75178979
chr11	75178979	75179286
chr11	75184104	75184383
chr11	75184383	75184542
chr11	75189815	75190087
chr11	75190087	75190398
chr11	75190398	75190831
chr11	75192860	75193111
chr11	75193552	75193715
chr11	75194964	75195180
chr11	75195180	75195398
chr11	75201063	75201257
chr11	75213512	75213709
chr11	75213709	75213923
chr11	75213923	75214293
chr11	75220195	75220431
chr11	75220585	75220831
chr11	75220856	75221023
chr11	75221173	75221491
chr11	75225370	75225613
chr11	75231848	75232100
chr11	75234810	75235142
chr11	75241043	75241260
chr11	75245720	75245997
chr11	75246694	75246858

chr11	75246880	75247034
chr11	75254408	75254585
chr11	75254592	75255005
chr11	75262986	75263136
chr11	75264630	75264910
chr11	75267855	75268055
chr11	75279196	75279551
chr11	75280560	75280710
chr11	75290946	75291250
chr11	75294163	75294320
chr11	75334935	75335235
chr11	75348089	75348566
chr11	75348566	75348916
chr11	75356946	75357132
chr11	75377209	75377359
chr11	75381951	75382131
chr11	75383415	75383886
chr11	75387527	75387768
chr11	75389213	75389437
chr11	75395570	75395884
chr11	75399311	75399774
chr11	75402254	75402540
chr11	75402545	75402716
chr11	75403501	75403652
chr11	75403656	75404092
chr11	75406070	75406483
chr11	75416360	75416587
chr11	75426617	75426788
chr11	75437111	75437358
chr11	75438356	75438508
chr11	75439802	75440052
chr11	75445374	75445524
chr11	75454133	75454360
chr11	75454360	75454595
chr11	75454595	75455035
chr11	75458752	75458923
chr11	75460495	75460757
chr11	75461047	75461495
chr11	75461495	75461805
chr11	75462290	75462802
chr11	75462910	75463126
chr11	75463126	75463397

chr11	75470698	75471069
chr11	75472819	75473270
chr11	75486360	75486510
chr11	75486512	75486888
chr11	75486888	75487124
chr11	75487124	75487308
chr11	75494072	75494258
chr11	75495213	75495608
chr11	75508829	75509340
chr11	75509753	75510143
chr11	75510143	75510606
chr11	75510606	75510995
chr11	75519901	75520194
chr11	75520271	75520483
chr11	75530408	75530677
chr11	75530677	75530880
chr11	75530880	75531064
chr11	75531094	75531282
chr11	75531282	75531501
chr11	75531501	75531837
chr11	75531837	75532266
chr11	75537814	75538099
chr11	75538099	75538267
chr11	75541146	75541554
chr11	75551884	75552202
chr11	75552202	75552518
chr11	75562690	75563234
chr11	75563734	75564259
chr11	75564259	75564670
chr11	75570647	75571132
chr11	75571265	75571756
chr11	75573209	75573707
chr11	75576532	75576734
chr11	75587875	75588211
chr11	75588211	75588557
chr11	75588557	75588977
chr11	75592694	75592982
chr11	75599897	75600094
chr11	75604917	75605069
chr11	75605388	75605798
chr11	75606264	75606593
chr11	75606593	75606929

chr11	75606992	75607247
chr11	75619999	75620279
chr11	75620299	75620831
chr11	75620944	75621202
chr11	75621235	75621732
chr11	75630575	75631428
chr11	75631694	75631883
chr11	75638484	75638734
chr11	75640232	75640415
chr11	75651393	75651703
chr11	75651807	75652061
chr11	75652245	75652510
chr11	75653288	75653659
chr11	75653839	75654416
chr11	75654501	75654719
chr11	75655756	75655993
chr11	75655993	75656239
chr11	75671928	75672078
chr11	75677866	75678206
chr11	75678585	75678946
chr11	75678986	75679377
chr11	75679377	75679678
chr11	75679678	75679966
chr11	75679966	75680217
chr11	75687466	75688129
chr11	75688180	75688440
chr11	75703804	75704113
chr11	75732549	75732951
chr11	75732951	75733248
chr11	75733248	75733455
chr11	75733455	75733748
chr11	75736891	75737403
chr11	75742118	75742388
chr11	75746024	75746192
chr11	75747586	75747745
chr11	75753822	75753972
chr11	75757610	75757829
chr11	75762782	75762968
chr11	75768069	75768243
chr11	75768265	75768417
chr11	75768478	75768630
chr11	75768636	75768856

chr11	75769189	75769616
chr11	75776004	75776279
chr11	75781850	75782388
chr11	75789228	75789431
chr11	75795151	75795364
chr11	75795944	75796411
chr11	75796593	75796758
chr11	75796758	75796844
chr11	75806295	75806471
chr11	75811741	75811904
chr11	75811911	75812264
chr11	75812981	75813554
chr11	75821759	75822078
chr11	75822078	75822538
chr11	75846796	75846983
chr11	75851663	75851864
chr11	75851998	75852303
chr11	75852675	75852830
chr11	75853153	75853333
chr11	75857754	75858115
chr11	75858129	75858318
chr11	75858370	75858773
chr11	75861171	75861595
chr11	75867003	75867308
chr11	75869034	75869321
chr11	75878562	75878777
chr11	75880066	75880266
chr11	75882624	75883117
chr11	75896205	75896355
chr11	75896611	75896871
chr11	75903554	75903812
chr11	75913326	75913593
chr11	75914027	75914308
chr11	75926947	75927109
chr11	75929497	75929656
chr11	75935752	75936308
chr11	75937371	75937936
chr11	75960872	75961026
chr11	75963817	75963997
chr11	75966366	75966813
chr11	75966813	75967068
chr11	75967068	75967423

chr11	75973186	75973383
chr11	75980740	75980895
chr11	75983320	75983538
chr11	75999026	75999188
chr11	75999553	75999761
chr11	75999766	76000258
chr11	76000309	76000459
chr11	76008185	76008539
chr11	76009169	76009329
chr11	76011308	76011733
chr11	76017431	76018049
chr11	76019405	76019600
chr11	76020456	76020748
chr11	76026881	76027115
chr11	76027115	76027355
chr11	76027355	76027654
chr11	76027654	76028092
chr11	76039358	76039617
chr11	76039617	76039835
chr11	76049659	76049985
chr11	76056550	76056790
chr11	76059890	76060155
chr11	76060155	76060374
chr11	76066996	76067235
chr11	76074132	76074294
chr11	76074353	76074634
chr11	76080297	76080754
chr11	76086041	76086201
chr11	76099768	76100067
chr11	76100178	76100368
chr11	76100438	76100625
chr11	76121075	76121620
chr11	76130210	76130459
chr11	76130700	76131192
chr11	76135454	76135604
chr11	76135679	76135994
chr11	76135994	76136327
chr11	76137255	76137427
chr11	76137952	76138315
chr11	76154855	76155153
chr11	76158162	76158765
chr11	76160316	76160615

chr11	76160615	76160848
chr11	76167472	76167632
chr11	76178661	76179083
chr11	76179107	76179514
chr11	76179514	76179765
chr11	76179765	76180165
chr11	76187001	76187217
chr11	76201724	76202259
chr11	76202259	76202752
chr11	76216736	76217059
chr11	76217098	76217993
chr11	76236094	76236422
chr11	76236422	76236706
chr11	76242622	76242863
chr11	76242985	76243145
chr11	76243218	76243607
chr11	76243607	76243857
chr11	76243857	76244092
chr11	76247330	76247511
chr11	76253212	76253459
chr11	76253459	76253681
chr11	76261111	76261718
chr11	76261792	76262172
chr11	76262417	76262671
chr11	76269181	76269423
chr11	76283886	76284116
chr11	76285412	76285803
chr11	76285827	76286036
chr11	76286036	76286258
chr11	76286539	76286858
chr11	76286874	76287310
chr11	76296495	76296972
chr11	76314831	76315123
chr11	76326714	76326962
chr11	76328925	76329227
chr11	76330290	76330465
chr11	76337302	76337582
chr11	76337799	76338034
chr11	76351498	76351969
chr11	76374877	76375041
chr11	76387190	76387343
chr11	76398378	76398802

chr11	76398802	76399071
chr11	76399071	76399333
chr11	76399333	76399561
chr11	76399561	76399826
chr11	76400063	76400271
chr11	76406619	76407025
chr11	76407025	76407292
chr11	76407292	76407514
chr11	76415117	76415358
chr11	76421385	76421598
chr11	76422050	76422596
chr11	76425741	76426278
chr11	76436817	76437072
chr11	76437233	76437427
chr11	76445615	76445792
chr11	76446342	76446551
chr11	76454567	76454838
chr11	76456227	76456729
chr11	76462568	76462772
chr11	76462772	76462985
chr11	76464468	76464697
chr11	76469137	76469336
chr11	76469411	76469903
chr11	76469903	76470299
chr11	76476845	76477263
chr11	76477263	76477606
chr11	76479714	76480417
chr11	76493157	76493518
chr11	76493805	76494242
chr11	76494386	76494726
chr11	76494726	76495066
chr11	76495066	76495353
chr11	76495353	76495606
chr11	76495606	76495747
chr11	76496160	76496402
chr11	76497766	76498081
chr11	76506722	76506920
chr11	76506920	76507158
chr11	76510339	76510632
chr11	76511101	76511265
chr11	76524989	76525285
chr11	76529707	76530060

chr11	76530969	76531377
chr11	76531428	76531645
chr11	76537057	76537300
chr11	76537316	76537480
chr11	76541007	76541441
chr11	76543359	76543630
chr11	76562177	76562328
chr11	76570857	76571103
chr11	76571191	76571505
chr11	76571505	76571825
chr11	76572934	76573133
chr11	76577158	76577346
chr11	76577762	76577912
chr11	76577921	76578203
chr11	76587340	76587734
chr11	76588160	76588489
chr11	76589001	76589480
chr11	76589480	76589692
chr11	76589801	76590241
chr11	76598318	76598748
chr11	76598748	76599037
chr11	76599165	76599590
chr11	76600932	76601098
chr11	76604379	76604702
chr11	76605223	76605819
chr11	76607102	76607283
chr11	76615250	76615546
chr11	76615546	76615826
chr11	76615910	76616060
chr11	76622083	76622451
chr11	76622451	76622693
chr11	76622693	76622867
chr11	76622867	76623106
chr11	76623106	76623410
chr11	76623416	76623849
chr11	76630105	76630284
chr11	76637742	76637966
chr11	76642230	76642628
chr11	76646018	76646327
chr11	76647316	76647573
chr11	76647573	76647907
chr11	76652530	76652902

chr11	76652902	76653160
chr11	76655127	76655348
chr11	76659381	76659535
chr11	76659563	76659923
chr11	76662317	76662675
chr11	76663015	76663184
chr11	76664824	76664976
chr11	76669052	76669280
chr11	76670560	76670866
chr11	76671329	76671549
chr11	76671904	76672059
chr11	76672852	76673091
chr11	76677050	76677443
chr11	76681296	76681668
chr11	76692038	76692259
chr11	76695960	76696119
chr11	76699735	76699982
chr11	76709785	76709992
chr11	76710110	76710349
chr11	76716738	76716922
chr11	76717651	76717807
chr11	76734646	76734844
chr11	76739210	76739431
chr11	76739538	76739721
chr11	76763038	76763460
chr11	76763460	76763825
chr11	76789354	76789574
chr11	76823718	76824038
chr11	76838185	76838342
chr11	76845547	76845957
chr11	76846020	76846528
chr11	76846528	76846906
chr11	76846906	76847262
chr11	76855156	76855362
chr11	76857180	76857367
chr11	76857367	76857542
chr11	76894308	76894610
chr11	76900433	76900671
chr11	76911279	76911739
chr11	76912881	76913033
chr11	76914425	76914719
chr11	76916549	76916905

chr11	76917007	76917200
chr11	76917200	76917404
chr11	76924830	76925185
chr11	76925561	76925787
chr11	76925915	76926207
chr11	76942775	76942940
chr11	76945335	76945858
chr11	76945858	76946240
chr11	76946302	76946468
chr11	76953588	76953748
chr11	76982023	76982772
chr11	76990896	76991225
chr11	76992920	76993187
chr11	76997314	76997812
chr11	76997890	76998115
chr11	76998115	76998423
chr11	76998475	76998666
chr11	77005651	77005849
chr11	77005961	77006218
chr11	77006228	77006627
chr11	77011673	77012055
chr11	77012055	77012435
chr11	77018100	77018338
chr11	77026681	77026869
chr11	77041300	77041465
chr11	77077864	77078114
chr11	77078145	77078819
chr11	77093372	77093562
chr11	77100145	77100466
chr11	77100466	77100652
chr11	77101008	77101586
chr11	77117021	77117178
chr11	77132736	77133049
chr11	77145477	77145767
chr11	77160353	77161054
chr11	77180886	77181247
chr11	77184966	77185314
chr11	77196821	77197117
chr11	77200678	77201171
chr11	77201442	77201777
chr11	77214782	77215162
chr11	77215494	77215858

chr11	77215930	77216346
chr11	77216346	77216701
chr11	77216701	77216899
chr11	77216899	77217114
chr11	77225320	77225533
chr11	77225533	77225711
chr11	77229803	77229965
chr11	77230865	77231074
chr11	77234821	77235123
chr11	77241733	77241937
chr11	77256044	77256324
chr11	77261190	77261436
chr11	77277311	77277473
chr11	77277967	77278232
chr11	77278561	77278911
chr11	77278911	77279338
chr11	77299272	77299422
chr11	77308289	77308614
chr11	77308620	77308866
chr11	77311406	77311556
chr11	77317763	77317927
chr11	77347904	77348451
chr11	77348451	77348895
chr11	77355908	77356083
chr11	77381395	77381565
chr11	77382782	77382932
chr11	77383919	77384587
chr11	77384913	77385126
chr11	77385669	77385959
chr11	77386413	77386643
chr11	77397645	77397830
chr11	77431201	77431412
chr11	77440757	77441091
chr11	77441091	77441386
chr11	77445153	77445340
chr11	77446418	77447076
chr11	77459038	77459196
chr11	77462459	77462701
chr11	77464660	77465312
chr11	77465672	77465866
chr11	77466103	77466400
chr11	77466400	77466600

chr11	77466600	77466873
chr11	77467069	77467421
chr11	77467921	77468086
chr11	77477447	77477784
chr11	77482988	77483408
chr11	77483461	77483617
chr11	77483627	77484184
chr11	77488813	77489043
chr11	77489971	77490179
chr11	77491109	77491344
chr11	77491438	77491761
chr11	77491761	77492024
chr11	77492851	77493044
chr11	77493097	77493400
chr11	77494115	77494355
chr11	77495566	77495866
chr11	77497097	77497373
chr11	77497373	77497660
chr11	77500455	77500911
chr11	77500911	77501253
chr11	77501480	77501709
chr11	77506499	77506657
chr11	77507501	77507776
chr11	77507776	77508042
chr11	77508094	77508402
chr11	77512839	77513175
chr11	77513175	77513784
chr11	77513856	77514030
chr11	77514855	77515355
chr11	77515355	77515705
chr11	77525847	77526139
chr11	77526187	77526498
chr11	77577873	77578038
chr11	77606815	77607079
chr11	77607079	77607317
chr11	77607317	77607532
chr11	77607532	77607819
chr11	77607819	77608335
chr11	77613413	77613904
chr11	77624441	77624705
chr11	77624721	77624923
chr11	77625111	77625450

chr11	77629591	77629844
chr11	77630030	77630217
chr11	77630217	77630426
chr11	77630426	77630700
chr11	77646628	77646801
chr11	77648981	77649233
chr11	77649323	77649670
chr11	77649670	77650026
chr11	77650026	77650232
chr11	77650232	77650459
chr11	77672364	77672699
chr11	77672772	77673276
chr11	77673489	77673751
chr11	77685687	77686259
chr11	77686259	77686564
chr11	77686564	77686817
chr11	77686876	77687103
chr11	77687138	77687365
chr11	77687365	77687620
chr11	77704120	77704309
chr11	77724119	77724269
chr11	77737347	77737612
chr11	77739467	77739619
chr11	77744027	77744409
chr11	77758454	77758703
chr11	77763087	77763414
chr11	77763414	77763668
chr11	77763799	77763996
chr11	77763996	77764330
chr11	77764660	77764862
chr11	77765134	77765447
chr11	77765447	77765775
chr11	77766351	77766581
chr11	77766581	77766905
chr11	77771742	77772146
chr11	77772146	77772823
chr11	77783217	77783749
chr11	77783749	77784018
chr11	77784018	77784297
chr11	77784343	77784575
chr11	77785812	77786105
chr11	77786105	77786355

chr11	77786355	77786652
chr11	77791212	77791473
chr11	77791476	77791636
chr11	77791829	77792263
chr11	77792798	77792950
chr11	77793175	77793608
chr11	77801080	77801469
chr11	77801483	77801633
chr11	77807961	77808250
chr11	77808250	77808618
chr11	77810811	77811157
chr11	77815884	77816093
chr11	77816093	77816356
chr11	77821722	77821951
chr11	77822751	77823150
chr11	77825909	77826165
chr11	77827278	77827575
chr11	77835191	77835415
chr11	77836779	77837097
chr11	77840041	77840198
chr11	77849222	77849427
chr11	77850337	77850518
chr11	77850518	77850707
chr11	77854331	77854499
chr11	77857860	77858015
chr11	77859583	77859747
chr11	77864760	77864914
chr11	77872462	77872668
chr11	77872709	77873206
chr11	77873216	77873459
chr11	77874762	77874921
chr11	77884546	77884953
chr11	77893869	77894048
chr11	77894099	77894290
chr11	77927771	77928240
chr11	77930717	77931035
chr11	77931035	77931319
chr11	77941422	77941667
chr11	77943009	77943161
chr11	77943708	77943858
chr11	77948185	77948554
chr11	77951348	77951499

chr11	77953735	77953979
chr11	77959096	77959311
chr11	77961033	77961237
chr11	77964442	77964776
chr11	77967205	77967456
chr11	77971525	77971725
chr11	77974205	77974406
chr11	77975164	77975428
chr11	77975523	77975765
chr11	77981615	77982071
chr11	77982345	77983104
chr11	77983609	77983809
chr11	77984364	77984651
chr11	77989484	77989697
chr11	78019559	78019718
chr11	78019754	78020005
chr11	78021768	78021896
chr11	78021896	78022047
chr11	78023690	78023927
chr11	78031828	78032211
chr11	78032211	78032433
chr11	78032433	78032864
chr11	78037687	78038058
chr11	78038058	78038290
chr11	78038290	78038554
chr11	78038569	78038761
chr11	78039286	78039546
chr11	78044786	78044983
chr11	78060228	78060447
chr11	78060447	78060728
chr11	78079930	78080533
chr11	78080533	78080918
chr11	78094480	78094839
chr11	78094839	78095270
chr11	78102191	78102493
chr11	78115174	78115631
chr11	78115631	78115909
chr11	78121271	78121470
chr11	78135433	78135598
chr11	78146262	78146479
chr11	78146516	78146671
chr11	78146674	78146961

chr11	78146961	78147326
chr11	78151258	78151520
chr11	78154093	78154281
chr11	78154338	78154704
chr11	78159115	78159457
chr11	78159510	78159651
chr11	78159651	78159791
chr11	78163212	78163501
chr11	78164930	78165356
chr11	78165378	78165790
chr11	78165790	78166161
chr11	78166314	78166464
chr11	78172128	78172353
chr11	78176278	78176529
chr11	78176593	78176876
chr11	78176886	78177279
chr11	78177393	78177767
chr11	78177802	78178355
chr11	78178355	78178806
chr11	78178806	78179111
chr11	78179111	78179383
chr11	78183203	78183460
chr11	78183460	78183765
chr11	78188251	78188522
chr11	78188522	78188829
chr11	78188829	78189208
chr11	78189208	78189528
chr11	78189528	78189739
chr11	78189739	78189962
chr11	78193185	78193484
chr11	78194692	78194850
chr11	78194948	78195142
chr11	78200652	78200807
chr11	78203730	78203890
chr11	78204034	78204336
chr11	78210012	78210186
chr11	78210754	78210998
chr11	78222624	78222921
chr11	78236312	78236463
chr11	78237926	78238217
chr11	78245373	78245593
chr11	78245593	78245851

chr11	78245851	78246113
chr11	78246113	78246429
chr11	78246845	78247363
chr11	78247890	78248089
chr11	78254647	78254889
chr11	78260484	78260796
chr11	78260815	78261005
chr11	78261282	78261619
chr11	78261619	78261845
chr11	78261845	78262106
chr11	78262106	78262348
chr11	78262671	78262882
chr11	78271426	78271581
chr11	78276264	78276467
chr11	78301258	78301655
chr11	78301655	78301891
chr11	78301891	78302086
chr11	78322703	78323332
chr11	78328148	78328522
chr11	78328522	78329103
chr11	78343318	78343607
chr11	78343607	78343911
chr11	78344041	78344244
chr11	78347424	78347680
chr11	78348446	78348833
chr11	78355571	78355721
chr11	78356731	78356920
chr11	78360151	78360359
chr11	78360565	78360988
chr11	78363015	78363316
chr11	78363316	78363622
chr11	78363643	78364011
chr11	78364132	78364316
chr11	78370115	78370416
chr11	78370416	78370716
chr11	78370716	78370991
chr11	78372204	78372558
chr11	78377630	78377865
chr11	78377865	78378069
chr11	78381489	78381697
chr11	78382342	78382593
chr11	78382708	78383118

chr11	78386499	78387016
chr11	78392829	78392993
chr11	78395164	78395371
chr11	78396949	78397379
chr11	78397379	78397766
chr11	78397766	78398029
chr11	78398488	78398727
chr11	78398727	78399005
chr11	78399544	78399733
chr11	78399767	78400020
chr11	78411645	78411971
chr11	78411971	78412350
chr11	78415897	78416094
chr11	78418536	78418719
chr11	78421548	78421902
chr11	78421902	78422347
chr11	78434116	78434349
chr11	78435769	78436016
chr11	78436016	78436295
chr11	78437217	78437463
chr11	78447148	78447564
chr11	78448280	78448622
chr11	78453842	78454006
chr11	78454237	78454717
chr11	78455331	78455645
chr11	78462778	78462976
chr11	78463005	78463360
chr11	78463360	78463610
chr11	78463704	78464029
chr11	78465543	78465795
chr11	78465875	78466240
chr11	78472993	78473546
chr11	78477602	78477760
chr11	78480736	78480952
chr11	78486227	78486582
chr11	78488220	78488626
chr11	78490800	78491096
chr11	78491111	78491350
chr11	78491829	78492203
chr11	78497152	78497526
chr11	78497583	78498011
chr11	78498015	78498207

chr11	78509250	78509473
chr11	78511828	78512362
chr11	78512362	78512581
chr11	78512581	78512762
chr11	78522657	78522886
chr11	78522886	78523075
chr11	78523612	78523918
chr11	78524021	78524325
chr11	78528470	78528596
chr11	78528596	78528750
chr11	78535436	78535723
chr11	78535844	78536723
chr11	78544723	78544921
chr11	78550239	78551050
chr11	78552414	78552636
chr11	78557341	78557768
chr11	78560499	78560777
chr11	78611390	78611613
chr11	78648369	78648748
chr11	78657796	78658184
chr11	78658184	78658564
chr11	78658564	78658987
chr11	78658989	78659258
chr11	78659352	78659668
chr11	78659668	78659976
chr11	78697000	78697366
chr11	78697366	78697757
chr11	78697757	78698214
chr11	78703134	78703532
chr11	78706057	78706333
chr11	78730948	78731124
chr11	78747293	78747813
chr11	78751239	78751545
chr11	78751545	78751887
chr11	78800322	78800542
chr11	78802274	78802677
chr11	78826363	78826565
chr11	78826565	78826713
chr11	78826713	78827040
chr11	78828426	78828706
chr11	78829494	78829766
chr11	78833093	78833354

chr11	78838127	78838365
chr11	78846673	78846844
chr11	78856265	78856637
chr11	78856637	78856938
chr11	78860187	78860449
chr11	78864826	78865283
chr11	78865424	78865805
chr11	78866528	78866836
chr11	78872688	78872855
chr11	78875932	78876590
chr11	78882303	78882710
chr11	78886803	78887036
chr11	78887040	78887458
chr11	78889170	78889444
chr11	78896767	78897077
chr11	78915500	78915699
chr11	78916539	78916734
chr11	78920532	78920814
chr11	78923083	78923355
chr11	78924994	78925402
chr11	78929406	78929614
chr11	78943085	78943352
chr11	78952554	78952850
chr11	78956358	78956595
chr11	78956622	78956794
chr11	78959957	78960113
chr11	78967450	78967719
chr11	78968170	78968329
chr11	78982059	78982363
chr11	78984465	78984822
chr11	78984822	78985180
chr11	78985180	78985386
chr11	79002422	79002718
chr11	79002718	79002986
chr11	79019026	79019304
chr11	79019340	79019792
chr11	79019925	79020169
chr11	79030633	79030944
chr11	79030944	79031456
chr11	79039665	79039820
chr11	79039986	79040131
chr11	79040131	79040293

chr11	79059751	79060312
chr11	79064289	79064675
chr11	79064822	79065030
chr11	79071249	79071550
chr11	79071550	79072095
chr11	79083229	79083587
chr11	79085031	79085622
chr11	79087684	79088032
chr11	79093921	79094293
chr11	79094293	79094614
chr11	79096312	79096547
chr11	79097582	79097773
chr11	79103170	79103431
chr11	79103431	79103733
chr11	79104942	79105373
chr11	79105715	79105948
chr11	79111872	79112251
chr11	79112251	79112547
chr11	79112547	79112892
chr11	79112960	79113178
chr11	79130071	79130247
chr11	79131678	79132559
chr11	79140813	79140999
chr11	79141387	79141542
chr11	79145659	79145877
chr11	79145900	79146256
chr11	79146256	79146755
chr11	79151898	79152271
chr11	79152753	79152920
chr11	79166462	79166612
chr11	79176791	79176972
chr11	79180221	79180519
chr11	79181864	79182260
chr11	79182260	79182585
chr11	79188084	79188498
chr11	79188498	79188752
chr11	79196123	79196508
chr11	79196524	79197023
chr11	79197191	79197462
chr11	79197470	79197791
chr11	79213799	79214527
chr11	79224490	79225161

chr11	79231507	79231776
chr11	79232152	79232312
chr11	79237578	79237749
chr11	79237749	79237930
chr11	79238070	79238273
chr11	79245501	79245777
chr11	79245777	79246114
chr11	79251644	79251795
chr11	79253170	79253485
chr11	79253485	79253764
chr11	79253984	79254255
chr11	79254322	79254917
chr11	79263547	79263883
chr11	79264380	79264736
chr11	79264770	79265023
chr11	79265102	79265253
chr11	79271549	79271725
chr11	79273109	79273321
chr11	79276208	79276488
chr11	79278743	79278979
chr11	79287136	79287389
chr11	79287389	79287843
chr11	79290553	79290813
chr11	79296243	79296433
chr11	79303957	79304457
chr11	79306604	79306877
chr11	79306998	79307265
chr11	79308240	79308670
chr11	79315719	79316063
chr11	79323301	79323472
chr11	79339192	79339777
chr11	79339777	79340083
chr11	79340083	79340387
chr11	79357491	79357713
chr11	79366463	79366614
chr11	79398420	79398695
chr11	79409433	79409768
chr11	79413241	79413623
chr11	79413658	79413844
chr11	79413844	79414066
chr11	79414247	79414397
chr11	79427728	79427986

chr11	79485392	79485552
chr11	79489517	79489937
chr11	79530480	79530675
chr11	79534085	79534343
chr11	79534343	79534686
chr11	79577781	79578081
chr11	79583968	79584355
chr11	79584355	79584544
chr11	79585173	79585379
chr11	79585379	79585586
chr11	79590701	79590858
chr11	79590912	79591090
chr11	79591116	79591506
chr11	79593581	79593827
chr11	79611022	79611745
chr11	79623139	79623341
chr11	79624714	79624946
chr11	79624946	79625330
chr11	79628795	79629021
chr11	79633174	79633747
chr11	79635051	79635222
chr11	79640062	79640247
chr11	79642929	79643095
chr11	79653319	79653795
chr11	79654003	79654168
chr11	79654392	79654889
chr11	79655720	79655874
chr11	79660291	79661116
chr11	79666494	79666874
chr11	79669157	79669307
chr11	79671812	79672024
chr11	79680649	79680801
chr11	79686632	79686836
chr11	79686836	79687252
chr11	79689999	79690244
chr11	79698106	79698540
chr11	79703484	79703848
chr11	79703920	79704265
chr11	79704265	79704706
chr11	79704706	79705112
chr11	79710797	79711133
chr11	79711133	79711443

chr11	79711443	79711746
chr11	79711769	79712001
chr11	79712834	79712988
chr11	79729449	79729983
chr11	79736751	79736968
chr11	79738079	79738420
chr11	79738789	79739005
chr11	79739041	79739771
chr11	79746632	79746955
chr11	79750028	79750365
chr11	79752287	79752685
chr11	79752744	79753067
chr11	79753067	79753394
chr11	79753394	79753623
chr11	79761309	79761473
chr11	79761880	79762467
chr11	79771303	79771860
chr11	79773877	79774063
chr11	79778512	79778919
chr11	79779700	79780054
chr11	79780189	79780499
chr11	79781211	79781566
chr11	79784969	79785129
chr11	79786217	79786603
chr11	79790188	79790524
chr11	79793426	79793578
chr11	79793900	79794213
chr11	79794213	79794548
chr11	79804034	79804328
chr11	79804389	79804539
chr11	79815335	79815485
chr11	79825966	79826177
chr11	79843097	79843277
chr11	79854876	79855159
chr11	79866605	79866755
chr11	79871820	79872140
chr11	79872328	79872562
chr11	79883105	79883285
chr11	79884919	79885135
chr11	79889609	79889863
chr11	79901149	79901572
chr11	79901671	79901956

chr11	79908645	79908884
chr11	79913150	79913324
chr11	79916040	79916359
chr11	79916435	79916638
chr11	79919820	79920230
chr11	79920255	79920425
chr11	79943566	79944054
chr11	79961450	79961605
chr11	79961854	79962063
chr11	79962063	79962275
chr11	79962275	79962580
chr11	79962580	79962872
chr11	79962872	79963024
chr11	79973685	79973862
chr11	79974172	79974483
chr11	79979786	79980043
chr11	79982192	79982366
chr11	79992471	79992814
chr11	79992814	79993902
chr11	80009158	80009356
chr11	80019928	80020101
chr11	80041667	80041817
chr11	80042518	80042929
chr11	80042932	80043182
chr11	80050015	80050428
chr11	80078857	80079055
chr11	80080467	80080781
chr11	80080781	80081231
chr11	80081231	80081662
chr11	80085364	80085896
chr11	80086930	80087180
chr11	80088161	80088353
chr11	80089309	80089517
chr11	80089517	80089885
chr11	80091580	80091745
chr11	80136850	80137029
chr11	80141631	80142026
chr11	80142026	80142296
chr11	80142296	80142574
chr11	80142951	80143204
chr11	80144225	80144466
chr11	80145660	80145892

chr11	80146039	80146328
chr11	80152082	80152613
chr11	80153174	80153429
chr11	80153832	80154161
chr11	80154161	80154549
chr11	80171903	80172270
chr11	80175632	80175784
chr11	80176095	80176353
chr11	80183333	80183682
chr11	80183682	80184324
chr11	80196927	80197131
chr11	80199876	80200047
chr11	80200439	80200654
chr11	80208653	80209029
chr11	80209029	80209362
chr11	80209362	80209808
chr11	80224790	80224973
chr11	80234182	80234455
chr11	80234455	80234713
chr11	80264237	80264411
chr11	80284673	80284823
chr11	80287189	80287491
chr11	80290046	80290403
chr11	80300612	80300818
chr11	80300818	80301043
chr11	80301043	80301324
chr11	80301473	80301739
chr11	80301739	80302073
chr11	80302073	80302385
chr11	80302641	80302931
chr11	80311074	80311300
chr11	80319037	80319187
chr11	80333846	80334332
chr11	80336610	80336766
chr11	80351335	80351485
chr11	80351853	80352159
chr11	80353887	80354139
chr11	80354931	80355206
chr11	80376614	80376936
chr11	80377608	80377938
chr11	80377938	80378174
chr11	80378174	80378394

chr11	80382837	80383031
chr11	80383064	80383374
chr11	80383374	80383621
chr11	80383621	80383975
chr11	80384637	80384795
chr11	80405275	80405462
chr11	80407555	80407737
chr11	80408195	80408413
chr11	80417102	80417594
chr11	80420197	80420359
chr11	80428238	80428722
chr11	80428722	80429117
chr11	80429117	80429511
chr11	80430439	80430815
chr11	80437519	80437674
chr11	80453293	80453472
chr11	80457389	80457916
chr11	80460736	80460902
chr11	80476436	80476880
chr11	80476880	80477267
chr11	80477267	80477597
chr11	80484297	80484810
chr11	80489487	80489655
chr11	80489655	80489917
chr11	80490881	80491172
chr11	80496034	80496391
chr11	80496689	80497052
chr11	80499562	80500088
chr11	80504870	80505441
chr11	80521185	80521380
chr11	80529228	80529500
chr11	80529500	80529937
chr11	80532791	80533352
chr11	80539042	80539473
chr11	80554046	80554296
chr11	80554329	80554711
chr11	80562553	80562774
chr11	80595621	80595810
chr11	80598576	80598745
chr11	80599176	80599390
chr11	80602071	80602227
chr11	80602709	80602927

chr11	80613643	80613825
chr11	80629104	80629911
chr11	80634013	80634229
chr11	80655224	80655511
chr11	80656290	80656499
chr11	80658882	80659116
chr11	80659126	80659285
chr11	80664404	80664663
chr11	80670473	80670771
chr11	80672713	80673093
chr11	80696208	80696358
chr11	80696424	80696707
chr11	80700623	80700973
chr11	80701637	80702279
chr11	80711355	80711725
chr11	80712494	80712673
chr11	80712693	80713028
chr11	80713696	80714110
chr11	80714721	80714942
chr11	80716674	80716851
chr11	80719015	80719458
chr11	80719543	80720049
chr11	80721692	80721880
chr11	80723502	80723669
chr11	80733474	80733709
chr11	80734624	80734817
chr11	80742684	80742880
chr11	80742978	80743172
chr11	80743316	80743602
chr11	80752708	80753005
chr11	80753731	80754067
chr11	80759101	80759383
chr11	80759419	80759790
chr11	80759790	80760077
chr11	80760091	80760258
chr11	80760258	80760456
chr11	80766316	80766640
chr11	80766680	80766900
chr11	80767196	80767550
chr11	80772581	80772891
chr11	80774648	80774886
chr11	80775086	80775353

chr11	80775353	80775655
chr11	80777816	80778013
chr11	80778269	80778526
chr11	80779329	80779563
chr11	80779563	80779848
chr11	80779848	80780327
chr11	80784191	80784574
chr11	80785749	80786104
chr11	80800045	80800321
chr11	80808523	80808857
chr11	80809876	80810230
chr11	80810230	80810672
chr11	80818146	80818458
chr11	80818458	80818705
chr11	80819877	80820178
chr11	80820178	80820673
chr11	80835479	80835794
chr11	80835946	80836178
chr11	80851996	80852146
chr11	80852971	80853253
chr11	80857741	80857900
chr11	80872541	80872838
chr11	80873146	80873453
chr11	80877451	80877626
chr11	80906533	80906775
chr11	80918383	80918692
chr11	80949037	80949379
chr11	80949379	80949725
chr11	80967617	80967933
chr11	80976077	80976711
chr11	81151917	81152067
chr11	81152209	81152419
chr11	81263241	81263592
chr11	81374917	81375149
chr11	81391808	81391984
chr11	81402287	81402623
chr11	81411199	81411636
chr11	81411700	81411984
chr11	81412406	81412743
chr11	81414653	81414885
chr11	81419101	81419297
chr11	81419297	81419507

chr11	81426625	81426813
chr11	81446032	81446351
chr11	81446351	81446845
chr11	81534765	81535037
chr11	81588705	81588948
chr11	81613320	81613509
chr11	81714426	81714631
chr11	81908821	81909176
chr11	81930960	81931112
chr11	81931233	81931495
chr11	81942352	81942510
chr11	82015198	82015409
chr11	82035474	82035646
chr11	82168418	82168601
chr11	82174861	82175278
chr11	82177850	82178091
chr11	82235672	82235892
chr11	82612068	82612340
chr11	82613587	82613826
chr11	82739121	82739332
chr11	82746211	82746430
chr11	82754159	82754469
chr11	82763740	82764085
chr11	82764085	82764439
chr11	82764439	82764644
chr11	82764644	82764860
chr11	82780936	82781234
chr11	82781234	82781481
chr11	82781481	82781873
chr11	82794326	82794499
chr11	82802417	82802640
chr11	82806603	82807170
chr11	82819419	82819733
chr11	82830767	82831039
chr11	82831405	82831562
chr11	82840462	82840799
chr11	82845625	82846044
chr11	82846044	82846270
chr11	82860845	82861128
chr11	82861549	82861864
chr11	82870088	82870301
chr11	82870420	82870774

chr11	82870774	82871097
chr11	82871097	82871281
chr11	82871281	82871484
chr11	82884934	82885115
chr11	82886642	82886795
chr11	82889601	82890060
chr11	82890060	82890376
chr11	82890376	82890839
chr11	82907525	82907707
chr11	82907715	82907910
chr11	82907910	82908193
chr11	82908193	82908592
chr11	82911079	82911278
chr11	82914843	82914993
chr11	82915007	82915329
chr11	82928913	82929252
chr11	82929367	82929634
chr11	82930712	82930886
chr11	82931036	82931343
chr11	82931894	82932191
chr11	82932191	82932495
chr11	82932957	82933132
chr11	82959726	82959914
chr11	83019898	83020169
chr11	83027449	83027666
chr11	83039233	83039490
chr11	83045734	83045916
chr11	83057940	83058150
chr11	83063125	83063411
chr11	83063853	83064268
chr11	83064365	83064527
chr11	83078190	83078417
chr11	83079409	83079807
chr11	83079807	83080099
chr11	83107137	83107474
chr11	83107474	83107709
chr11	83192160	83192365
chr11	83193197	83193383
chr11	83220985	83221313
chr11	83221464	83221712
chr11	83231050	83231201
chr11	83239799	83240080

chr11	83263282	83263462
chr11	83270954	83271105
chr11	83271144	83271339
chr11	83271416	83271787
chr11	83271787	83272053
chr11	83272053	83272441
chr11	83272441	83272801
chr11	83272801	83273008
chr11	83291435	83291875
chr11	83292002	83292152
chr11	83298588	83298869
chr11	83298869	83299195
chr11	83301594	83301753
chr11	83302161	83302313
chr11	83302491	83302911
chr11	83302911	83303227
chr11	83303762	83304152
chr11	83326831	83326981
chr11	83327560	83327740
chr11	83338924	83339116
chr11	83341972	83342376
chr11	83342451	83342684
chr11	83357518	83357727
chr11	83403254	83403477
chr11	83403941	83404161
chr11	83405751	83406039
chr11	83409102	83409498
chr11	83417149	83417411
chr11	83418666	83418986
chr11	83421384	83421607
chr11	83424758	83425020
chr11	83425650	83425847
chr11	83426028	83426184
chr11	83427088	83427308
chr11	83440122	83440386
chr11	83440386	83440631
chr11	83440631	83440869
chr11	83440869	83441126
chr11	83442938	83443224
chr11	83454633	83454797
chr11	83455530	83456073
chr11	83457945	83458254

chr11	83458254	83458496
chr11	83462436	83462650
chr11	83462697	83463218
chr11	83463236	83463400
chr11	83463430	83463694
chr11	83467267	83467646
chr11	83468814	83469080
chr11	83472306	83472550
chr11	83472550	83472836
chr11	83472836	83473179
chr11	83473179	83473508
chr11	83473508	83473791
chr11	83491612	83491776
chr11	83509614	83509880
chr11	83510991	83511327
chr11	83516910	83517123
chr11	83517168	83517503
chr11	83517618	83517778
chr11	83522156	83522367
chr11	83527145	83527426
chr11	83527961	83528381
chr11	83534105	83534413
chr11	83534461	83534993
chr11	83534993	83535347
chr11	83538133	83538338
chr11	83544301	83544474
chr11	83550070	83550661
chr11	83555530	83555820
chr11	83555874	83556196
chr11	83556214	83556409
chr11	83556409	83556885
chr11	83556896	83557263
chr11	83563729	83564059
chr11	83564059	83564306
chr11	83564306	83564521
chr11	83568828	83569048
chr11	83577170	83577469
chr11	83578117	83578339
chr11	83580861	83581248
chr11	83581930	83582168
chr11	83582306	83582505
chr11	83583147	83583641

chr11	83597619	83597862
chr11	83597862	83598219
chr11	83598219	83598782
chr11	83606217	83606718
chr11	83606718	83607199
chr11	83607233	83607443
chr11	83616469	83617073
chr11	83620895	83621053
chr11	83623389	83623634
chr11	83623634	83623991
chr11	83623993	83624338
chr11	83628864	83629147
chr11	83630686	83630983
chr11	83632114	83632370
chr11	83632849	83633098
chr11	83636786	83637180
chr11	83639036	83639245
chr11	83639618	83639808
chr11	83660167	83660362
chr11	83665684	83666197
chr11	83674268	83674418
chr11	83712737	83713054
chr11	83728804	83729075
chr11	83729099	83729596
chr11	83730557	83730744
chr11	83731043	83731527
chr11	83739110	83739273
chr11	83739295	83739567
chr11	83753401	83753822
chr11	83753822	83754177
chr11	83754177	83754483
chr11	83785439	83785692
chr11	83786910	83787128
chr11	83787299	83787685
chr11	83789112	83789790
chr11	83802215	83802416
chr11	83805407	83805634
chr11	83815767	83815948
chr11	83817011	83817256
chr11	83818032	83818285
chr11	83818483	83818637
chr11	83819839	83820267

chr11	83820267	83820608
chr11	83822355	83822610
chr11	83832441	83832727
chr11	83832727	83832921
chr11	83833100	83833364
chr11	83834237	83834490
chr11	83843517	83844146
chr11	83845802	83846018
chr11	83849565	83849827
chr11	83849870	83850289
chr11	83850333	83850484
chr11	83850712	83850926
chr11	83850984	83851470
chr11	83851470	83851711
chr11	83851948	83852346
chr11	83852626	83852821
chr11	83852882	83853056
chr11	83853056	83853261
chr11	83853276	83853504
chr11	83853504	83853720
chr11	83859648	83859870
chr11	83869236	83869562
chr11	83877552	83877798
chr11	83877798	83878178
chr11	83879307	83879498
chr11	83882526	83883057
chr11	83883872	83884280
chr11	83891169	83891924
chr11	83893440	83893635
chr11	83895385	83895654
chr11	83896289	83896511
chr11	83896511	83896800
chr11	83896800	83897143
chr11	83902928	83903741
chr11	83920196	83920591
chr11	83922635	83922886
chr11	83926499	83926714
chr11	83926719	83927015
chr11	83927170	83927433
chr11	83935786	83936271
chr11	83936271	83936673
chr11	83941916	83942458

chr11	83956946	83957099
chr11	83963931	83964263
chr11	83964263	83964540
chr11	83964540	83964818
chr11	83990765	83990933
chr11	83994544	83994712
chr11	83995822	83996038
chr11	84013502	84013771
chr11	84016090	84016519
chr11	84032041	84032367
chr11	84032609	84033007
chr11	84033495	84034221
chr11	84042815	84043291
chr11	84043291	84043814
chr11	84055628	84055944
chr11	84055944	84056405
chr11	84061631	84061843
chr11	84061984	84062266
chr11	84062805	84063107
chr11	84064615	84064773
chr11	84067039	84067363
chr11	84067833	84068014
chr11	84068014	84068232
chr11	84068232	84068502
chr11	84068502	84068755
chr11	84068755	84068972
chr11	84069033	84069385
chr11	84074226	84074468
chr11	84074468	84074692
chr11	84078466	84078696
chr11	84079378	84079771
chr11	84079793	84080292
chr11	84080292	84080682
chr11	84087904	84088373
chr11	84088373	84088647
chr11	84101047	84101613
chr11	84101613	84101922
chr11	84107210	84107394
chr11	84116360	84116510
chr11	84128924	84129076
chr11	84129103	84129479
chr11	84129479	84129757

chr11	84129757	84129997
chr11	84134435	84134625
chr11	84141838	84142068
chr11	84150975	84151185
chr11	84165755	84165948
chr11	84166078	84166275
chr11	84168908	84169207
chr11	84179170	84179925
chr11	84179925	84180478
chr11	84243662	84243889
chr11	84395152	84395354
chr11	84411295	84411605
chr11	84411623	84411850
chr11	84414389	84414608
chr11	84416396	84416753
chr11	84428720	84428922
chr11	84429617	84429921
chr11	84430508	84430978
chr11	84435021	84435171
chr11	84436745	84436976
chr11	84442261	84442678
chr11	84442678	84442958
chr11	84442958	84443323
chr11	84512775	84512962
chr11	84513054	84513428
chr11	84513428	84513747
chr11	84513747	84514053
chr11	84520764	84521170
chr11	84522608	84523025
chr11	84524969	84525123
chr11	84525374	84525580
chr11	84529268	84529560
chr11	84529764	84530097
chr11	84530362	84530677
chr11	84539753	84539989
chr11	84560794	84561008
chr11	84561170	84561659
chr11	84561691	84562098
chr11	84568313	84568556
chr11	84618644	84618809
chr11	84677829	84678034
chr11	84706030	84706441

chr11	84706503	84706712
chr11	84722098	84722274
chr11	84724201	84724435
chr11	84729263	84729469
chr11	84750045	84750246
chr11	84753861	84754082
chr11	84789173	84789424
chr11	84794541	84794859
chr11	84797805	84797989
chr11	84797989	84798276
chr11	84813791	84814049
chr11	84814197	84814413
chr11	84818080	84818374
chr11	84818675	84818950
chr11	84818950	84819296
chr11	84819296	84819745
chr11	84828591	84829332
chr11	84829332	84829664
chr11	84845248	84845518
chr11	84846752	84847086
chr11	84847729	84848012
chr11	84869664	84870300
chr11	84870300	84870598
chr11	84870598	84871043
chr11	84871045	84871346
chr11	84879721	84880096
chr11	84880096	84880510
chr11	84880510	84880972
chr11	84880985	84881267
chr11	84895293	84895456
chr11	84895652	84895814
chr11	84900914	84901114
chr11	84901629	84901823
chr11	84909454	84909615
chr11	84916044	84916671
chr11	84916984	84917276
chr11	84918511	84918772
chr11	84919415	84919578
chr11	84929693	84929803
chr11	84929803	84929855
chr11	84935149	84935325
chr11	84936139	84936418

chr11	84943985	84944399
chr11	84955302	84955509
chr11	84957687	84957873
chr11	84957991	84958172
chr11	84978162	84978441
chr11	85126432	85126975
chr11	85139580	85139966
chr11	85139966	85140610
chr11	85141282	85141481
chr11	85142937	85143122
chr11	85152292	85152533
chr11	85157116	85157391
chr11	85157391	85157816
chr11	85169913	85170129
chr11	85170129	85170568
chr11	85170936	85171126
chr11	85177833	85178355
chr11	85181436	85181640
chr11	85183022	85183337
chr11	85184208	85184451
chr11	85213340	85213604
chr11	85214662	85215138
chr11	85223387	85223593
chr11	85234364	85234733
chr11	85234733	85234987
chr11	85234987	85235282
chr11	85235282	85235542
chr11	85235542	85235733
chr11	85244778	85244963
chr11	85248197	85248498
chr11	85250470	85250960
chr11	85261284	85261487
chr11	85264641	85265038
chr11	85265863	85266050
chr11	85266182	85266782
chr11	85273826	85274172
chr11	85274187	85274542
chr11	85277617	85278094
chr11	85308879	85309077
chr11	85310643	85310924
chr11	85310929	85311215
chr11	85311215	85311535

chr11	85311535	85311879
chr11	85311914	85312193
chr11	85312261	85312494
chr11	85323631	85323909
chr11	85323909	85324253
chr11	85324253	85324559
chr11	85334536	85334864
chr11	85334864	85335169
chr11	85352537	85352905
chr11	85352905	85353470
chr11	85359642	85359972
chr11	85367694	85368211
chr11	85374807	85375121
chr11	85379037	85379424
chr11	85382120	85382385
chr11	85384376	85384531
chr11	85404954	85405206
chr11	85482817	85483100
chr11	85483190	85483412
chr11	85504960	85505245
chr11	85518162	85518312
chr11	85603745	85604050
chr11	85609311	85609610
chr11	85609610	85609834
chr11	85615487	85615654
chr11	85615776	85616006
chr11	85626149	85626351
chr11	85626945	85627095
chr11	85634979	85635132
chr11	85672883	85673036
chr11	85673957	85674503
chr11	85686157	85686631
chr11	85686765	85687003
chr11	85733708	85733885
chr11	85740804	85740980
chr11	85775421	85775840
chr11	85786201	85786450
chr11	85786450	85786774
chr11	85789831	85790043
chr11	85792502	85792897
chr11	85792897	85793256
chr11	85798516	85798747

chr11	85801461	85801718
chr11	85804093	85804252
chr11	85811877	85812255
chr11	85812255	85812578
chr11	85812578	85813011
chr11	85818550	85818747
chr11	85823260	85823533
chr11	85831136	85831362
chr11	85832066	85832493
chr11	85832493	85832778
chr11	85832778	85833028
chr11	85833132	85833572
chr11	85841907	85842343
chr11	85843302	85843641
chr11	85844151	85844458
chr11	85844490	85844640
chr11	85844681	85844946
chr11	85849123	85849486
chr11	85850914	85851254
chr11	85864671	85864874
chr11	85865012	85865267
chr11	85865267	85865495
chr11	85894697	85894934
chr11	85894934	85895157
chr11	85925300	85925772
chr11	85929691	85930133
chr11	85942304	85942488
chr11	85953052	85953282
chr11	85963063	85963243
chr11	85970471	85970712
chr11	85970799	85970982
chr11	85972444	85972716
chr11	85986201	85986880
chr11	85990038	85990592
chr11	86032848	86033346
chr11	86050391	86050791
chr11	86106744	86106913
chr11	86174319	86174488
chr11	86195162	86195389
chr11	86200625	86200985
chr11	86200985	86201468
chr11	86201477	86201805

chr11	86246594	86247030
chr11	86247344	86247543
chr11	86256763	86257244
chr11	86257244	86257458
chr11	86257458	86257733
chr11	86257733	86258102
chr11	86265916	86266455
chr11	86266484	86266853
chr11	86315079	86315341
chr11	86317077	86317389
chr11	86317389	86317687
chr11	86317687	86317925
chr11	86323947	86324136
chr11	86324224	86324885
chr11	86326003	86326163
chr11	86339046	86339343
chr11	86346489	86346981
chr11	86347133	86347345
chr11	86347472	86347689
chr11	86347777	86347927
chr11	86351774	86352061
chr11	86356335	86356514
chr11	86357193	86357504
chr11	86357504	86357787
chr11	86357953	86358217
chr11	86358217	86358590
chr11	86380421	86380817
chr11	86380817	86381130
chr11	86381241	86381553
chr11	86381553	86381982
chr11	86395842	86396121
chr11	86397139	86397323
chr11	86407341	86407760
chr11	86426873	86427031
chr11	86436166	86436481
chr11	86436870	86437185
chr11	86454099	86454254
chr11	86464352	86464592
chr11	86484095	86484896
chr11	86484986	86485220
chr11	86490836	86491087
chr11	86491379	86491604

chr11	86497990	86498238
chr11	86506355	86506664
chr11	86506664	86507019
chr11	86507091	86507267
chr11	86508080	86508313
chr11	86527565	86527908
chr11	86532927	86533152
chr11	86533827	86533984
chr11	86544003	86544584
chr11	86544584	86545062
chr11	86545099	86545308
chr11	86564455	86564621
chr11	86568442	86568757
chr11	86568821	86569043
chr11	86570162	86570700
chr11	86571365	86571525
chr11	86577224	86577624
chr11	86578183	86578450
chr11	86578789	86579047
chr11	86579047	86579448
chr11	86580181	86580483
chr11	86580483	86580794
chr11	86580794	86581027
chr11	86581161	86581665
chr11	86582161	86582347
chr11	86582347	86582517
chr11	86586752	86587011
chr11	86587011	86587233
chr11	86587233	86587681
chr11	86588028	86588244
chr11	86590148	86590365
chr11	86590668	86590861
chr11	86591313	86591466
chr11	86592719	86592923
chr11	86592985	86593569
chr11	86598149	86598546
chr11	86598565	86598944
chr11	86602222	86602401
chr11	86604798	86605119
chr11	86607833	86608141
chr11	86608141	86608403
chr11	86610294	86610616

chr11	86614145	86614421
chr11	86623723	86623956
chr11	86623968	86624287
chr11	86624287	86624601
chr11	86624601	86624901
chr11	86625388	86625601
chr11	86641228	86641455
chr11	86646228	86646378
chr11	86646423	86646897
chr11	86648170	86648448
chr11	86650798	86651065
chr11	86652136	86652306
chr11	86652332	86652538
chr11	86652538	86653052
chr11	86653509	86653742
chr11	86655810	86655960
chr11	86683411	86683741
chr11	86683741	86683937
chr11	86683937	86684354
chr11	86687407	86687790
chr11	86691443	86691828
chr11	86704000	86704290
chr11	86724112	86724331
chr11	86731560	86731787
chr11	86732764	86732965
chr11	86737669	86737890
chr11	86745287	86745817
chr11	86747275	86747524
chr11	86749429	86749851
chr11	86752508	86752740
chr11	86756954	86757151
chr11	86757225	86757715
chr11	86757715	86758017
chr11	86758017	86758326
chr11	86760438	86760729
chr11	86781289	86781510
chr11	86806945	86807378
chr11	86807378	86808012
chr11	86811886	86812196
chr11	86813276	86813994
chr11	86814414	86814574
chr11	86816024	86816352

chr11	86827500	86827813
chr11	86828240	86828742
chr11	86836439	86836745
chr11	86837086	86837255
chr11	86839737	86840115
chr11	86840115	86840444
chr11	86840553	86840867
chr11	86851792	86851979
chr11	86852711	86853087
chr11	86853159	86853401
chr11	86856365	86856822
chr11	86859501	86859663
chr11	86860340	86860491
chr11	86872622	86872815
chr11	86874994	86875192
chr11	86879487	86879666
chr11	86894053	86894637
chr11	86906832	86907087
chr11	86907087	86907339
chr11	86907339	86907679
chr11	86907936	86908112
chr11	86908275	86908618
chr11	86911693	86911987
chr11	86913110	86913411
chr11	86914402	86914638
chr11	86919533	86919835
chr11	86920053	86920749
chr11	86932285	86932442
chr11	86932690	86933120
chr11	86944556	86944886
chr11	86945641	86946039
chr11	86946039	86946298
chr11	86952649	86952974
chr11	86952974	86953218
chr11	86953218	86953496
chr11	86961873	86962383
chr11	86962399	86962720
chr11	86978758	86978965
chr11	86993037	86993481
chr11	86993481	86994094
chr11	86995691	86995877
chr11	87007401	87007707

chr11	87010491	87010852
chr11	87025172	87025422
chr11	87025482	87025671
chr11	87026262	87026506
chr11	87026561	87027010
chr11	87030318	87030468
chr11	87031806	87032153
chr11	87032442	87032682
chr11	87036299	87036468
chr11	87051457	87051683
chr11	87073230	87073639
chr11	87073639	87073937
chr11	87073937	87074226
chr11	87074226	87074462
chr11	87085604	87085763
chr11	87086095	87086249
chr11	87086258	87086594
chr11	87086594	87087120
chr11	87108562	87108878
chr11	87108878	87109053
chr11	87109053	87109383
chr11	87109404	87109615
chr11	87126698	87127018
chr11	87127018	87127356
chr11	87127356	87127738
chr11	87214266	87214557
chr11	87237904	87238193
chr11	87241451	87241613
chr11	87254249	87254455
chr11	87261794	87261999
chr11	87262785	87263004
chr11	87274959	87275260
chr11	87279412	87279570
chr11	87294582	87294830
chr11	87295632	87295991
chr11	87309402	87310118
chr11	87310372	87310785
chr11	87346738	87346978
chr11	87358092	87358376
chr11	87358376	87358669
chr11	87358669	87358930
chr11	87358930	87359487

chr11	87393963	87394131
chr11	87398498	87398685
chr11	87404008	87404409
chr11	87404409	87404864
chr11	87413510	87413743
chr11	87422543	87422704
chr11	87422807	87423908
chr11	87426636	87426912
chr11	87426912	87427227
chr11	87427227	87427907
chr11	87442809	87443110
chr11	87443149	87443928
chr11	87448219	87448500
chr11	87448544	87448913
chr11	87448913	87449231
chr11	87449231	87449668
chr11	87454073	87454233
chr11	87455555	87455901
chr11	87461870	87462159
chr11	87462198	87462631
chr11	87462631	87463163
chr11	87470957	87471242
chr11	87471280	87471649
chr11	87471649	87471946
chr11	87471946	87472439
chr11	87483162	87483555
chr11	87483887	87484093
chr11	87485933	87486093
chr11	87488983	87489175
chr11	87506474	87506700
chr11	87510302	87510725
chr11	87510949	87511425
chr11	87518008	87518219
chr11	87519152	87519641
chr11	87548597	87548877
chr11	87555160	87555435
chr11	87574741	87575320
chr11	87578277	87578695
chr11	87581991	87582259
chr11	87591675	87592632
chr11	87593348	87593586
chr11	87595438	87595768

chr11	87599416	87599985
chr11	87609968	87610253
chr11	87610253	87610778
chr11	87620629	87620781
chr11	87621612	87621898
chr11	87621927	87622131
chr11	87622131	87622346
chr11	87623190	87623510
chr11	87629936	87630161
chr11	87638900	87639189
chr11	87639189	87639655
chr11	87651774	87651936
chr11	87662372	87662965
chr11	87662983	87663182
chr11	87664528	87664742
chr11	87678444	87678875
chr11	87678896	87679231
chr11	87682110	87682394
chr11	87689210	87689474
chr11	87689474	87689987
chr11	87695730	87696028
chr11	87696745	87696920
chr11	87696934	87697621
chr11	87702521	87702689
chr11	87707603	87707781
chr11	87709603	87709774
chr11	87709810	87710537
chr11	87722936	87723253
chr11	87736527	87736727
chr11	87736727	87737127
chr11	87737142	87737409
chr11	87737409	87737845
chr11	87748776	87748994
chr11	87749542	87749697
chr11	87759672	87759854
chr11	87763499	87763919
chr11	87763919	87764154
chr11	87764154	87764496
chr11	87764496	87764869
chr11	87770936	87771350
chr11	87772455	87772829
chr11	87778290	87779165

chr11	87782805	87782992
chr11	87783590	87783872
chr11	87785363	87785533
chr11	87802545	87802941
chr11	87805538	87805749
chr11	87817019	87817258
chr11	87817258	87817530
chr11	87840771	87841023
chr11	87847956	87848243
chr11	87852055	87852255
chr11	87852798	87852956
chr11	87853026	87853348
chr11	87854541	87854916
chr11	87854916	87855245
chr11	87939641	87939882
chr11	87955222	87955466
chr11	87966204	87966393
chr11	87967671	87968007
chr11	87968073	87968430
chr11	87968443	87968650
chr11	87968792	87969145
chr11	87973976	87974389
chr11	87974549	87974981
chr11	87974981	87975195
chr11	87986188	87986343
chr11	87986391	87986767
chr11	87986893	87987170
chr11	87987170	87987439
chr11	87987439	87987893
chr11	88002860	88003068
chr11	88004169	88004443
chr11	88011013	88011581
chr11	88011650	88011909
chr11	88015974	88016131
chr11	88017871	88018200
chr11	88031488	88031640
chr11	88045925	88046153
chr11	88046599	88046805
chr11	88047106	88047493
chr11	88047493	88047886
chr11	88047886	88048215
chr11	88048215	88048677

chr11	88053926	88054270
chr11	88054475	88054699
chr11	88054699	88054971
chr11	88059098	88059455
chr11	88062313	88062557
chr11	88067294	88067448
chr11	88067700	88068115
chr11	88068115	88068388
chr11	88068388	88068611
chr11	88068611	88068927
chr11	88068927	88069295
chr11	88070518	88070689
chr11	88086873	88087275
chr11	88090358	88090614
chr11	88092014	88092226
chr11	88098660	88098838
chr11	88098838	88099065
chr11	88099065	88099318
chr11	88099318	88099686
chr11	88099686	88100061
chr11	88107489	88107791
chr11	88107992	88108178
chr11	88113643	88113825
chr11	88117146	88117300
chr11	88117676	88117946
chr11	88137815	88138063
chr11	88151274	88151611
chr11	88151611	88151837
chr11	88151837	88152058
chr11	88152644	88152924
chr11	88155107	88155332
chr11	88161058	88161284
chr11	88161392	88161596
chr11	88161617	88162054
chr11	88162054	88162421
chr11	88164341	88164617
chr11	88164713	88165410
chr11	88168687	88168848
chr11	88171974	88172226
chr11	88172871	88173370
chr11	88173414	88174226
chr11	88179472	88179664

chr11	88180234	88180627
chr11	88180627	88180945
chr11	88180945	88181276
chr11	88181276	88181846
chr11	88187456	88187962
chr11	88194850	88195072
chr11	88197155	88197445
chr11	88197502	88197659
chr11	88203708	88203961
chr11	88203961	88204215
chr11	88204215	88204660
chr11	88204664	88204894
chr11	88204962	88205112
chr11	88213777	88214156
chr11	88214301	88214495
chr11	88214568	88214782
chr11	88214921	88215119
chr11	88215164	88215573
chr11	88215573	88215859
chr11	88221625	88221838
chr11	88235221	88235650
chr11	88235650	88236120
chr11	88247559	88247807
chr11	88250576	88250860
chr11	88252114	88252305
chr11	88252305	88252584
chr11	88252590	88252783
chr11	88252981	88253201
chr11	88253302	88253464
chr11	88261289	88261456
chr11	88262688	88263316
chr11	88268155	88268578
chr11	88268578	88268913
chr11	88271941	88272189
chr11	88280359	88280770
chr11	88285966	88286635
chr11	88291752	88291998
chr11	88297563	88297804
chr11	88307976	88308367
chr11	88317601	88317760
chr11	88327114	88327348
chr11	88330523	88330694

chr11	88330857	88331233
chr11	88337983	88338244
chr11	88346047	88346419
chr11	88352555	88352780
chr11	88352780	88353116
chr11	88353139	88353292
chr11	88356807	88357297
chr11	88365955	88366406
chr11	88370101	88370376
chr11	88380716	88380890
chr11	88394358	88394627
chr11	88399520	88399670
chr11	88400037	88400226
chr11	88400239	88400718
chr11	88401413	88401731
chr11	88412498	88412764
chr11	88421024	88421382
chr11	88425706	88425858
chr11	88430304	88430499
chr11	88449227	88449525
chr11	88461519	88461966
chr11	88461966	88462348
chr11	88467738	88468390
chr11	88477550	88477831
chr11	88486121	88486296
chr11	88488228	88488489
chr11	88488629	88488828
chr11	88495672	88496067
chr11	88496067	88496385
chr11	88498500	88498833
chr11	88512571	88513015
chr11	88522132	88522692
chr11	88524499	88524749
chr11	88524749	88525279
chr11	88550627	88550796
chr11	88550802	88551398
chr11	88553125	88553346
chr11	88553649	88553832
chr11	88557087	88557454
chr11	88557454	88557782
chr11	88557811	88558181
chr11	88558181	88558518

chr11	88560729	88560981
chr11	88561160	88561455
chr11	88570585	88570965
chr11	88570965	88571282
chr11	88593919	88594163
chr11	88594912	88595092
chr11	88599571	88600024
chr11	88612239	88612478
chr11	88612644	88612809
chr11	88613694	88613973
chr11	88613973	88614303
chr11	88614385	88614558
chr11	88614741	88614969
chr11	88614969	88615318
chr11	88618748	88619044
chr11	88619044	88619334
chr11	88628939	88629821
chr11	88634640	88635164
chr11	88636386	88636780
chr11	88637904	88638251
chr11	88638251	88638653
chr11	88683011	88683231
chr11	88683336	88683748
chr11	88687084	88687356
chr11	88691092	88691315
chr11	88691807	88692004
chr11	88692440	88692795
chr11	88693389	88693678
chr11	88717736	88718113
chr11	88718113	88718448
chr11	88718448	88718820
chr11	88718820	88719102
chr11	88728645	88728799
chr11	88758471	88758621
chr11	88773927	88774309
chr11	88782525	88782890
chr11	88796274	88796618
chr11	88797623	88797778
chr11	88817113	88817367
chr11	88817367	88817857
chr11	88817949	88818215
chr11	88822620	88822858

chr11	88822858	88823090
chr11	88823090	88823367
chr11	88830486	88830741
chr11	88833008	88833470
chr11	88841461	88841698
chr11	88848662	88849087
chr11	88849853	88850123
chr11	88851377	88851706
chr11	88856433	88856688
chr11	88863865	88864345
chr11	88864345	88864648
chr11	88864648	88864875
chr11	88864875	88865104
chr11	88873101	88873294
chr11	88873344	88873494
chr11	88881136	88881593
chr11	88888500	88888689
chr11	88888936	88889326
chr11	88890533	88890828
chr11	88896616	88896955
chr11	88902667	88903101
chr11	88908322	88908787
chr11	88908787	88908998
chr11	88918285	88918435
chr11	88920047	88920206
chr11	88937921	88938270
chr11	88939693	88939929
chr11	88944471	88944621
chr11	88952235	88952438
chr11	88954183	88954333
chr11	88954864	88955145
chr11	88955230	88955721
chr11	88967781	88968155
chr11	88968216	88968467
chr11	88968467	88968748
chr11	88968748	88968982
chr11	88968990	88969266
chr11	88970174	88970374
chr11	88970374	88970588
chr11	88973733	88974045
chr11	88974045	88974358
chr11	88974358	88974769

chr11	88989600	88989869
chr11	88989869	88990128
chr11	88993618	88994032
chr11	88996376	88996738
chr11	88999062	88999599
chr11	88999599	88999969
chr11	88999969	89000310
chr11	89000521	89000688
chr11	89017726	89018036
chr11	89023241	89023899
chr11	89024074	89024882
chr11	89031228	89031552
chr11	89031578	89031730
chr11	89038397	89038949
chr11	89038949	89039184
chr11	89039375	89039636
chr11	89058612	89058841
chr11	89059009	89059213
chr11	89059887	89060156
chr11	89060156	89060517
chr11	89060517	89061137
chr11	89069457	89069643
chr11	89072453	89072607
chr11	89081624	89082151
chr11	89084742	89084910
chr11	89094966	89095285
chr11	89095543	89095762
chr11	89100368	89100820
chr11	89100886	89101056
chr11	89127634	89127784
chr11	89134244	89134485
chr11	89165323	89165613
chr11	89168947	89169229
chr11	89175579	89175855
chr11	89175855	89176161
chr11	89185908	89186319
chr11	89186871	89187275
chr11	89187275	89187832
chr11	89203520	89203685
chr11	89206456	89206757
chr11	89206757	89207050
chr11	89207050	89207321

chr11	89208212	89208507
chr11	89226842	89227091
chr11	89229942	89230108
chr11	89235519	89236057
chr11	89236232	89236445
chr11	89239847	89240212
chr11	89243424	89243586
chr11	89245927	89246134
chr11	89248695	89248906
chr11	89249041	89249201
chr11	89265188	89265374
chr11	89297515	89297850
chr11	89298249	89298534
chr11	89299293	89299488
chr11	89299499	89299678
chr11	89301345	89301546
chr11	89301586	89301751
chr11	89301819	89302021
chr11	89302021	89302311
chr11	89302311	89302630
chr11	89302658	89302909
chr11	89302909	89303266
chr11	89303326	89303764
chr11	89356588	89356745
chr11	89539807	89540091
chr11	89546266	89546521
chr11	89579410	89579635
chr11	89579810	89580309
chr11	89592486	89592689
chr11	89601246	89601457
chr11	89602428	89602578
chr11	89616641	89616882
chr11	89620658	89620939
chr11	89627316	89627560
chr11	89631260	89631786
chr11	89720986	89721151
chr11	89969688	89969858
chr11	89970785	89971149
chr11	89971280	89971653
chr11	89985307	89985520
chr11	89985520	89985756
chr11	89988953	89989208

chr11	89996849	89997138
chr11	89997446	89997708
chr11	90002504	90002764
chr11	90002764	90003057
chr11	90003057	90003244
chr11	90035587	90035908
chr11	90170061	90170312
chr11	90172431	90172666
chr11	90196284	90196519
chr11	90196519	90197094
chr11	90220379	90220547
chr11	90224151	90224363
chr11	90224756	90225194
chr11	90225194	90225464
chr11	90225761	90226125
chr11	90228328	90228905
chr11	90249087	90250111
chr11	90254298	90254519
chr11	90270593	90270835
chr11	90307027	90307307
chr11	90352590	90352950
chr11	90352950	90353258
chr11	90355155	90355574
chr11	90355574	90355863
chr11	90371567	90371797
chr11	90389561	90389793
chr11	90389849	90390115
chr11	90390172	90390378
chr11	90390560	90390779
chr11	90390779	90391150
chr11	90417767	90418330
chr11	90442329	90442545
chr11	90456537	90456822
chr11	90475412	90475680
chr11	90502036	90502194
chr11	90521122	90521300
chr11	90525366	90525661
chr11	90525661	90525956
chr11	90558216	90558702
chr11	90558702	90558947
chr11	90559466	90559818
chr11	90563117	90563422

chr11	90570698	90570848
chr11	90570918	90571211
chr11	90576182	90576431
chr11	90576431	90576678
chr11	90576678	90576982
chr11	90577425	90577672
chr11	90599456	90599699
chr11	90623636	90623793
chr11	90637519	90637975
chr11	90637975	90638444
chr11	90638444	90638967
chr11	90649794	90649965
chr11	90650226	90650499
chr11	90650499	90650903
chr11	90651788	90652040
chr11	90654552	90654749
chr11	90678116	90678603
chr11	90678603	90679031
chr11	90679111	90679336
chr11	90686711	90687051
chr11	90687150	90687468
chr11	90687468	90687884
chr11	90687884	90688145
chr11	90697365	90697838
chr11	90697838	90698248
chr11	90732612	90732801
chr11	90814565	90814817
chr11	90816797	90817018
chr11	90833592	90833763
chr11	90950419	90950576
chr11	90951580	90951826
chr11	91065359	91065749
chr11	91071828	91072003
chr11	91121413	91121570
chr11	91692089	91692241
chr11	92018534	92018729
chr11	92334829	92335075
chr11	92335185	92335598
chr11	92405918	92406070
chr11	92406891	92407080
chr11	92489603	92490014
chr11	92495336	92495602

chr11	92518386	92518571
chr11	92551499	92551819
chr11	92552845	92553037
chr11	92592686	92593185
chr11	92593185	92593380
chr11	92716475	92716671
chr11	92774904	92775092
chr11	92797484	92797665
chr11	92890081	92890420
chr11	92959575	92959725
chr11	93099003	93099223
chr11	93099545	93099743
chr11	93109495	93109976
chr11	93126511	93126758
chr11	93158955	93159175
chr11	93217248	93217542
chr11	93217542	93217884
chr11	93263515	93263953
chr11	93264014	93264283
chr11	93273982	93274241
chr11	93277675	93277829
chr11	93471923	93472160
chr11	93569194	93569406
chr11	93668181	93668513
chr11	93763148	93763353
chr11	93768797	93769030
chr11	93785797	93786046
chr11	93812545	93812826
chr11	93817251	93817406
chr11	93821267	93821437
chr11	93821440	93821831
chr11	93856370	93856643
chr11	93856645	93856824
chr11	93873743	93873930
chr11	93884860	93885345
chr11	93885345	93885610
chr11	93885610	93885940
chr11	93885940	93886283
chr11	93886306	93886460
chr11	93886812	93886966
chr11	93898318	93898532
chr11	93903440	93903966

chr11	93908833	93909310
chr11	93927903	93928091
chr11	93929200	93929351
chr11	93940590	93940766
chr11	93940766	93940986
chr11	93940986	93941297
chr11	93945031	93945284
chr11	93948716	93948918
chr11	93949132	93949534
chr11	93954906	93955116
chr11	93955116	93955464
chr11	93955464	93955837
chr11	93955837	93956174
chr11	93956205	93956376
chr11	93956376	93956540
chr11	93963026	93963386
chr11	93967579	93967742
chr11	93967922	93968171
chr11	93968171	93968373
chr11	93968373	93968730
chr11	93987326	93987485
chr11	93987491	93988043
chr11	93988043	93988438
chr11	93988517	93988785
chr11	93988785	93988998
chr11	93995023	93995523
chr11	93995670	93995999
chr11	93995999	93996317
chr11	93996317	93996622
chr11	93996665	93996971
chr11	94004802	94004977
chr11	94014305	94014546
chr11	94014546	94014787
chr11	94014787	94014995
chr11	94026319	94026641
chr11	94032845	94033042
chr11	94033042	94033559
chr11	94073379	94073639
chr11	94087026	94087320
chr11	94109605	94109869
chr11	94109869	94110145
chr11	94120943	94121158

chr11	94121158	94121621
chr11	94121623	94121778
chr11	94121931	94122134
chr11	94126880	94127045
chr11	94127968	94128299
chr11	94129699	94129957
chr11	94133288	94133662
chr11	94133662	94134007
chr11	94141528	94141959
chr11	94143464	94143683
chr11	94145162	94145587
chr11	94150851	94151001
chr11	94151010	94151245
chr11	94151427	94151725
chr11	94152622	94152808
chr11	94160310	94160735
chr11	94160735	94161059
chr11	94161135	94161736
chr11	94169808	94170120
chr11	94170968	94171331
chr11	94171331	94171538
chr11	94172210	94172563
chr11	94175294	94175561
chr11	94175796	94175955
chr11	94189347	94189573
chr11	94189573	94190015
chr11	94192634	94192795
chr11	94193090	94193392
chr11	94209983	94210576
chr11	94210620	94210996
chr11	94210996	94211350
chr11	94211350	94211629
chr11	94211629	94211886
chr11	94211886	94212124
chr11	94226041	94226373
chr11	94227669	94228158
chr11	94231569	94231807
chr11	94231807	94232136
chr11	94232227	94232549
chr11	94236075	94236375
chr11	94240663	94240985
chr11	94241039	94241423

chr11	94253551	94253802
chr11	94253802	94254174
chr11	94259164	94259449
chr11	94259920	94260100
chr11	94260484	94260644
chr11	94265341	94265553
chr11	94265553	94265723
chr11	94266304	94266545
chr11	94268900	94269076
chr11	94274418	94274619
chr11	94274619	94274970
chr11	94275236	94275613
chr11	94275666	94276175
chr11	94277028	94277329
chr11	94282341	94282702
chr11	94284073	94284223
chr11	94286258	94286469
chr11	94287258	94287520
chr11	94311078	94311271
chr11	94315385	94315642
chr11	94321099	94321279
chr11	94321396	94321849
chr11	94321849	94322425
chr11	94327190	94327388
chr11	94327743	94328180
chr11	94328180	94328721
chr11	94329605	94329857
chr11	94334649	94334915
chr11	94336593	94336926
chr11	94337712	94337953
chr11	94340264	94340560
chr11	94340987	94341299
chr11	94341402	94341636
chr11	94341636	94341882
chr11	94346427	94346670
chr11	94346670	94347172
chr11	94352064	94352310
chr11	94352310	94352571
chr11	94352647	94353087
chr11	94355346	94355549
chr11	94355937	94356117
chr11	94356386	94356556

chr11	94365117	94365806
chr11	94370019	94370380
chr11	94370469	94371294
chr11	94386027	94386178
chr11	94386443	94386735
chr11	94386763	94386913
chr11	94387920	94388216
chr11	94390964	94391160
chr11	94391710	94391984
chr11	94392256	94392443
chr11	94392560	94392869
chr11	94392869	94393135
chr11	94401142	94401321
chr11	94403781	94404141
chr11	94404141	94404524
chr11	94404524	94404779
chr11	94404779	94405037
chr11	94410765	94411126
chr11	94412998	94413248
chr11	94414129	94414350
chr11	94416028	94416270
chr11	94420233	94420588
chr11	94428593	94428757
chr11	94429108	94429261
chr11	94429699	94429863
chr11	94432320	94432497
chr11	94433878	94434056
chr11	94438116	94438357
chr11	94441196	94441405
chr11	94445201	94445712
chr11	94455470	94455733
chr11	94456181	94456368
chr11	94456424	94456587
chr11	94463239	94463389
chr11	94466097	94466279
chr11	94468134	94468568
chr11	94468568	94468859
chr11	94468859	94469118
chr11	94473775	94473935
chr11	94474070	94474750
chr11	94483590	94483751
chr11	94486050	94486280

chr11	94488808	94489138
chr11	94489138	94489583
chr11	94489594	94489840
chr11	94489840	94490113
chr11	94495919	94496070
chr11	94498106	94498525
chr11	94498531	94498796
chr11	94499216	94499392
chr11	94499591	94499824
chr11	94499824	94500180
chr11	94508268	94508534
chr11	94508534	94508877
chr11	94508877	94509495
chr11	94513926	94514189
chr11	94521172	94521439
chr11	94521439	94521660
chr11	94521660	94521905
chr11	94526613	94526802
chr11	94531969	94532533
chr11	94532586	94532806
chr11	94538136	94538435
chr11	94538683	94539231
chr11	94539265	94539608
chr11	94543818	94544281
chr11	94546545	94546802
chr11	94547994	94548305
chr11	94548399	94548720
chr11	94548822	94549599
chr11	94555263	94555581
chr11	94563793	94564131
chr11	94565244	94565481
chr11	94597372	94597542
chr11	94601385	94601728
chr11	94601728	94602052
chr11	94602052	94602395
chr11	94602395	94602744
chr11	94614458	94614688
chr11	94614688	94614929
chr11	94621570	94621772
chr11	94629544	94629887
chr11	94629887	94630124
chr11	94630124	94630337

chr11	94630348	94630565
chr11	94630565	94630769
chr11	94636915	94637220
chr11	94644849	94644996
chr11	94644996	94645162
chr11	94645269	94645583
chr11	94645583	94645983
chr11	94646522	94646707
chr11	94648166	94648323
chr11	94653467	94653880
chr11	94653880	94654122
chr11	94654122	94654377
chr11	94661013	94661164
chr11	94662832	94662982
chr11	94676653	94676981
chr11	94676981	94677348
chr11	94677348	94677624
chr11	94677624	94677813
chr11	94700587	94700846
chr11	94703420	94703572
chr11	94718141	94718291
chr11	94718596	94719039
chr11	94719253	94719596
chr11	94729717	94729872
chr11	94730322	94730571
chr11	94732843	94733105
chr11	94737661	94737846
chr11	94738557	94738708
chr11	94742001	94742158
chr11	94750152	94750313
chr11	94750325	94750492
chr11	94750667	94750863
chr11	94753386	94753609
chr11	94761417	94761684
chr11	94762862	94763099
chr11	94785283	94785643
chr11	94785805	94785945
chr11	94785945	94786043
chr11	94786102	94786471
chr11	94786471	94786859
chr11	94803553	94803810
chr11	94819050	94819327

chr11	94819506	94819727
chr11	94832644	94832882
chr11	94832882	94833076
chr11	94838212	94838378
chr11	94839270	94839450
chr11	94865528	94865745
chr11	94866599	94867004
chr11	94869712	94869879
chr11	94869922	94870307
chr11	94870307	94870677
chr11	94874164	94874316
chr11	94880773	94881095
chr11	94885598	94885751
chr11	94911025	94911243
chr11	94913781	94914018
chr11	94915738	94916022
chr11	94916311	94916474
chr11	94916492	94916910
chr11	94917845	94918057
chr11	94927559	94927967
chr11	94927967	94928415
chr11	94928729	94929216
chr11	94936701	94937043
chr11	94937043	94937323
chr11	94937323	94937620
chr11	94937652	94937939
chr11	94937939	94938127
chr11	94939381	94939580
chr11	94942368	94942623
chr11	94945803	94946145
chr11	94952312	94952503
chr11	94957925	94958263
chr11	94958263	94958793
chr11	94960793	94961057
chr11	94967498	94967658
chr11	94969760	94969935
chr11	94970213	94970602
chr11	94970649	94970882
chr11	94972182	94972520
chr11	94978914	94979343
chr11	94982936	94983171
chr11	94989757	94990045

chr11	94990770	94991065
chr11	94991065	94991310
chr11	94991689	94992251
chr11	94992492	94992717
chr11	94992881	94993376
chr11	94997707	94998062
chr11	95000686	95000884
chr11	95001061	95001416
chr11	95006611	95006857
chr11	95007610	95007768
chr11	95009432	95010082
chr11	95010216	95010447
chr11	95025454	95025654
chr11	95026102	95026551
chr11	95035031	95035191
chr11	95041127	95041776
chr11	95042202	95042494
chr11	95046998	95047365
chr11	95052307	95052621
chr11	95060356	95060643
chr11	95060643	95060828
chr11	95060828	95060974
chr11	95060980	95061315
chr11	95066087	95066300
chr11	95070204	95070574
chr11	95072874	95073262
chr11	95075485	95075700
chr11	95076023	95076242
chr11	95076310	95076576
chr11	95076576	95077122
chr11	95077930	95078467
chr11	95079571	95079889
chr11	95085569	95085827
chr11	95085827	95086178
chr11	95086178	95086703
chr11	95087327	95087609
chr11	95087609	95087971
chr11	95088876	95089171
chr11	95089497	95089789
chr11	95092983	95093199
chr11	95093199	95093494
chr11	95096585	95097007

chr11	95097007	95097409
chr11	95098631	95098881
chr11	95099603	95099946
chr11	95105239	95105627
chr11	95105627	95105944
chr11	95105944	95106242
chr11	95106695	95106943
chr11	95106943	95107221
chr11	95109018	95109452
chr11	95117024	95117525
chr11	95118587	95118837
chr11	95120003	95120290
chr11	95120290	95120594
chr11	95125426	95125707
chr11	95125707	95126009
chr11	95126291	95126480
chr11	95126483	95126957
chr11	95130727	95130994
chr11	95137339	95137508
chr11	95145297	95145583
chr11	95145769	95146532
chr11	95153168	95153568
chr11	95153802	95153971
chr11	95157523	95157821
chr11	95157839	95158405
chr11	95159704	95160262
chr11	95160262	95160664
chr11	95165896	95166252
chr11	95184684	95185050
chr11	95185789	95186017
chr11	95186850	95187141
chr11	95196960	95197131
chr11	95206199	95206634
chr11	95210278	95210429
chr11	95210761	95211094
chr11	95213635	95213832
chr11	95219266	95219520
chr11	95224227	95224447
chr11	95224564	95224824
chr11	95225168	95225421
chr11	95225427	95225845
chr11	95231860	95232339

chr11	95232443	95232752
chr11	95241299	95241654
chr11	95245531	95245939
chr11	95251353	95251645
chr11	95251883	95252064
chr11	95252064	95252309
chr11	95258514	95258904
chr11	95259065	95259407
chr11	95259407	95259874
chr11	95261306	95261456
chr11	95266337	95266497
chr11	95266935	95267153
chr11	95267980	95268148
chr11	95273730	95274002
chr11	95274705	95274893
chr11	95279136	95279333
chr11	95298221	95298641
chr11	95300020	95300249
chr11	95309475	95310039
chr11	95310063	95310597
chr11	95318629	95318779
chr11	95318861	95319125
chr11	95319577	95319775
chr11	95325813	95325963
chr11	95326323	95326474
chr11	95330571	95331023
chr11	95336553	95336824
chr11	95336824	95337084
chr11	95337084	95337315
chr11	95337315	95337509
chr11	95337509	95337728
chr11	95356792	95356979
chr11	95358345	95358561
chr11	95366564	95366849
chr11	95375996	95376162
chr11	95383344	95383525
chr11	95384131	95384505
chr11	95384505	95384736
chr11	95384775	95385017
chr11	95385017	95385229
chr11	95397339	95397506
chr11	95397506	95397691

chr11	95397907	95398143
chr11	95400875	95401047
chr11	95402887	95403125
chr11	95403517	95403853
chr11	95403853	95404338
chr11	95413738	95414240
chr11	95414240	95414604
chr11	95414604	95415065
chr11	95415831	95416056
chr11	95416134	95416363
chr11	95430466	95430795
chr11	95430835	95430989
chr11	95432273	95432439
chr11	95432743	95433071
chr11	95499364	95499589
chr11	95499589	95499931
chr11	95507044	95507562
chr11	95507562	95507886
chr11	95514047	95514986
chr11	95520723	95521078
chr11	95521303	95521571
chr11	95521571	95521936
chr11	95533598	95533792
chr11	95533799	95534230
chr11	95540740	95541023
chr11	95541069	95541219
chr11	95546041	95546333
chr11	95550388	95550675
chr11	95550675	95550885
chr11	95557309	95557600
chr11	95560784	95561110
chr11	95564714	95565167
chr11	95570834	95571121
chr11	95575615	95576271
chr11	95577810	95578110
chr11	95578110	95578404
chr11	95585142	95585401
chr11	95586182	95586551
chr11	95587515	95587978
chr11	95630438	95630721
chr11	95632517	95632695
chr11	95644235	95644800

chr11	95645616	95645848
chr11	95648903	95649161
chr11	95650152	95650369
chr11	95651043	95651489
chr11	95666555	95667129
chr11	95667129	95667539
chr11	95668181	95668392
chr11	95675238	95675465
chr11	95676382	95676612
chr11	95686011	95686374
chr11	95689584	95689747
chr11	95702706	95703037
chr11	95712099	95712337
chr11	95712512	95712857
chr11	95712857	95713186
chr11	95713186	95713500
chr11	95713500	95713807
chr11	95713965	95714123
chr11	95744544	95744721
chr11	95751062	95751303
chr11	95759951	95760160
chr11	95760160	95760439
chr11	95760439	95760714
chr11	95770781	95770987
chr11	95777338	95777596
chr11	95777596	95777861
chr11	95777861	95778267
chr11	95779376	95779721
chr11	95799017	95799238
chr11	95799238	95799446
chr11	95799446	95799727
chr11	95800365	95800969
chr11	95805703	95805952
chr11	95808426	95808588
chr11	95809471	95809695
chr11	95810465	95810679
chr11	95823958	95824622
chr11	95827530	95827731
chr11	95828016	95828255
chr11	95834200	95834356
chr11	95836531	95836865
chr11	95836917	95837438

chr11	95853798	95854058
chr11	95858319	95858479
chr11	95858537	95859036
chr11	95859036	95859413
chr11	95859456	95859860
chr11	95859960	95860147
chr11	95860147	95860381
chr11	95875949	95876101
chr11	95876366	95876612
chr11	95890546	95890697
chr11	95914235	95914503
chr11	95914567	95915088
chr11	95915376	95915546
chr11	95915930	95916090
chr11	95926564	95926715
chr11	95940235	95940403
chr11	95948548	95948806
chr11	95952948	95953520
chr11	95959277	95959505
chr11	95964422	95964616
chr11	95975861	95976011
chr11	95976429	95976597
chr11	95981088	95981440
chr11	95981440	95981658
chr11	95981810	95982027
chr11	95982027	95982252
chr11	95982310	95982472
chr11	95982510	95982816
chr11	95982816	95983081
chr11	95988239	95988595
chr11	95988595	95989186
chr11	95994777	95994927
chr11	96004709	96005035
chr11	96005035	96005367
chr11	96005367	96005594
chr11	96005594	96005804
chr11	96005804	96006031
chr11	96006517	96006820
chr11	96006836	96007130
chr11	96007130	96007460
chr11	96007460	96007743
chr11	96017773	96018610

chr11	96021323	96021510
chr11	96028457	96028620
chr11	96028701	96029299
chr11	96031805	96031966
chr11	96034182	96034388
chr11	96034607	96035021
chr11	96035021	96035250
chr11	96035250	96035448
chr11	96045206	96045391
chr11	96045391	96045512
chr11	96058369	96058531
chr11	96064453	96064870
chr11	96064870	96065235
chr11	96065235	96065545
chr11	96065545	96065796
chr11	96075309	96075462
chr11	96075494	96075860
chr11	96075860	96076224
chr11	96081115	96081265
chr11	96091138	96091408
chr11	96093212	96093388
chr11	96093411	96093714
chr11	96111858	96112083
chr11	96114075	96114320
chr11	96124354	96124596
chr11	96124596	96124829
chr11	96127036	96127302
chr11	96130757	96130954
chr11	96132842	96133101
chr11	96133586	96133818
chr11	96140696	96140991
chr11	96140991	96141268
chr11	96141268	96141598
chr11	96145659	96145937
chr11	96147562	96147714
chr11	96151211	96151426
chr11	96195770	96195920
chr11	96231465	96231648
chr11	96234996	96235241
chr11	96260356	96260658
chr11	96261627	96261873
chr11	96270993	96271365

chr11	96271432	96272013
chr11	96278835	96279100
chr11	96279519	96279677
chr11	96279775	96279947
chr11	96283090	96283412
chr11	96284140	96284432
chr11	96284432	96284753
chr11	96294594	96294836
chr11	96294836	96295039
chr11	96299211	96299580
chr11	96303118	96303356
chr11	96311618	96311856
chr11	96353179	96353446
chr11	96383734	96383884
chr11	96391783	96392066
chr11	96394307	96394500
chr11	96397466	96397616
chr11	96400062	96400214
chr11	96407794	96408026
chr11	96408383	96408543
chr11	96413537	96414018
chr11	96414018	96414344
chr11	96435743	96436031
chr11	96438823	96439145
chr11	96458966	96459141
chr11	96560154	96560504
chr11	96572609	96572862
chr11	96582085	96582337
chr11	96602781	96603107
chr11	96603107	96603391
chr11	96604282	96604611
chr11	96621276	96621494
chr11	96622758	96623163
chr11	96629877	96630069
chr11	96639917	96640079
chr11	96673918	96674078
chr11	96704238	96704491
chr11	96721742	96721924
chr11	96725801	96726263
chr11	96730603	96730793
chr11	96732747	96732917
chr11	96735992	96736233

chr11	96736385	96736751
chr11	96736751	96737099
chr11	96742081	96742364
chr11	96747138	96747602
chr11	96747602	96748060
chr11	96749149	96749493
chr11	96760054	96760482
chr11	96765768	96765920
chr11	96766264	96766586
chr11	96766586	96767065
chr11	96777313	96777990
chr11	96784914	96785151
chr11	96788956	96789252
chr11	96789252	96789724
chr11	96790124	96790350
chr11	96795550	96795701
chr11	96796688	96796871
chr11	96805020	96805270
chr11	96808133	96808363
chr11	96811092	96811358
chr11	96813505	96813668
chr11	96813668	96813840
chr11	96816011	96816178
chr11	96816366	96816606
chr11	96822637	96822975
chr11	96822975	96823224
chr11	96823908	96824614
chr11	96829344	96829843
chr11	96829843	96830311
chr11	96830311	96830627
chr11	96837250	96837695
chr11	96840503	96840708
chr11	96847062	96847298
chr11	96848781	96848992
chr11	96849571	96850018
chr11	96850021	96850268
chr11	96857519	96857877
chr11	96860535	96860889
chr11	96861280	96861514
chr11	96861514	96861713
chr11	96861713	96861961
chr11	96861961	96862325

chr11	96863549	96863987
chr11	96867193	96867472
chr11	96875464	96875823
chr11	96875823	96876112
chr11	96895978	96896247
chr11	96896247	96896471
chr11	96909941	96910379
chr11	96916013	96916358
chr11	96916358	96916856
chr11	96919021	96919576
chr11	96928741	96929002
chr11	96929047	96929621
chr11	96929639	96930045
chr11	96934858	96935471
chr11	96936380	96936530
chr11	96937502	96937862
chr11	96943598	96943949
chr11	96943949	96944431
chr11	96950879	96951045
chr11	96954294	96954466
chr11	96954874	96955054
chr11	96955172	96955422
chr11	96958074	96958232
chr11	96963612	96963901
chr11	96966924	96967205
chr11	96967205	96967453
chr11	96967499	96967944
chr11	96977051	96977286
chr11	96977286	96977668
chr11	96977668	96978272
chr11	96982465	96982698
chr11	96987969	96988127
chr11	96988334	96988627
chr11	96989481	96989639
chr11	96991497	96991741
chr11	96995127	96995324
chr11	96997509	96997706
chr11	97007129	97007652
chr11	97012862	97013066
chr11	97018581	97019088
chr11	97021476	97021759
chr11	97021759	97022154

chr11	97022154	97022495
chr11	97026129	97026434
chr11	97029708	97029999
chr11	97029999	97030236
chr11	97030236	97030459
chr11	97030476	97030626
chr11	97041261	97041487
chr11	97041487	97041699
chr11	97041699	97041953
chr11	97048051	97048282
chr11	97048282	97048592
chr11	97050628	97051030
chr11	97051264	97051511
chr11	97051975	97052269
chr11	97052269	97052894
chr11	97058014	97058181
chr11	97058434	97058813
chr11	97065273	97065488
chr11	97065586	97065974
chr11	97065974	97066256
chr11	97067049	97067226
chr11	97072568	97073068
chr11	97078889	97079040
chr11	97083637	97084201
chr11	97096033	97096504
chr11	97097508	97097695
chr11	97101754	97102207
chr11	97112684	97112834
chr11	97114601	97114891
chr11	97114891	97115135
chr11	97115218	97115402
chr11	97123374	97123577
chr11	97126888	97127192
chr11	97127192	97127466
chr11	97127611	97127772
chr11	97130175	97130351
chr11	97136625	97136816
chr11	97140763	97141226
chr11	97146820	97147051
chr11	97148336	97148677
chr11	97150634	97150862
chr11	97152198	97152447

chr11	97152735	97152885
chr11	97152921	97153219
chr11	97159160	97159379
chr11	97165685	97165920
chr11	97168809	97169006
chr11	97175580	97175841
chr11	97176897	97177324
chr11	97180840	97181036
chr11	97186926	97187361
chr11	97187361	97187578
chr11	97187686	97188760
chr11	97192234	97192735
chr11	97195160	97195600
chr11	97198686	97198843
chr11	97199549	97199699
chr11	97200788	97201055
chr11	97206911	97207214
chr11	97207214	97207453
chr11	97207453	97207676
chr11	97252314	97252536
chr11	97270049	97270353
chr11	97272891	97273129
chr11	97273649	97273830
chr11	97279280	97279572
chr11	97279572	97279915
chr11	97279915	97280243
chr11	97280243	97280490
chr11	97280490	97280898
chr11	97285971	97286312
chr11	97286312	97286598
chr11	97298230	97298480
chr11	97302119	97302742
chr11	97308943	97309172
chr11	97309306	97309822
chr11	97309822	97310197
chr11	97310214	97310540
chr11	97315458	97315723
chr11	97315723	97315957
chr11	97315957	97316164
chr11	97321811	97322224
chr11	97322224	97322562
chr11	97328119	97328501

chr11	97334229	97334434
chr11	97335398	97335558
chr11	97343454	97343772
chr11	97343772	97344030
chr11	97347751	97347908
chr11	97349934	97350397
chr11	97351187	97351363
chr11	97361891	97362472
chr11	97362472	97362940
chr11	97362940	97363278
chr11	97363457	97363707
chr11	97400199	97400714
chr11	97400718	97400878
chr11	97401010	97401488
chr11	97401488	97401807
chr11	97401807	97402027
chr11	97408264	97408536
chr11	97411023	97411185
chr11	97412579	97412763
chr11	97415356	97415706
chr11	97415706	97416018
chr11	97416018	97416321
chr11	97419380	97419986
chr11	97420834	97421102
chr11	97427724	97428038
chr11	97428038	97428787
chr11	97429672	97429943
chr11	97434671	97435127
chr11	97435127	97435464
chr11	97435592	97435882
chr11	97435882	97436180
chr11	97440063	97440229
chr11	97440498	97440828
chr11	97440997	97441384
chr11	97441454	97441609
chr11	97441865	97442088
chr11	97449514	97449917
chr11	97449917	97450244
chr11	97450244	97450531
chr11	97450531	97451028
chr11	97456859	97457145
chr11	97457145	97457530

chr11	97462626	97462838
chr11	97463922	97464224
chr11	97466544	97466707
chr11	97468344	97468690
chr11	97468690	97468982
chr11	97468982	97469295
chr11	97476321	97476707
chr11	97477061	97477442
chr11	97477442	97477821
chr11	97487480	97487819
chr11	97489565	97489973
chr11	97489973	97490253
chr11	97496723	97497008
chr11	97502292	97503028
chr11	97505783	97505912
chr11	97505912	97506050
chr11	97507900	97508293
chr11	97508293	97508738
chr11	97513107	97513391
chr11	97513391	97513825
chr11	97523521	97523850
chr11	97523850	97524088
chr11	97524088	97524339
chr11	97524339	97524633
chr11	97531691	97531994
chr11	97531994	97532421
chr11	97533134	97533368
chr11	97540206	97540397
chr11	97541017	97541184
chr11	97546411	97546590
chr11	97546728	97547393
chr11	97554165	97554354
chr11	97557070	97557319
chr11	97562603	97562874
chr11	97563368	97563689
chr11	97563689	97564164
chr11	97564283	97564688
chr11	97574278	97574556
chr11	97574722	97574874
chr11	97574980	97575621
chr11	97575621	97576147
chr11	97576254	97576537

chr11	97576537	97576719
chr11	97578358	97578599
chr11	97591975	97592241
chr11	97603925	97604179
chr11	97611054	97611528
chr11	97612859	97613028
chr11	97619377	97619611
chr11	97619611	97619860
chr11	97622489	97622769
chr11	97623258	97623564
chr11	97623726	97624065
chr11	97626871	97627167
chr11	97628567	97628956
chr11	97628956	97629243
chr11	97629272	97629553
chr11	97629600	97629988
chr11	97632007	97632255
chr11	97639151	97639336
chr11	97639547	97639864
chr11	97644868	97645285
chr11	97645506	97645709
chr11	97650412	97650755
chr11	97653334	97653631
chr11	97657305	97657620
chr11	97660185	97660529
chr11	97660529	97660765
chr11	97661690	97662047
chr11	97662231	97662658
chr11	97662947	97663332
chr11	97664229	97664396
chr11	97664763	97664936
chr11	97670844	97671173
chr11	97671864	97672073
chr11	97677908	97678123
chr11	97685523	97686051
chr11	97695175	97695434
chr11	97698839	97699085
chr11	97699679	97699907
chr11	97699950	97700200
chr11	97700200	97700429
chr11	97700429	97700658
chr11	97702412	97702645

chr11	97702645	97702873
chr11	97703029	97703414
chr11	97703414	97703714
chr11	97712959	97713119
chr11	97714536	97714707
chr11	97714809	97715124
chr11	97715432	97715684
chr11	97717727	97717920
chr11	97719164	97719319
chr11	97726514	97726912
chr11	97727788	97728126
chr11	97728260	97728609
chr11	97728609	97728880
chr11	97728880	97729157
chr11	97735586	97736007
chr11	97736608	97737063
chr11	97737063	97737433
chr11	97744081	97744545
chr11	97744545	97744784
chr11	97744784	97745030
chr11	97745030	97745474
chr11	97748840	97749061
chr11	97751605	97751918
chr11	97751918	97752480
chr11	97766257	97766470
chr11	97766470	97766948
chr11	97769604	97770125
chr11	97775133	97775294
chr11	97775416	97775584
chr11	97775760	97775989
chr11	97775989	97776285
chr11	97776479	97776749
chr11	97776796	97777097
chr11	97777097	97777407
chr11	97781732	97781994
chr11	97781994	97782288
chr11	97782288	97782790
chr11	97782860	97783024
chr11	97791011	97791161
chr11	97793229	97793552
chr11	97798884	97799332
chr11	97799361	97799773

chr11	97799773	97800039
chr11	97800039	97800383
chr11	97801189	97801367
chr11	97801511	97801953
chr11	97803926	97804230
chr11	97806152	97806346
chr11	97808085	97808287
chr11	97809081	97809290
chr11	97809353	97809584
chr11	97810290	97810648
chr11	97811949	97812373
chr11	97813193	97813422
chr11	97813773	97814173
chr11	97814173	97814431
chr11	97815812	97816034
chr11	97820737	97821220
chr11	97821293	97821738
chr11	97821819	97822053
chr11	97827722	97828021
chr11	97829418	97829688
chr11	97830077	97830227
chr11	97834081	97834277
chr11	97838867	97839185
chr11	97839185	97839500
chr11	97839500	97839770
chr11	97840561	97840819
chr11	97843972	97844293
chr11	97850465	97850626
chr11	97850710	97850887
chr11	97855492	97855902
chr11	97857451	97857933
chr11	97862211	97862378
chr11	97862556	97863025
chr11	97883536	97883868
chr11	97884006	97884383
chr11	97884383	97884654
chr11	97889640	97889848
chr11	97899848	97900126
chr11	97900126	97900437
chr11	97901866	97902255
chr11	97904913	97905077
chr11	97909075	97909255

chr11	97920911	97921151
chr11	97935731	97936337
chr11	97936850	97937143
chr11	97943314	97943555
chr11	97947520	97947752
chr11	97949622	97949994
chr11	97955968	97956125
chr11	97958527	97958847
chr11	97960964	97961260
chr11	97971126	97971772
chr11	97977867	97978133
chr11	97986369	97986519
chr11	97987576	97987911
chr11	97987911	97988164
chr11	97988164	97988577
chr11	97988577	97989117
chr11	97993945	97994202
chr11	97994202	97994542
chr11	97994542	97994872
chr11	97994872	97995214
chr11	97998200	97998406
chr11	98002697	98003068
chr11	98003074	98003397
chr11	98003397	98003732
chr11	98003788	98004136
chr11	98004209	98004462
chr11	98013942	98014097
chr11	98019276	98019580
chr11	98020814	98021096
chr11	98022406	98022764
chr11	98026520	98026749
chr11	98026879	98027074
chr11	98027074	98027451
chr11	98027459	98027609
chr11	98030777	98031049
chr11	98031140	98031484
chr11	98031533	98031710
chr11	98034034	98034387
chr11	98035831	98036181
chr11	98036181	98036422
chr11	98043746	98043897
chr11	98045692	98045901

chr11	98046501	98046662
chr11	98050313	98050468
chr11	98052239	98052736
chr11	98052748	98052920
chr11	98057407	98057716
chr11	98057716	98057923
chr11	98057972	98058366
chr11	98061983	98062217
chr11	98062274	98062445
chr11	98063885	98064078
chr11	98068386	98068581
chr11	98075271	98075492
chr11	98075492	98075711
chr11	98075711	98075900
chr11	98080573	98080813
chr11	98086719	98086919
chr11	98088249	98088479
chr11	98090622	98090877
chr11	98090877	98091254
chr11	98139088	98139434
chr11	98148913	98149207
chr11	98149207	98149517
chr11	98149517	98149781
chr11	98149781	98150069
chr11	98150069	98150383
chr11	98150383	98150717
chr11	98182235	98182491
chr11	98182491	98182790
chr11	98192808	98193185
chr11	98193185	98193483
chr11	98193483	98193680
chr11	98202148	98202300
chr11	98202503	98203001
chr11	98203001	98203394
chr11	98203394	98203725
chr11	98204203	98204402
chr11	98209468	98209665
chr11	98209866	98210016
chr11	98264933	98265398
chr11	98286279	98286493
chr11	98294281	98295119
chr11	98296368	98296624

chr11	98300476	98300687
chr11	98313932	98314130
chr11	98314130	98314353
chr11	98322177	98322343
chr11	98324097	98324278
chr11	98327283	98327779
chr11	98327784	98328062
chr11	98328215	98328412
chr11	98328425	98328586
chr11	98334409	98334585
chr11	98334585	98334790
chr11	98335349	98335499
chr11	98336286	98336549
chr11	98342055	98342292
chr11	98342476	98342929
chr11	98342959	98343327
chr11	98345300	98345585
chr11	98348135	98348358
chr11	98348500	98348748
chr11	98348748	98349075
chr11	98355807	98356099
chr11	98357121	98357364
chr11	98357942	98358464
chr11	98358464	98358762
chr11	98371832	98372077
chr11	98372135	98372312
chr11	98372491	98372728
chr11	98384460	98384661
chr11	98389484	98389744
chr11	98391584	98391796
chr11	98392032	98392335
chr11	98392615	98392924
chr11	98393267	98393608
chr11	98400076	98400354
chr11	98400354	98400639
chr11	98400849	98401173
chr11	98404773	98405192
chr11	98412233	98412659
chr11	98412659	98412978
chr11	98412978	98413163
chr11	98413772	98413991
chr11	98418309	98418522

chr11	98418671	98419193
chr11	98419193	98419516
chr11	98438415	98439207
chr11	98445722	98446024
chr11	98446664	98447167
chr11	98447228	98447449
chr11	98447449	98447677
chr11	98447882	98448083
chr11	98448144	98448383
chr11	98448383	98448613
chr11	98448613	98448844
chr11	98449427	98449685
chr11	98449685	98449988
chr11	98457084	98457355
chr11	98457355	98457729
chr11	98457729	98458352
chr11	98461437	98461642
chr11	98469842	98470008
chr11	98470142	98470536
chr11	98470536	98470792
chr11	98520502	98520661
chr11	98520669	98520870
chr11	98550809	98551216
chr11	98551216	98551669
chr11	98586914	98587158
chr11	98587158	98587537
chr11	98588106	98588609
chr11	98588867	98589053
chr11	98596014	98596198
chr11	98597423	98597639
chr11	98598113	98598263
chr11	98599072	98599421
chr11	98599421	98599642
chr11	98601018	98601175
chr11	98602131	98602409
chr11	98604081	98604253
chr11	98604970	98605131
chr11	98606807	98607040
chr11	98635328	98635858
chr11	98653031	98653303
chr11	98654973	98655679
chr11	98660596	98660890

chr11	98660927	98661090
chr11	98662385	98662583
chr11	98664247	98664442
chr11	98671930	98672210
chr11	98673079	98673296
chr11	98673296	98673892
chr11	98682095	98682451
chr11	98682451	98682755
chr11	98682755	98682980
chr11	98682980	98683195
chr11	98688057	98688350
chr11	98699951	98700295
chr11	98700295	98700854
chr11	98705952	98706252
chr11	98706311	98706514
chr11	98708113	98708288
chr11	98708484	98708877
chr11	98709042	98709267
chr11	98709267	98709523
chr11	98709523	98709919
chr11	98710435	98710770
chr11	98717949	98718117
chr11	98719616	98719794
chr11	98721369	98722121
chr11	98729132	98729645
chr11	98740526	98740766
chr11	98741609	98741897
chr11	98741897	98742148
chr11	98742148	98742444
chr11	98744045	98744234
chr11	98747490	98747694
chr11	98750498	98750953
chr11	98750953	98751372
chr11	98754093	98754554
chr11	98754712	98755162
chr11	98762698	98762926
chr11	98762926	98763207
chr11	98763207	98763548
chr11	98763548	98763869
chr11	98772634	98773239
chr11	98773627	98773909
chr11	98773909	98774284

chr11	98775186	98775719
chr11	98775817	98776092
chr11	98783234	98783699
chr11	98785811	98786050
chr11	98786369	98786592
chr11	98786592	98786869
chr11	98786869	98787254
chr11	98788768	98789222
chr11	98789267	98789440
chr11	98794419	98794703
chr11	98794703	98794995
chr11	98794995	98795276
chr11	98795276	98795517
chr11	98795517	98795737
chr11	98795737	98796071
chr11	98796071	98796517
chr11	98796554	98796757
chr11	98809503	98809942
chr11	98809942	98810330
chr11	98826111	98826395
chr11	98829455	98829612
chr11	98836443	98836598
chr11	98836604	98836890
chr11	98836890	98837156
chr11	98839465	98839734
chr11	98846125	98846348
chr11	98846918	98847141
chr11	98851556	98851708
chr11	98857577	98857752
chr11	98858766	98859094
chr11	98859133	98859421
chr11	98863078	98863231
chr11	98863404	98863727
chr11	98863727	98863995
chr11	98863995	98864568
chr11	98870847	98871016
chr11	98873855	98874333
chr11	98887801	98888023
chr11	98895223	98895499
chr11	98902621	98902900
chr11	98905025	98905486
chr11	98907548	98908199

chr11	98916438	98916684
chr11	98918346	98918565
chr11	98921871	98922075
chr11	98922333	98922613
chr11	98930886	98931135
chr11	98932478	98932801
chr11	98932953	98933194
chr11	98933194	98933599
chr11	98935949	98936159
chr11	98936159	98936447
chr11	98937446	98937782
chr11	98941389	98941671
chr11	98941710	98942264
chr11	98942264	98942634
chr11	98942664	98942848
chr11	98942869	98943220
chr11	98947932	98948283
chr11	98948283	98948522
chr11	98950018	98950173
chr11	98953912	98954192
chr11	98954271	98954572
chr11	98959492	98960091
chr11	98960141	98960538
chr11	98960538	98960865
chr11	98961063	98961240
chr11	98961420	98961613
chr11	98961879	98962122
chr11	98963119	98963580
chr11	98963636	98964063
chr11	98966704	98966913
chr11	98972122	98972300
chr11	98972300	98972580
chr11	98975184	98975657
chr11	98981910	98982434
chr11	98982434	98983043
chr11	98990987	98991269
chr11	98991771	98991913
chr11	98991913	98992008
chr11	98994016	98994305
chr11	98994305	98994622
chr11	99018700	99018861
chr11	99023524	99023688

chr11	99023688	99023978
chr11	99023978	99024300
chr11	99024300	99024559
chr11	99029466	99029666
chr11	99031265	99031437
chr11	99035462	99035622
chr11	99039797	99040065
chr11	99040065	99040266
chr11	99040310	99040605
chr11	99040680	99040905
chr11	99040922	99041387
chr11	99041387	99041958
chr11	99047876	99048105
chr11	99052327	99052495
chr11	99054710	99054874
chr11	99059941	99060164
chr11	99060166	99060452
chr11	99061771	99061995
chr11	99062239	99062478
chr11	99062998	99063217
chr11	99063223	99063436
chr11	99063463	99063639
chr11	99066051	99066363
chr11	99069280	99069543
chr11	99070308	99070463
chr11	99076515	99077000
chr11	99077000	99077396
chr11	99082122	99082442
chr11	99082592	99082943
chr11	99088530	99088775
chr11	99088791	99089214
chr11	99089214	99089739
chr11	99089783	99089948
chr11	99095022	99095206
chr11	99098867	99099323
chr11	99101847	99102008
chr11	99102954	99103290
chr11	99104602	99105028
chr11	99105028	99105308
chr11	99106061	99106258
chr11	99106345	99106780
chr11	99106780	99107126

chr11	99107126	99107441
chr11	99108257	99108754
chr11	99115371	99115523
chr11	99115912	99116163
chr11	99123986	99124254
chr11	99126541	99126731
chr11	99126926	99127076
chr11	99127795	99128181
chr11	99128181	99128499
chr11	99137433	99137734
chr11	99145810	99146161
chr11	99149936	99150273
chr11	99150891	99151260
chr11	99155095	99155329
chr11	99162993	99163311
chr11	99189121	99189358
chr11	99189358	99189793
chr11	99196218	99196383
chr11	99199967	99200243
chr11	99200243	99200609
chr11	99207162	99207388
chr11	99207618	99207768
chr11	99210976	99211135
chr11	99221875	99222101
chr11	99229639	99230044
chr11	99230044	99230340
chr11	99230340	99230588
chr11	99230588	99230828
chr11	99230828	99231035
chr11	99231035	99231273
chr11	99249170	99249394
chr11	99268303	99268455
chr11	99268654	99268892
chr11	99268921	99269266
chr11	99269266	99269613
chr11	99269641	99269793
chr11	99283467	99283649
chr11	99292889	99293148
chr11	99295186	99295369
chr11	99295701	99295897
chr11	99304680	99304846
chr11	99307596	99307913

chr11	99310891	99311208
chr11	99311784	99311955
chr11	99323733	99324046
chr11	99331397	99331549
chr11	99341780	99342425
chr11	99345981	99346448
chr11	99364530	99364762
chr11	99366900	99367162
chr11	99379659	99379921
chr11	99385847	99386263
chr11	99386263	99386734
chr11	99409243	99409398
chr11	99414875	99415030
chr11	99424620	99424829
chr11	99438024	99438319
chr11	99454084	99454234
chr11	99454864	99455057
chr11	99458811	99459261
chr11	99463243	99463469
chr11	99464873	99465354
chr11	99465664	99465935
chr11	99468605	99468902
chr11	99471922	99472377
chr11	99472377	99472753
chr11	99473379	99473776
chr11	99490803	99491048
chr11	99492082	99492360
chr11	99493164	99493314
chr11	99496392	99496861
chr11	99507835	99508608
chr11	99514044	99514647
chr11	99516511	99516751
chr11	99516895	99517175
chr11	99517175	99517574
chr11	99524232	99524441
chr11	99524441	99524782
chr11	99526171	99526357
chr11	99530015	99530335
chr11	99554201	99554482
chr11	99556828	99557151
chr11	99558474	99558925
chr11	99559200	99559457

chr11	99562502	99562922
chr11	99574031	99574489
chr11	99575672	99576007
chr11	99580627	99580988
chr11	99581100	99581432
chr11	99581576	99582025
chr11	99587375	99587573
chr11	99590957	99591107
chr11	99597760	99597910
chr11	99603065	99603226
chr11	99614780	99615444
chr11	99616028	99616265
chr11	99620727	99621043
chr11	99625795	99626025
chr11	99626095	99626274
chr11	99627623	99627856
chr11	99630547	99630734
chr11	99630951	99631285
chr11	99634461	99634765
chr11	99634765	99635053
chr11	99636273	99636493
chr11	99636493	99636679
chr11	99640889	99641285
chr11	99657528	99657775
chr11	99658310	99658683
chr11	99658683	99658978
chr11	99659083	99659644
chr11	99659793	99660042
chr11	99768271	99768486
chr11	99768486	99768765
chr11	99773051	99773382
chr11	99773382	99773590
chr11	99784810	99784994
chr11	99809647	99809802
chr11	99825064	99825227
chr11	99825925	99826080
chr11	99851071	99851374
chr11	99851374	99851686
chr11	99851686	99852055
chr11	99858031	99858414
chr11	99858414	99858620
chr11	99858620	99858857

chr11	99858857	99859110
chr11	99859110	99859363
chr11	99873578	99873782
chr11	99879345	99879559
chr11	99937667	99937972
chr11	99989780	99990082
chr11	99990082	99990359
chr11	99994362	99994573
chr11	100004956	100005126
chr11	100005904	100006263
chr11	100013771	100013947
chr11	100013947	100014196
chr11	100046404	100046599
chr11	100046599	100046773
chr11	100055591	100056179
chr11	100056179	100056683
chr11	100065994	100066267
chr11	100067392	100067683
chr11	100067683	100068139
chr11	100073791	100074349
chr11	100085364	100085646
chr11	100090015	100090399
chr11	100113026	100113484
chr11	100113484	100114113
chr11	100120404	100120821
chr11	100132139	100132435
chr11	100138509	100138818
chr11	100138818	100139195
chr11	100139195	100139501
chr11	100139501	100139775
chr11	100139775	100140058
chr11	100144952	100145186
chr11	100145186	100145427
chr11	100145427	100145842
chr11	100145842	100146538
chr11	100147597	100147939
chr11	100151143	100151293
chr11	100153838	100154197
chr11	100157420	100157717
chr11	100157717	100158051
chr11	100158051	100158376
chr11	100159388	100159972

chr11	100166684	100167049
chr11	100168065	100168656
chr11	100168799	100169043
chr11	100169043	100169348
chr11	100169422	100169697
chr11	100170092	100170271
chr11	100182693	100182863
chr11	100198541	100199110
chr11	100200172	100200347
chr11	100201479	100201639
chr11	100203564	100203714
chr11	100207347	100207864
chr11	100207864	100208105
chr11	100208347	100208603
chr11	100208905	100209113
chr11	100209851	100210006
chr11	100215210	100215511
chr11	100215511	100215874
chr11	100221026	100221186
chr11	100232167	100232352
chr11	100248836	100249522
chr11	100254238	100255090
chr11	100255632	100256065
chr11	100261178	100261523
chr11	100277711	100278086
chr11	100278086	100278424
chr11	100278424	100278881
chr11	100282057	100282227
chr11	100282668	100282885
chr11	100286922	100287465
chr11	100288919	100289373
chr11	100289967	100290258
chr11	100290258	100290600
chr11	100295381	100295671
chr11	100296404	100296748
chr11	100298347	100298639
chr11	100298639	100299216
chr11	100305287	100305800
chr11	100306556	100306862
chr11	100306953	100307259
chr11	100312037	100312262
chr11	100312504	100312669

chr11	100319575	100319821
chr11	100319821	100320203
chr11	100320203	100320713
chr11	100322896	100323065
chr11	100323536	100323869
chr11	100326892	100327146
chr11	100333228	100333397
chr11	100334033	100334417
chr11	100335816	100336097
chr11	100346857	100347227
chr11	100355076	100355339
chr11	100355339	100355649
chr11	100355649	100355973
chr11	100355973	100356267
chr11	100356298	100356482
chr11	100356482	100356689
chr11	100361145	100361383
chr11	100362499	100362926
chr11	100362980	100363233
chr11	100363247	100363531
chr11	100364251	100364475
chr11	100365539	100365823
chr11	100372264	100372424
chr11	100372827	100373065
chr11	100376824	100377002
chr11	100386684	100387028
chr11	100387598	100387976
chr11	100387976	100388365
chr11	100391118	100391316
chr11	100393669	100393898
chr11	100393898	100394169
chr11	100395286	100395649
chr11	100395730	100396144
chr11	100396920	100397083
chr11	100397083	100397267
chr11	100397267	100397657
chr11	100397657	100398028
chr11	100398028	100398339
chr11	100402720	100403077
chr11	100406315	100406474
chr11	100408136	100408581
chr11	100411935	100412155

chr11	100414126	100414628
chr11	100414628	100414935
chr11	100414935	100415202
chr11	100420390	100420622
chr11	100420689	100420843
chr11	100425620	100425843
chr11	100425843	100426301
chr11	100440575	100440956
chr11	100440956	100441414
chr11	100471976	100472347
chr11	100472347	100472645
chr11	100472645	100472939
chr11	100472939	100473186
chr11	100486209	100486447
chr11	100491043	100491237
chr11	100491291	100491447
chr11	100493751	100494004
chr11	100494092	100494337
chr11	100503377	100503631
chr11	100512425	100512759
chr11	100525382	100525534
chr11	100527033	100527461
chr11	100527461	100527821
chr11	100527821	100528410
chr11	100528424	100528610
chr11	100531307	100531547
chr11	100535723	100535881
chr11	100535955	100536380
chr11	100536441	100536874
chr11	100545281	100545492
chr11	100545492	100545757
chr11	100545757	100546004
chr11	100555470	100555932
chr11	100556104	100556284
chr11	100569765	100570187
chr11	100570253	100570468
chr11	100570483	100570762
chr11	100574209	100574419
chr11	100574614	100575115
chr11	100575115	100575476
chr11	100619011	100619412
chr11	100619412	100619778

chr11	100619778	100620032
chr11	100620077	100620272
chr11	100622699	100623251
chr11	100626714	100626960
chr11	100626960	100627218
chr11	100640082	100640359
chr11	100640359	100640615
chr11	100646165	100646345
chr11	100646345	100646738
chr11	100651805	100652247
chr11	100660009	100660264
chr11	100661848	100662027
chr11	100673206	100673436
chr11	100680314	100680643
chr11	100692430	100692587
chr11	100694987	100695283
chr11	100702150	100702434
chr11	100702434	100702794
chr11	100711739	100711927
chr11	100711927	100712143
chr11	100712143	100712355
chr11	100712355	100712606
chr11	100714622	100714897
chr11	100720401	100720674
chr11	100722313	100722676
chr11	100722831	100723088
chr11	100738041	100738735
chr11	100745090	100745414
chr11	100758896	100759122
chr11	100759160	100759483
chr11	100759511	100759859
chr11	100759915	100760170
chr11	100761538	100761776
chr11	100761776	100762212
chr11	100770134	100770502
chr11	100770502	100770863
chr11	100770863	100771118
chr11	100771118	100771356
chr11	100789740	100790114
chr11	100795427	100795685
chr11	100833443	100833823
chr11	100850027	100850322

chr11	100850322	100850785
chr11	100850785	100851198
chr11	100851198	100851495
chr11	100860075	100860905
chr11	100867245	100867475
chr11	100870693	100870878
chr11	100886116	100886305
chr11	100887300	100887689
chr11	100887819	100887977
chr11	100893452	100894104
chr11	100900336	100900913
chr11	100901995	100902200
chr11	100908176	100908420
chr11	100912810	100913106
chr11	100924556	100924778
chr11	100930763	100931111
chr11	100931677	100931901
chr11	100931958	100932175
chr11	100938737	100938974
chr11	100938985	100939652
chr11	100939652	100940027
chr11	100946995	100947161
chr11	100947614	100947867
chr11	100949646	100949800
chr11	100955239	100955514
chr11	100957303	100957466
chr11	100960686	100961035
chr11	100962258	100962727
chr11	100962727	100962998
chr11	100963760	100963957
chr11	100967935	100968193
chr11	100968935	100969085
chr11	100969089	100969385
chr11	100969385	100969624
chr11	100969681	100969991
chr11	100969991	100970438
chr11	100970438	100970940
chr11	100974380	100974662
chr11	100979951	100980279
chr11	100980539	100980772
chr11	100983182	100983468
chr11	100983468	100983734

chr11	100985721	100985920
chr11	100993254	100993802
chr11	100994150	100994307
chr11	100995072	100995378
chr11	100996578	100996840
chr11	101001480	101001688
chr11	101002957	101003438
chr11	101008139	101008480
chr11	101009232	101009583
chr11	101009583	101009828
chr11	101010071	101010338
chr11	101010530	101010690
chr11	101045393	101045674
chr11	101052853	101053086
chr11	101053086	101053303
chr11	101053303	101053571
chr11	101055529	101055772
chr11	101056883	101057429
chr11	101057925	101058189
chr11	101060283	101060481
chr11	101062353	101062748
chr11	101063284	101063638
chr11	101063656	101064029
chr11	101064029	101064477
chr11	101067109	101067288
chr11	101069792	101070199
chr11	101070199	101070420
chr11	101070420	101070636
chr11	101076818	101076981
chr11	101082137	101082456
chr11	101082456	101082854
chr11	101082854	101083260
chr11	101086886	101087046
chr11	101087120	101087477
chr11	101087477	101087792
chr11	101087792	101088018
chr11	101089922	101090155
chr11	101094943	101095643
chr11	101095643	101096069
chr11	101111258	101111469
chr11	101119479	101119717
chr11	101119717	101119952

chr11	101119952	101120230
chr11	101120230	101120504
chr11	101128908	101129428
chr11	101130009	101130215
chr11	101138431	101138845
chr11	101138845	101139224
chr11	101140015	101140383
chr11	101140383	101140815
chr11	101142080	101142243
chr11	101152735	101153249
chr11	101155733	101155988
chr11	101155988	101156224
chr11	101162268	101162641
chr11	101170320	101170637
chr11	101170845	101171265
chr11	101171265	101171706
chr11	101171999	101172252
chr11	101172252	101172431
chr11	101173169	101173399
chr11	101173602	101173802
chr11	101173823	101174104
chr11	101180880	101181113
chr11	101189573	101189786
chr11	101191513	101191686
chr11	101201147	101201381
chr11	101211951	101212286
chr11	101221065	101221233
chr11	101221350	101221501
chr11	101225932	101226302
chr11	101226302	101226600
chr11	101226600	101226888
chr11	101233283	101233460
chr11	101236801	101237125
chr11	101246038	101246353
chr11	101246353	101246790
chr11	101248707	101248913
chr11	101252897	101253298
chr11	101253366	101253584
chr11	101253584	101253837
chr11	101253837	101254309
chr11	101254455	101254687
chr11	101259914	101260109

chr11	101260109	101260371
chr11	101260371	101260676
chr11	101260676	101260919
chr11	101260919	101261132
chr11	101261132	101261312
chr11	101269657	101270058
chr11	101270800	101271119
chr11	101277528	101277818
chr11	101277818	101278219
chr11	101278219	101278561
chr11	101278624	101278860
chr11	101278860	101279188
chr11	101287351	101287659
chr11	101301663	101301930
chr11	101301930	101302149
chr11	101302149	101302494
chr11	101302525	101302683
chr11	101307833	101308117
chr11	101315665	101316080
chr11	101316080	101316511
chr11	101316511	101316942
chr11	101323569	101323949
chr11	101327385	101327581
chr11	101328816	101329267
chr11	101329387	101329562
chr11	101335727	101336071
chr11	101338236	101338386
chr11	101338410	101338901
chr11	101347151	101347366
chr11	101351312	101351679
chr11	101351679	101352009
chr11	101362333	101362700
chr11	101363201	101363356
chr11	101363358	101363794
chr11	101366632	101366818
chr11	101386605	101386888
chr11	101394868	101395140
chr11	101395191	101395486
chr11	101410606	101410822
chr11	101415282	101415695
chr11	101416505	101416742
chr11	101416763	101417068

chr11	101417068	101417317
chr11	101417317	101417535
chr11	101417535	101417736
chr11	101424248	101424424
chr11	101424519	101424841
chr11	101424888	101425225
chr11	101425225	101425519
chr11	101425519	101425805
chr11	101433248	101433638
chr11	101441930	101442501
chr11	101442501	101442893
chr11	101446354	101446566
chr11	101448114	101448525
chr11	101448525	101448803
chr11	101448992	101449289
chr11	101454438	101454617
chr11	101455153	101455303
chr11	101462971	101463280
chr11	101464771	101465087
chr11	101465087	101465573
chr11	101465598	101465947
chr11	101465953	101466412
chr11	101467465	101467886
chr11	101467886	101468351
chr11	101484800	101484969
chr11	101484969	101485126
chr11	101493042	101493216
chr11	101493216	101493391
chr11	101494322	101494619
chr11	101494652	101494803
chr11	101507165	101507350
chr11	101521280	101521545
chr11	101536808	101536981
chr11	101536981	101537083
chr11	101540523	101540962
chr11	101543634	101543863
chr11	101551760	101552028
chr11	101552028	101552233
chr11	101552233	101552451
chr11	101552471	101552898
chr11	101552898	101553209
chr11	101562326	101562799

chr11	101565230	101565380
chr11	101568523	101568799
chr11	101576174	101576487
chr11	101581958	101582404
chr11	101582532	101582692
chr11	101589472	101589711
chr11	101604493	101604750
chr11	101604752	101605128
chr11	101605128	101605717
chr11	101614978	101615373
chr11	101616166	101616410
chr11	101616410	101616643
chr11	101617084	101617320
chr11	101617936	101618175
chr11	101619977	101620421
chr11	101620436	101620586
chr11	101627017	101627209
chr11	101627283	101627606
chr11	101627606	101627940
chr11	101627940	101628326
chr11	101628326	101628656
chr11	101628764	101628947
chr11	101641695	101642020
chr11	101642020	101642267
chr11	101642267	101642739
chr11	101644894	101645144
chr11	101645146	101645424
chr11	101645424	101645660
chr11	101645660	101645977
chr11	101658160	101658547
chr11	101658547	101658824
chr11	101658824	101659013
chr11	101659013	101659174
chr11	101659174	101659458
chr11	101659458	101659749
chr11	101659749	101659953
chr11	101659953	101660206
chr11	101665225	101665991
chr11	101668219	101668500
chr11	101680562	101680792
chr11	101687720	101688409
chr11	101700203	101700804

chr11	101700804	101701203
chr11	101720080	101720506
chr11	101732577	101732855
chr11	101732855	101733129
chr11	101733129	101733410
chr11	101735015	101735299
chr11	101739841	101740696
chr11	101741148	101741325
chr11	101756786	101756955
chr11	101763764	101764176
chr11	101772465	101772715
chr11	101776856	101777347
chr11	101783713	101783909
chr11	101784258	101784448
chr11	101784448	101784676
chr11	101784676	101784901
chr11	101784901	101785150
chr11	101785150	101785547
chr11	101785594	101785775
chr11	101798052	101798215
chr11	101798223	101798397
chr11	101806894	101807052
chr11	101807139	101807790
chr11	101813321	101813570
chr11	101814829	101815115
chr11	101815152	101815310
chr11	101830204	101830533
chr11	101832680	101832853
chr11	101839377	101839797
chr11	101849099	101849757
chr11	101874010	101874286
chr11	101878801	101879088
chr11	101885216	101885424
chr11	101885784	101885960
chr11	101905161	101905449
chr11	101910147	101910566
chr11	101912268	101912490
chr11	101917949	101918212
chr11	101918957	101919197
chr11	101919696	101920072
chr11	101928465	101928670
chr11	101932921	101933274

chr11	101944877	101945071
chr11	101958717	101958961
chr11	101963751	101963988
chr11	101963988	101964315
chr11	101968384	101968574
chr11	101968738	101969161
chr11	101971512	101971691
chr11	101984071	101984576
chr11	101984576	101985115
chr11	101986081	101986289
chr11	101997973	101998427
chr11	101999477	101999709
chr11	102026632	102027117
chr11	102027117	102027415
chr11	102027716	102028082
chr11	102031284	102031517
chr11	102038619	102038769
chr11	102039583	102039828
chr11	102040161	102040559
chr11	102053966	102054147
chr11	102061216	102061650
chr11	102069064	102069225
chr11	102082380	102082752
chr11	102083278	102083498
chr11	102086956	102087525
chr11	102087525	102087804
chr11	102087804	102088026
chr11	102088046	102088334
chr11	102088369	102088676
chr11	102089160	102089359
chr11	102096810	102097376
chr11	102102570	102102817
chr11	102107112	102107359
chr11	102110360	102110667
chr11	102110667	102111005
chr11	102111712	102111886
chr11	102127677	102128069
chr11	102131450	102131717
chr11	102138267	102138429
chr11	102138459	102138766
chr11	102140590	102140834
chr11	102145389	102145594

chr11	102145594	102145834
chr11	102145834	102146137
chr11	102147427	102147577
chr11	102148524	102148681
chr11	102155681	102155925
chr11	102156055	102156527
chr11	102156527	102156822
chr11	102184488	102184856
chr11	102184856	102185137
chr11	102185137	102185482
chr11	102189505	102189771
chr11	102189771	102190004
chr11	102190004	102190202
chr11	102190216	102190400
chr11	102198337	102198507
chr11	102201241	102201606
chr11	102201606	102201890
chr11	102208771	102209357
chr11	102209793	102210095
chr11	102216037	102216347
chr11	102216347	102216567
chr11	102216636	102216920
chr11	102216920	102217208
chr11	102218615	102218820
chr11	102218820	102219200
chr11	102223221	102223462
chr11	102224739	102224951
chr11	102225258	102225465
chr11	102229344	102229548
chr11	102229691	102230091
chr11	102230091	102230405
chr11	102230405	102230615
chr11	102238183	102238593
chr11	102244898	102245506
chr11	102248633	102248829
chr11	102248829	102249009
chr11	102249009	102249500
chr11	102257582	102257739
chr11	102258615	102258797
chr11	102267360	102267806
chr11	102275519	102275791
chr11	102276338	102276870

chr11	102284496	102284786
chr11	102284786	102285045
chr11	102285045	102285596
chr11	102295290	102295591
chr11	102295591	102295782
chr11	102295875	102296286
chr11	102296319	102296516
chr11	102296524	102296931
chr11	102296931	102297365
chr11	102297365	102297619
chr11	102297619	102297781
chr11	102308908	102309385
chr11	102309385	102309744
chr11	102309909	102310170
chr11	102316584	102317123
chr11	102317123	102317563
chr11	102317563	102317910
chr11	102317910	102318210
chr11	102318210	102318544
chr11	102318681	102318833
chr11	102318925	102319150
chr11	102319339	102319714
chr11	102320279	102320474
chr11	102323625	102323875
chr11	102327245	102327602
chr11	102327962	102328115
chr11	102338560	102338908
chr11	102338908	102339138
chr11	102344643	102344802
chr11	102346913	102347360
chr11	102351443	102351798
chr11	102358352	102358540
chr11	102365088	102365283
chr11	102375303	102375577
chr11	102375591	102375829
chr11	102375829	102376154
chr11	102376531	102376809
chr11	102382865	102383316
chr11	102383316	102383732
chr11	102384540	102384765
chr11	102391538	102391758
chr11	102393210	102393622

chr11	102393994	102394144
chr11	102394192	102394411
chr11	102394411	102394722
chr11	102398520	102398759
chr11	102407039	102407312
chr11	102407312	102407600
chr11	102407600	102407892
chr11	102407926	102408130
chr11	102408344	102408643
chr11	102408643	102408900
chr11	102411761	102412024
chr11	102415100	102415299
chr11	102417367	102417544
chr11	102418920	102419204
chr11	102424646	102425230
chr11	102430090	102430642
chr11	102430788	102431030
chr11	102431036	102431321
chr11	102434469	102434737
chr11	102436446	102436723
chr11	102445344	102445791
chr11	102445791	102446275
chr11	102446381	102446577
chr11	102446577	102446812
chr11	102446843	102447184
chr11	102447240	102447476
chr11	102447476	102447825
chr11	102455655	102456036
chr11	102456036	102456531
chr11	102465986	102466502
chr11	102466866	102467328
chr11	102467328	102467664
chr11	102487054	102487348
chr11	102496287	102496575
chr11	102536248	102536551
chr11	102555627	102555841
chr11	102555841	102556064
chr11	102556064	102556255
chr11	102556255	102556595
chr11	102565199	102565559
chr11	102568837	102569053
chr11	102572860	102573136

chr11	102576112	102576400
chr11	102576482	102576726
chr11	102578604	102579046
chr11	102582354	102582616
chr11	102586512	102586681
chr11	102603627	102603808
chr11	102604025	102604535
chr11	102604535	102604925
chr11	102604925	102605157
chr11	102605157	102605427
chr11	102623344	102623584
chr11	102630285	102630683
chr11	102633511	102633749
chr11	102635398	102635583
chr11	102643701	102644187
chr11	102644187	102644563
chr11	102651507	102651661
chr11	102654445	102654628
chr11	102654806	102655002
chr11	102655499	102656395
chr11	102656419	102656603
chr11	102656603	102656801
chr11	102690258	102690541
chr11	102697243	102697402
chr11	102697419	102697883
chr11	102697883	102698348
chr11	102714092	102714331
chr11	102714667	102714850
chr11	102720472	102720767
chr11	102725671	102726067
chr11	102728569	102729066
chr11	102729682	102730320
chr11	102735059	102735275
chr11	102739166	102739504
chr11	102739504	102739835
chr11	102749500	102749798
chr11	102749798	102750141
chr11	102761365	102761727
chr11	102761727	102762012
chr11	102762012	102762287
chr11	102767083	102767459
chr11	102767491	102767652

chr11	102773905	102774182
chr11	102774182	102774395
chr11	102774395	102774600
chr11	102774615	102775166
chr11	102783158	102783470
chr11	102783470	102783695
chr11	102783842	102784070
chr11	102788434	102788598
chr11	102810724	102811007
chr11	102811212	102811407
chr11	102818528	102818970
chr11	102818970	102819353
chr11	102819353	102819775
chr11	102821332	102821527
chr11	102832380	102832622
chr11	102832814	102833047
chr11	102833047	102833299
chr11	102846683	102846870
chr11	102851912	102852218
chr11	102853832	102854095
chr11	102864373	102864584
chr11	102865196	102865346
chr11	102880513	102880814
chr11	102880814	102881111
chr11	102881122	102881448
chr11	102881448	102881894
chr11	102888032	102888304
chr11	102889784	102889958
chr11	102892948	102893281
chr11	102894613	102895091
chr11	102900645	102901089
chr11	102901089	102901323
chr11	102901323	102901521
chr11	102909972	102910160
chr11	102924491	102924744
chr11	102924744	102925012
chr11	102925012	102925348
chr11	102925348	102925691
chr11	102946191	102946351
chr11	102946438	102947089
chr11	102948501	102948788
chr11	102948788	102949023

chr11	102955735	102955926
chr11	102958480	102958862
chr11	102959049	102959197
chr11	102959197	102959368
chr11	102964634	102964818
chr11	102964818	102964994
chr11	102966923	102967073
chr11	102978154	102978346
chr11	102978389	102978608
chr11	102980991	102981229
chr11	102983870	102984047
chr11	102990165	102990466
chr11	102992977	102993317
chr11	102996218	102996914
chr11	102996985	102997244
chr11	103003086	103003783
chr11	103008583	103008893
chr11	103009354	103009608
chr11	103016898	103017333
chr11	103022240	103022412
chr11	103022558	103022720
chr11	103027952	103028207
chr11	103028207	103028440
chr11	103028440	103028663
chr11	103028663	103028960
chr11	103037645	103037852
chr11	103050296	103050567
chr11	103051263	103051812
chr11	103051997	103052167
chr11	103056518	103057208
chr11	103066705	103066954
chr11	103067018	103067195
chr11	103067219	103067752
chr11	103072628	103072836
chr11	103093860	103094278
chr11	103094280	103095014
chr11	103097034	103097260
chr11	103097298	103097760
chr11	103099961	103100234
chr11	103100300	103100571
chr11	103101535	103101824
chr11	103101992	103102201

chr11	103102201	103102426
chr11	103102652	103103231
chr11	103115260	103115617
chr11	103115864	103116186
chr11	103116186	103116675
chr11	103116936	103117356
chr11	103132210	103132667
chr11	103132667	103133135
chr11	103133135	103133483
chr11	103134309	103134612
chr11	103134706	103134885
chr11	103140453	103140905
chr11	103140905	103141338
chr11	103141338	103141572
chr11	103141572	103141783
chr11	103142431	103142590
chr11	103142763	103142913
chr11	103144363	103144545
chr11	103150263	103150432
chr11	103158570	103158857
chr11	103158857	103159101
chr11	103159101	103159379
chr11	103168625	103168984
chr11	103171036	103171220
chr11	103174294	103174768
chr11	103174768	103175349
chr11	103184975	103185223
chr11	103193098	103193281
chr11	103193438	103193766
chr11	103195422	103195578
chr11	103199392	103199857
chr11	103201387	103201708
chr11	103201710	103201966
chr11	103210142	103210311
chr11	103217778	103218075
chr11	103218146	103218511
chr11	103218511	103218789
chr11	103232995	103233295
chr11	103234250	103234437
chr11	103235697	103236054
chr11	103237859	103238064
chr11	103246123	103246758

chr11	103246758	103247243
chr11	103247567	103247735
chr11	103257996	103258389
chr11	103262083	103262386
chr11	103262386	103262689
chr11	103266899	103267299
chr11	103267299	103267937
chr11	103267952	103268131
chr11	103272978	103273227
chr11	103273438	103273669
chr11	103281895	103282273
chr11	103307123	103307370
chr11	103311904	103312643
chr11	103322850	103323172
chr11	103325257	103325570
chr11	103326991	103327265
chr11	103332512	103333057
chr11	103334039	103334296
chr11	103340637	103341113
chr11	103346353	103346597
chr11	103346705	103347124
chr11	103350733	103351484
chr11	103360270	103360615
chr11	103361420	103361762
chr11	103361794	103361971
chr11	103362074	103362413
chr11	103362570	103362916
chr11	103362916	103363313
chr11	103363313	103363756
chr11	103365528	103365728
chr11	103371041	103371625
chr11	103372661	103372870
chr11	103373061	103373229
chr11	103375029	103375242
chr11	103378081	103378585
chr11	103378585	103378846
chr11	103381992	103382213
chr11	103382213	103382339
chr11	103382339	103382717
chr11	103383628	103383828
chr11	103385656	103385819
chr11	103386823	103387185

chr11	103387335	103387758
chr11	103387758	103388194
chr11	103388247	103388502
chr11	103394044	103394417
chr11	103394577	103394823
chr11	103396698	103397237
chr11	103399134	103399363
chr11	103400105	103400385
chr11	103402247	103402727
chr11	103406621	103406923
chr11	103407221	103407504
chr11	103410998	103411255
chr11	103411405	103411588
chr11	103412208	103412635
chr11	103412635	103412953
chr11	103412953	103413232
chr11	103413245	103413451
chr11	103418588	103418753
chr11	103424283	103424616
chr11	103443825	103444073
chr11	103449775	103449976
chr11	103464358	103464775
chr11	103468351	103468568
chr11	103497945	103498141
chr11	103530716	103530878
chr11	103578517	103578668
chr11	103638877	103639027
chr11	103639462	103639691
chr11	103649246	103649453
chr11	103649462	103649614
chr11	103649738	103649948
chr11	103650052	103650292
chr11	103656609	103656838
chr11	103673815	103674015
chr11	103675630	103675885
chr11	103675885	103676049
chr11	103677060	103677371
chr11	103678572	103678828
chr11	103683732	103684077
chr11	103684835	103685034
chr11	103685151	103685803
chr11	103689103	103689386

chr11	103697201	103697565
chr11	103697565	103697853
chr11	103697853	103698225
chr11	103700252	103700587
chr11	103721505	103721657
chr11	103733134	103733428
chr11	103749345	103749537
chr11	103749603	103750168
chr11	103758796	103759075
chr11	103763801	103764037
chr11	103764037	103764237
chr11	103764647	103764809
chr11	103766058	103766260
chr11	103772671	103772830
chr11	103773515	103773684
chr11	103773906	103774150
chr11	103774274	103774473
chr11	103780250	103780507
chr11	103790099	103790309
chr11	103798737	103798934
chr11	103800976	103801562
chr11	103801596	103801746
chr11	103809719	103810014
chr11	103810014	103810296
chr11	103810296	103810564
chr11	103810564	103810756
chr11	103836671	103836928
chr11	103839068	103839379
chr11	103849411	103849647
chr11	103879532	103879755
chr11	103953139	103953432
chr11	103953432	103953813
chr11	103953813	103954277
chr11	103961299	103961693
chr11	103961693	103961944
chr11	103966241	103966767
chr11	103966767	103967143
chr11	103967240	103967493
chr11	103967493	103967783
chr11	103984405	103984582
chr11	103987438	103987729
chr11	103990125	103990600

chr11	103997462	103997690
chr11	104017584	104017846
chr11	104043164	104043370
chr11	104055941	104056301
chr11	104057230	104057429
chr11	104072738	104072894
chr11	104072897	104073134
chr11	104083873	104084027
chr11	104114731	104115074
chr11	104118324	104118622
chr11	104131860	104132073
chr11	104157924	104158252
chr11	104171572	104171820
chr11	104186171	104186324
chr11	104195727	104196192
chr11	104221777	104222074
chr11	104224625	104224888
chr11	104230964	104231881
chr11	104232264	104232496
chr11	104237264	104237451
chr11	104246207	104246417
chr11	104278274	104278624
chr11	104279595	104279912
chr11	104288896	104289139
chr11	104289139	104289447
chr11	104289447	104289774
chr11	104289774	104290203
chr11	104295755	104295985
chr11	104299230	104299440
chr11	104303380	104303641
chr11	104303641	104303937
chr11	104309814	104309964
chr11	104310462	104310795
chr11	104315785	104316017
chr11	104318943	104319445
chr11	104319445	104319793
chr11	104325513	104325716
chr11	104329254	104329451
chr11	104339561	104339768
chr11	104361596	104362207
chr11	104363711	104363958
chr11	104363958	104364218

chr11	104364218	104364513
chr11	104379717	104379872
chr11	104383496	104383708
chr11	104412303	104412619
chr11	104435192	104435488
chr11	104441602	104441903
chr11	104441903	104442219
chr11	104442219	104442640
chr11	104442640	104443122
chr11	104448535	104448899
chr11	104461710	104461861
chr11	104466988	104467196
chr11	104468847	104469263
chr11	104469263	104469828
chr11	104492172	104492416
chr11	104510701	104510852
chr11	104549511	104549661
chr11	104549976	104550350
chr11	104550350	104550629
chr11	104550629	104551012
chr11	104561051	104561338
chr11	104571478	104571764
chr11	104590003	104590587
chr11	104604212	104604378
chr11	104604431	104604631
chr11	104606577	104606761
chr11	104607643	104607924
chr11	104607924	104608734
chr11	104611610	104611967
chr11	104614234	104614448
chr11	104615623	104615790
chr11	104617850	104618165
chr11	104618521	104618909
chr11	104619454	104619868
chr11	104622581	104622849
chr11	104624619	104625107
chr11	104625107	104625517
chr11	104625517	104625815
chr11	104630230	104630590
chr11	104631178	104631737
chr11	104638859	104639333
chr11	104639456	104639606

chr11	104640901	104641215
chr11	104648607	104648881
chr11	104667750	104668129
chr11	104673762	104673912
chr11	104688312	104688770
chr11	104692220	104692468
chr11	104692468	104692823
chr11	104721007	104721297
chr11	104732711	104732919
chr11	104748808	104749137
chr11	104768149	104768368
chr11	104789948	104790246
chr11	104852004	104852177
chr11	104856288	104856538
chr11	104872814	104872975
chr11	104912667	104912817
chr11	104942941	104943192
chr11	104952338	104952931
chr11	104953046	104953303
chr11	104975008	104975164
chr11	105021130	105021406
chr11	105028868	105029532
chr11	105034989	105035410
chr11	105066507	105066657
chr11	105066660	105066938
chr11	105112444	105112821
chr11	105120521	105120984
chr11	105123998	105124336
chr11	105125693	105125933
chr11	105126017	105126379
chr11	105126379	105126619
chr11	105126622	105126790
chr11	105126790	105126968
chr11	105128900	105129062
chr11	105145232	105145438
chr11	105150234	105150543
chr11	105153259	105153646
chr11	105154298	105154567
chr11	105159754	105160035
chr11	105160035	105160355
chr11	105180623	105180825
chr11	105180825	105181097

chr11	105181097	105181449
chr11	105181449	105181773
chr11	105181773	105182104
chr11	105182104	105182393
chr11	105189250	105189456
chr11	105195825	105196336
chr11	105196339	105196489
chr11	105210049	105210334
chr11	105265057	105265300
chr11	105270228	105270385
chr11	105289142	105289385
chr11	105292161	105292511
chr11	105292511	105292979
chr11	105315390	105315543
chr11	105330077	105330337
chr11	105331802	105332130
chr11	105332540	105332779
chr11	105340102	105340357
chr11	105345360	105345608
chr11	105347609	105347864
chr11	105347864	105348149
chr11	105361911	105362640
chr11	105362742	105363347
chr11	105402264	105402767
chr11	105402852	105403148
chr11	105403260	105403482
chr11	105408796	105409167
chr11	105409167	105409402
chr11	105424651	105425143
chr11	105439828	105440125
chr11	105441858	105442187
chr11	105443552	105443802
chr11	105448918	105449190
chr11	105449544	105449944
chr11	105449944	105450309
chr11	105451433	105451645
chr11	105451654	105452026
chr11	105453471	105453787
chr11	105460011	105460231
chr11	105467285	105467573
chr11	105507961	105508175
chr11	105525640	105525790

chr11	105530961	105531129
chr11	105570729	105570887
chr11	105589205	105589568
chr11	105589568	105589876
chr11	105589876	105590138
chr11	105590138	105590444
chr11	105592006	105592216
chr11	105625864	105626095
chr11	105666915	105667160
chr11	105743249	105743491
chr11	105823189	105823608
chr11	105823815	105824160
chr11	105867835	105868136
chr11	105883538	105883769
chr11	105883769	105884041
chr11	105884041	105884291
chr11	105899215	105899576
chr11	105899629	105899894
chr11	105901343	105901985
chr11	105905532	105905874
chr11	105907441	105907803
chr11	105911647	105911943
chr11	105912042	105912333
chr11	105920938	105921092
chr11	105927088	105927479
chr11	105927574	105927935
chr11	105934279	105934507
chr11	105936819	105937132
chr11	105943183	105943442
chr11	105943479	105943817
chr11	105943817	105944124
chr11	105944124	105944368
chr11	105944368	105944679
chr11	105946972	105947165
chr11	105948270	105948556
chr11	105950609	105950827
chr11	105952952	105953141
chr11	105953141	105953421
chr11	105953421	105953894
chr11	105958662	105958838
chr11	105964878	105965028
chr11	105967551	105967792

chr11	105967792	105968088
chr11	105968088	105968568
chr11	105969456	105969665
chr11	105969665	105969884
chr11	105975055	105975240
chr11	105986828	105986978
chr11	105989476	105989703
chr11	106001851	106002021
chr11	106005459	106005702
chr11	106007854	106008290
chr11	106012715	106012886
chr11	106017258	106017525
chr11	106020018	106020360
chr11	106020360	106020854
chr11	106026591	106026747
chr11	106026750	106027074
chr11	106031900	106032155
chr11	106032155	106032428
chr11	106033356	106033634
chr11	106036545	106036978
chr11	106036978	106037406
chr11	106037464	106037796
chr11	106060639	106060874
chr11	106061353	106061780
chr11	106065789	106066020
chr11	106066020	106066226
chr11	106066226	106066800
chr11	106076359	106076677
chr11	106083808	106084109
chr11	106084109	106084650
chr11	106084650	106085139
chr11	106101610	106102068
chr11	106125852	106126158
chr11	106135273	106135455
chr11	106142021	106142242
chr11	106151112	106151263
chr11	106152292	106152450
chr11	106158141	106158371
chr11	106158961	106159559
chr11	106159559	106159981
chr11	106159981	106160392
chr11	106160392	106160705

chr11	106165561	106166049
chr11	106167510	106167693
chr11	106170875	106171128
chr11	106178126	106178432
chr11	106178507	106178906
chr11	106179724	106180041
chr11	106180137	106180459
chr11	106192993	106193152
chr11	106193156	106193398
chr11	106193398	106193637
chr11	106193637	106193989
chr11	106195324	106195756
chr11	106215977	106216293
chr11	106216293	106216654
chr11	106216690	106216904
chr11	106216904	106217153
chr11	106217153	106217455
chr11	106234064	106234267
chr11	106246920	106247148
chr11	106255226	106255478
chr11	106255488	106255714
chr11	106255714	106255948
chr11	106255970	106256248
chr11	106256248	106256620
chr11	106272254	106272757
chr11	106272757	106273310
chr11	106276485	106276792
chr11	106282518	106282772
chr11	106284016	106284266
chr11	106284266	106284562
chr11	106286899	106287120
chr11	106289431	106289701
chr11	106302523	106302716
chr11	106302716	106302976
chr11	106313634	106313854
chr11	106325897	106326259
chr11	106329782	106330077
chr11	106339039	106339209
chr11	106348406	106348614
chr11	106350887	106351210
chr11	106351210	106351448
chr11	106363998	106364175

chr11	106364839	106365195
chr11	106366397	106366689
chr11	106366712	106366862
chr11	106371385	106371596
chr11	106374486	106374716
chr11	106374716	106374940
chr11	106380224	106380374
chr11	106391884	106392152
chr11	106392152	106392435
chr11	106393067	106393500
chr11	106397700	106397945
chr11	106410115	106410374
chr11	106418639	106419084
chr11	106419436	106419586
chr11	106420247	106420569
chr11	106426514	106426726
chr11	106437647	106437912
chr11	106438576	106438878
chr11	106438878	106439249
chr11	106470267	106470556
chr11	106475256	106475630
chr11	106480370	106480636
chr11	106480636	106480897
chr11	106483956	106484232
chr11	106487239	106487485
chr11	106487485	106487737
chr11	106487737	106488097
chr11	106488112	106488323
chr11	106488323	106488577
chr11	106491493	106491643
chr11	106500435	106500674
chr11	106500674	106501183
chr11	106501183	106501683
chr11	106507496	106508034
chr11	106513245	106513462
chr11	106540093	106540251
chr11	106542420	106542761
chr11	106555823	106556273
chr11	106564292	106564662
chr11	106572830	106573274
chr11	106573274	106573551
chr11	106573551	106573868

chr11	106579946	106580234
chr11	106580234	106580493
chr11	106580493	106580797
chr11	106583361	106583587
chr11	106588133	106588499
chr11	106588577	106589121
chr11	106589121	106589495
chr11	106598044	106598274
chr11	106601869	106602081
chr11	106612153	106612472
chr11	106612472	106612836
chr11	106612836	106613218
chr11	106613218	106613578
chr11	106614479	106614640
chr11	106625637	106625860
chr11	106627629	106627812
chr11	106627812	106628101
chr11	106637794	106638109
chr11	106642016	106642166
chr11	106648363	106648796
chr11	106658028	106658311
chr11	106658311	106658559
chr11	106658559	106658853
chr11	106659435	106659633
chr11	106659633	106659923
chr11	106665444	106665598
chr11	106666277	106667001
chr11	106674400	106674608
chr11	106674608	106674806
chr11	106679917	106680117
chr11	106713630	106713963
chr11	106716624	106716779
chr11	106722771	106723281
chr11	106741301	106741563
chr11	106742627	106742787
chr11	106751063	106751374
chr11	106779005	106779427
chr11	106779427	106780287
chr11	106787573	106787813
chr11	106788183	106788549
chr11	106788549	106788766
chr11	106788766	106789052

chr11	106789052	106789518
chr11	106789842	106790000
chr11	106806523	106806894
chr11	106808406	106808606
chr11	106808606	106808834
chr11	106812447	106812734
chr11	106812859	106813064
chr11	106821041	106821250
chr11	106855514	106855669
chr11	106855672	106855832
chr11	106890526	106890859
chr11	106891501	106891827
chr11	106896617	106896850
chr11	106896989	106897176
chr11	106911420	106911570
chr11	106919576	106920107
chr11	106920107	106920585
chr11	106920585	106920789
chr11	106920789	106921020
chr11	106921020	106921289
chr11	106927977	106928357
chr11	106930568	106930790
chr11	106945562	106945785
chr11	106947147	106947325
chr11	106947887	106948127
chr11	106948275	106948618
chr11	106954245	106954468
chr11	106954691	106954859
chr11	106957344	106958095
chr11	106967650	106968002
chr11	106971107	106971393
chr11	106976317	106976796
chr11	106980498	106980772
chr11	106982226	106982732
chr11	106984249	106984627
chr11	106985583	106985823
chr11	106985951	106986274
chr11	106987061	106987215
chr11	106987386	106987545
chr11	106987688	106987990
chr11	106998762	106999080
chr11	106999080	106999394

chr11	106999394	106999604
chr11	106999604	106999873
chr11	107005398	107005917
chr11	107011830	107012066
chr11	107012215	107012596
chr11	107012596	107012802
chr11	107012802	107013043
chr11	107018688	107018941
chr11	107022402	107022579
chr11	107027377	107027574
chr11	107027619	107028065
chr11	107028065	107028289
chr11	107028289	107028577
chr11	107037371	107037526
chr11	107044170	107044703
chr11	107044712	107044886
chr11	107045247	107045481
chr11	107050486	107050646
chr11	107069934	107070185
chr11	107070218	107070386
chr11	107102596	107102819
chr11	107102898	107103263
chr11	107103263	107103627
chr11	107103766	107103981
chr11	107105375	107105540
chr11	107107767	107107984
chr11	107108564	107108734
chr11	107113102	107113640
chr11	107117932	107118096
chr11	107124255	107124968
chr11	107131119	107131368
chr11	107131377	107131606
chr11	107131606	107131790
chr11	107131790	107132054
chr11	107132054	107132422
chr11	107132507	107132800
chr11	107138794	107138947
chr11	107148559	107148856
chr11	107149348	107149690
chr11	107149690	107150056
chr11	107150056	107150493
chr11	107156772	107157210

chr11	107157210	107157722
chr11	107157736	107157969
chr11	107157969	107158202
chr11	107183959	107184199
chr11	107188600	107188972
chr11	107188972	107189198
chr11	107189198	107189562
chr11	107195386	107195605
chr11	107215262	107215424
chr11	107252635	107252849
chr11	107255438	107255673
chr11	107275951	107276280
chr11	107276386	107276658
chr11	107287091	107287790
chr11	107287790	107288074
chr11	107295412	107295699
chr11	107295744	107296042
chr11	107296256	107296800
chr11	107296800	107297276
chr11	107298974	107299240
chr11	107321866	107322016
chr11	107333042	107333208
chr11	107341250	107341776
chr11	107347628	107347873
chr11	107347975	107348574
chr11	107351311	107351601
chr11	107358725	107359062
chr11	107370561	107370863
chr11	107370863	107371134
chr11	107376272	107376600
chr11	107379560	107379721
chr11	107385361	107385627
chr11	107410380	107410720
chr11	107416996	107417702
chr11	107424763	107424989
chr11	107427942	107428462
chr11	107428477	107428633
chr11	107431281	107431534
chr11	107436016	107436285
chr11	107448053	107448318
chr11	107448592	107448852
chr11	107449214	107449432

chr11	107451148	107451698
chr11	107457329	107457571
chr11	107457717	107457894
chr11	107458002	107458656
chr11	107458656	107459017
chr11	107468926	107469406
chr11	107469517	107470010
chr11	107470010	107470308
chr11	107470509	107470985
chr11	107473121	107473466
chr11	107479234	107479624
chr11	107479624	107479850
chr11	107479850	107480045
chr11	107491381	107491580
chr11	107502464	107502646
chr11	107503256	107503413
chr11	107505983	107506137
chr11	107506387	107506643
chr11	107509685	107509931
chr11	107510922	107511199
chr11	107512028	107512254
chr11	107527386	107527577
chr11	107539080	107539314
chr11	107546048	107546420
chr11	107546577	107547194
chr11	107547319	107547664
chr11	107547664	107547970
chr11	107547970	107548242
chr11	107552418	107552614
chr11	107567923	107568170
chr11	107570698	107570914
chr11	107573895	107574215
chr11	107574776	107575172
chr11	107616042	107616192
chr11	107629546	107629831
chr11	107636197	107636448
chr11	107647204	107647442
chr11	107688470	107688620
chr11	107705143	107705391
chr11	107705526	107705714
chr11	107711453	107711640
chr11	107714322	107714486

chr11	107714520	107714723
chr11	107728608	107728920
chr11	107731584	107731829
chr11	107737917	107738149
chr11	107741142	107741326
chr11	107748278	107748625
chr11	107748868	107749112
chr11	107749114	107749406
chr11	107752562	107752807
chr11	107760776	107760938
chr11	107784514	107784748
chr11	107784748	107784967
chr11	107785025	107785215
chr11	107786003	107786186
chr11	107793186	107793435
chr11	107793435	107793712
chr11	107794605	107794876
chr11	107852403	107852589
chr11	107859353	107859586
chr11	107860081	107860253
chr11	107864116	107864282
chr11	107865211	107865460
chr11	107900558	107900780
chr11	107914909	107915133
chr11	107920211	107920410
chr11	107982625	107982782
chr11	107985949	107986281
chr11	107991868	107992362
chr11	107994597	107994810
chr11	107997460	107997610
chr11	108009853	108010109
chr11	108010109	108010481
chr11	108010615	108010766
chr11	108011167	108011646
chr11	108019246	108019573
chr11	108020053	108020578
chr11	108025677	108026122
chr11	108026122	108026465
chr11	108026465	108026772
chr11	108031028	108031264
chr11	108031264	108031637
chr11	108064515	108064773

chr11	108072474	108073176
chr11	108098380	108098620
chr11	108101408	108102064
chr11	108103258	108103408
chr11	108114764	108115031
chr11	108119007	108119193
chr11	108120331	108120769
chr11	108120769	108121190
chr11	108128625	108129054
chr11	108135487	108135701
chr11	108135771	108136072
chr11	108136947	108137223
chr11	108137274	108137594
chr11	108140124	108140388
chr11	108160192	108160418
chr11	108178314	108178582
chr11	108197859	108198102
chr11	108214914	108215256
chr11	108215423	108215890
chr11	108216911	108217131
chr11	108217131	108217465
chr11	108225283	108225640
chr11	108247270	108247506
chr11	108248115	108248368
chr11	108258775	108258990
chr11	108259401	108259667
chr11	108259667	108259980
chr11	108259980	108260344
chr11	108267741	108268196
chr11	108268196	108268651
chr11	108268735	108268960
chr11	108268960	108269219
chr11	108273965	108274142
chr11	108279936	108280305
chr11	108282787	108283124
chr11	108283124	108283396
chr11	108283396	108283719
chr11	108306768	108307190
chr11	108307190	108307785
chr11	108318069	108318303
chr11	108342891	108343295
chr11	108343295	108343654

chr11	108343654	108343998
chr11	108343998	108344495
chr11	108345425	108345625
chr11	108357398	108357579
chr11	108364871	108365271
chr11	108365271	108365638
chr11	108365673	108365950
chr11	108374997	108375346
chr11	108378051	108378438
chr11	108378454	108378637
chr11	108403102	108403280
chr11	108418631	108418865
chr11	108424711	108424979
chr11	108424979	108425318
chr11	108425318	108425586
chr11	108425586	108425793
chr11	108445215	108445682
chr11	108449856	108450013
chr11	108459497	108460183
chr11	108484779	108485061
chr11	108491633	108491832
chr11	108525672	108525862
chr11	108525867	108526018
chr11	108526574	108526739
chr11	108526739	108526900
chr11	108752153	108752686
chr11	108753575	108753866
chr11	108757814	108758092
chr11	108758092	108758384
chr11	108920053	108920559
chr11	108920559	108920923
chr11	108921102	108921310
chr11	108925259	108925546
chr11	108980478	108980693
chr11	108980693	108980973
chr11	108981131	108981290
chr11	109011572	109011818
chr11	109011818	109012185
chr11	109030536	109030761
chr11	109114017	109114167
chr11	109149627	109149784
chr11	109177604	109178156

chr11	109193275	109193513
chr11	109220941	109221215
chr11	109241010	109241193
chr11	109244611	109245004
chr11	109245332	109245517
chr11	109297808	109298048
chr11	109298048	109298408
chr11	109312199	109312459
chr11	109316208	109316540
chr11	109321841	109322059
chr11	109331128	109331363
chr11	109331363	109331626
chr11	109331626	109331879
chr11	109332706	109333078
chr11	109341421	109341602
chr11	109343933	109344323
chr11	109344323	109344653
chr11	109347734	109347932
chr11	109349693	109349860
chr11	109350017	109350260
chr11	109350346	109350646
chr11	109361650	109362143
chr11	109362297	109362756
chr11	109362756	109363074
chr11	109363074	109363374
chr11	109363374	109363615
chr11	109368946	109369395
chr11	109382469	109383073
chr11	109417020	109417236
chr11	109417417	109417922
chr11	109425365	109425901
chr11	109425901	109426503
chr11	109431566	109431716
chr11	109434289	109434639
chr11	109439252	109439577
chr11	109439784	109440404
chr11	109441245	109441471
chr11	109444713	109444907
chr11	109445670	109446132
chr11	109472220	109472449
chr11	109472937	109473488
chr11	109473488	109473836

chr11	109473836	109474119
chr11	109474119	109474372
chr11	109487771	109487942
chr11	109487990	109488367
chr11	109488367	109488627
chr11	109494780	109495290
chr11	109496280	109496495
chr11	109502574	109502828
chr11	109507637	109507848
chr11	109514152	109514526
chr11	109554479	109554829
chr11	109560734	109561022
chr11	109561954	109562104
chr11	109584983	109585417
chr11	109585453	109585604
chr11	109598873	109599023
chr11	109610257	109610474
chr11	109610788	109611059
chr11	109611059	109611277
chr11	109611277	109611517
chr11	109611517	109611754
chr11	109611754	109612157
chr11	109619103	109619401
chr11	109621113	109621544
chr11	109622622	109622772
chr11	109635868	109636213
chr11	109637178	109637477
chr11	109642122	109642314
chr11	109650262	109650637
chr11	109650748	109651050
chr11	109651050	109651496
chr11	109651679	109651845
chr11	109656829	109657151
chr11	109670431	109670715
chr11	109670715	109670997
chr11	109671755	109672017
chr11	109672017	109672317
chr11	109681608	109681905
chr11	109697202	109697360
chr11	109698824	109699042
chr11	109721118	109721435
chr11	109721435	109721861

chr11	109721861	109722207
chr11	109722207	109722468
chr11	109816959	109817218
chr11	109852641	109852872
chr11	109860193	109860564
chr11	109860564	109860940
chr11	109863409	109863633
chr11	109991090	109991292
chr11	109991379	109991585
chr11	109995068	109995400
chr11	109995684	109995992
chr11	110029014	110029265
chr11	110029265	110029648
chr11	110053740	110053980
chr11	110061197	110061382
chr11	110062012	110062391
chr11	110171313	110171513
chr11	110268372	110268568
chr11	110268568	110268830
chr11	110271819	110272056
chr11	110288501	110288723
chr11	110290751	110290906
chr11	110306423	110306825
chr11	110306825	110307227
chr11	110307707	110308088
chr11	110336973	110337346
chr11	110337346	110337533
chr11	110337533	110337723
chr11	110337723	110338168
chr11	110357824	110358198
chr11	110358198	110358654
chr11	110365127	110365315
chr11	110379059	110379363
chr11	110379591	110379929
chr11	110379929	110380328
chr11	110394890	110395045
chr11	110395149	110395323
chr11	110398787	110399237
chr11	110402764	110402972
chr11	110405399	110405688
chr11	110405688	110406048
chr11	110413780	110414243

chr11	110414243	110414676
chr11	110414821	110415021
chr11	110422204	110422389
chr11	110438906	110439231
chr11	110439317	110439725
chr11	110448803	110449020
chr11	110450109	110450815
chr11	110451572	110451853
chr11	110466820	110467135
chr11	110469097	110469356
chr11	110471833	110472046
chr11	110472064	110472475
chr11	110478985	110479413
chr11	110479621	110479771
chr11	110483260	110483520
chr11	110485084	110485481
chr11	110485930	110486122
chr11	110496084	110496458
chr11	110498676	110498945
chr11	110498982	110499132
chr11	110511324	110511980
chr11	110537587	110537864
chr11	110537867	110538027
chr11	110541531	110541681
chr11	110544516	110544883
chr11	110633431	110633591
chr11	110698021	110698409
chr11	110698409	110698636
chr11	110698636	110698852
chr11	110698918	110699161
chr11	110781935	110782236
chr11	111065620	111066261
chr11	111515542	111515861
chr11	111536190	111536341
chr11	111688422	111688842
chr11	111702477	111702825
chr11	111763676	111763835
chr11	111764081	111764232
chr11	111832323	111832617
chr11	111856672	111856943
chr11	111858139	111858425
chr11	111880108	111880455

chr11	111919565	111919715
chr11	111919717	111920112
chr11	111971212	111971410
chr11	112003141	112003331
chr11	112003331	112003539
chr11	112005280	112005490
chr11	112029047	112029254
chr11	112140833	112141010
chr11	112142830	112143039
chr11	112153267	112153563
chr11	112154848	112155474
chr11	112155561	112155854
chr11	112156611	112156969
chr11	112163861	112164048
chr11	112173293	112173595
chr11	112173595	112174087
chr11	112232405	112232688
chr11	112253005	112253418
chr11	112303088	112303430
chr11	112339533	112339700
chr11	112406279	112406808
chr11	112406808	112407245
chr11	112442716	112442918
chr11	112460198	112460391
chr11	112470464	112470739
chr11	112548737	112548887
chr11	112588150	112588343
chr11	112605081	112605258
chr11	112615509	112616099
chr11	112621181	112621350
chr11	112621487	112621759
chr11	112622003	112622198
chr11	112623607	112623829
chr11	112640424	112640596
chr11	112660024	112660238
chr11	112661499	112661671
chr11	112661671	112661859
chr11	112686129	112686298
chr11	112730467	112731207
chr11	112737187	112737416
chr11	112755261	112755518
chr11	112757995	112758462

chr11	112758566	112758768
chr11	112767163	112767317
chr11	112781404	112781611
chr11	112781611	112781928
chr11	112781928	112782300
chr11	112782300	112782689
chr11	112782689	112783142
chr11	112787979	112788277
chr11	112788352	112788687
chr11	112794803	112795034
chr11	112800768	112801042
chr11	112806483	112806706
chr11	112811563	112811903
chr11	112824175	112824503
chr11	112847230	112847447
chr11	112850562	112850881
chr11	112856477	112856868
chr11	112856868	112857167
chr11	112891656	112891920
chr11	112897612	112897983
chr11	112921669	112922029
chr11	112947153	112947433
chr11	112974933	112975157
chr11	112988372	112988641
chr11	112996887	112997217
chr11	113000468	113000620
chr11	113000853	113001613
chr11	113008511	113009047
chr11	113009232	113009637
chr11	113010848	113011318
chr11	113015876	113016241
chr11	113016273	113016423
chr11	113019161	113019459
chr11	113026819	113027310
chr11	113027310	113027554
chr11	113032177	113033012
chr11	113039027	113039213
chr11	113042792	113042956
chr11	113045590	113045857
chr11	113050765	113051070
chr11	113059976	113060172
chr11	113060797	113061017

chr11	113061017	113061532
chr11	113062858	113063439
chr11	113063439	113063768
chr11	113074175	113074970
chr11	113080256	113080464
chr11	113080972	113081174
chr11	113087155	113087351
chr11	113087351	113087524
chr11	113093918	113094181
chr11	113094181	113094382
chr11	113100916	113101080
chr11	113101080	113101259
chr11	113101533	113101982
chr11	113102590	113103073
chr11	113103073	113103512
chr11	113103512	113103902
chr11	113109223	113109451
chr11	113113022	113113287
chr11	113113287	113113707
chr11	113114545	113114744
chr11	113121483	113121641
chr11	113131191	113131400
chr11	113132146	113132328
chr11	113132475	113132697
chr11	113135297	113135609
chr11	113143176	113143355
chr11	113143848	113144124
chr11	113146586	113146846
chr11	113146846	113147332
chr11	113153416	113153756
chr11	113155440	113155969
chr11	113157383	113157552
chr11	113162468	113162642
chr11	113164988	113165420
chr11	113165420	113165798
chr11	113172491	113172719
chr11	113172880	113173095
chr11	113173976	113174172
chr11	113174172	113174280
chr11	113175439	113175773
chr11	113176233	113176465
chr11	113179898	113180569

chr11	113182550	113182742
chr11	113184284	113184434
chr11	113191776	113191933
chr11	113192014	113192427
chr11	113192564	113192848
chr11	113198254	113198553
chr11	113198559	113198904
chr11	113198904	113199128
chr11	113200879	113201036
chr11	113201449	113201703
chr11	113201703	113202037
chr11	113208351	113208801
chr11	113208801	113209186
chr11	113209186	113209525
chr11	113209525	113209750
chr11	113214791	113215365
chr11	113220665	113221000
chr11	113232523	113232673
chr11	113246459	113246903
chr11	113246903	113247144
chr11	113247630	113247835
chr11	113254959	113255199
chr11	113258165	113258329
chr11	113265326	113265559
chr11	113265605	113265826
chr11	113265826	113266060
chr11	113268848	113269322
chr11	113283212	113283606
chr11	113292017	113292408
chr11	113292408	113292717
chr11	113296241	113296465
chr11	113297369	113297562
chr11	113297656	113297806
chr11	113297834	113298115
chr11	113303328	113303551
chr11	113305760	113305915
chr11	113319669	113319821
chr11	113319860	113320151
chr11	113321726	113321939
chr11	113321939	113322118
chr11	113322177	113322465
chr11	113326016	113326167

chr11	113326406	113326684
chr11	113329830	113330024
chr11	113334461	113334635
chr11	113352122	113352821
chr11	113353968	113354451
chr11	113383898	113384190
chr11	113458695	113458876
chr11	113469558	113470000
chr11	113470000	113470461
chr11	113478262	113478478
chr11	113487333	113487956
chr11	113487956	113488262
chr11	113498965	113499203
chr11	113507385	113507565
chr11	113508129	113508552
chr11	113508795	113509045
chr11	113510420	113510572
chr11	113516283	113516489
chr11	113527500	113527756
chr11	113534383	113534780
chr11	113536378	113536825
chr11	113536825	113537208
chr11	113537208	113537418
chr11	113537730	113538146
chr11	113543779	113544076
chr11	113554774	113554992
chr11	113565241	113565571
chr11	113565571	113566017
chr11	113602774	113602972
chr11	113609705	113609968
chr11	113612056	113612575
chr11	113617700	113617855
chr11	113618648	113618829
chr11	113618829	113619102
chr11	113619102	113619780
chr11	113630754	113630918
chr11	113646727	113646877
chr11	113648025	113648183
chr11	113648244	113648505
chr11	113649218	113649663
chr11	113649663	113649879
chr11	113649879	113650188

chr11	113651371	113651525
chr11	113651927	113652104
chr11	113658835	113659193
chr11	113660287	113660489
chr11	113666757	113667075
chr11	113667142	113667671
chr11	113667788	113667939
chr11	113674033	113674268
chr11	113683162	113683576
chr11	113683576	113683926
chr11	113683926	113684264
chr11	113684351	113684552
chr11	113684552	113684882
chr11	113691393	113691543
chr11	113703587	113703793
chr11	113703793	113704254
chr11	113708655	113708862
chr11	113713969	113714249
chr11	113714298	113714627
chr11	113715156	113715348
chr11	113715361	113715574
chr11	113716213	113716597
chr11	113729913	113730375
chr11	113732371	113732538
chr11	113733415	113733567
chr11	113733578	113733997
chr11	113733997	113734434
chr11	113735190	113735515
chr11	113739222	113739521
chr11	113749844	113749998
chr11	113750590	113750978
chr11	113750978	113751220
chr11	113751220	113751598
chr11	113751598	113752130
chr11	113756784	113756969
chr11	113758976	113759193
chr11	113766366	113766921
chr11	113803570	113803862
chr11	113809746	113809896
chr11	113811088	113811242
chr11	113811267	113811536
chr11	113815737	113816023

chr11	113816973	113817448
chr11	113826190	113826352
chr11	113828374	113828529
chr11	113835119	113835473
chr11	113865645	113866060
chr11	113927416	113927588
chr11	113939215	113939370
chr11	114038108	114038367
chr11	114198401	114198553
chr11	114387192	114387400
chr11	114412118	114412383
chr11	114412383	114412602
chr11	114412602	114412825
chr11	114446801	114446958
chr11	114478853	114479053
chr11	114502228	114502496
chr11	114502839	114503007
chr11	114523555	114523710
chr11	114636231	114636652
chr11	114636915	114637169
chr11	114649091	114649290
chr11	114654676	114654917
chr11	114657814	114658253
chr11	114663066	114663319
chr11	114667974	114668169
chr11	114668331	114668704
chr11	114668704	114668948
chr11	114668948	114669268
chr11	114673283	114673567
chr11	114675164	114675811
chr11	114676146	114676326
chr11	114687307	114687578
chr11	114687578	114687857
chr11	114687857	114688286
chr11	114694425	114694818
chr11	114722977	114723387
chr11	114724986	114725188
chr11	114725188	114725571
chr11	114727242	114727572
chr11	114727861	114728060
chr11	114729941	114730181
chr11	114736897	114737117

chr11	114745143	114745448
chr11	114747825	114748008
chr11	114758528	114759142
chr11	114764810	114765156
chr11	114765156	114765612
chr11	114773368	114773611
chr11	114783371	114783584
chr11	114786263	114786473
chr11	114786473	114786676
chr11	114789075	114789423
chr11	114793863	114794231
chr11	114795287	114795510
chr11	114795561	114795756
chr11	114802965	114803188
chr11	114807611	114808195
chr11	114812694	114812941
chr11	114812958	114813126
chr11	114829531	114829806
chr11	114829818	114830098
chr11	114830098	114830454
chr11	114850346	114850964
chr11	114851010	114851225
chr11	114851479	114851801
chr11	114851801	114852112
chr11	114852112	114852378
chr11	114858720	114858943
chr11	114875279	114875531
chr11	114876192	114876497
chr11	114876787	114877245
chr11	114877245	114877631
chr11	114882970	114883120
chr11	114883455	114883605
chr11	114884030	114884355
chr11	114884385	114884668
chr11	114885163	114885341
chr11	114903404	114903596
chr11	114907669	114907967
chr11	114912589	114912872
chr11	114912872	114913120
chr11	114929431	114929670
chr11	114929670	114929998
chr11	114933662	114933871

chr11	114934020	114934254
chr11	114934254	114934620
chr11	114956899	114957671
chr11	114962911	114963303
chr11	114965228	114965809
chr11	114969286	114969473
chr11	114981901	114982201
chr11	114986000	114986215
chr11	114996484	114996715
chr11	114997593	114998021
chr11	114998128	114998341
chr11	115023227	115023552
chr11	115033985	115034329
chr11	115044635	115044955
chr11	115052276	115052529
chr11	115071671	115072031
chr11	115072031	115072457
chr11	115078642	115078808
chr11	115080940	115081198
chr11	115083369	115083621
chr11	115083621	115083815
chr11	115083815	115084064
chr11	115087163	115087330
chr11	115091593	115091902
chr11	115097539	115097693
chr11	115103629	115103779
chr11	115107626	115107815
chr11	115152160	115152316
chr11	115153632	115153962
chr11	115153962	115154403
chr11	115159691	115160211
chr11	115162988	115163523
chr11	115163658	115163883
chr11	115164522	115164802
chr11	115164948	115165337
chr11	115172556	115172816
chr11	115172816	115173112
chr11	115173116	115173383
chr11	115173497	115173914
chr11	115173914	115174347
chr11	115179669	115179969
chr11	115182383	115182644

chr11	115185012	115185283
chr11	115187050	115187699
chr11	115187725	115187958
chr11	115202218	115202622
chr11	115202622	115202831
chr11	115218373	115218639
chr11	115219050	115219893
chr11	115222649	115222805
chr11	115224980	115225146
chr11	115234820	115235209
chr11	115249736	115249934
chr11	115249934	115250158
chr11	115250158	115250386
chr11	115250452	115250713
chr11	115255942	115256109
chr11	115258025	115258467
chr11	115265339	115265590
chr11	115266983	115267325
chr11	115270192	115270368
chr11	115270368	115270574
chr11	115270574	115270856
chr11	115276626	115277086
chr11	115277086	115277456
chr11	115277500	115277660
chr11	115279721	115280095
chr11	115283554	115283804
chr11	115287417	115287772
chr11	115297188	115297528
chr11	115297528	115297844
chr11	115303230	115303486
chr11	115309230	115309439
chr11	115314308	115314564
chr11	115319797	115319959
chr11	115319969	115320309
chr11	115321964	115322175
chr11	115322349	115322653
chr11	115326788	115327163
chr11	115327163	115327479
chr11	115334988	115335138
chr11	115340850	115341047
chr11	115341875	115342232
chr11	115345053	115345287

chr11	115354232	115354652
chr11	115358533	115359050
chr11	115366787	115367309
chr11	115367309	115367616
chr11	115367616	115367906
chr11	115373868	115374145
chr11	115374145	115374774
chr11	115376741	115377038
chr11	115381682	115382093
chr11	115382093	115382594
chr11	115393791	115393982
chr11	115403402	115403881
chr11	115403881	115404221
chr11	115412336	115412487
chr11	115412625	115413092
chr11	115413092	115413553
chr11	115413553	115414042
chr11	115419570	115419867
chr11	115419867	115420257
chr11	115420257	115420660
chr11	115427899	115428333
chr11	115435467	115435706
chr11	115435723	115435909
chr11	115437609	115437851
chr11	115439495	115439687
chr11	115444837	115445129
chr11	115445180	115445330
chr11	115451755	115451948
chr11	115461776	115461936
chr11	115461995	115462158
chr11	115462161	115462667
chr11	115462856	115463011
chr11	115463346	115463738
chr11	115463738	115464065
chr11	115473996	115474265
chr11	115475335	115475772
chr11	115475772	115475995
chr11	115475995	115476177
chr11	115483809	115484044
chr11	115491107	115491262
chr11	115491275	115491530
chr11	115491530	115491763

chr11	115491763	115492062
chr11	115492068	115492234
chr11	115496184	115496339
chr11	115509993	115510178
chr11	115513288	115513541
chr11	115513541	115513901
chr11	115513901	115514313
chr11	115514313	115514701
chr11	115535356	115535694
chr11	115535694	115536048
chr11	115536048	115536637
chr11	115552878	115553099
chr11	115563444	115563667
chr11	115564005	115564320
chr11	115564320	115564571
chr11	115564571	115564763
chr11	115564763	115564966
chr11	115564998	115565181
chr11	115577736	115577925
chr11	115578035	115578197
chr11	115581091	115581289
chr11	115584802	115585008
chr11	115590669	115590861
chr11	115590900	115591106
chr11	115603373	115604015
chr11	115604015	115604285
chr11	115604285	115604485
chr11	115604485	115604703
chr11	115611975	115612408
chr11	115612408	115612802
chr11	115612802	115613080
chr11	115613080	115613296
chr11	115613420	115613608
chr11	115620548	115620745
chr11	115627627	115627994
chr11	115627994	115628507
chr11	115631117	115631318
chr11	115640658	115640819
chr11	115640870	115641210
chr11	115641210	115641475
chr11	115641475	115641780
chr11	115641792	115642104

chr11	115647512	115647755
chr11	115653395	115653590
chr11	115660356	115660763
chr11	115661354	115661509
chr11	115673759	115673956
chr11	115673956	115674187
chr11	115677658	115677815
chr11	115684975	115685166
chr11	115696503	115696984
chr11	115697825	115698017
chr11	115699266	115699706
chr11	115707855	115708328
chr11	115708328	115708905
chr11	115708952	115709114
chr11	115709165	115709361
chr11	115714484	115714882
chr11	115716890	115717041
chr11	115718113	115718334
chr11	115722621	115723093
chr11	115741003	115741452
chr11	115746216	115746457
chr11	115746620	115747072
chr11	115747484	115747860
chr11	115753361	115753590
chr11	115753590	115753873
chr11	115765154	115765548
chr11	115765548	115765856
chr11	115765856	115766156
chr11	115766256	115766451
chr11	115775933	115776235
chr11	115776464	115776826
chr11	115779164	115779614
chr11	115780134	115780411
chr11	115799487	115799670
chr11	115813511	115813921
chr11	115813921	115814274
chr11	115814519	115814821
chr11	115814821	115815171
chr11	115823715	115824109
chr11	115824109	115824410
chr11	115824410	115824640
chr11	115824891	115825046

chr11	115827411	115827665
chr11	115827665	115828020
chr11	115828058	115828209
chr11	115830990	115831389
chr11	115838637	115839275
chr11	115859207	115859397
chr11	115859397	115859629
chr11	115859629	115859964
chr11	115869037	115869238
chr11	115869300	115869834
chr11	115872405	115872831
chr11	115873378	115873648
chr11	115878222	115878596
chr11	115881038	115881357
chr11	115886336	115886486
chr11	115888305	115888630
chr11	115889374	115889524
chr11	115894800	115895083
chr11	115898903	115899155
chr11	115899915	115900227
chr11	115904097	115904400
chr11	115904639	115905330
chr11	115905353	115905667
chr11	115911238	115911655
chr11	115911655	115912084
chr11	115912084	115912410
chr11	115912410	115912754
chr11	115920005	115920155
chr11	115920190	115920430
chr11	115920455	115920769
chr11	115933005	115933194
chr11	115933202	115933703
chr11	115933703	115933901
chr11	115933901	115934089
chr11	115939742	115940061
chr11	115940197	115940364
chr11	115942060	115942319
chr11	115945206	115945366
chr11	115945368	115945592
chr11	115948322	115948652
chr11	115954680	115955096
chr11	115955104	115955409

chr11	115955409	115955916
chr11	115956517	115956779
chr11	115957946	115958692
chr11	115964671	115964961
chr11	115964961	115965391
chr11	115970368	115970640
chr11	115971002	115971403
chr11	115974394	115974725
chr11	115974725	115975131
chr11	115975131	115975380
chr11	115975577	115975885
chr11	115976491	115976716
chr11	115986613	115986799
chr11	115987110	115987276
chr11	115993009	115993310
chr11	115998407	115998632
chr11	115999232	115999403
chr11	115999559	115999866
chr11	115999883	116000052
chr11	116000299	116000531
chr11	116005559	116005812
chr11	116006262	116006479
chr11	116006481	116006645
chr11	116012144	116012561
chr11	116012561	116012876
chr11	116012876	116013148
chr11	116018137	116018548
chr11	116020148	116020311
chr11	116023744	116024059
chr11	116024059	116024423
chr11	116024423	116024871
chr11	116029999	116030396
chr11	116030396	116030838
chr11	116030907	116031143
chr11	116042434	116042713
chr11	116042850	116043027
chr11	116043375	116043598
chr11	116044365	116044575
chr11	116049679	116050015
chr11	116050015	116050318
chr11	116050768	116050923
chr11	116057274	116057458

chr11	116058068	116058275
chr11	116059295	116059678
chr11	116059799	116059986
chr11	116060697	116060962
chr11	116070284	116070560
chr11	116071481	116071832
chr11	116072581	116072953
chr11	116077427	116077825
chr11	116077825	116078266
chr11	116078266	116078631
chr11	116079559	116079851
chr11	116085841	116086023
chr11	116086531	116086877
chr11	116086877	116087318
chr11	116087461	116087914
chr11	116096841	116097063
chr11	116098653	116099014
chr11	116103599	116103884
chr11	116103976	116104590
chr11	116108520	116108670
chr11	116108707	116109124
chr11	116109124	116109538
chr11	116109538	116109982
chr11	116110080	116110434
chr11	116113921	116114245
chr11	116121860	116122238
chr11	116123941	116124390
chr11	116130159	116130464
chr11	116130464	116130871
chr11	116130871	116131295
chr11	116131319	116131477
chr11	116132334	116132546
chr11	116132546	116132819
chr11	116132819	116133067
chr11	116133067	116133278
chr11	116138465	116138784
chr11	116138784	116139192
chr11	116142598	116143007
chr11	116145294	116145646
chr11	116145646	116146010
chr11	116146202	116146703
chr11	116154125	116154524

chr11	116162349	116162518
chr11	116167318	116167505
chr11	116167838	116168099
chr11	116168099	116168458
chr11	116174790	116175133
chr11	116176046	116176393
chr11	116185920	116186107
chr11	116198563	116198951
chr11	116198951	116199257
chr11	116199257	116199523
chr11	116216581	116217045
chr11	116217927	116218527
chr11	116218527	116219253
chr11	116219981	116220219
chr11	116226480	116226788
chr11	116228604	116229364
chr11	116237063	116237236
chr11	116237764	116238029
chr11	116238029	116238595
chr11	116247630	116247800
chr11	116251372	116251601
chr11	116251601	116251817
chr11	116254628	116255017
chr11	116255017	116255384
chr11	116256061	116256349
chr11	116256349	116256642
chr11	116260341	116260761
chr11	116273013	116273216
chr11	116273542	116273779
chr11	116273779	116274023
chr11	116274023	116274361
chr11	116274361	116274723
chr11	116281086	116281321
chr11	116281321	116281559
chr11	116281559	116281934
chr11	116282183	116282408
chr11	116282957	116283333
chr11	116286749	116287015
chr11	116287015	116287317
chr11	116294294	116294709
chr11	116294753	116294904
chr11	116306081	116306568

chr11	116306568	116307031
chr11	116307888	116308126
chr11	116318848	116319044
chr11	116319044	116319368
chr11	116319368	116319773
chr11	116325423	116325625
chr11	116327300	116327555
chr11	116332565	116332896
chr11	116333419	116333998
chr11	116333998	116334332
chr11	116335302	116335676
chr11	116335676	116336012
chr11	116344625	116344848
chr11	116346705	116346884
chr11	116347706	116347924
chr11	116347926	116348239
chr11	116351356	116351614
chr11	116368139	116368468
chr11	116372004	116372204
chr11	116377425	116377707
chr11	116387713	116388065
chr11	116388771	116388962
chr11	116388962	116389222
chr11	116389222	116389651
chr11	116390519	116390782
chr11	116390782	116391038
chr11	116394391	116394558
chr11	116394855	116395110
chr11	116412274	116412561
chr11	116412561	116412881
chr11	116412881	116413234
chr11	116413234	116413686
chr11	116416231	116416412
chr11	116420490	116420746
chr11	116421190	116421625
chr11	116426879	116427046
chr11	116431822	116432018
chr11	116433639	116434420
chr11	116434610	116434846
chr11	116439646	116439989
chr11	116440377	116440626
chr11	116440760	116440929

chr11	116445727	116446063
chr11	116446063	116446301
chr11	116446301	116446586
chr11	116455817	116456028
chr11	116456028	116456137
chr11	116456245	116456928
chr11	116457018	116457184
chr11	116457328	116457718
chr11	116457756	116458005
chr11	116463610	116463895
chr11	116463895	116464238
chr11	116471070	116471223
chr11	116489645	116490124
chr11	116490124	116490511
chr11	116490517	116490758
chr11	116490758	116490962
chr11	116493900	116494138
chr11	116500590	116500751
chr11	116500751	116500996
chr11	116500996	116501323
chr11	116501323	116501595
chr11	116513066	116513418
chr11	116513622	116513890
chr11	116515755	116515988
chr11	116518498	116518850
chr11	116521912	116522139
chr11	116522139	116522451
chr11	116522463	116522649
chr11	116522796	116523153
chr11	116523399	116524189
chr11	116531784	116531939
chr11	116532294	116532514
chr11	116532514	116532744
chr11	116532802	116533367
chr11	116533367	116533612
chr11	116533612	116533826
chr11	116533826	116534186
chr11	116534186	116534569
chr11	116541185	116541524
chr11	116543195	116543494
chr11	116543880	116544107
chr11	116549206	116549468

chr11	116554543	116554810
chr11	116555741	116556015
chr11	116565342	116565580
chr11	116571707	116571857
chr11	116571900	116572118
chr11	116572118	116572337
chr11	116572710	116572867
chr11	116573225	116573454
chr11	116575226	116575461
chr11	116575461	116575702
chr11	116580048	116580338
chr11	116580747	116581236
chr11	116581236	116581782
chr11	116586818	116587121
chr11	116587121	116587448
chr11	116592066	116592305
chr11	116592430	116592795
chr11	116603363	116603662
chr11	116605487	116605701
chr11	116615662	116616112
chr11	116616112	116616618
chr11	116616618	116617038
chr11	116617196	116617346
chr11	116621180	116621741
chr11	116623361	116623548
chr11	116623904	116624141
chr11	116624141	116624472
chr11	116625731	116625901
chr11	116628884	116629140
chr11	116632330	116632607
chr11	116632996	116633275
chr11	116634319	116634651
chr11	116635719	116635911
chr11	116643280	116643627
chr11	116646184	116646495
chr11	116653671	116653875
chr11	116653875	116654068
chr11	116657636	116657895
chr11	116657895	116658221
chr11	116671077	116671389
chr11	116671389	116671716
chr11	116671716	116671981

chr11	116672692	116672890
chr11	116677644	116678204
chr11	116681964	116682407
chr11	116682423	116682628
chr11	116682717	116683081
chr11	116683276	116683511
chr11	116683822	116684064
chr11	116694288	116694558
chr11	116694558	116695075
chr11	116695231	116695440
chr11	116701304	116701618
chr11	116701618	116701981
chr11	116712676	116712873
chr11	116745706	116746070
chr11	116746070	116746288
chr11	116751110	116751260
chr11	116754146	116754412
chr11	116755215	116755503
chr11	116782690	116783019
chr11	116807232	116807566
chr11	116808135	116808529
chr11	116808529	116808747
chr11	116811337	116811529
chr11	116816824	116817198
chr11	116818812	116819091
chr11	116821138	116821579
chr11	116826574	116826872
chr11	116827494	116827842
chr11	116827842	116828213
chr11	116835451	116835603
chr11	116836366	116836595
chr11	116836595	116836788
chr11	116837537	116837699
chr11	116842779	116843198
chr11	116843198	116843999
chr11	116852310	116852769
chr11	116852769	116853196
chr11	116853196	116853546
chr11	116853546	116853907
chr11	116853933	116854089
chr11	116862784	116862977
chr11	116869191	116869613

chr11	116869659	116869947
chr11	116878517	116878810
chr11	116885262	116885700
chr11	116885700	116886073
chr11	116893745	116893964
chr11	116893964	116894302
chr11	116902553	116902947
chr11	116905830	116906064
chr11	116906121	116906355
chr11	116906401	116906592
chr11	116913094	116913407
chr11	116918633	116918975
chr11	116922097	116922661
chr11	116940519	116940916
chr11	116940916	116941251
chr11	116949752	116950149
chr11	116958727	116958899
chr11	116968189	116968366
chr11	116968484	116968652
chr11	116973565	116973736
chr11	116974579	116974851
chr11	116982616	116982767
chr11	116986044	116986216
chr11	116987039	116987260
chr11	117004398	117004565
chr11	117018336	117018838
chr11	117031418	117031621
chr11	117038303	117038593
chr11	117043402	117043565
chr11	117043573	117043855
chr11	117043871	117044073
chr11	117052856	117053170
chr11	117056802	117057192
chr11	117057192	117057380
chr11	117057542	117057804
chr11	117062797	117063201
chr11	117072162	117072633
chr11	117075116	117075306
chr11	117076481	117076885
chr11	117076885	117077071
chr11	117077131	117077292
chr11	117080417	117080610

chr11	117080987	117081137
chr11	117081193	117081599
chr11	117081643	117082137
chr11	117082465	117082710
chr11	117089954	117090490
chr11	117091489	117091794
chr11	117091794	117092161
chr11	117094253	117094477
chr11	117098104	117098443
chr11	117098623	117098891
chr11	117098891	117099151
chr11	117099380	117099685
chr11	117100277	117100483
chr11	117103645	117103822
chr11	117104034	117104323
chr11	117105017	117105241
chr11	117108681	117108905
chr11	117109240	117109501
chr11	117114105	117114492
chr11	117114492	117114828
chr11	117114828	117115093
chr11	117115093	117115410
chr11	117115410	117115731
chr11	117115897	117116084
chr11	117122880	117123256
chr11	117123897	117124215
chr11	117134549	117134765
chr11	117141726	117141983
chr11	117145882	117146035
chr11	117153933	117154464
chr11	117154464	117154690
chr11	117159353	117159565
chr11	117160068	117160228
chr11	117163004	117163289
chr11	117170255	117170537
chr11	117176247	117176715
chr11	117176897	117177302
chr11	117177302	117177649
chr11	117194479	117194647
chr11	117198125	117198590
chr11	117199302	117199791
chr11	117199791	117200133

chr11	117205004	117205424
chr11	117205424	117205810
chr11	117207845	117208348
chr11	117211896	117212067
chr11	117216791	117217165
chr11	117217241	117217443
chr11	117224889	117225117
chr11	117225117	117225261
chr11	117231471	117231996
chr11	117238207	117238510
chr11	117239647	117239946
chr11	117240982	117241132
chr11	117241413	117241578
chr11	117248731	117249014
chr11	117251771	117252154
chr11	117254736	117255071
chr11	117255878	117256312
chr11	117256312	117256578
chr11	117259531	117259990
chr11	117260659	117260955
chr11	117263123	117263360
chr11	117264615	117264765
chr11	117265235	117265583
chr11	117265718	117265873
chr11	117265991	117266419
chr11	117267510	117267715
chr11	117268759	117268959
chr11	117269399	117269680
chr11	117269908	117270207
chr11	117275892	117276445
chr11	117284641	117284800
chr11	117285043	117285327
chr11	117292427	117292954
chr11	117299755	117300154
chr11	117305513	117305853
chr11	117305853	117306075
chr11	117307965	117308252
chr11	117310847	117311117
chr11	117311117	117311417
chr11	117312734	117313003
chr11	117313070	117313280
chr11	117314027	117314353

chr11	117314982	117315229
chr11	117315229	117315589
chr11	117316635	117316827
chr11	117318830	117319211
chr11	117320155	117320305
chr11	117320580	117320922
chr11	117322842	117323048
chr11	117324201	117324383
chr11	117330291	117330473
chr11	117331209	117331779
chr11	117334176	117334376
chr11	117338307	117338532
chr11	117338532	117338799
chr11	117338799	117339008
chr11	117350651	117350886
chr11	117352952	117353511
chr11	117357577	117357727
chr11	117358262	117358622
chr11	117359210	117359638
chr11	117359960	117360188
chr11	117365463	117365669
chr11	117366520	117366756
chr11	117366934	117367192
chr11	117374403	117374630
chr11	117374641	117374933
chr11	117376650	117376849
chr11	117384729	117384950
chr11	117384950	117385242
chr11	117390007	117390553
chr11	117391153	117391329
chr11	117394490	117394826
chr11	117397810	117398093
chr11	117407700	117407869
chr11	117413340	117413496
chr11	117414412	117414635
chr11	117414894	117415158
chr11	117416031	117416233
chr11	117417332	117417487
chr11	117425558	117425772
chr11	117425898	117426274
chr11	117431054	117431268
chr11	117435897	117436168

chr11	117436757	117437014
chr11	117442449	117442756
chr11	117446774	117447038
chr11	117460066	117460264
chr11	117463595	117463949
chr11	117475716	117475962
chr11	117482523	117483034
chr11	117483034	117483513
chr11	117489463	117489807
chr11	117490405	117490628
chr11	117495228	117495387
chr11	117498922	117499192
chr11	117534962	117535190
chr11	117536554	117536941
chr11	117536941	117537273
chr11	117541691	117541919
chr11	117545117	117545271
chr11	117554554	117554772
chr11	117557435	117557641
chr11	117558086	117558295
chr11	117568312	117568639
chr11	117568639	117569155
chr11	117596245	117596535
chr11	117596981	117597133
chr11	117598136	117598397
chr11	117598638	117598788
chr11	117598878	117599169
chr11	117599543	117599705
chr11	117611068	117611525
chr11	117628409	117628588
chr11	117631242	117631549
chr11	117632805	117632992
chr11	117632992	117633164
chr11	117642317	117642499
chr11	117653967	117654430
chr11	117654430	117654840
chr11	117654840	117655214
chr11	117655214	117655503
chr11	117655503	117655685
chr11	117655969	117656126
chr11	117660958	117661339
chr11	117672517	117672757

chr11	117674618	117674784
chr11	117678861	117679050
chr11	117685390	117685940
chr11	117685949	117686273
chr11	117686273	117686519
chr11	117709430	117709654
chr11	117712072	117712382
chr11	117723415	117723783
chr11	117735994	117736314
chr11	117736314	117736687
chr11	117740103	117740328
chr11	117755256	117755600
chr11	117768751	117769049
chr11	117769049	117769289
chr11	117778928	117779197
chr11	117779552	117779836
chr11	117779997	117780249
chr11	117780249	117780570
chr11	117780570	117780875
chr11	117780875	117781349
chr11	117781349	117781889
chr11	117788464	117788749
chr11	117790309	117790644
chr11	117790644	117791183
chr11	117791232	117791415
chr11	117792187	117792602
chr11	117797988	117798205
chr11	117801260	117801528
chr11	117808699	117808872
chr11	117809539	117809913
chr11	117809913	117810355
chr11	117810355	117810754
chr11	117810754	117811066
chr11	117816413	117816765
chr11	117816765	117817110
chr11	117822704	117823369
chr11	117824119	117824523
chr11	117824523	117824882
chr11	117825418	117825582
chr11	117825723	117826090
chr11	117826232	117826485
chr11	117827508	117827829

chr11	117828229	117828441
chr11	117830478	117830667
chr11	117831645	117831802
chr11	117832110	117832431
chr11	117832431	117832849
chr11	117838547	117838821
chr11	117843136	117843486
chr11	117843486	117843793
chr11	117843793	117844031
chr11	117846679	117846994
chr11	117848579	117848799
chr11	117848799	117849054
chr11	117849054	117849400
chr11	117849400	117849761
chr11	117857610	117858122
chr11	117859067	117859548
chr11	117870714	117871006
chr11	117872987	117873286
chr11	117873286	117873608
chr11	117877963	117878210
chr11	117879676	117880029
chr11	117890584	117890879
chr11	117890879	117891134
chr11	117896714	117896999
chr11	117904942	117905205
chr11	117912711	117913005
chr11	117913005	117913537
chr11	117914559	117914884
chr11	117924525	117925000
chr11	117932079	117932786
chr11	117934034	117934575
chr11	117936295	117936576
chr11	117939730	117940038
chr11	117941381	117941863
chr11	117941863	117942273
chr11	117948939	117949167
chr11	117949321	117949711
chr11	117949711	117950206
chr11	117951163	117951417
chr11	117952238	117952523
chr11	117954262	117954422
chr11	117958032	117958616

chr11	117962388	117962909
chr11	117963157	117963598
chr11	117963598	117963999
chr11	117966479	117966761
chr11	117968797	117969132
chr11	117969132	117969446
chr11	117969446	117969793
chr11	117969793	117970201
chr11	117976098	117976394
chr11	117979415	117979660
chr11	117983803	117983965
chr11	117986693	117986980
chr11	117987043	117987422
chr11	117987843	117988246
chr11	117989904	117990212
chr11	117990212	117990409
chr11	117990618	117990935
chr11	117991661	117992139
chr11	117995312	117995617
chr11	117998388	117998691
chr11	117999649	117999853
chr11	118000571	118000836
chr11	118003006	118003508
chr11	118003792	118004065
chr11	118007386	118007834
chr11	118009965	118010131
chr11	118010314	118010496
chr11	118012421	118013146
chr11	118021476	118021634
chr11	118021634	118021790
chr11	118021851	118022113
chr11	118032971	118033263
chr11	118033263	118033677
chr11	118034027	118034320
chr11	118034375	118034642
chr11	118038317	118038524
chr11	118043207	118043563
chr11	118043911	118044087
chr11	118049645	118049966
chr11	118052126	118052339
chr11	118064528	118064678
chr11	118069787	118069941

chr11	118072685	118072918
chr11	118075714	118075924
chr11	118083872	118084133
chr11	118084202	118084399
chr11	118091071	118091318
chr11	118102807	118103268
chr11	118103952	118104360
chr11	118115999	118116159
chr11	118118905	118119073
chr11	118120728	118120900
chr11	118126958	118127146
chr11	118130015	118130267
chr11	118133694	118134073
chr11	118134751	118134923
chr11	118135402	118135593
chr11	118135694	118136069
chr11	118136069	118136357
chr11	118145695	118146378
chr11	118152805	118153508
chr11	118153624	118153918
chr11	118160580	118161077
chr11	118161077	118161320
chr11	118161320	118161548
chr11	118166671	118166859
chr11	118180294	118180489
chr11	118187398	118187993
chr11	118193282	118193782
chr11	118193782	118194144
chr11	118194340	118194490
chr11	118194894	118195142
chr11	118200275	118200467
chr11	118201327	118201556
chr11	118201556	118201789
chr11	118202629	118202779
chr11	118211853	118212061
chr11	118212620	118213122
chr11	118215857	118216460
chr11	118216566	118216751
chr11	118232285	118232550
chr11	118233710	118233959
chr11	118233959	118234409
chr11	118247457	118247697

chr11	118247697	118247918
chr11	118248125	118248695
chr11	118248695	118249049
chr11	118249049	118249359
chr11	118257877	118258148
chr11	118264341	118264747
chr11	118269927	118270155
chr11	118274443	118274605
chr11	118276295	118276536
chr11	118279390	118279556
chr11	118279556	118279697
chr11	118280281	118280590
chr11	118289034	118289223
chr11	118289653	118289998
chr11	118289998	118290595
chr11	118298818	118299242
chr11	118299242	118299617
chr11	118300723	118300966
chr11	118317148	118317487
chr11	118318697	118318877
chr11	118322099	118322320
chr11	118327115	118327580
chr11	118327580	118328006
chr11	118328556	118328813
chr11	118329412	118329591
chr11	118330057	118330344
chr11	118337579	118337744
chr11	118337852	118338160
chr11	118342450	118342852
chr11	118346629	118347173
chr11	118348870	118349197
chr11	118349197	118349478
chr11	118349478	118349898
chr11	118354849	118355260
chr11	118355260	118355730
chr11	118360524	118360996
chr11	118360996	118361453
chr11	118362956	118363245
chr11	118363245	118363567
chr11	118365140	118365583
chr11	118366197	118366378
chr11	118376391	118376713

chr11	118379209	118379572
chr11	118379572	118379981
chr11	118401696	118401971
chr11	118401971	118402337
chr11	118409537	118410354
chr11	118410456	118410815
chr11	118416061	118416526
chr11	118418495	118418803
chr11	118418803	118419050
chr11	118419050	118419298
chr11	118419342	118419520
chr11	118423353	118423565
chr11	118427386	118427588
chr11	118430247	118430509
chr11	118430509	118430797
chr11	118430797	118431228
chr11	118431228	118431730
chr11	118433309	118433769
chr11	118436449	118436622
chr11	118436622	118436807
chr11	118443229	118443703
chr11	118445913	118446174
chr11	118454198	118454585
chr11	118457148	118457309
chr11	118470193	118470475
chr11	118476662	118477386
chr11	118483390	118483570
chr11	118488105	118488511
chr11	118490889	118491138
chr11	118499890	118500176
chr11	118507923	118508239
chr11	118515716	118515892
chr11	118517978	118518292
chr11	118523744	118524277
chr11	118534737	118535032
chr11	118540193	118541062
chr11	118546320	118546578
chr11	118550892	118551062
chr11	118570636	118570920
chr11	118573619	118573841
chr11	118584832	118585121
chr11	118599382	118599581

chr11	118599581	118599805
chr11	118603185	118603448
chr11	118631647	118631922
chr11	118632570	118632758
chr11	118634738	118634888
chr11	118648423	118648600
chr11	118648600	118648795
chr11	118659385	118659644
chr11	118724339	118724597
chr11	118729430	118729672
chr11	118737917	118738161
chr11	118738406	118738624
chr11	118738781	118738972
chr11	118738972	118739191
chr11	118739518	118739720
chr11	118740027	118740296
chr11	118742905	118743057
chr11	118744905	118745062
chr11	118748783	118749051
chr11	118752373	118752831
chr11	118754589	118754877
chr11	118773021	118773246
chr11	118775682	118775832
chr11	118775844	118776050
chr11	118801443	118801628
chr11	118826727	118826916
chr11	118832843	118833122
chr11	118833122	118833420
chr11	118851360	118851981
chr11	118856412	118856651
chr11	118874846	118875155
chr11	118875188	118875520
chr11	118887230	118887428
chr11	118906710	118906903
chr11	118911400	118911593
chr11	118927212	118927363
chr11	118962507	118962917
chr11	118971551	118971781
chr11	118975499	118975793
chr11	118976034	118976288
chr11	118981063	118981247
chr11	118983959	118984114

chr11	118985576	118985785
chr11	118985785	118985999
chr11	118987394	118987579
chr11	118988382	118988532
chr11	118997290	118997485
chr11	118998526	118998944
chr11	118998944	118999147
chr11	118999634	118999854
chr11	118999854	119000061
chr11	119010256	119010446
chr11	119011752	119012019
chr11	119021164	119021509
chr11	119022677	119023126
chr11	119023126	119023373
chr11	119023373	119023768
chr11	119033997	119034216
chr11	119034216	119034576
chr11	119040551	119040928
chr11	119040928	119041302
chr11	119041302	119041723
chr11	119046979	119047308
chr11	119047308	119047658
chr11	119051984	119052186
chr11	119052628	119052987
chr11	119053200	119053614
chr11	119054583	119054801
chr11	119057969	119058385
chr11	119058397	119058550
chr11	119058662	119058879
chr11	119065741	119065920
chr11	119074303	119074482
chr11	119085491	119086083
chr11	119086083	119086510
chr11	119086510	119086826
chr11	119090936	119091392
chr11	119091425	119091594
chr11	119091604	119091895
chr11	119098362	119098549
chr11	119105245	119105622
chr11	119105777	119105968
chr11	119107529	119108013
chr11	119111450	119111672

chr11	119112768	119113342
chr11	119115479	119115677
chr11	119126197	119126841
chr11	119127128	119127677
chr11	119127754	119128138
chr11	119128138	119128450
chr11	119134021	119134323
chr11	119136305	119136468
chr11	119141885	119142077
chr11	119144744	119145062
chr11	119151856	119152142
chr11	119152142	119152370
chr11	119152370	119152766
chr11	119159514	119159812
chr11	119165181	119165644
chr11	119165809	119165971
chr11	119166051	119166563
chr11	119172516	119172700
chr11	119186395	119186748
chr11	119187000	119187338
chr11	119187607	119187835
chr11	119190141	119190446
chr11	119190446	119190936
chr11	119191776	119192082
chr11	119198983	119199187
chr11	119203499	119203802
chr11	119205873	119206068
chr11	119206594	119206924
chr11	119206930	119207118
chr11	119208068	119208291
chr11	119213197	119213431
chr11	119214984	119215328
chr11	119215328	119215912
chr11	119216053	119216461
chr11	119228043	119228392
chr11	119228392	119228838
chr11	119228852	119229373
chr11	119235296	119235469
chr11	119235656	119235961
chr11	119238071	119238245
chr11	119238397	119238680
chr11	119243913	119244090

chr11	119248717	119249022
chr11	119249022	119249562
chr11	119253382	119253558
chr11	119255667	119255934
chr11	119256605	119256802
chr11	119258536	119258833
chr11	119259033	119259334
chr11	119267580	119268296
chr11	119268352	119268563
chr11	119273257	119273518
chr11	119273690	119273853
chr11	119274211	119274361
chr11	119278539	119278790
chr11	119281099	119281377
chr11	119285947	119286696
chr11	119287147	119287476
chr11	119299608	119299953
chr11	119299953	119300533
chr11	119300648	119300874
chr11	119304976	119305320
chr11	119305320	119305656
chr11	119316546	119317081
chr11	119317081	119317493
chr11	119325158	119325540
chr11	119330326	119331287
chr11	119338603	119339120
chr11	119339120	119339504
chr11	119339504	119339714
chr11	119339746	119340049
chr11	119345003	119345286
chr11	119350534	119351086
chr11	119354895	119355071
chr11	119355243	119355847
chr11	119355869	119356075
chr11	119356142	119356431
chr11	119363384	119363821
chr11	119363821	119364061
chr11	119378607	119379015
chr11	119389736	119390194
chr11	119390194	119390515
chr11	119390515	119390769
chr11	119390859	119391011

chr11	119391879	119392074
chr11	119392074	119392349
chr11	119392968	119393345
chr11	119394263	119394506
chr11	119394842	119395071
chr11	119402389	119402801
chr11	119402801	119403242
chr11	119405750	119405913
chr11	119408052	119408353
chr11	119417360	119417605
chr11	119418292	119418450
chr11	119428628	119428899
chr11	119428899	119429256
chr11	119435980	119436197
chr11	119436377	119436594
chr11	119442267	119442501
chr11	119444689	119444863
chr11	119458847	119459019
chr11	119459251	119459588
chr11	119459588	119459906
chr11	119483379	119483600
chr11	119490950	119491471
chr11	119491471	119491740
chr11	119491740	119491949
chr11	119492932	119493087
chr11	119503040	119503256
chr11	119503516	119503666
chr11	119503717	119504188
chr11	119511799	119511950
chr11	119516897	119517290
chr11	119517290	119517547
chr11	119524508	119524987
chr11	119526655	119526908
chr11	119532394	119532570
chr11	119533889	119534090
chr11	119534358	119534785
chr11	119534785	119535047
chr11	119535627	119535824
chr11	119540112	119540325
chr11	119545481	119545769
chr11	119545973	119546128
chr11	119546482	119546692

chr11	119546834	119547050
chr11	119547166	119547498
chr11	119547498	119547915
chr11	119551429	119551830
chr11	119553186	119553549
chr11	119554844	119555053
chr11	119555143	119555615
chr11	119565775	119566023
chr11	119571802	119571968
chr11	119583252	119583402
chr11	119585399	119585569
chr11	119594087	119594270
chr11	119602784	119603081
chr11	119603081	119603327
chr11	119603327	119603655
chr11	119611560	119611925
chr11	119617677	119617827
chr11	119618740	119619129
chr11	119623112	119623399
chr11	119629515	119629760
chr11	119631560	119631720
chr11	119636191	119636383
chr11	119637617	119637840
chr11	119644249	119644461
chr11	119650978	119651517
chr11	119653311	119653691
chr11	119657491	119657861
chr11	119659317	119659547
chr11	119661520	119661790
chr11	119665430	119665593
chr11	119666401	119666718
chr11	119666718	119667028
chr11	119675597	119675890
chr11	119676448	119677155
chr11	119677155	119677447
chr11	119683936	119684096
chr11	119685090	119685276
chr11	119685535	119685692
chr11	119685958	119686144
chr11	119694626	119694822
chr11	119696029	119696395
chr11	119696395	119696774

chr11	119696876	119697136
chr11	119705431	119705706
chr11	119706998	119707259
chr11	119707259	119707563
chr11	119715802	119716484
chr11	119717941	119718481
chr11	119728293	119728476
chr11	119730581	119730796
chr11	119732336	119732740
chr11	119733741	119733954
chr11	119733954	119734178
chr11	119747251	119747483
chr11	119762194	119762373
chr11	119769755	119770026
chr11	119777109	119777648
chr11	119777831	119778110
chr11	119788757	119788978
chr11	119788978	119789277
chr11	119789285	119789581
chr11	119795892	119796441
chr11	119796441	119796797
chr11	119796797	119797397
chr11	119806316	119806537
chr11	119806572	119806762
chr11	119807716	119808047
chr11	119813519	119813679
chr11	119813930	119814080
chr11	119814107	119814533
chr11	119817404	119817848
chr11	119817848	119818146
chr11	119818168	119818413
chr11	119823842	119824004
chr11	119824220	119824511
chr11	119829446	119829751
chr11	119829883	119830240
chr11	119834532	119834743
chr11	119835925	119836080
chr11	119839490	119839656
chr11	119840995	119841245
chr11	119846657	119846967
chr11	119847154	119847374
chr11	119856065	119856579

chr11	119856618	119857252
chr11	119863111	119863338
chr11	119863338	119863857
chr11	119872851	119873001
chr11	119873451	119874023
chr11	119894667	119895066
chr11	119895066	119895360
chr11	119904651	119904927
chr11	119904927	119905267
chr11	119910281	119910582
chr11	119913252	119913594
chr11	119913594	119913894
chr11	119915646	119916048
chr11	119916110	119916475
chr11	119916475	119916811
chr11	119921532	119921760
chr11	119925249	119925504
chr11	119933152	119933728
chr11	119933728	119934171
chr11	119934171	119934511
chr11	119935519	119935704
chr11	119942146	119942393
chr11	119942393	119942698
chr11	119942698	119942999
chr11	119942999	119943331
chr11	119943331	119943624
chr11	119945160	119945539
chr11	119948022	119948248
chr11	119948256	119948858
chr11	119954442	119954840
chr11	119954840	119955331
chr11	119967716	119968326
chr11	119969770	119970457
chr11	119973830	119974092
chr11	119975716	119976102
chr11	119976102	119976629
chr11	119979585	119979877
chr11	119986950	119987157
chr11	119987157	119987554
chr11	119988393	119988699
chr11	119988774	119989583
chr11	119991129	119991628

chr11	119998713	119999371
chr11	120005876	120006080
chr11	120007553	120007855
chr11	120007879	120008116
chr11	120008116	120008362
chr11	120008447	120008624
chr11	120009185	120009429
chr11	120011870	120012027
chr11	120012913	120013066
chr11	120013122	120013394
chr11	120013394	120013781
chr11	120022299	120022613
chr11	120022750	120023230
chr11	120024932	120025082
chr11	120029715	120030062
chr11	120030247	120030682
chr11	120039638	120040039
chr11	120040470	120040860
chr11	120041327	120041741
chr11	120042245	120042507
chr11	120042560	120042823
chr11	120043435	120043758
chr11	120045889	120046093
chr11	120049114	120049391
chr11	120054633	120054816
chr11	120055505	120055677
chr11	120056020	120056182
chr11	120056526	120056750
chr11	120057097	120057247
chr11	120063076	120063322
chr11	120065269	120065692
chr11	120067545	120067740
chr11	120067904	120068075
chr11	120069731	120069925
chr11	120073284	120073595
chr11	120086068	120086318
chr11	120086318	120086661
chr11	120086661	120087153
chr11	120092941	120093134
chr11	120093219	120093411
chr11	120098403	120099006
chr11	120099006	120099425

chr11	120101139	120101366
chr11	120110562	120110822
chr11	120111091	120111319
chr11	120114486	120114683
chr11	120114683	120114854
chr11	120116539	120116743
chr11	120120821	120120987
chr11	120121961	120122391
chr11	120122391	120122660
chr11	120126957	120127131
chr11	120134745	120135087
chr11	120135247	120135498
chr11	120144294	120144467
chr11	120149855	120150131
chr11	120150396	120150586
chr11	120150712	120151087
chr11	120151087	120151629
chr11	120159321	120159481
chr11	120160384	120160581
chr11	120160581	120160821
chr11	120163321	120163543
chr11	120169205	120169402
chr11	120169402	120169652
chr11	120169665	120169947
chr11	120175836	120176261
chr11	120181495	120181738
chr11	120189090	120189375
chr11	120189375	120189630
chr11	120189630	120189926
chr11	120189926	120190292
chr11	120200690	120200934
chr11	120204415	120204613
chr11	120204613	120204877
chr11	120204887	120205181
chr11	120215674	120215871
chr11	120222599	120222820
chr11	120231058	120231431
chr11	120231431	120231809
chr11	120232578	120233062
chr11	120236539	120236716
chr11	120237251	120237464
chr11	120239121	120239318

chr11	120240361	120240532
chr11	120252485	120252698
chr11	120252810	120253266
chr11	120253364	120253607
chr11	120255095	120255569
chr11	120256147	120256318
chr11	120256580	120256902
chr11	120256902	120257300
chr11	120261409	120261783
chr11	120261799	120262078
chr11	120262078	120262385
chr11	120262385	120262653
chr11	120262653	120262928
chr11	120267331	120267479
chr11	120267479	120267667
chr11	120267714	120267999
chr11	120269421	120269723
chr11	120271057	120271252
chr11	120280241	120280577
chr11	120292409	120292571
chr11	120293792	120294038
chr11	120294427	120294614
chr11	120296801	120297150
chr11	120297876	120298110
chr11	120304857	120305145
chr11	120305145	120305590
chr11	120307652	120307892
chr11	120308135	120308347
chr11	120310607	120311291
chr11	120315347	120315678
chr11	120315842	120316085
chr11	120316085	120316392
chr11	120316392	120316704
chr11	120316704	120316935
chr11	120317019	120317239
chr11	120334804	120335041
chr11	120340088	120340621
chr11	120343872	120344035
chr11	120345169	120345344
chr11	120348372	120348722
chr11	120349502	120349819
chr11	120349850	120350358

chr11	120350358	120350664
chr11	120353479	120354054
chr11	120354069	120354229
chr11	120354997	120355232
chr11	120360089	120360450
chr11	120360689	120361058
chr11	120362462	120362612
chr11	120368623	120368899
chr11	120369560	120369939
chr11	120369939	120370188
chr11	120377632	120377823
chr11	120378039	120378576
chr11	120378576	120379199
chr11	120386088	120386273
chr11	120392537	120392823
chr11	120423657	120423926
chr11	120426749	120427087
chr11	120437037	120437548
chr11	120437548	120437900
chr11	120442836	120443107
chr11	120446468	120446649
chr11	120450030	120450229
chr11	120457485	120457753
chr11	120457753	120457991
chr11	120457991	120458249
chr11	120458249	120458572
chr11	120459279	120459431
chr11	120467007	120467392
chr11	120467392	120468131
chr11	120468357	120468539
chr11	120475341	120475507
chr11	120477120	120477360
chr11	120484064	120484229
chr11	120484237	120484472
chr11	120484472	120484744
chr11	120484744	120485140
chr11	120489334	120489634
chr11	120491651	120491986
chr11	120491986	120492233
chr11	120492233	120492475
chr11	120492475	120492828
chr11	120492928	120493101

chr11	120498226	120498573
chr11	120500185	120500342
chr11	120500349	120500775
chr11	120506969	120507220
chr11	120507471	120507839
chr11	120507839	120508174
chr11	120508174	120508562
chr11	120508562	120508883
chr11	120510043	120510194
chr11	120515463	120515956
chr11	120518181	120518429
chr11	120518654	120518827
chr11	120519748	120520079
chr11	120528845	120528995
chr11	120530340	120530633
chr11	120530660	120530918
chr11	120536705	120536973
chr11	120539202	120539705
chr11	120539708	120539859
chr11	120540148	120540373
chr11	120541907	120542117
chr11	120542117	120542351
chr11	120547588	120547786
chr11	120549113	120549973
chr11	120552827	120553051
chr11	120554746	120555242
chr11	120555265	120555614
chr11	120561392	120561604
chr11	120565187	120565468
chr11	120571094	120571417
chr11	120572475	120572713
chr11	120572713	120572948
chr11	120572948	120573157
chr11	120573157	120573456
chr11	120573456	120573734
chr11	120579753	120580055
chr11	120580055	120580227
chr11	120580286	120580542
chr11	120581107	120581401
chr11	120581401	120581733
chr11	120581733	120582001
chr11	120582001	120582398

chr11	120588607	120588872
chr11	120597633	120597866
chr11	120597873	120598299
chr11	120598299	120598810
chr11	120605240	120605409
chr11	120607183	120607390
chr11	120608017	120608368
chr11	120608436	120608596
chr11	120612750	120613033
chr11	120615629	120615974
chr11	120617549	120617828
chr11	120617828	120618209
chr11	120624340	120624619
chr11	120624619	120624879
chr11	120624879	120625153
chr11	120625200	120625356
chr11	120630101	120630369
chr11	120630375	120631133
chr11	120632513	120632808
chr11	120632808	120633221
chr11	120633240	120633533
chr11	120633533	120633865
chr11	120641706	120641981
chr11	120643086	120643413
chr11	120643413	120643661
chr11	120643661	120643992
chr11	120651966	120652174
chr11	120654023	120654387
chr11	120658904	120659164
chr11	120659164	120659375
chr11	120659829	120660197
chr11	120660197	120660478
chr11	120660478	120660687
chr11	120660687	120661199
chr11	120672642	120673141
chr11	120673141	120673534
chr11	120673534	120673989
chr11	120679856	120680093
chr11	120682708	120683086
chr11	120683588	120683912
chr11	120685436	120685598
chr11	120692299	120692731

chr11	120692731	120692925
chr11	120697663	120698474
chr11	120703737	120704491
chr11	120706145	120706432
chr11	120712760	120713205
chr11	120713217	120713567
chr11	120713567	120714082
chr11	120714102	120714429
chr11	120720481	120720769
chr11	120721200	120721604
chr11	120721604	120721832
chr11	120721832	120722374
chr11	120726872	120727191
chr11	120727191	120727573
chr11	120729532	120729769
chr11	120733601	120733814
chr11	120741862	120742058
chr11	120742583	120742927
chr11	120743935	120744286
chr11	120744608	120744849
chr11	120749571	120749738
chr11	120753422	120753713
chr11	120753713	120753965
chr11	120762214	120762670
chr11	120762907	120763057
chr11	120763102	120763559
chr11	120763559	120763967
chr11	120768187	120768630
chr11	120773498	120773784
chr11	120775913	120776482
chr11	120783544	120783775
chr11	120783824	120784123
chr11	120784123	120784860
chr11	120784871	120785049
chr11	120789908	120790060
chr11	120790777	120790989
chr11	120794994	120795153
chr11	120795901	120796277
chr11	120796277	120796586
chr11	120796586	120796871
chr11	120800061	120800353
chr11	120800353	120800637

chr11	120800640	120800795
chr11	120806557	120806974
chr11	120813538	120813762
chr11	120822288	120822588
chr11	120823554	120824004
chr11	120824004	120824275
chr11	120824275	120824514
chr11	120824514	120824983
chr11	120834803	120834974
chr11	120850704	120851091
chr11	120851201	120851446
chr11	120855833	120856179
chr11	120880035	120880258
chr11	120894493	120894646
chr11	120895091	120895254
chr11	120898073	120898363
chr11	120898363	120898683
chr11	120905427	120905693
chr11	120905693	120905937
chr11	120932493	120933249
chr11	120936340	120936531
chr11	120938805	120939339
chr11	120939429	120939621
chr11	120948823	120949223
chr11	120950606	120950867
chr11	120950867	120951205
chr11	120954970	120955322
chr11	120955577	120955829
chr11	120957685	120957879
chr11	120960408	120960558
chr11	120965673	120965866
chr11	120973379	120973542
chr11	120984363	120984758
chr11	120984758	120985259
chr11	120990157	120990347
chr11	120990347	120990565
chr11	120990565	120990866
chr11	120990866	120991187
chr11	120991215	120991546
chr11	120995737	120996037
chr11	120996549	120997125
chr11	121004275	121004810

chr11	121014736	121015362
chr11	121017760	121018034
chr11	121039262	121039573
chr11	121041207	121041684
chr11	121048099	121048283
chr11	121053597	121054030
chr11	121063489	121063714
chr11	121063794	121064151
chr11	121064209	121064574
chr11	121084937	121085654
chr11	121086319	121086629
chr11	121089078	121089274
chr11	121091000	121091376
chr11	121112161	121112345
chr11	121112411	121112807
chr11	121118354	121118509
chr11	121126464	121126709
chr11	121129750	121130159
chr11	121136233	121136469
chr11	121143185	121143732
chr11	121145875	121146158
chr11	121156473	121157011
chr11	121176252	121176865
chr11	121181630	121182004
chr11	121184558	121184806
chr11	121184806	121185095
chr11	121185095	121185410
chr11	121204004	121204568
chr11	121204568	121205044
chr11	121220796	121220950
chr11	121221577	121221988
chr11	121228758	121228958
chr11	121228958	121229219
chr11	121229219	121229660
chr11	121236569	121236923
chr11	121236923	121237289
chr11	121237289	121237588
chr11	121237621	121237817
chr11	121259246	121259463
chr11	121259480	121260192
chr11	121260243	121260488
chr11	121260570	121260882

chr11	121266295	121266462
chr11	121280003	121280158
chr11	121280381	121280745
chr11	121280826	121280976
chr11	121281143	121281428
chr11	121289966	121290122
chr11	121294916	121295122
chr11	121298558	121298845
chr11	121320922	121321144
chr11	121327779	121328130
chr11	121328231	121328695
chr11	121353740	121354207
chr11	121354207	121354812
chr11	121362327	121362542
chr11	121372729	121373093
chr11	121373341	121373658
chr11	121373658	121373921
chr11	121387806	121388160
chr11	121388160	121388547
chr11	121388547	121388823
chr11	121390834	121391012
chr11	121396301	121396560
chr11	121402979	121403214
chr11	121403214	121403463
chr11	121407425	121407621
chr11	121411843	121412157
chr11	121415452	121415656
chr11	121420837	121421206
chr11	121421206	121421582
chr11	121421582	121421910
chr11	121434246	121434406
chr11	121434720	121435279
chr11	121441820	121441970
chr11	121451522	121451929
chr11	121451929	121452220
chr11	121452220	121452565
chr11	121475567	121475757
chr11	121475757	121476034
chr11	121496168	121496324
chr11	121522391	121522602
chr11	121536649	121536833
chr11	121543327	121543806

chr11	121543806	121544303
chr11	121544303	121544530
chr11	121544788	121545086
chr11	121550277	121550607
chr11	121551208	121551574
chr11	121552214	121552540
chr11	121558064	121558262
chr11	121558405	121558933
chr11	121560314	121560603
chr11	121576068	121576546
chr11	121576546	121576879
chr11	121597089	121597731
chr11	121604108	121604523
chr11	121607731	121607948
chr11	121607948	121608275
chr11	121609990	121610177
chr11	121610410	121610588
chr11	121621805	121622138
chr11	121626898	121627235
chr11	121629605	121629898
chr11	121630414	121630747
chr11	121631774	121632120
chr11	121641586	121641736
chr11	121646336	121646857
chr11	121647165	121647390
chr11	121647390	121647681
chr11	121650856	121651036
chr11	121651863	121652403
chr11	121652679	121652928
chr11	121654047	121654277
chr11	121655237	121655441
chr11	121672663	121672986
chr11	121672986	121673549
chr11	121679628	121680174
chr11	121680737	121680949
chr11	121685611	121686036
chr11	121686178	121686412
chr11	121689365	121689579
chr11	121693817	121693967
chr11	121697491	121698008
chr11	121702131	121702483
chr11	121702483	121702911

chr11	121703222	121703518
chr11	121706510	121706955
chr11	121710769	121711022
chr11	121712329	121712579
chr11	121712622	121713013
chr11	121713013	121713349
chr11	121717678	121718131
chr11	121720795	121721207
chr11	121722520	121722865
chr11	121725280	121725533
chr11	121740670	121740944
chr11	121740944	121741329
chr11	121741391	121741997
chr11	121768881	121769161
chr11	121781900	121782081
chr11	121808212	121808560
chr11	121808560	121808775
chr11	121808777	121809093
chr11	121810793	121810948
chr11	121846023	121846210
chr11	121847745	121847998
chr11	121847998	121848385
chr11	121925556	121925721
chr11	121934931	121935253
chr11	121951765	121952146
chr11	121956589	121956915
chr11	121956946	121957138
chr11	121966031	121966184
chr11	121981734	121982010
chr11	121982294	121982610
chr12	3082173	3082391
chr12	3082400	3082556
chr12	3082556	3082728
chr12	3098259	3098519
chr12	3109802	3110169
chr12	3110568	3110797
chr12	3110797	3111009
chr12	3119951	3120189
chr12	3142820	3143072
chr12	3143917	3144107
chr12	3172241	3172426
chr12	3179850	3180056

chr12	3180843	3181284
chr12	3188820	3189184
chr12	3199098	3199318
chr12	3209225	3209403
chr12	3210247	3210533
chr12	3229405	3229555
chr12	3234964	3235209
chr12	3235209	3235556
chr12	3235556	3235870
chr12	3235870	3236129
chr12	3245897	3246144
chr12	3246184	3246410
chr12	3253668	3253898
chr12	3256288	3256536
chr12	3283066	3283295
chr12	3283918	3284133
chr12	3284364	3284746
chr12	3292276	3292430
chr12	3299450	3299802
chr12	3309349	3309711
chr12	3309711	3310048
chr12	3310048	3310643
chr12	3317083	3317296
chr12	3317296	3317521
chr12	3319456	3319774
chr12	3323200	3323561
chr12	3323561	3323822
chr12	3349403	3349750
chr12	3349750	3349994
chr12	3350061	3350393
chr12	3352212	3352741
chr12	3357419	3357590
chr12	3359257	3359502
chr12	3359502	3359732
chr12	3364826	3365591
chr12	3365591	3366300
chr12	3371109	3371606
chr12	3371606	3371950
chr12	3377216	3377521
chr12	3389281	3389776
chr12	3390958	3391211
chr12	3396088	3396218

chr12	3396218	3396348
chr12	3396517	3396711
chr12	3405764	3406084
chr12	3413955	3414487
chr12	3419400	3419709
chr12	3422650	3422893
chr12	3422893	3423190
chr12	3426033	3426266
chr12	3426489	3426931
chr12	3426931	3427333
chr12	3427333	3427675
chr12	3427675	3427912
chr12	3436434	3436632
chr12	3436632	3436840
chr12	3437236	3437795
chr12	3512206	3512554
chr12	3537673	3537827
chr12	3547484	3547685
chr12	3548273	3548461
chr12	3548498	3548720
chr12	3548720	3548985
chr12	3549396	3549662
chr12	3556181	3556356
chr12	3557917	3558258
chr12	3558858	3559043
chr12	3559700	3559888
chr12	3560662	3560965
chr12	3570607	3570830
chr12	3570905	3571081
chr12	3571842	3572522
chr12	3572522	3573092
chr12	3582397	3582547
chr12	3582593	3583231
chr12	3583655	3583805
chr12	3589001	3589263
chr12	3596936	3597130
chr12	3602871	3603113
chr12	3642045	3642265
chr12	3659572	3659775
chr12	3662626	3663125
chr12	3691149	3691377
chr12	3695819	3696057

chr12	3700649	3700829
chr12	3702647	3702797
chr12	3704770	3705066
chr12	3711174	3711956
chr12	3718262	3718559
chr12	3718559	3718763
chr12	3719035	3719891
chr12	3729622	3730061
chr12	3746076	3746368
chr12	3746368	3746738
chr12	3747619	3748022
chr12	3764067	3764490
chr12	3764490	3764900
chr12	3775859	3776034
chr12	3780404	3780631
chr12	3781034	3781355
chr12	3781551	3781860
chr12	3787817	3788132
chr12	3791771	3792188
chr12	3795588	3795902
chr12	3806247	3806578
chr12	3806578	3806882
chr12	3806882	3807265
chr12	3807311	3807575
chr12	3807575	3807815
chr12	3807815	3807992
chr12	3808780	3808973
chr12	3817132	3817310
chr12	3823458	3823807
chr12	3824868	3825160
chr12	3825191	3825440
chr12	3825793	3826039
chr12	3828758	3828945
chr12	3839459	3839693
chr12	3841103	3841284
chr12	3842903	3843058
chr12	3846240	3846604
chr12	3854296	3854822
chr12	3856181	3856408
chr12	3860768	3861021
chr12	3861658	3862020
chr12	3862329	3862517

chr12	3866629	3867047
chr12	3868649	3868870
chr12	3894681	3894863
chr12	3910811	3910991
chr12	3926201	3926434
chr12	3928940	3929180
chr12	3942194	3942584
chr12	3959791	3960155
chr12	3960155	3960417
chr12	3972796	3973283
chr12	3973283	3973637
chr12	3981188	3981786
chr12	3981786	3982120
chr12	3983819	3983971
chr12	4007245	4007499
chr12	4038103	4038376
chr12	4038407	4038719
chr12	4038719	4039403
chr12	4054473	4054641
chr12	4054900	4055131
chr12	4065579	4065746
chr12	4066725	4067018
chr12	4082259	4082500
chr12	4082500	4082774
chr12	4082774	4083079
chr12	4107427	4107846
chr12	4118320	4118529
chr12	4123001	4123248
chr12	4132939	4133247
chr12	4133247	4133798
chr12	4133953	4134137
chr12	4158199	4158395
chr12	4171015	4171580
chr12	4173337	4173902
chr12	4179865	4180066
chr12	4180550	4180792
chr12	4183943	4184103
chr12	4188126	4188697
chr12	4198633	4199161
chr12	4208710	4209119
chr12	4219308	4219567
chr12	4219567	4219934

chr12	4219934	4220517
chr12	4220517	4221040
chr12	4221040	4221378
chr12	4221378	4221711
chr12	4233633	4233898
chr12	4233898	4234185
chr12	4234185	4234670
chr12	4239366	4239547
chr12	4240078	4240300
chr12	4255684	4255908
chr12	4285379	4285579
chr12	4355436	4355622
chr12	4369240	4369533
chr12	4393507	4393734
chr12	4394465	4394637
chr12	4424455	4424754
chr12	4424754	4425132
chr12	4430763	4431094
chr12	4431094	4431393
chr12	4441525	4441862
chr12	4461068	4461276
chr12	4476195	4476611
chr12	4476611	4477041
chr12	4477041	4477609
chr12	4488632	4488782
chr12	4497374	4497835
chr12	4505305	4505578
chr12	4507532	4507704
chr12	4534392	4534542
chr12	4548701	4548984
chr12	4557577	4557761
chr12	4558577	4559218
chr12	4569176	4569389
chr12	4571960	4572221
chr12	4574101	4574531
chr12	4588593	4589011
chr12	4590574	4591006
chr12	4592712	4593245
chr12	4593245	4593565
chr12	4593616	4593849
chr12	4599155	4599441
chr12	4599744	4600021

chr12	4600021	4600258
chr12	4636811	4637151
chr12	4694652	4694815
chr12	4694913	4695390
chr12	4697338	4697491
chr12	4709206	4709422
chr12	4709488	4709638
chr12	4716861	4717028
chr12	4720439	4720685
chr12	4734065	4734223
chr12	4734223	4734383
chr12	4734439	4734596
chr12	4744589	4744758
chr12	4768818	4769144
chr12	4769144	4769577
chr12	4778464	4778756
chr12	4781383	4781552
chr12	4782172	4782448
chr12	4783213	4783397
chr12	4792790	4793081
chr12	4793470	4793781
chr12	4793781	4794217
chr12	4816963	4817343
chr12	4817343	4817629
chr12	4817629	4817882
chr12	4817882	4818096
chr12	4827354	4827595
chr12	4841326	4841855
chr12	4843162	4843628
chr12	4843833	4844086
chr12	4844382	4844612
chr12	4856306	4856466
chr12	4856915	4857106
chr12	4857325	4857475
chr12	4862765	4862993
chr12	4863687	4864034
chr12	4864074	4864444
chr12	4868983	4869204
chr12	4874082	4874705
chr12	4878412	4878665
chr12	4879176	4879326
chr12	4906962	4907424

chr12	4907424	4907667
chr12	4907667	4907921
chr12	4911856	4912122
chr12	4913199	4913369
chr12	4916942	4917456
chr12	4917456	4917756
chr12	4917756	4918127
chr12	4918185	4918357
chr12	4918360	4918548
chr12	4930955	4931142
chr12	4931344	4931656
chr12	4973035	4973241
chr12	5055079	5055282
chr12	5055282	5055490
chr12	5055543	5055693
chr12	5055938	5056338
chr12	5058405	5058585
chr12	5058585	5058701
chr12	5058701	5058864
chr12	5059166	5059496
chr12	5063587	5063775
chr12	5066829	5066979
chr12	5067242	5067688
chr12	5108657	5109062
chr12	5119760	5120052
chr12	5123346	5123577
chr12	5123577	5123829
chr12	5136335	5136662
chr12	5147746	5147898
chr12	5176813	5177053
chr12	5177053	5177334
chr12	5182214	5182417
chr12	5213626	5213851
chr12	5213851	5214290
chr12	5276939	5277646
chr12	5282501	5282967
chr12	5282967	5283259
chr12	5325416	5325566
chr12	5374149	5374326
chr12	5374839	5375063
chr12	5375063	5375286
chr12	5375286	5375561

chr12	5375561	5376107
chr12	5376109	5376316
chr12	5410459	5410681
chr12	5417533	5417717
chr12	5506056	5506451
chr12	5506451	5506675
chr12	5506675	5506922
chr12	6110823	6111085
chr12	6219780	6219942
chr12	6353237	6353505
chr12	6411791	6412069
chr12	6760247	6760537
chr12	6780679	6780872
chr12	6782480	6782817
chr12	6786139	6786528
chr12	6790365	6790675
chr12	6842753	6843121
chr12	6843257	6843602
chr12	6857835	6858088
chr12	7023093	7023350
chr12	7074245	7074540
chr12	7085216	7085505
chr12	7086993	7087195
chr12	7093506	7093687
chr12	7758226	7758438
chr12	7777743	7777986
chr12	7816615	7816967
chr12	7820370	7820520
chr12	7830930	7831175
chr12	7857216	7857544
chr12	7905410	7905634
chr12	7914016	7914210
chr12	7918464	7918946
chr12	7918946	7919432
chr12	7919432	7919766
chr12	7922322	7922525
chr12	7954767	7954994
chr12	7967139	7967361
chr12	7967361	7967996
chr12	7968110	7968708
chr12	7976028	7976227
chr12	7978211	7978473

chr12	7978473	7978792
chr12	7990881	7991115
chr12	8042716	8042894
chr12	8054752	8055032
chr12	8059660	8059902
chr12	8070427	8070751
chr12	8070751	8071386
chr12	8071388	8071637
chr12	8076047	8076470
chr12	8076508	8076675
chr12	8089980	8090191
chr12	8090191	8090397
chr12	8099184	8099449
chr12	8099921	8100128
chr12	8181606	8182182
chr12	8182981	8183141
chr12	8207956	8208500
chr12	8222984	8223663
chr12	8224379	8224529
chr12	8232788	8233031
chr12	8243096	8243660
chr12	8243736	8243949
chr12	8295481	8295824
chr12	8297946	8298192
chr12	8298192	8298421
chr12	8298454	8298840
chr12	8301826	8302008
chr12	8306441	8306629
chr12	8306629	8306857
chr12	8306974	8307276
chr12	8313192	8313537
chr12	8313537	8314099
chr12	8319920	8320139
chr12	8320139	8320403
chr12	8326693	8326896
chr12	8326896	8327317
chr12	8328323	8328534
chr12	8331985	8332194
chr12	8333537	8333994
chr12	8337739	8337923
chr12	8338338	8338574
chr12	8338574	8338875

chr12	8338875	8339155
chr12	8339235	8339439
chr12	8348810	8349095
chr12	8349893	8350046
chr12	8351060	8351704
chr12	8357356	8357819
chr12	8358559	8358875
chr12	8364464	8364980
chr12	8365946	8366242
chr12	8371799	8372247
chr12	8372247	8372496
chr12	8372776	8373127
chr12	8373511	8373688
chr12	8378941	8379084
chr12	8379084	8379229
chr12	8379731	8380351
chr12	8386346	8386973
chr12	8386973	8387455
chr12	8387455	8387785
chr12	8399008	8399158
chr12	8402121	8402333
chr12	8404397	8404609
chr12	8418821	8419550
chr12	8430928	8431274
chr12	8431777	8432087
chr12	8433477	8433778
chr12	8439975	8440228
chr12	8450049	8450296
chr12	8450553	8451043
chr12	8452931	8453110
chr12	8457041	8457424
chr12	8457454	8457654
chr12	8462637	8462837
chr12	8462985	8463229
chr12	8475952	8476102
chr12	8487061	8487319
chr12	8487319	8487648
chr12	8487983	8488234
chr12	8488340	8488638
chr12	8488638	8488874
chr12	8499065	8499773
chr12	8499773	8500461

chr12	8509696	8510047
chr12	8510992	8511427
chr12	8511427	8511757
chr12	8519416	8519805
chr12	8521352	8522008
chr12	8522095	8522260
chr12	8525001	8525290
chr12	8527839	8528096
chr12	8545288	8545538
chr12	8547318	8547859
chr12	8552155	8552332
chr12	8555320	8555844
chr12	8556325	8556626
chr12	8571274	8571534
chr12	8571595	8571818
chr12	8571897	8572322
chr12	8582998	8583297
chr12	8585224	8585386
chr12	8585987	8586536
chr12	8587736	8588032
chr12	8597411	8597580
chr12	8607226	8607472
chr12	8607807	8608063
chr12	8608075	8608226
chr12	8608474	8608624
chr12	8608657	8608842
chr12	8616426	8616669
chr12	8638771	8639057
chr12	8640685	8640952
chr12	8640952	8641395
chr12	8645453	8645627
chr12	8661376	8661760
chr12	8661782	8662099
chr12	8662205	8662722
chr12	8667858	8668144
chr12	8673769	8674392
chr12	8674392	8674864
chr12	8675376	8675581
chr12	8690277	8690682
chr12	8698967	8699212
chr12	8716159	8716407
chr12	8726982	8727172

chr12	8730820	8730985
chr12	8746677	8746958
chr12	8753329	8753530
chr12	8754146	8754555
chr12	8754555	8754935
chr12	8754935	8755249
chr12	8760722	8760905
chr12	8760905	8761172
chr12	8761172	8761798
chr12	8762785	8762948
chr12	8770897	8771228
chr12	8771228	8771546
chr12	8771546	8771908
chr12	8772508	8772668
chr12	8772738	8773201
chr12	8777597	8777858
chr12	8778041	8778640
chr12	8782079	8782337
chr12	8784011	8784240
chr12	8784240	8784691
chr12	8785234	8785394
chr12	8786377	8786698
chr12	8787018	8787338
chr12	8787338	8787571
chr12	8795246	8795435
chr12	8797070	8797373
chr12	8797914	8798197
chr12	8798197	8798452
chr12	8798912	8799312
chr12	8800034	8800193
chr12	8805370	8805685
chr12	8805982	8806265
chr12	8807229	8807447
chr12	8809023	8809614
chr12	8811018	8811357
chr12	8811357	8811645
chr12	8811645	8811957
chr12	8821743	8822228
chr12	8825381	8825641
chr12	8826814	8827352
chr12	8827352	8827717
chr12	8829641	8829818

chr12	8829818	8830047
chr12	8842131	8842296
chr12	8848149	8848470
chr12	8848635	8848876
chr12	8850221	8850446
chr12	8853946	8854214
chr12	8854227	8854924
chr12	8860191	8860364
chr12	8860688	8861037
chr12	8864731	8865255
chr12	8865255	8865606
chr12	8866828	8867016
chr12	8875528	8876074
chr12	8876317	8876855
chr12	8876855	8877215
chr12	8877549	8877746
chr12	8881812	8882057
chr12	8882603	8882898
chr12	8883149	8883414
chr12	8885981	8886203
chr12	8889201	8889614
chr12	8889621	8889793
chr12	8900956	8901163
chr12	8908344	8908497
chr12	8921061	8921282
chr12	8921448	8921771
chr12	8921771	8922119
chr12	8922119	8922445
chr12	8927977	8928192
chr12	8928731	8929005
chr12	8931549	8931802
chr12	8935154	8935723
chr12	8935952	8936181
chr12	8947622	8947984
chr12	8947984	8948456
chr12	8955906	8956092
chr12	8963909	8964135
chr12	8964243	8964463
chr12	8964463	8964694
chr12	8964694	8964993
chr12	8969814	8970215
chr12	8973739	8974126

chr12	8974126	8974333
chr12	8978744	8979038
chr12	8980875	8981190
chr12	9000244	9000563
chr12	9002093	9002343
chr12	9004813	9005081
chr12	9013417	9013653
chr12	9021410	9021678
chr12	9021678	9022062
chr12	9029646	9029796
chr12	9029866	9030294
chr12	9030294	9030593
chr12	9032039	9032238
chr12	9032925	9033203
chr12	9033235	9033440
chr12	9050942	9051181
chr12	9051265	9051633
chr12	9051633	9051903
chr12	9052097	9052446
chr12	9066056	9066325
chr12	9066424	9066917
chr12	9090253	9090620
chr12	9094708	9094868
chr12	9095105	9095496
chr12	9112023	9112187
chr12	9112190	9112604
chr12	9112604	9112850
chr12	9113676	9113989
chr12	9166244	9166516
chr12	9166516	9166744
chr12	9170319	9170539
chr12	9172539	9172730
chr12	9174353	9174654
chr12	9176231	9176586
chr12	9232474	9232768
chr12	9251843	9252103
chr12	9274336	9274488
chr12	9296721	9297013
chr12	9297453	9297691
chr12	9298854	9299476
chr12	9304213	9304498
chr12	9368776	9369084

chr12	9425135	9425462
chr12	9425462	9425727
chr12	9425727	9426012
chr12	9426012	9426243
chr12	9426243	9426493
chr12	9426493	9426758
chr12	9426758	9426950
chr12	9426950	9427130
chr12	9427130	9427344
chr12	9444163	9444347
chr12	9444890	9445186
chr12	9569974	9570375
chr12	9574293	9574613
chr12	9574613	9574831
chr12	9584569	9585145
chr12	9585169	9585419
chr12	9589556	9589749
chr12	9589749	9590004
chr12	9598296	9598594
chr12	9650245	9650475
chr12	9688077	9688682
chr12	9689565	9690063
chr12	9733075	9733233
chr12	9762900	9763188
chr12	9770334	9770772
chr12	9833428	9833618
chr12	9839196	9839480
chr12	9839480	9839868
chr12	9839868	9840539
chr12	9847499	9847814
chr12	9855746	9856195
chr12	9856195	9856557
chr12	9874483	9874850
chr12	9874850	9875165
chr12	9875165	9875535
chr12	9898365	9898906
chr12	9900818	9901095
chr12	9901396	9901608
chr12	9912412	9912670
chr12	9944632	9944807
chr12	9945888	9946082
chr12	9946171	9946389

chr12	9946389	9946596
chr12	9978455	9979031
chr12	9979031	9979593
chr12	9979632	9979790
chr12	10004863	10005096
chr12	10016827	10017046
chr12	10017046	10017266
chr12	10105757	10105944
chr12	10135866	10136078
chr12	10136078	10136283
chr12	10197344	10197540
chr12	10221914	10222080
chr12	10223921	10224170
chr12	10327883	10328033
chr12	10329437	10329872
chr12	10390589	10390921
chr12	10390921	10391167
chr12	10391167	10391481
chr12	10404694	10404856
chr12	10405223	10405561
chr12	10410761	10411171
chr12	10411465	10411800
chr12	10411864	10412016
chr12	10422399	10422684
chr12	10438075	10438300
chr12	10537171	10537418
chr12	10583278	10583450
chr12	10589470	10589970
chr12	10593532	10593714
chr12	10594667	10594902
chr12	10598431	10598594
chr12	10625245	10625445
chr12	10865854	10866087
chr12	10866087	10866488
chr12	10867647	10867976
chr12	10868196	10868694
chr12	10881686	10881909
chr12	10899264	10899458
chr12	10961495	10961776
chr12	10962503	10962728
chr12	10962728	10962932
chr12	11005997	11006309

chr12	11007884	11008089
chr12	11120857	11121024
chr12	11149908	11150058
chr12	11150203	11150522
chr12	11178618	11178800
chr12	11178805	11179106
chr12	11194240	11194460
chr12	11194460	11194710
chr12	11196310	11196554
chr12	11213120	11213397
chr12	11223158	11223374
chr12	11265412	11265759
chr12	11265759	11266232
chr12	11266232	11266644
chr12	11273216	11273490
chr12	11317641	11318091
chr12	11327842	11328042
chr12	11332421	11332649
chr12	11337007	11337193
chr12	11341973	11342564
chr12	11350889	11351189
chr12	11370093	11370453
chr12	11389059	11389693
chr12	11390614	11390830
chr12	11391530	11391867
chr12	11436530	11436780
chr12	11436780	11436972
chr12	11436972	11437151
chr12	11437640	11437896
chr12	11455747	11456190
chr12	11456190	11456604
chr12	11551225	11551393
chr12	11554007	11554319
chr12	11577102	11577320
chr12	11673362	11673540
chr12	11717508	11717966
chr12	11729163	11729377
chr12	11796795	11796984
chr12	12176309	12176473
chr12	12261132	12261609
chr12	12261671	12261987
chr12	12261987	12262492

chr12	12262646	12262863
chr12	12323586	12323861
chr12	12323861	12324157
chr12	12351172	12351484
chr12	12390328	12390562
chr12	12401083	12401312
chr12	12412750	12413008
chr12	12419598	12419927
chr12	12420344	12420739
chr12	12420858	12421337
chr12	12427411	12427636
chr12	12437428	12437857
chr12	12492210	12492531
chr12	12492627	12492779
chr12	12493231	12493492
chr12	12493492	12493736
chr12	12496847	12497151
chr12	12498115	12498271
chr12	12505676	12505936
chr12	12509153	12509573
chr12	12509573	12509808
chr12	12510165	12510399
chr12	12511053	12511271
chr12	12517126	12517276
chr12	12527353	12527770
chr12	12530757	12531224
chr12	12531233	12531441
chr12	12542364	12542721
chr12	12542897	12543234
chr12	12545383	12545895
chr12	12549372	12549866
chr12	12551901	12552087
chr12	12619597	12619789
chr12	12623024	12623232
chr12	12647594	12647937
chr12	12680554	12680714
chr12	12680714	12680877
chr12	12712670	12712820
chr12	12713471	12713806
chr12	12733515	12733688
chr12	12796851	12797050
chr12	12810098	12810712

chr12	12810712	12811248
chr12	12811336	12811626
chr12	12898812	12899008
chr12	12902527	12902794
chr12	12904649	12904952
chr12	12909958	12910163
chr12	12910411	12910939
chr12	12920917	12921221
chr12	12931184	12931344
chr12	12941098	12941289
chr12	12941297	12941452
chr12	12941521	12942226
chr12	12942226	12942684
chr12	12942684	12942901
chr12	12981361	12981511
chr12	12982536	12983000
chr12	12983033	12983308
chr12	12983308	12983506
chr12	13015126	13015337
chr12	13060051	13060490
chr12	13076987	13077345
chr12	13077540	13077695
chr12	13077763	13078298
chr12	13092590	13092995
chr12	13093248	13093398
chr12	13094710	13095214
chr12	13106998	13107209
chr12	13113771	13114187
chr12	13114187	13114607
chr12	13114607	13114944
chr12	13115047	13115298
chr12	13133874	13134565
chr12	13138004	13138255
chr12	13144587	13145026
chr12	13145026	13145270
chr12	13145355	13145844
chr12	13145934	13146106
chr12	13150508	13151116
chr12	13151460	13151915
chr12	13167048	13167459
chr12	13175846	13176027
chr12	13176157	13176470

chr12	13176954	13177270
chr12	13177453	13177692
chr12	13177692	13177935
chr12	13202080	13202827
chr12	13204639	13204792
chr12	13212108	13212409
chr12	13213308	13213616
chr12	13214941	13215216
chr12	13217327	13218042
chr12	13218042	13218566
chr12	13240554	13240763
chr12	13248647	13248982
chr12	13248982	13249542
chr12	13249630	13249851
chr12	13255036	13255555
chr12	13260873	13261058
chr12	13268543	13268765
chr12	13268765	13268954
chr12	13268961	13269250
chr12	13269250	13269575
chr12	13269575	13269937
chr12	13397218	13397453
chr12	13410188	13410519
chr12	13462392	13462642
chr12	13487085	13487291
chr12	13518143	13518465
chr12	13575480	13575657
chr12	13575737	13576478
chr12	13602525	13602675
chr12	13682994	13683207
chr12	13748735	13748971
chr12	13809339	13809666
chr12	13878510	13878891
chr12	13891301	13891757
chr12	14027362	14027708
chr12	14027732	14027906
chr12	14068948	14069377
chr12	14069377	14069702
chr12	14091248	14091516
chr12	14106462	14106625
chr12	14125959	14126168
chr12	14150045	14150301

chr12	14150301	14150552
chr12	14150552	14150870
chr12	14150949	14151183
chr12	14151287	14151712
chr12	14151812	14152380
chr12	14160303	14160735
chr12	14166937	14167151
chr12	14167562	14167783
chr12	14174381	14174560
chr12	14189656	14189835
chr12	14195228	14195385
chr12	14206030	14206539
chr12	14206580	14206840
chr12	14210070	14210227
chr12	14212681	14212975
chr12	14213173	14213403
chr12	14213459	14213619
chr12	14244557	14244890
chr12	14286156	14286465
chr12	14286465	14286749
chr12	14286749	14287063
chr12	14301735	14302177
chr12	14302382	14302647
chr12	14302647	14302985
chr12	14304145	14304387
chr12	14315724	14316002
chr12	14316002	14316413
chr12	14375984	14376218
chr12	14415297	14415737
chr12	14415737	14416162
chr12	14494487	14494685
chr12	14578248	14578500
chr12	14659305	14659647
chr12	14659647	14659865
chr12	14659871	14660085
chr12	14744792	14744990
chr12	14756375	14756525
chr12	14880140	14880343
chr12	14880570	14880781
chr12	14994982	14995275
chr12	14996381	14996531
chr12	15011080	15011429

chr12	15064454	15064612
chr12	15153678	15153915
chr12	15488423	15488609
chr12	15535948	15536110
chr12	15566163	15566398
chr12	15702161	15702468
chr12	15704578	15704952
chr12	15707729	15708025
chr12	15718001	15718252
chr12	15778588	15778773
chr12	15800361	15800717
chr12	15800881	15801492
chr12	15811667	15812056
chr12	15815762	15815963
chr12	15816236	15816417
chr12	15816648	15817291
chr12	15830898	15831048
chr12	15832377	15832623
chr12	15832737	15833021
chr12	15838040	15838600
chr12	15848493	15848879
chr12	15849877	15850195
chr12	15850502	15850970
chr12	15855598	15855827
chr12	15862010	15862178
chr12	15904571	15904983
chr12	15912375	15912751
chr12	15912842	15913078
chr12	15913078	15913345
chr12	16006642	16007097
chr12	16012135	16012332
chr12	16015450	16015694
chr12	16145486	16145995
chr12	16151163	16151457
chr12	16152246	16152427
chr12	16154216	16154635
chr12	16161947	16162275
chr12	16167869	16168093
chr12	16170158	16170552
chr12	16190821	16190978
chr12	16210698	16210874
chr12	16344079	16344301

chr12	16477677	16477883
chr12	16507484	16507701
chr12	16509424	16509700
chr12	16526112	16526541
chr12	16526681	16526890
chr12	16532093	16532267
chr12	16532427	16532827
chr12	16532827	16533173
chr12	16533272	16533641
chr12	16539187	16539337
chr12	16550257	16550407
chr12	16554623	16554834
chr12	16563478	16563773
chr12	16565010	16565188
chr12	16568575	16568838
chr12	16574183	16574412
chr12	16580407	16580828
chr12	16589509	16589951
chr12	16591443	16592002
chr12	16618616	16618898
chr12	16618898	16619343
chr12	16620228	16620494
chr12	16622193	16622343
chr12	16632376	16632590
chr12	16634482	16634729
chr12	16635836	16636185
chr12	16636844	16637080
chr12	16653442	16654046
chr12	16664921	16665513
chr12	16717706	16717968
chr12	16718970	16719239
chr12	16720566	16720756
chr12	16747747	16747987
chr12	16753179	16753365
chr12	16754396	16754600
chr12	16778995	16779208
chr12	16779208	16779509
chr12	16788676	16788929
chr12	16790461	16790793
chr12	16790793	16790994
chr12	16794663	16794833
chr12	16795802	16796163

chr12	16800465	16800774
chr12	16805573	16805862
chr12	16808118	16808376
chr12	16810676	16811095
chr12	16811095	16811466
chr12	16811485	16811672
chr12	16811744	16811894
chr12	16822507	16822664
chr12	16828473	16828844
chr12	16829841	16830120
chr12	16831172	16831392
chr12	16833767	16834033
chr12	16840845	16841411
chr12	16841411	16841729
chr12	16847558	16847788
chr12	16849696	16850154
chr12	16850539	16850892
chr12	16850892	16851211
chr12	16853348	16853785
chr12	16853785	16854192
chr12	16856067	16856217
chr12	16860004	16860295
chr12	16860715	16860907
chr12	16864653	16864817
chr12	16868974	16869167
chr12	16869930	16870114
chr12	16870114	16870320
chr12	16870320	16870912
chr12	16872087	16872369
chr12	16872983	16873226
chr12	16873266	16873539
chr12	16873573	16873725
chr12	16874149	16874665
chr12	16875372	16875563
chr12	16886460	16886956
chr12	16890558	16890725
chr12	16893981	16894332
chr12	16894332	16894679
chr12	16894679	16894978
chr12	16894978	16895227
chr12	16895447	16895670
chr12	16896138	16896461

chr12	16900071	16900301
chr12	16900858	16901257
chr12	16908005	16908220
chr12	16908379	16908598
chr12	16920036	16920262
chr12	16920262	16920552
chr12	16920552	16920865
chr12	16928033	16928236
chr12	16930822	16930972
chr12	16958916	16959185
chr12	16985894	16986074
chr12	16990939	16991101
chr12	16999333	16999489
chr12	16999761	17000260
chr12	17000260	17000605
chr12	17001218	17001545
chr12	17011425	17011639
chr12	17011639	17011888
chr12	17012366	17012575
chr12	17012725	17013061
chr12	17033216	17033437
chr12	17035275	17035488
chr12	17040357	17040519
chr12	17040565	17040952
chr12	17044306	17044548
chr12	17047547	17047888
chr12	17055767	17056160
chr12	17057033	17057409
chr12	17058653	17058960
chr12	17075776	17075983
chr12	17076448	17076879
chr12	17084982	17085163
chr12	17085179	17085580
chr12	17085614	17085764
chr12	17105104	17105386
chr12	17134392	17134668
chr12	17134668	17135168
chr12	17135701	17135947
chr12	17136521	17136909
chr12	17152494	17152648
chr12	17152876	17153323
chr12	17175666	17175816

chr12	17176062	17176493
chr12	17176493	17176968
chr12	17183901	17184051
chr12	17184216	17184560
chr12	17184560	17184922
chr12	17209586	17210052
chr12	17220176	17220539
chr12	17220539	17221157
chr12	17230789	17230943
chr12	17236060	17236560
chr12	17242124	17242344
chr12	17250542	17250805
chr12	17250988	17251288
chr12	17253093	17253363
chr12	17256955	17257399
chr12	17258066	17258322
chr12	17258322	17258548
chr12	17266239	17266673
chr12	17266673	17267108
chr12	17271401	17271583
chr12	17288574	17288911
chr12	17288911	17289280
chr12	17289280	17289642
chr12	17289663	17289901
chr12	17289932	17290272
chr12	17298366	17298566
chr12	17302611	17302765
chr12	17303378	17303848
chr12	17303848	17304218
chr12	17304889	17305132
chr12	17311953	17312190
chr12	17314013	17314292
chr12	17315091	17315252
chr12	17324084	17324470
chr12	17324470	17325013
chr12	17325013	17325345
chr12	17326729	17326986
chr12	17334312	17334479
chr12	17335912	17336141
chr12	17338412	17338808
chr12	17339194	17339437
chr12	17339470	17339649

chr12	17339649	17339823
chr12	17347903	17348082
chr12	17348084	17348248
chr12	17348266	17348635
chr12	17348635	17348932
chr12	17355628	17355854
chr12	17356949	17357158
chr12	17367029	17367381
chr12	17372791	17372985
chr12	17373557	17373966
chr12	17373966	17374432
chr12	17381058	17381394
chr12	17388061	17388377
chr12	17390154	17390501
chr12	17390501	17390852
chr12	17414822	17415243
chr12	17420065	17420429
chr12	17420429	17420721
chr12	17420762	17420927
chr12	17436542	17436716
chr12	17437922	17438129
chr12	17438185	17438363
chr12	17438431	17438584
chr12	17439282	17439479
chr12	17440448	17440789
chr12	17449662	17450069
chr12	17450069	17450529
chr12	17455080	17455278
chr12	17481732	17482085
chr12	17492207	17492464
chr12	17492564	17492996
chr12	17495868	17496103
chr12	17496103	17496376
chr12	17507020	17507260
chr12	17507260	17507553
chr12	17510087	17510673
chr12	17510977	17511331
chr12	17530928	17531078
chr12	17537125	17537300
chr12	17540785	17540985
chr12	17542569	17542937
chr12	17544288	17544536

chr12	17544536	17544796
chr12	17544796	17545057
chr12	17545057	17545453
chr12	17549307	17549482
chr12	17549482	17549666
chr12	17551135	17551487
chr12	17551602	17551755
chr12	17552800	17553073
chr12	17553073	17553466
chr12	17553611	17553884
chr12	17553884	17554054
chr12	17554105	17554403
chr12	17554410	17554587
chr12	17574296	17574468
chr12	17576129	17576336
chr12	17577628	17577803
chr12	17578692	17578927
chr12	17594015	17594339
chr12	17607136	17607341
chr12	17610554	17610705
chr12	17622872	17623120
chr12	17641358	17641626
chr12	17644648	17644862
chr12	17665865	17666017
chr12	17666680	17666924
chr12	17667395	17667545
chr12	17680302	17680534
chr12	17681030	17681280
chr12	17688952	17689222
chr12	17690257	17690625
chr12	17690625	17690944
chr12	17690944	17691288
chr12	17691343	17691520
chr12	17691548	17691745
chr12	17691942	17692114
chr12	17692194	17692424
chr12	17694559	17694779
chr12	17694779	17695072
chr12	17702024	17702189
chr12	17709601	17709785
chr12	17710358	17710556
chr12	17710884	17711162

chr12	17711453	17711793
chr12	17711793	17712077
chr12	17712077	17712500
chr12	17721023	17721355
chr12	17722640	17723338
chr12	17726571	17726811
chr12	17729078	17729257
chr12	17729332	17729689
chr12	17729689	17729953
chr12	17732846	17733092
chr12	17733092	17733484
chr12	17738348	17739182
chr12	17746712	17747026
chr12	17747315	17747525
chr12	17747581	17747790
chr12	17749709	17750126
chr12	17750372	17750894
chr12	17751743	17751901
chr12	17754900	17755053
chr12	17756434	17756625
chr12	17756689	17757111
chr12	17757111	17757395
chr12	17762373	17762600
chr12	17772747	17773095
chr12	17773095	17773432
chr12	17773511	17774142
chr12	17778446	17778658
chr12	17795011	17795289
chr12	17795427	17795657
chr12	17795776	17795978
chr12	17795978	17796221
chr12	17808493	17808680
chr12	17808680	17808971
chr12	17852234	17852445
chr12	17872339	17872976
chr12	17873796	17873950
chr12	17881337	17881626
chr12	17881626	17882057
chr12	17882057	17882568
chr12	17882811	17882961
chr12	17887255	17887472
chr12	17887472	17887824

chr12	17913042	17913440
chr12	17914332	17914629
chr12	17992537	17993039
chr12	17993039	17993504
chr12	17997829	17998098
chr12	17998102	17998727
chr12	18004302	18004485
chr12	18054524	18054759
chr12	18393792	18394002
chr12	18514131	18514621
chr12	18514621	18515124
chr12	18515262	18515412
chr12	18519782	18520019
chr12	18546257	18546542
chr12	18549593	18549790
chr12	18609788	18610272
chr12	18610272	18610698
chr12	18610908	18611145
chr12	18615274	18615754
chr12	18622531	18622714
chr12	18672193	18672389
chr12	18689809	18690014
chr12	18766095	18766264
chr12	18788619	18788822
chr12	18853387	18853582
chr12	18853874	18854086
chr12	18859355	18859601
chr12	18884490	18884696
chr12	19049276	19049472
chr12	19057271	19057427
chr12	19061638	19061867
chr12	19120309	19120504
chr12	19120504	19120736
chr12	19122102	19122312
chr12	19175672	19175830
chr12	19181226	19181522
chr12	19207536	19207813
chr12	19208822	19209031
chr12	19211091	19211276
chr12	19243302	19243458
chr12	19288273	19288707
chr12	19288794	19289017

chr12	19387248	19387529
chr12	19387529	19387977
chr12	19387977	19388398
chr12	19388628	19388813
chr12	19393642	19393851
chr12	19418522	19418813
chr12	19606539	19606737
chr12	19612390	19612712
chr12	19643221	19643371
chr12	19647488	19647750
chr12	19743748	19743978
chr12	19953924	19954218
chr12	19959652	19959892
chr12	20196372	20196804
chr12	20196804	20197215
chr12	20197459	20197613
chr12	20202154	20202437
chr12	20226940	20227090
chr12	20228447	20228700
chr12	20228700	20228956
chr12	20230985	20231158
chr12	20231411	20231597
chr12	20231684	20231874
chr12	20231899	20232104
chr12	20233223	20233442
chr12	20233551	20233729
chr12	20233860	20234069
chr12	20234071	20234280
chr12	20298356	20298506
chr12	20312082	20312343
chr12	20415413	20415697
chr12	20422012	20422239
chr12	20444350	20444554
chr12	20451497	20451657
chr12	20493014	20493345
chr12	20614472	20614687
chr12	20614696	20615182
chr12	20615182	20615627
chr12	20620365	20620822
chr12	20646214	20646547
chr12	20698946	20699240
chr12	20704020	20704273

chr12	20727492	20727642
chr12	20782135	20782310
chr12	20809744	20809945
chr12	20815175	20815606
chr12	20815606	20816079
chr12	20816098	20816303
chr12	20844975	20845181
chr12	20870125	20870300
chr12	20884183	20884412
chr12	20884894	20885230
chr12	20914726	20914995
chr12	20919519	20919867
chr12	20920137	20920553
chr12	20920553	20920945
chr12	20920945	20921197
chr12	20921197	20921621
chr12	20929036	20929294
chr12	20943022	20943183
chr12	20989909	20990213
chr12	20990213	20990637
chr12	20990637	20991156
chr12	20999004	20999303
chr12	21030956	21031196
chr12	21075814	21076074
chr12	21087644	21087794
chr12	21090240	21090467
chr12	21104917	21105067
chr12	21111173	21111463
chr12	21111463	21111896
chr12	21111896	21112467
chr12	21112562	21112872
chr12	21116828	21117466
chr12	21118589	21118758
chr12	21119635	21119904
chr12	21131635	21131941
chr12	21132074	21132326
chr12	21132691	21132931
chr12	21133059	21133358
chr12	21135708	21136532
chr12	21142516	21142844
chr12	21142859	21143184
chr12	21143795	21143970

chr12	21144587	21144999
chr12	21144999	21145432
chr12	21151365	21151905
chr12	21155245	21155395
chr12	21159755	21159920
chr12	21160155	21160405
chr12	21160405	21160845
chr12	21165087	21165330
chr12	21165814	21166117
chr12	21166117	21166454
chr12	21166892	21167221
chr12	21167252	21167624
chr12	21174979	21175170
chr12	21175401	21175827
chr12	21176477	21176737
chr12	21180618	21180819
chr12	21182085	21182249
chr12	21182883	21183070
chr12	21191639	21191805
chr12	21194560	21194712
chr12	21195544	21195791
chr12	21200647	21200960
chr12	21201305	21201540
chr12	21204718	21204996
chr12	21205926	21206076
chr12	21207395	21207776
chr12	21208148	21208386
chr12	21225497	21225664
chr12	21226305	21226726
chr12	21227013	21227468
chr12	21227468	21227946
chr12	21234668	21234936
chr12	21243310	21243460
chr12	21247831	21247981
chr12	21250652	21250875
chr12	21252778	21253019
chr12	21260937	21261180
chr12	21264340	21264559
chr12	21264649	21264981
chr12	21265953	21266249
chr12	21266425	21266861
chr12	21271153	21271318

chr12	21274533	21274715
chr12	21278139	21278341
chr12	21278973	21279190
chr12	21285518	21285838
chr12	21285838	21286061
chr12	21286061	21286239
chr12	21286239	21286657
chr12	21286803	21286994
chr12	21287046	21287291
chr12	21316105	21316483
chr12	21316483	21316771
chr12	21318224	21318468
chr12	21335858	21336197
chr12	21336197	21336478
chr12	21336569	21336740
chr12	21373135	21373446
chr12	21373446	21373803
chr12	21373803	21374180
chr12	21406234	21406457
chr12	21407785	21407946
chr12	21409848	21409998
chr12	21417031	21417417
chr12	21417417	21417728
chr12	21417728	21418054
chr12	21424642	21425024
chr12	21425371	21425717
chr12	21426174	21426397
chr12	21426533	21426868
chr12	21430099	21430449
chr12	21431167	21431344
chr12	21436783	21437056
chr12	21437056	21437425
chr12	21437505	21437771
chr12	21441940	21442187
chr12	21463551	21463738
chr12	21467484	21467729
chr12	21471299	21471493
chr12	21472418	21472917
chr12	21480244	21480600
chr12	21494064	21494279
chr12	21518338	21518494
chr12	21525646	21525885

chr12	21526925	21527291
chr12	21543160	21543354
chr12	21543354	21543605
chr12	21565260	21565534
chr12	21580010	21580259
chr12	21613845	21614093
chr12	21696081	21696384
chr12	21743835	21744009
chr12	21748495	21748704
chr12	21749348	21749556
chr12	21749822	21750058
chr12	21772231	21772522
chr12	21778291	21778732
chr12	21778732	21779161
chr12	21783946	21784379
chr12	21784379	21784865
chr12	21898200	21898422
chr12	21899231	21899419
chr12	21940356	21940513
chr12	21965049	21965199
chr12	22024934	22025245
chr12	22232192	22232377
chr12	22257294	22257548
chr12	22261731	22261986
chr12	22269702	22269964
chr12	22385741	22385902
chr12	22460994	22461305
chr12	22466507	22466657
chr12	22467001	22467189
chr12	22617338	22617602
chr12	22622335	22622495
chr12	22622812	22622974
chr12	22623189	22623644
chr12	22708125	22708311
chr12	22739860	22740159
chr12	22745582	22745875
chr12	22811392	22811570
chr12	22900486	22900775
chr12	22905404	22905601
chr12	22906368	22906657
chr12	23004512	23004758
chr12	23009696	23009858

chr12	23009926	23010250
chr12	23010250	23010494
chr12	23023728	23024106
chr12	23105113	23105295
chr12	23122827	23122997
chr12	23125011	23125193
chr12	23183898	23184237
chr12	23189193	23189374
chr12	23189600	23189896
chr12	23217436	23217586
chr12	23384679	23384857
chr12	23409660	23409878
chr12	23414102	23414351
chr12	23422108	23422303
chr12	23597723	23598246
chr12	23605327	23605559
chr12	23605727	23606081
chr12	23628721	23629008
chr12	23634609	23634800
chr12	23720509	23720662
chr12	23743749	23743902
chr12	23758744	23758907
chr12	23765683	23765874
chr12	23794927	23795167
chr12	23800748	23800968
chr12	23800968	23801186
chr12	23963088	23963348
chr12	23968128	23968281
chr12	23968618	23968798
chr12	23968961	23969425
chr12	24054070	24054240
chr12	24082258	24082761
chr12	24091379	24091591
chr12	24096243	24096428
chr12	24096731	24097071
chr12	24097071	24097488
chr12	24186585	24186749
chr12	24246380	24246686
chr12	24246686	24246883
chr12	24251417	24251642
chr12	24251810	24252293
chr12	24252293	24252755

chr12	24408959	24409319
chr12	24462178	24462455
chr12	24463459	24463933
chr12	24467701	24467899
chr12	24471017	24471258
chr12	24471336	24471927
chr12	24473218	24473649
chr12	24487092	24487278
chr12	24488287	24488607
chr12	24498266	24498885
chr12	24498885	24499118
chr12	24499158	24499310
chr12	24525095	24525474
chr12	24564618	24564971
chr12	24565350	24565551
chr12	24565551	24565738
chr12	24571793	24572746
chr12	24592035	24592255
chr12	24592895	24593173
chr12	24593173	24593469
chr12	24593469	24593810
chr12	24600830	24601191
chr12	24603358	24603580
chr12	24603995	24604351
chr12	24612314	24612464
chr12	24613427	24613644
chr12	24623770	24624120
chr12	24630278	24630565
chr12	24633865	24634042
chr12	24642355	24642566
chr12	24642669	24642819
chr12	24642831	24643038
chr12	24643040	24643228
chr12	24650938	24651521
chr12	24651521	24651955
chr12	24651955	24652470
chr12	24660036	24660281
chr12	24661220	24661391
chr12	24663254	24663447
chr12	24674508	24674663
chr12	24675435	24675798
chr12	24680526	24680763

chr12	24680763	24681083
chr12	24681742	24681946
chr12	24685955	24686182
chr12	24686182	24686432
chr12	24686432	24686724
chr12	24693743	24693928
chr12	24696467	24696721
chr12	24697119	24697432
chr12	24697625	24697890
chr12	24705964	24706116
chr12	24706850	24707121
chr12	24707352	24707836
chr12	24707867	24708426
chr12	24708426	24708947
chr12	24710194	24710479
chr12	24716534	24716732
chr12	24716937	24717382
chr12	24723073	24723290
chr12	24723393	24723653
chr12	24723858	24724328
chr12	24724328	24724786
chr12	24734702	24735165
chr12	24735165	24735600
chr12	24738852	24739029
chr12	24741677	24741834
chr12	24745366	24745592
chr12	24750617	24750840
chr12	24753069	24753219
chr12	24766892	24767263
chr12	24767637	24767816
chr12	24779150	24779390
chr12	24789772	24789972
chr12	24793336	24793607
chr12	24793607	24794024
chr12	24798559	24798729
chr12	24803337	24803970
chr12	24822889	24823142
chr12	24830722	24831132
chr12	24831132	24831502
chr12	24831502	24831706
chr12	24831706	24831921
chr12	24831921	24832176

chr12	24841619	24841773
chr12	24843461	24844184
chr12	24844233	24844591
chr12	24845475	24845846
chr12	24856098	24856313
chr12	24856937	24857112
chr12	24859450	24859608
chr12	24891108	24891665
chr12	24894079	24894375
chr12	24894413	24894563
chr12	24895353	24895856
chr12	24928620	24928836
chr12	24940542	24940889
chr12	24940995	24941219
chr12	24948345	24948716
chr12	24950635	24950978
chr12	24952098	24952515
chr12	24974616	24975133
chr12	24975133	24975440
chr12	24975480	24975750
chr12	25005351	25005754
chr12	25012074	25012530
chr12	25012612	25012912
chr12	25012914	25013232
chr12	25013232	25013485
chr12	25015894	25016062
chr12	25025258	25025466
chr12	25027411	25027645
chr12	25057910	25058067
chr12	25059715	25060299
chr12	25092190	25092641
chr12	25092641	25093211
chr12	25095951	25096518
chr12	25098549	25098785
chr12	25098785	25099102
chr12	25099463	25099774
chr12	25101559	25101709
chr12	25108839	25109070
chr12	25110081	25110325
chr12	25110325	25110670
chr12	25110670	25111039
chr12	25117688	25117854

chr12	25117888	25118309
chr12	25131832	25132678
chr12	25141129	25141290
chr12	25143592	25143809
chr12	25144558	25144810
chr12	25155247	25155775
chr12	25155775	25156096
chr12	25162094	25162244
chr12	25163558	25163806
chr12	25164711	25165136
chr12	25165585	25166231
chr12	25166233	25166393
chr12	25169600	25169975
chr12	25175960	25176136
chr12	25176136	25176314
chr12	25180342	25180665
chr12	25180936	25181196
chr12	25181327	25181907
chr12	25189217	25189478
chr12	25189478	25189762
chr12	25190396	25190775
chr12	25193798	25193992
chr12	25203578	25204066
chr12	25208929	25209179
chr12	25209179	25209411
chr12	25209411	25209986
chr12	25216600	25217250
chr12	25226353	25226555
chr12	25227799	25228145
chr12	25232978	25233458
chr12	25239247	25239397
chr12	25240082	25240397
chr12	25240657	25240851
chr12	25244621	25245139
chr12	25245139	25245722
chr12	25251571	25251850
chr12	25258449	25258727
chr12	25266024	25266224
chr12	25268193	25268746
chr12	25268882	25269073
chr12	25269162	25269440
chr12	25270777	25271049

chr12	25277484	25277685
chr12	25281449	25281921
chr12	25282263	25282729
chr12	25283769	25284109
chr12	25298905	25299079
chr12	25306090	25306361
chr12	25310309	25310477
chr12	25311677	25312141
chr12	25318853	25319006
chr12	25342360	25342558
chr12	25360272	25360466
chr12	25372573	25372797
chr12	25378089	25378475
chr12	25383068	25383387
chr12	25383401	25383765
chr12	25385244	25385449
chr12	25389025	25389281
chr12	25396090	25396315
chr12	25438457	25438747
chr12	25438747	25439307
chr12	25475518	25475693
chr12	25505654	25505817
chr12	25530621	25530854
chr12	25531190	25531417
chr12	25543139	25543491
chr12	25585870	25586121
chr12	25609188	25609349
chr12	25613555	25613779
chr12	25617609	25617777
chr12	25627652	25627930
chr12	25628005	25628305
chr12	25628305	25628637
chr12	25641959	25642349
chr12	25648340	25648596
chr12	25650074	25650289
chr12	25657614	25657949
chr12	25683784	25684147
chr12	25742278	25742574
chr12	25784365	25784646
chr12	25785761	25786256
chr12	25786256	25786567
chr12	25792501	25793137

chr12	25805333	25805561
chr12	25820095	25820257
chr12	25851694	25851875
chr12	25858479	25858718
chr12	25858718	25858979
chr12	25878214	25878377
chr12	25886902	25887275
chr12	25890844	25891028
chr12	25924726	25924902
chr12	25983317	25983993
chr12	25984991	25985332
chr12	25993628	25993812
chr12	25993934	25994470
chr12	25996745	25996938
chr12	26071610	26071884
chr12	26079016	26079211
chr12	26079214	26079573
chr12	26174855	26175012
chr12	26191633	26191963
chr12	26194147	26194305
chr12	26206821	26207128
chr12	26296170	26296360
chr12	26315077	26315276
chr12	26340732	26340934
chr12	26365998	26366219
chr12	26414615	26414827
chr12	26414831	26415148
chr12	26415148	26415470
chr12	26454718	26455095
chr12	26456267	26456712
chr12	26468976	26469761
chr12	26469788	26470136
chr12	26533019	26533282
chr12	26598595	26598821
chr12	26955080	26955284
chr12	27068457	27068632
chr12	27164534	27164898
chr12	27165744	27166074
chr12	27200301	27200516
chr12	27200549	27200739
chr12	27318982	27319239
chr12	27341395	27341800

chr12	27341800	27342144
chr12	27342144	27342499
chr12	27342522	27342932
chr12	27342932	27343262
chr12	27343320	27343549
chr12	27374867	27375105
chr12	27379956	27380168
chr12	27386633	27387004
chr12	27390342	27390852
chr12	27392058	27392286
chr12	27468094	27468498
chr12	27479498	27479793
chr12	27485538	27485716
chr12	27485888	27486158
chr12	27489839	27490216
chr12	27490550	27490730
chr12	27491521	27491730
chr12	27491730	27491916
chr12	27491916	27492077
chr12	27492274	27492784
chr12	27495481	27495693
chr12	27503107	27503284
chr12	27507574	27507901
chr12	27507901	27508476
chr12	27508520	27508797
chr12	27521450	27521856
chr12	27532643	27532922
chr12	27533261	27533411
chr12	27546895	27547120
chr12	27547357	27547696
chr12	27553007	27553232
chr12	27586328	27586588
chr12	27599249	27599399
chr12	27604348	27604667
chr12	27623106	27623305
chr12	27724865	27725142
chr12	27740177	27740493
chr12	27759142	27759382
chr12	27769059	27769252
chr12	27780875	27781209
chr12	27786164	27786623
chr12	27786662	27786931

chr12	27796366	27796642
chr12	27796642	27797160
chr12	27821598	27821785
chr12	27821792	27822031
chr12	27826006	27826265
chr12	27826265	27826678
chr12	27827221	27827493
chr12	27829410	27829642
chr12	27839557	27839870
chr12	27896699	27897018
chr12	27906638	27906818
chr12	27940582	27940752
chr12	27977975	27978135
chr12	27999158	27999508
chr12	28055511	28055760
chr12	28081941	28082150
chr12	28171304	28171669
chr12	28174185	28174472
chr12	28235545	28235752
chr12	28344759	28344936
chr12	28432501	28432686
chr12	28476884	28477340
chr12	28501795	28502388
chr12	28514417	28514760
chr12	28531761	28532224
chr12	28533337	28533575
chr12	28533575	28533870
chr12	28534350	28534500
chr12	28554858	28555052
chr12	28574008	28574244
chr12	28574267	28574878
chr12	28579218	28579368
chr12	28588092	28588289
chr12	28589203	28589364
chr12	28589371	28589907
chr12	28589924	28590074
chr12	28598490	28598648
chr12	28598783	28599057
chr12	28602391	28602589
chr12	28614583	28614878
chr12	28621848	28622001
chr12	28622253	28622450

chr12	28626262	28626662
chr12	28626700	28626949
chr12	28626949	28627506
chr12	28628079	28628379
chr12	28628487	28628685
chr12	28635241	28635535
chr12	28635535	28635786
chr12	28635786	28636099
chr12	28636099	28636370
chr12	28638867	28639071
chr12	28649260	28649809
chr12	28649809	28650089
chr12	28658169	28658461
chr12	28667446	28667629
chr12	28675014	28675628
chr12	28675628	28676234
chr12	28678404	28678574
chr12	28689037	28689383
chr12	28689389	28689561
chr12	28691137	28691287
chr12	28697793	28698202
chr12	28698299	28698595
chr12	28698595	28698846
chr12	28699105	28699420
chr12	28699737	28699963
chr12	28706538	28706812
chr12	28707239	28707438
chr12	28715359	28715526
chr12	28722969	28723381
chr12	28727051	28727370
chr12	28727370	28727621
chr12	28727621	28727871
chr12	28738341	28738822
chr12	28739153	28739650
chr12	28739650	28739938
chr12	28749890	28750336
chr12	28750336	28750652
chr12	28751332	28751628
chr12	28751628	28751913
chr12	28751913	28752245
chr12	28773902	28774129
chr12	28784382	28784665

chr12	28804830	28805151
chr12	28805151	28805647
chr12	28829533	28830044
chr12	28831099	28831456
chr12	28848260	28848469
chr12	28848640	28849200
chr12	28867610	28867893
chr12	28886939	28887435
chr12	28898390	28898560
chr12	28898684	28898857
chr12	28899857	28900095
chr12	28902233	28902420
chr12	28902539	28902753
chr12	28903079	28903335
chr12	28906747	28906918
chr12	28958879	28959059
chr12	28975481	28975631
chr12	29015286	29015854
chr12	29018544	29018776
chr12	29021214	29021573
chr12	29022845	29023428
chr12	29047901	29048251
chr12	29048273	29048733
chr12	29054390	29054735
chr12	29068252	29068486
chr12	29073440	29073736
chr12	29073736	29074043
chr12	29078998	29079307
chr12	29099708	29100031
chr12	29100031	29100571
chr12	29108907	29109113
chr12	29128693	29128917
chr12	29128917	29129165
chr12	29139126	29139317
chr12	29157352	29157582
chr12	29162365	29162793
chr12	29175812	29176014
chr12	29178229	29178553
chr12	29191288	29191438
chr12	29213707	29213943
chr12	29242136	29242341
chr12	29282657	29282807

chr12	29290721	29291455
chr12	29358693	29359019
chr12	29465303	29465503
chr12	29621860	29622057
chr12	29676026	29676231
chr12	29691230	29691438
chr12	29691563	29691851
chr12	29749130	29749483
chr12	29749483	29749960
chr12	29778996	29779161
chr12	29795143	29795357
chr12	29814383	29814548
chr12	29837568	29837783
chr12	29855102	29855344
chr12	29872526	29872730
chr12	29878627	29878838
chr12	29881756	29881975
chr12	29882941	29883192
chr12	29883284	29883573
chr12	29894348	29894616
chr12	29894727	29895001
chr12	29895719	29896434
chr12	29905243	29905559
chr12	29906921	29907073
chr12	29907076	29907450
chr12	29907450	29907833
chr12	29919878	29920230
chr12	29921578	29921824
chr12	29926952	29927283
chr12	29932955	29933339
chr12	29933464	29933806
chr12	29933806	29934220
chr12	29934335	29934682
chr12	29937802	29938126
chr12	29938126	29938398
chr12	29938398	29938667
chr12	29938667	29939061
chr12	29954096	29954283
chr12	29961447	29961772
chr12	29961780	29961937
chr12	29964943	29965511
chr12	29965577	29965759

chr12	29992332	29992482
chr12	29998963	29999269
chr12	29999391	29999681
chr12	30003444	30004054
chr12	30006422	30006787
chr12	30016239	30016472
chr12	30020496	30020781
chr12	30023139	30023289
chr12	30023611	30023763
chr12	30024450	30024768
chr12	30031966	30032237
chr12	30032709	30033247
chr12	30033355	30033550
chr12	30036670	30036830
chr12	30037603	30037901
chr12	30039420	30039824
chr12	30039824	30040014
chr12	30040057	30040232
chr12	30040262	30040528
chr12	30050014	30050192
chr12	30050285	30050525
chr12	30055246	30055454
chr12	30062199	30062525
chr12	30071110	30071440
chr12	30102995	30103164
chr12	30112274	30112431
chr12	30128633	30128889
chr12	30142188	30142341
chr12	30369897	30370122
chr12	30373212	30373578
chr12	30504978	30505135
chr12	30578101	30578470
chr12	30578470	30578836
chr12	30578836	30579133
chr12	30583882	30584070
chr12	30584117	30584579
chr12	30584579	30584969
chr12	30584973	30585182
chr12	30597050	30597306
chr12	30618385	30618558
chr12	30635134	30635527
chr12	30635547	30636082

chr12	30647165	30647335
chr12	30651274	30651466
chr12	30660400	30660563
chr12	30663996	30664258
chr12	30767630	30767942
chr12	30846715	30847203
chr12	30850831	30851162
chr12	30865623	30865878
chr12	30865878	30866216
chr12	30868525	30868760
chr12	30868841	30869087
chr12	30884057	30884610
chr12	30884700	30884900
chr12	30890127	30890379
chr12	30910838	30911096
chr12	30911166	30911529
chr12	30911529	30911935
chr12	30911935	30912373
chr12	30922437	30922768
chr12	30933398	30933582
chr12	30943350	30943553
chr12	31001939	31002091
chr12	31003861	31004141
chr12	31005086	31005356
chr12	31006785	31007016
chr12	31008223	31008550
chr12	31011976	31012141
chr12	31056353	31056951
chr12	31057049	31057483
chr12	31059354	31059968
chr12	31060271	31060474
chr12	31061784	31061995
chr12	31062089	31062256
chr12	31066206	31066467
chr12	31066467	31066726
chr12	31066958	31067118
chr12	31067361	31067578
chr12	31073518	31074229
chr12	31074229	31074736
chr12	31074736	31075201
chr12	31075230	31075524
chr12	31083377	31083851

chr12	31083851	31084361
chr12	31090450	31090691
chr12	31097226	31097599
chr12	31104319	31104469
chr12	31115827	31116160
chr12	31116160	31116427
chr12	31128901	31129171
chr12	31132272	31132486
chr12	31145093	31145349
chr12	31145349	31145799
chr12	31149954	31150237
chr12	31159058	31159221
chr12	31170780	31171068
chr12	31185148	31185553
chr12	31186475	31186774
chr12	31186774	31187090
chr12	31194654	31194827
chr12	31236858	31237034
chr12	31237318	31237478
chr12	31239278	31239474
chr12	31258595	31258824
chr12	31264680	31265092
chr12	31265092	31265384
chr12	31265384	31265678
chr12	31265678	31265918
chr12	31266047	31266383
chr12	31270889	31271152
chr12	31271152	31271432
chr12	31271432	31271764
chr12	31271764	31272075
chr12	31272328	31272613
chr12	31278441	31278633
chr12	31292155	31292413
chr12	31292413	31292740
chr12	31292804	31292984
chr12	31294504	31294951
chr12	31302853	31303243
chr12	31303243	31303506
chr12	31333125	31333291
chr12	31350336	31350510
chr12	31350844	31351288
chr12	31351288	31351630

chr12	31370224	31370519
chr12	31374636	31374989
chr12	31376389	31376623
chr12	31415894	31416342
chr12	31444142	31444548
chr12	31444808	31445004
chr12	31453869	31454062
chr12	31460351	31460503
chr12	31499037	31499198
chr12	31499254	31499478
chr12	31499478	31499755
chr12	31499755	31500265
chr12	31505556	31505712
chr12	31507269	31507481
chr12	31508072	31508222
chr12	31508503	31508862
chr12	31513783	31514098
chr12	31514098	31514345
chr12	31519925	31520146
chr12	31554789	31555014
chr12	31558964	31559409
chr12	31559409	31559953
chr12	31563960	31564313
chr12	31567564	31567947
chr12	31571695	31571853
chr12	31573833	31574071
chr12	31574486	31574901
chr12	31574964	31575192
chr12	31578177	31578342
chr12	31581108	31581313
chr12	31582177	31582669
chr12	31583798	31584159
chr12	31587340	31587545
chr12	31591064	31591232
chr12	31591417	31591713
chr12	31591843	31592053
chr12	31592087	31592334
chr12	31605873	31606085
chr12	31606085	31606377
chr12	31633980	31634286
chr12	31634302	31634837
chr12	31634837	31635245

chr12	31653725	31653923
chr12	31654305	31654465
chr12	31654469	31654718
chr12	31654718	31654964
chr12	31654964	31655204
chr12	31655204	31655430
chr12	31691826	31691982
chr12	31695416	31695587
chr12	31695587	31695742
chr12	31726787	31726978
chr12	31818125	31818363
chr12	31838368	31838897
chr12	31848965	31849421
chr12	31849485	31849975
chr12	31851175	31851325
chr12	31858559	31858735
chr12	31871786	31872000
chr12	31872000	31872216
chr12	31882728	31883065
chr12	31895959	31896243
chr12	31902593	31902892
chr12	31913594	31913986
chr12	31914819	31915186
chr12	31926620	31927079
chr12	31929763	31929914
chr12	31949191	31949678
chr12	31949678	31950090
chr12	31950090	31950451
chr12	31950451	31951021
chr12	31967612	31967899
chr12	31969240	31969390
chr12	31969900	31970313
chr12	31973476	31974053
chr12	31996731	31997299
chr12	31997384	31997574
chr12	32001617	32001847
chr12	32009174	32009610
chr12	32009610	32010192
chr12	32015330	32015792
chr12	32017547	32017963
chr12	32018111	32018311
chr12	32018421	32018700

chr12	32022513	32022761
chr12	32044575	32044730
chr12	32057544	32057792
chr12	32060651	32061145
chr12	32061145	32061710
chr12	32091144	32091294
chr12	32094559	32094715
chr12	32095061	32095499
chr12	32095518	32095678
chr12	32099678	32099923
chr12	32099923	32100166
chr12	32114561	32114740
chr12	32137622	32137837
chr12	32144446	32144877
chr12	32144877	32145133
chr12	32163765	32164049
chr12	32227680	32227840
chr12	32228766	32229017
chr12	32234244	32234502
chr12	32241305	32241656
chr12	32241656	32242050
chr12	32242214	32242383
chr12	32242846	32243402
chr12	32243402	32244036
chr12	32280024	32280186
chr12	32290943	32291340
chr12	32291340	32291589
chr12	32291960	32292379
chr12	32292920	32293081
chr12	32297486	32297772
chr12	32299552	32300140
chr12	32300161	32300349
chr12	32303564	32303715
chr12	32306424	32306777
chr12	32336827	32337247
chr12	32342076	32342301
chr12	32343531	32343860
chr12	32343860	32344106
chr12	32344184	32344530
chr12	32351090	32351333
chr12	32351733	32352319
chr12	32366684	32367009

chr12	32367036	32367265
chr12	32378457	32378757
chr12	32378757	32378981
chr12	32378981	32379324
chr12	32379324	32379958
chr12	32397812	32397962
chr12	32409071	32409299
chr12	32427607	32427875
chr12	32427875	32428348
chr12	32434600	32434962
chr12	32434962	32435224
chr12	32435224	32435459
chr12	32442146	32442357
chr12	32451172	32451593
chr12	32460319	32460569
chr12	32465138	32465289
chr12	32465306	32465986
chr12	32488327	32488644
chr12	32533580	32533987
chr12	32546436	32546754
chr12	32546754	32547063
chr12	32555561	32555859
chr12	32560818	32561171
chr12	32579048	32579604
chr12	32586041	32586219
chr12	32586219	32586557
chr12	32586619	32586945
chr12	32617066	32617292
chr12	32617292	32617503
chr12	32621710	32621860
chr12	32624173	32624345
chr12	32724512	32724753
chr12	32729485	32729705
chr12	32745812	32746068
chr12	32819820	32820232
chr12	32820232	32820591
chr12	32820591	32820904
chr12	32820904	32821164
chr12	32827035	32827306
chr12	32848084	32848602
chr12	32854894	32855151
chr12	32895813	32896125

chr12	32897520	32897807
chr12	32897807	32897992
chr12	32910115	32910482
chr12	32910571	32910842
chr12	32910915	32911176
chr12	32920243	32920463
chr12	32921597	32921914
chr12	32921914	32922205
chr12	32923064	32923262
chr12	32923520	32923705
chr12	32924359	32924534
chr12	32929909	32930065
chr12	32952601	32952757
chr12	32953504	32953836
chr12	32953836	32954257
chr12	32954257	32954751
chr12	32965424	32965629
chr12	32975609	32975778
chr12	32984856	32985439
chr12	32989705	32990055
chr12	32990055	32990309
chr12	32994731	32995602
chr12	33000322	33000788
chr12	33000810	33000960
chr12	33008305	33008911
chr12	33008911	33009140
chr12	33009722	33009911
chr12	33009911	33010120
chr12	33010120	33010377
chr12	33014990	33015372
chr12	33019853	33020096
chr12	33020096	33020401
chr12	33020401	33020832
chr12	33021270	33021536
chr12	33027040	33027543
chr12	33027641	33027814
chr12	33028395	33028611
chr12	33036270	33036457
chr12	33040512	33040662
chr12	33040685	33041169
chr12	33048448	33048623
chr12	33057409	33057559

chr12	33061550	33061767
chr12	33074184	33074362
chr12	33075244	33075440
chr12	33078980	33079164
chr12	33083458	33083608
chr12	33084111	33084289
chr12	33084748	33084998
chr12	33100528	33100898
chr12	33101751	33101965
chr12	33101965	33102183
chr12	33102258	33102631
chr12	33102631	33102906
chr12	33103557	33103724
chr12	33109279	33109508
chr12	33109578	33109905
chr12	33129536	33129760
chr12	33140450	33140630
chr12	33144921	33145085
chr12	33146784	33147133
chr12	33147133	33147496
chr12	33147496	33147826
chr12	33147826	33148141
chr12	33154869	33155050
chr12	33157132	33157316
chr12	33158035	33158697
chr12	33159421	33159683
chr12	33160492	33160803
chr12	33169886	33170222
chr12	33170821	33171310
chr12	33173713	33174072
chr12	33174288	33174480
chr12	33174588	33175072
chr12	33175072	33175365
chr12	33175961	33176312
chr12	33180524	33180696
chr12	33181269	33181469
chr12	33181732	33182328
chr12	33182328	33182804
chr12	33182998	33183163
chr12	33190222	33190432
chr12	33193399	33193952
chr12	33201640	33202311

chr12	33202434	33203042
chr12	33217043	33217301
chr12	33217301	33217580
chr12	33217685	33217900
chr12	33220479	33220702
chr12	33220702	33220888
chr12	33222290	33222536
chr12	33222592	33222770
chr12	33235027	33235227
chr12	33235300	33235639
chr12	33235694	33236159
chr12	33245461	33245857
chr12	33263270	33263482
chr12	33269600	33269832
chr12	33269832	33270069
chr12	33279780	33279959
chr12	33285305	33285766
chr12	33285766	33286327
chr12	33290138	33290550
chr12	33302069	33302560
chr12	33302786	33303096
chr12	33303096	33303319
chr12	33310806	33311183
chr12	33333301	33333742
chr12	33333779	33333983
chr12	33333983	33334446
chr12	33334595	33334831
chr12	33344121	33344309
chr12	33359962	33360255
chr12	33368929	33369135
chr12	33369592	33369867
chr12	33371675	33372250
chr12	33379742	33379919
chr12	33379934	33380456
chr12	33387555	33387776
chr12	33388698	33388848
chr12	33388874	33389182
chr12	33391224	33391469
chr12	33394832	33395207
chr12	33395214	33395504
chr12	33407679	33408078
chr12	33408078	33408388

chr12	33408388	33408626
chr12	33408626	33408922
chr12	33408922	33409240
chr12	33409272	33409484
chr12	33409484	33409734
chr12	33409734	33410116
chr12	33424436	33424631
chr12	33429353	33429740
chr12	33429740	33429955
chr12	33429955	33430230
chr12	33434895	33435050
chr12	33455557	33455801
chr12	33638035	33638286
chr12	33823780	33823963
chr12	33864565	33864725
chr12	33875499	33875661
chr12	33898874	33899050
chr12	33956931	33957159
chr12	33957159	33957339
chr12	33957339	33957541
chr12	33957541	33957810
chr12	33957810	33958249
chr12	33958249	33958670
chr12	33958670	33958908
chr12	33967511	33967693
chr12	33967693	33967874
chr12	33968150	33968356
chr12	34040096	34040533
chr12	34040628	34040921
chr12	34040921	34041365
chr12	34048633	34048785
chr12	34075614	34076172
chr12	34076172	34076463
chr12	34087239	34087577
chr12	34087577	34087962
chr12	34091853	34092118
chr12	34144320	34144609
chr12	34144617	34144793
chr12	34209140	34209290
chr12	34224221	34224378
chr12	34224556	34224833
chr12	34225512	34225667

chr12	34225710	34226077
chr12	34226889	34227102
chr12	34273959	34274131
chr12	34326496	34326972
chr12	34337100	34337371
chr12	34337380	34337798
chr12	34348307	34348476
chr12	34372871	34373117
chr12	34432551	34432778
chr12	34469401	34469676
chr12	34521565	34521715
chr12	34553870	34554092
chr12	34579311	34579500
chr12	34612576	34612813
chr12	34664901	34665069
chr12	34674336	34674517
chr12	34752194	34752409
chr12	34852007	34852157
chr12	34901571	34901843
chr12	34906910	34907493
chr12	34990223	34990625
chr12	34990625	34990957
chr12	34991232	34991533
chr12	34991934	34992381
chr12	35046647	35046971
chr12	35046971	35047369
chr12	35047369	35047698
chr12	35047698	35047992
chr12	35055182	35055410
chr12	35064829	35065135
chr12	35066549	35066862
chr12	35067660	35068005
chr12	35072863	35073013
chr12	35123497	35123857
chr12	35154265	35154532
chr12	35158103	35158440
chr12	35182727	35183009
chr12	35192261	35192411
chr12	35192513	35192718
chr12	35192937	35193349
chr12	35193349	35194044
chr12	35199503	35199668

chr12	35204979	35205193
chr12	35209212	35209870
chr12	35216060	35216323
chr12	35217768	35218048
chr12	35220024	35220421
chr12	35220421	35220780
chr12	35224461	35224790
chr12	35228697	35229047
chr12	35244362	35244554
chr12	35246179	35246678
chr12	35246678	35246983
chr12	35247226	35247502
chr12	35283935	35284119
chr12	35286759	35287043
chr12	35287043	35287277
chr12	35291617	35291844
chr12	35291844	35292278
chr12	35294536	35294831
chr12	35294831	35295095
chr12	35331567	35331837
chr12	35333522	35334021
chr12	35337045	35337394
chr12	35338408	35338929
chr12	35339190	35339395
chr12	35376460	35376641
chr12	35376641	35376823
chr12	35397661	35397845
chr12	35418790	35419029
chr12	35423785	35424129
chr12	35459762	35460185
chr12	35470943	35471244
chr12	35473047	35473322
chr12	35479881	35480149
chr12	35493244	35493660
chr12	35503676	35503886
chr12	35506407	35506682
chr12	35507063	35507330
chr12	35515626	35515904
chr12	35517279	35517479
chr12	35517479	35517697
chr12	35517697	35518067
chr12	35518147	35518363

chr12	35518363	35518658
chr12	35518658	35518851
chr12	35520447	35520817
chr12	35527530	35527908
chr12	35534033	35534255
chr12	35534255	35534509
chr12	35534509	35534894
chr12	35534894	35535372
chr12	35538836	35539139
chr12	35544526	35544830
chr12	35546070	35546344
chr12	35553180	35553399
chr12	35562801	35563400
chr12	35564596	35564896
chr12	35573924	35574235
chr12	35576219	35576422
chr12	35578879	35579184
chr12	35605472	35605698
chr12	35607462	35608224
chr12	35652351	35652649
chr12	35656208	35656579
chr12	35656579	35656939
chr12	35667175	35667325
chr12	35670977	35671229
chr12	35671340	35671490
chr12	35671868	35672067
chr12	35672242	35672529
chr12	35672529	35672757
chr12	35672757	35673033
chr12	35675586	35675799
chr12	35677031	35677267
chr12	35677267	35677608
chr12	35685322	35685553
chr12	35687744	35688032
chr12	35694258	35694635
chr12	35694832	35695156
chr12	35700334	35700495
chr12	35702625	35702942
chr12	35712169	35712463
chr12	35712519	35712847
chr12	35712847	35713118
chr12	35713204	35713469

chr12	35716700	35716850
chr12	35717325	35717475
chr12	35726999	35727249
chr12	35733647	35733981
chr12	35734374	35734813
chr12	35735089	35735312
chr12	35736323	35736555
chr12	35736555	35736802
chr12	35738029	35738254
chr12	35741959	35742328
chr12	35742616	35743080
chr12	35751401	35751851
chr12	35760495	35760778
chr12	35764818	35765237
chr12	35770564	35770944
chr12	35824534	35824985
chr12	35826829	35827022
chr12	35827049	35827395
chr12	35831234	35831632
chr12	35832967	35833318
chr12	35833318	35833650
chr12	35833685	35833853
chr12	35837017	35837392
chr12	35843051	35843253
chr12	35850162	35850383
chr12	35853172	35853538
chr12	35853773	35853991
chr12	35884963	35885180
chr12	35894414	35894589
chr12	35934920	35935232
chr12	35957315	35957480
chr12	35981791	35982045
chr12	35984823	35985109
chr12	35995900	35996118
chr12	35997734	35998096
chr12	35999114	35999309
chr12	36002320	36002610
chr12	36036825	36036994
chr12	36037070	36037230
chr12	36041809	36042096
chr12	36042096	36042407
chr12	36042407	36042889

chr12	36064544	36064767
chr12	36074328	36074906
chr12	36081697	36081865
chr12	36093345	36093557
chr12	36119144	36119433
chr12	36134192	36134455
chr12	36145451	36145653
chr12	36156258	36156976
chr12	36156985	36157310
chr12	36157332	36157588
chr12	36157591	36157946
chr12	36157946	36158232
chr12	36178356	36178570
chr12	36187229	36187394
chr12	36205702	36205867
chr12	36215221	36215378
chr12	36231490	36231700
chr12	36241250	36241430
chr12	36317180	36317501
chr12	36373721	36373948
chr12	36381392	36381701
chr12	36381701	36381999
chr12	36381999	36382376
chr12	36406083	36406251
chr12	36577860	36578098
chr12	36714115	36714292
chr12	36722830	36723017
chr12	36880022	36880304
chr12	36976738	36977196
chr12	36988322	36988770
chr12	37005203	37005547
chr12	37019075	37019700
chr12	37072272	37072510
chr12	37072510	37072739
chr12	37072739	37072998
chr12	37108951	37109342
chr12	37142052	37142330
chr12	37181407	37181589
chr12	37183772	37183993
chr12	37231191	37231356
chr12	37233580	37233784
chr12	37450868	37451051

chr12	37614832	37615111
chr12	37769913	37770127
chr12	38778277	38778458
chr12	38778521	38778870
chr12	38778870	38779227
chr12	38779227	38779750
chr12	38779874	38780036
chr12	38780131	38780383
chr12	38788512	38789015
chr12	38843755	38844315
chr12	38904641	38904803
chr12	38928913	38929304
chr12	38931101	38931301
chr12	39457506	39457699
chr12	39510013	39510285
chr12	39572909	39573065
chr12	39665514	39665770
chr12	39695401	39695748
chr12	39724210	39724671
chr12	39725242	39725473
chr12	39726273	39726572
chr12	39761109	39761260
chr12	39761353	39761652
chr12	39761965	39762409
chr12	39831601	39831815
chr12	39858857	39859052
chr12	39872344	39872631
chr12	39894043	39894503
chr12	39907660	39907820
chr12	39912798	39913098
chr12	39913098	39913554
chr12	39919430	39919826
chr12	39927206	39927400
chr12	39930865	39931015
chr12	39935025	39935186
chr12	39935877	39936188
chr12	39939053	39939353
chr12	39950351	39950573
chr12	39950573	39950769
chr12	39956575	39956731
chr12	39978207	39978506
chr12	39979179	39979382

chr12	39990203	39990512
chr12	40009851	40010281
chr12	40023391	40023553
chr12	40027371	40027780
chr12	40029267	40029549
chr12	40030164	40030403
chr12	40037139	40037738
chr12	40037738	40038074
chr12	40038074	40038415
chr12	40082524	40082784
chr12	40090067	40090315
chr12	40096564	40096714
chr12	40125957	40126123
chr12	40133453	40133996
chr12	40133996	40134321
chr12	40140720	40141105
chr12	40159760	40159991
chr12	40164685	40164965
chr12	40177227	40177377
chr12	40198118	40198268
chr12	40198452	40198764
chr12	40199495	40199754
chr12	40199844	40200014
chr12	40200017	40200513
chr12	40200551	40200779
chr12	40202406	40202594
chr12	40205424	40205774
chr12	40206646	40206890
chr12	40210777	40211045
chr12	40212666	40212949
chr12	40222254	40222583
chr12	40222583	40223051
chr12	40223051	40223688
chr12	40223886	40224090
chr12	40229738	40230014
chr12	40231163	40231388
chr12	40233399	40233560
chr12	40233802	40234136
chr12	40239245	40239605
chr12	40243746	40244241
chr12	40247982	40248326
chr12	40253144	40253641

chr12	40257300	40257530
chr12	40263127	40263305
chr12	40274694	40275302
chr12	40285744	40286055
chr12	40335412	40335562
chr12	40341551	40341759
chr12	40347861	40348019
chr12	40348167	40348680
chr12	40383719	40384093
chr12	40434200	40434466
chr12	40445269	40445592
chr12	40445666	40446357
chr12	40446357	40446960
chr12	40458588	40458936
chr12	40458936	40459363
chr12	40459756	40459933
chr12	40464279	40464547
chr12	40470460	40470610
chr12	40471523	40471683
chr12	40471712	40471925
chr12	40472060	40472417
chr12	40472462	40472634
chr12	40472733	40473123
chr12	40473335	40473678
chr12	40473678	40474006
chr12	40480276	40480613
chr12	40483508	40483740
chr12	40486498	40486674
chr12	40490498	40491107
chr12	40491162	40491329
chr12	40497821	40498065
chr12	40499368	40499570
chr12	40499611	40499906
chr12	40499906	40500300
chr12	40516279	40516601
chr12	40532429	40532660
chr12	40532660	40532882
chr12	40532882	40533151
chr12	40535749	40536026
chr12	40536026	40536297
chr12	40536297	40536589
chr12	40551415	40551572

chr12	40558719	40558869
chr12	40558886	40559549
chr12	40562695	40562958
chr12	40568982	40569621
chr12	40574086	40574333
chr12	40581146	40581296
chr12	40582086	40582258
chr12	40584531	40584955
chr12	40584955	40585340
chr12	40587961	40588203
chr12	40626617	40626913
chr12	40651536	40651811
chr12	40678354	40678754
chr12	40678769	40678943
chr12	40680596	40680886
chr12	40712335	40712553
chr12	40750917	40751136
chr12	40773194	40773386
chr12	40795846	40796061
chr12	40798129	40798480
chr12	40823840	40824021
chr12	40837702	40838059
chr12	40842693	40842860
chr12	40888081	40888860
chr12	40888909	40889094
chr12	40893643	40893802
chr12	40897101	40897266
chr12	40898777	40898938
chr12	40899031	40899399
chr12	40902750	40902995
chr12	40903858	40904062
chr12	40906936	40907162
chr12	40908133	40908428
chr12	40908693	40909153
chr12	40909211	40909477
chr12	40909491	40909676
chr12	40917484	40917831
chr12	41023783	41024146
chr12	41024146	41024583
chr12	41080317	41080510
chr12	41081731	41082256
chr12	41085790	41086188

chr12	41089948	41090370
chr12	41097474	41097727
chr12	41146805	41146959
chr12	41171541	41171873
chr12	41192363	41192548
chr12	41192921	41193154
chr12	41193154	41193403
chr12	41349482	41349679
chr12	41400498	41400662
chr12	41401209	41401435
chr12	41402081	41402337
chr12	41445181	41445341
chr12	41445462	41445931
chr12	41485839	41486175
chr12	41487107	41487272
chr12	41515047	41515263
chr12	41521758	41521938
chr12	41552114	41552324
chr12	41565680	41565957
chr12	41655280	41655501
chr12	41693858	41694009
chr12	41695458	41695624
chr12	41820105	41820608
chr12	41879512	41879712
chr12	41884060	41884231
chr12	41896956	41897163
chr12	41925140	41925399
chr12	41927587	41927868
chr12	41935456	41935909
chr12	41948243	41948419
chr12	41949495	41949793
chr12	41949793	41950074
chr12	41950074	41950364
chr12	41952462	41952723
chr12	41997432	41997676
chr12	42121481	42121638
chr12	42136038	42136409
chr12	42136882	42137164
chr12	42137316	42137591
chr12	42795860	42796112
chr12	42843456	42844083
chr12	42891114	42891293

chr12	42891293	42891479
chr12	42891482	42891841
chr12	42892294	42892602
chr12	42905797	42905961
chr12	44143800	44144058
chr12	44165264	44165424
chr12	44181384	44181615
chr12	44209453	44209925
chr12	44209925	44210687
chr12	44246674	44246945
chr12	44248428	44249003
chr12	44268592	44268968
chr12	44268968	44269312
chr12	44269312	44269631
chr12	44269631	44269888
chr12	44280210	44280459
chr12	44292359	44292590
chr12	44304158	44304316
chr12	44328606	44328761
chr12	44328785	44329043
chr12	44329043	44329330
chr12	44329330	44329647
chr12	44329667	44330200
chr12	44332090	44332444
chr12	44344210	44344365
chr12	44453832	44454136
chr12	44524532	44524909
chr12	44524909	44525177
chr12	44525177	44525744
chr12	44528482	44528711
chr12	44528711	44528931
chr12	44536236	44536569
chr12	44588203	44588361
chr12	44639584	44639946
chr12	44640024	44640282
chr12	44641838	44642311
chr12	44642313	44642634
chr12	44657808	44657983
chr12	44660014	44660348
chr12	44661386	44661694
chr12	44710105	44710418
chr12	44848584	44848805

chr12	44848805	44849015
chr12	44849015	44849230
chr12	44858049	44858497
chr12	44858511	44859040
chr12	44924686	44924896
chr12	44930820	44931103
chr12	44931103	44931321
chr12	44935376	44935551
chr12	44943198	44943573
chr12	44969159	44969392
chr12	44974305	44974466
chr12	45003739	45003900
chr12	45006089	45006254
chr12	45006599	45006917
chr12	45008576	45008726
chr12	45010065	45010284
chr12	45010284	45010583
chr12	45029666	45030030
chr12	45030040	45030240
chr12	45031447	45031792
chr12	45034025	45034208
chr12	45034208	45034399
chr12	45040615	45041030
chr12	45046891	45047161
chr12	45047161	45047604
chr12	45059872	45060224
chr12	45060224	45060565
chr12	45060611	45061189
chr12	45061249	45061614
chr12	45067082	45067517
chr12	45073778	45074007
chr12	45074007	45074298
chr12	45074298	45074668
chr12	45074668	45074981
chr12	45075034	45075350
chr12	45080135	45080339
chr12	45080424	45080737
chr12	45080737	45081175
chr12	45103069	45103315
chr12	45132950	45133133
chr12	45137740	45138345
chr12	45161728	45161903

chr12	45162075	45162355
chr12	45172011	45172276
chr12	45183326	45183503
chr12	45187240	45187448
chr12	45319794	45320006
chr12	45500380	45500765
chr12	45519673	45519870
chr12	45644220	45644640
chr12	45657394	45657737
chr12	45837462	45837739
chr12	46707608	46707879
chr12	46707879	46708158
chr12	46708158	46708476
chr12	46781559	46781770
chr12	46818860	46819350
chr12	46819350	46819884
chr12	46931070	46931429
chr12	46997235	46997514
chr12	46997514	46997811
chr12	47954134	47954886
chr12	48020429	48020770
chr12	48435567	48435781
chr12	48446199	48446370
chr12	48465378	48465729
chr12	48496628	48496874
chr12	48605404	48605614
chr12	48654552	48654914
chr12	48805997	48806231
chr12	48900459	48900707
chr12	49046046	49046278
chr12	49046317	49046710
chr12	49098966	49099130
chr12	49099588	49099969
chr12	49144229	49144495
chr12	49148571	49148772
chr12	49155242	49155444
chr12	49170195	49170345
chr12	49374601	49374758
chr12	49374785	49375020
chr12	49383269	49383594
chr12	49383626	49384245
chr12	49384264	49384501

chr12	49384501	49384722
chr12	49384722	49384967
chr12	49384967	49385253
chr12	49385253	49385704
chr12	49389740	49389929
chr12	49390307	49390537
chr12	49390996	49391161
chr12	49391210	49391486
chr12	49391486	49391755
chr12	49391755	49392023
chr12	49394864	49395091
chr12	49398297	49398483
chr12	49400617	49400783
chr12	49400818	49401239
chr12	49401239	49401534
chr12	49401534	49401899
chr12	49401987	49402139
chr12	49408310	49408471
chr12	49426199	49426617
chr12	49472808	49473096
chr12	49473352	49473644
chr12	49475706	49475933
chr12	49477020	49477381
chr12	49477381	49477786
chr12	49493447	49493831
chr12	49502791	49503141
chr12	49503141	49503467
chr12	49512773	49513087
chr12	49513087	49513584
chr12	49534659	49534885
chr12	49535269	49535524
chr12	49739950	49740119
chr12	49836531	49836727
chr12	49887329	49887547
chr12	49888654	49889144
chr12	49889272	49889422
chr12	49906752	49907012
chr12	49932296	49932504
chr12	49944999	49945149
chr12	49951879	49952140
chr12	49967897	49968421
chr12	49968421	49968691

chr12	49969547	49969876
chr12	49971214	49971429
chr12	49975078	49975339
chr12	50025827	50025986
chr12	50038860	50039512
chr12	50064636	50064836
chr12	50064865	50065114
chr12	50066006	50066312
chr12	50066472	50066755
chr12	50066759	50066936
chr12	50073724	50074114
chr12	50074172	50074378
chr12	50172524	50172686
chr12	50292965	50293125
chr12	50301464	50301614
chr12	50506591	50506857
chr12	50514661	50514846
chr12	50544354	50544656
chr12	50544712	50544923
chr12	50544923	50545204
chr12	50549848	50550078
chr12	50588447	50588812
chr12	50588858	50589097
chr12	50589097	50589291
chr12	50598175	50598374
chr12	50615607	50615826
chr12	50615925	50616149
chr12	50621101	50621283
chr12	50624217	50624550
chr12	50648240	50648467
chr12	50648520	50648782
chr12	50648782	50649132
chr12	50649132	50649594
chr12	50943707	50943958
chr12	50999703	50999964
chr12	51024119	51024304
chr12	51024484	51024737
chr12	51188325	51188530
chr12	51196253	51196679
chr12	51219279	51219550
chr12	51241225	51241505
chr12	51283765	51284019

chr12	51296080	51296470
chr12	51308554	51308910
chr12	51309897	51310127
chr12	51346226	51346471
chr12	51347888	51348671
chr12	51353103	51353439
chr12	51355354	51355622
chr12	51368534	51368850
chr12	51377257	51377878
chr12	51402167	51402355
chr12	51488517	51488669
chr12	51489973	51490215
chr12	51501428	51501793
chr12	51563975	51564171
chr12	51577860	51578173
chr12	51578218	51578451
chr12	51592014	51592229
chr12	51592967	51593237
chr12	51593237	51593444
chr12	51593444	51593637
chr12	51593637	51593896
chr12	51593896	51594208
chr12	51606228	51606379
chr12	51641927	51642188
chr12	51653342	51653523
chr12	51653791	51653947
chr12	51663983	51664149
chr12	51664364	51664529
chr12	51664835	51664992
chr12	51666196	51666348
chr12	51666789	51667129
chr12	51667129	51667430
chr12	51668142	51668327
chr12	51673311	51673579
chr12	51673579	51673912
chr12	51678919	51679186
chr12	51682043	51682211
chr12	51682482	51682638
chr12	51690905	51691098
chr12	51691163	51691560
chr12	51691560	51691858
chr12	51691858	51692184

chr12	51692184	51692606
chr12	51693578	51693728
chr12	51694145	51694464
chr12	51694513	51694708
chr12	51699928	51700484
chr12	51700484	51700817
chr12	51703784	51704187
chr12	51706713	51707178
chr12	51711446	51711885
chr12	51712732	51712969
chr12	51721217	51721645
chr12	51721670	51722298
chr12	51722454	51722672
chr12	51722672	51722859
chr12	51734319	51734594
chr12	51734594	51734901
chr12	51734901	51735185
chr12	51735195	51735355
chr12	51735854	51736175
chr12	51741019	51741181
chr12	51741644	51742063
chr12	51742088	51742257
chr12	51747148	51747657
chr12	51767556	51767731
chr12	51772227	51772384
chr12	51773276	51773482
chr12	51784858	51785159
chr12	51805671	51805893
chr12	51811343	51811724
chr12	51811724	51812021
chr12	51819494	51819709
chr12	51819767	51820075
chr12	51821420	51821666
chr12	51822942	51823189
chr12	51825391	51825654
chr12	51828660	51829068
chr12	51829068	51829483
chr12	51829483	51830140
chr12	51838149	51838323
chr12	51844797	51845327
chr12	51847835	51848064
chr12	51848064	51848339

chr12	51850371	51850792
chr12	51864828	51865116
chr12	51865116	51865387
chr12	51865480	51865676
chr12	51865762	51866335
chr12	51866335	51866834
chr12	51873773	51874091
chr12	51875453	51875605
chr12	51882610	51882945
chr12	51883015	51883276
chr12	51887608	51888164
chr12	51888493	51889023
chr12	51939874	51940039
chr12	51940039	51940215
chr12	51947349	51948020
chr12	51952381	51952666
chr12	51965042	51965338
chr12	51970787	51971102
chr12	51971102	51971701
chr12	51977464	51977748
chr12	51977839	51978170
chr12	51985101	51985309
chr12	51987262	51987443
chr12	51988557	51988984
chr12	52001510	52001896
chr12	52005939	52006271
chr12	52006271	52006679
chr12	52006699	52006920
chr12	52030034	52030244
chr12	52030823	52031078
chr12	52031078	52031395
chr12	52038383	52038669
chr12	52042899	52043094
chr12	52097575	52098070
chr12	52098432	52098582
chr12	52119662	52120083
chr12	52126428	52127035
chr12	52142529	52142873
chr12	52142873	52143221
chr12	52152195	52152436
chr12	52190066	52190322
chr12	52228792	52229071

chr12	52229105	52229305
chr12	52231154	52231333
chr12	52284567	52284796
chr12	52289276	52289441
chr12	52289642	52289795
chr12	52295895	52296105
chr12	52298021	52298307
chr12	52308710	52308877
chr12	52337182	52337499
chr12	52337591	52337793
chr12	52338102	52338541
chr12	52338541	52338881
chr12	52348685	52348972
chr12	52374105	52374307
chr12	52376746	52377162
chr12	52377162	52377558
chr12	52382911	52383207
chr12	52383304	52383454
chr12	52394475	52394640
chr12	52394640	52394869
chr12	52396285	52396816
chr12	52399947	52400508
chr12	52402413	52402724
chr12	52402777	52403163
chr12	52408487	52408715
chr12	52433891	52434424
chr12	52437005	52437276
chr12	52439167	52439729
chr12	52439865	52440050
chr12	52448004	52448625
chr12	52475823	52476070
chr12	52476141	52476357
chr12	52478440	52479157
chr12	52479379	52479681
chr12	52493279	52493446
chr12	52493446	52493622
chr12	52497338	52497548
chr12	52503168	52503500
chr12	52503500	52503962
chr12	52503962	52504380
chr12	52504380	52504589
chr12	52504589	52504787

chr12	52504787	52504971
chr12	52505041	52505192
chr12	52581359	52581692
chr12	52602794	52603284
chr12	52603284	52603662
chr12	52603707	52603993
chr12	52603993	52604245
chr12	52604459	52604646
chr12	52604779	52605036
chr12	52618489	52618653
chr12	52625485	52625810
chr12	52628819	52629020
chr12	52636159	52636505
chr12	52636505	52636779
chr12	52637972	52638139
chr12	52643999	52644532
chr12	52645037	52645187
chr12	52665024	52665240
chr12	52701998	52702339
chr12	52702339	52702639
chr12	52702639	52703002
chr12	52730239	52730438
chr12	52733839	52734291
chr12	52735063	52735420
chr12	52749885	52750448
chr12	52781187	52781403
chr12	52782777	52783445
chr12	52785195	52785389
chr12	52792359	52792733
chr12	52802513	52802752
chr12	52818638	52819026
chr12	52819157	52819307
chr12	52824816	52825088
chr12	52832317	52832545
chr12	52834993	52835166
chr12	52835235	52835794
chr12	52835856	52836017
chr12	52836163	52836381
chr12	52836381	52836617
chr12	52847036	52847468
chr12	52853620	52853794
chr12	52858057	52858579

chr12	52893059	52893397
chr12	52895744	52895972
chr12	52896058	52896359
chr12	52896359	52896683
chr12	52907245	52907428
chr12	52919004	52919242
chr12	52928165	52928416
chr12	52928416	52928727
chr12	52928909	52929130
chr12	52929196	52929604
chr12	52929665	52929870
chr12	52929996	52930361
chr12	52930659	52930821
chr12	52930964	52931135
chr12	52932079	52932319
chr12	52932319	52932471
chr12	52950688	52951249
chr12	53027393	53027710
chr12	53027710	53028009
chr12	53032792	53032993
chr12	53042873	53043026
chr12	53061409	53061762
chr12	53146429	53146639
chr12	53146639	53146897
chr12	53177839	53178228
chr12	53181591	53181805
chr12	53248387	53248549
chr12	53248588	53248798
chr12	53251780	53252003
chr12	53252003	53252122
chr12	53252474	53252966
chr12	53253195	53253347
chr12	53253549	53253699
chr12	53257762	53257941
chr12	53294034	53294184
chr12	53865743	53865993
chr12	53888638	53889020
chr12	53889336	53889518
chr12	53896003	53896179
chr12	53908789	53909038
chr12	53948774	53949464
chr12	53970764	53971128

chr12	53984180	53984348
chr12	54005714	54005869
chr12	54031316	54031536
chr12	54037053	54037234
chr12	54060847	54061082
chr12	54065782	54065995
chr12	54067967	54068241
chr12	54068307	54068474
chr12	54098836	54099117
chr12	54162614	54163193
chr12	54164264	54164416
chr12	54187728	54188181
chr12	54188224	54188507
chr12	54191804	54192360
chr12	54192379	54192578
chr12	54197342	54197929
chr12	54197986	54198176
chr12	54198335	54198585
chr12	54198585	54198806
chr12	54203155	54203335
chr12	54203335	54203508
chr12	54203508	54203739
chr12	54203739	54204040
chr12	54283685	54283932
chr12	54313045	54313404
chr12	54313404	54313641
chr12	54334481	54334790
chr12	54334790	54335162
chr12	54415143	54415333
chr12	54463891	54464103
chr12	54489828	54489987
chr12	54516611	54516792
chr12	54558214	54558364
chr12	54576902	54577155
chr12	54595571	54595992
chr12	54596832	54596990
chr12	54597983	54598325
chr12	54598408	54598736
chr12	54598736	54599003
chr12	54612266	54612429
chr12	54620050	54620392
chr12	54620392	54620741

chr12	54621981	54622287
chr12	54640321	54640731
chr12	54641348	54641807
chr12	54656007	54656460
chr12	54656460	54656754
chr12	54656754	54657008
chr12	54683308	54683475
chr12	54695443	54695731
chr12	54695731	54696025
chr12	54696429	54696634
chr12	54696710	54697009
chr12	54697009	54697352
chr12	54697352	54697814
chr12	54697814	54698314
chr12	54710242	54711545
chr12	54714162	54714387
chr12	54714387	54715042
chr12	54718471	54718806
chr12	54718806	54719400
chr12	54719469	54719703
chr12	54729224	54729456
chr12	54729530	54730131
chr12	54730131	54730562
chr12	54733870	54734352
chr12	54734352	54734774
chr12	54737373	54738199
chr12	54738199	54738693
chr12	54744140	54744301
chr12	54744493	54744658
chr12	54758997	54759147
chr12	54774058	54774279
chr12	54774279	54774572
chr12	54782557	54782823
chr12	54789369	54789699
chr12	54789819	54790130
chr12	54795117	54795512
chr12	54795512	54796105
chr12	54796568	54796775
chr12	54796775	54796970
chr12	54805665	54805925
chr12	54805925	54806213
chr12	54806213	54806535

chr12	54810200	54810552
chr12	54819299	54819455
chr12	54825261	54825544
chr12	54830663	54830890
chr12	54834076	54834263
chr12	54834824	54835185
chr12	54835185	54835487
chr12	54837335	54837585
chr12	54839925	54840227
chr12	54858253	54858490
chr12	54861985	54862237
chr12	54862356	54863200
chr12	54863270	54863468
chr12	54874671	54875297
chr12	54881411	54881574
chr12	54890548	54890714
chr12	54903051	54903280
chr12	54937581	54938042
chr12	54940062	54940265
chr12	54940937	54941247
chr12	54952653	54952752
chr12	54952752	54952803
chr12	54984735	54984974
chr12	54984974	54985285
chr12	54985285	54985616
chr12	54985623	54985852
chr12	54985852	54986177
chr12	54986177	54986590
chr12	54996009	54996353
chr12	54998728	54998885
chr12	54998988	54999332
chr12	54999332	54999738
chr12	55007448	55007692
chr12	55007713	55008011
chr12	55008011	55008313
chr12	55008390	55008577
chr12	55016298	55016928
chr12	55026780	55026970
chr12	55026988	55027574
chr12	55078779	55078990
chr12	55079800	55080034
chr12	55080034	55080329

chr12	55080329	55080628
chr12	55080628	55080886
chr12	55112696	55113010
chr12	55124193	55124626
chr12	55124626	55125185
chr12	55132050	55132359
chr12	55132359	55132623
chr12	55132623	55132839
chr12	55140458	55140616
chr12	55154637	55154873
chr12	55154873	55155163
chr12	55155163	55155468
chr12	55155468	55155760
chr12	55187755	55188025
chr12	55199256	55199692
chr12	55199692	55200175
chr12	55207151	55207432
chr12	55207432	55207694
chr12	55207694	55207910
chr12	55215522	55215686
chr12	55229700	55229938
chr12	55229938	55230230
chr12	55230230	55230530
chr12	55230530	55230810
chr12	55262021	55262325
chr12	55272382	55272577
chr12	55280153	55280366
chr12	55302324	55302868
chr12	55302879	55303086
chr12	55303131	55303360
chr12	55311971	55312138
chr12	55313979	55314148
chr12	55315176	55315571
chr12	55324320	55324574
chr12	55324574	55324805
chr12	55324878	55325208
chr12	55333787	55333953
chr12	55334365	55334721
chr12	55343135	55343506
chr12	55343506	55343849
chr12	55343849	55344152
chr12	55347879	55348084

chr12	55348276	55348552
chr12	55348865	55349073
chr12	55349073	55349215
chr12	55350578	55350818
chr12	55354976	55355236
chr12	55355473	55355688
chr12	55384069	55384325
chr12	55387794	55387979
chr12	55388665	55388876
chr12	55392078	55392376
chr12	55393114	55393393
chr12	55393393	55393642
chr12	55398225	55398385
chr12	55398425	55398903
chr12	55398903	55399132
chr12	55399132	55399319
chr12	55407866	55408325
chr12	55427075	55427275
chr12	55432389	55432584
chr12	55433841	55434177
chr12	55436857	55437136
chr12	55437136	55437556
chr12	55437559	55437715
chr12	55438915	55439136
chr12	55443055	55443697
chr12	55457693	55457941
chr12	55464413	55464639
chr12	55464818	55465129
chr12	55465129	55465351
chr12	55465625	55465915
chr12	55466308	55466729
chr12	55466879	55467196
chr12	55471469	55471761
chr12	55473655	55474231
chr12	55480573	55480874
chr12	55480874	55481200
chr12	55491634	55492299
chr12	55492299	55492942
chr12	55495135	55495527
chr12	55497803	55498121
chr12	55499095	55499401
chr12	55506028	55506178

chr12	55509679	55509846
chr12	55518029	55518273
chr12	55538386	55538630
chr12	55538630	55538889
chr12	55552718	55552868
chr12	55573969	55574131
chr12	55574460	55574646
chr12	55590475	55590625
chr12	55598981	55599225
chr12	55599225	55599596
chr12	55599606	55599831
chr12	55599831	55600028
chr12	55600028	55600267
chr12	55600267	55600736
chr12	55642464	55642685
chr12	55720414	55720627
chr12	55769160	55769420
chr12	55778364	55778580
chr12	55820130	55820423
chr12	55820423	55820715
chr12	55820715	55820989
chr12	55820989	55821351
chr12	55831155	55831410
chr12	55835957	55836520
chr12	55836520	55837040
chr12	55846451	55846610
chr12	55846822	55847071
chr12	55906459	55906625
chr12	55930490	55930756
chr12	55954148	55954399
chr12	56057204	56057360
chr12	56058529	56058786
chr12	56062073	56062477
chr12	56068419	56068599
chr12	56087592	56087748
chr12	56090175	56090348
chr12	56144176	56144429
chr12	56144429	56144965
chr12	56241772	56242079
chr12	56247726	56247912
chr12	56250388	56250574
chr12	56250632	56250959

chr12	56275448	56275937
chr12	56277143	56277299
chr12	56288097	56288319
chr12	56288343	56288900
chr12	56296486	56296785
chr12	56298734	56298984
chr12	56298984	56299191
chr12	56299191	56299427
chr12	56299427	56299695
chr12	56299707	56299881
chr12	56318896	56319389
chr12	56339400	56339550
chr12	56339714	56339913
chr12	56341810	56341985
chr12	56345383	56345732
chr12	56345732	56346095
chr12	56350758	56351003
chr12	56357517	56358147
chr12	56378294	56378484
chr12	56378484	56378763
chr12	56404939	56405178
chr12	56414588	56414843
chr12	56414843	56415166
chr12	56415166	56415449
chr12	56426959	56427162
chr12	56508451	56508792
chr12	56513599	56513893
chr12	56513893	56514199
chr12	56518256	56518517
chr12	56519513	56519711
chr12	56519711	56520191
chr12	56521042	56521358
chr12	56525296	56525794
chr12	56527964	56528124
chr12	56533042	56533385
chr12	56533385	56533917
chr12	56534153	56534389
chr12	56534514	56534807
chr12	56534807	56535035
chr12	56535129	56535386
chr12	56535552	56535714
chr12	56535739	56536340

chr12	56541107	56541476
chr12	56555671	56555876
chr12	56578101	56578280
chr12	56587714	56588094
chr12	56589199	56589358
chr12	56611841	56612043
chr12	56612043	56612238
chr12	56612238	56612405
chr12	56612718	56613168
chr12	56613294	56613444
chr12	56636884	56637122
chr12	56674864	56675047
chr12	56681019	56681278
chr12	56681278	56681552
chr12	56681552	56681864
chr12	56695349	56695747
chr12	56695747	56696092
chr12	56696394	56696563
chr12	56696563	56696727
chr12	56696729	56697193
chr12	56707397	56707652
chr12	56779464	56779679
chr12	56779679	56780196
chr12	56780278	56780658
chr12	56781346	56781567
chr12	56785367	56785589
chr12	56794642	56794895
chr12	56799110	56799335
chr12	56801694	56801924
chr12	56804401	56804938
chr12	56829501	56829810
chr12	56830407	56830772
chr12	56830820	56831089
chr12	56831407	56831747
chr12	56903088	56903448
chr12	56905757	56905960
chr12	56905960	56906167
chr12	56918204	56918358
chr12	56933986	56934179
chr12	56944743	56944904
chr12	56956173	56956433
chr12	57087182	57087390

chr12	57157810	57158000
chr12	57159028	57159393
chr12	57159902	57160304
chr12	57160770	57161186
chr12	57179445	57179867
chr12	57179867	57180188
chr12	57185567	57185784
chr12	57189649	57189944
chr12	57194746	57194979
chr12	57201491	57201914
chr12	57202124	57202395
chr12	57230085	57230434
chr12	57230434	57230863
chr12	57231130	57231350
chr12	57285746	57285982
chr12	57339083	57339457
chr12	57340082	57340487
chr12	57478533	57478710
chr12	57482392	57482556
chr12	57487993	57488429
chr12	57490454	57490853
chr12	57505435	57505650
chr12	57532339	57532629
chr12	57534270	57534482
chr12	57534524	57534811
chr12	57534811	57535187
chr12	57535346	57535577
chr12	57535996	57536394
chr12	57539059	57539274
chr12	57542034	57542268
chr12	57542365	57542591
chr12	57543837	57543987
chr12	57545578	57545928
chr12	57545928	57546231
chr12	57546350	57547182
chr12	57551606	57551858
chr12	57567936	57568230
chr12	57569016	57569212
chr12	57571839	57572104
chr12	57572452	57572773
chr12	57575994	57576203
chr12	57587609	57587894

chr12	57607736	57607916
chr12	57622192	57622366
chr12	57697709	57698002
chr12	57710074	57710261
chr12	57787536	57787801
chr12	58213709	58213947
chr12	58445532	58445685
chr12	58454925	58455147
chr12	58516935	58517143
chr12	58722175	58722368
chr12	58802511	58802728
chr12	58821319	58821637
chr12	58827883	58828253
chr12	58832053	58832262
chr12	58851989	58852486
chr12	58852636	58853021
chr12	58877470	58877952
chr12	58924129	58924279
chr12	58964727	58964924
chr12	59010609	59010790
chr12	59011392	59011735
chr12	59011735	59012357
chr12	59013251	59013533
chr12	59013533	59013740
chr12	59027526	59027783
chr12	59032217	59032395
chr12	59039941	59040198
chr12	59040254	59040596
chr12	59040596	59040852
chr12	59040852	59041182
chr12	59041182	59041476
chr12	59041476	59041779
chr12	59041921	59042104
chr12	59060923	59061274
chr12	59061274	59061646
chr12	59066423	59066702
chr12	59066702	59067043
chr12	59067043	59067488
chr12	59077043	59077438
chr12	59077438	59077806
chr12	59077882	59078227
chr12	59084472	59084686

chr12	59084988	59085349
chr12	59085930	59086114
chr12	59086190	59086555
chr12	59095210	59095670
chr12	59095670	59095962
chr12	59097112	59097368
chr12	59098350	59098761
chr12	59106494	59106644
chr12	59129044	59129410
chr12	59129640	59129838
chr12	59130764	59130995
chr12	59130995	59131226
chr12	59131226	59131915
chr12	59133039	59133199
chr12	59139432	59139683
chr12	59140397	59140633
chr12	59177065	59177264
chr12	59198721	59199178
chr12	59218689	59219180
chr12	59219180	59219699
chr12	59219699	59220010
chr12	59220010	59220269
chr12	59224934	59225475
chr12	59226729	59227032
chr12	59228493	59228724
chr12	59382894	59383102
chr12	59582386	59583030
chr12	59583030	59583392
chr12	59616029	59616272
chr12	59623297	59623513
chr12	59623934	59624263
chr12	59691802	59691975
chr12	59736582	59736752
chr12	59773818	59774135
chr12	59774135	59774328
chr12	59775010	59775420
chr12	59939781	59940054
chr12	60292678	60292944
chr12	60967553	60967712
chr12	61323320	61323479
chr12	61523021	61523406
chr12	63103792	63104053

chr12	63152703	63152953
chr12	63164617	63164904
chr12	63187270	63187514
chr12	63204660	63204874
chr12	63205239	63205500
chr12	63240387	63240845
chr12	63449869	63450089
chr12	64435102	64435272
chr12	64829731	64829967
chr12	64871218	64871431
chr12	64897411	64897643
chr12	64900315	64900484
chr12	64917588	64917966
chr12	64917966	64918291
chr12	64918291	64918547
chr12	64937600	64937822
chr12	64965216	64965483
chr12	64965483	64965931
chr12	64965931	64966458
chr12	64967696	64967877
chr12	64970653	64970885
chr12	64974996	64975166
chr12	64975595	64975935
chr12	64976152	64976544
chr12	64976855	64977005
chr12	65022908	65023188
chr12	65036148	65036463
chr12	65036463	65036855
chr12	65037257	65037410
chr12	65037590	65037904
chr12	65039266	65039520
chr12	65061175	65061381
chr12	65073361	65073845
chr12	65073845	65074350
chr12	65075406	65075763
chr12	65075763	65076067
chr12	65100642	65100793
chr12	65114106	65114271
chr12	65115215	65115525
chr12	65131196	65131347
chr12	65171910	65172118
chr12	65172147	65172558

chr12	65172558	65172934
chr12	65172934	65173308
chr12	65194232	65194521
chr12	65195780	65195944
chr12	65195946	65196140
chr12	65357920	65358077
chr12	65478809	65479014
chr12	65502054	65502352
chr12	66118199	66118512
chr12	66118636	66118786
chr12	66483013	66483209
chr12	66601325	66601497
chr12	66707510	66707716
chr12	66734582	66734928
chr12	66784372	66784638
chr12	67059101	67059769
chr12	67127545	67127789
chr12	67862641	67862868
chr12	67963541	67963868
chr12	67993318	67993468
chr12	67993481	67993835
chr12	68005353	68005923
chr12	68007887	68008309
chr12	68092078	68092465
chr12	68139818	68140244
chr12	68493117	68493274
chr12	68543426	68543658
chr12	68672182	68672372
chr12	68746347	68746536
chr12	68766083	68766302
chr12	68782526	68782943
chr12	68783064	68783240
chr12	68794433	68794731
chr12	68877319	68877529
chr12	69027950	69028169
chr12	69119997	69120258
chr12	69127223	69127473
chr12	69132073	69132280
chr12	69158589	69159041
chr12	69159041	69159286
chr12	69159286	69159682
chr12	69168254	69168644

chr12	69168663	69168938
chr12	69168938	69169207
chr12	69183637	69183956
chr12	69183956	69184359
chr12	69184359	69184695
chr12	69184695	69184898
chr12	69184898	69185119
chr12	69190581	69190823
chr12	69196698	69197017
chr12	69197017	69197318
chr12	69197318	69197727
chr12	69197727	69198204
chr12	69198204	69198531
chr12	69208610	69208821
chr12	69213658	69213808
chr12	69214850	69215040
chr12	69215054	69215327
chr12	69227744	69228083
chr12	69228083	69228572
chr12	69231143	69231352
chr12	69235507	69235725
chr12	69235820	69236156
chr12	69239598	69239754
chr12	69241532	69242408
chr12	69253863	69254313
chr12	69270834	69270984
chr12	69296490	69296940
chr12	69296940	69297591
chr12	69298311	69298620
chr12	69304111	69304405
chr12	69333644	69333864
chr12	69356971	69357235
chr12	69361119	69361607
chr12	69361607	69361925
chr12	69362534	69362684
chr12	69364169	69364427
chr12	69371439	69371658
chr12	69371658	69371911
chr12	69371911	69372320
chr12	69372320	69372877
chr12	69373812	69374090
chr12	69379468	69379618

chr12	69380861	69381027
chr12	69385902	69386346
chr12	69386709	69386940
chr12	69388282	69388565
chr12	69388565	69388842
chr12	69389643	69389793
chr12	69390024	69390533
chr12	69402199	69402425
chr12	69404194	69404349
chr12	69410864	69411203
chr12	69411988	69412452
chr12	69414936	69415144
chr12	69416791	69417319
chr12	69417972	69418136
chr12	69419087	69419285
chr12	69419694	69419945
chr12	69422646	69423151
chr12	69423175	69423440
chr12	69424917	69425203
chr12	69428052	69428506
chr12	69432056	69432314
chr12	69436805	69436955
chr12	69437127	69437544
chr12	69442349	69442703
chr12	69446515	69446992
chr12	69447295	69447616
chr12	69447938	69448257
chr12	69450350	69450718
chr12	69451066	69451435
chr12	69452987	69453247
chr12	69453247	69453524
chr12	69456647	69457054
chr12	69458045	69458317
chr12	69458385	69458625
chr12	69458625	69458853
chr12	69468022	69468279
chr12	69474436	69474608
chr12	69474839	69475650
chr12	69501387	69501547
chr12	69501808	69502006
chr12	69502079	69502385
chr12	69502385	69502743

chr12	69502743	69503066
chr12	69510673	69511209
chr12	69513177	69513561
chr12	69513615	69513801
chr12	69516493	69516976
chr12	69520305	69520608
chr12	69526877	69527154
chr12	69531661	69532050
chr12	69537261	69537675
chr12	69537675	69538043
chr12	69538043	69538416
chr12	69539381	69539680
chr12	69542351	69542887
chr12	69544304	69544606
chr12	69568348	69568597
chr12	69569993	69570258
chr12	69573363	69573734
chr12	69573734	69574065
chr12	69574065	69574277
chr12	69574384	69574545
chr12	69574591	69574822
chr12	69582213	69582365
chr12	69582575	69582867
chr12	69582867	69583390
chr12	69597485	69597824
chr12	69608573	69608797
chr12	69611832	69611989
chr12	69634853	69635078
chr12	69653987	69654408
chr12	69680658	69680865
chr12	69680865	69681108
chr12	69681108	69681296
chr12	69681298	69681671
chr12	69681671	69682080
chr12	69682080	69682457
chr12	69682461	69682611
chr12	69683648	69683919
chr12	69686601	69686794
chr12	69688373	69688556
chr12	69688556	69688731
chr12	69696815	69697060
chr12	69705171	69705417

chr12	69724342	69724922
chr12	69724922	69725114
chr12	69725114	69725454
chr12	69733586	69733829
chr12	69736492	69736784
chr12	69741975	69742414
chr12	69743824	69744034
chr12	69744104	69744559
chr12	69753748	69754385
chr12	69757561	69757852
chr12	69757852	69758208
chr12	69765665	69765830
chr12	69766192	69766401
chr12	69790237	69790530
chr12	69790530	69791012
chr12	69791012	69791406
chr12	69814189	69814633
chr12	69824898	69825237
chr12	69825285	69825525
chr12	69828302	69828566
chr12	69828582	69828824
chr12	69829858	69830169
chr12	69833993	69834297
chr12	69834297	69834646
chr12	69843638	69843804
chr12	69869507	69869811
chr12	69871077	69871228
chr12	69879109	69879695
chr12	69884115	69884407
chr12	69884915	69885185
chr12	69885185	69885528
chr12	69887797	69888152
chr12	69892484	69892727
chr12	69892727	69892991
chr12	69892991	69893544
chr12	69898174	69898465
chr12	69924150	69924307
chr12	69941584	69941924
chr12	69945974	69946139
chr12	69962542	69962798
chr12	69967202	69967468
chr12	69971471	69971693

chr12	69975975	69976170
chr12	69985592	69985828
chr12	69986126	69986395
chr12	69986395	69986824
chr12	69986824	69987176
chr12	69987176	69987513
chr12	69997642	69997876
chr12	69998179	69998570
chr12	69998608	69998988
chr12	69999914	70000127
chr12	70011806	70011959
chr12	70029973	70030133
chr12	70059553	70059716
chr12	70062185	70062421
chr12	70077959	70078185
chr12	70088422	70088715
chr12	70089902	70090289
chr12	70097364	70097693
chr12	70098914	70099064
chr12	70099160	70099463
chr12	70100527	70100824
chr12	70103013	70103465
chr12	70104182	70104566
chr12	70110949	70111126
chr12	70111166	70111471
chr12	70111471	70111634
chr12	70111634	70111832
chr12	70111832	70112236
chr12	70117048	70117500
chr12	70117529	70117719
chr12	70126044	70126261
chr12	70126516	70127029
chr12	70127036	70127186
chr12	70148991	70149165
chr12	70161120	70161402
chr12	70173858	70174350
chr12	70194858	70195207
chr12	70195207	70195665
chr12	70196363	70196670
chr12	70204426	70204779
chr12	70227187	70227433
chr12	70227436	70227924

chr12	70233069	70233220
chr12	70234446	70234702
chr12	70234951	70235283
chr12	70235283	70235474
chr12	70239865	70240031
chr12	70284970	70285410
chr12	70291994	70292303
chr12	70300811	70301032
chr12	70319208	70319408
chr12	70347106	70347643
chr12	70360402	70360663
chr12	70367709	70367886
chr12	70440099	70440304
chr12	70452691	70453247
chr12	70453247	70453604
chr12	70476405	70476556
chr12	70476955	70477129
chr12	70477610	70477818
chr12	70494872	70495271
chr12	70495271	70495587
chr12	70513865	70514259
chr12	70523538	70523749
chr12	70532913	70533169
chr12	70543285	70543565
chr12	70547061	70547630
chr12	70553869	70554337
chr12	70554588	70555011
chr12	70555013	70555196
chr12	70555791	70556027
chr12	70564149	70564420
chr12	70577893	70578043
chr12	70590480	70591135
chr12	70614752	70615006
chr12	70615770	70616055
chr12	70616541	70616988
chr12	70616988	70617396
chr12	70618486	70618682
chr12	70619552	70619779
chr12	70624305	70624716
chr12	70628432	70629246
chr12	70633149	70633582
chr12	70633582	70633975

chr12	70633982	70634222
chr12	70634222	70634427
chr12	70649488	70649727
chr12	70649727	70650235
chr12	70655384	70655910
chr12	70655952	70656172
chr12	70660324	70660772
chr12	70681596	70681764
chr12	70695038	70695390
chr12	70695390	70695804
chr12	70696295	70696474
chr12	70705789	70706046
chr12	70706874	70707422
chr12	70707498	70707951
chr12	70712362	70712658
chr12	70712658	70712950
chr12	70712950	70713392
chr12	70720223	70720589
chr12	70720589	70720926
chr12	70721273	70721645
chr12	70730061	70730316
chr12	70739815	70740088
chr12	70740361	70740642
chr12	70740724	70740949
chr12	70757941	70758600
chr12	70769403	70770176
chr12	70778684	70779058
chr12	70779058	70779350
chr12	70794905	70795630
chr12	70799446	70799790
chr12	70799790	70800016
chr12	70800365	70800526
chr12	70805044	70805318
chr12	70812889	70813158
chr12	70813158	70813471
chr12	70824731	70824912
chr12	70824912	70825169
chr12	70825169	70825427
chr12	70825427	70825736
chr12	70825736	70826130
chr12	70830996	70831366
chr12	70833133	70833332

chr12	70835016	70835428
chr12	70845463	70845665
chr12	70848705	70849056
chr12	70849056	70849415
chr12	70851925	70852138
chr12	70854607	70854801
chr12	70877692	70877975
chr12	70879395	70879626
chr12	70881140	70881648
chr12	70881910	70882179
chr12	70885043	70885201
chr12	70890779	70891493
chr12	70891598	70891920
chr12	70897332	70897631
chr12	70901395	70901719
chr12	70903347	70903510
chr12	70909953	70910481
chr12	70910481	70910749
chr12	70910952	70911360
chr12	70913548	70913766
chr12	70914534	70914684
chr12	70937276	70937580
chr12	70937580	70937832
chr12	70937832	70938025
chr12	70938025	70938505
chr12	70948805	70948958
chr12	70968054	70968448
chr12	70968448	70968820
chr12	70968850	70969058
chr12	70969058	70969273
chr12	70969847	70970109
chr12	70974388	70974813
chr12	70974813	70975049
chr12	70975049	70975311
chr12	71003999	71004371
chr12	71009811	71010134
chr12	71015103	71015554
chr12	71015554	71016348
chr12	71016454	71016664
chr12	71016664	71016880
chr12	71029787	71030001
chr12	71030180	71030737

chr12	71036772	71037074
chr12	71048174	71048520
chr12	71049686	71049837
chr12	71051064	71051510
chr12	71073186	71073413
chr12	71073445	71073717
chr12	71090954	71091144
chr12	71091520	71091789
chr12	71106218	71106407
chr12	71109725	71110058
chr12	71111452	71111664
chr12	71113969	71114279
chr12	71116507	71116769
chr12	71136130	71136533
chr12	71136533	71136777
chr12	71136831	71137055
chr12	71137055	71137307
chr12	71137325	71137537
chr12	71144486	71144680
chr12	71147721	71147889
chr12	71157222	71157408
chr12	71170460	71170659
chr12	71217765	71218387
chr12	71258679	71258894
chr12	71260247	71260455
chr12	71274602	71274762
chr12	71281381	71281588
chr12	71285544	71285735
chr12	71309464	71309984
chr12	71309984	71310409
chr12	71332201	71332459
chr12	71333545	71333874
chr12	71350182	71350420
chr12	71417019	71417416
chr12	71440970	71441186
chr12	71487849	71488172
chr12	71513949	71514342
chr12	71535289	71535498
chr12	71535900	71536120
chr12	71577363	71577706
chr12	71637369	71637543
chr12	71703566	71703858

chr12	71705400	71705604
chr12	71707837	71708045
chr12	71725287	71725691
chr12	71725732	71725992
chr12	71737783	71738032
chr12	71743191	71743351
chr12	71747334	71747673
chr12	71747922	71748397
chr12	71748607	71749147
chr12	71749147	71749443
chr12	71767930	71768209
chr12	71768523	71768731
chr12	71815322	71815628
chr12	71820124	71820470
chr12	71821049	71821215
chr12	71830609	71831316
chr12	71831316	71831901
chr12	71832401	71832642
chr12	71836631	71836924
chr12	71842974	71843139
chr12	71846781	71846994
chr12	71855955	71856161
chr12	71859189	71859418
chr12	71860717	71861140
chr12	71861168	71861436
chr12	71867376	71867587
chr12	71870722	71871017
chr12	71872429	71872601
chr12	71883085	71883430
chr12	71885085	71885572
chr12	71886976	71887239
chr12	71888312	71888792
chr12	71889331	71889554
chr12	71897436	71897677
chr12	71897677	71897931
chr12	71897984	71898605
chr12	71898992	71899161
chr12	71899624	71899978
chr12	71903278	71903598
chr12	71923183	71923335
chr12	71926066	71926311
chr12	71932803	71932988

chr12	71933094	71933288
chr12	71936415	71937100
chr12	71949783	71949947
chr12	71985134	71985284
chr12	72007768	72007926
chr12	72014491	72014983
chr12	72069940	72070316
chr12	72070316	72070559
chr12	72070559	72070802
chr12	72070802	72071044
chr12	72071044	72071316
chr12	72072249	72072498
chr12	72084939	72085464
chr12	72085464	72085923
chr12	72085923	72086428
chr12	72096254	72096509
chr12	72104570	72104864
chr12	72236484	72236741
chr12	72236741	72236985
chr12	72240901	72241271
chr12	72251898	72252537
chr12	72274147	72274469
chr12	72284765	72284941
chr12	72298217	72298508
chr12	72300943	72301376
chr12	72324476	72324799
chr12	72343770	72343968
chr12	72363422	72363729
chr12	72363788	72364138
chr12	72408480	72408901
chr12	72427851	72428126
chr12	72461591	72461754
chr12	72461939	72462167
chr12	72487621	72487781
chr12	72490067	72490296
chr12	72515584	72515734
chr12	72519938	72520163
chr12	72520735	72520892
chr12	72523346	72523674
chr12	72534123	72534294
chr12	72535876	72536656
chr12	72654783	72654984

chr12	72663988	72664249
chr12	72664249	72664589
chr12	72664589	72665013
chr12	72665013	72665328
chr12	72674503	72674749
chr12	72694019	72694233
chr12	72694582	72694732
chr12	72697944	72698312
chr12	72710706	72711079
chr12	72711698	72711971
chr12	72731702	72732065
chr12	72746896	72747161
chr12	72748395	72748673
chr12	72748896	72749186
chr12	72760305	72760635
chr12	72760635	72760947
chr12	72760947	72761417
chr12	72761417	72761948
chr12	72821121	72821443
chr12	72831995	72832356
chr12	72835311	72835509
chr12	72837303	72837655
chr12	72844292	72844503
chr12	72939695	72939887
chr12	72976779	72977063
chr12	72986084	72986472
chr12	73002323	73002534
chr12	73021022	73021242
chr12	73039785	73039964
chr12	73044495	73044682
chr12	73044682	73044875
chr12	73045147	73045362
chr12	73045362	73045592
chr12	73045680	73045884
chr12	73045884	73046084
chr12	73046084	73046302
chr12	73046309	73046611
chr12	73046611	73047097
chr12	73053580	73053860
chr12	73053860	73054246
chr12	73088656	73088812
chr12	73088929	73089207

chr12	73098641	73098848
chr12	73111607	73111779
chr12	73111879	73112149
chr12	73112170	73112320
chr12	73112392	73112758
chr12	73112758	73113193
chr12	73113193	73113682
chr12	73123238	73123390
chr12	73123548	73123842
chr12	73123842	73124105
chr12	73124483	73124665
chr12	73125378	73125535
chr12	73140779	73141044
chr12	73146925	73147119
chr12	73147138	73147637
chr12	73206318	73206608
chr12	73228917	73229331
chr12	73229394	73229700
chr12	73230072	73230286
chr12	73230286	73230565
chr12	73233232	73233385
chr12	73265605	73265856
chr12	73286394	73286622
chr12	73286622	73286975
chr12	73286975	73287356
chr12	73294931	73295241
chr12	73295241	73295565
chr12	73295565	73296057
chr12	73296222	73296380
chr12	73307656	73308141
chr12	73324824	73325077
chr12	73325610	73325960
chr12	73344465	73344673
chr12	73356903	73357055
chr12	73367202	73367531
chr12	73371214	73371471
chr12	73378107	73378382
chr12	73378702	73379039
chr12	73382925	73383249
chr12	73383249	73383816
chr12	73384788	73384997
chr12	73399426	73399802

chr12	73416140	73416442
chr12	73416442	73416977
chr12	73428879	73429089
chr12	73433894	73434113
chr12	73434113	73434383
chr12	73448987	73449539
chr12	73449539	73449807
chr12	73462131	73462400
chr12	73517055	73517210
chr12	73517433	73517657
chr12	73517883	73518077
chr12	73528197	73528477
chr12	73531918	73532342
chr12	73534747	73534902
chr12	73537788	73538167
chr12	73545735	73546023
chr12	73546023	73546339
chr12	73546339	73546795
chr12	73547801	73547955
chr12	73554173	73554544
chr12	73562949	73563286
chr12	73583931	73584092
chr12	73584209	73584892
chr12	73584892	73585251
chr12	73585251	73585550
chr12	73585550	73585763
chr12	73585868	73586071
chr12	73610698	73611255
chr12	73615002	73615196
chr12	73616607	73616787
chr12	73616813	73617144
chr12	73617462	73618146
chr12	73618775	73618955
chr12	73663251	73663924
chr12	73664012	73664181
chr12	73667667	73667850
chr12	73669231	73669507
chr12	73687365	73687887
chr12	73692356	73692665
chr12	73693415	73693741
chr12	73705367	73705628
chr12	73708853	73709245

chr12	73719977	73720140
chr12	73725716	73726172
chr12	73726365	73726515
chr12	73746097	73746334
chr12	73755545	73755934
chr12	73755934	73756222
chr12	73764580	73764882
chr12	73776601	73776839
chr12	73777776	73778045
chr12	73779212	73779563
chr12	73779563	73779851
chr12	73779851	73780214
chr12	73790382	73791152
chr12	73791165	73791380
chr12	73794167	73794448
chr12	73802643	73802985
chr12	73807833	73808010
chr12	73809987	73810303
chr12	73810422	73810670
chr12	73814958	73815189
chr12	73818779	73819287
chr12	73819347	73819597
chr12	73824995	73825282
chr12	73838462	73838750
chr12	73842090	73842469
chr12	73842469	73842774
chr12	73846668	73847004
chr12	73867784	73867995
chr12	73873883	73874110
chr12	73880272	73880756
chr12	73887877	73888351
chr12	73888351	73888819
chr12	73888832	73889076
chr12	73894716	73895314
chr12	73901141	73901501
chr12	73907427	73907669
chr12	73907669	73907903
chr12	73907903	73908305
chr12	73908392	73908718
chr12	73931409	73931563
chr12	73949694	73950063
chr12	73955978	73956535

chr12	73961825	73962203
chr12	73964276	73964639
chr12	73964639	73964966
chr12	73965011	73965182
chr12	73967649	73967889
chr12	73974597	73974978
chr12	73981290	73981499
chr12	73982588	73982856
chr12	73989694	73989925
chr12	73997547	73997726
chr12	74009439	74009706
chr12	74011918	74012206
chr12	74013794	74014086
chr12	74014819	74015038
chr12	74055746	74055984
chr12	74056139	74056526
chr12	74075636	74075980
chr12	74076005	74076171
chr12	74076174	74076417
chr12	74078056	74078277
chr12	74093021	74093555
chr12	74093928	74094194
chr12	74094765	74095140
chr12	74141934	74142156
chr12	74142211	74142407
chr12	74143755	74143980
chr12	74160734	74161415
chr12	74173201	74173684
chr12	74192527	74193014
chr12	74211976	74212196
chr12	74212229	74212493
chr12	74219143	74219348
chr12	74220694	74220987
chr12	74238678	74239168
chr12	74283847	74284624
chr12	74284803	74285021
chr12	74293752	74293902
chr12	74316744	74317028
chr12	74317028	74317420
chr12	74317492	74317665
chr12	74320398	74320628
chr12	74333085	74333319

chr12	74430940	74431090
chr12	74477629	74477966
chr12	74488980	74489131
chr12	74490717	74490917
chr12	74523906	74524384
chr12	74535971	74536276
chr12	74536276	74536815
chr12	74747411	74747635
chr12	74753410	74753616
chr12	74803850	74804025
chr12	74804169	74804554
chr12	74807710	74807903
chr12	74824756	74825061
chr12	74825061	74825577
chr12	74854357	74854546
chr12	75034565	75034854
chr12	75042025	75042214
chr12	75053797	75054052
chr12	75061354	75061620
chr12	75171114	75171285
chr12	75176685	75176862
chr12	75182755	75182905
chr12	75283139	75283374
chr12	75283374	75283614
chr12	75307918	75308151
chr12	75308151	75308367
chr12	75308618	75308867
chr12	75389500	75389653
chr12	75425924	75426231
chr12	75453090	75453284
chr12	75464112	75464293
chr12	75486962	75487258
chr12	75487329	75487530
chr12	75487608	75487766
chr12	75528432	75528583
chr12	75528814	75529332
chr12	75529332	75529787
chr12	75531486	75531702
chr12	75531740	75532318
chr12	75544334	75544611
chr12	75544698	75545028
chr12	75545862	75546199

chr12	75546199	75546517
chr12	75555003	75555292
chr12	75555292	75555518
chr12	75555665	75555915
chr12	75557855	75558314
chr12	75558372	75558718
chr12	75559239	75559476
chr12	75561040	75561250
chr12	75564591	75564778
chr12	75572348	75572581
chr12	75573226	75573376
chr12	75573683	75573901
chr12	75573974	75574250
chr12	75595458	75595646
chr12	75595682	75595904
chr12	75595904	75596182
chr12	75596182	75596944
chr12	75645948	75646105
chr12	75656685	75656873
chr12	75659519	75659712
chr12	75659712	75659941
chr12	75668956	75669339
chr12	75669339	75670179
chr12	75682895	75683228
chr12	75701124	75701331
chr12	75702158	75702459
chr12	75702459	75702769
chr12	75702769	75703112
chr12	75703112	75703360
chr12	75734241	75734433
chr12	75734836	75735151
chr12	75735151	75735521
chr12	75735521	75735919
chr12	75747772	75748025
chr12	75772712	75772907
chr12	75786752	75787009
chr12	75796589	75796818
chr12	75817955	75818423
chr12	75818423	75818672
chr12	75818672	75819088
chr12	75819170	75819478
chr12	75820180	75820701

chr12	75826758	75826955
chr12	75826976	75827546
chr12	75827550	75827800
chr12	75827800	75828108
chr12	75828108	75828342
chr12	75828342	75828497
chr12	75833314	75833487
chr12	75833602	75833785
chr12	75833785	75833979
chr12	75834009	75834200
chr12	75834321	75834608
chr12	75843244	75843465
chr12	75843640	75843794
chr12	75852465	75852748
chr12	75853025	75853177
chr12	75853277	75853651
chr12	75853706	75853920
chr12	75856260	75856410
chr12	75856468	75857062
chr12	75864834	75865051
chr12	75865051	75865286
chr12	75865286	75865559
chr12	75866820	75866995
chr12	75867001	75867303
chr12	75867303	75867554
chr12	75876995	75877163
chr12	75893495	75893754
chr12	75913227	75913497
chr12	75919020	75919230
chr12	75927695	75928010
chr12	75935041	75935227
chr12	75951437	75951643
chr12	75976395	75976608
chr12	75978933	75979710
chr12	75990816	75991090
chr12	75992196	75992526
chr12	75992526	75992821
chr12	76004283	76004460
chr12	76004639	76004981
chr12	76004981	76005309
chr12	76013507	76013844
chr12	76013943	76014149

chr12	76015001	76015351
chr12	76015541	76016019
chr12	76016019	76016617
chr12	76016702	76016947
chr12	76042390	76042540
chr12	76043333	76043647
chr12	76063086	76063293
chr12	76063646	76063799
chr12	76083012	76083179
chr12	76083997	76084249
chr12	76085038	76085711
chr12	76090257	76090557
chr12	76104148	76104423
chr12	76115721	76115985
chr12	76164219	76164400
chr12	76176956	76177215
chr12	76177224	76177520
chr12	76177895	76178151
chr12	76179147	76179446
chr12	76179667	76179856
chr12	76179865	76180127
chr12	76180127	76180377
chr12	76187923	76188164
chr12	76190218	76190391
chr12	76190949	76191317
chr12	76195887	76196372
chr12	76196372	76196669
chr12	76196669	76196943
chr12	76230684	76230886
chr12	76249997	76250151
chr12	76254858	76255501
chr12	76255501	76255864
chr12	76256154	76256326
chr12	76266889	76267150
chr12	76293282	76293542
chr12	76303518	76303807
chr12	76308134	76308305
chr12	76311729	76312445
chr12	76318216	76318595
chr12	76318595	76318989
chr12	76318989	76319332
chr12	76323801	76324039

chr12	76324624	76324966
chr12	76324966	76325249
chr12	76341191	76341355
chr12	76345918	76346068
chr12	76369003	76369300
chr12	76369300	76369854
chr12	76369900	76370264
chr12	76370264	76370648
chr12	76370648	76370879
chr12	76370976	76371227
chr12	76371334	76371500
chr12	76376557	76377015
chr12	76403460	76403648
chr12	76403648	76403913
chr12	76403913	76404172
chr12	76404172	76404727
chr12	76409293	76409505
chr12	76417427	76417673
chr12	76417673	76417893
chr12	76435698	76435869
chr12	76458879	76459645
chr12	76463411	76463724
chr12	76463758	76464009
chr12	76467126	76467347
chr12	76467347	76467634
chr12	76472100	76472410
chr12	76474307	76474500
chr12	76476273	76476423
chr12	76479313	76479505
chr12	76479808	76480609
chr12	76484666	76484821
chr12	76493398	76493980
chr12	76496160	76496328
chr12	76496380	76496751
chr12	76500883	76501336
chr12	76513564	76513722
chr12	76528942	76529183
chr12	76530869	76531081
chr12	76532089	76532254
chr12	76532980	76533324
chr12	76533324	76533685
chr12	76533690	76534157

chr12	76536813	76537103
chr12	76537428	76537794
chr12	76540701	76541063
chr12	76541063	76541332
chr12	76546029	76546244
chr12	76546273	76546603
chr12	76546603	76546814
chr12	76547008	76547337
chr12	76547337	76547618
chr12	76547618	76547862
chr12	76547862	76548246
chr12	76552172	76552850
chr12	76558185	76558638
chr12	76561743	76562231
chr12	76562231	76562516
chr12	76563264	76563482
chr12	76563482	76563870
chr12	76571147	76571583
chr12	76578470	76578674
chr12	76579764	76580151
chr12	76582169	76582346
chr12	76582962	76583168
chr12	76585127	76585334
chr12	76593597	76593833
chr12	76593833	76594098
chr12	76594098	76594359
chr12	76594394	76594603
chr12	76596402	76596678
chr12	76600934	76601121
chr12	76601508	76601696
chr12	76603722	76604070
chr12	76613774	76613910
chr12	76613910	76614071
chr12	76614167	76614327
chr12	76635166	76635371
chr12	76640612	76641093
chr12	76650096	76650378
chr12	76666610	76666937
chr12	76666937	76667319
chr12	76669366	76669516
chr12	76669750	76669916
chr12	76682033	76682331

chr12	76695191	76695341
chr12	76709362	76709547
chr12	76709658	76710072
chr12	76710072	76710423
chr12	76710423	76710977
chr12	76752703	76752994
chr12	76765356	76765637
chr12	76765637	76765965
chr12	76793795	76793956
chr12	76795027	76795757
chr12	76803455	76803652
chr12	76803906	76804222
chr12	76805107	76805398
chr12	76805468	76805750
chr12	76805750	76806119
chr12	76809892	76810169
chr12	76813723	76813959
chr12	76817552	76817776
chr12	76818108	76818453
chr12	76821981	76822297
chr12	76822297	76822750
chr12	76837211	76837536
chr12	76837536	76838099
chr12	76863572	76864084
chr12	76867675	76867939
chr12	76879343	76879587
chr12	76883904	76884221
chr12	76918178	76918414
chr12	76920484	76920734
chr12	76920734	76921011
chr12	76921106	76921310
chr12	76926683	76926953
chr12	76927004	76927418
chr12	76927418	76927869
chr12	76941700	76941878
chr12	76961509	76961727
chr12	76961727	76962091
chr12	76962091	76962527
chr12	76962527	76962808
chr12	76981346	76981699
chr12	76981699	76982023
chr12	76982023	76982389

chr12	76999687	76999971
chr12	77000110	77000401
chr12	77000433	77000674
chr12	77000674	77000995
chr12	77001092	77001293
chr12	77010554	77010746
chr12	77019236	77019472
chr12	77019625	77019986
chr12	77019996	77020495
chr12	77022049	77022293
chr12	77025524	77026026
chr12	77026565	77026732
chr12	77032629	77032860
chr12	77039987	77040194
chr12	77045172	77045668
chr12	77045992	77046259
chr12	77052802	77053089
chr12	77078849	77078999
chr12	77079331	77080034
chr12	77085254	77085660
chr12	77085884	77086268
chr12	77092845	77093261
chr12	77099414	77100001
chr12	77130483	77130960
chr12	77131183	77131399
chr12	77131399	77131705
chr12	77131769	77131931
chr12	77138744	77139314
chr12	77139685	77140174
chr12	77147390	77147550
chr12	77168645	77168901
chr12	77168901	77169238
chr12	77170322	77170489
chr12	77179401	77179777
chr12	77179968	77180206
chr12	77190763	77191124
chr12	77193413	77193563
chr12	77195007	77195464
chr12	77197030	77197265
chr12	77211904	77212201
chr12	77216513	77216897
chr12	77216897	77217235

chr12	77223604	77224143
chr12	77237861	77238257
chr12	77238257	77238567
chr12	77238567	77238841
chr12	77238841	77239139
chr12	77239139	77239410
chr12	77239626	77239888
chr12	77252940	77253122
chr12	77263879	77264113
chr12	77425173	77425344
chr12	77531378	77531536
chr12	77568422	77568596
chr12	77579846	77580007
chr12	77647530	77647891
chr12	77647891	77648181
chr12	77667911	77668061
chr12	77674888	77675051
chr12	77781468	77781702
chr12	77852677	77853010
chr12	77887408	77887708
chr12	77899126	77899324
chr12	77948932	77949504
chr12	77949504	77950154
chr12	77956939	77957422
chr12	77959224	77959374
chr12	77962512	77963102
chr12	77969208	77969358
chr12	77969419	77969626
chr12	77969962	77970376
chr12	77971642	77971837
chr12	77972232	77972522
chr12	77980073	77980311
chr12	77985152	77985361
chr12	78000512	78000672
chr12	78056040	78056303
chr12	78089755	78089985
chr12	78099879	78100121
chr12	78119365	78119552
chr12	78205910	78206148
chr12	78225731	78226084
chr12	78226084	78226484
chr12	78226484	78226927

chr12	78227070	78227288
chr12	78227288	78227504
chr12	78332198	78332390
chr12	78350799	78351245
chr12	78365297	78365463
chr12	78471500	78471668
chr12	78483614	78483871
chr12	78526096	78526387
chr12	78526416	78526688
chr12	78635777	78636070
chr12	78636070	78636383
chr12	78640074	78640476
chr12	78641353	78641832
chr12	78661806	78662137
chr12	78662912	78663094
chr12	78678886	78679067
chr12	78682291	78682629
chr12	78682629	78682930
chr12	78723850	78724485
chr12	78739797	78740045
chr12	78740131	78740657
chr12	78748539	78749026
chr12	78749026	78749385
chr12	78749385	78749646
chr12	78749646	78749866
chr12	78768917	78769134
chr12	78769134	78769380
chr12	78769380	78769651
chr12	78860861	78861223
chr12	78861231	78861883
chr12	78861883	78862178
chr12	78862178	78862446
chr12	78868217	78868481
chr12	78874697	78874915
chr12	78875515	78875704
chr12	78891244	78891449
chr12	78906700	78907204
chr12	78907594	78907803
chr12	78908073	78908602
chr12	78910283	78910529
chr12	78910722	78911007
chr12	78911091	78911282

chr12	78914428	78914790
chr12	78918256	78918577
chr12	78918579	78918734
chr12	78919356	78919701
chr12	78925972	78926269
chr12	78928840	78929005
chr12	78929418	78929640
chr12	78929971	78930259
chr12	78940096	78940613
chr12	78940809	78941092
chr12	78941092	78941522
chr12	78947271	78947469
chr12	78952059	78952337
chr12	78953363	78953801
chr12	78953872	78954484
chr12	78954510	78954753
chr12	78957811	78957961
chr12	78960389	78960625
chr12	78980857	78981020
chr12	78981331	78981526
chr12	78981843	78982246
chr12	78982246	78982521
chr12	78987890	78988078
chr12	78992394	78992908
chr12	79006471	79006803
chr12	79006803	79007173
chr12	79007173	79007640
chr12	79022906	79023187
chr12	79023187	79023445
chr12	79028756	79029334
chr12	79029334	79029868
chr12	79033790	79034037
chr12	79038752	79038986
chr12	79039657	79040132
chr12	79041437	79041764
chr12	79046992	79047236
chr12	79047236	79047764
chr12	79050579	79050803
chr12	79055317	79055645
chr12	79089109	79089579
chr12	79089579	79089855
chr12	79092573	79093003

chr12	79097479	79097631
chr12	79103772	79104076
chr12	79122901	79123119
chr12	79130382	79131205
chr12	79132088	79132508
chr12	79171967	79172292
chr12	79172292	79172600
chr12	79172600	79173009
chr12	79181438	79181601
chr12	79190846	79191165
chr12	79191165	79191392
chr12	79191392	79191728
chr12	79191752	79192088
chr12	79193586	79193751
chr12	79206217	79206464
chr12	79248025	79248534
chr12	79250015	79250415
chr12	79253225	79253660
chr12	79254492	79254811
chr12	79259490	79259691
chr12	79295910	79296701
chr12	79297155	79297404
chr12	79297404	79297622
chr12	79308017	79308260
chr12	79330120	79330345
chr12	79331710	79332045
chr12	79332045	79332279
chr12	79333950	79334160
chr12	79347623	79348154
chr12	79348920	79349104
chr12	79352162	79352535
chr12	79352535	79353055
chr12	79354741	79354933
chr12	79356865	79357020
chr12	79358877	79359182
chr12	79359476	79359719
chr12	79383582	79383915
chr12	79387067	79387329
chr12	79387939	79388180
chr12	79404128	79404281
chr12	79404582	79404817
chr12	79405175	79405351

chr12	79410422	79410572
chr12	79419240	79419598
chr12	79420752	79420904
chr12	79426943	79427095
chr12	79454109	79454265
chr12	79471670	79471897
chr12	79471897	79472549
chr12	79477369	79477549
chr12	79481996	79482351
chr12	79488651	79489287
chr12	79493587	79493737
chr12	79501916	79502203
chr12	79504012	79504473
chr12	79507078	79507425
chr12	79507464	79507614
chr12	79514708	79515044
chr12	79524570	79524835
chr12	79527274	79527579
chr12	79531704	79532287
chr12	79532287	79532774
chr12	79534839	79535301
chr12	79541516	79542005
chr12	79545114	79545416
chr12	79545927	79546124
chr12	79547572	79547765
chr12	79551389	79551548
chr12	79551887	79552597
chr12	79552656	79552878
chr12	79553130	79553447
chr12	79555184	79555445
chr12	79558143	79558329
chr12	79558854	79559079
chr12	79559079	79559321
chr12	79559374	79559933
chr12	79560130	79560412
chr12	79560957	79561332
chr12	79598369	79598817
chr12	79600098	79600330
chr12	79600330	79600764
chr12	79601082	79601386
chr12	79611027	79611416
chr12	79611433	79611583

chr12	79612749	79613185
chr12	79639536	79639794
chr12	79639794	79640058
chr12	79641662	79641828
chr12	79641979	79642378
chr12	79645705	79646079
chr12	79649033	79649428
chr12	79676957	79677283
chr12	79686984	79687283
chr12	79711132	79711360
chr12	79751815	79752011
chr12	79793429	79793785
chr12	79832723	79832893
chr12	79864170	79864470
chr12	79864470	79864760
chr12	79864760	79865183
chr12	79865201	79865368
chr12	79874247	79874542
chr12	79874542	79874791
chr12	79896006	79896385
chr12	79904299	79904544
chr12	79904891	79905273
chr12	79907082	79907381
chr12	79907398	79907687
chr12	79907687	79908106
chr12	79919083	79919555
chr12	79929773	79930354
chr12	79936619	79937012
chr12	79937012	79937304
chr12	79950627	79951118
chr12	79954950	79955134
chr12	79970965	79971291
chr12	79975922	79976204
chr12	79990412	79991072
chr12	79995455	79995750
chr12	79997036	79997186
chr12	79997296	79997449
chr12	79997943	79998118
chr12	80001951	80002221
chr12	80026127	80026813
chr12	80032336	80032846
chr12	80032846	80033142

chr12	80036378	80036603
chr12	80036603	80036798
chr12	80046641	80047095
chr12	80048630	80049054
chr12	80079909	80080353
chr12	80083896	80084259
chr12	80090770	80091408
chr12	80091408	80091654
chr12	80091654	80091883
chr12	80092816	80093042
chr12	80098863	80099214
chr12	80103205	80103503
chr12	80103503	80103808
chr12	80103808	80104284
chr12	80109002	80109275
chr12	80109847	80110012
chr12	80112832	80113234
chr12	80113294	80113475
chr12	80113543	80113720
chr12	80114296	80114683
chr12	80115155	80115356
chr12	80115406	80115622
chr12	80115622	80116086
chr12	80116192	80116342
chr12	80127111	80127384
chr12	80127384	80127686
chr12	80130621	80130926
chr12	80132101	80132285
chr12	80132285	80132618
chr12	80132618	80133071
chr12	80133076	80133226
chr12	80140890	80141454
chr12	80141454	80141827
chr12	80146502	80146775
chr12	80149605	80149797
chr12	80154036	80154292
chr12	80157799	80157949
chr12	80158738	80159169
chr12	80159169	80159571
chr12	80160120	80160292
chr12	80161031	80161248
chr12	80161678	80161873

chr12	80162555	80162756
chr12	80164447	80164953
chr12	80171026	80171368
chr12	80177034	80177217
chr12	80177217	80177377
chr12	80177377	80177529
chr12	80177529	80177723
chr12	80183921	80184454
chr12	80185317	80185569
chr12	80189306	80189530
chr12	80190917	80191075
chr12	80191273	80191465
chr12	80191465	80191654
chr12	80197450	80197691
chr12	80198575	80199005
chr12	80199048	80199448
chr12	80203517	80203669
chr12	80209675	80209975
chr12	80209975	80210357
chr12	80210357	80210709
chr12	80210918	80211103
chr12	80211103	80211288
chr12	80211360	80211544
chr12	80216187	80216355
chr12	80216492	80216743
chr12	80220205	80220502
chr12	80220502	80220925
chr12	80223567	80224057
chr12	80228527	80228867
chr12	80228977	80229359
chr12	80229359	80229827
chr12	80230053	80230313
chr12	80232184	80232375
chr12	80233030	80233243
chr12	80234255	80234644
chr12	80234644	80234977
chr12	80235388	80235660
chr12	80239366	80239797
chr12	80240595	80240745
chr12	80244113	80244449
chr12	80244685	80245094
chr12	80246014	80246223

chr12	80246734	80247013
chr12	80247053	80247388
chr12	80251305	80251527
chr12	80252138	80252315
chr12	80253869	80254068
chr12	80257114	80257270
chr12	80259412	80259660
chr12	80259671	80260203
chr12	80260203	80260740
chr12	80260740	80261085
chr12	80268061	80268421
chr12	80270594	80270947
chr12	80270947	80271347
chr12	80271914	80272173
chr12	80272980	80273131
chr12	80273307	80273661
chr12	80276130	80276352
chr12	80277204	80277921
chr12	80279512	80279814
chr12	80282496	80282727
chr12	80284321	80284554
chr12	80284835	80285035
chr12	80291402	80292494
chr12	80301098	80301361
chr12	80315525	80315695
chr12	80318912	80319489
chr12	80329691	80330207
chr12	80331707	80332141
chr12	80344583	80345306
chr12	80346532	80346687
chr12	80351526	80351753
chr12	80358522	80358809
chr12	80358853	80359340
chr12	80365967	80366219
chr12	80366219	80366614
chr12	80367414	80367587
chr12	80368186	80368459
chr12	80371989	80372269
chr12	80374681	80375104
chr12	80377700	80377901
chr12	80379795	80380215
chr12	80380215	80380782

chr12	80387361	80387615
chr12	80389442	80389816
chr12	80406734	80406992
chr12	80407909	80408151
chr12	80408357	80408664
chr12	80433425	80433645
chr12	80434725	80435105
chr12	80435105	80435402
chr12	80435402	80435730
chr12	80435986	80436114
chr12	80436114	80436267
chr12	80436371	80436738
chr12	80436738	80437025
chr12	80438513	80438711
chr12	80441359	80441514
chr12	80462996	80463328
chr12	80463328	80463747
chr12	80518490	80518748
chr12	80518748	80518966
chr12	80519141	80519413
chr12	80521934	80522289
chr12	80538164	80538391
chr12	80553371	80553522
chr12	80577203	80577354
chr12	80585051	80585270
chr12	80602627	80602793
chr12	80603308	80603632
chr12	80603632	80603921
chr12	80604886	80605069
chr12	80608577	80608912
chr12	80642715	80642970
chr12	80643299	80643501
chr12	80643501	80643723
chr12	80643723	80644044
chr12	80644071	80644339
chr12	80644339	80644568
chr12	80644614	80644764
chr12	80673244	80673506
chr12	80680701	80680943
chr12	80681406	80681658
chr12	80691839	80692001
chr12	80692231	80693022

chr12	80698649	80698801
chr12	80706674	80706888
chr12	80707371	80707481
chr12	80707481	80707526
chr12	80724897	80725143
chr12	80728936	80729112
chr12	80730595	80730912
chr12	80739627	80739795
chr12	80751629	80751866
chr12	80757717	80758127
chr12	80758127	80758519
chr12	80758519	80758938
chr12	80758951	80759311
chr12	80760381	80760604
chr12	80760604	80760850
chr12	80769651	80770104
chr12	80773648	80773816
chr12	80782275	80782451
chr12	80782993	80783143
chr12	80783936	80784165
chr12	80790248	80790688
chr12	80790688	80791184
chr12	80800193	80800492
chr12	80809613	80809902
chr12	80813902	80814195
chr12	80816813	80816963
chr12	80818397	80818614
chr12	80825971	80826350
chr12	80832286	80832790
chr12	80834173	80834546
chr12	80834563	80834758
chr12	80834780	80834930
chr12	80854007	80854168
chr12	80885240	80885638
chr12	80893805	80894003
chr12	80894063	80894260
chr12	80894959	80895283
chr12	80895283	80895563
chr12	80917953	80918105
chr12	80922836	80922986
chr12	80930261	80930445
chr12	80936814	80937051

chr12	80937690	80938116
chr12	80939342	80939786
chr12	80945089	80945249
chr12	80945280	80945848
chr12	80945848	80946411
chr12	80951781	80951931
chr12	80951946	80952128
chr12	80952383	80952586
chr12	80952586	80952847
chr12	80954204	80954479
chr12	80954769	80955029
chr12	80958071	80958228
chr12	80966344	80966520
chr12	80982399	80982638
chr12	80988382	80988611
chr12	81027048	81027219
chr12	81033806	81033987
chr12	81097803	81097986
chr12	81099220	81099448
chr12	81108744	81108943
chr12	81123914	81124086
chr12	81124202	81124590
chr12	81150999	81151275
chr12	81155842	81155992
chr12	81156863	81157162
chr12	81163086	81163327
chr12	81163327	81163551
chr12	81182179	81182412
chr12	81199625	81199904
chr12	81237196	81237424
chr12	81278656	81278846
chr12	81332001	81332228
chr12	81332228	81332450
chr12	81332530	81332828
chr12	81332828	81333153
chr12	81402260	81402478
chr12	81408983	81409336
chr12	81438915	81439137
chr12	81484711	81484979
chr12	81484979	81485264
chr12	81497083	81497269
chr12	81532183	81532661

chr12	81532661	81533138
chr12	81594881	81595534
chr12	81607809	81608163
chr12	81608513	81608675
chr12	81616860	81617569
chr12	81619121	81619389
chr12	81619389	81619676
chr12	81630435	81630931
chr12	81630931	81631313
chr12	81631313	81631656
chr12	81631656	81632001
chr12	81653376	81653612
chr12	81668149	81668431
chr12	81668431	81668810
chr12	81683429	81683705
chr12	81688462	81688971
chr12	81690528	81690727
chr12	81691177	81691708
chr12	81691994	81692249
chr12	81692313	81692494
chr12	81697122	81697351
chr12	81697864	81698451
chr12	81698451	81698790
chr12	81712034	81712215
chr12	81712527	81713048
chr12	81719044	81719426
chr12	81728919	81729069
chr12	81748101	81748748
chr12	81748821	81749394
chr12	81762500	81762660
chr12	81779931	81780160
chr12	81780650	81780810
chr12	81780813	81781615
chr12	81782693	81782874
chr12	81783018	81783336
chr12	81783336	81783714
chr12	81788673	81789055
chr12	81789634	81789827
chr12	81790818	81791210
chr12	81791210	81791528
chr12	81796613	81796868
chr12	81807280	81807513

chr12	81830430	81830757
chr12	81831129	81831457
chr12	81845051	81845211
chr12	81859701	81860083
chr12	81860083	81860330
chr12	81860330	81860576
chr12	81860576	81860820
chr12	81860820	81861233
chr12	81915649	81915880
chr12	81919072	81919472
chr12	81933575	81933767
chr12	81937491	81937641
chr12	81971211	81971693
chr12	81971693	81972284
chr12	81985672	81985977
chr12	81985986	81986157
chr12	81986159	81986402
chr12	82002690	82003111
chr12	82009178	82009356
chr12	82014113	82014294
chr12	82025696	82025980
chr12	82026018	82026342
chr12	82026342	82026567
chr12	82033548	82033898
chr12	82040023	82040187
chr12	82044939	82045140
chr12	82056475	82056882
chr12	82056882	82057172
chr12	82057172	82057396
chr12	82065089	82065357
chr12	82067901	82068083
chr12	82072793	82073131
chr12	82085774	82086040
chr12	82086040	82086420
chr12	82086420	82086764
chr12	82121445	82121612
chr12	82122230	82122605
chr12	82122605	82122877
chr12	82123000	82123219
chr12	82124669	82124864
chr12	82154545	82154988
chr12	82154988	82155331

chr12	82163578	82163892
chr12	82163892	82164184
chr12	82169068	82169736
chr12	82169736	82170184
chr12	82170184	82170435
chr12	82187512	82187769
chr12	82187886	82188146
chr12	82188447	82189358
chr12	82189404	82189637
chr12	82209651	82210101
chr12	82220798	82221016
chr12	82221079	82221320
chr12	82226052	82226212
chr12	82226780	82227047
chr12	82227367	82227587
chr12	82247644	82247901
chr12	82247901	82248129
chr12	82251383	82251631
chr12	82256904	82257064
chr12	82258067	82258315
chr12	82284586	82285371
chr12	82303090	82303412
chr12	82319790	82320114
chr12	82322819	82323041
chr12	82323136	82323401
chr12	82387813	82387969
chr12	82392360	82392665
chr12	82397455	82397605
chr12	82397849	82398104
chr12	82412289	82412483
chr12	82412483	82412689
chr12	82412689	82412974
chr12	82427434	82427755
chr12	82465055	82465219
chr12	82470017	82470524
chr12	82470590	82470946
chr12	82493357	82493572
chr12	82568477	82568684
chr12	82591824	82592133
chr12	82616781	82617012
chr12	82816025	82816293
chr12	82893390	82893571

chr12	82996293	82996447
chr12	83033270	83033443
chr12	83085256	83085406
chr12	83105599	83105799
chr12	83114501	83114656
chr12	83309890	83310071
chr12	83328962	83329310
chr12	83486734	83486908
chr12	83486930	83487435
chr12	83487510	83488018
chr12	83502309	83502552
chr12	83520229	83520575
chr12	83520575	83520871
chr12	83525908	83526098
chr12	83527575	83527725
chr12	83527891	83528125
chr12	83549267	83549435
chr12	83557594	83557952
chr12	83560238	83560581
chr12	83586737	83587005
chr12	83592089	83592388
chr12	83596784	83597564
chr12	83631817	83632162
chr12	83632162	83632389
chr12	83632389	83632671
chr12	83632671	83632993
chr12	83643008	83643480
chr12	83670003	83670158
chr12	83687840	83688393
chr12	83688393	83688975
chr12	83704913	83705291
chr12	83705337	83705662
chr12	83705662	83706089
chr12	83713835	83714076
chr12	83749247	83749425
chr12	83754727	83754935
chr12	83759491	83759696
chr12	83759696	83759875
chr12	83763419	83763656
chr12	83763656	83763891
chr12	83763904	83764147
chr12	83764147	83764381

chr12	83765926	83766187
chr12	83770267	83770517
chr12	83770517	83770784
chr12	83770927	83771190
chr12	83777070	83777222
chr12	83780312	83780565
chr12	83780602	83780783
chr12	83833989	83834323
chr12	83854305	83854616
chr12	83864546	83864780
chr12	83874458	83874965
chr12	83875112	83875645
chr12	83882579	83882792
chr12	83884935	83885301
chr12	83885301	83885623
chr12	83902356	83902835
chr12	83921376	83922016
chr12	83922016	83922321
chr12	83950461	83950833
chr12	83950833	83951221
chr12	83951221	83951692
chr12	83959085	83959245
chr12	83972078	83972487
chr12	83987268	83987617
chr12	83987617	83987950
chr12	83987950	83988204
chr12	83988204	83988553
chr12	83998579	83998980
chr12	83999091	83999606
chr12	83999606	83999892
chr12	84009361	84009644
chr12	84009644	84009944
chr12	84011599	84011884
chr12	84020482	84020904
chr12	84030771	84031005
chr12	84031005	84031299
chr12	84038248	84038463
chr12	84038463	84038763
chr12	84038763	84039113
chr12	84046936	84047252
chr12	84053641	84053933
chr12	84057232	84057676

chr12	84061035	84061201
chr12	84067996	84068291
chr12	84068962	84069328
chr12	84069328	84069659
chr12	84069659	84069957
chr12	84100692	84100911
chr12	84100911	84101146
chr12	84101146	84101490
chr12	84114108	84114467
chr12	84114467	84114880
chr12	84126926	84127268
chr12	84127301	84127551
chr12	84132341	84132570
chr12	84141256	84141488
chr12	84145729	84145891
chr12	84147930	84148085
chr12	84152558	84152719
chr12	84153535	84153696
chr12	84155235	84155397
chr12	84160541	84160754
chr12	84161633	84161854
chr12	84161854	84162136
chr12	84163129	84163698
chr12	84164099	84164285
chr12	84174893	84175229
chr12	84175546	84176134
chr12	84178117	84178382
chr12	84178651	84178878
chr12	84179138	84179360
chr12	84180972	84181366
chr12	84187186	84187336
chr12	84190133	84190348
chr12	84190348	84190618
chr12	84192777	84192986
chr12	84192986	84193274
chr12	84193665	84193929
chr12	84193929	84194397
chr12	84201304	84201572
chr12	84201575	84201915
chr12	84203263	84203516
chr12	84203608	84204170
chr12	84212403	84212632

chr12	84212831	84213085
chr12	84216955	84217202
chr12	84217202	84217482
chr12	84217543	84217883
chr12	84218253	84218695
chr12	84218695	84219088
chr12	84222597	84223033
chr12	84232869	84233165
chr12	84233229	84233536
chr12	84233536	84233756
chr12	84235672	84235939
chr12	84249254	84249545
chr12	84260795	84261008
chr12	84278917	84279568
chr12	84284861	84285160
chr12	84285160	84285662
chr12	84288581	84288751
chr12	84316630	84316960
chr12	84316960	84317316
chr12	84317316	84317721
chr12	84347266	84347516
chr12	84361517	84361803
chr12	84361803	84362510
chr12	84367133	84367310
chr12	84384639	84384833
chr12	84384833	84385026
chr12	84393929	84394194
chr12	84400794	84400985
chr12	84408086	84408330
chr12	84408330	84408750
chr12	84408750	84409367
chr12	84417135	84417370
chr12	84417370	84417733
chr12	84440528	84440678
chr12	84450654	84450892
chr12	84450892	84451131
chr12	84451131	84451304
chr12	84451304	84451617
chr12	84451653	84451803
chr12	84468784	84468946
chr12	84533854	84534009
chr12	84539690	84539915

chr12	84540812	84541078
chr12	84545877	84546027
chr12	84546070	84546366
chr12	84570080	84570342
chr12	84571685	84571862
chr12	84571862	84572067
chr12	84578158	84578315
chr12	84578445	84578797
chr12	84589777	84590106
chr12	84590603	84590753
chr12	84591239	84591422
chr12	84606003	84606167
chr12	84614399	84614608
chr12	84614608	84614892
chr12	84617070	84617317
chr12	84617317	84617671
chr12	84617844	84618013
chr12	84620952	84621194
chr12	84637447	84637665
chr12	84653822	84653972
chr12	84653983	84654249
chr12	84670626	84670856
chr12	84698227	84699062
chr12	84723503	84723655
chr12	84729664	84729905
chr12	84750765	84750985
chr12	84755375	84755632
chr12	84756019	84756169
chr12	84759112	84759364
chr12	84762569	84762729
chr12	84764679	84765083
chr12	84766330	84766830
chr12	84767499	84767817
chr12	84767817	84767996
chr12	84772707	84773432
chr12	84773618	84773768
chr12	84774485	84774675
chr12	84780984	84781294
chr12	84784536	84784925
chr12	84784925	84785214
chr12	84792592	84792844
chr12	84801640	84802254

chr12	84805644	84805917
chr12	84810938	84811277
chr12	84813840	84814118
chr12	84815449	84815765
chr12	84819353	84819643
chr12	84827551	84828059
chr12	84835230	84835533
chr12	84835642	84836010
chr12	84837219	84837369
chr12	84845811	84845999
chr12	84846467	84846804
chr12	84846804	84847190
chr12	84847190	84847571
chr12	84847571	84847858
chr12	84857865	84858015
chr12	84864932	84865192
chr12	84871442	84871673
chr12	84875840	84876137
chr12	84876162	84876331
chr12	84876776	84877135
chr12	84878659	84878848
chr12	84879704	84879854
chr12	84881085	84881547
chr12	84884612	84885011
chr12	84885011	84885423
chr12	84885423	84885741
chr12	84900139	84900301
chr12	84904504	84904751
chr12	84905159	84905449
chr12	84906790	84906968
chr12	84907714	84907880
chr12	84908630	84908873
chr12	84909510	84909717
chr12	84920278	84920599
chr12	84920616	84921012
chr12	84926583	84926866
chr12	84929462	84929890
chr12	84958920	84959211
chr12	84959211	84959481
chr12	84969438	84969600
chr12	84969824	84970005
chr12	84970043	84970204

chr12	84970252	84970506
chr12	84970526	84970778
chr12	84970778	84971157
chr12	84971235	84971385
chr12	84971421	84971605
chr12	84987054	84987238
chr12	84994334	84994491
chr12	84996005	84996543
chr12	84996543	84996887
chr12	84996887	84997176
chr12	84997176	84997427
chr12	85009861	85010175
chr12	85032770	85033030
chr12	85050922	85051189
chr12	85051189	85051528
chr12	85051528	85051851
chr12	85092319	85092524
chr12	85093966	85094608
chr12	85110154	85110323
chr12	85110442	85110940
chr12	85110940	85111162
chr12	85111162	85111368
chr12	85111439	85111589
chr12	85121472	85121638
chr12	85135891	85136263
chr12	85136263	85136578
chr12	85136578	85137017
chr12	85142846	85143129
chr12	85150966	85151492
chr12	85153060	85153335
chr12	85156366	85156630
chr12	85162987	85163147
chr12	85169479	85169815
chr12	85169907	85170100
chr12	85170142	85170292
chr12	85170298	85170507
chr12	85170507	85170698
chr12	85176507	85176663
chr12	85176677	85177025
chr12	85177025	85177248
chr12	85177248	85177715
chr12	85185874	85186295

chr12	85186295	85186786
chr12	85186795	85186958
chr12	85192296	85192686
chr12	85192686	85193198
chr12	85200508	85200841
chr12	85200894	85201060
chr12	85201528	85201822
chr12	85201822	85202189
chr12	85203663	85204138
chr12	85208906	85209058
chr12	85209058	85209254
chr12	85209463	85209686
chr12	85219110	85219542
chr12	85219542	85219811
chr12	85219811	85220001
chr12	85220001	85220189
chr12	85232851	85233138
chr12	85233422	85233986
chr12	85270256	85270766
chr12	85271562	85271712
chr12	85271878	85272148
chr12	85279794	85280190
chr12	85280190	85280580
chr12	85280580	85280820
chr12	85281209	85281454
chr12	85285062	85285307
chr12	85287048	85287251
chr12	85288094	85288447
chr12	85288447	85288815
chr12	85288815	85289177
chr12	85305383	85305622
chr12	85305622	85305875
chr12	85327305	85327471
chr12	85330109	85330785
chr12	85337390	85337589
chr12	85337986	85338197
chr12	85338808	85339222
chr12	85339222	85339478
chr12	85339478	85339723
chr12	85341213	85341535
chr12	85346470	85346703
chr12	85348314	85348961

chr12	85348961	85349340
chr12	85350649	85350857
chr12	85362448	85362981
chr12	85373864	85374140
chr12	85374140	85374504
chr12	85374504	85374827
chr12	85374859	85375232
chr12	85380332	85380682
chr12	85381044	85381255
chr12	85388597	85388905
chr12	85388931	85389100
chr12	85397894	85398310
chr12	85417853	85418397
chr12	85418978	85419228
chr12	85423917	85424136
chr12	85429963	85430402
chr12	85430402	85430915
chr12	85436238	85436647
chr12	85437686	85437892
chr12	85439384	85439602
chr12	85441626	85441912
chr12	85449819	85450300
chr12	85451559	85451882
chr12	85451882	85452198
chr12	85452200	85452495
chr12	85454901	85455292
chr12	85455292	85455805
chr12	85462341	85462560
chr12	85472023	85472413
chr12	85472413	85472801
chr12	85473384	85473728
chr12	85473728	85474110
chr12	85474110	85474390
chr12	85477894	85478393
chr12	85478769	85479017
chr12	85484769	85484987
chr12	85485375	85485726
chr12	85485726	85486093
chr12	85486093	85486410
chr12	85486817	85487092
chr12	85487535	85487722
chr12	85487772	85488032

chr12	85492130	85492470
chr12	85496469	85496654
chr12	85496780	85497074
chr12	85497428	85497866
chr12	85497866	85498209
chr12	85510579	85510864
chr12	85519601	85519937
chr12	85520803	85521036
chr12	85523531	85523754
chr12	85527128	85527349
chr12	85537869	85538106
chr12	85541058	85541615
chr12	85569546	85569751
chr12	85569751	85570113
chr12	85570113	85570642
chr12	85571298	85571653
chr12	85572206	85572452
chr12	85592945	85593289
chr12	85593490	85593736
chr12	85594483	85594655
chr12	85598628	85599565
chr12	85599565	85600139
chr12	85612123	85612339
chr12	85615609	85615813
chr12	85623584	85624080
chr12	85624110	85624408
chr12	85624588	85624901
chr12	85639330	85639513
chr12	85642692	85642898
chr12	85644754	85644961
chr12	85645762	85645974
chr12	85647393	85647653
chr12	85661020	85661280
chr12	85662940	85663187
chr12	85698134	85698608
chr12	85705076	85705305
chr12	85705305	85705547
chr12	85705547	85705827
chr12	85710855	85711124
chr12	85712587	85712780
chr12	85729443	85729746
chr12	85729929	85730222

chr12	85733125	85733425
chr12	85736241	85736562
chr12	85736821	85737275
chr12	85745750	85746056
chr12	85746056	85746345
chr12	85746345	85746669
chr12	85746679	85747033
chr12	85752841	85753074
chr12	85754681	85755010
chr12	85762896	85763124
chr12	85769966	85770244
chr12	85777535	85777706
chr12	85779768	85779992
chr12	85783968	85784249
chr12	85784249	85784591
chr12	85792353	85792859
chr12	85793079	85793414
chr12	85793512	85793732
chr12	85818456	85818850
chr12	85824411	85824818
chr12	85824818	85825335
chr12	85825383	85825570
chr12	85837845	85837995
chr12	85840693	85840903
chr12	85848220	85848488
chr12	85869120	85869398
chr12	85869398	85869747
chr12	85869747	85870123
chr12	85903959	85904388
chr12	85937513	85937794
chr12	85942739	85942965
chr12	85943042	85943253
chr12	85943253	85943439
chr12	85954249	85954513
chr12	85956305	85956564
chr12	85958560	85958837
chr12	85959798	85960124
chr12	85960124	85960557
chr12	85963718	85963994
chr12	85967876	85968095
chr12	85969559	85970015
chr12	85970222	85970507

chr12	85971714	85972185
chr12	85972565	85972766
chr12	85973545	85973948
chr12	85987287	85987659
chr12	85992073	85992349
chr12	86000330	86000624
chr12	86000624	86001035
chr12	86012144	86012575
chr12	86014687	86014946
chr12	86015103	86015397
chr12	86024535	86024685
chr12	86039919	86040089
chr12	86047898	86048252
chr12	86057705	86057887
chr12	86066717	86067045
chr12	86069075	86069297
chr12	86069341	86069491
chr12	86078859	86079200
chr12	86079236	86079744
chr12	86082404	86082685
chr12	86082685	86083093
chr12	86085920	86086122
chr12	86104933	86105339
chr12	86106153	86106341
chr12	86108591	86108810
chr12	86110723	86110958
chr12	86128715	86129065
chr12	86129065	86129524
chr12	86132927	86133104
chr12	86142199	86142779
chr12	86142819	86142980
chr12	86143277	86143429
chr12	86166363	86166587
chr12	86168813	86169073
chr12	86181997	86182262
chr12	86198232	86198658
chr12	86213167	86213378
chr12	86217140	86217294
chr12	86228394	86228803
chr12	86229769	86229966
chr12	86230314	86230608
chr12	86231451	86231799

chr12	86234364	86234702
chr12	86234702	86235041
chr12	86241228	86241493
chr12	86241590	86242058
chr12	86242058	86242372
chr12	86242372	86242550
chr12	86329028	86329201
chr12	86336300	86336450
chr12	86361353	86361559
chr12	86364209	86364705
chr12	86365546	86366017
chr12	86366043	86366347
chr12	86385793	86386094
chr12	86395379	86395648
chr12	86395648	86395840
chr12	86398727	86399095
chr12	86401856	86402356
chr12	86402440	86402774
chr12	86402774	86403053
chr12	86421568	86421833
chr12	86422462	86422650
chr12	86426368	86426550
chr12	86433300	86433590
chr12	86433590	86433824
chr12	86435306	86435585
chr12	86435585	86435893
chr12	86435893	86436148
chr12	86470007	86470157
chr12	86483987	86484143
chr12	86525602	86525773
chr12	86578004	86578294
chr12	86592100	86592296
chr12	86612852	86613027
chr12	86614271	86614474
chr12	86648627	86648780
chr12	86652460	86653110
chr12	86678548	86678826
chr12	86678826	86679058
chr12	86679058	86679309
chr12	86679309	86679556
chr12	86689448	86689728
chr12	86689869	86690116

chr12	86693086	86693292
chr12	86694422	86694848
chr12	86698567	86698900
chr12	86701038	86701206
chr12	86710309	86710770
chr12	86710779	86711025
chr12	86714169	86714374
chr12	86715451	86715734
chr12	86725717	86725870
chr12	86725922	86726693
chr12	86749501	86749723
chr12	86750468	86750694
chr12	86767923	86768159
chr12	86769078	86769327
chr12	86771914	86772426
chr12	86774045	86774279
chr12	86779681	86779953
chr12	86786799	86787188
chr12	86787188	86787617
chr12	86790369	86790737
chr12	86790737	86791053
chr12	86793966	86794295
chr12	86795439	86795800
chr12	86795862	86796051
chr12	86800790	86801168
chr12	86801168	86801597
chr12	86801597	86802121
chr12	86802121	86802683
chr12	86806401	86806551
chr12	86807473	86807678
chr12	86810846	86811121
chr12	86816953	86817103
chr12	86819231	86819529
chr12	86823815	86824227
chr12	86824227	86824575
chr12	86824816	86825370
chr12	86826365	86827198
chr12	86827253	86827408
chr12	86832067	86832499
chr12	86832499	86832963
chr12	86833588	86834107
chr12	86834499	86834854

chr12	86836697	86837039
chr12	86851346	86851760
chr12	86855834	86856001
chr12	86857499	86857671
chr12	86859361	86859722
chr12	86860216	86860609
chr12	86860661	86860816
chr12	86867228	86867545
chr12	86867672	86867968
chr12	86867968	86868270
chr12	86872269	86872715
chr12	86872715	86873020
chr12	86878397	86878654
chr12	86878762	86878974
chr12	86879113	86879304
chr12	86879506	86879657
chr12	86882269	86882604
chr12	86882821	86883027
chr12	86883344	86883538
chr12	86884004	86884237
chr12	86884295	86885132
chr12	86888149	86888395
chr12	86888550	86888946
chr12	86889424	86889654
chr12	86892458	86892708
chr12	86893971	86894147
chr12	86894219	86894534
chr12	86894534	86894890
chr12	86894971	86895183
chr12	86895183	86895475
chr12	86896417	86896698
chr12	86899558	86899791
chr12	86905272	86905527
chr12	86905723	86905887
chr12	86905933	86906207
chr12	86906207	86906444
chr12	86906444	86906761
chr12	86912788	86913112
chr12	86913358	86913628
chr12	86913628	86913853
chr12	86919110	86919303
chr12	86923704	86924181

chr12	86924492	86924675
chr12	86940899	86941606
chr12	86942154	86942419
chr12	86946858	86947195
chr12	86947195	86947597
chr12	86947744	86948171
chr12	86948174	86948334
chr12	86948700	86948967
chr12	86955730	86956324
chr12	86956439	86956704
chr12	86972994	86973736
chr12	86974337	86974683
chr12	86974683	86974947
chr12	86978032	86978205
chr12	86983529	86983767
chr12	86984246	86984461
chr12	86989929	86990509
chr12	86994281	86994504
chr12	86999247	86999802
chr12	87000187	87000363
chr12	87000688	87000915
chr12	87001526	87001678
chr12	87002288	87002446
chr12	87006587	87006758
chr12	87026052	87026319
chr12	87026319	87026572
chr12	87026572	87026916
chr12	87031510	87031703
chr12	87047649	87047799
chr12	87052798	87053253
chr12	87091225	87091577
chr12	87091577	87092180
chr12	87101718	87101936
chr12	87130281	87130609
chr12	87146957	87147381
chr12	87147381	87147798
chr12	87147798	87148087
chr12	87154105	87154288
chr12	87199608	87200021
chr12	87200021	87200396
chr12	87200396	87200642
chr12	87203923	87204220

chr12	87205969	87206153
chr12	87226837	87227034
chr12	87256669	87257004
chr12	87265944	87266540
chr12	87266540	87266829
chr12	87266829	87267064
chr12	87267064	87267280
chr12	87267280	87267445
chr12	87276106	87276261
chr12	87276667	87276825
chr12	87279423	87279620
chr12	87279794	87280245
chr12	87285631	87285786
chr12	87293960	87294275
chr12	87299182	87299625
chr12	87299625	87299967
chr12	87299967	87300293
chr12	87300293	87300525
chr12	87300525	87300727
chr12	87387749	87388043
chr12	87388083	87388403
chr12	87388403	87388761
chr12	87398147	87398398
chr12	87399078	87399404
chr12	87399404	87399736
chr12	87405237	87405509
chr12	87405778	87406153
chr12	87407818	87408447
chr12	87409170	87409458
chr12	87413241	87413675
chr12	87416763	87417018
chr12	87419934	87420385
chr12	87423803	87424029
chr12	87433870	87434156
chr12	87434689	87434913
chr12	87434920	87435367
chr12	87435451	87435762
chr12	87443357	87443737
chr12	87443737	87444061
chr12	87471836	87472148
chr12	87472148	87472455
chr12	87472455	87472669

chr12	87477191	87477372
chr12	87524152	87524394
chr12	87566950	87567100
chr12	87647245	87647562
chr12	87844565	87844744
chr12	87844972	87845331
chr12	87857868	87858051
chr12	87861989	87862152
chr12	87862337	87862577
chr12	87863598	87863898
chr12	87866446	87866606
chr12	87901751	87901901
chr12	87903112	87903616
chr12	87907525	87907632
chr12	87907632	87907677
chr12	87998479	87998858
chr12	88159541	88159701
chr12	88163550	88164122
chr12	88182795	88182980
chr12	88216456	88216660
chr12	88245709	88246190
chr12	88250078	88250270
chr12	88263264	88263561
chr12	88281932	88282082
chr12	88285354	88285536
chr12	88360376	88360673
chr12	88360673	88361136
chr12	88405192	88405419
chr12	88443204	88443517
chr12	88467426	88467741
chr12	88523120	88523270
chr12	88523323	88523493
chr12	88709384	88709536
chr12	88966273	88966668
chr12	88967428	88967633
chr12	88971221	88971471
chr12	89134531	89134688
chr12	89513871	89514081
chr12	89592588	89592787
chr12	89812147	89812598
chr12	90166091	90166565
chr12	90216677	90216967

chr12	90729741	90729895
chr12	90740526	90740777
chr12	90781092	90781335
chr12	90781354	90781909
chr12	90781909	90782255
chr12	90905075	90905269
chr12	90986927	90987077
chr12	90987742	90988351
chr12	90991001	90991262
chr12	91029081	91029588
chr12	91029588	91029918
chr12	91070310	91070527
chr12	91070530	91070984
chr12	91081763	91082055
chr12	91100226	91100394
chr12	91208566	91208765
chr12	91250505	91250825
chr12	91250827	91251023
chr12	91254920	91255129
chr12	91291129	91291473
chr12	91292566	91293057
chr12	91293057	91293625
chr12	91305623	91306153
chr12	91312829	91313266
chr12	91314280	91314509
chr12	91326235	91326392
chr12	91383885	91384251
chr12	91384251	91384686
chr12	91384709	91384923
chr12	91386319	91386580
chr12	91400247	91400674
chr12	91400674	91401065
chr12	91401065	91401292
chr12	91403849	91404041
chr12	91418954	91419111
chr12	91423200	91423433
chr12	91552068	91552294
chr12	91557746	91557920
chr12	91564533	91564683
chr12	91579709	91580086
chr12	91580091	91580259
chr12	91580276	91580468

chr12	91585263	91585464
chr12	91588245	91588660
chr12	91589321	91589487
chr12	91589777	91590120
chr12	91590120	91590396
chr12	91590396	91590685
chr12	91590803	91591103
chr12	91612242	91612392
chr12	91612971	91613131
chr12	91622551	91622773
chr12	91625113	91625268
chr12	91635710	91635889
chr12	91640795	91640947
chr12	91667545	91668065
chr12	91668065	91668371
chr12	91668477	91668963
chr12	91675990	91676266
chr12	91685832	91686330
chr12	91691515	91691840
chr12	91691840	91692084
chr12	91710938	91711122
chr12	91712324	91712525
chr12	91736651	91736907
chr12	91745019	91745331
chr12	91745861	91746342
chr12	91748208	91748854
chr12	91752698	91753054
chr12	91756634	91756788
chr12	91765547	91765710
chr12	91765710	91765915
chr12	91774776	91775012
chr12	91778236	91778422
chr12	91778464	91778804
chr12	91778804	91779050
chr12	91779050	91779489
chr12	91780674	91780854
chr12	91789788	91790080
chr12	91793372	91793560
chr12	91816059	91816353
chr12	91821486	91821653
chr12	91832187	91832383
chr12	91848564	91848769

chr12	91848769	91849074
chr12	91849074	91849535
chr12	91876688	91876978
chr12	91877767	91877959
chr12	91878401	91878573
chr12	91878708	91878919
chr12	91878919	91879204
chr12	91904390	91904828
chr12	93576913	93577315
chr12	93928528	93928722
chr12	93928722	93928972
chr12	93928972	93929251
chr12	93929251	93929486
chr12	94042330	94042578
chr12	94640236	94640454
chr12	95489519	95489669
chr12	95491579	95491794
chr12	95491893	95492380
chr12	95520125	95520288
chr12	95533923	95534192
chr12	95538685	95538922
chr12	95559956	95560122
chr12	95563422	95563804
chr12	95599324	95599627
chr12	95641667	95641961
chr12	95652278	95652449
chr12	95692156	95692364
chr12	95694956	95695263
chr12	95695263	95695520
chr12	95695520	95695883
chr12	95695883	95696200
chr12	95703862	95704062
chr12	95734118	95734397
chr12	95743931	95744199
chr12	95744350	95744587
chr12	95746932	95747155
chr12	95748752	95749082
chr12	95749320	95749639
chr12	95759229	95759398
chr12	95764593	95764808
chr12	95765071	95765229
chr12	95765236	95765610

chr12	95790171	95790509
chr12	95895822	95896096
chr12	95940589	95940938
chr12	96045938	96046098
chr12	96065925	96066091
chr12	96066555	96066708
chr12	96066761	96067210
chr12	96106343	96106505
chr12	96154200	96154379
chr12	96522742	96522956
chr12	96849421	96849587
chr12	96924720	96924957
chr12	97061365	97061852
chr12	97080459	97080617
chr12	97872509	97872713
chr12	97957394	97957620
chr12	98053539	98053827
chr12	98212514	98212785
chr12	98212908	98213058
chr12	98239014	98239164
chr12	98244800	98245014
chr12	98249694	98250068
chr12	98250070	98250234
chr12	98250265	98250541
chr12	98252396	98252731
chr12	98255455	98255953
chr12	98258615	98259321
chr12	98259321	98259732
chr12	98282766	98283125
chr12	98283125	98283479
chr12	98283479	98283915
chr12	98293867	98294017
chr12	98318392	98318797
chr12	98342254	98342471
chr12	98444934	98445154
chr12	98471326	98471517
chr12	98501630	98501822
chr12	98574006	98574156
chr12	98574536	98574874
chr12	98574874	98575174
chr12	98577816	98578031
chr12	98578031	98578354

chr12	98587084	98587365
chr12	98588510	98588817
chr12	98592135	98592286
chr12	98627817	98628248
chr12	98628248	98628563
chr12	98628563	98628869
chr12	98637999	98638240
chr12	98674591	98674891
chr12	98674891	98675232
chr12	98681926	98682229
chr12	98682281	98682512
chr12	98689237	98689550
chr12	98708381	98708652
chr12	98712895	98713140
chr12	98713556	98713858
chr12	98713858	98714314
chr12	98714502	98714656
chr12	98714722	98715161
chr12	98724812	98725510
chr12	98725536	98725691
chr12	98734377	98734857
chr12	98734897	98735096
chr12	98735112	98735309
chr12	98737272	98737767
chr12	98737879	98738131
chr12	98741071	98741658
chr12	98746152	98746469
chr12	98746701	98746920
chr12	98746920	98747121
chr12	98747121	98747424
chr12	98757721	98757896
chr12	98779236	98779446
chr12	98850842	98851088
chr12	98872195	98872565
chr12	98881838	98882062
chr12	98900594	98901043
chr12	98901043	98901347
chr12	98901347	98901850
chr12	98920129	98920456
chr12	98920456	98920711
chr12	98920711	98921027
chr12	98949951	98950105

chr12	98965619	98966174
chr12	98974056	98974740
chr12	98977108	98977385
chr12	98991157	98991456
chr12	98991456	98991673
chr12	98993763	98994058
chr12	98994402	98994744
chr12	98994751	98994901
chr12	98995985	98996200
chr12	98996539	98996855
chr12	99040186	99040582
chr12	99050505	99050685
chr12	99052400	99052550
chr12	99052556	99052983
chr12	99059491	99059835
chr12	99063807	99064319
chr12	99084130	99084582
chr12	99090135	99090345
chr12	99090345	99090632
chr12	99096934	99097253
chr12	99097268	99097572
chr12	99183306	99183728
chr12	99197004	99197159
chr12	99213458	99213756
chr12	99267685	99268184
chr12	99329375	99329555
chr12	99336483	99337070
chr12	99338421	99338672
chr12	99338801	99339285
chr12	99339996	99340232
chr12	99340764	99341280
chr12	99347048	99347310
chr12	99351777	99352049
chr12	99352183	99352494
chr12	99352726	99353003
chr12	99353047	99353206
chr12	99356481	99357035
chr12	99357035	99357399
chr12	99358353	99358527
chr12	99362772	99362977
chr12	99366124	99366326
chr12	99367707	99367864

chr12	99370885	99371072
chr12	99371721	99371899
chr12	99375378	99375552
chr12	99375909	99376159
chr12	99376954	99377153
chr12	99377173	99377744
chr12	99386562	99386712
chr12	99391953	99392530
chr12	99392552	99392878
chr12	99392878	99393121
chr12	99393121	99393382
chr12	99393382	99393679
chr12	99393679	99393967
chr12	99394246	99394405
chr12	99406350	99406604
chr12	99407449	99407635
chr12	99426649	99426840
chr12	99464221	99464593
chr12	99504873	99505025
chr12	99505154	99505337
chr12	99506475	99506693
chr12	99506693	99506808
chr12	99562229	99562445
chr12	99562445	99562679
chr12	99563338	99563787
chr12	99563787	99564011
chr12	99627637	99628347
chr12	99638038	99638509
chr12	99638994	99639621
chr12	99645056	99645392
chr12	99645392	99645746
chr12	99681729	99682021
chr12	99686716	99686968
chr12	99705294	99705454
chr12	99705454	99705632
chr12	99738197	99738623
chr12	99739130	99739375
chr12	99749361	99749706
chr12	99836509	99836659
chr12	99842854	99843039
chr12	99883110	99883337
chr12	99883337	99883692

chr12	99883806	99884065
chr12	99884304	99885049
chr12	99895167	99895372
chr12	99900133	99900385
chr12	99904313	99904490
chr12	99906147	99906456
chr12	99906469	99906853
chr12	99917818	99918062
chr12	99921210	99921372
chr12	99925268	99925656
chr12	99926989	99927241
chr12	99927852	99928256
chr12	99932243	99932547
chr12	99941726	99942060
chr12	99942650	99943252
chr12	99943252	99943521
chr12	99953044	99953217
chr12	99964319	99964591
chr12	99964591	99964835
chr12	99964886	99965488
chr12	99965694	99965930
chr12	99965930	99966163
chr12	99979641	99979952
chr12	100016639	100016966
chr12	100025942	100026135
chr12	100026216	100026525
chr12	100026923	100027109
chr12	100036111	100036286
chr12	100044798	100045121
chr12	100045426	100045728
chr12	100056080	100056408
chr12	100096843	100097235
chr12	100100456	100100619
chr12	100106387	100106537
chr12	100107460	100107695
chr12	100109979	100110509
chr12	100110509	100110989
chr12	100117026	100117194
chr12	100122931	100123130
chr12	100125032	100125287
chr12	100129876	100130392
chr12	100131767	100131944

chr12	100159281	100159539
chr12	100159539	100159962
chr12	100166233	100166637
chr12	100166961	100167196
chr12	100167931	100168254
chr12	100168256	100168514
chr12	100175166	100175663
chr12	100175761	100175951
chr12	100183765	100184090
chr12	100184090	100184381
chr12	100187149	100187328
chr12	100187348	100187878
chr12	100193198	100193410
chr12	100194251	100194461
chr12	100199053	100199362
chr12	100199362	100199803
chr12	100199865	100200196
chr12	100200196	100200590
chr12	100200590	100200978
chr12	100201843	100202197
chr12	100210784	100211431
chr12	100219883	100220351
chr12	100220351	100220639
chr12	100225317	100225525
chr12	100235004	100235384
chr12	100235459	100235878
chr12	100238116	100238332
chr12	100242389	100242643
chr12	100255552	100255873
chr12	100257783	100258206
chr12	100262995	100263188
chr12	100268095	100268316
chr12	100270335	100270575
chr12	100272944	100273457
chr12	100275838	100276207
chr12	100276950	100277249
chr12	100279822	100280324
chr12	100281132	100281852
chr12	100282293	100282685
chr12	100288822	100289034
chr12	100289647	100290079
chr12	100296925	100297075

chr12	100299114	100299721
chr12	100302122	100302567
chr12	100304334	100304542
chr12	100305108	100305356
chr12	100307558	100307823
chr12	100312988	100313296
chr12	100313296	100313627
chr12	100313646	100313874
chr12	100313878	100314028
chr12	100315188	100315423
chr12	100326596	100327086
chr12	100327086	100327324
chr12	100337278	100337713
chr12	100337713	100338042
chr12	100338166	100338427
chr12	100349643	100349838
chr12	100353513	100353784
chr12	100353916	100354409
chr12	100355703	100356062
chr12	100361574	100361724
chr12	100369246	100369594
chr12	100369884	100370065
chr12	100372125	100372294
chr12	100372758	100373204
chr12	100378293	100378622
chr12	100378650	100378933
chr12	100379310	100379482
chr12	100379807	100380138
chr12	100396875	100397274
chr12	100420082	100420288
chr12	100421615	100421832
chr12	100426286	100426498
chr12	100431890	100432213
chr12	100432213	100432500
chr12	100439173	100439473
chr12	100439520	100439884
chr12	100439962	100440204
chr12	100445003	100445373
chr12	100445412	100445622
chr12	100447184	100447448
chr12	100455497	100455887
chr12	100456281	100456562

chr12	100465206	100465622
chr12	100465834	100466162
chr12	100479947	100480265
chr12	100486320	100486478
chr12	100494281	100494568
chr12	100519574	100520119
chr12	100520282	100520519
chr12	100520519	100520766
chr12	100520766	100521205
chr12	100524789	100525097
chr12	100525983	100526462
chr12	100529263	100529488
chr12	100538544	100538780
chr12	100578364	100578520
chr12	100604612	100604872
chr12	100606842	100607037
chr12	100724627	100724957
chr12	100724957	100725270
chr12	100725270	100725622
chr12	100735349	100735514
chr12	100778967	100779251
chr12	100841296	100841538
chr12	100841538	100841727
chr12	100849327	100849941
chr12	100855533	100856015
chr12	100857869	100858077
chr12	100862361	100862539
chr12	100882470	100882662
chr12	100883274	100883440
chr12	100898206	100898628
chr12	100898907	100899643
chr12	100903807	100904128
chr12	100907993	100908306
chr12	100926302	100926586
chr12	100927108	100927551
chr12	100928352	100928668
chr12	100936123	100936566
chr12	100936649	100936907
chr12	100937661	100937847
chr12	100951937	100952172
chr12	100952195	100952494
chr12	100952583	100952989

chr12	100958035	100958474
chr12	100958723	100958965
chr12	100959274	100959428
chr12	100964034	100964224
chr12	100975885	100976102
chr12	100982123	100982688
chr12	100985225	100985398
chr12	100993186	100993392
chr12	100994192	100994464
chr12	100994592	100995013
chr12	100996600	100996750
chr12	100997769	100998015
chr12	100998015	100998546
chr12	101002787	101003062
chr12	101003186	101003392
chr12	101004866	101005045
chr12	101008249	101008452
chr12	101016058	101016214
chr12	101016495	101016843
chr12	101016843	101017062
chr12	101017062	101017247
chr12	101017977	101018268
chr12	101026638	101026844
chr12	101027430	101027854
chr12	101028704	101029235
chr12	101029235	101029499
chr12	101029499	101029763
chr12	101063385	101063546
chr12	101078059	101078234
chr12	101082584	101083058
chr12	101083058	101083465
chr12	101083465	101083854
chr12	101083854	101084136
chr12	101102668	101102990
chr12	101109699	101110099
chr12	101245810	101246014
chr12	101268142	101268516
chr12	101284203	101284400
chr12	101300029	101300252
chr12	101343197	101343375
chr12	101343592	101343791
chr12	101344403	101344680

chr12	101344680	101345015
chr12	101463267	101463486
chr12	101477981	101478492
chr12	101483428	101483964
chr12	101717991	101718342
chr12	101718342	101718738
chr12	101718738	101719070
chr12	101764839	101765246
chr12	101765439	101765725
chr12	101767364	101767656
chr12	101782949	101783194
chr12	101816885	101817146
chr12	101818713	101818962
chr12	101818962	101819303
chr12	101839854	101840085
chr12	101840085	101840277
chr12	101840277	101840476
chr12	101840711	101841097
chr12	101841210	101841714
chr12	101841714	101841974
chr12	101875258	101875408
chr12	101876395	101876825
chr12	101892904	101893250
chr12	101907434	101907584
chr12	101912404	101912806
chr12	101912806	101913053
chr12	101913053	101913545
chr12	101957742	101958127
chr12	101958127	101958505
chr12	101975324	101975603
chr12	101975603	101975851
chr12	101975851	101976091
chr12	101976091	101976664
chr12	102036055	102036264
chr12	102036264	102036479
chr12	102066616	102066794
chr12	102069951	102070109
chr12	102080796	102081041
chr12	102128937	102129218
chr12	102134776	102134927
chr12	102148823	102149075
chr12	102157108	102157343

chr12	102181882	102182112
chr12	102182112	102182315
chr12	102190728	102191449
chr12	102201963	102202409
chr12	102202858	102203322
chr12	102223536	102223793
chr12	102242119	102242605
chr12	102261776	102262188
chr12	102262188	102262490
chr12	102262706	102263195
chr12	102263807	102263999
chr12	102266733	102266953
chr12	102271434	102271685
chr12	102280403	102280658
chr12	102281366	102281563
chr12	102282722	102283296
chr12	102283296	102283512
chr12	102283549	102283846
chr12	102283846	102284068
chr12	102284068	102284264
chr12	102286649	102287038
chr12	102295204	102295672
chr12	102299164	102299664
chr12	102299664	102300043
chr12	102309733	102310181
chr12	102319982	102320221
chr12	102326163	102326632
chr12	102326956	102327179
chr12	102327694	102327874
chr12	102328068	102328500
chr12	102331915	102332169
chr12	102337875	102338133
chr12	102339117	102339308
chr12	102340437	102340701
chr12	102341596	102341775
chr12	102346585	102346814
chr12	102359511	102359742
chr12	102375638	102375907
chr12	102381558	102381717
chr12	102384935	102385177
chr12	102387279	102387576
chr12	102390136	102390338

chr12	102392028	102392298
chr12	102394453	102394849
chr12	102396020	102396170
chr12	102397433	102397724
chr12	102413393	102413640
chr12	102416343	102416641
chr12	102416649	102416809
chr12	102417743	102418053
chr12	102420569	102420738
chr12	102422058	102422420
chr12	102426288	102426701
chr12	102431931	102432581
chr12	102432581	102432908
chr12	102433214	102433559
chr12	102438225	102438389
chr12	102439362	102439610
chr12	102439610	102440095
chr12	102461303	102461629
chr12	102468812	102469127
chr12	102469127	102469365
chr12	102469365	102469718
chr12	102499819	102500023
chr12	102524810	102525088
chr12	102550603	102550848
chr12	102554945	102555366
chr12	102566837	102567184
chr12	102570792	102571018
chr12	102576804	102576965
chr12	102577032	102577582
chr12	102579123	102579355
chr12	102588490	102588712
chr12	102590777	102591024
chr12	102593862	102594036
chr12	102596546	102596721
chr12	102630564	102630760
chr12	102640580	102640791
chr12	102643450	102643617
chr12	102647838	102648266
chr12	102648314	102648472
chr12	102652553	102652845
chr12	102653113	102653312
chr12	102665846	102666104

chr12	102668326	102668619
chr12	102680901	102681069
chr12	102684348	102684634
chr12	102704271	102704421
chr12	102704437	102704810
chr12	102704810	102705216
chr12	102705216	102705820
chr12	102719611	102719936
chr12	102723131	102723281
chr12	102724791	102725019
chr12	102725113	102725485
chr12	102725689	102726089
chr12	102730295	102730817
chr12	102730888	102731164
chr12	102731183	102731608
chr12	102731608	102731854
chr12	102733446	102733708
chr12	102737625	102737947
chr12	102743285	102743573
chr12	102743573	102743848
chr12	102743848	102744130
chr12	102744264	102744455
chr12	102744918	102745162
chr12	102757415	102758022
chr12	102758022	102758424
chr12	102764909	102765148
chr12	102769148	102769304
chr12	102779515	102779741
chr12	102877092	102877308
chr12	102877308	102877518
chr12	102877518	102877804
chr12	102877804	102878089
chr12	102878089	102878330
chr12	102878330	102878839
chr12	102885854	102886005
chr12	102892897	102893088
chr12	102949586	102949869
chr12	102957364	102957569
chr12	102960956	102961396
chr12	103000301	103000463
chr12	103065039	103065272
chr12	103099196	103099561

chr12	103144194	103144486
chr12	103183129	103183327
chr12	103281426	103281659
chr12	103305228	103305420
chr12	103322864	103323097
chr12	103325282	103325868
chr12	103327670	103327841
chr12	103328838	103329157
chr12	103340594	103340931
chr12	103340931	103341322
chr12	103341322	103341710
chr12	103347033	103347308
chr12	103348396	103348603
chr12	103348603	103348868
chr12	103353693	103353927
chr12	103366256	103366533
chr12	103367928	103368167
chr12	103368167	103368373
chr12	103371803	103372045
chr12	103376707	103377173
chr12	103380378	103380637
chr12	103380730	103381058
chr12	103383387	103383779
chr12	103388464	103389139
chr12	103393725	103393940
chr12	103395533	103395907
chr12	103402918	103403086
chr12	103408643	103409052
chr12	103425464	103425775
chr12	103425775	103426389
chr12	103434084	103434339
chr12	103452621	103452856
chr12	103453407	103453610
chr12	103456823	103457025
chr12	103457038	103457443
chr12	103492836	103493173
chr12	103494529	103494786
chr12	103507210	103507441
chr12	103508418	103508570
chr12	103518614	103519015
chr12	103531980	103532593
chr12	103532593	103533012

chr12	103533012	103533292
chr12	103542419	103542585
chr12	103544198	103544432
chr12	103548647	103548855
chr12	103561148	103561324
chr12	103564742	103565011
chr12	103565121	103565510
chr12	103567279	103567429
chr12	103568347	103568521
chr12	103590774	103590982
chr12	103643420	103643655
chr12	103665140	103665476
chr12	103681998	103682202
chr12	103683785	103684219
chr12	103711679	103711829
chr12	103887288	103887536
chr12	103888276	103888556
chr12	103985064	103985239
chr12	103993026	103993259
chr12	103995381	103995612
chr12	103996094	103996294
chr12	103996404	103996827
chr12	104000567	104000774
chr12	104000774	104000984
chr12	104002528	104003162
chr12	104003204	104003595
chr12	104030086	104030251
chr12	104054021	104054192
chr12	104055724	104056180
chr12	104057077	104057377
chr12	104057620	104057869
chr12	104061740	104061993
chr12	104061993	104062245
chr12	104063319	104063494
chr12	104064574	104064881
chr12	104065003	104065421
chr12	104069745	104069959
chr12	104076169	104076634
chr12	104123891	104124295
chr12	104186297	104186683
chr12	104190906	104191162
chr12	104223030	104223315

chr12	104223315	104223617
chr12	104223687	104224060
chr12	104224302	104224578
chr12	104256621	104256802
chr12	104270302	104270452
chr12	104271282	104271555
chr12	104271555	104271903
chr12	104415481	104416165
chr12	104419238	104419388
chr12	104422722	104423043
chr12	104427059	104427234
chr12	104440107	104440392
chr12	104442300	104442775
chr12	104472048	104472257
chr12	104472683	104473161
chr12	104473168	104473493
chr12	104548623	104548915
chr12	104548915	104549174
chr12	104573791	104573994
chr12	104608694	104608844
chr12	104608986	104609298
chr12	104644798	104645059
chr12	104666117	104666282
chr12	104680899	104681085
chr12	104684601	104684754
chr12	104697904	104698157
chr12	104751341	104751824
chr12	104751824	104752459
chr12	104752477	104752725
chr12	104811020	104811249
chr12	104811866	104812029
chr12	104812800	104812967
chr12	104823758	104823945
chr12	104824575	104824977
chr12	104829866	104830191
chr12	104833542	104833695
chr12	104835447	104835873
chr12	104844436	104844590
chr12	104844747	104845047
chr12	104845047	104845333
chr12	104845335	104845701
chr12	104845701	104846258

chr12	104850712	104851068
chr12	104855548	104855784
chr12	104856133	104856326
chr12	104856625	104856885
chr12	104864368	104864763
chr12	104864763	104865240
chr12	104866780	104867000
chr12	104867400	104867658
chr12	104875870	104876087
chr12	104876087	104876423
chr12	104881583	104881899
chr12	104882098	104882417
chr12	104888733	104888932
chr12	104896839	104897021
chr12	104897049	104897367
chr12	104897367	104897795
chr12	104898750	104899059
chr12	104903930	104904642
chr12	104907237	104907434
chr12	104917346	104917765
chr12	104919338	104919613
chr12	104933312	104933545
chr12	104936317	104936540
chr12	104938924	104939171
chr12	104939743	104940061
chr12	104940076	104940227
chr12	104951336	104951816
chr12	104952504	104952774
chr12	104953976	104954249
chr12	104954963	104955374
chr12	104960076	104960308
chr12	104968216	104968479
chr12	104969249	104969410
chr12	104969516	104970032
chr12	104972885	104973235
chr12	104978474	104978627
chr12	104983213	104983730
chr12	104983730	104983995
chr12	104983995	104984317
chr12	104986549	104986778
chr12	104986778	104987088
chr12	104990727	104991151

chr12	104991151	104991492
chr12	104992414	104992669
chr12	104996027	104996361
chr12	104999266	104999433
chr12	105000111	105000408
chr12	105000573	105000908
chr12	105000908	105001331
chr12	105001367	105001623
chr12	105005472	105005835
chr12	105008736	105009205
chr12	105009462	105009704
chr12	105009704	105009944
chr12	105009944	105010306
chr12	105016657	105016842
chr12	105019933	105020278
chr12	105025308	105025569
chr12	105026293	105026566
chr12	105027792	105027958
chr12	105032120	105032720
chr12	105032720	105033169
chr12	105033179	105033358
chr12	105039602	105039767
chr12	105039960	105040110
chr12	105043154	105043358
chr12	105044792	105044980
chr12	105047572	105047838
chr12	105048587	105048757
chr12	105048757	105048931
chr12	105050979	105051211
chr12	105068995	105069229
chr12	105073769	105073976
chr12	105104137	105104358
chr12	105129605	105129807
chr12	105145033	105145240
chr12	105174382	105174639
chr12	105189041	105189277
chr12	105322172	105322592
chr12	105404739	105404928
chr12	105420534	105420814
chr12	105426629	105426901
chr12	105427420	105427735
chr12	105436045	105436300

chr12	105451896	105452088
chr12	105453736	105454124
chr12	105455165	105455615
chr12	105455766	105455967
chr12	105466952	105467261
chr12	105467955	105468156
chr12	105475531	105475776
chr12	105476538	105476815
chr12	105483592	105483820
chr12	105488942	105489116
chr12	105489751	105489902
chr12	105492377	105492614
chr12	105497744	105497970
chr12	105497970	105498205
chr12	105498205	105498392
chr12	105498500	105498729
chr12	105498828	105499207
chr12	105499207	105499518
chr12	105510196	105510382
chr12	105510703	105510917
chr12	105511163	105511463
chr12	105514199	105514472
chr12	105517092	105517276
chr12	105523737	105524202
chr12	105524671	105524897
chr12	105525085	105525368
chr12	105525368	105525625
chr12	105529638	105529892
chr12	105534948	105535135
chr12	105536886	105537163
chr12	105537163	105537719
chr12	105543782	105544142
chr12	105546104	105546560
chr12	105546637	105546876
chr12	105547227	105547387
chr12	105548011	105548262
chr12	105548262	105548555
chr12	105548984	105549273
chr12	105549941	105550097
chr12	105551392	105551684
chr12	105551684	105551980
chr12	105554104	105554456

chr12	105554456	105554778
chr12	105555277	105555531
chr12	105557249	105557614
chr12	105557669	105557871
chr12	105562093	105562395
chr12	105562932	105563301
chr12	105563307	105563457
chr12	105563854	105564226
chr12	105564558	105564740
chr12	105565249	105565606
chr12	105571688	105572163
chr12	105572734	105573036
chr12	105573036	105573283
chr12	105573854	105574024
chr12	105583387	105583832
chr12	105584904	105585204
chr12	105590443	105590746
chr12	105595730	105595905
chr12	105596214	105596490
chr12	105596719	105596905
chr12	105597015	105597169
chr12	105597278	105597430
chr12	105602485	105602739
chr12	105603650	105603826
chr12	105611790	105612042
chr12	105614782	105614937
chr12	105684048	105684198
chr12	105684300	105684555
chr12	105684555	105684975
chr12	105684975	105685429
chr12	105685429	105685792
chr12	105685871	105686036
chr12	105687569	105687740
chr12	105705862	105706154
chr12	105718519	105718989
chr12	105722747	105722995
chr12	105730860	105731132
chr12	105731132	105731412
chr12	105734238	105734565
chr12	105748136	105748361
chr12	105784150	105784594
chr12	105784594	105784816

chr12	105784816	105785049
chr12	105785049	105785375
chr12	105793915	105794156
chr12	105805800	105806085
chr12	105809082	105809234
chr12	105814044	105814283
chr12	105839186	105839346
chr12	105840633	105840833
chr12	105843701	105843904
chr12	105847659	105847813
chr12	105854687	105854990
chr12	105882454	105882606
chr12	105882903	105883167
chr12	105906683	105906988
chr12	105923940	105924115
chr12	105962551	105963024
chr12	105963024	105963375
chr12	105976602	105976773
chr12	106001878	106002120
chr12	106009973	106010846
chr12	106025574	106025797
chr12	106046048	106046249
chr12	106046249	106046476
chr12	106046476	106046734
chr12	106055857	106056147
chr12	106094935	106095085
chr12	106098963	106099198
chr12	106105480	106105673
chr12	106115906	106116199
chr12	106168547	106169141
chr12	106174142	106174408
chr12	106177024	106177345
chr12	106177345	106177814
chr12	106300876	106301028
chr12	106513136	106513379
chr12	106513379	106513810
chr12	106568907	106569179
chr12	106600885	106601050
chr12	106764482	106764811
chr12	106849753	106849913
chr12	106972370	106972531
chr12	107530769	107530991

chr12	107531079	107531352
chr12	107561194	107561534
chr12	107617268	107617550
chr12	107645164	107645468
chr12	107645468	107645865
chr12	107646737	107647046
chr12	107669967	107670235
chr12	107693708	107694086
chr12	107712288	107712438
chr12	107752230	107752488
chr12	107752672	107752895
chr12	107753118	107753298
chr12	107807508	107808087
chr12	107856550	107856748
chr12	107856748	107857004
chr12	107878464	107878621
chr12	107880105	107880316
chr12	107998820	107999063
chr12	108003913	108004326
chr12	108006187	108006442
chr12	108041630	108041898
chr12	108042012	108042205
chr12	108059522	108059701
chr12	108078179	108078473
chr12	108083275	108083430
chr12	108094769	108095133
chr12	108098235	108098496
chr12	108105237	108105506
chr12	108109800	108110124
chr12	108110212	108110395
chr12	108117545	108117824
chr12	108121374	108121621
chr12	108152875	108153035
chr12	108159961	108160147
chr12	108162135	108162350
chr12	108178383	108178604
chr12	108178740	108179150
chr12	108179150	108180087
chr12	108199629	108199784
chr12	108200170	108200325
chr12	108206701	108206967
chr12	108206967	108207162

chr12	108208360	108208515
chr12	108209060	108209347
chr12	108217484	108217873
chr12	108217873	108218043
chr12	108221261	108221540
chr12	108226087	108226258
chr12	108234865	108235242
chr12	108235242	108235532
chr12	108239317	108240036
chr12	108244716	108245247
chr12	108245512	108245956
chr12	108252251	108252721
chr12	108253190	108253401
chr12	108258910	108259134
chr12	108269037	108269383
chr12	108274918	108275674
chr12	108275674	108275973
chr12	108275973	108276315
chr12	108276381	108276601
chr12	108284717	108284919
chr12	108285002	108285572
chr12	108292451	108292797
chr12	108292961	108293314
chr12	108294592	108294988
chr12	108297117	108297318
chr12	108306432	108306648
chr12	108308296	108308665
chr12	108308665	108308953
chr12	108309393	108309744
chr12	108309789	108310042
chr12	108310527	108310793
chr12	108317479	108317635
chr12	108321888	108322295
chr12	108325457	108325667
chr12	108327852	108328198
chr12	108328324	108328508
chr12	108333070	108333357
chr12	108333581	108333983
chr12	108333983	108334620
chr12	108334624	108334839
chr12	108338743	108338917
chr12	108349743	108349963

chr12	108351614	108351841
chr12	108352765	108353127
chr12	108354399	108354711
chr12	108362531	108362747
chr12	108363805	108364306
chr12	108365463	108365647
chr12	108370415	108370882
chr12	108370882	108371291
chr12	108380273	108380462
chr12	108383986	108384201
chr12	108384201	108384486
chr12	108392154	108392630
chr12	108393148	108393440
chr12	108393665	108394118
chr12	108396070	108396492
chr12	108401250	108401680
chr12	108401680	108401965
chr12	108403965	108404417
chr12	108406752	108406979
chr12	108422562	108422898
chr12	108422898	108423675
chr12	108442243	108442396
chr12	108443381	108443593
chr12	108444423	108444805
chr12	108448448	108448647
chr12	108453404	108453815
chr12	108453815	108454134
chr12	108469099	108469350
chr12	108471332	108471490
chr12	108473091	108473270
chr12	108485630	108485801
chr12	108485825	108486178
chr12	108486178	108486528
chr12	108487889	108488279
chr12	108491072	108491295
chr12	108494202	108494372
chr12	108494372	108494706
chr12	108495500	108495910
chr12	108529282	108529545
chr12	108536540	108536778
chr12	108541125	108541360
chr12	108546958	108547108

chr12	108554115	108554319
chr12	108554337	108554822
chr12	108554822	108555084
chr12	108555084	108555281
chr12	108555281	108555485
chr12	108555485	108555689
chr12	108583989	108584217
chr12	108613160	108613441
chr12	108652103	108652284
chr12	108657460	108657683
chr12	108671383	108671533
chr12	108675500	108676066
chr12	108676096	108676312
chr12	108677223	108677493
chr12	108678453	108678676
chr12	108686982	108687250
chr12	108689197	108689576
chr12	108689576	108689897
chr12	108690557	108690865
chr12	108694822	108695078
chr12	108700184	108700400
chr12	108702171	108702613
chr12	108704360	108704644
chr12	108704644	108704945
chr12	108706222	108706372
chr12	108709478	108709695
chr12	108710883	108711285
chr12	108749645	108750016
chr12	108750302	108750463
chr12	108757547	108758420
chr12	108760611	108760818
chr12	108763603	108763846
chr12	108765669	108766023
chr12	108766023	108766241
chr12	108766241	108766483
chr12	108766656	108766813
chr12	108787076	108787226
chr12	108792197	108792449
chr12	108792503	108792854
chr12	108792854	108793444
chr12	108794105	108794449
chr12	108794449	108794770

chr12	108808542	108808692
chr12	108811245	108811471
chr12	108819433	108819671
chr12	108820496	108820679
chr12	108829109	108829327
chr12	108829596	108829843
chr12	108835369	108836094
chr12	108836094	108836386
chr12	108836386	108836613
chr12	108841258	108841507
chr12	108842800	108842969
chr12	108844903	108845165
chr12	108846483	108846841
chr12	108848025	108848449
chr12	108855931	108856165
chr12	108856240	108856523
chr12	108856523	108856736
chr12	108859085	108859321
chr12	108859321	108859554
chr12	108859554	108859780
chr12	108869693	108869928
chr12	108880726	108881068
chr12	108881349	108881544
chr12	108885953	108886303
chr12	108891195	108891371
chr12	108893405	108893717
chr12	108893784	108894074
chr12	108894074	108894617
chr12	108902971	108903332
chr12	108903534	108903882
chr12	108914348	108914537
chr12	108931150	108931368
chr12	108942625	108942801
chr12	108949861	108950107
chr12	108952821	108953065
chr12	108959749	108959935
chr12	108964936	108965213
chr12	108965263	108965418
chr12	108965445	108965711
chr12	108965863	108966052
chr12	108966121	108966531
chr12	108966531	108966877

chr12	108968040	108968220
chr12	108984488	108984895
chr12	108984895	108985173
chr12	108985173	108985409
chr12	108994731	108995017
chr12	109003055	109003281
chr12	109042943	109043138
chr12	109044388	109045182
chr12	109067920	109068238
chr12	109068308	109068459
chr12	109375652	109375833
chr12	109393853	109394140
chr12	109402213	109402693
chr12	109405876	109406033
chr12	109417563	109418277
chr12	109418317	109418616
chr12	109423615	109423926
chr12	109425125	109425351
chr12	109436321	109436532
chr12	109437785	109438062
chr12	109441484	109441739
chr12	109441916	109442149
chr12	109444930	109445321
chr12	109445587	109445738
chr12	109446113	109446312
chr12	109453186	109453362
chr12	109542883	109543191
chr12	109583302	109583498
chr12	109617234	109617434
chr12	109623305	109623525
chr12	109626937	109627355
chr12	109628974	109629216
chr12	109634695	109634866
chr12	109694116	109694516
chr12	109694774	109695287
chr12	109749698	109750285
chr12	109764300	109764566
chr12	109790393	109790779
chr12	109798822	109799202
chr12	109799202	109799475
chr12	109799475	109799910
chr12	109811080	109811292

chr12	109830374	109830565
chr12	109845693	109845897
chr12	109856490	109856884
chr12	109856884	109857279
chr12	109895576	109895833
chr12	109917888	109918152
chr12	109923132	109923871
chr12	109958391	109958693
chr12	109958693	109959077
chr12	110009231	110009548
chr12	110012615	110012811
chr12	110012989	110013173
chr12	110031399	110031629
chr12	110033195	110033670
chr12	110041304	110041741
chr12	110041741	110042199
chr12	110043041	110043293
chr12	110043293	110043623
chr12	110055764	110056030
chr12	110062921	110063367
chr12	110066490	110066832
chr12	110071870	110072282
chr12	110074500	110074911
chr12	110074911	110075310
chr12	110086662	110086874
chr12	110087444	110087684
chr12	110087846	110088137
chr12	110088189	110088424
chr12	110088424	110088668
chr12	110109140	110109419
chr12	110109421	110109674
chr12	110112537	110112717
chr12	110112717	110112997
chr12	110120214	110120564
chr12	110127025	110127453
chr12	110145517	110145714
chr12	110156457	110156759
chr12	110169071	110169555
chr12	110169555	110169978
chr12	110200934	110201270
chr12	110203415	110203638
chr12	110203739	110203945

chr12	110203959	110204116
chr12	110204286	110204661
chr12	110206327	110206579
chr12	110207581	110207912
chr12	110214935	110215160
chr12	110215403	110215747
chr12	110278022	110278293
chr12	110278782	110279194
chr12	110373437	110373892
chr12	110377838	110378070
chr12	110381133	110381418
chr12	110381418	110381689
chr12	110382254	110382451
chr12	110389432	110389792
chr12	110389856	110390187
chr12	110391793	110391944
chr12	110398483	110398730
chr12	110399166	110399653
chr12	110417880	110418501
chr12	110418761	110419106
chr12	110419930	110420151
chr12	110424064	110424312
chr12	110424312	110424530
chr12	110424530	110424718
chr12	110429468	110429870
chr12	110431817	110432201
chr12	110433528	110433970
chr12	110437404	110437580
chr12	110438443	110438729
chr12	110438762	110439043
chr12	110446202	110446460
chr12	110446460	110446788
chr12	110446788	110447266
chr12	110447407	110447686
chr12	110454207	110454526
chr12	110474657	110474948
chr12	110480790	110481053
chr12	110485161	110485327
chr12	110485327	110485482
chr12	110485555	110485985
chr12	110485985	110486546
chr12	110497164	110497356

chr12	110497465	110497731
chr12	110497731	110497975
chr12	110498168	110498359
chr12	110498359	110498631
chr12	110498631	110498914
chr12	110498914	110499216
chr12	110499216	110499530
chr12	110503536	110503721
chr12	110504691	110504841
chr12	110506070	110506401
chr12	110515236	110515397
chr12	110523705	110523857
chr12	110532771	110533046
chr12	110533353	110533553
chr12	110533918	110534070
chr12	110535270	110535517
chr12	110549522	110549814
chr12	110551631	110551788
chr12	110553430	110553692
chr12	110555542	110555804
chr12	110566531	110566728
chr12	110576753	110576915
chr12	110580146	110580306
chr12	110590840	110591021
chr12	110591021	110591226
chr12	110591226	110591520
chr12	110591520	110592070
chr12	110598628	110598841
chr12	110599879	110600105
chr12	110601206	110601543
chr12	110601543	110601944
chr12	110601944	110602262
chr12	110606791	110606999
chr12	110608512	110608823
chr12	110608823	110609270
chr12	110652643	110652821
chr12	110659408	110659594
chr12	110667499	110667838
chr12	110673798	110674045
chr12	110674325	110674566
chr12	110677503	110677701
chr12	110681229	110681530

chr12	110682548	110682832
chr12	110687811	110688136
chr12	110688136	110688632
chr12	110689235	110689386
chr12	110695643	110696113
chr12	110696113	110696478
chr12	110696478	110696939
chr12	110701444	110701779
chr12	110702982	110703649
chr12	110706814	110707029
chr12	110711300	110711665
chr12	110713799	110714020
chr12	110725272	110725475
chr12	110725481	110725920
chr12	110736743	110737071
chr12	110737074	110737236
chr12	110737313	110737492
chr12	110737492	110737686
chr12	110737686	110737888
chr12	110737888	110738118
chr12	110738118	110738490
chr12	110738490	110738849
chr12	110770009	110770200
chr12	110779805	110779964
chr12	110780148	110780472
chr12	110780490	110780797
chr12	110790714	110790869
chr12	110796808	110796979
chr12	110797324	110797815
chr12	110797815	110798261
chr12	110798261	110798542
chr12	110805850	110806051
chr12	110806380	110806600
chr12	110808936	110809192
chr12	110814992	110815202
chr12	110819182	110819430
chr12	110819430	110819768
chr12	110821625	110821801
chr12	110823540	110823814
chr12	110830475	110830688
chr12	110833948	110834195
chr12	110836092	110836337

chr12	110838386	110838589
chr12	110840455	110840609
chr12	110840669	110841138
chr12	110843736	110844018
chr12	110844031	110844194
chr12	110844826	110845055
chr12	110845099	110845631
chr12	110850124	110850383
chr12	110850383	110850766
chr12	110850766	110851122
chr12	110851374	110851524
chr12	110866338	110866591
chr12	110871294	110871456
chr12	110882394	110882671
chr12	110883649	110884003
chr12	110888627	110889058
chr12	110889058	110889380
chr12	110889380	110889832
chr12	110917166	110917316
chr12	110918699	110918887
chr12	110919381	110919564
chr12	110920946	110921096
chr12	110932938	110933101
chr12	110936137	110936339
chr12	110936339	110936647
chr12	110946355	110946679
chr12	110953077	110953329
chr12	110953441	110953813
chr12	110954408	110954625
chr12	110954625	110954882
chr12	110967046	110967500
chr12	110973207	110973507
chr12	110978386	110978902
chr12	110978902	110979561
chr12	110986597	110986824
chr12	110987797	110988146
chr12	110991334	110991936
chr12	110991936	110992310
chr12	110992310	110992613
chr12	111004176	111004562
chr12	111005981	111006252
chr12	111006290	111006517

chr12	111007763	111008070
chr12	111008701	111008869
chr12	111016825	111017125
chr12	111038319	111038482
chr12	111038523	111038684
chr12	111038698	111039649
chr12	111039844	111039994
chr12	111041364	111041517
chr12	111046975	111047164
chr12	111060201	111060391
chr12	111064577	111064955
chr12	111066652	111066883
chr12	111069331	111069680
chr12	111087900	111088120
chr12	111088120	111088333
chr12	111088333	111088597
chr12	111089224	111089386
chr12	111094250	111094463
chr12	111094931	111095168
chr12	111096118	111096554
chr12	111096554	111096850
chr12	111097128	111097382
chr12	111104917	111105067
chr12	111113542	111114008
chr12	111122406	111122846
chr12	111135624	111135869
chr12	111137204	111137426
chr12	111150287	111150494
chr12	111153200	111153374
chr12	111164256	111164542
chr12	111164542	111164819
chr12	111165161	111165466
chr12	111165750	111166007
chr12	111166007	111166297
chr12	111166297	111166717
chr12	111166717	111167130
chr12	111186672	111186859
chr12	111186871	111187477
chr12	111207028	111207190
chr12	111210735	111210966
chr12	111211346	111211669
chr12	111211793	111212092

chr12	111218625	111218796
chr12	111234419	111234669
chr12	111247219	111247383
chr12	111249223	111249384
chr12	111258107	111258287
chr12	111264578	111264757
chr12	111266336	111266536
chr12	111269791	111269951
chr12	111272499	111272767
chr12	111273305	111273455
chr12	111275217	111275520
chr12	111275520	111275813
chr12	111283638	111283797
chr12	111283892	111284042
chr12	111285330	111285480
chr12	111288025	111288194
chr12	111288194	111288379
chr12	111288806	111289078
chr12	111293108	111293269
chr12	111294226	111294465
chr12	111303046	111303341
chr12	111303341	111303553
chr12	111303647	111304089
chr12	111304089	111304503
chr12	111309968	111310182
chr12	111310182	111310553
chr12	111310568	111310765
chr12	111310765	111310984
chr12	111316982	111317172
chr12	111323387	111323804
chr12	111328192	111328533
chr12	111328533	111328774
chr12	111334286	111334492
chr12	111340540	111341177
chr12	111341243	111341394
chr12	111353114	111353410
chr12	111353410	111354118
chr12	111355468	111355618
chr12	111361571	111361748
chr12	111364356	111364584
chr12	111366083	111366270
chr12	111367006	111367158

chr12	111370236	111370771
chr12	111376841	111377073
chr12	111377073	111377385
chr12	111377385	111377867
chr12	111377867	111378269
chr12	111378269	111378513
chr12	111385872	111386412
chr12	111386412	111386871
chr12	111386871	111387196
chr12	111387223	111387513
chr12	111394778	111394933
chr12	111398864	111399383
chr12	111401453	111401823
chr12	111411645	111412028
chr12	111412031	111412288
chr12	111413179	111413365
chr12	111413365	111413701
chr12	111425307	111425567
chr12	111426134	111426402
chr12	111427414	111427564
chr12	111429622	111429977
chr12	111430137	111430394
chr12	111430394	111430713
chr12	111430713	111431086
chr12	111432079	111432571
chr12	111434243	111434475
chr12	111442250	111442896
chr12	111443366	111443852
chr12	111446470	111446651
chr12	111446658	111446988
chr12	111446988	111447264
chr12	111447916	111448249
chr12	111448434	111448586
chr12	111448913	111449322
chr12	111449322	111449772
chr12	111455205	111455631
chr12	111455631	111455862
chr12	111455862	111456045
chr12	111456045	111456363
chr12	111461419	111461841
chr12	111469579	111469852
chr12	111471036	111471534

chr12	111474823	111475181
chr12	111479968	111480177
chr12	111481434	111481666
chr12	111485311	111485625
chr12	111485625	111485902
chr12	111485902	111486112
chr12	111504958	111505280
chr12	111508503	111508754
chr12	111516891	111517102
chr12	111531097	111531399
chr12	111531520	111531887
chr12	111535436	111535655
chr12	111535815	111536209
chr12	111537426	111537593
chr12	111537703	111538472
chr12	111538531	111538889
chr12	111547436	111547621
chr12	111547621	111547712
chr12	111547862	111548058
chr12	111548203	111548400
chr12	111548400	111548588
chr12	111548598	111548952
chr12	111549009	111549224
chr12	111550806	111550992
chr12	111553077	111553387
chr12	111553387	111553657
chr12	111553657	111553907
chr12	111554139	111554308
chr12	111559671	111559939
chr12	111560072	111560264
chr12	111565123	111565375
chr12	111565375	111565669
chr12	111568674	111569284
chr12	111573486	111573664
chr12	111574033	111574264
chr12	111574264	111574563
chr12	111574563	111574818
chr12	111574818	111575003
chr12	111575119	111575352
chr12	111580189	111580400
chr12	111599608	111599953
chr12	111643852	111644058

chr12	111648169	111648370
chr12	111648370	111648643
chr12	111653493	111653774
chr12	111661962	111662211
chr12	111665106	111665402
chr12	111665856	111666010
chr12	111668409	111668663
chr12	111670370	111671149
chr12	111671172	111671408
chr12	111671452	111671721
chr12	111671726	111671946
chr12	111672127	111672493
chr12	111677444	111677599
chr12	111677602	111677880
chr12	111677880	111678201
chr12	111678201	111678422
chr12	111678422	111678624
chr12	111678624	111678940
chr12	111678940	111679286
chr12	111685448	111685614
chr12	111686193	111686348
chr12	111686419	111686806
chr12	111686808	111687004
chr12	111689584	111689805
chr12	111691204	111691610
chr12	111691610	111691934
chr12	111696394	111696644
chr12	111697431	111697739
chr12	111697739	111698044
chr12	111698044	111698268
chr12	111698297	111698674
chr12	111704398	111704548
chr12	111704584	111705042
chr12	111705042	111705683
chr12	111712516	111712878
chr12	111712878	111713304
chr12	111713304	111713592
chr12	111713592	111713787
chr12	111722759	111722925
chr12	111726392	111726609
chr12	111731412	111731603
chr12	111731813	111732101

chr12	111732499	111732868
chr12	111732875	111733027
chr12	111736751	111736962
chr12	111738802	111739106
chr12	111741508	111741658
chr12	111745352	111745643
chr12	111745743	111745908
chr12	111747624	111747923
chr12	111749753	111750039
chr12	111752531	111752898
chr12	111753096	111753341
chr12	111753366	111753701
chr12	111756837	111757057
chr12	111758352	111758730
chr12	111758730	111759179
chr12	111759218	111759415
chr12	111759674	111760120
chr12	111760311	111760504
chr12	111760534	111760816
chr12	111762785	111762935
chr12	111768077	111768273
chr12	111770967	111771154
chr12	111779628	111779849
chr12	111785248	111785546
chr12	111791075	111791295
chr12	111803531	111803783
chr12	111809832	111810145
chr12	111813092	111813255
chr12	111813409	111813615
chr12	111813743	111814269
chr12	111814269	111814654
chr12	111822388	111822551
chr12	111846123	111846326
chr12	111848287	111848541
chr12	111849001	111849303
chr12	111851340	111851495
chr12	111853660	111853993
chr12	111863878	111864182
chr12	111864182	111864505
chr12	111864505	111864774
chr12	111878318	111878612
chr12	111881747	111882037

chr12	111888201	111888496
chr12	111891793	111891970
chr12	111892085	111892270
chr12	111895904	111896156
chr12	111896836	111897055
chr12	111897055	111897266
chr12	111901515	111901742
chr12	111907562	111907922
chr12	111907922	111908342
chr12	111908342	111908701
chr12	111926010	111926263
chr12	111936621	111937187
chr12	111937187	111937607
chr12	111937607	111937906
chr12	111938764	111939110
chr12	111943827	111943977
chr12	111944021	111944442
chr12	111944442	111944833
chr12	111945760	111946170
chr12	111946170	111946518
chr12	111946765	111947054
chr12	111958050	111958480
chr12	111958502	111958657
chr12	111958700	111958855
chr12	111959048	111959387
chr12	111966321	111966578
chr12	111966578	111966910
chr12	111966910	111967238
chr12	111982607	111982882
chr12	111992030	111992307
chr12	111992609	111992991
chr12	111992991	111993262
chr12	112001204	112001640
chr12	112018522	112018984
chr12	112035531	112035810
chr12	112077656	112077967
chr12	112085724	112086004
chr12	112086004	112086361
chr12	112091152	112091445
chr12	112103284	112103491
chr12	112106570	112106981
chr12	112112726	112113033

chr12	112113033	112113364
chr12	112113773	112114147
chr12	112115581	112115758
chr12	112117721	112117975
chr12	112125839	112126426
chr12	112129576	112129858
chr12	112130090	112130375
chr12	112130449	112130878
chr12	112140164	112140566
chr12	112141378	112141588
chr12	112145235	112145503
chr12	112145538	112145778
chr12	112146104	112146347
chr12	112146347	112146633
chr12	112150262	112150468
chr12	112151546	112151837
chr12	112152830	112152992
chr12	112164160	112164353
chr12	112169338	112169511
chr12	112172299	112172654
chr12	112183726	112183971
chr12	112186859	112187169
chr12	112191929	112192080
chr12	112226618	112226797
chr12	112255330	112255567
chr12	112265330	112265578
chr12	112267839	112268019
chr12	112268019	112268123
chr12	112268397	112268799
chr12	112295152	112295536
chr12	112295536	112295932
chr12	112297144	112297402
chr12	112297402	112297688
chr12	112297688	112298009
chr12	112309808	112310037
chr12	112329273	112329454
chr12	112339860	112340164
chr12	112407760	112407898
chr12	112407898	112408048
chr12	112421585	112421781
chr12	112423074	112423359
chr12	112426654	112427007

chr12	112427007	112427225
chr12	112431730	112432204
chr12	112432828	112433066
chr12	112437384	112437539
chr12	112450980	112451261
chr12	112458008	112458168
chr12	112463943	112464293
chr12	112473800	112474119
chr12	112481637	112481903
chr12	112481903	112482301
chr12	112489973	112490384
chr12	112490384	112491093
chr12	112491144	112491348
chr12	112491348	112491667
chr12	112497763	112498112
chr12	112498662	112499025
chr12	112507778	112508191
chr12	112508191	112508602
chr12	112511157	112511360
chr12	112516645	112517511
chr12	112534988	112535407
chr12	112537360	112537683
chr12	112538414	112538705
chr12	112542822	112543267
chr12	112551997	112552382
chr12	112552382	112552706
chr12	112552706	112553159
chr12	112555010	112555281
chr12	112560135	112560691
chr12	112562527	112562713
chr12	112568375	112568864
chr12	112570037	112570306
chr12	112571152	112571396
chr12	112576463	112576689
chr12	112576776	112577096
chr12	112578345	112578686
chr12	112578686	112579138
chr12	112579635	112579866
chr12	112580055	112580290
chr12	112587294	112588045
chr12	112588337	112588701
chr12	112588701	112588933

chr12	112588933	112589302
chr12	112594365	112594586
chr12	112595262	112595534
chr12	112595563	112596056
chr12	112599158	112599537
chr12	112604798	112605078
chr12	112605078	112605383
chr12	112605383	112605722
chr12	112605722	112606077
chr12	112607048	112607384
chr12	112616661	112616863
chr12	112617230	112617604
chr12	112618035	112618249
chr12	112619758	112620110
chr12	112620110	112620413
chr12	112620793	112620943
chr12	112621931	112622212
chr12	112622331	112622491
chr12	112627535	112627893
chr12	112628279	112628567
chr12	112628903	112629348
chr12	112629348	112629824
chr12	112635398	112635563
chr12	112643169	112643365
chr12	112644312	112644944
chr12	112644944	112645225
chr12	112645225	112645457
chr12	112648185	112648432
chr12	112651906	112652142
chr12	112653172	112653475
chr12	112653475	112653716
chr12	112659203	112659424
chr12	112660669	112660959
chr12	112671999	112672178
chr12	112672906	112673154
chr12	112673211	112673414
chr12	112673849	112674002
chr12	112674220	112674576
chr12	112674576	112674979
chr12	112675079	112675349
chr12	112675349	112675707
chr12	112678495	112678978

chr12	112678978	112679587
chr12	112681002	112681152
chr12	112693264	112693955
chr12	112694413	112694620
chr12	112694620	112694876
chr12	112701732	112701923
chr12	112702258	112702720
chr12	112702720	112702962
chr12	112706851	112707074
chr12	112707074	112707358
chr12	112707628	112707800
chr12	112713396	112713559
chr12	112713828	112713996
chr12	112714375	112714538
chr12	112717212	112717372
chr12	112721273	112721828
chr12	112721828	112722367
chr12	112722367	112722793
chr12	112722793	112723053
chr12	112723053	112723330
chr12	112724029	112724254
chr12	112724296	112724470
chr12	112727880	112728165
chr12	112728325	112728669
chr12	112728685	112728914
chr12	112742785	112742968
chr12	112743015	112743165
chr12	112743206	112743492
chr12	112743509	112743667
chr12	112745902	112746068
chr12	112746687	112746984
chr12	112747614	112747848
chr12	112751114	112751453
chr12	112752424	112752761
chr12	112758022	112758184
chr12	112759683	112759908
chr12	112764016	112764210
chr12	112764210	112764539
chr12	112771418	112771689
chr12	112774015	112774224
chr12	112779081	112779233
chr12	112780414	112780612

chr12	112780775	112781045
chr12	112781969	112782119
chr12	112782726	112782907
chr12	112795689	112796126
chr12	112796933	112797235
chr12	112797235	112797514
chr12	112797514	112797760
chr12	112797760	112798010
chr12	112798010	112798472
chr12	112802608	112802800
chr12	112804076	112804391
chr12	112806055	112806458
chr12	112808699	112809042
chr12	112809042	112809456
chr12	112816305	112816570
chr12	112816982	112817144
chr12	112817512	112817692
chr12	112822794	112823000
chr12	112823582	112823756
chr12	112828960	112829290
chr12	112829290	112829584
chr12	112829584	112830011
chr12	112835095	112835402
chr12	112836019	112836233
chr12	112843691	112843841
chr12	112843862	112844145
chr12	112849213	112849446
chr12	112849477	112849640
chr12	112850513	112850769
chr12	112850993	112851577
chr12	112851652	112851883
chr12	112851883	112852181
chr12	112853243	112853436
chr12	112856351	112856626
chr12	112864850	112865254
chr12	112866522	112867191
chr12	112868612	112868950
chr12	112873600	112873851
chr12	112875241	112875604
chr12	112875604	112876069
chr12	112876277	112876734
chr12	112880851	112881029

chr12	112881194	112881412
chr12	112890660	112890821
chr12	112897530	112897819
chr12	112897819	112898337
chr12	112905527	112905684
chr12	112908164	112908414
chr12	112913799	112913967
chr12	112914062	112914219
chr12	112922641	112922889
chr12	112928416	112928664
chr12	112928664	112928920
chr12	112928920	112929261
chr12	112929342	112929642
chr12	112929642	112929962
chr12	112929962	112930202
chr12	112930202	112930403
chr12	112937872	112938398
chr12	112938943	112939209
chr12	112941762	112942034
chr12	112942034	112942336
chr12	112947383	112947799
chr12	112950198	112950759
chr12	112952049	112952281
chr12	112952434	112952897
chr12	112955873	112956165
chr12	112958315	112958791
chr12	112966922	112967082
chr12	112976314	112976737
chr12	112976737	112977255
chr12	112980870	112981112
chr12	112985430	112985657
chr12	112990982	112991199
chr12	112994289	112994524
chr12	113000063	113000314
chr12	113000314	113000642
chr12	113000642	113000936
chr12	113000936	113001374
chr12	113005043	113005303
chr12	113012029	113012299
chr12	113013773	113014112
chr12	113014112	113014588
chr12	113014588	113015087

chr12	113023065	113023473
chr12	113030190	113030426
chr12	113032665	113032826
chr12	113038501	113038745
chr12	113039760	113040278
chr12	113045378	113045636
chr12	113047621	113047920
chr12	113049833	113050076
chr12	113051726	113051851
chr12	113051851	113051982
chr12	113052150	113052301
chr12	113058963	113059303
chr12	113059330	113059621
chr12	113061679	113061854
chr12	113073734	113073993
chr12	113074330	113074672
chr12	113074672	113074960
chr12	113074960	113075165
chr12	113075165	113075486
chr12	113097740	113098026
chr12	113098026	113098388
chr12	113098530	113098880
chr12	113098880	113099345
chr12	113099498	113099780
chr12	113102531	113102782
chr12	113103987	113104214
chr12	113106288	113106709
chr12	113106709	113107010
chr12	113113923	113114073
chr12	113119121	113119305
chr12	113119342	113119542
chr12	113119542	113119824
chr12	113119830	113120036
chr12	113132387	113132932
chr12	113139191	113139508
chr12	113139901	113140296
chr12	113140296	113140712
chr12	113140712	113141158
chr12	113144500	113144723
chr12	113146220	113146414
chr12	113146619	113147137
chr12	113147269	113147530

chr12	113148455	113148750
chr12	113149322	113149723
chr12	113155142	113155433
chr12	113155491	113155685
chr12	113155685	113156052
chr12	113156052	113156527
chr12	113156527	113156906
chr12	113168062	113168304
chr12	113177263	113177669
chr12	113177669	113177952
chr12	113180893	113181337
chr12	113185637	113185824
chr12	113185824	113186022
chr12	113187512	113187982
chr12	113187982	113188304
chr12	113193340	113193550
chr12	113193550	113193776
chr12	113207838	113208019
chr12	113210630	113210942
chr12	113215950	113216284
chr12	113217354	113217606
chr12	113217951	113218228
chr12	113219927	113220175
chr12	113220558	113220824
chr12	113221371	113221577
chr12	113222073	113222346
chr12	113222346	113222659
chr12	113222825	113223079
chr12	113227723	113227877
chr12	113230821	113231081
chr12	113238031	113238181
chr12	113240032	113240185
chr12	113261907	113262176
chr12	113262380	113263061
chr12	113263061	113263456
chr12	113264423	113264647
chr12	113274916	113275071
chr12	113275285	113275659
chr12	113275668	113275843
chr12	113276075	113276226
chr12	113276254	113276569
chr12	113276569	113277046

chr12	113293171	113293358
chr12	113293358	113293643
chr12	113293643	113294027
chr12	113294027	113294300
chr12	113294300	113294538
chr12	113294538	113294820
chr12	113294820	113295047
chr12	113295186	113295417
chr12	113305725	113306030
chr12	113306030	113306334
chr12	113311202	113311357
chr12	113327935	113328133
chr12	113335017	113335343
chr12	113335579	113335797
chr12	113335940	113336187
chr12	113336264	113336477
chr12	113337613	113337815
chr12	113424238	113424672
chr12	113424672	113425033
chr12	113425033	113425458
chr12	113487775	113488033
chr12	114048094	114048254
chr12	114087651	114087893
chr12	114207368	114207554
chr12	114378675	114379026
chr12	114379052	114379313
chr12	114935816	114936029
chr12	114990932	114991236
chr12	114991236	114991455
chr12	115034499	115034757
chr12	115034757	115034968
chr12	115044739	115045198
chr12	115045211	115045612
chr12	115088784	115089142
chr12	115089197	115089545
chr12	115089545	115089766
chr12	115105562	115105731
chr12	115109734	115110087
chr12	115110490	115110718
chr12	115119024	115119272
chr12	115119686	115119851
chr12	115311975	115312493

chr12	115320733	115320939
chr12	115924117	115924280
chr12	115967644	115967809
chr12	116047394	116047808
chr12	116047808	116048297
chr12	116071024	116071479
chr12	116077246	116077431
chr12	116077431	116077655
chr12	116077655	116077975
chr12	116077975	116078284
chr12	116095578	116095752
chr12	116096563	116096830
chr12	116132997	116133193
chr12	116138902	116139134
chr12	116146447	116146603
chr12	116152947	116153098
chr12	116159334	116159542
chr12	116165155	116165403
chr12	116165483	116165643
chr12	116167338	116167497
chr12	116168210	116168400
chr12	116169703	116170074
chr12	116172414	116172567
chr12	116181353	116181729
chr12	116181729	116182053
chr12	116183414	116183722
chr12	116187640	116187790
chr12	116196710	116196867
chr12	116202241	116202416
chr12	116202416	116202596
chr12	116202675	116202938
chr12	116203184	116203345
chr12	116203793	116203943
chr12	116205713	116206148
chr12	116213870	116214023
chr12	116224549	116224826
chr12	116231579	116231802
chr12	116231802	116231989
chr12	116235376	116235660
chr12	116244616	116244934
chr12	116250201	116250371
chr12	116254719	116255035

chr12	116258258	116258687
chr12	116262540	116262884
chr12	116262884	116263132
chr12	116263132	116263356
chr12	116269093	116269529
chr12	116269529	116270069
chr12	116280591	116281063
chr12	116281063	116281342
chr12	116281342	116281739
chr12	116281866	116282120
chr12	116283606	116283794
chr12	116288474	116288733
chr12	116288830	116289040
chr12	116289040	116289237
chr12	116293646	116293885
chr12	116293885	116294126
chr12	116294126	116294369
chr12	116294369	116294635
chr12	116299211	116299417
chr12	116299844	116300335
chr12	116308051	116308266
chr12	116313938	116314090
chr12	116315267	116315467
chr12	116363484	116363834
chr12	116363834	116364241
chr12	116389782	116390238
chr12	116390329	116390596
chr12	116396077	116396608
chr12	116404597	116404881
chr12	116404914	116405279
chr12	116405279	116405697
chr12	116405697	116406094
chr12	116433870	116434048
chr12	116441757	116441908
chr12	116455805	116456043
chr12	116467026	116467486
chr12	116478832	116479055
chr12	116485471	116486094
chr12	116486113	116486327
chr12	116503113	116503330
chr12	116512523	116512817
chr12	116539385	116539619

chr12	116553203	116553801
chr12	116569585	116569899
chr12	116576716	116577116
chr12	116577116	116577518
chr12	116577518	116577824
chr12	116577824	116578043
chr12	116581927	116582231
chr12	116585057	116585400
chr12	116587527	116587890
chr12	116620007	116620203
chr12	116621535	116621720
chr12	116621720	116621970
chr12	116622623	116623006
chr12	116623006	116623253
chr12	116625673	116625882
chr12	116653782	116653979
chr12	116762855	116763057
chr12	116772459	116772641
chr12	116776473	116776764
chr12	117035317	117035536
chr12	117077843	117078130
chr12	117092590	117093043
chr12	117110687	117110863
chr12	117132173	117132323
chr12	117153686	117154085
chr12	117240075	117240282
chr12	117315966	117316310
chr12	117353084	117353234
chr12	117385356	117385607
chr12	117389573	117390205
chr12	117394424	117394670
chr12	117394698	117394961
chr12	117394961	117395294
chr12	117395372	117395587
chr12	117395587	117395784
chr12	117403574	117403825
chr12	117403866	117404079
chr12	117406978	117407200
chr12	117410528	117410901
chr12	117412910	117413084
chr12	117427450	117427746
chr12	117430665	117430832

chr12	117430866	117431345
chr12	117432328	117432553
chr12	117432553	117432849
chr12	117432849	117433137
chr12	117436551	117436836
chr12	117441960	117442216
chr12	117442216	117442456
chr12	117443440	117443750
chr12	117448276	117448472
chr12	117448472	117448735
chr12	117448735	117448985
chr12	117450083	117450551
chr12	117462505	117462715
chr12	117468316	117468707
chr12	117472582	117472906
chr12	117472906	117473162
chr12	117475656	117476001
chr12	117479684	117479952
chr12	117480904	117481177
chr12	117481177	117481490
chr12	117487689	117487839
chr12	117496151	117496509
chr12	117503325	117503478
chr12	117505277	117505467
chr12	117507867	117508048
chr12	117523649	117523806
chr12	117533538	117534031
chr12	117534031	117534340
chr12	117534340	117534873
chr12	117539876	117540113
chr12	117545238	117545724
chr12	117569145	117569365
chr12	117586168	117586448
chr12	117588306	117588704
chr12	117588939	117589091
chr12	117667215	117667522
chr12	117668546	117669462
chr12	117673281	117673599
chr12	117675916	117676093
chr12	117676205	117676445
chr12	117679698	117679948
chr12	117690661	117690932

chr12	117691330	117691519
chr12	117700053	117700270
chr12	117709753	117709903
chr12	117727499	117727871
chr12	117727935	117728120
chr12	117728120	117728312
chr12	117728312	117728521
chr12	117742554	117742858
chr12	117761701	117762165
chr12	117764877	117765185
chr12	117767358	117767654
chr12	117769204	117769443
chr12	117772685	117772955
chr12	117778727	117778976
chr12	117820183	117820437
chr12	117820631	117820783
chr12	117820880	117821165
chr12	117828462	117828635
chr12	117834792	117834942
chr12	117843201	117843699
chr12	117843699	117844262
chr12	117853416	117853606
chr12	117861336	117861608
chr12	117861608	117861958
chr12	117861958	117862320
chr12	117868108	117868258
chr12	117888264	117888423
chr12	117993475	117993830
chr12	118073205	118073441
chr12	118073441	118073622
chr12	118073622	118073998
chr12	118078429	118078831
chr12	118095000	118095294
chr12	118118930	118119105
chr12	118177207	118177447
chr12	118177447	118177714
chr12	118177824	118178051
chr12	118189568	118189874
chr12	118193712	118193914
chr12	118196309	118196589
chr12	118198446	118198841
chr12	118198841	118199115

chr12	118213809	118213976
chr12	118263912	118264255
chr12	118300867	118301485
chr12	118301485	118301762
chr12	118301847	118302168
chr12	118302168	118302511
chr12	118359313	118359503
chr12	118367432	118367681
chr12	118367696	118368048
chr12	118368815	118369421
chr12	118373568	118373747
chr12	118439748	118439921
chr12	118457825	118458067
chr12	118548051	118548239
chr12	118656584	118656852
chr12	118656852	118657137
chr12	118657760	118658032
chr12	118658192	118658535
chr12	118694453	118694841
chr12	118694841	118695216
chr12	118695307	118695678
chr12	118701890	118702234
chr12	118702339	118702531
chr12	118724347	118724644
chr12	118728196	118728471
chr12	118728543	118728749
chr12	118732597	118732768
chr12	118732768	118732973
chr12	118732981	118733341
chr12	118747003	118747212
chr12	118754164	118754349
chr12	118775791	118776018
chr12	118805927	118806310
chr12	118806310	118806813
chr12	118814552	118814973
chr12	118815307	118815616
chr12	118816117	118816533
chr12	118824277	118824447
chr12	118826277	118826558
chr12	118836424	118837083
chr12	118837083	118837429
chr12	118846635	118846965

chr12	118846965	118847313
chr12	118848109	118848329
chr12	118848329	118848557
chr12	118848557	118848858
chr12	118848858	118849346
chr12	118849370	118849580
chr12	118854879	118855479
chr12	118926792	118926942
chr12	118930640	118930867
chr12	119026461	119026611
chr12	119070543	119070742
chr12	119091534	119091725
chr12	119099860	119100057
chr12	119135033	119135241
chr12	119230599	119230810
chr12	119238200	119238514
chr12	119238514	119238718
chr12	119238718	119238991
chr12	119249638	119249788
chr12	119262828	119263184
chr12	119265044	119265360
chr12	119268213	119268530
chr12	119308525	119308807
chr12	119325104	119325284
chr12	119330651	119330945
chr12	119364967	119365549
chr12	119376857	119377404
chr12	119386660	119386963
chr12	119387450	119387630
chr12	119387770	119387946
chr12	119387954	119388273
chr12	119390114	119390438
chr12	119390438	119390777
chr12	119397921	119398578
chr12	119404936	119405098
chr12	119425918	119426150
chr12	119426217	119426569
chr12	119426569	119426816
chr12	119426859	119427119
chr12	119438998	119439492
chr12	119440283	119440552
chr12	119440555	119440772

chr12	119441211	119441418
chr12	119441418	119441758
chr12	119451233	119451430
chr12	119470325	119470604
chr12	119565976	119566267
chr12	120028593	120029091
chr13	3036665	3036846
chr13	3037050	3037444
chr13	3128247	3128495
chr13	3216167	3216405
chr13	3341756	3342049
chr13	3355497	3355665
chr13	3359709	3359917
chr13	3360317	3360927
chr13	3360927	3361198
chr13	3361198	3361484
chr13	3367369	3367807
chr13	3372646	3372807
chr13	3372821	3373353
chr13	3373353	3373771
chr13	3406182	3406590
chr13	3406590	3406967
chr13	3414349	3414514
chr13	3417029	3417393
chr13	3429579	3429729
chr13	3473510	3473675
chr13	3477986	3478361
chr13	3478361	3478825
chr13	3515479	3515639
chr13	3531942	3532314
chr13	3537518	3537890
chr13	3537890	3538260
chr13	3538260	3538607
chr13	3538607	3538880
chr13	3544166	3544367
chr13	3544818	3545374
chr13	3556112	3556306
chr13	3564046	3564426
chr13	3568140	3568290
chr13	3610219	3610453
chr13	3610453	3610727
chr13	3610727	3611033

chr13	3611033	3611433
chr13	3633864	3634202
chr13	3634202	3634489
chr13	3634489	3634747
chr13	3635206	3635448
chr13	3636774	3637147
chr13	3642142	3642437
chr13	3645023	3645186
chr13	3665258	3665872
chr13	3667652	3667802
chr13	3678957	3679432
chr13	3679432	3679760
chr13	3680403	3680611
chr13	3694675	3694957
chr13	3694957	3695455
chr13	3700425	3700705
chr13	3700705	3701219
chr13	3723709	3724006
chr13	3730271	3730692
chr13	3730716	3730866
chr13	3743028	3743255
chr13	3743255	3743455
chr13	3743455	3743652
chr13	3743819	3744093
chr13	3744093	3744500
chr13	3745336	3745787
chr13	3746511	3746799
chr13	3750720	3750996
chr13	3756720	3756958
chr13	3759541	3759705
chr13	3769522	3769701
chr13	3773492	3773744
chr13	3776945	3777269
chr13	3777269	3777706
chr13	3779027	3779293
chr13	3779749	3779949
chr13	3779949	3780191
chr13	3780563	3780760
chr13	3782446	3783002
chr13	3783119	3783292
chr13	3807209	3807435
chr13	3807518	3807700

chr13	3818511	3818695
chr13	3821577	3821789
chr13	3821796	3822029
chr13	3822104	3822502
chr13	3825573	3825766
chr13	3831684	3832021
chr13	3832021	3832427
chr13	3832427	3832813
chr13	3854561	3854791
chr13	3862394	3862793
chr13	3865386	3866268
chr13	3879974	3880124
chr13	3886669	3887158
chr13	3890106	3890334
chr13	3893214	3893450
chr13	3893450	3893744
chr13	3898492	3898742
chr13	3901455	3901715
chr13	3901715	3901983
chr13	3901983	3902185
chr13	3902185	3902439
chr13	3902783	3903003
chr13	3907269	3907446
chr13	3917516	3917862
chr13	3917862	3918075
chr13	3918075	3918379
chr13	3924587	3924782
chr13	3947348	3947619
chr13	3964358	3964572
chr13	3964572	3964821
chr13	3992106	3992525
chr13	4001786	4002021
chr13	4032645	4032811
chr13	4059580	4059730
chr13	4110575	4110821
chr13	4129186	4129510
chr13	4133127	4133357
chr13	4150600	4150883
chr13	4160370	4160747
chr13	4183218	4183401
chr13	4186733	4186942
chr13	4190881	4191101

chr13	4191101	4191473
chr13	4193522	4193719
chr13	4206224	4206451
chr13	4233329	4234079
chr13	4238416	4238787
chr13	4245006	4245335
chr13	4245335	4245634
chr13	4248161	4248311
chr13	4276724	4276896
chr13	4279270	4279686
chr13	4281590	4281794
chr13	4310958	4311160
chr13	4313568	4313726
chr13	4318932	4319116
chr13	4364123	4364474
chr13	4381919	4382192
chr13	4415597	4415754
chr13	4508342	4508550
chr13	4537677	4537902
chr13	4605905	4606220
chr13	4608660	4608980
chr13	4608980	4609469
chr13	4662393	4662774
chr13	4662774	4663252
chr13	4666878	4667028
chr13	4667226	4667383
chr13	4667383	4667547
chr13	4672384	4672793
chr13	4707356	4707715
chr13	4712743	4713094
chr13	4714672	4715086
chr13	4715169	4715394
chr13	4771262	4772068
chr13	4778335	4778556
chr13	4778556	4778776
chr13	4824543	4824721
chr13	4826641	4827196
chr13	4831227	4831470
chr13	4936067	4936261
chr13	4942575	4942989
chr13	5005709	5005875
chr13	5008428	5008714

chr13	5079316	5079469
chr13	5106315	5106497
chr13	5186434	5186689
chr13	5211223	5211389
chr13	5212522	5212856
chr13	5240502	5240917
chr13	5248463	5249001
chr13	5249001	5249296
chr13	5284659	5284848
chr13	5289703	5289956
chr13	5302182	5302664
chr13	5318497	5318798
chr13	5319048	5319486
chr13	5343235	5343448
chr13	5345780	5346110
chr13	5346732	5346978
chr13	5347098	5347374
chr13	5347950	5348321
chr13	5350194	5350391
chr13	5373280	5373475
chr13	5394215	5394395
chr13	5412187	5412441
chr13	5421995	5422227
chr13	5423218	5423523
chr13	5424027	5424459
chr13	5424540	5424718
chr13	5425415	5425742
chr13	5444869	5445091
chr13	5445091	5445372
chr13	5545127	5545446
chr13	5546830	5547089
chr13	5549210	5549548
chr13	5549575	5549725
chr13	5549757	5550097
chr13	5551067	5551362
chr13	5558091	5558434
chr13	5614744	5615278
chr13	5616201	5616351
chr13	5621928	5622142
chr13	5623947	5624256
chr13	5624256	5624619
chr13	5629404	5629584

chr13	5657649	5657799
chr13	5666630	5666968
chr13	5674535	5675001
chr13	5677994	5678359
chr13	5680628	5680834
chr13	5704304	5704547
chr13	5714332	5714623
chr13	5714868	5715018
chr13	5724338	5724746
chr13	5726475	5726630
chr13	5726651	5726884
chr13	5760091	5760335
chr13	5769375	5769651
chr13	5769651	5770195
chr13	5773871	5774137
chr13	5779471	5779725
chr13	5791867	5792362
chr13	5792362	5792650
chr13	5802189	5802366
chr13	5802821	5803205
chr13	5803321	5803676
chr13	5803676	5803927
chr13	5803927	5804253
chr13	5804369	5804600
chr13	5822602	5822984
chr13	5823198	5823676
chr13	5825692	5826092
chr13	5826092	5826555
chr13	5837200	5837805
chr13	5841464	5841800
chr13	5843965	5844141
chr13	5844270	5844795
chr13	5844804	5845147
chr13	5845147	5845423
chr13	5845423	5845677
chr13	5845677	5845948
chr13	5859129	5859405
chr13	5859668	5859858
chr13	5859858	5860124
chr13	5860714	5860958
chr13	5861004	5861455
chr13	5861455	5861856

chr13	5864188	5864566
chr13	5866162	5866528
chr13	5869986	5870197
chr13	5870374	5870682
chr13	5870682	5871025
chr13	5871033	5871281
chr13	5871390	5871751
chr13	5874794	5874984
chr13	5882010	5882201
chr13	5886641	5887350
chr13	5892565	5893090
chr13	5893090	5893406
chr13	5894977	5895189
chr13	5913045	5913551
chr13	5913551	5913844
chr13	5950324	5950665
chr13	5971100	5971571
chr13	5993734	5993943
chr13	5994119	5994648
chr13	5994687	5994982
chr13	6009399	6009777
chr13	6009777	6010180
chr13	6012318	6012597
chr13	6023010	6023291
chr13	6023322	6023673
chr13	6023673	6023980
chr13	6029460	6029747
chr13	6030081	6030538
chr13	6043389	6043942
chr13	6077394	6077763
chr13	6085751	6086441
chr13	6089520	6089766
chr13	6089766	6090106
chr13	6109022	6109334
chr13	6109334	6109863
chr13	6111877	6112147
chr13	6131376	6131895
chr13	6153700	6153953
chr13	6165178	6165527
chr13	6165542	6165709
chr13	6165731	6166263
chr13	6167138	6167533

chr13	6180831	6180987
chr13	6185980	6186214
chr13	6191936	6192234
chr13	6193023	6193292
chr13	6193292	6193571
chr13	6193603	6194119
chr13	6200540	6200871
chr13	6230336	6230577
chr13	6230577	6230849
chr13	6230849	6231137
chr13	6236522	6236948
chr13	6237149	6237626
chr13	6245784	6246127
chr13	6255330	6255735
chr13	6255735	6256153
chr13	6256153	6256515
chr13	6261389	6261550
chr13	6291087	6291347
chr13	6315443	6315644
chr13	6319181	6319346
chr13	6357804	6358018
chr13	6366046	6366342
chr13	6367761	6367979
chr13	6377929	6378262
chr13	6379545	6379721
chr13	6417171	6417471
chr13	6419399	6419907
chr13	6434812	6435086
chr13	6447050	6447262
chr13	6456656	6456841
chr13	6467310	6467513
chr13	6469067	6469222
chr13	6471726	6471973
chr13	6473401	6473647
chr13	6473922	6474072
chr13	6476156	6476665
chr13	6484979	6485412
chr13	6492601	6492875
chr13	6506290	6506671
chr13	6508470	6509051
chr13	6515658	6515813
chr13	6516870	6517285

chr13	6529182	6529360
chr13	6532784	6532990
chr13	6547463	6547652
chr13	6547678	6548297
chr13	6548297	6548601
chr13	6553808	6553984
chr13	6576154	6576432
chr13	6595434	6595681
chr13	6606263	6606440
chr13	6607787	6607937
chr13	6614079	6614432
chr13	6618759	6619043
chr13	6624056	6624577
chr13	6624577	6624950
chr13	6626639	6626866
chr13	6626866	6627117
chr13	6635752	6635976
chr13	6635976	6636321
chr13	6637066	6637250
chr13	6646359	6646546
chr13	6647011	6647407
chr13	6647407	6647658
chr13	6647687	6647965
chr13	6647965	6648223
chr13	6648249	6648442
chr13	6648445	6649023
chr13	6659450	6659600
chr13	6689135	6689343
chr13	6690018	6690296
chr13	6807757	6807953
chr13	6836822	6836983
chr13	6897697	6898010
chr13	6898051	6898230
chr13	7057672	7057860
chr13	7059064	7059342
chr13	7059440	7059618
chr13	7535327	7535477
chr13	7593699	7593907
chr13	7629164	7629408
chr13	7634156	7634335
chr13	7640795	7641193
chr13	7648090	7648295

chr13	7721355	7721622
chr13	7721622	7721960
chr13	7900233	7900383
chr13	8133980	8134203
chr13	8406122	8406436
chr13	8456848	8457388
chr13	8645207	8645632
chr13	8645686	8646036
chr13	8727580	8727851
chr13	8731291	8731654
chr13	8744625	8744901
chr13	8755061	8755407
chr13	8755407	8755754
chr13	8755754	8755994
chr13	8780474	8780814
chr13	8782515	8782869
chr13	8785319	8785545
chr13	8805804	8806295
chr13	8807006	8807180
chr13	8818666	8818895
chr13	8828989	8829299
chr13	8829888	8830038
chr13	8830154	8830404
chr13	8831402	8831633
chr13	8831633	8831939
chr13	8831939	8832203
chr13	8832901	8833086
chr13	8841038	8841300
chr13	8847806	8848040
chr13	8870522	8870727
chr13	8870727	8870950
chr13	8870950	8871188
chr13	8871188	8871526
chr13	8871526	8871934
chr13	8885397	8885717
chr13	8885717	8886259
chr13	8893567	8893847
chr13	8893847	8894070
chr13	8894649	8894878
chr13	8894956	8895182
chr13	8895425	8895627
chr13	8943914	8944164

chr13	8944905	8945404
chr13	8949742	8950194
chr13	8955864	8956188
chr13	8956958	8957166
chr13	8960029	8960212
chr13	8960231	8960562
chr13	8961072	8961248
chr13	8961248	8961449
chr13	8961574	8961849
chr13	8962818	8963090
chr13	8965537	8965735
chr13	8966688	8966867
chr13	8995506	8995878
chr13	8995878	8996371
chr13	8996371	8996733
chr13	9002110	9002319
chr13	9011710	9011884
chr13	9013500	9013830
chr13	9014313	9014934
chr13	9017180	9017454
chr13	9026762	9027172
chr13	9027172	9027591
chr13	9032104	9032358
chr13	9032375	9032571
chr13	9037475	9037684
chr13	9055024	9055209
chr13	9061333	9061921
chr13	9073803	9074244
chr13	9080559	9080759
chr13	9082256	9082506
chr13	9093376	9093666
chr13	9093666	9094019
chr13	9094019	9094355
chr13	9094355	9094616
chr13	9094618	9094836
chr13	9099369	9099621
chr13	9100451	9100832
chr13	9107538	9107798
chr13	9113821	9114100
chr13	9128639	9129351
chr13	9139533	9139924
chr13	9139924	9140252

chr13	9140252	9140576
chr13	9162234	9162516
chr13	9167539	9167782
chr13	9167908	9168428
chr13	9176672	9176883
chr13	9181944	9182196
chr13	9184361	9184558
chr13	9184558	9184768
chr13	9190901	9191615
chr13	9198231	9198500
chr13	9199185	9199444
chr13	9199444	9199809
chr13	9203693	9203947
chr13	9239120	9239387
chr13	9239387	9239664
chr13	9239664	9239950
chr13	9239950	9240189
chr13	9255401	9255625
chr13	9271895	9272123
chr13	9276319	9276929
chr13	9276929	9277379
chr13	9277379	9277741
chr13	9297860	9298023
chr13	9314187	9314383
chr13	9325344	9325580
chr13	9340262	9340671
chr13	9340733	9340884
chr13	9342833	9343022
chr13	9353879	9354227
chr13	9450354	9450587
chr13	9454913	9455071
chr13	9467329	9467552
chr13	9482809	9483148
chr13	9494787	9495085
chr13	9507949	9508101
chr13	9562993	9563164
chr13	9589246	9589497
chr13	9636045	9636301
chr13	9636591	9636802
chr13	9695446	9695771
chr13	9695771	9696223
chr13	9696364	9696598

chr13	9696598	9696847
chr13	9720130	9720283
chr13	9749685	9749917
chr13	9749925	9750263
chr13	9750399	9750606
chr13	9763092	9763444
chr13	9763444	9763721
chr13	9763721	9764011
chr13	9764011	9764301
chr13	9764301	9764575
chr13	9764575	9764840
chr13	9764840	9765313
chr13	9767439	9767627
chr13	9772735	9772946
chr13	9773184	9773628
chr13	9773674	9773839
chr13	9773839	9773943
chr13	9834232	9834589
chr13	9844110	9844313
chr13	9846857	9847138
chr13	9869053	9869215
chr13	9882350	9882652
chr13	9884824	9885052
chr13	9929975	9930258
chr13	10040800	10040971
chr13	10049641	10049857
chr13	10354806	10355145
chr13	10357389	10357636
chr13	10375640	10375798
chr13	10636889	10637118
chr13	10710676	10710976
chr13	10732681	10732946
chr13	10938804	10938977
chr13	10947685	10947993
chr13	11191342	11191670
chr13	11193220	11193590
chr13	11218187	11218359
chr13	11290030	11290294
chr13	11291638	11291871
chr13	11291898	11292371
chr13	11432494	11432736
chr13	11432770	11433054

chr13	11433054	11433288
chr13	11453080	11453375
chr13	11532077	11532309
chr13	11532997	11533152
chr13	11533168	11533652
chr13	11543943	11544195
chr13	11549125	11549610
chr13	11563722	11563944
chr13	11564573	11564781
chr13	11566307	11566683
chr13	11566683	11566913
chr13	11566913	11567169
chr13	11807960	11808236
chr13	11809479	11809658
chr13	11905037	11905495
chr13	11908237	11908476
chr13	11959850	11960196
chr13	12106610	12106880
chr13	12106880	12107123
chr13	12109060	12109247
chr13	12132229	12132485
chr13	12133545	12133880
chr13	12150388	12150707
chr13	12210348	12210622
chr13	12223684	12223906
chr13	12242985	12243254
chr13	12257590	12257814
chr13	12257814	12258056
chr13	12258056	12258400
chr13	12264637	12265023
chr13	12265023	12265233
chr13	12267166	12267408
chr13	12293775	12294070
chr13	12307352	12307600
chr13	12331501	12331781
chr13	12340450	12340601
chr13	12340640	12340998
chr13	12394733	12395008
chr13	12395008	12395284
chr13	12395284	12395591
chr13	12395591	12395945
chr13	12442080	12442266

chr13	12442308	12442567
chr13	12460437	12460787
chr13	12460911	12461359
chr13	12461359	12461745
chr13	12461745	12462048
chr13	12468556	12468767
chr13	12469644	12469840
chr13	12473734	12474083
chr13	12474113	12474272
chr13	12488129	12488305
chr13	12506888	12507066
chr13	12519988	12520182
chr13	12520187	12520559
chr13	12542059	12542629
chr13	12550518	12550712
chr13	12565431	12565979
chr13	12565979	12566411
chr13	12589102	12589326
chr13	12608687	12608967
chr13	12626294	12626531
chr13	12629955	12630243
chr13	12630243	12630433
chr13	12634891	12635248
chr13	12635248	12635656
chr13	12637373	12637543
chr13	12638443	12638690
chr13	12640832	12641187
chr13	12642064	12642319
chr13	12642319	12642571
chr13	12645497	12645750
chr13	12649393	12649552
chr13	12649838	12650046
chr13	12650046	12650302
chr13	12650302	12650491
chr13	12650491	12650659
chr13	12650659	12650887
chr13	12650887	12651115
chr13	12667802	12668061
chr13	12668061	12668309
chr13	12680643	12680840
chr13	12702910	12703597
chr13	12703653	12703876

chr13	12703881	12704218
chr13	12705758	12705997
chr13	12705997	12706294
chr13	12781199	12781349
chr13	12781489	12781811
chr13	12784823	12784994
chr13	12788603	12788755
chr13	12789099	12789302
chr13	12793811	12793963
chr13	12799936	12800454
chr13	12802358	12802899
chr13	12808529	12809168
chr13	12809195	12809364
chr13	12812796	12813018
chr13	12813383	12813545
chr13	12813869	12814239
chr13	12814239	12814544
chr13	12818601	12819032
chr13	12848358	12848575
chr13	12851212	12851424
chr13	12859658	12859809
chr13	12864274	12864641
chr13	12889038	12889321
chr13	12889683	12889896
chr13	12902422	12902618
chr13	12906244	12906445
chr13	12908546	12908696
chr13	12908780	12909027
chr13	12911170	12911403
chr13	12917649	12917799
chr13	12925614	12925935
chr13	12925935	12926144
chr13	12926194	12926346
chr13	12931567	12931763
chr13	12931904	12932402
chr13	12935256	12935428
chr13	12935778	12936024
chr13	12937782	12937941
chr13	12986535	12986841
chr13	12986841	12987182
chr13	12990129	12990291
chr13	12990422	12990609

chr13	12993847	12994105
chr13	12994435	12994617
chr13	12994929	12995089
chr13	12995092	12995253
chr13	12999007	12999252
chr13	13005248	13005782
chr13	13007765	13008208
chr13	13013818	13014708
chr13	13024676	13025011
chr13	13025691	13026165
chr13	13026165	13026460
chr13	13030553	13030979
chr13	13067298	13067498
chr13	13076262	13076682
chr13	13093648	13093949
chr13	13126791	13126967
chr13	13162874	13163484
chr13	13168385	13168752
chr13	13168752	13169118
chr13	13169814	13170012
chr13	13173446	13173743
chr13	13173743	13174075
chr13	13174075	13174310
chr13	13179927	13180438
chr13	13182385	13182565
chr13	13216367	13216730
chr13	13232753	13232958
chr13	13273779	13273965
chr13	13328973	13329202
chr13	13349430	13349581
chr13	13352109	13352624
chr13	13352723	13352936
chr13	13362363	13362581
chr13	13368973	13369169
chr13	13369914	13370243
chr13	13378163	13378510
chr13	13378510	13378911
chr13	13380631	13380814
chr13	13381709	13381956
chr13	13384098	13384450
chr13	13385322	13385585
chr13	13385585	13385838

chr13	13388721	13389005
chr13	13392603	13392766
chr13	13392855	13393005
chr13	13393046	13393372
chr13	13393372	13393917
chr13	13393917	13394322
chr13	13404206	13404412
chr13	13404564	13404913
chr13	13404924	13405099
chr13	13415456	13415673
chr13	13438204	13438906
chr13	13438989	13439207
chr13	13439212	13439549
chr13	13441079	13441323
chr13	13441323	13441628
chr13	13460494	13460968
chr13	13463953	13464328
chr13	13471485	13471868
chr13	13472824	13473268
chr13	13474586	13474826
chr13	13474865	13475463
chr13	13482049	13482229
chr13	13491354	13491638
chr13	13493427	13493964
chr13	13515074	13515269
chr13	13516711	13516872
chr13	13516898	13517227
chr13	13517802	13517952
chr13	13529060	13529328
chr13	13529328	13529761
chr13	13537699	13537919
chr13	13538445	13538911
chr13	13538911	13539185
chr13	13539427	13539719
chr13	13551922	13552426
chr13	13562701	13562976
chr13	13564175	13564460
chr13	13572446	13572691
chr13	13572958	13573291
chr13	13573508	13573838
chr13	13573838	13574228
chr13	13590061	13590316

chr13	13590316	13590601
chr13	13590601	13591080
chr13	13601498	13601732
chr13	13612936	13613208
chr13	13637659	13637898
chr13	13745962	13746330
chr13	13752927	13753253
chr13	13753253	13753533
chr13	13753533	13753721
chr13	13753727	13754002
chr13	13754959	13755315
chr13	13783808	13784126
chr13	13784126	13784444
chr13	13784444	13784721
chr13	13784721	13784946
chr13	13785079	13785333
chr13	13792082	13792302
chr13	13824402	13824557
chr13	13845028	13845374
chr13	13845374	13845561
chr13	13860540	13860816
chr13	13860816	13861056
chr13	13862912	13863315
chr13	13867165	13867414
chr13	13867414	13867663
chr13	13868022	13868229
chr13	13889437	13889768
chr13	13889768	13890058
chr13	13953947	13954314
chr13	13954314	13954860
chr13	13954860	13955364
chr13	13968434	13968820
chr13	13968820	13969187
chr13	13981946	13982140
chr13	14020394	14020668
chr13	14022094	14022288
chr13	14022905	14023275
chr13	14024703	14024915
chr13	14033820	14034365
chr13	14039262	14039547
chr13	14039547	14040027
chr13	14040306	14040658

chr13	14049864	14050180
chr13	14056920	14057124
chr13	14057124	14057332
chr13	14057332	14057556
chr13	14062982	14063325
chr13	14063325	14063782
chr13	14063782	14064256
chr13	14064855	14065005
chr13	14095262	14095620
chr13	14115486	14115746
chr13	14165174	14165389
chr13	14225152	14225402
chr13	14225615	14225772
chr13	14355417	14355589
chr13	14418290	14418562
chr13	14424170	14424611
chr13	14428826	14429210
chr13	14485068	14485296
chr13	14496918	14497536
chr13	14497673	14497900
chr13	14514049	14514214
chr13	14522136	14522538
chr13	14522538	14522943
chr13	14522943	14523278
chr13	14557549	14557763
chr13	14557763	14557961
chr13	14585031	14585369
chr13	14589732	14590029
chr13	14612804	14613110
chr13	14613110	14613321
chr13	14613321	14613660
chr13	14618757	14619112
chr13	14619305	14619473
chr13	14629851	14630102
chr13	14630102	14630717
chr13	14669091	14669413
chr13	14671974	14672124
chr13	15384235	15384451
chr13	15462838	15463432
chr13	15463432	15463751
chr13	15463751	15464159
chr13	15464166	15464533

chr13	15514104	15514409
chr13	15537209	15537588
chr13	15537698	15538068
chr13	15538111	15538282
chr13	15538450	15538739
chr13	15550732	15551068
chr13	15561507	15561727
chr13	15624897	15625066
chr13	15638228	15638480
chr13	15660350	15660593
chr13	15660593	15660822
chr13	15660822	15661065
chr13	15707474	15707683
chr13	15715292	15715453
chr13	15741780	15742178
chr13	15759144	15759455
chr13	15759495	15759676
chr13	15762392	15762701
chr13	15763101	15763329
chr13	15767397	15767587
chr13	15773463	15773695
chr13	15776504	15776658
chr13	15776845	15777009
chr13	15777676	15777912
chr13	15786398	15786624
chr13	15802468	15802732
chr13	15803971	15804284
chr13	15806624	15806989
chr13	15806989	15807423
chr13	15830586	15830968
chr13	15845682	15846156
chr13	15846156	15846579
chr13	15846602	15846842
chr13	15848070	15848255
chr13	15853046	15853545
chr13	15854041	15854203
chr13	15863009	15863256
chr13	15863313	15863806
chr13	15875351	15875515
chr13	15891290	15891581
chr13	15907745	15908159
chr13	15982520	15982755

chr13	15985120	15985362
chr13	15992417	15992623
chr13	15993939	15994165
chr13	16005609	16005822
chr13	16007750	16008038
chr13	16009189	16009408
chr13	16009901	16010051
chr13	16011119	16011406
chr13	16011406	16011642
chr13	16011820	16012161
chr13	16012224	16012533
chr13	16012533	16012773
chr13	16014190	16014494
chr13	16016535	16016789
chr13	16017332	16017542
chr13	16017542	16017771
chr13	16017771	16017975
chr13	16019118	16019374
chr13	16020788	16020938
chr13	16030684	16030961
chr13	16156756	16157111
chr13	16175933	16176188
chr13	16191103	16191253
chr13	16208389	16208648
chr13	16208698	16209256
chr13	16211403	16211722
chr13	16216256	16216777
chr13	16216887	16217043
chr13	16219881	16220218
chr13	16222762	16223276
chr13	16239215	16239565
chr13	16242377	16242712
chr13	16296269	16296764
chr13	16299835	16300168
chr13	16334364	16334578
chr13	16414803	16415073
chr13	16433613	16434016
chr13	16435544	16435924
chr13	16453041	16453997
chr13	16456286	16456557
chr13	16486966	16487564
chr13	16490764	16490959

chr13	16498107	16498362
chr13	16499125	16499360
chr13	16529761	16530068
chr13	16532207	16532384
chr13	16543035	16543220
chr13	16549803	16550463
chr13	16564345	16564621
chr13	16564971	16565291
chr13	16571284	16571736
chr13	16578282	16578555
chr13	16641634	16641944
chr13	16641944	16642395
chr13	16657405	16657806
chr13	16659225	16659612
chr13	16659612	16659861
chr13	16659861	16660327
chr13	16668676	16669065
chr13	16670129	16670427
chr13	16670427	16670724
chr13	16670799	16671049
chr13	16676934	16677187
chr13	16678363	16678517
chr13	16696340	16696696
chr13	16715362	16715610
chr13	16715610	16716059
chr13	16717463	16717706
chr13	16718024	16718199
chr13	16732409	16732560
chr13	16738266	16738729
chr13	16738794	16738944
chr13	16739356	16740002
chr13	16746338	16746899
chr13	16756274	16756583
chr13	16760054	16760404
chr13	16761037	16761795
chr13	16763085	16763564
chr13	16763824	16764087
chr13	16801432	16801598
chr13	16805546	16806028
chr13	16828491	16828720
chr13	16835135	16835552
chr13	16838227	16838392

chr13	16838413	16838809
chr13	16846623	16846808
chr13	16846808	16847013
chr13	16847203	16847604
chr13	16847604	16848138
chr13	17000047	17000248
chr13	17000390	17000566
chr13	17028697	17028878
chr13	17049291	17049530
chr13	17211768	17211924
chr13	17212092	17212297
chr13	17212450	17212677
chr13	17212682	17213120
chr13	17213120	17213565
chr13	17228407	17228600
chr13	17354861	17355130
chr13	17406338	17406488
chr13	17419635	17420019
chr13	17420108	17420260
chr13	17428405	17428561
chr13	17432612	17432772
chr13	17458058	17458245
chr13	17492837	17493266
chr13	17494019	17494570
chr13	17495023	17495191
chr13	17511119	17511510
chr13	17511510	17511869
chr13	17581747	17581980
chr13	17584892	17585127
chr13	17603650	17604221
chr13	17604681	17605098
chr13	17606834	17607028
chr13	17614102	17614379
chr13	17614682	17615065
chr13	17636590	17636872
chr13	17660466	17660710
chr13	17670127	17670569
chr13	17670689	17670921
chr13	17685838	17686165
chr13	17687044	17687314
chr13	17689800	17690068
chr13	17693822	17694149

chr13	17694525	17694854
chr13	17694854	17695265
chr13	17695265	17695539
chr13	17695539	17695745
chr13	17697848	17698116
chr13	17704492	17705040
chr13	17705040	17705467
chr13	17705467	17705810
chr13	17705854	17706103
chr13	17714209	17714554
chr13	17746225	17746444
chr13	17781906	17782162
chr13	17784117	17784486
chr13	17795843	17796036
chr13	17803321	17804094
chr13	17804132	17804354
chr13	17804432	17804904
chr13	17804904	17805296
chr13	17805296	17805694
chr13	17837607	17838099
chr13	17838099	17838509
chr13	17838539	17838807
chr13	17839113	17839313
chr13	17839313	17839525
chr13	17870718	17870935
chr13	17884305	17884467
chr13	17900591	17901046
chr13	17901046	17901357
chr13	17901487	17901834
chr13	17902255	17902512
chr13	17943274	17943449
chr13	17943449	17943614
chr13	17943659	17944072
chr13	17944072	17944692
chr13	17967368	17967570
chr13	17969847	17969997
chr13	17975516	17975791
chr13	17975811	17976331
chr13	17983240	17983517
chr13	17992779	17993003
chr13	17993003	17993254
chr13	17993254	17993590

chr13	18012846	18013123
chr13	18056585	18056753
chr13	18056942	18057122
chr13	18057299	18057539
chr13	18174085	18174271
chr13	18195705	18196127
chr13	18196127	18196386
chr13	18196842	18197323
chr13	18303049	18303233
chr13	18303618	18303869
chr13	18306793	18307158
chr13	18309699	18309967
chr13	18325836	18326054
chr13	18330379	18330804
chr13	18400568	18400723
chr13	18717134	18717596
chr13	18730728	18730906
chr13	18737794	18738143
chr13	18743926	18744219
chr13	18757431	18757649
chr13	18757715	18757875
chr13	18810080	18810302
chr13	18820747	18820932
chr13	18843553	18843944
chr13	18847417	18847744
chr13	18849632	18849785
chr13	18868071	18868285
chr13	18868285	18868706
chr13	18868708	18868879
chr13	18869695	18869853
chr13	18875507	18875806
chr13	18875806	18876284
chr13	18876368	18876575
chr13	18892092	18892354
chr13	18948101	18948484
chr13	18948538	18948722
chr13	18948722	18948941
chr13	19026972	19027222
chr13	19133933	19134138
chr13	19156533	19156777
chr13	19156777	19156990
chr13	19158599	19158751

chr13	19177666	19178004
chr13	19242693	19242876
chr13	19304352	19304570
chr13	19308905	19309221
chr13	19322781	19323109
chr13	19330441	19330743
chr13	19332383	19332703
chr13	19336970	19337306
chr13	19338700	19338977
chr13	19339828	19340060
chr13	19342033	19342186
chr13	19343241	19343592
chr13	19350807	19351328
chr13	19351350	19351500
chr13	19355306	19355640
chr13	19358013	19358253
chr13	19364220	19364758
chr13	19365680	19365915
chr13	19394229	19394494
chr13	19395274	19395673
chr13	19395673	19396086
chr13	19396094	19396255
chr13	19411897	19412187
chr13	19438129	19438336
chr13	19512176	19512672
chr13	19517377	19517790
chr13	19517996	19518303
chr13	19518428	19518756
chr13	19580433	19580818
chr13	19591062	19591424
chr13	19591450	19591692
chr13	19592087	19592363
chr13	19604508	19604851
chr13	19618965	19619152
chr13	19619207	19619456
chr13	19619456	19619682
chr13	19619682	19619978
chr13	19622960	19623205
chr13	19626518	19626851
chr13	19628196	19628357
chr13	19636225	19636445
chr13	19644297	19644462

chr13	19653451	19653807
chr13	19683386	19683576
chr13	19689135	19689434
chr13	19692534	19692781
chr13	19692781	19693217
chr13	19698866	19699055
chr13	19703751	19704405
chr13	19704790	19705344
chr13	19761913	19762332
chr13	19764244	19764803
chr13	19824140	19824340
chr13	19840265	19840468
chr13	19841699	19841870
chr13	19851967	19852155
chr13	19854667	19854948
chr13	19855044	19855264
chr13	19883984	19884231
chr13	19897249	19897615
chr13	19918497	19918683
chr13	19920972	19921477
chr13	19921518	19921763
chr13	19963118	19963393
chr13	20048297	20048681
chr13	20090155	20090417
chr13	20090417	20090809
chr13	20090809	20091270
chr13	20091270	20091600
chr13	20111311	20111647
chr13	20111651	20111966
chr13	20115289	20115620
chr13	20115620	20115988
chr13	20120769	20121035
chr13	20131563	20132030
chr13	20133825	20134227
chr13	20136853	20137011
chr13	20163164	20163348
chr13	20163364	20164001
chr13	20290466	20290967
chr13	20292532	20292686
chr13	20295668	20295887
chr13	20297910	20298169
chr13	20299500	20299726

chr13	20300179	20300491
chr13	20306165	20306515
chr13	20306515	20306824
chr13	20311662	20311946
chr13	20386602	20386757
chr13	20428162	20428442
chr13	20432982	20433197
chr13	20433203	20433736
chr13	20438735	20438975
chr13	20461116	20461279
chr13	20462948	20463222
chr13	20471836	20472030
chr13	20489782	20490008
chr13	20736407	20736579
chr13	21066023	21066274
chr13	21158836	21159126
chr13	21159185	21159377
chr13	21159381	21159793
chr13	21160358	21160508
chr13	21160715	21161011
chr13	21161050	21161264
chr13	21161264	21161529
chr13	21167522	21167739
chr13	21167960	21168173
chr13	21168714	21168901
chr13	21169643	21169873
chr13	21169873	21170136
chr13	21170136	21170402
chr13	21171654	21171844
chr13	21172291	21172529
chr13	21172549	21172997
chr13	21173103	21173405
chr13	21177755	21178000
chr13	21179448	21179728
chr13	21179728	21180016
chr13	21180016	21180292
chr13	21180292	21180630
chr13	21180630	21181269
chr13	21190348	21190508
chr13	21198605	21198790
chr13	21198943	21199290
chr13	21199767	21200024

chr13	21200055	21200446
chr13	21200446	21200832
chr13	21215246	21215673
chr13	21215688	21215873
chr13	21221999	21222157
chr13	21222163	21222477
chr13	21222477	21222741
chr13	21222741	21222995
chr13	21223187	21223634
chr13	21228573	21228969
chr13	21233629	21234307
chr13	21234307	21234676
chr13	21240850	21241216
chr13	21241634	21241892
chr13	21242503	21242743
chr13	21249054	21249439
chr13	21250227	21250377
chr13	21254052	21254353
chr13	21259061	21259358
chr13	21259380	21259558
chr13	21259695	21259922
chr13	21259922	21260178
chr13	21260223	21260408
chr13	21260460	21260640
chr13	21268062	21268602
chr13	21274949	21275361
chr13	21275366	21275534
chr13	21275600	21275789
chr13	21275882	21276143
chr13	21276188	21276365
chr13	21279548	21280143
chr13	21322759	21322933
chr13	21325535	21325702
chr13	21326229	21326509
chr13	21326568	21326917
chr13	21326917	21327265
chr13	21327512	21327729
chr13	21357665	21357931
chr13	21362600	21363171
chr13	21363830	21364036
chr13	21369546	21369778
chr13	21384957	21385300

chr13	21396042	21396382
chr13	21401736	21401901
chr13	21402308	21402619
chr13	21402619	21402877
chr13	21407940	21408229
chr13	21408274	21408889
chr13	21417389	21418046
chr13	21418924	21419172
chr13	21440506	21440899
chr13	21440899	21441243
chr13	21453404	21453633
chr13	21453633	21453899
chr13	21456349	21456727
chr13	21468038	21468422
chr13	21468422	21468914
chr13	21478851	21479175
chr13	21479175	21479613
chr13	21479644	21479808
chr13	21492787	21492974
chr13	21506146	21506454
chr13	21506454	21506736
chr13	21508517	21508874
chr13	21530731	21531018
chr13	21531018	21531412
chr13	21550412	21550725
chr13	21550725	21551205
chr13	21709963	21710216
chr13	21710216	21710460
chr13	21710509	21710766
chr13	21710856	21711131
chr13	21714897	21715047
chr13	21715667	21715936
chr13	21715936	21716233
chr13	21716233	21716551
chr13	21716551	21716821
chr13	21716821	21717041
chr13	21717506	21717789
chr13	21717789	21718031
chr13	21721408	21721798
chr13	21721798	21722240
chr13	21722240	21722591
chr13	21733975	21734189

chr13	21734189	21734416
chr13	21734439	21734629
chr13	21734664	21734880
chr13	21734882	21735210
chr13	21735210	21735433
chr13	21735433	21735767
chr13	21750150	21750393
chr13	21750393	21750626
chr13	21753410	21753763
chr13	21753763	21754081
chr13	21754081	21754567
chr13	21764431	21765094
chr13	21765643	21765902
chr13	21767845	21768009
chr13	21779097	21779346
chr13	21779346	21779729
chr13	21779729	21780044
chr13	21780155	21780514
chr13	21780514	21780764
chr13	21782349	21782524
chr13	21782972	21783555
chr13	21786807	21787222
chr13	21787222	21787663
chr13	21787663	21787981
chr13	21797224	21797492
chr13	21802974	21803430
chr13	21808627	21808822
chr13	21808970	21809150
chr13	21809250	21809400
chr13	21809548	21809753
chr13	21809753	21810068
chr13	21810068	21810362
chr13	21810362	21810598
chr13	21810598	21810878
chr13	21811161	21811402
chr13	21811609	21812156
chr13	21812160	21812446
chr13	21819620	21819897
chr13	21819969	21820151
chr13	21820176	21820543
chr13	21820597	21820851
chr13	21828607	21828794

chr13	21831494	21831741
chr13	21831796	21832046
chr13	21832046	21832345
chr13	21832548	21832772
chr13	21833064	21833349
chr13	21833349	21833584
chr13	21833584	21834210
chr13	21834227	21834394
chr13	21834542	21834692
chr13	21834792	21834969
chr13	21835160	21835315
chr13	21840513	21840856
chr13	21846415	21846694
chr13	21847896	21848238
chr13	21852289	21852550
chr13	21855190	21855622
chr13	21855653	21855869
chr13	21855929	21856153
chr13	21862128	21862511
chr13	21881791	21882208
chr13	21882294	21882621
chr13	21882803	21883075
chr13	21883075	21883323
chr13	21892498	21892734
chr13	21892839	21893196
chr13	21893930	21894269
chr13	21896836	21897226
chr13	21897226	21897525
chr13	21907054	21907291
chr13	21907335	21907522
chr13	21907574	21907752
chr13	21907773	21908110
chr13	21911253	21911467
chr13	21913830	21914191
chr13	21914581	21914764
chr13	21914764	21914961
chr13	21917069	21917287
chr13	21918805	21919118
chr13	21919118	21919690
chr13	21919839	21920134
chr13	21920134	21920350
chr13	21924706	21925100

chr13	21927690	21928215
chr13	21928619	21928877
chr13	21931068	21931282
chr13	21933928	21934094
chr13	21935423	21935658
chr13	21935882	21936144
chr13	21936951	21937435
chr13	21940257	21940644
chr13	21940793	21941038
chr13	21944688	21944967
chr13	21944967	21945414
chr13	21945414	21945895
chr13	21959324	21959474
chr13	21973999	21974185
chr13	21974185	21974443
chr13	21974443	21974693
chr13	21974735	21974966
chr13	21974966	21975377
chr13	21975494	21975761
chr13	21981277	21981551
chr13	21981551	21981859
chr13	21993953	21994346
chr13	21995405	21995994
chr13	21996117	21996271
chr13	21996271	21996423
chr13	21996550	21996788
chr13	21996868	21997221
chr13	22005878	22006283
chr13	22006283	22006610
chr13	22006610	22006875
chr13	22006875	22007253
chr13	22015607	22016137
chr13	22020548	22020758
chr13	22020824	22021060
chr13	22021060	22021297
chr13	22021589	22021755
chr13	22021768	22021996
chr13	22021996	22022275
chr13	22022275	22022668
chr13	22026472	22026641
chr13	22027936	22028130
chr13	22028685	22029058

chr13	22029155	22029381
chr13	22033383	22033625
chr13	22035123	22035398
chr13	22035398	22035615
chr13	22035615	22036310
chr13	22040953	22041529
chr13	22042510	22042732
chr13	22042732	22042872
chr13	22042872	22043098
chr13	22043098	22043693
chr13	22049696	22050023
chr13	22050440	22050916
chr13	22050916	22051233
chr13	22051233	22051496
chr13	22272429	22272638
chr13	22277226	22277442
chr13	22342035	22342229
chr13	22347547	22347823
chr13	22397689	22397879
chr13	22404228	22404513
chr13	22413494	22413729
chr13	22415981	22416614
chr13	22418979	22419591
chr13	22498779	22498991
chr13	22573144	22573472
chr13	22608935	22609285
chr13	22650256	22650406
chr13	22714145	22714482
chr13	22747658	22747808
chr13	22747993	22748172
chr13	22793959	22794330
chr13	22830329	22830505
chr13	22830852	22831374
chr13	22831374	22831708
chr13	22837532	22837703
chr13	22861103	22861352
chr13	22871767	22871981
chr13	22896917	22897078
chr13	22898502	22898941
chr13	22898951	22899213
chr13	22914364	22914637
chr13	22914840	22914991

chr13	22938386	22938563
chr13	22939976	22940416
chr13	22940428	22940693
chr13	22978423	22978686
chr13	22992328	22992738
chr13	23087525	23087877
chr13	23157170	23157392
chr13	23157750	23158228
chr13	23177076	23177226
chr13	23234345	23234560
chr13	23258475	23259083
chr13	23260606	23260875
chr13	23267365	23267535
chr13	23269118	23269333
chr13	23270402	23270654
chr13	23285081	23285567
chr13	23285567	23285912
chr13	23285919	23286169
chr13	23286169	23286410
chr13	23286506	23286675
chr13	23286675	23286778
chr13	23286847	23287001
chr13	23287001	23287141
chr13	23298438	23298899
chr13	23298899	23299341
chr13	23299341	23299694
chr13	23299694	23299979
chr13	23299979	23300238
chr13	23307043	23307251
chr13	23307276	23307518
chr13	23307518	23307926
chr13	23308133	23308336
chr13	23308413	23308579
chr13	23308608	23308909
chr13	23308909	23309192
chr13	23309293	23309819
chr13	23315585	23315865
chr13	23317387	23317615
chr13	23317615	23317902
chr13	23324694	23324849
chr13	23329812	23330109
chr13	23365633	23365843

chr13	23368599	23368998
chr13	23368998	23369454
chr13	23369478	23369641
chr13	23375708	23375882
chr13	23395830	23395994
chr13	23396037	23396208
chr13	23396304	23396678
chr13	23396678	23396885
chr13	23397007	23397267
chr13	23400726	23400949
chr13	23401014	23401170
chr13	23401289	23401493
chr13	23401660	23401880
chr13	23401880	23402104
chr13	23402104	23402357
chr13	23402719	23402929
chr13	23411690	23411935
chr13	23411935	23412266
chr13	23412266	23412567
chr13	23412567	23412822
chr13	23412822	23413163
chr13	23413202	23413401
chr13	23414088	23414526
chr13	23423553	23423708
chr13	23423767	23424146
chr13	23424494	23424875
chr13	23425504	23425813
chr13	23426460	23426696
chr13	23426925	23427228
chr13	23430713	23431132
chr13	23431327	23431817
chr13	23432530	23432905
chr13	23432927	23433214
chr13	23436957	23437109
chr13	23437498	23437678
chr13	23437678	23437900
chr13	23437930	23438319
chr13	23438652	23438825
chr13	23445515	23445799
chr13	23445799	23446079
chr13	23446079	23446362
chr13	23446437	23446669

chr13	23446712	23446950
chr13	23449277	23449487
chr13	23450568	23450800
chr13	23453361	23453628
chr13	23453628	23453923
chr13	23463402	23463760
chr13	23464016	23464199
chr13	23464222	23464512
chr13	23465585	23465739
chr13	23465772	23466240
chr13	23466321	23466655
chr13	23466929	23467248
chr13	23469085	23469467
chr13	23479073	23479525
chr13	23488374	23488519
chr13	23488519	23488667
chr13	23488727	23489020
chr13	23489020	23489233
chr13	23495650	23495847
chr13	23497787	23497946
chr13	23497963	23498288
chr13	23500030	23500221
chr13	23500237	23500387
chr13	23500462	23500732
chr13	23500830	23501172
chr13	23503911	23504115
chr13	23505996	23506269
chr13	23506443	23506668
chr13	23507112	23507362
chr13	23510234	23510436
chr13	23517141	23517343
chr13	23517445	23517936
chr13	23517936	23518327
chr13	23518953	23519243
chr13	23519526	23519789
chr13	23521808	23522054
chr13	23524683	23524975
chr13	23526451	23526662
chr13	23530311	23530461
chr13	23530867	23531246
chr13	23531246	23531524
chr13	23531603	23531896

chr13	23533664	23534011
chr13	23534011	23534311
chr13	23535118	23535473
chr13	23535473	23535836
chr13	23542913	23543171
chr13	23543171	23543389
chr13	23543389	23543680
chr13	23544210	23544604
chr13	23544604	23544987
chr13	23551273	23551577
chr13	23551577	23551931
chr13	23554893	23555152
chr13	23555152	23555486
chr13	23555486	23555941
chr13	23555941	23556382
chr13	23561716	23562202
chr13	23562202	23562448
chr13	23562448	23562736
chr13	23563136	23563479
chr13	23570701	23571062
chr13	23571062	23571389
chr13	23571389	23571910
chr13	23573937	23574513
chr13	23574513	23574878
chr13	23575624	23575877
chr13	23575877	23576155
chr13	23581346	23581848
chr13	23581848	23582173
chr13	23584984	23585221
chr13	23585484	23585773
chr13	23585773	23586174
chr13	23598020	23598290
chr13	23609233	23609539
chr13	23610853	23611106
chr13	23611106	23611389
chr13	23620723	23621021
chr13	23621021	23621461
chr13	23621461	23621893
chr13	23622035	23622427
chr13	23622427	23622717
chr13	23630730	23630995
chr13	23659588	23659898

chr13	23660005	23660337
chr13	23665154	23665338
chr13	23682191	23682457
chr13	23683499	23683800
chr13	23683800	23684068
chr13	23684068	23684360
chr13	23684360	23684699
chr13	23691929	23692257
chr13	23697466	23697699
chr13	23697699	23697905
chr13	23697905	23698127
chr13	23698127	23698321
chr13	23698321	23698718
chr13	23700871	23701085
chr13	23701205	23701497
chr13	23708278	23708428
chr13	23710149	23710341
chr13	23710341	23710548
chr13	23710548	23710768
chr13	23710768	23710975
chr13	23712910	23713084
chr13	23730475	23730897
chr13	23732289	23732439
chr13	23732567	23732759
chr13	23738659	23738912
chr13	23738912	23739363
chr13	23739363	23740166
chr13	23745109	23745526
chr13	23745526	23745729
chr13	23746139	23746420
chr13	23746420	23746709
chr13	23746709	23747194
chr13	23749623	23750062
chr13	23750946	23751311
chr13	23751311	23751562
chr13	23752104	23752476
chr13	23752476	23752778
chr13	23756785	23757053
chr13	23757053	23757392
chr13	23757394	23757555
chr13	23760797	23761049
chr13	23761049	23761313

chr13	23761313	23761555
chr13	23761555	23761781
chr13	23761877	23762239
chr13	23762239	23762564
chr13	23763543	23763695
chr13	23792569	23792789
chr13	23800274	23800591
chr13	23819014	23819164
chr13	23819958	23820371
chr13	23820371	23820728
chr13	23833196	23833346
chr13	23885619	23885798
chr13	23913821	23914230
chr13	23926365	23926561
chr13	23928734	23928986
chr13	23931029	23931231
chr13	23933712	23934001
chr13	23942944	23943164
chr13	23950917	23951174
chr13	23990908	23991175
chr13	24018528	24018850
chr13	24053530	24053777
chr13	24058291	24058529
chr13	24065592	24065888
chr13	24095762	24096045
chr13	24096045	24096363
chr13	24096639	24097013
chr13	24103995	24104372
chr13	24104690	24104916
chr13	24104920	24105328
chr13	24105328	24105745
chr13	24108322	24108576
chr13	24110224	24110447
chr13	24112760	24113004
chr13	24113004	24113337
chr13	24114061	24114213
chr13	24135359	24135559
chr13	24155915	24156071
chr13	24169727	24169898
chr13	24198511	24198771
chr13	24247603	24247792
chr13	24254129	24254386

chr13	24261021	24261346
chr13	24261346	24261581
chr13	24269248	24269434
chr13	24272835	24273063
chr13	24273063	24273275
chr13	24279948	24280181
chr13	24280181	24280403
chr13	24280418	24280615
chr13	24280849	24281190
chr13	24281190	24281506
chr13	24297386	24297555
chr13	24316598	24316936
chr13	24318717	24318868
chr13	24322336	24322708
chr13	24332530	24332884
chr13	24337947	24338163
chr13	24370047	24370216
chr13	24374777	24374951
chr13	24375032	24375293
chr13	24375350	24375671
chr13	24375671	24375892
chr13	24416507	24416752
chr13	24453859	24454110
chr13	24481950	24482191
chr13	24487654	24487814
chr13	24499641	24499791
chr13	24514891	24515180
chr13	24518951	24519236
chr13	24521441	24521767
chr13	24522356	24522774
chr13	24522922	24523079
chr13	24526991	24527241
chr13	24536642	24537194
chr13	24537317	24537500
chr13	24538696	24538992
chr13	24538992	24539315
chr13	24547445	24547635
chr13	24548396	24548665
chr13	24548981	24549236
chr13	24553197	24553737
chr13	24564781	24564990
chr13	24565167	24565455

chr13	24565455	24565862
chr13	24565862	24566428
chr13	24576470	24576702
chr13	24576787	24576940
chr13	24580871	24581030
chr13	24584443	24584640
chr13	24601736	24601943
chr13	24604192	24604349
chr13	24623708	24623912
chr13	24638354	24638782
chr13	24638782	24639126
chr13	24639126	24639452
chr13	24642904	24643180
chr13	24646271	24646810
chr13	24668600	24668855
chr13	24674022	24674362
chr13	24674362	24674778
chr13	24678745	24679023
chr13	24681471	24681804
chr13	24681804	24682070
chr13	24687385	24687653
chr13	24713488	24713954
chr13	24721613	24721815
chr13	24724463	24724636
chr13	24726151	24726331
chr13	24735585	24736291
chr13	24750813	24751118
chr13	24751334	24751837
chr13	24761174	24761751
chr13	24761751	24762147
chr13	24768494	24768828
chr13	24771650	24772047
chr13	24776225	24776492
chr13	24776492	24776708
chr13	24776708	24776857
chr13	24781947	24782253
chr13	24782253	24782471
chr13	24786042	24786581
chr13	24791993	24792444
chr13	24792444	24792742
chr13	24792742	24793117
chr13	24799487	24799646

chr13	24799932	24800331
chr13	24800606	24800930
chr13	24801040	24801376
chr13	24801376	24801675
chr13	24801675	24801914
chr13	24802417	24802615
chr13	24814896	24815082
chr13	24816263	24816665
chr13	24816665	24817023
chr13	24817023	24817428
chr13	24825457	24826074
chr13	24826074	24826511
chr13	24829080	24829300
chr13	24831305	24831796
chr13	24831796	24832362
chr13	24844907	24845378
chr13	24849406	24849761
chr13	24850103	24850339
chr13	24854453	24854768
chr13	24904029	24904236
chr13	24914744	24915159
chr13	24915159	24915406
chr13	24920377	24920694
chr13	24920707	24920869
chr13	24936997	24937620
chr13	24937620	24937842
chr13	24937842	24938065
chr13	24938065	24938266
chr13	24938686	24939043
chr13	24948787	24949209
chr13	24949209	24949474
chr13	24949474	24949719
chr13	24952993	24953226
chr13	24955183	24955333
chr13	24955518	24955997
chr13	24964359	24964581
chr13	24965270	24965467
chr13	24965520	24965833
chr13	24965865	24966017
chr13	24971903	24972100
chr13	24977601	24977772
chr13	24977889	24978366

chr13	24988465	24988737
chr13	25003081	25003291
chr13	25007303	25007703
chr13	25007703	25008102
chr13	25015170	25015628
chr13	25019890	25020230
chr13	25020230	25020539
chr13	25050340	25050738
chr13	25055087	25055340
chr13	25055340	25055590
chr13	25055819	25056049
chr13	25056049	25056363
chr13	25064377	25064562
chr13	25069933	25070260
chr13	25070612	25070767
chr13	25070827	25071209
chr13	25071248	25071398
chr13	25085159	25085581
chr13	25105421	25105868
chr13	25105868	25106260
chr13	25115648	25115972
chr13	25116056	25116261
chr13	25131197	25131363
chr13	25159693	25159953
chr13	25160042	25160199
chr13	25192839	25193224
chr13	25193224	25193609
chr13	25205998	25206307
chr13	25208188	25208526
chr13	25264817	25264987
chr13	25265286	25265522
chr13	25269121	25269355
chr13	25269898	25270215
chr13	25309647	25309996
chr13	25310036	25310414
chr13	25311013	25311389
chr13	25311524	25311773
chr13	25312130	25312535
chr13	25314697	25314898
chr13	25337498	25337924
chr13	25341918	25342220
chr13	25350811	25351023

chr13	25357652	25357857
chr13	25369850	25370014
chr13	25370514	25370720
chr13	25388290	25388593
chr13	25453135	25453319
chr13	25456270	25456517
chr13	25470749	25471067
chr13	25475526	25475942
chr13	25475942	25476293
chr13	25562756	25562982
chr13	25639958	25640221
chr13	25677838	25678058
chr13	25785200	25785384
chr13	25785556	25786050
chr13	25808936	25809129
chr13	25834087	25834328
chr13	25834361	25834519
chr13	25837531	25837976
chr13	25844593	25844832
chr13	25909234	25909422
chr13	25932905	25933064
chr13	25955421	25955736
chr13	25958632	25958962
chr13	26009227	26009477
chr13	26088835	26088987
chr13	26216643	26216862
chr13	26217343	26217686
chr13	26217698	26217851
chr13	26218345	26218712
chr13	26237520	26237732
chr13	26274347	26274522
chr13	26338826	26339106
chr13	26354484	26354747
chr13	26387919	26388140
chr13	26412536	26412745
chr13	26412823	26413039
chr13	26457889	26458282
chr13	26640791	26641055
chr13	26693054	26693276
chr13	26694242	26694413
chr13	26930967	26931478
chr13	26939798	26940158

chr13	26940170	26940454
chr13	26940454	26940857
chr13	26953689	26954211
chr13	26976053	26976656
chr13	26980511	26980839
chr13	26980839	26981116
chr13	26997803	26998046
chr13	27022118	27022316
chr13	27026024	27026227
chr13	27026687	27027006
chr13	27027257	27027587
chr13	27027587	27027890
chr13	27028598	27028822
chr13	27028970	27029205
chr13	27030020	27030237
chr13	27033029	27033388
chr13	27046108	27046687
chr13	27057927	27058201
chr13	27059223	27059597
chr13	27080320	27080900
chr13	27080955	27081350
chr13	27083122	27083288
chr13	27113165	27113702
chr13	27113752	27114232
chr13	27146511	27146736
chr13	27148272	27148781
chr13	27148871	27149355
chr13	27281950	27282204
chr13	27312467	27312721
chr13	27386132	27386407
chr13	27403821	27404009
chr13	27497705	27498009
chr13	27553355	27553773
chr13	27660484	27660795
chr13	27660795	27661087
chr13	27706445	27707028
chr13	27724783	27725479
chr13	27725483	27725648
chr13	27730237	27730650
chr13	27730650	27730984
chr13	27734546	27734953
chr13	27771059	27771301

chr13	27798870	27799112
chr13	27799171	27799741
chr13	27815476	27815723
chr13	27816632	27816881
chr13	27822755	27823393
chr13	27837768	27838079
chr13	27838643	27838805
chr13	27842236	27842532
chr13	27847800	27847973
chr13	27904325	27904476
chr13	27921836	27921991
chr13	27923660	27923824
chr13	27976853	27977088
chr13	27996498	27996971
chr13	28068988	28069812
chr13	28109435	28109611
chr13	28110886	28111268
chr13	28132649	28133390
chr13	28289068	28289218
chr13	28297584	28297938
chr13	28310154	28310364
chr13	28311162	28311501
chr13	28312107	28312414
chr13	28312479	28312632
chr13	28313498	28313842
chr13	28391458	28391812
chr13	28408229	28408428
chr13	28417444	28417594
chr13	28417600	28417880
chr13	28417880	28418188
chr13	28418188	28418571
chr13	28424670	28424833
chr13	28457367	28457865
chr13	28458062	28458260
chr13	28492085	28492390
chr13	28519060	28519512
chr13	28524395	28524891
chr13	28526382	28526655
chr13	28532138	28532760
chr13	28533189	28533406
chr13	28544050	28544370
chr13	28544412	28545018

chr13	28545416	28545660
chr13	28563651	28563804
chr13	28565970	28566473
chr13	28589094	28589349
chr13	28592258	28592468
chr13	28600827	28600977
chr13	28605943	28606101
chr13	28607633	28607851
chr13	28607885	28608053
chr13	28617445	28617806
chr13	28626653	28626911
chr13	28629468	28629698
chr13	28633091	28633499
chr13	28633499	28634019
chr13	28646003	28646304
chr13	28651873	28652115
chr13	28652455	28652688
chr13	28652688	28652971
chr13	28654964	28655196
chr13	28655777	28655988
chr13	28659675	28659992
chr13	28680140	28680828
chr13	28685608	28686054
chr13	28690989	28691163
chr13	28692585	28692987
chr13	28693283	28693433
chr13	28699746	28700036
chr13	28718604	28719230
chr13	28730195	28730570
chr13	28731717	28731872
chr13	28737276	28737640
chr13	28737810	28738125
chr13	28738125	28738597
chr13	28738597	28739111
chr13	28739111	28739486
chr13	28749712	28750194
chr13	28750861	28751353
chr13	28757774	28758082
chr13	28758082	28758390
chr13	28758390	28758637
chr13	28758637	28758841
chr13	28758878	28759072

chr13	28759072	28759332
chr13	28765134	28765408
chr13	28823430	28823881
chr13	28824144	28824294
chr13	28830295	28830611
chr13	28832796	28833222
chr13	28833411	28833601
chr13	28835876	28836039
chr13	28839196	28839390
chr13	28842813	28843230
chr13	28843497	28843701
chr13	28850644	28850947
chr13	28850947	28851214
chr13	28854202	28854764
chr13	28869195	28869395
chr13	28873905	28874373
chr13	28874373	28874730
chr13	28881042	28881399
chr13	28883546	28883696
chr13	28883741	28884022
chr13	28884163	28884413
chr13	28884413	28884638
chr13	28885050	28885301
chr13	28885301	28885550
chr13	28885550	28885808
chr13	28892980	28893250
chr13	28896825	28897117
chr13	28915347	28915560
chr13	28916947	28917368
chr13	28917368	28917812
chr13	28917812	28918272
chr13	28923227	28923467
chr13	28924179	28924491
chr13	28930632	28930790
chr13	28931230	28931761
chr13	28931814	28932175
chr13	28938843	28939052
chr13	28945823	28946009
chr13	28946625	28946812
chr13	28946905	28947055
chr13	28948057	28948408
chr13	28948601	28948815

chr13	28950811	28950962
chr13	28952239	28952538
chr13	28952582	28953013
chr13	28953013	28953262
chr13	28953262	28953573
chr13	28953573	28954054
chr13	28957834	28958128
chr13	28958191	28958722
chr13	28958722	28959068
chr13	28959503	28959934
chr13	29014466	29014876
chr13	29057293	29057543
chr13	29148690	29149004
chr13	29163161	29163404
chr13	29193912	29194171
chr13	29196840	29197268
chr13	29201999	29202492
chr13	29202492	29203209
chr13	29210504	29210788
chr13	29210788	29211068
chr13	29218409	29219190
chr13	29477549	29477699
chr13	29502706	29503171
chr13	29503493	29503968
chr13	29504868	29505237
chr13	29505237	29505634
chr13	29505730	29505880
chr13	29574743	29575183
chr13	29597317	29597603
chr13	29601126	29601304
chr13	29604331	29604562
chr13	29606174	29606340
chr13	29625080	29625230
chr13	29627453	29627676
chr13	29635025	29635182
chr13	29640037	29640611
chr13	29641263	29641507
chr13	29663599	29663754
chr13	29701485	29701733
chr13	29710665	29710902
chr13	29710902	29711090
chr13	29738086	29738288

chr13	29738300	29738458
chr13	29750161	29750591
chr13	29750591	29751156
chr13	29777285	29777530
chr13	29778222	29778471
chr13	29779191	29779463
chr13	29780623	29780928
chr13	29780928	29781185
chr13	29781209	29781362
chr13	29792147	29792432
chr13	29805334	29805659
chr13	29817071	29817373
chr13	29830733	29831028
chr13	29831057	29831563
chr13	29831908	29832068
chr13	29837918	29838246
chr13	29838743	29838975
chr13	29855178	29855502
chr13	29855502	29855764
chr13	29857419	29857741
chr13	29857796	29857950
chr13	29870707	29870926
chr13	29878165	29878316
chr13	29901720	29901972
chr13	29903192	29903806
chr13	29915543	29915709
chr13	29921869	29922020
chr13	29938859	29939070
chr13	29949108	29949259
chr13	29973703	29973923
chr13	29984138	29984685
chr13	29984685	29985084
chr13	29985147	29985345
chr13	29985345	29985623
chr13	29985925	29986493
chr13	29986516	29986749
chr13	29996674	29996824
chr13	30038554	30038811
chr13	30041539	30041816
chr13	30062394	30062621
chr13	30070956	30071198
chr13	30074567	30074754

chr13	30085045	30085358
chr13	30085575	30085760
chr13	30085796	30085991
chr13	30093213	30093392
chr13	30093762	30093912
chr13	30095872	30096356
chr13	30098546	30098696
chr13	30108886	30109276
chr13	30109276	30109475
chr13	30109475	30109710
chr13	30109963	30110200
chr13	30136232	30136850
chr13	30138004	30138271
chr13	30140231	30140494
chr13	30141552	30141860
chr13	30145481	30145683
chr13	30145768	30145940
chr13	30146988	30147143
chr13	30149068	30149553
chr13	30149604	30149950
chr13	30157824	30158052
chr13	30161078	30161270
chr13	30161926	30162368
chr13	30169418	30169741
chr13	30177456	30177687
chr13	30183073	30183248
chr13	30192400	30192607
chr13	30194145	30194420
chr13	30194784	30195312
chr13	30197972	30198566
chr13	30205471	30205920
chr13	30251505	30251776
chr13	30259925	30260259
chr13	30263483	30263646
chr13	30263646	30263871
chr13	30272353	30272614
chr13	30336231	30336479
chr13	30336479	30336758
chr13	30358864	30359089
chr13	30368631	30369103
chr13	30452079	30452491
chr13	30460601	30460978

chr13	30530862	30531087
chr13	30533262	30533489
chr13	30544801	30545012
chr13	30545012	30545250
chr13	30545250	30545568
chr13	30548893	30549192
chr13	30550387	30550708
chr13	30550812	30551045
chr13	30553138	30553361
chr13	30565000	30565265
chr13	30565265	30565477
chr13	30566863	30567090
chr13	30604023	30604299
chr13	30619754	30620047
chr13	30621772	30622080
chr13	30628522	30628710
chr13	30642014	30642498
chr13	30646949	30647446
chr13	30652252	30652481
chr13	30659542	30659848
chr13	30659848	30660486
chr13	30665345	30665497
chr13	30691945	30692184
chr13	30704681	30704863
chr13	30713585	30713960
chr13	30725076	30725282
chr13	30728848	30729090
chr13	30729428	30729613
chr13	30730125	30730348
chr13	30749035	30749268
chr13	30749337	30749477
chr13	30749477	30749630
chr13	30752660	30752871
chr13	30769328	30769478
chr13	30781852	30782300
chr13	30782300	30782700
chr13	30786197	30786530
chr13	30786636	30786927
chr13	30792192	30792782
chr13	30792893	30793064
chr13	30793332	30794042
chr13	30796236	30796444

chr13	30811644	30811806
chr13	30814320	30814500
chr13	30888547	30888808
chr13	30931670	30931952
chr13	30972157	30972477
chr13	30973627	30974286
chr13	30974340	30974492
chr13	30981745	30981985
chr13	30990308	30990587
chr13	30993914	30994096
chr13	31033269	31033524
chr13	31060733	31061353
chr13	31062199	31062553
chr13	31062630	31063036
chr13	31082552	31082706
chr13	31084963	31085155
chr13	31107224	31107426
chr13	31107437	31107790
chr13	31125693	31126002
chr13	31184865	31185092
chr13	31186935	31187154
chr13	31189206	31189484
chr13	31197669	31198040
chr13	31206271	31206665
chr13	31207779	31208163
chr13	31209229	31209460
chr13	31215047	31215575
chr13	31216327	31216527
chr13	31216527	31216683
chr13	31251881	31252169
chr13	31257790	31257965
chr13	31257965	31258104
chr13	31275498	31275910
chr13	31275910	31276137
chr13	31279933	31280306
chr13	31302463	31302659
chr13	31305211	31305414
chr13	31308929	31309248
chr13	31309248	31309575
chr13	31311304	31311516
chr13	31327115	31327459
chr13	31327459	31327717

chr13	31327717	31328297
chr13	31334931	31335355
chr13	31341266	31341464
chr13	31342076	31342353
chr13	31351583	31351822
chr13	31405349	31405690
chr13	31408732	31408991
chr13	31429248	31430130
chr13	31439895	31440055
chr13	31440138	31440351
chr13	31458857	31459104
chr13	31465199	31465378
chr13	31468078	31468446
chr13	31468446	31468655
chr13	31468807	31469007
chr13	31473399	31473675
chr13	31476487	31476662
chr13	31477313	31477496
chr13	31491662	31492113
chr13	31492505	31492718
chr13	31501125	31501361
chr13	31504393	31504661
chr13	31506203	31506794
chr13	31506794	31507110
chr13	31518290	31519012
chr13	31519528	31519702
chr13	31531059	31531530
chr13	31531530	31531880
chr13	31531880	31532183
chr13	31538847	31539024
chr13	31547673	31547934
chr13	31557780	31557991
chr13	31557991	31558323
chr13	31558323	31558665
chr13	31558730	31558897
chr13	31558900	31559138
chr13	31559138	31559311
chr13	31559311	31559612
chr13	31559612	31559925
chr13	31559925	31560196
chr13	31567158	31567554
chr13	31567554	31567984

chr13	31567984	31568378
chr13	31572938	31573171
chr13	31573171	31573531
chr13	31577737	31577940
chr13	31578366	31578894
chr13	31579924	31580224
chr13	31584159	31584421
chr13	31618234	31618579
chr13	31618579	31618833
chr13	31619644	31619905
chr13	31625066	31625270
chr13	31625409	31625727
chr13	31625727	31626020
chr13	31626020	31626254
chr13	31626254	31626590
chr13	31626592	31626790
chr13	31626790	31627179
chr13	31629477	31629810
chr13	31632470	31632791
chr13	31639321	31640000
chr13	31640145	31640433
chr13	31659175	31659335
chr13	31689420	31689629
chr13	31696472	31696715
chr13	31714108	31714259
chr13	31738431	31738621
chr13	31768742	31768892
chr13	31781366	31781578
chr13	31792519	31792797
chr13	31800641	31800804
chr13	31801047	31801255
chr13	31801255	31801653
chr13	31806173	31806401
chr13	31806401	31806862
chr13	31806972	31807571
chr13	31807581	31807984
chr13	31807987	31808450
chr13	31808450	31808738
chr13	31826739	31826947
chr13	31835934	31836187
chr13	31877188	31877340
chr13	31884479	31884753

chr13	31915800	31916071
chr13	31955207	31955397
chr13	31956678	31956890
chr13	31969973	31970158
chr13	32025716	32025984
chr13	32055450	32055609
chr13	32091316	32091637
chr13	32092490	32092868
chr13	32116230	32116415
chr13	32134515	32134749
chr13	32134749	32135045
chr13	32135045	32135345
chr13	32169309	32169539
chr13	32193066	32193307
chr13	32214154	32214391
chr13	32219987	32220327
chr13	32242211	32242591
chr13	32251141	32251360
chr13	32267189	32267423
chr13	32268309	32268523
chr13	32297460	32297786
chr13	32298547	32298833
chr13	32312739	32312996
chr13	32318323	32318605
chr13	32318632	32318782
chr13	32329839	32330061
chr13	32337789	32338150
chr13	32338150	32338510
chr13	32338510	32338968
chr13	32348969	32349200
chr13	32370793	32371243
chr13	32371910	32372228
chr13	32372228	32372534
chr13	32375704	32375926
chr13	32411672	32411904
chr13	32414591	32414818
chr13	32415269	32415569
chr13	32428160	32428465
chr13	32428465	32428912
chr13	32478925	32479411
chr13	32479411	32479668
chr13	32493421	32493598

chr13	32493853	32494134
chr13	32496995	32497161
chr13	32509797	32509947
chr13	32515100	32515360
chr13	32545003	32545336
chr13	32545336	32545714
chr13	32549996	32550351
chr13	32555662	32555834
chr13	32555834	32556005
chr13	32556016	32556360
chr13	32556362	32556901
chr13	32565689	32565956
chr13	32581308	32581574
chr13	32583238	32583408
chr13	32592853	32593139
chr13	32593255	32593405
chr13	32604292	32604476
chr13	32605632	32605909
chr13	32610761	32611410
chr13	32611491	32611759
chr13	32630293	32630642
chr13	32660444	32660706
chr13	32662108	32662345
chr13	32678951	32679302
chr13	32681204	32681466
chr13	32707911	32708233
chr13	32721303	32721689
chr13	32721689	32722174
chr13	32734389	32734655
chr13	32769963	32770197
chr13	32801731	32802190
chr13	32802190	32802443
chr13	32802443	32802763
chr13	32802763	32803094
chr13	32809993	32810302
chr13	32828013	32828246
chr13	32829001	32829205
chr13	32831669	32831835
chr13	32846248	32846729
chr13	32852041	32852284
chr13	32860922	32861105
chr13	32861923	32862073

chr13	32862537	32862815
chr13	32865671	32865868
chr13	32866511	32866819
chr13	32884108	32884413
chr13	32884803	32885139
chr13	32900721	32900948
chr13	32902540	32902837
chr13	32903239	32903727
chr13	32903736	32903887
chr13	32905005	32905229
chr13	32912810	32913064
chr13	32913131	32913291
chr13	32923926	32924157
chr13	32924894	32925407
chr13	32926732	32926966
chr13	32927481	32927658
chr13	32928042	32928484
chr13	32933820	32933979
chr13	32934737	32935065
chr13	32935065	32935417
chr13	32938398	32938594
chr13	32939090	32939956
chr13	32957952	32958289
chr13	32960338	32960497
chr13	32963991	32964397
chr13	32964397	32964808
chr13	32964912	32965479
chr13	32965479	32965841
chr13	32978683	32978843
chr13	32982679	32983111
chr13	32995058	32995265
chr13	33001022	33001183
chr13	33003583	33003750
chr13	33003830	33004041
chr13	33004331	33005019
chr13	33005786	33006015
chr13	33006613	33006948
chr13	33013379	33013535
chr13	33026561	33026721
chr13	33026807	33027119
chr13	33028158	33028519
chr13	33028519	33029043

chr13	33029570	33029744
chr13	33030365	33030607
chr13	33050820	33051222
chr13	33051222	33051651
chr13	33051651	33052225
chr13	33063132	33063367
chr13	33065961	33066204
chr13	33074079	33074366
chr13	33078393	33078696
chr13	33079505	33079766
chr13	33080389	33080702
chr13	33085725	33085973
chr13	33089692	33089974
chr13	33090563	33090906
chr13	33109068	33109300
chr13	33120448	33121267
chr13	33134072	33134271
chr13	33141104	33141254
chr13	33141324	33141775
chr13	33158484	33158699
chr13	33182259	33182581
chr13	33182581	33182834
chr13	33193349	33193709
chr13	33193709	33194167
chr13	33194236	33194418
chr13	33249413	33249622
chr13	33249970	33250157
chr13	33250157	33250391
chr13	33250391	33250659
chr13	33250659	33250897
chr13	33253807	33254026
chr13	33289197	33289406
chr13	33323846	33324063
chr13	33324448	33324805
chr13	33324846	33325341
chr13	33363834	33364041
chr13	33409216	33409374
chr13	33448992	33449187
chr13	33484591	33484807
chr13	33485171	33485577
chr13	33485577	33486053
chr13	33514772	33515089

chr13	33518375	33518668
chr13	33522256	33522473
chr13	33544778	33545017
chr13	33559674	33560056
chr13	33561634	33562040
chr13	33562040	33562328
chr13	33569323	33569693
chr13	33570837	33571369
chr13	33577986	33578136
chr13	33585882	33586119
chr13	33586119	33586593
chr13	33688512	33688781
chr13	33762814	33763097
chr13	33764684	33765169
chr13	33788856	33789085
chr13	33789868	33790203
chr13	33821060	33821218
chr13	33821261	33821558
chr13	33821558	33821854
chr13	33823373	33823708
chr13	33823708	33823985
chr13	33894263	33894505
chr13	33903343	33903627
chr13	33908296	33908561
chr13	33934664	33935067
chr13	33935413	33935680
chr13	33935680	33935911
chr13	33935911	33936221
chr13	33951195	33951681
chr13	33951681	33952058
chr13	33952058	33952434
chr13	33956882	33957108
chr13	33957147	33957299
chr13	33964354	33965201
chr13	33965233	33965440
chr13	33965734	33965915
chr13	33995268	33995555
chr13	34001999	34002247
chr13	34002247	34002478
chr13	34002478	34002715
chr13	34002715	34003038
chr13	34028354	34028504

chr13	34032186	34032382
chr13	34032625	34032921
chr13	34032963	34033354
chr13	34037153	34037491
chr13	34037491	34037732
chr13	34037732	34038022
chr13	34043900	34044107
chr13	34046425	34046723
chr13	34050602	34050849
chr13	34054455	34054722
chr13	34066345	34066646
chr13	34075934	34076233
chr13	34077653	34078414
chr13	34079795	34080234
chr13	34084053	34084247
chr13	34087092	34087501
chr13	34088194	34088697
chr13	34095971	34096445
chr13	34096480	34096898
chr13	34096955	34097426
chr13	34109776	34110014
chr13	34112310	34112914
chr13	34113142	34113438
chr13	34115871	34116052
chr13	34129785	34129993
chr13	34129993	34130308
chr13	34130308	34130894
chr13	34131031	34131352
chr13	34147051	34147270
chr13	34147659	34147908
chr13	34153983	34154277
chr13	34154277	34154700
chr13	34154725	34154930
chr13	34154957	34155192
chr13	34155256	34155411
chr13	34156232	34156453
chr13	34162646	34163078
chr13	34163078	34163590
chr13	34178240	34178495
chr13	34187568	34187794
chr13	34190406	34190752
chr13	34191869	34192191

chr13	34192309	34192695
chr13	34192695	34192908
chr13	34192908	34193159
chr13	34200818	34200968
chr13	34201580	34201961
chr13	34208127	34208423
chr13	34209945	34210243
chr13	34211403	34211718
chr13	34212524	34212970
chr13	34212970	34213444
chr13	34214151	34214303
chr13	34224274	34224858
chr13	34233020	34233336
chr13	34234058	34234344
chr13	34234362	34234789
chr13	34234789	34235061
chr13	34235063	34235450
chr13	34246576	34246887
chr13	34246887	34247229
chr13	34247229	34247529
chr13	34260391	34260541
chr13	34267221	34267432
chr13	34271124	34271471
chr13	34275067	34275231
chr13	34275453	34275710
chr13	34277644	34278039
chr13	34278140	34278527
chr13	34281894	34282044
chr13	34289689	34289921
chr13	34290360	34290909
chr13	34292104	34292336
chr13	34299608	34299905
chr13	34303619	34303958
chr13	34305004	34305215
chr13	34307789	34307939
chr13	34308270	34308646
chr13	34313304	34313513
chr13	34314515	34314681
chr13	34317067	34317423
chr13	34326802	34327187
chr13	34329709	34329872
chr13	34330727	34330966

chr13	34332575	34332816
chr13	34332816	34333168
chr13	34334283	34334630
chr13	34343885	34344056
chr13	34344113	34344501
chr13	34344501	34344750
chr13	34344750	34345072
chr13	34345072	34345628
chr13	34348572	34348785
chr13	34365308	34365561
chr13	34369883	34370396
chr13	34370396	34370850
chr13	34371844	34372509
chr13	34390802	34391068
chr13	34391068	34391323
chr13	34394547	34394916
chr13	34397858	34398082
chr13	34398082	34398452
chr13	34398458	34398754
chr13	34398912	34399143
chr13	34399551	34399820
chr13	34402606	34403043
chr13	34408059	34408355
chr13	34408355	34408647
chr13	34408647	34408953
chr13	34419209	34419465
chr13	34419465	34419691
chr13	34419774	34419924
chr13	34420268	34420623
chr13	34420677	34420914
chr13	34420914	34421173
chr13	34425099	34425443
chr13	34460824	34461413
chr13	34462906	34463269
chr13	34465866	34466175
chr13	34504936	34505278
chr13	34537032	34537191
chr13	34540732	34540999
chr13	34553810	34553973
chr13	34565111	34565499
chr13	34566014	34566200
chr13	34566690	34567091

chr13	34568283	34568539
chr13	34572147	34572384
chr13	34572459	34572784
chr13	34572784	34573025
chr13	34575859	34576202
chr13	34600139	34600445
chr13	34611486	34611646
chr13	34629263	34629421
chr13	34629473	34629877
chr13	34635972	34636330
chr13	34640992	34641452
chr13	34641600	34641766
chr13	34643084	34643234
chr13	34650943	34651271
chr13	34651446	34651779
chr13	34652133	34652360
chr13	34652360	34652607
chr13	34652607	34652890
chr13	34652890	34653394
chr13	34653470	34653679
chr13	34659329	34659706
chr13	34659706	34660026
chr13	34677248	34677446
chr13	34677446	34677681
chr13	34683177	34683681
chr13	34683695	34683850
chr13	34685316	34685551
chr13	34689954	34690251
chr13	34691100	34691342
chr13	34691342	34691629
chr13	34696758	34697164
chr13	34697164	34697636
chr13	34708944	34709458
chr13	34709559	34709922
chr13	34711448	34711749
chr13	34715107	34715402
chr13	34715402	34715991
chr13	34725819	34726112
chr13	34726129	34726495
chr13	34726495	34726761
chr13	34731921	34732198
chr13	34733676	34734164

chr13	34736059	34736427
chr13	34736675	34736937
chr13	34748522	34748889
chr13	34761972	34762189
chr13	34762189	34762456
chr13	34763634	34763874
chr13	34765326	34765653
chr13	34766644	34766902
chr13	34767697	34767896
chr13	34784653	34785237
chr13	34793656	34793973
chr13	34794016	34794209
chr13	34794209	34794435
chr13	34794450	34794652
chr13	34806815	34807016
chr13	34828341	34828567
chr13	34861305	34861491
chr13	34864444	34864766
chr13	34874138	34874645
chr13	34875159	34875721
chr13	34875721	34876203
chr13	34888888	34889043
chr13	34896707	34896982
chr13	34898204	34898823
chr13	34973867	34974029
chr13	34993594	34993960
chr13	34993960	34994448
chr13	34994448	34994829
chr13	35027044	35027208
chr13	35031825	35031984
chr13	35038167	35038533
chr13	35038533	35038814
chr13	35038814	35039251
chr13	35086423	35086622
chr13	35131253	35132105
chr13	35147948	35148399
chr13	35166226	35166434
chr13	35167792	35168017
chr13	35208171	35208647
chr13	35209040	35209255
chr13	35214998	35215167
chr13	35215167	35215356

chr13	35217443	35218027
chr13	35227571	35227822
chr13	35255306	35255538
chr13	35255538	35255732
chr13	35257449	35257734
chr13	35263428	35263807
chr13	35283933	35284373
chr13	35291529	35291687
chr13	35328176	35328776
chr13	35328806	35329246
chr13	35329339	35329523
chr13	35330206	35330550
chr13	35346764	35347033
chr13	35386290	35386618
chr13	35395068	35395592
chr13	35395592	35396086
chr13	35403767	35404020
chr13	35418873	35419180
chr13	35433778	35434045
chr13	35434045	35434420
chr13	35435195	35435499
chr13	35446587	35446808
chr13	35450628	35450795
chr13	35451146	35451322
chr13	35452523	35453090
chr13	35454978	35455297
chr13	35463964	35464241
chr13	35464518	35464677
chr13	35467452	35467655
chr13	35469315	35469556
chr13	35469556	35469985
chr13	35474351	35474592
chr13	35474622	35475191
chr13	35476168	35476568
chr13	35476568	35476894
chr13	35481202	35481698
chr13	35501687	35502119
chr13	35502119	35502518
chr13	35502793	35503132
chr13	35507162	35507518
chr13	35512453	35512740
chr13	35518296	35518490

chr13	35519472	35519982
chr13	35524511	35524991
chr13	35541407	35541623
chr13	35563922	35564127
chr13	35604044	35604247
chr13	35732225	35732547
chr13	35740532	35741014
chr13	35741014	35741243
chr13	35741243	35741506
chr13	35741506	35741927
chr13	35741989	35742185
chr13	35755984	35756177
chr13	35760442	35760693
chr13	35760872	35761074
chr13	35765851	35766192
chr13	35773815	35774329
chr13	35774823	35775217
chr13	35781861	35782026
chr13	35782959	35783241
chr13	35798753	35798909
chr13	35816074	35816229
chr13	35817709	35817963
chr13	35820195	35820551
chr13	35860783	35861167
chr13	35871314	35871508
chr13	35886482	35886649
chr13	35905774	35906213
chr13	35906213	35906563
chr13	35947429	35947657
chr13	35947696	35947990
chr13	35968177	35968465
chr13	35968465	35968760
chr13	35968760	35969085
chr13	35969085	35969419
chr13	35969419	35969705
chr13	35991283	35991496
chr13	35994064	35994478
chr13	36000068	36000362
chr13	36000820	36001187
chr13	36001335	36001548
chr13	36009859	36010078
chr13	36010615	36010829

chr13	36013571	36013896
chr13	36028850	36029156
chr13	36052618	36053054
chr13	36081443	36081721
chr13	36081721	36082003
chr13	36086596	36086936
chr13	36090908	36091249
chr13	36116835	36117062
chr13	36117240	36117520
chr13	36117520	36117731
chr13	36117731	36118108
chr13	36139599	36139754
chr13	36152839	36153149
chr13	36153149	36153433
chr13	36162949	36163405
chr13	36163405	36163758
chr13	36196722	36197069
chr13	36200545	36200782
chr13	36203019	36203438
chr13	36204594	36204831
chr13	36211032	36211235
chr13	36214146	36214404
chr13	36282159	36282381
chr13	36282870	36283102
chr13	36283132	36283619
chr13	36315560	36315750
chr13	36316186	36316647
chr13	36317222	36317508
chr13	36317576	36317746
chr13	36317961	36318306
chr13	36368390	36368580
chr13	36377452	36377664
chr13	36452231	36452457
chr13	36463307	36463516
chr13	36463631	36464063
chr13	36465403	36465660
chr13	36513060	36513504
chr13	36516334	36516801
chr13	36520344	36520642
chr13	36522053	36522272
chr13	36522272	36522650
chr13	36524343	36524621

chr13	36529578	36529773
chr13	36529773	36529959
chr13	36530420	36530797
chr13	36530797	36531263
chr13	36534125	36534486
chr13	36534486	36534928
chr13	36546685	36546931
chr13	36547066	36547338
chr13	36547533	36547703
chr13	36548538	36548868
chr13	36575152	36575381
chr13	36576894	36577071
chr13	36578514	36578894
chr13	36578894	36579219
chr13	36590220	36590385
chr13	36610696	36610851
chr13	36695804	36696271
chr13	36696271	36696776
chr13	36703322	36703508
chr13	36720771	36720921
chr13	36726505	36726938
chr13	36726938	36727429
chr13	36727429	36727858
chr13	36734114	36734321
chr13	36734397	36734631
chr13	36734687	36734976
chr13	36734976	36735259
chr13	36736028	36736516
chr13	36740746	36741002
chr13	36802999	36803280
chr13	36804232	36804430
chr13	36821762	36821913
chr13	36828356	36828840
chr13	36828840	36829272
chr13	36829305	36829456
chr13	36839175	36839447
chr13	36873959	36874216
chr13	36940345	36940696
chr13	36947725	36948108
chr13	36948373	36948669
chr13	36958491	36958671
chr13	36958712	36959025

chr13	36962204	36962709
chr13	36962991	36963312
chr13	36963925	36964120
chr13	36978494	36978741
chr13	36978741	36979004
chr13	36986558	36986896
chr13	37010478	37010804
chr13	37010804	37011090
chr13	37011717	37012262
chr13	37019460	37019720
chr13	37045136	37045303
chr13	37052001	37052444
chr13	37052444	37052907
chr13	37106137	37106334
chr13	37107917	37108136
chr13	37108887	37109309
chr13	37109543	37109693
chr13	37176570	37176830
chr13	37193870	37194225
chr13	37194225	37194525
chr13	37234866	37235088
chr13	37238090	37238267
chr13	37241219	37241457
chr13	37290647	37290963
chr13	37298913	37299199
chr13	37312268	37312619
chr13	37312619	37313088
chr13	37325558	37325804
chr13	37358205	37358440
chr13	37364372	37364827
chr13	37417476	37417659
chr13	37428544	37428827
chr13	37431082	37431437
chr13	37431568	37431792
chr13	37434116	37434356
chr13	37441029	37441207
chr13	37452448	37452736
chr13	37453191	37453481
chr13	37454208	37454857
chr13	37456949	37457326
chr13	37465542	37466099
chr13	37474421	37474644

chr13	37474963	37475251
chr13	37477705	37478213
chr13	37478316	37478466
chr13	37506754	37507066
chr13	37507088	37507516
chr13	37508997	37509208
chr13	37520034	37520295
chr13	37529069	37529454
chr13	37531598	37531814
chr13	37541650	37542052
chr13	37546251	37546739
chr13	37546739	37547151
chr13	37547151	37547448
chr13	37550373	37550564
chr13	37556954	37557814
chr13	37561437	37561749
chr13	37591963	37592601
chr13	37599990	37600185
chr13	37601207	37601372
chr13	37615690	37615844
chr13	37619843	37620183
chr13	37622376	37622754
chr13	37622754	37623061
chr13	37624843	37625062
chr13	37625122	37625396
chr13	37642843	37643034
chr13	37643396	37643563
chr13	37651329	37651537
chr13	37666144	37666482
chr13	37666482	37667067
chr13	37671176	37671491
chr13	37672676	37672826
chr13	37678789	37679010
chr13	37680826	37681264
chr13	37681272	37681519
chr13	37690324	37690492
chr13	37690527	37690730
chr13	37703399	37703918
chr13	37704538	37705166
chr13	37705556	37705782
chr13	37707636	37707835
chr13	37712957	37713199

chr13	37714998	37715776
chr13	37715879	37716036
chr13	37716208	37716648
chr13	37725879	37726044
chr13	37732517	37732667
chr13	37751324	37751479
chr13	37761037	37761241
chr13	37777357	37777730
chr13	37777762	37778034
chr13	37778086	37778324
chr13	37778324	37778760
chr13	37778760	37779380
chr13	37783608	37783851
chr13	37783851	37784134
chr13	37784219	37784659
chr13	37801094	37801419
chr13	37818273	37818557
chr13	37825079	37825298
chr13	37825763	37826150
chr13	37826398	37827099
chr13	37827176	37827441
chr13	37827561	37827741
chr13	37827774	37827992
chr13	37828947	37829211
chr13	37830807	37831082
chr13	37831082	37831427
chr13	37846962	37847465
chr13	37847566	37848133
chr13	37852921	37853129
chr13	37853635	37853793
chr13	37853947	37854438
chr13	37854581	37854761
chr13	37857382	37858053
chr13	37864409	37864624
chr13	37864765	37864918
chr13	37866339	37866782
chr13	37866973	37867148
chr13	37880143	37880322
chr13	37895399	37895644
chr13	37895644	37895909
chr13	37895920	37896340
chr13	37926979	37927282

chr13	37936446	37936708
chr13	37944377	37944653
chr13	37945441	37945693
chr13	37946462	37946752
chr13	37946752	37947059
chr13	37953065	37953312
chr13	37953312	37953561
chr13	37963295	37963708
chr13	37963708	37963939
chr13	37966760	37966979
chr13	37969413	37969676
chr13	37970943	37971194
chr13	37973999	37974155
chr13	37992977	37993155
chr13	37993547	37993967
chr13	37993967	37994451
chr13	37999840	38000028
chr13	38012568	38012718
chr13	38016660	38016816
chr13	38024935	38025092
chr13	38036763	38037076
chr13	38037076	38037343
chr13	38037343	38037659
chr13	38037887	38038100
chr13	38064859	38065301
chr13	38088116	38088302
chr13	38089809	38089961
chr13	38089964	38090167
chr13	38091549	38091917
chr13	38096328	38096583
chr13	38098736	38099143
chr13	38104586	38104754
chr13	38110801	38111042
chr13	38123602	38124084
chr13	38130248	38130590
chr13	38132759	38132966
chr13	38144990	38145325
chr13	38145325	38145624
chr13	38150581	38150788
chr13	38151020	38151606
chr13	38151606	38152178
chr13	38152244	38152496

chr13	38152496	38152769
chr13	38152769	38153014
chr13	38153765	38154031
chr13	38204259	38204490
chr13	38204699	38205060
chr13	38205060	38205496
chr13	38229471	38229818
chr13	38233027	38233182
chr13	38235461	38235631
chr13	38251098	38251255
chr13	38251297	38251573
chr13	38257464	38257699
chr13	38276453	38276926
chr13	38277478	38277744
chr13	38288341	38288649
chr13	38288649	38288949
chr13	38311964	38312372
chr13	38312372	38312672
chr13	38312672	38312960
chr13	38320303	38320766
chr13	38335002	38335194
chr13	38338115	38338416
chr13	38338416	38338684
chr13	38342728	38342878
chr13	38343377	38343544
chr13	38344905	38345247
chr13	38345505	38345744
chr13	38348613	38348936
chr13	38349739	38350193
chr13	38350376	38350542
chr13	38360794	38361100
chr13	38361183	38361510
chr13	38361510	38361747
chr13	38376171	38376344
chr13	38387731	38387933
chr13	38390808	38391317
chr13	38398751	38398909
chr13	38410309	38410617
chr13	38410617	38410940
chr13	38411492	38411720
chr13	38412587	38412843
chr13	38419801	38420090

chr13	38422528	38422949
chr13	38424483	38424682
chr13	38426903	38427163
chr13	38427163	38427647
chr13	38438020	38438205
chr13	38450285	38450632
chr13	38450632	38451124
chr13	38454371	38454638
chr13	38482168	38482416
chr13	38505732	38505933
chr13	38506380	38506634
chr13	38515539	38515736
chr13	38515965	38516115
chr13	38527603	38527864
chr13	38527864	38528253
chr13	38528253	38528693
chr13	38535424	38536222
chr13	38554668	38554925
chr13	38565591	38565754
chr13	38574726	38574991
chr13	38580608	38581085
chr13	38589632	38589784
chr13	38598480	38598789
chr13	38603041	38603310
chr13	38618333	38618487
chr13	38634304	38634501
chr13	38634531	38635014
chr13	38635014	38635360
chr13	38658461	38658826
chr13	38658826	38659299
chr13	38668588	38668817
chr13	38710011	38710286
chr13	38736360	38736986
chr13	38759139	38759296
chr13	38761514	38761690
chr13	38786168	38786526
chr13	38793574	38793790
chr13	38850058	38850341
chr13	38914888	38915195
chr13	38957999	38958177
chr13	38960035	38960354
chr13	38960354	38960721

chr13	38960721	38961040
chr13	38967187	38967377
chr13	38971090	38971407
chr13	38999127	38999281
chr13	39010362	39010607
chr13	39011717	39012147
chr13	39163406	39163574
chr13	39226923	39227141
chr13	39252663	39252940
chr13	39361592	39361826
chr13	39441015	39441185
chr13	39698332	39698542
chr13	39787562	39787733
chr13	39798924	39799140
chr13	39836184	39836376
chr13	39849050	39849210
chr13	39849412	39849851
chr13	39851873	39852194
chr13	39865299	39865623
chr13	39898523	39898679
chr13	39898679	39898832
chr13	39919662	39919868
chr13	39923152	39923461
chr13	39929438	39929623
chr13	39970874	39971146
chr13	40000142	40000351
chr13	40043998	40044221
chr13	40137160	40137422
chr13	40139117	40139377
chr13	40166301	40166587
chr13	40166587	40166867
chr13	40182446	40182649
chr13	40182649	40182832
chr13	40232728	40232918
chr13	40233035	40233205
chr13	40321131	40321360
chr13	40360698	40361059
chr13	40361391	40361645
chr13	40361686	40361884
chr13	40368651	40368832
chr13	40460477	40460714
chr13	40463104	40463275

chr13	40463412	40463580
chr13	40472602	40473141
chr13	40473258	40473443
chr13	40564641	40564801
chr13	40569495	40569767
chr13	40577386	40577577
chr13	40597024	40597221
chr13	40635934	40636315
chr13	40637286	40637798
chr13	40638563	40638788
chr13	40639717	40640015
chr13	40651139	40651350
chr13	40656113	40656382
chr13	40656382	40656698
chr13	40659538	40659952
chr13	40659952	40660278
chr13	40660278	40660650
chr13	40663527	40663917
chr13	40669228	40669378
chr13	40683559	40684194
chr13	40704000	40704172
chr13	40719091	40719421
chr13	40722533	40722975
chr13	40722975	40723482
chr13	40730645	40730929
chr13	40730929	40731211
chr13	40731211	40731521
chr13	40731521	40731861
chr13	40732897	40733201
chr13	40733675	40734148
chr13	40749218	40749329
chr13	40749329	40749374
chr13	40774831	40774981
chr13	40784114	40784362
chr13	40843485	40843750
chr13	40843799	40843980
chr13	40854544	40854852
chr13	40859580	40860324
chr13	40868086	40868276
chr13	40868558	40869473
chr13	40877961	40878140
chr13	40879278	40879561

chr13	40900718	40900891
chr13	40904110	40904411
chr13	40913721	40913997
chr13	40916518	40916915
chr13	40916915	40917343
chr13	40917395	40917799
chr13	40921279	40921691
chr13	40921691	40921965
chr13	40929010	40929169
chr13	40930449	40930816
chr13	40931815	40932205
chr13	40937755	40937921
chr13	40941435	40941587
chr13	40953548	40953698
chr13	40955663	40955871
chr13	40971238	40971645
chr13	40977698	40977919
chr13	40982761	40983194
chr13	40985851	40986154
chr13	40986154	40986423
chr13	41000097	41000371
chr13	41000371	41000625
chr13	41000625	41000851
chr13	41000851	41001107
chr13	41001107	41001561
chr13	41002112	41002286
chr13	41009116	41009266
chr13	41015916	41016407
chr13	41016407	41016733
chr13	41016870	41017081
chr13	41033313	41033615
chr13	41045928	41046226
chr13	41054863	41055111
chr13	41076727	41076930
chr13	41077223	41077488
chr13	41079137	41079843
chr13	41220014	41220812
chr13	41238174	41238341
chr13	41249545	41249917
chr13	41249917	41250297
chr13	41250297	41250592
chr13	41280772	41281010

chr13	41282355	41282531
chr13	41283275	41283707
chr13	41284548	41284787
chr13	41285446	41285814
chr13	41300123	41300385
chr13	41300731	41300944
chr13	41302726	41302998
chr13	41314095	41314311
chr13	41321268	41321852
chr13	41330955	41331193
chr13	41331193	41331413
chr13	41331698	41332284
chr13	41334719	41334927
chr13	41336223	41336405
chr13	41338002	41338287
chr13	41339317	41340159
chr13	41345759	41345964
chr13	41345964	41346131
chr13	41349737	41349902
chr13	41352106	41352324
chr13	41352324	41352743
chr13	41355676	41355866
chr13	41358467	41358805
chr13	41358805	41359227
chr13	41366774	41367135
chr13	41367135	41367714
chr13	41381934	41382124
chr13	41384502	41384807
chr13	41384978	41385242
chr13	41386569	41386746
chr13	41386746	41386925
chr13	41401739	41402078
chr13	41403796	41404409
chr13	41405059	41405510
chr13	41410271	41410555
chr13	41416066	41416440
chr13	41419029	41419340
chr13	41419340	41419590
chr13	41423842	41424018
chr13	41445947	41446149
chr13	41453301	41454012
chr13	41457827	41457982

chr13	41469442	41469923
chr13	41471421	41471796
chr13	41476214	41476599
chr13	41476604	41477214
chr13	41519864	41520172
chr13	41530716	41530998
chr13	41531636	41531786
chr13	41532673	41533002
chr13	41569821	41570058
chr13	41572068	41572309
chr13	41583895	41584078
chr13	41605182	41605465
chr13	41605465	41605774
chr13	41605774	41606166
chr13	41606166	41606518
chr13	41606522	41606760
chr13	41608631	41608814
chr13	41612060	41612417
chr13	41622489	41622639
chr13	41622788	41623005
chr13	41623030	41623395
chr13	41624243	41624416
chr13	41634269	41634621
chr13	41634621	41634883
chr13	41642961	41643187
chr13	41643433	41643622
chr13	41643648	41644233
chr13	41650785	41651006
chr13	41651706	41652152
chr13	41653923	41654156
chr13	41662101	41662482
chr13	41662482	41662782
chr13	41662782	41663255
chr13	41673699	41674058
chr13	41679430	41679603
chr13	41680855	41681170
chr13	41683691	41683914
chr13	41705420	41706151
chr13	41709164	41709314
chr13	41709578	41709735
chr13	41747030	41747241
chr13	41748962	41749199

chr13	41750611	41750852
chr13	41773787	41774195
chr13	41784950	41785201
chr13	41785201	41785431
chr13	41785431	41785697
chr13	41801112	41801347
chr13	41801402	41801690
chr13	41802891	41803246
chr13	41803925	41804294
chr13	41805553	41805741
chr13	41820058	41820253
chr13	41820998	41821259
chr13	41823197	41823489
chr13	41827576	41827889
chr13	41831024	41831179
chr13	41846612	41846830
chr13	41847432	41847781
chr13	41847781	41848173
chr13	41848497	41848647
chr13	41925696	41925966
chr13	41929613	41930140
chr13	41930949	41931231
chr13	41940850	41941021
chr13	41941066	41941336
chr13	41941336	41941559
chr13	41941559	41941830
chr13	41980129	41980281
chr13	41988415	41988636
chr13	42035282	42035547
chr13	42049453	42049769
chr13	42051164	42051441
chr13	42051492	42051919
chr13	42051919	42052237
chr13	42052237	42052588
chr13	42052588	42053019
chr13	42053069	42053224
chr13	42064330	42064667
chr13	42064768	42065122
chr13	42066032	42066324
chr13	42067029	42067251
chr13	42073217	42073629
chr13	42073849	42074182

chr13	42077658	42077851
chr13	42088273	42088835
chr13	42090616	42090947
chr13	42094701	42094852
chr13	42095228	42095515
chr13	42101455	42101765
chr13	42102982	42103420
chr13	42108304	42109014
chr13	42109257	42109662
chr13	42130713	42131061
chr13	42131629	42131831
chr13	42144741	42144900
chr13	42152785	42153028
chr13	42153028	42153242
chr13	42155931	42156283
chr13	42175101	42175323
chr13	42221766	42221975
chr13	42228864	42229194
chr13	42233852	42234086
chr13	42234086	42234342
chr13	42234342	42234681
chr13	42238290	42238910
chr13	42244284	42244642
chr13	42244642	42245055
chr13	42245698	42245972
chr13	42255145	42255575
chr13	42260647	42260964
chr13	42260964	42261283
chr13	42261802	42262040
chr13	42265765	42266332
chr13	42276660	42276998
chr13	42277117	42277389
chr13	42277597	42277811
chr13	42277811	42278101
chr13	42278128	42278671
chr13	42278681	42278953
chr13	42285835	42286266
chr13	42286363	42286644
chr13	42298880	42299072
chr13	42299459	42299629
chr13	42299699	42299942
chr13	42300604	42300918

chr13	42301121	42301771
chr13	42302320	42302587
chr13	42302691	42302861
chr13	42303625	42303845
chr13	42307048	42307319
chr13	42308374	42308597
chr13	42310468	42310808
chr13	42310808	42311130
chr13	42317882	42318082
chr13	42322801	42323136
chr13	42323136	42323663
chr13	42328440	42328925
chr13	42329172	42329447
chr13	42351522	42352322
chr13	42357704	42358231
chr13	42358545	42358926
chr13	42361250	42361405
chr13	42374189	42374654
chr13	42374694	42374961
chr13	42376129	42376418
chr13	42383162	42383449
chr13	42390068	42390312
chr13	42390424	42390723
chr13	42394491	42394775
chr13	42398297	42398657
chr13	42424544	42424723
chr13	42424744	42424907
chr13	42430401	42430674
chr13	42436686	42436877
chr13	42447011	42447237
chr13	42457885	42458113
chr13	42495168	42495426
chr13	42538270	42538420
chr13	42544798	42545182
chr13	42548302	42548562
chr13	42548615	42548963
chr13	42548974	42549194
chr13	42549203	42549542
chr13	42553755	42553909
chr13	42554960	42555279
chr13	42557976	42558265
chr13	42558573	42558830

chr13	42565123	42565451
chr13	42569721	42569983
chr13	42570558	42570921
chr13	42574231	42574427
chr13	42586286	42586651
chr13	42586937	42587147
chr13	42587147	42587393
chr13	42589712	42589879
chr13	42592983	42593304
chr13	42600104	42600429
chr13	42602035	42602195
chr13	42607144	42607483
chr13	42617896	42618111
chr13	42651894	42652206
chr13	42657056	42657299
chr13	42689703	42690033
chr13	42724124	42724324
chr13	42749410	42749587
chr13	42792388	42792595
chr13	42818466	42818630
chr13	42822627	42822896
chr13	42866737	42866962
chr13	42916439	42916594
chr13	42961565	42961846
chr13	43004118	43004467
chr13	43032258	43032755
chr13	43032755	43032987
chr13	43042865	43043084
chr13	43068662	43069079
chr13	43069079	43069333
chr13	43069333	43069564
chr13	43085251	43085582
chr13	43085582	43085827
chr13	43086167	43086317
chr13	43148706	43149057
chr13	43149509	43150082
chr13	43154104	43154391
chr13	43159601	43159779
chr13	43171097	43171370
chr13	43171370	43171780
chr13	43172169	43172347
chr13	43172364	43172739

chr13	43173330	43173606
chr13	43181115	43181349
chr13	43182175	43182407
chr13	43182407	43182662
chr13	43182976	43183200
chr13	43183261	43183417
chr13	43183773	43184029
chr13	43193014	43193258
chr13	43193258	43193756
chr13	43201092	43201407
chr13	43202431	43202729
chr13	43205604	43205824
chr13	43211025	43211265
chr13	43212175	43212785
chr13	43222461	43222885
chr13	43231923	43232482
chr13	43234918	43235109
chr13	43241815	43242105
chr13	43243272	43243591
chr13	43261293	43261518
chr13	43261518	43261757
chr13	43261757	43262068
chr13	43262068	43262403
chr13	43262403	43262712
chr13	43263676	43263992
chr13	43268924	43269397
chr13	43271512	43271972
chr13	43274913	43275238
chr13	43277574	43277779
chr13	43284361	43284521
chr13	43284696	43284957
chr13	43284957	43285215
chr13	43286836	43287178
chr13	43292846	43293393
chr13	43293871	43294068
chr13	43296578	43296760
chr13	43297671	43298112
chr13	43303359	43303578
chr13	43303578	43303797
chr13	43303797	43304090
chr13	43304090	43304578
chr13	43304730	43305051

chr13	43306573	43306956
chr13	43317439	43317824
chr13	43319428	43319731
chr13	43319731	43319946
chr13	43326499	43326776
chr13	43349227	43349469
chr13	43349469	43349753
chr13	43365098	43365340
chr13	43365340	43365960
chr13	43365982	43366178
chr13	43379436	43379704
chr13	43398150	43398522
chr13	43398522	43398775
chr13	43398775	43399036
chr13	43411763	43412068
chr13	43412068	43412356
chr13	43412356	43412602
chr13	43434839	43435139
chr13	43464730	43464919
chr13	43469164	43469454
chr13	43471553	43471811
chr13	43478604	43479011
chr13	43480098	43480281
chr13	43480281	43480482
chr13	43480482	43480722
chr13	43480848	43481166
chr13	43481166	43481698
chr13	43488574	43488903
chr13	43493004	43493154
chr13	43493332	43493774
chr13	43493854	43494110
chr13	43501676	43501910
chr13	43506807	43506957
chr13	43524771	43525398
chr13	43525431	43525614
chr13	43531410	43531732
chr13	43535449	43535637
chr13	43546922	43547162
chr13	43559591	43560091
chr13	43560091	43560498
chr13	43565443	43565694
chr13	43579787	43580080

chr13	43592979	43593166
chr13	43595896	43596152
chr13	43602497	43602774
chr13	43615621	43616312
chr13	43629782	43630134
chr13	43725762	43726042
chr13	43739711	43739965
chr13	43773926	43774517
chr13	43774785	43774973
chr13	43775099	43775483
chr13	43785013	43785264
chr13	43864405	43864960
chr13	43991919	43992118
chr13	43993072	43993293
chr13	44085521	44086042
chr13	44087056	44087362
chr13	44130527	44130693
chr13	44176203	44176359
chr13	44185115	44185335
chr13	44280393	44280634
chr13	44301933	44302322
chr13	44306297	44306497
chr13	44315353	44315503
chr13	44455301	44455554
chr13	44622816	44622992
chr13	44642148	44642326
chr13	44642379	44642859
chr13	44659818	44660227
chr13	44660227	44660526
chr13	44673415	44673869
chr13	44673869	44674128
chr13	44674128	44674380
chr13	44674660	44674848
chr13	44704143	44704310
chr13	44730163	44730461
chr13	44730461	44730813
chr13	44730813	44731098
chr13	44731185	44731412
chr13	44733372	44733797
chr13	44733797	44734222
chr13	44758827	44759037
chr13	44770880	44771262

chr13	44775762	44776147
chr13	44781219	44781436
chr13	44784561	44784782
chr13	44784782	44784936
chr13	44784936	44785161
chr13	44786353	44786662
chr13	44804964	44805131
chr13	44833485	44833682
chr13	44839975	44840162
chr13	44845787	44845987
chr13	44859268	44859549
chr13	44869494	44869785
chr13	44869785	44870094
chr13	44904202	44904898
chr13	44916033	44916251
chr13	44917269	44917504
chr13	44924071	44924491
chr13	44939205	44939670
chr13	44939705	44939972
chr13	44940467	44940869
chr13	44943417	44943633
chr13	44968650	44969055
chr13	44971439	44971755
chr13	44971755	44972296
chr13	44977946	44978096
chr13	44985502	44985713
chr13	44985713	44985913
chr13	44990150	44990335
chr13	45000885	45001157
chr13	45001164	45001591
chr13	45001591	45001921
chr13	45001921	45002456
chr13	45005024	45005341
chr13	45030845	45031170
chr13	45078770	45078973
chr13	45078973	45079144
chr13	45097986	45098136
chr13	45387316	45387592
chr13	45389395	45389809
chr13	45389809	45390260
chr13	45390262	45390455
chr13	45398568	45398736

chr13	45402327	45402714
chr13	45402714	45403039
chr13	45507222	45507772
chr13	45507772	45508077
chr13	45508658	45508889
chr13	45509320	45509581
chr13	45512543	45512861
chr13	45512933	45513509
chr13	45515292	45515484
chr13	45516778	45517153
chr13	45521615	45521866
chr13	45530754	45531077
chr13	45538747	45538942
chr13	45538942	45539206
chr13	45539287	45539737
chr13	45546958	45547140
chr13	45560638	45560849
chr13	45562057	45562286
chr13	45569942	45570374
chr13	45571780	45571992
chr13	45577909	45578073
chr13	45578282	45578627
chr13	45613435	45613661
chr13	45614841	45615239
chr13	45623773	45623929
chr13	45627009	45627442
chr13	45627442	45627726
chr13	45647253	45647635
chr13	45649303	45649710
chr13	45657684	45657972
chr13	45657972	45658295
chr13	45658295	45658664
chr13	45667141	45667420
chr13	45684281	45684536
chr13	45684591	45685148
chr13	45685148	45685543
chr13	45694849	45695586
chr13	45706667	45707046
chr13	45707046	45707411
chr13	45707766	45708060
chr13	45711122	45711304
chr13	45718637	45719432

chr13	45744949	45745177
chr13	45748596	45749112
chr13	45750020	45750206
chr13	45751187	45751581
chr13	45792678	45792857
chr13	45796284	45796686
chr13	45803160	45803594
chr13	45803618	45803830
chr13	45806310	45806625
chr13	45806625	45806875
chr13	45820406	45820832
chr13	45820832	45821223
chr13	45821310	45821543
chr13	45827412	45827619
chr13	45827619	45827956
chr13	45827956	45828478
chr13	45829109	45829311
chr13	45829432	45829686
chr13	45837571	45837961
chr13	45839709	45840330
chr13	45850186	45850558
chr13	45850558	45850949
chr13	45851888	45852058
chr13	45857430	45857666
chr13	45867316	45867773
chr13	45872188	45872483
chr13	45883075	45883496
chr13	45884976	45885213
chr13	45906323	45906813
chr13	45906852	45907179
chr13	45907179	45907388
chr13	45907435	45907848
chr13	45908445	45908664
chr13	45911914	45912380
chr13	45915125	45915303
chr13	45917326	45917641
chr13	45922557	45922958
chr13	45922981	45923499
chr13	45924662	45924882
chr13	45925390	45925581
chr13	45928386	45928677
chr13	45928677	45928982

chr13	45931183	45931480
chr13	45938698	45938987
chr13	45942457	45942607
chr13	45963453	45963789
chr13	45963789	45964133
chr13	45964497	45964669
chr13	45964848	45965244
chr13	45965244	45965554
chr13	45965554	45965819
chr13	45965819	45966154
chr13	45966154	45966495
chr13	45966626	45966831
chr13	45971186	45971547
chr13	45974688	45975103
chr13	45976921	45977259
chr13	46011325	46011565
chr13	46030818	46031025
chr13	46043509	46044090
chr13	46044990	46045333
chr13	46047028	46047263
chr13	46047289	46047663
chr13	46054504	46054691
chr13	46097056	46097267
chr13	46161199	46161627
chr13	46161627	46161976
chr13	46161976	46162342
chr13	46166598	46166855
chr13	46255773	46255934
chr13	46257937	46258125
chr13	46271267	46271417
chr13	46626711	46626863
chr13	46662592	46662779
chr13	46669013	46669336
chr13	46669336	46669705
chr13	46669705	46670370
chr13	46674715	46674981
chr13	46683430	46683709
chr13	46690613	46690876
chr13	46690876	46691084
chr13	46691146	46691380
chr13	46696809	46697266
chr13	46703247	46703407

chr13	46716009	46716484
chr13	46726517	46726756
chr13	46727082	46727576
chr13	46727576	46728000
chr13	46728000	46728257
chr13	46735566	46735778
chr13	46735778	46736018
chr13	46736018	46736241
chr13	46736241	46736476
chr13	46736476	46736700
chr13	46736700	46736987
chr13	46742448	46742736
chr13	46745030	46745393
chr13	46748552	46748726
chr13	46776995	46777201
chr13	46788400	46788617
chr13	46788617	46788810
chr13	46798241	46798467
chr13	46810199	46810444
chr13	46812542	46812842
chr13	46812842	46813272
chr13	46838714	46838864
chr13	46840635	46841076
chr13	46842158	46842708
chr13	46849594	46849744
chr13	46883418	46883803
chr13	46888330	46888633
chr13	46907792	46908226
chr13	46908279	46908439
chr13	46908979	46909429
chr13	46928929	46929156
chr13	46929160	46929535
chr13	46929629	46930038
chr13	46930038	46930366
chr13	46930366	46930717
chr13	46930717	46931089
chr13	46937101	46937322
chr13	46955836	46955987
chr13	46959087	46959584
chr13	46976027	46976282
chr13	46983839	46984261
chr13	46986239	46986697

chr13	47014255	47014685
chr13	47014685	47015170
chr13	47017365	47017608
chr13	47042987	47043392
chr13	47043392	47043749
chr13	47043749	47044073
chr13	47063053	47063524
chr13	47081007	47081240
chr13	47089353	47089631
chr13	47105653	47106060
chr13	47106060	47106455
chr13	47106455	47106785
chr13	47112786	47112962
chr13	47121858	47122071
chr13	47122528	47122754
chr13	47122754	47122975
chr13	47124564	47124726
chr13	47152400	47152668
chr13	47162036	47162329
chr13	47170872	47171237
chr13	47171237	47171661
chr13	47178182	47178505
chr13	47193559	47194288
chr13	47194334	47194610
chr13	47194623	47195130
chr13	47195220	47195371
chr13	47212999	47213528
chr13	47213815	47214224
chr13	47216293	47216561
chr13	47217048	47217458
chr13	47224175	47224417
chr13	47262082	47262273
chr13	47272766	47272968
chr13	47272968	47273327
chr13	47273327	47273757
chr13	47288499	47288788
chr13	47301139	47301342
chr13	47335834	47336069
chr13	47336069	47336446
chr13	47347957	47348145
chr13	47391687	47391837
chr13	47394472	47394745

chr13	47394981	47395224
chr13	47462165	47462488
chr13	47483339	47483538
chr13	47502008	47502163
chr13	47532957	47533339
chr13	47667427	47667660
chr13	47894372	47894684
chr13	48026783	48027125
chr13	48074458	48074608
chr13	48074659	48074998
chr13	48097433	48097935
chr13	48104595	48105130
chr13	48105130	48105390
chr13	48109942	48110328
chr13	48110338	48110623
chr13	48110623	48110951
chr13	48116097	48116288
chr13	48120882	48121153
chr13	48130916	48131107
chr13	48138821	48139096
chr13	48140649	48140842
chr13	48193807	48194493
chr13	48194532	48194840
chr13	48195448	48195734
chr13	48214174	48214324
chr13	48227690	48228140
chr13	48228140	48228431
chr13	48233992	48234217
chr13	48260668	48261235
chr13	48261371	48261701
chr13	48261701	48261962
chr13	48261965	48262200
chr13	48262200	48262390
chr13	48273589	48273764
chr13	48273767	48274064
chr13	48314533	48314948
chr13	48338568	48338813
chr13	48398348	48398626
chr13	48398658	48399002
chr13	48437056	48437209
chr13	48464358	48464530
chr13	48475625	48475800

chr13	48490895	48491197
chr13	48491442	48491863
chr13	48492681	48492848
chr13	48492964	48493192
chr13	48493220	48493559
chr13	48502155	48502445
chr13	48502445	48502906
chr13	48512970	48513289
chr13	48513289	48513666
chr13	48515946	48516174
chr13	48528166	48528356
chr13	48528395	48528599
chr13	48528599	48528853
chr13	48530728	48531089
chr13	48531471	48531728
chr13	48533467	48533854
chr13	48539110	48539346
chr13	48545533	48546275
chr13	48546275	48546922
chr13	48552164	48552498
chr13	48552498	48552974
chr13	48564894	48565196
chr13	48566052	48566335
chr13	48566335	48566602
chr13	48567716	48568031
chr13	48571226	48571672
chr13	48572222	48572450
chr13	48576634	48576854
chr13	48587309	48587803
chr13	48602575	48602733
chr13	48602766	48603366
chr13	48609052	48609654
chr13	48614529	48614880
chr13	48614904	48615054
chr13	48625160	48625477
chr13	48625477	48625794
chr13	48625794	48626034
chr13	48639654	48639905
chr13	48641764	48641937
chr13	48644242	48644413
chr13	48653492	48653828
chr13	48653860	48654106

chr13	48655610	48655931
chr13	48656146	48656347
chr13	48659995	48660145
chr13	48662797	48663001
chr13	48663213	48663386
chr13	48665236	48665405
chr13	48669021	48669210
chr13	48669210	48669389
chr13	48671705	48671895
chr13	48674524	48674735
chr13	48679140	48679299
chr13	48679305	48679714
chr13	48697020	48697176
chr13	48697326	48697511
chr13	48697517	48697830
chr13	48711387	48711729
chr13	48726536	48726734
chr13	48727913	48728181
chr13	48731625	48731960
chr13	48732621	48732939
chr13	48745201	48745445
chr13	48748198	48748385
chr13	48782882	48783050
chr13	48797005	48797230
chr13	48800756	48801180
chr13	48801228	48801578
chr13	48807448	48807725
chr13	48809784	48809951
chr13	48815686	48815891
chr13	48822343	48822612
chr13	48822822	48823040
chr13	48832281	48832546
chr13	48840661	48840892
chr13	48841782	48842287
chr13	48842317	48842731
chr13	48852935	48853127
chr13	48853996	48854341
chr13	48857947	48858181
chr13	48858875	48859094
chr13	48861968	48862248
chr13	48869603	48869901
chr13	48869901	48870192

chr13	48870192	48870406
chr13	48870420	48870643
chr13	48870643	48870827
chr13	48871058	48871250
chr13	48871250	48871413
chr13	48871596	48871824
chr13	48871957	48872145
chr13	48872804	48873109
chr13	48873109	48873334
chr13	48878401	48879105
chr13	48890337	48890487
chr13	48891710	48891954
chr13	48906848	48907117
chr13	48913738	48913928
chr13	48921330	48921658
chr13	48921658	48922020
chr13	48922086	48922258
chr13	48954968	48955138
chr13	48962566	48962923
chr13	48965577	48965928
chr13	48966098	48966263
chr13	48966836	48967010
chr13	48967322	48967529
chr13	48967529	48967809
chr13	48967852	48968168
chr13	48968168	48968428
chr13	48968428	48968864
chr13	48974760	48975046
chr13	48979079	48979624
chr13	48983844	48984013
chr13	48990032	48990487
chr13	48990487	48990749
chr13	48996204	48996540
chr13	48997420	48997604
chr13	48998603	48998915
chr13	49001077	49001396
chr13	49013322	49013673
chr13	49013673	49014006
chr13	49020272	49020429
chr13	49021758	49022018
chr13	49024828	49025085
chr13	49025601	49025756

chr13	49036567	49036759
chr13	49047157	49047349
chr13	49052485	49052763
chr13	49058583	49058824
chr13	49076158	49076308
chr13	49088921	49089210
chr13	49103970	49104198
chr13	49117638	49117871
chr13	49130668	49130852
chr13	49141931	49142221
chr13	49146301	49146518
chr13	49146953	49147201
chr13	49147201	49147444
chr13	49147444	49147671
chr13	49147671	49147925
chr13	49147925	49148213
chr13	49159382	49159532
chr13	49168774	49168984
chr13	49169838	49170136
chr13	49172952	49173263
chr13	49173263	49173560
chr13	49173565	49173723
chr13	49177925	49178194
chr13	49181520	49181762
chr13	49187286	49187621
chr13	49187621	49187904
chr13	49187904	49188197
chr13	49188197	49188434
chr13	49199951	49200254
chr13	49205210	49205429
chr13	49205429	49205748
chr13	49205748	49206042
chr13	49213379	49214016
chr13	49215459	49215758
chr13	49215790	49216207
chr13	49216207	49216436
chr13	49242712	49242862
chr13	49248025	49248322
chr13	49249563	49249960
chr13	49252178	49252432
chr13	49258482	49258813
chr13	49258908	49259186

chr13	49259261	49259420
chr13	49264248	49264533
chr13	49264533	49264823
chr13	49271892	49272042
chr13	49272348	49272645
chr13	49272814	49273181
chr13	49273955	49274127
chr13	49286826	49287029
chr13	49294121	49294467
chr13	49298314	49298889
chr13	49298889	49299147
chr13	49299147	49299463
chr13	49300757	49301231
chr13	49306942	49307276
chr13	49307372	49308013
chr13	49311705	49311911
chr13	49313773	49313956
chr13	49316349	49316823
chr13	49316874	49317049
chr13	49317154	49317306
chr13	49320158	49320435
chr13	49321500	49321745
chr13	49321745	49321946
chr13	49322231	49322513
chr13	49326361	49326520
chr13	49326890	49327136
chr13	49328774	49328956
chr13	49330967	49331209
chr13	49338985	49339170
chr13	49340979	49341297
chr13	49341297	49341848
chr13	49345499	49345826
chr13	49346078	49346332
chr13	49352168	49352389
chr13	49361025	49361218
chr13	49362326	49362595
chr13	49362595	49362798
chr13	49362798	49363096
chr13	49364814	49365041
chr13	49368103	49368327
chr13	49371447	49371886
chr13	49380718	49381076

chr13	49381111	49381600
chr13	49391455	49391674
chr13	49394939	49395386
chr13	49396498	49396791
chr13	49396791	49397184
chr13	49400244	49400452
chr13	49400865	49401182
chr13	49402038	49402369
chr13	49402369	49402663
chr13	49402663	49403083
chr13	49410220	49410436
chr13	49410436	49410831
chr13	49420684	49420977
chr13	49420977	49421322
chr13	49421322	49421616
chr13	49421616	49421912
chr13	49422064	49422285
chr13	49441110	49441279
chr13	49446840	49447164
chr13	49448975	49449129
chr13	49449304	49449523
chr13	49449523	49449788
chr13	49460356	49460553
chr13	49481615	49481782
chr13	49549432	49549706
chr13	49549706	49550006
chr13	49556138	49556525
chr13	49556947	49557289
chr13	49559272	49559450
chr13	49560813	49561047
chr13	49561047	49561274
chr13	49561723	49562069
chr13	49577496	49577741
chr13	49577852	49578158
chr13	49581188	49581380
chr13	49581531	49581794
chr13	49583851	49584282
chr13	49584426	49584639
chr13	49623334	49623484
chr13	49626021	49626249
chr13	49642733	49642951
chr13	49644705	49644963

chr13	49645005	49645178
chr13	49645291	49645446
chr13	49652328	49652723
chr13	49652723	49653201
chr13	49653253	49653518
chr13	49678537	49678763
chr13	49681888	49682512
chr13	49682526	49682732
chr13	49700460	49700637
chr13	49706700	49706950
chr13	49708460	49708624
chr13	49725419	49725570
chr13	49751080	49751312
chr13	49768046	49768426
chr13	49768426	49768743
chr13	49768743	49769109
chr13	49807275	49807434
chr13	50007766	50007916
chr13	50183203	50183427
chr13	50323847	50324034
chr13	50342097	50342294
chr13	50417675	50417977
chr13	50417977	50418325
chr13	50418626	50418931
chr13	50763501	50763788
chr13	50780170	50780345
chr13	50829995	50830145
chr13	50942539	50942984
chr13	50942984	50943387
chr13	50949595	50949933
chr13	50957950	50958796
chr13	50961010	50961212
chr13	50965023	50965278
chr13	50977474	50977726
chr13	50977726	50977963
chr13	50977963	50978262
chr13	51010852	51011023
chr13	51017925	51018083
chr13	51019105	51019608
chr13	51043217	51043421
chr13	51063053	51063334
chr13	51073464	51073815

chr13	51073815	51074108
chr13	51085700	51085850
chr13	51090024	51090293
chr13	51090293	51090561
chr13	51099872	51100092
chr13	51100121	51100584
chr13	51100584	51100788
chr13	51100788	51101006
chr13	51101006	51101363
chr13	51101383	51101595
chr13	51106096	51106295
chr13	51114093	51114333
chr13	51114388	51114794
chr13	51115799	51116193
chr13	51128053	51128286
chr13	51128345	51128763
chr13	51128763	51129064
chr13	51131236	51131437
chr13	51150977	51151187
chr13	51152858	51153549
chr13	51153665	51153885
chr13	51170641	51170874
chr13	51170874	51171095
chr13	51171095	51171304
chr13	51171304	51171495
chr13	51171495	51171757
chr13	51202581	51202872
chr13	51202872	51203133
chr13	51227768	51227987
chr13	51227987	51228205
chr13	51269740	51269900
chr13	51299143	51299310
chr13	51329043	51329255
chr13	51333018	51333168
chr13	51333549	51333783
chr13	51373176	51373338
chr13	51418620	51418915
chr13	51420527	51420677
chr13	51424825	51425011
chr13	51434602	51434752
chr13	51518974	51519298
chr13	51526532	51526831

chr13	51539811	51540193
chr13	51569084	51569269
chr13	51569390	51569630
chr13	51594922	51595072
chr13	51603338	51603501
chr13	51634113	51634423
chr13	51635500	51636193
chr13	51637001	51637165
chr13	51638710	51638995
chr13	51644815	51645447
chr13	51645447	51645826
chr13	51645968	51646160
chr13	51651339	51651802
chr13	51651802	51652134
chr13	51663999	51664236
chr13	51664236	51664489
chr13	51676549	51677089
chr13	51677089	51677456
chr13	51678791	51678995
chr13	51679424	51679683
chr13	51686051	51686206
chr13	51695806	51696201
chr13	51697243	51697466
chr13	51697466	51697784
chr13	51704944	51705149
chr13	51706572	51706808
chr13	51706808	51706986
chr13	51710861	51711083
chr13	51711250	51711413
chr13	51717015	51717413
chr13	51717413	51717631
chr13	51717631	51717822
chr13	51720353	51720515
chr13	51726186	51726593
chr13	51729907	51730064
chr13	51731274	51731582
chr13	51731678	51731867
chr13	51731867	51732149
chr13	51739593	51740001
chr13	51740001	51740340
chr13	51748076	51748472
chr13	51748472	51748793

chr13	51753734	51753915
chr13	51754085	51754396
chr13	51754396	51754737
chr13	51754737	51755063
chr13	51755634	51755845
chr13	51756490	51756858
chr13	51762870	51763801
chr13	51781546	51781765
chr13	51790778	51791200
chr13	51791663	51791920
chr13	51792277	51792570
chr13	51792783	51793032
chr13	51793032	51793331
chr13	51793331	51793622
chr13	51793622	51793905
chr13	51806379	51806625
chr13	51806627	51807010
chr13	51813632	51813925
chr13	51813925	51814257
chr13	51814257	51814475
chr13	51815916	51816196
chr13	51819032	51819279
chr13	51830773	51830952
chr13	51833661	51834016
chr13	51834105	51834463
chr13	51838726	51838876
chr13	51839712	51839972
chr13	51845546	51845903
chr13	51845903	51846186
chr13	51846400	51846794
chr13	51846854	51847506
chr13	51847922	51848144
chr13	51848144	51848409
chr13	51848919	51849135
chr13	51852866	51853184
chr13	51853240	51853626
chr13	51853626	51854072
chr13	51858575	51858786
chr13	51859268	51859488
chr13	51859816	51860085
chr13	51860739	51860913
chr13	51866436	51866773

chr13	51868465	51868727
chr13	51870829	51871074
chr13	51871294	51871444
chr13	51886127	51886414
chr13	51886758	51887257
chr13	51892039	51892515
chr13	51897674	51897925
chr13	51898507	51898776
chr13	51899626	51899786
chr13	51906194	51906523
chr13	51908749	51909468
chr13	51936424	51936574
chr13	51945943	51946242
chr13	51946242	51946520
chr13	51955929	51956345
chr13	51958525	51958726
chr13	51962664	51963095
chr13	51963770	51964044
chr13	51964353	51964624
chr13	51964624	51964858
chr13	51968468	51968817
chr13	51968817	51969219
chr13	51970077	51970454
chr13	51972674	51973014
chr13	51975516	51975851
chr13	51975851	51976412
chr13	51979391	51979568
chr13	51983072	51983283
chr13	51986561	51986833
chr13	51994181	51994509
chr13	51995985	51996272
chr13	51996272	51996562
chr13	52001846	52002118
chr13	52002118	52002385
chr13	52003232	52003423
chr13	52003423	52003622
chr13	52003622	52003973
chr13	52013600	52014171
chr13	52014617	52014861
chr13	52017705	52018253
chr13	52024189	52024386
chr13	52024386	52024722

chr13	52025213	52025466
chr13	52025466	52025808
chr13	52030797	52031068
chr13	52036452	52036610
chr13	52038866	52039202
chr13	52041908	52042127
chr13	52043525	52043805
chr13	52044888	52045255
chr13	52048011	52048287
chr13	52048743	52049185
chr13	52055156	52055520
chr13	52061879	52062291
chr13	52064637	52064975
chr13	52065278	52065689
chr13	52069760	52070097
chr13	52078645	52078947
chr13	52078956	52079154
chr13	52079244	52079404
chr13	52079633	52079935
chr13	52079935	52080338
chr13	52101730	52101880
chr13	52109766	52109952
chr13	52114965	52115283
chr13	52151910	52152128
chr13	52161759	52162168
chr13	52175624	52176045
chr13	52188342	52188577
chr13	52188577	52188888
chr13	52189646	52189857
chr13	52189857	52190066
chr13	52190247	52190686
chr13	52199606	52199871
chr13	52206468	52206618
chr13	52217103	52217258
chr13	52223466	52223903
chr13	52234068	52234311
chr13	52235580	52235902
chr13	52236103	52236766
chr13	52239736	52239956
chr13	52241816	52242105
chr13	52243395	52243830
chr13	52249369	52249946

chr13	52250007	52250187
chr13	52250469	52250701
chr13	52275718	52276059
chr13	52277198	52277628
chr13	52293488	52293707
chr13	52305331	52305729
chr13	52307109	52307405
chr13	52308725	52308888
chr13	52323773	52324014
chr13	52324376	52324968
chr13	52333937	52334151
chr13	52336685	52336980
chr13	52339017	52339676
chr13	52343242	52343397
chr13	52344442	52344742
chr13	52344831	52345359
chr13	52345650	52346014
chr13	52361521	52361736
chr13	52449052	52449238
chr13	52455179	52455448
chr13	52455448	52455741
chr13	52455843	52456146
chr13	52469682	52470178
chr13	52477985	52478292
chr13	52478292	52478614
chr13	52481777	52482294
chr13	52488577	52489191
chr13	52489545	52489853
chr13	52495558	52496091
chr13	52510175	52510459
chr13	52515997	52516403
chr13	52518407	52518609
chr13	52530251	52530679
chr13	52530679	52530984
chr13	52535619	52535875
chr13	52563897	52564052
chr13	52565492	52565703
chr13	52568053	52568230
chr13	52580409	52580705
chr13	52580876	52581185
chr13	52582585	52582891
chr13	52582920	52583175

chr13	52583175	52583691
chr13	52587772	52588204
chr13	52602913	52603089
chr13	52623143	52623455
chr13	52623537	52623957
chr13	52624086	52624375
chr13	52628611	52628926
chr13	52629059	52629229
chr13	52651391	52651670
chr13	52651849	52652307
chr13	52658579	52658812
chr13	52658812	52659089
chr13	52659089	52659371
chr13	52659371	52659856
chr13	52669817	52670335
chr13	52673359	52673645
chr13	52675569	52675927
chr13	52675952	52676307
chr13	52676609	52677001
chr13	52695013	52695203
chr13	52702049	52702316
chr13	52707449	52707664
chr13	52711756	52712033
chr13	52712033	52712340
chr13	52726163	52726334
chr13	52765510	52765773
chr13	52765773	52766083
chr13	52770512	52770733
chr13	52807683	52807898
chr13	52809067	52809447
chr13	52818812	52819235
chr13	52821557	52822031
chr13	52828016	52828166
chr13	52828274	52828437
chr13	52828920	52829451
chr13	52829574	52829746
chr13	52833307	52833532
chr13	52850398	52850649
chr13	52860406	52860569
chr13	52866049	52866273
chr13	52873919	52874377
chr13	52874501	52874704

chr13	52876055	52876205
chr13	52883171	52883417
chr13	52891364	52891555
chr13	52891555	52891746
chr13	52891813	52891978
chr13	52892000	52892315
chr13	52892315	52892659
chr13	52892876	52893187
chr13	52893187	52893568
chr13	52900249	52900516
chr13	52903823	52904016
chr13	52929197	52929813
chr13	52929813	52930112
chr13	52966135	52966323
chr13	52971175	52971453
chr13	52973370	52973967
chr13	52978421	52978633
chr13	52980202	52980505
chr13	52980505	52980881
chr13	52980881	52981306
chr13	52981306	52981652
chr13	52981652	52981947
chr13	52981947	52982227
chr13	52982727	52982923
chr13	52982928	52983112
chr13	52987472	52987817
chr13	52987900	52988304
chr13	52988444	52988732
chr13	52994281	52994528
chr13	52995278	52995474
chr13	53001435	53001909
chr13	53001909	53002269
chr13	53008530	53008794
chr13	53008794	53009403
chr13	53018131	53018421
chr13	53019045	53019279
chr13	53019345	53019613
chr13	53019613	53019857
chr13	53026623	53026799
chr13	53033549	53034189
chr13	53060616	53060804
chr13	53064764	53065168

chr13	53065242	53065494
chr13	53079647	53079800
chr13	53088495	53088660
chr13	53093367	53093784
chr13	53093789	53093976
chr13	53096733	53096966
chr13	53099691	53100074
chr13	53109541	53109964
chr13	53118442	53118701
chr13	53119792	53120031
chr13	53125697	53125868
chr13	53151272	53151531
chr13	53152888	53153171
chr13	53160475	53160865
chr13	53187410	53187583
chr13	53211948	53212168
chr13	53232183	53232377
chr13	53232416	53232785
chr13	53240863	53241044
chr13	53247455	53247662
chr13	53248644	53249012
chr13	53249012	53249554
chr13	53261486	53261640
chr13	53267991	53268166
chr13	53269800	53270064
chr13	53279394	53279719
chr13	53285599	53286043
chr13	53286043	53286389
chr13	53298614	53298933
chr13	53299094	53299410
chr13	53315843	53316058
chr13	53316462	53316959
chr13	53338700	53338908
chr13	53338919	53339198
chr13	53339198	53339566
chr13	53339566	53339898
chr13	53342283	53342572
chr13	53356199	53356440
chr13	53356440	53356712
chr13	53361324	53361557
chr13	53377038	53377821
chr13	53390151	53390679

chr13	53398979	53399324
chr13	53400024	53400369
chr13	53402252	53402418
chr13	53402421	53402572
chr13	53403601	53403787
chr13	53404248	53404535
chr13	53404535	53404887
chr13	53407309	53407476
chr13	53412907	53413099
chr13	53413174	53413741
chr13	53414707	53415044
chr13	53426538	53426859
chr13	53430975	53431142
chr13	53431273	53431511
chr13	53439563	53439879
chr13	53439951	53440363
chr13	53442666	53442865
chr13	53443687	53444162
chr13	53445754	53446034
chr13	53447640	53447955
chr13	53461733	53462176
chr13	53462176	53462473
chr13	53462473	53462799
chr13	53463047	53463251
chr13	53471460	53471610
chr13	53472225	53472698
chr13	53472698	53473007
chr13	53475658	53475874
chr13	53475874	53476268
chr13	53496653	53496862
chr13	53501158	53501319
chr13	53501947	53502162
chr13	53502270	53502799
chr13	53503005	53503178
chr13	53503178	53503360
chr13	53511403	53511711
chr13	53514516	53514965
chr13	53535200	53535752
chr13	53551499	53551677
chr13	53558893	53559275
chr13	53567531	53568011
chr13	53584709	53585095

chr13	53585095	53585459
chr13	53588361	53588728
chr13	53592851	53593075
chr13	53598132	53598318
chr13	53614880	53615219
chr13	53627776	53628092
chr13	53652867	53653283
chr13	53654568	53655139
chr13	53658235	53658512
chr13	53659174	53659651
chr13	53662804	53663289
chr13	53664729	53664940
chr13	53680531	53680723
chr13	53684784	53685042
chr13	53685042	53685410
chr13	53688522	53688821
chr13	53694573	53695006
chr13	53695432	53695740
chr13	53699473	53699743
chr13	53717271	53717458
chr13	53717565	53717896
chr13	53721085	53721626
chr13	53729954	53730338
chr13	53730368	53730785
chr13	53731306	53731534
chr13	53734577	53734849
chr13	53734849	53735106
chr13	53764736	53765214
chr13	53770133	53770660
chr13	53775733	53776061
chr13	53778674	53778876
chr13	53779214	53779400
chr13	53783538	53783791
chr13	53815704	53816052
chr13	53920151	53920381
chr13	54019671	54020211
chr13	54020211	54020550
chr13	54055408	54055894
chr13	54069716	54070057
chr13	54071631	54071995
chr13	54071995	54072502
chr13	54075525	54075724

chr13	54078677	54078854
chr13	54100779	54101146
chr13	54101146	54101450
chr13	54101450	54101815
chr13	54102151	54102389
chr13	54104006	54104176
chr13	54113855	54114137
chr13	54117993	54118346
chr13	54152522	54152694
chr13	54189970	54190224
chr13	54231860	54232359
chr13	54247625	54247920
chr13	54296090	54296299
chr13	54302656	54303044
chr13	54303044	54303346
chr13	54303346	54303662
chr13	54303770	54303987
chr13	54323069	54323331
chr13	54371350	54371625
chr13	54371625	54372032
chr13	54372843	54373087
chr13	54415212	54415392
chr13	54446859	54447009
chr13	54447044	54447416
chr13	54447950	54448177
chr13	54457135	54457395
chr13	54468277	54468629
chr13	54468629	54469043
chr13	54469063	54469237
chr13	54483918	54484189
chr13	54486265	54486425
chr13	54488531	54488735
chr13	54503313	54503599
chr13	54503599	54503874
chr13	54503874	54504108
chr13	54504108	54504312
chr13	54504312	54504563
chr13	54520349	54520525
chr13	54524908	54525300
chr13	54525300	54525790
chr13	54525790	54526293
chr13	54527165	54527501

chr13	54535931	54536523
chr13	54564306	54564486
chr13	54564707	54564892
chr13	54564892	54565180
chr13	54565180	54565597
chr13	54565630	54565889
chr13	54569900	54570065
chr13	54574828	54575192
chr13	54575192	54575530
chr13	54575552	54575746
chr13	54575746	54575929
chr13	54589619	54589771
chr13	54589771	54589936
chr13	54589945	54590348
chr13	54590348	54590958
chr13	54596104	54596423
chr13	54605477	54605882
chr13	54608152	54608504
chr13	54608504	54608848
chr13	54608848	54609080
chr13	54610631	54610921
chr13	54610921	54611205
chr13	54611205	54611474
chr13	54611474	54611706
chr13	54617102	54617316
chr13	54621554	54621925
chr13	54621925	54622138
chr13	54622138	54622328
chr13	54622328	54622592
chr13	54625608	54625794
chr13	54647023	54647905
chr13	54660987	54661150
chr13	54661217	54661700
chr13	54661700	54661976
chr13	54670379	54670823
chr13	54671772	54671934
chr13	54675790	54676274
chr13	54678442	54678666
chr13	54678669	54678873
chr13	54686369	54686607
chr13	54687108	54687486
chr13	54687486	54687742

chr13	54687742	54688003
chr13	54688003	54688472
chr13	54693505	54694136
chr13	54694136	54694425
chr13	54694425	54694698
chr13	54701291	54701546
chr13	54703797	54704316
chr13	54708586	54708777
chr13	54708965	54709292
chr13	54709292	54709605
chr13	54710944	54711212
chr13	54716062	54716486
chr13	54716981	54717348
chr13	54719602	54719961
chr13	54737639	54737913
chr13	54737913	54738350
chr13	54748885	54749257
chr13	54749257	54749829
chr13	54754203	54754420
chr13	54757120	54757421
chr13	54758043	54758339
chr13	54758339	54758917
chr13	54766036	54766207
chr13	54766267	54766745
chr13	54768875	54769055
chr13	54780718	54781029
chr13	54788956	54789285
chr13	54789285	54789570
chr13	54789570	54789837
chr13	54789837	54790128
chr13	54797476	54797656
chr13	54799496	54799750
chr13	54799750	54799992
chr13	54800011	54800188
chr13	54813222	54813735
chr13	54813735	54814082
chr13	54821217	54821418
chr13	54828115	54828340
chr13	54828641	54828794
chr13	54868013	54868214
chr13	54911781	54911940
chr13	54945432	54945647

chr13	54948929	54949294
chr13	54949294	54949559
chr13	54949559	54949978
chr13	54951302	54951469
chr13	54955754	54955959
chr13	54955977	54956485
chr13	54970943	54971161
chr13	54973830	54974020
chr13	54997962	54998113
chr13	55004593	55004826
chr13	55004826	55005044
chr13	55005139	55005365
chr13	55005533	55005705
chr13	55005705	55005812
chr13	55011938	55012263
chr13	55087639	55087815
chr13	55099388	55099757
chr13	55099757	55100085
chr13	55100085	55100460
chr13	55105075	55105423
chr13	55105423	55105824
chr13	55107867	55108046
chr13	55135262	55135422
chr13	55135422	55135606
chr13	55149188	55149415
chr13	55149832	55150179
chr13	55151196	55151573
chr13	55171588	55171908
chr13	55171908	55172196
chr13	55172581	55172790
chr13	55178667	55178896
chr13	55183347	55183796
chr13	55184297	55184506
chr13	55184518	55184917
chr13	55187056	55187400
chr13	55188784	55189096
chr13	55189278	55189428
chr13	55189453	55189672
chr13	55189672	55190060
chr13	55190060	55190530
chr13	55190530	55190797
chr13	55192785	55192937

chr13	55211125	55211402
chr13	55211402	55211690
chr13	55211690	55211994
chr13	55211994	55212332
chr13	55212332	55212596
chr13	55212596	55212844
chr13	55212891	55213095
chr13	55230781	55231002
chr13	55269559	55269966
chr13	55319953	55320492
chr13	55321436	55321670
chr13	55321670	55321894
chr13	55321894	55322140
chr13	55322140	55322360
chr13	55322501	55322738
chr13	55327388	55327644
chr13	55327675	55328065
chr13	55329187	55329427
chr13	55329427	55329687
chr13	55329687	55329950
chr13	55329950	55330134
chr13	55330134	55330374
chr13	55330374	55330705
chr13	55339294	55339691
chr13	55343301	55343452
chr13	55350483	55350745
chr13	55351251	55351422
chr13	55352831	55352989
chr13	55354587	55354775
chr13	55362075	55362605
chr13	55362605	55363206
chr13	55368481	55368710
chr13	55369068	55369363
chr13	55369363	55369589
chr13	55369589	55369809
chr13	55369912	55370195
chr13	55378932	55379290
chr13	55379290	55379557
chr13	55380143	55380349
chr13	55386273	55386435
chr13	55388163	55388321
chr13	55393015	55393183

chr13	55399863	55400023
chr13	55404478	55404722
chr13	55405440	55405771
chr13	55417948	55418568
chr13	55420621	55420800
chr13	55421194	55421425
chr13	55422251	55422468
chr13	55425968	55426211
chr13	55429266	55429554
chr13	55430677	55430878
chr13	55444453	55444865
chr13	55444865	55445152
chr13	55445152	55445648
chr13	55450199	55450459
chr13	55450459	55450987
chr13	55464031	55464328
chr13	55464328	55464610
chr13	55464610	55464940
chr13	55465163	55465816
chr13	55469280	55469442
chr13	55471218	55471445
chr13	55471445	55471803
chr13	55471803	55472214
chr13	55472214	55472525
chr13	55472525	55472747
chr13	55472747	55473014
chr13	55475893	55476072
chr13	55482600	55482774
chr13	55487082	55487321
chr13	55487321	55487556
chr13	55487556	55487804
chr13	55487808	55488620
chr13	55493752	55493946
chr13	55493946	55494126
chr13	55494277	55494554
chr13	55494644	55494818
chr13	55496759	55496948
chr13	55498002	55498242
chr13	55498453	55498627
chr13	55498770	55499016
chr13	55499749	55499932
chr13	55510368	55510639

chr13	55510639	55511007
chr13	55511007	55511402
chr13	55511402	55511853
chr13	55512697	55512917
chr13	55513113	55513390
chr13	55513390	55513708
chr13	55517063	55517213
chr13	55522866	55523048
chr13	55524696	55524857
chr13	55526115	55526348
chr13	55527149	55527553
chr13	55528720	55528952
chr13	55535994	55536217
chr13	55536217	55536524
chr13	55536524	55536944
chr13	55536966	55537132
chr13	55548472	55548784
chr13	55569748	55569950
chr13	55570194	55570416
chr13	55570416	55570615
chr13	55570615	55570922
chr13	55570922	55571275
chr13	55571275	55571536
chr13	55576041	55576246
chr13	55578906	55579089
chr13	55592805	55593258
chr13	55593258	55593634
chr13	55599732	55600256
chr13	55600709	55601025
chr13	55604594	55605066
chr13	55611245	55611468
chr13	55611802	55611952
chr13	55612703	55612951
chr13	55619093	55619243
chr13	55622404	55622852
chr13	55622852	55623130
chr13	55623130	55623334
chr13	55623334	55623545
chr13	55634587	55634849
chr13	55634849	55635128
chr13	55635128	55635547
chr13	55640529	55640826

chr13	55644614	55644887
chr13	55650192	55650403
chr13	55668633	55668883
chr13	55670695	55670845
chr13	55675450	55675604
chr13	55683625	55683904
chr13	55692181	55692409
chr13	55692409	55692789
chr13	55692789	55693630
chr13	55704386	55704619
chr13	55714285	55714471
chr13	55714486	55714772
chr13	55714772	55715283
chr13	55727034	55727594
chr13	55727594	55727863
chr13	55727925	55728242
chr13	55737977	55738197
chr13	55757717	55757926
chr13	55786204	55786409
chr13	55789966	55790181
chr13	55824174	55824462
chr13	55828599	55828794
chr13	55830940	55831109
chr13	55831109	55831304
chr13	55831304	55831585
chr13	55835738	55835898
chr13	55835901	55836162
chr13	55836850	55837018
chr13	55837110	55837395
chr13	55862052	55862321
chr13	55866977	55867148
chr13	55867453	55867979
chr13	55868591	55868793
chr13	55894734	55894921
chr13	55895432	55895853
chr13	55897990	55898187
chr13	55902384	55902599
chr13	55911018	55911305
chr13	55912118	55912311
chr13	55919876	55920160
chr13	55920445	55920824
chr13	55923593	55923783

chr13	55934058	55934349
chr13	55936998	55937279
chr13	55954417	55954685
chr13	55963140	55963334
chr13	55963334	55963586
chr13	55974215	55974434
chr13	55977065	55977374
chr13	55992628	55993069
chr13	55993069	55993326
chr13	55996510	55996775
chr13	56004048	56004306
chr13	56005781	56005994
chr13	56006919	56007074
chr13	56007406	56007591
chr13	56008126	56008448
chr13	56021448	56022099
chr13	56026761	56027061
chr13	56027648	56027862
chr13	56028037	56028351
chr13	56029092	56029398
chr13	56039678	56040030
chr13	56040030	56040349
chr13	56045132	56045450
chr13	56067301	56067677
chr13	56072348	56072577
chr13	56077012	56077164
chr13	56080640	56080838
chr13	56084112	56084267
chr13	56090701	56090888
chr13	56092550	56092929
chr13	56094293	56094454
chr13	56113517	56114052
chr13	56115399	56115677
chr13	56120200	56120350
chr13	56134548	56134796
chr13	56134796	56135246
chr13	56135246	56136033
chr13	56137520	56137745
chr13	56142468	56142729
chr13	56145640	56145790
chr13	56151797	56152172
chr13	56159423	56159588

chr13	56167551	56167754
chr13	56172655	56172815
chr13	56197507	56197792
chr13	56197792	56198016
chr13	56201400	56201716
chr13	56251785	56251940
chr13	56251942	56252092
chr13	56266536	56266774
chr13	56275224	56275414
chr13	56276537	56276708
chr13	56279485	56279936
chr13	56279968	56280135
chr13	56295851	56296194
chr13	56296194	56296826
chr13	56303745	56304097
chr13	56304097	56304246
chr13	56304246	56304451
chr13	56304718	56304914
chr13	56311835	56312069
chr13	56327860	56328059
chr13	56330810	56331154
chr13	56334311	56334461
chr13	56335887	56336153
chr13	56345865	56346222
chr13	56346475	56347128
chr13	56352398	56352744
chr13	56362212	56362651
chr13	56362682	56362961
chr13	56365685	56365919
chr13	56370664	56370817
chr13	56387624	56388041
chr13	56396670	56397097
chr13	56403983	56404243
chr13	56404432	56404866
chr13	56404866	56405350
chr13	56412498	56412864
chr13	56418616	56418811
chr13	56420411	56420758
chr13	56422398	56422634
chr13	56425724	56425905
chr13	56425965	56426698
chr13	56429139	56429481

chr13	56429495	56429691
chr13	56437832	56438172
chr13	56438172	56438507
chr13	56438507	56438747
chr13	56438747	56438976
chr13	56440872	56441130
chr13	56443467	56443784
chr13	56443784	56444039
chr13	56444083	56444566
chr13	56449566	56450513
chr13	56456493	56456715
chr13	56475112	56475357
chr13	56475405	56475693
chr13	56476544	56476724
chr13	56476795	56477216
chr13	56487931	56488321
chr13	56501932	56502110
chr13	56503011	56503427
chr13	56508375	56508599
chr13	56511041	56511211
chr13	56516244	56516623
chr13	56516840	56517091
chr13	56522062	56522353
chr13	56522353	56522809
chr13	56522900	56523050
chr13	56535724	56535938
chr13	56535938	56536172
chr13	56539627	56539836
chr13	56549367	56549920
chr13	56554467	56554704
chr13	56556058	56556264
chr13	56567161	56567311
chr13	56575654	56575926
chr13	56581407	56581625
chr13	56582214	56582469
chr13	56582653	56583140
chr13	56585788	56586062
chr13	56586147	56586426
chr13	56588660	56588830
chr13	56589152	56589454
chr13	56601151	56601609
chr13	56607339	56607569

chr13	56607834	56608148
chr13	56608148	56608375
chr13	56608981	56609400
chr13	56609400	56609695
chr13	56609709	56609894
chr13	56614368	56614878
chr13	56626336	56626947
chr13	56627469	56627619
chr13	56634902	56635172
chr13	56635172	56635495
chr13	56641201	56641378
chr13	56642729	56642934
chr13	56647948	56648420
chr13	56648420	56649209
chr13	56658142	56658403
chr13	56658403	56658657
chr13	56664057	56664242
chr13	56664295	56664863
chr13	56664863	56665172
chr13	56668088	56668238
chr13	56670891	56671078
chr13	56679121	56679503
chr13	56682224	56682900
chr13	56692021	56692434
chr13	56702647	56702875
chr13	56702875	56703197
chr13	56703197	56703520
chr13	56703520	56703905
chr13	56703905	56704339
chr13	56718696	56718930
chr13	56734732	56734929
chr13	56735635	56735790
chr13	56744629	56744846
chr13	56744846	56745102
chr13	56745102	56745328
chr13	56745328	56745512
chr13	56745512	56745707
chr13	56758559	56758888
chr13	56761134	56761704
chr13	56766239	56766810
chr13	56772683	56772915
chr13	56786072	56786455

chr13	56792970	56793188
chr13	56794045	56794302
chr13	56794302	56794632
chr13	56801612	56801812
chr13	56806794	56807129
chr13	56987180	56987438
chr13	57017725	57018028
chr13	57228349	57228514
chr13	57229190	57229350
chr13	57259990	57260240
chr13	57269717	57269981
chr13	57598081	57598261
chr13	57908090	57908272
chr13	57908272	57908534
chr13	57909333	57909483
chr13	57910426	57910858
chr13	57970760	57970953
chr13	57970953	57971238
chr13	57971272	57971526
chr13	57971614	57971874
chr13	57972097	57972298
chr13	58012185	58012454
chr13	58031848	58032017
chr13	58035565	58035752
chr13	58035752	58035986
chr13	58067847	58068079
chr13	58113046	58113312
chr13	58113429	58113740
chr13	58115267	58115588
chr13	58117301	58117451
chr13	58120602	58120853
chr13	58128021	58128173
chr13	58128176	58128440
chr13	58128440	58128764
chr13	58128764	58129174
chr13	58129174	58129544
chr13	58146757	58147092
chr13	58148333	58148570
chr13	58148570	58148859
chr13	58149073	58149337
chr13	58149337	58149639
chr13	58149681	58150027

chr13	58157463	58157766
chr13	58157766	58158106
chr13	58158151	58158386
chr13	58158386	58158612
chr13	58158612	58158923
chr13	58168976	58169126
chr13	58188460	58188778
chr13	58189121	58189285
chr13	58189559	58189720
chr13	58191974	58192148
chr13	58215055	58215304
chr13	58215304	58215544
chr13	58215544	58215808
chr13	58215808	58216081
chr13	58216081	58216383
chr13	58232692	58232944
chr13	58247439	58247589
chr13	58273456	58273797
chr13	58273797	58274087
chr13	58274087	58274358
chr13	58274358	58274688
chr13	58274688	58274993
chr13	58282018	58282264
chr13	58287885	58288165
chr13	58292863	58293056
chr13	58298834	58299082
chr13	58311442	58311670
chr13	58334201	58334434
chr13	58335977	58336161
chr13	58346324	58346666
chr13	58346666	58346990
chr13	58346990	58347326
chr13	58347326	58347603
chr13	58347603	58347846
chr13	58347846	58348207
chr13	58348207	58348623
chr13	58348623	58348936
chr13	58348936	58349334
chr13	58349334	58349730
chr13	58349730	58349934
chr13	58358668	58359354
chr13	58359354	58359611

chr13	58384740	58385057
chr13	58385057	58385555
chr13	58387955	58388243
chr13	58388243	58388477
chr13	58388477	58388662
chr13	58389158	58389497
chr13	58389497	58389768
chr13	58401365	58401676
chr13	58401676	58401974
chr13	58401974	58402350
chr13	58402350	58402738
chr13	58402738	58403051
chr13	58403051	58403324
chr13	58407599	58407777
chr13	58418655	58418805
chr13	58443900	58444130
chr13	58458726	58459393
chr13	58459909	58460444
chr13	58465253	58465554
chr13	58465554	58465792
chr13	58465792	58466033
chr13	58466033	58466267
chr13	58466640	58466990
chr13	58474803	58475080
chr13	58475330	58475913
chr13	58476053	58476251
chr13	58480996	58481146
chr13	58481245	58481692
chr13	58500618	58500844
chr13	58516292	58516903
chr13	58520039	58520375
chr13	58534230	58534722
chr13	58534722	58535214
chr13	58541716	58541911
chr13	58543207	58543680
chr13	58544097	58544284
chr13	58545299	58545537
chr13	58547584	58547885
chr13	58552005	58552331
chr13	58556102	58556317
chr13	58588031	58588300
chr13	58589171	58589547

chr13	58589547	58589843
chr13	58590707	58590935
chr13	58590935	58591104
chr13	58593218	58593476
chr13	58606476	58606926
chr13	58622491	58622993
chr13	58622993	58623312
chr13	58624829	58625063
chr13	58629460	58629679
chr13	58630693	58631073
chr13	58631073	58631412
chr13	58631412	58631730
chr13	58631730	58632088
chr13	58637408	58637657
chr13	58639037	58639195
chr13	58640482	58640660
chr13	58647164	58647343
chr13	58653971	58654632
chr13	58654632	58655085
chr13	58668795	58668983
chr13	58675751	58675906
chr13	58716650	58716874
chr13	58794178	58794352
chr13	58806408	58806571
chr13	58912682	58912837
chr13	58971406	58971591
chr13	59014967	59015136
chr13	59073699	59073859
chr13	59228143	59228616
chr13	59308428	59308712
chr13	59365547	59365751
chr13	59385899	59386130
chr13	59387944	59388229
chr13	59392163	59392441
chr13	59392868	59393282
chr13	59393357	59393522
chr13	59434687	59434866
chr13	59452512	59452971
chr13	59452971	59453533
chr13	59453548	59453890
chr13	59454111	59454320
chr13	59455901	59456132

chr13	59456306	59456513
chr13	59460767	59461292
chr13	59461292	59461654
chr13	59467712	59467990
chr13	59468303	59468502
chr13	59494981	59495132
chr13	59509799	59510045
chr13	59510045	59510322
chr13	59515137	59515460
chr13	59556592	59557108
chr13	59557108	59557427
chr13	59557427	59557656
chr13	59557656	59557842
chr13	59560893	59561048
chr13	59561504	59561944
chr13	59562571	59562794
chr13	59584465	59584760
chr13	59584970	59585418
chr13	59585418	59586009
chr13	59604272	59604537
chr13	59604537	59604914
chr13	59624428	59624812
chr13	59624824	59625240
chr13	59632575	59632727
chr13	59633618	59633752
chr13	59633752	59633892
chr13	59647103	59647297
chr13	59647906	59648096
chr13	59656458	59656807
chr13	59656809	59656964
chr13	59660460	59660622
chr13	59663291	59663571
chr13	59663571	59663930
chr13	59663984	59664240
chr13	59668369	59668948
chr13	59668948	59669417
chr13	59674213	59674436
chr13	59674759	59675123
chr13	59675331	59676054
chr13	59679430	59679732
chr13	59683761	59684048
chr13	59718816	59719121

chr13	59728315	59728755
chr13	59728765	59729098
chr13	59735272	59735520
chr13	59738165	59738431
chr13	59738431	59738609
chr13	59746247	59746460
chr13	59746758	59747159
chr13	59753532	59753733
chr13	59769269	59769485
chr13	59769485	59769734
chr13	59769734	59770119
chr13	59770119	59770573
chr13	59779321	59779525
chr13	59822508	59822758
chr13	59822758	59823018
chr13	59823018	59823454
chr13	59829040	59829285
chr13	59834756	59835010
chr13	59835213	59835415
chr13	59836053	59836310
chr13	59840482	59840716
chr13	59840797	59841134
chr13	59842270	59842455
chr13	59842553	59842808
chr13	59842877	59843143
chr13	59853543	59853793
chr13	59856370	59856603
chr13	59870060	59870316
chr13	59875640	59875843
chr13	59889827	59890018
chr13	60019624	60019781
chr13	60019871	60020131
chr13	60096704	60097644
chr13	60098526	60098685
chr13	60110494	60110794
chr13	60166484	60166755
chr13	60176108	60176455
chr13	60176455	60176890
chr13	60176890	60177364
chr13	60177448	60177664
chr13	60177664	60177890
chr13	60178176	60178347

chr13	60178498	60178774
chr13	60195866	60196081
chr13	60196146	60196302
chr13	60221692	60221894
chr13	60427168	60427472
chr13	60427737	60428083
chr13	60428083	60428410
chr13	60431479	60431845
chr13	60431890	60432120
chr13	60438300	60438511
chr13	60453883	60454158
chr13	60454158	60454627
chr13	60455039	60455195
chr13	60455313	60455487
chr13	60460665	60460866
chr13	60464542	60464736
chr13	60479036	60479292
chr13	60479478	60479944
chr13	60548150	60548300
chr13	60558475	60558656
chr13	60601693	60602024
chr13	60602024	60602355
chr13	60602355	60602878
chr13	60608539	60608700
chr13	60617852	60618071
chr13	60618441	60618707
chr13	60644675	60645005
chr13	60662533	60662882
chr13	60662882	60663256
chr13	60664251	60664614
chr13	60672291	60672743
chr13	60672815	60673163
chr13	60676526	60676723
chr13	60680963	60681180
chr13	60681180	60681483
chr13	60681483	60681815
chr13	60681815	60682209
chr13	60683145	60683498
chr13	60684197	60684614
chr13	60728281	60728783
chr13	60728857	60729122
chr13	60737711	60737922

chr13	60737922	60738422
chr13	60738429	60738771
chr13	60744013	60744231
chr13	60744231	60744477
chr13	60748615	60748946
chr13	60752355	60752724
chr13	60757188	60757576
chr13	60759255	60759406
chr13	60799723	60799997
chr13	60812941	60813152
chr13	60813760	60814129
chr13	60820515	60820804
chr13	60820804	60821336
chr13	60848300	60848516
chr13	60854328	60854721
chr13	60855076	60855358
chr13	60855451	60855613
chr13	60864328	60864655
chr13	60864732	60864917
chr13	60864917	60865249
chr13	60866291	60866529
chr13	60897256	60897712
chr13	60897712	60898074
chr13	60932388	60932852
chr13	60936391	60937042
chr13	60953378	60953601
chr13	60953880	60954130
chr13	60956735	60956902
chr13	60956960	60957398
chr13	60959204	60959535
chr13	60959535	60959784
chr13	60963919	60964110
chr13	60964112	60964392
chr13	60972458	60972638
chr13	60987012	60987249
chr13	61018056	61018304
chr13	61241594	61241861
chr13	61265875	61266168
chr13	61266339	61266520
chr13	61375487	61375917
chr13	61375917	61376210
chr13	61433176	61433579

chr13	61451569	61452238
chr13	61535054	61535384
chr13	61542419	61542623
chr13	61710903	61711223
chr13	61725652	61725883
chr13	61725883	61726164
chr13	61803878	61804108
chr13	61804108	61804355
chr13	61804355	61804562
chr13	61804562	61804759
chr13	61998457	61998612
chr13	61998789	61999127
chr13	62015515	62015752
chr13	62015752	62016000
chr13	62016000	62016203
chr13	62016203	62016398
chr13	62037473	62038057
chr13	62082263	62082503
chr13	62083691	62084239
chr13	62093262	62093472
chr13	62129706	62130063
chr13	62130063	62130371
chr13	62153238	62153492
chr13	62153500	62153661
chr13	62285706	62285864
chr13	62291870	62292080
chr13	62293465	62293905
chr13	62296433	62296679
chr13	62382735	62383138
chr13	62383138	62383370
chr13	62383370	62383592
chr13	62466372	62466754
chr13	62466754	62467006
chr13	62467006	62467238
chr13	62558152	62558444
chr13	62558444	62558783
chr13	62558783	62559024
chr13	62623881	62624358
chr13	62652768	62653070
chr13	62653070	62653403
chr13	62653403	62653637
chr13	62684486	62685121

chr13	62685121	62685364
chr13	62760252	62760407
chr13	62760420	62760901
chr13	62785299	62785476
chr13	62785488	62785996
chr13	62785998	62786212
chr13	62807993	62808218
chr13	62812933	62813114
chr13	62834523	62834719
chr13	62856220	62856486
chr13	62856486	62856729
chr13	62858295	62858581
chr13	62858581	62858869
chr13	62877346	62877540
chr13	62919698	62919909
chr13	62920020	62920246
chr13	62926547	62926801
chr13	62964640	62965433
chr13	62980447	62980746
chr13	63031394	63031658
chr13	63031658	63031909
chr13	63038541	63038691
chr13	63045887	63046254
chr13	63046254	63046577
chr13	63046577	63046980
chr13	63047050	63047295
chr13	63066368	63066663
chr13	63066663	63066926
chr13	63066926	63067137
chr13	63072388	63072735
chr13	63082626	63082776
chr13	63087944	63088362
chr13	63088362	63088669
chr13	63088838	63089152
chr13	63089152	63089516
chr13	63092359	63092642
chr13	63093002	63093217
chr13	63093508	63093946
chr13	63106603	63106936
chr13	63109494	63109746
chr13	63117883	63118053
chr13	63120391	63120557

chr13	63121133	63121364
chr13	63134811	63135442
chr13	63140820	63141261
chr13	63141447	63141657
chr13	63146060	63146275
chr13	63154605	63154875
chr13	63154875	63155416
chr13	63164199	63164349
chr13	63165412	63165567
chr13	63165733	63166175
chr13	63176356	63176513
chr13	63177354	63177510
chr13	63182062	63182238
chr13	63186847	63187552
chr13	63187552	63187977
chr13	63193439	63193982
chr13	63205723	63206069
chr13	63214792	63215209
chr13	63216452	63216871
chr13	63216871	63217229
chr13	63221972	63222155
chr13	63230925	63231286
chr13	63231286	63231685
chr13	63231731	63231967
chr13	63239546	63239797
chr13	63239808	63240254
chr13	63240268	63240478
chr13	63240481	63240663
chr13	63240879	63241187
chr13	63241187	63241492
chr13	63243599	63243961
chr13	63251583	63251805
chr13	63253128	63253532
chr13	63254002	63254204
chr13	63254419	63254605
chr13	63255442	63255644
chr13	63255644	63255787
chr13	63255804	63256146
chr13	63256794	63257147
chr13	63258171	63258693
chr13	63266168	63266667
chr13	63270336	63270493

chr13	63272375	63273323
chr13	63274002	63274202
chr13	63284435	63284590
chr13	63297089	63297420
chr13	63303460	63303728
chr13	63304004	63304427
chr13	63304427	63304773
chr13	63304778	63305089
chr13	63305089	63305504
chr13	63311184	63311497
chr13	63316456	63316670
chr13	63318675	63318970
chr13	63319037	63319460
chr13	63324895	63325209
chr13	63325256	63325584
chr13	63331484	63331701
chr13	63332678	63332899
chr13	63335827	63336077
chr13	63341626	63341841
chr13	63347763	63347951
chr13	63356627	63356861
chr13	63365362	63365617
chr13	63421293	63421518
chr13	63430766	63431041
chr13	63431041	63431364
chr13	63431364	63431651
chr13	63431651	63432071
chr13	63432851	63433025
chr13	63480137	63480369
chr13	63500649	63500801
chr13	63507965	63508115
chr13	63513243	63513403
chr13	63513549	63513699
chr13	63520952	63521400
chr13	63548425	63549066
chr13	63556540	63556690
chr13	63563061	63563249
chr13	63563278	63563585
chr13	63563782	63563973
chr13	63564178	63564409
chr13	63564852	63565262
chr13	63565338	63565747

chr13	63565918	63566182
chr13	63566184	63566497
chr13	63567809	63568297
chr13	63572986	63573228
chr13	63573549	63573699
chr13	63578811	63579317
chr13	63581205	63581436
chr13	63614513	63615004
chr13	63623832	63624134
chr13	63642624	63642958
chr13	63642958	63643310
chr13	63643343	63643764
chr13	63644312	63644635
chr13	63654568	63654840
chr13	63657186	63657567
chr13	63672465	63672702
chr13	63682288	63682696
chr13	63696497	63696711
chr13	63700774	63701159
chr13	63701855	63702108
chr13	63716522	63716745
chr13	63724237	63724687
chr13	63724699	63725049
chr13	63737239	63737818
chr13	63737818	63738175
chr13	63748881	63749215
chr13	63749215	63749506
chr13	63749960	63750480
chr13	63756137	63756346
chr13	63770928	63771212
chr13	63771212	63771469
chr13	63814771	63815088
chr13	63815088	63815411
chr13	63815411	63815732
chr13	63845823	63846065
chr13	63847069	63847479
chr13	63865440	63865675
chr13	63879148	63879534
chr13	63915978	63916183
chr13	63916595	63916933
chr13	63916969	63917620
chr13	63929492	63929652

chr13	63954380	63954617
chr13	63975150	63975322
chr13	63994998	63995175
chr13	63999426	63999728
chr13	64002970	64003145
chr13	64004292	64004527
chr13	64011881	64012048
chr13	64030860	64031054
chr13	64036206	64036389
chr13	64036411	64036673
chr13	64038256	64038434
chr13	64049634	64049844
chr13	64052125	64052293
chr13	64052383	64052533
chr13	64076155	64076408
chr13	64076408	64076715
chr13	64077692	64077902
chr13	64093399	64093627
chr13	64100240	64100546
chr13	64105031	64105215
chr13	64105693	64106143
chr13	64106145	64106312
chr13	64106312	64106410
chr13	64115498	64115753
chr13	64123974	64124150
chr13	64129087	64129560
chr13	64129631	64129878
chr13	64129916	64130068
chr13	64152223	64152601
chr13	64152601	64152874
chr13	64153088	64153471
chr13	64153471	64153809
chr13	64153849	64154297
chr13	64155178	64155602
chr13	64161420	64162011
chr13	64162011	64162489
chr13	64180369	64180597
chr13	64180900	64181272
chr13	64191200	64191370
chr13	64191370	64191583
chr13	64204835	64205039
chr13	64205257	64205470

chr13	64248314	64248529
chr13	64248529	64248836
chr13	64248836	64249164
chr13	64252179	64252521
chr13	64258021	64258224
chr13	64267234	64267434
chr13	64274137	64274338
chr13	64274368	64274833
chr13	64274833	64275182
chr13	64275182	64275440
chr13	64275512	64275669
chr13	64284779	64284935
chr13	64294584	64294781
chr13	64295252	64295417
chr13	64306015	64306438
chr13	64310727	64310968
chr13	64311351	64311679
chr13	64312086	64312419
chr13	64312419	64313029
chr13	64314890	64315098
chr13	64315618	64315783
chr13	64320138	64320316
chr13	64327120	64327353
chr13	64332080	64332302
chr13	64332368	64332710
chr13	64337291	64337507
chr13	64337663	64338035
chr13	64338035	64338298
chr13	64349470	64349875
chr13	64350298	64350522
chr13	64350798	64351127
chr13	64360661	64360873
chr13	64360873	64361086
chr13	64361086	64361489
chr13	64361540	64361789
chr13	64362004	64362175
chr13	64364316	64364584
chr13	64369802	64370165
chr13	64370165	64370547
chr13	64370547	64370878
chr13	64377409	64377634
chr13	64377634	64377972

chr13	64382828	64383168
chr13	64388221	64388840
chr13	64388961	64389169
chr13	64396116	64396370
chr13	64396998	64397171
chr13	64398107	64398403
chr13	64401134	64401386
chr13	64405185	64405365
chr13	64406635	64407107
chr13	64420899	64421165
chr13	64421354	64421788
chr13	64421992	64422276
chr13	64422276	64422555
chr13	64422655	64422901
chr13	64432122	64432610
chr13	64432610	64432954
chr13	64432954	64433162
chr13	64438979	64439448
chr13	64439451	64439857
chr13	64443362	64443587
chr13	64447987	64448287
chr13	64489812	64489999
chr13	64490020	64490223
chr13	64495728	64495932
chr13	64559730	64559923
chr13	64573827	64574003
chr13	64685202	64685532
chr13	64685532	64685792
chr13	64989592	64989821
chr13	65058177	65058450
chr13	65112104	65112288
chr13	65112410	65112803
chr13	65112803	65113241
chr13	65176243	65176505
chr13	65218334	65218521
chr13	65240401	65240992
chr13	65240992	65241525
chr13	65241525	65241768
chr13	65241928	65242186
chr13	65258082	65258609
chr13	65278138	65278290
chr13	65278354	65278633

chr13	65278633	65278903
chr13	65278903	65279140
chr13	65279140	65279412
chr13	65484998	65485204
chr13	65530729	65530903
chr13	65586328	65586510
chr13	65620403	65620611
chr13	65990952	65991494
chr13	66007593	66007800
chr13	66385843	66386226
chr13	66457960	66458137
chr13	66556561	66556757
chr13	66625383	66626018
chr13	66768696	66769199
chr13	66771233	66771406
chr13	66904755	66905213
chr13	66905213	66905503
chr13	66905503	66905700
chr13	66932654	66932928
chr13	66932928	66933223
chr13	66933223	66933583
chr13	66933583	66934045
chr13	66984395	66984877
chr13	66984877	66985160
chr13	66991730	66991889
chr13	67060099	67060619
chr13	67060619	67061339
chr13	67080972	67081454
chr13	67081454	67081723
chr13	67082090	67082256
chr13	67108075	67108507
chr13	67108507	67108874
chr13	67108885	67109111
chr13	67113365	67113629
chr13	67113711	67113967
chr13	67128008	67128288
chr13	67128288	67128536
chr13	67128536	67128807
chr13	67167821	67168028
chr13	67172960	67173260
chr13	67173260	67173495
chr13	67173495	67173758

chr13	67194322	67194579
chr13	67194579	67195047
chr13	67268673	67269066
chr13	67269066	67269419
chr13	67269431	67269700
chr13	67269700	67269929
chr13	67278362	67278694
chr13	67305926	67306385
chr13	67306385	67306659
chr13	67332084	67332510
chr13	67332510	67332851
chr13	67353551	67353909
chr13	67354403	67354580
chr13	67359897	67360104
chr13	67360104	67360300
chr13	67360300	67360519
chr13	67360519	67360828
chr13	67375201	67375465
chr13	67375465	67375704
chr13	67375704	67376000
chr13	67376400	67376569
chr13	67399131	67399461
chr13	67399461	67399694
chr13	67399694	67399989
chr13	67413762	67413953
chr13	67420947	67421354
chr13	67421354	67421571
chr13	67421571	67421774
chr13	67451125	67451801
chr13	67483725	67483949
chr13	67483949	67484167
chr13	67484167	67484449
chr13	67500013	67500400
chr13	67500400	67500730
chr13	67525626	67526073
chr13	67526073	67526381
chr13	67552592	67553052
chr13	67553052	67553375
chr13	67572527	67573185
chr13	67579914	67580064
chr13	67609116	67609530
chr13	67609530	67609971

chr13	67637193	67637609
chr13	67637609	67637996
chr13	67668975	67669192
chr13	67683011	67683462
chr13	67683462	67683756
chr13	67683917	67684165
chr13	67723778	67724152
chr13	67724152	67724513
chr13	67728696	67728907
chr13	67728907	67729113
chr13	67729113	67729369
chr13	67729504	67729805
chr13	67754616	67754868
chr13	67754868	67755085
chr13	67755085	67755356
chr13	67766983	67767278
chr13	67779388	67779812
chr13	67779812	67780052
chr13	67780052	67780315
chr13	67787994	67788225
chr13	67791270	67791586
chr13	67813625	67813910
chr13	67813910	67814343
chr13	67814402	67814581
chr13	67818773	67818958
chr13	67830953	67831108
chr13	67831153	67831379
chr13	67833216	67833453
chr13	67843127	67843440
chr13	67844057	67844466
chr13	67844466	67844726
chr13	67870695	67871005
chr13	67876411	67876771
chr13	67887442	67887691
chr13	67889064	67889253
chr13	67891875	67892025
chr13	67996427	67996577
chr13	68015377	68015974
chr13	68043769	68044089
chr13	68044089	68044458
chr13	68049814	68049964
chr13	68093218	68093683

chr13	68094951	68095101
chr13	68116875	68117184
chr13	68173604	68173812
chr13	68213368	68213618
chr13	68214917	68215421
chr13	68217811	68218055
chr13	68218351	68218559
chr13	68233147	68233396
chr13	68234600	68235094
chr13	68235994	68236226
chr13	68238047	68238356
chr13	68258055	68258711
chr13	68258842	68259039
chr13	68259329	68259479
chr13	68261579	68261909
chr13	68296182	68296332
chr13	68299028	68299534
chr13	68300682	68300982
chr13	68451568	68451732
chr13	68530466	68530620
chr13	68553472	68553722
chr13	68558637	68558819
chr13	68569657	68569942
chr13	68581710	68582580
chr13	68582585	68582892
chr13	68597714	68598043
chr13	68601305	68601473
chr13	68605636	68606145
chr13	68606937	68607097
chr13	68608630	68608879
chr13	68622429	68622713
chr13	68655676	68655881
chr13	68669517	68669677
chr13	69293986	69294174
chr13	69339843	69340028
chr13	69386500	69386694
chr13	69386767	69386981
chr13	69386981	69387184
chr13	69427214	69427536
chr13	69431607	69431972
chr13	69460205	69460357
chr13	69463952	69464102

chr13	69488636	69489415
chr13	69489573	69490083
chr13	69509472	69509682
chr13	69509682	69509930
chr13	69509930	69510285
chr13	69516893	69517175
chr13	69517175	69517490
chr13	69517490	69517828
chr13	69517873	69518338
chr13	69523983	69524530
chr13	69524530	69524809
chr13	69531554	69531755
chr13	69533471	69533640
chr13	69534262	69534639
chr13	69534639	69535089
chr13	69535089	69535555
chr13	69538455	69538605
chr13	69540913	69541215
chr13	69541215	69541634
chr13	69541634	69542080
chr13	69543098	69543312
chr13	69545057	69545314
chr13	69553163	69553367
chr13	69563228	69563420
chr13	69585558	69585850
chr13	69585850	69586189
chr13	69586189	69586434
chr13	69586532	69586746
chr13	69592980	69593376
chr13	69593376	69593919
chr13	69594878	69595237
chr13	69598868	69599342
chr13	69599422	69599678
chr13	69604189	69604687
chr13	69604687	69604986
chr13	69610665	69610903
chr13	69610954	69611223
chr13	69611223	69611487
chr13	69611487	69611862
chr13	69611945	69612175
chr13	69614959	69615114
chr13	69621697	69621847

chr13	69628098	69628284
chr13	69629157	69629330
chr13	69629493	69629800
chr13	69629836	69629998
chr13	69635892	69636115
chr13	69637766	69637945
chr13	69642350	69642592
chr13	69642615	69643288
chr13	69643403	69643648
chr13	69655406	69655772
chr13	69657807	69658084
chr13	69682551	69682718
chr13	69687130	69687332
chr13	69690018	69690378
chr13	69691147	69691330
chr13	69696402	69696641
chr13	69738565	69738754
chr13	69738858	69739008
chr13	69739391	69739740
chr13	69739740	69740171
chr13	69767401	69767708
chr13	69767708	69768032
chr13	69768148	69768572
chr13	69770546	69770968
chr13	69780401	69780716
chr13	69791820	69792053
chr13	69792471	69792729
chr13	69809665	69810152
chr13	69810152	69810695
chr13	69821284	69821445
chr13	69821520	69821757
chr13	69896676	69896831
chr13	69963761	69964031
chr13	69991317	69991604
chr13	70003505	70003956
chr13	70032302	70032481
chr13	70168694	70168868
chr13	70195710	70196207
chr13	70212919	70213380
chr13	70213380	70213599
chr13	70213708	70213953
chr13	70230242	70230421

chr13	70234415	70234751
chr13	70439538	70439688
chr13	70459094	70459297
chr13	70479320	70479508
chr13	70497298	70497629
chr13	70508694	70509199
chr13	70536024	70536323
chr13	70567241	70567408
chr13	70574292	70574598
chr13	70587067	70587307
chr13	70590201	70590488
chr13	70590488	70590876
chr13	70600065	70600698
chr13	70604810	70605002
chr13	70611097	70611382
chr13	70621466	70621924
chr13	70626590	70626811
chr13	70635808	70636082
chr13	70637272	70637491
chr13	70637491	70637675
chr13	70637675	70638003
chr13	70638003	70638408
chr13	70641432	70641635
chr13	70643504	70643705
chr13	70643739	70644092
chr13	70644133	70644372
chr13	70645287	70645498
chr13	70654229	70654778
chr13	70655553	70655928
chr13	70657613	70658088
chr13	70658088	70658362
chr13	70667007	70667161
chr13	70669862	70670587
chr13	70680981	70681391
chr13	70681391	70681734
chr13	70681844	70682259
chr13	70686529	70686682
chr13	70688578	70688866
chr13	70688933	70689162
chr13	70689258	70689520
chr13	70689520	70689852
chr13	70690048	70690336

chr13	70690541	70690771
chr13	70698842	70699008
chr13	70713301	70713774
chr13	70716111	70716307
chr13	70724287	70724614
chr13	70724614	70724892
chr13	70724892	70725313
chr13	70730098	70730397
chr13	70731329	70731815
chr13	70731890	70732275
chr13	70732837	70733091
chr13	70733965	70734343
chr13	70734731	70734885
chr13	70744376	70744543
chr13	70744978	70745166
chr13	70767577	70767745
chr13	70772545	70772696
chr13	70773311	70773621
chr13	70784278	70784428
chr13	70789164	70789319
chr13	70792969	70793307
chr13	70794027	70794643
chr13	70819020	70819348
chr13	70836269	70836503
chr13	70837622	70837999
chr13	70837999	70838343
chr13	70840261	70840487
chr13	70853608	70853826
chr13	70854219	70854370
chr13	70862458	70862840
chr13	70863410	70863579
chr13	70867022	70867371
chr13	70874540	70874693
chr13	70874711	70875188
chr13	70886791	70887023
chr13	70887158	70887542
chr13	70888301	70888464
chr13	70902729	70903193
chr13	70916306	70916456
chr13	71001403	71001653
chr13	71006206	71006441
chr13	71018378	71018520

chr13	71018520	71018686
chr13	71018686	71019048
chr13	71019065	71019349
chr13	71019349	71019627
chr13	71019627	71019856
chr13	71019881	71020159
chr13	71020159	71020477
chr13	71105303	71105519
chr13	71106399	71106630
chr13	71107922	71108193
chr13	71108193	71108563
chr13	71111463	71111875
chr13	71150000	71150468
chr13	71247360	71247650
chr13	71248754	71248924
chr13	71270368	71270596
chr13	71272305	71272518
chr13	71272518	71273076
chr13	71273433	71273961
chr13	71314914	71315098
chr13	71437452	71437810
chr13	71437810	71438117
chr13	71447467	71447692
chr13	71458456	71458724
chr13	71519823	71520070
chr13	71529806	71529999
chr13	71587647	71587863
chr13	71649055	71649208
chr13	71653556	71653713
chr13	71658007	71658201
chr13	71659150	71659339
chr13	71688672	71688892
chr13	71845617	71845917
chr13	71845977	71846132
chr13	71863182	71863428
chr13	71910395	71910738
chr13	71935912	71936062
chr13	71945114	71945500
chr13	71945500	71945792
chr13	71945792	71946084
chr13	71946084	71946367
chr13	71946367	71946626

chr13	71956495	71956819
chr13	71956819	71957162
chr13	71957266	71957417
chr13	71958636	71958830
chr13	71959270	71959843
chr13	71962745	71962914
chr13	71963125	71963544
chr13	71963544	71964010
chr13	71964051	71964223
chr13	71964439	71964698
chr13	71986825	71987136
chr13	72006157	72006390
chr13	72007633	72007947
chr13	72045165	72045393
chr13	72071110	72071428
chr13	72073853	72074151
chr13	72074263	72074487
chr13	72074487	72074780
chr13	72076391	72076817
chr13	72076820	72077157
chr13	72077322	72077771
chr13	72093190	72093340
chr13	72097343	72097552
chr13	72100466	72100753
chr13	72100753	72101099
chr13	72106151	72106577
chr13	72131714	72131876
chr13	72141673	72141864
chr13	72166327	72166516
chr13	72167456	72167742
chr13	72197850	72198114
chr13	72198114	72198324
chr13	72198359	72198686
chr13	72198686	72199220
chr13	72208157	72208391
chr13	72208391	72208633
chr13	72208633	72208914
chr13	72209892	72210267
chr13	72230827	72231001
chr13	72231400	72231596
chr13	72231596	72231956
chr13	72231956	72232482

chr13	72263887	72264297
chr13	72264297	72264949
chr13	72272415	72272717
chr13	72280155	72280823
chr13	72280851	72281312
chr13	72282767	72283137
chr13	72287215	72287568
chr13	72299792	72300057
chr13	72301933	72302212
chr13	72349382	72349564
chr13	72352188	72352342
chr13	72352618	72352918
chr13	72352918	72353277
chr13	72370275	72370536
chr13	72378798	72379033
chr13	72419500	72419727
chr13	72419727	72419947
chr13	72429205	72429419
chr13	72437799	72438091
chr13	72455978	72456574
chr13	72485280	72485700
chr13	72505861	72506288
chr13	72508438	72508634
chr13	72530518	72530715
chr13	72541862	72542089
chr13	72544398	72544660
chr13	72546864	72547032
chr13	72547304	72547454
chr13	72551964	72552247
chr13	72552325	72552924
chr13	72561931	72562269
chr13	72562269	72562592
chr13	72594771	72594976
chr13	72620445	72620655
chr13	72624043	72624202
chr13	72625282	72625504
chr13	72627425	72627728
chr13	72628019	72628418
chr13	72628418	72629160
chr13	72638415	72638660
chr13	72638719	72639007
chr13	72639214	72639455

chr13	72639455	72639671
chr13	72639671	72639909
chr13	72639909	72640172
chr13	72640172	72640462
chr13	72656443	72656808
chr13	72687927	72688094
chr13	72708784	72709101
chr13	72709997	72710464
chr13	72714815	72715081
chr13	72723103	72723287
chr13	72735913	72736116
chr13	72749224	72749381
chr13	72756382	72756599
chr13	72783367	72783517
chr13	72825162	72825494
chr13	72834906	72835082
chr13	72835579	72835850
chr13	72848234	72848395
chr13	72978163	72978380
chr13	72979044	72979532
chr13	73020280	73020839
chr13	73031311	73031550
chr13	73033128	73033541
chr13	73033541	73034085
chr13	73034085	73034590
chr13	73044264	73044497
chr13	73071846	73072137
chr13	73077156	73077853
chr13	73077895	73078215
chr13	73087901	73088183
chr13	73090560	73090729
chr13	73109858	73110116
chr13	73122797	73123347
chr13	73137560	73137803
chr13	73160177	73160770
chr13	73251284	73251539
chr13	73254534	73254763
chr13	73264437	73264619
chr13	73265487	73265663
chr13	73265663	73265831
chr13	73268367	73268670
chr13	73268720	73269080

chr13	73271851	73272022
chr13	73284977	73285334
chr13	73290058	73290676
chr13	73290676	73291010
chr13	73303037	73303216
chr13	73310269	73310586
chr13	73310589	73310941
chr13	73311081	73311233
chr13	73327925	73328113
chr13	73328158	73328409
chr13	73328409	73328716
chr13	73330849	73331121
chr13	73331121	73331636
chr13	73339215	73339665
chr13	73345219	73345417
chr13	73345541	73345815
chr13	73346192	73346464
chr13	73346775	73347093
chr13	73347162	73347470
chr13	73355091	73355368
chr13	73355368	73355711
chr13	73364973	73365159
chr13	73365289	73365531
chr13	73368588	73368794
chr13	73373933	73374826
chr13	73383674	73383894
chr13	73384769	73385096
chr13	73386178	73386376
chr13	73396642	73396824
chr13	73402535	73402693
chr13	73405764	73406080
chr13	73417910	73418119
chr13	73420967	73421265
chr13	73423165	73423378
chr13	73425898	73426085
chr13	73429560	73429932
chr13	73429932	73430234
chr13	73431040	73431624
chr13	73431624	73431958
chr13	73432547	73432821
chr13	73432821	73433128
chr13	73433443	73433612

chr13	73441675	73441965
chr13	73442381	73442647
chr13	73445133	73445423
chr13	73447127	73447277
chr13	73453606	73453760
chr13	73453880	73454178
chr13	73454178	73454596
chr13	73454750	73454993
chr13	73455828	73456195
chr13	73456195	73456501
chr13	73457871	73458241
chr13	73459515	73459734
chr13	73466994	73467384
chr13	73467384	73467789
chr13	73467789	73468168
chr13	73469923	73470077
chr13	73472607	73472795
chr13	73476237	73476502
chr13	73480002	73480274
chr13	73480274	73480611
chr13	73486067	73486522
chr13	73489902	73490060
chr13	73495599	73495859
chr13	73499980	73500305
chr13	73500305	73500672
chr13	73500947	73501158
chr13	73505481	73505729
chr13	73505761	73506062
chr13	73512506	73512656
chr13	73513053	73513262
chr13	73513292	73513741
chr13	73516993	73517177
chr13	73519112	73519352
chr13	73520616	73521050
chr13	73536050	73536434
chr13	73536434	73537102
chr13	73538983	73539339
chr13	73575053	73575314
chr13	73575314	73575580
chr13	73577435	73577607
chr13	73580344	73580504
chr13	73603687	73604155

chr13	73604155	73604531
chr13	73604531	73604906
chr13	73620726	73620946
chr13	73626470	73626957
chr13	73626957	73627275
chr13	73627275	73627555
chr13	73631929	73632088
chr13	73646485	73646704
chr13	73646714	73647124
chr13	73659041	73659281
chr13	73667596	73667746
chr13	73667815	73668024
chr13	73672565	73672764
chr13	73680305	73681003
chr13	73682633	73682837
chr13	73683733	73683930
chr13	73692330	73692887
chr13	73695402	73695625
chr13	73696960	73697293
chr13	73699772	73700059
chr13	73714033	73714415
chr13	73718305	73718614
chr13	73722980	73723346
chr13	73723349	73723691
chr13	73724019	73724207
chr13	73726714	73726949
chr13	73732173	73732360
chr13	73732994	73733150
chr13	73737330	73737538
chr13	73742950	73743210
chr13	73743898	73744185
chr13	73748675	73749211
chr13	73750604	73750766
chr13	73754949	73755435
chr13	73755515	73755665
chr13	73763284	73763637
chr13	73763637	73763829
chr13	73763829	73764072
chr13	73764072	73764422
chr13	73769315	73769527
chr13	73779511	73779661
chr13	73803409	73803760

chr13	73803760	73804341
chr13	73808561	73808811
chr13	73814856	73815057
chr13	73822257	73822528
chr13	73822528	73822727
chr13	73822727	73822904
chr13	73831221	73831400
chr13	73831400	73831583
chr13	73831604	73831846
chr13	73846921	73847441
chr13	73847441	73847839
chr13	73860461	73860705
chr13	73879911	73880141
chr13	73886253	73886403
chr13	73887589	73888048
chr13	73888048	73888343
chr13	73888343	73888676
chr13	73888966	73889242
chr13	73907742	73908028
chr13	73910552	73910732
chr13	73918417	73918701
chr13	73937051	73937321
chr13	73937321	73937624
chr13	73937624	73937901
chr13	73937901	73938201
chr13	73943671	73943909
chr13	73961281	73961471
chr13	73962369	73962526
chr13	73965899	73966062
chr13	73970556	73970957
chr13	73970957	73971633
chr13	73981553	73981831
chr13	73990378	73990610
chr13	74008956	74009768
chr13	74009774	74009931
chr13	74025049	74025301
chr13	74025301	74025591
chr13	74025591	74026136
chr13	74026205	74026599
chr13	74031259	74031473
chr13	74061915	74062225
chr13	74062225	74062641

chr13	74096827	74097085
chr13	74098329	74098631
chr13	74117272	74117446
chr13	74119199	74119349
chr13	74121179	74121630
chr13	74125322	74125588
chr13	74126862	74127173
chr13	74157524	74157807
chr13	74159263	74159514
chr13	74166774	74167119
chr13	74167268	74167455
chr13	74167515	74167859
chr13	74169930	74170214
chr13	74192668	74192940
chr13	74208345	74208815
chr13	74208815	74209058
chr13	74209058	74209212
chr13	74245566	74245899
chr13	74245955	74246105
chr13	74259789	74260157
chr13	74260291	74260459
chr13	74265431	74265705
chr13	74270543	74270696
chr13	74292271	74292443
chr13	74296332	74296801
chr13	74301241	74301577
chr13	74301577	74301999
chr13	74301999	74302366
chr13	74316450	74316736
chr13	74316785	74317522
chr13	74317522	74317787
chr13	74324932	74325124
chr13	74341084	74341449
chr13	74349616	74349857
chr13	74349857	74350172
chr13	74350172	74350472
chr13	74350472	74350824
chr13	74363343	74363933
chr13	74363933	74364244
chr13	74390286	74390640
chr13	74390640	74390966
chr13	74399328	74399706

chr13	74406361	74406564
chr13	74406564	74406857
chr13	74457168	74457354
chr13	74462399	74462642
chr13	74462642	74463045
chr13	74463045	74463436
chr13	74493673	74494134
chr13	74494134	74494674
chr13	74522428	74522748
chr13	74522831	74523017
chr13	74528407	74528614
chr13	74528614	74528854
chr13	74528854	74529135
chr13	74529155	74529476
chr13	74529482	74529809
chr13	74529842	74530212
chr13	74530315	74530690
chr13	74563580	74564073
chr13	74564073	74564354
chr13	74564354	74564596
chr13	74591585	74591899
chr13	74617039	74617547
chr13	74617547	74617825
chr13	74617825	74618072
chr13	74632403	74632644
chr13	74639023	74639357
chr13	74639444	74639702
chr13	74639702	74640008
chr13	74640008	74640534
chr13	74736095	74736266
chr13	74762061	74762226
chr13	74762788	74763159
chr13	74764900	74765166
chr13	74767651	74767881
chr13	74769921	74770225
chr13	74770396	74770749
chr13	74770749	74771032
chr13	74771318	74771577
chr13	74772147	74772480
chr13	74773035	74773198
chr13	74807524	74807719
chr13	74807740	74808194

chr13	74808194	74808538
chr13	74808538	74808763
chr13	74808763	74808952
chr13	74815716	74815911
chr13	74822068	74822508
chr13	74825103	74825416
chr13	74854364	74854701
chr13	74863600	74864175
chr13	74865352	74865610
chr13	74903763	74903981
chr13	75088682	75088832
chr13	75215826	75215976
chr13	75223294	75223536
chr13	75227680	75227937
chr13	75231969	75232245
chr13	75232591	75232895
chr13	75236523	75236678
chr13	75270965	75271299
chr13	75271399	75271674
chr13	75315907	75316174
chr13	75337084	75337238
chr13	75337308	75337563
chr13	75366776	75367075
chr13	75367079	75367463
chr13	75368302	75368733
chr13	75375990	75376168
chr13	75377221	75377423
chr13	75389306	75389479
chr13	75390191	75390592
chr13	75412846	75413054
chr13	75420783	75421020
chr13	75460437	75460768
chr13	75501622	75501862
chr13	75503261	75503681
chr13	75503681	75503979
chr13	75514232	75514406
chr13	75543594	75543847
chr13	75574499	75574750
chr13	75579007	75579255
chr13	75584379	75584570
chr13	75598277	75598572
chr13	75605555	75605788

chr13	75623243	75623603
chr13	75627666	75627821
chr13	75707057	75707730
chr13	75707730	75708115
chr13	75708185	75708625
chr13	75720726	75721042
chr13	75739017	75739355
chr13	75740064	75740286
chr13	75740286	75740488
chr13	75752142	75752317
chr13	75752317	75752555
chr13	75752555	75752903
chr13	75754389	75754740
chr13	75760987	75761248
chr13	75786312	75786620
chr13	75788967	75789547
chr13	75789547	75790020
chr13	75797647	75798211
chr13	75798345	75798688
chr13	75798688	75799017
chr13	75799037	75799236
chr13	75824666	75824967
chr13	75824967	75825344
chr13	75825344	75825672
chr13	75833507	75833751
chr13	75839656	75839988
chr13	75839988	75840247
chr13	75840247	75840487
chr13	75841179	75841375
chr13	75841475	75841733
chr13	75844081	75844322
chr13	75852234	75852384
chr13	75852452	75852885
chr13	75866807	75866980
chr13	75875065	75875320
chr13	75875902	75876085
chr13	75876310	75876559
chr13	75877982	75878223
chr13	75894234	75894429
chr13	75895675	75896000
chr13	75901798	75901962
chr13	75942783	75943006

chr13	75943129	75943385
chr13	75943385	75943593
chr13	75943593	75943791
chr13	75943791	75944072
chr13	75944339	75944604
chr13	75944731	75944881
chr13	76017449	76017700
chr13	76018037	76018379
chr13	76018379	76018881
chr13	76037547	76037704
chr13	76055489	76055740
chr13	76055740	76056126
chr13	76056126	76056789
chr13	76098162	76098499
chr13	76098499	76098865
chr13	76098865	76099297
chr13	76102070	76102388
chr13	76180171	76180416
chr13	76284096	76284320
chr13	76284587	76284965
chr13	76358751	76359130
chr13	76383833	76384316
chr13	76384316	76384690
chr13	76384690	76385083
chr13	76385083	76385379
chr13	76429589	76429821
chr13	76481728	76481898
chr13	76515913	76516243
chr13	76579462	76579729
chr13	76591219	76591557
chr13	76591690	76592041
chr13	76593065	76593220
chr13	76639777	76640032
chr13	76939340	76939668
chr13	77043425	77043685
chr13	77071187	77071480
chr13	77081647	77081870
chr13	77109558	77109843
chr13	77128377	77128570
chr13	77134768	77134918
chr13	77134933	77135171
chr13	77135171	77135392

chr13	77135392	77135650
chr13	77135650	77135949
chr13	77438842	77439106
chr13	77695399	77695549
chr13	77708474	77709161
chr13	77748441	77748671
chr13	77838823	77839070
chr13	78010263	78010427
chr13	78032951	78033134
chr13	78162303	78162466
chr13	78163001	78163424
chr13	78163424	78163763
chr13	78163763	78164032
chr13	78171346	78171496
chr13	78186393	78186600
chr13	78198093	78198280
chr13	78198280	78198565
chr13	78199011	78199409
chr13	78199409	78199672
chr13	78200457	78200743
chr13	78201377	78201877
chr13	78201918	78202068
chr13	78203187	78203448
chr13	78203811	78204082
chr13	78210382	78210555
chr13	78456700	78456885
chr13	78466850	78467089
chr13	78474170	78474419
chr13	78483188	78483577
chr13	78483577	78483949
chr13	78522379	78522689
chr13	78543354	78543591
chr13	78548806	78548967
chr13	78602726	78603123
chr13	78603123	78603310
chr13	78679312	78679955
chr13	78685274	78685501
chr13	78704191	78704410
chr13	78704452	78704680
chr13	78704730	78705071
chr13	78715388	78715868
chr13	78809567	78810083

chr13	78812090	78812342
chr13	78875105	78875529
chr13	78956668	78956838
chr13	78998687	78999221
chr13	79020583	79020733
chr13	79028209	79028396
chr13	79029977	79030302
chr13	79243281	79243526
chr13	79670979	79671137
chr13	80557276	80557479
chr13	80747125	80747286
chr13	80806262	80806485
chr13	80862334	80862512
chr13	80864245	80864522
chr13	80881865	80882015
chr13	80882970	80883169
chr13	80883169	80883506
chr13	80884751	80884929
chr13	80885378	80885835
chr13	80885835	80886114
chr13	80886114	80886449
chr13	80902842	80903082
chr13	80915155	80915388
chr13	80928362	80928589
chr13	80935210	80935545
chr13	80936327	80936822
chr13	80939847	80940108
chr13	80940500	80940796
chr13	80940801	80941220
chr13	80941220	80941587
chr13	80949105	80949323
chr13	80951278	80951800
chr13	80951976	80952176
chr13	80953349	80953509
chr13	80957068	80957305
chr13	80962759	80963241
chr13	80963241	80963622
chr13	80971691	80972021
chr13	80986794	80986951
chr13	80996724	80997136
chr13	81047583	81047787
chr13	81050926	81051345

chr13	81051736	81052140
chr13	81065619	81065850
chr13	81096244	81096753
chr13	81096781	81096976
chr13	81103856	81104233
chr13	81136622	81137016
chr13	81151060	81151241
chr13	81155374	81155677
chr13	81182012	81182250
chr13	81182250	81182457
chr13	81238091	81238274
chr13	81238904	81239216
chr13	81312221	81312375
chr13	81320215	81320388
chr13	81342762	81342914
chr13	81343080	81343565
chr13	81401686	81402150
chr13	81413796	81414062
chr13	81424037	81424427
chr13	81439450	81439673
chr13	81441485	81441753
chr13	81473887	81474081
chr13	81487017	81487442
chr13	81495612	81495877
chr13	81526777	81527003
chr13	81534724	81535021
chr13	81535021	81535565
chr13	81602005	81602188
chr13	81632764	81633101
chr13	81633101	81633485
chr13	81639672	81639979
chr13	81640721	81641218
chr13	81641218	81641542
chr13	81641631	81641807
chr13	81641858	81642016
chr13	81657221	81657542
chr13	81657542	81658125
chr13	81662757	81663078
chr13	81663121	81663397
chr13	81664369	81664698
chr13	81683070	81683538
chr13	81683993	81684152

chr13	81684152	81684359
chr13	81684359	81684704
chr13	81684904	81685221
chr13	81701056	81701383
chr13	81710719	81711215
chr13	81711215	81711637
chr13	81711637	81712156
chr13	81760446	81760697
chr13	81761937	81762094
chr13	81776691	81776853
chr13	81782921	81783427
chr13	81783427	81783988
chr13	81807100	81807347
chr13	81807611	81807811
chr13	81852643	81852943
chr13	81942058	81942325
chr13	81944157	81944312
chr13	81966670	81966866
chr13	81982241	81982880
chr13	82177713	82177863
chr13	82361472	82361682
chr13	82540087	82540356
chr13	82656735	82656935
chr13	82678484	82678849
chr13	82777310	82777639
chr13	82779604	82779802
chr13	82781228	82781407
chr13	82781454	82781700
chr13	82865861	82866027
chr13	82887414	82887700
chr13	82892730	82893328
chr13	82973685	82973913
chr13	83042170	83042356
chr13	83164041	83164292
chr13	83193522	83193799
chr13	83200371	83200570
chr13	83246191	83246485
chr13	83378681	83378898
chr13	83593103	83593296
chr13	83601767	83602065
chr13	83603756	83603916
chr13	83603918	83604219

chr13	83604393	83604628
chr13	83608215	83608676
chr13	83652016	83652183
chr13	83652276	83652491
chr13	83652617	83652983
chr13	83654972	83655200
chr13	83715042	83715296
chr13	83715324	83715482
chr13	83720886	83721056
chr13	83721114	83721301
chr13	83725058	83725307
chr13	83729695	83729970
chr13	83730421	83730683
chr13	83730693	83730960
chr13	83730960	83731257
chr13	83731257	83731590
chr13	83744923	83745288
chr13	84221780	84222449
chr13	84222449	84222813
chr13	84251349	84251644
chr13	84347854	84348587
chr13	84348624	84348934
chr13	84371144	84371295
chr13	84569440	84569595
chr13	84612976	84613331
chr13	84621489	84621749
chr13	84633024	84633375
chr13	84634015	84634200
chr13	84646938	84647350
chr13	84703152	84703302
chr13	84758613	84758868
chr13	84791175	84791364
chr13	85126660	85127031
chr13	85127031	85127258
chr13	85127258	85127454
chr13	85169320	85169593
chr13	85188991	85189226
chr13	85189226	85189588
chr13	85189588	85190109
chr13	85279587	85279822
chr13	85282207	85282413
chr13	85288345	85288765

chr13	85288765	85289138
chr13	85289138	85289492
chr13	85289492	85289799
chr13	85306360	85306649
chr13	85333189	85333339
chr13	85333444	85333606
chr13	85337014	85337202
chr13	85337216	85337505
chr13	85359164	85359544
chr13	85359665	85359889
chr13	85376598	85376948
chr13	85390251	85390414
chr13	85390439	85390870
chr13	85398752	85399157
chr13	85399157	85399433
chr13	85415063	85415282
chr13	85418703	85419086
chr13	85419119	85419509
chr13	85419509	85419950
chr13	85425193	85425759
chr13	85430026	85430415
chr13	85430826	85431313
chr13	85431398	85431625
chr13	85438526	85438920
chr13	85458695	85458898
chr13	85471172	85471547
chr13	85473170	85473559
chr13	85480896	85481208
chr13	85481208	85481756
chr13	85509660	85509843
chr13	85545012	85545164
chr13	85556227	85556377
chr13	85692348	85692543
chr13	85766805	85767026
chr13	85791285	85791527
chr13	85791589	85791777
chr13	85791777	85792056
chr13	85894749	85894985
chr13	85921183	85921478
chr13	85955435	85955744
chr13	86046198	86046677
chr13	86046677	86047034

chr13	86148163	86148420
chr13	86728589	86728883
chr13	87101696	87101931
chr13	87352892	87353070
chr13	87375003	87375163
chr13	87631985	87632474
chr13	87928694	87928982
chr13	87929478	87929865
chr13	88017515	88017685
chr13	88201750	88202307
chr13	88212430	88212660
chr13	88400403	88400592
chr13	88799029	88799552
chr13	88801052	88801347
chr13	88805762	88806200
chr13	88806422	88806592
chr13	88810111	88810508
chr13	88816573	88816758
chr13	88816758	88817135
chr13	88821365	88821621
chr13	88821621	88821945
chr13	88821945	88822239
chr13	88823693	88823863
chr13	88938615	88938895
chr13	88998243	88998505
chr13	89029752	89030087
chr13	89065084	89065323
chr13	89068281	89068481
chr13	89189479	89189748
chr13	89324408	89324609
chr13	89383859	89384031
chr13	89384850	89385021
chr13	89395330	89395525
chr13	89439248	89439469
chr13	89443675	89443911
chr13	89444611	89445350
chr13	89453677	89454134
chr13	89454751	89454975
chr13	89477897	89478096
chr13	89485918	89486102
chr13	89539169	89539460
chr13	89539472	89539686

chr13	89543109	89543318
chr13	89599459	89599651
chr13	89634214	89634411
chr13	89636955	89637427
chr13	89653305	89653583
chr13	89668093	89668322
chr13	89709911	89710224
chr13	89720609	89720780
chr13	89739287	89739751
chr13	89739810	89739978
chr13	89740010	89740213
chr13	89740247	89740685
chr13	89740685	89741082
chr13	89779330	89779491
chr13	89828396	89828868
chr13	89889061	89889480
chr13	89899153	89899354
chr13	89933076	89933415
chr13	90005670	90005837
chr13	90009879	90010099
chr13	90089008	90089411
chr13	90089411	90089815
chr13	90089815	90090114
chr13	90117266	90117463
chr13	90129536	90129728
chr13	90144486	90144906
chr13	90144973	90145170
chr13	90150159	90150335
chr13	90151917	90152208
chr13	90152264	90152466
chr13	90167274	90167425
chr13	90171392	90171900
chr13	90216855	90217106
chr13	90217106	90217479
chr13	90230591	90230876
chr13	90230876	90231156
chr13	90233843	90234594
chr13	90337422	90337645
chr13	90337645	90337941
chr13	90337948	90338184
chr13	90338923	90339199
chr13	90394961	90395205

chr13	90397756	90398084
chr13	90473056	90473383
chr13	90473458	90473769
chr13	90502273	90502437
chr13	90513829	90514320
chr13	90515833	90516376
chr13	90519103	90519268
chr13	90521124	90521305
chr13	90559570	90559775
chr13	90560094	90560424
chr13	90572652	90572910
chr13	90575173	90575831
chr13	90599453	90599610
chr13	90685133	90685401
chr13	90770962	90771170
chr13	90782103	90782310
chr13	90796806	90797168
chr13	90797215	90797713
chr13	90799168	90799565
chr13	90826576	90826869
chr13	90841009	90841257
chr13	90871628	90871919
chr13	90875007	90875306
chr13	90885937	90886097
chr13	90886245	90886402
chr13	90886570	90886742
chr13	90886798	90887015
chr13	90922693	90922999
chr13	90922999	90923381
chr13	90923381	90923717
chr13	90929293	90929481
chr13	90929481	90929669
chr13	90929907	90930154
chr13	90935906	90936113
chr13	90941105	90941757
chr13	90967506	90967798
chr13	90977095	90977494
chr13	90992977	90993311
chr13	91038066	91038379
chr13	91038747	91038976
chr13	91039247	91039496
chr13	91040534	91040736

chr13	91041349	91041585
chr13	91091360	91091792
chr13	91091951	91092122
chr13	91115026	91115213
chr13	91137830	91138023
chr13	91164899	91165721
chr13	91173425	91173586
chr13	91173685	91173859
chr13	91174711	91175204
chr13	91186661	91186909
chr13	91189768	91189983
chr13	91202948	91203162
chr13	91206197	91206754
chr13	91215323	91215495
chr13	91223626	91224389
chr13	91224594	91224753
chr13	91243753	91244042
chr13	91246788	91247062
chr13	91267458	91267674
chr13	91306053	91306341
chr13	91306552	91306927
chr13	91311295	91311678
chr13	91313304	91313481
chr13	91365076	91365669
chr13	91366594	91366919
chr13	91370868	91371231
chr13	91371231	91371523
chr13	91372043	91372213
chr13	91372213	91372434
chr13	91448978	91449151
chr13	91455524	91455683
chr13	91455745	91455951
chr13	91456108	91456335
chr13	91460368	91460625
chr13	91460625	91460901
chr13	91460901	91461388
chr13	91546413	91546638
chr13	91601925	91602168
chr13	91636902	91637141
chr13	91648399	91648584
chr13	91680293	91680602
chr13	91681142	91681409

chr13	91687716	91687968
chr13	91697126	91697290
chr13	91702420	91702585
chr13	91723191	91723431
chr13	91724006	91724281
chr13	91724281	91724551
chr13	91741278	91741494
chr13	91741494	91741713
chr13	91741713	91741954
chr13	91756222	91756541
chr13	91779615	91779914
chr13	91779914	91780279
chr13	91786165	91786431
chr13	91801431	91801589
chr13	91801818	91802079
chr13	91807123	91807494
chr13	91807494	91807990
chr13	91808134	91808327
chr13	91888598	91888822
chr13	91893195	91893432
chr13	91904232	91904471
chr13	91904640	91904866
chr13	91908290	91908561
chr13	91911241	91911565
chr13	91911709	91911911
chr13	91915965	91916263
chr13	91916408	91916921
chr13	91929994	91930243
chr13	91930253	91930598
chr13	91935949	91936387
chr13	91937298	91937555
chr13	91940228	91940468
chr13	91940544	91940794
chr13	92067840	92068208
chr13	92069705	92069930
chr13	92072155	92072341
chr13	92073014	92073549
chr13	92095138	92095389
chr13	92095389	92095655
chr13	92122616	92122850
chr13	92126116	92126270
chr13	92131010	92131476

chr13	92131476	92132130
chr13	92150780	92151104
chr13	92151306	92151507
chr13	92151507	92151745
chr13	92198073	92198417
chr13	92202455	92202678
chr13	92213886	92214165
chr13	92214300	92214737
chr13	92228029	92228331
chr13	92228356	92228726
chr13	92229345	92229623
chr13	92236106	92236348
chr13	92241353	92241776
chr13	92241776	92242011
chr13	92259115	92259352
chr13	92274349	92274889
chr13	92315915	92316119
chr13	92316123	92316324
chr13	92316937	92317097
chr13	92336805	92337050
chr13	92337050	92337294
chr13	92353925	92354112
chr13	92354459	92354866
chr13	92354866	92355265
chr13	92355265	92355633
chr13	92369543	92369698
chr13	92402104	92402387
chr13	92406823	92407095
chr13	92425675	92426289
chr13	92426289	92426740
chr13	92426740	92426989
chr13	92483358	92483617
chr13	92483786	92484361
chr13	92506625	92506823
chr13	92530371	92530679
chr13	92530679	92530861
chr13	92530861	92531258
chr13	92532343	92532658
chr13	92537167	92537465
chr13	92554925	92555175
chr13	92580302	92580688
chr13	92610565	92610894

chr13	92610894	92611154
chr13	92611154	92611377
chr13	92611377	92611758
chr13	92613186	92613497
chr13	92617054	92617237
chr13	92625104	92625279
chr13	92629484	92630121
chr13	92630121	92630464
chr13	92636878	92637276
chr13	92642235	92642748
chr13	92642748	92643012
chr13	92643012	92643213
chr13	92656893	92657111
chr13	92660217	92660408
chr13	92664460	92664685
chr13	92664714	92665261
chr13	92667678	92667958
chr13	92668167	92668503
chr13	92675371	92675657
chr13	92675956	92676205
chr13	92679487	92679642
chr13	92679696	92680152
chr13	92693096	92693384
chr13	92694269	92694535
chr13	92722401	92722914
chr13	92733429	92733579
chr13	92740424	92740602
chr13	92740904	92741128
chr13	92750947	92751310
chr13	92751363	92751528
chr13	92751768	92752076
chr13	92789110	92789272
chr13	92794618	92794837
chr13	92797752	92798134
chr13	92799560	92800262
chr13	92804410	92804635
chr13	92844585	92844889
chr13	92844889	92845238
chr13	92858670	92859075
chr13	92901995	92902150
chr13	92917849	92918179
chr13	92918386	92918641

chr13	92919403	92919622
chr13	92919735	92920204
chr13	92963494	92963804
chr13	92986782	92987022
chr13	93021013	93021336
chr13	93061175	93061457
chr13	93061457	93061678
chr13	93061678	93061882
chr13	93062156	93062347
chr13	93063303	93063699
chr13	93064566	93064866
chr13	93115502	93115683
chr13	93144037	93144275
chr13	93144275	93144575
chr13	93144575	93144881
chr13	93144881	93145096
chr13	93191815	93192090
chr13	93192090	93192369
chr13	93192369	93192738
chr13	93214738	93214965
chr13	93243077	93243254
chr13	93243254	93243486
chr13	93303298	93303588
chr13	93303588	93303871
chr13	93303871	93304189
chr13	93304189	93304524
chr13	93304524	93304937
chr13	93313089	93313458
chr13	93330801	93330983
chr13	93351464	93351763
chr13	93351960	93352385
chr13	93362935	93363292
chr13	93418342	93418580
chr13	93422925	93423264
chr13	93423280	93423443
chr13	93440281	93440484
chr13	93484228	93484598
chr13	93488542	93488823
chr13	93492723	93492988
chr13	93492988	93493304
chr13	93493323	93493562
chr13	93493600	93493759

chr13	93498243	93498442
chr13	93498442	93498634
chr13	93498634	93498870
chr13	93498870	93499272
chr13	93499272	93499685
chr13	93499685	93500171
chr13	93517332	93517489
chr13	93526767	93527063
chr13	93589233	93589413
chr13	93604820	93605155
chr13	93605245	93605526
chr13	93605526	93605799
chr13	93613026	93613246
chr13	93723613	93723795
chr13	93749780	93749980
chr13	93771515	93771906
chr13	93771906	93772287
chr13	93772287	93772532
chr13	93793398	93793625
chr13	93841437	93841603
chr13	93842308	93842556
chr13	93842840	93843101
chr13	93843101	93843347
chr13	93882227	93882480
chr13	93892109	93892500
chr13	93898554	93898818
chr13	93941141	93941381
chr13	93941381	93941686
chr13	93944039	93944218
chr13	93952501	93952745
chr13	93975616	93976044
chr13	93984472	93984684
chr13	93984831	93985229
chr13	93985229	93985595
chr13	93985668	93985826
chr13	93989032	93989202
chr13	93989277	93989515
chr13	93992356	93992739
chr13	93997834	93998385
chr13	93998385	93998707
chr13	94024662	94024975
chr13	94025228	94025387

chr13	94050812	94051188
chr13	94057385	94057810
chr13	94057810	94058018
chr13	94058018	94058342
chr13	94062608	94063110
chr13	94063297	94063456
chr13	94090696	94091067
chr13	94094705	94095026
chr13	94098386	94098889
chr13	94109153	94109350
chr13	94111421	94111646
chr13	94132420	94132673
chr13	94132673	94133056
chr13	94134494	94134798
chr13	94134798	94135022
chr13	94136511	94136748
chr13	94140132	94140382
chr13	94140982	94141186
chr13	94152457	94152743
chr13	94152883	94153245
chr13	94153641	94153836
chr13	94154502	94154850
chr13	94154859	94155049
chr13	94155049	94155221
chr13	94165492	94165744
chr13	94172706	94172861
chr13	94173695	94173911
chr13	94173911	94174154
chr13	94174154	94174337
chr13	94174337	94174520
chr13	94184614	94185026
chr13	94185026	94185297
chr13	94190403	94190859
chr13	94193270	94193802
chr13	94205308	94205553
chr13	94205959	94206109
chr13	94212799	94212954
chr13	94225660	94225815
chr13	94242041	94242203
chr13	94250770	94250920
chr13	94257646	94257827
chr13	94261036	94261277

chr13	94262579	94263053
chr13	94264071	94264233
chr13	94271451	94271637
chr13	94284571	94284789
chr13	94284837	94285200
chr13	94285200	94285577
chr13	94285577	94285941
chr13	94297772	94297992
chr13	94298451	94298812
chr13	94312770	94313181
chr13	94327997	94328505
chr13	94358229	94358529
chr13	94358529	94358787
chr13	94358787	94359506
chr13	94368037	94368187
chr13	94391864	94392162
chr13	94435061	94435220
chr13	94438778	94439068
chr13	94451321	94451523
chr13	94468577	94468878
chr13	94504561	94504831
chr13	94515058	94515339
chr13	94523299	94523511
chr13	94559725	94559875
chr13	94560091	94560321
chr13	94567779	94568009
chr13	94702583	94702845
chr13	94702864	94703078
chr13	94709857	94710067
chr13	94788702	94789048
chr13	94789048	94789267
chr13	94789267	94789673
chr13	94806380	94806565
chr13	94812430	94812610
chr13	94827943	94828173
chr13	94828350	94828527
chr13	94828700	94828900
chr13	94828900	94829076
chr13	94829446	94829690
chr13	94829690	94829921
chr13	94829929	94830267
chr13	94837346	94837541

chr13	94838117	94838390
chr13	94838852	94839007
chr13	94843092	94843378
chr13	94844207	94844382
chr13	94857091	94857403
chr13	94868484	94868985
chr13	94873682	94873873
chr13	94882676	94882983
chr13	94883087	94883694
chr13	94941332	94941548
chr13	94950793	94951369
chr13	94955756	94955969
chr13	94956051	94956277
chr13	94975878	94976457
chr13	94976457	94976770
chr13	95018124	95018279
chr13	95038214	95038694
chr13	95038694	95038988
chr13	95043445	95043601
chr13	95049379	95049710
chr13	95075616	95075948
chr13	95103094	95103291
chr13	95135150	95135310
chr13	95135498	95135648
chr13	95145989	95146227
chr13	95187299	95187498
chr13	95207179	95207330
chr13	95215684	95215946
chr13	95215946	95216235
chr13	95222698	95222897
chr13	95261515	95261724
chr13	95287054	95287340
chr13	95290197	95290347
chr13	95324267	95324539
chr13	95324610	95324992
chr13	95324992	95325502
chr13	95325502	95325953
chr13	95326608	95326843
chr13	95347034	95347636
chr13	95370712	95370914
chr13	95374666	95375006
chr13	95375006	95375233

chr13	95375233	95375535
chr13	95376899	95377198
chr13	95381300	95381590
chr13	95384230	95384498
chr13	95388073	95388297
chr13	95388324	95388484
chr13	95389121	95389420
chr13	95390249	95390529
chr13	95390529	95390827
chr13	95412353	95412553
chr13	95413590	95413929
chr13	95413929	95414256
chr13	95424873	95425198
chr13	95427234	95427547
chr13	95427547	95427970
chr13	95443476	95443671
chr13	95443704	95444010
chr13	95444010	95444243
chr13	95444765	95445029
chr13	95450534	95450830
chr13	95450830	95451170
chr13	95451462	95451883
chr13	95469700	95470030
chr13	95470896	95471328
chr13	95481490	95481774
chr13	95484659	95485250
chr13	95491912	95492236
chr13	95506798	95507200
chr13	95507387	95507581
chr13	95514775	95515088
chr13	95515088	95515372
chr13	95524168	95524460
chr13	95524470	95524693
chr13	95524693	95525095
chr13	95525095	95525651
chr13	95529285	95529529
chr13	95535245	95535504
chr13	95535504	95535888
chr13	95545631	95546121
chr13	95546121	95546557
chr13	95546557	95546813
chr13	95548333	95548851

chr13	95551944	95552310
chr13	95553840	95554471
chr13	95563056	95563405
chr13	95563747	95564128
chr13	95583682	95583966
chr13	95584879	95585217
chr13	95585217	95585573
chr13	95595955	95596196
chr13	95598344	95598695
chr13	95598695	95598979
chr13	95599005	95599160
chr13	95599630	95600021
chr13	95600021	95600248
chr13	95600886	95601103
chr13	95601411	95601717
chr13	95602631	95602785
chr13	95608309	95608492
chr13	95608502	95608776
chr13	95608776	95609243
chr13	95611216	95611539
chr13	95611779	95612098
chr13	95617990	95618188
chr13	95618246	95618557
chr13	95618557	95618852
chr13	95622895	95623426
chr13	95630781	95631014
chr13	95633313	95633934
chr13	95633971	95634188
chr13	95634425	95634797
chr13	95640190	95640364
chr13	95640463	95640957
chr13	95640957	95641274
chr13	95641274	95641718
chr13	95643716	95644110
chr13	95650905	95651108
chr13	95667177	95667570
chr13	95667570	95667780
chr13	95679768	95680114
chr13	95684761	95685163
chr13	95685811	95686033
chr13	95686106	95686667
chr13	95696791	95697225

chr13	95700128	95700372
chr13	95705320	95705653
chr13	95705653	95706019
chr13	95706172	95706439
chr13	95719335	95719686
chr13	95719686	95719955
chr13	95719955	95720250
chr13	95722585	95722737
chr13	95746574	95746791
chr13	95776937	95777120
chr13	95777263	95777596
chr13	95783056	95783216
chr13	95794360	95794680
chr13	95805771	95805975
chr13	95813687	95813946
chr13	95820243	95820414
chr13	95828833	95829076
chr13	95831456	95831615
chr13	95831764	95832160
chr13	95843504	95844075
chr13	95849387	95849796
chr13	95888266	95888499
chr13	95889871	95890022
chr13	95890029	95890479
chr13	95890942	95891248
chr13	95891248	95891509
chr13	95891594	95891933
chr13	95891933	95892326
chr13	95921460	95922039
chr13	95922669	95923070
chr13	95924226	95924506
chr13	95924506	95924713
chr13	95938077	95938384
chr13	95939264	95939539
chr13	95941273	95941495
chr13	95948894	95949197
chr13	95955248	95955554
chr13	95955554	95955790
chr13	95968079	95968383
chr13	95973361	95973750
chr13	95988169	95988522
chr13	96010751	96011147

chr13	96011147	96011672
chr13	96015577	96015889
chr13	96030769	96030931
chr13	96039436	96039921
chr13	96049930	96050119
chr13	96131114	96131429
chr13	96132502	96132782
chr13	96346612	96346899
chr13	96351365	96351645
chr13	96387925	96388567
chr13	96388577	96388787
chr13	96407084	96407304
chr13	96431779	96432096
chr13	96432360	96432809
chr13	96436189	96436518
chr13	96441455	96441690
chr13	96451467	96451709
chr13	96460713	96460863
chr13	96470599	96470986
chr13	96479547	96480025
chr13	96480185	96480641
chr13	96487241	96487908
chr13	96510278	96510450
chr13	96510479	96511005
chr13	96511005	96511304
chr13	96511304	96511640
chr13	96521806	96522291
chr13	96533557	96534199
chr13	96538185	96538413
chr13	96542237	96542473
chr13	96542473	96542788
chr13	96542788	96543148
chr13	96543148	96543485
chr13	96543485	96543707
chr13	96574268	96574499
chr13	96576126	96576302
chr13	96591371	96591585
chr13	96593715	96593889
chr13	96617908	96618207
chr13	96644260	96644460
chr13	96645912	96646131
chr13	96647167	96647398

chr13	96647398	96647654
chr13	96647925	96648151
chr13	96648154	96648529
chr13	96655064	96655294
chr13	96658476	96658626
chr13	96670333	96671275
chr13	96677145	96677426
chr13	96680519	96680790
chr13	96680790	96681038
chr13	96681090	96681373
chr13	96681373	96681665
chr13	96681738	96681903
chr13	96696967	96697124
chr13	96697712	96698126
chr13	96743023	96743184
chr13	96792221	96792385
chr13	96792684	96793004
chr13	96793142	96793463
chr13	96793463	96793757
chr13	96793770	96794099
chr13	96808009	96808439
chr13	96808439	96808696
chr13	96855270	96855499
chr13	96870908	96871088
chr13	96906776	96907143
chr13	96924128	96924473
chr13	96924473	96924992
chr13	96925000	96925280
chr13	96925280	96925594
chr13	96926462	96926612
chr13	96928534	96928751
chr13	96937879	96938440
chr13	96939378	96939543
chr13	96939650	96940102
chr13	96962790	96963383
chr13	96971077	96971427
chr13	96973429	96973711
chr13	96997144	96997405
chr13	96999069	96999593
chr13	97001315	97001798
chr13	97001866	97002018
chr13	97007469	97007720

chr13	97007952	97008261
chr13	97008261	97008529
chr13	97008533	97008736
chr13	97008736	97009087
chr13	97021911	97022176
chr13	97022176	97022430
chr13	97050332	97050531
chr13	97052683	97053034
chr13	97064971	97065121
chr13	97071169	97071578
chr13	97071578	97071837
chr13	97071837	97072205
chr13	97075584	97075982
chr13	97082464	97082826
chr13	97082826	97083093
chr13	97099543	97099779
chr13	97137525	97137800
chr13	97137800	97138074
chr13	97138074	97138357
chr13	97138509	97138694
chr13	97145898	97146138
chr13	97172795	97173202
chr13	97186434	97186686
chr13	97190470	97190703
chr13	97192694	97192844
chr13	97197831	97198222
chr13	97198222	97198598
chr13	97201225	97201432
chr13	97205090	97205433
chr13	97205433	97205708
chr13	97227705	97227964
chr13	97235106	97235380
chr13	97238285	97238535
chr13	97240250	97240521
chr13	97240521	97240901
chr13	97240901	97241290
chr13	97241290	97241541
chr13	97241541	97241774
chr13	97248534	97248768
chr13	97248853	97249563
chr13	97249563	97250110
chr13	97250177	97250416

chr13	97254188	97254341
chr13	97256951	97257326
chr13	97271461	97272240
chr13	97275019	97275228
chr13	97282212	97282571
chr13	97292121	97292356
chr13	97299877	97300248
chr13	97300248	97300556
chr13	97300556	97300961
chr13	97305503	97305696
chr13	97305696	97305910
chr13	97309765	97310062
chr13	97310236	97310427
chr13	97310514	97310873
chr13	97311497	97311750
chr13	97311935	97312398
chr13	97318333	97318516
chr13	97321369	97321731
chr13	97355554	97355759
chr13	97357055	97357205
chr13	97362711	97363466
chr13	97363745	97364382
chr13	97372443	97372593
chr13	97385657	97385864
chr13	97386470	97386863
chr13	97386863	97387148
chr13	97387670	97387839
chr13	97390414	97390693
chr13	97395389	97395621
chr13	97398635	97398949
chr13	97404964	97405152
chr13	97406846	97407143
chr13	97419039	97419426
chr13	97429681	97429965
chr13	97442827	97443170
chr13	97443354	97443551
chr13	97443684	97444100
chr13	97444100	97444422
chr13	97462358	97462556
chr13	97471838	97472006
chr13	97482526	97482748
chr13	97485978	97486296

chr13	97497203	97497493
chr13	97497493	97497885
chr13	97503116	97503381
chr13	97503489	97503852
chr13	97504984	97505434
chr13	97510900	97511456
chr13	97512637	97512863
chr13	97524151	97524359
chr13	97524923	97525073
chr13	97526784	97526943
chr13	97530575	97530925
chr13	97533836	97534177
chr13	97534177	97534617
chr13	97539784	97540007
chr13	97545984	97546134
chr13	97546139	97546512
chr13	97550886	97551168
chr13	97551168	97551441
chr13	97587176	97587404
chr13	97594861	97595106
chr13	97604472	97604842
chr13	97613709	97613975
chr13	97621378	97621688
chr13	97626688	97627143
chr13	97638497	97638829
chr13	97644791	97644984
chr13	97678267	97678943
chr13	97678943	97679223
chr13	97764117	97764354
chr13	97781629	97781931
chr13	97825487	97825661
chr13	97841530	97841857
chr13	97842730	97842982
chr13	97849123	97849468
chr13	97853003	97853164
chr13	97882580	97882730
chr13	97915611	97915910
chr13	97916184	97916527
chr13	97916527	97916775
chr13	97927840	97928112
chr13	97928112	97928376
chr13	97938928	97939274

chr13	97939342	97939834
chr13	97945430	97945630
chr13	97976562	97977093
chr13	97990886	97991409
chr13	98002352	98002508
chr13	98002644	98002924
chr13	98004744	98004950
chr13	98005618	98005926
chr13	98007366	98007536
chr13	98008683	98008907
chr13	98013635	98014131
chr13	98015988	98016280
chr13	98016280	98016574
chr13	98016581	98016747
chr13	98016986	98017183
chr13	98027221	98027435
chr13	98027455	98027673
chr13	98027673	98027955
chr13	98027955	98028459
chr13	98031334	98031590
chr13	98038807	98038957
chr13	98040711	98040923
chr13	98076952	98077233
chr13	98077311	98077523
chr13	98093018	98093308
chr13	98105186	98105466
chr13	98111099	98111317
chr13	98113111	98113371
chr13	98120608	98120949
chr13	98121894	98122364
chr13	98124229	98124451
chr13	98124451	98124685
chr13	98132821	98133376
chr13	98133376	98133708
chr13	98140579	98141054
chr13	98146543	98147085
chr13	98147558	98147779
chr13	98148416	98148655
chr13	98149501	98149755
chr13	98152692	98152992
chr13	98176529	98176701
chr13	98180611	98180834

chr13	98183201	98183395
chr13	98187346	98187612
chr13	98192332	98192679
chr13	98192679	98192905
chr13	98192905	98193210
chr13	98198933	98199225
chr13	98199677	98199856
chr13	98200754	98201077
chr13	98202770	98203015
chr13	98205570	98206028
chr13	98206028	98206335
chr13	98206335	98206706
chr13	98222915	98223243
chr13	98239799	98239966
chr13	98240109	98240264
chr13	98240322	98240495
chr13	98255039	98255305
chr13	98262629	98263057
chr13	98263057	98263257
chr13	98263257	98263673
chr13	98275429	98276140
chr13	98281087	98281524
chr13	98290807	98291013
chr13	98295965	98296470
chr13	98298892	98299119
chr13	98299119	98299394
chr13	98305325	98305607
chr13	98307239	98307392
chr13	98307673	98307980
chr13	98308099	98308327
chr13	98316322	98316662
chr13	98316662	98316868
chr13	98316868	98317347
chr13	98317408	98317558
chr13	98332173	98332428
chr13	98351606	98351821
chr13	98354092	98354354
chr13	98354354	98354754
chr13	98354754	98355101
chr13	98355101	98355299
chr13	98355299	98355536
chr13	98355536	98355794

chr13	98355822	98356007
chr13	98365778	98366108
chr13	98366148	98366597
chr13	98377825	98378064
chr13	98391142	98391330
chr13	98391330	98391556
chr13	98427870	98428021
chr13	98435283	98435575
chr13	98435660	98435888
chr13	98453346	98453516
chr13	98456397	98456878
chr13	98468196	98468439
chr13	98477748	98478039
chr13	98478039	98478379
chr13	98478379	98478774
chr13	98491832	98492134
chr13	98494907	98495245
chr13	98495275	98495505
chr13	98495505	98495699
chr13	98497540	98497936
chr13	98498049	98498463
chr13	98504522	98505213
chr13	98518438	98518642
chr13	98533418	98533763
chr13	98534157	98534595
chr13	98534631	98534856
chr13	98534856	98535136
chr13	98588529	98588889
chr13	98588889	98589244
chr13	98589244	98589569
chr13	98596787	98596948
chr13	98598166	98598318
chr13	98600902	98601086
chr13	98611833	98612107
chr13	98622547	98622968
chr13	98622994	98623261
chr13	98623261	98623695
chr13	98632302	98632872
chr13	98635232	98635559
chr13	98648280	98648476
chr13	98648757	98648907
chr13	98651064	98651527

chr13	98651527	98651844
chr13	98668195	98668367
chr13	98679253	98679650
chr13	98679650	98680233
chr13	98684332	98684687
chr13	98691930	98692142
chr13	98692142	98692494
chr13	98692683	98692980
chr13	98693010	98693268
chr13	98693289	98693605
chr13	98694746	98695074
chr13	98701095	98701335
chr13	98701881	98702140
chr13	98704428	98704702
chr13	98715542	98716199
chr13	98722343	98722623
chr13	98726568	98726804
chr13	98747026	98747212
chr13	98768473	98768637
chr13	98773006	98773209
chr13	98809185	98809335
chr13	98814707	98815045
chr13	98815089	98815525
chr13	98815525	98815807
chr13	98818109	98818646
chr13	98819158	98819388
chr13	98826069	98826265
chr13	98831148	98831398
chr13	98834031	98834213
chr13	98834312	98834496
chr13	98836736	98837053
chr13	98867905	98868170
chr13	98871236	98871598
chr13	98890123	98890635
chr13	98890635	98890896
chr13	98890896	98891221
chr13	98891472	98891771
chr13	98894021	98894302
chr13	98896562	98896740
chr13	98896787	98896988
chr13	98912698	98912962
chr13	98915642	98915974

chr13	98916287	98916606
chr13	98917968	98918137
chr13	98921978	98922196
chr13	98925550	98925828
chr13	98925833	98926187
chr13	98926187	98926547
chr13	98926547	98926880
chr13	98931629	98931874
chr13	98941875	98942335
chr13	98944643	98944866
chr13	98946534	98946816
chr13	98954732	98954910
chr13	98955249	98955658
chr13	98955684	98955936
chr13	98971047	98971307
chr13	98971307	98971632
chr13	98971632	98971970
chr13	98986656	98986954
chr13	98994912	98995247
chr13	99037289	99037593
chr13	99038331	99038703
chr13	99046460	99046630
chr13	99055644	99055869
chr13	99058186	99058470
chr13	99058477	99059020
chr13	99063085	99063243
chr13	99067489	99067790
chr13	99070556	99070706
chr13	99077142	99077322
chr13	99085035	99085288
chr13	99085336	99085493
chr13	99085575	99085768
chr13	99085768	99085964
chr13	99087836	99088181
chr13	99088316	99088639
chr13	99093377	99093667
chr13	99094090	99094608
chr13	99100431	99100801
chr13	99100867	99101020
chr13	99105298	99105474
chr13	99108584	99108950
chr13	99116946	99117156

chr13	99124849	99125283
chr13	99125843	99126056
chr13	99126056	99126262
chr13	99128467	99128840
chr13	99142448	99142977
chr13	99143322	99143574
chr13	99150675	99150939
chr13	99150942	99151094
chr13	99152262	99152618
chr13	99152618	99152992
chr13	99157808	99158012
chr13	99158528	99158705
chr13	99158712	99159288
chr13	99163891	99164041
chr13	99164137	99164301
chr13	99169460	99169789
chr13	99170359	99170829
chr13	99173292	99173630
chr13	99194233	99194540
chr13	99194540	99194909
chr13	99216479	99216835
chr13	99218043	99218334
chr13	99220490	99220648
chr13	99220836	99221209
chr13	99223523	99223778
chr13	99236876	99237071
chr13	99237121	99237458
chr13	99238203	99238428
chr13	99238428	99238665
chr13	99238665	99238949
chr13	99238949	99239240
chr13	99307483	99307633
chr13	99307737	99308406
chr13	99308406	99308857
chr13	99314104	99314292
chr13	99317196	99317346
chr13	99317596	99317937
chr13	99331311	99331566
chr13	99331748	99331907
chr13	99344245	99345070
chr13	99352772	99353155
chr13	99353155	99353563

chr13	99360255	99360616
chr13	99375592	99375955
chr13	99379386	99379536
chr13	99384617	99384922
chr13	99384922	99385219
chr13	99385219	99385491
chr13	99391082	99391471
chr13	99391471	99391779
chr13	99391779	99392086
chr13	99395696	99395980
chr13	99397423	99397547
chr13	99397547	99397614
chr13	99397881	99398130
chr13	99398130	99398342
chr13	99399414	99399701
chr13	99403291	99403480
chr13	99410428	99410649
chr13	99410649	99410856
chr13	99410856	99411389
chr13	99412864	99413030
chr13	99414417	99414625
chr13	99416688	99416919
chr13	99418505	99418690
chr13	99431381	99431698
chr13	99439414	99439681
chr13	99441742	99442011
chr13	99447415	99447859
chr13	99449138	99449323
chr13	99466137	99466289
chr13	99475235	99475963
chr13	99481477	99481764
chr13	99502990	99503309
chr13	99503639	99504027
chr13	99514903	99515196
chr13	99515196	99515501
chr13	99515501	99515829
chr13	99515829	99516116
chr13	99516116	99516436
chr13	99516436	99517136
chr13	99528169	99528733
chr13	99528733	99529009
chr13	99534933	99535140

chr13	99540226	99540378
chr13	99553074	99553224
chr13	99553904	99554090
chr13	99592484	99592881
chr13	99592881	99593289
chr13	99606733	99606895
chr13	99607343	99607494
chr13	99610467	99610887
chr13	99647158	99647441
chr13	99647607	99648343
chr13	99761865	99762112
chr13	99775999	99776285
chr13	99781254	99781408
chr13	99790878	99791214
chr13	99802844	99802994
chr13	99830139	99830658
chr13	99830999	99831263
chr13	99834467	99834688
chr13	99837854	99838035
chr13	99842732	99842891
chr13	99857897	99858263
chr13	99859136	99859371
chr13	99900436	99900822
chr13	99902871	99903251
chr13	99920843	99921124
chr13	99921201	99921911
chr13	99937913	99938111
chr13	99996967	99997180
chr13	100001834	100002206
chr13	100014999	100015151
chr13	100015258	100015532
chr13	100015532	100015922
chr13	100023943	100024094
chr13	100035010	100035203
chr13	100078223	100078501
chr13	100101132	100101282
chr13	100103597	100103928
chr13	100103928	100104237
chr13	100107746	100108195
chr13	100108195	100108545
chr13	100112390	100112579
chr13	100124689	100125202

chr13	100125316	100125691
chr13	100125742	100125981
chr13	100152159	100152349
chr13	100152349	100152557
chr13	100152712	100152897
chr13	100173478	100173854
chr13	100210614	100210885
chr13	100341749	100342240
chr13	100398733	100398953
chr13	100411868	100412368
chr13	100419610	100419760
chr13	100419828	100420133
chr13	100438622	100439055
chr13	100439143	100439300
chr13	100440284	100440495
chr13	100449514	100449995
chr13	100451722	100451906
chr13	100453732	100454023
chr13	100457717	100458072
chr13	100461248	100461831
chr13	100463454	100463855
chr13	100463855	100464147
chr13	100492062	100492284
chr13	100492284	100492554
chr13	100492554	100492831
chr13	100493313	100493694
chr13	100494838	100494990
chr13	100496015	100496215
chr13	100510831	100511070
chr13	100511133	100511505
chr13	100511505	100511786
chr13	100514058	100514365
chr13	100516635	100517169
chr13	100521769	100522032
chr13	100522205	100522635
chr13	100522833	100522989
chr13	100523192	100523387
chr13	100532927	100533332
chr13	100534280	100534440
chr13	100535216	100535625
chr13	100535908	100536169
chr13	100536169	100536371

chr13	100536371	100536582
chr13	100536822	100536992
chr13	100543079	100543367
chr13	100543367	100543729
chr13	100547536	100547937
chr13	100551285	100551565
chr13	100551565	100551835
chr13	100551973	100552138
chr13	100552176	100552837
chr13	100552837	100553075
chr13	100553115	100553349
chr13	100568476	100568728
chr13	100576087	100576283
chr13	100580043	100580254
chr13	100581479	100581757
chr13	100581757	100581981
chr13	100581981	100582238
chr13	100582238	100582523
chr13	100589070	100589273
chr13	100592595	100592857
chr13	100592857	100593168
chr13	100593974	100594514
chr13	100600591	100600791
chr13	100602661	100602878
chr13	100607832	100608042
chr13	100609225	100609529
chr13	100616263	100616549
chr13	100616549	100616869
chr13	100616869	100617141
chr13	100628247	100628510
chr13	100650598	100650917
chr13	100650917	100651183
chr13	100651183	100651473
chr13	100651473	100651730
chr13	100656257	100656409
chr13	100657376	100657594
chr13	100659457	100659711
chr13	100659916	100660203
chr13	100662389	100662710
chr13	100662838	100663044
chr13	100668362	100668625
chr13	100669285	100669584

chr13	100669584	100669830
chr13	100669830	100670087
chr13	100670410	100670564
chr13	100670585	100670803
chr13	100684428	100684688
chr13	100690545	100690813
chr13	100690813	100691078
chr13	100703612	100703797
chr13	100730427	100731296
chr13	100743811	100744053
chr13	100744053	100744436
chr13	100744436	100744836
chr13	100756742	100756979
chr13	100767315	100767541
chr13	100775342	100775572
chr13	100775572	100775799
chr13	100775799	100776054
chr13	100776054	100776272
chr13	100776272	100776467
chr13	100776522	100776672
chr13	100786194	100786510
chr13	100786510	100786839
chr13	100786839	100787072
chr13	100787411	100787661
chr13	100804921	100805222
chr13	100813923	100814182
chr13	100832771	100833157
chr13	100833157	100833718
chr13	100843386	100843691
chr13	100844016	100844249
chr13	100846343	100846657
chr13	100849183	100849436
chr13	100853202	100853415
chr13	100854060	100854285
chr13	100854285	100854496
chr13	100892225	100892471
chr13	100940871	100941082
chr13	100990756	100990906
chr13	101017153	101017303
chr13	101127849	101128121
chr13	101161811	101162119
chr13	101465487	101465694

chr13	101478590	101478999
chr13	101479056	101479298
chr13	101510044	101510194
chr13	101510693	101510843
chr13	101540392	101541067
chr13	101547962	101548181
chr13	101632857	101633093
chr13	101634555	101634708
chr13	101658355	101658585
chr13	101659083	101659304
chr13	101664099	101664262
chr13	101692373	101692558
chr13	101694009	101694324
chr13	101694463	101694771
chr13	101694823	101695014
chr13	101695014	101695218
chr13	101695218	101695397
chr13	101695946	101696181
chr13	101712832	101712982
chr13	101713068	101713406
chr13	101717790	101718320
chr13	101719304	101719456
chr13	101721869	101722259
chr13	101726796	101727492
chr13	101735854	101736013
chr13	101736753	101736913
chr13	101740135	101740400
chr13	101752260	101752412
chr13	101753636	101754103
chr13	101759257	101759596
chr13	101760321	101760698
chr13	101760698	101761007
chr13	101767278	101767536
chr13	101767569	101767821
chr13	101767821	101768068
chr13	101768068	101768282
chr13	101768282	101768545
chr13	101768545	101769005
chr13	101945707	101945962
chr13	102052862	102053194
chr13	102053314	102053588
chr13	102089243	102089440

chr13	102089552	102089911
chr13	102103230	102103398
chr13	102106362	102106728
chr13	102107107	102107488
chr13	102109003	102109389
chr13	102109638	102109859
chr13	102110114	102110272
chr13	102122422	102122621
chr13	102161231	102161519
chr13	102167317	102167542
chr13	102202393	102202690
chr13	102216551	102216822
chr13	102231108	102231419
chr13	102257458	102257707
chr13	102258228	102258466
chr13	102482433	102482858
chr13	102526852	102527067
chr13	102573211	102573461
chr13	102573461	102573724
chr13	102573924	102574110
chr13	102582376	102582674
chr13	102583934	102584320
chr13	102584320	102584558
chr13	102639550	102639928
chr13	102645010	102645164
chr13	102674177	102674453
chr13	102678512	102678854
chr13	102697736	102697891
chr13	102705900	102706205
chr13	102727015	102727352
chr13	102728081	102728233
chr13	102728480	102728665
chr13	102729834	102730109
chr13	102730109	102730354
chr13	102745079	102745252
chr13	102745263	102745570
chr13	102745570	102745962
chr13	102745962	102746252
chr13	102753741	102754158
chr13	102756120	102756411
chr13	102786771	102786952
chr13	102794432	102794692

chr13	102820568	102820730
chr13	102821248	102821648
chr13	102827033	102827252
chr13	102844000	102844595
chr13	102844630	102844841
chr13	102849098	102849367
chr13	102850608	102850847
chr13	102856931	102857380
chr13	102857380	102857623
chr13	102857623	102857869
chr13	102857869	102858166
chr13	102868449	102868828
chr13	102869490	102869953
chr13	102877087	102877563
chr13	102880382	102880999
chr13	102889085	102889245
chr13	102894297	102894598
chr13	102895854	102896291
chr13	102897891	102898051
chr13	102898721	102899250
chr13	102899250	102899838
chr13	102900641	102900907
chr13	102905925	102906315
chr13	102906315	102906594
chr13	102906594	102907001
chr13	102910116	102910343
chr13	102914415	102914698
chr13	102915334	102915487
chr13	102931979	102932275
chr13	102942531	102942996
chr13	102943613	102943793
chr13	102957408	102957644
chr13	102957644	102957944
chr13	102957944	102958368
chr13	102958511	102958689
chr13	102958689	102958889
chr13	102958919	102959144
chr13	102959144	102959418
chr13	102963295	102963824
chr13	102970232	102970480
chr13	102981448	102981754
chr13	102985657	102985911

chr13	102989754	102990027
chr13	102990167	102990392
chr13	103037871	103038099
chr13	103049428	103049762
chr13	103051408	103051589
chr13	103071878	103072104
chr13	103073394	103073854
chr13	103083037	103083201
chr13	103089169	103089350
chr13	103096672	103097113
chr13	103102511	103103274
chr13	103109603	103109846
chr13	103109846	103110247
chr13	103110277	103110448
chr13	103120937	103121203
chr13	103151131	103151304
chr13	103173850	103174094
chr13	103186426	103186792
chr13	103186792	103187130
chr13	103201005	103201385
chr13	103201385	103202006
chr13	103207252	103207709
chr13	103207839	103208044
chr13	103211559	103211760
chr13	103212626	103212781
chr13	103225260	103225554
chr13	103225587	103225780
chr13	103226431	103226779
chr13	103226779	103227002
chr13	103238113	103238368
chr13	103238368	103238687
chr13	103239489	103240088
chr13	103244116	103244339
chr13	103245446	103245872
chr13	103247253	103247409
chr13	103260027	103260377
chr13	103260421	103260747
chr13	103260747	103260974
chr13	103261476	103261692
chr13	103271149	103271479
chr13	103271883	103272183
chr13	103279751	103279973

chr13	103290999	103291247
chr13	103291247	103291530
chr13	103303604	103303938
chr13	103303938	103304303
chr13	103304303	103304780
chr13	103317612	103318453
chr13	103319261	103319588
chr13	103319673	103319831
chr13	103321278	103321430
chr13	103324761	103325003
chr13	103327606	103328514
chr13	103333612	103333932
chr13	103333932	103334289
chr13	103334289	103334782
chr13	103344086	103344351
chr13	103374516	103374806
chr13	103386872	103387466
chr13	103409327	103409593
chr13	103410808	103411134
chr13	103459562	103459830
chr13	103491962	103492112
chr13	103494472	103494695
chr13	103539673	103539943
chr13	103540686	103540978
chr13	103609524	103609758
chr13	103644455	103644808
chr13	103645756	103645913
chr13	103646095	103646332
chr13	103670594	103670968
chr13	103680764	103681197
chr13	103681197	103681709
chr13	103686418	103686832
chr13	103686832	103687130
chr13	103687130	103687519
chr13	103695548	103695714
chr13	103695957	103696269
chr13	103696269	103696553
chr13	103703398	103703731
chr13	103711304	103711687
chr13	103733372	103733553
chr13	103735630	103735877
chr13	103751475	103751700

chr13	103752148	103752459
chr13	103756200	103756577
chr13	103756633	103756903
chr13	103759597	103759832
chr13	103773836	103774115
chr13	103774115	103774419
chr13	103774419	103774937
chr13	103795108	103795258
chr13	103821480	103821769
chr13	103838738	103838997
chr13	103841305	103841656
chr13	103841656	103841995
chr13	103842233	103842415
chr13	103847426	103847608
chr13	103870936	103871103
chr13	103884466	103884812
chr13	103909990	103910176
chr13	103920085	103920553
chr13	103920553	103920915
chr13	103920915	103921339
chr13	103921339	103921849
chr13	103962073	103962282
chr13	103966415	103966565
chr13	103977821	103978193
chr13	103978193	103978573
chr13	103978573	103978924
chr13	103990821	103990971
chr13	103991154	103991359
chr13	104005486	104005663
chr13	104015365	104015732
chr13	104015732	104016315
chr13	104021099	104021359
chr13	104023334	104023696
chr13	104023696	104024024
chr13	104024857	104025008
chr13	104029176	104029363
chr13	104036254	104036420
chr13	104036523	104036827
chr13	104037035	104037374
chr13	104037507	104037899
chr13	104037899	104038171
chr13	104041118	104041365

chr13	104048931	104049171
chr13	104065651	104065989
chr13	104065989	104066322
chr13	104074593	104075214
chr13	104082070	104082817
chr13	104082817	104083078
chr13	104089247	104089679
chr13	104089679	104089967
chr13	104094634	104095043
chr13	104095043	104095432
chr13	104109008	104109333
chr13	104109333	104109851
chr13	104109851	104110288
chr13	104110327	104110789
chr13	104139623	104139948
chr13	104155753	104156057
chr13	104157458	104157737
chr13	104177898	104178113
chr13	104178113	104178335
chr13	104178335	104178645
chr13	104178645	104178936
chr13	104178936	104179216
chr13	104187467	104187641
chr13	104207112	104207517
chr13	104228422	104228755
chr13	104228755	104229227
chr13	104229291	104229448
chr13	104253047	104253255
chr13	104285051	104285242
chr13	104285315	104285457
chr13	104285457	104285606
chr13	104286034	104286233
chr13	104286695	104287342
chr13	104287740	104287956
chr13	104288143	104288483
chr13	104288564	104288836
chr13	104295500	104295825
chr13	104318176	104318377
chr13	104323583	104323818
chr13	104428854	104429027
chr13	104432778	104433257
chr13	104433257	104433517

chr13	104443242	104443433
chr13	104447651	104447822
chr13	104448238	104448697
chr13	104448912	104449077
chr13	104460286	104460892
chr13	104467501	104468003
chr13	104468003	104468403
chr13	104506036	104506567
chr13	104507353	104507592
chr13	104513939	104514329
chr13	104515158	104515363
chr13	104517091	104517272
chr13	104520287	104520560
chr13	104520560	104520929
chr13	104521538	104522007
chr13	104526670	104526981
chr13	104554916	104555118
chr13	104562119	104562273
chr13	104588439	104588648
chr13	104622216	104622797
chr13	104630864	104631125
chr13	104662086	104662343
chr13	104662425	104662883
chr13	104662887	104663068
chr13	104789754	104790014
chr13	104805786	104805938
chr13	104816332	104816803
chr13	104816803	104817067
chr13	104817067	104817532
chr13	104846537	104846729
chr13	104863313	104863812
chr13	104863812	104864218
chr13	104869695	104869929
chr13	104883590	104883740
chr13	104894840	104895086
chr13	104910262	104910550
chr13	104914622	104914824
chr13	104918823	104918986
chr13	104924218	104924404
chr13	104934187	104934378
chr13	104939111	104939676
chr13	104939950	104940183

chr13	104948014	104948283
chr13	104985743	104986032
chr13	105022280	105022638
chr13	105054136	105054379
chr13	105054379	105054602
chr13	105054602	105054939
chr13	105293977	105294355
chr13	105294355	105294548
chr13	105377078	105377387
chr13	105735065	105735228
chr13	106427268	106427500
chr13	106551285	106551594
chr13	106551644	106551962
chr13	106562670	106562933
chr13	106584872	106585054
chr13	106819810	106820106
chr13	106828058	106828208
chr13	106830497	106830657
chr13	106880956	106881114
chr13	106894378	106894583
chr13	106936195	106936772
chr13	106936772	106937251
chr13	106941650	106941834
chr13	106946652	106946951
chr13	106946951	106947213
chr13	106947213	106947525
chr13	106960598	106960913
chr13	106961401	106961826
chr13	106994655	106994857
chr13	106994857	106995091
chr13	106998734	106999030
chr13	107003127	107003643
chr13	107015711	107015913
chr13	107019351	107019821
chr13	107021466	107021704
chr13	107021704	107021987
chr13	107021987	107022279
chr13	107022279	107022750
chr13	107022750	107023193
chr13	107024352	107024514
chr13	107035365	107035542
chr13	107036658	107037069

chr13	107037069	107037453
chr13	107056405	107056591
chr13	107063522	107063775
chr13	107094600	107094821
chr13	107094939	107095317
chr13	107095317	107095998
chr13	107096941	107097270
chr13	107101662	107101917
chr13	107113968	107114196
chr13	107114196	107114384
chr13	107121637	107121820
chr13	107130018	107130221
chr13	107134280	107134558
chr13	107168589	107168953
chr13	107168953	107169191
chr13	107182625	107182830
chr13	107207957	107208208
chr13	107325786	107325941
chr13	107329064	107329388
chr13	107333645	107333860
chr13	107371657	107372227
chr13	107377567	107377786
chr13	107378201	107378363
chr13	107461003	107461347
chr13	107461347	107461721
chr13	107469617	107470105
chr13	107485481	107486128
chr13	107536438	107536856
chr13	107536970	107537282
chr13	107537418	107537772
chr13	107548597	107548832
chr13	107562481	107562889
chr13	107574974	107575420
chr13	107598572	107598771
chr13	107600672	107600904
chr13	107607398	107607704
chr13	107616895	107617058
chr13	107619557	107619799
chr13	107668459	107668808
chr13	107668868	107669118
chr13	107670971	107671144
chr13	107676289	107676816

chr13	107676816	107677191
chr13	107682517	107682904
chr13	107698858	107699014
chr13	107700284	107700591
chr13	107700659	107700954
chr13	107711315	107711903
chr13	107712366	107712591
chr13	107714049	107714460
chr13	107718268	107718514
chr13	107723171	107723321
chr13	107736679	107736898
chr13	107743807	107744276
chr13	107798091	107798486
chr13	107813536	107814295
chr13	107814356	107814523
chr13	107814531	107814691
chr13	107829992	107830153
chr13	107831138	107831587
chr13	107838098	107838612
chr13	107838920	107839070
chr13	107844128	107844397
chr13	107844949	107845143
chr13	107845494	107845754
chr13	107845854	107846139
chr13	107846759	107846958
chr13	107846996	107847202
chr13	107847214	107847554
chr13	107847554	107847947
chr13	107848148	107848336
chr13	107854857	107855020
chr13	107858202	107858404
chr13	107858404	107858711
chr13	107858711	107859059
chr13	107859059	107859348
chr13	107859348	107859764
chr13	107859764	107860140
chr13	107865928	107866363
chr13	107866392	107866675
chr13	107866675	107866900
chr13	107866900	107867156
chr13	107873121	107873759
chr13	107888826	107889095

chr13	107889095	107889300
chr13	107889300	107889501
chr13	107889889	107890359
chr13	107890359	107890768
chr13	107890768	107891151
chr13	107891151	107891456
chr13	107923373	107923582
chr13	107925463	107925892
chr13	107933712	107934050
chr13	107940041	107940200
chr13	107955812	107956067
chr13	107961962	107962464
chr13	107963495	107963805
chr13	107967982	107968356
chr13	108015287	108015482
chr13	108025919	108026098
chr13	108041522	108041773
chr13	108043934	108044210
chr13	108044210	108044573
chr13	108044573	108044937
chr13	108046046	108046411
chr13	108074494	108074825
chr13	108097085	108097384
chr13	108098791	108099037
chr13	108127249	108127453
chr13	108153056	108153300
chr13	108158097	108158445
chr13	108158445	108158917
chr13	108158917	108159249
chr13	108188010	108188354
chr13	108191402	108191645
chr13	108204967	108205127
chr13	108206397	108206588
chr13	108214007	108214520
chr13	108214520	108214797
chr13	108214797	108215133
chr13	108228658	108229134
chr13	108229134	108229629
chr13	108229629	108229930
chr13	108229979	108230314
chr13	108253915	108254386
chr13	108263125	108263474

chr13	108277434	108277619
chr13	108297307	108297623
chr13	108297623	108297906
chr13	108297906	108298110
chr13	108302957	108303306
chr13	108303306	108303728
chr13	108303728	108304189
chr13	108315818	108316544
chr13	108316544	108316976
chr13	108329549	108330084
chr13	108338074	108338336
chr13	108338336	108338654
chr13	108347071	108347360
chr13	108373321	108373472
chr13	108390629	108390925
chr13	108411377	108411763
chr13	108415540	108415790
chr13	108480495	108480720
chr13	108485064	108485411
chr13	108500485	108500641
chr13	108553120	108553270
chr13	108599480	108599892
chr13	108603465	108603920
chr13	108628528	108629020
chr13	108636215	108636408
chr13	108658092	108658452
chr13	108677522	108677695
chr13	108688268	108688506
chr13	108692947	108693511
chr13	108693511	108693804
chr13	108695359	108695623
chr13	108710829	108711051
chr13	108713956	108714434
chr13	108716589	108716824
chr13	108746678	108746919
chr13	108751212	108751454
chr13	108751526	108751789
chr13	108752015	108752165
chr13	108752305	108752683
chr13	108775704	108775907
chr13	108775909	108776207
chr13	108812958	108813384

chr13	108813384	108813702
chr13	108817818	108818108
chr13	108819852	108820447
chr13	108834366	108834977
chr13	108900242	108900452
chr13	108964286	108964499
chr13	109116037	109116373
chr13	109116373	109116746
chr13	109116792	109117050
chr13	109158841	109158991
chr13	109166359	109166577
chr13	109170655	109170868
chr13	109175329	109175656
chr13	109204752	109204957
chr13	109209651	109209921
chr13	109210494	109210716
chr13	109211531	109211779
chr13	109211826	109211992
chr13	109212000	109212417
chr13	109212417	109212629
chr13	109212673	109212832
chr13	109213140	109213290
chr13	109213312	109213478
chr13	109229313	109229575
chr13	109229575	109229837
chr13	109229837	109230138
chr13	109230138	109230485
chr13	109230485	109230773
chr13	109230773	109230996
chr13	109236000	109236633
chr13	109242429	109243042
chr13	109243116	109243556
chr13	109243721	109243968
chr13	109260170	109260714
chr13	109260714	109261023
chr13	109261023	109261222
chr13	109261222	109261458
chr13	109261964	109262200
chr13	109265287	109265492
chr13	109268293	109268629
chr13	109268917	109269313
chr13	109278769	109278919

chr13	109279048	109279737
chr13	109305123	109305284
chr13	109315510	109315888
chr13	109315888	109316172
chr13	109317303	109317459
chr13	109318505	109319022
chr13	109319038	109319235
chr13	109343659	109343950
chr13	109348901	109349125
chr13	109357807	109357992
chr13	109360037	109360379
chr13	109384080	109384241
chr13	109408374	109408618
chr13	109440370	109440799
chr13	109440799	109441287
chr13	109441505	109441674
chr13	109481355	109482020
chr13	109486837	109487156
chr13	109487325	109487776
chr13	109502760	109503354
chr13	109508551	109508772
chr13	109510799	109511092
chr13	109536554	109536976
chr13	109539815	109540043
chr13	109547775	109548090
chr13	109554430	109554635
chr13	109555274	109555556
chr13	109555556	109555889
chr13	109558363	109558525
chr13	109590111	109590310
chr13	109609876	109610126
chr13	109616011	109616241
chr13	109623266	109623416
chr13	109631140	109631394
chr13	109632527	109632844
chr13	109633202	109633646
chr13	109643383	109643737
chr13	109647868	109648178
chr13	109648178	109648520
chr13	109651742	109652159
chr13	109652217	109652396
chr13	109658687	109658968

chr13	109658981	109659472
chr13	109659607	109659801
chr13	109669601	109669849
chr13	109669962	109670380
chr13	109683065	109683226
chr13	109685293	109685855
chr13	109685855	109686135
chr13	109686135	109686467
chr13	109693037	109693227
chr13	109693697	109694163
chr13	109694163	109694588
chr13	109703523	109703764
chr13	109710730	109710910
chr13	109732519	109732744
chr13	109736397	109736547
chr13	109754971	109755228
chr13	109759286	109759493
chr13	109759873	109760158
chr13	109775938	109776481
chr13	109789183	109789568
chr13	109802263	109802537
chr13	109806659	109806826
chr13	109819068	109819404
chr13	109822152	109822426
chr13	109822426	109822926
chr13	109839320	109839581
chr13	109840335	109840707
chr13	109841220	109841494
chr13	109841494	109841703
chr13	109842977	109843400
chr13	109874843	109875086
chr13	109881069	109881552
chr13	109881552	109881868
chr13	109897887	109898253
chr13	109899796	109900024
chr13	109900162	109900463
chr13	109902451	109902803
chr13	109902803	109903120
chr13	109903120	109903531
chr13	109907700	109907904
chr13	109917428	109917733
chr13	109922311	109922556

chr13	110018410	110018658
chr13	110018658	110018881
chr13	110019536	110019710
chr13	110023173	110023349
chr13	110040224	110040398
chr13	110062209	110062626
chr13	110062794	110063045
chr13	110211766	110212023
chr13	110218577	110218937
chr13	110229823	110230072
chr13	110230072	110230636
chr13	110237673	110237907
chr13	110280027	110280275
chr13	110283902	110284107
chr13	110333748	110333927
chr13	110368679	110369021
chr13	110380460	110380711
chr13	110382618	110382852
chr13	110394273	110394600
chr13	110394600	110394834
chr13	110394834	110395013
chr13	110395013	110395282
chr13	110395282	110395609
chr13	110401177	110401327
chr13	110401482	110401666
chr13	110401666	110401855
chr13	110401947	110402235
chr13	110402742	110403100
chr13	110403151	110403319
chr13	110403319	110403511
chr13	110403587	110403761
chr13	110403761	110403979
chr13	110404068	110404394
chr13	110406247	110406579
chr13	110409957	110410252
chr13	110410354	110410699
chr13	110412892	110413146
chr13	110418694	110418994
chr13	110419569	110419998
chr13	110428655	110429211
chr13	110435737	110435954
chr13	110435977	110436473

chr13	110436625	110437131
chr13	110444918	110445187
chr13	110459936	110460349
chr13	110466512	110466832
chr13	110469123	110469277
chr13	110470825	110471142
chr13	110490142	110490686
chr13	110490796	110491056
chr13	110492690	110492987
chr13	110502887	110503101
chr13	110506352	110506588
chr13	110507784	110508250
chr13	110540611	110540902
chr13	110541602	110541851
chr13	110541851	110542072
chr13	110545332	110545897
chr13	110550160	110550459
chr13	110561890	110562085
chr13	110562827	110563357
chr13	110600063	110600269
chr13	110600413	110600681
chr13	110601344	110601699
chr13	110601703	110601929
chr13	110602987	110603179
chr13	110609354	110609532
chr13	110609554	110609712
chr13	110633088	110633847
chr13	110643481	110644139
chr13	110656900	110657246
chr13	110672723	110672909
chr13	110678528	110678808
chr13	110679815	110680181
chr13	110680765	110681032
chr13	110700205	110700583
chr13	110720282	110720757
chr13	110724061	110724595
chr13	110724617	110724938
chr13	110725847	110726209
chr13	110735348	110735557
chr13	110735599	110736016
chr13	110739152	110739400
chr13	110741058	110741377

chr13	110741582	110741894
chr13	110743899	110744305
chr13	110744305	110744615
chr13	110748668	110749014
chr13	110749014	110749376
chr13	110755063	110755408
chr13	110756945	110757159
chr13	110768666	110768855
chr13	110769245	110769542
chr13	110771271	110771442
chr13	110780795	110780994
chr13	110798389	110798544
chr13	110804621	110804921
chr13	110817108	110817506
chr13	110824852	110825312
chr13	110827914	110828183
chr13	110854269	110854544
chr13	110859138	110859564
chr13	110874383	110874628
chr13	110877216	110877422
chr13	110877873	110878055
chr13	110878123	110878375
chr13	110882680	110883340
chr13	110896613	110897186
chr13	110903335	110903569
chr13	110907655	110908068
chr13	110911246	110911593
chr13	110911865	110912257
chr13	110913654	110914127
chr13	110917566	110917786
chr13	110924991	110925301
chr13	110925301	110925613
chr13	110929539	110929977
chr13	110941603	110941970
chr13	110941970	110942212
chr13	110943020	110943213
chr13	110943213	110943416
chr13	110967052	110967592
chr13	110967592	110968065
chr13	110968113	110968429
chr13	110972176	110972454
chr13	111031722	111032037

chr13	111050165	111050610
chr13	111068992	111069147
chr13	111092071	111092345
chr13	111095514	111095764
chr13	111099261	111099583
chr13	111099955	111100119
chr13	111100496	111100844
chr13	111116490	111116722
chr13	111160287	111160489
chr13	111233261	111233731
chr13	111237941	111238207
chr13	111256195	111256528
chr13	111258199	111258560
chr13	111294729	111295060
chr13	111295060	111295507
chr13	111295952	111296241
chr13	111345317	111345779
chr13	111345798	111346409
chr13	111411052	111411214
chr13	111418128	111418443
chr13	111461467	111461709
chr13	111489363	111489779
chr13	111489779	111490177
chr13	111490177	111490467
chr13	111490467	111490774
chr13	111542187	111542469
chr13	111542469	111542703
chr13	111545325	111545652
chr13	111548366	111548579
chr13	111570091	111570242
chr13	111601496	111601651
chr13	111623125	111623423
chr13	111685727	111686095
chr13	111686095	111686397
chr13	111686397	111686672
chr13	111686672	111686946
chr13	111686946	111687230
chr13	111687230	111687524
chr13	111687524	111687828
chr13	111725024	111725398
chr13	111725398	111725680
chr13	111725680	111725918

chr13	111725918	111726140
chr13	111744422	111744705
chr13	111744705	111745058
chr13	111771283	111771600
chr13	111772111	111772539
chr13	111788186	111788445
chr13	111788445	111788807
chr13	111789198	111789438
chr13	111796663	111796853
chr13	111797044	111797362
chr13	111797583	111797924
chr13	111797924	111798325
chr13	111802942	111803230
chr13	111807729	111807986
chr13	111807986	111808259
chr13	111808288	111808629
chr13	111808629	111808977
chr13	111808977	111809446
chr13	111809446	111809944
chr13	111826676	111826852
chr13	111850519	111850780
chr13	111856031	111856210
chr13	111862425	111862660
chr13	111919490	111919697
chr13	111941264	111941498
chr13	111952091	111952247
chr13	111966126	111966572
chr13	111966572	111966867
chr13	111967553	111967824
chr13	111967824	111968055
chr13	111984642	111984802
chr13	111986715	111987023
chr13	111999704	112000075
chr13	112001061	112001297
chr13	112001297	112001574
chr13	112002673	112003011
chr13	112003011	112003472
chr13	112018606	112018955
chr13	112018955	112019280
chr13	112024915	112025254
chr13	112030673	112031397
chr13	112043628	112043857

chr13	112052123	112052328
chr13	112052721	112052926
chr13	112054565	112054728
chr13	112054776	112055378
chr13	112062059	112062512
chr13	112063504	112064255
chr13	112071180	112071693
chr13	112071997	112072250
chr13	112076251	112076406
chr13	112100654	112100995
chr13	112110112	112110465
chr13	112144919	112145305
chr13	112145473	112145729
chr13	112150718	112151080
chr13	112152981	112153407
chr13	112185217	112185389
chr13	112188305	112188713
chr13	112222406	112222818
chr13	112251532	112251818
chr13	112252125	112252346
chr13	112253954	112254315
chr13	112254315	112254676
chr13	112258495	112258877
chr13	112259477	112259747
chr13	112264754	112264982
chr13	112271953	112272223
chr13	112286811	112287168
chr13	112287168	112287530
chr13	112308721	112308937
chr13	112311670	112311958
chr13	112316729	112316949
chr13	112317145	112317371
chr13	112318102	112318527
chr13	112318527	112318792
chr13	112326255	112326480
chr13	112326480	112326738
chr13	112340800	112341084
chr13	112343131	112343288
chr13	112343496	112343974
chr13	112349198	112349391
chr13	112363190	112363630
chr13	112365760	112366121

chr13	112371189	112371537
chr13	112375867	112376067
chr13	112379308	112379590
chr13	112379590	112379856
chr13	112379856	112380215
chr13	112380215	112380517
chr13	112380517	112380726
chr13	112389860	112390082
chr13	112390260	112390653
chr13	112390912	112391138
chr13	112391184	112391435
chr13	112391435	112391704
chr13	112425181	112425406
chr13	112425406	112425646
chr13	112430521	112430715
chr13	112430715	112430939
chr13	112430939	112431339
chr13	112431339	112431711
chr13	112431711	112432007
chr13	112432007	112432319
chr13	112440083	112440276
chr13	112447236	112447439
chr13	112454159	112454331
chr13	112461214	112461571
chr13	112463573	112463873
chr13	112463873	112464210
chr13	112464210	112464628
chr13	112509546	112509828
chr13	112517283	112517530
chr13	112529926	112530345
chr13	112541887	112542041
chr13	112551718	112551926
chr13	112556648	112556923
chr13	112578106	112578400
chr13	112593861	112594060
chr13	112594933	112595156
chr13	112619745	112620072
chr13	112645635	112645785
chr13	112660321	112660555
chr13	112660555	112660836
chr13	112660836	112661124
chr13	112661124	112661413

chr13	112672870	112673021
chr13	112674066	112674395
chr13	112678243	112678459
chr13	112683417	112683714
chr13	112683717	112683878
chr13	112706259	112706796
chr13	112711707	112712167
chr13	112712167	112712500
chr13	112724955	112725155
chr13	112725155	112725405
chr13	112726028	112726402
chr13	112739834	112739986
chr13	112740184	112740566
chr13	112744394	112744642
chr13	112754988	112755361
chr13	112762739	112762986
chr13	112763405	112763565
chr13	112768863	112769013
chr13	112770380	112770798
chr13	112770798	112771251
chr13	112771251	112771646
chr13	112772910	112773215
chr13	112774128	112774356
chr13	112787202	112787438
chr13	112800564	112800992
chr13	112800992	112801339
chr13	112801339	112801674
chr13	112803544	112803694
chr13	112803728	112803984
chr13	112810333	112810726
chr13	112810726	112811300
chr13	112811606	112811766
chr13	112822442	112822924
chr13	112861724	112862130
chr13	112862130	112862374
chr13	112862374	112862627
chr13	112863229	112863446
chr13	112909762	112909968
chr13	112926864	112927076
chr13	112927288	112927775
chr13	112927775	112928152
chr13	112987371	112987945

chr13	112987945	112988230
chr13	112988230	112988431
chr13	112993499	112993875
chr13	112993875	112994082
chr13	112994082	112994288
chr13	112994288	112994547
chr13	112996500	112996663
chr13	112998451	112998661
chr13	112998661	112998907
chr13	112998907	112999269
chr13	113000678	113000865
chr13	113013189	113013339
chr13	113013590	113013799
chr13	113023482	113024011
chr13	113033890	113034248
chr13	113034303	113034508
chr13	113034938	113035234
chr13	113035234	113035463
chr13	113035891	113036242
chr13	113046298	113046560
chr13	113046560	113046842
chr13	113047744	113047894
chr13	113048503	113048717
chr13	113049416	113049912
chr13	113056743	113057008
chr13	113119040	113119190
chr13	113120780	113120948
chr13	113129599	113129808
chr13	113168295	113168512
chr13	113168512	113168754
chr13	113170255	113170787
chr13	113183264	113183721
chr13	113202147	113202489
chr13	113202489	113202770
chr13	113203017	113203451
chr13	113218860	113219054
chr13	113223961	113224572
chr13	113225747	113226434
chr13	113227215	113227404
chr13	113229527	113229797
chr13	113252713	113252951
chr13	113256815	113257121

chr13	113259326	113259476
chr13	113262856	113263318
chr13	113263676	113263907
chr13	113265322	113265539
chr13	113266506	113266662
chr13	113266686	113266943
chr13	113268631	113268786
chr13	113269847	113270295
chr13	113270763	113271125
chr13	113287942	113288113
chr13	113288888	113289055
chr13	113293008	113293297
chr13	113293297	113293591
chr13	113322024	113322280
chr13	113337328	113337489
chr13	113340679	113340995
chr13	113341242	113341432
chr13	113344687	113344838
chr13	113350819	113351102
chr13	113351102	113351312
chr13	113353953	113354414
chr13	113355019	113355253
chr13	113403721	113404134
chr13	113414064	113414364
chr13	113417525	113417709
chr13	113418195	113418412
chr13	113443147	113443328
chr13	113448466	113448647
chr13	113454457	113455044
chr13	113465254	113465420
chr13	113466564	113466817
chr13	113466817	113467036
chr13	113483995	113484194
chr13	113484194	113484422
chr13	113485634	113485861
chr13	113487254	113487425
chr13	113490082	113490379
chr13	113490379	113490756
chr13	113495546	113495893
chr13	113495893	113496370
chr13	113502067	113502866
chr13	113507498	113507734

chr13	113509332	113509754
chr13	113509754	113510058
chr13	113517862	113518487
chr13	113550842	113551406
chr13	113553622	113554018
chr13	113573433	113573591
chr13	113574297	113574539
chr13	113580039	113580339
chr13	113582530	113582774
chr13	113582774	113582986
chr13	113583221	113583371
chr13	113597270	113597505
chr13	113601095	113601423
chr13	113602828	113603424
chr13	113603642	113603877
chr13	113603877	113604166
chr13	113606236	113606465
chr13	113614717	113614922
chr13	113615026	113615334
chr13	113615805	113615973
chr13	113616252	113616507
chr13	113616782	113616960
chr13	113617831	113618205
chr13	113618205	113618433
chr13	113618433	113618822
chr13	113622457	113622749
chr13	113622922	113623171
chr13	113634828	113635032
chr13	113645014	113645256
chr13	113688262	113688440
chr13	113693467	113693695
chr13	113725554	113725735
chr13	113774866	113775088
chr13	113793950	113794302
chr13	113794302	113794609
chr13	113794609	113794824
chr13	113794824	113795041
chr13	113795041	113795305
chr13	113806528	113806678
chr13	113806750	113806915
chr13	113806915	113807074
chr13	113829127	113829398

chr13	113842419	113842713
chr13	113864996	113865222
chr13	113865960	113866113
chr13	113867360	113868051
chr13	113869284	113869481
chr13	113877506	113877730
chr13	113918801	113918952
chr13	113933259	113933550
chr13	113972312	113972838
chr13	113983803	113984040
chr13	113984602	113984837
chr13	113999626	113999917
chr13	114001605	114001937
chr13	114003198	114003376
chr13	114003526	114003834
chr13	114076118	114076316
chr13	114086357	114086800
chr13	114092444	114092817
chr13	114103080	114103473
chr13	114103473	114103764
chr13	114109560	114109752
chr13	114113597	114113855
chr13	114113855	114114113
chr13	114203207	114203614
chr13	114254724	114255113
chr13	114260541	114260776
chr13	114260776	114261002
chr13	114261002	114261252
chr13	114304737	114304921
chr13	114319181	114319577
chr13	114360554	114360948
chr13	114360948	114361296
chr13	114362139	114362494
chr13	114379378	114379562
chr13	114387177	114387529
chr13	114387551	114387887
chr13	114387887	114388225
chr13	114393684	114393995
chr13	114405890	114406177
chr13	114420055	114420909
chr13	114447268	114447561
chr13	114457316	114457540

chr13	114458398	114458781
chr13	114458975	114459393
chr13	114459438	114459674
chr13	114460327	114460582
chr13	114460666	114460931
chr13	114462579	114463211
chr13	114490537	114490738
chr13	114493707	114493929
chr13	114493929	114494172
chr13	114494172	114494411
chr13	114494438	114494653
chr13	114500885	114501365
chr13	114513108	114513382
chr13	114529380	114529548
chr13	114578280	114578468
chr13	114584368	114585073
chr13	114592808	114593120
chr13	114599677	114600061
chr13	114607529	114607779
chr13	114618456	114618742
chr13	114619079	114619546
chr13	114626215	114626376
chr13	114632300	114632603
chr13	114635783	114636179
chr13	114638746	114639147
chr13	114639147	114639400
chr13	114639572	114639792
chr13	114639816	114640200
chr13	114654111	114654885
chr13	114655009	114655257
chr13	114661337	114661734
chr13	114668803	114669030
chr13	114678859	114679101
chr13	114679101	114679328
chr13	114679823	114680012
chr13	114680068	114680370
chr13	114680370	114680786
chr13	114698791	114699517
chr13	114699573	114699802
chr13	114702193	114702390
chr13	114722052	114722258
chr13	114726349	114726586

chr13	114726831	114727220
chr13	114727220	114727595
chr13	114735027	114735496
chr13	114735638	114735811
chr13	114735892	114736104
chr13	114736104	114736605
chr13	114742565	114742948
chr13	114757536	114757789
chr13	114781841	114782093
chr13	114793346	114793603
chr13	114795573	114795985
chr13	114801281	114801525
chr13	114809674	114809937
chr13	114809937	114810241
chr13	114817697	114818114
chr13	114818114	114818440
chr13	114818552	114818764
chr13	114818764	114818972
chr13	114824131	114824340
chr13	114827045	114827257
chr13	114827398	114827722
chr13	114827756	114827968
chr13	114828448	114828614
chr13	114830394	114830576
chr13	114830643	114830805
chr13	114830999	114831293
chr13	114831315	114831537
chr13	114831537	114831774
chr13	114836555	114836705
chr13	114841623	114841797
chr13	114841797	114842015
chr13	114844639	114844914
chr13	114848443	114848744
chr13	114849035	114849233
chr13	114852300	114852579
chr13	114852579	114852830
chr13	114852830	114853112
chr13	114853170	114853545
chr13	114859991	114860246
chr13	114860256	114860755
chr13	114865225	114865422
chr13	114876089	114876350

chr13	114876365	114876644
chr13	114886540	114886788
chr13	114886788	114887029
chr13	114887161	114887311
chr13	114890877	114891181
chr13	114891639	114892057
chr13	114895852	114896269
chr13	114896892	114897062
chr13	114904085	114904322
chr13	114909657	114909829
chr13	114914628	114914943
chr13	114914943	114915289
chr13	114915289	114915552
chr13	114915799	114915954
chr13	114916363	114916576
chr13	114918071	114918347
chr13	114918389	114918660
chr13	114923367	114923678
chr13	114924919	114925084
chr13	114926643	114926885
chr13	114931706	114932038
chr13	114932038	114932486
chr13	114933365	114933951
chr13	114933951	114934183
chr13	114935497	114935885
chr13	114944764	114944973
chr13	114945331	114945857
chr13	114945857	114946495
chr13	114949159	114949309
chr13	114963930	114964088
chr13	114964177	114964618
chr13	114968151	114968316
chr13	114984722	114984884
chr13	114985169	114985434
chr13	115005396	115005800
chr13	115006242	115006392
chr13	115033191	115033466
chr13	115064289	115064498
chr13	115077837	115078137
chr13	115089289	115089651
chr13	115089651	115090030
chr13	115090030	115090402

chr13	115090402	115090662
chr13	115090662	115090912
chr13	115101323	115101832
chr13	115101832	115102259
chr13	115107503	115107788
chr13	115230551	115230780
chr13	115345566	115345721
chr13	115346247	115346397
chr13	115423792	115424209
chr13	115491500	115491688
chr13	115556097	115556261
chr13	115567405	115567641
chr13	115571999	115572484
chr13	115606502	115606739
chr13	115742180	115742402
chr13	115757861	115758232
chr13	115814242	115814610
chr13	115890342	115890561
chr13	115986345	115986576
chr13	115987069	115987257
chr13	115988157	115988346
chr13	115989120	115989447
chr13	115990576	115990963
chr13	116003802	116004026
chr13	116017955	116018174
chr13	116030252	116030414
chr13	116152723	116153007
chr13	116166716	116166885
chr13	116192826	116193060
chr13	116309547	116310054
chr13	116310095	116310278
chr13	116410735	116411101
chr13	116443253	116443478
chr13	116509144	116509393
chr13	116526939	116527140
chr13	116527140	116527626
chr13	116535257	116535497
chr13	116535592	116535855
chr13	116596469	116596625
chr13	116712302	116712639
chr13	116788468	116788788
chr13	116828984	116829334

chr13	116926883	116927142
chr13	116988779	116988970
chr13	116990413	116990591
chr13	117000578	117000834
chr13	117000881	117001156
chr13	117024085	117024310
chr13	117024310	117024587
chr13	117024587	117024920
chr13	117024920	117025204
chr13	117025293	117025600
chr13	117025600	117025844
chr13	117025844	117026088
chr13	117127308	117127585
chr13	117143713	117144229
chr13	117202774	117202936
chr13	117220459	117220745
chr13	117220745	117220983
chr13	117220983	117221265
chr13	117223100	117223313
chr13	117223313	117223564
chr13	117311564	117311714
chr13	117413676	117414010
chr13	117426065	117426302
chr13	117467495	117467692
chr13	117602231	117602779
chr13	117669402	117669588
chr13	117710822	117711151
chr13	117847398	117847567
chr13	117848881	117849101
chr13	117898106	117898308
chr13	117907873	117908117
chr13	117931838	117932074
chr13	117953760	117954053
chr13	117959146	117959447
chr13	118021308	118021503
chr13	118027928	118028195
chr13	118028195	118028401
chr13	118035566	118035946
chr13	118058620	118058862
chr13	118124955	118125244
chr13	118339114	118339411
chr13	118339662	118339911

chr13	118379260	118379428
chr13	118386507	118386920
chr13	118386920	118387176
chr13	118387176	118387656
chr13	118555213	118555467
chr13	118560408	118560612
chr13	118570921	118571302
chr13	118571361	118571603
chr13	118739632	118739790
chr13	118763778	118764066
chr13	118772648	118772803
chr13	118773058	118773565
chr13	118803818	118803995
chr13	118917286	118917517
chr13	119143416	119143665
chr13	119177777	119177976
chr13	119177976	119178171
chr13	119258657	119258816
chr13	119283556	119283739
chr13	119341923	119342167
chr13	119378531	119378698
chr13	119388899	119389129
chr13	119408511	119409127
chr13	119409127	119409410
chr13	119409410	119409709
chr13	119428197	119428630
chr13	119428630	119429094
chr13	119429094	119429469
chr13	119429469	119429918
chr13	119442359	119442545
chr13	119461970	119462120
chr13	119462348	119462573
chr13	119462573	119462877
chr13	119462925	119463135
chr13	119463266	119463504
chr13	119464676	119464827
chr13	119471970	119472221
chr13	119477846	119478127
chr13	119487689	119487922
chr13	119487922	119488196
chr13	119488202	119488704
chr13	119488704	119489110

chr13	119494093	119494293
chr13	119596364	119596581
chr13	119596637	119596899
chr13	119596899	119597119
chr13	119597573	119597785
chr13	119598054	119598401
chr13	119598565	119598769
chr13	119598933	119599391
chr13	119599796	119599988
chr13	119600228	119600456
chr13	119600456	119600649
chr13	119601240	119601558
chr13	119601558	119601813
chr13	119601813	119602222
chr13	119603302	119603478
chr13	119609636	119609828
chr13	119613493	119613650
chr13	119613654	119613911
chr13	119614462	119614631
chr13	119615415	119615771
chr13	119616091	119616258
chr13	119616335	119616790
chr13	119616919	119617129
chr13	119617189	119617571
chr13	119623679	119623978
chr13	119626035	119626267
chr13	119629143	119629392
chr13	119629392	119629636
chr13	119631616	119632129
chr13	119635511	119635692
chr13	119635692	119635868
chr13	119640636	119640814
chr13	119661444	119661887
chr13	119661887	119662308
chr13	119669361	119669868
chr13	119673690	119674098
chr13	119682253	119682514
chr13	119683902	119684141
chr13	119689909	119690262
chr13	119690262	119690659
chr13	119690659	119691022
chr13	119703036	119703230

chr13	119704421	119704615
chr13	119706012	119706307
chr13	119707065	119707279
chr13	119709801	119710033
chr13	119710040	119710276
chr13	119710276	119710528
chr13	119710528	119710789
chr13	119710789	119710993
chr13	119712344	119712563
chr13	119716741	119716979
chr13	119722161	119722522
chr13	119722522	119722839
chr13	119724618	119724773
chr13	119729578	119729728
chr13	119737001	119737226
chr13	119755168	119755687
chr13	119755687	119756073
chr13	119756073	119756405
chr13	119766586	119766782
chr13	119774282	119774497
chr13	119783646	119783913
chr13	119789948	119790100
chr13	119790331	119790674
chr13	119790674	119791002
chr13	119791002	119791340
chr13	119791340	119791671
chr13	119800994	119801308
chr13	119805686	119805998
chr13	119835472	119835943
chr13	119836305	119836486
chr13	119836486	119836782
chr13	119837845	119838009
chr13	119838033	119838262
chr13	119840048	119840277
chr13	119965422	119965635
chr13	119965635	119965924
chr13	119965924	119966231
chr13	119966279	119966460
chr13	119991367	119991587
chr13	119998761	119998949
chr13	120021582	120021814
chr13	120034737	120034902

chr13	120040308	120040458
chr13	120041213	120041440
chr13	120063582	120063761
chr13	120070141	120070300
chr13	120070941	120071159
chr13	120096105	120096260
chr13	120096336	120096645
chr13	120096645	120096945
chr13	120096945	120097163
chr13	120183180	120183376
chr13	120231946	120232101
chr13	120321330	120321709
chr14	3271877	3272298
chr14	3279996	3280364
chr14	3332989	3333190
chr14	3333508	3333715
chr14	3413508	3413721
chr14	3521701	3522047
chr14	3545466	3546190
chr14	3652919	3653113
chr14	3761082	3761432
chr14	3893940	3894100
chr14	3901932	3902177
chr14	3922324	3922670
chr14	4595276	4595438
chr14	4603226	4603492
chr14	4856453	4856658
chr14	4940178	4940450
chr14	4948211	4948485
chr14	4968826	4969062
chr14	4969062	4969410
chr14	5459271	5459428
chr14	5811765	5811915
chr14	6107568	6107779
chr14	6108042	6108331
chr14	6108331	6108596
chr14	6108596	6108857
chr14	6108857	6109064
chr14	6312860	6313081
chr14	6334025	6334359
chr14	6342128	6342419
chr14	6604141	6604294

chr14	6604617	6604907
chr14	6605147	6605326
chr14	6636899	6637180
chr14	6637180	6637688
chr14	6658129	6658560
chr14	6666229	6666568
chr14	6674327	6674626
chr14	6755934	6756173
chr14	6756404	6756708
chr14	6756708	6757093
chr14	6757093	6757445
chr14	6757445	6757659
chr14	7104369	7104574
chr14	7173496	7173653
chr14	7174450	7174675
chr14	7313956	7314106
chr14	7394424	7394826
chr14	7402511	7402824
chr14	7487535	7487742
chr14	7567411	7567563
chr14	7568457	7568704
chr14	7657516	7657725
chr14	7657997	7658243
chr14	7658294	7658480
chr14	7658480	7658690
chr14	7749576	7749731
chr14	7765884	7766247
chr14	7773926	7774215
chr14	7791394	7791775
chr14	7791775	7792119
chr14	7801957	7802145
chr14	7808317	7808757
chr14	7808757	7809094
chr14	7809514	7809986
chr14	7812530	7812688
chr14	7817733	7818115
chr14	7818115	7818430
chr14	7818430	7818736
chr14	7818736	7819082
chr14	7819580	7819782
chr14	7824226	7824462
chr14	7824703	7825078

chr14	7826108	7826369
chr14	7826369	7826647
chr14	7826647	7826912
chr14	7826912	7827218
chr14	7832777	7832932
chr14	7833316	7834013
chr14	7842967	7843241
chr14	7846689	7847014
chr14	7847699	7848283
chr14	7850948	7851323
chr14	7851438	7851778
chr14	7851778	7852158
chr14	7853690	7854149
chr14	7857970	7858166
chr14	7860609	7860788
chr14	7861409	7861564
chr14	7862345	7862603
chr14	7863246	7863524
chr14	7864060	7864229
chr14	7864896	7865112
chr14	7867339	7867526
chr14	7870483	7870844
chr14	7870844	7871187
chr14	7871187	7871388
chr14	7871388	7871709
chr14	7873499	7873947
chr14	7880354	7880640
chr14	7896148	7896502
chr14	7905863	7906054
chr14	7912412	7912661
chr14	7912661	7912889
chr14	7922047	7922239
chr14	7924052	7924259
chr14	7926941	7927113
chr14	7929207	7929380
chr14	7932774	7933050
chr14	7933412	7933692
chr14	7935320	7935605
chr14	7940663	7941133
chr14	7941187	7941587
chr14	7943102	7943457
chr14	7944788	7944971

chr14	7945381	7945621
chr14	7951925	7952245
chr14	7952686	7953423
chr14	7953982	7954568
chr14	7961215	7961372
chr14	7961468	7961810
chr14	7961810	7962302
chr14	7962486	7962768
chr14	7962793	7962943
chr14	7963129	7963617
chr14	7964544	7964774
chr14	7975303	7975485
chr14	7989469	7989632
chr14	7994219	7994527
chr14	7994527	7994838
chr14	7997298	7997457
chr14	7998786	7999093
chr14	7999093	7999434
chr14	7999559	7999794
chr14	8002731	8003078
chr14	8005947	8006271
chr14	8016300	8016644
chr14	8016977	8017253
chr14	8028629	8028812
chr14	8042148	8042360
chr14	8044433	8044587
chr14	8045283	8045517
chr14	8059996	8060170
chr14	8080127	8080443
chr14	8080443	8080761
chr14	8080857	8081112
chr14	8092594	8092744
chr14	8097160	8097343
chr14	8097917	8098293
chr14	8098293	8098643
chr14	8098755	8098905
chr14	8099753	8100023
chr14	8111296	8111498
chr14	8111511	8111856
chr14	8111856	8112146
chr14	8112165	8112320
chr14	8122276	8122564

chr14	8125278	8125736
chr14	8128013	8128312
chr14	8135114	8135565
chr14	8154128	8154458
chr14	8157081	8157321
chr14	8161825	8162117
chr14	8172646	8172871
chr14	8172871	8173148
chr14	8173148	8173543
chr14	8173723	8173874
chr14	8201863	8202098
chr14	8208823	8209115
chr14	8213666	8214642
chr14	8214699	8214963
chr14	8215533	8215726
chr14	8220460	8220627
chr14	8232038	8232673
chr14	8233298	8233612
chr14	8242473	8242741
chr14	8251184	8251363
chr14	8257120	8257385
chr14	8258339	8258600
chr14	8258681	8259310
chr14	8268723	8268965
chr14	8274735	8274956
chr14	8277498	8277660
chr14	8286574	8286923
chr14	8294202	8294819
chr14	8297945	8298161
chr14	8300374	8300686
chr14	8304972	8305156
chr14	8305156	8305358
chr14	8305366	8305668
chr14	8306920	8307443
chr14	8309636	8309913
chr14	8309913	8310329
chr14	8311125	8311385
chr14	8314695	8314853
chr14	8319644	8319838
chr14	8325381	8325541
chr14	8332262	8332665
chr14	8332665	8333019

chr14	8346663	8346924
chr14	8348979	8349277
chr14	8356048	8356259
chr14	8356267	8356507
chr14	8356670	8357459
chr14	8367074	8367867
chr14	8387687	8387947
chr14	8415229	8415408
chr14	8418445	8419126
chr14	8419598	8419788
chr14	8429868	8430046
chr14	8430640	8430926
chr14	8430926	8431237
chr14	8444469	8444687
chr14	8460569	8460798
chr14	8486333	8486502
chr14	8486619	8486823
chr14	8489146	8489334
chr14	8608640	8608859
chr14	8610218	8610440
chr14	8651818	8651968
chr14	8665789	8665939
chr14	8665944	8666518
chr14	8680445	8680769
chr14	8682878	8683092
chr14	8779184	8779370
chr14	8789322	8789721
chr14	8789879	8790046
chr14	8799818	8800007
chr14	8800338	8800554
chr14	8909805	8909987
chr14	8985546	8985698
chr14	9015974	9016206
chr14	9239475	9239652
chr14	9245401	9245615
chr14	9284493	9284665
chr14	9284937	9285518
chr14	9285948	9286290
chr14	9289530	9289750
chr14	9548011	9548237
chr14	9618535	9618728
chr14	9619978	9620166

chr14	9620363	9620543
chr14	9669821	9670276
chr14	9670392	9670745
chr14	9766722	9766995
chr14	9785896	9786160
chr14	9805157	9805334
chr14	9805989	9806192
chr14	9828778	9829024
chr14	9990588	9990982
chr14	10129187	10129504
chr14	10372355	10372529
chr14	10445210	10445488
chr14	10452985	10453253
chr14	10453507	10453674
chr14	10548595	10549051
chr14	10551986	10552309
chr14	10554796	10555006
chr14	10658311	10658491
chr14	10764012	10764263
chr14	10777864	10778175
chr14	10811615	10811915
chr14	10822058	10822282
chr14	10824170	10824328
chr14	10882874	10883105
chr14	10979970	10980155
chr14	11148677	11148940
chr14	11160787	11161058
chr14	11161725	11161993
chr14	11161993	11162300
chr14	11397300	11397703
chr14	11445979	11446134
chr14	11553243	11553457
chr14	11553457	11553687
chr14	11553687	11553985
chr14	11553985	11554225
chr14	11554278	11554684
chr14	11554684	11555020
chr14	11556406	11556619
chr14	11612280	11612514
chr14	11612514	11612819
chr14	11612864	11613154
chr14	11613154	11613498

chr14	11615632	11616080
chr14	11617511	11617773
chr14	11618207	11618569
chr14	11622901	11623095
chr14	11641051	11641489
chr14	11658716	11659054
chr14	11680318	11680713
chr14	11680713	11681030
chr14	11681412	11681576
chr14	11682528	11682866
chr14	11683840	11684105
chr14	11685486	11685660
chr14	11811371	11811759
chr14	11825913	11826247
chr14	11826247	11826566
chr14	11826566	11826899
chr14	11826899	11827122
chr14	11858089	11858413
chr14	11915850	11916127
chr14	12070900	12071413
chr14	12072530	12072726
chr14	12073498	12073786
chr14	12116905	12117297
chr14	12124807	12125013
chr14	12139981	12140227
chr14	12152018	12152239
chr14	12242217	12242423
chr14	12284058	12284394
chr14	12284394	12284689
chr14	12284689	12285049
chr14	12537626	12537818
chr14	12591904	12592175
chr14	12915125	12915331
chr14	12966500	12966712
chr14	13001165	13001551
chr14	13022714	13023222
chr14	13090949	13091162
chr14	13357975	13358445
chr14	13361202	13361593
chr14	13416681	13417170
chr14	13551211	13551480
chr14	13553622	13553946

chr14	13555534	13555741
chr14	13597894	13598125
chr14	13901676	13901848
chr14	13901880	13902098
chr14	13917744	13917949
chr14	13950491	13950697
chr14	13957903	13958078
chr14	13960127	13960367
chr14	13960586	13960912
chr14	13960912	13961122
chr14	13961122	13961377
chr14	13961377	13961850
chr14	14012214	14012865
chr14	14012865	14013335
chr14	14013335	14013661
chr14	14019567	14019824
chr14	14036418	14036626
chr14	14037414	14037665
chr14	14054475	14054693
chr14	14058374	14058575
chr14	14063464	14063677
chr14	14088653	14088872
chr14	14091285	14091513
chr14	14091513	14091697
chr14	14098677	14098919
chr14	14119830	14120032
chr14	14120225	14120428
chr14	14120428	14120624
chr14	14120624	14120832
chr14	14120832	14121150
chr14	14197166	14197524
chr14	14237183	14237625
chr14	14238264	14238585
chr14	14294424	14294774
chr14	14345438	14345982
chr14	14345982	14346377
chr14	14346377	14346710
chr14	14347076	14347406
chr14	14348083	14348275
chr14	14348275	14348472
chr14	14423381	14423641
chr14	14596477	14596643

chr14	14604400	14604738
chr14	14607145	14607358
chr14	14628517	14628667
chr14	14628744	14629013
chr14	14632555	14632737
chr14	14633025	14633472
chr14	14633472	14633768
chr14	14633768	14633941
chr14	14639262	14639447
chr14	14642723	14642987
chr14	14643602	14643863
chr14	14650426	14650647
chr14	14702467	14702723
chr14	14702723	14702973
chr14	14702973	14703166
chr14	14703166	14703398
chr14	14703398	14703848
chr14	14803876	14804214
chr14	14833007	14833207
chr14	14843258	14843462
chr14	14863873	14864055
chr14	14890537	14890935
chr14	14905065	14905282
chr14	14915394	14915545
chr14	14985319	14985597
chr14	15421064	15421412
chr14	16191943	16192104
chr14	16242862	16243652
chr14	16243736	16244206
chr14	16249096	16249439
chr14	16249439	16249675
chr14	16249675	16249900
chr14	16256112	16256566
chr14	16364157	16364349
chr14	16364533	16364917
chr14	16364917	16365418
chr14	16365418	16365861
chr14	16365861	16366170
chr14	16366174	16366364
chr14	16380213	16380533
chr14	16384939	16385165
chr14	16433296	16433497

chr14	16434457	16434695
chr14	16447615	16447971
chr14	16448602	16449052
chr14	16483594	16483956
chr14	16484024	16484291
chr14	16484291	16484503
chr14	16509933	16510113
chr14	16510594	16510992
chr14	16513475	16513659
chr14	16526163	16526422
chr14	16541712	16541957
chr14	16545009	16545555
chr14	16545593	16545806
chr14	16545806	16546089
chr14	16551147	16551302
chr14	16551487	16551665
chr14	16551665	16551891
chr14	16551899	16552220
chr14	16556936	16557217
chr14	16557405	16557557
chr14	16557919	16558507
chr14	16566216	16566547
chr14	16575207	16575929
chr14	16577179	16577380
chr14	16578805	16578999
chr14	16587981	16588197
chr14	16594524	16594973
chr14	16598239	16598470
chr14	16603301	16603487
chr14	16605541	16605728
chr14	16663173	16663502
chr14	16677092	16677457
chr14	16681769	16682123
chr14	16682189	16682375
chr14	16732133	16732337
chr14	16765989	16766155
chr14	16813814	16813964
chr14	16818668	16818963
chr14	16874985	16875191
chr14	16883557	16883784
chr14	16944555	16944710
chr14	16954608	16954760

chr14	16957860	16958171
chr14	16958412	16958588
chr14	17012180	17012587
chr14	17012786	17013178
chr14	17023581	17023873
chr14	17024310	17024556
chr14	17063327	17063538
chr14	17172745	17173066
chr14	17207457	17207639
chr14	17211578	17211848
chr14	17212169	17212362
chr14	17352759	17352996
chr14	17388645	17388924
chr14	17397034	17397413
chr14	17402782	17403019
chr14	17404157	17404382
chr14	17406065	17406266
chr14	17409542	17409801
chr14	17443140	17443328
chr14	17589645	17589871
chr14	17659049	17659275
chr14	17659324	17659481
chr14	17660068	17660390
chr14	17660390	17660737
chr14	17660737	17661258
chr14	17675051	17675532
chr14	17675532	17675836
chr14	17735514	17735818
chr14	17737327	17737477
chr14	17777024	17777214
chr14	17784351	17784712
chr14	17822814	17823054
chr14	17857025	17857534
chr14	17862335	17862633
chr14	17862714	17863034
chr14	17903216	17903459
chr14	17911570	17911783
chr14	17916682	17916999
chr14	17917940	17918339
chr14	17918410	17918691
chr14	17918691	17919027
chr14	17920168	17920638

chr14	17927561	17927752
chr14	17967187	17967421
chr14	17967421	17967656
chr14	18175775	18175953
chr14	18201111	18201358
chr14	18204307	18204652
chr14	18210873	18211089
chr14	18219529	18219772
chr14	18234075	18234287
chr14	18238160	18238470
chr14	18238470	18238845
chr14	18238845	18239209
chr14	18239209	18239625
chr14	18265528	18265687
chr14	18270514	18270686
chr14	18270686	18270950
chr14	18270950	18271553
chr14	18280401	18280736
chr14	18282007	18282203
chr14	18329390	18329625
chr14	18330814	18331061
chr14	18331061	18331500
chr14	18331500	18332169
chr14	18355606	18355807
chr14	18355879	18356117
chr14	18375270	18375420
chr14	18378046	18378237
chr14	18387256	18387593
chr14	18443084	18443306
chr14	18443306	18443588
chr14	18448729	18449029
chr14	18455450	18455917
chr14	18456238	18456631
chr14	18460539	18460881
chr14	18461210	18461386
chr14	18462330	18462816
chr14	18462816	18463268
chr14	18463268	18463560
chr14	18468279	18468438
chr14	18468551	18468980
chr14	18468980	18469229
chr14	18480132	18480349

chr14	18494173	18494323
chr14	18511145	18511393
chr14	18530150	18530312
chr14	18554677	18554870
chr14	18555377	18555540
chr14	18564965	18565275
chr14	18574474	18574637
chr14	18592221	18592527
chr14	18619404	18619577
chr14	18676330	18676502
chr14	18678465	18678822
chr14	18773746	18774175
chr14	18774311	18774523
chr14	18774564	18774897
chr14	18778999	18779341
chr14	18779597	18779750
chr14	18820651	18820910
chr14	18879343	18879712
chr14	18879712	18880007
chr14	18882600	18882840
chr14	18882840	18883101
chr14	18893082	18893304
chr14	18893304	18893651
chr14	18893651	18894103
chr14	18894103	18894420
chr14	18896744	18896978
chr14	18939878	18940595
chr14	18978404	18978587
chr14	19036070	19036249
chr14	19038932	19039155
chr14	19040962	19041218
chr14	19042021	19042319
chr14	19042403	19042883
chr14	19111708	19111874
chr14	19151028	19151280
chr14	19175399	19175549
chr14	19178132	19178313
chr14	19180280	19180630
chr14	19189733	19189883
chr14	19278071	19278297
chr14	19415655	19415986
chr14	19416119	19416272

chr14	19416866	19417126
chr14	19417126	19417446
chr14	19417446	19417723
chr14	19417749	19418004
chr14	19418103	19418371
chr14	19418536	19418987
chr14	19419206	19419432
chr14	19486932	19487218
chr14	19494988	19495292
chr14	19584041	19584279
chr14	19584515	19584815
chr14	19584815	19585202
chr14	19585202	19585563
chr14	19585563	19585784
chr14	19610522	19610681
chr14	19610851	19611091
chr14	19664810	19664960
chr14	19665756	19666094
chr14	19667009	19667200
chr14	19705737	19705965
chr14	19706585	19706754
chr14	19707151	19707316
chr14	19750937	19751388
chr14	19803704	19803949
chr14	19823290	19823485
chr14	19823485	19823711
chr14	19823711	19824088
chr14	19841086	19841358
chr14	19881392	19881590
chr14	19964093	19964483
chr14	19976610	19976823
chr14	19976880	19977259
chr14	19985007	19985225
chr14	19986813	19987027
chr14	19987098	19987337
chr14	20032690	20033006
chr14	20033006	20033362
chr14	20033382	20033552
chr14	20034448	20034598
chr14	20043233	20043436
chr14	20046030	20046222
chr14	20067330	20067665

chr14	20070742	20070924
chr14	20082601	20082952
chr14	20082952	20083242
chr14	20083242	20083513
chr14	20098181	20098364
chr14	20114058	20114403
chr14	20117410	20117676
chr14	20129867	20130053
chr14	20135110	20135261
chr14	20135282	20135601
chr14	20135601	20136033
chr14	20138994	20139497
chr14	20139595	20139804
chr14	20149050	20149479
chr14	20153895	20154150
chr14	20154248	20154485
chr14	20154742	20155217
chr14	20155217	20155658
chr14	20155849	20156111
chr14	20162472	20163039
chr14	20165285	20165915
chr14	20168617	20168779
chr14	20169328	20169546
chr14	20175231	20175491
chr14	20180388	20180584
chr14	20180586	20181090
chr14	20181090	20181434
chr14	20181434	20181688
chr14	20181688	20182031
chr14	20196915	20197082
chr14	20208459	20208813
chr14	20214245	20214561
chr14	20229454	20229708
chr14	20246794	20247317
chr14	20249501	20249745
chr14	20253242	20253643
chr14	20256999	20257200
chr14	20257265	20257508
chr14	20258481	20258791
chr14	20291221	20291639
chr14	20294465	20294824
chr14	20297684	20297932

chr14	20298182	20298485
chr14	20311629	20311928
chr14	20319542	20319694
chr14	20328512	20328895
chr14	20340224	20340441
chr14	20345878	20346335
chr14	20347757	20348030
chr14	20348030	20348605
chr14	20378350	20378643
chr14	20388313	20388736
chr14	20388736	20388923
chr14	20388923	20389146
chr14	20393215	20393981
chr14	20400118	20400394
chr14	20422915	20423600
chr14	20428714	20428912
chr14	20478449	20479158
chr14	20479454	20479785
chr14	20479785	20480253
chr14	20502942	20503324
chr14	20503324	20503555
chr14	20545752	20546092
chr14	20546092	20546392
chr14	20546392	20546720
chr14	20576206	20576483
chr14	20607884	20608060
chr14	20608699	20609131
chr14	20609457	20609850
chr14	20609850	20610125
chr14	20610140	20610578
chr14	20616516	20616690
chr14	20629482	20629774
chr14	20630853	20631146
chr14	20631258	20631688
chr14	20640030	20640232
chr14	20640269	20640686
chr14	20640686	20641207
chr14	20655024	20655341
chr14	20658403	20658826
chr14	20660798	20661296
chr14	20661296	20661713
chr14	20667573	20667835

chr14	20673838	20674055
chr14	20674094	20674495
chr14	20674495	20674872
chr14	20682492	20682701
chr14	20682701	20682913
chr14	20694292	20694463
chr14	20694586	20694754
chr14	20694805	20695100
chr14	20695100	20695490
chr14	20695490	20695993
chr14	20697105	20697295
chr14	20702045	20702259
chr14	20702485	20702817
chr14	20702817	20703156
chr14	20703156	20703362
chr14	20707142	20707419
chr14	20707419	20707880
chr14	20707940	20708231
chr14	20708474	20708649
chr14	20721500	20721743
chr14	20724146	20724375
chr14	20733929	20734177
chr14	20734177	20734469
chr14	20734469	20734799
chr14	20737336	20737664
chr14	20741511	20741680
chr14	20757709	20757891
chr14	20765562	20765949
chr14	20765949	20766365
chr14	20780245	20780529
chr14	20785279	20785508
chr14	20791541	20791722
chr14	20793225	20793531
chr14	20793531	20793843
chr14	20793843	20794513
chr14	20798850	20799100
chr14	20803613	20803870
chr14	20804989	20805333
chr14	20805333	20805719
chr14	20806161	20806563
chr14	20806563	20806990
chr14	20810394	20810548

chr14	20811252	20811678
chr14	20818808	20819314
chr14	20819314	20819744
chr14	20827554	20827859
chr14	20828876	20829089
chr14	20829834	20830034
chr14	20831174	20831570
chr14	20834140	20834444
chr14	20834444	20834682
chr14	20836168	20836506
chr14	20836506	20836992
chr14	20844171	20844378
chr14	20844796	20844990
chr14	20846366	20846658
chr14	20846665	20846815
chr14	20847729	20847925
chr14	20850316	20851009
chr14	20863336	20863644
chr14	20863778	20864145
chr14	20865225	20865415
chr14	20865557	20865754
chr14	20865802	20866234
chr14	20866387	20866677
chr14	20871602	20871774
chr14	20873366	20873649
chr14	20874426	20874602
chr14	20885181	20885365
chr14	20885603	20885815
chr14	20891687	20892413
chr14	20902995	20903234
chr14	20903234	20903515
chr14	20914416	20914794
chr14	20919330	20919540
chr14	20923852	20924062
chr14	20924120	20924380
chr14	20924892	20925164
chr14	20928863	20929250
chr14	20929250	20929511
chr14	20929511	20929775
chr14	20929954	20930110
chr14	20930639	20930807
chr14	20936654	20936921

chr14	20938123	20938300
chr14	20938393	20938985
chr14	20947328	20947689
chr14	20967313	20967622
chr14	20969146	20969397
chr14	20970417	20970580
chr14	20973590	20974459
chr14	20976786	20977000
chr14	20985559	20985796
chr14	20990163	20990419
chr14	20993419	20993634
chr14	21019078	21019305
chr14	21031973	21032583
chr14	21051689	21051846
chr14	21051874	21052310
chr14	21052310	21052685
chr14	21052685	21053107
chr14	21064026	21064302
chr14	21065473	21065644
chr14	21065644	21065865
chr14	21065865	21066239
chr14	21066545	21066729
chr14	21069691	21069845
chr14	21075722	21076083
chr14	21076083	21076514
chr14	21076559	21076764
chr14	21082781	21083246
chr14	21087423	21087639
chr14	21087677	21088106
chr14	21088870	21089124
chr14	21112214	21112527
chr14	21112701	21112924
chr14	21112945	21113208
chr14	21116030	21116280
chr14	21135009	21135244
chr14	21135356	21135634
chr14	21136984	21137220
chr14	21177916	21178083
chr14	21203950	21204256
chr14	21268127	21268703
chr14	21272722	21272945
chr14	21278449	21278626

chr14	21278918	21279283
chr14	21279283	21279514
chr14	21284165	21284385
chr14	21307631	21307796
chr14	21336086	21336353
chr14	21337949	21338420
chr14	21339031	21339195
chr14	21350674	21350824
chr14	21350872	21351254
chr14	21389907	21390191
chr14	21453831	21454325
chr14	21472760	21473065
chr14	21477216	21477449
chr14	21499690	21499878
chr14	21499878	21500098
chr14	21500098	21500384
chr14	21500384	21500658
chr14	21500658	21500984
chr14	21500984	21501448
chr14	21525741	21526098
chr14	21547988	21548311
chr14	21549722	21550154
chr14	21552996	21553478
chr14	21553877	21554146
chr14	21554146	21554369
chr14	21585554	21585862
chr14	21586359	21586694
chr14	21606248	21606543
chr14	21610321	21610543
chr14	21659739	21659979
chr14	21662537	21662845
chr14	21672548	21672737
chr14	21682640	21682887
chr14	21682887	21683193
chr14	21685558	21685768
chr14	21695028	21695447
chr14	21709639	21709818
chr14	21721038	21721262
chr14	21741844	21742178
chr14	21742515	21742728
chr14	21746901	21747200
chr14	21747794	21748163

chr14	21750403	21750840
chr14	21750910	21751313
chr14	21751344	21751571
chr14	21766480	21766872
chr14	21806611	21807062
chr14	21808226	21808574
chr14	21808634	21808967
chr14	21812893	21813136
chr14	21817493	21817808
chr14	21820878	21821441
chr14	21822804	21822967
chr14	21822967	21823153
chr14	21826167	21826349
chr14	21830697	21831089
chr14	21831089	21831394
chr14	21831394	21831745
chr14	21831745	21832023
chr14	21832212	21832387
chr14	21841962	21842133
chr14	21847581	21847858
chr14	21847858	21848232
chr14	21848232	21848669
chr14	21848669	21849177
chr14	21853426	21853676
chr14	21853921	21854081
chr14	21869964	21870200
chr14	21932810	21933024
chr14	21969043	21969265
chr14	21981478	21981795
chr14	21982730	21982955
chr14	21986040	21986275
chr14	21987415	21987669
chr14	21987707	21988164
chr14	21988346	21988590
chr14	21988906	21989138
chr14	21989336	21989570
chr14	21989570	21989794
chr14	21995118	21995506
chr14	21995506	21995823
chr14	22003747	22003949
chr14	22019738	22019896
chr14	22171781	22172143

chr14	22578347	22578512
chr14	22578671	22578932
chr14	22587285	22587648
chr14	22587665	22588221
chr14	22700354	22700535
chr14	22727650	22727863
chr14	23401165	23401389
chr14	23561483	23561639
chr14	23751249	23751517
chr14	23885585	23885974
chr14	23886050	23886525
chr14	24059807	24060007
chr14	24182004	24182465
chr14	24183003	24183392
chr14	24194018	24194168
chr14	24199793	24200046
chr14	24206303	24206706
chr14	24206706	24207324
chr14	24207436	24207660
chr14	24212890	24213549
chr14	24213659	24213867
chr14	24222451	24222647
chr14	24222789	24223372
chr14	24228411	24228895
chr14	24232526	24232939
chr14	24232982	24233153
chr14	24244256	24244580
chr14	24244686	24245012
chr14	24245012	24245319
chr14	24245319	24245577
chr14	24245577	24245809
chr14	24245809	24246035
chr14	24273142	24273353
chr14	24273653	24273845
chr14	24275698	24275848
chr14	24307245	24307460
chr14	24311197	24311431
chr14	24358372	24358579
chr14	24468818	24469132
chr14	24469175	24469327
chr14	24486524	24486838
chr14	24486838	24487324

chr14	24490462	24490830
chr14	24490830	24491035
chr14	24491035	24491249
chr14	24491321	24491500
chr14	24497732	24497894
chr14	24498788	24499248
chr14	24499635	24499868
chr14	24500350	24500539
chr14	24500539	24500835
chr14	24500850	24501016
chr14	24778872	24779097
chr14	24779097	24779396
chr14	24779396	24779695
chr14	24793638	24794013
chr14	24794044	24794194
chr14	25116492	25116724
chr14	25155087	25155380
chr14	25155380	25155743
chr14	25155743	25156110
chr14	25156110	25156504
chr14	25160738	25161098
chr14	25186071	25186480
chr14	25186480	25186864
chr14	25187860	25188083
chr14	25189419	25189596
chr14	25226561	25226968
chr14	25248995	25249176
chr14	25268027	25268243
chr14	25304043	25304212
chr14	25304212	25304427
chr14	25304427	25304655
chr14	25304655	25304869
chr14	25313650	25314150
chr14	25342059	25342292
chr14	25345978	25346367
chr14	25354496	25354800
chr14	25365266	25365622
chr14	25371573	25371974
chr14	25377811	25378301
chr14	25378301	25378705
chr14	25378874	25379226
chr14	25380607	25380859

chr14	25397016	25397216
chr14	25397289	25397478
chr14	25405346	25405518
chr14	25451630	25452049
chr14	25452049	25452361
chr14	25457356	25457521
chr14	25457587	25457949
chr14	25458209	25458380
chr14	25458380	25458556
chr14	25458664	25459027
chr14	25459027	25459371
chr14	25459371	25459651
chr14	25459692	25459857
chr14	25461533	25461724
chr14	25464552	25464893
chr14	25464893	25465192
chr14	25466370	25466648
chr14	25467670	25467873
chr14	25472591	25472840
chr14	25473064	25473253
chr14	25474029	25474405
chr14	25474849	25475079
chr14	25475954	25476469
chr14	25481314	25481673
chr14	25484064	25484449
chr14	25486428	25486643
chr14	25486771	25487059
chr14	25487059	25487534
chr14	25488106	25488425
chr14	25489423	25489662
chr14	25496060	25496582
chr14	25496675	25496929
chr14	25497040	25497400
chr14	25497672	25498037
chr14	25504907	25505784
chr14	25515891	25516116
chr14	25516640	25517137
chr14	25517249	25517494
chr14	25519610	25520216
chr14	25526836	25527288
chr14	25527288	25527810
chr14	25532711	25532861

chr14	25532932	25533536
chr14	25533667	25533858
chr14	25533858	25534016
chr14	25534054	25534426
chr14	25538562	25538946
chr14	25539794	25539986
chr14	25551665	25552157
chr14	25554665	25555115
chr14	25561020	25561213
chr14	25564207	25564404
chr14	25565556	25566052
chr14	25576723	25576990
chr14	25576990	25577236
chr14	25586695	25586938
chr14	25586938	25587237
chr14	25589419	25589579
chr14	25600337	25600570
chr14	25603519	25603928
chr14	25606631	25606961
chr14	25607245	25607553
chr14	25627632	25628014
chr14	25628550	25628786
chr14	25629876	25630235
chr14	25630235	25630638
chr14	25630641	25630847
chr14	25640346	25640946
chr14	25646197	25646676
chr14	25660756	25660938
chr14	25663684	25663862
chr14	25666782	25667023
chr14	25667188	25667431
chr14	25674002	25674571
chr14	25676202	25676714
chr14	25676714	25677015
chr14	25677592	25677863
chr14	25681872	25682483
chr14	25693894	25694545
chr14	25694549	25694854
chr14	25700774	25701123
chr14	25701123	25701515
chr14	25701527	25701708
chr14	25707180	25707476

chr14	25707768	25707936
chr14	25707998	25708196
chr14	25708279	25708797
chr14	25711307	25711457
chr14	25713514	25713730
chr14	25714576	25714861
chr14	25719482	25719716
chr14	25726311	25726606
chr14	25735280	25735609
chr14	25747218	25747373
chr14	25750703	25750932
chr14	25755109	25755512
chr14	25755790	25755977
chr14	25768253	25768588
chr14	25768588	25768922
chr14	25768922	25769306
chr14	25774211	25774389
chr14	25776039	25776290
chr14	25776290	25776624
chr14	25780655	25780898
chr14	25785865	25786088
chr14	25800244	25800526
chr14	25807283	25807470
chr14	25811168	25811368
chr14	25816471	25816768
chr14	25832275	25832742
chr14	25839630	25840196
chr14	25841954	25842310
chr14	25842310	25842520
chr14	25842520	25842760
chr14	25842881	25843326
chr14	25848234	25848519
chr14	25849262	25849422
chr14	25855325	25855520
chr14	25855549	25855846
chr14	25855883	25856089
chr14	25856937	25857265
chr14	25857265	25857849
chr14	25861745	25862176
chr14	25862185	25862432
chr14	25869235	25869400
chr14	25873189	25873374

chr14	25876359	25876553
chr14	25876569	25877094
chr14	25881789	25882162
chr14	25882933	25883152
chr14	25883152	25883513
chr14	25885988	25886197
chr14	25886826	25886986
chr14	25889021	25889340
chr14	25891848	25892195
chr14	25892195	25892455
chr14	25895788	25896020
chr14	25902391	25902567
chr14	25902780	25902995
chr14	25907358	25907635
chr14	25918359	25918612
chr14	25918612	25918889
chr14	25926826	25927126
chr14	25927126	25927454
chr14	25927454	25927836
chr14	25940238	25940472
chr14	25954636	25954853
chr14	25957776	25957971
chr14	25958049	25958199
chr14	26007492	26007761
chr14	26013706	26013913
chr14	26013996	26014530
chr14	26016353	26016855
chr14	26021571	26021934
chr14	26022704	26023369
chr14	26025758	26025947
chr14	26026565	26026840
chr14	26028797	26029102
chr14	26031607	26031970
chr14	26031970	26032224
chr14	26042157	26042319
chr14	26047109	26047359
chr14	26058111	26058354
chr14	26058354	26058649
chr14	26066567	26066876
chr14	26066876	26067208
chr14	26067208	26067589
chr14	26079990	26080225

chr14	26094399	26094622
chr14	26097520	26097718
chr14	26097782	26097965
chr14	26147115	26147378
chr14	26153316	26153527
chr14	26153610	26154041
chr14	26155948	26156476
chr14	26161186	26161598
chr14	26162317	26162874
chr14	26165372	26165590
chr14	26166199	26166370
chr14	26168405	26168725
chr14	26171253	26171586
chr14	26171586	26171837
chr14	26175226	26175451
chr14	26181771	26181934
chr14	26182188	26182343
chr14	26186722	26186973
chr14	26197711	26197957
chr14	26197957	26198259
chr14	26206186	26206498
chr14	26206498	26206824
chr14	26206824	26207205
chr14	26219606	26219802
chr14	26234016	26234225
chr14	26237126	26237371
chr14	26286764	26287030
chr14	26292992	26293207
chr14	26293240	26293695
chr14	26303193	26303396
chr14	26376735	26376961
chr14	26436420	26436580
chr14	26468002	26468246
chr14	26491185	26491527
chr14	26507410	26507750
chr14	26510573	26510876
chr14	26527694	26527849
chr14	26533352	26533539
chr14	26533791	26534222
chr14	26534222	26534717
chr14	26534717	26535167
chr14	26535196	26535427

chr14	26540656	26540940
chr14	26540940	26541224
chr14	26547350	26547526
chr14	26547868	26548087
chr14	26562190	26562938
chr14	26563079	26563281
chr14	26579354	26579725
chr14	26579725	26580069
chr14	26580069	26580336
chr14	26617740	26617894
chr14	26637728	26638570
chr14	26638700	26639047
chr14	26640088	26640260
chr14	26657090	26657240
chr14	26659038	26659459
chr14	26668334	26668545
chr14	26668545	26668736
chr14	26668736	26668995
chr14	26668995	26669294
chr14	26669294	26669542
chr14	26669542	26669769
chr14	26669805	26670160
chr14	26687739	26687910
chr14	26687910	26688147
chr14	26688147	26688501
chr14	26695258	26695551
chr14	26695575	26695842
chr14	26782401	26782614
chr14	26794044	26794485
chr14	26794516	26794777
chr14	26878789	26879041
chr14	26911226	26911497
chr14	26969827	26970305
chr14	26970305	26970954
chr14	26981602	26981842
chr14	26981851	26982111
chr14	26982118	26982534
chr14	27019785	27020117
chr14	27022961	27023194
chr14	27029846	27030077
chr14	27031211	27031500
chr14	27038763	27039291

chr14	27039291	27039718
chr14	27045076	27045344
chr14	27054042	27054230
chr14	27058465	27058900
chr14	27059268	27059418
chr14	27069536	27069753
chr14	27069795	27070111
chr14	27070132	27070756
chr14	27070867	27071094
chr14	27071941	27072177
chr14	27072177	27072381
chr14	27096820	27097130
chr14	27108791	27109057
chr14	27109185	27109418
chr14	27114226	27114660
chr14	27114660	27115001
chr14	27115001	27115323
chr14	27115332	27115499
chr14	27131346	27131856
chr14	27131856	27132314
chr14	27140081	27140235
chr14	27149519	27149725
chr14	27150052	27150309
chr14	27150347	27150497
chr14	27172952	27173213
chr14	27173213	27173538
chr14	27173541	27173719
chr14	27174705	27175395
chr14	27182028	27182233
chr14	27182233	27182420
chr14	27182420	27182666
chr14	27183777	27183928
chr14	27185183	27185560
chr14	27187069	27187367
chr14	27192555	27192857
chr14	27196988	27197367
chr14	27198347	27198534
chr14	27198825	27199182
chr14	27199210	27199405
chr14	27199441	27199934
chr14	27213956	27214161
chr14	27214203	27214353

chr14	27223847	27224098
chr14	27224670	27224829
chr14	27225363	27225557
chr14	27229427	27229776
chr14	27234370	27234630
chr14	27239580	27239950
chr14	27242275	27242593
chr14	27242808	27243090
chr14	27243520	27243670
chr14	27243766	27243959
chr14	27249139	27249716
chr14	27270806	27270966
chr14	27271305	27271981
chr14	27299460	27299625
chr14	27299649	27300097
chr14	27335793	27336137
chr14	27336137	27336694
chr14	27340778	27341009
chr14	27341150	27341791
chr14	27341791	27342078
chr14	27347239	27347693
chr14	27347754	27347910
chr14	27371326	27371658
chr14	27373481	27373870
chr14	27376089	27376537
chr14	27414065	27414259
chr14	27428497	27429021
chr14	27429021	27429377
chr14	27429377	27429741
chr14	27507993	27508284
chr14	27508284	27508682
chr14	27508685	27508878
chr14	27512430	27512688
chr14	27521083	27521601
chr14	27521601	27522001
chr14	27528234	27528456
chr14	27558835	27559167
chr14	27560530	27560793
chr14	27589740	27589895
chr14	27596383	27596611
chr14	27622032	27622592
chr14	27622592	27622931

chr14	27623425	27623701
chr14	27628709	27629100
chr14	27635735	27635946
chr14	27636573	27636943
chr14	27741669	27741821
chr14	27754369	27754691
chr14	27756981	27757250
chr14	27808175	27808329
chr14	27810811	27811158
chr14	27811522	27812014
chr14	27822570	27822904
chr14	27823014	27823576
chr14	27823824	27824291
chr14	27860389	27860712
chr14	27860712	27861015
chr14	27862281	27862711
chr14	27870460	27870828
chr14	27943502	27943712
chr14	27945391	27945661
chr14	27948458	27948628
chr14	27956815	27957078
chr14	27990692	27990890
chr14	28034103	28034369
chr14	28035085	28035354
chr14	28040440	28040797
chr14	28113203	28113416
chr14	28359555	28359828
chr14	28369306	28369508
chr14	28417052	28417381
chr14	28430572	28430888
chr14	28456360	28456666
chr14	28490463	28490816
chr14	28504717	28504941
chr14	28504941	28505223
chr14	28505225	28505409
chr14	28505409	28505591
chr14	28505722	28505992
chr14	28507597	28507824
chr14	28584344	28584577
chr14	28731306	28731568
chr14	28808145	28808527
chr14	28884666	28884828

chr14	28884845	28885228
chr14	28885228	28885450
chr14	28894582	28894897
chr14	28984284	28984500
chr14	29080621	29081025
chr14	29081977	29082819
chr14	29090151	29090399
chr14	29132191	29132351
chr14	29136361	29136650
chr14	29137701	29137851
chr14	29178315	29178497
chr14	29221115	29221507
chr14	29230829	29231052
chr14	29278440	29278630
chr14	29362487	29362775
chr14	29521371	29521781
chr14	29722025	29722193
chr14	29722501	29722688
chr14	29865048	29865340
chr14	29865340	29865727
chr14	29865766	29865937
chr14	29876372	29876611
chr14	29946983	29947185
chr14	29968020	29968760
chr14	29978230	29978540
chr14	29978540	29978893
chr14	29978919	29979084
chr14	29979084	29979241
chr14	30008670	30009333
chr14	30052904	30053199
chr14	30056590	30056837
chr14	30080217	30080405
chr14	30119120	30119274
chr14	30129541	30129696
chr14	30137044	30137391
chr14	30196355	30196569
chr14	30218211	30218394
chr14	30459941	30460175
chr14	30464549	30464937
chr14	30465108	30465219
chr14	30465219	30465264
chr14	30473627	30474110

chr14	30479211	30479728
chr14	30479728	30480103
chr14	30497696	30498012
chr14	30498012	30498268
chr14	30498268	30498597
chr14	30516359	30516614
chr14	30531499	30531766
chr14	30531782	30532239
chr14	30532818	30533110
chr14	30541552	30541834
chr14	30548087	30548420
chr14	30548420	30548715
chr14	30548836	30549285
chr14	30549285	30549699
chr14	30556600	30556986
chr14	30561070	30561376
chr14	30561376	30561636
chr14	30561636	30562128
chr14	30564449	30564776
chr14	30570324	30570836
chr14	30573277	30573447
chr14	30573447	30573531
chr14	30574092	30574268
chr14	30574270	30574532
chr14	30576060	30576220
chr14	30576228	30576550
chr14	30584034	30584457
chr14	30586734	30586951
chr14	30586951	30587150
chr14	30587293	30587510
chr14	30591489	30591724
chr14	30592985	30593524
chr14	30593524	30593977
chr14	30599794	30600244
chr14	30612545	30612805
chr14	30612805	30613004
chr14	30616852	30617029
chr14	30618942	30619155
chr14	30624099	30624593
chr14	30625073	30625269
chr14	30625295	30625697
chr14	30625900	30626111

chr14	30626111	30626390
chr14	30626390	30626727
chr14	30630860	30631032
chr14	30631251	30631541
chr14	30635228	30635600
chr14	30635600	30635925
chr14	30637211	30637409
chr14	30638079	30638353
chr14	30642523	30642870
chr14	30642870	30643252
chr14	30643860	30644386
chr14	30648433	30648661
chr14	30648661	30648909
chr14	30650974	30651397
chr14	30654218	30654699
chr14	30671120	30671482
chr14	30671807	30672022
chr14	30672022	30672302
chr14	30699744	30700020
chr14	30714932	30715276
chr14	30715276	30715541
chr14	30715541	30715808
chr14	30715808	30716043
chr14	30716043	30716335
chr14	30716335	30716743
chr14	30716839	30717270
chr14	30789556	30789816
chr14	30790602	30790821
chr14	30791123	30791368
chr14	30823181	30823586
chr14	30825363	30825775
chr14	30825775	30826191
chr14	30827437	30827661
chr14	30856730	30857432
chr14	30865386	30865618
chr14	30867084	30867377
chr14	30875454	30875939
chr14	30877807	30878176
chr14	30878276	30878445
chr14	30879152	30879558
chr14	30888252	30888627
chr14	30904258	30904757

chr14	30931923	30932132
chr14	30938643	30938793
chr14	30943963	30944243
chr14	30944313	30944635
chr14	30951021	30951539
chr14	30951539	30951880
chr14	30982219	30982467
chr14	30991009	30991314
chr14	31001264	31001519
chr14	31001519	31001758
chr14	31001758	31002032
chr14	31002032	31002288
chr14	31010232	31010536
chr14	31011265	31011425
chr14	31011653	31011933
chr14	31017895	31018137
chr14	31018790	31019229
chr14	31019229	31019687
chr14	31025895	31026158
chr14	31062343	31062539
chr14	31088519	31088908
chr14	31090007	31090259
chr14	31128354	31128580
chr14	31128675	31129269
chr14	31130809	31131401
chr14	31139683	31139961
chr14	31160024	31160224
chr14	31160357	31160971
chr14	31166488	31166681
chr14	31188922	31189288
chr14	31196088	31196274
chr14	31197848	31198152
chr14	31198152	31198475
chr14	31198746	31198976
chr14	31205223	31205517
chr14	31205517	31205856
chr14	31205856	31206174
chr14	31206174	31206516
chr14	31206516	31207107
chr14	31212605	31212789
chr14	31215267	31215487
chr14	31215509	31215786

chr14	31217608	31218110
chr14	31234043	31234294
chr14	31250909	31251193
chr14	31251193	31251392
chr14	31251392	31251585
chr14	31251585	31251928
chr14	31269497	31269712
chr14	31272210	31272493
chr14	31316631	31316946
chr14	31323830	31324192
chr14	31327220	31327524
chr14	31327796	31327970
chr14	31336309	31336594
chr14	31336594	31336841
chr14	31336841	31337206
chr14	31365437	31365659
chr14	31367675	31367852
chr14	31386039	31386500
chr14	31386540	31386727
chr14	31387025	31387384
chr14	31387636	31387803
chr14	31403747	31404014
chr14	31404014	31404417
chr14	31404427	31404622
chr14	31404738	31404916
chr14	31405767	31406126
chr14	31414693	31414945
chr14	31416954	31417153
chr14	31417176	31417463
chr14	31417494	31417659
chr14	31417659	31417821
chr14	31420723	31420897
chr14	31423228	31423421
chr14	31423564	31423719
chr14	31428769	31428955
chr14	31435168	31435519
chr14	31435674	31436376
chr14	31462339	31462577
chr14	31471996	31472276
chr14	31494525	31494934
chr14	31494934	31495225
chr14	31495225	31495503

chr14	31496134	31496505
chr14	31510065	31510217
chr14	31533243	31533559
chr14	31573123	31573279
chr14	31613797	31614031
chr14	31640442	31640612
chr14	31640651	31641147
chr14	31641147	31641464
chr14	31656537	31656782
chr14	31656905	31657159
chr14	31657957	31658194
chr14	31659585	31659786
chr14	31661673	31661827
chr14	31662022	31662362
chr14	31662362	31662755
chr14	31662755	31663221
chr14	31663945	31664376
chr14	31669101	31669723
chr14	31669723	31670162
chr14	31686403	31686705
chr14	31700419	31700784
chr14	31726283	31726522
chr14	31746321	31746528
chr14	31768202	31768517
chr14	31768517	31768895
chr14	31769049	31769291
chr14	31774248	31774445
chr14	31781367	31781590
chr14	31781590	31781831
chr14	31781831	31782121
chr14	31785650	31786057
chr14	31786562	31786840
chr14	31786986	31787243
chr14	31794523	31794732
chr14	31800925	31801256
chr14	31829895	31830398
chr14	31830398	31830822
chr14	31830822	31831241
chr14	31857682	31857862
chr14	31868695	31868916
chr14	31896619	31896805
chr14	31902003	31902307

chr14	31902319	31902471
chr14	31929300	31929688
chr14	31929724	31929907
chr14	31930296	31930802
chr14	31931778	31932060
chr14	31932078	31932246
chr14	31934862	31935062
chr14	31941647	31941805
chr14	31944831	31945052
chr14	31945090	31945619
chr14	31946101	31946447
chr14	31947220	31947614
chr14	31947660	31947815
chr14	31954966	31955242
chr14	31962985	31963263
chr14	31963263	31963555
chr14	31963555	31963979
chr14	31964034	31964429
chr14	31970494	31970745
chr14	31974498	31974683
chr14	32009864	32010047
chr14	32014867	32015479
chr14	32015479	32015762
chr14	32015857	32016324
chr14	32022542	32022759
chr14	32032562	32032999
chr14	32037903	32038158
chr14	32042283	32042445
chr14	32049666	32049934
chr14	32049934	32050228
chr14	32050228	32050631
chr14	32073192	32073477
chr14	32073477	32073754
chr14	32076868	32077233
chr14	32085176	32085468
chr14	32085468	32086133
chr14	32086408	32086712
chr14	32113995	32114375
chr14	32117608	32117776
chr14	32118329	32118877
chr14	32121973	32122266
chr14	32140440	32140592

chr14	32159210	32159438
chr14	32159438	32159751
chr14	32159751	32160064
chr14	32160064	32160293
chr14	32160293	32160477
chr14	32165212	32165545
chr14	32165545	32165985
chr14	32177806	32178147
chr14	32192522	32192752
chr14	32193641	32193857
chr14	32201386	32201770
chr14	32201770	32202057
chr14	32202057	32202298
chr14	32202298	32202666
chr14	32233870	32234059
chr14	32304822	32305175
chr14	32305175	32305454
chr14	32309869	32310158
chr14	32317957	32318175
chr14	32321766	32322279
chr14	32344719	32344919
chr14	32351320	32351540
chr14	32351540	32351760
chr14	32388237	32388585
chr14	32437452	32437625
chr14	32482157	32482328
chr14	32492553	32493011
chr14	32493504	32494001
chr14	32496162	32496347
chr14	32496570	32496748
chr14	32506432	32506793
chr14	32513304	32513617
chr14	32513617	32514096
chr14	32516086	32516503
chr14	32524138	32524396
chr14	32530972	32531189
chr14	32537376	32537557
chr14	32537859	32538490
chr14	32538643	32539142
chr14	32587067	32587339
chr14	32597244	32597501
chr14	32609424	32609690

chr14	32629335	32629584
chr14	32878603	32878823
chr14	33084337	33084579
chr14	33207086	33207470
chr14	33212610	33212937
chr14	33212950	33213170
chr14	33213811	33214176
chr14	33214176	33214506
chr14	33214506	33214789
chr14	33214789	33215011
chr14	33235016	33235197
chr14	33247532	33248091
chr14	33250139	33250502
chr14	33250502	33250720
chr14	33250720	33250943
chr14	33251062	33251229
chr14	33257101	33257294
chr14	33257453	33258040
chr14	33258257	33258708
chr14	33258708	33259125
chr14	33264840	33265131
chr14	33276310	33276677
chr14	33277676	33277826
chr14	33282407	33282749
chr14	33282749	33283241
chr14	33287881	33288363
chr14	33302777	33302994
chr14	33303329	33303578
chr14	33303578	33303899
chr14	33313488	33313990
chr14	33314140	33314508
chr14	33319351	33319576
chr14	33319576	33319784
chr14	33319784	33320105
chr14	33320105	33320548
chr14	33322747	33322911
chr14	33329072	33329427
chr14	33329427	33329786
chr14	33335557	33336201
chr14	33336201	33336626
chr14	33338516	33338750
chr14	33340934	33341214

chr14	33347674	33348168
chr14	33348168	33348546
chr14	33350011	33350204
chr14	33350918	33351135
chr14	33351135	33351413
chr14	33352356	33352846
chr14	33355294	33355622
chr14	33357748	33357958
chr14	33358143	33358348
chr14	33358698	33359223
chr14	33359223	33359540
chr14	33363900	33364408
chr14	33364923	33365073
chr14	33370099	33370325
chr14	33370755	33370910
chr14	33370923	33371100
chr14	33371275	33371614
chr14	33443802	33444319
chr14	33446843	33447304
chr14	33447304	33447608
chr14	33471546	33471725
chr14	33498816	33499011
chr14	33499011	33499259
chr14	33673746	33673997
chr14	33695460	33695618
chr14	33701811	33701969
chr14	33825102	33825317
chr14	33825317	33825569
chr14	33959451	33959623
chr14	33974767	33974949
chr14	33980383	33980649
chr14	33987030	33987320
chr14	34022464	34022666
chr14	34042718	34042940
chr14	34056971	34057195
chr14	34080277	34080650
chr14	34080650	34080860
chr14	34085371	34085550
chr14	34085666	34086123
chr14	34087711	34088273
chr14	34089877	34090243
chr14	34097908	34098206

chr14	34099025	34099300
chr14	34099300	34099534
chr14	34103629	34104212
chr14	34104835	34105280
chr14	34106782	34106969
chr14	34142095	34142279
chr14	34142303	34142763
chr14	34142871	34143053
chr14	34152410	34152697
chr14	34154640	34154950
chr14	34154981	34155242
chr14	34173657	34173830
chr14	34188708	34188945
chr14	34190461	34190658
chr14	34198682	34198952
chr14	34201004	34201200
chr14	34201331	34201674
chr14	34216680	34216830
chr14	34219931	34220274
chr14	34229078	34229535
chr14	34235043	34235391
chr14	34247456	34247751
chr14	34249380	34249562
chr14	34297167	34297509
chr14	34297736	34297886
chr14	34310202	34310597
chr14	34310597	34311107
chr14	34311156	34311500
chr14	34317932	34318498
chr14	34346101	34346457
chr14	34355373	34355600
chr14	34358493	34358679
chr14	34362886	34363054
chr14	34363064	34363433
chr14	34363433	34363740
chr14	34363740	34364089
chr14	34366434	34366688
chr14	34372718	34372966
chr14	34373844	34374042
chr14	34376401	34376573
chr14	34401210	34401475
chr14	34447658	34448001

chr14	34458989	34459208
chr14	34464514	34464690
chr14	34464891	34465041
chr14	34467567	34467779
chr14	34475000	34475209
chr14	34492816	34493001
chr14	34493791	34493953
chr14	34501898	34502332
chr14	34502332	34502713
chr14	34502713	34503038
chr14	34503038	34503424
chr14	34508163	34508541
chr14	34514204	34514398
chr14	34532664	34533035
chr14	34540658	34540863
chr14	34548450	34548716
chr14	34548716	34548966
chr14	34548966	34549224
chr14	34549224	34549440
chr14	34549440	34549741
chr14	34562535	34562769
chr14	34570360	34570644
chr14	34574187	34574402
chr14	34580861	34581078
chr14	34583993	34584217
chr14	34584217	34584425
chr14	34615181	34615538
chr14	34616840	34617155
chr14	34658046	34658286
chr14	34661855	34662126
chr14	34673258	34673680
chr14	34673680	34674114
chr14	34674114	34674488
chr14	34674605	34674782
chr14	34763747	34764101
chr14	34764101	34764644
chr14	34768437	34768721
chr14	34768994	34769257
chr14	34769292	34769469
chr14	34769596	34769866
chr14	34777917	34778175
chr14	34781414	34781582

chr14	34784826	34785022
chr14	34795454	34795642
chr14	34806973	34807162
chr14	34816398	34816555
chr14	34820051	34820256
chr14	34822653	34822803
chr14	35051526	35051815
chr14	35067177	35067412
chr14	35692484	35692760
chr14	36137768	36138127
chr14	36145164	36145653
chr14	36147266	36147669
chr14	36316472	36316752
chr14	36316752	36317021
chr14	36340922	36341153
chr14	36393090	36393487
chr14	36495213	36495510
chr14	36527107	36527725
chr14	36538429	36538722
chr14	36559704	36560078
chr14	36583710	36584041
chr14	36584041	36584297
chr14	36584297	36584537
chr14	36586022	36586204
chr14	36603181	36603331
chr14	36610309	36610626
chr14	36611313	36611640
chr14	36611640	36611928
chr14	36691343	36691556
chr14	36698458	36698662
chr14	36698917	36699399
chr14	36699399	36699709
chr14	36726248	36726512
chr14	36755443	36755913
chr14	36756054	36756212
chr14	36774401	36774690
chr14	36779018	36779354
chr14	36799824	36800235
chr14	36802232	36802488
chr14	36802488	36802828
chr14	36805858	36806201
chr14	36809054	36809334

chr14	36855127	36855306
chr14	36862439	36862728
chr14	36863445	36863692
chr14	36863716	36863871
chr14	36863943	36864427
chr14	36867039	36867259
chr14	36919204	36919544
chr14	36952222	36952450
chr14	36962428	36962975
chr14	36968058	36968530
chr14	36968530	36969195
chr14	37033336	37033575
chr14	37049473	37049694
chr14	37098148	37098542
chr14	37110674	37111065
chr14	37111465	37111673
chr14	37115828	37116094
chr14	37117143	37117474
chr14	37117711	37117879
chr14	37119503	37119779
chr14	37134644	37134868
chr14	37134874	37135637
chr14	37184820	37185186
chr14	37234957	37235233
chr14	37503096	37503424
chr14	38206300	38206495
chr14	40791714	40791864
chr14	40793642	40793836
chr14	40851815	40852020
chr14	40885216	40885618
chr14	40886897	40887105
chr14	40894567	40894928
chr14	40894928	40895154
chr14	40895801	40895963
chr14	40898041	40898240
chr14	40902198	40902672
chr14	40902672	40903132
chr14	40903132	40903611
chr14	40908426	40908615
chr14	40914677	40915118
chr14	40915118	40915435
chr14	40915600	40915830

chr14	40917711	40917977
chr14	40917977	40918167
chr14	40918251	40918428
chr14	40918428	40918649
chr14	40926887	40927430
chr14	40931196	40931376
chr14	40935597	40935969
chr14	40935969	40936376
chr14	40937133	40937423
chr14	40938356	40938506
chr14	40947479	40947718
chr14	40947796	40948065
chr14	40948065	40948292
chr14	40948478	40948950
chr14	40949716	40950020
chr14	40950069	40950353
chr14	40956039	40956530
chr14	40956530	40956858
chr14	40958618	40958800
chr14	40959300	40959613
chr14	40965904	40966064
chr14	40966361	40966753
chr14	40966753	40967077
chr14	40967077	40967609
chr14	40972403	40972757
chr14	40975208	40975481
chr14	40979052	40979358
chr14	40987087	40987573
chr14	40995266	40995615
chr14	40995615	40995869
chr14	40995869	40996129
chr14	40996172	40996705
chr14	40999911	41000319
chr14	41008138	41008418
chr14	41013522	41013993
chr14	41014018	41014215
chr14	41017171	41017431
chr14	41017431	41017849
chr14	41018372	41018568
chr14	41019215	41019574
chr14	41031097	41031307
chr14	41031307	41031622

chr14	41033501	41033655
chr14	41035239	41035478
chr14	41035511	41035761
chr14	41040677	41040892
chr14	41053300	41053684
chr14	41053684	41054099
chr14	41061327	41061624
chr14	41072649	41072999
chr14	41104893	41105177
chr14	41105177	41105468
chr14	41141494	41141733
chr14	41162775	41162983
chr14	41167215	41167625
chr14	41185246	41185396
chr14	41232167	41232317
chr14	41265277	41265466
chr14	41336634	41336788
chr14	41337574	41337861
chr14	41421613	41421951
chr14	41421951	41422172
chr14	41422172	41422461
chr14	41425638	41426006
chr14	41486985	41487198
chr14	41489285	41489678
chr14	41501157	41501396
chr14	41644025	41644240
chr14	41646421	41646660
chr14	41699142	41699308
chr14	41715039	41715303
chr14	41723850	41724017
chr14	41772305	41772634
chr14	41983197	41983422
chr14	42130697	42131046
chr14	42131046	42131419
chr14	42134750	42135086
chr14	42172764	42173021
chr14	42188317	42188530
chr14	42190726	42190942
chr14	42243895	42244103
chr14	42452836	42453018
chr14	42583643	42583854
chr14	42797852	42798165

chr14	42850741	42850902
chr14	42866594	42866904
chr14	43013085	43013254
chr14	43015496	43015806
chr14	43073784	43074223
chr14	43084888	43085119
chr14	43085119	43085386
chr14	43085386	43085595
chr14	43089627	43089849
chr14	43153803	43153970
chr14	43164281	43164658
chr14	43164658	43165080
chr14	43168869	43169101
chr14	43238877	43239158
chr14	43245775	43245983
chr14	43246735	43247088
chr14	43247088	43247335
chr14	43247335	43247586
chr14	43249808	43249972
chr14	43250638	43250898
chr14	43250898	43251152
chr14	43256862	43257060
chr14	43269350	43269603
chr14	43270660	43270813
chr14	43277857	43278044
chr14	43278072	43278518
chr14	43278518	43278855
chr14	43291045	43291384
chr14	43296461	43296889
chr14	43300449	43300663
chr14	43300663	43300897
chr14	43318898	43319195
chr14	43319563	43319717
chr14	43329697	43329984
chr14	43333461	43333700
chr14	43345231	43345660
chr14	43347088	43347341
chr14	43347478	43347692
chr14	43347692	43347985
chr14	43350504	43350817
chr14	43361211	43361376
chr14	43361782	43362089

chr14	43368127	43368320
chr14	43368416	43368608
chr14	43368893	43369332
chr14	43377849	43378115
chr14	43379924	43380339
chr14	43413466	43413686
chr14	43413686	43413805
chr14	43419138	43419300
chr14	43470050	43470212
chr14	43478720	43478953
chr14	43480467	43480646
chr14	43480744	43481219
chr14	43500893	43501045
chr14	43514298	43514627
chr14	43519970	43520137
chr14	43520771	43521062
chr14	43525195	43525605
chr14	43527702	43527863
chr14	43528565	43529202
chr14	43529226	43529407
chr14	43531702	43531910
chr14	43532109	43532439
chr14	43532439	43532950
chr14	43605541	43605739
chr14	43607933	43608229
chr14	43610838	43610990
chr14	43611246	43611525
chr14	43804547	43804813
chr14	43812145	43812324
chr14	43812450	43812786
chr14	43918249	43918443
chr14	43925035	43925449
chr14	43925449	43925799
chr14	43933054	43933382
chr14	43983989	43984224
chr14	43987521	43987715
chr14	44139642	44139856
chr14	44182918	44183159
chr14	44183159	44183408
chr14	44186593	44186877
chr14	44256680	44256837
chr14	44256910	44257172

chr14	44361500	44361670
chr14	44500022	44500188
chr14	44503289	44503492
chr14	44547846	44548176
chr14	44703214	44703390
chr14	44977381	44977575
chr14	45211481	45211873
chr14	45219179	45219884
chr14	45219884	45220254
chr14	45220254	45220468
chr14	45220468	45220675
chr14	45220675	45220861
chr14	45317898	45318050
chr14	45318060	45318688
chr14	45318688	45318942
chr14	45318942	45319144
chr14	45325328	45325799
chr14	45329149	45329306
chr14	45329503	45329884
chr14	45329884	45330324
chr14	45350845	45351405
chr14	45351405	45351879
chr14	45387594	45387795
chr14	45388420	45389311
chr14	45398579	45398801
chr14	45422411	45422700
chr14	45423295	45423501
chr14	45443194	45443506
chr14	45463475	45463660
chr14	45463682	45464006
chr14	45477677	45478038
chr14	45478918	45479125
chr14	45479212	45479364
chr14	45479375	45479730
chr14	45492010	45492592
chr14	45493033	45493245
chr14	45528897	45529111
chr14	45529288	45529684
chr14	45529684	45529996
chr14	45529996	45530382
chr14	45530382	45530688
chr14	45540279	45540518

chr14	45543322	45543786
chr14	45561807	45561986
chr14	45563502	45563715
chr14	45566425	45566628
chr14	45566628	45566863
chr14	45629870	45630224
chr14	45632945	45633109
chr14	45634067	45634391
chr14	45642043	45642322
chr14	45642322	45642683
chr14	45652766	45653069
chr14	45656938	45657129
chr14	45657132	45657392
chr14	45657392	45657685
chr14	45657685	45657990
chr14	45657990	45658301
chr14	45658301	45658750
chr14	45690619	45691021
chr14	45691101	45691251
chr14	45692922	45693081
chr14	45732458	45732813
chr14	45764341	45764661
chr14	45769409	45769811
chr14	45772664	45772895
chr14	45812736	45812905
chr14	45842573	45842842
chr14	45849893	45850492
chr14	45850492	45850991
chr14	45859676	45860071
chr14	45878406	45878638
chr14	45878731	45878969
chr14	45895667	45896026
chr14	45958091	45958513
chr14	45963204	45963796
chr14	45963796	45964092
chr14	45995690	45995854
chr14	45996615	45997287
chr14	46002105	46002443
chr14	46060935	46061421
chr14	46079502	46079747
chr14	46084033	46084368
chr14	46084368	46084594

chr14	46084594	46084832
chr14	46144738	46145150
chr14	46172639	46172833
chr14	46227507	46227726
chr14	46338832	46339142
chr14	46357863	46358028
chr14	46363287	46363575
chr14	46387014	46387181
chr14	46387188	46387745
chr14	46387745	46388165
chr14	46388775	46388990
chr14	46394282	46394515
chr14	46408357	46408864
chr14	46409588	46409783
chr14	46439722	46439907
chr14	46478854	46479066
chr14	46481046	46481261
chr14	46500800	46501255
chr14	46501468	46501672
chr14	46512141	46512479
chr14	46516056	46516626
chr14	46516626	46516945
chr14	46517316	46517571
chr14	46519091	46519331
chr14	46574347	46574732
chr14	46577985	46578390
chr14	46578468	46578817
chr14	46596434	46596595
chr14	46613806	46614053
chr14	46634287	46634642
chr14	46634672	46635178
chr14	46635306	46635715
chr14	46636161	46636770
chr14	46660662	46661129
chr14	46661183	46661523
chr14	46670929	46671446
chr14	46702832	46703068
chr14	46703261	46703417
chr14	46704063	46704737
chr14	46721045	46721267
chr14	46721267	46721502
chr14	46722074	46722485

chr14	46723951	46724265
chr14	46729139	46729652
chr14	46760239	46760728
chr14	46760728	46761075
chr14	46770322	46770533
chr14	46787806	46788125
chr14	46788125	46788529
chr14	46790454	46790682
chr14	46790727	46791086
chr14	46791108	46791265
chr14	46798984	46799139
chr14	46800637	46801011
chr14	46801051	46801278
chr14	46801278	46801504
chr14	46818785	46818962
chr14	46820108	46820285
chr14	46821451	46821631
chr14	46821709	46822089
chr14	46822089	46822433
chr14	46831911	46832341
chr14	46832341	46832702
chr14	46881030	46881204
chr14	46881976	46882210
chr14	46882210	46882430
chr14	46882430	46882643
chr14	46882643	46882843
chr14	46882843	46883240
chr14	46883398	46883613
chr14	46883710	46883871
chr14	46884065	46884310
chr14	46884880	46885109
chr14	46885953	46886212
chr14	46887703	46887875
chr14	46888021	46888183
chr14	46908915	46909152
chr14	46909152	46909393
chr14	46909510	46910000
chr14	46912505	46912761
chr14	46924102	46924312
chr14	46924462	46924682
chr14	46925048	46925254
chr14	46944446	46944708

chr14	46965846	46966190
chr14	46966190	46966564
chr14	46980463	46980661
chr14	46980805	46981086
chr14	46981089	46981451
chr14	46988772	46989162
chr14	46989162	46989563
chr14	46989563	46990075
chr14	46999042	46999266
chr14	46999509	46999674
chr14	47003725	47004136
chr14	47004136	47004465
chr14	47007217	47007698
chr14	47011290	47011461
chr14	47020905	47021296
chr14	47022145	47022500
chr14	47023513	47023942
chr14	47024505	47024807
chr14	47024960	47025110
chr14	47031584	47031859
chr14	47033850	47034103
chr14	47034103	47034370
chr14	47036784	47037109
chr14	47037109	47037405
chr14	47037463	47037711
chr14	47043036	47043221
chr14	47059093	47059276
chr14	47075412	47075620
chr14	47077495	47077985
chr14	47078131	47078296
chr14	47086551	47087169
chr14	47087169	47087580
chr14	47107465	47107851
chr14	47108454	47108626
chr14	47113412	47113764
chr14	47116028	47116399
chr14	47128114	47128320
chr14	47134032	47134272
chr14	47135319	47135540
chr14	47161238	47161492
chr14	47177821	47178249
chr14	47179563	47179858

chr14	47186564	47186872
chr14	47188685	47189197
chr14	47189197	47189622
chr14	47189648	47189847
chr14	47208623	47208820
chr14	47209181	47209436
chr14	47242811	47243080
chr14	47249808	47250172
chr14	47250230	47250429
chr14	47259049	47259201
chr14	47276122	47276323
chr14	47276323	47276646
chr14	47276646	47277058
chr14	47277058	47277412
chr14	47297812	47298082
chr14	47298082	47298447
chr14	47298447	47298742
chr14	47299053	47299203
chr14	47302914	47303148
chr14	47303306	47303804
chr14	47308896	47309065
chr14	47309102	47309393
chr14	47311242	47311529
chr14	47323215	47323382
chr14	47327366	47327707
chr14	47327751	47328088
chr14	47334871	47335121
chr14	47349954	47350131
chr14	47351017	47351380
chr14	47351468	47351618
chr14	47352292	47352636
chr14	47353985	47354361
chr14	47354361	47354619
chr14	47361719	47361944
chr14	47366941	47367205
chr14	47367464	47367690
chr14	47367690	47367893
chr14	47368267	47368557
chr14	47371476	47372158
chr14	47373672	47374102
chr14	47374106	47374298
chr14	47374359	47374701

chr14	47375202	47375419
chr14	47377049	47377299
chr14	47378490	47378672
chr14	47382717	47383042
chr14	47383895	47384086
chr14	47384089	47384415
chr14	47385480	47385790
chr14	47385891	47386073
chr14	47386215	47386474
chr14	47387090	47387527
chr14	47396153	47396555
chr14	47417376	47417679
chr14	47417796	47418134
chr14	47418134	47418491
chr14	47421711	47421944
chr14	47433079	47433269
chr14	47441373	47441572
chr14	47472335	47472713
chr14	47472713	47472942
chr14	47472942	47473223
chr14	47474655	47474918
chr14	47482306	47482456
chr14	47488735	47489047
chr14	47496011	47496218
chr14	47503987	47504355
chr14	47504634	47504894
chr14	47514915	47515090
chr14	47515149	47515410
chr14	47516708	47517127
chr14	47518639	47519031
chr14	47519181	47519369
chr14	47524787	47525302
chr14	47529119	47529339
chr14	47533436	47534222
chr14	47541656	47541923
chr14	47548388	47548557
chr14	47567944	47568326
chr14	47568326	47568744
chr14	47581391	47581594
chr14	47597901	47598159
chr14	47629393	47629600
chr14	47634137	47634744

chr14	47638086	47638350
chr14	47638529	47638858
chr14	47648954	47649578
chr14	47649578	47650110
chr14	47654297	47654509
chr14	47664540	47664812
chr14	47665179	47665341
chr14	47665433	47665758
chr14	47703286	47703472
chr14	47751854	47752131
chr14	47753624	47753940
chr14	47754189	47754633
chr14	47766453	47766731
chr14	47771682	47771837
chr14	47776265	47776560
chr14	47776560	47776777
chr14	47795745	47796006
chr14	47808522	47808791
chr14	47813956	47814172
chr14	47863081	47863448
chr14	47868477	47868689
chr14	47878147	47878366
chr14	47944706	47945008
chr14	47945802	47946062
chr14	48120738	48121166
chr14	48121258	48121728
chr14	48189426	48189597
chr14	48197410	48197566
chr14	48212953	48213137
chr14	48224821	48225114
chr14	48225121	48225287
chr14	48237113	48237583
chr14	48241428	48241704
chr14	48246276	48246555
chr14	48249506	48249770
chr14	48282829	48283116
chr14	48283133	48283656
chr14	48288397	48288605
chr14	48288616	48288996
chr14	48289668	48289927
chr14	48290004	48290299
chr14	48290299	48290586

chr14	48295491	48295757
chr14	48295803	48296387
chr14	48367183	48367336
chr14	48370423	48370573
chr14	48371869	48372079
chr14	48421967	48422440
chr14	48423058	48423259
chr14	48423918	48424088
chr14	48443807	48444089
chr14	48445935	48446254
chr14	48446254	48446746
chr14	48458621	48458925
chr14	48462466	48462866
chr14	48679168	48679357
chr14	48679395	48679654
chr14	48679654	48680141
chr14	48842191	48842347
chr14	48949311	48949542
chr14	48998823	48999001
chr14	49033581	49033805
chr14	49065902	49066259
chr14	49066259	49066559
chr14	49066559	49066977
chr14	49074212	49074688
chr14	49171353	49171611
chr14	49171821	49172239
chr14	49172239	49172540
chr14	49172540	49172746
chr14	49172746	49172952
chr14	49172952	49173287
chr14	49174686	49174881
chr14	49238308	49238481
chr14	49373010	49373265
chr14	49373556	49373783
chr14	49373783	49374124
chr14	49376582	49376970
chr14	49441722	49441910
chr14	49458522	49458866
chr14	49458973	49459170
chr14	49464388	49464565
chr14	49525177	49525488
chr14	49783117	49783543

chr14	50061991	50062256
chr14	50062256	50062533
chr14	50062533	50062786
chr14	50785015	50785214
chr14	50785214	50785434
chr14	50785434	50785718
chr14	50788252	50788469
chr14	50798590	50798815
chr14	50799943	50800135
chr14	50807268	50807780
chr14	50807780	50808066
chr14	50834100	50834345
chr14	50859347	50859536
chr14	50870009	50870219
chr14	50870219	50870476
chr14	50870476	50870822
chr14	50870822	50871121
chr14	50871231	50871404
chr14	50896280	50896537
chr14	50903983	50904233
chr14	50904233	50904553
chr14	50924478	50925012
chr14	50925012	50925249
chr14	50925249	50925425
chr14	50930410	50930811
chr14	50930811	50931557
chr14	50939069	50939219
chr14	50944248	50944639
chr14	50944639	50944948
chr14	50945138	50945359
chr14	50947962	50948146
chr14	50948146	50948360
chr14	50955874	50956274
chr14	50956274	50956561
chr14	50956785	50957018
chr14	50959816	50960061
chr14	51026798	51027053
chr14	51036309	51036480
chr14	51063360	51063716
chr14	51063727	51064083
chr14	51064138	51064323
chr14	51064489	51064757

chr14	51064757	51065061
chr14	51065061	51065356
chr14	51065356	51065619
chr14	51069698	51069937
chr14	51081173	51081407
chr14	51082625	51083008
chr14	51083008	51083260
chr14	51083260	51083586
chr14	51088610	51088836
chr14	51088836	51089087
chr14	51089087	51089395
chr14	51089395	51089700
chr14	51089700	51089927
chr14	51090247	51090439
chr14	51090543	51090797
chr14	51090949	51091228
chr14	51096108	51096495
chr14	51098346	51098647
chr14	51103414	51103576
chr14	51132530	51132686
chr14	51140554	51140714
chr14	51147698	51147860
chr14	51176325	51176559
chr14	51187196	51187347
chr14	51214175	51214343
chr14	51218499	51218688
chr14	51266190	51266426
chr14	51633196	51633346
chr14	51635574	51635780
chr14	51735021	51735263
chr14	51827926	51828145
chr14	51838592	51838759
chr14	51841770	51842111
chr14	51842360	51842625
chr14	51851093	51851310
chr14	51863701	51863928
chr14	51870793	51871115
chr14	51877508	51878017
chr14	51883561	51883948
chr14	51884376	51884658
chr14	51884658	51884996
chr14	51884996	51885256

chr14	51893375	51893733
chr14	51902876	51903108
chr14	51904761	51904975
chr14	51912652	51912942
chr14	51912942	51913279
chr14	51913279	51913640
chr14	51913643	51913941
chr14	51916496	51916791
chr14	51930897	51931161
chr14	51932184	51932608
chr14	51932629	51933226
chr14	51939128	51939339
chr14	51954303	51954680
chr14	51959521	51959731
chr14	51960074	51960364
chr14	51960364	51960790
chr14	51963714	51964032
chr14	51965311	51965558
chr14	51984386	51984616
chr14	51984616	51984783
chr14	51984783	51985221
chr14	51985345	51985548
chr14	51985579	51985829
chr14	51985829	51986206
chr14	51987150	51987596
chr14	52003998	52004337
chr14	52004337	52004640
chr14	52004870	52005154
chr14	52008847	52009185
chr14	52012208	52012393
chr14	52013877	52014280
chr14	52014590	52014814
chr14	52014814	52015040
chr14	52019351	52019608
chr14	52019608	52019954
chr14	52019954	52020432
chr14	52020527	52020921
chr14	52023081	52023426
chr14	52045393	52045603
chr14	52052716	52053064
chr14	52053261	52053493
chr14	52057871	52058174

chr14	52067528	52067678
chr14	52069150	52069558
chr14	52103880	52104322
chr14	52104322	52104593
chr14	52106898	52107119
chr14	52118811	52119048
chr14	52119048	52119287
chr14	52119287	52119563
chr14	52124009	52124235
chr14	52164045	52164285
chr14	52190747	52191005
chr14	52196675	52197077
chr14	52197077	52197377
chr14	52197377	52197786
chr14	52232340	52232555
chr14	52234368	52234671
chr14	52244072	52244389
chr14	52244389	52244688
chr14	52244688	52245053
chr14	52246222	52246476
chr14	52257365	52258244
chr14	52278863	52279297
chr14	52279297	52279740
chr14	52279806	52280090
chr14	52295718	52295948
chr14	52304394	52304999
chr14	52304999	52305479
chr14	52311884	52312040
chr14	52315704	52316030
chr14	52316030	52316370
chr14	52316397	52316584
chr14	52316650	52316802
chr14	52325995	52326162
chr14	52338724	52338972
chr14	52343058	52343279
chr14	52343731	52343897
chr14	52403191	52403375
chr14	52768338	52768530
chr14	52821748	52821905
chr14	52918661	52918867
chr14	52941816	52942112
chr14	52942511	52942770

chr14	52948681	52948936
chr14	52956692	52956875
chr14	53032692	53032848
chr14	53229630	53229845
chr14	53252846	53253128
chr14	53253539	53253797
chr14	53259696	53259904
chr14	53336882	53337032
chr14	53516755	53516921
chr14	53559584	53559746
chr14	53582584	53582852
chr14	53598691	53598882
chr14	53604186	53604532
chr14	53614418	53614648
chr14	53615458	53615724
chr14	53616119	53616348
chr14	53619168	53619433
chr14	53620862	53621104
chr14	53621459	53621852
chr14	53621893	53622048
chr14	53633349	53633536
chr14	53633653	53634130
chr14	53634130	53634519
chr14	53634607	53634891
chr14	53634891	53635098
chr14	53640655	53641053
chr14	53649324	53649480
chr14	53649664	53649814
chr14	53683461	53683648
chr14	53703692	53703889
chr14	53710089	53710466
chr14	53729327	53729504
chr14	53738060	53738276
chr14	53804313	53804585
chr14	53896125	53896345
chr14	53943355	53943689
chr14	54032168	54032366
chr14	54059853	54060090
chr14	54061658	54061992
chr14	54064575	54064862
chr14	54064862	54065279
chr14	54067925	54068193

chr14	54074719	54075203
chr14	54097090	54097324
chr14	54097475	54097673
chr14	54097730	54098183
chr14	54098183	54098878
chr14	54105639	54106026
chr14	54106142	54106333
chr14	54106993	54107253
chr14	54107253	54107720
chr14	54108669	54109090
chr14	54121075	54121689
chr14	54122909	54123078
chr14	54127521	54127717
chr14	54128489	54128743
chr14	54146414	54146808
chr14	54152913	54153148
chr14	54179344	54179590
chr14	54184804	54184989
chr14	54185049	54185265
chr14	54185302	54185904
chr14	54185904	54186329
chr14	54186365	54186566
chr14	54222112	54222583
chr14	54222583	54222805
chr14	54223369	54223620
chr14	54223782	54223937
chr14	54224082	54224457
chr14	54224457	54224836
chr14	54224836	54225217
chr14	54225232	54225396
chr14	54227254	54227854
chr14	54230711	54230985
chr14	54231124	54231396
chr14	54232024	54232525
chr14	54238909	54239394
chr14	54239400	54239571
chr14	54253533	54254300
chr14	54254313	54254482
chr14	54254907	54255097
chr14	54259021	54259359
chr14	54259359	54259784
chr14	54259804	54259996

chr14	54282877	54283160
chr14	54297109	54297286
chr14	54340947	54341106
chr14	54350330	54350480
chr14	54360634	54360914
chr14	54360914	54361161
chr14	54361161	54361541
chr14	54371089	54371397
chr14	54383315	54383657
chr14	54412835	54413077
chr14	54413969	54414330
chr14	54414345	54414622
chr14	54414658	54414839
chr14	54415267	54415523
chr14	54423448	54423667
chr14	54426276	54426507
chr14	54426656	54427014
chr14	54427014	54427254
chr14	54431468	54431652
chr14	54432262	54432489
chr14	54433552	54433730
chr14	54434059	54434511
chr14	54435073	54435493
chr14	54435493	54435882
chr14	54448830	54449062
chr14	54449062	54449325
chr14	54449325	54449635
chr14	54462691	54463107
chr14	54463735	54463886
chr14	54463986	54464262
chr14	54464262	54464593
chr14	54464593	54465027
chr14	54475814	54476485
chr14	54481282	54481548
chr14	54481548	54481943
chr14	54482646	54482796
chr14	54487833	54488032
chr14	54492367	54492629
chr14	54492769	54493099
chr14	54494258	54494561
chr14	54501430	54501819
chr14	54517082	54517883

chr14	54528494	54528759
chr14	54528823	54528996
chr14	54553533	54553683
chr14	54553872	54554198
chr14	54554198	54554511
chr14	54554511	54554813
chr14	54562681	54562937
chr14	54569081	54569260
chr14	54572757	54573202
chr14	54573202	54573511
chr14	54574194	54574602
chr14	54575532	54575999
chr14	54577460	54577727
chr14	54578219	54578414
chr14	54583745	54584007
chr14	54585150	54585313
chr14	54589054	54589289
chr14	54596432	54596835
chr14	54597138	54597399
chr14	54603034	54603335
chr14	54603335	54603686
chr14	54605881	54606156
chr14	54610033	54610294
chr14	54616193	54616449
chr14	54617478	54617814
chr14	54617814	54618157
chr14	54618157	54618404
chr14	54634273	54634575
chr14	54641047	54641276
chr14	54641276	54641652
chr14	54641652	54642020
chr14	54644346	54644641
chr14	54650646	54650921
chr14	54651104	54651411
chr14	54653573	54653920
chr14	54654244	54654399
chr14	54669492	54669731
chr14	54678706	54678964
chr14	54685670	54685891
chr14	54686002	54686270
chr14	54686270	54686526
chr14	54686526	54686820

chr14	54686820	54687152
chr14	54694985	54695174
chr14	54701383	54701648
chr14	54713698	54713853
chr14	54714005	54714463
chr14	54714622	54715211
chr14	54767534	54767757
chr14	54781106	54781347
chr14	54781759	54782031
chr14	54786008	54786235
chr14	54817927	54818077
chr14	54835710	54835864
chr14	54835958	54836118
chr14	54843558	54843730
chr14	54863475	54863775
chr14	54863775	54864061
chr14	54864061	54864387
chr14	54864416	54864635
chr14	54870939	54871432
chr14	54871434	54871614
chr14	54876200	54876572
chr14	54876572	54877004
chr14	54877004	54877390
chr14	54877390	54877686
chr14	54883143	54883553
chr14	54883553	54883839
chr14	54883839	54884086
chr14	54884451	54884647
chr14	54886755	54886938
chr14	54893583	54894226
chr14	54894226	54894674
chr14	54900469	54900735
chr14	54903589	54904155
chr14	54912203	54912429
chr14	54912616	54912810
chr14	54912993	54913181
chr14	54925896	54926074
chr14	54926074	54926289
chr14	54929861	54930376
chr14	54930408	54930804
chr14	54931402	54931747
chr14	54939395	54939809

chr14	54945741	54945935
chr14	54945946	54946224
chr14	55014609	55015043
chr14	55015043	55015307
chr14	55015307	55015599
chr14	55015599	55015976
chr14	55029099	55029278
chr14	55054966	55055225
chr14	55055225	55055527
chr14	55055527	55055748
chr14	55079306	55079584
chr14	55091993	55092250
chr14	55092250	55092654
chr14	55094653	55094891
chr14	55094891	55095297
chr14	55098166	55098381
chr14	55101834	55101989
chr14	55105876	55106040
chr14	55106166	55106456
chr14	55106456	55106764
chr14	55114050	55114238
chr14	55114690	55115182
chr14	55115182	55115461
chr14	55115461	55115659
chr14	55121983	55122294
chr14	55124547	55124859
chr14	55125775	55126037
chr14	55153003	55153432
chr14	55157464	55157625
chr14	55161203	55161671
chr14	55166639	55167024
chr14	55167024	55167467
chr14	55177398	55177576
chr14	55194802	55194982
chr14	55205124	55205386
chr14	55208006	55208336
chr14	55208336	55208672
chr14	55208703	55208899
chr14	55279345	55279640
chr14	55458341	55458601
chr14	55459963	55460129
chr14	55469136	55469291

chr14	55477494	55477662
chr14	55478624	55479052
chr14	55479052	55479445
chr14	55490981	55491264
chr14	55491264	55491532
chr14	55491532	55491844
chr14	55497121	55497804
chr14	55520463	55520946
chr14	55520946	55521294
chr14	55540042	55540622
chr14	55540622	55541038
chr14	55554473	55554828
chr14	55557065	55557361
chr14	55559733	55560140
chr14	55560157	55560614
chr14	55560614	55561031
chr14	55578048	55578303
chr14	55578303	55578637
chr14	55578637	55578962
chr14	55578981	55579181
chr14	55584650	55584821
chr14	55584924	55585497
chr14	55585607	55585772
chr14	55590844	55591251
chr14	55591251	55591561
chr14	55591561	55591892
chr14	55591892	55592141
chr14	55603759	55604280
chr14	55604280	55604594
chr14	55629387	55629622
chr14	55634996	55635506
chr14	55635506	55635909
chr14	55642825	55643238
chr14	55643238	55643463
chr14	55643463	55643713
chr14	55643713	55644005
chr14	55659939	55660276
chr14	55660276	55660679
chr14	55660727	55660877
chr14	55671578	55671786
chr14	55671786	55672048
chr14	55672048	55672471

chr14	55672517	55672687
chr14	55681218	55681439
chr14	55681439	55681733
chr14	55681733	55682220
chr14	55702448	55702725
chr14	55702744	55703309
chr14	55706254	55706597
chr14	55709385	55709885
chr14	55711122	55711343
chr14	55713338	55713680
chr14	55713848	55714147
chr14	55714207	55714447
chr14	55714817	55715367
chr14	55715367	55715707
chr14	55716925	55717150
chr14	55721689	55722118
chr14	55722118	55722432
chr14	55724278	55724928
chr14	55727864	55728226
chr14	55730078	55730563
chr14	55737725	55738010
chr14	55743749	55744054
chr14	55744889	55745206
chr14	55745206	55745507
chr14	55745507	55746025
chr14	55758349	55758551
chr14	55758567	55758849
chr14	55760030	55760489
chr14	55762162	55762420
chr14	55767762	55768133
chr14	55768133	55768506
chr14	55768609	55768865
chr14	55781141	55781731
chr14	55795685	55796249
chr14	55797177	55797448
chr14	55804129	55804362
chr14	55814934	55815204
chr14	55815287	55815750
chr14	55817645	55818181
chr14	55822505	55822801
chr14	55822904	55823234
chr14	55823234	55823495

chr14	55823495	55823711
chr14	55863836	55864109
chr14	55873770	55874098
chr14	55874098	55874395
chr14	55874395	55874658
chr14	55879716	55879875
chr14	55882061	55882707
chr14	55884743	55885404
chr14	55899651	55899886
chr14	55899886	55900108
chr14	55900108	55900479
chr14	55901497	55901697
chr14	55903528	55904081
chr14	55905596	55905807
chr14	55905807	55905992
chr14	55918357	55918533
chr14	55918533	55918735
chr14	55918740	55919014
chr14	55923578	55923800
chr14	55946343	55946592
chr14	55946624	55946945
chr14	55950336	55950691
chr14	55997385	55997561
chr14	55998092	55998292
chr14	56013108	56013348
chr14	56019537	56019766
chr14	56034307	56034917
chr14	56039862	56040080
chr14	56043911	56044184
chr14	56055430	56055597
chr14	56057644	56058280
chr14	56078233	56078812
chr14	56084256	56084505
chr14	56084977	56085250
chr14	56085250	56085559
chr14	56098993	56099268
chr14	56120333	56120634
chr14	56120634	56120938
chr14	56120949	56121234
chr14	56152130	56152525
chr14	56155981	56156207
chr14	56156449	56156622

chr14	56168018	56168291
chr14	56171731	56171958
chr14	56172029	56172237
chr14	56186991	56187410
chr14	56202092	56202250
chr14	56205109	56205269
chr14	56261988	56262173
chr14	56262229	56262544
chr14	56265553	56265853
chr14	56304335	56304489
chr14	56314481	56314704
chr14	56352018	56352286
chr14	56352313	56352608
chr14	56364882	56365081
chr14	56375799	56376078
chr14	56468195	56468487
chr14	56512236	56512435
chr14	56571481	56572135
chr14	56575421	56575895
chr14	56581173	56581327
chr14	56598845	56598998
chr14	56667814	56668370
chr14	56668370	56668768
chr14	56689783	56690067
chr14	56693925	56694156
chr14	56708305	56708466
chr14	56777360	56777545
chr14	56777545	56777773
chr14	56777773	56777984
chr14	56777984	56778248
chr14	56778248	56778533
chr14	56778533	56778891
chr14	56793315	56793558
chr14	56811177	56811635
chr14	56811635	56811990
chr14	56811990	56812407
chr14	56812407	56812742
chr14	56812742	56812997
chr14	56820546	56820719
chr14	56832171	56832610
chr14	56832662	56833072
chr14	56833433	56833613

chr14	56875299	56875581
chr14	56875581	56875806
chr14	56875806	56876134
chr14	56885335	56885545
chr14	56885545	56885830
chr14	56885859	56886287
chr14	56886287	56886650
chr14	56886650	56887142
chr14	56887164	56887398
chr14	56888017	56888197
chr14	56895011	56895292
chr14	56964458	56964609
chr14	57016827	57017274
chr14	57057602	57057791
chr14	57057895	57058065
chr14	57060079	57060367
chr14	57067986	57068218
chr14	57070012	57070241
chr14	57093112	57093349
chr14	57104104	57105030
chr14	57107920	57108255
chr14	57109346	57109496
chr14	57122708	57122893
chr14	57133224	57133487
chr14	57138369	57138594
chr14	57138594	57138854
chr14	57138854	57139234
chr14	57195421	57195696
chr14	57197241	57197408
chr14	57249861	57250074
chr14	57251230	57251410
chr14	57257939	57258097
chr14	57266881	57267077
chr14	57268318	57268735
chr14	57268760	57268983
chr14	57271968	57272120
chr14	57274055	57274205
chr14	57291325	57292083
chr14	57297616	57298205
chr14	57301971	57302121
chr14	57316781	57317032
chr14	57317042	57317695

chr14	57317890	57318159
chr14	57318159	57318393
chr14	57338711	57339008
chr14	57339008	57339330
chr14	57339367	57339608
chr14	57348190	57348487
chr14	57362827	57363085
chr14	57372442	57372726
chr14	57376760	57377253
chr14	57385785	57385943
chr14	57398162	57398750
chr14	57398818	57399111
chr14	57407436	57407732
chr14	57423541	57423841
chr14	57423841	57424198
chr14	57424198	57424484
chr14	57459889	57460178
chr14	57524640	57524874
chr14	57524874	57525280
chr14	57525280	57525739
chr14	57525739	57526120
chr14	57533254	57533469
chr14	57554689	57554962
chr14	57571237	57571479
chr14	57571479	57571780
chr14	57607081	57607263
chr14	57607369	57607883
chr14	57619563	57619786
chr14	57656511	57656764
chr14	57656764	57656971
chr14	57658438	57658596
chr14	57664283	57664492
chr14	57664492	57664654
chr14	57664654	57664885
chr14	57664885	57665515
chr14	57679995	57680425
chr14	57684658	57684910
chr14	57684910	57685196
chr14	57689155	57689329
chr14	57695673	57695846
chr14	57698056	57698270
chr14	57702774	57703023

chr14	57704890	57705477
chr14	57707145	57707305
chr14	57709357	57709641
chr14	57711438	57711859
chr14	57711862	57712330
chr14	57718141	57718349
chr14	57718349	57718549
chr14	57729805	57730222
chr14	57730281	57731145
chr14	57736648	57736923
chr14	57736923	57737348
chr14	57745403	57745783
chr14	57745783	57746026
chr14	57746026	57746303
chr14	57746303	57746532
chr14	57746532	57746732
chr14	57749392	57749607
chr14	57753763	57753940
chr14	57759894	57760159
chr14	57760229	57761133
chr14	57762751	57762945
chr14	57777351	57777675
chr14	57777675	57778033
chr14	57778044	57778194
chr14	57791186	57791473
chr14	57797478	57797747
chr14	57797771	57798334
chr14	57798334	57798567
chr14	57798567	57798832
chr14	57798832	57799035
chr14	57808628	57808822
chr14	57810760	57811008
chr14	57811008	57811311
chr14	57811311	57811568
chr14	57825815	57826097
chr14	57826097	57826448
chr14	57826448	57826866
chr14	57839706	57840342
chr14	57840697	57840852
chr14	57850404	57850911
chr14	57850927	57851131
chr14	57851137	57851398

chr14	57851936	57852319
chr14	57852319	57852534
chr14	57853074	57853271
chr14	57881803	57882144
chr14	57882799	57883032
chr14	57883383	57883696
chr14	57889532	57889728
chr14	57889728	57889952
chr14	57889952	57890230
chr14	57890230	57890761
chr14	57894359	57894625
chr14	57896124	57896352
chr14	57896958	57897219
chr14	57910480	57911027
chr14	57911923	57912272
chr14	57912272	57912595
chr14	57959367	57959740
chr14	57980294	57980519
chr14	57997586	57997774
chr14	57998741	57999118
chr14	57999118	57999597
chr14	58019013	58019165
chr14	58027984	58028269
chr14	58029355	58029771
chr14	58030087	58030455
chr14	58037214	58037431
chr14	58045870	58046125
chr14	58046166	58046402
chr14	58071070	58071254
chr14	58071254	58071429
chr14	58071700	58072030
chr14	58072030	58072389
chr14	58074972	58075138
chr14	58075550	58075714
chr14	58076313	58076576
chr14	58096868	58097120
chr14	58175695	58175880
chr14	58183059	58183248
chr14	58189894	58190367
chr14	58253321	58253663
chr14	58253663	58254040
chr14	58281857	58282266

chr14	58306674	58307044
chr14	58308717	58309198
chr14	58324237	58324498
chr14	58343354	58343744
chr14	58445768	58445971
chr14	58487946	58488126
chr14	58658650	58658836
chr14	59025891	59026058
chr14	59116111	59116605
chr14	59200821	59201196
chr14	59201196	59201533
chr14	59201533	59201843
chr14	59330574	59330804
chr14	59440415	59441366
chr14	59463555	59463881
chr14	59467907	59468068
chr14	59470882	59471202
chr14	59478687	59478932
chr14	59547686	59548070
chr14	59550929	59551197
chr14	59551438	59551627
chr14	59551929	59552435
chr14	59552435	59552849
chr14	59569452	59569603
chr14	59571912	59572175
chr14	59572175	59572416
chr14	59573418	59573643
chr14	59573965	59574209
chr14	59588039	59588244
chr14	59590671	59590914
chr14	59597359	59597739
chr14	59597739	59598074
chr14	59598074	59598311
chr14	59604080	59604294
chr14	59605792	59605987
chr14	59609516	59609668
chr14	59625138	59625396
chr14	59625399	59625730
chr14	59994959	59995182
chr14	60015994	60016216
chr14	60059711	60059896
chr14	60086790	60087040

chr14	60163516	60163689
chr14	60176836	60177413
chr14	60177413	60177779
chr14	60188310	60188470
chr14	60188519	60188748
chr14	60240172	60240521
chr14	60245342	60245613
chr14	60250894	60251272
chr14	60251272	60251717
chr14	60251717	60252005
chr14	60252005	60252222
chr14	60264922	60265455
chr14	60265455	60265778
chr14	60286274	60286464
chr14	60286591	60286741
chr14	60318746	60318915
chr14	60372123	60372335
chr14	60378100	60378417
chr14	60378514	60378782
chr14	60378782	60379078
chr14	60379078	60379283
chr14	60409592	60409931
chr14	60409931	60410242
chr14	60418044	60418368
chr14	60418903	60419315
chr14	60425680	60425866
chr14	60427948	60428439
chr14	60428439	60429012
chr14	60430323	60430528
chr14	60431824	60432252
chr14	60432252	60432625
chr14	60435640	60435843
chr14	60485189	60485343
chr14	60485354	60485542
chr14	60485545	60485933
chr14	60485942	60486235
chr14	60486589	60486863
chr14	60525369	60525616
chr14	60526841	60527168
chr14	60529084	60529534
chr14	60529882	60530269
chr14	60541279	60541594

chr14	60541594	60542030
chr14	60569810	60570024
chr14	60572512	60572791
chr14	60572887	60573037
chr14	60592905	60593215
chr14	60593218	60593392
chr14	60606229	60606618
chr14	60621042	60621367
chr14	60621367	60621607
chr14	60634002	60634287
chr14	60634287	60634567
chr14	60634567	60634860
chr14	60634860	60635287
chr14	60657944	60658227
chr14	60667500	60667716
chr14	60668036	60668252
chr14	60688865	60689105
chr14	60694750	60694900
chr14	60697330	60697801
chr14	60712537	60712908
chr14	60726080	60726486
chr14	60726486	60726814
chr14	60732760	60733205
chr14	60736564	60736807
chr14	60736807	60737066
chr14	60740579	60740800
chr14	60741057	60741334
chr14	60741519	60741789
chr14	60741789	60742215
chr14	60759947	60760462
chr14	60765077	60765273
chr14	60771010	60771240
chr14	60771240	60771488
chr14	60784380	60784699
chr14	60784699	60784985
chr14	60784985	60785237
chr14	60790194	60790449
chr14	60935631	60935935
chr14	60947056	60947444
chr14	60993154	60993463
chr14	60993496	60993885
chr14	61037866	61038072

chr14	61046397	61046758
chr14	61060454	61060607
chr14	61108728	61108900
chr14	61112563	61112723
chr14	61144006	61144156
chr14	61171461	61171894
chr14	61172453	61173214
chr14	61173214	61173621
chr14	61238788	61239188
chr14	61261849	61262010
chr14	61294491	61294711
chr14	61294801	61294957
chr14	61302479	61303067
chr14	61303369	61303631
chr14	61305240	61305449
chr14	61309628	61309914
chr14	61310100	61310413
chr14	61310413	61310731
chr14	61310878	61311051
chr14	61316915	61317334
chr14	61324596	61324860
chr14	61327089	61327310
chr14	61327765	61327947
chr14	61328052	61328202
chr14	61331162	61331367
chr14	61331367	61331658
chr14	61354997	61355155
chr14	61360043	61360591
chr14	61360652	61360825
chr14	61421328	61421483
chr14	61439188	61439453
chr14	61439453	61439851
chr14	61439851	61440388
chr14	61447759	61448312
chr14	61452599	61452803
chr14	61468902	61469152
chr14	61471724	61471971
chr14	61472886	61473081
chr14	61473663	61473846
chr14	61479692	61479849
chr14	61484070	61484232
chr14	61498098	61498286

chr14	61498286	61498545
chr14	61510388	61510592
chr14	61530383	61530799
chr14	61556083	61556623
chr14	61556623	61556934
chr14	61556934	61557294
chr14	61559954	61560136
chr14	61571463	61571665
chr14	61599367	61599840
chr14	61647966	61648251
chr14	61648251	61648608
chr14	61648608	61649045
chr14	61649045	61649445
chr14	61680785	61681280
chr14	61681280	61681750
chr14	61682077	61682347
chr14	61682347	61682652
chr14	61682652	61683085
chr14	61686889	61687086
chr14	61687204	61687354
chr14	61706522	61706727
chr14	61723666	61724022
chr14	61737499	61738045
chr14	61739518	61739836
chr14	61739860	61740010
chr14	61744138	61744339
chr14	61751806	61752090
chr14	61757122	61757413
chr14	61757413	61757819
chr14	61758647	61758902
chr14	61770618	61770965
chr14	61773931	61774085
chr14	61774792	61775168
chr14	61775801	61775987
chr14	61797343	61797562
chr14	61809363	61809587
chr14	61825209	61825415
chr14	61863425	61863890
chr14	61863890	61864258
chr14	61880564	61880910
chr14	61880910	61881340
chr14	61930919	61931069

chr14	62023095	62023423
chr14	62170416	62170696
chr14	62252093	62252334
chr14	62300037	62300274
chr14	62331909	62332216
chr14	62332216	62332460
chr14	62332460	62332764
chr14	62339288	62339444
chr14	62353406	62353697
chr14	62353711	62354190
chr14	62361491	62361771
chr14	62370933	62371164
chr14	62389568	62389723
chr14	62443786	62443975
chr14	62446144	62446304
chr14	62446371	62446797
chr14	62454308	62454584
chr14	62457494	62457784
chr14	62489285	62489478
chr14	62492515	62492800
chr14	62493719	62493996
chr14	62506709	62507032
chr14	62509099	62509449
chr14	62509449	62509785
chr14	62512946	62513104
chr14	62530785	62531267
chr14	62531267	62531569
chr14	62533841	62534091
chr14	62555302	62556223
chr14	62556226	62556389
chr14	62580990	62581286
chr14	62627400	62627552
chr14	62638202	62638381
chr14	62641973	62642169
chr14	62730482	62730636
chr14	62759856	62760064
chr14	62760064	62760290
chr14	62760290	62760521
chr14	62760521	62760958
chr14	62760958	62761382
chr14	62761382	62761855
chr14	62774377	62774617

chr14	62775014	62775381
chr14	62775381	62775624
chr14	62784642	62784997
chr14	62785875	62786148
chr14	62786710	62786945
chr14	62787506	62787829
chr14	62796586	62796747
chr14	62798585	62799124
chr14	62806372	62806647
chr14	62806689	62806907
chr14	62806907	62807119
chr14	62807119	62807397
chr14	62812760	62813121
chr14	62814556	62814885
chr14	62825419	62825943
chr14	62825943	62826292
chr14	62837088	62837441
chr14	62837441	62837866
chr14	62837866	62838270
chr14	62843225	62843400
chr14	62855178	62855375
chr14	62858459	62858609
chr14	62870492	62870708
chr14	62872096	62872393
chr14	62879475	62879924
chr14	62880030	62880306
chr14	62880306	62880805
chr14	62885274	62885439
chr14	62888348	62888498
chr14	62949997	62950242
chr14	62957360	62957707
chr14	62957707	62957989
chr14	62987532	62987707
chr14	62987750	62988134
chr14	62991260	62991609
chr14	62991769	62992231
chr14	62992243	62992498
chr14	63013225	63013416
chr14	63013931	63014222
chr14	63014744	63015072
chr14	63020752	63021155
chr14	63054743	63055058

chr14	63066642	63066855
chr14	63067008	63067227
chr14	63073270	63073542
chr14	63076203	63076692
chr14	63088537	63088814
chr14	63093677	63094027
chr14	63102911	63103454
chr14	63103863	63104119
[truncated: 216,636 more chars]
